# Plasma metabolomic signatures of all and cause-specific cancers: a multi-platform population-based study

**DOI:** 10.1007/s11306-026-02397-6

**Published:** 2026-02-20

**Authors:** Yu Shuai, Rikje Ruiter, Bruno H. Stricker, M. Arfan Ikram, Mohsen Ghanbari

**Affiliations:** 1 Department of Epidemiology, Erasmus MC University Medical Center, Rotterdam, The Netherlands; 2 Department of Internal Medicine, Maasstad hospital, Rotterdam, Netherlands

**Keywords:** Metabolomic signatures, Circulating metabolites, Biomarker, All cancers, Cause-specific cancers, Population-based study

## Abstract

**Introduction:**

Early diagnosis of cancer is essential for improving patient outcomes. Metabolomics analysis has shown promise in detecting cancer and distinguishing its metastatic burdens in previous studies.

**Objectives:**

We hypothesized that metabolomics data can differentiate between people with and without cancer at a population level, uncovering new biomarkers and deepening our understanding of cancer metabolism.

**Methods:**

A total of 1,386 metabolites were measured by two commonly used metabolomics platforms: Nightingale and Metabolon, in baseline plasma samples from participants in the population-based Rotterdam Study, with sample sizes of 2,538 and 5,057, respectively. Logistic regression and competing risk Cox proportional hazards models were employed to examine associations between these metabolites and both baseline prevalent and incident during follow-up of all and cause-specific cancers. Statistical significance was defined by a false discovery rate (FDR) < 0.05.

**Results:**

There were 654 cancer cases at baseline, and 618 new cases also occurred during follow-up of nearly 10 years. In the cross-sectional study, 68, 7, and 10 metabolites were significantly associated with prevalent blood, colorectal, and all cancer, after multivariate adjustment. In the longitudinal study, 19, 11, 2, 3, and 1 metabolites were significantly associated with incident blood, colorectal, lung, prostate, and all cancer, respectively. Among these, 17 and 2 metabolites were associated with both prevalent and incident blood and colorectal cancer.

**Conclusions:**

This study indicates several circulating metabolites that are associated with different cancers. These metabolites may contribute to better understanding of the metabolic pathways of cancer and serve as biomarkers for early cancer diagnosis.

**Supplementary Information:**

The online version contains supplementary material available at 10.1007/s11306-026-02397-6.

## Introduction

Metabolomics analysis aims to detect, identify, and quantify a diverse range of low molecular weight biochemicals present in biological fluids, tissues, and cells (Johnson et al., 2016). It reflects end-stage alterations in the genome, transcriptome, and proteome, thereby offering more insights into the pathological processes underlying diseases (Adam et al., 2021; Li et al., 2020; Liu et al., 2022). Substantial evidence suggests that the reprogramming of cellular energy metabolism is a central characteristic of cancer, effectively supporting the demands of uncontrolled tumor proliferation (Schiliro & Firestein, 2021). This metabolic dysregulation gives rise to a characteristic metabolic phenotype, emerging as a novel diagnostic approach for various human cancers, including breast (Xiao et al., 2022), liver (Liu et al., 2022), and colorectal (Yachida et al., 2019) cancers, among others (Wang et al., 2021). Moreover, it can aid in tumor staging and prognosis, or serve as a biomarker for therapeutic response (Li et al., 2022a; Luo et al., 2020). Previously, altered expression of numerous plasma metabolites has been shown in various types of cancer, rather than a singular type. For instance, mutations in the isocitrate dehydrogenase (IDH), IDH-1 and IDH-2 genes occur in patients with malignant glioma (60–90%), chondrosarcoma (50–70%), cholangiocarcinoma (10–20%) and acute myeloid leukemia (10–20%) (Cairns & Mak, 2013). These mutations result in the excessive production of 2-hydroxyglutaric acid (2-HG), a metabolite whose accumulation interferes with normal cellular metabolic and signaling pathways implicated in tumorigenesis and progression (Dang et al., 2009). Furthermore, in a cohort study of prostate cancer, elevated levels of omega-6 polyunsaturated fatty acids were positively correlated with an increased risk of cancer, while omega-3 polyunsaturated fatty acids were inversely associated with cancer risk (Leitzmann et al., 2004). Similarly, certain polyunsaturated fatty acids were associated with an increased risk of breast cancer in a nested case-control study (Saadatian-Elahi et al., 2002).

Despite extensive body of research examining metabolites as potential biomarkers of cancer, previous studies typically employed case-control designs or focus solely on specific subclasses of metabolites, such as free amino acids in plasma, while lacking replication datasets (Adam et al., 2021; Huang et al., 2021; Stevens et al., 2023). Hence there is a need to systematically explore the association between numerous circulatory metabolites involved in different metabolic pathways and different type of cancers in order to discover novel biomarkers, including testing their causal role in the cancer development and progression. To this end, we included a wide-range of plasma metabolites from two commonly used metabolomics platforms and a prospective population-based cohort study for cancer assessment.

## Methods

### Study population

**Fig. 1:**
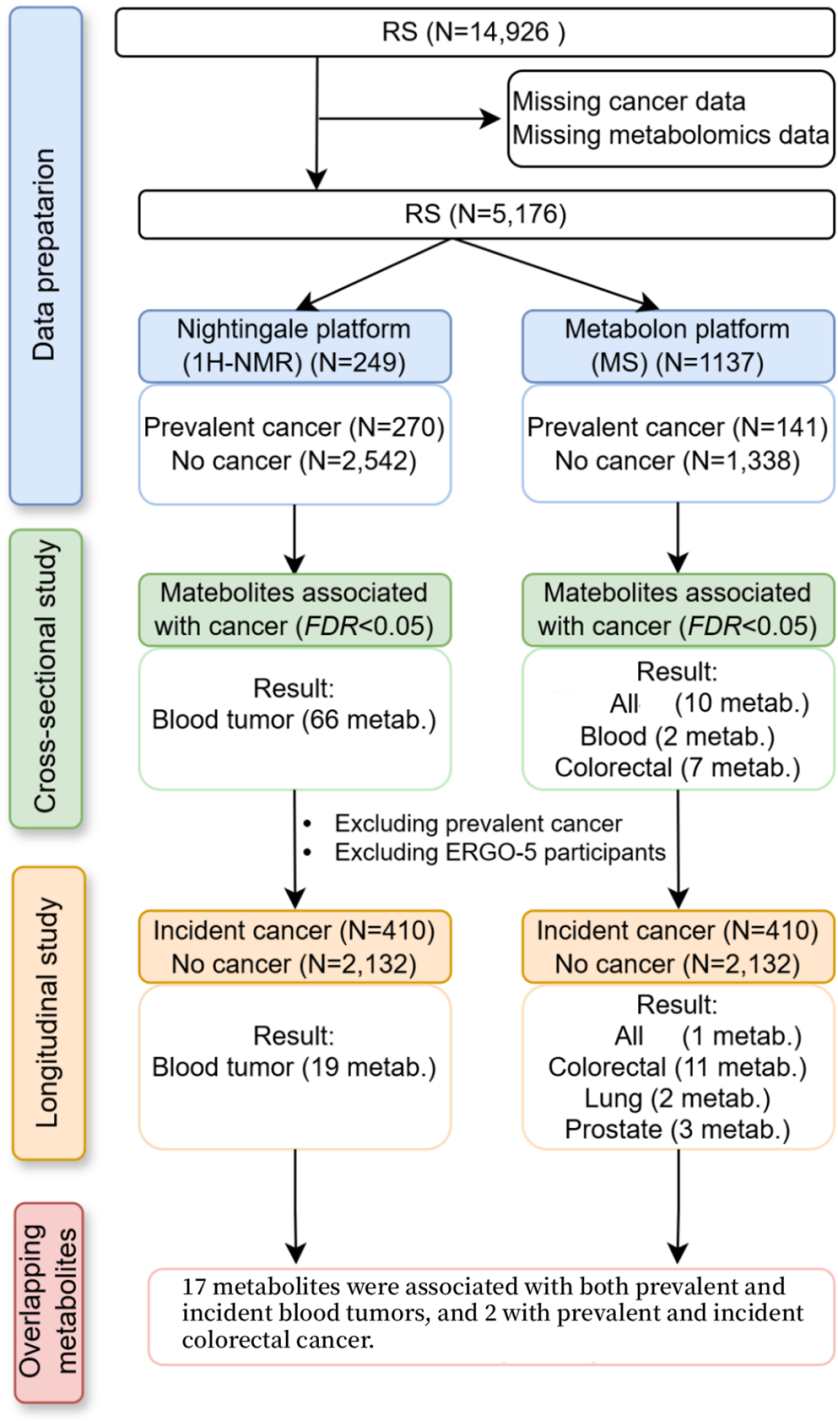
Graphical abstract and flowchart of the study participants. The observational analysis included 5,176 participants from the Rotterdam Study, based on the fourth (ERGO-4) and fifth (ERGO-5) cohort visits. The Metabolon platform measured 1,137 metabolites, with 939 metabolites overlapped between the two Rotterdam Study visits. N, sample sizes; metab., metabolites. ^*^ refers to the correlation between prevalent cancer and 939 metabolites from meta-analysis of ERGO-4 and ERGO-5. ^#^ refers to the correlation between incident cancer and 1086 metabolites from ERGO-4

The study was carried out in the Rotterdam Study (RS), which is a prospective ongoing population-based elderly cohort. In 1990 (RS-I), all residents aged 55 and older residing in Ommoord, a district of Rotterdam in the Netherlands, were invited to participate in the study. Of the 10,215 invited inhabitants, 7,983 agreed to participate in the baseline examinations. In 2000 (RS-II), 3,011 participants (out of 4,472 invitees) who had become 55 years of age or moved into the study district since the start of the study were added to the cohort. In 2006 (RS-III) a further extension of the cohort was initiated in which 3,932 subjects were included. In 2016 (RS-IV), the most recent extension of the cohort was set up with 3,005 persons aged 40 years. By the start of 2021, the Rotterdam Study overall comprised 17,931 participants aged 40 years or over. Further details regarding the objectives and design of the study have been reported previously (Ikram et al., 2020). The current study examined the expression profiles of subsets of the RS from the fourth (ERGO-4) and fifth (ERGO-5) visits based on the baseline cohort using two metabolomics platforms. Plasma samples were evaluated using the Nightingale metabolomics platform consisting of a group of 2,735 participants from the fourth visit to RS-I (RS-I-4), and a group of 77 participants from the second visit to RS-II (RS-II-2) in ERGO-4, and a group of 496 participants from the third visit to RS-II (RS-II-3) and a group of 1,749 participants from the second visit to RS-Ⅲ (RS-Ⅲ-2) in ERGO-5. In addition, plasma metabolites measured by the Metabolon platform were available in subsets from the RS-I-4 (*n* = 1,479) in ERGO-4 and RS-III-2 (*n* = 1,509) in ERGO-5. The respondents in the Rotterdam Study received regular follow-ups at intervals of approximately 3 to 5 years. At each visit, participants completed questionnaires, underwent physical examinations, and provided fasting blood samples. We included participants for whom data on circulating metabolites and cancer information were available at baseline. Given the length of follow-up time and sample size, the longitudinal analyses were conducted only in the ERGO-4 visit (RS-I-4 and RS-II-2), excluding participants with prevalent cancer. A comprehensive illustration of the study approach and design is depicted in overview Fig. 1.

## Assessment of plasma metabolites

Metabolic biomarkers in the Nightingale platform were quantified by analyzing ethylenediaminetetraacetic acid (EDTA) fasting plasma samples using high-throughput proton nuclear magnetic resonance (NMR) metabolomics (Nightingale Ltd, Helsinki, Finland). The platform simultaneously quantified 249 metabolites (168 individual metabolites and 81 ratios), including conventional lipids, lipoprotein subclass analysis of lipid concentrations within 14 subclasses, fatty acid composition, and a range of low molecular weight metabolites such as amino acids, ketone bodies, and metabolites related to sugar metabolism. Further information on the experiments and applications of this NMR metabolomics platform has been previously described (Ikram et al., 2020; Soininen et al., 2009).

In addition, 1,387 circulating metabolites were measured by the untargeted Metabolon HD4 platform. These metabolites are from different biochemical pathways, including lipids, amino acids, xenobiotics, nucleotides, cofactors and vitamins, peptides, carbohydrates, energy-related metabolites, and uncharacterized metabolites. Quantification on this platform was conducted using ultra-high performance liquid chromatography linked to tandem mass spectrometry (UHPLC-MS/MS), a technique developed by Metabolon, Inc. Pre-processing of the data was performed before data-analyses. First, 14 participants with missingness greater than 5 times the standard deviation (SD) of the mean missingness in all participants were excluded. Then all metabolites with missingness greater than 5 times SD of the mean missingness in all metabolites and with a coefficient of variation greater than 30% in internal control samples (NIST Standard Reference Material) were excluded. The remaining metabolites were log-transformed using a log_2_ transformation. Finally, we removed metabolites with missingness greater than 30% and then imputed the missing data in the remaining metabolites (*n* = 1,137) with the lowest limit of detection. Overall, the remaining metabolites from this platform included in our study were *n* = 1,086 in ERGO-4 subcohort and *n* = 990 in ERGO-5 subcohort (for more details see supplementary material). Of these, the number of overlapping plasma metabolites was 939.

## Assessment of cancer

Data on cancer cases were obtained through the general practitioners data and linked with a local pathology laboratory in Rotterdam (PATHAN), a national database of pathology (PALGA), and a database of hospital discharges. Cancer was defined as any primary malignant tumor, excluding non-melanoma skin cancer cases. Only pathology-confirmed cancer cases were included in the analysis to exclude the possibility of false-positive cancer diagnoses. Cancer diagnoses were independently coded and classified by two physicians according to the International Classification of Diseases, 10th revision (ICD-10). In the case of discrepancies, consensus was sought through consultation with a physician specialized in internal medicine. The date of diagnosis was based on the date of biopsy (for solid tumors) and laboratory assessment (for hematologic tumors), or if unavailable, the date of hospital admission or discharge letter. In the case of multiple cancers within one participant, only the first diagnosis was included for analysis.

To align with the study objectives and accommodate statistical power constraints, incident all-cancer cases were defined as the primary outcome. Cause-specific cancers were designated as secondary outcomes and were analyzed only for cancer types with ≥ 25 cases. Cancer types with fewer cases were not evaluated separately due to insufficient statistical power. Additionally, baseline prevalent all-cancer and cause-specific cancers were also considered as secondary outcomes.

## Assessment of covariates

Home administered interviews were used to assess participants’ age, sex, and smoking status (current/former/never). Body mass index was calculated based on weight in kilograms divided by the height in meters squared. Participants were categorized as primary education (primary), lower/intermediate general education or lower vocational education (lower), intermediate vocational education or higher general education (intermediate), and higher vocational education or university (higher). Alcohol consumption was assessed in grams of ethanol per day. In addition, participants’ lipid-lowering medication use history was assessed by interview (yes/no). Blood samples of participants were obtained during the visit to the research center.

## Statistical analyses

Baseline characteristics were described using median and interquartile range (IQR) for continuous variables and proportions for categorical variables. Values of individual biomarkers were natural log-transformed to approximate normality and then standardized to the same scale by z-scores [mean = 0, SD = 1].

Participants contributed person-time to the study from the date of blood draw until the occurrence of cancer, death, the last health status update when they were known to be cancer-free, or January 1, 2015, whichever came first. The competing risk Cox proportional hazards regression model was used to estimate hazard ratios (HRs) and 95% confidence intervals (CIs) for metabolites and cancer risk associations, taking into account that the Rotterdam Study was an elderly cohort study. The Schoenfeld residuals were used to test the assumption of proportional hazards. Subsequently, we tested for potential nonlinear associations using multivariate restricted cubic spline and found no evidence of deviation from linearity. To reduce the false-positive results, the Benjamini-Hochberg procedure was used to adjust the *P* values into false discovery rate (FDR). In this study, an FDR-corrected *P* < 0.05 was considered statistically significant. Meanwhile, multivariable logistic regression models were used to investigate the association between metabolite levels and prevalent cancer at baseline. Specifically, participants diagnosed with cancer at the baseline were compared with healthy (cancer-free) participants. The basic model (Model 1) was adjusted for age, sex, time differences, sub-cohort, and lipid lowering medication, whereas the multivariable model (Model 2) was further adjusted for body mass index (BMI), smoking status, alcohol consumption, and education level. Importantly, sex was not adjusted for in models 1 and 2 for breast and prostate cancers. In addition, we performed subgroup analyses using the ERGO-4 and ERGO-5 sub-cohorts separately in the cross-sectional analyses. When using ERGO-5 or ERGO-4 alone, no adjustment was made for time differences. Notably subgroup analyses were performed on the Nightingale platform, and meta-analysis of the results from the Metabolon platform in two sub-cohorts (overlapping plasma metabolites *n* = 939) was performed using a fixed effects model. The effect of heterogeneity was estimated by I^2^, which describes the percentage of the total variation in the meta-analysis attributable to study heterogeneity. Moreover, hematological tumors constitute a heterogeneous group of malignancies. Categorizing them as a single entity may have introduced misclassification bias due to sample size limitations. To address this, a sensitivity analysis was performed, excluding participants diagnosed with hematological cancers in both the cross-sectional study (*n* = 28 [Nightingale platform], *n* = 17 [Metabolon platform]) and the longitudinal study (*n* = 42 [Nightingale platform], *n* = 21 [Metabolon platform]). The association between circulating metabolites and all cancer risk was subsequently reassessed using the same model. To approximate a preclinical window, we conducted additional longitudinal analyses restricted to a follow-up period of ≤ 5 years. These analyses were performed separately for each cancer type and applied the same competing risk Cox models and covariate adjustments as in the full follow-up longitudinal analyses. To mitigate potential bias resulting from missing values, missing covariate data (ranging from 0.04% to 1.8%) were imputed using a multiple imputation technique (*n* = 5 imputations).

All analyses were performed in SPSS statistical software (SPSS, version 25; IBM Crop) and R software version 4.1.1 (Statistical Computing R Foundation). We used different R packages for data manipulation and processing, “MICE” for multiple interpolation, “survival” for longitudinal analyses, and “ggplot2”, “pheatmap” for visualisation.

## Results

 At baseline, the study included 5,057 and 2,538 participants with cancer data and plasma metabolomics data drawn by the Nightingale and the Metabolon platform, respectively (Table 1). These participants showed partial overlap, meaning that a subject may have circulating metabolite data from the two platforms (Fig. S1). Baseline characteristics were not significantly different between the platforms. Notably, the cancer subgroups had a higher mean age compared to the participants without cancer in both platforms, consistent with aging being a known risk factor for cancer. Additionally, Tables S1-2 present the baseline characteristics of the ERGO-4 and ERGO-5 subgroups in different platforms. Participants in ERGO-5 exhibited a slightly younger mean age compared to those in ERGO-4.

**Fig. 2:**
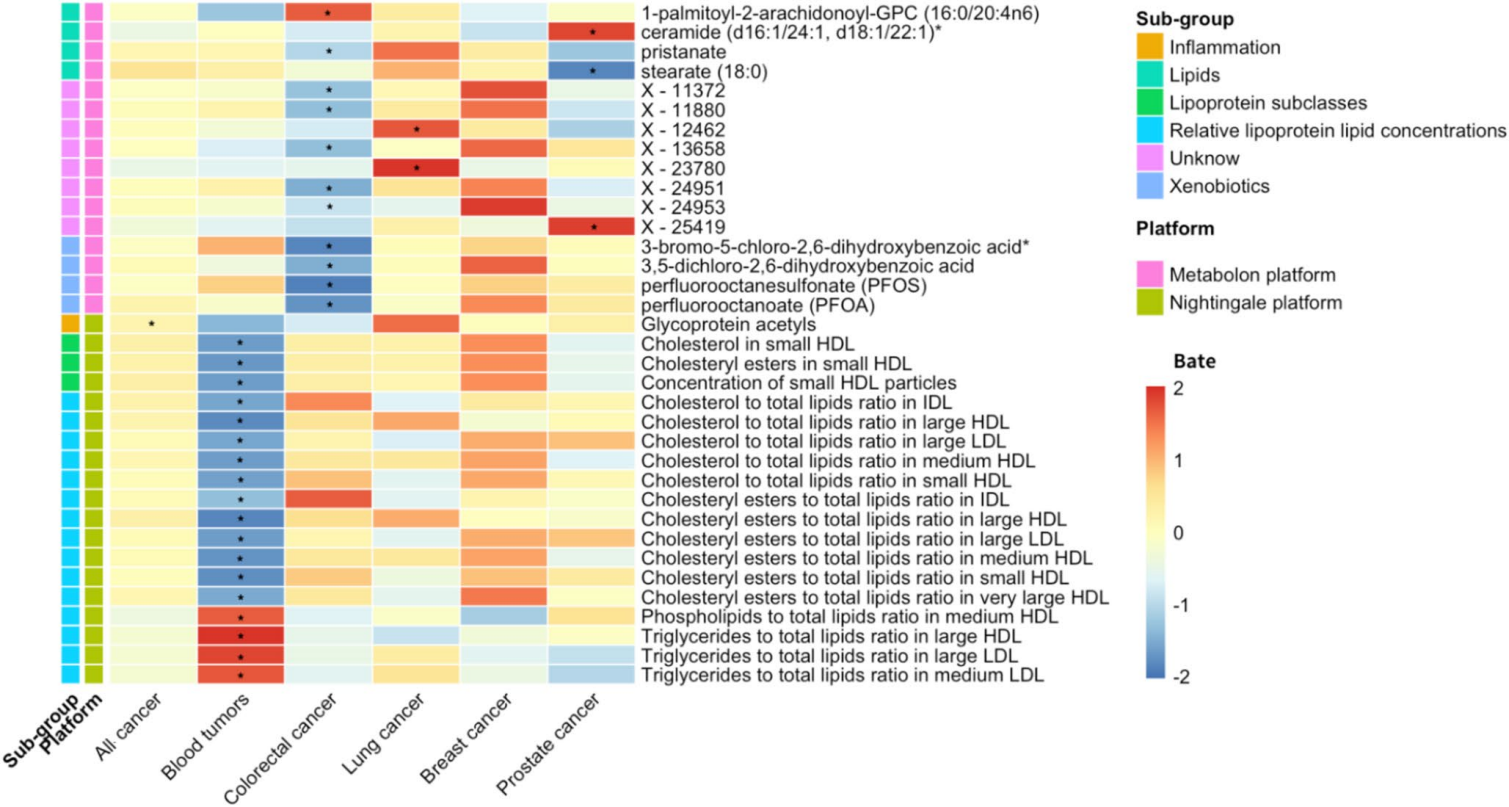
Heatmap visualization of metabolites associated with prevalent all and cause-specific cancers in the cross-sectional analysis. The figure shows metabolites from the Nightingale and Metabolon platforms that were significantly associated with prevalent all, hematological, colorectal, breast, and prostate cancers. The significant threshold is corrected for a false discovery rate (*FDR*) adjusted *P* < 0.05 using * labeled. The colors in columns represent the effect estimates (betas) red indicates a positive relationship, whereas blue indicates a negative relationship. Abbreviations: HDL, high-density lipoprotein; LDL, low-density lipoprotein; IDL, intermediate-density lipoprotein; VLDL, very low-density lipoprotein

 Through a cross-sectional analysis of metabolomics data from both platforms in the Rotterdam Study sub-cohorts, we identified 10, 68, and 7 metabolites, as well as their ratios, significantly associated with prevalent all cancers (Tables S3-4), haematological tumors (Tables S5-6), and colorectal cancers (Tables S7-8), respectively. Figure 2 displays these significant metabolites in the multivariate model 2, with *FDR* < 0.05. Among these, perfluorooctanesulfonate (PFOS) and an the unannotated circulating metabolite X-12,816 showed significant and consistently inverse associations with both colorectal and all cancers. No metabolites were significantly associated with prevalent breast or prostate cancer (Tables S9-12). In subgroup analyses of the Metabolon platform data from ERGO-4 and ERGO-5 visits, which included 147 and 51 non-overlapping metabolites, three unique metabolites from the ERGO-5 visit showed significant association with prevalent breast cancer (Table S11).

**Table 1:** Characteristics of study participants

Characteristic	Nightingale platform (*n* = 5057)			Metabolon platform (*n* = 2538)		
	Prevalent (*n* = 443)	Incident (*n* = 459)	No (*n* = 4155)	Prevalent (*n* = 211)	Incident (*n* = 221)	No (*n* = 2106)
**Age**,** mean (± SD)**,** y**	72.79 (7.54)	73.95 (6.00)	69.86 (8.47)	72.93 (7.86)	75.33 (6.00)	70.31 (9.28)
**Sex**,** Male**,** n (%)**	208 (47.0)	235 (51.2)	1670 (40.2)	102 (48.3)	118 (53.4)	861 (40.9)
**Difference in time**,** ERGO-4**,** n (%)**	270 (60.9)	410 (89.3)	2132 (51.3)	141 (66.8)	208 (94.1)	1130 (53.7)
**Rotterdam Study cohort**,** n (%)**						
RS-I	260 (58.7)	407 (88.7)	2068 (49.8)	141 (66.8)	208 (94.1)	1130 (53.7)
RS-II	71 (16.0)	33 (7.2)	469 (11.3)	-	-	-
RS-III	112 (25.3)	19 (4.1)	1618 (38.9)	70 (33.2)	13 (5.9)	976 (46.3)
**Smoking status**,** n (%)**						
Current	52 (12.0)	68 (15.1)	487 (11.9)	24 (11.5)	29 (13.3)	260 (12.4)
Former	260 (59.9)	271 (60.4)	2206 (53.8)	128 (61.2)	136 (62.4)	1124 (53.7)
Never	122 (28.1)	110 (24.5)	1411 (34.4)	57 (27.3)	53 (24.3)	711 (33.9)
**BMI**,** kg/m2**,** mean (± SD)**	27.56 (4.17)	27.63 (4.00)	27.43 (4.26)	27.65 (4.11)	27.45 (3.52)	27.35 (4.26)
**Education level**,** n(%)**						
Primary	49 (11.2)	59 (13.0)	467 (11.3)	26 (12.4)	30 (13.7)	265 (12.6)
Lower	187 (42.7)	184 (40.4)	1649 (39.9)	93 (44.5)	95 (43.4)	799 (38.1)
Intermediate	131 (29.9)	151 (33.2)	1210 (29.3)	64 (30.6)	72 (32.9)	599 (28.6)
Higher	71 (16.2)	61 (13.4)	809 (19.6)	26 (12.4)	22 (10.0)	433 (20.7)
**Lipid lowering treatment**,** yes**,** n (%)**	102 (23.1)	104 (22.8)	1049 (25.3)	56 (26.5)	52 (23.5)	515 (24.5)
**Alcohol intake (g/day)**,** Median (IQR)**	7.14 (0.54–15.21)	7.85 (0.71–17.86)	6.43 (0.54–14.29)	8.57 (0.54–17.14)	7.29 (0.71–17.14)	6.43 (0.54–14.29)
**Cancer types**,** yes**,** n (%)**						
Lung	9 (2.0)	61 (13.3)	-	3 (1.4)	30 (13.6)	-
Breast	120 (27.1)	59 (12.9)	-	57 (27.0)	29 (13.1)	-
Prostate	120 (27.1)	58 (12.6)	-	58 (27.5)	22 (10.0)	-
Blood	28 (6.3)	47 (10.2)	-	17 (8.1)	24 (10.9)	-
Colorectal	60 (13.5)	85 (18.5)	-	30 (14.2)	41 (18.6)	-
Other	106 (24.0)	149 (32.5)	-	46 (21.8)	75 (33.9)	-

The table shows characteristics of the study participants within two metabolomics platforms. Values are represented as mean (± SD), sample sizes (%), or median (inter-quartile range) for characteristics with skewed distributions. Values are shown for non-imputed data. The number of missing values in the Nightingale and Metabolon platform study populations was 70 (1.4%) and 16 (0.6%) for smoking status; 91 (1.8%) and 23 (0.9%) for BMI; 29 (0.6%) and 14 (0.6%) for education; 70 (1.4%) and 16 (0.6%) for alcohol; 12 (0.2%) and 0 (0%) for lipid lowering treatment, respectively. Other means the sum of rest cancer samples. ERGO-4 and 5 refer to the fourth and fifth visits from the baseline cohort, respectively. Prevalent, incident, and no mean the prevalent cancer, incident cancer, and no cancer, respectively. *RS* Rotterdam study cohort, *BMI* Body mass index, *SD* Standard deviation, *IQR* Interquartile range, *y* years, *n* number

**Fig. 3:**
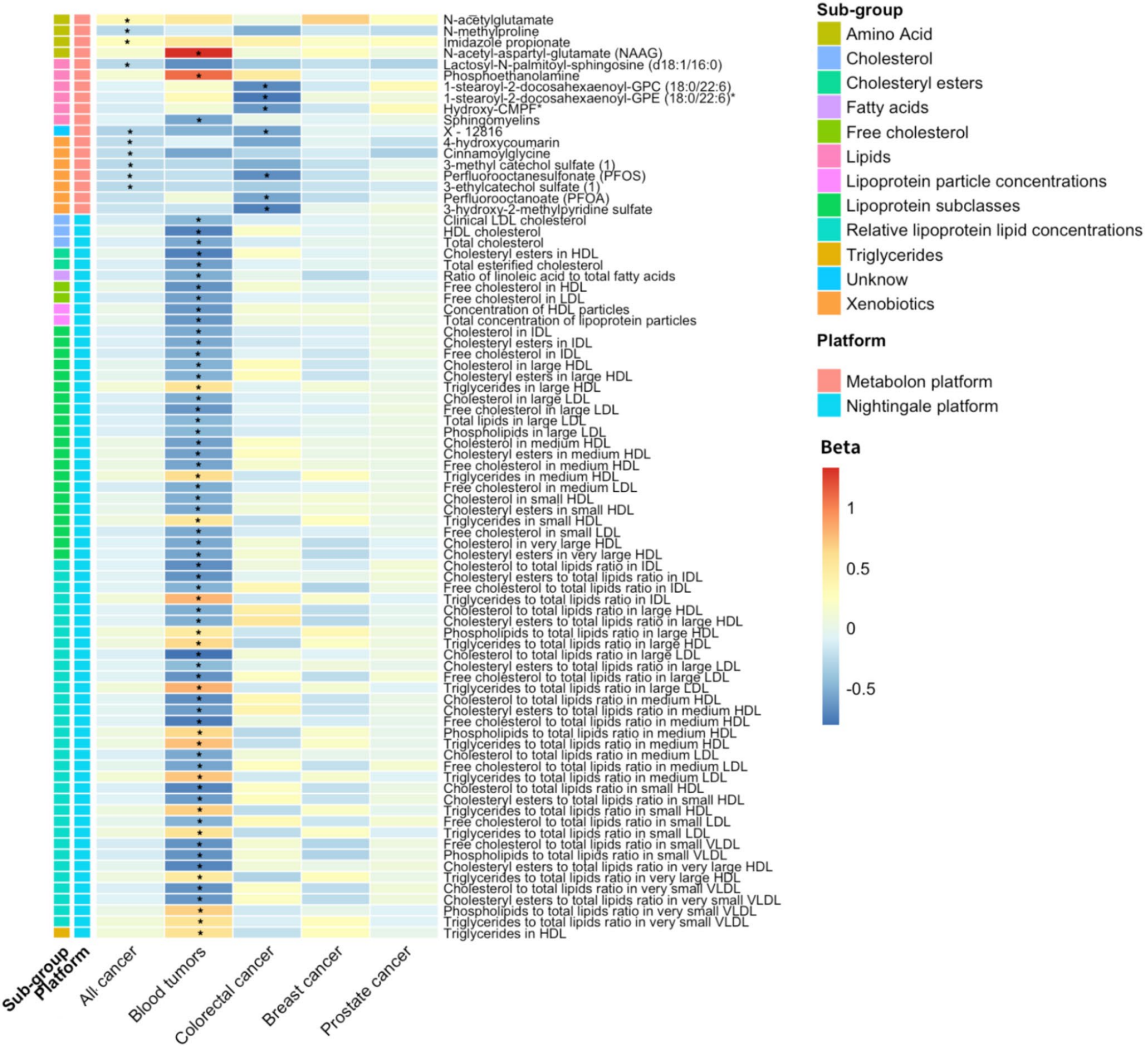
Heatmap visualization of metabolites associated with incident all and cause-specific cancers in the longitudinal analysis. The figure shows metabolites from the Nightingale and Metabolone platform that were significantly associated with incident all, blood, colorectal, breast, lung, and prostate cancers in ERGO-4 subgroup. The significant threshold is corrected for a false discovery rate (*FDR*) adjusted *P* < 0.05 using * labeled. The colors in columns represent the standardized HR, red indicates a positive relationship, whereas blue indicates a negative relationship. ERGO-4 refer to the fourth visits of the baseline cohort. Abbreviations: HDL, high-density lipoprotein; LDL, low-density lipoprotein; IDL, intermediate-density lipoprotein; HR, hazard ratio

 In the longitudinal analysis, the follow-up period for the ERGO-4 subgroup was 8.13 (± 3.2) years for participants with the Nightingale platform and 8.05 (± 3.2) years for the Metabolon platform. A total of 410 (14.6%) participants in the Nightingale group and 208 (18.4%) in the Metabolon group were diagnosed with incident cancers. This included 85 cases of lung cancer, 72 of breast cancer, 71 of prostate cancer, 63 of hematologic malignancies, and 118 of colorectal cancer. Competing risk Cox proportional hazards regression analysis identified 19 and 11 metabolites significantly associated with incident hematologic malignancies and colorectal cancer (*FDR* < 0.05, model 2) across both platforms, respectively (Tables S13-16). Furthermore, we identified a positive correlation between the expression levels of glycoprotein acetyls (GlycA) and incident all cancers (HR, 1.24 [95% CI, 1.11–1.37], *FDR* = 2.22 × 10^− 2^) (Tables S17-18). Also, two and three circulating metabolites were significantly associated with incident lung and prostate cancers, respectively (Tables S19-22). No metabolites were associated with incident breast cancer (Tables S23-24). An overview of the metabolites significantly associated with incident cancers of different types is shown in Fig. 3.

 To explore potential early preclinical metabolic alterations, we conducted an additional longitudinal analysis restricting follow-up to ≤ 5 years. Although the number of incident cancer cases was reduced across both platforms (*N* = 342, Table S25), several patterns remained significant. Of note, 56 metabolites were significantly associated with incident hematologic malignancies within the 5-year window (*FDR* < 0.05, Model 2; Tables S26–S27). These metabolites substantially overlapped with those identified in the prevalent hematologic malignancy analysis and the full follow-up analysis, primarily involving HDL-related lipid components, cholesterol esters, and triglyceride-to-lipid ratios across multiple lipoprotein subclasses (Table 2, Figure S2A). Likewise, 15 metabolites were significantly associated with incident colorectal cancer in the 5-year analysis (*FDR* < 0.05, Model 2, Tables S28–S29). Notably, PFOS (HR, 0.44 [95% CI, 0.34–0.56]; *FDR* = 1.67 × 10^−7^) and perfluorooctanoate (PFOA) (HR, 0.48 [95% CI, 0.35–0.66]; *FDR* = 1.62 × 10^−2^) overlapped with findings from both the cross-sectional and full longitudinal analyses (Table 2, Figure S2B). GlycA also remained significantly associated with all cancer incidence (Tables S30–S31). Also, 3 and 15 metabolites were associated with incident lung and prostate cancer, respectively (Tables S32–S35); however, none overlapped with the corresponding cross-sectional or full longitudinal analyses, although several effect directions were consistent. No significant associations were observed for breast cancer (Tables S36–S37). In our sensitivity analyses, we examined the associations between plasma metabolites and all cancer after excluding participants with hematological tumors. With this exclusion, the statistical significance of results decreased in the cross-sectional study, 9 of the 10 metabolites previously associated with prevalent all cancer lost statistical significance (Tables S38–S39). However, despite this loss of significance, these 9 metabolites exhibited a consistent direction of effect. In the longitudinal study, the association for glycoprotein acetyls remained significant (*FDR* = 1.29 × 10⁻², Tables S40–S41).

**Table 2:** Circulating metabolites significantly associated with cancer in cross-sectional and longitudinal (≤ 5-year or full follow-up) analyses

Metabolite	Cross-sectional		Longitudinal (≤ 5-year)		Longitudinal (full follow-up)	
	Beta ± SE	FDR value	HR (95% CI)	FDR value	HR (95% CI)	FDR value
Blood tumors						
Cholesterol to total lipids ratio in IDL^(N)^	−0.67 ± 0.15	**4.02E-04**	0.71 (0.54–0.94)	8.79E-02	0.68 (0.54–0.84)	**1.51E-02**
Cholesteryl esters to total lipids ratio in IDL^(N)^	−0.65 ± 0.15	**6.68E-04**	0.73 (0.51–1.05)	2.68E-01	0.67 (0.52–0.86)	**2.59E-02**
Cholesterol to total lipids ratio in large HDL^(N)^	−0.52 ± 0.12	**5.08E-04**	0.66 (0.46–0.93)	1.03E-01	0.68 (0.54–0.86)	**2.26E-02**
Cholesteryl esters to total lipids ratio in large HDL^(N)^	−0.52 ± 0.12	**4.02E-04**	0.65 (0.48–0.87)	**4.41E-02**	0.68 (0.56–0.83)	**7.70E-03**
Triglycerides to total lipids ratio in large HDL^(N)^	0.64 ± 0.17	**1.81E-03**	1.58 (1.04–2.4)	1.35E-01	1.59 (1.19–2.12)	**2.59E-02**
Cholesterol to total lipids ratio in large LDL^(N)^	−0.81 ± 0.17	**3.96E-04**	0,64 (0.45–0.91)	8.73E-02	0.61 (0.47–0.8)	**1.05E-02**
Cholesteryl esters to total lipids ratio in large LDL^(N)^	−0.44 ± 0.17	**4.26E-02**	0.65 (0.46–0.91)	7.86E-02	0.66 (0.52–0.85)	**2.07E-02**
Triglycerides to total lipids ratio in large LDL^(N)^	0.81 ± 0.18	**4.02E-04**	1.59 (1.13–2.24)	6.60E-02	1.6 (1.21–2.11)	**1.91E-02**
Cholesterol to total lipids ratio in medium HDL^(N)^	−0.66 ± 0.15	**5.08E-04**	0.62 (0.44–0.87)	**4.85E-02**	0.63 (0.49–0.81)	**1.05E-02**
Cholesteryl esters to total lipids ratio in medium HDL^(N)^	−0.6 ± 0.15	**1.01E-03**	0.68 (0.50–0.92)	8.56E-02	0.66 (0.53–0.83)	**1.05E-02**
Phospholipids to total lipids ratio in medium HDL^(N)^	0.64 ± 0.17	**1.72E-03**	1.78 (1.22–2.6)	**3.35E-02**	1.57 (1.18–2.09)	**2.83E-02**
Triglycerides to total lipids ratio in medium LDL^(N)^	0.72 ± 0.17	**7.32E-04**	1.57 (1.16–2.13)	**4.06E-02**	1.52 (1.18–1.96)	**2.14E-02**
Cholesterol in small HDL^(N)^	−0.51 ± 0.2	**4.55E-02**	0.41 (0.28–0.58)	**1.32E-04**	0.55 (0.39–0.78)	**1.51E-02**
Cholesterol to total lipids ratio in small HDL^(N)^	−0.71 ± 0.16	**4.02E-04**	0.73 (0.49–1.07)	3.16E-01	0.64 (0.51–0.82)	**1.20E-02**
Cholesteryl esters in small HDL^(N)^	−0.54 ± 0.19	**3.15E-02**	0.41 (0.31–0.55)	**8.27E-07**	0.53 (0.38–0.74)	**1.05E-02**
Cholesteryl esters to total lipids ratio in small HDL^(N)^	−0.61 ± 0.15	**1.38E-03**	0.75 (0.57–1.00.57.00)	1.86E-01	0.66 (0.54–0.81)	**7.70E-03**
Cholesteryl esters to total lipids ratio in very large HDL^(N)^	−0.71 ± 0.19	**3.28E-03**	0.45 (0.28–0.73)	**1.63E-02**	0.48 (0.36–0.64)	**1.20E-04**
Concentration of small HDL particles^(N)^	−0.44 ± 0.20	8.67E-02	0.41 (0.3–0.57)	**2.93E-06**	0.57 (0.41–0.80)	**2.07E-02**
Cholesteryl esters in HDL^(N)^	−0.74 ± 0.22	**9.17E-03**	0.40 (0.23–0.71)	**2.26E-02**	0,65 (0.44–0.95)	2.12E-01
Cholesterol to total lipids ratio in medium LDL^(N)^	0.56 ± 0.16	**7.04E-03**	0.61 (0.44–0.83)	**2.49E-02**	0.89 (0.61–1.29)	7.85E-01
HDL cholesterol^(N)^	−0.74 ± 0.23	**1.08E-02**	0.40 (0.22–0.73)	**3.35E-02**	0.66 (0.45–0.98)	2.39E-01
Total concentration of lipoprotein particles^(N)^	−0.63 ± 0.22	**2.48E-02**	0.37 (0.25–0.55)	**4.25E-05**	0.62 (0.43–0.89)	1.10E-01
Concentration of HDL particles^(N)^	−0.63 ± 0.22	**2.74E-02**	0.35 (0.23–0.53)	**4.25E-05**	0.61 (0.42–0.88)	1.05E-01
Cholesteryl esters in medium HDL^(N)^	−0.58 ± 0.21	**2.92E-02**	0.41 (0.27–0.61)	**2.55E-04**	0.64 (0,46-0.91.91)	1.33E-01
Cholesterol in medium HDL^(N)^	−0.58 ± 0.21	**3.07E-02**	0.40 (0.26–0.61)	**3.62E-04**	0.65 (0.46–0.92)	1.46E-01
Free cholesterol in medium HDL^(N)^	−0.56 ± 0.22	**4.20E-02**	0.41 (0.27–0.63)	**9.76E-04**	0.69 (0.49–0.97)	2.19E-01
**Colorectal cancer**						
Perfluorooctanesulfonate (PFOS)	−0.66 ± 0.13	**4.10E-04**	0.44 (0,34-0.56.56)	**1.67E-07**	0.47 (0.37–0.59)	**1.50E-07**
Perfluorooctanoate (PFOA)	−0.57 ± 0.13	**5.35E-03**	0.48 (0.35–0.66)	**1.62E-03**	0.52 (0.4–0.66)	**7.20E-05**
X − 11,372	−0.36 ± 0.17	4.21E-01	0.45 (0.32–0.64)	**1.62E-03**	0.52 (0.39–0.69)	**1.33E-03**
X − 24,953	−0.47 ± 0.16	1.42E-01	0.60 (0.48–0.75)	**1.62E-03**	0.64 (0.52–0.79)	**4.32E-03**
X − 24,951	−0.47 ± 0.15	1.26E-01	0.51 (0.38–0.69)	**2.47E-03**	0.52 (0.42–0.65)	**5.39E-06**
3-bromo-5-chloro-2,6-dihydroxybenzoic acid*	−0.41 ± 0.14	1.82E-01	0.49 (0.35–0.69)	**6.07E-03**	0.53 (0.41–0.69)	**7.73E-04**
3,5-dichloro-2,6-dihydroxybenzoic acid	−0.39 ± 0.13	1.74E-01	0.59 (0.45–0.76)	**6.64E-03**	0.60 (0.48–0.75)	**1.33E-03**
X − 11,880	−0.29 ± 0.17	5.88E-01	0.52 (0.38–0.73)	**1.24E-02**	0.60 (0.45–0.79)	**3.69E-02**

The table shows circulating metabolites that were significantly associated with cancer in at least two of the three analytic approaches—the cross-sectional (logistic regression analysis), ≤ 5-year longitudinal analysis, and full follow-up longitudinal (competing risk Cox proportional hazards regression analysis). The metabolites are from the Nightingale (N) and Metabolon (M) platforms. The results are from model 2: adjusted for age, sex, RS-cohort, time differences (only in cross-sectional analysis), lipid-lowering medication, smoking status, alcohol consumption, BMI, and education level. The significance threshold was set at FDR-adjusted *P*-value < 0.05. Bold text indicates *FDR* < 0.05. *SE* Standard error, *IDL* Intermediate-density lipoprotein, *LDL* Low-density lipoprotein, *HDL* High-density lipoprotein, *VLDL* Very-low-density lipoprotein, *BMI* Body mass index, *HR *Hazard ratio, *CI* Confidence interval, *FDR* False discovery rate

## Discussion

In this population-based study, we investigated the association between 1,386 plasma circulating metabolites, assessed by two widely used metabolomic platforms, and different cancer types. Our cross-sectional analysis identified several circulating metabolites significantly associated with prevalent all, blood, and colorectal cancers. Furthermore, several metabolites were significantly associated with incident all, blood, colorectal, lung, and prostate cancer in the longitudinal study. Notably, 17 and 2 of the identified metabolites were commonly associated with blood and colorectal cancers in both cross-sectional and longituidinal studies. These findings suggest the aberrant expression of circulating metabolites as potential biomarkers several years before a cancer diagnosis, which could also provide an improved understanding of cancer pathogenesis.

Circulating metabolites in plasma can respond to changes in cancer pathophysiology and also offer early warning of the evolution of precancerous metabolic profiles (Breeur et al., 2022; Harewood et al., 2024; Huang et al., 2021). For example, Ozturk et al.(Ozturk, 2021) reported higher triglyceride (TG) and lower total cholesterol (TC) levels in the haematological tumour group (*n* = 98) compared to normal controls (*n* = 40). Although the study did not explore the various density lipoprotein subclasses further, we were able to replicate the above findings in a number of subgroups. We found that TG in high-density lipoprotein (HDL) and small/medium/large HDL were significantly and positively associated with prevalent haematological tumours, while TC and cholesterol in intermediate-density lipoprotein/low-density lipoprotein/HDL were negatively associated. Moreover, a large population-based study with 15,864 hematologic malignancies, within a follow-up of 8.3 years, showed the significant association of lower high-density lipoprotein cholesterol (HDL-C) levels with increased hematologic cancer risk, aligning with our results (Jeong et al., 2021). Similar metabolic abnormalities were observed in different hematologic tumor subclasses, as Alford et al.(Alford et al., 2018) showed declining cholesterol levels before lymphoma diagnosis, and Pedersen et al.(Pedersen et al., 2020) linked lower HDL-C levels to non-Hodgkin lymphoma (NHL) and multiple myeloma risk. Previous studies have also shown that high levels of TG and low levels of HDL-C are associated with acute lymphoblastic leukaemia and NHL and suggested them as potential biomarkers for blood cancers (Dessì et al., 1995; Kuliszkiewicz-Janus et al., 2008).

Despite these findings, the mechanism underlying the aberrant metabolome in patients with haematological tumours is not yet clear. In general, reduced HDL-C levels may be a secondary phenomenon driven by cancer cell metabolism, as malignant cells induce lipogenesis and accumulate cholesterol esters for the biosynthesis of new membranes (Choi et al., 2021; Jeong et al., 2021; Pedersen et al., 2020). HDL-C may also influence cellular microenvironment processes, affecting the redistribution of other lipids in the periphery (Lazaris et al., 2021). However, recent studies have shown that different subclasses of HDL differ in their function and ability to promote cholesterol excretion (Akinkuolie et al., 2014; de Leeuw et al., 2021; von Eckardstein et al., 2023). Camont et al.(Camont et al., 2011) found that small HDL has stronger antioxidant and anti-inflammatory properties than large HDL rich in lipids, which could explain the association we found between lower small HDL-C/cholesteryl esters (CE) levels and higher blood tumor risk. Our study identified 17 lipid metabolites significantly associated with hematologic tumor risk, with abnormal expressions occurring up to eight years before diagnosis, particularly in small HDL-C/CE. These findings suggest HDL subclasses may be informative for studying blood tumor pathophysiology, with shifts in lipid metabolism requiring further research.

We also found the association between PFOS and colorectal cancer. However, epidemiological findings on the association between PFOS/PFOA and colorectal cancer are still inconclusive (Barry et al., 2013; Innes et al., 2014; Li et al., 2022b; Rhee et al., 2023). PFOS and PFOA are ubiquitous and persistent environmental pollutants that can affect metabolic regulation, inflammation, and factors related to the development of colorectal cancer precursors (Durham et al., 2023). A large cross-sectional study by Innes et al.(Innes et al., 2014) found a significant inverse association between PFOS and PFOA and colorectal cancer, consistent with our results. Wimsatt et al.(Wimsatt et al., 2016) found that in a familial adenomatous polyposis mouse model, PFOS significantly inhibited the spontaneous tumor growth of both male and female mice in a dose-dependent manner. However, a Swedish cohort study found a positive association between rectal cancer and PFOS exposure (Li et al., 2022b). The American C8 Science Panel study found no association between PFOA exposure and colorectal cancer (Barry et al., 2013). These discrepancies could stem from differences in study design or exposure modeling (like dose, exposure window), highlighting the need for well-designed studies to elucidate the effects of PFOS and PFOA on gastrointestinal tract pathology.

This study has several major strengths. First, we conducted comprehensive observational analyses using data from a prospective population-based cohort study to explore the biomarker potential of circulating metabolites and common cancers. Second, the availability of various covariates allowed us to make relatively rigorous adjustments for potential confounders. Nonetheless, this study has some limitations that must be considered. First and foremost, we were unable to analyze subgroups of cancer subtypes, such as haematological tumors and lung cancer, due to the limited sample size of cancers. Second, we do not connect the metabolite differences to specific defined metabolic pathways and validate our findings in extra cohorts. Third, although the analyses carefully controlled for potential confounders, residual and unmeasured confounding might have influenced the associations. Finally, the majority of Rotterdam participants are European, which may limit the generalizability of our results to other ethnicities. Therefore, future studies should assess the causal relationship between plasma levels of identified metabolites and the risk of cancer development by incorporating additional independent cohorts, preferably from different ethnic groups.

## Conclusion

This study identified several circulating metabolites associated with hematological tumors and colorectal cancer in both cross-sectional and longitudinal analyses, which have the potential to serve as biomarkers. Our results also provide insights into the metabolic underpinnings of cancer pathophysiology. These findings may lay the groundwork for the ongoing search for specific biomarkers that could aid in the early detection and management of cancer.

## Supplementary Information

Below is the link to the electronic supplementary material.

Supplementary Material 1

Supplementary Material 2

## Data Availability

Data can be obtained upon request. Requests should be directed towards the management team of the Rotterdam Study(datamanagement.ergo@erasmusmc.nl), which has a protocol for approving data requests. Because of restrictions based on privacy regulations and informed consent of the participants, data cannot be made freely available in a public repository.

## Abbreviations

FDR: False discovery rate
RS: Rotterdam study
SD: Standard deviation
CIs: Confidence intervals
BMI: Body mass index
PFOS: Perfluorooctanesulfonate
PFOA: Perfluorooctanoate
CE: Cholesteryl esters
TG: Triglyceride
TC: Total cholesterol
HDL: High-density lipoprotein
HDL-C: High-density lipoprotein cholesterol
