# Supplementary material for: Plasma metabolomic signatures of all and cause-specific cancers: a multi-platform population-based study
Source: Metabolomics. 2026 Feb 20;22(2):27. doi: 10.1007/s11306-026-02397-6 (PMC12923424; doi:10.1007/s11306-026-02397-6)

**Figure. S1. Venn diagram showing the overlap of cohort participants with metabolomics data using the Nightingale and Metabolon platforms**

**
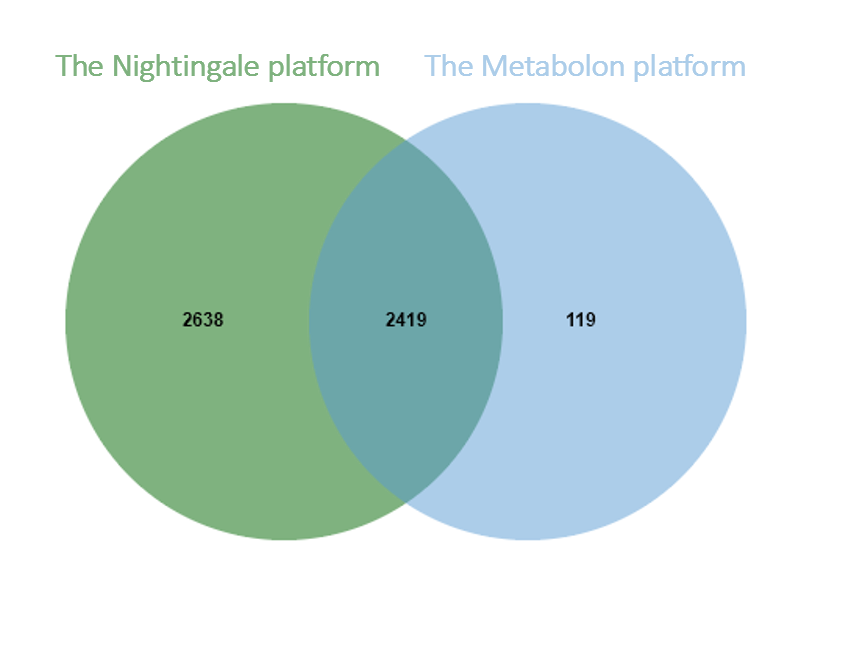
**

**Figure S2. Overlap of significant metabolites identified in the cross-sectional, full follow-up, and ≤5-year follow-up analyses for hematologic malignancies (A) and colorectal cancer (B).**


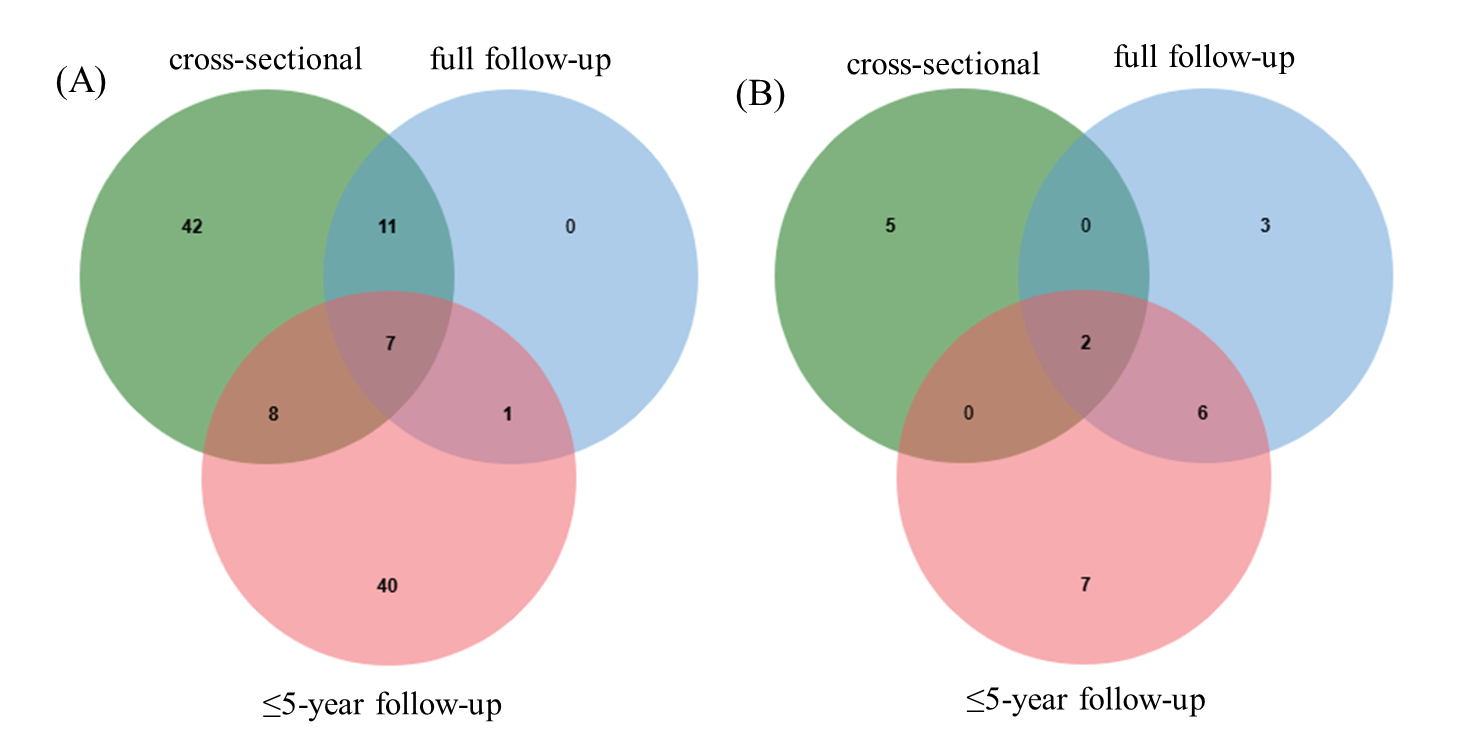

Supplement: Supplementary file 1 — Supplementary Material 1 [file 11306_2026_2397_MOESM1_ESM.docx]
