# Supplementary material for: Plasma metabolomic signatures of all and cause-specific cancers: a multi-platform population-based study
Source: Metabolomics. 2026 Feb 20;22(2):27. doi: 10.1007/s11306-026-02397-6 (PMC12923424; doi:10.1007/s11306-026-02397-6)
Supplement: Supplementary file 2 — Supplementary Material 2 [file 11306_2026_2397_MOESM2_ESM.docx]

| **Supplemental Table/ File** | **Title** |
| --- | --- |
| Supl. table 1 | Baseline characteristics of participants in the ERGO-4 and ERGO-5 subcohorts of the Nightingale platform. |
| Supl. table 2 | Baseline characteristics of participants in the ERGO-4 and ERGO-5 subcohorts of the Metabolon platform. |
| Supl. table 3 | Serum metabolites associated with prevalent all cancer from the Nightingale platform at baseline. |
| Supl. table 4 | Serum metabolites associated with prevalent all cancer from the Metabolon platform at baseline. |
| Supl. table 5 | Serum metabolites associated with prevalent hematological malignancy from the Nightingale platform at baseline. |
| Supl. table 6 | Serum metabolites associated with prevalent hematological malignancy from the Metabolon platform at baseline. |
| Supl. table 7 | Serum metabolites associated with prevalent colorectal cancer from the Nightingale platform at baseline. |
| Supl. table 8 | Serum metabolites associated with prevalent colorectal cancer from the Metabolon platform at baseline. |
| Supl. table 9 | Serum metabolites associated with prevalent breast cancer from the Nightingale platform at baseline. |
| Supl. table 10 | Serum metabolites associated with prevalent breast cancer from the Metabolon platform at baseline. |
| Supl. table 11 | Serum metabolites associated with prevalent prostate cancer from the Nightingale platform at baseline. |
| Supl. table 12 | Serum metabolites associated with prevalent prostate cancer from the Metabolon platform at baseline. |
| Supl. table 13 | Serum metabolites associated with incident hematological malignancy from the Nightingale platform. |
| Supl. table 14 | Serum metabolites associated with incident hematological malignancy from the Metabolon platform. |
| Supl. table 15 | Serum metabolites associated with incident colorectal cancer from the Nightingale platform. |
| Supl. table 16 | Serum metabolites associated with incident colorectal cancer from the Metabolon platform. |
| Supl. table 17 | Serum metabolites associated with incident all cancer from the Nightingale platform. |
| Supl. table 18 | Serum metabolites associated with incident all cancer from the Metabolon platform. |
| Supl. table 19 | Serum metabolites associated with incident lung cancer from the Nightingale platform. |
| Supl. table 20 | Serum metabolites associated with incident lung cancer from the Metabolon platform. |
| Supl. table 21 | Serum metabolites associated with incident prostate cancer from the Nightingale platform. |
| Supl. table 22 | Serum metabolites associated with incident prostate cancer from the Metabolon platform. |
| Supl. table 23 | Serum metabolites associated with incident breast cancer from the Nightingale platform. |
| Supl. table 24 | Serum metabolites associated with incident breast cancer from the Metabolon platform. |
| Supl. table 25 | Counts of incident cancer cases within ≤5 years of follow-up across the Nightingale and Metabolon metabolomics platforms |
| Supl. table 26 | Serum metabolites associated with 5-year incident hematologic malignancies in the Nightingale platform. |
| Supl. table 27 | Serum metaboloites associated with 5-year incident hematological malignancy from the Metabolon platfor |
| Supl. table 28 | Serum metaboloites associated with5-year incident colorectal cancer from the Nightingale platform |
| Supl. table 29 | Serum metaboloites associated with 5-year incident colorectal cancer from the Metabolon platform |
| Supl. table 30 | Serum metaboloites associated with 5-year incident all cancer from the Nightingale platform |
| Supl. table 31 | Serum metaboloites associated with 5-year incident all from the Metabolon platform |
| Supl. table 32 | Serum metaboloites associated with 5-year incident lung cancer from the Nightingale platform |
| Supl. table 33 | Serum metaboloites associated with 5-year incident lung cancer from the Metabolon platform |
| Supl. table 34 | Serum metaboloites associated with 5-year incident prostate cancer from the Nightingale platform |
| Supl. table 35 | Serum metaboloites associated with 5-year incident prostate cancer from the Metabolon platform |
| Supl. table 36 | Serum metaboloites associated with 5-year incident breast cancer from the Nightingale platform |
| Supl. table 37 | Serum metaboloites associated with 5-year incident breast cancer from the Metabolon platform |
| Supl. table 28 | Sensitivity analysis of serum metabolites associated with prevalent all cancer at baseline from the Nightingale platform, excluding hematological cancers. |
| Supl. table 29 | Sensitivity analysis of serum metabolites associated with prevalent all cancer at baseline from the Metabolon platform, excluding hematological cancers. |
| Supl. table 40 | Sensitivity analysis of serum metabolites associated with incident all cancer from the Nightingale platform, excluding hematological cancers. |
| Supl. table 41 | Sensitivity analysis of serum metabolites associated with incident all cancer from the Metabolon platform, excluding hematological cancers. |

**Supplementary Table 1. Baseline characteristics of participants in the ERGO-4 and ERGO-5 subcohorts of the Nightingale platform.**

| **Characteristic** | **ERGO-4** | | | | | **ERGO-5** | | | | |  |
| --- | --- | --- | --- | --- | --- | --- | --- | --- | --- | --- | --- |
|  | **Prevalent cancer (n =270)** | **Incident cancer (n=410)** | **No cancer (n=2,132)** | **All (n= 2,812)** | **Missing** | **Prevalent cancer (n =173)** | **Incident cancer (n=49)** | **No cancer (n=2,023)** | **All (n= 2,245)** | **Missing** | |
| Age, mean (±SD), y | 75.99 (6.11) | 74.77 (5.41) | 75.43 (6.28) | 75.39 (6.15) | 0 | 67.79 (6.81) | 67.11 (6.38) | 63.98 (6.19) | 64.34 (6.34) | 0 | |
| Sex, Male, n (%) | 144 (53.3) | 221 (53.9) | 810 (38.0) | 1175(41.8) | 0 | 64 (37.0) | 21 (42.9) | 860 (42.5) | 945 (42.1) | 0 | |
| Rotterdam Study cohort, n (%) |  |  |  |  | 0 |  |  |  |  | 0 | |
| RS-I | 260 (96.3) | 407 (99.3) | 2068 (97.0) | 2,735 (97.3) |  | - | - | - |  |  | |
| RS-II | 10 (3.7) | 2 (0.7) | 64 (3.0) | 77 (2.7) |  | 61 (35.3) | 30 (61.2) | 405 (20.0) | 496 (22.1) |  | |
| RS-III | - | - | - |  |  | 112 (64.7) | 19 (38.8) | 1618 (80.0) | 1749 (77.9) |  | |
| Smoking status, n (%) |  |  |  |  | 60 |  |  |  |  | 10 | |
| Current | 34 (12.9) | 61 (15.2) | 231 (11.1) | 326 (11.8) |  | 18 (10.5) | 7 (14.3) | 256 (12.7) | 281 (12.6) |  | |
| Former | 161 (61.2) | 242 (60.5) | 1168 (55.9) | 1,571 (57.1) |  | 99 (57.9) | 29 (59.2) | 1038 (51.5) | 1,166 (52.2) |  | |
| Never | 68 (25.9) | 97 (24.2) | 690 (33.0) | 855 (31.1) |  | 54 (31.6) | 13 (26.5) | 721 (35.8) | 788 (35.3) |  | |
| BMI, kg/m2, mean (±SD) | 27.04 (4.04) | 27.55 (4.07) | 27.00 (4.11) | 27.40 (4.10) | 90 | 28.34 (4.26) | 28.28 (3.36) | 27.00 (4.41) | 27.53 (4.39) | 1 | |
| Education level, n(%) |  |  |  |  | 22 |  |  |  |  | 7 | |
| Primary | 39 (14.6) | 55 (13.5) | 313 (14.8) | 407 (14.6) |  | 10 (5.8) | 4 (8.2) | 154 (7.6) | 168 (7.5) |  | |
| Lower | 107 (40.1) | 168 (41.4) | 919 (43.4) | 1194 (42.8) |  | 80 (46.8) | 16 (32.7) | 730 (36.2) | 826 (36.9) |  | |
| Intermediate | 81 (30.3) | 135 (33.3) | 653 (30.8) | 869 (31.1) |  | 50 (29.2) | 16 (32.7) | 557 (27.6) | 623 (27.8) |  | |
| Higher | 40 (15.0) | 48 (11.8) | 232 (11.0) | 320 (11.5) |  | 31 (18.1) | 13 (26.5) | 577 (28.6) | 621 (27.7) |  | |
| Lipid lowering treatment, yes, n (%) | 60 (22.2) | 90 (22.1) | 465 (21.9) | 615 (21.9) | 10 | 42 (24.4) | 14 (28.6) | 584 (28.9) | 640 (28.5) | 2 | |
| Alcohol intake (g/day), Median (IQR) | 9.29 (0.71-20.21) | 8.57 (1.00-18.75) | 5.71 (0.29-16.43) | 5.86 (0.57-17.43) | 60 | 6.43 (0.54-8.57) | 6.43 (0.54-8.57) | 6.43 (1.61-8.57) | 6.42 (0.54-8.57) | 10 | |
| Cancer types, yes, n (%) |  |  |  |  | - |  |  |  |  | - | |
| Lung | 3 (1.1) | 56 (13.7) | - | - |  | 6 (3.5) | 5 (10.2) | - | - |  | |
| Breast | 61 (22.6) | 48 (11.7) | - | - |  | 59 (34.1) | 11 (22.4) | - | - |  | |
| Prostate | 97 (35.9) | 50 (12.2) | - | - |  | 23 (13.3) | 8 (16.3) | - | - |  | |
| Blood | 16 (5.9) | 42 (10.2) | - | - |  | 12 (6.9) | 5 (10.2) | - | - |  | |
| Colorectal | 41 (15.2) | 79 (19.3) | - | - |  | 19 (11.0) | 6 (12.2) | - | - |  |  |
| Other | 52 (19.3) | 135 (32.9) | - | - |  | 60 (31.2) | 14 (28.5) | - | - |  |  |

Note: Values are represented as mean (±standard deviation), sample sizes (%), or median (inter-quartile range) for characteristics with skewed distributions. Other means the sum of rest cancer samples. ERGO-4, ERGO-5 refer to the fourth and fifth visits from the baseline cohort, respectively. Abbreviations: RS, Rotterdam study cohort I; BMI, body mass index; SD, standard deviation; IQR, interquartile range; y, years; n, number.

**Supplementary Table 2. Baseline characteristics of participants in the ERGO-4 and ERGO-5 subcohorts of the Metabolon platform.**

| **Characteristic** | **ERGO-4** | | | | | **ERGO-5** | | | | |
| --- | --- | --- | --- | --- | --- | --- | --- | --- | --- | --- |
|  | **Prevalent cancer (n =141)** | **Incident cancer (n=208)** | **No cancer (n=1130)** | **All (n= 1479)** | **Missing** | **Prevalent cancer (n =70)** | **Incident cancer (n=13)** | **No cancer (n= 976)** | **All (n= 1509)** | **Missing** |
| Age, mean (±SD), y | 76.60 (4.95) | 76.31 (4.53) | 77.09 (5.7) | 76.94 (5.49) | 0 | 65.53 (7.42) | 59.60 (4.57) | 62.47 (5.76) | 62.63 (5.93) | 0 |
| Sex, Male, n (%) | 79 (56.0) | 114 (54.8) | 422 (37.3) | 615 (41.6) | 0 | 23 (32.9) | 4 (30.8) | 439 (45.0) | 466 (44.0) | 0 |
| Rotterdam Study cohort, n (%) |  |  |  |  | 0 |  |  |  |  | 0 |
| RS-I | - | - | - | 1479 (100.0) |  | - | - | - | 0 (0) |  |
| RS-III | - | - | - | 0 (0) |  | - | - | - | 1059 (100.0) |  |
| Smoking status, n (%) |  |  |  |  | 12 |  |  |  |  | 4 |
| Current | 18 (12.9) | 26 (12.7) | 122 (10.9) | 166 (11.3) |  | 6 (8.7) | 3 (23.1) | 138 (14.2) | 147 (13.9) |  |
| Former | 87 (62.1) | 132 (64.4) | 642 (57.2) | 861 (58.7) |  | 41 (59.4) | 4 (30.8) | 482 (49.5) | 527 (50.0) |  |
| Never | 35 (25.0) | 47 (22.9) | 358 (31.9) | 440 (30.0) |  | 22 (31.9) | 6 (46.2) | 353 (36.3) | 381 (36.1) |  |
| BMI, kg/m2, mean (±SD) | 27.18 (3.90) | 27.48 (3.60) | 27.00 (4.20) | 27.41 (4.09) | 22 | 28.60 (4.36) | 27.06 (1.98) | 27.00 (4.32) | 27.34 (4.31) | 1 |
| Education level, n(%) |  |  |  |  | 11 |  |  |  |  | 3 |
| Primary | 20 (14.3) | 28 (13.6) | 188 (16.8) | 236 (16.1) |  | 6 (8.7) | 2 (15.4) | 77 (7.9) | 85 (8.0) |  |
| Lower | 64 (45.7) | 93 (45.1) | 496 (44.2) | 653 (44.5) |  | 29 (42.0) | 2 (15.4) | 303 (31.1) | 334 (31.6) |  |
| Intermediate | 43 (30.7) | 68 (33.0) | 320 (28.5) | 421 (29.4) |  | 21 (330.4) | 4 (30.8) | 279 (28.6) | 304 (28.8) |  |
| Higher | 13 (9.3) | 17 (8.3) | 118 (10.5) | 148 (10.1) |  | 113 (18.8) | 5 (38.5) | 315 (32.3) | 333 (31.5) |  |
| Lipid lowering treatment, yes, n (%) | 37 (26.2) | 50 (24.0) | 251 (26.9) | 338 (22.9) | 0 | 19 (27.1) | 2 (15.4) | 264 (27.0) | 285 (26.9) | 0 |
| Alcohol intake (g/day), Median (IQR) | 10.00 (0.71-20.00) | 7.86 (0.71-18.57) | 5.71 (0.29-15.71) | 6.00 (0.42-16.79) | 12 | 3.75 (0.54-8.57) | 3.75 (0.54-8.7) | 6.43 (1.61-8.57) | 6.23 (1.25-8.57) | 4 |
| Cancer types, yes, n (%) |  |  |  |  |  |  |  |  |  |  |
| Lung | 2 (1.4) | 29 (13.9) | - | - | - | 1 (1.4) | 1 (7.7) | - | - | - |
| Breast | 27 (19.2) | 24 (11.5) | - | - |  | 30 (42.8) | 5 (38.5) | - | - |  |
| Prostate | 52 (36.9) | 21 (10.1) | - | - |  | 6 (8.6) | 1 (7.7) | - | - |  |
| Blood | 11 (7.8) | 21 (10.1) | - | - |  | 6 (8.6) | 3 (23.1) | - | - |  |
| Colorectal | 24 (17.0) | 39 (18.8) | - | - |  | 6 (8.6) | 2 (15.4) | - | - |  |
| Other | 25 (17.7) | 74 (35.6) | - | - |  | 21 (30.0) | 1 (7.7) | - | - |  |

Note: Values are represented as mean (±standard deviation), sample sizes (%), or median (inter-quartile range) for characteristics with skewed distributions. Other means the sum of rest cancer samples. ERGO-4, ERGO-5 refer to the fourth and fifth visits from the baseline cohort, respectively. Abbreviations: RS, Rotterdam study cohort I; BMI, body mass index; SD, standard deviation; IQR, interquartile range; y, years; n, number.

**Supplementary Table 3. Serum metabolites associated with prevalent all cancer from the Nightingale platform at baseline.**

| **Metabolite** | **ERGO-4&5** | | | | | | **ERGO-4** | | | | | | **ERGO-5** | | | | | |
| --- | --- | --- | --- | --- | --- | --- | --- | --- | --- | --- | --- | --- | --- | --- | --- | --- | --- | --- |
|  | **Model 1** | | | **Model 2** | | | **Model 1** | | | **Model 2** | | | **Model 1** | | | **Model 2** | | |
|  | **Beta** | **SE** | ***FDR* value** | **Beta** | **SE** | ***FDR* value** | **Beta** | **SE** | ***FDR* value** | **Beta** | **SE** | ***FDR* value** | **Beta** | **SE** | ***FDR* value** | **Beta** | **SE** | ***FDR* value** |
| Free cholesterol in small LDL | -0.14 | 0.05 | 2.02E-01 | -0.14 | 0.05 | 2.00E-01 | -0.10 | 0.07 | 9.37E-01 | -0.09 | 0.07 | 9.95E-01 | -0.23 | 0.08 | 1.44E-01 | -0.23 | 0.08 | 2.33E-01 |
| Cholesteryl esters in medium VLDL | -0.15 | 0.05 | 2.02E-01 | -0.14 | 0.05 | 2.00E-01 | -0.10 | 0.07 | 9.37E-01 | -0.10 | 0.07 | 9.95E-01 | -0.21 | 0.09 | 1.44E-01 | -0.19 | 0.09 | 2.33E-01 |
| Ratio of linoleic acid to total fatty acids | -0.15 | 0.05 | 2.02E-01 | -0.14 | 0.05 | 2.00E-01 | -0.14 | 0.06 | 9.37E-01 | -0.15 | 0.07 | 9.95E-01 | -0.18 | 0.09 | 2.18E-01 | -0.17 | 0.09 | 2.63E-01 |
| Concentration of small LDL particles | -0.13 | 0.05 | 2.02E-01 | -0.14 | 0.05 | 2.00E-01 | -0.10 | 0.07 | 9.37E-01 | -0.09 | 0.07 | 9.95E-01 | -0.20 | 0.08 | 1.47E-01 | -0.20 | 0.08 | 2.33E-01 |
| Phospholipids in small LDL | -0.13 | 0.05 | 2.02E-01 | -0.13 | 0.05 | 2.00E-01 | -0.09 | 0.07 | 9.37E-01 | -0.08 | 0.07 | 9.95E-01 | -0.23 | 0.08 | 1.44E-01 | -0.22 | 0.08 | 2.33E-01 |
| Cholesterol in small LDL | -0.12 | 0.05 | 2.02E-01 | -0.13 | 0.05 | 2.00E-01 | -0.09 | 0.07 | 9.37E-01 | -0.08 | 0.07 | 9.95E-01 | -0.20 | 0.08 | 1.44E-01 | -0.20 | 0.08 | 2.33E-01 |
| Total lipids in small LDL | -0.12 | 0.05 | 2.02E-01 | -0.13 | 0.05 | 2.00E-01 | -0.08 | 0.07 | 9.37E-01 | -0.08 | 0.07 | 9.95E-01 | -0.21 | 0.08 | 1.44E-01 | -0.20 | 0.08 | 2.33E-01 |
| Cholesterol in medium VLDL | -0.13 | 0.05 | 2.02E-01 | -0.13 | 0.05 | 2.00E-01 | -0.09 | 0.07 | 9.37E-01 | -0.08 | 0.07 | 9.95E-01 | -0.20 | 0.08 | 1.44E-01 | -0.20 | 0.08 | 2.33E-01 |
| Concentration of LDL particles | -0.13 | 0.05 | 2.02E-01 | -0.13 | 0.05 | 2.00E-01 | -0.09 | 0.07 | 9.37E-01 | -0.08 | 0.07 | 9.95E-01 | -0.21 | 0.09 | 1.44E-01 | -0.20 | 0.09 | 2.33E-01 |
| Free cholesterol in LDL | -0.13 | 0.05 | 2.02E-01 | -0.13 | 0.05 | 2.00E-01 | -0.08 | 0.07 | 9.37E-01 | -0.08 | 0.07 | 9.95E-01 | -0.23 | 0.09 | 1.44E-01 | -0.21 | 0.09 | 2.33E-01 |
| Concentration of large LDL particles | -0.13 | 0.05 | 2.02E-01 | -0.13 | 0.05 | 2.00E-01 | -0.09 | 0.07 | 9.37E-01 | -0.09 | 0.07 | 9.95E-01 | -0.22 | 0.09 | 1.44E-01 | -0.21 | 0.09 | 2.33E-01 |
| Free cholesterol in medium LDL | -0.12 | 0.05 | 2.02E-01 | -0.12 | 0.05 | 2.00E-01 | -0.07 | 0.07 | 9.37E-01 | -0.07 | 0.07 | 9.95E-01 | -0.22 | 0.08 | 1.44E-01 | -0.21 | 0.08 | 2.33E-01 |
| Clinical LDL cholesterol | -0.13 | 0.05 | 2.02E-01 | -0.13 | 0.05 | 2.00E-01 | -0.08 | 0.07 | 9.37E-01 | -0.08 | 0.07 | 9.95E-01 | -0.22 | 0.09 | 1.44E-01 | -0.20 | 0.09 | 2.33E-01 |
| Phospholipids to total lipids ratio in small VLDL | -0.14 | 0.05 | 2.02E-01 | -0.13 | 0.06 | 2.00E-01 | -0.10 | 0.07 | 9.37E-01 | -0.12 | 0.07 | 9.95E-01 | -0.21 | 0.09 | 1.48E-01 | -0.19 | 0.09 | 2.33E-01 |
| Cholesteryl esters in small LDL | -0.12 | 0.05 | 2.02E-01 | -0.12 | 0.05 | 2.00E-01 | -0.08 | 0.07 | 9.37E-01 | -0.08 | 0.07 | 9.95E-01 | -0.19 | 0.08 | 1.49E-01 | -0.18 | 0.08 | 2.33E-01 |
| Free cholesterol to total lipids ratio in very small VLDL | -0.13 | 0.05 | 2.02E-01 | -0.12 | 0.05 | 2.00E-01 | -0.08 | 0.07 | 9.37E-01 | -0.07 | 0.07 | 9.95E-01 | -0.22 | 0.09 | 1.44E-01 | -0.23 | 0.09 | 2.33E-01 |
| Triglycerides to total lipids ratio in medium LDL | 0.13 | 0.05 | 2.02E-01 | 0.12 | 0.05 | 2.00E-01 | 0.07 | 0.07 | 9.37E-01 | 0.07 | 0.07 | 9.95E-01 | 0.25 | 0.09 | 1.44E-01 | 0.25 | 0.09 | 2.33E-01 |
| Apolipoprotein B | -0.12 | 0.05 | 2.02E-01 | -0.12 | 0.05 | 2.00E-01 | -0.08 | 0.07 | 9.37E-01 | -0.07 | 0.07 | 9.95E-01 | -0.20 | 0.09 | 1.47E-01 | -0.20 | 0.09 | 2.33E-01 |
| Free cholesterol to total lipids ratio in small VLDL | -0.13 | 0.05 | 2.02E-01 | -0.13 | 0.06 | 2.00E-01 | -0.10 | 0.07 | 9.37E-01 | -0.12 | 0.07 | 9.95E-01 | -0.20 | 0.09 | 1.79E-01 | -0.17 | 0.09 | 2.65E-01 |
| Ratio of apolipoprotein B to apolipoprotein A1 | -0.11 | 0.05 | 2.02E-01 | -0.12 | 0.05 | 2.00E-01 | -0.11 | 0.07 | 9.37E-01 | -0.09 | 0.07 | 9.95E-01 | -0.16 | 0.09 | 2.26E-01 | -0.17 | 0.08 | 2.33E-01 |
| Phospholipids in LDL | -0.12 | 0.05 | 2.02E-01 | -0.12 | 0.05 | 2.00E-01 | -0.07 | 0.07 | 9.37E-01 | -0.07 | 0.07 | 9.95E-01 | -0.22 | 0.09 | 1.44E-01 | -0.20 | 0.09 | 2.33E-01 |
| Phospholipids to total lipids ratio in small HDL | 0.12 | 0.05 | 2.02E-01 | 0.12 | 0.05 | 2.00E-01 | 0.10 | 0.07 | 9.37E-01 | 0.09 | 0.07 | 9.95E-01 | 0.17 | 0.08 | 2.05E-01 | 0.17 | 0.08 | 2.33E-01 |
| Free cholesterol in large LDL | -0.12 | 0.05 | 2.02E-01 | -0.12 | 0.05 | 2.00E-01 | -0.07 | 0.07 | 9.37E-01 | -0.08 | 0.07 | 9.95E-01 | -0.23 | 0.09 | 1.44E-01 | -0.20 | 0.09 | 2.33E-01 |
| Cholesterol to total lipids ratio in small HDL | -0.12 | 0.05 | 2.02E-01 | -0.11 | 0.05 | 2.00E-01 | -0.08 | 0.06 | 9.37E-01 | -0.09 | 0.07 | 9.95E-01 | -0.18 | 0.08 | 1.48E-01 | -0.18 | 0.08 | 2.33E-01 |
| Free cholesterol in IDL | -0.13 | 0.06 | 2.02E-01 | -0.13 | 0.06 | 2.00E-01 | -0.07 | 0.07 | 9.37E-01 | -0.08 | 0.07 | 9.95E-01 | -0.23 | 0.10 | 1.44E-01 | -0.18 | 0.09 | 2.33E-01 |
| Phospholipids in large LDL | -0.12 | 0.05 | 2.03E-01 | -0.12 | 0.05 | 2.00E-01 | -0.06 | 0.07 | 9.37E-01 | -0.07 | 0.07 | 9.95E-01 | -0.22 | 0.09 | 1.44E-01 | -0.19 | 0.09 | 2.33E-01 |
| LDL cholesterol | -0.11 | 0.05 | 2.03E-01 | -0.12 | 0.05 | 2.00E-01 | -0.07 | 0.07 | 9.37E-01 | -0.07 | 0.07 | 9.95E-01 | -0.20 | 0.09 | 1.47E-01 | -0.18 | 0.08 | 2.33E-01 |
| Cholesterol in large LDL | -0.11 | 0.05 | 2.03E-01 | -0.11 | 0.05 | 2.00E-01 | -0.07 | 0.07 | 9.37E-01 | -0.07 | 0.07 | 9.95E-01 | -0.21 | 0.09 | 1.44E-01 | -0.19 | 0.09 | 2.33E-01 |
| Phospholipids to total lipids ratio in very large VLDL | -0.10 | 0.04 | 2.02E-01 | -0.10 | 0.05 | 2.00E-01 | -0.06 | 0.06 | 9.37E-01 | -0.05 | 0.06 | 9.95E-01 | -0.13 | 0.06 | 1.86E-01 | -0.14 | 0.06 | 2.33E-01 |
| Cholesterol in medium LDL | -0.10 | 0.05 | 2.10E-01 | -0.11 | 0.05 | 2.00E-01 | -0.07 | 0.07 | 9.37E-01 | -0.06 | 0.07 | 9.95E-01 | -0.18 | 0.08 | 1.75E-01 | -0.17 | 0.08 | 2.33E-01 |
| Total cholesterol minus HDL-C | -0.11 | 0.05 | 2.10E-01 | -0.11 | 0.05 | 2.00E-01 | -0.07 | 0.07 | 9.37E-01 | -0.07 | 0.07 | 9.95E-01 | -0.20 | 0.09 | 1.48E-01 | -0.18 | 0.09 | 2.33E-01 |
| Linoleic acid | -0.12 | 0.05 | 2.02E-01 | -0.11 | 0.05 | 2.00E-01 | -0.08 | 0.07 | 9.37E-01 | -0.08 | 0.07 | 9.95E-01 | -0.19 | 0.09 | 1.63E-01 | -0.16 | 0.08 | 2.48E-01 |
| Cholesterol in IDL | -0.12 | 0.06 | 2.03E-01 | -0.12 | 0.06 | 2.00E-01 | -0.06 | 0.07 | 9.37E-01 | -0.07 | 0.07 | 9.95E-01 | -0.24 | 0.09 | 1.44E-01 | -0.19 | 0.09 | 2.33E-01 |
| Total free cholesterol | -0.11 | 0.05 | 2.10E-01 | -0.12 | 0.05 | 2.00E-01 | -0.06 | 0.07 | 9.37E-01 | -0.06 | 0.07 | 9.95E-01 | -0.21 | 0.09 | 1.47E-01 | -0.17 | 0.09 | 2.33E-01 |
| Phospholipids to total lipids ratio in large HDL | 0.12 | 0.05 | 2.02E-01 | 0.11 | 0.05 | 2.00E-01 | 0.09 | 0.06 | 9.37E-01 | 0.11 | 0.07 | 9.95E-01 | 0.15 | 0.09 | 2.91E-01 | 0.13 | 0.09 | 4.02E-01 |
| Total lipids in LDL | -0.11 | 0.05 | 2.10E-01 | -0.11 | 0.05 | 2.00E-01 | -0.06 | 0.07 | 9.37E-01 | -0.06 | 0.07 | 9.95E-01 | -0.19 | 0.09 | 1.48E-01 | -0.18 | 0.08 | 2.33E-01 |
| Cholesteryl esters in LDL | -0.11 | 0.05 | 2.10E-01 | -0.11 | 0.05 | 2.00E-01 | -0.07 | 0.07 | 9.37E-01 | -0.06 | 0.07 | 9.95E-01 | -0.19 | 0.08 | 1.52E-01 | -0.17 | 0.08 | 2.33E-01 |
| Phospholipids in medium LDL | -0.10 | 0.05 | 2.10E-01 | -0.11 | 0.05 | 2.00E-01 | -0.07 | 0.07 | 9.37E-01 | -0.06 | 0.07 | 9.95E-01 | -0.18 | 0.08 | 1.50E-01 | -0.18 | 0.08 | 2.33E-01 |
| Triglycerides to total lipids ratio in small VLDL | 0.12 | 0.05 | 2.02E-01 | 0.12 | 0.06 | 2.00E-01 | 0.09 | 0.07 | 9.37E-01 | 0.11 | 0.07 | 9.95E-01 | 0.17 | 0.09 | 2.26E-01 | 0.15 | 0.09 | 3.31E-01 |
| Concentration of medium LDL particles | -0.10 | 0.05 | 2.13E-01 | -0.11 | 0.05 | 2.00E-01 | -0.06 | 0.07 | 9.37E-01 | -0.05 | 0.07 | 9.95E-01 | -0.17 | 0.08 | 2.05E-01 | -0.16 | 0.08 | 2.40E-01 |
| Triglycerides in large HDL | 0.12 | 0.05 | 2.02E-01 | 0.12 | 0.06 | 2.00E-01 | 0.14 | 0.07 | 9.37E-01 | 0.12 | 0.07 | 9.95E-01 | 0.12 | 0.09 | 4.33E-01 | 0.14 | 0.09 | 3.75E-01 |
| Total cholesterol | -0.11 | 0.06 | 2.10E-01 | -0.12 | 0.06 | 2.00E-01 | -0.05 | 0.07 | 9.37E-01 | -0.06 | 0.07 | 9.95E-01 | -0.23 | 0.09 | 1.44E-01 | -0.18 | 0.09 | 2.33E-01 |
| Cholesteryl esters in IDL | -0.12 | 0.06 | 2.10E-01 | -0.12 | 0.06 | 2.00E-01 | -0.06 | 0.07 | 9.37E-01 | -0.07 | 0.07 | 9.95E-01 | -0.24 | 0.09 | 1.44E-01 | -0.19 | 0.09 | 2.33E-01 |
| Total lipids in large LDL | -0.11 | 0.05 | 2.10E-01 | -0.11 | 0.05 | 2.00E-01 | -0.06 | 0.07 | 9.37E-01 | -0.06 | 0.07 | 9.95E-01 | -0.21 | 0.09 | 1.47E-01 | -0.18 | 0.09 | 2.33E-01 |
| Triglycerides to total lipids ratio in large LDL | 0.12 | 0.05 | 2.02E-01 | 0.11 | 0.05 | 2.00E-01 | 0.06 | 0.07 | 9.37E-01 | 0.06 | 0.07 | 9.95E-01 | 0.25 | 0.09 | 1.44E-01 | 0.24 | 0.09 | 2.33E-01 |
| Cholesterol to total lipids ratio in medium LDL | -0.11 | 0.05 | 2.02E-01 | -0.10 | 0.05 | 2.00E-01 | -0.04 | 0.07 | 9.37E-01 | -0.04 | 0.07 | 9.95E-01 | -0.18 | 0.08 | 1.50E-01 | -0.19 | 0.08 | 2.33E-01 |
| Remnant cholesterol (non-HDL | -0.11 | 0.05 | 2.10E-01 | -0.11 | 0.05 | 2.00E-01 | -0.06 | 0.07 | 9.37E-01 | -0.06 | 0.07 | 9.95E-01 | -0.20 | 0.09 | 1.48E-01 | -0.18 | 0.09 | 2.33E-01 |
| Glutamine | -0.11 | 0.05 | 2.02E-01 | -0.11 | 0.05 | 2.00E-01 | -0.13 | 0.06 | 9.37E-01 | -0.15 | 0.07 | 9.95E-01 | -0.05 | 0.09 | 8.10E-01 | -0.05 | 0.09 | 8.07E-01 |
| Total esterified cholesterol | -0.11 | 0.06 | 2.10E-01 | -0.11 | 0.06 | 2.00E-01 | -0.05 | 0.07 | 9.37E-01 | -0.06 | 0.07 | 9.95E-01 | -0.23 | 0.09 | 1.44E-01 | -0.18 | 0.09 | 2.33E-01 |
| Total lipids in medium LDL | -0.10 | 0.05 | 2.19E-01 | -0.11 | 0.05 | 2.00E-01 | -0.06 | 0.07 | 9.37E-01 | -0.06 | 0.07 | 9.95E-01 | -0.17 | 0.08 | 1.86E-01 | -0.17 | 0.08 | 2.33E-01 |
| Concentration of IDL particles | -0.11 | 0.06 | 2.10E-01 | -0.11 | 0.06 | 2.00E-01 | -0.05 | 0.07 | 9.37E-01 | -0.05 | 0.07 | 9.95E-01 | -0.22 | 0.09 | 1.44E-01 | -0.19 | 0.09 | 2.33E-01 |
| Cholesteryl esters in large LDL | -0.10 | 0.05 | 2.10E-01 | -0.11 | 0.05 | 2.04E-01 | -0.06 | 0.07 | 9.37E-01 | -0.06 | 0.07 | 9.95E-01 | -0.20 | 0.09 | 1.48E-01 | -0.18 | 0.09 | 2.33E-01 |
| Cholesterol to total lipids ratio in large LDL | -0.11 | 0.05 | 2.02E-01 | -0.10 | 0.05 | 2.04E-01 | -0.07 | 0.07 | 9.37E-01 | -0.07 | 0.07 | 9.95E-01 | -0.20 | 0.09 | 1.44E-01 | -0.20 | 0.08 | 2.33E-01 |
| Cholesteryl esters in medium LDL | -0.09 | 0.05 | 2.39E-01 | -0.10 | 0.05 | 2.12E-01 | -0.06 | 0.07 | 9.37E-01 | -0.06 | 0.07 | 9.95E-01 | -0.16 | 0.08 | 2.18E-01 | -0.15 | 0.08 | 2.63E-01 |
| Ratio of saturated fatty acids to total fatty acids | 0.12 | 0.05 | 2.02E-01 | 0.10 | 0.05 | 2.19E-01 | 0.12 | 0.06 | 9.37E-01 | 0.12 | 0.07 | 9.95E-01 | 0.10 | 0.09 | 5.05E-01 | 0.11 | 0.08 | 5.03E-01 |
| Triglycerides in medium HDL | 0.12 | 0.05 | 2.02E-01 | 0.10 | 0.05 | 2.26E-01 | 0.11 | 0.07 | 9.37E-01 | 0.12 | 0.07 | 9.95E-01 | 0.12 | 0.09 | 4.16E-01 | 0.13 | 0.09 | 4.02E-01 |
| Free cholesterol in medium VLDL | -0.09 | 0.05 | 2.32E-01 | -0.10 | 0.05 | 2.26E-01 | -0.06 | 0.07 | 9.37E-01 | -0.05 | 0.07 | 9.95E-01 | -0.16 | 0.08 | 2.05E-01 | -0.16 | 0.08 | 2.40E-01 |
| Total lipids in IDL | -0.11 | 0.06 | 2.19E-01 | -0.11 | 0.06 | 2.26E-01 | -0.05 | 0.07 | 9.37E-01 | -0.06 | 0.07 | 9.95E-01 | -0.22 | 0.09 | 1.48E-01 | -0.17 | 0.09 | 2.63E-01 |
| Cholesterol to total lipids ratio in very small VLDL | -0.11 | 0.05 | 2.03E-01 | -0.10 | 0.05 | 2.35E-01 | -0.07 | 0.07 | 9.37E-01 | -0.09 | 0.07 | 9.95E-01 | -0.18 | 0.09 | 2.05E-01 | -0.16 | 0.09 | 3.04E-01 |
| Triglycerides to total lipids ratio in very small VLDL | 0.12 | 0.06 | 2.02E-01 | 0.11 | 0.06 | 2.35E-01 | 0.07 | 0.07 | 9.37E-01 | 0.09 | 0.07 | 9.95E-01 | 0.19 | 0.10 | 1.98E-01 | 0.17 | 0.09 | 2.67E-01 |
| Phospholipids in IDL | -0.11 | 0.06 | 2.20E-01 | -0.11 | 0.06 | 2.39E-01 | -0.06 | 0.07 | 9.37E-01 | -0.07 | 0.07 | 9.95E-01 | -0.19 | 0.09 | 1.89E-01 | -0.15 | 0.09 | 3.25E-01 |
| Cholesteryl esters in VLDL | -0.09 | 0.05 | 2.49E-01 | -0.10 | 0.05 | 2.40E-01 | -0.06 | 0.07 | 9.37E-01 | -0.05 | 0.07 | 9.95E-01 | -0.16 | 0.08 | 2.21E-01 | -0.16 | 0.08 | 2.63E-01 |
| Triglycerides to total lipids ratio in IDL | 0.11 | 0.05 | 2.10E-01 | 0.10 | 0.05 | 2.46E-01 | 0.05 | 0.07 | 9.37E-01 | 0.07 | 0.07 | 9.95E-01 | 0.23 | 0.09 | 1.44E-01 | 0.21 | 0.09 | 2.33E-01 |
| Free cholesterol in small VLDL | -0.09 | 0.05 | 2.59E-01 | -0.10 | 0.05 | 2.46E-01 | -0.06 | 0.07 | 9.37E-01 | -0.05 | 0.07 | 9.95E-01 | -0.16 | 0.08 | 2.12E-01 | -0.16 | 0.08 | 2.63E-01 |
| Glycine | -0.11 | 0.05 | 2.10E-01 | -0.10 | 0.05 | 2.50E-01 | -0.06 | 0.07 | 9.37E-01 | -0.07 | 0.07 | 9.95E-01 | -0.17 | 0.09 | 2.26E-01 | -0.13 | 0.09 | 4.04E-01 |
| Phospholipids in medium VLDL | -0.09 | 0.05 | 2.97E-01 | -0.09 | 0.05 | 2.60E-01 | -0.06 | 0.07 | 9.37E-01 | -0.05 | 0.07 | 9.95E-01 | -0.15 | 0.08 | 2.27E-01 | -0.15 | 0.08 | 2.63E-01 |
| Concentration of medium VLDL particles | -0.08 | 0.05 | 3.19E-01 | -0.09 | 0.05 | 2.93E-01 | -0.05 | 0.07 | 9.37E-01 | -0.04 | 0.07 | 9.95E-01 | -0.15 | 0.08 | 2.52E-01 | -0.15 | 0.08 | 2.76E-01 |
| Cholesterol to total lipids ratio in small LDL | -0.10 | 0.05 | 2.19E-01 | -0.09 | 0.05 | 2.93E-01 | -0.05 | 0.07 | 9.37E-01 | -0.06 | 0.07 | 9.95E-01 | -0.16 | 0.09 | 2.27E-01 | -0.14 | 0.09 | 3.25E-01 |
| Triglycerides in HDL | 0.10 | 0.05 | 2.10E-01 | 0.09 | 0.05 | 2.93E-01 | 0.10 | 0.07 | 9.37E-01 | 0.10 | 0.07 | 9.95E-01 | 0.11 | 0.09 | 4.73E-01 | 0.12 | 0.09 | 4.49E-01 |
| Cholesterol to total lipids ratio in small VLDL | -0.10 | 0.05 | 2.15E-01 | -0.09 | 0.05 | 2.93E-01 | -0.08 | 0.07 | 9.37E-01 | -0.10 | 0.07 | 9.95E-01 | -0.12 | 0.09 | 4.57E-01 | -0.10 | 0.09 | 5.88E-01 |
| Cholesteryl esters to total lipids ratio in small HDL | -0.10 | 0.05 | 2.13E-01 | -0.09 | 0.05 | 2.93E-01 | -0.07 | 0.06 | 9.37E-01 | -0.07 | 0.06 | 9.95E-01 | -0.14 | 0.08 | 2.88E-01 | -0.14 | 0.08 | 3.02E-01 |
| Cholesteryl esters to total lipids ratio in very small VLDL | -0.10 | 0.05 | 2.13E-01 | -0.09 | 0.05 | 2.95E-01 | -0.07 | 0.07 | 9.37E-01 | -0.09 | 0.07 | 9.95E-01 | -0.15 | 0.09 | 2.88E-01 | -0.13 | 0.09 | 4.25E-01 |
| Omega-6 fatty acids | -0.09 | 0.05 | 3.14E-01 | -0.09 | 0.05 | 2.99E-01 | -0.05 | 0.07 | 9.37E-01 | -0.05 | 0.07 | 9.95E-01 | -0.18 | 0.09 | 1.86E-01 | -0.15 | 0.08 | 3.02E-01 |
| Cholesterol to total lipids ratio in IDL | -0.09 | 0.05 | 2.51E-01 | -0.09 | 0.05 | 3.19E-01 | -0.03 | 0.07 | 9.37E-01 | -0.04 | 0.07 | 9.95E-01 | -0.21 | 0.08 | 1.44E-01 | -0.19 | 0.08 | 2.33E-01 |
| Cholesteryl esters in very small VLDL | -0.10 | 0.06 | 3.02E-01 | -0.09 | 0.06 | 3.19E-01 | -0.05 | 0.07 | 9.37E-01 | -0.06 | 0.07 | 9.95E-01 | -0.16 | 0.09 | 2.83E-01 | -0.13 | 0.09 | 4.44E-01 |
| VLDL cholesterol | -0.08 | 0.05 | 3.52E-01 | -0.09 | 0.05 | 3.19E-01 | -0.05 | 0.07 | 9.37E-01 | -0.04 | 0.07 | 9.95E-01 | -0.14 | 0.08 | 2.88E-01 | -0.14 | 0.08 | 3.23E-01 |
| Phenylalanine | 0.09 | 0.05 | 3.14E-01 | 0.09 | 0.05 | 3.49E-01 | -0.05 | 0.07 | 9.37E-01 | -0.04 | 0.07 | 9.95E-01 | 0.32 | 0.09 | 1.00E-01 | 0.28 | 0.09 | 2.33E-01 |
| Triglycerides to total lipids ratio in small LDL | 0.10 | 0.05 | 2.31E-01 | 0.09 | 0.05 | 3.49E-01 | 0.05 | 0.07 | 9.37E-01 | 0.06 | 0.07 | 9.95E-01 | 0.18 | 0.09 | 2.05E-01 | 0.17 | 0.09 | 2.63E-01 |
| Cholesterol in small VLDL | -0.08 | 0.05 | 3.74E-01 | -0.08 | 0.05 | 3.67E-01 | -0.05 | 0.07 | 9.37E-01 | -0.04 | 0.07 | 9.95E-01 | -0.14 | 0.08 | 3.14E-01 | -0.13 | 0.08 | 3.61E-01 |
| Phospholipids in small VLDL | -0.07 | 0.05 | 3.84E-01 | -0.08 | 0.05 | 3.74E-01 | -0.04 | 0.07 | 9.37E-01 | -0.03 | 0.07 | 9.95E-01 | -0.14 | 0.08 | 2.83E-01 | -0.14 | 0.08 | 3.16E-01 |
| Cholesterol in very small VLDL | -0.08 | 0.06 | 3.54E-01 | -0.09 | 0.06 | 3.86E-01 | -0.04 | 0.07 | 9.37E-01 | -0.05 | 0.07 | 9.95E-01 | -0.15 | 0.09 | 3.21E-01 | -0.12 | 0.09 | 4.79E-01 |
| Total lipids in medium VLDL | -0.07 | 0.05 | 4.07E-01 | -0.08 | 0.05 | 3.95E-01 | -0.05 | 0.06 | 9.37E-01 | -0.03 | 0.07 | 9.95E-01 | -0.13 | 0.08 | 3.55E-01 | -0.13 | 0.08 | 3.61E-01 |
| Cholesterol in very large HDL | -0.09 | 0.05 | 3.19E-01 | -0.08 | 0.06 | 4.18E-01 | -0.04 | 0.07 | 9.37E-01 | -0.07 | 0.07 | 9.95E-01 | -0.13 | 0.09 | 4.16E-01 | -0.09 | 0.09 | 6.27E-01 |
| Total lipids in lipoprotein particles | -0.07 | 0.05 | 4.55E-01 | -0.08 | 0.05 | 4.18E-01 | -0.02 | 0.07 | 9.58E-01 | -0.02 | 0.07 | 9.95E-01 | -0.16 | 0.09 | 2.26E-01 | -0.13 | 0.08 | 3.87E-01 |
| Cholesteryl esters in very large VLDL | -0.06 | 0.05 | 4.34E-01 | -0.07 | 0.05 | 4.18E-01 | -0.05 | 0.06 | 9.37E-01 | -0.03 | 0.07 | 9.95E-01 | -0.12 | 0.08 | 4.14E-01 | -0.13 | 0.08 | 3.88E-01 |
| Free cholesterol in very large HDL | -0.09 | 0.05 | 3.14E-01 | -0.08 | 0.05 | 4.18E-01 | -0.05 | 0.07 | 9.37E-01 | -0.07 | 0.07 | 9.95E-01 | -0.11 | 0.09 | 4.73E-01 | -0.09 | 0.09 | 6.11E-01 |
| Triglycerides to total lipids ratio in medium VLDL | 0.09 | 0.05 | 3.15E-01 | 0.08 | 0.06 | 4.18E-01 | 0.05 | 0.07 | 9.37E-01 | 0.07 | 0.07 | 9.95E-01 | 0.14 | 0.09 | 3.96E-01 | 0.11 | 0.09 | 5.33E-01 |
| Free cholesterol to total lipids ratio in very large VLDL | -0.09 | 0.05 | 3.12E-01 | -0.08 | 0.05 | 4.18E-01 | -0.03 | 0.07 | 9.58E-01 | -0.04 | 0.07 | 9.95E-01 | -0.15 | 0.08 | 2.29E-01 | -0.14 | 0.08 | 3.04E-01 |
| Cholesteryl esters in very large HDL | -0.08 | 0.05 | 3.34E-01 | -0.08 | 0.06 | 4.18E-01 | -0.04 | 0.07 | 9.37E-01 | -0.07 | 0.07 | 9.95E-01 | -0.13 | 0.09 | 4.16E-01 | -0.09 | 0.09 | 6.27E-01 |
| Phospholipids to total lipids ratio in medium VLDL | -0.09 | 0.05 | 3.30E-01 | -0.08 | 0.05 | 4.18E-01 | -0.05 | 0.07 | 9.37E-01 | -0.06 | 0.07 | 9.95E-01 | -0.13 | 0.09 | 3.99E-01 | -0.11 | 0.09 | 5.41E-01 |
| Sphingomyelins | -0.07 | 0.06 | 4.34E-01 | -0.08 | 0.06 | 4.35E-01 | 0.00 | 0.07 | 9.96E-01 | -0.02 | 0.07 | 9.95E-01 | -0.19 | 0.10 | 2.05E-01 | -0.12 | 0.09 | 4.34E-01 |
| Cholesteryl esters to total lipids ratio in large HDL | -0.08 | 0.05 | 3.19E-01 | -0.07 | 0.05 | 4.42E-01 | -0.08 | 0.06 | 9.37E-01 | -0.10 | 0.06 | 9.95E-01 | -0.07 | 0.08 | 6.53E-01 | -0.05 | 0.08 | 7.78E-01 |
| Free cholesterol to total lipids ratio in IDL | -0.08 | 0.05 | 3.31E-01 | -0.07 | 0.05 | 4.42E-01 | -0.04 | 0.06 | 9.37E-01 | -0.05 | 0.06 | 9.95E-01 | -0.12 | 0.09 | 4.16E-01 | -0.11 | 0.09 | 4.97E-01 |
| Cholesteryl esters to total lipids ratio in IDL | -0.07 | 0.05 | 3.84E-01 | -0.07 | 0.05 | 4.42E-01 | -0.03 | 0.07 | 9.58E-01 | -0.03 | 0.07 | 9.95E-01 | -0.17 | 0.08 | 1.66E-01 | -0.16 | 0.08 | 2.33E-01 |
| Triglycerides to total lipids ratio in large HDL | 0.07 | 0.05 | 3.74E-01 | 0.07 | 0.05 | 4.42E-01 | 0.07 | 0.06 | 9.37E-01 | 0.09 | 0.07 | 9.95E-01 | 0.09 | 0.09 | 5.19E-01 | 0.08 | 0.09 | 6.59E-01 |
| Cholesterol to total lipids ratio in large HDL | -0.08 | 0.05 | 3.34E-01 | -0.07 | 0.05 | 4.42E-01 | -0.08 | 0.06 | 9.37E-01 | -0.10 | 0.06 | 9.95E-01 | -0.06 | 0.08 | 6.76E-01 | -0.05 | 0.08 | 8.28E-01 |
| Free cholesterol to total lipids ratio in small HDL | -0.07 | 0.05 | 3.96E-01 | -0.07 | 0.05 | 4.42E-01 | -0.03 | 0.07 | 9.58E-01 | -0.04 | 0.07 | 9.95E-01 | -0.13 | 0.09 | 3.97E-01 | -0.11 | 0.09 | 5.08E-01 |
| Free cholesterol to total lipids ratio in medium VLDL | -0.08 | 0.05 | 3.47E-01 | -0.07 | 0.05 | 4.42E-01 | -0.04 | 0.07 | 9.37E-01 | -0.06 | 0.07 | 9.95E-01 | -0.13 | 0.09 | 3.99E-01 | -0.11 | 0.09 | 5.41E-01 |
| Polyunsaturated fatty acids | -0.07 | 0.05 | 4.56E-01 | -0.07 | 0.05 | 4.42E-01 | -0.02 | 0.07 | 9.58E-01 | -0.02 | 0.07 | 9.95E-01 | -0.16 | 0.09 | 2.26E-01 | -0.13 | 0.08 | 3.87E-01 |
| Cholesteryl esters in small VLDL | -0.06 | 0.05 | 4.56E-01 | -0.07 | 0.05 | 4.42E-01 | -0.04 | 0.07 | 9.37E-01 | -0.03 | 0.07 | 9.95E-01 | -0.11 | 0.08 | 4.21E-01 | -0.11 | 0.08 | 4.69E-01 |
| Free cholesterol in VLDL | -0.06 | 0.05 | 4.96E-01 | -0.07 | 0.05 | 4.42E-01 | -0.04 | 0.06 | 9.37E-01 | -0.03 | 0.07 | 9.95E-01 | -0.12 | 0.08 | 4.16E-01 | -0.12 | 0.08 | 4.34E-01 |
| Cholesteryl esters to total lipids ratio in large LDL | -0.07 | 0.05 | 4.14E-01 | -0.07 | 0.05 | 4.42E-01 | -0.05 | 0.06 | 9.37E-01 | -0.04 | 0.06 | 9.95E-01 | -0.11 | 0.08 | 4.16E-01 | -0.12 | 0.08 | 4.07E-01 |
| Ratio of omega-6 fatty acids to total fatty acids | -0.09 | 0.05 | 2.60E-01 | -0.07 | 0.05 | 4.42E-01 | -0.08 | 0.06 | 9.37E-01 | -0.10 | 0.07 | 9.95E-01 | -0.08 | 0.09 | 5.80E-01 | -0.08 | 0.09 | 6.37E-01 |
| Cholesteryl esters in large VLDL | -0.06 | 0.05 | 4.86E-01 | -0.07 | 0.05 | 4.61E-01 | -0.04 | 0.06 | 9.37E-01 | -0.02 | 0.07 | 9.95E-01 | -0.11 | 0.08 | 4.16E-01 | -0.12 | 0.08 | 4.02E-01 |
| Phospholipids in medium HDL | 0.08 | 0.06 | 3.84E-01 | 0.08 | 0.06 | 4.65E-01 | 0.11 | 0.07 | 9.37E-01 | 0.09 | 0.07 | 9.95E-01 | 0.04 | 0.10 | 8.81E-01 | 0.07 | 0.08 | 7.07E-01 |
| Cholesterol to total lipids ratio in medium VLDL | -0.08 | 0.05 | 3.62E-01 | -0.07 | 0.05 | 4.68E-01 | -0.05 | 0.07 | 9.37E-01 | -0.07 | 0.07 | 9.95E-01 | -0.11 | 0.09 | 5.05E-01 | -0.08 | 0.09 | 6.37E-01 |
| Free cholesterol to total lipids ratio in medium HDL | -0.06 | 0.05 | 4.80E-01 | -0.07 | 0.06 | 4.99E-01 | 0.01 | 0.07 | 9.70E-01 | -0.02 | 0.07 | 9.95E-01 | -0.20 | 0.10 | 1.75E-01 | -0.13 | 0.09 | 4.04E-01 |
| Lactate | -0.06 | 0.05 | 5.11E-01 | -0.06 | 0.05 | 5.01E-01 | -0.16 | 0.07 | 9.37E-01 | -0.15 | 0.07 | 9.95E-01 | 0.10 | 0.08 | 5.05E-01 | 0.09 | 0.08 | 6.27E-01 |
| Alanine | 0.07 | 0.05 | 4.34E-01 | 0.06 | 0.05 | 5.08E-01 | -0.01 | 0.06 | 9.58E-01 | 0.00 | 0.07 | 9.95E-01 | 0.17 | 0.09 | 2.05E-01 | 0.17 | 0.09 | 2.44E-01 |
| Cholesteryl esters to total lipids ratio in small VLDL | -0.07 | 0.05 | 3.96E-01 | -0.06 | 0.05 | 5.08E-01 | -0.07 | 0.07 | 9.37E-01 | -0.08 | 0.07 | 9.95E-01 | -0.06 | 0.09 | 6.89E-01 | -0.05 | 0.09 | 8.07E-01 |
| Triglycerides to total lipids ratio in medium HDL | 0.07 | 0.05 | 4.14E-01 | 0.06 | 0.05 | 5.08E-01 | 0.05 | 0.06 | 9.37E-01 | 0.07 | 0.07 | 9.95E-01 | 0.10 | 0.09 | 5.05E-01 | 0.09 | 0.09 | 6.27E-01 |
| Cholesteryl esters to total lipids ratio in medium VLDL | -0.07 | 0.05 | 3.84E-01 | -0.06 | 0.05 | 5.08E-01 | -0.05 | 0.07 | 9.37E-01 | -0.07 | 0.07 | 9.95E-01 | -0.09 | 0.09 | 5.40E-01 | -0.07 | 0.09 | 7.07E-01 |
| Free cholesterol to total lipids ratio in large LDL | -0.07 | 0.05 | 3.84E-01 | -0.06 | 0.05 | 5.08E-01 | -0.04 | 0.07 | 9.37E-01 | -0.06 | 0.07 | 9.95E-01 | -0.13 | 0.09 | 3.97E-01 | -0.11 | 0.09 | 5.33E-01 |
| Triglycerides to total lipids ratio in very large HDL | 0.07 | 0.05 | 3.96E-01 | 0.06 | 0.05 | 5.29E-01 | 0.05 | 0.06 | 9.37E-01 | 0.07 | 0.07 | 9.95E-01 | 0.10 | 0.09 | 5.19E-01 | 0.08 | 0.09 | 6.40E-01 |
| Cholesterol in very large VLDL | -0.05 | 0.05 | 5.96E-01 | -0.06 | 0.05 | 5.35E-01 | -0.04 | 0.06 | 9.37E-01 | -0.02 | 0.07 | 9.95E-01 | -0.09 | 0.08 | 5.18E-01 | -0.10 | 0.08 | 5.33E-01 |
| Cholesteryl esters to total lipids ratio in large VLDL | -0.08 | 0.05 | 3.82E-01 | -0.06 | 0.05 | 5.35E-01 | -0.01 | 0.07 | 9.58E-01 | -0.03 | 0.07 | 9.95E-01 | -0.13 | 0.09 | 3.97E-01 | -0.12 | 0.09 | 4.79E-01 |
| Concentration of VLDL particles | -0.05 | 0.05 | 5.95E-01 | -0.06 | 0.05 | 5.35E-01 | -0.03 | 0.07 | 9.58E-01 | -0.01 | 0.07 | 9.95E-01 | -0.11 | 0.08 | 4.73E-01 | -0.11 | 0.08 | 5.07E-01 |
| Phospholipids in VLDL | -0.05 | 0.05 | 6.09E-01 | -0.06 | 0.05 | 5.35E-01 | -0.04 | 0.06 | 9.37E-01 | -0.02 | 0.07 | 9.95E-01 | -0.10 | 0.08 | 5.05E-01 | -0.10 | 0.08 | 5.33E-01 |
| Triglycerides in small HDL | 0.07 | 0.05 | 3.96E-01 | 0.06 | 0.05 | 5.49E-01 | 0.05 | 0.07 | 9.37E-01 | 0.07 | 0.07 | 9.95E-01 | 0.08 | 0.09 | 5.80E-01 | 0.08 | 0.09 | 6.59E-01 |
| Total lipids in very large HDL | -0.07 | 0.05 | 4.51E-01 | -0.06 | 0.06 | 5.55E-01 | -0.02 | 0.07 | 9.58E-01 | -0.05 | 0.07 | 9.95E-01 | -0.10 | 0.09 | 5.18E-01 | -0.07 | 0.09 | 7.09E-01 |
| Cholesterol to total lipids ratio in medium HDL | -0.06 | 0.05 | 4.58E-01 | -0.06 | 0.05 | 5.63E-01 | -0.03 | 0.06 | 9.37E-01 | -0.06 | 0.07 | 9.95E-01 | -0.10 | 0.08 | 4.95E-01 | -0.08 | 0.08 | 6.27E-01 |
| Phospholipids to total lipids ratio in medium HDL | 0.06 | 0.05 | 4.56E-01 | 0.06 | 0.05 | 5.63E-01 | 0.02 | 0.06 | 9.58E-01 | 0.05 | 0.07 | 9.95E-01 | 0.11 | 0.09 | 4.73E-01 | 0.08 | 0.08 | 6.37E-01 |
| Free cholesterol in very small VLDL | -0.05 | 0.05 | 5.91E-01 | -0.06 | 0.05 | 5.63E-01 | -0.02 | 0.07 | 9.58E-01 | -0.02 | 0.07 | 9.95E-01 | -0.11 | 0.09 | 4.73E-01 | -0.09 | 0.09 | 6.11E-01 |
| Total lipids in medium HDL | 0.07 | 0.06 | 4.81E-01 | 0.06 | 0.06 | 5.65E-01 | 0.10 | 0.07 | 9.37E-01 | 0.08 | 0.07 | 9.95E-01 | 0.02 | 0.10 | 9.41E-01 | 0.05 | 0.08 | 7.78E-01 |
| Concentration of very large HDL particles | -0.07 | 0.05 | 4.77E-01 | -0.06 | 0.06 | 5.65E-01 | -0.02 | 0.07 | 9.58E-01 | -0.05 | 0.07 | 9.95E-01 | -0.10 | 0.09 | 5.18E-01 | -0.06 | 0.09 | 7.35E-01 |
| Creatinine | 0.07 | 0.06 | 5.15E-01 | 0.06 | 0.06 | 5.71E-01 | 0.02 | 0.07 | 9.58E-01 | 0.04 | 0.07 | 9.95E-01 | -0.04 | 0.13 | 9.33E-01 | -0.04 | 0.13 | 8.98E-01 |
| Cholesteryl esters to total lipids ratio in chylomicrons and extremely large VLDL | -0.07 | 0.05 | 4.34E-01 | -0.06 | 0.05 | 5.72E-01 | 0.00 | 0.07 | 9.96E-01 | -0.02 | 0.07 | 9.95E-01 | -0.09 | 0.09 | 5.45E-01 | -0.08 | 0.09 | 6.37E-01 |
| Free cholesterol to total lipids ratio in large HDL | -0.06 | 0.05 | 5.11E-01 | -0.05 | 0.05 | 5.78E-01 | -0.05 | 0.06 | 9.37E-01 | -0.07 | 0.07 | 9.95E-01 | -0.04 | 0.09 | 8.78E-01 | -0.02 | 0.09 | 9.25E-01 |
| Phospholipids in small HDL | 0.07 | 0.05 | 4.07E-01 | 0.06 | 0.06 | 5.83E-01 | 0.10 | 0.07 | 9.37E-01 | 0.10 | 0.07 | 9.95E-01 | 0.01 | 0.09 | 9.63E-01 | 0.04 | 0.08 | 8.46E-01 |
| Concentration of very small VLDL particles | -0.05 | 0.05 | 6.24E-01 | -0.05 | 0.05 | 5.87E-01 | -0.02 | 0.07 | 9.58E-01 | -0.02 | 0.07 | 9.95E-01 | -0.10 | 0.09 | 5.05E-01 | -0.08 | 0.09 | 6.27E-01 |
| Cholesterol in large VLDL | -0.04 | 0.05 | 6.81E-01 | -0.05 | 0.05 | 5.89E-01 | -0.03 | 0.06 | 9.39E-01 | -0.01 | 0.07 | 9.95E-01 | -0.09 | 0.08 | 5.19E-01 | -0.10 | 0.08 | 5.41E-01 |
| Phospholipids in very large HDL | -0.06 | 0.05 | 4.80E-01 | -0.05 | 0.05 | 5.90E-01 | -0.01 | 0.07 | 9.58E-01 | -0.04 | 0.07 | 9.95E-01 | -0.09 | 0.09 | 5.23E-01 | -0.06 | 0.08 | 7.09E-01 |
| Phospholipids in very large VLDL | -0.04 | 0.05 | 7.17E-01 | -0.05 | 0.05 | 6.02E-01 | -0.04 | 0.06 | 9.37E-01 | -0.02 | 0.07 | 9.95E-01 | -0.08 | 0.08 | 5.56E-01 | -0.09 | 0.08 | 5.87E-01 |
| Total phospholipids in lipoprotein particles | -0.04 | 0.06 | 7.13E-01 | -0.06 | 0.06 | 6.07E-01 | 0.01 | 0.07 | 9.58E-01 | 0.00 | 0.07 | 9.99E-01 | -0.15 | 0.09 | 3.14E-01 | -0.09 | 0.09 | 6.14E-01 |
| Triglycerides to total lipids ratio in small HDL | 0.06 | 0.05 | 5.11E-01 | 0.05 | 0.05 | 6.20E-01 | 0.03 | 0.06 | 9.58E-01 | 0.05 | 0.07 | 9.95E-01 | 0.10 | 0.09 | 5.05E-01 | 0.08 | 0.09 | 6.56E-01 |
| Cholesterol to total lipids ratio in large VLDL | -0.06 | 0.05 | 4.65E-01 | -0.05 | 0.05 | 6.20E-01 | -0.01 | 0.07 | 9.58E-01 | -0.02 | 0.07 | 9.95E-01 | -0.10 | 0.08 | 4.96E-01 | -0.10 | 0.09 | 5.87E-01 |
| Ratio of polyunsaturated fatty acids to total fatty acids | -0.07 | 0.05 | 4.07E-01 | -0.05 | 0.05 | 6.23E-01 | -0.05 | 0.06 | 9.37E-01 | -0.06 | 0.07 | 9.95E-01 | -0.08 | 0.09 | 5.90E-01 | -0.07 | 0.09 | 6.59E-01 |
| Cholesteryl esters to total lipids ratio in medium HDL | -0.05 | 0.05 | 5.15E-01 | -0.05 | 0.05 | 6.44E-01 | -0.04 | 0.06 | 9.37E-01 | -0.05 | 0.07 | 9.95E-01 | -0.08 | 0.08 | 5.83E-01 | -0.06 | 0.08 | 7.07E-01 |
| Total lipids in VLDL | -0.03 | 0.05 | 7.67E-01 | -0.05 | 0.05 | 6.52E-01 | -0.03 | 0.06 | 9.58E-01 | -0.01 | 0.07 | 9.95E-01 | -0.08 | 0.08 | 5.80E-01 | -0.09 | 0.08 | 6.27E-01 |
| Free cholesterol in very large VLDL | -0.03 | 0.05 | 7.69E-01 | -0.05 | 0.05 | 6.52E-01 | -0.04 | 0.06 | 9.37E-01 | -0.02 | 0.07 | 9.95E-01 | -0.07 | 0.08 | 6.10E-01 | -0.08 | 0.08 | 6.27E-01 |
| Phospholipids in HDL | 0.05 | 0.06 | 5.95E-01 | 0.05 | 0.06 | 6.52E-01 | 0.09 | 0.07 | 9.37E-01 | 0.07 | 0.07 | 9.95E-01 | 0.01 | 0.10 | 9.71E-01 | 0.05 | 0.08 | 8.28E-01 |
| Total lipids in small VLDL | -0.04 | 0.05 | 7.49E-01 | -0.05 | 0.05 | 6.52E-01 | -0.01 | 0.06 | 9.58E-01 | 0.00 | 0.07 | 9.99E-01 | -0.09 | 0.08 | 5.19E-01 | -0.09 | 0.08 | 5.87E-01 |
| Cholesteryl esters to total lipids ratio in small LDL | -0.04 | 0.05 | 6.84E-01 | -0.05 | 0.05 | 6.52E-01 | -0.03 | 0.07 | 9.58E-01 | -0.02 | 0.07 | 9.95E-01 | -0.06 | 0.09 | 7.16E-01 | -0.05 | 0.08 | 8.26E-01 |
| Total lipids in very small VLDL | -0.04 | 0.05 | 7.15E-01 | -0.05 | 0.05 | 6.52E-01 | -0.01 | 0.07 | 9.58E-01 | -0.01 | 0.07 | 9.95E-01 | -0.09 | 0.09 | 5.19E-01 | -0.07 | 0.09 | 6.59E-01 |
| Phospholipids to total lipids ratio in very large HDL | -0.05 | 0.05 | 5.42E-01 | -0.04 | 0.05 | 6.52E-01 | -0.01 | 0.07 | 9.75E-01 | -0.02 | 0.07 | 9.95E-01 | -0.08 | 0.08 | 5.49E-01 | -0.06 | 0.08 | 6.81E-01 |
| Concentration of medium HDL particles | 0.05 | 0.06 | 5.96E-01 | 0.05 | 0.06 | 6.52E-01 | 0.09 | 0.07 | 9.37E-01 | 0.07 | 0.07 | 9.95E-01 | 0.00 | 0.10 | 9.88E-01 | 0.04 | 0.08 | 8.46E-01 |
| Cholesterol to total lipids ratio in very large VLDL | -0.06 | 0.05 | 4.96E-01 | -0.05 | 0.06 | 6.79E-01 | -0.01 | 0.07 | 9.58E-01 | -0.03 | 0.07 | 9.95E-01 | -0.10 | 0.09 | 5.19E-01 | -0.08 | 0.09 | 6.59E-01 |
| Acetone | 0.04 | 0.05 | 6.84E-01 | 0.04 | 0.05 | 6.94E-01 | 0.03 | 0.06 | 9.58E-01 | 0.02 | 0.06 | 9.95E-01 | 0.09 | 0.08 | 5.05E-01 | 0.08 | 0.08 | 6.27E-01 |
| Acetoacetate | 0.05 | 0.05 | 5.95E-01 | 0.04 | 0.05 | 7.02E-01 | 0.11 | 0.06 | 9.37E-01 | 0.10 | 0.06 | 9.95E-01 | -0.03 | 0.08 | 8.79E-01 | -0.04 | 0.08 | 8.46E-01 |
| Triglycerides in medium VLDL | -0.03 | 0.05 | 8.15E-01 | -0.04 | 0.05 | 7.08E-01 | -0.02 | 0.06 | 9.58E-01 | 0.00 | 0.07 | 9.95E-01 | -0.07 | 0.08 | 6.10E-01 | -0.08 | 0.08 | 6.27E-01 |
| Monounsaturated fatty acids | -0.02 | 0.05 | 8.48E-01 | -0.04 | 0.05 | 7.08E-01 | -0.02 | 0.07 | 9.58E-01 | -0.01 | 0.07 | 9.95E-01 | -0.07 | 0.09 | 6.53E-01 | -0.06 | 0.09 | 7.57E-01 |
| Triglycerides to total lipids ratio in very large VLDL | 0.06 | 0.05 | 5.45E-01 | 0.04 | 0.05 | 7.08E-01 | 0.01 | 0.07 | 9.70E-01 | 0.02 | 0.07 | 9.95E-01 | 0.11 | 0.09 | 5.05E-01 | 0.09 | 0.09 | 6.27E-01 |
| Cholesteryl esters to total lipids ratio in very large VLDL | -0.06 | 0.05 | 5.24E-01 | -0.04 | 0.05 | 7.10E-01 | -0.01 | 0.07 | 9.58E-01 | -0.03 | 0.07 | 9.95E-01 | -0.09 | 0.09 | 5.69E-01 | -0.07 | 0.09 | 7.07E-01 |
| Total cholines | -0.03 | 0.06 | 8.15E-01 | -0.04 | 0.06 | 7.10E-01 | 0.02 | 0.07 | 9.58E-01 | 0.01 | 0.07 | 9.95E-01 | -0.14 | 0.10 | 4.14E-01 | -0.07 | 0.08 | 6.82E-01 |
| Free cholesterol in large VLDL | -0.03 | 0.05 | 8.19E-01 | -0.04 | 0.05 | 7.10E-01 | -0.03 | 0.06 | 9.58E-01 | -0.01 | 0.07 | 9.95E-01 | -0.07 | 0.08 | 6.53E-01 | -0.08 | 0.08 | 6.40E-01 |
| Phospholipids in very small VLDL | -0.03 | 0.05 | 7.74E-01 | -0.04 | 0.05 | 7.10E-01 | -0.01 | 0.07 | 9.58E-01 | -0.01 | 0.07 | 9.95E-01 | -0.08 | 0.08 | 5.83E-01 | -0.06 | 0.08 | 7.12E-01 |
| Total fatty acids | -0.02 | 0.05 | 8.37E-01 | -0.04 | 0.05 | 7.10E-01 | 0.00 | 0.07 | 9.96E-01 | 0.01 | 0.07 | 9.95E-01 | -0.10 | 0.09 | 5.05E-01 | -0.08 | 0.08 | 6.37E-01 |
| Cholesterol to total lipids ratio in chylomicrons and extremely large VLDL | -0.05 | 0.05 | 5.95E-01 | -0.04 | 0.05 | 7.20E-01 | 0.01 | 0.07 | 9.58E-01 | 0.00 | 0.07 | 9.95E-01 | -0.07 | 0.09 | 6.48E-01 | -0.07 | 0.09 | 7.09E-01 |
| Concentration of small VLDL particles | -0.03 | 0.05 | 8.15E-01 | -0.04 | 0.05 | 7.25E-01 | -0.01 | 0.06 | 9.58E-01 | 0.01 | 0.07 | 9.95E-01 | -0.08 | 0.08 | 5.80E-01 | -0.08 | 0.08 | 6.27E-01 |
| Apolipoprotein A1 | 0.05 | 0.06 | 6.84E-01 | 0.04 | 0.06 | 7.25E-01 | 0.09 | 0.07 | 9.37E-01 | 0.07 | 0.07 | 9.95E-01 | -0.01 | 0.10 | 9.63E-01 | 0.03 | 0.08 | 8.71E-01 |
| Total lipids in large VLDL | -0.02 | 0.05 | 8.35E-01 | -0.04 | 0.05 | 7.25E-01 | -0.03 | 0.06 | 9.58E-01 | -0.01 | 0.07 | 9.95E-01 | -0.06 | 0.08 | 6.76E-01 | -0.07 | 0.08 | 6.59E-01 |
| Cholesteryl esters to total lipids ratio in medium LDL | -0.03 | 0.05 | 8.35E-01 | -0.04 | 0.05 | 7.50E-01 | -0.02 | 0.07 | 9.58E-01 | -0.01 | 0.07 | 9.95E-01 | -0.02 | 0.09 | 9.41E-01 | -0.03 | 0.08 | 8.73E-01 |
| Free cholesterol to total lipids ratio in small LDL | -0.05 | 0.05 | 6.23E-01 | -0.03 | 0.05 | 7.55E-01 | -0.01 | 0.06 | 9.58E-01 | -0.03 | 0.07 | 9.95E-01 | -0.09 | 0.08 | 5.34E-01 | -0.08 | 0.08 | 6.27E-01 |
| Triglycerides to total lipids ratio in large VLDL | 0.04 | 0.05 | 6.84E-01 | 0.03 | 0.05 | 7.61E-01 | 0.01 | 0.07 | 9.58E-01 | 0.01 | 0.07 | 9.95E-01 | 0.06 | 0.08 | 7.03E-01 | 0.06 | 0.08 | 7.57E-01 |
| Phospholipids in large VLDL | -0.02 | 0.05 | 8.48E-01 | -0.03 | 0.05 | 7.61E-01 | -0.02 | 0.06 | 9.58E-01 | 0.00 | 0.07 | 9.95E-01 | -0.05 | 0.08 | 7.31E-01 | -0.07 | 0.08 | 7.07E-01 |
| Total lipids in small HDL | 0.05 | 0.05 | 5.91E-01 | 0.04 | 0.06 | 7.61E-01 | 0.08 | 0.07 | 9.37E-01 | 0.08 | 0.07 | 9.95E-01 | -0.02 | 0.09 | 9.41E-01 | 0.01 | 0.08 | 9.62E-01 |
| Histidine | -0.03 | 0.05 | 7.74E-01 | -0.03 | 0.05 | 7.64E-01 | -0.07 | 0.06 | 9.37E-01 | -0.07 | 0.06 | 9.95E-01 | 0.01 | 0.08 | 9.56E-01 | 0.01 | 0.08 | 9.62E-01 |
| Ratio of docosahexaenoic acid to total fatty acids | 0.03 | 0.05 | 7.67E-01 | 0.03 | 0.05 | 7.65E-01 | 0.05 | 0.06 | 9.37E-01 | 0.04 | 0.07 | 9.95E-01 | 0.05 | 0.08 | 7.93E-01 | 0.06 | 0.08 | 7.63E-01 |
| Cholesteryl esters to total lipids ratio in very large HDL | -0.03 | 0.05 | 8.15E-01 | -0.03 | 0.05 | 7.66E-01 | -0.04 | 0.07 | 9.37E-01 | -0.04 | 0.07 | 9.95E-01 | -0.05 | 0.08 | 7.65E-01 | -0.05 | 0.08 | 7.98E-01 |
| Cholesteryl esters in medium HDL | 0.04 | 0.05 | 7.67E-01 | 0.04 | 0.06 | 7.66E-01 | 0.07 | 0.07 | 9.37E-01 | 0.05 | 0.07 | 9.95E-01 | -0.01 | 0.10 | 9.63E-01 | 0.03 | 0.08 | 8.85E-01 |
| Triglycerides in large VLDL | -0.02 | 0.05 | 8.67E-01 | -0.03 | 0.05 | 7.66E-01 | -0.02 | 0.06 | 9.58E-01 | -0.01 | 0.07 | 9.95E-01 | -0.05 | 0.08 | 7.34E-01 | -0.06 | 0.08 | 7.09E-01 |
| Cholesterol in medium HDL | 0.04 | 0.05 | 7.67E-01 | 0.04 | 0.06 | 7.66E-01 | 0.07 | 0.07 | 9.37E-01 | 0.05 | 0.07 | 9.95E-01 | -0.02 | 0.10 | 9.56E-01 | 0.03 | 0.08 | 8.98E-01 |
| Ratio of monounsaturated fatty acids to total fatty acids | -0.01 | 0.05 | 9.01E-01 | -0.03 | 0.05 | 7.66E-01 | -0.05 | 0.07 | 9.37E-01 | -0.04 | 0.07 | 9.95E-01 | 0.01 | 0.09 | 9.63E-01 | 0.00 | 0.09 | 9.99E-01 |
| Ratio of omega-6 fatty acids to omega-3 fatty acids | -0.05 | 0.05 | 6.44E-01 | -0.03 | 0.05 | 7.66E-01 | -0.05 | 0.07 | 9.37E-01 | -0.05 | 0.07 | 9.95E-01 | -0.03 | 0.09 | 8.83E-01 | -0.04 | 0.09 | 8.46E-01 |
| Total lipids in very large VLDL | -0.02 | 0.05 | 8.80E-01 | -0.03 | 0.05 | 7.72E-01 | -0.03 | 0.06 | 9.58E-01 | -0.01 | 0.07 | 9.95E-01 | -0.05 | 0.09 | 8.02E-01 | -0.06 | 0.08 | 7.57E-01 |
| Concentration of large VLDL particles | -0.02 | 0.05 | 8.71E-01 | -0.03 | 0.05 | 7.72E-01 | -0.02 | 0.06 | 9.58E-01 | 0.00 | 0.07 | 9.95E-01 | -0.05 | 0.08 | 7.58E-01 | -0.06 | 0.08 | 7.23E-01 |
| 3-Hydroxybutyrate | -0.03 | 0.05 | 8.33E-01 | -0.03 | 0.05 | 7.72E-01 | 0.02 | 0.07 | 9.58E-01 | 0.02 | 0.07 | 9.95E-01 | -0.07 | 0.08 | 6.10E-01 | -0.08 | 0.08 | 6.27E-01 |
| Total lipids in HDL | 0.04 | 0.06 | 7.74E-01 | 0.03 | 0.06 | 7.72E-01 | 0.08 | 0.07 | 9.37E-01 | 0.05 | 0.07 | 9.95E-01 | -0.02 | 0.10 | 9.56E-01 | 0.03 | 0.09 | 8.85E-01 |
| Average diameter for VLDL particles | -0.02 | 0.05 | 8.80E-01 | -0.03 | 0.05 | 8.06E-01 | -0.04 | 0.07 | 9.37E-01 | -0.02 | 0.07 | 9.95E-01 | -0.03 | 0.09 | 8.95E-01 | -0.05 | 0.09 | 8.24E-01 |
| Phospholipids in large HDL | 0.03 | 0.05 | 8.37E-01 | 0.03 | 0.06 | 8.06E-01 | 0.05 | 0.07 | 9.37E-01 | 0.02 | 0.07 | 9.95E-01 | 0.01 | 0.10 | 9.63E-01 | 0.04 | 0.09 | 8.43E-01 |
| Free cholesterol in medium HDL | 0.04 | 0.06 | 7.74E-01 | 0.03 | 0.06 | 8.06E-01 | 0.08 | 0.07 | 9.37E-01 | 0.06 | 0.07 | 9.95E-01 | -0.04 | 0.10 | 8.78E-01 | 0.01 | 0.09 | 9.55E-01 |
| Triglycerides in very small VLDL | 0.04 | 0.05 | 6.84E-01 | 0.03 | 0.05 | 8.11E-01 | 0.03 | 0.06 | 9.37E-01 | 0.04 | 0.07 | 9.95E-01 | 0.03 | 0.08 | 8.78E-01 | 0.04 | 0.08 | 8.46E-01 |
| Free cholesterol to total lipids ratio in medium LDL | -0.04 | 0.05 | 7.15E-01 | -0.03 | 0.05 | 8.35E-01 | -0.01 | 0.06 | 9.58E-01 | -0.02 | 0.07 | 9.95E-01 | -0.10 | 0.09 | 5.18E-01 | -0.08 | 0.09 | 6.37E-01 |
| Triglycerides in chylomicrons and extremely large VLDL | -0.02 | 0.05 | 8.90E-01 | -0.02 | 0.05 | 8.36E-01 | -0.05 | 0.06 | 9.37E-01 | -0.03 | 0.06 | 9.95E-01 | -0.03 | 0.08 | 9.20E-01 | -0.03 | 0.08 | 8.63E-01 |
| Glucose | 0.04 | 0.05 | 6.89E-01 | 0.03 | 0.05 | 8.36E-01 | -0.01 | 0.06 | 9.58E-01 | 0.00 | 0.07 | 9.99E-01 | 0.07 | 0.09 | 6.53E-01 | 0.06 | 0.09 | 7.64E-01 |
| Triglycerides to total lipids ratio in chylomicrons and extremely large VLDL | -0.02 | 0.05 | 8.71E-01 | -0.02 | 0.05 | 8.36E-01 | -0.04 | 0.06 | 9.37E-01 | -0.03 | 0.06 | 9.95E-01 | -0.03 | 0.08 | 8.79E-01 | -0.04 | 0.08 | 8.46E-01 |
| Cholesteryl esters in chylomicrons and extremely large VLDL | -0.01 | 0.05 | 9.05E-01 | -0.03 | 0.05 | 8.36E-01 | -0.02 | 0.06 | 9.58E-01 | 0.00 | 0.07 | 9.95E-01 | -0.04 | 0.09 | 8.32E-01 | -0.05 | 0.09 | 7.98E-01 |
| Concentration of very large VLDL particles | -0.01 | 0.05 | 9.16E-01 | -0.03 | 0.05 | 8.36E-01 | -0.02 | 0.06 | 9.58E-01 | 0.00 | 0.07 | 9.95E-01 | -0.03 | 0.09 | 8.81E-01 | -0.05 | 0.08 | 8.28E-01 |
| Free cholesterol to total lipids ratio in chylomicrons and extremely large VLDL | -0.03 | 0.05 | 8.15E-01 | -0.02 | 0.05 | 8.48E-01 | 0.03 | 0.06 | 9.58E-01 | 0.02 | 0.07 | 9.95E-01 | -0.08 | 0.09 | 5.56E-01 | -0.08 | 0.09 | 6.37E-01 |
| Isoleucine | 0.03 | 0.06 | 8.15E-01 | 0.03 | 0.06 | 8.48E-01 | -0.04 | 0.07 | 9.37E-01 | -0.01 | 0.07 | 9.95E-01 | 0.11 | 0.10 | 5.18E-01 | 0.05 | 0.09 | 8.28E-01 |
| Phosphoglycerides | -0.01 | 0.06 | 9.45E-01 | -0.02 | 0.06 | 8.86E-01 | 0.04 | 0.07 | 9.37E-01 | 0.03 | 0.07 | 9.95E-01 | -0.11 | 0.09 | 5.05E-01 | -0.05 | 0.08 | 7.98E-01 |
| Phosphatidylcholines | -0.01 | 0.06 | 9.45E-01 | -0.02 | 0.06 | 8.86E-01 | 0.04 | 0.07 | 9.39E-01 | 0.02 | 0.07 | 9.95E-01 | -0.10 | 0.09 | 5.18E-01 | -0.05 | 0.08 | 8.26E-01 |
| Triglycerides in very large HDL | 0.03 | 0.05 | 8.15E-01 | 0.02 | 0.05 | 8.90E-01 | 0.04 | 0.07 | 9.37E-01 | 0.04 | 0.07 | 9.95E-01 | 0.02 | 0.09 | 9.41E-01 | 0.03 | 0.08 | 8.80E-01 |
| Albumin | -0.02 | 0.05 | 8.37E-01 | -0.02 | 0.05 | 8.92E-01 | -0.01 | 0.07 | 9.58E-01 | -0.01 | 0.07 | 9.95E-01 | -0.02 | 0.09 | 9.33E-01 | -0.02 | 0.09 | 9.06E-01 |
| Degree of unsaturation | -0.03 | 0.05 | 8.01E-01 | -0.02 | 0.05 | 8.94E-01 | 0.01 | 0.06 | 9.70E-01 | 0.00 | 0.07 | 9.95E-01 | -0.09 | 0.08 | 5.19E-01 | -0.07 | 0.08 | 6.59E-01 |
| Ratio of omega-3 fatty acids to total fatty acids | 0.03 | 0.05 | 8.25E-01 | 0.02 | 0.05 | 9.08E-01 | 0.04 | 0.07 | 9.37E-01 | 0.04 | 0.07 | 9.95E-01 | 0.01 | 0.09 | 9.56E-01 | 0.02 | 0.08 | 9.06E-01 |
| Phospholipids to total lipids ratio in very small VLDL | 0.02 | 0.05 | 8.67E-01 | 0.02 | 0.05 | 9.08E-01 | -0.01 | 0.06 | 9.58E-01 | 0.01 | 0.07 | 9.95E-01 | 0.04 | 0.08 | 8.33E-01 | 0.03 | 0.08 | 8.73E-01 |
| Cholesteryl esters in large HDL | -0.02 | 0.05 | 8.37E-01 | -0.02 | 0.06 | 9.08E-01 | -0.01 | 0.07 | 9.58E-01 | -0.04 | 0.07 | 9.95E-01 | -0.03 | 0.10 | 9.33E-01 | 0.01 | 0.09 | 9.55E-01 |
| Triglycerides in small LDL | 0.00 | 0.05 | 9.79E-01 | -0.02 | 0.05 | 9.08E-01 | -0.01 | 0.06 | 9.58E-01 | 0.00 | 0.07 | 9.95E-01 | -0.02 | 0.09 | 9.41E-01 | -0.02 | 0.09 | 9.06E-01 |
| Total concentration of branched-chain amino acids (leucine + isoleucine + valine) | 0.03 | 0.05 | 8.37E-01 | 0.02 | 0.06 | 9.08E-01 | -0.04 | 0.07 | 9.37E-01 | -0.01 | 0.07 | 9.95E-01 | 0.08 | 0.10 | 6.10E-01 | 0.03 | 0.09 | 8.71E-01 |
| Pyruvate | -0.01 | 0.05 | 9.60E-01 | -0.02 | 0.05 | 9.08E-01 | -0.09 | 0.06 | 9.37E-01 | -0.09 | 0.07 | 9.95E-01 | 0.10 | 0.08 | 5.05E-01 | 0.10 | 0.08 | 5.68E-01 |
| Triglycerides in very large VLDL | 0.00 | 0.05 | 9.78E-01 | -0.02 | 0.05 | 9.08E-01 | -0.02 | 0.06 | 9.58E-01 | 0.00 | 0.07 | 9.99E-01 | -0.02 | 0.09 | 9.33E-01 | -0.04 | 0.09 | 8.46E-01 |
| Triglycerides in VLDL | 0.00 | 0.05 | 9.78E-01 | -0.02 | 0.05 | 9.08E-01 | -0.01 | 0.06 | 9.58E-01 | 0.01 | 0.07 | 9.95E-01 | -0.03 | 0.09 | 9.11E-01 | -0.04 | 0.08 | 8.46E-01 |
| Valine | 0.03 | 0.05 | 8.37E-01 | 0.02 | 0.06 | 9.09E-01 | -0.04 | 0.07 | 9.37E-01 | -0.01 | 0.07 | 9.95E-01 | 0.07 | 0.09 | 6.53E-01 | 0.04 | 0.09 | 8.46E-01 |
| Phospholipids in chylomicrons and extremely large VLDL | 0.03 | 0.05 | 8.19E-01 | 0.02 | 0.05 | 9.09E-01 | -0.01 | 0.06 | 9.58E-01 | 0.01 | 0.07 | 9.95E-01 | 0.05 | 0.09 | 8.10E-01 | 0.03 | 0.09 | 8.82E-01 |
| Triglycerides in IDL | 0.03 | 0.05 | 8.01E-01 | 0.02 | 0.05 | 9.16E-01 | 0.03 | 0.07 | 9.58E-01 | 0.03 | 0.07 | 9.95E-01 | 0.02 | 0.09 | 9.41E-01 | 0.03 | 0.08 | 8.73E-01 |
| Average diameter for HDL particles | 0.01 | 0.05 | 9.45E-01 | 0.02 | 0.06 | 9.18E-01 | 0.03 | 0.07 | 9.37E-01 | 0.01 | 0.07 | 9.95E-01 | 0.01 | 0.09 | 9.63E-01 | 0.04 | 0.09 | 8.46E-01 |
| Phospholipids to total lipids ratio in chylomicrons and extremely large VLDL | 0.02 | 0.05 | 8.37E-01 | 0.02 | 0.05 | 9.18E-01 | 0.00 | 0.06 | 9.96E-01 | 0.01 | 0.07 | 9.95E-01 | 0.02 | 0.09 | 9.41E-01 | 0.01 | 0.09 | 9.71E-01 |
| Cholesterol in large HDL | -0.02 | 0.05 | 8.67E-01 | -0.02 | 0.06 | 9.26E-01 | 0.00 | 0.07 | 9.83E-01 | -0.03 | 0.07 | 9.95E-01 | -0.02 | 0.10 | 9.41E-01 | 0.02 | 0.09 | 9.42E-01 |
| Cholesterol to total lipids ratio in very large HDL | -0.01 | 0.05 | 9.45E-01 | -0.02 | 0.05 | 9.26E-01 | -0.04 | 0.07 | 9.37E-01 | -0.02 | 0.07 | 9.95E-01 | 0.00 | 0.09 | 9.72E-01 | -0.02 | 0.08 | 8.98E-01 |
| Total lipids in large HDL | 0.01 | 0.05 | 9.45E-01 | 0.01 | 0.06 | 9.41E-01 | 0.04 | 0.07 | 9.37E-01 | 0.01 | 0.07 | 9.95E-01 | -0.01 | 0.10 | 9.65E-01 | 0.03 | 0.09 | 8.85E-01 |
| Concentration of HDL particles | 0.02 | 0.06 | 8.67E-01 | 0.01 | 0.06 | 9.41E-01 | 0.07 | 0.07 | 9.37E-01 | 0.05 | 0.07 | 9.95E-01 | -0.06 | 0.10 | 7.21E-01 | -0.01 | 0.08 | 9.62E-01 |
| Phospholipids to total lipids ratio in large VLDL | 0.02 | 0.05 | 8.80E-01 | 0.01 | 0.05 | 9.64E-01 | 0.00 | 0.06 | 9.75E-01 | 0.01 | 0.07 | 9.95E-01 | 0.04 | 0.09 | 8.60E-01 | 0.03 | 0.09 | 8.98E-01 |
| Free cholesterol in small HDL | 0.03 | 0.05 | 8.35E-01 | 0.01 | 0.06 | 9.64E-01 | 0.08 | 0.07 | 9.37E-01 | 0.07 | 0.07 | 9.95E-01 | -0.07 | 0.09 | 6.76E-01 | -0.03 | 0.08 | 8.85E-01 |
| Leucine | 0.02 | 0.06 | 8.80E-01 | 0.01 | 0.06 | 9.64E-01 | -0.04 | 0.07 | 9.37E-01 | -0.02 | 0.07 | 9.95E-01 | 0.07 | 0.10 | 6.76E-01 | 0.02 | 0.09 | 9.37E-01 |
| Average diameter for LDL particles | -0.02 | 0.05 | 8.67E-01 | -0.01 | 0.05 | 9.64E-01 | -0.02 | 0.06 | 9.58E-01 | -0.03 | 0.07 | 9.95E-01 | -0.06 | 0.09 | 7.19E-01 | -0.04 | 0.08 | 8.40E-01 |
| Free cholesterol to total lipids ratio in large VLDL | -0.01 | 0.05 | 9.05E-01 | -0.01 | 0.05 | 9.64E-01 | 0.00 | 0.06 | 9.82E-01 | -0.01 | 0.06 | 9.95E-01 | -0.01 | 0.08 | 9.56E-01 | -0.01 | 0.08 | 9.43E-01 |
| Cholesterol in chylomicrons and extremely large VLDL | 0.00 | 0.05 | 9.78E-01 | -0.01 | 0.05 | 9.64E-01 | -0.01 | 0.06 | 9.58E-01 | 0.01 | 0.07 | 9.95E-01 | -0.01 | 0.09 | 9.63E-01 | -0.02 | 0.09 | 9.09E-01 |
| Docosahexaenoic acid | 0.02 | 0.05 | 8.67E-01 | 0.01 | 0.05 | 9.64E-01 | 0.05 | 0.07 | 9.37E-01 | 0.04 | 0.07 | 9.95E-01 | -0.02 | 0.08 | 9.33E-01 | 0.00 | 0.08 | 9.80E-01 |
| Phospholipids to total lipids ratio in small LDL | 0.00 | 0.05 | 9.78E-01 | 0.01 | 0.05 | 9.64E-01 | 0.00 | 0.07 | 9.92E-01 | -0.01 | 0.07 | 9.95E-01 | 0.00 | 0.08 | 9.72E-01 | -0.01 | 0.08 | 9.62E-01 |
| Acetate | 0.00 | 0.05 | 9.78E-01 | 0.01 | 0.05 | 9.64E-01 | 0.06 | 0.06 | 9.37E-01 | 0.06 | 0.06 | 9.95E-01 | -0.13 | 0.09 | 4.16E-01 | -0.13 | 0.09 | 4.44E-01 |
| Triglycerides in small VLDL | 0.02 | 0.05 | 8.67E-01 | 0.01 | 0.05 | 9.64E-01 | 0.02 | 0.06 | 9.58E-01 | 0.04 | 0.07 | 9.95E-01 | -0.01 | 0.08 | 9.63E-01 | -0.02 | 0.08 | 9.28E-01 |
| Total triglycerides | 0.01 | 0.05 | 9.67E-01 | -0.01 | 0.05 | 9.64E-01 | 0.00 | 0.06 | 9.92E-01 | 0.02 | 0.07 | 9.95E-01 | -0.02 | 0.09 | 9.41E-01 | -0.03 | 0.09 | 8.98E-01 |
| Free cholesterol to total lipids ratio in very large HDL | 0.01 | 0.05 | 9.05E-01 | 0.01 | 0.06 | 9.66E-01 | -0.02 | 0.07 | 9.58E-01 | 0.00 | 0.07 | 9.95E-01 | 0.03 | 0.10 | 9.07E-01 | -0.01 | 0.09 | 9.71E-01 |
| Saturated fatty acids | 0.01 | 0.05 | 9.16E-01 | -0.01 | 0.05 | 9.76E-01 | 0.04 | 0.07 | 9.37E-01 | 0.04 | 0.07 | 9.95E-01 | -0.06 | 0.09 | 6.76E-01 | -0.04 | 0.08 | 8.38E-01 |
| Triglycerides in medium LDL | 0.01 | 0.05 | 9.30E-01 | -0.01 | 0.05 | 9.76E-01 | 0.01 | 0.06 | 9.75E-01 | 0.01 | 0.07 | 9.95E-01 | -0.01 | 0.09 | 9.63E-01 | 0.00 | 0.08 | 9.71E-01 |
| Cholesteryl esters in small HDL | 0.00 | 0.05 | 9.78E-01 | -0.01 | 0.05 | 9.76E-01 | 0.03 | 0.07 | 9.37E-01 | 0.03 | 0.07 | 9.95E-01 | -0.07 | 0.09 | 6.53E-01 | -0.04 | 0.08 | 8.46E-01 |
| Total concentration of lipoprotein particles | 0.00 | 0.06 | 9.78E-01 | -0.01 | 0.06 | 9.76E-01 | 0.06 | 0.07 | 9.37E-01 | 0.04 | 0.07 | 9.95E-01 | -0.10 | 0.10 | 5.45E-01 | -0.04 | 0.08 | 8.46E-01 |
| Free cholesterol in HDL | 0.01 | 0.06 | 9.45E-01 | 0.01 | 0.06 | 9.87E-01 | 0.06 | 0.07 | 9.37E-01 | 0.03 | 0.07 | 9.95E-01 | -0.06 | 0.10 | 7.32E-01 | -0.01 | 0.09 | 9.71E-01 |
| Phospholipids to total lipids ratio in IDL | 0.00 | 0.05 | 9.78E-01 | 0.01 | 0.05 | 9.87E-01 | -0.06 | 0.07 | 9.37E-01 | -0.05 | 0.07 | 9.95E-01 | 0.10 | 0.08 | 4.74E-01 | 0.09 | 0.08 | 5.90E-01 |
| Ratio of polyunsaturated fatty acids to monounsaturated fatty acids | -0.03 | 0.05 | 8.35E-01 | -0.01 | 0.05 | 9.87E-01 | 0.00 | 0.06 | 9.96E-01 | -0.01 | 0.07 | 9.95E-01 | -0.05 | 0.09 | 8.10E-01 | -0.04 | 0.09 | 8.63E-01 |
| Concentration of chylomicrons and extremely large VLDL particles | 0.02 | 0.05 | 8.80E-01 | 0.00 | 0.05 | 9.90E-01 | -0.01 | 0.06 | 9.58E-01 | 0.01 | 0.07 | 9.95E-01 | 0.01 | 0.09 | 9.63E-01 | 0.00 | 0.09 | 9.80E-01 |
| Free cholesterol in chylomicrons and extremely large VLDL | 0.02 | 0.05 | 8.80E-01 | 0.00 | 0.05 | 9.90E-01 | -0.02 | 0.06 | 9.58E-01 | 0.00 | 0.07 | 9.95E-01 | 0.02 | 0.09 | 9.33E-01 | 0.01 | 0.09 | 9.55E-01 |
| Phospholipids to total lipids ratio in medium LDL | -0.01 | 0.05 | 9.67E-01 | 0.00 | 0.05 | 9.90E-01 | -0.02 | 0.07 | 9.58E-01 | -0.02 | 0.07 | 9.95E-01 | -0.03 | 0.08 | 9.12E-01 | -0.02 | 0.08 | 9.15E-01 |
| Triglycerides in large LDL | 0.02 | 0.05 | 8.67E-01 | 0.00 | 0.05 | 9.90E-01 | 0.02 | 0.07 | 9.58E-01 | 0.02 | 0.07 | 9.95E-01 | 0.00 | 0.09 | 9.71E-01 | 0.02 | 0.08 | 9.24E-01 |
| Citrate | -0.02 | 0.05 | 8.80E-01 | 0.00 | 0.05 | 9.90E-01 | -0.08 | 0.07 | 9.37E-01 | -0.08 | 0.07 | 9.95E-01 | 0.11 | 0.08 | 4.47E-01 | 0.11 | 0.08 | 4.79E-01 |
| Cholesterol in small HDL | 0.01 | 0.05 | 9.30E-01 | 0.00 | 0.05 | 9.90E-01 | 0.05 | 0.07 | 9.37E-01 | 0.04 | 0.07 | 9.95E-01 | -0.07 | 0.09 | 6.53E-01 | -0.04 | 0.08 | 8.46E-01 |
| HDL cholesterol | 0.00 | 0.05 | 9.78E-01 | 0.00 | 0.06 | 9.90E-01 | 0.05 | 0.07 | 9.37E-01 | 0.02 | 0.07 | 9.95E-01 | -0.06 | 0.10 | 7.65E-01 | 0.00 | 0.09 | 9.71E-01 |
| Triglycerides in LDL | 0.02 | 0.05 | 8.90E-01 | 0.00 | 0.05 | 9.90E-01 | 0.01 | 0.07 | 9.58E-01 | 0.01 | 0.07 | 9.95E-01 | 0.00 | 0.09 | 9.77E-01 | 0.01 | 0.08 | 9.71E-01 |
| Phospholipids to total lipids ratio in large LDL | 0.00 | 0.05 | 9.78E-01 | 0.00 | 0.05 | 9.90E-01 | 0.01 | 0.06 | 9.58E-01 | 0.01 | 0.06 | 9.95E-01 | 0.01 | 0.08 | 9.63E-01 | 0.00 | 0.08 | 9.71E-01 |
| Omega-3 fatty acids | 0.02 | 0.05 | 8.90E-01 | 0.00 | 0.05 | 9.90E-01 | 0.03 | 0.07 | 9.37E-01 | 0.03 | 0.07 | 9.95E-01 | -0.03 | 0.09 | 8.78E-01 | -0.02 | 0.08 | 9.24E-01 |
| Ratio of triglycerides to phosphoglycerides | 0.01 | 0.05 | 9.45E-01 | 0.00 | 0.05 | 9.90E-01 | -0.02 | 0.06 | 9.58E-01 | 0.01 | 0.07 | 9.95E-01 | 0.02 | 0.09 | 9.41E-01 | -0.01 | 0.09 | 9.71E-01 |
| Glycoprotein acetyls | 0.01 | 0.05 | 9.07E-01 | 0.00 | 0.05 | 9.90E-01 | -0.01 | 0.06 | 9.58E-01 | 0.00 | 0.07 | 9.95E-01 | 0.02 | 0.09 | 9.33E-01 | 0.03 | 0.09 | 8.98E-01 |
| Tyrosine | 0.01 | 0.05 | 9.16E-01 | 0.00 | 0.05 | 9.90E-01 | -0.03 | 0.06 | 9.56E-01 | -0.02 | 0.07 | 9.95E-01 | 0.01 | 0.09 | 9.56E-01 | 0.01 | 0.09 | 9.55E-01 |
| Total lipids in chylomicrons and extremely large VLDL | 0.01 | 0.05 | 9.05E-01 | 0.00 | 0.05 | 9.90E-01 | -0.02 | 0.06 | 9.58E-01 | 0.00 | 0.07 | 9.95E-01 | 0.01 | 0.09 | 9.71E-01 | -0.01 | 0.09 | 9.71E-01 |
| Cholesteryl esters in HDL | 0.00 | 0.05 | 9.96E-01 | 0.00 | 0.06 | 9.90E-01 | 0.04 | 0.07 | 9.37E-01 | 0.02 | 0.07 | 9.95E-01 | -0.06 | 0.10 | 7.80E-01 | 0.00 | 0.09 | 9.71E-01 |
| Concentration of large HDL particles | 0.00 | 0.05 | 9.75E-01 | 0.00 | 0.06 | 9.90E-01 | 0.02 | 0.07 | 9.58E-01 | 0.00 | 0.07 | 9.95E-01 | -0.02 | 0.10 | 9.41E-01 | 0.02 | 0.09 | 9.25E-01 |
| Concentration of small HDL particles | 0.01 | 0.05 | 9.08E-01 | 0.00 | 0.05 | 9.90E-01 | 0.05 | 0.07 | 9.37E-01 | 0.04 | 0.07 | 9.95E-01 | -0.07 | 0.09 | 6.72E-01 | -0.04 | 0.08 | 8.46E-01 |
| Free cholesterol in large HDL | -0.01 | 0.05 | 9.67E-01 | 0.00 | 0.06 | 9.90E-01 | 0.02 | 0.07 | 9.58E-01 | -0.01 | 0.07 | 9.95E-01 | -0.01 | 0.10 | 9.56E-01 | 0.02 | 0.09 | 9.10E-01 |

Note: The table shows associations from the logistic regression analysis where plasms metabolomics level from the Nightingale platform was main exposure and prevalent all cancer as the main outcome. Model 1 : adjusted for age, sex, cohort, time differences (ERGO), and lipid lowering medication. Model 2: adjusted for model1, smoking status, BMI, alcohol and education. No adjustment for time differences was made in the analyses of the subcohorts (ERGO-4 and ERGO-5). The table is ranked based on the *FDR* values in model 2 within the E4&E5 cohort, using bold text indicates metabolites are surpassing *FDR* adjusted *P* value treshold (*P*<0.05). ERGO-4 and ERGO-5 refer to the fourth and fifth visits subcohort from the baseline cohort. ERGO-4&5 refers to the combined data from the fourth and fifth visits of the baseline cohort. Abbreviations: SE, standard erorr; IDL, intermediate-density lipoprotein; LDL, low-density lipoprotein; HDL, high-density lipoprotein; VLDL, very-low-density lipoprotein; BMI, body mass index; FDR, false discovery rate.

**Supplementary Table 4. Serum metabolites associated with prevalent all cancer from the Metabolon platform at baseline.**

| **Metabolite** | **ERGO-4&5** | | | | | | | | **ERGO-4** | | | | **ERGO-5** | | | |
| --- | --- | --- | --- | --- | --- | --- | --- | --- | --- | --- | --- | --- | --- | --- | --- | --- |
|  | **Model 1** | | | | **Model 2** | | | | **Model 1** | | **Model 2** | | **Model 1** | | **Model 2** | |
|  | **Beta±SE** | **I^2^** | **Het.*P-*value** | ***FDR* value** | **Beta±SE** | **I^2^** | **Het.*P-*value** | ***FDR* value** | **Beta±SE** | ***FDR* value** | **Beta±SE** | ***FDR* value** | **Beta±SE** | ***FDR* value** | **Beta±SE** | ***FDR* value** |
| X - 12816 | -0.31±0.06 | 0 | 9.20E-01 | **1.72E-03** | -0.31±0.06 | 0 | 8.39E-01 | **2.13E-03** | -0.31±0.08 | 6.10E-02 | -0.32±0.08 | 5.11E-02 | -0.3±0.12 | 2.03E-01 | -0.29±0.12 | 4.03E-01 |
| imidazole propionate | 0.28±0.07 | 72.53 | 5.64E-02 | **2.11E-02** | 0.27±0.07 | 60.99 | 1.09E-01 | **2.68E-02** | 0.17±0.09 | 6.10E-01 | 0.18±0.09 | 5.40E-01 | 0.44±0.11 | **1.28E-02** | 0.41±0.11 | 6.54E-02 |
| 3-ethylcatechol sulfate (1) | -0.25±0.07 | 0 | 4.81E-01 | **2.46E-02** | -0.27±0.07 | 0 | 5.45E-01 | **2.68E-02** | -0.22±0.08 | 2.84E-01 | -0.24±0.09 | 2.58E-01 | -0.32±0.12 | 1.99E-01 | -0.34±0.13 | 3.58E-01 |
| lactosyl-N-palmitoyl-sphingosine (d18:1/16:0) | -0.26±0.07 | 0 | 4.13E-01 | **2.11E-02** | -0.26±0.07 | 29.98 | 2.32E-01 | **2.68E-02** | -0.32±0.09 | 9.01E-02 | -0.34±0.1 | 7.50E-02 | -0.21±0.09 | 2.96E-01 | -0.18±0.1 | 4.63E-01 |
| perfluorooctanesulfonate (PFOS) | -0.26±0.06 | 0 | 3.91E-01 | **2.11E-02** | -0.25±0.07 | 29.99 | 2.32E-01 | **2.68E-02** | -0.29±0.08 | 8.83E-02 | -0.3±0.08 | 6.27E-02 | -0.17±0.12 | 5.51E-01 | -0.12±0.12 | 6.78E-01 |
| cinnamoylglycine | -0.25±0.06 | 0 | 5.33E-01 | **2.24E-02** | -0.25±0.07 | 0 | 6.65E-01 | **2.98E-02** | -0.22±0.08 | 2.66E-01 | -0.23±0.08 | 2.58E-01 | -0.3±0.11 | 1.99E-01 | -0.29±0.12 | 4.03E-01 |
| N-methylproline | -0.27±0.07 | 0 | 7.50E-01 | **2.24E-02** | -0.26±0.07 | 0 | 6.90E-01 | **2.98E-02** | -0.29±0.09 | 9.45E-02 | -0.28±0.09 | 1.30E-01 | -0.24±0.12 | 3.42E-01 | -0.22±0.13 | 4.65E-01 |
| 3-methyl catechol sulfate (1) | -0.26±0.07 | 0 | 4.65E-01 | **3.01E-02** | -0.27±0.07 | 0 | 4.12E-01 | **2.98E-02** | -0.3±0.09 | 9.01E-02 | -0.31±0.09 | 7.50E-02 | -0.18±0.13 | 5.23E-01 | -0.18±0.13 | 5.81E-01 |
| N-acetylglutamate | 0.34±0.09 | 57.28 | 1.26E-01 | **2.46E-02** | 0.34±0.09 | 39.18 | 2.00E-01 | **3.74E-02** | 0.25±0.11 | 3.95E-01 | 0.26±0.11 | 3.68E-01 | 0.57±0.17 | 9.87E-02 | 0.52±0.17 | 2.50E-01 |
| 4-hydroxycoumarin | -0.26±0.07 | 0 | 5.19E-01 | **2.46E-02** | -0.24±0.07 | 0 | 3.53E-01 | **4.61E-02** | -0.28±0.08 | 9.01E-02 | -0.29±0.08 | 7.50E-02 | -0.19±0.12 | 4.98E-01 | -0.14±0.13 | 6.46E-01 |
| X - 24344 | -0.23±0.07 | 0 | 6.85E-01 | 9.38E-02 | -0.28±0.08 | 0 | 4.98E-01 | 6.08E-02 | -0.25±0.09 | 2.66E-01 | -0.31±0.1 | 1.32E-01 | -0.18±0.13 | 5.43E-01 | -0.19±0.15 | 6.07E-01 |
| adenosine 5'-monophosphate (AMP) | 0.22±0.07 | 64.48 | 9.33E-02 | 8.47E-02 | 0.23±0.07 | 62.9 | 1.01E-01 | 8.06E-02 | 0.31±0.09 | 9.01E-02 | 0.32±0.09 | 7.50E-02 | 0.05±0.12 | 8.80E-01 | 0.07±0.12 | 8.22E-01 |
| N-methylhydroxyproline** | -0.24±0.07 | 0 | 3.70E-01 | 5.35E-02 | -0.23±0.07 | 0 | 3.73E-01 | 8.09E-02 | -0.28±0.09 | 9.98E-02 | -0.28±0.09 | 1.32E-01 | -0.15±0.12 | 5.89E-01 | -0.14±0.13 | 6.51E-01 |
| X - 21442 | -0.23±0.07 | 18.85 | 2.67E-01 | 5.35E-02 | -0.22±0.07 | 47.23 | 1.69E-01 | 8.37E-02 | -0.28±0.08 | 9.01E-02 | -0.28±0.08 | 7.50E-02 | -0.12±0.12 | 6.66E-01 | -0.07±0.13 | 8.09E-01 |
| N1-methylinosine | 0.27±0.08 | 0 | 4.94E-01 | 5.35E-02 | 0.26±0.08 | 0 | 8.21E-01 | 8.80E-02 | 0.23±0.1 | 3.41E-01 | 0.24±0.1 | 3.18E-01 | 0.35±0.14 | 2.19E-01 | 0.28±0.14 | 4.52E-01 |
| eicosanedioate (C20-DC) | -0.23±0.07 | 0 | 6.14E-01 | 8.92E-02 | -0.24±0.08 | 0 | 6.24E-01 | 8.80E-02 | -0.26±0.09 | 2.66E-01 | -0.27±0.09 | 2.58E-01 | -0.18±0.13 | 5.23E-01 | -0.19±0.13 | 5.47E-01 |
| X - 12111 | -0.24±0.07 | 0 | 4.89E-01 | 6.47E-02 | -0.24±0.07 | 0 | 4.72E-01 | 8.80E-02 | -0.28±0.09 | 1.82E-01 | -0.27±0.09 | 2.24E-01 | -0.17±0.13 | 5.53E-01 | -0.16±0.13 | 6.23E-01 |
| perfluorooctanoate (PFOA) | -0.21±0.07 | 41.68 | 1.90E-01 | 8.06E-02 | -0.21±0.07 | 65.88 | 8.69E-02 | 9.08E-02 | -0.27±0.08 | 9.01E-02 | -0.28±0.08 | 7.50E-02 | -0.08±0.12 | 8.02E-01 | -0.03±0.12 | 9.34E-01 |
| lactosyl-N-nervonoyl-sphingosine (d18:1/24:1)* | -0.22±0.07 | 0 | 6.54E-01 | 8.47E-02 | -0.22±0.07 | 0 | 9.81E-01 | 1.29E-01 | -0.19±0.09 | 5.29E-01 | -0.22±0.1 | 4.05E-01 | -0.25±0.1 | 2.19E-01 | -0.22±0.11 | 4.45E-01 |
| glycohyocholate | -0.22±0.07 | 0 | 8.64E-01 | 1.13E-01 | -0.23±0.07 | 0 | 9.85E-01 | 1.29E-01 | -0.21±0.09 | 3.75E-01 | -0.23±0.09 | 3.26E-01 | -0.24±0.13 | 3.57E-01 | -0.22±0.13 | 4.74E-01 |
| 2-piperidinone | 0.23±0.07 | 68.56 | 7.45E-02 | 8.92E-02 | 0.21±0.07 | 53.07 | 1.44E-01 | 1.38E-01 | 0.13±0.09 | 7.90E-01 | 0.13±0.09 | 7.51E-01 | 0.4±0.12 | 9.87E-02 | 0.36±0.12 | 3.19E-01 |
| gamma-glutamylisoleucine* | 0.24±0.08 | 69.58 | 6.98E-02 | 1.20E-01 | 0.25±0.09 | 28.11 | 2.38E-01 | 1.38E-01 | 0.16±0.09 | 6.71E-01 | 0.2±0.1 | 5.26E-01 | 0.53±0.18 | 1.81E-01 | 0.44±0.18 | 4.03E-01 |
| N-stearoyl-sphinganine (d18:0/18:0)* | 0.22±0.08 | 21.75 | 2.58E-01 | 1.20E-01 | 0.23±0.08 | 0 | 5.13E-01 | 1.38E-01 | 0.16±0.09 | 6.71E-01 | 0.2±0.1 | 5.64E-01 | 0.35±0.13 | 2.03E-01 | 0.31±0.14 | 4.18E-01 |
| o-cresol sulfate | -0.19±0.08 | 24.73 | 2.49E-01 | 3.02E-01 | -0.26±0.09 | 0 | 4.07E-01 | 1.38E-01 | -0.13±0.09 | 7.90E-01 | -0.21±0.11 | 5.98E-01 | -0.32±0.14 | 2.68E-01 | -0.36±0.16 | 4.18E-01 |
| gamma-glutamylthreonine | 0.25±0.08 | 0 | 3.80E-01 | 1.19E-01 | 0.25±0.09 | 0 | 3.75E-01 | 1.38E-01 | 0.22±0.09 | 3.61E-01 | 0.22±0.09 | 3.52E-01 | 0.42±0.21 | 3.40E-01 | 0.42±0.21 | 4.45E-01 |
| 2-hydroxy-3-methylvalerate | 0.24±0.08 | 0 | 6.69E-01 | 8.92E-02 | 0.23±0.08 | 0 | 8.90E-01 | 1.38E-01 | 0.22±0.09 | 3.64E-01 | 0.23±0.1 | 3.52E-01 | 0.29±0.14 | 3.25E-01 | 0.25±0.14 | 4.74E-01 |
| stachydrine | -0.21±0.07 | 0 | 6.76E-01 | 8.92E-02 | -0.2±0.07 | 0 | 5.59E-01 | 1.38E-01 | -0.23±0.08 | 2.66E-01 | -0.22±0.08 | 2.61E-01 | -0.17±0.12 | 5.32E-01 | -0.14±0.12 | 6.62E-01 |
| X - 12544 | -0.24±0.08 | 0 | 3.48E-01 | 1.20E-01 | -0.24±0.08 | 13.91 | 2.81E-01 | 1.38E-01 | -0.3±0.1 | 2.66E-01 | -0.3±0.1 | 2.24E-01 | -0.14±0.14 | 6.60E-01 | -0.12±0.14 | 7.24E-01 |
| 1-ribosyl-imidazoleacetate* | 0.19±0.07 | 75.58 | 4.30E-02 | 2.17E-01 | 0.2±0.07 | 76.34 | 3.98E-02 | 1.52E-01 | 0.09±0.09 | 8.69E-01 | 0.1±0.09 | 8.36E-01 | 0.39±0.12 | 1.11E-01 | 0.42±0.13 | 1.34E-01 |
| glutarate (C5-DC) | 0.23±0.08 | 32.84 | 2.22E-01 | 1.20E-01 | 0.22±0.08 | 8.82 | 2.95E-01 | 1.52E-01 | 0.16±0.1 | 7.16E-01 | 0.16±0.1 | 6.80E-01 | 0.36±0.14 | 1.99E-01 | 0.34±0.14 | 4.03E-01 |
| formiminoglutamate | 0.22±0.07 | 41.76 | 1.90E-01 | 1.20E-01 | 0.22±0.08 | 0 | 4.44E-01 | 1.52E-01 | 0.15±0.09 | 7.50E-01 | 0.18±0.1 | 6.15E-01 | 0.35±0.13 | 1.99E-01 | 0.3±0.13 | 4.18E-01 |
| N2,N2-dimethylguanosine | 0.23±0.08 | 0 | 3.85E-01 | 1.78E-01 | 0.23±0.08 | 0 | 7.53E-01 | 1.52E-01 | 0.19±0.09 | 5.29E-01 | 0.22±0.1 | 3.64E-01 | 0.37±0.19 | 3.34E-01 | 0.28±0.18 | 4.95E-01 |
| glycine conjugate of C10H12O2* | -0.17±0.07 | 56.83 | 1.28E-01 | 2.81E-01 | -0.2±0.07 | 0.24 | 3.17E-01 | 1.52E-01 | -0.25±0.09 | 2.66E-01 | -0.25±0.09 | 2.58E-01 | -0.02±0.12 | 9.70E-01 | -0.09±0.13 | 7.64E-01 |
| 3-phosphoglycerate | 0.19±0.07 | 0 | 8.42E-01 | 2.13E-01 | 0.19±0.07 | 0 | 8.66E-01 | 1.74E-01 | 0.2±0.09 | 3.79E-01 | 0.2±0.09 | 3.52E-01 | 0.17±0.12 | 5.53E-01 | 0.18±0.12 | 5.74E-01 |
| sphingomyelin (d18:1/22:1, d18:2/22:0, d16:1/24:1)* | -0.2±0.07 | 0 | 3.72E-01 | 1.51E-01 | -0.19±0.07 | 5.8 | 3.03E-01 | 1.94E-01 | -0.25±0.1 | 2.84E-01 | -0.26±0.1 | 2.63E-01 | -0.13±0.1 | 5.71E-01 | -0.11±0.1 | 6.66E-01 |
| X - 14939 | -0.17±0.07 | 0 | 8.70E-01 | 3.02E-01 | -0.19±0.07 | 0 | 9.40E-01 | 2.22E-01 | -0.18±0.09 | 5.29E-01 | -0.18±0.09 | 4.98E-01 | -0.16±0.12 | 5.71E-01 | -0.2±0.12 | 4.95E-01 |
| guaiacol sulfate | -0.19±0.07 | 31.33 | 2.28E-01 | 2.24E-01 | -0.19±0.07 | 12.95 | 2.84E-01 | 2.40E-01 | -0.26±0.09 | 2.66E-01 | -0.25±0.09 | 2.60E-01 | -0.06±0.13 | 8.47E-01 | -0.08±0.13 | 8.03E-01 |
| X - 17654 | -0.18±0.07 | 0 | 4.46E-01 | 2.24E-01 | -0.17±0.07 | 0 | 3.52E-01 | 2.43E-01 | -0.21±0.08 | 2.84E-01 | -0.22±0.08 | 2.63E-01 | -0.1±0.12 | 7.29E-01 | -0.08±0.12 | 7.97E-01 |
| N-(2-furoyl)glycine | -0.2±0.08 | 40.94 | 1.93E-01 | 2.81E-01 | -0.21±0.08 | 23.89 | 2.52E-01 | 2.43E-01 | -0.27±0.1 | 2.66E-01 | -0.28±0.1 | 2.60E-01 | -0.06±0.14 | 8.81E-01 | -0.08±0.14 | 8.08E-01 |
| X - 23739 | 0.19±0.08 | 0 | 5.45E-01 | 2.81E-01 | 0.19±0.08 | 0 | 4.88E-01 | 2.73E-01 | 0.16±0.09 | 6.56E-01 | 0.16±0.09 | 6.48E-01 | 0.26±0.14 | 3.67E-01 | 0.28±0.14 | 4.52E-01 |
| glycine conjugate of C10H14O2 (1)* | -0.16±0.07 | 0 | 5.71E-01 | 3.66E-01 | -0.18±0.07 | 0 | 8.64E-01 | 2.73E-01 | -0.19±0.09 | 5.24E-01 | -0.19±0.09 | 4.69E-01 | -0.1±0.12 | 7.29E-01 | -0.16±0.13 | 5.97E-01 |
| 3-phenylpropionate (hydrocinnamate) | -0.18±0.07 | 0 | 8.86E-01 | 2.17E-01 | -0.17±0.07 | 0 | 7.13E-01 | 2.73E-01 | -0.18±0.08 | 4.10E-01 | -0.19±0.08 | 3.68E-01 | -0.16±0.12 | 5.36E-01 | -0.13±0.12 | 6.66E-01 |
| 3-hydroxy-2-methylpyridine sulfate | -0.19±0.08 | 0 | 5.33E-01 | 3.01E-01 | -0.2±0.08 | 0 | 5.25E-01 | 2.73E-01 | -0.22±0.09 | 3.71E-01 | -0.23±0.1 | 3.52E-01 | -0.12±0.13 | 6.82E-01 | -0.13±0.14 | 7.06E-01 |
| alpha-hydroxyisovalerate | 0.19±0.07 | 0 | 7.41E-01 | 1.94E-01 | 0.18±0.07 | 0 | 5.32E-01 | 2.78E-01 | 0.21±0.09 | 3.61E-01 | 0.22±0.09 | 3.52E-01 | 0.16±0.13 | 5.71E-01 | 0.12±0.13 | 7.11E-01 |
| tyramine O-sulfate | 0.18±0.07 | 58.01 | 1.23E-01 | 2.32E-01 | 0.17±0.07 | 52.58 | 1.46E-01 | 2.84E-01 | 0.1±0.09 | 8.65E-01 | 0.09±0.09 | 8.36E-01 | 0.32±0.12 | 1.99E-01 | 0.3±0.12 | 3.58E-01 |
| N-acetyl-beta-alanine | 0.19±0.08 | 44.53 | 1.79E-01 | 3.02E-01 | 0.2±0.08 | 33.85 | 2.19E-01 | 2.84E-01 | 0.12±0.09 | 7.90E-01 | 0.14±0.1 | 7.54E-01 | 0.36±0.15 | 2.30E-01 | 0.35±0.15 | 4.18E-01 |
| 5-hydroxylysine | 0.18±0.07 | 49.85 | 1.58E-01 | 2.24E-01 | 0.17±0.07 | 0 | 3.57E-01 | 2.84E-01 | 0.12±0.08 | 7.90E-01 | 0.13±0.09 | 7.26E-01 | 0.33±0.12 | 2.03E-01 | 0.28±0.13 | 4.21E-01 |
| isoleucylglycine | 0.18±0.08 | 0 | 6.50E-01 | 3.37E-01 | 0.2±0.08 | 0 | 4.07E-01 | 2.84E-01 | 0.16±0.1 | 7.50E-01 | 0.15±0.1 | 7.43E-01 | 0.23±0.13 | 3.99E-01 | 0.29±0.14 | 4.21E-01 |
| sulfate* | 0.18±0.07 | 16.91 | 2.73E-01 | 2.51E-01 | 0.18±0.07 | 0 | 4.43E-01 | 2.84E-01 | 0.13±0.09 | 7.85E-01 | 0.14±0.09 | 7.26E-01 | 0.29±0.12 | 2.42E-01 | 0.25±0.12 | 4.30E-01 |
| X - 21310 | 0.2±0.08 | 0 | 7.62E-01 | 3.02E-01 | 0.2±0.08 | 0 | 9.29E-01 | 2.84E-01 | 0.18±0.1 | 6.33E-01 | 0.19±0.1 | 5.64E-01 | 0.24±0.15 | 4.64E-01 | 0.21±0.15 | 5.70E-01 |
| X - 11372 | -0.16±0.07 | 0 | 3.79E-01 | 3.45E-01 | -0.17±0.07 | 0 | 3.87E-01 | 2.84E-01 | -0.2±0.09 | 3.61E-01 | -0.22±0.09 | 3.18E-01 | -0.07±0.12 | 8.14E-01 | -0.09±0.12 | 7.82E-01 |
| quinate | -0.17±0.07 | 0 | 4.29E-01 | 2.81E-01 | -0.17±0.07 | 0 | 3.89E-01 | 2.84E-01 | -0.2±0.08 | 3.13E-01 | -0.2±0.08 | 3.18E-01 | -0.09±0.12 | 7.53E-01 | -0.08±0.12 | 7.96E-01 |
| X - 11308 | -0.16±0.07 | 0 | 6.66E-01 | 3.51E-01 | -0.17±0.07 | 0 | 6.38E-01 | 2.90E-01 | -0.18±0.09 | 5.29E-01 | -0.2±0.09 | 4.10E-01 | -0.12±0.12 | 6.69E-01 | -0.13±0.12 | 6.77E-01 |
| X - 11880 | -0.15±0.07 | 52.84 | 1.45E-01 | 3.68E-01 | -0.17±0.07 | 25.81 | 2.46E-01 | 2.99E-01 | -0.22±0.09 | 2.89E-01 | -0.23±0.09 | 2.78E-01 | 0±0.12 | 9.96E-01 | -0.05±0.13 | 8.85E-01 |
| N-acetyl-isoputreanine | 0.18±0.08 | 0 | 7.20E-01 | 3.27E-01 | 0.18±0.08 | 0 | 5.81E-01 | 3.06E-01 | 0.16±0.09 | 6.54E-01 | 0.16±0.09 | 6.44E-01 | 0.22±0.15 | 4.94E-01 | 0.26±0.15 | 4.79E-01 |
| 1-palmitoyl-2-palmitoleoyl-GPC (16:0/16:1)* | 0.19±0.08 | 53.03 | 1.45E-01 | 2.81E-01 | 0.19±0.08 | 44.3 | 1.80E-01 | 3.24E-01 | 0.11±0.09 | 8.31E-01 | 0.11±0.1 | 8.36E-01 | 0.35±0.14 | 2.03E-01 | 0.34±0.14 | 4.03E-01 |
| 3-hydroxypyridine sulfate | -0.17±0.07 | 50.37 | 1.56E-01 | 3.27E-01 | -0.17±0.07 | 56.76 | 1.28E-01 | 3.24E-01 | -0.25±0.09 | 2.66E-01 | -0.25±0.09 | 2.58E-01 | -0.02±0.13 | 9.60E-01 | -0.01±0.13 | 9.85E-01 |
| 4-vinylguaiacol sulfate | -0.16±0.08 | 0 | 9.00E-01 | 3.73E-01 | -0.19±0.08 | 0 | 7.93E-01 | 3.29E-01 | -0.16±0.09 | 7.15E-01 | -0.17±0.1 | 6.15E-01 | -0.18±0.14 | 5.71E-01 | -0.22±0.15 | 5.48E-01 |
| N-acetylglucosamine/N-acetylgalactosamine | 0.19±0.08 | 0 | 8.75E-01 | 3.02E-01 | 0.18±0.08 | 0 | 9.12E-01 | 3.29E-01 | 0.18±0.09 | 6.10E-01 | 0.19±0.09 | 5.25E-01 | 0.2±0.14 | 5.31E-01 | 0.17±0.14 | 6.45E-01 |
| 5-acetylamino-6-amino-3-methyluracil | -0.16±0.07 | 26.89 | 2.42E-01 | 3.27E-01 | -0.16±0.07 | 38.26 | 2.03E-01 | 3.53E-01 | -0.22±0.08 | 2.84E-01 | -0.22±0.08 | 2.78E-01 | -0.04±0.12 | 9.05E-01 | -0.03±0.12 | 9.34E-01 |
| pyrraline | -0.16±0.07 | 41.77 | 1.90E-01 | 3.51E-01 | -0.16±0.07 | 16.61 | 2.73E-01 | 3.54E-01 | -0.22±0.08 | 2.84E-01 | -0.21±0.08 | 3.18E-01 | -0.02±0.13 | 9.68E-01 | -0.04±0.13 | 9.01E-01 |
| N-acetyl-aspartyl-glutamate (NAAG) | 0.16±0.08 | 0 | 8.14E-01 | 3.68E-01 | 0.17±0.08 | 0 | 6.46E-01 | 3.59E-01 | 0.15±0.09 | 7.50E-01 | 0.15±0.09 | 6.96E-01 | 0.19±0.13 | 5.33E-01 | 0.23±0.14 | 4.92E-01 |
| 1,5-anhydroglucitol (1,5-AG) | -0.14±0.07 | 32.19 | 2.25E-01 | 3.66E-01 | -0.15±0.07 | 41.39 | 1.91E-01 | 3.62E-01 | -0.2±0.08 | 3.41E-01 | -0.21±0.08 | 2.78E-01 | -0.02±0.12 | 9.54E-01 | -0.03±0.12 | 9.41E-01 |
| X - 25172 | 0.17±0.08 | 34.86 | 2.15E-01 | 3.51E-01 | 0.17±0.08 | 42.33 | 1.88E-01 | 3.62E-01 | 0.12±0.09 | 7.90E-01 | 0.12±0.09 | 8.25E-01 | 0.34±0.16 | 3.02E-01 | 0.36±0.16 | 4.18E-01 |
| X - 18921 | -0.15±0.07 | 0 | 7.51E-01 | 3.73E-01 | -0.16±0.07 | 0 | 8.24E-01 | 3.62E-01 | -0.17±0.09 | 6.26E-01 | -0.17±0.09 | 5.61E-01 | -0.12±0.12 | 6.66E-01 | -0.14±0.13 | 6.66E-01 |
| 4-ethylphenylsulfate | -0.18±0.08 | 0 | 9.70E-01 | 3.27E-01 | -0.18±0.08 | 0 | 7.42E-01 | 3.62E-01 | -0.18±0.09 | 6.17E-01 | -0.2±0.1 | 5.16E-01 | -0.18±0.14 | 5.53E-01 | -0.14±0.14 | 6.91E-01 |
| 1-(1-enyl-palmitoyl)-2-linoleoyl-GPC (P-16:0/18:2)* | -0.17±0.07 | 0 | 5.61E-01 | 3.27E-01 | -0.17±0.08 | 23.28 | 2.54E-01 | 3.62E-01 | -0.2±0.09 | 4.50E-01 | -0.23±0.1 | 3.18E-01 | -0.11±0.12 | 6.66E-01 | -0.05±0.13 | 8.77E-01 |
| sphingomyelin (d18:0/18:0, d19:0/17:0)* | 0.17±0.08 | 0 | 4.56E-01 | 3.49E-01 | 0.18±0.08 | 0 | 7.06E-01 | 3.63E-01 | 0.14±0.09 | 7.71E-01 | 0.16±0.1 | 6.96E-01 | 0.26±0.14 | 3.57E-01 | 0.22±0.14 | 4.95E-01 |
| 3-hydroxy-3-methylglutarate | 0.18±0.08 | 88.83 | 2.77E-03 | 3.66E-01 | 0.19±0.09 | 87.94 | 3.97E-03 | 3.71E-01 | 0.04±0.1 | 9.47E-01 | 0.05±0.1 | 9.18E-01 | 0.62±0.17 | **4.17E-02** | 0.63±0.17 | 6.54E-02 |
| androstenediol (3beta,17beta) disulfate (1) | 0.18±0.08 | 0 | 4.01E-01 | 3.53E-01 | 0.19±0.09 | 11.55 | 2.88E-01 | 3.71E-01 | 0.13±0.1 | 7.90E-01 | 0.12±0.1 | 8.36E-01 | 0.28±0.14 | 3.38E-01 | 0.32±0.15 | 4.21E-01 |
| 1-palmitoleoyl-GPC (16:1)* | 0.17±0.08 | 0 | 4.49E-01 | 3.66E-01 | 0.17±0.08 | 0 | 3.49E-01 | 3.71E-01 | 0.13±0.09 | 7.90E-01 | 0.13±0.09 | 7.69E-01 | 0.26±0.15 | 3.77E-01 | 0.29±0.15 | 4.52E-01 |
| X - 21842 | -0.17±0.07 | 0 | 4.96E-01 | 3.37E-01 | -0.16±0.07 | 0 | 5.41E-01 | 3.71E-01 | -0.14±0.09 | 7.50E-01 | -0.13±0.09 | 7.37E-01 | -0.25±0.13 | 3.66E-01 | -0.23±0.14 | 4.74E-01 |
| 1-stearoyl-2-linoleoyl-GPC (18:0/18:2)* | -0.16±0.07 | 36.78 | 2.09E-01 | 3.37E-01 | -0.16±0.07 | 52.65 | 1.46E-01 | 3.71E-01 | -0.23±0.09 | 2.84E-01 | -0.24±0.09 | 2.78E-01 | -0.05±0.12 | 8.90E-01 | -0.02±0.12 | 9.56E-01 |
| erythritol | 0.16±0.08 | 72.11 | 5.83E-02 | 3.73E-01 | 0.17±0.08 | 67.14 | 8.11E-02 | 3.81E-01 | 0.07±0.09 | 9.03E-01 | 0.08±0.09 | 8.63E-01 | 0.39±0.14 | 1.99E-01 | 0.39±0.15 | 3.58E-01 |
| X - 11795 | 0.16±0.07 | 52.49 | 1.47E-01 | 3.66E-01 | 0.16±0.07 | 68.7 | 7.39E-02 | 3.81E-01 | 0.08±0.09 | 8.73E-01 | 0.07±0.09 | 8.94E-01 | 0.31±0.13 | 2.30E-01 | 0.36±0.13 | 3.58E-01 |
| X - 21467 | 0.17±0.08 | 67.28 | 8.04E-02 | 3.66E-01 | 0.18±0.09 | 41.62 | 1.91E-01 | 3.81E-01 | 0.09±0.1 | 8.80E-01 | 0.12±0.1 | 8.36E-01 | 0.41±0.16 | 2.03E-01 | 0.36±0.16 | 4.18E-01 |
| 11beta-hydroxyetiocholanolone glucuronide* | -0.16±0.07 | 0 | 3.46E-01 | 3.27E-01 | -0.15±0.07 | 0 | 5.09E-01 | 3.81E-01 | -0.12±0.09 | 7.90E-01 | -0.12±0.09 | 7.63E-01 | -0.26±0.12 | 3.23E-01 | -0.22±0.12 | 4.65E-01 |
| X - 12740 | 0.16±0.07 | 0 | 4.64E-01 | 3.66E-01 | 0.16±0.07 | 0 | 6.72E-01 | 3.81E-01 | 0.12±0.09 | 7.90E-01 | 0.14±0.09 | 7.37E-01 | 0.23±0.13 | 3.66E-01 | 0.2±0.13 | 4.95E-01 |
| N2-acetyl,N6,N6-dimethyllysine | 0.16±0.07 | 0 | 6.73E-01 | 3.66E-01 | 0.16±0.07 | 0 | 6.30E-01 | 3.81E-01 | 0.14±0.09 | 7.50E-01 | 0.14±0.09 | 7.26E-01 | 0.2±0.13 | 4.72E-01 | 0.22±0.13 | 4.95E-01 |
| X - 10458 | 0.17±0.08 | 0 | 7.89E-01 | 3.51E-01 | 0.17±0.08 | 0 | 8.67E-01 | 3.81E-01 | 0.16±0.09 | 6.88E-01 | 0.16±0.09 | 6.80E-01 | 0.2±0.14 | 5.19E-01 | 0.19±0.14 | 5.98E-01 |
| arachidate (20:0) | -0.16±0.08 | 0 | 9.47E-01 | 3.66E-01 | -0.17±0.08 | 0 | 8.39E-01 | 3.81E-01 | -0.16±0.09 | 6.98E-01 | -0.18±0.1 | 6.01E-01 | -0.17±0.13 | 5.65E-01 | -0.14±0.13 | 6.66E-01 |
| 1-linoleoyl-2-linolenoyl-GPC (18:2/18:3)* | -0.16±0.07 | 0 | 4.79E-01 | 3.66E-01 | -0.16±0.08 | 0 | 3.77E-01 | 3.81E-01 | -0.19±0.09 | 4.60E-01 | -0.21±0.09 | 4.01E-01 | -0.09±0.12 | 7.53E-01 | -0.07±0.13 | 8.28E-01 |
| citraconate/glutaconate | -0.16±0.08 | 57.12 | 1.27E-01 | 3.66E-01 | -0.16±0.08 | 53.09 | 1.44E-01 | 3.81E-01 | -0.24±0.09 | 2.84E-01 | -0.24±0.09 | 2.81E-01 | 0±0.13 | 9.99E-01 | -0.01±0.13 | 9.91E-01 |
| gamma-glutamylvaline | 0.17±0.08 | 73.16 | 5.36E-02 | 3.76E-01 | 0.18±0.09 | 44.73 | 1.79E-01 | 3.93E-01 | 0.09±0.09 | 8.69E-01 | 0.12±0.1 | 8.32E-01 | 0.5±0.19 | 2.03E-01 | 0.4±0.19 | 4.18E-01 |
| N-palmitoyl-sphinganine (d18:0/16:0) | 0.16±0.08 | 50.32 | 1.56E-01 | 3.66E-01 | 0.16±0.08 | 40.04 | 1.97E-01 | 3.93E-01 | 0.09±0.09 | 8.72E-01 | 0.09±0.09 | 8.38E-01 | 0.32±0.13 | 2.41E-01 | 0.3±0.14 | 4.18E-01 |
| campesterol | -0.16±0.07 | 0 | 4.24E-01 | 3.66E-01 | -0.16±0.07 | 37.26 | 2.07E-01 | 3.93E-01 | -0.2±0.09 | 4.42E-01 | -0.22±0.09 | 3.30E-01 | -0.07±0.12 | 8.03E-01 | -0.02±0.13 | 9.51E-01 |
| 1-myristoyl-2-palmitoyl-GPC (14:0/16:0) | 0.16±0.08 | 58.2 | 1.22E-01 | 3.83E-01 | 0.16±0.08 | 59.86 | 1.14E-01 | 4.04E-01 | 0.08±0.09 | 8.90E-01 | 0.08±0.09 | 8.68E-01 | 0.34±0.14 | 2.30E-01 | 0.34±0.14 | 4.03E-01 |
| X - 24422 | 0.17±0.08 | 43.98 | 1.82E-01 | 3.66E-01 | 0.17±0.08 | 32.26 | 2.24E-01 | 4.04E-01 | 0.25±0.1 | 3.13E-01 | 0.24±0.1 | 3.52E-01 | 0.04±0.13 | 9.20E-01 | 0.04±0.13 | 9.11E-01 |
| 7-methylguanine | 0.17±0.08 | 19.67 | 2.65E-01 | 3.66E-01 | 0.17±0.08 | 0 | 3.35E-01 | 4.28E-01 | 0.13±0.09 | 7.90E-01 | 0.13±0.09 | 7.80E-01 | 0.35±0.17 | 3.38E-01 | 0.32±0.17 | 4.65E-01 |
| trans-urocanate | -0.14±0.07 | 0 | 4.35E-01 | 4.06E-01 | -0.15±0.07 | 0 | 4.59E-01 | 4.33E-01 | -0.1±0.09 | 8.65E-01 | -0.11±0.09 | 8.36E-01 | -0.22±0.12 | 3.71E-01 | -0.22±0.12 | 4.65E-01 |
| N4-acetylcytidine | 0.16±0.08 | 83.4 | 1.41E-02 | 3.68E-01 | 0.16±0.08 | 77.32 | 3.57E-02 | 4.34E-01 | 0.05±0.09 | 9.27E-01 | 0.06±0.09 | 8.94E-01 | 0.49±0.15 | 1.11E-01 | 0.45±0.16 | 3.28E-01 |
| cis-4-decenoylcarnitine (C10:1) | -0.15±0.07 | 69.95 | 6.81E-02 | 3.76E-01 | -0.14±0.07 | 65.59 | 8.82E-02 | 4.34E-01 | -0.05±0.09 | 9.27E-01 | -0.05±0.09 | 9.04E-01 | -0.32±0.12 | 1.99E-01 | -0.31±0.12 | 3.78E-01 |
| N-acetyl-2-aminoadipate | 0.17±0.08 | 71.82 | 5.96E-02 | 3.66E-01 | 0.16±0.08 | 35.7 | 2.12E-01 | 4.34E-01 | 0.07±0.09 | 9.01E-01 | 0.1±0.09 | 8.36E-01 | 0.39±0.14 | 1.99E-01 | 0.31±0.15 | 4.21E-01 |
| linolenate [alpha or gamma; (18:3n3 or 6)] | -0.15±0.07 | 7.01 | 3.00E-01 | 4.21E-01 | -0.15±0.08 | 11.83 | 2.87E-01 | 4.34E-01 | -0.09±0.09 | 8.69E-01 | -0.1±0.09 | 8.36E-01 | -0.26±0.13 | 3.40E-01 | -0.27±0.13 | 4.30E-01 |
| taurochenodeoxycholic acid 3-sulfate | -0.14±0.07 | 0 | 8.51E-01 | 4.21E-01 | -0.15±0.07 | 0 | 9.26E-01 | 4.34E-01 | -0.15±0.09 | 7.12E-01 | -0.16±0.09 | 6.64E-01 | -0.12±0.12 | 6.60E-01 | -0.14±0.13 | 6.66E-01 |
| lignoceroylcarnitine (C24)* | -0.15±0.07 | 0 | 5.07E-01 | 3.78E-01 | -0.15±0.07 | 0 | 4.80E-01 | 4.34E-01 | -0.19±0.09 | 5.29E-01 | -0.19±0.09 | 5.16E-01 | -0.09±0.12 | 7.46E-01 | -0.08±0.12 | 7.91E-01 |
| nicotinamide | 0.15±0.07 | 61.44 | 1.07E-01 | 4.21E-01 | 0.15±0.07 | 64.48 | 9.34E-02 | 4.34E-01 | 0.23±0.09 | 3.13E-01 | 0.24±0.09 | 2.78E-01 | -0.03±0.13 | 9.53E-01 | -0.02±0.13 | 9.50E-01 |
| EDTA | -0.15±0.08 | 74.1 | 4.94E-02 | 4.21E-01 | -0.16±0.08 | 74.86 | 4.61E-02 | 4.49E-01 | -0.23±0.09 | 2.84E-01 | -0.23±0.09 | 2.63E-01 | 0.16±0.18 | 6.82E-01 | 0.16±0.18 | 7.13E-01 |
| tetradecadienedioate (C14:2-DC)* | 0.16±0.07 | 49.51 | 1.59E-01 | 3.66E-01 | 0.15±0.08 | 56.02 | 1.32E-01 | 4.50E-01 | 0.24±0.09 | 3.03E-01 | 0.23±0.09 | 3.18E-01 | 0.01±0.13 | 9.80E-01 | 0±0.13 | 9.92E-01 |
| 3,4-dihydroxybutyrate | 0.15±0.08 | 79.33 | 2.78E-02 | 4.21E-01 | 0.15±0.08 | 75.58 | 4.30E-02 | 4.52E-01 | 0.04±0.09 | 9.34E-01 | 0.05±0.09 | 9.04E-01 | 0.4±0.14 | 1.99E-01 | 0.4±0.14 | 3.48E-01 |
| N-methylpipecolate | 0.15±0.08 | 0 | 5.23E-01 | 4.21E-01 | 0.15±0.08 | 0 | 5.19E-01 | 4.52E-01 | 0.11±0.09 | 8.31E-01 | 0.12±0.09 | 8.25E-01 | 0.22±0.14 | 4.52E-01 | 0.22±0.14 | 4.95E-01 |
| X - 21364 | 0.16±0.08 | 69.35 | 7.09E-02 | 4.06E-01 | 0.16±0.08 | 63.31 | 9.87E-02 | 4.56E-01 | 0.07±0.1 | 9.03E-01 | 0.07±0.1 | 8.94E-01 | 0.4±0.15 | 2.03E-01 | 0.38±0.16 | 4.03E-01 |
| N-formylmethionine | 0.16±0.08 | 67.04 | 8.16E-02 | 3.83E-01 | 0.15±0.08 | 50.96 | 1.53E-01 | 4.56E-01 | 0.07±0.09 | 9.01E-01 | 0.08±0.09 | 8.68E-01 | 0.38±0.15 | 2.03E-01 | 0.33±0.15 | 4.18E-01 |
| 1-(1-enyl-palmitoyl)-GPE (P-16:0)* | 0.14±0.07 | 0 | 8.33E-01 | 4.32E-01 | 0.15±0.08 | 0 | 9.63E-01 | 4.56E-01 | 0.15±0.09 | 6.98E-01 | 0.15±0.09 | 6.96E-01 | 0.12±0.13 | 6.92E-01 | 0.15±0.14 | 6.62E-01 |
| dimethylarginine (SDMA + ADMA) | 0.16±0.08 | 0 | 8.05E-01 | 4.21E-01 | 0.16±0.08 | 0 | 7.72E-01 | 4.57E-01 | 0.14±0.09 | 7.50E-01 | 0.15±0.09 | 7.19E-01 | 0.19±0.17 | 6.10E-01 | 0.2±0.17 | 6.38E-01 |
| cystathionine | 0.15±0.07 | 70.68 | 6.48E-02 | 3.76E-01 | 0.14±0.07 | 44.6 | 1.79E-01 | 4.66E-01 | 0.06±0.09 | 9.06E-01 | 0.07±0.09 | 8.91E-01 | 0.35±0.13 | 1.99E-01 | 0.28±0.13 | 4.18E-01 |
| 1-arachidonoyl-GPE (20:4n6)* | 0.16±0.08 | 9.61 | 2.93E-01 | 4.06E-01 | 0.15±0.08 | 20.41 | 2.62E-01 | 4.66E-01 | 0.11±0.09 | 8.31E-01 | 0.1±0.09 | 8.36E-01 | 0.3±0.16 | 3.53E-01 | 0.31±0.16 | 4.52E-01 |
| phenyllactate (PLA) | 0.17±0.08 | 30.75 | 2.29E-01 | 3.66E-01 | 0.15±0.08 | 0 | 4.03E-01 | 4.66E-01 | 0.1±0.1 | 8.65E-01 | 0.1±0.1 | 8.36E-01 | 0.3±0.14 | 3.02E-01 | 0.24±0.14 | 4.65E-01 |
| 5-methylthioadenosine (MTA) | 0.15±0.08 | 39.67 | 1.98E-01 | 3.93E-01 | 0.15±0.08 | 0 | 4.79E-01 | 4.66E-01 | 0.09±0.09 | 8.65E-01 | 0.11±0.09 | 8.36E-01 | 0.31±0.14 | 3.08E-01 | 0.23±0.14 | 4.95E-01 |
| choline phosphate | 0.12±0.07 | 0 | 7.76E-01 | 5.51E-01 | 0.14±0.07 | 0 | 5.50E-01 | 4.66E-01 | 0.1±0.09 | 8.65E-01 | 0.11±0.09 | 8.36E-01 | 0.15±0.13 | 6.04E-01 | 0.21±0.13 | 4.95E-01 |
| sphinganine | 0.15±0.08 | 0 | 9.98E-01 | 3.93E-01 | 0.14±0.08 | 0 | 9.49E-01 | 4.66E-01 | 0.15±0.09 | 7.16E-01 | 0.15±0.1 | 7.19E-01 | 0.15±0.13 | 6.00E-01 | 0.14±0.13 | 6.66E-01 |
| tetrahydrocortisone glucuronide (5) | -0.12±0.07 | 86.72 | 6.07E-03 | 5.47E-01 | -0.13±0.07 | 80.86 | 2.23E-02 | 4.66E-01 | -0.22±0.08 | 2.66E-01 | -0.22±0.08 | 2.58E-01 | 0.22±0.14 | 4.64E-01 | 0.15±0.14 | 6.67E-01 |
| taurolithocholate 3-sulfate | -0.14±0.07 | 0 | 9.59E-01 | 4.06E-01 | -0.14±0.07 | 0 | 8.51E-01 | 4.66E-01 | -0.14±0.09 | 7.16E-01 | -0.14±0.09 | 6.80E-01 | -0.13±0.12 | 6.14E-01 | -0.12±0.12 | 6.93E-01 |
| phosphoethanolamine | 0.12±0.07 | 0 | 6.98E-01 | 5.48E-01 | 0.14±0.08 | 0 | 9.36E-01 | 4.66E-01 | 0.14±0.09 | 7.50E-01 | 0.15±0.09 | 6.92E-01 | 0.08±0.13 | 8.13E-01 | 0.14±0.14 | 6.96E-01 |
| 11-ketoetiocholanolone glucuronide | -0.14±0.07 | 0 | 9.44E-01 | 3.93E-01 | -0.14±0.07 | 0 | 8.21E-01 | 4.66E-01 | -0.14±0.08 | 6.92E-01 | -0.15±0.09 | 6.48E-01 | -0.14±0.13 | 6.26E-01 | -0.11±0.13 | 7.25E-01 |
| trigonelline (N'-methylnicotinate) | -0.14±0.07 | 78.55 | 3.08E-02 | 4.21E-01 | -0.14±0.07 | 79.2 | 2.83E-02 | 4.66E-01 | -0.24±0.09 | 2.66E-01 | -0.24±0.09 | 2.58E-01 | 0.1±0.13 | 7.38E-01 | 0.11±0.13 | 7.49E-01 |
| glycochenodeoxycholate 3-sulfate | -0.13±0.07 | 36.92 | 2.08E-01 | 4.93E-01 | -0.14±0.07 | 17.87 | 2.70E-01 | 4.66E-01 | -0.19±0.09 | 4.42E-01 | -0.19±0.09 | 4.14E-01 | 0±0.12 | 9.96E-01 | -0.02±0.13 | 9.50E-01 |
| isoleucine | 0.17±0.09 | 77.88 | 3.35E-02 | 4.48E-01 | 0.17±0.09 | 60.2 | 1.13E-01 | 4.73E-01 | 0.08±0.1 | 9.03E-01 | 0.1±0.1 | 8.40E-01 | 0.56±0.2 | 1.99E-01 | 0.47±0.21 | 4.18E-01 |
| N2-acetyl,N6-methyllysine | 0.14±0.07 | 0 | 9.43E-01 | 4.79E-01 | 0.14±0.07 | 0 | 9.90E-01 | 4.73E-01 | 0.14±0.09 | 7.50E-01 | 0.14±0.09 | 7.20E-01 | 0.13±0.13 | 6.66E-01 | 0.14±0.14 | 6.67E-01 |
| X - 24811 | -0.14±0.07 | 21.08 | 2.60E-01 | 4.46E-01 | -0.14±0.07 | 34.36 | 2.17E-01 | 4.80E-01 | -0.19±0.09 | 4.50E-01 | -0.2±0.09 | 4.05E-01 | -0.02±0.13 | 9.73E-01 | 0±0.13 | 9.98E-01 |
| hippurate | -0.14±0.07 | 0 | 5.93E-01 | 4.12E-01 | -0.14±0.07 | 0 | 5.25E-01 | 4.80E-01 | -0.17±0.09 | 6.10E-01 | -0.17±0.09 | 5.98E-01 | -0.09±0.13 | 7.66E-01 | -0.07±0.13 | 8.26E-01 |
| X - 12112 | 0.14±0.08 | 0 | 8.33E-01 | 4.29E-01 | 0.14±0.08 | 0 | 8.37E-01 | 4.87E-01 | 0.16±0.09 | 7.15E-01 | 0.15±0.09 | 6.96E-01 | 0.12±0.13 | 6.66E-01 | 0.12±0.13 | 7.06E-01 |
| X - 21471 | 0.16±0.08 | 64.75 | 9.21E-02 | 4.21E-01 | 0.15±0.08 | 46.94 | 1.70E-01 | 4.91E-01 | 0.07±0.1 | 9.03E-01 | 0.08±0.1 | 8.92E-01 | 0.37±0.15 | 2.19E-01 | 0.33±0.15 | 4.18E-01 |
| octadecanedioate (C18-DC) | -0.14±0.07 | 33.56 | 2.20E-01 | 4.21E-01 | -0.14±0.07 | 7.49 | 2.98E-01 | 5.07E-01 | -0.07±0.09 | 9.01E-01 | -0.08±0.09 | 8.68E-01 | -0.26±0.12 | 3.14E-01 | -0.24±0.12 | 4.52E-01 |
| hydroxy-CMPF* | -0.15±0.07 | 53.64 | 1.42E-01 | 3.66E-01 | -0.13±0.07 | 24.99 | 2.48E-01 | 5.10E-01 | -0.07±0.09 | 9.00E-01 | -0.07±0.09 | 8.91E-01 | -0.29±0.12 | 2.19E-01 | -0.24±0.12 | 4.44E-01 |
| pseudouridine | 0.14±0.07 | 0 | 3.39E-01 | 4.28E-01 | 0.14±0.07 | 0 | 4.28E-01 | 5.11E-01 | 0.09±0.09 | 8.65E-01 | 0.1±0.09 | 8.36E-01 | 0.24±0.13 | 3.55E-01 | 0.22±0.13 | 4.79E-01 |
| 2-methylbutyrylcarnitine (C5) | 0.14±0.08 | 72.9 | 5.47E-02 | 4.68E-01 | 0.14±0.08 | 57.92 | 1.23E-01 | 5.14E-01 | 0.05±0.09 | 9.27E-01 | 0.06±0.09 | 8.94E-01 | 0.38±0.15 | 2.03E-01 | 0.33±0.14 | 4.18E-01 |
| N-acetylvaline | 0.14±0.07 | 44.97 | 1.78E-01 | 4.93E-01 | 0.14±0.08 | 0 | 3.28E-01 | 5.14E-01 | 0.07±0.09 | 9.02E-01 | 0.09±0.09 | 8.47E-01 | 0.29±0.14 | 3.22E-01 | 0.25±0.14 | 4.65E-01 |
| X - 21319 | -0.12±0.07 | 0 | 4.18E-01 | 5.47E-01 | -0.13±0.07 | 0 | 3.75E-01 | 5.14E-01 | -0.08±0.09 | 8.80E-01 | -0.08±0.09 | 8.63E-01 | -0.2±0.12 | 4.30E-01 | -0.21±0.12 | 4.65E-01 |
| valine | 0.17±0.1 | 36.83 | 2.08E-01 | 5.39E-01 | 0.18±0.1 | 0 | 4.71E-01 | 5.14E-01 | 0.1±0.11 | 8.80E-01 | 0.13±0.12 | 8.36E-01 | 0.38±0.2 | 3.42E-01 | 0.29±0.18 | 4.95E-01 |
| N-stearoyl-sphingosine (d18:1/18:0)* | 0.14±0.08 | 0 | 7.62E-01 | 4.93E-01 | 0.14±0.08 | 0 | 9.75E-01 | 5.14E-01 | 0.13±0.09 | 7.90E-01 | 0.14±0.1 | 7.26E-01 | 0.18±0.14 | 5.82E-01 | 0.14±0.14 | 6.91E-01 |
| citramalate | -0.12±0.07 | 78.26 | 3.20E-02 | 5.47E-01 | -0.13±0.07 | 72.44 | 5.68E-02 | 5.14E-01 | -0.22±0.09 | 2.84E-01 | -0.22±0.09 | 3.03E-01 | 0.11±0.13 | 7.03E-01 | 0.08±0.13 | 8.03E-01 |
| X - 17653 | -0.12±0.07 | 0 | 4.08E-01 | 5.47E-01 | -0.13±0.07 | 0 | 4.10E-01 | 5.14E-01 | -0.16±0.09 | 6.33E-01 | -0.17±0.09 | 5.64E-01 | -0.04±0.12 | 9.20E-01 | -0.05±0.13 | 8.99E-01 |
| catechol sulfate | -0.14±0.07 | 44.81 | 1.78E-01 | 4.21E-01 | -0.13±0.07 | 52.46 | 1.47E-01 | 5.17E-01 | -0.21±0.09 | 3.61E-01 | -0.2±0.09 | 3.68E-01 | 0±0.13 | 9.99E-01 | 0.02±0.13 | 9.49E-01 |
| N-acetylneuraminate | 0.13±0.07 | 0 | 9.08E-01 | 5.47E-01 | 0.14±0.08 | 0 | 9.00E-01 | 5.17E-01 | 0.13±0.09 | 7.71E-01 | 0.14±0.09 | 7.19E-01 | 0.11±0.14 | 7.29E-01 | 0.12±0.14 | 7.37E-01 |
| 1,3-dimethylurate | -0.12±0.07 | 0 | 5.00E-01 | 5.36E-01 | -0.12±0.07 | 0 | 4.85E-01 | 5.17E-01 | -0.15±0.08 | 6.37E-01 | -0.16±0.08 | 6.01E-01 | -0.05±0.12 | 8.75E-01 | -0.05±0.12 | 8.71E-01 |
| X - 23655 | -0.12±0.07 | 29.56 | 2.33E-01 | 5.47E-01 | -0.13±0.07 | 18.39 | 2.68E-01 | 5.17E-01 | -0.19±0.09 | 5.29E-01 | -0.19±0.09 | 4.93E-01 | 0±0.13 | 9.96E-01 | -0.01±0.13 | 9.72E-01 |
| anthranilate | 0.13±0.08 | 56.22 | 1.31E-01 | 5.47E-01 | 0.14±0.08 | 45.8 | 1.74E-01 | 5.21E-01 | 0.06±0.09 | 9.23E-01 | 0.07±0.1 | 8.94E-01 | 0.32±0.15 | 3.04E-01 | 0.32±0.15 | 4.21E-01 |
| N-acetylphenylalanine | 0.15±0.08 | 27.88 | 2.39E-01 | 4.21E-01 | 0.14±0.08 | 0 | 4.05E-01 | 5.21E-01 | 0.08±0.09 | 8.80E-01 | 0.09±0.1 | 8.52E-01 | 0.27±0.13 | 3.23E-01 | 0.23±0.13 | 4.74E-01 |
| 4-hydroxyglutamate | 0.16±0.08 | 86.17 | 7.17E-03 | 3.71E-01 | 0.14±0.08 | 73.41 | 5.24E-02 | 5.29E-01 | 0.03±0.09 | 9.54E-01 | 0.04±0.1 | 9.18E-01 | 0.48±0.14 | 8.35E-02 | 0.39±0.15 | 3.78E-01 |
| quinolinate | 0.13±0.07 | 47.18 | 1.69E-01 | 5.17E-01 | 0.13±0.08 | 0 | 3.76E-01 | 5.29E-01 | 0.06±0.09 | 9.06E-01 | 0.09±0.09 | 8.52E-01 | 0.28±0.13 | 3.08E-01 | 0.23±0.14 | 4.74E-01 |
| 3-hydroxy-2-ethylpropionate | 0.14±0.08 | 0 | 7.35E-01 | 5.17E-01 | 0.14±0.08 | 0 | 8.80E-01 | 5.29E-01 | 0.12±0.1 | 7.93E-01 | 0.13±0.1 | 7.69E-01 | 0.18±0.14 | 5.71E-01 | 0.16±0.14 | 6.66E-01 |
| 4-methoxyphenol sulfate | 0.13±0.08 | 0 | 8.93E-01 | 5.47E-01 | 0.14±0.08 | 0 | 9.60E-01 | 5.29E-01 | 0.14±0.1 | 7.81E-01 | 0.14±0.1 | 7.54E-01 | 0.12±0.13 | 6.77E-01 | 0.13±0.13 | 6.77E-01 |
| sulfate of piperine metabolite C18H21NO3 (3)* | -0.1±0.07 | 0 | 6.13E-01 | 6.05E-01 | -0.13±0.07 | 0 | 7.36E-01 | 5.29E-01 | -0.13±0.09 | 7.81E-01 | -0.15±0.09 | 6.96E-01 | -0.05±0.12 | 8.78E-01 | -0.1±0.13 | 7.64E-01 |
| corticosterone | -0.12±0.07 | 0 | 4.88E-01 | 5.36E-01 | -0.12±0.07 | 5.53 | 3.04E-01 | 5.29E-01 | -0.16±0.08 | 6.33E-01 | -0.17±0.09 | 5.20E-01 | -0.05±0.12 | 8.90E-01 | -0.01±0.13 | 9.72E-01 |
| X - 11478 | -0.11±0.07 | 0 | 6.14E-01 | 5.64E-01 | -0.13±0.07 | 0 | 8.15E-01 | 5.42E-01 | -0.14±0.09 | 7.50E-01 | -0.14±0.09 | 7.20E-01 | -0.06±0.13 | 8.57E-01 | -0.1±0.13 | 7.50E-01 |
| X - 13695 | -0.13±0.07 | 0 | 9.73E-01 | 5.39E-01 | -0.13±0.07 | 0 | 9.32E-01 | 5.45E-01 | -0.13±0.09 | 7.81E-01 | -0.12±0.09 | 7.69E-01 | -0.12±0.13 | 6.66E-01 | -0.14±0.13 | 6.67E-01 |
| 4-hydroxyhippurate | 0.11±0.07 | 64.62 | 9.27E-02 | 5.50E-01 | 0.12±0.07 | 59.72 | 1.15E-01 | 5.47E-01 | 0.02±0.09 | 9.67E-01 | 0.04±0.09 | 9.18E-01 | 0.27±0.12 | 2.66E-01 | 0.27±0.12 | 4.18E-01 |
| hydroxyasparagine** | 0.14±0.09 | 15.04 | 2.78E-01 | 5.47E-01 | 0.14±0.09 | 0 | 5.43E-01 | 5.53E-01 | 0.1±0.09 | 8.65E-01 | 0.12±0.1 | 8.36E-01 | 0.33±0.2 | 4.10E-01 | 0.24±0.18 | 5.97E-01 |
| X - 11315 | 0.11±0.08 | 0 | 3.25E-01 | 6.01E-01 | 0.14±0.08 | 48.09 | 1.65E-01 | 5.53E-01 | 0.06±0.09 | 9.06E-01 | 0.06±0.09 | 8.94E-01 | 0.23±0.14 | 4.51E-01 | 0.3±0.14 | 4.21E-01 |
| X - 21286 | 0.13±0.08 | 0 | 9.12E-01 | 5.48E-01 | 0.13±0.08 | 0 | 7.22E-01 | 5.56E-01 | 0.13±0.09 | 7.81E-01 | 0.15±0.09 | 7.04E-01 | 0.12±0.13 | 6.96E-01 | 0.09±0.13 | 7.82E-01 |
| gamma-glutamyltyrosine | 0.13±0.08 | 83.72 | 1.32E-02 | 5.48E-01 | 0.14±0.08 | 77.55 | 3.48E-02 | 5.60E-01 | 0.04±0.09 | 9.27E-01 | 0.06±0.09 | 9.00E-01 | 0.58±0.2 | 1.97E-01 | 0.52±0.2 | 3.58E-01 |
| X - 25419 | 0.12±0.07 | 0 | 3.35E-01 | 5.48E-01 | 0.12±0.07 | 32.12 | 2.25E-01 | 5.60E-01 | 0.07±0.09 | 9.03E-01 | 0.06±0.09 | 9.00E-01 | 0.21±0.12 | 3.97E-01 | 0.25±0.12 | 4.52E-01 |
| 1-methyl-5-imidazoleacetate | -0.12±0.07 | 0 | 3.87E-01 | 5.50E-01 | -0.13±0.08 | 7.25 | 2.99E-01 | 5.60E-01 | -0.07±0.09 | 9.03E-01 | -0.07±0.1 | 8.94E-01 | -0.2±0.12 | 4.36E-01 | -0.23±0.12 | 4.63E-01 |
| X - 15728 | -0.13±0.07 | 7.19 | 2.99E-01 | 4.69E-01 | -0.12±0.07 | 0 | 4.44E-01 | 5.60E-01 | -0.08±0.09 | 8.80E-01 | -0.08±0.09 | 8.63E-01 | -0.23±0.12 | 3.42E-01 | -0.2±0.12 | 4.95E-01 |
| spermidine | 0.1±0.07 | 0 | 6.14E-01 | 6.01E-01 | 0.12±0.07 | 0 | 4.48E-01 | 5.60E-01 | 0.08±0.09 | 8.73E-01 | 0.09±0.09 | 8.38E-01 | 0.16±0.13 | 5.85E-01 | 0.21±0.14 | 5.08E-01 |
| dimethylglycine | 0.13±0.07 | 0 | 6.36E-01 | 5.41E-01 | 0.12±0.07 | 0 | 6.95E-01 | 5.60E-01 | 0.1±0.09 | 8.34E-01 | 0.1±0.09 | 8.36E-01 | 0.18±0.14 | 5.53E-01 | 0.17±0.14 | 6.22E-01 |
| sulfate of piperine metabolite C18H21NO3 (1)* | -0.1±0.07 | 0 | 5.63E-01 | 6.13E-01 | -0.13±0.07 | 0 | 6.22E-01 | 5.60E-01 | -0.14±0.09 | 7.71E-01 | -0.15±0.09 | 6.85E-01 | -0.04±0.13 | 9.05E-01 | -0.08±0.13 | 8.03E-01 |
| cis-3,4-methyleneheptanoate | -0.1±0.07 | 0 | 3.61E-01 | 6.05E-01 | -0.12±0.07 | 0 | 6.39E-01 | 5.60E-01 | -0.14±0.09 | 6.98E-01 | -0.14±0.09 | 6.92E-01 | -0.01±0.12 | 9.92E-01 | -0.07±0.12 | 8.22E-01 |
| glucuronide of C10H18O2 (7)* | -0.12±0.07 | 68.05 | 7.69E-02 | 5.50E-01 | -0.12±0.07 | 63.75 | 9.67E-02 | 5.60E-01 | -0.21±0.09 | 3.71E-01 | -0.21±0.09 | 3.54E-01 | 0.06±0.12 | 8.47E-01 | 0.05±0.13 | 8.95E-01 |
| X - 15486 | -0.1±0.07 | 33.31 | 2.21E-01 | 6.57E-01 | -0.12±0.07 | 0 | 4.84E-01 | 5.60E-01 | -0.16±0.09 | 6.65E-01 | -0.16±0.09 | 6.39E-01 | 0.03±0.13 | 9.28E-01 | -0.05±0.13 | 8.98E-01 |
| gamma-carboxyglutamate | 0.12±0.07 | 18.7 | 2.67E-01 | 5.48E-01 | 0.13±0.07 | 37.29 | 2.07E-01 | 5.60E-01 | 0.18±0.09 | 5.86E-01 | 0.2±0.09 | 4.93E-01 | 0.01±0.13 | 9.86E-01 | 0±0.13 | 9.97E-01 |
| 1-methyl-4-imidazoleacetate | 0.13±0.07 | 79.77 | 2.62E-02 | 5.47E-01 | 0.13±0.08 | 77.43 | 3.53E-02 | 5.61E-01 | 0.02±0.09 | 9.74E-01 | 0.03±0.09 | 9.55E-01 | 0.38±0.14 | 1.99E-01 | 0.39±0.15 | 3.58E-01 |
| ceramide (d16:1/24:1, d18:1/22:1)* | 0.14±0.08 | 8.78 | 2.95E-01 | 5.48E-01 | 0.14±0.08 | 0 | 3.52E-01 | 5.61E-01 | 0.09±0.09 | 8.80E-01 | 0.09±0.1 | 8.47E-01 | 0.28±0.16 | 3.97E-01 | 0.27±0.16 | 4.95E-01 |
| X - 23593 | 0.14±0.08 | 0 | 4.16E-01 | 5.48E-01 | 0.14±0.08 | 0 | 4.41E-01 | 5.61E-01 | 0.1±0.09 | 8.65E-01 | 0.1±0.09 | 8.36E-01 | 0.26±0.17 | 4.98E-01 | 0.25±0.17 | 5.48E-01 |
| X - 12411 | 0.12±0.07 | 42.74 | 1.86E-01 | 5.48E-01 | 0.12±0.07 | 44.52 | 1.79E-01 | 5.62E-01 | 0.05±0.09 | 9.27E-01 | 0.05±0.09 | 9.18E-01 | 0.25±0.12 | 3.25E-01 | 0.26±0.12 | 4.30E-01 |
| lanthionine | 0.12±0.08 | 8.39 | 2.96E-01 | 5.50E-01 | 0.12±0.08 | 9.56 | 2.93E-01 | 5.62E-01 | 0.07±0.09 | 9.03E-01 | 0.07±0.09 | 8.94E-01 | 0.24±0.13 | 3.85E-01 | 0.24±0.13 | 4.65E-01 |
| 2-methylserine | 0.12±0.08 | 0 | 6.70E-01 | 5.74E-01 | 0.13±0.08 | 0 | 5.27E-01 | 5.62E-01 | 0.1±0.09 | 8.65E-01 | 0.1±0.09 | 8.36E-01 | 0.17±0.15 | 6.10E-01 | 0.21±0.16 | 5.81E-01 |
| 5,6-dihydrouridine | 0.14±0.07 | 0 | 3.92E-01 | 5.17E-01 | 0.12±0.08 | 0 | 5.85E-01 | 5.71E-01 | 0.09±0.09 | 8.72E-01 | 0.1±0.09 | 8.36E-01 | 0.23±0.13 | 4.01E-01 | 0.18±0.13 | 5.80E-01 |
| 4-allylcatechol sulfate | -0.13±0.07 | 65.36 | 8.93E-02 | 5.47E-01 | -0.12±0.07 | 68.64 | 7.42E-02 | 5.71E-01 | -0.22±0.09 | 3.61E-01 | -0.22±0.09 | 3.52E-01 | 0.05±0.13 | 8.93E-01 | 0.07±0.13 | 8.32E-01 |
| cytidine | 0.12±0.07 | 74.64 | 4.71E-02 | 5.48E-01 | 0.12±0.07 | 69.2 | 7.16E-02 | 5.72E-01 | 0.01±0.09 | 9.78E-01 | 0.02±0.09 | 9.57E-01 | 0.32±0.12 | 2.03E-01 | 0.31±0.13 | 4.03E-01 |
| carboxyethyl-GABA | 0.12±0.08 | 0 | 5.49E-01 | 5.85E-01 | 0.13±0.08 | 0 | 4.91E-01 | 5.72E-01 | 0.08±0.09 | 8.80E-01 | 0.09±0.09 | 8.38E-01 | 0.18±0.14 | 5.61E-01 | 0.21±0.14 | 5.54E-01 |
| phenylacetylcarnitine | 0.12±0.08 | 0 | 5.10E-01 | 5.50E-01 | 0.12±0.08 | 0 | 5.97E-01 | 5.72E-01 | 0.09±0.09 | 8.72E-01 | 0.1±0.09 | 8.36E-01 | 0.2±0.13 | 5.23E-01 | 0.18±0.14 | 5.86E-01 |
| 3-bromo-5-chloro-2,6-dihydroxybenzoic acid* | -0.11±0.07 | 77.08 | 3.67E-02 | 5.55E-01 | -0.11±0.07 | 75.81 | 4.20E-02 | 5.72E-01 | -0.19±0.08 | 3.61E-01 | -0.2±0.08 | 3.52E-01 | 0.16±0.15 | 6.23E-01 | 0.14±0.14 | 6.91E-01 |
| decadienedioic acid (C10:2-DC)** | 0.14±0.07 | 0 | 8.79E-01 | 4.93E-01 | 0.12±0.08 | 0 | 7.15E-01 | 5.72E-01 | 0.15±0.09 | 7.50E-01 | 0.14±0.1 | 7.26E-01 | 0.12±0.13 | 6.66E-01 | 0.09±0.13 | 7.94E-01 |
| N6-methyladenosine | 0.13±0.08 | 0 | 7.65E-01 | 5.47E-01 | 0.12±0.08 | 0 | 6.25E-01 | 5.72E-01 | 0.15±0.09 | 7.50E-01 | 0.15±0.09 | 7.04E-01 | 0.1±0.13 | 7.44E-01 | 0.07±0.13 | 8.26E-01 |
| isoursodeoxycholate | 0.13±0.07 | 0 | 8.32E-01 | 5.36E-01 | 0.12±0.07 | 0 | 6.05E-01 | 5.72E-01 | 0.14±0.09 | 7.50E-01 | 0.14±0.09 | 6.96E-01 | 0.1±0.13 | 7.11E-01 | 0.06±0.13 | 8.50E-01 |
| sphingosine | 0.12±0.07 | 0 | 8.07E-01 | 5.48E-01 | 0.12±0.07 | 0 | 7.05E-01 | 5.77E-01 | 0.14±0.09 | 7.71E-01 | 0.14±0.09 | 7.26E-01 | 0.1±0.13 | 7.38E-01 | 0.08±0.13 | 7.96E-01 |
| beta-citrylglutamate | 0.13±0.08 | 72.53 | 5.64E-02 | 5.50E-01 | 0.13±0.08 | 57.31 | 1.26E-01 | 5.79E-01 | 0.04±0.09 | 9.27E-01 | 0.06±0.09 | 9.00E-01 | 0.4±0.16 | 2.27E-01 | 0.34±0.16 | 4.21E-01 |
| X - 15503 | 0.13±0.07 | 72.57 | 5.62E-02 | 5.47E-01 | 0.13±0.08 | 38.66 | 2.02E-01 | 5.79E-01 | 0.02±0.09 | 9.59E-01 | 0.05±0.1 | 9.18E-01 | 0.32±0.13 | 2.03E-01 | 0.27±0.14 | 4.52E-01 |
| ectoine | 0.12±0.07 | 0 | 3.44E-01 | 5.50E-01 | 0.12±0.07 | 0 | 3.88E-01 | 5.79E-01 | 0.06±0.09 | 9.03E-01 | 0.07±0.09 | 8.94E-01 | 0.21±0.12 | 4.02E-01 | 0.2±0.12 | 4.95E-01 |
| retinol (Vitamin A) | 0.12±0.08 | 0 | 4.79E-01 | 5.64E-01 | 0.12±0.08 | 0 | 4.81E-01 | 5.79E-01 | 0.09±0.09 | 8.72E-01 | 0.09±0.09 | 8.47E-01 | 0.21±0.15 | 5.31E-01 | 0.21±0.15 | 5.70E-01 |
| N6-methyllysine | 0.11±0.07 | 0 | 7.62E-01 | 5.76E-01 | 0.12±0.07 | 0 | 6.37E-01 | 5.79E-01 | 0.09±0.09 | 8.65E-01 | 0.09±0.09 | 8.36E-01 | 0.14±0.13 | 6.14E-01 | 0.17±0.13 | 6.07E-01 |
| X - 12221 | -0.12±0.07 | 84.51 | 1.11E-02 | 5.50E-01 | -0.12±0.07 | 84.76 | 1.04E-02 | 5.79E-01 | -0.24±0.09 | 2.66E-01 | -0.24±0.09 | 2.60E-01 | 0.16±0.13 | 5.85E-01 | 0.16±0.13 | 6.22E-01 |
| etiocholanolone glucuronide | -0.12±0.07 | 0 | 7.47E-01 | 5.48E-01 | -0.12±0.07 | 0 | 4.80E-01 | 5.79E-01 | -0.14±0.09 | 7.50E-01 | -0.15±0.09 | 6.64E-01 | -0.09±0.13 | 7.74E-01 | -0.04±0.13 | 9.17E-01 |
| tetradecanedioate (C14-DC) | 0.11±0.07 | 9.52 | 2.93E-01 | 6.01E-01 | 0.12±0.07 | 0 | 3.77E-01 | 5.79E-01 | 0.16±0.09 | 6.54E-01 | 0.16±0.09 | 6.15E-01 | 0±0.13 | 9.96E-01 | 0.03±0.13 | 9.43E-01 |
| 1-linoleoyl-GPI (18:2)* | -0.12±0.07 | 0 | 3.68E-01 | 5.50E-01 | -0.12±0.07 | 1.87 | 3.13E-01 | 5.79E-01 | -0.17±0.09 | 6.37E-01 | -0.17±0.09 | 6.01E-01 | -0.03±0.13 | 9.44E-01 | -0.01±0.13 | 9.72E-01 |
| N-acetylputrescine | 0.12±0.08 | 67.57 | 7.91E-02 | 5.68E-01 | 0.12±0.08 | 61.65 | 1.06E-01 | 5.88E-01 | 0.04±0.09 | 9.27E-01 | 0.05±0.09 | 9.18E-01 | 0.37±0.16 | 2.71E-01 | 0.35±0.16 | 4.18E-01 |
| linoleate (18:2n6) | -0.11±0.07 | 57.11 | 1.27E-01 | 5.90E-01 | -0.11±0.07 | 58.76 | 1.19E-01 | 5.88E-01 | -0.03±0.09 | 9.58E-01 | -0.03±0.09 | 9.44E-01 | -0.26±0.13 | 3.23E-01 | -0.27±0.13 | 4.18E-01 |
| hydantoin-5-propionate | 0.12±0.08 | 74.1 | 4.94E-02 | 5.50E-01 | 0.12±0.08 | 48.73 | 1.63E-01 | 5.88E-01 | 0.02±0.09 | 9.74E-01 | 0.05±0.1 | 9.18E-01 | 0.34±0.13 | 2.03E-01 | 0.28±0.14 | 4.30E-01 |
| N-acetylkynurenine (2) | 0.13±0.08 | 67.26 | 8.05E-02 | 5.48E-01 | 0.12±0.08 | 49.18 | 1.61E-01 | 5.88E-01 | 0.03±0.1 | 9.48E-01 | 0.04±0.1 | 9.18E-01 | 0.32±0.14 | 2.42E-01 | 0.28±0.14 | 4.30E-01 |
| N2,N5-diacetylornithine | 0.1±0.07 | 0 | 3.26E-01 | 6.59E-01 | 0.12±0.07 | 52.62 | 1.46E-01 | 5.88E-01 | 0.04±0.09 | 9.27E-01 | 0.04±0.09 | 9.18E-01 | 0.19±0.12 | 4.74E-01 | 0.28±0.13 | 4.30E-01 |
| X - 21353 | -0.1±0.07 | 26.14 | 2.45E-01 | 6.13E-01 | -0.11±0.07 | 39.58 | 1.98E-01 | 5.88E-01 | -0.04±0.09 | 9.27E-01 | -0.05±0.09 | 9.18E-01 | -0.22±0.13 | 3.92E-01 | -0.25±0.13 | 4.52E-01 |
| N1-methyladenosine | 0.14±0.09 | 58.51 | 1.21E-01 | 5.63E-01 | 0.14±0.09 | 34.49 | 2.17E-01 | 5.88E-01 | 0.08±0.09 | 8.80E-01 | 0.09±0.1 | 8.52E-01 | 0.48±0.24 | 3.34E-01 | 0.4±0.23 | 4.74E-01 |
| X - 24518 | 0.13±0.07 | 43.82 | 1.82E-01 | 5.47E-01 | 0.12±0.08 | 0 | 4.55E-01 | 5.88E-01 | 0.05±0.09 | 9.27E-01 | 0.08±0.1 | 8.87E-01 | 0.26±0.13 | 3.23E-01 | 0.2±0.13 | 5.37E-01 |
| pregnenediol disulfate (C21H34O8S2)* | 0.13±0.09 | 0 | 5.79E-01 | 5.64E-01 | 0.13±0.09 | 0 | 5.20E-01 | 5.88E-01 | 0.1±0.1 | 8.72E-01 | 0.09±0.11 | 8.68E-01 | 0.2±0.15 | 5.55E-01 | 0.21±0.15 | 5.77E-01 |
| gamma-tocopherol/beta-tocopherol | 0.13±0.08 | 0 | 5.15E-01 | 5.47E-01 | 0.12±0.08 | 0 | 6.75E-01 | 5.88E-01 | 0.1±0.09 | 8.65E-01 | 0.09±0.09 | 8.36E-01 | 0.2±0.13 | 4.98E-01 | 0.16±0.13 | 6.29E-01 |
| linoleoyl ethanolamide | -0.1±0.07 | 0 | 8.57E-01 | 6.53E-01 | -0.11±0.07 | 0 | 7.16E-01 | 5.88E-01 | -0.09±0.09 | 8.72E-01 | -0.09±0.09 | 8.36E-01 | -0.11±0.12 | 6.73E-01 | -0.15±0.12 | 6.30E-01 |
| ethyl beta-glucopyranoside | -0.13±0.07 | 0 | 4.72E-01 | 5.39E-01 | -0.12±0.08 | 0 | 6.98E-01 | 5.88E-01 | -0.09±0.09 | 8.65E-01 | -0.1±0.09 | 8.36E-01 | -0.2±0.13 | 4.64E-01 | -0.16±0.13 | 6.36E-01 |
| N6,N6-dimethyllysine | 0.12±0.07 | 0 | 8.34E-01 | 5.68E-01 | 0.12±0.07 | 0 | 8.48E-01 | 5.88E-01 | 0.1±0.09 | 8.48E-01 | 0.11±0.09 | 8.36E-01 | 0.14±0.14 | 6.49E-01 | 0.14±0.14 | 6.83E-01 |
| adipoylcarnitine (C6-DC) | 0.12±0.07 | 0 | 6.80E-01 | 5.50E-01 | 0.11±0.07 | 0 | 8.96E-01 | 5.88E-01 | 0.1±0.09 | 8.65E-01 | 0.11±0.09 | 8.36E-01 | 0.16±0.13 | 5.71E-01 | 0.13±0.13 | 6.91E-01 |
| ethyl alpha-glucopyranoside | 0.1±0.07 | 0 | 6.94E-01 | 6.17E-01 | 0.13±0.09 | 0 | 8.98E-01 | 5.88E-01 | 0.12±0.09 | 7.90E-01 | 0.12±0.1 | 8.36E-01 | 0.06±0.13 | 8.59E-01 | 0.15±0.15 | 6.91E-01 |
| 4-vinylphenol sulfate | -0.11±0.07 | 0 | 7.88E-01 | 5.89E-01 | -0.12±0.08 | 0 | 9.93E-01 | 5.88E-01 | -0.1±0.09 | 8.65E-01 | -0.12±0.1 | 8.25E-01 | -0.14±0.13 | 6.23E-01 | -0.12±0.14 | 7.24E-01 |
| N-palmitoyl-sphingosine (d18:1/16:0) | 0.12±0.08 | 0 | 9.30E-01 | 5.72E-01 | 0.12±0.08 | 0 | 9.58E-01 | 5.88E-01 | 0.12±0.09 | 7.90E-01 | 0.12±0.09 | 8.25E-01 | 0.11±0.14 | 7.39E-01 | 0.13±0.14 | 7.24E-01 |
| beta-cryptoxanthin | -0.14±0.07 | 0 | 8.03E-01 | 3.93E-01 | -0.11±0.07 | 0 | 8.85E-01 | 5.88E-01 | -0.13±0.09 | 7.71E-01 | -0.12±0.09 | 7.89E-01 | -0.17±0.12 | 5.33E-01 | -0.1±0.13 | 7.64E-01 |
| nonadecanoate (19:0) | -0.12±0.07 | 0 | 9.30E-01 | 5.50E-01 | -0.12±0.08 | 0 | 7.83E-01 | 5.88E-01 | -0.13±0.09 | 7.90E-01 | -0.13±0.1 | 7.62E-01 | -0.11±0.13 | 6.91E-01 | -0.09±0.13 | 7.82E-01 |
| 3-hydroxystachydrine* | -0.12±0.07 | 0 | 4.31E-01 | 5.41E-01 | -0.11±0.07 | 0 | 3.75E-01 | 5.88E-01 | -0.16±0.08 | 6.26E-01 | -0.15±0.08 | 6.41E-01 | -0.04±0.13 | 9.20E-01 | -0.01±0.13 | 9.80E-01 |
| X - 18901 | -0.13±0.07 | 0 | 4.01E-01 | 5.36E-01 | -0.11±0.07 | 22.13 | 2.57E-01 | 5.88E-01 | -0.17±0.09 | 6.10E-01 | -0.17±0.09 | 5.98E-01 | -0.04±0.13 | 9.20E-01 | 0.01±0.13 | 9.85E-01 |
| aspartate | 0.12±0.08 | 65.76 | 8.74E-02 | 5.76E-01 | 0.13±0.08 | 20.06 | 2.63E-01 | 5.94E-01 | 0.04±0.09 | 9.38E-01 | 0.06±0.1 | 8.94E-01 | 0.35±0.16 | 2.92E-01 | 0.27±0.15 | 4.65E-01 |
| ergothioneine | 0.09±0.08 | 55.94 | 1.32E-01 | 6.88E-01 | 0.12±0.08 | 20.54 | 2.62E-01 | 5.94E-01 | 0.18±0.1 | 6.26E-01 | 0.18±0.1 | 6.01E-01 | -0.06±0.12 | 8.66E-01 | 0±0.13 | 9.98E-01 |
| paraxanthine | -0.1±0.07 | 46.49 | 1.72E-01 | 6.49E-01 | -0.11±0.07 | 26.5 | 2.43E-01 | 5.98E-01 | -0.16±0.08 | 6.26E-01 | -0.16±0.09 | 5.98E-01 | 0.06±0.13 | 8.80E-01 | 0.02±0.13 | 9.64E-01 |
| gamma-glutamyl-alpha-lysine | 0.12±0.08 | 0 | 4.27E-01 | 5.89E-01 | 0.12±0.08 | 0 | 4.78E-01 | 5.98E-01 | 0.09±0.09 | 8.69E-01 | 0.09±0.09 | 8.36E-01 | 0.24±0.18 | 5.43E-01 | 0.23±0.17 | 5.97E-01 |
| gamma-glutamylphenylalanine | 0.13±0.08 | 91.42 | 6.40E-04 | 5.48E-01 | 0.12±0.08 | 88.24 | 3.54E-03 | 6.02E-01 | -0.03±0.09 | 9.59E-01 | -0.01±0.09 | 9.80E-01 | 0.61±0.16 | **3.13E-02** | 0.55±0.17 | 1.34E-01 |
| palmitoylcarnitine (C16) | 0.12±0.08 | 0 | 7.55E-01 | 5.89E-01 | 0.12±0.08 | 0 | 7.61E-01 | 6.06E-01 | 0.1±0.09 | 8.65E-01 | 0.1±0.1 | 8.36E-01 | 0.15±0.14 | 6.23E-01 | 0.15±0.14 | 6.67E-01 |
| X - 24473 | 0.09±0.07 | 0.26 | 3.17E-01 | 6.91E-01 | 0.11±0.08 | 56.11 | 1.31E-01 | 6.13E-01 | 0.03±0.09 | 9.48E-01 | 0.03±0.09 | 9.24E-01 | 0.19±0.12 | 5.09E-01 | 0.28±0.13 | 4.30E-01 |
| gamma-glutamylleucine | 0.12±0.08 | 68.42 | 7.51E-02 | 5.64E-01 | 0.12±0.08 | 41.57 | 1.91E-01 | 6.13E-01 | 0.03±0.1 | 9.48E-01 | 0.05±0.1 | 9.18E-01 | 0.35±0.15 | 2.55E-01 | 0.28±0.15 | 4.52E-01 |
| 5,6-dihydrothymine | -0.12±0.07 | 52.8 | 1.46E-01 | 5.52E-01 | -0.11±0.07 | 57.22 | 1.26E-01 | 6.13E-01 | -0.19±0.09 | 4.91E-01 | -0.18±0.09 | 4.98E-01 | 0.04±0.13 | 9.20E-01 | 0.05±0.12 | 8.85E-01 |
| 3-methyl catechol sulfate (2) | -0.1±0.07 | 0 | 5.37E-01 | 6.29E-01 | -0.11±0.07 | 0 | 5.56E-01 | 6.13E-01 | -0.13±0.09 | 7.71E-01 | -0.14±0.09 | 7.20E-01 | -0.04±0.13 | 9.26E-01 | -0.04±0.14 | 9.11E-01 |
| adenosine 3',5'-cyclic monophosphate (cAMP) | -0.11±0.07 | 32.62 | 2.23E-01 | 6.05E-01 | -0.11±0.07 | 20.98 | 2.61E-01 | 6.13E-01 | -0.17±0.09 | 6.26E-01 | -0.17±0.09 | 6.01E-01 | 0.02±0.12 | 9.75E-01 | 0.01±0.13 | 9.85E-01 |
| X - 18888 | -0.1±0.07 | 0 | 9.75E-01 | 6.55E-01 | -0.11±0.08 | 0 | 8.13E-01 | 6.15E-01 | -0.1±0.09 | 8.65E-01 | -0.1±0.09 | 8.36E-01 | -0.1±0.13 | 7.35E-01 | -0.14±0.14 | 6.77E-01 |
| X - 15461 | 0.12±0.08 | 74.02 | 4.98E-02 | 5.52E-01 | 0.11±0.08 | 64.89 | 9.15E-02 | 6.17E-01 | 0.04±0.09 | 9.38E-01 | 0.04±0.09 | 9.18E-01 | 0.4±0.16 | 2.19E-01 | 0.35±0.16 | 4.18E-01 |
| androstenediol (3beta,17beta) monosulfate (1) | 0.12±0.08 | 68.54 | 7.46E-02 | 5.58E-01 | 0.12±0.08 | 70.6 | 6.52E-02 | 6.17E-01 | 0.03±0.1 | 9.48E-01 | 0.02±0.1 | 9.71E-01 | 0.34±0.14 | 2.50E-01 | 0.34±0.15 | 4.18E-01 |
| cis-3,4-methyleneheptanoylcarnitine | -0.09±0.07 | 0 | 8.33E-01 | 6.88E-01 | -0.11±0.07 | 0 | 7.78E-01 | 6.17E-01 | -0.1±0.09 | 8.65E-01 | -0.09±0.09 | 8.36E-01 | -0.06±0.12 | 8.41E-01 | -0.13±0.12 | 6.66E-01 |
| indolelactate | 0.11±0.08 | 0 | 5.91E-01 | 6.05E-01 | 0.12±0.08 | 0 | 7.81E-01 | 6.17E-01 | 0.09±0.09 | 8.80E-01 | 0.1±0.1 | 8.36E-01 | 0.18±0.15 | 5.82E-01 | 0.15±0.14 | 6.69E-01 |
| X - 12026 | 0.12±0.08 | 0 | 9.53E-01 | 5.76E-01 | 0.12±0.08 | 0 | 5.94E-01 | 6.17E-01 | 0.12±0.1 | 8.31E-01 | 0.14±0.1 | 7.37E-01 | 0.11±0.13 | 7.03E-01 | 0.06±0.14 | 8.81E-01 |
| behenoyl sphingomyelin (d18:1/22:0)* | -0.11±0.07 | 40.8 | 1.94E-01 | 5.90E-01 | -0.11±0.07 | 55.33 | 1.35E-01 | 6.17E-01 | -0.18±0.09 | 6.03E-01 | -0.19±0.09 | 5.16E-01 | 0.02±0.13 | 9.59E-01 | 0.05±0.13 | 8.95E-01 |
| sphingomyelin (d18:2/16:0, d18:1/16:1)* | -0.11±0.08 | 34.52 | 2.17E-01 | 5.90E-01 | -0.11±0.08 | 44.42 | 1.80E-01 | 6.17E-01 | -0.18±0.1 | 6.10E-01 | -0.19±0.1 | 5.53E-01 | 0.02±0.13 | 9.79E-01 | 0.03±0.13 | 9.43E-01 |
| 1,2-dilinoleoyl-GPC (18:2/18:2) | -0.11±0.07 | 0 | 4.67E-01 | 5.58E-01 | -0.11±0.08 | 29.33 | 2.34E-01 | 6.17E-01 | -0.15±0.09 | 6.98E-01 | -0.17±0.09 | 6.01E-01 | -0.04±0.12 | 9.11E-01 | 0.02±0.14 | 9.58E-01 |
| sphingosine 1-phosphate | 0.11±0.08 | 0 | 5.95E-01 | 6.04E-01 | 0.11±0.08 | 0 | 4.92E-01 | 6.24E-01 | 0.09±0.09 | 8.69E-01 | 0.08±0.09 | 8.61E-01 | 0.18±0.16 | 6.00E-01 | 0.21±0.16 | 6.07E-01 |
| 2'-O-methylcytidine | 0.1±0.07 | 0 | 5.04E-01 | 6.48E-01 | 0.11±0.08 | 0 | 6.45E-01 | 6.24E-01 | 0.14±0.09 | 7.71E-01 | 0.13±0.09 | 7.54E-01 | 0.03±0.13 | 9.40E-01 | 0.06±0.13 | 8.66E-01 |
| 3-methylglutaconate | 0.11±0.08 | 58.6 | 1.20E-01 | 6.29E-01 | 0.12±0.08 | 62.08 | 1.04E-01 | 6.29E-01 | 0.03±0.1 | 9.50E-01 | 0.03±0.1 | 9.25E-01 | 0.3±0.15 | 3.25E-01 | 0.33±0.15 | 4.21E-01 |
| 1-arachidonoyl-GPC (20:4n6)* | 0.11±0.08 | 0 | 5.13E-01 | 6.29E-01 | 0.11±0.08 | 1.55 | 3.14E-01 | 6.29E-01 | 0.08±0.09 | 8.95E-01 | 0.06±0.09 | 8.94E-01 | 0.19±0.15 | 5.71E-01 | 0.24±0.15 | 4.95E-01 |
| 3-hydroxyhexanoate | 0.12±0.08 | 0 | 3.98E-01 | 5.52E-01 | 0.11±0.08 | 0 | 5.10E-01 | 6.29E-01 | 0.08±0.09 | 8.80E-01 | 0.08±0.09 | 8.68E-01 | 0.23±0.14 | 4.72E-01 | 0.19±0.15 | 5.97E-01 |
| 1-methylurate | -0.1±0.07 | 63.01 | 1.00E-01 | 6.29E-01 | -0.1±0.07 | 60.27 | 1.13E-01 | 6.29E-01 | -0.18±0.09 | 5.29E-01 | -0.18±0.09 | 5.12E-01 | 0.08±0.13 | 7.97E-01 | 0.07±0.13 | 8.25E-01 |
| palmitoyl-sphingosine-phosphoethanolamine (d18:1/16:0) | -0.12±0.07 | 0 | 9.92E-01 | 5.48E-01 | -0.1±0.07 | 0 | 5.88E-01 | 6.29E-01 | -0.11±0.09 | 7.90E-01 | -0.13±0.09 | 7.42E-01 | -0.12±0.12 | 6.66E-01 | -0.04±0.13 | 9.02E-01 |
| 3beta-hydroxy-5-cholestenoate | -0.11±0.07 | 0 | 5.44E-01 | 5.89E-01 | -0.11±0.08 | 43.92 | 1.82E-01 | 6.29E-01 | -0.14±0.09 | 7.50E-01 | -0.18±0.09 | 5.69E-01 | -0.05±0.12 | 8.97E-01 | 0.03±0.13 | 9.29E-01 |
| urate | 0.13±0.08 | 59.47 | 1.16E-01 | 5.48E-01 | 0.11±0.08 | 0 | 3.26E-01 | 6.30E-01 | 0.05±0.09 | 9.27E-01 | 0.06±0.1 | 9.00E-01 | 0.32±0.14 | 3.02E-01 | 0.23±0.15 | 4.95E-01 |
| ascorbic acid 2-sulfate | 0.1±0.08 | 0 | 3.45E-01 | 6.72E-01 | 0.12±0.08 | 9.01 | 2.94E-01 | 6.30E-01 | 0.05±0.1 | 9.27E-01 | 0.06±0.1 | 9.01E-01 | 0.22±0.15 | 5.15E-01 | 0.25±0.15 | 4.95E-01 |
| 3-hydroxysebacate | 0.12±0.08 | 0 | 5.20E-01 | 5.52E-01 | 0.11±0.08 | 0 | 3.72E-01 | 6.30E-01 | 0.16±0.09 | 7.15E-01 | 0.16±0.1 | 6.80E-01 | 0.05±0.13 | 8.90E-01 | 0.01±0.14 | 9.85E-01 |
| pro-hydroxy-pro | 0.12±0.08 | 43.69 | 1.83E-01 | 6.05E-01 | 0.12±0.08 | 45.61 | 1.75E-01 | 6.30E-01 | 0.06±0.09 | 9.04E-01 | 0.06±0.09 | 8.94E-01 | 0.33±0.18 | 3.67E-01 | 0.34±0.18 | 4.63E-01 |
| betaine | -0.11±0.08 | 74.04 | 4.97E-02 | 6.05E-01 | -0.11±0.08 | 79.02 | 2.90E-02 | 6.30E-01 | -0.22±0.1 | 3.95E-01 | -0.23±0.1 | 3.52E-01 | 0.12±0.14 | 7.20E-01 | 0.16±0.15 | 6.67E-01 |
| glycosyl-N-behenoyl-sphingadienine (d18:2/22:0)* | -0.11±0.07 | 0 | 9.49E-01 | 5.55E-01 | -0.1±0.07 | 0 | 6.65E-01 | 6.30E-01 | -0.12±0.09 | 8.25E-01 | -0.13±0.1 | 7.69E-01 | -0.11±0.12 | 6.72E-01 | -0.06±0.12 | 8.34E-01 |
| 1-stearoyl-2-docosahexaenoyl-GPE (18:0/22:6)* | -0.09±0.07 | 0 | 8.69E-01 | 6.89E-01 | -0.11±0.08 | 0 | 8.33E-01 | 6.32E-01 | -0.1±0.09 | 8.65E-01 | -0.1±0.1 | 8.36E-01 | -0.07±0.13 | 8.31E-01 | -0.13±0.14 | 6.91E-01 |
| 1-lignoceroyl-GPC (24:0) | -0.11±0.07 | 0 | 7.71E-01 | 5.63E-01 | -0.11±0.08 | 18.73 | 2.67E-01 | 6.32E-01 | -0.13±0.09 | 7.81E-01 | -0.16±0.09 | 6.39E-01 | -0.08±0.13 | 7.87E-01 | 0.02±0.14 | 9.56E-01 |
| X - 16935 | -0.1±0.07 | 0 | 3.37E-01 | 6.57E-01 | -0.1±0.07 | 0 | 3.89E-01 | 6.33E-01 | -0.14±0.09 | 7.34E-01 | -0.14±0.09 | 6.93E-01 | 0.01±0.13 | 9.92E-01 | -0.01±0.13 | 9.85E-01 |
| fumarate | 0.11±0.09 | 15.08 | 2.78E-01 | 6.59E-01 | 0.12±0.09 | 28.67 | 2.36E-01 | 6.34E-01 | 0.06±0.1 | 9.27E-01 | 0.06±0.1 | 9.04E-01 | 0.26±0.16 | 4.63E-01 | 0.29±0.17 | 4.74E-01 |
| glutamine_degradant* | 0.11±0.09 | 89.17 | 2.38E-03 | 6.72E-01 | 0.12±0.09 | 90.8 | 9.76E-04 | 6.35E-01 | -0.07±0.11 | 9.03E-01 | -0.08±0.11 | 8.94E-01 | 0.5±0.16 | 1.11E-01 | 0.55±0.16 | 7.79E-02 |
| valylglycine | 0.1±0.07 | 0 | 3.28E-01 | 6.29E-01 | 0.1±0.08 | 33.26 | 2.21E-01 | 6.35E-01 | 0.05±0.09 | 9.27E-01 | 0.04±0.09 | 9.18E-01 | 0.21±0.13 | 4.64E-01 | 0.24±0.14 | 4.65E-01 |
| ceramide (d18:1/17:0, d17:1/18:0)* | 0.1±0.08 | 0 | 6.76E-01 | 6.53E-01 | 0.11±0.08 | 0 | 8.49E-01 | 6.35E-01 | 0.08±0.09 | 8.80E-01 | 0.1±0.1 | 8.36E-01 | 0.15±0.14 | 6.23E-01 | 0.13±0.14 | 7.06E-01 |
| lactosyl-N-behenoyl-sphingosine (d18:1/22:0)* | -0.1±0.07 | 0 | 6.58E-01 | 6.17E-01 | -0.11±0.08 | 0 | 4.98E-01 | 6.35E-01 | -0.13±0.1 | 7.90E-01 | -0.14±0.1 | 7.31E-01 | -0.06±0.12 | 8.47E-01 | -0.04±0.13 | 9.26E-01 |
| X - 25422 | 0.11±0.08 | 0 | 3.33E-01 | 5.89E-01 | 0.11±0.08 | 18.52 | 2.68E-01 | 6.35E-01 | 0.17±0.1 | 6.65E-01 | 0.17±0.1 | 6.39E-01 | 0.02±0.12 | 9.67E-01 | 0±0.12 | 9.97E-01 |
| 1-palmitoyl-GPE (16:0) | 0.09±0.08 | 0 | 8.63E-01 | 6.89E-01 | 0.11±0.08 | 0 | 5.70E-01 | 6.35E-01 | 0.08±0.09 | 8.80E-01 | 0.08±0.09 | 8.68E-01 | 0.11±0.14 | 7.29E-01 | 0.17±0.14 | 6.29E-01 |
| X - 25810 | 0.1±0.07 | 0 | 9.15E-01 | 6.29E-01 | 0.1±0.07 | 0 | 8.33E-01 | 6.35E-01 | 0.09±0.09 | 8.65E-01 | 0.09±0.09 | 8.36E-01 | 0.11±0.12 | 6.96E-01 | 0.12±0.13 | 7.03E-01 |
| cis-4-decenoate (10:1n6)* | -0.09±0.07 | 0 | 3.20E-01 | 6.72E-01 | -0.1±0.07 | 32.47 | 2.24E-01 | 6.36E-01 | -0.04±0.09 | 9.38E-01 | -0.04±0.09 | 9.19E-01 | -0.19±0.12 | 4.83E-01 | -0.22±0.12 | 4.65E-01 |
| 3,5-dichloro-2,6-dihydroxybenzoic acid | -0.09±0.07 | 83.33 | 1.43E-02 | 6.72E-01 | -0.09±0.07 | 80.62 | 2.31E-02 | 6.42E-01 | -0.18±0.08 | 3.75E-01 | -0.19±0.08 | 3.52E-01 | 0.21±0.14 | 5.09E-01 | 0.17±0.13 | 6.23E-01 |
| sphingomyelin (d18:1/20:1, d18:2/20:0)* | -0.11±0.07 | 0 | 8.38E-01 | 5.72E-01 | -0.1±0.07 | 0 | 7.62E-01 | 6.42E-01 | -0.12±0.09 | 7.90E-01 | -0.12±0.1 | 8.35E-01 | -0.09±0.12 | 7.29E-01 | -0.07±0.12 | 7.96E-01 |
| gamma-glutamylmethionine | 0.12±0.09 | 77.75 | 3.40E-02 | 6.13E-01 | 0.12±0.09 | 75.64 | 4.28E-02 | 6.45E-01 | 0.04±0.09 | 9.38E-01 | 0.04±0.09 | 9.19E-01 | 0.52±0.21 | 2.13E-01 | 0.49±0.2 | 4.03E-01 |
| X - 12847 | -0.11±0.07 | 0 | 6.00E-01 | 5.72E-01 | -0.1±0.08 | 0 | 3.19E-01 | 6.45E-01 | -0.14±0.09 | 7.50E-01 | -0.16±0.09 | 6.80E-01 | -0.06±0.13 | 8.66E-01 | 0±0.13 | 9.92E-01 |
| erucate (22:1n9) | -0.1±0.08 | 0 | 8.07E-01 | 6.53E-01 | -0.1±0.08 | 0 | 9.81E-01 | 6.49E-01 | -0.09±0.09 | 8.72E-01 | -0.1±0.09 | 8.36E-01 | -0.13±0.13 | 6.66E-01 | -0.1±0.14 | 7.64E-01 |
| trans-4-hydroxyproline | 0.12±0.07 | 48.77 | 1.62E-01 | 5.48E-01 | 0.1±0.07 | 31.46 | 2.27E-01 | 6.52E-01 | 0.05±0.09 | 9.27E-01 | 0.04±0.09 | 9.19E-01 | 0.27±0.13 | 3.23E-01 | 0.23±0.13 | 4.65E-01 |
| cyclo(leu-pro) | 0.12±0.08 | 82.07 | 1.82E-02 | 5.55E-01 | 0.11±0.08 | 68.61 | 7.43E-02 | 6.54E-01 | 0±0.09 | 9.93E-01 | 0.01±0.1 | 9.80E-01 | 0.39±0.14 | 1.99E-01 | 0.32±0.14 | 4.18E-01 |
| 4-ethylcatechol sulfate | -0.11±0.07 | 0 | 4.29E-01 | 6.04E-01 | -0.1±0.07 | 0 | 5.95E-01 | 6.57E-01 | -0.06±0.09 | 9.03E-01 | -0.07±0.09 | 8.94E-01 | -0.19±0.13 | 5.09E-01 | -0.16±0.13 | 6.30E-01 |
| 1-myristoyl-2-arachidonoyl-GPC (14:0/20:4)* | 0.12±0.08 | 64.15 | 9.49E-02 | 6.05E-01 | 0.11±0.08 | 57.06 | 1.27E-01 | 6.63E-01 | 0.03±0.1 | 9.55E-01 | 0.03±0.1 | 9.56E-01 | 0.33±0.15 | 3.04E-01 | 0.3±0.15 | 4.44E-01 |
| 5alpha-pregnan-3beta,20alpha-diol disulfate | 0.1±0.08 | 0.53 | 3.16E-01 | 6.55E-01 | 0.1±0.08 | 11.45 | 2.88E-01 | 6.63E-01 | 0.04±0.1 | 9.27E-01 | 0.04±0.1 | 9.18E-01 | 0.21±0.13 | 4.64E-01 | 0.21±0.13 | 4.95E-01 |
| stearidonate (18:4n3) | -0.09±0.08 | 0 | 9.49E-01 | 6.79E-01 | -0.1±0.08 | 0 | 9.54E-01 | 6.63E-01 | -0.1±0.09 | 8.65E-01 | -0.1±0.1 | 8.36E-01 | -0.09±0.13 | 7.83E-01 | -0.1±0.14 | 7.79E-01 |
| lignoceroyl sphingomyelin (d18:1/24:0) | -0.1±0.07 | 0 | 5.47E-01 | 6.29E-01 | -0.1±0.07 | 14.03 | 2.81E-01 | 6.63E-01 | -0.13±0.09 | 7.81E-01 | -0.16±0.09 | 6.64E-01 | -0.04±0.12 | 9.04E-01 | 0.01±0.12 | 9.84E-01 |
| glutamate | 0.11±0.08 | 77.47 | 3.51E-02 | 6.10E-01 | 0.11±0.08 | 46.85 | 1.70E-01 | 6.68E-01 | 0.01±0.09 | 9.86E-01 | 0.04±0.1 | 9.24E-01 | 0.38±0.15 | 2.03E-01 | 0.29±0.16 | 4.62E-01 |
| 3-methyl-2-oxovalerate | 0.11±0.08 | 60.17 | 1.13E-01 | 6.29E-01 | 0.1±0.08 | 36.29 | 2.10E-01 | 6.68E-01 | 0.02±0.1 | 9.71E-01 | 0.03±0.1 | 9.29E-01 | 0.29±0.14 | 3.23E-01 | 0.25±0.14 | 4.65E-01 |
| X - 18886 | 0.13±0.08 | 14.7 | 2.79E-01 | 5.52E-01 | 0.11±0.08 | 0 | 5.40E-01 | 6.68E-01 | 0.06±0.1 | 9.07E-01 | 0.07±0.1 | 8.94E-01 | 0.25±0.14 | 3.73E-01 | 0.18±0.14 | 6.23E-01 |
| stearate (18:0) | -0.09±0.07 | 0 | 9.25E-01 | 6.91E-01 | -0.1±0.08 | 0 | 8.25E-01 | 6.68E-01 | -0.08±0.09 | 8.80E-01 | -0.09±0.09 | 8.60E-01 | -0.1±0.13 | 7.38E-01 | -0.12±0.13 | 7.02E-01 |
| X - 12100 | 0.1±0.08 | 0 | 6.80E-01 | 6.72E-01 | 0.1±0.08 | 0 | 9.31E-01 | 6.68E-01 | 0.07±0.09 | 9.01E-01 | 0.09±0.09 | 8.36E-01 | 0.14±0.14 | 6.39E-01 | 0.11±0.13 | 7.50E-01 |
| X - 23680 | -0.08±0.07 | 0 | 8.11E-01 | 6.91E-01 | -0.09±0.07 | 0 | 9.87E-01 | 6.68E-01 | -0.09±0.09 | 8.65E-01 | -0.09±0.09 | 8.36E-01 | -0.06±0.12 | 8.53E-01 | -0.1±0.12 | 7.53E-01 |
| sphingomyelin (d18:2/24:2)* | -0.11±0.07 | 0 | 9.02E-01 | 5.72E-01 | -0.1±0.08 | 0 | 8.87E-01 | 6.68E-01 | -0.11±0.1 | 8.65E-01 | -0.11±0.1 | 8.36E-01 | -0.12±0.12 | 6.33E-01 | -0.08±0.12 | 7.82E-01 |
| octadecadienedioate (C18:2-DC)* | -0.11±0.08 | 0 | 9.54E-01 | 6.17E-01 | -0.1±0.08 | 0 | 8.76E-01 | 6.68E-01 | -0.11±0.09 | 8.41E-01 | -0.11±0.1 | 8.36E-01 | -0.1±0.13 | 7.39E-01 | -0.08±0.14 | 8.03E-01 |
| gamma-CEHC | -0.1±0.07 | 0 | 5.51E-01 | 6.50E-01 | -0.09±0.07 | 0 | 7.02E-01 | 6.68E-01 | -0.13±0.09 | 7.81E-01 | -0.11±0.09 | 8.25E-01 | -0.04±0.12 | 9.20E-01 | -0.06±0.12 | 8.62E-01 |
| dodecanedioate (C12-DC) | 0.09±0.07 | 0 | 4.98E-01 | 6.72E-01 | 0.1±0.08 | 0 | 5.53E-01 | 6.68E-01 | 0.13±0.09 | 7.90E-01 | 0.13±0.1 | 7.62E-01 | 0.03±0.12 | 9.47E-01 | 0.04±0.13 | 9.16E-01 |
| N-acetyl-1-methylhistidine* | 0.1±0.07 | 0 | 4.93E-01 | 6.10E-01 | 0.1±0.07 | 0 | 3.45E-01 | 6.68E-01 | 0.14±0.09 | 7.50E-01 | 0.15±0.09 | 7.04E-01 | 0.04±0.12 | 9.20E-01 | 0±0.13 | 9.99E-01 |
| myristoyl dihydrosphingomyelin (d18:0/14:0)* | 0.1±0.08 | 64.75 | 9.21E-02 | 6.72E-01 | 0.11±0.08 | 70.48 | 6.57E-02 | 6.69E-01 | 0.03±0.1 | 9.50E-01 | 0.03±0.1 | 9.55E-01 | 0.37±0.18 | 3.23E-01 | 0.4±0.18 | 4.18E-01 |
| X - 16087 | 0.11±0.08 | 55.27 | 1.35E-01 | 6.10E-01 | 0.1±0.08 | 17.35 | 2.71E-01 | 6.69E-01 | 0.03±0.09 | 9.48E-01 | 0.04±0.09 | 9.18E-01 | 0.27±0.13 | 3.25E-01 | 0.22±0.13 | 4.95E-01 |
| eicosenoate (20:1) | -0.08±0.07 | 0 | 3.23E-01 | 6.91E-01 | -0.1±0.07 | 0.37 | 3.16E-01 | 6.69E-01 | -0.03±0.09 | 9.48E-01 | -0.04±0.09 | 9.18E-01 | -0.18±0.13 | 5.19E-01 | -0.2±0.13 | 4.97E-01 |
| myo-inositol | 0.08±0.07 | 0 | 4.88E-01 | 6.97E-01 | 0.1±0.08 | 0 | 4.20E-01 | 6.69E-01 | 0.05±0.09 | 9.27E-01 | 0.06±0.09 | 9.01E-01 | 0.16±0.13 | 5.92E-01 | 0.19±0.14 | 5.79E-01 |
| N6-acetyllysine | 0.1±0.08 | 0 | 3.75E-01 | 6.53E-01 | 0.1±0.08 | 0 | 5.38E-01 | 6.69E-01 | 0.06±0.09 | 9.06E-01 | 0.07±0.09 | 8.94E-01 | 0.22±0.15 | 5.23E-01 | 0.18±0.15 | 6.32E-01 |
| cis-3,4-methyleneheptanoylglycine | -0.08±0.07 | 0 | 9.32E-01 | 6.92E-01 | -0.09±0.07 | 0 | 9.50E-01 | 6.69E-01 | -0.09±0.09 | 8.72E-01 | -0.09±0.09 | 8.36E-01 | -0.07±0.12 | 8.09E-01 | -0.1±0.13 | 7.50E-01 |
| deoxycholic acid glucuronide | 0.11±0.08 | 0 | 7.86E-01 | 5.90E-01 | 0.1±0.08 | 0 | 9.41E-01 | 6.69E-01 | 0.1±0.09 | 8.65E-01 | 0.1±0.09 | 8.36E-01 | 0.14±0.13 | 6.26E-01 | 0.09±0.14 | 7.93E-01 |
| acisoga | 0.11±0.07 | 0 | 9.47E-01 | 6.05E-01 | 0.1±0.07 | 0 | 8.14E-01 | 6.69E-01 | 0.11±0.09 | 8.33E-01 | 0.11±0.09 | 8.36E-01 | 0.1±0.13 | 7.38E-01 | 0.07±0.13 | 8.26E-01 |
| 2-hydroxybehenate | -0.09±0.07 | 0 | 5.23E-01 | 6.72E-01 | -0.09±0.07 | 0 | 4.89E-01 | 6.69E-01 | -0.13±0.09 | 7.90E-01 | -0.13±0.09 | 7.54E-01 | -0.03±0.12 | 9.40E-01 | -0.03±0.12 | 9.43E-01 |
| linolenoylcarnitine (C18:3)* | -0.1±0.07 | 0 | 7.07E-01 | 6.57E-01 | -0.09±0.07 | 0 | 7.04E-01 | 6.76E-01 | -0.12±0.09 | 8.16E-01 | -0.11±0.09 | 8.32E-01 | -0.06±0.13 | 8.64E-01 | -0.05±0.13 | 8.79E-01 |
| 2-aminophenol sulfate | 0.08±0.07 | 42.94 | 1.86E-01 | 7.20E-01 | 0.1±0.07 | 54.23 | 1.39E-01 | 6.80E-01 | 0.01±0.09 | 9.86E-01 | 0.02±0.09 | 9.66E-01 | 0.22±0.13 | 4.19E-01 | 0.26±0.13 | 4.52E-01 |
| indolin-2-one | 0.09±0.08 | 0 | 4.62E-01 | 6.91E-01 | 0.1±0.08 | 0 | 5.32E-01 | 6.80E-01 | 0.05±0.09 | 9.27E-01 | 0.07±0.09 | 8.94E-01 | 0.18±0.14 | 5.77E-01 | 0.17±0.14 | 6.30E-01 |
| octanoylcarnitine (C8) | -0.09±0.07 | 57.79 | 1.24E-01 | 6.72E-01 | -0.09±0.07 | 49.73 | 1.58E-01 | 6.89E-01 | -0.01±0.09 | 9.87E-01 | -0.01±0.09 | 9.80E-01 | -0.24±0.12 | 3.38E-01 | -0.23±0.12 | 4.54E-01 |
| citrate | -0.09±0.07 | 66.31 | 8.49E-02 | 6.74E-01 | -0.09±0.07 | 66.78 | 8.27E-02 | 6.89E-01 | -0.18±0.09 | 5.91E-01 | -0.18±0.09 | 5.26E-01 | 0.1±0.14 | 7.38E-01 | 0.1±0.14 | 7.64E-01 |
| 5alpha-androstan-3beta,17beta-diol disulfate | 0.11±0.09 | 55.59 | 1.33E-01 | 6.91E-01 | 0.12±0.1 | 70.28 | 6.66E-02 | 7.00E-01 | 0±0.11 | 9.89E-01 | 0±0.12 | 9.93E-01 | 0.3±0.16 | 3.57E-01 | 0.37±0.17 | 4.18E-01 |
| N-acetylthreonine | 0.09±0.08 | 49.71 | 1.59E-01 | 6.91E-01 | 0.1±0.08 | 40.82 | 1.94E-01 | 7.00E-01 | 0.03±0.09 | 9.48E-01 | 0.04±0.09 | 9.18E-01 | 0.3±0.16 | 3.75E-01 | 0.29±0.16 | 4.74E-01 |
| docosahexaenoate (DHA; 22:6n3) | -0.1±0.07 | 0 | 3.78E-01 | 6.72E-01 | -0.09±0.07 | 0 | 4.69E-01 | 7.00E-01 | -0.05±0.09 | 9.27E-01 | -0.05±0.09 | 9.18E-01 | -0.18±0.12 | 5.15E-01 | -0.16±0.12 | 5.97E-01 |
| S-adenosylhomocysteine (SAH) | 0.1±0.08 | 0 | 5.38E-01 | 6.53E-01 | 0.1±0.08 | 0 | 8.23E-01 | 7.00E-01 | 0.07±0.1 | 9.03E-01 | 0.09±0.1 | 8.68E-01 | 0.17±0.14 | 5.71E-01 | 0.12±0.14 | 7.18E-01 |
| 2-ketocaprylate | -0.09±0.07 | 0 | 9.88E-01 | 6.74E-01 | -0.09±0.07 | 0 | 9.60E-01 | 7.00E-01 | -0.09±0.09 | 8.69E-01 | -0.09±0.09 | 8.36E-01 | -0.09±0.12 | 7.39E-01 | -0.09±0.12 | 7.82E-01 |
| X - 12851 | 0.08±0.07 | 0 | 7.08E-01 | 7.16E-01 | 0.09±0.07 | 0 | 8.34E-01 | 7.00E-01 | 0.1±0.09 | 8.65E-01 | 0.1±0.09 | 8.36E-01 | 0.04±0.13 | 9.11E-01 | 0.07±0.13 | 8.26E-01 |
| X - 25936 | 0.08±0.07 | 0 | 4.75E-01 | 6.97E-01 | 0.09±0.07 | 0 | 6.02E-01 | 7.00E-01 | 0.12±0.09 | 7.90E-01 | 0.12±0.09 | 8.25E-01 | 0.01±0.12 | 9.84E-01 | 0.04±0.12 | 9.19E-01 |
| X - 11632 | 0.11±0.07 | 5 | 3.05E-01 | 6.05E-01 | 0.09±0.07 | 25.7 | 2.46E-01 | 7.00E-01 | 0.16±0.09 | 6.65E-01 | 0.15±0.09 | 6.80E-01 | 0±0.13 | 9.96E-01 | -0.03±0.13 | 9.33E-01 |
| 2-acetamidophenol sulfate | 0.07±0.07 | 57.83 | 1.24E-01 | 7.59E-01 | 0.09±0.08 | 70.8 | 6.42E-02 | 7.01E-01 | -0.01±0.09 | 9.87E-01 | 0±0.09 | 9.93E-01 | 0.24±0.13 | 3.76E-01 | 0.3±0.14 | 4.18E-01 |
| margaroylcarnitine (C17)* | 0.08±0.08 | 23.67 | 2.52E-01 | 7.37E-01 | 0.09±0.08 | 44.54 | 1.79E-01 | 7.01E-01 | 0.02±0.09 | 9.74E-01 | 0.03±0.09 | 9.55E-01 | 0.21±0.14 | 4.98E-01 | 0.26±0.14 | 4.65E-01 |
| decanoylcarnitine (C10) | -0.09±0.07 | 51.23 | 1.52E-01 | 6.72E-01 | -0.09±0.07 | 36.89 | 2.08E-01 | 7.01E-01 | -0.02±0.09 | 9.77E-01 | -0.02±0.09 | 9.62E-01 | -0.24±0.13 | 3.53E-01 | -0.22±0.12 | 4.74E-01 |
| glyco-beta-muricholate** | -0.08±0.07 | 30.5 | 2.30E-01 | 6.91E-01 | -0.09±0.07 | 39.21 | 2.00E-01 | 7.01E-01 | -0.02±0.09 | 9.67E-01 | -0.02±0.09 | 9.58E-01 | -0.21±0.13 | 4.40E-01 | -0.22±0.13 | 4.74E-01 |
| eicosenedioate (C20:1-DC)* | -0.09±0.07 | 40.08 | 1.96E-01 | 6.72E-01 | -0.09±0.07 | 33.23 | 2.21E-01 | 7.01E-01 | -0.02±0.09 | 9.74E-01 | -0.02±0.09 | 9.70E-01 | -0.21±0.12 | 3.74E-01 | -0.2±0.12 | 4.74E-01 |
| dopamine 3-O-sulfate | 0.08±0.07 | 0 | 4.55E-01 | 6.97E-01 | 0.09±0.07 | 0 | 3.39E-01 | 7.01E-01 | 0.04±0.09 | 9.27E-01 | 0.04±0.09 | 9.19E-01 | 0.15±0.12 | 5.71E-01 | 0.18±0.12 | 5.48E-01 |
| 2-hydroxynervonate* | -0.07±0.07 | 0 | 4.61E-01 | 7.33E-01 | -0.08±0.07 | 0 | 4.39E-01 | 7.01E-01 | -0.03±0.09 | 9.48E-01 | -0.04±0.09 | 9.18E-01 | -0.13±0.11 | 5.82E-01 | -0.15±0.11 | 5.87E-01 |
| p-cresol sulfate | 0.09±0.08 | 0 | 5.73E-01 | 6.91E-01 | 0.1±0.08 | 0 | 4.34E-01 | 7.01E-01 | 0.06±0.09 | 9.11E-01 | 0.06±0.1 | 9.04E-01 | 0.16±0.14 | 6.23E-01 | 0.19±0.14 | 5.97E-01 |
| phenylacetylglutamine | 0.09±0.08 | 0 | 4.71E-01 | 6.91E-01 | 0.09±0.08 | 0 | 4.63E-01 | 7.01E-01 | 0.05±0.09 | 9.27E-01 | 0.06±0.09 | 9.04E-01 | 0.17±0.14 | 5.80E-01 | 0.18±0.14 | 6.09E-01 |
| X - 24418 | -0.09±0.07 | 0 | 6.60E-01 | 6.72E-01 | -0.09±0.07 | 0 | 7.52E-01 | 7.01E-01 | -0.07±0.09 | 9.03E-01 | -0.07±0.09 | 8.91E-01 | -0.14±0.12 | 6.23E-01 | -0.12±0.13 | 7.03E-01 |
| homocitrulline | 0.09±0.07 | 0 | 6.59E-01 | 6.88E-01 | 0.09±0.07 | 0 | 7.86E-01 | 7.01E-01 | 0.06±0.09 | 9.03E-01 | 0.07±0.09 | 8.91E-01 | 0.13±0.13 | 6.29E-01 | 0.12±0.13 | 7.06E-01 |
| N-delta-acetylornithine | 0.07±0.07 | 0 | 9.50E-01 | 7.53E-01 | 0.09±0.08 | 0 | 7.78E-01 | 7.01E-01 | 0.07±0.09 | 9.01E-01 | 0.07±0.09 | 8.91E-01 | 0.06±0.13 | 8.47E-01 | 0.12±0.13 | 7.06E-01 |
| pristanate | -0.09±0.07 | 0 | 7.41E-01 | 6.72E-01 | -0.09±0.07 | 0 | 8.91E-01 | 7.01E-01 | -0.07±0.09 | 9.01E-01 | -0.08±0.09 | 8.63E-01 | -0.12±0.12 | 6.54E-01 | -0.1±0.12 | 7.47E-01 |
| X - 13844 | -0.09±0.07 | 64.63 | 9.27E-02 | 6.88E-01 | -0.09±0.07 | 55.51 | 1.34E-01 | 7.01E-01 | -0.17±0.09 | 6.10E-01 | -0.16±0.09 | 6.11E-01 | 0.1±0.13 | 7.43E-01 | 0.08±0.13 | 8.14E-01 |
| X - 12812 | -0.1±0.07 | 0 | 7.97E-01 | 6.53E-01 | -0.09±0.07 | 0 | 8.60E-01 | 7.01E-01 | -0.11±0.09 | 8.31E-01 | -0.1±0.09 | 8.36E-01 | -0.07±0.12 | 8.14E-01 | -0.07±0.12 | 8.22E-01 |
| sphingomyelin (d18:1/20:2, d18:2/20:1, d16:1/22:2)* | 0.08±0.08 | 0 | 3.37E-01 | 7.40E-01 | 0.09±0.08 | 6.42 | 3.01E-01 | 7.01E-01 | 0.13±0.1 | 7.90E-01 | 0.16±0.1 | 7.19E-01 | -0.02±0.13 | 9.61E-01 | -0.01±0.13 | 9.80E-01 |
| 1-(1-enyl-stearoyl)-GPE (P-18:0)* | 0.08±0.07 | 0 | 6.53E-01 | 7.34E-01 | 0.09±0.08 | 0 | 3.30E-01 | 7.02E-01 | 0.05±0.09 | 9.27E-01 | 0.04±0.09 | 9.18E-01 | 0.13±0.13 | 6.69E-01 | 0.21±0.14 | 5.61E-01 |
| ursodeoxycholate | 0.1±0.07 | 37.62 | 2.05E-01 | 6.29E-01 | 0.09±0.07 | 0 | 3.45E-01 | 7.02E-01 | 0.04±0.09 | 9.38E-01 | 0.04±0.09 | 9.18E-01 | 0.24±0.13 | 3.67E-01 | 0.19±0.13 | 5.64E-01 |
| X - 24456 | 0.08±0.07 | 0 | 4.87E-01 | 7.20E-01 | 0.09±0.08 | 0 | 4.27E-01 | 7.02E-01 | 0.04±0.09 | 9.36E-01 | 0.04±0.1 | 9.18E-01 | 0.15±0.13 | 5.98E-01 | 0.17±0.13 | 5.92E-01 |
| X - 21441 | 0.1±0.08 | 0 | 6.05E-01 | 6.72E-01 | 0.09±0.08 | 0 | 5.52E-01 | 7.02E-01 | 0.07±0.09 | 9.03E-01 | 0.06±0.1 | 9.00E-01 | 0.15±0.13 | 6.05E-01 | 0.16±0.14 | 6.45E-01 |
| sphinganine-1-phosphate | 0.09±0.07 | 0 | 5.97E-01 | 6.88E-01 | 0.09±0.07 | 0 | 6.12E-01 | 7.02E-01 | 0.06±0.09 | 9.03E-01 | 0.06±0.09 | 8.94E-01 | 0.15±0.14 | 6.21E-01 | 0.15±0.14 | 6.67E-01 |
| X - 24588 | 0.1±0.08 | 0 | 5.15E-01 | 6.72E-01 | 0.09±0.08 | 0 | 8.46E-01 | 7.02E-01 | 0.07±0.09 | 9.03E-01 | 0.08±0.1 | 8.68E-01 | 0.18±0.15 | 5.83E-01 | 0.12±0.14 | 7.44E-01 |
| dodecadienoate (12:2)* | -0.08±0.07 | 77.22 | 3.62E-02 | 6.91E-01 | -0.09±0.07 | 76.77 | 3.80E-02 | 7.06E-01 | 0.03±0.09 | 9.58E-01 | 0.03±0.09 | 9.55E-01 | -0.29±0.12 | 2.42E-01 | -0.3±0.13 | 4.18E-01 |
| 6-bromotryptophan | 0.08±0.07 | 0 | 8.31E-01 | 7.06E-01 | 0.09±0.07 | 0 | 8.88E-01 | 7.06E-01 | 0.07±0.09 | 9.03E-01 | 0.08±0.09 | 8.68E-01 | 0.1±0.13 | 7.28E-01 | 0.1±0.13 | 7.50E-01 |
| 4-hydroxyphenylacetylglutamine | 0.1±0.07 | 55.11 | 1.36E-01 | 6.53E-01 | 0.09±0.08 | 14.11 | 2.81E-01 | 7.07E-01 | 0.02±0.09 | 9.77E-01 | 0.03±0.09 | 9.44E-01 | 0.25±0.12 | 3.38E-01 | 0.2±0.13 | 5.04E-01 |
| X - 24462 | 0.08±0.07 | 0 | 5.29E-01 | 7.33E-01 | 0.09±0.07 | 0 | 4.95E-01 | 7.07E-01 | 0.04±0.09 | 9.27E-01 | 0.05±0.09 | 9.04E-01 | 0.14±0.13 | 6.22E-01 | 0.16±0.14 | 6.29E-01 |
| N-acetylarginine | 0.09±0.07 | 0 | 5.71E-01 | 6.89E-01 | 0.09±0.07 | 0 | 6.98E-01 | 7.07E-01 | 0.06±0.09 | 9.06E-01 | 0.07±0.09 | 8.94E-01 | 0.15±0.14 | 6.14E-01 | 0.13±0.13 | 6.91E-01 |
| C-glycosyltryptophan | 0.09±0.08 | 0 | 7.64E-01 | 6.91E-01 | 0.09±0.08 | 0 | 8.79E-01 | 7.07E-01 | 0.07±0.09 | 9.03E-01 | 0.08±0.1 | 8.68E-01 | 0.12±0.13 | 6.77E-01 | 0.1±0.13 | 7.50E-01 |
| X - 17685 | -0.08±0.07 | 51.65 | 1.50E-01 | 6.91E-01 | -0.09±0.07 | 42.63 | 1.87E-01 | 7.07E-01 | -0.16±0.09 | 6.65E-01 | -0.16±0.09 | 6.64E-01 | 0.06±0.13 | 8.47E-01 | 0.05±0.13 | 8.85E-01 |
| 3-(4-hydroxyphenyl)lactate | 0.1±0.08 | 20.86 | 2.61E-01 | 6.55E-01 | 0.09±0.08 | 0 | 4.49E-01 | 7.08E-01 | 0.04±0.1 | 9.34E-01 | 0.05±0.1 | 9.18E-01 | 0.23±0.14 | 4.32E-01 | 0.18±0.14 | 6.09E-01 |
| pregnenetriol disulfate* | 0.1±0.08 | 0 | 4.69E-01 | 6.88E-01 | 0.1±0.08 | 0 | 4.47E-01 | 7.09E-01 | 0.06±0.1 | 9.27E-01 | 0.05±0.1 | 9.18E-01 | 0.18±0.14 | 5.71E-01 | 0.19±0.14 | 6.09E-01 |
| indole-3-carboxylate | 0.09±0.08 | 75.58 | 4.30E-02 | 6.74E-01 | 0.09±0.08 | 72.8 | 5.52E-02 | 7.14E-01 | -0.01±0.09 | 9.87E-01 | -0.01±0.09 | 9.80E-01 | 0.32±0.14 | 2.42E-01 | 0.31±0.14 | 4.18E-01 |
| androsterone glucuronide | -0.09±0.08 | 73.94 | 5.01E-02 | 6.89E-01 | -0.09±0.08 | 73.97 | 5.00E-02 | 7.14E-01 | -0.19±0.09 | 5.29E-01 | -0.19±0.09 | 5.16E-01 | 0.14±0.14 | 6.66E-01 | 0.14±0.14 | 6.86E-01 |
| methyl glucopyranoside (alpha + beta) | -0.1±0.07 | 0 | 7.26E-01 | 6.29E-01 | -0.09±0.08 | 0 | 8.73E-01 | 7.14E-01 | -0.08±0.09 | 8.80E-01 | -0.08±0.09 | 8.68E-01 | -0.14±0.13 | 6.23E-01 | -0.1±0.13 | 7.50E-01 |
| X - 12456 | 0.09±0.08 | 84.58 | 1.09E-02 | 6.87E-01 | 0.09±0.08 | 72.3 | 5.74E-02 | 7.17E-01 | -0.03±0.09 | 9.48E-01 | -0.01±0.1 | 9.80E-01 | 0.41±0.14 | 1.99E-01 | 0.34±0.15 | 4.18E-01 |
| tetradecadienoate (14:2)* | -0.07±0.07 | 75.62 | 4.28E-02 | 7.40E-01 | -0.08±0.08 | 76.63 | 3.86E-02 | 7.17E-01 | 0.04±0.09 | 9.38E-01 | 0.03±0.09 | 9.31E-01 | -0.28±0.13 | 3.02E-01 | -0.3±0.13 | 4.18E-01 |
| 1-palmitoyl-2-oleoyl-GPE (16:0/18:1) | 0.1±0.08 | 78.99 | 2.91E-02 | 6.46E-01 | 0.09±0.08 | 69.47 | 7.03E-02 | 7.17E-01 | 0±0.09 | 9.92E-01 | 0±0.09 | 9.98E-01 | 0.37±0.14 | 2.03E-01 | 0.31±0.15 | 4.21E-01 |
| N-acetylglutamine | 0.08±0.07 | 60.83 | 1.10E-01 | 6.97E-01 | 0.08±0.08 | 58.6 | 1.20E-01 | 7.17E-01 | 0±0.09 | 9.89E-01 | 0±0.09 | 9.93E-01 | 0.26±0.14 | 3.42E-01 | 0.26±0.14 | 4.52E-01 |
| ethylmalonate | 0.08±0.07 | 56.07 | 1.31E-01 | 7.06E-01 | 0.08±0.07 | 46.98 | 1.70E-01 | 7.17E-01 | 0±0.09 | 9.92E-01 | 0.01±0.09 | 9.80E-01 | 0.23±0.13 | 3.61E-01 | 0.22±0.13 | 4.65E-01 |
| androstenediol (3beta,17beta) disulfate (2) | 0.09±0.08 | 53.01 | 1.45E-01 | 6.91E-01 | 0.09±0.08 | 51.04 | 1.53E-01 | 7.17E-01 | 0.01±0.1 | 9.86E-01 | 0.01±0.1 | 9.80E-01 | 0.27±0.15 | 3.66E-01 | 0.27±0.15 | 4.65E-01 |
| phenylacetylglutamate | 0.08±0.08 | 51.98 | 1.49E-01 | 7.34E-01 | 0.08±0.08 | 52.46 | 1.47E-01 | 7.17E-01 | 0±0.09 | 9.89E-01 | 0.01±0.09 | 9.80E-01 | 0.24±0.14 | 3.92E-01 | 0.25±0.14 | 4.65E-01 |
| X - 24328 | 0.11±0.09 | 51.15 | 1.53E-01 | 6.88E-01 | 0.1±0.09 | 42.71 | 1.86E-01 | 7.17E-01 | 0.01±0.11 | 9.87E-01 | 0.01±0.11 | 9.80E-01 | 0.28±0.15 | 3.65E-01 | 0.27±0.15 | 4.74E-01 |
| 1-oleoyl-2-arachidonoyl-GPE (18:1/20:4)* | 0.1±0.08 | 48.05 | 1.65E-01 | 6.74E-01 | 0.09±0.08 | 38.81 | 2.01E-01 | 7.17E-01 | 0.03±0.09 | 9.48E-01 | 0.02±0.09 | 9.62E-01 | 0.27±0.15 | 3.67E-01 | 0.24±0.15 | 4.92E-01 |
| kynurenine | 0.09±0.08 | 65.11 | 9.04E-02 | 6.88E-01 | 0.09±0.08 | 26.85 | 2.42E-01 | 7.17E-01 | 0.01±0.09 | 9.87E-01 | 0.03±0.1 | 9.45E-01 | 0.3±0.15 | 3.25E-01 | 0.23±0.15 | 4.95E-01 |
| heneicosapentaenoate (21:5n3) | -0.08±0.07 | 36.5 | 2.10E-01 | 6.97E-01 | -0.08±0.08 | 13.24 | 2.83E-01 | 7.17E-01 | -0.02±0.09 | 9.74E-01 | -0.03±0.09 | 9.55E-01 | -0.22±0.13 | 4.32E-01 | -0.2±0.13 | 5.37E-01 |
| sphingomyelin (d18:0/20:0, d16:0/22:0)* | 0.09±0.08 | 27.17 | 2.41E-01 | 6.91E-01 | 0.09±0.08 | 0 | 3.38E-01 | 7.17E-01 | 0.03±0.09 | 9.48E-01 | 0.04±0.1 | 9.19E-01 | 0.23±0.15 | 4.56E-01 | 0.2±0.14 | 5.77E-01 |
| 2-aminoheptanoate | 0.09±0.07 | 0 | 3.74E-01 | 6.88E-01 | 0.08±0.07 | 0 | 4.60E-01 | 7.17E-01 | 0.04±0.09 | 9.34E-01 | 0.04±0.09 | 9.18E-01 | 0.18±0.13 | 5.30E-01 | 0.16±0.13 | 6.22E-01 |
| mannitol/sorbitol | 0.08±0.07 | 0 | 4.24E-01 | 7.05E-01 | 0.08±0.07 | 0 | 4.74E-01 | 7.17E-01 | 0.04±0.09 | 9.38E-01 | 0.04±0.09 | 9.18E-01 | 0.15±0.12 | 5.70E-01 | 0.14±0.12 | 6.23E-01 |
| glycerophosphorylcholine (GPC) | 0.07±0.08 | 0 | 7.66E-01 | 7.65E-01 | 0.09±0.08 | 0 | 4.34E-01 | 7.17E-01 | 0.05±0.09 | 9.27E-01 | 0.05±0.09 | 9.18E-01 | 0.1±0.14 | 7.39E-01 | 0.19±0.15 | 6.23E-01 |
| 1-palmitoyl-2-dihomo-linolenoyl-GPC (16:0/20:3n3 or 6)* | -0.09±0.08 | 74.01 | 4.98E-02 | 7.06E-01 | -0.09±0.08 | 72.4 | 5.70E-02 | 7.17E-01 | -0.17±0.09 | 6.26E-01 | -0.17±0.09 | 6.01E-01 | 0.22±0.17 | 5.80E-01 | 0.19±0.17 | 6.46E-01 |
| 3-methylcytidine | 0.09±0.08 | 0 | 5.34E-01 | 6.91E-01 | 0.09±0.08 | 0 | 5.32E-01 | 7.17E-01 | 0.06±0.09 | 9.27E-01 | 0.05±0.09 | 9.04E-01 | 0.16±0.14 | 6.04E-01 | 0.16±0.14 | 6.46E-01 |
| 1-oleoyl-GPI (18:1) | -0.08±0.07 | 74.76 | 4.66E-02 | 7.20E-01 | -0.08±0.07 | 76.86 | 3.76E-02 | 7.17E-01 | -0.18±0.09 | 5.82E-01 | -0.18±0.09 | 5.12E-01 | 0.14±0.13 | 6.31E-01 | 0.15±0.13 | 6.56E-01 |
| p-cresol glucuronide* | 0.08±0.08 | 0 | 6.44E-01 | 7.05E-01 | 0.08±0.08 | 0 | 5.92E-01 | 7.17E-01 | 0.06±0.09 | 9.06E-01 | 0.06±0.09 | 9.04E-01 | 0.14±0.14 | 6.54E-01 | 0.14±0.14 | 6.67E-01 |
| 1-stearoyl-2-docosahexaenoyl-GPC (18:0/22:6) | -0.09±0.07 | 0 | 5.98E-01 | 6.72E-01 | -0.08±0.07 | 0 | 6.64E-01 | 7.17E-01 | -0.06±0.09 | 9.06E-01 | -0.05±0.09 | 9.04E-01 | -0.14±0.12 | 6.00E-01 | -0.12±0.12 | 6.77E-01 |
| X - 24812 | -0.06±0.07 | 0 | 5.75E-01 | 7.92E-01 | -0.1±0.09 | 0 | 6.88E-01 | 7.17E-01 | -0.03±0.09 | 9.49E-01 | -0.07±0.11 | 8.94E-01 | -0.12±0.13 | 6.82E-01 | -0.15±0.15 | 6.88E-01 |
| 3-(3-amino-3-carboxypropyl)uridine* | 0.09±0.08 | 0 | 5.99E-01 | 6.87E-01 | 0.09±0.08 | 0 | 7.51E-01 | 7.17E-01 | 0.06±0.09 | 9.06E-01 | 0.07±0.09 | 8.94E-01 | 0.15±0.13 | 6.14E-01 | 0.12±0.13 | 7.13E-01 |
| taurocholate | -0.07±0.07 | 0 | 9.93E-01 | 7.44E-01 | -0.08±0.07 | 0 | 7.59E-01 | 7.17E-01 | -0.07±0.09 | 9.01E-01 | -0.06±0.09 | 8.94E-01 | -0.07±0.12 | 8.14E-01 | -0.11±0.13 | 7.24E-01 |
| 1-methyl-5-imidazolelactate | -0.06±0.07 | 0 | 9.90E-01 | 7.86E-01 | -0.08±0.08 | 0 | 7.62E-01 | 7.17E-01 | -0.06±0.09 | 9.13E-01 | -0.06±0.1 | 8.94E-01 | -0.06±0.12 | 8.51E-01 | -0.11±0.13 | 7.24E-01 |
| X - 12906 | -0.09±0.07 | 0 | 6.52E-01 | 6.72E-01 | -0.08±0.07 | 0 | 8.28E-01 | 7.17E-01 | -0.07±0.09 | 9.03E-01 | -0.07±0.09 | 8.94E-01 | -0.13±0.12 | 6.14E-01 | -0.1±0.12 | 7.41E-01 |
| X - 21470 | 0.09±0.08 | 0 | 9.08E-01 | 6.88E-01 | 0.09±0.08 | 0 | 9.35E-01 | 7.17E-01 | 0.09±0.1 | 8.80E-01 | 0.09±0.1 | 8.68E-01 | 0.1±0.13 | 7.29E-01 | 0.1±0.13 | 7.64E-01 |
| glycerophosphoethanolamine | 0.07±0.07 | 0 | 9.50E-01 | 7.40E-01 | 0.08±0.07 | 0 | 8.28E-01 | 7.17E-01 | 0.07±0.09 | 8.80E-01 | 0.07±0.09 | 8.92E-01 | 0.06±0.14 | 8.51E-01 | 0.11±0.14 | 7.64E-01 |
| cholesterol sulfate | -0.09±0.08 | 14.72 | 2.79E-01 | 7.05E-01 | -0.09±0.08 | 58.78 | 1.19E-01 | 7.17E-01 | -0.15±0.1 | 7.50E-01 | -0.18±0.1 | 6.15E-01 | 0.03±0.13 | 9.40E-01 | 0.08±0.14 | 8.03E-01 |
| arginine | -0.08±0.07 | 15.08 | 2.78E-01 | 7.08E-01 | -0.08±0.07 | 46.54 | 1.71E-01 | 7.17E-01 | -0.13±0.09 | 7.71E-01 | -0.15±0.09 | 6.80E-01 | 0.04±0.13 | 9.20E-01 | 0.07±0.14 | 8.25E-01 |
| N-stearoyl-sphingadienine (d18:2/18:0)* | 0.09±0.08 | 0 | 9.80E-01 | 6.91E-01 | 0.09±0.08 | 0 | 7.76E-01 | 7.17E-01 | 0.09±0.1 | 8.73E-01 | 0.1±0.1 | 8.36E-01 | 0.09±0.14 | 7.66E-01 | 0.06±0.13 | 8.79E-01 |
| hexanoylglutamine | 0.09±0.08 | 0 | 8.59E-01 | 6.75E-01 | 0.08±0.08 | 0 | 7.59E-01 | 7.17E-01 | 0.1±0.1 | 8.65E-01 | 0.1±0.1 | 8.36E-01 | 0.08±0.13 | 8.13E-01 | 0.05±0.13 | 8.95E-01 |
| docosapentaenoate (n6 DPA; 22:5n6) | -0.07±0.07 | 0 | 5.44E-01 | 7.48E-01 | -0.08±0.07 | 0 | 6.66E-01 | 7.17E-01 | -0.1±0.09 | 8.65E-01 | -0.1±0.09 | 8.36E-01 | -0.01±0.12 | 9.84E-01 | -0.04±0.12 | 9.13E-01 |
| alpha-ketobutyrate | 0.1±0.08 | 0 | 9.61E-01 | 6.57E-01 | 0.09±0.08 | 0 | 6.28E-01 | 7.17E-01 | 0.1±0.09 | 8.65E-01 | 0.12±0.1 | 8.36E-01 | 0.1±0.13 | 7.28E-01 | 0.04±0.13 | 9.28E-01 |
| 1,7-dimethylurate | -0.07±0.07 | 37.34 | 2.06E-01 | 7.34E-01 | -0.08±0.07 | 0 | 3.20E-01 | 7.17E-01 | -0.13±0.08 | 7.50E-01 | -0.13±0.09 | 7.37E-01 | 0.07±0.13 | 8.31E-01 | 0.03±0.13 | 9.34E-01 |
| arachidoylcarnitine (C20)* | -0.09±0.07 | 0 | 5.17E-01 | 6.72E-01 | -0.08±0.08 | 0.68 | 3.16E-01 | 7.17E-01 | -0.13±0.09 | 7.90E-01 | -0.14±0.09 | 7.37E-01 | -0.02±0.13 | 9.54E-01 | 0.03±0.14 | 9.43E-01 |
| theophylline | -0.07±0.07 | 19.51 | 2.65E-01 | 7.39E-01 | -0.08±0.07 | 0 | 3.36E-01 | 7.17E-01 | -0.12±0.09 | 7.81E-01 | -0.13±0.09 | 7.37E-01 | 0.05±0.13 | 8.98E-01 | 0.02±0.13 | 9.58E-01 |
| N-palmitoyl-sphingadienine (d18:2/16:0)* | -0.08±0.07 | 0 | 4.83E-01 | 6.97E-01 | -0.08±0.07 | 0 | 4.19E-01 | 7.17E-01 | -0.11±0.09 | 7.90E-01 | -0.12±0.09 | 7.76E-01 | -0.01±0.12 | 9.86E-01 | 0±0.12 | 9.92E-01 |
| 2,3-dihydroxypyridine | -0.08±0.07 | 0 | 4.10E-01 | 7.17E-01 | -0.08±0.07 | 0 | 4.71E-01 | 7.17E-01 | -0.12±0.09 | 7.90E-01 | -0.12±0.09 | 7.89E-01 | 0.01±0.13 | 9.86E-01 | 0±0.13 | 9.92E-01 |
| sphingomyelin (d18:1/19:0, d19:1/18:0)* | 0.07±0.08 | 0 | 4.30E-01 | 7.84E-01 | 0.09±0.08 | 0 | 4.95E-01 | 7.20E-01 | 0.03±0.1 | 9.59E-01 | 0.05±0.1 | 9.18E-01 | 0.17±0.15 | 6.14E-01 | 0.17±0.15 | 6.45E-01 |
| X - 25520 | 0.06±0.07 | 78.39 | 3.14E-02 | 7.92E-01 | 0.08±0.07 | 83.73 | 1.32E-02 | 7.27E-01 | -0.05±0.09 | 9.27E-01 | -0.05±0.09 | 9.18E-01 | 0.28±0.13 | 3.02E-01 | 0.34±0.13 | 3.58E-01 |
| 1-palmitoyl-2-docosahexaenoyl-GPE (16:0/22:6)* | -0.08±0.07 | 0 | 8.21E-01 | 7.33E-01 | -0.08±0.07 | 0 | 8.61E-01 | 7.27E-01 | -0.09±0.09 | 8.69E-01 | -0.09±0.09 | 8.47E-01 | -0.05±0.13 | 8.90E-01 | -0.06±0.13 | 8.53E-01 |
| 1-methylxanthine | -0.07±0.07 | 17.09 | 2.72E-01 | 7.53E-01 | -0.08±0.07 | 26.41 | 2.44E-01 | 7.27E-01 | -0.12±0.09 | 7.90E-01 | -0.13±0.09 | 7.26E-01 | 0.05±0.13 | 9.00E-01 | 0.05±0.13 | 8.95E-01 |
| X - 13431 | -0.09±0.07 | 13.27 | 2.83E-01 | 6.72E-01 | -0.08±0.07 | 32.59 | 2.23E-01 | 7.27E-01 | -0.15±0.09 | 7.16E-01 | -0.14±0.09 | 7.19E-01 | 0.02±0.13 | 9.75E-01 | 0.05±0.13 | 8.98E-01 |
| X - 24970 | -0.08±0.07 | 0 | 5.38E-01 | 7.10E-01 | -0.08±0.07 | 0 | 5.08E-01 | 7.27E-01 | -0.11±0.09 | 8.31E-01 | -0.11±0.09 | 8.35E-01 | -0.01±0.13 | 9.81E-01 | -0.01±0.13 | 9.85E-01 |
| X - 23780 | -0.07±0.07 | 0 | 4.95E-01 | 7.34E-01 | -0.08±0.07 | 0 | 4.65E-01 | 7.27E-01 | -0.1±0.09 | 8.31E-01 | -0.11±0.09 | 8.25E-01 | 0±0.12 | 9.96E-01 | 0±0.13 | 9.98E-01 |
| X - 16397 | -0.07±0.07 | 0 | 6.80E-01 | 7.44E-01 | -0.07±0.07 | 0 | 5.96E-01 | 7.31E-01 | -0.05±0.09 | 9.27E-01 | -0.05±0.09 | 9.18E-01 | -0.11±0.12 | 6.82E-01 | -0.13±0.12 | 6.67E-01 |
| X - 21834 | -0.07±0.07 | 0 | 8.17E-01 | 7.33E-01 | -0.08±0.07 | 0 | 9.35E-01 | 7.31E-01 | -0.06±0.09 | 9.03E-01 | -0.07±0.09 | 8.91E-01 | -0.1±0.12 | 7.29E-01 | -0.08±0.12 | 7.82E-01 |
| glycolithocholate | 0.08±0.07 | 0 | 8.53E-01 | 7.12E-01 | 0.08±0.07 | 0 | 7.79E-01 | 7.31E-01 | 0.09±0.09 | 8.69E-01 | 0.09±0.09 | 8.36E-01 | 0.06±0.12 | 8.47E-01 | 0.05±0.12 | 8.85E-01 |
| heptenedioate (C7:1-DC)* | 0.07±0.08 | 13.02 | 2.84E-01 | 7.53E-01 | 0.08±0.08 | 0 | 3.27E-01 | 7.33E-01 | 0.02±0.09 | 9.77E-01 | 0.03±0.09 | 9.32E-01 | 0.2±0.14 | 5.31E-01 | 0.2±0.14 | 5.77E-01 |
| acetylcarnitine (C2) | 0.09±0.08 | 0 | 6.37E-01 | 6.91E-01 | 0.08±0.08 | 0 | 7.70E-01 | 7.33E-01 | 0.06±0.09 | 9.03E-01 | 0.06±0.09 | 8.94E-01 | 0.14±0.14 | 6.48E-01 | 0.11±0.14 | 7.44E-01 |
| ximenoylcarnitine (C26:1)* | -0.08±0.07 | 0 | 7.06E-01 | 7.06E-01 | -0.08±0.07 | 0 | 6.39E-01 | 7.33E-01 | -0.1±0.09 | 8.65E-01 | -0.1±0.09 | 8.36E-01 | -0.04±0.12 | 9.05E-01 | -0.03±0.13 | 9.34E-01 |
| sphingomyelin (d18:2/18:1)* | -0.1±0.08 | 0 | 3.92E-01 | 6.74E-01 | -0.08±0.08 | 0 | 3.68E-01 | 7.33E-01 | -0.15±0.1 | 7.71E-01 | -0.14±0.1 | 7.69E-01 | -0.01±0.13 | 9.84E-01 | 0±0.13 | 9.92E-01 |
| hexadecadienoate (16:2n6) | -0.08±0.08 | 56.43 | 1.30E-01 | 7.33E-01 | -0.08±0.08 | 61.26 | 1.08E-01 | 7.35E-01 | 0±0.1 | 9.89E-01 | 0.01±0.1 | 9.80E-01 | -0.25±0.13 | 3.67E-01 | -0.26±0.14 | 4.52E-01 |
| 5-hydroxyindole sulfate | 0.07±0.08 | 62.97 | 1.00E-01 | 7.59E-01 | 0.08±0.08 | 60.13 | 1.13E-01 | 7.35E-01 | -0.01±0.09 | 9.79E-01 | 0±0.09 | 9.94E-01 | 0.26±0.14 | 3.57E-01 | 0.26±0.14 | 4.54E-01 |
| 1-palmitoyl-GPC (16:0) | 0.07±0.08 | 0 | 8.08E-01 | 7.62E-01 | 0.08±0.08 | 0 | 5.19E-01 | 7.35E-01 | 0.06±0.09 | 9.10E-01 | 0.05±0.09 | 9.13E-01 | 0.1±0.14 | 7.66E-01 | 0.17±0.16 | 6.67E-01 |
| X - 21607 | -0.07±0.07 | 0 | 6.20E-01 | 7.33E-01 | -0.07±0.07 | 0 | 6.92E-01 | 7.35E-01 | -0.05±0.09 | 9.27E-01 | -0.05±0.09 | 9.04E-01 | -0.12±0.12 | 6.54E-01 | -0.11±0.12 | 7.12E-01 |
| glycoursodeoxycholic acid sulfate (1) | 0.09±0.07 | 0 | 5.90E-01 | 6.88E-01 | 0.08±0.08 | 0 | 7.66E-01 | 7.35E-01 | 0.06±0.09 | 9.06E-01 | 0.06±0.09 | 8.94E-01 | 0.14±0.13 | 6.11E-01 | 0.11±0.13 | 7.41E-01 |
| sebacate (C10-DC) | 0.08±0.07 | 0 | 7.29E-01 | 6.97E-01 | 0.08±0.07 | 0 | 7.79E-01 | 7.35E-01 | 0.06±0.09 | 9.03E-01 | 0.06±0.09 | 8.94E-01 | 0.12±0.12 | 6.68E-01 | 0.11±0.13 | 7.47E-01 |
| tridecenedioate (C13:1-DC)* | 0.07±0.08 | 0 | 7.97E-01 | 7.48E-01 | 0.08±0.08 | 0 | 8.00E-01 | 7.35E-01 | 0.06±0.09 | 9.20E-01 | 0.06±0.09 | 8.94E-01 | 0.1±0.13 | 7.38E-01 | 0.1±0.13 | 7.50E-01 |
| taurodeoxycholic acid 3-sulfate | -0.07±0.07 | 0 | 9.83E-01 | 7.39E-01 | -0.07±0.07 | 0 | 8.78E-01 | 7.35E-01 | -0.07±0.09 | 9.01E-01 | -0.07±0.09 | 8.94E-01 | -0.07±0.13 | 8.09E-01 | -0.09±0.13 | 7.81E-01 |
| X - 11852 | -0.07±0.07 | 0 | 8.41E-01 | 7.59E-01 | -0.08±0.07 | 0 | 9.00E-01 | 7.35E-01 | -0.08±0.09 | 8.80E-01 | -0.08±0.09 | 8.63E-01 | -0.05±0.13 | 9.04E-01 | -0.06±0.13 | 8.50E-01 |
| cysteine sulfinic acid | 0.07±0.07 | 0 | 6.26E-01 | 7.48E-01 | 0.08±0.08 | 0 | 7.61E-01 | 7.35E-01 | 0.1±0.09 | 8.65E-01 | 0.1±0.1 | 8.36E-01 | 0.02±0.12 | 9.61E-01 | 0.05±0.13 | 9.01E-01 |
| N-stearoyltaurine | -0.06±0.07 | 0 | 7.15E-01 | 7.85E-01 | -0.07±0.07 | 0 | 7.50E-01 | 7.35E-01 | -0.08±0.09 | 8.80E-01 | -0.09±0.09 | 8.36E-01 | -0.02±0.12 | 9.54E-01 | -0.04±0.12 | 9.08E-01 |
| glycine | -0.09±0.07 | 0 | 9.94E-01 | 6.89E-01 | -0.08±0.08 | 0 | 6.14E-01 | 7.35E-01 | -0.09±0.1 | 8.80E-01 | -0.11±0.1 | 8.36E-01 | -0.09±0.11 | 7.38E-01 | -0.03±0.12 | 9.28E-01 |
| 5-hydroxyhexanoate | 0.08±0.07 | 0 | 6.28E-01 | 7.33E-01 | 0.08±0.07 | 0 | 6.69E-01 | 7.35E-01 | 0.1±0.09 | 8.65E-01 | 0.1±0.09 | 8.36E-01 | 0.03±0.12 | 9.40E-01 | 0.03±0.13 | 9.28E-01 |
| isovalerylcarnitine (C5) | -0.07±0.08 | 1.76 | 3.13E-01 | 7.62E-01 | -0.08±0.08 | 0 | 4.58E-01 | 7.35E-01 | -0.12±0.09 | 7.90E-01 | -0.12±0.1 | 8.25E-01 | 0.04±0.13 | 9.20E-01 | -0.01±0.12 | 9.90E-01 |
| 1-stearoyl-GPE (18:0) | 0.06±0.08 | 0 | 7.85E-01 | 7.85E-01 | 0.08±0.08 | 0 | 5.32E-01 | 7.38E-01 | 0.05±0.09 | 9.27E-01 | 0.05±0.09 | 9.18E-01 | 0.09±0.14 | 7.66E-01 | 0.16±0.14 | 6.67E-01 |
| creatine | -0.06±0.08 | 77.89 | 3.34E-02 | 7.85E-01 | -0.08±0.08 | 82.01 | 1.84E-02 | 7.40E-01 | 0.07±0.1 | 9.06E-01 | 0.07±0.1 | 8.94E-01 | -0.28±0.13 | 3.04E-01 | -0.31±0.13 | 4.03E-01 |
| aconitate [cis or trans] | 0.09±0.08 | 82.71 | 1.62E-02 | 6.91E-01 | 0.08±0.08 | 71.83 | 5.95E-02 | 7.40E-01 | -0.02±0.09 | 9.71E-01 | -0.01±0.09 | 9.80E-01 | 0.42±0.16 | 2.03E-01 | 0.34±0.16 | 4.21E-01 |
| indoleacetoylcarnitine* | 0.09±0.07 | 69.27 | 7.12E-02 | 6.88E-01 | 0.07±0.07 | 62.16 | 1.04E-01 | 7.40E-01 | 0±0.09 | 9.89E-01 | -0.01±0.09 | 9.80E-01 | 0.28±0.13 | 3.08E-01 | 0.24±0.13 | 4.52E-01 |
| 1-oleoyl-GPE (18:1) | 0.07±0.08 | 53.04 | 1.44E-01 | 7.44E-01 | 0.08±0.08 | 61.94 | 1.05E-01 | 7.40E-01 | 0±0.09 | 9.89E-01 | 0±0.09 | 9.98E-01 | 0.25±0.14 | 3.97E-01 | 0.28±0.15 | 4.52E-01 |
| metabolonic lactone sulfate | 0.09±0.07 | 74.88 | 4.60E-02 | 6.87E-01 | 0.08±0.08 | 40.68 | 1.94E-01 | 7.40E-01 | -0.01±0.09 | 9.84E-01 | 0.01±0.1 | 9.86E-01 | 0.3±0.13 | 2.55E-01 | 0.22±0.14 | 4.95E-01 |
| 1-palmitoyl-2-oleoyl-GPC (16:0/18:1) | 0.08±0.08 | 4.06 | 3.07E-01 | 7.08E-01 | 0.08±0.08 | 30.57 | 2.30E-01 | 7.40E-01 | 0.04±0.09 | 9.38E-01 | 0.02±0.09 | 9.62E-01 | 0.21±0.15 | 5.23E-01 | 0.24±0.15 | 5.10E-01 |
| 3-hydroxylaurate | -0.06±0.07 | 0 | 3.89E-01 | 7.85E-01 | -0.08±0.08 | 10.28 | 2.91E-01 | 7.40E-01 | -0.01±0.09 | 9.78E-01 | -0.02±0.09 | 9.63E-01 | -0.15±0.13 | 6.04E-01 | -0.19±0.13 | 5.70E-01 |
| 5-oxoproline | 0.06±0.07 | 0 | 8.44E-01 | 7.74E-01 | 0.07±0.07 | 0 | 7.16E-01 | 7.40E-01 | 0.05±0.09 | 9.27E-01 | 0.06±0.09 | 9.04E-01 | 0.09±0.13 | 7.78E-01 | 0.11±0.13 | 7.25E-01 |
| 1-(1-enyl-palmitoyl)-2-linoleoyl-GPE (P-16:0/18:2)* | -0.07±0.07 | 57.22 | 1.26E-01 | 7.34E-01 | -0.07±0.07 | 62.6 | 1.02E-01 | 7.40E-01 | -0.15±0.09 | 7.01E-01 | -0.15±0.09 | 6.48E-01 | 0.1±0.13 | 7.57E-01 | 0.1±0.13 | 7.50E-01 |
| carotene diol (2) | -0.09±0.07 | 0 | 3.52E-01 | 6.72E-01 | -0.08±0.08 | 58.04 | 1.23E-01 | 7.40E-01 | -0.14±0.09 | 7.50E-01 | -0.16±0.09 | 6.80E-01 | 0±0.13 | 9.96E-01 | 0.1±0.14 | 7.64E-01 |
| 2-hydroxyoctanoate | -0.07±0.07 | 19.77 | 2.64E-01 | 7.44E-01 | -0.07±0.08 | 7.29 | 2.99E-01 | 7.40E-01 | -0.14±0.09 | 7.81E-01 | -0.13±0.09 | 7.62E-01 | 0.04±0.12 | 9.20E-01 | 0.03±0.13 | 9.31E-01 |
| 3-hydroxyphenylacetoylglutamine | 0.06±0.07 | 0 | 5.66E-01 | 7.79E-01 | 0.07±0.07 | 0 | 5.18E-01 | 7.40E-01 | 0.09±0.09 | 8.65E-01 | 0.11±0.09 | 8.36E-01 | 0.01±0.12 | 9.92E-01 | 0.01±0.13 | 9.85E-01 |
| 2'-deoxyuridine | 0.07±0.07 | 0 | 8.75E-01 | 7.44E-01 | 0.07±0.07 | 0 | 8.49E-01 | 7.40E-01 | 0.06±0.09 | 9.06E-01 | 0.06±0.09 | 8.94E-01 | 0.09±0.12 | 7.66E-01 | 0.09±0.12 | 7.64E-01 |
| 3-indoxyl sulfate | 0.07±0.07 | 0 | 6.61E-01 | 7.40E-01 | 0.07±0.08 | 0 | 8.79E-01 | 7.40E-01 | 0.05±0.09 | 9.27E-01 | 0.07±0.09 | 8.94E-01 | 0.12±0.13 | 6.77E-01 | 0.09±0.13 | 7.82E-01 |
| guanidinoacetate | -0.08±0.07 | 0 | 5.32E-01 | 7.33E-01 | -0.07±0.08 | 12.87 | 2.84E-01 | 7.40E-01 | -0.11±0.09 | 8.31E-01 | -0.13±0.09 | 7.62E-01 | -0.01±0.12 | 9.84E-01 | 0.04±0.13 | 9.11E-01 |
| sulfate of piperine metabolite C16H19NO3 (3)* | -0.05±0.07 | 0 | 4.92E-01 | 8.16E-01 | -0.08±0.08 | 0 | 7.43E-01 | 7.40E-01 | -0.08±0.09 | 8.80E-01 | -0.09±0.1 | 8.38E-01 | 0.03±0.13 | 9.54E-01 | -0.04±0.13 | 9.19E-01 |
| isobutyrylglycine | -0.07±0.07 | 0 | 6.81E-01 | 7.33E-01 | -0.07±0.07 | 0 | 6.10E-01 | 7.47E-01 | -0.09±0.09 | 8.65E-01 | -0.1±0.09 | 8.36E-01 | -0.03±0.12 | 9.32E-01 | -0.02±0.13 | 9.72E-01 |
| dihydroorotate | -0.07±0.07 | 87.74 | 4.29E-03 | 7.67E-01 | -0.07±0.07 | 84.67 | 1.06E-02 | 7.47E-01 | -0.2±0.09 | 3.95E-01 | -0.2±0.09 | 4.14E-01 | 0.27±0.14 | 3.42E-01 | 0.22±0.14 | 4.95E-01 |
| N-acetylserine | 0.07±0.08 | 67.89 | 7.76E-02 | 7.74E-01 | 0.08±0.08 | 61.4 | 1.08E-01 | 7.51E-01 | -0.01±0.09 | 9.86E-01 | 0±0.1 | 9.95E-01 | 0.32±0.16 | 3.42E-01 | 0.31±0.17 | 4.54E-01 |
| picolinoylglycine | 0.09±0.07 | 75.63 | 4.28E-02 | 6.88E-01 | 0.07±0.08 | 50.7 | 1.54E-01 | 7.51E-01 | -0.02±0.09 | 9.70E-01 | 0±0.1 | 9.93E-01 | 0.29±0.12 | 2.50E-01 | 0.23±0.13 | 4.74E-01 |
| N-acetylcitrulline | 0.08±0.07 | 61.02 | 1.09E-01 | 7.06E-01 | 0.07±0.07 | 23.09 | 2.54E-01 | 7.51E-01 | 0±0.09 | 9.92E-01 | 0.01±0.09 | 9.80E-01 | 0.26±0.13 | 3.42E-01 | 0.2±0.14 | 5.48E-01 |
| gluconate | 0.06±0.08 | 0 | 4.44E-01 | 7.85E-01 | 0.08±0.08 | 0 | 5.93E-01 | 7.51E-01 | 0.03±0.09 | 9.48E-01 | 0.05±0.09 | 9.18E-01 | 0.17±0.16 | 6.25E-01 | 0.15±0.16 | 7.00E-01 |
| X - 22162 | 0.06±0.07 | 0 | 7.83E-01 | 7.85E-01 | 0.07±0.07 | 0 | 7.32E-01 | 7.51E-01 | 0.05±0.09 | 9.27E-01 | 0.05±0.09 | 9.04E-01 | 0.09±0.13 | 7.66E-01 | 0.11±0.13 | 7.44E-01 |
| urea | 0.08±0.07 | 0 | 5.99E-01 | 7.33E-01 | 0.07±0.07 | 0 | 8.24E-01 | 7.51E-01 | 0.05±0.09 | 9.27E-01 | 0.06±0.09 | 8.94E-01 | 0.13±0.13 | 6.44E-01 | 0.1±0.13 | 7.64E-01 |
| 7-methylxanthine | -0.07±0.07 | 45.55 | 1.75E-01 | 7.48E-01 | -0.07±0.07 | 47.45 | 1.68E-01 | 7.51E-01 | -0.13±0.09 | 7.50E-01 | -0.14±0.09 | 7.20E-01 | 0.07±0.13 | 8.14E-01 | 0.08±0.13 | 8.03E-01 |
| X - 23639 | -0.07±0.07 | 0 | 5.23E-01 | 7.33E-01 | -0.07±0.07 | 0 | 3.18E-01 | 7.60E-01 | -0.11±0.09 | 8.31E-01 | -0.12±0.09 | 7.76E-01 | -0.01±0.12 | 9.84E-01 | 0.03±0.13 | 9.29E-01 |
| threonate | -0.09±0.07 | 0 | 4.79E-01 | 6.88E-01 | -0.07±0.07 | 0 | 4.03E-01 | 7.62E-01 | -0.12±0.09 | 7.90E-01 | -0.11±0.09 | 8.32E-01 | -0.01±0.12 | 9.84E-01 | 0.02±0.13 | 9.59E-01 |
| cerotoylcarnitine (C26)* | -0.08±0.07 | 8.87 | 2.95E-01 | 7.08E-01 | -0.07±0.07 | 30.08 | 2.32E-01 | 7.67E-01 | -0.13±0.09 | 7.71E-01 | -0.13±0.09 | 7.54E-01 | 0.03±0.13 | 9.38E-01 | 0.06±0.13 | 8.60E-01 |
| X - 25420 | 0.07±0.08 | 83.95 | 1.25E-02 | 7.53E-01 | 0.08±0.08 | 87.22 | 5.16E-03 | 7.68E-01 | -0.06±0.1 | 9.20E-01 | -0.08±0.1 | 8.87E-01 | 0.37±0.14 | 2.03E-01 | 0.42±0.15 | 3.28E-01 |
| N-acetylasparagine | 0.07±0.07 | 86.52 | 6.45E-03 | 7.55E-01 | 0.07±0.07 | 86.7 | 6.11E-03 | 7.68E-01 | -0.07±0.09 | 9.03E-01 | -0.07±0.09 | 8.94E-01 | 0.36±0.13 | 1.99E-01 | 0.37±0.13 | 3.42E-01 |
| 1-linoleoyl-GPE (18:2)* | 0.06±0.07 | 0 | 4.86E-01 | 7.92E-01 | 0.07±0.08 | 0 | 3.35E-01 | 7.68E-01 | 0.02±0.09 | 9.59E-01 | 0.02±0.09 | 9.58E-01 | 0.14±0.14 | 6.54E-01 | 0.19±0.14 | 5.97E-01 |
| 6-hydroxyindole sulfate | 0.07±0.07 | 0 | 6.93E-01 | 7.65E-01 | 0.07±0.07 | 0 | 8.89E-01 | 7.68E-01 | 0.05±0.09 | 9.27E-01 | 0.06±0.09 | 8.94E-01 | 0.11±0.13 | 7.08E-01 | 0.09±0.13 | 7.92E-01 |
| glycocholate | -0.06±0.07 | 0 | 8.10E-01 | 7.85E-01 | -0.07±0.07 | 0 | 9.27E-01 | 7.68E-01 | -0.07±0.09 | 9.03E-01 | -0.06±0.09 | 8.94E-01 | -0.04±0.12 | 9.20E-01 | -0.08±0.13 | 8.00E-01 |
| X - 12844 | -0.06±0.07 | 0 | 5.91E-01 | 7.67E-01 | -0.06±0.07 | 0 | 6.52E-01 | 7.71E-01 | -0.09±0.09 | 8.65E-01 | -0.09±0.09 | 8.36E-01 | -0.01±0.12 | 9.86E-01 | -0.02±0.12 | 9.56E-01 |
| pantothenate | 0.06±0.07 | 0 | 5.33E-01 | 7.85E-01 | 0.07±0.07 | 0 | 6.41E-01 | 7.73E-01 | 0.03±0.09 | 9.59E-01 | 0.04±0.09 | 9.18E-01 | 0.12±0.12 | 6.60E-01 | 0.11±0.12 | 7.06E-01 |
| N6-carbamoylthreonyladenosine | 0.08±0.08 | 50.22 | 1.56E-01 | 7.48E-01 | 0.07±0.08 | 29.96 | 2.32E-01 | 7.73E-01 | 0.01±0.09 | 9.89E-01 | 0.01±0.1 | 9.80E-01 | 0.26±0.15 | 4.12E-01 | 0.22±0.15 | 5.37E-01 |
| N-acetylproline | 0.08±0.08 | 38.19 | 2.03E-01 | 7.44E-01 | 0.07±0.08 | 18.66 | 2.68E-01 | 7.74E-01 | 0.01±0.09 | 9.86E-01 | 0.01±0.09 | 9.80E-01 | 0.23±0.14 | 4.63E-01 | 0.21±0.14 | 5.70E-01 |
| xanthurenate | 0.08±0.08 | 83.45 | 1.40E-02 | 7.39E-01 | 0.07±0.08 | 76.91 | 3.74E-02 | 7.85E-01 | -0.05±0.09 | 9.27E-01 | -0.04±0.1 | 9.19E-01 | 0.37±0.14 | 2.03E-01 | 0.32±0.14 | 4.18E-01 |
| methyl indole-3-acetate | 0.08±0.07 | 71.83 | 5.96E-02 | 7.20E-01 | 0.07±0.07 | 62.19 | 1.04E-01 | 7.90E-01 | -0.02±0.09 | 9.71E-01 | -0.02±0.09 | 9.66E-01 | 0.28±0.13 | 3.08E-01 | 0.24±0.13 | 4.63E-01 |
| asparagine | 0.06±0.08 | 0 | 4.65E-01 | 7.85E-01 | 0.07±0.08 | 18.87 | 2.67E-01 | 7.90E-01 | 0.03±0.09 | 9.48E-01 | 0.02±0.09 | 9.58E-01 | 0.16±0.15 | 6.35E-01 | 0.23±0.16 | 5.77E-01 |
| X - 12680 | -0.07±0.08 | 84.82 | 1.03E-02 | 7.59E-01 | -0.07±0.08 | 83.88 | 1.28E-02 | 7.91E-01 | -0.2±0.09 | 4.52E-01 | -0.2±0.09 | 4.89E-01 | 0.23±0.14 | 4.40E-01 | 0.22±0.14 | 4.95E-01 |
| N-palmitoylserine | 0.08±0.08 | 0 | 7.54E-01 | 7.34E-01 | 0.07±0.08 | 0 | 7.12E-01 | 7.91E-01 | 0.06±0.09 | 9.11E-01 | 0.05±0.09 | 9.18E-01 | 0.11±0.13 | 7.04E-01 | 0.11±0.13 | 7.44E-01 |
| oleate/vaccenate (18:1) | -0.06±0.07 | 33.93 | 2.19E-01 | 7.92E-01 | -0.07±0.07 | 44.81 | 1.78E-01 | 7.91E-01 | 0.01±0.1 | 9.78E-01 | 0.01±0.1 | 9.80E-01 | -0.17±0.12 | 5.23E-01 | -0.2±0.12 | 4.95E-01 |
| hyocholate | -0.06±0.07 | 0 | 7.08E-01 | 7.92E-01 | -0.06±0.07 | 0 | 6.95E-01 | 7.91E-01 | -0.04±0.09 | 9.38E-01 | -0.04±0.09 | 9.18E-01 | -0.09±0.13 | 7.41E-01 | -0.11±0.13 | 7.44E-01 |
| X - 21339 | -0.05±0.07 | 0 | 4.08E-01 | 8.11E-01 | -0.06±0.07 | 0 | 5.84E-01 | 7.96E-01 | -0.09±0.09 | 8.65E-01 | -0.1±0.09 | 8.36E-01 | 0.03±0.12 | 9.23E-01 | -0.01±0.13 | 9.85E-01 |
| arabitol/xylitol | 0.06±0.08 | 84.86 | 1.02E-02 | 8.06E-01 | 0.06±0.08 | 85.09 | 9.61E-03 | 7.98E-01 | -0.07±0.09 | 9.03E-01 | -0.06±0.09 | 8.94E-01 | 0.39±0.15 | 2.03E-01 | 0.4±0.15 | 3.58E-01 |
| X - 15245 | 0.06±0.08 | 83.18 | 1.48E-02 | 7.92E-01 | 0.06±0.08 | 77.26 | 3.60E-02 | 7.98E-01 | -0.06±0.09 | 9.11E-01 | -0.04±0.09 | 9.18E-01 | 0.35±0.14 | 2.19E-01 | 0.31±0.14 | 4.18E-01 |
| pentose acid* | 0.04±0.07 | 66.1 | 8.59E-02 | 8.40E-01 | 0.07±0.08 | 75.84 | 4.19E-02 | 7.98E-01 | -0.04±0.09 | 9.27E-01 | -0.04±0.09 | 9.19E-01 | 0.23±0.13 | 3.97E-01 | 0.3±0.14 | 4.18E-01 |
| methionine | 0.07±0.08 | 70.92 | 6.37E-02 | 7.74E-01 | 0.07±0.08 | 71.02 | 6.32E-02 | 7.98E-01 | -0.01±0.09 | 9.78E-01 | -0.02±0.09 | 9.70E-01 | 0.32±0.16 | 3.25E-01 | 0.32±0.16 | 4.30E-01 |
| methionine sulfoxide | 0.06±0.08 | 75.13 | 4.49E-02 | 7.92E-01 | 0.07±0.08 | 70.27 | 6.67E-02 | 7.98E-01 | -0.04±0.09 | 9.27E-01 | -0.03±0.09 | 9.35E-01 | 0.29±0.14 | 3.23E-01 | 0.27±0.14 | 4.44E-01 |
| tetrahydrocortisol glucuronide | -0.04±0.07 | 88.4 | 3.33E-03 | 8.43E-01 | -0.05±0.07 | 85.18 | 9.40E-03 | 7.98E-01 | -0.15±0.08 | 6.10E-01 | -0.15±0.08 | 5.69E-01 | 0.32±0.14 | 2.71E-01 | 0.27±0.14 | 4.52E-01 |
| androstenediol (3alpha, 17alpha) monosulfate (2) | -0.06±0.07 | 85.99 | 7.56E-03 | 7.85E-01 | -0.06±0.07 | 86.22 | 7.07E-03 | 7.98E-01 | -0.18±0.09 | 5.29E-01 | -0.18±0.09 | 5.09E-01 | 0.26±0.14 | 3.65E-01 | 0.27±0.14 | 4.52E-01 |
| 3-carboxy-4-methyl-5-propyl-2-furanpropanoate (CMPF) | -0.06±0.07 | 71.62 | 6.05E-02 | 7.65E-01 | -0.06±0.07 | 67.39 | 7.99E-02 | 7.98E-01 | 0.04±0.09 | 9.38E-01 | 0.04±0.09 | 9.18E-01 | -0.24±0.12 | 3.25E-01 | -0.23±0.12 | 4.54E-01 |
| N-acetylalanine | 0.07±0.09 | 72.21 | 5.78E-02 | 7.92E-01 | 0.07±0.09 | 58.71 | 1.20E-01 | 7.98E-01 | -0.01±0.09 | 9.89E-01 | 0.01±0.1 | 9.80E-01 | 0.42±0.21 | 3.25E-01 | 0.35±0.2 | 4.65E-01 |
| threonine | 0.07±0.08 | 51.18 | 1.52E-01 | 7.85E-01 | 0.07±0.08 | 59.69 | 1.15E-01 | 7.98E-01 | 0.01±0.09 | 9.89E-01 | 0±0.09 | 9.98E-01 | 0.28±0.17 | 4.40E-01 | 0.31±0.17 | 4.65E-01 |
| 1-palmitoyl-2-oleoyl-GPI (16:0/18:1)* | 0.07±0.08 | 58.81 | 1.19E-01 | 7.53E-01 | 0.06±0.08 | 58.97 | 1.18E-01 | 7.98E-01 | 0±0.09 | 9.89E-01 | -0.01±0.09 | 9.80E-01 | 0.27±0.15 | 3.71E-01 | 0.26±0.15 | 4.73E-01 |
| X - 25267 | 0.06±0.08 | 51.7 | 1.50E-01 | 7.85E-01 | 0.06±0.08 | 57.99 | 1.23E-01 | 7.98E-01 | 0±0.09 | 9.89E-01 | 0±0.09 | 9.96E-01 | 0.27±0.16 | 4.38E-01 | 0.29±0.17 | 4.74E-01 |
| lactate | -0.06±0.08 | 87.2 | 5.19E-03 | 7.85E-01 | -0.06±0.08 | 84.99 | 9.85E-03 | 7.98E-01 | -0.2±0.09 | 4.41E-01 | -0.2±0.09 | 4.81E-01 | 0.26±0.14 | 3.57E-01 | 0.23±0.14 | 4.80E-01 |
| arachidonoylcarnitine (C20:4) | 0.07±0.08 | 48.48 | 1.64E-01 | 7.79E-01 | 0.06±0.08 | 53.88 | 1.41E-01 | 7.98E-01 | 0±0.09 | 9.89E-01 | -0.01±0.09 | 9.80E-01 | 0.23±0.14 | 4.45E-01 | 0.24±0.14 | 4.82E-01 |
| glycocholenate sulfate* | 0.06±0.08 | 34.96 | 2.15E-01 | 7.92E-01 | 0.07±0.08 | 49.67 | 1.59E-01 | 7.98E-01 | -0.01±0.09 | 9.89E-01 | -0.01±0.09 | 9.83E-01 | 0.19±0.13 | 5.23E-01 | 0.22±0.14 | 4.92E-01 |
| methylsuccinate | 0.06±0.08 | 47.43 | 1.68E-01 | 7.92E-01 | 0.06±0.08 | 44.05 | 1.81E-01 | 7.98E-01 | -0.01±0.09 | 9.89E-01 | 0±0.09 | 9.98E-01 | 0.23±0.15 | 4.64E-01 | 0.23±0.15 | 4.97E-01 |
| dihomo-linoleate (20:2n6) | -0.05±0.07 | 25.64 | 2.46E-01 | 8.16E-01 | -0.06±0.07 | 38.74 | 2.01E-01 | 7.98E-01 | 0.01±0.09 | 9.78E-01 | 0.01±0.09 | 9.80E-01 | -0.16±0.12 | 5.61E-01 | -0.19±0.12 | 5.21E-01 |
| 3-carboxy-4-methyl-5-pentyl-2-furanpropionate (3-CMPFP)** | 0.05±0.07 | 35.03 | 2.15E-01 | 8.09E-01 | 0.06±0.07 | 24.3 | 2.50E-01 | 7.98E-01 | -0.01±0.09 | 9.86E-01 | 0±0.09 | 9.93E-01 | 0.19±0.13 | 5.31E-01 | 0.19±0.13 | 5.69E-01 |
| 2R,3R-dihydroxybutyrate | 0.06±0.07 | 53.14 | 1.44E-01 | 7.74E-01 | 0.06±0.07 | 25.42 | 2.47E-01 | 7.98E-01 | -0.01±0.09 | 9.89E-01 | 0±0.09 | 9.97E-01 | 0.23±0.14 | 4.16E-01 | 0.19±0.13 | 5.77E-01 |
| S-carboxyethylcysteine | 0.06±0.07 | 0.38 | 3.16E-01 | 7.74E-01 | 0.06±0.08 | 23.71 | 2.52E-01 | 7.98E-01 | 0.01±0.09 | 9.87E-01 | 0±0.1 | 9.94E-01 | 0.16±0.12 | 5.61E-01 | 0.18±0.13 | 5.77E-01 |
| androstenediol (3beta,17beta) monosulfate (2) | 0.07±0.08 | 0 | 3.38E-01 | 7.59E-01 | 0.07±0.08 | 20.2 | 2.63E-01 | 7.98E-01 | 0.02±0.09 | 9.59E-01 | 0.01±0.09 | 9.80E-01 | 0.19±0.14 | 5.70E-01 | 0.21±0.15 | 5.77E-01 |
| 1-palmitoyl-GPG (16:0)* | 0.07±0.08 | 36.69 | 2.09E-01 | 7.70E-01 | 0.06±0.08 | 2.2 | 3.12E-01 | 7.98E-01 | 0±0.09 | 9.89E-01 | 0.01±0.09 | 9.80E-01 | 0.21±0.14 | 4.86E-01 | 0.18±0.14 | 6.16E-01 |
| X - 12306 | -0.07±0.07 | 69.01 | 7.24E-02 | 7.34E-01 | -0.06±0.07 | 76.1 | 4.08E-02 | 7.98E-01 | -0.16±0.09 | 6.33E-01 | -0.16±0.09 | 6.15E-01 | 0.12±0.13 | 6.81E-01 | 0.17±0.13 | 6.22E-01 |
| 1H-indole-7-acetic acid | -0.06±0.07 | 0 | 3.18E-01 | 7.92E-01 | -0.06±0.07 | 0 | 3.66E-01 | 7.98E-01 | 0±0.09 | 9.89E-01 | -0.01±0.09 | 9.80E-01 | -0.16±0.12 | 5.77E-01 | -0.15±0.12 | 6.36E-01 |
| succinylcarnitine (C4-DC) | 0.07±0.08 | 29.3 | 2.34E-01 | 7.74E-01 | 0.06±0.08 | 0 | 4.16E-01 | 7.98E-01 | 0.01±0.09 | 9.89E-01 | 0.02±0.09 | 9.58E-01 | 0.21±0.14 | 5.19E-01 | 0.16±0.14 | 6.56E-01 |
| lysine | 0.06±0.08 | 0 | 4.27E-01 | 8.07E-01 | 0.06±0.08 | 0 | 4.38E-01 | 7.98E-01 | 0.02±0.09 | 9.59E-01 | 0.03±0.09 | 9.45E-01 | 0.17±0.17 | 6.40E-01 | 0.17±0.17 | 6.67E-01 |
| docosadienoate (22:2n6) | -0.05±0.07 | 0 | 4.70E-01 | 8.24E-01 | -0.06±0.07 | 0 | 4.79E-01 | 7.98E-01 | -0.01±0.09 | 9.89E-01 | -0.02±0.09 | 9.58E-01 | -0.12±0.12 | 6.66E-01 | -0.13±0.13 | 6.67E-01 |
| 1-stearoyl-GPI (18:0) | -0.06±0.07 | 63.96 | 9.58E-02 | 7.74E-01 | -0.06±0.07 | 68.73 | 7.37E-02 | 7.98E-01 | -0.15±0.09 | 7.16E-01 | -0.15±0.09 | 6.80E-01 | 0.12±0.14 | 6.82E-01 | 0.14±0.14 | 6.67E-01 |
| glutamine | -0.06±0.08 | 49.07 | 1.61E-01 | 7.92E-01 | -0.06±0.08 | 62.66 | 1.02E-01 | 7.98E-01 | -0.12±0.09 | 7.90E-01 | -0.13±0.09 | 7.37E-01 | 0.13±0.16 | 7.03E-01 | 0.18±0.17 | 6.67E-01 |
| X - 22776 | -0.06±0.07 | 0 | 4.50E-01 | 7.85E-01 | -0.06±0.07 | 0 | 4.99E-01 | 7.98E-01 | -0.02±0.09 | 9.77E-01 | -0.02±0.09 | 9.63E-01 | -0.13±0.12 | 6.21E-01 | -0.12±0.12 | 6.77E-01 |
| X - 24949 | -0.05±0.07 | 0 | 6.61E-01 | 8.11E-01 | -0.06±0.07 | 0 | 5.28E-01 | 7.98E-01 | -0.03±0.09 | 9.58E-01 | -0.03±0.09 | 9.45E-01 | -0.09±0.12 | 7.38E-01 | -0.12±0.12 | 6.78E-01 |
| X - 26111 | 0.06±0.07 | 74.25 | 4.88E-02 | 8.06E-01 | 0.07±0.08 | 71.46 | 6.12E-02 | 7.98E-01 | 0.17±0.1 | 6.56E-01 | 0.17±0.1 | 6.15E-01 | -0.13±0.12 | 6.21E-01 | -0.12±0.13 | 6.91E-01 |
| oleoyl-linoleoyl-glycerol (18:1/18:2) [2] | -0.05±0.07 | 0 | 6.82E-01 | 8.16E-01 | -0.06±0.07 | 0 | 5.27E-01 | 7.98E-01 | -0.03±0.09 | 9.58E-01 | -0.02±0.09 | 9.58E-01 | -0.09±0.12 | 7.57E-01 | -0.12±0.12 | 6.91E-01 |
| X - 12410 | 0.04±0.07 | 0 | 6.87E-01 | 8.28E-01 | 0.06±0.07 | 0 | 5.51E-01 | 7.98E-01 | 0.02±0.09 | 9.59E-01 | 0.03±0.09 | 9.44E-01 | 0.09±0.13 | 7.70E-01 | 0.12±0.13 | 7.05E-01 |
| 4-hydroxyphenylacetate | 0.08±0.07 | 0 | 3.71E-01 | 7.33E-01 | 0.06±0.07 | 0 | 5.84E-01 | 7.98E-01 | 0.03±0.09 | 9.48E-01 | 0.03±0.09 | 9.24E-01 | 0.17±0.13 | 5.61E-01 | 0.12±0.13 | 7.12E-01 |
| pyridoxate | 0.05±0.07 | 0 | 6.22E-01 | 8.06E-01 | 0.06±0.07 | 0 | 6.31E-01 | 7.98E-01 | 0.02±0.09 | 9.59E-01 | 0.03±0.09 | 9.19E-01 | 0.1±0.11 | 7.11E-01 | 0.1±0.11 | 7.12E-01 |
| 1-palmitoleoylglycerol (16:1)* | 0.08±0.07 | 0 | 4.73E-01 | 7.33E-01 | 0.06±0.08 | 0 | 6.21E-01 | 7.98E-01 | 0.04±0.09 | 9.34E-01 | 0.04±0.09 | 9.19E-01 | 0.16±0.13 | 6.00E-01 | 0.12±0.13 | 7.24E-01 |
| 1-stearoyl-2-arachidonoyl-GPI (18:0/20:4) | 0.06±0.08 | 0 | 6.17E-01 | 7.85E-01 | 0.06±0.08 | 0 | 6.32E-01 | 7.98E-01 | 0.04±0.09 | 9.38E-01 | 0.04±0.09 | 9.19E-01 | 0.12±0.14 | 7.01E-01 | 0.12±0.14 | 7.44E-01 |
| pentadecanoate (15:0) | -0.06±0.08 | 0 | 6.93E-01 | 7.84E-01 | -0.06±0.08 | 0 | 6.51E-01 | 7.98E-01 | -0.04±0.1 | 9.34E-01 | -0.04±0.1 | 9.19E-01 | -0.11±0.13 | 7.28E-01 | -0.11±0.13 | 7.44E-01 |
| mannose | -0.06±0.08 | 72.9 | 5.47E-02 | 8.09E-01 | -0.07±0.08 | 56.38 | 1.30E-01 | 7.98E-01 | -0.15±0.09 | 7.50E-01 | -0.15±0.1 | 7.21E-01 | 0.2±0.15 | 5.71E-01 | 0.11±0.14 | 7.52E-01 |
| 3b-hydroxy-5-cholenoic acid | -0.07±0.07 | 0 | 4.23E-01 | 7.44E-01 | -0.06±0.07 | 0 | 7.22E-01 | 7.98E-01 | -0.03±0.09 | 9.48E-01 | -0.04±0.09 | 9.18E-01 | -0.15±0.12 | 5.85E-01 | -0.1±0.13 | 7.53E-01 |
| gamma-glutamylcitrulline* | -0.06±0.07 | 0 | 6.14E-01 | 7.48E-01 | -0.06±0.07 | 0 | 7.89E-01 | 7.98E-01 | -0.04±0.09 | 9.38E-01 | -0.04±0.09 | 9.18E-01 | -0.11±0.11 | 6.58E-01 | -0.08±0.11 | 7.64E-01 |
| 3-hydroxyisobutyrate | 0.06±0.07 | 0 | 5.52E-01 | 7.85E-01 | 0.06±0.07 | 0 | 7.38E-01 | 7.98E-01 | 0.03±0.09 | 9.48E-01 | 0.04±0.09 | 9.18E-01 | 0.12±0.13 | 6.66E-01 | 0.09±0.13 | 7.64E-01 |
| 13-HODE + 9-HODE | -0.07±0.07 | 0 | 5.82E-01 | 7.44E-01 | -0.06±0.07 | 0 | 7.48E-01 | 7.98E-01 | -0.04±0.09 | 9.27E-01 | -0.04±0.09 | 9.18E-01 | -0.13±0.13 | 6.53E-01 | -0.09±0.13 | 7.73E-01 |
| bilirubin degradation product, C17H18N2O4 (3)** | -0.07±0.07 | 0 | 3.19E-01 | 7.34E-01 | -0.06±0.07 | 43.6 | 1.83E-01 | 7.98E-01 | -0.12±0.09 | 7.90E-01 | -0.13±0.09 | 7.62E-01 | 0.03±0.13 | 9.28E-01 | 0.09±0.14 | 7.90E-01 |
| 17alpha-hydroxypregnanolone glucuronide | -0.07±0.09 | 0 | 6.48E-01 | 8.06E-01 | -0.07±0.09 | 0 | 8.05E-01 | 7.98E-01 | -0.04±0.11 | 9.48E-01 | -0.05±0.12 | 9.18E-01 | -0.12±0.15 | 7.29E-01 | -0.1±0.16 | 7.93E-01 |
| fructosyllysine | 0.07±0.07 | 0 | 4.90E-01 | 7.53E-01 | 0.06±0.07 | 0 | 8.34E-01 | 7.98E-01 | 0.04±0.09 | 9.41E-01 | 0.05±0.09 | 9.18E-01 | 0.15±0.13 | 6.23E-01 | 0.08±0.13 | 7.96E-01 |
| 3-methylhistidine | -0.05±0.07 | 0 | 9.96E-01 | 8.16E-01 | -0.06±0.07 | 0 | 8.58E-01 | 7.98E-01 | -0.05±0.09 | 9.27E-01 | -0.05±0.09 | 9.18E-01 | -0.05±0.12 | 8.98E-01 | -0.08±0.13 | 8.03E-01 |
| 1-(1-enyl-stearoyl)-2-linoleoyl-GPE (P-18:0/18:2)* | -0.06±0.07 | 0 | 3.35E-01 | 7.74E-01 | -0.06±0.07 | 38.03 | 2.04E-01 | 7.98E-01 | -0.11±0.09 | 8.31E-01 | -0.13±0.09 | 7.62E-01 | 0.04±0.13 | 9.20E-01 | 0.08±0.13 | 8.08E-01 |
| N,N-dimethylalanine | -0.06±0.07 | 0 | 9.03E-01 | 7.85E-01 | -0.06±0.07 | 0 | 9.33E-01 | 7.98E-01 | -0.05±0.09 | 9.27E-01 | -0.06±0.09 | 8.94E-01 | -0.07±0.12 | 8.14E-01 | -0.07±0.13 | 8.13E-01 |
| 2-hydroxyphenylacetate | 0.05±0.08 | 22.24 | 2.57E-01 | 8.09E-01 | 0.06±0.08 | 43.06 | 1.85E-01 | 7.98E-01 | 0.12±0.1 | 8.31E-01 | 0.14±0.1 | 7.54E-01 | -0.06±0.12 | 8.59E-01 | -0.07±0.12 | 8.18E-01 |
| bilirubin degradation product, C17H18N2O4 (1)** | -0.07±0.07 | 0 | 4.21E-01 | 7.33E-01 | -0.06±0.07 | 26.55 | 2.43E-01 | 7.98E-01 | -0.11±0.09 | 7.95E-01 | -0.11±0.09 | 8.25E-01 | 0.01±0.13 | 9.84E-01 | 0.07±0.14 | 8.22E-01 |
| 3-methoxycatechol sulfate (1) | -0.06±0.07 | 39.87 | 1.97E-01 | 7.74E-01 | -0.06±0.07 | 35.44 | 2.13E-01 | 7.98E-01 | -0.13±0.09 | 7.81E-01 | -0.13±0.09 | 7.62E-01 | 0.07±0.13 | 8.28E-01 | 0.07±0.13 | 8.26E-01 |
| 3-methoxytyramine sulfate | 0.06±0.07 | 0 | 9.05E-01 | 7.92E-01 | 0.06±0.07 | 0 | 9.15E-01 | 7.98E-01 | 0.06±0.09 | 9.03E-01 | 0.05±0.09 | 9.04E-01 | 0.04±0.13 | 9.05E-01 | 0.07±0.13 | 8.27E-01 |
| X - 24757 | -0.06±0.07 | 34.31 | 2.17E-01 | 7.74E-01 | -0.06±0.07 | 30.62 | 2.30E-01 | 7.98E-01 | -0.13±0.09 | 7.87E-01 | -0.12±0.09 | 7.69E-01 | 0.06±0.13 | 8.47E-01 | 0.06±0.13 | 8.46E-01 |
| X - 16580 | 0.06±0.07 | 0 | 8.69E-01 | 7.92E-01 | 0.06±0.08 | 0 | 9.70E-01 | 7.98E-01 | 0.05±0.09 | 9.27E-01 | 0.06±0.09 | 9.04E-01 | 0.08±0.13 | 8.13E-01 | 0.06±0.13 | 8.50E-01 |
| stearoyl sphingomyelin (d18:1/18:0) | 0.05±0.07 | 0 | 9.39E-01 | 8.19E-01 | 0.06±0.07 | 0 | 9.65E-01 | 7.98E-01 | 0.04±0.09 | 9.27E-01 | 0.06±0.09 | 9.04E-01 | 0.06±0.13 | 8.80E-01 | 0.06±0.13 | 8.50E-01 |
| taurochenodeoxycholate | -0.06±0.07 | 0 | 8.83E-01 | 7.85E-01 | -0.06±0.07 | 0 | 9.98E-01 | 7.98E-01 | -0.07±0.09 | 9.03E-01 | -0.06±0.09 | 8.94E-01 | -0.04±0.12 | 9.05E-01 | -0.06±0.13 | 8.50E-01 |
| alpha-tocopherol | 0.05±0.07 | 0 | 8.92E-01 | 8.06E-01 | 0.06±0.07 | 0 | 1.00E+00 | 7.98E-01 | 0.06±0.09 | 9.06E-01 | 0.06±0.09 | 8.94E-01 | 0.04±0.13 | 9.20E-01 | 0.06±0.13 | 8.50E-01 |
| 16a-hydroxy DHEA 3-sulfate | 0.07±0.08 | 0 | 9.05E-01 | 7.65E-01 | 0.06±0.08 | 0 | 9.88E-01 | 7.98E-01 | 0.06±0.09 | 9.06E-01 | 0.06±0.1 | 8.94E-01 | 0.08±0.13 | 7.92E-01 | 0.06±0.13 | 8.54E-01 |
| 7-alpha-hydroxy-3-oxo-4-cholestenoate (7-Hoca) | -0.05±0.07 | 47.27 | 1.68E-01 | 8.16E-01 | -0.06±0.08 | 23.52 | 2.53E-01 | 7.98E-01 | -0.12±0.09 | 7.90E-01 | -0.12±0.09 | 8.09E-01 | 0.1±0.13 | 7.46E-01 | 0.06±0.13 | 8.60E-01 |
| eicosapentaenoate (EPA; 20:5n3) | -0.06±0.07 | 0 | 8.54E-01 | 7.84E-01 | -0.06±0.08 | 0 | 9.58E-01 | 7.98E-01 | -0.05±0.09 | 9.27E-01 | -0.06±0.09 | 8.94E-01 | -0.08±0.13 | 7.89E-01 | -0.06±0.13 | 8.74E-01 |
| X - 12822 | -0.06±0.07 | 0 | 7.22E-01 | 8.06E-01 | -0.06±0.08 | 0 | 9.42E-01 | 7.98E-01 | -0.07±0.09 | 9.01E-01 | -0.06±0.1 | 8.94E-01 | -0.02±0.13 | 9.71E-01 | -0.05±0.13 | 8.85E-01 |
| 3-hydroxydodecanedioate* | 0.07±0.07 | 0 | 4.00E-01 | 7.44E-01 | 0.06±0.08 | 5.54 | 3.04E-01 | 7.98E-01 | 0.12±0.09 | 8.19E-01 | 0.12±0.09 | 8.35E-01 | -0.01±0.13 | 9.84E-01 | -0.05±0.13 | 8.98E-01 |
| X - 12798 | -0.06±0.07 | 12.72 | 2.84E-01 | 7.74E-01 | -0.06±0.07 | 0 | 3.54E-01 | 7.98E-01 | -0.11±0.08 | 7.93E-01 | -0.1±0.09 | 8.36E-01 | 0.06±0.13 | 8.76E-01 | 0.04±0.13 | 9.11E-01 |
| 4-methylguaiacol sulfate | -0.05±0.07 | 0 | 3.55E-01 | 8.06E-01 | -0.06±0.07 | 0 | 3.49E-01 | 7.98E-01 | -0.1±0.09 | 8.65E-01 | -0.1±0.09 | 8.36E-01 | 0.04±0.13 | 9.05E-01 | 0.04±0.13 | 9.11E-01 |
| octadecenedioate (C18:1-DC) | -0.07±0.07 | 0 | 9.55E-01 | 7.33E-01 | -0.06±0.07 | 0 | 7.91E-01 | 7.98E-01 | -0.08±0.09 | 8.86E-01 | -0.08±0.09 | 8.68E-01 | -0.07±0.12 | 8.14E-01 | -0.04±0.12 | 9.17E-01 |
| phenol sulfate | -0.06±0.07 | 0 | 5.49E-01 | 7.92E-01 | -0.06±0.07 | 0 | 7.72E-01 | 7.98E-01 | -0.09±0.09 | 8.69E-01 | -0.08±0.09 | 8.68E-01 | 0±0.13 | 9.94E-01 | -0.04±0.12 | 9.24E-01 |
| beta-hydroxyisovaleroylcarnitine | -0.07±0.08 | 5.29 | 3.04E-01 | 7.67E-01 | -0.06±0.08 | 0 | 3.44E-01 | 7.98E-01 | -0.12±0.09 | 7.90E-01 | -0.12±0.1 | 8.35E-01 | 0.04±0.13 | 9.20E-01 | 0.03±0.13 | 9.29E-01 |
| X - 21383 | -0.05±0.07 | 0 | 5.89E-01 | 8.09E-01 | -0.06±0.07 | 0 | 7.57E-01 | 7.98E-01 | -0.08±0.09 | 8.80E-01 | -0.08±0.09 | 8.68E-01 | 0±0.12 | 9.94E-01 | -0.03±0.13 | 9.33E-01 |
| sphingomyelin (d18:2/24:1, d18:1/24:2)* | -0.06±0.07 | 0 | 9.40E-01 | 7.74E-01 | -0.06±0.07 | 0 | 7.11E-01 | 7.98E-01 | -0.07±0.1 | 9.03E-01 | -0.08±0.1 | 8.68E-01 | -0.06±0.11 | 8.47E-01 | -0.03±0.12 | 9.41E-01 |
| hydroxypalmitoyl sphingomyelin (d18:1/16:0(OH))** | -0.08±0.07 | 0 | 8.75E-01 | 7.12E-01 | -0.06±0.07 | 0 | 6.82E-01 | 7.98E-01 | -0.09±0.09 | 8.76E-01 | -0.09±0.1 | 8.63E-01 | -0.06±0.11 | 8.14E-01 | -0.03±0.12 | 9.43E-01 |
| laurate (12:0) | -0.06±0.08 | 0 | 7.64E-01 | 7.85E-01 | -0.06±0.08 | 0 | 7.30E-01 | 7.98E-01 | -0.08±0.09 | 8.99E-01 | -0.08±0.1 | 8.68E-01 | -0.03±0.13 | 9.40E-01 | -0.03±0.14 | 9.43E-01 |
| tartronate (hydroxymalonate) | -0.08±0.07 | 0 | 8.68E-01 | 6.91E-01 | -0.06±0.07 | 0 | 6.97E-01 | 7.98E-01 | -0.09±0.09 | 8.65E-01 | -0.08±0.09 | 8.60E-01 | -0.06±0.12 | 8.24E-01 | -0.02±0.13 | 9.56E-01 |
| 1-oleoyl-2-docosahexaenoyl-GPC (18:1/22:6)* | -0.08±0.07 | 0 | 9.22E-01 | 6.91E-01 | -0.06±0.07 | 0 | 6.58E-01 | 7.98E-01 | -0.09±0.09 | 8.72E-01 | -0.09±0.09 | 8.60E-01 | -0.07±0.12 | 7.89E-01 | -0.02±0.12 | 9.65E-01 |
| 1-palmitoyl-2-linoleoyl-GPC (16:0/18:2) | -0.06±0.07 | 0 | 6.30E-01 | 7.85E-01 | -0.06±0.07 | 0 | 4.65E-01 | 7.98E-01 | -0.09±0.09 | 8.72E-01 | -0.1±0.09 | 8.36E-01 | -0.01±0.12 | 9.82E-01 | 0.02±0.13 | 9.71E-01 |
| 1-(1-enyl-palmitoyl)-2-oleoyl-GPC (P-16:0/18:1)* | -0.07±0.07 | 0 | 9.73E-01 | 7.44E-01 | -0.06±0.08 | 0 | 4.74E-01 | 7.98E-01 | -0.07±0.09 | 9.03E-01 | -0.1±0.1 | 8.36E-01 | -0.07±0.12 | 8.14E-01 | 0.01±0.13 | 9.76E-01 |
| X - 17612 | -0.05±0.07 | 0 | 5.17E-01 | 8.09E-01 | -0.06±0.07 | 0 | 5.04E-01 | 7.98E-01 | -0.08±0.09 | 8.73E-01 | -0.09±0.09 | 8.36E-01 | 0.02±0.13 | 9.73E-01 | 0.01±0.13 | 9.79E-01 |
| methylsuccinoylcarnitine | 0.06±0.07 | 0 | 6.45E-01 | 7.92E-01 | 0.06±0.07 | 0 | 6.38E-01 | 7.98E-01 | 0.08±0.09 | 8.80E-01 | 0.08±0.09 | 8.52E-01 | 0.01±0.12 | 9.86E-01 | 0.01±0.13 | 9.80E-01 |
| sulfate of piperine metabolite C16H19NO3 (2)* | -0.04±0.07 | 0 | 3.48E-01 | 8.55E-01 | -0.06±0.08 | 0 | 5.36E-01 | 7.98E-01 | -0.09±0.09 | 8.73E-01 | -0.1±0.1 | 8.36E-01 | 0.07±0.13 | 8.47E-01 | 0.01±0.14 | 9.85E-01 |
| X - 25271 | 0.05±0.07 | 0 | 6.04E-01 | 8.16E-01 | 0.06±0.07 | 0 | 6.32E-01 | 7.98E-01 | 0.08±0.09 | 8.95E-01 | 0.08±0.09 | 8.63E-01 | 0±0.13 | 9.94E-01 | 0.01±0.13 | 9.89E-01 |
| X - 23665 | 0.07±0.07 | 0 | 7.10E-01 | 7.40E-01 | 0.06±0.07 | 0 | 5.31E-01 | 7.98E-01 | 0.09±0.09 | 8.65E-01 | 0.1±0.09 | 8.36E-01 | 0.04±0.13 | 9.20E-01 | 0±0.13 | 9.98E-01 |
| methionine sulfone | 0.04±0.08 | 0 | 9.08E-01 | 8.43E-01 | 0.06±0.08 | 0 | 7.27E-01 | 8.01E-01 | 0.04±0.09 | 9.46E-01 | 0.04±0.1 | 9.19E-01 | 0.05±0.13 | 8.80E-01 | 0.1±0.13 | 7.69E-01 |
| butyrylglycine | 0.06±0.07 | 0 | 5.85E-01 | 7.85E-01 | 0.06±0.07 | 0 | 6.91E-01 | 8.01E-01 | 0.09±0.09 | 8.69E-01 | 0.08±0.09 | 8.68E-01 | 0.01±0.12 | 9.92E-01 | 0.02±0.13 | 9.71E-01 |
| leucine | 0.06±0.09 | 70.37 | 6.62E-02 | 8.09E-01 | 0.07±0.09 | 51.71 | 1.50E-01 | 8.02E-01 | -0.02±0.1 | 9.74E-01 | 0±0.1 | 9.93E-01 | 0.38±0.2 | 3.42E-01 | 0.3±0.19 | 4.95E-01 |
| 2-hydroxy-4-(methylthio)butanoic acid | 0.07±0.08 | 0 | 5.67E-01 | 7.44E-01 | 0.06±0.08 | 0 | 7.58E-01 | 8.02E-01 | 0.04±0.09 | 9.30E-01 | 0.04±0.1 | 9.18E-01 | 0.14±0.13 | 6.44E-01 | 0.09±0.13 | 7.82E-01 |
| argininate* | 0.06±0.07 | 0 | 5.45E-01 | 8.00E-01 | 0.06±0.08 | 0 | 8.22E-01 | 8.02E-01 | 0.03±0.09 | 9.59E-01 | 0.04±0.09 | 9.18E-01 | 0.12±0.13 | 6.77E-01 | 0.08±0.13 | 8.03E-01 |
| bilirubin degradation product, C17H18N2O4 (2)** | -0.07±0.07 | 0 | 5.97E-01 | 7.40E-01 | -0.06±0.07 | 0 | 3.93E-01 | 8.02E-01 | -0.1±0.09 | 8.65E-01 | -0.1±0.09 | 8.36E-01 | -0.02±0.13 | 9.79E-01 | 0.04±0.13 | 9.22E-01 |
| 3-acetylphenol sulfate | -0.05±0.07 | 57.95 | 1.23E-01 | 8.11E-01 | -0.06±0.07 | 52.28 | 1.48E-01 | 8.04E-01 | -0.13±0.09 | 7.81E-01 | -0.13±0.09 | 7.54E-01 | 0.11±0.13 | 7.01E-01 | 0.1±0.13 | 7.64E-01 |
| androsterone sulfate | -0.07±0.07 | 0 | 4.72E-01 | 7.40E-01 | -0.06±0.07 | 26.2 | 2.44E-01 | 8.06E-01 | -0.11±0.09 | 8.31E-01 | -0.12±0.09 | 8.25E-01 | 0±0.13 | 9.96E-01 | 0.07±0.13 | 8.26E-01 |
| dehydroepiandrosterone sulfate (DHEA-S) | 0.06±0.08 | 21.47 | 2.59E-01 | 8.09E-01 | 0.06±0.08 | 52.05 | 1.49E-01 | 8.09E-01 | 0±0.09 | 9.89E-01 | -0.02±0.1 | 9.67E-01 | 0.19±0.14 | 5.61E-01 | 0.23±0.14 | 4.95E-01 |
| dodecenedioate (C12:1-DC)* | 0.05±0.07 | 0 | 4.21E-01 | 8.09E-01 | 0.06±0.08 | 0 | 4.24E-01 | 8.09E-01 | 0.1±0.09 | 8.65E-01 | 0.1±0.09 | 8.36E-01 | -0.03±0.13 | 9.40E-01 | -0.03±0.13 | 9.41E-01 |
| malate | 0.04±0.07 | 0 | 3.60E-01 | 8.43E-01 | 0.05±0.07 | 16.43 | 2.74E-01 | 8.12E-01 | 0±0.09 | 9.89E-01 | 0±0.09 | 9.98E-01 | 0.14±0.13 | 6.25E-01 | 0.17±0.13 | 5.97E-01 |
| 5-dodecenoylcarnitine (C12:1) | 0.06±0.07 | 77.12 | 3.66E-02 | 7.92E-01 | 0.06±0.08 | 76.76 | 3.81E-02 | 8.12E-01 | 0.17±0.09 | 6.33E-01 | 0.17±0.09 | 6.15E-01 | -0.16±0.13 | 5.77E-01 | -0.16±0.13 | 6.20E-01 |
| umbelliferone sulfate | -0.05±0.07 | 55.57 | 1.34E-01 | 8.06E-01 | -0.05±0.07 | 59.66 | 1.15E-01 | 8.12E-01 | -0.13±0.09 | 7.77E-01 | -0.14±0.09 | 7.31E-01 | 0.1±0.12 | 7.30E-01 | 0.11±0.13 | 7.32E-01 |
| glucuronate | -0.05±0.07 | 59.17 | 1.18E-01 | 8.11E-01 | -0.05±0.07 | 53.78 | 1.41E-01 | 8.12E-01 | -0.13±0.09 | 7.81E-01 | -0.13±0.09 | 7.54E-01 | 0.12±0.13 | 6.77E-01 | 0.1±0.13 | 7.50E-01 |
| X - 21736 | 0.07±0.07 | 0 | 7.22E-01 | 7.67E-01 | 0.06±0.07 | 0 | 9.71E-01 | 8.12E-01 | 0.05±0.09 | 9.27E-01 | 0.05±0.09 | 9.04E-01 | 0.1±0.13 | 7.28E-01 | 0.06±0.13 | 8.61E-01 |
| 2-palmitoyl-GPC (16:0)* | -0.06±0.07 | 0 | 4.24E-01 | 7.84E-01 | -0.05±0.07 | 13.54 | 2.82E-01 | 8.12E-01 | -0.1±0.09 | 8.65E-01 | -0.11±0.09 | 8.36E-01 | 0.02±0.12 | 9.67E-01 | 0.06±0.13 | 8.60E-01 |
| sphingomyelin (d17:1/16:0, d18:1/15:0, d16:1/17:0)* | -0.07±0.08 | 56.34 | 1.30E-01 | 7.62E-01 | -0.06±0.08 | 64.47 | 9.34E-02 | 8.17E-01 | -0.15±0.1 | 7.50E-01 | -0.15±0.1 | 7.37E-01 | 0.11±0.15 | 7.38E-01 | 0.16±0.16 | 6.67E-01 |
| 12,13-DiHOME | -0.07±0.07 | 16.19 | 2.75E-01 | 7.44E-01 | -0.05±0.07 | 46.03 | 1.73E-01 | 8.17E-01 | -0.12±0.09 | 7.90E-01 | -0.12±0.09 | 7.89E-01 | 0.05±0.13 | 9.01E-01 | 0.11±0.14 | 7.60E-01 |
| citrulline | -0.06±0.07 | 0 | 9.38E-01 | 7.84E-01 | -0.05±0.07 | 0 | 7.83E-01 | 8.17E-01 | -0.06±0.09 | 9.03E-01 | -0.07±0.09 | 8.94E-01 | -0.05±0.12 | 8.66E-01 | -0.03±0.12 | 9.41E-01 |
| ribitol | -0.05±0.07 | 86.11 | 7.30E-03 | 8.26E-01 | -0.05±0.08 | 83.48 | 1.39E-02 | 8.18E-01 | -0.17±0.09 | 5.98E-01 | -0.17±0.09 | 5.64E-01 | 0.27±0.14 | 3.42E-01 | 0.23±0.14 | 4.85E-01 |
| 1-stearoyl-2-oleoyl-GPE (18:0/18:1) | 0.07±0.08 | 78.11 | 3.26E-02 | 7.62E-01 | 0.06±0.08 | 65.57 | 8.83E-02 | 8.18E-01 | -0.03±0.09 | 9.47E-01 | -0.03±0.09 | 9.42E-01 | 0.32±0.14 | 2.68E-01 | 0.26±0.14 | 4.65E-01 |
| phenylacetate | 0.04±0.07 | 0 | 3.28E-01 | 8.39E-01 | 0.05±0.08 | 10.5 | 2.90E-01 | 8.18E-01 | 0±0.09 | 9.89E-01 | 0±0.09 | 9.98E-01 | 0.15±0.13 | 6.10E-01 | 0.17±0.14 | 6.16E-01 |
| 4-vinylcatechol sulfate | -0.05±0.07 | 0 | 5.64E-01 | 8.06E-01 | -0.06±0.08 | 0 | 4.23E-01 | 8.18E-01 | -0.09±0.09 | 8.80E-01 | -0.1±0.09 | 8.36E-01 | 0±0.13 | 9.94E-01 | 0.03±0.13 | 9.33E-01 |
| X - 19438 | 0.05±0.07 | 88.03 | 3.85E-03 | 8.09E-01 | 0.05±0.07 | 85.7 | 8.19E-03 | 8.23E-01 | -0.07±0.09 | 9.01E-01 | -0.06±0.09 | 8.94E-01 | 0.43±0.15 | 1.99E-01 | 0.4±0.15 | 3.58E-01 |
| X - 17357 | 0.05±0.07 | 71.83 | 5.95E-02 | 8.06E-01 | 0.05±0.07 | 66.08 | 8.60E-02 | 8.23E-01 | -0.03±0.09 | 9.48E-01 | -0.03±0.09 | 9.45E-01 | 0.28±0.14 | 3.38E-01 | 0.26±0.14 | 4.65E-01 |
| 1-palmitoyl-2-arachidonoyl-GPC (16:0/20:4n6) | 0.07±0.08 | 54.63 | 1.38E-01 | 7.65E-01 | 0.06±0.08 | 52.24 | 1.48E-01 | 8.23E-01 | 0±0.09 | 9.89E-01 | -0.01±0.09 | 9.80E-01 | 0.27±0.16 | 3.97E-01 | 0.25±0.16 | 4.95E-01 |
| X - 26107 | 0.07±0.08 | 25.02 | 2.48E-01 | 7.62E-01 | 0.05±0.08 | 0 | 3.89E-01 | 8.23E-01 | 0.01±0.09 | 9.85E-01 | 0.01±0.09 | 9.80E-01 | 0.21±0.14 | 5.19E-01 | 0.16±0.14 | 6.66E-01 |
| cysteine | 0.04±0.07 | 0 | 4.95E-01 | 8.45E-01 | 0.05±0.07 | 0 | 5.52E-01 | 8.23E-01 | 0.01±0.09 | 9.89E-01 | 0.02±0.09 | 9.57E-01 | 0.12±0.14 | 7.01E-01 | 0.12±0.14 | 7.24E-01 |
| piperine | -0.03±0.07 | 0 | 7.74E-01 | 9.08E-01 | -0.05±0.08 | 0 | 9.31E-01 | 8.23E-01 | -0.04±0.09 | 9.34E-01 | -0.05±0.1 | 9.18E-01 | 0±0.13 | 9.96E-01 | -0.06±0.13 | 8.50E-01 |
| glycolithocholate sulfate* | -0.05±0.07 | 0 | 4.11E-01 | 8.06E-01 | -0.05±0.07 | 0 | 3.98E-01 | 8.23E-01 | -0.09±0.09 | 8.65E-01 | -0.09±0.09 | 8.36E-01 | 0.04±0.13 | 9.20E-01 | 0.04±0.13 | 9.13E-01 |
| carotene diol (3) | -0.08±0.07 | 0 | 6.86E-01 | 7.08E-01 | -0.05±0.07 | 0 | 9.12E-01 | 8.23E-01 | -0.06±0.09 | 9.09E-01 | -0.06±0.09 | 9.00E-01 | -0.12±0.12 | 6.66E-01 | -0.04±0.13 | 9.19E-01 |
| X - 24307 | -0.06±0.07 | 0 | 8.22E-01 | 7.92E-01 | -0.05±0.07 | 0 | 7.18E-01 | 8.23E-01 | -0.07±0.09 | 9.03E-01 | -0.07±0.09 | 8.94E-01 | -0.03±0.12 | 9.23E-01 | -0.01±0.12 | 9.72E-01 |
| N-acetyltyrosine | 0.06±0.07 | 54.55 | 1.38E-01 | 7.92E-01 | 0.05±0.08 | 27.93 | 2.39E-01 | 8.24E-01 | -0.02±0.09 | 9.77E-01 | -0.01±0.09 | 9.80E-01 | 0.22±0.13 | 4.36E-01 | 0.18±0.13 | 5.85E-01 |
| 10-undecenoate (11:1n1) | -0.05±0.07 | 0 | 4.58E-01 | 8.11E-01 | -0.05±0.07 | 0 | 3.48E-01 | 8.28E-01 | -0.09±0.09 | 8.69E-01 | -0.1±0.09 | 8.36E-01 | 0.03±0.13 | 9.40E-01 | 0.05±0.13 | 8.95E-01 |
| 3-methylglutarylcarnitine (2) | 0.05±0.08 | 0 | 5.76E-01 | 8.09E-01 | 0.05±0.08 | 0 | 4.37E-01 | 8.28E-01 | 0.08±0.09 | 8.80E-01 | 0.1±0.1 | 8.36E-01 | -0.01±0.13 | 9.92E-01 | -0.03±0.13 | 9.32E-01 |
| margarate (17:0) | -0.05±0.07 | 0 | 7.57E-01 | 8.19E-01 | -0.05±0.08 | 0 | 6.66E-01 | 8.30E-01 | -0.03±0.09 | 9.48E-01 | -0.03±0.1 | 9.50E-01 | -0.08±0.13 | 7.93E-01 | -0.1±0.13 | 7.64E-01 |
| gamma-glutamyltryptophan | 0.06±0.08 | 85.87 | 7.81E-03 | 7.88E-01 | 0.05±0.08 | 79.67 | 2.66E-02 | 8.33E-01 | -0.06±0.09 | 9.06E-01 | -0.05±0.09 | 9.04E-01 | 0.41±0.15 | 1.99E-01 | 0.34±0.15 | 4.18E-01 |
| X - 25790 | 0.04±0.08 | 0 | 3.26E-01 | 8.43E-01 | 0.05±0.08 | 40.13 | 1.96E-01 | 8.33E-01 | -0.01±0.1 | 9.87E-01 | -0.02±0.1 | 9.80E-01 | 0.16±0.14 | 6.14E-01 | 0.21±0.15 | 5.65E-01 |
| X - 23659 | 0.05±0.08 | 42.1 | 1.89E-01 | 8.16E-01 | 0.05±0.08 | 24.63 | 2.49E-01 | 8.33E-01 | -0.02±0.09 | 9.74E-01 | -0.01±0.09 | 9.80E-01 | 0.2±0.14 | 5.23E-01 | 0.18±0.14 | 5.97E-01 |
| X - 12407 | -0.05±0.07 | 79.42 | 2.75E-02 | 8.11E-01 | -0.05±0.07 | 77.08 | 3.67E-02 | 8.33E-01 | -0.17±0.09 | 6.43E-01 | -0.16±0.09 | 6.39E-01 | 0.18±0.13 | 5.31E-01 | 0.17±0.13 | 5.97E-01 |
| N-palmitoyl-heptadecasphingosine (d17:1/16:0)* | 0.04±0.08 | 0 | 4.27E-01 | 8.43E-01 | 0.05±0.08 | 0 | 3.45E-01 | 8.33E-01 | 0±0.09 | 9.92E-01 | 0±0.09 | 9.93E-01 | 0.13±0.14 | 6.66E-01 | 0.17±0.14 | 6.45E-01 |
| orotidine | 0.06±0.07 | 0 | 6.71E-01 | 7.85E-01 | 0.05±0.07 | 0 | 9.08E-01 | 8.33E-01 | 0.04±0.09 | 9.38E-01 | 0.04±0.09 | 9.18E-01 | 0.1±0.13 | 7.28E-01 | 0.06±0.13 | 8.50E-01 |
| 2-aminobutyrate | 0.05±0.07 | 0 | 9.76E-01 | 8.11E-01 | 0.05±0.07 | 0 | 9.92E-01 | 8.33E-01 | 0.05±0.09 | 9.27E-01 | 0.05±0.09 | 9.18E-01 | 0.05±0.13 | 9.04E-01 | 0.05±0.13 | 8.95E-01 |
| imidazole lactate | 0.05±0.07 | 0 | 3.86E-01 | 8.11E-01 | 0.05±0.07 | 0 | 3.72E-01 | 8.33E-01 | 0.1±0.09 | 8.65E-01 | 0.1±0.09 | 8.36E-01 | -0.03±0.12 | 9.20E-01 | -0.04±0.12 | 9.19E-01 |
| taurocholenate sulfate* | -0.06±0.07 | 0 | 6.72E-01 | 7.92E-01 | -0.05±0.07 | 0 | 4.99E-01 | 8.33E-01 | -0.08±0.09 | 8.80E-01 | -0.09±0.09 | 8.60E-01 | -0.01±0.13 | 9.84E-01 | 0.02±0.13 | 9.56E-01 |
| 1-linoleoyl-GPG (18:2)* | -0.04±0.07 | 0 | 4.39E-01 | 8.50E-01 | -0.05±0.07 | 0 | 6.15E-01 | 8.33E-01 | -0.08±0.09 | 8.80E-01 | -0.07±0.09 | 8.76E-01 | 0.04±0.12 | 9.06E-01 | 0±0.13 | 9.92E-01 |
| serine | 0.04±0.07 | 0 | 9.92E-01 | 8.40E-01 | 0.05±0.08 | 0 | 7.33E-01 | 8.33E-01 | 0.04±0.09 | 9.27E-01 | 0.03±0.09 | 9.24E-01 | 0.04±0.13 | 9.05E-01 | 0.09±0.14 | 7.92E-01 |
| X - 12283 | 0.04±0.07 | 76.44 | 3.94E-02 | 8.73E-01 | 0.05±0.08 | 78.56 | 3.08E-02 | 8.33E-01 | -0.07±0.09 | 9.03E-01 | -0.06±0.09 | 8.94E-01 | 0.27±0.14 | 3.40E-01 | 0.3±0.14 | 4.18E-01 |
| X - 17676 | -0.04±0.07 | 78.85 | 2.97E-02 | 8.41E-01 | -0.05±0.08 | 74.65 | 4.70E-02 | 8.33E-01 | -0.16±0.09 | 6.65E-01 | -0.16±0.1 | 6.64E-01 | 0.17±0.12 | 5.33E-01 | 0.15±0.13 | 6.32E-01 |
| laurylcarnitine (C12) | -0.05±0.07 | 0 | 7.96E-01 | 8.16E-01 | -0.05±0.07 | 0 | 9.00E-01 | 8.33E-01 | -0.04±0.09 | 9.46E-01 | -0.04±0.09 | 9.18E-01 | -0.08±0.13 | 8.09E-01 | -0.06±0.13 | 8.50E-01 |
| tyrosine | -0.05±0.08 | 80.77 | 2.26E-02 | 8.11E-01 | -0.05±0.08 | 77.01 | 3.70E-02 | 8.33E-01 | -0.16±0.09 | 6.65E-01 | -0.15±0.09 | 6.82E-01 | 0.24±0.15 | 4.63E-01 | 0.21±0.15 | 5.71E-01 |
| deoxycholate | 0.06±0.07 | 0 | 7.42E-01 | 7.74E-01 | 0.05±0.07 | 0 | 9.01E-01 | 8.33E-01 | 0.05±0.09 | 9.27E-01 | 0.06±0.09 | 9.04E-01 | 0.1±0.13 | 7.38E-01 | 0.04±0.13 | 9.28E-01 |
| glycerate | -0.07±0.07 | 31.54 | 2.27E-01 | 7.44E-01 | -0.05±0.08 | 58.48 | 1.21E-01 | 8.34E-01 | -0.13±0.09 | 7.72E-01 | -0.12±0.09 | 7.69E-01 | 0.06±0.13 | 8.52E-01 | 0.14±0.14 | 6.91E-01 |
| X - 24306 | -0.04±0.07 | 0 | 7.18E-01 | 8.32E-01 | -0.05±0.07 | 0 | 6.60E-01 | 8.34E-01 | -0.03±0.09 | 9.58E-01 | -0.02±0.09 | 9.55E-01 | -0.08±0.12 | 7.88E-01 | -0.09±0.13 | 7.64E-01 |
| 3-methylxanthine | -0.05±0.07 | 30.11 | 2.32E-01 | 8.16E-01 | -0.05±0.07 | 21.02 | 2.60E-01 | 8.34E-01 | -0.11±0.09 | 8.31E-01 | -0.11±0.09 | 8.36E-01 | 0.07±0.12 | 8.14E-01 | 0.07±0.13 | 8.26E-01 |
| glycochenodeoxycholate | -0.04±0.07 | 0 | 8.86E-01 | 8.43E-01 | -0.05±0.07 | 0 | 9.33E-01 | 8.34E-01 | -0.05±0.09 | 9.27E-01 | -0.04±0.09 | 9.18E-01 | -0.03±0.12 | 9.47E-01 | -0.06±0.12 | 8.61E-01 |
| N-oleoylserine | 0.05±0.07 | 0 | 9.25E-01 | 8.06E-01 | 0.05±0.07 | 0 | 9.94E-01 | 8.34E-01 | 0.06±0.09 | 9.11E-01 | 0.05±0.09 | 9.18E-01 | 0.04±0.12 | 9.05E-01 | 0.05±0.13 | 8.97E-01 |
| 9,10-DiHOME | -0.06±0.07 | 0 | 5.33E-01 | 7.85E-01 | -0.05±0.07 | 0 | 4.05E-01 | 8.34E-01 | -0.09±0.09 | 8.65E-01 | -0.09±0.09 | 8.36E-01 | 0.01±0.13 | 9.92E-01 | 0.04±0.13 | 9.11E-01 |
| pantoate | 0.05±0.07 | 0 | 5.87E-01 | 8.16E-01 | 0.05±0.08 | 0 | 5.20E-01 | 8.35E-01 | 0.02±0.09 | 9.71E-01 | 0.01±0.09 | 9.80E-01 | 0.11±0.13 | 7.25E-01 | 0.12±0.13 | 7.18E-01 |
| 2-oxoarginine* | 0.05±0.07 | 0 | 3.23E-01 | 8.09E-01 | 0.05±0.08 | 0 | 6.32E-01 | 8.35E-01 | 0±0.09 | 9.89E-01 | 0.02±0.09 | 9.57E-01 | 0.16±0.13 | 5.85E-01 | 0.1±0.14 | 7.64E-01 |
| 2-hydroxystearate | -0.04±0.07 | 35.25 | 2.14E-01 | 8.40E-01 | -0.05±0.07 | 36.25 | 2.10E-01 | 8.35E-01 | -0.11±0.09 | 8.34E-01 | -0.11±0.09 | 8.32E-01 | 0.08±0.13 | 7.80E-01 | 0.08±0.13 | 7.96E-01 |
| 2-hydroxybutyrate/2-hydroxyisobutyrate | 0.05±0.07 | 0 | 9.08E-01 | 8.03E-01 | 0.05±0.08 | 0 | 6.13E-01 | 8.35E-01 | 0.06±0.09 | 9.06E-01 | 0.08±0.09 | 8.91E-01 | 0.04±0.13 | 9.06E-01 | 0±0.13 | 9.92E-01 |
| glycosyl-N-palmitoyl-sphingosine (d18:1/16:0) | 0.03±0.07 | 18.31 | 2.69E-01 | 8.90E-01 | 0.05±0.08 | 0 | 4.59E-01 | 8.37E-01 | 0.1±0.1 | 8.65E-01 | 0.1±0.1 | 8.47E-01 | -0.06±0.11 | 8.14E-01 | -0.02±0.12 | 9.56E-01 |
| X - 24334 | 0.05±0.08 | 81.33 | 2.07E-02 | 8.40E-01 | 0.05±0.08 | 77.88 | 3.35E-02 | 8.42E-01 | -0.08±0.1 | 9.01E-01 | -0.07±0.1 | 8.94E-01 | 0.32±0.14 | 2.92E-01 | 0.31±0.15 | 4.21E-01 |
| behenoyl dihydrosphingomyelin (d18:0/22:0)* | 0.06±0.07 | 0 | 3.76E-01 | 8.01E-01 | 0.05±0.08 | 0 | 5.28E-01 | 8.43E-01 | 0.01±0.09 | 9.87E-01 | 0.01±0.1 | 9.80E-01 | 0.15±0.13 | 6.05E-01 | 0.12±0.13 | 7.24E-01 |
| phytanate | -0.05±0.07 | 28.39 | 2.37E-01 | 8.09E-01 | -0.05±0.07 | 39.42 | 1.99E-01 | 8.43E-01 | -0.11±0.09 | 8.31E-01 | -0.11±0.09 | 8.32E-01 | 0.07±0.12 | 8.23E-01 | 0.09±0.13 | 7.82E-01 |
| uracil | -0.04±0.07 | 0 | 9.58E-01 | 8.16E-01 | -0.04±0.07 | 0 | 9.98E-01 | 8.44E-01 | -0.04±0.09 | 9.27E-01 | -0.04±0.09 | 9.18E-01 | -0.05±0.12 | 8.81E-01 | -0.04±0.12 | 8.98E-01 |
| X - 24475 | 0.03±0.07 | 0 | 8.97E-01 | 9.08E-01 | 0.05±0.07 | 0 | 9.46E-01 | 8.44E-01 | 0.03±0.09 | 9.48E-01 | 0.04±0.09 | 9.18E-01 | 0.01±0.13 | 9.84E-01 | 0.05±0.13 | 8.84E-01 |
| N-acetylcarnosine | 0.06±0.09 | 77.95 | 3.32E-02 | 8.36E-01 | 0.06±0.09 | 69.56 | 6.99E-02 | 8.46E-01 | -0.06±0.1 | 9.27E-01 | -0.04±0.11 | 9.19E-01 | 0.39±0.18 | 3.09E-01 | 0.34±0.18 | 4.54E-01 |
| proline | 0.05±0.08 | 71.74 | 5.99E-02 | 8.20E-01 | 0.05±0.08 | 64.92 | 9.14E-02 | 8.46E-01 | -0.04±0.09 | 9.28E-01 | -0.03±0.09 | 9.19E-01 | 0.28±0.15 | 3.42E-01 | 0.26±0.15 | 4.65E-01 |
| 11beta-hydroxyandrosterone glucuronide | 0.05±0.07 | 76.4 | 3.95E-02 | 8.16E-01 | 0.04±0.07 | 71.52 | 6.09E-02 | 8.47E-01 | -0.04±0.09 | 9.27E-01 | -0.04±0.09 | 9.18E-01 | 0.3±0.14 | 3.23E-01 | 0.27±0.14 | 4.52E-01 |
| xanthine | 0.04±0.07 | 48.64 | 1.63E-01 | 8.53E-01 | 0.05±0.07 | 29.37 | 2.34E-01 | 8.47E-01 | -0.03±0.09 | 9.48E-01 | -0.01±0.09 | 9.80E-01 | 0.2±0.14 | 5.23E-01 | 0.18±0.14 | 5.97E-01 |
| 3-ureidopropionate | -0.06±0.08 | 45.57 | 1.75E-01 | 7.85E-01 | -0.05±0.08 | 58.58 | 1.20E-01 | 8.47E-01 | -0.14±0.1 | 7.81E-01 | -0.13±0.1 | 7.62E-01 | 0.08±0.13 | 7.92E-01 | 0.12±0.13 | 7.12E-01 |
| 2-hydroxydecanoate | -0.04±0.07 | 0 | 3.21E-01 | 8.39E-01 | -0.05±0.07 | 10.98 | 2.89E-01 | 8.47E-01 | -0.1±0.09 | 8.65E-01 | -0.1±0.09 | 8.36E-01 | 0.06±0.13 | 8.66E-01 | 0.06±0.13 | 8.48E-01 |
| uridine | 0.04±0.07 | 0 | 3.89E-01 | 8.43E-01 | 0.04±0.07 | 0 | 3.40E-01 | 8.47E-01 | 0.09±0.09 | 8.72E-01 | 0.1±0.09 | 8.36E-01 | -0.04±0.12 | 9.05E-01 | -0.04±0.12 | 8.98E-01 |
| 1-oleoyl-GPC (18:1) | 0.03±0.07 | 0 | 7.69E-01 | 8.73E-01 | 0.05±0.08 | 0 | 3.89E-01 | 8.48E-01 | 0.02±0.09 | 9.74E-01 | 0.01±0.09 | 9.88E-01 | 0.07±0.13 | 8.47E-01 | 0.16±0.15 | 6.67E-01 |
| dihomo-linolenate (20:3n3 or n6) | -0.04±0.07 | 0 | 6.18E-01 | 8.73E-01 | -0.05±0.08 | 0 | 7.45E-01 | 8.48E-01 | -0.06±0.09 | 9.06E-01 | -0.06±0.09 | 8.94E-01 | 0.02±0.13 | 9.75E-01 | -0.01±0.13 | 9.80E-01 |
| 1-palmitoyl-2-linoleoyl-GPI (16:0/18:2) | 0.05±0.08 | 35.79 | 2.12E-01 | 8.11E-01 | 0.05±0.08 | 12 | 2.86E-01 | 8.48E-01 | -0.01±0.09 | 9.86E-01 | -0.01±0.09 | 9.80E-01 | 0.2±0.14 | 5.31E-01 | 0.17±0.14 | 6.29E-01 |
| sphingomyelin (d18:2/21:0, d16:2/23:0)* | 0.03±0.09 | 0 | 5.60E-01 | 9.03E-01 | 0.05±0.09 | 0 | 6.79E-01 | 8.48E-01 | 0±0.1 | 9.89E-01 | 0.03±0.11 | 9.58E-01 | 0.1±0.15 | 7.68E-01 | 0.1±0.15 | 7.82E-01 |
| X - 18899 | -0.05±0.07 | 0 | 6.09E-01 | 8.09E-01 | -0.04±0.07 | 0 | 7.08E-01 | 8.48E-01 | -0.02±0.09 | 9.59E-01 | -0.02±0.09 | 9.57E-01 | -0.1±0.12 | 7.18E-01 | -0.08±0.12 | 7.91E-01 |
| linoleoylcarnitine (C18:2)* | -0.05±0.07 | 0 | 6.63E-01 | 8.03E-01 | -0.04±0.07 | 0 | 7.69E-01 | 8.48E-01 | -0.03±0.09 | 9.48E-01 | -0.03±0.09 | 9.46E-01 | -0.09±0.12 | 7.29E-01 | -0.07±0.12 | 8.03E-01 |
| beta-alanine | -0.04±0.08 | 43.74 | 1.82E-01 | 8.68E-01 | -0.04±0.08 | 15.44 | 2.77E-01 | 8.48E-01 | -0.1±0.09 | 8.65E-01 | -0.1±0.09 | 8.36E-01 | 0.13±0.14 | 6.95E-01 | 0.08±0.14 | 8.16E-01 |
| 1-stearoyl-2-linoleoyl-GPI (18:0/18:2) | -0.05±0.07 | 0 | 7.36E-01 | 8.09E-01 | -0.04±0.07 | 0 | 5.93E-01 | 8.48E-01 | -0.07±0.09 | 9.03E-01 | -0.07±0.09 | 8.91E-01 | -0.02±0.13 | 9.76E-01 | 0.01±0.13 | 9.80E-01 |
| maleate | 0.06±0.07 | 71.78 | 5.98E-02 | 7.85E-01 | 0.04±0.07 | 58.4 | 1.21E-01 | 8.50E-01 | -0.05±0.09 | 9.27E-01 | -0.05±0.09 | 9.18E-01 | 0.22±0.11 | 3.40E-01 | 0.18±0.12 | 4.95E-01 |
| vanillylmandelate (VMA) | 0.04±0.08 | 60.86 | 1.10E-01 | 8.43E-01 | 0.04±0.08 | 55.78 | 1.33E-01 | 8.50E-01 | -0.05±0.1 | 9.27E-01 | -0.04±0.1 | 9.19E-01 | 0.22±0.14 | 4.56E-01 | 0.22±0.14 | 5.00E-01 |
| 5-methyluridine (ribothymidine) | 0.04±0.08 | 23.08 | 2.54E-01 | 8.73E-01 | 0.04±0.08 | 38.01 | 2.04E-01 | 8.50E-01 | -0.02±0.09 | 9.59E-01 | -0.02±0.09 | 9.58E-01 | 0.16±0.14 | 5.92E-01 | 0.19±0.14 | 5.80E-01 |
| palmitate (16:0) | -0.03±0.07 | 9.87 | 2.92E-01 | 8.73E-01 | -0.04±0.08 | 30.42 | 2.31E-01 | 8.50E-01 | 0.03±0.09 | 9.59E-01 | 0.02±0.1 | 9.58E-01 | -0.14±0.13 | 6.14E-01 | -0.17±0.13 | 5.97E-01 |
| beta-hydroxyisovalerate | 0.05±0.08 | 39.75 | 1.98E-01 | 8.16E-01 | 0.04±0.08 | 21.52 | 2.59E-01 | 8.50E-01 | -0.01±0.09 | 9.78E-01 | -0.01±0.09 | 9.80E-01 | 0.21±0.14 | 5.29E-01 | 0.18±0.14 | 6.20E-01 |
| X - 11849 | 0.04±0.07 | 16.54 | 2.74E-01 | 8.41E-01 | 0.04±0.07 | 8.24 | 2.97E-01 | 8.50E-01 | -0.01±0.09 | 9.80E-01 | -0.01±0.09 | 9.80E-01 | 0.16±0.13 | 5.83E-01 | 0.16±0.13 | 6.36E-01 |
| X - 26109 | 0.05±0.07 | 47.67 | 1.67E-01 | 8.11E-01 | 0.04±0.08 | 0 | 3.54E-01 | 8.50E-01 | -0.02±0.09 | 9.71E-01 | 0±0.09 | 9.93E-01 | 0.2±0.13 | 4.95E-01 | 0.15±0.13 | 6.66E-01 |
| vanillactate | 0.05±0.08 | 17.82 | 2.70E-01 | 8.11E-01 | 0.04±0.08 | 0 | 3.75E-01 | 8.50E-01 | 0±0.09 | 9.89E-01 | 0±0.09 | 9.96E-01 | 0.18±0.14 | 5.71E-01 | 0.15±0.14 | 6.67E-01 |
| X - 26119 | -0.04±0.08 | 62.08 | 1.04E-01 | 8.62E-01 | -0.04±0.08 | 61.81 | 1.06E-01 | 8.50E-01 | -0.12±0.09 | 7.93E-01 | -0.13±0.09 | 7.69E-01 | 0.15±0.14 | 6.23E-01 | 0.14±0.14 | 6.73E-01 |
| sphingomyelin (d17:2/16:0, d18:2/15:0)* | -0.06±0.09 | 64.55 | 9.30E-02 | 7.92E-01 | -0.05±0.09 | 60.63 | 1.11E-01 | 8.50E-01 | -0.16±0.1 | 7.50E-01 | -0.15±0.11 | 7.62E-01 | 0.14±0.15 | 6.66E-01 | 0.14±0.15 | 6.93E-01 |
| creatinine | 0.05±0.09 | 0 | 5.54E-01 | 8.43E-01 | 0.05±0.09 | 0 | 5.38E-01 | 8.50E-01 | 0.01±0.1 | 9.87E-01 | 0.01±0.1 | 9.80E-01 | 0.12±0.15 | 7.29E-01 | 0.13±0.15 | 7.39E-01 |
| 3-hydroxybutyrate (BHBA) | 0.04±0.07 | 41.79 | 1.90E-01 | 8.41E-01 | 0.04±0.07 | 31.17 | 2.28E-01 | 8.50E-01 | 0.11±0.09 | 8.31E-01 | 0.11±0.09 | 8.36E-01 | -0.09±0.13 | 7.44E-01 | -0.09±0.13 | 7.91E-01 |
| X - 17325 | -0.04±0.07 | 25.5 | 2.47E-01 | 8.42E-01 | -0.04±0.07 | 16.93 | 2.73E-01 | 8.50E-01 | -0.1±0.09 | 8.65E-01 | -0.1±0.09 | 8.36E-01 | 0.08±0.13 | 8.00E-01 | 0.07±0.13 | 8.18E-01 |
| 2-aminooctanoate | -0.06±0.07 | 0 | 7.24E-01 | 7.92E-01 | -0.04±0.07 | 0 | 9.96E-01 | 8.50E-01 | -0.04±0.09 | 9.38E-01 | -0.04±0.09 | 9.18E-01 | -0.09±0.13 | 7.44E-01 | -0.04±0.13 | 9.02E-01 |
| (S)-3-hydroxybutyrylcarnitine | 0.06±0.07 | 0 | 8.01E-01 | 7.80E-01 | 0.04±0.07 | 0 | 4.55E-01 | 8.50E-01 | 0.08±0.09 | 8.94E-01 | 0.08±0.09 | 8.63E-01 | 0.04±0.13 | 9.20E-01 | -0.04±0.13 | 9.26E-01 |
| 4-methylhexanoylglutamine | 0.05±0.08 | 0 | 9.43E-01 | 8.39E-01 | 0.04±0.08 | 0 | 9.22E-01 | 8.50E-01 | 0.05±0.1 | 9.27E-01 | 0.05±0.1 | 9.18E-01 | 0.04±0.13 | 9.20E-01 | 0.04±0.13 | 9.28E-01 |
| 5alpha-androstan-3beta,17alpha-diol disulfate | -0.07±0.08 | 0 | 6.99E-01 | 7.84E-01 | -0.05±0.08 | 0 | 5.21E-01 | 8.50E-01 | -0.09±0.1 | 8.80E-01 | -0.09±0.1 | 8.68E-01 | -0.03±0.13 | 9.47E-01 | 0.02±0.13 | 9.56E-01 |
| glycoursodeoxycholate | 0.05±0.07 | 0 | 7.74E-01 | 8.09E-01 | 0.04±0.07 | 0 | 6.57E-01 | 8.50E-01 | 0.07±0.09 | 9.03E-01 | 0.07±0.09 | 8.94E-01 | 0.02±0.12 | 9.61E-01 | 0±0.13 | 9.94E-01 |
| arachidonate (20:4n6) | -0.03±0.07 | 0 | 7.04E-01 | 8.82E-01 | -0.04±0.07 | 0 | 6.93E-01 | 8.50E-01 | -0.05±0.09 | 9.27E-01 | -0.06±0.09 | 8.94E-01 | 0.01±0.12 | 9.92E-01 | 0±0.12 | 9.95E-01 |
| hypotaurine | 0.03±0.08 | 65.71 | 8.77E-02 | 9.00E-01 | 0.04±0.08 | 78.19 | 3.23E-02 | 8.56E-01 | -0.06±0.09 | 9.11E-01 | -0.07±0.1 | 8.94E-01 | 0.22±0.14 | 4.46E-01 | 0.3±0.14 | 4.21E-01 |
| androstenediol (3alpha, 17alpha) monosulfate (3) | 0.03±0.08 | 66 | 8.64E-02 | 8.93E-01 | 0.05±0.08 | 77.28 | 3.59E-02 | 8.56E-01 | -0.06±0.1 | 9.20E-01 | -0.07±0.1 | 8.94E-01 | 0.25±0.15 | 4.40E-01 | 0.31±0.15 | 4.30E-01 |
| 1-stearoyl-2-oleoyl-GPC (18:0/18:1) | 0.05±0.08 | 68.64 | 7.41E-02 | 8.20E-01 | 0.05±0.08 | 67.83 | 7.79E-02 | 8.56E-01 | -0.03±0.1 | 9.48E-01 | -0.04±0.1 | 9.19E-01 | 0.31±0.17 | 3.61E-01 | 0.3±0.17 | 4.65E-01 |
| 1-palmitoyl-2-arachidonoyl-GPI (16:0/20:4)* | 0.06±0.08 | 70.68 | 6.48E-02 | 8.09E-01 | 0.04±0.08 | 62.21 | 1.04E-01 | 8.56E-01 | -0.04±0.09 | 9.46E-01 | -0.04±0.1 | 9.19E-01 | 0.29±0.15 | 3.42E-01 | 0.25±0.15 | 4.86E-01 |
| 1-stearoyl-2-oleoyl-GPI (18:0/18:1)* | 0.04±0.08 | 37.54 | 2.06E-01 | 8.43E-01 | 0.04±0.08 | 51.73 | 1.50E-01 | 8.56E-01 | -0.02±0.09 | 9.78E-01 | -0.02±0.09 | 9.57E-01 | 0.21±0.15 | 5.43E-01 | 0.24±0.16 | 5.28E-01 |
| X - 18887 | 0.03±0.08 | 23.85 | 2.52E-01 | 8.90E-01 | 0.04±0.08 | 51.91 | 1.49E-01 | 8.56E-01 | -0.03±0.09 | 9.48E-01 | -0.04±0.09 | 9.19E-01 | 0.15±0.13 | 6.00E-01 | 0.2±0.13 | 5.37E-01 |
| cysteinylglycine disulfide* | 0.04±0.08 | 24.18 | 2.51E-01 | 8.43E-01 | 0.04±0.08 | 2.78 | 3.10E-01 | 8.56E-01 | 0±0.09 | 9.89E-01 | 0±0.09 | 9.98E-01 | 0.23±0.18 | 5.71E-01 | 0.19±0.17 | 6.45E-01 |
| 2,3-dihydroxyisovalerate | 0.02±0.07 | 0 | 8.05E-01 | 9.18E-01 | 0.04±0.08 | 0 | 3.91E-01 | 8.56E-01 | 0.01±0.09 | 9.87E-01 | 0±0.09 | 9.94E-01 | 0.05±0.13 | 9.01E-01 | 0.14±0.14 | 6.77E-01 |
| 1-palmitoyl-2-docosahexaenoyl-GPC (16:0/22:6) | -0.05±0.07 | 0 | 4.08E-01 | 7.99E-01 | -0.04±0.07 | 0 | 6.12E-01 | 8.56E-01 | 0±0.09 | 9.89E-01 | -0.01±0.09 | 9.80E-01 | -0.12±0.11 | 6.14E-01 | -0.08±0.11 | 7.64E-01 |
| 3-hydroxydecanoylcarnitine | -0.04±0.07 | 0 | 6.07E-01 | 8.43E-01 | -0.04±0.08 | 0 | 6.98E-01 | 8.56E-01 | -0.01±0.09 | 9.80E-01 | -0.02±0.09 | 9.63E-01 | -0.1±0.13 | 7.44E-01 | -0.08±0.13 | 7.96E-01 |
| hexadecenedioate (C16:1-DC)* | 0.04±0.07 | 0 | 9.39E-01 | 8.46E-01 | 0.04±0.07 | 0 | 9.85E-01 | 8.56E-01 | 0.04±0.09 | 9.27E-01 | 0.04±0.09 | 9.18E-01 | 0.03±0.12 | 9.38E-01 | 0.04±0.13 | 9.06E-01 |
| X - 23974 | 0.04±0.07 | 0 | 4.31E-01 | 8.40E-01 | 0.04±0.07 | 0 | 4.73E-01 | 8.56E-01 | 0.09±0.09 | 8.73E-01 | 0.08±0.09 | 8.68E-01 | -0.04±0.12 | 9.20E-01 | -0.03±0.13 | 9.29E-01 |
| X - 25957 | -0.04±0.07 | 0 | 5.26E-01 | 8.43E-01 | -0.04±0.07 | 0 | 4.94E-01 | 8.56E-01 | -0.07±0.09 | 9.01E-01 | -0.07±0.09 | 8.68E-01 | 0.03±0.13 | 9.40E-01 | 0.03±0.13 | 9.29E-01 |
| X - 26106 | -0.03±0.07 | 0 | 7.70E-01 | 8.73E-01 | -0.04±0.08 | 0 | 9.14E-01 | 8.56E-01 | -0.05±0.09 | 9.27E-01 | -0.05±0.09 | 9.18E-01 | 0±0.13 | 9.96E-01 | -0.03±0.13 | 9.35E-01 |
| pyruvate | -0.04±0.08 | 87.15 | 5.28E-03 | 8.47E-01 | -0.04±0.08 | 83.92 | 1.26E-02 | 8.59E-01 | -0.17±0.09 | 6.26E-01 | -0.16±0.09 | 6.39E-01 | 0.32±0.15 | 3.16E-01 | 0.28±0.15 | 4.63E-01 |
| 3-hydroxybutyroylglycine** | 0.04±0.08 | 81.23 | 2.10E-02 | 8.43E-01 | 0.04±0.08 | 79.34 | 2.78E-02 | 8.59E-01 | 0.2±0.1 | 6.10E-01 | 0.19±0.1 | 6.15E-01 | -0.17±0.12 | 5.33E-01 | -0.18±0.13 | 5.85E-01 |
| myristoylcarnitine (C14) | 0.04±0.08 | 0 | 8.78E-01 | 8.43E-01 | 0.04±0.08 | 0 | 8.94E-01 | 8.59E-01 | 0.03±0.09 | 9.48E-01 | 0.03±0.09 | 9.24E-01 | 0.06±0.13 | 8.66E-01 | 0.06±0.13 | 8.79E-01 |
| tartarate | 0.01±0.07 | 0 | 3.46E-01 | 9.49E-01 | 0.04±0.07 | 0 | 5.50E-01 | 8.59E-01 | 0.07±0.09 | 9.03E-01 | 0.07±0.09 | 8.93E-01 | -0.08±0.12 | 7.89E-01 | -0.02±0.12 | 9.58E-01 |
| 3-aminoisobutyrate | 0.04±0.07 | 0 | 4.53E-01 | 8.57E-01 | 0.04±0.07 | 0 | 5.67E-01 | 8.59E-01 | 0.08±0.09 | 8.94E-01 | 0.07±0.09 | 8.94E-01 | -0.04±0.13 | 9.15E-01 | -0.02±0.13 | 9.59E-01 |
| 5-(galactosylhydroxy)-L-lysine | 0.05±0.08 | 19.05 | 2.66E-01 | 8.16E-01 | 0.04±0.08 | 0 | 4.68E-01 | 8.60E-01 | 0±0.09 | 9.89E-01 | 0±0.09 | 9.93E-01 | 0.18±0.14 | 5.71E-01 | 0.13±0.14 | 7.17E-01 |
| arabonate/xylonate | 0.02±0.08 | 79.71 | 2.64E-02 | 9.18E-01 | 0.04±0.08 | 82.14 | 1.80E-02 | 8.60E-01 | -0.09±0.09 | 8.72E-01 | -0.08±0.09 | 8.68E-01 | 0.28±0.14 | 3.34E-01 | 0.33±0.14 | 4.18E-01 |
| sphingomyelin (d17:1/14:0, d16:1/15:0)* | 0.02±0.08 | 46.55 | 1.71E-01 | 9.47E-01 | 0.05±0.09 | 59.86 | 1.14E-01 | 8.60E-01 | -0.06±0.1 | 9.27E-01 | -0.05±0.1 | 9.18E-01 | 0.18±0.15 | 5.77E-01 | 0.25±0.16 | 4.95E-01 |
| 1-linoleoyl-2-arachidonoyl-GPC (18:2/20:4n6)* | -0.04±0.08 | 68.55 | 7.46E-02 | 8.75E-01 | -0.04±0.08 | 75.47 | 4.35E-02 | 8.60E-01 | -0.11±0.09 | 8.31E-01 | -0.12±0.09 | 7.62E-01 | 0.22±0.16 | 5.51E-01 | 0.27±0.17 | 5.04E-01 |
| N-acetylmethionine | 0.04±0.07 | 41.75 | 1.90E-01 | 8.43E-01 | 0.04±0.08 | 16.74 | 2.73E-01 | 8.60E-01 | -0.03±0.09 | 9.59E-01 | -0.02±0.09 | 9.66E-01 | 0.18±0.13 | 5.33E-01 | 0.16±0.13 | 6.34E-01 |
| deoxycarnitine | 0.04±0.08 | 0 | 4.51E-01 | 8.43E-01 | 0.04±0.08 | 0 | 4.48E-01 | 8.60E-01 | 0.01±0.09 | 9.89E-01 | 0.01±0.09 | 9.88E-01 | 0.14±0.15 | 6.77E-01 | 0.14±0.15 | 7.10E-01 |
| glycosyl ceramide (d18:2/24:1, d18:1/24:2)* | -0.06±0.07 | 25.68 | 2.46E-01 | 7.92E-01 | -0.04±0.07 | 0 | 4.56E-01 | 8.60E-01 | 0.02±0.1 | 9.78E-01 | 0.01±0.1 | 9.80E-01 | -0.15±0.11 | 5.39E-01 | -0.1±0.12 | 7.13E-01 |
| oxalate (ethanedioate) | -0.06±0.07 | 0 | 7.60E-01 | 7.70E-01 | -0.04±0.07 | 0 | 9.56E-01 | 8.60E-01 | -0.05±0.09 | 9.27E-01 | -0.04±0.09 | 9.19E-01 | -0.09±0.12 | 7.35E-01 | -0.04±0.12 | 8.99E-01 |
| indolebutyrate | 0.05±0.07 | 88.48 | 3.21E-03 | 8.09E-01 | 0.04±0.07 | 86.11 | 7.30E-03 | 8.61E-01 | -0.11±0.09 | 8.52E-01 | -0.1±0.09 | 8.36E-01 | 0.35±0.12 | 1.99E-01 | 0.31±0.12 | 4.03E-01 |
| 2,3-dihydroxy-5-methylthio-4-pentenoate (DMTPA)* | 0.05±0.08 | 28.66 | 2.36E-01 | 8.16E-01 | 0.04±0.08 | 0 | 4.80E-01 | 8.61E-01 | -0.01±0.09 | 9.80E-01 | 0±0.1 | 9.98E-01 | 0.18±0.13 | 5.53E-01 | 0.12±0.13 | 7.24E-01 |
| 2-hydroxysebacate | 0.05±0.07 | 0 | 3.94E-01 | 8.20E-01 | 0.04±0.07 | 0 | 4.87E-01 | 8.61E-01 | 0±0.09 | 9.89E-01 | 0±0.09 | 9.94E-01 | 0.14±0.13 | 6.26E-01 | 0.12±0.13 | 7.25E-01 |
| N-carbamoylvaline | 0.05±0.08 | 0 | 4.29E-01 | 8.11E-01 | 0.04±0.08 | 4.32 | 3.07E-01 | 8.61E-01 | 0.1±0.1 | 8.65E-01 | 0.1±0.1 | 8.36E-01 | -0.03±0.13 | 9.40E-01 | -0.07±0.13 | 8.35E-01 |
| 1-(1-enyl-palmitoyl)-2-palmitoyl-GPC (P-16:0/16:0)* | -0.04±0.07 | 0 | 8.51E-01 | 8.16E-01 | -0.04±0.07 | 0 | 7.89E-01 | 8.61E-01 | -0.03±0.09 | 9.47E-01 | -0.05±0.1 | 9.04E-01 | -0.06±0.11 | 8.18E-01 | -0.01±0.12 | 9.72E-01 |
| branched-chain, straight-chain, or cyclopropyl 12:1 fatty acid* | -0.04±0.08 | 76.9 | 3.75E-02 | 8.46E-01 | -0.04±0.08 | 76.33 | 3.98E-02 | 8.62E-01 | 0.08±0.1 | 8.80E-01 | 0.08±0.1 | 8.68E-01 | -0.24±0.12 | 3.42E-01 | -0.24±0.12 | 4.52E-01 |
| choline | 0.04±0.08 | 42.94 | 1.86E-01 | 8.47E-01 | 0.04±0.08 | 25.79 | 2.46E-01 | 8.64E-01 | -0.02±0.09 | 9.77E-01 | -0.01±0.09 | 9.80E-01 | 0.23±0.16 | 5.31E-01 | 0.2±0.16 | 6.21E-01 |
| 1-palmitoyl-2-stearoyl-GPC (16:0/18:0) | 0.03±0.08 | 0 | 4.56E-01 | 8.90E-01 | 0.04±0.08 | 17.99 | 2.69E-01 | 8.64E-01 | 0±0.09 | 9.89E-01 | -0.01±0.09 | 9.80E-01 | 0.13±0.15 | 7.03E-01 | 0.2±0.17 | 6.29E-01 |
| retinal | 0.03±0.07 | 0 | 5.87E-01 | 8.90E-01 | 0.04±0.07 | 0 | 5.14E-01 | 8.64E-01 | 0±0.09 | 9.89E-01 | 0.01±0.09 | 9.88E-01 | 0.09±0.13 | 7.73E-01 | 0.11±0.14 | 7.44E-01 |
| sphingomyelin (d18:1/17:0, d17:1/18:0, d19:1/16:0) | 0.02±0.08 | 0 | 5.85E-01 | 9.45E-01 | 0.04±0.08 | 0 | 4.91E-01 | 8.64E-01 | -0.01±0.1 | 9.86E-01 | 0±0.1 | 9.93E-01 | 0.08±0.14 | 8.14E-01 | 0.13±0.15 | 7.32E-01 |
| N-palmitoylglycine | 0.04±0.07 | 0 | 3.77E-01 | 8.41E-01 | 0.04±0.08 | 0 | 4.16E-01 | 8.64E-01 | 0.09±0.1 | 8.69E-01 | 0.09±0.1 | 8.63E-01 | -0.04±0.12 | 9.05E-01 | -0.04±0.12 | 9.02E-01 |
| oleoylcarnitine (C18:1) | 0.04±0.07 | 0 | 8.83E-01 | 8.61E-01 | 0.04±0.07 | 0 | 9.14E-01 | 8.64E-01 | 0.04±0.09 | 9.27E-01 | 0.04±0.09 | 9.18E-01 | 0.02±0.13 | 9.61E-01 | 0.03±0.13 | 9.41E-01 |
| gamma-glutamylglutamine | -0.04±0.07 | 0 | 4.46E-01 | 8.49E-01 | -0.04±0.08 | 13.97 | 2.81E-01 | 8.66E-01 | -0.07±0.09 | 8.90E-01 | -0.09±0.09 | 8.36E-01 | 0.05±0.13 | 9.05E-01 | 0.09±0.14 | 7.96E-01 |
| homovanillate (HVA) | -0.03±0.07 | 71.16 | 6.26E-02 | 9.03E-01 | -0.04±0.07 | 67.32 | 8.02E-02 | 8.66E-01 | -0.12±0.09 | 7.90E-01 | -0.13±0.09 | 7.62E-01 | 0.16±0.13 | 5.68E-01 | 0.15±0.13 | 6.46E-01 |
| N-acetyltryptophan | 0.04±0.08 | 0 | 4.61E-01 | 8.44E-01 | 0.04±0.08 | 0 | 6.18E-01 | 8.66E-01 | 0±0.1 | 9.92E-01 | 0.01±0.1 | 9.80E-01 | 0.13±0.14 | 6.82E-01 | 0.1±0.14 | 7.82E-01 |
| glycerol | 0.05±0.08 | 0 | 8.22E-01 | 8.39E-01 | 0.04±0.08 | 0 | 6.04E-01 | 8.66E-01 | 0.06±0.1 | 9.20E-01 | 0.07±0.1 | 8.94E-01 | 0.02±0.14 | 9.67E-01 | -0.02±0.14 | 9.62E-01 |
| X - 23644 | -0.04±0.07 | 0 | 6.49E-01 | 8.68E-01 | -0.04±0.07 | 0 | 6.60E-01 | 8.66E-01 | -0.06±0.09 | 9.08E-01 | -0.06±0.09 | 8.94E-01 | 0.01±0.12 | 9.84E-01 | 0.01±0.13 | 9.85E-01 |
| 4-allylphenol sulfate | -0.06±0.07 | 0 | 9.68E-01 | 7.74E-01 | -0.04±0.07 | 0 | 7.01E-01 | 8.66E-01 | -0.06±0.09 | 9.03E-01 | -0.06±0.09 | 9.00E-01 | -0.06±0.12 | 8.59E-01 | 0±0.13 | 9.92E-01 |
| sphingomyelin (d18:1/14:0, d16:1/16:0)* | 0.03±0.08 | 43.72 | 1.83E-01 | 9.00E-01 | 0.04±0.08 | 51.73 | 1.50E-01 | 8.67E-01 | -0.03±0.1 | 9.48E-01 | -0.03±0.1 | 9.55E-01 | 0.22±0.16 | 5.53E-01 | 0.26±0.17 | 5.37E-01 |
| 2-naphthol sulfate | -0.01±0.07 | 0 | 6.25E-01 | 9.49E-01 | -0.05±0.1 | 37.2 | 2.07E-01 | 8.68E-01 | -0.04±0.09 | 9.34E-01 | -0.14±0.12 | 8.36E-01 | 0.04±0.13 | 9.20E-01 | 0.11±0.16 | 7.82E-01 |
| indoleacetate | 0.04±0.07 | 76.51 | 3.91E-02 | 8.20E-01 | 0.04±0.07 | 72.7 | 5.56E-02 | 8.69E-01 | -0.07±0.09 | 9.03E-01 | -0.07±0.09 | 8.91E-01 | 0.24±0.12 | 3.34E-01 | 0.21±0.12 | 4.65E-01 |
| thioproline | 0.04±0.07 | 28.01 | 2.39E-01 | 8.40E-01 | 0.04±0.07 | 3.18 | 3.09E-01 | 8.69E-01 | -0.03±0.09 | 9.59E-01 | -0.02±0.09 | 9.55E-01 | 0.15±0.12 | 5.71E-01 | 0.12±0.11 | 6.66E-01 |
| cortolone glucuronide (1) | -0.01±0.07 | 80.1 | 2.50E-02 | 9.51E-01 | -0.04±0.07 | 58.17 | 1.22E-01 | 8.69E-01 | -0.11±0.08 | 7.90E-01 | -0.11±0.09 | 8.36E-01 | 0.25±0.14 | 3.69E-01 | 0.15±0.14 | 6.67E-01 |
| X - 12216 | 0.02±0.07 | 0 | 7.58E-01 | 9.45E-01 | 0.04±0.08 | 0 | 9.86E-01 | 8.69E-01 | 0.03±0.09 | 9.46E-01 | 0.04±0.09 | 9.19E-01 | -0.01±0.13 | 9.82E-01 | 0.04±0.13 | 9.22E-01 |
| O-sulfo-L-tyrosine | 0.04±0.07 | 0 | 9.22E-01 | 8.43E-01 | 0.04±0.07 | 0 | 8.68E-01 | 8.69E-01 | 0.04±0.09 | 9.27E-01 | 0.04±0.09 | 9.18E-01 | 0.03±0.13 | 9.40E-01 | 0.02±0.13 | 9.65E-01 |
| X - 12707 | -0.04±0.07 | 71.18 | 6.25E-02 | 8.39E-01 | -0.04±0.08 | 77.28 | 3.59E-02 | 8.70E-01 | -0.14±0.09 | 7.50E-01 | -0.14±0.09 | 7.19E-01 | 0.16±0.13 | 5.89E-01 | 0.2±0.14 | 5.48E-01 |
| sphingomyelin (d18:2/23:1)* | 0.02±0.08 | 0 | 6.31E-01 | 9.45E-01 | 0.04±0.09 | 0 | 6.15E-01 | 8.71E-01 | -0.01±0.1 | 9.87E-01 | 0.01±0.11 | 9.80E-01 | 0.07±0.14 | 8.40E-01 | 0.1±0.14 | 7.82E-01 |
| 3-formylindole | 0.03±0.08 | 0 | 5.61E-01 | 8.95E-01 | 0.04±0.08 | 0 | 7.01E-01 | 8.71E-01 | 0±0.1 | 9.94E-01 | 0.02±0.1 | 9.80E-01 | 0.1±0.14 | 7.66E-01 | 0.08±0.14 | 8.08E-01 |
| glutamine conjugate of C6H10O2 (1)* | -0.03±0.08 | 0 | 7.19E-01 | 9.16E-01 | -0.04±0.08 | 0 | 9.87E-01 | 8.71E-01 | -0.04±0.1 | 9.27E-01 | -0.04±0.1 | 9.19E-01 | 0.01±0.13 | 9.84E-01 | -0.04±0.13 | 9.20E-01 |
| hexadecanedioate (C16-DC) | 0.03±0.07 | 0 | 4.61E-01 | 8.91E-01 | 0.04±0.08 | 0 | 5.56E-01 | 8.71E-01 | 0.07±0.09 | 9.03E-01 | 0.07±0.09 | 8.94E-01 | -0.05±0.13 | 9.04E-01 | -0.03±0.13 | 9.43E-01 |
| alanine | 0.04±0.08 | 77.93 | 3.33E-02 | 8.79E-01 | 0.04±0.08 | 74.01 | 4.98E-02 | 8.71E-01 | -0.06±0.09 | 9.07E-01 | -0.05±0.09 | 9.12E-01 | 0.33±0.16 | 3.23E-01 | 0.31±0.16 | 4.52E-01 |
| N-acetylglycine | 0.02±0.07 | 86 | 7.52E-03 | 9.47E-01 | 0.04±0.08 | 75.38 | 4.39E-02 | 8.71E-01 | 0.15±0.09 | 6.98E-01 | 0.14±0.09 | 7.26E-01 | -0.26±0.13 | 3.23E-01 | -0.19±0.14 | 5.77E-01 |
| pipecolate | 0.03±0.07 | 29.65 | 2.33E-01 | 8.73E-01 | 0.03±0.07 | 33.7 | 2.19E-01 | 8.71E-01 | -0.03±0.09 | 9.48E-01 | -0.03±0.09 | 9.25E-01 | 0.15±0.12 | 5.80E-01 | 0.16±0.12 | 6.17E-01 |
| N-carbamoylalanine | -0.04±0.07 | 0 | 6.18E-01 | 8.43E-01 | -0.03±0.07 | 0 | 6.20E-01 | 8.71E-01 | -0.06±0.09 | 9.03E-01 | -0.06±0.09 | 8.94E-01 | 0.01±0.13 | 9.83E-01 | 0.02±0.13 | 9.58E-01 |
| X - 12830 | -0.04±0.07 | 0 | 6.80E-01 | 8.60E-01 | -0.03±0.07 | 0 | 6.99E-01 | 8.74E-01 | -0.01±0.09 | 9.78E-01 | -0.01±0.09 | 9.80E-01 | -0.08±0.12 | 7.97E-01 | -0.07±0.12 | 8.09E-01 |
| N-acetylhistidine | 0.02±0.08 | 58.69 | 1.20E-01 | 9.45E-01 | 0.04±0.08 | 74.52 | 4.76E-02 | 8.75E-01 | -0.06±0.09 | 9.06E-01 | -0.07±0.09 | 8.94E-01 | 0.19±0.14 | 5.31E-01 | 0.26±0.14 | 4.52E-01 |
| N,N-dimethyl-pro-pro | 0.04±0.09 | 65.31 | 8.95E-02 | 8.90E-01 | 0.04±0.09 | 64.34 | 9.40E-02 | 8.75E-01 | -0.02±0.1 | 9.71E-01 | -0.01±0.1 | 9.80E-01 | 0.46±0.26 | 3.99E-01 | 0.47±0.27 | 4.74E-01 |
| X - 26108 | 0.04±0.07 | 57.19 | 1.26E-01 | 8.43E-01 | 0.03±0.07 | 47.51 | 1.68E-01 | 8.75E-01 | -0.03±0.09 | 9.48E-01 | -0.03±0.09 | 9.19E-01 | 0.22±0.14 | 4.64E-01 | 0.19±0.14 | 5.77E-01 |
| 5alpha-androstan-3beta,17beta-diol monosulfate (2) | 0.03±0.08 | 5.94 | 3.03E-01 | 8.90E-01 | 0.04±0.08 | 39.43 | 1.99E-01 | 8.75E-01 | -0.03±0.1 | 9.59E-01 | -0.04±0.1 | 9.24E-01 | 0.15±0.14 | 6.23E-01 | 0.19±0.14 | 5.97E-01 |
| pregnenediol sulfate (C21H34O5S)* | 0.03±0.08 | 0 | 6.29E-01 | 8.99E-01 | 0.04±0.08 | 0 | 4.67E-01 | 8.75E-01 | 0±0.1 | 9.89E-01 | 0±0.1 | 9.95E-01 | 0.09±0.15 | 7.97E-01 | 0.12±0.15 | 7.32E-01 |
| octadecanedioylcarnitine (C18-DC)* | -0.05±0.08 | 42.25 | 1.88E-01 | 8.16E-01 | -0.04±0.08 | 48.26 | 1.64E-01 | 8.75E-01 | -0.12±0.1 | 7.93E-01 | -0.12±0.1 | 8.36E-01 | 0.09±0.13 | 7.78E-01 | 0.11±0.13 | 7.39E-01 |
| sphingomyelin (d18:1/22:2, d18:2/22:1, d16:1/24:2)* | 0.02±0.08 | 0 | 9.54E-01 | 9.45E-01 | 0.04±0.08 | 0 | 9.94E-01 | 8.75E-01 | 0.02±0.1 | 9.71E-01 | 0.04±0.1 | 9.19E-01 | 0.01±0.13 | 9.84E-01 | 0.04±0.13 | 9.28E-01 |
| X - 17690 | -0.05±0.07 | 0 | 9.56E-01 | 8.16E-01 | -0.03±0.07 | 0 | 8.42E-01 | 8.75E-01 | -0.04±0.09 | 9.27E-01 | -0.04±0.09 | 9.18E-01 | -0.05±0.13 | 8.81E-01 | -0.01±0.13 | 9.80E-01 |
| 3-hydroxyoleoylcarnitine | -0.03±0.07 | 0 | 6.49E-01 | 9.08E-01 | -0.03±0.07 | 0 | 7.90E-01 | 8.75E-01 | -0.05±0.09 | 9.27E-01 | -0.05±0.09 | 9.18E-01 | 0.02±0.13 | 9.55E-01 | 0±0.13 | 9.92E-01 |
| palmitoleoylcarnitine (C16:1)* | 0.04±0.07 | 0 | 6.54E-01 | 8.50E-01 | 0.03±0.08 | 0 | 6.32E-01 | 8.75E-01 | 0.06±0.09 | 9.06E-01 | 0.06±0.1 | 8.94E-01 | -0.01±0.13 | 9.92E-01 | -0.01±0.13 | 9.72E-01 |
| myristoleoylcarnitine (C14:1)* | -0.03±0.07 | 21.5 | 2.59E-01 | 8.90E-01 | -0.03±0.07 | 8.08 | 2.97E-01 | 8.76E-01 | 0.03±0.09 | 9.48E-01 | 0.03±0.09 | 9.55E-01 | -0.14±0.12 | 6.05E-01 | -0.14±0.12 | 6.66E-01 |
| indolepropionate | -0.05±0.07 | 12.88 | 2.84E-01 | 8.06E-01 | -0.03±0.08 | 50.58 | 1.55E-01 | 8.76E-01 | -0.1±0.09 | 8.41E-01 | -0.1±0.09 | 8.36E-01 | 0.07±0.14 | 8.36E-01 | 0.14±0.15 | 6.90E-01 |
| 1-arachidonoyl-GPI (20:4)* | -0.03±0.07 | 56.64 | 1.29E-01 | 9.03E-01 | -0.03±0.07 | 53.71 | 1.42E-01 | 8.76E-01 | -0.1±0.09 | 8.65E-01 | -0.11±0.09 | 8.36E-01 | 0.14±0.13 | 6.26E-01 | 0.13±0.13 | 6.91E-01 |
| undecenoylcarnitine (C11:1) | -0.04±0.07 | 56.29 | 1.30E-01 | 8.43E-01 | -0.03±0.07 | 55.82 | 1.32E-01 | 8.76E-01 | -0.12±0.09 | 7.90E-01 | -0.11±0.09 | 8.36E-01 | 0.12±0.13 | 6.77E-01 | 0.13±0.13 | 6.91E-01 |
| trimethylamine N-oxide | 0.03±0.07 | 0 | 4.83E-01 | 8.89E-01 | 0.03±0.07 | 0 | 5.07E-01 | 8.76E-01 | -0.01±0.09 | 9.89E-01 | 0±0.09 | 9.95E-01 | 0.1±0.12 | 7.26E-01 | 0.1±0.13 | 7.50E-01 |
| glycochenodeoxycholate glucuronide (1) | 0.04±0.08 | 0 | 6.06E-01 | 8.43E-01 | 0.04±0.08 | 0 | 7.07E-01 | 8.76E-01 | 0.01±0.1 | 9.82E-01 | 0.01±0.1 | 9.80E-01 | 0.1±0.13 | 7.44E-01 | 0.08±0.14 | 8.18E-01 |
| palmitoyl sphingomyelin (d18:1/16:0) | -0.04±0.07 | 0 | 8.20E-01 | 8.39E-01 | -0.03±0.08 | 0 | 5.10E-01 | 8.76E-01 | -0.06±0.09 | 9.23E-01 | -0.07±0.1 | 8.94E-01 | -0.02±0.12 | 9.54E-01 | 0.04±0.13 | 9.28E-01 |
| 1-(1-enyl-oleoyl)-GPE (P-18:1)* | 0.03±0.07 | 0 | 8.65E-01 | 9.03E-01 | 0.03±0.07 | 0 | 9.81E-01 | 8.76E-01 | 0.04±0.09 | 9.38E-01 | 0.03±0.09 | 9.25E-01 | 0.01±0.13 | 9.86E-01 | 0.04±0.13 | 9.28E-01 |
| epiandrosterone sulfate | -0.05±0.08 | 0 | 7.06E-01 | 8.16E-01 | -0.03±0.08 | 0 | 3.52E-01 | 8.76E-01 | -0.07±0.1 | 9.03E-01 | -0.09±0.1 | 8.64E-01 | -0.01±0.13 | 9.84E-01 | 0.07±0.14 | 8.40E-01 |
| X - 13866 | -0.04±0.07 | 0 | 7.65E-01 | 8.53E-01 | -0.03±0.07 | 0 | 7.52E-01 | 8.78E-01 | -0.05±0.09 | 9.27E-01 | -0.05±0.09 | 9.18E-01 | 0±0.13 | 9.94E-01 | 0±0.13 | 9.98E-01 |
| (2 or 3)-decenoate (10:1n7 or n8) | -0.02±0.07 | 58.64 | 1.20E-01 | 9.13E-01 | -0.03±0.07 | 65.55 | 8.84E-02 | 8.80E-01 | 0.06±0.09 | 9.03E-01 | 0.07±0.09 | 8.94E-01 | -0.17±0.12 | 5.30E-01 | -0.2±0.12 | 4.95E-01 |
| propionylcarnitine (C3) | -0.03±0.07 | 50.67 | 1.55E-01 | 8.97E-01 | -0.03±0.08 | 22.34 | 2.56E-01 | 8.80E-01 | -0.1±0.09 | 8.65E-01 | -0.09±0.09 | 8.36E-01 | 0.13±0.14 | 6.66E-01 | 0.09±0.13 | 7.82E-01 |
| 2-O-methylascorbic acid | 0.04±0.07 | 0 | 3.44E-01 | 8.43E-01 | 0.03±0.07 | 0 | 6.24E-01 | 8.80E-01 | -0.01±0.09 | 9.87E-01 | 0.01±0.09 | 9.88E-01 | 0.14±0.13 | 6.23E-01 | 0.08±0.13 | 7.94E-01 |
| 1-(1-enyl-palmitoyl)-2-arachidonoyl-GPC (P-16:0/20:4)* | -0.03±0.07 | 0 | 7.52E-01 | 9.08E-01 | -0.03±0.07 | 0 | 5.84E-01 | 8.80E-01 | -0.04±0.09 | 9.27E-01 | -0.06±0.09 | 8.94E-01 | 0.01±0.12 | 9.92E-01 | 0.03±0.13 | 9.43E-01 |
| cysteinylglycine | 0.03±0.07 | 44.42 | 1.80E-01 | 8.88E-01 | 0.03±0.07 | 27.84 | 2.39E-01 | 8.82E-01 | -0.03±0.09 | 9.48E-01 | -0.03±0.09 | 9.52E-01 | 0.19±0.14 | 5.51E-01 | 0.17±0.14 | 6.29E-01 |
| X - 24556 | -0.05±0.07 | 0 | 7.52E-01 | 8.11E-01 | -0.03±0.07 | 0 | 6.61E-01 | 8.82E-01 | -0.06±0.09 | 9.03E-01 | -0.05±0.09 | 9.04E-01 | -0.02±0.12 | 9.75E-01 | 0.01±0.12 | 9.72E-01 |
| bilirubin (E,E)* | -0.05±0.07 | 0.59 | 3.16E-01 | 8.16E-01 | -0.03±0.07 | 40.67 | 1.94E-01 | 8.82E-01 | -0.1±0.09 | 8.65E-01 | -0.1±0.09 | 8.36E-01 | 0.06±0.13 | 8.66E-01 | 0.11±0.13 | 7.44E-01 |
| succinate | -0.04±0.07 | 10.4 | 2.91E-01 | 8.41E-01 | -0.03±0.07 | 41.88 | 1.90E-01 | 8.92E-01 | -0.1±0.09 | 8.65E-01 | -0.1±0.09 | 8.36E-01 | 0.07±0.13 | 8.31E-01 | 0.12±0.14 | 7.25E-01 |
| X - 11850 | -0.04±0.07 | 0 | 6.91E-01 | 8.60E-01 | -0.03±0.07 | 0 | 8.49E-01 | 8.95E-01 | -0.02±0.09 | 9.78E-01 | -0.02±0.09 | 9.62E-01 | -0.08±0.13 | 8.02E-01 | -0.05±0.13 | 8.94E-01 |
| indoleacetylglutamine | 0.05±0.07 | 72.36 | 5.72E-02 | 8.26E-01 | 0.03±0.07 | 62.28 | 1.03E-01 | 8.99E-01 | -0.05±0.09 | 9.27E-01 | -0.05±0.09 | 9.04E-01 | 0.25±0.13 | 3.43E-01 | 0.21±0.13 | 4.97E-01 |
| N1-Methyl-2-pyridone-5-carboxamide | -0.03±0.07 | 20.52 | 2.62E-01 | 9.01E-01 | -0.03±0.07 | 56.18 | 1.31E-01 | 8.99E-01 | 0.03±0.09 | 9.46E-01 | 0.06±0.09 | 9.04E-01 | -0.14±0.12 | 6.14E-01 | -0.18±0.12 | 5.65E-01 |
| tiglylcarnitine (C5:1-DC) | 0.02±0.07 | 21.43 | 2.59E-01 | 9.18E-01 | 0.03±0.07 | 7.05 | 3.00E-01 | 8.99E-01 | -0.03±0.09 | 9.46E-01 | -0.02±0.09 | 9.55E-01 | 0.15±0.13 | 6.21E-01 | 0.14±0.13 | 6.67E-01 |
| X - 24947 | -0.04±0.07 | 0 | 3.44E-01 | 8.40E-01 | -0.03±0.07 | 41.59 | 1.91E-01 | 8.99E-01 | -0.09±0.09 | 8.65E-01 | -0.1±0.09 | 8.36E-01 | 0.06±0.13 | 8.74E-01 | 0.11±0.13 | 7.32E-01 |
| pimeloylcarnitine/3-methyladipoylcarnitine (C7-DC) | 0±0.07 | 0 | 9.35E-01 | 9.88E-01 | 0.03±0.07 | 0 | 6.13E-01 | 8.99E-01 | 0±0.09 | 9.89E-01 | 0±0.09 | 9.95E-01 | 0.01±0.12 | 9.86E-01 | 0.08±0.13 | 7.96E-01 |
| salicylate | -0.03±0.07 | 0 | 4.87E-01 | 8.88E-01 | -0.03±0.07 | 0 | 4.25E-01 | 8.99E-01 | -0.07±0.09 | 9.03E-01 | -0.07±0.09 | 8.91E-01 | 0.04±0.12 | 9.20E-01 | 0.05±0.12 | 8.85E-01 |
| 5alpha-androstan-3alpha,17beta-diol monosulfate (2) | 0.02±0.11 | 11.87 | 2.87E-01 | 9.49E-01 | 0.04±0.11 | 25.68 | 2.46E-01 | 8.99E-01 | -0.05±0.13 | 9.38E-01 | -0.04±0.13 | 9.45E-01 | 0.21±0.21 | 6.54E-01 | 0.25±0.21 | 6.39E-01 |
| glutamine conjugate of C6H10O2 (2)* | -0.01±0.08 | 0 | 4.67E-01 | 9.54E-01 | -0.03±0.08 | 21.78 | 2.58E-01 | 8.99E-01 | 0.03±0.09 | 9.58E-01 | 0.03±0.09 | 9.31E-01 | -0.09±0.13 | 7.67E-01 | -0.16±0.14 | 6.46E-01 |
| ornithine | -0.03±0.08 | 58.41 | 1.21E-01 | 9.08E-01 | -0.03±0.08 | 56.11 | 1.31E-01 | 8.99E-01 | -0.1±0.09 | 8.65E-01 | -0.1±0.09 | 8.36E-01 | 0.18±0.16 | 6.00E-01 | 0.16±0.15 | 6.66E-01 |
| nonanoylcarnitine (C9) | -0.03±0.07 | 0.07 | 3.17E-01 | 8.73E-01 | -0.03±0.07 | 0 | 3.64E-01 | 8.99E-01 | 0.02±0.09 | 9.71E-01 | 0.02±0.09 | 9.58E-01 | -0.14±0.13 | 6.23E-01 | -0.12±0.13 | 6.96E-01 |
| sphingomyelin (d18:1/25:0, d19:0/24:1, d20:1/23:0, d19:1/24:0)* | -0.04±0.07 | 13.28 | 2.83E-01 | 8.43E-01 | -0.03±0.07 | 45.22 | 1.77E-01 | 8.99E-01 | -0.1±0.09 | 8.65E-01 | -0.09±0.09 | 8.36E-01 | 0.07±0.13 | 8.14E-01 | 0.13±0.14 | 7.06E-01 |
| X - 11847 | 0.02±0.07 | 0 | 4.72E-01 | 9.19E-01 | 0.03±0.07 | 0 | 4.12E-01 | 8.99E-01 | -0.01±0.09 | 9.78E-01 | -0.01±0.09 | 9.80E-01 | 0.1±0.13 | 7.38E-01 | 0.12±0.13 | 7.18E-01 |
| X - 11470 | -0.03±0.07 | 17.52 | 2.71E-01 | 8.73E-01 | -0.03±0.07 | 31.16 | 2.28E-01 | 8.99E-01 | -0.08±0.08 | 8.69E-01 | -0.08±0.08 | 8.47E-01 | 0.09±0.13 | 7.71E-01 | 0.11±0.13 | 7.49E-01 |
| 3-(3-hydroxyphenyl)propionate | -0.02±0.07 | 56.47 | 1.30E-01 | 9.41E-01 | -0.03±0.07 | 28.34 | 2.37E-01 | 8.99E-01 | -0.1±0.09 | 8.65E-01 | -0.09±0.09 | 8.38E-01 | 0.14±0.13 | 6.23E-01 | 0.1±0.13 | 7.64E-01 |
| cortisol | -0.03±0.07 | 0 | 4.49E-01 | 8.73E-01 | -0.03±0.07 | 0 | 3.20E-01 | 8.99E-01 | -0.07±0.08 | 9.01E-01 | -0.07±0.09 | 8.68E-01 | 0.05±0.12 | 9.04E-01 | 0.08±0.13 | 8.01E-01 |
| glutarylcarnitine (C5-DC) | -0.04±0.08 | 13.85 | 2.81E-01 | 8.69E-01 | -0.03±0.08 | 0 | 3.34E-01 | 8.99E-01 | -0.09±0.09 | 8.69E-01 | -0.08±0.09 | 8.68E-01 | 0.09±0.14 | 7.89E-01 | 0.09±0.14 | 8.03E-01 |
| sarcosine | -0.03±0.07 | 0 | 4.15E-01 | 9.13E-01 | -0.03±0.07 | 0 | 3.31E-01 | 8.99E-01 | -0.06±0.09 | 9.03E-01 | -0.08±0.09 | 8.68E-01 | 0.07±0.14 | 8.47E-01 | 0.08±0.14 | 8.03E-01 |
| X - 23787 | 0.03±0.07 | 0 | 9.96E-01 | 9.08E-01 | 0.03±0.08 | 0 | 8.83E-01 | 8.99E-01 | 0.03±0.09 | 9.59E-01 | 0.02±0.1 | 9.64E-01 | 0.03±0.12 | 9.47E-01 | 0.04±0.13 | 9.02E-01 |
| gamma-glutamylglycine | -0.04±0.07 | 0 | 9.73E-01 | 8.47E-01 | -0.03±0.08 | 0 | 6.64E-01 | 8.99E-01 | -0.04±0.09 | 9.46E-01 | -0.06±0.1 | 9.07E-01 | -0.04±0.12 | 9.05E-01 | 0.01±0.13 | 9.72E-01 |
| andro steroid monosulfate C19H28O6S (1)* | 0.03±0.07 | 0 | 8.99E-01 | 8.73E-01 | 0.03±0.08 | 0 | 8.60E-01 | 8.99E-01 | 0.04±0.09 | 9.34E-01 | 0.04±0.1 | 9.19E-01 | 0.02±0.13 | 9.61E-01 | 0.01±0.13 | 9.80E-01 |
| X - 18913 | -0.02±0.08 | 20.14 | 2.63E-01 | 9.31E-01 | -0.03±0.08 | 11.57 | 2.88E-01 | 8.99E-01 | -0.08±0.09 | 8.80E-01 | -0.09±0.09 | 8.63E-01 | 0.1±0.13 | 7.38E-01 | 0.09±0.14 | 7.87E-01 |
| taurodeoxycholate | -0.02±0.07 | 0 | 8.53E-01 | 9.45E-01 | -0.03±0.07 | 0 | 5.88E-01 | 8.99E-01 | -0.01±0.09 | 9.89E-01 | 0±0.09 | 9.98E-01 | -0.04±0.12 | 9.20E-01 | -0.08±0.13 | 7.91E-01 |
| thymol sulfate | -0.04±0.07 | 0 | 8.21E-01 | 8.47E-01 | -0.03±0.08 | 0 | 9.60E-01 | 9.01E-01 | -0.03±0.09 | 9.57E-01 | -0.03±0.09 | 9.55E-01 | -0.06±0.13 | 8.51E-01 | -0.03±0.13 | 9.29E-01 |
| X - 11444 | -0.03±0.07 | 65.15 | 9.03E-02 | 8.91E-01 | -0.03±0.07 | 55.97 | 1.32E-01 | 9.03E-01 | -0.1±0.08 | 8.31E-01 | -0.09±0.09 | 8.36E-01 | 0.17±0.14 | 5.77E-01 | 0.15±0.14 | 6.66E-01 |
| 1,2-dipalmitoyl-GPC (16:0/16:0) | 0.03±0.08 | 0 | 5.76E-01 | 9.14E-01 | 0.03±0.08 | 0 | 3.68E-01 | 9.03E-01 | 0±0.09 | 9.89E-01 | -0.02±0.09 | 9.79E-01 | 0.09±0.14 | 7.88E-01 | 0.14±0.15 | 6.96E-01 |
| 3-amino-2-piperidone | 0.03±0.08 | 52.25 | 1.48E-01 | 8.90E-01 | 0.03±0.08 | 38.67 | 2.02E-01 | 9.04E-01 | -0.04±0.09 | 9.38E-01 | -0.04±0.09 | 9.19E-01 | 0.22±0.15 | 5.23E-01 | 0.19±0.15 | 6.18E-01 |
| N-acetylleucine | 0.04±0.07 | 28.99 | 2.35E-01 | 8.46E-01 | 0.03±0.08 | 0 | 5.01E-01 | 9.04E-01 | -0.02±0.09 | 9.59E-01 | -0.01±0.09 | 9.80E-01 | 0.16±0.13 | 5.71E-01 | 0.1±0.13 | 7.64E-01 |
| X - 11787 | 0.03±0.07 | 0 | 4.02E-01 | 8.91E-01 | 0.03±0.07 | 0 | 5.33E-01 | 9.04E-01 | -0.01±0.09 | 9.82E-01 | -0.01±0.09 | 9.86E-01 | 0.12±0.13 | 6.78E-01 | 0.09±0.13 | 7.78E-01 |
| X - 13728 | 0.03±0.07 | 0 | 8.35E-01 | 8.90E-01 | 0.03±0.07 | 0 | 9.27E-01 | 9.04E-01 | 0.02±0.09 | 9.71E-01 | 0.02±0.09 | 9.58E-01 | 0.05±0.13 | 8.86E-01 | 0.04±0.13 | 9.26E-01 |
| (14 or 15)-methylpalmitate (a17:0 or i17:0) | 0.02±0.08 | 0 | 9.50E-01 | 9.45E-01 | 0.03±0.08 | 0 | 9.47E-01 | 9.04E-01 | 0.02±0.1 | 9.68E-01 | 0.02±0.1 | 9.58E-01 | 0.01±0.13 | 9.84E-01 | 0.04±0.13 | 9.28E-01 |
| 1-linoleoylglycerol (18:2) | -0.02±0.07 | 0 | 6.24E-01 | 9.18E-01 | -0.03±0.07 | 0 | 6.27E-01 | 9.04E-01 | -0.05±0.09 | 9.27E-01 | -0.05±0.09 | 9.04E-01 | 0.03±0.13 | 9.40E-01 | 0.03±0.13 | 9.47E-01 |
| caproate (6:0) | -0.02±0.07 | 0 | 7.51E-01 | 9.26E-01 | -0.03±0.07 | 0 | 8.09E-01 | 9.04E-01 | -0.04±0.09 | 9.38E-01 | -0.04±0.09 | 9.18E-01 | 0.01±0.13 | 9.84E-01 | 0±0.13 | 9.98E-01 |
| 4-chlorobenzoic acid | -0.01±0.07 | 0 | 6.03E-01 | 9.68E-01 | -0.03±0.07 | 0 | 7.81E-01 | 9.04E-01 | -0.04±0.09 | 9.42E-01 | -0.04±0.09 | 9.18E-01 | 0.05±0.13 | 9.05E-01 | 0±0.13 | 9.92E-01 |
| alpha-hydroxyisocaproate | 0.04±0.08 | 70.27 | 6.67E-02 | 8.79E-01 | 0.03±0.08 | 62.86 | 1.01E-01 | 9.07E-01 | -0.05±0.1 | 9.27E-01 | -0.06±0.1 | 9.04E-01 | 0.29±0.16 | 3.74E-01 | 0.25±0.16 | 4.95E-01 |
| hexadecasphingosine (d16:1)* | 0.04±0.07 | 47.93 | 1.66E-01 | 8.43E-01 | 0.03±0.07 | 9.02 | 2.94E-01 | 9.07E-01 | -0.03±0.09 | 9.48E-01 | -0.03±0.09 | 9.42E-01 | 0.18±0.13 | 5.23E-01 | 0.14±0.13 | 6.67E-01 |
| cholesterol | -0.03±0.07 | 0 | 7.25E-01 | 9.00E-01 | -0.03±0.08 | 0 | 5.18E-01 | 9.07E-01 | -0.05±0.09 | 9.27E-01 | -0.06±0.1 | 8.94E-01 | 0.01±0.12 | 9.92E-01 | 0.04±0.13 | 9.13E-01 |
| 3-hydroxyhippurate | -0.02±0.07 | 42.42 | 1.88E-01 | 9.13E-01 | -0.03±0.07 | 13.94 | 2.81E-01 | 9.09E-01 | -0.09±0.09 | 8.65E-01 | -0.08±0.09 | 8.63E-01 | 0.11±0.13 | 6.91E-01 | 0.09±0.13 | 7.82E-01 |
| X - 23636 | -0.03±0.07 | 0 | 4.15E-01 | 9.09E-01 | -0.03±0.07 | 0 | 4.16E-01 | 9.09E-01 | -0.07±0.09 | 9.03E-01 | -0.07±0.09 | 8.94E-01 | 0.06±0.13 | 8.51E-01 | 0.06±0.13 | 8.50E-01 |
| 4-hydroxychlorothalonil | -0.04±0.07 | 82.36 | 1.73E-02 | 8.47E-01 | -0.03±0.07 | 84.37 | 1.14E-02 | 9.11E-01 | -0.14±0.08 | 7.16E-01 | -0.13±0.09 | 7.20E-01 | 0.26±0.14 | 3.74E-01 | 0.3±0.15 | 4.36E-01 |
| 2-pyrrolidinone | 0.02±0.07 | 37.03 | 2.08E-01 | 9.45E-01 | 0.03±0.07 | 42.36 | 1.88E-01 | 9.11E-01 | -0.04±0.09 | 9.27E-01 | -0.04±0.09 | 9.19E-01 | 0.17±0.14 | 5.98E-01 | 0.19±0.15 | 6.05E-01 |
| 1-(1-enyl-palmitoyl)-2-oleoyl-GPE (P-16:0/18:1)* | -0.01±0.08 | 56.68 | 1.29E-01 | 9.49E-01 | -0.03±0.08 | 58.99 | 1.18E-01 | 9.11E-01 | -0.08±0.09 | 8.76E-01 | -0.1±0.09 | 8.36E-01 | 0.19±0.15 | 5.85E-01 | 0.17±0.15 | 6.45E-01 |
| pregnenetriol sulfate* | 0.02±0.08 | 0.35 | 3.16E-01 | 9.44E-01 | 0.03±0.08 | 26.19 | 2.44E-01 | 9.11E-01 | -0.04±0.1 | 9.48E-01 | -0.04±0.1 | 9.19E-01 | 0.14±0.14 | 6.66E-01 | 0.16±0.14 | 6.46E-01 |
| X - 07765 | -0.03±0.07 | 40.75 | 1.94E-01 | 9.06E-01 | -0.02±0.07 | 51.83 | 1.50E-01 | 9.11E-01 | -0.1±0.09 | 8.65E-01 | -0.1±0.09 | 8.36E-01 | 0.09±0.12 | 7.29E-01 | 0.11±0.12 | 7.05E-01 |
| X - 16576 | -0.03±0.07 | 0 | 6.94E-01 | 8.73E-01 | -0.03±0.07 | 0 | 5.59E-01 | 9.11E-01 | -0.05±0.09 | 9.27E-01 | -0.05±0.09 | 9.04E-01 | 0.01±0.13 | 9.90E-01 | 0.04±0.13 | 9.22E-01 |
| branched-chain, straight-chain, or cyclopropyl 10:1 fatty acid (1)* | -0.02±0.07 | 0 | 9.12E-01 | 9.18E-01 | -0.03±0.08 | 0 | 7.33E-01 | 9.11E-01 | -0.03±0.09 | 9.48E-01 | -0.04±0.09 | 9.18E-01 | -0.01±0.12 | 9.84E-01 | 0.01±0.13 | 9.80E-01 |
| 4-methyl-2-oxopentanoate | 0.03±0.08 | 67.28 | 8.04E-02 | 9.08E-01 | 0.03±0.08 | 60.01 | 1.14E-01 | 9.14E-01 | -0.07±0.09 | 9.03E-01 | -0.06±0.1 | 8.94E-01 | 0.22±0.14 | 4.45E-01 | 0.2±0.14 | 5.48E-01 |
| 3beta,7alpha-dihydroxy-5-cholestenoate | -0.02±0.07 | 0 | 3.27E-01 | 9.18E-01 | -0.03±0.08 | 0 | 4.45E-01 | 9.14E-01 | 0.03±0.09 | 9.48E-01 | 0.02±0.09 | 9.71E-01 | -0.12±0.12 | 6.66E-01 | -0.1±0.13 | 7.49E-01 |
| 2-methoxyhydroquinone sulfate (1) | -0.02±0.07 | 84.4 | 1.13E-02 | 9.45E-01 | -0.02±0.07 | 80.62 | 2.31E-02 | 9.15E-01 | -0.15±0.09 | 7.16E-01 | -0.14±0.09 | 7.21E-01 | 0.25±0.13 | 3.42E-01 | 0.22±0.13 | 4.79E-01 |
| homoarginine | 0.03±0.08 | 60.59 | 1.11E-01 | 8.93E-01 | 0.03±0.08 | 38.63 | 2.02E-01 | 9.15E-01 | -0.04±0.09 | 9.27E-01 | -0.04±0.1 | 9.19E-01 | 0.25±0.16 | 4.66E-01 | 0.19±0.15 | 6.22E-01 |
| phenylpyruvate | -0.02±0.07 | 52.02 | 1.49E-01 | 9.45E-01 | -0.02±0.07 | 40.38 | 1.95E-01 | 9.15E-01 | -0.09±0.09 | 8.65E-01 | -0.09±0.09 | 8.38E-01 | 0.14±0.13 | 6.26E-01 | 0.12±0.13 | 7.19E-01 |
| glutamine conjugate of C7H12O2* | -0.02±0.08 | 0 | 5.55E-01 | 9.45E-01 | -0.03±0.08 | 0 | 3.94E-01 | 9.15E-01 | 0.01±0.1 | 9.78E-01 | 0.02±0.1 | 9.58E-01 | -0.08±0.13 | 7.97E-01 | -0.11±0.13 | 7.24E-01 |
| theobromine | -0.02±0.07 | 51.08 | 1.53E-01 | 9.45E-01 | -0.02±0.07 | 20.52 | 2.62E-01 | 9.15E-01 | -0.09±0.09 | 8.65E-01 | -0.08±0.09 | 8.63E-01 | 0.13±0.13 | 6.40E-01 | 0.1±0.13 | 7.64E-01 |
| oleoyl ethanolamide | -0.01±0.07 | 0 | 6.35E-01 | 9.61E-01 | -0.02±0.08 | 0 | 5.76E-01 | 9.15E-01 | 0.02±0.09 | 9.78E-01 | 0.01±0.1 | 9.80E-01 | -0.06±0.12 | 8.60E-01 | -0.08±0.13 | 7.96E-01 |
| 4-cholesten-3-one | 0.03±0.07 | 0 | 4.40E-01 | 8.81E-01 | 0.02±0.07 | 0 | 6.15E-01 | 9.15E-01 | -0.01±0.09 | 9.89E-01 | 0±0.09 | 9.94E-01 | 0.11±0.13 | 6.92E-01 | 0.07±0.13 | 8.08E-01 |
| X - 24243 | 0.04±0.07 | 83.74 | 1.31E-02 | 8.40E-01 | 0.02±0.07 | 76.54 | 3.90E-02 | 9.15E-01 | -0.07±0.09 | 9.01E-01 | -0.07±0.09 | 8.91E-01 | 0.34±0.14 | 2.30E-01 | 0.28±0.14 | 4.52E-01 |
| 1-linolenoyl-GPC (18:3)* | 0.01±0.08 | 48.27 | 1.64E-01 | 9.49E-01 | 0.02±0.08 | 64.96 | 9.12E-02 | 9.15E-01 | -0.05±0.09 | 9.27E-01 | -0.06±0.09 | 8.94E-01 | 0.18±0.14 | 5.71E-01 | 0.23±0.14 | 4.95E-01 |
| 3-(3-hydroxyphenyl)propionate sulfate | -0.03±0.07 | 67.11 | 8.12E-02 | 8.90E-01 | -0.02±0.07 | 65.58 | 8.83E-02 | 9.15E-01 | -0.12±0.09 | 7.90E-01 | -0.11±0.09 | 8.36E-01 | 0.15±0.13 | 5.89E-01 | 0.16±0.13 | 6.23E-01 |
| 1-stearoyl-2-arachidonoyl-GPE (18:0/20:4) | 0.04±0.08 | 56.53 | 1.29E-01 | 8.49E-01 | 0.02±0.08 | 33.12 | 2.21E-01 | 9.15E-01 | -0.03±0.09 | 9.47E-01 | -0.04±0.09 | 9.19E-01 | 0.23±0.15 | 4.72E-01 | 0.17±0.14 | 6.36E-01 |
| dihomo-linolenoylcarnitine (C20:3n3 or 6)* | 0.02±0.07 | 8 | 2.97E-01 | 9.19E-01 | 0.02±0.07 | 14.52 | 2.79E-01 | 9.15E-01 | -0.03±0.09 | 9.48E-01 | -0.03±0.09 | 9.25E-01 | 0.14±0.13 | 6.40E-01 | 0.14±0.14 | 6.67E-01 |
| myristate (14:0) | -0.02±0.08 | 0 | 6.22E-01 | 9.18E-01 | -0.02±0.08 | 0 | 6.23E-01 | 9.15E-01 | 0±0.1 | 9.89E-01 | 0±0.1 | 9.94E-01 | -0.08±0.13 | 8.14E-01 | -0.08±0.14 | 8.11E-01 |
| glucuronide of piperine metabolite C17H21NO3 (4)* | 0±0.07 | 0 | 7.42E-01 | 9.75E-01 | -0.02±0.08 | 0 | 9.18E-01 | 9.15E-01 | -0.01±0.09 | 9.84E-01 | -0.02±0.1 | 9.70E-01 | 0.04±0.13 | 9.20E-01 | -0.04±0.14 | 9.28E-01 |
| eicosenoylcarnitine (C20:1)* | -0.03±0.07 | 0 | 7.87E-01 | 9.08E-01 | -0.02±0.08 | 0 | 6.42E-01 | 9.15E-01 | -0.04±0.09 | 9.36E-01 | -0.05±0.09 | 9.18E-01 | 0±0.13 | 9.96E-01 | 0.03±0.13 | 9.43E-01 |
| taurine | -0.04±0.07 | 0 | 9.41E-01 | 8.60E-01 | -0.02±0.07 | 0 | 8.29E-01 | 9.15E-01 | -0.03±0.09 | 9.48E-01 | -0.03±0.09 | 9.19E-01 | -0.04±0.11 | 9.05E-01 | 0±0.12 | 9.97E-01 |
| 6-oxopiperidine-2-carboxylate | 0.03±0.07 | 72.87 | 5.49E-02 | 9.08E-01 | 0.02±0.08 | 54.14 | 1.40E-01 | 9.16E-01 | -0.07±0.09 | 9.03E-01 | -0.05±0.09 | 9.11E-01 | 0.25±0.14 | 3.73E-01 | 0.2±0.14 | 5.77E-01 |
| 5-dodecenoate (12:1n7) | -0.02±0.08 | 44.87 | 1.78E-01 | 9.45E-01 | -0.02±0.08 | 40.42 | 1.95E-01 | 9.16E-01 | 0.06±0.1 | 9.27E-01 | 0.05±0.1 | 9.18E-01 | -0.17±0.14 | 5.80E-01 | -0.17±0.14 | 6.25E-01 |
| sphingomyelin (d18:1/21:0, d17:1/22:0, d16:1/23:0)* | -0.04±0.08 | 53.58 | 1.42E-01 | 8.49E-01 | -0.03±0.08 | 58.84 | 1.19E-01 | 9.16E-01 | -0.12±0.1 | 8.31E-01 | -0.11±0.1 | 8.36E-01 | 0.14±0.15 | 6.66E-01 | 0.18±0.16 | 6.45E-01 |
| X - 13507 | 0.03±0.07 | 29.06 | 2.35E-01 | 9.09E-01 | 0.02±0.07 | 19.08 | 2.66E-01 | 9.16E-01 | -0.04±0.09 | 9.38E-01 | -0.04±0.09 | 9.19E-01 | 0.15±0.13 | 6.04E-01 | 0.14±0.13 | 6.67E-01 |
| palmitoyl dihydrosphingomyelin (d18:0/16:0)* | 0.02±0.08 | 0 | 5.41E-01 | 9.41E-01 | 0.02±0.08 | 0 | 3.31E-01 | 9.16E-01 | -0.01±0.09 | 9.87E-01 | -0.03±0.1 | 9.55E-01 | 0.09±0.14 | 7.81E-01 | 0.15±0.15 | 6.91E-01 |
| 1,3,7-trimethylurate | -0.01±0.07 | 36.26 | 2.10E-01 | 9.61E-01 | -0.02±0.08 | 0 | 3.79E-01 | 9.16E-01 | -0.07±0.09 | 9.01E-01 | -0.07±0.09 | 8.94E-01 | 0.13±0.13 | 6.66E-01 | 0.07±0.13 | 8.18E-01 |
| 10-nonadecenoate (19:1n9) | -0.02±0.08 | 0 | 7.67E-01 | 9.45E-01 | -0.02±0.08 | 0 | 7.01E-01 | 9.16E-01 | 0±0.1 | 9.97E-01 | 0±0.1 | 9.98E-01 | -0.05±0.13 | 9.04E-01 | -0.06±0.13 | 8.50E-01 |
| 1-linoleoyl-GPC (18:2) | -0.04±0.07 | 0 | 9.60E-01 | 8.43E-01 | -0.02±0.08 | 0 | 5.30E-01 | 9.16E-01 | -0.04±0.09 | 9.33E-01 | -0.06±0.1 | 9.04E-01 | -0.03±0.12 | 9.20E-01 | 0.05±0.14 | 9.02E-01 |
| X - 12117 | 0.04±0.07 | 0 | 9.63E-01 | 8.73E-01 | 0.02±0.08 | 0 | 5.64E-01 | 9.16E-01 | 0.03±0.09 | 9.48E-01 | 0.06±0.1 | 9.04E-01 | 0.04±0.13 | 9.20E-01 | -0.04±0.13 | 9.19E-01 |
| 2-stearoyl-GPE (18:0)* | -0.03±0.07 | 0 | 7.83E-01 | 8.90E-01 | -0.02±0.07 | 0 | 6.38E-01 | 9.16E-01 | -0.04±0.09 | 9.27E-01 | -0.05±0.09 | 9.18E-01 | 0±0.13 | 9.96E-01 | 0.03±0.13 | 9.41E-01 |
| 1-(1-enyl-palmitoyl)-2-palmitoleoyl-GPC (P-16:0/16:1)* | -0.03±0.07 | 0 | 9.05E-01 | 9.06E-01 | -0.02±0.08 | 0 | 7.79E-01 | 9.16E-01 | -0.02±0.09 | 9.71E-01 | -0.04±0.1 | 9.19E-01 | -0.04±0.12 | 9.20E-01 | 0.01±0.13 | 9.90E-01 |
| glycosyl-N-(2-hydroxynervonoyl)-sphingosine (d18:1/24:1(2OH))* | -0.02±0.07 | 0 | 6.39E-01 | 9.48E-01 | -0.02±0.07 | 0 | 8.47E-01 | 9.16E-01 | -0.04±0.09 | 9.34E-01 | -0.03±0.09 | 9.25E-01 | 0.03±0.13 | 9.28E-01 | 0±0.13 | 9.98E-01 |
| X - 17010 | 0.02±0.07 | 0 | 7.59E-01 | 9.45E-01 | 0.02±0.07 | 0 | 8.22E-01 | 9.16E-01 | 0.03±0.09 | 9.47E-01 | 0.03±0.09 | 9.19E-01 | -0.01±0.12 | 9.81E-01 | 0±0.12 | 9.98E-01 |
| alpha-ketoglutarate | -0.02±0.08 | 90.12 | 1.46E-03 | 9.45E-01 | -0.02±0.08 | 87.61 | 4.49E-03 | 9.17E-01 | -0.13±0.08 | 7.50E-01 | -0.12±0.09 | 7.51E-01 | 0.51±0.18 | 1.99E-01 | 0.45±0.18 | 4.03E-01 |
| X - 12101 | 0.03±0.07 | 0 | 9.93E-01 | 9.00E-01 | 0.02±0.07 | 0 | 8.94E-01 | 9.17E-01 | 0.03±0.09 | 9.54E-01 | 0.03±0.09 | 9.45E-01 | 0.03±0.13 | 9.40E-01 | 0.01±0.13 | 9.85E-01 |
| X - 17367 | -0.02±0.07 | 56.01 | 1.32E-01 | 9.45E-01 | -0.02±0.07 | 54.68 | 1.37E-01 | 9.22E-01 | -0.09±0.09 | 8.65E-01 | -0.09±0.09 | 8.36E-01 | 0.14±0.13 | 6.23E-01 | 0.14±0.13 | 6.67E-01 |
| N6,N6,N6-trimethyllysine | 0.02±0.08 | 0 | 3.52E-01 | 9.18E-01 | 0.02±0.08 | 0 | 4.43E-01 | 9.22E-01 | -0.02±0.09 | 9.71E-01 | -0.02±0.09 | 9.67E-01 | 0.14±0.15 | 6.66E-01 | 0.11±0.14 | 7.50E-01 |
| X - 21821 | 0.01±0.07 | 51.53 | 1.51E-01 | 9.71E-01 | 0.02±0.07 | 56.19 | 1.31E-01 | 9.23E-01 | -0.06±0.09 | 9.03E-01 | -0.06±0.09 | 9.00E-01 | 0.16±0.13 | 5.77E-01 | 0.19±0.13 | 5.77E-01 |
| stearoylcarnitine (C18) | 0.01±0.08 | 0 | 3.57E-01 | 9.61E-01 | 0.02±0.08 | 34.54 | 2.16E-01 | 9.23E-01 | -0.04±0.09 | 9.38E-01 | -0.04±0.09 | 9.18E-01 | 0.12±0.14 | 7.11E-01 | 0.17±0.14 | 6.38E-01 |
| N-acetylglucosaminylasparagine | 0.02±0.08 | 0 | 5.39E-01 | 9.31E-01 | 0.02±0.08 | 0 | 5.82E-01 | 9.23E-01 | -0.01±0.09 | 9.86E-01 | -0.01±0.09 | 9.80E-01 | 0.09±0.13 | 7.80E-01 | 0.08±0.13 | 8.03E-01 |
| cortisone | -0.02±0.07 | 15.35 | 2.77E-01 | 9.08E-01 | -0.02±0.07 | 50.34 | 1.56E-01 | 9.23E-01 | -0.07±0.08 | 8.80E-01 | -0.08±0.08 | 8.47E-01 | 0.09±0.13 | 7.47E-01 | 0.14±0.13 | 6.67E-01 |
| glycosyl-N-stearoyl-sphingosine (d18:1/18:0) | 0±0.08 | 0 | 9.78E-01 | 9.75E-01 | 0.02±0.08 | 0 | 7.48E-01 | 9.25E-01 | 0.01±0.09 | 9.89E-01 | 0±0.1 | 9.95E-01 | 0±0.13 | 9.96E-01 | 0.06±0.13 | 8.81E-01 |
| X - 11299 | -0.03±0.07 | 58.85 | 1.19E-01 | 8.88E-01 | -0.02±0.07 | 48.99 | 1.61E-01 | 9.30E-01 | 0.06±0.09 | 9.27E-01 | 0.06±0.09 | 9.01E-01 | -0.18±0.12 | 5.02E-01 | -0.16±0.13 | 6.09E-01 |
| 3-hydroxydecanoate | 0±0.07 | 0 | 9.46E-01 | 9.85E-01 | -0.02±0.08 | 0 | 8.82E-01 | 9.31E-01 | -0.01±0.09 | 9.89E-01 | -0.01±0.09 | 9.80E-01 | 0±0.13 | 9.94E-01 | -0.04±0.13 | 9.28E-01 |
| 5alpha-androstan-3alpha,17beta-diol monosulfate (1) | 0.01±0.08 | 56.47 | 1.30E-01 | 9.61E-01 | 0.02±0.08 | 63.71 | 9.69E-02 | 9.35E-01 | -0.07±0.09 | 9.03E-01 | -0.07±0.09 | 8.94E-01 | 0.18±0.14 | 5.61E-01 | 0.21±0.14 | 5.34E-01 |
| butyrate/isobutyrate (4:0) | 0.02±0.07 | 72.77 | 5.53E-02 | 9.18E-01 | 0.02±0.07 | 56.03 | 1.32E-01 | 9.35E-01 | -0.08±0.09 | 8.90E-01 | -0.06±0.09 | 8.94E-01 | 0.22±0.13 | 3.97E-01 | 0.17±0.13 | 5.85E-01 |
| 1-stearoyl-2-linoleoyl-GPE (18:0/18:2)* | -0.01±0.07 | 64.12 | 9.50E-02 | 9.61E-01 | -0.02±0.07 | 48.1 | 1.65E-01 | 9.35E-01 | -0.09±0.09 | 8.65E-01 | -0.09±0.09 | 8.47E-01 | 0.18±0.14 | 5.61E-01 | 0.14±0.14 | 6.77E-01 |
| oleoyl-linoleoyl-glycerol (18:1/18:2) [1] | 0±0.07 | 0 | 5.64E-01 | 9.78E-01 | -0.02±0.07 | 0 | 4.16E-01 | 9.35E-01 | 0.03±0.09 | 9.55E-01 | 0.03±0.09 | 9.45E-01 | -0.06±0.12 | 8.47E-01 | -0.09±0.12 | 7.50E-01 |
| docosapentaenoate (n3 DPA; 22:5n3) | -0.01±0.08 | 0 | 5.59E-01 | 9.55E-01 | -0.02±0.08 | 0 | 5.83E-01 | 9.35E-01 | 0.02±0.1 | 9.71E-01 | 0.01±0.1 | 9.80E-01 | -0.07±0.13 | 8.14E-01 | -0.07±0.13 | 8.10E-01 |
| X - 23587 | -0.01±0.07 | 0 | 8.75E-01 | 9.75E-01 | -0.02±0.07 | 0 | 9.93E-01 | 9.35E-01 | -0.01±0.09 | 9.78E-01 | -0.02±0.09 | 9.66E-01 | 0.01±0.13 | 9.84E-01 | -0.02±0.13 | 9.71E-01 |
| erythronate* | 0.01±0.08 | 83.09 | 1.50E-02 | 9.57E-01 | 0.02±0.08 | 78.84 | 2.97E-02 | 9.39E-01 | -0.1±0.1 | 8.65E-01 | -0.09±0.1 | 8.68E-01 | 0.36±0.16 | 3.04E-01 | 0.34±0.17 | 4.44E-01 |
| orotate | -0.03±0.08 | 56.78 | 1.28E-01 | 9.18E-01 | -0.02±0.08 | 61.06 | 1.09E-01 | 9.39E-01 | -0.12±0.1 | 8.41E-01 | -0.12±0.1 | 8.36E-01 | 0.13±0.13 | 6.51E-01 | 0.14±0.13 | 6.62E-01 |
| X - 19141 | -0.01±0.07 | 0 | 3.55E-01 | 9.49E-01 | -0.02±0.07 | 0 | 4.35E-01 | 9.39E-01 | -0.06±0.09 | 9.03E-01 | -0.06±0.09 | 9.00E-01 | 0.08±0.13 | 7.89E-01 | 0.06±0.13 | 8.40E-01 |
| glycodeoxycholate 3-sulfate | 0.02±0.07 | 0 | 6.40E-01 | 9.29E-01 | 0.02±0.07 | 0 | 7.74E-01 | 9.39E-01 | 0±0.09 | 9.89E-01 | 0±0.09 | 9.95E-01 | 0.07±0.13 | 8.24E-01 | 0.05±0.13 | 8.98E-01 |
| 16-hydroxypalmitate | 0.01±0.07 | 0 | 5.55E-01 | 9.49E-01 | 0.02±0.08 | 0 | 6.38E-01 | 9.39E-01 | 0.05±0.09 | 9.27E-01 | 0.04±0.09 | 9.18E-01 | -0.05±0.13 | 9.05E-01 | -0.03±0.13 | 9.29E-01 |
| glycosyl ceramide (d18:1/20:0, d16:1/22:0)* | 0±0.07 | 0 | 7.30E-01 | 9.88E-01 | 0.02±0.08 | 0 | 9.81E-01 | 9.39E-01 | 0.02±0.1 | 9.71E-01 | 0.02±0.1 | 9.66E-01 | -0.03±0.12 | 9.28E-01 | 0.01±0.13 | 9.72E-01 |
| X - 13553 | 0.01±0.08 | 80.64 | 2.31E-02 | 9.51E-01 | 0.02±0.08 | 80.59 | 2.32E-02 | 9.41E-01 | -0.12±0.1 | 8.31E-01 | -0.11±0.1 | 8.36E-01 | 0.25±0.13 | 3.42E-01 | 0.26±0.13 | 4.52E-01 |
| carnitine | -0.01±0.08 | 59.79 | 1.15E-01 | 9.61E-01 | -0.02±0.08 | 53.72 | 1.42E-01 | 9.41E-01 | -0.08±0.09 | 8.80E-01 | -0.08±0.09 | 8.60E-01 | 0.21±0.16 | 5.66E-01 | 0.18±0.15 | 6.45E-01 |
| 1-(1-enyl-stearoyl)-2-arachidonoyl-GPE (P-18:0/20:4)* | 0±0.08 | 51.13 | 1.53E-01 | 9.75E-01 | -0.02±0.08 | 57.05 | 1.27E-01 | 9.42E-01 | -0.07±0.09 | 9.01E-01 | -0.09±0.09 | 8.36E-01 | 0.17±0.14 | 6.01E-01 | 0.17±0.14 | 6.45E-01 |
| X - 25265 | 0.01±0.08 | 22.76 | 2.55E-01 | 9.68E-01 | 0.02±0.08 | 0 | 3.48E-01 | 9.43E-01 | 0.07±0.1 | 9.03E-01 | 0.07±0.1 | 8.94E-01 | -0.11±0.13 | 7.03E-01 | -0.08±0.13 | 7.98E-01 |
| X - 24337 | 0.04±0.08 | 86.71 | 6.08E-03 | 8.43E-01 | 0.02±0.08 | 81.39 | 2.04E-02 | 9.44E-01 | -0.11±0.09 | 8.65E-01 | -0.11±0.1 | 8.36E-01 | 0.34±0.13 | 2.03E-01 | 0.28±0.14 | 4.30E-01 |
| pregnenolone sulfate | 0.01±0.08 | 13.94 | 2.81E-01 | 9.49E-01 | 0.02±0.08 | 46.47 | 1.72E-01 | 9.44E-01 | -0.04±0.09 | 9.34E-01 | -0.06±0.09 | 9.04E-01 | 0.14±0.14 | 6.58E-01 | 0.17±0.14 | 6.22E-01 |
| allantoin | -0.02±0.07 | 60 | 1.14E-01 | 9.45E-01 | -0.02±0.07 | 57.25 | 1.26E-01 | 9.44E-01 | -0.1±0.09 | 8.65E-01 | -0.09±0.09 | 8.36E-01 | 0.16±0.14 | 6.00E-01 | 0.16±0.14 | 6.45E-01 |
| 4-acetylphenol sulfate | -0.04±0.07 | 0 | 4.70E-01 | 8.43E-01 | -0.02±0.07 | 20.12 | 2.63E-01 | 9.44E-01 | -0.08±0.09 | 8.80E-01 | -0.07±0.09 | 8.91E-01 | 0.03±0.13 | 9.28E-01 | 0.1±0.13 | 7.50E-01 |
| X - 11483 | -0.02±0.07 | 0 | 4.60E-01 | 9.45E-01 | -0.02±0.07 | 0 | 4.49E-01 | 9.44E-01 | 0.02±0.09 | 9.59E-01 | 0.03±0.09 | 9.55E-01 | -0.09±0.12 | 7.47E-01 | -0.09±0.13 | 7.64E-01 |
| 3-methoxycatechol sulfate (2) | -0.02±0.07 | 14.97 | 2.78E-01 | 9.45E-01 | -0.01±0.07 | 0 | 3.30E-01 | 9.44E-01 | -0.07±0.09 | 9.01E-01 | -0.06±0.09 | 8.94E-01 | 0.1±0.13 | 7.38E-01 | 0.09±0.13 | 7.82E-01 |
| 5alpha-pregnan-3beta,20beta-diol monosulfate (1) | 0.01±0.07 | 0 | 5.62E-01 | 9.61E-01 | 0.01±0.07 | 0 | 4.79E-01 | 9.44E-01 | -0.02±0.09 | 9.71E-01 | -0.02±0.09 | 9.58E-01 | 0.07±0.13 | 8.23E-01 | 0.09±0.13 | 7.82E-01 |
| histidine | -0.02±0.07 | 0 | 4.29E-01 | 9.23E-01 | -0.02±0.08 | 0 | 3.32E-01 | 9.44E-01 | -0.06±0.09 | 9.03E-01 | -0.07±0.09 | 8.94E-01 | 0.06±0.13 | 8.57E-01 | 0.09±0.13 | 7.86E-01 |
| dihomo-linoleoylcarnitine (C20:2)* | -0.02±0.07 | 0 | 8.34E-01 | 9.45E-01 | -0.02±0.08 | 0 | 7.14E-01 | 9.44E-01 | -0.03±0.09 | 9.48E-01 | -0.04±0.09 | 9.19E-01 | 0±0.13 | 9.95E-01 | 0.02±0.13 | 9.52E-01 |
| 2-hydroxyhippurate (salicylurate) | -0.01±0.07 | 0 | 3.94E-01 | 9.57E-01 | -0.01±0.07 | 0 | 3.76E-01 | 9.45E-01 | -0.06±0.09 | 9.09E-01 | -0.06±0.09 | 8.94E-01 | 0.07±0.12 | 8.14E-01 | 0.07±0.12 | 8.10E-01 |
| 9-hydroxystearate | 0.02±0.08 | 0 | 8.11E-01 | 9.45E-01 | 0.02±0.08 | 0 | 7.64E-01 | 9.45E-01 | 0.01±0.09 | 9.89E-01 | 0±0.09 | 9.98E-01 | 0.05±0.13 | 9.05E-01 | 0.05±0.14 | 8.98E-01 |
| X - 25343 | -0.02±0.08 | 81.57 | 1.98E-02 | 9.45E-01 | -0.02±0.08 | 77.6 | 3.46E-02 | 9.45E-01 | -0.13±0.09 | 7.90E-01 | -0.12±0.09 | 8.25E-01 | 0.28±0.15 | 3.58E-01 | 0.25±0.15 | 4.80E-01 |
| biliverdin | -0.03±0.07 | 0 | 8.47E-01 | 9.03E-01 | -0.01±0.08 | 0 | 6.46E-01 | 9.46E-01 | -0.04±0.09 | 9.38E-01 | -0.04±0.09 | 9.18E-01 | -0.01±0.13 | 9.92E-01 | 0.03±0.13 | 9.29E-01 |
| homostachydrine* | 0.01±0.07 | 0 | 5.15E-01 | 9.49E-01 | 0.01±0.07 | 0 | 4.31E-01 | 9.46E-01 | -0.02±0.09 | 9.71E-01 | -0.03±0.09 | 9.45E-01 | 0.08±0.12 | 7.89E-01 | 0.1±0.13 | 7.64E-01 |
| 4-guanidinobutanoate | 0.02±0.08 | 91.55 | 5.82E-04 | 9.45E-01 | 0.02±0.08 | 91.98 | 4.14E-04 | 9.46E-01 | -0.17±0.1 | 6.97E-01 | -0.18±0.1 | 6.28E-01 | 0.44±0.15 | 1.81E-01 | 0.45±0.15 | 2.50E-01 |
| bilirubin (Z,Z) | -0.03±0.08 | 47.5 | 1.68E-01 | 9.08E-01 | -0.01±0.08 | 67.38 | 8.00E-02 | 9.48E-01 | -0.1±0.09 | 8.65E-01 | -0.1±0.09 | 8.36E-01 | 0.14±0.14 | 6.66E-01 | 0.21±0.15 | 5.77E-01 |
| gamma-glutamyl-2-aminobutyrate | -0.01±0.07 | 0 | 3.52E-01 | 9.49E-01 | -0.01±0.07 | 0 | 3.44E-01 | 9.48E-01 | -0.06±0.09 | 9.06E-01 | -0.06±0.09 | 8.94E-01 | 0.09±0.13 | 7.80E-01 | 0.09±0.13 | 7.82E-01 |
| palmitoleate (16:1n7) | 0.02±0.08 | 47.38 | 1.68E-01 | 9.25E-01 | 0.02±0.08 | 49.03 | 1.61E-01 | 9.48E-01 | 0.1±0.1 | 8.65E-01 | 0.1±0.1 | 8.47E-01 | -0.13±0.14 | 6.66E-01 | -0.14±0.14 | 6.74E-01 |
| S-methylcysteine sulfoxide | 0±0.07 | 0 | 3.25E-01 | 9.99E-01 | 0.01±0.07 | 41.39 | 1.91E-01 | 9.51E-01 | -0.05±0.09 | 9.27E-01 | -0.06±0.09 | 9.00E-01 | 0.1±0.12 | 7.29E-01 | 0.14±0.12 | 6.45E-01 |
| X - 12193 | 0.01±0.07 | 0 | 3.45E-01 | 9.58E-01 | 0.01±0.08 | 6.29 | 3.02E-01 | 9.52E-01 | -0.04±0.09 | 9.46E-01 | -0.04±0.09 | 9.19E-01 | 0.12±0.14 | 6.96E-01 | 0.14±0.14 | 6.93E-01 |
| X - 21258 | 0.01±0.07 | 33.59 | 2.20E-01 | 9.61E-01 | 0.01±0.07 | 9.45 | 2.93E-01 | 9.52E-01 | 0.07±0.09 | 8.95E-01 | 0.07±0.09 | 8.94E-01 | -0.11±0.12 | 6.82E-01 | -0.09±0.12 | 7.64E-01 |
| thyroxine | 0.02±0.08 | 0.26 | 3.17E-01 | 9.45E-01 | 0.01±0.08 | 0 | 4.60E-01 | 9.52E-01 | -0.03±0.09 | 9.48E-01 | -0.03±0.09 | 9.55E-01 | 0.13±0.14 | 6.66E-01 | 0.1±0.13 | 7.79E-01 |
| sphingomyelin (d18:2/23:0, d18:1/23:1, d17:1/24:1)* | -0.03±0.08 | 0 | 5.63E-01 | 8.91E-01 | -0.01±0.08 | 0 | 4.12E-01 | 9.52E-01 | -0.07±0.1 | 9.06E-01 | -0.06±0.1 | 9.00E-01 | 0.03±0.13 | 9.40E-01 | 0.08±0.14 | 8.18E-01 |
| N-acetyl-2-aminooctanoate* | -0.02±0.08 | 0 | 5.30E-01 | 9.45E-01 | -0.01±0.08 | 0 | 4.85E-01 | 9.52E-01 | -0.05±0.09 | 9.27E-01 | -0.05±0.1 | 9.13E-01 | 0.04±0.13 | 9.05E-01 | 0.06±0.13 | 8.58E-01 |
| 1-oleoyl-2-linoleoyl-GPE (18:1/18:2)* | 0.01±0.07 | 42.61 | 1.87E-01 | 9.49E-01 | 0.01±0.07 | 34.98 | 2.15E-01 | 9.53E-01 | -0.05±0.09 | 9.27E-01 | -0.05±0.09 | 9.18E-01 | 0.17±0.14 | 5.85E-01 | 0.16±0.14 | 6.49E-01 |
| hexanoylcarnitine (C6) | -0.01±0.07 | 0 | 4.05E-01 | 9.75E-01 | -0.01±0.07 | 0 | 3.55E-01 | 9.53E-01 | 0.04±0.09 | 9.34E-01 | 0.04±0.09 | 9.18E-01 | -0.09±0.12 | 7.58E-01 | -0.1±0.12 | 7.41E-01 |
| 10-heptadecenoate (17:1n7) | -0.01±0.08 | 0 | 4.36E-01 | 9.71E-01 | -0.01±0.08 | 0 | 3.84E-01 | 9.53E-01 | 0.04±0.1 | 9.46E-01 | 0.04±0.1 | 9.19E-01 | -0.09±0.13 | 7.70E-01 | -0.11±0.13 | 7.50E-01 |
| sphingomyelin (d18:1/24:1, d18:2/24:0)* | -0.01±0.07 | 0 | 9.51E-01 | 9.57E-01 | -0.01±0.08 | 0 | 6.57E-01 | 9.53E-01 | -0.01±0.09 | 9.78E-01 | -0.04±0.09 | 9.19E-01 | -0.01±0.12 | 9.92E-01 | 0.03±0.13 | 9.29E-01 |
| octadecenedioylcarnitine (C18:1-DC)* | -0.01±0.07 | 0 | 9.92E-01 | 9.49E-01 | -0.01±0.08 | 0 | 9.65E-01 | 9.53E-01 | -0.01±0.09 | 9.78E-01 | -0.02±0.09 | 9.79E-01 | -0.01±0.13 | 9.84E-01 | -0.01±0.13 | 9.85E-01 |
| glucose | -0.01±0.08 | 73.7 | 5.12E-02 | 9.57E-01 | -0.01±0.08 | 57.91 | 1.23E-01 | 9.53E-01 | -0.1±0.1 | 8.65E-01 | -0.09±0.1 | 8.60E-01 | 0.29±0.18 | 4.40E-01 | 0.2±0.16 | 6.23E-01 |
| isovalerylglycine | 0.01±0.07 | 0 | 7.49E-01 | 9.75E-01 | 0.01±0.07 | 0 | 7.46E-01 | 9.53E-01 | -0.01±0.09 | 9.87E-01 | 0±0.09 | 9.93E-01 | 0.04±0.13 | 9.20E-01 | 0.05±0.13 | 8.98E-01 |
| X - 21733 | -0.01±0.07 | 0 | 7.23E-01 | 9.68E-01 | 0.01±0.08 | 0 | 7.11E-01 | 9.56E-01 | 0.01±0.09 | 9.86E-01 | 0.03±0.1 | 9.25E-01 | -0.04±0.12 | 9.05E-01 | -0.03±0.13 | 9.43E-01 |
| X - 17438 | -0.01±0.07 | 0 | 3.93E-01 | 9.54E-01 | -0.01±0.07 | 0 | 4.11E-01 | 9.56E-01 | 0.03±0.09 | 9.48E-01 | 0.03±0.09 | 9.25E-01 | -0.1±0.13 | 7.29E-01 | -0.1±0.13 | 7.64E-01 |
| 4-methylcatechol sulfate | -0.01±0.07 | 26.23 | 2.44E-01 | 9.57E-01 | -0.01±0.07 | 30.27 | 2.31E-01 | 9.56E-01 | -0.07±0.09 | 9.03E-01 | -0.07±0.09 | 8.91E-01 | 0.11±0.13 | 6.96E-01 | 0.12±0.13 | 7.13E-01 |
| ascorbic acid 3-sulfate* | -0.03±0.07 | 58.31 | 1.21E-01 | 9.00E-01 | -0.01±0.07 | 64.46 | 9.35E-02 | 9.59E-01 | -0.11±0.09 | 8.41E-01 | -0.1±0.09 | 8.36E-01 | 0.14±0.13 | 6.25E-01 | 0.17±0.13 | 6.05E-01 |
| X - 18922 | 0.03±0.07 | 0 | 5.18E-01 | 9.08E-01 | 0.01±0.07 | 0 | 6.98E-01 | 9.60E-01 | -0.01±0.09 | 9.89E-01 | -0.01±0.09 | 9.80E-01 | 0.1±0.13 | 7.47E-01 | 0.05±0.13 | 8.85E-01 |
| 12-HETE | -0.01±0.07 | 78.38 | 3.15E-02 | 9.68E-01 | -0.01±0.07 | 79.34 | 2.78E-02 | 9.61E-01 | 0.11±0.09 | 8.31E-01 | 0.11±0.09 | 8.36E-01 | -0.22±0.12 | 3.79E-01 | -0.22±0.12 | 4.63E-01 |
| cys-gly, oxidized | -0.01±0.08 | 59.51 | 1.16E-01 | 9.53E-01 | -0.01±0.08 | 62.47 | 1.03E-01 | 9.61E-01 | -0.09±0.09 | 8.72E-01 | -0.09±0.09 | 8.52E-01 | 0.19±0.15 | 5.71E-01 | 0.2±0.15 | 5.95E-01 |
| sphingomyelin (d18:2/14:0, d18:1/14:1)* | -0.02±0.09 | 54.14 | 1.40E-01 | 9.49E-01 | -0.01±0.09 | 45.84 | 1.74E-01 | 9.61E-01 | -0.12±0.11 | 8.65E-01 | -0.11±0.12 | 8.60E-01 | 0.17±0.16 | 6.23E-01 | 0.16±0.16 | 6.83E-01 |
| lithocholate sulfate (1) | 0.01±0.07 | 0 | 5.25E-01 | 9.49E-01 | 0.01±0.07 | 0 | 6.37E-01 | 9.61E-01 | -0.02±0.09 | 9.77E-01 | -0.01±0.09 | 9.80E-01 | 0.09±0.14 | 7.89E-01 | 0.06±0.13 | 8.51E-01 |
| 8-methoxykynurenate | 0.01±0.07 | 17.76 | 2.70E-01 | 9.73E-01 | -0.01±0.08 | 0 | 6.35E-01 | 9.61E-01 | -0.05±0.09 | 9.27E-01 | -0.04±0.09 | 9.19E-01 | 0.13±0.14 | 6.66E-01 | 0.04±0.14 | 9.13E-01 |
| 2-palmitoleoyl-GPC (16:1)* | -0.01±0.07 | 0 | 9.22E-01 | 9.61E-01 | -0.01±0.07 | 0 | 7.64E-01 | 9.61E-01 | -0.02±0.09 | 9.77E-01 | -0.03±0.09 | 9.46E-01 | 0±0.12 | 9.96E-01 | 0.02±0.12 | 9.58E-01 |
| pregnanediol-3-glucuronide | -0.02±0.07 | 0 | 8.34E-01 | 9.45E-01 | -0.01±0.07 | 0 | 8.63E-01 | 9.61E-01 | -0.01±0.09 | 9.89E-01 | -0.02±0.09 | 9.63E-01 | -0.04±0.13 | 9.20E-01 | 0.01±0.13 | 9.85E-01 |
| X - 23782 | 0±0.07 | 0 | 7.90E-01 | 9.88E-01 | 0.01±0.07 | 0 | 9.89E-01 | 9.61E-01 | 0.02±0.09 | 9.78E-01 | 0.01±0.09 | 9.80E-01 | -0.03±0.13 | 9.47E-01 | 0.01±0.13 | 9.85E-01 |
| 1-stearoyl-GPG (18:0) | 0.01±0.07 | 0 | 5.52E-01 | 9.49E-01 | 0.01±0.07 | 0 | 6.84E-01 | 9.63E-01 | -0.02±0.09 | 9.77E-01 | -0.01±0.09 | 9.80E-01 | 0.08±0.13 | 8.09E-01 | 0.05±0.13 | 8.81E-01 |
| 1-palmitoyl-2-linoleoyl-GPE (16:0/18:2) | 0.02±0.07 | 51.3 | 1.52E-01 | 9.48E-01 | 0.01±0.07 | 39.82 | 1.97E-01 | 9.65E-01 | -0.06±0.09 | 9.20E-01 | -0.05±0.09 | 9.04E-01 | 0.17±0.13 | 5.67E-01 | 0.15±0.13 | 6.46E-01 |
| X - 24544 | 0.01±0.08 | 0 | 5.67E-01 | 9.61E-01 | 0.01±0.08 | 0 | 5.15E-01 | 9.65E-01 | -0.02±0.09 | 9.71E-01 | -0.02±0.09 | 9.57E-01 | 0.07±0.13 | 8.24E-01 | 0.08±0.13 | 8.03E-01 |
| 2,6-dihydroxybenzoic acid | -0.01±0.07 | 9.52 | 2.93E-01 | 9.61E-01 | 0.01±0.07 | 40.21 | 1.96E-01 | 9.69E-01 | -0.06±0.09 | 9.03E-01 | -0.06±0.09 | 9.00E-01 | 0.1±0.13 | 7.35E-01 | 0.15±0.13 | 6.51E-01 |
| caffeine | 0±0.07 | 36.72 | 2.09E-01 | 9.95E-01 | -0.01±0.08 | 0 | 3.62E-01 | 9.69E-01 | -0.06±0.09 | 9.03E-01 | -0.06±0.09 | 9.01E-01 | 0.14±0.13 | 6.35E-01 | 0.09±0.14 | 7.82E-01 |
| N,N,N-trimethyl-5-aminovalerate | 0.01±0.08 | 83.66 | 1.34E-02 | 9.68E-01 | 0.01±0.08 | 80.88 | 2.22E-02 | 9.70E-01 | -0.1±0.09 | 8.65E-01 | -0.09±0.09 | 8.36E-01 | 0.34±0.15 | 3.02E-01 | 0.32±0.16 | 4.30E-01 |
| 1-palmitoyl-GPI (16:0) | -0.01±0.08 | 80.68 | 2.29E-02 | 9.68E-01 | -0.01±0.08 | 81.29 | 2.08E-02 | 9.70E-01 | -0.13±0.09 | 7.90E-01 | -0.13±0.09 | 7.69E-01 | 0.26±0.14 | 3.67E-01 | 0.26±0.14 | 4.54E-01 |
| lyxonate | -0.02±0.08 | 67.39 | 7.99E-02 | 9.45E-01 | -0.01±0.08 | 72.02 | 5.87E-02 | 9.70E-01 | -0.1±0.09 | 8.65E-01 | -0.1±0.09 | 8.36E-01 | 0.21±0.15 | 5.43E-01 | 0.25±0.16 | 4.95E-01 |
| gamma-glutamylglutamate | 0.02±0.08 | 78.88 | 2.96E-02 | 9.47E-01 | 0.01±0.08 | 64.52 | 9.32E-02 | 9.70E-01 | -0.09±0.09 | 8.73E-01 | -0.08±0.1 | 8.86E-01 | 0.29±0.15 | 3.40E-01 | 0.21±0.15 | 5.50E-01 |
| 1-palmitoyl-2-arachidonoyl-GPE (16:0/20:4)* | 0.02±0.08 | 68.42 | 7.51E-02 | 9.20E-01 | 0.01±0.08 | 61.45 | 1.07E-01 | 9.70E-01 | -0.06±0.09 | 9.06E-01 | -0.07±0.09 | 8.94E-01 | 0.24±0.14 | 4.32E-01 | 0.2±0.14 | 5.77E-01 |
| butyrylcarnitine (C4) | 0±0.08 | 77.85 | 3.36E-02 | 9.85E-01 | -0.01±0.08 | 68.98 | 7.26E-02 | 9.70E-01 | -0.12±0.1 | 8.31E-01 | -0.11±0.1 | 8.36E-01 | 0.22±0.13 | 4.04E-01 | 0.17±0.13 | 5.85E-01 |
| isobutyrylcarnitine (C4) | -0.02±0.07 | 34.13 | 2.18E-01 | 9.45E-01 | -0.01±0.07 | 44.58 | 1.79E-01 | 9.70E-01 | -0.08±0.09 | 8.80E-01 | -0.08±0.09 | 8.68E-01 | 0.11±0.13 | 6.92E-01 | 0.14±0.13 | 6.67E-01 |
| 3-hydroxyoctanoylcarnitine (1) | -0.01±0.07 | 0.63 | 3.16E-01 | 9.73E-01 | -0.01±0.08 | 0 | 3.80E-01 | 9.70E-01 | 0.05±0.09 | 9.27E-01 | 0.04±0.09 | 9.18E-01 | -0.11±0.13 | 6.98E-01 | -0.1±0.13 | 7.59E-01 |
| kynurenate | 0.01±0.07 | 54.59 | 1.38E-01 | 9.73E-01 | -0.01±0.08 | 0 | 3.27E-01 | 9.70E-01 | -0.07±0.09 | 9.03E-01 | -0.06±0.1 | 8.94E-01 | 0.16±0.13 | 5.71E-01 | 0.1±0.13 | 7.64E-01 |
| 2S,3R-dihydroxybutyrate | -0.01±0.08 | 0 | 3.90E-01 | 9.75E-01 | -0.01±0.09 | 0 | 3.91E-01 | 9.70E-01 | -0.06±0.1 | 9.27E-01 | -0.06±0.1 | 9.04E-01 | 0.1±0.15 | 7.81E-01 | 0.09±0.15 | 7.96E-01 |
| ceramide (d18:2/24:1, d18:1/24:2)* | -0.01±0.07 | 0 | 6.32E-01 | 9.68E-01 | -0.01±0.07 | 0 | 5.83E-01 | 9.70E-01 | 0.02±0.09 | 9.71E-01 | 0.02±0.09 | 9.57E-01 | -0.05±0.12 | 8.71E-01 | -0.06±0.12 | 8.46E-01 |
| 3-hydroxyhexanoylcarnitine (1) | 0.01±0.08 | 0 | 9.91E-01 | 9.68E-01 | -0.01±0.08 | 0 | 7.19E-01 | 9.70E-01 | 0.01±0.09 | 9.87E-01 | 0.01±0.09 | 9.80E-01 | 0.01±0.13 | 9.92E-01 | -0.05±0.13 | 9.00E-01 |
| X - 17354 | -0.03±0.07 | 0 | 9.32E-01 | 9.06E-01 | -0.01±0.07 | 0 | 8.10E-01 | 9.70E-01 | -0.02±0.09 | 9.67E-01 | -0.02±0.09 | 9.58E-01 | -0.04±0.13 | 9.21E-01 | 0.02±0.13 | 9.71E-01 |
| N-acetylisoleucine | 0.01±0.08 | 69.35 | 7.09E-02 | 9.61E-01 | 0.01±0.08 | 49.28 | 1.60E-01 | 9.71E-01 | -0.08±0.09 | 8.80E-01 | -0.06±0.09 | 8.94E-01 | 0.23±0.14 | 4.64E-01 | 0.18±0.14 | 6.23E-01 |
| 1-(1-enyl-palmitoyl)-GPC (P-16:0)* | -0.01±0.07 | 0 | 9.03E-01 | 9.68E-01 | 0.01±0.08 | 0 | 6.97E-01 | 9.75E-01 | 0±0.09 | 9.92E-01 | -0.01±0.09 | 9.80E-01 | -0.02±0.12 | 9.61E-01 | 0.05±0.13 | 8.98E-01 |
| X - 12729 | -0.01±0.07 | 6.12 | 3.02E-01 | 9.75E-01 | 0.01±0.07 | 0 | 5.08E-01 | 9.75E-01 | 0.04±0.09 | 9.27E-01 | 0.04±0.09 | 9.18E-01 | -0.12±0.13 | 6.82E-01 | -0.07±0.13 | 8.46E-01 |
| eugenol sulfate | 0.02±0.07 | 78.88 | 2.96E-02 | 9.45E-01 | 0.01±0.07 | 76.94 | 3.73E-02 | 9.77E-01 | -0.1±0.09 | 8.65E-01 | -0.1±0.09 | 8.36E-01 | 0.24±0.13 | 3.47E-01 | 0.23±0.13 | 4.65E-01 |
| X - 24546 | 0±0.08 | 0 | 8.12E-01 | 9.80E-01 | 0.01±0.08 | 0 | 7.02E-01 | 9.78E-01 | -0.01±0.1 | 9.87E-01 | -0.01±0.1 | 9.80E-01 | 0.03±0.14 | 9.40E-01 | 0.05±0.14 | 8.98E-01 |
| phenylalanine | 0.01±0.09 | 85.64 | 8.33E-03 | 9.75E-01 | 0.01±0.09 | 80.42 | 2.38E-02 | 9.81E-01 | -0.08±0.09 | 8.80E-01 | -0.07±0.09 | 8.94E-01 | 0.56±0.22 | 2.19E-01 | 0.47±0.22 | 4.21E-01 |
| vanillic acid glycine | 0±0.07 | 64.15 | 9.49E-02 | 9.85E-01 | -0.01±0.07 | 47.21 | 1.69E-01 | 9.83E-01 | -0.09±0.09 | 8.69E-01 | -0.08±0.09 | 8.68E-01 | 0.17±0.13 | 5.53E-01 | 0.14±0.13 | 6.66E-01 |
| 1-(1-enyl-palmitoyl)-2-arachidonoyl-GPE (P-16:0/20:4)* | 0.01±0.07 | 37.7 | 2.05E-01 | 9.75E-01 | -0.01±0.07 | 32.02 | 2.25E-01 | 9.83E-01 | -0.06±0.09 | 9.18E-01 | -0.07±0.09 | 8.94E-01 | 0.15±0.14 | 6.21E-01 | 0.13±0.13 | 6.96E-01 |
| X - 22834 | 0.02±0.07 | 0 | 8.01E-01 | 9.18E-01 | 0.01±0.07 | 0 | 5.22E-01 | 9.83E-01 | 0.04±0.09 | 9.38E-01 | 0.04±0.09 | 9.18E-01 | 0±0.13 | 9.96E-01 | -0.06±0.13 | 8.50E-01 |
| (16 or 17)-methylstearate (a19:0 or i19:0) | 0±0.08 | 0 | 5.08E-01 | 9.88E-01 | -0.01±0.08 | 0 | 5.30E-01 | 9.83E-01 | -0.04±0.09 | 9.38E-01 | -0.04±0.09 | 9.18E-01 | 0.07±0.13 | 8.41E-01 | 0.06±0.13 | 8.58E-01 |
| carotene diol (1) | -0.03±0.07 | 0 | 9.87E-01 | 8.79E-01 | -0.01±0.08 | 0 | 5.41E-01 | 9.83E-01 | -0.03±0.09 | 9.47E-01 | -0.04±0.09 | 9.19E-01 | -0.03±0.13 | 9.38E-01 | 0.06±0.13 | 8.58E-01 |
| X - 11843 | -0.01±0.07 | 0 | 6.84E-01 | 9.57E-01 | -0.01±0.07 | 0 | 7.96E-01 | 9.83E-01 | 0.01±0.09 | 9.87E-01 | 0.01±0.09 | 9.80E-01 | -0.06±0.13 | 8.80E-01 | -0.03±0.13 | 9.29E-01 |
| X - 22509 | -0.03±0.07 | 0 | 5.99E-01 | 9.08E-01 | 0±0.07 | 0 | 3.45E-01 | 9.84E-01 | -0.05±0.09 | 9.27E-01 | -0.05±0.09 | 9.04E-01 | 0.03±0.13 | 9.40E-01 | 0.1±0.13 | 7.64E-01 |
| myristoleate (14:1n5) | 0±0.08 | 0 | 3.59E-01 | 9.78E-01 | -0.01±0.08 | 0 | 3.71E-01 | 9.85E-01 | 0.05±0.1 | 9.27E-01 | 0.05±0.1 | 9.18E-01 | -0.11±0.14 | 7.35E-01 | -0.11±0.14 | 7.58E-01 |
| 3-hydroxyoctanoate | 0.02±0.07 | 0 | 9.95E-01 | 9.45E-01 | 0±0.08 | 0 | 8.46E-01 | 9.87E-01 | 0.02±0.09 | 9.74E-01 | 0.02±0.09 | 9.79E-01 | 0.02±0.13 | 9.75E-01 | -0.02±0.13 | 9.72E-01 |
| 4-acetamidobutanoate | 0±0.07 | 80.88 | 2.22E-02 | 9.76E-01 | 0±0.07 | 79.5 | 2.72E-02 | 9.87E-01 | -0.1±0.09 | 8.31E-01 | -0.1±0.09 | 8.36E-01 | 0.28±0.14 | 3.42E-01 | 0.27±0.14 | 4.54E-01 |
| S-methylcysteine | -0.01±0.07 | 55.79 | 1.33E-01 | 9.61E-01 | 0±0.07 | 67.64 | 7.88E-02 | 9.87E-01 | -0.09±0.09 | 8.65E-01 | -0.1±0.09 | 8.36E-01 | 0.14±0.12 | 6.14E-01 | 0.17±0.12 | 5.85E-01 |
| picolinate | 0±0.08 | 48.4 | 1.64E-01 | 9.78E-01 | 0±0.08 | 34.96 | 2.15E-01 | 9.87E-01 | -0.07±0.09 | 9.03E-01 | -0.06±0.09 | 8.94E-01 | 0.15±0.13 | 6.04E-01 | 0.14±0.13 | 6.67E-01 |
| 3-methyl-2-oxobutyrate | 0.01±0.07 | 57.99 | 1.23E-01 | 9.61E-01 | 0±0.08 | 26.51 | 2.43E-01 | 9.87E-01 | -0.07±0.09 | 9.03E-01 | -0.06±0.09 | 9.00E-01 | 0.18±0.13 | 5.53E-01 | 0.13±0.13 | 6.91E-01 |
| hydroxy-N6,N6,N6-trimethyllysine* | 0.01±0.07 | 0 | 3.21E-01 | 9.68E-01 | 0±0.07 | 0 | 4.49E-01 | 9.87E-01 | -0.04±0.09 | 9.34E-01 | -0.03±0.09 | 9.19E-01 | 0.12±0.13 | 6.88E-01 | 0.09±0.13 | 7.91E-01 |
| isovalerate (i5:0) | 0±0.07 | 0 | 4.57E-01 | 9.79E-01 | 0±0.08 | 0 | 6.07E-01 | 9.87E-01 | -0.04±0.09 | 9.46E-01 | -0.03±0.09 | 9.25E-01 | 0.09±0.13 | 7.89E-01 | 0.05±0.13 | 8.95E-01 |
| N-oleoyltaurine | 0.01±0.07 | 0 | 8.62E-01 | 9.49E-01 | 0±0.07 | 0 | 7.43E-01 | 9.87E-01 | 0.03±0.09 | 9.59E-01 | 0.02±0.09 | 9.58E-01 | 0±0.12 | 9.96E-01 | -0.03±0.12 | 9.34E-01 |
| tryptophan betaine | 0±0.07 | 0 | 8.63E-01 | 9.75E-01 | 0±0.08 | 0 | 9.33E-01 | 9.87E-01 | 0.01±0.09 | 9.78E-01 | 0.01±0.1 | 9.80E-01 | -0.01±0.12 | 9.84E-01 | 0±0.13 | 9.92E-01 |
| caprate (10:0) | 0±0.08 | 0 | 9.93E-01 | 9.75E-01 | 0±0.08 | 0 | 7.39E-01 | 9.88E-01 | 0±0.09 | 9.89E-01 | -0.01±0.1 | 9.80E-01 | -0.01±0.13 | 9.92E-01 | 0.04±0.13 | 9.19E-01 |
| sphingomyelin (d18:1/20:0, d16:1/22:0)* | -0.01±0.08 | 0 | 5.80E-01 | 9.57E-01 | 0±0.08 | 0 | 5.39E-01 | 9.89E-01 | -0.04±0.09 | 9.34E-01 | -0.04±0.09 | 9.19E-01 | 0.05±0.13 | 9.05E-01 | 0.06±0.13 | 8.50E-01 |
| sphingomyelin (d18:1/18:1, d18:2/18:0) | 0±0.08 | 0 | 8.90E-01 | 9.78E-01 | 0±0.08 | 0 | 9.72E-01 | 9.89E-01 | -0.01±0.1 | 9.86E-01 | 0±0.1 | 9.97E-01 | 0.01±0.13 | 9.86E-01 | 0.01±0.13 | 9.85E-01 |
| 3,7-dimethylurate | 0.01±0.07 | 0 | 6.74E-01 | 9.75E-01 | 0±0.07 | 0 | 7.81E-01 | 9.93E-01 | -0.02±0.09 | 9.77E-01 | -0.01±0.09 | 9.80E-01 | 0.05±0.13 | 8.97E-01 | 0.03±0.12 | 9.29E-01 |
| hydroquinone sulfate | 0±0.07 | 66.21 | 8.54E-02 | 9.75E-01 | 0±0.07 | 56.94 | 1.28E-01 | 9.94E-01 | -0.09±0.09 | 8.65E-01 | -0.08±0.09 | 8.63E-01 | 0.18±0.13 | 5.43E-01 | 0.16±0.13 | 6.25E-01 |
| ceramide (d18:1/14:0, d16:1/16:0)* | 0±0.07 | 50.1 | 1.57E-01 | 9.75E-01 | 0±0.07 | 48.33 | 1.64E-01 | 9.94E-01 | -0.07±0.09 | 9.01E-01 | -0.07±0.09 | 8.94E-01 | 0.16±0.14 | 6.04E-01 | 0.16±0.14 | 6.45E-01 |
| X - 21796 | 0±0.08 | 22.06 | 2.57E-01 | 9.88E-01 | 0±0.08 | 34.54 | 2.16E-01 | 9.94E-01 | -0.06±0.09 | 9.13E-01 | -0.06±0.09 | 8.94E-01 | 0.13±0.14 | 6.69E-01 | 0.15±0.15 | 6.76E-01 |
| caprylate (8:0) | -0.01±0.07 | 0 | 4.27E-01 | 9.68E-01 | 0±0.07 | 1 | 3.15E-01 | 9.94E-01 | -0.05±0.09 | 9.27E-01 | -0.06±0.09 | 9.00E-01 | 0.07±0.12 | 8.14E-01 | 0.09±0.12 | 7.55E-01 |
| X - 11381 | -0.01±0.07 | 0 | 4.99E-01 | 9.67E-01 | 0±0.07 | 0 | 5.02E-01 | 9.94E-01 | -0.04±0.09 | 9.27E-01 | -0.04±0.09 | 9.19E-01 | 0.06±0.13 | 8.51E-01 | 0.07±0.13 | 8.27E-01 |
| X - 17335 | 0.01±0.07 | 0 | 9.14E-01 | 9.73E-01 | 0±0.08 | 0 | 7.81E-01 | 9.94E-01 | 0.01±0.09 | 9.82E-01 | 0.01±0.09 | 9.80E-01 | 0±0.13 | 9.94E-01 | -0.03±0.13 | 9.29E-01 |
| 1-stearoyl-GPC (18:0) | -0.01±0.07 | 0 | 6.22E-01 | 9.49E-01 | 0±0.08 | 0 | 4.03E-01 | 9.96E-01 | -0.04±0.09 | 9.34E-01 | -0.04±0.09 | 9.18E-01 | 0.04±0.13 | 9.20E-01 | 0.1±0.14 | 7.82E-01 |
| glycerol 3-phosphate | 0±0.07 | 81.58 | 1.98E-02 | 9.77E-01 | 0±0.07 | 81.82 | 1.90E-02 | 9.96E-01 | -0.11±0.09 | 7.90E-01 | -0.11±0.09 | 8.25E-01 | 0.26±0.13 | 3.42E-01 | 0.26±0.13 | 4.52E-01 |
| 1-stearoyl-2-arachidonoyl-GPC (18:0/20:4) | 0.01±0.08 | 67.02 | 8.16E-02 | 9.69E-01 | 0±0.08 | 62.04 | 1.05E-01 | 9.96E-01 | -0.07±0.09 | 9.03E-01 | -0.08±0.1 | 8.68E-01 | 0.27±0.17 | 4.64E-01 | 0.23±0.16 | 5.77E-01 |
| bilirubin (E,Z or Z,E)* | -0.01±0.07 | 63.45 | 9.81E-02 | 9.57E-01 | 0±0.08 | 74.83 | 4.62E-02 | 9.98E-01 | -0.09±0.09 | 8.65E-01 | -0.1±0.09 | 8.36E-01 | 0.18±0.14 | 5.71E-01 | 0.25±0.15 | 4.74E-01 |
| 1-methylnicotinamide | -0.01±0.07 | 67.45 | 7.97E-02 | 9.61E-01 | 0±0.07 | 62.18 | 1.04E-01 | 9.98E-01 | 0.08±0.09 | 8.80E-01 | 0.09±0.09 | 8.47E-01 | -0.19±0.12 | 4.98E-01 | -0.16±0.12 | 5.98E-01 |
| (N(1) + N(8))-acetylspermidine | 0±0.08 | 48.04 | 1.65E-01 | 9.88E-01 | 0±0.08 | 48.44 | 1.64E-01 | 9.98E-01 | -0.07±0.09 | 9.02E-01 | -0.07±0.09 | 8.94E-01 | 0.16±0.14 | 6.10E-01 | 0.16±0.14 | 6.45E-01 |
| 1-(1-enyl-stearoyl)-2-oleoyl-GPE (P-18:0/18:1) | 0.01±0.08 | 27.96 | 2.39E-01 | 9.61E-01 | 0±0.08 | 41.94 | 1.89E-01 | 9.98E-01 | -0.04±0.09 | 9.27E-01 | -0.06±0.09 | 8.94E-01 | 0.16±0.14 | 6.25E-01 | 0.16±0.14 | 6.56E-01 |
| 2-hydroxypalmitate | 0±0.07 | 0 | 5.39E-01 | 9.85E-01 | 0±0.07 | 0 | 4.32E-01 | 9.98E-01 | -0.03±0.09 | 9.48E-01 | -0.04±0.09 | 9.18E-01 | 0.06±0.13 | 8.41E-01 | 0.08±0.13 | 7.95E-01 |
| X - 24494 | 0±0.07 | 0 | 5.93E-01 | 9.88E-01 | 0±0.07 | 0 | 5.23E-01 | 9.98E-01 | -0.03±0.09 | 9.58E-01 | -0.03±0.09 | 9.19E-01 | 0.06±0.13 | 8.66E-01 | 0.06±0.13 | 8.39E-01 |
| 2-hydroxyarachidate* | 0.01±0.07 | 0 | 5.34E-01 | 9.49E-01 | 0±0.07 | 0 | 5.30E-01 | 9.98E-01 | -0.02±0.09 | 9.74E-01 | -0.03±0.09 | 9.21E-01 | 0.08±0.13 | 7.97E-01 | 0.07±0.13 | 8.40E-01 |
| X - 25371 | 0±0.07 | 5.77 | 3.03E-01 | 9.75E-01 | 0±0.07 | 0 | 6.62E-01 | 9.98E-01 | -0.05±0.09 | 9.27E-01 | -0.03±0.09 | 9.55E-01 | 0.11±0.13 | 6.92E-01 | 0.04±0.13 | 9.02E-01 |
| 3-methoxytyrosine | -0.01±0.08 | 0 | 7.89E-01 | 9.49E-01 | 0±0.08 | 0 | 6.43E-01 | 9.98E-01 | -0.03±0.1 | 9.48E-01 | -0.03±0.1 | 9.45E-01 | 0.01±0.12 | 9.84E-01 | 0.04±0.13 | 9.02E-01 |
| 3-hydroxyoctanoylcarnitine (2) | 0±0.07 | 0 | 9.95E-01 | 9.88E-01 | 0±0.08 | 0 | 9.90E-01 | 9.98E-01 | 0±0.09 | 9.89E-01 | 0±0.09 | 9.96E-01 | 0±0.13 | 9.96E-01 | 0±0.13 | 9.99E-01 |
| tryptophan | 0±0.08 | 69.84 | 6.86E-02 | 9.80E-01 | 0±0.08 | 61.24 | 1.08E-01 | 1.00E+00 | -0.08±0.09 | 8.80E-01 | -0.08±0.09 | 8.91E-01 | 0.26±0.17 | 4.64E-01 | 0.22±0.16 | 5.77E-01 |
| cytosine | 0.01±0.07 | 68.21 | 7.61E-02 | 9.68E-01 | 0±0.07 | 62.69 | 1.02E-01 | 1.00E+00 | -0.08±0.09 | 8.80E-01 | -0.08±0.09 | 8.60E-01 | 0.19±0.13 | 4.94E-01 | 0.17±0.13 | 5.87E-01 |
| 5alpha-pregnan-3beta,20alpha-diol monosulfate (2) | -0.01±0.07 | 32.6 | 2.23E-01 | 9.73E-01 | 0±0.07 | 50.86 | 1.54E-01 | 1.00E+00 | -0.07±0.09 | 9.03E-01 | -0.07±0.09 | 8.91E-01 | 0.12±0.13 | 6.66E-01 | 0.15±0.13 | 6.45E-01 |
| N,N,N-trimethyl-alanylproline betaine (TMAP) | 0±0.09 | 46.97 | 1.70E-01 | 9.85E-01 | 0±0.09 | 40.75 | 1.94E-01 | 1.00E+00 | -0.07±0.1 | 9.03E-01 | -0.07±0.1 | 8.94E-01 | 0.19±0.17 | 6.04E-01 | 0.18±0.16 | 6.62E-01 |
| X - 26097 | 0±0.07 | 47.15 | 1.69E-01 | 9.75E-01 | 0±0.07 | 36.75 | 2.09E-01 | 1.00E+00 | -0.08±0.09 | 8.80E-01 | -0.07±0.09 | 8.94E-01 | 0.14±0.12 | 6.23E-01 | 0.13±0.12 | 6.78E-01 |
| 1-palmitoleoyl-2-linolenoyl-GPC (16:1/18:3)* | 0±0.08 | 0 | 3.95E-01 | 9.82E-01 | 0±0.08 | 0 | 3.45E-01 | 1.00E+00 | -0.05±0.09 | 9.27E-01 | -0.05±0.09 | 9.18E-01 | 0.09±0.14 | 7.70E-01 | 0.11±0.14 | 7.51E-01 |
| tricosanoyl sphingomyelin (d18:1/23:0)* | -0.01±0.08 | 0 | 5.68E-01 | 9.49E-01 | 0±0.08 | 0 | 3.89E-01 | 1.00E+00 | -0.05±0.1 | 9.27E-01 | -0.05±0.1 | 9.18E-01 | 0.05±0.13 | 9.05E-01 | 0.1±0.14 | 7.74E-01 |
| X - 12462 | 0±0.07 | 0 | 7.61E-01 | 9.95E-01 | 0±0.07 | 0 | 9.04E-01 | 1.00E+00 | 0.02±0.09 | 9.77E-01 | 0.01±0.09 | 9.86E-01 | -0.03±0.12 | 9.38E-01 | -0.01±0.12 | 9.80E-01 |
| X - 14082 |  |  |  |  |  |  |  |  | -0.3±0.09 | 9.01E-02 | -0.31±0.09 | 7.50E-02 |  |  |  |  |
| 5-hydroxy-2-methylpyridine sulfate |  |  |  |  |  |  |  |  | -0.26±0.1 | 2.66E-01 | -0.28±0.1 | 2.58E-01 |  |  |  |  |
| X - 24951 |  |  |  |  |  |  |  |  | -0.21±0.08 | 2.66E-01 | -0.22±0.08 | 2.58E-01 |  |  |  |  |
| trans-3,4-methyleneheptanoate |  |  |  |  |  |  |  |  | -0.23±0.09 | 2.66E-01 | -0.24±0.09 | 2.61E-01 |  |  |  |  |
| X - 12738 |  |  |  |  |  |  |  |  | -0.25±0.09 | 2.84E-01 | -0.25±0.09 | 2.63E-01 |  |  |  |  |
| 3-hydroxypyridine glucuronide |  |  |  |  |  |  |  |  | -0.23±0.09 | 3.41E-01 | -0.24±0.1 | 3.28E-01 |  |  |  |  |
| catechol glucuronide |  |  |  |  |  |  |  |  | -0.22±0.09 | 3.61E-01 | -0.22±0.09 | 3.52E-01 |  |  |  |  |
| X - 21312 |  |  |  |  |  |  |  |  | -0.21±0.09 | 3.61E-01 | -0.21±0.09 | 3.52E-01 |  |  |  |  |
| X - 24728 |  |  |  |  |  |  |  |  | 0.2±0.09 | 4.52E-01 | 0.2±0.09 | 4.14E-01 |  |  |  |  |
| X - 24953 |  |  |  |  |  |  |  |  | -0.17±0.08 | 5.29E-01 | -0.18±0.08 | 4.14E-01 |  |  |  |  |
| X - 17346 |  |  |  |  |  |  |  |  | -0.2±0.09 | 4.52E-01 | -0.2±0.09 | 4.69E-01 |  |  |  |  |
| propionylglycine |  |  |  |  |  |  |  |  | -0.17±0.08 | 5.29E-01 | -0.18±0.09 | 4.93E-01 |  |  |  |  |
| X - 16124 |  |  |  |  |  |  |  |  | -0.18±0.09 | 6.10E-01 | -0.18±0.09 | 5.25E-01 |  |  |  |  |
| iminodiacetate (IDA) |  |  |  |  |  |  |  |  | -0.16±0.09 | 6.54E-01 | -0.17±0.09 | 6.01E-01 |  |  |  |  |
| (2,4 or 2,5)-dimethylphenol sulfate |  |  |  |  |  |  |  |  | -0.11±0.09 | 8.31E-01 | -0.21±0.11 | 6.01E-01 |  |  |  |  |
| 1-palmitoylglycerol (16:0) |  |  |  |  |  |  |  |  | -0.16±0.09 | 6.44E-01 | -0.16±0.09 | 6.15E-01 |  |  |  |  |
| malonylcarnitine |  |  |  |  |  |  |  |  | 0.16±0.09 | 6.54E-01 | 0.17±0.09 | 6.15E-01 |  |  |  |  |
| sucrose |  |  |  |  |  |  |  |  | -0.16±0.1 | 6.88E-01 | -0.17±0.1 | 6.39E-01 |  |  |  |  |
| X - 12127 |  |  |  |  |  |  |  |  | 0.15±0.09 | 7.21E-01 | 0.16±0.09 | 6.56E-01 |  |  |  |  |
| histidine betaine (hercynine)* |  |  |  |  |  |  |  |  | 0.15±0.09 | 7.16E-01 | 0.15±0.09 | 6.82E-01 |  |  |  |  |
| fructose |  |  |  |  |  |  |  |  | -0.16±0.09 | 6.98E-01 | -0.15±0.09 | 6.85E-01 |  |  |  |  |
| 1-linolenoylglycerol (18:3) |  |  |  |  |  |  |  |  | -0.14±0.09 | 7.33E-01 | -0.14±0.09 | 6.93E-01 |  |  |  |  |
| isoeugenol sulfate |  |  |  |  |  |  |  |  | -0.12±0.09 | 7.90E-01 | -0.14±0.09 | 7.26E-01 |  |  |  |  |
| X - 13658 |  |  |  |  |  |  |  |  | -0.12±0.09 | 7.90E-01 | -0.14±0.09 | 7.26E-01 |  |  |  |  |
| dihydrocaffeate sulfate (2) |  |  |  |  |  |  |  |  | -0.14±0.09 | 7.50E-01 | -0.14±0.09 | 7.37E-01 |  |  |  |  |
| cystine |  |  |  |  |  |  |  |  | 0.14±0.09 | 7.71E-01 | 0.14±0.09 | 7.51E-01 |  |  |  |  |
| X - 24411 |  |  |  |  |  |  |  |  | 0.15±0.1 | 7.71E-01 | 0.14±0.1 | 7.51E-01 |  |  |  |  |
| 2-hydroxyfluorene sulfate |  |  |  |  |  |  |  |  | -0.06±0.09 | 9.06E-01 | -0.18±0.12 | 7.54E-01 |  |  |  |  |
| S-methylmethionine |  |  |  |  |  |  |  |  | -0.14±0.09 | 7.71E-01 | -0.13±0.09 | 7.57E-01 |  |  |  |  |
| X - 26058 |  |  |  |  |  |  |  |  | 0.11±0.09 | 8.31E-01 | 0.12±0.09 | 7.65E-01 |  |  |  |  |
| 2,2'-Methylenebis(6-tert-butyl-p-cresol) |  |  |  |  |  |  |  |  | -0.12±0.09 | 7.95E-01 | -0.13±0.09 | 7.69E-01 |  |  |  |  |
| branched chain 14:0 dicarboxylic acid** |  |  |  |  |  |  |  |  | -0.12±0.08 | 7.87E-01 | -0.11±0.08 | 7.76E-01 |  |  |  |  |
| 3-hydroxyhippurate sulfate |  |  |  |  |  |  |  |  | -0.13±0.09 | 7.81E-01 | -0.12±0.09 | 7.79E-01 |  |  |  |  |
| (R)-3-hydroxybutyrylcarnitine |  |  |  |  |  |  |  |  | 0.12±0.09 | 7.90E-01 | 0.12±0.09 | 7.89E-01 |  |  |  |  |
| X - 21327 |  |  |  |  |  |  |  |  | -0.11±0.08 | 7.90E-01 | -0.11±0.08 | 7.91E-01 |  |  |  |  |
| 4-hydroxyphenylacetoylcarnitine |  |  |  |  |  |  |  |  | 0.11±0.09 | 8.31E-01 | 0.12±0.09 | 8.16E-01 |  |  |  |  |
| gamma-CEHC glucuronide* |  |  |  |  |  |  |  |  | -0.12±0.09 | 7.90E-01 | -0.11±0.09 | 8.25E-01 |  |  |  |  |
| behenoylcarnitine (C22)* |  |  |  |  |  |  |  |  | -0.11±0.09 | 7.90E-01 | -0.11±0.09 | 8.25E-01 |  |  |  |  |
| palmitoyl ethanolamide |  |  |  |  |  |  |  |  | -0.11±0.09 | 8.41E-01 | -0.12±0.09 | 8.25E-01 |  |  |  |  |
| X - 14056 |  |  |  |  |  |  |  |  | -0.12±0.09 | 7.90E-01 | -0.11±0.09 | 8.36E-01 |  |  |  |  |
| dihydroferulate |  |  |  |  |  |  |  |  | -0.11±0.09 | 8.31E-01 | -0.1±0.09 | 8.36E-01 |  |  |  |  |
| X - 12726 |  |  |  |  |  |  |  |  | -0.11±0.09 | 8.31E-01 | -0.11±0.09 | 8.36E-01 |  |  |  |  |
| alpha-CEHC sulfate |  |  |  |  |  |  |  |  | -0.11±0.09 | 8.34E-01 | -0.09±0.09 | 8.36E-01 |  |  |  |  |
| dopamine 4-sulfate |  |  |  |  |  |  |  |  | 0.11±0.09 | 8.34E-01 | 0.11±0.09 | 8.36E-01 |  |  |  |  |
| X - 12007 |  |  |  |  |  |  |  |  | -0.11±0.09 | 8.34E-01 | -0.1±0.09 | 8.36E-01 |  |  |  |  |
| X - 18345 |  |  |  |  |  |  |  |  | 0.11±0.09 | 8.41E-01 | 0.11±0.09 | 8.36E-01 |  |  |  |  |
| gentisate |  |  |  |  |  |  |  |  | 0.1±0.09 | 8.65E-01 | 0.1±0.09 | 8.36E-01 |  |  |  |  |
| ferulic acid 4-sulfate |  |  |  |  |  |  |  |  | -0.1±0.09 | 8.65E-01 | -0.1±0.09 | 8.36E-01 |  |  |  |  |
| suberoylcarnitine (C8-DC) |  |  |  |  |  |  |  |  | 0.1±0.09 | 8.65E-01 | 0.11±0.09 | 8.36E-01 |  |  |  |  |
| dihydroferulic acid sulfate |  |  |  |  |  |  |  |  | -0.1±0.09 | 8.65E-01 | -0.09±0.09 | 8.36E-01 |  |  |  |  |
| X - 12126 |  |  |  |  |  |  |  |  | 0.09±0.09 | 8.65E-01 | 0.1±0.09 | 8.36E-01 |  |  |  |  |
| X - 11979 |  |  |  |  |  |  |  |  | 0.09±0.09 | 8.69E-01 | 0.1±0.09 | 8.36E-01 |  |  |  |  |
| 5alpha-androstan-3alpha,17alpha-diol monosulfate | |  |  |  |  |  |  |  | -0.1±0.1 | 8.65E-01 | -0.1±0.1 | 8.38E-01 |  |  |  |  |
| X - 12104 |  |  |  |  |  |  |  |  | 0.09±0.09 | 8.72E-01 | 0.09±0.09 | 8.38E-01 |  |  |  |  |
| X - 12015 |  |  |  |  |  |  |  |  | 0.08±0.09 | 8.80E-01 | 0.09±0.09 | 8.38E-01 |  |  |  |  |
| trans-2-hexenoylglycine |  |  |  |  |  |  |  |  | 0.1±0.1 | 8.65E-01 | 0.09±0.1 | 8.47E-01 |  |  |  |  |
| methyl-4-hydroxybenzoate sulfate |  |  |  |  |  |  |  |  | -0.09±0.09 | 8.69E-01 | -0.09±0.09 | 8.48E-01 |  |  |  |  |
| delta-CEHC |  |  |  |  |  |  |  |  | -0.09±0.09 | 8.65E-01 | -0.08±0.09 | 8.52E-01 |  |  |  |  |
| 2,4-di-tert-butylphenol |  |  |  |  |  |  |  |  | -0.08±0.09 | 8.99E-01 | -0.09±0.09 | 8.60E-01 |  |  |  |  |
| X - 17351 |  |  |  |  |  |  |  |  | -0.09±0.09 | 8.65E-01 | -0.08±0.09 | 8.63E-01 |  |  |  |  |
| X - 24295 |  |  |  |  |  |  |  |  | -0.1±0.09 | 8.65E-01 | -0.09±0.09 | 8.63E-01 |  |  |  |  |
| cholic acid glucuronide |  |  |  |  |  |  |  |  | 0.08±0.09 | 8.80E-01 | 0.08±0.09 | 8.63E-01 |  |  |  |  |
| X - 25948 |  |  |  |  |  |  |  |  | 0.07±0.09 | 9.01E-01 | 0.08±0.09 | 8.63E-01 |  |  |  |  |
| gulonate* |  |  |  |  |  |  |  |  | -0.08±0.09 | 8.80E-01 | -0.08±0.09 | 8.68E-01 |  |  |  |  |
| bilirubin degradation product, C17H20N2O5 (1)** | |  |  |  |  |  |  |  | 0.08±0.09 | 8.80E-01 | 0.08±0.09 | 8.68E-01 |  |  |  |  |
| 1-oleoyl-GPG (18:1)* |  |  |  |  |  |  |  |  | 0.08±0.09 | 9.01E-01 | 0.08±0.09 | 8.68E-01 |  |  |  |  |
| hexanoylglycine |  |  |  |  |  |  |  |  | 0.09±0.09 | 8.72E-01 | 0.07±0.1 | 8.94E-01 |  |  |  |  |
| succinoyltaurine |  |  |  |  |  |  |  |  | -0.08±0.09 | 8.80E-01 | -0.07±0.09 | 8.94E-01 |  |  |  |  |
| mannonate* |  |  |  |  |  |  |  |  | -0.08±0.09 | 8.80E-01 | -0.07±0.09 | 8.94E-01 |  |  |  |  |
| bilirubin degradation product, C17H20N2O5 (2)** | |  |  |  |  |  |  |  | 0.07±0.09 | 9.01E-01 | 0.07±0.09 | 8.94E-01 |  |  |  |  |
| 1-myristoylglycerol (14:0) |  |  |  |  |  |  |  |  | -0.07±0.09 | 9.02E-01 | -0.07±0.09 | 8.94E-01 |  |  |  |  |
| suberate (C8-DC) |  |  |  |  |  |  |  |  | -0.06±0.09 | 9.03E-01 | -0.06±0.09 | 8.94E-01 |  |  |  |  |
| 1-stearoyl-2-oleoyl-GPS (18:0/18:1) |  |  |  |  |  |  |  |  | 0.06±0.09 | 9.03E-01 | 0.06±0.09 | 8.94E-01 |  |  |  |  |
| 1-oleoylglycerol (18:1) |  |  |  |  |  |  |  |  | -0.06±0.09 | 9.03E-01 | -0.06±0.09 | 8.94E-01 |  |  |  |  |
| gamma-glutamylalanine |  |  |  |  |  |  |  |  | 0.07±0.09 | 9.03E-01 | 0.07±0.09 | 8.94E-01 |  |  |  |  |
| X - 13007 |  |  |  |  |  |  |  |  | 0.07±0.09 | 9.03E-01 | 0.07±0.09 | 8.94E-01 |  |  |  |  |
| X - 13729 |  |  |  |  |  |  |  |  | -0.07±0.09 | 9.03E-01 | -0.07±0.09 | 8.94E-01 |  |  |  |  |
| X - 24531 |  |  |  |  |  |  |  |  | 0.07±0.09 | 9.03E-01 | 0.06±0.09 | 8.94E-01 |  |  |  |  |
| inosine |  |  |  |  |  |  |  |  | 0.06±0.09 | 9.06E-01 | 0.07±0.09 | 8.94E-01 |  |  |  |  |
| X - 23641 |  |  |  |  |  |  |  |  | 0.07±0.1 | 9.06E-01 | 0.07±0.1 | 8.94E-01 |  |  |  |  |
| cysteine s-sulfate |  |  |  |  |  |  |  |  | 0.06±0.09 | 9.11E-01 | 0.06±0.09 | 8.94E-01 |  |  |  |  |
| X - 18935 |  |  |  |  |  |  |  |  | 0.06±0.09 | 9.20E-01 | 0.06±0.09 | 8.94E-01 |  |  |  |  |
| N-formylanthranilic acid |  |  |  |  |  |  |  |  | 0.05±0.09 | 9.27E-01 | 0.06±0.09 | 8.94E-01 |  |  |  |  |
| hypoxanthine |  |  |  |  |  |  |  |  | 0.04±0.09 | 9.27E-01 | 0.06±0.09 | 8.94E-01 |  |  |  |  |
| dihomo-linolenoyl-choline |  |  |  |  |  |  |  |  | 0.06±0.09 | 9.03E-01 | 0.06±0.09 | 9.00E-01 |  |  |  |  |
| 5-hydroxyindoleacetate |  |  |  |  |  |  |  |  | -0.06±0.09 | 9.06E-01 | -0.06±0.09 | 9.00E-01 |  |  |  |  |
| dimethyl sulfone |  |  |  |  |  |  |  |  | -0.05±0.09 | 9.27E-01 | -0.06±0.09 | 9.01E-01 |  |  |  |  |
| 1,2,3-benzenetriol sulfate (2) |  |  |  |  |  |  |  |  | -0.06±0.09 | 9.03E-01 | -0.05±0.09 | 9.04E-01 |  |  |  |  |
| X - 21959 |  |  |  |  |  |  |  |  | 0.05±0.09 | 9.27E-01 | 0.05±0.09 | 9.04E-01 |  |  |  |  |
| 1-dihomo-linolenylglycerol (20:3) |  |  |  |  |  |  |  |  | -0.05±0.09 | 9.27E-01 | -0.05±0.09 | 9.18E-01 |  |  |  |  |
| X - 12261 |  |  |  |  |  |  |  |  | -0.04±0.09 | 9.27E-01 | -0.05±0.09 | 9.18E-01 |  |  |  |  |
| ribonate |  |  |  |  |  |  |  |  | -0.06±0.09 | 9.23E-01 | -0.04±0.09 | 9.18E-01 |  |  |  |  |
| 2-oleoylglycerol (18:1) |  |  |  |  |  |  |  |  | -0.05±0.09 | 9.27E-01 | -0.04±0.09 | 9.18E-01 |  |  |  |  |
| adrenate (22:4n6) |  |  |  |  |  |  |  |  | 0.05±0.09 | 9.27E-01 | 0.04±0.09 | 9.18E-01 |  |  |  |  |
| phenol glucuronide |  |  |  |  |  |  |  |  | -0.04±0.09 | 9.27E-01 | -0.04±0.09 | 9.18E-01 |  |  |  |  |
| oleoylcholine |  |  |  |  |  |  |  |  | 0.05±0.09 | 9.27E-01 | 0.04±0.09 | 9.18E-01 |  |  |  |  |
| arachidonoylcholine |  |  |  |  |  |  |  |  | 0.05±0.09 | 9.27E-01 | 0.04±0.09 | 9.18E-01 |  |  |  |  |
| docosahexaenoylcholine |  |  |  |  |  |  |  |  | 0.05±0.09 | 9.27E-01 | 0.04±0.09 | 9.18E-01 |  |  |  |  |
| glucuronide of piperine metabolite C17H21NO3 (3)* | |  |  |  |  |  |  |  | -0.05±0.09 | 9.27E-01 | -0.05±0.09 | 9.18E-01 |  |  |  |  |
| glucuronide of piperine metabolite C17H21NO3 (5)* | |  |  |  |  |  |  |  | -0.04±0.09 | 9.27E-01 | -0.05±0.09 | 9.18E-01 |  |  |  |  |
| cholate |  |  |  |  |  |  |  |  | 0.05±0.09 | 9.27E-01 | 0.05±0.09 | 9.18E-01 |  |  |  |  |
| X - 23997 |  |  |  |  |  |  |  |  | -0.04±0.09 | 9.27E-01 | -0.04±0.09 | 9.18E-01 |  |  |  |  |
| 2,3-dihydroxy-2-methylbutyrate |  |  |  |  |  |  |  |  | 0.04±0.09 | 9.34E-01 | 0.05±0.09 | 9.18E-01 |  |  |  |  |
| N,N-dimethyl-5-aminovalerate |  |  |  |  |  |  |  |  | -0.04±0.09 | 9.38E-01 | -0.04±0.09 | 9.18E-01 |  |  |  |  |
| X - 21410 |  |  |  |  |  |  |  |  | 0.04±0.1 | 9.38E-01 | 0.05±0.1 | 9.18E-01 |  |  |  |  |
| X - 23648 |  |  |  |  |  |  |  |  | 0.04±0.09 | 9.38E-01 | 0.05±0.09 | 9.18E-01 |  |  |  |  |
| X - 26054 |  |  |  |  |  |  |  |  | 0.04±0.09 | 9.38E-01 | 0.04±0.09 | 9.18E-01 |  |  |  |  |
| prolylglycine |  |  |  |  |  |  |  |  | 0.03±0.09 | 9.46E-01 | 0.05±0.09 | 9.18E-01 |  |  |  |  |
| 17alpha-hydroxypregnenolone 3-sulfate |  |  |  |  |  |  |  |  | -0.03±0.09 | 9.48E-01 | -0.04±0.09 | 9.18E-01 |  |  |  |  |
| xanthosine |  |  |  |  |  |  |  |  | -0.05±0.09 | 9.27E-01 | -0.04±0.09 | 9.19E-01 |  |  |  |  |
| 1-arachidonylglycerol (20:4) |  |  |  |  |  |  |  |  | 0.04±0.09 | 9.27E-01 | 0.04±0.09 | 9.19E-01 |  |  |  |  |
| palmitoylcholine |  |  |  |  |  |  |  |  | 0.04±0.09 | 9.27E-01 | 0.03±0.09 | 9.19E-01 |  |  |  |  |
| GlcNAc sulfate conjugate of C21H34O2 steroid** | |  |  |  |  |  |  |  | -0.03±0.09 | 9.47E-01 | -0.04±0.09 | 9.19E-01 |  |  |  |  |
| tetrahydrocortisol sulfate (1) |  |  |  |  |  |  |  |  | 0.02±0.09 | 9.77E-01 | 0.04±0.09 | 9.19E-01 |  |  |  |  |
| X - 23666 |  |  |  |  |  |  |  |  | -0.04±0.09 | 9.35E-01 | -0.03±0.09 | 9.24E-01 |  |  |  |  |
| 3-indoleglyoxylic acid |  |  |  |  |  |  |  |  | 0.02±0.09 | 9.60E-01 | 0.03±0.09 | 9.31E-01 |  |  |  |  |
| 2-linoleoylglycerol (18:2) |  |  |  |  |  |  |  |  | -0.03±0.09 | 9.59E-01 | -0.03±0.09 | 9.32E-01 |  |  |  |  |
| 5-acetylamino-6-formylamino-3-methyluracil |  |  |  |  |  |  |  |  | -0.04±0.09 | 9.38E-01 | -0.03±0.09 | 9.45E-01 |  |  |  |  |
| docosatrienoate (22:3n3) |  |  |  |  |  |  |  |  | 0.02±0.09 | 9.78E-01 | 0.02±0.09 | 9.55E-01 |  |  |  |  |
| bilirubin degradation product, C16H18N2O5 (4)** | |  |  |  |  |  |  |  | 0.02±0.09 | 9.59E-01 | 0.02±0.09 | 9.58E-01 |  |  |  |  |
| X - 23276 |  |  |  |  |  |  |  |  | 0.01±0.09 | 9.78E-01 | 0.02±0.09 | 9.58E-01 |  |  |  |  |
| cyclo(gly-pro) |  |  |  |  |  |  |  |  | 0.03±0.09 | 9.51E-01 | 0.02±0.09 | 9.62E-01 |  |  |  |  |
| X - 23583 |  |  |  |  |  |  |  |  | -0.02±0.09 | 9.67E-01 | -0.02±0.09 | 9.62E-01 |  |  |  |  |
| nisinate (24:6n3) |  |  |  |  |  |  |  |  | -0.02±0.09 | 9.77E-01 | -0.02±0.09 | 9.63E-01 |  |  |  |  |
| N-acetyltaurine |  |  |  |  |  |  |  |  | 0.02±0.09 | 9.59E-01 | 0.02±0.09 | 9.66E-01 |  |  |  |  |
| phosphate |  |  |  |  |  |  |  |  | 0.03±0.09 | 9.59E-01 | 0.02±0.1 | 9.66E-01 |  |  |  |  |
| stearoylcholine* |  |  |  |  |  |  |  |  | 0.02±0.09 | 9.59E-01 | 0.02±0.09 | 9.66E-01 |  |  |  |  |
| bilirubin degradation product, C16H18N2O5 (3)** | |  |  |  |  |  |  |  | -0.02±0.09 | 9.77E-01 | -0.02±0.09 | 9.67E-01 |  |  |  |  |
| X - 12096 |  |  |  |  |  |  |  |  | 0±0.09 | 9.89E-01 | 0.02±0.09 | 9.70E-01 |  |  |  |  |
| nervonoylcarnitine (C24:1)* |  |  |  |  |  |  |  |  | -0.01±0.09 | 9.78E-01 | -0.02±0.09 | 9.73E-01 |  |  |  |  |
| docosatrienoate (22:3n6)* |  |  |  |  |  |  |  |  | -0.01±0.09 | 9.89E-01 | -0.02±0.09 | 9.73E-01 |  |  |  |  |
| 4-hydroxy-2-oxoglutaric acid |  |  |  |  |  |  |  |  | 0.01±0.09 | 9.87E-01 | 0.02±0.09 | 9.79E-01 |  |  |  |  |
| X - 12713 |  |  |  |  |  |  |  |  | -0.02±0.09 | 9.71E-01 | -0.02±0.09 | 9.80E-01 |  |  |  |  |
| docosahexaenoylcarnitine (C22:6)* |  |  |  |  |  |  |  |  | -0.02±0.09 | 9.77E-01 | -0.01±0.09 | 9.80E-01 |  |  |  |  |
| gamma-glutamylhistidine |  |  |  |  |  |  |  |  | -0.01±0.09 | 9.78E-01 | -0.01±0.09 | 9.80E-01 |  |  |  |  |
| bilirubin degradation product, C16H18N2O5 (1)** | |  |  |  |  |  |  |  | -0.01±0.09 | 9.87E-01 | -0.01±0.09 | 9.80E-01 |  |  |  |  |
| X - 23890 |  |  |  |  |  |  |  |  | 0.01±0.09 | 9.87E-01 | 0.02±0.09 | 9.80E-01 |  |  |  |  |
| X - 12839 |  |  |  |  |  |  |  |  | -0.01±0.09 | 9.87E-01 | -0.01±0.09 | 9.80E-01 |  |  |  |  |
| oxindolylalanine |  |  |  |  |  |  |  |  | 0.01±0.09 | 9.89E-01 | 0.01±0.09 | 9.80E-01 |  |  |  |  |
| lithocholate |  |  |  |  |  |  |  |  | 0.01±0.09 | 9.89E-01 | 0.01±0.09 | 9.80E-01 |  |  |  |  |
| X - 12013 |  |  |  |  |  |  |  |  | -0.01±0.09 | 9.89E-01 | -0.01±0.09 | 9.80E-01 |  |  |  |  |
| X - 25519 |  |  |  |  |  |  |  |  | 0±0.09 | 9.89E-01 | 0.01±0.09 | 9.80E-01 |  |  |  |  |
| galactonate |  |  |  |  |  |  |  |  | -0.01±0.09 | 9.89E-01 | 0.01±0.09 | 9.89E-01 |  |  |  |  |
| linoleoylcholine* |  |  |  |  |  |  |  |  | 0.01±0.09 | 9.80E-01 | 0.01±0.09 | 9.93E-01 |  |  |  |  |
| bilirubin degradation product, C16H18N2O5 (2)** | |  |  |  |  |  |  |  | 0±0.09 | 9.89E-01 | 0±0.09 | 9.93E-01 |  |  |  |  |
| N-acetylaspartate (NAA) |  |  |  |  |  |  |  |  | 0±0.09 | 9.92E-01 | 0±0.09 | 9.96E-01 |  |  |  |  |
| 4-hydroxyphenylpyruvate |  |  |  |  |  |  |  |  | -0.01±0.09 | 9.89E-01 | 0±0.09 | 9.98E-01 |  |  |  |  |
| 1-carboxyethylphenylalanine |  |  |  |  |  |  |  |  |  |  |  |  | 0.53±0.12 | 7.51E-03 | 0.49±0.13 | 6.54E-02 |
| 1-carboxyethyltyrosine |  |  |  |  |  |  |  |  |  |  |  |  | 0.57±0.13 | 7.51E-03 | 0.52±0.14 | 6.54E-02 |
| 1-carboxyethylisoleucine |  |  |  |  |  |  |  |  |  |  |  |  | 0.57±0.14 | 7.51E-03 | 0.52±0.14 | 6.54E-02 |
| 1-carboxyethylvaline |  |  |  |  |  |  |  |  |  |  |  |  | 0.47±0.13 | 5.01E-02 | 0.41±0.14 | 3.03E-01 |
| 1-carboxyethylleucine |  |  |  |  |  |  |  |  |  |  |  |  | 0.42±0.14 | 1.48E-01 | 0.35±0.14 | 4.03E-01 |
| X - 24241 |  |  |  |  |  |  |  |  |  |  |  |  | 0.44±0.2 | 2.92E-01 | 0.44±0.2 | 4.18E-01 |
| saccharin |  |  |  |  |  |  |  |  |  |  |  |  | 0.22±0.12 | 3.72E-01 | 0.23±0.12 | 4.52E-01 |
| linoleoyl-linolenoyl-glycerol (18:2/18:3) [2]* |  |  |  |  |  |  |  |  |  |  |  |  | -0.18±0.12 | 4.94E-01 | -0.21±0.12 | 4.65E-01 |
| dibutyl sulfosuccinate |  |  |  |  |  |  |  |  |  |  |  |  | 0.2±0.11 | 3.97E-01 | 0.19±0.11 | 4.80E-01 |
| palmitoyl-oleoyl-glycerol (16:0/18:1) [2]* |  |  |  |  |  |  |  |  |  |  |  |  | 0.29±0.14 | 3.08E-01 | 0.23±0.14 | 4.95E-01 |
| palmitoyl-arachidonoyl-glycerol (16:0/20:4) [2]* |  |  |  |  |  |  |  |  |  |  |  |  | 0.29±0.14 | 3.25E-01 | 0.24±0.15 | 4.95E-01 |
| myristoyl-linoleoyl-glycerol (14:0/18:2) [2]* |  |  |  |  |  |  |  |  |  |  |  |  | 0.28±0.13 | 3.23E-01 | 0.21±0.14 | 4.95E-01 |
| 3-methylcrotonylglycine |  |  |  |  |  |  |  |  |  |  |  |  | 0.17±0.13 | 5.71E-01 | 0.21±0.13 | 5.04E-01 |
| palmitoyl-oleoyl-glycerol (16:0/18:1) [1]* |  |  |  |  |  |  |  |  |  |  |  |  | 0.28±0.14 | 3.38E-01 | 0.21±0.14 | 5.74E-01 |
| stearoyl-arachidonoyl-glycerol (18:0/20:4) [1]* |  |  |  |  |  |  |  |  |  |  |  |  | 0.19±0.14 | 5.36E-01 | 0.19±0.14 | 5.85E-01 |
| beta-sitosterol |  |  |  |  |  |  |  |  |  |  |  |  | -0.25±0.13 | 3.25E-01 | -0.18±0.13 | 5.85E-01 |
| 2-hydroxyglutarate |  |  |  |  |  |  |  |  |  |  |  |  | 0.16±0.14 | 6.00E-01 | 0.16±0.14 | 6.30E-01 |
| palmitoleoyl-oleoyl-glycerol (16:1/18:1) [2]* |  |  |  |  |  |  |  |  |  |  |  |  | 0.2±0.14 | 5.31E-01 | 0.15±0.14 | 6.67E-01 |
| palmitoleoyl-arachidonoyl-glycerol (16:1/20:4) [2]* | |  |  |  |  |  |  |  |  |  |  |  | 0.21±0.14 | 5.14E-01 | 0.15±0.14 | 6.67E-01 |
| ceramide (d18:1/20:0, d16:1/22:0, d20:1/18:0)* |  |  |  |  |  |  |  |  |  |  |  |  | 0.18±0.15 | 6.04E-01 | 0.15±0.15 | 6.81E-01 |
| palmitoyl-docosahexaenoyl-glycerol (16:0/22:6) [1]* | |  |  |  |  |  |  |  |  |  |  |  | 0.15±0.13 | 6.14E-01 | 0.12±0.13 | 7.05E-01 |
| oleoyl-arachidonoyl-glycerol (18:1/20:4) [2]* |  |  |  |  |  |  |  |  |  |  |  |  | 0.18±0.14 | 5.71E-01 | 0.12±0.14 | 7.12E-01 |
| oleoyl-arachidonoyl-glycerol (18:1/20:4) [1]* |  |  |  |  |  |  |  |  |  |  |  |  | 0.18±0.14 | 5.76E-01 | 0.12±0.14 | 7.24E-01 |
| stearoyl-arachidonoyl-glycerol (18:0/20:4) [2]* |  |  |  |  |  |  |  |  |  |  |  |  | -0.09±0.12 | 7.38E-01 | -0.11±0.12 | 7.24E-01 |
| X - 22771 |  |  |  |  |  |  |  |  |  |  |  |  | 0.08±0.13 | 7.89E-01 | 0.1±0.13 | 7.64E-01 |
| X - 16964 |  |  |  |  |  |  |  |  |  |  |  |  | 0.09±0.13 | 7.80E-01 | 0.09±0.13 | 7.82E-01 |
| palmitoleoyl-linoleoyl-glycerol (16:1/18:2) [1]* |  |  |  |  |  |  |  |  |  |  |  |  | 0.15±0.13 | 6.13E-01 | 0.09±0.13 | 7.91E-01 |
| X - 21829 |  |  |  |  |  |  |  |  |  |  |  |  | 0.15±0.13 | 6.00E-01 | 0.09±0.14 | 7.91E-01 |
| diacylglycerol (16:1/18:2 [2], 16:0/18:3 [1])* |  |  |  |  |  |  |  |  |  |  |  |  | 0.15±0.13 | 6.04E-01 | 0.08±0.13 | 7.96E-01 |
| N1-Methyl-4-pyridone-3-carboxamide |  |  |  |  |  |  |  |  |  |  |  |  | 0.11±0.13 | 6.98E-01 | 0.08±0.13 | 8.03E-01 |
| 1-stearoyl-2-arachidonoyl-GPS (18:0/20:4) |  |  |  |  |  |  |  |  |  |  |  |  | 0.07±0.13 | 8.24E-01 | 0.08±0.13 | 8.03E-01 |
| linoleoyl-linoleoyl-glycerol (18:2/18:2) [1]* |  |  |  |  |  |  |  |  |  |  |  |  | -0.07±0.12 | 8.26E-01 | -0.07±0.12 | 8.15E-01 |
| palmitoyl-linoleoyl-glycerol (16:0/18:2) [2]* |  |  |  |  |  |  |  |  |  |  |  |  | 0.14±0.13 | 6.48E-01 | 0.07±0.13 | 8.25E-01 |
| X - 13684 |  |  |  |  |  |  |  |  |  |  |  |  | 0.14±0.14 | 6.49E-01 | 0.07±0.14 | 8.26E-01 |
| 3-hydroxymyristate |  |  |  |  |  |  |  |  |  |  |  |  | -0.03±0.13 | 9.28E-01 | -0.07±0.13 | 8.45E-01 |
| thymidine |  |  |  |  |  |  |  |  |  |  |  |  | -0.04±0.12 | 9.05E-01 | -0.06±0.12 | 8.46E-01 |
| serotonin |  |  |  |  |  |  |  |  |  |  |  |  | -0.11±0.12 | 6.92E-01 | -0.06±0.12 | 8.50E-01 |
| oleoyl-oleoyl-glycerol (18:1/18:1) [1]* |  |  |  |  |  |  |  |  |  |  |  |  | 0.1±0.13 | 7.38E-01 | 0.06±0.13 | 8.77E-01 |
| gamma-glutamyl-epsilon-lysine |  |  |  |  |  |  |  |  |  |  |  |  | 0.08±0.13 | 8.14E-01 | 0.05±0.13 | 8.92E-01 |
| (2-butoxyethoxy)acetic acid |  |  |  |  |  |  |  |  |  |  |  |  | -0.04±0.13 | 9.13E-01 | -0.04±0.13 | 9.02E-01 |
| X - 25828 |  |  |  |  |  |  |  |  |  |  |  |  | -0.07±0.13 | 8.46E-01 | 0.04±0.14 | 9.28E-01 |
| X - 16938 |  |  |  |  |  |  |  |  |  |  |  |  | -0.05±0.13 | 8.81E-01 | -0.03±0.13 | 9.37E-01 |
| glycosyl ceramide (d18:1/23:1, d17:1/24:1)* |  |  |  |  |  |  |  |  |  |  |  |  | -0.03±0.13 | 9.47E-01 | 0.02±0.13 | 9.50E-01 |
| stearoyl ethanolamide |  |  |  |  |  |  |  |  |  |  |  |  | 0.02±0.13 | 9.74E-01 | -0.02±0.13 | 9.57E-01 |
| linoleoyl-linoleoyl-glycerol (18:2/18:2) [2]* |  |  |  |  |  |  |  |  |  |  |  |  | 0.02±0.13 | 9.72E-01 | -0.02±0.13 | 9.71E-01 |
| linoleoyl-arachidonoyl-glycerol (18:2/20:4) [1]* |  |  |  |  |  |  |  |  |  |  |  |  | 0.03±0.13 | 9.32E-01 | -0.02±0.13 | 9.72E-01 |
| N-behenoyl-sphingadienine (d18:2/22:0)* |  |  |  |  |  |  |  |  |  |  |  |  | 0.02±0.13 | 9.70E-01 | 0.01±0.12 | 9.72E-01 |
| 1-methylhistidine |  |  |  |  |  |  |  |  |  |  |  |  | 0.02±0.12 | 9.68E-01 | -0.01±0.12 | 9.80E-01 |
| 2-aminoadipate |  |  |  |  |  |  |  |  |  |  |  |  | 0.07±0.13 | 8.28E-01 | 0.01±0.13 | 9.85E-01 |
| X - 12849 |  |  |  |  |  |  |  |  |  |  |  |  | -0.04±0.12 | 9.06E-01 | -0.01±0.12 | 9.85E-01 |
| linoleoyl-arachidonoyl-glycerol (18:2/20:4) [2]* |  |  |  |  |  |  |  |  |  |  |  |  | 0.02±0.13 | 9.54E-01 | -0.01±0.12 | 9.91E-01 |

Note: The table shows associations from the logistic regression analysis where plasms metabolomics level from the Metabolon platform was main exposure and prevalent all cancer as the main outcome in ERGO-4 and ERGO-5, respectively. ERGO-4 and ERGO-5 have 939 overlapping circulating metabolites, and ERGO-4&5 refers to the correlation between prevalent all cancer and 939 metabolites derived from meta-analysis of ERGO-4 and ERGO-5. Model 1: adjusted for age, sex, cohort, and lipid lowering medication. Model 2: adjusted for model1, smoking status, BMI, alcohol and education. The table is ranked based on the *FDR* values in model 2 within the E4&E5 cohort, using bold text indicates metabolites are surpassing *FDR* adjusted *P* value treshold (*P*<0.05). ERGO-4 and ERGO-5 refer to the fourth and fifth visits subcohort from the baseline cohort. Abbreviations: SE, standard erorr; I^2^: heterogeneity parameter, Het. *P*-value: significance of the heterogeneity; BMI, body mass index; FDR, false discovery rate.

**Supplementary Table 5. Serum metabolites associated with prevalent hematological malignancy from the Nightingale platform at baseline.**

| **Metabolite** | **ERGO-4&5** | | | | | | **ERGO-4** | | | | | | **ERGO-5** | | | | | |
| --- | --- | --- | --- | --- | --- | --- | --- | --- | --- | --- | --- | --- | --- | --- | --- | --- | --- | --- |
|  | **Model 1** | | | **Model 2** | | | **Model 1** | | | **Model 2** | | | **Model 1** | | | **Model 2** | | |
|  | **Beta** | **SE** | ***FDR* value** | **Beta** | **SE** | ***FDR* value** | **Beta** | **SE** | ***FDR* value** | **Beta** | **SE** | ***FDR* value** | **Beta** | **SE** | ***FDR* value** | **Beta** | **SE** | ***FDR* value** |
| Cholesterol to total lipids ratio in large LDL | -0.61 | 0.16 | **1.20E-02** | -0.81 | 0.17 | **3.96E-04** | -0.81 | 0.21 | **1.31E-02** | -0.87 | 0.22 | **6.33E-03** | -0.67 | 0.26 | 9.27E-02 | -0.81 | 0.27 | **4.54E-02** |
| Cholesterol to total lipids ratio in small HDL | -0.60 | 0.15 | **1.20E-02** | -0.71 | 0.16 | **4.02E-04** | -0.65 | 0.21 | **3.08E-02** | -0.72 | 0.22 | **2.02E-02** | -0.67 | 0.23 | 7.02E-02 | -0.76 | 0.24 | **4.43E-02** |
| Cholesterol to total lipids ratio in IDL | -0.48 | 0.14 | **1.36E-02** | -0.67 | 0.15 | **4.02E-04** | -0.62 | 0.19 | **2.66E-02** | -0.66 | 0.19 | **1.62E-02** | -0.64 | 0.23 | 7.70E-02 | -0.75 | 0.24 | **4.43E-02** |
| Triglycerides to total lipids ratio in large LDL | 0.58 | 0.17 | **1.36E-02** | 0.81 | 0.18 | **4.02E-04** | 0.79 | 0.23 | **2.35E-02** | 0.85 | 0.23 | **1.48E-02** | 0.70 | 0.28 | 9.27E-02 | 0.85 | 0.29 | **4.54E-02** |
| Cholesteryl esters to total lipids ratio in large HDL | -0.41 | 0.11 | **1.20E-02** | -0.52 | 0.12 | **4.02E-04** | -0.63 | 0.16 | **8.41E-03** | -0.71 | 0.17 | **4.55E-03** | -0.36 | 0.18 | 1.80E-01 | -0.38 | 0.19 | 1.68E-01 |
| Triglycerides to total lipids ratio in IDL | 0.52 | 0.16 | **2.41E-02** | 0.79 | 0.18 | **4.64E-04** | 0.80 | 0.23 | **2.35E-02** | 0.86 | 0.24 | **1.48E-02** | 0.65 | 0.27 | 1.05E-01 | 0.76 | 0.29 | 7.52E-02 |
| Cholesterol to total lipids ratio in medium HDL | -0.55 | 0.14 | **1.20E-02** | -0.66 | 0.15 | **5.08E-04** | -0.64 | 0.20 | **2.76E-02** | -0.72 | 0.21 | **1.62E-02** | -0.58 | 0.23 | 9.27E-02 | -0.63 | 0.25 | 7.81E-02 |
| Cholesterol to total lipids ratio in large HDL | -0.39 | 0.11 | **1.36E-02** | -0.52 | 0.12 | **5.08E-04** | -0.67 | 0.17 | **8.41E-03** | -0.76 | 0.18 | **4.55E-03** | -0.34 | 0.18 | 2.17E-01 | -0.36 | 0.20 | 2.03E-01 |
| Cholesteryl esters to total lipids ratio in IDL | -0.49 | 0.15 | **1.36E-02** | -0.65 | 0.15 | **6.68E-04** | -0.60 | 0.19 | **3.08E-02** | -0.65 | 0.20 | **2.02E-02** | -0.63 | 0.24 | 9.27E-02 | -0.73 | 0.25 | **4.54E-02** |
| Free cholesterol to total lipids ratio in medium HDL | -0.61 | 0.17 | **1.36E-02** | -0.75 | 0.18 | **7.32E-04** | -0.69 | 0.23 | **3.49E-02** | -0.73 | 0.24 | **2.61E-02** | -0.83 | 0.28 | 7.02E-02 | -0.94 | 0.32 | **4.54E-02** |
| Triglycerides to total lipids ratio in medium LDL | 0.51 | 0.17 | **3.12E-02** | 0.72 | 0.17 | **7.32E-04** | 0.72 | 0.21 | **2.62E-02** | 0.78 | 0.22 | **1.53E-02** | 0.61 | 0.28 | 1.44E-01 | 0.76 | 0.29 | 7.81E-02 |
| Free cholesterol to total lipids ratio in large LDL | -0.44 | 0.14 | **2.89E-02** | -0.65 | 0.16 | **8.87E-04** | -0.60 | 0.19 | **3.08E-02** | -0.67 | 0.21 | **2.02E-02** | -0.57 | 0.24 | 1.05E-01 | -0.63 | 0.26 | 8.96E-02 |
| Cholesteryl esters to total lipids ratio in medium HDL | -0.51 | 0.14 | **1.27E-02** | -0.60 | 0.15 | **1.01E-03** | -0.58 | 0.19 | **3.49E-02** | -0.63 | 0.20 | **2.31E-02** | -0.53 | 0.22 | 1.05E-01 | -0.59 | 0.24 | 8.49E-02 |
| Cholesteryl esters to total lipids ratio in small HDL | -0.52 | 0.15 | **1.36E-02** | -0.61 | 0.15 | **1.38E-03** | -0.54 | 0.20 | **4.54E-02** | -0.58 | 0.20 | **3.19E-02** | -0.60 | 0.24 | 9.27E-02 | -0.72 | 0.26 | 5.71E-02 |
| Phospholipids to total lipids ratio in medium HDL | 0.53 | 0.16 | **1.36E-02** | 0.64 | 0.17 | **1.72E-03** | 0.56 | 0.21 | 5.61E-02 | 0.64 | 0.22 | **3.30E-02** | 0.65 | 0.25 | 9.27E-02 | 0.69 | 0.27 | 7.81E-02 |
| Triglycerides to total lipids ratio in large HDL | 0.55 | 0.16 | **1.36E-02** | 0.64 | 0.17 | **1.81E-03** | 0.80 | 0.23 | **2.35E-02** | 0.85 | 0.24 | **1.48E-02** | 0.43 | 0.26 | 2.86E-01 | 0.45 | 0.27 | 2.73E-01 |
| Cholesteryl esters to total lipids ratio in very small VLDL | -0.39 | 0.15 | 8.87E-02 | -0.63 | 0.17 | **2.34E-03** | -0.69 | 0.22 | **3.08E-02** | -0.72 | 0.22 | **2.02E-02** | -0.52 | 0.25 | 1.73E-01 | -0.56 | 0.27 | 1.56E-01 |
| Cholesterol to total lipids ratio in very small VLDL | -0.39 | 0.16 | 9.57E-02 | -0.65 | 0.17 | **2.58E-03** | -0.72 | 0.22 | **2.76E-02** | -0.76 | 0.23 | **2.02E-02** | -0.52 | 0.27 | 2.10E-01 | -0.56 | 0.29 | 1.81E-01 |
| Cholesteryl esters to total lipids ratio in very large HDL | -0.61 | 0.18 | **1.36E-02** | -0.71 | 0.19 | **3.28E-03** | -0.55 | 0.28 | 1.77E-01 | -0.63 | 0.29 | 1.28E-01 | -0.80 | 0.26 | 7.02E-02 | -0.90 | 0.27 | **4.43E-02** |
| Triglycerides to total lipids ratio in medium HDL | 0.65 | 0.20 | **2.12E-02** | 0.74 | 0.21 | **4.97E-03** | 0.84 | 0.28 | **3.49E-02** | 0.91 | 0.29 | **2.31E-02** | 0.51 | 0.32 | 3.18E-01 | 0.53 | 0.33 | 2.86E-01 |
| Free cholesterol to total lipids ratio in medium LDL | -0.42 | 0.15 | 6.58E-02 | -0.56 | 0.16 | **7.04E-03** | -0.55 | 0.21 | 5.74E-02 | -0.61 | 0.22 | **4.26E-02** | -0.46 | 0.23 | 1.92E-01 | -0.49 | 0.25 | 1.81E-01 |
| Free cholesterol in large LDL | -0.42 | 0.18 | 1.16E-01 | -0.62 | 0.19 | **9.17E-03** | -0.50 | 0.25 | 1.66E-01 | -0.53 | 0.25 | 1.39E-01 | -0.76 | 0.26 | 7.02E-02 | -0.85 | 0.28 | **4.43E-02** |
| Cholesteryl esters in HDL | -0.62 | 0.21 | **3.72E-02** | -0.74 | 0.22 | **9.17E-03** | -0.61 | 0.28 | 1.28E-01 | -0.69 | 0.29 | 1.05E-01 | -0.86 | 0.35 | 1.05E-01 | -0.88 | 0.37 | 9.85E-02 |
| Free cholesterol in LDL | -0.41 | 0.18 | 1.17E-01 | -0.59 | 0.18 | **1.08E-02** | -0.47 | 0.25 | 1.93E-01 | -0.49 | 0.25 | 1.65E-01 | -0.75 | 0.26 | 7.02E-02 | -0.83 | 0.27 | **4.43E-02** |
| Phospholipids to total lipids ratio in very small VLDL | 0.57 | 0.20 | 5.34E-02 | 0.67 | 0.21 | **1.08E-02** | 0.48 | 0.26 | 2.16E-01 | 0.50 | 0.26 | 1.89E-01 | 1.16 | 0.40 | 7.02E-02 | 1.28 | 0.41 | **4.43E-02** |
| Cholesterol to total lipids ratio in medium LDL | -0.40 | 0.16 | 9.27E-02 | -0.54 | 0.16 | **1.08E-02** | -0.44 | 0.22 | 1.78E-01 | -0.47 | 0.23 | 1.47E-01 | -0.68 | 0.25 | 9.27E-02 | -0.77 | 0.25 | **4.43E-02** |
| HDL cholesterol | -0.61 | 0.21 | **4.86E-02** | -0.74 | 0.23 | **1.08E-02** | -0.61 | 0.28 | 1.44E-01 | -0.68 | 0.30 | 1.11E-01 | -0.87 | 0.37 | 1.05E-01 | -0.89 | 0.38 | 1.08E-01 |
| Free cholesterol to total lipids ratio in small VLDL | -0.40 | 0.18 | 1.42E-01 | -0.64 | 0.20 | **1.08E-02** | -0.71 | 0.25 | **3.88E-02** | -0.75 | 0.25 | **2.77E-02** | -0.46 | 0.31 | 3.70E-01 | -0.48 | 0.33 | 3.45E-01 |
| Free cholesterol to total lipids ratio in small LDL | -0.40 | 0.15 | 6.64E-02 | -0.50 | 0.15 | **1.08E-02** | -0.57 | 0.19 | **3.49E-02** | -0.60 | 0.20 | **2.61E-02** | -0.33 | 0.25 | 4.28E-01 | -0.37 | 0.26 | 3.63E-01 |
| Triglycerides to total lipids ratio in small HDL | 0.56 | 0.20 | 5.97E-02 | 0.68 | 0.21 | **1.08E-02** | 0.75 | 0.27 | **4.39E-02** | 0.82 | 0.28 | **3.00E-02** | 0.45 | 0.33 | 4.18E-01 | 0.48 | 0.36 | 3.84E-01 |
| Triglycerides to total lipids ratio in small LDL | 0.40 | 0.17 | 1.23E-01 | 0.58 | 0.18 | **1.17E-02** | 0.66 | 0.22 | **3.64E-02** | 0.72 | 0.24 | **2.61E-02** | 0.33 | 0.29 | 5.22E-01 | 0.39 | 0.31 | 4.55E-01 |
| Phospholipids to total lipids ratio in small VLDL | -0.41 | 0.19 | 1.45E-01 | -0.64 | 0.21 | **1.51E-02** | -0.70 | 0.26 | **4.52E-02** | -0.76 | 0.27 | **3.30E-02** | -0.46 | 0.32 | 3.89E-01 | -0.47 | 0.34 | 3.72E-01 |
| Free cholesterol in small LDL | -0.40 | 0.18 | 1.23E-01 | -0.54 | 0.18 | **1.74E-02** | -0.46 | 0.23 | 1.80E-01 | -0.48 | 0.23 | 1.47E-01 | -0.67 | 0.26 | 9.27E-02 | -0.73 | 0.27 | 6.60E-02 |
| Free cholesterol in medium LDL | -0.39 | 0.17 | 1.37E-01 | -0.53 | 0.18 | **1.99E-02** | -0.38 | 0.24 | 3.08E-01 | -0.40 | 0.24 | 2.72E-01 | -0.74 | 0.25 | 7.02E-02 | -0.81 | 0.26 | **4.43E-02** |
| Total esterified cholesterol | -0.40 | 0.19 | 1.54E-01 | -0.59 | 0.20 | **2.15E-02** | -0.46 | 0.27 | 2.50E-01 | -0.48 | 0.27 | 2.36E-01 | -0.77 | 0.28 | 9.27E-02 | -0.87 | 0.30 | **4.97E-02** |
| Total concentration of lipoprotein particles | -0.52 | 0.21 | 9.09E-02 | -0.63 | 0.22 | **2.48E-02** | -0.35 | 0.28 | 3.89E-01 | -0.39 | 0.29 | 3.55E-01 | -1.06 | 0.35 | 7.02E-02 | -1.19 | 0.37 | **4.43E-02** |
| Concentration of HDL particles | -0.54 | 0.21 | 8.90E-02 | -0.63 | 0.22 | **2.74E-02** | -0.34 | 0.28 | 3.97E-01 | -0.38 | 0.29 | 3.64E-01 | -1.06 | 0.36 | 7.02E-02 | -1.15 | 0.37 | **4.43E-02** |
| Cholesterol in large LDL | -0.37 | 0.18 | 1.76E-01 | -0.52 | 0.18 | **2.74E-02** | -0.41 | 0.25 | 2.86E-01 | -0.43 | 0.25 | 2.55E-01 | -0.70 | 0.26 | 9.27E-02 | -0.76 | 0.27 | 5.71E-02 |
| Cholesteryl esters in large HDL | -0.40 | 0.16 | 1.06E-01 | -0.51 | 0.18 | **2.74E-02** | -0.60 | 0.23 | 5.61E-02 | -0.65 | 0.24 | **4.26E-02** | -0.36 | 0.28 | 4.49E-01 | -0.33 | 0.30 | 5.45E-01 |
| Triglycerides in medium HDL | 0.53 | 0.21 | 9.09E-02 | 0.62 | 0.22 | **2.74E-02** | 0.87 | 0.29 | **3.49E-02** | 0.92 | 0.30 | **2.61E-02** | 0.17 | 0.31 | 8.45E-01 | 0.20 | 0.34 | 7.42E-01 |
| Phospholipids to total lipids ratio in large HDL | 0.32 | 0.16 | 1.77E-01 | 0.49 | 0.17 | **2.74E-02** | 0.68 | 0.21 | **2.76E-02** | 0.75 | 0.22 | **1.62E-02** | 0.11 | 0.30 | 8.65E-01 | 0.05 | 0.33 | 9.49E-01 |
| Triglycerides in HDL | 0.49 | 0.20 | 9.57E-02 | 0.57 | 0.20 | **2.88E-02** | 0.78 | 0.27 | **3.85E-02** | 0.83 | 0.28 | **2.77E-02** | 0.16 | 0.31 | 8.45E-01 | 0.20 | 0.33 | 7.42E-01 |
| Cholesteryl esters in medium HDL | -0.51 | 0.20 | 8.39E-02 | -0.58 | 0.21 | **2.92E-02** | -0.37 | 0.26 | 3.66E-01 | -0.42 | 0.28 | 3.27E-01 | -0.82 | 0.31 | 9.27E-02 | -0.84 | 0.32 | 7.81E-02 |
| Cholesterol in medium HDL | -0.52 | 0.20 | 8.78E-02 | -0.58 | 0.21 | **3.07E-02** | -0.38 | 0.27 | 3.66E-01 | -0.42 | 0.28 | 3.31E-01 | -0.84 | 0.32 | 9.27E-02 | -0.86 | 0.33 | 7.81E-02 |
| Cholesteryl esters in small HDL | -0.48 | 0.19 | 9.27E-02 | -0.54 | 0.19 | **3.15E-02** | -0.16 | 0.26 | 6.62E-01 | -0.19 | 0.27 | 5.88E-01 | -1.09 | 0.30 | 5.79E-02 | -1.17 | 0.29 | **1.72E-02** |
| Free cholesterol in HDL | -0.53 | 0.22 | 1.09E-01 | -0.65 | 0.24 | **3.25E-02** | -0.54 | 0.29 | 2.16E-01 | -0.58 | 0.31 | 1.97E-01 | -0.81 | 0.38 | 1.58E-01 | -0.82 | 0.41 | 1.69E-01 |
| Cholesterol in large HDL | -0.38 | 0.17 | 1.23E-01 | -0.50 | 0.18 | **3.25E-02** | -0.59 | 0.23 | 6.67E-02 | -0.64 | 0.24 | 5.40E-02 | -0.35 | 0.28 | 4.78E-01 | -0.31 | 0.31 | 5.87E-01 |
| Total cholesterol | -0.36 | 0.19 | 1.98E-01 | -0.54 | 0.20 | **3.43E-02** | -0.42 | 0.27 | 3.08E-01 | -0.44 | 0.27 | 2.76E-01 | -0.71 | 0.28 | 9.27E-02 | -0.80 | 0.30 | 7.27E-02 |
| Cholesteryl esters in IDL | -0.32 | 0.18 | 2.57E-01 | -0.54 | 0.20 | **3.43E-02** | -0.45 | 0.26 | 2.50E-01 | -0.46 | 0.27 | 2.41E-01 | -0.68 | 0.29 | 1.05E-01 | -0.76 | 0.31 | 8.72E-02 |
| Cholesterol in IDL | -0.31 | 0.18 | 2.67E-01 | -0.53 | 0.20 | **3.58E-02** | -0.46 | 0.26 | 2.50E-01 | -0.47 | 0.27 | 2.41E-01 | -0.68 | 0.29 | 1.06E-01 | -0.75 | 0.31 | 9.10E-02 |
| Sphingomyelins | -0.40 | 0.20 | 1.77E-01 | -0.56 | 0.21 | **3.71E-02** | -0.48 | 0.28 | 2.50E-01 | -0.48 | 0.28 | 2.47E-01 | -0.71 | 0.31 | 1.32E-01 | -0.76 | 0.34 | 1.26E-01 |
| Triglycerides to total lipids ratio in very small VLDL | 0.29 | 0.19 | 3.16E-01 | 0.57 | 0.22 | **3.71E-02** | 0.76 | 0.28 | **4.52E-02** | 0.80 | 0.28 | **3.47E-02** | 0.26 | 0.34 | 7.59E-01 | 0.27 | 0.36 | 6.96E-01 |
| Cholesteryl esters in very large HDL | -0.38 | 0.19 | 1.77E-01 | -0.56 | 0.21 | **3.71E-02** | -0.81 | 0.28 | **3.85E-02** | -0.88 | 0.30 | **2.77E-02** | -0.17 | 0.35 | 8.45E-01 | -0.08 | 0.37 | 9.12E-01 |
| Free cholesterol to total lipids ratio in IDL | -0.34 | 0.17 | 1.77E-01 | -0.50 | 0.19 | **3.73E-02** | -0.48 | 0.25 | 1.80E-01 | -0.49 | 0.25 | 1.69E-01 | -0.54 | 0.29 | 2.23E-01 | -0.52 | 0.30 | 2.42E-01 |
| Ratio of linoleic acid to total fatty acids | -0.28 | 0.18 | 3.05E-01 | -0.53 | 0.20 | **3.78E-02** | -0.65 | 0.23 | **3.87E-02** | -0.79 | 0.25 | **2.02E-02** | 0.00 | 0.32 | 1.00E+00 | -0.01 | 0.35 | 9.83E-01 |
| Free cholesterol in medium HDL | -0.49 | 0.20 | 1.09E-01 | -0.56 | 0.22 | **4.20E-02** | -0.37 | 0.27 | 3.71E-01 | -0.40 | 0.29 | 3.46E-01 | -0.84 | 0.34 | 1.04E-01 | -0.89 | 0.36 | 8.86E-02 |
| Cholesteryl esters to total lipids ratio in large LDL | -0.38 | 0.17 | 1.39E-01 | -0.44 | 0.17 | **4.26E-02** | -0.45 | 0.23 | 1.79E-01 | -0.50 | 0.23 | 1.28E-01 | -0.42 | 0.26 | 3.18E-01 | -0.48 | 0.26 | 2.03E-01 |
| Triglycerides in small HDL | 0.44 | 0.20 | 1.45E-01 | 0.53 | 0.21 | **4.42E-02** | 0.76 | 0.27 | **4.05E-02** | 0.82 | 0.28 | **3.00E-02** | 0.08 | 0.32 | 9.09E-01 | 0.05 | 0.34 | 9.43E-01 |
| Clinical LDL cholesterol | -0.31 | 0.18 | 2.57E-01 | -0.47 | 0.18 | **4.43E-02** | -0.38 | 0.25 | 3.30E-01 | -0.40 | 0.25 | 2.89E-01 | -0.64 | 0.27 | 1.05E-01 | -0.70 | 0.28 | 8.21E-02 |
| Cholesterol in small HDL | -0.45 | 0.20 | 1.23E-01 | -0.51 | 0.20 | **4.55E-02** | -0.12 | 0.27 | 7.47E-01 | -0.15 | 0.28 | 6.93E-01 | -1.07 | 0.31 | 5.79E-02 | -1.18 | 0.32 | **2.24E-02** |
| Phospholipids in large LDL | -0.32 | 0.18 | 2.57E-01 | -0.48 | 0.19 | **4.55E-02** | -0.32 | 0.26 | 3.97E-01 | -0.34 | 0.26 | 3.67E-01 | -0.72 | 0.27 | 9.27E-02 | -0.79 | 0.28 | 5.71E-02 |
| Total lipids in large LDL | -0.33 | 0.18 | 2.48E-01 | -0.47 | 0.19 | **4.55E-02** | -0.34 | 0.26 | 3.71E-01 | -0.36 | 0.26 | 3.45E-01 | -0.68 | 0.27 | 9.27E-02 | -0.73 | 0.28 | 7.27E-02 |
| Free cholesterol in IDL | -0.29 | 0.18 | 3.15E-01 | -0.52 | 0.20 | **4.55E-02** | -0.46 | 0.27 | 2.57E-01 | -0.47 | 0.27 | 2.52E-01 | -0.66 | 0.29 | 1.33E-01 | -0.69 | 0.31 | 1.32E-01 |
| Triglycerides in large HDL | 0.50 | 0.22 | 1.23E-01 | 0.56 | 0.22 | **4.76E-02** | 0.60 | 0.29 | 1.47E-01 | 0.66 | 0.29 | 1.11E-01 | 0.34 | 0.33 | 5.93E-01 | 0.47 | 0.36 | 4.11E-01 |
| Cholesterol in very large HDL | -0.35 | 0.19 | 2.14E-01 | -0.54 | 0.22 | **4.80E-02** | -0.80 | 0.29 | **4.39E-02** | -0.85 | 0.30 | **3.30E-02** | -0.14 | 0.36 | 8.55E-01 | -0.04 | 0.38 | 9.53E-01 |
| Triglycerides to total lipids ratio in very large HDL | 0.35 | 0.18 | 1.79E-01 | 0.48 | 0.19 | **4.80E-02** | 0.72 | 0.25 | **3.85E-02** | 0.76 | 0.26 | **2.77E-02** | 0.06 | 0.32 | 9.23E-01 | 0.00 | 0.34 | 9.91E-01 |
| Cholesteryl esters in large LDL | -0.31 | 0.18 | 2.71E-01 | -0.45 | 0.19 | 5.84E-02 | -0.37 | 0.25 | 3.53E-01 | -0.39 | 0.25 | 3.05E-01 | -0.61 | 0.28 | 1.40E-01 | -0.66 | 0.28 | 1.04E-01 |
| Ratio of omega-6 fatty acids to total fatty acids | -0.28 | 0.16 | 2.67E-01 | -0.43 | 0.18 | 5.84E-02 | -0.56 | 0.20 | **4.51E-02** | -0.63 | 0.21 | **2.77E-02** | 0.03 | 0.32 | 9.63E-01 | 0.04 | 0.35 | 9.50E-01 |
| Free cholesterol in large HDL | -0.33 | 0.17 | 1.79E-01 | -0.44 | 0.18 | 5.89E-02 | -0.55 | 0.24 | 1.18E-01 | -0.57 | 0.25 | 1.11E-01 | -0.33 | 0.29 | 5.21E-01 | -0.28 | 0.31 | 6.44E-01 |
| Triglycerides in very small VLDL | 0.38 | 0.19 | 1.76E-01 | 0.46 | 0.19 | 5.95E-02 | 0.59 | 0.25 | 1.05E-01 | 0.62 | 0.25 | 8.39E-02 | 0.15 | 0.30 | 8.45E-01 | 0.19 | 0.32 | 7.42E-01 |
| Cholesterol to total lipids ratio in small LDL | -0.26 | 0.17 | 3.38E-01 | -0.42 | 0.18 | 6.58E-02 | -0.10 | 0.26 | 7.86E-01 | -0.13 | 0.26 | 7.06E-01 | -0.77 | 0.25 | 7.02E-02 | -0.90 | 0.27 | **4.20E-02** |
| Concentration of large HDL particles | -0.36 | 0.18 | 1.79E-01 | -0.46 | 0.20 | 6.96E-02 | -0.55 | 0.25 | 1.21E-01 | -0.60 | 0.26 | 1.09E-01 | -0.30 | 0.31 | 6.31E-01 | -0.23 | 0.34 | 7.23E-01 |
| Phospholipids in LDL | -0.29 | 0.18 | 3.05E-01 | -0.43 | 0.19 | 7.04E-02 | -0.29 | 0.26 | 4.26E-01 | -0.31 | 0.26 | 4.03E-01 | -0.65 | 0.27 | 1.05E-01 | -0.71 | 0.27 | 7.81E-02 |
| LDL cholesterol | -0.29 | 0.18 | 3.05E-01 | -0.43 | 0.19 | 7.04E-02 | -0.34 | 0.25 | 3.72E-01 | -0.36 | 0.26 | 3.45E-01 | -0.60 | 0.28 | 1.40E-01 | -0.66 | 0.28 | 1.04E-01 |
| Total lipids in HDL | -0.47 | 0.22 | 1.65E-01 | -0.54 | 0.24 | 7.04E-02 | -0.40 | 0.29 | 3.66E-01 | -0.44 | 0.31 | 3.40E-01 | -0.74 | 0.38 | 2.05E-01 | -0.74 | 0.41 | 2.03E-01 |
| Triglycerides in IDL | 0.38 | 0.18 | 1.73E-01 | 0.43 | 0.19 | 7.04E-02 | 0.56 | 0.24 | 1.11E-01 | 0.59 | 0.24 | 8.87E-02 | 0.11 | 0.30 | 8.65E-01 | 0.16 | 0.32 | 7.62E-01 |
| Isoleucine | 0.43 | 0.20 | 1.64E-01 | 0.50 | 0.22 | 7.11E-02 | 0.70 | 0.25 | **4.46E-02** | 0.74 | 0.26 | **3.46E-02** | 0.06 | 0.36 | 9.27E-01 | -0.05 | 0.39 | 9.50E-01 |
| Total lipids in large HDL | -0.37 | 0.18 | 1.77E-01 | -0.45 | 0.20 | 7.16E-02 | -0.52 | 0.25 | 1.59E-01 | -0.56 | 0.26 | 1.39E-01 | -0.35 | 0.31 | 5.31E-01 | -0.28 | 0.33 | 6.56E-01 |
| Phospholipids to total lipids ratio in small HDL | 0.38 | 0.20 | 2.00E-01 | 0.45 | 0.20 | 7.49E-02 | 0.32 | 0.25 | 3.89E-01 | 0.37 | 0.26 | 3.34E-01 | 0.56 | 0.31 | 2.41E-01 | 0.67 | 0.34 | 1.69E-01 |
| Phospholipids to total lipids ratio in large VLDL | 0.64 | 0.31 | 1.66E-01 | 0.73 | 0.32 | 7.49E-02 | 1.18 | 0.41 | **3.87E-02** | 1.19 | 0.41 | **3.00E-02** | -0.03 | 0.36 | 9.63E-01 | -0.03 | 0.36 | 9.62E-01 |
| Apolipoprotein A1 | -0.46 | 0.22 | 1.73E-01 | -0.53 | 0.23 | 7.77E-02 | -0.31 | 0.29 | 4.36E-01 | -0.34 | 0.30 | 4.17E-01 | -0.85 | 0.38 | 1.37E-01 | -0.88 | 0.40 | 1.32E-01 |
| Phospholipids in small LDL | -0.29 | 0.18 | 3.05E-01 | -0.41 | 0.19 | 8.45E-02 | -0.38 | 0.25 | 3.20E-01 | -0.40 | 0.25 | 2.85E-01 | -0.50 | 0.28 | 2.48E-01 | -0.54 | 0.28 | 1.81E-01 |
| Cholesteryl esters to total lipids ratio in medium VLDL | -0.24 | 0.16 | 3.77E-01 | -0.40 | 0.18 | 8.48E-02 | -0.50 | 0.22 | 1.11E-01 | -0.53 | 0.22 | 1.01E-01 | -0.20 | 0.29 | 7.80E-01 | -0.16 | 0.31 | 7.62E-01 |
| Concentration of small HDL particles | -0.39 | 0.20 | 1.79E-01 | -0.44 | 0.20 | 8.67E-02 | -0.06 | 0.27 | 8.77E-01 | -0.08 | 0.28 | 8.25E-01 | -1.00 | 0.31 | 7.02E-02 | -1.14 | 0.33 | **3.70E-02** |
| Concentration of medium HDL particles | -0.42 | 0.21 | 1.76E-01 | -0.47 | 0.22 | 9.20E-02 | -0.26 | 0.28 | 4.99E-01 | -0.28 | 0.29 | 4.75E-01 | -0.76 | 0.34 | 1.33E-01 | -0.79 | 0.36 | 1.29E-01 |
| Free cholesterol to total lipids ratio in large HDL | -0.20 | 0.14 | 3.62E-01 | -0.31 | 0.14 | 9.20E-02 | -0.46 | 0.20 | 1.11E-01 | -0.44 | 0.20 | 1.18E-01 | -0.22 | 0.23 | 6.31E-01 | -0.19 | 0.25 | 6.90E-01 |
| Phospholipids in large HDL | -0.35 | 0.18 | 2.01E-01 | -0.40 | 0.19 | 1.06E-01 | -0.45 | 0.25 | 2.16E-01 | -0.47 | 0.26 | 2.14E-01 | -0.36 | 0.31 | 5.22E-01 | -0.29 | 0.33 | 6.44E-01 |
| Total lipids in IDL | -0.23 | 0.19 | 4.56E-01 | -0.43 | 0.21 | 1.08E-01 | -0.35 | 0.28 | 3.89E-01 | -0.35 | 0.28 | 3.83E-01 | -0.60 | 0.30 | 1.80E-01 | -0.65 | 0.32 | 1.66E-01 |
| Total cholines | -0.34 | 0.21 | 3.05E-01 | -0.44 | 0.22 | 1.20E-01 | -0.25 | 0.29 | 5.11E-01 | -0.26 | 0.29 | 5.00E-01 | -0.73 | 0.32 | 1.32E-01 | -0.83 | 0.35 | 1.04E-01 |
| Total lipids in LDL | -0.25 | 0.18 | 3.97E-01 | -0.38 | 0.19 | 1.20E-01 | -0.28 | 0.26 | 4.35E-01 | -0.30 | 0.26 | 4.12E-01 | -0.57 | 0.28 | 1.78E-01 | -0.62 | 0.28 | 1.34E-01 |
| Cholesteryl esters in LDL | -0.25 | 0.18 | 3.97E-01 | -0.38 | 0.19 | 1.20E-01 | -0.29 | 0.26 | 4.29E-01 | -0.31 | 0.26 | 4.04E-01 | -0.55 | 0.28 | 1.88E-01 | -0.60 | 0.28 | 1.45E-01 |
| Total free cholesterol | -0.25 | 0.19 | 4.15E-01 | -0.40 | 0.20 | 1.20E-01 | -0.31 | 0.27 | 4.26E-01 | -0.32 | 0.28 | 4.11E-01 | -0.57 | 0.28 | 1.80E-01 | -0.62 | 0.29 | 1.53E-01 |
| Cholesterol to total lipids ratio in medium VLDL | -0.22 | 0.17 | 4.38E-01 | -0.39 | 0.19 | 1.20E-01 | -0.48 | 0.24 | 1.62E-01 | -0.51 | 0.24 | 1.39E-01 | -0.22 | 0.30 | 7.65E-01 | -0.18 | 0.32 | 7.50E-01 |
| Free cholesterol to total lipids ratio in very small VLDL | -0.19 | 0.18 | 5.27E-01 | -0.39 | 0.20 | 1.20E-01 | -0.56 | 0.24 | 1.11E-01 | -0.59 | 0.25 | 9.59E-02 | -0.10 | 0.34 | 8.81E-01 | -0.14 | 0.34 | 8.05E-01 |
| Phospholipids to total lipids ratio in IDL | 0.34 | 0.19 | 2.49E-01 | 0.38 | 0.20 | 1.43E-01 | 0.27 | 0.26 | 4.51E-01 | 0.28 | 0.26 | 4.29E-01 | 0.60 | 0.35 | 2.77E-01 | 0.69 | 0.36 | 1.83E-01 |
| Ratio of omega-6 fatty acids to omega-3 fatty acids | -0.26 | 0.19 | 3.97E-01 | -0.38 | 0.20 | 1.43E-01 | -0.55 | 0.26 | 1.47E-01 | -0.56 | 0.25 | 1.22E-01 | -0.09 | 0.30 | 8.81E-01 | -0.08 | 0.30 | 8.85E-01 |
| Tyrosine | -0.37 | 0.20 | 2.09E-01 | -0.39 | 0.20 | 1.47E-01 | -0.38 | 0.26 | 3.55E-01 | -0.41 | 0.27 | 3.16E-01 | -0.31 | 0.31 | 6.04E-01 | -0.44 | 0.34 | 4.13E-01 |
| Total lipids in medium HDL | -0.39 | 0.21 | 2.26E-01 | -0.42 | 0.22 | 1.52E-01 | -0.20 | 0.28 | 5.95E-01 | -0.21 | 0.29 | 5.88E-01 | -0.73 | 0.35 | 1.61E-01 | -0.76 | 0.36 | 1.56E-01 |
| Phospholipids in HDL | -0.39 | 0.22 | 2.49E-01 | -0.44 | 0.23 | 1.52E-01 | -0.31 | 0.29 | 4.47E-01 | -0.32 | 0.30 | 4.35E-01 | -0.65 | 0.38 | 2.70E-01 | -0.64 | 0.40 | 2.82E-01 |
| Free cholesterol to total lipids ratio in medium VLDL | -0.21 | 0.18 | 4.86E-01 | -0.38 | 0.20 | 1.52E-01 | -0.42 | 0.26 | 2.86E-01 | -0.44 | 0.27 | 2.76E-01 | -0.35 | 0.31 | 5.30E-01 | -0.31 | 0.32 | 6.07E-01 |
| Omega-6 fatty acids | -0.27 | 0.20 | 3.97E-01 | -0.38 | 0.20 | 1.53E-01 | -0.33 | 0.27 | 3.97E-01 | -0.37 | 0.28 | 3.55E-01 | -0.43 | 0.30 | 3.89E-01 | -0.49 | 0.31 | 2.86E-01 |
| Linoleic acid | -0.24 | 0.19 | 4.56E-01 | -0.37 | 0.20 | 1.58E-01 | -0.37 | 0.27 | 3.68E-01 | -0.43 | 0.28 | 2.98E-01 | -0.34 | 0.30 | 5.22E-01 | -0.40 | 0.31 | 4.13E-01 |
| Concentration of very large HDL particles | -0.28 | 0.20 | 3.97E-01 | -0.42 | 0.23 | 1.59E-01 | -0.69 | 0.30 | 1.11E-01 | -0.72 | 0.31 | 1.09E-01 | -0.02 | 0.37 | 9.68E-01 | 0.10 | 0.38 | 8.85E-01 |
| Total lipids in small LDL | -0.23 | 0.19 | 4.56E-01 | -0.34 | 0.19 | 1.69E-01 | -0.23 | 0.26 | 5.11E-01 | -0.26 | 0.26 | 4.74E-01 | -0.52 | 0.27 | 2.10E-01 | -0.56 | 0.27 | 1.56E-01 |
| Total phospholipids in lipoprotein particles | -0.27 | 0.20 | 4.02E-01 | -0.39 | 0.21 | 1.70E-01 | -0.22 | 0.28 | 5.61E-01 | -0.23 | 0.29 | 5.59E-01 | -0.65 | 0.31 | 1.63E-01 | -0.74 | 0.34 | 1.34E-01 |
| Phospholipids in IDL | -0.18 | 0.19 | 5.79E-01 | -0.37 | 0.21 | 1.76E-01 | -0.31 | 0.28 | 4.26E-01 | -0.31 | 0.28 | 4.19E-01 | -0.53 | 0.30 | 2.54E-01 | -0.56 | 0.32 | 2.42E-01 |
| Free cholesterol in very large HDL | -0.22 | 0.19 | 4.65E-01 | -0.38 | 0.22 | 1.78E-01 | -0.63 | 0.27 | 1.11E-01 | -0.63 | 0.28 | 1.11E-01 | 0.00 | 0.36 | 1.00E+00 | 0.10 | 0.38 | 8.85E-01 |
| Total lipids in very large HDL | -0.24 | 0.19 | 4.56E-01 | -0.37 | 0.22 | 2.04E-01 | -0.64 | 0.28 | 1.11E-01 | -0.66 | 0.29 | 1.09E-01 | 0.06 | 0.37 | 9.27E-01 | 0.19 | 0.38 | 7.63E-01 |
| Triglycerides in large LDL | 0.28 | 0.19 | 3.45E-01 | 0.32 | 0.19 | 2.04E-01 | 0.51 | 0.24 | 1.45E-01 | 0.54 | 0.24 | 1.17E-01 | -0.10 | 0.31 | 8.81E-01 | -0.07 | 0.32 | 9.12E-01 |
| Free cholesterol to total lipids ratio in large VLDL | 0.33 | 0.18 | 2.49E-01 | 0.33 | 0.19 | 2.09E-01 | 0.47 | 0.23 | 1.66E-01 | 0.48 | 0.24 | 1.47E-01 | -0.07 | 0.33 | 9.16E-01 | -0.05 | 0.34 | 9.43E-01 |
| Cholesterol in small LDL | -0.20 | 0.19 | 5.17E-01 | -0.32 | 0.19 | 2.11E-01 | -0.23 | 0.26 | 5.11E-01 | -0.26 | 0.26 | 4.67E-01 | -0.48 | 0.29 | 2.86E-01 | -0.53 | 0.29 | 2.03E-01 |
| Triglycerides in very large HDL | 0.29 | 0.19 | 3.40E-01 | 0.33 | 0.20 | 2.17E-01 | 0.41 | 0.26 | 3.08E-01 | 0.43 | 0.26 | 2.76E-01 | 0.13 | 0.30 | 8.45E-01 | 0.19 | 0.31 | 7.42E-01 |
| Ratio of triglycerides to phosphoglycerides | 0.26 | 0.19 | 3.97E-01 | 0.33 | 0.20 | 2.17E-01 | 0.42 | 0.25 | 2.72E-01 | 0.46 | 0.26 | 2.48E-01 | 0.15 | 0.32 | 8.45E-01 | 0.09 | 0.33 | 8.85E-01 |
| Phosphoglycerides | -0.28 | 0.21 | 3.97E-01 | -0.36 | 0.22 | 2.19E-01 | -0.14 | 0.29 | 7.31E-01 | -0.14 | 0.30 | 7.30E-01 | -0.69 | 0.32 | 1.40E-01 | -0.79 | 0.35 | 1.17E-01 |
| Phosphatidylcholines | -0.28 | 0.21 | 3.97E-01 | -0.35 | 0.22 | 2.19E-01 | -0.19 | 0.28 | 6.27E-01 | -0.19 | 0.29 | 6.32E-01 | -0.63 | 0.31 | 1.80E-01 | -0.71 | 0.34 | 1.56E-01 |
| Average diameter for HDL particles | -0.34 | 0.22 | 3.45E-01 | -0.39 | 0.24 | 2.21E-01 | -0.59 | 0.32 | 2.13E-01 | -0.62 | 0.33 | 1.99E-01 | -0.07 | 0.36 | 9.16E-01 | 0.04 | 0.37 | 9.50E-01 |
| Cholesterol in medium LDL | -0.20 | 0.18 | 5.16E-01 | -0.30 | 0.19 | 2.23E-01 | -0.18 | 0.26 | 6.16E-01 | -0.20 | 0.26 | 5.70E-01 | -0.52 | 0.28 | 2.17E-01 | -0.58 | 0.28 | 1.56E-01 |
| Phospholipids in medium LDL | -0.19 | 0.18 | 5.17E-01 | -0.30 | 0.19 | 2.23E-01 | -0.18 | 0.26 | 6.11E-01 | -0.20 | 0.26 | 5.59E-01 | -0.51 | 0.28 | 2.24E-01 | -0.57 | 0.28 | 1.64E-01 |
| Free cholesterol to total lipids ratio in small HDL | -0.26 | 0.20 | 4.02E-01 | -0.34 | 0.21 | 2.23E-01 | -0.39 | 0.27 | 3.60E-01 | -0.39 | 0.28 | 3.45E-01 | -0.29 | 0.32 | 6.58E-01 | -0.24 | 0.33 | 7.15E-01 |
| Ratio of omega-3 fatty acids to total fatty acids | 0.22 | 0.19 | 4.83E-01 | 0.31 | 0.19 | 2.24E-01 | 0.45 | 0.26 | 2.50E-01 | 0.45 | 0.25 | 2.38E-01 | 0.11 | 0.30 | 8.65E-01 | 0.10 | 0.30 | 8.59E-01 |
| Free cholesterol in small HDL | -0.30 | 0.20 | 3.49E-01 | -0.34 | 0.21 | 2.27E-01 | 0.03 | 0.28 | 9.43E-01 | 0.03 | 0.29 | 9.46E-01 | -0.90 | 0.32 | 9.27E-02 | -1.03 | 0.35 | **4.54E-02** |
| Triglycerides in LDL | 0.25 | 0.18 | 3.97E-01 | 0.30 | 0.19 | 2.27E-01 | 0.50 | 0.24 | 1.47E-01 | 0.53 | 0.24 | 1.22E-01 | -0.12 | 0.31 | 8.55E-01 | -0.11 | 0.32 | 8.59E-01 |
| Triglycerides to total lipids ratio in large VLDL | -0.29 | 0.17 | 2.67E-01 | -0.29 | 0.18 | 2.37E-01 | -0.38 | 0.23 | 2.69E-01 | -0.39 | 0.23 | 2.55E-01 | -0.03 | 0.32 | 9.64E-01 | -0.07 | 0.33 | 9.12E-01 |
| Total cholesterol minus HDL-C | -0.16 | 0.19 | 6.25E-01 | -0.30 | 0.20 | 2.42E-01 | -0.24 | 0.27 | 5.11E-01 | -0.25 | 0.27 | 4.79E-01 | -0.45 | 0.29 | 3.33E-01 | -0.49 | 0.29 | 2.57E-01 |
| Concentration of large LDL particles | -0.16 | 0.19 | 6.25E-01 | -0.30 | 0.19 | 2.42E-01 | -0.27 | 0.26 | 4.51E-01 | -0.30 | 0.26 | 4.17E-01 | -0.38 | 0.29 | 4.37E-01 | -0.40 | 0.29 | 3.73E-01 |
| Ratio of polyunsaturated fatty acids to total fatty acids | -0.19 | 0.17 | 5.16E-01 | -0.28 | 0.18 | 2.42E-01 | -0.38 | 0.21 | 2.29E-01 | -0.44 | 0.22 | 1.54E-01 | 0.07 | 0.32 | 9.16E-01 | 0.08 | 0.35 | 9.04E-01 |
| Phospholipids in medium HDL | -0.32 | 0.21 | 3.39E-01 | -0.33 | 0.22 | 2.64E-01 | -0.12 | 0.28 | 7.53E-01 | -0.12 | 0.30 | 7.62E-01 | -0.66 | 0.34 | 2.10E-01 | -0.68 | 0.36 | 1.91E-01 |
| Total concentration of branched-chain amino acids (leucine + isoleucine + valine) | 0.27 | 0.20 | 3.97E-01 | 0.31 | 0.22 | 2.89E-01 | 0.58 | 0.25 | 1.11E-01 | 0.61 | 0.26 | 1.07E-01 | -0.17 | 0.35 | 8.45E-01 | -0.34 | 0.38 | 6.44E-01 |
| Phospholipids in very small VLDL | 0.32 | 0.19 | 2.90E-01 | 0.29 | 0.20 | 2.94E-01 | 0.32 | 0.27 | 4.09E-01 | 0.33 | 0.27 | 3.83E-01 | 0.13 | 0.31 | 8.48E-01 | 0.17 | 0.32 | 7.62E-01 |
| Total lipids in medium LDL | -0.17 | 0.19 | 5.93E-01 | -0.27 | 0.19 | 2.96E-01 | -0.14 | 0.26 | 7.07E-01 | -0.16 | 0.26 | 6.52E-01 | -0.49 | 0.28 | 2.54E-01 | -0.55 | 0.28 | 1.81E-01 |
| Cholesterol to total lipids ratio in very large HDL | -0.31 | 0.23 | 3.97E-01 | -0.34 | 0.24 | 2.98E-01 | -0.01 | 0.28 | 9.80E-01 | -0.05 | 0.29 | 8.99E-01 | -0.90 | 0.44 | 1.72E-01 | -1.10 | 0.45 | 9.09E-02 |
| Total lipids in lipoprotein particles | -0.18 | 0.19 | 5.88E-01 | -0.28 | 0.20 | 3.07E-01 | -0.12 | 0.28 | 7.47E-01 | -0.13 | 0.28 | 7.22E-01 | -0.52 | 0.29 | 2.43E-01 | -0.58 | 0.30 | 1.81E-01 |
| Concentration of LDL particles | -0.14 | 0.19 | 6.64E-01 | -0.27 | 0.19 | 3.15E-01 | -0.20 | 0.26 | 5.73E-01 | -0.22 | 0.27 | 5.25E-01 | -0.40 | 0.29 | 3.95E-01 | -0.44 | 0.29 | 3.08E-01 |
| Triglycerides in medium LDL | 0.21 | 0.18 | 4.85E-01 | 0.26 | 0.19 | 3.20E-01 | 0.49 | 0.24 | 1.62E-01 | 0.51 | 0.24 | 1.39E-01 | -0.19 | 0.31 | 8.32E-01 | -0.20 | 0.32 | 7.42E-01 |
| Cholesteryl esters in medium VLDL | -0.09 | 0.18 | 7.75E-01 | -0.26 | 0.20 | 3.30E-01 | -0.31 | 0.26 | 3.99E-01 | -0.34 | 0.26 | 3.67E-01 | -0.25 | 0.29 | 6.87E-01 | -0.27 | 0.30 | 6.46E-01 |
| Cholesterol to total lipids ratio in small VLDL | -0.11 | 0.18 | 7.15E-01 | -0.26 | 0.20 | 3.34E-01 | -0.31 | 0.25 | 3.97E-01 | -0.32 | 0.26 | 3.83E-01 | -0.23 | 0.30 | 7.59E-01 | -0.19 | 0.32 | 7.42E-01 |
| Ratio of polyunsaturated fatty acids to monounsaturated fatty acids | -0.16 | 0.18 | 6.25E-01 | -0.25 | 0.19 | 3.34E-01 | -0.31 | 0.23 | 3.71E-01 | -0.36 | 0.24 | 3.16E-01 | -0.03 | 0.32 | 9.63E-01 | -0.01 | 0.34 | 9.83E-01 |
| Triglycerides to total lipids ratio in small VLDL | 0.12 | 0.20 | 7.15E-01 | 0.28 | 0.22 | 3.55E-01 | 0.34 | 0.28 | 3.97E-01 | 0.36 | 0.29 | 3.83E-01 | 0.20 | 0.34 | 8.40E-01 | 0.17 | 0.34 | 7.63E-01 |
| Phospholipids to total lipids ratio in medium VLDL | -0.12 | 0.19 | 7.03E-01 | -0.26 | 0.21 | 3.59E-01 | -0.24 | 0.27 | 5.07E-01 | -0.25 | 0.27 | 5.00E-01 | -0.33 | 0.31 | 5.45E-01 | -0.29 | 0.32 | 6.44E-01 |
| Triglycerides in small VLDL | 0.20 | 0.19 | 5.17E-01 | 0.25 | 0.20 | 3.61E-01 | 0.37 | 0.26 | 3.60E-01 | 0.38 | 0.26 | 3.34E-01 | 0.03 | 0.30 | 9.60E-01 | -0.01 | 0.31 | 9.87E-01 |
| Valine | 0.24 | 0.20 | 4.56E-01 | 0.27 | 0.21 | 3.63E-01 | 0.59 | 0.25 | 1.11E-01 | 0.64 | 0.27 | 1.01E-01 | -0.24 | 0.34 | 7.65E-01 | -0.42 | 0.37 | 5.07E-01 |
| Free cholesterol to total lipids ratio in very large HDL | 0.21 | 0.20 | 5.17E-01 | 0.27 | 0.22 | 3.69E-01 | 0.49 | 0.27 | 2.13E-01 | 0.53 | 0.28 | 1.97E-01 | -0.11 | 0.36 | 8.81E-01 | -0.26 | 0.38 | 7.23E-01 |
| Cholesteryl esters in small LDL | -0.13 | 0.19 | 6.86E-01 | -0.24 | 0.19 | 3.78E-01 | -0.13 | 0.26 | 7.31E-01 | -0.15 | 0.27 | 6.71E-01 | -0.42 | 0.29 | 3.78E-01 | -0.47 | 0.29 | 2.73E-01 |
| Triglycerides to total lipids ratio in medium VLDL | 0.12 | 0.20 | 7.03E-01 | 0.26 | 0.22 | 3.87E-01 | 0.31 | 0.27 | 4.26E-01 | 0.33 | 0.28 | 4.11E-01 | 0.19 | 0.33 | 8.45E-01 | 0.15 | 0.34 | 8.05E-01 |
| Polyunsaturated fatty acids | -0.17 | 0.20 | 6.25E-01 | -0.24 | 0.20 | 3.93E-01 | -0.13 | 0.27 | 7.31E-01 | -0.16 | 0.27 | 6.61E-01 | -0.38 | 0.30 | 4.49E-01 | -0.43 | 0.30 | 3.49E-01 |
| Phospholipids in very large HDL | -0.14 | 0.18 | 6.51E-01 | -0.24 | 0.20 | 4.05E-01 | -0.48 | 0.24 | 1.79E-01 | -0.49 | 0.25 | 1.82E-01 | 0.21 | 0.42 | 8.45E-01 | 0.36 | 0.45 | 6.86E-01 |
| Triglycerides in small LDL | 0.18 | 0.18 | 5.59E-01 | 0.22 | 0.19 | 4.09E-01 | 0.41 | 0.23 | 2.50E-01 | 0.43 | 0.24 | 2.35E-01 | -0.14 | 0.31 | 8.45E-01 | -0.17 | 0.32 | 7.57E-01 |
| Creatinine | 0.25 | 0.22 | 4.86E-01 | 0.26 | 0.22 | 4.11E-01 | 0.36 | 0.26 | 3.68E-01 | 0.37 | 0.27 | 3.46E-01 | 7.76 | 5.38 | 3.89E-01 | 9.85 | 5.38 | 2.03E-01 |
| Cholesteryl esters in medium LDL | -0.13 | 0.19 | 6.74E-01 | -0.22 | 0.19 | 4.11E-01 | -0.08 | 0.26 | 8.10E-01 | -0.10 | 0.27 | 7.65E-01 | -0.45 | 0.28 | 3.18E-01 | -0.51 | 0.28 | 2.11E-01 |
| Omega-3 fatty acids | 0.16 | 0.19 | 6.27E-01 | 0.22 | 0.20 | 4.21E-01 | 0.43 | 0.27 | 2.86E-01 | 0.43 | 0.26 | 2.72E-01 | -0.07 | 0.29 | 9.12E-01 | -0.09 | 0.29 | 8.80E-01 |
| 3-Hydroxybutyrate | -0.24 | 0.20 | 4.65E-01 | -0.23 | 0.20 | 4.22E-01 | -0.19 | 0.26 | 5.91E-01 | -0.19 | 0.26 | 5.78E-01 | -0.34 | 0.32 | 5.45E-01 | -0.31 | 0.34 | 6.34E-01 |
| Total lipids in small HDL | -0.22 | 0.21 | 5.16E-01 | -0.24 | 0.21 | 4.36E-01 | 0.18 | 0.28 | 6.37E-01 | 0.18 | 0.29 | 6.41E-01 | -0.87 | 0.33 | 9.27E-02 | -1.02 | 0.36 | **4.97E-02** |
| Concentration of small LDL particles | -0.10 | 0.19 | 7.45E-01 | -0.22 | 0.20 | 4.36E-01 | -0.10 | 0.27 | 7.86E-01 | -0.12 | 0.27 | 7.32E-01 | -0.40 | 0.29 | 4.02E-01 | -0.45 | 0.29 | 3.00E-01 |
| Phospholipids to total lipids ratio in large LDL | 0.19 | 0.18 | 5.17E-01 | 0.20 | 0.18 | 4.36E-01 | 0.31 | 0.23 | 3.74E-01 | 0.32 | 0.23 | 3.46E-01 | 0.06 | 0.29 | 9.16E-01 | 0.07 | 0.30 | 9.04E-01 |
| Phospholipids to total lipids ratio in small LDL | -0.22 | 0.18 | 4.65E-01 | -0.21 | 0.19 | 4.46E-01 | -0.61 | 0.22 | **4.46E-02** | -0.60 | 0.22 | **4.26E-02** | 0.50 | 0.32 | 3.31E-01 | 0.61 | 0.33 | 2.03E-01 |
| Concentration of small VLDL particles | 0.21 | 0.19 | 4.92E-01 | 0.21 | 0.20 | 4.49E-01 | 0.33 | 0.26 | 3.86E-01 | 0.34 | 0.26 | 3.68E-01 | -0.02 | 0.30 | 9.64E-01 | -0.06 | 0.31 | 9.22E-01 |
| Total lipids in very small VLDL | 0.26 | 0.19 | 4.02E-01 | 0.22 | 0.20 | 4.64E-01 | 0.27 | 0.27 | 4.57E-01 | 0.29 | 0.27 | 4.29E-01 | 0.01 | 0.31 | 9.91E-01 | 0.04 | 0.32 | 9.51E-01 |
| Leucine | 0.20 | 0.21 | 5.75E-01 | 0.23 | 0.22 | 4.75E-01 | 0.43 | 0.26 | 2.72E-01 | 0.43 | 0.26 | 2.76E-01 | -0.15 | 0.36 | 8.48E-01 | -0.29 | 0.38 | 6.92E-01 |
| Average diameter for LDL particles | -0.11 | 0.18 | 7.16E-01 | -0.19 | 0.19 | 4.75E-01 | -0.33 | 0.22 | 3.31E-01 | -0.36 | 0.23 | 2.89E-01 | 0.08 | 0.33 | 9.09E-01 | 0.14 | 0.34 | 8.05E-01 |
| Apolipoprotein B | -0.08 | 0.19 | 8.07E-01 | -0.20 | 0.20 | 4.77E-01 | -0.12 | 0.27 | 7.40E-01 | -0.14 | 0.27 | 6.96E-01 | -0.36 | 0.29 | 4.71E-01 | -0.39 | 0.29 | 3.85E-01 |
| Glycoprotein acetyls | 0.16 | 0.19 | 6.25E-01 | 0.19 | 0.19 | 4.89E-01 | 0.06 | 0.26 | 8.77E-01 | 0.05 | 0.26 | 8.69E-01 | 0.42 | 0.28 | 3.70E-01 | 0.43 | 0.30 | 3.46E-01 |
| Ratio of monounsaturated fatty acids to total fatty acids | 0.11 | 0.19 | 7.31E-01 | 0.20 | 0.20 | 4.89E-01 | 0.20 | 0.25 | 5.54E-01 | 0.23 | 0.26 | 5.00E-01 | 0.10 | 0.32 | 8.80E-01 | 0.08 | 0.34 | 9.02E-01 |
| Total triglycerides | 0.16 | 0.19 | 6.25E-01 | 0.19 | 0.19 | 5.05E-01 | 0.35 | 0.25 | 3.64E-01 | 0.37 | 0.26 | 3.34E-01 | -0.10 | 0.31 | 8.81E-01 | -0.16 | 0.32 | 7.62E-01 |
| Ratio of docosahexaenoic acid to total fatty acids | 0.12 | 0.19 | 7.03E-01 | 0.18 | 0.19 | 5.07E-01 | 0.25 | 0.24 | 4.57E-01 | 0.26 | 0.24 | 4.42E-01 | 0.09 | 0.29 | 8.81E-01 | 0.12 | 0.30 | 8.05E-01 |
| Concentration of medium LDL particles | -0.10 | 0.19 | 7.54E-01 | -0.19 | 0.20 | 5.09E-01 | -0.05 | 0.27 | 8.97E-01 | -0.06 | 0.27 | 8.69E-01 | -0.43 | 0.29 | 3.70E-01 | -0.49 | 0.29 | 2.57E-01 |
| Cholesterol in medium VLDL | -0.05 | 0.19 | 8.81E-01 | -0.19 | 0.19 | 5.12E-01 | -0.22 | 0.26 | 5.28E-01 | -0.25 | 0.26 | 4.79E-01 | -0.19 | 0.30 | 8.10E-01 | -0.22 | 0.30 | 6.94E-01 |
| Phenylalanine | -0.17 | 0.20 | 6.25E-01 | -0.19 | 0.20 | 5.13E-01 | -0.15 | 0.25 | 6.64E-01 | -0.17 | 0.26 | 6.29E-01 | -0.18 | 0.34 | 8.45E-01 | -0.21 | 0.36 | 7.42E-01 |
| Remnant cholesterol (non-HDL | -0.05 | 0.19 | 8.78E-01 | -0.19 | 0.20 | 5.16E-01 | -0.09 | 0.28 | 7.95E-01 | -0.11 | 0.28 | 7.69E-01 | -0.37 | 0.29 | 4.59E-01 | -0.40 | 0.29 | 3.82E-01 |
| Glycine | 0.16 | 0.21 | 6.52E-01 | 0.20 | 0.22 | 5.16E-01 | 0.12 | 0.27 | 7.54E-01 | 0.15 | 0.28 | 6.93E-01 | 0.15 | 0.33 | 8.45E-01 | 0.27 | 0.35 | 6.90E-01 |
| Cholesteryl esters in small VLDL | 0.24 | 0.19 | 4.56E-01 | 0.19 | 0.20 | 5.16E-01 | 0.33 | 0.27 | 3.97E-01 | 0.33 | 0.27 | 3.95E-01 | -0.09 | 0.31 | 8.81E-01 | -0.10 | 0.31 | 8.59E-01 |
| Total lipids in small VLDL | 0.20 | 0.19 | 5.27E-01 | 0.18 | 0.20 | 5.16E-01 | 0.31 | 0.26 | 3.99E-01 | 0.31 | 0.26 | 4.03E-01 | -0.05 | 0.30 | 9.23E-01 | -0.09 | 0.31 | 8.80E-01 |
| Ratio of saturated fatty acids to total fatty acids | 0.14 | 0.19 | 6.62E-01 | 0.18 | 0.20 | 5.21E-01 | 0.48 | 0.25 | 1.86E-01 | 0.56 | 0.26 | 1.22E-01 | -0.39 | 0.31 | 4.60E-01 | -0.39 | 0.32 | 4.74E-01 |
| Concentration of very small VLDL particles | 0.23 | 0.19 | 4.57E-01 | 0.18 | 0.20 | 5.33E-01 | 0.26 | 0.27 | 4.77E-01 | 0.28 | 0.27 | 4.43E-01 | -0.05 | 0.31 | 9.23E-01 | -0.03 | 0.32 | 9.53E-01 |
| Free cholesterol in very small VLDL | 0.24 | 0.19 | 4.56E-01 | 0.18 | 0.21 | 5.48E-01 | 0.22 | 0.27 | 5.53E-01 | 0.24 | 0.27 | 5.17E-01 | 0.00 | 0.31 | 1.00E+00 | 0.02 | 0.32 | 9.62E-01 |
| Phospholipids in large VLDL | 0.16 | 0.20 | 6.45E-01 | 0.17 | 0.21 | 6.02E-01 | 0.36 | 0.28 | 3.88E-01 | 0.38 | 0.29 | 3.67E-01 | -0.15 | 0.31 | 8.45E-01 | -0.21 | 0.31 | 7.23E-01 |
| Histidine | -0.11 | 0.18 | 7.11E-01 | -0.14 | 0.19 | 6.24E-01 | -0.29 | 0.23 | 3.89E-01 | -0.30 | 0.23 | 3.64E-01 | 0.16 | 0.31 | 8.45E-01 | 0.12 | 0.31 | 8.43E-01 |
| Phospholipids in small HDL | -0.16 | 0.21 | 6.51E-01 | -0.16 | 0.22 | 6.38E-01 | 0.23 | 0.28 | 5.40E-01 | 0.25 | 0.29 | 5.20E-01 | -0.77 | 0.34 | 1.31E-01 | -0.90 | 0.36 | 8.34E-02 |
| Concentration of IDL particles | -0.01 | 0.19 | 9.79E-01 | -0.15 | 0.21 | 6.38E-01 | -0.08 | 0.28 | 8.40E-01 | -0.07 | 0.28 | 8.48E-01 | -0.35 | 0.32 | 5.36E-01 | -0.38 | 0.32 | 4.76E-01 |
| Concentration of very large VLDL particles | 0.14 | 0.20 | 6.78E-01 | 0.16 | 0.21 | 6.38E-01 | 0.38 | 0.28 | 3.68E-01 | 0.40 | 0.29 | 3.45E-01 | -0.21 | 0.31 | 7.80E-01 | -0.29 | 0.32 | 6.32E-01 |
| Cholesteryl esters in very large VLDL | 0.19 | 0.20 | 5.85E-01 | 0.15 | 0.21 | 6.38E-01 | 0.35 | 0.28 | 3.89E-01 | 0.35 | 0.29 | 3.88E-01 | -0.16 | 0.30 | 8.45E-01 | -0.22 | 0.30 | 6.97E-01 |
| Degree of unsaturation | -0.12 | 0.19 | 7.03E-01 | -0.14 | 0.20 | 6.38E-01 | -0.09 | 0.26 | 7.89E-01 | -0.12 | 0.26 | 7.32E-01 | -0.15 | 0.30 | 8.45E-01 | -0.18 | 0.31 | 7.46E-01 |
| Concentration of VLDL particles | 0.18 | 0.19 | 5.91E-01 | 0.15 | 0.20 | 6.38E-01 | 0.27 | 0.26 | 4.57E-01 | 0.27 | 0.27 | 4.54E-01 | -0.08 | 0.30 | 8.81E-01 | -0.12 | 0.30 | 8.07E-01 |
| Cholesterol in very large VLDL | 0.17 | 0.20 | 6.25E-01 | 0.15 | 0.21 | 6.45E-01 | 0.37 | 0.27 | 3.71E-01 | 0.38 | 0.28 | 3.56E-01 | -0.20 | 0.30 | 8.02E-01 | -0.27 | 0.31 | 6.44E-01 |
| Cholesteryl esters in chylomicrons and extremely large VLDL | 0.14 | 0.20 | 6.86E-01 | 0.15 | 0.21 | 6.50E-01 | 0.49 | 0.30 | 2.92E-01 | 0.51 | 0.31 | 2.76E-01 | -0.30 | 0.30 | 6.12E-01 | -0.37 | 0.31 | 4.62E-01 |
| Cholesteryl esters in very small VLDL | 0.00 | 0.19 | 9.98E-01 | -0.15 | 0.22 | 6.50E-01 | -0.15 | 0.29 | 7.24E-01 | -0.13 | 0.29 | 7.32E-01 | -0.28 | 0.32 | 6.61E-01 | -0.26 | 0.33 | 6.86E-01 |
| Free cholesterol in large VLDL | 0.14 | 0.20 | 6.64E-01 | 0.14 | 0.21 | 6.50E-01 | 0.33 | 0.27 | 3.97E-01 | 0.34 | 0.28 | 3.93E-01 | -0.16 | 0.31 | 8.45E-01 | -0.24 | 0.31 | 6.92E-01 |
| Citrate | -0.11 | 0.20 | 7.54E-01 | -0.14 | 0.20 | 6.51E-01 | -0.04 | 0.27 | 9.23E-01 | -0.06 | 0.27 | 8.69E-01 | -0.17 | 0.31 | 8.45E-01 | -0.21 | 0.31 | 7.23E-01 |
| Cholesteryl esters to total lipids ratio in small LDL | 0.17 | 0.18 | 5.88E-01 | 0.13 | 0.19 | 6.54E-01 | 0.50 | 0.23 | 1.44E-01 | 0.48 | 0.23 | 1.42E-01 | -0.42 | 0.31 | 4.18E-01 | -0.50 | 0.32 | 2.88E-01 |
| Phospholipids in VLDL | 0.16 | 0.19 | 6.25E-01 | 0.14 | 0.20 | 6.54E-01 | 0.30 | 0.26 | 4.26E-01 | 0.30 | 0.27 | 4.17E-01 | -0.13 | 0.30 | 8.45E-01 | -0.19 | 0.30 | 7.42E-01 |
| Cholesterol in large VLDL | 0.15 | 0.20 | 6.51E-01 | 0.14 | 0.20 | 6.60E-01 | 0.30 | 0.27 | 4.26E-01 | 0.31 | 0.28 | 4.19E-01 | -0.14 | 0.31 | 8.45E-01 | -0.21 | 0.31 | 7.23E-01 |
| Free cholesterol in very large VLDL | 0.14 | 0.20 | 6.77E-01 | 0.14 | 0.21 | 6.68E-01 | 0.38 | 0.27 | 3.68E-01 | 0.40 | 0.29 | 3.45E-01 | -0.25 | 0.30 | 6.92E-01 | -0.32 | 0.30 | 5.49E-01 |
| Total lipids in VLDL | 0.14 | 0.19 | 6.64E-01 | 0.13 | 0.20 | 6.71E-01 | 0.29 | 0.26 | 4.26E-01 | 0.30 | 0.26 | 4.17E-01 | -0.14 | 0.30 | 8.45E-01 | -0.21 | 0.31 | 7.23E-01 |
| Phospholipids to total lipids ratio in very large VLDL | -0.06 | 0.18 | 8.38E-01 | -0.11 | 0.17 | 6.75E-01 | 0.42 | 0.34 | 3.97E-01 | 0.42 | 0.34 | 3.95E-01 | -0.36 | 0.14 | 9.27E-02 | -0.37 | 0.15 | 8.49E-02 |
| Total lipids in very large VLDL | 0.12 | 0.20 | 7.15E-01 | 0.13 | 0.21 | 6.79E-01 | 0.36 | 0.28 | 3.80E-01 | 0.39 | 0.29 | 3.57E-01 | -0.24 | 0.31 | 7.46E-01 | -0.33 | 0.32 | 5.69E-01 |
| Triglycerides in VLDL | 0.10 | 0.19 | 7.45E-01 | 0.12 | 0.20 | 6.79E-01 | 0.28 | 0.25 | 4.29E-01 | 0.30 | 0.26 | 4.17E-01 | -0.13 | 0.31 | 8.45E-01 | -0.21 | 0.32 | 7.23E-01 |
| Cholesteryl esters in large VLDL | 0.16 | 0.20 | 6.45E-01 | 0.13 | 0.20 | 6.79E-01 | 0.27 | 0.27 | 4.75E-01 | 0.27 | 0.28 | 4.76E-01 | -0.11 | 0.30 | 8.65E-01 | -0.17 | 0.30 | 7.46E-01 |
| Glucose | -0.17 | 0.21 | 6.33E-01 | -0.13 | 0.22 | 6.84E-01 | -0.03 | 0.26 | 9.39E-01 | -0.02 | 0.27 | 9.46E-01 | -0.32 | 0.35 | 6.48E-01 | -0.35 | 0.36 | 5.87E-01 |
| Concentration of large VLDL particles | 0.12 | 0.19 | 7.15E-01 | 0.12 | 0.20 | 6.91E-01 | 0.29 | 0.26 | 4.33E-01 | 0.30 | 0.27 | 4.24E-01 | -0.15 | 0.31 | 8.45E-01 | -0.23 | 0.31 | 6.97E-01 |
| Cholesterol to total lipids ratio in large VLDL | 0.16 | 0.19 | 6.25E-01 | 0.12 | 0.20 | 7.00E-01 | 0.06 | 0.26 | 8.77E-01 | 0.06 | 0.27 | 8.69E-01 | 0.14 | 0.32 | 8.45E-01 | 0.20 | 0.33 | 7.42E-01 |
| Docosahexaenoic acid | 0.07 | 0.19 | 8.38E-01 | 0.11 | 0.19 | 7.11E-01 | 0.28 | 0.25 | 4.26E-01 | 0.29 | 0.25 | 4.17E-01 | -0.14 | 0.30 | 8.45E-01 | -0.14 | 0.30 | 7.80E-01 |
| Cholesteryl esters to total lipids ratio in medium LDL | 0.08 | 0.19 | 7.96E-01 | 0.11 | 0.19 | 7.12E-01 | 0.23 | 0.24 | 4.90E-01 | 0.25 | 0.25 | 4.65E-01 | -0.11 | 0.31 | 8.65E-01 | -0.19 | 0.32 | 7.42E-01 |
| Cholesterol in chylomicrons and extremely large VLDL | 0.09 | 0.20 | 7.88E-01 | 0.11 | 0.21 | 7.45E-01 | 0.46 | 0.30 | 3.13E-01 | 0.48 | 0.31 | 2.89E-01 | -0.37 | 0.30 | 4.71E-01 | -0.46 | 0.31 | 3.31E-01 |
| Triglycerides in very large VLDL | 0.09 | 0.20 | 7.98E-01 | 0.11 | 0.21 | 7.45E-01 | 0.32 | 0.28 | 4.26E-01 | 0.35 | 0.29 | 4.05E-01 | -0.23 | 0.31 | 7.65E-01 | -0.32 | 0.32 | 5.87E-01 |
| Cholesterol in small VLDL | 0.16 | 0.19 | 6.25E-01 | 0.10 | 0.20 | 7.71E-01 | 0.22 | 0.27 | 5.43E-01 | 0.22 | 0.27 | 5.53E-01 | -0.15 | 0.30 | 8.45E-01 | -0.17 | 0.30 | 7.50E-01 |
| Total lipids in large VLDL | 0.09 | 0.19 | 7.75E-01 | 0.09 | 0.20 | 7.82E-01 | 0.25 | 0.26 | 4.92E-01 | 0.26 | 0.27 | 4.79E-01 | -0.16 | 0.31 | 8.45E-01 | -0.24 | 0.31 | 6.92E-01 |
| Concentration of chylomicrons and extremely large VLDL particles | 0.06 | 0.20 | 8.42E-01 | 0.09 | 0.21 | 8.05E-01 | 0.43 | 0.32 | 3.71E-01 | 0.47 | 0.33 | 3.45E-01 | -0.35 | 0.29 | 4.79E-01 | -0.44 | 0.30 | 3.40E-01 |
| Free cholesterol in VLDL | 0.12 | 0.19 | 7.03E-01 | 0.08 | 0.20 | 8.21E-01 | 0.24 | 0.26 | 5.03E-01 | 0.24 | 0.27 | 5.00E-01 | -0.18 | 0.29 | 8.32E-01 | -0.24 | 0.30 | 6.86E-01 |
| Free cholesterol to total lipids ratio in very large VLDL | -0.01 | 0.20 | 9.91E-01 | -0.08 | 0.21 | 8.21E-01 | -0.11 | 0.27 | 7.58E-01 | -0.13 | 0.28 | 7.32E-01 | -0.09 | 0.34 | 8.81E-01 | -0.04 | 0.34 | 9.50E-01 |
| Ratio of apolipoprotein B to apolipoprotein A1 | 0.16 | 0.19 | 6.27E-01 | 0.08 | 0.21 | 8.21E-01 | 0.07 | 0.26 | 8.36E-01 | 0.06 | 0.27 | 8.69E-01 | 0.06 | 0.31 | 9.16E-01 | 0.01 | 0.32 | 9.83E-01 |
| Lactate | -0.09 | 0.19 | 7.88E-01 | -0.07 | 0.20 | 8.42E-01 | 0.25 | 0.24 | 4.57E-01 | 0.27 | 0.25 | 4.32E-01 | -0.51 | 0.30 | 2.89E-01 | -0.61 | 0.32 | 1.87E-01 |
| Phospholipids in very large VLDL | 0.08 | 0.20 | 8.07E-01 | 0.08 | 0.21 | 8.42E-01 | 0.40 | 0.29 | 3.66E-01 | 0.43 | 0.30 | 3.44E-01 | -0.36 | 0.27 | 4.18E-01 | -0.42 | 0.26 | 2.86E-01 |
| Total fatty acids | -0.05 | 0.20 | 8.90E-01 | -0.07 | 0.20 | 8.42E-01 | 0.11 | 0.27 | 7.71E-01 | 0.10 | 0.27 | 7.71E-01 | -0.35 | 0.31 | 5.22E-01 | -0.41 | 0.31 | 4.13E-01 |
| Phospholipids in chylomicrons and extremely large VLDL | 0.05 | 0.20 | 8.86E-01 | 0.08 | 0.21 | 8.48E-01 | 0.49 | 0.36 | 3.71E-01 | 0.52 | 0.38 | 3.46E-01 | -0.41 | 0.29 | 3.92E-01 | -0.51 | 0.31 | 2.63E-01 |
| Phospholipids to total lipids ratio in medium LDL | -0.02 | 0.19 | 9.56E-01 | -0.07 | 0.19 | 8.49E-01 | -0.22 | 0.23 | 4.99E-01 | -0.24 | 0.23 | 4.52E-01 | 0.26 | 0.34 | 7.59E-01 | 0.29 | 0.35 | 6.77E-01 |
| Triglycerides in chylomicrons and extremely large VLDL | 0.07 | 0.21 | 8.38E-01 | 0.08 | 0.22 | 8.49E-01 | 0.29 | 0.35 | 5.33E-01 | 0.30 | 0.36 | 5.25E-01 | -0.16 | 0.28 | 8.45E-01 | -0.22 | 0.29 | 6.94E-01 |
| VLDL cholesterol | 0.14 | 0.19 | 6.74E-01 | 0.07 | 0.20 | 8.49E-01 | 0.20 | 0.27 | 5.79E-01 | 0.20 | 0.27 | 5.88E-01 | -0.16 | 0.30 | 8.45E-01 | -0.20 | 0.30 | 7.23E-01 |
| Alanine | -0.09 | 0.19 | 7.88E-01 | -0.06 | 0.19 | 8.49E-01 | 0.28 | 0.25 | 4.26E-01 | 0.28 | 0.25 | 4.17E-01 | -0.53 | 0.31 | 2.70E-01 | -0.61 | 0.30 | 1.69E-01 |
| Triglycerides to total lipids ratio in chylomicrons and extremely large VLDL | 0.09 | 0.23 | 8.23E-01 | 0.07 | 0.23 | 8.52E-01 | 0.14 | 0.32 | 7.51E-01 | 0.13 | 0.32 | 7.61E-01 | 0.00 | 0.33 | 1.00E+00 | -0.03 | 0.33 | 9.53E-01 |
| Pyruvate | 0.04 | 0.19 | 9.16E-01 | 0.06 | 0.20 | 8.53E-01 | 0.43 | 0.26 | 2.73E-01 | 0.45 | 0.27 | 2.52E-01 | -0.42 | 0.29 | 3.92E-01 | -0.50 | 0.32 | 2.86E-01 |
| Free cholesterol in chylomicrons and extremely large VLDL | 0.04 | 0.20 | 9.16E-01 | 0.06 | 0.21 | 8.55E-01 | 0.41 | 0.30 | 3.68E-01 | 0.44 | 0.31 | 3.45E-01 | -0.39 | 0.29 | 4.18E-01 | -0.49 | 0.30 | 2.84E-01 |
| Triglycerides in medium VLDL | 0.06 | 0.19 | 8.40E-01 | 0.06 | 0.20 | 8.63E-01 | 0.18 | 0.26 | 6.06E-01 | 0.18 | 0.27 | 6.27E-01 | -0.13 | 0.30 | 8.48E-01 | -0.20 | 0.31 | 7.26E-01 |
| Cholesteryl esters to total lipids ratio in very large VLDL | 0.02 | 0.20 | 9.52E-01 | -0.06 | 0.22 | 8.87E-01 | -0.28 | 0.28 | 4.57E-01 | -0.32 | 0.29 | 4.17E-01 | 0.31 | 0.32 | 6.29E-01 | 0.40 | 0.33 | 4.62E-01 |
| Free cholesterol in medium VLDL | 0.04 | 0.19 | 9.10E-01 | -0.05 | 0.20 | 8.91E-01 | -0.02 | 0.26 | 9.43E-01 | -0.04 | 0.27 | 8.99E-01 | -0.13 | 0.29 | 8.45E-01 | -0.18 | 0.29 | 7.42E-01 |
| Cholesteryl esters in VLDL | 0.14 | 0.19 | 6.74E-01 | 0.05 | 0.20 | 8.98E-01 | 0.15 | 0.27 | 6.90E-01 | 0.14 | 0.28 | 6.96E-01 | -0.15 | 0.30 | 8.45E-01 | -0.18 | 0.30 | 7.42E-01 |
| Albumin | -0.02 | 0.19 | 9.52E-01 | -0.04 | 0.20 | 9.02E-01 | 0.36 | 0.26 | 3.66E-01 | 0.32 | 0.26 | 3.90E-01 | -0.48 | 0.31 | 3.31E-01 | -0.62 | 0.32 | 1.81E-01 |
| Cholesterol to total lipids ratio in very large VLDL | 0.03 | 0.20 | 9.30E-01 | -0.05 | 0.22 | 9.02E-01 | -0.25 | 0.28 | 5.11E-01 | -0.29 | 0.29 | 4.74E-01 | 0.27 | 0.32 | 6.92E-01 | 0.35 | 0.33 | 5.44E-01 |
| Phospholipids in small VLDL | 0.10 | 0.19 | 7.54E-01 | 0.04 | 0.20 | 9.02E-01 | 0.15 | 0.26 | 6.68E-01 | 0.14 | 0.27 | 6.93E-01 | -0.16 | 0.29 | 8.45E-01 | -0.19 | 0.29 | 7.26E-01 |
| Glutamine | -0.05 | 0.19 | 8.78E-01 | -0.04 | 0.20 | 9.03E-01 | -0.01 | 0.26 | 9.80E-01 | 0.00 | 0.26 | 9.96E-01 | -0.04 | 0.32 | 9.49E-01 | -0.09 | 0.33 | 8.85E-01 |
| Acetoacetate | -0.06 | 0.19 | 8.40E-01 | -0.04 | 0.19 | 9.05E-01 | 0.12 | 0.24 | 7.31E-01 | 0.13 | 0.25 | 6.96E-01 | -0.35 | 0.31 | 5.31E-01 | -0.28 | 0.32 | 6.46E-01 |
| Total lipids in chylomicrons and extremely large VLDL | 0.03 | 0.20 | 9.41E-01 | 0.04 | 0.21 | 9.12E-01 | 0.37 | 0.29 | 3.88E-01 | 0.40 | 0.30 | 3.64E-01 | -0.39 | 0.29 | 4.22E-01 | -0.49 | 0.31 | 2.82E-01 |
| Triglycerides in large VLDL | 0.04 | 0.19 | 9.10E-01 | 0.04 | 0.20 | 9.12E-01 | 0.17 | 0.26 | 6.27E-01 | 0.17 | 0.27 | 6.36E-01 | -0.16 | 0.31 | 8.45E-01 | -0.25 | 0.31 | 6.90E-01 |
| Phospholipids to total lipids ratio in very large HDL | 0.01 | 0.21 | 9.74E-01 | -0.04 | 0.22 | 9.20E-01 | -0.24 | 0.23 | 4.57E-01 | -0.23 | 0.24 | 4.75E-01 | 0.76 | 0.80 | 6.31E-01 | 1.05 | 0.87 | 4.66E-01 |
| Cholesteryl esters to total lipids ratio in large VLDL | 0.10 | 0.19 | 7.54E-01 | 0.04 | 0.21 | 9.20E-01 | -0.11 | 0.26 | 7.61E-01 | -0.12 | 0.27 | 7.35E-01 | 0.20 | 0.32 | 8.32E-01 | 0.28 | 0.33 | 6.56E-01 |
| Phospholipids to total lipids ratio in chylomicrons and extremely large VLDL | 0.01 | 0.20 | 9.74E-01 | 0.03 | 0.21 | 9.21E-01 | 0.46 | 0.44 | 4.51E-01 | 0.47 | 0.44 | 4.35E-01 | -0.43 | 0.28 | 3.40E-01 | -0.50 | 0.29 | 2.42E-01 |
| Cholesterol in very small VLDL | 0.08 | 0.20 | 8.04E-01 | -0.04 | 0.22 | 9.21E-01 | -0.02 | 0.29 | 9.60E-01 | 0.00 | 0.29 | 9.94E-01 | -0.18 | 0.32 | 8.45E-01 | -0.16 | 0.33 | 7.63E-01 |
| Total lipids in medium VLDL | 0.08 | 0.19 | 8.04E-01 | 0.03 | 0.20 | 9.21E-01 | 0.10 | 0.26 | 7.86E-01 | 0.08 | 0.27 | 8.25E-01 | -0.08 | 0.29 | 8.81E-01 | -0.15 | 0.30 | 7.63E-01 |
| Acetone | -0.05 | 0.19 | 8.90E-01 | -0.03 | 0.20 | 9.36E-01 | -0.26 | 0.28 | 5.03E-01 | -0.26 | 0.29 | 5.00E-01 | 0.25 | 0.27 | 6.57E-01 | 0.31 | 0.28 | 5.38E-01 |
| Monounsaturated fatty acids | 0.01 | 0.19 | 9.73E-01 | 0.02 | 0.20 | 9.47E-01 | 0.16 | 0.26 | 6.63E-01 | 0.16 | 0.26 | 6.48E-01 | -0.23 | 0.31 | 7.65E-01 | -0.28 | 0.32 | 6.46E-01 |
| Cholesterol to total lipids ratio in chylomicrons and extremely large VLDL | 0.03 | 0.19 | 9.47E-01 | 0.02 | 0.20 | 9.72E-01 | -0.22 | 0.28 | 5.61E-01 | -0.23 | 0.30 | 5.53E-01 | 0.27 | 0.29 | 6.31E-01 | 0.34 | 0.29 | 4.88E-01 |
| Average diameter for VLDL particles | 0.00 | 0.19 | 9.91E-01 | 0.01 | 0.20 | 9.83E-01 | 0.20 | 0.25 | 5.54E-01 | 0.20 | 0.27 | 5.78E-01 | -0.24 | 0.33 | 7.65E-01 | -0.35 | 0.33 | 5.54E-01 |
| Saturated fatty acids | 0.00 | 0.20 | 9.91E-01 | -0.01 | 0.21 | 9.86E-01 | 0.24 | 0.26 | 5.03E-01 | 0.26 | 0.27 | 4.74E-01 | -0.43 | 0.32 | 4.14E-01 | -0.49 | 0.33 | 3.20E-01 |
| Cholesteryl esters to total lipids ratio in chylomicrons and extremely large VLDL | 0.02 | 0.19 | 9.54E-01 | 0.00 | 0.21 | 9.96E-01 | -0.24 | 0.29 | 5.33E-01 | -0.26 | 0.30 | 5.17E-01 | 0.27 | 0.28 | 6.31E-01 | 0.33 | 0.29 | 4.88E-01 |
| Acetate | 0.03 | 0.20 | 9.41E-01 | 0.00 | 0.20 | 9.96E-01 | -0.19 | 0.26 | 5.79E-01 | -0.22 | 0.27 | 5.43E-01 | 0.29 | 0.34 | 6.73E-01 | 0.35 | 0.35 | 5.84E-01 |
| Phospholipids in medium VLDL | 0.07 | 0.19 | 8.37E-01 | 0.00 | 0.20 | 9.96E-01 | 0.05 | 0.27 | 8.90E-01 | 0.03 | 0.27 | 9.28E-01 | -0.12 | 0.29 | 8.51E-01 | -0.17 | 0.29 | 7.42E-01 |
| Concentration of medium VLDL particles | 0.07 | 0.19 | 8.37E-01 | 0.00 | 0.20 | 9.96E-01 | 0.05 | 0.26 | 8.92E-01 | 0.03 | 0.27 | 9.36E-01 | -0.10 | 0.29 | 8.80E-01 | -0.16 | 0.30 | 7.61E-01 |
| Free cholesterol in small VLDL | 0.08 | 0.19 | 8.07E-01 | -0.01 | 0.20 | 9.96E-01 | 0.03 | 0.27 | 9.43E-01 | 0.01 | 0.27 | 9.78E-01 | -0.12 | 0.30 | 8.55E-01 | -0.15 | 0.30 | 7.68E-01 |
| Triglycerides to total lipids ratio in very large VLDL | -0.06 | 0.19 | 8.40E-01 | 0.01 | 0.21 | 9.96E-01 | 0.05 | 0.27 | 8.92E-01 | 0.07 | 0.28 | 8.64E-01 | -0.06 | 0.33 | 9.23E-01 | -0.11 | 0.33 | 8.61E-01 |
| Cholesteryl esters to total lipids ratio in small VLDL | 0.10 | 0.20 | 7.54E-01 | 0.00 | 0.21 | 9.96E-01 | 0.01 | 0.28 | 9.65E-01 | 0.02 | 0.28 | 9.55E-01 | -0.09 | 0.31 | 8.81E-01 | -0.03 | 0.31 | 9.56E-01 |
| Free cholesterol to total lipids ratio in chylomicrons and extremely large VLDL | -0.02 | 0.19 | 9.52E-01 | 0.00 | 0.20 | 9.99E-01 | -0.08 | 0.26 | 8.17E-01 | -0.07 | 0.26 | 8.48E-01 | 0.02 | 0.31 | 9.64E-01 | 0.08 | 0.31 | 8.85E-01 |

Note: The table shows associations from the logistic regression analysis where plasms metabolomics level from the Nightingale platform was main exposure and prevalent hematological malignancy as the main outcome. Model 1 : adjusted for age, sex, cohort, time differences (ERGO), and lipid lowering medication. Model 2: adjusted for model1, smoking status, BMI, alcohol and education. No adjustment for time differences was made in the analyses of the subcohorts (ERGO-4 and ERGO-5). The table is ranked based on the *FDR* values in model 2 within the E4&E5 cohort, using bold text indicates metabolites are surpassing *FDR* adjusted *P* value treshold (*P*<0.05). ERGO-4 and ERGO-5 refer to the fourth and fifth visits subcohort from the baseline cohort. ERGO-4&5 refers to the combined data from the fourth and fifth visits of the baseline cohort. Abbreviations: SE, standard erorr; IDL, intermediate-density lipoprotein; LDL, low-density lipoprotein; HDL, high-density lipoprotein; VLDL, very-low-density lipoprotein; BMI, body mass index; FDR, false discovery rate.

**Supplementary Table 6. Serum metaboloites associated with prevalent hematological malignancy from the Metabolon platform at baseline.**

| **Metabolite** | **ERGO-4&5** | | | | | | | | **ERGO-4** | | | | **ERGO-5** | | | |
| --- | --- | --- | --- | --- | --- | --- | --- | --- | --- | --- | --- | --- | --- | --- | --- | --- |
|  | **Model 1** | | | | **Model 2** | | | | **Model 1** | | **Model 2** | | **Model 1** | | **Model 2** | |
|  | **Beta±SE** | **I^2^** | **Het.*P-*value** | ***FDR* value** | **Beta±SE** | **I^2^** | **Het.*P-*value** | ***FDR* value** | **Beta±SE** | ***FDR* value** | **Beta±SE** | ***FDR* value** | **Beta±SE** | ***FDR* value** | **Beta±SE** | ***FDR* value** |
| N-acetyl-aspartyl-glutamate (NAAG) | 1.22±0.29 | 0 | 4.27E-01 | **3.02E-02** | 1.33±0.32 | 0 | 5.64E-01 | **3.10E-02** | 1.41±0.38 | **2.87E-02** | 1.48±0.41 | **4.82E-02** | 0.94±0.45 | 9.98E-01 | 1.1±0.52 | 9.91E-01 |
| phosphoethanolamine | 0.96±0.25 | 59.18 | 1.18E-01 | 6.04E-02 | 1.09±0.28 | 37.76 | 2.05E-01 | **3.44E-02** | 1.18±0.29 | **1.45E-02** | 1.26±0.31 | **1.25E-02** | 0.26±0.51 | 9.98E-01 | 0.39±0.62 | 9.91E-01 |
| quinolinate | 0.69±0.2 | 0 | 5.77E-01 | 9.33E-02 | 0.78±0.21 | 0 | 7.66E-01 | 7.97E-02 | 0.76±0.23 | 6.79E-02 | 0.81±0.24 | 5.98E-02 | 0.51±0.38 | 9.98E-01 | 0.65±0.48 | 9.91E-01 |
| octadecadienedioate (C18:2-DC)* | -0.99±0.3 | 0 | 4.97E-01 | 1.02E-01 | -1.17±0.33 | 0 | 9.35E-01 | 9.34E-02 | -1.17±0.4 | 9.95E-02 | -1.19±0.41 | 1.28E-01 | -0.77±0.44 | 9.98E-01 | -1.13±0.55 | 9.91E-01 |
| gamma-glutamylisoleucine* | 0.95±0.28 | 0 | 4.03E-01 | 1.02E-01 | 1.03±0.3 | 12.52 | 2.85E-01 | 1.03E-01 | 1.08±0.32 | **4.65E-02** | 1.19±0.33 | **4.97E-02** | 0.5±0.61 | 9.98E-01 | 0.41±0.65 | 9.91E-01 |
| 2-hydroxy-3-methylvalerate | 0.84±0.23 | 0 | 4.84E-01 | 6.49E-02 | 0.79±0.23 | 0 | 4.57E-01 | 1.13E-01 | 0.92±0.25 | **2.87E-02** | 0.87±0.26 | 6.41E-02 | 0.51±0.53 | 9.98E-01 | 0.43±0.53 | 9.91E-01 |
| 7-methylguanine | 0.97±0.27 | 0 | 6.00E-01 | 7.04E-02 | 0.93±0.28 | 0 | 7.02E-01 | 1.29E-01 | 1.03±0.29 | **3.37E-02** | 0.98±0.31 | 8.82E-02 | 0.65±0.66 | 9.98E-01 | 0.68±0.71 | 9.91E-01 |
| lactosyl-N-palmitoyl-sphingosine (d18:1/16:0) | -0.56±0.18 | 88.75 | 2.87E-03 | 1.73E-01 | -0.66±0.21 | 88.05 | 3.82E-03 | 1.79E-01 | -1.36±0.32 | **1.45E-02** | -1.64±0.4 | **1.25E-02** | -0.19±0.22 | 9.98E-01 | -0.28±0.25 | 9.91E-01 |
| 1-palmitoyl-GPE (16:0) | 0.82±0.26 | 0 | 6.23E-01 | 1.73E-01 | 0.88±0.28 | 0 | 7.08E-01 | 1.79E-01 | 0.88±0.3 | 9.64E-02 | 0.93±0.32 | 1.23E-01 | 0.57±0.56 | 9.98E-01 | 0.69±0.57 | 9.91E-01 |
| adenosine 5'-monophosphate (AMP) | 0.6±0.2 | 76.35 | 3.98E-02 | 1.77E-01 | 0.64±0.21 | 73.59 | 5.17E-02 | 2.18E-01 | 0.81±0.23 | **2.88E-02** | 0.87±0.24 | **4.82E-02** | -0.17±0.42 | 9.98E-01 | -0.09±0.43 | 9.91E-01 |
| cinnamoylglycine | -0.58±0.18 | 46.66 | 1.71E-01 | 1.24E-01 | -0.56±0.18 | 37.59 | 2.06E-01 | 2.18E-01 | -0.68±0.19 | **3.37E-02** | -0.65±0.2 | 7.80E-02 | -0.01±0.45 | 9.98E-01 | 0.05±0.51 | 9.91E-01 |
| sphingomyelin (d18:1/22:1, d18:2/22:0, d16:1/24:1)* | -0.63±0.22 | 89.49 | 2.04E-03 | 2.32E-01 | -0.7±0.24 | 87.67 | 4.40E-03 | 2.46E-01 | -1.47±0.35 | **1.45E-02** | -1.51±0.37 | **1.25E-02** | -0.06±0.29 | 9.98E-01 | -0.14±0.31 | 9.91E-01 |
| 1-(1-enyl-palmitoyl)-2-linoleoyl-GPC (P-16:0/18:2)* | -0.77±0.28 | 91.05 | 8.30E-04 | 2.47E-01 | -0.86±0.31 | 91.74 | 5.01E-04 | 2.57E-01 | -1.22±0.31 | **1.68E-02** | -1.39±0.34 | **1.25E-02** | 1.15±0.64 | 9.98E-01 | 1.34±0.7 | 9.91E-01 |
| sphingomyelin (d18:1/20:1, d18:2/20:0)* | -0.7±0.24 | 81.7 | 1.94E-02 | 1.77E-01 | -0.68±0.24 | 75.89 | 4.17E-02 | 2.57E-01 | -1.08±0.29 | **2.52E-02** | -1.05±0.3 | 5.02E-02 | 0.09±0.41 | 9.98E-01 | -0.05±0.39 | 9.91E-01 |
| lactosyl-N-nervonoyl-sphingosine (d18:1/24:1)* | -0.53±0.2 | 74.59 | 4.73E-02 | 2.64E-01 | -0.67±0.23 | 69.07 | 7.21E-02 | 2.57E-01 | -1.07±0.34 | 7.25E-02 | -1.19±0.37 | 8.82E-02 | -0.24±0.25 | 9.98E-01 | -0.34±0.29 | 9.91E-01 |
| alpha-hydroxyisovalerate | 0.68±0.21 | 52.17 | 1.48E-01 | 1.02E-01 | 0.61±0.22 | 40.55 | 1.95E-01 | 2.57E-01 | 0.86±0.24 | **2.88E-02** | 0.77±0.25 | 1.10E-01 | 0.17±0.41 | 9.98E-01 | 0.08±0.46 | 9.91E-01 |
| 11beta-hydroxyetiocholanolone glucuronide* | -0.56±0.18 | 0 | 3.38E-01 | 1.73E-01 | -0.54±0.19 | 0 | 3.34E-01 | 2.57E-01 | -0.65±0.2 | 7.25E-02 | -0.63±0.21 | 1.23E-01 | -0.22±0.4 | 9.98E-01 | -0.18±0.42 | 9.91E-01 |
| androsterone sulfate | -0.56±0.19 | 0 | 5.86E-01 | 1.77E-01 | -0.54±0.19 | 0 | 3.87E-01 | 2.57E-01 | -0.62±0.22 | 1.25E-01 | -0.64±0.22 | 1.28E-01 | -0.39±0.36 | 9.98E-01 | -0.25±0.39 | 9.91E-01 |
| 1-stearoyl-GPE (18:0) | 0.74±0.27 | 0 | 4.41E-01 | 2.62E-01 | 0.79±0.29 | 0 | 4.16E-01 | 2.57E-01 | 0.86±0.32 | 1.49E-01 | 0.93±0.33 | 1.45E-01 | 0.39±0.53 | 9.98E-01 | 0.4±0.56 | 9.91E-01 |
| hexadecenedioate (C16:1-DC)* | -0.63±0.25 | 0 | 5.74E-01 | 2.73E-01 | -0.78±0.28 | 0 | 6.36E-01 | 2.57E-01 | -0.79±0.37 | 3.26E-01 | -0.89±0.37 | 2.30E-01 | -0.5±0.34 | 9.98E-01 | -0.63±0.42 | 9.91E-01 |
| cortisone | -0.31±0.13 | 0 | 9.53E-01 | 3.08E-01 | -0.39±0.14 | 0 | 8.12E-01 | 2.57E-01 | -0.32±0.15 | 3.30E-01 | -0.38±0.16 | 2.30E-01 | -0.3±0.28 | 9.98E-01 | -0.46±0.32 | 9.91E-01 |
| octadecenedioate (C18:1-DC) | -0.54±0.21 | 0 | 5.36E-01 | 2.73E-01 | -0.68±0.24 | 0 | 7.95E-01 | 2.57E-01 | -0.7±0.34 | 3.38E-01 | -0.75±0.34 | 2.97E-01 | -0.43±0.27 | 9.98E-01 | -0.62±0.35 | 9.91E-01 |
| palmitoyl dihydrosphingomyelin (d18:0/16:0)* | -0.57±0.26 | 71.24 | 6.22E-02 | 3.48E-01 | -0.72±0.28 | 67.05 | 8.15E-02 | 2.64E-01 | -0.89±0.31 | 1.17E-01 | -0.97±0.31 | 1.01E-01 | 0.17±0.47 | 9.98E-01 | 0.23±0.62 | 9.91E-01 |
| 1-(1-enyl-palmitoyl)-2-oleoyl-GPC (P-16:0/18:1)* | -0.45±0.19 | 51.88 | 1.49E-01 | 3.08E-01 | -0.57±0.21 | 69.58 | 6.98E-02 | 2.64E-01 | -0.54±0.2 | 1.49E-01 | -0.68±0.22 | 1.05E-01 | 0.35±0.58 | 9.98E-01 | 0.73±0.75 | 9.91E-01 |
| beta-citrylglutamate | 0.72±0.24 | 0 | 3.45E-01 | 1.77E-01 | 0.66±0.24 | 25.46 | 2.47E-01 | 2.64E-01 | 0.83±0.27 | 7.60E-02 | 0.83±0.28 | 1.23E-01 | 0.29±0.5 | 9.98E-01 | 0.18±0.49 | 9.91E-01 |
| orotidine | 0.38±0.14 | 28 | 2.39E-01 | 2.62E-01 | 0.41±0.16 | 21.67 | 2.59E-01 | 2.64E-01 | 0.44±0.15 | 9.89E-02 | 0.48±0.17 | 1.28E-01 | -0.04±0.38 | 9.98E-01 | -0.01±0.4 | 9.91E-01 |
| 9,10-DiHOME | -0.58±0.24 | 50.54 | 1.55E-01 | 2.87E-01 | -0.66±0.25 | 37.22 | 2.07E-01 | 2.64E-01 | -0.82±0.29 | 1.34E-01 | -0.89±0.31 | 1.28E-01 | -0.1±0.42 | 9.98E-01 | -0.2±0.45 | 9.91E-01 |
| lignoceroyl sphingomyelin (d18:1/24:0) | -0.42±0.18 | 61.94 | 1.05E-01 | 3.13E-01 | -0.52±0.2 | 53.02 | 1.45E-01 | 2.64E-01 | -0.78±0.29 | 1.49E-01 | -0.81±0.28 | 1.28E-01 | -0.17±0.24 | 9.98E-01 | -0.23±0.28 | 9.91E-01 |
| glutarate (C5-DC) | 0.76±0.29 | 27.65 | 2.40E-01 | 2.64E-01 | 0.81±0.31 | 19.55 | 2.65E-01 | 2.64E-01 | 1.05±0.37 | 1.34E-01 | 1.09±0.4 | 1.67E-01 | 0.36±0.44 | 9.98E-01 | 0.38±0.49 | 9.91E-01 |
| N2,N2-dimethylguanosine | 0.68±0.26 | 0 | 8.01E-01 | 2.64E-01 | 0.69±0.27 | 0 | 8.99E-01 | 2.64E-01 | 0.71±0.28 | 2.01E-01 | 0.7±0.29 | 2.21E-01 | 0.53±0.64 | 9.98E-01 | 0.6±0.75 | 9.91E-01 |
| 2-piperidinone | 0.7±0.25 | 0 | 7.50E-01 | 2.32E-01 | 0.69±0.26 | 0 | 6.27E-01 | 2.64E-01 | 0.76±0.31 | 2.30E-01 | 0.78±0.32 | 2.21E-01 | 0.59±0.41 | 9.98E-01 | 0.51±0.46 | 9.91E-01 |
| corticosterone | -0.49±0.2 | 0 | 9.34E-01 | 2.85E-01 | -0.55±0.21 | 0 | 9.81E-01 | 2.64E-01 | -0.51±0.24 | 3.26E-01 | -0.56±0.24 | 2.65E-01 | -0.47±0.38 | 9.98E-01 | -0.54±0.43 | 9.91E-01 |
| 11-ketoetiocholanolone glucuronide | -0.55±0.19 | 0 | 8.78E-01 | 1.81E-01 | -0.52±0.2 | 0 | 7.04E-01 | 2.64E-01 | -0.54±0.21 | 1.92E-01 | -0.48±0.22 | 2.89E-01 | -0.61±0.41 | 9.98E-01 | -0.68±0.45 | 9.91E-01 |
| cortisol | -0.37±0.15 | 0 | 6.91E-01 | 2.83E-01 | -0.44±0.16 | 0 | 5.56E-01 | 2.64E-01 | -0.34±0.17 | 3.63E-01 | -0.4±0.18 | 2.96E-01 | -0.48±0.31 | 9.98E-01 | -0.65±0.38 | 9.91E-01 |
| octadecanedioate (C18-DC) | -0.57±0.22 | 0 | 7.64E-01 | 2.73E-01 | -0.64±0.24 | 0 | 6.96E-01 | 2.64E-01 | -0.51±0.29 | 4.61E-01 | -0.58±0.3 | 3.90E-01 | -0.64±0.34 | 9.98E-01 | -0.77±0.41 | 9.91E-01 |
| isoleucine | 0.77±0.27 | 53.2 | 1.44E-01 | 1.98E-01 | 0.62±0.25 | 79.42 | 2.75E-02 | 2.79E-01 | 1.02±0.32 | 6.91E-02 | 1.08±0.32 | 6.68E-02 | 0.17±0.49 | 9.98E-01 | -0.04±0.39 | 9.91E-01 |
| histidine | -0.54±0.25 | 75.49 | 4.34E-02 | 3.48E-01 | -0.63±0.25 | 57.39 | 1.26E-01 | 2.79E-01 | -1.1±0.37 | 9.64E-02 | -1.08±0.38 | 1.36E-01 | -0.1±0.33 | 9.98E-01 | -0.3±0.33 | 9.91E-01 |
| androstenediol (3alpha, 17alpha) monosulfate (2) | -0.49±0.2 | 0 | 5.63E-01 | 2.85E-01 | -0.53±0.21 | 0 | 6.59E-01 | 2.79E-01 | -0.55±0.22 | 2.25E-01 | -0.57±0.23 | 2.21E-01 | -0.26±0.45 | 9.98E-01 | -0.34±0.47 | 9.91E-01 |
| phenyllactate (PLA) | 0.64±0.23 | 0 | 7.52E-01 | 2.59E-01 | 0.63±0.25 | 0 | 7.23E-01 | 2.79E-01 | 0.68±0.27 | 1.88E-01 | 0.67±0.28 | 2.30E-01 | 0.5±0.49 | 9.98E-01 | 0.47±0.51 | 9.91E-01 |
| epiandrosterone sulfate | -0.55±0.21 | 0 | 8.35E-01 | 2.69E-01 | -0.53±0.21 | 0 | 6.35E-01 | 2.79E-01 | -0.58±0.25 | 2.71E-01 | -0.59±0.25 | 2.30E-01 | -0.48±0.39 | 9.98E-01 | -0.36±0.42 | 9.91E-01 |
| imidazole propionate | 0.54±0.2 | 0 | 3.66E-01 | 2.62E-01 | 0.53±0.21 | 0 | 3.21E-01 | 2.79E-01 | 0.42±0.24 | 4.62E-01 | 0.39±0.25 | 5.26E-01 | 0.8±0.35 | 9.98E-01 | 0.85±0.39 | 9.91E-01 |
| glutamine_degradant* | 0.74±0.3 | 41.61 | 1.91E-01 | 2.85E-01 | 0.78±0.31 | 54.05 | 1.40E-01 | 2.79E-01 | 0.53±0.35 | 5.53E-01 | 0.53±0.35 | 5.61E-01 | 1.47±0.63 | 9.98E-01 | 1.64±0.66 | 9.91E-01 |
| 5alpha-pregnan-3beta,20beta-diol monosulfate (1) | -0.59±0.22 | 71.8 | 5.97E-02 | 2.64E-01 | -0.57±0.23 | 76.84 | 3.77E-02 | 2.83E-01 | -0.83±0.26 | 6.79E-02 | -0.87±0.27 | 8.82E-02 | 0.13±0.44 | 9.98E-01 | 0.2±0.44 | 9.91E-01 |
| X - 12216 | -0.63±0.24 | 66.44 | 8.43E-02 | 2.64E-01 | -0.62±0.25 | 62.89 | 1.01E-01 | 2.83E-01 | -0.88±0.28 | 7.46E-02 | -0.87±0.29 | 1.23E-01 | 0.05±0.46 | 9.98E-01 | 0.04±0.47 | 9.91E-01 |
| hexadecanedioate (C16-DC) | -0.63±0.27 | 0 | 7.71E-01 | 3.08E-01 | -0.73±0.29 | 0 | 6.96E-01 | 2.83E-01 | -0.7±0.36 | 3.72E-01 | -0.81±0.36 | 2.72E-01 | -0.54±0.42 | 9.98E-01 | -0.56±0.51 | 9.91E-01 |
| X - 15728 | -0.55±0.22 | 0 | 8.97E-01 | 2.73E-01 | -0.56±0.23 | 0 | 8.58E-01 | 2.83E-01 | -0.57±0.26 | 3.16E-01 | -0.59±0.28 | 3.22E-01 | -0.51±0.4 | 9.98E-01 | -0.5±0.41 | 9.91E-01 |
| sphingomyelin (d18:1/20:0, d16:1/22:0)* | -0.55±0.24 | 87.54 | 4.61E-03 | 3.08E-01 | -0.58±0.24 | 84.24 | 1.18E-02 | 3.09E-01 | -1.39±0.38 | **2.87E-02** | -1.35±0.39 | 5.21E-02 | -0.02±0.3 | 9.98E-01 | -0.1±0.31 | 9.91E-01 |
| lactosyl-N-behenoyl-sphingosine (d18:1/22:0)* | -0.49±0.21 | 0 | 3.89E-01 | 3.08E-01 | -0.52±0.22 | 0 | 3.86E-01 | 3.09E-01 | -0.61±0.25 | 2.33E-01 | -0.65±0.26 | 2.21E-01 | -0.22±0.37 | 9.98E-01 | -0.23±0.4 | 9.91E-01 |
| X - 12816 | -0.5±0.2 | 0 | 7.91E-01 | 2.85E-01 | -0.5±0.21 | 0 | 7.39E-01 | 3.09E-01 | -0.53±0.24 | 3.03E-01 | -0.54±0.24 | 2.72E-01 | -0.41±0.39 | 9.98E-01 | -0.38±0.42 | 9.91E-01 |
| indolelactate | 0.67±0.27 | 0 | 9.29E-01 | 2.85E-01 | 0.66±0.27 | 0 | 9.73E-01 | 3.09E-01 | 0.65±0.32 | 3.38E-01 | 0.65±0.32 | 3.52E-01 | 0.71±0.54 | 9.98E-01 | 0.67±0.54 | 9.91E-01 |
| dimethylglycine | 0.5±0.21 | 0 | 4.61E-01 | 3.04E-01 | 0.52±0.22 | 0 | 4.69E-01 | 3.13E-01 | 0.59±0.24 | 2.30E-01 | 0.6±0.25 | 2.21E-01 | 0.22±0.44 | 9.98E-01 | 0.21±0.49 | 9.91E-01 |
| 1-linoleoyl-GPE (18:2)* | 0.59±0.27 | 0 | 5.76E-01 | 3.48E-01 | 0.7±0.29 | 0 | 3.94E-01 | 3.13E-01 | 0.68±0.32 | 3.26E-01 | 0.86±0.35 | 2.21E-01 | 0.35±0.49 | 9.98E-01 | 0.31±0.54 | 9.91E-01 |
| bilirubin degradation product, C17H18N2O4 (1)** | -0.41±0.21 | 0 | 9.40E-01 | 4.63E-01 | -0.53±0.22 | 0 | 9.18E-01 | 3.13E-01 | -0.42±0.26 | 5.14E-01 | -0.51±0.28 | 4.24E-01 | -0.39±0.34 | 9.98E-01 | -0.56±0.37 | 9.91E-01 |
| X - 17690 | 0.54±0.24 | 0 | 7.38E-01 | 3.45E-01 | 0.59±0.25 | 0 | 4.90E-01 | 3.13E-01 | 0.48±0.3 | 5.09E-01 | 0.48±0.3 | 5.23E-01 | 0.65±0.42 | 9.98E-01 | 0.85±0.45 | 9.91E-01 |
| serine | -0.46±0.22 | 66.36 | 8.47E-02 | 4.20E-01 | -0.54±0.23 | 64.21 | 9.46E-02 | 3.13E-01 | -0.75±0.28 | 1.49E-01 | -0.83±0.29 | 1.28E-01 | 0.05±0.37 | 9.98E-01 | -0.04±0.38 | 9.91E-01 |
| 5alpha-androstan-3beta,17alpha-diol disulfate | -0.66±0.26 | 0 | 4.62E-01 | 2.73E-01 | -0.63±0.27 | 0 | 3.37E-01 | 3.13E-01 | -0.82±0.34 | 2.30E-01 | -0.83±0.34 | 2.21E-01 | -0.42±0.42 | 9.98E-01 | -0.3±0.43 | 9.91E-01 |
| N-palmitoyl-sphinganine (d18:0/16:0) | 0.61±0.26 | 0 | 4.22E-01 | 3.08E-01 | 0.67±0.29 | 0 | 5.60E-01 | 3.13E-01 | 0.78±0.34 | 2.69E-01 | 0.79±0.35 | 2.72E-01 | 0.34±0.43 | 9.98E-01 | 0.44±0.49 | 9.91E-01 |
| nicotinamide | 0.48±0.2 | 92.34 | 3.02E-04 | 3.04E-01 | 0.5±0.21 | 92.84 | 1.86E-04 | 3.14E-01 | 0.94±0.24 | **1.68E-02** | 1.04±0.26 | **1.25E-02** | -0.67±0.38 | 9.98E-01 | -0.68±0.38 | 9.91E-01 |
| sphingomyelin (d18:2/18:1)* | -0.69±0.27 | 77.99 | 3.31E-02 | 2.73E-01 | -0.62±0.27 | 69.66 | 6.94E-02 | 3.14E-01 | -1.06±0.32 | 5.55E-02 | -0.98±0.33 | 1.23E-01 | 0.18±0.49 | 9.98E-01 | 0.03±0.44 | 9.91E-01 |
| myristoyl dihydrosphingomyelin (d18:0/14:0)* | -0.65±0.26 | 77.09 | 3.67E-02 | 2.83E-01 | -0.61±0.27 | 75.75 | 4.23E-02 | 3.14E-01 | -0.9±0.29 | 7.53E-02 | -0.86±0.29 | 1.23E-01 | 0.53±0.62 | 9.98E-01 | 0.56±0.64 | 9.91E-01 |
| spermidine | 0.32±0.15 | 12.57 | 2.85E-01 | 3.48E-01 | 0.35±0.15 | 43.83 | 1.82E-01 | 3.14E-01 | 0.39±0.16 | 2.30E-01 | 0.43±0.17 | 2.02E-01 | -0.07±0.39 | 9.98E-01 | -0.17±0.42 | 9.91E-01 |
| choline phosphate | 0.52±0.25 | 0 | 4.53E-01 | 4.20E-01 | 0.61±0.26 | 0 | 4.71E-01 | 3.14E-01 | 0.66±0.31 | 3.26E-01 | 0.74±0.32 | 2.60E-01 | 0.26±0.43 | 9.98E-01 | 0.34±0.46 | 9.91E-01 |
| 1-palmitoyl-2-oleoyl-GPE (16:0/18:1) | 0.64±0.28 | 0 | 9.88E-01 | 3.20E-01 | 0.68±0.29 | 0 | 7.60E-01 | 3.14E-01 | 0.64±0.34 | 4.09E-01 | 0.63±0.34 | 4.10E-01 | 0.65±0.51 | 9.98E-01 | 0.85±0.62 | 9.91E-01 |
| eicosanedioate (C20-DC) | -0.55±0.25 | 0 | 7.96E-01 | 3.48E-01 | -0.61±0.26 | 0 | 9.91E-01 | 3.14E-01 | -0.6±0.32 | 4.10E-01 | -0.61±0.33 | 4.24E-01 | -0.47±0.39 | 9.98E-01 | -0.61±0.43 | 9.91E-01 |
| X - 18899 | -0.49±0.21 | 0 | 8.38E-01 | 3.08E-01 | -0.49±0.21 | 0 | 6.26E-01 | 3.14E-01 | -0.46±0.25 | 4.39E-01 | -0.42±0.26 | 5.15E-01 | -0.55±0.37 | 9.98E-01 | -0.64±0.37 | 9.91E-01 |
| 3-phenylpropionate (hydrocinnamate) | -0.5±0.19 | 59.21 | 1.17E-01 | 2.73E-01 | -0.47±0.2 | 49.14 | 1.61E-01 | 3.15E-01 | -0.6±0.2 | 9.89E-02 | -0.57±0.22 | 2.02E-01 | 0.36±0.58 | 9.98E-01 | 0.35±0.61 | 9.91E-01 |
| X - 25419 | 0.52±0.23 | 50.04 | 1.57E-01 | 3.20E-01 | 0.56±0.24 | 0 | 4.29E-01 | 3.15E-01 | 0.77±0.29 | 1.53E-01 | 0.69±0.3 | 2.51E-01 | 0.08±0.39 | 9.98E-01 | 0.29±0.42 | 9.91E-01 |
| 3-methyl-2-oxovalerate | 0.67±0.28 | 72.32 | 5.73E-02 | 2.85E-01 | 0.65±0.29 | 80.02 | 2.53E-02 | 3.48E-01 | 1.12±0.36 | 7.60E-02 | 1.21±0.38 | 9.23E-02 | 0.06±0.42 | 9.98E-01 | -0.11±0.45 | 9.91E-01 |
| sphingomyelin (d18:2/23:0, d18:1/23:1, d17:1/24:1)* | -0.65±0.27 | 76.29 | 4.00E-02 | 3.08E-01 | -0.66±0.3 | 67.93 | 7.74E-02 | 3.48E-01 | -1.03±0.33 | 7.60E-02 | -0.98±0.35 | 1.36E-01 | 0.2±0.49 | 9.98E-01 | 0.21±0.58 | 9.91E-01 |
| 1-(1-enyl-palmitoyl)-2-linoleoyl-GPE (P-16:0/18:2)* | -0.5±0.2 | 64.31 | 9.41E-02 | 2.73E-01 | -0.48±0.22 | 40.91 | 1.93E-01 | 3.48E-01 | -0.71±0.23 | 8.18E-02 | -0.66±0.26 | 2.21E-01 | 0.04±0.38 | 9.98E-01 | -0.04±0.4 | 9.91E-01 |
| X - 12707 | -0.42±0.19 | 24.64 | 2.49E-01 | 3.84E-01 | -0.48±0.21 | 0 | 3.67E-01 | 3.48E-01 | -0.54±0.22 | 2.30E-01 | -0.58±0.24 | 2.30E-01 | 0±0.41 | 9.98E-01 | -0.11±0.45 | 9.91E-01 |
| kynurenine | 0.5±0.25 | 0 | 5.13E-01 | 4.54E-01 | 0.58±0.26 | 0 | 3.40E-01 | 3.63E-01 | 0.61±0.3 | 3.38E-01 | 0.73±0.31 | 2.34E-01 | 0.25±0.45 | 9.98E-01 | 0.17±0.51 | 9.91E-01 |
| N-acetylisoleucine | 0.58±0.27 | 0 | 9.62E-01 | 3.86E-01 | 0.61±0.28 | 0 | 8.90E-01 | 3.63E-01 | 0.59±0.31 | 4.10E-01 | 0.59±0.31 | 3.93E-01 | 0.56±0.54 | 9.98E-01 | 0.69±0.64 | 9.91E-01 |
| 1-(1-enyl-stearoyl)-2-linoleoyl-GPE (P-18:0/18:2)* | -0.46±0.2 | 51.32 | 1.52E-01 | 3.08E-01 | -0.46±0.21 | 18.23 | 2.69E-01 | 3.71E-01 | -0.65±0.24 | 1.44E-01 | -0.61±0.25 | 2.22E-01 | -0.06±0.34 | 9.98E-01 | -0.11±0.38 | 9.91E-01 |
| 1-palmitoleoyl-GPC (16:1)* | 0.58±0.26 | 0 | 9.59E-01 | 3.16E-01 | 0.53±0.25 | 0 | 7.79E-01 | 3.73E-01 | 0.59±0.29 | 3.39E-01 | 0.5±0.28 | 4.36E-01 | 0.56±0.53 | 9.98E-01 | 0.67±0.54 | 9.91E-01 |
| sphingomyelin (d17:1/16:0, d18:1/15:0, d16:1/17:0)* | -0.7±0.27 | 76.02 | 4.11E-02 | 2.73E-01 | -0.61±0.29 | 66.68 | 8.32E-02 | 3.82E-01 | -0.97±0.3 | 7.25E-02 | -0.84±0.31 | 1.83E-01 | 0.45±0.62 | 9.98E-01 | 0.47±0.69 | 9.91E-01 |
| X - 12026 | 0.55±0.25 | 0 | 9.49E-01 | 3.48E-01 | 0.55±0.26 | 0 | 9.23E-01 | 3.82E-01 | 0.53±0.33 | 5.16E-01 | 0.53±0.31 | 4.75E-01 | 0.57±0.37 | 9.98E-01 | 0.59±0.45 | 9.91E-01 |
| 1-stearoyl-2-oleoyl-GPE (18:0/18:1) | 0.52±0.25 | 9.57 | 2.93E-01 | 4.38E-01 | 0.57±0.27 | 40.69 | 1.94E-01 | 3.99E-01 | 0.72±0.32 | 3.01E-01 | 0.81±0.33 | 2.21E-01 | 0.17±0.42 | 9.98E-01 | 0.06±0.48 | 9.91E-01 |
| 5alpha-pregnan-3beta,20alpha-diol monosulfate (2) | -0.66±0.26 | 90.17 | 1.42E-03 | 2.73E-01 | -0.55±0.26 | 90.08 | 1.50E-03 | 4.14E-01 | -1.16±0.3 | **2.34E-02** | -1.12±0.32 | **4.99E-02** | 0.71±0.5 | 9.98E-01 | 0.67±0.47 | 9.91E-01 |
| tetradecadienoate (14:2)* | -0.43±0.23 | 0 | 5.77E-01 | 5.07E-01 | -0.5±0.24 | 0 | 6.47E-01 | 4.16E-01 | -0.52±0.28 | 4.16E-01 | -0.57±0.28 | 3.75E-01 | -0.23±0.42 | 9.98E-01 | -0.32±0.45 | 9.91E-01 |
| ethyl alpha-glucopyranoside | 0.56±0.24 | 27.1 | 2.42E-01 | 3.13E-01 | 0.59±0.28 | 0 | 9.66E-01 | 4.16E-01 | 0.76±0.3 | 1.92E-01 | 0.59±0.34 | 4.69E-01 | 0.15±0.42 | 9.98E-01 | 0.57±0.49 | 9.91E-01 |
| X - 17685 | 0.49±0.25 | 0 | 4.54E-01 | 4.79E-01 | 0.52±0.25 | 0 | 4.05E-01 | 4.16E-01 | 0.34±0.32 | 7.33E-01 | 0.36±0.31 | 6.95E-01 | 0.73±0.41 | 9.98E-01 | 0.79±0.41 | 9.91E-01 |
| 1-(1-enyl-palmitoyl)-2-oleoyl-GPE (P-16:0/18:1)* | -0.4±0.21 | 58.69 | 1.20E-01 | 5.02E-01 | -0.49±0.23 | 49.48 | 1.59E-01 | 4.21E-01 | -0.74±0.3 | 2.33E-01 | -0.8±0.33 | 2.21E-01 | -0.07±0.3 | 9.98E-01 | -0.14±0.34 | 9.91E-01 |
| 1-lignoceroyl-GPC (24:0) | -0.46±0.25 | 48.35 | 1.64E-01 | 5.02E-01 | -0.55±0.27 | 52.2 | 1.48E-01 | 4.21E-01 | -0.7±0.3 | 2.67E-01 | -0.78±0.31 | 2.21E-01 | 0.03±0.43 | 9.98E-01 | 0.09±0.52 | 9.91E-01 |
| tetrahydrocortisone glucuronide (5) | -0.34±0.18 | 0 | 3.91E-01 | 5.02E-01 | -0.41±0.2 | 48.21 | 1.65E-01 | 4.21E-01 | -0.23±0.22 | 7.43E-01 | -0.21±0.24 | 7.65E-01 | -0.55±0.3 | 9.98E-01 | -0.8±0.34 | 9.91E-01 |
| sphingomyelin (d18:1/21:0, d17:1/22:0, d16:1/23:0)* | -0.57±0.24 | 72.65 | 5.59E-02 | 3.08E-01 | -0.54±0.26 | 57.26 | 1.26E-01 | 4.22E-01 | -0.85±0.28 | 8.71E-02 | -0.77±0.3 | 2.21E-01 | 0.19±0.46 | 9.98E-01 | 0.18±0.54 | 9.91E-01 |
| dimethylarginine (SDMA + ADMA) | 0.49±0.21 | 35.7 | 2.12E-01 | 3.13E-01 | 0.45±0.22 | 36.76 | 2.09E-01 | 4.22E-01 | 0.67±0.25 | 1.72E-01 | 0.62±0.26 | 2.30E-01 | 0.09±0.39 | 9.98E-01 | 0.01±0.41 | 9.91E-01 |
| 1-myristoyl-2-palmitoyl-GPC (14:0/16:0) | 0.54±0.28 | 0 | 4.57E-01 | 4.71E-01 | 0.58±0.28 | 0 | 5.27E-01 | 4.22E-01 | 0.41±0.33 | 6.71E-01 | 0.46±0.33 | 6.02E-01 | 0.86±0.51 | 9.98E-01 | 0.86±0.52 | 9.91E-01 |
| bilirubin degradation product, C17H18N2O4 (2)** | -0.38±0.22 | 0 | 8.92E-01 | 5.44E-01 | -0.47±0.23 | 0 | 7.11E-01 | 4.25E-01 | -0.35±0.28 | 6.70E-01 | -0.4±0.29 | 6.03E-01 | -0.41±0.34 | 9.98E-01 | -0.58±0.37 | 9.91E-01 |
| 1-linoleoyl-GPI (18:2)* | -0.49±0.26 | 75.37 | 4.39E-02 | 5.02E-01 | -0.53±0.27 | 72.36 | 5.72E-02 | 4.37E-01 | -0.87±0.32 | 1.49E-01 | -0.85±0.32 | 1.85E-01 | 0.24±0.45 | 9.98E-01 | 0.29±0.51 | 9.91E-01 |
| 1-oleoyl-GPI (18:1) | -0.49±0.24 | 68.25 | 7.60E-02 | 4.54E-01 | -0.51±0.26 | 58.96 | 1.19E-01 | 4.37E-01 | -0.8±0.3 | 1.53E-01 | -0.76±0.3 | 2.21E-01 | 0.11±0.42 | 9.98E-01 | 0.12±0.47 | 9.91E-01 |
| 3,5-dichloro-2,6-dihydroxybenzoic acid | -0.41±0.19 | 62.98 | 1.00E-01 | 3.91E-01 | -0.41±0.21 | 54.14 | 1.40E-01 | 4.37E-01 | -0.55±0.21 | 1.68E-01 | -0.59±0.24 | 2.21E-01 | 0.26±0.45 | 9.98E-01 | 0.11±0.41 | 9.91E-01 |
| 1-oleoyl-GPE (18:1) | 0.46±0.23 | 41.55 | 1.91E-01 | 4.63E-01 | 0.5±0.25 | 53 | 1.45E-01 | 4.37E-01 | 0.69±0.29 | 2.49E-01 | 0.74±0.3 | 2.21E-01 | 0.05±0.39 | 9.98E-01 | -0.05±0.45 | 9.91E-01 |
| sphingomyelin (d18:2/24:1, d18:1/24:2)* | -0.42±0.24 | 62.13 | 1.04E-01 | 5.52E-01 | -0.51±0.26 | 59.7 | 1.15E-01 | 4.37E-01 | -0.74±0.31 | 2.60E-01 | -0.82±0.33 | 2.21E-01 | 0.07±0.38 | 9.98E-01 | 0.04±0.43 | 9.91E-01 |
| sphingomyelin (d18:1/24:1, d18:2/24:0)* | -0.33±0.21 | 52.35 | 1.47E-01 | 6.11E-01 | -0.46±0.23 | 59.19 | 1.17E-01 | 4.37E-01 | -0.66±0.31 | 3.26E-01 | -0.82±0.33 | 2.21E-01 | -0.06±0.28 | 9.98E-01 | -0.1±0.33 | 9.91E-01 |
| 2'-O-methylcytidine | 0.62±0.27 | 28.84 | 2.36E-01 | 3.13E-01 | 0.57±0.28 | 28.47 | 2.37E-01 | 4.37E-01 | 0.84±0.33 | 1.88E-01 | 0.8±0.35 | 2.51E-01 | 0.15±0.48 | 9.98E-01 | 0.09±0.5 | 9.91E-01 |
| 2-O-methylascorbic acid | 0.44±0.25 | 24.29 | 2.50E-01 | 5.31E-01 | 0.53±0.26 | 22.91 | 2.55E-01 | 4.37E-01 | 0.67±0.32 | 3.26E-01 | 0.74±0.32 | 2.52E-01 | 0.09±0.39 | 9.98E-01 | 0.08±0.47 | 9.91E-01 |
| gamma-glutamylcitrulline* | -0.34±0.2 | 29.33 | 2.34E-01 | 5.52E-01 | -0.41±0.21 | 0 | 3.61E-01 | 4.37E-01 | -0.52±0.25 | 3.38E-01 | -0.56±0.26 | 3.22E-01 | -0.03±0.33 | 9.98E-01 | -0.17±0.34 | 9.91E-01 |
| 4-methoxyphenol sulfate | 0.57±0.32 | 0 | 3.74E-01 | 5.31E-01 | 0.68±0.34 | 0 | 3.28E-01 | 4.37E-01 | 0.9±0.49 | 4.16E-01 | 1.06±0.51 | 3.52E-01 | 0.33±0.42 | 9.98E-01 | 0.4±0.44 | 9.91E-01 |
| 3-(4-hydroxyphenyl)lactate | 0.55±0.26 | 0 | 5.82E-01 | 4.20E-01 | 0.52±0.27 | 0 | 4.76E-01 | 4.37E-01 | 0.65±0.32 | 3.38E-01 | 0.65±0.32 | 3.76E-01 | 0.33±0.48 | 9.98E-01 | 0.23±0.49 | 9.91E-01 |
| glycerophosphoethanolamine | 0.36±0.2 | 0 | 6.29E-01 | 5.35E-01 | 0.42±0.22 | 0 | 5.88E-01 | 4.37E-01 | 0.41±0.23 | 4.28E-01 | 0.47±0.23 | 3.78E-01 | 0.16±0.47 | 9.98E-01 | 0.14±0.55 | 9.91E-01 |
| 1-stearoyl-2-linoleoyl-GPE (18:0/18:2)* | 0.51±0.27 | 0 | 6.43E-01 | 5.02E-01 | 0.55±0.28 | 0 | 5.39E-01 | 4.37E-01 | 0.6±0.33 | 4.22E-01 | 0.66±0.34 | 3.86E-01 | 0.33±0.46 | 9.98E-01 | 0.28±0.52 | 9.91E-01 |
| N-acetylvaline | 0.42±0.23 | 0 | 5.91E-01 | 5.11E-01 | 0.49±0.25 | 0 | 6.77E-01 | 4.37E-01 | 0.5±0.27 | 4.19E-01 | 0.54±0.28 | 3.93E-01 | 0.23±0.42 | 9.98E-01 | 0.29±0.54 | 9.91E-01 |
| alpha-hydroxyisocaproate | 0.69±0.33 | 0 | 9.72E-01 | 4.20E-01 | 0.7±0.34 | 0 | 8.56E-01 | 4.37E-01 | 0.7±0.38 | 4.16E-01 | 0.73±0.4 | 4.24E-01 | 0.67±0.71 | 9.98E-01 | 0.59±0.68 | 9.91E-01 |
| X - 21442 | -0.4±0.19 | 0 | 9.94E-01 | 4.20E-01 | -0.4±0.2 | 0 | 8.23E-01 | 4.37E-01 | -0.4±0.23 | 4.57E-01 | -0.43±0.24 | 4.36E-01 | -0.4±0.37 | 9.98E-01 | -0.33±0.39 | 9.91E-01 |
| 1-linolenoyl-GPC (18:3)* | 0.48±0.26 | 0 | 9.99E-01 | 5.17E-01 | 0.54±0.27 | 0 | 9.35E-01 | 4.37E-01 | 0.48±0.31 | 5.42E-01 | 0.56±0.32 | 4.66E-01 | 0.48±0.49 | 9.98E-01 | 0.51±0.54 | 9.91E-01 |
| lanthionine | 0.51±0.29 | 0 | 7.11E-01 | 5.42E-01 | 0.6±0.3 | 0 | 7.29E-01 | 4.37E-01 | 0.6±0.38 | 5.19E-01 | 0.68±0.39 | 4.75E-01 | 0.38±0.46 | 9.98E-01 | 0.47±0.48 | 9.91E-01 |
| 3,4-dihydroxybutyrate | 0.33±0.16 | 0 | 5.60E-01 | 4.20E-01 | 0.37±0.18 | 0 | 3.91E-01 | 4.37E-01 | 0.29±0.18 | 5.13E-01 | 0.31±0.19 | 5.28E-01 | 0.53±0.38 | 9.98E-01 | 0.77±0.51 | 9.91E-01 |
| octadecanedioylcarnitine (C18-DC)* | -0.47±0.25 | 0 | 9.86E-01 | 5.02E-01 | -0.53±0.26 | 0 | 7.46E-01 | 4.37E-01 | -0.47±0.34 | 6.15E-01 | -0.46±0.33 | 6.02E-01 | -0.48±0.37 | 9.98E-01 | -0.64±0.44 | 9.91E-01 |
| X - 23644 | -0.47±0.27 | 0 | 7.27E-01 | 5.42E-01 | -0.56±0.28 | 0 | 5.23E-01 | 4.37E-01 | -0.4±0.33 | 6.76E-01 | -0.43±0.35 | 6.58E-01 | -0.59±0.45 | 9.98E-01 | -0.8±0.48 | 9.91E-01 |
| N-oleoylserine | -0.36±0.21 | 0 | 7.12E-01 | 5.42E-01 | -0.42±0.22 | 0 | 5.99E-01 | 4.37E-01 | -0.28±0.3 | 7.70E-01 | -0.32±0.3 | 7.13E-01 | -0.43±0.28 | 9.98E-01 | -0.54±0.31 | 9.91E-01 |
| 4-hydroxychlorothalonil | -0.39±0.2 | 82.67 | 1.63E-02 | 4.71E-01 | -0.4±0.21 | 78.48 | 3.11E-02 | 4.38E-01 | -0.59±0.22 | 1.44E-01 | -0.59±0.23 | 2.21E-01 | 0.7±0.49 | 9.98E-01 | 0.67±0.54 | 9.91E-01 |
| stearoyl sphingomyelin (d18:1/18:0) | -0.48±0.25 | 68.65 | 7.41E-02 | 5.02E-01 | -0.5±0.26 | 61.95 | 1.05E-01 | 4.38E-01 | -0.81±0.31 | 1.81E-01 | -0.79±0.32 | 2.21E-01 | 0.14±0.43 | 9.98E-01 | 0.12±0.46 | 9.91E-01 |
| behenoyl sphingomyelin (d18:1/22:0)* | -0.41±0.24 | 62.52 | 1.02E-01 | 5.44E-01 | -0.47±0.25 | 51.42 | 1.51E-01 | 4.38E-01 | -0.72±0.31 | 2.49E-01 | -0.73±0.31 | 2.39E-01 | 0.07±0.38 | 9.98E-01 | 0.03±0.43 | 9.91E-01 |
| 5-dodecenoate (12:1n7) | -0.45±0.26 | 36.83 | 2.08E-01 | 5.44E-01 | -0.52±0.28 | 45.21 | 1.77E-01 | 4.38E-01 | -0.67±0.31 | 3.26E-01 | -0.75±0.32 | 2.51E-01 | 0.04±0.47 | 9.98E-01 | 0.07±0.52 | 9.91E-01 |
| arginine | -0.34±0.2 | 41.94 | 1.89E-01 | 5.67E-01 | -0.4±0.21 | 33.57 | 2.20E-01 | 4.38E-01 | -0.55±0.26 | 3.26E-01 | -0.62±0.28 | 2.83E-01 | 0.01±0.33 | 9.98E-01 | -0.08±0.34 | 9.91E-01 |
| N-delta-acetylornithine | 0.46±0.27 | 51.96 | 1.49E-01 | 5.52E-01 | 0.56±0.29 | 28.13 | 2.38E-01 | 4.38E-01 | 0.85±0.38 | 3.03E-01 | 0.87±0.4 | 2.91E-01 | 0.06±0.39 | 9.98E-01 | 0.17±0.44 | 9.91E-01 |
| tyrosine | -0.41±0.23 | 58.13 | 1.22E-01 | 5.42E-01 | -0.44±0.23 | 11.79 | 2.87E-01 | 4.38E-01 | -0.77±0.33 | 2.67E-01 | -0.7±0.34 | 3.52E-01 | -0.05±0.33 | 9.98E-01 | -0.2±0.32 | 9.91E-01 |
| N-formylmethionine | 0.45±0.24 | 0 | 4.40E-01 | 5.02E-01 | 0.5±0.26 | 0 | 5.05E-01 | 4.38E-01 | 0.58±0.29 | 3.63E-01 | 0.6±0.3 | 3.85E-01 | 0.18±0.42 | 9.98E-01 | 0.19±0.52 | 9.91E-01 |
| (2 or 3)-decenoate (10:1n7 or n8) | -0.34±0.21 | 0 | 3.74E-01 | 5.77E-01 | -0.4±0.22 | 0 | 4.48E-01 | 4.38E-01 | -0.44±0.24 | 4.16E-01 | -0.49±0.25 | 3.85E-01 | -0.01±0.43 | 9.98E-01 | -0.1±0.46 | 9.91E-01 |
| glycosyl-N-palmitoyl-sphingosine (d18:1/16:0) | 0.52±0.27 | 22.61 | 2.56E-01 | 4.69E-01 | 0.56±0.3 | 0 | 5.71E-01 | 4.38E-01 | 0.76±0.34 | 3.03E-01 | 0.66±0.34 | 3.93E-01 | 0.14±0.43 | 9.98E-01 | 0.27±0.6 | 9.91E-01 |
| etiocholanolone glucuronide | -0.45±0.22 | 0 | 6.89E-01 | 4.20E-01 | -0.41±0.22 | 0 | 4.82E-01 | 4.38E-01 | -0.52±0.27 | 3.84E-01 | -0.51±0.27 | 3.93E-01 | -0.33±0.38 | 9.98E-01 | -0.18±0.39 | 9.91E-01 |
| N-acetylproline | 0.57±0.32 | 0 | 5.33E-01 | 5.31E-01 | 0.62±0.34 | 0 | 5.34E-01 | 4.38E-01 | 0.75±0.43 | 4.61E-01 | 0.8±0.43 | 4.24E-01 | 0.35±0.47 | 9.98E-01 | 0.37±0.53 | 9.91E-01 |
| N6-carbamoylthreonyladenosine | 0.46±0.25 | 0 | 6.16E-01 | 5.02E-01 | 0.49±0.26 | 0 | 7.58E-01 | 4.38E-01 | 0.54±0.29 | 4.10E-01 | 0.53±0.29 | 4.36E-01 | 0.26±0.48 | 9.98E-01 | 0.34±0.56 | 9.91E-01 |
| taurolithocholate 3-sulfate | -0.42±0.2 | 0 | 7.60E-01 | 4.20E-01 | -0.38±0.2 | 0 | 6.99E-01 | 4.38E-01 | -0.46±0.24 | 3.95E-01 | -0.43±0.24 | 4.55E-01 | -0.33±0.36 | 9.98E-01 | -0.26±0.38 | 9.91E-01 |
| quinate | -0.37±0.19 | 0 | 9.73E-01 | 4.69E-01 | -0.36±0.19 | 0 | 8.25E-01 | 4.38E-01 | -0.36±0.23 | 5.16E-01 | -0.39±0.23 | 5.01E-01 | -0.38±0.33 | 9.98E-01 | -0.3±0.34 | 9.91E-01 |
| X - 21353 | -0.4±0.23 | 0 | 8.61E-01 | 5.42E-01 | -0.46±0.24 | 0 | 9.67E-01 | 4.38E-01 | -0.43±0.28 | 5.29E-01 | -0.47±0.28 | 5.01E-01 | -0.34±0.42 | 9.98E-01 | -0.45±0.46 | 9.91E-01 |
| mannitol/sorbitol | 0.36±0.2 | 0 | 9.48E-01 | 5.32E-01 | 0.39±0.21 | 0 | 9.47E-01 | 4.38E-01 | 0.37±0.24 | 5.48E-01 | 0.4±0.24 | 5.15E-01 | 0.34±0.37 | 9.98E-01 | 0.37±0.38 | 9.91E-01 |
| X - 23639 | -0.3±0.2 | 0 | 7.88E-01 | 6.27E-01 | -0.41±0.22 | 0 | 7.60E-01 | 4.38E-01 | -0.35±0.28 | 6.55E-01 | -0.47±0.31 | 5.51E-01 | -0.24±0.3 | 9.98E-01 | -0.34±0.31 | 9.91E-01 |
| hexadecasphingosine (d16:1)* | -0.47±0.25 | 0 | 9.17E-01 | 5.02E-01 | -0.49±0.26 | 0 | 7.73E-01 | 4.38E-01 | -0.49±0.31 | 5.19E-01 | -0.44±0.32 | 6.02E-01 | -0.43±0.44 | 9.98E-01 | -0.6±0.46 | 9.91E-01 |
| bilirubin degradation product, C17H18N2O4 (3)** | -0.34±0.22 | 0 | 9.70E-01 | 6.18E-01 | -0.45±0.23 | 0 | 8.85E-01 | 4.38E-01 | -0.35±0.29 | 6.76E-01 | -0.42±0.3 | 6.02E-01 | -0.33±0.34 | 9.98E-01 | -0.49±0.37 | 9.91E-01 |
| pimeloylcarnitine/3-methyladipoylcarnitine (C7-DC) | 0.49±0.26 | 0 | 8.08E-01 | 5.02E-01 | 0.49±0.26 | 0 | 5.99E-01 | 4.38E-01 | 0.44±0.33 | 6.36E-01 | 0.38±0.33 | 6.95E-01 | 0.57±0.43 | 9.98E-01 | 0.67±0.43 | 9.91E-01 |
| 3-methoxycatechol sulfate (1) | 0.4±0.25 | 0 | 5.04E-01 | 5.76E-01 | 0.46±0.25 | 0 | 4.36E-01 | 4.38E-01 | 0.28±0.31 | 7.86E-01 | 0.32±0.31 | 7.13E-01 | 0.62±0.4 | 9.98E-01 | 0.72±0.41 | 9.91E-01 |
| X - 11372 | -0.35±0.21 | 0 | 8.60E-01 | 5.76E-01 | -0.42±0.22 | 0 | 4.36E-01 | 4.38E-01 | -0.32±0.28 | 7.03E-01 | -0.27±0.29 | 7.52E-01 | -0.4±0.33 | 9.98E-01 | -0.62±0.34 | 9.91E-01 |
| dopamine 3-O-sulfate | 0.4±0.2 | 4.58 | 3.06E-01 | 4.54E-01 | 0.39±0.21 | 0.4 | 3.16E-01 | 4.38E-01 | 0.24±0.25 | 7.59E-01 | 0.24±0.25 | 7.54E-01 | 0.67±0.33 | 9.98E-01 | 0.67±0.35 | 9.91E-01 |
| creatine | -0.45±0.22 | 55.94 | 1.32E-01 | 4.37E-01 | -0.46±0.24 | 63.03 | 1.00E-01 | 4.38E-01 | -0.07±0.34 | 9.55E-01 | -0.04±0.35 | 9.86E-01 | -0.74±0.29 | 9.98E-01 | -0.83±0.33 | 9.91E-01 |
| dehydroepiandrosterone sulfate (DHEA-S) | -0.39±0.2 | 0 | 3.78E-01 | 5.02E-01 | -0.37±0.2 | 25.53 | 2.47E-01 | 4.38E-01 | -0.47±0.22 | 3.38E-01 | -0.48±0.22 | 3.06E-01 | 0±0.49 | 9.99E-01 | 0.13±0.48 | 9.91E-01 |
| sphingomyelin (d18:2/16:0, d18:1/16:1)* | -0.49±0.27 | 50.42 | 1.56E-01 | 5.35E-01 | -0.51±0.28 | 47.6 | 1.67E-01 | 4.46E-01 | -0.66±0.3 | 3.05E-01 | -0.71±0.31 | 2.70E-01 | 0.37±0.66 | 9.98E-01 | 0.23±0.61 | 9.91E-01 |
| hydroxypalmitoyl sphingomyelin (d18:1/16:0(OH))** | -0.44±0.24 | 61 | 1.09E-01 | 5.17E-01 | -0.47±0.25 | 45.9 | 1.74E-01 | 4.54E-01 | -0.7±0.29 | 2.37E-01 | -0.67±0.29 | 2.72E-01 | 0.15±0.44 | 9.98E-01 | 0.13±0.51 | 9.91E-01 |
| 1-palmitoyl-2-linoleoyl-GPE (16:0/18:2) | 0.46±0.27 | 0 | 7.85E-01 | 5.47E-01 | 0.52±0.28 | 0 | 6.74E-01 | 4.54E-01 | 0.41±0.32 | 6.55E-01 | 0.44±0.33 | 6.11E-01 | 0.57±0.48 | 9.98E-01 | 0.71±0.55 | 9.91E-01 |
| X - 18901 | -0.57±0.26 | 79.79 | 2.61E-02 | 3.49E-01 | -0.51±0.28 | 82.2 | 1.78E-02 | 4.54E-01 | -1.03±0.33 | 7.60E-02 | -1.07±0.37 | 1.23E-01 | 0.18±0.43 | 9.98E-01 | 0.27±0.43 | 9.91E-01 |
| 1-(1-enyl-palmitoyl)-2-palmitoleoyl-GPC (P-16:0/16:1)* | -0.38±0.25 | 55.3 | 1.35E-01 | 6.18E-01 | -0.51±0.28 | 65.57 | 8.84E-02 | 4.57E-01 | -0.67±0.31 | 3.26E-01 | -0.84±0.34 | 2.21E-01 | 0.1±0.41 | 9.98E-01 | 0.19±0.5 | 9.91E-01 |
| 1H-indole-7-acetic acid | -0.38±0.22 | 11.42 | 2.88E-01 | 5.44E-01 | -0.4±0.22 | 0 | 3.48E-01 | 4.61E-01 | -0.52±0.25 | 3.38E-01 | -0.53±0.26 | 3.69E-01 | 0.01±0.43 | 9.98E-01 | -0.05±0.43 | 9.91E-01 |
| N-methylpipecolate | 0.41±0.25 | 31.07 | 2.28E-01 | 5.90E-01 | 0.5±0.27 | 0 | 3.23E-01 | 4.61E-01 | 0.68±0.34 | 3.59E-01 | 0.7±0.35 | 3.69E-01 | 0.06±0.38 | 9.98E-01 | 0.14±0.45 | 9.91E-01 |
| 1-(1-enyl-stearoyl)-2-arachidonoyl-GPE (P-18:0/20:4)* | -0.35±0.21 | 35.31 | 2.14E-01 | 5.78E-01 | -0.4±0.22 | 13.28 | 2.83E-01 | 4.61E-01 | -0.56±0.27 | 3.38E-01 | -0.58±0.28 | 3.49E-01 | -0.01±0.34 | 9.98E-01 | -0.08±0.37 | 9.91E-01 |
| gamma-glutamylvaline | 0.56±0.34 | 0 | 7.70E-01 | 5.76E-01 | 0.63±0.35 | 0 | 4.12E-01 | 4.61E-01 | 0.63±0.42 | 5.53E-01 | 0.85±0.44 | 3.93E-01 | 0.42±0.59 | 9.98E-01 | 0.26±0.57 | 9.91E-01 |
| 1-palmitoyl-GPC (16:0) | 0.51±0.28 | 0 | 5.53E-01 | 5.31E-01 | 0.53±0.3 | 0 | 5.28E-01 | 4.73E-01 | 0.61±0.33 | 4.19E-01 | 0.65±0.35 | 4.14E-01 | 0.24±0.53 | 9.98E-01 | 0.21±0.59 | 9.91E-01 |
| 4-ethylcatechol sulfate | -0.48±0.24 | 0 | 4.96E-01 | 4.57E-01 | -0.44±0.25 | 0 | 3.66E-01 | 4.75E-01 | -0.59±0.29 | 3.38E-01 | -0.58±0.29 | 3.85E-01 | -0.24±0.43 | 9.98E-01 | -0.08±0.47 | 9.91E-01 |
| 2,3-dihydroxyisovalerate | 0.39±0.21 | 0 | 8.89E-01 | 5.07E-01 | 0.4±0.23 | 22.3 | 2.57E-01 | 4.75E-01 | 0.37±0.25 | 5.70E-01 | 0.26±0.26 | 7.43E-01 | 0.44±0.39 | 9.98E-01 | 0.85±0.46 | 9.91E-01 |
| 1-palmitoyl-GPG (16:0)* | 0.54±0.31 | 0 | 7.97E-01 | 5.42E-01 | 0.54±0.3 | 0 | 8.70E-01 | 4.79E-01 | 0.49±0.35 | 6.04E-01 | 0.51±0.35 | 5.77E-01 | 0.68±0.63 | 9.98E-01 | 0.63±0.63 | 9.91E-01 |
| N-acetylcitrulline | -0.42±0.25 | 1.85 | 3.13E-01 | 5.52E-01 | -0.45±0.26 | 0 | 5.90E-01 | 4.90E-01 | -0.6±0.3 | 3.63E-01 | -0.55±0.31 | 4.69E-01 | -0.06±0.43 | 9.98E-01 | -0.25±0.45 | 9.91E-01 |
| X - 13431 | 0.43±0.25 | 62.74 | 1.01E-01 | 5.52E-01 | 0.46±0.26 | 62.59 | 1.02E-01 | 4.90E-01 | 0.17±0.3 | 8.79E-01 | 0.21±0.3 | 8.33E-01 | 1.1±0.48 | 9.98E-01 | 1.2±0.52 | 9.91E-01 |
| 3-bromo-5-chloro-2,6-dihydroxybenzoic acid* | -0.4±0.2 | 46.75 | 1.71E-01 | 4.78E-01 | -0.37±0.21 | 40 | 1.97E-01 | 4.92E-01 | -0.52±0.22 | 2.67E-01 | -0.51±0.24 | 3.17E-01 | 0.22±0.5 | 9.98E-01 | 0.13±0.44 | 9.91E-01 |
| 1-(1-enyl-stearoyl)-2-oleoyl-GPE (P-18:0/18:1) | -0.32±0.21 | 37.48 | 2.06E-01 | 6.11E-01 | -0.39±0.23 | 6.47 | 3.01E-01 | 4.92E-01 | -0.63±0.32 | 3.63E-01 | -0.64±0.33 | 3.90E-01 | -0.1±0.27 | 9.98E-01 | -0.18±0.31 | 9.91E-01 |
| campesterol | -0.41±0.25 | 67.29 | 8.04E-02 | 5.78E-01 | -0.46±0.27 | 76.63 | 3.86E-02 | 4.93E-01 | -0.69±0.3 | 2.71E-01 | -0.83±0.32 | 2.21E-01 | 0.27±0.46 | 9.98E-01 | 0.37±0.48 | 9.91E-01 |
| 6-bromotryptophan | 0.44±0.25 | 0 | 4.70E-01 | 5.39E-01 | 0.45±0.26 | 0.79 | 3.15E-01 | 4.93E-01 | 0.58±0.31 | 4.19E-01 | 0.63±0.32 | 3.85E-01 | 0.21±0.39 | 9.98E-01 | 0.08±0.45 | 9.91E-01 |
| glycerophosphorylcholine (GPC) | 0.4±0.25 | 0 | 5.38E-01 | 5.90E-01 | 0.47±0.28 | 0 | 3.81E-01 | 4.93E-01 | 0.51±0.31 | 4.92E-01 | 0.63±0.33 | 3.93E-01 | 0.17±0.45 | 9.98E-01 | 0.1±0.51 | 9.91E-01 |
| 1-oleoyl-2-linoleoyl-GPE (18:1/18:2)* | 0.51±0.32 | 0 | 4.31E-01 | 5.78E-01 | 0.6±0.35 | 0 | 3.49E-01 | 4.93E-01 | 0.76±0.44 | 4.62E-01 | 0.89±0.47 | 3.98E-01 | 0.26±0.45 | 9.98E-01 | 0.24±0.52 | 9.91E-01 |
| X - 12193 | 0.42±0.25 | 0 | 6.48E-01 | 5.52E-01 | 0.45±0.26 | 0 | 6.37E-01 | 4.93E-01 | 0.49±0.29 | 4.73E-01 | 0.52±0.3 | 4.75E-01 | 0.23±0.49 | 9.98E-01 | 0.24±0.53 | 9.91E-01 |
| linolenate [alpha or gamma; (18:3n3 or 6)] | -0.37±0.23 | 0 | 9.86E-01 | 5.76E-01 | -0.41±0.24 | 0 | 9.06E-01 | 4.93E-01 | -0.37±0.27 | 6.23E-01 | -0.4±0.28 | 6.02E-01 | -0.38±0.41 | 9.98E-01 | -0.46±0.45 | 9.91E-01 |
| 3-hydroxyphenylacetoylglutamine | 0.41±0.26 | 0 | 8.45E-01 | 6.11E-01 | 0.47±0.27 | 0 | 7.97E-01 | 4.93E-01 | 0.37±0.33 | 6.95E-01 | 0.42±0.33 | 6.56E-01 | 0.48±0.44 | 9.98E-01 | 0.56±0.45 | 9.91E-01 |
| eicosenoate (20:1) | -0.34±0.23 | 0 | 5.01E-01 | 6.23E-01 | -0.41±0.24 | 0 | 6.86E-01 | 4.93E-01 | -0.23±0.29 | 8.09E-01 | -0.35±0.29 | 6.81E-01 | -0.55±0.38 | 9.98E-01 | -0.55±0.42 | 9.91E-01 |
| octadecenedioylcarnitine (C18:1-DC)* | -0.35±0.23 | 0 | 4.21E-01 | 6.18E-01 | -0.42±0.24 | 0 | 3.50E-01 | 4.93E-01 | -0.2±0.3 | 8.54E-01 | -0.27±0.29 | 7.57E-01 | -0.58±0.36 | 9.98E-01 | -0.76±0.44 | 9.91E-01 |
| X - 13553 | -0.41±0.24 | 0 | 3.94E-01 | 5.52E-01 | -0.44±0.26 | 0 | 5.75E-01 | 4.95E-01 | -0.57±0.31 | 4.16E-01 | -0.55±0.32 | 4.75E-01 | -0.16±0.38 | 9.98E-01 | -0.24±0.45 | 9.91E-01 |
| erythritol | 0.36±0.26 | 0 | 8.21E-01 | 6.94E-01 | 0.48±0.28 | 0 | 7.67E-01 | 4.95E-01 | 0.32±0.31 | 7.46E-01 | 0.43±0.32 | 6.11E-01 | 0.44±0.45 | 9.98E-01 | 0.62±0.56 | 9.91E-01 |
| methionine sulfone | 0.38±0.24 | 52.76 | 1.46E-01 | 5.78E-01 | 0.44±0.26 | 46.37 | 1.72E-01 | 4.95E-01 | 0.69±0.32 | 3.16E-01 | 0.71±0.33 | 2.97E-01 | -0.01±0.36 | 9.98E-01 | 0±0.41 | 9.94E-01 |
| X - 11880 | -0.29±0.21 | 0 | 8.36E-01 | 6.96E-01 | -0.37±0.22 | 0 | 4.33E-01 | 5.01E-01 | -0.25±0.28 | 7.81E-01 | -0.23±0.28 | 7.97E-01 | -0.34±0.32 | 9.98E-01 | -0.58±0.34 | 9.91E-01 |
| N-acetylglutamine | -0.36±0.23 | 0 | 8.64E-01 | 6.18E-01 | -0.43±0.25 | 0 | 8.74E-01 | 5.02E-01 | -0.4±0.31 | 6.52E-01 | -0.46±0.32 | 5.99E-01 | -0.32±0.36 | 9.98E-01 | -0.38±0.4 | 9.91E-01 |
| sphingomyelin (d18:2/14:0, d18:1/14:1)* | -0.55±0.3 | 80.88 | 2.22E-02 | 5.31E-01 | -0.54±0.32 | 74.08 | 4.95E-02 | 5.06E-01 | -0.95±0.35 | 1.49E-01 | -0.92±0.37 | 2.21E-01 | 0.66±0.61 | 9.98E-01 | 0.51±0.62 | 9.91E-01 |
| 5-methyluridine (ribothymidine) | -0.27±0.17 | 0 | 8.71E-01 | 6.18E-01 | -0.31±0.18 | 0 | 7.43E-01 | 5.06E-01 | -0.29±0.23 | 6.64E-01 | -0.26±0.23 | 6.98E-01 | -0.23±0.26 | 9.98E-01 | -0.38±0.3 | 9.91E-01 |
| lignoceroylcarnitine (C24)* | -0.27±0.22 | 0 | 9.53E-01 | 7.66E-01 | -0.38±0.22 | 0 | 8.31E-01 | 5.06E-01 | -0.28±0.34 | 8.01E-01 | -0.33±0.34 | 7.43E-01 | -0.26±0.28 | 9.98E-01 | -0.42±0.3 | 9.91E-01 |
| 5alpha-androstan-3alpha,17beta-diol monosulfate (1) | -0.4±0.21 | 21.32 | 2.60E-01 | 5.02E-01 | -0.35±0.21 | 43.98 | 1.82E-01 | 5.07E-01 | -0.55±0.25 | 3.15E-01 | -0.53±0.25 | 3.24E-01 | -0.01±0.41 | 9.98E-01 | 0.09±0.39 | 9.91E-01 |
| sphingomyelin (d18:1/17:0, d17:1/18:0, d19:1/16:0) | -0.55±0.28 | 63.78 | 9.66E-02 | 4.63E-01 | -0.48±0.29 | 44.82 | 1.78E-01 | 5.07E-01 | -0.8±0.32 | 1.93E-01 | -0.67±0.32 | 3.41E-01 | 0.28±0.57 | 9.98E-01 | 0.3±0.65 | 9.91E-01 |
| X - 17654 | -0.34±0.21 | 55.75 | 1.33E-01 | 5.76E-01 | -0.36±0.22 | 40.61 | 1.94E-01 | 5.07E-01 | -0.5±0.23 | 3.26E-01 | -0.51±0.24 | 3.49E-01 | 0.28±0.46 | 9.98E-01 | 0.19±0.48 | 9.91E-01 |
| isobutyrylcarnitine (C4) | -0.38±0.21 | 1.71 | 3.13E-01 | 5.07E-01 | -0.37±0.22 | 0 | 6.29E-01 | 5.07E-01 | -0.56±0.27 | 3.38E-01 | -0.45±0.28 | 5.17E-01 | -0.14±0.32 | 9.98E-01 | -0.24±0.35 | 9.91E-01 |
| hyocholate | -0.32±0.23 | 0 | 8.78E-01 | 6.94E-01 | -0.4±0.24 | 0 | 9.57E-01 | 5.07E-01 | -0.35±0.28 | 6.71E-01 | -0.39±0.29 | 6.11E-01 | -0.27±0.41 | 9.98E-01 | -0.42±0.42 | 9.91E-01 |
| X - 21310 | 0.55±0.31 | 34.22 | 2.18E-01 | 5.42E-01 | 0.54±0.33 | 0 | 4.36E-01 | 5.24E-01 | 0.31±0.37 | 7.97E-01 | 0.38±0.39 | 7.43E-01 | 1.18±0.6 | 9.98E-01 | 0.94±0.61 | 9.91E-01 |
| N-acetyl-beta-alanine | 0.37±0.22 | 81.1 | 2.14E-02 | 5.52E-01 | 0.39±0.24 | 81.69 | 1.94E-02 | 5.26E-01 | 0.76±0.28 | 1.44E-01 | 0.82±0.3 | 1.61E-01 | -0.26±0.35 | 9.98E-01 | -0.32±0.38 | 9.91E-01 |
| 1-stearoyl-GPI (18:0) | -0.38±0.25 | 81.39 | 2.05E-02 | 6.23E-01 | -0.44±0.27 | 79.76 | 2.62E-02 | 5.26E-01 | -0.69±0.29 | 2.30E-01 | -0.73±0.3 | 2.21E-01 | 0.69±0.53 | 9.98E-01 | 0.75±0.59 | 9.91E-01 |
| 3-hydroxybutyrate (BHBA) | -0.3±0.24 | 0 | 3.47E-01 | 7.41E-01 | -0.43±0.26 | 0 | 3.69E-01 | 5.26E-01 | -0.12±0.3 | 8.96E-01 | -0.27±0.31 | 7.71E-01 | -0.58±0.38 | 9.98E-01 | -0.77±0.46 | 9.91E-01 |
| 4-acetylphenol sulfate | 0.32±0.25 | 0 | 7.00E-01 | 7.41E-01 | 0.43±0.26 | 0 | 7.56E-01 | 5.26E-01 | 0.39±0.3 | 6.52E-01 | 0.48±0.31 | 5.51E-01 | 0.18±0.45 | 9.98E-01 | 0.3±0.48 | 9.91E-01 |
| X - 24422 | 0.45±0.31 | 12.54 | 2.85E-01 | 6.48E-01 | 0.55±0.33 | 0 | 7.36E-01 | 5.26E-01 | 0.81±0.46 | 4.57E-01 | 0.63±0.42 | 5.65E-01 | 0.15±0.41 | 9.98E-01 | 0.4±0.54 | 9.91E-01 |
| linoleate (18:2n6) | -0.31±0.21 | 0 | 9.60E-01 | 6.51E-01 | -0.37±0.22 | 0 | 8.77E-01 | 5.26E-01 | -0.3±0.26 | 6.77E-01 | -0.34±0.27 | 6.28E-01 | -0.33±0.38 | 9.98E-01 | -0.42±0.42 | 9.91E-01 |
| 2-hydroxyhippurate (salicylurate) | 0.35±0.23 | 67.47 | 7.95E-02 | 6.07E-01 | 0.39±0.24 | 75.61 | 4.29E-02 | 5.26E-01 | -0.04±0.32 | 9.80E-01 | -0.05±0.32 | 9.75E-01 | 0.75±0.32 | 9.98E-01 | 0.92±0.35 | 9.91E-01 |
| arachidonate (20:4n6) | -0.31±0.22 | 0 | 7.44E-01 | 6.86E-01 | -0.37±0.23 | 0 | 8.45E-01 | 5.30E-01 | -0.38±0.3 | 6.55E-01 | -0.41±0.3 | 6.02E-01 | -0.23±0.33 | 9.98E-01 | -0.32±0.36 | 9.91E-01 |
| dihomo-linoleate (20:2n6) | -0.32±0.22 | 0 | 7.29E-01 | 6.73E-01 | -0.38±0.23 | 0 | 7.45E-01 | 5.30E-01 | -0.25±0.29 | 7.94E-01 | -0.32±0.3 | 7.13E-01 | -0.41±0.34 | 9.98E-01 | -0.48±0.38 | 9.91E-01 |
| sphingomyelin (d18:2/24:2)* | -0.44±0.27 | 62.22 | 1.04E-01 | 5.76E-01 | -0.45±0.28 | 56.91 | 1.28E-01 | 5.33E-01 | -0.69±0.31 | 3.03E-01 | -0.69±0.32 | 3.21E-01 | 0.34±0.55 | 9.98E-01 | 0.3±0.57 | 9.91E-01 |
| X - 26119 | -0.36±0.24 | 40.64 | 1.94E-01 | 6.23E-01 | -0.39±0.25 | 42.53 | 1.87E-01 | 5.33E-01 | -0.55±0.28 | 3.72E-01 | -0.57±0.28 | 3.69E-01 | 0.16±0.46 | 9.98E-01 | 0.2±0.52 | 9.91E-01 |
| 4-acetamidobutanoate | -0.3±0.2 | 30.96 | 2.29E-01 | 6.27E-01 | -0.35±0.22 | 29.95 | 2.32E-01 | 5.33E-01 | -0.44±0.23 | 4.02E-01 | -0.47±0.24 | 3.89E-01 | 0.1±0.38 | 9.98E-01 | 0.2±0.51 | 9.91E-01 |
| 1-palmitoyl-2-palmitoleoyl-GPC (16:0/16:1)* | 0.44±0.27 | 0 | 7.57E-01 | 5.76E-01 | 0.44±0.28 | 0 | 3.47E-01 | 5.33E-01 | 0.38±0.33 | 7.03E-01 | 0.29±0.32 | 7.65E-01 | 0.55±0.45 | 9.98E-01 | 0.87±0.54 | 9.91E-01 |
| 3-indoxyl sulfate | 0.37±0.25 | 0 | 4.16E-01 | 6.27E-01 | 0.44±0.27 | 0 | 4.14E-01 | 5.33E-01 | 0.21±0.31 | 8.54E-01 | 0.29±0.33 | 7.65E-01 | 0.63±0.4 | 9.98E-01 | 0.78±0.5 | 9.91E-01 |
| X - 24243 | 0.57±0.29 | 0 | 3.50E-01 | 4.71E-01 | 0.53±0.33 | 0.11 | 3.17E-01 | 5.33E-01 | 0.32±0.4 | 8.09E-01 | 0.32±0.39 | 7.97E-01 | 0.87±0.43 | 9.98E-01 | 1.03±0.6 | 9.91E-01 |
| thymol sulfate | 0.35±0.23 | 0 | 3.27E-01 | 6.23E-01 | 0.38±0.23 | 39.92 | 1.97E-01 | 5.33E-01 | 0.16±0.3 | 8.85E-01 | 0.14±0.3 | 8.91E-01 | 0.62±0.36 | 9.98E-01 | 0.77±0.38 | 9.91E-01 |
| 2-hydroxybehenate | -0.29±0.21 | 20.12 | 2.63E-01 | 6.96E-01 | -0.37±0.23 | 49.78 | 1.58E-01 | 5.33E-01 | -0.03±0.31 | 9.81E-01 | -0.06±0.32 | 9.68E-01 | -0.5±0.28 | 9.98E-01 | -0.71±0.34 | 9.91E-01 |
| X - 17653 | -0.34±0.22 | 0 | 3.21E-01 | 6.18E-01 | -0.36±0.23 | 0 | 5.10E-01 | 5.39E-01 | -0.5±0.27 | 4.19E-01 | -0.48±0.29 | 5.01E-01 | -0.04±0.38 | 9.98E-01 | -0.16±0.38 | 9.91E-01 |
| branched-chain, straight-chain, or cyclopropyl 12:1 fatty acid* | -0.33±0.21 | 0 | 5.84E-01 | 6.07E-01 | -0.34±0.22 | 0 | 6.26E-01 | 5.39E-01 | -0.4±0.24 | 5.08E-01 | -0.4±0.25 | 5.15E-01 | -0.13±0.43 | 9.98E-01 | -0.15±0.44 | 9.91E-01 |
| 3-hydroxy-2-ethylpropionate | 0.45±0.27 | 0 | 9.45E-01 | 5.76E-01 | 0.45±0.28 | 0 | 7.10E-01 | 5.39E-01 | 0.44±0.31 | 5.92E-01 | 0.4±0.31 | 6.26E-01 | 0.48±0.57 | 9.98E-01 | 0.69±0.71 | 9.91E-01 |
| cortolone glucuronide (1) | -0.23±0.18 | 0 | 8.45E-01 | 7.56E-01 | -0.32±0.2 | 0 | 8.33E-01 | 5.39E-01 | -0.25±0.22 | 6.87E-01 | -0.3±0.24 | 6.59E-01 | -0.17±0.34 | 9.98E-01 | -0.39±0.38 | 9.91E-01 |
| 2-stearoyl-GPE (18:0)* | 0.36±0.23 | 41.97 | 1.89E-01 | 6.18E-01 | 0.39±0.25 | 52.12 | 1.48E-01 | 5.60E-01 | 0.6±0.3 | 3.45E-01 | 0.64±0.31 | 3.38E-01 | -0.03±0.38 | 9.98E-01 | -0.11±0.42 | 9.91E-01 |
| tricosanoyl sphingomyelin (d18:1/23:0)* | -0.35±0.21 | 51.74 | 1.50E-01 | 5.76E-01 | -0.37±0.24 | 13.45 | 2.82E-01 | 5.60E-01 | -0.63±0.29 | 3.16E-01 | -0.55±0.29 | 4.10E-01 | -0.01±0.32 | 9.98E-01 | -0.02±0.4 | 9.91E-01 |
| 4-hydroxyhippurate | 0.3±0.22 | 0 | 8.01E-01 | 7.15E-01 | 0.36±0.23 | 0 | 9.53E-01 | 5.60E-01 | 0.34±0.28 | 6.71E-01 | 0.37±0.28 | 6.11E-01 | 0.22±0.37 | 9.98E-01 | 0.34±0.44 | 9.91E-01 |
| N1-methylinosine | 0.47±0.26 | 0 | 9.77E-01 | 5.31E-01 | 0.4±0.26 | 0 | 9.75E-01 | 5.60E-01 | 0.47±0.31 | 5.50E-01 | 0.4±0.31 | 6.26E-01 | 0.46±0.47 | 9.98E-01 | 0.42±0.5 | 9.91E-01 |
| 4-vinylphenol sulfate | -0.34±0.26 | 0 | 4.10E-01 | 7.28E-01 | -0.44±0.29 | 0 | 6.45E-01 | 5.60E-01 | -0.19±0.33 | 8.79E-01 | -0.35±0.35 | 7.42E-01 | -0.64±0.45 | 9.98E-01 | -0.63±0.5 | 9.91E-01 |
| pantoate | 0.35±0.25 | 0 | 7.85E-01 | 6.90E-01 | 0.43±0.28 | 0 | 4.90E-01 | 5.60E-01 | 0.3±0.31 | 7.59E-01 | 0.32±0.32 | 7.43E-01 | 0.45±0.42 | 9.98E-01 | 0.77±0.56 | 9.91E-01 |
| sphingomyelin (d18:1/25:0, d19:0/24:1, d20:1/23:0, d19:1/24:0)* | -0.33±0.21 | 43.65 | 1.83E-01 | 6.18E-01 | -0.36±0.23 | 0 | 3.29E-01 | 5.60E-01 | -0.58±0.28 | 3.38E-01 | -0.51±0.28 | 4.24E-01 | 0±0.33 | 9.99E-01 | -0.04±0.4 | 9.91E-01 |
| X - 12100 | 0.31±0.22 | 0 | 5.46E-01 | 6.92E-01 | 0.36±0.24 | 0 | 4.96E-01 | 5.61E-01 | 0.4±0.27 | 5.56E-01 | 0.45±0.27 | 5.01E-01 | 0.11±0.39 | 9.98E-01 | 0.08±0.48 | 9.91E-01 |
| imidazole lactate | 0.4±0.25 | 0 | 4.84E-01 | 5.76E-01 | 0.4±0.26 | 0 | 7.79E-01 | 5.65E-01 | 0.53±0.3 | 4.61E-01 | 0.44±0.3 | 5.80E-01 | 0.16±0.42 | 9.98E-01 | 0.28±0.5 | 9.91E-01 |
| 1-palmitoleoylglycerol (16:1)* | 0.44±0.27 | 0 | 4.12E-01 | 5.71E-01 | 0.41±0.27 | 0 | 4.97E-01 | 5.86E-01 | 0.61±0.34 | 4.22E-01 | 0.55±0.34 | 5.15E-01 | 0.16±0.43 | 9.98E-01 | 0.16±0.46 | 9.91E-01 |
| vanillactate | 0.25±0.17 | 0 | 6.66E-01 | 6.23E-01 | 0.27±0.18 | 0 | 5.58E-01 | 5.86E-01 | 0.22±0.18 | 6.71E-01 | 0.23±0.19 | 6.59E-01 | 0.43±0.45 | 9.98E-01 | 0.55±0.52 | 9.91E-01 |
| glycerol 3-phosphate | 0.36±0.26 | 42.59 | 1.87E-01 | 6.96E-01 | 0.4±0.27 | 31.02 | 2.29E-01 | 5.88E-01 | 0.13±0.31 | 8.96E-01 | 0.19±0.32 | 8.62E-01 | 0.88±0.47 | 9.98E-01 | 0.89±0.48 | 9.91E-01 |
| dodecadienoate (12:2)* | -0.3±0.23 | 0 | 4.76E-01 | 7.28E-01 | -0.35±0.24 | 0 | 6.42E-01 | 5.96E-01 | -0.41±0.27 | 5.56E-01 | -0.42±0.28 | 5.61E-01 | -0.05±0.41 | 9.98E-01 | -0.18±0.44 | 9.91E-01 |
| 5alpha-pregnan-3beta,20alpha-diol disulfate | -0.38±0.25 | 68.98 | 7.26E-02 | 6.18E-01 | -0.37±0.25 | 75.05 | 4.53E-02 | 6.00E-01 | -0.64±0.29 | 3.03E-01 | -0.68±0.29 | 2.51E-01 | 0.37±0.48 | 9.98E-01 | 0.43±0.47 | 9.91E-01 |
| N-acetylhistidine | -0.36±0.23 | 52.11 | 1.48E-01 | 6.23E-01 | -0.37±0.25 | 72.7 | 5.56E-02 | 6.00E-01 | -0.56±0.27 | 3.38E-01 | -0.62±0.28 | 2.89E-01 | 0.2±0.45 | 9.98E-01 | 0.5±0.52 | 9.91E-01 |
| palmitoleate (16:1n7) | -0.31±0.24 | 0 | 6.31E-01 | 7.31E-01 | -0.37±0.25 | 0 | 3.91E-01 | 6.00E-01 | -0.38±0.28 | 6.22E-01 | -0.5±0.29 | 4.76E-01 | -0.13±0.45 | 9.98E-01 | -0.01±0.49 | 9.91E-01 |
| lysine | -0.26±0.19 | 15.37 | 2.77E-01 | 7.08E-01 | -0.29±0.19 | 0 | 4.87E-01 | 6.00E-01 | -0.5±0.29 | 4.62E-01 | -0.44±0.3 | 5.65E-01 | -0.08±0.26 | 9.98E-01 | -0.17±0.26 | 9.91E-01 |
| X - 17010 | 0.35±0.23 | 0 | 5.79E-01 | 6.18E-01 | 0.34±0.23 | 0 | 4.88E-01 | 6.00E-01 | 0.26±0.28 | 7.75E-01 | 0.23±0.27 | 7.71E-01 | 0.53±0.4 | 9.98E-01 | 0.58±0.41 | 9.91E-01 |
| tryptophan betaine | -0.27±0.23 | 0 | 5.43E-01 | 7.66E-01 | -0.34±0.23 | 0 | 5.22E-01 | 6.00E-01 | -0.16±0.29 | 8.85E-01 | -0.23±0.29 | 8.04E-01 | -0.44±0.36 | 9.98E-01 | -0.53±0.38 | 9.91E-01 |
| lyxonate | -0.28±0.2 | 0 | 9.03E-01 | 6.72E-01 | -0.31±0.21 | 0 | 8.01E-01 | 6.00E-01 | -0.31±0.29 | 7.24E-01 | -0.26±0.29 | 7.57E-01 | -0.26±0.27 | 9.98E-01 | -0.37±0.31 | 9.91E-01 |
| 1-palmitoyl-2-arachidonoyl-GPI (16:0/20:4)* | 0.39±0.26 | 0 | 8.52E-01 | 6.23E-01 | 0.4±0.27 | 0 | 6.97E-01 | 6.05E-01 | 0.43±0.31 | 6.18E-01 | 0.46±0.32 | 5.84E-01 | 0.32±0.49 | 9.98E-01 | 0.23±0.51 | 9.91E-01 |
| 1-palmitoyl-2-arachidonoyl-GPC (16:0/20:4n6) | -0.26±0.23 | 14.83 | 2.79E-01 | 7.85E-01 | -0.35±0.24 | 25.78 | 2.46E-01 | 6.14E-01 | -0.47±0.3 | 5.30E-01 | -0.59±0.32 | 4.11E-01 | 0.04±0.36 | 9.98E-01 | -0.02±0.38 | 9.91E-01 |
| androsterone glucuronide | -0.37±0.22 | 0 | 6.59E-01 | 5.52E-01 | -0.32±0.22 | 0 | 4.52E-01 | 6.24E-01 | -0.44±0.26 | 4.92E-01 | -0.44±0.27 | 5.15E-01 | -0.23±0.39 | 9.98E-01 | -0.08±0.4 | 9.91E-01 |
| biliverdin | -0.27±0.23 | 0 | 6.96E-01 | 7.68E-01 | -0.35±0.25 | 0 | 7.22E-01 | 6.31E-01 | -0.36±0.32 | 7.13E-01 | -0.43±0.33 | 6.26E-01 | -0.17±0.33 | 9.98E-01 | -0.26±0.37 | 9.91E-01 |
| X - 22162 | 0.33±0.24 | 0 | 8.98E-01 | 7.14E-01 | 0.37±0.26 | 0 | 9.72E-01 | 6.31E-01 | 0.35±0.29 | 6.71E-01 | 0.37±0.29 | 6.47E-01 | 0.28±0.47 | 9.98E-01 | 0.39±0.57 | 9.91E-01 |
| N-acetylcarnosine | 0.46±0.32 | 31.11 | 2.28E-01 | 6.59E-01 | 0.47±0.32 | 0 | 3.93E-01 | 6.31E-01 | 0.25±0.36 | 8.45E-01 | 0.33±0.36 | 7.57E-01 | 1.18±0.67 | 9.98E-01 | 1.04±0.74 | 9.91E-01 |
| 4-hydroxyphenylacetylglutamine | 0.31±0.23 | 0 | 7.47E-01 | 7.14E-01 | 0.37±0.26 | 0 | 7.86E-01 | 6.32E-01 | 0.37±0.3 | 6.55E-01 | 0.41±0.31 | 6.11E-01 | 0.22±0.38 | 9.98E-01 | 0.26±0.47 | 9.91E-01 |
| 3-methoxytyramine sulfate | 0.34±0.23 | 0 | 8.08E-01 | 6.23E-01 | 0.33±0.23 | 0 | 9.75E-01 | 6.32E-01 | 0.38±0.27 | 6.06E-01 | 0.34±0.27 | 6.59E-01 | 0.25±0.42 | 9.98E-01 | 0.32±0.44 | 9.91E-01 |
| oleate/vaccenate (18:1) | -0.24±0.21 | 0 | 9.77E-01 | 7.66E-01 | -0.31±0.22 | 0 | 9.50E-01 | 6.32E-01 | -0.24±0.26 | 7.81E-01 | -0.3±0.27 | 6.98E-01 | -0.25±0.34 | 9.98E-01 | -0.33±0.39 | 9.91E-01 |
| 1-stearoyl-2-linoleoyl-GPC (18:0/18:2)* | -0.28±0.19 | 0 | 3.75E-01 | 6.27E-01 | -0.28±0.19 | 0 | 6.64E-01 | 6.32E-01 | -0.41±0.24 | 4.62E-01 | -0.34±0.25 | 6.02E-01 | -0.07±0.3 | 9.98E-01 | -0.17±0.31 | 9.91E-01 |
| 3-aminoisobutyrate | 0.33±0.21 | 52.27 | 1.48E-01 | 6.18E-01 | 0.31±0.22 | 47.65 | 1.67E-01 | 6.35E-01 | 0.55±0.26 | 3.38E-01 | 0.53±0.27 | 3.90E-01 | -0.1±0.36 | 9.98E-01 | -0.12±0.39 | 9.91E-01 |
| N-acetylneuraminate | 0.32±0.23 | 0 | 9.38E-01 | 6.88E-01 | 0.35±0.24 | 0 | 6.49E-01 | 6.37E-01 | 0.33±0.25 | 6.45E-01 | 0.31±0.26 | 6.84E-01 | 0.28±0.51 | 9.98E-01 | 0.65±0.71 | 9.91E-01 |
| carboxyethyl-GABA | -0.23±0.21 | 0 | 7.57E-01 | 7.93E-01 | -0.3±0.21 | 0 | 7.11E-01 | 6.42E-01 | -0.17±0.29 | 8.79E-01 | -0.22±0.3 | 8.12E-01 | -0.29±0.3 | 9.98E-01 | -0.38±0.31 | 9.91E-01 |
| X - 21842 | -0.36±0.25 | 88.82 | 2.78E-03 | 6.48E-01 | -0.36±0.26 | 85.54 | 8.54E-03 | 6.49E-01 | -0.99±0.32 | 8.18E-02 | -0.9±0.33 | 1.61E-01 | 0.51±0.38 | 9.98E-01 | 0.48±0.41 | 9.91E-01 |
| pregnenolone sulfate | -0.33±0.24 | 0 | 3.74E-01 | 7.03E-01 | -0.34±0.24 | 26.14 | 2.45E-01 | 6.53E-01 | -0.44±0.27 | 5.12E-01 | -0.51±0.28 | 4.47E-01 | 0.06±0.49 | 9.98E-01 | 0.15±0.49 | 9.91E-01 |
| margaroylcarnitine (C17)* | 0.35±0.27 | 44.95 | 1.78E-01 | 7.41E-01 | 0.38±0.27 | 47.45 | 1.68E-01 | 6.53E-01 | 0.16±0.31 | 8.88E-01 | 0.19±0.3 | 8.51E-01 | 1.06±0.59 | 9.98E-01 | 1.12±0.6 | 9.91E-01 |
| 3-methylcytidine | 0.47±0.29 | 37.06 | 2.07E-01 | 5.78E-01 | 0.43±0.31 | 4.08 | 3.07E-01 | 6.56E-01 | 0.81±0.39 | 3.38E-01 | 0.69±0.4 | 4.75E-01 | 0.08±0.42 | 9.98E-01 | 0.05±0.48 | 9.91E-01 |
| androstenediol (3alpha, 17alpha) monosulfate (3) | -0.36±0.24 | 0 | 3.67E-01 | 6.27E-01 | -0.33±0.24 | 46.18 | 1.73E-01 | 6.58E-01 | -0.49±0.28 | 4.61E-01 | -0.52±0.28 | 4.10E-01 | 0.02±0.49 | 9.98E-01 | 0.23±0.48 | 9.91E-01 |
| N4-acetylcytidine | 0.39±0.26 | 0 | 9.23E-01 | 6.34E-01 | 0.38±0.28 | 0 | 8.62E-01 | 6.63E-01 | 0.4±0.31 | 6.45E-01 | 0.41±0.32 | 6.42E-01 | 0.35±0.49 | 9.98E-01 | 0.3±0.54 | 9.91E-01 |
| 3-carboxy-4-methyl-5-propyl-2-furanpropanoate (CMPF) | 0.36±0.27 | 50.53 | 1.55E-01 | 7.28E-01 | 0.37±0.27 | 0 | 3.18E-01 | 6.73E-01 | 0.68±0.35 | 3.84E-01 | 0.59±0.35 | 4.90E-01 | -0.1±0.42 | 9.98E-01 | 0.04±0.43 | 9.91E-01 |
| phenol sulfate | -0.33±0.27 | 0 | 8.48E-01 | 7.66E-01 | -0.38±0.28 | 0 | 9.89E-01 | 6.73E-01 | -0.37±0.34 | 7.17E-01 | -0.39±0.35 | 7.05E-01 | -0.26±0.46 | 9.98E-01 | -0.38±0.46 | 9.91E-01 |
| sphingomyelin (d18:1/18:1, d18:2/18:0) | -0.36±0.27 | 55.29 | 1.35E-01 | 7.08E-01 | -0.36±0.27 | 41.01 | 1.93E-01 | 6.73E-01 | -0.62±0.32 | 3.74E-01 | -0.61±0.33 | 4.11E-01 | 0.25±0.49 | 9.98E-01 | 0.12±0.46 | 9.91E-01 |
| pregnanediol-3-glucuronide | -0.33±0.24 | 9.45 | 2.93E-01 | 6.94E-01 | -0.33±0.24 | 44.47 | 1.80E-01 | 6.73E-01 | -0.49±0.28 | 4.61E-01 | -0.53±0.28 | 4.11E-01 | 0.07±0.45 | 9.98E-01 | 0.18±0.45 | 9.91E-01 |
| 3-hydroxypyridine sulfate | -0.32±0.23 | 0 | 8.14E-01 | 6.88E-01 | -0.33±0.24 | 0 | 7.25E-01 | 6.73E-01 | -0.36±0.27 | 6.37E-01 | -0.38±0.28 | 6.11E-01 | -0.24±0.42 | 9.98E-01 | -0.19±0.46 | 9.91E-01 |
| isovalerylcarnitine (C5) | -0.24±0.2 | 0 | 6.67E-01 | 7.66E-01 | -0.29±0.21 | 0 | 4.19E-01 | 6.73E-01 | -0.14±0.31 | 8.96E-01 | -0.1±0.32 | 9.42E-01 | -0.32±0.27 | 9.98E-01 | -0.45±0.29 | 9.91E-01 |
| arachidate (20:0) | -0.29±0.25 | 0 | 8.21E-01 | 7.66E-01 | -0.36±0.26 | 0 | 9.53E-01 | 6.81E-01 | -0.25±0.31 | 8.09E-01 | -0.35±0.32 | 7.05E-01 | -0.37±0.43 | 9.98E-01 | -0.38±0.48 | 9.91E-01 |
| 5-dodecenoylcarnitine (C12:1) | -0.23±0.26 | 43.64 | 1.83E-01 | 8.44E-01 | -0.35±0.27 | 64.87 | 9.16E-02 | 6.81E-01 | -0.43±0.3 | 5.81E-01 | -0.6±0.31 | 3.90E-01 | 0.33±0.49 | 9.98E-01 | 0.5±0.57 | 9.91E-01 |
| sphingomyelin (d18:2/23:1)* | -0.5±0.3 | 66.68 | 8.32E-02 | 5.76E-01 | -0.42±0.32 | 55.87 | 1.32E-01 | 6.81E-01 | -0.79±0.35 | 2.82E-01 | -0.69±0.36 | 4.10E-01 | 0.43±0.61 | 9.98E-01 | 0.43±0.64 | 9.91E-01 |
| 2-methylserine | -0.34±0.26 | 54.37 | 1.39E-01 | 7.28E-01 | -0.36±0.27 | 55.7 | 1.33E-01 | 6.81E-01 | -0.47±0.28 | 4.62E-01 | -0.51±0.29 | 4.70E-01 | 0.82±0.83 | 9.98E-01 | 0.81±0.83 | 9.91E-01 |
| guanidinoacetate | -0.24±0.24 | 0 | 3.60E-01 | 8.24E-01 | -0.34±0.26 | 13.9 | 2.81E-01 | 6.81E-01 | -0.4±0.29 | 6.18E-01 | -0.52±0.3 | 4.90E-01 | 0.06±0.4 | 9.98E-01 | 0.11±0.49 | 9.91E-01 |
| retinol (Vitamin A) | 0.3±0.23 | 0 | 3.18E-01 | 7.41E-01 | 0.32±0.25 | 0 | 3.42E-01 | 6.81E-01 | 0.5±0.31 | 5.13E-01 | 0.5±0.31 | 5.15E-01 | 0.03±0.36 | 9.98E-01 | 0.01±0.41 | 9.91E-01 |
| 1-stearoyl-GPC (18:0) | 0.34±0.27 | 0 | 5.85E-01 | 7.56E-01 | 0.37±0.28 | 0 | 3.32E-01 | 6.81E-01 | 0.46±0.35 | 6.36E-01 | 0.59±0.36 | 5.15E-01 | 0.15±0.45 | 9.98E-01 | 0.03±0.45 | 9.91E-01 |
| X - 24544 | -0.29±0.22 | 0 | 3.70E-01 | 7.28E-01 | -0.3±0.23 | 0 | 3.33E-01 | 6.81E-01 | -0.39±0.25 | 5.22E-01 | -0.4±0.25 | 5.17E-01 | 0.09±0.48 | 9.98E-01 | 0.15±0.52 | 9.91E-01 |
| caprylate (8:0) | -0.28±0.25 | 0 | 3.78E-01 | 7.85E-01 | -0.32±0.24 | 0 | 3.67E-01 | 6.81E-01 | -0.46±0.32 | 5.90E-01 | -0.5±0.31 | 5.17E-01 | -0.02±0.39 | 9.98E-01 | -0.05±0.39 | 9.91E-01 |
| 1-arachidonoyl-GPI (20:4)* | -0.28±0.24 | 18.71 | 2.67E-01 | 7.66E-01 | -0.33±0.25 | 0 | 3.61E-01 | 6.81E-01 | -0.48±0.3 | 5.16E-01 | -0.48±0.3 | 5.23E-01 | 0.08±0.41 | 9.98E-01 | 0.02±0.45 | 9.91E-01 |
| 1-(1-enyl-palmitoyl)-GPE (P-16:0)* | 0.29±0.23 | 0 | 4.64E-01 | 7.41E-01 | 0.31±0.24 | 0 | 4.27E-01 | 6.81E-01 | 0.42±0.28 | 5.75E-01 | 0.43±0.28 | 5.57E-01 | 0.06±0.39 | 9.98E-01 | 0.01±0.44 | 9.91E-01 |
| 1,2-dilinoleoyl-GPC (18:2/18:2) | -0.29±0.23 | 0 | 3.27E-01 | 7.48E-01 | -0.32±0.24 | 0 | 5.06E-01 | 6.81E-01 | -0.46±0.29 | 5.16E-01 | -0.44±0.3 | 5.77E-01 | 0.01±0.39 | 9.98E-01 | -0.09±0.43 | 9.91E-01 |
| 3-hydroxylaurate | -0.27±0.24 | 0 | 6.56E-01 | 7.85E-01 | -0.33±0.25 | 0 | 6.50E-01 | 6.81E-01 | -0.35±0.29 | 6.77E-01 | -0.4±0.3 | 6.11E-01 | -0.12±0.43 | 9.98E-01 | -0.16±0.46 | 9.91E-01 |
| X - 24473 | 0.29±0.27 | 26.31 | 2.44E-01 | 8.08E-01 | 0.39±0.3 | 0 | 6.40E-01 | 6.81E-01 | 0.6±0.38 | 5.21E-01 | 0.49±0.37 | 6.16E-01 | -0.04±0.4 | 9.98E-01 | 0.2±0.5 | 9.91E-01 |
| hexadecadienoate (16:2n6) | -0.29±0.24 | 0 | 6.14E-01 | 7.66E-01 | -0.32±0.25 | 0 | 7.26E-01 | 6.81E-01 | -0.36±0.27 | 6.37E-01 | -0.37±0.29 | 6.28E-01 | -0.09±0.46 | 9.98E-01 | -0.17±0.51 | 9.91E-01 |
| N-acetylglutamate | 0.43±0.35 | 0 | 5.79E-01 | 7.66E-01 | 0.51±0.39 | 0 | 6.46E-01 | 6.81E-01 | 0.64±0.53 | 6.71E-01 | 0.69±0.55 | 6.57E-01 | 0.25±0.47 | 9.98E-01 | 0.33±0.55 | 9.91E-01 |
| 13-HODE + 9-HODE | -0.31±0.25 | 0 | 9.54E-01 | 7.66E-01 | -0.32±0.25 | 0 | 7.95E-01 | 6.81E-01 | -0.32±0.31 | 7.40E-01 | -0.37±0.3 | 6.59E-01 | -0.29±0.44 | 9.98E-01 | -0.23±0.44 | 9.91E-01 |
| sphinganine-1-phosphate | -0.27±0.21 | 0 | 7.57E-01 | 7.41E-01 | -0.29±0.22 | 0 | 8.97E-01 | 6.81E-01 | -0.33±0.28 | 6.77E-01 | -0.31±0.27 | 6.95E-01 | -0.19±0.34 | 9.98E-01 | -0.25±0.39 | 9.91E-01 |
| X - 15245 | 0.36±0.28 | 0 | 9.49E-01 | 7.31E-01 | 0.36±0.28 | 0 | 9.17E-01 | 6.81E-01 | 0.35±0.34 | 7.43E-01 | 0.39±0.35 | 7.05E-01 | 0.38±0.47 | 9.98E-01 | 0.32±0.48 | 9.91E-01 |
| stearidonate (18:4n3) | -0.31±0.25 | 0 | 9.32E-01 | 7.66E-01 | -0.36±0.26 | 0 | 9.75E-01 | 6.81E-01 | -0.3±0.31 | 7.59E-01 | -0.36±0.32 | 7.05E-01 | -0.34±0.44 | 9.98E-01 | -0.34±0.46 | 9.91E-01 |
| X - 21258 | -0.33±0.25 | 0 | 7.75E-01 | 7.28E-01 | -0.33±0.26 | 0 | 9.14E-01 | 6.81E-01 | -0.28±0.31 | 7.81E-01 | -0.31±0.32 | 7.43E-01 | -0.43±0.44 | 9.98E-01 | -0.37±0.43 | 9.91E-01 |
| X - 23739 | 0.38±0.25 | 0 | 6.59E-01 | 6.23E-01 | 0.33±0.25 | 0 | 7.91E-01 | 6.81E-01 | 0.31±0.29 | 7.23E-01 | 0.29±0.3 | 7.46E-01 | 0.57±0.5 | 9.98E-01 | 0.44±0.47 | 9.91E-01 |
| X - 24475 | 0.31±0.27 | 0 | 7.80E-01 | 7.85E-01 | 0.37±0.28 | 0 | 8.65E-01 | 6.81E-01 | 0.25±0.34 | 8.31E-01 | 0.34±0.35 | 7.52E-01 | 0.41±0.45 | 9.98E-01 | 0.44±0.47 | 9.91E-01 |
| gamma-CEHC | -0.26±0.22 | 0 | 7.86E-01 | 7.66E-01 | -0.32±0.24 | 0 | 7.02E-01 | 6.81E-01 | -0.31±0.3 | 7.30E-01 | -0.25±0.3 | 7.91E-01 | -0.19±0.34 | 9.98E-01 | -0.45±0.41 | 9.91E-01 |
| X - 23665 | -0.25±0.22 | 0 | 7.69E-01 | 7.85E-01 | -0.3±0.23 | 0 | 5.52E-01 | 6.81E-01 | -0.19±0.3 | 8.66E-01 | -0.19±0.29 | 8.41E-01 | -0.32±0.34 | 9.98E-01 | -0.48±0.37 | 9.91E-01 |
| X - 11308 | -0.24±0.22 | 0 | 8.30E-01 | 8.18E-01 | -0.3±0.23 | 0 | 5.20E-01 | 6.81E-01 | -0.2±0.29 | 8.54E-01 | -0.17±0.3 | 8.64E-01 | -0.3±0.35 | 9.98E-01 | -0.47±0.36 | 9.91E-01 |
| X - 25420 | 0.31±0.27 | 53.9 | 1.41E-01 | 7.66E-01 | 0.38±0.29 | 68.68 | 7.40E-02 | 6.81E-01 | 0.07±0.32 | 9.50E-01 | 0.05±0.34 | 9.76E-01 | 0.96±0.51 | 9.98E-01 | 1.18±0.54 | 9.91E-01 |
| sulfate of piperine metabolite C18H21NO3 (3)* | -0.28±0.24 | 0 | 4.47E-01 | 7.85E-01 | -0.34±0.26 | 0 | 4.80E-01 | 6.81E-01 | -0.4±0.3 | 6.22E-01 | -0.47±0.31 | 5.69E-01 | 0±0.44 | 9.99E-01 | -0.08±0.45 | 9.91E-01 |
| N-acetylarginine | -0.34±0.26 | 67.22 | 8.07E-02 | 7.25E-01 | -0.33±0.26 | 55.28 | 1.35E-01 | 6.82E-01 | -0.61±0.3 | 3.38E-01 | -0.57±0.3 | 4.11E-01 | 0.4±0.49 | 9.98E-01 | 0.31±0.5 | 9.91E-01 |
| myristoleate (14:1n5) | -0.31±0.25 | 32.58 | 2.23E-01 | 7.56E-01 | -0.33±0.26 | 52.98 | 1.45E-01 | 6.82E-01 | -0.5±0.29 | 4.67E-01 | -0.56±0.3 | 4.20E-01 | 0.17±0.47 | 9.98E-01 | 0.3±0.5 | 9.91E-01 |
| tiglylcarnitine (C5:1-DC) | -0.32±0.24 | 19.26 | 2.66E-01 | 7.14E-01 | -0.32±0.25 | 0 | 3.54E-01 | 6.82E-01 | -0.53±0.3 | 4.61E-01 | -0.49±0.31 | 5.26E-01 | 0.03±0.39 | 9.98E-01 | -0.01±0.41 | 9.91E-01 |
| citrate | -0.27±0.25 | 33.11 | 2.21E-01 | 7.87E-01 | -0.32±0.25 | 0 | 3.67E-01 | 6.82E-01 | -0.52±0.32 | 5.09E-01 | -0.5±0.32 | 5.35E-01 | 0.09±0.39 | 9.98E-01 | -0.05±0.38 | 9.91E-01 |
| 1-stearoyl-GPG (18:0) | 0.35±0.27 | 0 | 5.46E-01 | 7.48E-01 | 0.35±0.28 | 0 | 4.86E-01 | 6.82E-01 | 0.47±0.35 | 6.15E-01 | 0.5±0.35 | 5.97E-01 | 0.13±0.45 | 9.98E-01 | 0.11±0.45 | 9.91E-01 |
| 1-linoleoyl-2-arachidonoyl-GPC (18:2/20:4n6)* | -0.23±0.2 | 0 | 5.62E-01 | 7.85E-01 | -0.26±0.2 | 0 | 6.71E-01 | 6.82E-01 | -0.33±0.27 | 6.71E-01 | -0.33±0.27 | 6.58E-01 | -0.1±0.3 | 9.98E-01 | -0.16±0.31 | 9.91E-01 |
| hydroquinone sulfate | 0.29±0.26 | 0 | 9.27E-01 | 7.85E-01 | 0.34±0.26 | 0 | 7.54E-01 | 6.82E-01 | 0.31±0.33 | 7.65E-01 | 0.4±0.33 | 6.59E-01 | 0.26±0.43 | 9.98E-01 | 0.22±0.45 | 9.91E-01 |
| eugenol sulfate | 0.33±0.26 | 0 | 4.85E-01 | 7.31E-01 | 0.34±0.27 | 0 | 6.58E-01 | 6.82E-01 | 0.2±0.32 | 8.69E-01 | 0.25±0.34 | 8.12E-01 | 0.58±0.43 | 9.98E-01 | 0.5±0.45 | 9.91E-01 |
| salicylate | 0.27±0.23 | 62.5 | 1.02E-01 | 7.66E-01 | 0.31±0.24 | 71.39 | 6.16E-02 | 6.82E-01 | -0.12±0.34 | 9.15E-01 | -0.12±0.34 | 9.29E-01 | 0.64±0.33 | 9.98E-01 | 0.78±0.35 | 9.91E-01 |
| X - 23780 | 0.34±0.28 | 83.95 | 1.26E-02 | 7.66E-01 | 0.35±0.28 | 81.41 | 2.04E-02 | 6.88E-01 | 1.14±0.42 | 1.49E-01 | 1.07±0.42 | 2.21E-01 | -0.27±0.37 | 9.98E-01 | -0.25±0.39 | 9.91E-01 |
| N6,N6-dimethyllysine | 0.28±0.23 | 55.98 | 1.32E-01 | 7.66E-01 | 0.31±0.25 | 54.34 | 1.39E-01 | 6.88E-01 | 0.6±0.31 | 3.84E-01 | 0.61±0.32 | 3.99E-01 | -0.1±0.34 | 9.98E-01 | -0.14±0.39 | 9.91E-01 |
| 5alpha-androstan-3beta,17beta-diol monosulfate (2) | -0.32±0.24 | 0 | 3.43E-01 | 7.28E-01 | -0.3±0.24 | 41.97 | 1.89E-01 | 6.88E-01 | -0.46±0.29 | 5.15E-01 | -0.5±0.28 | 4.69E-01 | 0.05±0.45 | 9.98E-01 | 0.2±0.44 | 9.91E-01 |
| sphingomyelin (d18:1/19:0, d19:1/18:0)* | -0.44±0.28 | 63.72 | 9.68E-02 | 5.90E-01 | -0.36±0.29 | 40.24 | 1.96E-01 | 6.88E-01 | -0.71±0.32 | 3.03E-01 | -0.58±0.34 | 4.75E-01 | 0.35±0.55 | 9.98E-01 | 0.29±0.58 | 9.91E-01 |
| myristoleoylcarnitine (C14:1)* | -0.21±0.24 | 0 | 3.51E-01 | 8.44E-01 | -0.31±0.25 | 25.89 | 2.45E-01 | 6.88E-01 | -0.36±0.29 | 6.71E-01 | -0.5±0.3 | 5.01E-01 | 0.13±0.43 | 9.98E-01 | 0.15±0.48 | 9.91E-01 |
| 9-hydroxystearate | -0.26±0.22 | 0 | 3.34E-01 | 7.66E-01 | -0.28±0.23 | 17.84 | 2.70E-01 | 6.88E-01 | -0.38±0.25 | 5.63E-01 | -0.41±0.26 | 5.17E-01 | 0.15±0.48 | 9.98E-01 | 0.21±0.5 | 9.91E-01 |
| sulfate of piperine metabolite C18H21NO3 (1)* | -0.25±0.24 | 5.96 | 3.02E-01 | 8.19E-01 | -0.31±0.25 | 0 | 3.27E-01 | 6.88E-01 | -0.39±0.28 | 5.95E-01 | -0.46±0.29 | 5.35E-01 | 0.18±0.48 | 9.98E-01 | 0.1±0.49 | 9.91E-01 |
| X - 26111 | 0.28±0.26 | 0 | 3.66E-01 | 8.19E-01 | 0.33±0.27 | 0 | 5.66E-01 | 6.88E-01 | 0.46±0.33 | 6.08E-01 | 0.45±0.33 | 6.11E-01 | -0.03±0.42 | 9.98E-01 | 0.12±0.46 | 9.91E-01 |
| indoleacetylglutamine | 0.23±0.25 | 0 | 6.17E-01 | 8.44E-01 | 0.33±0.27 | 0 | 5.46E-01 | 6.88E-01 | 0.33±0.32 | 7.39E-01 | 0.46±0.34 | 6.11E-01 | 0.08±0.39 | 9.98E-01 | 0.13±0.44 | 9.91E-01 |
| X - 11849 | 0.34±0.26 | 0 | 6.44E-01 | 7.28E-01 | 0.34±0.27 | 0 | 6.75E-01 | 6.88E-01 | 0.43±0.33 | 6.34E-01 | 0.42±0.33 | 6.48E-01 | 0.18±0.43 | 9.98E-01 | 0.19±0.46 | 9.91E-01 |
| 1-(1-enyl-palmitoyl)-2-arachidonoyl-GPE (P-16:0/20:4)* | -0.23±0.23 | 0 | 5.37E-01 | 8.29E-01 | -0.29±0.24 | 0 | 6.40E-01 | 6.88E-01 | -0.35±0.31 | 6.91E-01 | -0.39±0.31 | 6.57E-01 | -0.08±0.33 | 9.98E-01 | -0.17±0.36 | 9.91E-01 |
| X - 13728 | 0.4±0.29 | 0 | 9.52E-01 | 6.96E-01 | 0.38±0.3 | 0 | 7.66E-01 | 6.88E-01 | 0.41±0.36 | 6.92E-01 | 0.45±0.37 | 6.76E-01 | 0.37±0.5 | 9.98E-01 | 0.26±0.51 | 9.91E-01 |
| taurocholenate sulfate* | -0.27±0.23 | 0 | 9.71E-01 | 7.66E-01 | -0.31±0.25 | 0 | 7.81E-01 | 6.88E-01 | -0.27±0.31 | 7.94E-01 | -0.37±0.32 | 6.95E-01 | -0.28±0.35 | 9.98E-01 | -0.23±0.4 | 9.91E-01 |
| N-acetylputrescine | 0.29±0.24 | 0 | 9.01E-01 | 7.66E-01 | 0.34±0.27 | 0 | 8.72E-01 | 6.88E-01 | 0.31±0.29 | 7.21E-01 | 0.31±0.3 | 7.13E-01 | 0.24±0.47 | 9.98E-01 | 0.42±0.61 | 9.91E-01 |
| methionine | -0.24±0.22 | 0 | 5.37E-01 | 8.08E-01 | -0.28±0.23 | 0 | 8.70E-01 | 6.88E-01 | -0.38±0.32 | 6.76E-01 | -0.32±0.32 | 7.43E-01 | -0.11±0.31 | 9.98E-01 | -0.25±0.31 | 9.91E-01 |
| 2-aminophenol sulfate | 0.26±0.26 | 0 | 3.38E-01 | 8.31E-01 | 0.32±0.26 | 0 | 4.34E-01 | 6.88E-01 | 0.08±0.32 | 9.49E-01 | 0.17±0.32 | 8.77E-01 | 0.61±0.45 | 9.98E-01 | 0.61±0.45 | 9.91E-01 |
| X - 16576 | -0.3±0.24 | 0 | 4.52E-01 | 7.66E-01 | -0.31±0.25 | 0 | 3.62E-01 | 6.88E-01 | -0.17±0.3 | 8.79E-01 | -0.15±0.3 | 8.82E-01 | -0.56±0.42 | 9.98E-01 | -0.63±0.44 | 9.91E-01 |
| uracil | -0.24±0.2 | 0 | 3.27E-01 | 7.66E-01 | -0.26±0.2 | 24.97 | 2.48E-01 | 6.88E-01 | -0.02±0.3 | 9.82E-01 | 0.01±0.3 | 9.95E-01 | -0.42±0.27 | 9.98E-01 | -0.47±0.28 | 9.91E-01 |
| X - 24546 | -0.31±0.26 | 0 | 4.43E-01 | 7.66E-01 | -0.33±0.27 | 13.05 | 2.84E-01 | 6.91E-01 | -0.45±0.32 | 6.03E-01 | -0.53±0.33 | 5.15E-01 | -0.02±0.46 | 9.98E-01 | 0.07±0.46 | 9.91E-01 |
| 1-palmitoyl-2-linoleoyl-GPC (16:0/18:2) | -0.22±0.22 | 0 | 4.11E-01 | 8.29E-01 | -0.28±0.23 | 0 | 5.08E-01 | 6.91E-01 | -0.4±0.31 | 6.49E-01 | -0.42±0.31 | 6.11E-01 | -0.03±0.32 | 9.98E-01 | -0.11±0.35 | 9.91E-01 |
| X - 21796 | -0.26±0.24 | 0 | 5.04E-01 | 8.08E-01 | -0.3±0.24 | 0 | 5.83E-01 | 6.91E-01 | -0.38±0.3 | 6.55E-01 | -0.39±0.29 | 6.16E-01 | -0.04±0.41 | 9.98E-01 | -0.09±0.44 | 9.91E-01 |
| glycochenodeoxycholate | 0.27±0.26 | 0 | 9.96E-01 | 8.08E-01 | 0.31±0.26 | 0 | 8.80E-01 | 6.91E-01 | 0.28±0.33 | 7.99E-01 | 0.29±0.32 | 7.65E-01 | 0.27±0.41 | 9.98E-01 | 0.37±0.43 | 9.91E-01 |
| 16a-hydroxy DHEA 3-sulfate | 0.33±0.26 | 0 | 4.98E-01 | 7.56E-01 | 0.34±0.28 | 0 | 5.25E-01 | 6.91E-01 | 0.21±0.32 | 8.68E-01 | 0.21±0.34 | 8.51E-01 | 0.59±0.46 | 9.98E-01 | 0.58±0.48 | 9.91E-01 |
| 3-(3-amino-3-carboxypropyl)uridine* | 0.27±0.22 | 31.99 | 2.25E-01 | 7.66E-01 | 0.29±0.24 | 17.18 | 2.72E-01 | 6.92E-01 | 0.47±0.28 | 4.77E-01 | 0.45±0.28 | 5.15E-01 | -0.1±0.37 | 9.98E-01 | -0.13±0.45 | 9.91E-01 |
| palmitoleoylcarnitine (C16:1)* | -0.23±0.25 | 22.76 | 2.55E-01 | 8.44E-01 | -0.32±0.26 | 35.65 | 2.13E-01 | 6.94E-01 | -0.42±0.31 | 6.12E-01 | -0.52±0.31 | 4.94E-01 | 0.21±0.46 | 9.98E-01 | 0.2±0.49 | 9.91E-01 |
| gamma-glutamylthreonine | 0.36±0.27 | 0 | 8.74E-01 | 7.03E-01 | 0.34±0.28 | 0 | 8.17E-01 | 6.94E-01 | 0.39±0.3 | 6.51E-01 | 0.37±0.31 | 6.81E-01 | 0.28±0.58 | 9.98E-01 | 0.21±0.61 | 9.91E-01 |
| arachidoylcarnitine (C20)* | -0.24±0.24 | 0 | 8.55E-01 | 8.29E-01 | -0.3±0.25 | 0 | 9.72E-01 | 6.95E-01 | -0.27±0.3 | 7.79E-01 | -0.3±0.31 | 7.46E-01 | -0.18±0.41 | 9.98E-01 | -0.32±0.43 | 9.91E-01 |
| methylsuccinate | 0.4±0.36 | 0 | 6.42E-01 | 7.87E-01 | 0.46±0.38 | 0 | 5.88E-01 | 6.96E-01 | 0.58±0.53 | 7.18E-01 | 0.68±0.55 | 6.59E-01 | 0.24±0.48 | 9.98E-01 | 0.26±0.53 | 9.91E-01 |
| X - 11381 | -0.27±0.23 | 0 | 4.52E-01 | 7.66E-01 | -0.29±0.24 | 0 | 8.56E-01 | 6.96E-01 | -0.39±0.28 | 6.04E-01 | -0.32±0.29 | 7.05E-01 | -0.03±0.4 | 9.98E-01 | -0.23±0.43 | 9.91E-01 |
| 2-pyrrolidinone | -0.3±0.23 | 45.18 | 1.77E-01 | 7.41E-01 | -0.3±0.25 | 54.4 | 1.39E-01 | 6.98E-01 | -0.45±0.26 | 4.61E-01 | -0.45±0.27 | 5.01E-01 | 0.35±0.54 | 9.98E-01 | 0.59±0.64 | 9.91E-01 |
| 1-palmitoyl-2-docosahexaenoyl-GPE (16:0/22:6)* | 0.3±0.27 | 0 | 6.01E-01 | 7.85E-01 | 0.32±0.27 | 0 | 7.22E-01 | 6.99E-01 | 0.41±0.34 | 6.71E-01 | 0.4±0.34 | 6.88E-01 | 0.13±0.43 | 9.98E-01 | 0.2±0.45 | 9.91E-01 |
| N-acetyl-isoputreanine | 0.36±0.26 | 48.41 | 1.64E-01 | 6.96E-01 | 0.31±0.26 | 67.01 | 8.17E-02 | 7.08E-01 | 0.16±0.29 | 8.79E-01 | 0.09±0.29 | 9.43E-01 | 1.03±0.55 | 9.98E-01 | 1.23±0.59 | 9.91E-01 |
| beta-hydroxyisovaleroylcarnitine | -0.26±0.23 | 0 | 7.11E-01 | 7.87E-01 | -0.28±0.24 | 0 | 8.57E-01 | 7.08E-01 | -0.33±0.31 | 7.23E-01 | -0.32±0.31 | 7.26E-01 | -0.16±0.34 | 9.98E-01 | -0.23±0.37 | 9.91E-01 |
| 1-stearoyl-2-arachidonoyl-GPC (18:0/20:4) | -0.21±0.22 | 0 | 5.01E-01 | 8.38E-01 | -0.25±0.21 | 0 | 5.40E-01 | 7.09E-01 | -0.37±0.32 | 6.95E-01 | -0.41±0.33 | 6.59E-01 | -0.08±0.29 | 9.98E-01 | -0.14±0.28 | 9.91E-01 |
| N-stearoyl-sphinganine (d18:0/18:0)* | 0.33±0.28 | 0 | 9.96E-01 | 7.66E-01 | 0.36±0.31 | 0 | 9.52E-01 | 7.10E-01 | 0.33±0.35 | 7.67E-01 | 0.35±0.38 | 7.62E-01 | 0.33±0.46 | 9.98E-01 | 0.39±0.51 | 9.91E-01 |
| X - 12844 | -0.21±0.21 | 0 | 7.65E-01 | 8.23E-01 | -0.25±0.21 | 0 | 7.05E-01 | 7.13E-01 | -0.16±0.28 | 8.79E-01 | -0.18±0.28 | 8.54E-01 | -0.28±0.31 | 9.98E-01 | -0.34±0.31 | 9.91E-01 |
| 1-oleoyl-GPC (18:1) | 0.29±0.24 | 0 | 3.74E-01 | 7.66E-01 | 0.31±0.27 | 0 | 4.23E-01 | 7.14E-01 | 0.47±0.32 | 5.56E-01 | 0.46±0.33 | 6.00E-01 | 0.04±0.37 | 9.98E-01 | 0.01±0.46 | 9.91E-01 |
| tetradecanedioate (C14-DC) | -0.27±0.26 | 0 | 9.79E-01 | 8.23E-01 | -0.33±0.28 | 0 | 8.75E-01 | 7.17E-01 | -0.26±0.33 | 8.09E-01 | -0.36±0.34 | 7.20E-01 | -0.28±0.43 | 9.98E-01 | -0.26±0.47 | 9.91E-01 |
| adenosine 3',5'-cyclic monophosphate (cAMP) | -0.27±0.24 | 7.35 | 2.99E-01 | 7.85E-01 | -0.29±0.25 | 0 | 3.73E-01 | 7.18E-01 | -0.43±0.28 | 5.53E-01 | -0.42±0.29 | 5.84E-01 | 0.12±0.44 | 9.98E-01 | 0.1±0.5 | 9.91E-01 |
| 1-stearoyl-2-docosahexaenoyl-GPE (18:0/22:6)* | 0.3±0.27 | 0 | 4.74E-01 | 7.87E-01 | 0.32±0.27 | 0 | 4.44E-01 | 7.18E-01 | 0.45±0.34 | 6.37E-01 | 0.49±0.35 | 6.02E-01 | 0.06±0.43 | 9.98E-01 | 0.06±0.43 | 9.91E-01 |
| hydroxy-N6,N6,N6-trimethyllysine* | -0.25±0.21 | 0 | 4.89E-01 | 7.66E-01 | -0.27±0.23 | 0 | 7.34E-01 | 7.18E-01 | -0.39±0.29 | 6.25E-01 | -0.34±0.3 | 7.05E-01 | -0.09±0.32 | 9.98E-01 | -0.17±0.38 | 9.91E-01 |
| 2-palmitoleoyl-GPC (16:1)* | -0.22±0.23 | 0 | 4.29E-01 | 8.44E-01 | -0.27±0.23 | 0.07 | 3.17E-01 | 7.18E-01 | -0.07±0.29 | 9.49E-01 | -0.1±0.29 | 9.39E-01 | -0.44±0.37 | 9.98E-01 | -0.58±0.39 | 9.91E-01 |
| 3beta-hydroxy-5-cholestenoate | -0.23±0.25 | 22.25 | 2.57E-01 | 8.44E-01 | -0.3±0.26 | 57.84 | 1.24E-01 | 7.26E-01 | -0.43±0.31 | 6.04E-01 | -0.57±0.31 | 4.36E-01 | 0.17±0.43 | 9.98E-01 | 0.3±0.47 | 9.91E-01 |
| sphingomyelin (d18:2/21:0, d16:2/23:0)* | -0.45±0.3 | 66.67 | 8.33E-02 | 6.23E-01 | -0.36±0.32 | 42.12 | 1.89E-01 | 7.28E-01 | -0.75±0.34 | 3.16E-01 | -0.62±0.37 | 5.01E-01 | 0.46±0.61 | 9.98E-01 | 0.32±0.6 | 9.91E-01 |
| behenoyl dihydrosphingomyelin (d18:0/22:0)* | -0.2±0.23 | 0 | 8.15E-01 | 8.44E-01 | -0.28±0.24 | 0 | 8.70E-01 | 7.33E-01 | -0.26±0.33 | 8.09E-01 | -0.31±0.34 | 7.55E-01 | -0.15±0.31 | 9.98E-01 | -0.24±0.35 | 9.91E-01 |
| N-acetylglucosaminylasparagine | 0.25±0.22 | 46.58 | 1.71E-01 | 7.85E-01 | 0.28±0.25 | 32.72 | 2.23E-01 | 7.39E-01 | 0.5±0.29 | 4.61E-01 | 0.49±0.3 | 5.15E-01 | -0.13±0.36 | 9.98E-01 | -0.15±0.43 | 9.91E-01 |
| stearate (18:0) | -0.25±0.24 | 0 | 9.35E-01 | 8.22E-01 | -0.28±0.25 | 0 | 8.70E-01 | 7.40E-01 | -0.23±0.29 | 8.09E-01 | -0.25±0.29 | 7.71E-01 | -0.27±0.41 | 9.98E-01 | -0.34±0.45 | 9.91E-01 |
| glucuronate | 0.22±0.24 | 0 | 6.74E-01 | 8.44E-01 | 0.28±0.25 | 0 | 5.44E-01 | 7.40E-01 | 0.15±0.3 | 8.92E-01 | 0.17±0.31 | 8.75E-01 | 0.37±0.41 | 9.98E-01 | 0.48±0.42 | 9.91E-01 |
| X - 24518 | 0.24±0.24 | 64.78 | 9.20E-02 | 8.34E-01 | 0.3±0.26 | 66.88 | 8.23E-02 | 7.40E-01 | -0.04±0.29 | 9.71E-01 | 0±0.31 | 9.95E-01 | 0.83±0.43 | 9.98E-01 | 1.01±0.49 | 9.91E-01 |
| nonanoylcarnitine (C9) | -0.22±0.23 | 76.7 | 3.83E-02 | 8.44E-01 | -0.27±0.24 | 79.75 | 2.63E-02 | 7.42E-01 | -0.54±0.28 | 3.84E-01 | -0.63±0.29 | 3.06E-01 | 0.51±0.43 | 9.98E-01 | 0.53±0.44 | 9.91E-01 |
| palmitoyl-sphingosine-phosphoethanolamine (d18:1/16:0) | -0.2±0.22 | 0 | 4.41E-01 | 8.44E-01 | -0.26±0.23 | 0 | 3.72E-01 | 7.43E-01 | -0.31±0.26 | 6.77E-01 | -0.37±0.26 | 6.02E-01 | 0.06±0.4 | 9.98E-01 | 0.14±0.51 | 9.91E-01 |
| 3-methyl-2-oxobutyrate | 0.3±0.25 | 46.26 | 1.73E-01 | 7.66E-01 | 0.29±0.26 | 64.98 | 9.11E-02 | 7.45E-01 | 0.63±0.34 | 4.22E-01 | 0.71±0.36 | 3.86E-01 | -0.04±0.35 | 9.98E-01 | -0.17±0.37 | 9.91E-01 |
| X - 23782 | -0.24±0.24 | 0 | 8.36E-01 | 8.23E-01 | -0.28±0.25 | 0 | 7.95E-01 | 7.45E-01 | -0.28±0.31 | 7.77E-01 | -0.33±0.31 | 7.13E-01 | -0.18±0.37 | 9.98E-01 | -0.19±0.43 | 9.91E-01 |
| sphingomyelin (d18:0/20:0, d16:0/22:0)* | -0.21±0.24 | 0 | 3.85E-01 | 8.44E-01 | -0.28±0.26 | 0 | 4.30E-01 | 7.51E-01 | -0.4±0.33 | 6.71E-01 | -0.46±0.34 | 6.11E-01 | 0.01±0.35 | 9.98E-01 | -0.06±0.39 | 9.91E-01 |
| andro steroid monosulfate C19H28O6S (1)* | 0.29±0.27 | 0 | 4.50E-01 | 8.08E-01 | 0.31±0.28 | 0 | 3.47E-01 | 7.51E-01 | 0.16±0.32 | 8.92E-01 | 0.14±0.33 | 9.16E-01 | 0.6±0.49 | 9.98E-01 | 0.7±0.5 | 9.91E-01 |
| cis-4-decenoate (10:1n6)* | -0.22±0.23 | 0 | 5.82E-01 | 8.38E-01 | -0.24±0.22 | 0 | 7.68E-01 | 7.51E-01 | -0.31±0.28 | 7.13E-01 | -0.28±0.26 | 7.05E-01 | -0.03±0.43 | 9.98E-01 | -0.14±0.41 | 9.91E-01 |
| X - 12680 | 0.2±0.25 | 36.23 | 2.10E-01 | 8.44E-01 | 0.29±0.27 | 23.12 | 2.54E-01 | 7.52E-01 | 0±0.3 | 9.96E-01 | 0.12±0.31 | 9.29E-01 | 0.68±0.46 | 9.98E-01 | 0.81±0.53 | 9.91E-01 |
| 1-methylurate | -0.26±0.23 | 0 | 3.54E-01 | 7.93E-01 | -0.26±0.24 | 6.45 | 3.01E-01 | 7.60E-01 | -0.39±0.27 | 5.95E-01 | -0.4±0.27 | 5.78E-01 | 0.1±0.45 | 9.98E-01 | 0.15±0.46 | 9.91E-01 |
| cis-4-decenoylcarnitine (C10:1) | -0.2±0.24 | 0 | 3.46E-01 | 8.44E-01 | -0.26±0.24 | 0 | 3.43E-01 | 7.60E-01 | -0.36±0.29 | 6.75E-01 | -0.41±0.29 | 5.97E-01 | 0.13±0.42 | 9.98E-01 | 0.08±0.43 | 9.91E-01 |
| 10-heptadecenoate (17:1n7) | -0.24±0.24 | 0 | 5.28E-01 | 8.29E-01 | -0.27±0.25 | 0 | 4.63E-01 | 7.60E-01 | -0.34±0.29 | 6.77E-01 | -0.39±0.3 | 6.22E-01 | -0.02±0.42 | 9.98E-01 | 0.01±0.47 | 9.91E-01 |
| X - 12830 | -0.24±0.24 | 0 | 5.64E-01 | 8.31E-01 | -0.27±0.25 | 0 | 6.28E-01 | 7.60E-01 | -0.35±0.31 | 7.03E-01 | -0.37±0.32 | 6.95E-01 | -0.07±0.39 | 9.98E-01 | -0.12±0.4 | 9.91E-01 |
| X - 11795 | 0.32±0.23 | 0 | 7.30E-01 | 6.94E-01 | 0.28±0.25 | 0 | 9.27E-01 | 7.60E-01 | 0.37±0.28 | 6.22E-01 | 0.29±0.3 | 7.43E-01 | 0.2±0.41 | 9.98E-01 | 0.24±0.49 | 9.91E-01 |
| 1,2-dipalmitoyl-GPC (16:0/16:0) | 0.3±0.27 | 0 | 9.61E-01 | 7.85E-01 | 0.3±0.27 | 0 | 7.46E-01 | 7.60E-01 | 0.29±0.3 | 7.59E-01 | 0.26±0.3 | 7.71E-01 | 0.33±0.57 | 9.98E-01 | 0.5±0.67 | 9.91E-01 |
| 2-aminooctanoate | 0.26±0.25 | 0 | 3.69E-01 | 8.19E-01 | 0.29±0.27 | 0 | 3.79E-01 | 7.61E-01 | 0.43±0.31 | 6.12E-01 | 0.46±0.33 | 6.02E-01 | -0.05±0.43 | 9.98E-01 | -0.05±0.47 | 9.91E-01 |
| butyrate/isobutyrate (4:0) | -0.26±0.25 | 0 | 6.62E-01 | 8.22E-01 | -0.28±0.25 | 0 | 9.92E-01 | 7.61E-01 | -0.33±0.3 | 7.14E-01 | -0.28±0.32 | 7.65E-01 | -0.1±0.43 | 9.98E-01 | -0.27±0.43 | 9.91E-01 |
| N6-methyllysine | 0.25±0.24 | 53.99 | 1.40E-01 | 8.19E-01 | 0.27±0.25 | 42.22 | 1.88E-01 | 7.66E-01 | 0.57±0.32 | 4.57E-01 | 0.54±0.32 | 5.01E-01 | -0.14±0.35 | 9.98E-01 | -0.15±0.41 | 9.91E-01 |
| X - 11852 | -0.25±0.28 | 0 | 3.29E-01 | 8.44E-01 | -0.3±0.28 | 0 | 4.07E-01 | 7.70E-01 | -0.49±0.37 | 6.36E-01 | -0.5±0.37 | 6.11E-01 | 0.06±0.43 | 9.98E-01 | -0.02±0.44 | 9.91E-01 |
| 5-acetylamino-6-amino-3-methyluracil | -0.26±0.22 | 0 | 4.46E-01 | 7.66E-01 | -0.24±0.22 | 0 | 6.23E-01 | 7.70E-01 | -0.12±0.28 | 8.96E-01 | -0.15±0.28 | 8.82E-01 | -0.47±0.35 | 9.98E-01 | -0.37±0.36 | 9.91E-01 |
| 3-methyl catechol sulfate (1) | -0.25±0.23 | 0 | 7.49E-01 | 8.04E-01 | -0.26±0.25 | 0 | 4.79E-01 | 7.72E-01 | -0.3±0.27 | 7.17E-01 | -0.36±0.28 | 6.42E-01 | -0.13±0.45 | 9.98E-01 | 0.05±0.51 | 9.91E-01 |
| sulfate of piperine metabolite C16H19NO3 (3)* | -0.2±0.24 | 0 | 5.12E-01 | 8.44E-01 | -0.27±0.25 | 0 | 7.27E-01 | 7.72E-01 | -0.29±0.28 | 7.33E-01 | -0.32±0.3 | 7.10E-01 | 0.05±0.45 | 9.98E-01 | -0.13±0.48 | 9.91E-01 |
| N-acetyl-2-aminoadipate | -0.2±0.24 | 55.9 | 1.32E-01 | 8.44E-01 | -0.27±0.26 | 26.2 | 2.44E-01 | 7.82E-01 | -0.44±0.29 | 5.56E-01 | -0.45±0.3 | 5.65E-01 | 0.38±0.46 | 9.98E-01 | 0.23±0.51 | 9.91E-01 |
| X - 12740 | 0.28±0.26 | 0 | 4.66E-01 | 7.91E-01 | 0.28±0.27 | 0 | 7.43E-01 | 7.84E-01 | 0.15±0.31 | 8.91E-01 | 0.22±0.32 | 8.29E-01 | 0.55±0.45 | 9.98E-01 | 0.41±0.49 | 9.91E-01 |
| 5-(galactosylhydroxy)-L-lysine | -0.3±0.26 | 52.67 | 1.46E-01 | 7.85E-01 | -0.3±0.28 | 37.5 | 2.06E-01 | 7.89E-01 | -0.57±0.32 | 4.57E-01 | -0.53±0.34 | 5.36E-01 | 0.25±0.46 | 9.98E-01 | 0.27±0.53 | 9.91E-01 |
| decanoylcarnitine (C10) | -0.16±0.24 | 0 | 4.10E-01 | 8.57E-01 | -0.26±0.25 | 0 | 3.18E-01 | 7.91E-01 | -0.29±0.29 | 7.46E-01 | -0.42±0.3 | 6.02E-01 | 0.14±0.44 | 9.98E-01 | 0.13±0.46 | 9.91E-01 |
| N6,N6,N6-trimethyllysine | 0.3±0.29 | 0 | 6.12E-01 | 8.23E-01 | 0.31±0.3 | 0 | 9.82E-01 | 7.91E-01 | 0.22±0.33 | 8.62E-01 | 0.31±0.35 | 7.65E-01 | 0.57±0.61 | 9.98E-01 | 0.32±0.58 | 9.91E-01 |
| sphingomyelin (d18:1/14:0, d16:1/16:0)* | -0.34±0.28 | 50.03 | 1.57E-01 | 7.66E-01 | -0.31±0.3 | 44.6 | 1.79E-01 | 7.92E-01 | -0.55±0.32 | 4.62E-01 | -0.51±0.33 | 5.57E-01 | 0.41±0.6 | 9.98E-01 | 0.51±0.68 | 9.91E-01 |
| phenylacetate | -0.31±0.23 | 73.68 | 5.13E-02 | 7.14E-01 | -0.25±0.24 | 63.77 | 9.66E-02 | 7.94E-01 | -0.53±0.26 | 3.38E-01 | -0.45±0.27 | 5.01E-01 | 0.62±0.53 | 9.98E-01 | 0.56±0.54 | 9.91E-01 |
| thyroxine | -0.27±0.25 | 40.96 | 1.93E-01 | 8.08E-01 | -0.27±0.26 | 0 | 3.18E-01 | 7.94E-01 | -0.44±0.29 | 5.29E-01 | -0.41±0.3 | 6.02E-01 | 0.36±0.55 | 9.98E-01 | 0.2±0.53 | 9.91E-01 |
| mannose | -0.17±0.24 | 0 | 4.37E-01 | 8.54E-01 | -0.25±0.24 | 0 | 4.21E-01 | 7.94E-01 | -0.33±0.32 | 7.33E-01 | -0.43±0.33 | 6.28E-01 | 0.04±0.36 | 9.98E-01 | -0.04±0.35 | 9.91E-01 |
| dihydroorotate | -0.21±0.23 | 0 | 4.39E-01 | 8.44E-01 | -0.24±0.24 | 0 | 6.87E-01 | 7.94E-01 | -0.33±0.28 | 6.77E-01 | -0.31±0.29 | 7.13E-01 | 0.05±0.4 | 9.98E-01 | -0.11±0.41 | 9.91E-01 |
| X - 16087 | 0.24±0.27 | 0 | 9.18E-01 | 8.44E-01 | 0.29±0.28 | 0 | 9.80E-01 | 7.94E-01 | 0.27±0.34 | 8.10E-01 | 0.29±0.35 | 7.74E-01 | 0.21±0.43 | 9.98E-01 | 0.28±0.47 | 9.91E-01 |
| argininate* | -0.26±0.22 | 0 | 6.42E-01 | 7.66E-01 | -0.24±0.24 | 0 | 8.43E-01 | 7.94E-01 | -0.35±0.29 | 6.76E-01 | -0.21±0.31 | 8.40E-01 | -0.14±0.35 | 9.98E-01 | -0.3±0.38 | 9.91E-01 |
| 1-palmitoyl-2-arachidonoyl-GPE (16:0/20:4)* | 0.31±0.28 | 0 | 8.33E-01 | 7.93E-01 | 0.29±0.29 | 0 | 7.22E-01 | 7.94E-01 | 0.27±0.34 | 8.10E-01 | 0.23±0.34 | 8.42E-01 | 0.4±0.5 | 9.98E-01 | 0.45±0.54 | 9.91E-01 |
| docosapentaenoate (n3 DPA; 22:5n3) | -0.2±0.23 | 0 | 6.93E-01 | 8.44E-01 | -0.25±0.24 | 0 | 7.34E-01 | 7.94E-01 | -0.11±0.31 | 9.12E-01 | -0.18±0.32 | 8.72E-01 | -0.3±0.34 | 9.98E-01 | -0.35±0.37 | 9.91E-01 |
| 6-hydroxyindole sulfate | 0.21±0.24 | 24.23 | 2.51E-01 | 8.44E-01 | 0.27±0.27 | 18.98 | 2.67E-01 | 7.94E-01 | 0±0.3 | 9.97E-01 | 0.08±0.32 | 9.53E-01 | 0.58±0.41 | 9.98E-01 | 0.73±0.49 | 9.91E-01 |
| laurate (12:0) | -0.26±0.26 | 73.92 | 5.02E-02 | 8.29E-01 | -0.27±0.27 | 66.9 | 8.22E-02 | 7.98E-01 | -0.63±0.32 | 3.72E-01 | -0.6±0.33 | 4.24E-01 | 0.45±0.45 | 9.98E-01 | 0.39±0.47 | 9.91E-01 |
| androstenediol (3beta,17beta) monosulfate (2) | -0.17±0.23 | 0 | 5.04E-01 | 8.54E-01 | -0.23±0.23 | 0 | 3.53E-01 | 7.98E-01 | -0.26±0.26 | 7.59E-01 | -0.34±0.25 | 6.20E-01 | 0.11±0.48 | 9.98E-01 | 0.2±0.52 | 9.91E-01 |
| gamma-glutamylglutamate | 0.29±0.25 | 0 | 6.56E-01 | 7.66E-01 | 0.26±0.26 | 0 | 3.76E-01 | 7.98E-01 | 0.37±0.3 | 6.71E-01 | 0.43±0.32 | 6.11E-01 | 0.13±0.44 | 9.98E-01 | -0.05±0.44 | 9.91E-01 |
| phenylacetylglutamate | -0.27±0.23 | 83.69 | 1.33E-02 | 7.66E-01 | -0.25±0.25 | 82.29 | 1.75E-02 | 7.99E-01 | -0.57±0.26 | 3.03E-01 | -0.52±0.27 | 3.98E-01 | 0.76±0.47 | 9.98E-01 | 1.03±0.59 | 9.91E-01 |
| sphingomyelin (d18:1/22:2, d18:2/22:1, d16:1/24:2)* | -0.34±0.29 | 48.53 | 1.63E-01 | 7.66E-01 | -0.3±0.3 | 35.24 | 2.14E-01 | 8.02E-01 | -0.59±0.34 | 4.61E-01 | -0.52±0.35 | 5.69E-01 | 0.3±0.54 | 9.98E-01 | 0.32±0.58 | 9.91E-01 |
| cholesterol sulfate | -0.2±0.25 | 0 | 4.84E-01 | 8.44E-01 | -0.27±0.27 | 0 | 4.59E-01 | 8.02E-01 | -0.35±0.33 | 7.23E-01 | -0.4±0.32 | 6.58E-01 | 0.01±0.4 | 9.98E-01 | 0.02±0.46 | 9.91E-01 |
| glycolithocholate | 0.21±0.25 | 0 | 7.16E-01 | 8.44E-01 | 0.25±0.25 | 0 | 6.39E-01 | 8.02E-01 | 0.29±0.32 | 7.86E-01 | 0.35±0.32 | 7.13E-01 | 0.1±0.41 | 9.98E-01 | 0.1±0.41 | 9.91E-01 |
| X - 25810 | 0.22±0.23 | 0 | 4.96E-01 | 8.31E-01 | 0.25±0.25 | 0 | 7.58E-01 | 8.02E-01 | 0.33±0.28 | 6.77E-01 | 0.29±0.29 | 7.42E-01 | 0.01±0.39 | 9.98E-01 | 0.12±0.47 | 9.91E-01 |
| 3-hydroxydecanoate | -0.21±0.25 | 0 | 7.51E-01 | 8.44E-01 | -0.26±0.26 | 0 | 7.47E-01 | 8.02E-01 | -0.27±0.31 | 7.99E-01 | -0.32±0.32 | 7.42E-01 | -0.1±0.42 | 9.98E-01 | -0.14±0.45 | 9.91E-01 |
| X - 21339 | -0.2±0.23 | 0 | 5.81E-01 | 8.44E-01 | -0.24±0.24 | 0 | 9.88E-01 | 8.02E-01 | -0.29±0.29 | 7.43E-01 | -0.24±0.29 | 7.97E-01 | -0.02±0.4 | 9.98E-01 | -0.23±0.41 | 9.91E-01 |
| N-carbamoylalanine | 0.23±0.27 | 0 | 6.53E-01 | 8.44E-01 | 0.29±0.29 | 0 | 6.20E-01 | 8.02E-01 | 0.15±0.33 | 8.96E-01 | 0.2±0.34 | 8.64E-01 | 0.41±0.48 | 9.98E-01 | 0.52±0.55 | 9.91E-01 |
| sphingomyelin (d18:1/20:2, d18:2/20:1, d16:1/22:2)* | -0.3±0.25 | 43.27 | 1.84E-01 | 7.66E-01 | -0.27±0.27 | 26.65 | 2.43E-01 | 8.02E-01 | -0.48±0.29 | 4.92E-01 | -0.44±0.31 | 5.97E-01 | 0.32±0.53 | 9.98E-01 | 0.29±0.55 | 9.91E-01 |
| phenylpyruvate | 0.28±0.27 | 0 | 7.38E-01 | 8.29E-01 | 0.28±0.28 | 0 | 8.78E-01 | 8.03E-01 | 0.21±0.33 | 8.69E-01 | 0.25±0.35 | 8.25E-01 | 0.41±0.48 | 9.98E-01 | 0.34±0.49 | 9.91E-01 |
| sulfate* | 0.2±0.23 | 0 | 6.93E-01 | 8.44E-01 | 0.25±0.25 | 0 | 5.32E-01 | 8.04E-01 | 0.27±0.3 | 7.79E-01 | 0.35±0.31 | 6.95E-01 | 0.08±0.39 | 9.98E-01 | 0.01±0.46 | 9.91E-01 |
| ascorbic acid 3-sulfate* | -0.21±0.2 | 0 | 7.23E-01 | 8.22E-01 | -0.21±0.22 | 0 | 8.20E-01 | 8.04E-01 | -0.26±0.25 | 7.40E-01 | -0.24±0.26 | 7.53E-01 | -0.11±0.32 | 9.98E-01 | -0.14±0.38 | 9.91E-01 |
| gamma-glutamyl-2-aminobutyrate | -0.19±0.22 | 0 | 8.18E-01 | 8.44E-01 | -0.22±0.22 | 0 | 9.05E-01 | 8.04E-01 | -0.23±0.27 | 7.99E-01 | -0.24±0.27 | 7.65E-01 | -0.12±0.38 | 9.98E-01 | -0.18±0.41 | 9.91E-01 |
| palmitate (16:0) | -0.18±0.22 | 0 | 9.23E-01 | 8.49E-01 | -0.23±0.24 | 0 | 8.91E-01 | 8.04E-01 | -0.19±0.27 | 8.45E-01 | -0.25±0.28 | 7.65E-01 | -0.15±0.39 | 9.98E-01 | -0.18±0.44 | 9.91E-01 |
| X - 12456 | 0.21±0.27 | 0 | 7.64E-01 | 8.49E-01 | 0.29±0.29 | 0 | 9.36E-01 | 8.04E-01 | 0.16±0.32 | 8.90E-01 | 0.3±0.35 | 7.71E-01 | 0.34±0.5 | 9.98E-01 | 0.25±0.53 | 9.91E-01 |
| N-palmitoyl-heptadecasphingosine (d17:1/16:0)* | 0.21±0.27 | 0 | 4.94E-01 | 8.49E-01 | 0.28±0.29 | 0 | 6.03E-01 | 8.04E-01 | 0.1±0.31 | 9.26E-01 | 0.2±0.32 | 8.55E-01 | 0.51±0.52 | 9.98E-01 | 0.55±0.6 | 9.91E-01 |
| X - 18922 | -0.16±0.22 | 61.17 | 1.09E-01 | 8.54E-01 | -0.22±0.23 | 66.1 | 8.59E-02 | 8.04E-01 | 0.19±0.31 | 8.72E-01 | 0.16±0.32 | 8.84E-01 | -0.5±0.3 | 9.98E-01 | -0.63±0.33 | 9.91E-01 |
| N-acetylmethionine | 0.21±0.24 | 0 | 4.37E-01 | 8.44E-01 | 0.25±0.26 | 0 | 5.15E-01 | 8.04E-01 | 0.37±0.31 | 6.77E-01 | 0.37±0.31 | 6.84E-01 | 0±0.36 | 9.99E-01 | 0±0.47 | 9.99E-01 |
| succinate | -0.2±0.24 | 0 | 3.32E-01 | 8.44E-01 | -0.25±0.26 | 1.79 | 3.13E-01 | 8.07E-01 | -0.38±0.3 | 6.58E-01 | -0.42±0.31 | 6.11E-01 | 0.1±0.39 | 9.98E-01 | 0.16±0.49 | 9.91E-01 |
| xanthine | -0.21±0.24 | 19.89 | 2.64E-01 | 8.44E-01 | -0.24±0.25 | 0 | 4.26E-01 | 8.07E-01 | -0.44±0.31 | 6.04E-01 | -0.42±0.33 | 6.56E-01 | 0.1±0.36 | 9.98E-01 | -0.03±0.36 | 9.91E-01 |
| 3-ethylcatechol sulfate (1) | -0.18±0.22 | 0 | 7.82E-01 | 8.44E-01 | -0.23±0.23 | 0 | 7.99E-01 | 8.07E-01 | -0.15±0.26 | 8.79E-01 | -0.26±0.27 | 7.46E-01 | -0.28±0.42 | 9.98E-01 | -0.12±0.47 | 9.91E-01 |
| tyramine O-sulfate | 0.21±0.23 | 0 | 9.45E-01 | 8.44E-01 | 0.23±0.23 | 0 | 1.00E+00 | 8.07E-01 | 0.22±0.27 | 8.07E-01 | 0.23±0.28 | 7.97E-01 | 0.19±0.41 | 9.98E-01 | 0.23±0.42 | 9.91E-01 |
| deoxycholic acid glucuronide | -0.28±0.23 | 84.13 | 1.21E-02 | 7.66E-01 | -0.25±0.26 | 88.27 | 3.51E-03 | 8.07E-01 | 0.25±0.31 | 8.10E-01 | 0.25±0.31 | 7.97E-01 | -0.92±0.34 | 9.98E-01 | -1.4±0.47 | 9.91E-01 |
| fumarate | 0.46±0.44 | 0 | 8.12E-01 | 8.23E-01 | 0.43±0.45 | 0 | 9.02E-01 | 8.07E-01 | 0.59±0.71 | 8.02E-01 | 0.49±0.66 | 8.12E-01 | 0.37±0.57 | 9.98E-01 | 0.38±0.61 | 9.91E-01 |
| 5-methylthioadenosine (MTA) | 0.2±0.22 | 58.42 | 1.21E-01 | 8.44E-01 | 0.21±0.23 | 75.68 | 4.26E-02 | 8.10E-01 | 0.49±0.28 | 4.62E-01 | 0.59±0.3 | 3.85E-01 | -0.2±0.34 | 9.98E-01 | -0.38±0.37 | 9.91E-01 |
| urate | 0.26±0.24 | 61.74 | 1.06E-01 | 8.08E-01 | 0.24±0.26 | 73.32 | 5.29E-02 | 8.10E-01 | 0.61±0.33 | 4.09E-01 | 0.67±0.34 | 3.89E-01 | -0.18±0.36 | 9.98E-01 | -0.35±0.4 | 9.91E-01 |
| orotate | -0.2±0.27 | 58.16 | 1.22E-01 | 8.54E-01 | -0.26±0.29 | 65.46 | 8.89E-02 | 8.10E-01 | -0.65±0.4 | 5.09E-01 | -0.8±0.43 | 4.11E-01 | 0.19±0.37 | 9.98E-01 | 0.18±0.39 | 9.91E-01 |
| 3-phosphoglycerate | 0.17±0.21 | 60.36 | 1.12E-01 | 8.44E-01 | 0.2±0.22 | 61.54 | 1.07E-01 | 8.10E-01 | 0.39±0.25 | 5.22E-01 | 0.44±0.27 | 5.14E-01 | -0.31±0.37 | 9.98E-01 | -0.35±0.41 | 9.91E-01 |
| 3-amino-2-piperidone | -0.21±0.27 | 72.08 | 5.84E-02 | 8.49E-01 | -0.27±0.29 | 62.84 | 1.01E-01 | 8.10E-01 | -0.49±0.3 | 5.18E-01 | -0.51±0.32 | 5.28E-01 | 0.73±0.57 | 9.98E-01 | 0.66±0.64 | 9.91E-01 |
| sphingomyelin (d17:2/16:0, d18:2/15:0)* | -0.34±0.28 | 66.01 | 8.63E-02 | 7.66E-01 | -0.28±0.3 | 43.5 | 1.83E-01 | 8.10E-01 | -0.64±0.33 | 3.84E-01 | -0.51±0.35 | 5.78E-01 | 0.44±0.54 | 9.98E-01 | 0.41±0.6 | 9.91E-01 |
| gamma-glutamyltryptophan | -0.23±0.27 | 60.44 | 1.12E-01 | 8.44E-01 | -0.26±0.28 | 42.13 | 1.89E-01 | 8.10E-01 | -0.46±0.31 | 5.56E-01 | -0.46±0.32 | 5.81E-01 | 0.56±0.57 | 9.98E-01 | 0.39±0.56 | 9.91E-01 |
| 1-methyl-5-imidazolelactate | 0.25±0.24 | 11.09 | 2.89E-01 | 8.23E-01 | 0.24±0.26 | 0 | 3.38E-01 | 8.10E-01 | 0.44±0.3 | 5.83E-01 | 0.4±0.31 | 6.28E-01 | -0.12±0.42 | 9.98E-01 | -0.13±0.46 | 9.91E-01 |
| pregnenetriol sulfate* | -0.22±0.22 | 0 | 3.48E-01 | 8.38E-01 | -0.21±0.23 | 18.26 | 2.69E-01 | 8.10E-01 | -0.31±0.25 | 6.55E-01 | -0.32±0.25 | 6.28E-01 | 0.25±0.54 | 9.98E-01 | 0.35±0.55 | 9.91E-01 |
| S-carboxyethylcysteine | 0.18±0.22 | 34.67 | 2.16E-01 | 8.44E-01 | 0.23±0.25 | 0 | 3.29E-01 | 8.10E-01 | 0.34±0.26 | 6.35E-01 | 0.35±0.28 | 6.53E-01 | -0.3±0.45 | 9.98E-01 | -0.24±0.54 | 9.91E-01 |
| X - 24811 | -0.19±0.22 | 0 | 5.47E-01 | 8.44E-01 | -0.21±0.23 | 0 | 3.78E-01 | 8.10E-01 | -0.27±0.26 | 7.40E-01 | -0.33±0.27 | 6.59E-01 | 0.03±0.43 | 9.98E-01 | 0.14±0.45 | 9.91E-01 |
| choline | 0.21±0.24 | 0 | 5.41E-01 | 8.44E-01 | 0.22±0.24 | 0 | 4.31E-01 | 8.10E-01 | 0.33±0.31 | 7.23E-01 | 0.37±0.31 | 6.74E-01 | 0.04±0.37 | 9.98E-01 | -0.03±0.39 | 9.91E-01 |
| 2-hydroxydecanoate | -0.24±0.27 | 0 | 4.41E-01 | 8.44E-01 | -0.26±0.28 | 0 | 5.19E-01 | 8.10E-01 | -0.41±0.35 | 6.77E-01 | -0.4±0.35 | 6.95E-01 | 0.01±0.42 | 9.98E-01 | -0.03±0.45 | 9.91E-01 |
| myo-inositol | -0.2±0.22 | 0 | 4.32E-01 | 8.44E-01 | -0.22±0.24 | 0 | 5.23E-01 | 8.10E-01 | -0.34±0.28 | 6.76E-01 | -0.32±0.29 | 7.05E-01 | 0.01±0.34 | 9.98E-01 | 0.01±0.43 | 9.91E-01 |
| dodecanedioate (C12-DC) | -0.22±0.26 | 0 | 4.70E-01 | 8.44E-01 | -0.25±0.27 | 0 | 5.56E-01 | 8.10E-01 | -0.39±0.34 | 7.03E-01 | -0.38±0.34 | 7.05E-01 | -0.01±0.4 | 9.98E-01 | -0.06±0.42 | 9.91E-01 |
| gamma-glutamylleucine | 0.27±0.27 | 0 | 9.70E-01 | 8.29E-01 | 0.26±0.27 | 0 | 6.01E-01 | 8.10E-01 | 0.27±0.32 | 7.94E-01 | 0.35±0.33 | 7.12E-01 | 0.25±0.5 | 9.98E-01 | 0.04±0.49 | 9.91E-01 |
| sulfate of piperine metabolite C16H19NO3 (2)* | -0.18±0.24 | 0 | 4.81E-01 | 8.54E-01 | -0.24±0.25 | 0 | 6.31E-01 | 8.10E-01 | -0.28±0.28 | 7.43E-01 | -0.32±0.3 | 7.13E-01 | 0.09±0.46 | 9.98E-01 | -0.05±0.47 | 9.91E-01 |
| malate | 0.23±0.25 | 0 | 5.55E-01 | 8.44E-01 | 0.24±0.26 | 0 | 6.47E-01 | 8.10E-01 | 0.36±0.32 | 7.11E-01 | 0.33±0.33 | 7.41E-01 | 0.06±0.38 | 9.98E-01 | 0.07±0.45 | 9.91E-01 |
| ursodeoxycholate | 0.27±0.26 | 0 | 7.99E-01 | 8.19E-01 | 0.24±0.26 | 0 | 6.55E-01 | 8.10E-01 | 0.32±0.32 | 7.53E-01 | 0.33±0.33 | 7.41E-01 | 0.18±0.45 | 9.98E-01 | 0.08±0.44 | 9.91E-01 |
| X - 21834 | -0.23±0.24 | 0 | 7.34E-01 | 8.38E-01 | -0.23±0.25 | 0 | 6.35E-01 | 8.10E-01 | -0.29±0.29 | 7.56E-01 | -0.32±0.31 | 7.41E-01 | -0.11±0.43 | 9.98E-01 | -0.07±0.42 | 9.91E-01 |
| N-oleoyltaurine | -0.19±0.25 | 0 | 7.18E-01 | 8.53E-01 | -0.23±0.25 | 0 | 6.92E-01 | 8.10E-01 | -0.26±0.31 | 8.02E-01 | -0.31±0.31 | 7.43E-01 | -0.07±0.41 | 9.98E-01 | -0.1±0.43 | 9.91E-01 |
| bilirubin (Z,Z) | -0.15±0.24 | 0 | 6.25E-01 | 8.65E-01 | -0.23±0.25 | 0 | 6.78E-01 | 8.10E-01 | -0.25±0.31 | 8.09E-01 | -0.31±0.31 | 7.43E-01 | -0.01±0.38 | 9.98E-01 | -0.1±0.4 | 9.91E-01 |
| 1-stearoyl-2-oleoyl-GPC (18:0/18:1) | 0.26±0.24 | 0 | 8.55E-01 | 8.08E-01 | 0.24±0.26 | 0 | 9.22E-01 | 8.10E-01 | 0.29±0.29 | 7.49E-01 | 0.25±0.29 | 7.65E-01 | 0.19±0.46 | 9.98E-01 | 0.19±0.55 | 9.91E-01 |
| X - 17612 | 0.26±0.29 | 0 | 8.29E-01 | 8.44E-01 | 0.29±0.3 | 0 | 8.43E-01 | 8.10E-01 | 0.31±0.37 | 7.99E-01 | 0.33±0.37 | 7.65E-01 | 0.18±0.48 | 9.98E-01 | 0.21±0.5 | 9.91E-01 |
| ornithine | -0.16±0.22 | 0 | 5.98E-01 | 8.54E-01 | -0.2±0.22 | 0 | 7.53E-01 | 8.10E-01 | -0.27±0.31 | 7.94E-01 | -0.27±0.32 | 7.71E-01 | -0.04±0.31 | 9.98E-01 | -0.13±0.31 | 9.91E-01 |
| perfluorooctanesulfonate (PFOS) | -0.18±0.23 | 0 | 9.18E-01 | 8.44E-01 | -0.21±0.22 | 0 | 9.35E-01 | 8.10E-01 | -0.2±0.29 | 8.47E-01 | -0.22±0.29 | 8.07E-01 | -0.16±0.35 | 9.98E-01 | -0.19±0.34 | 9.91E-01 |
| N-acetylglucosamine/N-acetylgalactosamine | 0.26±0.27 | 0 | 7.49E-01 | 8.31E-01 | 0.26±0.28 | 0 | 8.31E-01 | 8.10E-01 | 0.22±0.3 | 8.43E-01 | 0.23±0.31 | 8.12E-01 | 0.42±0.57 | 9.98E-01 | 0.38±0.61 | 9.91E-01 |
| gamma-glutamyltyrosine | 0.23±0.27 | 0 | 6.44E-01 | 8.44E-01 | 0.25±0.27 | 0 | 8.35E-01 | 8.10E-01 | 0.17±0.3 | 8.79E-01 | 0.23±0.3 | 8.12E-01 | 0.5±0.64 | 9.98E-01 | 0.38±0.66 | 9.91E-01 |
| 1-(1-enyl-palmitoyl)-GPC (P-16:0)* | 0.24±0.28 | 0 | 7.39E-01 | 8.44E-01 | 0.26±0.29 | 0 | 7.95E-01 | 8.10E-01 | 0.19±0.32 | 8.79E-01 | 0.22±0.33 | 8.41E-01 | 0.4±0.55 | 9.98E-01 | 0.4±0.6 | 9.91E-01 |
| 3-carboxy-4-methyl-5-pentyl-2-furanpropionate (3-CMPFP)** | 0.19±0.25 | 0 | 7.48E-01 | 8.54E-01 | 0.23±0.26 | 0 | 8.78E-01 | 8.10E-01 | 0.13±0.3 | 8.96E-01 | 0.21±0.31 | 8.42E-01 | 0.31±0.45 | 9.98E-01 | 0.3±0.47 | 9.91E-01 |
| dihomo-linolenoylcarnitine (C20:3n3 or 6)* | 0.24±0.26 | 0 | 7.54E-01 | 8.44E-01 | 0.24±0.26 | 0 | 7.57E-01 | 8.10E-01 | 0.18±0.32 | 8.79E-01 | 0.19±0.32 | 8.55E-01 | 0.36±0.46 | 9.98E-01 | 0.37±0.48 | 9.91E-01 |
| erucate (22:1n9) | -0.18±0.26 | 0 | 3.72E-01 | 8.57E-01 | -0.25±0.27 | 0 | 6.34E-01 | 8.10E-01 | -0.03±0.31 | 9.81E-01 | -0.16±0.33 | 8.85E-01 | -0.53±0.47 | 9.98E-01 | -0.44±0.5 | 9.91E-01 |
| X - 25371 | -0.18±0.23 | 0 | 9.80E-01 | 8.49E-01 | -0.22±0.24 | 0 | 4.66E-01 | 8.10E-01 | -0.19±0.31 | 8.79E-01 | -0.07±0.31 | 9.62E-01 | -0.17±0.35 | 9.98E-01 | -0.43±0.38 | 9.91E-01 |
| 2-methoxyhydroquinone sulfate (1) | 0.17±0.25 | 50.26 | 1.56E-01 | 8.57E-01 | 0.24±0.26 | 36.45 | 2.10E-01 | 8.10E-01 | -0.07±0.3 | 9.49E-01 | 0.03±0.31 | 9.86E-01 | 0.69±0.44 | 9.98E-01 | 0.74±0.47 | 9.91E-01 |
| 1-ribosyl-imidazoleacetate* | 0.13±0.24 | 0 | 4.59E-01 | 9.03E-01 | 0.25±0.27 | 30.07 | 2.32E-01 | 8.10E-01 | -0.04±0.33 | 9.81E-01 | 0.03±0.33 | 9.86E-01 | 0.32±0.35 | 9.98E-01 | 0.73±0.48 | 9.91E-01 |
| glucose | -0.14±0.22 | 0 | 6.09E-01 | 8.73E-01 | -0.2±0.23 | 0 | 6.80E-01 | 8.14E-01 | -0.27±0.34 | 8.10E-01 | -0.32±0.37 | 7.65E-01 | -0.04±0.29 | 9.98E-01 | -0.13±0.29 | 9.91E-01 |
| alpha-ketoglutarate | 0.27±0.27 | 0 | 8.75E-01 | 8.31E-01 | 0.26±0.29 | 0 | 9.68E-01 | 8.14E-01 | 0.3±0.34 | 7.86E-01 | 0.27±0.35 | 8.02E-01 | 0.21±0.47 | 9.98E-01 | 0.24±0.57 | 9.91E-01 |
| homostachydrine* | -0.17±0.23 | 0 | 8.86E-01 | 8.54E-01 | -0.22±0.25 | 0 | 9.96E-01 | 8.14E-01 | -0.14±0.3 | 8.96E-01 | -0.22±0.31 | 8.24E-01 | -0.2±0.35 | 9.98E-01 | -0.22±0.42 | 9.91E-01 |
| 1-methyl-4-imidazoleacetate | 0.15±0.26 | 22.58 | 2.56E-01 | 8.75E-01 | 0.25±0.28 | 62.01 | 1.05E-01 | 8.14E-01 | -0.08±0.33 | 9.50E-01 | -0.05±0.34 | 9.76E-01 | 0.52±0.41 | 9.98E-01 | 0.95±0.52 | 9.91E-01 |
| pentose acid* | 0.18±0.25 | 0 | 3.43E-01 | 8.54E-01 | 0.23±0.26 | 52.46 | 1.47E-01 | 8.14E-01 | 0.03±0.3 | 9.81E-01 | 0.02±0.3 | 9.95E-01 | 0.54±0.45 | 9.98E-01 | 0.89±0.52 | 9.91E-01 |
| 10-nonadecenoate (19:1n9) | -0.19±0.24 | 0 | 6.90E-01 | 8.44E-01 | -0.22±0.25 | 0 | 6.60E-01 | 8.19E-01 | -0.27±0.3 | 7.94E-01 | -0.3±0.31 | 7.43E-01 | -0.07±0.39 | 9.98E-01 | -0.06±0.44 | 9.91E-01 |
| 4-ethylphenylsulfate | -0.2±0.27 | 18.57 | 2.68E-01 | 8.54E-01 | -0.25±0.28 | 0 | 4.01E-01 | 8.19E-01 | -0.03±0.31 | 9.81E-01 | -0.1±0.33 | 9.42E-01 | -0.71±0.53 | 9.98E-01 | -0.62±0.53 | 9.91E-01 |
| 2-acetamidophenol sulfate | 0.17±0.26 | 50.7 | 1.54E-01 | 8.57E-01 | 0.23±0.26 | 26.08 | 2.45E-01 | 8.19E-01 | -0.07±0.31 | 9.50E-01 | 0.02±0.32 | 9.95E-01 | 0.73±0.47 | 9.98E-01 | 0.66±0.46 | 9.91E-01 |
| phytanate | 0.21±0.25 | 0 | 8.98E-01 | 8.44E-01 | 0.23±0.26 | 0 | 9.54E-01 | 8.20E-01 | 0.19±0.32 | 8.79E-01 | 0.22±0.32 | 8.37E-01 | 0.25±0.42 | 9.98E-01 | 0.25±0.47 | 9.91E-01 |
| ceramide (d16:1/24:1, d18:1/22:1)* | 0.28±0.31 | 0 | 8.96E-01 | 8.44E-01 | 0.29±0.33 | 0 | 9.38E-01 | 8.21E-01 | 0.32±0.4 | 8.10E-01 | 0.31±0.41 | 8.12E-01 | 0.23±0.48 | 9.98E-01 | 0.26±0.56 | 9.91E-01 |
| X - 25343 | 0.21±0.23 | 12.82 | 2.84E-01 | 8.44E-01 | 0.21±0.24 | 46.86 | 1.70E-01 | 8.22E-01 | 0.43±0.3 | 5.95E-01 | 0.49±0.32 | 5.41E-01 | -0.05±0.34 | 9.98E-01 | -0.18±0.37 | 9.91E-01 |
| X - 11787 | -0.17±0.21 | 0 | 8.21E-01 | 8.50E-01 | -0.19±0.22 | 0 | 7.03E-01 | 8.22E-01 | -0.22±0.31 | 8.45E-01 | -0.11±0.31 | 9.31E-01 | -0.12±0.3 | 9.98E-01 | -0.28±0.32 | 9.91E-01 |
| 12-HETE | -0.17±0.23 | 0 | 5.11E-01 | 8.54E-01 | -0.2±0.23 | 0 | 5.15E-01 | 8.22E-01 | -0.05±0.29 | 9.62E-01 | -0.08±0.3 | 9.52E-01 | -0.37±0.38 | 9.98E-01 | -0.39±0.37 | 9.91E-01 |
| 4-cholesten-3-one | 0.26±0.24 | 0 | 9.99E-01 | 8.08E-01 | 0.22±0.25 | 0 | 7.31E-01 | 8.23E-01 | 0.26±0.35 | 8.31E-01 | 0.31±0.36 | 7.74E-01 | 0.26±0.34 | 9.98E-01 | 0.13±0.34 | 9.91E-01 |
| hypotaurine | -0.24±0.25 | 60.52 | 1.12E-01 | 8.38E-01 | -0.22±0.25 | 65.9 | 8.68E-02 | 8.23E-01 | -0.43±0.27 | 5.24E-01 | -0.43±0.28 | 5.57E-01 | 0.56±0.56 | 9.98E-01 | 0.68±0.58 | 9.91E-01 |
| docosadienoate (22:2n6) | -0.15±0.22 | 0 | 4.11E-01 | 8.57E-01 | -0.21±0.24 | 0 | 5.26E-01 | 8.25E-01 | 0.01±0.29 | 9.92E-01 | -0.09±0.3 | 9.42E-01 | -0.37±0.34 | 9.98E-01 | -0.41±0.4 | 9.91E-01 |
| 3-acetylphenol sulfate | 0.21±0.25 | 0 | 8.42E-01 | 8.44E-01 | 0.22±0.26 | 0 | 9.46E-01 | 8.29E-01 | 0.17±0.31 | 8.79E-01 | 0.21±0.31 | 8.40E-01 | 0.28±0.45 | 9.98E-01 | 0.25±0.48 | 9.91E-01 |
| 2-hydroxyoctanoate | 0.21±0.23 | 0 | 3.91E-01 | 8.44E-01 | 0.2±0.24 | 0 | 3.70E-01 | 8.33E-01 | 0.34±0.28 | 6.71E-01 | 0.34±0.28 | 6.69E-01 | -0.08±0.41 | 9.98E-01 | -0.11±0.42 | 9.91E-01 |
| 5alpha-androstan-3beta,17beta-diol disulfate | -0.21±0.27 | 0 | 9.41E-01 | 8.53E-01 | -0.23±0.28 | 0 | 6.16E-01 | 8.33E-01 | -0.2±0.34 | 8.79E-01 | -0.33±0.33 | 7.43E-01 | -0.24±0.46 | 9.98E-01 | -0.03±0.49 | 9.91E-01 |
| 1-linoleoylglycerol (18:2) | 0.22±0.24 | 0 | 7.68E-01 | 8.44E-01 | 0.21±0.25 | 0 | 6.94E-01 | 8.33E-01 | 0.27±0.29 | 7.75E-01 | 0.27±0.3 | 7.57E-01 | 0.12±0.41 | 9.98E-01 | 0.06±0.46 | 9.91E-01 |
| bilirubin (E,Z or Z,E)* | -0.16±0.24 | 0 | 6.35E-01 | 8.57E-01 | -0.21±0.25 | 0 | 7.35E-01 | 8.33E-01 | -0.25±0.31 | 8.09E-01 | -0.27±0.31 | 7.65E-01 | -0.02±0.38 | 9.98E-01 | -0.1±0.42 | 9.91E-01 |
| dodecenedioate (C12:1-DC)* | -0.2±0.25 | 0 | 9.58E-01 | 8.44E-01 | -0.22±0.26 | 0 | 9.69E-01 | 8.33E-01 | -0.19±0.32 | 8.79E-01 | -0.23±0.32 | 8.24E-01 | -0.22±0.41 | 9.98E-01 | -0.2±0.46 | 9.91E-01 |
| margarate (17:0) | -0.2±0.24 | 0 | 8.75E-01 | 8.44E-01 | -0.21±0.25 | 0 | 9.86E-01 | 8.33E-01 | -0.22±0.29 | 8.17E-01 | -0.21±0.3 | 8.27E-01 | -0.15±0.4 | 9.98E-01 | -0.2±0.44 | 9.91E-01 |
| 3-methoxycatechol sulfate (2) | 0.17±0.26 | 0 | 7.56E-01 | 8.57E-01 | 0.23±0.27 | 0 | 9.10E-01 | 8.33E-01 | 0.12±0.32 | 9.12E-01 | 0.21±0.32 | 8.46E-01 | 0.29±0.47 | 9.98E-01 | 0.27±0.48 | 9.91E-01 |
| 2-hydroxynervonate* | -0.1±0.2 | 13.32 | 2.83E-01 | 9.12E-01 | -0.19±0.23 | 20.88 | 2.61E-01 | 8.33E-01 | 0.19±0.33 | 8.79E-01 | 0.09±0.34 | 9.55E-01 | -0.26±0.26 | 9.98E-01 | -0.44±0.31 | 9.91E-01 |
| ergothioneine | 0.2±0.24 | 64.56 | 9.30E-02 | 8.44E-01 | 0.21±0.25 | 53.28 | 1.43E-01 | 8.34E-01 | 0.57±0.33 | 4.61E-01 | 0.51±0.32 | 5.28E-01 | -0.24±0.35 | 9.98E-01 | -0.23±0.39 | 9.91E-01 |
| leucine | 0.24±0.25 | 0 | 5.24E-01 | 8.31E-01 | 0.2±0.23 | 40.14 | 1.96E-01 | 8.34E-01 | 0.37±0.32 | 6.87E-01 | 0.49±0.33 | 5.65E-01 | 0.05±0.38 | 9.98E-01 | -0.11±0.33 | 9.91E-01 |
| S-methylcysteine | -0.19±0.27 | 34.92 | 2.15E-01 | 8.57E-01 | -0.23±0.28 | 33.8 | 2.19E-01 | 8.34E-01 | -0.47±0.35 | 6.30E-01 | -0.53±0.37 | 5.97E-01 | 0.2±0.41 | 9.98E-01 | 0.16±0.42 | 9.91E-01 |
| deoxycholate | 0.18±0.26 | 0 | 7.85E-01 | 8.57E-01 | 0.21±0.26 | 0 | 5.01E-01 | 8.34E-01 | 0.23±0.31 | 8.32E-01 | 0.33±0.31 | 7.13E-01 | 0.08±0.44 | 9.98E-01 | -0.03±0.45 | 9.91E-01 |
| X - 17438 | -0.21±0.26 | 0 | 5.93E-01 | 8.44E-01 | -0.22±0.27 | 0 | 6.03E-01 | 8.34E-01 | -0.31±0.32 | 7.59E-01 | -0.32±0.32 | 7.43E-01 | -0.02±0.45 | 9.98E-01 | -0.02±0.46 | 9.91E-01 |
| X - 13507 | 0.24±0.24 | 0 | 7.80E-01 | 8.31E-01 | 0.21±0.25 | 0 | 6.98E-01 | 8.34E-01 | 0.28±0.3 | 7.59E-01 | 0.27±0.3 | 7.59E-01 | 0.14±0.42 | 9.98E-01 | 0.06±0.47 | 9.91E-01 |
| X - 25172 | 0.21±0.23 | 0 | 8.33E-01 | 8.44E-01 | 0.21±0.25 | 0 | 8.50E-01 | 8.34E-01 | 0.24±0.27 | 7.94E-01 | 0.23±0.29 | 7.97E-01 | 0.13±0.42 | 9.98E-01 | 0.13±0.49 | 9.91E-01 |
| X - 18913 | -0.18±0.25 | 0 | 6.53E-01 | 8.57E-01 | -0.23±0.27 | 0 | 5.37E-01 | 8.34E-01 | -0.08±0.33 | 9.49E-01 | -0.1±0.34 | 9.48E-01 | -0.31±0.39 | 9.98E-01 | -0.44±0.45 | 9.91E-01 |
| sphingomyelin (d17:1/14:0, d16:1/15:0)* | -0.35±0.28 | 82.32 | 1.74E-02 | 7.45E-01 | -0.24±0.29 | 78.86 | 2.96E-02 | 8.34E-01 | -0.74±0.32 | 2.71E-01 | -0.59±0.34 | 4.66E-01 | 0.76±0.54 | 9.98E-01 | 0.9±0.6 | 9.91E-01 |
| octanoylcarnitine (C8) | -0.12±0.25 | 28.51 | 2.37E-01 | 9.11E-01 | -0.21±0.26 | 42.9 | 1.86E-01 | 8.34E-01 | -0.33±0.31 | 7.23E-01 | -0.45±0.31 | 5.99E-01 | 0.3±0.44 | 9.98E-01 | 0.27±0.44 | 9.91E-01 |
| pyruvate | 0.2±0.23 | 0 | 3.64E-01 | 8.44E-01 | 0.2±0.24 | 31 | 2.29E-01 | 8.34E-01 | 0.38±0.3 | 6.55E-01 | 0.44±0.32 | 6.02E-01 | -0.03±0.34 | 9.98E-01 | -0.15±0.38 | 9.91E-01 |
| 2-methylbutyrylcarnitine (C5) | 0.15±0.21 | 0 | 6.02E-01 | 8.54E-01 | 0.17±0.21 | 0 | 4.89E-01 | 8.34E-01 | 0.22±0.25 | 7.90E-01 | 0.25±0.24 | 7.16E-01 | -0.02±0.39 | 9.98E-01 | -0.09±0.42 | 9.91E-01 |
| X - 24418 | -0.16±0.23 | 0 | 7.75E-01 | 8.57E-01 | -0.2±0.24 | 0 | 6.20E-01 | 8.34E-01 | -0.21±0.29 | 8.37E-01 | -0.27±0.29 | 7.51E-01 | -0.07±0.4 | 9.98E-01 | -0.01±0.44 | 9.91E-01 |
| (S)-3-hydroxybutyrylcarnitine | 0.22±0.23 | 0 | 6.73E-01 | 8.44E-01 | 0.19±0.24 | 0 | 6.94E-01 | 8.34E-01 | 0.29±0.28 | 7.43E-01 | 0.25±0.28 | 7.65E-01 | 0.08±0.4 | 9.98E-01 | 0.05±0.43 | 9.91E-01 |
| sphingosine | 0.19±0.24 | 0 | 8.20E-01 | 8.49E-01 | 0.21±0.25 | 0 | 8.00E-01 | 8.34E-01 | 0.23±0.3 | 8.17E-01 | 0.25±0.31 | 7.97E-01 | 0.11±0.42 | 9.98E-01 | 0.11±0.45 | 9.91E-01 |
| N-acetyltyrosine | -0.14±0.26 | 0 | 6.08E-01 | 8.94E-01 | -0.22±0.27 | 0 | 8.16E-01 | 8.34E-01 | -0.24±0.32 | 8.17E-01 | -0.26±0.34 | 8.02E-01 | 0.03±0.42 | 9.98E-01 | -0.13±0.46 | 9.91E-01 |
| glycine conjugate of C10H12O2* | -0.17±0.23 | 0 | 7.76E-01 | 8.54E-01 | -0.2±0.24 | 0 | 9.42E-01 | 8.34E-01 | -0.21±0.28 | 8.17E-01 | -0.21±0.29 | 8.15E-01 | -0.07±0.41 | 9.98E-01 | -0.17±0.46 | 9.91E-01 |
| N1-methyladenosine | 0.32±0.31 | 0 | 8.08E-01 | 8.29E-01 | 0.25±0.31 | 0 | 9.61E-01 | 8.34E-01 | 0.28±0.34 | 8.09E-01 | 0.24±0.34 | 8.25E-01 | 0.48±0.76 | 9.98E-01 | 0.28±0.74 | 9.91E-01 |
| erythronate* | 0.15±0.25 | 0 | 7.74E-01 | 8.73E-01 | 0.21±0.27 | 0 | 7.94E-01 | 8.34E-01 | 0.11±0.29 | 9.12E-01 | 0.18±0.29 | 8.49E-01 | 0.27±0.48 | 9.98E-01 | 0.37±0.66 | 9.91E-01 |
| N,N-dimethyl-pro-pro | 0.24±0.29 | 0 | 4.63E-01 | 8.44E-01 | 0.24±0.29 | 0 | 4.30E-01 | 8.34E-01 | 0.18±0.3 | 8.79E-01 | 0.18±0.3 | 8.55E-01 | 0.97±1.04 | 9.98E-01 | 1.18±1.23 | 9.91E-01 |
| piperine | -0.13±0.23 | 0 | 9.23E-01 | 8.94E-01 | -0.2±0.24 | 0 | 7.74E-01 | 8.34E-01 | -0.14±0.27 | 8.88E-01 | -0.15±0.3 | 8.82E-01 | -0.09±0.41 | 9.98E-01 | -0.3±0.43 | 9.91E-01 |
| sphinganine | 0.19±0.26 | 0 | 7.32E-01 | 8.54E-01 | 0.22±0.27 | 0 | 8.22E-01 | 8.34E-01 | 0.12±0.33 | 9.08E-01 | 0.18±0.33 | 8.82E-01 | 0.31±0.45 | 9.98E-01 | 0.31±0.47 | 9.91E-01 |
| ribitol | 0.21±0.27 | 0 | 3.19E-01 | 8.53E-01 | 0.21±0.26 | 0 | 3.83E-01 | 8.34E-01 | 0.06±0.31 | 9.54E-01 | 0.11±0.28 | 9.18E-01 | 0.72±0.58 | 9.98E-01 | 0.72±0.64 | 9.91E-01 |
| indolepropionate | -0.17±0.2 | 64.61 | 9.28E-02 | 8.44E-01 | -0.17±0.22 | 58.64 | 1.20E-01 | 8.35E-01 | -0.33±0.22 | 5.54E-01 | -0.31±0.24 | 6.11E-01 | 0.53±0.46 | 9.98E-01 | 0.61±0.54 | 9.91E-01 |
| X - 23587 | 0.17±0.24 | 32.81 | 2.22E-01 | 8.57E-01 | 0.2±0.26 | 17.32 | 2.71E-01 | 8.35E-01 | 0.4±0.31 | 6.45E-01 | 0.42±0.33 | 6.30E-01 | -0.21±0.4 | 9.98E-01 | -0.16±0.41 | 9.91E-01 |
| X - 24494 | -0.11±0.23 | 0 | 3.48E-01 | 9.11E-01 | -0.17±0.23 | 22.12 | 2.57E-01 | 8.35E-01 | -0.26±0.28 | 7.75E-01 | -0.35±0.28 | 6.48E-01 | 0.2±0.4 | 9.98E-01 | 0.21±0.41 | 9.91E-01 |
| X - 11299 | 0.15±0.26 | 40.48 | 1.95E-01 | 8.85E-01 | 0.2±0.27 | 16.76 | 2.73E-01 | 8.35E-01 | 0.41±0.33 | 6.55E-01 | 0.42±0.33 | 6.50E-01 | -0.27±0.41 | 9.98E-01 | -0.18±0.44 | 9.91E-01 |
| 4-methylcatechol sulfate | -0.22±0.23 | 13.39 | 2.83E-01 | 8.31E-01 | -0.18±0.23 | 0 | 3.51E-01 | 8.35E-01 | -0.37±0.26 | 6.04E-01 | -0.31±0.27 | 6.98E-01 | 0.18±0.44 | 9.98E-01 | 0.18±0.46 | 9.91E-01 |
| p-cresol glucuronide* | -0.2±0.23 | 15.57 | 2.76E-01 | 8.44E-01 | -0.18±0.24 | 0 | 3.66E-01 | 8.35E-01 | -0.34±0.26 | 6.52E-01 | -0.31±0.28 | 7.05E-01 | 0.26±0.48 | 9.98E-01 | 0.19±0.47 | 9.91E-01 |
| asparagine | -0.13±0.28 | 0 | 3.42E-01 | 9.12E-01 | -0.21±0.28 | 0 | 3.92E-01 | 8.35E-01 | -0.28±0.32 | 7.94E-01 | -0.36±0.33 | 7.12E-01 | 0.35±0.57 | 9.98E-01 | 0.21±0.57 | 9.91E-01 |
| gamma-tocopherol/beta-tocopherol | 0.23±0.26 | 0 | 5.70E-01 | 8.44E-01 | 0.2±0.27 | 0 | 4.71E-01 | 8.35E-01 | 0.36±0.34 | 7.40E-01 | 0.36±0.34 | 7.13E-01 | 0.06±0.4 | 9.98E-01 | -0.04±0.43 | 9.91E-01 |
| hexanoylcarnitine (C6) | -0.11±0.25 | 0 | 5.39E-01 | 9.21E-01 | -0.19±0.25 | 0 | 4.79E-01 | 8.35E-01 | -0.22±0.31 | 8.43E-01 | -0.33±0.32 | 7.21E-01 | 0.09±0.41 | 9.98E-01 | 0.04±0.42 | 9.91E-01 |
| 5,6-dihydrothymine | 0.25±0.25 | 0 | 6.99E-01 | 8.31E-01 | 0.19±0.24 | 0 | 5.72E-01 | 8.35E-01 | 0.32±0.32 | 7.43E-01 | 0.3±0.31 | 7.50E-01 | 0.12±0.41 | 9.98E-01 | 0.02±0.39 | 9.91E-01 |
| trigonelline (N'-methylnicotinate) | -0.17±0.22 | 0 | 6.64E-01 | 8.54E-01 | -0.17±0.23 | 0 | 5.79E-01 | 8.35E-01 | -0.23±0.26 | 7.94E-01 | -0.25±0.27 | 7.56E-01 | -0.01±0.43 | 9.98E-01 | 0.04±0.44 | 9.91E-01 |
| heneicosapentaenoate (21:5n3) | 0.19±0.25 | 0 | 4.14E-01 | 8.52E-01 | 0.2±0.25 | 0 | 6.62E-01 | 8.35E-01 | 0.34±0.31 | 7.13E-01 | 0.28±0.32 | 7.65E-01 | -0.08±0.42 | 9.98E-01 | 0.05±0.42 | 9.91E-01 |
| X - 11470 | -0.16±0.21 | 0 | 7.76E-01 | 8.54E-01 | -0.18±0.22 | 0 | 6.70E-01 | 8.35E-01 | -0.2±0.26 | 8.17E-01 | -0.24±0.27 | 7.65E-01 | -0.07±0.37 | 9.98E-01 | -0.04±0.38 | 9.91E-01 |
| X - 11847 | 0.21±0.25 | 0 | 7.23E-01 | 8.44E-01 | 0.2±0.26 | 0 | 7.73E-01 | 8.35E-01 | 0.27±0.32 | 7.94E-01 | 0.26±0.32 | 7.97E-01 | 0.08±0.43 | 9.98E-01 | 0.1±0.45 | 9.91E-01 |
| X - 13866 | 0.18±0.24 | 0 | 5.91E-01 | 8.54E-01 | 0.19±0.26 | 0 | 7.58E-01 | 8.35E-01 | 0.27±0.29 | 7.79E-01 | 0.25±0.31 | 8.00E-01 | -0.01±0.44 | 9.98E-01 | 0.08±0.45 | 9.91E-01 |
| glycine | -0.12±0.22 | 0 | 7.59E-01 | 8.91E-01 | -0.19±0.25 | 0 | 7.61E-01 | 8.35E-01 | -0.19±0.31 | 8.77E-01 | -0.26±0.34 | 8.02E-01 | -0.06±0.31 | 9.98E-01 | -0.11±0.37 | 9.91E-01 |
| 16-hydroxypalmitate | -0.16±0.25 | 0 | 9.27E-01 | 8.57E-01 | -0.2±0.27 | 0 | 7.84E-01 | 8.35E-01 | -0.18±0.31 | 8.79E-01 | -0.25±0.32 | 8.04E-01 | -0.13±0.42 | 9.98E-01 | -0.09±0.48 | 9.91E-01 |
| 3-hydroxy-2-methylpyridine sulfate | -0.25±0.26 | 0 | 8.15E-01 | 8.31E-01 | -0.2±0.27 | 0 | 8.42E-01 | 8.35E-01 | -0.29±0.31 | 7.66E-01 | -0.23±0.31 | 8.12E-01 | -0.16±0.47 | 9.98E-01 | -0.11±0.54 | 9.91E-01 |
| cystathionine | 0.14±0.24 | 0 | 7.15E-01 | 8.91E-01 | 0.19±0.24 | 0 | 8.75E-01 | 8.35E-01 | 0.08±0.29 | 9.44E-01 | 0.21±0.29 | 8.14E-01 | 0.26±0.42 | 9.98E-01 | 0.13±0.46 | 9.91E-01 |
| X - 12407 | 0.17±0.25 | 0 | 7.68E-01 | 8.57E-01 | 0.2±0.25 | 0 | 9.46E-01 | 8.35E-01 | 0.12±0.3 | 8.96E-01 | 0.21±0.31 | 8.33E-01 | 0.28±0.44 | 9.98E-01 | 0.18±0.45 | 9.91E-01 |
| 3-(3-hydroxyphenyl)propionate | 0.21±0.26 | 0 | 7.70E-01 | 8.44E-01 | 0.19±0.26 | 0 | 9.65E-01 | 8.35E-01 | 0.16±0.31 | 8.88E-01 | 0.2±0.3 | 8.45E-01 | 0.32±0.46 | 9.98E-01 | 0.17±0.48 | 9.91E-01 |
| X - 25267 | 0.22±0.26 | 0 | 8.38E-01 | 8.44E-01 | 0.21±0.27 | 0 | 9.14E-01 | 8.35E-01 | 0.26±0.33 | 8.10E-01 | 0.19±0.33 | 8.70E-01 | 0.15±0.43 | 9.98E-01 | 0.25±0.49 | 9.91E-01 |
| gamma-glutamylphenylalanine | 0.24±0.27 | 0 | 5.85E-01 | 8.44E-01 | 0.22±0.29 | 0 | 7.46E-01 | 8.35E-01 | 0.16±0.31 | 8.86E-01 | 0.17±0.32 | 8.82E-01 | 0.53±0.59 | 9.98E-01 | 0.4±0.62 | 9.91E-01 |
| N6-methyladenosine | 0.22±0.27 | 0 | 6.78E-01 | 8.44E-01 | 0.22±0.28 | 0 | 7.85E-01 | 8.35E-01 | 0.14±0.32 | 8.96E-01 | 0.17±0.33 | 8.82E-01 | 0.39±0.49 | 9.98E-01 | 0.35±0.54 | 9.91E-01 |
| methylsuccinoylcarnitine | 0.12±0.24 | 0 | 7.96E-01 | 9.08E-01 | 0.19±0.25 | 0 | 8.14E-01 | 8.35E-01 | 0.07±0.3 | 9.49E-01 | 0.15±0.3 | 8.84E-01 | 0.2±0.4 | 9.98E-01 | 0.28±0.46 | 9.91E-01 |
| 3-methylxanthine | 0.2±0.25 | 0 | 6.90E-01 | 8.44E-01 | 0.19±0.25 | 0 | 7.70E-01 | 8.35E-01 | 0.13±0.3 | 8.96E-01 | 0.14±0.3 | 8.92E-01 | 0.34±0.43 | 9.98E-01 | 0.31±0.46 | 9.91E-01 |
| kynurenate | -0.14±0.24 | 0 | 9.19E-01 | 8.82E-01 | -0.2±0.26 | 0 | 7.90E-01 | 8.35E-01 | -0.16±0.31 | 8.86E-01 | -0.15±0.32 | 8.98E-01 | -0.11±0.39 | 9.98E-01 | -0.29±0.44 | 9.91E-01 |
| indolebutyrate | 0.16±0.25 | 0 | 5.24E-01 | 8.57E-01 | 0.19±0.25 | 0 | 7.10E-01 | 8.35E-01 | 0.05±0.3 | 9.69E-01 | 0.12±0.31 | 9.17E-01 | 0.38±0.43 | 9.98E-01 | 0.32±0.44 | 9.91E-01 |
| S-adenosylhomocysteine (SAH) | 0.17±0.26 | 0 | 5.91E-01 | 8.57E-01 | 0.21±0.28 | 0 | 5.91E-01 | 8.35E-01 | 0.07±0.32 | 9.50E-01 | 0.12±0.32 | 9.29E-01 | 0.38±0.47 | 9.98E-01 | 0.46±0.54 | 9.91E-01 |
| X - 16935 | -0.17±0.23 | 33.26 | 2.21E-01 | 8.54E-01 | -0.18±0.24 | 54.46 | 1.38E-01 | 8.35E-01 | 0.07±0.31 | 9.49E-01 | 0.11±0.31 | 9.34E-01 | -0.5±0.36 | 9.98E-01 | -0.61±0.38 | 9.91E-01 |
| 3-hydroxyoctanoylcarnitine (2) | 0.26±0.26 | 0 | 5.11E-01 | 8.22E-01 | 0.2±0.26 | 0 | 3.71E-01 | 8.35E-01 | 0.15±0.31 | 8.94E-01 | 0.05±0.31 | 9.69E-01 | 0.51±0.46 | 9.98E-01 | 0.56±0.48 | 9.91E-01 |
| X - 12847 | 0.18±0.26 | 0 | 7.72E-01 | 8.57E-01 | 0.2±0.27 | 0 | 4.49E-01 | 8.35E-01 | 0.13±0.31 | 8.96E-01 | 0.06±0.34 | 9.70E-01 | 0.29±0.48 | 9.98E-01 | 0.49±0.47 | 9.91E-01 |
| propionylcarnitine (C3) | -0.15±0.2 | 0 | 8.50E-01 | 8.49E-01 | -0.16±0.21 | 0 | 4.30E-01 | 8.35E-01 | -0.11±0.3 | 9.08E-01 | 0.03±0.31 | 9.86E-01 | -0.19±0.26 | 9.98E-01 | -0.3±0.28 | 9.91E-01 |
| stachydrine | -0.21±0.22 | 0 | 5.55E-01 | 8.44E-01 | -0.17±0.23 | 0 | 3.80E-01 | 8.35E-01 | -0.11±0.28 | 9.04E-01 | -0.02±0.29 | 9.94E-01 | -0.38±0.36 | 9.98E-01 | -0.43±0.38 | 9.91E-01 |
| undecenoylcarnitine (C11:1) | 0.18±0.25 | 0 | 3.34E-01 | 8.54E-01 | 0.2±0.26 | 23.47 | 2.53E-01 | 8.35E-01 | 0.03±0.3 | 9.81E-01 | 0.01±0.3 | 9.95E-01 | 0.55±0.46 | 9.98E-01 | 0.66±0.48 | 9.91E-01 |
| N-palmitoyl-sphingosine (d18:1/16:0) | 0.2±0.23 | 0 | 3.97E-01 | 8.44E-01 | 0.2±0.25 | 0 | 4.13E-01 | 8.35E-01 | 0.39±0.32 | 6.71E-01 | 0.37±0.33 | 6.98E-01 | 0±0.33 | 9.99E-01 | -0.03±0.37 | 9.91E-01 |
| 1-arachidonoyl-GPE (20:4n6)* | 0.25±0.25 | 0 | 5.54E-01 | 8.29E-01 | 0.19±0.25 | 0 | 5.09E-01 | 8.35E-01 | 0.37±0.32 | 6.95E-01 | 0.33±0.32 | 7.28E-01 | 0.07±0.38 | 9.98E-01 | -0.01±0.39 | 9.93E-01 |
| 2-hydroxyarachidate* | 0.18±0.24 | 0 | 4.61E-01 | 8.54E-01 | 0.2±0.27 | 0 | 5.66E-01 | 8.35E-01 | 0.32±0.31 | 7.40E-01 | 0.32±0.34 | 7.52E-01 | -0.05±0.4 | 9.98E-01 | 0.01±0.43 | 9.93E-01 |
| 1-stearoyl-2-docosahexaenoyl-GPC (18:0/22:6) | 0.17±0.24 | 0 | 3.84E-01 | 8.54E-01 | 0.18±0.25 | 0 | 3.85E-01 | 8.36E-01 | 0.35±0.31 | 7.11E-01 | 0.36±0.32 | 7.05E-01 | -0.06±0.36 | 9.98E-01 | -0.08±0.39 | 9.91E-01 |
| aspartate | 0.2±0.19 | 88.47 | 3.23E-03 | 8.22E-01 | 0.14±0.2 | 91.52 | 5.94E-04 | 8.40E-01 | 0.85±0.29 | 1.02E-01 | 0.97±0.31 | 9.93E-02 | -0.28±0.25 | 9.98E-01 | -0.4±0.25 | 9.91E-01 |
| X - 24947 | -0.16±0.24 | 60.34 | 1.12E-01 | 8.57E-01 | -0.17±0.24 | 72.48 | 5.66E-02 | 8.40E-01 | -0.39±0.28 | 6.11E-01 | -0.47±0.29 | 5.15E-01 | 0.47±0.46 | 9.98E-01 | 0.56±0.45 | 9.91E-01 |
| X - 21286 | -0.21±0.24 | 80.9 | 2.21E-02 | 8.44E-01 | -0.19±0.26 | 70.22 | 6.69E-02 | 8.40E-01 | -0.52±0.28 | 4.09E-01 | -0.44±0.29 | 5.61E-01 | 0.79±0.5 | 9.98E-01 | 0.68±0.53 | 9.91E-01 |
| taurochenodeoxycholic acid 3-sulfate | -0.16±0.24 | 0 | 4.14E-01 | 8.57E-01 | -0.18±0.25 | 15.59 | 2.76E-01 | 8.40E-01 | -0.3±0.3 | 7.53E-01 | -0.37±0.31 | 6.65E-01 | 0.13±0.43 | 9.98E-01 | 0.22±0.45 | 9.91E-01 |
| 4-methyl-2-oxopentanoate | 0.22±0.26 | 0 | 4.04E-01 | 8.44E-01 | 0.2±0.28 | 0.59 | 3.16E-01 | 8.40E-01 | 0.42±0.35 | 6.77E-01 | 0.44±0.36 | 6.68E-01 | -0.02±0.4 | 9.98E-01 | -0.13±0.44 | 9.91E-01 |
| X - 24588 | 0.21±0.24 | 0 | 4.96E-01 | 8.44E-01 | 0.18±0.25 | 0 | 3.92E-01 | 8.40E-01 | 0.34±0.31 | 7.17E-01 | 0.35±0.32 | 7.05E-01 | 0.01±0.37 | 9.98E-01 | -0.08±0.39 | 9.91E-01 |
| 5-hydroxylysine | 0.17±0.21 | 0 | 5.35E-01 | 8.49E-01 | 0.16±0.23 | 0 | 3.81E-01 | 8.40E-01 | 0.26±0.26 | 7.53E-01 | 0.28±0.27 | 7.13E-01 | -0.04±0.4 | 9.98E-01 | -0.18±0.45 | 9.91E-01 |
| 7-alpha-hydroxy-3-oxo-4-cholestenoate (7-Hoca) | 0.16±0.23 | 0 | 6.82E-01 | 8.57E-01 | 0.17±0.24 | 0 | 3.95E-01 | 8.40E-01 | 0.24±0.29 | 8.09E-01 | 0.32±0.3 | 7.13E-01 | 0.04±0.39 | 9.98E-01 | -0.12±0.42 | 9.91E-01 |
| palmitoyl sphingomyelin (d18:1/16:0) | -0.14±0.26 | 0 | 4.48E-01 | 9.08E-01 | -0.2±0.28 | 0 | 4.42E-01 | 8.40E-01 | -0.28±0.33 | 7.94E-01 | -0.33±0.33 | 7.42E-01 | 0.13±0.44 | 9.98E-01 | 0.18±0.57 | 9.91E-01 |
| cysteinylglycine disulfide* | -0.16±0.26 | 0 | 4.01E-01 | 8.57E-01 | -0.19±0.25 | 0 | 4.91E-01 | 8.40E-01 | -0.28±0.29 | 7.59E-01 | -0.29±0.3 | 7.43E-01 | 0.23±0.54 | 9.98E-01 | 0.1±0.49 | 9.91E-01 |
| docosapentaenoate (n6 DPA; 22:5n6) | -0.18±0.24 | 0 | 4.64E-01 | 8.54E-01 | -0.17±0.24 | 0 | 5.29E-01 | 8.40E-01 | -0.29±0.28 | 7.40E-01 | -0.26±0.28 | 7.53E-01 | 0.09±0.44 | 9.98E-01 | 0.09±0.48 | 9.91E-01 |
| glutamine | -0.1±0.25 | 0 | 5.20E-01 | 9.26E-01 | -0.18±0.26 | 0 | 5.49E-01 | 8.40E-01 | -0.22±0.31 | 8.46E-01 | -0.3±0.32 | 7.57E-01 | 0.13±0.44 | 9.98E-01 | 0.02±0.43 | 9.91E-01 |
| glutamine conjugate of C6H10O2 (1)* | 0.21±0.23 | 0 | 7.98E-01 | 8.44E-01 | 0.18±0.24 | 0 | 7.13E-01 | 8.40E-01 | 0.25±0.29 | 7.94E-01 | 0.24±0.3 | 7.97E-01 | 0.13±0.39 | 9.98E-01 | 0.06±0.41 | 9.91E-01 |
| X - 26108 | 0.23±0.28 | 0 | 9.08E-01 | 8.44E-01 | 0.19±0.27 | 0 | 9.14E-01 | 8.40E-01 | 0.25±0.35 | 8.43E-01 | 0.22±0.33 | 8.45E-01 | 0.18±0.48 | 9.98E-01 | 0.15±0.47 | 9.91E-01 |
| proline | 0.17±0.25 | 0 | 6.36E-01 | 8.57E-01 | 0.18±0.26 | 0 | 9.64E-01 | 8.40E-01 | 0.1±0.3 | 9.23E-01 | 0.19±0.3 | 8.48E-01 | 0.36±0.48 | 9.98E-01 | 0.16±0.5 | 9.91E-01 |
| phenylacetylcarnitine | -0.2±0.22 | 0 | 5.83E-01 | 8.44E-01 | -0.17±0.24 | 0 | 9.39E-01 | 8.40E-01 | -0.28±0.26 | 7.26E-01 | -0.18±0.29 | 8.51E-01 | -0.02±0.39 | 9.98E-01 | -0.14±0.43 | 9.91E-01 |
| beta-hydroxyisovalerate | -0.12±0.22 | 0 | 9.63E-01 | 9.01E-01 | -0.16±0.22 | 0 | 9.74E-01 | 8.40E-01 | -0.13±0.27 | 8.96E-01 | -0.15±0.27 | 8.71E-01 | -0.11±0.38 | 9.98E-01 | -0.17±0.39 | 9.91E-01 |
| 1-stearoyl-2-linoleoyl-GPI (18:0/18:2) | 0.22±0.28 | 0 | 8.05E-01 | 8.53E-01 | 0.2±0.28 | 0 | 8.52E-01 | 8.40E-01 | 0.17±0.33 | 8.87E-01 | 0.17±0.33 | 8.82E-01 | 0.33±0.53 | 9.98E-01 | 0.3±0.59 | 9.91E-01 |
| sphingosine 1-phosphate | -0.1±0.2 | 0 | 9.00E-01 | 9.08E-01 | -0.16±0.22 | 0 | 9.71E-01 | 8.40E-01 | -0.13±0.31 | 8.96E-01 | -0.17±0.32 | 8.82E-01 | -0.08±0.26 | 9.98E-01 | -0.15±0.31 | 9.91E-01 |
| 1-oleoyl-2-arachidonoyl-GPE (18:1/20:4)* | 0.24±0.31 | 0 | 8.77E-01 | 8.49E-01 | 0.23±0.33 | 0 | 8.99E-01 | 8.40E-01 | 0.2±0.4 | 8.88E-01 | 0.2±0.42 | 8.94E-01 | 0.3±0.49 | 9.98E-01 | 0.28±0.52 | 9.91E-01 |
| X - 14939 | -0.12±0.24 | 0 | 9.27E-01 | 9.11E-01 | -0.17±0.24 | 0 | 8.10E-01 | 8.40E-01 | -0.1±0.29 | 9.19E-01 | -0.13±0.3 | 9.12E-01 | -0.15±0.4 | 9.98E-01 | -0.25±0.39 | 9.91E-01 |
| 1-stearoyl-2-oleoyl-GPI (18:0/18:1)* | 0.21±0.29 | 0 | 5.70E-01 | 8.54E-01 | 0.2±0.29 | 0 | 5.24E-01 | 8.40E-01 | 0.11±0.33 | 9.19E-01 | 0.11±0.32 | 9.31E-01 | 0.49±0.58 | 9.98E-01 | 0.58±0.66 | 9.91E-01 |
| X - 11315 | 0.15±0.27 | 0 | 5.64E-01 | 8.86E-01 | 0.21±0.29 | 0 | 5.29E-01 | 8.40E-01 | 0.06±0.32 | 9.59E-01 | 0.09±0.34 | 9.52E-01 | 0.41±0.51 | 9.98E-01 | 0.49±0.53 | 9.91E-01 |
| adipoylcarnitine (C6-DC) | -0.09±0.22 | 0 | 7.33E-01 | 9.26E-01 | -0.17±0.23 | 0 | 5.13E-01 | 8.40E-01 | -0.02±0.3 | 9.82E-01 | -0.05±0.29 | 9.69E-01 | -0.17±0.33 | 9.98E-01 | -0.37±0.39 | 9.91E-01 |
| 4-methylhexanoylglutamine | 0.23±0.27 | 0 | 4.66E-01 | 8.44E-01 | 0.19±0.27 | 20.22 | 2.63E-01 | 8.40E-01 | 0.11±0.32 | 9.21E-01 | 0.03±0.31 | 9.86E-01 | 0.53±0.49 | 9.98E-01 | 0.76±0.58 | 9.91E-01 |
| tetrahydrocortisol glucuronide | -0.13±0.23 | 0 | 4.53E-01 | 8.84E-01 | -0.17±0.23 | 0 | 3.65E-01 | 8.40E-01 | -0.01±0.28 | 9.90E-01 | -0.03±0.28 | 9.86E-01 | -0.36±0.38 | 9.98E-01 | -0.48±0.42 | 9.91E-01 |
| X - 17354 | -0.21±0.26 | 61.99 | 1.05E-01 | 8.44E-01 | -0.18±0.26 | 61.93 | 1.05E-01 | 8.43E-01 | -0.48±0.31 | 5.22E-01 | -0.47±0.32 | 5.70E-01 | 0.42±0.46 | 9.98E-01 | 0.45±0.47 | 9.91E-01 |
| laurylcarnitine (C12) | -0.08±0.25 | 48.69 | 1.63E-01 | 9.51E-01 | -0.18±0.26 | 59.86 | 1.14E-01 | 8.43E-01 | -0.3±0.3 | 7.53E-01 | -0.42±0.3 | 6.02E-01 | 0.49±0.47 | 9.98E-01 | 0.53±0.52 | 9.91E-01 |
| gamma-carboxyglutamate | 0.21±0.2 | 71.74 | 5.99E-02 | 8.19E-01 | 0.15±0.22 | 80.68 | 2.29E-02 | 8.44E-01 | 0.59±0.29 | 3.38E-01 | 0.6±0.3 | 3.78E-01 | -0.18±0.29 | 9.98E-01 | -0.42±0.34 | 9.91E-01 |
| 1-palmitoyl-2-docosahexaenoyl-GPC (16:0/22:6) | 0.16±0.22 | 13.09 | 2.83E-01 | 8.54E-01 | 0.17±0.25 | 0 | 3.98E-01 | 8.44E-01 | 0.4±0.32 | 6.55E-01 | 0.35±0.33 | 7.13E-01 | -0.08±0.32 | 9.98E-01 | -0.07±0.38 | 9.91E-01 |
| 4-methylguaiacol sulfate | -0.2±0.24 | 0 | 3.36E-01 | 8.44E-01 | -0.17±0.25 | 0 | 3.72E-01 | 8.44E-01 | -0.36±0.29 | 6.71E-01 | -0.31±0.3 | 7.13E-01 | 0.14±0.43 | 9.98E-01 | 0.17±0.45 | 9.91E-01 |
| 2-hydroxystearate | -0.16±0.25 | 11.01 | 2.89E-01 | 8.73E-01 | -0.17±0.26 | 0 | 3.82E-01 | 8.44E-01 | -0.35±0.31 | 7.07E-01 | -0.33±0.32 | 7.13E-01 | 0.21±0.43 | 9.98E-01 | 0.15±0.46 | 9.91E-01 |
| X - 21383 | -0.11±0.24 | 0 | 5.15E-01 | 9.12E-01 | -0.17±0.25 | 0 | 6.07E-01 | 8.44E-01 | -0.23±0.3 | 8.17E-01 | -0.26±0.3 | 7.71E-01 | 0.09±0.38 | 9.98E-01 | 0.01±0.43 | 9.91E-01 |
| dihomo-linolenate (20:3n3 or n6) | -0.13±0.24 | 0 | 6.09E-01 | 8.94E-01 | -0.17±0.25 | 0 | 6.72E-01 | 8.44E-01 | -0.23±0.3 | 8.17E-01 | -0.24±0.31 | 8.00E-01 | 0.03±0.39 | 9.98E-01 | -0.02±0.44 | 9.91E-01 |
| X - 22776 | -0.18±0.21 | 0 | 9.88E-01 | 8.44E-01 | -0.15±0.22 | 0 | 7.54E-01 | 8.44E-01 | -0.18±0.3 | 8.78E-01 | -0.22±0.3 | 8.22E-01 | -0.18±0.3 | 9.98E-01 | -0.08±0.33 | 9.91E-01 |
| X - 19438 | 0.21±0.29 | 0 | 9.88E-01 | 8.54E-01 | 0.2±0.3 | 0 | 8.08E-01 | 8.44E-01 | 0.21±0.36 | 8.79E-01 | 0.26±0.38 | 8.40E-01 | 0.22±0.48 | 9.98E-01 | 0.11±0.47 | 9.91E-01 |
| N-acetylserine | 0.13±0.22 | 0 | 8.26E-01 | 8.94E-01 | 0.17±0.25 | 0 | 8.55E-01 | 8.44E-01 | 0.17±0.29 | 8.79E-01 | 0.2±0.29 | 8.40E-01 | 0.06±0.36 | 9.98E-01 | 0.09±0.49 | 9.91E-01 |
| glucuronide of C10H18O2 (7)* | 0.09±0.24 | 0 | 9.36E-01 | 9.36E-01 | 0.17±0.25 | 0 | 8.71E-01 | 8.44E-01 | 0.1±0.3 | 9.22E-01 | 0.2±0.31 | 8.46E-01 | 0.06±0.4 | 9.98E-01 | 0.12±0.42 | 9.91E-01 |
| X - 24334 | 0.16±0.25 | 0 | 8.72E-01 | 8.68E-01 | 0.19±0.28 | 0 | 9.05E-01 | 8.44E-01 | 0.19±0.31 | 8.79E-01 | 0.21±0.33 | 8.48E-01 | 0.1±0.45 | 9.98E-01 | 0.13±0.52 | 9.91E-01 |
| glyco-beta-muricholate** | 0.17±0.25 | 0 | 8.47E-01 | 8.57E-01 | 0.17±0.25 | 0 | 9.70E-01 | 8.44E-01 | 0.14±0.31 | 8.96E-01 | 0.16±0.32 | 8.82E-01 | 0.24±0.42 | 9.98E-01 | 0.18±0.43 | 9.91E-01 |
| methionine sulfoxide | -0.16±0.23 | 0 | 8.00E-01 | 8.57E-01 | -0.16±0.23 | 0 | 8.08E-01 | 8.44E-01 | -0.21±0.3 | 8.54E-01 | -0.12±0.3 | 9.20E-01 | -0.09±0.36 | 9.98E-01 | -0.23±0.38 | 9.91E-01 |
| pregnenediol disulfate (C21H34O8S2)* | 0.22±0.29 | 0 | 9.53E-01 | 8.54E-01 | 0.2±0.3 | 0 | 7.47E-01 | 8.44E-01 | 0.23±0.35 | 8.62E-01 | 0.14±0.36 | 9.20E-01 | 0.19±0.54 | 9.98E-01 | 0.36±0.56 | 9.91E-01 |
| palmitoylcarnitine (C16) | 0.23±0.27 | 0 | 6.95E-01 | 8.44E-01 | 0.19±0.27 | 0 | 6.30E-01 | 8.44E-01 | 0.17±0.32 | 8.85E-01 | 0.11±0.31 | 9.29E-01 | 0.41±0.53 | 9.98E-01 | 0.42±0.55 | 9.91E-01 |
| X - 17367 | 0.14±0.26 | 0 | 5.04E-01 | 9.04E-01 | 0.18±0.26 | 0 | 6.59E-01 | 8.44E-01 | 0.03±0.3 | 9.81E-01 | 0.1±0.31 | 9.40E-01 | 0.4±0.47 | 9.98E-01 | 0.36±0.5 | 9.91E-01 |
| 5-hydroxyhexanoate | 0.16±0.25 | 0 | 5.59E-01 | 8.57E-01 | 0.18±0.26 | 0 | 6.25E-01 | 8.44E-01 | 0.06±0.3 | 9.55E-01 | 0.09±0.31 | 9.48E-01 | 0.37±0.44 | 9.98E-01 | 0.36±0.46 | 9.91E-01 |
| 3-hydroxydecanoylcarnitine | 0.25±0.26 | 0 | 3.60E-01 | 8.44E-01 | 0.18±0.27 | 28.95 | 2.35E-01 | 8.44E-01 | 0.09±0.31 | 9.35E-01 | -0.02±0.31 | 9.95E-01 | 0.61±0.47 | 9.98E-01 | 0.69±0.51 | 9.91E-01 |
| p-cresol sulfate | -0.17±0.2 | 51.28 | 1.52E-01 | 8.44E-01 | -0.14±0.22 | 45.04 | 1.77E-01 | 8.47E-01 | -0.28±0.22 | 6.36E-01 | -0.26±0.23 | 7.05E-01 | 0.56±0.55 | 9.98E-01 | 0.6±0.59 | 9.91E-01 |
| myristate (14:0) | -0.16±0.25 | 0 | 3.72E-01 | 8.57E-01 | -0.17±0.26 | 0 | 3.19E-01 | 8.47E-01 | -0.32±0.3 | 7.40E-01 | -0.34±0.31 | 7.05E-01 | 0.16±0.44 | 9.98E-01 | 0.22±0.47 | 9.91E-01 |
| lithocholate sulfate (1) | -0.21±0.23 | 42.87 | 1.86E-01 | 8.44E-01 | -0.15±0.24 | 18.92 | 2.67E-01 | 8.47E-01 | -0.37±0.26 | 5.92E-01 | -0.28±0.26 | 7.12E-01 | 0.38±0.5 | 9.98E-01 | 0.38±0.53 | 9.91E-01 |
| 1-stearoyl-2-arachidonoyl-GPE (18:0/20:4) | 0.23±0.27 | 0 | 8.13E-01 | 8.44E-01 | 0.18±0.27 | 0 | 7.34E-01 | 8.47E-01 | 0.28±0.35 | 8.09E-01 | 0.26±0.36 | 8.20E-01 | 0.15±0.42 | 9.98E-01 | 0.07±0.43 | 9.91E-01 |
| eicosenedioate (C20:1-DC)* | -0.15±0.23 | 0 | 7.25E-01 | 8.57E-01 | -0.16±0.25 | 0 | 9.65E-01 | 8.47E-01 | -0.21±0.29 | 8.25E-01 | -0.17±0.3 | 8.72E-01 | -0.04±0.41 | 9.98E-01 | -0.15±0.44 | 9.91E-01 |
| ectoine | 0.12±0.24 | 57 | 1.27E-01 | 9.08E-01 | 0.16±0.24 | 61.21 | 1.08E-01 | 8.47E-01 | -0.16±0.3 | 8.85E-01 | -0.16±0.31 | 8.82E-01 | 0.57±0.38 | 9.98E-01 | 0.62±0.38 | 9.91E-01 |
| N-acetylthreonine | 0.15±0.26 | 0 | 9.46E-01 | 8.84E-01 | 0.19±0.28 | 0 | 8.74E-01 | 8.47E-01 | 0.14±0.31 | 8.96E-01 | 0.16±0.32 | 8.82E-01 | 0.18±0.44 | 9.98E-01 | 0.27±0.61 | 9.91E-01 |
| X - 21471 | -0.11±0.25 | 0 | 3.72E-01 | 9.21E-01 | -0.17±0.26 | 18.57 | 2.68E-01 | 8.47E-01 | 0.07±0.32 | 9.50E-01 | 0.07±0.34 | 9.68E-01 | -0.38±0.39 | 9.98E-01 | -0.52±0.41 | 9.91E-01 |
| 1-palmitoleoyl-2-linolenoyl-GPC (16:1/18:3)* | 0.16±0.3 | 8.92 | 2.95E-01 | 9.03E-01 | 0.21±0.31 | 29.25 | 2.34E-01 | 8.47E-01 | 0.02±0.33 | 9.82E-01 | 0.05±0.34 | 9.76E-01 | 0.88±0.75 | 9.98E-01 | 1.07±0.79 | 9.91E-01 |
| 3-hydroxyoctanoylcarnitine (1) | 0.23±0.26 | 0 | 5.53E-01 | 8.44E-01 | 0.17±0.26 | 0 | 3.99E-01 | 8.47E-01 | 0.13±0.31 | 8.96E-01 | 0.03±0.31 | 9.86E-01 | 0.46±0.46 | 9.98E-01 | 0.52±0.49 | 9.91E-01 |
| X - 12117 | 0.14±0.27 | 43.39 | 1.84E-01 | 9.03E-01 | 0.19±0.29 | 45.47 | 1.76E-01 | 8.47E-01 | -0.05±0.3 | 9.69E-01 | 0.01±0.32 | 9.95E-01 | 0.79±0.55 | 9.98E-01 | 1.06±0.7 | 9.91E-01 |
| O-sulfo-L-tyrosine | 0.15±0.22 | 0 | 4.62E-01 | 8.57E-01 | 0.16±0.24 | 0 | 4.38E-01 | 8.50E-01 | 0.28±0.28 | 7.54E-01 | 0.28±0.29 | 7.46E-01 | -0.06±0.36 | 9.98E-01 | -0.14±0.45 | 9.91E-01 |
| 1-palmitoyl-2-dihomo-linolenoyl-GPC (16:0/20:3n3 or 6)* | -0.17±0.26 | 0 | 3.79E-01 | 8.57E-01 | -0.18±0.28 | 0 | 4.57E-01 | 8.50E-01 | -0.29±0.29 | 7.59E-01 | -0.28±0.31 | 7.61E-01 | 0.31±0.61 | 9.98E-01 | 0.23±0.62 | 9.91E-01 |
| cysteine sulfinic acid | -0.17±0.24 | 0 | 5.63E-01 | 8.54E-01 | -0.16±0.25 | 0 | 7.57E-01 | 8.51E-01 | -0.06±0.3 | 9.55E-01 | -0.1±0.31 | 9.42E-01 | -0.34±0.38 | 9.98E-01 | -0.26±0.4 | 9.91E-01 |
| 2-aminobutyrate | -0.1±0.22 | 0 | 3.26E-01 | 9.12E-01 | -0.15±0.23 | 0 | 3.19E-01 | 8.51E-01 | 0.12±0.31 | 9.03E-01 | 0.08±0.32 | 9.56E-01 | -0.3±0.3 | 9.98E-01 | -0.37±0.32 | 9.91E-01 |
| X - 12798 | -0.09±0.23 | 79.45 | 2.74E-02 | 9.36E-01 | -0.15±0.24 | 85.04 | 9.72E-03 | 8.54E-01 | 0.63±0.4 | 5.19E-01 | 0.72±0.42 | 4.75E-01 | -0.44±0.28 | 9.98E-01 | -0.61±0.3 | 9.91E-01 |
| valine | 0.26±0.29 | 0 | 3.77E-01 | 8.44E-01 | 0.17±0.27 | 62.24 | 1.04E-01 | 8.54E-01 | 0.57±0.46 | 6.55E-01 | 0.88±0.51 | 4.76E-01 | 0.05±0.37 | 9.98E-01 | -0.1±0.32 | 9.91E-01 |
| X - 18886 | 0.21±0.24 | 23.78 | 2.52E-01 | 8.44E-01 | 0.17±0.26 | 31.62 | 2.27E-01 | 8.54E-01 | 0.43±0.31 | 6.06E-01 | 0.38±0.32 | 6.69E-01 | -0.15±0.4 | 9.98E-01 | -0.31±0.47 | 9.91E-01 |
| lactate | 0.15±0.23 | 0 | 5.86E-01 | 8.57E-01 | 0.15±0.24 | 0 | 4.09E-01 | 8.54E-01 | 0.25±0.29 | 7.99E-01 | 0.3±0.3 | 7.42E-01 | -0.01±0.36 | 9.98E-01 | -0.12±0.41 | 9.91E-01 |
| N-acetylalanine | 0.14±0.26 | 0 | 8.75E-01 | 9.04E-01 | 0.17±0.27 | 0 | 9.93E-01 | 8.54E-01 | 0.11±0.3 | 9.08E-01 | 0.17±0.3 | 8.72E-01 | 0.21±0.51 | 9.98E-01 | 0.18±0.61 | 9.91E-01 |
| pristanate | 0.17±0.26 | 0 | 9.90E-01 | 8.57E-01 | 0.17±0.27 | 0 | 9.66E-01 | 8.54E-01 | 0.17±0.32 | 8.85E-01 | 0.16±0.33 | 8.83E-01 | 0.16±0.44 | 9.98E-01 | 0.19±0.5 | 9.91E-01 |
| beta-cryptoxanthin | -0.15±0.23 | 0 | 7.10E-01 | 8.57E-01 | -0.15±0.24 | 0 | 4.70E-01 | 8.54E-01 | -0.08±0.29 | 9.40E-01 | -0.01±0.3 | 9.95E-01 | -0.25±0.36 | 9.98E-01 | -0.36±0.37 | 9.91E-01 |
| caproate (6:0) | -0.12±0.27 | 8.68 | 2.95E-01 | 9.12E-01 | -0.17±0.27 | 17.66 | 2.70E-01 | 8.56E-01 | -0.27±0.3 | 7.94E-01 | -0.32±0.31 | 7.13E-01 | 0.44±0.6 | 9.98E-01 | 0.42±0.6 | 9.91E-01 |
| branched-chain, straight-chain, or cyclopropyl 10:1 fatty acid (1)* | -0.12±0.27 | 45.45 | 1.76E-01 | 9.21E-01 | -0.18±0.28 | 65.85 | 8.70E-02 | 8.57E-01 | -0.31±0.31 | 7.43E-01 | -0.4±0.31 | 6.42E-01 | 0.57±0.57 | 9.98E-01 | 0.89±0.69 | 9.91E-01 |
| 2-hydroxy-4-(methylthio)butanoic acid | 0.2±0.25 | 0 | 7.13E-01 | 8.44E-01 | 0.16±0.25 | 0 | 6.02E-01 | 8.57E-01 | 0.27±0.31 | 7.94E-01 | 0.25±0.31 | 7.97E-01 | 0.07±0.45 | 9.98E-01 | -0.04±0.46 | 9.91E-01 |
| cyclo(leu-pro) | 0.22±0.26 | 0 | 7.40E-01 | 8.44E-01 | 0.18±0.29 | 0 | 9.63E-01 | 8.57E-01 | 0.16±0.32 | 8.96E-01 | 0.19±0.35 | 8.82E-01 | 0.34±0.44 | 9.98E-01 | 0.16±0.49 | 9.91E-01 |
| X - 15486 | -0.11±0.23 | 0 | 9.76E-01 | 9.12E-01 | -0.15±0.24 | 0 | 9.10E-01 | 8.57E-01 | -0.11±0.28 | 9.00E-01 | -0.13±0.28 | 8.98E-01 | -0.1±0.4 | 9.98E-01 | -0.19±0.44 | 9.91E-01 |
| X - 12410 | 0.14±0.24 | 0 | 8.68E-01 | 8.91E-01 | 0.15±0.25 | 0 | 8.84E-01 | 8.57E-01 | 0.11±0.29 | 9.04E-01 | 0.13±0.29 | 8.98E-01 | 0.2±0.44 | 9.98E-01 | 0.21±0.48 | 9.91E-01 |
| 1-(1-enyl-oleoyl)-GPE (P-18:1)* | -0.15±0.23 | 0 | 5.59E-01 | 8.57E-01 | -0.15±0.24 | 0 | 4.20E-01 | 8.57E-01 | -0.03±0.3 | 9.81E-01 | 0.01±0.31 | 9.95E-01 | -0.3±0.34 | 9.98E-01 | -0.39±0.38 | 9.91E-01 |
| N-palmitoylserine | 0.22±0.27 | 0 | 5.66E-01 | 8.44E-01 | 0.16±0.27 | 0 | 4.36E-01 | 8.59E-01 | 0.34±0.34 | 7.53E-01 | 0.33±0.34 | 7.46E-01 | 0.03±0.43 | 9.98E-01 | -0.1±0.43 | 9.91E-01 |
| N-palmitoyl-sphingadienine (d18:2/16:0)* | -0.1±0.2 | 0 | 7.75E-01 | 9.12E-01 | -0.13±0.21 | 0 | 9.47E-01 | 8.59E-01 | -0.14±0.26 | 8.85E-01 | -0.14±0.26 | 8.82E-01 | -0.02±0.32 | 9.98E-01 | -0.11±0.34 | 9.91E-01 |
| N-palmitoylglycine | -0.12±0.23 | 0 | 8.45E-01 | 9.04E-01 | -0.15±0.25 | 0 | 8.13E-01 | 8.62E-01 | -0.08±0.31 | 9.49E-01 | -0.11±0.32 | 9.39E-01 | -0.17±0.33 | 9.98E-01 | -0.23±0.41 | 9.91E-01 |
| eicosenoylcarnitine (C20:1)* | -0.1±0.25 | 0 | 8.35E-01 | 9.26E-01 | -0.15±0.25 | 0 | 8.44E-01 | 8.63E-01 | -0.14±0.31 | 8.96E-01 | -0.19±0.31 | 8.55E-01 | -0.04±0.4 | 9.98E-01 | -0.08±0.43 | 9.91E-01 |
| X - 21319 | -0.09±0.23 | 0 | 7.59E-01 | 9.36E-01 | -0.14±0.24 | 0 | 7.13E-01 | 8.64E-01 | -0.14±0.28 | 8.96E-01 | -0.21±0.29 | 8.28E-01 | 0.02±0.42 | 9.98E-01 | -0.02±0.42 | 9.91E-01 |
| X - 23787 | -0.14±0.23 | 0 | 7.45E-01 | 8.76E-01 | -0.14±0.23 | 0 | 5.37E-01 | 8.68E-01 | -0.19±0.27 | 8.54E-01 | -0.23±0.27 | 7.81E-01 | -0.02±0.42 | 9.98E-01 | 0.09±0.43 | 9.91E-01 |
| cytosine | 0.19±0.26 | 0 | 9.41E-01 | 8.54E-01 | 0.16±0.26 | 0 | 8.74E-01 | 8.68E-01 | 0.2±0.32 | 8.72E-01 | 0.19±0.32 | 8.64E-01 | 0.16±0.44 | 9.98E-01 | 0.1±0.45 | 9.91E-01 |
| phenylacetylglutamine | -0.19±0.24 | 64.68 | 9.24E-02 | 8.49E-01 | -0.15±0.26 | 54.89 | 1.37E-01 | 8.70E-01 | -0.39±0.27 | 5.71E-01 | -0.34±0.28 | 6.81E-01 | 0.58±0.51 | 9.98E-01 | 0.64±0.59 | 9.91E-01 |
| 3-hydroxyisobutyrate | 0.16±0.21 | 42.31 | 1.88E-01 | 8.54E-01 | 0.13±0.23 | 46.86 | 1.70E-01 | 8.71E-01 | 0.42±0.3 | 5.92E-01 | 0.4±0.3 | 6.14E-01 | -0.15±0.31 | 9.98E-01 | -0.23±0.35 | 9.91E-01 |
| 2-ketocaprylate | 0.11±0.23 | 23.36 | 2.53E-01 | 9.12E-01 | 0.14±0.24 | 15.41 | 2.77E-01 | 8.71E-01 | 0.3±0.29 | 7.37E-01 | 0.32±0.29 | 7.05E-01 | -0.26±0.39 | 9.98E-01 | -0.23±0.41 | 9.91E-01 |
| glycocholate | 0.09±0.25 | 0 | 5.93E-01 | 9.36E-01 | 0.15±0.25 | 0 | 5.13E-01 | 8.71E-01 | 0.19±0.31 | 8.77E-01 | 0.27±0.31 | 7.71E-01 | -0.09±0.42 | 9.98E-01 | -0.09±0.44 | 9.91E-01 |
| 1-(1-enyl-palmitoyl)-2-palmitoyl-GPC (P-16:0/16:0)* | -0.07±0.25 | 0 | 5.90E-01 | 9.64E-01 | -0.17±0.29 | 0 | 4.75E-01 | 8.71E-01 | -0.17±0.32 | 8.85E-01 | -0.29±0.34 | 7.71E-01 | 0.1±0.4 | 9.98E-01 | 0.19±0.58 | 9.91E-01 |
| X - 23636 | -0.07±0.25 | 0 | 5.70E-01 | 9.64E-01 | -0.14±0.25 | 0 | 6.08E-01 | 8.71E-01 | -0.18±0.32 | 8.79E-01 | -0.25±0.32 | 8.04E-01 | 0.11±0.4 | 9.98E-01 | 0.01±0.39 | 9.91E-01 |
| cis-3,4-methyleneheptanoate | -0.11±0.22 | 0 | 6.72E-01 | 9.08E-01 | -0.13±0.22 | 0 | 8.11E-01 | 8.71E-01 | -0.18±0.27 | 8.62E-01 | -0.17±0.28 | 8.55E-01 | 0.02±0.37 | 9.98E-01 | -0.06±0.38 | 9.91E-01 |
| glycosyl-N-(2-hydroxynervonoyl)-sphingosine (d18:1/24:1(2OH))* | -0.13±0.24 | 0 | 7.29E-01 | 9.08E-01 | -0.15±0.26 | 0 | 8.67E-01 | 8.71E-01 | -0.19±0.31 | 8.77E-01 | -0.18±0.33 | 8.72E-01 | -0.02±0.39 | 9.98E-01 | -0.09±0.42 | 9.91E-01 |
| N-stearoyl-sphingosine (d18:1/18:0)* | 0.15±0.26 | 0 | 9.31E-01 | 8.85E-01 | 0.16±0.28 | 0 | 8.96E-01 | 8.71E-01 | 0.14±0.33 | 8.96E-01 | 0.19±0.35 | 8.73E-01 | 0.18±0.44 | 9.98E-01 | 0.11±0.48 | 9.91E-01 |
| oleoyl ethanolamide | -0.1±0.24 | 36.34 | 2.10E-01 | 9.21E-01 | -0.15±0.25 | 6.5 | 3.01E-01 | 8.71E-01 | 0.17±0.32 | 8.86E-01 | 0.06±0.32 | 9.69E-01 | -0.42±0.35 | 9.98E-01 | -0.47±0.4 | 9.91E-01 |
| linoleoylcarnitine (C18:2)* | -0.08±0.22 | 0 | 9.45E-01 | 9.36E-01 | -0.13±0.22 | 0 | 7.34E-01 | 8.71E-01 | -0.07±0.31 | 9.50E-01 | -0.06±0.31 | 9.69E-01 | -0.1±0.33 | 9.98E-01 | -0.21±0.32 | 9.91E-01 |
| citramalate | 0.13±0.26 | 0 | 5.26E-01 | 9.08E-01 | 0.15±0.26 | 0 | 5.36E-01 | 8.71E-01 | 0.02±0.31 | 9.82E-01 | 0.05±0.31 | 9.69E-01 | 0.37±0.46 | 9.98E-01 | 0.42±0.5 | 9.91E-01 |
| X - 11850 | 0.04±0.25 | 35.9 | 2.12E-01 | 9.76E-01 | 0.15±0.27 | 13.4 | 2.83E-01 | 8.71E-01 | -0.16±0.3 | 8.85E-01 | -0.03±0.32 | 9.86E-01 | 0.52±0.46 | 9.98E-01 | 0.59±0.48 | 9.91E-01 |
| glycine conjugate of C10H14O2 (1)* | -0.12±0.24 | 0 | 5.52E-01 | 9.08E-01 | -0.14±0.24 | 0 | 4.92E-01 | 8.71E-01 | -0.02±0.29 | 9.82E-01 | -0.03±0.3 | 9.86E-01 | -0.31±0.4 | 9.98E-01 | -0.39±0.43 | 9.91E-01 |
| X - 26097 | -0.11±0.24 | 0 | 4.44E-01 | 9.11E-01 | -0.15±0.25 | 0 | 6.59E-01 | 8.71E-01 | -0.26±0.3 | 7.99E-01 | -0.23±0.32 | 8.14E-01 | 0.13±0.4 | 9.98E-01 | 0±0.41 | 9.93E-01 |
| trans-urocanate | -0.12±0.24 | 0 | 9.09E-01 | 9.11E-01 | -0.14±0.25 | 0 | 8.07E-01 | 8.72E-01 | -0.09±0.3 | 9.28E-01 | -0.1±0.32 | 9.42E-01 | -0.15±0.38 | 9.98E-01 | -0.23±0.42 | 9.91E-01 |
| anthranilate | -0.13±0.23 | 86.41 | 6.67E-03 | 8.94E-01 | -0.14±0.25 | 80.92 | 2.20E-02 | 8.72E-01 | -0.44±0.25 | 4.62E-01 | -0.39±0.27 | 6.00E-01 | 1.09±0.5 | 9.98E-01 | 1.2±0.64 | 9.91E-01 |
| androstenediol (3beta,17beta) disulfate (2) | -0.12±0.26 | 0 | 9.52E-01 | 9.17E-01 | -0.15±0.26 | 0 | 8.89E-01 | 8.72E-01 | -0.11±0.3 | 9.15E-01 | -0.17±0.31 | 8.72E-01 | -0.14±0.49 | 9.98E-01 | -0.09±0.5 | 9.91E-01 |
| urea | -0.11±0.23 | 0 | 8.08E-01 | 9.11E-01 | -0.13±0.23 | 0 | 8.30E-01 | 8.72E-01 | -0.16±0.3 | 8.87E-01 | -0.09±0.3 | 9.42E-01 | -0.04±0.35 | 9.98E-01 | -0.19±0.37 | 9.91E-01 |
| 5alpha-androstan-3alpha,17beta-diol monosulfate (2) | 0.23±0.38 | 41.41 | 1.91E-01 | 8.73E-01 | 0.22±0.4 | 56.99 | 1.27E-01 | 8.75E-01 | -0.1±0.46 | 9.50E-01 | -0.19±0.48 | 9.17E-01 | 0.98±0.68 | 9.98E-01 | 1.1±0.7 | 9.91E-01 |
| 11beta-hydroxyandrosterone glucuronide | -0.14±0.21 | 0 | 5.56E-01 | 8.57E-01 | -0.12±0.21 | 0 | 4.18E-01 | 8.81E-01 | -0.21±0.24 | 7.94E-01 | -0.24±0.26 | 7.61E-01 | 0.06±0.4 | 9.98E-01 | 0.14±0.38 | 9.91E-01 |
| thioproline | -0.1±0.24 | 73.29 | 5.30E-02 | 9.22E-01 | -0.13±0.25 | 81.62 | 1.97E-02 | 8.81E-01 | 0.26±0.31 | 7.97E-01 | 0.27±0.3 | 7.65E-01 | -0.71±0.4 | 9.98E-01 | -0.96±0.43 | 9.91E-01 |
| sphingomyelin (d18:0/18:0, d19:0/17:0)* | -0.09±0.24 | 0 | 8.08E-01 | 9.36E-01 | -0.15±0.27 | 0 | 7.45E-01 | 8.81E-01 | -0.14±0.34 | 8.96E-01 | -0.23±0.36 | 8.49E-01 | -0.03±0.35 | 9.98E-01 | -0.05±0.41 | 9.91E-01 |
| X - 12101 | 0.17±0.23 | 0 | 7.76E-01 | 8.54E-01 | 0.13±0.25 | 0 | 7.77E-01 | 8.81E-01 | 0.22±0.28 | 8.17E-01 | 0.18±0.3 | 8.58E-01 | 0.07±0.41 | 9.98E-01 | 0.03±0.44 | 9.91E-01 |
| glucuronide of piperine metabolite C17H21NO3 (4)* | 0.19±0.27 | 0 | 8.46E-01 | 8.54E-01 | 0.15±0.28 | 0 | 8.12E-01 | 8.81E-01 | 0.23±0.33 | 8.47E-01 | 0.2±0.34 | 8.64E-01 | 0.12±0.47 | 9.98E-01 | 0.06±0.49 | 9.91E-01 |
| 1-methylnicotinamide | -0.13±0.24 | 0 | 9.63E-01 | 9.05E-01 | -0.14±0.25 | 0 | 8.74E-01 | 8.81E-01 | -0.14±0.3 | 8.96E-01 | -0.16±0.31 | 8.82E-01 | -0.11±0.42 | 9.98E-01 | -0.08±0.45 | 9.91E-01 |
| glycodeoxycholate 3-sulfate | 0.14±0.26 | 0 | 9.27E-01 | 9.04E-01 | 0.14±0.27 | 0 | 9.26E-01 | 8.81E-01 | 0.16±0.32 | 8.96E-01 | 0.16±0.33 | 8.90E-01 | 0.11±0.44 | 9.98E-01 | 0.11±0.45 | 9.91E-01 |
| X - 23659 | 0.14±0.28 | 75.72 | 4.24E-02 | 9.08E-01 | 0.15±0.28 | 78.24 | 3.20E-02 | 8.81E-01 | -0.14±0.31 | 8.96E-01 | -0.14±0.31 | 9.04E-01 | 1.27±0.62 | 9.98E-01 | 1.42±0.66 | 9.91E-01 |
| succinylcarnitine (C4-DC) | -0.16±0.24 | 0 | 6.41E-01 | 8.57E-01 | -0.13±0.25 | 0 | 9.89E-01 | 8.81E-01 | -0.24±0.3 | 8.09E-01 | -0.13±0.31 | 9.08E-01 | -0.01±0.4 | 9.98E-01 | -0.14±0.42 | 9.91E-01 |
| pyridoxate | 0.06±0.24 | 0 | 9.55E-01 | 9.65E-01 | 0.13±0.25 | 0 | 8.61E-01 | 8.81E-01 | 0.07±0.28 | 9.50E-01 | 0.11±0.29 | 9.29E-01 | 0.04±0.44 | 9.98E-01 | 0.21±0.51 | 9.91E-01 |
| glycolithocholate sulfate* | -0.16±0.23 | 0 | 9.45E-01 | 8.57E-01 | -0.12±0.23 | 0 | 8.98E-01 | 8.81E-01 | -0.14±0.28 | 8.87E-01 | -0.1±0.28 | 9.31E-01 | -0.18±0.39 | 9.98E-01 | -0.16±0.4 | 9.91E-01 |
| X - 18921 | -0.07±0.23 | 0 | 8.77E-01 | 9.49E-01 | -0.13±0.24 | 0 | 8.30E-01 | 8.81E-01 | -0.05±0.28 | 9.67E-01 | -0.1±0.29 | 9.34E-01 | -0.12±0.41 | 9.98E-01 | -0.22±0.47 | 9.91E-01 |
| guaiacol sulfate | -0.17±0.25 | 0 | 9.28E-01 | 8.57E-01 | -0.14±0.26 | 0 | 7.59E-01 | 8.81E-01 | -0.15±0.31 | 8.90E-01 | -0.09±0.31 | 9.48E-01 | -0.2±0.44 | 9.98E-01 | -0.26±0.47 | 9.91E-01 |
| X - 25790 | 0.17±0.26 | 30.69 | 2.30E-01 | 8.57E-01 | 0.15±0.27 | 61.61 | 1.07E-01 | 8.81E-01 | -0.03±0.31 | 9.81E-01 | -0.08±0.31 | 9.52E-01 | 0.66±0.48 | 9.98E-01 | 1.01±0.6 | 9.91E-01 |
| N-methylproline | -0.18±0.24 | 0 | 9.77E-01 | 8.54E-01 | -0.13±0.25 | 0 | 7.32E-01 | 8.81E-01 | -0.18±0.29 | 8.72E-01 | -0.07±0.3 | 9.62E-01 | -0.17±0.42 | 9.98E-01 | -0.25±0.43 | 9.91E-01 |
| X - 26106 | 0.19±0.27 | 14.03 | 2.81E-01 | 8.57E-01 | 0.15±0.27 | 47.77 | 1.66E-01 | 8.81E-01 | 0.01±0.31 | 9.90E-01 | -0.07±0.31 | 9.62E-01 | 0.64±0.5 | 9.98E-01 | 0.78±0.53 | 9.91E-01 |
| X - 24757 | 0.11±0.25 | 0 | 4.98E-01 | 9.21E-01 | 0.14±0.26 | 0 | 6.43E-01 | 8.81E-01 | 0±0.3 | 9.97E-01 | 0.07±0.3 | 9.63E-01 | 0.38±0.47 | 9.98E-01 | 0.35±0.51 | 9.91E-01 |
| 4-chlorobenzoic acid | -0.08±0.25 | 0 | 8.61E-01 | 9.42E-01 | -0.13±0.25 | 0 | 6.99E-01 | 8.81E-01 | -0.05±0.3 | 9.63E-01 | -0.07±0.31 | 9.65E-01 | -0.15±0.44 | 9.98E-01 | -0.27±0.44 | 9.91E-01 |
| X - 18887 | 0.1±0.25 | 0 | 6.22E-01 | 9.26E-01 | 0.14±0.26 | 0 | 5.70E-01 | 8.81E-01 | 0.02±0.31 | 9.85E-01 | 0.04±0.31 | 9.80E-01 | 0.28±0.44 | 9.98E-01 | 0.37±0.48 | 9.91E-01 |
| taurodeoxycholate | -0.17±0.24 | 0 | 5.89E-01 | 8.54E-01 | -0.14±0.26 | 0 | 5.23E-01 | 8.81E-01 | -0.08±0.3 | 9.42E-01 | -0.03±0.31 | 9.86E-01 | -0.36±0.42 | 9.98E-01 | -0.38±0.46 | 9.91E-01 |
| citrulline | -0.07±0.22 | 0 | 8.72E-01 | 9.51E-01 | -0.12±0.23 | 0 | 6.42E-01 | 8.81E-01 | -0.03±0.3 | 9.81E-01 | -0.02±0.32 | 9.95E-01 | -0.1±0.32 | 9.98E-01 | -0.23±0.32 | 9.91E-01 |
| X - 12112 | 0.09±0.25 | 65.89 | 8.68E-02 | 9.36E-01 | 0.14±0.27 | 56.89 | 1.28E-01 | 8.82E-01 | 0.55±0.36 | 5.56E-01 | 0.51±0.37 | 6.02E-01 | -0.31±0.34 | 9.98E-01 | -0.32±0.41 | 9.91E-01 |
| vanillylmandelate (VMA) | -0.12±0.24 | 68.81 | 7.34E-02 | 9.11E-01 | -0.13±0.25 | 58.31 | 1.21E-01 | 8.82E-01 | -0.41±0.29 | 6.06E-01 | -0.35±0.29 | 6.66E-01 | 0.53±0.44 | 9.98E-01 | 0.56±0.51 | 9.91E-01 |
| hydroxy-CMPF* | 0.11±0.26 | 4.09 | 3.07E-01 | 9.21E-01 | 0.13±0.26 | 0 | 4.30E-01 | 8.82E-01 | 0.33±0.33 | 7.56E-01 | 0.3±0.33 | 7.65E-01 | -0.21±0.41 | 9.98E-01 | -0.13±0.42 | 9.91E-01 |
| glycocholenate sulfate* | -0.14±0.25 | 0 | 6.54E-01 | 8.94E-01 | -0.13±0.26 | 0 | 5.83E-01 | 8.82E-01 | -0.23±0.33 | 8.43E-01 | -0.25±0.33 | 8.12E-01 | -0.01±0.38 | 9.98E-01 | 0.04±0.41 | 9.91E-01 |
| X - 17325 | 0.09±0.25 | 0 | 6.77E-01 | 9.36E-01 | 0.14±0.26 | 0 | 8.49E-01 | 8.82E-01 | 0.03±0.3 | 9.81E-01 | 0.11±0.31 | 9.34E-01 | 0.26±0.47 | 9.98E-01 | 0.22±0.52 | 9.91E-01 |
| allantoin | -0.11±0.23 | 0 | 8.74E-01 | 9.12E-01 | -0.12±0.23 | 0 | 8.32E-01 | 8.82E-01 | -0.13±0.29 | 8.96E-01 | -0.08±0.29 | 9.48E-01 | -0.06±0.37 | 9.98E-01 | -0.19±0.4 | 9.91E-01 |
| glycoursodeoxycholate | 0.12±0.24 | 0 | 9.34E-01 | 9.08E-01 | 0.13±0.25 | 0 | 8.00E-01 | 8.82E-01 | 0.11±0.3 | 9.13E-01 | 0.08±0.31 | 9.52E-01 | 0.15±0.42 | 9.98E-01 | 0.22±0.43 | 9.91E-01 |
| glutamine conjugate of C7H12O2* | 0.16±0.25 | 0 | 7.87E-01 | 8.57E-01 | 0.13±0.26 | 0 | 7.10E-01 | 8.82E-01 | 0.11±0.31 | 9.19E-01 | 0.06±0.32 | 9.68E-01 | 0.25±0.41 | 9.98E-01 | 0.26±0.44 | 9.91E-01 |
| methyl indole-3-acetate | 0.08±0.25 | 0 | 5.02E-01 | 9.44E-01 | 0.14±0.26 | 0 | 7.05E-01 | 8.82E-01 | -0.05±0.32 | 9.69E-01 | 0.06±0.33 | 9.69E-01 | 0.3±0.41 | 9.98E-01 | 0.27±0.45 | 9.91E-01 |
| 3-hydroxyhippurate | 0.12±0.26 | 0 | 3.85E-01 | 9.15E-01 | 0.13±0.25 | 0 | 5.67E-01 | 8.82E-01 | -0.03±0.3 | 9.81E-01 | 0.04±0.29 | 9.83E-01 | 0.46±0.47 | 9.98E-01 | 0.35±0.46 | 9.91E-01 |
| N-acetylglycine | -0.07±0.22 | 21.66 | 2.59E-01 | 9.42E-01 | -0.13±0.26 | 0 | 5.51E-01 | 8.82E-01 | 0.14±0.29 | 8.96E-01 | -0.03±0.32 | 9.86E-01 | -0.38±0.35 | 9.98E-01 | -0.36±0.47 | 9.91E-01 |
| trimethylamine N-oxide | 0.05±0.22 | 0 | 6.66E-01 | 9.65E-01 | 0.12±0.24 | 0 | 6.91E-01 | 8.82E-01 | 0.14±0.3 | 8.96E-01 | 0.19±0.3 | 8.45E-01 | -0.05±0.33 | 9.98E-01 | -0.01±0.41 | 9.93E-01 |
| X - 23655 | -0.14±0.24 | 0 | 3.74E-01 | 8.85E-01 | -0.13±0.25 | 33.45 | 2.20E-01 | 8.87E-01 | -0.27±0.28 | 7.59E-01 | -0.3±0.29 | 7.13E-01 | 0.2±0.45 | 9.98E-01 | 0.39±0.49 | 9.91E-01 |
| X - 12906 | -0.15±0.23 | 7.48 | 2.99E-01 | 8.57E-01 | -0.12±0.24 | 10.35 | 2.91E-01 | 8.87E-01 | -0.27±0.25 | 7.24E-01 | -0.23±0.26 | 7.65E-01 | 0.3±0.49 | 9.98E-01 | 0.44±0.58 | 9.91E-01 |
| X - 11632 | -0.13±0.24 | 0 | 8.91E-01 | 9.04E-01 | -0.12±0.25 | 0 | 8.99E-01 | 8.87E-01 | -0.15±0.3 | 8.89E-01 | -0.14±0.3 | 8.90E-01 | -0.09±0.39 | 9.98E-01 | -0.08±0.45 | 9.91E-01 |
| carotene diol (3) | -0.15±0.24 | 0 | 3.37E-01 | 8.68E-01 | -0.12±0.25 | 0 | 3.67E-01 | 8.87E-01 | 0.03±0.3 | 9.81E-01 | 0.05±0.31 | 9.75E-01 | -0.43±0.38 | 9.98E-01 | -0.42±0.41 | 9.91E-01 |
| phenylalanine | -0.08±0.25 | 0 | 5.86E-01 | 9.42E-01 | -0.12±0.24 | 0 | 8.32E-01 | 8.88E-01 | -0.18±0.31 | 8.79E-01 | -0.17±0.33 | 8.82E-01 | 0.09±0.4 | 9.98E-01 | -0.06±0.35 | 9.91E-01 |
| formiminoglutamate | 0.18±0.24 | 0 | 6.93E-01 | 8.54E-01 | 0.14±0.28 | 0 | 9.23E-01 | 8.88E-01 | 0.1±0.31 | 9.23E-01 | 0.12±0.32 | 9.29E-01 | 0.29±0.38 | 9.98E-01 | 0.18±0.54 | 9.91E-01 |
| androstenediol (3beta,17beta) disulfate (1) | 0.18±0.27 | 0 | 6.98E-01 | 8.57E-01 | 0.14±0.28 | 0 | 7.79E-01 | 8.94E-01 | 0.25±0.33 | 8.17E-01 | 0.09±0.33 | 9.53E-01 | 0.03±0.49 | 9.98E-01 | 0.26±0.51 | 9.91E-01 |
| 8-methoxykynurenate | -0.11±0.24 | 0 | 7.73E-01 | 9.11E-01 | -0.12±0.25 | 0 | 1.00E+00 | 8.97E-01 | -0.16±0.29 | 8.79E-01 | -0.12±0.29 | 9.12E-01 | -0.01±0.44 | 9.98E-01 | -0.12±0.5 | 9.91E-01 |
| eicosapentaenoate (EPA; 20:5n3) | -0.1±0.24 | 0 | 3.50E-01 | 9.22E-01 | -0.12±0.26 | 0 | 5.06E-01 | 8.97E-01 | 0.07±0.3 | 9.50E-01 | 0±0.32 | 9.95E-01 | -0.41±0.41 | 9.98E-01 | -0.36±0.43 | 9.91E-01 |
| dihomo-linoleoylcarnitine (C20:2)* | 0.17±0.25 | 0 | 6.48E-01 | 8.57E-01 | 0.12±0.26 | 0 | 6.58E-01 | 8.98E-01 | 0.25±0.32 | 8.09E-01 | 0.21±0.32 | 8.45E-01 | 0.01±0.42 | 9.98E-01 | -0.03±0.43 | 9.91E-01 |
| 1-palmitoyl-2-oleoyl-GPI (16:0/18:1)* | 0.14±0.28 | 0 | 3.98E-01 | 9.08E-01 | 0.14±0.28 | 16.76 | 2.73E-01 | 8.98E-01 | 0±0.33 | 9.97E-01 | -0.03±0.32 | 9.86E-01 | 0.52±0.53 | 9.98E-01 | 0.73±0.61 | 9.91E-01 |
| oleoyl-linoleoyl-glycerol (18:1/18:2) [1] | 0.18±0.26 | 0 | 3.20E-01 | 8.57E-01 | 0.12±0.26 | 49.13 | 1.61E-01 | 9.01E-01 | 0.45±0.37 | 6.76E-01 | 0.51±0.38 | 6.11E-01 | -0.07±0.36 | 9.98E-01 | -0.22±0.36 | 9.91E-01 |
| glycerol | -0.11±0.26 | 0 | 4.60E-01 | 9.26E-01 | -0.13±0.27 | 0 | 4.52E-01 | 9.01E-01 | -0.24±0.32 | 8.17E-01 | -0.26±0.33 | 7.97E-01 | 0.17±0.46 | 9.98E-01 | 0.18±0.49 | 9.91E-01 |
| X - 11483 | 0.09±0.25 | 0 | 6.44E-01 | 9.36E-01 | 0.13±0.27 | 0 | 7.00E-01 | 9.01E-01 | 0.18±0.31 | 8.79E-01 | 0.2±0.32 | 8.55E-01 | -0.07±0.42 | 9.98E-01 | -0.02±0.47 | 9.91E-01 |
| X - 15503 | 0.1±0.24 | 0 | 5.59E-01 | 9.21E-01 | 0.13±0.27 | 0 | 9.61E-01 | 9.01E-01 | 0±0.3 | 9.97E-01 | 0.12±0.33 | 9.29E-01 | 0.3±0.41 | 9.98E-01 | 0.15±0.49 | 9.91E-01 |
| alpha-ketobutyrate | -0.08±0.24 | 86.97 | 5.59E-03 | 9.42E-01 | -0.12±0.25 | 88.7 | 2.93E-03 | 9.01E-01 | 0.7±0.37 | 3.95E-01 | 0.8±0.4 | 3.78E-01 | -0.63±0.31 | 9.98E-01 | -0.73±0.33 | 9.91E-01 |
| X - 24328 | 0.15±0.31 | 21.07 | 2.60E-01 | 9.08E-01 | 0.15±0.33 | 34.16 | 2.18E-01 | 9.01E-01 | 0.48±0.42 | 7.03E-01 | 0.5±0.43 | 6.95E-01 | -0.22±0.45 | 9.98E-01 | -0.31±0.49 | 9.91E-01 |
| X - 21733 | -0.16±0.21 | 80.2 | 2.46E-02 | 8.50E-01 | -0.11±0.23 | 75.11 | 4.50E-02 | 9.01E-01 | -0.35±0.23 | 5.25E-01 | -0.27±0.25 | 7.05E-01 | 1±0.56 | 9.98E-01 | 1.19±0.68 | 9.91E-01 |
| pregnenediol sulfate (C21H34O5S)* | -0.12±0.25 | 30.39 | 2.31E-01 | 9.11E-01 | -0.12±0.26 | 48.03 | 1.65E-01 | 9.01E-01 | -0.29±0.29 | 7.53E-01 | -0.31±0.29 | 7.13E-01 | 0.44±0.53 | 9.98E-01 | 0.53±0.54 | 9.91E-01 |
| 3-methylglutaconate | -0.13±0.27 | 20.35 | 2.63E-01 | 9.11E-01 | -0.13±0.29 | 31.94 | 2.25E-01 | 9.01E-01 | -0.35±0.33 | 7.33E-01 | -0.34±0.34 | 7.41E-01 | 0.28±0.45 | 9.98E-01 | 0.47±0.58 | 9.91E-01 |
| 3-methylglutarylcarnitine (2) | -0.14±0.23 | 17.41 | 2.71E-01 | 8.73E-01 | -0.11±0.25 | 0 | 3.88E-01 | 9.01E-01 | -0.32±0.28 | 7.03E-01 | -0.24±0.29 | 7.81E-01 | 0.22±0.41 | 9.98E-01 | 0.24±0.48 | 9.91E-01 |
| X - 16580 | 0.15±0.22 | 0 | 4.47E-01 | 8.57E-01 | 0.11±0.25 | 0 | 6.86E-01 | 9.01E-01 | 0.3±0.3 | 7.43E-01 | 0.19±0.31 | 8.55E-01 | -0.04±0.34 | 9.98E-01 | -0.02±0.41 | 9.91E-01 |
| bilirubin (E,E)* | -0.05±0.25 | 0 | 9.13E-01 | 9.72E-01 | -0.12±0.26 | 0 | 9.05E-01 | 9.01E-01 | -0.07±0.31 | 9.50E-01 | -0.14±0.32 | 9.04E-01 | -0.01±0.41 | 9.98E-01 | -0.08±0.45 | 9.91E-01 |
| acetylcarnitine (C2) | 0.18±0.25 | 0 | 9.74E-01 | 8.54E-01 | 0.12±0.26 | 0 | 8.63E-01 | 9.01E-01 | 0.17±0.3 | 8.79E-01 | 0.1±0.3 | 9.42E-01 | 0.19±0.45 | 9.98E-01 | 0.2±0.53 | 9.91E-01 |
| tridecenedioate (C13:1-DC)* | 0.13±0.25 | 0 | 4.17E-01 | 9.08E-01 | 0.12±0.26 | 8.5 | 2.96E-01 | 9.01E-01 | -0.02±0.31 | 9.82E-01 | -0.05±0.31 | 9.70E-01 | 0.42±0.44 | 9.98E-01 | 0.56±0.49 | 9.91E-01 |
| N-stearoyltaurine | -0.09±0.24 | 0 | 7.80E-01 | 9.36E-01 | -0.11±0.24 | 0 | 6.42E-01 | 9.01E-01 | -0.04±0.3 | 9.80E-01 | -0.03±0.3 | 9.86E-01 | -0.18±0.39 | 9.98E-01 | -0.27±0.41 | 9.91E-01 |
| glycoursodeoxycholic acid sulfate (1) | 0.14±0.26 | 0 | 5.87E-01 | 8.94E-01 | 0.12±0.26 | 0 | 4.65E-01 | 9.01E-01 | 0.04±0.31 | 9.76E-01 | -0.01±0.32 | 9.95E-01 | 0.34±0.44 | 9.98E-01 | 0.39±0.45 | 9.91E-01 |
| 2-palmitoyl-GPC (16:0)* | -0.06±0.23 | 0 | 7.15E-01 | 9.65E-01 | -0.11±0.24 | 0 | 5.51E-01 | 9.01E-01 | 0.02±0.31 | 9.82E-01 | 0±0.31 | 9.95E-01 | -0.15±0.34 | 9.98E-01 | -0.29±0.39 | 9.91E-01 |
| pregnenetriol disulfate* | 0.14±0.28 | 0 | 9.22E-01 | 9.11E-01 | 0.13±0.28 | 0 | 8.10E-01 | 9.02E-01 | 0.15±0.33 | 8.96E-01 | 0.09±0.33 | 9.52E-01 | 0.1±0.5 | 9.98E-01 | 0.24±0.55 | 9.91E-01 |
| alanine | 0.12±0.23 | 0 | 6.77E-01 | 9.08E-01 | 0.1±0.23 | 0 | 3.67E-01 | 9.05E-01 | 0.2±0.31 | 8.62E-01 | 0.28±0.3 | 7.57E-01 | 0.01±0.35 | 9.98E-01 | -0.14±0.35 | 9.91E-01 |
| X - 24344 | -0.11±0.25 | 0 | 8.18E-01 | 9.21E-01 | -0.12±0.27 | 0 | 3.91E-01 | 9.05E-01 | -0.15±0.3 | 8.96E-01 | -0.28±0.33 | 7.71E-01 | -0.03±0.43 | 9.98E-01 | 0.22±0.48 | 9.91E-01 |
| 1-stearoyl-2-arachidonoyl-GPI (18:0/20:4) | 0.17±0.25 | 0 | 6.54E-01 | 8.57E-01 | 0.11±0.24 | 0 | 4.77E-01 | 9.05E-01 | 0.27±0.33 | 8.09E-01 | 0.27±0.33 | 7.97E-01 | 0.04±0.37 | 9.98E-01 | -0.08±0.35 | 9.91E-01 |
| N-acetylleucine | -0.07±0.24 | 0 | 6.70E-01 | 9.51E-01 | -0.11±0.25 | 0 | 3.68E-01 | 9.05E-01 | 0.02±0.32 | 9.82E-01 | 0.07±0.33 | 9.65E-01 | -0.19±0.36 | 9.98E-01 | -0.39±0.4 | 9.91E-01 |
| X - 12221 | 0.04±0.24 | 0 | 4.86E-01 | 9.76E-01 | 0.11±0.25 | 0 | 5.76E-01 | 9.05E-01 | -0.06±0.29 | 9.50E-01 | 0.02±0.3 | 9.91E-01 | 0.31±0.46 | 9.98E-01 | 0.33±0.47 | 9.91E-01 |
| X - 25265 | 0.07±0.25 | 0 | 7.44E-01 | 9.51E-01 | 0.11±0.24 | 0 | 5.90E-01 | 9.05E-01 | 0.01±0.32 | 9.92E-01 | 0±0.32 | 9.95E-01 | 0.17±0.38 | 9.98E-01 | 0.26±0.38 | 9.91E-01 |
| 10-undecenoate (11:1n1) | -0.06±0.24 | 67.79 | 7.81E-02 | 9.65E-01 | -0.11±0.25 | 76.52 | 3.91E-02 | 9.06E-01 | -0.32±0.28 | 7.03E-01 | -0.39±0.29 | 6.11E-01 | 0.63±0.46 | 9.98E-01 | 0.85±0.53 | 9.91E-01 |
| 4-hydroxycoumarin | -0.18±0.23 | 73.24 | 5.32E-02 | 8.54E-01 | -0.1±0.24 | 68.15 | 7.64E-02 | 9.06E-01 | -0.43±0.27 | 5.19E-01 | -0.33±0.27 | 6.65E-01 | 0.65±0.49 | 9.98E-01 | 0.69±0.51 | 9.91E-01 |
| citraconate/glutaconate | -0.16±0.25 | 24.26 | 2.51E-01 | 8.57E-01 | -0.11±0.26 | 45.91 | 1.74E-01 | 9.06E-01 | -0.36±0.31 | 6.77E-01 | -0.34±0.31 | 7.05E-01 | 0.24±0.43 | 9.98E-01 | 0.42±0.47 | 9.91E-01 |
| 1-linoleoyl-GPC (18:2) | 0.1±0.24 | 0 | 6.49E-01 | 9.21E-01 | 0.11±0.26 | 0 | 4.34E-01 | 9.06E-01 | 0.21±0.33 | 8.72E-01 | 0.29±0.35 | 7.81E-01 | -0.01±0.35 | 9.98E-01 | -0.11±0.38 | 9.91E-01 |
| X - 25422 | 0.08±0.22 | 0 | 5.22E-01 | 9.42E-01 | 0.11±0.25 | 0 | 4.69E-01 | 9.06E-01 | 0.22±0.31 | 8.47E-01 | 0.25±0.32 | 8.00E-01 | -0.07±0.32 | 9.98E-01 | -0.12±0.4 | 9.91E-01 |
| X - 21470 | -0.09±0.24 | 0 | 8.33E-01 | 9.33E-01 | -0.1±0.25 | 0 | 5.52E-01 | 9.06E-01 | -0.13±0.3 | 8.96E-01 | -0.21±0.3 | 8.36E-01 | -0.02±0.41 | 9.98E-01 | 0.1±0.43 | 9.91E-01 |
| perfluorooctanoate (PFOA) | 0.16±0.26 | 0 | 5.43E-01 | 8.74E-01 | 0.11±0.27 | 0 | 6.15E-01 | 9.06E-01 | 0.31±0.36 | 7.94E-01 | 0.24±0.37 | 8.45E-01 | -0.01±0.39 | 9.98E-01 | -0.03±0.39 | 9.91E-01 |
| 3,7-dimethylurate | 0.13±0.26 | 0 | 7.81E-01 | 9.08E-01 | 0.11±0.26 | 0 | 6.48E-01 | 9.06E-01 | 0.18±0.32 | 8.79E-01 | 0.21±0.33 | 8.54E-01 | 0.03±0.44 | 9.98E-01 | -0.04±0.43 | 9.91E-01 |
| aconitate [cis or trans] | 0.15±0.25 | 0 | 8.98E-01 | 8.79E-01 | 0.11±0.26 | 0 | 6.77E-01 | 9.06E-01 | 0.18±0.33 | 8.85E-01 | 0.2±0.33 | 8.55E-01 | 0.11±0.39 | 9.98E-01 | -0.02±0.42 | 9.91E-01 |
| xanthurenate | 0.11±0.24 | 0 | 8.24E-01 | 9.21E-01 | 0.11±0.26 | 0 | 9.03E-01 | 9.06E-01 | 0.07±0.3 | 9.50E-01 | 0.13±0.31 | 9.12E-01 | 0.19±0.44 | 9.98E-01 | 0.06±0.44 | 9.91E-01 |
| taurodeoxycholic acid 3-sulfate | -0.1±0.24 | 0 | 9.88E-01 | 9.22E-01 | -0.11±0.25 | 0 | 9.73E-01 | 9.06E-01 | -0.1±0.3 | 9.22E-01 | -0.12±0.31 | 9.29E-01 | -0.11±0.41 | 9.98E-01 | -0.1±0.45 | 9.91E-01 |
| N-methylhydroxyproline** | -0.16±0.24 | 0 | 6.87E-01 | 8.57E-01 | -0.11±0.25 | 0 | 9.95E-01 | 9.06E-01 | -0.22±0.29 | 8.17E-01 | -0.11±0.31 | 9.34E-01 | -0.01±0.43 | 9.98E-01 | -0.11±0.45 | 9.91E-01 |
| metabolonic lactone sulfate | 0.1±0.25 | 0 | 6.76E-01 | 9.26E-01 | 0.11±0.26 | 0 | 9.28E-01 | 9.06E-01 | 0.03±0.3 | 9.81E-01 | 0.1±0.33 | 9.45E-01 | 0.25±0.43 | 9.98E-01 | 0.15±0.45 | 9.91E-01 |
| X - 11843 | 0.01±0.25 | 38.89 | 2.01E-01 | 9.97E-01 | 0.11±0.27 | 8.04 | 2.97E-01 | 9.06E-01 | -0.19±0.29 | 8.63E-01 | -0.06±0.32 | 9.68E-01 | 0.53±0.48 | 9.98E-01 | 0.55±0.5 | 9.91E-01 |
| 2R,3R-dihydroxybutyrate | 0.09±0.25 | 0 | 6.81E-01 | 9.36E-01 | 0.11±0.26 | 0 | 7.33E-01 | 9.06E-01 | 0.02±0.3 | 9.82E-01 | 0.05±0.31 | 9.69E-01 | 0.23±0.43 | 9.98E-01 | 0.25±0.5 | 9.91E-01 |
| retinal | -0.09±0.24 | 0 | 6.14E-01 | 9.36E-01 | -0.11±0.25 | 0 | 6.83E-01 | 9.06E-01 | 0.01±0.32 | 9.90E-01 | -0.03±0.32 | 9.86E-01 | -0.24±0.38 | 9.98E-01 | -0.25±0.42 | 9.91E-01 |
| 1-palmitoyl-2-linoleoyl-GPI (16:0/18:2) | 0.12±0.27 | 2.85 | 3.10E-01 | 9.18E-01 | 0.12±0.28 | 0 | 4.09E-01 | 9.06E-01 | -0.05±0.32 | 9.69E-01 | -0.02±0.32 | 9.95E-01 | 0.57±0.52 | 9.98E-01 | 0.51±0.54 | 9.91E-01 |
| 1-palmitoyl-2-oleoyl-GPC (16:0/18:1) | 0.18±0.27 | 0 | 8.43E-01 | 8.57E-01 | 0.12±0.28 | 0 | 4.45E-01 | 9.06E-01 | 0.14±0.32 | 8.96E-01 | 0±0.32 | 9.95E-01 | 0.26±0.48 | 9.98E-01 | 0.53±0.62 | 9.91E-01 |
| 3b-hydroxy-5-cholenoic acid | -0.13±0.23 | 64.2 | 9.47E-02 | 8.85E-01 | -0.1±0.24 | 79.91 | 2.57E-02 | 9.07E-01 | -0.38±0.27 | 6.04E-01 | -0.42±0.28 | 5.65E-01 | 0.44±0.41 | 9.98E-01 | 0.78±0.46 | 9.91E-01 |
| androstenediol (3beta,17beta) monosulfate (1) | -0.06±0.24 | 64.47 | 9.34E-02 | 9.65E-01 | -0.1±0.24 | 70.7 | 6.47E-02 | 9.07E-01 | -0.28±0.28 | 7.53E-01 | -0.32±0.27 | 6.77E-01 | 0.72±0.53 | 9.98E-01 | 0.78±0.53 | 9.91E-01 |
| oleoyl-linoleoyl-glycerol (18:1/18:2) [2] | 0.14±0.25 | 0 | 4.26E-01 | 9.03E-01 | 0.1±0.25 | 34.21 | 2.18E-01 | 9.07E-01 | 0.32±0.34 | 7.71E-01 | 0.4±0.35 | 6.98E-01 | -0.08±0.37 | 9.98E-01 | -0.23±0.37 | 9.91E-01 |
| caprate (10:0) | -0.09±0.27 | 7.13 | 2.99E-01 | 9.42E-01 | -0.11±0.27 | 26.45 | 2.44E-01 | 9.07E-01 | -0.31±0.34 | 7.81E-01 | -0.36±0.35 | 7.26E-01 | 0.25±0.42 | 9.98E-01 | 0.29±0.44 | 9.91E-01 |
| hippurate | -0.15±0.24 | 62.86 | 1.01E-01 | 8.57E-01 | -0.1±0.25 | 51.44 | 1.51E-01 | 9.07E-01 | -0.35±0.27 | 6.37E-01 | -0.27±0.28 | 7.43E-01 | 0.63±0.54 | 9.98E-01 | 0.63±0.57 | 9.91E-01 |
| S-methylcysteine sulfoxide | -0.09±0.26 | 0 | 5.06E-01 | 9.36E-01 | -0.1±0.26 | 0 | 4.86E-01 | 9.07E-01 | -0.23±0.33 | 8.47E-01 | -0.25±0.34 | 8.12E-01 | 0.12±0.41 | 9.98E-01 | 0.12±0.41 | 9.91E-01 |
| 2-hydroxypalmitate | -0.07±0.25 | 0 | 4.61E-01 | 9.52E-01 | -0.11±0.26 | 0 | 4.72E-01 | 9.07E-01 | -0.21±0.31 | 8.54E-01 | -0.24±0.32 | 8.12E-01 | 0.17±0.42 | 9.98E-01 | 0.17±0.47 | 9.91E-01 |
| 3beta,7alpha-dihydroxy-5-cholestenoate | 0.16±0.27 | 0 | 4.72E-01 | 8.86E-01 | 0.11±0.28 | 0 | 5.13E-01 | 9.07E-01 | 0.33±0.36 | 7.81E-01 | 0.27±0.37 | 8.14E-01 | -0.06±0.41 | 9.98E-01 | -0.1±0.42 | 9.91E-01 |
| X - 18888 | -0.07±0.24 | 0 | 5.94E-01 | 9.61E-01 | -0.1±0.25 | 0 | 5.36E-01 | 9.07E-01 | -0.16±0.3 | 8.86E-01 | -0.2±0.3 | 8.40E-01 | 0.12±0.42 | 9.98E-01 | 0.15±0.48 | 9.91E-01 |
| indoleacetoylcarnitine* | 0.07±0.25 | 0 | 7.69E-01 | 9.60E-01 | 0.1±0.26 | 0 | 6.77E-01 | 9.07E-01 | 0.12±0.31 | 9.03E-01 | 0.18±0.32 | 8.72E-01 | -0.03±0.42 | 9.98E-01 | -0.04±0.43 | 9.91E-01 |
| cholesterol | -0.05±0.24 | 0 | 7.37E-01 | 9.72E-01 | -0.11±0.26 | 0 | 7.22E-01 | 9.07E-01 | -0.12±0.32 | 9.08E-01 | -0.18±0.33 | 8.77E-01 | 0.04±0.36 | 9.98E-01 | 0.01±0.42 | 9.91E-01 |
| sarcosine | -0.04±0.24 | 0 | 7.10E-01 | 9.78E-01 | -0.1±0.25 | 0 | 7.36E-01 | 9.07E-01 | -0.11±0.31 | 9.15E-01 | -0.16±0.32 | 8.82E-01 | 0.07±0.37 | 9.98E-01 | 0.02±0.43 | 9.91E-01 |
| EDTA | -0.04±0.27 | 0 | 6.28E-01 | 9.78E-01 | -0.11±0.28 | 0 | 6.53E-01 | 9.07E-01 | -0.1±0.3 | 9.22E-01 | -0.16±0.29 | 8.82E-01 | 0.25±0.66 | 9.98E-01 | 0.23±0.8 | 9.91E-01 |
| isovalerylglycine | 0±0.24 | 0 | 8.59E-01 | 9.97E-01 | 0.1±0.25 | 0 | 7.42E-01 | 9.07E-01 | 0.03±0.3 | 9.81E-01 | 0.16±0.32 | 8.82E-01 | -0.06±0.39 | 9.98E-01 | -0.01±0.42 | 9.91E-01 |
| X - 21364 | -0.05±0.24 | 0 | 7.92E-01 | 9.65E-01 | -0.1±0.26 | 0 | 7.96E-01 | 9.07E-01 | -0.09±0.28 | 9.22E-01 | -0.14±0.29 | 8.98E-01 | 0.05±0.45 | 9.98E-01 | 0.02±0.51 | 9.91E-01 |
| cis-3,4-methyleneheptanoylglycine | -0.09±0.23 | 0 | 8.59E-01 | 9.33E-01 | -0.09±0.23 | 0 | 8.26E-01 | 9.07E-01 | -0.12±0.28 | 8.96E-01 | -0.12±0.28 | 9.04E-01 | -0.03±0.4 | 9.98E-01 | -0.01±0.43 | 9.91E-01 |
| pyrraline | -0.1±0.24 | 0 | 3.84E-01 | 9.22E-01 | -0.09±0.24 | 23.41 | 2.53E-01 | 9.07E-01 | 0.08±0.32 | 9.49E-01 | 0.13±0.31 | 9.08E-01 | -0.35±0.37 | 9.98E-01 | -0.42±0.37 | 9.91E-01 |
| isovalerate (i5:0) | -0.09±0.23 | 0 | 8.31E-01 | 9.26E-01 | -0.1±0.23 | 0 | 8.89E-01 | 9.07E-01 | -0.13±0.27 | 8.96E-01 | -0.12±0.28 | 9.12E-01 | -0.02±0.43 | 9.98E-01 | -0.04±0.43 | 9.91E-01 |
| arabitol/xylitol | 0.1±0.23 | 0 | 9.42E-01 | 9.24E-01 | 0.1±0.26 | 0 | 8.31E-01 | 9.07E-01 | 0.09±0.29 | 9.35E-01 | 0.07±0.29 | 9.62E-01 | 0.12±0.4 | 9.98E-01 | 0.2±0.54 | 9.91E-01 |
| cis-3,4-methyleneheptanoylcarnitine | -0.08±0.23 | 0 | 6.08E-01 | 9.37E-01 | -0.09±0.24 | 0 | 3.89E-01 | 9.07E-01 | 0.01±0.29 | 9.85E-01 | 0.06±0.3 | 9.68E-01 | -0.23±0.37 | 9.98E-01 | -0.37±0.4 | 9.91E-01 |
| 4-vinylcatechol sulfate | 0.03±0.24 | 0 | 9.22E-01 | 9.78E-01 | 0.1±0.25 | 0 | 4.06E-01 | 9.07E-01 | 0.01±0.3 | 9.85E-01 | -0.06±0.31 | 9.69E-01 | 0.06±0.39 | 9.98E-01 | 0.39±0.43 | 9.91E-01 |
| cysteinylglycine | -0.02±0.23 | 0 | 9.18E-01 | 9.93E-01 | -0.1±0.25 | 0 | 5.05E-01 | 9.07E-01 | 0.01±0.31 | 9.93E-01 | 0.05±0.33 | 9.78E-01 | -0.04±0.35 | 9.98E-01 | -0.29±0.38 | 9.91E-01 |
| X - 25520 | 0.03±0.24 | 0 | 8.05E-01 | 9.78E-01 | 0.1±0.25 | 0 | 7.32E-01 | 9.07E-01 | -0.01±0.3 | 9.89E-01 | 0.04±0.31 | 9.85E-01 | 0.11±0.39 | 9.98E-01 | 0.22±0.44 | 9.91E-01 |
| X - 17676 | 0.09±0.24 | 0 | 3.56E-01 | 9.36E-01 | 0.1±0.26 | 0 | 4.45E-01 | 9.07E-01 | -0.09±0.3 | 9.37E-01 | -0.03±0.31 | 9.86E-01 | 0.37±0.39 | 9.98E-01 | 0.4±0.46 | 9.91E-01 |
| arabonate/xylonate | 0.04±0.21 | 0 | 6.06E-01 | 9.76E-01 | 0.09±0.24 | 0 | 5.25E-01 | 9.10E-01 | 0.14±0.29 | 8.96E-01 | 0.19±0.29 | 8.40E-01 | -0.08±0.31 | 9.98E-01 | -0.13±0.41 | 9.91E-01 |
| X - 24949 | -0.04±0.24 | 0 | 5.71E-01 | 9.76E-01 | -0.1±0.25 | 0 | 5.40E-01 | 9.10E-01 | 0.07±0.31 | 9.50E-01 | 0.03±0.33 | 9.86E-01 | -0.21±0.38 | 9.98E-01 | -0.28±0.4 | 9.91E-01 |
| X - 24970 | -0.02±0.24 | 0 | 4.03E-01 | 9.84E-01 | -0.09±0.25 | 0 | 5.60E-01 | 9.10E-01 | -0.18±0.31 | 8.79E-01 | -0.2±0.31 | 8.45E-01 | 0.23±0.39 | 9.98E-01 | 0.09±0.4 | 9.91E-01 |
| nonadecanoate (19:0) | -0.08±0.24 | 0 | 8.55E-01 | 9.44E-01 | -0.09±0.25 | 0 | 8.97E-01 | 9.10E-01 | -0.05±0.29 | 9.69E-01 | -0.07±0.31 | 9.62E-01 | -0.14±0.41 | 9.98E-01 | -0.14±0.44 | 9.91E-01 |
| 12,13-DiHOME | -0.11±0.22 | 23.18 | 2.54E-01 | 9.11E-01 | -0.09±0.24 | 11.36 | 2.88E-01 | 9.11E-01 | -0.24±0.25 | 7.66E-01 | -0.21±0.26 | 7.97E-01 | 0.38±0.48 | 9.98E-01 | 0.41±0.53 | 9.91E-01 |
| 3-hydroxysebacate | -0.03±0.24 | 0 | 4.08E-01 | 9.78E-01 | -0.1±0.26 | 0 | 4.31E-01 | 9.11E-01 | 0.12±0.31 | 9.03E-01 | 0.04±0.31 | 9.86E-01 | -0.3±0.41 | 9.98E-01 | -0.41±0.48 | 9.91E-01 |
| 1-methylxanthine | -0.05±0.24 | 0 | 8.78E-01 | 9.65E-01 | -0.09±0.24 | 0 | 7.63E-01 | 9.11E-01 | -0.08±0.3 | 9.42E-01 | -0.14±0.3 | 8.91E-01 | 0±0.43 | 9.99E-01 | 0.01±0.43 | 9.91E-01 |
| 2-oxoarginine* | 0.03±0.24 | 0 | 8.25E-01 | 9.78E-01 | 0.1±0.26 | 0 | 3.55E-01 | 9.12E-01 | 0.07±0.3 | 9.50E-01 | 0.28±0.33 | 7.71E-01 | -0.04±0.41 | 9.98E-01 | -0.22±0.43 | 9.91E-01 |
| glycosyl-N-behenoyl-sphingadienine (d18:2/22:0)* | 0.15±0.27 | 0 | 7.82E-01 | 9.04E-01 | 0.11±0.29 | 0 | 7.99E-01 | 9.13E-01 | 0.09±0.33 | 9.40E-01 | 0.06±0.35 | 9.72E-01 | 0.25±0.48 | 9.98E-01 | 0.22±0.52 | 9.91E-01 |
| 3-hydroxydodecanedioate* | -0.04±0.25 | 0 | 8.57E-01 | 9.78E-01 | -0.09±0.26 | 0 | 7.62E-01 | 9.16E-01 | -0.07±0.32 | 9.50E-01 | -0.15±0.32 | 8.94E-01 | 0.02±0.4 | 9.98E-01 | 0.02±0.45 | 9.91E-01 |
| 3-hydroxy-3-methylglutarate | 0.05±0.23 | 0 | 6.14E-01 | 9.66E-01 | 0.09±0.26 | 0 | 5.50E-01 | 9.16E-01 | -0.05±0.3 | 9.69E-01 | -0.02±0.32 | 9.95E-01 | 0.19±0.36 | 9.98E-01 | 0.32±0.46 | 9.91E-01 |
| homocitrulline | 0.08±0.24 | 0 | 5.33E-01 | 9.49E-01 | 0.09±0.26 | 0 | 3.71E-01 | 9.19E-01 | 0.18±0.3 | 8.77E-01 | 0.25±0.31 | 7.98E-01 | -0.14±0.42 | 9.98E-01 | -0.24±0.45 | 9.91E-01 |
| (16 or 17)-methylstearate (a19:0 or i19:0) | -0.08±0.25 | 0 | 6.57E-01 | 9.51E-01 | -0.09±0.26 | 0 | 5.97E-01 | 9.19E-01 | -0.16±0.31 | 8.88E-01 | -0.19±0.32 | 8.61E-01 | 0.07±0.4 | 9.98E-01 | 0.1±0.45 | 9.91E-01 |
| acisoga | -0.06±0.24 | 0 | 9.16E-01 | 9.65E-01 | -0.09±0.25 | 0 | 9.33E-01 | 9.19E-01 | -0.04±0.3 | 9.80E-01 | -0.07±0.3 | 9.62E-01 | -0.09±0.4 | 9.98E-01 | -0.11±0.42 | 9.91E-01 |
| X - 17335 | 0.16±0.26 | 0 | 9.27E-01 | 8.68E-01 | 0.09±0.27 | 0 | 7.64E-01 | 9.19E-01 | 0.15±0.31 | 8.96E-01 | 0.05±0.31 | 9.73E-01 | 0.2±0.48 | 9.98E-01 | 0.23±0.53 | 9.91E-01 |
| creatinine | 0.09±0.3 | 0 | 5.05E-01 | 9.49E-01 | 0.11±0.31 | 0 | 4.84E-01 | 9.19E-01 | -0.02±0.34 | 9.85E-01 | 0.01±0.34 | 9.95E-01 | 0.46±0.62 | 9.98E-01 | 0.57±0.73 | 9.91E-01 |
| homoarginine | 0±0.24 | 0 | 9.22E-01 | 1.00E+00 | -0.08±0.24 | 0 | 7.08E-01 | 9.22E-01 | -0.02±0.31 | 9.82E-01 | 0.01±0.33 | 9.95E-01 | 0.03±0.38 | 9.98E-01 | -0.17±0.34 | 9.91E-01 |
| paraxanthine | -0.06±0.25 | 0 | 6.85E-01 | 9.65E-01 | -0.09±0.25 | 0 | 7.03E-01 | 9.22E-01 | 0.01±0.31 | 9.87E-01 | -0.01±0.33 | 9.95E-01 | -0.19±0.41 | 9.98E-01 | -0.2±0.39 | 9.91E-01 |
| N2-acetyl,N6,N6-dimethyllysine | 0.07±0.24 | 0 | 4.66E-01 | 9.56E-01 | 0.08±0.25 | 0 | 6.24E-01 | 9.24E-01 | 0.21±0.3 | 8.54E-01 | 0.17±0.31 | 8.71E-01 | -0.15±0.39 | 9.98E-01 | -0.08±0.42 | 9.91E-01 |
| glycerate | -0.13±0.23 | 0 | 3.40E-01 | 8.86E-01 | -0.08±0.23 | 0 | 3.94E-01 | 9.25E-01 | -0.23±0.25 | 7.77E-01 | -0.16±0.25 | 8.48E-01 | 0.34±0.55 | 9.98E-01 | 0.41±0.62 | 9.91E-01 |
| X - 25936 | 0.09±0.24 | 0 | 9.81E-01 | 9.36E-01 | 0.09±0.26 | 0 | 9.97E-01 | 9.25E-01 | 0.09±0.31 | 9.35E-01 | 0.09±0.31 | 9.51E-01 | 0.08±0.39 | 9.98E-01 | 0.09±0.45 | 9.91E-01 |
| 5-hydroxyindole sulfate | 0.05±0.25 | 43.78 | 1.82E-01 | 9.76E-01 | 0.09±0.26 | 33.61 | 2.20E-01 | 9.25E-01 | -0.16±0.29 | 8.85E-01 | -0.09±0.3 | 9.42E-01 | 0.59±0.47 | 9.98E-01 | 0.67±0.54 | 9.91E-01 |
| ceramide (d18:1/14:0, d16:1/16:0)* | -0.08±0.23 | 0 | 4.51E-01 | 9.36E-01 | -0.08±0.25 | 0 | 5.53E-01 | 9.28E-01 | -0.19±0.27 | 8.45E-01 | -0.17±0.29 | 8.64E-01 | 0.19±0.43 | 9.98E-01 | 0.17±0.49 | 9.91E-01 |
| cytidine | -0.03±0.23 | 0 | 3.99E-01 | 9.78E-01 | -0.08±0.25 | 0 | 5.21E-01 | 9.29E-01 | -0.18±0.29 | 8.79E-01 | -0.2±0.31 | 8.46E-01 | 0.23±0.38 | 9.98E-01 | 0.15±0.44 | 9.91E-01 |
| X - 15461 | 0.03±0.24 | 2.18 | 3.12E-01 | 9.78E-01 | 0.08±0.25 | 31.55 | 2.27E-01 | 9.30E-01 | -0.12±0.29 | 8.96E-01 | -0.09±0.29 | 9.42E-01 | 0.42±0.45 | 9.98E-01 | 0.62±0.51 | 9.91E-01 |
| tartarate | 0.01±0.22 | 24.36 | 2.50E-01 | 9.93E-01 | 0.07±0.22 | 56.27 | 1.30E-01 | 9.31E-01 | -0.16±0.27 | 8.79E-01 | -0.15±0.27 | 8.70E-01 | 0.38±0.38 | 9.98E-01 | 0.58±0.41 | 9.91E-01 |
| N,N,N-trimethyl-5-aminovalerate | 0.04±0.27 | 0 | 4.54E-01 | 9.78E-01 | 0.09±0.27 | 0 | 5.98E-01 | 9.31E-01 | -0.08±0.31 | 9.47E-01 | 0±0.31 | 9.95E-01 | 0.37±0.51 | 9.98E-01 | 0.33±0.53 | 9.91E-01 |
| glycosyl-N-stearoyl-sphingosine (d18:1/18:0) | -0.05±0.25 | 0 | 5.70E-01 | 9.76E-01 | -0.08±0.26 | 0 | 4.60E-01 | 9.32E-01 | -0.15±0.31 | 8.92E-01 | -0.2±0.3 | 8.45E-01 | 0.14±0.41 | 9.98E-01 | 0.25±0.52 | 9.91E-01 |
| hexanoylglutamine | -0.01±0.25 | 0 | 9.03E-01 | 9.97E-01 | -0.08±0.26 | 0 | 9.05E-01 | 9.32E-01 | 0.01±0.31 | 9.85E-01 | -0.1±0.31 | 9.41E-01 | -0.05±0.41 | 9.98E-01 | -0.04±0.45 | 9.91E-01 |
| N-acetylphenylalanine | 0.12±0.25 | 0 | 8.87E-01 | 9.11E-01 | 0.09±0.27 | 0 | 9.12E-01 | 9.32E-01 | 0.15±0.32 | 8.96E-01 | 0.11±0.33 | 9.42E-01 | 0.08±0.39 | 9.98E-01 | 0.04±0.47 | 9.91E-01 |
| X - 24337 | -0.03±0.25 | 0 | 5.56E-01 | 9.84E-01 | -0.08±0.26 | 0 | 7.61E-01 | 9.32E-01 | -0.13±0.3 | 8.96E-01 | -0.13±0.31 | 9.08E-01 | 0.18±0.42 | 9.98E-01 | 0.04±0.47 | 9.91E-01 |
| 3-hydroxybutyroylglycine** | 0.1±0.27 | 73.97 | 5.00E-02 | 9.36E-01 | 0.1±0.31 | 65.95 | 8.66E-02 | 9.35E-01 | 0.71±0.41 | 4.62E-01 | 0.57±0.42 | 6.07E-01 | -0.36±0.36 | 9.98E-01 | -0.5±0.47 | 9.91E-01 |
| X - 26107 | 0.12±0.27 | 0 | 4.04E-01 | 9.21E-01 | 0.08±0.27 | 0 | 3.60E-01 | 9.36E-01 | -0.02±0.31 | 9.82E-01 | -0.05±0.3 | 9.69E-01 | 0.47±0.5 | 9.98E-01 | 0.52±0.55 | 9.91E-01 |
| 3-methoxytyrosine | 0.08±0.26 | 0 | 5.78E-01 | 9.51E-01 | 0.09±0.28 | 0 | 5.17E-01 | 9.36E-01 | -0.01±0.31 | 9.87E-01 | -0.02±0.33 | 9.95E-01 | 0.31±0.49 | 9.98E-01 | 0.41±0.57 | 9.91E-01 |
| 2-naphthol sulfate | 0.17±0.25 | 68.57 | 7.45E-02 | 8.57E-01 | 0.1±0.34 | 47.86 | 1.66E-01 | 9.38E-01 | 0.39±0.28 | 6.09E-01 | 0.4±0.41 | 7.42E-01 | -0.75±0.58 | 9.98E-01 | -0.65±0.64 | 9.91E-01 |
| 5,6-dihydrouridine | 0.1±0.22 | 0 | 4.99E-01 | 9.21E-01 | 0.07±0.24 | 0 | 3.92E-01 | 9.38E-01 | 0.23±0.29 | 8.15E-01 | 0.23±0.3 | 8.12E-01 | -0.08±0.34 | 9.98E-01 | -0.21±0.42 | 9.91E-01 |
| pro-hydroxy-pro | -0.09±0.27 | 0 | 3.62E-01 | 9.47E-01 | -0.08±0.28 | 0 | 4.48E-01 | 9.38E-01 | -0.21±0.31 | 8.47E-01 | -0.19±0.31 | 8.55E-01 | 0.41±0.61 | 9.98E-01 | 0.34±0.63 | 9.91E-01 |
| X - 12111 | -0.12±0.25 | 0 | 7.42E-01 | 9.08E-01 | -0.08±0.25 | 0 | 9.78E-01 | 9.38E-01 | -0.18±0.3 | 8.79E-01 | -0.08±0.31 | 9.52E-01 | -0.01±0.43 | 9.98E-01 | -0.07±0.43 | 9.91E-01 |
| 2-hydroxysebacate | 0.08±0.25 | 0 | 9.76E-01 | 9.42E-01 | 0.08±0.26 | 0 | 8.67E-01 | 9.38E-01 | 0.09±0.31 | 9.40E-01 | 0.05±0.32 | 9.73E-01 | 0.07±0.41 | 9.98E-01 | 0.14±0.46 | 9.91E-01 |
| X - 21441 | 0.13±0.26 | 0 | 6.85E-01 | 9.08E-01 | 0.08±0.27 | 0 | 4.62E-01 | 9.38E-01 | 0.07±0.31 | 9.50E-01 | -0.03±0.3 | 9.86E-01 | 0.3±0.49 | 9.98E-01 | 0.43±0.55 | 9.91E-01 |
| X - 12462 | 0.03±0.24 | 0 | 9.52E-01 | 9.81E-01 | -0.07±0.24 | 0 | 7.67E-01 | 9.38E-01 | 0.04±0.31 | 9.80E-01 | -0.01±0.32 | 9.95E-01 | 0.01±0.37 | 9.98E-01 | -0.15±0.37 | 9.91E-01 |
| pentadecanoate (15:0) | -0.1±0.25 | 0 | 3.41E-01 | 9.36E-01 | -0.07±0.25 | 0 | 3.26E-01 | 9.39E-01 | -0.26±0.3 | 7.94E-01 | -0.24±0.3 | 8.00E-01 | 0.25±0.44 | 9.98E-01 | 0.3±0.46 | 9.91E-01 |
| cysteine | -0.02±0.21 | 0 | 5.29E-01 | 9.84E-01 | -0.06±0.21 | 24.27 | 2.51E-01 | 9.40E-01 | 0.13±0.32 | 9.00E-01 | 0.21±0.32 | 8.45E-01 | -0.14±0.29 | 9.98E-01 | -0.28±0.29 | 9.91E-01 |
| X - 10458 | 0.07±0.24 | 0 | 6.31E-01 | 9.56E-01 | 0.07±0.25 | 0 | 6.38E-01 | 9.40E-01 | 0.16±0.31 | 8.88E-01 | 0.16±0.31 | 8.82E-01 | -0.08±0.4 | 9.98E-01 | -0.09±0.43 | 9.91E-01 |
| glycochenodeoxycholate glucuronide (1) | 0.08±0.27 | 0 | 7.03E-01 | 9.56E-01 | 0.08±0.28 | 0 | 7.11E-01 | 9.41E-01 | 0±0.33 | 9.96E-01 | 0.01±0.34 | 9.95E-01 | 0.22±0.47 | 9.98E-01 | 0.23±0.5 | 9.91E-01 |
| fructosyllysine | -0.04±0.2 | 13.92 | 2.81E-01 | 9.76E-01 | -0.06±0.21 | 47.84 | 1.66E-01 | 9.41E-01 | 0.19±0.29 | 8.63E-01 | 0.22±0.3 | 8.12E-01 | -0.25±0.28 | 9.98E-01 | -0.36±0.3 | 9.91E-01 |
| 1,5-anhydroglucitol (1,5-AG) | 0.03±0.24 | 0 | 3.19E-01 | 9.78E-01 | -0.07±0.23 | 39.66 | 1.98E-01 | 9.42E-01 | 0.29±0.36 | 8.09E-01 | 0.29±0.36 | 7.98E-01 | -0.2±0.33 | 9.98E-01 | -0.32±0.31 | 9.91E-01 |
| X - 12411 | 0.03±0.24 | 1.16 | 3.14E-01 | 9.81E-01 | 0.07±0.26 | 31.5 | 2.27E-01 | 9.42E-01 | -0.19±0.32 | 8.79E-01 | -0.17±0.33 | 8.82E-01 | 0.3±0.36 | 9.98E-01 | 0.49±0.43 | 9.91E-01 |
| beta-alanine | 0.11±0.24 | 0 | 9.14E-01 | 9.13E-01 | 0.06±0.23 | 0 | 6.43E-01 | 9.42E-01 | 0.13±0.3 | 8.96E-01 | 0.15±0.29 | 8.83E-01 | 0.08±0.41 | 9.98E-01 | -0.07±0.38 | 9.91E-01 |
| ethylmalonate | -0.04±0.25 | 0 | 5.95E-01 | 9.78E-01 | -0.07±0.26 | 0 | 6.96E-01 | 9.42E-01 | -0.15±0.33 | 8.96E-01 | -0.15±0.33 | 8.98E-01 | 0.13±0.4 | 9.98E-01 | 0.06±0.44 | 9.91E-01 |
| methyl glucopyranoside (alpha + beta) | 0.05±0.25 | 0 | 9.49E-01 | 9.74E-01 | 0.07±0.25 | 0 | 8.74E-01 | 9.42E-01 | 0.06±0.31 | 9.55E-01 | 0.1±0.31 | 9.42E-01 | 0.03±0.42 | 9.98E-01 | 0.01±0.42 | 9.91E-01 |
| carotene diol (2) | -0.08±0.23 | 0 | 8.70E-01 | 9.42E-01 | -0.07±0.26 | 0 | 9.16E-01 | 9.42E-01 | -0.11±0.3 | 9.12E-01 | -0.09±0.31 | 9.48E-01 | -0.03±0.37 | 9.98E-01 | -0.03±0.45 | 9.91E-01 |
| decadienedioic acid (C10:2-DC)** | 0.13±0.25 | 0 | 7.60E-01 | 9.08E-01 | 0.07±0.26 | 0 | 9.59E-01 | 9.42E-01 | 0.19±0.32 | 8.79E-01 | 0.08±0.31 | 9.55E-01 | 0.03±0.41 | 9.98E-01 | 0.05±0.47 | 9.91E-01 |
| betaine | -0.04±0.22 | 0 | 7.19E-01 | 9.76E-01 | -0.06±0.23 | 0 | 5.46E-01 | 9.42E-01 | 0.05±0.32 | 9.69E-01 | 0.07±0.32 | 9.63E-01 | -0.11±0.3 | 9.98E-01 | -0.2±0.33 | 9.91E-01 |
| N-stearoyl-sphingadienine (d18:2/18:0)* | 0.09±0.27 | 0 | 8.01E-01 | 9.42E-01 | 0.08±0.27 | 0 | 9.97E-01 | 9.42E-01 | 0.04±0.34 | 9.80E-01 | 0.08±0.35 | 9.66E-01 | 0.18±0.45 | 9.98E-01 | 0.08±0.45 | 9.91E-01 |
| 4-allylphenol sulfate | -0.11±0.25 | 0 | 9.49E-01 | 9.21E-01 | -0.07±0.25 | 0 | 9.59E-01 | 9.42E-01 | -0.12±0.3 | 9.04E-01 | -0.06±0.31 | 9.68E-01 | -0.08±0.43 | 9.98E-01 | -0.09±0.44 | 9.91E-01 |
| gluconate | 0.04±0.25 | 20.13 | 2.63E-01 | 9.78E-01 | 0.07±0.27 | 11.05 | 2.89E-01 | 9.42E-01 | -0.1±0.28 | 9.12E-01 | -0.05±0.29 | 9.69E-01 | 0.61±0.57 | 9.98E-01 | 0.72±0.67 | 9.91E-01 |
| 7-methylxanthine | 0.1±0.25 | 0 | 3.70E-01 | 9.26E-01 | 0.07±0.26 | 0 | 4.17E-01 | 9.42E-01 | -0.04±0.29 | 9.80E-01 | -0.05±0.3 | 9.70E-01 | 0.46±0.48 | 9.98E-01 | 0.44±0.53 | 9.91E-01 |
| theobromine | 0.1±0.25 | 32.65 | 2.23E-01 | 9.26E-01 | 0.07±0.26 | 0 | 4.21E-01 | 9.42E-01 | -0.09±0.29 | 9.35E-01 | -0.05±0.3 | 9.69E-01 | 0.59±0.47 | 9.98E-01 | 0.42±0.5 | 9.91E-01 |
| 1-(1-enyl-stearoyl)-GPE (P-18:0)* | 0.06±0.23 | 0 | 8.20E-01 | 9.65E-01 | 0.06±0.24 | 0 | 6.71E-01 | 9.46E-01 | 0.1±0.3 | 9.22E-01 | 0.14±0.3 | 8.98E-01 | 0±0.35 | 9.98E-01 | -0.08±0.41 | 9.91E-01 |
| 1-palmitoyl-2-stearoyl-GPC (16:0/18:0) | -0.04±0.24 | 0 | 7.48E-01 | 9.76E-01 | -0.06±0.25 | 0 | 9.05E-01 | 9.46E-01 | -0.11±0.33 | 9.22E-01 | -0.09±0.33 | 9.52E-01 | 0.04±0.35 | 9.98E-01 | -0.03±0.37 | 9.91E-01 |
| ethyl beta-glucopyranoside | -0.08±0.25 | 0 | 7.84E-01 | 9.42E-01 | -0.07±0.25 | 0 | 9.68E-01 | 9.46E-01 | -0.04±0.31 | 9.81E-01 | -0.07±0.31 | 9.62E-01 | -0.18±0.44 | 9.98E-01 | -0.05±0.43 | 9.91E-01 |
| taurocholate | -0.15±0.25 | 0 | 4.63E-01 | 8.85E-01 | -0.07±0.26 | 0 | 4.71E-01 | 9.46E-01 | -0.02±0.3 | 9.82E-01 | 0.05±0.31 | 9.69E-01 | -0.42±0.45 | 9.98E-01 | -0.37±0.49 | 9.91E-01 |
| 2,6-dihydroxybenzoic acid | -0.01±0.25 | 0 | 8.88E-01 | 9.97E-01 | 0.07±0.25 | 0 | 8.93E-01 | 9.46E-01 | -0.03±0.31 | 9.81E-01 | 0.04±0.3 | 9.76E-01 | 0.04±0.43 | 9.98E-01 | 0.12±0.45 | 9.91E-01 |
| 3-methylhistidine | -0.03±0.25 | 0 | 5.54E-01 | 9.84E-01 | -0.07±0.25 | 0 | 5.46E-01 | 9.46E-01 | 0.07±0.3 | 9.49E-01 | 0.03±0.3 | 9.86E-01 | -0.24±0.44 | 9.98E-01 | -0.31±0.48 | 9.91E-01 |
| X - 23593 | -0.06±0.26 | 0 | 4.91E-01 | 9.65E-01 | -0.07±0.28 | 0 | 3.87E-01 | 9.47E-01 | -0.16±0.3 | 8.84E-01 | -0.18±0.31 | 8.64E-01 | 0.24±0.5 | 9.98E-01 | 0.49±0.71 | 9.91E-01 |
| X - 12283 | -0.11±0.24 | 73.83 | 5.06E-02 | 9.17E-01 | -0.06±0.25 | 71.1 | 6.29E-02 | 9.50E-01 | -0.36±0.27 | 6.37E-01 | -0.29±0.28 | 7.22E-01 | 0.79±0.52 | 9.98E-01 | 0.9±0.58 | 9.91E-01 |
| indolin-2-one | 0.02±0.26 | 71.39 | 6.16E-02 | 9.93E-01 | 0.07±0.28 | 64.98 | 9.11E-02 | 9.50E-01 | -0.25±0.3 | 8.01E-01 | -0.16±0.31 | 8.82E-01 | 0.92±0.55 | 9.98E-01 | 1.01±0.62 | 9.91E-01 |
| X - 24462 | 0.02±0.26 | 32.28 | 2.24E-01 | 9.85E-01 | 0.07±0.27 | 45.74 | 1.75E-01 | 9.50E-01 | -0.18±0.31 | 8.79E-01 | -0.15±0.31 | 8.91E-01 | 0.49±0.46 | 9.98E-01 | 0.68±0.53 | 9.91E-01 |
| ceramide (d18:2/24:1, d18:1/24:2)* | 0.09±0.25 | 0 | 9.79E-01 | 9.36E-01 | 0.06±0.26 | 0 | 8.13E-01 | 9.50E-01 | 0.09±0.31 | 9.40E-01 | 0.11±0.32 | 9.37E-01 | 0.1±0.43 | 9.98E-01 | -0.02±0.44 | 9.91E-01 |
| X - 22509 | -0.1±0.25 | 18.81 | 2.67E-01 | 9.26E-01 | -0.06±0.26 | 41.43 | 1.91E-01 | 9.52E-01 | -0.28±0.3 | 7.67E-01 | -0.27±0.3 | 7.65E-01 | 0.32±0.46 | 9.98E-01 | 0.48±0.49 | 9.91E-01 |
| caffeine | 0.08±0.26 | 0 | 7.14E-01 | 9.56E-01 | 0.07±0.28 | 0 | 5.90E-01 | 9.54E-01 | 0.15±0.32 | 8.96E-01 | 0.18±0.35 | 8.82E-01 | -0.06±0.45 | 9.98E-01 | -0.14±0.47 | 9.91E-01 |
| 3-ureidopropionate | 0.07±0.25 | 0 | 6.97E-01 | 9.59E-01 | 0.06±0.27 | 0 | 6.25E-01 | 9.54E-01 | 0.15±0.32 | 8.96E-01 | 0.16±0.33 | 8.91E-01 | -0.05±0.4 | 9.98E-01 | -0.12±0.46 | 9.91E-01 |
| 1-(1-enyl-palmitoyl)-2-arachidonoyl-GPC (P-16:0/20:4)* | 0.02±0.25 | 0 | 7.66E-01 | 9.91E-01 | -0.06±0.26 | 0 | 7.23E-01 | 9.54E-01 | -0.04±0.32 | 9.80E-01 | -0.13±0.32 | 9.17E-01 | 0.12±0.41 | 9.98E-01 | 0.06±0.43 | 9.91E-01 |
| glycohyocholate | 0.07±0.25 | 0 | 7.52E-01 | 9.56E-01 | 0.06±0.26 | 0 | 9.57E-01 | 9.54E-01 | 0.13±0.31 | 8.96E-01 | 0.07±0.32 | 9.65E-01 | -0.03±0.41 | 9.98E-01 | 0.04±0.43 | 9.91E-01 |
| oleoylcarnitine (C18:1) | -0.01±0.24 | 0 | 9.34E-01 | 9.97E-01 | -0.06±0.24 | 0 | 9.93E-01 | 9.54E-01 | -0.02±0.32 | 9.82E-01 | -0.05±0.3 | 9.69E-01 | 0.02±0.37 | 9.98E-01 | -0.06±0.39 | 9.91E-01 |
| 1-palmitoyl-GPI (16:0) | -0.05±0.27 | 84.57 | 1.09E-02 | 9.76E-01 | -0.06±0.29 | 84.88 | 1.01E-02 | 9.55E-01 | -0.45±0.31 | 5.92E-01 | -0.44±0.32 | 6.11E-01 | 1.13±0.54 | 9.98E-01 | 1.4±0.64 | 9.91E-01 |
| 4-guanidinobutanoate | -0.11±0.29 | 75.59 | 4.30E-02 | 9.36E-01 | -0.06±0.28 | 75.68 | 4.26E-02 | 9.55E-01 | -0.51±0.35 | 5.81E-01 | -0.44±0.34 | 6.20E-01 | 0.73±0.5 | 9.98E-01 | 0.77±0.5 | 9.91E-01 |
| cerotoylcarnitine (C26)* | 0.07±0.23 | 0 | 6.09E-01 | 9.56E-01 | 0.05±0.24 | 0 | 4.06E-01 | 9.55E-01 | 0.19±0.33 | 8.79E-01 | 0.25±0.33 | 8.12E-01 | -0.05±0.33 | 9.98E-01 | -0.14±0.33 | 9.91E-01 |
| tetradecadienedioate (C14:2-DC)* | 0±0.26 | 0 | 4.72E-01 | 9.97E-01 | -0.06±0.26 | 0.03 | 3.17E-01 | 9.55E-01 | -0.12±0.31 | 9.04E-01 | -0.21±0.3 | 8.36E-01 | 0.28±0.46 | 9.98E-01 | 0.4±0.53 | 9.91E-01 |
| pantothenate | 0.01±0.23 | 0 | 4.03E-01 | 9.93E-01 | 0.05±0.24 | 0 | 3.22E-01 | 9.55E-01 | 0.14±0.27 | 8.88E-01 | 0.17±0.27 | 8.45E-01 | -0.28±0.42 | 9.98E-01 | -0.4±0.52 | 9.91E-01 |
| trans-4-hydroxyproline | -0.03±0.26 | 0 | 5.40E-01 | 9.78E-01 | -0.06±0.26 | 0 | 5.71E-01 | 9.55E-01 | -0.15±0.32 | 8.96E-01 | -0.17±0.32 | 8.82E-01 | 0.19±0.44 | 9.98E-01 | 0.15±0.45 | 9.91E-01 |
| N-acetyltryptophan | 0.04±0.26 | 0 | 5.39E-01 | 9.76E-01 | 0.06±0.27 | 0 | 5.80E-01 | 9.55E-01 | 0.16±0.32 | 8.91E-01 | 0.16±0.33 | 8.84E-01 | -0.17±0.43 | 9.98E-01 | -0.16±0.48 | 9.91E-01 |
| 6-oxopiperidine-2-carboxylate | -0.09±0.24 | 0 | 3.46E-01 | 9.36E-01 | -0.05±0.25 | 0 | 6.21E-01 | 9.55E-01 | -0.22±0.28 | 8.09E-01 | -0.13±0.29 | 9.04E-01 | 0.29±0.46 | 9.98E-01 | 0.15±0.48 | 9.91E-01 |
| N-(2-furoyl)glycine | 0.01±0.25 | 0 | 3.60E-01 | 9.97E-01 | 0.06±0.26 | 0 | 3.28E-01 | 9.55E-01 | -0.18±0.32 | 8.79E-01 | -0.12±0.32 | 9.29E-01 | 0.29±0.4 | 9.98E-01 | 0.43±0.46 | 9.91E-01 |
| maleate | 0.07±0.23 | 0 | 8.81E-01 | 9.56E-01 | 0.05±0.24 | 0 | 8.17E-01 | 9.55E-01 | 0.09±0.28 | 9.23E-01 | 0.09±0.29 | 9.42E-01 | 0.02±0.41 | 9.98E-01 | -0.03±0.43 | 9.91E-01 |
| threonine | -0.01±0.25 | 0 | 6.42E-01 | 9.93E-01 | -0.05±0.25 | 0 | 8.15E-01 | 9.55E-01 | -0.09±0.3 | 9.35E-01 | -0.09±0.3 | 9.42E-01 | 0.16±0.46 | 9.98E-01 | 0.03±0.43 | 9.91E-01 |
| X - 13695 | 0.02±0.25 | 0 | 3.76E-01 | 9.93E-01 | 0.05±0.26 | 0 | 4.61E-01 | 9.55E-01 | -0.13±0.3 | 8.96E-01 | -0.06±0.3 | 9.68E-01 | 0.36±0.46 | 9.98E-01 | 0.36±0.49 | 9.91E-01 |
| N-acetylkynurenine (2) | -0.01±0.26 | 0 | 8.29E-01 | 9.97E-01 | -0.06±0.27 | 0 | 9.83E-01 | 9.55E-01 | -0.05±0.32 | 9.69E-01 | -0.05±0.34 | 9.72E-01 | 0.07±0.43 | 9.98E-01 | -0.07±0.43 | 9.91E-01 |
| ascorbic acid 2-sulfate | 0.06±0.25 | 0 | 9.08E-01 | 9.65E-01 | 0.06±0.28 | 0 | 9.35E-01 | 9.55E-01 | 0.03±0.32 | 9.81E-01 | 0.05±0.34 | 9.76E-01 | 0.09±0.39 | 9.98E-01 | 0.1±0.49 | 9.91E-01 |
| 3-(3-hydroxyphenyl)propionate sulfate | 0.03±0.25 | 0 | 4.26E-01 | 9.78E-01 | 0.05±0.24 | 0 | 5.91E-01 | 9.55E-01 | -0.1±0.29 | 9.22E-01 | -0.03±0.29 | 9.86E-01 | 0.33±0.45 | 9.98E-01 | 0.26±0.45 | 9.91E-01 |
| 1-oleoyl-2-docosahexaenoyl-GPC (18:1/22:6)* | 0.04±0.25 | 0 | 9.00E-01 | 9.76E-01 | 0.06±0.27 | 0 | 8.91E-01 | 9.55E-01 | 0.02±0.32 | 9.84E-01 | 0.03±0.33 | 9.86E-01 | 0.08±0.41 | 9.98E-01 | 0.11±0.48 | 9.91E-01 |
| N2-acetyl,N6-methyllysine | -0.03±0.24 | 0 | 7.63E-01 | 9.78E-01 | -0.05±0.24 | 0 | 8.81E-01 | 9.55E-01 | 0.02±0.3 | 9.82E-01 | -0.03±0.3 | 9.86E-01 | -0.13±0.4 | 9.98E-01 | -0.1±0.41 | 9.91E-01 |
| docosahexaenoate (DHA; 22:6n3) | -0.04±0.23 | 0 | 5.81E-01 | 9.76E-01 | -0.06±0.25 | 0 | 6.89E-01 | 9.55E-01 | 0.07±0.31 | 9.50E-01 | 0.02±0.32 | 9.91E-01 | -0.19±0.36 | 9.98E-01 | -0.18±0.4 | 9.91E-01 |
| 1,3,7-trimethylurate | -0.04±0.25 | 0 | 8.60E-01 | 9.76E-01 | -0.06±0.26 | 0 | 7.71E-01 | 9.55E-01 | -0.01±0.31 | 9.87E-01 | 0±0.33 | 9.97E-01 | -0.11±0.43 | 9.98E-01 | -0.16±0.44 | 9.91E-01 |
| glycosyl ceramide (d18:2/24:1, d18:1/24:2)* | 0.01±0.27 | 0 | 7.08E-01 | 9.97E-01 | -0.06±0.3 | 0 | 5.80E-01 | 9.56E-01 | -0.07±0.34 | 9.55E-01 | -0.17±0.35 | 8.91E-01 | 0.14±0.43 | 9.98E-01 | 0.19±0.54 | 9.91E-01 |
| isobutyrylglycine | -0.11±0.23 | 0 | 5.91E-01 | 9.12E-01 | -0.05±0.25 | 0 | 6.09E-01 | 9.56E-01 | -0.18±0.27 | 8.54E-01 | -0.12±0.28 | 9.12E-01 | 0.1±0.45 | 9.98E-01 | 0.19±0.53 | 9.91E-01 |
| X - 23680 | 0.08±0.24 | 0 | 8.80E-01 | 9.42E-01 | 0.05±0.24 | 0 | 9.80E-01 | 9.56E-01 | 0.06±0.29 | 9.55E-01 | 0.05±0.29 | 9.69E-01 | 0.13±0.43 | 9.98E-01 | 0.04±0.45 | 9.91E-01 |
| glutamate | 0.1±0.21 | 82.38 | 1.72E-02 | 9.08E-01 | 0.04±0.22 | 88.69 | 2.95E-03 | 9.56E-01 | 0.71±0.33 | 3.26E-01 | 0.89±0.36 | 2.21E-01 | -0.31±0.27 | 9.98E-01 | -0.45±0.28 | 9.91E-01 |
| (14 or 15)-methylpalmitate (a17:0 or i17:0) | -0.07±0.25 | 0 | 4.71E-01 | 9.59E-01 | -0.05±0.26 | 0 | 3.63E-01 | 9.57E-01 | -0.19±0.3 | 8.72E-01 | -0.2±0.3 | 8.45E-01 | 0.2±0.45 | 9.98E-01 | 0.31±0.47 | 9.91E-01 |
| alpha-tocopherol | 0.05±0.23 | 0 | 6.20E-01 | 9.67E-01 | 0.05±0.23 | 0 | 5.25E-01 | 9.57E-01 | 0.17±0.33 | 8.88E-01 | 0.19±0.33 | 8.64E-01 | -0.05±0.31 | 9.98E-01 | -0.11±0.34 | 9.91E-01 |
| N1-Methyl-2-pyridone-5-carboxamide | -0.09±0.22 | 0 | 3.99E-01 | 9.32E-01 | -0.05±0.23 | 11.77 | 2.87E-01 | 9.57E-01 | 0.06±0.29 | 9.50E-01 | 0.13±0.29 | 9.00E-01 | -0.33±0.36 | 9.98E-01 | -0.4±0.41 | 9.91E-01 |
| 1-arachidonoyl-GPC (20:4n6)* | 0.1±0.24 | 0 | 8.40E-01 | 9.26E-01 | 0.05±0.24 | 0 | 7.68E-01 | 9.57E-01 | 0.14±0.32 | 8.96E-01 | 0.11±0.32 | 9.37E-01 | 0.04±0.38 | 9.98E-01 | -0.04±0.38 | 9.91E-01 |
| stearoylcarnitine (C18) | -0.02±0.25 | 0 | 6.60E-01 | 9.93E-01 | -0.05±0.26 | 0 | 7.72E-01 | 9.57E-01 | -0.1±0.31 | 9.24E-01 | -0.1±0.32 | 9.41E-01 | 0.13±0.42 | 9.98E-01 | 0.05±0.44 | 9.91E-01 |
| X - 21607 | 0.11±0.26 | 0 | 8.97E-01 | 9.26E-01 | 0.05±0.26 | 0 | 8.49E-01 | 9.57E-01 | 0.13±0.32 | 8.96E-01 | 0.09±0.32 | 9.52E-01 | 0.06±0.44 | 9.98E-01 | -0.02±0.45 | 9.91E-01 |
| indoleacetate | 0±0.24 | 0 | 8.49E-01 | 9.97E-01 | 0.05±0.25 | 0 | 9.75E-01 | 9.57E-01 | -0.04±0.32 | 9.76E-01 | 0.05±0.32 | 9.69E-01 | 0.05±0.38 | 9.98E-01 | 0.04±0.4 | 9.91E-01 |
| umbelliferone sulfate | -0.06±0.25 | 79.83 | 2.60E-02 | 9.65E-01 | -0.05±0.26 | 80.29 | 2.43E-02 | 9.61E-01 | -0.51±0.32 | 5.19E-01 | -0.51±0.33 | 5.57E-01 | 0.65±0.41 | 9.98E-01 | 0.71±0.43 | 9.91E-01 |
| sebacate (C10-DC) | 0.05±0.23 | 12.3 | 2.86E-01 | 9.65E-01 | 0.04±0.24 | 0 | 3.65E-01 | 9.62E-01 | 0.22±0.27 | 8.10E-01 | 0.17±0.28 | 8.55E-01 | -0.3±0.4 | 9.98E-01 | -0.32±0.47 | 9.91E-01 |
| 3-hydroxyoleoylcarnitine | -0.01±0.24 | 0 | 4.53E-01 | 9.97E-01 | -0.05±0.25 | 0 | 4.65E-01 | 9.62E-01 | -0.11±0.28 | 8.98E-01 | -0.15±0.29 | 8.82E-01 | 0.3±0.48 | 9.98E-01 | 0.27±0.5 | 9.91E-01 |
| 1-myristoyl-2-arachidonoyl-GPC (14:0/20:4)* | -0.05±0.27 | 0 | 4.21E-01 | 9.76E-01 | -0.05±0.27 | 0 | 5.86E-01 | 9.62E-01 | -0.21±0.33 | 8.72E-01 | -0.15±0.32 | 8.98E-01 | 0.25±0.46 | 9.98E-01 | 0.16±0.47 | 9.91E-01 |
| 5-oxoproline | 0.08±0.24 | 11.18 | 2.89E-01 | 9.42E-01 | 0.05±0.26 | 0 | 3.81E-01 | 9.62E-01 | -0.09±0.29 | 9.26E-01 | -0.09±0.3 | 9.42E-01 | 0.46±0.43 | 9.98E-01 | 0.42±0.49 | 9.91E-01 |
| valylglycine | -0.04±0.25 | 0 | 6.95E-01 | 9.76E-01 | -0.05±0.26 | 0 | 8.33E-01 | 9.62E-01 | -0.11±0.3 | 9.15E-01 | -0.08±0.31 | 9.52E-01 | 0.1±0.44 | 9.98E-01 | 0.04±0.47 | 9.91E-01 |
| X - 16397 | 0.13±0.26 | 0 | 6.61E-01 | 9.08E-01 | 0.05±0.26 | 0 | 5.83E-01 | 9.62E-01 | 0.06±0.31 | 9.55E-01 | -0.03±0.3 | 9.86E-01 | 0.31±0.48 | 9.98E-01 | 0.3±0.53 | 9.91E-01 |
| N,N,N-trimethyl-alanylproline betaine (TMAP) | -0.05±0.3 | 21.67 | 2.59E-01 | 9.78E-01 | -0.05±0.31 | 8.9 | 2.95E-01 | 9.62E-01 | -0.21±0.33 | 8.72E-01 | -0.18±0.33 | 8.73E-01 | 0.65±0.68 | 9.98E-01 | 0.75±0.83 | 9.91E-01 |
| X - 24812 | 0.06±0.24 | 0 | 4.96E-01 | 9.65E-01 | 0.05±0.3 | 0 | 9.03E-01 | 9.62E-01 | 0.17±0.29 | 8.79E-01 | 0.03±0.36 | 9.89E-01 | -0.18±0.42 | 9.98E-01 | 0.11±0.55 | 9.91E-01 |
| glutamine conjugate of C6H10O2 (2)* | 0.01±0.24 | 0 | 5.01E-01 | 9.97E-01 | -0.04±0.26 | 0 | 5.47E-01 | 9.64E-01 | 0.13±0.3 | 8.96E-01 | 0.06±0.31 | 9.69E-01 | -0.22±0.41 | 9.98E-01 | -0.28±0.47 | 9.91E-01 |
| myristoylcarnitine (C14) | 0±0.27 | 38.12 | 2.04E-01 | 9.97E-01 | -0.04±0.26 | 44.28 | 1.80E-01 | 9.66E-01 | -0.19±0.31 | 8.77E-01 | -0.22±0.3 | 8.12E-01 | 0.61±0.54 | 9.98E-01 | 0.64±0.58 | 9.91E-01 |
| cys-gly, oxidized | 0±0.26 | 0 | 3.84E-01 | 1.00E+00 | -0.04±0.28 | 0 | 4.46E-01 | 9.66E-01 | -0.13±0.3 | 8.96E-01 | -0.16±0.31 | 8.84E-01 | 0.41±0.54 | 9.98E-01 | 0.35±0.59 | 9.91E-01 |
| carotene diol (1) | -0.06±0.23 | 0 | 5.11E-01 | 9.65E-01 | -0.04±0.25 | 0 | 4.85E-01 | 9.66E-01 | 0.08±0.31 | 9.49E-01 | 0.1±0.32 | 9.42E-01 | -0.23±0.34 | 9.98E-01 | -0.25±0.39 | 9.91E-01 |
| heptenedioate (C7:1-DC)* | 0.04±0.25 | 0 | 5.04E-01 | 9.76E-01 | 0.04±0.26 | 0 | 4.11E-01 | 9.66E-01 | -0.07±0.3 | 9.50E-01 | -0.07±0.3 | 9.57E-01 | 0.3±0.47 | 9.98E-01 | 0.44±0.55 | 9.91E-01 |
| 3-hydroxyoctanoate | 0±0.24 | 0 | 7.42E-01 | 9.97E-01 | -0.04±0.26 | 0 | 7.90E-01 | 9.66E-01 | 0.06±0.3 | 9.52E-01 | 0.01±0.32 | 9.95E-01 | -0.1±0.39 | 9.98E-01 | -0.14±0.45 | 9.91E-01 |
| X - 23974 | 0.06±0.25 | 0 | 8.91E-01 | 9.65E-01 | 0.04±0.25 | 0 | 7.91E-01 | 9.66E-01 | 0.04±0.3 | 9.80E-01 | 0±0.3 | 9.95E-01 | 0.11±0.43 | 9.98E-01 | 0.15±0.47 | 9.91E-01 |
| indole-3-carboxylate | 0±0.26 | 64.25 | 9.44E-02 | 9.97E-01 | 0.04±0.27 | 46.86 | 1.70E-01 | 9.67E-01 | -0.29±0.31 | 7.75E-01 | -0.19±0.32 | 8.55E-01 | 0.64±0.46 | 9.98E-01 | 0.62±0.5 | 9.91E-01 |
| N,N-dimethylalanine | 0.06±0.23 | 0 | 6.87E-01 | 9.65E-01 | 0.04±0.24 | 0 | 5.82E-01 | 9.67E-01 | 0.13±0.28 | 8.96E-01 | 0.13±0.29 | 9.04E-01 | -0.07±0.41 | 9.98E-01 | -0.16±0.43 | 9.91E-01 |
| linolenoylcarnitine (C18:3)* | -0.02±0.24 | 0 | 7.19E-01 | 9.85E-01 | -0.04±0.25 | 0 | 9.00E-01 | 9.67E-01 | -0.08±0.29 | 9.40E-01 | -0.06±0.3 | 9.68E-01 | 0.11±0.43 | 9.98E-01 | 0.01±0.45 | 9.93E-01 |
| gamma-glutamylglutamine | 0.03±0.28 | 0 | 5.04E-01 | 9.81E-01 | -0.04±0.29 | 0 | 5.55E-01 | 9.68E-01 | -0.06±0.31 | 9.62E-01 | -0.13±0.32 | 9.20E-01 | 0.43±0.65 | 9.98E-01 | 0.31±0.67 | 9.91E-01 |
| oxalate (ethanedioate) | -0.01±0.23 | 0 | 6.26E-01 | 9.97E-01 | 0.04±0.24 | 0 | 7.66E-01 | 9.68E-01 | -0.07±0.27 | 9.42E-01 | 0±0.27 | 9.95E-01 | 0.19±0.47 | 9.98E-01 | 0.17±0.5 | 9.91E-01 |
| isoleucylglycine | 0.05±0.23 | 0 | 3.59E-01 | 9.72E-01 | 0.04±0.25 | 0 | 4.08E-01 | 9.69E-01 | -0.08±0.27 | 9.37E-01 | -0.08±0.28 | 9.49E-01 | 0.41±0.45 | 9.98E-01 | 0.4±0.5 | 9.91E-01 |
| 3-hydroxyhexanoate | 0.12±0.26 | 0 | 7.29E-01 | 9.11E-01 | 0.04±0.27 | 0 | 6.28E-01 | 9.69E-01 | 0.07±0.3 | 9.49E-01 | -0.03±0.31 | 9.86E-01 | 0.28±0.51 | 9.98E-01 | 0.28±0.56 | 9.91E-01 |
| ximenoylcarnitine (C26:1)* | 0.03±0.22 | 0 | 3.87E-01 | 9.78E-01 | -0.03±0.23 | 0 | 3.83E-01 | 9.70E-01 | 0.2±0.3 | 8.54E-01 | 0.13±0.3 | 9.04E-01 | -0.19±0.33 | 9.98E-01 | -0.28±0.36 | 9.91E-01 |
| arachidonoylcarnitine (C20:4) | 0.05±0.26 | 0 | 9.41E-01 | 9.76E-01 | 0.04±0.26 | 0 | 9.93E-01 | 9.70E-01 | 0.03±0.32 | 9.81E-01 | 0.04±0.31 | 9.86E-01 | 0.07±0.43 | 9.98E-01 | 0.04±0.46 | 9.91E-01 |
| X - 21821 | -0.07±0.24 | 69.14 | 7.18E-02 | 9.56E-01 | -0.03±0.25 | 66.84 | 8.25E-02 | 9.71E-01 | -0.29±0.27 | 7.21E-01 | -0.24±0.27 | 7.65E-01 | 0.74±0.51 | 9.98E-01 | 0.84±0.56 | 9.91E-01 |
| 2-hydroxybutyrate/2-hydroxyisobutyrate | -0.01±0.23 | 65.56 | 8.84E-02 | 9.97E-01 | -0.03±0.25 | 69.73 | 6.91E-02 | 9.73E-01 | 0.35±0.31 | 7.11E-01 | 0.37±0.33 | 7.05E-01 | -0.45±0.35 | 9.98E-01 | -0.55±0.38 | 9.91E-01 |
| 2,3-dihydroxypyridine | 0.01±0.26 | 42.25 | 1.88E-01 | 9.93E-01 | 0.04±0.26 | 33.83 | 2.19E-01 | 9.73E-01 | 0.3±0.33 | 7.92E-01 | 0.3±0.34 | 7.65E-01 | -0.39±0.4 | 9.98E-01 | -0.37±0.42 | 9.91E-01 |
| X - 24456 | -0.04±0.24 | 0 | 7.74E-01 | 9.76E-01 | -0.03±0.24 | 0 | 8.82E-01 | 9.73E-01 | -0.08±0.28 | 9.35E-01 | -0.06±0.3 | 9.68E-01 | 0.06±0.42 | 9.98E-01 | 0.02±0.41 | 9.91E-01 |
| gamma-glutamyl-alpha-lysine | 0±0.21 | 0 | 8.94E-01 | 1.00E+00 | -0.03±0.22 | 0 | 6.96E-01 | 9.73E-01 | 0.03±0.3 | 9.81E-01 | 0.06±0.31 | 9.69E-01 | -0.03±0.3 | 9.98E-01 | -0.11±0.3 | 9.91E-01 |
| X - 12544 | -0.03±0.28 | 73.19 | 5.34E-02 | 9.85E-01 | -0.04±0.29 | 66.24 | 8.52E-02 | 9.76E-01 | -0.75±0.47 | 5.16E-01 | -0.65±0.46 | 6.00E-01 | 0.38±0.35 | 9.98E-01 | 0.36±0.37 | 9.91E-01 |
| tartronate (hydroxymalonate) | -0.07±0.22 | 25.84 | 2.46E-01 | 9.44E-01 | -0.03±0.23 | 0 | 3.36E-01 | 9.76E-01 | -0.18±0.23 | 8.25E-01 | -0.11±0.24 | 8.96E-01 | 0.53±0.56 | 9.98E-01 | 0.53±0.63 | 9.91E-01 |
| X - 19141 | -0.04±0.23 | 84.76 | 1.04E-02 | 9.76E-01 | -0.03±0.25 | 87.99 | 3.91E-03 | 9.76E-01 | 0.55±0.33 | 4.75E-01 | 0.56±0.32 | 4.75E-01 | -0.62±0.32 | 9.98E-01 | -0.92±0.4 | 9.91E-01 |
| threonate | -0.08±0.23 | 15.48 | 2.77E-01 | 9.36E-01 | -0.03±0.24 | 0 | 3.29E-01 | 9.76E-01 | -0.19±0.25 | 8.17E-01 | -0.12±0.26 | 8.91E-01 | 0.51±0.59 | 9.98E-01 | 0.54±0.63 | 9.91E-01 |
| N-carbamoylvaline | 0.03±0.24 | 0 | 7.36E-01 | 9.78E-01 | 0.03±0.26 | 0 | 6.73E-01 | 9.76E-01 | 0.1±0.31 | 9.27E-01 | 0.11±0.31 | 9.37E-01 | -0.07±0.38 | 9.98E-01 | -0.13±0.45 | 9.91E-01 |
| 1-linoleoyl-GPG (18:2)* | 0.06±0.25 | 0 | 9.64E-01 | 9.65E-01 | 0.03±0.25 | 0 | 7.21E-01 | 9.76E-01 | 0.07±0.31 | 9.50E-01 | 0.09±0.3 | 9.42E-01 | 0.04±0.43 | 9.98E-01 | -0.1±0.43 | 9.91E-01 |
| 4-allylcatechol sulfate | -0.01±0.24 | 0 | 4.72E-01 | 9.93E-01 | 0.03±0.24 | 0 | 5.70E-01 | 9.76E-01 | -0.13±0.29 | 8.96E-01 | -0.06±0.29 | 9.65E-01 | 0.24±0.42 | 9.98E-01 | 0.23±0.43 | 9.91E-01 |
| glycochenodeoxycholate 3-sulfate | 0.06±0.25 | 0 | 7.88E-01 | 9.65E-01 | 0.03±0.25 | 0 | 8.36E-01 | 9.76E-01 | 0.12±0.32 | 9.12E-01 | 0.07±0.31 | 9.68E-01 | -0.02±0.4 | 9.98E-01 | -0.04±0.43 | 9.91E-01 |
| pseudouridine | 0.06±0.24 | 0 | 9.24E-01 | 9.65E-01 | 0.03±0.26 | 0 | 8.54E-01 | 9.76E-01 | 0.08±0.31 | 9.49E-01 | 0.07±0.32 | 9.68E-01 | 0.03±0.37 | 9.98E-01 | -0.04±0.47 | 9.91E-01 |
| hydroxyasparagine** | 0.02±0.24 | 0 | 8.30E-01 | 9.93E-01 | 0.03±0.24 | 0 | 9.60E-01 | 9.76E-01 | -0.02±0.31 | 9.82E-01 | 0.04±0.31 | 9.85E-01 | 0.08±0.38 | 9.98E-01 | 0.01±0.4 | 9.91E-01 |
| o-cresol sulfate | 0.05±0.25 | 0 | 6.00E-01 | 9.72E-01 | 0.04±0.3 | 0 | 7.65E-01 | 9.76E-01 | 0.14±0.3 | 8.96E-01 | -0.02±0.36 | 9.94E-01 | -0.15±0.45 | 9.98E-01 | 0.17±0.53 | 9.91E-01 |
| glycosyl ceramide (d18:1/20:0, d16:1/22:0)* | 0.06±0.25 | 0 | 9.21E-01 | 9.65E-01 | 0.03±0.27 | 0 | 8.61E-01 | 9.76E-01 | 0.04±0.33 | 9.80E-01 | 0±0.34 | 9.95E-01 | 0.09±0.38 | 9.98E-01 | 0.09±0.45 | 9.91E-01 |
| gamma-glutamylglycine | 0.05±0.24 | 0 | 8.48E-01 | 9.65E-01 | 0.03±0.28 | 0 | 7.01E-01 | 9.77E-01 | 0.02±0.3 | 9.82E-01 | -0.03±0.32 | 9.86E-01 | 0.11±0.4 | 9.98E-01 | 0.21±0.55 | 9.91E-01 |
| butyrylcarnitine (C4) | 0.03±0.21 | 0 | 6.70E-01 | 9.78E-01 | -0.02±0.22 | 0 | 5.18E-01 | 9.79E-01 | 0.11±0.28 | 9.04E-01 | 0.09±0.28 | 9.42E-01 | -0.08±0.33 | 9.98E-01 | -0.2±0.35 | 9.91E-01 |
| uridine | 0.08±0.23 | 0 | 4.27E-01 | 9.36E-01 | 0.02±0.23 | 46.14 | 1.73E-01 | 9.81E-01 | 0.26±0.32 | 8.09E-01 | 0.34±0.33 | 7.20E-01 | -0.1±0.32 | 9.98E-01 | -0.29±0.32 | 9.91E-01 |
| 2,3-dihydroxy-5-methylthio-4-pentenoate (DMTPA)* | 0.01±0.22 | 0 | 5.70E-01 | 9.93E-01 | 0.02±0.24 | 0 | 3.64E-01 | 9.81E-01 | 0.13±0.29 | 8.96E-01 | 0.19±0.3 | 8.48E-01 | -0.12±0.32 | 9.98E-01 | -0.26±0.39 | 9.91E-01 |
| pipecolate | 0.05±0.24 | 0 | 6.44E-01 | 9.75E-01 | 0.03±0.25 | 0 | 5.87E-01 | 9.81E-01 | 0.13±0.3 | 8.96E-01 | 0.12±0.3 | 9.20E-01 | -0.1±0.4 | 9.98E-01 | -0.17±0.43 | 9.91E-01 |
| deoxycarnitine | 0.07±0.29 | 0 | 3.24E-01 | 9.65E-01 | 0.03±0.3 | 0 | 4.16E-01 | 9.81E-01 | -0.07±0.32 | 9.54E-01 | -0.09±0.34 | 9.52E-01 | 0.69±0.7 | 9.98E-01 | 0.54±0.69 | 9.91E-01 |
| 17alpha-hydroxypregnanolone glucuronide | 0.01±0.29 | 40.81 | 1.94E-01 | 9.97E-01 | 0.03±0.3 | 8.24 | 2.97E-01 | 9.82E-01 | 0.3±0.36 | 8.03E-01 | 0.25±0.37 | 8.37E-01 | -0.47±0.47 | 9.98E-01 | -0.41±0.51 | 9.91E-01 |
| glutarylcarnitine (C5-DC) | 0.01±0.27 | 44.74 | 1.79E-01 | 9.97E-01 | -0.03±0.28 | 34.14 | 2.18E-01 | 9.82E-01 | -0.19±0.3 | 8.77E-01 | -0.19±0.31 | 8.55E-01 | 0.69±0.57 | 9.98E-01 | 0.73±0.67 | 9.91E-01 |
| X - 13844 | -0.06±0.24 | 18.68 | 2.67E-01 | 9.65E-01 | -0.02±0.24 | 0 | 3.26E-01 | 9.82E-01 | -0.22±0.28 | 8.17E-01 | -0.17±0.28 | 8.62E-01 | 0.4±0.48 | 9.98E-01 | 0.37±0.47 | 9.91E-01 |
| 4-vinylguaiacol sulfate | -0.02±0.26 | 0 | 5.53E-01 | 9.93E-01 | 0.03±0.28 | 0 | 8.18E-01 | 9.82E-01 | 0.09±0.31 | 9.40E-01 | 0.06±0.33 | 9.68E-01 | -0.25±0.47 | 9.98E-01 | -0.08±0.54 | 9.91E-01 |
| picolinoylglycine | -0.02±0.24 | 0 | 6.11E-01 | 9.84E-01 | -0.02±0.27 | 0 | 9.95E-01 | 9.82E-01 | -0.11±0.3 | 9.08E-01 | -0.02±0.32 | 9.91E-01 | 0.15±0.42 | 9.98E-01 | -0.03±0.5 | 9.91E-01 |
| X - 11478 | 0.02±0.24 | 0 | 9.85E-01 | 9.93E-01 | -0.02±0.25 | 0 | 8.74E-01 | 9.82E-01 | 0.01±0.29 | 9.85E-01 | 0±0.3 | 9.95E-01 | 0.02±0.43 | 9.98E-01 | -0.08±0.44 | 9.91E-01 |
| carnitine | 0.05±0.25 | 0 | 7.74E-01 | 9.76E-01 | 0.02±0.26 | 0 | 8.36E-01 | 9.82E-01 | 0±0.31 | 9.96E-01 | -0.01±0.31 | 9.95E-01 | 0.15±0.45 | 9.98E-01 | 0.11±0.47 | 9.91E-01 |
| 1,7-dimethylurate | -0.01±0.26 | 0 | 5.75E-01 | 9.97E-01 | -0.02±0.27 | 0 | 5.50E-01 | 9.84E-01 | 0.11±0.33 | 9.22E-01 | 0.11±0.35 | 9.42E-01 | -0.19±0.43 | 9.98E-01 | -0.21±0.41 | 9.91E-01 |
| ceramide (d18:1/17:0, d17:1/18:0)* | -0.05±0.25 | 0 | 3.93E-01 | 9.76E-01 | 0.02±0.27 | 0 | 6.35E-01 | 9.84E-01 | -0.18±0.3 | 8.79E-01 | -0.05±0.32 | 9.70E-01 | 0.31±0.48 | 9.98E-01 | 0.25±0.55 | 9.91E-01 |
| 2-aminoheptanoate | 0.03±0.23 | 39.94 | 1.97E-01 | 9.78E-01 | -0.02±0.24 | 60.88 | 1.10E-01 | 9.85E-01 | 0.29±0.3 | 7.70E-01 | 0.31±0.32 | 7.43E-01 | -0.31±0.35 | 9.98E-01 | -0.46±0.37 | 9.91E-01 |
| X - 17357 | 0±0.23 | 0 | 4.55E-01 | 1.00E+00 | -0.02±0.24 | 0 | 4.51E-01 | 9.85E-01 | -0.11±0.27 | 9.03E-01 | -0.12±0.28 | 9.04E-01 | 0.28±0.44 | 9.98E-01 | 0.3±0.48 | 9.91E-01 |
| taurochenodeoxycholate | -0.06±0.24 | 0 | 7.98E-01 | 9.65E-01 | 0.02±0.25 | 0 | 6.03E-01 | 9.85E-01 | -0.1±0.3 | 9.19E-01 | -0.07±0.3 | 9.62E-01 | 0.03±0.41 | 9.98E-01 | 0.2±0.43 | 9.91E-01 |
| picolinate | -0.01±0.25 | 0 | 9.06E-01 | 9.97E-01 | 0.02±0.25 | 0 | 8.00E-01 | 9.85E-01 | -0.03±0.3 | 9.81E-01 | 0.06±0.31 | 9.68E-01 | 0.03±0.42 | 9.98E-01 | -0.07±0.45 | 9.91E-01 |
| X - 12729 | -0.01±0.25 | 0 | 5.09E-01 | 9.93E-01 | -0.02±0.25 | 0 | 6.38E-01 | 9.85E-01 | 0.08±0.28 | 9.40E-01 | 0.04±0.29 | 9.73E-01 | -0.3±0.5 | 9.98E-01 | -0.24±0.53 | 9.91E-01 |
| 3-hydroxyhexanoylcarnitine (1) | 0.05±0.24 | 0 | 7.99E-01 | 9.65E-01 | 0.02±0.25 | 0 | 9.21E-01 | 9.85E-01 | 0.1±0.29 | 9.22E-01 | 0.04±0.3 | 9.85E-01 | -0.03±0.39 | 9.98E-01 | -0.02±0.43 | 9.91E-01 |
| C-glycosyltryptophan | -0.02±0.24 | 0 | 8.87E-01 | 9.84E-01 | -0.02±0.26 | 0 | 9.12E-01 | 9.85E-01 | -0.05±0.31 | 9.66E-01 | -0.04±0.32 | 9.85E-01 | 0.02±0.37 | 9.98E-01 | 0.02±0.47 | 9.91E-01 |
| X - 21467 | -0.02±0.25 | 0 | 7.78E-01 | 9.93E-01 | -0.02±0.26 | 0 | 8.80E-01 | 9.85E-01 | -0.07±0.3 | 9.50E-01 | 0.01±0.32 | 9.95E-01 | 0.08±0.43 | 9.98E-01 | -0.07±0.43 | 9.91E-01 |
| 2S,3R-dihydroxybutyrate | 0.01±0.26 | 0 | 8.57E-01 | 9.96E-01 | -0.02±0.29 | 0 | 9.74E-01 | 9.85E-01 | 0.05±0.34 | 9.69E-01 | -0.02±0.35 | 9.95E-01 | -0.04±0.41 | 9.98E-01 | -0.04±0.49 | 9.91E-01 |
| tryptophan | 0.04±0.24 | 0 | 9.67E-01 | 9.76E-01 | 0.02±0.24 | 0 | 6.71E-01 | 9.86E-01 | 0.03±0.3 | 9.81E-01 | 0.11±0.33 | 9.37E-01 | 0.05±0.38 | 9.98E-01 | -0.09±0.35 | 9.91E-01 |
| theophylline | 0±0.26 | 0 | 4.66E-01 | 1.00E+00 | -0.02±0.26 | 0 | 5.22E-01 | 9.87E-01 | 0.15±0.33 | 8.96E-01 | 0.12±0.34 | 9.31E-01 | -0.23±0.41 | 9.98E-01 | -0.22±0.4 | 9.91E-01 |
| X - 12812 | -0.06±0.24 | 0 | 7.54E-01 | 9.65E-01 | -0.02±0.24 | 0 | 9.33E-01 | 9.87E-01 | -0.11±0.3 | 9.08E-01 | -0.03±0.3 | 9.86E-01 | 0.04±0.4 | 9.98E-01 | 0.01±0.39 | 9.91E-01 |
| N2,N5-diacetylornithine | 0±0.24 | 0 | 4.45E-01 | 9.97E-01 | 0.02±0.26 | 0 | 6.87E-01 | 9.87E-01 | 0.14±0.3 | 8.96E-01 | 0.09±0.31 | 9.52E-01 | -0.24±0.4 | 9.98E-01 | -0.15±0.48 | 9.91E-01 |
| homovanillate (HVA) | 0.05±0.24 | 86.28 | 6.93E-03 | 9.72E-01 | 0.02±0.25 | 85.79 | 7.97E-03 | 9.87E-01 | -0.39±0.29 | 6.37E-01 | -0.41±0.3 | 6.11E-01 | 1.05±0.44 | 9.98E-01 | 1.09±0.48 | 9.91E-01 |
| 2'-deoxyuridine | 0.07±0.23 | 0 | 4.71E-01 | 9.44E-01 | -0.01±0.23 | 0 | 3.27E-01 | 9.87E-01 | 0.21±0.3 | 8.43E-01 | 0.16±0.29 | 8.72E-01 | -0.12±0.35 | 9.98E-01 | -0.3±0.37 | 9.91E-01 |
| butyrylglycine | -0.03±0.24 | 0 | 9.96E-01 | 9.78E-01 | -0.02±0.25 | 0 | 9.31E-01 | 9.87E-01 | -0.03±0.3 | 9.81E-01 | -0.03±0.3 | 9.86E-01 | -0.03±0.42 | 9.98E-01 | 0.02±0.44 | 9.91E-01 |
| N-acetylasparagine | -0.04±0.24 | 0 | 3.92E-01 | 9.76E-01 | -0.01±0.24 | 0 | 5.32E-01 | 9.87E-01 | -0.18±0.29 | 8.72E-01 | -0.11±0.29 | 9.20E-01 | 0.26±0.42 | 9.98E-01 | 0.22±0.44 | 9.91E-01 |
| X - 12851 | -0.03±0.24 | 0 | 7.22E-01 | 9.78E-01 | -0.01±0.25 | 0 | 9.14E-01 | 9.88E-01 | 0.03±0.3 | 9.81E-01 | 0±0.3 | 9.95E-01 | -0.15±0.41 | 9.98E-01 | -0.06±0.47 | 9.91E-01 |
| X - 12306 | -0.06±0.25 | 74.01 | 4.98E-02 | 9.65E-01 | 0.01±0.26 | 68.52 | 7.47E-02 | 9.89E-01 | -0.31±0.28 | 7.18E-01 | -0.22±0.29 | 8.07E-01 | 0.91±0.55 | 9.98E-01 | 0.9±0.56 | 9.91E-01 |
| N6-acetyllysine | 0.08±0.22 | 0 | 5.20E-01 | 9.42E-01 | 0.01±0.22 | 0 | 3.35E-01 | 9.90E-01 | 0.22±0.32 | 8.47E-01 | 0.24±0.33 | 8.12E-01 | -0.06±0.31 | 9.98E-01 | -0.18±0.3 | 9.91E-01 |
| isoursodeoxycholate | 0.02±0.24 | 0 | 7.06E-01 | 9.87E-01 | 0.01±0.26 | 0 | 5.03E-01 | 9.90E-01 | 0.08±0.29 | 9.37E-01 | 0.12±0.3 | 9.21E-01 | -0.11±0.42 | 9.98E-01 | -0.27±0.5 | 9.91E-01 |
| N-acetyl-1-methylhistidine* | 0.08±0.24 | 0 | 5.44E-01 | 9.42E-01 | -0.01±0.25 | 0 | 7.36E-01 | 9.90E-01 | -0.03±0.3 | 9.81E-01 | -0.07±0.3 | 9.64E-01 | 0.28±0.41 | 9.98E-01 | 0.11±0.45 | 9.91E-01 |
| X - 24307 | 0.02±0.24 | 0 | 7.49E-01 | 9.87E-01 | 0.01±0.25 | 0 | 7.86E-01 | 9.90E-01 | 0.08±0.3 | 9.47E-01 | 0.06±0.31 | 9.68E-01 | -0.08±0.39 | 9.98E-01 | -0.08±0.42 | 9.91E-01 |
| 3-formylindole | -0.06±0.25 | 0 | 7.76E-01 | 9.65E-01 | -0.01±0.27 | 0 | 9.71E-01 | 9.90E-01 | -0.11±0.32 | 9.15E-01 | -0.01±0.33 | 9.95E-01 | 0.03±0.39 | 9.98E-01 | -0.03±0.44 | 9.91E-01 |
| linoleoyl ethanolamide | 0.02±0.25 | 0 | 8.64E-01 | 9.89E-01 | 0.01±0.25 | 0 | 8.65E-01 | 9.90E-01 | -0.01±0.3 | 9.90E-01 | -0.01±0.29 | 9.95E-01 | 0.08±0.44 | 9.98E-01 | 0.08±0.47 | 9.91E-01 |
| X - 25957 | 0.07±0.25 | 0 | 5.97E-01 | 9.65E-01 | 0.01±0.26 | 0 | 4.02E-01 | 9.90E-01 | 0.18±0.34 | 8.85E-01 | 0.19±0.34 | 8.64E-01 | -0.09±0.39 | 9.98E-01 | -0.24±0.39 | 9.91E-01 |
| 1,3-dimethylurate | -0.05±0.25 | 0 | 3.33E-01 | 9.72E-01 | -0.01±0.25 | 0 | 5.71E-01 | 9.90E-01 | 0.18±0.34 | 8.88E-01 | 0.12±0.34 | 9.31E-01 | -0.3±0.36 | 9.98E-01 | -0.16±0.37 | 9.91E-01 |
| X - 07765 | -0.05±0.28 | 7.16 | 2.99E-01 | 9.76E-01 | 0.01±0.29 | 1.31 | 3.14E-01 | 9.90E-01 | 0.06±0.3 | 9.55E-01 | 0.11±0.3 | 9.34E-01 | -0.86±0.83 | 9.98E-01 | -0.88±0.93 | 9.91E-01 |
| N-acetyl-2-aminooctanoate* | 0.03±0.25 | 0 | 8.80E-01 | 9.78E-01 | 0.01±0.26 | 0 | 7.96E-01 | 9.90E-01 | 0.06±0.31 | 9.55E-01 | 0.06±0.32 | 9.69E-01 | -0.02±0.42 | 9.98E-01 | -0.08±0.43 | 9.91E-01 |
| X - 24556 | -0.01±0.24 | 0 | 9.84E-01 | 9.93E-01 | 0.01±0.25 | 0 | 9.91E-01 | 9.90E-01 | -0.02±0.3 | 9.83E-01 | 0.01±0.31 | 9.95E-01 | -0.01±0.39 | 9.98E-01 | 0.01±0.42 | 9.93E-01 |
| X - 26109 | -0.04±0.24 | 54.27 | 1.39E-01 | 9.76E-01 | -0.01±0.25 | 41.74 | 1.90E-01 | 9.91E-01 | -0.23±0.27 | 8.02E-01 | -0.18±0.29 | 8.45E-01 | 0.64±0.52 | 9.98E-01 | 0.62±0.54 | 9.91E-01 |
| 2-hydroxyphenylacetate | 0.03±0.24 | 83.07 | 1.51E-02 | 9.82E-01 | -0.01±0.25 | 86.67 | 6.17E-03 | 9.91E-01 | 0.71±0.37 | 3.84E-01 | 0.77±0.38 | 3.75E-01 | -0.46±0.31 | 9.98E-01 | -0.63±0.34 | 9.91E-01 |
| X - 21736 | 0.08±0.23 | 77.42 | 3.53E-02 | 9.42E-01 | -0.01±0.26 | 80.13 | 2.49E-02 | 9.91E-01 | 0.43±0.29 | 5.56E-01 | 0.36±0.31 | 6.81E-01 | -0.62±0.41 | 9.98E-01 | -0.92±0.48 | 9.91E-01 |
| X - 25271 | -0.03±0.24 | 43.13 | 1.85E-01 | 9.78E-01 | -0.01±0.25 | 49.78 | 1.58E-01 | 9.91E-01 | 0.22±0.31 | 8.43E-01 | 0.27±0.32 | 7.71E-01 | -0.43±0.39 | 9.98E-01 | -0.45±0.4 | 9.91E-01 |
| 4-hydroxyphenylacetate | 0.02±0.24 | 50.17 | 1.57E-01 | 9.84E-01 | 0.01±0.26 | 53.73 | 1.42E-01 | 9.91E-01 | -0.25±0.31 | 8.09E-01 | -0.25±0.31 | 7.97E-01 | 0.46±0.39 | 9.98E-01 | 0.57±0.46 | 9.91E-01 |
| 4-hydroxyglutamate | 0.02±0.26 | 0 | 8.94E-01 | 9.89E-01 | 0.01±0.29 | 0 | 3.83E-01 | 9.91E-01 | 0.04±0.31 | 9.72E-01 | 0.17±0.34 | 8.86E-01 | -0.03±0.46 | 9.98E-01 | -0.38±0.53 | 9.91E-01 |
| 3-hydroxystachydrine* | -0.04±0.25 | 0 | 6.11E-01 | 9.76E-01 | -0.01±0.26 | 0 | 4.17E-01 | 9.91E-01 | 0.06±0.31 | 9.62E-01 | 0.16±0.33 | 8.91E-01 | -0.2±0.4 | 9.98E-01 | -0.27±0.41 | 9.91E-01 |
| hydantoin-5-propionate | -0.02±0.24 | 48.19 | 1.65E-01 | 9.85E-01 | -0.01±0.25 | 0 | 4.15E-01 | 9.91E-01 | -0.25±0.29 | 7.99E-01 | -0.12±0.29 | 9.12E-01 | 0.48±0.44 | 9.98E-01 | 0.37±0.53 | 9.91E-01 |
| X - 22834 | 0±0.25 | 0 | 9.20E-01 | 9.99E-01 | 0.01±0.26 | 0 | 7.52E-01 | 9.91E-01 | -0.02±0.3 | 9.82E-01 | 0.06±0.32 | 9.68E-01 | 0.03±0.43 | 9.98E-01 | -0.11±0.47 | 9.91E-01 |
| taurine | 0±0.23 | 0 | 9.82E-01 | 1.00E+00 | -0.01±0.25 | 0 | 9.52E-01 | 9.91E-01 | 0±0.31 | 9.96E-01 | 0±0.32 | 9.95E-01 | -0.01±0.34 | 9.98E-01 | -0.03±0.4 | 9.91E-01 |
| 3-methyl catechol sulfate (2) | -0.01±0.24 | 69.29 | 7.12E-02 | 9.93E-01 | 0±0.26 | 78.4 | 3.14E-02 | 9.95E-01 | -0.27±0.28 | 7.59E-01 | -0.27±0.29 | 7.53E-01 | 0.73±0.48 | 9.98E-01 | 1.18±0.61 | 9.91E-01 |
| 1-methyl-5-imidazoleacetate | 0±0.25 | 0 | 9.05E-01 | 9.97E-01 | 0±0.26 | 0 | 8.85E-01 | 9.95E-01 | -0.03±0.3 | 9.81E-01 | -0.03±0.31 | 9.86E-01 | 0.04±0.41 | 9.98E-01 | 0.05±0.47 | 9.91E-01 |
| gamma-glutamylmethionine | 0.03±0.26 | 0 | 7.44E-01 | 9.81E-01 | 0±0.25 | 0 | 9.69E-01 | 9.95E-01 | -0.03±0.33 | 9.81E-01 | 0±0.32 | 9.95E-01 | 0.15±0.44 | 9.98E-01 | 0.02±0.42 | 9.91E-01 |
| catechol sulfate | -0.03±0.25 | 0 | 9.26E-01 | 9.78E-01 | 0±0.26 | 0 | 8.95E-01 | 9.96E-01 | -0.05±0.31 | 9.69E-01 | 0.02±0.31 | 9.95E-01 | 0±0.44 | 9.99E-01 | -0.06±0.48 | 9.91E-01 |
| vanillic acid glycine | -0.06±0.25 | 30.99 | 2.29E-01 | 9.65E-01 | 0±0.26 | 0 | 3.55E-01 | 9.97E-01 | -0.27±0.3 | 7.94E-01 | -0.16±0.31 | 8.82E-01 | 0.37±0.43 | 9.98E-01 | 0.37±0.48 | 9.91E-01 |
| 1-linoleoyl-2-linolenoyl-GPC (18:2/18:3)* | -0.04±0.26 | 0 | 4.36E-01 | 9.78E-01 | 0±0.28 | 0 | 5.17E-01 | 9.97E-01 | -0.16±0.31 | 8.86E-01 | -0.11±0.32 | 9.41E-01 | 0.29±0.5 | 9.98E-01 | 0.31±0.56 | 9.91E-01 |
| X - 24306 | 0.03±0.23 | 0 | 6.31E-01 | 9.78E-01 | 0±0.25 | 0 | 5.15E-01 | 9.99E-01 | 0.12±0.3 | 8.96E-01 | 0.13±0.31 | 9.17E-01 | -0.1±0.37 | 9.98E-01 | -0.2±0.4 | 9.91E-01 |
| X - 11444 | 0±0.24 | 0 | 7.28E-01 | 9.97E-01 | 0±0.25 | 0 | 8.00E-01 | 9.99E-01 | -0.06±0.29 | 9.53E-01 | -0.04±0.3 | 9.76E-01 | 0.12±0.42 | 9.98E-01 | 0.09±0.44 | 9.91E-01 |
| X - 12822 | 0±0.25 | 0 | 9.58E-01 | 1.00E+00 | 0±0.26 | 0 | 9.69E-01 | 9.99E-01 | -0.01±0.31 | 9.89E-01 | 0.01±0.32 | 9.95E-01 | 0.02±0.4 | 9.98E-01 | -0.01±0.45 | 9.91E-01 |
| (N(1) + N(8))-acetylspermidine | 0.04±0.25 | 0 | 8.15E-01 | 9.76E-01 | 0±0.26 | 0 | 9.86E-01 | 9.99E-01 | 0±0.31 | 9.98E-01 | 0±0.32 | 9.95E-01 | 0.12±0.43 | 9.98E-01 | 0.01±0.45 | 9.93E-01 |
| branched chain 14:0 dicarboxylic acid** |  |  |  |  |  |  |  |  | -0.53±0.16 | **4.65E-02** | -0.5±0.16 | 1.10E-01 |  |  |  |  |
| X - 24728 |  |  |  |  |  |  |  |  | 0.72±0.26 | 1.49E-01 | 0.67±0.28 | 2.21E-01 |  |  |  |  |
| X - 18345 |  |  |  |  |  |  |  |  | 0.71±0.32 | 3.03E-01 | 0.79±0.33 | 2.34E-01 |  |  |  |  |
| X - 16124 |  |  |  |  |  |  |  |  | -0.94±0.39 | 2.49E-01 | -0.98±0.41 | 2.37E-01 |  |  |  |  |
| X - 25948 |  |  |  |  |  |  |  |  | 0.65±0.31 | 3.26E-01 | 0.75±0.32 | 2.51E-01 |  |  |  |  |
| 5alpha-androstan-3alpha,17alpha-diol monosulfate |  |  |  |  |  |  |  |  | -0.8±0.37 | 3.26E-01 | -0.85±0.38 | 2.83E-01 |  |  |  |  |
| inosine |  |  |  |  |  |  |  |  | 0.64±0.31 | 3.38E-01 | 0.65±0.32 | 3.69E-01 |  |  |  |  |
| N-acetyltaurine |  |  |  |  |  |  |  |  | 0.5±0.22 | 3.03E-01 | 0.46±0.23 | 3.90E-01 |  |  |  |  |
| X - 23648 |  |  |  |  |  |  |  |  | 0.55±0.34 | 5.09E-01 | 0.67±0.34 | 3.90E-01 |  |  |  |  |
| X - 12126 |  |  |  |  |  |  |  |  | -0.55±0.27 | 3.38E-01 | -0.53±0.27 | 3.93E-01 |  |  |  |  |
| X - 13729 |  |  |  |  |  |  |  |  | -0.54±0.27 | 3.63E-01 | -0.52±0.28 | 4.11E-01 |  |  |  |  |
| S-methylmethionine |  |  |  |  |  |  |  |  | -0.69±0.37 | 4.06E-01 | -0.69±0.38 | 4.24E-01 |  |  |  |  |
| N-formylanthranilic acid |  |  |  |  |  |  |  |  | 0.41±0.25 | 4.89E-01 | 0.43±0.25 | 4.69E-01 |  |  |  |  |
| X - 21410 |  |  |  |  |  |  |  |  | 0.9±0.49 | 4.19E-01 | 0.87±0.5 | 4.75E-01 |  |  |  |  |
| 4-hydroxyphenylpyruvate |  |  |  |  |  |  |  |  | 0.61±0.37 | 5.08E-01 | 0.66±0.39 | 4.89E-01 |  |  |  |  |
| X - 12127 |  |  |  |  |  |  |  |  | 0.54±0.31 | 4.61E-01 | 0.54±0.32 | 5.01E-01 |  |  |  |  |
| X - 13658 |  |  |  |  |  |  |  |  | -0.5±0.33 | 5.53E-01 | -0.57±0.35 | 5.14E-01 |  |  |  |  |
| 1,2,3-benzenetriol sulfate (2) |  |  |  |  |  |  |  |  | 0.5±0.32 | 5.37E-01 | 0.52±0.32 | 5.15E-01 |  |  |  |  |
| 2,2'-Methylenebis(6-tert-butyl-p-cresol) |  |  |  |  |  |  |  |  | -0.52±0.34 | 5.53E-01 | -0.54±0.33 | 5.15E-01 |  |  |  |  |
| (R)-3-hydroxybutyrylcarnitine |  |  |  |  |  |  |  |  | 0.56±0.28 | 3.59E-01 | 0.45±0.29 | 5.29E-01 |  |  |  |  |
| X - 17351 |  |  |  |  |  |  |  |  | -0.43±0.24 | 4.45E-01 | -0.39±0.25 | 5.51E-01 |  |  |  |  |
| 1-oleoyl-GPG (18:1)* |  |  |  |  |  |  |  |  | 0.79±0.49 | 5.16E-01 | 0.73±0.48 | 5.57E-01 |  |  |  |  |
| X - 18935 |  |  |  |  |  |  |  |  | -0.5±0.31 | 5.09E-01 | -0.46±0.31 | 5.69E-01 |  |  |  |  |
| adrenate (22:4n6) |  |  |  |  |  |  |  |  | -0.39±0.29 | 6.30E-01 | -0.43±0.29 | 5.69E-01 |  |  |  |  |
| X - 14056 |  |  |  |  |  |  |  |  | 0.39±0.32 | 6.76E-01 | 0.5±0.34 | 5.77E-01 |  |  |  |  |
| X - 23997 |  |  |  |  |  |  |  |  | -0.42±0.25 | 4.75E-01 | -0.37±0.25 | 5.80E-01 |  |  |  |  |
| trans-3,4-methyleneheptanoate |  |  |  |  |  |  |  |  | -0.42±0.28 | 5.56E-01 | -0.4±0.29 | 6.02E-01 |  |  |  |  |
| X - 24411 |  |  |  |  |  |  |  |  | 0.57±0.4 | 5.92E-01 | 0.56±0.41 | 6.02E-01 |  |  |  |  |
| docosahexaenoylcholine |  |  |  |  |  |  |  |  | 0.62±0.46 | 6.25E-01 | 0.66±0.47 | 6.02E-01 |  |  |  |  |
| 17alpha-hydroxypregnenolone 3-sulfate |  |  |  |  |  |  |  |  | -0.4±0.31 | 6.55E-01 | -0.45±0.32 | 6.02E-01 |  |  |  |  |
| X - 21959 |  |  |  |  |  |  |  |  | 0.36±0.3 | 6.71E-01 | 0.42±0.3 | 6.02E-01 |  |  |  |  |
| 5-acetylamino-6-formylamino-3-methyluracil |  |  |  |  |  |  |  |  | 0.49±0.34 | 5.89E-01 | 0.45±0.34 | 6.11E-01 |  |  |  |  |
| methyl-4-hydroxybenzoate sulfate |  |  |  |  |  |  |  |  | -0.4±0.31 | 6.48E-01 | -0.4±0.31 | 6.26E-01 |  |  |  |  |
| X - 12738 |  |  |  |  |  |  |  |  | -0.49±0.34 | 5.89E-01 | -0.44±0.34 | 6.28E-01 |  |  |  |  |
| X - 21312 |  |  |  |  |  |  |  |  | 0.34±0.32 | 7.26E-01 | 0.4±0.33 | 6.59E-01 |  |  |  |  |
| sucrose |  |  |  |  |  |  |  |  | 0.32±0.33 | 7.57E-01 | 0.41±0.33 | 6.59E-01 |  |  |  |  |
| (2,4 or 2,5)-dimethylphenol sulfate |  |  |  |  |  |  |  |  | 0.44±0.29 | 5.48E-01 | 0.41±0.35 | 6.86E-01 |  |  |  |  |
| cyclo(gly-pro) |  |  |  |  |  |  |  |  | 0.41±0.35 | 6.81E-01 | 0.41±0.36 | 6.95E-01 |  |  |  |  |
| ferulic acid 4-sulfate |  |  |  |  |  |  |  |  | 0.3±0.31 | 7.57E-01 | 0.36±0.31 | 6.95E-01 |  |  |  |  |
| N-acetylaspartate (NAA) |  |  |  |  |  |  |  |  | 0.57±0.5 | 7.08E-01 | 0.56±0.5 | 7.04E-01 |  |  |  |  |
| bilirubin degradation product, C16H18N2O5 (1)** |  |  |  |  |  |  |  |  | -0.29±0.29 | 7.58E-01 | -0.34±0.3 | 7.04E-01 |  |  |  |  |
| X - 12726 |  |  |  |  |  |  |  |  | -0.4±0.29 | 6.06E-01 | -0.33±0.3 | 7.05E-01 |  |  |  |  |
| catechol glucuronide |  |  |  |  |  |  |  |  | -0.43±0.32 | 6.37E-01 | -0.36±0.33 | 7.05E-01 |  |  |  |  |
| 4-hydroxy-2-oxoglutaric acid |  |  |  |  |  |  |  |  | 0.42±0.36 | 6.93E-01 | 0.41±0.37 | 7.05E-01 |  |  |  |  |
| histidine betaine (hercynine)* |  |  |  |  |  |  |  |  | 0.39±0.33 | 6.76E-01 | 0.36±0.33 | 7.10E-01 |  |  |  |  |
| X - 26058 |  |  |  |  |  |  |  |  | 0.25±0.28 | 7.86E-01 | 0.3±0.28 | 7.10E-01 |  |  |  |  |
| gamma-glutamylhistidine |  |  |  |  |  |  |  |  | -0.4±0.35 | 6.87E-01 | -0.37±0.36 | 7.13E-01 |  |  |  |  |
| bilirubin degradation product, C16H18N2O5 (2)** |  |  |  |  |  |  |  |  | -0.28±0.31 | 7.75E-01 | -0.33±0.31 | 7.13E-01 |  |  |  |  |
| 2-hydroxyfluorene sulfate |  |  |  |  |  |  |  |  | 0.42±0.28 | 5.71E-01 | 0.42±0.41 | 7.16E-01 |  |  |  |  |
| 3-hydroxypyridine glucuronide |  |  |  |  |  |  |  |  | -0.35±0.32 | 7.23E-01 | -0.33±0.33 | 7.39E-01 |  |  |  |  |
| oleoylcholine |  |  |  |  |  |  |  |  | 0.33±0.34 | 7.59E-01 | 0.36±0.36 | 7.42E-01 |  |  |  |  |
| 1-dihomo-linolenylglycerol (20:3) |  |  |  |  |  |  |  |  | -0.29±0.3 | 7.57E-01 | -0.3±0.31 | 7.43E-01 |  |  |  |  |
| X - 23276 |  |  |  |  |  |  |  |  | 0.24±0.28 | 7.94E-01 | 0.28±0.28 | 7.43E-01 |  |  |  |  |
| glucuronide of piperine metabolite C17H21NO3 (3)* |  |  |  |  |  |  |  |  | -0.25±0.29 | 7.94E-01 | -0.28±0.29 | 7.43E-01 |  |  |  |  |
| delta-CEHC |  |  |  |  |  |  |  |  | -0.33±0.3 | 7.17E-01 | -0.3±0.31 | 7.46E-01 |  |  |  |  |
| 2,4-di-tert-butylphenol |  |  |  |  |  |  |  |  | -0.31±0.33 | 7.71E-01 | -0.31±0.33 | 7.52E-01 |  |  |  |  |
| GlcNAc sulfate conjugate of C21H34O2 steroid** |  |  |  |  |  |  |  |  | -0.25±0.32 | 8.15E-01 | -0.31±0.33 | 7.52E-01 |  |  |  |  |
| X - 14082 |  |  |  |  |  |  |  |  | -0.3±0.28 | 7.19E-01 | -0.26±0.28 | 7.52E-01 |  |  |  |  |
| dihomo-linolenoyl-choline |  |  |  |  |  |  |  |  | 0.28±0.35 | 8.09E-01 | 0.35±0.37 | 7.52E-01 |  |  |  |  |
| dopamine 4-sulfate |  |  |  |  |  |  |  |  | 0.26±0.3 | 7.99E-01 | 0.28±0.3 | 7.53E-01 |  |  |  |  |
| palmitoyl ethanolamide |  |  |  |  |  |  |  |  | -0.23±0.31 | 8.37E-01 | -0.29±0.31 | 7.57E-01 |  |  |  |  |
| phenol glucuronide |  |  |  |  |  |  |  |  | -0.31±0.33 | 7.70E-01 | -0.3±0.34 | 7.65E-01 |  |  |  |  |
| bilirubin degradation product, C17H20N2O5 (1)** |  |  |  |  |  |  |  |  | -0.24±0.28 | 8.04E-01 | -0.26±0.29 | 7.65E-01 |  |  |  |  |
| 4-hydroxyphenylacetoylcarnitine |  |  |  |  |  |  |  |  | 0.24±0.32 | 8.17E-01 | 0.29±0.33 | 7.65E-01 |  |  |  |  |
| fructose |  |  |  |  |  |  |  |  | -0.3±0.32 | 7.65E-01 | -0.28±0.33 | 7.71E-01 |  |  |  |  |
| hypoxanthine |  |  |  |  |  |  |  |  | 0.21±0.32 | 8.56E-01 | 0.27±0.33 | 7.97E-01 |  |  |  |  |
| cysteine s-sulfate |  |  |  |  |  |  |  |  | -0.26±0.32 | 8.09E-01 | -0.25±0.32 | 8.02E-01 |  |  |  |  |
| bilirubin degradation product, C17H20N2O5 (2)** |  |  |  |  |  |  |  |  | -0.21±0.29 | 8.45E-01 | -0.23±0.29 | 8.02E-01 |  |  |  |  |
| 5-hydroxyindoleacetate |  |  |  |  |  |  |  |  | -0.26±0.29 | 7.94E-01 | -0.23±0.31 | 8.12E-01 |  |  |  |  |
| 1-stearoyl-2-oleoyl-GPS (18:0/18:1) |  |  |  |  |  |  |  |  | 0.22±0.31 | 8.47E-01 | 0.24±0.32 | 8.12E-01 |  |  |  |  |
| 1-linolenoylglycerol (18:3) |  |  |  |  |  |  |  |  | 0.22±0.32 | 8.47E-01 | 0.25±0.33 | 8.12E-01 |  |  |  |  |
| 2,3-dihydroxy-2-methylbutyrate |  |  |  |  |  |  |  |  | 0.19±0.33 | 8.79E-01 | 0.25±0.33 | 8.12E-01 |  |  |  |  |
| galactonate |  |  |  |  |  |  |  |  | 0.16±0.34 | 8.96E-01 | 0.26±0.36 | 8.12E-01 |  |  |  |  |
| X - 12096 |  |  |  |  |  |  |  |  | -0.32±0.27 | 6.90E-01 | -0.22±0.3 | 8.13E-01 |  |  |  |  |
| gamma-CEHC glucuronide* |  |  |  |  |  |  |  |  | -0.26±0.28 | 7.75E-01 | -0.21±0.29 | 8.13E-01 |  |  |  |  |
| palmitoylcholine |  |  |  |  |  |  |  |  | 0.22±0.33 | 8.54E-01 | 0.26±0.35 | 8.13E-01 |  |  |  |  |
| dihydroferulic acid sulfate |  |  |  |  |  |  |  |  | 0.18±0.3 | 8.79E-01 | 0.22±0.3 | 8.14E-01 |  |  |  |  |
| stearoylcholine* |  |  |  |  |  |  |  |  | 0.18±0.34 | 8.85E-01 | 0.26±0.36 | 8.20E-01 |  |  |  |  |
| 2-oleoylglycerol (18:1) |  |  |  |  |  |  |  |  | -0.17±0.26 | 8.54E-01 | -0.19±0.27 | 8.27E-01 |  |  |  |  |
| iminodiacetate (IDA) |  |  |  |  |  |  |  |  | 0.28±0.31 | 7.94E-01 | 0.23±0.33 | 8.33E-01 |  |  |  |  |
| malonylcarnitine |  |  |  |  |  |  |  |  | 0.23±0.33 | 8.47E-01 | 0.22±0.33 | 8.40E-01 |  |  |  |  |
| X - 12015 |  |  |  |  |  |  |  |  | 0.12±0.31 | 9.03E-01 | 0.21±0.31 | 8.40E-01 |  |  |  |  |
| 1-oleoylglycerol (18:1) |  |  |  |  |  |  |  |  | 0.25±0.32 | 8.15E-01 | 0.21±0.32 | 8.45E-01 |  |  |  |  |
| X - 23666 |  |  |  |  |  |  |  |  | 0.18±0.31 | 8.79E-01 | 0.21±0.32 | 8.45E-01 |  |  |  |  |
| X - 24531 |  |  |  |  |  |  |  |  | -0.24±0.29 | 8.04E-01 | -0.2±0.31 | 8.45E-01 |  |  |  |  |
| 1-palmitoylglycerol (16:0) |  |  |  |  |  |  |  |  | -0.2±0.3 | 8.56E-01 | -0.19±0.29 | 8.45E-01 |  |  |  |  |
| dihydroferulate |  |  |  |  |  |  |  |  | 0.15±0.31 | 8.90E-01 | 0.2±0.3 | 8.45E-01 |  |  |  |  |
| bilirubin degradation product, C16H18N2O5 (3)** |  |  |  |  |  |  |  |  | -0.15±0.31 | 8.95E-01 | -0.21±0.32 | 8.45E-01 |  |  |  |  |
| trans-2-hexenoylglycine |  |  |  |  |  |  |  |  | -0.11±0.31 | 9.17E-01 | -0.21±0.33 | 8.49E-01 |  |  |  |  |
| tetrahydrocortisol sulfate (1) |  |  |  |  |  |  |  |  | 0.13±0.3 | 8.96E-01 | 0.2±0.32 | 8.51E-01 |  |  |  |  |
| X - 12104 |  |  |  |  |  |  |  |  | 0.2±0.33 | 8.79E-01 | 0.2±0.34 | 8.64E-01 |  |  |  |  |
| 5-hydroxy-2-methylpyridine sulfate |  |  |  |  |  |  |  |  | -0.21±0.32 | 8.62E-01 | -0.18±0.33 | 8.73E-01 |  |  |  |  |
| suberoylcarnitine (C8-DC) |  |  |  |  |  |  |  |  | 0.24±0.31 | 8.17E-01 | 0.16±0.31 | 8.82E-01 |  |  |  |  |
| X - 25519 |  |  |  |  |  |  |  |  | 0.17±0.3 | 8.79E-01 | 0.16±0.31 | 8.82E-01 |  |  |  |  |
| X - 23583 |  |  |  |  |  |  |  |  | -0.13±0.3 | 8.96E-01 | -0.16±0.31 | 8.82E-01 |  |  |  |  |
| X - 23890 |  |  |  |  |  |  |  |  | -0.23±0.29 | 8.17E-01 | -0.15±0.3 | 8.89E-01 |  |  |  |  |
| 2-linoleoylglycerol (18:2) |  |  |  |  |  |  |  |  | 0.17±0.34 | 8.88E-01 | 0.16±0.33 | 8.91E-01 |  |  |  |  |
| succinoyltaurine |  |  |  |  |  |  |  |  | -0.14±0.29 | 8.96E-01 | -0.14±0.3 | 8.98E-01 |  |  |  |  |
| X - 24953 |  |  |  |  |  |  |  |  | -0.08±0.28 | 9.39E-01 | -0.13±0.3 | 9.04E-01 |  |  |  |  |
| hexanoylglycine |  |  |  |  |  |  |  |  | 0±0.32 | 9.96E-01 | -0.14±0.33 | 9.04E-01 |  |  |  |  |
| lithocholate |  |  |  |  |  |  |  |  | -0.17±0.3 | 8.79E-01 | -0.13±0.31 | 9.12E-01 |  |  |  |  |
| 3-indoleglyoxylic acid |  |  |  |  |  |  |  |  | -0.21±0.3 | 8.43E-01 | -0.13±0.32 | 9.12E-01 |  |  |  |  |
| nisinate (24:6n3) |  |  |  |  |  |  |  |  | -0.1±0.3 | 9.23E-01 | -0.12±0.29 | 9.12E-01 |  |  |  |  |
| X - 11979 |  |  |  |  |  |  |  |  | 0.08±0.33 | 9.49E-01 | 0.14±0.33 | 9.16E-01 |  |  |  |  |
| docosahexaenoylcarnitine (C22:6)* |  |  |  |  |  |  |  |  | 0.17±0.31 | 8.79E-01 | 0.12±0.3 | 9.17E-01 |  |  |  |  |
| X - 21327 |  |  |  |  |  |  |  |  | -0.17±0.26 | 8.56E-01 | -0.1±0.26 | 9.20E-01 |  |  |  |  |
| docosatrienoate (22:3n3) |  |  |  |  |  |  |  |  | -0.12±0.28 | 8.96E-01 | -0.1±0.28 | 9.29E-01 |  |  |  |  |
| X - 23641 |  |  |  |  |  |  |  |  | -0.12±0.29 | 8.96E-01 | -0.11±0.3 | 9.29E-01 |  |  |  |  |
| isoeugenol sulfate |  |  |  |  |  |  |  |  | 0.13±0.31 | 8.96E-01 | 0.12±0.32 | 9.29E-01 |  |  |  |  |
| 3-hydroxyhippurate sulfate |  |  |  |  |  |  |  |  | 0.06±0.31 | 9.55E-01 | 0.11±0.3 | 9.29E-01 |  |  |  |  |
| cholate |  |  |  |  |  |  |  |  | -0.13±0.31 | 8.96E-01 | -0.11±0.31 | 9.34E-01 |  |  |  |  |
| 1-myristoylglycerol (14:0) |  |  |  |  |  |  |  |  | -0.12±0.3 | 8.96E-01 | -0.1±0.29 | 9.36E-01 |  |  |  |  |
| X - 12013 |  |  |  |  |  |  |  |  | -0.2±0.3 | 8.54E-01 | -0.1±0.32 | 9.42E-01 |  |  |  |  |
| suberate (C8-DC) |  |  |  |  |  |  |  |  | 0.14±0.3 | 8.96E-01 | 0.09±0.3 | 9.42E-01 |  |  |  |  |
| 1-arachidonylglycerol (20:4) |  |  |  |  |  |  |  |  | 0.12±0.31 | 9.00E-01 | 0.1±0.31 | 9.42E-01 |  |  |  |  |
| X - 26054 |  |  |  |  |  |  |  |  | 0.09±0.31 | 9.37E-01 | 0.1±0.31 | 9.42E-01 |  |  |  |  |
| bilirubin degradation product, C16H18N2O5 (4)** |  |  |  |  |  |  |  |  | -0.05±0.31 | 9.66E-01 | -0.1±0.31 | 9.42E-01 |  |  |  |  |
| linoleoylcholine* |  |  |  |  |  |  |  |  | 0.05±0.32 | 9.69E-01 | 0.09±0.33 | 9.48E-01 |  |  |  |  |
| alpha-CEHC sulfate |  |  |  |  |  |  |  |  | 0.03±0.3 | 9.81E-01 | 0.09±0.31 | 9.48E-01 |  |  |  |  |
| dimethyl sulfone |  |  |  |  |  |  |  |  | 0.08±0.28 | 9.35E-01 | 0.08±0.27 | 9.48E-01 |  |  |  |  |
| cholic acid glucuronide |  |  |  |  |  |  |  |  | 0.08±0.31 | 9.49E-01 | 0.09±0.31 | 9.49E-01 |  |  |  |  |
| gentisate |  |  |  |  |  |  |  |  | -0.12±0.29 | 8.96E-01 | -0.08±0.3 | 9.52E-01 |  |  |  |  |
| gulonate* |  |  |  |  |  |  |  |  | 0.02±0.31 | 9.84E-01 | 0.08±0.33 | 9.53E-01 |  |  |  |  |
| cystine |  |  |  |  |  |  |  |  | -0.07±0.29 | 9.49E-01 | -0.07±0.31 | 9.63E-01 |  |  |  |  |
| X - 12713 |  |  |  |  |  |  |  |  | 0.06±0.32 | 9.59E-01 | 0.07±0.33 | 9.65E-01 |  |  |  |  |
| arachidonoylcholine |  |  |  |  |  |  |  |  | 0.06±0.32 | 9.62E-01 | 0.07±0.33 | 9.68E-01 |  |  |  |  |
| N,N-dimethyl-5-aminovalerate |  |  |  |  |  |  |  |  | 0.04±0.3 | 9.80E-01 | 0.06±0.32 | 9.68E-01 |  |  |  |  |
| X - 17346 |  |  |  |  |  |  |  |  | 0.03±0.31 | 9.81E-01 | 0.07±0.31 | 9.68E-01 |  |  |  |  |
| nervonoylcarnitine (C24:1)* |  |  |  |  |  |  |  |  | 0.16±0.31 | 8.87E-01 | 0.05±0.31 | 9.69E-01 |  |  |  |  |
| docosatrienoate (22:3n6)* |  |  |  |  |  |  |  |  | 0.16±0.31 | 8.89E-01 | 0.05±0.31 | 9.69E-01 |  |  |  |  |
| glucuronide of piperine metabolite C17H21NO3 (5)* |  |  |  |  |  |  |  |  | -0.03±0.3 | 9.81E-01 | -0.05±0.31 | 9.69E-01 |  |  |  |  |
| prolylglycine |  |  |  |  |  |  |  |  | -0.02±0.3 | 9.82E-01 | 0.05±0.31 | 9.69E-01 |  |  |  |  |
| gamma-glutamylalanine |  |  |  |  |  |  |  |  | -0.05±0.29 | 9.63E-01 | -0.05±0.31 | 9.76E-01 |  |  |  |  |
| propionylglycine |  |  |  |  |  |  |  |  | -0.04±0.3 | 9.80E-01 | 0.04±0.31 | 9.83E-01 |  |  |  |  |
| X - 12261 |  |  |  |  |  |  |  |  | -0.15±0.31 | 8.90E-01 | -0.04±0.33 | 9.86E-01 |  |  |  |  |
| dihydrocaffeate sulfate (2) |  |  |  |  |  |  |  |  | -0.06±0.3 | 9.55E-01 | -0.02±0.29 | 9.89E-01 |  |  |  |  |
| oxindolylalanine |  |  |  |  |  |  |  |  | -0.05±0.3 | 9.69E-01 | 0.02±0.31 | 9.91E-01 |  |  |  |  |
| X - 24951 |  |  |  |  |  |  |  |  | -0.08±0.31 | 9.40E-01 | -0.02±0.33 | 9.92E-01 |  |  |  |  |
| ribonate |  |  |  |  |  |  |  |  | -0.09±0.3 | 9.35E-01 | -0.02±0.3 | 9.95E-01 |  |  |  |  |
| phosphate |  |  |  |  |  |  |  |  | 0.05±0.34 | 9.69E-01 | -0.02±0.36 | 9.95E-01 |  |  |  |  |
| X - 24295 |  |  |  |  |  |  |  |  | -0.02±0.31 | 9.82E-01 | -0.02±0.32 | 9.95E-01 |  |  |  |  |
| xanthosine |  |  |  |  |  |  |  |  | -0.09±0.29 | 9.33E-01 | -0.01±0.31 | 9.95E-01 |  |  |  |  |
| X - 12839 |  |  |  |  |  |  |  |  | 0.06±0.31 | 9.55E-01 | -0.01±0.31 | 9.95E-01 |  |  |  |  |
| X - 12007 |  |  |  |  |  |  |  |  | -0.05±0.29 | 9.69E-01 | 0±0.3 | 9.95E-01 |  |  |  |  |
| behenoylcarnitine (C22)* |  |  |  |  |  |  |  |  | 0.03±0.33 | 9.82E-01 | 0.01±0.32 | 9.95E-01 |  |  |  |  |
| mannonate* |  |  |  |  |  |  |  |  | -0.02±0.31 | 9.82E-01 | -0.01±0.32 | 9.95E-01 |  |  |  |  |
| X - 13007 |  |  |  |  |  |  |  |  | 0.02±0.3 | 9.84E-01 | 0.01±0.31 | 9.95E-01 |  |  |  |  |
| saccharin |  |  |  |  |  |  |  |  |  |  |  |  | 0.06±0.4 | 9.98E-01 | 0.07±0.41 | 9.91E-01 |
| 3-hydroxymyristate |  |  |  |  |  |  |  |  |  |  |  |  | -0.07±0.43 | 9.98E-01 | -0.08±0.44 | 9.91E-01 |
| beta-sitosterol |  |  |  |  |  |  |  |  |  |  |  |  | 0.05±0.42 | 9.98E-01 | 0.12±0.46 | 9.91E-01 |
| 1-methylhistidine |  |  |  |  |  |  |  |  |  |  |  |  | 0.05±0.39 | 9.98E-01 | 0.09±0.44 | 9.91E-01 |
| gamma-glutamyl-epsilon-lysine |  |  |  |  |  |  |  |  |  |  |  |  | 0.26±0.56 | 9.98E-01 | 0.14±0.57 | 9.91E-01 |
| N1-Methyl-4-pyridone-3-carboxamide |  |  |  |  |  |  |  |  |  |  |  |  | -0.25±0.37 | 9.98E-01 | -0.3±0.4 | 9.91E-01 |
| 1-stearoyl-2-arachidonoyl-GPS (18:0/20:4) |  |  |  |  |  |  |  |  |  |  |  |  | -0.47±0.43 | 9.98E-01 | -0.47±0.44 | 9.91E-01 |
| 2-hydroxyglutarate |  |  |  |  |  |  |  |  |  |  |  |  | -0.53±0.27 | 9.98E-01 | -0.59±0.29 | 9.91E-01 |
| stearoyl ethanolamide |  |  |  |  |  |  |  |  |  |  |  |  | 0.08±0.41 | 9.98E-01 | 0.06±0.46 | 9.91E-01 |
| palmitoleoyl-oleoyl-glycerol (16:1/18:1) [2]* |  |  |  |  |  |  |  |  |  |  |  |  | -0.03±0.42 | 9.98E-01 | -0.05±0.43 | 9.91E-01 |
| palmitoyl-oleoyl-glycerol (16:0/18:1) [1]* |  |  |  |  |  |  |  |  |  |  |  |  | 0.08±0.43 | 9.98E-01 | -0.14±0.47 | 9.91E-01 |
| palmitoyl-oleoyl-glycerol (16:0/18:1) [2]* |  |  |  |  |  |  |  |  |  |  |  |  | 0.36±0.46 | 9.98E-01 | 0.29±0.51 | 9.91E-01 |
| oleoyl-oleoyl-glycerol (18:1/18:1) [1]* |  |  |  |  |  |  |  |  |  |  |  |  | 0.21±0.46 | 9.98E-01 | 0.1±0.48 | 9.91E-01 |
| linoleoyl-arachidonoyl-glycerol (18:2/20:4) [1]* |  |  |  |  |  |  |  |  |  |  |  |  | 0.02±0.37 | 9.98E-01 | -0.14±0.37 | 9.91E-01 |
| linoleoyl-arachidonoyl-glycerol (18:2/20:4) [2]* |  |  |  |  |  |  |  |  |  |  |  |  | -0.25±0.31 | 9.98E-01 | -0.36±0.32 | 9.91E-01 |
| palmitoyl-arachidonoyl-glycerol (16:0/20:4) [2]* |  |  |  |  |  |  |  |  |  |  |  |  | -0.05±0.41 | 9.98E-01 | -0.15±0.4 | 9.91E-01 |
| linoleoyl-linolenoyl-glycerol (18:2/18:3) [2]* |  |  |  |  |  |  |  |  |  |  |  |  | -0.23±0.37 | 9.98E-01 | -0.42±0.38 | 9.91E-01 |
| palmitoleoyl-linoleoyl-glycerol (16:1/18:2) [1]* |  |  |  |  |  |  |  |  |  |  |  |  | 0.14±0.43 | 9.98E-01 | 0.03±0.46 | 9.91E-01 |
| oleoyl-arachidonoyl-glycerol (18:1/20:4) [1]* |  |  |  |  |  |  |  |  |  |  |  |  | 0.06±0.38 | 9.98E-01 | -0.05±0.38 | 9.91E-01 |
| oleoyl-arachidonoyl-glycerol (18:1/20:4) [2]* |  |  |  |  |  |  |  |  |  |  |  |  | -0.02±0.35 | 9.98E-01 | -0.12±0.36 | 9.91E-01 |
| diacylglycerol (16:1/18:2 [2], 16:0/18:3 [1])* |  |  |  |  |  |  |  |  |  |  |  |  | 0.41±0.47 | 9.98E-01 | 0.3±0.48 | 9.91E-01 |
| linoleoyl-linoleoyl-glycerol (18:2/18:2) [1]* |  |  |  |  |  |  |  |  |  |  |  |  | -0.27±0.35 | 9.98E-01 | -0.36±0.35 | 9.91E-01 |
| linoleoyl-linoleoyl-glycerol (18:2/18:2) [2]* |  |  |  |  |  |  |  |  |  |  |  |  | 0.39±0.51 | 9.98E-01 | 0.2±0.5 | 9.91E-01 |
| stearoyl-arachidonoyl-glycerol (18:0/20:4) [1]* |  |  |  |  |  |  |  |  |  |  |  |  | 0.12±0.41 | 9.98E-01 | 0.13±0.44 | 9.91E-01 |
| stearoyl-arachidonoyl-glycerol (18:0/20:4) [2]* |  |  |  |  |  |  |  |  |  |  |  |  | 0.45±0.49 | 9.98E-01 | 0.31±0.52 | 9.91E-01 |
| palmitoyl-docosahexaenoyl-glycerol (16:0/22:6) [1]* |  |  |  |  |  |  |  |  |  |  |  |  | 0.56±0.47 | 9.98E-01 | 0.61±0.48 | 9.91E-01 |
| N-behenoyl-sphingadienine (d18:2/22:0)* |  |  |  |  |  |  |  |  |  |  |  |  | 0.14±0.42 | 9.98E-01 | -0.07±0.38 | 9.91E-01 |
| palmitoleoyl-arachidonoyl-glycerol (16:1/20:4) [2]* |  |  |  |  |  |  |  |  |  |  |  |  | 0.12±0.43 | 9.98E-01 | -0.01±0.4 | 9.91E-01 |
| myristoyl-linoleoyl-glycerol (14:0/18:2) [2]* |  |  |  |  |  |  |  |  |  |  |  |  | 0.7±0.48 | 9.98E-01 | 0.53±0.5 | 9.91E-01 |
| glycosyl ceramide (d18:1/23:1, d17:1/24:1)* |  |  |  |  |  |  |  |  |  |  |  |  | 0.34±0.5 | 9.98E-01 | 0.43±0.56 | 9.91E-01 |
| ceramide (d18:1/20:0, d16:1/22:0, d20:1/18:0)* |  |  |  |  |  |  |  |  |  |  |  |  | 0.14±0.42 | 9.98E-01 | -0.02±0.39 | 9.91E-01 |
| 1-carboxyethylphenylalanine |  |  |  |  |  |  |  |  |  |  |  |  | -0.05±0.43 | 9.98E-01 | -0.28±0.55 | 9.91E-01 |
| 1-carboxyethyltyrosine |  |  |  |  |  |  |  |  |  |  |  |  | 0.17±0.44 | 9.98E-01 | 0.1±0.51 | 9.91E-01 |
| 1-carboxyethylvaline |  |  |  |  |  |  |  |  |  |  |  |  | -0.01±0.42 | 9.98E-01 | -0.2±0.46 | 9.91E-01 |
| 1-carboxyethylleucine |  |  |  |  |  |  |  |  |  |  |  |  | -0.16±0.39 | 9.98E-01 | -0.44±0.45 | 9.91E-01 |
| 1-carboxyethylisoleucine |  |  |  |  |  |  |  |  |  |  |  |  | 0.3±0.43 | 9.98E-01 | 0.27±0.52 | 9.91E-01 |
| (2-butoxyethoxy)acetic acid |  |  |  |  |  |  |  |  |  |  |  |  | 0.05±0.43 | 9.98E-01 | 0.09±0.44 | 9.91E-01 |
| dibutyl sulfosuccinate |  |  |  |  |  |  |  |  |  |  |  |  | -0.13±0.4 | 9.98E-01 | -0.17±0.39 | 9.91E-01 |
| 2-aminoadipate |  |  |  |  |  |  |  |  |  |  |  |  | -0.04±0.42 | 9.98E-01 | -0.19±0.4 | 9.91E-01 |
| serotonin |  |  |  |  |  |  |  |  |  |  |  |  | -0.19±0.39 | 9.98E-01 | -0.04±0.39 | 9.91E-01 |
| thymidine |  |  |  |  |  |  |  |  |  |  |  |  | 0.15±0.44 | 9.98E-01 | 0.02±0.43 | 9.91E-01 |
| X - 12849 |  |  |  |  |  |  |  |  |  |  |  |  | 0.19±0.42 | 9.98E-01 | 0.34±0.48 | 9.91E-01 |
| X - 13684 |  |  |  |  |  |  |  |  |  |  |  |  | 0.17±0.45 | 9.98E-01 | 0.12±0.48 | 9.91E-01 |
| X - 16938 |  |  |  |  |  |  |  |  |  |  |  |  | 0.67±0.44 | 9.98E-01 | 0.65±0.47 | 9.91E-01 |
| X - 16964 |  |  |  |  |  |  |  |  |  |  |  |  | -0.03±0.41 | 9.98E-01 | -0.03±0.42 | 9.91E-01 |
| X - 21829 |  |  |  |  |  |  |  |  |  |  |  |  | -0.4±0.37 | 9.98E-01 | -0.61±0.44 | 9.91E-01 |
| X - 22771 |  |  |  |  |  |  |  |  |  |  |  |  | -0.23±0.36 | 9.98E-01 | -0.23±0.38 | 9.91E-01 |
| X - 24241 |  |  |  |  |  |  |  |  |  |  |  |  | 1.24±1.1 | 9.98E-01 | 1.01±1.01 | 9.91E-01 |
| X - 25828 |  |  |  |  |  |  |  |  |  |  |  |  | 0.91±0.52 | 9.98E-01 | 0.8±0.47 | 9.91E-01 |
| 3-methylcrotonylglycine |  |  |  |  |  |  |  |  |  |  |  |  | 0±0.4 | 9.99E-01 | 0.06±0.42 | 9.91E-01 |
| palmitoyl-linoleoyl-glycerol (16:0/18:2) [2]* |  |  |  |  |  |  |  |  |  |  |  |  | 0.16±0.43 | 9.98E-01 | 0±0.45 | 9.95E-01 |

Note: The table shows associations from the logistic regression analysis where plasms metabolomics level from the Metabolon platform was main exposure and prevalent hematological malignancy as the main outcome in ERGO-4 and ERGO-5, respectively. ERGO-4 and ERGO-5 have 939 overlapping circulating metabolites, and ERGO-4&5 refers to the correlation between prevalent hematological malignancy and 939 metabolites derived from meta-analysis of ERGO-4 and ERGO-5. Model 1: adjusted for age, sex, cohort, and lipid lowering medication. Model 2: adjusted for model1, smoking status, BMI, alcohol and education. The table is ranked based on the *FDR* values in model 2 within the E4&E5 cohort, using bold text indicates metabolites are surpassing *FDR* adjusted *P* value treshold (*P*<0.05). ERGO-4 and ERGO-5 refer to the fourth and fifth visits subcohort from the baseline cohort. Abbreviations: SE, standard erorr; I^2^: heterogeneity parameter, Het. *P*-value: significance of the heterogeneity; BMI, body mass index; FDR, false discovery rate.

**Supplementary Table 7. Serum metabolites associated with prevalent colorectal cancer from the Nightingale platform at baseline.**

| **Metabolite** | **ERGO-4&5** | | | | | | **ERGO-4** | | | | | | **ERGO-5** | | | | | |
| --- | --- | --- | --- | --- | --- | --- | --- | --- | --- | --- | --- | --- | --- | --- | --- | --- | --- | --- |
|  | **Model 1** | | | **Model 2** | | | **Model 1** | | | **Model 2** | | | **Model 1** | | | **Model 2** | | |
|  | **Beta** | **SE** | ***FDR* value** | **Beta** | **SE** | ***FDR* value** | **Beta** | **SE** | ***FDR* value** | **Beta** | **SE** | ***FDR* value** | **Beta** | **SE** | ***FDR* value** | **Beta** | **SE** | ***FDR* value** |
| Triglycerides in small LDL | -0.38 | 0.15 | 1.71E-01 | -0.34 | 0.15 | 3.38E-01 | -0.42 | 0.18 | 2.01E-01 | -0.35 | 0.19 | 4.85E-01 | -0.32 | 0.26 | 8.70E-01 | -0.37 | 0.27 | 7.11E-01 |
| Cholesteryl esters to total lipids ratio in medium HDL | 0.41 | 0.16 | 1.71E-01 | 0.37 | 0.17 | 3.38E-01 | 0.41 | 0.19 | 2.01E-01 | 0.32 | 0.20 | 4.85E-01 | 0.46 | 0.30 | 8.70E-01 | 0.55 | 0.32 | 7.11E-01 |
| Cholesteryl esters to total lipids ratio in large HDL | 0.57 | 0.23 | 1.71E-01 | 0.52 | 0.23 | 3.38E-01 | 0.57 | 0.26 | 2.01E-01 | 0.46 | 0.27 | 4.85E-01 | 0.65 | 0.48 | 8.70E-01 | 0.81 | 0.52 | 7.11E-01 |
| Phospholipids in VLDL | -0.34 | 0.13 | 1.71E-01 | -0.30 | 0.14 | 3.38E-01 | -0.39 | 0.16 | 2.01E-01 | -0.32 | 0.17 | 4.85E-01 | -0.27 | 0.24 | 8.70E-01 | -0.32 | 0.24 | 7.11E-01 |
| Total lipids in VLDL | -0.34 | 0.14 | 1.71E-01 | -0.30 | 0.14 | 3.38E-01 | -0.41 | 0.17 | 2.01E-01 | -0.32 | 0.17 | 4.85E-01 | -0.25 | 0.24 | 8.70E-01 | -0.31 | 0.25 | 7.11E-01 |
| Monounsaturated fatty acids | -0.36 | 0.15 | 1.71E-01 | -0.32 | 0.15 | 3.38E-01 | -0.42 | 0.18 | 2.01E-01 | -0.35 | 0.18 | 4.85E-01 | -0.28 | 0.26 | 8.70E-01 | -0.33 | 0.27 | 7.11E-01 |
| Free cholesterol in VLDL | -0.33 | 0.13 | 1.71E-01 | -0.29 | 0.14 | 3.38E-01 | -0.39 | 0.16 | 2.01E-01 | -0.31 | 0.17 | 4.85E-01 | -0.25 | 0.23 | 8.70E-01 | -0.30 | 0.24 | 7.11E-01 |
| Concentration of VLDL particles | -0.33 | 0.13 | 1.71E-01 | -0.29 | 0.14 | 3.38E-01 | -0.38 | 0.16 | 2.01E-01 | -0.31 | 0.17 | 4.85E-01 | -0.25 | 0.24 | 8.70E-01 | -0.29 | 0.24 | 7.11E-01 |
| Cholesterol to total lipids ratio in medium HDL | 0.38 | 0.15 | 1.71E-01 | 0.34 | 0.16 | 3.38E-01 | 0.36 | 0.18 | 2.01E-01 | 0.26 | 0.19 | 4.85E-01 | 0.46 | 0.29 | 8.70E-01 | 0.56 | 0.31 | 7.11E-01 |
| Total triglycerides | -0.34 | 0.14 | 1.71E-01 | -0.30 | 0.15 | 3.38E-01 | -0.40 | 0.17 | 2.01E-01 | -0.31 | 0.18 | 4.85E-01 | -0.24 | 0.25 | 8.70E-01 | -0.31 | 0.26 | 7.11E-01 |
| VLDL cholesterol | -0.31 | 0.13 | 1.71E-01 | -0.27 | 0.13 | 3.38E-01 | -0.36 | 0.16 | 2.01E-01 | -0.29 | 0.17 | 4.85E-01 | -0.23 | 0.23 | 8.70E-01 | -0.27 | 0.24 | 7.11E-01 |
| Phospholipids in medium VLDL | -0.30 | 0.13 | 1.71E-01 | -0.26 | 0.13 | 3.38E-01 | -0.37 | 0.16 | 2.01E-01 | -0.30 | 0.16 | 4.85E-01 | -0.19 | 0.23 | 8.70E-01 | -0.22 | 0.23 | 7.11E-01 |
| Triglycerides in medium LDL | -0.32 | 0.14 | 1.71E-01 | -0.29 | 0.15 | 3.38E-01 | -0.36 | 0.17 | 2.01E-01 | -0.31 | 0.18 | 4.85E-01 | -0.25 | 0.25 | 8.70E-01 | -0.28 | 0.26 | 7.11E-01 |
| Total lipids in medium VLDL | -0.30 | 0.13 | 1.71E-01 | -0.26 | 0.13 | 3.38E-01 | -0.38 | 0.16 | 2.01E-01 | -0.31 | 0.16 | 4.85E-01 | -0.17 | 0.23 | 8.76E-01 | -0.21 | 0.24 | 7.11E-01 |
| Cholesterol to total lipids ratio in large HDL | 0.51 | 0.22 | 1.71E-01 | 0.44 | 0.23 | 3.38E-01 | 0.49 | 0.25 | 2.01E-01 | 0.37 | 0.25 | 4.85E-01 | 0.60 | 0.49 | 8.70E-01 | 0.75 | 0.53 | 7.11E-01 |
| Cholesteryl esters in large VLDL | -0.29 | 0.12 | 1.71E-01 | -0.25 | 0.13 | 3.38E-01 | -0.33 | 0.15 | 2.01E-01 | -0.25 | 0.15 | 4.85E-01 | -0.24 | 0.23 | 8.70E-01 | -0.28 | 0.23 | 7.11E-01 |
| Free cholesterol in very large VLDL | -0.29 | 0.12 | 1.71E-01 | -0.25 | 0.13 | 3.38E-01 | -0.33 | 0.15 | 2.01E-01 | -0.24 | 0.16 | 4.85E-01 | -0.26 | 0.22 | 8.70E-01 | -0.32 | 0.22 | 7.11E-01 |
| Phospholipids in very large VLDL | -0.28 | 0.11 | 1.71E-01 | -0.24 | 0.12 | 3.38E-01 | -0.31 | 0.14 | 2.01E-01 | -0.22 | 0.15 | 4.85E-01 | -0.26 | 0.21 | 8.70E-01 | -0.31 | 0.21 | 7.11E-01 |
| Total lipids in small VLDL | -0.30 | 0.13 | 1.71E-01 | -0.26 | 0.13 | 3.38E-01 | -0.35 | 0.16 | 2.01E-01 | -0.27 | 0.16 | 4.85E-01 | -0.24 | 0.23 | 8.70E-01 | -0.28 | 0.24 | 7.11E-01 |
| Concentration of medium VLDL particles | -0.30 | 0.13 | 1.71E-01 | -0.26 | 0.13 | 3.38E-01 | -0.37 | 0.16 | 2.01E-01 | -0.29 | 0.16 | 4.85E-01 | -0.19 | 0.23 | 8.70E-01 | -0.22 | 0.24 | 7.11E-01 |
| Ratio of apolipoprotein B to apolipoprotein A1 | -0.29 | 0.13 | 1.71E-01 | -0.26 | 0.14 | 3.38E-01 | -0.35 | 0.16 | 2.01E-01 | -0.29 | 0.17 | 4.85E-01 | -0.19 | 0.24 | 8.70E-01 | -0.23 | 0.25 | 7.11E-01 |
| Triglycerides in LDL | -0.31 | 0.14 | 1.71E-01 | -0.28 | 0.15 | 3.38E-01 | -0.34 | 0.17 | 2.01E-01 | -0.30 | 0.18 | 4.85E-01 | -0.26 | 0.25 | 8.70E-01 | -0.28 | 0.26 | 7.11E-01 |
| Ratio of monounsaturated fatty acids to total fatty acids | -0.32 | 0.14 | 1.71E-01 | -0.29 | 0.15 | 3.38E-01 | -0.34 | 0.17 | 2.01E-01 | -0.28 | 0.18 | 4.85E-01 | -0.29 | 0.27 | 8.70E-01 | -0.36 | 0.28 | 7.11E-01 |
| Concentration of small VLDL particles | -0.30 | 0.13 | 1.71E-01 | -0.26 | 0.14 | 3.38E-01 | -0.34 | 0.16 | 2.01E-01 | -0.26 | 0.17 | 4.85E-01 | -0.25 | 0.24 | 8.70E-01 | -0.29 | 0.24 | 7.11E-01 |
| Total fatty acids | -0.31 | 0.14 | 1.71E-01 | -0.28 | 0.15 | 3.38E-01 | -0.37 | 0.18 | 2.01E-01 | -0.32 | 0.18 | 4.85E-01 | -0.23 | 0.26 | 8.70E-01 | -0.26 | 0.26 | 7.11E-01 |
| Triglycerides to total lipids ratio in very large HDL | -0.34 | 0.15 | 1.71E-01 | -0.30 | 0.16 | 3.38E-01 | -0.37 | 0.18 | 2.01E-01 | -0.27 | 0.19 | 4.85E-01 | -0.31 | 0.28 | 8.70E-01 | -0.38 | 0.29 | 7.11E-01 |
| Cholesterol in large VLDL | -0.28 | 0.12 | 1.71E-01 | -0.24 | 0.13 | 3.38E-01 | -0.33 | 0.15 | 2.01E-01 | -0.25 | 0.16 | 4.85E-01 | -0.22 | 0.23 | 8.70E-01 | -0.28 | 0.23 | 7.11E-01 |
| Triglycerides in VLDL | -0.31 | 0.14 | 1.71E-01 | -0.27 | 0.14 | 3.38E-01 | -0.38 | 0.17 | 2.01E-01 | -0.29 | 0.17 | 4.85E-01 | -0.20 | 0.25 | 8.70E-01 | -0.26 | 0.25 | 7.11E-01 |
| Cholesterol in very large VLDL | -0.28 | 0.12 | 1.71E-01 | -0.24 | 0.13 | 3.38E-01 | -0.32 | 0.15 | 2.01E-01 | -0.24 | 0.16 | 4.85E-01 | -0.24 | 0.22 | 8.70E-01 | -0.29 | 0.23 | 7.11E-01 |
| Lactate | -0.29 | 0.14 | 1.71E-01 | -0.26 | 0.14 | 3.38E-01 | -0.44 | 0.17 | 2.01E-01 | -0.38 | 0.18 | 4.85E-01 | 0.00 | 0.24 | 9.88E-01 | -0.05 | 0.25 | 9.36E-01 |
| Free cholesterol in medium VLDL | -0.28 | 0.13 | 1.71E-01 | -0.25 | 0.13 | 3.38E-01 | -0.35 | 0.16 | 2.01E-01 | -0.28 | 0.16 | 4.85E-01 | -0.18 | 0.23 | 8.70E-01 | -0.21 | 0.23 | 7.11E-01 |
| Ratio of triglycerides to phosphoglycerides | -0.31 | 0.14 | 1.71E-01 | -0.27 | 0.15 | 3.38E-01 | -0.36 | 0.17 | 2.01E-01 | -0.26 | 0.18 | 4.85E-01 | -0.24 | 0.25 | 8.70E-01 | -0.32 | 0.27 | 7.11E-01 |
| Phospholipids in small VLDL | -0.28 | 0.13 | 1.71E-01 | -0.24 | 0.13 | 3.38E-01 | -0.33 | 0.16 | 2.01E-01 | -0.27 | 0.16 | 4.85E-01 | -0.21 | 0.23 | 8.70E-01 | -0.23 | 0.24 | 7.11E-01 |
| Total lipids in large VLDL | -0.29 | 0.13 | 1.71E-01 | -0.24 | 0.13 | 3.38E-01 | -0.36 | 0.15 | 2.01E-01 | -0.27 | 0.16 | 4.85E-01 | -0.17 | 0.24 | 8.76E-01 | -0.23 | 0.24 | 7.11E-01 |
| Cholesteryl esters in VLDL | -0.28 | 0.13 | 1.71E-01 | -0.24 | 0.13 | 3.38E-01 | -0.33 | 0.16 | 2.01E-01 | -0.28 | 0.17 | 4.85E-01 | -0.20 | 0.24 | 8.70E-01 | -0.23 | 0.24 | 7.11E-01 |
| Cholesteryl esters in large HDL | 0.35 | 0.16 | 1.71E-01 | 0.30 | 0.17 | 3.38E-01 | 0.38 | 0.19 | 2.01E-01 | 0.26 | 0.20 | 4.85E-01 | 0.31 | 0.30 | 8.70E-01 | 0.42 | 0.32 | 7.11E-01 |
| Concentration of large VLDL particles | -0.29 | 0.13 | 1.71E-01 | -0.24 | 0.13 | 3.38E-01 | -0.35 | 0.16 | 2.01E-01 | -0.26 | 0.16 | 4.85E-01 | -0.18 | 0.24 | 8.70E-01 | -0.24 | 0.24 | 7.11E-01 |
| Cholesteryl esters in very large VLDL | -0.27 | 0.12 | 1.71E-01 | -0.23 | 0.13 | 3.38E-01 | -0.31 | 0.15 | 2.01E-01 | -0.23 | 0.15 | 4.85E-01 | -0.22 | 0.23 | 8.70E-01 | -0.27 | 0.23 | 7.11E-01 |
| Free cholesterol in large VLDL | -0.28 | 0.12 | 1.71E-01 | -0.23 | 0.13 | 3.38E-01 | -0.32 | 0.15 | 2.01E-01 | -0.23 | 0.16 | 4.85E-01 | -0.21 | 0.23 | 8.70E-01 | -0.26 | 0.24 | 7.11E-01 |
| Triglycerides in medium VLDL | -0.29 | 0.13 | 1.71E-01 | -0.24 | 0.14 | 3.38E-01 | -0.37 | 0.16 | 2.01E-01 | -0.28 | 0.17 | 4.85E-01 | -0.13 | 0.24 | 9.29E-01 | -0.18 | 0.24 | 7.90E-01 |
| Total lipids in very large VLDL | -0.27 | 0.12 | 1.71E-01 | -0.22 | 0.13 | 3.38E-01 | -0.32 | 0.14 | 2.01E-01 | -0.23 | 0.15 | 4.85E-01 | -0.20 | 0.23 | 8.70E-01 | -0.26 | 0.24 | 7.11E-01 |
| Remnant cholesterol (non-HDL | -0.27 | 0.14 | 1.95E-01 | -0.25 | 0.14 | 3.38E-01 | -0.34 | 0.17 | 2.01E-01 | -0.31 | 0.17 | 4.85E-01 | -0.16 | 0.25 | 8.76E-01 | -0.18 | 0.25 | 7.98E-01 |
| Triglycerides in large VLDL | -0.28 | 0.13 | 1.71E-01 | -0.24 | 0.14 | 3.38E-01 | -0.37 | 0.16 | 2.01E-01 | -0.28 | 0.17 | 4.85E-01 | -0.12 | 0.24 | 9.30E-01 | -0.18 | 0.25 | 7.95E-01 |
| Cholesterol in small VLDL | -0.26 | 0.13 | 1.82E-01 | -0.23 | 0.13 | 3.38E-01 | -0.31 | 0.16 | 2.01E-01 | -0.25 | 0.16 | 4.85E-01 | -0.20 | 0.23 | 8.70E-01 | -0.22 | 0.24 | 7.11E-01 |
| Phospholipids in small LDL | -0.25 | 0.13 | 1.99E-01 | -0.23 | 0.13 | 3.38E-01 | -0.33 | 0.16 | 2.01E-01 | -0.30 | 0.16 | 4.85E-01 | -0.09 | 0.24 | 9.30E-01 | -0.11 | 0.24 | 9.04E-01 |
| Free cholesterol in small VLDL | -0.26 | 0.13 | 1.95E-01 | -0.23 | 0.13 | 3.38E-01 | -0.32 | 0.16 | 2.01E-01 | -0.26 | 0.16 | 4.85E-01 | -0.17 | 0.23 | 8.76E-01 | -0.19 | 0.24 | 7.54E-01 |
| Saturated fatty acids | -0.29 | 0.15 | 1.95E-01 | -0.26 | 0.15 | 3.38E-01 | -0.36 | 0.18 | 2.01E-01 | -0.30 | 0.19 | 4.85E-01 | -0.19 | 0.26 | 8.76E-01 | -0.22 | 0.27 | 7.51E-01 |
| Concentration of very large VLDL particles | -0.26 | 0.12 | 1.71E-01 | -0.22 | 0.13 | 3.38E-01 | -0.30 | 0.14 | 2.01E-01 | -0.21 | 0.15 | 4.85E-01 | -0.20 | 0.23 | 8.70E-01 | -0.27 | 0.24 | 7.11E-01 |
| Phospholipids in large VLDL | -0.25 | 0.12 | 1.71E-01 | -0.21 | 0.13 | 3.38E-01 | -0.29 | 0.14 | 2.01E-01 | -0.21 | 0.15 | 4.85E-01 | -0.20 | 0.23 | 8.70E-01 | -0.25 | 0.23 | 7.11E-01 |
| Apolipoprotein B | -0.25 | 0.14 | 1.99E-01 | -0.23 | 0.14 | 3.38E-01 | -0.34 | 0.16 | 2.01E-01 | -0.30 | 0.17 | 4.85E-01 | -0.11 | 0.25 | 9.30E-01 | -0.13 | 0.25 | 8.73E-01 |
| Concentration of small LDL particles | -0.26 | 0.14 | 1.99E-01 | -0.23 | 0.14 | 3.38E-01 | -0.37 | 0.17 | 2.01E-01 | -0.32 | 0.17 | 4.85E-01 | -0.05 | 0.25 | 9.53E-01 | -0.07 | 0.25 | 9.32E-01 |
| Average diameter for VLDL particles | -0.30 | 0.14 | 1.82E-01 | -0.25 | 0.15 | 3.38E-01 | -0.36 | 0.18 | 2.01E-01 | -0.26 | 0.18 | 4.85E-01 | -0.18 | 0.26 | 8.76E-01 | -0.25 | 0.27 | 7.11E-01 |
| Total lipids in small LDL | -0.25 | 0.13 | 1.99E-01 | -0.22 | 0.13 | 3.38E-01 | -0.34 | 0.16 | 2.01E-01 | -0.30 | 0.16 | 4.85E-01 | -0.07 | 0.24 | 9.40E-01 | -0.09 | 0.25 | 9.18E-01 |
| Triglycerides in very large VLDL | -0.25 | 0.12 | 1.71E-01 | -0.21 | 0.13 | 3.38E-01 | -0.30 | 0.14 | 2.01E-01 | -0.21 | 0.15 | 4.85E-01 | -0.18 | 0.23 | 8.70E-01 | -0.24 | 0.24 | 7.11E-01 |
| Cholesteryl esters in small VLDL | -0.26 | 0.13 | 1.82E-01 | -0.23 | 0.13 | 3.38E-01 | -0.30 | 0.16 | 2.01E-01 | -0.24 | 0.16 | 4.85E-01 | -0.22 | 0.23 | 8.70E-01 | -0.24 | 0.24 | 7.11E-01 |
| Triglycerides to total lipids ratio in large HDL | -0.31 | 0.14 | 1.71E-01 | -0.27 | 0.15 | 3.38E-01 | -0.32 | 0.17 | 2.29E-01 | -0.24 | 0.18 | 4.85E-01 | -0.32 | 0.27 | 8.70E-01 | -0.38 | 0.27 | 7.11E-01 |
| Ratio of polyunsaturated fatty acids to monounsaturated fatty acids | 0.31 | 0.15 | 1.82E-01 | 0.27 | 0.16 | 3.38E-01 | 0.34 | 0.18 | 2.29E-01 | 0.27 | 0.19 | 4.85E-01 | 0.26 | 0.28 | 8.70E-01 | 0.34 | 0.29 | 7.11E-01 |
| Triglycerides in small VLDL | -0.27 | 0.13 | 1.82E-01 | -0.23 | 0.14 | 3.38E-01 | -0.30 | 0.16 | 2.29E-01 | -0.22 | 0.17 | 4.85E-01 | -0.23 | 0.24 | 8.70E-01 | -0.28 | 0.25 | 7.11E-01 |
| Phospholipids in very small VLDL | -0.27 | 0.14 | 1.95E-01 | -0.25 | 0.14 | 3.38E-01 | -0.30 | 0.17 | 2.38E-01 | -0.27 | 0.18 | 4.85E-01 | -0.25 | 0.24 | 8.70E-01 | -0.26 | 0.24 | 7.11E-01 |
| Triglycerides in large LDL | -0.27 | 0.14 | 1.96E-01 | -0.25 | 0.14 | 3.38E-01 | -0.30 | 0.17 | 2.38E-01 | -0.28 | 0.18 | 4.85E-01 | -0.23 | 0.25 | 8.70E-01 | -0.24 | 0.26 | 7.11E-01 |
| Total lipids in very small VLDL | -0.27 | 0.14 | 1.99E-01 | -0.25 | 0.14 | 3.38E-01 | -0.30 | 0.17 | 2.38E-01 | -0.28 | 0.18 | 4.85E-01 | -0.23 | 0.25 | 8.70E-01 | -0.24 | 0.25 | 7.11E-01 |
| Free cholesterol in very small VLDL | -0.26 | 0.14 | 1.99E-01 | -0.24 | 0.14 | 3.38E-01 | -0.30 | 0.17 | 2.38E-01 | -0.27 | 0.18 | 4.85E-01 | -0.22 | 0.24 | 8.70E-01 | -0.23 | 0.25 | 7.11E-01 |
| Triglycerides in very small VLDL | -0.29 | 0.14 | 1.71E-01 | -0.26 | 0.14 | 3.38E-01 | -0.28 | 0.17 | 2.41E-01 | -0.22 | 0.17 | 4.85E-01 | -0.33 | 0.25 | 8.70E-01 | -0.37 | 0.26 | 7.11E-01 |
| Linoleic acid | -0.25 | 0.14 | 2.04E-01 | -0.25 | 0.14 | 3.38E-01 | -0.28 | 0.17 | 2.41E-01 | -0.27 | 0.17 | 4.85E-01 | -0.20 | 0.26 | 8.70E-01 | -0.20 | 0.26 | 7.63E-01 |
| Concentration of very small VLDL particles | -0.26 | 0.14 | 1.99E-01 | -0.24 | 0.14 | 3.38E-01 | -0.30 | 0.17 | 2.41E-01 | -0.27 | 0.18 | 4.85E-01 | -0.23 | 0.25 | 8.70E-01 | -0.23 | 0.25 | 7.11E-01 |
| Triglycerides in IDL | -0.28 | 0.14 | 1.95E-01 | -0.25 | 0.15 | 3.38E-01 | -0.28 | 0.17 | 2.46E-01 | -0.25 | 0.18 | 4.85E-01 | -0.29 | 0.26 | 8.70E-01 | -0.31 | 0.26 | 7.11E-01 |
| Free cholesterol to total lipids ratio in IDL | 0.36 | 0.15 | 1.71E-01 | 0.34 | 0.15 | 3.38E-01 | 0.28 | 0.18 | 2.56E-01 | 0.25 | 0.18 | 4.85E-01 | 0.53 | 0.27 | 8.70E-01 | 0.57 | 0.28 | 7.11E-01 |
| Triglycerides in very large HDL | -0.26 | 0.14 | 1.99E-01 | -0.26 | 0.14 | 3.38E-01 | -0.25 | 0.17 | 2.81E-01 | -0.26 | 0.17 | 4.85E-01 | -0.29 | 0.25 | 8.70E-01 | -0.30 | 0.25 | 7.11E-01 |
| Free cholesterol to total lipids ratio in small LDL | 0.34 | 0.16 | 1.82E-01 | 0.30 | 0.17 | 3.38E-01 | 0.32 | 0.20 | 2.46E-01 | 0.25 | 0.20 | 4.87E-01 | 0.39 | 0.30 | 8.70E-01 | 0.43 | 0.31 | 7.11E-01 |
| Cholesterol in large HDL | 0.34 | 0.16 | 1.71E-01 | 0.29 | 0.17 | 3.38E-01 | 0.36 | 0.19 | 2.01E-01 | 0.25 | 0.20 | 4.99E-01 | 0.30 | 0.30 | 8.70E-01 | 0.40 | 0.32 | 7.11E-01 |
| Triglycerides in small HDL | -0.27 | 0.13 | 1.71E-01 | -0.22 | 0.13 | 3.38E-01 | -0.28 | 0.15 | 2.29E-01 | -0.19 | 0.16 | 5.31E-01 | -0.27 | 0.23 | 8.70E-01 | -0.33 | 0.24 | 7.11E-01 |
| Triglycerides to total lipids ratio in small HDL | -0.27 | 0.13 | 1.71E-01 | -0.24 | 0.13 | 3.38E-01 | -0.26 | 0.15 | 2.38E-01 | -0.18 | 0.16 | 5.31E-01 | -0.33 | 0.24 | 8.70E-01 | -0.40 | 0.25 | 7.11E-01 |
| Cholesteryl esters to total lipids ratio in small HDL | 0.28 | 0.15 | 1.99E-01 | 0.27 | 0.15 | 3.38E-01 | 0.22 | 0.18 | 3.58E-01 | 0.20 | 0.18 | 5.34E-01 | 0.44 | 0.29 | 8.70E-01 | 0.47 | 0.30 | 7.11E-01 |
| Triglycerides to total lipids ratio in medium HDL | -0.26 | 0.12 | 1.74E-01 | -0.22 | 0.13 | 3.38E-01 | -0.25 | 0.15 | 2.42E-01 | -0.17 | 0.16 | 5.44E-01 | -0.32 | 0.23 | 8.70E-01 | -0.39 | 0.24 | 7.11E-01 |
| Phospholipids to total lipids ratio in medium HDL | -0.32 | 0.15 | 1.71E-01 | -0.27 | 0.15 | 3.38E-01 | -0.29 | 0.17 | 2.41E-01 | -0.18 | 0.18 | 5.59E-01 | -0.43 | 0.28 | 8.70E-01 | -0.54 | 0.30 | 7.11E-01 |
| Cholesteryl esters in medium HDL | 0.27 | 0.14 | 1.99E-01 | 0.25 | 0.15 | 3.52E-01 | 0.26 | 0.17 | 2.76E-01 | 0.20 | 0.18 | 5.44E-01 | 0.29 | 0.27 | 8.70E-01 | 0.38 | 0.29 | 7.11E-01 |
| Total lipids in lipoprotein particles | -0.25 | 0.14 | 2.09E-01 | -0.23 | 0.14 | 3.55E-01 | -0.34 | 0.17 | 2.01E-01 | -0.31 | 0.17 | 4.85E-01 | -0.09 | 0.26 | 9.30E-01 | -0.10 | 0.26 | 9.18E-01 |
| Cholesteryl esters in small LDL | -0.24 | 0.13 | 1.99E-01 | -0.21 | 0.14 | 3.55E-01 | -0.35 | 0.16 | 2.01E-01 | -0.29 | 0.16 | 4.85E-01 | -0.05 | 0.25 | 9.53E-01 | -0.07 | 0.25 | 9.31E-01 |
| Polyunsaturated fatty acids | -0.24 | 0.14 | 2.26E-01 | -0.22 | 0.14 | 3.55E-01 | -0.28 | 0.17 | 2.42E-01 | -0.26 | 0.17 | 4.85E-01 | -0.18 | 0.26 | 8.76E-01 | -0.19 | 0.26 | 7.90E-01 |
| Ratio of omega-6 fatty acids to total fatty acids | 0.31 | 0.16 | 1.95E-01 | 0.26 | 0.16 | 3.55E-01 | 0.34 | 0.19 | 2.38E-01 | 0.26 | 0.20 | 4.87E-01 | 0.27 | 0.29 | 8.70E-01 | 0.35 | 0.31 | 7.11E-01 |
| Cholesteryl esters in HDL | 0.27 | 0.14 | 1.96E-01 | 0.24 | 0.15 | 3.55E-01 | 0.27 | 0.17 | 2.46E-01 | 0.18 | 0.18 | 5.44E-01 | 0.30 | 0.27 | 8.70E-01 | 0.40 | 0.29 | 7.11E-01 |
| Tyrosine | -0.26 | 0.14 | 1.95E-01 | -0.22 | 0.14 | 3.57E-01 | -0.34 | 0.16 | 2.01E-01 | -0.25 | 0.17 | 4.85E-01 | -0.11 | 0.24 | 9.30E-01 | -0.16 | 0.25 | 8.38E-01 |
| Concentration of LDL particles | -0.23 | 0.14 | 2.18E-01 | -0.21 | 0.14 | 3.57E-01 | -0.33 | 0.16 | 2.01E-01 | -0.29 | 0.17 | 4.85E-01 | -0.05 | 0.25 | 9.53E-01 | -0.07 | 0.25 | 9.33E-01 |
| Total lipids in large HDL | 0.29 | 0.15 | 1.95E-01 | 0.25 | 0.16 | 3.57E-01 | 0.33 | 0.18 | 2.29E-01 | 0.21 | 0.19 | 5.31E-01 | 0.24 | 0.28 | 8.70E-01 | 0.34 | 0.30 | 7.11E-01 |
| Phospholipids in large HDL | 0.28 | 0.15 | 1.99E-01 | 0.24 | 0.16 | 3.57E-01 | 0.32 | 0.18 | 2.36E-01 | 0.21 | 0.19 | 5.31E-01 | 0.22 | 0.28 | 8.70E-01 | 0.31 | 0.30 | 7.11E-01 |
| Glucose | -0.30 | 0.16 | 1.99E-01 | -0.25 | 0.16 | 3.57E-01 | -0.32 | 0.20 | 2.46E-01 | -0.23 | 0.20 | 5.31E-01 | -0.28 | 0.28 | 8.70E-01 | -0.35 | 0.29 | 7.11E-01 |
| Concentration of large HDL particles | 0.29 | 0.15 | 1.96E-01 | 0.24 | 0.16 | 3.57E-01 | 0.32 | 0.18 | 2.38E-01 | 0.20 | 0.19 | 5.44E-01 | 0.26 | 0.29 | 8.70E-01 | 0.37 | 0.31 | 7.11E-01 |
| Triglycerides in HDL | -0.25 | 0.14 | 2.00E-01 | -0.21 | 0.14 | 3.57E-01 | -0.23 | 0.16 | 3.10E-01 | -0.17 | 0.17 | 5.44E-01 | -0.31 | 0.25 | 8.70E-01 | -0.35 | 0.26 | 7.11E-01 |
| Cholesterol in medium HDL | 0.25 | 0.14 | 2.12E-01 | 0.23 | 0.15 | 3.57E-01 | 0.25 | 0.17 | 2.99E-01 | 0.18 | 0.18 | 5.62E-01 | 0.28 | 0.27 | 8.70E-01 | 0.37 | 0.29 | 7.11E-01 |
| Free cholesterol to total lipids ratio in medium LDL | 0.29 | 0.15 | 1.99E-01 | 0.24 | 0.16 | 3.57E-01 | 0.25 | 0.18 | 3.10E-01 | 0.17 | 0.19 | 6.02E-01 | 0.42 | 0.31 | 8.70E-01 | 0.50 | 0.32 | 7.11E-01 |
| Triglycerides to total lipids ratio in small LDL | -0.27 | 0.15 | 2.04E-01 | -0.24 | 0.16 | 3.57E-01 | -0.22 | 0.18 | 3.58E-01 | -0.16 | 0.18 | 6.36E-01 | -0.40 | 0.28 | 8.70E-01 | -0.46 | 0.30 | 7.11E-01 |
| Cholesterol to total lipids ratio in small HDL | 0.26 | 0.15 | 2.04E-01 | 0.23 | 0.15 | 3.57E-01 | 0.20 | 0.18 | 3.92E-01 | 0.15 | 0.18 | 6.39E-01 | 0.43 | 0.28 | 8.70E-01 | 0.47 | 0.29 | 7.11E-01 |
| Cholesterol to total lipids ratio in small LDL | 0.20 | 0.15 | 3.13E-01 | 0.22 | 0.15 | 3.74E-01 | 0.10 | 0.17 | 6.39E-01 | 0.13 | 0.17 | 6.59E-01 | 0.44 | 0.29 | 8.70E-01 | 0.46 | 0.30 | 7.11E-01 |
| Cholesteryl esters to total lipids ratio in medium VLDL | 0.29 | 0.16 | 2.04E-01 | 0.24 | 0.17 | 3.74E-01 | 0.34 | 0.20 | 2.41E-01 | 0.24 | 0.20 | 5.13E-01 | 0.19 | 0.28 | 8.76E-01 | 0.25 | 0.30 | 7.40E-01 |
| Cholesterol in small LDL | -0.22 | 0.13 | 2.26E-01 | -0.19 | 0.13 | 3.77E-01 | -0.32 | 0.16 | 2.01E-01 | -0.28 | 0.16 | 4.85E-01 | -0.03 | 0.25 | 9.59E-01 | -0.05 | 0.25 | 9.41E-01 |
| Cholesteryl esters in medium LDL | -0.23 | 0.13 | 2.09E-01 | -0.19 | 0.13 | 3.77E-01 | -0.33 | 0.16 | 2.01E-01 | -0.26 | 0.16 | 4.85E-01 | -0.06 | 0.24 | 9.40E-01 | -0.09 | 0.24 | 9.18E-01 |
| Concentration of large LDL particles | -0.21 | 0.13 | 2.51E-01 | -0.19 | 0.14 | 3.77E-01 | -0.30 | 0.16 | 2.27E-01 | -0.28 | 0.16 | 4.85E-01 | -0.02 | 0.25 | 9.71E-01 | -0.03 | 0.25 | 9.70E-01 |
| Cholesterol in medium VLDL | -0.22 | 0.13 | 2.26E-01 | -0.19 | 0.13 | 3.77E-01 | -0.28 | 0.16 | 2.38E-01 | -0.24 | 0.16 | 4.85E-01 | -0.12 | 0.24 | 9.30E-01 | -0.14 | 0.24 | 8.61E-01 |
| Concentration of IDL particles | -0.22 | 0.14 | 2.51E-01 | -0.21 | 0.15 | 3.77E-01 | -0.25 | 0.17 | 2.98E-01 | -0.24 | 0.18 | 4.85E-01 | -0.20 | 0.26 | 8.70E-01 | -0.21 | 0.26 | 7.56E-01 |
| Free cholesterol to total lipids ratio in large LDL | 0.29 | 0.16 | 2.04E-01 | 0.24 | 0.17 | 3.77E-01 | 0.31 | 0.19 | 2.57E-01 | 0.21 | 0.20 | 5.44E-01 | 0.28 | 0.30 | 8.70E-01 | 0.34 | 0.32 | 7.11E-01 |
| HDL cholesterol | 0.25 | 0.14 | 2.04E-01 | 0.22 | 0.15 | 3.77E-01 | 0.25 | 0.17 | 2.78E-01 | 0.16 | 0.18 | 6.10E-01 | 0.28 | 0.27 | 8.70E-01 | 0.38 | 0.29 | 7.11E-01 |
| Free cholesterol in large HDL | 0.29 | 0.16 | 1.99E-01 | 0.23 | 0.16 | 3.81E-01 | 0.31 | 0.19 | 2.41E-01 | 0.19 | 0.20 | 5.69E-01 | 0.25 | 0.30 | 8.70E-01 | 0.35 | 0.33 | 7.11E-01 |
| Pyruvate | -0.21 | 0.13 | 2.26E-01 | -0.18 | 0.13 | 3.86E-01 | -0.27 | 0.15 | 2.38E-01 | -0.22 | 0.16 | 4.85E-01 | -0.08 | 0.23 | 9.30E-01 | -0.13 | 0.24 | 8.63E-01 |
| Total lipids in medium LDL | -0.22 | 0.13 | 2.26E-01 | -0.18 | 0.13 | 3.88E-01 | -0.31 | 0.16 | 2.01E-01 | -0.26 | 0.16 | 4.85E-01 | -0.04 | 0.24 | 9.59E-01 | -0.06 | 0.24 | 9.33E-01 |
| Total cholesterol minus HDL-C | -0.21 | 0.14 | 2.50E-01 | -0.19 | 0.14 | 3.88E-01 | -0.30 | 0.16 | 2.29E-01 | -0.27 | 0.17 | 4.85E-01 | -0.05 | 0.25 | 9.56E-01 | -0.06 | 0.26 | 9.33E-01 |
| Omega-6 fatty acids | -0.20 | 0.14 | 2.88E-01 | -0.20 | 0.14 | 3.88E-01 | -0.25 | 0.17 | 2.97E-01 | -0.23 | 0.17 | 4.85E-01 | -0.14 | 0.26 | 9.30E-01 | -0.15 | 0.26 | 8.63E-01 |
| Free cholesterol in chylomicrons and extremely large VLDL | -0.22 | 0.12 | 1.99E-01 | -0.18 | 0.13 | 3.88E-01 | -0.20 | 0.15 | 3.10E-01 | -0.11 | 0.16 | 6.99E-01 | -0.30 | 0.23 | 8.70E-01 | -0.38 | 0.24 | 7.11E-01 |
| Cholesteryl esters to total lipids ratio in very small VLDL | 0.28 | 0.17 | 2.26E-01 | 0.24 | 0.17 | 3.88E-01 | 0.22 | 0.19 | 3.92E-01 | 0.14 | 0.20 | 6.99E-01 | 0.42 | 0.33 | 8.70E-01 | 0.50 | 0.34 | 7.11E-01 |
| Concentration of medium LDL particles | -0.22 | 0.13 | 2.26E-01 | -0.18 | 0.14 | 4.10E-01 | -0.30 | 0.16 | 2.29E-01 | -0.23 | 0.17 | 4.85E-01 | -0.11 | 0.24 | 9.30E-01 | -0.13 | 0.24 | 8.73E-01 |
| Cholesterol to total lipids ratio in very small VLDL | 0.26 | 0.16 | 2.39E-01 | 0.22 | 0.17 | 4.11E-01 | 0.20 | 0.19 | 4.12E-01 | 0.13 | 0.20 | 7.24E-01 | 0.40 | 0.32 | 8.70E-01 | 0.47 | 0.34 | 7.11E-01 |
| Phospholipids to total lipids ratio in very large VLDL | -0.16 | 0.10 | 2.47E-01 | -0.14 | 0.10 | 4.11E-01 | -0.11 | 0.14 | 5.34E-01 | -0.07 | 0.14 | 7.75E-01 | -0.25 | 0.14 | 8.70E-01 | -0.26 | 0.15 | 7.11E-01 |
| Cholesterol in medium LDL | -0.21 | 0.13 | 2.39E-01 | -0.17 | 0.13 | 4.11E-01 | -0.31 | 0.16 | 2.01E-01 | -0.25 | 0.16 | 4.85E-01 | -0.03 | 0.24 | 9.59E-01 | -0.05 | 0.25 | 9.36E-01 |
| Phospholipids in medium LDL | -0.20 | 0.13 | 2.51E-01 | -0.17 | 0.13 | 4.11E-01 | -0.29 | 0.16 | 2.27E-01 | -0.25 | 0.16 | 4.85E-01 | -0.03 | 0.24 | 9.59E-01 | -0.05 | 0.24 | 9.42E-01 |
| Total free cholesterol | -0.20 | 0.14 | 3.12E-01 | -0.19 | 0.14 | 4.21E-01 | -0.28 | 0.17 | 2.41E-01 | -0.28 | 0.17 | 4.85E-01 | -0.03 | 0.26 | 9.59E-01 | -0.03 | 0.27 | 9.70E-01 |
| Glycine | 0.23 | 0.14 | 2.26E-01 | 0.18 | 0.14 | 4.25E-01 | 0.45 | 0.17 | 2.01E-01 | 0.37 | 0.18 | 4.85E-01 | -0.24 | 0.26 | 8.70E-01 | -0.21 | 0.26 | 7.54E-01 |
| Cholesteryl esters to total lipids ratio in very large VLDL | 0.22 | 0.13 | 2.17E-01 | 0.17 | 0.13 | 4.41E-01 | 0.27 | 0.15 | 2.38E-01 | 0.18 | 0.16 | 5.44E-01 | 0.13 | 0.24 | 9.30E-01 | 0.19 | 0.25 | 7.88E-01 |
| Cholesterol in very small VLDL | -0.19 | 0.15 | 3.25E-01 | -0.19 | 0.15 | 4.43E-01 | -0.24 | 0.18 | 3.10E-01 | -0.25 | 0.18 | 4.85E-01 | -0.12 | 0.26 | 9.30E-01 | -0.12 | 0.27 | 9.01E-01 |
| Ratio of polyunsaturated fatty acids to total fatty acids | 0.24 | 0.15 | 2.39E-01 | 0.20 | 0.16 | 4.43E-01 | 0.27 | 0.19 | 2.96E-01 | 0.20 | 0.19 | 5.44E-01 | 0.19 | 0.28 | 8.76E-01 | 0.26 | 0.29 | 7.11E-01 |
| Free cholesterol to total lipids ratio in very large HDL | -0.25 | 0.16 | 2.30E-01 | -0.20 | 0.16 | 4.43E-01 | -0.31 | 0.19 | 2.42E-01 | -0.20 | 0.20 | 5.44E-01 | -0.15 | 0.29 | 9.30E-01 | -0.24 | 0.30 | 7.57E-01 |
| Concentration of chylomicrons and extremely large VLDL particles | -0.21 | 0.12 | 2.18E-01 | -0.16 | 0.13 | 4.43E-01 | -0.22 | 0.14 | 2.78E-01 | -0.12 | 0.15 | 6.42E-01 | -0.21 | 0.22 | 8.70E-01 | -0.29 | 0.24 | 7.11E-01 |
| Cholesterol to total lipids ratio in medium VLDL | 0.24 | 0.15 | 2.47E-01 | 0.19 | 0.16 | 4.44E-01 | 0.29 | 0.19 | 2.56E-01 | 0.21 | 0.19 | 5.44E-01 | 0.11 | 0.27 | 9.30E-01 | 0.17 | 0.28 | 8.55E-01 |
| Triglycerides in medium HDL | -0.21 | 0.13 | 2.39E-01 | -0.17 | 0.13 | 4.48E-01 | -0.19 | 0.16 | 3.68E-01 | -0.13 | 0.16 | 6.57E-01 | -0.26 | 0.24 | 8.70E-01 | -0.30 | 0.24 | 7.11E-01 |
| Concentration of medium HDL particles | 0.21 | 0.15 | 2.99E-01 | 0.19 | 0.15 | 4.55E-01 | 0.20 | 0.18 | 3.87E-01 | 0.14 | 0.19 | 6.68E-01 | 0.23 | 0.27 | 8.70E-01 | 0.31 | 0.29 | 7.11E-01 |
| Phospholipids to total lipids ratio in very small VLDL | -0.18 | 0.13 | 3.07E-01 | -0.16 | 0.13 | 4.55E-01 | -0.13 | 0.17 | 5.31E-01 | -0.09 | 0.17 | 7.75E-01 | -0.26 | 0.21 | 8.70E-01 | -0.28 | 0.21 | 7.11E-01 |
| Phospholipids in chylomicrons and extremely large VLDL | -0.19 | 0.12 | 2.26E-01 | -0.15 | 0.12 | 4.57E-01 | -0.18 | 0.14 | 3.39E-01 | -0.09 | 0.15 | 7.48E-01 | -0.27 | 0.22 | 8.70E-01 | -0.35 | 0.24 | 7.11E-01 |
| Total lipids in chylomicrons and extremely large VLDL | -0.21 | 0.13 | 2.26E-01 | -0.16 | 0.13 | 4.62E-01 | -0.23 | 0.15 | 2.78E-01 | -0.13 | 0.16 | 6.42E-01 | -0.19 | 0.23 | 8.70E-01 | -0.26 | 0.24 | 7.11E-01 |
| Total lipids in medium HDL | 0.20 | 0.15 | 3.20E-01 | 0.18 | 0.15 | 4.67E-01 | 0.20 | 0.18 | 3.91E-01 | 0.14 | 0.19 | 6.59E-01 | 0.21 | 0.27 | 8.70E-01 | 0.28 | 0.29 | 7.11E-01 |
| Phospholipids in LDL | -0.18 | 0.14 | 3.33E-01 | -0.16 | 0.14 | 4.75E-01 | -0.26 | 0.16 | 2.42E-01 | -0.24 | 0.16 | 4.85E-01 | -0.01 | 0.25 | 9.81E-01 | -0.02 | 0.26 | 9.82E-01 |
| Isoleucine | -0.23 | 0.14 | 2.30E-01 | -0.17 | 0.15 | 4.75E-01 | -0.16 | 0.17 | 4.55E-01 | -0.06 | 0.17 | 8.41E-01 | -0.45 | 0.27 | 8.70E-01 | -0.59 | 0.29 | 7.11E-01 |
| Total lipids in HDL | 0.20 | 0.14 | 2.91E-01 | 0.17 | 0.15 | 4.95E-01 | 0.21 | 0.17 | 3.58E-01 | 0.12 | 0.18 | 7.04E-01 | 0.21 | 0.27 | 8.70E-01 | 0.29 | 0.29 | 7.11E-01 |
| Total lipids in LDL | -0.17 | 0.14 | 3.39E-01 | -0.15 | 0.14 | 4.98E-01 | -0.27 | 0.16 | 2.41E-01 | -0.24 | 0.16 | 4.85E-01 | 0.02 | 0.25 | 9.71E-01 | 0.01 | 0.26 | 9.91E-01 |
| Average diameter for HDL particles | 0.21 | 0.13 | 2.27E-01 | 0.15 | 0.14 | 4.98E-01 | 0.25 | 0.15 | 2.41E-01 | 0.13 | 0.16 | 6.41E-01 | 0.14 | 0.25 | 9.23E-01 | 0.21 | 0.26 | 7.50E-01 |
| Cholesteryl esters in LDL | -0.17 | 0.13 | 3.39E-01 | -0.15 | 0.14 | 5.01E-01 | -0.28 | 0.16 | 2.41E-01 | -0.24 | 0.16 | 4.85E-01 | 0.03 | 0.25 | 9.59E-01 | 0.02 | 0.25 | 9.74E-01 |
| Phosphatidylcholines | -0.16 | 0.15 | 4.23E-01 | -0.17 | 0.15 | 5.01E-01 | -0.19 | 0.18 | 4.12E-01 | -0.22 | 0.18 | 5.18E-01 | -0.11 | 0.27 | 9.30E-01 | -0.10 | 0.28 | 9.18E-01 |
| Phospholipids to total lipids ratio in large HDL | -0.24 | 0.16 | 2.71E-01 | -0.18 | 0.17 | 5.01E-01 | -0.20 | 0.19 | 4.12E-01 | -0.10 | 0.20 | 7.75E-01 | -0.34 | 0.31 | 8.70E-01 | -0.45 | 0.32 | 7.11E-01 |
| Cholesteryl esters to total lipids ratio in chylomicrons and extremely large VLDL | 0.18 | 0.12 | 2.72E-01 | 0.14 | 0.13 | 5.13E-01 | 0.25 | 0.15 | 2.41E-01 | 0.16 | 0.16 | 5.44E-01 | 0.04 | 0.23 | 9.59E-01 | 0.08 | 0.24 | 9.19E-01 |
| Apolipoprotein A1 | 0.19 | 0.14 | 3.33E-01 | 0.16 | 0.15 | 5.13E-01 | 0.18 | 0.17 | 4.12E-01 | 0.11 | 0.18 | 7.48E-01 | 0.21 | 0.27 | 8.70E-01 | 0.29 | 0.29 | 7.11E-01 |
| Valine | -0.23 | 0.14 | 2.26E-01 | -0.16 | 0.15 | 5.13E-01 | -0.21 | 0.16 | 3.37E-01 | -0.07 | 0.18 | 8.13E-01 | -0.31 | 0.26 | 8.70E-01 | -0.43 | 0.28 | 7.11E-01 |
| Acetate | -0.14 | 0.14 | 4.52E-01 | -0.15 | 0.14 | 5.21E-01 | -0.17 | 0.16 | 4.22E-01 | -0.19 | 0.17 | 5.31E-01 | -0.06 | 0.27 | 9.53E-01 | -0.04 | 0.27 | 9.63E-01 |
| Phenylalanine | 0.09 | 0.13 | 6.27E-01 | 0.14 | 0.14 | 5.24E-01 | 0.09 | 0.16 | 6.56E-01 | 0.17 | 0.16 | 5.44E-01 | 0.10 | 0.27 | 9.30E-01 | 0.07 | 0.28 | 9.33E-01 |
| Cholesterol to total lipids ratio in very large VLDL | 0.19 | 0.13 | 2.65E-01 | 0.14 | 0.13 | 5.24E-01 | 0.25 | 0.15 | 2.41E-01 | 0.16 | 0.16 | 5.44E-01 | 0.04 | 0.25 | 9.59E-01 | 0.09 | 0.26 | 9.18E-01 |
| Phospholipids in medium HDL | 0.17 | 0.15 | 3.86E-01 | 0.16 | 0.15 | 5.24E-01 | 0.18 | 0.18 | 4.37E-01 | 0.13 | 0.19 | 7.04E-01 | 0.17 | 0.27 | 8.80E-01 | 0.23 | 0.29 | 7.54E-01 |
| Phospholipids in HDL | 0.19 | 0.14 | 3.37E-01 | 0.16 | 0.15 | 5.24E-01 | 0.20 | 0.17 | 3.87E-01 | 0.12 | 0.18 | 7.24E-01 | 0.17 | 0.27 | 8.80E-01 | 0.25 | 0.29 | 7.37E-01 |
| Cholesteryl esters in very large HDL | 0.22 | 0.14 | 2.54E-01 | 0.15 | 0.15 | 5.24E-01 | 0.24 | 0.17 | 3.10E-01 | 0.10 | 0.18 | 7.66E-01 | 0.21 | 0.28 | 8.76E-01 | 0.30 | 0.30 | 7.11E-01 |
| Cholesterol in chylomicrons and extremely large VLDL | -0.19 | 0.13 | 2.66E-01 | -0.14 | 0.13 | 5.24E-01 | -0.19 | 0.15 | 3.55E-01 | -0.09 | 0.16 | 7.66E-01 | -0.22 | 0.24 | 8.70E-01 | -0.29 | 0.25 | 7.11E-01 |
| Free cholesterol in medium HDL | 0.19 | 0.15 | 3.37E-01 | 0.16 | 0.16 | 5.24E-01 | 0.18 | 0.18 | 4.39E-01 | 0.10 | 0.19 | 7.75E-01 | 0.24 | 0.28 | 8.70E-01 | 0.32 | 0.30 | 7.11E-01 |
| Triglycerides to total lipids ratio in large LDL | -0.17 | 0.15 | 3.88E-01 | -0.15 | 0.15 | 5.24E-01 | -0.09 | 0.17 | 7.00E-01 | -0.06 | 0.18 | 8.23E-01 | -0.37 | 0.28 | 8.70E-01 | -0.39 | 0.29 | 7.11E-01 |
| Glycoprotein acetyls | -0.18 | 0.14 | 3.25E-01 | -0.14 | 0.14 | 5.25E-01 | -0.29 | 0.16 | 2.38E-01 | -0.22 | 0.17 | 4.85E-01 | 0.08 | 0.24 | 9.30E-01 | 0.06 | 0.26 | 9.35E-01 |
| LDL cholesterol | -0.15 | 0.14 | 3.95E-01 | -0.14 | 0.14 | 5.25E-01 | -0.26 | 0.16 | 2.55E-01 | -0.23 | 0.16 | 4.85E-01 | 0.05 | 0.25 | 9.56E-01 | 0.04 | 0.26 | 9.53E-01 |
| Cholesteryl esters in very small VLDL | -0.15 | 0.15 | 4.36E-01 | -0.15 | 0.15 | 5.25E-01 | -0.20 | 0.18 | 3.87E-01 | -0.22 | 0.18 | 5.10E-01 | -0.07 | 0.27 | 9.40E-01 | -0.06 | 0.27 | 9.35E-01 |
| Phospholipids to total lipids ratio in very large HDL | 0.27 | 0.21 | 3.39E-01 | 0.21 | 0.21 | 5.25E-01 | 0.35 | 0.26 | 3.28E-01 | 0.20 | 0.25 | 6.41E-01 | 0.11 | 0.34 | 9.30E-01 | 0.17 | 0.37 | 9.01E-01 |
| Degree of unsaturation | 0.16 | 0.14 | 3.89E-01 | 0.14 | 0.14 | 5.25E-01 | 0.16 | 0.16 | 4.42E-01 | 0.14 | 0.17 | 6.41E-01 | 0.13 | 0.25 | 9.30E-01 | 0.16 | 0.26 | 8.35E-01 |
| Triglycerides to total lipids ratio in medium LDL | -0.16 | 0.15 | 4.28E-01 | -0.15 | 0.15 | 5.25E-01 | -0.08 | 0.17 | 7.20E-01 | -0.07 | 0.18 | 8.16E-01 | -0.33 | 0.28 | 8.70E-01 | -0.35 | 0.30 | 7.11E-01 |
| Total lipids in IDL | -0.14 | 0.15 | 4.68E-01 | -0.15 | 0.15 | 5.25E-01 | -0.21 | 0.17 | 3.71E-01 | -0.24 | 0.18 | 4.85E-01 | -0.02 | 0.27 | 9.71E-01 | -0.01 | 0.28 | 9.92E-01 |
| Free cholesterol in small LDL | -0.14 | 0.13 | 4.23E-01 | -0.13 | 0.13 | 5.27E-01 | -0.23 | 0.16 | 2.84E-01 | -0.22 | 0.16 | 4.85E-01 | 0.04 | 0.25 | 9.59E-01 | 0.03 | 0.25 | 9.70E-01 |
| Cholesteryl esters in IDL | -0.13 | 0.15 | 4.84E-01 | -0.15 | 0.15 | 5.27E-01 | -0.20 | 0.17 | 3.91E-01 | -0.23 | 0.18 | 4.87E-01 | -0.02 | 0.27 | 9.71E-01 | -0.01 | 0.28 | 9.92E-01 |
| Triglycerides to total lipids ratio in IDL | -0.18 | 0.15 | 3.73E-01 | -0.15 | 0.15 | 5.27E-01 | -0.12 | 0.18 | 6.08E-01 | -0.07 | 0.18 | 8.21E-01 | -0.33 | 0.29 | 8.70E-01 | -0.37 | 0.30 | 7.11E-01 |
| Clinical LDL cholesterol | -0.14 | 0.14 | 4.28E-01 | -0.13 | 0.14 | 5.27E-01 | -0.23 | 0.16 | 2.97E-01 | -0.22 | 0.16 | 4.85E-01 | 0.03 | 0.26 | 9.59E-01 | 0.03 | 0.26 | 9.71E-01 |
| Total phospholipids in lipoprotein particles | -0.14 | 0.15 | 4.77E-01 | -0.14 | 0.15 | 5.27E-01 | -0.20 | 0.18 | 3.87E-01 | -0.22 | 0.18 | 5.09E-01 | -0.02 | 0.28 | 9.71E-01 | 0.00 | 0.29 | 9.92E-01 |
| Phospholipids to total lipids ratio in small VLDL | 0.18 | 0.14 | 3.40E-01 | 0.14 | 0.15 | 5.27E-01 | 0.16 | 0.17 | 4.69E-01 | 0.09 | 0.18 | 7.78E-01 | 0.23 | 0.27 | 8.70E-01 | 0.28 | 0.28 | 7.11E-01 |
| Free cholesterol to total lipids ratio in small VLDL | 0.18 | 0.15 | 3.41E-01 | 0.14 | 0.15 | 5.27E-01 | 0.16 | 0.17 | 4.80E-01 | 0.08 | 0.18 | 8.08E-01 | 0.24 | 0.28 | 8.70E-01 | 0.30 | 0.29 | 7.11E-01 |
| Phospholipids to total lipids ratio in chylomicrons and extremely large VLDL | -0.15 | 0.11 | 3.25E-01 | -0.11 | 0.12 | 5.27E-01 | -0.10 | 0.14 | 5.75E-01 | -0.03 | 0.14 | 9.03E-01 | -0.31 | 0.21 | 8.70E-01 | -0.39 | 0.22 | 7.11E-01 |
| Free cholesterol in HDL | 0.18 | 0.14 | 3.37E-01 | 0.14 | 0.15 | 5.44E-01 | 0.18 | 0.17 | 4.16E-01 | 0.07 | 0.18 | 8.15E-01 | 0.22 | 0.27 | 8.70E-01 | 0.31 | 0.29 | 7.11E-01 |
| Total concentration of branched-chain amino acids (leucine + isoleucine + valine) | -0.21 | 0.14 | 2.72E-01 | -0.14 | 0.15 | 5.53E-01 | -0.16 | 0.17 | 4.55E-01 | -0.02 | 0.18 | 9.37E-01 | -0.38 | 0.27 | 8.70E-01 | -0.51 | 0.29 | 7.11E-01 |
| Phospholipids in large LDL | -0.14 | 0.14 | 4.52E-01 | -0.13 | 0.14 | 5.55E-01 | -0.22 | 0.16 | 3.14E-01 | -0.21 | 0.17 | 4.87E-01 | 0.03 | 0.26 | 9.59E-01 | 0.03 | 0.27 | 9.70E-01 |
| Concentration of HDL particles | 0.15 | 0.15 | 4.36E-01 | 0.14 | 0.15 | 5.55E-01 | 0.12 | 0.17 | 6.08E-01 | 0.07 | 0.18 | 8.21E-01 | 0.24 | 0.27 | 8.70E-01 | 0.30 | 0.29 | 7.11E-01 |
| Omega-3 fatty acids | -0.16 | 0.14 | 3.89E-01 | -0.12 | 0.14 | 5.56E-01 | -0.16 | 0.17 | 4.47E-01 | -0.11 | 0.17 | 7.34E-01 | -0.16 | 0.24 | 8.76E-01 | -0.18 | 0.24 | 7.92E-01 |
| Phospholipids in very large HDL | 0.21 | 0.16 | 3.25E-01 | 0.15 | 0.16 | 5.56E-01 | 0.26 | 0.19 | 3.23E-01 | 0.12 | 0.19 | 7.48E-01 | 0.13 | 0.30 | 9.30E-01 | 0.20 | 0.32 | 8.38E-01 |
| Total lipids in large LDL | -0.13 | 0.14 | 4.79E-01 | -0.12 | 0.14 | 5.59E-01 | -0.23 | 0.16 | 3.10E-01 | -0.22 | 0.17 | 4.85E-01 | 0.07 | 0.26 | 9.40E-01 | 0.07 | 0.26 | 9.33E-01 |
| Total cholesterol | -0.12 | 0.14 | 5.28E-01 | -0.12 | 0.15 | 5.59E-01 | -0.21 | 0.17 | 3.59E-01 | -0.22 | 0.17 | 4.87E-01 | 0.06 | 0.27 | 9.53E-01 | 0.07 | 0.28 | 9.33E-01 |
| Phospholipids in IDL | -0.12 | 0.15 | 5.36E-01 | -0.13 | 0.15 | 5.59E-01 | -0.19 | 0.17 | 4.02E-01 | -0.22 | 0.18 | 5.10E-01 | 0.02 | 0.27 | 9.71E-01 | 0.03 | 0.28 | 9.70E-01 |
| Cholesterol in IDL | -0.12 | 0.15 | 5.42E-01 | -0.13 | 0.15 | 5.59E-01 | -0.18 | 0.17 | 4.12E-01 | -0.22 | 0.18 | 5.13E-01 | 0.01 | 0.28 | 9.80E-01 | 0.02 | 0.29 | 9.76E-01 |
| Cholesterol to total lipids ratio in chylomicrons and extremely large VLDL | 0.16 | 0.13 | 3.46E-01 | 0.11 | 0.13 | 5.59E-01 | 0.26 | 0.15 | 2.38E-01 | 0.17 | 0.15 | 5.31E-01 | -0.09 | 0.25 | 9.30E-01 | -0.05 | 0.25 | 9.36E-01 |
| Cholesteryl esters in medium VLDL | -0.13 | 0.14 | 4.84E-01 | -0.12 | 0.14 | 5.59E-01 | -0.19 | 0.16 | 3.92E-01 | -0.17 | 0.17 | 5.44E-01 | -0.03 | 0.26 | 9.59E-01 | -0.02 | 0.26 | 9.71E-01 |
| Phosphoglycerides | -0.13 | 0.15 | 5.28E-01 | -0.13 | 0.15 | 5.59E-01 | -0.16 | 0.18 | 4.69E-01 | -0.19 | 0.18 | 5.44E-01 | -0.06 | 0.27 | 9.53E-01 | -0.05 | 0.29 | 9.53E-01 |
| Cholesteryl esters to total lipids ratio in medium LDL | -0.17 | 0.13 | 3.39E-01 | -0.12 | 0.14 | 5.59E-01 | -0.18 | 0.16 | 4.02E-01 | -0.09 | 0.17 | 7.66E-01 | -0.19 | 0.25 | 8.70E-01 | -0.25 | 0.26 | 7.11E-01 |
| Cholesteryl esters in chylomicrons and extremely large VLDL | -0.16 | 0.12 | 3.33E-01 | -0.11 | 0.13 | 5.59E-01 | -0.17 | 0.15 | 3.78E-01 | -0.08 | 0.16 | 7.75E-01 | -0.15 | 0.24 | 8.76E-01 | -0.22 | 0.25 | 7.11E-01 |
| Cholesterol in very large HDL | 0.20 | 0.14 | 3.05E-01 | 0.13 | 0.15 | 5.59E-01 | 0.22 | 0.17 | 3.39E-01 | 0.08 | 0.18 | 8.08E-01 | 0.18 | 0.27 | 8.76E-01 | 0.26 | 0.29 | 7.11E-01 |
| Cholesterol to total lipids ratio in large LDL | 0.15 | 0.15 | 4.36E-01 | 0.12 | 0.15 | 5.59E-01 | 0.02 | 0.17 | 9.39E-01 | -0.02 | 0.17 | 9.48E-01 | 0.48 | 0.29 | 8.70E-01 | 0.50 | 0.30 | 7.11E-01 |
| Cholesteryl esters in large LDL | -0.12 | 0.14 | 4.91E-01 | -0.11 | 0.14 | 5.62E-01 | -0.23 | 0.16 | 3.05E-01 | -0.22 | 0.16 | 4.85E-01 | 0.09 | 0.26 | 9.30E-01 | 0.09 | 0.26 | 9.18E-01 |
| Free cholesterol in medium LDL | -0.12 | 0.13 | 4.84E-01 | -0.11 | 0.13 | 5.62E-01 | -0.22 | 0.16 | 3.07E-01 | -0.20 | 0.16 | 4.87E-01 | 0.08 | 0.26 | 9.30E-01 | 0.08 | 0.26 | 9.33E-01 |
| Phospholipids to total lipids ratio in IDL | 0.11 | 0.13 | 5.28E-01 | 0.11 | 0.13 | 5.62E-01 | 0.09 | 0.16 | 6.56E-01 | 0.09 | 0.16 | 7.66E-01 | 0.16 | 0.24 | 8.76E-01 | 0.18 | 0.25 | 7.93E-01 |
| Cholesteryl esters to total lipids ratio in very large HDL | 0.10 | 0.14 | 6.01E-01 | 0.11 | 0.14 | 5.84E-01 | 0.02 | 0.17 | 9.35E-01 | 0.06 | 0.18 | 8.41E-01 | 0.24 | 0.22 | 8.70E-01 | 0.22 | 0.22 | 7.11E-01 |
| Total lipids in very large HDL | 0.19 | 0.14 | 3.37E-01 | 0.12 | 0.15 | 5.89E-01 | 0.22 | 0.17 | 3.47E-01 | 0.08 | 0.18 | 8.08E-01 | 0.13 | 0.27 | 9.30E-01 | 0.20 | 0.29 | 7.97E-01 |
| Cholesterol to total lipids ratio in medium LDL | 0.07 | 0.13 | 7.03E-01 | 0.10 | 0.13 | 5.89E-01 | 0.02 | 0.16 | 9.34E-01 | 0.07 | 0.16 | 8.13E-01 | 0.17 | 0.27 | 8.76E-01 | 0.14 | 0.27 | 8.73E-01 |
| Cholesterol in large LDL | -0.11 | 0.14 | 5.39E-01 | -0.11 | 0.14 | 5.95E-01 | -0.21 | 0.16 | 3.29E-01 | -0.21 | 0.17 | 4.87E-01 | 0.10 | 0.26 | 9.30E-01 | 0.10 | 0.27 | 9.18E-01 |
| Triglycerides to total lipids ratio in very large VLDL | -0.14 | 0.12 | 3.85E-01 | -0.09 | 0.12 | 5.95E-01 | -0.19 | 0.13 | 2.86E-01 | -0.12 | 0.14 | 6.36E-01 | 0.04 | 0.26 | 9.59E-01 | 0.01 | 0.26 | 9.92E-01 |
| Triglycerides to total lipids ratio in very small VLDL | -0.15 | 0.15 | 4.36E-01 | -0.11 | 0.15 | 5.95E-01 | -0.12 | 0.17 | 6.08E-01 | -0.05 | 0.18 | 8.80E-01 | -0.23 | 0.28 | 8.70E-01 | -0.27 | 0.29 | 7.11E-01 |
| Alanine | -0.15 | 0.13 | 4.03E-01 | -0.10 | 0.14 | 5.97E-01 | -0.22 | 0.16 | 3.20E-01 | -0.13 | 0.16 | 6.47E-01 | 0.00 | 0.24 | 9.88E-01 | -0.06 | 0.25 | 9.33E-01 |
| 3-Hydroxybutyrate | -0.09 | 0.13 | 6.28E-01 | -0.10 | 0.13 | 5.97E-01 | -0.06 | 0.16 | 7.90E-01 | -0.08 | 0.16 | 7.75E-01 | -0.15 | 0.23 | 8.76E-01 | -0.14 | 0.24 | 8.58E-01 |
| Phospholipids to total lipids ratio in large VLDL | -0.12 | 0.11 | 3.89E-01 | -0.08 | 0.11 | 5.97E-01 | -0.09 | 0.14 | 6.22E-01 | -0.01 | 0.15 | 9.58E-01 | -0.21 | 0.17 | 8.70E-01 | -0.23 | 0.18 | 7.11E-01 |
| Free cholesterol to total lipids ratio in medium VLDL | 0.15 | 0.15 | 4.36E-01 | 0.11 | 0.15 | 5.98E-01 | 0.23 | 0.18 | 3.39E-01 | 0.15 | 0.18 | 6.41E-01 | -0.03 | 0.26 | 9.59E-01 | 0.00 | 0.27 | 9.99E-01 |
| Cholesterol to total lipids ratio in small VLDL | 0.14 | 0.15 | 4.84E-01 | 0.11 | 0.15 | 6.04E-01 | 0.13 | 0.18 | 5.61E-01 | 0.08 | 0.18 | 8.12E-01 | 0.12 | 0.27 | 9.30E-01 | 0.16 | 0.27 | 8.61E-01 |
| Cholesteryl esters in small HDL | 0.08 | 0.14 | 6.80E-01 | 0.10 | 0.14 | 6.04E-01 | 0.00 | 0.17 | 9.88E-01 | 0.03 | 0.17 | 9.03E-01 | 0.24 | 0.25 | 8.70E-01 | 0.26 | 0.26 | 7.11E-01 |
| Total cholines | -0.09 | 0.15 | 6.41E-01 | -0.11 | 0.15 | 6.24E-01 | -0.14 | 0.18 | 5.46E-01 | -0.17 | 0.18 | 5.77E-01 | -0.01 | 0.28 | 9.78E-01 | 0.01 | 0.29 | 9.92E-01 |
| Free cholesterol in LDL | -0.09 | 0.14 | 6.11E-01 | -0.09 | 0.14 | 6.43E-01 | -0.19 | 0.16 | 3.87E-01 | -0.19 | 0.17 | 5.31E-01 | 0.11 | 0.26 | 9.30E-01 | 0.11 | 0.27 | 9.18E-01 |
| Triglycerides in chylomicrons and extremely large VLDL | -0.12 | 0.12 | 4.35E-01 | -0.08 | 0.12 | 6.43E-01 | -0.21 | 0.13 | 2.42E-01 | -0.15 | 0.14 | 5.44E-01 | 0.12 | 0.26 | 9.30E-01 | 0.09 | 0.26 | 9.19E-01 |
| Total esterified cholesterol | -0.09 | 0.14 | 6.48E-01 | -0.10 | 0.15 | 6.47E-01 | -0.18 | 0.17 | 4.17E-01 | -0.20 | 0.17 | 5.31E-01 | 0.10 | 0.28 | 9.30E-01 | 0.12 | 0.28 | 9.17E-01 |
| Docosahexaenoic acid | -0.10 | 0.14 | 5.86E-01 | -0.09 | 0.14 | 6.47E-01 | -0.11 | 0.17 | 6.08E-01 | -0.10 | 0.18 | 7.66E-01 | -0.07 | 0.24 | 9.34E-01 | -0.06 | 0.24 | 9.33E-01 |
| Free cholesterol to total lipids ratio in small HDL | -0.03 | 0.13 | 8.65E-01 | -0.08 | 0.14 | 6.79E-01 | -0.06 | 0.16 | 7.84E-01 | -0.16 | 0.16 | 5.69E-01 | 0.05 | 0.24 | 9.56E-01 | 0.09 | 0.25 | 9.18E-01 |
| Total concentration of lipoprotein particles | 0.10 | 0.15 | 6.01E-01 | 0.09 | 0.15 | 6.93E-01 | 0.05 | 0.18 | 8.21E-01 | 0.01 | 0.18 | 9.79E-01 | 0.21 | 0.27 | 8.70E-01 | 0.27 | 0.29 | 7.11E-01 |
| Cholesterol to total lipids ratio in IDL | 0.11 | 0.15 | 5.73E-01 | 0.09 | 0.15 | 7.01E-01 | 0.08 | 0.18 | 7.34E-01 | 0.03 | 0.18 | 9.03E-01 | 0.20 | 0.30 | 8.76E-01 | 0.21 | 0.30 | 7.98E-01 |
| Triglycerides in large HDL | -0.06 | 0.15 | 7.55E-01 | -0.08 | 0.15 | 7.21E-01 | -0.02 | 0.18 | 9.35E-01 | -0.06 | 0.18 | 8.21E-01 | -0.15 | 0.25 | 9.02E-01 | -0.13 | 0.26 | 8.73E-01 |
| Ratio of omega-6 fatty acids to omega-3 fatty acids | 0.11 | 0.14 | 5.41E-01 | 0.07 | 0.14 | 7.25E-01 | 0.10 | 0.17 | 6.37E-01 | 0.04 | 0.17 | 8.74E-01 | 0.14 | 0.24 | 9.23E-01 | 0.15 | 0.25 | 8.45E-01 |
| Free cholesterol in large LDL | -0.07 | 0.14 | 7.22E-01 | -0.08 | 0.14 | 7.32E-01 | -0.16 | 0.17 | 4.47E-01 | -0.18 | 0.17 | 5.44E-01 | 0.13 | 0.27 | 9.30E-01 | 0.14 | 0.28 | 8.73E-01 |
| Cholesterol in small HDL | 0.05 | 0.14 | 7.95E-01 | 0.07 | 0.14 | 7.40E-01 | -0.03 | 0.17 | 8.80E-01 | 0.00 | 0.17 | 9.96E-01 | 0.22 | 0.25 | 8.70E-01 | 0.24 | 0.26 | 7.11E-01 |
| Concentration of very large HDL particles | 0.14 | 0.14 | 4.42E-01 | 0.07 | 0.15 | 7.52E-01 | 0.15 | 0.17 | 4.72E-01 | 0.01 | 0.17 | 9.65E-01 | 0.14 | 0.27 | 9.30E-01 | 0.21 | 0.28 | 7.75E-01 |
| Cholesteryl esters to total lipids ratio in small VLDL | 0.08 | 0.14 | 6.63E-01 | 0.07 | 0.15 | 7.55E-01 | 0.10 | 0.17 | 6.56E-01 | 0.07 | 0.18 | 8.21E-01 | 0.04 | 0.25 | 9.59E-01 | 0.06 | 0.26 | 9.33E-01 |
| Triglycerides to total lipids ratio in medium VLDL | -0.11 | 0.14 | 5.35E-01 | -0.07 | 0.14 | 7.64E-01 | -0.18 | 0.16 | 4.12E-01 | -0.09 | 0.17 | 7.68E-01 | 0.04 | 0.26 | 9.59E-01 | 0.01 | 0.27 | 9.92E-01 |
| Cholesteryl esters to total lipids ratio in large VLDL | 0.11 | 0.14 | 5.41E-01 | 0.06 | 0.14 | 7.90E-01 | 0.23 | 0.17 | 3.14E-01 | 0.14 | 0.17 | 6.41E-01 | -0.16 | 0.25 | 8.76E-01 | -0.13 | 0.26 | 8.73E-01 |
| Free cholesterol in IDL | -0.06 | 0.15 | 7.83E-01 | -0.07 | 0.15 | 7.92E-01 | -0.14 | 0.18 | 5.45E-01 | -0.17 | 0.18 | 6.02E-01 | 0.10 | 0.28 | 9.30E-01 | 0.13 | 0.29 | 9.04E-01 |
| Ratio of docosahexaenoic acid to total fatty acids | 0.06 | 0.13 | 7.34E-01 | 0.06 | 0.14 | 7.92E-01 | 0.07 | 0.16 | 7.53E-01 | 0.06 | 0.17 | 8.21E-01 | 0.07 | 0.24 | 9.40E-01 | 0.10 | 0.24 | 9.04E-01 |
| Histidine | 0.03 | 0.13 | 8.45E-01 | 0.05 | 0.13 | 8.02E-01 | 0.09 | 0.16 | 6.56E-01 | 0.14 | 0.16 | 6.41E-01 | -0.09 | 0.23 | 9.30E-01 | -0.09 | 0.24 | 9.18E-01 |
| Cholesteryl esters to total lipids ratio in IDL | -0.03 | 0.14 | 8.48E-01 | -0.05 | 0.14 | 8.02E-01 | -0.04 | 0.16 | 8.51E-01 | -0.07 | 0.17 | 8.08E-01 | -0.03 | 0.25 | 9.59E-01 | -0.04 | 0.25 | 9.62E-01 |
| Phospholipids to total lipids ratio in medium LDL | 0.10 | 0.14 | 6.01E-01 | 0.05 | 0.14 | 8.02E-01 | 0.10 | 0.16 | 6.22E-01 | 0.05 | 0.17 | 8.49E-01 | 0.10 | 0.25 | 9.30E-01 | 0.13 | 0.26 | 8.73E-01 |
| Glutamine | -0.01 | 0.14 | 9.51E-01 | -0.05 | 0.14 | 8.06E-01 | -0.04 | 0.16 | 8.51E-01 | -0.12 | 0.16 | 6.59E-01 | 0.07 | 0.27 | 9.40E-01 | 0.09 | 0.27 | 9.19E-01 |
| Acetoacetate | -0.04 | 0.13 | 8.05E-01 | -0.05 | 0.13 | 8.06E-01 | -0.01 | 0.16 | 9.75E-01 | -0.02 | 0.16 | 9.36E-01 | -0.13 | 0.23 | 9.23E-01 | -0.11 | 0.24 | 8.86E-01 |
| Free cholesterol to total lipids ratio in medium HDL | 0.11 | 0.15 | 5.77E-01 | 0.06 | 0.15 | 8.14E-01 | 0.06 | 0.17 | 8.15E-01 | -0.06 | 0.18 | 8.41E-01 | 0.26 | 0.29 | 8.70E-01 | 0.34 | 0.31 | 7.11E-01 |
| Albumin | 0.06 | 0.13 | 7.35E-01 | 0.05 | 0.13 | 8.24E-01 | -0.01 | 0.16 | 9.53E-01 | -0.02 | 0.16 | 9.36E-01 | 0.23 | 0.24 | 8.70E-01 | 0.24 | 0.25 | 7.11E-01 |
| Leucine | -0.12 | 0.14 | 5.28E-01 | -0.05 | 0.15 | 8.29E-01 | -0.04 | 0.17 | 8.48E-01 | 0.08 | 0.18 | 8.08E-01 | -0.40 | 0.28 | 8.70E-01 | -0.48 | 0.29 | 7.11E-01 |
| Free cholesterol in very large HDL | 0.12 | 0.14 | 5.28E-01 | 0.05 | 0.14 | 8.37E-01 | 0.14 | 0.17 | 5.31E-01 | 0.00 | 0.17 | 9.96E-01 | 0.09 | 0.26 | 9.30E-01 | 0.14 | 0.28 | 8.73E-01 |
| Citrate | 0.05 | 0.14 | 7.72E-01 | 0.04 | 0.14 | 8.40E-01 | -0.05 | 0.17 | 8.40E-01 | -0.07 | 0.17 | 8.14E-01 | 0.27 | 0.25 | 8.70E-01 | 0.28 | 0.25 | 7.11E-01 |
| Triglycerides to total lipids ratio in small VLDL | -0.08 | 0.14 | 6.80E-01 | -0.05 | 0.14 | 8.40E-01 | -0.07 | 0.17 | 7.53E-01 | -0.01 | 0.17 | 9.79E-01 | -0.09 | 0.26 | 9.30E-01 | -0.12 | 0.26 | 9.01E-01 |
| Cholesterol to total lipids ratio in large VLDL | 0.08 | 0.13 | 6.63E-01 | 0.04 | 0.14 | 8.44E-01 | 0.21 | 0.16 | 3.25E-01 | 0.15 | 0.16 | 6.15E-01 | -0.24 | 0.24 | 8.70E-01 | -0.23 | 0.25 | 7.11E-01 |
| Free cholesterol to total lipids ratio in very large VLDL | 0.01 | 0.13 | 9.44E-01 | -0.04 | 0.13 | 8.53E-01 | 0.20 | 0.15 | 3.14E-01 | 0.11 | 0.15 | 6.59E-01 | -0.39 | 0.20 | 8.70E-01 | -0.41 | 0.21 | 7.11E-01 |
| Creatinine | -0.06 | 0.16 | 7.72E-01 | -0.04 | 0.16 | 8.59E-01 | 0.00 | 0.17 | 9.88E-01 | 0.06 | 0.17 | 8.21E-01 | -0.28 | 0.39 | 8.76E-01 | -0.26 | 0.39 | 8.13E-01 |
| Ratio of omega-3 fatty acids to total fatty acids | -0.06 | 0.14 | 7.42E-01 | -0.03 | 0.14 | 8.84E-01 | -0.05 | 0.16 | 8.19E-01 | -0.01 | 0.17 | 9.79E-01 | -0.09 | 0.24 | 9.30E-01 | -0.09 | 0.24 | 9.18E-01 |
| Phospholipids to total lipids ratio in large LDL | 0.01 | 0.13 | 9.48E-01 | 0.03 | 0.13 | 8.90E-01 | 0.14 | 0.15 | 4.69E-01 | 0.18 | 0.16 | 5.34E-01 | -0.31 | 0.25 | 8.70E-01 | -0.31 | 0.25 | 7.11E-01 |
| Cholesterol to total lipids ratio in very large HDL | -0.08 | 0.15 | 7.02E-01 | -0.03 | 0.15 | 8.97E-01 | -0.17 | 0.19 | 4.82E-01 | -0.07 | 0.19 | 8.21E-01 | 0.08 | 0.25 | 9.30E-01 | 0.04 | 0.26 | 9.63E-01 |
| Ratio of saturated fatty acids to total fatty acids | 0.00 | 0.14 | 9.81E-01 | 0.03 | 0.14 | 8.97E-01 | -0.04 | 0.17 | 8.49E-01 | -0.01 | 0.17 | 9.79E-01 | 0.08 | 0.25 | 9.30E-01 | 0.08 | 0.26 | 9.25E-01 |
| Free cholesterol in small HDL | -0.04 | 0.14 | 8.21E-01 | -0.03 | 0.15 | 8.97E-01 | -0.12 | 0.17 | 5.93E-01 | -0.11 | 0.18 | 7.24E-01 | 0.11 | 0.26 | 9.30E-01 | 0.13 | 0.27 | 8.84E-01 |
| Phospholipids to total lipids ratio in medium VLDL | 0.07 | 0.14 | 7.22E-01 | 0.03 | 0.15 | 8.97E-01 | 0.15 | 0.17 | 5.09E-01 | 0.07 | 0.18 | 8.13E-01 | -0.11 | 0.25 | 9.30E-01 | -0.08 | 0.26 | 9.19E-01 |
| Triglycerides to total lipids ratio in large VLDL | 0.02 | 0.13 | 9.08E-01 | 0.03 | 0.13 | 8.99E-01 | -0.11 | 0.15 | 5.75E-01 | -0.09 | 0.15 | 7.48E-01 | 0.35 | 0.23 | 8.70E-01 | 0.35 | 0.24 | 7.11E-01 |
| Free cholesterol to total lipids ratio in large HDL | 0.08 | 0.17 | 7.35E-01 | 0.03 | 0.17 | 8.99E-01 | 0.05 | 0.20 | 8.49E-01 | -0.04 | 0.19 | 8.96E-01 | 0.15 | 0.34 | 9.30E-01 | 0.19 | 0.36 | 8.73E-01 |
| Concentration of small HDL particles | 0.00 | 0.14 | 9.84E-01 | 0.03 | 0.14 | 9.03E-01 | -0.09 | 0.17 | 6.88E-01 | -0.04 | 0.17 | 8.80E-01 | 0.16 | 0.25 | 8.76E-01 | 0.17 | 0.26 | 8.30E-01 |
| Cholesteryl esters to total lipids ratio in large LDL | -0.04 | 0.13 | 8.31E-01 | -0.02 | 0.13 | 9.12E-01 | -0.17 | 0.16 | 4.03E-01 | -0.13 | 0.16 | 6.39E-01 | 0.25 | 0.25 | 8.70E-01 | 0.22 | 0.25 | 7.11E-01 |
| Cholesteryl esters to total lipids ratio in small LDL | -0.07 | 0.14 | 7.22E-01 | -0.02 | 0.14 | 9.19E-01 | -0.14 | 0.16 | 5.23E-01 | -0.06 | 0.17 | 8.21E-01 | 0.07 | 0.25 | 9.40E-01 | 0.05 | 0.26 | 9.36E-01 |
| Free cholesterol to total lipids ratio in chylomicrons and extremely large VLDL | 0.02 | 0.13 | 9.20E-01 | -0.02 | 0.13 | 9.41E-01 | 0.24 | 0.15 | 2.56E-01 | 0.18 | 0.15 | 5.31E-01 | -0.60 | 0.26 | 8.70E-01 | -0.59 | 0.26 | 7.11E-01 |
| Free cholesterol to total lipids ratio in large VLDL | 0.01 | 0.13 | 9.47E-01 | 0.02 | 0.13 | 9.41E-01 | 0.17 | 0.15 | 4.02E-01 | 0.15 | 0.15 | 5.44E-01 | -0.32 | 0.20 | 8.70E-01 | -0.34 | 0.20 | 7.11E-01 |
| Acetone | 0.04 | 0.13 | 8.42E-01 | 0.01 | 0.13 | 9.44E-01 | 0.16 | 0.15 | 4.12E-01 | 0.12 | 0.15 | 6.57E-01 | -0.27 | 0.26 | 8.70E-01 | -0.23 | 0.26 | 7.19E-01 |
| Triglycerides to total lipids ratio in chylomicrons and extremely large VLDL | -0.04 | 0.12 | 7.95E-01 | -0.01 | 0.13 | 9.49E-01 | -0.18 | 0.13 | 3.05E-01 | -0.13 | 0.13 | 5.44E-01 | 0.99 | 0.90 | 8.70E-01 | 0.95 | 0.88 | 7.11E-01 |
| Sphingomyelins | 0.04 | 0.15 | 8.55E-01 | 0.01 | 0.15 | 9.50E-01 | -0.04 | 0.18 | 8.51E-01 | -0.10 | 0.18 | 7.66E-01 | 0.20 | 0.27 | 8.76E-01 | 0.24 | 0.28 | 7.19E-01 |
| Free cholesterol to total lipids ratio in very small VLDL | -0.01 | 0.14 | 9.44E-01 | 0.01 | 0.14 | 9.50E-01 | -0.04 | 0.17 | 8.48E-01 | 0.00 | 0.17 | 9.96E-01 | 0.02 | 0.26 | 9.71E-01 | 0.00 | 0.27 | 9.92E-01 |
| Phospholipids to total lipids ratio in small HDL | 0.02 | 0.14 | 9.12E-01 | 0.01 | 0.14 | 9.56E-01 | 0.10 | 0.16 | 6.22E-01 | 0.07 | 0.16 | 8.13E-01 | -0.15 | 0.24 | 8.76E-01 | -0.14 | 0.25 | 8.58E-01 |
| Average diameter for LDL particles | 0.04 | 0.14 | 8.16E-01 | 0.01 | 0.14 | 9.59E-01 | 0.02 | 0.16 | 9.35E-01 | -0.04 | 0.17 | 8.96E-01 | 0.11 | 0.26 | 9.30E-01 | 0.14 | 0.26 | 8.73E-01 |
| Phospholipids to total lipids ratio in small LDL | 0.04 | 0.14 | 8.14E-01 | -0.01 | 0.14 | 9.76E-01 | 0.11 | 0.17 | 6.08E-01 | 0.03 | 0.17 | 9.20E-01 | -0.09 | 0.25 | 9.30E-01 | -0.07 | 0.26 | 9.33E-01 |
| Phospholipids in small HDL | -0.03 | 0.15 | 8.69E-01 | 0.00 | 0.15 | 9.84E-01 | -0.08 | 0.18 | 7.14E-01 | -0.04 | 0.18 | 8.95E-01 | 0.07 | 0.26 | 9.44E-01 | 0.08 | 0.27 | 9.33E-01 |
| Ratio of linoleic acid to total fatty acids | 0.06 | 0.14 | 7.74E-01 | 0.00 | 0.15 | 9.84E-01 | 0.06 | 0.17 | 7.96E-01 | -0.02 | 0.17 | 9.47E-01 | 0.05 | 0.26 | 9.56E-01 | 0.10 | 0.28 | 9.18E-01 |
| Total lipids in small HDL | -0.03 | 0.14 | 8.55E-01 | 0.00 | 0.15 | 9.89E-01 | -0.10 | 0.17 | 6.45E-01 | -0.05 | 0.18 | 8.50E-01 | 0.09 | 0.26 | 9.30E-01 | 0.10 | 0.27 | 9.18E-01 |

Note: The table shows associations from the logistic regression analysis where plasms metabolomics level from the Nightingale platform was main exposure and prevalent colorectal cancer as the main outcome. Model 1 : adjusted for age, sex, cohort, time differences (ERGO), and lipid lowering medication. Model 2: adjusted for model1, smoking status, BMI, alcohol and education. No adjustment for time differences was made in the analyses of the subcohorts (ERGO-4 and ERGO-5). The table is ranked based on the *FDR* values in model 2 within the E4&E5 cohort, using bold text indicates metabolites are surpassing *FDR* adjusted *P* value threshold (*P*<0.05). ERGO-4 and ERGO-5 refer to the fourth and fifth visits subcohort from the baseline cohort. ERGO-4&5 refers to the combined data from the fourth and fifth visits of the baseline cohort. Abbreviations: SE, standard error; IDL, intermediate-density lipoprotein; LDL, low-density lipoprotein; HDL, high-density lipoprotein; VLDL, very-low-density lipoprotein; BMI, body mass index; FDR, false discovery rate.

**Supplementary Table 8. Serum metabolites associated with prevalent colorectal cancer from the Metabolon platform at baseline.**

| **Metabolite** | **ERGO-4&5** | | | | | | | | **ERGO-4** | | | | **ERGO-5** | | | |
| --- | --- | --- | --- | --- | --- | --- | --- | --- | --- | --- | --- | --- | --- | --- | --- | --- |
|  | **Model 1** | | | | **Model 2** | | | | **Model 1** | | **Model 2** | | **Model 1** | | **Model 2** | |
|  | **Beta±SE** | **I^2^** | **Het.*P-*value** | ***FDR* value** | **Beta±SE** | **I^2^** | **Het.*P-*value** | ***FDR* value** | **Beta±SE** | ***FDR* value** | **Beta±SE** | ***FDR* value** | **Beta±SE** | ***FDR* value** | **Beta±SE** | ***FDR* value** |
| perfluorooctanesulfonate (PFOS) | -0.68±0.13 | 50.88 | 1.54E-01 | **1.24E-04** | -0.66±0.13 | 54.35 | 1.39E-01 | **4.10E-04** | -0.75±0.14 | **5.69E-05** | -0.73±0.14 | **1.62E-04** | -0.18±0.37 | 9.96E-01 | -0.13±0.38 | 9.96E-01 |
| perfluorooctanoate (PFOA) | -0.57±0.12 | 0 | 3.25E-01 | **2.19E-03** | -0.57±0.13 | 0 | 4.47E-01 | **5.35E-03** | -0.62±0.13 | **2.16E-03** | -0.61±0.14 | **6.43E-03** | -0.27±0.33 | 9.96E-01 | -0.3±0.37 | 9.96E-01 |
| 1-stearoyl-2-docosahexaenoyl-GPE (18:0/22:6)* | -0.81±0.19 | 0 | 5.92E-01 | **3.77E-03** | -0.77±0.2 | 0 | 5.36E-01 | **2.57E-02** | -0.76±0.21 | **3.81E-02** | -0.71±0.22 | 1.18E-01 | -1.02±0.43 | 9.96E-01 | -1.03±0.47 | 9.96E-01 |
| hydroxy-CMPF* | -0.66±0.16 | 0 | 8.19E-01 | **5.75E-03** | -0.62±0.16 | 0 | 7.29E-01 | **2.57E-02** | -0.64±0.18 | **3.86E-02** | -0.59±0.18 | 1.18E-01 | -0.73±0.35 | 9.96E-01 | -0.73±0.36 | 9.96E-01 |
| 1-stearoyl-2-docosahexaenoyl-GPC (18:0/22:6) | -0.69±0.17 | 42.61 | 1.87E-01 | **8.22E-03** | -0.68±0.18 | 0 | 3.43E-01 | **2.91E-02** | -0.85±0.21 | **1.50E-02** | -0.79±0.21 | **4.77E-02** | -0.38±0.28 | 9.96E-01 | -0.41±0.33 | 9.96E-01 |
| X - 12816 | -0.57±0.15 | 47.63 | 1.67E-01 | **2.38E-02** | -0.56±0.15 | 37.09 | 2.07E-01 | **4.00E-02** | -0.65±0.16 | **1.50E-02** | -0.63±0.16 | **3.85E-02** | -0.01±0.43 | 9.96E-01 | -0.03±0.44 | 9.96E-01 |
| 3-hydroxy-2-methylpyridine sulfate | -0.69±0.2 | 6.03 | 3.02E-01 | **4.75E-02** | -0.74±0.21 | 32.9 | 2.22E-01 | **4.15E-02** | -0.61±0.21 | 1.70E-01 | -0.65±0.22 | 1.66E-01 | -1.19±0.52 | 9.96E-01 | -1.41±0.58 | 9.96E-01 |
| 4-hydroxycoumarin | -0.53±0.16 | 64.84 | 9.17E-02 | 7.00E-02 | -0.56±0.16 | 65.7 | 8.78E-02 | 7.41E-02 | -0.64±0.17 | **3.81E-02** | -0.67±0.18 | **3.85E-02** | 0.13±0.42 | 9.96E-01 | 0.15±0.45 | 9.96E-01 |
| N-methylhydroxyproline** | -0.55±0.17 | 0 | 7.14E-01 | 9.06E-02 | -0.59±0.18 | 0 | 8.79E-01 | 1.07E-01 | -0.58±0.19 | 1.26E-01 | -0.6±0.2 | 1.42E-01 | -0.41±0.4 | 9.96E-01 | -0.53±0.43 | 9.96E-01 |
| X - 11795 | 0.46±0.15 | 0 | 3.76E-01 | 9.88E-02 | 0.51±0.16 | 0 | 4.98E-01 | 1.15E-01 | 0.51±0.16 | 7.79E-02 | 0.55±0.17 | 1.18E-01 | 0.12±0.41 | 9.96E-01 | 0.22±0.46 | 9.96E-01 |
| 1-palmitoyl-2-docosahexaenoyl-GPC (16:0/22:6) | -0.53±0.15 | 27.35 | 2.41E-01 | **4.98E-02** | -0.53±0.17 | 0 | 3.97E-01 | 1.36E-01 | -0.67±0.19 | 5.77E-02 | -0.62±0.2 | 1.26E-01 | -0.3±0.25 | 9.96E-01 | -0.31±0.3 | 9.96E-01 |
| X - 21442 | -0.45±0.14 | 38.08 | 2.04E-01 | 8.75E-02 | -0.45±0.15 | 35.31 | 2.14E-01 | 1.42E-01 | -0.52±0.15 | 5.77E-02 | -0.51±0.15 | 1.18E-01 | 0.05±0.42 | 9.96E-01 | 0.08±0.45 | 9.96E-01 |
| 1-arachidonoyl-GPE (20:4n6)* | 0.73±0.21 | 0 | 9.37E-01 | **4.98E-02** | 0.67±0.22 | 0 | 8.67E-01 | 1.42E-01 | 0.74±0.23 | 7.35E-02 | 0.68±0.23 | 1.69E-01 | 0.69±0.58 | 9.96E-01 | 0.58±0.56 | 9.96E-01 |
| 3-carboxy-4-methyl-5-propyl-2-furanpropanoate (CMPF) | -0.64±0.17 | 57.02 | 1.27E-01 | **3.63E-02** | -0.56±0.18 | 64.82 | 9.18E-02 | 1.42E-01 | -0.5±0.2 | 2.45E-01 | -0.42±0.2 | 4.11E-01 | -1.17±0.39 | 9.96E-01 | -1.24±0.44 | 9.96E-01 |
| 4-hydroxychlorothalonil | -0.45±0.15 | 67.19 | 8.08E-02 | 9.88E-02 | -0.47±0.16 | 72.81 | 5.51E-02 | 1.57E-01 | -0.54±0.15 | 5.77E-02 | -0.57±0.16 | 9.17E-02 | 0.3±0.45 | 9.96E-01 | 0.41±0.48 | 9.96E-01 |
| N-acetyl-isoputreanine | 0.53±0.17 | 32.34 | 2.24E-01 | 9.88E-02 | 0.51±0.17 | 21.55 | 2.59E-01 | 1.74E-01 | 0.62±0.19 | 6.96E-02 | 0.6±0.19 | 1.26E-01 | 0.06±0.42 | 9.96E-01 | 0.06±0.43 | 9.96E-01 |
| 1-palmitoyl-2-docosahexaenoyl-GPE (16:0/22:6)* | -0.58±0.18 | 0 | 8.51E-01 | 8.43E-02 | -0.55±0.19 | 0 | 7.60E-01 | 1.74E-01 | -0.56±0.21 | 2.29E-01 | -0.52±0.21 | 2.97E-01 | -0.63±0.34 | 9.96E-01 | -0.65±0.39 | 9.96E-01 |
| 3-methyl catechol sulfate (1) | -0.51±0.17 | 17.32 | 2.71E-01 | 9.90E-02 | -0.51±0.17 | 37.21 | 2.07E-01 | 1.74E-01 | -0.42±0.18 | 3.30E-01 | -0.42±0.19 | 3.74E-01 | -0.88±0.38 | 9.96E-01 | -1.02±0.44 | 9.96E-01 |
| 3,5-dichloro-2,6-dihydroxybenzoic acid | -0.42±0.13 | 25.09 | 2.48E-01 | 8.75E-02 | -0.39±0.13 | 23.56 | 2.53E-01 | 1.74E-01 | -0.48±0.14 | 5.77E-02 | -0.45±0.14 | 1.26E-01 | 0±0.39 | 9.96E-01 | 0±0.36 | 9.98E-01 |
| 3-bromo-5-chloro-2,6-dihydroxybenzoic acid* | -0.45±0.14 | 35.48 | 2.13E-01 | 8.75E-02 | -0.41±0.14 | 42.79 | 1.86E-01 | 1.82E-01 | -0.52±0.15 | 5.77E-02 | -0.5±0.16 | 1.26E-01 | 0±0.39 | 9.96E-01 | 0.02±0.36 | 9.96E-01 |
| 4-vinylguaiacol sulfate | -0.57±0.2 | 63.36 | 9.85E-02 | 1.76E-01 | -0.6±0.21 | 73.83 | 5.06E-02 | 1.82E-01 | -0.47±0.21 | 3.30E-01 | -0.49±0.21 | 3.21E-01 | -1.69±0.71 | 9.96E-01 | -2.19±0.84 | 9.96E-01 |
| 2,3-dihydroxypyridine | -0.52±0.18 | 62.64 | 1.02E-01 | 1.75E-01 | -0.53±0.19 | 56.63 | 1.29E-01 | 1.90E-01 | -0.65±0.2 | 7.32E-02 | -0.65±0.2 | 1.26E-01 | 0.15±0.45 | 9.96E-01 | 0.15±0.49 | 9.96E-01 |
| N-methylproline | -0.48±0.18 | 0 | 6.30E-01 | 2.25E-01 | -0.52±0.18 | 0 | 7.15E-01 | 1.90E-01 | -0.52±0.2 | 2.34E-01 | -0.56±0.21 | 2.35E-01 | -0.3±0.41 | 9.96E-01 | -0.39±0.42 | 9.96E-01 |
| 1-arachidonoyl-GPC (20:4n6)* | 0.63±0.21 | 0 | 7.27E-01 | 1.08E-01 | 0.6±0.21 | 0 | 7.68E-01 | 1.99E-01 | 0.61±0.22 | 2.04E-01 | 0.58±0.23 | 2.75E-01 | 0.84±0.63 | 9.96E-01 | 0.77±0.62 | 9.96E-01 |
| erucate (22:1n9) | -0.57±0.22 | 0 | 6.88E-01 | 2.65E-01 | -0.63±0.23 | 0 | 6.90E-01 | 2.37E-01 | -0.62±0.25 | 2.64E-01 | -0.68±0.26 | 2.36E-01 | -0.4±0.48 | 9.96E-01 | -0.45±0.51 | 9.96E-01 |
| 1-oleoyl-2-docosahexaenoyl-GPC (18:1/22:6)* | -0.43±0.17 | 22.02 | 2.57E-01 | 2.67E-01 | -0.48±0.18 | 21.46 | 2.59E-01 | 2.51E-01 | -0.55±0.2 | 2.04E-01 | -0.58±0.2 | 1.69E-01 | -0.11±0.33 | 9.96E-01 | -0.1±0.38 | 9.96E-01 |
| X - 12221 | -0.41±0.16 | 0 | 6.30E-01 | 2.65E-01 | -0.45±0.17 | 0 | 6.93E-01 | 2.55E-01 | -0.45±0.18 | 2.45E-01 | -0.48±0.18 | 2.35E-01 | -0.22±0.43 | 9.96E-01 | -0.28±0.45 | 9.96E-01 |
| cholesterol | -0.47±0.18 | 52.28 | 1.48E-01 | 2.65E-01 | -0.52±0.2 | 55.78 | 1.33E-01 | 2.57E-01 | -0.61±0.21 | 1.45E-01 | -0.65±0.22 | 1.42E-01 | 0.04±0.4 | 9.96E-01 | 0.12±0.47 | 9.96E-01 |
| EDTA | -0.4±0.15 | 0 | 4.13E-01 | 2.31E-01 | -0.4±0.15 | 0 | 4.21E-01 | 2.57E-01 | -0.42±0.15 | 2.04E-01 | -0.42±0.15 | 2.35E-01 | 0.44±1.04 | 9.96E-01 | 0.55±1.19 | 9.96E-01 |
| androstenediol (3beta,17beta) monosulfate (2) | 0.49±0.23 | 0 | 6.92E-01 | 4.03E-01 | 0.67±0.26 | 0 | 7.62E-01 | 2.57E-01 | 0.54±0.26 | 3.92E-01 | 0.7±0.28 | 2.84E-01 | 0.31±0.53 | 9.96E-01 | 0.51±0.59 | 9.96E-01 |
| glycochenodeoxycholate glucuronide (1) | 0.47±0.2 | 0 | 8.89E-01 | 3.48E-01 | 0.53±0.2 | 0 | 8.77E-01 | 2.57E-01 | 0.48±0.22 | 3.69E-01 | 0.55±0.23 | 2.97E-01 | 0.41±0.45 | 9.96E-01 | 0.47±0.46 | 9.96E-01 |
| androstenediol (3beta,17beta) disulfate (1) | 0.38±0.21 | 0 | 7.36E-01 | 5.85E-01 | 0.59±0.23 | 0 | 8.70E-01 | 2.57E-01 | 0.41±0.23 | 5.53E-01 | 0.61±0.25 | 2.97E-01 | 0.24±0.46 | 9.96E-01 | 0.51±0.53 | 9.96E-01 |
| 3-hydroxypyridine sulfate | -0.43±0.17 | 0 | 9.30E-01 | 2.65E-01 | -0.45±0.17 | 0 | 8.28E-01 | 2.57E-01 | -0.44±0.19 | 3.01E-01 | -0.44±0.19 | 3.21E-01 | -0.4±0.36 | 9.96E-01 | -0.54±0.42 | 9.96E-01 |
| X - 21441 | 0.48±0.2 | 0 | 9.77E-01 | 3.32E-01 | 0.54±0.21 | 0 | 8.38E-01 | 2.59E-01 | 0.48±0.23 | 3.84E-01 | 0.52±0.23 | 3.37E-01 | 0.49±0.48 | 9.96E-01 | 0.63±0.5 | 9.96E-01 |
| imidazole propionate | 0.43±0.16 | 0 | 8.74E-01 | 2.25E-01 | 0.4±0.16 | 0 | 7.65E-01 | 2.99E-01 | 0.44±0.17 | 2.45E-01 | 0.42±0.17 | 2.90E-01 | 0.37±0.39 | 9.96E-01 | 0.29±0.41 | 9.96E-01 |
| X - 18901 | -0.48±0.19 | 0 | 4.15E-01 | 2.90E-01 | -0.5±0.2 | 0 | 4.04E-01 | 3.06E-01 | -0.56±0.22 | 2.45E-01 | -0.57±0.22 | 2.35E-01 | -0.16±0.44 | 9.96E-01 | -0.15±0.46 | 9.96E-01 |
| X - 12111 | -0.45±0.19 | 0 | 7.61E-01 | 3.22E-01 | -0.48±0.19 | 0 | 8.95E-01 | 3.12E-01 | -0.48±0.21 | 3.30E-01 | -0.49±0.21 | 3.21E-01 | -0.34±0.42 | 9.96E-01 | -0.43±0.43 | 9.96E-01 |
| citraconate/glutaconate | -0.5±0.19 | 0 | 4.65E-01 | 2.65E-01 | -0.48±0.19 | 0 | 4.36E-01 | 3.15E-01 | -0.43±0.21 | 4.40E-01 | -0.41±0.21 | 5.01E-01 | -0.79±0.44 | 9.96E-01 | -0.81±0.46 | 9.96E-01 |
| N-acetylglycine | 0.42±0.16 | 65.62 | 8.81E-02 | 2.65E-01 | 0.43±0.18 | 53.61 | 1.42E-01 | 3.49E-01 | 0.55±0.18 | 1.20E-01 | 0.55±0.2 | 2.01E-01 | -0.19±0.39 | 9.96E-01 | -0.14±0.43 | 9.96E-01 |
| sphinganine | 0.58±0.24 | 0 | 4.82E-01 | 3.31E-01 | 0.62±0.25 | 0 | 4.41E-01 | 3.49E-01 | 0.69±0.29 | 2.94E-01 | 0.74±0.3 | 2.90E-01 | 0.31±0.45 | 9.96E-01 | 0.32±0.46 | 9.96E-01 |
| 3-hydroxystachydrine* | -0.38±0.16 | 0 | 7.33E-01 | 3.17E-01 | -0.4±0.16 | 0 | 8.18E-01 | 3.49E-01 | -0.41±0.17 | 3.01E-01 | -0.41±0.18 | 3.21E-01 | -0.26±0.39 | 9.96E-01 | -0.32±0.39 | 9.96E-01 |
| hippurate | -0.36±0.17 | 0 | 4.74E-01 | 4.11E-01 | -0.43±0.18 | 0 | 4.87E-01 | 3.57E-01 | -0.42±0.19 | 3.50E-01 | -0.48±0.19 | 2.84E-01 | -0.08±0.43 | 9.96E-01 | -0.14±0.45 | 9.96E-01 |
| docosahexaenoate (DHA; 22:6n3) | -0.46±0.18 | 0 | 8.26E-01 | 2.75E-01 | -0.46±0.19 | 0 | 9.42E-01 | 3.57E-01 | -0.48±0.21 | 3.30E-01 | -0.46±0.22 | 3.97E-01 | -0.39±0.37 | 9.96E-01 | -0.43±0.4 | 9.96E-01 |
| campesterol | -0.31±0.18 | 67.12 | 8.12E-02 | 6.22E-01 | -0.43±0.19 | 73.69 | 5.12E-02 | 3.70E-01 | -0.43±0.2 | 3.61E-01 | -0.58±0.2 | 1.92E-01 | 0.53±0.52 | 9.96E-01 | 0.51±0.52 | 9.96E-01 |
| gamma-glutamylglutamate | -0.53±0.2 | 63.2 | 9.93E-02 | 2.25E-01 | -0.48±0.21 | 52.56 | 1.47E-01 | 3.70E-01 | -0.69±0.22 | 9.13E-02 | -0.63±0.23 | 2.35E-01 | 0.12±0.44 | 9.96E-01 | 0.14±0.48 | 9.96E-01 |
| 11-ketoetiocholanolone glucuronide | -0.34±0.15 | 54 | 1.40E-01 | 3.50E-01 | -0.36±0.15 | 39.64 | 1.98E-01 | 3.70E-01 | -0.43±0.16 | 2.29E-01 | -0.42±0.16 | 2.35E-01 | 0.24±0.43 | 9.96E-01 | 0.23±0.48 | 9.96E-01 |
| sphingomyelin (d18:1/21:0, d17:1/22:0, d16:1/23:0)* | -0.44±0.19 | 51.83 | 1.50E-01 | 3.51E-01 | -0.46±0.2 | 54.38 | 1.39E-01 | 3.70E-01 | -0.54±0.21 | 2.34E-01 | -0.56±0.21 | 2.35E-01 | 0.32±0.57 | 9.96E-01 | 0.45±0.65 | 9.96E-01 |
| sphingomyelin (d18:1/22:1, d18:2/22:0, d16:1/24:1)* | -0.43±0.2 | 39.13 | 2.00E-01 | 3.79E-01 | -0.46±0.2 | 40.57 | 1.95E-01 | 3.70E-01 | -0.57±0.22 | 2.45E-01 | -0.59±0.22 | 2.35E-01 | 0.03±0.41 | 9.96E-01 | 0.04±0.43 | 9.96E-01 |
| 2-hydroxy-3-methylvalerate | 0.34±0.18 | 36.96 | 2.08E-01 | 5.73E-01 | 0.43±0.18 | 48.27 | 1.64E-01 | 3.70E-01 | 0.45±0.2 | 3.50E-01 | 0.54±0.2 | 2.35E-01 | -0.14±0.43 | 9.96E-01 | -0.14±0.45 | 9.96E-01 |
| tricosanoyl sphingomyelin (d18:1/23:0)* | -0.39±0.18 | 31.97 | 2.25E-01 | 3.82E-01 | -0.43±0.19 | 41.01 | 1.93E-01 | 3.70E-01 | -0.5±0.2 | 2.63E-01 | -0.54±0.21 | 2.36E-01 | 0.05±0.4 | 9.96E-01 | 0.13±0.47 | 9.96E-01 |
| sphingomyelin (d18:2/16:0, d18:1/16:1)* | -0.45±0.19 | 48.27 | 1.64E-01 | 3.50E-01 | -0.44±0.19 | 51.43 | 1.51E-01 | 3.70E-01 | -0.51±0.2 | 2.45E-01 | -0.52±0.2 | 2.37E-01 | 0.62±0.79 | 9.96E-01 | 0.61±0.76 | 9.96E-01 |
| X - 24418 | -0.33±0.16 | 40.7 | 1.94E-01 | 4.67E-01 | -0.37±0.16 | 40.14 | 1.96E-01 | 3.70E-01 | -0.41±0.17 | 3.01E-01 | -0.45±0.17 | 2.41E-01 | 0.2±0.44 | 9.96E-01 | 0.2±0.47 | 9.96E-01 |
| tetrahydrocortisone glucuronide (5) | -0.36±0.13 | 30.23 | 2.31E-01 | 2.25E-01 | -0.32±0.14 | 0 | 3.82E-01 | 3.70E-01 | -0.4±0.14 | 1.43E-01 | -0.35±0.14 | 2.84E-01 | 0.17±0.46 | 9.96E-01 | 0.09±0.49 | 9.96E-01 |
| butyrylglycine | 0.56±0.22 | 0 | 3.85E-01 | 2.65E-01 | 0.5±0.22 | 0 | 4.03E-01 | 3.70E-01 | 0.67±0.25 | 2.34E-01 | 0.6±0.25 | 2.97E-01 | 0.22±0.45 | 9.96E-01 | 0.17±0.45 | 9.96E-01 |
| stachydrine | -0.34±0.16 | 0 | 7.59E-01 | 4.03E-01 | -0.37±0.16 | 0 | 8.25E-01 | 3.70E-01 | -0.36±0.17 | 4.03E-01 | -0.39±0.18 | 3.80E-01 | -0.23±0.39 | 9.96E-01 | -0.3±0.39 | 9.96E-01 |
| 4-vinylcatechol sulfate | -0.39±0.16 | 0 | 9.16E-01 | 3.32E-01 | -0.39±0.17 | 0 | 9.25E-01 | 3.70E-01 | -0.4±0.18 | 3.61E-01 | -0.4±0.18 | 3.93E-01 | -0.35±0.38 | 9.96E-01 | -0.36±0.41 | 9.96E-01 |
| phosphoethanolamine | 0.55±0.21 | 0 | 7.70E-01 | 2.25E-01 | 0.49±0.21 | 0 | 8.22E-01 | 3.70E-01 | 0.53±0.22 | 2.63E-01 | 0.47±0.22 | 4.11E-01 | 0.73±0.65 | 9.96E-01 | 0.62±0.63 | 9.96E-01 |
| 4-ethylcatechol sulfate | -0.4±0.18 | 0 | 8.74E-01 | 3.82E-01 | -0.42±0.19 | 0 | 8.88E-01 | 3.70E-01 | -0.41±0.2 | 4.40E-01 | -0.43±0.21 | 4.27E-01 | -0.34±0.4 | 9.96E-01 | -0.36±0.43 | 9.96E-01 |
| glycerophosphorylcholine (GPC) | 0.55±0.2 | 0 | 9.70E-01 | 2.25E-01 | 0.47±0.21 | 0 | 8.32E-01 | 3.70E-01 | 0.55±0.21 | 2.45E-01 | 0.46±0.22 | 4.30E-01 | 0.53±0.59 | 9.96E-01 | 0.6±0.65 | 9.96E-01 |
| X - 21470 | 0.38±0.21 | 0 | 9.08E-01 | 5.98E-01 | 0.5±0.22 | 0 | 8.80E-01 | 3.70E-01 | 0.39±0.24 | 6.01E-01 | 0.51±0.25 | 4.42E-01 | 0.33±0.46 | 9.96E-01 | 0.43±0.47 | 9.96E-01 |
| gamma-CEHC | -0.31±0.17 | 0 | 9.52E-01 | 5.74E-01 | -0.39±0.17 | 0 | 6.93E-01 | 3.70E-01 | -0.3±0.2 | 6.15E-01 | -0.35±0.2 | 5.66E-01 | -0.33±0.33 | 9.96E-01 | -0.51±0.35 | 9.96E-01 |
| o-cresol sulfate | -0.49±0.21 | 80.68 | 2.29E-02 | 3.51E-01 | -0.53±0.24 | 82.62 | 1.65E-02 | 3.70E-01 | -0.29±0.23 | 7.24E-01 | -0.32±0.25 | 7.70E-01 | -1.7±0.57 | 9.96E-01 | -2.06±0.68 | 9.96E-01 |
| ribitol | -0.41±0.18 | 45.31 | 1.76E-01 | 3.50E-01 | -0.42±0.18 | 62.47 | 1.03E-01 | 3.70E-01 | -0.28±0.2 | 6.96E-01 | -0.25±0.21 | 8.05E-01 | -0.84±0.36 | 9.96E-01 | -0.95±0.38 | 9.96E-01 |
| glycine | 0.5±0.18 | 0 | 5.59E-01 | 2.25E-01 | 0.43±0.19 | 0 | 6.94E-01 | 3.76E-01 | 0.54±0.2 | 2.04E-01 | 0.46±0.21 | 3.71E-01 | 0.24±0.48 | 9.96E-01 | 0.24±0.5 | 9.96E-01 |
| sphingomyelin (d18:2/21:0, d16:2/23:0)* | -0.48±0.21 | 51.34 | 1.52E-01 | 3.51E-01 | -0.49±0.22 | 51.27 | 1.52E-01 | 3.78E-01 | -0.61±0.23 | 2.29E-01 | -0.62±0.24 | 2.36E-01 | 0.2±0.52 | 9.96E-01 | 0.29±0.59 | 9.96E-01 |
| asparagine | 0.48±0.19 | 0 | 9.75E-01 | 2.65E-01 | 0.42±0.19 | 0 | 9.23E-01 | 4.01E-01 | 0.48±0.2 | 2.64E-01 | 0.42±0.2 | 4.42E-01 | 0.46±0.6 | 9.96E-01 | 0.48±0.61 | 9.96E-01 |
| N-(2-furoyl)glycine | -0.46±0.2 | 0 | 8.68E-01 | 3.77E-01 | -0.46±0.21 | 0 | 8.35E-01 | 4.03E-01 | -0.44±0.22 | 4.49E-01 | -0.44±0.23 | 4.92E-01 | -0.53±0.51 | 9.96E-01 | -0.57±0.58 | 9.96E-01 |
| alpha-hydroxyisovalerate | 0.26±0.17 | 66.64 | 8.34E-02 | 6.22E-01 | 0.38±0.17 | 71.18 | 6.25E-02 | 4.05E-01 | 0.4±0.19 | 3.70E-01 | 0.51±0.19 | 2.35E-01 | -0.36±0.4 | 9.96E-01 | -0.38±0.44 | 9.96E-01 |
| glycocholate | 0.37±0.18 | 82.97 | 1.54E-02 | 4.42E-01 | 0.39±0.18 | 81.51 | 2.00E-02 | 4.13E-01 | 0.57±0.2 | 1.73E-01 | 0.57±0.2 | 1.71E-01 | -0.62±0.45 | 9.96E-01 | -0.59±0.46 | 9.96E-01 |
| glycoursodeoxycholate | 0.4±0.18 | 69.09 | 7.21E-02 | 3.77E-01 | 0.4±0.19 | 66.03 | 8.62E-02 | 4.13E-01 | 0.55±0.2 | 2.04E-01 | 0.55±0.2 | 2.35E-01 | -0.3±0.43 | 9.96E-01 | -0.28±0.44 | 9.96E-01 |
| 2-hydroxybehenate | -0.41±0.19 | 37.92 | 2.04E-01 | 3.79E-01 | -0.41±0.19 | 32.16 | 2.25E-01 | 4.13E-01 | -0.54±0.21 | 2.45E-01 | -0.53±0.22 | 2.90E-01 | 0.03±0.4 | 9.96E-01 | 0.03±0.41 | 9.96E-01 |
| glycosyl-N-(2-hydroxynervonoyl)-sphingosine (d18:1/24:1(2OH))* | -0.4±0.16 | 0 | 5.50E-01 | 2.75E-01 | -0.36±0.17 | 0 | 5.52E-01 | 4.13E-01 | -0.45±0.18 | 2.49E-01 | -0.41±0.19 | 3.76E-01 | -0.2±0.37 | 9.96E-01 | -0.15±0.4 | 9.96E-01 |
| hydroquinone sulfate | -0.38±0.19 | 0 | 6.83E-01 | 4.42E-01 | -0.41±0.19 | 0 | 7.22E-01 | 4.13E-01 | -0.34±0.21 | 5.98E-01 | -0.37±0.21 | 5.66E-01 | -0.53±0.4 | 9.96E-01 | -0.54±0.41 | 9.96E-01 |
| orotidine | -0.43±0.2 | 0 | 5.85E-01 | 3.77E-01 | -0.42±0.2 | 0 | 5.79E-01 | 4.13E-01 | -0.36±0.24 | 6.18E-01 | -0.35±0.24 | 7.00E-01 | -0.59±0.35 | 9.96E-01 | -0.58±0.34 | 9.96E-01 |
| 1-(1-enyl-stearoyl)-GPE (P-18:0)* | 0.47±0.19 | 0 | 4.53E-01 | 2.90E-01 | 0.43±0.2 | 0 | 3.94E-01 | 4.16E-01 | 0.43±0.2 | 3.89E-01 | 0.37±0.21 | 5.66E-01 | 0.9±0.59 | 9.96E-01 | 0.94±0.64 | 9.96E-01 |
| sphingomyelin (d17:2/16:0, d18:2/15:0)* | -0.46±0.21 | 75.15 | 4.49E-02 | 3.77E-01 | -0.46±0.22 | 76.37 | 3.97E-02 | 4.21E-01 | -0.62±0.22 | 2.04E-01 | -0.6±0.23 | 2.35E-01 | 0.59±0.56 | 9.96E-01 | 0.94±0.72 | 9.96E-01 |
| hydroxypalmitoyl sphingomyelin (d18:1/16:0(OH))** | -0.31±0.19 | 0 | 3.39E-01 | 6.22E-01 | -0.42±0.2 | 31.89 | 2.26E-01 | 4.21E-01 | -0.41±0.21 | 4.67E-01 | -0.54±0.22 | 2.97E-01 | 0.01±0.39 | 9.96E-01 | 0.04±0.43 | 9.96E-01 |
| quinate | -0.32±0.15 | 0 | 6.73E-01 | 3.82E-01 | -0.31±0.15 | 0 | 6.68E-01 | 4.21E-01 | -0.34±0.16 | 3.61E-01 | -0.34±0.16 | 4.11E-01 | -0.17±0.39 | 9.96E-01 | -0.16±0.39 | 9.96E-01 |
| X - 12680 | -0.29±0.18 | 0 | 5.55E-01 | 6.22E-01 | -0.38±0.18 | 0 | 7.28E-01 | 4.21E-01 | -0.35±0.2 | 5.74E-01 | -0.42±0.21 | 4.66E-01 | -0.1±0.36 | 9.96E-01 | -0.27±0.38 | 9.96E-01 |
| X - 11372 | -0.3±0.17 | 0 | 6.06E-01 | 5.74E-01 | -0.36±0.17 | 0 | 9.86E-01 | 4.21E-01 | -0.34±0.18 | 5.02E-01 | -0.36±0.19 | 5.16E-01 | -0.12±0.39 | 9.96E-01 | -0.36±0.4 | 9.96E-01 |
| pregnenediol disulfate (C21H34O8S2)* | 0.39±0.22 | 0 | 6.03E-01 | 6.07E-01 | 0.48±0.23 | 0 | 5.20E-01 | 4.21E-01 | 0.34±0.25 | 6.96E-01 | 0.42±0.25 | 6.05E-01 | 0.64±0.52 | 9.96E-01 | 0.81±0.55 | 9.96E-01 |
| 1-(1-enyl-palmitoyl)-GPE (P-16:0)* | 0.41±0.19 | 0 | 5.32E-01 | 3.82E-01 | 0.41±0.2 | 0 | 5.40E-01 | 4.37E-01 | 0.37±0.2 | 5.19E-01 | 0.37±0.21 | 5.65E-01 | 0.76±0.59 | 9.96E-01 | 0.77±0.62 | 9.96E-01 |
| 1-(1-enyl-palmitoyl)-GPC (P-16:0)* | 0.5±0.21 | 0 | 8.73E-01 | 3.29E-01 | 0.45±0.22 | 0 | 6.83E-01 | 4.53E-01 | 0.49±0.22 | 3.61E-01 | 0.42±0.23 | 5.64E-01 | 0.59±0.61 | 9.96E-01 | 0.7±0.64 | 9.96E-01 |
| glycerophosphoethanolamine | 0.38±0.17 | 0 | 5.10E-01 | 3.51E-01 | 0.36±0.18 | 0 | 5.30E-01 | 4.54E-01 | 0.35±0.17 | 4.03E-01 | 0.33±0.18 | 5.63E-01 | 0.84±0.72 | 9.96E-01 | 0.8±0.72 | 9.96E-01 |
| X - 24546 | 0.33±0.21 | 0 | 4.12E-01 | 6.22E-01 | 0.45±0.22 | 0 | 4.46E-01 | 4.54E-01 | 0.26±0.22 | 7.61E-01 | 0.37±0.24 | 6.55E-01 | 0.72±0.53 | 9.96E-01 | 0.8±0.51 | 9.96E-01 |
| citramalate | -0.39±0.17 | 38.9 | 2.01E-01 | 3.58E-01 | -0.37±0.18 | 29.95 | 2.32E-01 | 4.55E-01 | -0.49±0.19 | 2.45E-01 | -0.45±0.19 | 3.17E-01 | 0.12±0.44 | 9.96E-01 | 0.17±0.48 | 9.96E-01 |
| 1,5-anhydroglucitol (1,5-AG) | -0.28±0.15 | 56.73 | 1.28E-01 | 5.85E-01 | -0.32±0.16 | 49.23 | 1.60E-01 | 4.65E-01 | -0.36±0.16 | 3.50E-01 | -0.4±0.17 | 3.03E-01 | 0.36±0.45 | 9.96E-01 | 0.27±0.45 | 9.96E-01 |
| pyrraline | -0.3±0.16 | 23.73 | 2.52E-01 | 5.73E-01 | -0.33±0.17 | 0 | 3.43E-01 | 4.66E-01 | -0.38±0.18 | 3.61E-01 | -0.4±0.18 | 3.71E-01 | 0.15±0.43 | 9.96E-01 | 0.03±0.42 | 9.96E-01 |
| 5-hydroxyindole sulfate | -0.32±0.17 | 29.12 | 2.35E-01 | 5.74E-01 | -0.35±0.18 | 0 | 3.43E-01 | 4.75E-01 | -0.41±0.19 | 3.61E-01 | -0.42±0.19 | 3.71E-01 | 0.13±0.41 | 9.96E-01 | 0.03±0.44 | 9.96E-01 |
| 3-ethylcatechol sulfate (1) | -0.36±0.16 | 79.55 | 2.70E-02 | 3.70E-01 | -0.34±0.17 | 81.85 | 1.89E-02 | 4.75E-01 | -0.18±0.18 | 7.95E-01 | -0.17±0.19 | 8.79E-01 | -1.04±0.35 | 9.96E-01 | -1.26±0.43 | 9.96E-01 |
| isovalerylcarnitine (C5) | -0.44±0.18 | 0 | 3.58E-01 | 3.22E-01 | -0.39±0.19 | 0 | 5.83E-01 | 4.76E-01 | -0.54±0.21 | 2.45E-01 | -0.45±0.23 | 4.66E-01 | -0.15±0.36 | 9.96E-01 | -0.21±0.38 | 9.96E-01 |
| X - 26097 | -0.33±0.18 | 1.96 | 3.13E-01 | 5.74E-01 | -0.36±0.18 | 0 | 3.53E-01 | 4.94E-01 | -0.42±0.2 | 3.85E-01 | -0.45±0.21 | 3.78E-01 | 0.04±0.42 | 9.96E-01 | -0.03±0.4 | 9.96E-01 |
| X - 13695 | -0.35±0.18 | 0 | 6.02E-01 | 5.29E-01 | -0.37±0.19 | 0 | 5.48E-01 | 4.95E-01 | -0.31±0.2 | 6.15E-01 | -0.32±0.2 | 6.28E-01 | -0.57±0.45 | 9.96E-01 | -0.64±0.49 | 9.96E-01 |
| glycerol | 0.36±0.2 | 41.65 | 1.91E-01 | 6.07E-01 | 0.4±0.21 | 40.07 | 1.96E-01 | 5.24E-01 | 0.5±0.23 | 3.61E-01 | 0.54±0.23 | 3.21E-01 | -0.15±0.44 | 9.96E-01 | -0.12±0.45 | 9.96E-01 |
| 16-hydroxypalmitate | 0.36±0.17 | 51.15 | 1.53E-01 | 4.40E-01 | 0.35±0.18 | 43.81 | 1.82E-01 | 5.26E-01 | 0.47±0.19 | 2.63E-01 | 0.45±0.2 | 3.21E-01 | -0.2±0.43 | 9.96E-01 | -0.18±0.44 | 9.96E-01 |
| N1-methylinosine | 0.35±0.19 | 23.28 | 2.54E-01 | 5.74E-01 | 0.37±0.19 | 45.76 | 1.75E-01 | 5.26E-01 | 0.47±0.22 | 3.61E-01 | 0.52±0.22 | 3.13E-01 | -0.04±0.39 | 9.96E-01 | -0.08±0.38 | 9.96E-01 |
| X - 13866 | -0.44±0.2 | 0 | 6.18E-01 | 3.77E-01 | -0.38±0.2 | 0 | 4.62E-01 | 5.26E-01 | -0.39±0.22 | 5.41E-01 | -0.32±0.21 | 6.72E-01 | -0.66±0.48 | 9.96E-01 | -0.72±0.5 | 9.96E-01 |
| glucuronide of piperine metabolite C17H21NO3 (4)* | 0.32±0.22 | 0 | 5.22E-01 | 6.48E-01 | 0.44±0.24 | 0 | 3.52E-01 | 5.39E-01 | 0.4±0.25 | 6.15E-01 | 0.57±0.27 | 4.21E-01 | 0.06±0.47 | 9.96E-01 | 0.05±0.48 | 9.96E-01 |
| gamma-glutamylglycine | 0.43±0.18 | 0 | 4.81E-01 | 3.09E-01 | 0.35±0.19 | 0 | 6.24E-01 | 5.39E-01 | 0.49±0.19 | 2.49E-01 | 0.39±0.2 | 5.01E-01 | 0.15±0.44 | 9.96E-01 | 0.14±0.46 | 9.96E-01 |
| guaiacol sulfate | -0.34±0.19 | 0 | 9.38E-01 | 5.74E-01 | -0.36±0.19 | 0 | 8.39E-01 | 5.39E-01 | -0.33±0.21 | 6.15E-01 | -0.34±0.21 | 6.10E-01 | -0.37±0.41 | 9.96E-01 | -0.44±0.44 | 9.96E-01 |
| X - 24344 | -0.39±0.2 | 35.59 | 2.13E-01 | 4.73E-01 | -0.4±0.21 | 20.76 | 2.61E-01 | 5.39E-01 | -0.31±0.21 | 6.23E-01 | -0.32±0.22 | 6.96E-01 | -1.14±0.63 | 9.96E-01 | -1.1±0.66 | 9.96E-01 |
| X - 21383 | -0.36±0.18 | 0 | 7.35E-01 | 4.67E-01 | -0.35±0.18 | 0 | 3.80E-01 | 5.39E-01 | -0.32±0.2 | 6.15E-01 | -0.27±0.2 | 7.61E-01 | -0.46±0.36 | 9.96E-01 | -0.67±0.41 | 9.96E-01 |
| sphingomyelin (d18:1/19:0, d19:1/18:0)* | -0.41±0.21 | 58.14 | 1.22E-01 | 4.73E-01 | -0.41±0.22 | 66.61 | 8.35E-02 | 5.41E-01 | -0.53±0.22 | 2.84E-01 | -0.54±0.23 | 3.13E-01 | 0.43±0.58 | 9.96E-01 | 0.62±0.63 | 9.96E-01 |
| sphingomyelin (d18:2/23:0, d18:1/23:1, d17:1/24:1)* | -0.34±0.21 | 25.4 | 2.47E-01 | 6.22E-01 | -0.4±0.21 | 41.98 | 1.89E-01 | 5.41E-01 | -0.44±0.23 | 4.63E-01 | -0.49±0.22 | 3.76E-01 | 0.27±0.57 | 9.96E-01 | 0.41±0.65 | 9.96E-01 |
| carotene diol (3) | -0.27±0.16 | 0 | 4.52E-01 | 6.22E-01 | -0.31±0.17 | 0 | 3.91E-01 | 5.41E-01 | -0.33±0.18 | 5.41E-01 | -0.37±0.18 | 4.42E-01 | 0.02±0.42 | 9.96E-01 | 0.05±0.46 | 9.96E-01 |
| X - 11308 | -0.26±0.17 | 0 | 5.57E-01 | 6.22E-01 | -0.32±0.17 | 0 | 7.97E-01 | 5.41E-01 | -0.31±0.19 | 6.08E-01 | -0.34±0.19 | 5.64E-01 | -0.05±0.4 | 9.96E-01 | -0.23±0.4 | 9.96E-01 |
| 2-hydroxynervonate* | -0.24±0.13 | 0 | 9.18E-01 | 5.98E-01 | -0.27±0.15 | 0 | 9.92E-01 | 5.41E-01 | -0.24±0.15 | 5.91E-01 | -0.27±0.16 | 5.68E-01 | -0.21±0.31 | 9.96E-01 | -0.28±0.38 | 9.96E-01 |
| 3-phosphoglycerate | 0.3±0.17 | 0 | 9.74E-01 | 5.95E-01 | 0.31±0.17 | 0 | 9.75E-01 | 5.41E-01 | 0.3±0.18 | 5.95E-01 | 0.32±0.18 | 5.68E-01 | 0.31±0.44 | 9.96E-01 | 0.3±0.43 | 9.96E-01 |
| 1-(1-enyl-palmitoyl)-2-arachidonoyl-GPC (P-16:0/20:4)* | 0.35±0.2 | 0 | 4.31E-01 | 6.22E-01 | 0.39±0.21 | 0 | 4.57E-01 | 5.41E-01 | 0.29±0.22 | 7.07E-01 | 0.33±0.22 | 6.84E-01 | 0.77±0.57 | 9.96E-01 | 0.78±0.56 | 9.96E-01 |
| X - 25419 | -0.43±0.21 | 21.57 | 2.59E-01 | 4.30E-01 | -0.4±0.22 | 41.7 | 1.90E-01 | 5.52E-01 | -0.57±0.24 | 2.94E-01 | -0.57±0.25 | 3.50E-01 | -0.03±0.41 | 9.96E-01 | 0.07±0.42 | 9.96E-01 |
| X - 24811 | -0.31±0.16 | 0 | 8.26E-01 | 5.52E-01 | -0.31±0.17 | 0 | 8.44E-01 | 5.52E-01 | -0.3±0.18 | 5.95E-01 | -0.29±0.18 | 6.10E-01 | -0.4±0.41 | 9.96E-01 | -0.38±0.43 | 9.96E-01 |
| androstenediol (3beta,17beta) monosulfate (1) | 0.33±0.21 | 0 | 6.77E-01 | 6.22E-01 | 0.4±0.22 | 0 | 5.94E-01 | 5.52E-01 | 0.29±0.23 | 7.32E-01 | 0.34±0.24 | 6.86E-01 | 0.51±0.49 | 9.96E-01 | 0.65±0.53 | 9.96E-01 |
| glycoursodeoxycholic acid sulfate (1) | 0.31±0.19 | 46.87 | 1.70E-01 | 6.22E-01 | 0.36±0.2 | 50.24 | 1.56E-01 | 5.88E-01 | 0.44±0.22 | 4.38E-01 | 0.5±0.22 | 3.71E-01 | -0.24±0.44 | 9.96E-01 | -0.25±0.47 | 9.96E-01 |
| glycosyl-N-behenoyl-sphingadienine (d18:2/22:0)* | -0.26±0.19 | 0 | 3.48E-01 | 6.70E-01 | -0.34±0.19 | 17.67 | 2.70E-01 | 5.88E-01 | -0.35±0.21 | 5.88E-01 | -0.43±0.21 | 4.27E-01 | 0.11±0.44 | 9.96E-01 | 0.11±0.45 | 9.96E-01 |
| X - 12844 | -0.29±0.15 | 25.41 | 2.47E-01 | 4.84E-01 | -0.26±0.15 | 0 | 3.85E-01 | 5.88E-01 | -0.35±0.15 | 3.46E-01 | -0.31±0.16 | 4.92E-01 | 0.18±0.43 | 9.96E-01 | 0.11±0.46 | 9.96E-01 |
| cinnamoylglycine | -0.24±0.16 | 0 | 3.92E-01 | 6.22E-01 | -0.28±0.15 | 0 | 4.24E-01 | 5.88E-01 | -0.29±0.17 | 5.80E-01 | -0.32±0.16 | 4.92E-01 | 0.15±0.48 | 9.96E-01 | 0.1±0.49 | 9.96E-01 |
| X - 12216 | -0.27±0.18 | 0 | 6.57E-01 | 6.22E-01 | -0.32±0.18 | 0 | 6.01E-01 | 5.88E-01 | -0.31±0.2 | 6.15E-01 | -0.36±0.2 | 5.63E-01 | -0.11±0.4 | 9.96E-01 | -0.12±0.41 | 9.96E-01 |
| sulfate of piperine metabolite C16H19NO3 (2)* | 0.29±0.21 | 0 | 9.90E-01 | 6.94E-01 | 0.38±0.22 | 0 | 8.49E-01 | 5.88E-01 | 0.29±0.23 | 7.26E-01 | 0.4±0.24 | 5.75E-01 | 0.29±0.52 | 9.96E-01 | 0.29±0.52 | 9.96E-01 |
| piperine | 0.27±0.21 | 0 | 8.56E-01 | 7.10E-01 | 0.38±0.21 | 0 | 9.29E-01 | 5.88E-01 | 0.26±0.23 | 7.70E-01 | 0.39±0.24 | 6.06E-01 | 0.36±0.49 | 9.96E-01 | 0.34±0.49 | 9.96E-01 |
| X - 26106 | -0.37±0.19 | 0 | 9.89E-01 | 4.73E-01 | -0.34±0.19 | 0 | 8.75E-01 | 5.88E-01 | -0.37±0.21 | 5.53E-01 | -0.32±0.21 | 6.55E-01 | -0.37±0.4 | 9.96E-01 | -0.39±0.41 | 9.96E-01 |
| guanidinoacetate | 0.45±0.22 | 0 | 7.58E-01 | 4.50E-01 | 0.39±0.22 | 0 | 5.74E-01 | 5.88E-01 | 0.42±0.23 | 5.41E-01 | 0.34±0.23 | 6.84E-01 | 0.62±0.61 | 9.96E-01 | 0.72±0.63 | 9.96E-01 |
| X - 11880 | -0.26±0.16 | 0 | 8.03E-01 | 6.22E-01 | -0.29±0.17 | 0 | 7.59E-01 | 5.88E-01 | -0.28±0.18 | 6.17E-01 | -0.27±0.19 | 7.05E-01 | -0.18±0.37 | 9.96E-01 | -0.4±0.37 | 9.96E-01 |
| 1-stearoyl-2-linoleoyl-GPE (18:0/18:2)* | -0.31±0.17 | 0 | 9.72E-01 | 6.03E-01 | -0.32±0.18 | 0 | 9.44E-01 | 5.88E-01 | -0.3±0.21 | 6.55E-01 | -0.31±0.22 | 7.11E-01 | -0.32±0.3 | 9.96E-01 | -0.34±0.33 | 9.96E-01 |
| 2-O-methylascorbic acid | -0.33±0.17 | 0 | 3.76E-01 | 4.97E-01 | -0.32±0.18 | 32.69 | 2.23E-01 | 5.88E-01 | -0.24±0.19 | 7.32E-01 | -0.2±0.21 | 8.64E-01 | -0.57±0.32 | 9.96E-01 | -0.7±0.36 | 9.96E-01 |
| homoarginine | -0.39±0.2 | 49.63 | 1.59E-01 | 4.87E-01 | -0.35±0.2 | 40.77 | 1.94E-01 | 6.24E-01 | -0.5±0.21 | 3.01E-01 | -0.45±0.22 | 4.32E-01 | 0.36±0.57 | 9.96E-01 | 0.35±0.58 | 9.96E-01 |
| X - 12544 | -0.34±0.22 | 0 | 7.30E-01 | 6.22E-01 | -0.39±0.23 | 0 | 6.69E-01 | 6.24E-01 | -0.38±0.26 | 6.25E-01 | -0.45±0.26 | 5.72E-01 | -0.21±0.44 | 9.96E-01 | -0.23±0.45 | 9.96E-01 |
| 1-oleoyl-GPI (18:1) | -0.3±0.18 | 0 | 4.95E-01 | 6.22E-01 | -0.32±0.19 | 0 | 4.28E-01 | 6.24E-01 | -0.37±0.21 | 5.53E-01 | -0.39±0.21 | 5.13E-01 | -0.07±0.39 | 9.96E-01 | -0.01±0.44 | 9.98E-01 |
| sulfate of piperine metabolite C16H19NO3 (3)* | 0.28±0.21 | 0 | 7.39E-01 | 7.04E-01 | 0.37±0.21 | 0 | 5.35E-01 | 6.29E-01 | 0.32±0.23 | 6.96E-01 | 0.43±0.24 | 5.64E-01 | 0.14±0.47 | 9.96E-01 | 0.09±0.49 | 9.96E-01 |
| phenol sulfate | -0.32±0.2 | 0 | 6.27E-01 | 6.22E-01 | -0.35±0.2 | 0 | 5.25E-01 | 6.29E-01 | -0.27±0.22 | 7.32E-01 | -0.29±0.22 | 7.70E-01 | -0.52±0.47 | 9.96E-01 | -0.62±0.47 | 9.96E-01 |
| sphingomyelin (d18:1/24:1, d18:2/24:0)* | -0.29±0.19 | 0 | 3.43E-01 | 6.22E-01 | -0.33±0.2 | 7.68 | 2.98E-01 | 6.35E-01 | -0.38±0.21 | 5.41E-01 | -0.42±0.21 | 4.93E-01 | 0.08±0.43 | 9.96E-01 | 0.17±0.53 | 9.96E-01 |
| andro steroid monosulfate C19H28O6S (1)* | 0.31±0.21 | 0 | 5.91E-01 | 6.30E-01 | 0.36±0.21 | 0 | 5.68E-01 | 6.35E-01 | 0.37±0.24 | 6.15E-01 | 0.42±0.24 | 5.64E-01 | 0.11±0.42 | 9.96E-01 | 0.12±0.48 | 9.96E-01 |
| biliverdin | 0.29±0.18 | 0 | 6.46E-01 | 6.22E-01 | 0.32±0.19 | 0 | 6.36E-01 | 6.35E-01 | 0.33±0.2 | 5.98E-01 | 0.36±0.21 | 5.68E-01 | 0.11±0.43 | 9.96E-01 | 0.13±0.44 | 9.96E-01 |
| 16a-hydroxy DHEA 3-sulfate | 0.29±0.2 | 0 | 9.96E-01 | 6.54E-01 | 0.35±0.21 | 0 | 9.70E-01 | 6.35E-01 | 0.29±0.23 | 7.24E-01 | 0.34±0.23 | 6.62E-01 | 0.29±0.45 | 9.96E-01 | 0.36±0.48 | 9.96E-01 |
| 12-HETE | 0.35±0.2 | 0 | 4.73E-01 | 6.22E-01 | 0.35±0.2 | 0 | 5.34E-01 | 6.35E-01 | 0.28±0.22 | 7.26E-01 | 0.29±0.23 | 7.70E-01 | 0.65±0.47 | 9.96E-01 | 0.62±0.48 | 9.96E-01 |
| X - 12407 | -0.3±0.18 | 0 | 6.22E-01 | 6.22E-01 | -0.32±0.19 | 0 | 5.97E-01 | 6.35E-01 | -0.26±0.2 | 7.26E-01 | -0.27±0.21 | 7.70E-01 | -0.48±0.42 | 9.96E-01 | -0.52±0.43 | 9.96E-01 |
| eugenol sulfate | -0.34±0.19 | 38.73 | 2.01E-01 | 5.98E-01 | -0.32±0.19 | 55.47 | 1.34E-01 | 6.35E-01 | -0.24±0.2 | 7.34E-01 | -0.22±0.2 | 8.32E-01 | -0.96±0.52 | 9.96E-01 | -1.08±0.54 | 9.96E-01 |
| trigonelline (N'-methylnicotinate) | -0.26±0.17 | 0 | 4.63E-01 | 6.22E-01 | -0.28±0.17 | 0 | 5.38E-01 | 6.43E-01 | -0.31±0.18 | 5.68E-01 | -0.32±0.18 | 5.64E-01 | 0.02±0.42 | 9.96E-01 | -0.02±0.45 | 9.96E-01 |
| N-stearoyl-sphingadienine (d18:2/18:0)* | -0.38±0.18 | 39.55 | 1.98E-01 | 4.52E-01 | -0.31±0.19 | 38.23 | 2.03E-01 | 6.44E-01 | -0.48±0.2 | 2.91E-01 | -0.42±0.21 | 4.50E-01 | 0.16±0.46 | 9.96E-01 | 0.24±0.48 | 9.96E-01 |
| X - 13507 | -0.26±0.19 | 0 | 9.46E-01 | 7.04E-01 | -0.34±0.2 | 0 | 6.90E-01 | 6.44E-01 | -0.25±0.22 | 7.61E-01 | -0.29±0.23 | 7.70E-01 | -0.28±0.4 | 9.96E-01 | -0.49±0.44 | 9.96E-01 |
| 3-hydroxydecanoylcarnitine | -0.28±0.18 | 0 | 7.45E-01 | 6.22E-01 | -0.31±0.19 | 0 | 6.96E-01 | 6.44E-01 | -0.25±0.21 | 7.52E-01 | -0.26±0.21 | 7.72E-01 | -0.39±0.36 | 9.96E-01 | -0.43±0.37 | 9.96E-01 |
| 1-methyl-5-imidazolelactate | -0.37±0.18 | 0 | 5.42E-01 | 4.47E-01 | -0.3±0.18 | 0 | 4.30E-01 | 6.44E-01 | -0.32±0.21 | 6.16E-01 | -0.23±0.2 | 8.10E-01 | -0.59±0.39 | 9.96E-01 | -0.58±0.4 | 9.96E-01 |
| cis-3,4-methyleneheptanoylcarnitine | -0.26±0.17 | 0 | 4.49E-01 | 6.31E-01 | -0.29±0.18 | 43.74 | 1.82E-01 | 6.44E-01 | -0.18±0.2 | 8.32E-01 | -0.16±0.2 | 8.88E-01 | -0.49±0.35 | 9.96E-01 | -0.72±0.36 | 9.96E-01 |
| isoursodeoxycholate | 0.28±0.17 | 79.37 | 2.77E-02 | 6.22E-01 | 0.27±0.17 | 80.62 | 2.31E-02 | 6.45E-01 | 0.44±0.18 | 2.64E-01 | 0.43±0.18 | 3.13E-01 | -0.53±0.4 | 9.96E-01 | -0.63±0.43 | 9.96E-01 |
| choline phosphate | 0.36±0.19 | 53.39 | 1.43E-01 | 5.73E-01 | 0.31±0.19 | 44.01 | 1.81E-01 | 6.45E-01 | 0.51±0.22 | 3.01E-01 | 0.44±0.22 | 4.51E-01 | -0.17±0.41 | 9.96E-01 | -0.19±0.42 | 9.96E-01 |
| X - 24970 | -0.3±0.19 | 31.25 | 2.28E-01 | 6.22E-01 | -0.3±0.19 | 5.82 | 3.03E-01 | 6.45E-01 | -0.42±0.21 | 4.40E-01 | -0.39±0.21 | 5.13E-01 | 0.15±0.42 | 9.96E-01 | 0.1±0.43 | 9.96E-01 |
| lactosyl-N-palmitoyl-sphingosine (d18:1/16:0) | -0.22±0.21 | 18.38 | 2.68E-01 | 8.14E-01 | -0.36±0.23 | 39.4 | 1.99E-01 | 6.45E-01 | -0.3±0.22 | 6.96E-01 | -0.46±0.24 | 5.13E-01 | 0.45±0.64 | 9.96E-01 | 0.5±0.71 | 9.96E-01 |
| mannose | -0.4±0.19 | 35.45 | 2.13E-01 | 4.03E-01 | -0.31±0.19 | 0 | 3.20E-01 | 6.45E-01 | -0.53±0.21 | 2.63E-01 | -0.42±0.22 | 5.26E-01 | 0.04±0.4 | 9.96E-01 | 0.02±0.39 | 9.96E-01 |
| cys-gly, oxidized | -0.29±0.18 | 0 | 4.31E-01 | 6.22E-01 | -0.29±0.18 | 0 | 4.30E-01 | 6.45E-01 | -0.37±0.2 | 5.41E-01 | -0.37±0.2 | 5.64E-01 | -0.04±0.36 | 9.96E-01 | -0.03±0.38 | 9.96E-01 |
| ursodeoxycholate | 0.31±0.2 | 0 | 4.33E-01 | 6.22E-01 | 0.32±0.2 | 0 | 4.44E-01 | 6.45E-01 | 0.4±0.23 | 5.63E-01 | 0.41±0.23 | 5.64E-01 | 0.03±0.41 | 9.96E-01 | 0.04±0.42 | 9.96E-01 |
| glutarylcarnitine (C5-DC) | -0.33±0.19 | 0 | 4.03E-01 | 6.07E-01 | -0.31±0.2 | 0 | 4.74E-01 | 6.45E-01 | -0.42±0.22 | 4.65E-01 | -0.38±0.22 | 5.66E-01 | -0.05±0.38 | 9.96E-01 | -0.04±0.43 | 9.96E-01 |
| X - 14939 | -0.29±0.17 | 0 | 4.66E-01 | 6.22E-01 | -0.28±0.17 | 0 | 6.55E-01 | 6.45E-01 | -0.34±0.19 | 5.41E-01 | -0.32±0.19 | 5.89E-01 | -0.01±0.42 | 9.96E-01 | -0.12±0.41 | 9.96E-01 |
| X - 21467 | 0.33±0.21 | 0 | 8.64E-01 | 6.22E-01 | 0.36±0.22 | 0 | 6.28E-01 | 6.45E-01 | 0.34±0.24 | 6.55E-01 | 0.42±0.25 | 6.05E-01 | 0.25±0.49 | 9.96E-01 | 0.15±0.48 | 9.96E-01 |
| 4-ethylphenylsulfate | -0.31±0.2 | 0 | 8.88E-01 | 6.22E-01 | -0.33±0.21 | 0 | 7.15E-01 | 6.45E-01 | -0.33±0.22 | 6.37E-01 | -0.37±0.23 | 6.13E-01 | -0.25±0.48 | 9.96E-01 | -0.17±0.49 | 9.96E-01 |
| X - 13844 | -0.23±0.18 | 0 | 5.53E-01 | 7.14E-01 | -0.29±0.18 | 0 | 7.44E-01 | 6.45E-01 | -0.28±0.19 | 6.55E-01 | -0.31±0.19 | 6.18E-01 | -0.01±0.41 | 9.96E-01 | -0.16±0.43 | 9.96E-01 |
| choline | 0.32±0.2 | 0 | 9.41E-01 | 6.22E-01 | 0.32±0.2 | 0 | 7.41E-01 | 6.45E-01 | 0.33±0.21 | 6.15E-01 | 0.35±0.22 | 6.24E-01 | 0.29±0.56 | 9.96E-01 | 0.17±0.49 | 9.96E-01 |
| 1-stearoyl-2-oleoyl-GPE (18:0/18:1) | -0.33±0.19 | 0 | 6.33E-01 | 5.97E-01 | -0.31±0.19 | 0 | 7.35E-01 | 6.45E-01 | -0.39±0.22 | 5.59E-01 | -0.35±0.23 | 6.55E-01 | -0.2±0.34 | 9.96E-01 | -0.2±0.37 | 9.96E-01 |
| 4-allylphenol sulfate | -0.3±0.19 | 0 | 7.19E-01 | 6.22E-01 | -0.3±0.19 | 0 | 6.99E-01 | 6.45E-01 | -0.26±0.21 | 7.25E-01 | -0.26±0.21 | 7.70E-01 | -0.43±0.43 | 9.96E-01 | -0.45±0.44 | 9.96E-01 |
| ascorbic acid 3-sulfate* | -0.26±0.16 | 0 | 8.59E-01 | 6.22E-01 | -0.27±0.17 | 0 | 8.39E-01 | 6.45E-01 | -0.24±0.19 | 7.26E-01 | -0.25±0.19 | 7.70E-01 | -0.3±0.31 | 9.96E-01 | -0.32±0.33 | 9.96E-01 |
| eicosanedioate (C20-DC) | -0.25±0.19 | 0 | 9.70E-01 | 7.10E-01 | -0.31±0.19 | 0 | 8.30E-01 | 6.45E-01 | -0.25±0.21 | 7.53E-01 | -0.28±0.22 | 7.70E-01 | -0.23±0.39 | 9.96E-01 | -0.38±0.4 | 9.96E-01 |
| androstenediol (3beta,17beta) disulfate (2) | 0.27±0.22 | 0 | 4.41E-01 | 7.61E-01 | 0.36±0.23 | 0 | 5.10E-01 | 6.45E-01 | 0.19±0.24 | 8.77E-01 | 0.3±0.25 | 8.04E-01 | 0.65±0.54 | 9.96E-01 | 0.69±0.54 | 9.96E-01 |
| 1-methyl-5-imidazoleacetate | -0.34±0.19 | 0 | 4.08E-01 | 6.17E-01 | -0.32±0.19 | 0 | 3.61E-01 | 6.45E-01 | -0.25±0.22 | 7.70E-01 | -0.23±0.22 | 8.47E-01 | -0.62±0.39 | 9.96E-01 | -0.66±0.42 | 9.96E-01 |
| dehydroepiandrosterone sulfate (DHEA-S) | 0.31±0.21 | 0 | 3.80E-01 | 6.31E-01 | 0.34±0.21 | 0 | 3.22E-01 | 6.45E-01 | 0.23±0.23 | 8.07E-01 | 0.24±0.24 | 8.47E-01 | 0.71±0.5 | 9.96E-01 | 0.8±0.51 | 9.96E-01 |
| X - 12822 | -0.31±0.18 | 0 | 4.94E-01 | 6.03E-01 | -0.3±0.18 | 0 | 3.50E-01 | 6.45E-01 | -0.23±0.21 | 7.77E-01 | -0.18±0.22 | 8.79E-01 | -0.49±0.32 | 9.96E-01 | -0.55±0.32 | 9.96E-01 |
| 5alpha-pregnan-3beta,20alpha-diol disulfate | 0.3±0.2 | 43.59 | 1.83E-01 | 6.22E-01 | 0.33±0.2 | 51.88 | 1.49E-01 | 6.45E-01 | 0.18±0.22 | 8.73E-01 | 0.2±0.22 | 8.79E-01 | 0.88±0.48 | 9.96E-01 | 1±0.51 | 9.96E-01 |
| tyramine O-sulfate | 0.26±0.17 | 0 | 5.61E-01 | 6.30E-01 | 0.29±0.18 | 0 | 4.97E-01 | 6.45E-01 | 0.3±0.19 | 6.15E-01 | 0.34±0.19 | 5.64E-01 | 0.02±0.45 | 9.96E-01 | 0±0.46 | 9.98E-01 |
| X - 12306 | -0.25±0.18 | 0 | 4.48E-01 | 6.69E-01 | -0.28±0.18 | 0 | 4.63E-01 | 6.45E-01 | -0.31±0.2 | 6.15E-01 | -0.34±0.2 | 5.66E-01 | 0.04±0.42 | 9.96E-01 | 0.01±0.44 | 9.98E-01 |
| 3-phenylpropionate (hydrocinnamate) | -0.25±0.16 | 32.71 | 2.23E-01 | 6.22E-01 | -0.25±0.16 | 32.32 | 2.24E-01 | 6.47E-01 | -0.3±0.17 | 5.41E-01 | -0.3±0.17 | 5.63E-01 | 0.44±0.58 | 9.96E-01 | 0.46±0.6 | 9.96E-01 |
| tetrahydrocortisol glucuronide | -0.26±0.13 | 0 | 4.02E-01 | 4.72E-01 | -0.21±0.14 | 0 | 4.95E-01 | 6.47E-01 | -0.3±0.14 | 3.61E-01 | -0.24±0.14 | 5.76E-01 | 0.08±0.43 | 9.96E-01 | 0.08±0.45 | 9.96E-01 |
| heneicosapentaenoate (21:5n3) | -0.35±0.19 | 0 | 8.53E-01 | 5.73E-01 | -0.3±0.19 | 0 | 8.77E-01 | 6.47E-01 | -0.34±0.21 | 6.08E-01 | -0.29±0.21 | 7.56E-01 | -0.43±0.45 | 9.96E-01 | -0.36±0.45 | 9.96E-01 |
| mannitol/sorbitol | -0.38±0.23 | 0 | 4.06E-01 | 6.22E-01 | -0.37±0.24 | 0 | 4.01E-01 | 6.47E-01 | -0.29±0.26 | 7.70E-01 | -0.29±0.26 | 8.10E-01 | -0.8±0.55 | 9.96E-01 | -0.86±0.63 | 9.96E-01 |
| adenosine 5'-monophosphate (AMP) | 0.27±0.18 | 0 | 4.06E-01 | 6.30E-01 | 0.28±0.18 | 0 | 5.09E-01 | 6.47E-01 | 0.2±0.2 | 7.95E-01 | 0.22±0.2 | 8.22E-01 | 0.58±0.41 | 9.96E-01 | 0.53±0.41 | 9.96E-01 |
| 8-methoxykynurenate | -0.3±0.17 | 0 | 4.83E-01 | 6.19E-01 | -0.29±0.18 | 17.5 | 2.71E-01 | 6.47E-01 | -0.24±0.19 | 7.25E-01 | -0.2±0.2 | 8.60E-01 | -0.56±0.41 | 9.96E-01 | -0.74±0.45 | 9.96E-01 |
| X - 18888 | -0.24±0.19 | 40.89 | 1.93E-01 | 7.53E-01 | -0.29±0.19 | 61.67 | 1.06E-01 | 6.47E-01 | -0.12±0.21 | 9.06E-01 | -0.16±0.21 | 8.90E-01 | -0.77±0.45 | 9.96E-01 | -0.97±0.46 | 9.96E-01 |
| 1-linoleoyl-GPC (18:2) | 0.47±0.21 | 0 | 6.68E-01 | 3.79E-01 | 0.35±0.23 | 0 | 8.37E-01 | 6.49E-01 | 0.5±0.23 | 3.61E-01 | 0.37±0.25 | 6.61E-01 | 0.25±0.55 | 9.96E-01 | 0.24±0.58 | 9.96E-01 |
| sphingomyelin (d18:2/18:1)* | -0.28±0.22 | 41.54 | 1.91E-01 | 7.18E-01 | -0.35±0.22 | 50.84 | 1.54E-01 | 6.56E-01 | -0.38±0.23 | 5.95E-01 | -0.46±0.24 | 5.01E-01 | 0.54±0.67 | 9.96E-01 | 0.54±0.66 | 9.96E-01 |
| X - 25371 | -0.26±0.18 | 0 | 8.87E-01 | 6.69E-01 | -0.3±0.19 | 0 | 7.44E-01 | 6.56E-01 | -0.27±0.21 | 7.24E-01 | -0.26±0.22 | 8.10E-01 | -0.21±0.36 | 9.96E-01 | -0.4±0.37 | 9.96E-01 |
| phenylpyruvate | -0.26±0.16 | 37.91 | 2.04E-01 | 6.22E-01 | -0.25±0.16 | 4.36 | 3.07E-01 | 6.58E-01 | -0.34±0.17 | 4.49E-01 | -0.32±0.18 | 5.64E-01 | 0.29±0.47 | 9.96E-01 | 0.2±0.47 | 9.96E-01 |
| 1,3-dimethylurate | -0.3±0.16 | 24.24 | 2.51E-01 | 5.74E-01 | -0.25±0.17 | 18.73 | 2.67E-01 | 6.62E-01 | -0.37±0.17 | 3.84E-01 | -0.32±0.18 | 5.63E-01 | 0.2±0.46 | 9.96E-01 | 0.23±0.46 | 9.96E-01 |
| 17alpha-hydroxypregnanolone glucuronide | -0.33±0.21 | 26.43 | 2.44E-01 | 6.22E-01 | -0.33±0.21 | 24.61 | 2.49E-01 | 6.65E-01 | -0.44±0.23 | 4.86E-01 | -0.43±0.23 | 5.62E-01 | 0.25±0.54 | 9.96E-01 | 0.28±0.57 | 9.96E-01 |
| taurochenodeoxycholate | 0.26±0.19 | 25.87 | 2.45E-01 | 6.74E-01 | 0.3±0.19 | 9.73 | 2.93E-01 | 6.65E-01 | 0.38±0.21 | 5.53E-01 | 0.4±0.22 | 5.63E-01 | -0.15±0.4 | 9.96E-01 | -0.09±0.42 | 9.96E-01 |
| X - 23655 | -0.28±0.18 | 0 | 3.22E-01 | 6.22E-01 | -0.27±0.18 | 0 | 3.40E-01 | 6.65E-01 | -0.36±0.19 | 5.24E-01 | -0.34±0.19 | 5.64E-01 | 0.09±0.41 | 9.96E-01 | 0.12±0.44 | 9.96E-01 |
| tetradecanedioate (C14-DC) | 0.32±0.17 | 0 | 4.39E-01 | 5.74E-01 | 0.26±0.17 | 0 | 4.72E-01 | 6.65E-01 | 0.38±0.19 | 4.44E-01 | 0.32±0.19 | 5.89E-01 | 0.05±0.39 | 9.96E-01 | 0.02±0.38 | 9.96E-01 |
| 9-hydroxystearate | 0.33±0.22 | 0 | 4.82E-01 | 6.22E-01 | 0.33±0.22 | 0 | 5.33E-01 | 6.65E-01 | 0.41±0.24 | 5.80E-01 | 0.4±0.25 | 6.06E-01 | 0.04±0.47 | 9.96E-01 | 0.05±0.5 | 9.96E-01 |
| X - 15461 | 0.26±0.17 | 0 | 6.71E-01 | 6.22E-01 | 0.26±0.17 | 0 | 7.52E-01 | 6.65E-01 | 0.24±0.19 | 7.26E-01 | 0.24±0.18 | 7.61E-01 | 0.45±0.48 | 9.96E-01 | 0.41±0.5 | 9.96E-01 |
| 3-hydroxyhexanoylcarnitine (1) | -0.27±0.19 | 0 | 6.79E-01 | 6.43E-01 | -0.28±0.19 | 0 | 4.18E-01 | 6.65E-01 | -0.23±0.21 | 7.90E-01 | -0.2±0.21 | 8.60E-01 | -0.41±0.38 | 9.96E-01 | -0.58±0.41 | 9.96E-01 |
| lignoceroyl sphingomyelin (d18:1/24:0) | -0.24±0.18 | 0 | 4.97E-01 | 7.04E-01 | -0.28±0.18 | 0 | 3.88E-01 | 6.65E-01 | -0.31±0.21 | 6.32E-01 | -0.37±0.21 | 5.66E-01 | -0.03±0.35 | 9.96E-01 | 0±0.37 | 9.98E-01 |
| undecenoylcarnitine (C11:1) | -0.3±0.17 | 0 | 9.91E-01 | 6.07E-01 | -0.26±0.17 | 0 | 9.10E-01 | 6.74E-01 | -0.3±0.2 | 6.23E-01 | -0.24±0.2 | 7.70E-01 | -0.29±0.33 | 9.96E-01 | -0.29±0.35 | 9.96E-01 |
| dimethylarginine (SDMA + ADMA) | 0.31±0.18 | 0 | 4.98E-01 | 6.19E-01 | 0.27±0.18 | 0 | 4.57E-01 | 6.79E-01 | 0.36±0.2 | 5.19E-01 | 0.34±0.2 | 5.92E-01 | 0.05±0.41 | 9.96E-01 | 0.02±0.38 | 9.96E-01 |
| sulfate of piperine metabolite C18H21NO3 (1)* | 0.22±0.21 | 0 | 4.95E-01 | 8.13E-01 | 0.32±0.22 | 0 | 5.86E-01 | 6.79E-01 | 0.17±0.23 | 8.78E-01 | 0.27±0.24 | 8.09E-01 | 0.6±0.58 | 9.96E-01 | 0.61±0.57 | 9.96E-01 |
| X - 11483 | -0.3±0.17 | 0 | 4.71E-01 | 6.22E-01 | -0.27±0.18 | 0 | 4.79E-01 | 6.79E-01 | -0.23±0.2 | 7.52E-01 | -0.21±0.2 | 8.33E-01 | -0.54±0.38 | 9.96E-01 | -0.54±0.42 | 9.96E-01 |
| sphingomyelin (d18:1/17:0, d17:1/18:0, d19:1/16:0) | -0.29±0.21 | 55.37 | 1.34E-01 | 6.89E-01 | -0.32±0.22 | 68.91 | 7.29E-02 | 6.89E-01 | -0.38±0.22 | 5.68E-01 | -0.44±0.23 | 5.01E-01 | 0.74±0.72 | 9.96E-01 | 0.93±0.73 | 9.96E-01 |
| 11beta-hydroxyetiocholanolone glucuronide* | -0.21±0.16 | 0 | 6.29E-01 | 7.58E-01 | -0.25±0.17 | 0 | 5.94E-01 | 6.89E-01 | -0.24±0.18 | 6.98E-01 | -0.29±0.19 | 6.49E-01 | -0.02±0.42 | 9.96E-01 | -0.03±0.44 | 9.96E-01 |
| glucose | -0.32±0.19 | 0 | 3.73E-01 | 6.21E-01 | -0.28±0.19 | 0 | 5.48E-01 | 6.89E-01 | -0.48±0.26 | 5.17E-01 | -0.39±0.26 | 6.81E-01 | -0.15±0.27 | 9.96E-01 | -0.16±0.27 | 9.96E-01 |
| N-palmitoylglycine | 0.31±0.18 | 0 | 7.02E-01 | 6.22E-01 | 0.27±0.19 | 0 | 7.81E-01 | 6.89E-01 | 0.35±0.21 | 5.80E-01 | 0.3±0.21 | 7.11E-01 | 0.17±0.41 | 9.96E-01 | 0.17±0.41 | 9.96E-01 |
| taurine | 0.37±0.21 | 71.65 | 6.04E-02 | 6.21E-01 | 0.32±0.22 | 67.98 | 7.72E-02 | 6.89E-01 | 0.25±0.22 | 7.70E-01 | 0.2±0.23 | 8.79E-01 | 1.7±0.74 | 9.96E-01 | 1.58±0.74 | 9.96E-01 |
| 3-methyl catechol sulfate (2) | -0.28±0.19 | 63.62 | 9.73E-02 | 6.30E-01 | -0.28±0.19 | 66.49 | 8.41E-02 | 6.89E-01 | -0.17±0.2 | 8.72E-01 | -0.17±0.2 | 8.87E-01 | -1.11±0.54 | 9.96E-01 | -1.22±0.58 | 9.96E-01 |
| deoxycarnitine | 0.38±0.24 | 0 | 9.74E-01 | 6.22E-01 | 0.35±0.24 | 0 | 9.39E-01 | 6.89E-01 | 0.39±0.25 | 6.21E-01 | 0.36±0.26 | 7.43E-01 | 0.36±0.66 | 9.96E-01 | 0.31±0.64 | 9.96E-01 |
| X - 21364 | 0.23±0.21 | 0 | 4.96E-01 | 7.97E-01 | 0.32±0.22 | 0 | 5.56E-01 | 6.90E-01 | 0.17±0.23 | 8.78E-01 | 0.26±0.23 | 8.18E-01 | 0.56±0.52 | 9.96E-01 | 0.62±0.56 | 9.96E-01 |
| 1-linoleoyl-GPE (18:2)* | 0.4±0.19 | 0 | 4.36E-01 | 4.56E-01 | 0.29±0.2 | 0 | 5.51E-01 | 6.91E-01 | 0.47±0.22 | 3.61E-01 | 0.35±0.23 | 6.42E-01 | 0.09±0.44 | 9.96E-01 | 0.06±0.44 | 9.96E-01 |
| (S)-3-hydroxybutyrylcarnitine | -0.27±0.19 | 0 | 8.04E-01 | 6.70E-01 | -0.28±0.19 | 0 | 4.82E-01 | 6.93E-01 | -0.24±0.22 | 7.77E-01 | -0.21±0.22 | 8.63E-01 | -0.36±0.4 | 9.96E-01 | -0.54±0.41 | 9.96E-01 |
| X - 25172 | 0.28±0.18 | 0 | 4.28E-01 | 6.22E-01 | 0.27±0.19 | 6.04 | 3.02E-01 | 6.93E-01 | 0.23±0.19 | 7.40E-01 | 0.21±0.2 | 8.32E-01 | 0.71±0.57 | 9.96E-01 | 0.89±0.63 | 9.96E-01 |
| ethyl alpha-glucopyranoside | 0.14±0.19 | 75.83 | 4.19E-02 | 8.91E-01 | 0.31±0.22 | 75.92 | 4.16E-02 | 6.99E-01 | 0.33±0.21 | 6.15E-01 | 0.53±0.24 | 3.76E-01 | -0.62±0.42 | 9.96E-01 | -0.58±0.49 | 9.96E-01 |
| sphingomyelin (d18:1/20:1, d18:2/20:0)* | -0.26±0.2 | 39.42 | 1.99E-01 | 7.04E-01 | -0.28±0.2 | 47.67 | 1.67E-01 | 6.99E-01 | -0.36±0.21 | 5.80E-01 | -0.38±0.21 | 5.64E-01 | 0.43±0.58 | 9.96E-01 | 0.46±0.57 | 9.96E-01 |
| arachidate (20:0) | -0.19±0.19 | 9.44 | 2.93E-01 | 8.24E-01 | -0.29±0.2 | 18.72 | 2.67E-01 | 6.99E-01 | -0.3±0.22 | 6.96E-01 | -0.4±0.22 | 5.64E-01 | 0.19±0.41 | 9.96E-01 | 0.15±0.44 | 9.96E-01 |
| N-acetylglucosaminylasparagine | -0.28±0.18 | 0 | 9.61E-01 | 6.22E-01 | -0.26±0.18 | 0 | 8.97E-01 | 6.99E-01 | -0.27±0.21 | 7.21E-01 | -0.25±0.21 | 8.09E-01 | -0.29±0.34 | 9.96E-01 | -0.3±0.37 | 9.96E-01 |
| 1-linoleoyl-GPI (18:2)* | -0.22±0.18 | 0 | 7.04E-01 | 7.58E-01 | -0.27±0.19 | 0 | 7.55E-01 | 6.99E-01 | -0.18±0.21 | 8.70E-01 | -0.24±0.22 | 8.27E-01 | -0.33±0.34 | 9.96E-01 | -0.37±0.38 | 9.96E-01 |
| sulfate of piperine metabolite C18H21NO3 (3)* | 0.21±0.2 | 0 | 6.45E-01 | 8.24E-01 | 0.3±0.21 | 0 | 7.70E-01 | 7.15E-01 | 0.17±0.22 | 8.78E-01 | 0.27±0.23 | 8.06E-01 | 0.43±0.52 | 9.96E-01 | 0.43±0.52 | 9.96E-01 |
| sphingomyelin (d18:1/14:0, d16:1/16:0)* | -0.3±0.2 | 23.7 | 2.52E-01 | 6.45E-01 | -0.29±0.21 | 33.88 | 2.19E-01 | 7.15E-01 | -0.39±0.22 | 5.59E-01 | -0.37±0.22 | 5.68E-01 | 0.32±0.58 | 9.96E-01 | 0.51±0.69 | 9.96E-01 |
| X - 23782 | 0.25±0.18 | 0 | 6.94E-01 | 7.04E-01 | 0.26±0.19 | 0 | 7.23E-01 | 7.17E-01 | 0.28±0.2 | 6.93E-01 | 0.29±0.21 | 7.18E-01 | 0.1±0.43 | 9.96E-01 | 0.13±0.42 | 9.96E-01 |
| X - 11299 | -0.29±0.17 | 0 | 6.97E-01 | 6.22E-01 | -0.24±0.17 | 0 | 8.29E-01 | 7.17E-01 | -0.25±0.19 | 7.12E-01 | -0.23±0.19 | 8.04E-01 | -0.42±0.38 | 9.96E-01 | -0.33±0.42 | 9.96E-01 |
| 3-methylhistidine | -0.31±0.2 | 0 | 9.46E-01 | 6.22E-01 | -0.27±0.19 | 0 | 8.72E-01 | 7.20E-01 | -0.3±0.22 | 6.96E-01 | -0.26±0.21 | 8.04E-01 | -0.33±0.43 | 9.96E-01 | -0.33±0.44 | 9.96E-01 |
| myo-inositol | 0.26±0.18 | 0 | 3.50E-01 | 6.63E-01 | 0.25±0.18 | 0 | 3.62E-01 | 7.20E-01 | 0.35±0.21 | 5.80E-01 | 0.34±0.21 | 6.04E-01 | -0.03±0.36 | 9.96E-01 | -0.06±0.38 | 9.96E-01 |
| (N(1) + N(8))-acetylspermidine | 0.27±0.16 | 0 | 3.72E-01 | 6.22E-01 | 0.23±0.16 | 0 | 3.87E-01 | 7.20E-01 | 0.34±0.18 | 5.09E-01 | 0.29±0.18 | 6.09E-01 | -0.03±0.38 | 9.96E-01 | -0.07±0.38 | 9.96E-01 |
| arachidonoylcarnitine (C20:4) | 0.32±0.22 | 0 | 4.29E-01 | 6.31E-01 | 0.31±0.22 | 0 | 4.20E-01 | 7.20E-01 | 0.42±0.25 | 5.80E-01 | 0.42±0.26 | 6.20E-01 | 0.03±0.43 | 9.96E-01 | 0.02±0.42 | 9.96E-01 |
| glyco-beta-muricholate** | 0.31±0.19 | 0 | 5.15E-01 | 6.22E-01 | 0.27±0.19 | 0 | 5.32E-01 | 7.20E-01 | 0.37±0.22 | 5.80E-01 | 0.34±0.22 | 6.55E-01 | 0.07±0.41 | 9.96E-01 | 0.04±0.42 | 9.96E-01 |
| N-acetylmethionine | -0.2±0.15 | 0 | 5.13E-01 | 7.07E-01 | -0.21±0.15 | 0 | 6.09E-01 | 7.20E-01 | -0.25±0.17 | 6.37E-01 | -0.24±0.17 | 6.81E-01 | 0.03±0.39 | 9.96E-01 | -0.02±0.41 | 9.96E-01 |
| docosapentaenoate (n6 DPA; 22:5n6) | 0.29±0.21 | 0 | 7.96E-01 | 7.04E-01 | 0.3±0.22 | 0 | 8.42E-01 | 7.20E-01 | 0.27±0.23 | 7.53E-01 | 0.28±0.23 | 7.78E-01 | 0.43±0.59 | 9.96E-01 | 0.41±0.6 | 9.96E-01 |
| X - 24307 | -0.25±0.18 | 0 | 7.67E-01 | 6.84E-01 | -0.24±0.18 | 0 | 8.05E-01 | 7.20E-01 | -0.21±0.21 | 7.95E-01 | -0.22±0.21 | 8.33E-01 | -0.33±0.34 | 9.96E-01 | -0.32±0.35 | 9.96E-01 |
| X - 15486 | -0.21±0.17 | 0 | 4.73E-01 | 7.61E-01 | -0.24±0.17 | 45.31 | 1.76E-01 | 7.20E-01 | -0.15±0.2 | 8.78E-01 | -0.13±0.19 | 9.26E-01 | -0.46±0.38 | 9.96E-01 | -0.73±0.4 | 9.96E-01 |
| N-acetylglucosamine/N-acetylgalactosamine | 0.23±0.18 | 0 | 6.45E-01 | 7.59E-01 | 0.26±0.19 | 0 | 5.05E-01 | 7.20E-01 | 0.27±0.21 | 7.07E-01 | 0.33±0.21 | 6.53E-01 | 0.05±0.42 | 9.96E-01 | 0.01±0.42 | 9.98E-01 |
| glycolithocholate | 0.29±0.2 | 40.3 | 1.96E-01 | 6.31E-01 | 0.27±0.2 | 36.12 | 2.11E-01 | 7.21E-01 | 0.43±0.23 | 4.76E-01 | 0.4±0.22 | 5.64E-01 | -0.17±0.41 | 9.96E-01 | -0.2±0.42 | 9.96E-01 |
| myristoleate (14:1n5) | 0.28±0.21 | 0 | 8.55E-01 | 7.04E-01 | 0.29±0.21 | 0 | 9.70E-01 | 7.21E-01 | 0.3±0.23 | 7.24E-01 | 0.29±0.23 | 7.70E-01 | 0.2±0.49 | 9.96E-01 | 0.27±0.5 | 9.96E-01 |
| sphingosine | 0.23±0.19 | 0 | 6.24E-01 | 7.61E-01 | 0.26±0.19 | 0 | 7.20E-01 | 7.21E-01 | 0.19±0.21 | 8.44E-01 | 0.23±0.21 | 8.22E-01 | 0.44±0.48 | 9.96E-01 | 0.42±0.48 | 9.96E-01 |
| erythronate* | -0.22±0.17 | 0 | 9.65E-01 | 7.10E-01 | -0.24±0.17 | 0 | 9.63E-01 | 7.21E-01 | -0.23±0.23 | 7.95E-01 | -0.23±0.23 | 8.60E-01 | -0.21±0.25 | 9.96E-01 | -0.25±0.26 | 9.96E-01 |
| 2-oxoarginine* | -0.25±0.18 | 0 | 7.09E-01 | 6.86E-01 | -0.26±0.19 | 0 | 4.86E-01 | 7.21E-01 | -0.21±0.21 | 7.95E-01 | -0.19±0.22 | 8.79E-01 | -0.37±0.36 | 9.96E-01 | -0.5±0.39 | 9.96E-01 |
| decanoylcarnitine (C10) | -0.26±0.18 | 9.86 | 2.92E-01 | 6.43E-01 | -0.25±0.18 | 7.18 | 2.99E-01 | 7.21E-01 | -0.15±0.2 | 8.78E-01 | -0.14±0.21 | 9.20E-01 | -0.58±0.36 | 9.96E-01 | -0.58±0.37 | 9.96E-01 |
| 3,4-dihydroxybutyrate | -0.26±0.18 | 0 | 9.30E-01 | 6.67E-01 | -0.26±0.19 | 0 | 8.78E-01 | 7.31E-01 | -0.24±0.24 | 7.95E-01 | -0.23±0.25 | 8.63E-01 | -0.28±0.28 | 9.96E-01 | -0.29±0.3 | 9.96E-01 |
| hexadecanedioate (C16-DC) | 0.26±0.18 | 4.37 | 3.06E-01 | 6.31E-01 | 0.23±0.18 | 0 | 3.31E-01 | 7.37E-01 | 0.35±0.19 | 5.53E-01 | 0.32±0.2 | 6.06E-01 | -0.12±0.41 | 9.96E-01 | -0.1±0.39 | 9.96E-01 |
| behenoyl sphingomyelin (d18:1/22:0)* | -0.26±0.19 | 0 | 3.99E-01 | 6.89E-01 | -0.26±0.19 | 0 | 3.99E-01 | 7.37E-01 | -0.34±0.21 | 6.15E-01 | -0.34±0.22 | 6.35E-01 | 0.04±0.4 | 9.96E-01 | 0.07±0.44 | 9.96E-01 |
| 1-palmitoyl-2-oleoyl-GPE (16:0/18:1) | -0.28±0.17 | 0 | 6.92E-01 | 6.22E-01 | -0.24±0.18 | 0 | 7.09E-01 | 7.37E-01 | -0.33±0.21 | 6.15E-01 | -0.29±0.22 | 7.56E-01 | -0.18±0.32 | 9.96E-01 | -0.14±0.34 | 9.96E-01 |
| sphingomyelin (d18:1/18:1, d18:2/18:0) | -0.34±0.2 | 31.13 | 2.28E-01 | 6.22E-01 | -0.27±0.21 | 32.9 | 2.22E-01 | 7.42E-01 | -0.45±0.22 | 4.30E-01 | -0.38±0.23 | 5.72E-01 | 0.21±0.5 | 9.96E-01 | 0.3±0.51 | 9.96E-01 |
| N-palmitoyl-sphingosine (d18:1/16:0) | -0.25±0.19 | 0 | 4.27E-01 | 7.10E-01 | -0.25±0.19 | 0 | 3.73E-01 | 7.42E-01 | -0.33±0.22 | 6.17E-01 | -0.34±0.22 | 6.35E-01 | 0.02±0.39 | 9.96E-01 | 0.09±0.43 | 9.96E-01 |
| X - 24757 | -0.2±0.18 | 0 | 8.78E-01 | 8.00E-01 | -0.24±0.18 | 0 | 8.26E-01 | 7.42E-01 | -0.21±0.2 | 7.91E-01 | -0.26±0.2 | 7.70E-01 | -0.14±0.41 | 9.96E-01 | -0.16±0.42 | 9.96E-01 |
| 1-palmitoleoyl-GPC (16:1)* | 0.22±0.19 | 0 | 8.93E-01 | 7.79E-01 | 0.27±0.2 | 0 | 9.69E-01 | 7.42E-01 | 0.24±0.21 | 7.77E-01 | 0.26±0.22 | 7.91E-01 | 0.17±0.45 | 9.96E-01 | 0.28±0.5 | 9.96E-01 |
| X - 25790 | -0.17±0.17 | 0 | 9.30E-01 | 8.24E-01 | -0.23±0.17 | 0 | 9.74E-01 | 7.42E-01 | -0.16±0.21 | 8.78E-01 | -0.22±0.21 | 8.27E-01 | -0.2±0.3 | 9.96E-01 | -0.24±0.31 | 9.96E-01 |
| isobutyrylcarnitine (C4) | -0.19±0.17 | 0 | 7.59E-01 | 7.92E-01 | -0.23±0.18 | 0 | 7.16E-01 | 7.42E-01 | -0.16±0.2 | 8.78E-01 | -0.19±0.21 | 8.65E-01 | -0.27±0.31 | 9.96E-01 | -0.34±0.33 | 9.96E-01 |
| 2R,3R-dihydroxybutyrate | -0.25±0.16 | 0 | 3.46E-01 | 6.22E-01 | -0.23±0.17 | 29.8 | 2.33E-01 | 7.42E-01 | -0.13±0.21 | 9.00E-01 | -0.08±0.21 | 9.63E-01 | -0.45±0.27 | 9.96E-01 | -0.52±0.3 | 9.96E-01 |
| serine | 0.33±0.2 | 0 | 4.69E-01 | 6.22E-01 | 0.27±0.2 | 0 | 4.21E-01 | 7.44E-01 | 0.28±0.21 | 7.21E-01 | 0.22±0.22 | 8.60E-01 | 0.77±0.65 | 9.96E-01 | 0.77±0.66 | 9.96E-01 |
| cysteinylglycine disulfide* | -0.23±0.17 | 0 | 5.27E-01 | 6.70E-01 | -0.22±0.17 | 0 | 5.93E-01 | 7.64E-01 | -0.31±0.2 | 6.21E-01 | -0.28±0.2 | 7.43E-01 | -0.08±0.29 | 9.96E-01 | -0.08±0.3 | 9.96E-01 |
| sphingomyelin (d18:2/14:0, d18:1/14:1)* | -0.35±0.23 | 47.23 | 1.69E-01 | 6.22E-01 | -0.3±0.23 | 42.13 | 1.89E-01 | 7.80E-01 | -0.49±0.25 | 4.58E-01 | -0.42±0.25 | 5.89E-01 | 0.35±0.56 | 9.96E-01 | 0.46±0.62 | 9.96E-01 |
| beta-cryptoxanthin | -0.19±0.18 | 9.77 | 2.92E-01 | 8.13E-01 | -0.23±0.18 | 8.64 | 2.95E-01 | 7.80E-01 | -0.27±0.19 | 6.88E-01 | -0.31±0.2 | 6.38E-01 | 0.25±0.46 | 9.96E-01 | 0.23±0.47 | 9.96E-01 |
| 4-acetamidobutanoate | -0.21±0.16 | 0 | 8.28E-01 | 7.09E-01 | -0.2±0.16 | 0 | 9.60E-01 | 7.80E-01 | -0.23±0.18 | 7.26E-01 | -0.21±0.18 | 8.10E-01 | -0.15±0.32 | 9.96E-01 | -0.19±0.34 | 9.96E-01 |
| tetradecadienedioate (C14:2-DC)* | 0.26±0.2 | 0 | 9.93E-01 | 7.04E-01 | 0.25±0.2 | 0 | 9.51E-01 | 7.80E-01 | 0.26±0.22 | 7.33E-01 | 0.25±0.22 | 8.10E-01 | 0.27±0.47 | 9.96E-01 | 0.28±0.48 | 9.96E-01 |
| N-acetyltryptophan | -0.32±0.19 | 0 | 5.44E-01 | 6.22E-01 | -0.25±0.2 | 0 | 3.30E-01 | 7.80E-01 | -0.25±0.22 | 7.61E-01 | -0.15±0.23 | 9.26E-01 | -0.51±0.36 | 9.96E-01 | -0.61±0.42 | 9.96E-01 |
| 2,3-dihydroxy-5-methylthio-4-pentenoate (DMTPA)* | -0.25±0.17 | 0 | 5.39E-01 | 6.67E-01 | -0.23±0.18 | 4.54 | 3.06E-01 | 7.80E-01 | -0.16±0.22 | 8.78E-01 | -0.11±0.22 | 9.41E-01 | -0.38±0.28 | 9.96E-01 | -0.51±0.32 | 9.96E-01 |
| S-methylcysteine | 0.27±0.18 | 67.44 | 7.97E-02 | 6.22E-01 | 0.23±0.18 | 66.32 | 8.48E-02 | 7.80E-01 | 0.11±0.2 | 9.14E-01 | 0.06±0.21 | 9.66E-01 | 0.87±0.38 | 9.96E-01 | 0.82±0.39 | 9.96E-01 |
| S-methylcysteine sulfoxide | 0.27±0.18 | 71.96 | 5.90E-02 | 6.31E-01 | 0.23±0.18 | 71.23 | 6.23E-02 | 7.80E-01 | 0.1±0.2 | 9.14E-01 | 0.06±0.2 | 9.66E-01 | 0.95±0.4 | 9.96E-01 | 0.91±0.41 | 9.96E-01 |
| branched-chain, straight-chain, or cyclopropyl 10:1 fatty acid (1)* | 0.3±0.19 | 0 | 7.29E-01 | 6.22E-01 | 0.25±0.2 | 0 | 8.30E-01 | 7.81E-01 | 0.33±0.21 | 6.15E-01 | 0.27±0.21 | 7.70E-01 | 0.15±0.46 | 9.96E-01 | 0.15±0.51 | 9.96E-01 |
| eicosapentaenoate (EPA; 20:5n3) | -0.28±0.2 | 0 | 8.19E-01 | 6.60E-01 | -0.25±0.19 | 0 | 8.08E-01 | 7.81E-01 | -0.3±0.22 | 6.91E-01 | -0.27±0.22 | 7.70E-01 | -0.19±0.44 | 9.96E-01 | -0.15±0.44 | 9.96E-01 |
| p-cresol glucuronide* | 0.32±0.21 | 0 | 8.96E-01 | 6.22E-01 | 0.26±0.21 | 0 | 8.38E-01 | 7.81E-01 | 0.33±0.24 | 6.67E-01 | 0.28±0.23 | 7.79E-01 | 0.27±0.47 | 9.96E-01 | 0.18±0.47 | 9.96E-01 |
| threonate | -0.17±0.16 | 0 | 8.44E-01 | 8.13E-01 | -0.21±0.17 | 0 | 9.12E-01 | 7.81E-01 | -0.19±0.18 | 7.95E-01 | -0.22±0.19 | 8.05E-01 | -0.11±0.37 | 9.96E-01 | -0.18±0.39 | 9.96E-01 |
| 2S,3R-dihydroxybutyrate | -0.34±0.2 | 0 | 6.92E-01 | 6.22E-01 | -0.26±0.21 | 0 | 6.14E-01 | 7.81E-01 | -0.28±0.25 | 7.67E-01 | -0.19±0.25 | 8.98E-01 | -0.45±0.34 | 9.96E-01 | -0.42±0.38 | 9.96E-01 |
| sphingomyelin (d18:1/20:0, d16:1/22:0)* | -0.27±0.22 | 0 | 3.63E-01 | 7.61E-01 | -0.27±0.22 | 0 | 3.55E-01 | 7.83E-01 | -0.34±0.23 | 6.37E-01 | -0.35±0.23 | 6.70E-01 | 0.22±0.58 | 9.96E-01 | 0.29±0.65 | 9.96E-01 |
| 3-hydroxyisobutyrate | -0.32±0.19 | 0 | 4.02E-01 | 6.22E-01 | -0.24±0.19 | 0 | 5.57E-01 | 7.83E-01 | -0.4±0.21 | 5.12E-01 | -0.3±0.22 | 7.43E-01 | -0.02±0.4 | 9.96E-01 | -0.04±0.39 | 9.96E-01 |
| caprate (10:0) | 0.28±0.18 | 68.43 | 7.51E-02 | 6.22E-01 | 0.23±0.19 | 62.66 | 1.02E-01 | 7.96E-01 | 0.41±0.2 | 3.85E-01 | 0.36±0.2 | 5.64E-01 | -0.5±0.47 | 9.96E-01 | -0.49±0.48 | 9.96E-01 |
| N,N,N-trimethyl-5-aminovalerate | -0.21±0.17 | 0 | 9.50E-01 | 7.59E-01 | -0.21±0.17 | 0 | 9.89E-01 | 7.96E-01 | -0.22±0.2 | 7.95E-01 | -0.21±0.21 | 8.47E-01 | -0.19±0.3 | 9.96E-01 | -0.22±0.32 | 9.96E-01 |
| dodecadienoate (12:2)* | 0.29±0.2 | 42.79 | 1.86E-01 | 6.30E-01 | 0.24±0.19 | 55.18 | 1.35E-01 | 7.97E-01 | 0.43±0.22 | 4.58E-01 | 0.4±0.22 | 5.64E-01 | -0.18±0.41 | 9.96E-01 | -0.3±0.41 | 9.96E-01 |
| X - 24812 | -0.27±0.2 | 0.78 | 3.15E-01 | 6.86E-01 | -0.27±0.22 | 0 | 3.21E-01 | 7.99E-01 | -0.17±0.22 | 8.78E-01 | -0.16±0.25 | 9.26E-01 | -0.65±0.43 | 9.96E-01 | -0.7±0.48 | 9.96E-01 |
| 5alpha-androstan-3beta,17beta-diol disulfate | 0.15±0.24 | 0 | 5.16E-01 | 9.03E-01 | 0.32±0.26 | 0 | 3.85E-01 | 8.00E-01 | 0.07±0.26 | 9.49E-01 | 0.22±0.28 | 8.89E-01 | 0.48±0.56 | 9.96E-01 | 0.84±0.66 | 9.96E-01 |
| X - 07765 | -0.25±0.2 | 0 | 5.53E-01 | 7.59E-01 | -0.24±0.2 | 0 | 4.95E-01 | 8.08E-01 | -0.21±0.21 | 7.95E-01 | -0.2±0.2 | 8.60E-01 | -0.65±0.72 | 9.96E-01 | -0.73±0.74 | 9.96E-01 |
| phenyllactate (PLA) | 0.19±0.19 | 0 | 7.17E-01 | 8.24E-01 | 0.24±0.2 | 0 | 4.81E-01 | 8.10E-01 | 0.22±0.21 | 7.95E-01 | 0.31±0.22 | 7.39E-01 | 0.04±0.44 | 9.96E-01 | -0.05±0.45 | 9.96E-01 |
| 4-vinylphenol sulfate | -0.24±0.19 | 0 | 8.34E-01 | 7.61E-01 | -0.25±0.2 | 0 | 7.57E-01 | 8.10E-01 | -0.21±0.22 | 8.07E-01 | -0.22±0.23 | 8.65E-01 | -0.31±0.42 | 9.96E-01 | -0.37±0.45 | 9.96E-01 |
| 2-stearoyl-GPE (18:0)* | -0.15±0.18 | 0 | 5.03E-01 | 8.72E-01 | -0.22±0.19 | 0 | 4.33E-01 | 8.17E-01 | -0.21±0.2 | 7.95E-01 | -0.29±0.21 | 7.18E-01 | 0.1±0.42 | 9.96E-01 | 0.08±0.44 | 9.96E-01 |
| glucuronide of C10H18O2 (7)* | -0.25±0.19 | 0 | 9.58E-01 | 7.10E-01 | -0.23±0.2 | 0 | 8.84E-01 | 8.17E-01 | -0.25±0.21 | 7.52E-01 | -0.22±0.21 | 8.47E-01 | -0.27±0.46 | 9.96E-01 | -0.3±0.48 | 9.96E-01 |
| 3-hydroxyoctanoylcarnitine (2) | -0.23±0.19 | 0 | 9.99E-01 | 7.61E-01 | -0.23±0.19 | 0 | 9.23E-01 | 8.17E-01 | -0.23±0.21 | 7.80E-01 | -0.22±0.21 | 8.47E-01 | -0.23±0.41 | 9.96E-01 | -0.26±0.42 | 9.96E-01 |
| pregnenetriol disulfate* | 0.17±0.21 | 0 | 6.08E-01 | 8.82E-01 | 0.27±0.22 | 0 | 5.44E-01 | 8.17E-01 | 0.12±0.23 | 9.14E-01 | 0.2±0.24 | 8.79E-01 | 0.4±0.5 | 9.96E-01 | 0.56±0.54 | 9.96E-01 |
| trimethylamine N-oxide | -0.24±0.18 | 16.55 | 2.74E-01 | 7.07E-01 | -0.22±0.18 | 0 | 3.59E-01 | 8.18E-01 | -0.35±0.21 | 5.80E-01 | -0.31±0.21 | 6.72E-01 | 0.1±0.36 | 9.96E-01 | 0.1±0.39 | 9.96E-01 |
| X - 21471 | 0.23±0.21 | 0 | 6.28E-01 | 8.14E-01 | 0.26±0.22 | 0 | 7.88E-01 | 8.18E-01 | 0.18±0.23 | 8.78E-01 | 0.24±0.25 | 8.63E-01 | 0.46±0.52 | 9.96E-01 | 0.39±0.51 | 9.96E-01 |
| 1-stearoyl-2-linoleoyl-GPC (18:0/18:2)* | -0.15±0.19 | 0 | 5.96E-01 | 8.77E-01 | -0.22±0.18 | 0 | 5.17E-01 | 8.26E-01 | -0.2±0.21 | 8.09E-01 | -0.27±0.2 | 7.56E-01 | 0.06±0.44 | 9.96E-01 | 0.04±0.44 | 9.96E-01 |
| 1-palmitoyl-2-oleoyl-GPC (16:0/18:1) | -0.25±0.17 | 0 | 6.49E-01 | 6.43E-01 | -0.21±0.18 | 0 | 6.43E-01 | 8.26E-01 | -0.3±0.21 | 6.53E-01 | -0.27±0.22 | 7.70E-01 | -0.14±0.29 | 9.96E-01 | -0.08±0.33 | 9.96E-01 |
| 1-linolenoyl-GPC (18:3)* | 0.31±0.2 | 0 | 7.56E-01 | 6.22E-01 | 0.24±0.2 | 0 | 8.20E-01 | 8.26E-01 | 0.34±0.22 | 6.15E-01 | 0.26±0.22 | 8.09E-01 | 0.18±0.47 | 9.96E-01 | 0.14±0.48 | 9.96E-01 |
| X - 24243 | -0.2±0.17 | 0 | 9.80E-01 | 7.62E-01 | -0.2±0.17 | 0 | 9.84E-01 | 8.26E-01 | -0.2±0.18 | 7.80E-01 | -0.2±0.19 | 8.33E-01 | -0.19±0.38 | 9.96E-01 | -0.2±0.39 | 9.96E-01 |
| tiglylcarnitine (C5:1-DC) | -0.25±0.18 | 0 | 8.98E-01 | 6.70E-01 | -0.22±0.18 | 0 | 9.65E-01 | 8.26E-01 | -0.26±0.21 | 7.25E-01 | -0.22±0.21 | 8.40E-01 | -0.21±0.36 | 9.96E-01 | -0.2±0.37 | 9.96E-01 |
| hypotaurine | 0.34±0.21 | 0 | 8.08E-01 | 6.22E-01 | 0.25±0.22 | 0 | 8.26E-01 | 8.32E-01 | 0.36±0.23 | 6.15E-01 | 0.27±0.24 | 8.10E-01 | 0.23±0.47 | 9.96E-01 | 0.15±0.48 | 9.96E-01 |
| N2-acetyl,N6-methyllysine | 0.22±0.19 | 0 | 9.11E-01 | 7.77E-01 | 0.22±0.19 | 0 | 9.10E-01 | 8.32E-01 | 0.23±0.21 | 7.77E-01 | 0.23±0.21 | 8.27E-01 | 0.18±0.44 | 9.96E-01 | 0.18±0.45 | 9.96E-01 |
| X - 11478 | -0.17±0.17 | 0 | 6.36E-01 | 8.24E-01 | -0.2±0.17 | 0 | 9.56E-01 | 8.32E-01 | -0.21±0.19 | 7.77E-01 | -0.21±0.19 | 8.27E-01 | 0.01±0.42 | 9.96E-01 | -0.18±0.45 | 9.96E-01 |
| 1-methyl-4-imidazoleacetate | 0.2±0.17 | 0 | 9.47E-01 | 7.97E-01 | 0.21±0.18 | 0 | 9.63E-01 | 8.32E-01 | 0.19±0.19 | 7.95E-01 | 0.22±0.2 | 8.27E-01 | 0.22±0.43 | 9.96E-01 | 0.19±0.47 | 9.96E-01 |
| glycodeoxycholate 3-sulfate | 0.22±0.2 | 0 | 6.98E-01 | 8.14E-01 | 0.23±0.2 | 0 | 6.90E-01 | 8.32E-01 | 0.18±0.22 | 8.78E-01 | 0.2±0.22 | 8.79E-01 | 0.4±0.51 | 9.96E-01 | 0.41±0.49 | 9.96E-01 |
| X - 17676 | -0.18±0.19 | 0 | 7.79E-01 | 8.24E-01 | -0.23±0.2 | 0 | 6.73E-01 | 8.32E-01 | -0.16±0.21 | 8.78E-01 | -0.19±0.22 | 8.79E-01 | -0.29±0.41 | 9.96E-01 | -0.4±0.45 | 9.96E-01 |
| laurylcarnitine (C12) | -0.22±0.18 | 0 | 5.32E-01 | 7.61E-01 | -0.21±0.18 | 0 | 5.42E-01 | 8.32E-01 | -0.15±0.21 | 8.85E-01 | -0.14±0.21 | 9.20E-01 | -0.4±0.35 | 9.96E-01 | -0.4±0.36 | 9.96E-01 |
| glycine conjugate of C10H14O2 (1)* | -0.19±0.18 | 10.54 | 2.90E-01 | 8.15E-01 | -0.21±0.18 | 62.16 | 1.04E-01 | 8.32E-01 | -0.09±0.2 | 9.22E-01 | -0.08±0.2 | 9.63E-01 | -0.55±0.39 | 9.96E-01 | -0.86±0.44 | 9.96E-01 |
| 5-acetylamino-6-amino-3-methyluracil | -0.2±0.17 | 0 | 4.56E-01 | 7.64E-01 | -0.19±0.17 | 0 | 4.81E-01 | 8.33E-01 | -0.25±0.18 | 6.93E-01 | -0.24±0.18 | 7.61E-01 | 0.12±0.46 | 9.96E-01 | 0.11±0.46 | 9.96E-01 |
| 9,10-DiHOME | 0.28±0.18 | 0 | 7.42E-01 | 6.22E-01 | 0.21±0.18 | 0 | 6.70E-01 | 8.33E-01 | 0.3±0.19 | 6.15E-01 | 0.24±0.2 | 7.78E-01 | 0.14±0.45 | 9.96E-01 | 0.04±0.45 | 9.96E-01 |
| urate | -0.27±0.19 | 0 | 5.55E-01 | 6.31E-01 | -0.22±0.19 | 0 | 7.89E-01 | 8.33E-01 | -0.33±0.21 | 6.15E-01 | -0.24±0.21 | 8.10E-01 | -0.05±0.42 | 9.96E-01 | -0.11±0.44 | 9.96E-01 |
| isovalerylglycine | 0.22±0.19 | 0 | 9.99E-01 | 7.97E-01 | 0.22±0.2 | 0 | 9.68E-01 | 8.33E-01 | 0.22±0.21 | 7.95E-01 | 0.22±0.21 | 8.47E-01 | 0.21±0.46 | 9.96E-01 | 0.24±0.48 | 9.96E-01 |
| ergothioneine | 0.12±0.19 | 21.01 | 2.61E-01 | 9.03E-01 | 0.22±0.2 | 6.04 | 3.02E-01 | 8.39E-01 | 0.25±0.22 | 7.70E-01 | 0.33±0.22 | 6.70E-01 | -0.23±0.37 | 9.96E-01 | -0.15±0.42 | 9.96E-01 |
| catechol sulfate | -0.18±0.18 | 0 | 9.05E-01 | 8.24E-01 | -0.21±0.19 | 0 | 8.70E-01 | 8.43E-01 | -0.17±0.21 | 8.73E-01 | -0.2±0.21 | 8.68E-01 | -0.22±0.39 | 9.96E-01 | -0.27±0.41 | 9.96E-01 |
| 1-oleoyl-GPC (18:1) | 0.31±0.2 | 0 | 7.27E-01 | 6.22E-01 | 0.23±0.21 | 0 | 9.84E-01 | 8.44E-01 | 0.34±0.21 | 6.15E-01 | 0.24±0.22 | 8.33E-01 | 0.15±0.49 | 9.96E-01 | 0.22±0.55 | 9.96E-01 |
| gamma-glutamylmethionine | 0.22±0.2 | 0 | 5.30E-01 | 8.13E-01 | 0.24±0.21 | 0 | 4.80E-01 | 8.44E-01 | 0.18±0.21 | 8.70E-01 | 0.19±0.22 | 8.79E-01 | 0.65±0.71 | 9.96E-01 | 0.72±0.72 | 9.96E-01 |
| 3beta-hydroxy-5-cholestenoate | -0.13±0.19 | 0 | 3.18E-01 | 8.99E-01 | -0.21±0.19 | 41.77 | 1.90E-01 | 8.53E-01 | -0.22±0.21 | 7.95E-01 | -0.34±0.21 | 6.35E-01 | 0.27±0.44 | 9.96E-01 | 0.31±0.44 | 9.96E-01 |
| bilirubin (E,Z or Z,E)* | 0.21±0.19 | 0 | 9.75E-01 | 8.13E-01 | 0.21±0.19 | 0 | 9.90E-01 | 8.54E-01 | 0.21±0.21 | 7.95E-01 | 0.22±0.21 | 8.47E-01 | 0.19±0.45 | 9.96E-01 | 0.21±0.46 | 9.96E-01 |
| caprylate (8:0) | 0.15±0.16 | 52.12 | 1.48E-01 | 8.27E-01 | 0.18±0.17 | 46.24 | 1.73E-01 | 8.74E-01 | 0.25±0.18 | 6.55E-01 | 0.28±0.18 | 6.53E-01 | -0.4±0.42 | 9.96E-01 | -0.35±0.42 | 9.96E-01 |
| branched-chain, straight-chain, or cyclopropyl 12:1 fatty acid* | 0.25±0.21 | 0 | 3.54E-01 | 7.75E-01 | 0.23±0.21 | 0 | 3.73E-01 | 8.74E-01 | 0.37±0.25 | 6.37E-01 | 0.34±0.24 | 7.43E-01 | -0.1±0.43 | 9.96E-01 | -0.11±0.44 | 9.96E-01 |
| glycosyl-N-palmitoyl-sphingosine (d18:1/16:0) | -0.17±0.2 | 0 | 5.18E-01 | 8.72E-01 | -0.23±0.21 | 0 | 3.84E-01 | 8.74E-01 | -0.24±0.23 | 7.95E-01 | -0.33±0.24 | 7.50E-01 | 0.06±0.42 | 9.96E-01 | 0.11±0.44 | 9.96E-01 |
| 1-linoleoyl-2-linolenoyl-GPC (18:2/18:3)* | -0.09±0.19 | 0 | 4.58E-01 | 9.22E-01 | -0.22±0.2 | 0 | 4.04E-01 | 8.74E-01 | -0.15±0.21 | 8.85E-01 | -0.28±0.21 | 7.61E-01 | 0.26±0.51 | 9.96E-01 | 0.21±0.55 | 9.96E-01 |
| ceramide (d18:2/24:1, d18:1/24:2)* | -0.22±0.18 | 0 | 5.09E-01 | 7.69E-01 | -0.21±0.19 | 0 | 4.91E-01 | 8.74E-01 | -0.28±0.21 | 6.96E-01 | -0.27±0.21 | 7.70E-01 | 0.02±0.4 | 9.96E-01 | 0.06±0.43 | 9.96E-01 |
| 5-hydroxylysine | 0.12±0.17 | 0 | 8.68E-01 | 9.00E-01 | 0.19±0.17 | 0 | 8.75E-01 | 8.74E-01 | 0.11±0.19 | 9.14E-01 | 0.2±0.19 | 8.33E-01 | 0.18±0.41 | 9.96E-01 | 0.13±0.44 | 9.96E-01 |
| X - 23739 | 0.24±0.18 | 0 | 9.15E-01 | 7.14E-01 | 0.2±0.19 | 0 | 9.35E-01 | 8.74E-01 | 0.23±0.2 | 7.61E-01 | 0.19±0.2 | 8.63E-01 | 0.29±0.46 | 9.96E-01 | 0.23±0.45 | 9.96E-01 |
| 3-hydroxyoctanoylcarnitine (1) | -0.17±0.18 | 0 | 7.63E-01 | 8.27E-01 | -0.2±0.19 | 0 | 7.57E-01 | 8.74E-01 | -0.14±0.21 | 8.89E-01 | -0.17±0.21 | 8.88E-01 | -0.28±0.39 | 9.96E-01 | -0.31±0.39 | 9.96E-01 |
| 6-hydroxyindole sulfate | -0.15±0.18 | 0 | 9.02E-01 | 8.72E-01 | -0.19±0.18 | 0 | 6.72E-01 | 8.74E-01 | -0.14±0.2 | 8.89E-01 | -0.16±0.19 | 8.88E-01 | -0.19±0.4 | 9.96E-01 | -0.35±0.42 | 9.96E-01 |
| sphingomyelin (d17:1/16:0, d18:1/15:0, d16:1/17:0)* | -0.17±0.21 | 47.53 | 1.67E-01 | 8.84E-01 | -0.23±0.22 | 61.49 | 1.07E-01 | 8.81E-01 | -0.26±0.22 | 7.52E-01 | -0.34±0.23 | 6.62E-01 | 0.73±0.69 | 9.96E-01 | 0.88±0.72 | 9.96E-01 |
| hydantoin-5-propionate | -0.18±0.18 | 8.95 | 2.95E-01 | 8.24E-01 | -0.21±0.2 | 0 | 4.56E-01 | 8.81E-01 | -0.27±0.2 | 6.98E-01 | -0.28±0.22 | 7.70E-01 | 0.22±0.42 | 9.96E-01 | 0.1±0.47 | 9.96E-01 |
| oleoyl-linoleoyl-glycerol (18:1/18:2) [2] | -0.19±0.17 | 0 | 7.74E-01 | 8.13E-01 | -0.19±0.18 | 0 | 6.95E-01 | 8.81E-01 | -0.16±0.2 | 8.78E-01 | -0.15±0.21 | 9.16E-01 | -0.27±0.34 | 9.96E-01 | -0.31±0.35 | 9.96E-01 |
| X - 12729 | -0.19±0.2 | 62.23 | 1.04E-01 | 8.27E-01 | -0.22±0.21 | 62.68 | 1.02E-01 | 8.82E-01 | -0.06±0.21 | 9.49E-01 | -0.08±0.22 | 9.63E-01 | -0.97±0.52 | 9.96E-01 | -1.05±0.55 | 9.96E-01 |
| 5-dodecenoate (12:1n7) | 0.24±0.2 | 0 | 5.70E-01 | 7.61E-01 | 0.22±0.2 | 0 | 6.59E-01 | 8.82E-01 | 0.29±0.22 | 6.99E-01 | 0.25±0.22 | 8.10E-01 | -0.01±0.48 | 9.96E-01 | 0.01±0.51 | 9.98E-01 |
| oleoyl ethanolamide | -0.2±0.19 | 0 | 3.28E-01 | 8.24E-01 | -0.21±0.19 | 11.17 | 2.89E-01 | 8.84E-01 | -0.29±0.21 | 6.96E-01 | -0.31±0.22 | 7.11E-01 | 0.18±0.43 | 9.96E-01 | 0.2±0.43 | 9.96E-01 |
| taurocholate | 0.14±0.18 | 82.53 | 1.67E-02 | 8.87E-01 | 0.19±0.19 | 80.99 | 2.18E-02 | 8.85E-01 | 0.33±0.2 | 5.90E-01 | 0.37±0.2 | 5.63E-01 | -0.88±0.46 | 9.96E-01 | -0.86±0.5 | 9.96E-01 |
| 13-HODE + 9-HODE | 0.23±0.19 | 65.1 | 9.05E-02 | 7.61E-01 | 0.2±0.19 | 67.3 | 8.03E-02 | 8.85E-01 | 0.37±0.2 | 5.41E-01 | 0.36±0.21 | 5.72E-01 | -0.44±0.44 | 9.96E-01 | -0.49±0.44 | 9.96E-01 |
| 1-stearoyl-2-oleoyl-GPC (18:0/18:1) | -0.22±0.19 | 0 | 6.37E-01 | 7.62E-01 | -0.21±0.2 | 0 | 6.41E-01 | 8.85E-01 | -0.29±0.23 | 7.26E-01 | -0.27±0.23 | 8.10E-01 | -0.1±0.32 | 9.96E-01 | -0.06±0.36 | 9.96E-01 |
| 1-stearoyl-2-oleoyl-GPI (18:0/18:1)* | -0.19±0.18 | 0 | 6.89E-01 | 8.13E-01 | -0.19±0.18 | 0 | 6.76E-01 | 8.85E-01 | -0.23±0.21 | 7.70E-01 | -0.23±0.21 | 8.17E-01 | -0.07±0.34 | 9.96E-01 | -0.05±0.39 | 9.96E-01 |
| 1-methylxanthine | 0.2±0.21 | 0 | 4.36E-01 | 8.24E-01 | 0.22±0.21 | 0 | 4.77E-01 | 8.85E-01 | 0.13±0.22 | 9.06E-01 | 0.16±0.22 | 9.16E-01 | 0.57±0.52 | 9.96E-01 | 0.57±0.54 | 9.96E-01 |
| 1-linoleoylglycerol (18:2) | -0.17±0.18 | 0 | 6.82E-01 | 8.27E-01 | -0.2±0.19 | 0 | 5.81E-01 | 8.85E-01 | -0.13±0.21 | 9.00E-01 | -0.15±0.21 | 9.20E-01 | -0.31±0.38 | 9.96E-01 | -0.4±0.42 | 9.96E-01 |
| pregnenediol sulfate (C21H34O5S)* | 0.23±0.21 | 28.61 | 2.37E-01 | 8.13E-01 | 0.22±0.21 | 42.52 | 1.87E-01 | 8.85E-01 | 0.11±0.23 | 9.14E-01 | 0.1±0.23 | 9.53E-01 | 0.76±0.5 | 9.96E-01 | 0.84±0.51 | 9.96E-01 |
| O-sulfo-L-tyrosine | -0.16±0.17 | 0 | 4.60E-01 | 8.38E-01 | -0.18±0.17 | 0 | 4.09E-01 | 8.85E-01 | -0.07±0.21 | 9.49E-01 | -0.09±0.21 | 9.58E-01 | -0.33±0.29 | 9.96E-01 | -0.39±0.31 | 9.96E-01 |
| X - 24306 | -0.18±0.18 | 0 | 8.71E-01 | 8.14E-01 | -0.18±0.18 | 0 | 8.92E-01 | 8.86E-01 | -0.2±0.2 | 7.95E-01 | -0.2±0.2 | 8.60E-01 | -0.13±0.39 | 9.96E-01 | -0.14±0.38 | 9.96E-01 |
| S-adenosylhomocysteine (SAH) | 0.19±0.19 | 0 | 8.35E-01 | 8.24E-01 | 0.2±0.19 | 0 | 6.79E-01 | 8.89E-01 | 0.21±0.21 | 8.07E-01 | 0.24±0.22 | 8.18E-01 | 0.11±0.43 | 9.96E-01 | 0.04±0.44 | 9.96E-01 |
| sphingomyelin (d18:2/24:1, d18:1/24:2)* | -0.17±0.2 | 0 | 5.15E-01 | 8.72E-01 | -0.22±0.21 | 0 | 4.17E-01 | 8.89E-01 | -0.23±0.22 | 7.95E-01 | -0.28±0.22 | 7.70E-01 | 0.13±0.5 | 9.96E-01 | 0.22±0.58 | 9.96E-01 |
| umbelliferone sulfate | -0.23±0.19 | 76.79 | 3.79E-02 | 7.64E-01 | -0.2±0.19 | 73.59 | 5.17E-02 | 8.89E-01 | -0.44±0.22 | 4.39E-01 | -0.39±0.22 | 5.64E-01 | 0.53±0.41 | 9.96E-01 | 0.53±0.42 | 9.96E-01 |
| glycochenodeoxycholate | 0.21±0.2 | 0 | 3.91E-01 | 8.13E-01 | 0.21±0.2 | 0 | 4.28E-01 | 8.89E-01 | 0.31±0.23 | 6.97E-01 | 0.29±0.23 | 7.70E-01 | -0.1±0.41 | 9.96E-01 | -0.08±0.42 | 9.96E-01 |
| methylsuccinate | -0.15±0.15 | 0 | 8.59E-01 | 8.24E-01 | -0.16±0.15 | 0 | 9.06E-01 | 8.89E-01 | -0.16±0.17 | 8.07E-01 | -0.16±0.17 | 8.60E-01 | -0.09±0.35 | 9.96E-01 | -0.12±0.35 | 9.96E-01 |
| 3-methylglutarylcarnitine (2) | 0.23±0.19 | 0 | 9.36E-01 | 7.61E-01 | 0.21±0.2 | 0 | 9.23E-01 | 8.89E-01 | 0.22±0.21 | 7.95E-01 | 0.21±0.22 | 8.63E-01 | 0.26±0.4 | 9.96E-01 | 0.17±0.45 | 9.96E-01 |
| 4-chlorobenzoic acid | -0.15±0.19 | 0 | 7.76E-01 | 8.77E-01 | -0.19±0.18 | 0 | 7.86E-01 | 8.89E-01 | -0.13±0.21 | 9.02E-01 | -0.16±0.21 | 8.89E-01 | -0.27±0.44 | 9.96E-01 | -0.29±0.41 | 9.96E-01 |
| ximenoylcarnitine (C26:1)* | -0.21±0.18 | 0 | 6.20E-01 | 7.61E-01 | -0.19±0.18 | 0 | 6.48E-01 | 8.89E-01 | -0.15±0.21 | 8.86E-01 | -0.14±0.22 | 9.26E-01 | -0.34±0.31 | 9.96E-01 | -0.32±0.35 | 9.96E-01 |
| glucuronate | -0.19±0.16 | 0 | 3.19E-01 | 7.75E-01 | -0.17±0.17 | 15.33 | 2.77E-01 | 8.89E-01 | -0.07±0.2 | 9.47E-01 | -0.04±0.21 | 9.79E-01 | -0.4±0.27 | 9.96E-01 | -0.42±0.29 | 9.96E-01 |
| X - 24588 | 0.1±0.18 | 30.25 | 2.31E-01 | 9.03E-01 | 0.18±0.18 | 61.17 | 1.09E-01 | 8.90E-01 | 0.25±0.21 | 7.52E-01 | 0.39±0.22 | 5.66E-01 | -0.19±0.3 | 9.96E-01 | -0.23±0.31 | 9.96E-01 |
| gamma-glutamyl-2-aminobutyrate | -0.2±0.17 | 0 | 9.76E-01 | 7.62E-01 | -0.18±0.18 | 0 | 9.26E-01 | 8.90E-01 | -0.2±0.19 | 7.95E-01 | -0.19±0.2 | 8.63E-01 | -0.21±0.36 | 9.96E-01 | -0.15±0.39 | 9.96E-01 |
| glycocholenate sulfate* | 0.18±0.2 | 0 | 6.27E-01 | 8.52E-01 | 0.2±0.2 | 0 | 6.06E-01 | 8.91E-01 | 0.13±0.22 | 9.06E-01 | 0.15±0.22 | 9.26E-01 | 0.37±0.44 | 9.96E-01 | 0.4±0.43 | 9.96E-01 |
| dihomo-linoleoylcarnitine (C20:2)* | 0.26±0.2 | 13.85 | 2.81E-01 | 7.04E-01 | 0.2±0.2 | 1.95 | 3.13E-01 | 8.93E-01 | 0.38±0.22 | 5.80E-01 | 0.32±0.23 | 7.43E-01 | -0.12±0.41 | 9.96E-01 | -0.15±0.41 | 9.96E-01 |
| palmitoyl sphingomyelin (d18:1/16:0) | -0.11±0.2 | 0 | 5.13E-01 | 9.03E-01 | -0.22±0.22 | 0 | 3.47E-01 | 8.93E-01 | -0.17±0.22 | 8.78E-01 | -0.29±0.23 | 7.70E-01 | 0.23±0.55 | 9.96E-01 | 0.38±0.68 | 9.96E-01 |
| bilirubin (Z,Z) | 0.17±0.19 | 0 | 9.55E-01 | 8.46E-01 | 0.19±0.19 | 0 | 9.90E-01 | 8.93E-01 | 0.18±0.21 | 8.70E-01 | 0.19±0.21 | 8.77E-01 | 0.15±0.46 | 9.96E-01 | 0.19±0.48 | 9.96E-01 |
| taurodeoxycholic acid 3-sulfate | 0.14±0.19 | 0 | 7.64E-01 | 8.91E-01 | 0.19±0.19 | 0 | 7.30E-01 | 8.93E-01 | 0.11±0.21 | 9.14E-01 | 0.16±0.21 | 9.03E-01 | 0.25±0.41 | 9.96E-01 | 0.32±0.42 | 9.96E-01 |
| 3-methoxycatechol sulfate (2) | -0.16±0.18 | 0 | 7.66E-01 | 8.67E-01 | -0.18±0.18 | 0 | 7.10E-01 | 8.93E-01 | -0.13±0.2 | 9.00E-01 | -0.15±0.2 | 9.12E-01 | -0.26±0.39 | 9.96E-01 | -0.32±0.41 | 9.96E-01 |
| myristoleoylcarnitine (C14:1)* | -0.19±0.18 | 0 | 4.15E-01 | 8.13E-01 | -0.18±0.18 | 0 | 4.54E-01 | 8.93E-01 | -0.1±0.21 | 9.14E-01 | -0.1±0.21 | 9.41E-01 | -0.43±0.35 | 9.96E-01 | -0.41±0.35 | 9.96E-01 |
| taurocholenate sulfate* | -0.22±0.18 | 0 | 3.31E-01 | 7.61E-01 | -0.18±0.19 | 0.31 | 3.17E-01 | 8.97E-01 | -0.3±0.2 | 6.30E-01 | -0.27±0.2 | 7.61E-01 | 0.16±0.43 | 9.96E-01 | 0.23±0.46 | 9.96E-01 |
| 3-aminoisobutyrate | 0.19±0.17 | 0 | 3.72E-01 | 8.13E-01 | 0.18±0.18 | 0 | 3.70E-01 | 8.97E-01 | 0.26±0.2 | 6.96E-01 | 0.26±0.2 | 7.70E-01 | -0.12±0.39 | 9.96E-01 | -0.14±0.4 | 9.96E-01 |
| 1-oleoyl-GPE (18:1) | 0.26±0.19 | 0 | 6.50E-01 | 6.70E-01 | 0.19±0.19 | 0 | 7.91E-01 | 8.97E-01 | 0.3±0.21 | 6.48E-01 | 0.21±0.21 | 8.60E-01 | 0.08±0.44 | 9.96E-01 | 0.08±0.43 | 9.96E-01 |
| gamma-glutamyl-alpha-lysine | 0.18±0.2 | 0 | 8.67E-01 | 8.42E-01 | 0.19±0.2 | 0 | 9.07E-01 | 8.97E-01 | 0.17±0.21 | 8.78E-01 | 0.18±0.21 | 8.79E-01 | 0.27±0.59 | 9.96E-01 | 0.25±0.57 | 9.96E-01 |
| N-acetylisoleucine | -0.22±0.19 | 0 | 8.89E-01 | 7.77E-01 | -0.19±0.19 | 0 | 8.80E-01 | 8.97E-01 | -0.23±0.21 | 7.77E-01 | -0.17±0.22 | 8.89E-01 | -0.17±0.4 | 9.96E-01 | -0.24±0.4 | 9.96E-01 |
| X - 16576 | 0.22±0.2 | 0 | 7.37E-01 | 8.00E-01 | 0.19±0.19 | 0 | 7.09E-01 | 8.97E-01 | 0.19±0.22 | 8.70E-01 | 0.16±0.21 | 9.06E-01 | 0.36±0.46 | 9.96E-01 | 0.35±0.46 | 9.96E-01 |
| X - 15245 | 0.16±0.2 | 0 | 4.62E-01 | 8.87E-01 | 0.2±0.21 | 0 | 6.17E-01 | 8.97E-01 | 0.09±0.22 | 9.24E-01 | 0.16±0.23 | 9.16E-01 | 0.5±0.51 | 9.96E-01 | 0.45±0.54 | 9.96E-01 |
| uridine | 0.24±0.2 | 0 | 4.47E-01 | 7.62E-01 | 0.2±0.2 | 0 | 5.52E-01 | 8.97E-01 | 0.17±0.22 | 8.78E-01 | 0.15±0.22 | 9.26E-01 | 0.59±0.5 | 9.96E-01 | 0.49±0.52 | 9.96E-01 |
| 1-palmitoyl-2-linoleoyl-GPE (16:0/18:2) | -0.17±0.18 | 0 | 6.30E-01 | 8.24E-01 | -0.18±0.18 | 0 | 7.08E-01 | 8.97E-01 | -0.12±0.21 | 9.14E-01 | -0.14±0.21 | 9.26E-01 | -0.31±0.33 | 9.96E-01 | -0.29±0.34 | 9.96E-01 |
| X - 26109 | -0.22±0.18 | 0 | 4.92E-01 | 7.37E-01 | -0.19±0.19 | 0 | 3.46E-01 | 8.97E-01 | -0.17±0.2 | 8.70E-01 | -0.1±0.21 | 9.48E-01 | -0.47±0.4 | 9.96E-01 | -0.54±0.42 | 9.96E-01 |
| pregnenolone sulfate | 0.2±0.2 | 63.18 | 9.94E-02 | 8.24E-01 | 0.2±0.2 | 68.16 | 7.64E-02 | 8.97E-01 | 0.07±0.22 | 9.49E-01 | 0.05±0.22 | 9.78E-01 | 1±0.53 | 9.96E-01 | 1.06±0.53 | 9.96E-01 |
| sphingomyelin (d18:1/25:0, d19:0/24:1, d20:1/23:0, d19:1/24:0)* | -0.19±0.19 | 72.53 | 5.64E-02 | 8.24E-01 | -0.18±0.19 | 78.41 | 3.14E-02 | 9.01E-01 | -0.31±0.2 | 6.15E-01 | -0.31±0.2 | 6.42E-01 | 0.93±0.62 | 9.96E-01 | 1.12±0.64 | 9.96E-01 |
| dodecanedioate (C12-DC) | 0.29±0.18 | 25.98 | 2.45E-01 | 6.22E-01 | 0.18±0.18 | 22.91 | 2.55E-01 | 9.01E-01 | 0.39±0.2 | 4.49E-01 | 0.28±0.2 | 7.43E-01 | -0.13±0.4 | 9.96E-01 | -0.23±0.4 | 9.96E-01 |
| lactate | -0.22±0.2 | 50.2 | 1.56E-01 | 8.13E-01 | -0.2±0.21 | 31.02 | 2.29E-01 | 9.01E-01 | -0.35±0.22 | 6.15E-01 | -0.31±0.23 | 7.54E-01 | 0.42±0.5 | 9.96E-01 | 0.36±0.51 | 9.96E-01 |
| stearoyl sphingomyelin (d18:1/18:0) | -0.23±0.19 | 0 | 3.47E-01 | 7.64E-01 | -0.19±0.2 | 10.08 | 2.92E-01 | 9.01E-01 | -0.31±0.21 | 6.40E-01 | -0.28±0.22 | 7.70E-01 | 0.17±0.47 | 9.96E-01 | 0.33±0.54 | 9.96E-01 |
| N-stearoyl-sphingosine (d18:1/18:0)* | -0.26±0.18 | 0 | 4.07E-01 | 6.43E-01 | -0.18±0.19 | 0 | 4.38E-01 | 9.01E-01 | -0.34±0.2 | 5.81E-01 | -0.25±0.21 | 7.87E-01 | 0.03±0.39 | 9.96E-01 | 0.14±0.46 | 9.96E-01 |
| N-acetylcarnosine | -0.25±0.2 | 0 | 4.10E-01 | 7.61E-01 | -0.21±0.22 | 0 | 5.06E-01 | 9.01E-01 | -0.32±0.22 | 6.53E-01 | -0.27±0.24 | 8.10E-01 | 0.16±0.53 | 9.96E-01 | 0.12±0.55 | 9.96E-01 |
| 12,13-DiHOME | 0.27±0.2 | 0 | 8.61E-01 | 7.05E-01 | 0.2±0.21 | 0 | 7.82E-01 | 9.01E-01 | 0.29±0.23 | 7.26E-01 | 0.22±0.23 | 8.60E-01 | 0.2±0.45 | 9.96E-01 | 0.08±0.45 | 9.96E-01 |
| alpha-tocopherol | -0.14±0.17 | 0 | 8.42E-01 | 8.72E-01 | -0.17±0.17 | 0 | 7.74E-01 | 9.01E-01 | -0.16±0.19 | 8.72E-01 | -0.19±0.2 | 8.60E-01 | -0.08±0.33 | 9.96E-01 | -0.08±0.35 | 9.96E-01 |
| kynurenine | -0.23±0.18 | 0 | 6.21E-01 | 7.47E-01 | -0.18±0.19 | 0 | 9.21E-01 | 9.01E-01 | -0.28±0.21 | 6.98E-01 | -0.19±0.22 | 8.79E-01 | -0.07±0.36 | 9.96E-01 | -0.15±0.35 | 9.96E-01 |
| X - 26108 | -0.18±0.17 | 0 | 9.42E-01 | 8.24E-01 | -0.17±0.18 | 0 | 9.87E-01 | 9.01E-01 | -0.17±0.19 | 8.67E-01 | -0.17±0.2 | 8.79E-01 | -0.2±0.42 | 9.96E-01 | -0.17±0.41 | 9.96E-01 |
| octadecenedioate (C18:1-DC) | 0.22±0.18 | 0 | 9.13E-01 | 7.61E-01 | 0.18±0.19 | 0 | 9.16E-01 | 9.01E-01 | 0.21±0.2 | 7.95E-01 | 0.17±0.21 | 8.89E-01 | 0.26±0.4 | 9.96E-01 | 0.22±0.41 | 9.96E-01 |
| palmitoleate (16:1n7) | 0.16±0.2 | 0 | 8.49E-01 | 8.88E-01 | 0.2±0.2 | 0 | 7.07E-01 | 9.01E-01 | 0.14±0.22 | 9.02E-01 | 0.16±0.22 | 9.16E-01 | 0.24±0.47 | 9.96E-01 | 0.36±0.48 | 9.96E-01 |
| 3-acetylphenol sulfate | -0.18±0.18 | 0 | 5.97E-01 | 8.24E-01 | -0.18±0.19 | 0 | 5.37E-01 | 9.01E-01 | -0.13±0.21 | 9.00E-01 | -0.12±0.21 | 9.27E-01 | -0.38±0.42 | 9.96E-01 | -0.43±0.45 | 9.96E-01 |
| N-palmitoyl-heptadecasphingosine (d17:1/16:0)* | 0.21±0.2 | 0 | 4.67E-01 | 8.14E-01 | 0.2±0.21 | 4.31 | 3.07E-01 | 9.01E-01 | 0.15±0.22 | 8.90E-01 | 0.12±0.22 | 9.30E-01 | 0.55±0.51 | 9.96E-01 | 0.78±0.61 | 9.96E-01 |
| cis-4-decenoylcarnitine (C10:1) | -0.16±0.17 | 77.79 | 3.38E-02 | 8.39E-01 | -0.16±0.17 | 77.19 | 3.63E-02 | 9.01E-01 | 0.08±0.2 | 9.35E-01 | 0.06±0.2 | 9.66E-01 | -0.71±0.31 | 9.96E-01 | -0.74±0.33 | 9.96E-01 |
| N-acetylleucine | -0.2±0.18 | 0 | 5.28E-01 | 8.13E-01 | -0.17±0.18 | 0.36 | 3.16E-01 | 9.01E-01 | -0.12±0.22 | 9.14E-01 | -0.04±0.22 | 9.79E-01 | -0.37±0.33 | 9.96E-01 | -0.44±0.32 | 9.96E-01 |
| X - 21607 | -0.16±0.17 | 46.52 | 1.71E-01 | 8.50E-01 | -0.17±0.17 | 55.86 | 1.32E-01 | 9.01E-01 | -0.03±0.2 | 9.83E-01 | -0.02±0.2 | 9.93E-01 | -0.59±0.36 | 9.96E-01 | -0.65±0.36 | 9.96E-01 |
| glutamate | -0.3±0.19 | 0 | 4.07E-01 | 6.22E-01 | -0.19±0.2 | 0 | 6.15E-01 | 9.01E-01 | -0.37±0.21 | 5.41E-01 | -0.25±0.23 | 8.32E-01 | 0.02±0.44 | 9.96E-01 | 0.01±0.45 | 9.98E-01 |
| 7-alpha-hydroxy-3-oxo-4-cholestenoate (7-Hoca) | 0.14±0.16 | 54.5 | 1.38E-01 | 8.72E-01 | 0.15±0.17 | 60.6 | 1.11E-01 | 9.05E-01 | 0.29±0.19 | 6.37E-01 | 0.31±0.2 | 6.53E-01 | -0.27±0.32 | 9.96E-01 | -0.32±0.34 | 9.96E-01 |
| dihomo-linolenoylcarnitine (C20:3n3 or 6)* | 0.2±0.2 | 55.91 | 1.32E-01 | 8.24E-01 | 0.19±0.2 | 52.72 | 1.46E-01 | 9.05E-01 | 0.37±0.23 | 6.01E-01 | 0.36±0.24 | 6.54E-01 | -0.3±0.39 | 9.96E-01 | -0.31±0.4 | 9.96E-01 |
| taurodeoxycholate | 0.15±0.19 | 64.61 | 9.28E-02 | 8.87E-01 | 0.17±0.2 | 62.54 | 1.02E-01 | 9.05E-01 | 0.32±0.22 | 6.42E-01 | 0.34±0.22 | 6.55E-01 | -0.47±0.42 | 9.96E-01 | -0.46±0.43 | 9.96E-01 |
| corticosterone | -0.1±0.17 | 48.57 | 1.63E-01 | 9.03E-01 | -0.15±0.17 | 56.97 | 1.27E-01 | 9.05E-01 | -0.2±0.18 | 7.80E-01 | -0.26±0.18 | 7.18E-01 | 0.45±0.43 | 9.96E-01 | 0.45±0.43 | 9.96E-01 |
| betaine | -0.13±0.21 | 58.45 | 1.21E-01 | 9.03E-01 | -0.19±0.21 | 55.24 | 1.35E-01 | 9.05E-01 | -0.25±0.22 | 7.70E-01 | -0.31±0.23 | 7.43E-01 | 0.73±0.59 | 9.96E-01 | 0.65±0.6 | 9.96E-01 |
| ceramide (d18:1/17:0, d17:1/18:0)* | -0.18±0.18 | 40.72 | 1.94E-01 | 8.24E-01 | -0.16±0.19 | 48.61 | 1.63E-01 | 9.05E-01 | -0.26±0.19 | 6.91E-01 | -0.24±0.19 | 7.70E-01 | 0.45±0.51 | 9.96E-01 | 0.66±0.61 | 9.96E-01 |
| X - 12411 | 0.14±0.17 | 23.53 | 2.53E-01 | 8.72E-01 | 0.16±0.18 | 11.04 | 2.89E-01 | 9.05E-01 | 0.24±0.19 | 7.26E-01 | 0.24±0.19 | 7.70E-01 | -0.27±0.4 | 9.96E-01 | -0.25±0.42 | 9.96E-01 |
| sphingomyelin (d18:2/23:1)* | -0.2±0.22 | 16.86 | 2.73E-01 | 8.51E-01 | -0.2±0.23 | 38.42 | 2.03E-01 | 9.05E-01 | -0.29±0.24 | 7.27E-01 | -0.3±0.24 | 7.70E-01 | 0.43±0.62 | 9.96E-01 | 0.61±0.67 | 9.96E-01 |
| 1-ribosyl-imidazoleacetate* | 0.18±0.17 | 0 | 4.77E-01 | 8.14E-01 | 0.16±0.18 | 0 | 4.60E-01 | 9.05E-01 | 0.25±0.19 | 7.26E-01 | 0.23±0.2 | 8.10E-01 | -0.07±0.39 | 9.96E-01 | -0.12±0.42 | 9.96E-01 |
| acisoga | 0.19±0.19 | 0 | 6.04E-01 | 8.24E-01 | 0.16±0.18 | 0 | 4.98E-01 | 9.05E-01 | 0.24±0.21 | 7.67E-01 | 0.22±0.2 | 8.22E-01 | -0.01±0.42 | 9.96E-01 | -0.09±0.41 | 9.96E-01 |
| glycerate | -0.13±0.18 | 0 | 4.44E-01 | 8.96E-01 | -0.16±0.18 | 0 | 4.34E-01 | 9.05E-01 | -0.17±0.18 | 8.25E-01 | -0.21±0.19 | 8.32E-01 | 0.27±0.55 | 9.96E-01 | 0.27±0.58 | 9.96E-01 |
| X - 19141 | -0.13±0.18 | 0 | 3.46E-01 | 8.96E-01 | -0.16±0.19 | 0 | 5.25E-01 | 9.05E-01 | -0.22±0.2 | 7.95E-01 | -0.22±0.21 | 8.33E-01 | 0.23±0.42 | 9.96E-01 | 0.08±0.43 | 9.96E-01 |
| glutamine_degradant* | -0.12±0.22 | 0 | 5.68E-01 | 9.03E-01 | -0.19±0.22 | 0 | 5.15E-01 | 9.05E-01 | -0.19±0.24 | 8.78E-01 | -0.26±0.24 | 8.33E-01 | 0.12±0.48 | 9.96E-01 | 0.09±0.48 | 9.96E-01 |
| beta-alanine | -0.2±0.2 | 0 | 3.94E-01 | 8.24E-01 | -0.17±0.19 | 0 | 5.36E-01 | 9.05E-01 | -0.27±0.21 | 7.26E-01 | -0.22±0.21 | 8.42E-01 | 0.22±0.53 | 9.96E-01 | 0.12±0.5 | 9.96E-01 |
| tyrosine | -0.22±0.18 | 0 | 6.01E-01 | 7.61E-01 | -0.17±0.19 | 0 | 7.06E-01 | 9.05E-01 | -0.27±0.21 | 7.12E-01 | -0.2±0.21 | 8.63E-01 | -0.06±0.36 | 9.96E-01 | -0.04±0.39 | 9.96E-01 |
| 1-palmitoyl-GPC (16:0) | 0.23±0.2 | 0 | 6.86E-01 | 7.79E-01 | 0.18±0.2 | 0 | 8.37E-01 | 9.05E-01 | 0.27±0.22 | 7.34E-01 | 0.2±0.22 | 8.79E-01 | 0.07±0.43 | 9.96E-01 | 0.1±0.47 | 9.96E-01 |
| 4-cholesten-3-one | -0.17±0.18 | 0 | 6.33E-01 | 8.27E-01 | -0.16±0.18 | 0 | 8.26E-01 | 9.05E-01 | -0.21±0.2 | 7.95E-01 | -0.18±0.2 | 8.79E-01 | -0.01±0.38 | 9.96E-01 | -0.08±0.38 | 9.96E-01 |
| eicosenoate (20:1) | -0.17±0.18 | 0 | 7.55E-01 | 8.45E-01 | -0.16±0.18 | 0 | 8.01E-01 | 9.05E-01 | -0.19±0.2 | 8.12E-01 | -0.18±0.2 | 8.79E-01 | -0.05±0.42 | 9.96E-01 | -0.06±0.44 | 9.96E-01 |
| 4-hydroxyhippurate | 0.18±0.17 | 0 | 9.32E-01 | 8.14E-01 | 0.16±0.18 | 0 | 9.34E-01 | 9.05E-01 | 0.17±0.2 | 8.66E-01 | 0.17±0.2 | 8.79E-01 | 0.21±0.37 | 9.96E-01 | 0.13±0.4 | 9.96E-01 |
| 4-allylcatechol sulfate | -0.18±0.18 | 0 | 9.82E-01 | 8.24E-01 | -0.17±0.19 | 0 | 9.42E-01 | 9.05E-01 | -0.18±0.2 | 8.67E-01 | -0.17±0.2 | 8.79E-01 | -0.17±0.43 | 9.96E-01 | -0.14±0.46 | 9.96E-01 |
| X - 24337 | -0.14±0.19 | 0 | 6.85E-01 | 8.91E-01 | -0.17±0.19 | 0 | 8.70E-01 | 9.05E-01 | -0.18±0.21 | 8.70E-01 | -0.19±0.21 | 8.79E-01 | 0.01±0.41 | 9.96E-01 | -0.11±0.43 | 9.96E-01 |
| pimeloylcarnitine/3-methyladipoylcarnitine (C7-DC) | -0.16±0.17 | 0 | 8.80E-01 | 8.24E-01 | -0.15±0.17 | 0 | 9.92E-01 | 9.05E-01 | -0.15±0.19 | 8.78E-01 | -0.16±0.19 | 8.81E-01 | -0.22±0.38 | 9.96E-01 | -0.15±0.41 | 9.96E-01 |
| cortolone glucuronide (1) | -0.21±0.14 | 0 | 6.54E-01 | 6.60E-01 | -0.14±0.16 | 0 | 9.73E-01 | 9.05E-01 | -0.23±0.16 | 6.33E-01 | -0.14±0.18 | 8.88E-01 | -0.05±0.38 | 9.96E-01 | -0.16±0.4 | 9.96E-01 |
| vanillactate | -0.17±0.19 | 0 | 8.74E-01 | 8.72E-01 | -0.18±0.2 | 0 | 9.99E-01 | 9.05E-01 | -0.18±0.23 | 8.77E-01 | -0.18±0.23 | 8.89E-01 | -0.11±0.38 | 9.96E-01 | -0.18±0.39 | 9.96E-01 |
| 5-oxoproline | 0.21±0.19 | 0 | 9.29E-01 | 8.13E-01 | 0.18±0.2 | 0 | 9.10E-01 | 9.05E-01 | 0.22±0.22 | 7.95E-01 | 0.17±0.22 | 9.00E-01 | 0.18±0.4 | 9.96E-01 | 0.22±0.45 | 9.96E-01 |
| thioproline | -0.17±0.19 | 0 | 9.23E-01 | 8.52E-01 | -0.17±0.19 | 0 | 9.04E-01 | 9.05E-01 | -0.18±0.21 | 8.70E-01 | -0.16±0.21 | 9.12E-01 | -0.13±0.46 | 9.96E-01 | -0.22±0.46 | 9.96E-01 |
| 2-methylbutyrylcarnitine (C5) | -0.21±0.18 | 0 | 9.35E-01 | 7.77E-01 | -0.17±0.19 | 0 | 8.76E-01 | 9.05E-01 | -0.22±0.21 | 7.95E-01 | -0.15±0.21 | 9.16E-01 | -0.18±0.37 | 9.96E-01 | -0.22±0.39 | 9.96E-01 |
| pyridoxate | 0.14±0.16 | 0 | 8.59E-01 | 8.72E-01 | 0.15±0.17 | 0 | 8.46E-01 | 9.05E-01 | 0.13±0.18 | 8.89E-01 | 0.13±0.18 | 9.16E-01 | 0.2±0.36 | 9.96E-01 | 0.21±0.39 | 9.96E-01 |
| ethylmalonate | 0.15±0.17 | 0 | 6.89E-01 | 8.72E-01 | 0.15±0.17 | 0 | 8.16E-01 | 9.05E-01 | 0.11±0.19 | 9.06E-01 | 0.13±0.19 | 9.20E-01 | 0.29±0.4 | 9.96E-01 | 0.23±0.39 | 9.96E-01 |
| X - 12847 | -0.24±0.19 | 0 | 5.52E-01 | 7.14E-01 | -0.17±0.19 | 0 | 7.64E-01 | 9.05E-01 | -0.19±0.21 | 8.41E-01 | -0.14±0.21 | 9.26E-01 | -0.48±0.43 | 9.96E-01 | -0.29±0.44 | 9.96E-01 |
| X - 13431 | -0.18±0.18 | 0 | 7.49E-01 | 8.24E-01 | -0.17±0.18 | 0 | 7.70E-01 | 9.05E-01 | -0.14±0.21 | 8.89E-01 | -0.14±0.21 | 9.26E-01 | -0.27±0.35 | 9.96E-01 | -0.26±0.37 | 9.96E-01 |
| X - 24462 | 0.16±0.19 | 0 | 6.85E-01 | 8.77E-01 | 0.17±0.19 | 0 | 7.02E-01 | 9.05E-01 | 0.12±0.21 | 9.14E-01 | 0.14±0.21 | 9.26E-01 | 0.33±0.46 | 9.96E-01 | 0.34±0.47 | 9.96E-01 |
| X - 23780 | -0.17±0.17 | 0 | 7.00E-01 | 8.24E-01 | -0.16±0.18 | 0 | 7.00E-01 | 9.05E-01 | -0.14±0.19 | 8.89E-01 | -0.13±0.2 | 9.26E-01 | -0.3±0.38 | 9.96E-01 | -0.3±0.39 | 9.96E-01 |
| X - 19438 | 0.16±0.21 | 0 | 7.19E-01 | 8.91E-01 | 0.19±0.22 | 0 | 7.97E-01 | 9.05E-01 | 0.12±0.23 | 9.14E-01 | 0.16±0.25 | 9.26E-01 | 0.31±0.48 | 9.96E-01 | 0.3±0.47 | 9.96E-01 |
| indoleacetoylcarnitine* | 0.14±0.19 | 0 | 7.01E-01 | 8.96E-01 | 0.17±0.19 | 0 | 7.18E-01 | 9.05E-01 | 0.1±0.21 | 9.14E-01 | 0.13±0.21 | 9.26E-01 | 0.28±0.43 | 9.96E-01 | 0.3±0.42 | 9.96E-01 |
| X - 23974 | 0.14±0.19 | 0 | 6.21E-01 | 8.96E-01 | 0.16±0.19 | 0 | 5.91E-01 | 9.05E-01 | 0.1±0.21 | 9.14E-01 | 0.12±0.21 | 9.26E-01 | 0.35±0.47 | 9.96E-01 | 0.42±0.52 | 9.96E-01 |
| 2,6-dihydroxybenzoic acid | 0.2±0.19 | 0 | 5.85E-01 | 8.13E-01 | 0.16±0.19 | 0 | 5.79E-01 | 9.05E-01 | 0.15±0.21 | 8.78E-01 | 0.11±0.21 | 9.30E-01 | 0.42±0.44 | 9.96E-01 | 0.38±0.44 | 9.96E-01 |
| X - 18886 | -0.22±0.19 | 0 | 9.94E-01 | 7.79E-01 | -0.17±0.2 | 0 | 5.82E-01 | 9.05E-01 | -0.22±0.22 | 7.95E-01 | -0.12±0.22 | 9.39E-01 | -0.22±0.4 | 9.96E-01 | -0.38±0.42 | 9.96E-01 |
| arachidonate (20:4n6) | 0.18±0.19 | 0 | 4.21E-01 | 8.27E-01 | 0.17±0.2 | 0 | 4.70E-01 | 9.05E-01 | 0.11±0.21 | 9.14E-01 | 0.11±0.22 | 9.40E-01 | 0.53±0.47 | 9.96E-01 | 0.5±0.49 | 9.96E-01 |
| X - 15728 | -0.17±0.18 | 0 | 4.91E-01 | 8.24E-01 | -0.15±0.18 | 0 | 5.37E-01 | 9.05E-01 | -0.11±0.2 | 9.14E-01 | -0.1±0.2 | 9.41E-01 | -0.41±0.39 | 9.96E-01 | -0.37±0.4 | 9.96E-01 |
| X - 11852 | -0.2±0.2 | 32.45 | 2.24E-01 | 8.24E-01 | -0.18±0.2 | 23.59 | 2.53E-01 | 9.05E-01 | -0.1±0.22 | 9.14E-01 | -0.09±0.22 | 9.63E-01 | -0.78±0.51 | 9.96E-01 | -0.75±0.53 | 9.96E-01 |
| argininate* | -0.16±0.18 | 0 | 6.29E-01 | 8.54E-01 | -0.16±0.18 | 0 | 4.92E-01 | 9.05E-01 | -0.1±0.21 | 9.14E-01 | -0.08±0.21 | 9.63E-01 | -0.3±0.34 | 9.96E-01 | -0.37±0.36 | 9.96E-01 |
| X - 18913 | -0.13±0.19 | 0 | 4.91E-01 | 8.96E-01 | -0.18±0.2 | 0 | 3.73E-01 | 9.05E-01 | -0.06±0.22 | 9.49E-01 | -0.08±0.23 | 9.66E-01 | -0.37±0.4 | 9.96E-01 | -0.5±0.42 | 9.96E-01 |
| 1-palmitoyl-GPI (16:0) | -0.15±0.19 | 0 | 7.41E-01 | 8.82E-01 | -0.17±0.2 | 0 | 6.62E-01 | 9.05E-01 | -0.19±0.22 | 8.70E-01 | -0.21±0.22 | 8.60E-01 | -0.04±0.4 | 9.96E-01 | 0±0.43 | 9.98E-01 |
| trans-4-hydroxyproline | 0.15±0.19 | 0 | 8.13E-01 | 8.82E-01 | 0.16±0.19 | 0 | 6.65E-01 | 9.05E-01 | 0.17±0.21 | 8.73E-01 | 0.2±0.21 | 8.63E-01 | 0.06±0.43 | 9.96E-01 | -0.01±0.44 | 9.98E-01 |
| N1-methyladenosine | 0.16±0.2 | 0 | 6.70E-01 | 8.82E-01 | 0.18±0.2 | 0 | 5.60E-01 | 9.05E-01 | 0.21±0.23 | 8.45E-01 | 0.26±0.24 | 8.32E-01 | 0.02±0.38 | 9.96E-01 | 0±0.37 | 1.00E+00 |
| (2 or 3)-decenoate (10:1n7 or n8) | 0.21±0.2 | 13.21 | 2.83E-01 | 8.24E-01 | 0.17±0.2 | 32.71 | 2.23E-01 | 9.05E-01 | 0.34±0.24 | 6.55E-01 | 0.31±0.23 | 7.56E-01 | -0.15±0.39 | 9.96E-01 | -0.25±0.4 | 9.96E-01 |
| 3-methoxytyramine sulfate | 0.16±0.18 | 47.4 | 1.68E-01 | 8.35E-01 | 0.15±0.19 | 45.55 | 1.75E-01 | 9.05E-01 | 0.28±0.19 | 6.55E-01 | 0.28±0.21 | 7.56E-01 | -0.34±0.41 | 9.96E-01 | -0.35±0.41 | 9.96E-01 |
| 1-stearoyl-GPI (18:0) | -0.12±0.19 | 0 | 3.45E-01 | 9.03E-01 | -0.16±0.19 | 16.42 | 2.74E-01 | 9.05E-01 | -0.2±0.21 | 8.07E-01 | -0.25±0.21 | 8.05E-01 | 0.29±0.48 | 9.96E-01 | 0.37±0.52 | 9.96E-01 |
| X - 23639 | -0.05±0.19 | 0 | 5.82E-01 | 9.61E-01 | -0.16±0.19 | 0 | 4.33E-01 | 9.05E-01 | -0.1±0.2 | 9.14E-01 | -0.23±0.21 | 8.32E-01 | 0.18±0.45 | 9.96E-01 | 0.17±0.47 | 9.96E-01 |
| 3-(3-hydroxyphenyl)propionate sulfate | -0.13±0.18 | 0 | 5.44E-01 | 8.96E-01 | -0.15±0.18 | 0 | 5.51E-01 | 9.05E-01 | -0.18±0.2 | 8.53E-01 | -0.2±0.2 | 8.60E-01 | 0.1±0.42 | 9.96E-01 | 0.08±0.42 | 9.96E-01 |
| cholesterol sulfate | -0.12±0.2 | 0 | 8.20E-01 | 9.03E-01 | -0.18±0.21 | 0 | 6.17E-01 | 9.05E-01 | -0.14±0.23 | 9.00E-01 | -0.23±0.23 | 8.60E-01 | -0.04±0.4 | 9.96E-01 | 0.03±0.45 | 9.96E-01 |
| creatine | 0.16±0.21 | 0 | 7.99E-01 | 8.91E-01 | 0.18±0.21 | 0 | 7.04E-01 | 9.05E-01 | 0.19±0.24 | 8.78E-01 | 0.22±0.24 | 8.77E-01 | 0.06±0.45 | 9.96E-01 | 0.03±0.44 | 9.96E-01 |
| 1-palmitoyl-GPG (16:0)* | -0.18±0.17 | 0 | 8.53E-01 | 8.14E-01 | -0.15±0.18 | 0 | 8.30E-01 | 9.05E-01 | -0.2±0.19 | 7.95E-01 | -0.17±0.2 | 8.79E-01 | -0.11±0.39 | 9.96E-01 | -0.07±0.41 | 9.96E-01 |
| X - 25810 | -0.2±0.2 | 0 | 7.75E-01 | 8.24E-01 | -0.17±0.2 | 0 | 7.64E-01 | 9.05E-01 | -0.23±0.23 | 7.95E-01 | -0.2±0.22 | 8.79E-01 | -0.1±0.41 | 9.96E-01 | -0.04±0.45 | 9.96E-01 |
| malate | -0.14±0.18 | 0 | 9.98E-01 | 8.87E-01 | -0.15±0.18 | 0 | 9.64E-01 | 9.05E-01 | -0.14±0.21 | 8.90E-01 | -0.14±0.21 | 9.24E-01 | -0.14±0.35 | 9.96E-01 | -0.16±0.35 | 9.96E-01 |
| 3-(4-hydroxyphenyl)lactate | -0.22±0.19 | 0 | 9.87E-01 | 7.88E-01 | -0.17±0.2 | 0 | 8.59E-01 | 9.05E-01 | -0.22±0.22 | 7.95E-01 | -0.15±0.23 | 9.26E-01 | -0.22±0.4 | 9.96E-01 | -0.24±0.43 | 9.96E-01 |
| leucine | -0.2±0.18 | 0 | 8.28E-01 | 8.10E-01 | -0.15±0.19 | 0 | 9.62E-01 | 9.05E-01 | -0.23±0.23 | 7.95E-01 | -0.15±0.24 | 9.26E-01 | -0.15±0.29 | 9.96E-01 | -0.17±0.3 | 9.96E-01 |
| lysine | 0.15±0.21 | 0 | 5.70E-01 | 8.91E-01 | 0.17±0.2 | 0 | 5.94E-01 | 9.05E-01 | 0.12±0.21 | 9.14E-01 | 0.14±0.21 | 9.26E-01 | 0.59±0.79 | 9.96E-01 | 0.57±0.79 | 9.96E-01 |
| cis-3,4-methyleneheptanoate | -0.1±0.18 | 0 | 8.55E-01 | 9.03E-01 | -0.15±0.18 | 0 | 7.23E-01 | 9.05E-01 | -0.11±0.2 | 9.14E-01 | -0.12±0.2 | 9.26E-01 | -0.03±0.39 | 9.96E-01 | -0.28±0.4 | 9.96E-01 |
| behenoyl dihydrosphingomyelin (d18:0/22:0)* | -0.22±0.18 | 0 | 9.81E-01 | 7.61E-01 | -0.15±0.19 | 0 | 8.35E-01 | 9.05E-01 | -0.22±0.22 | 7.95E-01 | -0.13±0.23 | 9.30E-01 | -0.23±0.32 | 9.96E-01 | -0.21±0.33 | 9.96E-01 |
| X - 18921 | -0.08±0.18 | 0 | 9.77E-01 | 9.31E-01 | -0.15±0.18 | 0 | 6.11E-01 | 9.05E-01 | -0.07±0.2 | 9.37E-01 | -0.11±0.2 | 9.30E-01 | -0.09±0.4 | 9.96E-01 | -0.36±0.46 | 9.96E-01 |
| indolebutyrate | -0.11±0.18 | 0 | 7.74E-01 | 9.03E-01 | -0.16±0.19 | 0 | 5.79E-01 | 9.05E-01 | -0.09±0.21 | 9.28E-01 | -0.11±0.21 | 9.30E-01 | -0.22±0.42 | 9.96E-01 | -0.38±0.45 | 9.96E-01 |
| 1-(1-enyl-oleoyl)-GPE (P-18:1)* | 0.19±0.19 | 0 | 4.72E-01 | 8.24E-01 | 0.17±0.2 | 0 | 4.95E-01 | 9.05E-01 | 0.13±0.21 | 9.00E-01 | 0.11±0.22 | 9.39E-01 | 0.53±0.51 | 9.96E-01 | 0.49±0.51 | 9.96E-01 |
| N-carbamoylalanine | -0.09±0.17 | 0 | 6.84E-01 | 9.03E-01 | -0.15±0.17 | 0 | 5.16E-01 | 9.05E-01 | -0.06±0.19 | 9.49E-01 | -0.09±0.19 | 9.44E-01 | -0.23±0.38 | 9.96E-01 | -0.38±0.4 | 9.96E-01 |
| X - 24475 | 0.19±0.19 | 0 | 3.69E-01 | 8.24E-01 | 0.16±0.19 | 0 | 3.89E-01 | 9.05E-01 | 0.11±0.22 | 9.14E-01 | 0.08±0.21 | 9.63E-01 | 0.56±0.45 | 9.96E-01 | 0.52±0.46 | 9.96E-01 |
| 4-hydroxyphenylacetylglutamine | -0.15±0.18 | 42.28 | 1.88E-01 | 8.83E-01 | -0.16±0.19 | 66.01 | 8.63E-02 | 9.05E-01 | 0.01±0.22 | 9.97E-01 | 0.05±0.22 | 9.79E-01 | -0.53±0.34 | 9.96E-01 | -0.67±0.35 | 9.96E-01 |
| trans-urocanate | -0.13±0.19 | 0 | 7.49E-01 | 9.03E-01 | -0.15±0.18 | 0 | 7.79E-01 | 9.05E-01 | -0.16±0.21 | 8.78E-01 | -0.17±0.2 | 8.79E-01 | 0±0.43 | 9.97E-01 | -0.04±0.42 | 9.96E-01 |
| 7-methylguanine | 0.11±0.17 | 0 | 3.27E-01 | 9.03E-01 | 0.14±0.18 | 11.62 | 2.87E-01 | 9.06E-01 | 0.23±0.21 | 7.80E-01 | 0.27±0.21 | 7.70E-01 | -0.13±0.3 | 9.96E-01 | -0.13±0.32 | 9.96E-01 |
| dopamine 3-O-sulfate | 0.16±0.16 | 58.08 | 1.22E-01 | 8.24E-01 | 0.13±0.17 | 64.14 | 9.49E-02 | 9.13E-01 | 0.25±0.17 | 6.37E-01 | 0.24±0.18 | 7.56E-01 | -0.45±0.42 | 9.96E-01 | -0.58±0.46 | 9.96E-01 |
| pseudouridine | 0.16±0.17 | 32.1 | 2.25E-01 | 8.24E-01 | 0.14±0.18 | 42.76 | 1.86E-01 | 9.13E-01 | 0.29±0.2 | 6.51E-01 | 0.27±0.2 | 7.56E-01 | -0.18±0.33 | 9.96E-01 | -0.27±0.36 | 9.96E-01 |
| cis-3,4-methyleneheptanoylglycine | 0.22±0.19 | 1.12 | 3.15E-01 | 7.88E-01 | 0.15±0.19 | 37.67 | 2.05E-01 | 9.13E-01 | 0.32±0.22 | 6.37E-01 | 0.28±0.21 | 7.70E-01 | -0.12±0.38 | 9.96E-01 | -0.3±0.41 | 9.96E-01 |
| 6-bromotryptophan | 0.16±0.19 | 0 | 9.92E-01 | 8.72E-01 | 0.16±0.19 | 0 | 8.81E-01 | 9.13E-01 | 0.16±0.22 | 8.78E-01 | 0.17±0.22 | 8.94E-01 | 0.16±0.41 | 9.96E-01 | 0.1±0.42 | 9.96E-01 |
| X - 13728 | 0.17±0.2 | 0 | 8.38E-01 | 8.72E-01 | 0.17±0.21 | 0 | 9.39E-01 | 9.13E-01 | 0.15±0.22 | 8.90E-01 | 0.16±0.23 | 9.20E-01 | 0.26±0.45 | 9.96E-01 | 0.2±0.49 | 9.96E-01 |
| X - 26107 | -0.16±0.17 | 0 | 9.23E-01 | 8.24E-01 | -0.14±0.17 | 0 | 8.16E-01 | 9.13E-01 | -0.15±0.18 | 8.70E-01 | -0.12±0.19 | 9.26E-01 | -0.2±0.4 | 9.96E-01 | -0.22±0.41 | 9.96E-01 |
| 1-palmitoyl-2-arachidonoyl-GPC (16:0/20:4n6) | 0.09±0.21 | 0 | 6.02E-01 | 9.25E-01 | 0.17±0.21 | 0 | 6.19E-01 | 9.13E-01 | 0.05±0.22 | 9.57E-01 | 0.13±0.23 | 9.30E-01 | 0.37±0.57 | 9.96E-01 | 0.43±0.56 | 9.96E-01 |
| erythritol | -0.13±0.18 | 0 | 7.57E-01 | 8.91E-01 | -0.15±0.19 | 0 | 5.84E-01 | 9.13E-01 | -0.1±0.21 | 9.14E-01 | -0.1±0.21 | 9.43E-01 | -0.22±0.33 | 9.96E-01 | -0.35±0.42 | 9.96E-01 |
| glycine conjugate of C10H12O2* | -0.13±0.18 | 0 | 5.49E-01 | 8.96E-01 | -0.14±0.18 | 21.64 | 2.59E-01 | 9.13E-01 | -0.07±0.2 | 9.39E-01 | -0.05±0.2 | 9.78E-01 | -0.33±0.39 | 9.96E-01 | -0.57±0.42 | 9.96E-01 |
| N-acetylcitrulline | -0.16±0.19 | 50.22 | 1.56E-01 | 8.72E-01 | -0.15±0.19 | 58.93 | 1.19E-01 | 9.13E-01 | -0.02±0.21 | 9.94E-01 | 0.01±0.22 | 9.96E-01 | -0.66±0.4 | 9.96E-01 | -0.72±0.41 | 9.96E-01 |
| 1-palmitoleoylglycerol (16:1)* | -0.23±0.19 | 0 | 8.81E-01 | 7.61E-01 | -0.15±0.19 | 0 | 9.06E-01 | 9.14E-01 | -0.25±0.21 | 7.61E-01 | -0.16±0.22 | 9.02E-01 | -0.18±0.4 | 9.96E-01 | -0.11±0.42 | 9.96E-01 |
| 2-hydroxysebacate | 0.16±0.18 | 0 | 9.84E-01 | 8.54E-01 | 0.14±0.18 | 0 | 9.77E-01 | 9.14E-01 | 0.16±0.2 | 8.78E-01 | 0.15±0.2 | 9.12E-01 | 0.17±0.41 | 9.96E-01 | 0.13±0.43 | 9.96E-01 |
| salicylate | -0.15±0.2 | 0 | 6.47E-01 | 8.91E-01 | -0.16±0.2 | 0 | 5.95E-01 | 9.14E-01 | -0.1±0.23 | 9.14E-01 | -0.1±0.23 | 9.52E-01 | -0.33±0.45 | 9.96E-01 | -0.37±0.44 | 9.96E-01 |
| lactosyl-N-nervonoyl-sphingosine (d18:1/24:1)* | -0.06±0.2 | 0 | 7.81E-01 | 9.57E-01 | -0.16±0.21 | 0 | 5.46E-01 | 9.16E-01 | -0.09±0.22 | 9.35E-01 | -0.23±0.24 | 8.60E-01 | 0.04±0.42 | 9.96E-01 | 0.07±0.44 | 9.96E-01 |
| X - 21339 | -0.13±0.17 | 0 | 4.95E-01 | 8.96E-01 | -0.14±0.18 | 15.7 | 2.76E-01 | 9.18E-01 | -0.06±0.2 | 9.49E-01 | -0.03±0.2 | 9.92E-01 | -0.34±0.35 | 9.96E-01 | -0.47±0.35 | 9.96E-01 |
| X - 22162 | 0.13±0.18 | 0 | 8.46E-01 | 8.96E-01 | 0.14±0.18 | 0 | 8.29E-01 | 9.18E-01 | 0.15±0.2 | 8.78E-01 | 0.16±0.21 | 8.89E-01 | 0.06±0.39 | 9.96E-01 | 0.06±0.41 | 9.96E-01 |
| bilirubin degradation product, C17H18N2O4 (2)** | 0.14±0.19 | 0 | 9.54E-01 | 8.96E-01 | 0.15±0.2 | 0 | 9.71E-01 | 9.18E-01 | 0.14±0.21 | 8.91E-01 | 0.15±0.22 | 9.16E-01 | 0.12±0.43 | 9.96E-01 | 0.14±0.44 | 9.96E-01 |
| deoxycholate | 0.13±0.19 | 0 | 7.84E-01 | 9.03E-01 | 0.15±0.19 | 0 | 6.94E-01 | 9.18E-01 | 0.15±0.21 | 8.80E-01 | 0.18±0.21 | 8.79E-01 | 0.02±0.42 | 9.96E-01 | 0±0.43 | 9.98E-01 |
| 2,3-dihydroxyisovalerate | 0.08±0.18 | 60.37 | 1.12E-01 | 9.24E-01 | 0.15±0.19 | 47.33 | 1.68E-01 | 9.18E-01 | 0.18±0.19 | 8.06E-01 | 0.24±0.2 | 8.04E-01 | -0.67±0.51 | 9.96E-01 | -0.6±0.57 | 9.96E-01 |
| 1-stearoyl-GPE (18:0) | 0.23±0.2 | 0 | 8.89E-01 | 7.86E-01 | 0.16±0.21 | 0 | 7.17E-01 | 9.20E-01 | 0.22±0.22 | 7.95E-01 | 0.13±0.23 | 9.30E-01 | 0.3±0.52 | 9.96E-01 | 0.34±0.53 | 9.96E-01 |
| X - 23787 | 0.07±0.19 | 0 | 8.28E-01 | 9.49E-01 | 0.15±0.2 | 0 | 6.81E-01 | 9.20E-01 | 0.04±0.21 | 9.63E-01 | 0.11±0.22 | 9.41E-01 | 0.15±0.41 | 9.96E-01 | 0.32±0.44 | 9.96E-01 |
| 3-hydroxy-3-methylglutarate | 0.17±0.21 | 2.34 | 3.12E-01 | 8.77E-01 | 0.16±0.21 | 0 | 3.64E-01 | 9.20E-01 | 0.08±0.23 | 9.39E-01 | 0.08±0.23 | 9.64E-01 | 0.66±0.53 | 9.96E-01 | 0.64±0.56 | 9.96E-01 |
| phenylacetylglutamine | -0.07±0.18 | 0 | 8.77E-01 | 9.40E-01 | -0.14±0.18 | 0 | 7.78E-01 | 9.22E-01 | -0.06±0.21 | 9.49E-01 | -0.11±0.21 | 9.30E-01 | -0.13±0.4 | 9.96E-01 | -0.24±0.41 | 9.96E-01 |
| stearidonate (18:4n3) | -0.13±0.19 | 0 | 4.88E-01 | 9.00E-01 | -0.15±0.2 | 0 | 4.15E-01 | 9.22E-01 | -0.07±0.21 | 9.48E-01 | -0.08±0.21 | 9.63E-01 | -0.42±0.46 | 9.96E-01 | -0.51±0.48 | 9.96E-01 |
| cortisol | -0.11±0.15 | 49.74 | 1.58E-01 | 8.96E-01 | -0.11±0.15 | 50.52 | 1.55E-01 | 9.23E-01 | -0.18±0.16 | 7.70E-01 | -0.18±0.16 | 8.09E-01 | 0.52±0.47 | 9.96E-01 | 0.51±0.46 | 9.96E-01 |
| X - 12112 | 0.12±0.19 | 0 | 3.40E-01 | 9.03E-01 | 0.15±0.2 | 9.84 | 2.92E-01 | 9.23E-01 | 0.22±0.22 | 7.95E-01 | 0.26±0.22 | 8.10E-01 | -0.2±0.39 | 9.96E-01 | -0.23±0.41 | 9.96E-01 |
| picolinate | -0.15±0.19 | 24.25 | 2.51E-01 | 8.87E-01 | -0.13±0.19 | 12.74 | 2.84E-01 | 9.23E-01 | -0.26±0.21 | 7.33E-01 | -0.23±0.21 | 8.27E-01 | 0.28±0.41 | 9.96E-01 | 0.27±0.42 | 9.96E-01 |
| X - 22509 | -0.12±0.18 | 0 | 3.89E-01 | 9.03E-01 | -0.13±0.19 | 0.01 | 3.17E-01 | 9.23E-01 | -0.19±0.2 | 8.19E-01 | -0.22±0.2 | 8.33E-01 | 0.22±0.43 | 9.96E-01 | 0.27±0.44 | 9.96E-01 |
| gamma-carboxyglutamate | 0.1±0.18 | 0 | 5.39E-01 | 9.08E-01 | 0.13±0.19 | 0 | 3.94E-01 | 9.23E-01 | 0.16±0.21 | 8.78E-01 | 0.22±0.22 | 8.47E-01 | -0.1±0.37 | 9.96E-01 | -0.15±0.38 | 9.96E-01 |
| X - 17612 | -0.13±0.18 | 0 | 3.61E-01 | 8.91E-01 | -0.13±0.18 | 0 | 3.85E-01 | 9.23E-01 | -0.2±0.19 | 7.95E-01 | -0.19±0.19 | 8.60E-01 | 0.28±0.49 | 9.96E-01 | 0.27±0.5 | 9.96E-01 |
| X - 16397 | -0.18±0.16 | 0 | 3.33E-01 | 7.97E-01 | -0.11±0.17 | 0 | 3.77E-01 | 9.23E-01 | -0.24±0.17 | 6.91E-01 | -0.17±0.18 | 8.63E-01 | 0.23±0.45 | 9.96E-01 | 0.26±0.45 | 9.96E-01 |
| X - 17357 | -0.14±0.16 | 21.05 | 2.60E-01 | 8.72E-01 | -0.12±0.17 | 0 | 4.15E-01 | 9.23E-01 | -0.2±0.17 | 7.52E-01 | -0.17±0.18 | 8.63E-01 | 0.36±0.47 | 9.96E-01 | 0.28±0.52 | 9.96E-01 |
| citrulline | -0.04±0.19 | 0 | 4.85E-01 | 9.64E-01 | -0.13±0.19 | 0 | 4.30E-01 | 9.23E-01 | -0.1±0.2 | 9.14E-01 | -0.2±0.21 | 8.63E-01 | 0.27±0.48 | 9.96E-01 | 0.21±0.47 | 9.96E-01 |
| glycosyl ceramide (d18:1/20:0, d16:1/22:0)* | -0.08±0.2 | 0 | 6.27E-01 | 9.35E-01 | -0.14±0.2 | 0 | 4.70E-01 | 9.23E-01 | -0.13±0.22 | 9.06E-01 | -0.21±0.23 | 8.77E-01 | 0.1±0.42 | 9.96E-01 | 0.17±0.47 | 9.96E-01 |
| N-carbamoylvaline | 0.15±0.19 | 0 | 6.54E-01 | 8.77E-01 | 0.14±0.19 | 0 | 5.98E-01 | 9.23E-01 | 0.2±0.21 | 8.31E-01 | 0.19±0.21 | 8.79E-01 | -0.01±0.42 | 9.96E-01 | -0.06±0.42 | 9.96E-01 |
| X - 24473 | 0.11±0.2 | 0 | 3.55E-01 | 9.03E-01 | 0.14±0.21 | 0 | 5.34E-01 | 9.23E-01 | 0.21±0.23 | 8.33E-01 | 0.21±0.24 | 8.79E-01 | -0.22±0.41 | 9.96E-01 | -0.12±0.47 | 9.96E-01 |
| X - 21319 | -0.09±0.18 | 0 | 3.56E-01 | 9.13E-01 | -0.12±0.18 | 0 | 5.32E-01 | 9.23E-01 | -0.16±0.2 | 8.72E-01 | -0.17±0.19 | 8.79E-01 | 0.28±0.44 | 9.96E-01 | 0.14±0.45 | 9.96E-01 |
| X - 21796 | 0.12±0.2 | 0 | 6.20E-01 | 9.03E-01 | 0.14±0.2 | 0 | 6.36E-01 | 9.23E-01 | 0.17±0.22 | 8.78E-01 | 0.19±0.23 | 8.79E-01 | -0.07±0.42 | 9.96E-01 | -0.04±0.43 | 9.96E-01 |
| bilirubin (E,E)* | 0.11±0.19 | 0 | 7.84E-01 | 9.03E-01 | 0.14±0.19 | 0 | 6.76E-01 | 9.23E-01 | 0.14±0.21 | 8.92E-01 | 0.18±0.22 | 8.86E-01 | 0.01±0.41 | 9.96E-01 | -0.02±0.42 | 9.96E-01 |
| gamma-glutamyltyrosine | -0.2±0.18 | 0 | 5.02E-01 | 7.77E-01 | -0.13±0.19 | 0 | 6.28E-01 | 9.23E-01 | -0.26±0.19 | 6.98E-01 | -0.17±0.21 | 8.86E-01 | 0.05±0.41 | 9.96E-01 | 0.07±0.45 | 9.96E-01 |
| gamma-glutamylphenylalanine | 0.07±0.19 | 0 | 8.76E-01 | 9.40E-01 | 0.13±0.19 | 0 | 6.68E-01 | 9.23E-01 | 0.08±0.21 | 9.35E-01 | 0.17±0.21 | 8.88E-01 | 0.01±0.4 | 9.96E-01 | -0.02±0.4 | 9.96E-01 |
| 3-hydroxyoleoylcarnitine | -0.13±0.17 | 0 | 6.85E-01 | 8.91E-01 | -0.12±0.17 | 0 | 7.25E-01 | 9.23E-01 | -0.16±0.18 | 8.70E-01 | -0.14±0.19 | 8.99E-01 | 0.04±0.44 | 9.96E-01 | 0.02±0.44 | 9.96E-01 |
| propionylcarnitine (C3) | -0.17±0.18 | 0 | 7.59E-01 | 8.24E-01 | -0.14±0.18 | 0 | 8.79E-01 | 9.23E-01 | -0.2±0.2 | 7.95E-01 | -0.15±0.21 | 9.12E-01 | -0.08±0.35 | 9.96E-01 | -0.09±0.37 | 9.96E-01 |
| X - 21310 | 0.17±0.21 | 0 | 9.78E-01 | 8.72E-01 | 0.14±0.21 | 0 | 8.25E-01 | 9.23E-01 | 0.18±0.23 | 8.78E-01 | 0.17±0.23 | 9.14E-01 | 0.16±0.48 | 9.96E-01 | 0.05±0.48 | 9.96E-01 |
| 6-oxopiperidine-2-carboxylate | 0.18±0.2 | 0 | 9.01E-01 | 8.52E-01 | 0.15±0.21 | 0 | 9.48E-01 | 9.23E-01 | 0.17±0.22 | 8.78E-01 | 0.15±0.23 | 9.26E-01 | 0.23±0.45 | 9.96E-01 | 0.12±0.46 | 9.96E-01 |
| X - 12740 | 0.13±0.19 | 0 | 9.28E-01 | 8.96E-01 | 0.13±0.19 | 0 | 9.15E-01 | 9.23E-01 | 0.14±0.21 | 8.90E-01 | 0.14±0.21 | 9.26E-01 | 0.1±0.41 | 9.96E-01 | 0.09±0.42 | 9.96E-01 |
| 1H-indole-7-acetic acid | 0.13±0.2 | 0 | 9.39E-01 | 9.03E-01 | 0.15±0.2 | 0 | 9.73E-01 | 9.23E-01 | 0.12±0.22 | 9.14E-01 | 0.15±0.23 | 9.26E-01 | 0.16±0.45 | 9.96E-01 | 0.13±0.47 | 9.96E-01 |
| stearoylcarnitine (C18) | 0.17±0.2 | 0 | 9.40E-01 | 8.60E-01 | 0.14±0.2 | 0 | 9.68E-01 | 9.23E-01 | 0.18±0.22 | 8.72E-01 | 0.14±0.22 | 9.26E-01 | 0.14±0.46 | 9.96E-01 | 0.16±0.47 | 9.96E-01 |
| lyxonate | -0.13±0.18 | 0 | 8.94E-01 | 8.96E-01 | -0.14±0.19 | 0 | 9.66E-01 | 9.23E-01 | -0.11±0.21 | 9.14E-01 | -0.14±0.22 | 9.26E-01 | -0.17±0.36 | 9.96E-01 | -0.16±0.38 | 9.96E-01 |
| oxalate (ethanedioate) | -0.09±0.17 | 0 | 9.88E-01 | 9.03E-01 | -0.12±0.18 | 0 | 9.53E-01 | 9.23E-01 | -0.09±0.19 | 9.14E-01 | -0.12±0.2 | 9.26E-01 | -0.09±0.39 | 9.96E-01 | -0.14±0.41 | 9.96E-01 |
| dimethylglycine | -0.11±0.19 | 0 | 9.56E-01 | 9.03E-01 | -0.14±0.19 | 0 | 9.81E-01 | 9.23E-01 | -0.12±0.22 | 9.14E-01 | -0.14±0.23 | 9.26E-01 | -0.09±0.37 | 9.96E-01 | -0.13±0.36 | 9.96E-01 |
| valine | -0.12±0.13 | 0 | 9.42E-01 | 8.33E-01 | -0.1±0.15 | 0 | 9.48E-01 | 9.23E-01 | -0.13±0.14 | 8.67E-01 | -0.1±0.17 | 9.30E-01 | -0.1±0.31 | 9.96E-01 | -0.12±0.31 | 9.96E-01 |
| 2-hydroxybutyrate/2-hydroxyisobutyrate | -0.25±0.19 | 0 | 9.45E-01 | 7.10E-01 | -0.14±0.2 | 0 | 8.95E-01 | 9.23E-01 | -0.25±0.21 | 7.52E-01 | -0.12±0.22 | 9.30E-01 | -0.22±0.4 | 9.96E-01 | -0.19±0.42 | 9.96E-01 |
| homostachydrine* | 0.15±0.18 | 0 | 8.99E-01 | 8.72E-01 | 0.13±0.19 | 0 | 8.68E-01 | 9.23E-01 | 0.14±0.2 | 8.89E-01 | 0.12±0.21 | 9.30E-01 | 0.2±0.41 | 9.96E-01 | 0.2±0.42 | 9.96E-01 |
| 5,6-dihydrothymine | 0.12±0.19 | 0 | 8.00E-01 | 9.03E-01 | 0.14±0.19 | 0 | 8.34E-01 | 9.23E-01 | 0.09±0.21 | 9.18E-01 | 0.12±0.21 | 9.30E-01 | 0.23±0.47 | 9.96E-01 | 0.23±0.48 | 9.96E-01 |
| gamma-glutamylthreonine | 0.15±0.2 | 0 | 7.27E-01 | 8.91E-01 | 0.14±0.2 | 0 | 7.02E-01 | 9.23E-01 | 0.13±0.21 | 9.05E-01 | 0.11±0.21 | 9.30E-01 | 0.37±0.65 | 9.96E-01 | 0.37±0.65 | 9.96E-01 |
| caproate (6:0) | 0.11±0.2 | 0 | 4.57E-01 | 9.03E-01 | 0.15±0.2 | 0 | 5.26E-01 | 9.23E-01 | 0.06±0.21 | 9.49E-01 | 0.11±0.21 | 9.31E-01 | 0.6±0.69 | 9.96E-01 | 0.56±0.67 | 9.96E-01 |
| X - 23644 | 0.17±0.19 | 0 | 5.52E-01 | 8.54E-01 | 0.14±0.19 | 0 | 7.22E-01 | 9.23E-01 | 0.11±0.21 | 9.14E-01 | 0.1±0.21 | 9.41E-01 | 0.39±0.42 | 9.96E-01 | 0.28±0.44 | 9.96E-01 |
| 1-myristoyl-2-arachidonoyl-GPC (14:0/20:4)* | 0.1±0.21 | 0 | 5.87E-01 | 9.24E-01 | 0.15±0.22 | 0 | 6.62E-01 | 9.23E-01 | 0.04±0.23 | 9.71E-01 | 0.11±0.24 | 9.42E-01 | 0.35±0.52 | 9.96E-01 | 0.36±0.53 | 9.96E-01 |
| alpha-ketobutyrate | -0.23±0.18 | 0 | 7.77E-01 | 7.21E-01 | -0.13±0.19 | 0 | 6.63E-01 | 9.23E-01 | -0.2±0.2 | 7.95E-01 | -0.09±0.22 | 9.63E-01 | -0.32±0.38 | 9.96E-01 | -0.29±0.4 | 9.96E-01 |
| 2-naphthol sulfate | -0.19±0.21 | 0 | 3.81E-01 | 8.37E-01 | -0.18±0.26 | 0 | 5.53E-01 | 9.23E-01 | -0.12±0.22 | 9.14E-01 | -0.11±0.28 | 9.63E-01 | -0.65±0.57 | 9.96E-01 | -0.52±0.63 | 9.96E-01 |
| X - 11787 | -0.09±0.18 | 0 | 7.66E-01 | 9.13E-01 | -0.13±0.18 | 0 | 6.07E-01 | 9.23E-01 | -0.05±0.21 | 9.49E-01 | -0.07±0.21 | 9.66E-01 | -0.17±0.32 | 9.96E-01 | -0.28±0.34 | 9.96E-01 |
| 1-linoleoyl-2-arachidonoyl-GPC (18:2/20:4n6)* | 0.18±0.22 | 27.72 | 2.40E-01 | 8.77E-01 | 0.15±0.22 | 32.12 | 2.25E-01 | 9.23E-01 | 0.1±0.23 | 9.25E-01 | 0.07±0.23 | 9.66E-01 | 1.01±0.74 | 9.96E-01 | 1.08±0.8 | 9.96E-01 |
| sulfate* | -0.12±0.17 | 0 | 6.68E-01 | 8.96E-01 | -0.13±0.17 | 0 | 6.19E-01 | 9.23E-01 | -0.07±0.21 | 9.49E-01 | -0.07±0.21 | 9.66E-01 | -0.22±0.28 | 9.96E-01 | -0.25±0.3 | 9.96E-01 |
| N-delta-acetylornithine | -0.12±0.17 | 0 | 5.30E-01 | 8.96E-01 | -0.13±0.18 | 0 | 4.69E-01 | 9.23E-01 | -0.06±0.2 | 9.49E-01 | -0.06±0.2 | 9.66E-01 | -0.31±0.35 | 9.96E-01 | -0.37±0.38 | 9.96E-01 |
| 3-indoxyl sulfate | -0.09±0.18 | 0 | 7.22E-01 | 9.08E-01 | -0.12±0.18 | 0 | 4.91E-01 | 9.23E-01 | -0.06±0.2 | 9.49E-01 | -0.07±0.2 | 9.66E-01 | -0.22±0.4 | 9.96E-01 | -0.38±0.42 | 9.96E-01 |
| X - 23680 | -0.1±0.17 | 52.93 | 1.45E-01 | 9.03E-01 | -0.12±0.17 | 70.23 | 6.68E-02 | 9.23E-01 | 0.06±0.2 | 9.49E-01 | 0.07±0.2 | 9.66E-01 | -0.48±0.31 | 9.96E-01 | -0.65±0.33 | 9.96E-01 |
| (14 or 15)-methylpalmitate (a17:0 or i17:0) | 0.16±0.2 | 0 | 4.19E-01 | 8.87E-01 | 0.14±0.2 | 0 | 3.65E-01 | 9.23E-01 | 0.08±0.22 | 9.37E-01 | 0.06±0.22 | 9.66E-01 | 0.51±0.48 | 9.96E-01 | 0.54±0.48 | 9.96E-01 |
| 2-hydroxyhippurate (salicylurate) | -0.14±0.21 | 0 | 3.46E-01 | 9.00E-01 | -0.15±0.21 | 2.19 | 3.12E-01 | 9.23E-01 | -0.07±0.22 | 9.49E-01 | -0.07±0.22 | 9.66E-01 | -0.64±0.57 | 9.96E-01 | -0.69±0.58 | 9.96E-01 |
| 7-methylxanthine | 0.15±0.19 | 9.63 | 2.93E-01 | 8.82E-01 | 0.15±0.2 | 16.31 | 2.74E-01 | 9.23E-01 | 0.06±0.21 | 9.49E-01 | 0.06±0.21 | 9.67E-01 | 0.6±0.47 | 9.96E-01 | 0.68±0.53 | 9.96E-01 |
| 3-hydroxyoctanoate | -0.12±0.19 | 0 | 5.81E-01 | 9.03E-01 | -0.14±0.19 | 0 | 4.12E-01 | 9.23E-01 | -0.07±0.22 | 9.49E-01 | -0.06±0.22 | 9.75E-01 | -0.32±0.4 | 9.96E-01 | -0.44±0.41 | 9.96E-01 |
| X - 15503 | -0.14±0.19 | 0 | 7.71E-01 | 8.96E-01 | -0.14±0.2 | 0 | 3.58E-01 | 9.23E-01 | -0.11±0.21 | 9.14E-01 | -0.05±0.22 | 9.78E-01 | -0.24±0.39 | 9.96E-01 | -0.52±0.46 | 9.96E-01 |
| quinolinate | -0.14±0.18 | 0 | 7.80E-01 | 8.91E-01 | -0.13±0.18 | 0 | 4.84E-01 | 9.23E-01 | -0.11±0.22 | 9.14E-01 | -0.05±0.22 | 9.79E-01 | -0.22±0.33 | 9.96E-01 | -0.32±0.33 | 9.96E-01 |
| X - 21736 | -0.21±0.18 | 0 | 4.52E-01 | 7.79E-01 | -0.13±0.19 | 8.19 | 2.97E-01 | 9.23E-01 | -0.14±0.2 | 8.89E-01 | -0.04±0.21 | 9.80E-01 | -0.49±0.41 | 9.96E-01 | -0.54±0.44 | 9.96E-01 |
| sphingomyelin (d18:0/20:0, d16:0/22:0)* | -0.21±0.19 | 0 | 5.74E-01 | 7.97E-01 | -0.14±0.2 | 0 | 6.47E-01 | 9.23E-01 | -0.28±0.22 | 7.26E-01 | -0.19±0.23 | 8.79E-01 | -0.04±0.36 | 9.96E-01 | 0.01±0.38 | 9.98E-01 |
| 3-methylglutaconate | 0.17±0.18 | 0 | 7.35E-01 | 8.38E-01 | 0.13±0.18 | 0 | 6.96E-01 | 9.23E-01 | 0.2±0.21 | 8.07E-01 | 0.17±0.21 | 8.86E-01 | 0.05±0.39 | 9.96E-01 | 0±0.38 | 9.98E-01 |
| 5alpha-androstan-3beta,17beta-diol monosulfate (2) | 0.07±0.2 | 0 | 9.11E-01 | 9.42E-01 | 0.14±0.21 | 0 | 8.73E-01 | 9.25E-01 | 0.06±0.23 | 9.49E-01 | 0.13±0.24 | 9.30E-01 | 0.12±0.45 | 9.96E-01 | 0.21±0.48 | 9.96E-01 |
| N-acetylthreonine | 0.13±0.17 | 0 | 3.71E-01 | 8.91E-01 | 0.11±0.17 | 0 | 3.85E-01 | 9.26E-01 | 0.22±0.2 | 7.70E-01 | 0.2±0.2 | 8.47E-01 | -0.11±0.31 | 9.96E-01 | -0.12±0.32 | 9.96E-01 |
| thymol sulfate | 0.06±0.18 | 0 | 6.81E-01 | 9.55E-01 | 0.13±0.19 | 0 | 9.68E-01 | 9.26E-01 | 0.09±0.2 | 9.14E-01 | 0.13±0.21 | 9.26E-01 | -0.1±0.41 | 9.96E-01 | 0.11±0.44 | 9.96E-01 |
| X - 12101 | -0.11±0.19 | 0 | 9.10E-01 | 9.03E-01 | -0.12±0.18 | 0 | 7.33E-01 | 9.26E-01 | -0.09±0.21 | 9.22E-01 | -0.09±0.21 | 9.58E-01 | -0.14±0.38 | 9.96E-01 | -0.23±0.37 | 9.96E-01 |
| linoleoyl ethanolamide | -0.08±0.18 | 0 | 8.03E-01 | 9.29E-01 | -0.12±0.18 | 0 | 8.20E-01 | 9.27E-01 | -0.1±0.2 | 9.14E-01 | -0.14±0.2 | 9.20E-01 | 0.02±0.42 | 9.96E-01 | -0.04±0.41 | 9.96E-01 |
| X - 16935 | -0.09±0.18 | 0 | 5.48E-01 | 9.13E-01 | -0.12±0.18 | 0 | 6.94E-01 | 9.30E-01 | -0.13±0.19 | 8.89E-01 | -0.15±0.2 | 8.99E-01 | 0.15±0.43 | 9.96E-01 | 0.03±0.42 | 9.96E-01 |
| creatinine | -0.11±0.19 | 0 | 9.55E-01 | 9.03E-01 | -0.13±0.19 | 0 | 9.45E-01 | 9.30E-01 | -0.1±0.24 | 9.23E-01 | -0.12±0.24 | 9.41E-01 | -0.13±0.31 | 9.96E-01 | -0.15±0.33 | 9.96E-01 |
| 3-hydroxylaurate | -0.11±0.19 | 0 | 5.95E-01 | 9.03E-01 | -0.13±0.19 | 0 | 4.41E-01 | 9.30E-01 | -0.07±0.21 | 9.49E-01 | -0.06±0.21 | 9.68E-01 | -0.32±0.43 | 9.96E-01 | -0.45±0.46 | 9.96E-01 |
| 1-lignoceroyl-GPC (24:0) | 0.22±0.2 | 0 | 4.73E-01 | 7.97E-01 | 0.14±0.21 | 17.34 | 2.71E-01 | 9.30E-01 | 0.16±0.22 | 8.78E-01 | 0.05±0.22 | 9.79E-01 | 0.55±0.5 | 9.96E-01 | 0.73±0.57 | 9.96E-01 |
| bilirubin degradation product, C17H18N2O4 (1)** | 0.11±0.19 | 0 | 7.91E-01 | 9.03E-01 | 0.13±0.2 | 0 | 7.73E-01 | 9.30E-01 | 0.08±0.21 | 9.35E-01 | 0.1±0.22 | 9.43E-01 | 0.22±0.46 | 9.96E-01 | 0.25±0.46 | 9.96E-01 |
| X - 22776 | -0.14±0.18 | 0 | 9.42E-01 | 8.87E-01 | -0.12±0.18 | 0 | 9.81E-01 | 9.33E-01 | -0.13±0.21 | 9.00E-01 | -0.12±0.21 | 9.30E-01 | -0.16±0.35 | 9.96E-01 | -0.13±0.36 | 9.96E-01 |
| alpha-ketoglutarate | -0.14±0.15 | 0 | 9.91E-01 | 8.37E-01 | -0.11±0.16 | 0 | 8.81E-01 | 9.33E-01 | -0.13±0.17 | 8.78E-01 | -0.09±0.2 | 9.53E-01 | -0.14±0.26 | 9.96E-01 | -0.14±0.28 | 9.96E-01 |
| linoleoylcarnitine (C18:2)* | 0.17±0.18 | 59.31 | 1.17E-01 | 8.24E-01 | 0.1±0.18 | 58.71 | 1.20E-01 | 9.34E-01 | 0.36±0.21 | 5.80E-01 | 0.3±0.22 | 7.50E-01 | -0.23±0.31 | 9.96E-01 | -0.28±0.3 | 9.96E-01 |
| N-acetyl-1-methylhistidine* | 0.09±0.19 | 58.81 | 1.19E-01 | 9.22E-01 | 0.11±0.18 | 63.58 | 9.75E-02 | 9.34E-01 | 0.25±0.21 | 7.52E-01 | 0.28±0.21 | 7.56E-01 | -0.42±0.38 | 9.96E-01 | -0.42±0.37 | 9.96E-01 |
| X - 11849 | -0.11±0.18 | 73.47 | 5.22E-02 | 9.03E-01 | -0.1±0.19 | 69.74 | 6.91E-02 | 9.34E-01 | -0.25±0.2 | 7.26E-01 | -0.23±0.2 | 8.10E-01 | 0.81±0.51 | 9.96E-01 | 0.83±0.54 | 9.96E-01 |
| sphingomyelin (d17:1/14:0, d16:1/15:0)* | -0.1±0.21 | 28.65 | 2.36E-01 | 9.20E-01 | -0.13±0.22 | 49.74 | 1.58E-01 | 9.34E-01 | -0.22±0.23 | 8.31E-01 | -0.24±0.24 | 8.43E-01 | 0.44±0.5 | 9.96E-01 | 0.73±0.65 | 9.96E-01 |
| sphingosine 1-phosphate | -0.11±0.21 | 6.75 | 3.00E-01 | 9.03E-01 | -0.13±0.21 | 17.75 | 2.70E-01 | 9.34E-01 | -0.18±0.22 | 8.72E-01 | -0.21±0.22 | 8.64E-01 | 0.55±0.67 | 9.96E-01 | 0.6±0.7 | 9.96E-01 |
| dodecenedioate (C12:1-DC)* | 0.19±0.18 | 0 | 3.85E-01 | 8.24E-01 | 0.11±0.18 | 0 | 3.87E-01 | 9.34E-01 | 0.27±0.2 | 7.19E-01 | 0.18±0.2 | 8.79E-01 | -0.13±0.41 | 9.96E-01 | -0.22±0.42 | 9.96E-01 |
| laurate (12:0) | 0.2±0.19 | 0 | 4.28E-01 | 8.24E-01 | 0.11±0.2 | 0 | 3.89E-01 | 9.34E-01 | 0.27±0.21 | 7.26E-01 | 0.19±0.22 | 8.79E-01 | -0.13±0.46 | 9.96E-01 | -0.25±0.47 | 9.96E-01 |
| alpha-hydroxyisocaproate | -0.18±0.2 | 8.15 | 2.97E-01 | 8.30E-01 | -0.12±0.2 | 0 | 3.24E-01 | 9.34E-01 | -0.26±0.21 | 7.27E-01 | -0.19±0.21 | 8.79E-01 | 0.39±0.59 | 9.96E-01 | 0.43±0.6 | 9.96E-01 |
| paraxanthine | -0.15±0.17 | 25.66 | 2.46E-01 | 8.60E-01 | -0.11±0.18 | 0 | 3.52E-01 | 9.34E-01 | -0.22±0.18 | 7.34E-01 | -0.17±0.19 | 8.79E-01 | 0.43±0.53 | 9.96E-01 | 0.36±0.53 | 9.96E-01 |
| linolenoylcarnitine (C18:3)* | 0.18±0.19 | 0 | 5.31E-01 | 8.24E-01 | 0.12±0.19 | 0 | 4.88E-01 | 9.34E-01 | 0.24±0.21 | 7.61E-01 | 0.19±0.22 | 8.79E-01 | -0.05±0.41 | 9.96E-01 | -0.14±0.41 | 9.96E-01 |
| X - 26119 | -0.1±0.19 | 0 | 4.43E-01 | 9.12E-01 | -0.11±0.19 | 0 | 4.33E-01 | 9.34E-01 | -0.16±0.21 | 8.78E-01 | -0.17±0.21 | 8.79E-01 | 0.25±0.49 | 9.96E-01 | 0.26±0.52 | 9.96E-01 |
| threonine | -0.09±0.2 | 0 | 3.37E-01 | 9.24E-01 | -0.11±0.19 | 1.64 | 3.13E-01 | 9.34E-01 | -0.16±0.21 | 8.78E-01 | -0.17±0.2 | 8.79E-01 | 0.46±0.6 | 9.96E-01 | 0.47±0.61 | 9.96E-01 |
| tartarate | 0.08±0.19 | 0 | 4.16E-01 | 9.35E-01 | 0.11±0.19 | 0 | 3.92E-01 | 9.34E-01 | 0.16±0.21 | 8.78E-01 | 0.2±0.22 | 8.79E-01 | -0.19±0.38 | 9.96E-01 | -0.18±0.38 | 9.96E-01 |
| indolin-2-one | -0.08±0.19 | 0 | 4.29E-01 | 9.35E-01 | -0.12±0.19 | 0 | 4.71E-01 | 9.34E-01 | -0.14±0.2 | 8.89E-01 | -0.18±0.21 | 8.79E-01 | 0.28±0.49 | 9.96E-01 | 0.21±0.5 | 9.96E-01 |
| retinal | -0.13±0.19 | 0 | 4.25E-01 | 8.96E-01 | -0.1±0.19 | 0 | 3.95E-01 | 9.34E-01 | -0.19±0.2 | 8.25E-01 | -0.17±0.2 | 8.86E-01 | 0.25±0.51 | 9.96E-01 | 0.31±0.52 | 9.96E-01 |
| hexadecenedioate (C16:1-DC)* | 0.14±0.18 | 0 | 5.63E-01 | 8.87E-01 | 0.11±0.18 | 0 | 5.77E-01 | 9.34E-01 | 0.19±0.2 | 8.16E-01 | 0.16±0.2 | 8.88E-01 | -0.06±0.38 | 9.96E-01 | -0.07±0.37 | 9.96E-01 |
| N2-acetyl,N6,N6-dimethyllysine | 0.12±0.19 | 0 | 5.63E-01 | 9.03E-01 | 0.11±0.19 | 0 | 5.09E-01 | 9.34E-01 | 0.18±0.21 | 8.70E-01 | 0.17±0.21 | 8.88E-01 | -0.09±0.41 | 9.96E-01 | -0.14±0.42 | 9.96E-01 |
| hydroxyasparagine** | 0.07±0.17 | 0 | 6.96E-01 | 9.36E-01 | 0.09±0.17 | 0 | 5.28E-01 | 9.34E-01 | 0.11±0.21 | 9.14E-01 | 0.17±0.2 | 8.88E-01 | -0.04±0.33 | 9.96E-01 | -0.07±0.31 | 9.96E-01 |
| carnitine | -0.15±0.18 | 0 | 5.31E-01 | 8.72E-01 | -0.11±0.19 | 0 | 5.34E-01 | 9.34E-01 | -0.2±0.2 | 7.95E-01 | -0.16±0.2 | 8.89E-01 | 0.12±0.46 | 9.96E-01 | 0.17±0.5 | 9.96E-01 |
| 2-aminobutyrate | -0.19±0.19 | 0 | 6.30E-01 | 8.24E-01 | -0.11±0.2 | 0 | 6.08E-01 | 9.34E-01 | -0.24±0.21 | 7.67E-01 | -0.16±0.22 | 9.03E-01 | -0.02±0.4 | 9.96E-01 | 0.09±0.45 | 9.96E-01 |
| glutarate (C5-DC) | 0.15±0.2 | 0 | 7.04E-01 | 8.96E-01 | 0.12±0.21 | 0 | 6.40E-01 | 9.34E-01 | 0.19±0.23 | 8.72E-01 | 0.17±0.23 | 9.14E-01 | 0.01±0.43 | 9.96E-01 | -0.06±0.43 | 9.96E-01 |
| 3-methyl-2-oxobutyrate | -0.2±0.18 | 0 | 6.70E-01 | 8.00E-01 | -0.12±0.19 | 0 | 9.22E-01 | 9.34E-01 | -0.24±0.2 | 7.52E-01 | -0.13±0.21 | 9.26E-01 | -0.06±0.37 | 9.96E-01 | -0.09±0.39 | 9.96E-01 |
| X - 16580 | -0.17±0.18 | 0 | 6.76E-01 | 8.27E-01 | -0.1±0.19 | 0 | 8.23E-01 | 9.34E-01 | -0.22±0.21 | 7.95E-01 | -0.13±0.21 | 9.26E-01 | -0.04±0.37 | 9.96E-01 | -0.03±0.37 | 9.96E-01 |
| vanillylmandelate (VMA) | 0.18±0.19 | 0 | 9.97E-01 | 8.24E-01 | 0.12±0.2 | 0 | 9.37E-01 | 9.34E-01 | 0.18±0.22 | 8.70E-01 | 0.13±0.22 | 9.26E-01 | 0.19±0.41 | 9.96E-01 | 0.09±0.43 | 9.96E-01 |
| X - 23587 | -0.16±0.18 | 0 | 8.67E-01 | 8.72E-01 | -0.11±0.18 | 0 | 8.43E-01 | 9.34E-01 | -0.17±0.2 | 8.70E-01 | -0.13±0.2 | 9.26E-01 | -0.1±0.4 | 9.96E-01 | -0.04±0.4 | 9.96E-01 |
| X - 25271 | -0.12±0.18 | 0 | 7.34E-01 | 9.03E-01 | -0.11±0.19 | 0 | 8.48E-01 | 9.34E-01 | -0.15±0.2 | 8.84E-01 | -0.12±0.21 | 9.26E-01 | 0.01±0.42 | 9.96E-01 | -0.03±0.44 | 9.96E-01 |
| pantoate | 0.12±0.19 | 0 | 8.61E-01 | 9.03E-01 | 0.12±0.2 | 0 | 8.75E-01 | 9.34E-01 | 0.14±0.21 | 9.00E-01 | 0.13±0.22 | 9.26E-01 | 0.05±0.45 | 9.96E-01 | 0.05±0.48 | 9.96E-01 |
| deoxycholic acid glucuronide | 0.1±0.19 | 0 | 9.31E-01 | 9.13E-01 | 0.12±0.2 | 0 | 8.18E-01 | 9.34E-01 | 0.11±0.22 | 9.14E-01 | 0.14±0.22 | 9.26E-01 | 0.06±0.43 | 9.96E-01 | 0.02±0.45 | 9.96E-01 |
| allantoin | -0.09±0.18 | 0 | 8.77E-01 | 9.20E-01 | -0.11±0.18 | 0 | 9.45E-01 | 9.34E-01 | -0.1±0.2 | 9.14E-01 | -0.12±0.2 | 9.26E-01 | -0.03±0.41 | 9.96E-01 | -0.09±0.41 | 9.96E-01 |
| 2'-O-methylcytidine | 0.16±0.19 | 0 | 8.21E-01 | 8.72E-01 | 0.11±0.2 | 0 | 8.84E-01 | 9.34E-01 | 0.19±0.22 | 8.70E-01 | 0.12±0.22 | 9.30E-01 | 0.08±0.43 | 9.96E-01 | 0.05±0.44 | 9.96E-01 |
| indolelactate | -0.12±0.2 | 0 | 6.98E-01 | 9.03E-01 | -0.11±0.2 | 0 | 8.76E-01 | 9.34E-01 | -0.16±0.22 | 8.78E-01 | -0.13±0.23 | 9.30E-01 | 0.02±0.42 | 9.96E-01 | -0.06±0.41 | 9.96E-01 |
| lithocholate sulfate (1) | 0.11±0.19 | 0 | 9.21E-01 | 9.03E-01 | 0.13±0.2 | 0 | 9.73E-01 | 9.34E-01 | 0.1±0.22 | 9.14E-01 | 0.12±0.22 | 9.30E-01 | 0.15±0.46 | 9.96E-01 | 0.14±0.49 | 9.96E-01 |
| X - 11381 | -0.08±0.18 | 0 | 8.14E-01 | 9.29E-01 | -0.1±0.18 | 0 | 8.94E-01 | 9.34E-01 | -0.1±0.2 | 9.14E-01 | -0.11±0.19 | 9.30E-01 | 0.01±0.41 | 9.96E-01 | -0.05±0.44 | 9.96E-01 |
| 1-myristoyl-2-palmitoyl-GPC (14:0/16:0) | 0.07±0.19 | 0 | 8.61E-01 | 9.40E-01 | 0.11±0.19 | 0 | 9.14E-01 | 9.34E-01 | 0.09±0.22 | 9.28E-01 | 0.13±0.22 | 9.30E-01 | 0.01±0.4 | 9.96E-01 | 0.07±0.42 | 9.96E-01 |
| N-acetyl-aspartyl-glutamate (NAAG) | 0.11±0.19 | 0 | 9.89E-01 | 9.03E-01 | 0.11±0.19 | 0 | 9.72E-01 | 9.34E-01 | 0.11±0.21 | 9.14E-01 | 0.1±0.21 | 9.41E-01 | 0.11±0.42 | 9.96E-01 | 0.12±0.44 | 9.96E-01 |
| glycolithocholate sulfate* | 0.11±0.19 | 0 | 9.34E-01 | 9.03E-01 | 0.11±0.19 | 0 | 8.98E-01 | 9.34E-01 | 0.1±0.21 | 9.14E-01 | 0.1±0.21 | 9.41E-01 | 0.14±0.45 | 9.96E-01 | 0.17±0.45 | 9.96E-01 |
| 1-stearoyl-GPC (18:0) | 0.23±0.21 | 0 | 9.44E-01 | 8.06E-01 | 0.13±0.21 | 0 | 8.23E-01 | 9.34E-01 | 0.23±0.23 | 8.01E-01 | 0.11±0.23 | 9.43E-01 | 0.27±0.54 | 9.96E-01 | 0.24±0.54 | 9.96E-01 |
| X - 23659 | 0.12±0.19 | 0 | 6.95E-01 | 9.03E-01 | 0.11±0.19 | 0 | 7.92E-01 | 9.34E-01 | 0.09±0.2 | 9.14E-01 | 0.09±0.2 | 9.47E-01 | 0.3±0.48 | 9.96E-01 | 0.23±0.5 | 9.96E-01 |
| 1-palmitoyl-GPE (16:0) | 0.16±0.19 | 0 | 8.75E-01 | 8.72E-01 | 0.11±0.2 | 0 | 9.19E-01 | 9.34E-01 | 0.18±0.21 | 8.72E-01 | 0.1±0.22 | 9.48E-01 | 0.1±0.43 | 9.96E-01 | 0.15±0.46 | 9.96E-01 |
| hexadecasphingosine (d16:1)* | -0.13±0.18 | 0 | 9.56E-01 | 8.96E-01 | -0.11±0.19 | 0 | 8.70E-01 | 9.34E-01 | -0.12±0.2 | 9.05E-01 | -0.09±0.21 | 9.53E-01 | -0.15±0.42 | 9.96E-01 | -0.17±0.43 | 9.96E-01 |
| X - 11843 | -0.1±0.19 | 0 | 7.88E-01 | 9.03E-01 | -0.12±0.19 | 0 | 7.78E-01 | 9.34E-01 | -0.08±0.21 | 9.37E-01 | -0.09±0.21 | 9.53E-01 | -0.21±0.43 | 9.96E-01 | -0.22±0.43 | 9.96E-01 |
| cysteinylglycine | -0.1±0.18 | 0 | 9.02E-01 | 9.03E-01 | -0.11±0.18 | 0 | 7.65E-01 | 9.34E-01 | -0.09±0.2 | 9.23E-01 | -0.08±0.21 | 9.63E-01 | -0.14±0.35 | 9.96E-01 | -0.2±0.37 | 9.96E-01 |
| N-acetylserine | -0.09±0.17 | 0 | 8.40E-01 | 9.13E-01 | -0.11±0.18 | 0 | 8.36E-01 | 9.34E-01 | -0.06±0.22 | 9.49E-01 | -0.08±0.22 | 9.63E-01 | -0.13±0.28 | 9.96E-01 | -0.16±0.3 | 9.96E-01 |
| N-acetylarginine | 0.15±0.19 | 0 | 4.83E-01 | 8.87E-01 | 0.12±0.2 | 0 | 4.79E-01 | 9.34E-01 | 0.1±0.21 | 9.15E-01 | 0.07±0.21 | 9.66E-01 | 0.47±0.5 | 9.96E-01 | 0.47±0.53 | 9.96E-01 |
| N-acetylalanine | -0.11±0.17 | 0 | 8.64E-01 | 9.03E-01 | -0.11±0.18 | 0 | 8.20E-01 | 9.34E-01 | -0.08±0.22 | 9.37E-01 | -0.08±0.22 | 9.66E-01 | -0.14±0.28 | 9.96E-01 | -0.16±0.28 | 9.96E-01 |
| X - 21834 | -0.1±0.18 | 0 | 5.91E-01 | 9.03E-01 | -0.11±0.18 | 0 | 6.25E-01 | 9.34E-01 | -0.05±0.21 | 9.49E-01 | -0.06±0.2 | 9.66E-01 | -0.3±0.4 | 9.96E-01 | -0.28±0.4 | 9.96E-01 |
| arabitol/xylitol | -0.1±0.17 | 0 | 7.37E-01 | 9.03E-01 | -0.1±0.17 | 0 | 7.55E-01 | 9.34E-01 | -0.06±0.21 | 9.49E-01 | -0.06±0.21 | 9.66E-01 | -0.18±0.29 | 9.96E-01 | -0.18±0.32 | 9.96E-01 |
| 5alpha-androstan-3alpha,17beta-diol monosulfate (2) | 0.08±0.29 | 0 | 4.46E-01 | 9.58E-01 | 0.19±0.29 | 0 | 4.75E-01 | 9.34E-01 | -0.02±0.32 | 9.95E-01 | 0.09±0.32 | 9.67E-01 | 0.55±0.68 | 9.96E-01 | 0.64±0.7 | 9.96E-01 |
| isoleucine | -0.15±0.18 | 0 | 9.73E-01 | 8.72E-01 | -0.11±0.19 | 0 | 7.77E-01 | 9.34E-01 | -0.15±0.23 | 9.00E-01 | -0.07±0.24 | 9.68E-01 | -0.16±0.29 | 9.96E-01 | -0.18±0.3 | 9.96E-01 |
| X - 12851 | 0.15±0.18 | 0 | 5.74E-01 | 8.82E-01 | 0.12±0.19 | 0 | 4.72E-01 | 9.34E-01 | 0.1±0.21 | 9.14E-01 | 0.05±0.21 | 9.78E-01 | 0.35±0.41 | 9.96E-01 | 0.39±0.42 | 9.96E-01 |
| androstenediol (3alpha, 17alpha) monosulfate (3) | 0.09±0.21 | 0 | 5.99E-01 | 9.28E-01 | 0.12±0.22 | 0 | 4.75E-01 | 9.34E-01 | 0.04±0.23 | 9.75E-01 | 0.06±0.24 | 9.78E-01 | 0.34±0.51 | 9.96E-01 | 0.48±0.54 | 9.96E-01 |
| X - 12456 | -0.01±0.19 | 20.31 | 2.63E-01 | 9.82E-01 | 0.12±0.21 | 0 | 3.56E-01 | 9.34E-01 | -0.09±0.21 | 9.14E-01 | 0.05±0.23 | 9.79E-01 | 0.53±0.52 | 9.96E-01 | 0.6±0.56 | 9.96E-01 |
| 2-methoxyhydroquinone sulfate (1) | -0.1±0.19 | 0 | 4.54E-01 | 9.03E-01 | -0.12±0.2 | 0 | 4.30E-01 | 9.34E-01 | -0.03±0.21 | 9.83E-01 | -0.04±0.22 | 9.79E-01 | -0.4±0.44 | 9.96E-01 | -0.45±0.47 | 9.96E-01 |
| 2-hydroxyarachidate* | -0.12±0.18 | 0 | 5.09E-01 | 9.01E-01 | -0.1±0.18 | 0 | 5.10E-01 | 9.34E-01 | -0.06±0.2 | 9.49E-01 | -0.04±0.21 | 9.80E-01 | -0.33±0.36 | 9.96E-01 | -0.32±0.37 | 9.96E-01 |
| N-acetylkynurenine (2) | -0.19±0.19 | 0 | 5.91E-01 | 8.24E-01 | -0.11±0.19 | 0 | 5.03E-01 | 9.34E-01 | -0.14±0.21 | 8.93E-01 | -0.04±0.22 | 9.80E-01 | -0.39±0.41 | 9.96E-01 | -0.35±0.42 | 9.96E-01 |
| 3-hydroxydecanoate | -0.09±0.19 | 0 | 5.59E-01 | 9.20E-01 | -0.11±0.19 | 0 | 4.16E-01 | 9.34E-01 | -0.03±0.21 | 9.83E-01 | -0.03±0.21 | 9.86E-01 | -0.31±0.42 | 9.96E-01 | -0.42±0.43 | 9.96E-01 |
| 3-methylxanthine | 0.12±0.18 | 2.7 | 3.11E-01 | 9.03E-01 | 0.11±0.19 | 0 | 3.32E-01 | 9.34E-01 | 0.02±0.2 | 9.94E-01 | 0.03±0.2 | 9.86E-01 | 0.48±0.4 | 9.96E-01 | 0.5±0.44 | 9.96E-01 |
| octanoylcarnitine (C8) | -0.13±0.18 | 35.54 | 2.13E-01 | 8.96E-01 | -0.11±0.18 | 29.77 | 2.33E-01 | 9.34E-01 | 0.01±0.21 | 9.97E-01 | 0.02±0.21 | 9.94E-01 | -0.49±0.34 | 9.96E-01 | -0.48±0.35 | 9.96E-01 |
| oleoyl-linoleoyl-glycerol (18:1/18:2) [1] | -0.09±0.18 | 0 | 4.94E-01 | 9.10E-01 | -0.11±0.18 | 0 | 4.21E-01 | 9.34E-01 | -0.02±0.21 | 9.94E-01 | -0.01±0.22 | 9.94E-01 | -0.28±0.33 | 9.96E-01 | -0.33±0.33 | 9.96E-01 |
| 4-methoxyphenol sulfate | 0.16±0.19 | 30.01 | 2.32E-01 | 8.72E-01 | 0.11±0.19 | 20.11 | 2.63E-01 | 9.34E-01 | 0.06±0.21 | 9.49E-01 | 0.01±0.21 | 9.95E-01 | 0.66±0.46 | 9.96E-01 | 0.6±0.48 | 9.96E-01 |
| pregnenetriol sulfate* | 0.08±0.2 | 8.19 | 2.97E-01 | 9.40E-01 | 0.11±0.2 | 23.97 | 2.51E-01 | 9.34E-01 | -0.01±0.22 | 9.95E-01 | 0.01±0.22 | 9.95E-01 | 0.56±0.5 | 9.96E-01 | 0.65±0.51 | 9.96E-01 |
| 1-palmitoyl-2-linoleoyl-GPI (16:0/18:2) | -0.09±0.19 | 0 | 3.43E-01 | 9.22E-01 | -0.11±0.19 | 0 | 3.70E-01 | 9.34E-01 | 0.02±0.22 | 9.93E-01 | -0.01±0.23 | 9.96E-01 | -0.37±0.35 | 9.96E-01 | -0.39±0.36 | 9.96E-01 |
| X - 16087 | 0.05±0.18 | 60.41 | 1.12E-01 | 9.63E-01 | 0.11±0.19 | 57.24 | 1.26E-01 | 9.34E-01 | -0.07±0.2 | 9.37E-01 | 0±0.2 | 9.99E-01 | 0.76±0.49 | 9.96E-01 | 0.84±0.51 | 9.96E-01 |
| theobromine | 0.1±0.18 | 63.23 | 9.91E-02 | 9.10E-01 | 0.12±0.19 | 54.45 | 1.38E-01 | 9.34E-01 | -0.03±0.2 | 9.80E-01 | 0±0.2 | 9.99E-01 | 0.79±0.46 | 9.96E-01 | 0.79±0.49 | 9.96E-01 |
| N-acetylglutamine | 0.13±0.19 | 0 | 8.08E-01 | 9.03E-01 | 0.11±0.2 | 0 | 7.81E-01 | 9.34E-01 | 0.15±0.22 | 8.89E-01 | 0.14±0.22 | 9.26E-01 | 0.04±0.41 | 9.96E-01 | 0±0.44 | 9.98E-01 |
| X - 21286 | -0.07±0.19 | 0 | 6.62E-01 | 9.40E-01 | -0.12±0.19 | 0 | 7.98E-01 | 9.34E-01 | -0.11±0.21 | 9.14E-01 | -0.14±0.22 | 9.26E-01 | 0.11±0.44 | 9.96E-01 | -0.02±0.44 | 9.98E-01 |
| 2-aminoheptanoate | 0.1±0.19 | 0 | 5.10E-01 | 9.03E-01 | 0.1±0.19 | 0 | 3.83E-01 | 9.36E-01 | 0.17±0.21 | 8.78E-01 | 0.19±0.21 | 8.79E-01 | -0.13±0.4 | 9.96E-01 | -0.22±0.41 | 9.96E-01 |
| sarcosine | -0.09±0.2 | 0 | 5.69E-01 | 9.24E-01 | -0.11±0.2 | 0 | 5.36E-01 | 9.36E-01 | -0.14±0.21 | 9.00E-01 | -0.16±0.22 | 9.12E-01 | 0.19±0.54 | 9.96E-01 | 0.21±0.56 | 9.96E-01 |
| 4-acetylphenol sulfate | -0.07±0.19 | 0 | 6.20E-01 | 9.40E-01 | -0.1±0.19 | 0 | 5.77E-01 | 9.36E-01 | -0.12±0.21 | 9.14E-01 | -0.15±0.21 | 9.12E-01 | 0.11±0.42 | 9.96E-01 | 0.13±0.46 | 9.96E-01 |
| N-acetyl-2-aminoadipate | 0.01±0.18 | 0 | 8.92E-01 | 9.87E-01 | 0.11±0.2 | 0 | 7.33E-01 | 9.36E-01 | 0.02±0.21 | 9.94E-01 | 0.14±0.22 | 9.26E-01 | -0.04±0.4 | 9.96E-01 | -0.03±0.44 | 9.96E-01 |
| 5-methylthioadenosine (MTA) | -0.12±0.17 | 0 | 4.81E-01 | 9.00E-01 | -0.1±0.18 | 9.94 | 2.92E-01 | 9.36E-01 | -0.04±0.21 | 9.71E-01 | 0.02±0.21 | 9.94E-01 | -0.31±0.32 | 9.96E-01 | -0.39±0.33 | 9.96E-01 |
| 1-stearoyl-2-linoleoyl-GPI (18:0/18:2) | -0.07±0.17 | 0 | 4.16E-01 | 9.40E-01 | -0.1±0.18 | 0 | 4.68E-01 | 9.36E-01 | 0.05±0.22 | 9.65E-01 | 0±0.22 | 9.99E-01 | -0.24±0.28 | 9.96E-01 | -0.28±0.31 | 9.96E-01 |
| dihomo-linolenate (20:3n3 or n6) | 0.09±0.19 | 0 | 5.82E-01 | 9.20E-01 | 0.11±0.2 | 0 | 6.78E-01 | 9.37E-01 | 0.04±0.21 | 9.65E-01 | 0.07±0.22 | 9.66E-01 | 0.33±0.46 | 9.96E-01 | 0.29±0.48 | 9.96E-01 |
| N-acetyl-beta-alanine | 0.05±0.18 | 54.66 | 1.37E-01 | 9.60E-01 | 0.08±0.19 | 67.55 | 7.92E-02 | 9.37E-01 | 0.22±0.21 | 7.95E-01 | 0.27±0.21 | 7.70E-01 | -0.38±0.34 | 9.96E-01 | -0.49±0.38 | 9.96E-01 |
| N6-acetyllysine | 0.11±0.17 | 45.94 | 1.74E-01 | 9.03E-01 | 0.08±0.17 | 50.25 | 1.56E-01 | 9.37E-01 | 0.29±0.22 | 6.98E-01 | 0.27±0.22 | 7.72E-01 | -0.19±0.28 | 9.96E-01 | -0.24±0.28 | 9.96E-01 |
| X - 24494 | -0.07±0.18 | 75.24 | 4.45E-02 | 9.40E-01 | -0.08±0.18 | 72.06 | 5.85E-02 | 9.37E-01 | -0.22±0.19 | 7.70E-01 | -0.23±0.19 | 8.09E-01 | 0.77±0.45 | 9.96E-01 | 0.7±0.45 | 9.96E-01 |
| X - 25422 | 0.05±0.18 | 56.94 | 1.28E-01 | 9.61E-01 | 0.07±0.18 | 56.43 | 1.30E-01 | 9.37E-01 | 0.24±0.22 | 7.74E-01 | 0.25±0.22 | 8.10E-01 | -0.33±0.31 | 9.96E-01 | -0.35±0.33 | 9.96E-01 |
| ethyl beta-glucopyranoside | 0.06±0.19 | 63.99 | 9.56E-02 | 9.55E-01 | 0.1±0.2 | 61.46 | 1.07E-01 | 9.37E-01 | 0.21±0.21 | 7.95E-01 | 0.24±0.22 | 8.27E-01 | -0.62±0.45 | 9.96E-01 | -0.63±0.5 | 9.96E-01 |
| 2-ketocaprylate | 0.14±0.18 | 12.23 | 2.86E-01 | 8.87E-01 | 0.09±0.18 | 25.76 | 2.46E-01 | 9.37E-01 | 0.24±0.2 | 7.52E-01 | 0.2±0.2 | 8.60E-01 | -0.24±0.4 | 9.96E-01 | -0.31±0.39 | 9.96E-01 |
| 2'-deoxyuridine | 0.11±0.18 | 25.53 | 2.47E-01 | 9.03E-01 | 0.07±0.18 | 31.82 | 2.26E-01 | 9.37E-01 | 0.24±0.21 | 7.61E-01 | 0.21±0.21 | 8.60E-01 | -0.21±0.33 | 9.96E-01 | -0.26±0.33 | 9.96E-01 |
| homovanillate (HVA) | -0.06±0.18 | 56.64 | 1.29E-01 | 9.55E-01 | -0.08±0.19 | 53.8 | 1.41E-01 | 9.37E-01 | -0.19±0.2 | 8.31E-01 | -0.21±0.21 | 8.60E-01 | 0.54±0.44 | 9.96E-01 | 0.56±0.47 | 9.96E-01 |
| pregnanediol-3-glucuronide | -0.06±0.18 | 53.57 | 1.42E-01 | 9.55E-01 | -0.07±0.19 | 58.52 | 1.21E-01 | 9.37E-01 | -0.18±0.2 | 8.66E-01 | -0.2±0.21 | 8.60E-01 | 0.54±0.45 | 9.96E-01 | 0.58±0.46 | 9.96E-01 |
| 1-stearoyl-GPG (18:0) | -0.12±0.18 | 12.66 | 2.85E-01 | 9.03E-01 | -0.09±0.18 | 4.62 | 3.06E-01 | 9.37E-01 | -0.2±0.19 | 7.95E-01 | -0.17±0.2 | 8.79E-01 | 0.36±0.48 | 9.96E-01 | 0.37±0.49 | 9.96E-01 |
| glycohyocholate | 0.13±0.19 | 2.87 | 3.10E-01 | 9.03E-01 | 0.1±0.2 | 0 | 3.81E-01 | 9.37E-01 | 0.23±0.22 | 7.95E-01 | 0.2±0.23 | 8.79E-01 | -0.24±0.41 | 9.96E-01 | -0.22±0.42 | 9.96E-01 |
| butyrate/isobutyrate (4:0) | -0.12±0.19 | 20.06 | 2.63E-01 | 9.03E-01 | -0.1±0.19 | 0 | 3.45E-01 | 9.37E-01 | -0.22±0.21 | 7.95E-01 | -0.19±0.21 | 8.79E-01 | 0.34±0.45 | 9.96E-01 | 0.28±0.45 | 9.96E-01 |
| nonadecanoate (19:0) | -0.03±0.19 | 6.76 | 3.00E-01 | 9.69E-01 | -0.09±0.18 | 11.59 | 2.88E-01 | 9.37E-01 | -0.13±0.21 | 9.02E-01 | -0.19±0.21 | 8.79E-01 | 0.35±0.41 | 9.96E-01 | 0.31±0.42 | 9.96E-01 |
| 2-hydroxystearate | -0.04±0.19 | 0 | 3.39E-01 | 9.68E-01 | -0.08±0.19 | 0 | 3.29E-01 | 9.37E-01 | -0.12±0.21 | 9.06E-01 | -0.17±0.21 | 8.88E-01 | 0.35±0.45 | 9.96E-01 | 0.31±0.45 | 9.96E-01 |
| sphingomyelin (d18:1/22:2, d18:2/22:1, d16:1/24:2)* | -0.1±0.21 | 0 | 5.18E-01 | 9.20E-01 | -0.11±0.21 | 0 | 4.06E-01 | 9.37E-01 | -0.16±0.22 | 8.89E-01 | -0.18±0.22 | 8.89E-01 | 0.21±0.52 | 9.96E-01 | 0.32±0.56 | 9.96E-01 |
| glycosyl ceramide (d18:2/24:1, d18:1/24:2)* | -0.02±0.21 | 0 | 5.66E-01 | 9.76E-01 | -0.11±0.22 | 0 | 3.94E-01 | 9.37E-01 | -0.07±0.23 | 9.49E-01 | -0.18±0.23 | 8.89E-01 | 0.26±0.52 | 9.96E-01 | 0.33±0.55 | 9.96E-01 |
| pyruvate | -0.17±0.2 | 1.53 | 3.14E-01 | 8.72E-01 | -0.11±0.21 | 0 | 4.46E-01 | 9.37E-01 | -0.25±0.21 | 7.52E-01 | -0.17±0.22 | 8.98E-01 | 0.31±0.52 | 9.96E-01 | 0.28±0.55 | 9.96E-01 |
| gamma-glutamylcitrulline* | -0.01±0.19 | 0 | 5.00E-01 | 9.82E-01 | -0.1±0.19 | 0 | 4.25E-01 | 9.37E-01 | -0.06±0.21 | 9.49E-01 | -0.16±0.21 | 9.00E-01 | 0.32±0.53 | 9.96E-01 | 0.31±0.54 | 9.96E-01 |
| taurolithocholate 3-sulfate | -0.11±0.18 | 0 | 5.32E-01 | 9.03E-01 | -0.08±0.18 | 0 | 4.65E-01 | 9.37E-01 | -0.15±0.19 | 8.78E-01 | -0.14±0.19 | 9.16E-01 | 0.15±0.45 | 9.96E-01 | 0.23±0.46 | 9.96E-01 |
| indoleacetate | 0.08±0.18 | 0 | 6.36E-01 | 9.25E-01 | 0.08±0.18 | 0 | 4.95E-01 | 9.37E-01 | 0.12±0.2 | 9.02E-01 | 0.14±0.2 | 9.20E-01 | -0.09±0.41 | 9.96E-01 | -0.18±0.42 | 9.96E-01 |
| 1-(1-enyl-palmitoyl)-2-oleoyl-GPC (P-16:0/18:1)* | 0±0.2 | 0 | 4.73E-01 | 9.89E-01 | -0.08±0.2 | 20.48 | 2.62E-01 | 9.37E-01 | -0.05±0.21 | 9.50E-01 | -0.15±0.21 | 9.20E-01 | 0.41±0.61 | 9.96E-01 | 0.69±0.71 | 9.96E-01 |
| arabonate/xylonate | -0.07±0.19 | 0 | 6.16E-01 | 9.40E-01 | -0.09±0.19 | 0 | 5.71E-01 | 9.37E-01 | -0.12±0.21 | 9.14E-01 | -0.14±0.22 | 9.26E-01 | 0.11±0.4 | 9.96E-01 | 0.14±0.44 | 9.96E-01 |
| 3-hydroxysebacate | 0.12±0.19 | 0 | 7.20E-01 | 9.03E-01 | 0.08±0.19 | 0 | 6.22E-01 | 9.37E-01 | 0.16±0.21 | 8.78E-01 | 0.13±0.21 | 9.26E-01 | -0.01±0.41 | 9.96E-01 | -0.11±0.43 | 9.96E-01 |
| eicosenedioate (C20:1-DC)* | 0.12±0.19 | 0 | 6.93E-01 | 9.03E-01 | 0.07±0.19 | 0 | 5.71E-01 | 9.37E-01 | 0.16±0.21 | 8.78E-01 | 0.13±0.21 | 9.26E-01 | -0.02±0.4 | 9.96E-01 | -0.13±0.4 | 9.96E-01 |
| X - 12798 | -0.1±0.18 | 0 | 5.11E-01 | 9.03E-01 | -0.08±0.18 | 0 | 5.63E-01 | 9.37E-01 | -0.15±0.2 | 8.78E-01 | -0.12±0.19 | 9.26E-01 | 0.19±0.48 | 9.96E-01 | 0.18±0.49 | 9.96E-01 |
| hyocholate | 0.11±0.2 | 0 | 7.56E-01 | 9.03E-01 | 0.1±0.2 | 0 | 7.17E-01 | 9.37E-01 | 0.14±0.22 | 9.00E-01 | 0.14±0.23 | 9.26E-01 | -0.01±0.43 | 9.96E-01 | -0.05±0.45 | 9.96E-01 |
| pipecolate | 0.07±0.18 | 0 | 5.95E-01 | 9.40E-01 | 0.08±0.18 | 0 | 6.26E-01 | 9.37E-01 | 0.11±0.2 | 9.14E-01 | 0.12±0.2 | 9.26E-01 | -0.14±0.43 | 9.96E-01 | -0.12±0.44 | 9.96E-01 |
| X - 24949 | -0.07±0.19 | 64.14 | 9.49E-02 | 9.40E-01 | -0.07±0.19 | 72.91 | 5.47E-02 | 9.37E-01 | 0.11±0.22 | 9.14E-01 | 0.14±0.22 | 9.26E-01 | -0.6±0.37 | 9.96E-01 | -0.71±0.38 | 9.96E-01 |
| stearate (18:0) | -0.05±0.18 | 0 | 4.94E-01 | 9.63E-01 | -0.08±0.19 | 0 | 5.58E-01 | 9.37E-01 | -0.11±0.21 | 9.14E-01 | -0.13±0.2 | 9.26E-01 | 0.21±0.42 | 9.96E-01 | 0.15±0.44 | 9.96E-01 |
| X - 11315 | -0.03±0.18 | 0 | 5.33E-01 | 9.69E-01 | -0.08±0.18 | 0 | 5.32E-01 | 9.37E-01 | -0.07±0.19 | 9.35E-01 | -0.12±0.2 | 9.26E-01 | 0.23±0.46 | 9.96E-01 | 0.21±0.5 | 9.96E-01 |
| N-acetylasparagine | -0.03±0.18 | 0 | 5.56E-01 | 9.70E-01 | -0.08±0.19 | 0 | 5.06E-01 | 9.37E-01 | -0.08±0.2 | 9.35E-01 | -0.13±0.21 | 9.26E-01 | 0.19±0.41 | 9.96E-01 | 0.2±0.45 | 9.96E-01 |
| eicosenoylcarnitine (C20:1)* | -0.03±0.19 | 0 | 6.68E-01 | 9.70E-01 | -0.08±0.2 | 0 | 5.51E-01 | 9.37E-01 | -0.06±0.21 | 9.49E-01 | -0.13±0.22 | 9.26E-01 | 0.15±0.46 | 9.96E-01 | 0.17±0.47 | 9.96E-01 |
| linoleate (18:2n6) | 0.13±0.19 | 0 | 7.00E-01 | 9.03E-01 | 0.08±0.19 | 0 | 6.07E-01 | 9.37E-01 | 0.17±0.22 | 8.78E-01 | 0.13±0.21 | 9.27E-01 | -0.02±0.43 | 9.96E-01 | -0.13±0.44 | 9.96E-01 |
| N6,N6-dimethyllysine | -0.03±0.17 | 33.49 | 2.20E-01 | 9.69E-01 | -0.06±0.17 | 59.5 | 1.16E-01 | 9.37E-01 | 0.12±0.2 | 9.14E-01 | 0.12±0.2 | 9.27E-01 | -0.32±0.29 | 9.96E-01 | -0.44±0.29 | 9.96E-01 |
| margarate (17:0) | -0.03±0.19 | 0 | 5.17E-01 | 9.69E-01 | -0.07±0.19 | 0 | 5.70E-01 | 9.37E-01 | -0.1±0.21 | 9.15E-01 | -0.12±0.21 | 9.27E-01 | 0.21±0.42 | 9.96E-01 | 0.15±0.43 | 9.96E-01 |
| N6-methyllysine | 0.07±0.18 | 0 | 6.66E-01 | 9.40E-01 | 0.07±0.18 | 0 | 6.12E-01 | 9.37E-01 | 0.11±0.21 | 9.14E-01 | 0.12±0.21 | 9.28E-01 | -0.08±0.39 | 9.96E-01 | -0.11±0.4 | 9.96E-01 |
| octadecadienedioate (C18:2-DC)* | 0.12±0.17 | 0 | 7.29E-01 | 8.96E-01 | 0.08±0.18 | 0 | 7.05E-01 | 9.37E-01 | 0.15±0.19 | 8.78E-01 | 0.11±0.2 | 9.30E-01 | 0±0.41 | 9.96E-01 | -0.07±0.44 | 9.96E-01 |
| epiandrosterone sulfate | -0.1±0.19 | 0 | 7.34E-01 | 9.03E-01 | -0.07±0.19 | 0 | 6.01E-01 | 9.37E-01 | -0.13±0.21 | 9.00E-01 | -0.12±0.21 | 9.30E-01 | 0.03±0.44 | 9.96E-01 | 0.16±0.48 | 9.96E-01 |
| androsterone sulfate | -0.11±0.18 | 0 | 8.68E-01 | 9.03E-01 | -0.09±0.18 | 0 | 7.86E-01 | 9.37E-01 | -0.13±0.2 | 9.00E-01 | -0.11±0.2 | 9.30E-01 | -0.05±0.4 | 9.96E-01 | 0.02±0.43 | 9.96E-01 |
| X - 12410 | -0.07±0.17 | 72.77 | 5.53E-02 | 9.31E-01 | -0.07±0.18 | 74.18 | 4.91E-02 | 9.37E-01 | 0.1±0.2 | 9.14E-01 | 0.11±0.2 | 9.30E-01 | -0.69±0.36 | 9.96E-01 | -0.74±0.38 | 9.96E-01 |
| X - 17690 | 0.03±0.19 | 0 | 5.25E-01 | 9.70E-01 | 0.09±0.19 | 0 | 7.37E-01 | 9.37E-01 | 0.08±0.21 | 9.35E-01 | 0.11±0.21 | 9.30E-01 | -0.22±0.43 | 9.96E-01 | -0.06±0.46 | 9.96E-01 |
| cystathionine | 0.07±0.18 | 0 | 7.68E-01 | 9.40E-01 | 0.07±0.19 | 0 | 6.49E-01 | 9.37E-01 | 0.09±0.2 | 9.14E-01 | 0.11±0.2 | 9.30E-01 | -0.04±0.41 | 9.96E-01 | -0.11±0.44 | 9.96E-01 |
| p-cresol sulfate | 0.18±0.21 | 0 | 9.79E-01 | 8.75E-01 | 0.11±0.21 | 0 | 9.20E-01 | 9.37E-01 | 0.18±0.24 | 8.78E-01 | 0.12±0.23 | 9.39E-01 | 0.16±0.47 | 9.96E-01 | 0.07±0.47 | 9.96E-01 |
| 2-aminooctanoate | 0±0.18 | 59.34 | 1.17E-01 | 9.94E-01 | -0.07±0.19 | 67.95 | 7.73E-02 | 9.37E-01 | 0.16±0.21 | 8.78E-01 | 0.11±0.21 | 9.40E-01 | -0.51±0.37 | 9.96E-01 | -0.67±0.39 | 9.96E-01 |
| sphingomyelin (d18:0/18:0, d19:0/17:0)* | -0.2±0.19 | 0 | 6.85E-01 | 8.13E-01 | -0.08±0.21 | 0 | 7.50E-01 | 9.37E-01 | -0.25±0.23 | 7.70E-01 | -0.12±0.24 | 9.41E-01 | -0.08±0.36 | 9.96E-01 | 0.03±0.41 | 9.96E-01 |
| succinate | 0.13±0.19 | 0 | 8.69E-01 | 8.96E-01 | 0.09±0.18 | 0 | 9.54E-01 | 9.37E-01 | 0.15±0.21 | 8.85E-01 | 0.1±0.21 | 9.41E-01 | 0.07±0.41 | 9.96E-01 | 0.07±0.41 | 9.96E-01 |
| dihomo-linoleate (20:2n6) | 0.11±0.19 | 0 | 9.98E-01 | 9.03E-01 | 0.09±0.19 | 0 | 9.26E-01 | 9.37E-01 | 0.11±0.21 | 9.14E-01 | 0.1±0.21 | 9.41E-01 | 0.11±0.42 | 9.96E-01 | 0.06±0.43 | 9.96E-01 |
| 4-methylhexanoylglutamine | 0.08±0.19 | 0 | 8.09E-01 | 9.35E-01 | 0.08±0.19 | 0 | 7.82E-01 | 9.37E-01 | 0.1±0.22 | 9.14E-01 | 0.11±0.21 | 9.41E-01 | -0.01±0.43 | 9.96E-01 | -0.03±0.45 | 9.96E-01 |
| adenosine 3',5'-cyclic monophosphate (cAMP) | -0.04±0.19 | 0 | 8.51E-01 | 9.64E-01 | -0.09±0.18 | 0 | 9.24E-01 | 9.37E-01 | -0.06±0.21 | 9.49E-01 | -0.1±0.2 | 9.41E-01 | 0.03±0.43 | 9.96E-01 | -0.05±0.41 | 9.96E-01 |
| taurochenodeoxycholic acid 3-sulfate | 0.04±0.19 | 0 | 8.11E-01 | 9.68E-01 | 0.08±0.19 | 0 | 8.67E-01 | 9.37E-01 | 0.06±0.21 | 9.49E-01 | 0.1±0.21 | 9.43E-01 | -0.05±0.41 | 9.96E-01 | 0.02±0.43 | 9.96E-01 |
| myristate (14:0) | 0.12±0.2 | 0 | 9.72E-01 | 9.03E-01 | 0.09±0.19 | 0 | 9.81E-01 | 9.37E-01 | 0.12±0.22 | 9.14E-01 | 0.09±0.21 | 9.53E-01 | 0.1±0.45 | 9.96E-01 | 0.1±0.46 | 9.96E-01 |
| hydroxy-N6,N6,N6-trimethyllysine* | -0.08±0.18 | 0 | 9.06E-01 | 9.22E-01 | -0.09±0.18 | 0 | 9.54E-01 | 9.37E-01 | -0.09±0.2 | 9.14E-01 | -0.09±0.2 | 9.53E-01 | -0.05±0.36 | 9.96E-01 | -0.11±0.36 | 9.96E-01 |
| X - 10458 | 0.08±0.19 | 0 | 9.58E-01 | 9.27E-01 | 0.09±0.2 | 0 | 9.97E-01 | 9.37E-01 | 0.09±0.21 | 9.28E-01 | 0.09±0.22 | 9.53E-01 | 0.06±0.42 | 9.96E-01 | 0.09±0.46 | 9.96E-01 |
| sphinganine-1-phosphate | 0.09±0.19 | 0 | 9.13E-01 | 9.27E-01 | 0.1±0.2 | 0 | 9.46E-01 | 9.37E-01 | 0.08±0.21 | 9.39E-01 | 0.09±0.22 | 9.53E-01 | 0.13±0.45 | 9.96E-01 | 0.13±0.45 | 9.96E-01 |
| 5alpha-androstan-3alpha,17beta-diol monosulfate (1) | 0.04±0.19 | 0 | 9.77E-01 | 9.64E-01 | 0.1±0.2 | 0 | 9.56E-01 | 9.37E-01 | 0.04±0.22 | 9.74E-01 | 0.1±0.23 | 9.53E-01 | 0.05±0.42 | 9.96E-01 | 0.12±0.44 | 9.96E-01 |
| octadecenedioylcarnitine (C18:1-DC)* | 0.1±0.19 | 0 | 9.82E-01 | 9.03E-01 | 0.09±0.19 | 0 | 9.89E-01 | 9.37E-01 | 0.1±0.22 | 9.14E-01 | 0.09±0.21 | 9.54E-01 | 0.11±0.4 | 9.96E-01 | 0.08±0.4 | 9.96E-01 |
| glycochenodeoxycholate 3-sulfate | 0.06±0.2 | 0 | 9.97E-01 | 9.57E-01 | 0.1±0.2 | 0 | 9.81E-01 | 9.37E-01 | 0.06±0.22 | 9.49E-01 | 0.09±0.23 | 9.60E-01 | 0.06±0.43 | 9.96E-01 | 0.1±0.45 | 9.96E-01 |
| 1,2-dipalmitoyl-GPC (16:0/16:0) | 0.09±0.18 | 0 | 8.52E-01 | 9.13E-01 | 0.08±0.19 | 0 | 9.66E-01 | 9.37E-01 | 0.11±0.21 | 9.14E-01 | 0.08±0.22 | 9.63E-01 | 0.03±0.38 | 9.96E-01 | 0.1±0.44 | 9.96E-01 |
| 2-piperidinone | 0.11±0.19 | 0 | 7.56E-01 | 9.03E-01 | 0.09±0.19 | 0 | 9.09E-01 | 9.37E-01 | 0.08±0.21 | 9.36E-01 | 0.08±0.21 | 9.63E-01 | 0.23±0.41 | 9.96E-01 | 0.14±0.45 | 9.96E-01 |
| X - 12193 | -0.07±0.18 | 0 | 9.99E-01 | 9.40E-01 | -0.09±0.18 | 0 | 8.89E-01 | 9.37E-01 | -0.07±0.21 | 9.45E-01 | -0.08±0.2 | 9.63E-01 | -0.07±0.4 | 9.96E-01 | -0.14±0.42 | 9.96E-01 |
| 5-(galactosylhydroxy)-L-lysine | 0.08±0.19 | 0 | 9.52E-01 | 9.40E-01 | 0.08±0.19 | 0 | 9.87E-01 | 9.37E-01 | 0.07±0.21 | 9.48E-01 | 0.08±0.21 | 9.63E-01 | 0.1±0.44 | 9.96E-01 | 0.07±0.45 | 9.96E-01 |
| X - 13553 | -0.06±0.19 | 0 | 9.04E-01 | 9.55E-01 | -0.1±0.19 | 0 | 9.15E-01 | 9.37E-01 | -0.05±0.22 | 9.59E-01 | -0.08±0.22 | 9.63E-01 | -0.1±0.39 | 9.96E-01 | -0.13±0.41 | 9.96E-01 |
| X - 12906 | -0.03±0.18 | 0 | 9.92E-01 | 9.69E-01 | -0.09±0.18 | 0 | 9.04E-01 | 9.37E-01 | -0.03±0.2 | 9.83E-01 | -0.08±0.2 | 9.63E-01 | -0.02±0.44 | 9.96E-01 | -0.14±0.46 | 9.96E-01 |
| X - 21258 | -0.07±0.18 | 0 | 9.84E-01 | 9.40E-01 | -0.08±0.18 | 0 | 9.15E-01 | 9.37E-01 | -0.07±0.21 | 9.49E-01 | -0.07±0.2 | 9.64E-01 | -0.08±0.4 | 9.96E-01 | -0.12±0.4 | 9.96E-01 |
| glutamine conjugate of C7H12O2* | 0.12±0.19 | 0 | 8.21E-01 | 9.03E-01 | 0.08±0.19 | 0 | 9.71E-01 | 9.37E-01 | 0.1±0.21 | 9.14E-01 | 0.07±0.21 | 9.66E-01 | 0.21±0.42 | 9.96E-01 | 0.09±0.46 | 9.96E-01 |
| 2-hydroxyphenylacetate | -0.1±0.19 | 0 | 9.83E-01 | 9.03E-01 | -0.1±0.19 | 0 | 8.43E-01 | 9.37E-01 | -0.1±0.21 | 9.14E-01 | -0.08±0.22 | 9.66E-01 | -0.11±0.41 | 9.96E-01 | -0.17±0.41 | 9.96E-01 |
| N,N,N-trimethyl-alanylproline betaine (TMAP) | -0.07±0.19 | 0 | 9.42E-01 | 9.42E-01 | -0.08±0.19 | 0 | 9.93E-01 | 9.37E-01 | -0.08±0.22 | 9.45E-01 | -0.08±0.23 | 9.66E-01 | -0.05±0.34 | 9.96E-01 | -0.08±0.34 | 9.96E-01 |
| cytidine | 0.08±0.19 | 0 | 7.32E-01 | 9.27E-01 | 0.1±0.19 | 0 | 7.45E-01 | 9.37E-01 | 0.05±0.21 | 9.57E-01 | 0.07±0.2 | 9.66E-01 | 0.21±0.41 | 9.96E-01 | 0.23±0.45 | 9.96E-01 |
| X - 11850 | -0.04±0.19 | 0 | 9.19E-01 | 9.64E-01 | -0.08±0.19 | 0 | 9.32E-01 | 9.37E-01 | -0.03±0.21 | 9.83E-01 | -0.07±0.21 | 9.66E-01 | -0.08±0.43 | 9.96E-01 | -0.11±0.45 | 9.96E-01 |
| xanthurenate | -0.13±0.18 | 30.6 | 2.30E-01 | 8.96E-01 | -0.09±0.19 | 59.08 | 1.18E-01 | 9.37E-01 | -0.01±0.2 | 9.95E-01 | 0.07±0.22 | 9.66E-01 | -0.53±0.38 | 9.96E-01 | -0.66±0.42 | 9.96E-01 |
| gamma-glutamylvaline | -0.12±0.18 | 0 | 8.89E-01 | 9.00E-01 | -0.07±0.18 | 0 | 9.53E-01 | 9.37E-01 | -0.14±0.2 | 8.92E-01 | -0.07±0.22 | 9.66E-01 | -0.08±0.35 | 9.96E-01 | -0.09±0.34 | 9.96E-01 |
| 1-palmitoyl-2-oleoyl-GPI (16:0/18:1)* | -0.12±0.18 | 0 | 7.83E-01 | 9.03E-01 | -0.09±0.19 | 0 | 8.23E-01 | 9.37E-01 | -0.08±0.22 | 9.35E-01 | -0.07±0.22 | 9.66E-01 | -0.19±0.31 | 9.96E-01 | -0.16±0.34 | 9.96E-01 |
| bilirubin degradation product, C17H18N2O4 (3)** | 0.09±0.19 | 0 | 7.47E-01 | 9.21E-01 | 0.1±0.19 | 0 | 7.27E-01 | 9.37E-01 | 0.06±0.21 | 9.49E-01 | 0.07±0.22 | 9.66E-01 | 0.22±0.45 | 9.96E-01 | 0.24±0.45 | 9.96E-01 |
| 3beta,7alpha-dihydroxy-5-cholestenoate | -0.04±0.18 | 0 | 8.92E-01 | 9.64E-01 | -0.09±0.18 | 0 | 8.02E-01 | 9.37E-01 | -0.03±0.21 | 9.83E-01 | -0.06±0.21 | 9.66E-01 | -0.09±0.4 | 9.96E-01 | -0.18±0.4 | 9.96E-01 |
| gamma-glutamylglutamine | 0.17±0.2 | 0 | 8.37E-01 | 8.72E-01 | 0.09±0.21 | 0 | 6.81E-01 | 9.37E-01 | 0.16±0.22 | 8.78E-01 | 0.06±0.22 | 9.67E-01 | 0.29±0.61 | 9.96E-01 | 0.35±0.66 | 9.96E-01 |
| N-stearoyl-sphinganine (d18:0/18:0)* | -0.08±0.19 | 0 | 8.05E-01 | 9.36E-01 | 0.09±0.21 | 0 | 8.25E-01 | 9.37E-01 | -0.1±0.21 | 9.14E-01 | 0.07±0.23 | 9.67E-01 | 0.02±0.42 | 9.96E-01 | 0.18±0.47 | 9.96E-01 |
| nicotinamide | -0.09±0.19 | 0 | 7.61E-01 | 9.13E-01 | -0.09±0.19 | 0 | 7.61E-01 | 9.37E-01 | -0.07±0.21 | 9.49E-01 | -0.06±0.21 | 9.67E-01 | -0.21±0.43 | 9.96E-01 | -0.2±0.42 | 9.96E-01 |
| phenylalanine | 0.01±0.2 | 0 | 7.03E-01 | 9.87E-01 | 0.07±0.19 | 0 | 8.49E-01 | 9.37E-01 | -0.02±0.21 | 9.94E-01 | 0.06±0.2 | 9.67E-01 | 0.22±0.59 | 9.96E-01 | 0.17±0.55 | 9.96E-01 |
| 1,2-dilinoleoyl-GPC (18:2/18:2) | 0.23±0.21 | 0 | 7.91E-01 | 8.00E-01 | 0.1±0.22 | 0 | 6.79E-01 | 9.37E-01 | 0.21±0.23 | 8.31E-01 | 0.06±0.23 | 9.68E-01 | 0.38±0.57 | 9.96E-01 | 0.33±0.6 | 9.96E-01 |
| 1-(1-enyl-palmitoyl)-2-linoleoyl-GPE (P-16:0/18:2)* | 0.13±0.2 | 0 | 6.55E-01 | 9.03E-01 | 0.1±0.19 | 0 | 6.59E-01 | 9.37E-01 | 0.09±0.22 | 9.25E-01 | 0.06±0.21 | 9.68E-01 | 0.33±0.48 | 9.96E-01 | 0.28±0.46 | 9.96E-01 |
| X - 24334 | -0.14±0.2 | 0 | 9.93E-01 | 8.96E-01 | -0.08±0.21 | 0 | 8.41E-01 | 9.37E-01 | -0.14±0.23 | 9.02E-01 | -0.06±0.23 | 9.75E-01 | -0.14±0.43 | 9.96E-01 | -0.16±0.45 | 9.96E-01 |
| 3-ureidopropionate | 0.04±0.2 | 0 | 7.79E-01 | 9.68E-01 | 0.08±0.2 | 0 | 7.72E-01 | 9.37E-01 | 0.01±0.22 | 9.95E-01 | 0.06±0.22 | 9.75E-01 | 0.16±0.46 | 9.96E-01 | 0.21±0.48 | 9.96E-01 |
| X - 25957 | -0.06±0.18 | 0 | 8.56E-01 | 9.49E-01 | -0.07±0.18 | 0 | 8.04E-01 | 9.37E-01 | -0.05±0.21 | 9.57E-01 | -0.05±0.21 | 9.78E-01 | -0.13±0.41 | 9.96E-01 | -0.16±0.41 | 9.96E-01 |
| 1-(1-enyl-palmitoyl)-2-arachidonoyl-GPE (P-16:0/20:4)* | 0.02±0.19 | 0 | 6.85E-01 | 9.76E-01 | 0.07±0.19 | 0 | 7.93E-01 | 9.37E-01 | -0.02±0.21 | 9.94E-01 | 0.05±0.21 | 9.78E-01 | 0.18±0.46 | 9.96E-01 | 0.18±0.45 | 9.96E-01 |
| aspartate | -0.06±0.2 | 0 | 3.92E-01 | 9.55E-01 | 0.09±0.21 | 0 | 5.90E-01 | 9.37E-01 | -0.13±0.22 | 9.06E-01 | 0.04±0.23 | 9.79E-01 | 0.38±0.56 | 9.96E-01 | 0.38±0.58 | 9.96E-01 |
| indolepropionate | 0.12±0.2 | 0 | 7.96E-01 | 9.03E-01 | 0.08±0.2 | 0 | 6.87E-01 | 9.37E-01 | 0.09±0.22 | 9.28E-01 | 0.05±0.22 | 9.79E-01 | 0.22±0.46 | 9.96E-01 | 0.28±0.54 | 9.96E-01 |
| methionine | 0.11±0.2 | 0 | 6.87E-01 | 9.03E-01 | 0.09±0.21 | 0 | 6.09E-01 | 9.37E-01 | 0.08±0.22 | 9.37E-01 | 0.05±0.22 | 9.79E-01 | 0.31±0.52 | 9.96E-01 | 0.35±0.55 | 9.96E-01 |
| androstenediol (3alpha, 17alpha) monosulfate (2) | 0.08±0.2 | 76.46 | 3.93E-02 | 9.40E-01 | 0.09±0.2 | 74.62 | 4.72E-02 | 9.37E-01 | -0.05±0.21 | 9.49E-01 | -0.04±0.21 | 9.79E-01 | 1.35±0.65 | 9.96E-01 | 1.29±0.64 | 9.96E-01 |
| glutamine conjugate of C6H10O2 (2)* | -0.06±0.19 | 0 | 7.40E-01 | 9.52E-01 | -0.1±0.19 | 0 | 4.66E-01 | 9.37E-01 | -0.03±0.21 | 9.83E-01 | -0.04±0.21 | 9.79E-01 | -0.19±0.42 | 9.96E-01 | -0.43±0.49 | 9.96E-01 |
| glutamine conjugate of C6H10O2 (1)* | -0.04±0.19 | 0 | 9.16E-01 | 9.68E-01 | -0.09±0.19 | 0 | 6.20E-01 | 9.37E-01 | -0.03±0.22 | 9.85E-01 | -0.05±0.21 | 9.79E-01 | -0.08±0.4 | 9.96E-01 | -0.3±0.46 | 9.96E-01 |
| 1-(1-enyl-palmitoyl)-2-palmitoleoyl-GPC (P-16:0/16:1)* | 0.09±0.2 | 57.7 | 1.24E-01 | 9.31E-01 | 0.11±0.22 | 75.57 | 4.31E-02 | 9.37E-01 | -0.03±0.22 | 9.86E-01 | -0.04±0.23 | 9.79E-01 | 0.94±0.59 | 9.96E-01 | 1.37±0.66 | 9.96E-01 |
| X - 25420 | 0.14±0.2 | 42.75 | 1.86E-01 | 8.96E-01 | 0.1±0.21 | 56.23 | 1.31E-01 | 9.37E-01 | 0.03±0.22 | 9.90E-01 | -0.04±0.23 | 9.79E-01 | 0.76±0.51 | 9.96E-01 | 0.8±0.51 | 9.96E-01 |
| 1-(1-enyl-stearoyl)-2-arachidonoyl-GPE (P-18:0/20:4)* | 0.08±0.2 | 0 | 3.66E-01 | 9.35E-01 | 0.11±0.2 | 0 | 4.07E-01 | 9.37E-01 | 0.02±0.21 | 9.94E-01 | 0.04±0.21 | 9.79E-01 | 0.56±0.57 | 9.96E-01 | 0.55±0.58 | 9.96E-01 |
| 3-carboxy-4-methyl-5-pentyl-2-furanpropionate (3-CMPFP)** | 0.05±0.19 | 0 | 6.26E-01 | 9.64E-01 | 0.07±0.19 | 0 | 7.32E-01 | 9.37E-01 | 0±0.21 | 9.98E-01 | 0.04±0.21 | 9.79E-01 | 0.23±0.43 | 9.96E-01 | 0.21±0.45 | 9.96E-01 |
| 4-hydroxyglutamate | -0.11±0.18 | 0 | 9.74E-01 | 9.03E-01 | -0.08±0.2 | 0 | 6.06E-01 | 9.37E-01 | -0.11±0.2 | 9.14E-01 | -0.03±0.22 | 9.86E-01 | -0.12±0.45 | 9.96E-01 | -0.31±0.49 | 9.96E-01 |
| 1-oleoyl-2-arachidonoyl-GPE (18:1/20:4)* | 0.12±0.21 | 0 | 6.55E-01 | 9.03E-01 | 0.08±0.21 | 0 | 6.30E-01 | 9.37E-01 | 0.08±0.23 | 9.49E-01 | 0.04±0.23 | 9.86E-01 | 0.32±0.49 | 9.96E-01 | 0.29±0.48 | 9.96E-01 |
| N-acetylproline | -0.06±0.18 | 0 | 7.37E-01 | 9.42E-01 | -0.08±0.18 | 0 | 6.41E-01 | 9.37E-01 | -0.03±0.2 | 9.83E-01 | -0.03±0.21 | 9.86E-01 | -0.17±0.35 | 9.96E-01 | -0.23±0.37 | 9.96E-01 |
| myristoylcarnitine (C14) | -0.1±0.18 | 0 | 6.84E-01 | 9.03E-01 | -0.07±0.18 | 0 | 7.03E-01 | 9.37E-01 | -0.05±0.21 | 9.57E-01 | -0.03±0.21 | 9.89E-01 | -0.21±0.32 | 9.96E-01 | -0.18±0.33 | 9.96E-01 |
| margaroylcarnitine (C17)* | 0.11±0.19 | 0 | 5.11E-01 | 9.03E-01 | 0.09±0.2 | 0 | 3.85E-01 | 9.37E-01 | 0.05±0.21 | 9.49E-01 | 0.03±0.21 | 9.92E-01 | 0.41±0.5 | 9.96E-01 | 0.53±0.53 | 9.96E-01 |
| N-palmitoyl-sphingadienine (d18:2/16:0)* | 0.06±0.2 | 0 | 4.42E-01 | 9.55E-01 | 0.08±0.19 | 0 | 3.86E-01 | 9.37E-01 | 0.01±0.21 | 9.95E-01 | 0.02±0.2 | 9.93E-01 | 0.53±0.65 | 9.96E-01 | 0.64±0.68 | 9.96E-01 |
| maleate | 0.06±0.18 | 0 | 5.22E-01 | 9.54E-01 | 0.08±0.19 | 0 | 5.67E-01 | 9.37E-01 | -0.02±0.22 | 9.94E-01 | 0.02±0.21 | 9.94E-01 | 0.24±0.33 | 9.96E-01 | 0.27±0.37 | 9.96E-01 |
| X - 17654 | 0.2±0.21 | 6.36 | 3.01E-01 | 8.27E-01 | 0.1±0.2 | 0 | 3.44E-01 | 9.37E-01 | 0.11±0.23 | 9.14E-01 | 0.02±0.22 | 9.94E-01 | 0.71±0.53 | 9.96E-01 | 0.58±0.55 | 9.96E-01 |
| gamma-glutamylleucine | -0.13±0.18 | 0 | 7.27E-01 | 8.96E-01 | -0.07±0.19 | 0 | 4.95E-01 | 9.37E-01 | -0.09±0.22 | 9.34E-01 | 0.02±0.23 | 9.94E-01 | -0.22±0.32 | 9.96E-01 | -0.26±0.34 | 9.96E-01 |
| caffeine | -0.01±0.19 | 0 | 3.47E-01 | 9.83E-01 | 0.08±0.2 | 0 | 4.45E-01 | 9.37E-01 | -0.08±0.2 | 9.35E-01 | 0.02±0.22 | 9.94E-01 | 0.43±0.5 | 9.96E-01 | 0.47±0.55 | 9.96E-01 |
| 4-hydroxyphenylacetate | -0.08±0.19 | 0 | 7.97E-01 | 9.31E-01 | -0.07±0.19 | 0 | 5.29E-01 | 9.37E-01 | -0.06±0.21 | 9.49E-01 | -0.02±0.22 | 9.94E-01 | -0.18±0.44 | 9.96E-01 | -0.33±0.45 | 9.96E-01 |
| octadecanedioylcarnitine (C18-DC)* | 0.12±0.18 | 0 | 4.72E-01 | 9.03E-01 | 0.09±0.18 | 0 | 5.20E-01 | 9.37E-01 | 0.04±0.22 | 9.82E-01 | 0.02±0.22 | 9.94E-01 | 0.32±0.33 | 9.96E-01 | 0.28±0.35 | 9.96E-01 |
| 2-hydroxypalmitate | 0.12±0.19 | 11.57 | 2.88E-01 | 9.03E-01 | 0.08±0.19 | 19.66 | 2.65E-01 | 9.37E-01 | 0.03±0.21 | 9.83E-01 | -0.02±0.21 | 9.94E-01 | 0.57±0.46 | 9.96E-01 | 0.56±0.47 | 9.96E-01 |
| 2-acetamidophenol sulfate | 0.12±0.19 | 19.47 | 2.65E-01 | 9.03E-01 | 0.08±0.19 | 26.45 | 2.44E-01 | 9.37E-01 | 0.03±0.21 | 9.83E-01 | -0.02±0.21 | 9.94E-01 | 0.61±0.48 | 9.96E-01 | 0.59±0.48 | 9.96E-01 |
| fructosyllysine | -0.13±0.18 | 0 | 8.21E-01 | 8.96E-01 | -0.07±0.19 | 0 | 5.71E-01 | 9.37E-01 | -0.1±0.22 | 9.14E-01 | -0.01±0.22 | 9.96E-01 | -0.19±0.33 | 9.96E-01 | -0.24±0.34 | 9.96E-01 |
| kynurenate | -0.13±0.18 | 0 | 5.89E-01 | 8.96E-01 | -0.09±0.19 | 2 | 3.12E-01 | 9.37E-01 | -0.08±0.21 | 9.37E-01 | 0.01±0.22 | 9.96E-01 | -0.31±0.38 | 9.96E-01 | -0.46±0.41 | 9.96E-01 |
| methionine sulfoxide | 0.07±0.19 | 9.19 | 2.94E-01 | 9.40E-01 | 0.07±0.2 | 0 | 3.59E-01 | 9.37E-01 | -0.02±0.21 | 9.94E-01 | -0.01±0.21 | 9.96E-01 | 0.51±0.46 | 9.96E-01 | 0.48±0.48 | 9.96E-01 |
| 5,6-dihydrouridine | -0.06±0.18 | 0 | 5.37E-01 | 9.55E-01 | -0.07±0.18 | 0 | 4.23E-01 | 9.37E-01 | 0.01±0.21 | 9.95E-01 | 0.01±0.21 | 9.96E-01 | -0.23±0.33 | 9.96E-01 | -0.35±0.39 | 9.96E-01 |
| anthranilate | 0.09±0.2 | 39.77 | 1.98E-01 | 9.20E-01 | 0.09±0.21 | 6.79 | 3.00E-01 | 9.37E-01 | -0.01±0.21 | 9.97E-01 | 0.01±0.22 | 9.96E-01 | 0.73±0.53 | 9.96E-01 | 0.67±0.6 | 9.96E-01 |
| N2,N5-diacetylornithine | -0.07±0.19 | 0 | 4.34E-01 | 9.40E-01 | -0.07±0.19 | 0 | 4.48E-01 | 9.37E-01 | 0±0.21 | 9.98E-01 | -0.01±0.21 | 9.96E-01 | -0.36±0.41 | 9.96E-01 | -0.37±0.44 | 9.96E-01 |
| 3,7-dimethylurate | 0.07±0.18 | 42.36 | 1.88E-01 | 9.40E-01 | 0.09±0.19 | 29.86 | 2.32E-01 | 9.37E-01 | -0.03±0.2 | 9.80E-01 | 0±0.2 | 9.99E-01 | 0.62±0.46 | 9.96E-01 | 0.63±0.48 | 9.96E-01 |
| 3-hydroxybutyrate (BHBA) | -0.07±0.19 | 0 | 3.89E-01 | 9.40E-01 | -0.07±0.18 | 0 | 4.04E-01 | 9.37E-01 | 0.01±0.21 | 9.97E-01 | 0±0.2 | 9.99E-01 | -0.41±0.44 | 9.96E-01 | -0.41±0.45 | 9.96E-01 |
| isobutyrylglycine | 0.1±0.19 | 0 | 8.08E-01 | 9.03E-01 | 0.1±0.19 | 0 | 7.88E-01 | 9.37E-01 | 0.13±0.21 | 9.06E-01 | 0.12±0.21 | 9.30E-01 | 0.01±0.43 | 9.96E-01 | -0.02±0.46 | 9.98E-01 |
| 2-palmitoleoyl-GPC (16:1)* | 0.1±0.19 | 0 | 6.54E-01 | 9.10E-01 | 0.09±0.19 | 0 | 8.16E-01 | 9.37E-01 | 0.14±0.21 | 8.90E-01 | 0.12±0.22 | 9.30E-01 | -0.06±0.39 | 9.96E-01 | 0.01±0.42 | 9.98E-01 |
| N,N-dimethyl-pro-pro | -0.05±0.2 | 0 | 8.09E-01 | 9.64E-01 | -0.08±0.2 | 0 | 8.09E-01 | 9.37E-01 | -0.07±0.22 | 9.49E-01 | -0.11±0.23 | 9.43E-01 | 0.05±0.44 | 9.96E-01 | 0.01±0.43 | 9.98E-01 |
| pro-hydroxy-pro | 0.15±0.19 | 0 | 8.67E-01 | 8.87E-01 | 0.08±0.19 | 0 | 8.32E-01 | 9.37E-01 | 0.16±0.21 | 8.78E-01 | 0.1±0.21 | 9.41E-01 | 0.08±0.47 | 9.96E-01 | 0±0.43 | 1.00E+00 |
| N-acetyl-2-aminooctanoate* | 0.11±0.19 | 62.02 | 1.05E-01 | 9.03E-01 | 0.07±0.19 | 65.75 | 8.75E-02 | 9.39E-01 | 0.28±0.21 | 7.12E-01 | 0.24±0.21 | 8.10E-01 | -0.44±0.39 | 9.96E-01 | -0.53±0.4 | 9.96E-01 |
| homocitrulline | 0.1±0.19 | 44.42 | 1.80E-01 | 9.13E-01 | 0.07±0.19 | 53.44 | 1.43E-01 | 9.39E-01 | 0.22±0.21 | 7.95E-01 | 0.19±0.21 | 8.77E-01 | -0.44±0.44 | 9.96E-01 | -0.56±0.47 | 9.96E-01 |
| indoleacetylglutamine | 0.06±0.19 | 4.1 | 3.07E-01 | 9.55E-01 | 0.07±0.19 | 26.99 | 2.42E-01 | 9.39E-01 | 0.16±0.21 | 8.78E-01 | 0.19±0.22 | 8.79E-01 | -0.3±0.4 | 9.96E-01 | -0.37±0.42 | 9.96E-01 |
| tetradecadienoate (14:2)* | 0.12±0.19 | 0 | 3.59E-01 | 9.03E-01 | 0.07±0.19 | 0 | 3.19E-01 | 9.39E-01 | 0.21±0.22 | 8.07E-01 | 0.16±0.22 | 8.99E-01 | -0.23±0.43 | 9.96E-01 | -0.33±0.44 | 9.96E-01 |
| methyl indole-3-acetate | 0.07±0.19 | 0 | 4.98E-01 | 9.40E-01 | 0.07±0.19 | 0 | 3.61E-01 | 9.39E-01 | 0.14±0.21 | 9.00E-01 | 0.15±0.21 | 9.12E-01 | -0.17±0.4 | 9.96E-01 | -0.27±0.41 | 9.96E-01 |
| androsterone glucuronide | -0.09±0.2 | 0 | 3.40E-01 | 9.20E-01 | -0.07±0.2 | 0 | 3.53E-01 | 9.39E-01 | -0.17±0.21 | 8.77E-01 | -0.15±0.21 | 9.20E-01 | 0.35±0.5 | 9.96E-01 | 0.36±0.51 | 9.96E-01 |
| X - 24456 | 0.1±0.2 | 0 | 7.45E-01 | 9.13E-01 | 0.07±0.2 | 0 | 7.38E-01 | 9.39E-01 | 0.14±0.23 | 9.06E-01 | 0.11±0.23 | 9.41E-01 | -0.02±0.42 | 9.96E-01 | -0.05±0.41 | 9.96E-01 |
| 10-heptadecenoate (17:1n7) | 0.06±0.2 | 0 | 6.97E-01 | 9.60E-01 | 0.07±0.19 | 0 | 6.68E-01 | 9.39E-01 | 0.02±0.22 | 9.94E-01 | 0.03±0.21 | 9.86E-01 | 0.22±0.46 | 9.96E-01 | 0.25±0.47 | 9.96E-01 |
| X - 25520 | -0.03±0.18 | 0 | 7.11E-01 | 9.70E-01 | -0.06±0.18 | 0 | 7.10E-01 | 9.39E-01 | 0.01±0.21 | 9.97E-01 | -0.03±0.2 | 9.86E-01 | -0.15±0.39 | 9.96E-01 | -0.19±0.39 | 9.96E-01 |
| X - 21842 | -0.04±0.19 | 5.45 | 3.04E-01 | 9.68E-01 | -0.07±0.19 | 16.14 | 2.75E-01 | 9.39E-01 | 0.05±0.21 | 9.57E-01 | 0.02±0.21 | 9.94E-01 | -0.49±0.47 | 9.96E-01 | -0.55±0.48 | 9.96E-01 |
| carboxyethyl-GABA | -0.03±0.18 | 0 | 8.21E-01 | 9.69E-01 | -0.06±0.18 | 0 | 8.44E-01 | 9.41E-01 | -0.01±0.21 | 9.97E-01 | -0.04±0.21 | 9.79E-01 | -0.11±0.37 | 9.96E-01 | -0.13±0.39 | 9.96E-01 |
| carotene diol (1) | 0.02±0.19 | 0 | 7.03E-01 | 9.70E-01 | -0.07±0.2 | 0 | 5.42E-01 | 9.43E-01 | -0.01±0.21 | 9.95E-01 | -0.12±0.22 | 9.30E-01 | 0.18±0.46 | 9.96E-01 | 0.2±0.49 | 9.96E-01 |
| 5-methyluridine (ribothymidine) | 0.12±0.19 | 0 | 9.38E-01 | 9.03E-01 | 0.07±0.19 | 0 | 9.27E-01 | 9.43E-01 | 0.13±0.22 | 9.06E-01 | 0.08±0.22 | 9.66E-01 | 0.09±0.42 | 9.96E-01 | 0.03±0.4 | 9.96E-01 |
| methyl glucopyranoside (alpha + beta) | 0.08±0.19 | 27.41 | 2.41E-01 | 9.35E-01 | 0.07±0.2 | 44.13 | 1.81E-01 | 9.47E-01 | 0.18±0.21 | 8.67E-01 | 0.18±0.21 | 8.79E-01 | -0.39±0.44 | 9.96E-01 | -0.55±0.5 | 9.96E-01 |
| X - 12830 | 0.01±0.18 | 0 | 9.86E-01 | 9.87E-01 | 0.06±0.18 | 0 | 9.59E-01 | 9.48E-01 | 0±0.21 | 9.97E-01 | 0.07±0.21 | 9.66E-01 | 0.01±0.39 | 9.96E-01 | 0.05±0.38 | 9.96E-01 |
| 3-hydroxyhexanoate | 0.03±0.19 | 0 | 6.90E-01 | 9.69E-01 | 0.06±0.19 | 0 | 8.44E-01 | 9.49E-01 | 0±0.21 | 9.98E-01 | 0.05±0.2 | 9.78E-01 | 0.21±0.48 | 9.96E-01 | 0.15±0.48 | 9.96E-01 |
| 1-palmitoleoyl-2-linolenoyl-GPC (16:1/18:3)* | 0.12±0.21 | 0 | 7.58E-01 | 9.03E-01 | 0.07±0.22 | 0 | 7.12E-01 | 9.50E-01 | 0.09±0.24 | 9.35E-01 | 0.04±0.24 | 9.86E-01 | 0.27±0.51 | 9.96E-01 | 0.26±0.55 | 9.96E-01 |
| isoleucylglycine | 0.14±0.21 | 70.9 | 6.38E-02 | 9.03E-01 | 0.07±0.21 | 67.24 | 8.06E-02 | 9.50E-01 | 0.41±0.26 | 6.15E-01 | 0.31±0.25 | 7.78E-01 | -0.44±0.38 | 9.96E-01 | -0.51±0.4 | 9.96E-01 |
| cysteine sulfinic acid | 0.01±0.2 | 42.33 | 1.88E-01 | 9.76E-01 | 0.07±0.21 | 29.39 | 2.34E-01 | 9.50E-01 | 0.16±0.23 | 8.85E-01 | 0.21±0.24 | 8.79E-01 | -0.44±0.39 | 9.96E-01 | -0.37±0.42 | 9.96E-01 |
| etiocholanolone glucuronide | -0.05±0.19 | 31.05 | 2.28E-01 | 9.62E-01 | -0.06±0.19 | 41.29 | 1.92E-01 | 9.50E-01 | -0.15±0.2 | 8.78E-01 | -0.17±0.21 | 8.88E-01 | 0.46±0.46 | 9.96E-01 | 0.5±0.47 | 9.96E-01 |
| palmitoyl dihydrosphingomyelin (d18:0/16:0)* | -0.03±0.21 | 0 | 5.94E-01 | 9.70E-01 | -0.07±0.22 | 0 | 4.57E-01 | 9.50E-01 | -0.07±0.22 | 9.48E-01 | -0.12±0.23 | 9.30E-01 | 0.25±0.57 | 9.96E-01 | 0.41±0.67 | 9.96E-01 |
| 1-arachidonoyl-GPI (20:4)* | -0.06±0.19 | 0 | 6.67E-01 | 9.57E-01 | -0.06±0.19 | 0 | 6.21E-01 | 9.50E-01 | -0.09±0.21 | 9.19E-01 | -0.1±0.21 | 9.41E-01 | 0.12±0.45 | 9.96E-01 | 0.15±0.46 | 9.96E-01 |
| 1-stearoyl-2-arachidonoyl-GPE (18:0/20:4) | -0.1±0.2 | 0 | 7.20E-01 | 9.13E-01 | -0.06±0.2 | 0 | 7.42E-01 | 9.50E-01 | -0.14±0.22 | 9.05E-01 | -0.1±0.23 | 9.53E-01 | 0.03±0.41 | 9.96E-01 | 0.06±0.41 | 9.96E-01 |
| X - 21733 | -0.01±0.18 | 0 | 6.58E-01 | 9.80E-01 | -0.06±0.19 | 0 | 8.06E-01 | 9.50E-01 | -0.04±0.2 | 9.57E-01 | -0.08±0.21 | 9.63E-01 | 0.18±0.48 | 9.96E-01 | 0.05±0.5 | 9.96E-01 |
| X - 24518 | -0.07±0.18 | 0 | 9.34E-01 | 9.35E-01 | -0.06±0.18 | 0 | 8.61E-01 | 9.50E-01 | -0.08±0.2 | 9.28E-01 | -0.04±0.2 | 9.79E-01 | -0.05±0.39 | 9.96E-01 | -0.12±0.41 | 9.96E-01 |
| histidine | 0.1±0.19 | 0 | 8.65E-01 | 9.13E-01 | 0.06±0.19 | 0 | 8.31E-01 | 9.50E-01 | 0.08±0.21 | 9.36E-01 | 0.04±0.21 | 9.79E-01 | 0.17±0.46 | 9.96E-01 | 0.15±0.45 | 9.96E-01 |
| 2-hydroxy-4-(methylthio)butanoic acid | 0.03±0.19 | 0 | 8.07E-01 | 9.70E-01 | 0.07±0.2 | 0 | 8.50E-01 | 9.50E-01 | 0.01±0.21 | 9.97E-01 | 0.05±0.22 | 9.79E-01 | 0.13±0.44 | 9.96E-01 | 0.15±0.46 | 9.96E-01 |
| decadienedioic acid (C10:2-DC)** | 0.01±0.19 | 0 | 8.30E-01 | 9.79E-01 | 0.06±0.19 | 0 | 8.43E-01 | 9.50E-01 | -0.01±0.21 | 9.97E-01 | 0.05±0.21 | 9.79E-01 | 0.09±0.42 | 9.96E-01 | 0.15±0.46 | 9.96E-01 |
| lignoceroylcarnitine (C24)* | -0.05±0.19 | 0 | 8.56E-01 | 9.64E-01 | -0.06±0.19 | 0 | 7.50E-01 | 9.50E-01 | -0.02±0.22 | 9.92E-01 | -0.02±0.22 | 9.94E-01 | -0.1±0.36 | 9.96E-01 | -0.16±0.36 | 9.96E-01 |
| X - 25936 | 0.07±0.18 | 0 | 8.07E-01 | 9.36E-01 | 0.06±0.18 | 0 | 8.70E-01 | 9.50E-01 | 0.1±0.21 | 9.14E-01 | 0.08±0.21 | 9.65E-01 | -0.01±0.37 | 9.96E-01 | 0.01±0.37 | 9.98E-01 |
| spermidine | 0.08±0.16 | 0 | 5.11E-01 | 9.24E-01 | 0.06±0.18 | 0 | 5.67E-01 | 9.51E-01 | 0.13±0.18 | 8.89E-01 | 0.11±0.2 | 9.30E-01 | -0.15±0.39 | 9.96E-01 | -0.16±0.41 | 9.96E-01 |
| N-acetylvaline | -0.08±0.17 | 0 | 4.46E-01 | 9.22E-01 | -0.06±0.18 | 21.9 | 2.58E-01 | 9.53E-01 | 0.01±0.21 | 9.95E-01 | 0.07±0.21 | 9.66E-01 | -0.27±0.3 | 9.96E-01 | -0.37±0.33 | 9.96E-01 |
| myristoyl dihydrosphingomyelin (d18:0/14:0)* | 0.06±0.21 | 0 | 3.98E-01 | 9.57E-01 | 0.07±0.21 | 16.27 | 2.74E-01 | 9.53E-01 | 0±0.22 | 9.98E-01 | -0.01±0.22 | 9.96E-01 | 0.55±0.62 | 9.96E-01 | 0.76±0.67 | 9.96E-01 |
| palmitoyl-sphingosine-phosphoethanolamine (d18:1/16:0) | 0.12±0.2 | 0 | 4.46E-01 | 9.03E-01 | 0.06±0.2 | 10.95 | 2.89E-01 | 9.55E-01 | 0.05±0.22 | 9.57E-01 | -0.01±0.21 | 9.94E-01 | 0.5±0.55 | 9.96E-01 | 0.65±0.59 | 9.96E-01 |
| S-carboxyethylcysteine | -0.09±0.19 | 19.41 | 2.65E-01 | 9.20E-01 | -0.06±0.2 | 6.11 | 3.02E-01 | 9.55E-01 | 0±0.21 | 9.99E-01 | 0.02±0.22 | 9.94E-01 | -0.6±0.49 | 9.96E-01 | -0.59±0.55 | 9.96E-01 |
| 1,3,7-trimethylurate | -0.13±0.18 | 0 | 5.58E-01 | 8.96E-01 | -0.06±0.19 | 0 | 7.13E-01 | 9.55E-01 | -0.18±0.2 | 8.53E-01 | -0.09±0.21 | 9.54E-01 | 0.11±0.45 | 9.96E-01 | 0.1±0.46 | 9.96E-01 |
| tryptophan betaine | 0.05±0.19 | 0 | 7.40E-01 | 9.63E-01 | 0.06±0.2 | 0 | 9.22E-01 | 9.55E-01 | 0.02±0.21 | 9.94E-01 | 0.05±0.22 | 9.78E-01 | 0.18±0.43 | 9.96E-01 | 0.1±0.45 | 9.96E-01 |
| 3b-hydroxy-5-cholenoic acid | 0.09±0.18 | 0 | 8.55E-01 | 9.13E-01 | 0.05±0.18 | 0 | 8.73E-01 | 9.55E-01 | 0.11±0.2 | 9.14E-01 | 0.04±0.2 | 9.79E-01 | 0.03±0.39 | 9.96E-01 | 0.11±0.38 | 9.96E-01 |
| metabolonic lactone sulfate | -0.15±0.18 | 0 | 5.85E-01 | 8.77E-01 | -0.06±0.19 | 0 | 3.69E-01 | 9.55E-01 | -0.1±0.2 | 9.14E-01 | 0.04±0.22 | 9.86E-01 | -0.34±0.4 | 9.96E-01 | -0.38±0.41 | 9.96E-01 |
| 10-undecenoate (11:1n1) | 0.02±0.19 | 0 | 6.75E-01 | 9.70E-01 | 0.06±0.19 | 0 | 6.53E-01 | 9.55E-01 | -0.01±0.21 | 9.95E-01 | 0.02±0.21 | 9.94E-01 | 0.19±0.45 | 9.96E-01 | 0.24±0.46 | 9.96E-01 |
| 1-palmitoyl-2-linoleoyl-GPC (16:0/18:2) | -0.02±0.19 | 0 | 9.50E-01 | 9.70E-01 | -0.06±0.19 | 0 | 9.44E-01 | 9.56E-01 | -0.01±0.22 | 9.95E-01 | -0.06±0.22 | 9.67E-01 | -0.04±0.36 | 9.96E-01 | -0.03±0.38 | 9.96E-01 |
| lanthionine | -0.04±0.18 | 0 | 7.43E-01 | 9.64E-01 | -0.05±0.18 | 0 | 7.77E-01 | 9.56E-01 | -0.01±0.2 | 9.95E-01 | -0.03±0.2 | 9.91E-01 | -0.15±0.39 | 9.96E-01 | -0.15±0.39 | 9.96E-01 |
| retinol (Vitamin A) | -0.08±0.17 | 0 | 7.97E-01 | 9.27E-01 | -0.05±0.18 | 0 | 7.43E-01 | 9.56E-01 | -0.05±0.2 | 9.57E-01 | -0.02±0.21 | 9.94E-01 | -0.14±0.31 | 9.96E-01 | -0.14±0.33 | 9.96E-01 |
| docosapentaenoate (n3 DPA; 22:5n3) | -0.06±0.19 | 0 | 8.28E-01 | 9.55E-01 | -0.06±0.19 | 0 | 8.64E-01 | 9.56E-01 | -0.08±0.21 | 9.37E-01 | -0.07±0.21 | 9.66E-01 | 0.03±0.43 | 9.96E-01 | 0.01±0.43 | 9.98E-01 |
| X - 12117 | -0.05±0.18 | 0 | 9.73E-01 | 9.62E-01 | -0.06±0.19 | 0 | 7.02E-01 | 9.57E-01 | -0.05±0.21 | 9.59E-01 | -0.02±0.22 | 9.94E-01 | -0.06±0.39 | 9.96E-01 | -0.2±0.41 | 9.96E-01 |
| N,N-dimethylalanine | 0.1±0.19 | 73.06 | 5.40E-02 | 9.10E-01 | 0.06±0.19 | 72.3 | 5.74E-02 | 9.58E-01 | -0.07±0.21 | 9.37E-01 | -0.1±0.21 | 9.41E-01 | 0.84±0.43 | 9.96E-01 | 0.9±0.49 | 9.96E-01 |
| N-acetylneuraminate | 0.07±0.18 | 0 | 8.70E-01 | 9.40E-01 | 0.05±0.18 | 0 | 9.23E-01 | 9.58E-01 | 0.08±0.2 | 9.28E-01 | 0.06±0.21 | 9.66E-01 | 0.01±0.36 | 9.96E-01 | 0.02±0.37 | 9.96E-01 |
| 2-palmitoyl-GPC (16:0)* | -0.02±0.19 | 0 | 4.10E-01 | 9.70E-01 | -0.05±0.19 | 0 | 3.68E-01 | 9.62E-01 | -0.09±0.21 | 9.22E-01 | -0.13±0.21 | 9.26E-01 | 0.32±0.45 | 9.96E-01 | 0.32±0.46 | 9.96E-01 |
| alanine | -0.09±0.19 | 0 | 6.63E-01 | 9.20E-01 | -0.05±0.19 | 0 | 7.78E-01 | 9.62E-01 | -0.13±0.21 | 9.02E-01 | -0.08±0.21 | 9.63E-01 | 0.07±0.42 | 9.96E-01 | 0.05±0.4 | 9.96E-01 |
| imidazole lactate | 0.05±0.19 | 0 | 8.56E-01 | 9.63E-01 | 0.06±0.2 | 0 | 9.89E-01 | 9.62E-01 | 0.03±0.22 | 9.83E-01 | 0.06±0.23 | 9.77E-01 | 0.12±0.43 | 9.96E-01 | 0.05±0.41 | 9.96E-01 |
| lactosyl-N-behenoyl-sphingosine (d18:1/22:0)* | 0.02±0.21 | 19.51 | 2.65E-01 | 9.70E-01 | -0.05±0.2 | 35.41 | 2.13E-01 | 9.67E-01 | -0.07±0.22 | 9.49E-01 | -0.15±0.21 | 9.16E-01 | 0.6±0.56 | 9.96E-01 | 0.56±0.53 | 9.96E-01 |
| citrate | 0.02±0.21 | 51.31 | 1.52E-01 | 9.76E-01 | -0.06±0.21 | 24.5 | 2.50E-01 | 9.67E-01 | -0.08±0.22 | 9.36E-01 | -0.14±0.22 | 9.26E-01 | 0.91±0.66 | 9.96E-01 | 0.67±0.66 | 9.96E-01 |
| methylsuccinoylcarnitine | -0.02±0.18 | 0 | 5.75E-01 | 9.70E-01 | -0.05±0.19 | 0 | 6.16E-01 | 9.67E-01 | -0.07±0.2 | 9.37E-01 | -0.1±0.21 | 9.47E-01 | 0.17±0.39 | 9.96E-01 | 0.15±0.43 | 9.96E-01 |
| butyrylcarnitine (C4) | 0.01±0.18 | 0 | 9.38E-01 | 9.83E-01 | 0.05±0.18 | 0 | 7.35E-01 | 9.67E-01 | 0.01±0.2 | 9.94E-01 | 0.08±0.2 | 9.63E-01 | -0.02±0.38 | 9.96E-01 | -0.06±0.37 | 9.96E-01 |
| 3-amino-2-piperidone | 0.08±0.19 | 0 | 9.52E-01 | 9.35E-01 | 0.05±0.19 | 0 | 9.65E-01 | 9.67E-01 | 0.08±0.22 | 9.45E-01 | 0.06±0.22 | 9.75E-01 | 0.1±0.44 | 9.96E-01 | 0.04±0.42 | 9.96E-01 |
| 3-(3-hydroxyphenyl)propionate | -0.02±0.19 | 0 | 4.95E-01 | 9.70E-01 | -0.05±0.19 | 0 | 5.98E-01 | 9.68E-01 | -0.08±0.21 | 9.37E-01 | -0.1±0.21 | 9.47E-01 | 0.25±0.44 | 9.96E-01 | 0.16±0.45 | 9.96E-01 |
| X - 12462 | 0.07±0.19 | 0 | 7.88E-01 | 9.40E-01 | 0.05±0.19 | 0 | 7.97E-01 | 9.68E-01 | 0.05±0.21 | 9.57E-01 | 0.03±0.21 | 9.88E-01 | 0.19±0.48 | 9.96E-01 | 0.17±0.49 | 9.96E-01 |
| cysteine | 0.03±0.19 | 0 | 9.81E-01 | 9.69E-01 | 0.05±0.19 | 0 | 8.60E-01 | 9.68E-01 | 0.03±0.21 | 9.83E-01 | 0.07±0.21 | 9.66E-01 | 0.04±0.41 | 9.96E-01 | -0.01±0.39 | 9.98E-01 |
| beta-citrylglutamate | -0.02±0.17 | 30.61 | 2.30E-01 | 9.70E-01 | 0.04±0.17 | 57.59 | 1.25E-01 | 9.68E-01 | 0.14±0.21 | 9.00E-01 | 0.25±0.22 | 8.10E-01 | -0.28±0.27 | 9.96E-01 | -0.29±0.27 | 9.96E-01 |
| X - 22834 | 0.06±0.2 | 62.95 | 1.00E-01 | 9.55E-01 | 0.05±0.2 | 66.39 | 8.45E-02 | 9.68E-01 | 0.22±0.22 | 7.95E-01 | 0.21±0.22 | 8.63E-01 | -0.6±0.45 | 9.96E-01 | -0.67±0.46 | 9.96E-01 |
| X - 11847 | -0.03±0.19 | 70.15 | 6.72E-02 | 9.70E-01 | -0.04±0.19 | 65.78 | 8.74E-02 | 9.68E-01 | -0.17±0.2 | 8.72E-01 | -0.16±0.2 | 8.88E-01 | 0.8±0.49 | 9.96E-01 | 0.85±0.56 | 9.96E-01 |
| N2,N2-dimethylguanosine | 0.04±0.17 | 0 | 5.50E-01 | 9.64E-01 | 0.04±0.17 | 0 | 4.26E-01 | 9.68E-01 | 0.12±0.21 | 9.14E-01 | 0.15±0.22 | 9.20E-01 | -0.1±0.29 | 9.96E-01 | -0.13±0.28 | 9.96E-01 |
| theophylline | -0.1±0.18 | 36.42 | 2.10E-01 | 9.03E-01 | -0.04±0.19 | 12.3 | 2.86E-01 | 9.68E-01 | -0.18±0.19 | 8.07E-01 | -0.12±0.2 | 9.26E-01 | 0.51±0.52 | 9.96E-01 | 0.48±0.52 | 9.96E-01 |
| N-acetylglutamate | 0.06±0.19 | 0 | 4.83E-01 | 9.55E-01 | 0.05±0.2 | 0 | 4.72E-01 | 9.68E-01 | 0.16±0.24 | 8.92E-01 | 0.14±0.24 | 9.26E-01 | -0.13±0.34 | 9.96E-01 | -0.17±0.36 | 9.96E-01 |
| hexanoylcarnitine (C6) | -0.09±0.17 | 51.85 | 1.50E-01 | 9.13E-01 | -0.04±0.18 | 54.34 | 1.39E-01 | 9.68E-01 | 0.07±0.21 | 9.37E-01 | 0.12±0.21 | 9.26E-01 | -0.46±0.31 | 9.96E-01 | -0.45±0.33 | 9.96E-01 |
| 1-methylurate | -0.06±0.18 | 12.08 | 2.86E-01 | 9.55E-01 | -0.04±0.19 | 0 | 3.20E-01 | 9.68E-01 | -0.14±0.2 | 8.89E-01 | -0.12±0.2 | 9.27E-01 | 0.43±0.49 | 9.96E-01 | 0.42±0.5 | 9.96E-01 |
| orotate | 0.03±0.17 | 0 | 5.10E-01 | 9.69E-01 | 0.04±0.17 | 0 | 5.13E-01 | 9.68E-01 | 0.09±0.19 | 9.14E-01 | 0.1±0.2 | 9.39E-01 | -0.17±0.35 | 9.96E-01 | -0.16±0.36 | 9.96E-01 |
| hexanoylglutamine | -0.04±0.19 | 51.86 | 1.50E-01 | 9.65E-01 | -0.05±0.19 | 61.27 | 1.08E-01 | 9.68E-01 | 0.1±0.21 | 9.14E-01 | 0.11±0.21 | 9.40E-01 | -0.55±0.4 | 9.96E-01 | -0.64±0.42 | 9.96E-01 |
| linolenate [alpha or gamma; (18:3n3 or 6)] | 0.1±0.19 | 0 | 6.15E-01 | 9.03E-01 | 0.04±0.19 | 0 | 5.25E-01 | 9.68E-01 | 0.15±0.21 | 8.89E-01 | 0.1±0.21 | 9.43E-01 | -0.09±0.44 | 9.96E-01 | -0.23±0.47 | 9.96E-01 |
| X - 23636 | -0.05±0.2 | 0 | 4.04E-01 | 9.62E-01 | -0.05±0.2 | 0 | 4.27E-01 | 9.68E-01 | -0.11±0.21 | 9.14E-01 | -0.1±0.21 | 9.47E-01 | 0.5±0.69 | 9.96E-01 | 0.5±0.72 | 9.96E-01 |
| beta-hydroxyisovaleroylcarnitine | -0.1±0.19 | 0 | 5.79E-01 | 9.03E-01 | -0.05±0.19 | 0 | 6.28E-01 | 9.68E-01 | -0.16±0.21 | 8.78E-01 | -0.09±0.21 | 9.53E-01 | 0.1±0.41 | 9.96E-01 | 0.13±0.42 | 9.96E-01 |
| 3-hydroxy-2-ethylpropionate | -0.1±0.19 | 0 | 5.02E-01 | 9.10E-01 | -0.04±0.19 | 0 | 6.29E-01 | 9.68E-01 | -0.16±0.21 | 8.78E-01 | -0.08±0.21 | 9.63E-01 | 0.19±0.47 | 9.96E-01 | 0.17±0.48 | 9.96E-01 |
| X - 12812 | -0.05±0.18 | 0 | 5.63E-01 | 9.63E-01 | -0.05±0.19 | 0 | 7.15E-01 | 9.68E-01 | -0.1±0.21 | 9.14E-01 | -0.08±0.21 | 9.63E-01 | 0.16±0.4 | 9.96E-01 | 0.09±0.42 | 9.96E-01 |
| adipoylcarnitine (C6-DC) | -0.06±0.18 | 0 | 4.29E-01 | 9.55E-01 | -0.04±0.18 | 0.95 | 3.15E-01 | 9.68E-01 | 0.03±0.21 | 9.83E-01 | 0.06±0.21 | 9.66E-01 | -0.29±0.34 | 9.96E-01 | -0.36±0.37 | 9.96E-01 |
| X - 17685 | 0.08±0.19 | 0 | 8.65E-01 | 9.31E-01 | 0.05±0.19 | 0 | 9.61E-01 | 9.68E-01 | 0.07±0.21 | 9.49E-01 | 0.04±0.21 | 9.79E-01 | 0.14±0.41 | 9.96E-01 | 0.07±0.42 | 9.96E-01 |
| beta-hydroxyisovalerate | -0.02±0.19 | 0 | 7.57E-01 | 9.70E-01 | 0.05±0.2 | 0 | 8.87E-01 | 9.68E-01 | -0.05±0.21 | 9.57E-01 | 0.04±0.22 | 9.81E-01 | 0.11±0.46 | 9.96E-01 | 0.11±0.47 | 9.96E-01 |
| sphingomyelin (d18:1/20:2, d18:2/20:1, d16:1/22:2)* | 0.05±0.2 | 0 | 9.99E-01 | 9.63E-01 | 0.05±0.2 | 0 | 8.76E-01 | 9.68E-01 | 0.05±0.22 | 9.57E-01 | 0.03±0.22 | 9.86E-01 | 0.05±0.43 | 9.96E-01 | 0.11±0.46 | 9.96E-01 |
| 3-hydroxyhippurate | -0.01±0.19 | 0 | 9.75E-01 | 9.83E-01 | -0.04±0.19 | 0 | 8.41E-01 | 9.68E-01 | -0.01±0.21 | 9.97E-01 | -0.02±0.21 | 9.94E-01 | -0.02±0.43 | 9.96E-01 | -0.12±0.43 | 9.96E-01 |
| nonanoylcarnitine (C9) | -0.05±0.19 | 0 | 5.06E-01 | 9.61E-01 | -0.05±0.19 | 0 | 5.37E-01 | 9.68E-01 | 0.02±0.22 | 9.94E-01 | 0.02±0.22 | 9.94E-01 | -0.27±0.38 | 9.96E-01 | -0.26±0.39 | 9.96E-01 |
| oleoylcarnitine (C18:1) | -0.03±0.18 | 0 | 7.50E-01 | 9.69E-01 | -0.04±0.19 | 0 | 8.30E-01 | 9.68E-01 | 0.01±0.21 | 9.97E-01 | -0.02±0.22 | 9.94E-01 | -0.12±0.34 | 9.96E-01 | -0.11±0.35 | 9.96E-01 |
| carotene diol (2) | 0.14±0.19 | 0 | 5.25E-01 | 8.96E-01 | 0.05±0.2 | 0 | 4.11E-01 | 9.68E-01 | 0.1±0.21 | 9.14E-01 | -0.01±0.21 | 9.96E-01 | 0.47±0.55 | 9.96E-01 | 0.5±0.58 | 9.96E-01 |
| 4-methyl-2-oxopentanoate | -0.13±0.19 | 0 | 7.83E-01 | 9.03E-01 | -0.05±0.2 | 0 | 6.09E-01 | 9.68E-01 | -0.09±0.22 | 9.28E-01 | 0.01±0.23 | 9.96E-01 | -0.21±0.37 | 9.96E-01 | -0.22±0.39 | 9.96E-01 |
| X - 17335 | -0.09±0.18 | 0 | 7.85E-01 | 9.13E-01 | -0.04±0.18 | 0 | 7.03E-01 | 9.68E-01 | -0.07±0.2 | 9.49E-01 | -0.01±0.21 | 9.96E-01 | -0.19±0.39 | 9.96E-01 | -0.17±0.39 | 9.96E-01 |
| ornithine | 0.06±0.2 | 0 | 5.61E-01 | 9.55E-01 | 0.05±0.2 | 0 | 6.60E-01 | 9.68E-01 | 0.02±0.21 | 9.92E-01 | 0.01±0.22 | 9.96E-01 | 0.41±0.63 | 9.96E-01 | 0.28±0.58 | 9.96E-01 |
| ectoine | -0.05±0.19 | 0 | 5.85E-01 | 9.64E-01 | -0.04±0.19 | 0 | 5.80E-01 | 9.68E-01 | 0±0.21 | 9.97E-01 | 0.01±0.22 | 9.96E-01 | -0.25±0.42 | 9.96E-01 | -0.27±0.45 | 9.96E-01 |
| 1-stearoyl-2-arachidonoyl-GPI (18:0/20:4) | -0.05±0.19 | 0 | 9.26E-01 | 9.62E-01 | -0.05±0.19 | 0 | 9.16E-01 | 9.68E-01 | -0.06±0.22 | 9.49E-01 | -0.06±0.22 | 9.73E-01 | -0.02±0.38 | 9.96E-01 | -0.01±0.4 | 9.98E-01 |
| 1-methylnicotinamide | 0±0.18 | 45.27 | 1.76E-01 | 9.91E-01 | 0.04±0.18 | 46.71 | 1.71E-01 | 9.69E-01 | 0.12±0.2 | 9.02E-01 | 0.16±0.2 | 8.89E-01 | -0.47±0.39 | 9.96E-01 | -0.45±0.4 | 9.96E-01 |
| palmitoylcarnitine (C16) | -0.01±0.18 | 0 | 5.83E-01 | 9.87E-01 | 0.04±0.18 | 0 | 5.70E-01 | 9.69E-01 | 0.06±0.21 | 9.49E-01 | 0.1±0.21 | 9.41E-01 | -0.15±0.32 | 9.96E-01 | -0.12±0.33 | 9.96E-01 |
| thyroxine | 0.01±0.2 | 0 | 4.23E-01 | 9.80E-01 | -0.04±0.19 | 0 | 4.88E-01 | 9.69E-01 | -0.05±0.21 | 9.59E-01 | -0.09±0.21 | 9.52E-01 | 0.44±0.57 | 9.96E-01 | 0.3±0.53 | 9.96E-01 |
| tryptophan | -0.12±0.19 | 0 | 4.70E-01 | 9.03E-01 | -0.04±0.19 | 0 | 6.07E-01 | 9.69E-01 | -0.17±0.2 | 8.70E-01 | -0.08±0.21 | 9.63E-01 | 0.27±0.57 | 9.96E-01 | 0.23±0.55 | 9.96E-01 |
| X - 26111 | -0.03±0.18 | 0 | 4.86E-01 | 9.68E-01 | 0.04±0.19 | 0 | 6.69E-01 | 9.69E-01 | 0.03±0.21 | 9.83E-01 | 0.08±0.21 | 9.63E-01 | -0.28±0.4 | 9.96E-01 | -0.13±0.45 | 9.96E-01 |
| octadecanedioate (C18-DC) | 0.07±0.19 | 0 | 8.11E-01 | 9.50E-01 | 0.04±0.19 | 0 | 7.63E-01 | 9.69E-01 | 0.09±0.21 | 9.28E-01 | 0.07±0.21 | 9.66E-01 | -0.02±0.42 | 9.96E-01 | -0.07±0.41 | 9.96E-01 |
| X - 11632 | 0.05±0.19 | 0 | 7.78E-01 | 9.60E-01 | 0.04±0.2 | 0 | 7.07E-01 | 9.69E-01 | 0.08±0.21 | 9.36E-01 | 0.08±0.22 | 9.66E-01 | -0.05±0.41 | 9.96E-01 | -0.11±0.45 | 9.96E-01 |
| X - 23665 | -0.07±0.18 | 0 | 4.56E-01 | 9.40E-01 | -0.04±0.18 | 0 | 3.92E-01 | 9.69E-01 | 0±0.21 | 9.98E-01 | 0.05±0.21 | 9.78E-01 | -0.31±0.37 | 9.96E-01 | -0.31±0.37 | 9.96E-01 |
| tartronate (hydroxymalonate) | 0±0.18 | 0 | 9.19E-01 | 9.89E-01 | -0.04±0.19 | 0 | 9.75E-01 | 9.69E-01 | 0±0.2 | 9.97E-01 | -0.04±0.2 | 9.79E-01 | 0.04±0.43 | 9.96E-01 | -0.03±0.48 | 9.96E-01 |
| N-oleoyltaurine | 0.06±0.19 | 3.39 | 3.09E-01 | 9.55E-01 | 0.04±0.2 | 0 | 3.22E-01 | 9.69E-01 | -0.02±0.21 | 9.94E-01 | -0.04±0.21 | 9.80E-01 | 0.54±0.51 | 9.96E-01 | 0.52±0.52 | 9.96E-01 |
| X - 11444 | -0.04±0.18 | 0 | 8.65E-01 | 9.64E-01 | -0.04±0.18 | 0 | 9.18E-01 | 9.69E-01 | -0.06±0.2 | 9.49E-01 | -0.03±0.2 | 9.86E-01 | 0.02±0.39 | 9.96E-01 | -0.08±0.4 | 9.96E-01 |
| pentadecanoate (15:0) | 0.06±0.19 | 0 | 4.59E-01 | 9.55E-01 | 0.04±0.19 | 0 | 4.47E-01 | 9.69E-01 | -0.01±0.21 | 9.97E-01 | -0.03±0.21 | 9.90E-01 | 0.35±0.44 | 9.96E-01 | 0.34±0.44 | 9.96E-01 |
| formiminoglutamate | -0.12±0.2 | 0 | 8.29E-01 | 9.03E-01 | -0.04±0.21 | 0 | 5.34E-01 | 9.69E-01 | -0.1±0.22 | 9.14E-01 | 0.02±0.23 | 9.94E-01 | -0.21±0.43 | 9.96E-01 | -0.31±0.48 | 9.96E-01 |
| X - 25265 | -0.04±0.2 | 0 | 6.45E-01 | 9.68E-01 | -0.04±0.2 | 0 | 8.00E-01 | 9.69E-01 | 0±0.22 | 9.97E-01 | -0.02±0.22 | 9.94E-01 | -0.23±0.46 | 9.96E-01 | -0.14±0.44 | 9.96E-01 |
| 3-methyl-2-oxovalerate | -0.11±0.19 | 0 | 9.29E-01 | 9.03E-01 | -0.04±0.2 | 0 | 7.35E-01 | 9.69E-01 | -0.1±0.22 | 9.14E-01 | 0±0.23 | 9.96E-01 | -0.14±0.39 | 9.96E-01 | -0.16±0.41 | 9.96E-01 |
| X - 25343 | -0.03±0.2 | 0 | 3.61E-01 | 9.70E-01 | 0.04±0.2 | 0 | 4.95E-01 | 9.69E-01 | -0.1±0.21 | 9.14E-01 | -0.01±0.22 | 9.96E-01 | 0.43±0.54 | 9.96E-01 | 0.41±0.58 | 9.96E-01 |
| X - 17354 | 0.05±0.19 | 0 | 7.33E-01 | 9.63E-01 | 0.04±0.19 | 0 | 6.76E-01 | 9.69E-01 | 0.02±0.21 | 9.94E-01 | 0±0.21 | 9.99E-01 | 0.18±0.43 | 9.96E-01 | 0.21±0.45 | 9.96E-01 |
| 2-hydroxydecanoate | -0.02±0.19 | 0 | 7.06E-01 | 9.70E-01 | -0.04±0.19 | 0 | 6.65E-01 | 9.69E-01 | 0.02±0.21 | 9.94E-01 | 0±0.22 | 9.99E-01 | -0.16±0.42 | 9.96E-01 | -0.2±0.42 | 9.96E-01 |
| N-formylmethionine | 0.04±0.18 | 0 | 5.50E-01 | 9.64E-01 | 0.04±0.18 | 0 | 4.48E-01 | 9.69E-01 | 0.11±0.22 | 9.14E-01 | 0.12±0.22 | 9.30E-01 | -0.12±0.33 | 9.96E-01 | -0.19±0.34 | 9.96E-01 |
| indole-3-carboxylate | 0.01±0.19 | 0 | 8.27E-01 | 9.82E-01 | -0.04±0.19 | 0 | 8.56E-01 | 9.69E-01 | -0.01±0.21 | 9.95E-01 | -0.05±0.21 | 9.75E-01 | 0.09±0.43 | 9.96E-01 | 0.03±0.44 | 9.96E-01 |
| N6-methyladenosine | 0.06±0.19 | 70.94 | 6.36E-02 | 9.57E-01 | 0.04±0.19 | 72.14 | 5.82E-02 | 9.74E-01 | 0.3±0.23 | 7.19E-01 | 0.28±0.23 | 7.78E-01 | -0.45±0.33 | 9.96E-01 | -0.5±0.34 | 9.96E-01 |
| 2-methylserine | 0.05±0.17 | 61.67 | 1.06E-01 | 9.54E-01 | 0.03±0.17 | 45.04 | 1.77E-01 | 9.74E-01 | 0.26±0.21 | 7.26E-01 | 0.21±0.21 | 8.60E-01 | -0.29±0.27 | 9.96E-01 | -0.26±0.27 | 9.96E-01 |
| glycerol 3-phosphate | 0.05±0.19 | 23.77 | 2.52E-01 | 9.63E-01 | 0.04±0.19 | 32.39 | 2.24E-01 | 9.74E-01 | 0.17±0.21 | 8.78E-01 | 0.16±0.22 | 9.00E-01 | -0.32±0.37 | 9.96E-01 | -0.38±0.39 | 9.96E-01 |
| X - 12707 | 0.04±0.19 | 0 | 6.71E-01 | 9.64E-01 | -0.04±0.19 | 0 | 6.43E-01 | 9.74E-01 | 0±0.21 | 9.98E-01 | -0.08±0.22 | 9.63E-01 | 0.2±0.42 | 9.96E-01 | 0.14±0.43 | 9.96E-01 |
| (16 or 17)-methylstearate (a19:0 or i19:0) | 0.05±0.19 | 0 | 3.18E-01 | 9.63E-01 | 0.03±0.19 | 0 | 3.17E-01 | 9.74E-01 | -0.04±0.21 | 9.71E-01 | -0.05±0.21 | 9.78E-01 | 0.46±0.45 | 9.96E-01 | 0.46±0.46 | 9.96E-01 |
| gluconate | -0.01±0.18 | 0 | 9.95E-01 | 9.76E-01 | -0.03±0.18 | 0 | 8.39E-01 | 9.74E-01 | -0.01±0.2 | 9.94E-01 | -0.01±0.21 | 9.95E-01 | -0.01±0.36 | 9.96E-01 | -0.1±0.38 | 9.96E-01 |
| palmitoleoylcarnitine (C16:1)* | -0.06±0.19 | 0 | 9.13E-01 | 9.50E-01 | -0.03±0.19 | 0 | 9.88E-01 | 9.74E-01 | -0.05±0.21 | 9.56E-01 | -0.03±0.22 | 9.86E-01 | -0.1±0.38 | 9.96E-01 | -0.04±0.4 | 9.96E-01 |
| cortisone | -0.07±0.15 | 4.5 | 3.06E-01 | 9.28E-01 | -0.03±0.15 | 17.79 | 2.70E-01 | 9.75E-01 | -0.12±0.16 | 8.78E-01 | -0.08±0.16 | 9.41E-01 | 0.4±0.48 | 9.96E-01 | 0.52±0.52 | 9.96E-01 |
| phenylacetate | 0.01±0.19 | 0 | 5.69E-01 | 9.82E-01 | -0.03±0.19 | 0 | 6.41E-01 | 9.75E-01 | -0.04±0.21 | 9.71E-01 | -0.07±0.21 | 9.66E-01 | 0.25±0.46 | 9.96E-01 | 0.17±0.48 | 9.96E-01 |
| 2-aminophenol sulfate | 0.07±0.19 | 3.94 | 3.08E-01 | 9.49E-01 | 0.03±0.19 | 7.42 | 2.99E-01 | 9.75E-01 | -0.02±0.21 | 9.93E-01 | -0.06±0.21 | 9.68E-01 | 0.49±0.45 | 9.96E-01 | 0.47±0.46 | 9.96E-01 |
| sebacate (C10-DC) | 0.02±0.18 | 0 | 9.05E-01 | 9.70E-01 | -0.03±0.18 | 0 | 9.77E-01 | 9.75E-01 | 0.01±0.21 | 9.95E-01 | -0.03±0.2 | 9.85E-01 | 0.06±0.4 | 9.96E-01 | -0.02±0.4 | 9.96E-01 |
| acetylcarnitine (C2) | -0.05±0.17 | 0 | 7.89E-01 | 9.61E-01 | -0.03±0.17 | 0 | 7.00E-01 | 9.75E-01 | -0.02±0.21 | 9.94E-01 | 0.01±0.21 | 9.94E-01 | -0.12±0.31 | 9.96E-01 | -0.13±0.31 | 9.96E-01 |
| urea | -0.04±0.18 | 0 | 9.50E-01 | 9.68E-01 | -0.03±0.18 | 0 | 7.79E-01 | 9.75E-01 | -0.03±0.2 | 9.83E-01 | -0.01±0.2 | 9.96E-01 | -0.06±0.38 | 9.96E-01 | -0.13±0.38 | 9.96E-01 |
| X - 17010 | -0.06±0.19 | 0 | 5.68E-01 | 9.50E-01 | -0.03±0.19 | 0 | 6.06E-01 | 9.76E-01 | -0.12±0.21 | 9.14E-01 | -0.08±0.21 | 9.63E-01 | 0.14±0.41 | 9.96E-01 | 0.16±0.41 | 9.96E-01 |
| 3-methoxycatechol sulfate (1) | 0±0.19 | 0 | 9.79E-01 | 9.93E-01 | -0.03±0.19 | 0 | 8.93E-01 | 9.76E-01 | 0±0.21 | 9.99E-01 | -0.02±0.21 | 9.94E-01 | 0.01±0.42 | 9.96E-01 | -0.08±0.43 | 9.96E-01 |
| 1-(1-enyl-stearoyl)-2-linoleoyl-GPE (P-18:0/18:2)* | 0.09±0.2 | 0 | 6.47E-01 | 9.31E-01 | 0.03±0.2 | 0 | 5.75E-01 | 9.76E-01 | 0.05±0.22 | 9.57E-01 | -0.01±0.21 | 9.96E-01 | 0.31±0.53 | 9.96E-01 | 0.31±0.53 | 9.96E-01 |
| 3-methoxytyrosine | -0.02±0.21 | 0 | 6.77E-01 | 9.76E-01 | -0.04±0.22 | 0 | 6.66E-01 | 9.76E-01 | -0.06±0.24 | 9.49E-01 | -0.09±0.25 | 9.64E-01 | 0.15±0.45 | 9.96E-01 | 0.12±0.43 | 9.96E-01 |
| X - 11470 | -0.02±0.18 | 0 | 4.77E-01 | 9.70E-01 | -0.03±0.18 | 0 | 5.45E-01 | 9.76E-01 | -0.08±0.2 | 9.35E-01 | -0.08±0.19 | 9.63E-01 | 0.24±0.41 | 9.96E-01 | 0.21±0.43 | 9.96E-01 |
| X - 21821 | 0.06±0.19 | 0 | 6.88E-01 | 9.50E-01 | 0.03±0.19 | 0 | 6.84E-01 | 9.76E-01 | 0.1±0.21 | 9.14E-01 | 0.07±0.22 | 9.66E-01 | -0.08±0.42 | 9.96E-01 | -0.12±0.42 | 9.96E-01 |
| 1-oleoyl-2-linoleoyl-GPE (18:1/18:2)* | 0.01±0.19 | 0 | 3.95E-01 | 9.87E-01 | -0.03±0.19 | 0 | 4.49E-01 | 9.78E-01 | 0.12±0.23 | 9.14E-01 | 0.06±0.23 | 9.68E-01 | -0.23±0.33 | 9.96E-01 | -0.25±0.34 | 9.96E-01 |
| X - 21353 | 0.02±0.19 | 44.54 | 1.79E-01 | 9.70E-01 | -0.03±0.19 | 64.11 | 9.51E-02 | 9.82E-01 | 0.15±0.21 | 8.89E-01 | 0.12±0.21 | 9.30E-01 | -0.48±0.42 | 9.96E-01 | -0.73±0.46 | 9.96E-01 |
| picolinoylglycine | -0.1±0.19 | 0 | 6.08E-01 | 9.03E-01 | -0.03±0.2 | 0 | 3.70E-01 | 9.82E-01 | -0.05±0.21 | 9.49E-01 | 0.05±0.22 | 9.76E-01 | -0.29±0.42 | 9.96E-01 | -0.4±0.45 | 9.96E-01 |
| 4-methylguaiacol sulfate | -0.02±0.19 | 0 | 7.04E-01 | 9.76E-01 | -0.03±0.19 | 0 | 6.95E-01 | 9.82E-01 | 0.02±0.21 | 9.94E-01 | 0.01±0.21 | 9.96E-01 | -0.16±0.42 | 9.96E-01 | -0.18±0.43 | 9.96E-01 |
| vanillic acid glycine | 0.02±0.19 | 49.74 | 1.58E-01 | 9.70E-01 | -0.03±0.19 | 51.31 | 1.52E-01 | 9.83E-01 | -0.11±0.21 | 9.14E-01 | -0.16±0.22 | 9.06E-01 | 0.53±0.4 | 9.96E-01 | 0.55±0.45 | 9.96E-01 |
| dihydroorotate | -0.04±0.18 | 2 | 3.12E-01 | 9.64E-01 | -0.03±0.19 | 0 | 4.57E-01 | 9.83E-01 | -0.12±0.2 | 9.02E-01 | -0.09±0.2 | 9.53E-01 | 0.38±0.46 | 9.96E-01 | 0.28±0.46 | 9.96E-01 |
| X - 18922 | -0.01±0.18 | 0 | 7.44E-01 | 9.87E-01 | 0.02±0.18 | 0 | 5.24E-01 | 9.83E-01 | 0.03±0.21 | 9.84E-01 | 0.09±0.21 | 9.53E-01 | -0.12±0.38 | 9.96E-01 | -0.18±0.37 | 9.96E-01 |
| sphingomyelin (d18:2/24:2)* | 0.04±0.21 | 0 | 4.89E-01 | 9.69E-01 | -0.03±0.21 | 0 | 4.10E-01 | 9.83E-01 | -0.02±0.22 | 9.94E-01 | -0.09±0.22 | 9.54E-01 | 0.43±0.6 | 9.96E-01 | 0.43±0.59 | 9.96E-01 |
| N-methylpipecolate | 0.02±0.18 | 0 | 7.12E-01 | 9.70E-01 | 0.02±0.18 | 0 | 6.58E-01 | 9.83E-01 | 0.06±0.21 | 9.49E-01 | 0.07±0.21 | 9.66E-01 | -0.09±0.36 | 9.96E-01 | -0.11±0.36 | 9.96E-01 |
| gamma-glutamylisoleucine* | -0.03±0.19 | 0 | 8.81E-01 | 9.69E-01 | 0.03±0.19 | 0 | 6.58E-01 | 9.83E-01 | -0.02±0.22 | 9.94E-01 | 0.08±0.22 | 9.66E-01 | -0.08±0.36 | 9.96E-01 | -0.11±0.37 | 9.96E-01 |
| 5alpha-pregnan-3beta,20beta-diol monosulfate (1) | 0.04±0.19 | 0 | 3.20E-01 | 9.64E-01 | 0.03±0.19 | 14.39 | 2.80E-01 | 9.83E-01 | -0.04±0.21 | 9.71E-01 | -0.06±0.21 | 9.66E-01 | 0.46±0.46 | 9.96E-01 | 0.5±0.48 | 9.96E-01 |
| aconitate [cis or trans] | 0.02±0.19 | 0 | 9.67E-01 | 9.70E-01 | 0.03±0.19 | 0 | 7.85E-01 | 9.83E-01 | 0.01±0.22 | 9.95E-01 | 0.06±0.22 | 9.74E-01 | 0.03±0.38 | 9.96E-01 | -0.05±0.35 | 9.96E-01 |
| 1-(1-enyl-palmitoyl)-2-linoleoyl-GPC (P-16:0/18:2)* | 0.13±0.2 | 4.21 | 3.07E-01 | 9.03E-01 | 0.03±0.21 | 18.13 | 2.69E-01 | 9.83E-01 | 0.06±0.21 | 9.49E-01 | -0.05±0.22 | 9.78E-01 | 0.72±0.61 | 9.96E-01 | 0.67±0.62 | 9.96E-01 |
| 3-(3-amino-3-carboxypropyl)uridine* | -0.02±0.19 | 0 | 7.61E-01 | 9.70E-01 | -0.03±0.19 | 0 | 6.07E-01 | 9.83E-01 | 0.01±0.21 | 9.95E-01 | 0.03±0.21 | 9.93E-01 | -0.12±0.38 | 9.96E-01 | -0.21±0.4 | 9.96E-01 |
| X - 17325 | -0.01±0.19 | 0 | 9.98E-01 | 9.87E-01 | -0.03±0.19 | 0 | 9.88E-01 | 9.83E-01 | -0.01±0.21 | 9.97E-01 | -0.02±0.21 | 9.94E-01 | -0.01±0.43 | 9.96E-01 | -0.03±0.43 | 9.96E-01 |
| gamma-glutamyltryptophan | -0.02±0.19 | 0 | 5.55E-01 | 9.70E-01 | 0.03±0.2 | 0 | 7.11E-01 | 9.83E-01 | -0.07±0.21 | 9.45E-01 | -0.01±0.22 | 9.96E-01 | 0.25±0.5 | 9.96E-01 | 0.2±0.5 | 9.96E-01 |
| phenylacetylglutamate | 0.03±0.19 | 0 | 9.88E-01 | 9.70E-01 | -0.03±0.19 | 0 | 8.55E-01 | 9.83E-01 | 0.03±0.21 | 9.84E-01 | -0.01±0.21 | 9.96E-01 | 0.02±0.42 | 9.96E-01 | -0.1±0.44 | 9.96E-01 |
| 1-(1-enyl-palmitoyl)-2-oleoyl-GPE (P-16:0/18:1)* | 0.02±0.2 | 0 | 7.77E-01 | 9.70E-01 | 0.03±0.2 | 0 | 7.42E-01 | 9.83E-01 | 0±0.21 | 9.99E-01 | 0±0.21 | 9.99E-01 | 0.15±0.49 | 9.96E-01 | 0.18±0.5 | 9.96E-01 |
| cis-4-decenoate (10:1n6)* | 0.03±0.18 | 55.66 | 1.33E-01 | 9.69E-01 | -0.02±0.18 | 67.03 | 8.16E-02 | 9.84E-01 | 0.2±0.21 | 8.45E-01 | 0.18±0.22 | 8.88E-01 | -0.43±0.36 | 9.96E-01 | -0.54±0.35 | 9.96E-01 |
| N-stearoyltaurine | 0±0.19 | 0 | 4.46E-01 | 9.89E-01 | -0.02±0.19 | 0 | 4.24E-01 | 9.84E-01 | -0.06±0.2 | 9.49E-01 | -0.09±0.21 | 9.53E-01 | 0.33±0.47 | 9.96E-01 | 0.32±0.48 | 9.96E-01 |
| 1-palmitoyl-2-dihomo-linolenoyl-GPC (16:0/20:3n3 or 6)* | -0.07±0.2 | 0 | 6.12E-01 | 9.49E-01 | -0.03±0.2 | 0 | 6.65E-01 | 9.84E-01 | -0.11±0.21 | 9.14E-01 | -0.06±0.22 | 9.73E-01 | 0.2±0.57 | 9.96E-01 | 0.22±0.59 | 9.96E-01 |
| N-acetylputrescine | 0±0.17 | 0 | 6.15E-01 | 9.90E-01 | -0.02±0.18 | 0 | 5.94E-01 | 9.84E-01 | 0.06±0.21 | 9.49E-01 | 0.04±0.22 | 9.79E-01 | -0.14±0.32 | 9.96E-01 | -0.17±0.33 | 9.96E-01 |
| 1-palmitoyl-2-arachidonoyl-GPI (16:0/20:4)* | -0.01±0.19 | 0 | 9.20E-01 | 9.80E-01 | 0.02±0.19 | 0 | 8.61E-01 | 9.84E-01 | 0±0.22 | 9.99E-01 | 0.04±0.23 | 9.79E-01 | -0.04±0.36 | 9.96E-01 | -0.03±0.37 | 9.96E-01 |
| N-acetylhistidine | 0.04±0.19 | 78.99 | 2.91E-02 | 9.64E-01 | 0.02±0.2 | 76.14 | 4.06E-02 | 9.86E-01 | 0.29±0.22 | 7.24E-01 | 0.26±0.23 | 8.13E-01 | -0.68±0.39 | 9.96E-01 | -0.67±0.39 | 9.96E-01 |
| ascorbic acid 2-sulfate | 0.04±0.21 | 0 | 5.35E-01 | 9.68E-01 | 0.02±0.21 | 0 | 5.27E-01 | 9.86E-01 | 0.15±0.27 | 9.14E-01 | 0.13±0.26 | 9.42E-01 | -0.12±0.33 | 9.96E-01 | -0.15±0.34 | 9.96E-01 |
| uracil | -0.03±0.18 | 0 | 3.73E-01 | 9.69E-01 | -0.02±0.18 | 0 | 4.10E-01 | 9.86E-01 | -0.1±0.2 | 9.14E-01 | -0.08±0.2 | 9.57E-01 | 0.38±0.49 | 9.96E-01 | 0.34±0.47 | 9.96E-01 |
| 5alpha-androstan-3beta,17alpha-diol disulfate | -0.07±0.2 | 0 | 4.81E-01 | 9.55E-01 | -0.02±0.2 | 0 | 4.50E-01 | 9.86E-01 | -0.13±0.22 | 9.06E-01 | -0.09±0.22 | 9.60E-01 | 0.23±0.46 | 9.96E-01 | 0.31±0.48 | 9.96E-01 |
| 1-palmitoyl-2-arachidonoyl-GPE (16:0/20:4)* | -0.04±0.19 | 0 | 8.02E-01 | 9.68E-01 | -0.02±0.2 | 0 | 7.28E-01 | 9.86E-01 | -0.07±0.22 | 9.49E-01 | -0.06±0.22 | 9.75E-01 | 0.05±0.41 | 9.96E-01 | 0.11±0.43 | 9.96E-01 |
| valylglycine | 0.04±0.19 | 0 | 7.74E-01 | 9.64E-01 | -0.02±0.19 | 0 | 7.80E-01 | 9.86E-01 | 0.02±0.21 | 9.94E-01 | -0.05±0.21 | 9.79E-01 | 0.16±0.44 | 9.96E-01 | 0.09±0.45 | 9.96E-01 |
| X - 24544 | 0.01±0.19 | 0 | 4.62E-01 | 9.76E-01 | 0.02±0.2 | 0 | 4.20E-01 | 9.86E-01 | -0.04±0.21 | 9.62E-01 | -0.04±0.21 | 9.79E-01 | 0.36±0.5 | 9.96E-01 | 0.42±0.53 | 9.96E-01 |
| 3-formylindole | -0.05±0.2 | 0 | 7.13E-01 | 9.64E-01 | -0.02±0.2 | 0 | 8.99E-01 | 9.86E-01 | -0.08±0.22 | 9.37E-01 | -0.04±0.23 | 9.86E-01 | 0.11±0.47 | 9.96E-01 | 0.03±0.46 | 9.96E-01 |
| 2-pyrrolidinone | -0.02±0.18 | 0 | 6.56E-01 | 9.70E-01 | -0.02±0.18 | 0 | 6.53E-01 | 9.86E-01 | 0.03±0.21 | 9.83E-01 | 0.03±0.21 | 9.86E-01 | -0.15±0.34 | 9.96E-01 | -0.15±0.34 | 9.96E-01 |
| X - 12026 | 0.04±0.19 | 0 | 8.81E-01 | 9.68E-01 | -0.02±0.2 | 0 | 6.32E-01 | 9.86E-01 | 0.05±0.22 | 9.57E-01 | 0.03±0.23 | 9.94E-01 | -0.02±0.4 | 9.96E-01 | -0.21±0.44 | 9.96E-01 |
| cytosine | -0.04±0.19 | 0 | 7.02E-01 | 9.68E-01 | -0.02±0.19 | 0 | 6.27E-01 | 9.86E-01 | 0±0.21 | 9.98E-01 | 0.02±0.21 | 9.94E-01 | -0.18±0.43 | 9.96E-01 | -0.22±0.44 | 9.96E-01 |
| 1-(1-enyl-palmitoyl)-2-palmitoyl-GPC (P-16:0/16:0)* | 0.06±0.2 | 0 | 8.49E-01 | 9.55E-01 | 0.02±0.21 | 0 | 6.69E-01 | 9.86E-01 | 0.04±0.22 | 9.70E-01 | -0.01±0.23 | 9.96E-01 | 0.15±0.49 | 9.96E-01 | 0.26±0.6 | 9.96E-01 |
| tridecenedioate (C13:1-DC)* | 0.04±0.19 | 0 | 8.26E-01 | 9.68E-01 | 0.02±0.18 | 0 | 9.12E-01 | 9.86E-01 | 0.02±0.21 | 9.94E-01 | 0.01±0.21 | 9.96E-01 | 0.12±0.41 | 9.96E-01 | 0.06±0.41 | 9.96E-01 |
| oleate/vaccenate (18:1) | -0.02±0.19 | 0 | 8.87E-01 | 9.70E-01 | -0.02±0.18 | 0 | 9.44E-01 | 9.86E-01 | -0.03±0.21 | 9.83E-01 | -0.03±0.2 | 9.93E-01 | 0.04±0.43 | 9.96E-01 | 0.01±0.44 | 9.98E-01 |
| X - 24422 | 0.01±0.18 | 41.92 | 1.89E-01 | 9.80E-01 | 0.02±0.19 | 33.54 | 2.20E-01 | 9.87E-01 | 0.19±0.23 | 8.71E-01 | 0.19±0.24 | 8.88E-01 | -0.3±0.3 | 9.96E-01 | -0.3±0.33 | 9.96E-01 |
| X - 24328 | -0.06±0.22 | 0 | 3.30E-01 | 9.58E-01 | -0.02±0.22 | 0 | 4.18E-01 | 9.87E-01 | -0.17±0.25 | 8.89E-01 | -0.11±0.25 | 9.53E-01 | 0.37±0.5 | 9.96E-01 | 0.34±0.5 | 9.96E-01 |
| 5-dodecenoylcarnitine (C12:1) | -0.05±0.18 | 0 | 3.34E-01 | 9.63E-01 | -0.02±0.19 | 0 | 3.96E-01 | 9.87E-01 | 0.06±0.21 | 9.49E-01 | 0.07±0.21 | 9.66E-01 | -0.35±0.36 | 9.96E-01 | -0.31±0.4 | 9.96E-01 |
| 5-hydroxyhexanoate | 0.02±0.18 | 0 | 3.36E-01 | 9.70E-01 | 0.02±0.18 | 0 | 4.19E-01 | 9.87E-01 | -0.06±0.2 | 9.49E-01 | -0.05±0.2 | 9.75E-01 | 0.38±0.42 | 9.96E-01 | 0.32±0.42 | 9.96E-01 |
| pentose acid* | -0.02±0.18 | 0 | 6.36E-01 | 9.70E-01 | 0.02±0.18 | 0 | 7.65E-01 | 9.87E-01 | 0.03±0.2 | 9.83E-01 | 0.04±0.21 | 9.79E-01 | -0.17±0.37 | 9.96E-01 | -0.09±0.41 | 9.96E-01 |
| gamma-tocopherol/beta-tocopherol | -0.03±0.18 | 0 | 7.94E-01 | 9.69E-01 | -0.02±0.18 | 0 | 8.78E-01 | 9.87E-01 | -0.06±0.2 | 9.49E-01 | -0.03±0.2 | 9.86E-01 | 0.07±0.41 | 9.96E-01 | 0.04±0.41 | 9.96E-01 |
| palmitate (16:0) | 0.01±0.19 | 0 | 9.58E-01 | 9.76E-01 | 0.02±0.19 | 0 | 9.11E-01 | 9.87E-01 | 0.02±0.21 | 9.94E-01 | 0.03±0.21 | 9.92E-01 | -0.01±0.42 | 9.96E-01 | -0.03±0.44 | 9.96E-01 |
| isovalerate (i5:0) | -0.03±0.18 | 0 | 8.84E-01 | 9.68E-01 | 0.02±0.19 | 0 | 9.91E-01 | 9.87E-01 | -0.05±0.2 | 9.57E-01 | 0.02±0.21 | 9.94E-01 | 0.02±0.44 | 9.96E-01 | 0.02±0.44 | 9.96E-01 |
| N-palmitoyl-sphinganine (d18:0/16:0) | -0.08±0.18 | 0 | 6.59E-01 | 9.24E-01 | -0.02±0.19 | 0 | 6.95E-01 | 9.87E-01 | -0.04±0.21 | 9.71E-01 | 0.02±0.21 | 9.94E-01 | -0.23±0.38 | 9.96E-01 | -0.16±0.41 | 9.96E-01 |
| methionine sulfone | 0.03±0.19 | 0 | 9.72E-01 | 9.69E-01 | -0.02±0.19 | 0 | 9.37E-01 | 9.87E-01 | 0.03±0.21 | 9.83E-01 | -0.01±0.22 | 9.96E-01 | 0.02±0.39 | 9.96E-01 | -0.04±0.4 | 9.96E-01 |
| docosadienoate (22:2n6) | 0.03±0.18 | 0 | 8.99E-01 | 9.69E-01 | 0.02±0.19 | 0 | 8.91E-01 | 9.87E-01 | 0.02±0.21 | 9.94E-01 | 0.01±0.21 | 9.96E-01 | 0.08±0.41 | 9.96E-01 | 0.07±0.43 | 9.96E-01 |
| X - 23593 | 0.01±0.18 | 0 | 8.47E-01 | 9.82E-01 | -0.02±0.18 | 0 | 8.77E-01 | 9.87E-01 | 0.03±0.21 | 9.83E-01 | 0±0.21 | 9.99E-01 | -0.05±0.34 | 9.96E-01 | -0.06±0.34 | 9.96E-01 |
| 1-palmitoyl-2-palmitoleoyl-GPC (16:0/16:1)* | -0.1±0.19 | 0 | 9.11E-01 | 9.13E-01 | 0.02±0.2 | 0 | 9.48E-01 | 9.87E-01 | -0.09±0.22 | 9.36E-01 | 0.02±0.23 | 9.94E-01 | -0.13±0.38 | 9.96E-01 | -0.01±0.43 | 9.98E-01 |
| N-acetylphenylalanine | -0.02±0.19 | 44.97 | 1.78E-01 | 9.70E-01 | 0.02±0.2 | 58.15 | 1.22E-01 | 9.87E-01 | 0.12±0.22 | 9.14E-01 | 0.19±0.23 | 8.86E-01 | -0.47±0.38 | 9.96E-01 | -0.53±0.4 | 9.96E-01 |
| hexadecadienoate (16:2n6) | 0.03±0.2 | 32.9 | 2.22E-01 | 9.69E-01 | 0.01±0.2 | 33.88 | 2.19E-01 | 9.87E-01 | 0.16±0.23 | 8.89E-01 | 0.13±0.22 | 9.26E-01 | -0.46±0.45 | 9.96E-01 | -0.5±0.47 | 9.96E-01 |
| X - 12100 | -0.03±0.16 | 0 | 3.74E-01 | 9.69E-01 | -0.01±0.17 | 23.31 | 2.53E-01 | 9.87E-01 | 0.09±0.21 | 9.28E-01 | 0.13±0.21 | 9.26E-01 | -0.21±0.27 | 9.96E-01 | -0.26±0.28 | 9.96E-01 |
| X - 17367 | 0.02±0.19 | 0 | 6.02E-01 | 9.76E-01 | -0.01±0.19 | 0 | 5.85E-01 | 9.87E-01 | -0.03±0.2 | 9.83E-01 | -0.06±0.21 | 9.66E-01 | 0.22±0.44 | 9.96E-01 | 0.21±0.44 | 9.96E-01 |
| 1-(1-enyl-stearoyl)-2-oleoyl-GPE (P-18:0/18:1) | -0.02±0.2 | 0 | 5.92E-01 | 9.70E-01 | -0.01±0.2 | 0 | 5.61E-01 | 9.87E-01 | -0.06±0.21 | 9.49E-01 | -0.06±0.21 | 9.67E-01 | 0.25±0.53 | 9.96E-01 | 0.27±0.53 | 9.96E-01 |
| 10-nonadecenoate (19:1n9) | 0.02±0.19 | 0 | 4.37E-01 | 9.70E-01 | 0.01±0.19 | 0 | 4.45E-01 | 9.87E-01 | -0.05±0.21 | 9.57E-01 | -0.05±0.21 | 9.78E-01 | 0.34±0.45 | 9.96E-01 | 0.34±0.47 | 9.96E-01 |
| N-palmitoylserine | 0.02±0.18 | 0 | 8.46E-01 | 9.70E-01 | -0.01±0.18 | 0 | 7.93E-01 | 9.87E-01 | 0.01±0.2 | 9.97E-01 | -0.04±0.2 | 9.80E-01 | 0.1±0.42 | 9.96E-01 | 0.09±0.43 | 9.96E-01 |
| X - 18899 | 0.03±0.19 | 0 | 6.35E-01 | 9.69E-01 | 0.01±0.19 | 0 | 6.14E-01 | 9.87E-01 | -0.01±0.21 | 9.97E-01 | -0.03±0.21 | 9.86E-01 | 0.22±0.44 | 9.96E-01 | 0.21±0.44 | 9.96E-01 |
| X - 25267 | -0.04±0.18 | 0 | 9.72E-01 | 9.64E-01 | -0.01±0.18 | 0 | 8.97E-01 | 9.87E-01 | -0.04±0.2 | 9.61E-01 | -0.02±0.21 | 9.93E-01 | -0.03±0.37 | 9.96E-01 | 0.03±0.39 | 9.96E-01 |
| cerotoylcarnitine (C26)* | -0.02±0.19 | 0 | 9.59E-01 | 9.70E-01 | -0.01±0.19 | 0 | 9.09E-01 | 9.87E-01 | -0.03±0.21 | 9.83E-01 | -0.03±0.22 | 9.94E-01 | -0.01±0.38 | 9.96E-01 | 0.03±0.4 | 9.96E-01 |
| 4-methylcatechol sulfate | 0.02±0.19 | 0 | 7.91E-01 | 9.70E-01 | -0.01±0.19 | 0 | 7.67E-01 | 9.87E-01 | 0.05±0.21 | 9.57E-01 | 0.01±0.21 | 9.94E-01 | -0.07±0.41 | 9.96E-01 | -0.13±0.44 | 9.96E-01 |
| X - 24947 | -0.04±0.19 | 0 | 9.36E-01 | 9.68E-01 | 0.01±0.19 | 0 | 8.29E-01 | 9.87E-01 | -0.05±0.21 | 9.61E-01 | -0.01±0.21 | 9.96E-01 | -0.01±0.41 | 9.96E-01 | 0.1±0.44 | 9.96E-01 |
| succinylcarnitine (C4-DC) | -0.02±0.19 | 0 | 9.23E-01 | 9.70E-01 | -0.01±0.19 | 0 | 8.40E-01 | 9.87E-01 | -0.01±0.21 | 9.95E-01 | 0.01±0.22 | 9.96E-01 | -0.05±0.4 | 9.96E-01 | -0.08±0.4 | 9.96E-01 |
| glutamine | 0.11±0.2 | 0 | 8.16E-01 | 9.10E-01 | 0.01±0.21 | 0 | 6.94E-01 | 9.89E-01 | 0.09±0.22 | 9.28E-01 | -0.02±0.22 | 9.94E-01 | 0.24±0.62 | 9.96E-01 | 0.24±0.61 | 9.96E-01 |
| cyclo(leu-pro) | -0.1±0.17 | 68.5 | 7.48E-02 | 9.03E-01 | -0.01±0.19 | 60.1 | 1.13E-01 | 9.90E-01 | -0.22±0.18 | 7.32E-01 | -0.13±0.21 | 9.26E-01 | 0.66±0.46 | 9.96E-01 | 0.74±0.51 | 9.96E-01 |
| 5alpha-pregnan-3beta,20alpha-diol monosulfate (2) | 0.05±0.19 | 56.44 | 1.30E-01 | 9.64E-01 | 0.01±0.19 | 61.8 | 1.06E-01 | 9.90E-01 | -0.07±0.21 | 9.39E-01 | -0.11±0.21 | 9.30E-01 | 0.75±0.5 | 9.96E-01 | 0.8±0.52 | 9.96E-01 |
| C-glycosyltryptophan | 0.02±0.18 | 0 | 6.93E-01 | 9.70E-01 | 0.01±0.19 | 0 | 5.83E-01 | 9.90E-01 | 0.06±0.21 | 9.49E-01 | 0.07±0.21 | 9.66E-01 | -0.1±0.35 | 9.96E-01 | -0.17±0.37 | 9.96E-01 |
| phytanate | 0.04±0.19 | 18.29 | 2.69E-01 | 9.64E-01 | 0.01±0.19 | 7.13 | 2.99E-01 | 9.91E-01 | -0.05±0.21 | 9.49E-01 | -0.08±0.21 | 9.63E-01 | 0.49±0.45 | 9.96E-01 | 0.46±0.47 | 9.96E-01 |
| 1-stearoyl-2-arachidonoyl-GPC (18:0/20:4) | -0.02±0.22 | 14.56 | 2.79E-01 | 9.70E-01 | 0.01±0.22 | 0 | 3.51E-01 | 9.91E-01 | -0.1±0.23 | 9.16E-01 | -0.06±0.23 | 9.72E-01 | 0.67±0.68 | 9.96E-01 | 0.57±0.63 | 9.96E-01 |
| X - 17653 | 0.11±0.19 | 0 | 8.22E-01 | 9.03E-01 | 0.01±0.19 | 0 | 9.32E-01 | 9.91E-01 | 0.09±0.21 | 9.28E-01 | 0±0.21 | 9.99E-01 | 0.2±0.44 | 9.96E-01 | 0.04±0.44 | 9.96E-01 |
| arachidoylcarnitine (C20)* | 0.07±0.19 | 0 | 8.59E-01 | 9.40E-01 | 0.01±0.19 | 0 | 9.40E-01 | 9.91E-01 | 0.09±0.21 | 9.28E-01 | 0.02±0.21 | 9.94E-01 | 0±0.43 | 9.97E-01 | -0.02±0.45 | 9.96E-01 |
| fumarate | 0±0.18 | 0 | 9.53E-01 | 9.94E-01 | -0.01±0.19 | 0 | 9.73E-01 | 9.93E-01 | 0±0.21 | 9.97E-01 | -0.01±0.21 | 9.96E-01 | -0.02±0.37 | 9.96E-01 | -0.02±0.38 | 9.96E-01 |
| ceramide (d16:1/24:1, d18:1/22:1)* | -0.06±0.17 | 0 | 8.94E-01 | 9.50E-01 | -0.01±0.18 | 0 | 8.73E-01 | 9.94E-01 | -0.07±0.2 | 9.37E-01 | -0.02±0.2 | 9.94E-01 | -0.02±0.37 | 9.96E-01 | 0.05±0.43 | 9.96E-01 |
| arginine | 0.07±0.2 | 0 | 4.73E-01 | 9.52E-01 | -0.01±0.2 | 0 | 3.81E-01 | 9.95E-01 | 0.03±0.21 | 9.90E-01 | -0.06±0.21 | 9.68E-01 | 0.56±0.72 | 9.96E-01 | 0.62±0.75 | 9.96E-01 |
| 2-hydroxyoctanoate | 0.03±0.17 | 63.07 | 9.99E-02 | 9.69E-01 | 0.01±0.17 | 68.81 | 7.33E-02 | 9.99E-01 | 0.2±0.2 | 8.03E-01 | 0.19±0.2 | 8.63E-01 | -0.44±0.34 | 9.96E-01 | -0.5±0.33 | 9.96E-01 |
| 1-linoleoyl-GPG (18:2)* | 0.02±0.19 | 23.41 | 2.53E-01 | 9.70E-01 | 0±0.19 | 42.19 | 1.88E-01 | 9.99E-01 | 0.15±0.22 | 8.90E-01 | 0.15±0.22 | 9.26E-01 | -0.33±0.35 | 9.96E-01 | -0.44±0.38 | 9.96E-01 |
| 3-hydroxydodecanedioate* | 0.06±0.18 | 0 | 3.32E-01 | 9.55E-01 | 0±0.18 | 7.92 | 2.97E-01 | 9.99E-01 | 0.16±0.21 | 8.78E-01 | 0.11±0.21 | 9.41E-01 | -0.27±0.39 | 9.96E-01 | -0.36±0.39 | 9.96E-01 |
| heptenedioate (C7:1-DC)* | -0.01±0.18 | 66.01 | 8.63E-02 | 9.82E-01 | 0±0.2 | 63.36 | 9.85E-02 | 9.99E-01 | -0.16±0.2 | 8.78E-01 | -0.1±0.21 | 9.41E-01 | 0.65±0.43 | 9.96E-01 | 1.06±0.67 | 9.96E-01 |
| pristanate | 0.04±0.19 | 0 | 3.66E-01 | 9.68E-01 | 0±0.19 | 0 | 3.28E-01 | 9.99E-01 | 0.13±0.22 | 9.02E-01 | 0.11±0.22 | 9.41E-01 | -0.26±0.38 | 9.96E-01 | -0.33±0.39 | 9.96E-01 |
| X - 17438 | 0.06±0.18 | 0 | 4.50E-01 | 9.55E-01 | 0±0.19 | 0 | 4.06E-01 | 9.99E-01 | 0.13±0.2 | 9.02E-01 | 0.08±0.21 | 9.63E-01 | -0.22±0.41 | 9.96E-01 | -0.32±0.42 | 9.96E-01 |
| 3-hydroxybutyroylglycine** | 0.04±0.2 | 0 | 5.35E-01 | 9.66E-01 | 0±0.2 | 0 | 4.68E-01 | 9.99E-01 | 0.12±0.23 | 9.14E-01 | 0.08±0.22 | 9.66E-01 | -0.17±0.39 | 9.96E-01 | -0.27±0.42 | 9.96E-01 |
| ceramide (d18:1/14:0, d16:1/16:0)* | -0.02±0.18 | 0 | 3.34E-01 | 9.70E-01 | 0±0.19 | 18.32 | 2.69E-01 | 9.99E-01 | -0.09±0.2 | 9.14E-01 | -0.07±0.2 | 9.66E-01 | 0.44±0.51 | 9.96E-01 | 0.63±0.6 | 9.96E-01 |
| 3-hydroxyphenylacetoylglutamine | -0.01±0.19 | 0 | 5.49E-01 | 9.82E-01 | -0.01±0.19 | 0 | 4.54E-01 | 9.99E-01 | 0.05±0.21 | 9.57E-01 | 0.07±0.22 | 9.66E-01 | -0.22±0.4 | 9.96E-01 | -0.29±0.42 | 9.96E-01 |
| glycosyl-N-stearoyl-sphingosine (d18:1/18:0) | 0.03±0.2 | 0 | 6.12E-01 | 9.69E-01 | 0±0.2 | 0 | 4.00E-01 | 9.99E-01 | -0.01±0.22 | 9.95E-01 | -0.07±0.22 | 9.66E-01 | 0.24±0.45 | 9.96E-01 | 0.39±0.51 | 9.96E-01 |
| pantothenate | -0.03±0.19 | 0 | 5.40E-01 | 9.69E-01 | 0±0.2 | 0 | 6.03E-01 | 9.99E-01 | -0.1±0.22 | 9.23E-01 | -0.06±0.22 | 9.75E-01 | 0.18±0.39 | 9.96E-01 | 0.19±0.42 | 9.96E-01 |
| N-acetyltyrosine | -0.08±0.19 | 0 | 7.26E-01 | 9.24E-01 | 0±0.19 | 0 | 6.03E-01 | 9.99E-01 | -0.05±0.21 | 9.57E-01 | 0.06±0.22 | 9.75E-01 | -0.2±0.38 | 9.96E-01 | -0.19±0.41 | 9.96E-01 |
| X - 24556 | -0.03±0.18 | 0 | 4.48E-01 | 9.70E-01 | 0.01±0.18 | 0 | 5.34E-01 | 9.99E-01 | -0.09±0.2 | 9.14E-01 | -0.05±0.2 | 9.78E-01 | 0.25±0.4 | 9.96E-01 | 0.24±0.42 | 9.96E-01 |
| 3-methylcytidine | -0.01±0.19 | 0 | 5.63E-01 | 9.79E-01 | 0±0.18 | 0 | 6.02E-01 | 9.99E-01 | -0.06±0.2 | 9.49E-01 | -0.04±0.2 | 9.79E-01 | 0.23±0.46 | 9.96E-01 | 0.22±0.47 | 9.96E-01 |
| 4-guanidinobutanoate | 0±0.19 | 0 | 7.49E-01 | 9.88E-01 | 0.01±0.2 | 0 | 7.23E-01 | 9.99E-01 | 0.03±0.21 | 9.83E-01 | 0.04±0.22 | 9.79E-01 | -0.12±0.44 | 9.96E-01 | -0.14±0.46 | 9.96E-01 |
| phenylacetylcarnitine | 0.03±0.19 | 0 | 7.64E-01 | 9.70E-01 | 0±0.2 | 0 | 6.83E-01 | 9.99E-01 | 0.06±0.22 | 9.49E-01 | 0.04±0.22 | 9.80E-01 | -0.08±0.4 | 9.96E-01 | -0.16±0.43 | 9.96E-01 |
| 1-palmitoyl-2-stearoyl-GPC (16:0/18:0) | 0.03±0.19 | 0 | 7.77E-01 | 9.69E-01 | 0±0.2 | 0 | 6.69E-01 | 9.99E-01 | 0.01±0.2 | 9.95E-01 | -0.04±0.21 | 9.80E-01 | 0.16±0.5 | 9.96E-01 | 0.21±0.55 | 9.96E-01 |
| N-oleoylserine | 0.04±0.19 | 0 | 7.19E-01 | 9.68E-01 | 0±0.2 | 0 | 6.90E-01 | 9.99E-01 | 0.01±0.21 | 9.97E-01 | -0.04±0.22 | 9.81E-01 | 0.2±0.48 | 9.96E-01 | 0.18±0.49 | 9.96E-01 |
| 1,7-dimethylurate | -0.05±0.18 | 0 | 4.65E-01 | 9.63E-01 | 0±0.19 | 0 | 6.33E-01 | 9.99E-01 | -0.1±0.2 | 9.14E-01 | -0.03±0.2 | 9.86E-01 | 0.32±0.53 | 9.96E-01 | 0.24±0.53 | 9.96E-01 |
| xanthine | -0.04±0.19 | 0 | 6.54E-01 | 9.68E-01 | 0±0.2 | 0 | 7.63E-01 | 9.99E-01 | -0.08±0.21 | 9.35E-01 | -0.03±0.22 | 9.91E-01 | 0.13±0.44 | 9.96E-01 | 0.11±0.42 | 9.96E-01 |
| N1-Methyl-2-pyridone-5-carboxamide | -0.04±0.18 | 0 | 9.30E-01 | 9.64E-01 | -0.01±0.19 | 0 | 7.46E-01 | 9.99E-01 | -0.03±0.2 | 9.81E-01 | 0.02±0.21 | 9.94E-01 | -0.07±0.4 | 9.96E-01 | -0.13±0.42 | 9.96E-01 |
| N6-carbamoylthreonyladenosine | -0.03±0.18 | 0 | 9.74E-01 | 9.70E-01 | 0±0.18 | 0 | 8.46E-01 | 9.99E-01 | -0.02±0.21 | 9.93E-01 | 0.02±0.21 | 9.94E-01 | -0.04±0.38 | 9.96E-01 | -0.06±0.36 | 9.96E-01 |
| N6,N6,N6-trimethyllysine | 0.01±0.2 | 0 | 7.93E-01 | 9.81E-01 | 0±0.2 | 0 | 8.54E-01 | 9.99E-01 | -0.01±0.22 | 9.95E-01 | -0.02±0.22 | 9.94E-01 | 0.12±0.48 | 9.96E-01 | 0.07±0.44 | 9.96E-01 |
| X - 18887 | 0.06±0.19 | 0 | 9.25E-01 | 9.55E-01 | 0±0.19 | 0 | 9.59E-01 | 9.99E-01 | 0.07±0.21 | 9.49E-01 | 0±0.21 | 9.99E-01 | 0.02±0.41 | 9.96E-01 | -0.02±0.39 | 9.96E-01 |
| X - 12283 | 0.03±0.19 | 0 | 9.79E-01 | 9.69E-01 | 0±0.19 | 0 | 9.91E-01 | 9.99E-01 | 0.03±0.21 | 9.83E-01 | 0±0.21 | 9.99E-01 | 0.04±0.43 | 9.96E-01 | 0±0.43 | 9.98E-01 |
| proline | -0.03±0.19 | 0 | 9.39E-01 | 9.70E-01 | 0±0.19 | 0 | 9.70E-01 | 9.99E-01 | -0.03±0.21 | 9.83E-01 | -0.01±0.21 | 9.96E-01 | 0±0.4 | 9.97E-01 | 0.01±0.43 | 9.98E-01 |
| 11beta-hydroxyandrosterone glucuronide | -0.06±0.17 | 0 | 3.27E-01 | 9.40E-01 | 0±0.17 | 0 | 3.92E-01 | 1.00E+00 | -0.13±0.18 | 8.78E-01 | -0.06±0.19 | 9.66E-01 | 0.32±0.43 | 9.96E-01 | 0.37±0.47 | 9.96E-01 |
| N4-acetylcytidine | -0.04±0.19 | 0 | 3.79E-01 | 9.64E-01 | 0±0.2 | 0 | 3.98E-01 | 1.00E+00 | -0.11±0.21 | 9.14E-01 | -0.06±0.21 | 9.66E-01 | 0.35±0.49 | 9.96E-01 | 0.44±0.56 | 9.96E-01 |
| docosahexaenoylcarnitine (C22:6)* |  |  |  |  |  |  |  |  | -0.68±0.2 | 5.77E-02 | -0.65±0.21 | 1.26E-01 |  |  |  |  |
| X - 24951 |  |  |  |  |  |  |  |  | -0.48±0.15 | 7.35E-02 | -0.47±0.15 | 1.26E-01 |  |  |  |  |
| X - 24953 |  |  |  |  |  |  |  |  | -0.49±0.15 | 7.76E-02 | -0.47±0.16 | 1.42E-01 |  |  |  |  |
| X - 14082 |  |  |  |  |  |  |  |  | -0.51±0.19 | 2.29E-01 | -0.51±0.19 | 2.35E-01 |  |  |  |  |
| glucuronide of piperine metabolite C17H21NO3 (3)* | |  |  |  |  |  |  |  | 0.6±0.27 | 3.50E-01 | 0.78±0.29 | 2.35E-01 |  |  |  |  |
| X - 12738 |  |  |  |  |  |  |  |  | -0.57±0.23 | 2.63E-01 | -0.58±0.23 | 2.84E-01 |  |  |  |  |
| 5-hydroxy-2-methylpyridine sulfate |  |  |  |  |  |  |  |  | -0.58±0.24 | 2.91E-01 | -0.59±0.24 | 2.97E-01 |  |  |  |  |
| 1-palmitoylglycerol (16:0) |  |  |  |  |  |  |  |  | -0.43±0.19 | 3.30E-01 | -0.47±0.2 | 2.99E-01 |  |  |  |  |
| hexanoylglycine |  |  |  |  |  |  |  |  | 0.59±0.23 | 2.45E-01 | 0.55±0.23 | 3.09E-01 |  |  |  |  |
| X - 13729 |  |  |  |  |  |  |  |  | -0.4±0.19 | 4.03E-01 | -0.46±0.2 | 3.13E-01 |  |  |  |  |
| bilirubin degradation product, C16H18N2O5 (4)** | |  |  |  |  |  |  |  | 0.44±0.21 | 3.89E-01 | 0.44±0.21 | 4.27E-01 |  |  |  |  |
| glucuronide of piperine metabolite C17H21NO3 (5)* | |  |  |  |  |  |  |  | 0.36±0.23 | 6.15E-01 | 0.5±0.24 | 4.46E-01 |  |  |  |  |
| 3-hydroxypyridine glucuronide |  |  |  |  |  |  |  |  | -0.47±0.23 | 4.32E-01 | -0.48±0.24 | 4.50E-01 |  |  |  |  |
| cyclo(gly-pro) |  |  |  |  |  |  |  |  | -0.4±0.2 | 4.49E-01 | -0.41±0.2 | 4.51E-01 |  |  |  |  |
| 2-hydroxyfluorene sulfate |  |  |  |  |  |  |  |  | -0.41±0.26 | 6.15E-01 | -0.64±0.32 | 4.66E-01 |  |  |  |  |
| dihydrocaffeate sulfate (2) |  |  |  |  |  |  |  |  | -0.42±0.21 | 4.53E-01 | -0.43±0.22 | 4.77E-01 |  |  |  |  |
| X - 13658 |  |  |  |  |  |  |  |  | -0.43±0.22 | 4.67E-01 | -0.44±0.23 | 5.13E-01 |  |  |  |  |
| docosatrienoate (22:3n3) |  |  |  |  |  |  |  |  | 0.4±0.24 | 5.80E-01 | 0.46±0.25 | 5.43E-01 |  |  |  |  |
| trans-2-hexenoylglycine |  |  |  |  |  |  |  |  | 0.52±0.23 | 3.48E-01 | 0.43±0.23 | 5.62E-01 |  |  |  |  |
| bilirubin degradation product, C17H20N2O5 (1)** | |  |  |  |  |  |  |  | 0.44±0.24 | 5.41E-01 | 0.43±0.24 | 5.66E-01 |  |  |  |  |
| X - 21312 |  |  |  |  |  |  |  |  | -0.38±0.21 | 5.43E-01 | -0.37±0.22 | 5.66E-01 |  |  |  |  |
| bilirubin degradation product, C16H18N2O5 (2)** | |  |  |  |  |  |  |  | 0.37±0.21 | 5.72E-01 | 0.37±0.21 | 5.66E-01 |  |  |  |  |
| arachidonoylcholine |  |  |  |  |  |  |  |  | 0.48±0.24 | 4.49E-01 | 0.43±0.25 | 5.68E-01 |  |  |  |  |
| X - 11979 |  |  |  |  |  |  |  |  | 0.4±0.24 | 5.80E-01 | 0.42±0.24 | 5.68E-01 |  |  |  |  |
| adrenate (22:4n6) |  |  |  |  |  |  |  |  | 0.35±0.22 | 6.15E-01 | 0.39±0.23 | 5.68E-01 |  |  |  |  |
| palmitoyl ethanolamide |  |  |  |  |  |  |  |  | -0.37±0.21 | 5.62E-01 | -0.38±0.22 | 5.71E-01 |  |  |  |  |
| dihydroferulate |  |  |  |  |  |  |  |  | -0.37±0.22 | 5.80E-01 | -0.37±0.22 | 5.79E-01 |  |  |  |  |
| cystine |  |  |  |  |  |  |  |  | 0.32±0.21 | 6.17E-01 | 0.36±0.22 | 5.89E-01 |  |  |  |  |
| bilirubin degradation product, C17H20N2O5 (2)** | |  |  |  |  |  |  |  | 0.42±0.24 | 5.77E-01 | 0.4±0.24 | 5.98E-01 |  |  |  |  |
| GlcNAc sulfate conjugate of C21H34O2 steroid** | |  |  |  |  |  |  |  | 0.31±0.23 | 6.91E-01 | 0.39±0.24 | 5.98E-01 |  |  |  |  |
| bilirubin degradation product, C16H18N2O5 (3)** | |  |  |  |  |  |  |  | 0.32±0.2 | 6.15E-01 | 0.34±0.21 | 6.10E-01 |  |  |  |  |
| cholate |  |  |  |  |  |  |  |  | 0.29±0.2 | 6.55E-01 | 0.32±0.21 | 6.53E-01 |  |  |  |  |
| bilirubin degradation product, C16H18N2O5 (1)** | |  |  |  |  |  |  |  | 0.33±0.21 | 6.15E-01 | 0.33±0.22 | 6.54E-01 |  |  |  |  |
| dihomo-linolenoyl-choline |  |  |  |  |  |  |  |  | 0.42±0.25 | 5.80E-01 | 0.39±0.26 | 6.55E-01 |  |  |  |  |
| dopamine 4-sulfate |  |  |  |  |  |  |  |  | 0.33±0.2 | 6.15E-01 | 0.32±0.21 | 6.55E-01 |  |  |  |  |
| X - 17346 |  |  |  |  |  |  |  |  | -0.32±0.21 | 6.15E-01 | -0.31±0.21 | 6.68E-01 |  |  |  |  |
| dihydroferulic acid sulfate |  |  |  |  |  |  |  |  | -0.34±0.22 | 6.15E-01 | -0.33±0.22 | 6.70E-01 |  |  |  |  |
| gulonate* |  |  |  |  |  |  |  |  | -0.23±0.18 | 7.26E-01 | -0.26±0.18 | 7.24E-01 |  |  |  |  |
| succinoyltaurine |  |  |  |  |  |  |  |  | -0.28±0.19 | 6.55E-01 | -0.27±0.19 | 7.43E-01 |  |  |  |  |
| 4-hydroxyphenylpyruvate |  |  |  |  |  |  |  |  | -0.28±0.19 | 6.17E-01 | -0.26±0.19 | 7.56E-01 |  |  |  |  |
| X - 21410 |  |  |  |  |  |  |  |  | 0.25±0.24 | 7.95E-01 | 0.33±0.25 | 7.56E-01 |  |  |  |  |
| inosine |  |  |  |  |  |  |  |  | 0.29±0.23 | 7.26E-01 | 0.33±0.24 | 7.56E-01 |  |  |  |  |
| malonylcarnitine |  |  |  |  |  |  |  |  | 0.27±0.22 | 7.32E-01 | 0.3±0.23 | 7.56E-01 |  |  |  |  |
| delta-CEHC |  |  |  |  |  |  |  |  | -0.25±0.2 | 7.26E-01 | -0.27±0.2 | 7.58E-01 |  |  |  |  |
| X - 12013 |  |  |  |  |  |  |  |  | -0.25±0.2 | 7.40E-01 | -0.27±0.2 | 7.59E-01 |  |  |  |  |
| ferulic acid 4-sulfate |  |  |  |  |  |  |  |  | -0.3±0.21 | 6.93E-01 | -0.29±0.22 | 7.61E-01 |  |  |  |  |
| 2,4-di-tert-butylphenol |  |  |  |  |  |  |  |  | -0.28±0.22 | 7.26E-01 | -0.31±0.23 | 7.61E-01 |  |  |  |  |
| propionylglycine |  |  |  |  |  |  |  |  | 0.34±0.23 | 6.55E-01 | 0.29±0.23 | 7.70E-01 |  |  |  |  |
| X - 26054 |  |  |  |  |  |  |  |  | 0.32±0.23 | 6.96E-01 | 0.29±0.23 | 7.70E-01 |  |  |  |  |
| fructose |  |  |  |  |  |  |  |  | -0.29±0.21 | 6.98E-01 | -0.28±0.22 | 7.70E-01 |  |  |  |  |
| 5-acetylamino-6-formylamino-3-methyluracil | |  |  |  |  |  |  |  | -0.27±0.21 | 7.07E-01 | -0.26±0.21 | 7.70E-01 |  |  |  |  |
| alpha-CEHC sulfate |  |  |  |  |  |  |  |  | -0.25±0.2 | 7.26E-01 | -0.26±0.21 | 7.70E-01 |  |  |  |  |
| histidine betaine (hercynine)* |  |  |  |  |  |  |  |  | 0.25±0.22 | 7.52E-01 | 0.27±0.22 | 7.70E-01 |  |  |  |  |
| (2,4 or 2,5)-dimethylphenol sulfate |  |  |  |  |  |  |  |  | -0.26±0.22 | 7.53E-01 | -0.32±0.26 | 7.70E-01 |  |  |  |  |
| palmitoylcholine |  |  |  |  |  |  |  |  | 0.38±0.23 | 6.15E-01 | 0.3±0.24 | 7.78E-01 |  |  |  |  |
| linoleoylcholine* |  |  |  |  |  |  |  |  | 0.41±0.24 | 5.80E-01 | 0.3±0.25 | 7.79E-01 |  |  |  |  |
| iminodiacetate (IDA) |  |  |  |  |  |  |  |  | -0.24±0.21 | 7.67E-01 | -0.24±0.21 | 8.10E-01 |  |  |  |  |
| hypoxanthine |  |  |  |  |  |  |  |  | 0.24±0.22 | 7.80E-01 | 0.25±0.22 | 8.10E-01 |  |  |  |  |
| 1-oleoylglycerol (18:1) |  |  |  |  |  |  |  |  | -0.29±0.21 | 6.91E-01 | -0.24±0.21 | 8.17E-01 |  |  |  |  |
| X - 24728 |  |  |  |  |  |  |  |  | 0.15±0.2 | 8.78E-01 | 0.23±0.21 | 8.22E-01 |  |  |  |  |
| gamma-CEHC glucuronide* |  |  |  |  |  |  |  |  | -0.19±0.19 | 8.07E-01 | -0.21±0.19 | 8.27E-01 |  |  |  |  |
| tetrahydrocortisol sulfate (1) |  |  |  |  |  |  |  |  | 0.17±0.21 | 8.78E-01 | 0.24±0.22 | 8.27E-01 |  |  |  |  |
| X - 18935 |  |  |  |  |  |  |  |  | -0.18±0.21 | 8.67E-01 | -0.22±0.21 | 8.32E-01 |  |  |  |  |
| X - 23890 |  |  |  |  |  |  |  |  | -0.23±0.21 | 7.74E-01 | -0.22±0.2 | 8.33E-01 |  |  |  |  |
| stearoylcholine* |  |  |  |  |  |  |  |  | 0.35±0.24 | 6.48E-01 | 0.26±0.25 | 8.42E-01 |  |  |  |  |
| X - 12007 |  |  |  |  |  |  |  |  | -0.2±0.2 | 7.95E-01 | -0.21±0.2 | 8.47E-01 |  |  |  |  |
| 3-hydroxyhippurate sulfate |  |  |  |  |  |  |  |  | -0.22±0.2 | 7.95E-01 | -0.21±0.21 | 8.54E-01 |  |  |  |  |
| oleoylcholine |  |  |  |  |  |  |  |  | 0.32±0.23 | 6.96E-01 | 0.23±0.24 | 8.60E-01 |  |  |  |  |
| 1-oleoyl-GPG (18:1)* |  |  |  |  |  |  |  |  | -0.21±0.18 | 7.52E-01 | -0.18±0.19 | 8.63E-01 |  |  |  |  |
| 2,2'-Methylenebis(6-tert-butyl-p-cresol) |  |  |  |  |  |  |  |  | -0.21±0.22 | 8.32E-01 | -0.22±0.23 | 8.63E-01 |  |  |  |  |
| 1-stearoyl-2-oleoyl-GPS (18:0/18:1) |  |  |  |  |  |  |  |  | 0.19±0.21 | 8.54E-01 | 0.21±0.22 | 8.64E-01 |  |  |  |  |
| ribonate |  |  |  |  |  |  |  |  | -0.18±0.2 | 8.67E-01 | -0.19±0.21 | 8.79E-01 |  |  |  |  |
| cholic acid glucuronide |  |  |  |  |  |  |  |  | 0.19±0.22 | 8.67E-01 | 0.19±0.22 | 8.79E-01 |  |  |  |  |
| 17alpha-hydroxypregnenolone 3-sulfate |  |  |  |  |  |  |  |  | 0.18±0.21 | 8.70E-01 | 0.18±0.21 | 8.79E-01 |  |  |  |  |
| X - 12726 |  |  |  |  |  |  |  |  | -0.17±0.2 | 8.71E-01 | -0.18±0.2 | 8.79E-01 |  |  |  |  |
| 1-myristoylglycerol (14:0) |  |  |  |  |  |  |  |  | -0.15±0.2 | 8.78E-01 | -0.18±0.2 | 8.79E-01 |  |  |  |  |
| catechol glucuronide |  |  |  |  |  |  |  |  | -0.16±0.21 | 8.78E-01 | -0.18±0.21 | 8.79E-01 |  |  |  |  |
| suberate (C8-DC) |  |  |  |  |  |  |  |  | 0.14±0.21 | 8.90E-01 | 0.17±0.21 | 8.79E-01 |  |  |  |  |
| X - 25948 |  |  |  |  |  |  |  |  | 0.14±0.21 | 8.90E-01 | 0.18±0.21 | 8.79E-01 |  |  |  |  |
| X - 23997 |  |  |  |  |  |  |  |  | -0.13±0.19 | 8.92E-01 | -0.18±0.2 | 8.79E-01 |  |  |  |  |
| trans-3,4-methyleneheptanoate |  |  |  |  |  |  |  |  | -0.12±0.2 | 9.02E-01 | -0.17±0.2 | 8.79E-01 |  |  |  |  |
| docosahexaenoylcholine |  |  |  |  |  |  |  |  | -0.12±0.19 | 9.05E-01 | -0.17±0.2 | 8.79E-01 |  |  |  |  |
| X - 25519 |  |  |  |  |  |  |  |  | -0.17±0.21 | 8.73E-01 | -0.17±0.22 | 8.89E-01 |  |  |  |  |
| dimethyl sulfone |  |  |  |  |  |  |  |  | -0.18±0.22 | 8.78E-01 | -0.18±0.22 | 8.89E-01 |  |  |  |  |
| oxindolylalanine |  |  |  |  |  |  |  |  | -0.17±0.2 | 8.72E-01 | -0.15±0.2 | 8.90E-01 |  |  |  |  |
| X - 12104 |  |  |  |  |  |  |  |  | 0.15±0.22 | 8.90E-01 | 0.17±0.22 | 8.99E-01 |  |  |  |  |
| gentisate |  |  |  |  |  |  |  |  | -0.12±0.2 | 9.06E-01 | -0.16±0.21 | 8.99E-01 |  |  |  |  |
| phenol glucuronide |  |  |  |  |  |  |  |  | -0.13±0.21 | 9.00E-01 | -0.17±0.22 | 8.99E-01 |  |  |  |  |
| 4-hydroxyphenylacetoylcarnitine |  |  |  |  |  |  |  |  | 0.14±0.21 | 8.90E-01 | 0.17±0.22 | 9.02E-01 |  |  |  |  |
| X - 12127 |  |  |  |  |  |  |  |  | 0.12±0.21 | 9.14E-01 | 0.16±0.21 | 9.07E-01 |  |  |  |  |
| X - 12096 |  |  |  |  |  |  |  |  | -0.16±0.2 | 8.78E-01 | -0.15±0.21 | 9.16E-01 |  |  |  |  |
| X - 12261 |  |  |  |  |  |  |  |  | -0.12±0.21 | 9.14E-01 | -0.15±0.21 | 9.19E-01 |  |  |  |  |
| gamma-glutamylhistidine |  |  |  |  |  |  |  |  | 0.13±0.21 | 9.02E-01 | 0.14±0.21 | 9.24E-01 |  |  |  |  |
| X - 24531 |  |  |  |  |  |  |  |  | 0.19±0.22 | 8.70E-01 | 0.15±0.22 | 9.26E-01 |  |  |  |  |
| X - 26058 |  |  |  |  |  |  |  |  | 0.14±0.2 | 8.80E-01 | 0.14±0.21 | 9.26E-01 |  |  |  |  |
| X - 16124 |  |  |  |  |  |  |  |  | -0.14±0.21 | 9.00E-01 | -0.14±0.21 | 9.26E-01 |  |  |  |  |
| mannonate* |  |  |  |  |  |  |  |  | -0.21±0.22 | 8.07E-01 | -0.14±0.22 | 9.26E-01 |  |  |  |  |
| 3-indoleglyoxylic acid |  |  |  |  |  |  |  |  | 0.16±0.22 | 8.78E-01 | 0.14±0.22 | 9.26E-01 |  |  |  |  |
| cysteine s-sulfate |  |  |  |  |  |  |  |  | -0.15±0.21 | 8.89E-01 | -0.13±0.22 | 9.26E-01 |  |  |  |  |
| 1-arachidonylglycerol (20:4) |  |  |  |  |  |  |  |  | 0.13±0.21 | 9.00E-01 | 0.13±0.22 | 9.26E-01 |  |  |  |  |
| lithocholate |  |  |  |  |  |  |  |  | 0.1±0.21 | 9.14E-01 | 0.13±0.21 | 9.26E-01 |  |  |  |  |
| 5-hydroxyindoleacetate |  |  |  |  |  |  |  |  | -0.12±0.21 | 9.14E-01 | -0.13±0.21 | 9.26E-01 |  |  |  |  |
| 1-dihomo-linolenylglycerol (20:3) |  |  |  |  |  |  |  |  | -0.13±0.21 | 9.05E-01 | -0.13±0.22 | 9.27E-01 |  |  |  |  |
| nervonoylcarnitine (C24:1)* |  |  |  |  |  |  |  |  | -0.14±0.21 | 8.90E-01 | -0.12±0.21 | 9.29E-01 |  |  |  |  |
| X - 17351 |  |  |  |  |  |  |  |  | 0.14±0.21 | 8.92E-01 | 0.12±0.22 | 9.30E-01 |  |  |  |  |
| X - 12015 |  |  |  |  |  |  |  |  | -0.08±0.2 | 9.35E-01 | -0.12±0.21 | 9.30E-01 |  |  |  |  |
| X - 13007 |  |  |  |  |  |  |  |  | 0.1±0.21 | 9.14E-01 | 0.11±0.21 | 9.30E-01 |  |  |  |  |
| docosatrienoate (22:3n6)* |  |  |  |  |  |  |  |  | -0.1±0.21 | 9.14E-01 | -0.11±0.21 | 9.40E-01 |  |  |  |  |
| branched chain 14:0 dicarboxylic acid** |  |  |  |  |  |  |  |  | -0.06±0.2 | 9.49E-01 | -0.1±0.2 | 9.41E-01 |  |  |  |  |
| sucrose |  |  |  |  |  |  |  |  | -0.07±0.22 | 9.49E-01 | -0.11±0.23 | 9.41E-01 |  |  |  |  |
| X - 23641 |  |  |  |  |  |  |  |  | 0.06±0.23 | 9.49E-01 | 0.12±0.24 | 9.41E-01 |  |  |  |  |
| X - 24295 |  |  |  |  |  |  |  |  | -0.02±0.21 | 9.93E-01 | 0.11±0.22 | 9.41E-01 |  |  |  |  |
| isoeugenol sulfate |  |  |  |  |  |  |  |  | -0.12±0.21 | 9.14E-01 | -0.09±0.22 | 9.53E-01 |  |  |  |  |
| X - 21327 |  |  |  |  |  |  |  |  | -0.09±0.19 | 9.14E-01 | -0.09±0.2 | 9.53E-01 |  |  |  |  |
| 1,2,3-benzenetriol sulfate (2) |  |  |  |  |  |  |  |  | -0.09±0.21 | 9.28E-01 | -0.09±0.21 | 9.54E-01 |  |  |  |  |
| (R)-3-hydroxybutyrylcarnitine |  |  |  |  |  |  |  |  | -0.18±0.21 | 8.70E-01 | -0.08±0.21 | 9.63E-01 |  |  |  |  |
| prolylglycine |  |  |  |  |  |  |  |  | -0.09±0.21 | 9.15E-01 | -0.08±0.21 | 9.63E-01 |  |  |  |  |
| N,N-dimethyl-5-aminovalerate |  |  |  |  |  |  |  |  | -0.08±0.2 | 9.36E-01 | -0.07±0.2 | 9.63E-01 |  |  |  |  |
| 1-linolenoylglycerol (18:3) |  |  |  |  |  |  |  |  | -0.07±0.2 | 9.39E-01 | -0.08±0.21 | 9.63E-01 |  |  |  |  |
| suberoylcarnitine (C8-DC) |  |  |  |  |  |  |  |  | 0.04±0.21 | 9.71E-01 | 0.08±0.21 | 9.63E-01 |  |  |  |  |
| 2-linoleoylglycerol (18:2) |  |  |  |  |  |  |  |  | -0.07±0.19 | 9.45E-01 | -0.06±0.19 | 9.66E-01 |  |  |  |  |
| behenoylcarnitine (C22)* |  |  |  |  |  |  |  |  | 0.07±0.22 | 9.47E-01 | 0.07±0.22 | 9.66E-01 |  |  |  |  |
| 4-hydroxy-2-oxoglutaric acid |  |  |  |  |  |  |  |  | -0.07±0.2 | 9.48E-01 | -0.06±0.21 | 9.66E-01 |  |  |  |  |
| S-methylmethionine |  |  |  |  |  |  |  |  | 0.1±0.21 | 9.14E-01 | 0.05±0.2 | 9.78E-01 |  |  |  |  |
| 2-oleoylglycerol (18:1) |  |  |  |  |  |  |  |  | 0.01±0.21 | 9.94E-01 | 0.05±0.21 | 9.78E-01 |  |  |  |  |
| X - 23276 |  |  |  |  |  |  |  |  | -0.07±0.21 | 9.45E-01 | -0.05±0.21 | 9.78E-01 |  |  |  |  |
| nisinate (24:6n3) |  |  |  |  |  |  |  |  | -0.08±0.2 | 9.35E-01 | -0.04±0.21 | 9.79E-01 |  |  |  |  |
| X - 12839 |  |  |  |  |  |  |  |  | 0.06±0.21 | 9.49E-01 | 0.04±0.21 | 9.79E-01 |  |  |  |  |
| X - 23648 |  |  |  |  |  |  |  |  | 0.04±0.21 | 9.80E-01 | 0.04±0.21 | 9.79E-01 |  |  |  |  |
| galactonate |  |  |  |  |  |  |  |  | 0.02±0.21 | 9.94E-01 | 0.04±0.21 | 9.79E-01 |  |  |  |  |
| phosphate |  |  |  |  |  |  |  |  | 0.1±0.23 | 9.28E-01 | 0.04±0.23 | 9.86E-01 |  |  |  |  |
| X - 14056 |  |  |  |  |  |  |  |  | -0.04±0.21 | 9.69E-01 | 0.03±0.22 | 9.86E-01 |  |  |  |  |
| X - 12126 |  |  |  |  |  |  |  |  | 0.02±0.21 | 9.94E-01 | -0.03±0.21 | 9.86E-01 |  |  |  |  |
| X - 23583 |  |  |  |  |  |  |  |  | 0.04±0.21 | 9.81E-01 | 0.02±0.21 | 9.94E-01 |  |  |  |  |
| X - 18345 |  |  |  |  |  |  |  |  | -0.03±0.21 | 9.84E-01 | -0.02±0.21 | 9.94E-01 |  |  |  |  |
| 2,3-dihydroxy-2-methylbutyrate |  |  |  |  |  |  |  |  | 0.02±0.21 | 9.92E-01 | 0.02±0.21 | 9.94E-01 |  |  |  |  |
| 5alpha-androstan-3alpha,17alpha-diol monosulfate | |  |  |  |  |  |  |  | -0.02±0.22 | 9.94E-01 | -0.02±0.23 | 9.94E-01 |  |  |  |  |
| X - 21959 |  |  |  |  |  |  |  |  | -0.01±0.21 | 9.97E-01 | 0.02±0.21 | 9.94E-01 |  |  |  |  |
| N-acetyltaurine |  |  |  |  |  |  |  |  | 0±0.2 | 9.97E-01 | 0.02±0.2 | 9.94E-01 |  |  |  |  |
| methyl-4-hydroxybenzoate sulfate |  |  |  |  |  |  |  |  | 0.04±0.22 | 9.75E-01 | 0.01±0.23 | 9.95E-01 |  |  |  |  |
| xanthosine |  |  |  |  |  |  |  |  | -0.03±0.21 | 9.83E-01 | 0±0.21 | 9.96E-01 |  |  |  |  |
| gamma-glutamylalanine |  |  |  |  |  |  |  |  | -0.03±0.2 | 9.84E-01 | 0.01±0.2 | 9.96E-01 |  |  |  |  |
| X - 23666 |  |  |  |  |  |  |  |  | 0.01±0.21 | 9.95E-01 | 0.01±0.21 | 9.96E-01 |  |  |  |  |
| N-acetylaspartate (NAA) |  |  |  |  |  |  |  |  | -0.01±0.2 | 9.97E-01 | -0.01±0.21 | 9.96E-01 |  |  |  |  |
| X - 12713 |  |  |  |  |  |  |  |  | 0±0.21 | 9.98E-01 | 0.01±0.21 | 9.96E-01 |  |  |  |  |
| N-formylanthranilic acid |  |  |  |  |  |  |  |  | 0.02±0.21 | 9.94E-01 | 0±0.21 | 9.99E-01 |  |  |  |  |
| X - 24411 |  |  |  |  |  |  |  |  | 0±0.21 | 9.97E-01 | 0±0.21 | 9.99E-01 |  |  |  |  |
| 3-hydroxymyristate |  |  |  |  |  |  |  |  |  |  |  |  | -0.3±0.44 | 9.96E-01 | -0.4±0.46 | 9.96E-01 |
| beta-sitosterol |  |  |  |  |  |  |  |  |  |  |  |  | -0.13±0.41 | 9.96E-01 | -0.14±0.44 | 9.96E-01 |
| 1-methylhistidine |  |  |  |  |  |  |  |  |  |  |  |  | -0.37±0.32 | 9.96E-01 | -0.47±0.36 | 9.96E-01 |
| gamma-glutamyl-epsilon-lysine |  |  |  |  |  |  |  |  |  |  |  |  | -0.11±0.35 | 9.96E-01 | -0.14±0.36 | 9.96E-01 |
| N1-Methyl-4-pyridone-3-carboxamide |  |  |  |  |  |  |  |  |  |  |  |  | -0.04±0.41 | 9.96E-01 | -0.11±0.41 | 9.96E-01 |
| 1-stearoyl-2-arachidonoyl-GPS (18:0/20:4) |  |  |  |  |  |  |  |  |  |  |  |  | 1.2±0.57 | 9.96E-01 | 1.27±0.64 | 9.96E-01 |
| 2-hydroxyglutarate |  |  |  |  |  |  |  |  |  |  |  |  | 0.11±0.42 | 9.96E-01 | 0.16±0.41 | 9.96E-01 |
| palmitoleoyl-oleoyl-glycerol (16:1/18:1) [2]* |  |  |  |  |  |  |  |  |  |  |  |  | -0.29±0.4 | 9.96E-01 | -0.16±0.41 | 9.96E-01 |
| palmitoyl-linoleoyl-glycerol (16:0/18:2) [2]* |  |  |  |  |  |  |  |  |  |  |  |  | -0.18±0.35 | 9.96E-01 | -0.2±0.38 | 9.96E-01 |
| palmitoyl-oleoyl-glycerol (16:0/18:1) [1]* |  |  |  |  |  |  |  |  |  |  |  |  | -0.36±0.4 | 9.96E-01 | -0.38±0.44 | 9.96E-01 |
| palmitoyl-oleoyl-glycerol (16:0/18:1) [2]* |  |  |  |  |  |  |  |  |  |  |  |  | -0.27±0.38 | 9.96E-01 | -0.25±0.43 | 9.96E-01 |
| oleoyl-oleoyl-glycerol (18:1/18:1) [1]* |  |  |  |  |  |  |  |  |  |  |  |  | -0.19±0.31 | 9.96E-01 | -0.17±0.33 | 9.96E-01 |
| linoleoyl-arachidonoyl-glycerol (18:2/20:4) [1]* | |  |  |  |  |  |  |  |  |  |  |  | -0.29±0.33 | 9.96E-01 | -0.37±0.34 | 9.96E-01 |
| linoleoyl-arachidonoyl-glycerol (18:2/20:4) [2]* | |  |  |  |  |  |  |  |  |  |  |  | -0.34±0.32 | 9.96E-01 | -0.37±0.33 | 9.96E-01 |
| palmitoyl-arachidonoyl-glycerol (16:0/20:4) [2]* | |  |  |  |  |  |  |  |  |  |  |  | -0.32±0.37 | 9.96E-01 | -0.3±0.39 | 9.96E-01 |
| linoleoyl-linolenoyl-glycerol (18:2/18:3) [2]* |  |  |  |  |  |  |  |  |  |  |  |  | -0.1±0.41 | 9.96E-01 | -0.24±0.42 | 9.96E-01 |
| palmitoleoyl-linoleoyl-glycerol (16:1/18:2) [1]* | |  |  |  |  |  |  |  |  |  |  |  | -0.1±0.41 | 9.96E-01 | -0.12±0.43 | 9.96E-01 |
| oleoyl-arachidonoyl-glycerol (18:1/20:4) [1]* |  |  |  |  |  |  |  |  |  |  |  |  | -0.16±0.32 | 9.96E-01 | -0.16±0.34 | 9.96E-01 |
| oleoyl-arachidonoyl-glycerol (18:1/20:4) [2]* |  |  |  |  |  |  |  |  |  |  |  |  | -0.14±0.34 | 9.96E-01 | -0.15±0.36 | 9.96E-01 |
| diacylglycerol (16:1/18:2 [2], 16:0/18:3 [1])* |  |  |  |  |  |  |  |  |  |  |  |  | -0.06±0.41 | 9.96E-01 | -0.06±0.44 | 9.96E-01 |
| linoleoyl-linoleoyl-glycerol (18:2/18:2) [1]* |  |  |  |  |  |  |  |  |  |  |  |  | -0.1±0.39 | 9.96E-01 | -0.25±0.39 | 9.96E-01 |
| linoleoyl-linoleoyl-glycerol (18:2/18:2) [2]* |  |  |  |  |  |  |  |  |  |  |  |  | -0.05±0.41 | 9.96E-01 | -0.05±0.4 | 9.96E-01 |
| stearoyl-arachidonoyl-glycerol (18:0/20:4) [1]* | |  |  |  |  |  |  |  |  |  |  |  | 0.25±0.44 | 9.96E-01 | 0.34±0.47 | 9.96E-01 |
| stearoyl-arachidonoyl-glycerol (18:0/20:4) [2]* | |  |  |  |  |  |  |  |  |  |  |  | -0.05±0.41 | 9.96E-01 | -0.09±0.42 | 9.96E-01 |
| palmitoyl-docosahexaenoyl-glycerol (16:0/22:6) [1]* | |  |  |  |  |  |  |  |  |  |  |  | -0.62±0.42 | 9.96E-01 | -0.57±0.45 | 9.96E-01 |
| N-behenoyl-sphingadienine (d18:2/22:0)* |  |  |  |  |  |  |  |  |  |  |  |  | 0.29±0.52 | 9.96E-01 | 0.22±0.52 | 9.96E-01 |
| myristoyl-linoleoyl-glycerol (14:0/18:2) [2]* |  |  |  |  |  |  |  |  |  |  |  |  | -0.56±0.43 | 9.96E-01 | -0.64±0.46 | 9.96E-01 |
| glycosyl ceramide (d18:1/23:1, d17:1/24:1)* |  |  |  |  |  |  |  |  |  |  |  |  | 0.57±0.54 | 9.96E-01 | 0.72±0.61 | 9.96E-01 |
| ceramide (d18:1/20:0, d16:1/22:0, d20:1/18:0)* | |  |  |  |  |  |  |  |  |  |  |  | 0.13±0.46 | 9.96E-01 | 0.14±0.47 | 9.96E-01 |
| 1-carboxyethylphenylalanine |  |  |  |  |  |  |  |  |  |  |  |  | -0.17±0.43 | 9.96E-01 | -0.33±0.47 | 9.96E-01 |
| 1-carboxyethyltyrosine |  |  |  |  |  |  |  |  |  |  |  |  | -0.04±0.44 | 9.96E-01 | -0.14±0.48 | 9.96E-01 |
| 1-carboxyethylvaline |  |  |  |  |  |  |  |  |  |  |  |  | -0.28±0.4 | 9.96E-01 | -0.35±0.4 | 9.96E-01 |
| 1-carboxyethylleucine |  |  |  |  |  |  |  |  |  |  |  |  | -0.27±0.4 | 9.96E-01 | -0.41±0.44 | 9.96E-01 |
| 1-carboxyethylisoleucine |  |  |  |  |  |  |  |  |  |  |  |  | -0.2±0.42 | 9.96E-01 | -0.4±0.47 | 9.96E-01 |
| (2-butoxyethoxy)acetic acid |  |  |  |  |  |  |  |  |  |  |  |  | -0.06±0.43 | 9.96E-01 | -0.03±0.43 | 9.96E-01 |
| dibutyl sulfosuccinate |  |  |  |  |  |  |  |  |  |  |  |  | 0.2±0.31 | 9.96E-01 | 0.11±0.31 | 9.96E-01 |
| 2-aminoadipate |  |  |  |  |  |  |  |  |  |  |  |  | -0.21±0.39 | 9.96E-01 | -0.28±0.41 | 9.96E-01 |
| serotonin |  |  |  |  |  |  |  |  |  |  |  |  | 1.59±0.77 | 9.96E-01 | 1.62±0.78 | 9.96E-01 |
| thymidine |  |  |  |  |  |  |  |  |  |  |  |  | 0±0.41 | 9.97E-01 | -0.09±0.42 | 9.96E-01 |
| X - 12849 |  |  |  |  |  |  |  |  |  |  |  |  | 0.03±0.37 | 9.96E-01 | 0.09±0.4 | 9.96E-01 |
| X - 13684 |  |  |  |  |  |  |  |  |  |  |  |  | 0.11±0.43 | 9.96E-01 | 0.04±0.42 | 9.96E-01 |
| X - 16938 |  |  |  |  |  |  |  |  |  |  |  |  | 0.1±0.42 | 9.96E-01 | 0.19±0.44 | 9.96E-01 |
| X - 16964 |  |  |  |  |  |  |  |  |  |  |  |  | 0.27±0.48 | 9.96E-01 | 0.27±0.49 | 9.96E-01 |
| X - 21829 |  |  |  |  |  |  |  |  |  |  |  |  | -0.59±0.37 | 9.96E-01 | -0.68±0.39 | 9.96E-01 |
| X - 22771 |  |  |  |  |  |  |  |  |  |  |  |  | 0.56±0.64 | 9.96E-01 | 0.59±0.67 | 9.96E-01 |
| X - 24241 |  |  |  |  |  |  |  |  |  |  |  |  | 0.9±0.97 | 9.96E-01 | 0.85±0.95 | 9.96E-01 |
| X - 25828 |  |  |  |  |  |  |  |  |  |  |  |  | 0.06±0.46 | 9.96E-01 | 0.16±0.47 | 9.96E-01 |
| saccharin |  |  |  |  |  |  |  |  |  |  |  |  | -0.08±0.4 | 9.96E-01 | 0.01±0.41 | 9.98E-01 |
| 3-methylcrotonylglycine |  |  |  |  |  |  |  |  |  |  |  |  | -0.02±0.4 | 9.96E-01 | 0.01±0.41 | 9.98E-01 |
| stearoyl ethanolamide |  |  |  |  |  |  |  |  |  |  |  |  | 0±0.42 | 9.97E-01 | 0.01±0.44 | 9.98E-01 |
| palmitoleoyl-arachidonoyl-glycerol (16:1/20:4) [2]* | |  |  |  |  |  |  |  |  |  |  |  | 0.07±0.45 | 9.96E-01 | -0.01±0.42 | 9.98E-01 |

Note: The table shows associations from the logistic regression analysis where plasms metabolomics level from the Metabolon platform was main exposure and prevalent colorectal cancer as the main outcome in ERGO-4 and ERGO-5, respectively. ERGO-4 and ERGO-5 have 939 overlapping circulating metabolites, and ERGO-4&5 refers to the correlation between prevalent colorectal cancer and 939 metabolites derived from meta-analysis of ERGO-4 and ERGO-5. Model 1: adjusted for age, sex, cohort, and lipid lowering medication. Model 2: adjusted for model1, smoking status, BMI, alcohol and education. The table is ranked based on the *FDR* values in model 2 within the E4&E5 cohort, using bold text indicates metabolites are surpassing *FDR* adjusted *P* value treshold (*P*<0.05). ERGO-4 and ERGO-5 refer to the fourth and fifth visits subcohort from the baseline cohort. Abbreviations: SE, standard erorr; I^2^: heterogeneity parameter, Het. *P*-value: significance of the heterogeneity; BMI, body mass index; FDR, false discovery rate.

**Supplementary Table 9. Serum metabolites associated with prevalent breast cancer from the Nightingale platform at baseline.**

| **Metabolite** | **ERGO-4&5** | | | | | | **ERGO-4** | | | | | | **ERGO-5** | | | | | |
| --- | --- | --- | --- | --- | --- | --- | --- | --- | --- | --- | --- | --- | --- | --- | --- | --- | --- | --- |
|  | **Model 1** | | | **Model 2** | | | **Model 1** | | | **Model 2** | | | **Model 1** | | | **Model 2** | | |
|  | **Beta** | **SE** | ***FDR* value** | **Beta** | **SE** | ***FDR* value** | **Beta** | **SE** | ***FDR* value** | **Beta** | **SE** | ***FDR* value** | **Beta** | **SE** | ***FDR* value** | **Beta** | **SE** | ***FDR* value** |
| Glycine | -0.34 | 0.09 | **4.15E-02** | -0.35 | 0.09 | 5.80E-02 | -0.34 | 0.12 | 2.40E-01 | -0.35 | 0.13 | 3.17E-01 | -0.34 | 0.14 | 1.30E-01 | -0.34 | 0.14 | 1.26E-01 |
| Phospholipids to total lipids ratio in large HDL | 0.33 | 0.10 | 6.57E-02 | 0.34 | 0.10 | 6.75E-02 | 0.24 | 0.13 | 3.97E-01 | 0.24 | 0.14 | 4.71E-01 | 0.42 | 0.15 | 1.01E-01 | 0.46 | 0.16 | 8.06E-02 |
| Glucose | 0.28 | 0.08 | 6.57E-02 | 0.30 | 0.09 | 6.75E-02 | 0.23 | 0.11 | 3.26E-01 | 0.25 | 0.12 | 3.17E-01 | 0.33 | 0.14 | 1.30E-01 | 0.36 | 0.15 | 1.18E-01 |
| Phospholipids to total lipids ratio in small VLDL | -0.30 | 0.10 | 6.57E-02 | -0.30 | 0.10 | 8.85E-02 | -0.22 | 0.14 | 4.77E-01 | -0.21 | 0.14 | 5.21E-01 | -0.40 | 0.15 | 1.01E-01 | -0.44 | 0.15 | 8.06E-02 |
| Cholesteryl esters to total lipids ratio in medium VLDL | -0.29 | 0.10 | 6.57E-02 | -0.29 | 0.10 | 8.85E-02 | -0.20 | 0.14 | 5.06E-01 | -0.19 | 0.14 | 5.59E-01 | -0.38 | 0.14 | 1.01E-01 | -0.41 | 0.14 | 8.06E-02 |
| Cholesterol to total lipids ratio in medium VLDL | -0.28 | 0.10 | 6.57E-02 | -0.29 | 0.10 | 8.85E-02 | -0.18 | 0.14 | 5.44E-01 | -0.17 | 0.14 | 5.91E-01 | -0.40 | 0.14 | 1.01E-01 | -0.44 | 0.15 | 8.06E-02 |
| Triglycerides to total lipids ratio in small VLDL | 0.29 | 0.10 | 6.83E-02 | 0.29 | 0.11 | 8.85E-02 | 0.18 | 0.14 | 5.44E-01 | 0.16 | 0.14 | 6.15E-01 | 0.44 | 0.16 | 1.01E-01 | 0.47 | 0.16 | 8.06E-02 |
| Triglycerides to total lipids ratio in medium VLDL | 0.27 | 0.10 | 7.30E-02 | 0.28 | 0.10 | 8.85E-02 | 0.12 | 0.14 | 6.73E-01 | 0.11 | 0.14 | 6.92E-01 | 0.46 | 0.15 | 1.01E-01 | 0.49 | 0.16 | 8.06E-02 |
| Alanine | 0.27 | 0.10 | 6.57E-02 | 0.27 | 0.10 | 8.85E-02 | 0.12 | 0.13 | 6.90E-01 | 0.11 | 0.14 | 6.92E-01 | 0.44 | 0.14 | 1.01E-01 | 0.45 | 0.15 | 8.06E-02 |
| Free cholesterol to total lipids ratio in small VLDL | -0.28 | 0.10 | 6.57E-02 | -0.29 | 0.10 | 8.85E-02 | -0.21 | 0.14 | 4.77E-01 | -0.20 | 0.14 | 5.32E-01 | -0.37 | 0.15 | 1.23E-01 | -0.41 | 0.15 | 9.57E-02 |
| Cholesterol to total lipids ratio in small VLDL | -0.28 | 0.10 | 6.57E-02 | -0.28 | 0.10 | 8.85E-02 | -0.21 | 0.14 | 4.77E-01 | -0.20 | 0.14 | 5.47E-01 | -0.37 | 0.15 | 1.23E-01 | -0.39 | 0.15 | 9.97E-02 |
| Triglycerides to total lipids ratio in very large HDL | 0.26 | 0.09 | 6.91E-02 | 0.27 | 0.10 | 8.85E-02 | 0.19 | 0.13 | 5.06E-01 | 0.20 | 0.13 | 5.20E-01 | 0.32 | 0.14 | 1.43E-01 | 0.33 | 0.14 | 1.26E-01 |
| Ratio of monounsaturated fatty acids to total fatty acids | 0.26 | 0.09 | 6.91E-02 | 0.27 | 0.10 | 8.85E-02 | 0.22 | 0.13 | 4.77E-01 | 0.23 | 0.13 | 5.20E-01 | 0.30 | 0.14 | 1.70E-01 | 0.34 | 0.15 | 1.39E-01 |
| Free cholesterol to total lipids ratio in IDL | -0.31 | 0.10 | 6.57E-02 | -0.30 | 0.10 | 8.85E-02 | -0.30 | 0.14 | 2.55E-01 | -0.28 | 0.14 | 3.38E-01 | -0.30 | 0.14 | 1.71E-01 | -0.31 | 0.14 | 1.61E-01 |
| Ratio of polyunsaturated fatty acids to monounsaturated fatty acids | -0.27 | 0.09 | 6.57E-02 | -0.27 | 0.09 | 8.85E-02 | -0.27 | 0.12 | 2.57E-01 | -0.27 | 0.13 | 3.17E-01 | -0.27 | 0.14 | 2.00E-01 | -0.31 | 0.15 | 1.63E-01 |
| Cholesterol to total lipids ratio in large HDL | -0.25 | 0.09 | 6.91E-02 | -0.25 | 0.09 | 8.85E-02 | -0.23 | 0.12 | 4.07E-01 | -0.23 | 0.13 | 4.71E-01 | -0.26 | 0.14 | 2.05E-01 | -0.28 | 0.14 | 1.90E-01 |
| Triglycerides in small HDL | 0.26 | 0.09 | 6.91E-02 | 0.26 | 0.10 | 8.85E-02 | 0.26 | 0.13 | 3.83E-01 | 0.26 | 0.14 | 4.18E-01 | 0.24 | 0.13 | 2.50E-01 | 0.25 | 0.14 | 2.34E-01 |
| Ratio of linoleic acid to total fatty acids | -0.28 | 0.10 | 6.57E-02 | -0.27 | 0.10 | 8.85E-02 | -0.29 | 0.13 | 2.40E-01 | -0.27 | 0.14 | 3.88E-01 | -0.26 | 0.14 | 2.55E-01 | -0.27 | 0.16 | 2.56E-01 |
| Cholesteryl esters to total lipids ratio in large HDL | -0.26 | 0.09 | 6.57E-02 | -0.26 | 0.09 | 8.85E-02 | -0.26 | 0.12 | 2.40E-01 | -0.26 | 0.12 | 3.17E-01 | -0.24 | 0.14 | 2.70E-01 | -0.25 | 0.15 | 2.57E-01 |
| Ratio of omega-6 fatty acids to total fatty acids | -0.27 | 0.09 | 6.57E-02 | -0.26 | 0.09 | 8.85E-02 | -0.30 | 0.12 | 2.40E-01 | -0.29 | 0.13 | 3.17E-01 | -0.23 | 0.14 | 2.88E-01 | -0.24 | 0.15 | 2.82E-01 |
| Ratio of polyunsaturated fatty acids to total fatty acids | -0.26 | 0.09 | 6.57E-02 | -0.26 | 0.09 | 8.85E-02 | -0.30 | 0.12 | 2.40E-01 | -0.30 | 0.12 | 3.17E-01 | -0.22 | 0.14 | 3.20E-01 | -0.24 | 0.15 | 2.92E-01 |
| Triglycerides in medium HDL | 0.29 | 0.10 | 6.57E-02 | 0.27 | 0.10 | 8.85E-02 | 0.34 | 0.14 | 2.40E-01 | 0.33 | 0.14 | 3.17E-01 | 0.22 | 0.14 | 3.22E-01 | 0.21 | 0.14 | 3.21E-01 |
| Triglycerides in HDL | 0.27 | 0.10 | 6.57E-02 | 0.26 | 0.10 | 8.85E-02 | 0.34 | 0.14 | 2.40E-01 | 0.33 | 0.14 | 3.17E-01 | 0.19 | 0.14 | 3.63E-01 | 0.19 | 0.14 | 3.75E-01 |
| Phospholipids in small HDL | 0.30 | 0.10 | 6.57E-02 | 0.28 | 0.11 | 8.85E-02 | 0.36 | 0.15 | 2.40E-01 | 0.37 | 0.15 | 3.17E-01 | 0.20 | 0.15 | 3.76E-01 | 0.19 | 0.15 | 4.15E-01 |
| Phenylalanine | 0.25 | 0.10 | 7.92E-02 | 0.26 | 0.10 | 8.85E-02 | 0.00 | 0.13 | 9.92E-01 | -0.01 | 0.14 | 9.88E-01 | 0.54 | 0.14 | **1.81E-02** | 0.55 | 0.14 | **2.13E-02** |
| Monounsaturated fatty acids | 0.26 | 0.10 | 6.91E-02 | 0.25 | 0.10 | 9.23E-02 | 0.32 | 0.13 | 2.40E-01 | 0.32 | 0.13 | 3.17E-01 | 0.17 | 0.14 | 4.25E-01 | 0.17 | 0.14 | 4.27E-01 |
| Free cholesterol to total lipids ratio in medium VLDL | -0.26 | 0.10 | 8.03E-02 | -0.26 | 0.10 | 9.88E-02 | -0.13 | 0.14 | 6.68E-01 | -0.12 | 0.14 | 6.72E-01 | -0.41 | 0.15 | 1.01E-01 | -0.44 | 0.15 | 8.06E-02 |
| Cholesteryl esters in very large HDL | -0.23 | 0.10 | 1.09E-01 | -0.26 | 0.10 | 1.00E-01 | -0.05 | 0.14 | 8.48E-01 | -0.07 | 0.14 | 8.13E-01 | -0.42 | 0.14 | 1.01E-01 | -0.45 | 0.15 | 8.06E-02 |
| Cholesterol in very large HDL | -0.23 | 0.10 | 1.13E-01 | -0.25 | 0.10 | 1.00E-01 | -0.04 | 0.14 | 8.62E-01 | -0.06 | 0.14 | 8.22E-01 | -0.40 | 0.14 | 1.01E-01 | -0.43 | 0.14 | 8.06E-02 |
| Cholesteryl esters to total lipids ratio in large VLDL | -0.25 | 0.10 | 8.03E-02 | -0.24 | 0.10 | 1.00E-01 | -0.14 | 0.13 | 6.28E-01 | -0.12 | 0.14 | 6.54E-01 | -0.37 | 0.14 | 1.09E-01 | -0.38 | 0.14 | 9.57E-02 |
| Cholesterol to total lipids ratio in very large VLDL | -0.26 | 0.10 | 8.09E-02 | -0.26 | 0.10 | 1.00E-01 | -0.14 | 0.14 | 6.28E-01 | -0.13 | 0.14 | 6.49E-01 | -0.38 | 0.15 | 1.23E-01 | -0.40 | 0.16 | 1.02E-01 |
| Cholesteryl esters to total lipids ratio in very large VLDL | -0.26 | 0.10 | 8.03E-02 | -0.26 | 0.10 | 1.00E-01 | -0.15 | 0.14 | 6.26E-01 | -0.14 | 0.14 | 6.22E-01 | -0.37 | 0.15 | 1.23E-01 | -0.40 | 0.16 | 1.13E-01 |
| Triglycerides to total lipids ratio in very small VLDL | 0.27 | 0.11 | 8.38E-02 | 0.27 | 0.11 | 1.00E-01 | 0.20 | 0.15 | 5.27E-01 | 0.19 | 0.15 | 5.79E-01 | 0.35 | 0.16 | 1.66E-01 | 0.39 | 0.16 | 1.26E-01 |
| Cholesteryl esters to total lipids ratio in small VLDL | -0.25 | 0.10 | 8.33E-02 | -0.24 | 0.10 | 1.00E-01 | -0.19 | 0.14 | 5.27E-01 | -0.17 | 0.14 | 5.91E-01 | -0.32 | 0.14 | 1.66E-01 | -0.33 | 0.15 | 1.39E-01 |
| Triglycerides to total lipids ratio in small LDL | 0.24 | 0.10 | 8.38E-02 | 0.24 | 0.10 | 1.00E-01 | 0.24 | 0.13 | 4.07E-01 | 0.23 | 0.13 | 5.03E-01 | 0.24 | 0.14 | 2.74E-01 | 0.27 | 0.15 | 2.34E-01 |
| Free cholesterol to total lipids ratio in large LDL | -0.24 | 0.09 | 8.53E-02 | -0.23 | 0.10 | 1.00E-01 | -0.23 | 0.13 | 4.07E-01 | -0.22 | 0.13 | 5.03E-01 | -0.24 | 0.14 | 2.74E-01 | -0.28 | 0.15 | 2.34E-01 |
| Cholesterol to total lipids ratio in small HDL | -0.24 | 0.09 | 7.92E-02 | -0.23 | 0.09 | 1.00E-01 | -0.29 | 0.12 | 2.40E-01 | -0.27 | 0.12 | 3.17E-01 | -0.17 | 0.14 | 4.18E-01 | -0.18 | 0.14 | 4.12E-01 |
| Total lipids in small HDL | 0.27 | 0.10 | 7.92E-02 | 0.26 | 0.11 | 1.00E-01 | 0.33 | 0.15 | 2.55E-01 | 0.33 | 0.15 | 3.17E-01 | 0.19 | 0.14 | 4.08E-01 | 0.18 | 0.15 | 4.37E-01 |
| Cholesterol to total lipids ratio in very small VLDL | -0.25 | 0.10 | 1.09E-01 | -0.25 | 0.11 | 1.29E-01 | -0.20 | 0.14 | 5.27E-01 | -0.20 | 0.15 | 5.75E-01 | -0.31 | 0.15 | 2.00E-01 | -0.34 | 0.16 | 1.61E-01 |
| Free cholesterol in very large HDL | -0.21 | 0.09 | 1.33E-01 | -0.22 | 0.10 | 1.39E-01 | -0.05 | 0.13 | 8.52E-01 | -0.06 | 0.14 | 8.21E-01 | -0.35 | 0.13 | 1.01E-01 | -0.36 | 0.13 | 9.57E-02 |
| Triglycerides to total lipids ratio in very large VLDL | 0.25 | 0.11 | 1.09E-01 | 0.24 | 0.11 | 1.39E-01 | 0.10 | 0.14 | 7.50E-01 | 0.08 | 0.14 | 7.85E-01 | 0.43 | 0.16 | 1.05E-01 | 0.43 | 0.16 | 9.57E-02 |
| Cholesteryl esters to total lipids ratio in very small VLDL | -0.24 | 0.10 | 1.19E-01 | -0.24 | 0.11 | 1.39E-01 | -0.19 | 0.14 | 5.42E-01 | -0.18 | 0.15 | 5.79E-01 | -0.30 | 0.15 | 2.04E-01 | -0.33 | 0.16 | 1.63E-01 |
| Total triglycerides | 0.22 | 0.09 | 1.18E-01 | 0.22 | 0.10 | 1.39E-01 | 0.20 | 0.13 | 4.77E-01 | 0.20 | 0.13 | 5.20E-01 | 0.21 | 0.14 | 3.21E-01 | 0.23 | 0.14 | 2.94E-01 |
| Glutamine | -0.23 | 0.10 | 1.02E-01 | -0.22 | 0.10 | 1.39E-01 | -0.35 | 0.13 | 2.40E-01 | -0.37 | 0.13 | 3.17E-01 | -0.08 | 0.15 | 7.83E-01 | -0.06 | 0.15 | 8.11E-01 |
| Free cholesterol to total lipids ratio in medium LDL | -0.22 | 0.09 | 1.18E-01 | -0.22 | 0.10 | 1.40E-01 | -0.18 | 0.13 | 5.27E-01 | -0.17 | 0.13 | 5.86E-01 | -0.27 | 0.14 | 2.05E-01 | -0.30 | 0.14 | 1.69E-01 |
| Total lipids in very large HDL | -0.21 | 0.10 | 1.47E-01 | -0.22 | 0.10 | 1.42E-01 | 0.00 | 0.14 | 9.92E-01 | -0.02 | 0.14 | 9.36E-01 | -0.41 | 0.14 | 1.01E-01 | -0.43 | 0.14 | 8.06E-02 |
| Free cholesterol to total lipids ratio in very small VLDL | -0.23 | 0.10 | 1.09E-01 | -0.22 | 0.10 | 1.42E-01 | -0.22 | 0.13 | 4.77E-01 | -0.21 | 0.14 | 5.20E-01 | -0.25 | 0.14 | 2.70E-01 | -0.26 | 0.15 | 2.34E-01 |
| Average diameter for VLDL particles | 0.21 | 0.10 | 1.43E-01 | 0.22 | 0.10 | 1.45E-01 | 0.08 | 0.14 | 7.83E-01 | 0.09 | 0.14 | 7.57E-01 | 0.33 | 0.14 | 1.42E-01 | 0.36 | 0.15 | 1.18E-01 |
| Saturated fatty acids | 0.24 | 0.10 | 1.02E-01 | 0.22 | 0.10 | 1.45E-01 | 0.39 | 0.14 | 2.40E-01 | 0.38 | 0.14 | 3.17E-01 | 0.05 | 0.15 | 8.48E-01 | 0.04 | 0.15 | 8.78E-01 |
| Phospholipids to total lipids ratio in medium VLDL | -0.22 | 0.10 | 1.37E-01 | -0.22 | 0.10 | 1.45E-01 | -0.09 | 0.14 | 7.52E-01 | -0.08 | 0.14 | 7.85E-01 | -0.38 | 0.15 | 1.21E-01 | -0.40 | 0.15 | 9.57E-02 |
| Triglycerides in VLDL | 0.21 | 0.09 | 1.43E-01 | 0.21 | 0.10 | 1.52E-01 | 0.16 | 0.13 | 5.44E-01 | 0.17 | 0.13 | 5.91E-01 | 0.23 | 0.14 | 2.74E-01 | 0.25 | 0.14 | 2.56E-01 |
| Triglycerides to total lipids ratio in large HDL | 0.22 | 0.10 | 1.43E-01 | 0.22 | 0.10 | 1.52E-01 | 0.18 | 0.14 | 5.44E-01 | 0.18 | 0.14 | 5.79E-01 | 0.26 | 0.15 | 2.70E-01 | 0.27 | 0.15 | 2.54E-01 |
| Cholesterol to total lipids ratio in medium HDL | -0.20 | 0.09 | 1.43E-01 | -0.21 | 0.10 | 1.52E-01 | -0.20 | 0.13 | 4.77E-01 | -0.20 | 0.13 | 5.20E-01 | -0.20 | 0.14 | 3.60E-01 | -0.22 | 0.15 | 3.24E-01 |
| Triglycerides in small VLDL | 0.20 | 0.10 | 1.43E-01 | 0.20 | 0.10 | 1.60E-01 | 0.21 | 0.13 | 4.77E-01 | 0.20 | 0.13 | 5.20E-01 | 0.19 | 0.14 | 3.71E-01 | 0.20 | 0.14 | 3.39E-01 |
| Phospholipids in very large HDL | -0.19 | 0.09 | 1.65E-01 | -0.20 | 0.10 | 1.63E-01 | 0.02 | 0.14 | 9.42E-01 | 0.00 | 0.15 | 9.88E-01 | -0.34 | 0.12 | 1.01E-01 | -0.35 | 0.12 | 8.06E-02 |
| Triglycerides in very large VLDL | 0.20 | 0.10 | 1.51E-01 | 0.21 | 0.10 | 1.63E-01 | 0.14 | 0.14 | 6.26E-01 | 0.14 | 0.14 | 6.15E-01 | 0.25 | 0.14 | 2.55E-01 | 0.27 | 0.15 | 2.34E-01 |
| Total fatty acids | 0.22 | 0.10 | 1.31E-01 | 0.21 | 0.10 | 1.63E-01 | 0.34 | 0.14 | 2.40E-01 | 0.34 | 0.14 | 3.17E-01 | 0.07 | 0.15 | 7.83E-01 | 0.06 | 0.15 | 8.02E-01 |
| Pyruvate | 0.20 | 0.09 | 1.48E-01 | 0.20 | 0.10 | 1.66E-01 | 0.05 | 0.13 | 8.33E-01 | 0.04 | 0.13 | 8.65E-01 | 0.34 | 0.13 | 1.23E-01 | 0.34 | 0.14 | 1.18E-01 |
| Cholesterol to total lipids ratio in large VLDL | -0.20 | 0.09 | 1.43E-01 | -0.19 | 0.09 | 1.66E-01 | -0.12 | 0.13 | 6.77E-01 | -0.10 | 0.14 | 7.17E-01 | -0.28 | 0.13 | 1.66E-01 | -0.29 | 0.13 | 1.61E-01 |
| Triglycerides to total lipids ratio in medium HDL | 0.20 | 0.10 | 1.59E-01 | 0.21 | 0.10 | 1.66E-01 | 0.18 | 0.14 | 5.44E-01 | 0.18 | 0.14 | 5.79E-01 | 0.22 | 0.14 | 3.29E-01 | 0.23 | 0.15 | 2.96E-01 |
| Triglycerides to total lipids ratio in small HDL | 0.19 | 0.10 | 1.67E-01 | 0.20 | 0.10 | 1.66E-01 | 0.17 | 0.13 | 5.44E-01 | 0.18 | 0.14 | 5.79E-01 | 0.21 | 0.14 | 3.34E-01 | 0.23 | 0.15 | 2.96E-01 |
| Degree of unsaturation | -0.20 | 0.09 | 1.48E-01 | -0.20 | 0.10 | 1.66E-01 | -0.21 | 0.13 | 4.77E-01 | -0.21 | 0.13 | 5.20E-01 | -0.21 | 0.14 | 3.40E-01 | -0.23 | 0.14 | 2.99E-01 |
| Cholesteryl esters to total lipids ratio in small HDL | -0.20 | 0.09 | 1.37E-01 | -0.19 | 0.09 | 1.66E-01 | -0.31 | 0.12 | 2.40E-01 | -0.28 | 0.12 | 3.17E-01 | -0.07 | 0.14 | 7.83E-01 | -0.07 | 0.14 | 7.69E-01 |
| Cholesteryl esters to total lipids ratio in medium HDL | -0.19 | 0.09 | 1.59E-01 | -0.19 | 0.10 | 1.78E-01 | -0.23 | 0.13 | 4.07E-01 | -0.22 | 0.13 | 5.17E-01 | -0.14 | 0.14 | 5.36E-01 | -0.15 | 0.15 | 5.10E-01 |
| Phospholipids to total lipids ratio in small LDL | -0.18 | 0.09 | 1.72E-01 | -0.18 | 0.09 | 1.80E-01 | -0.14 | 0.13 | 6.26E-01 | -0.15 | 0.13 | 6.15E-01 | -0.23 | 0.13 | 2.70E-01 | -0.22 | 0.13 | 2.75E-01 |
| Free cholesterol to total lipids ratio in very large VLDL | -0.19 | 0.09 | 1.48E-01 | -0.18 | 0.09 | 1.84E-01 | -0.12 | 0.14 | 6.74E-01 | -0.11 | 0.14 | 6.98E-01 | -0.27 | 0.12 | 1.70E-01 | -0.26 | 0.12 | 1.61E-01 |
| Concentration of very large HDL particles | -0.18 | 0.10 | 2.11E-01 | -0.20 | 0.10 | 1.85E-01 | 0.04 | 0.14 | 8.62E-01 | 0.03 | 0.14 | 9.12E-01 | -0.41 | 0.14 | 1.01E-01 | -0.43 | 0.15 | 8.06E-02 |
| Cholesteryl esters in large HDL | -0.19 | 0.10 | 2.02E-01 | -0.20 | 0.11 | 1.85E-01 | -0.08 | 0.14 | 7.86E-01 | -0.09 | 0.14 | 7.57E-01 | -0.31 | 0.15 | 1.85E-01 | -0.35 | 0.16 | 1.61E-01 |
| Ratio of triglycerides to phosphoglycerides | 0.18 | 0.10 | 1.96E-01 | 0.19 | 0.10 | 1.85E-01 | 0.10 | 0.13 | 7.37E-01 | 0.10 | 0.14 | 7.12E-01 | 0.26 | 0.14 | 2.37E-01 | 0.30 | 0.15 | 1.90E-01 |
| Total lipids in chylomicrons and extremely large VLDL | 0.18 | 0.10 | 1.94E-01 | 0.19 | 0.10 | 1.85E-01 | 0.11 | 0.13 | 6.93E-01 | 0.11 | 0.13 | 6.92E-01 | 0.26 | 0.14 | 2.55E-01 | 0.28 | 0.15 | 2.34E-01 |
| Triglycerides in large VLDL | 0.19 | 0.10 | 1.84E-01 | 0.19 | 0.10 | 1.85E-01 | 0.13 | 0.13 | 6.34E-01 | 0.13 | 0.14 | 6.22E-01 | 0.23 | 0.14 | 2.99E-01 | 0.24 | 0.14 | 2.75E-01 |
| Cholesterol in chylomicrons and extremely large VLDL | 0.19 | 0.10 | 1.85E-01 | 0.20 | 0.10 | 1.85E-01 | 0.14 | 0.13 | 6.26E-01 | 0.15 | 0.14 | 6.15E-01 | 0.22 | 0.14 | 3.22E-01 | 0.24 | 0.15 | 2.92E-01 |
| Concentration of large VLDL particles | 0.18 | 0.10 | 1.85E-01 | 0.19 | 0.10 | 1.85E-01 | 0.15 | 0.13 | 6.26E-01 | 0.15 | 0.14 | 6.15E-01 | 0.20 | 0.14 | 3.40E-01 | 0.21 | 0.14 | 3.17E-01 |
| Concentration of very large VLDL particles | 0.19 | 0.10 | 1.85E-01 | 0.19 | 0.10 | 1.85E-01 | 0.16 | 0.14 | 6.06E-01 | 0.16 | 0.14 | 6.07E-01 | 0.20 | 0.14 | 3.52E-01 | 0.21 | 0.14 | 3.31E-01 |
| Free cholesterol to total lipids ratio in small LDL | -0.19 | 0.09 | 1.72E-01 | -0.18 | 0.10 | 1.85E-01 | -0.21 | 0.13 | 4.77E-01 | -0.20 | 0.13 | 5.20E-01 | -0.15 | 0.14 | 4.85E-01 | -0.17 | 0.15 | 4.48E-01 |
| Triglycerides in small LDL | 0.19 | 0.09 | 1.59E-01 | 0.19 | 0.10 | 1.85E-01 | 0.25 | 0.13 | 3.94E-01 | 0.24 | 0.13 | 4.41E-01 | 0.11 | 0.14 | 6.25E-01 | 0.12 | 0.14 | 6.12E-01 |
| Total lipids in very large VLDL | 0.18 | 0.10 | 1.96E-01 | 0.19 | 0.10 | 1.88E-01 | 0.14 | 0.14 | 6.26E-01 | 0.15 | 0.14 | 6.15E-01 | 0.20 | 0.14 | 3.48E-01 | 0.22 | 0.15 | 3.24E-01 |
| Cholesteryl esters to total lipids ratio in medium LDL | 0.18 | 0.09 | 1.85E-01 | 0.18 | 0.09 | 1.94E-01 | 0.06 | 0.13 | 8.04E-01 | 0.05 | 0.13 | 8.34E-01 | 0.31 | 0.13 | 1.42E-01 | 0.34 | 0.14 | 1.21E-01 |
| Triglycerides in medium VLDL | 0.18 | 0.10 | 1.96E-01 | 0.18 | 0.10 | 1.96E-01 | 0.15 | 0.13 | 6.26E-01 | 0.15 | 0.14 | 6.15E-01 | 0.19 | 0.14 | 3.63E-01 | 0.20 | 0.14 | 3.39E-01 |
| Phospholipids to total lipids ratio in medium LDL | -0.17 | 0.09 | 1.80E-01 | -0.17 | 0.09 | 1.98E-01 | -0.12 | 0.12 | 6.32E-01 | -0.11 | 0.13 | 6.76E-01 | -0.25 | 0.12 | 2.00E-01 | -0.27 | 0.13 | 1.67E-01 |
| Phospholipids to total lipids ratio in medium HDL | 0.18 | 0.10 | 1.96E-01 | 0.19 | 0.10 | 2.00E-01 | 0.14 | 0.13 | 6.26E-01 | 0.14 | 0.14 | 6.22E-01 | 0.22 | 0.15 | 3.33E-01 | 0.26 | 0.16 | 2.92E-01 |
| Total lipids in large VLDL | 0.17 | 0.10 | 2.03E-01 | 0.18 | 0.10 | 2.02E-01 | 0.14 | 0.13 | 6.30E-01 | 0.14 | 0.14 | 6.15E-01 | 0.20 | 0.14 | 3.60E-01 | 0.21 | 0.14 | 3.39E-01 |
| Cholesteryl esters in chylomicrons and extremely large VLDL | 0.17 | 0.10 | 2.11E-01 | 0.18 | 0.10 | 2.02E-01 | 0.16 | 0.14 | 5.77E-01 | 0.17 | 0.14 | 5.91E-01 | 0.18 | 0.14 | 4.13E-01 | 0.19 | 0.15 | 4.03E-01 |
| Free cholesterol in small HDL | 0.21 | 0.11 | 1.65E-01 | 0.20 | 0.11 | 2.13E-01 | 0.37 | 0.15 | 2.40E-01 | 0.37 | 0.16 | 3.17E-01 | 0.04 | 0.15 | 8.88E-01 | 0.02 | 0.16 | 9.33E-01 |
| Triglycerides to total lipids ratio in IDL | 0.19 | 0.10 | 2.00E-01 | 0.18 | 0.11 | 2.44E-01 | 0.21 | 0.14 | 4.89E-01 | 0.20 | 0.14 | 5.51E-01 | 0.18 | 0.15 | 4.64E-01 | 0.20 | 0.16 | 4.15E-01 |
| Cholesterol in large HDL | -0.17 | 0.11 | 2.50E-01 | -0.19 | 0.11 | 2.45E-01 | -0.03 | 0.15 | 8.80E-01 | -0.05 | 0.15 | 8.48E-01 | -0.31 | 0.15 | 1.85E-01 | -0.34 | 0.16 | 1.61E-01 |
| Free cholesterol to total lipids ratio in very large HDL | 0.16 | 0.10 | 2.76E-01 | 0.18 | 0.11 | 2.51E-01 | -0.07 | 0.15 | 7.91E-01 | -0.06 | 0.15 | 8.43E-01 | 0.40 | 0.15 | 1.01E-01 | 0.43 | 0.15 | 8.32E-02 |
| Total concentration of branched-chain amino acids (leucine + isoleucine + valine) | 0.17 | 0.10 | 2.47E-01 | 0.18 | 0.10 | 2.52E-01 | 0.03 | 0.14 | 9.09E-01 | 0.03 | 0.14 | 9.04E-01 | 0.31 | 0.14 | 1.84E-01 | 0.32 | 0.15 | 1.63E-01 |
| Free cholesterol in large VLDL | 0.16 | 0.10 | 2.50E-01 | 0.16 | 0.10 | 2.52E-01 | 0.14 | 0.14 | 6.30E-01 | 0.14 | 0.14 | 6.15E-01 | 0.17 | 0.14 | 4.33E-01 | 0.17 | 0.14 | 4.27E-01 |
| Concentration of small HDL particles | 0.19 | 0.10 | 1.96E-01 | 0.18 | 0.10 | 2.52E-01 | 0.21 | 0.15 | 5.27E-01 | 0.22 | 0.15 | 5.47E-01 | 0.14 | 0.14 | 5.44E-01 | 0.13 | 0.15 | 5.98E-01 |
| Triglycerides in medium LDL | 0.18 | 0.10 | 1.96E-01 | 0.16 | 0.10 | 2.52E-01 | 0.31 | 0.13 | 2.40E-01 | 0.30 | 0.13 | 3.17E-01 | 0.02 | 0.14 | 9.17E-01 | 0.02 | 0.14 | 9.32E-01 |
| Phospholipids in large VLDL | 0.16 | 0.10 | 2.50E-01 | 0.16 | 0.10 | 2.58E-01 | 0.14 | 0.14 | 6.26E-01 | 0.15 | 0.14 | 6.15E-01 | 0.15 | 0.14 | 4.68E-01 | 0.16 | 0.14 | 4.48E-01 |
| Total lipids in VLDL | 0.16 | 0.10 | 2.47E-01 | 0.16 | 0.10 | 2.58E-01 | 0.17 | 0.13 | 5.44E-01 | 0.18 | 0.13 | 5.79E-01 | 0.13 | 0.14 | 5.60E-01 | 0.13 | 0.14 | 5.55E-01 |
| Leucine | 0.17 | 0.10 | 2.47E-01 | 0.18 | 0.11 | 2.61E-01 | 0.06 | 0.14 | 8.23E-01 | 0.06 | 0.14 | 8.22E-01 | 0.28 | 0.15 | 2.37E-01 | 0.29 | 0.16 | 2.34E-01 |
| Valine | 0.16 | 0.10 | 2.50E-01 | 0.17 | 0.10 | 2.66E-01 | 0.02 | 0.13 | 9.29E-01 | 0.03 | 0.14 | 9.19E-01 | 0.29 | 0.14 | 1.85E-01 | 0.29 | 0.15 | 1.86E-01 |
| Phospholipids in chylomicrons and extremely large VLDL | 0.16 | 0.10 | 2.58E-01 | 0.16 | 0.10 | 2.66E-01 | 0.03 | 0.13 | 8.72E-01 | 0.04 | 0.13 | 8.81E-01 | 0.28 | 0.14 | 2.16E-01 | 0.30 | 0.15 | 1.87E-01 |
| Phospholipids in very large VLDL | 0.16 | 0.10 | 2.62E-01 | 0.16 | 0.10 | 2.72E-01 | 0.14 | 0.14 | 6.30E-01 | 0.14 | 0.14 | 6.15E-01 | 0.16 | 0.14 | 4.81E-01 | 0.16 | 0.15 | 4.57E-01 |
| Free cholesterol in very large VLDL | 0.15 | 0.10 | 2.62E-01 | 0.16 | 0.10 | 2.74E-01 | 0.13 | 0.14 | 6.30E-01 | 0.14 | 0.14 | 6.15E-01 | 0.15 | 0.14 | 4.83E-01 | 0.16 | 0.14 | 4.57E-01 |
| Tyrosine | 0.16 | 0.09 | 2.30E-01 | 0.15 | 0.09 | 2.74E-01 | 0.16 | 0.13 | 5.44E-01 | 0.15 | 0.13 | 6.07E-01 | 0.13 | 0.14 | 5.45E-01 | 0.12 | 0.14 | 5.98E-01 |
| Triglycerides to total lipids ratio in medium LDL | 0.17 | 0.10 | 2.26E-01 | 0.16 | 0.10 | 2.74E-01 | 0.27 | 0.13 | 3.36E-01 | 0.25 | 0.13 | 4.61E-01 | 0.07 | 0.15 | 7.83E-01 | 0.08 | 0.15 | 7.46E-01 |
| Acetone | 0.15 | 0.09 | 2.50E-01 | 0.15 | 0.09 | 2.75E-01 | 0.20 | 0.13 | 4.77E-01 | 0.21 | 0.13 | 5.20E-01 | 0.13 | 0.13 | 5.59E-01 | 0.13 | 0.13 | 5.50E-01 |
| Free cholesterol to total lipids ratio in small HDL | -0.15 | 0.10 | 2.82E-01 | -0.16 | 0.10 | 2.83E-01 | 0.08 | 0.14 | 7.83E-01 | 0.08 | 0.14 | 7.85E-01 | -0.40 | 0.15 | 1.01E-01 | -0.42 | 0.15 | 9.57E-02 |
| Free cholesterol in IDL | -0.17 | 0.11 | 2.78E-01 | -0.18 | 0.11 | 2.83E-01 | -0.01 | 0.16 | 9.71E-01 | 0.00 | 0.16 | 9.89E-01 | -0.37 | 0.16 | 1.43E-01 | -0.39 | 0.16 | 1.18E-01 |
| Cholesteryl esters in medium VLDL | -0.17 | 0.10 | 2.50E-01 | -0.16 | 0.10 | 2.83E-01 | -0.07 | 0.14 | 7.91E-01 | -0.05 | 0.15 | 8.48E-01 | -0.29 | 0.14 | 2.01E-01 | -0.30 | 0.14 | 1.69E-01 |
| Free cholesterol in chylomicrons and extremely large VLDL | 0.15 | 0.10 | 2.76E-01 | 0.16 | 0.10 | 2.83E-01 | 0.07 | 0.13 | 7.91E-01 | 0.07 | 0.13 | 8.12E-01 | 0.23 | 0.14 | 3.07E-01 | 0.25 | 0.15 | 2.75E-01 |
| Average diameter for LDL particles | -0.16 | 0.09 | 2.50E-01 | -0.15 | 0.10 | 2.83E-01 | -0.15 | 0.13 | 5.89E-01 | -0.13 | 0.13 | 6.22E-01 | -0.17 | 0.14 | 4.25E-01 | -0.19 | 0.14 | 4.03E-01 |
| Concentration of chylomicrons and extremely large VLDL particles | 0.14 | 0.10 | 2.93E-01 | 0.15 | 0.10 | 3.05E-01 | 0.06 | 0.13 | 8.19E-01 | 0.06 | 0.13 | 8.34E-01 | 0.23 | 0.14 | 3.10E-01 | 0.25 | 0.15 | 2.79E-01 |
| Triglycerides in very small VLDL | 0.16 | 0.10 | 2.50E-01 | 0.15 | 0.10 | 3.05E-01 | 0.27 | 0.14 | 3.36E-01 | 0.27 | 0.14 | 4.18E-01 | 0.03 | 0.13 | 9.03E-01 | 0.03 | 0.14 | 9.10E-01 |
| Phospholipids to total lipids ratio in very large HDL | -0.13 | 0.09 | 3.02E-01 | -0.13 | 0.09 | 3.20E-01 | 0.05 | 0.16 | 8.68E-01 | 0.03 | 0.16 | 9.14E-01 | -0.23 | 0.10 | 1.71E-01 | -0.22 | 0.11 | 1.61E-01 |
| Isoleucine | 0.14 | 0.10 | 3.31E-01 | 0.16 | 0.11 | 3.20E-01 | -0.02 | 0.14 | 9.42E-01 | 0.00 | 0.15 | 9.88E-01 | 0.32 | 0.15 | 1.85E-01 | 0.34 | 0.16 | 1.61E-01 |
| Phosphoglycerides | 0.19 | 0.11 | 2.47E-01 | 0.17 | 0.12 | 3.20E-01 | 0.44 | 0.16 | 2.40E-01 | 0.43 | 0.17 | 3.17E-01 | -0.08 | 0.16 | 7.83E-01 | -0.11 | 0.16 | 7.11E-01 |
| Triglycerides in LDL | 0.16 | 0.10 | 2.50E-01 | 0.14 | 0.10 | 3.26E-01 | 0.32 | 0.13 | 2.40E-01 | 0.31 | 0.13 | 3.17E-01 | -0.03 | 0.14 | 9.03E-01 | -0.04 | 0.14 | 8.75E-01 |
| Cholesterol in large VLDL | 0.13 | 0.10 | 3.32E-01 | 0.14 | 0.10 | 3.27E-01 | 0.12 | 0.14 | 6.68E-01 | 0.13 | 0.14 | 6.22E-01 | 0.13 | 0.14 | 5.62E-01 | 0.13 | 0.14 | 5.55E-01 |
| Phosphatidylcholines | 0.19 | 0.11 | 2.50E-01 | 0.16 | 0.12 | 3.35E-01 | 0.43 | 0.16 | 2.40E-01 | 0.42 | 0.17 | 3.17E-01 | -0.08 | 0.16 | 7.83E-01 | -0.10 | 0.16 | 7.11E-01 |
| Phospholipids to total lipids ratio in small HDL | 0.16 | 0.10 | 2.50E-01 | 0.14 | 0.10 | 3.42E-01 | 0.25 | 0.13 | 4.07E-01 | 0.21 | 0.14 | 5.20E-01 | 0.07 | 0.14 | 7.83E-01 | 0.06 | 0.15 | 8.02E-01 |
| Cholesterol in small HDL | 0.16 | 0.10 | 2.68E-01 | 0.15 | 0.11 | 3.43E-01 | 0.18 | 0.15 | 5.81E-01 | 0.19 | 0.15 | 5.91E-01 | 0.12 | 0.15 | 6.39E-01 | 0.10 | 0.15 | 6.97E-01 |
| Cholesterol in very large VLDL | 0.13 | 0.10 | 3.47E-01 | 0.13 | 0.10 | 3.48E-01 | 0.13 | 0.14 | 6.53E-01 | 0.14 | 0.14 | 6.18E-01 | 0.11 | 0.13 | 6.30E-01 | 0.11 | 0.14 | 6.15E-01 |
| Triglycerides to total lipids ratio in large LDL | 0.16 | 0.10 | 2.76E-01 | 0.14 | 0.10 | 3.48E-01 | 0.23 | 0.14 | 4.67E-01 | 0.21 | 0.14 | 5.20E-01 | 0.08 | 0.15 | 7.83E-01 | 0.09 | 0.16 | 7.46E-01 |
| Free cholesterol to total lipids ratio in large HDL | -0.16 | 0.11 | 3.41E-01 | -0.16 | 0.12 | 3.49E-01 | 0.04 | 0.17 | 8.72E-01 | 0.04 | 0.18 | 9.12E-01 | -0.35 | 0.14 | 1.36E-01 | -0.36 | 0.15 | 1.21E-01 |
| Cholesteryl esters to total lipids ratio in small LDL | 0.13 | 0.10 | 3.47E-01 | 0.13 | 0.10 | 3.65E-01 | 0.09 | 0.13 | 7.52E-01 | 0.10 | 0.13 | 7.05E-01 | 0.17 | 0.14 | 4.41E-01 | 0.16 | 0.14 | 4.57E-01 |
| Free cholesterol in large HDL | -0.13 | 0.11 | 4.19E-01 | -0.15 | 0.11 | 3.69E-01 | 0.06 | 0.15 | 8.33E-01 | 0.04 | 0.16 | 8.82E-01 | -0.34 | 0.15 | 1.66E-01 | -0.37 | 0.16 | 1.34E-01 |
| Phospholipids in VLDL | 0.13 | 0.10 | 3.48E-01 | 0.13 | 0.10 | 3.74E-01 | 0.17 | 0.14 | 5.44E-01 | 0.18 | 0.14 | 5.79E-01 | 0.07 | 0.14 | 7.83E-01 | 0.06 | 0.14 | 7.89E-01 |
| Acetoacetate | 0.13 | 0.09 | 3.06E-01 | 0.12 | 0.09 | 3.74E-01 | 0.23 | 0.13 | 4.07E-01 | 0.23 | 0.13 | 4.71E-01 | 0.04 | 0.13 | 8.81E-01 | 0.03 | 0.13 | 8.98E-01 |
| Phospholipids to total lipids ratio in large LDL | -0.13 | 0.10 | 3.41E-01 | -0.13 | 0.10 | 3.75E-01 | -0.16 | 0.13 | 5.77E-01 | -0.16 | 0.13 | 5.91E-01 | -0.08 | 0.14 | 7.70E-01 | -0.09 | 0.14 | 7.11E-01 |
| Concentration of large HDL particles | -0.13 | 0.11 | 4.46E-01 | -0.15 | 0.11 | 3.89E-01 | 0.05 | 0.15 | 8.42E-01 | 0.04 | 0.16 | 8.94E-01 | -0.33 | 0.16 | 1.85E-01 | -0.37 | 0.17 | 1.61E-01 |
| Cholesterol to total lipids ratio in IDL | -0.13 | 0.10 | 3.48E-01 | -0.13 | 0.10 | 3.97E-01 | -0.16 | 0.13 | 5.44E-01 | -0.15 | 0.13 | 6.07E-01 | -0.11 | 0.15 | 6.69E-01 | -0.13 | 0.15 | 6.08E-01 |
| Concentration of small VLDL particles | 0.12 | 0.09 | 3.80E-01 | 0.12 | 0.10 | 3.99E-01 | 0.17 | 0.13 | 5.44E-01 | 0.18 | 0.13 | 5.75E-01 | 0.05 | 0.13 | 8.19E-01 | 0.05 | 0.14 | 8.13E-01 |
| Average diameter for HDL particles | -0.11 | 0.10 | 4.49E-01 | -0.13 | 0.10 | 4.02E-01 | 0.06 | 0.13 | 8.16E-01 | 0.04 | 0.14 | 8.71E-01 | -0.30 | 0.15 | 2.04E-01 | -0.32 | 0.16 | 1.78E-01 |
| Triglycerides in very large HDL | 0.14 | 0.10 | 3.41E-01 | 0.13 | 0.10 | 4.04E-01 | 0.32 | 0.14 | 2.40E-01 | 0.31 | 0.14 | 3.17E-01 | -0.06 | 0.14 | 8.14E-01 | -0.06 | 0.15 | 8.13E-01 |
| Total lipids in large HDL | -0.12 | 0.11 | 4.72E-01 | -0.14 | 0.11 | 4.14E-01 | 0.06 | 0.15 | 8.35E-01 | 0.04 | 0.15 | 8.85E-01 | -0.31 | 0.16 | 2.00E-01 | -0.35 | 0.16 | 1.63E-01 |
| Phospholipids in medium HDL | 0.16 | 0.11 | 3.02E-01 | 0.14 | 0.11 | 4.14E-01 | 0.30 | 0.15 | 3.36E-01 | 0.29 | 0.16 | 4.41E-01 | -0.02 | 0.16 | 9.49E-01 | -0.04 | 0.17 | 8.98E-01 |
| Free cholesterol to total lipids ratio in medium HDL | -0.12 | 0.11 | 4.82E-01 | -0.14 | 0.12 | 4.23E-01 | 0.11 | 0.16 | 7.52E-01 | 0.10 | 0.17 | 7.68E-01 | -0.38 | 0.16 | 1.43E-01 | -0.43 | 0.17 | 1.18E-01 |
| Triglycerides in large HDL | 0.14 | 0.11 | 3.41E-01 | 0.13 | 0.11 | 4.26E-01 | 0.32 | 0.15 | 2.97E-01 | 0.30 | 0.15 | 4.18E-01 | -0.03 | 0.14 | 9.07E-01 | -0.03 | 0.15 | 8.98E-01 |
| Total lipids in small VLDL | 0.11 | 0.09 | 4.19E-01 | 0.11 | 0.10 | 4.31E-01 | 0.18 | 0.14 | 5.44E-01 | 0.18 | 0.13 | 5.73E-01 | 0.04 | 0.13 | 8.81E-01 | 0.03 | 0.14 | 8.78E-01 |
| Triglycerides in large LDL | 0.13 | 0.10 | 3.41E-01 | 0.12 | 0.10 | 4.44E-01 | 0.33 | 0.14 | 2.40E-01 | 0.32 | 0.14 | 3.17E-01 | -0.08 | 0.14 | 7.62E-01 | -0.10 | 0.14 | 7.04E-01 |
| Cholesteryl esters in small HDL | 0.13 | 0.10 | 3.66E-01 | 0.12 | 0.10 | 4.46E-01 | 0.10 | 0.15 | 7.52E-01 | 0.11 | 0.15 | 7.17E-01 | 0.14 | 0.14 | 5.60E-01 | 0.12 | 0.15 | 6.13E-01 |
| Free cholesterol in VLDL | 0.11 | 0.10 | 4.46E-01 | 0.11 | 0.10 | 4.57E-01 | 0.16 | 0.14 | 5.89E-01 | 0.17 | 0.14 | 5.91E-01 | 0.04 | 0.14 | 8.74E-01 | 0.04 | 0.14 | 8.75E-01 |
| Total lipids in medium VLDL | 0.11 | 0.10 | 4.49E-01 | 0.11 | 0.10 | 4.60E-01 | 0.14 | 0.14 | 6.30E-01 | 0.15 | 0.14 | 6.15E-01 | 0.06 | 0.14 | 8.00E-01 | 0.06 | 0.14 | 8.02E-01 |
| Cholesteryl esters in large VLDL | 0.10 | 0.10 | 4.73E-01 | 0.11 | 0.10 | 4.60E-01 | 0.11 | 0.14 | 6.96E-01 | 0.12 | 0.14 | 6.54E-01 | 0.08 | 0.13 | 7.62E-01 | 0.08 | 0.14 | 7.31E-01 |
| Phospholipids to total lipids ratio in large VLDL | 0.10 | 0.09 | 4.73E-01 | 0.11 | 0.10 | 4.69E-01 | 0.12 | 0.14 | 6.92E-01 | 0.14 | 0.15 | 6.35E-01 | 0.08 | 0.12 | 7.45E-01 | 0.08 | 0.13 | 7.31E-01 |
| Cholesterol in IDL | -0.11 | 0.11 | 4.83E-01 | -0.12 | 0.11 | 4.76E-01 | 0.03 | 0.16 | 9.01E-01 | 0.04 | 0.16 | 8.82E-01 | -0.31 | 0.16 | 2.04E-01 | -0.33 | 0.16 | 1.63E-01 |
| Cholesteryl esters to total lipids ratio in chylomicrons and extremely large VLDL | -0.11 | 0.10 | 4.49E-01 | -0.11 | 0.10 | 4.76E-01 | -0.01 | 0.13 | 9.49E-01 | 0.00 | 0.13 | 9.88E-01 | -0.21 | 0.15 | 3.52E-01 | -0.21 | 0.15 | 3.39E-01 |
| Free cholesterol in small LDL | -0.11 | 0.10 | 4.62E-01 | -0.11 | 0.10 | 4.76E-01 | -0.08 | 0.14 | 7.83E-01 | -0.06 | 0.14 | 8.22E-01 | -0.17 | 0.14 | 4.36E-01 | -0.17 | 0.14 | 4.15E-01 |
| Cholesteryl esters in very large VLDL | 0.10 | 0.09 | 4.83E-01 | 0.10 | 0.10 | 4.76E-01 | 0.11 | 0.14 | 6.92E-01 | 0.13 | 0.14 | 6.35E-01 | 0.06 | 0.13 | 7.83E-01 | 0.06 | 0.14 | 7.87E-01 |
| Concentration of VLDL particles | 0.11 | 0.10 | 4.51E-01 | 0.11 | 0.10 | 4.76E-01 | 0.19 | 0.14 | 5.27E-01 | 0.19 | 0.14 | 5.47E-01 | 0.01 | 0.14 | 9.53E-01 | 0.01 | 0.14 | 9.73E-01 |
| Phospholipids to total lipids ratio in chylomicrons and extremely large VLDL | 0.10 | 0.09 | 4.87E-01 | 0.10 | 0.10 | 4.90E-01 | -0.02 | 0.12 | 9.35E-01 | -0.01 | 0.12 | 9.67E-01 | 0.21 | 0.14 | 3.34E-01 | 0.23 | 0.15 | 3.09E-01 |
| Creatinine | -0.13 | 0.12 | 4.82E-01 | -0.13 | 0.12 | 4.90E-01 | -0.27 | 0.16 | 4.77E-01 | -0.26 | 0.17 | 5.20E-01 | 0.09 | 0.22 | 8.00E-01 | 0.09 | 0.22 | 8.11E-01 |
| Lactate | 0.09 | 0.09 | 4.87E-01 | 0.10 | 0.09 | 5.00E-01 | -0.10 | 0.13 | 7.31E-01 | -0.10 | 0.14 | 7.05E-01 | 0.28 | 0.13 | 1.70E-01 | 0.28 | 0.13 | 1.61E-01 |
| Total cholines | 0.14 | 0.11 | 4.13E-01 | 0.12 | 0.11 | 5.00E-01 | 0.39 | 0.16 | 2.40E-01 | 0.38 | 0.16 | 3.17E-01 | -0.14 | 0.16 | 5.94E-01 | -0.16 | 0.16 | 5.32E-01 |
| Triglycerides in IDL | 0.12 | 0.10 | 4.19E-01 | 0.10 | 0.10 | 5.00E-01 | 0.32 | 0.14 | 2.40E-01 | 0.30 | 0.14 | 3.17E-01 | -0.10 | 0.14 | 7.00E-01 | -0.11 | 0.14 | 6.48E-01 |
| Ratio of saturated fatty acids to total fatty acids | 0.14 | 0.10 | 3.41E-01 | 0.11 | 0.10 | 5.00E-01 | 0.30 | 0.13 | 2.40E-01 | 0.27 | 0.14 | 4.11E-01 | -0.06 | 0.15 | 8.16E-01 | -0.08 | 0.15 | 7.59E-01 |
| Cholesterol to total lipids ratio in very large HDL | 0.09 | 0.10 | 5.37E-01 | 0.10 | 0.10 | 5.03E-01 | -0.19 | 0.15 | 5.44E-01 | -0.16 | 0.15 | 6.15E-01 | 0.33 | 0.13 | 1.23E-01 | 0.32 | 0.13 | 1.18E-01 |
| Cholesterol to total lipids ratio in chylomicrons and extremely large VLDL | -0.10 | 0.09 | 4.73E-01 | -0.10 | 0.10 | 5.03E-01 | -0.02 | 0.13 | 9.23E-01 | -0.01 | 0.13 | 9.86E-01 | -0.18 | 0.14 | 4.10E-01 | -0.18 | 0.14 | 4.12E-01 |
| Total lipids in lipoprotein particles | 0.12 | 0.11 | 4.46E-01 | 0.11 | 0.11 | 5.03E-01 | 0.29 | 0.15 | 3.83E-01 | 0.29 | 0.15 | 4.29E-01 | -0.08 | 0.15 | 7.83E-01 | -0.09 | 0.15 | 7.25E-01 |
| Ratio of docosahexaenoic acid to total fatty acids | -0.08 | 0.10 | 6.10E-01 | -0.10 | 0.10 | 5.03E-01 | -0.09 | 0.14 | 7.52E-01 | -0.12 | 0.14 | 6.54E-01 | -0.07 | 0.14 | 7.95E-01 | -0.08 | 0.15 | 7.61E-01 |
| Polyunsaturated fatty acids | 0.11 | 0.10 | 4.82E-01 | 0.10 | 0.10 | 5.10E-01 | 0.21 | 0.15 | 5.12E-01 | 0.22 | 0.15 | 5.20E-01 | -0.03 | 0.15 | 8.96E-01 | -0.04 | 0.15 | 8.75E-01 |
| Free cholesterol in LDL | -0.10 | 0.10 | 5.21E-01 | -0.10 | 0.11 | 5.34E-01 | -0.04 | 0.15 | 8.72E-01 | -0.02 | 0.15 | 9.36E-01 | -0.20 | 0.15 | 3.90E-01 | -0.21 | 0.15 | 3.39E-01 |
| Cholesteryl esters in very small VLDL | -0.10 | 0.11 | 5.42E-01 | -0.10 | 0.11 | 5.37E-01 | 0.12 | 0.15 | 7.18E-01 | 0.12 | 0.15 | 6.97E-01 | -0.35 | 0.15 | 1.66E-01 | -0.36 | 0.16 | 1.34E-01 |
| Phospholipids in IDL | -0.10 | 0.11 | 5.45E-01 | -0.10 | 0.11 | 5.37E-01 | 0.09 | 0.15 | 7.83E-01 | 0.10 | 0.16 | 7.57E-01 | -0.33 | 0.16 | 1.85E-01 | -0.35 | 0.16 | 1.61E-01 |
| Phospholipids in large HDL | -0.09 | 0.11 | 6.12E-01 | -0.11 | 0.11 | 5.37E-01 | 0.09 | 0.15 | 7.59E-01 | 0.07 | 0.15 | 8.13E-01 | -0.29 | 0.16 | 2.44E-01 | -0.32 | 0.16 | 1.91E-01 |
| Cholesteryl esters in IDL | -0.10 | 0.11 | 5.63E-01 | -0.10 | 0.11 | 5.37E-01 | 0.04 | 0.15 | 8.72E-01 | 0.05 | 0.16 | 8.48E-01 | -0.28 | 0.16 | 2.50E-01 | -0.31 | 0.16 | 1.91E-01 |
| Free cholesterol in large LDL | -0.10 | 0.11 | 5.38E-01 | -0.10 | 0.11 | 5.37E-01 | -0.01 | 0.15 | 9.71E-01 | 0.01 | 0.15 | 9.84E-01 | -0.23 | 0.15 | 3.34E-01 | -0.25 | 0.16 | 2.92E-01 |
| Total lipids in medium HDL | 0.13 | 0.11 | 4.46E-01 | 0.11 | 0.11 | 5.37E-01 | 0.27 | 0.15 | 4.27E-01 | 0.26 | 0.16 | 5.20E-01 | -0.04 | 0.16 | 8.81E-01 | -0.07 | 0.17 | 8.13E-01 |
| Acetate | 0.10 | 0.10 | 4.87E-01 | 0.09 | 0.10 | 5.37E-01 | 0.17 | 0.13 | 5.42E-01 | 0.17 | 0.13 | 5.79E-01 | -0.01 | 0.14 | 9.65E-01 | -0.01 | 0.15 | 9.47E-01 |
| Free cholesterol to total lipids ratio in chylomicrons and extremely large VLDL | -0.09 | 0.09 | 5.08E-01 | -0.08 | 0.09 | 5.55E-01 | -0.09 | 0.13 | 7.52E-01 | -0.07 | 0.13 | 7.85E-01 | -0.09 | 0.13 | 7.04E-01 | -0.09 | 0.13 | 6.97E-01 |
| Triglycerides to total lipids ratio in large VLDL | 0.09 | 0.09 | 4.87E-01 | 0.08 | 0.09 | 5.57E-01 | 0.02 | 0.13 | 9.35E-01 | -0.01 | 0.13 | 9.88E-01 | 0.16 | 0.12 | 3.97E-01 | 0.16 | 0.12 | 3.92E-01 |
| Cholesterol to total lipids ratio in large LDL | -0.10 | 0.10 | 4.94E-01 | -0.09 | 0.10 | 5.57E-01 | -0.18 | 0.13 | 5.27E-01 | -0.16 | 0.14 | 5.91E-01 | -0.02 | 0.15 | 9.39E-01 | -0.02 | 0.15 | 9.33E-01 |
| Cholesteryl esters to total lipids ratio in large LDL | 0.08 | 0.10 | 5.88E-01 | 0.09 | 0.10 | 5.64E-01 | -0.04 | 0.14 | 8.72E-01 | -0.02 | 0.14 | 9.36E-01 | 0.19 | 0.15 | 4.14E-01 | 0.19 | 0.15 | 4.08E-01 |
| Omega-6 fatty acids | 0.09 | 0.10 | 5.67E-01 | 0.09 | 0.10 | 5.64E-01 | 0.20 | 0.15 | 5.27E-01 | 0.22 | 0.15 | 5.20E-01 | -0.06 | 0.15 | 8.00E-01 | -0.07 | 0.15 | 7.90E-01 |
| Concentration of HDL particles | 0.11 | 0.11 | 4.82E-01 | 0.10 | 0.11 | 5.75E-01 | 0.25 | 0.15 | 4.77E-01 | 0.25 | 0.16 | 5.20E-01 | -0.05 | 0.16 | 8.74E-01 | -0.07 | 0.17 | 8.02E-01 |
| Total lipids in IDL | -0.09 | 0.11 | 6.15E-01 | -0.09 | 0.11 | 5.81E-01 | 0.09 | 0.16 | 7.74E-01 | 0.10 | 0.16 | 7.48E-01 | -0.31 | 0.16 | 2.01E-01 | -0.33 | 0.16 | 1.63E-01 |
| Total phospholipids in lipoprotein particles | 0.11 | 0.11 | 4.87E-01 | 0.09 | 0.11 | 6.03E-01 | 0.36 | 0.16 | 2.40E-01 | 0.36 | 0.16 | 3.17E-01 | -0.17 | 0.16 | 4.95E-01 | -0.20 | 0.16 | 4.27E-01 |
| Concentration of medium VLDL particles | 0.08 | 0.10 | 6.11E-01 | 0.08 | 0.10 | 6.04E-01 | 0.12 | 0.14 | 6.92E-01 | 0.13 | 0.14 | 6.49E-01 | 0.01 | 0.14 | 9.49E-01 | 0.01 | 0.14 | 9.50E-01 |
| Total concentration of lipoprotein particles | 0.11 | 0.11 | 4.98E-01 | 0.09 | 0.11 | 6.14E-01 | 0.26 | 0.16 | 4.77E-01 | 0.27 | 0.16 | 5.20E-01 | -0.08 | 0.16 | 7.83E-01 | -0.10 | 0.17 | 7.11E-01 |
| Concentration of IDL particles | -0.07 | 0.10 | 6.75E-01 | -0.08 | 0.10 | 6.61E-01 | 0.10 | 0.15 | 7.53E-01 | 0.10 | 0.15 | 7.38E-01 | -0.27 | 0.15 | 2.37E-01 | -0.29 | 0.15 | 1.91E-01 |
| Phospholipids in medium VLDL | 0.07 | 0.10 | 6.67E-01 | 0.07 | 0.10 | 6.71E-01 | 0.13 | 0.14 | 6.74E-01 | 0.14 | 0.14 | 6.22E-01 | -0.01 | 0.14 | 9.49E-01 | -0.01 | 0.14 | 9.45E-01 |
| Concentration of medium HDL particles | 0.10 | 0.11 | 5.38E-01 | 0.08 | 0.12 | 6.71E-01 | 0.24 | 0.15 | 4.77E-01 | 0.24 | 0.16 | 5.22E-01 | -0.07 | 0.16 | 8.00E-01 | -0.09 | 0.17 | 7.48E-01 |
| Apolipoprotein A1 | 0.10 | 0.11 | 5.42E-01 | 0.08 | 0.11 | 6.77E-01 | 0.26 | 0.15 | 4.27E-01 | 0.25 | 0.15 | 5.20E-01 | -0.09 | 0.16 | 7.57E-01 | -0.12 | 0.17 | 6.82E-01 |
| Albumin | -0.06 | 0.09 | 6.75E-01 | -0.07 | 0.10 | 6.77E-01 | -0.05 | 0.14 | 8.33E-01 | -0.04 | 0.14 | 8.65E-01 | -0.09 | 0.13 | 7.07E-01 | -0.10 | 0.14 | 6.97E-01 |
| Cholesteryl esters in medium LDL | 0.07 | 0.10 | 6.44E-01 | 0.07 | 0.10 | 6.77E-01 | 0.10 | 0.14 | 7.50E-01 | 0.11 | 0.14 | 6.98E-01 | 0.02 | 0.14 | 9.17E-01 | 0.02 | 0.14 | 9.45E-01 |
| VLDL cholesterol | 0.06 | 0.10 | 6.80E-01 | 0.07 | 0.10 | 6.94E-01 | 0.14 | 0.14 | 6.26E-01 | 0.16 | 0.14 | 6.15E-01 | -0.04 | 0.14 | 8.81E-01 | -0.04 | 0.14 | 8.55E-01 |
| Cholesterol in very small VLDL | -0.07 | 0.11 | 7.12E-01 | -0.07 | 0.11 | 7.01E-01 | 0.15 | 0.15 | 6.30E-01 | 0.16 | 0.15 | 6.15E-01 | -0.31 | 0.15 | 1.85E-01 | -0.33 | 0.15 | 1.61E-01 |
| Cholesteryl esters in HDL | -0.05 | 0.10 | 7.97E-01 | -0.07 | 0.11 | 7.01E-01 | 0.08 | 0.14 | 7.83E-01 | 0.07 | 0.15 | 8.22E-01 | -0.20 | 0.15 | 4.10E-01 | -0.24 | 0.17 | 3.39E-01 |
| Concentration of large LDL particles | -0.07 | 0.10 | 6.71E-01 | -0.06 | 0.10 | 7.01E-01 | 0.00 | 0.14 | 9.92E-01 | 0.03 | 0.14 | 9.14E-01 | -0.17 | 0.14 | 4.30E-01 | -0.18 | 0.14 | 4.12E-01 |
| Free cholesterol in medium LDL | -0.06 | 0.10 | 7.04E-01 | -0.06 | 0.10 | 7.10E-01 | -0.02 | 0.14 | 9.35E-01 | 0.00 | 0.15 | 9.95E-01 | -0.14 | 0.14 | 5.44E-01 | -0.15 | 0.14 | 4.97E-01 |
| Histidine | -0.05 | 0.09 | 7.54E-01 | -0.06 | 0.09 | 7.20E-01 | -0.19 | 0.13 | 5.06E-01 | -0.18 | 0.13 | 5.47E-01 | 0.10 | 0.14 | 7.04E-01 | 0.10 | 0.14 | 6.97E-01 |
| Glycoprotein acetyls | 0.06 | 0.10 | 7.45E-01 | 0.06 | 0.10 | 7.27E-01 | 0.09 | 0.14 | 7.70E-01 | 0.10 | 0.14 | 7.20E-01 | 0.02 | 0.14 | 9.23E-01 | 0.01 | 0.15 | 9.48E-01 |
| HDL cholesterol | -0.04 | 0.11 | 8.30E-01 | -0.06 | 0.11 | 7.45E-01 | 0.11 | 0.15 | 7.18E-01 | 0.10 | 0.15 | 7.48E-01 | -0.22 | 0.16 | 3.74E-01 | -0.26 | 0.17 | 3.14E-01 |
| Clinical LDL cholesterol | -0.06 | 0.10 | 7.53E-01 | -0.06 | 0.10 | 7.45E-01 | 0.02 | 0.15 | 9.42E-01 | 0.03 | 0.15 | 9.02E-01 | -0.16 | 0.15 | 4.75E-01 | -0.18 | 0.15 | 4.27E-01 |
| Phospholipids to total lipids ratio in IDL | -0.07 | 0.10 | 6.71E-01 | -0.05 | 0.10 | 7.45E-01 | -0.05 | 0.13 | 8.33E-01 | -0.05 | 0.14 | 8.48E-01 | -0.05 | 0.14 | 8.49E-01 | -0.04 | 0.14 | 8.75E-01 |
| Sphingomyelins | -0.04 | 0.11 | 8.22E-01 | -0.06 | 0.11 | 7.57E-01 | 0.17 | 0.16 | 6.26E-01 | 0.17 | 0.16 | 6.15E-01 | -0.28 | 0.16 | 2.50E-01 | -0.31 | 0.16 | 1.91E-01 |
| Phospholipids in small VLDL | 0.05 | 0.10 | 7.58E-01 | 0.05 | 0.10 | 7.68E-01 | 0.14 | 0.14 | 6.30E-01 | 0.15 | 0.14 | 6.15E-01 | -0.05 | 0.13 | 8.16E-01 | -0.06 | 0.14 | 8.02E-01 |
| Cholesterol in medium VLDL | -0.06 | 0.10 | 7.45E-01 | -0.05 | 0.10 | 7.69E-01 | 0.04 | 0.14 | 8.72E-01 | 0.06 | 0.15 | 8.34E-01 | -0.18 | 0.14 | 4.15E-01 | -0.18 | 0.14 | 4.03E-01 |
| Ratio of apolipoprotein B to apolipoprotein A1 | -0.06 | 0.10 | 6.85E-01 | -0.05 | 0.10 | 7.69E-01 | -0.08 | 0.13 | 7.83E-01 | -0.05 | 0.14 | 8.34E-01 | -0.06 | 0.14 | 7.88E-01 | -0.07 | 0.14 | 7.87E-01 |
| Ratio of omega-3 fatty acids to total fatty acids | -0.03 | 0.10 | 8.98E-01 | -0.05 | 0.10 | 7.73E-01 | -0.05 | 0.14 | 8.42E-01 | -0.09 | 0.14 | 7.54E-01 | -0.02 | 0.14 | 9.18E-01 | -0.04 | 0.14 | 8.75E-01 |
| Phospholipids in HDL | 0.08 | 0.11 | 6.44E-01 | 0.06 | 0.11 | 7.76E-01 | 0.26 | 0.15 | 4.08E-01 | 0.25 | 0.15 | 5.20E-01 | -0.14 | 0.16 | 5.94E-01 | -0.17 | 0.17 | 5.24E-01 |
| Triglycerides to total lipids ratio in chylomicrons and extremely large VLDL | -0.04 | 0.08 | 7.97E-01 | -0.04 | 0.08 | 7.78E-01 | -0.06 | 0.11 | 7.83E-01 | -0.07 | 0.11 | 7.63E-01 | -0.02 | 0.12 | 9.39E-01 | -0.02 | 0.13 | 9.33E-01 |
| Phospholipids in large LDL | -0.05 | 0.10 | 7.99E-01 | -0.05 | 0.11 | 7.89E-01 | 0.05 | 0.15 | 8.62E-01 | 0.07 | 0.15 | 8.22E-01 | -0.18 | 0.15 | 4.33E-01 | -0.19 | 0.15 | 4.03E-01 |
| Linoleic acid | 0.04 | 0.10 | 8.35E-01 | 0.05 | 0.10 | 7.97E-01 | 0.12 | 0.15 | 6.93E-01 | 0.15 | 0.15 | 6.15E-01 | -0.08 | 0.15 | 7.83E-01 | -0.08 | 0.15 | 7.61E-01 |
| Concentration of medium LDL particles | 0.05 | 0.10 | 7.74E-01 | 0.05 | 0.10 | 7.97E-01 | 0.11 | 0.14 | 6.96E-01 | 0.12 | 0.14 | 6.68E-01 | -0.03 | 0.14 | 8.99E-01 | -0.04 | 0.14 | 8.75E-01 |
| Phospholipids in small LDL | -0.04 | 0.10 | 8.05E-01 | -0.05 | 0.10 | 7.99E-01 | 0.03 | 0.14 | 8.93E-01 | 0.04 | 0.14 | 8.65E-01 | -0.15 | 0.14 | 5.05E-01 | -0.15 | 0.14 | 4.79E-01 |
| Free cholesterol in medium HDL | 0.08 | 0.11 | 6.78E-01 | 0.05 | 0.12 | 8.00E-01 | 0.25 | 0.16 | 4.77E-01 | 0.24 | 0.16 | 5.22E-01 | -0.13 | 0.16 | 6.38E-01 | -0.16 | 0.17 | 5.54E-01 |
| Free cholesterol in medium VLDL | 0.04 | 0.10 | 8.22E-01 | 0.04 | 0.10 | 8.22E-01 | 0.11 | 0.14 | 7.08E-01 | 0.12 | 0.14 | 6.61E-01 | -0.05 | 0.14 | 8.16E-01 | -0.06 | 0.14 | 8.02E-01 |
| Total lipids in medium LDL | 0.05 | 0.10 | 7.97E-01 | 0.04 | 0.10 | 8.22E-01 | 0.09 | 0.14 | 7.52E-01 | 0.11 | 0.15 | 7.10E-01 | -0.03 | 0.14 | 9.07E-01 | -0.04 | 0.14 | 8.75E-01 |
| Cholesterol in large LDL | -0.04 | 0.10 | 8.34E-01 | -0.04 | 0.11 | 8.34E-01 | 0.04 | 0.15 | 8.72E-01 | 0.06 | 0.15 | 8.35E-01 | -0.16 | 0.15 | 5.09E-01 | -0.17 | 0.15 | 4.57E-01 |
| Cholesterol to total lipids ratio in small LDL | -0.05 | 0.10 | 7.74E-01 | -0.04 | 0.10 | 8.34E-01 | -0.12 | 0.14 | 6.92E-01 | -0.09 | 0.14 | 7.57E-01 | 0.01 | 0.15 | 9.65E-01 | -0.01 | 0.15 | 9.68E-01 |
| Cholesterol in medium LDL | 0.04 | 0.10 | 8.16E-01 | 0.04 | 0.10 | 8.42E-01 | 0.08 | 0.14 | 7.87E-01 | 0.09 | 0.15 | 7.57E-01 | -0.02 | 0.14 | 9.40E-01 | -0.03 | 0.14 | 9.10E-01 |
| Total esterified cholesterol | -0.03 | 0.11 | 8.98E-01 | -0.04 | 0.11 | 8.55E-01 | 0.14 | 0.16 | 6.85E-01 | 0.15 | 0.16 | 6.49E-01 | -0.23 | 0.16 | 3.34E-01 | -0.26 | 0.16 | 2.92E-01 |
| Free cholesterol in HDL | -0.01 | 0.11 | 9.79E-01 | -0.03 | 0.11 | 9.04E-01 | 0.21 | 0.15 | 5.15E-01 | 0.20 | 0.15 | 5.79E-01 | -0.26 | 0.16 | 2.99E-01 | -0.31 | 0.17 | 2.34E-01 |
| Total cholesterol | -0.02 | 0.11 | 9.31E-01 | -0.03 | 0.11 | 9.04E-01 | 0.15 | 0.16 | 6.56E-01 | 0.16 | 0.16 | 6.22E-01 | -0.23 | 0.16 | 3.45E-01 | -0.25 | 0.16 | 2.96E-01 |
| Total lipids in large LDL | -0.03 | 0.10 | 9.02E-01 | -0.03 | 0.11 | 9.04E-01 | 0.07 | 0.15 | 8.04E-01 | 0.09 | 0.15 | 7.67E-01 | -0.16 | 0.15 | 4.92E-01 | -0.17 | 0.15 | 4.44E-01 |
| Phospholipids in LDL | -0.03 | 0.10 | 9.02E-01 | -0.03 | 0.10 | 9.04E-01 | 0.06 | 0.15 | 8.42E-01 | 0.07 | 0.15 | 8.12E-01 | -0.14 | 0.14 | 5.45E-01 | -0.15 | 0.14 | 4.97E-01 |
| Triglycerides in chylomicrons and extremely large VLDL | 0.03 | 0.09 | 8.74E-01 | 0.03 | 0.09 | 9.04E-01 | -0.03 | 0.12 | 8.72E-01 | -0.04 | 0.12 | 8.48E-01 | 0.10 | 0.14 | 7.04E-01 | 0.10 | 0.15 | 6.97E-01 |
| Omega-3 fatty acids | 0.06 | 0.10 | 7.47E-01 | 0.03 | 0.10 | 9.04E-01 | 0.07 | 0.14 | 8.03E-01 | 0.04 | 0.15 | 8.82E-01 | 0.01 | 0.14 | 9.49E-01 | 0.00 | 0.14 | 9.81E-01 |
| Concentration of LDL particles | -0.03 | 0.10 | 8.85E-01 | -0.03 | 0.10 | 9.08E-01 | 0.04 | 0.14 | 8.66E-01 | 0.06 | 0.14 | 8.22E-01 | -0.13 | 0.14 | 5.60E-01 | -0.14 | 0.14 | 5.38E-01 |
| Cholesterol in medium HDL | 0.05 | 0.11 | 7.99E-01 | 0.03 | 0.11 | 9.11E-01 | 0.16 | 0.15 | 6.26E-01 | 0.15 | 0.16 | 6.22E-01 | -0.09 | 0.16 | 7.70E-01 | -0.12 | 0.17 | 6.97E-01 |
| Cholesteryl esters in small VLDL | 0.02 | 0.09 | 9.07E-01 | 0.02 | 0.09 | 9.11E-01 | 0.11 | 0.14 | 6.96E-01 | 0.12 | 0.14 | 6.49E-01 | -0.08 | 0.13 | 7.62E-01 | -0.08 | 0.13 | 7.11E-01 |
| Citrate | -0.05 | 0.10 | 7.58E-01 | -0.03 | 0.10 | 9.11E-01 | -0.09 | 0.14 | 7.52E-01 | -0.05 | 0.14 | 8.35E-01 | 0.01 | 0.14 | 9.62E-01 | 0.01 | 0.14 | 9.48E-01 |
| Free cholesterol to total lipids ratio in large VLDL | -0.03 | 0.09 | 8.82E-01 | -0.02 | 0.09 | 9.19E-01 | -0.04 | 0.13 | 8.72E-01 | -0.02 | 0.13 | 9.29E-01 | -0.03 | 0.12 | 9.03E-01 | -0.03 | 0.12 | 8.75E-01 |
| Cholesteryl esters in VLDL | 0.02 | 0.10 | 9.22E-01 | 0.02 | 0.10 | 9.25E-01 | 0.12 | 0.14 | 6.74E-01 | 0.14 | 0.14 | 6.22E-01 | -0.10 | 0.14 | 6.66E-01 | -0.11 | 0.14 | 6.28E-01 |
| Cholesterol in small VLDL | 0.02 | 0.10 | 9.31E-01 | 0.02 | 0.10 | 9.32E-01 | 0.11 | 0.14 | 6.96E-01 | 0.13 | 0.14 | 6.49E-01 | -0.09 | 0.13 | 7.13E-01 | -0.09 | 0.13 | 6.97E-01 |
| Cholesteryl esters in medium HDL | 0.04 | 0.11 | 8.22E-01 | 0.02 | 0.11 | 9.32E-01 | 0.14 | 0.15 | 6.40E-01 | 0.13 | 0.15 | 6.68E-01 | -0.08 | 0.16 | 7.83E-01 | -0.11 | 0.17 | 7.11E-01 |
| Cholesteryl esters in small LDL | 0.02 | 0.10 | 9.13E-01 | 0.02 | 0.10 | 9.32E-01 | 0.08 | 0.14 | 7.83E-01 | 0.09 | 0.14 | 7.48E-01 | -0.06 | 0.14 | 8.07E-01 | -0.07 | 0.14 | 7.87E-01 |
| Total lipids in HDL | 0.04 | 0.11 | 8.22E-01 | 0.02 | 0.11 | 9.42E-01 | 0.22 | 0.15 | 4.77E-01 | 0.21 | 0.15 | 5.47E-01 | -0.17 | 0.16 | 4.95E-01 | -0.20 | 0.17 | 4.27E-01 |
| Cholesteryl esters in large LDL | -0.02 | 0.10 | 9.43E-01 | -0.02 | 0.10 | 9.42E-01 | 0.06 | 0.15 | 8.29E-01 | 0.08 | 0.15 | 7.86E-01 | -0.13 | 0.15 | 5.91E-01 | -0.14 | 0.15 | 5.50E-01 |
| Docosahexaenoic acid | 0.05 | 0.10 | 7.99E-01 | 0.02 | 0.10 | 9.42E-01 | 0.08 | 0.14 | 7.83E-01 | 0.06 | 0.15 | 8.34E-01 | -0.01 | 0.14 | 9.49E-01 | -0.03 | 0.15 | 9.10E-01 |
| Apolipoprotein B | -0.02 | 0.10 | 9.39E-01 | -0.02 | 0.10 | 9.44E-01 | 0.08 | 0.14 | 7.83E-01 | 0.10 | 0.14 | 7.46E-01 | -0.14 | 0.14 | 5.23E-01 | -0.15 | 0.14 | 4.84E-01 |
| 3-Hydroxybutyrate | 0.02 | 0.09 | 9.37E-01 | 0.01 | 0.09 | 9.50E-01 | 0.08 | 0.13 | 7.52E-01 | 0.10 | 0.13 | 7.17E-01 | -0.04 | 0.13 | 8.67E-01 | -0.04 | 0.13 | 8.45E-01 |
| LDL cholesterol | -0.01 | 0.10 | 9.64E-01 | -0.01 | 0.10 | 9.56E-01 | 0.05 | 0.15 | 8.42E-01 | 0.07 | 0.15 | 8.13E-01 | -0.11 | 0.14 | 6.66E-01 | -0.12 | 0.14 | 6.13E-01 |
| Phospholipids to total lipids ratio in very large VLDL | -0.02 | 0.08 | 8.98E-01 | -0.01 | 0.09 | 9.56E-01 | 0.00 | 0.13 | 9.92E-01 | 0.02 | 0.14 | 9.30E-01 | -0.05 | 0.11 | 7.88E-01 | -0.05 | 0.11 | 7.87E-01 |
| Cholesteryl esters to total lipids ratio in IDL | -0.02 | 0.10 | 9.43E-01 | -0.02 | 0.10 | 9.56E-01 | -0.08 | 0.14 | 7.83E-01 | -0.07 | 0.14 | 8.05E-01 | 0.02 | 0.15 | 9.39E-01 | 0.01 | 0.15 | 9.75E-01 |
| Phospholipids in medium LDL | 0.02 | 0.10 | 9.43E-01 | 0.01 | 0.10 | 9.60E-01 | 0.07 | 0.14 | 8.04E-01 | 0.09 | 0.14 | 7.65E-01 | -0.07 | 0.14 | 7.83E-01 | -0.08 | 0.14 | 7.48E-01 |
| Free cholesterol in very small VLDL | 0.00 | 0.10 | 9.79E-01 | -0.01 | 0.10 | 9.60E-01 | 0.20 | 0.14 | 5.27E-01 | 0.20 | 0.14 | 5.47E-01 | -0.23 | 0.14 | 3.10E-01 | -0.24 | 0.14 | 2.75E-01 |
| Total free cholesterol | 0.00 | 0.11 | 9.85E-01 | -0.01 | 0.11 | 9.60E-01 | 0.16 | 0.15 | 6.26E-01 | 0.17 | 0.15 | 6.15E-01 | -0.21 | 0.15 | 3.87E-01 | -0.22 | 0.15 | 3.39E-01 |
| Cholesteryl esters to total lipids ratio in very large HDL | 0.01 | 0.10 | 9.79E-01 | 0.01 | 0.10 | 9.60E-01 | -0.19 | 0.14 | 5.42E-01 | -0.15 | 0.14 | 6.15E-01 | 0.18 | 0.13 | 3.97E-01 | 0.17 | 0.14 | 4.08E-01 |
| Free cholesterol in small VLDL | 0.01 | 0.10 | 9.70E-01 | 0.01 | 0.10 | 9.60E-01 | 0.11 | 0.14 | 7.18E-01 | 0.12 | 0.14 | 6.62E-01 | -0.11 | 0.14 | 6.41E-01 | -0.11 | 0.14 | 6.13E-01 |
| Cholesterol in small LDL | -0.01 | 0.10 | 9.75E-01 | -0.01 | 0.10 | 9.60E-01 | 0.04 | 0.14 | 8.72E-01 | 0.06 | 0.14 | 8.34E-01 | -0.09 | 0.14 | 7.48E-01 | -0.09 | 0.14 | 6.98E-01 |
| Cholesteryl esters in LDL | 0.02 | 0.10 | 9.43E-01 | 0.01 | 0.10 | 9.60E-01 | 0.08 | 0.15 | 7.83E-01 | 0.10 | 0.15 | 7.48E-01 | -0.08 | 0.14 | 7.78E-01 | -0.09 | 0.14 | 7.11E-01 |
| Ratio of omega-6 fatty acids to omega-3 fatty acids | -0.04 | 0.10 | 8.22E-01 | -0.01 | 0.10 | 9.60E-01 | -0.02 | 0.14 | 9.42E-01 | 0.03 | 0.15 | 9.20E-01 | -0.04 | 0.14 | 8.81E-01 | -0.02 | 0.15 | 9.29E-01 |
| Phospholipids in very small VLDL | 0.01 | 0.10 | 9.45E-01 | 0.01 | 0.10 | 9.68E-01 | 0.21 | 0.14 | 4.77E-01 | 0.21 | 0.14 | 5.20E-01 | -0.20 | 0.14 | 3.52E-01 | -0.22 | 0.14 | 3.16E-01 |
| Total lipids in very small VLDL | 0.01 | 0.10 | 9.45E-01 | 0.01 | 0.10 | 9.75E-01 | 0.22 | 0.14 | 4.77E-01 | 0.22 | 0.14 | 5.20E-01 | -0.22 | 0.14 | 3.34E-01 | -0.23 | 0.14 | 2.96E-01 |
| Concentration of small LDL particles | -0.01 | 0.10 | 9.79E-01 | -0.01 | 0.10 | 9.78E-01 | 0.08 | 0.14 | 7.74E-01 | 0.10 | 0.14 | 7.31E-01 | -0.13 | 0.14 | 5.79E-01 | -0.13 | 0.14 | 5.59E-01 |
| Total cholesterol minus HDL-C | 0.00 | 0.10 | 9.95E-01 | 0.00 | 0.10 | 9.82E-01 | 0.10 | 0.15 | 7.51E-01 | 0.12 | 0.15 | 6.95E-01 | -0.14 | 0.15 | 5.59E-01 | -0.15 | 0.15 | 5.10E-01 |
| Total lipids in LDL | 0.00 | 0.10 | 9.97E-01 | 0.00 | 0.10 | 9.82E-01 | 0.08 | 0.15 | 7.83E-01 | 0.10 | 0.15 | 7.48E-01 | -0.11 | 0.14 | 6.41E-01 | -0.12 | 0.14 | 5.98E-01 |
| Concentration of very small VLDL particles | 0.00 | 0.10 | 9.79E-01 | 0.00 | 0.10 | 9.90E-01 | 0.22 | 0.14 | 4.77E-01 | 0.22 | 0.14 | 5.20E-01 | -0.23 | 0.14 | 3.14E-01 | -0.24 | 0.15 | 2.79E-01 |
| Remnant cholesterol (non-HDL | 0.01 | 0.10 | 9.79E-01 | 0.00 | 0.10 | 9.90E-01 | 0.15 | 0.15 | 6.30E-01 | 0.16 | 0.15 | 6.15E-01 | -0.17 | 0.15 | 4.68E-01 | -0.18 | 0.15 | 4.27E-01 |
| Total lipids in small LDL | 0.00 | 0.10 | 9.97E-01 | 0.00 | 0.10 | 9.90E-01 | 0.06 | 0.14 | 8.16E-01 | 0.08 | 0.14 | 7.85E-01 | -0.09 | 0.14 | 7.20E-01 | -0.10 | 0.14 | 6.97E-01 |
| Phospholipids to total lipids ratio in very small VLDL | -0.01 | 0.10 | 9.79E-01 | 0.00 | 0.10 | 9.90E-01 | 0.04 | 0.15 | 8.68E-01 | 0.06 | 0.15 | 8.22E-01 | -0.05 | 0.14 | 8.61E-01 | -0.05 | 0.15 | 8.43E-01 |
| Cholesterol to total lipids ratio in medium LDL | -0.01 | 0.10 | 9.79E-01 | 0.00 | 0.10 | 9.99E-01 | -0.14 | 0.13 | 6.26E-01 | -0.14 | 0.13 | 6.15E-01 | 0.19 | 0.15 | 4.25E-01 | 0.19 | 0.15 | 4.27E-01 |

Note: The table shows associations from the logistic regression analysis where plasms metabolomics level from the Nightingale platform was main exposure and prevalent breast cancer as the main outcome. Model 1 : adjusted for age, cohort, time differences (ERGO), and lipid lowering medication. Model 2: adjusted for model1, smoking status, BMI, alcohol and education. No adjustment for time differences was made in the analyses of the subcohorts (ERGO-4 and ERGO-5). The table is ranked based on the *FDR* values in model 2 within the E4&E5 cohort, using bold text indicates metabolites are surpassing *FDR* adjusted *P* value threshold (*P*<0.05). ERGO-4 and ERGO-5 refer to the fourth and fifth visits subcohort from the baseline cohort. ERGO-4&5 refers to the combined data from the fourth and fifth visits of the baseline cohort. Abbreviations: SE, standard error; IDL, intermediate-density lipoprotein; LDL, low-density lipoprotein; HDL, high-density lipoprotein; VLDL, very-low-density lipoprotein; BMI, body mass index; FDR, false discovery rate.

**Supplementary Table 10. Serum metabolites associated with prevalent breast cancer from the Metabolon platform at baseline.**

| **Metabolite** | **ERGO-4&5** | | | | | | | | **ERGO-4** | | | | **ERGO-5** | | | |
| --- | --- | --- | --- | --- | --- | --- | --- | --- | --- | --- | --- | --- | --- | --- | --- | --- |
|  | **Model 1** | | | | **Model 2** | | | | **Model 1** | | **Model 2** | | **Model 1** | | **Model 2** | |
|  | **Beta±SE** | **I^2^** | **Het.*P-*value** | ***FDR* value** | **Beta±SE** | **I^2^** | **Het.*P-*value** | ***FDR* value** | **Beta±SE** | ***FDR* value** | **Beta±SE** | ***FDR* value** | **Beta±SE** | ***FDR* value** | **Beta±SE** | ***FDR* value** |
| N-acetylglutamate | 0.88±0.21 | 84.53 | 1.10E-02 | **2.21E-02** | 0.68±0.18 | 87.19 | 5.21E-03 | 1.78E-01 | 0.31±0.24 | 8.40E-01 | 0.26±0.24 | 8.44E-01 | 1.37±0.28 | **1.34E-03** | 1.3±0.29 | **5.81E-03** |
| N-acetyl-2-aminoadipate | 0.53±0.16 | 76.74 | 3.81E-02 | 3.69E-01 | 0.46±0.16 | 76.03 | 4.11E-02 | 5.83E-01 | 0.22±0.2 | 8.40E-01 | 0.18±0.21 | 8.45E-01 | 0.88±0.23 | **2.67E-02** | 0.84±0.24 | 1.04E-01 |
| X - 25520 | 0.43±0.15 | 29.09 | 2.35E-01 | 6.01E-01 | 0.48±0.15 | 43.92 | 1.82E-01 | 5.83E-01 | 0.23±0.2 | 8.40E-01 | 0.27±0.22 | 8.44E-01 | 0.59±0.2 | 1.13E-01 | 0.67±0.21 | 1.20E-01 |
| cystathionine | 0.28±0.11 | 70.22 | 6.69E-02 | 6.01E-01 | 0.39±0.14 | 52.87 | 1.45E-01 | 5.83E-01 | 0.14±0.2 | 8.55E-01 | 0.18±0.2 | 8.45E-01 | 0.57±0.19 | 1.13E-01 | 0.6±0.2 | 1.69E-01 |
| N-acetylcitrulline | 0.49±0.17 | 0 | 4.89E-01 | 6.01E-01 | 0.45±0.15 | 0 | 3.61E-01 | 5.83E-01 | 0.31±0.21 | 8.19E-01 | 0.31±0.21 | 8.44E-01 | 0.56±0.21 | 1.58E-01 | 0.58±0.21 | 2.06E-01 |
| 2-piperidinone | 0.43±0.16 | 0 | 7.31E-01 | 6.01E-01 | 0.42±0.14 | 0 | 6.34E-01 | 5.83E-01 | 0.33±0.21 | 8.16E-01 | 0.34±0.22 | 8.44E-01 | 0.46±0.18 | 1.73E-01 | 0.47±0.18 | 2.28E-01 |
| N-acetylphenylalanine | 0.52±0.17 | 0 | 6.20E-01 | 6.01E-01 | 0.41±0.15 | 0 | 5.35E-01 | 5.83E-01 | 0.36±0.22 | 8.16E-01 | 0.31±0.22 | 8.44E-01 | 0.56±0.19 | 1.13E-01 | 0.49±0.2 | 2.39E-01 |
| methionine | 0.48±0.2 | 0 | 5.84E-01 | 6.01E-01 | 0.45±0.16 | 0 | 3.51E-01 | 5.83E-01 | 0.34±0.21 | 8.16E-01 | 0.32±0.21 | 8.44E-01 | 0.57±0.25 | 2.44E-01 | 0.64±0.26 | 2.53E-01 |
| glyco-beta-muricholate** | -0.45±0.17 | 0 | 4.97E-01 | 6.01E-01 | -0.4±0.15 | 0 | 5.34E-01 | 5.83E-01 | -0.29±0.2 | 8.19E-01 | -0.31±0.2 | 8.44E-01 | -0.53±0.21 | 1.75E-01 | -0.49±0.21 | 2.75E-01 |
| 5alpha-androstan-3beta,17beta-diol disulfate | 0.58±0.22 | 0 | 8.17E-01 | 6.01E-01 | 0.52±0.18 | 0 | 7.06E-01 | 5.83E-01 | 0.48±0.25 | 8.16E-01 | 0.45±0.26 | 8.44E-01 | 0.61±0.24 | 1.85E-01 | 0.59±0.26 | 2.92E-01 |
| hydantoin-5-propionate | 0.48±0.19 | 0 | 9.50E-01 | 6.01E-01 | 0.47±0.15 | 0 | 6.55E-01 | 5.83E-01 | 0.51±0.22 | 8.16E-01 | 0.54±0.22 | 8.44E-01 | 0.48±0.21 | 2.27E-01 | 0.4±0.21 | 3.70E-01 |
| gamma-glutamylmethionine | 0.58±0.25 | 0 | 4.38E-01 | 6.01E-01 | 0.48±0.18 | 2.86 | 3.10E-01 | 5.87E-01 | 0.37±0.2 | 8.16E-01 | 0.36±0.21 | 8.44E-01 | 0.75±0.33 | 2.40E-01 | 0.76±0.33 | 2.84E-01 |
| glycohyocholate | -0.4±0.18 | 0 | 9.68E-01 | 6.01E-01 | -0.38±0.14 | 0 | 9.94E-01 | 6.18E-01 | -0.39±0.2 | 8.16E-01 | -0.38±0.2 | 8.44E-01 | -0.41±0.2 | 2.90E-01 | -0.38±0.2 | 3.97E-01 |
| indolebutyrate | -0.01±0.01 | 90.64 | 1.08E-03 | 7.39E-01 | 0.35±0.14 | 80.66 | 2.30E-02 | 6.93E-01 | -0.01±0.2 | 9.92E-01 | 0.01±0.2 | 9.90E-01 | 0.6±0.19 | 8.33E-02 | 0.65±0.19 | 1.09E-01 |
| glutamate | 0.27±0.11 | 86.99 | 5.56E-03 | 6.01E-01 | 0.41±0.17 | 76.35 | 3.97E-02 | 6.93E-01 | 0.12±0.2 | 8.75E-01 | 0.13±0.22 | 8.70E-01 | 0.87±0.24 | **3.97E-02** | 0.84±0.27 | 1.47E-01 |
| xanthurenate | 0.47±0.17 | 6.47 | 3.01E-01 | 6.01E-01 | 0.41±0.17 | 28.99 | 2.35E-01 | 6.93E-01 | 0.26±0.23 | 8.40E-01 | 0.2±0.24 | 8.45E-01 | 0.63±0.23 | 1.58E-01 | 0.6±0.23 | 2.28E-01 |
| glucose | 0.44±0.18 | 69.09 | 7.21E-02 | 6.01E-01 | 0.36±0.15 | 63.13 | 9.96E-02 | 6.93E-01 | 0.22±0.17 | 8.40E-01 | 0.23±0.17 | 8.44E-01 | 0.9±0.31 | 1.22E-01 | 0.84±0.33 | 2.34E-01 |
| X - 21364 | 0.41±0.16 | 30.82 | 2.29E-01 | 6.01E-01 | 0.39±0.16 | 14.78 | 2.79E-01 | 6.93E-01 | 0.22±0.21 | 8.40E-01 | 0.23±0.22 | 8.44E-01 | 0.6±0.23 | 1.64E-01 | 0.58±0.24 | 2.53E-01 |
| X - 11315 | 0.39±0.17 | 0 | 6.99E-01 | 6.01E-01 | 0.4±0.16 | 0 | 4.48E-01 | 6.93E-01 | 0.29±0.24 | 8.40E-01 | 0.26±0.24 | 8.44E-01 | 0.43±0.21 | 2.82E-01 | 0.51±0.22 | 2.75E-01 |
| N2-acetyl,N6,N6-dimethyllysine | 0.37±0.17 | 0 | 6.49E-01 | 6.01E-01 | 0.38±0.16 | 0 | 5.25E-01 | 6.93E-01 | 0.27±0.22 | 8.40E-01 | 0.28±0.22 | 8.44E-01 | 0.43±0.22 | 3.18E-01 | 0.48±0.22 | 3.24E-01 |
| methionine sulfoxide | 0.36±0.17 | 0 | 8.08E-01 | 6.44E-01 | 0.34±0.14 | 0 | 5.39E-01 | 6.93E-01 | 0.3±0.19 | 8.16E-01 | 0.26±0.19 | 8.44E-01 | 0.39±0.21 | 3.68E-01 | 0.43±0.21 | 3.42E-01 |
| X - 21471 | 0.42±0.19 | 0 | 6.77E-01 | 6.01E-01 | 0.39±0.17 | 0 | 6.40E-01 | 6.93E-01 | 0.32±0.23 | 8.27E-01 | 0.31±0.23 | 8.44E-01 | 0.48±0.23 | 2.93E-01 | 0.47±0.24 | 3.66E-01 |
| 1-myristoyl-2-arachidonoyl-GPC (14:0/20:4)* | 0.47±0.21 | 0 | 8.24E-01 | 6.01E-01 | 0.41±0.17 | 0 | 8.39E-01 | 6.93E-01 | 0.4±0.24 | 8.16E-01 | 0.37±0.24 | 8.44E-01 | 0.5±0.25 | 3.07E-01 | 0.44±0.24 | 4.09E-01 |
| 1-palmitoyl-2-palmitoleoyl-GPC (16:0/16:1)* | 0.42±0.19 | 0 | 8.90E-01 | 6.01E-01 | 0.4±0.16 | 0 | 6.01E-01 | 6.93E-01 | 0.48±0.21 | 8.16E-01 | 0.48±0.23 | 8.44E-01 | 0.41±0.21 | 3.49E-01 | 0.32±0.22 | 5.08E-01 |
| X - 23665 | 0.2±0.19 | 0 | 4.69E-01 | 7.36E-01 | 0.36±0.14 | 74.59 | 4.73E-02 | 6.93E-01 | 0.67±0.21 | 8.16E-01 | 0.67±0.21 | 8.44E-01 | 0.17±0.19 | 7.15E-01 | 0.1±0.19 | 8.29E-01 |
| X - 15503 | 0.05±0.03 | 90.82 | 9.65E-04 | 7.36E-01 | 0.33±0.16 | 81.71 | 1.94E-02 | 7.00E-01 | 0.03±0.21 | 9.70E-01 | -0.05±0.22 | 9.63E-01 | 0.73±0.21 | 5.06E-02 | 0.7±0.22 | 1.47E-01 |
| N-acetylkynurenine (2) | 0.3±0.12 | 74.51 | 4.76E-02 | 6.01E-01 | 0.36±0.16 | 69.12 | 7.19E-02 | 7.00E-01 | 0.14±0.22 | 8.55E-01 | 0.08±0.23 | 9.33E-01 | 0.66±0.22 | 1.08E-01 | 0.65±0.23 | 1.84E-01 |
| picolinoylglycine | 0.16±0.08 | 83.94 | 1.26E-02 | 6.87E-01 | 0.34±0.16 | 71.87 | 5.94E-02 | 7.00E-01 | 0.09±0.22 | 9.19E-01 | 0.05±0.23 | 9.61E-01 | 0.69±0.23 | 1.08E-01 | 0.66±0.24 | 1.87E-01 |
| ribitol | 0.22±0.1 | 77.67 | 3.43E-02 | 6.32E-01 | 0.36±0.16 | 68.12 | 7.65E-02 | 7.00E-01 | 0.12±0.21 | 8.81E-01 | 0.13±0.2 | 8.65E-01 | 0.69±0.24 | 1.42E-01 | 0.69±0.24 | 1.87E-01 |
| metabolonic lactone sulfate | 0.18±0.08 | 85.21 | 9.32E-03 | 6.01E-01 | 0.34±0.16 | 64.59 | 9.29E-02 | 7.00E-01 | 0.09±0.2 | 9.06E-01 | 0.09±0.22 | 9.27E-01 | 0.65±0.2 | 7.84E-02 | 0.61±0.22 | 2.06E-01 |
| X - 19438 | 0.06±0.04 | 84.31 | 1.16E-02 | 7.36E-01 | 0.33±0.16 | 70.53 | 6.55E-02 | 7.00E-01 | 0.04±0.21 | 9.54E-01 | 0.08±0.21 | 9.28E-01 | 0.67±0.24 | 1.58E-01 | 0.67±0.25 | 2.06E-01 |
| N2,N5-diacetylornithine | 0.24±0.11 | 56.95 | 1.27E-01 | 6.01E-01 | 0.32±0.16 | 60.16 | 1.13E-01 | 7.00E-01 | 0.13±0.22 | 8.75E-01 | 0.05±0.23 | 9.58E-01 | 0.49±0.2 | 1.85E-01 | 0.55±0.21 | 2.28E-01 |
| 5-methylthioadenosine (MTA) | 0.19±0.09 | 75.9 | 4.16E-02 | 6.48E-01 | 0.32±0.15 | 59.34 | 1.17E-01 | 7.00E-01 | 0.1±0.2 | 8.98E-01 | 0.09±0.21 | 9.27E-01 | 0.58±0.21 | 1.58E-01 | 0.57±0.22 | 2.34E-01 |
| N-acetyl-beta-alanine | 0.39±0.16 | 37.24 | 2.07E-01 | 6.01E-01 | 0.36±0.16 | 51.01 | 1.53E-01 | 7.00E-01 | 0.21±0.21 | 8.55E-01 | 0.15±0.22 | 8.59E-01 | 0.61±0.23 | 1.73E-01 | 0.61±0.24 | 2.34E-01 |
| phenylalanine | 0.34±0.16 | 75.33 | 4.41E-02 | 6.01E-01 | 0.37±0.18 | 63.31 | 9.88E-02 | 7.00E-01 | 0.18±0.2 | 8.55E-01 | 0.19±0.21 | 8.45E-01 | 0.95±0.34 | 1.52E-01 | 0.86±0.35 | 2.50E-01 |
| gamma-glutamylglutamate | 0.2±0.1 | 77.04 | 3.69E-02 | 6.87E-01 | 0.34±0.16 | 46.78 | 1.70E-01 | 7.00E-01 | 0.11±0.2 | 8.91E-01 | 0.15±0.21 | 8.59E-01 | 0.67±0.25 | 1.58E-01 | 0.61±0.25 | 2.62E-01 |
| N-acetylglutamine | 0.29±0.13 | 37.97 | 2.04E-01 | 6.01E-01 | 0.32±0.15 | 36.52 | 2.09E-01 | 7.00E-01 | 0.16±0.2 | 8.55E-01 | 0.14±0.2 | 8.59E-01 | 0.5±0.21 | 2.05E-01 | 0.51±0.21 | 2.62E-01 |
| sulfate* | 0.23±0.11 | 58.61 | 1.20E-01 | 6.01E-01 | 0.31±0.15 | 29.47 | 2.34E-01 | 7.00E-01 | 0.13±0.21 | 8.72E-01 | 0.13±0.21 | 8.65E-01 | 0.5±0.2 | 1.85E-01 | 0.49±0.22 | 2.84E-01 |
| 4-hydroxyglutamate | 0.27±0.12 | 50.36 | 1.56E-01 | 6.01E-01 | 0.32±0.16 | 20.03 | 2.63E-01 | 7.00E-01 | 0.15±0.21 | 8.55E-01 | 0.15±0.22 | 8.61E-01 | 0.5±0.2 | 1.85E-01 | 0.5±0.23 | 2.95E-01 |
| X - 26109 | 0.37±0.15 | 15.83 | 2.76E-01 | 6.01E-01 | 0.36±0.16 | 0 | 4.21E-01 | 7.00E-01 | 0.21±0.22 | 8.55E-01 | 0.23±0.22 | 8.44E-01 | 0.53±0.21 | 1.75E-01 | 0.49±0.22 | 3.03E-01 |
| formiminoglutamate | 0.3±0.12 | 37.26 | 2.07E-01 | 6.01E-01 | 0.3±0.13 | 0 | 4.13E-01 | 7.00E-01 | 0.16±0.19 | 8.55E-01 | 0.18±0.19 | 8.45E-01 | 0.47±0.18 | 1.73E-01 | 0.4±0.19 | 3.24E-01 |
| retinal | 0.4±0.18 | 0 | 6.16E-01 | 6.01E-01 | 0.37±0.16 | 0 | 4.87E-01 | 7.00E-01 | 0.29±0.22 | 8.40E-01 | 0.27±0.21 | 8.44E-01 | 0.48±0.23 | 3.03E-01 | 0.49±0.24 | 3.41E-01 |
| androstenediol (3alpha, 17alpha) monosulfate (3) | 0.33±0.16 | 0 | 5.16E-01 | 6.78E-01 | 0.38±0.17 | 0 | 5.27E-01 | 7.00E-01 | 0.23±0.24 | 8.55E-01 | 0.27±0.25 | 8.44E-01 | 0.44±0.23 | 3.59E-01 | 0.49±0.24 | 3.41E-01 |
| 3-phosphoglycerate | 0.3±0.14 | 0 | 7.45E-01 | 6.01E-01 | 0.28±0.12 | 0 | 5.77E-01 | 7.00E-01 | 0.24±0.17 | 8.40E-01 | 0.21±0.18 | 8.44E-01 | 0.33±0.17 | 3.39E-01 | 0.35±0.17 | 3.66E-01 |
| X - 21736 | 0.37±0.15 | 0 | 5.75E-01 | 6.01E-01 | 0.29±0.14 | 0 | 4.69E-01 | 7.00E-01 | 0.26±0.18 | 8.33E-01 | 0.19±0.19 | 8.44E-01 | 0.43±0.19 | 2.25E-01 | 0.39±0.2 | 3.67E-01 |
| 11beta-hydroxyandrosterone glucuronide | 0.38±0.17 | 0 | 4.33E-01 | 6.01E-01 | 0.36±0.17 | 0 | 5.17E-01 | 7.00E-01 | 0.24±0.23 | 8.47E-01 | 0.24±0.24 | 8.44E-01 | 0.51±0.23 | 2.56E-01 | 0.46±0.23 | 3.69E-01 |
| N2-acetyl,N6-methyllysine | 0.34±0.17 | 0 | 9.08E-01 | 7.23E-01 | 0.35±0.15 | 0 | 7.89E-01 | 7.00E-01 | 0.31±0.2 | 8.16E-01 | 0.31±0.21 | 8.44E-01 | 0.35±0.21 | 4.18E-01 | 0.39±0.22 | 4.18E-01 |
| tyrosine | 0.37±0.18 | 0 | 5.80E-01 | 6.61E-01 | 0.33±0.16 | 0 | 5.48E-01 | 7.00E-01 | 0.27±0.21 | 8.40E-01 | 0.25±0.21 | 8.44E-01 | 0.47±0.25 | 3.63E-01 | 0.45±0.25 | 4.19E-01 |
| 5alpha-androstan-3alpha,17beta-diol monosulfate (1) | 0.35±0.18 | 0 | 7.65E-01 | 6.87E-01 | 0.33±0.16 | 0 | 7.69E-01 | 7.00E-01 | 0.28±0.22 | 8.40E-01 | 0.28±0.23 | 8.44E-01 | 0.39±0.22 | 4.03E-01 | 0.38±0.22 | 4.40E-01 |
| androstenediol (3beta,17beta) disulfate (1) | 0.39±0.18 | 0 | 9.42E-01 | 6.01E-01 | 0.34±0.16 | 0 | 9.65E-01 | 7.00E-01 | 0.36±0.21 | 8.16E-01 | 0.35±0.23 | 8.44E-01 | 0.4±0.2 | 3.39E-01 | 0.33±0.22 | 4.73E-01 |
| N-oleoylserine | 0.34±0.18 | 0 | 8.37E-01 | 7.36E-01 | 0.34±0.16 | 0 | 8.74E-01 | 7.00E-01 | 0.29±0.21 | 8.27E-01 | 0.31±0.21 | 8.44E-01 | 0.37±0.23 | 4.50E-01 | 0.37±0.24 | 4.73E-01 |
| 5alpha-androstan-3alpha,17beta-diol monosulfate (2) | 0.53±0.27 | 0 | 9.40E-01 | 6.87E-01 | 0.49±0.24 | 0 | 9.27E-01 | 7.00E-01 | 0.5±0.33 | 8.16E-01 | 0.47±0.34 | 8.44E-01 | 0.54±0.31 | 4.06E-01 | 0.51±0.33 | 4.74E-01 |
| sphinganine | 0.3±0.18 | 0 | 7.88E-01 | 7.36E-01 | 0.32±0.15 | 0 | 5.72E-01 | 7.00E-01 | 0.4±0.23 | 8.16E-01 | 0.42±0.23 | 8.44E-01 | 0.28±0.2 | 4.81E-01 | 0.25±0.2 | 5.62E-01 |
| homoarginine | 0.34±0.21 | 0 | 8.89E-01 | 7.36E-01 | 0.34±0.16 | 0 | 7.29E-01 | 7.00E-01 | 0.39±0.21 | 8.16E-01 | 0.39±0.22 | 8.44E-01 | 0.32±0.24 | 5.17E-01 | 0.28±0.24 | 6.03E-01 |
| adenosine 5'-monophosphate (AMP) | 0.18±0.16 | 0 | 5.44E-01 | 7.36E-01 | 0.28±0.13 | 0 | 3.30E-01 | 7.00E-01 | 0.41±0.17 | 8.16E-01 | 0.4±0.18 | 8.44E-01 | 0.14±0.18 | 7.57E-01 | 0.16±0.18 | 7.09E-01 |
| X - 24344 | -0.2±0.19 | 0 | 4.85E-01 | 7.36E-01 | -0.35±0.16 | 64.87 | 9.16E-02 | 7.00E-01 | -0.59±0.21 | 8.16E-01 | -0.61±0.22 | 8.44E-01 | -0.15±0.2 | 7.57E-01 | -0.08±0.22 | 8.86E-01 |
| urate | 0.34±0.14 | 48.88 | 1.62E-01 | 6.01E-01 | 0.31±0.15 | 30.22 | 2.31E-01 | 7.07E-01 | 0.18±0.19 | 8.55E-01 | 0.14±0.21 | 8.59E-01 | 0.57±0.21 | 1.63E-01 | 0.51±0.22 | 2.94E-01 |
| tetrahydrocortisol glucuronide | 0.32±0.13 | 46.95 | 1.70E-01 | 6.01E-01 | 0.33±0.16 | 11.04 | 2.89E-01 | 7.07E-01 | 0.17±0.23 | 8.55E-01 | 0.14±0.24 | 8.70E-01 | 0.55±0.22 | 1.75E-01 | 0.49±0.23 | 3.20E-01 |
| arachidonoylcarnitine (C20:4) | 0.32±0.16 | 0 | 6.20E-01 | 6.87E-01 | 0.31±0.15 | 0 | 5.76E-01 | 7.07E-01 | 0.24±0.21 | 8.40E-01 | 0.23±0.21 | 8.44E-01 | 0.4±0.22 | 3.77E-01 | 0.4±0.22 | 4.18E-01 |
| sphingomyelin (d18:2/24:2)* | -0.3±0.14 | 0 | 9.52E-01 | 6.01E-01 | -0.26±0.13 | 0 | 9.78E-01 | 7.07E-01 | -0.28±0.2 | 8.34E-01 | -0.26±0.21 | 8.44E-01 | -0.3±0.16 | 3.39E-01 | -0.26±0.16 | 4.67E-01 |
| N6-methyllysine | 0.26±0.15 | 0 | 9.53E-01 | 7.36E-01 | 0.28±0.14 | 0 | 8.11E-01 | 7.07E-01 | 0.24±0.2 | 8.40E-01 | 0.25±0.2 | 8.44E-01 | 0.26±0.19 | 4.99E-01 | 0.31±0.2 | 4.67E-01 |
| 1-palmitoleoylglycerol (16:1)* | 0.36±0.17 | 0 | 8.28E-01 | 6.44E-01 | 0.29±0.15 | 0 | 9.40E-01 | 7.07E-01 | 0.31±0.21 | 8.19E-01 | 0.28±0.21 | 8.44E-01 | 0.39±0.21 | 3.68E-01 | 0.31±0.21 | 5.10E-01 |
| tryptophan betaine | 0.32±0.18 | 0 | 8.37E-01 | 7.36E-01 | 0.32±0.16 | 0 | 9.20E-01 | 7.07E-01 | 0.39±0.24 | 8.16E-01 | 0.34±0.25 | 8.44E-01 | 0.3±0.21 | 4.76E-01 | 0.31±0.21 | 5.22E-01 |
| X - 24462 | 0.24±0.18 | 0 | 6.78E-01 | 7.36E-01 | 0.3±0.15 | 0 | 6.59E-01 | 7.07E-01 | 0.37±0.21 | 8.16E-01 | 0.37±0.22 | 8.44E-01 | 0.19±0.21 | 6.75E-01 | 0.24±0.21 | 6.14E-01 |
| gamma-glutamylphenylalanine | 0.01±0.01 | 92.18 | 3.49E-04 | 7.36E-01 | 0.31±0.16 | 82.45 | 1.70E-02 | 7.12E-01 | 0.01±0.21 | 9.92E-01 | -0.04±0.22 | 9.67E-01 | 0.81±0.23 | **3.97E-02** | 0.72±0.24 | 1.47E-01 |
| X - 11795 | -0.01±0.03 | 89.68 | 1.85E-03 | 8.19E-01 | 0.28±0.14 | 81.51 | 2.00E-02 | 7.12E-01 | -0.03±0.21 | 9.70E-01 | -0.1±0.22 | 9.13E-01 | 0.53±0.18 | 1.10E-01 | 0.58±0.19 | 1.62E-01 |
| X - 16087 | 0.3±0.13 | 41.89 | 1.90E-01 | 6.01E-01 | 0.29±0.15 | 18 | 2.69E-01 | 7.12E-01 | 0.17±0.21 | 8.55E-01 | 0.13±0.21 | 8.65E-01 | 0.52±0.21 | 1.88E-01 | 0.46±0.21 | 3.24E-01 |
| gamma-glutamyltyrosine | 0.39±0.17 | 45.44 | 1.76E-01 | 6.01E-01 | 0.34±0.17 | 22.02 | 2.57E-01 | 7.12E-01 | 0.21±0.2 | 8.40E-01 | 0.2±0.22 | 8.45E-01 | 0.71±0.3 | 2.11E-01 | 0.62±0.3 | 3.41E-01 |
| fructosyllysine | 0.3±0.14 | 36.91 | 2.08E-01 | 6.01E-01 | 0.29±0.15 | 0 | 3.54E-01 | 7.12E-01 | 0.17±0.19 | 8.55E-01 | 0.17±0.19 | 8.45E-01 | 0.54±0.23 | 2.31E-01 | 0.45±0.23 | 3.70E-01 |
| 8-methoxykynurenate | 0.36±0.17 | 0 | 6.73E-01 | 6.01E-01 | 0.3±0.16 | 0 | 6.37E-01 | 7.12E-01 | 0.27±0.21 | 8.40E-01 | 0.23±0.22 | 8.44E-01 | 0.42±0.22 | 3.39E-01 | 0.38±0.23 | 4.43E-01 |
| N4-acetylcytidine | 0.3±0.14 | 30.33 | 2.31E-01 | 6.01E-01 | 0.31±0.16 | 18.86 | 2.67E-01 | 7.14E-01 | 0.17±0.21 | 8.55E-01 | 0.14±0.22 | 8.59E-01 | 0.52±0.23 | 2.31E-01 | 0.5±0.23 | 3.29E-01 |
| tetrahydrocortisone glucuronide (5) | 0.32±0.15 | 0 | 3.50E-01 | 6.01E-01 | 0.32±0.17 | 0 | 4.44E-01 | 7.14E-01 | 0.2±0.23 | 8.55E-01 | 0.19±0.24 | 8.54E-01 | 0.48±0.23 | 2.81E-01 | 0.44±0.23 | 3.78E-01 |
| 1-palmitoyl-2-oleoyl-GPE (16:0/18:1) | 0.3±0.14 | 0 | 3.57E-01 | 6.01E-01 | 0.29±0.15 | 0 | 4.12E-01 | 7.14E-01 | 0.19±0.21 | 8.55E-01 | 0.17±0.21 | 8.45E-01 | 0.45±0.21 | 2.81E-01 | 0.43±0.22 | 3.78E-01 |
| 3-hydroxyhexanoate | 0.3±0.18 | 0 | 9.52E-01 | 7.36E-01 | 0.28±0.15 | 0 | 9.81E-01 | 7.14E-01 | 0.32±0.19 | 8.16E-01 | 0.28±0.2 | 8.44E-01 | 0.29±0.22 | 5.18E-01 | 0.28±0.22 | 5.62E-01 |
| 1-palmitoyl-2-arachidonoyl-GPC (16:0/20:4n6) | 0.3±0.21 | 0 | 7.04E-01 | 7.36E-01 | 0.31±0.16 | 0 | 5.29E-01 | 7.14E-01 | 0.46±0.23 | 8.16E-01 | 0.41±0.23 | 8.44E-01 | 0.26±0.24 | 6.03E-01 | 0.21±0.23 | 6.92E-01 |
| methylsuccinoylcarnitine | 0.11±0.18 | 0 | 4.11E-01 | 7.76E-01 | 0.27±0.14 | 41.43 | 1.91E-01 | 7.14E-01 | 0.48±0.21 | 8.16E-01 | 0.47±0.21 | 8.44E-01 | 0.06±0.19 | 9.20E-01 | 0.1±0.2 | 8.52E-01 |
| X - 17685 | -0.16±0.17 | 0 | 4.99E-01 | 7.36E-01 | -0.25±0.13 | 42.24 | 1.88E-01 | 7.14E-01 | -0.41±0.18 | 8.16E-01 | -0.42±0.18 | 8.44E-01 | -0.1±0.19 | 8.25E-01 | -0.07±0.19 | 8.98E-01 |
| pregnenetriol disulfate* | 0.3±0.15 | 0 | 4.90E-01 | 6.87E-01 | 0.3±0.16 | 0 | 6.31E-01 | 7.17E-01 | 0.2±0.22 | 8.55E-01 | 0.22±0.23 | 8.45E-01 | 0.41±0.22 | 3.63E-01 | 0.37±0.22 | 4.36E-01 |
| X - 17357 | 0.29±0.17 | 0 | 9.42E-01 | 7.36E-01 | 0.32±0.17 | 0 | 9.39E-01 | 7.18E-01 | 0.27±0.25 | 8.40E-01 | 0.3±0.26 | 8.44E-01 | 0.3±0.22 | 4.99E-01 | 0.33±0.22 | 5.04E-01 |
| sphingosine 1-phosphate | 0.25±0.19 | 0 | 8.95E-01 | 7.36E-01 | 0.3±0.16 | 0 | 9.00E-01 | 7.26E-01 | 0.28±0.19 | 8.19E-01 | 0.31±0.2 | 8.44E-01 | 0.23±0.26 | 7.13E-01 | 0.27±0.27 | 6.59E-01 |
| 1,5-anhydroglucitol (1,5-AG) | -0.11±0.17 | 0 | 4.25E-01 | 7.75E-01 | -0.25±0.13 | 63.02 | 1.00E-01 | 7.26E-01 | -0.41±0.17 | 8.16E-01 | -0.46±0.18 | 8.44E-01 | -0.05±0.19 | 9.38E-01 | -0.02±0.19 | 9.81E-01 |
| lactosyl-N-palmitoyl-sphingosine (d18:1/16:0) | -0.26±0.12 | 0 | 8.86E-01 | 6.01E-01 | -0.22±0.12 | 0 | 9.10E-01 | 7.27E-01 | -0.24±0.21 | 8.40E-01 | -0.2±0.21 | 8.45E-01 | -0.27±0.13 | 2.86E-01 | -0.23±0.14 | 4.67E-01 |
| N-acetyl-aspartyl-glutamate (NAAG) | 0.1±0.06 | 71.35 | 6.17E-02 | 7.36E-01 | 0.28±0.15 | 61.29 | 1.08E-01 | 7.39E-01 | 0.06±0.2 | 9.38E-01 | 0.06±0.2 | 9.38E-01 | 0.48±0.22 | 2.43E-01 | 0.54±0.22 | 2.62E-01 |
| X - 12112 | 0.21±0.11 | 0 | 3.22E-01 | 7.36E-01 | 0.27±0.15 | 0 | 4.33E-01 | 7.39E-01 | 0.14±0.2 | 8.55E-01 | 0.16±0.2 | 8.55E-01 | 0.38±0.21 | 3.68E-01 | 0.39±0.21 | 4.03E-01 |
| palmitoyl-sphingosine-phosphoethanolamine (d18:1/16:0) | -0.28±0.14 | 0 | 9.84E-01 | 6.87E-01 | -0.23±0.12 | 0 | 8.61E-01 | 7.52E-01 | -0.28±0.17 | 8.16E-01 | -0.25±0.18 | 8.44E-01 | -0.27±0.16 | 4.06E-01 | -0.21±0.18 | 6.04E-01 |
| palmitoyl sphingomyelin (d18:1/16:0) | -0.19±0.14 | 0 | 5.54E-01 | 7.36E-01 | -0.23±0.13 | 31.5 | 2.27E-01 | 7.52E-01 | -0.44±0.2 | 8.16E-01 | -0.43±0.21 | 8.44E-01 | -0.17±0.15 | 5.99E-01 | -0.11±0.16 | 7.81E-01 |
| lyxonate | 0.38±0.17 | 32.94 | 2.22E-01 | 6.01E-01 | 0.3±0.16 | 59.18 | 1.18E-01 | 7.67E-01 | 0.21±0.2 | 8.40E-01 | 0.11±0.2 | 8.86E-01 | 0.63±0.27 | 2.12E-01 | 0.65±0.28 | 2.77E-01 |
| aspartate | 0.25±0.12 | 64.53 | 9.31E-02 | 6.49E-01 | 0.29±0.16 | 37.78 | 2.05E-01 | 7.67E-01 | 0.14±0.2 | 8.55E-01 | 0.12±0.21 | 8.70E-01 | 0.61±0.25 | 1.85E-01 | 0.54±0.25 | 3.29E-01 |
| N-acetyltyrosine | 0.31±0.14 | 0 | 3.90E-01 | 6.01E-01 | 0.26±0.14 | 0 | 3.70E-01 | 7.74E-01 | 0.19±0.2 | 8.55E-01 | 0.13±0.2 | 8.65E-01 | 0.43±0.2 | 2.65E-01 | 0.38±0.2 | 3.78E-01 |
| arabitol/xylitol | 0.04±0.1 | 90.96 | 8.79E-04 | 8.49E-01 | 0.29±0.17 | 84.83 | 1.02E-02 | 7.94E-01 | -0.11±0.23 | 9.04E-01 | -0.12±0.23 | 8.95E-01 | 0.76±0.24 | 8.53E-02 | 0.74±0.24 | 1.47E-01 |
| thioproline | 0.16±0.11 | 85.13 | 9.51E-03 | 7.36E-01 | 0.21±0.12 | 76.28 | 4.01E-02 | 7.94E-01 | -0.16±0.2 | 8.55E-01 | -0.11±0.2 | 8.70E-01 | 0.39±0.14 | 1.42E-01 | 0.39±0.15 | 2.28E-01 |
| N,N,N-trimethyl-5-aminovalerate | 0.04±0.03 | 82.83 | 1.58E-02 | 7.36E-01 | 0.27±0.16 | 70.51 | 6.56E-02 | 7.94E-01 | 0.03±0.2 | 9.70E-01 | 0.04±0.2 | 9.63E-01 | 0.65±0.25 | 1.75E-01 | 0.64±0.26 | 2.40E-01 |
| X - 24473 | 0.07±0.05 | 78.64 | 3.05E-02 | 7.36E-01 | 0.24±0.14 | 69.94 | 6.82E-02 | 7.94E-01 | 0.05±0.2 | 9.54E-01 | -0.02±0.2 | 9.84E-01 | 0.45±0.18 | 1.85E-01 | 0.51±0.2 | 2.50E-01 |
| 1-stearoyl-2-oleoyl-GPE (18:0/18:1) | 0.06±0.04 | 82.96 | 1.54E-02 | 7.36E-01 | 0.28±0.16 | 66.89 | 8.23E-02 | 7.94E-01 | 0.04±0.21 | 9.57E-01 | 0.02±0.22 | 9.82E-01 | 0.6±0.23 | 1.64E-01 | 0.58±0.23 | 2.54E-01 |
| phenylacetylcarnitine | 0.06±0.04 | 75.52 | 4.33E-02 | 7.36E-01 | 0.26±0.15 | 52.8 | 1.46E-01 | 7.94E-01 | 0.04±0.2 | 9.54E-01 | 0.05±0.21 | 9.49E-01 | 0.5±0.22 | 2.37E-01 | 0.5±0.22 | 2.94E-01 |
| N-acetylhistidine | 0.16±0.09 | 38.29 | 2.03E-01 | 7.36E-01 | 0.26±0.15 | 41.77 | 1.90E-01 | 7.94E-01 | 0.1±0.21 | 9.03E-01 | 0.08±0.2 | 9.27E-01 | 0.41±0.22 | 3.63E-01 | 0.48±0.23 | 3.30E-01 |
| N-acetyltryptophan | 0.32±0.15 | 0 | 3.22E-01 | 6.01E-01 | 0.28±0.16 | 0 | 3.27E-01 | 7.94E-01 | 0.19±0.22 | 8.55E-01 | 0.13±0.22 | 8.77E-01 | 0.49±0.23 | 2.61E-01 | 0.44±0.23 | 3.75E-01 |
| 1-myristoyl-2-palmitoyl-GPC (14:0/16:0) | 0.21±0.12 | 0 | 3.37E-01 | 7.36E-01 | 0.26±0.15 | 0 | 3.84E-01 | 7.94E-01 | 0.14±0.21 | 8.55E-01 | 0.13±0.21 | 8.70E-01 | 0.38±0.21 | 3.77E-01 | 0.39±0.22 | 4.09E-01 |
| androstenediol (3beta,17beta) disulfate (2) | 0.25±0.13 | 0 | 4.87E-01 | 7.36E-01 | 0.26±0.15 | 0 | 6.29E-01 | 7.94E-01 | 0.18±0.21 | 8.55E-01 | 0.18±0.22 | 8.45E-01 | 0.37±0.21 | 4.01E-01 | 0.33±0.21 | 4.67E-01 |
| 2,6-dihydroxybenzoic acid | 0.22±0.14 | 0 | 7.64E-01 | 7.36E-01 | 0.25±0.15 | 0 | 5.98E-01 | 7.94E-01 | 0.18±0.2 | 8.55E-01 | 0.17±0.2 | 8.45E-01 | 0.27±0.2 | 5.23E-01 | 0.32±0.21 | 4.67E-01 |
| epiandrosterone sulfate | 0.25±0.16 | 0 | 9.87E-01 | 7.36E-01 | 0.27±0.16 | 0 | 8.61E-01 | 7.94E-01 | 0.25±0.23 | 8.40E-01 | 0.25±0.23 | 8.44E-01 | 0.25±0.21 | 5.71E-01 | 0.3±0.22 | 5.44E-01 |
| androsterone sulfate | 0.23±0.16 | 0 | 9.90E-01 | 7.36E-01 | 0.27±0.16 | 0 | 8.41E-01 | 7.94E-01 | 0.23±0.22 | 8.55E-01 | 0.24±0.23 | 8.44E-01 | 0.23±0.21 | 6.00E-01 | 0.3±0.22 | 5.44E-01 |
| undecenoylcarnitine (C11:1) | 0.23±0.15 | 0 | 9.10E-01 | 7.36E-01 | 0.25±0.15 | 0 | 8.49E-01 | 7.94E-01 | 0.21±0.2 | 8.40E-01 | 0.22±0.21 | 8.44E-01 | 0.24±0.2 | 5.66E-01 | 0.27±0.21 | 5.47E-01 |
| 1-palmitoyl-2-oleoyl-GPC (16:0/18:1) | 0.32±0.19 | 0 | 8.91E-01 | 7.36E-01 | 0.28±0.17 | 0 | 8.71E-01 | 7.94E-01 | 0.29±0.21 | 8.27E-01 | 0.26±0.22 | 8.44E-01 | 0.35±0.25 | 4.97E-01 | 0.31±0.25 | 5.77E-01 |
| cortolone glucuronide (1) | 0.33±0.19 | 0 | 8.58E-01 | 7.36E-01 | 0.29±0.17 | 0 | 5.09E-01 | 7.94E-01 | 0.39±0.24 | 8.16E-01 | 0.43±0.27 | 8.44E-01 | 0.31±0.21 | 4.81E-01 | 0.2±0.22 | 6.88E-01 |
| glycosyl ceramide (d18:2/24:1, d18:1/24:2)* | -0.23±0.14 | 0 | 7.70E-01 | 7.36E-01 | -0.23±0.13 | 0 | 5.28E-01 | 7.94E-01 | -0.31±0.21 | 8.16E-01 | -0.33±0.21 | 8.44E-01 | -0.21±0.16 | 5.17E-01 | -0.16±0.17 | 6.92E-01 |
| hydroxypalmitoyl sphingomyelin (d18:1/16:0(OH))** | -0.21±0.14 | 0 | 6.43E-01 | 7.36E-01 | -0.22±0.13 | 0 | 4.27E-01 | 7.94E-01 | -0.37±0.2 | 8.16E-01 | -0.34±0.21 | 8.44E-01 | -0.19±0.15 | 5.56E-01 | -0.14±0.16 | 7.16E-01 |
| valine | 0.19±0.11 | 64.4 | 9.37E-02 | 7.36E-01 | 0.35±0.21 | 24.55 | 2.50E-01 | 7.97E-01 | 0.12±0.24 | 8.98E-01 | 0.16±0.26 | 8.65E-01 | 0.7±0.32 | 2.59E-01 | 0.66±0.34 | 3.70E-01 |
| acetylcarnitine (C2) | 0.31±0.18 | 0 | 9.15E-01 | 7.36E-01 | 0.25±0.15 | 0 | 9.68E-01 | 7.97E-01 | 0.28±0.19 | 8.19E-01 | 0.25±0.2 | 8.44E-01 | 0.32±0.23 | 4.97E-01 | 0.26±0.23 | 6.06E-01 |
| taurine | -0.09±0.16 | 0 | 3.97E-01 | 7.91E-01 | -0.22±0.13 | 73.38 | 5.26E-02 | 7.97E-01 | -0.43±0.18 | 8.16E-01 | -0.49±0.19 | 8.44E-01 | -0.04±0.17 | 9.43E-01 | 0.02±0.18 | 9.75E-01 |
| 1-stearoyl-2-arachidonoyl-GPE (18:0/20:4) | 0.32±0.18 | 0 | 8.53E-01 | 7.36E-01 | 0.28±0.17 | 0 | 8.92E-01 | 8.00E-01 | 0.28±0.23 | 8.40E-01 | 0.26±0.23 | 8.44E-01 | 0.35±0.24 | 4.86E-01 | 0.3±0.24 | 5.78E-01 |
| androstenediol (3beta,17beta) monosulfate (1) | 0.3±0.17 | 0 | 9.06E-01 | 7.36E-01 | 0.26±0.16 | 0 | 9.81E-01 | 8.04E-01 | 0.27±0.22 | 8.40E-01 | 0.26±0.23 | 8.44E-01 | 0.31±0.21 | 4.70E-01 | 0.26±0.21 | 5.98E-01 |
| 1-oleoyl-2-linoleoyl-GPE (18:1/18:2)* | 0.14±0.09 | 2.25 | 3.12E-01 | 7.36E-01 | 0.27±0.16 | 1.43 | 3.14E-01 | 8.10E-01 | 0.1±0.21 | 9.03E-01 | 0.12±0.22 | 8.82E-01 | 0.36±0.23 | 4.50E-01 | 0.45±0.24 | 3.97E-01 |
| erythritol | 0.18±0.14 | 90.7 | 1.04E-03 | 7.36E-01 | 0.25±0.16 | 89.28 | 2.25E-03 | 8.16E-01 | -0.18±0.21 | 8.55E-01 | -0.19±0.22 | 8.45E-01 | 0.78±0.23 | 6.93E-02 | 0.79±0.24 | 1.15E-01 |
| 3-methyl-2-oxovalerate | -0.01±0.06 | 88.08 | 3.78E-03 | 9.56E-01 | 0.23±0.15 | 76.55 | 3.89E-02 | 8.16E-01 | -0.07±0.21 | 9.38E-01 | -0.09±0.22 | 9.27E-01 | 0.56±0.21 | 1.58E-01 | 0.54±0.21 | 2.34E-01 |
| N-acetylasparagine | 0.02±0.02 | 78.86 | 2.96E-02 | 7.36E-01 | 0.23±0.14 | 71.06 | 6.31E-02 | 8.16E-01 | 0.02±0.19 | 9.84E-01 | -0.04±0.2 | 9.64E-01 | 0.45±0.2 | 2.31E-01 | 0.49±0.2 | 2.54E-01 |
| 4-methyl-2-oxopentanoate | 0.01±0.01 | 82.91 | 1.56E-02 | 7.36E-01 | 0.25±0.15 | 63.56 | 9.76E-02 | 8.16E-01 | 0.01±0.21 | 9.92E-01 | -0.02±0.22 | 9.84E-01 | 0.52±0.21 | 1.85E-01 | 0.49±0.21 | 2.77E-01 |
| X - 26108 | 0.13±0.07 | 69.96 | 6.81E-02 | 7.36E-01 | 0.24±0.15 | 51.9 | 1.49E-01 | 8.16E-01 | 0.08±0.2 | 9.19E-01 | 0.06±0.2 | 9.40E-01 | 0.5±0.22 | 2.27E-01 | 0.49±0.23 | 3.24E-01 |
| phenyllactate (PLA) | 0.09±0.06 | 76.74 | 3.81E-02 | 7.36E-01 | 0.24±0.15 | 50.3 | 1.56E-01 | 8.16E-01 | 0.06±0.21 | 9.42E-01 | 0.02±0.21 | 9.76E-01 | 0.51±0.21 | 1.97E-01 | 0.45±0.21 | 3.24E-01 |
| orotate | 0.04±0.03 | 72.58 | 5.62E-02 | 7.36E-01 | 0.24±0.15 | 46.44 | 1.72E-01 | 8.16E-01 | 0.03±0.21 | 9.66E-01 | 0.03±0.22 | 9.76E-01 | 0.46±0.22 | 2.83E-01 | 0.45±0.22 | 3.41E-01 |
| 3-(4-hydroxyphenyl)lactate | 0.13±0.07 | 70.62 | 6.50E-02 | 7.36E-01 | 0.25±0.16 | 37.28 | 2.07E-01 | 8.16E-01 | 0.08±0.22 | 9.30E-01 | 0.05±0.22 | 9.58E-01 | 0.5±0.22 | 2.25E-01 | 0.45±0.22 | 3.42E-01 |
| imidazole propionate | 0.04±0.03 | 65.61 | 8.81E-02 | 7.36E-01 | 0.21±0.14 | 34.74 | 2.16E-01 | 8.16E-01 | 0.03±0.19 | 9.61E-01 | 0.03±0.2 | 9.71E-01 | 0.34±0.17 | 3.39E-01 | 0.37±0.19 | 3.66E-01 |
| X - 24328 | 0.08±0.06 | 66.03 | 8.62E-02 | 7.36E-01 | 0.27±0.18 | 33.56 | 2.20E-01 | 8.16E-01 | 0.06±0.25 | 9.54E-01 | 0.05±0.25 | 9.63E-01 | 0.48±0.24 | 3.07E-01 | 0.49±0.25 | 3.70E-01 |
| tyramine O-sulfate | 0.19±0.1 | 0 | 4.39E-01 | 7.36E-01 | 0.2±0.13 | 0 | 3.70E-01 | 8.16E-01 | 0.13±0.19 | 8.55E-01 | 0.07±0.19 | 9.27E-01 | 0.29±0.17 | 3.97E-01 | 0.3±0.17 | 4.17E-01 |
| 4-acetamidobutanoate | 0.29±0.15 | 0 | 3.94E-01 | 7.36E-01 | 0.27±0.17 | 0 | 3.85E-01 | 8.16E-01 | 0.19±0.23 | 8.55E-01 | 0.14±0.23 | 8.65E-01 | 0.46±0.25 | 3.77E-01 | 0.43±0.25 | 4.33E-01 |
| 2'-deoxyuridine | 0.17±0.1 | 0 | 4.05E-01 | 7.36E-01 | 0.24±0.15 | 0 | 3.71E-01 | 8.16E-01 | 0.12±0.21 | 8.80E-01 | 0.11±0.21 | 8.98E-01 | 0.32±0.21 | 4.51E-01 | 0.38±0.22 | 4.33E-01 |
| N-acetylleucine | 0.23±0.12 | 0 | 3.94E-01 | 7.36E-01 | 0.24±0.15 | 0 | 5.01E-01 | 8.16E-01 | 0.15±0.21 | 8.55E-01 | 0.14±0.21 | 8.63E-01 | 0.37±0.2 | 3.77E-01 | 0.34±0.21 | 4.67E-01 |
| N-acetylputrescine | 0.32±0.17 | 0 | 4.66E-01 | 7.36E-01 | 0.26±0.16 | 0 | 4.43E-01 | 8.16E-01 | 0.22±0.2 | 8.40E-01 | 0.17±0.2 | 8.45E-01 | 0.47±0.26 | 3.81E-01 | 0.42±0.26 | 4.67E-01 |
| spermidine | 0.17±0.13 | 0 | 9.95E-01 | 7.36E-01 | 0.19±0.12 | 0 | 7.01E-01 | 8.16E-01 | 0.17±0.14 | 8.40E-01 | 0.16±0.15 | 8.44E-01 | 0.17±0.2 | 7.27E-01 | 0.26±0.21 | 5.88E-01 |
| 5alpha-androstan-3beta,17beta-diol monosulfate (2) | 0.26±0.16 | 0 | 9.29E-01 | 7.36E-01 | 0.24±0.16 | 0 | 9.15E-01 | 8.16E-01 | 0.25±0.23 | 8.40E-01 | 0.22±0.23 | 8.45E-01 | 0.28±0.21 | 5.28E-01 | 0.26±0.21 | 5.96E-01 |
| X - 26107 | 0.31±0.17 | 0 | 9.54E-01 | 7.36E-01 | 0.26±0.16 | 0 | 9.88E-01 | 8.16E-01 | 0.29±0.24 | 8.40E-01 | 0.26±0.24 | 8.44E-01 | 0.32±0.22 | 4.76E-01 | 0.26±0.22 | 6.03E-01 |
| sphinganine-1-phosphate | 0.22±0.16 | 0 | 8.98E-01 | 7.36E-01 | 0.24±0.15 | 0 | 8.16E-01 | 8.16E-01 | 0.25±0.21 | 8.40E-01 | 0.27±0.21 | 8.44E-01 | 0.21±0.21 | 6.61E-01 | 0.2±0.22 | 6.88E-01 |
| glycoursodeoxycholic acid sulfate (1) | -0.19±0.16 | 0 | 7.07E-01 | 7.36E-01 | -0.22±0.14 | 0 | 7.46E-01 | 8.16E-01 | -0.26±0.2 | 8.40E-01 | -0.27±0.2 | 8.44E-01 | -0.14±0.2 | 7.79E-01 | -0.18±0.2 | 7.11E-01 |
| 3-ethylcatechol sulfate (1) | -0.2±0.14 | 0 | 8.77E-01 | 7.36E-01 | -0.2±0.13 | 0 | 7.05E-01 | 8.16E-01 | -0.22±0.16 | 8.19E-01 | -0.25±0.17 | 8.44E-01 | -0.18±0.18 | 6.46E-01 | -0.15±0.2 | 7.71E-01 |
| 3-hydroxyisobutyrate | 0.23±0.17 | 0 | 7.50E-01 | 7.36E-01 | 0.22±0.14 | 0 | 5.69E-01 | 8.16E-01 | 0.32±0.19 | 8.16E-01 | 0.29±0.19 | 8.44E-01 | 0.2±0.21 | 6.63E-01 | 0.14±0.2 | 7.81E-01 |
| glycosyl-N-palmitoyl-sphingosine (d18:1/16:0) | -0.19±0.14 | 0 | 5.96E-01 | 7.36E-01 | -0.22±0.14 | 20.27 | 2.63E-01 | 8.16E-01 | -0.37±0.22 | 8.16E-01 | -0.43±0.24 | 8.44E-01 | -0.16±0.15 | 6.31E-01 | -0.11±0.17 | 8.07E-01 |
| 3-hydroxyoctanoate | 0.17±0.17 | 0 | 5.78E-01 | 7.36E-01 | 0.21±0.13 | 0 | 4.39E-01 | 8.16E-01 | 0.33±0.17 | 8.16E-01 | 0.3±0.18 | 8.44E-01 | 0.11±0.2 | 8.22E-01 | 0.09±0.21 | 8.64E-01 |
| citraconate/glutaconate | 0±0.18 | 9.08 | 2.94E-01 | 9.99E-01 | -0.22±0.14 | 81.99 | 1.85E-02 | 8.16E-01 | -0.62±0.21 | 8.16E-01 | -0.6±0.22 | 8.44E-01 | 0.06±0.19 | 9.16E-01 | 0.08±0.19 | 8.65E-01 |
| trans-urocanate | -0.13±0.18 | 0 | 4.46E-01 | 7.60E-01 | -0.22±0.14 | 35.89 | 2.12E-01 | 8.16E-01 | -0.41±0.2 | 8.16E-01 | -0.4±0.2 | 8.44E-01 | -0.06±0.2 | 9.15E-01 | -0.05±0.2 | 9.27E-01 |
| glycine | -0.17±0.15 | 0 | 5.10E-01 | 7.36E-01 | -0.22±0.14 | 56.7 | 1.29E-01 | 8.16E-01 | -0.45±0.2 | 8.16E-01 | -0.47±0.21 | 8.44E-01 | -0.13±0.16 | 7.27E-01 | -0.05±0.18 | 9.28E-01 |
| perfluorooctanoate (PFOA) | -0.07±0.19 | 0 | 3.54E-01 | 8.61E-01 | -0.24±0.15 | 74.86 | 4.61E-02 | 8.16E-01 | -0.46±0.21 | 8.16E-01 | -0.55±0.21 | 8.44E-01 | 0.01±0.21 | 9.91E-01 | 0.05±0.21 | 9.31E-01 |
| 3-methyl catechol sulfate (1) | -0.1±0.17 | 0 | 4.13E-01 | 7.83E-01 | -0.21±0.14 | 62.41 | 1.03E-01 | 8.16E-01 | -0.4±0.18 | 8.16E-01 | -0.43±0.19 | 8.44E-01 | -0.04±0.19 | 9.45E-01 | 0.02±0.2 | 9.81E-01 |
| glycoursodeoxycholate | -0.05±0.18 | 0 | 3.34E-01 | 9.11E-01 | -0.23±0.14 | 68.04 | 7.69E-02 | 8.16E-01 | -0.5±0.21 | 8.16E-01 | -0.5±0.21 | 8.44E-01 | 0.02±0.19 | 9.82E-01 | 0.01±0.19 | 9.94E-01 |
| 3-hydroxy-3-methylglutarate | -0.02±0.05 | 90.09 | 1.49E-03 | 8.51E-01 | 0.24±0.16 | 84.92 | 1.00E-02 | 8.22E-01 | -0.05±0.2 | 9.47E-01 | -0.04±0.19 | 9.58E-01 | 0.8±0.26 | 1.08E-01 | 0.82±0.28 | 1.67E-01 |
| X - 21467 | 0.08±0.06 | 73.29 | 5.30E-02 | 7.36E-01 | 0.24±0.16 | 53.67 | 1.42E-01 | 8.22E-01 | 0.06±0.2 | 9.38E-01 | 0.06±0.2 | 9.44E-01 | 0.54±0.24 | 2.44E-01 | 0.53±0.25 | 3.38E-01 |
| lactosyl-N-nervonoyl-sphingosine (d18:1/24:1)* | -0.24±0.12 | 0 | 7.64E-01 | 6.96E-01 | -0.21±0.14 | 0 | 9.67E-01 | 8.22E-01 | -0.19±0.22 | 8.55E-01 | -0.2±0.22 | 8.45E-01 | -0.27±0.16 | 4.06E-01 | -0.21±0.17 | 5.88E-01 |
| N-stearoyl-sphinganine (d18:0/18:0)* | 0.29±0.16 | 0 | 9.10E-01 | 7.36E-01 | 0.23±0.15 | 0 | 8.98E-01 | 8.22E-01 | 0.27±0.22 | 8.40E-01 | 0.26±0.24 | 8.44E-01 | 0.31±0.2 | 4.50E-01 | 0.22±0.2 | 6.23E-01 |
| X - 12111 | -0.19±0.15 | 0 | 7.12E-01 | 7.36E-01 | -0.21±0.14 | 0 | 5.11E-01 | 8.22E-01 | -0.27±0.2 | 8.40E-01 | -0.31±0.2 | 8.44E-01 | -0.15±0.19 | 7.57E-01 | -0.13±0.2 | 8.07E-01 |
| 5-methyluridine (ribothymidine) | 0.17±0.12 | 0 | 7.32E-01 | 7.36E-01 | 0.23±0.15 | 0 | 5.88E-01 | 8.23E-01 | 0.14±0.21 | 8.55E-01 | 0.15±0.21 | 8.59E-01 | 0.23±0.21 | 6.00E-01 | 0.31±0.22 | 5.22E-01 |
| retinol (Vitamin A) | 0.17±0.1 | 66.27 | 8.51E-02 | 7.36E-01 | 0.25±0.17 | 58.05 | 1.23E-01 | 8.23E-01 | 0.1±0.21 | 9.03E-01 | 0.04±0.22 | 9.71E-01 | 0.59±0.26 | 2.43E-01 | 0.56±0.26 | 3.24E-01 |
| gamma-glutamylglycine | -0.15±0.16 | 0 | 4.89E-01 | 7.36E-01 | -0.21±0.14 | 55.94 | 1.32E-01 | 8.37E-01 | -0.41±0.19 | 8.16E-01 | -0.45±0.21 | 8.44E-01 | -0.1±0.17 | 8.00E-01 | -0.02±0.19 | 9.71E-01 |
| 6-bromotryptophan | 0.09±0.06 | 59.29 | 1.17E-01 | 7.36E-01 | 0.22±0.15 | 52.02 | 1.49E-01 | 8.42E-01 | 0.06±0.2 | 9.38E-01 | 0.01±0.21 | 9.98E-01 | 0.4±0.21 | 3.39E-01 | 0.44±0.21 | 3.41E-01 |
| heneicosapentaenoate (21:5n3) | -0.01±0.01 | 56.93 | 1.28E-01 | 7.36E-01 | -0.21±0.14 | 0 | 3.37E-01 | 8.42E-01 | -0.01±0.19 | 9.92E-01 | -0.07±0.2 | 9.27E-01 | -0.3±0.19 | 4.49E-01 | -0.35±0.2 | 4.33E-01 |
| N,N-dimethylalanine | -0.23±0.13 | 0 | 7.06E-01 | 7.36E-01 | -0.21±0.14 | 0 | 7.46E-01 | 8.42E-01 | -0.18±0.22 | 8.55E-01 | -0.16±0.22 | 8.59E-01 | -0.28±0.18 | 4.51E-01 | -0.25±0.19 | 5.46E-01 |
| (S)-3-hydroxybutyrylcarnitine | 0.23±0.14 | 0 | 7.35E-01 | 7.36E-01 | 0.21±0.14 | 0 | 8.96E-01 | 8.42E-01 | 0.19±0.19 | 8.55E-01 | 0.19±0.2 | 8.45E-01 | 0.28±0.2 | 4.95E-01 | 0.23±0.2 | 6.13E-01 |
| X - 12740 | 0.21±0.14 | 0 | 9.02E-01 | 7.36E-01 | 0.2±0.14 | 0 | 8.45E-01 | 8.42E-01 | 0.23±0.2 | 8.40E-01 | 0.23±0.2 | 8.44E-01 | 0.2±0.18 | 6.08E-01 | 0.18±0.19 | 6.82E-01 |
| glutamine | 0±0.23 | 22.36 | 2.56E-01 | 9.99E-01 | -0.26±0.18 | 77.15 | 3.64E-02 | 8.42E-01 | -0.48±0.2 | 8.16E-01 | -0.53±0.22 | 8.44E-01 | 0.14±0.26 | 8.25E-01 | 0.26±0.31 | 7.11E-01 |
| sphingomyelin (d18:1/22:2, d18:2/22:1, d16:1/24:2)* | -0.2±0.15 | 0 | 6.82E-01 | 7.36E-01 | -0.19±0.13 | 0 | 5.85E-01 | 8.42E-01 | -0.31±0.21 | 8.19E-01 | -0.29±0.22 | 8.44E-01 | -0.17±0.17 | 6.46E-01 | -0.14±0.17 | 7.17E-01 |
| 3-methoxycatechol sulfate (1) | -0.11±0.17 | 0 | 4.32E-01 | 7.71E-01 | -0.2±0.14 | 49.34 | 1.60E-01 | 8.42E-01 | -0.38±0.19 | 8.16E-01 | -0.4±0.2 | 8.44E-01 | -0.05±0.19 | 9.34E-01 | -0.01±0.19 | 9.88E-01 |
| caprylate (8:0) | 0.16±0.1 | 0 | 6.17E-01 | 7.36E-01 | 0.2±0.13 | 0 | 5.39E-01 | 8.44E-01 | 0.12±0.19 | 8.71E-01 | 0.1±0.2 | 8.97E-01 | 0.23±0.17 | 5.26E-01 | 0.27±0.18 | 4.95E-01 |
| gamma-glutamyltryptophan | 0.26±0.15 | 0 | 7.39E-01 | 7.36E-01 | 0.21±0.14 | 0 | 8.68E-01 | 8.52E-01 | 0.21±0.19 | 8.40E-01 | 0.18±0.2 | 8.45E-01 | 0.31±0.21 | 4.76E-01 | 0.23±0.2 | 6.11E-01 |
| 1-(1-enyl-palmitoyl)-2-linoleoyl-GPC (P-16:0/18:2)* | -0.17±0.09 | 0 | 4.96E-01 | 7.36E-01 | -0.19±0.13 | 0 | 9.48E-01 | 8.54E-01 | -0.12±0.2 | 8.75E-01 | -0.18±0.21 | 8.45E-01 | -0.25±0.15 | 4.18E-01 | -0.19±0.17 | 6.03E-01 |
| 4-vinylguaiacol sulfate | -0.2±0.17 | 0 | 6.44E-01 | 7.36E-01 | -0.21±0.15 | 0 | 4.63E-01 | 8.54E-01 | -0.32±0.19 | 8.16E-01 | -0.31±0.2 | 8.44E-01 | -0.15±0.21 | 7.79E-01 | -0.09±0.22 | 8.65E-01 |
| N-acetylglycine | -0.13±0.08 | 12.51 | 2.85E-01 | 7.36E-01 | -0.2±0.14 | 0 | 5.84E-01 | 8.54E-01 | -0.09±0.2 | 9.06E-01 | -0.12±0.21 | 8.72E-01 | -0.31±0.18 | 4.12E-01 | -0.28±0.19 | 5.22E-01 |
| leucine | 0.02±0.02 | 81.67 | 1.95E-02 | 7.36E-01 | 0.27±0.19 | 64.93 | 9.13E-02 | 8.58E-01 | 0.02±0.22 | 9.90E-01 | 0.05±0.23 | 9.58E-01 | 0.76±0.32 | 2.11E-01 | 0.74±0.34 | 3.06E-01 |
| 2-methylbutyrylcarnitine (C5) | 0.06±0.1 | 90.43 | 1.23E-03 | 7.86E-01 | 0.22±0.16 | 85.75 | 8.06E-03 | 8.62E-01 | -0.12±0.21 | 8.80E-01 | -0.18±0.22 | 8.45E-01 | 0.69±0.22 | 9.55E-02 | 0.65±0.22 | 1.81E-01 |
| X - 17654 | -0.2±0.15 | 0 | 8.30E-01 | 7.36E-01 | -0.19±0.13 | 0 | 7.79E-01 | 8.62E-01 | -0.25±0.17 | 8.19E-01 | -0.22±0.18 | 8.44E-01 | -0.18±0.19 | 6.61E-01 | -0.15±0.19 | 7.60E-01 |
| sphingosine | 0.18±0.16 | 0 | 6.37E-01 | 7.36E-01 | 0.2±0.14 | 0 | 5.24E-01 | 8.62E-01 | 0.3±0.19 | 8.16E-01 | 0.28±0.19 | 8.44E-01 | 0.13±0.19 | 7.87E-01 | 0.11±0.19 | 8.29E-01 |
| 1-stearoyl-GPC (18:0) | -0.1±0.17 | 0 | 4.08E-01 | 7.83E-01 | -0.2±0.14 | 65.13 | 9.04E-02 | 8.62E-01 | -0.43±0.2 | 8.16E-01 | -0.46±0.21 | 8.44E-01 | -0.05±0.18 | 9.34E-01 | 0.02±0.2 | 9.69E-01 |
| 1-lignoceroyl-GPC (24:0) | -0.13±0.17 | 0 | 4.63E-01 | 7.50E-01 | -0.22±0.15 | 57.11 | 1.27E-01 | 8.62E-01 | -0.38±0.2 | 8.16E-01 | -0.44±0.21 | 8.44E-01 | -0.07±0.19 | 9.15E-01 | 0.03±0.22 | 9.73E-01 |
| hyocholate | -0.09±0.17 | 0 | 3.97E-01 | 8.02E-01 | -0.19±0.14 | 51.59 | 1.51E-01 | 8.62E-01 | -0.38±0.19 | 8.16E-01 | -0.39±0.19 | 8.44E-01 | -0.02±0.19 | 9.74E-01 | 0.01±0.2 | 9.94E-01 |
| X - 25957 | 0.02±0.02 | 52.92 | 1.45E-01 | 7.36E-01 | 0.19±0.14 | 13.59 | 2.82E-01 | 8.73E-01 | 0.02±0.21 | 9.90E-01 | 0.02±0.21 | 9.82E-01 | 0.28±0.18 | 4.50E-01 | 0.31±0.18 | 4.20E-01 |
| gamma-glutamyl-2-aminobutyrate | 0.27±0.15 | 0 | 8.05E-01 | 7.36E-01 | 0.22±0.15 | 0 | 7.39E-01 | 8.73E-01 | 0.23±0.23 | 8.55E-01 | 0.16±0.22 | 8.59E-01 | 0.31±0.21 | 4.69E-01 | 0.26±0.21 | 5.78E-01 |
| sphingomyelin (d18:1/20:1, d18:2/20:0)* | -0.21±0.13 | 0 | 8.68E-01 | 7.36E-01 | -0.18±0.13 | 0 | 8.22E-01 | 8.73E-01 | -0.24±0.21 | 8.40E-01 | -0.22±0.22 | 8.44E-01 | -0.19±0.15 | 5.57E-01 | -0.16±0.16 | 6.59E-01 |
| sphingomyelin (d18:2/24:1, d18:1/24:2)* | -0.19±0.12 | 0 | 7.89E-01 | 7.36E-01 | -0.17±0.12 | 0 | 7.01E-01 | 8.73E-01 | -0.25±0.21 | 8.40E-01 | -0.24±0.21 | 8.44E-01 | -0.17±0.15 | 5.81E-01 | -0.14±0.15 | 6.95E-01 |
| N-palmitoylserine | 0.18±0.12 | 0 | 8.04E-01 | 7.36E-01 | 0.2±0.15 | 0 | 8.51E-01 | 8.74E-01 | 0.16±0.21 | 8.55E-01 | 0.17±0.22 | 8.52E-01 | 0.22±0.2 | 6.00E-01 | 0.23±0.2 | 6.04E-01 |
| hydroxy-CMPF* | -0.16±0.12 | 0 | 9.64E-01 | 7.36E-01 | -0.18±0.13 | 0 | 8.77E-01 | 8.77E-01 | -0.16±0.19 | 8.55E-01 | -0.21±0.19 | 8.44E-01 | -0.17±0.18 | 6.61E-01 | -0.16±0.19 | 7.06E-01 |
| isoleucine | 0.01±0.11 | 90.06 | 1.52E-03 | 9.56E-01 | 0.25±0.19 | 83.81 | 1.29E-02 | 8.83E-01 | -0.11±0.22 | 8.97E-01 | -0.08±0.23 | 9.33E-01 | 0.9±0.3 | 1.08E-01 | 0.92±0.33 | 1.87E-01 |
| picolinate | 0.02±0.02 | 69.66 | 6.94E-02 | 7.36E-01 | 0.19±0.14 | 49.96 | 1.57E-01 | 8.83E-01 | 0.02±0.18 | 9.90E-01 | 0.01±0.19 | 9.84E-01 | 0.41±0.22 | 3.58E-01 | 0.42±0.21 | 3.70E-01 |
| 2-pyrrolidinone | 0.07±0.05 | 57.7 | 1.24E-01 | 7.36E-01 | 0.22±0.16 | 48.45 | 1.64E-01 | 8.83E-01 | 0.05±0.21 | 9.54E-01 | 0.04±0.21 | 9.71E-01 | 0.45±0.25 | 3.88E-01 | 0.5±0.26 | 3.74E-01 |
| X - 24456 | 0.08±0.06 | 0.1 | 3.17E-01 | 7.36E-01 | 0.19±0.14 | 10.62 | 2.90E-01 | 8.83E-01 | 0.06±0.21 | 9.38E-01 | 0.02±0.21 | 9.76E-01 | 0.26±0.19 | 4.97E-01 | 0.33±0.19 | 4.36E-01 |
| 11beta-hydroxyetiocholanolone glucuronide* | -0.05±0.04 | 65.01 | 9.09E-02 | 7.36E-01 | -0.18±0.14 | 2.27 | 3.12E-01 | 8.83E-01 | -0.04±0.2 | 9.57E-01 | -0.03±0.2 | 9.71E-01 | -0.35±0.18 | 3.39E-01 | -0.31±0.18 | 4.43E-01 |
| X - 18886 | 0.21±0.11 | 38.96 | 2.01E-01 | 7.09E-01 | 0.21±0.15 | 0 | 3.26E-01 | 8.83E-01 | 0.13±0.21 | 8.75E-01 | 0.05±0.22 | 9.55E-01 | 0.43±0.21 | 2.81E-01 | 0.35±0.22 | 4.62E-01 |
| ergothioneine | 0.22±0.13 | 0 | 6.29E-01 | 7.36E-01 | 0.21±0.16 | 0 | 3.98E-01 | 8.83E-01 | 0.17±0.22 | 8.55E-01 | 0.08±0.22 | 9.33E-01 | 0.3±0.2 | 4.76E-01 | 0.34±0.22 | 4.73E-01 |
| fumarate | 0.21±0.14 | 0 | 6.49E-01 | 7.36E-01 | 0.25±0.18 | 0 | 5.58E-01 | 8.83E-01 | 0.17±0.25 | 8.55E-01 | 0.15±0.25 | 8.70E-01 | 0.32±0.26 | 5.69E-01 | 0.36±0.27 | 5.44E-01 |
| X - 24588 | 0.31±0.17 | 0 | 6.51E-01 | 7.36E-01 | 0.23±0.17 | 0 | 6.38E-01 | 8.83E-01 | 0.23±0.22 | 8.40E-01 | 0.16±0.23 | 8.59E-01 | 0.39±0.25 | 4.50E-01 | 0.32±0.25 | 5.62E-01 |
| heptenedioate (C7:1-DC)* | 0.25±0.16 | 0 | 9.56E-01 | 7.36E-01 | 0.21±0.16 | 0 | 9.50E-01 | 8.83E-01 | 0.24±0.22 | 8.40E-01 | 0.2±0.22 | 8.45E-01 | 0.26±0.23 | 5.97E-01 | 0.22±0.23 | 6.72E-01 |
| 1-stearoyl-2-arachidonoyl-GPI (18:0/20:4) | 0.22±0.16 | 0 | 8.64E-01 | 7.36E-01 | 0.22±0.17 | 0 | 8.72E-01 | 8.83E-01 | 0.2±0.22 | 8.55E-01 | 0.2±0.22 | 8.45E-01 | 0.26±0.26 | 6.49E-01 | 0.25±0.25 | 6.73E-01 |
| 2-palmitoleoyl-GPC (16:1)* | 0.18±0.14 | 0 | 9.39E-01 | 7.36E-01 | 0.19±0.14 | 0 | 9.50E-01 | 8.83E-01 | 0.19±0.2 | 8.55E-01 | 0.2±0.2 | 8.45E-01 | 0.17±0.2 | 7.18E-01 | 0.18±0.2 | 6.95E-01 |
| cortisone | 0.14±0.17 | 0 | 4.93E-01 | 7.37E-01 | 0.21±0.16 | 0 | 5.34E-01 | 8.83E-01 | 0.37±0.28 | 8.40E-01 | 0.36±0.28 | 8.44E-01 | 0.08±0.19 | 8.69E-01 | 0.14±0.2 | 7.73E-01 |
| N-methylproline | -0.19±0.14 | 0 | 8.64E-01 | 7.36E-01 | -0.18±0.14 | 0 | 7.06E-01 | 8.83E-01 | -0.22±0.19 | 8.40E-01 | -0.24±0.19 | 8.44E-01 | -0.17±0.18 | 6.67E-01 | -0.14±0.19 | 7.79E-01 |
| N-stearoyl-sphingosine (d18:1/18:0)* | 0.23±0.18 | 0 | 7.92E-01 | 7.36E-01 | 0.22±0.16 | 0 | 5.36E-01 | 8.83E-01 | 0.29±0.22 | 8.40E-01 | 0.32±0.23 | 8.44E-01 | 0.19±0.23 | 7.29E-01 | 0.13±0.22 | 8.22E-01 |
| X - 12462 | -0.16±0.16 | 0 | 5.52E-01 | 7.36E-01 | -0.19±0.14 | 0 | 3.28E-01 | 8.83E-01 | -0.33±0.22 | 8.16E-01 | -0.36±0.22 | 8.44E-01 | -0.11±0.18 | 8.02E-01 | -0.08±0.18 | 8.60E-01 |
| 1,3-dimethylurate | 0±0.17 | 27.63 | 2.40E-01 | 9.91E-01 | -0.18±0.13 | 69.24 | 7.14E-02 | 8.83E-01 | -0.33±0.17 | 8.16E-01 | -0.39±0.18 | 8.44E-01 | 0.12±0.2 | 8.00E-01 | 0.08±0.19 | 8.66E-01 |
| 3-hydroxydecanoate | 0.14±0.17 | 0 | 4.97E-01 | 7.39E-01 | 0.19±0.14 | 8.95 | 2.95E-01 | 8.83E-01 | 0.36±0.19 | 8.16E-01 | 0.33±0.2 | 8.44E-01 | 0.08±0.19 | 8.83E-01 | 0.04±0.19 | 9.41E-01 |
| 1-linoleoyl-GPC (18:2) | -0.13±0.15 | 0 | 4.81E-01 | 7.36E-01 | -0.19±0.14 | 60.16 | 1.13E-01 | 8.83E-01 | -0.38±0.21 | 8.16E-01 | -0.47±0.23 | 8.44E-01 | -0.09±0.16 | 8.17E-01 | -0.01±0.19 | 9.94E-01 |
| perfluorooctanesulfonate (PFOS) | -0.13±0.19 | 0 | 4.43E-01 | 7.65E-01 | -0.21±0.16 | 51.79 | 1.50E-01 | 8.83E-01 | -0.39±0.23 | 8.16E-01 | -0.46±0.23 | 8.44E-01 | -0.05±0.21 | 9.38E-01 | 0±0.22 | 9.96E-01 |
| 5,6-dihydrouridine | 0.13±0.08 | 19.52 | 2.65E-01 | 7.36E-01 | 0.2±0.15 | 0 | 4.60E-01 | 8.85E-01 | 0.09±0.22 | 9.13E-01 | 0.08±0.22 | 9.27E-01 | 0.34±0.21 | 4.35E-01 | 0.31±0.21 | 5.18E-01 |
| nicotinamide | 0.15±0.16 | 0 | 5.74E-01 | 7.36E-01 | 0.18±0.14 | 0 | 4.40E-01 | 8.85E-01 | 0.28±0.19 | 8.19E-01 | 0.29±0.2 | 8.44E-01 | 0.09±0.19 | 8.58E-01 | 0.08±0.19 | 8.84E-01 |
| glycochenodeoxycholate 3-sulfate | -0.14±0.16 | 0 | 5.41E-01 | 7.36E-01 | -0.18±0.13 | 0 | 5.83E-01 | 8.97E-01 | -0.28±0.19 | 8.16E-01 | -0.25±0.19 | 8.44E-01 | -0.07±0.19 | 9.03E-01 | -0.1±0.19 | 8.41E-01 |
| alpha-ketoglutarate | -0.01±0.02 | 89.42 | 2.11E-03 | 7.40E-01 | 0.2±0.16 | 82.07 | 1.82E-02 | 8.98E-01 | -0.02±0.19 | 9.90E-01 | -0.04±0.19 | 9.61E-01 | 0.84±0.28 | 1.08E-01 | 0.78±0.29 | 2.25E-01 |
| X - 13553 | 0.06±0.12 | 84.26 | 1.17E-02 | 8.02E-01 | 0.2±0.15 | 78.33 | 3.17E-02 | 8.98E-01 | -0.15±0.21 | 8.55E-01 | -0.14±0.22 | 8.63E-01 | 0.5±0.21 | 2.11E-01 | 0.52±0.21 | 2.62E-01 |
| indoleacetate | 0.16±0.14 | 81.08 | 2.15E-02 | 7.36E-01 | 0.18±0.15 | 79.66 | 2.66E-02 | 8.98E-01 | -0.21±0.23 | 8.55E-01 | -0.24±0.24 | 8.44E-01 | 0.43±0.18 | 2.12E-01 | 0.44±0.19 | 2.77E-01 |
| 3,4-dihydroxybutyrate | -0.01±0.08 | 82.92 | 1.55E-02 | 9.46E-01 | 0.21±0.16 | 68.15 | 7.64E-02 | 8.98E-01 | -0.08±0.22 | 9.28E-01 | -0.06±0.23 | 9.44E-01 | 0.5±0.23 | 2.46E-01 | 0.52±0.24 | 3.24E-01 |
| vanillylmandelate (VMA) | -0.03±0.04 | 74.23 | 4.89E-02 | 7.66E-01 | 0.21±0.17 | 63.94 | 9.58E-02 | 8.98E-01 | -0.04±0.22 | 9.61E-01 | -0.05±0.23 | 9.57E-01 | 0.42±0.23 | 3.77E-01 | 0.5±0.24 | 3.41E-01 |
| gamma-glutamylleucine | -0.01±0.01 | 82.39 | 1.72E-02 | 7.39E-01 | 0.2±0.16 | 59.54 | 1.16E-01 | 8.98E-01 | -0.01±0.21 | 9.90E-01 | -0.05±0.23 | 9.61E-01 | 0.51±0.22 | 2.25E-01 | 0.45±0.22 | 3.51E-01 |
| glutarate (C5-DC) | 0.11±0.07 | 49.24 | 1.60E-01 | 7.36E-01 | 0.19±0.15 | 27.31 | 2.41E-01 | 8.98E-01 | 0.07±0.2 | 9.30E-01 | 0.02±0.2 | 9.75E-01 | 0.39±0.21 | 3.70E-01 | 0.37±0.21 | 4.32E-01 |
| malate | 0.04±0.03 | 41.16 | 1.92E-01 | 7.36E-01 | 0.19±0.15 | 23.9 | 2.52E-01 | 8.98E-01 | 0.03±0.2 | 9.65E-01 | 0.02±0.21 | 9.80E-01 | 0.31±0.21 | 4.74E-01 | 0.36±0.21 | 4.33E-01 |
| ascorbic acid 2-sulfate | 0.1±0.07 | 0 | 3.34E-01 | 7.36E-01 | 0.2±0.16 | 25.74 | 2.46E-01 | 8.98E-01 | 0.08±0.21 | 9.30E-01 | 0.05±0.21 | 9.54E-01 | 0.34±0.26 | 5.34E-01 | 0.45±0.27 | 4.50E-01 |
| X - 22509 | 0.07±0.05 | 0 | 3.91E-01 | 7.36E-01 | 0.18±0.14 | 7.82 | 2.98E-01 | 8.98E-01 | 0.06±0.2 | 9.38E-01 | 0.02±0.2 | 9.76E-01 | 0.23±0.19 | 5.71E-01 | 0.32±0.2 | 4.67E-01 |
| 2-hydroxyphenylacetate | 0.12±0.09 | 0 | 4.02E-01 | 7.36E-01 | 0.2±0.16 | 0 | 3.91E-01 | 8.98E-01 | 0.09±0.21 | 9.09E-01 | 0.09±0.21 | 9.27E-01 | 0.31±0.25 | 5.53E-01 | 0.37±0.25 | 5.18E-01 |
| argininate* | 0.11±0.07 | 21.26 | 2.60E-01 | 7.36E-01 | 0.19±0.15 | 0 | 4.26E-01 | 8.98E-01 | 0.08±0.2 | 9.19E-01 | 0.07±0.21 | 9.33E-01 | 0.33±0.21 | 4.45E-01 | 0.31±0.21 | 5.19E-01 |
| X - 12410 | 0.12±0.09 | 0 | 5.69E-01 | 7.36E-01 | 0.19±0.14 | 0 | 5.11E-01 | 8.98E-01 | 0.09±0.2 | 9.04E-01 | 0.09±0.21 | 9.21E-01 | 0.22±0.19 | 5.97E-01 | 0.28±0.2 | 5.39E-01 |
| deoxycholic acid glucuronide | 0.16±0.1 | 7.37 | 2.99E-01 | 7.36E-01 | 0.19±0.15 | 0 | 4.93E-01 | 8.98E-01 | 0.11±0.21 | 8.93E-01 | 0.09±0.21 | 9.23E-01 | 0.35±0.21 | 4.12E-01 | 0.29±0.21 | 5.44E-01 |
| androstenediol (3beta,17beta) monosulfate (2) | 0.18±0.11 | 0 | 3.26E-01 | 7.36E-01 | 0.2±0.16 | 0 | 4.98E-01 | 8.98E-01 | 0.12±0.21 | 8.75E-01 | 0.11±0.21 | 8.90E-01 | 0.38±0.23 | 4.27E-01 | 0.32±0.24 | 5.44E-01 |
| trimethylamine N-oxide | 0.13±0.09 | 0 | 4.47E-01 | 7.36E-01 | 0.18±0.14 | 0 | 5.13E-01 | 8.98E-01 | 0.1±0.19 | 8.99E-01 | 0.08±0.2 | 9.27E-01 | 0.26±0.19 | 5.10E-01 | 0.26±0.2 | 5.44E-01 |
| 1-oleoyl-2-arachidonoyl-GPE (18:1/20:4)* | 0.15±0.1 | 0 | 5.21E-01 | 7.36E-01 | 0.22±0.17 | 0 | 5.63E-01 | 8.98E-01 | 0.11±0.24 | 9.03E-01 | 0.11±0.25 | 9.16E-01 | 0.28±0.23 | 5.66E-01 | 0.31±0.23 | 5.47E-01 |
| mannitol/sorbitol | 0.12±0.08 | 0 | 4.67E-01 | 7.36E-01 | 0.16±0.13 | 0 | 5.67E-01 | 8.98E-01 | 0.09±0.2 | 9.04E-01 | 0.07±0.2 | 9.28E-01 | 0.23±0.17 | 4.99E-01 | 0.22±0.17 | 5.60E-01 |
| N6,N6-dimethyllysine | 0.13±0.09 | 0 | 4.89E-01 | 7.36E-01 | 0.19±0.16 | 0 | 5.50E-01 | 8.98E-01 | 0.1±0.21 | 8.98E-01 | 0.11±0.21 | 8.86E-01 | 0.28±0.23 | 5.65E-01 | 0.3±0.23 | 5.62E-01 |
| 2-O-methylascorbic acid | 0.24±0.13 | 0 | 5.69E-01 | 7.36E-01 | 0.18±0.15 | 0 | 6.26E-01 | 8.98E-01 | 0.18±0.21 | 8.55E-01 | 0.1±0.22 | 9.13E-01 | 0.33±0.21 | 4.35E-01 | 0.25±0.2 | 5.85E-01 |
| X - 24518 | 0.18±0.11 | 0 | 3.90E-01 | 7.36E-01 | 0.2±0.16 | 0 | 7.14E-01 | 8.98E-01 | 0.12±0.21 | 8.75E-01 | 0.14±0.23 | 8.65E-01 | 0.33±0.2 | 4.37E-01 | 0.25±0.22 | 6.03E-01 |
| X - 10458 | 0.18±0.12 | 0 | 6.58E-01 | 7.36E-01 | 0.18±0.15 | 0 | 6.56E-01 | 8.98E-01 | 0.15±0.2 | 8.55E-01 | 0.12±0.2 | 8.70E-01 | 0.26±0.21 | 5.66E-01 | 0.25±0.21 | 6.03E-01 |
| lysine | 0.23±0.16 | 0 | 6.02E-01 | 7.36E-01 | 0.23±0.19 | 0 | 5.40E-01 | 8.98E-01 | 0.18±0.21 | 8.55E-01 | 0.16±0.22 | 8.59E-01 | 0.39±0.35 | 6.00E-01 | 0.42±0.36 | 6.03E-01 |
| 2-aminobutyrate | 0.23±0.14 | 0 | 7.86E-01 | 7.36E-01 | 0.18±0.15 | 0 | 7.34E-01 | 8.98E-01 | 0.19±0.19 | 8.55E-01 | 0.13±0.2 | 8.62E-01 | 0.27±0.21 | 5.35E-01 | 0.23±0.21 | 6.19E-01 |
| o-cresol sulfate | -0.24±0.14 | 0 | 7.05E-01 | 7.36E-01 | -0.21±0.17 | 0 | 8.64E-01 | 8.98E-01 | -0.19±0.21 | 8.55E-01 | -0.18±0.25 | 8.59E-01 | -0.3±0.21 | 4.81E-01 | -0.24±0.24 | 6.56E-01 |
| 1-palmitoleoyl-GPC (16:1)* | 0.22±0.15 | 0 | 9.46E-01 | 7.36E-01 | 0.18±0.15 | 0 | 9.10E-01 | 8.98E-01 | 0.21±0.2 | 8.40E-01 | 0.17±0.2 | 8.45E-01 | 0.23±0.23 | 6.31E-01 | 0.2±0.22 | 6.92E-01 |
| dehydroepiandrosterone sulfate (DHEA-S) | 0.18±0.14 | 0 | 8.74E-01 | 7.36E-01 | 0.19±0.15 | 0 | 8.98E-01 | 8.98E-01 | 0.2±0.22 | 8.55E-01 | 0.21±0.23 | 8.45E-01 | 0.16±0.21 | 7.57E-01 | 0.17±0.21 | 7.35E-01 |
| palmitoylcarnitine (C16) | 0.23±0.17 | 0 | 8.23E-01 | 7.36E-01 | 0.19±0.15 | 0 | 7.95E-01 | 8.98E-01 | 0.27±0.2 | 8.35E-01 | 0.23±0.2 | 8.44E-01 | 0.19±0.23 | 7.15E-01 | 0.15±0.22 | 7.89E-01 |
| tridecenedioate (C13:1-DC)* | 0.18±0.16 | 0 | 6.58E-01 | 7.36E-01 | 0.18±0.14 | 0 | 6.46E-01 | 8.98E-01 | 0.28±0.2 | 8.35E-01 | 0.25±0.21 | 8.44E-01 | 0.13±0.2 | 7.94E-01 | 0.12±0.2 | 8.13E-01 |
| sulfate of piperine metabolite C18H21NO3 (1)* | -0.11±0.1 | 0 | 8.13E-01 | 7.36E-01 | -0.18±0.14 | 0 | 5.73E-01 | 8.98E-01 | -0.13±0.2 | 8.65E-01 | -0.27±0.21 | 8.44E-01 | -0.07±0.18 | 8.93E-01 | -0.11±0.19 | 8.20E-01 |
| sulfate of piperine metabolite C18H21NO3 (3)* | -0.11±0.12 | 0 | 7.24E-01 | 7.36E-01 | -0.18±0.14 | 0 | 5.58E-01 | 8.98E-01 | -0.15±0.2 | 8.55E-01 | -0.27±0.21 | 8.44E-01 | -0.06±0.19 | 9.15E-01 | -0.1±0.19 | 8.29E-01 |
| 1-(1-enyl-palmitoyl)-GPC (P-16:0)* | -0.16±0.12 | 0 | 8.06E-01 | 7.36E-01 | -0.16±0.13 | 0 | 5.00E-01 | 8.98E-01 | -0.2±0.19 | 8.55E-01 | -0.26±0.2 | 8.44E-01 | -0.13±0.16 | 7.13E-01 | -0.08±0.17 | 8.47E-01 |
| biliverdin | 0.14±0.16 | 0 | 5.23E-01 | 7.40E-01 | 0.18±0.15 | 0 | 5.91E-01 | 8.98E-01 | 0.28±0.2 | 8.19E-01 | 0.25±0.21 | 8.44E-01 | 0.06±0.2 | 9.16E-01 | 0.1±0.21 | 8.57E-01 |
| 10-undecenoate (11:1n1) | 0.15±0.16 | 0 | 5.67E-01 | 7.36E-01 | 0.17±0.14 | 0 | 5.14E-01 | 8.98E-01 | 0.29±0.2 | 8.19E-01 | 0.27±0.21 | 8.44E-01 | 0.09±0.19 | 8.58E-01 | 0.09±0.19 | 8.59E-01 |
| sphingomyelin (d18:1/22:1, d18:2/22:0, d16:1/24:1)* | -0.13±0.15 | 0 | 5.09E-01 | 7.36E-01 | -0.16±0.13 | 27.89 | 2.39E-01 | 8.98E-01 | -0.32±0.21 | 8.16E-01 | -0.35±0.21 | 8.44E-01 | -0.08±0.17 | 8.48E-01 | -0.03±0.18 | 9.58E-01 |
| X - 24306 | 0.07±0.17 | 0 | 3.60E-01 | 8.46E-01 | 0.17±0.14 | 24.34 | 2.50E-01 | 8.98E-01 | 0.34±0.2 | 8.16E-01 | 0.34±0.2 | 8.44E-01 | -0.02±0.2 | 9.79E-01 | 0.02±0.2 | 9.81E-01 |
| hexadecenedioate (C16:1-DC)* | 0.11±0.16 | 0 | 4.47E-01 | 7.71E-01 | 0.16±0.13 | 1.95 | 3.13E-01 | 8.98E-01 | 0.3±0.17 | 8.16E-01 | 0.28±0.18 | 8.44E-01 | 0.03±0.2 | 9.68E-01 | 0.01±0.2 | 9.88E-01 |
| aconitate [cis or trans] | 0.01±0.09 | 89.38 | 2.15E-03 | 9.58E-01 | 0.19±0.16 | 83.49 | 1.38E-02 | 9.00E-01 | -0.09±0.2 | 9.04E-01 | -0.13±0.21 | 8.63E-01 | 0.69±0.24 | 1.22E-01 | 0.66±0.25 | 2.28E-01 |
| X - 18922 | 0.04±0.03 | 72.48 | 5.66E-02 | 7.36E-01 | 0.18±0.15 | 45.73 | 1.75E-01 | 9.00E-01 | 0.03±0.2 | 9.61E-01 | 0±0.2 | 9.99E-01 | 0.48±0.23 | 2.86E-01 | 0.42±0.23 | 4.10E-01 |
| dimethylglycine | -0.03±0.04 | 61.26 | 1.08E-01 | 7.65E-01 | 0.18±0.16 | 9.42 | 2.93E-01 | 9.00E-01 | -0.04±0.23 | 9.57E-01 | 0±0.24 | 9.99E-01 | 0.29±0.2 | 4.81E-01 | 0.33±0.21 | 4.67E-01 |
| 2-acetamidophenol sulfate | 0.02±0.02 | 8.69 | 2.95E-01 | 7.36E-01 | 0.17±0.15 | 0 | 3.59E-01 | 9.00E-01 | 0.02±0.19 | 9.90E-01 | 0.05±0.2 | 9.54E-01 | 0.23±0.2 | 5.97E-01 | 0.32±0.21 | 5.10E-01 |
| X - 12216 | 0.1±0.08 | 0 | 4.12E-01 | 7.36E-01 | 0.18±0.15 | 0 | 4.05E-01 | 9.00E-01 | 0.08±0.2 | 9.18E-01 | 0.06±0.21 | 9.38E-01 | 0.27±0.22 | 5.56E-01 | 0.32±0.23 | 5.31E-01 |
| 1-stearoyl-2-oleoyl-GPC (18:0/18:1) | 0.16±0.11 | 0 | 3.46E-01 | 7.36E-01 | 0.19±0.16 | 0 | 4.26E-01 | 9.00E-01 | 0.11±0.19 | 8.75E-01 | 0.1±0.19 | 9.02E-01 | 0.39±0.27 | 4.79E-01 | 0.35±0.26 | 5.44E-01 |
| N-acetylarginine | 0.14±0.09 | 0 | 5.03E-01 | 7.36E-01 | 0.17±0.15 | 0 | 5.20E-01 | 9.00E-01 | 0.1±0.2 | 8.93E-01 | 0.08±0.2 | 9.27E-01 | 0.26±0.21 | 5.56E-01 | 0.27±0.21 | 5.60E-01 |
| 1-stearoyl-2-arachidonoyl-GPC (18:0/20:4) | 0.23±0.16 | 0 | 7.98E-01 | 7.36E-01 | 0.21±0.17 | 0 | 8.86E-01 | 9.00E-01 | 0.2±0.23 | 8.55E-01 | 0.18±0.24 | 8.58E-01 | 0.28±0.27 | 6.22E-01 | 0.23±0.25 | 6.92E-01 |
| ceramide (d18:1/14:0, d16:1/16:0)* | 0.17±0.15 | 0 | 8.65E-01 | 7.36E-01 | 0.18±0.15 | 0 | 8.05E-01 | 9.00E-01 | 0.19±0.21 | 8.55E-01 | 0.21±0.21 | 8.44E-01 | 0.14±0.22 | 7.96E-01 | 0.14±0.22 | 8.05E-01 |
| X - 23787 | 0.17±0.15 | 0 | 6.68E-01 | 7.36E-01 | 0.17±0.14 | 0 | 6.22E-01 | 9.00E-01 | 0.27±0.21 | 8.40E-01 | 0.25±0.22 | 8.44E-01 | 0.13±0.19 | 7.79E-01 | 0.11±0.19 | 8.22E-01 |
| palmitoleoylcarnitine (C16:1)* | 0.01±0.18 | 10.35 | 2.91E-01 | 9.91E-01 | 0.16±0.13 | 77.04 | 3.69E-02 | 9.00E-01 | 0.49±0.2 | 8.16E-01 | 0.47±0.2 | 8.44E-01 | -0.06±0.19 | 9.15E-01 | -0.09±0.18 | 8.45E-01 |
| X - 17010 | 0.07±0.15 | 0 | 3.61E-01 | 8.32E-01 | 0.15±0.13 | 0 | 4.10E-01 | 9.00E-01 | 0.27±0.17 | 8.16E-01 | 0.25±0.17 | 8.44E-01 | -0.03±0.19 | 9.64E-01 | 0.03±0.19 | 9.49E-01 |
| glycochenodeoxycholate | 0.02±0.17 | 25.73 | 2.46E-01 | 9.60E-01 | -0.15±0.13 | 67.09 | 8.13E-02 | 9.04E-01 | -0.4±0.17 | 8.16E-01 | -0.35±0.17 | 8.44E-01 | 0.12±0.19 | 8.06E-01 | 0.11±0.2 | 8.27E-01 |
| glycosyl-N-behenoyl-sphingadienine (d18:2/22:0)* | -0.16±0.16 | 0 | 5.79E-01 | 7.36E-01 | -0.17±0.15 | 22.45 | 2.56E-01 | 9.04E-01 | -0.3±0.21 | 8.19E-01 | -0.36±0.22 | 8.44E-01 | -0.11±0.18 | 8.12E-01 | -0.02±0.2 | 9.81E-01 |
| proline | 0.05±0.12 | 85.8 | 7.96E-03 | 8.32E-01 | 0.18±0.16 | 82.78 | 1.59E-02 | 9.06E-01 | -0.14±0.21 | 8.55E-01 | -0.15±0.21 | 8.59E-01 | 0.57±0.23 | 1.85E-01 | 0.63±0.24 | 2.28E-01 |
| myristoylcarnitine (C14) | 0.03±0.17 | 3.16 | 3.10E-01 | 9.47E-01 | 0.16±0.14 | 68.24 | 7.60E-02 | 9.12E-01 | 0.45±0.2 | 8.16E-01 | 0.43±0.21 | 8.44E-01 | -0.05±0.19 | 9.38E-01 | -0.05±0.18 | 9.15E-01 |
| X - 11850 | -0.15±0.16 | 0 | 5.95E-01 | 7.36E-01 | -0.16±0.14 | 0 | 4.22E-01 | 9.12E-01 | -0.27±0.19 | 8.35E-01 | -0.28±0.2 | 8.44E-01 | -0.09±0.19 | 8.50E-01 | -0.06±0.19 | 9.16E-01 |
| 4-guanidinobutanoate | 0.31±0.18 | 81.43 | 2.03E-02 | 7.36E-01 | 0.19±0.16 | 88.35 | 3.39E-03 | 9.13E-01 | -0.33±0.24 | 8.35E-01 | -0.33±0.24 | 8.44E-01 | 0.59±0.22 | 1.63E-01 | 0.63±0.22 | 1.87E-01 |
| N-acetylvaline | 0.17±0.15 | 87.27 | 5.06E-03 | 7.36E-01 | 0.18±0.16 | 84.77 | 1.04E-02 | 9.13E-01 | -0.2±0.22 | 8.55E-01 | -0.23±0.23 | 8.44E-01 | 0.66±0.23 | 1.41E-01 | 0.61±0.23 | 2.28E-01 |
| 2R,3R-dihydroxybutyrate | -0.01±0.07 | 87.65 | 4.43E-03 | 9.47E-01 | 0.18±0.15 | 78.73 | 3.01E-02 | 9.13E-01 | -0.07±0.2 | 9.32E-01 | -0.12±0.21 | 8.70E-01 | 0.6±0.23 | 1.63E-01 | 0.55±0.23 | 2.62E-01 |
| alpha-hydroxyisovalerate | 0.07±0.1 | 83.77 | 1.31E-02 | 7.62E-01 | 0.16±0.14 | 70.62 | 6.51E-02 | 9.13E-01 | -0.13±0.23 | 8.75E-01 | -0.2±0.24 | 8.45E-01 | 0.4±0.17 | 2.11E-01 | 0.35±0.17 | 3.66E-01 |
| 3-methylglutaconate | -0.02±0.03 | 73.03 | 5.42E-02 | 7.44E-01 | 0.18±0.16 | 62.41 | 1.03E-01 | 9.13E-01 | -0.03±0.21 | 9.75E-01 | -0.06±0.22 | 9.48E-01 | 0.44±0.24 | 3.77E-01 | 0.48±0.25 | 3.70E-01 |
| 4-hydroxyhippurate | 0.01±0.01 | 53.77 | 1.41E-01 | 7.36E-01 | 0.15±0.14 | 37.75 | 2.05E-01 | 9.13E-01 | 0.01±0.21 | 9.92E-01 | -0.06±0.22 | 9.47E-01 | 0.27±0.17 | 4.50E-01 | 0.3±0.18 | 4.50E-01 |
| acisoga | 0.02±0.01 | 59.46 | 1.16E-01 | 7.36E-01 | 0.16±0.14 | 25.75 | 2.46E-01 | 9.13E-01 | 0.01±0.19 | 9.90E-01 | 0.01±0.19 | 9.98E-01 | 0.34±0.2 | 4.20E-01 | 0.33±0.21 | 4.67E-01 |
| bilirubin (Z,Z) | 0.14±0.1 | 0 | 5.92E-01 | 7.36E-01 | 0.18±0.16 | 0 | 4.25E-01 | 9.13E-01 | 0.11±0.2 | 8.81E-01 | 0.07±0.21 | 9.33E-01 | 0.25±0.23 | 6.04E-01 | 0.33±0.25 | 5.46E-01 |
| butyrylcarnitine (C4) | 0.07±0.05 | 28.91 | 2.36E-01 | 7.36E-01 | 0.15±0.14 | 0 | 4.40E-01 | 9.13E-01 | 0.05±0.19 | 9.46E-01 | 0.04±0.19 | 9.61E-01 | 0.28±0.19 | 4.65E-01 | 0.25±0.19 | 5.47E-01 |
| N-acetyl-2-aminooctanoate* | 0.16±0.12 | 0 | 9.87E-01 | 7.36E-01 | 0.16±0.14 | 0 | 7.63E-01 | 9.13E-01 | 0.16±0.2 | 8.55E-01 | 0.12±0.2 | 8.70E-01 | 0.16±0.19 | 7.15E-01 | 0.2±0.2 | 6.52E-01 |
| 1-stearoyl-2-docosahexaenoyl-GPC (18:0/22:6) | -0.11±0.08 | 0 | 6.43E-01 | 7.36E-01 | -0.15±0.14 | 0 | 8.45E-01 | 9.13E-01 | -0.09±0.2 | 9.06E-01 | -0.12±0.21 | 8.70E-01 | -0.18±0.18 | 6.36E-01 | -0.17±0.18 | 6.81E-01 |
| ethylmalonate | 0.11±0.08 | 0 | 6.60E-01 | 7.36E-01 | 0.15±0.13 | 0 | 8.12E-01 | 9.13E-01 | 0.09±0.19 | 9.04E-01 | 0.11±0.19 | 8.66E-01 | 0.18±0.19 | 6.61E-01 | 0.18±0.19 | 6.88E-01 |
| X - 25172 | 0.15±0.12 | 0 | 8.38E-01 | 7.36E-01 | 0.16±0.14 | 0 | 8.00E-01 | 9.13E-01 | 0.14±0.19 | 8.55E-01 | 0.13±0.19 | 8.59E-01 | 0.19±0.22 | 7.13E-01 | 0.2±0.22 | 6.95E-01 |
| 1-palmitoyl-2-arachidonoyl-GPE (16:0/20:4)* | 0.19±0.14 | 0 | 9.43E-01 | 7.36E-01 | 0.18±0.16 | 0 | 9.19E-01 | 9.13E-01 | 0.18±0.22 | 8.55E-01 | 0.16±0.23 | 8.59E-01 | 0.2±0.22 | 6.87E-01 | 0.19±0.21 | 7.02E-01 |
| 2-ketocaprylate | -0.13±0.11 | 0 | 9.61E-01 | 7.36E-01 | -0.15±0.13 | 0 | 8.95E-01 | 9.13E-01 | -0.14±0.19 | 8.55E-01 | -0.16±0.19 | 8.45E-01 | -0.13±0.17 | 7.75E-01 | -0.13±0.18 | 7.79E-01 |
| 3b-hydroxy-5-cholenoic acid | -0.14±0.11 | 0 | 9.75E-01 | 7.36E-01 | -0.15±0.14 | 0 | 9.13E-01 | 9.13E-01 | -0.14±0.19 | 8.55E-01 | -0.17±0.19 | 8.45E-01 | -0.15±0.19 | 7.54E-01 | -0.14±0.2 | 7.81E-01 |
| N-carbamoylvaline | -0.15±0.12 | 0 | 9.00E-01 | 7.36E-01 | -0.15±0.14 | 0 | 8.80E-01 | 9.13E-01 | -0.17±0.2 | 8.55E-01 | -0.17±0.2 | 8.45E-01 | -0.14±0.19 | 7.75E-01 | -0.13±0.19 | 7.81E-01 |
| 5alpha-androstan-3beta,17alpha-diol disulfate | 0.16±0.15 | 0 | 6.19E-01 | 7.36E-01 | 0.17±0.15 | 0 | 6.29E-01 | 9.13E-01 | 0.26±0.22 | 8.40E-01 | 0.25±0.22 | 8.44E-01 | 0.1±0.19 | 8.37E-01 | 0.1±0.2 | 8.45E-01 |
| oleoylcarnitine (C18:1) | 0.17±0.16 | 0 | 6.60E-01 | 7.36E-01 | 0.16±0.14 | 0 | 6.33E-01 | 9.13E-01 | 0.25±0.2 | 8.40E-01 | 0.23±0.2 | 8.44E-01 | 0.11±0.21 | 8.32E-01 | 0.09±0.21 | 8.60E-01 |
| X - 21383 | -0.14±0.13 | 0 | 7.48E-01 | 7.36E-01 | -0.16±0.14 | 0 | 5.61E-01 | 9.13E-01 | -0.17±0.2 | 8.55E-01 | -0.25±0.2 | 8.44E-01 | -0.09±0.19 | 8.44E-01 | -0.08±0.19 | 8.64E-01 |
| 3-methylcytidine | -0.03±0.17 | 18.98 | 2.67E-01 | 9.48E-01 | -0.15±0.13 | 57.75 | 1.24E-01 | 9.13E-01 | -0.29±0.18 | 8.16E-01 | -0.34±0.18 | 8.44E-01 | 0.1±0.2 | 8.37E-01 | 0.08±0.2 | 8.86E-01 |
| X - 13695 | -0.15±0.14 | 0 | 6.78E-01 | 7.36E-01 | -0.15±0.14 | 0 | 5.93E-01 | 9.13E-01 | -0.22±0.19 | 8.40E-01 | -0.22±0.19 | 8.44E-01 | -0.1±0.19 | 8.47E-01 | -0.08±0.2 | 8.86E-01 |
| N-methylhydroxyproline** | -0.14±0.14 | 0 | 6.34E-01 | 7.36E-01 | -0.15±0.13 | 0 | 4.60E-01 | 9.13E-01 | -0.23±0.19 | 8.40E-01 | -0.26±0.19 | 8.44E-01 | -0.09±0.18 | 8.37E-01 | -0.06±0.18 | 9.10E-01 |
| 1-linoleoyl-2-linolenoyl-GPC (18:2/18:3)* | -0.17±0.14 | 0 | 7.08E-01 | 7.36E-01 | -0.16±0.14 | 0 | 4.80E-01 | 9.13E-01 | -0.24±0.18 | 8.40E-01 | -0.26±0.2 | 8.44E-01 | -0.13±0.18 | 7.78E-01 | -0.06±0.2 | 9.14E-01 |
| X - 23655 | -0.06±0.17 | 0 | 3.48E-01 | 8.71E-01 | -0.15±0.14 | 59.3 | 1.17E-01 | 9.13E-01 | -0.38±0.19 | 8.16E-01 | -0.38±0.2 | 8.44E-01 | 0.02±0.19 | 9.79E-01 | 0.06±0.19 | 9.16E-01 |
| imidazole lactate | -0.08±0.17 | 0 | 3.71E-01 | 8.32E-01 | -0.16±0.14 | 47.81 | 1.66E-01 | 9.13E-01 | -0.33±0.21 | 8.16E-01 | -0.39±0.22 | 8.44E-01 | 0.01±0.19 | 9.82E-01 | 0±0.19 | 9.95E-01 |
| X - 19141 | -0.02±0.06 | 78.5 | 3.10E-02 | 8.35E-01 | 0.16±0.15 | 68.61 | 7.43E-02 | 9.14E-01 | -0.06±0.2 | 9.38E-01 | -0.08±0.2 | 9.27E-01 | 0.41±0.21 | 3.35E-01 | 0.44±0.22 | 3.42E-01 |
| X - 23639 | 0.03±0.19 | 24.12 | 2.51E-01 | 9.56E-01 | -0.16±0.15 | 82.82 | 1.58E-02 | 9.14E-01 | -0.47±0.19 | 8.16E-01 | -0.49±0.2 | 8.44E-01 | 0.12±0.21 | 8.13E-01 | 0.23±0.22 | 6.42E-01 |
| dihomo-linolenoylcarnitine (C20:3n3 or 6)* | 0.16±0.11 | 0 | 7.73E-01 | 7.36E-01 | 0.16±0.15 | 0 | 7.64E-01 | 9.14E-01 | 0.14±0.2 | 8.55E-01 | 0.12±0.2 | 8.70E-01 | 0.21±0.21 | 6.50E-01 | 0.21±0.21 | 6.82E-01 |
| X - 17325 | -0.06±0.15 | 0 | 3.52E-01 | 8.51E-01 | -0.14±0.13 | 30.52 | 2.30E-01 | 9.18E-01 | -0.29±0.18 | 8.16E-01 | -0.29±0.18 | 8.44E-01 | 0.03±0.18 | 9.67E-01 | 0.02±0.18 | 9.80E-01 |
| N-formylmethionine | 0.37±0.19 | 86.95 | 5.63E-03 | 6.94E-01 | 0.16±0.16 | 90.56 | 1.14E-03 | 9.19E-01 | -0.31±0.22 | 8.30E-01 | -0.32±0.22 | 8.44E-01 | 0.77±0.24 | 8.23E-02 | 0.74±0.24 | 1.47E-01 |
| erythronate* | 0.21±0.19 | 88.16 | 3.66E-03 | 7.36E-01 | 0.21±0.19 | 88.38 | 3.35E-03 | 9.19E-01 | -0.24±0.24 | 8.55E-01 | -0.25±0.25 | 8.44E-01 | 0.87±0.3 | 1.13E-01 | 0.92±0.31 | 1.68E-01 |
| beta-citrylglutamate | 0.05±0.12 | 91.36 | 6.67E-04 | 8.32E-01 | 0.17±0.17 | 88.1 | 3.75E-03 | 9.19E-01 | -0.13±0.21 | 8.62E-01 | -0.2±0.22 | 8.45E-01 | 0.96±0.29 | 7.84E-02 | 0.86±0.3 | 1.77E-01 |
| gamma-glutamylisoleucine* | 0.2±0.17 | 89.57 | 1.95E-03 | 7.36E-01 | 0.17±0.18 | 87.64 | 4.44E-03 | 9.19E-01 | -0.22±0.22 | 8.55E-01 | -0.26±0.24 | 8.44E-01 | 0.84±0.27 | 8.69E-02 | 0.79±0.28 | 1.87E-01 |
| 1-ribosyl-imidazoleacetate* | 0.34±0.18 | 75.6 | 4.29E-02 | 7.36E-01 | 0.15±0.16 | 89 | 2.57E-03 | 9.19E-01 | -0.41±0.25 | 8.16E-01 | -0.43±0.25 | 8.44E-01 | 0.51±0.19 | 1.73E-01 | 0.53±0.2 | 2.28E-01 |
| 2,3-dihydroxy-5-methylthio-4-pentenoate (DMTPA)* | 0.1±0.13 | 87 | 5.55E-03 | 7.60E-01 | 0.17±0.17 | 82.18 | 1.78E-02 | 9.19E-01 | -0.16±0.23 | 8.55E-01 | -0.23±0.24 | 8.45E-01 | 0.63±0.23 | 1.63E-01 | 0.59±0.25 | 2.62E-01 |
| carboxyethyl-GABA | -0.02±0.07 | 77.89 | 3.35E-02 | 8.77E-01 | 0.15±0.15 | 69.41 | 7.06E-02 | 9.19E-01 | -0.07±0.19 | 9.25E-01 | -0.09±0.2 | 9.17E-01 | 0.42±0.22 | 3.49E-01 | 0.45±0.22 | 3.47E-01 |
| cyclo(leu-pro) | -0.01±0.01 | 85.35 | 8.98E-03 | 7.40E-01 | 0.16±0.15 | 66.79 | 8.27E-02 | 9.19E-01 | -0.01±0.19 | 9.90E-01 | -0.1±0.21 | 9.13E-01 | 0.52±0.21 | 1.75E-01 | 0.43±0.21 | 3.66E-01 |
| N-acetylcarnosine | 0.01±0.01 | 76.84 | 3.77E-02 | 7.36E-01 | 0.2±0.19 | 60.93 | 1.10E-01 | 9.19E-01 | 0.01±0.24 | 9.94E-01 | -0.03±0.24 | 9.73E-01 | 0.63±0.3 | 2.81E-01 | 0.6±0.31 | 3.75E-01 |
| dopamine 3-O-sulfate | 0.02±0.11 | 68.77 | 7.36E-02 | 9.42E-01 | 0.14±0.14 | 65.63 | 8.81E-02 | 9.19E-01 | -0.15±0.22 | 8.55E-01 | -0.17±0.23 | 8.59E-01 | 0.27±0.18 | 4.69E-01 | 0.33±0.18 | 4.03E-01 |
| N-methylpipecolate | -0.03±0.05 | 71.35 | 6.17E-02 | 7.91E-01 | 0.16±0.15 | 49.26 | 1.60E-01 | 9.19E-01 | -0.05±0.2 | 9.47E-01 | -0.03±0.2 | 9.73E-01 | 0.36±0.22 | 4.10E-01 | 0.39±0.22 | 4.20E-01 |
| hexadecadienoate (16:2n6) | 0.03±0.06 | 71.44 | 6.13E-02 | 8.27E-01 | -0.16±0.15 | 47.13 | 1.69E-01 | 9.19E-01 | 0.06±0.21 | 9.38E-01 | 0.05±0.21 | 9.52E-01 | -0.33±0.2 | 4.19E-01 | -0.35±0.21 | 4.33E-01 |
| propionylcarnitine (C3) | 0.03±0.02 | 64.91 | 9.14E-02 | 7.36E-01 | 0.16±0.15 | 41.33 | 1.92E-01 | 9.19E-01 | 0.02±0.2 | 9.77E-01 | -0.02±0.21 | 9.84E-01 | 0.42±0.23 | 3.77E-01 | 0.39±0.23 | 4.49E-01 |
| 1-stearoyl-2-linoleoyl-GPE (18:0/18:2)* | -0.01±0.01 | 60.48 | 1.12E-01 | 7.36E-01 | 0.17±0.16 | 33.86 | 2.19E-01 | 9.19E-01 | -0.01±0.21 | 9.92E-01 | -0.02±0.22 | 9.84E-01 | 0.35±0.23 | 4.50E-01 | 0.38±0.24 | 4.67E-01 |
| N1-methylinosine | -0.01±0.01 | 70.21 | 6.69E-02 | 7.36E-01 | 0.16±0.16 | 25.76 | 2.46E-01 | 9.19E-01 | -0.01±0.2 | 9.94E-01 | 0±0.21 | 9.99E-01 | 0.4±0.22 | 3.77E-01 | 0.36±0.23 | 4.67E-01 |
| succinylcarnitine (C4-DC) | -0.03±0.05 | 63.75 | 9.67E-02 | 7.65E-01 | 0.16±0.16 | 14.29 | 2.80E-01 | 9.19E-01 | -0.05±0.21 | 9.54E-01 | 0.01±0.21 | 9.98E-01 | 0.34±0.23 | 4.70E-01 | 0.35±0.23 | 5.04E-01 |
| bilirubin (E,Z or Z,E)* | 0.05±0.04 | 0 | 3.60E-01 | 7.36E-01 | 0.15±0.16 | 6.03 | 3.02E-01 | 9.19E-01 | 0.05±0.2 | 9.54E-01 | 0±0.22 | 9.99E-01 | 0.25±0.22 | 5.89E-01 | 0.32±0.23 | 5.31E-01 |
| hexadecasphingosine (d16:1)* | 0.03±0.03 | 58.08 | 1.22E-01 | 7.36E-01 | 0.15±0.14 | 0 | 3.79E-01 | 9.19E-01 | 0.03±0.2 | 9.76E-01 | 0.02±0.2 | 9.76E-01 | 0.34±0.2 | 4.10E-01 | 0.27±0.2 | 5.44E-01 |
| 2-oxoarginine* | 0±0 | 49.59 | 1.59E-01 | 7.36E-01 | 0.15±0.15 | 0 | 3.31E-01 | 9.19E-01 | 0±0.21 | 9.99E-01 | -0.01±0.22 | 9.97E-01 | 0.29±0.2 | 4.89E-01 | 0.28±0.21 | 5.44E-01 |
| cinnamoylglycine | -0.05±0.04 | 35.03 | 2.15E-01 | 7.36E-01 | -0.14±0.13 | 0 | 4.13E-01 | 9.19E-01 | -0.04±0.19 | 9.54E-01 | -0.01±0.2 | 9.84E-01 | -0.25±0.16 | 4.54E-01 | -0.23±0.18 | 5.49E-01 |
| X - 15461 | 0.1±0.07 | 9.98 | 2.92E-01 | 7.36E-01 | 0.17±0.16 | 0 | 4.05E-01 | 9.19E-01 | 0.08±0.21 | 9.30E-01 | 0.05±0.22 | 9.51E-01 | 0.35±0.25 | 4.95E-01 | 0.33±0.25 | 5.52E-01 |
| X - 11787 | 0.08±0.06 | 0 | 4.60E-01 | 7.36E-01 | 0.14±0.15 | 0 | 4.21E-01 | 9.19E-01 | 0.07±0.21 | 9.38E-01 | 0.02±0.22 | 9.82E-01 | 0.23±0.22 | 6.08E-01 | 0.27±0.21 | 5.79E-01 |
| X - 23593 | 0.12±0.09 | 0 | 4.08E-01 | 7.36E-01 | 0.18±0.17 | 0 | 4.48E-01 | 9.19E-01 | 0.1±0.21 | 9.04E-01 | 0.09±0.21 | 9.27E-01 | 0.35±0.29 | 5.69E-01 | 0.36±0.29 | 5.84E-01 |
| ascorbic acid 3-sulfate* | 0.11±0.09 | 0 | 6.58E-01 | 7.36E-01 | 0.16±0.16 | 0 | 5.77E-01 | 9.19E-01 | 0.1±0.22 | 9.06E-01 | 0.07±0.23 | 9.38E-01 | 0.2±0.22 | 6.77E-01 | 0.25±0.22 | 6.09E-01 |
| 1-palmitoyl-GPG (16:0)* | 0.07±0.05 | 0 | 3.46E-01 | 7.36E-01 | 0.14±0.14 | 0 | 5.57E-01 | 9.19E-01 | 0.06±0.19 | 9.38E-01 | 0.06±0.19 | 9.33E-01 | 0.26±0.2 | 5.53E-01 | 0.23±0.2 | 6.11E-01 |
| cysteinylglycine disulfide* | 0.12±0.09 | 0 | 3.67E-01 | 7.36E-01 | 0.18±0.17 | 0 | 5.04E-01 | 9.19E-01 | 0.09±0.2 | 9.03E-01 | 0.11±0.2 | 8.84E-01 | 0.4±0.33 | 5.62E-01 | 0.37±0.33 | 6.11E-01 |
| arachidate (20:0) | -0.04±0.03 | 1.55 | 3.14E-01 | 7.36E-01 | -0.14±0.14 | 0 | 5.69E-01 | 9.19E-01 | -0.04±0.2 | 9.59E-01 | -0.06±0.2 | 9.40E-01 | -0.23±0.19 | 5.66E-01 | -0.22±0.2 | 6.16E-01 |
| xanthine | 0.07±0.06 | 0 | 4.98E-01 | 7.36E-01 | 0.16±0.16 | 0 | 5.44E-01 | 9.19E-01 | 0.06±0.21 | 9.46E-01 | 0.07±0.22 | 9.38E-01 | 0.22±0.23 | 6.67E-01 | 0.26±0.24 | 6.16E-01 |
| X - 23659 | 0.15±0.1 | 0 | 5.84E-01 | 7.36E-01 | 0.15±0.14 | 0 | 6.21E-01 | 9.19E-01 | 0.11±0.21 | 8.85E-01 | 0.08±0.2 | 9.28E-01 | 0.24±0.21 | 5.71E-01 | 0.22±0.2 | 6.29E-01 |
| homocitrulline | 0.1±0.08 | 0 | 8.29E-01 | 7.36E-01 | 0.15±0.15 | 0 | 8.35E-01 | 9.19E-01 | 0.09±0.21 | 9.12E-01 | 0.12±0.22 | 8.87E-01 | 0.13±0.2 | 7.79E-01 | 0.18±0.2 | 6.95E-01 |
| ximenoylcarnitine (C26:1)* | 0.19±0.14 | 0 | 9.06E-01 | 7.36E-01 | 0.15±0.14 | 0 | 8.65E-01 | 9.19E-01 | 0.17±0.19 | 8.55E-01 | 0.13±0.19 | 8.59E-01 | 0.21±0.22 | 6.67E-01 | 0.18±0.22 | 7.17E-01 |
| X - 12906 | -0.14±0.09 | 0 | 5.83E-01 | 7.36E-01 | -0.13±0.13 | 0 | 9.46E-01 | 9.19E-01 | -0.11±0.2 | 8.93E-01 | -0.12±0.2 | 8.69E-01 | -0.21±0.16 | 5.36E-01 | -0.14±0.17 | 7.34E-01 |
| pregnenetriol sulfate* | 0.12±0.09 | 0 | 7.89E-01 | 7.36E-01 | 0.15±0.15 | 0 | 9.09E-01 | 9.19E-01 | 0.11±0.21 | 8.99E-01 | 0.13±0.22 | 8.70E-01 | 0.17±0.2 | 7.29E-01 | 0.16±0.2 | 7.34E-01 |
| hydroxy-N6,N6,N6-trimethyllysine* | 0.05±0.19 | 34.81 | 2.16E-01 | 9.18E-01 | -0.14±0.15 | 75.54 | 4.32E-02 | 9.19E-01 | -0.44±0.21 | 8.16E-01 | -0.44±0.21 | 8.44E-01 | 0.17±0.21 | 7.57E-01 | 0.16±0.21 | 7.62E-01 |
| 2-aminophenol sulfate | 0.1±0.09 | 0 | 9.45E-01 | 7.36E-01 | 0.14±0.14 | 0 | 9.32E-01 | 9.19E-01 | 0.1±0.2 | 9.01E-01 | 0.13±0.2 | 8.65E-01 | 0.11±0.2 | 8.13E-01 | 0.15±0.21 | 7.71E-01 |
| allantoin | 0.16±0.14 | 0 | 7.74E-01 | 7.36E-01 | 0.15±0.14 | 0 | 9.44E-01 | 9.19E-01 | 0.2±0.2 | 8.55E-01 | 0.16±0.21 | 8.53E-01 | 0.12±0.2 | 8.00E-01 | 0.14±0.2 | 7.73E-01 |
| tetradecanedioate (C14-DC) | -0.02±0.17 | 29.17 | 2.35E-01 | 9.58E-01 | 0.13±0.14 | 71.27 | 6.21E-02 | 9.19E-01 | 0.38±0.18 | 8.16E-01 | 0.37±0.19 | 8.44E-01 | -0.13±0.19 | 7.91E-01 | -0.14±0.2 | 7.83E-01 |
| 2-aminooctanoate | -0.13±0.1 | 0 | 8.53E-01 | 7.36E-01 | -0.13±0.14 | 0 | 9.98E-01 | 9.19E-01 | -0.12±0.19 | 8.72E-01 | -0.13±0.19 | 8.59E-01 | -0.16±0.19 | 7.19E-01 | -0.13±0.2 | 7.89E-01 |
| X - 11444 | 0.16±0.13 | 0 | 9.31E-01 | 7.36E-01 | 0.17±0.17 | 0 | 9.06E-01 | 9.19E-01 | 0.16±0.24 | 8.55E-01 | 0.19±0.25 | 8.54E-01 | 0.14±0.22 | 7.96E-01 | 0.15±0.23 | 7.89E-01 |
| X - 12117 | -0.13±0.12 | 0 | 7.31E-01 | 7.36E-01 | -0.14±0.14 | 0 | 9.24E-01 | 9.19E-01 | -0.16±0.2 | 8.55E-01 | -0.16±0.21 | 8.58E-01 | -0.07±0.19 | 9.00E-01 | -0.13±0.2 | 7.89E-01 |
| docosapentaenoate (n6 DPA; 22:5n6) | 0.14±0.11 | 0 | 8.94E-01 | 7.36E-01 | 0.14±0.14 | 0 | 9.57E-01 | 9.19E-01 | 0.13±0.19 | 8.55E-01 | 0.14±0.19 | 8.56E-01 | 0.17±0.21 | 7.54E-01 | 0.13±0.21 | 8.13E-01 |
| pregnenediol disulfate (C21H34O8S2)* | 0.16±0.13 | 0 | 9.30E-01 | 7.36E-01 | 0.16±0.16 | 0 | 8.57E-01 | 9.19E-01 | 0.17±0.23 | 8.55E-01 | 0.19±0.24 | 8.53E-01 | 0.15±0.21 | 7.79E-01 | 0.13±0.21 | 8.13E-01 |
| X - 11308 | -0.17±0.13 | 0 | 9.40E-01 | 7.36E-01 | -0.15±0.15 | 0 | 7.51E-01 | 9.19E-01 | -0.18±0.21 | 8.55E-01 | -0.2±0.22 | 8.45E-01 | -0.16±0.18 | 7.15E-01 | -0.11±0.19 | 8.27E-01 |
| serine | 0.11±0.13 | 0 | 6.09E-01 | 7.36E-01 | 0.15±0.15 | 0 | 8.78E-01 | 9.19E-01 | 0.17±0.2 | 8.55E-01 | 0.17±0.21 | 8.45E-01 | 0.03±0.2 | 9.64E-01 | 0.13±0.22 | 8.27E-01 |
| (N(1) + N(8))-acetylspermidine | -0.01±0.18 | 14.66 | 2.79E-01 | 9.81E-01 | -0.16±0.15 | 72.08 | 5.84E-02 | 9.19E-01 | -0.41±0.22 | 8.16E-01 | -0.47±0.23 | 8.44E-01 | 0.09±0.21 | 8.81E-01 | 0.1±0.2 | 8.47E-01 |
| isoursodeoxycholate | -0.11±0.13 | 0 | 5.58E-01 | 7.40E-01 | -0.15±0.14 | 0 | 6.90E-01 | 9.19E-01 | -0.19±0.21 | 8.55E-01 | -0.21±0.21 | 8.44E-01 | -0.03±0.19 | 9.67E-01 | -0.1±0.2 | 8.47E-01 |
| sphingomyelin (d18:2/23:1)* | -0.16±0.15 | 0 | 6.39E-01 | 7.36E-01 | -0.14±0.14 | 0 | 5.70E-01 | 9.19E-01 | -0.27±0.23 | 8.40E-01 | -0.25±0.24 | 8.44E-01 | -0.12±0.18 | 7.79E-01 | -0.08±0.18 | 8.59E-01 |
| campesterol | -0.15±0.11 | 0 | 9.72E-01 | 7.36E-01 | -0.14±0.14 | 0 | 6.70E-01 | 9.19E-01 | -0.15±0.19 | 8.55E-01 | -0.2±0.2 | 8.44E-01 | -0.15±0.18 | 7.17E-01 | -0.09±0.19 | 8.60E-01 |
| N-stearoyltaurine | -0.13±0.12 | 0 | 7.70E-01 | 7.36E-01 | -0.13±0.13 | 0 | 7.30E-01 | 9.19E-01 | -0.16±0.18 | 8.55E-01 | -0.17±0.19 | 8.45E-01 | -0.09±0.18 | 8.53E-01 | -0.08±0.19 | 8.60E-01 |
| 3-methylglutarylcarnitine (2) | 0.13±0.14 | 0 | 6.56E-01 | 7.36E-01 | 0.15±0.15 | 0 | 6.55E-01 | 9.19E-01 | 0.19±0.21 | 8.55E-01 | 0.22±0.21 | 8.44E-01 | 0.07±0.21 | 9.15E-01 | 0.08±0.21 | 8.86E-01 |
| beta-alanine | -0.04±0.18 | 5.82 | 3.03E-01 | 9.25E-01 | -0.14±0.14 | 57.06 | 1.27E-01 | 9.19E-01 | -0.33±0.2 | 8.16E-01 | -0.36±0.2 | 8.44E-01 | 0.07±0.21 | 9.15E-01 | 0.07±0.2 | 8.91E-01 |
| sphingomyelin (d18:1/24:1, d18:2/24:0)* | -0.14±0.13 | 0 | 6.29E-01 | 7.36E-01 | -0.13±0.13 | 0 | 4.87E-01 | 9.19E-01 | -0.22±0.21 | 8.40E-01 | -0.24±0.21 | 8.44E-01 | -0.09±0.16 | 8.20E-01 | -0.06±0.17 | 8.98E-01 |
| 3-acetylphenol sulfate | -0.08±0.16 | 0 | 3.77E-01 | 8.27E-01 | -0.13±0.13 | 50.34 | 1.56E-01 | 9.19E-01 | -0.32±0.19 | 8.16E-01 | -0.32±0.19 | 8.44E-01 | 0.01±0.19 | 9.91E-01 | 0.06±0.19 | 8.98E-01 |
| X - 24811 | -0.1±0.16 | 0 | 4.23E-01 | 7.86E-01 | -0.13±0.13 | 37.14 | 2.07E-01 | 9.19E-01 | -0.3±0.17 | 8.16E-01 | -0.28±0.18 | 8.44E-01 | -0.02±0.19 | 9.81E-01 | 0.06±0.2 | 9.16E-01 |
| 3-(3-hydroxyphenyl)propionate | -0.04±0.16 | 6.47 | 3.01E-01 | 9.25E-01 | -0.13±0.14 | 48.24 | 1.65E-01 | 9.19E-01 | -0.3±0.19 | 8.16E-01 | -0.32±0.19 | 8.44E-01 | 0.06±0.18 | 9.15E-01 | 0.05±0.19 | 9.21E-01 |
| S-methylcysteine | -0.07±0.17 | 0 | 3.63E-01 | 8.46E-01 | -0.14±0.14 | 60.85 | 1.10E-01 | 9.19E-01 | -0.38±0.22 | 8.16E-01 | -0.42±0.22 | 8.44E-01 | 0±0.18 | 9.95E-01 | 0.05±0.18 | 9.26E-01 |
| 3-carboxy-4-methyl-5-propyl-2-furanpropanoate (CMPF) | -0.09±0.11 | 0 | 5.67E-01 | 7.42E-01 | -0.14±0.14 | 0 | 5.38E-01 | 9.19E-01 | -0.14±0.2 | 8.55E-01 | -0.23±0.2 | 8.44E-01 | -0.01±0.19 | 9.91E-01 | -0.05±0.2 | 9.27E-01 |
| N-(2-furoyl)glycine | -0.07±0.17 | 0 | 3.57E-01 | 8.55E-01 | -0.14±0.14 | 53.24 | 1.44E-01 | 9.19E-01 | -0.42±0.21 | 8.16E-01 | -0.38±0.22 | 8.44E-01 | 0±0.19 | 9.95E-01 | 0.04±0.19 | 9.38E-01 |
| 1,2-dilinoleoyl-GPC (18:2/18:2) | -0.13±0.16 | 0 | 5.16E-01 | 7.36E-01 | -0.15±0.15 | 35.51 | 2.13E-01 | 9.19E-01 | -0.31±0.19 | 8.16E-01 | -0.32±0.2 | 8.44E-01 | -0.07±0.18 | 8.85E-01 | 0.04±0.21 | 9.45E-01 |
| glycerophosphoethanolamine | -0.12±0.13 | 0 | 5.08E-01 | 7.36E-01 | -0.13±0.13 | 31.04 | 2.29E-01 | 9.19E-01 | -0.28±0.21 | 8.35E-01 | -0.35±0.22 | 8.44E-01 | -0.07±0.15 | 8.53E-01 | -0.03±0.15 | 9.47E-01 |
| 1-linoleoyl-GPI (18:2)* | -0.12±0.16 | 0 | 4.97E-01 | 7.45E-01 | -0.15±0.14 | 0 | 3.82E-01 | 9.19E-01 | -0.27±0.21 | 8.40E-01 | -0.29±0.22 | 8.44E-01 | -0.05±0.19 | 9.38E-01 | -0.04±0.19 | 9.47E-01 |
| 3-hydroxydodecanedioate* | 0.1±0.16 | 0 | 4.16E-01 | 7.91E-01 | 0.14±0.13 | 29.31 | 2.34E-01 | 9.19E-01 | 0.31±0.18 | 8.16E-01 | 0.29±0.19 | 8.44E-01 | 0.01±0.19 | 9.82E-01 | -0.03±0.2 | 9.58E-01 |
| pimeloylcarnitine/3-methyladipoylcarnitine (C7-DC) | -0.13±0.15 | 0 | 5.80E-01 | 7.36E-01 | -0.14±0.14 | 0 | 4.37E-01 | 9.19E-01 | -0.23±0.18 | 8.40E-01 | -0.24±0.19 | 8.44E-01 | -0.06±0.19 | 9.15E-01 | -0.03±0.2 | 9.69E-01 |
| cytosine | -0.06±0.16 | 0 | 3.50E-01 | 8.55E-01 | -0.14±0.13 | 28.15 | 2.38E-01 | 9.19E-01 | -0.33±0.19 | 8.16E-01 | -0.3±0.19 | 8.44E-01 | 0.03±0.19 | 9.69E-01 | 0.02±0.19 | 9.81E-01 |
| X - 17612 | -0.09±0.15 | 0 | 4.23E-01 | 7.86E-01 | -0.14±0.13 | 6.3 | 3.02E-01 | 9.19E-01 | -0.24±0.17 | 8.19E-01 | -0.25±0.17 | 8.44E-01 | 0.01±0.19 | 9.91E-01 | 0.02±0.19 | 9.81E-01 |
| N-acetylthreonine | 0±0.11 | 86.11 | 7.28E-03 | 9.91E-01 | 0.17±0.18 | 80.84 | 2.23E-02 | 9.21E-01 | -0.11±0.23 | 8.98E-01 | -0.14±0.22 | 8.65E-01 | 0.73±0.29 | 1.85E-01 | 0.72±0.3 | 2.62E-01 |
| octanoylcarnitine (C8) | -0.05±0.11 | 81.78 | 1.91E-02 | 8.27E-01 | -0.12±0.13 | 70.12 | 6.73E-02 | 9.21E-01 | 0.13±0.18 | 8.55E-01 | 0.13±0.19 | 8.59E-01 | -0.39±0.18 | 2.59E-01 | -0.35±0.18 | 3.70E-01 |
| threonine | 0.01±0.01 | 58.61 | 1.20E-01 | 7.36E-01 | 0.16±0.17 | 55.44 | 1.34E-01 | 9.21E-01 | 0.01±0.2 | 9.94E-01 | -0.01±0.2 | 9.84E-01 | 0.46±0.29 | 4.49E-01 | 0.53±0.3 | 4.20E-01 |
| X - 12456 | -0.02±0.04 | 81.84 | 1.89E-02 | 8.02E-01 | 0.14±0.15 | 54.01 | 1.40E-01 | 9.21E-01 | -0.04±0.2 | 9.54E-01 | -0.07±0.21 | 9.33E-01 | 0.46±0.21 | 2.52E-01 | 0.38±0.22 | 4.33E-01 |
| dihydroorotate | -0.01±0.01 | 68.09 | 7.67E-02 | 7.36E-01 | 0.13±0.15 | 41.44 | 1.91E-01 | 9.21E-01 | -0.01±0.2 | 9.92E-01 | -0.03±0.19 | 9.71E-01 | 0.38±0.22 | 4.06E-01 | 0.35±0.22 | 4.67E-01 |
| caproate (6:0) | 0.01±0.08 | 65.35 | 8.94E-02 | 9.47E-01 | -0.12±0.13 | 22.08 | 2.57E-01 | 9.21E-01 | 0.09±0.19 | 8.99E-01 | 0.06±0.2 | 9.42E-01 | -0.22±0.16 | 4.97E-01 | -0.24±0.16 | 5.22E-01 |
| 2-hydroxy-4-(methylthio)butanoic acid | -0.02±0.02 | 53.81 | 1.41E-01 | 7.40E-01 | 0.14±0.15 | 0 | 4.29E-01 | 9.21E-01 | -0.02±0.21 | 9.78E-01 | 0.01±0.21 | 9.84E-01 | 0.27±0.2 | 5.03E-01 | 0.25±0.2 | 5.88E-01 |
| X - 21842 | -0.02±0.02 | 37.89 | 2.04E-01 | 7.36E-01 | -0.13±0.15 | 0 | 4.50E-01 | 9.21E-01 | -0.02±0.2 | 9.83E-01 | -0.03±0.2 | 9.73E-01 | -0.28±0.21 | 4.97E-01 | -0.25±0.21 | 6.03E-01 |
| 1-methylxanthine | 0.07±0.17 | 53.56 | 1.42E-01 | 8.52E-01 | -0.12±0.13 | 79.63 | 2.67E-02 | 9.21E-01 | -0.3±0.16 | 8.16E-01 | -0.34±0.16 | 8.44E-01 | 0.23±0.2 | 5.92E-01 | 0.25±0.21 | 6.03E-01 |
| X - 11478 | 0±0 | 23.64 | 2.52E-01 | 7.36E-01 | 0.14±0.15 | 0 | 4.62E-01 | 9.21E-01 | 0±0.21 | 9.99E-01 | 0.02±0.21 | 9.76E-01 | 0.22±0.19 | 5.96E-01 | 0.24±0.2 | 6.03E-01 |
| adipoylcarnitine (C6-DC) | 0.04±0.03 | 3.43 | 3.09E-01 | 7.36E-01 | 0.13±0.14 | 0 | 5.11E-01 | 9.21E-01 | 0.03±0.2 | 9.63E-01 | 0.03±0.2 | 9.71E-01 | 0.23±0.19 | 5.66E-01 | 0.22±0.2 | 6.11E-01 |
| stearoyl sphingomyelin (d18:1/18:0) | -0.14±0.11 | 0 | 8.37E-01 | 7.36E-01 | -0.12±0.13 | 0 | 8.66E-01 | 9.21E-01 | -0.16±0.21 | 8.55E-01 | -0.15±0.22 | 8.59E-01 | -0.11±0.16 | 7.79E-01 | -0.11±0.16 | 7.93E-01 |
| X - 24556 | -0.04±0.16 | 6.17 | 3.02E-01 | 9.11E-01 | -0.12±0.13 | 60.45 | 1.12E-01 | 9.21E-01 | -0.27±0.18 | 8.16E-01 | -0.32±0.18 | 8.44E-01 | 0.07±0.2 | 9.06E-01 | 0.11±0.2 | 8.27E-01 |
| EDTA | 0.14±0.14 | 0 | 7.30E-01 | 7.36E-01 | 0.14±0.16 | 0 | 8.73E-01 | 9.21E-01 | 0.18±0.2 | 8.55E-01 | 0.17±0.21 | 8.53E-01 | 0.08±0.23 | 9.15E-01 | 0.11±0.24 | 8.57E-01 |
| 3-methoxytyrosine | -0.08±0.17 | 0 | 3.71E-01 | 8.32E-01 | -0.14±0.15 | 62.08 | 1.04E-01 | 9.21E-01 | -0.44±0.24 | 8.16E-01 | -0.45±0.24 | 8.44E-01 | -0.01±0.19 | 9.82E-01 | 0.06±0.2 | 9.14E-01 |
| X - 12847 | -0.08±0.16 | 0 | 3.86E-01 | 8.19E-01 | -0.14±0.14 | 49.7 | 1.59E-01 | 9.21E-01 | -0.29±0.2 | 8.19E-01 | -0.37±0.22 | 8.44E-01 | 0.01±0.19 | 9.88E-01 | 0.04±0.19 | 9.40E-01 |
| decadienedioic acid (C10:2-DC)** | 0.15±0.16 | 0 | 5.47E-01 | 7.36E-01 | 0.13±0.14 | 0 | 4.30E-01 | 9.21E-01 | 0.31±0.21 | 8.19E-01 | 0.26±0.22 | 8.44E-01 | 0.09±0.19 | 8.42E-01 | 0.03±0.19 | 9.53E-01 |
| 1-(1-enyl-palmitoyl)-GPE (P-16:0)* | -0.09±0.15 | 0 | 4.42E-01 | 7.76E-01 | -0.13±0.14 | 27.15 | 2.41E-01 | 9.21E-01 | -0.23±0.2 | 8.40E-01 | -0.3±0.21 | 8.44E-01 | 0±0.19 | 9.97E-01 | 0.03±0.19 | 9.63E-01 |
| 5-oxoproline | -0.12±0.15 | 0 | 5.10E-01 | 7.44E-01 | -0.12±0.13 | 12.81 | 2.84E-01 | 9.21E-01 | -0.26±0.19 | 8.19E-01 | -0.27±0.19 | 8.44E-01 | -0.05±0.18 | 9.17E-01 | 0.02±0.19 | 9.75E-01 |
| X - 24757 | -0.07±0.14 | 0 | 3.94E-01 | 8.02E-01 | -0.12±0.13 | 0 | 3.28E-01 | 9.21E-01 | -0.23±0.18 | 8.40E-01 | -0.24±0.18 | 8.44E-01 | 0.02±0.18 | 9.74E-01 | 0.01±0.18 | 9.91E-01 |
| isobutyrylglycine | -0.11±0.13 | 0 | 5.71E-01 | 7.36E-01 | -0.12±0.13 | 0 | 3.62E-01 | 9.21E-01 | -0.2±0.18 | 8.40E-01 | -0.24±0.18 | 8.44E-01 | -0.05±0.18 | 9.30E-01 | 0±0.18 | 9.99E-01 |
| 1-(1-enyl-stearoyl)-GPE (P-18:0)* | 0±0.18 | 19.21 | 2.66E-01 | 9.99E-01 | -0.14±0.16 | 80.06 | 2.51E-02 | 9.22E-01 | -0.42±0.21 | 8.16E-01 | -0.5±0.22 | 8.44E-01 | 0.1±0.2 | 8.48E-01 | 0.21±0.22 | 6.88E-01 |
| glycerophosphorylcholine (GPC) | -0.09±0.15 | 0 | 4.16E-01 | 7.91E-01 | -0.13±0.14 | 43.03 | 1.85E-01 | 9.25E-01 | -0.28±0.19 | 8.19E-01 | -0.32±0.2 | 8.44E-01 | -0.01±0.19 | 9.91E-01 | 0.06±0.2 | 9.16E-01 |
| 6-hydroxyindole sulfate | 0.05±0.04 | 15.93 | 2.75E-01 | 7.36E-01 | 0.13±0.15 | 0 | 3.53E-01 | 9.29E-01 | 0.04±0.2 | 9.57E-01 | -0.01±0.21 | 9.98E-01 | 0.27±0.21 | 5.36E-01 | 0.27±0.21 | 5.62E-01 |
| ceramide (d16:1/24:1, d18:1/22:1)* | 0.03±0.03 | 37.19 | 2.07E-01 | 7.36E-01 | 0.14±0.16 | 0 | 3.97E-01 | 9.29E-01 | 0.03±0.2 | 9.70E-01 | 0.04±0.2 | 9.61E-01 | 0.38±0.28 | 4.97E-01 | 0.32±0.27 | 5.92E-01 |
| citramalate | 0.14±0.18 | 52.1 | 1.48E-01 | 7.53E-01 | -0.13±0.14 | 81.81 | 1.90E-02 | 9.29E-01 | -0.44±0.19 | 8.16E-01 | -0.44±0.19 | 8.44E-01 | 0.26±0.2 | 5.38E-01 | 0.23±0.21 | 6.16E-01 |
| N1-Methyl-2-pyridone-5-carboxamide | 0.01±0.01 | 0 | 4.10E-01 | 7.36E-01 | -0.12±0.13 | 0 | 6.10E-01 | 9.29E-01 | 0.01±0.2 | 9.92E-01 | -0.04±0.2 | 9.63E-01 | -0.13±0.17 | 7.57E-01 | -0.17±0.17 | 6.58E-01 |
| cys-gly, oxidized | 0.06±0.05 | 0 | 4.90E-01 | 7.36E-01 | 0.14±0.16 | 0 | 5.98E-01 | 9.29E-01 | 0.05±0.2 | 9.48E-01 | 0.07±0.21 | 9.33E-01 | 0.23±0.25 | 6.82E-01 | 0.24±0.25 | 6.76E-01 |
| pro-hydroxy-pro | -0.01±0.21 | 31.31 | 2.28E-01 | 9.81E-01 | -0.14±0.16 | 68.4 | 7.53E-02 | 9.29E-01 | -0.34±0.2 | 8.16E-01 | -0.37±0.21 | 8.44E-01 | 0.18±0.26 | 7.79E-01 | 0.23±0.27 | 7.09E-01 |
| 2-hydroxybutyrate/2-hydroxyisobutyrate | 0.17±0.14 | 0 | 7.81E-01 | 7.36E-01 | 0.13±0.14 | 0 | 6.88E-01 | 9.29E-01 | 0.21±0.2 | 8.40E-01 | 0.19±0.21 | 8.45E-01 | 0.13±0.19 | 7.79E-01 | 0.07±0.2 | 8.91E-01 |
| X - 26106 | 0.16±0.15 | 0 | 7.10E-01 | 7.36E-01 | 0.13±0.15 | 0 | 7.15E-01 | 9.31E-01 | 0.23±0.2 | 8.40E-01 | 0.18±0.21 | 8.45E-01 | 0.11±0.2 | 8.17E-01 | 0.08±0.2 | 8.86E-01 |
| X - 17690 | -0.11±0.11 | 0 | 6.97E-01 | 7.36E-01 | -0.13±0.14 | 0 | 5.42E-01 | 9.33E-01 | -0.14±0.21 | 8.55E-01 | -0.23±0.22 | 8.44E-01 | -0.05±0.19 | 9.30E-01 | -0.05±0.19 | 9.26E-01 |
| linoleoyl ethanolamide | -0.08±0.06 | 0 | 6.24E-01 | 7.36E-01 | -0.12±0.13 | 0 | 5.97E-01 | 9.35E-01 | -0.07±0.2 | 9.30E-01 | -0.04±0.2 | 9.64E-01 | -0.16±0.18 | 6.79E-01 | -0.18±0.18 | 6.58E-01 |
| X - 26097 | 0.05±0.04 | 0 | 5.06E-01 | 7.36E-01 | 0.12±0.14 | 0 | 5.13E-01 | 9.37E-01 | 0.04±0.2 | 9.57E-01 | 0.02±0.2 | 9.75E-01 | 0.17±0.19 | 6.98E-01 | 0.21±0.19 | 6.21E-01 |
| ethyl beta-glucopyranoside | -0.08±0.09 | 0 | 7.22E-01 | 7.36E-01 | -0.13±0.14 | 0 | 7.27E-01 | 9.37E-01 | -0.1±0.19 | 8.98E-01 | -0.18±0.2 | 8.45E-01 | -0.02±0.19 | 9.74E-01 | -0.07±0.21 | 8.93E-01 |
| 1-methyl-4-imidazoleacetate | 0.18±0.16 | 75.15 | 4.48E-02 | 7.36E-01 | 0.14±0.16 | 78.46 | 3.12E-02 | 9.38E-01 | -0.28±0.25 | 8.40E-01 | -0.28±0.25 | 8.44E-01 | 0.4±0.2 | 2.93E-01 | 0.41±0.2 | 3.45E-01 |
| 1-palmitoyl-2-linoleoyl-GPE (16:0/18:2) | 0.01±0.01 | 0 | 3.42E-01 | 7.36E-01 | 0.13±0.15 | 0 | 4.20E-01 | 9.38E-01 | 0.01±0.21 | 9.90E-01 | 0.01±0.21 | 9.97E-01 | 0.21±0.21 | 6.36E-01 | 0.25±0.21 | 6.03E-01 |
| 1-arachidonoyl-GPE (20:4n6)* | 0.06±0.05 | 0 | 3.76E-01 | 7.36E-01 | 0.14±0.16 | 0 | 4.45E-01 | 9.38E-01 | 0.05±0.21 | 9.54E-01 | 0.03±0.22 | 9.73E-01 | 0.27±0.24 | 6.00E-01 | 0.28±0.24 | 6.06E-01 |
| 5-hydroxylysine | -0.03±0.04 | 71.72 | 6.00E-02 | 7.76E-01 | 0.13±0.15 | 31.25 | 2.28E-01 | 9.39E-01 | -0.04±0.21 | 9.57E-01 | -0.08±0.23 | 9.33E-01 | 0.34±0.2 | 4.06E-01 | 0.3±0.21 | 5.22E-01 |
| catechol sulfate | 0.03±0.17 | 35.1 | 2.14E-01 | 9.35E-01 | -0.11±0.13 | 79.65 | 2.67E-02 | 9.39E-01 | -0.36±0.17 | 8.16E-01 | -0.39±0.18 | 8.44E-01 | 0.14±0.19 | 7.57E-01 | 0.19±0.19 | 6.58E-01 |
| taurolithocholate 3-sulfate | -0.06±0.05 | 0 | 4.96E-01 | 7.36E-01 | -0.11±0.13 | 0 | 7.03E-01 | 9.39E-01 | -0.05±0.2 | 9.54E-01 | -0.06±0.2 | 9.40E-01 | -0.17±0.18 | 6.61E-01 | -0.16±0.18 | 7.00E-01 |
| hexadecanedioate (C16-DC) | 0.04±0.17 | 4.96 | 3.05E-01 | 9.14E-01 | 0.12±0.14 | 50.35 | 1.56E-01 | 9.39E-01 | 0.3±0.18 | 8.16E-01 | 0.29±0.18 | 8.44E-01 | -0.07±0.2 | 9.15E-01 | -0.1±0.21 | 8.47E-01 |
| 2-hydroxystearate | -0.08±0.16 | 0 | 3.85E-01 | 8.19E-01 | -0.12±0.14 | 18.43 | 2.68E-01 | 9.39E-01 | -0.29±0.2 | 8.19E-01 | -0.28±0.2 | 8.44E-01 | 0.01±0.19 | 9.84E-01 | 0.02±0.18 | 9.73E-01 |
| anthranilate | 0.15±0.12 | 0 | 9.31E-01 | 7.36E-01 | 0.13±0.15 | 0 | 9.44E-01 | 9.40E-01 | 0.15±0.21 | 8.55E-01 | 0.12±0.22 | 8.84E-01 | 0.17±0.21 | 7.47E-01 | 0.14±0.21 | 7.94E-01 |
| pentose acid* | 0.09±0.13 | 83.69 | 1.33E-02 | 7.62E-01 | 0.13±0.15 | 84.73 | 1.05E-02 | 9.45E-01 | -0.16±0.2 | 8.55E-01 | -0.26±0.21 | 8.44E-01 | 0.48±0.2 | 2.11E-01 | 0.52±0.22 | 2.62E-01 |
| phenol sulfate | 0.04±0.03 | 48.68 | 1.63E-01 | 7.36E-01 | 0.11±0.13 | 6.42 | 3.01E-01 | 9.45E-01 | 0.03±0.2 | 9.65E-01 | -0.05±0.2 | 9.58E-01 | 0.29±0.18 | 4.45E-01 | 0.23±0.18 | 5.52E-01 |
| androsterone glucuronide | 0.06±0.05 | 0 | 4.75E-01 | 7.36E-01 | 0.13±0.15 | 0 | 6.59E-01 | 9.45E-01 | 0.05±0.21 | 9.51E-01 | 0.06±0.21 | 9.38E-01 | 0.21±0.21 | 6.50E-01 | 0.19±0.22 | 6.95E-01 |
| margaroylcarnitine (C17)* | 0.13±0.12 | 0 | 8.85E-01 | 7.36E-01 | 0.12±0.15 | 0 | 9.97E-01 | 9.45E-01 | 0.14±0.21 | 8.55E-01 | 0.12±0.21 | 8.70E-01 | 0.1±0.21 | 8.37E-01 | 0.12±0.21 | 8.13E-01 |
| dodecanedioate (C12-DC) | 0.03±0.16 | 15.39 | 2.77E-01 | 9.47E-01 | 0.12±0.14 | 52.66 | 1.46E-01 | 9.45E-01 | 0.3±0.2 | 8.16E-01 | 0.34±0.21 | 8.44E-01 | -0.09±0.19 | 8.63E-01 | -0.07±0.19 | 8.87E-01 |
| palmitoyl dihydrosphingomyelin (d18:0/16:0)* | -0.12±0.12 | 0 | 6.88E-01 | 7.36E-01 | -0.11±0.13 | 0 | 5.17E-01 | 9.46E-01 | -0.17±0.2 | 8.55E-01 | -0.22±0.21 | 8.44E-01 | -0.08±0.17 | 8.65E-01 | -0.04±0.18 | 9.38E-01 |
| cis-4-decenoylcarnitine (C10:1) | -0.05±0.11 | 81.33 | 2.06E-02 | 8.19E-01 | -0.11±0.13 | 69.81 | 6.87E-02 | 9.47E-01 | 0.14±0.19 | 8.55E-01 | 0.15±0.2 | 8.58E-01 | -0.39±0.18 | 2.67E-01 | -0.34±0.18 | 4.03E-01 |
| sphingomyelin (d18:1/20:2, d18:2/20:1, d16:1/22:2)* | 0.01±0.01 | 39.88 | 1.97E-01 | 7.36E-01 | -0.11±0.13 | 0 | 4.12E-01 | 9.47E-01 | 0.01±0.21 | 9.94E-01 | 0.03±0.21 | 9.71E-01 | -0.2±0.16 | 5.53E-01 | -0.19±0.16 | 6.05E-01 |
| 1-(1-enyl-stearoyl)-2-oleoyl-GPE (P-18:0/18:1) | 0.1±0.08 | 0 | 5.58E-01 | 7.36E-01 | 0.13±0.16 | 0 | 4.74E-01 | 9.47E-01 | 0.09±0.2 | 9.09E-01 | 0.04±0.2 | 9.64E-01 | 0.24±0.25 | 6.61E-01 | 0.27±0.25 | 6.21E-01 |
| bilirubin degradation product, C17H18N2O4 (3)** | 0.12±0.1 | 0 | 9.99E-01 | 7.36E-01 | 0.12±0.15 | 0 | 6.97E-01 | 9.47E-01 | 0.12±0.21 | 8.80E-01 | 0.06±0.22 | 9.38E-01 | 0.12±0.2 | 8.12E-01 | 0.18±0.21 | 7.11E-01 |
| X - 12812 | 0.12±0.09 | 0 | 8.98E-01 | 7.36E-01 | 0.12±0.15 | 0 | 8.83E-01 | 9.47E-01 | 0.11±0.21 | 8.93E-01 | 0.1±0.21 | 9.13E-01 | 0.14±0.19 | 7.79E-01 | 0.14±0.2 | 7.79E-01 |
| 1H-indole-7-acetic acid | -0.13±0.1 | 0 | 9.43E-01 | 7.36E-01 | -0.11±0.13 | 0 | 9.08E-01 | 9.47E-01 | -0.12±0.18 | 8.55E-01 | -0.12±0.18 | 8.59E-01 | -0.14±0.19 | 7.77E-01 | -0.09±0.19 | 8.47E-01 |
| S-methylcysteine sulfoxide | -0.11±0.15 | 0 | 4.62E-01 | 7.62E-01 | -0.12±0.14 | 27.01 | 2.42E-01 | 9.47E-01 | -0.28±0.21 | 8.40E-01 | -0.31±0.22 | 8.44E-01 | -0.03±0.18 | 9.61E-01 | 0.03±0.19 | 9.59E-01 |
| 1-methyl-5-imidazoleacetate | -0.1±0.13 | 0 | 5.39E-01 | 7.44E-01 | -0.12±0.14 | 0 | 4.65E-01 | 9.47E-01 | -0.18±0.21 | 8.55E-01 | -0.23±0.22 | 8.44E-01 | -0.02±0.18 | 9.74E-01 | -0.03±0.19 | 9.65E-01 |
| methyl glucopyranoside (alpha + beta) | -0.1±0.13 | 0 | 5.49E-01 | 7.44E-01 | -0.12±0.14 | 0 | 4.17E-01 | 9.47E-01 | -0.17±0.2 | 8.55E-01 | -0.25±0.22 | 8.44E-01 | -0.02±0.19 | 9.74E-01 | -0.02±0.19 | 9.81E-01 |
| X - 17367 | 0±0.15 | 28.32 | 2.38E-01 | 9.99E-01 | -0.1±0.13 | 63.17 | 9.94E-02 | 9.49E-01 | -0.28±0.17 | 8.16E-01 | -0.3±0.18 | 8.44E-01 | 0.11±0.18 | 7.97E-01 | 0.11±0.18 | 8.13E-01 |
| 1-linoleoyl-2-arachidonoyl-GPC (18:2/20:4n6)* | 0.04±0.04 | 20.73 | 2.61E-01 | 7.36E-01 | 0.14±0.17 | 28.68 | 2.36E-01 | 9.54E-01 | 0.04±0.21 | 9.57E-01 | 0±0.21 | 9.99E-01 | 0.37±0.29 | 5.52E-01 | 0.43±0.3 | 5.22E-01 |
| 2-hydroxyarachidate* | -0.03±0.04 | 47.57 | 1.67E-01 | 7.56E-01 | 0.12±0.15 | 0 | 3.98E-01 | 9.54E-01 | -0.04±0.2 | 9.57E-01 | -0.01±0.22 | 9.98E-01 | 0.25±0.21 | 5.66E-01 | 0.25±0.22 | 6.03E-01 |
| X - 21310 | 0.06±0.05 | 0 | 3.52E-01 | 7.36E-01 | 0.12±0.15 | 0 | 4.07E-01 | 9.54E-01 | 0.05±0.2 | 9.54E-01 | 0±0.21 | 9.99E-01 | 0.26±0.22 | 5.79E-01 | 0.25±0.22 | 6.04E-01 |
| 3-hydroxy-2-ethylpropionate | 0.17±0.12 | 0 | 6.81E-01 | 7.36E-01 | 0.12±0.15 | 0 | 6.81E-01 | 9.54E-01 | 0.14±0.2 | 8.55E-01 | 0.06±0.21 | 9.38E-01 | 0.25±0.22 | 5.99E-01 | 0.19±0.22 | 7.11E-01 |
| indoleacetylglutamine | 0.09±0.18 | 49.64 | 1.59E-01 | 8.26E-01 | -0.12±0.15 | 76.7 | 3.83E-02 | 9.56E-01 | -0.37±0.2 | 8.16E-01 | -0.42±0.21 | 8.44E-01 | 0.23±0.2 | 6.00E-01 | 0.19±0.21 | 6.95E-01 |
| carotene diol (3) | -0.02±0.02 | 7.8 | 2.98E-01 | 7.36E-01 | -0.11±0.14 | 0 | 8.24E-01 | 9.56E-01 | -0.02±0.2 | 9.84E-01 | -0.08±0.21 | 9.28E-01 | -0.2±0.18 | 5.81E-01 | -0.14±0.19 | 7.71E-01 |
| stearidonate (18:4n3) | -0.13±0.13 | 0 | 7.69E-01 | 7.36E-01 | -0.12±0.15 | 0 | 8.59E-01 | 9.56E-01 | -0.16±0.21 | 8.55E-01 | -0.14±0.21 | 8.59E-01 | -0.08±0.2 | 8.81E-01 | -0.09±0.2 | 8.59E-01 |
| X - 17335 | 0.14±0.15 | 0 | 6.48E-01 | 7.36E-01 | 0.11±0.14 | 0 | 6.00E-01 | 9.56E-01 | 0.22±0.2 | 8.40E-01 | 0.19±0.2 | 8.45E-01 | 0.08±0.2 | 8.90E-01 | 0.04±0.2 | 9.47E-01 |
| 1-(1-enyl-palmitoyl)-2-palmitoleoyl-GPC (P-16:0/16:1)* | -0.03±0.11 | 67.6 | 7.90E-02 | 9.21E-01 | -0.1±0.13 | 28.57 | 2.37E-01 | 9.58E-01 | 0.15±0.2 | 8.55E-01 | 0.09±0.21 | 9.18E-01 | -0.24±0.16 | 4.76E-01 | -0.22±0.17 | 5.44E-01 |
| indole-3-carboxylate | 0.2±0.17 | 80.29 | 2.43E-02 | 7.36E-01 | 0.12±0.16 | 84.49 | 1.11E-02 | 9.58E-01 | -0.27±0.21 | 8.40E-01 | -0.26±0.22 | 8.44E-01 | 0.52±0.22 | 2.21E-01 | 0.56±0.24 | 2.62E-01 |
| eugenol sulfate | 0.11±0.13 | 80.92 | 2.21E-02 | 7.39E-01 | 0.1±0.14 | 84.74 | 1.05E-02 | 9.58E-01 | -0.18±0.19 | 8.55E-01 | -0.25±0.2 | 8.44E-01 | 0.42±0.19 | 2.44E-01 | 0.46±0.2 | 2.77E-01 |
| X - 24418 | -0.3±0.17 | 76.26 | 4.01E-02 | 7.36E-01 | -0.11±0.15 | 86.92 | 5.69E-03 | 9.58E-01 | 0.37±0.23 | 8.16E-01 | 0.4±0.24 | 8.44E-01 | -0.48±0.19 | 1.75E-01 | -0.45±0.19 | 2.83E-01 |
| 4-methylcatechol sulfate | 0.13±0.14 | 80.36 | 2.40E-02 | 7.36E-01 | 0.1±0.14 | 82.61 | 1.65E-02 | 9.58E-01 | -0.21±0.19 | 8.40E-01 | -0.21±0.19 | 8.44E-01 | 0.44±0.2 | 2.44E-01 | 0.44±0.2 | 2.95E-01 |
| 3-carboxy-4-methyl-5-pentyl-2-furanpropionate (3-CMPFP)** | 0.09±0.13 | 79.83 | 2.60E-02 | 7.72E-01 | 0.1±0.14 | 81.96 | 1.85E-02 | 9.58E-01 | -0.17±0.2 | 8.55E-01 | -0.25±0.21 | 8.44E-01 | 0.4±0.19 | 2.81E-01 | 0.41±0.19 | 3.29E-01 |
| eicosenedioate (C20:1-DC)* | -0.26±0.15 | 73.33 | 5.28E-02 | 7.36E-01 | -0.1±0.13 | 83.42 | 1.41E-02 | 9.58E-01 | 0.34±0.21 | 8.16E-01 | 0.32±0.22 | 8.44E-01 | -0.39±0.17 | 2.11E-01 | -0.36±0.17 | 3.38E-01 |
| lactate | 0.08±0.14 | 78.14 | 3.24E-02 | 8.02E-01 | 0.11±0.15 | 76.25 | 4.02E-02 | 9.58E-01 | -0.19±0.21 | 8.55E-01 | -0.2±0.21 | 8.45E-01 | 0.41±0.21 | 3.29E-01 | 0.41±0.21 | 3.70E-01 |
| 1-oleoyl-GPE (18:1) | -0.01±0.1 | 72.76 | 5.53E-02 | 9.53E-01 | 0.12±0.16 | 69.73 | 6.91E-02 | 9.58E-01 | -0.12±0.21 | 8.81E-01 | -0.15±0.22 | 8.59E-01 | 0.37±0.23 | 4.23E-01 | 0.42±0.23 | 4.07E-01 |
| taurochenodeoxycholic acid 3-sulfate | -0.04±0.12 | 75.49 | 4.34E-02 | 8.94E-01 | -0.1±0.14 | 71.57 | 6.07E-02 | 9.58E-01 | 0.15±0.2 | 8.55E-01 | 0.17±0.2 | 8.45E-01 | -0.35±0.2 | 3.83E-01 | -0.36±0.2 | 4.09E-01 |
| cysteinylglycine | -0.03±0.08 | 73.49 | 5.21E-02 | 8.51E-01 | 0.12±0.15 | 63.44 | 9.82E-02 | 9.58E-01 | -0.08±0.19 | 9.12E-01 | -0.09±0.2 | 9.12E-01 | 0.41±0.24 | 4.06E-01 | 0.42±0.24 | 4.20E-01 |
| octadecanedioate (C18-DC) | -0.07±0.13 | 73.27 | 5.31E-02 | 8.02E-01 | -0.1±0.13 | 68.42 | 7.52E-02 | 9.58E-01 | 0.18±0.2 | 8.55E-01 | 0.17±0.2 | 8.45E-01 | -0.31±0.18 | 4.02E-01 | -0.31±0.18 | 4.28E-01 |
| tryptophan | -0.04±0.06 | 74.46 | 4.79E-02 | 7.88E-01 | 0.14±0.17 | 57.99 | 1.23E-01 | 9.58E-01 | -0.06±0.21 | 9.38E-01 | -0.06±0.22 | 9.38E-01 | 0.52±0.29 | 3.77E-01 | 0.49±0.29 | 4.34E-01 |
| methylsuccinate | -0.03±0.05 | 63.47 | 9.80E-02 | 7.76E-01 | 0.1±0.14 | 54.45 | 1.38E-01 | 9.58E-01 | -0.05±0.17 | 9.38E-01 | -0.06±0.18 | 9.33E-01 | 0.34±0.23 | 4.76E-01 | 0.38±0.24 | 4.67E-01 |
| 3-methoxycatechol sulfate (2) | 0±0.12 | 65.67 | 8.79E-02 | 9.91E-01 | 0.1±0.14 | 58.79 | 1.19E-01 | 9.58E-01 | -0.15±0.2 | 8.55E-01 | -0.12±0.2 | 8.70E-01 | 0.29±0.21 | 4.97E-01 | 0.34±0.21 | 4.67E-01 |
| ornithine | 0.14±0.23 | 63.74 | 9.68E-02 | 7.80E-01 | -0.13±0.16 | 83.51 | 1.38E-02 | 9.58E-01 | -0.4±0.18 | 8.16E-01 | -0.4±0.19 | 8.44E-01 | 0.41±0.28 | 4.76E-01 | 0.44±0.28 | 4.67E-01 |
| cysteine | -0.02±0.02 | 53.67 | 1.42E-01 | 7.36E-01 | 0.11±0.16 | 51.35 | 1.52E-01 | 9.58E-01 | -0.02±0.2 | 9.78E-01 | -0.07±0.2 | 9.33E-01 | 0.35±0.25 | 4.97E-01 | 0.4±0.26 | 4.67E-01 |
| 3-methyl-2-oxobutyrate | -0.02±0.08 | 75.19 | 4.47E-02 | 9.32E-01 | 0.1±0.14 | 56.33 | 1.30E-01 | 9.58E-01 | -0.09±0.19 | 9.06E-01 | -0.12±0.21 | 8.70E-01 | 0.35±0.2 | 3.97E-01 | 0.31±0.2 | 4.68E-01 |
| linolenate [alpha or gamma; (18:3n3 or 6)] | 0.02±0.08 | 70.24 | 6.68E-02 | 9.02E-01 | -0.11±0.14 | 53.32 | 1.43E-01 | 9.58E-01 | 0.08±0.2 | 9.12E-01 | 0.12±0.21 | 8.84E-01 | -0.3±0.19 | 4.50E-01 | -0.31±0.2 | 4.73E-01 |
| tartronate (hydroxymalonate) | -0.19±0.13 | 57.75 | 1.24E-01 | 7.36E-01 | -0.1±0.14 | 70.16 | 6.72E-02 | 9.58E-01 | 0.42±0.28 | 8.16E-01 | 0.36±0.29 | 8.44E-01 | -0.26±0.14 | 3.68E-01 | -0.25±0.16 | 4.74E-01 |
| N-palmitoyl-sphinganine (d18:0/16:0) | -0.03±0.06 | 75.57 | 4.31E-02 | 8.32E-01 | 0.1±0.14 | 50.4 | 1.56E-01 | 9.58E-01 | -0.06±0.19 | 9.38E-01 | -0.07±0.18 | 9.27E-01 | 0.38±0.21 | 3.77E-01 | 0.33±0.21 | 4.74E-01 |
| gamma-glutamyl-alpha-lysine | 0.01±0.01 | 51.2 | 1.52E-01 | 7.36E-01 | 0.13±0.18 | 33.23 | 2.21E-01 | 9.58E-01 | 0.01±0.21 | 9.92E-01 | -0.01±0.21 | 9.97E-01 | 0.48±0.33 | 4.76E-01 | 0.48±0.33 | 5.23E-01 |
| X - 24949 | -0.01±0.01 | 58.96 | 1.19E-01 | 7.36E-01 | 0.11±0.15 | 31.59 | 2.27E-01 | 9.58E-01 | -0.01±0.19 | 9.91E-01 | -0.05±0.2 | 9.46E-01 | 0.32±0.21 | 4.65E-01 | 0.3±0.21 | 5.36E-01 |
| gamma-tocopherol/beta-tocopherol | -0.03±0.06 | 72.3 | 5.75E-02 | 8.04E-01 | 0.1±0.14 | 24.36 | 2.50E-01 | 9.58E-01 | -0.06±0.18 | 9.38E-01 | -0.04±0.19 | 9.63E-01 | 0.37±0.22 | 4.07E-01 | 0.29±0.22 | 5.46E-01 |
| 2-hydroxy-3-methylvalerate | -0.02±0.03 | 64.12 | 9.50E-02 | 7.44E-01 | 0.12±0.15 | 19.65 | 2.65E-01 | 9.58E-01 | -0.03±0.22 | 9.76E-01 | -0.07±0.23 | 9.38E-01 | 0.33±0.21 | 4.50E-01 | 0.28±0.21 | 5.47E-01 |
| X - 25267 | -0.02±0.02 | 38.44 | 2.02E-01 | 7.36E-01 | 0.13±0.16 | 0 | 3.30E-01 | 9.58E-01 | -0.02±0.2 | 9.84E-01 | 0±0.21 | 9.99E-01 | 0.32±0.27 | 5.67E-01 | 0.33±0.26 | 5.79E-01 |
| X - 18913 | -0.02±0.02 | 27.5 | 2.40E-01 | 7.36E-01 | 0.1±0.15 | 0 | 3.24E-01 | 9.58E-01 | -0.02±0.2 | 9.84E-01 | -0.04±0.21 | 9.61E-01 | 0.22±0.2 | 6.08E-01 | 0.25±0.21 | 6.00E-01 |
| 3-indoxyl sulfate | 0.03±0.02 | 7.95 | 2.97E-01 | 7.36E-01 | 0.11±0.15 | 0 | 3.86E-01 | 9.58E-01 | 0.03±0.21 | 9.77E-01 | -0.03±0.21 | 9.73E-01 | 0.24±0.21 | 5.78E-01 | 0.23±0.2 | 6.13E-01 |
| glycocholate | 0.12±0.17 | 39.34 | 1.99E-01 | 7.60E-01 | -0.11±0.14 | 85.17 | 9.40E-03 | 9.58E-01 | -0.55±0.21 | 8.16E-01 | -0.54±0.22 | 8.44E-01 | 0.19±0.18 | 6.08E-01 | 0.19±0.18 | 6.29E-01 |
| tiglylcarnitine (C5:1-DC) | 0.08±0.06 | 0 | 4.68E-01 | 7.36E-01 | 0.11±0.15 | 0 | 4.73E-01 | 9.58E-01 | 0.07±0.2 | 9.38E-01 | 0±0.21 | 9.98E-01 | 0.23±0.21 | 6.11E-01 | 0.22±0.21 | 6.42E-01 |
| 1-palmitoyl-2-oleoyl-GPI (16:0/18:1)* | 0.08±0.06 | 0 | 4.39E-01 | 7.36E-01 | 0.12±0.16 | 0 | 5.49E-01 | 9.58E-01 | 0.07±0.21 | 9.38E-01 | 0.03±0.22 | 9.73E-01 | 0.26±0.24 | 6.08E-01 | 0.22±0.23 | 6.82E-01 |
| 1-arachidonoyl-GPI (20:4)* | 0.05±0.04 | 0 | 3.68E-01 | 7.36E-01 | 0.1±0.14 | 0 | 5.96E-01 | 9.58E-01 | 0.04±0.21 | 9.54E-01 | 0.02±0.21 | 9.76E-01 | 0.23±0.21 | 5.95E-01 | 0.18±0.2 | 7.00E-01 |
| O-sulfo-L-tyrosine | 0.05±0.19 | 38.6 | 2.02E-01 | 9.19E-01 | -0.11±0.15 | 75.32 | 4.41E-02 | 9.58E-01 | -0.39±0.22 | 8.16E-01 | -0.43±0.22 | 8.44E-01 | 0.18±0.21 | 7.27E-01 | 0.18±0.21 | 7.12E-01 |
| 1-(1-enyl-palmitoyl)-2-oleoyl-GPC (P-16:0/18:1)* | 0.02±0.03 | 54.16 | 1.40E-01 | 7.62E-01 | -0.09±0.13 | 0 | 6.46E-01 | 9.58E-01 | 0.03±0.2 | 9.65E-01 | -0.02±0.21 | 9.84E-01 | -0.19±0.15 | 5.36E-01 | -0.14±0.16 | 7.14E-01 |
| pristanate | 0.02±0.02 | 0 | 3.28E-01 | 7.36E-01 | -0.1±0.14 | 0 | 6.73E-01 | 9.58E-01 | 0.02±0.21 | 9.90E-01 | -0.03±0.21 | 9.71E-01 | -0.16±0.18 | 7.06E-01 | -0.15±0.19 | 7.29E-01 |
| andro steroid monosulfate C19H28O6S (1)* | -0.03±0.03 | 0 | 5.83E-01 | 7.36E-01 | -0.1±0.13 | 0 | 6.79E-01 | 9.58E-01 | -0.03±0.19 | 9.70E-01 | -0.04±0.2 | 9.64E-01 | -0.13±0.18 | 7.79E-01 | -0.15±0.18 | 7.34E-01 |
| myristoleoylcarnitine (C14:1)* | -0.04±0.16 | 34.43 | 2.17E-01 | 9.14E-01 | 0.1±0.13 | 73.82 | 5.06E-02 | 9.58E-01 | 0.38±0.19 | 8.16E-01 | 0.37±0.19 | 8.44E-01 | -0.14±0.18 | 7.57E-01 | -0.15±0.18 | 7.36E-01 |
| 5alpha-pregnan-3beta,20alpha-diol disulfate | 0.04±0.04 | 0 | 5.58E-01 | 7.36E-01 | 0.11±0.14 | 0 | 7.81E-01 | 9.58E-01 | 0.04±0.21 | 9.57E-01 | 0.06±0.22 | 9.40E-01 | 0.15±0.19 | 7.41E-01 | 0.14±0.18 | 7.60E-01 |
| X - 16397 | 0±0 | 0 | 6.55E-01 | 7.36E-01 | -0.1±0.14 | 0 | 7.45E-01 | 9.58E-01 | 0±0.19 | 9.99E-01 | -0.06±0.19 | 9.38E-01 | -0.09±0.2 | 8.69E-01 | -0.15±0.2 | 7.71E-01 |
| 3-hydroxylaurate | -0.01±0.18 | 21.24 | 2.60E-01 | 9.84E-01 | 0.11±0.14 | 71.96 | 5.90E-02 | 9.58E-01 | 0.43±0.21 | 8.16E-01 | 0.41±0.21 | 8.44E-01 | -0.1±0.2 | 8.32E-01 | -0.14±0.2 | 7.81E-01 |
| cysteine sulfinic acid | 0±0 | 0 | 6.14E-01 | 7.36E-01 | 0.11±0.15 | 0 | 8.02E-01 | 9.58E-01 | 0±0.21 | 9.97E-01 | 0.07±0.22 | 9.38E-01 | 0.1±0.19 | 8.32E-01 | 0.14±0.2 | 7.85E-01 |
| 3-formylindole | 0.12±0.1 | 0 | 9.95E-01 | 7.36E-01 | 0.11±0.16 | 0 | 8.50E-01 | 9.58E-01 | 0.12±0.23 | 8.97E-01 | 0.08±0.23 | 9.33E-01 | 0.12±0.21 | 8.23E-01 | 0.14±0.21 | 7.93E-01 |
| 9-hydroxystearate | -0.05±0.04 | 0 | 6.69E-01 | 7.36E-01 | -0.1±0.14 | 0 | 8.89E-01 | 9.58E-01 | -0.04±0.2 | 9.54E-01 | -0.08±0.21 | 9.27E-01 | -0.13±0.19 | 7.79E-01 | -0.12±0.19 | 8.13E-01 |
| trigonelline (N'-methylnicotinate) | -0.06±0.15 | 0 | 3.38E-01 | 8.55E-01 | -0.09±0.13 | 37.43 | 2.06E-01 | 9.58E-01 | -0.25±0.17 | 8.16E-01 | -0.24±0.17 | 8.44E-01 | 0.05±0.19 | 9.33E-01 | 0.09±0.19 | 8.59E-01 |
| 3-hydroxybutyrate (BHBA) | 0.05±0.16 | 0 | 3.26E-01 | 9.02E-01 | 0.1±0.14 | 47.86 | 1.66E-01 | 9.58E-01 | 0.33±0.19 | 8.16E-01 | 0.3±0.19 | 8.44E-01 | -0.04±0.19 | 9.41E-01 | -0.08±0.19 | 8.64E-01 |
| sphingomyelin (d18:2/16:0, d18:1/16:1)* | -0.12±0.11 | 0 | 8.33E-01 | 7.36E-01 | -0.1±0.13 | 0 | 6.94E-01 | 9.58E-01 | -0.14±0.21 | 8.58E-01 | -0.17±0.21 | 8.53E-01 | -0.09±0.17 | 8.23E-01 | -0.06±0.17 | 8.97E-01 |
| cortisol | 0.1±0.14 | 0 | 5.15E-01 | 7.45E-01 | 0.11±0.15 | 0 | 6.99E-01 | 9.58E-01 | 0.2±0.23 | 8.55E-01 | 0.18±0.24 | 8.54E-01 | 0.02±0.18 | 9.73E-01 | 0.06±0.19 | 8.98E-01 |
| erucate (22:1n9) | -0.1±0.1 | 0 | 8.27E-01 | 7.36E-01 | -0.1±0.15 | 0 | 7.34E-01 | 9.58E-01 | -0.11±0.21 | 8.91E-01 | -0.16±0.22 | 8.59E-01 | -0.06±0.2 | 9.15E-01 | -0.06±0.2 | 9.16E-01 |
| X - 12851 | 0.1±0.12 | 0 | 6.21E-01 | 7.36E-01 | 0.1±0.14 | 0 | 6.85E-01 | 9.58E-01 | 0.15±0.2 | 8.55E-01 | 0.16±0.2 | 8.53E-01 | 0.03±0.19 | 9.64E-01 | 0.05±0.19 | 9.26E-01 |
| sebacate (C10-DC) | 0.08±0.15 | 0 | 3.92E-01 | 8.02E-01 | 0.1±0.14 | 0 | 3.19E-01 | 9.58E-01 | 0.23±0.18 | 8.40E-01 | 0.23±0.18 | 8.44E-01 | -0.03±0.2 | 9.67E-01 | -0.05±0.2 | 9.38E-01 |
| X - 21353 | 0.05±0.16 | 0 | 3.33E-01 | 8.77E-01 | 0.11±0.14 | 19.84 | 2.64E-01 | 9.58E-01 | 0.29±0.2 | 8.19E-01 | 0.28±0.21 | 8.44E-01 | -0.04±0.19 | 9.38E-01 | -0.04±0.19 | 9.42E-01 |
| glycosyl-N-stearoyl-sphingosine (d18:1/18:0) | -0.11±0.14 | 0 | 5.10E-01 | 7.49E-01 | -0.11±0.14 | 3.58 | 3.08E-01 | 9.58E-01 | -0.21±0.2 | 8.40E-01 | -0.26±0.2 | 8.44E-01 | -0.02±0.2 | 9.74E-01 | 0.04±0.21 | 9.49E-01 |
| X - 11843 | -0.12±0.13 | 0 | 6.56E-01 | 7.36E-01 | -0.11±0.14 | 0 | 5.83E-01 | 9.58E-01 | -0.18±0.19 | 8.55E-01 | -0.18±0.2 | 8.45E-01 | -0.06±0.19 | 9.15E-01 | -0.03±0.19 | 9.53E-01 |
| glycine conjugate of C10H12O2* | -0.08±0.14 | 0 | 3.93E-01 | 8.02E-01 | -0.1±0.14 | 0 | 3.45E-01 | 9.58E-01 | -0.21±0.19 | 8.40E-01 | -0.24±0.2 | 8.44E-01 | 0.03±0.19 | 9.67E-01 | 0.02±0.19 | 9.69E-01 |
| X - 21258 | 0.1±0.14 | 0 | 4.68E-01 | 7.62E-01 | 0.11±0.14 | 0 | 4.90E-01 | 9.58E-01 | 0.23±0.2 | 8.40E-01 | 0.22±0.21 | 8.44E-01 | 0.01±0.19 | 9.82E-01 | 0.02±0.19 | 9.73E-01 |
| N-stearoyl-sphingadienine (d18:2/18:0)* | 0.12±0.16 | 0 | 4.77E-01 | 7.60E-01 | 0.11±0.15 | 3.57 | 3.09E-01 | 9.58E-01 | 0.28±0.23 | 8.40E-01 | 0.29±0.24 | 8.44E-01 | 0.04±0.2 | 9.57E-01 | -0.02±0.2 | 9.74E-01 |
| 1-stearoyl-2-linoleoyl-GPC (18:0/18:2)* | -0.12±0.14 | 0 | 5.60E-01 | 7.36E-01 | -0.1±0.14 | 0 | 3.77E-01 | 9.58E-01 | -0.22±0.2 | 8.40E-01 | -0.24±0.21 | 8.44E-01 | -0.05±0.18 | 9.20E-01 | 0.01±0.19 | 9.87E-01 |
| X - 13844 | -0.1±0.15 | 0 | 4.55E-01 | 7.71E-01 | -0.1±0.14 | 0 | 4.36E-01 | 9.58E-01 | -0.23±0.19 | 8.40E-01 | -0.21±0.19 | 8.44E-01 | 0±0.19 | 9.95E-01 | 0.01±0.2 | 9.93E-01 |
| 3-hydroxyoctanoylcarnitine (2) | 0.09±0.16 | 0 | 4.13E-01 | 7.91E-01 | 0.11±0.14 | 0 | 4.08E-01 | 9.58E-01 | 0.27±0.2 | 8.40E-01 | 0.23±0.21 | 8.44E-01 | 0±0.19 | 9.97E-01 | 0±0.2 | 9.97E-01 |
| sphingomyelin (d18:2/18:1)* | -0.12±0.09 | 0 | 9.16E-01 | 7.36E-01 | -0.09±0.13 | 0 | 9.78E-01 | 9.58E-01 | -0.12±0.22 | 8.91E-01 | -0.09±0.23 | 9.27E-01 | -0.14±0.16 | 7.13E-01 | -0.09±0.16 | 8.21E-01 |
| phenylacetylglutamate | 0.11±0.16 | 77.61 | 3.46E-02 | 7.62E-01 | 0.11±0.16 | 83.11 | 1.50E-02 | 9.59E-01 | -0.22±0.2 | 8.40E-01 | -0.26±0.22 | 8.44E-01 | 0.44±0.22 | 3.17E-01 | 0.52±0.23 | 2.95E-01 |
| 2S,3R-dihydroxybutyrate | 0.06±0.05 | 16.06 | 2.75E-01 | 7.36E-01 | 0.12±0.17 | 0 | 3.71E-01 | 9.59E-01 | 0.05±0.24 | 9.57E-01 | -0.04±0.25 | 9.71E-01 | 0.31±0.24 | 5.28E-01 | 0.27±0.24 | 6.14E-01 |
| etiocholanolone glucuronide | 0±0 | 13.11 | 2.83E-01 | 7.36E-01 | -0.09±0.14 | 0 | 5.29E-01 | 9.59E-01 | 0±0.2 | 9.94E-01 | 0±0.2 | 9.99E-01 | -0.2±0.18 | 6.00E-01 | -0.17±0.19 | 6.89E-01 |
| 3-hydroxypyridine sulfate | 0.01±0.17 | 29.69 | 2.33E-01 | 9.76E-01 | -0.09±0.14 | 71.94 | 5.90E-02 | 9.59E-01 | -0.34±0.18 | 8.16E-01 | -0.33±0.19 | 8.44E-01 | 0.13±0.19 | 7.91E-01 | 0.18±0.2 | 6.95E-01 |
| X - 11483 | 0.06±0.05 | 0 | 5.44E-01 | 7.36E-01 | 0.1±0.14 | 0 | 6.42E-01 | 9.59E-01 | 0.05±0.2 | 9.54E-01 | 0.03±0.21 | 9.73E-01 | 0.17±0.19 | 7.11E-01 | 0.17±0.2 | 7.29E-01 |
| oleoyl-linoleoyl-glycerol (18:1/18:2) [2] | 0.1±0.09 | 0 | 8.41E-01 | 7.36E-01 | 0.1±0.15 | 0 | 8.25E-01 | 9.59E-01 | 0.11±0.21 | 8.98E-01 | 0.13±0.21 | 8.63E-01 | 0.06±0.2 | 9.16E-01 | 0.07±0.2 | 8.98E-01 |
| ceramide (d18:2/24:1, d18:1/24:2)* | 0.09±0.08 | 0 | 8.95E-01 | 7.36E-01 | 0.1±0.15 | 0 | 8.60E-01 | 9.59E-01 | 0.09±0.21 | 9.06E-01 | 0.13±0.2 | 8.65E-01 | 0.06±0.21 | 9.17E-01 | 0.07±0.21 | 8.98E-01 |
| glycerate | -0.04±0.15 | 24.37 | 2.50E-01 | 9.25E-01 | 0.11±0.15 | 38.78 | 2.01E-01 | 9.59E-01 | 0.45±0.27 | 8.16E-01 | 0.41±0.29 | 8.44E-01 | -0.1±0.16 | 8.00E-01 | -0.02±0.18 | 9.71E-01 |
| phenylacetylglutamine | 0.08±0.15 | 80.47 | 2.36E-02 | 8.02E-01 | 0.1±0.16 | 82.29 | 1.75E-02 | 9.60E-01 | -0.19±0.19 | 8.55E-01 | -0.22±0.21 | 8.44E-01 | 0.49±0.24 | 2.81E-01 | 0.53±0.24 | 2.96E-01 |
| glycerol 3-phosphate | 0.07±0.14 | 74.85 | 4.62E-02 | 8.14E-01 | 0.1±0.15 | 76.74 | 3.81E-02 | 9.60E-01 | -0.19±0.21 | 8.55E-01 | -0.23±0.21 | 8.44E-01 | 0.36±0.2 | 3.77E-01 | 0.38±0.2 | 3.79E-01 |
| nonanoylcarnitine (C9) | -0.13±0.14 | 78.59 | 3.07E-02 | 7.36E-01 | -0.09±0.13 | 76.64 | 3.85E-02 | 9.60E-01 | 0.21±0.19 | 8.40E-01 | 0.21±0.2 | 8.44E-01 | -0.39±0.18 | 2.74E-01 | -0.34±0.18 | 3.83E-01 |
| X - 25419 | 0.03±0.11 | 73.17 | 5.35E-02 | 9.14E-01 | 0.09±0.14 | 70.3 | 6.65E-02 | 9.60E-01 | -0.14±0.21 | 8.55E-01 | -0.2±0.21 | 8.45E-01 | 0.3±0.18 | 4.12E-01 | 0.32±0.19 | 4.33E-01 |
| X - 12798 | -0.03±0.1 | 67.93 | 7.74E-02 | 9.14E-01 | 0.11±0.16 | 45.09 | 1.77E-01 | 9.60E-01 | -0.11±0.21 | 8.93E-01 | -0.09±0.22 | 9.27E-01 | 0.34±0.23 | 4.74E-01 | 0.34±0.24 | 5.19E-01 |
| isovalerylcarnitine (C5) | -0.03±0.04 | 66.48 | 8.41E-02 | 7.60E-01 | 0.1±0.15 | 34.87 | 2.15E-01 | 9.60E-01 | -0.04±0.2 | 9.59E-01 | -0.08±0.21 | 9.27E-01 | 0.35±0.22 | 4.48E-01 | 0.3±0.22 | 5.44E-01 |
| lanthionine | 0±0 | 0 | 3.30E-01 | 7.36E-01 | 0.1±0.15 | 0 | 4.37E-01 | 9.60E-01 | 0±0.2 | 9.99E-01 | -0.01±0.21 | 9.84E-01 | 0.2±0.2 | 6.61E-01 | 0.21±0.21 | 6.56E-01 |
| oleoyl-linoleoyl-glycerol (18:1/18:2) [1] | 0.07±0.06 | 0 | 8.74E-01 | 7.36E-01 | 0.09±0.14 | 0 | 9.56E-01 | 9.60E-01 | 0.06±0.2 | 9.38E-01 | 0.09±0.2 | 9.19E-01 | 0.1±0.2 | 8.48E-01 | 0.1±0.2 | 8.47E-01 |
| X - 23636 | 0.11±0.11 | 0 | 8.14E-01 | 7.36E-01 | 0.1±0.15 | 0 | 9.93E-01 | 9.60E-01 | 0.12±0.21 | 8.75E-01 | 0.1±0.21 | 9.06E-01 | 0.06±0.22 | 9.16E-01 | 0.1±0.22 | 8.57E-01 |
| pantothenate | 0.13±0.11 | 0 | 8.56E-01 | 7.36E-01 | 0.08±0.12 | 0 | 9.70E-01 | 9.60E-01 | 0.15±0.17 | 8.55E-01 | 0.09±0.18 | 8.98E-01 | 0.1±0.18 | 8.12E-01 | 0.08±0.17 | 8.59E-01 |
| X - 12844 | -0.03±0.16 | 20.93 | 2.61E-01 | 9.47E-01 | 0.1±0.15 | 68 | 7.71E-02 | 9.60E-01 | 0.48±0.27 | 8.16E-01 | 0.51±0.28 | 8.44E-01 | -0.09±0.17 | 8.25E-01 | -0.07±0.18 | 8.73E-01 |
| (16 or 17)-methylstearate (a19:0 or i19:0) | 0.12±0.13 | 0 | 6.72E-01 | 7.36E-01 | 0.09±0.14 | 0 | 6.83E-01 | 9.60E-01 | 0.17±0.2 | 8.55E-01 | 0.16±0.21 | 8.58E-01 | 0.07±0.19 | 9.15E-01 | 0.04±0.19 | 9.42E-01 |
| taurocholenate sulfate* | 0.08±0.13 | 0 | 4.09E-01 | 7.86E-01 | 0.1±0.15 | 0 | 5.05E-01 | 9.60E-01 | 0.18±0.2 | 8.55E-01 | 0.19±0.21 | 8.45E-01 | -0.04±0.19 | 9.45E-01 | 0±0.2 | 9.97E-01 |
| butyrate/isobutyrate (4:0) | -0.03±0.06 | 68.68 | 7.39E-02 | 8.26E-01 | 0.09±0.14 | 32.38 | 2.24E-01 | 9.61E-01 | -0.06±0.2 | 9.38E-01 | -0.09±0.2 | 9.13E-01 | 0.28±0.18 | 4.50E-01 | 0.24±0.18 | 5.56E-01 |
| arabonate/xylonate | 0.33±0.21 | 79.78 | 2.62E-02 | 7.36E-01 | 0.11±0.17 | 89.3 | 2.24E-03 | 9.64E-01 | -0.38±0.23 | 8.16E-01 | -0.39±0.24 | 8.44E-01 | 0.62±0.25 | 1.75E-01 | 0.66±0.25 | 2.28E-01 |
| S-carboxyethylcysteine | 0.31±0.16 | 47.29 | 1.68E-01 | 7.36E-01 | 0.09±0.15 | 93.83 | 5.71E-05 | 9.64E-01 | -0.87±0.28 | 8.16E-01 | -0.92±0.29 | 8.44E-01 | 0.35±0.17 | 2.81E-01 | 0.46±0.18 | 2.28E-01 |
| gamma-glutamylthreonine | -0.03±0.1 | 84.59 | 1.08E-02 | 9.02E-01 | 0.11±0.18 | 80.76 | 2.26E-02 | 9.64E-01 | -0.1±0.2 | 8.93E-01 | -0.12±0.21 | 8.70E-01 | 0.84±0.36 | 2.12E-01 | 0.82±0.36 | 2.84E-01 |
| N-acetylmethionine | 0.18±0.17 | 80.81 | 2.24E-02 | 7.36E-01 | 0.09±0.14 | 83.57 | 1.36E-02 | 9.64E-01 | -0.25±0.19 | 8.40E-01 | -0.23±0.19 | 8.44E-01 | 0.51±0.22 | 2.23E-01 | 0.49±0.22 | 2.94E-01 |
| 5-hydroxyindole sulfate | 0.04±0.12 | 81.32 | 2.07E-02 | 9.00E-01 | 0.09±0.15 | 79.91 | 2.57E-02 | 9.64E-01 | -0.14±0.19 | 8.55E-01 | -0.18±0.19 | 8.45E-01 | 0.47±0.22 | 2.81E-01 | 0.48±0.23 | 3.29E-01 |
| N-acetyl-isoputreanine | 0.01±0.12 | 80.21 | 2.46E-02 | 9.81E-01 | 0.1±0.16 | 76.83 | 3.77E-02 | 9.64E-01 | -0.14±0.2 | 8.55E-01 | -0.15±0.2 | 8.58E-01 | 0.52±0.26 | 3.07E-01 | 0.53±0.26 | 3.45E-01 |
| caffeine | -0.02±0.06 | 85.22 | 9.30E-03 | 9.11E-01 | 0.09±0.15 | 76.16 | 4.05E-02 | 9.64E-01 | -0.07±0.19 | 9.30E-01 | -0.17±0.19 | 8.45E-01 | 0.55±0.23 | 2.04E-01 | 0.45±0.23 | 3.70E-01 |
| 2-aminoheptanoate | 0.02±0.11 | 74.26 | 4.87E-02 | 9.27E-01 | 0.08±0.14 | 75.21 | 4.46E-02 | 9.64E-01 | -0.14±0.19 | 8.55E-01 | -0.19±0.19 | 8.44E-01 | 0.32±0.19 | 4.06E-01 | 0.36±0.2 | 4.05E-01 |
| X - 23739 | 0.02±0.14 | 70.33 | 6.64E-02 | 9.51E-01 | 0.09±0.16 | 72.87 | 5.49E-02 | 9.64E-01 | -0.18±0.22 | 8.55E-01 | -0.21±0.22 | 8.45E-01 | 0.36±0.23 | 4.50E-01 | 0.41±0.23 | 4.18E-01 |
| linoleate (18:2n6) | -0.1±0.14 | 71.89 | 5.93E-02 | 7.62E-01 | -0.08±0.14 | 73.77 | 5.09E-02 | 9.64E-01 | 0.22±0.21 | 8.40E-01 | 0.24±0.22 | 8.44E-01 | -0.32±0.19 | 4.01E-01 | -0.32±0.19 | 4.33E-01 |
| methyl indole-3-acetate | 0.09±0.15 | 72.76 | 5.54E-02 | 7.91E-01 | 0.09±0.15 | 68.95 | 7.27E-02 | 9.64E-01 | -0.22±0.23 | 8.55E-01 | -0.22±0.23 | 8.45E-01 | 0.35±0.2 | 4.04E-01 | 0.33±0.2 | 4.67E-01 |
| urea | 0.03±0.13 | 73.03 | 5.42E-02 | 9.35E-01 | 0.09±0.15 | 65.97 | 8.65E-02 | 9.64E-01 | -0.16±0.21 | 8.55E-01 | -0.18±0.22 | 8.50E-01 | 0.36±0.22 | 4.18E-01 | 0.35±0.22 | 4.67E-01 |
| glycocholenate sulfate* | 0.02±0.14 | 65.09 | 9.05E-02 | 9.45E-01 | 0.09±0.15 | 63.45 | 9.81E-02 | 9.64E-01 | -0.19±0.21 | 8.55E-01 | -0.15±0.21 | 8.59E-01 | 0.29±0.21 | 4.97E-01 | 0.35±0.21 | 4.67E-01 |
| X - 24334 | -0.01±0.1 | 72.19 | 5.79E-02 | 9.58E-01 | 0.1±0.16 | 62.58 | 1.02E-01 | 9.64E-01 | -0.12±0.22 | 8.93E-01 | -0.17±0.23 | 8.58E-01 | 0.35±0.22 | 4.37E-01 | 0.34±0.22 | 4.67E-01 |
| pipecolate | 0.05±0.12 | 72.13 | 5.82E-02 | 8.36E-01 | 0.09±0.14 | 65.64 | 8.80E-02 | 9.64E-01 | -0.17±0.23 | 8.55E-01 | -0.24±0.24 | 8.45E-01 | 0.3±0.18 | 4.14E-01 | 0.27±0.18 | 4.74E-01 |
| 1-stearoyl-2-oleoyl-GPI (18:0/18:1)* | -0.03±0.04 | 40.28 | 1.96E-01 | 7.44E-01 | 0.1±0.17 | 31.14 | 2.28E-01 | 9.64E-01 | -0.04±0.21 | 9.57E-01 | -0.06±0.21 | 9.46E-01 | 0.3±0.26 | 5.87E-01 | 0.36±0.27 | 5.49E-01 |
| X - 21319 | 0.01±0.1 | 63.03 | 1.00E-01 | 9.84E-01 | -0.08±0.14 | 37.8 | 2.05E-01 | 9.64E-01 | 0.12±0.21 | 8.81E-01 | 0.13±0.22 | 8.70E-01 | -0.22±0.17 | 5.28E-01 | -0.23±0.18 | 5.62E-01 |
| X - 13507 | 0.14±0.18 | 35.46 | 2.13E-01 | 7.44E-01 | -0.08±0.15 | 88.73 | 2.89E-03 | 9.64E-01 | -0.7±0.25 | 8.16E-01 | -0.71±0.26 | 8.44E-01 | 0.2±0.18 | 6.00E-01 | 0.23±0.18 | 5.77E-01 |
| beta-cryptoxanthin | 0.02±0.08 | 70.05 | 6.76E-02 | 9.25E-01 | -0.09±0.14 | 1.68 | 3.13E-01 | 9.64E-01 | 0.08±0.21 | 9.13E-01 | 0.08±0.22 | 9.33E-01 | -0.27±0.18 | 4.50E-01 | -0.22±0.19 | 6.11E-01 |
| N1-methyladenosine | -0.04±0.19 | 58.62 | 1.20E-01 | 9.35E-01 | -0.1±0.17 | 60.96 | 1.09E-01 | 9.64E-01 | -0.23±0.19 | 8.40E-01 | -0.25±0.2 | 8.44E-01 | 0.44±0.36 | 5.66E-01 | 0.4±0.36 | 6.11E-01 |
| adenosine 3',5'-cyclic monophosphate (cAMP) | 0.02±0.07 | 58.43 | 1.21E-01 | 8.77E-01 | -0.07±0.12 | 0 | 3.68E-01 | 9.64E-01 | 0.08±0.19 | 9.18E-01 | 0.06±0.19 | 9.33E-01 | -0.19±0.15 | 5.57E-01 | -0.16±0.16 | 6.56E-01 |
| 1-(1-enyl-palmitoyl)-2-oleoyl-GPE (P-16:0/18:1)* | 0.05±0.04 | 0 | 4.15E-01 | 7.36E-01 | 0.1±0.16 | 0 | 4.42E-01 | 9.64E-01 | 0.04±0.2 | 9.54E-01 | 0±0.21 | 9.99E-01 | 0.26±0.27 | 6.50E-01 | 0.25±0.25 | 6.72E-01 |
| 2-stearoyl-GPE (18:0)* | -0.04±0.05 | 0 | 3.62E-01 | 7.44E-01 | 0.08±0.14 | 0 | 4.32E-01 | 9.64E-01 | -0.05±0.2 | 9.54E-01 | -0.03±0.2 | 9.73E-01 | 0.14±0.2 | 7.79E-01 | 0.2±0.2 | 6.81E-01 |
| 2-hydroxyhippurate (salicylurate) | 0±0 | 0 | 3.55E-01 | 7.36E-01 | 0.09±0.14 | 0 | 4.72E-01 | 9.64E-01 | 0±0.21 | 9.99E-01 | -0.02±0.22 | 9.76E-01 | 0.18±0.2 | 6.83E-01 | 0.19±0.19 | 6.83E-01 |
| 3beta,7alpha-dihydroxy-5-cholestenoate | 0±0 | 45.45 | 1.76E-01 | 7.36E-01 | -0.08±0.14 | 0 | 4.71E-01 | 9.64E-01 | 0±0.19 | 9.99E-01 | 0.01±0.19 | 9.87E-01 | -0.25±0.19 | 5.09E-01 | -0.18±0.2 | 6.88E-01 |
| salicylate | 0.01±0.01 | 0 | 4.66E-01 | 7.36E-01 | 0.09±0.14 | 0 | 5.46E-01 | 9.64E-01 | 0.01±0.21 | 9.92E-01 | 0±0.21 | 9.99E-01 | 0.16±0.2 | 7.53E-01 | 0.17±0.2 | 7.00E-01 |
| 4-chlorobenzoic acid | 0.02±0.02 | 0 | 4.07E-01 | 7.36E-01 | 0.08±0.14 | 0 | 5.46E-01 | 9.64E-01 | 0.02±0.2 | 9.90E-01 | 0±0.2 | 9.99E-01 | 0.18±0.19 | 6.83E-01 | 0.16±0.19 | 7.10E-01 |
| X - 24422 | 0.02±0.02 | 0 | 3.46E-01 | 7.36E-01 | 0.09±0.15 | 0 | 5.83E-01 | 9.64E-01 | 0.02±0.21 | 9.90E-01 | 0.01±0.21 | 9.95E-01 | 0.21±0.2 | 6.31E-01 | 0.17±0.21 | 7.25E-01 |
| sphingomyelin (d18:1/18:1, d18:2/18:0) | 0.02±0.03 | 0 | 3.55E-01 | 7.36E-01 | -0.08±0.14 | 0 | 5.77E-01 | 9.64E-01 | 0.03±0.23 | 9.77E-01 | 0.03±0.24 | 9.73E-01 | -0.13±0.17 | 7.57E-01 | -0.13±0.17 | 7.44E-01 |
| deoxycarnitine | 0.02±0.22 | 28.59 | 2.37E-01 | 9.76E-01 | -0.12±0.19 | 68.52 | 7.47E-02 | 9.64E-01 | -0.47±0.28 | 8.16E-01 | -0.49±0.28 | 8.44E-01 | 0.16±0.26 | 7.96E-01 | 0.19±0.26 | 7.66E-01 |
| phytanate | 0.06±0.06 | 0 | 7.84E-01 | 7.36E-01 | 0.09±0.14 | 0 | 6.96E-01 | 9.64E-01 | 0.06±0.2 | 9.38E-01 | 0.03±0.21 | 9.73E-01 | 0.11±0.19 | 8.06E-01 | 0.14±0.19 | 7.73E-01 |
| 3-hydroxysebacate | 0.03±0.17 | 11.99 | 2.86E-01 | 9.35E-01 | 0.08±0.14 | 57.05 | 1.27E-01 | 9.64E-01 | 0.3±0.18 | 8.16E-01 | 0.27±0.19 | 8.44E-01 | -0.09±0.2 | 8.81E-01 | -0.15±0.2 | 7.73E-01 |
| gamma-glutamylcitrulline* | -0.07±0.05 | 0 | 4.71E-01 | 7.36E-01 | -0.08±0.13 | 0 | 7.32E-01 | 9.64E-01 | -0.06±0.19 | 9.38E-01 | -0.03±0.2 | 9.71E-01 | -0.18±0.16 | 5.96E-01 | -0.12±0.17 | 7.79E-01 |
| umbelliferone sulfate | 0.08±0.07 | 0 | 7.69E-01 | 7.36E-01 | 0.09±0.14 | 0 | 7.43E-01 | 9.64E-01 | 0.07±0.21 | 9.32E-01 | 0.04±0.21 | 9.64E-01 | 0.13±0.19 | 7.79E-01 | 0.13±0.19 | 7.81E-01 |
| pantoate | 0.03±0.03 | 0 | 3.28E-01 | 7.39E-01 | -0.07±0.13 | 0 | 7.54E-01 | 9.64E-01 | 0.03±0.18 | 9.65E-01 | -0.03±0.19 | 9.71E-01 | -0.14±0.18 | 7.33E-01 | -0.11±0.17 | 8.09E-01 |
| pyrraline | -0.06±0.05 | 0 | 6.61E-01 | 7.36E-01 | -0.09±0.14 | 0 | 8.17E-01 | 9.64E-01 | -0.05±0.2 | 9.46E-01 | -0.05±0.2 | 9.49E-01 | -0.14±0.19 | 7.72E-01 | -0.12±0.19 | 8.13E-01 |
| 1-arachidonoyl-GPC (20:4n6)* | 0.07±0.07 | 0 | 9.04E-01 | 7.36E-01 | 0.08±0.15 | 0 | 7.84E-01 | 9.64E-01 | 0.07±0.21 | 9.32E-01 | 0.04±0.21 | 9.61E-01 | 0.1±0.21 | 8.58E-01 | 0.13±0.21 | 8.13E-01 |
| bilirubin degradation product, C17H18N2O4 (1)** | 0.09±0.09 | 0 | 9.04E-01 | 7.36E-01 | 0.09±0.15 | 0 | 7.87E-01 | 9.64E-01 | 0.1±0.21 | 9.04E-01 | 0.05±0.21 | 9.58E-01 | 0.07±0.2 | 9.15E-01 | 0.13±0.21 | 8.13E-01 |
| bilirubin degradation product, C17H18N2O4 (2)** | 0.1±0.11 | 0 | 7.36E-01 | 7.36E-01 | 0.09±0.15 | 0 | 9.20E-01 | 9.64E-01 | 0.13±0.21 | 8.72E-01 | 0.08±0.22 | 9.33E-01 | 0.05±0.2 | 9.38E-01 | 0.11±0.21 | 8.45E-01 |
| arachidoylcarnitine (C20)* | 0.08±0.08 | 0 | 7.14E-01 | 7.36E-01 | 0.09±0.15 | 0 | 9.62E-01 | 9.64E-01 | 0.09±0.21 | 9.06E-01 | 0.08±0.21 | 9.27E-01 | 0.01±0.19 | 9.82E-01 | 0.1±0.21 | 8.56E-01 |
| X - 17354 | 0.08±0.08 | 0 | 7.48E-01 | 7.36E-01 | 0.08±0.14 | 0 | 9.34E-01 | 9.64E-01 | 0.09±0.2 | 9.06E-01 | 0.07±0.21 | 9.33E-01 | 0.02±0.19 | 9.74E-01 | 0.09±0.2 | 8.57E-01 |
| arginine | -0.09±0.15 | 0 | 4.11E-01 | 7.91E-01 | -0.08±0.15 | 20.52 | 2.62E-01 | 9.64E-01 | -0.23±0.19 | 8.40E-01 | -0.24±0.2 | 8.44E-01 | 0.02±0.19 | 9.79E-01 | 0.09±0.22 | 8.64E-01 |
| 1-stearoyl-2-docosahexaenoyl-GPE (18:0/22:6)* | 0.13±0.11 | 0 | 9.86E-01 | 7.36E-01 | 0.09±0.16 | 0 | 9.85E-01 | 9.64E-01 | 0.13±0.23 | 8.80E-01 | 0.1±0.23 | 9.27E-01 | 0.14±0.21 | 7.88E-01 | 0.09±0.21 | 8.65E-01 |
| sphingomyelin (d18:0/20:0, d16:0/22:0)* | 0.14±0.12 | 0 | 9.47E-01 | 7.36E-01 | 0.09±0.15 | 0 | 9.74E-01 | 9.64E-01 | 0.15±0.21 | 8.55E-01 | 0.09±0.22 | 9.21E-01 | 0.13±0.21 | 8.01E-01 | 0.08±0.2 | 8.65E-01 |
| gamma-carboxyglutamate | -0.07±0.15 | 0 | 3.58E-01 | 8.27E-01 | -0.08±0.15 | 4.91 | 3.05E-01 | 9.64E-01 | -0.23±0.21 | 8.40E-01 | -0.23±0.21 | 8.44E-01 | 0.06±0.21 | 9.30E-01 | 0.07±0.21 | 9.10E-01 |
| hexanoylglutamine | 0.1±0.15 | 0 | 4.34E-01 | 7.80E-01 | 0.08±0.14 | 9.36 | 2.94E-01 | 9.64E-01 | 0.26±0.19 | 8.40E-01 | 0.22±0.19 | 8.44E-01 | 0.01±0.19 | 9.94E-01 | -0.06±0.19 | 9.10E-01 |
| N-carbamoylalanine | -0.11±0.14 | 0 | 5.30E-01 | 7.44E-01 | -0.08±0.13 | 0 | 3.54E-01 | 9.64E-01 | -0.2±0.18 | 8.40E-01 | -0.19±0.18 | 8.44E-01 | -0.03±0.19 | 9.69E-01 | 0.06±0.19 | 9.16E-01 |
| sphingomyelin (d18:1/17:0, d17:1/18:0, d19:1/16:0) | -0.13±0.12 | 0 | 7.19E-01 | 7.36E-01 | -0.08±0.14 | 0 | 7.27E-01 | 9.64E-01 | -0.18±0.22 | 8.55E-01 | -0.14±0.23 | 8.65E-01 | -0.1±0.16 | 8.11E-01 | -0.04±0.17 | 9.35E-01 |
| 3-hydroxyhippurate | -0.07±0.14 | 0 | 3.75E-01 | 8.13E-01 | -0.08±0.13 | 0 | 3.42E-01 | 9.64E-01 | -0.2±0.19 | 8.40E-01 | -0.21±0.19 | 8.44E-01 | 0.04±0.18 | 9.43E-01 | 0.04±0.19 | 9.38E-01 |
| lignoceroyl sphingomyelin (d18:1/24:0) | -0.1±0.14 | 0 | 4.83E-01 | 7.61E-01 | -0.09±0.15 | 0 | 3.20E-01 | 9.64E-01 | -0.21±0.21 | 8.55E-01 | -0.25±0.22 | 8.44E-01 | -0.02±0.18 | 9.79E-01 | 0.05±0.2 | 9.38E-01 |
| sphingomyelin (d18:2/23:0, d18:1/23:1, d17:1/24:1)* | -0.14±0.12 | 0 | 8.16E-01 | 7.36E-01 | -0.09±0.14 | 0 | 6.97E-01 | 9.64E-01 | -0.16±0.22 | 8.55E-01 | -0.15±0.22 | 8.59E-01 | -0.11±0.17 | 8.00E-01 | -0.04±0.18 | 9.39E-01 |
| sphingomyelin (d17:1/16:0, d18:1/15:0, d16:1/17:0)* | -0.11±0.15 | 0 | 4.82E-01 | 7.60E-01 | -0.09±0.15 | 0 | 3.40E-01 | 9.64E-01 | -0.27±0.22 | 8.40E-01 | -0.25±0.22 | 8.44E-01 | -0.04±0.19 | 9.57E-01 | 0.04±0.21 | 9.47E-01 |
| eicosapentaenoate (EPA; 20:5n3) | -0.08±0.09 | 0 | 7.17E-01 | 7.36E-01 | -0.08±0.14 | 0 | 7.27E-01 | 9.64E-01 | -0.1±0.2 | 8.99E-01 | -0.13±0.2 | 8.61E-01 | -0.02±0.19 | 9.74E-01 | -0.03±0.2 | 9.49E-01 |
| cholesterol | -0.11±0.12 | 0 | 6.95E-01 | 7.36E-01 | -0.08±0.14 | 0 | 5.74E-01 | 9.64E-01 | -0.15±0.23 | 8.55E-01 | -0.19±0.23 | 8.52E-01 | -0.06±0.18 | 9.15E-01 | -0.02±0.18 | 9.73E-01 |
| arachidonate (20:4n6) | 0.09±0.14 | 0 | 4.67E-01 | 7.65E-01 | 0.08±0.14 | 0 | 4.70E-01 | 9.64E-01 | 0.21±0.2 | 8.55E-01 | 0.19±0.21 | 8.45E-01 | 0±0.19 | 9.95E-01 | -0.01±0.18 | 9.88E-01 |
| thymol sulfate | -0.07±0.08 | 0 | 6.33E-01 | 7.36E-01 | -0.09±0.14 | 0 | 5.70E-01 | 9.64E-01 | -0.08±0.2 | 9.06E-01 | -0.17±0.21 | 8.45E-01 | 0.01±0.19 | 9.82E-01 | -0.01±0.2 | 9.90E-01 |
| stachydrine | -0.11±0.11 | 0 | 7.70E-01 | 7.36E-01 | -0.08±0.14 | 0 | 5.67E-01 | 9.64E-01 | -0.13±0.2 | 8.55E-01 | -0.17±0.2 | 8.47E-01 | -0.07±0.19 | 9.11E-01 | -0.01±0.19 | 9.94E-01 |
| ceramide (d18:1/17:0, d17:1/18:0)* | 0.1±0.13 | 0 | 5.35E-01 | 7.44E-01 | 0.09±0.15 | 0 | 4.71E-01 | 9.64E-01 | 0.18±0.23 | 8.55E-01 | 0.23±0.24 | 8.45E-01 | 0.01±0.2 | 9.82E-01 | 0±0.2 | 9.96E-01 |
| dihomo-linoleoylcarnitine (C20:2)* | 0.09±0.13 | 0 | 4.94E-01 | 7.60E-01 | 0.08±0.15 | 0 | 5.82E-01 | 9.64E-01 | 0.17±0.21 | 8.55E-01 | 0.16±0.21 | 8.53E-01 | -0.01±0.2 | 9.83E-01 | 0±0.21 | 9.96E-01 |
| X - 12729 | -0.12±0.13 | 0 | 6.83E-01 | 7.36E-01 | -0.09±0.15 | 0 | 4.57E-01 | 9.64E-01 | -0.17±0.22 | 8.55E-01 | -0.23±0.24 | 8.45E-01 | -0.06±0.19 | 9.15E-01 | 0±0.19 | 9.96E-01 |
| alanine | 0.12±0.17 | 81.42 | 2.04E-02 | 7.62E-01 | 0.08±0.16 | 83.72 | 1.32E-02 | 9.75E-01 | -0.23±0.2 | 8.40E-01 | -0.23±0.21 | 8.44E-01 | 0.57±0.26 | 2.46E-01 | 0.61±0.27 | 2.84E-01 |
| isobutyrylcarnitine (C4) | 0.11±0.17 | 72.51 | 5.65E-02 | 7.62E-01 | 0.08±0.15 | 81.2 | 2.11E-02 | 9.75E-01 | -0.25±0.2 | 8.40E-01 | -0.26±0.21 | 8.44E-01 | 0.38±0.22 | 3.91E-01 | 0.43±0.21 | 3.51E-01 |
| X - 12306 | -0.03±0.08 | 58.86 | 1.19E-01 | 8.49E-01 | 0.07±0.14 | 63.25 | 9.90E-02 | 9.75E-01 | -0.09±0.19 | 9.03E-01 | -0.13±0.19 | 8.59E-01 | 0.25±0.2 | 5.53E-01 | 0.32±0.21 | 4.67E-01 |
| 3-methylxanthine | -0.02±0.08 | 60.71 | 1.11E-01 | 9.02E-01 | 0.07±0.13 | 47.24 | 1.69E-01 | 9.75E-01 | -0.1±0.18 | 8.93E-01 | -0.11±0.19 | 8.70E-01 | 0.23±0.18 | 5.45E-01 | 0.25±0.18 | 5.46E-01 |
| guaiacol sulfate | 0.13±0.18 | 50.31 | 1.56E-01 | 7.62E-01 | -0.08±0.14 | 83.86 | 1.28E-02 | 9.75E-01 | -0.43±0.2 | 8.16E-01 | -0.43±0.2 | 8.44E-01 | 0.24±0.2 | 5.59E-01 | 0.26±0.19 | 5.46E-01 |
| paraxanthine | 0.07±0.16 | 69.71 | 6.92E-02 | 8.33E-01 | -0.07±0.14 | 77.07 | 3.68E-02 | 9.75E-01 | -0.23±0.17 | 8.40E-01 | -0.29±0.17 | 8.44E-01 | 0.35±0.22 | 4.49E-01 | 0.29±0.22 | 5.50E-01 |
| vanillactate | -0.04±0.08 | 56.37 | 1.30E-01 | 8.19E-01 | 0.09±0.16 | 31.71 | 2.26E-01 | 9.75E-01 | -0.09±0.22 | 9.13E-01 | -0.1±0.22 | 9.21E-01 | 0.29±0.23 | 5.56E-01 | 0.29±0.23 | 5.77E-01 |
| glycine conjugate of C10H14O2 (1)* | -0.03±0.09 | 59.63 | 1.16E-01 | 8.85E-01 | 0.07±0.14 | 19.86 | 2.64E-01 | 9.75E-01 | -0.1±0.2 | 8.98E-01 | -0.08±0.2 | 9.27E-01 | 0.25±0.2 | 5.53E-01 | 0.24±0.2 | 6.04E-01 |
| X - 23644 | -0.03±0.04 | 29.27 | 2.34E-01 | 7.45E-01 | 0.07±0.14 | 24.64 | 2.49E-01 | 9.75E-01 | -0.04±0.2 | 9.57E-01 | -0.1±0.2 | 8.97E-01 | 0.19±0.19 | 6.46E-01 | 0.22±0.19 | 6.04E-01 |
| 1-linolenoyl-GPC (18:3)* | 0.02±0.18 | 36.85 | 2.08E-01 | 9.58E-01 | -0.08±0.15 | 73.25 | 5.32E-02 | 9.75E-01 | -0.34±0.19 | 8.16E-01 | -0.33±0.2 | 8.44E-01 | 0.16±0.21 | 7.57E-01 | 0.25±0.22 | 6.14E-01 |
| X - 12544 | -0.03±0.12 | 40.64 | 1.94E-01 | 9.25E-01 | 0.08±0.15 | 0 | 3.30E-01 | 9.75E-01 | -0.15±0.22 | 8.55E-01 | -0.1±0.23 | 9.24E-01 | 0.16±0.19 | 7.15E-01 | 0.19±0.19 | 6.56E-01 |
| 1-(1-enyl-palmitoyl)-2-palmitoyl-GPC (P-16:0/16:0)* | 0.03±0.04 | 39.48 | 1.99E-01 | 7.72E-01 | -0.07±0.12 | 0 | 6.24E-01 | 9.75E-01 | 0.04±0.21 | 9.55E-01 | 0.02±0.21 | 9.84E-01 | -0.15±0.14 | 6.31E-01 | -0.11±0.15 | 7.79E-01 |
| 3-methyl catechol sulfate (2) | -0.06±0.16 | 0 | 3.47E-01 | 8.50E-01 | -0.07±0.14 | 45.49 | 1.76E-01 | 9.75E-01 | -0.27±0.19 | 8.19E-01 | -0.27±0.2 | 8.44E-01 | 0.04±0.19 | 9.45E-01 | 0.12±0.2 | 8.21E-01 |
| 2-hydroxynervonate* | -0.07±0.07 | 0 | 9.45E-01 | 7.36E-01 | -0.07±0.13 | 0 | 8.59E-01 | 9.75E-01 | -0.07±0.2 | 9.30E-01 | -0.04±0.21 | 9.64E-01 | -0.08±0.17 | 8.37E-01 | -0.09±0.17 | 8.44E-01 |
| 1,2-dipalmitoyl-GPC (16:0/16:0) | -0.07±0.15 | 0 | 3.58E-01 | 8.26E-01 | -0.08±0.15 | 27.91 | 2.39E-01 | 9.75E-01 | -0.22±0.2 | 8.40E-01 | -0.24±0.2 | 8.44E-01 | 0.06±0.21 | 9.26E-01 | 0.11±0.22 | 8.47E-01 |
| sphingomyelin (d18:1/14:0, d16:1/16:0)* | -0.08±0.16 | 0 | 3.46E-01 | 8.28E-01 | -0.09±0.16 | 30.28 | 2.31E-01 | 9.75E-01 | -0.23±0.21 | 8.40E-01 | -0.27±0.22 | 8.44E-01 | 0.07±0.22 | 9.15E-01 | 0.11±0.22 | 8.56E-01 |
| 3-hydroxyhexanoylcarnitine (1) | 0.06±0.05 | 0 | 7.62E-01 | 7.36E-01 | 0.08±0.14 | 0 | 9.06E-01 | 9.75E-01 | 0.05±0.19 | 9.46E-01 | 0.06±0.2 | 9.38E-01 | 0.11±0.2 | 8.13E-01 | 0.09±0.2 | 8.59E-01 |
| 1-palmitoyl-2-linoleoyl-GPC (16:0/18:2) | 0.09±0.09 | 0 | 7.53E-01 | 7.36E-01 | 0.08±0.15 | 0 | 9.94E-01 | 9.75E-01 | 0.1±0.2 | 8.97E-01 | 0.08±0.21 | 9.27E-01 | 0.03±0.21 | 9.72E-01 | 0.08±0.21 | 8.88E-01 |
| N-acetylneuraminate | -0.06±0.18 | 0 | 3.35E-01 | 8.94E-01 | -0.09±0.16 | 37.29 | 2.07E-01 | 9.75E-01 | -0.39±0.27 | 8.19E-01 | -0.36±0.27 | 8.44E-01 | 0.03±0.2 | 9.64E-01 | 0.06±0.2 | 9.10E-01 |
| linoleoylcarnitine (C18:2)* | 0.07±0.07 | 0 | 7.25E-01 | 7.36E-01 | 0.08±0.15 | 0 | 9.30E-01 | 9.75E-01 | 0.08±0.2 | 9.26E-01 | 0.09±0.2 | 9.22E-01 | 0±0.2 | 9.97E-01 | 0.06±0.21 | 9.15E-01 |
| X - 13866 | 0.12±0.12 | 0 | 7.23E-01 | 7.36E-01 | 0.07±0.14 | 0 | 8.88E-01 | 9.75E-01 | 0.15±0.19 | 8.55E-01 | 0.09±0.2 | 9.08E-01 | 0.06±0.19 | 9.15E-01 | 0.05±0.19 | 9.16E-01 |
| thyroxine | 0.09±0.11 | 0 | 6.11E-01 | 7.36E-01 | 0.07±0.14 | 0 | 7.93E-01 | 9.75E-01 | 0.13±0.21 | 8.72E-01 | 0.12±0.22 | 8.86E-01 | 0.01±0.19 | 9.82E-01 | 0.04±0.19 | 9.43E-01 |
| glutamine conjugate of C6H10O2 (1)* | -0.08±0.15 | 0 | 3.76E-01 | 8.14E-01 | -0.08±0.15 | 0 | 3.87E-01 | 9.75E-01 | -0.22±0.22 | 8.55E-01 | -0.22±0.22 | 8.44E-01 | 0.04±0.2 | 9.47E-01 | 0.04±0.2 | 9.47E-01 |
| X - 26111 | 0.12±0.12 | 0 | 7.09E-01 | 7.36E-01 | 0.08±0.14 | 0 | 7.32E-01 | 9.75E-01 | 0.16±0.2 | 8.55E-01 | 0.13±0.21 | 8.65E-01 | 0.07±0.19 | 9.11E-01 | 0.03±0.2 | 9.58E-01 |
| X - 18899 | 0.04±0.16 | 8.57 | 2.96E-01 | 9.25E-01 | 0.08±0.15 | 0 | 3.79E-01 | 9.75E-01 | 0.29±0.24 | 8.40E-01 | 0.24±0.24 | 8.44E-01 | -0.07±0.19 | 9.03E-01 | -0.03±0.19 | 9.60E-01 |
| N-palmitoyl-sphingadienine (d18:2/16:0)* | 0.08±0.13 | 0 | 4.20E-01 | 7.83E-01 | 0.07±0.14 | 0 | 4.76E-01 | 9.75E-01 | 0.17±0.22 | 8.55E-01 | 0.19±0.21 | 8.45E-01 | -0.03±0.18 | 9.66E-01 | -0.01±0.18 | 9.88E-01 |
| 4-hydroxyphenylacetylglutamine | 0.22±0.18 | 77.53 | 3.49E-02 | 7.36E-01 | 0.08±0.16 | 84.55 | 1.09E-02 | 9.75E-01 | -0.3±0.24 | 8.40E-01 | -0.37±0.24 | 8.44E-01 | 0.48±0.21 | 2.43E-01 | 0.45±0.22 | 3.41E-01 |
| hydroquinone sulfate | 0.12±0.15 | 73.74 | 5.10E-02 | 7.44E-01 | 0.07±0.14 | 80.55 | 2.34E-02 | 9.75E-01 | -0.24±0.2 | 8.40E-01 | -0.25±0.2 | 8.44E-01 | 0.37±0.2 | 3.68E-01 | 0.39±0.2 | 3.70E-01 |
| hypotaurine | 0.07±0.16 | 69.72 | 6.92E-02 | 8.32E-01 | 0.08±0.16 | 77.97 | 3.31E-02 | 9.75E-01 | -0.23±0.2 | 8.40E-01 | -0.23±0.21 | 8.44E-01 | 0.34±0.22 | 4.48E-01 | 0.43±0.23 | 3.82E-01 |
| X - 12407 | 0.11±0.16 | 69.54 | 7.00E-02 | 7.62E-01 | 0.07±0.14 | 78.87 | 2.96E-02 | 9.75E-01 | -0.25±0.2 | 8.40E-01 | -0.24±0.2 | 8.44E-01 | 0.33±0.2 | 4.14E-01 | 0.38±0.2 | 3.83E-01 |
| myo-inositol | 0±0.12 | 77.24 | 3.61E-02 | 9.91E-01 | 0.08±0.15 | 73.9 | 5.03E-02 | 9.75E-01 | -0.13±0.19 | 8.55E-01 | -0.17±0.2 | 8.45E-01 | 0.44±0.24 | 3.68E-01 | 0.45±0.25 | 4.04E-01 |
| orotidine | 0.07±0.15 | 78.98 | 2.92E-02 | 8.32E-01 | 0.08±0.17 | 71.07 | 6.30E-02 | 9.75E-01 | -0.19±0.22 | 8.55E-01 | -0.2±0.23 | 8.45E-01 | 0.48±0.24 | 3.17E-01 | 0.42±0.25 | 4.34E-01 |
| laurate (12:0) | 0.01±0.11 | 68.39 | 7.53E-02 | 9.85E-01 | -0.07±0.15 | 54.25 | 1.39E-01 | 9.75E-01 | 0.12±0.2 | 8.72E-01 | 0.15±0.21 | 8.59E-01 | -0.29±0.2 | 4.76E-01 | -0.29±0.21 | 5.39E-01 |
| 1-palmitoyl-2-dihomo-linolenoyl-GPC (16:0/20:3n3 or 6)* | 0.02±0.02 | 0 | 3.46E-01 | 7.36E-01 | 0.09±0.17 | 0 | 4.08E-01 | 9.75E-01 | 0.02±0.2 | 9.84E-01 | -0.02±0.21 | 9.84E-01 | 0.32±0.32 | 6.46E-01 | 0.29±0.3 | 6.82E-01 |
| X - 12221 | -0.01±0.16 | 28.5 | 2.37E-01 | 9.86E-01 | -0.06±0.13 | 56.55 | 1.29E-01 | 9.75E-01 | -0.27±0.17 | 8.16E-01 | -0.24±0.17 | 8.44E-01 | 0.12±0.19 | 7.96E-01 | 0.16±0.2 | 7.29E-01 |
| glutamine conjugate of C7H12O2* | 0.01±0.01 | 0 | 4.81E-01 | 7.36E-01 | -0.07±0.14 | 0 | 5.83E-01 | 9.75E-01 | 0.01±0.2 | 9.90E-01 | 0.01±0.21 | 9.91E-01 | -0.12±0.19 | 7.96E-01 | -0.14±0.19 | 7.71E-01 |
| 3beta-hydroxy-5-cholestenoate | 0.01±0.01 | 0 | 9.44E-01 | 7.36E-01 | 0.07±0.15 | 0 | 7.90E-01 | 9.75E-01 | 0.01±0.19 | 9.92E-01 | 0.03±0.21 | 9.72E-01 | 0±0.19 | 9.97E-01 | 0.11±0.2 | 8.34E-01 |
| homovanillate (HVA) | 0.09±0.09 | 0 | 7.51E-01 | 7.36E-01 | 0.07±0.14 | 0 | 9.23E-01 | 9.75E-01 | 0.11±0.2 | 8.91E-01 | 0.05±0.21 | 9.49E-01 | 0.04±0.19 | 9.45E-01 | 0.08±0.19 | 8.65E-01 |
| octadecenedioylcarnitine (C18:1-DC)* | 0.06±0.14 | 0 | 3.39E-01 | 8.32E-01 | 0.07±0.14 | 0 | 3.42E-01 | 9.75E-01 | 0.2±0.19 | 8.40E-01 | 0.2±0.19 | 8.44E-01 | -0.07±0.19 | 9.15E-01 | -0.07±0.2 | 8.98E-01 |
| lignoceroylcarnitine (C24)* | 0.1±0.11 | 0 | 5.74E-01 | 7.40E-01 | 0.07±0.14 | 0 | 9.52E-01 | 9.75E-01 | 0.14±0.2 | 8.55E-01 | 0.08±0.2 | 9.27E-01 | 0.01±0.19 | 9.91E-01 | 0.06±0.2 | 9.10E-01 |
| threonate | 0.07±0.15 | 0 | 3.70E-01 | 8.27E-01 | 0.08±0.16 | 0 | 6.07E-01 | 9.75E-01 | 0.24±0.26 | 8.55E-01 | 0.19±0.27 | 8.59E-01 | -0.03±0.18 | 9.64E-01 | 0.02±0.19 | 9.74E-01 |
| X - 21796 | 0.08±0.15 | 0 | 3.83E-01 | 8.04E-01 | 0.07±0.14 | 0 | 5.00E-01 | 9.75E-01 | 0.22±0.21 | 8.50E-01 | 0.18±0.21 | 8.45E-01 | -0.04±0.2 | 9.53E-01 | -0.02±0.2 | 9.78E-01 |
| N-acetylserine | 0.37±0.23 | 82.06 | 1.82E-02 | 7.36E-01 | 0.09±0.19 | 89.07 | 2.49E-03 | 9.76E-01 | -0.4±0.25 | 8.16E-01 | -0.4±0.25 | 8.44E-01 | 0.76±0.29 | 1.64E-01 | 0.76±0.29 | 2.28E-01 |
| pyruvate | 0.26±0.18 | 81.98 | 1.85E-02 | 7.36E-01 | 0.07±0.15 | 86.67 | 6.16E-03 | 9.76E-01 | -0.3±0.2 | 8.16E-01 | -0.29±0.2 | 8.44E-01 | 0.6±0.23 | 1.75E-01 | 0.56±0.23 | 2.62E-01 |
| gamma-glutamylvaline | 0.11±0.17 | 86.31 | 6.88E-03 | 7.66E-01 | 0.08±0.17 | 83.29 | 1.44E-02 | 9.76E-01 | -0.21±0.19 | 8.40E-01 | -0.18±0.2 | 8.45E-01 | 0.76±0.29 | 1.73E-01 | 0.72±0.31 | 2.83E-01 |
| 1,3,7-trimethylurate | 0.02±0.1 | 86.24 | 7.01E-03 | 9.41E-01 | 0.06±0.14 | 80.69 | 2.29E-02 | 9.76E-01 | -0.11±0.18 | 8.72E-01 | -0.21±0.19 | 8.44E-01 | 0.58±0.23 | 1.85E-01 | 0.47±0.23 | 3.45E-01 |
| N,N-dimethyl-pro-pro | -0.01±0.27 | 70.18 | 6.71E-02 | 9.91E-01 | -0.11±0.23 | 76.61 | 3.87E-02 | 9.76E-01 | -0.32±0.25 | 8.40E-01 | -0.33±0.25 | 8.44E-01 | 0.78±0.51 | 4.51E-01 | 0.91±0.55 | 4.48E-01 |
| 3-amino-2-piperidone | 0.2±0.23 | 64.59 | 9.29E-02 | 7.36E-01 | -0.08±0.17 | 84.33 | 1.15E-02 | 9.76E-01 | -0.45±0.21 | 8.16E-01 | -0.44±0.22 | 8.44E-01 | 0.43±0.27 | 4.41E-01 | 0.44±0.27 | 4.61E-01 |
| N6-acetyllysine | 0.14±0.2 | 73.82 | 5.07E-02 | 7.65E-01 | -0.07±0.16 | 81.8 | 1.91E-02 | 9.76E-01 | -0.3±0.19 | 8.16E-01 | -0.34±0.2 | 8.44E-01 | 0.48±0.27 | 3.77E-01 | 0.43±0.26 | 4.67E-01 |
| N2,N2-dimethylguanosine | -0.04±0.06 | 75.98 | 4.13E-02 | 7.83E-01 | 0.08±0.19 | 59.36 | 1.17E-01 | 9.76E-01 | -0.07±0.21 | 9.38E-01 | -0.1±0.22 | 9.11E-01 | 0.61±0.32 | 3.62E-01 | 0.53±0.34 | 4.67E-01 |
| dodecenedioate (C12:1-DC)* | -0.03±0.13 | 63.92 | 9.59E-02 | 9.32E-01 | -0.07±0.14 | 63.1 | 9.97E-02 | 9.76E-01 | 0.18±0.2 | 8.55E-01 | 0.18±0.2 | 8.45E-01 | -0.25±0.19 | 5.10E-01 | -0.29±0.19 | 5.08E-01 |
| asparagine | -0.04±0.06 | 23.54 | 2.53E-01 | 7.44E-01 | 0.08±0.17 | 50.19 | 1.57E-01 | 9.76E-01 | -0.06±0.19 | 9.38E-01 | -0.1±0.21 | 9.14E-01 | 0.25±0.26 | 6.65E-01 | 0.43±0.3 | 5.31E-01 |
| 7-alpha-hydroxy-3-oxo-4-cholestenoate (7-Hoca) | 0±0.12 | 68.12 | 7.65E-02 | 9.91E-01 | 0.06±0.15 | 56.97 | 1.27E-01 | 9.76E-01 | -0.15±0.2 | 8.55E-01 | -0.15±0.21 | 8.58E-01 | 0.31±0.21 | 4.76E-01 | 0.3±0.21 | 5.38E-01 |
| ethyl alpha-glucopyranoside | 0.09±0.15 | 68.68 | 7.39E-02 | 8.02E-01 | -0.07±0.17 | 83.28 | 1.45E-02 | 9.76E-01 | -0.24±0.21 | 8.40E-01 | -0.51±0.25 | 8.44E-01 | 0.32±0.2 | 4.48E-01 | 0.31±0.23 | 5.44E-01 |
| 3-methylhistidine | -0.02±0.08 | 65.03 | 9.08E-02 | 9.02E-01 | 0.06±0.14 | 54.25 | 1.39E-01 | 9.76E-01 | -0.09±0.19 | 9.06E-01 | -0.16±0.2 | 8.52E-01 | 0.25±0.18 | 4.97E-01 | 0.24±0.19 | 5.55E-01 |
| X - 25371 | 0.14±0.19 | 60.34 | 1.12E-01 | 7.45E-01 | -0.07±0.16 | 79.92 | 2.56E-02 | 9.76E-01 | -0.38±0.22 | 8.16E-01 | -0.46±0.24 | 8.44E-01 | 0.31±0.22 | 4.76E-01 | 0.27±0.22 | 5.91E-01 |
| 13-HODE + 9-HODE | 0±0.11 | 64.86 | 9.16E-02 | 9.97E-01 | -0.06±0.13 | 37.86 | 2.05E-01 | 9.76E-01 | 0.13±0.19 | 8.55E-01 | 0.12±0.19 | 8.65E-01 | -0.25±0.18 | 5.06E-01 | -0.22±0.19 | 6.03E-01 |
| alpha-ketobutyrate | -0.03±0.04 | 71.66 | 6.03E-02 | 7.83E-01 | 0.07±0.15 | 24.1 | 2.51E-01 | 9.76E-01 | -0.04±0.2 | 9.54E-01 | -0.1±0.21 | 9.12E-01 | 0.34±0.2 | 4.07E-01 | 0.24±0.21 | 6.04E-01 |
| cis-3,4-methyleneheptanoylcarnitine | -0.02±0.11 | 58.19 | 1.22E-01 | 9.42E-01 | 0.06±0.15 | 28.05 | 2.38E-01 | 9.76E-01 | -0.13±0.2 | 8.55E-01 | -0.11±0.2 | 8.87E-01 | 0.25±0.21 | 5.69E-01 | 0.24±0.21 | 6.11E-01 |
| X - 24947 | -0.04±0.08 | 46.92 | 1.70E-01 | 8.27E-01 | 0.06±0.15 | 25.48 | 2.47E-01 | 9.76E-01 | -0.09±0.2 | 9.06E-01 | -0.1±0.21 | 8.95E-01 | 0.22±0.2 | 6.25E-01 | 0.24±0.21 | 6.11E-01 |
| N-delta-acetylornithine | -0.04±0.05 | 33.43 | 2.20E-01 | 7.62E-01 | 0.06±0.14 | 20.5 | 2.62E-01 | 9.76E-01 | -0.05±0.2 | 9.42E-01 | -0.1±0.2 | 8.98E-01 | 0.2±0.2 | 6.49E-01 | 0.22±0.2 | 6.19E-01 |
| X - 12283 | -0.03±0.05 | 37.62 | 2.05E-01 | 7.62E-01 | 0.07±0.15 | 0 | 3.50E-01 | 9.76E-01 | -0.05±0.2 | 9.48E-01 | -0.07±0.21 | 9.33E-01 | 0.21±0.2 | 6.28E-01 | 0.2±0.2 | 6.59E-01 |
| hydroxyasparagine** | 0.03±0.23 | 58.55 | 1.20E-01 | 9.55E-01 | -0.1±0.2 | 62.65 | 1.02E-01 | 9.76E-01 | -0.33±0.24 | 8.40E-01 | -0.37±0.26 | 8.44E-01 | 0.39±0.33 | 5.69E-01 | 0.3±0.32 | 6.82E-01 |
| X - 15486 | -0.04±0.07 | 48.03 | 1.65E-01 | 7.91E-01 | 0.07±0.15 | 0 | 3.70E-01 | 9.76E-01 | -0.07±0.2 | 9.30E-01 | -0.06±0.2 | 9.38E-01 | 0.23±0.2 | 5.99E-01 | 0.2±0.21 | 6.82E-01 |
| betaine | -0.02±0.18 | 21.17 | 2.60E-01 | 9.58E-01 | -0.07±0.16 | 64.53 | 9.31E-02 | 9.76E-01 | -0.32±0.21 | 8.16E-01 | -0.33±0.22 | 8.44E-01 | 0.11±0.21 | 8.27E-01 | 0.21±0.23 | 6.95E-01 |
| carnitine | 0.01±0.01 | 0 | 3.40E-01 | 7.36E-01 | 0.07±0.15 | 0 | 4.41E-01 | 9.76E-01 | 0.01±0.2 | 9.92E-01 | -0.03±0.2 | 9.73E-01 | 0.26±0.26 | 6.46E-01 | 0.21±0.24 | 7.06E-01 |
| gluconate | -0.04±0.04 | 0 | 3.83E-01 | 7.36E-01 | 0.08±0.17 | 0 | 4.75E-01 | 9.76E-01 | -0.05±0.21 | 9.54E-01 | -0.01±0.22 | 9.87E-01 | 0.21±0.29 | 7.75E-01 | 0.24±0.29 | 7.17E-01 |
| laurylcarnitine (C12) | -0.02±0.16 | 36.39 | 2.10E-01 | 9.58E-01 | 0.06±0.13 | 55.85 | 1.32E-01 | 9.76E-01 | 0.29±0.19 | 8.16E-01 | 0.28±0.2 | 8.44E-01 | -0.14±0.18 | 7.57E-01 | -0.12±0.18 | 7.89E-01 |
| X - 23680 | -0.02±0.03 | 0 | 5.18E-01 | 7.36E-01 | 0.07±0.15 | 0 | 6.63E-01 | 9.76E-01 | -0.03±0.2 | 9.74E-01 | 0±0.21 | 9.99E-01 | 0.1±0.2 | 8.35E-01 | 0.13±0.21 | 8.10E-01 |
| 2,3-dihydroxyisovalerate | -0.03±0.12 | 34.1 | 2.18E-01 | 9.21E-01 | -0.06±0.15 | 48.91 | 1.62E-01 | 9.76E-01 | -0.16±0.21 | 8.55E-01 | -0.3±0.23 | 8.44E-01 | 0.14±0.18 | 7.57E-01 | 0.12±0.2 | 8.13E-01 |
| 1-linoleoylglycerol (18:2) | -0.02±0.02 | 0 | 5.76E-01 | 7.36E-01 | 0.06±0.14 | 0 | 6.89E-01 | 9.76E-01 | -0.02±0.2 | 9.83E-01 | 0±0.2 | 9.98E-01 | 0.09±0.19 | 8.65E-01 | 0.12±0.19 | 8.13E-01 |
| docosadienoate (22:2n6) | 0.04±0.15 | 5.98 | 3.02E-01 | 9.06E-01 | 0.06±0.14 | 41.92 | 1.89E-01 | 9.76E-01 | 0.26±0.2 | 8.40E-01 | 0.26±0.21 | 8.44E-01 | -0.07±0.19 | 9.03E-01 | -0.11±0.19 | 8.21E-01 |
| phosphoethanolamine | -0.08±0.13 | 0 | 3.97E-01 | 7.91E-01 | -0.07±0.15 | 36.38 | 2.10E-01 | 9.76E-01 | -0.18±0.19 | 8.55E-01 | -0.25±0.21 | 8.44E-01 | 0.05±0.2 | 9.38E-01 | 0.13±0.22 | 8.21E-01 |
| 1-stearoyl-GPG (18:0) | 0.01±0.01 | 0 | 5.13E-01 | 7.36E-01 | -0.06±0.13 | 0 | 6.93E-01 | 9.76E-01 | 0.01±0.19 | 9.92E-01 | 0±0.19 | 9.99E-01 | -0.11±0.18 | 8.00E-01 | -0.11±0.19 | 8.22E-01 |
| 1-oleoyl-GPC (18:1) | -0.08±0.13 | 0 | 4.18E-01 | 7.83E-01 | -0.07±0.15 | 24.07 | 2.51E-01 | 9.76E-01 | -0.18±0.19 | 8.55E-01 | -0.23±0.2 | 8.44E-01 | 0.04±0.2 | 9.61E-01 | 0.12±0.22 | 8.40E-01 |
| X - 25271 | 0.06±0.06 | 0 | 7.64E-01 | 7.36E-01 | 0.06±0.14 | 0 | 7.52E-01 | 9.76E-01 | 0.06±0.2 | 9.38E-01 | 0.01±0.2 | 9.88E-01 | 0.12±0.19 | 8.00E-01 | 0.1±0.2 | 8.44E-01 |
| X - 18921 | 0±0 | 0 | 6.79E-01 | 7.36E-01 | 0.07±0.14 | 0 | 8.12E-01 | 9.76E-01 | 0±0.2 | 9.94E-01 | 0.03±0.21 | 9.71E-01 | 0.07±0.19 | 8.99E-01 | 0.1±0.2 | 8.47E-01 |
| 1-(1-enyl-palmitoyl)-2-arachidonoyl-GPC (P-16:0/20:4)* | 0.02±0.02 | 0 | 5.70E-01 | 7.36E-01 | -0.06±0.14 | 0 | 8.84E-01 | 9.76E-01 | 0.02±0.2 | 9.77E-01 | -0.04±0.21 | 9.71E-01 | -0.08±0.18 | 8.73E-01 | -0.08±0.18 | 8.65E-01 |
| 12,13-DiHOME | 0.01±0.14 | 36.55 | 2.09E-01 | 9.91E-01 | 0.07±0.15 | 13.75 | 2.82E-01 | 9.76E-01 | 0.23±0.21 | 8.40E-01 | 0.24±0.22 | 8.44E-01 | -0.14±0.18 | 7.57E-01 | -0.08±0.2 | 8.86E-01 |
| glucuronide of piperine metabolite C17H21NO3 (4)* | -0.03±0.04 | 18.12 | 2.69E-01 | 7.44E-01 | -0.06±0.15 | 0 | 3.53E-01 | 9.76E-01 | -0.04±0.19 | 9.54E-01 | -0.19±0.2 | 8.45E-01 | 0.19±0.2 | 6.77E-01 | 0.08±0.21 | 8.88E-01 |
| X - 18887 | -0.09±0.13 | 0 | 4.73E-01 | 7.62E-01 | -0.06±0.14 | 0 | 3.26E-01 | 9.76E-01 | -0.17±0.2 | 8.55E-01 | -0.2±0.2 | 8.44E-01 | 0.01±0.19 | 9.83E-01 | 0.07±0.2 | 8.88E-01 |
| oleoyl ethanolamide | 0.08±0.14 | 0 | 4.13E-01 | 7.91E-01 | 0.06±0.14 | 6.16 | 3.02E-01 | 9.76E-01 | 0.21±0.2 | 8.55E-01 | 0.22±0.21 | 8.44E-01 | -0.02±0.19 | 9.74E-01 | -0.07±0.19 | 8.93E-01 |
| 4-ethylphenylsulfate | -0.09±0.14 | 0 | 4.48E-01 | 7.75E-01 | -0.06±0.14 | 5 | 3.05E-01 | 9.76E-01 | -0.21±0.21 | 8.55E-01 | -0.23±0.21 | 8.44E-01 | 0±0.18 | 9.97E-01 | 0.06±0.18 | 8.98E-01 |
| 2-hydroxypalmitate | -0.08±0.12 | 0 | 4.40E-01 | 7.72E-01 | -0.06±0.14 | 0 | 4.20E-01 | 9.76E-01 | -0.16±0.2 | 8.55E-01 | -0.19±0.21 | 8.45E-01 | 0.03±0.19 | 9.64E-01 | 0.04±0.19 | 9.42E-01 |
| X - 11299 | 0.1±0.1 | 0 | 7.45E-01 | 7.36E-01 | 0.06±0.14 | 0 | 8.71E-01 | 9.76E-01 | 0.11±0.2 | 8.81E-01 | 0.09±0.21 | 9.27E-01 | 0.04±0.19 | 9.41E-01 | 0.04±0.2 | 9.47E-01 |
| sphingomyelin (d18:1/20:0, d16:1/22:0)* | 0.08±0.1 | 0 | 6.10E-01 | 7.36E-01 | 0.07±0.14 | 0 | 8.36E-01 | 9.76E-01 | 0.11±0.2 | 8.82E-01 | 0.1±0.2 | 9.06E-01 | 0±0.19 | 9.97E-01 | 0.04±0.2 | 9.47E-01 |
| 1-(1-enyl-oleoyl)-GPE (P-18:1)* | -0.08±0.1 | 0 | 5.45E-01 | 7.44E-01 | -0.07±0.14 | 0 | 4.72E-01 | 9.76E-01 | -0.12±0.19 | 8.72E-01 | -0.17±0.2 | 8.45E-01 | 0.02±0.2 | 9.76E-01 | 0.04±0.2 | 9.49E-01 |
| glycosyl-N-(2-hydroxynervonoyl)-sphingosine (d18:1/24:1(2OH))* | 0.08±0.08 | 0 | 9.37E-01 | 7.36E-01 | 0.07±0.15 | 0 | 8.15E-01 | 9.76E-01 | 0.08±0.23 | 9.30E-01 | 0.11±0.24 | 9.14E-01 | 0.07±0.2 | 9.15E-01 | 0.03±0.2 | 9.53E-01 |
| 9,10-DiHOME | -0.09±0.08 | 0 | 9.36E-01 | 7.36E-01 | -0.06±0.14 | 0 | 7.88E-01 | 9.76E-01 | -0.08±0.2 | 9.09E-01 | -0.1±0.2 | 8.95E-01 | -0.1±0.18 | 8.22E-01 | -0.03±0.19 | 9.62E-01 |
| 3,7-dimethylurate | -0.07±0.1 | 0 | 4.86E-01 | 7.60E-01 | -0.06±0.13 | 0 | 4.75E-01 | 9.76E-01 | -0.12±0.19 | 8.62E-01 | -0.16±0.19 | 8.45E-01 | 0.03±0.18 | 9.64E-01 | 0.03±0.18 | 9.62E-01 |
| carotene diol (1) | -0.1±0.1 | 0 | 7.64E-01 | 7.36E-01 | -0.06±0.14 | 0 | 5.60E-01 | 9.76E-01 | -0.12±0.2 | 8.72E-01 | -0.15±0.21 | 8.59E-01 | -0.06±0.18 | 9.16E-01 | 0.01±0.19 | 9.88E-01 |
| sarcosine | -0.1±0.11 | 0 | 6.84E-01 | 7.36E-01 | -0.06±0.14 | 0 | 5.51E-01 | 9.76E-01 | -0.13±0.2 | 8.55E-01 | -0.16±0.21 | 8.58E-01 | -0.04±0.18 | 9.34E-01 | 0.01±0.19 | 9.88E-01 |
| glycochenodeoxycholate glucuronide (1) | -0.08±0.1 | 0 | 5.42E-01 | 7.44E-01 | -0.06±0.14 | 0 | 5.98E-01 | 9.76E-01 | -0.12±0.2 | 8.75E-01 | -0.14±0.2 | 8.59E-01 | 0.02±0.19 | 9.76E-01 | 0.01±0.2 | 9.88E-01 |
| glycerol | 0.11±0.12 | 0 | 7.05E-01 | 7.36E-01 | 0.06±0.15 | 0 | 6.34E-01 | 9.76E-01 | 0.15±0.21 | 8.55E-01 | 0.14±0.21 | 8.63E-01 | 0.05±0.21 | 9.38E-01 | -0.01±0.21 | 9.94E-01 |
| 2,3-dihydroxypyridine | -0.11±0.11 | 0 | 7.00E-01 | 7.36E-01 | -0.06±0.14 | 0 | 6.55E-01 | 9.76E-01 | -0.14±0.19 | 8.55E-01 | -0.12±0.2 | 8.65E-01 | -0.05±0.18 | 9.17E-01 | 0±0.19 | 9.97E-01 |
| stearate (18:0) | 0±0.12 | 60.79 | 1.10E-01 | 9.91E-01 | -0.06±0.14 | 56.11 | 1.31E-01 | 9.77E-01 | 0.16±0.19 | 8.55E-01 | 0.15±0.19 | 8.53E-01 | -0.24±0.19 | 5.56E-01 | -0.26±0.19 | 5.45E-01 |
| glycodeoxycholate 3-sulfate | 0.01±0.01 | 0 | 4.98E-01 | 7.36E-01 | -0.06±0.14 | 0 | 6.30E-01 | 9.77E-01 | 0.01±0.2 | 9.94E-01 | 0.02±0.2 | 9.84E-01 | -0.12±0.18 | 7.93E-01 | -0.12±0.18 | 8.13E-01 |
| N-acetylalanine | 0.4±0.25 | 86.79 | 5.94E-03 | 7.36E-01 | 0.06±0.2 | 90.21 | 1.39E-03 | 9.78E-01 | -0.38±0.24 | 8.16E-01 | -0.39±0.24 | 8.44E-01 | 1.02±0.34 | 1.08E-01 | 0.98±0.35 | 1.87E-01 |
| glutamine_degradant* | 0.23±0.21 | 63.17 | 9.94E-02 | 7.36E-01 | 0.05±0.17 | 87.26 | 5.08E-03 | 9.78E-01 | -0.48±0.24 | 8.16E-01 | -0.44±0.25 | 8.44E-01 | 0.39±0.23 | 4.06E-01 | 0.5±0.23 | 3.24E-01 |
| X - 25343 | 0.23±0.2 | 70.29 | 6.66E-02 | 7.36E-01 | -0.05±0.15 | 86.1 | 7.32E-03 | 9.78E-01 | -0.4±0.2 | 8.16E-01 | -0.4±0.2 | 8.44E-01 | 0.45±0.24 | 3.46E-01 | 0.43±0.24 | 4.09E-01 |
| glycolithocholate | 0.16±0.15 | 69.9 | 6.84E-02 | 7.36E-01 | 0.04±0.13 | 81.47 | 2.02E-02 | 9.78E-01 | -0.29±0.19 | 8.16E-01 | -0.29±0.19 | 8.44E-01 | 0.34±0.18 | 3.68E-01 | 0.32±0.18 | 4.18E-01 |
| ursodeoxycholate | 0.12±0.15 | 74.59 | 4.73E-02 | 7.44E-01 | 0.04±0.14 | 77.9 | 3.34E-02 | 9.78E-01 | -0.24±0.2 | 8.40E-01 | -0.25±0.2 | 8.44E-01 | 0.38±0.2 | 3.59E-01 | 0.36±0.2 | 4.32E-01 |
| theophylline | -0.01±0.09 | 80.47 | 2.36E-02 | 9.56E-01 | 0.04±0.14 | 72.49 | 5.66E-02 | 9.78E-01 | -0.1±0.18 | 8.91E-01 | -0.18±0.18 | 8.45E-01 | 0.48±0.24 | 3.03E-01 | 0.39±0.23 | 4.49E-01 |
| 5-dodecenoylcarnitine (C12:1) | -0.18±0.18 | 57.55 | 1.25E-01 | 7.36E-01 | 0.04±0.14 | 85.22 | 9.30E-03 | 9.78E-01 | 0.45±0.2 | 8.16E-01 | 0.43±0.21 | 8.44E-01 | -0.3±0.2 | 4.50E-01 | -0.32±0.2 | 4.67E-01 |
| indolin-2-one | -0.03±0.1 | 69.07 | 7.22E-02 | 9.14E-01 | 0.05±0.15 | 67.8 | 7.80E-02 | 9.78E-01 | -0.11±0.19 | 8.80E-01 | -0.16±0.19 | 8.47E-01 | 0.35±0.23 | 4.57E-01 | 0.38±0.24 | 4.67E-01 |
| kynurenine | 0.2±0.19 | 72.12 | 5.83E-02 | 7.36E-01 | -0.06±0.16 | 84.09 | 1.22E-02 | 9.78E-01 | -0.35±0.2 | 8.16E-01 | -0.43±0.22 | 8.44E-01 | 0.45±0.23 | 3.46E-01 | 0.38±0.24 | 4.67E-01 |
| dodecadienoate (12:2)* | -0.11±0.15 | 69.8 | 6.88E-02 | 7.44E-01 | -0.06±0.14 | 73.56 | 5.18E-02 | 9.78E-01 | 0.25±0.2 | 8.40E-01 | 0.25±0.21 | 8.44E-01 | -0.31±0.18 | 4.06E-01 | -0.3±0.19 | 4.67E-01 |
| X - 24243 | 0.01±0.12 | 74.44 | 4.79E-02 | 9.58E-01 | 0.05±0.14 | 70.11 | 6.74E-02 | 9.78E-01 | -0.14±0.18 | 8.55E-01 | -0.18±0.19 | 8.45E-01 | 0.36±0.21 | 4.06E-01 | 0.34±0.21 | 4.67E-01 |
| pentadecanoate (15:0) | -0.07±0.15 | 67.93 | 7.74E-02 | 8.26E-01 | -0.05±0.14 | 72.26 | 5.76E-02 | 9.78E-01 | 0.22±0.2 | 8.40E-01 | 0.23±0.2 | 8.44E-01 | -0.3±0.2 | 4.51E-01 | -0.32±0.2 | 4.67E-01 |
| choline | -0.03±0.13 | 69.56 | 6.99E-02 | 9.35E-01 | 0.05±0.17 | 67.37 | 8.00E-02 | 9.78E-01 | -0.14±0.21 | 8.55E-01 | -0.18±0.21 | 8.45E-01 | 0.44±0.29 | 4.54E-01 | 0.46±0.29 | 4.67E-01 |
| myristate (14:0) | -0.08±0.16 | 63.85 | 9.63E-02 | 8.08E-01 | -0.04±0.15 | 73.54 | 5.19E-02 | 9.78E-01 | 0.25±0.21 | 8.40E-01 | 0.26±0.21 | 8.44E-01 | -0.28±0.2 | 4.81E-01 | -0.31±0.2 | 4.74E-01 |
| X - 25810 | 0.06±0.14 | 65.63 | 8.81E-02 | 8.49E-01 | 0.04±0.14 | 73.82 | 5.06E-02 | 9.78E-01 | -0.21±0.21 | 8.55E-01 | -0.28±0.22 | 8.44E-01 | 0.27±0.19 | 4.81E-01 | 0.29±0.19 | 5.04E-01 |
| choline phosphate | -0.01±0.11 | 58.15 | 1.22E-01 | 9.58E-01 | 0.06±0.14 | 68.39 | 7.53E-02 | 9.78E-01 | -0.14±0.2 | 8.55E-01 | -0.22±0.21 | 8.44E-01 | 0.22±0.19 | 5.71E-01 | 0.29±0.19 | 5.04E-01 |
| decanoylcarnitine (C10) | -0.07±0.13 | 71.65 | 6.04E-02 | 8.02E-01 | -0.04±0.13 | 67.98 | 7.72E-02 | 9.78E-01 | 0.2±0.19 | 8.50E-01 | 0.21±0.2 | 8.44E-01 | -0.31±0.18 | 4.10E-01 | -0.27±0.18 | 5.19E-01 |
| 2-methylserine | -0.04±0.11 | 56.58 | 1.29E-01 | 8.52E-01 | 0.05±0.17 | 60 | 1.14E-01 | 9.78E-01 | -0.12±0.2 | 8.72E-01 | -0.14±0.21 | 8.59E-01 | 0.32±0.26 | 5.66E-01 | 0.41±0.28 | 5.19E-01 |
| 1-palmitoyl-2-arachidonoyl-GPI (16:0/20:4)* | 0.01±0.14 | 71.95 | 5.90E-02 | 9.68E-01 | 0.04±0.16 | 63.32 | 9.87E-02 | 9.78E-01 | -0.17±0.2 | 8.55E-01 | -0.18±0.21 | 8.45E-01 | 0.4±0.25 | 4.41E-01 | 0.36±0.25 | 5.22E-01 |
| glucuronate | -0.02±0.11 | 71.38 | 6.16E-02 | 9.47E-01 | 0.04±0.15 | 60.02 | 1.14E-01 | 9.78E-01 | -0.13±0.19 | 8.55E-01 | -0.14±0.19 | 8.55E-01 | 0.38±0.24 | 4.48E-01 | 0.34±0.24 | 5.36E-01 |
| 1-methylnicotinamide | -0.07±0.13 | 69.23 | 7.14E-02 | 8.02E-01 | -0.04±0.13 | 62.54 | 1.02E-01 | 9.78E-01 | 0.2±0.18 | 8.40E-01 | 0.17±0.18 | 8.45E-01 | -0.28±0.17 | 4.44E-01 | -0.25±0.18 | 5.41E-01 |
| taurodeoxycholic acid 3-sulfate | -0.07±0.15 | 67.64 | 7.88E-02 | 8.32E-01 | -0.04±0.14 | 65.34 | 8.94E-02 | 9.78E-01 | 0.22±0.2 | 8.40E-01 | 0.21±0.21 | 8.44E-01 | -0.3±0.2 | 4.57E-01 | -0.27±0.2 | 5.43E-01 |
| X - 12411 | 0.04±0.13 | 67.42 | 7.98E-02 | 9.02E-01 | 0.05±0.15 | 63.09 | 9.97E-02 | 9.78E-01 | -0.19±0.23 | 8.55E-01 | -0.24±0.23 | 8.44E-01 | 0.29±0.19 | 4.76E-01 | 0.26±0.2 | 5.46E-01 |
| 5-acetylamino-6-amino-3-methyluracil | 0.03±0.16 | 59.5 | 1.16E-01 | 9.32E-01 | -0.04±0.14 | 73.2 | 5.34E-02 | 9.78E-01 | -0.23±0.17 | 8.35E-01 | -0.25±0.17 | 8.44E-01 | 0.26±0.21 | 5.59E-01 | 0.29±0.22 | 5.49E-01 |
| N-oleoyltaurine | -0.09±0.16 | 48.76 | 1.62E-01 | 7.91E-01 | 0.04±0.14 | 80.77 | 2.26E-02 | 9.78E-01 | 0.37±0.2 | 8.16E-01 | 0.39±0.21 | 8.44E-01 | -0.21±0.18 | 5.96E-01 | -0.23±0.18 | 5.60E-01 |
| pseudouridine | 0±0.13 | 64.05 | 9.53E-02 | 9.91E-01 | 0.05±0.16 | 56.57 | 1.29E-01 | 9.78E-01 | -0.17±0.23 | 8.55E-01 | -0.21±0.23 | 8.45E-01 | 0.28±0.21 | 5.17E-01 | 0.27±0.21 | 5.62E-01 |
| X - 17676 | 0.12±0.18 | 41.19 | 1.92E-01 | 7.62E-01 | -0.05±0.15 | 83.55 | 1.37E-02 | 9.78E-01 | -0.54±0.23 | 8.16E-01 | -0.5±0.23 | 8.44E-01 | 0.21±0.19 | 6.06E-01 | 0.24±0.19 | 5.62E-01 |
| quinolinate | 0.02±0.13 | 71.87 | 5.94E-02 | 9.46E-01 | 0.05±0.16 | 54.64 | 1.38E-01 | 9.78E-01 | -0.16±0.22 | 8.55E-01 | -0.2±0.23 | 8.45E-01 | 0.34±0.21 | 4.41E-01 | 0.27±0.21 | 5.79E-01 |
| X - 15245 | 0±0.13 | 65.57 | 8.83E-02 | 9.99E-01 | 0.05±0.14 | 47.06 | 1.69E-01 | 9.78E-01 | -0.16±0.19 | 8.55E-01 | -0.13±0.19 | 8.59E-01 | 0.3±0.21 | 4.97E-01 | 0.26±0.21 | 5.84E-01 |
| indolelactate | 0.09±0.19 | 58.5 | 1.21E-01 | 8.32E-01 | -0.05±0.16 | 74 | 4.98E-02 | 9.78E-01 | -0.33±0.22 | 8.19E-01 | -0.35±0.22 | 8.44E-01 | 0.3±0.24 | 5.52E-01 | 0.29±0.24 | 5.85E-01 |
| piperine | -0.01±0.01 | 64.23 | 9.45E-02 | 7.36E-01 | 0.04±0.16 | 49.18 | 1.61E-01 | 9.78E-01 | -0.01±0.2 | 9.94E-01 | -0.15±0.21 | 8.59E-01 | 0.37±0.22 | 4.20E-01 | 0.28±0.23 | 5.88E-01 |
| 4-hydroxychlorothalonil | -0.04±0.09 | 57.59 | 1.25E-01 | 8.45E-01 | 0.07±0.17 | 40.91 | 1.93E-01 | 9.78E-01 | -0.1±0.23 | 9.06E-01 | -0.15±0.23 | 8.63E-01 | 0.29±0.23 | 5.56E-01 | 0.28±0.24 | 5.94E-01 |
| eicosenoate (20:1) | 0±0.14 | 47.35 | 1.68E-01 | 9.97E-01 | -0.04±0.14 | 52.42 | 1.47E-01 | 9.78E-01 | 0.19±0.2 | 8.55E-01 | 0.18±0.2 | 8.45E-01 | -0.18±0.19 | 6.66E-01 | -0.23±0.19 | 6.00E-01 |
| creatinine | -0.05±0.14 | 48.35 | 1.64E-01 | 8.61E-01 | 0.05±0.19 | 42.92 | 1.86E-01 | 9.78E-01 | -0.16±0.25 | 8.62E-01 | -0.16±0.25 | 8.63E-01 | 0.3±0.29 | 6.31E-01 | 0.36±0.3 | 6.02E-01 |
| 1-methyl-5-imidazolelactate | -0.03±0.08 | 65.92 | 8.67E-02 | 8.77E-01 | 0.04±0.14 | 48.24 | 1.65E-01 | 9.78E-01 | -0.08±0.19 | 9.06E-01 | -0.15±0.2 | 8.53E-01 | 0.27±0.19 | 4.81E-01 | 0.23±0.19 | 6.03E-01 |
| androstenediol (3alpha, 17alpha) monosulfate (2) | -0.02±0.11 | 56.2 | 1.31E-01 | 9.47E-01 | 0.03±0.14 | 45.53 | 1.75E-01 | 9.78E-01 | -0.14±0.18 | 8.55E-01 | -0.13±0.18 | 8.59E-01 | 0.23±0.2 | 5.92E-01 | 0.24±0.2 | 6.03E-01 |
| theobromine | -0.03±0.07 | 54.95 | 1.36E-01 | 8.32E-01 | 0.04±0.13 | 37.4 | 2.06E-01 | 9.78E-01 | -0.08±0.18 | 9.06E-01 | -0.12±0.18 | 8.62E-01 | 0.23±0.19 | 5.69E-01 | 0.22±0.19 | 6.11E-01 |
| 2-hydroxysebacate | 0.03±0.1 | 49.05 | 1.61E-01 | 8.82E-01 | -0.05±0.14 | 25.67 | 2.46E-01 | 9.78E-01 | 0.12±0.2 | 8.75E-01 | 0.1±0.19 | 8.86E-01 | -0.21±0.2 | 6.31E-01 | -0.22±0.2 | 6.18E-01 |
| X - 11632 | -0.11±0.17 | 45.45 | 1.76E-01 | 7.72E-01 | 0.05±0.14 | 77.72 | 3.41E-02 | 9.78E-01 | 0.43±0.21 | 8.16E-01 | 0.4±0.21 | 8.44E-01 | -0.2±0.18 | 5.97E-01 | -0.19±0.18 | 6.34E-01 |
| eicosanedioate (C20-DC) | 0.01±0.12 | 58.2 | 1.22E-01 | 9.81E-01 | -0.04±0.14 | 25.21 | 2.48E-01 | 9.78E-01 | 0.15±0.19 | 8.55E-01 | 0.11±0.19 | 8.70E-01 | -0.23±0.2 | 5.71E-01 | -0.21±0.2 | 6.42E-01 |
| 4-acetylphenol sulfate | 0.01±0.16 | 29.72 | 2.33E-01 | 9.91E-01 | -0.04±0.13 | 72.96 | 5.45E-02 | 9.78E-01 | -0.3±0.2 | 8.16E-01 | -0.33±0.2 | 8.44E-01 | 0.12±0.18 | 7.91E-01 | 0.19±0.18 | 6.42E-01 |
| 2-hydroxybehenate | -0.03±0.11 | 42.7 | 1.86E-01 | 9.14E-01 | 0.04±0.14 | 27.45 | 2.40E-01 | 9.78E-01 | -0.14±0.21 | 8.55E-01 | -0.14±0.21 | 8.59E-01 | 0.17±0.19 | 6.90E-01 | 0.2±0.2 | 6.58E-01 |
| stearoylcarnitine (C18) | -0.04±0.05 | 0 | 3.76E-01 | 7.40E-01 | 0.06±0.15 | 0 | 3.27E-01 | 9.78E-01 | -0.05±0.2 | 9.53E-01 | -0.07±0.2 | 9.33E-01 | 0.15±0.22 | 7.79E-01 | 0.23±0.23 | 6.67E-01 |
| 4-cholesten-3-one | -0.03±0.05 | 45.98 | 1.74E-01 | 7.49E-01 | 0.05±0.16 | 0 | 3.30E-01 | 9.78E-01 | -0.05±0.2 | 9.54E-01 | -0.08±0.21 | 9.27E-01 | 0.29±0.25 | 5.69E-01 | 0.23±0.24 | 6.82E-01 |
| X - 22776 | 0.03±0.11 | 43.1 | 1.85E-01 | 9.25E-01 | -0.04±0.13 | 13.49 | 2.82E-01 | 9.78E-01 | 0.14±0.19 | 8.55E-01 | 0.11±0.19 | 8.70E-01 | -0.16±0.18 | 6.91E-01 | -0.17±0.18 | 6.83E-01 |
| 1-(1-enyl-palmitoyl)-2-arachidonoyl-GPE (P-16:0/20:4)* | -0.02±0.03 | 27.85 | 2.39E-01 | 7.36E-01 | 0.04±0.15 | 8.36 | 2.96E-01 | 9.78E-01 | -0.03±0.2 | 9.70E-01 | -0.11±0.22 | 8.86E-01 | 0.24±0.23 | 6.19E-01 | 0.2±0.22 | 6.88E-01 |
| 1-linoleoyl-GPE (18:2)* | -0.04±0.16 | 14.62 | 2.79E-01 | 9.23E-01 | -0.04±0.16 | 57.86 | 1.23E-01 | 9.78E-01 | -0.26±0.22 | 8.40E-01 | -0.29±0.23 | 8.44E-01 | 0.1±0.21 | 8.50E-01 | 0.2±0.22 | 6.95E-01 |
| gamma-CEHC | -0.04±0.12 | 26.34 | 2.44E-01 | 8.77E-01 | 0.04±0.14 | 5.77 | 3.03E-01 | 9.78E-01 | -0.16±0.2 | 8.55E-01 | -0.12±0.21 | 8.70E-01 | 0.13±0.19 | 7.79E-01 | 0.18±0.2 | 6.98E-01 |
| 2-palmitoyl-GPC (16:0)* | -0.04±0.06 | 0 | 3.76E-01 | 7.44E-01 | 0.04±0.14 | 0 | 3.49E-01 | 9.78E-01 | -0.06±0.2 | 9.38E-01 | -0.1±0.21 | 9.03E-01 | 0.13±0.2 | 7.97E-01 | 0.17±0.2 | 7.17E-01 |
| 2-methoxyhydroquinone sulfate (1) | 0.03±0.16 | 43.68 | 1.83E-01 | 9.35E-01 | -0.05±0.14 | 58.52 | 1.20E-01 | 9.78E-01 | -0.29±0.2 | 8.19E-01 | -0.27±0.2 | 8.44E-01 | 0.17±0.19 | 6.90E-01 | 0.16±0.2 | 7.25E-01 |
| 10-nonadecenoate (19:1n9) | 0±0.16 | 29.9 | 2.32E-01 | 9.99E-01 | 0.04±0.14 | 58.14 | 1.22E-01 | 9.78E-01 | 0.29±0.21 | 8.19E-01 | 0.28±0.21 | 8.44E-01 | -0.12±0.19 | 7.91E-01 | -0.15±0.19 | 7.34E-01 |
| 5,6-dihydrothymine | -0.04±0.15 | 24.15 | 2.51E-01 | 9.02E-01 | -0.04±0.15 | 45.18 | 1.77E-01 | 9.78E-01 | -0.2±0.21 | 8.55E-01 | -0.24±0.21 | 8.44E-01 | 0.14±0.22 | 7.96E-01 | 0.17±0.22 | 7.46E-01 |
| X - 12193 | -0.03±0.16 | 21.85 | 2.58E-01 | 9.47E-01 | -0.05±0.15 | 53.44 | 1.43E-01 | 9.78E-01 | -0.27±0.2 | 8.40E-01 | -0.27±0.21 | 8.44E-01 | 0.11±0.2 | 8.22E-01 | 0.16±0.21 | 7.60E-01 |
| 3-hydroxyoctanoylcarnitine (1) | -0.04±0.16 | 41.88 | 1.90E-01 | 9.25E-01 | 0.05±0.14 | 55.26 | 1.35E-01 | 9.78E-01 | 0.31±0.21 | 8.16E-01 | 0.28±0.21 | 8.44E-01 | -0.17±0.19 | 7.08E-01 | -0.14±0.19 | 7.66E-01 |
| nonadecanoate (19:0) | 0.03±0.04 | 9.62 | 2.93E-01 | 7.44E-01 | -0.06±0.14 | 0 | 5.16E-01 | 9.78E-01 | 0.04±0.2 | 9.57E-01 | 0.04±0.2 | 9.63E-01 | -0.16±0.19 | 7.11E-01 | -0.14±0.19 | 7.71E-01 |
| 4-vinylphenol sulfate | -0.06±0.16 | 0 | 3.33E-01 | 8.71E-01 | -0.05±0.15 | 55.16 | 1.35E-01 | 9.78E-01 | -0.28±0.21 | 8.40E-01 | -0.29±0.22 | 8.44E-01 | 0.05±0.19 | 9.34E-01 | 0.15±0.2 | 7.71E-01 |
| citrulline | -0.07±0.09 | 0 | 4.49E-01 | 7.60E-01 | 0.04±0.15 | 0 | 4.48E-01 | 9.78E-01 | -0.1±0.19 | 8.93E-01 | -0.07±0.2 | 9.33E-01 | 0.07±0.21 | 9.15E-01 | 0.16±0.22 | 7.78E-01 |
| X - 21470 | 0.02±0.15 | 21.56 | 2.59E-01 | 9.58E-01 | 0.04±0.14 | 51.65 | 1.50E-01 | 9.78E-01 | 0.28±0.21 | 8.40E-01 | 0.28±0.22 | 8.44E-01 | -0.1±0.19 | 8.25E-01 | -0.13±0.19 | 7.79E-01 |
| 3-aminoisobutyrate | -0.02±0.02 | 0 | 6.40E-01 | 7.36E-01 | 0.06±0.15 | 0 | 5.39E-01 | 9.78E-01 | -0.02±0.21 | 9.86E-01 | -0.04±0.22 | 9.65E-01 | 0.07±0.2 | 9.07E-01 | 0.14±0.2 | 7.79E-01 |
| 1-palmitoyl-GPC (16:0) | -0.07±0.12 | 0 | 3.64E-01 | 7.85E-01 | -0.04±0.16 | 23.07 | 2.54E-01 | 9.78E-01 | -0.15±0.2 | 8.55E-01 | -0.2±0.21 | 8.45E-01 | 0.1±0.23 | 8.69E-01 | 0.17±0.25 | 7.81E-01 |
| X - 11372 | 0.04±0.06 | 40.12 | 1.96E-01 | 7.80E-01 | -0.06±0.15 | 0 | 5.45E-01 | 9.78E-01 | 0.06±0.22 | 9.42E-01 | 0.05±0.22 | 9.62E-01 | -0.19±0.19 | 6.31E-01 | -0.13±0.19 | 7.86E-01 |
| 4-methoxyphenol sulfate | 0.04±0.05 | 4.13 | 3.07E-01 | 7.44E-01 | -0.04±0.14 | 0 | 4.88E-01 | 9.78E-01 | 0.05±0.21 | 9.54E-01 | 0.07±0.21 | 9.33E-01 | -0.15±0.19 | 7.44E-01 | -0.13±0.19 | 7.93E-01 |
| X - 13431 | -0.03±0.03 | 0 | 6.30E-01 | 7.36E-01 | 0.04±0.14 | 0 | 5.23E-01 | 9.78E-01 | -0.03±0.19 | 9.61E-01 | -0.05±0.19 | 9.55E-01 | 0.06±0.19 | 9.16E-01 | 0.13±0.2 | 7.94E-01 |
| bilirubin (E,E)* | 0±0 | 0 | 6.17E-01 | 7.36E-01 | 0.05±0.14 | 0 | 5.49E-01 | 9.78E-01 | 0±0.2 | 9.99E-01 | -0.04±0.21 | 9.61E-01 | 0.1±0.19 | 8.46E-01 | 0.13±0.2 | 8.02E-01 |
| X - 12816 | 0.04±0.07 | 10.23 | 2.91E-01 | 7.76E-01 | -0.04±0.14 | 0 | 5.35E-01 | 9.78E-01 | 0.07±0.21 | 9.30E-01 | 0.06±0.22 | 9.45E-01 | -0.13±0.18 | 7.75E-01 | -0.12±0.19 | 8.10E-01 |
| N-acetylisoleucine | -0.04±0.05 | 0 | 3.32E-01 | 7.44E-01 | 0.04±0.15 | 0 | 5.50E-01 | 9.78E-01 | -0.05±0.22 | 9.54E-01 | -0.06±0.22 | 9.49E-01 | 0.15±0.21 | 7.59E-01 | 0.12±0.2 | 8.13E-01 |
| 3-hydroxybutyroylglycine** | -0.01±0.01 | 0 | 5.03E-01 | 7.36E-01 | -0.05±0.14 | 0 | 6.35E-01 | 9.78E-01 | -0.01±0.21 | 9.92E-01 | 0.02±0.21 | 9.76E-01 | -0.13±0.18 | 7.79E-01 | -0.11±0.19 | 8.13E-01 |
| myristoyl dihydrosphingomyelin (d18:0/14:0)* | -0.01±0.01 | 0 | 5.58E-01 | 7.36E-01 | 0.05±0.16 | 0 | 5.72E-01 | 9.78E-01 | -0.01±0.21 | 9.92E-01 | -0.04±0.22 | 9.64E-01 | 0.13±0.23 | 8.23E-01 | 0.14±0.23 | 8.13E-01 |
| 2-naphthol sulfate | -0.09±0.16 | 0 | 4.01E-01 | 8.02E-01 | -0.07±0.18 | 44.79 | 1.78E-01 | 9.78E-01 | -0.27±0.22 | 8.40E-01 | -0.34±0.27 | 8.44E-01 | 0.01±0.2 | 9.82E-01 | 0.15±0.24 | 8.13E-01 |
| 3-methoxytyramine sulfate | -0.05±0.14 | 1.87 | 3.13E-01 | 8.51E-01 | -0.05±0.14 | 33.29 | 2.21E-01 | 9.78E-01 | -0.2±0.19 | 8.55E-01 | -0.23±0.2 | 8.44E-01 | 0.08±0.19 | 8.82E-01 | 0.11±0.2 | 8.22E-01 |
| glutamine conjugate of C6H10O2 (2)* | 0.01±0.01 | 0 | 7.01E-01 | 7.36E-01 | -0.05±0.14 | 0 | 6.46E-01 | 9.78E-01 | 0.01±0.2 | 9.92E-01 | 0.02±0.2 | 9.84E-01 | -0.07±0.2 | 9.15E-01 | -0.11±0.21 | 8.27E-01 |
| 3-(3-hydroxyphenyl)propionate sulfate | -0.05±0.12 | 13.55 | 2.82E-01 | 8.51E-01 | -0.04±0.13 | 8.41 | 2.96E-01 | 9.78E-01 | -0.16±0.19 | 8.55E-01 | -0.18±0.19 | 8.45E-01 | 0.11±0.19 | 8.22E-01 | 0.11±0.19 | 8.29E-01 |
| N-palmitoyl-sphingosine (d18:1/16:0) | -0.01±0.01 | 0 | 5.97E-01 | 7.36E-01 | 0.05±0.15 | 0 | 6.76E-01 | 9.78E-01 | -0.01±0.21 | 9.90E-01 | -0.01±0.21 | 9.98E-01 | 0.11±0.23 | 8.57E-01 | 0.12±0.23 | 8.29E-01 |
| pregnanediol-3-glucuronide | 0.03±0.04 | 0 | 3.81E-01 | 7.40E-01 | -0.04±0.14 | 0 | 6.54E-01 | 9.78E-01 | 0.04±0.2 | 9.57E-01 | 0.03±0.21 | 9.73E-01 | -0.12±0.18 | 7.79E-01 | -0.1±0.19 | 8.41E-01 |
| X - 12100 | -0.04±0.15 | 27.85 | 2.39E-01 | 9.15E-01 | -0.05±0.16 | 25.89 | 2.45E-01 | 9.78E-01 | -0.21±0.23 | 8.55E-01 | -0.26±0.24 | 8.44E-01 | 0.15±0.22 | 7.81E-01 | 0.11±0.21 | 8.44E-01 |
| N6-carbamoylthreonyladenosine | -0.06±0.13 | 13.08 | 2.83E-01 | 8.32E-01 | -0.04±0.15 | 0 | 3.35E-01 | 9.78E-01 | -0.17±0.21 | 8.55E-01 | -0.18±0.21 | 8.45E-01 | 0.13±0.22 | 8.12E-01 | 0.11±0.22 | 8.47E-01 |
| pyridoxate | 0.06±0.07 | 0 | 5.94E-01 | 7.37E-01 | -0.04±0.13 | 0 | 6.98E-01 | 9.78E-01 | 0.08±0.18 | 9.06E-01 | 0.02±0.19 | 9.84E-01 | -0.03±0.18 | 9.69E-01 | -0.08±0.17 | 8.52E-01 |
| uridine | 0±0.14 | 32.45 | 2.24E-01 | 9.99E-01 | 0.04±0.14 | 25.99 | 2.45E-01 | 9.78E-01 | 0.25±0.21 | 8.40E-01 | 0.24±0.22 | 8.44E-01 | -0.12±0.17 | 7.79E-01 | -0.09±0.18 | 8.56E-01 |
| hexanoylcarnitine (C6) | 0.06±0.13 | 0 | 3.21E-01 | 8.32E-01 | 0.03±0.13 | 0 | 3.49E-01 | 9.78E-01 | 0.17±0.18 | 8.55E-01 | 0.16±0.19 | 8.45E-01 | -0.08±0.18 | 8.81E-01 | -0.08±0.18 | 8.57E-01 |
| cis-3,4-methyleneheptanoylglycine | -0.06±0.14 | 0 | 3.38E-01 | 8.32E-01 | -0.04±0.15 | 0 | 3.72E-01 | 9.78E-01 | -0.2±0.2 | 8.55E-01 | -0.17±0.21 | 8.47E-01 | 0.07±0.19 | 9.15E-01 | 0.09±0.2 | 8.60E-01 |
| X - 24307 | 0.01±0.14 | 30.11 | 2.32E-01 | 9.60E-01 | 0.05±0.13 | 12.45 | 2.85E-01 | 9.78E-01 | 0.22±0.19 | 8.40E-01 | 0.21±0.2 | 8.44E-01 | -0.12±0.18 | 7.85E-01 | -0.08±0.18 | 8.63E-01 |
| ectoine | -0.06±0.12 | 0 | 3.54E-01 | 8.08E-01 | -0.04±0.13 | 0 | 3.28E-01 | 9.78E-01 | -0.15±0.19 | 8.55E-01 | -0.18±0.2 | 8.45E-01 | 0.06±0.18 | 9.11E-01 | 0.08±0.18 | 8.64E-01 |
| X - 25422 | -0.06±0.14 | 10.99 | 2.89E-01 | 8.48E-01 | -0.04±0.14 | 0 | 3.83E-01 | 9.78E-01 | -0.18±0.18 | 8.55E-01 | -0.16±0.2 | 8.45E-01 | 0.12±0.21 | 8.25E-01 | 0.09±0.21 | 8.65E-01 |
| X - 12822 | -0.06±0.09 | 0 | 3.76E-01 | 7.65E-01 | -0.04±0.15 | 0 | 3.95E-01 | 9.78E-01 | -0.1±0.2 | 9.03E-01 | -0.16±0.21 | 8.53E-01 | 0.1±0.2 | 8.37E-01 | 0.09±0.21 | 8.67E-01 |
| taurocholate | -0.05±0.13 | 5.85 | 3.03E-01 | 8.62E-01 | -0.04±0.14 | 0 | 3.45E-01 | 9.78E-01 | -0.18±0.2 | 8.55E-01 | -0.19±0.21 | 8.45E-01 | 0.08±0.17 | 8.67E-01 | 0.07±0.18 | 8.86E-01 |
| glucuronide of C10H18O2 (7)* | -0.07±0.11 | 0 | 4.13E-01 | 7.75E-01 | -0.04±0.14 | 0 | 4.06E-01 | 9.78E-01 | -0.13±0.2 | 8.62E-01 | -0.16±0.2 | 8.53E-01 | 0.06±0.19 | 9.16E-01 | 0.07±0.2 | 8.86E-01 |
| sphingomyelin (d18:2/14:0, d18:1/14:1)* | 0.04±0.04 | 0 | 8.22E-01 | 7.36E-01 | 0.05±0.17 | 0 | 8.31E-01 | 9.78E-01 | 0.04±0.25 | 9.70E-01 | 0.01±0.26 | 9.98E-01 | 0.09±0.23 | 9.03E-01 | 0.08±0.22 | 8.93E-01 |
| S-adenosylhomocysteine (SAH) | 0.07±0.14 | 0 | 3.77E-01 | 8.02E-01 | 0.04±0.15 | 0 | 4.12E-01 | 9.78E-01 | 0.19±0.22 | 8.55E-01 | 0.17±0.22 | 8.53E-01 | -0.06±0.2 | 9.28E-01 | -0.07±0.2 | 8.93E-01 |
| X - 17653 | -0.03±0.03 | 0 | 6.44E-01 | 7.36E-01 | -0.05±0.14 | 0 | 8.66E-01 | 9.78E-01 | -0.03±0.2 | 9.70E-01 | -0.02±0.21 | 9.84E-01 | -0.11±0.18 | 7.96E-01 | -0.07±0.19 | 8.94E-01 |
| octadecenedioate (C18:1-DC) | 0.06±0.11 | 0 | 3.35E-01 | 8.04E-01 | 0.03±0.14 | 0 | 4.66E-01 | 9.78E-01 | 0.14±0.2 | 8.55E-01 | 0.14±0.2 | 8.59E-01 | -0.09±0.19 | 8.60E-01 | -0.07±0.2 | 8.98E-01 |
| 5alpha-pregnan-3beta,20alpha-diol monosulfate (2) | -0.08±0.14 | 0 | 3.92E-01 | 8.02E-01 | -0.05±0.14 | 0 | 4.08E-01 | 9.78E-01 | -0.2±0.2 | 8.55E-01 | -0.17±0.2 | 8.45E-01 | 0.03±0.19 | 9.61E-01 | 0.06±0.19 | 9.12E-01 |
| butyrylglycine | -0.03±0.03 | 0 | 8.13E-01 | 7.36E-01 | -0.03±0.14 | 0 | 8.74E-01 | 9.78E-01 | -0.03±0.2 | 9.70E-01 | -0.01±0.2 | 9.94E-01 | -0.07±0.18 | 8.90E-01 | -0.05±0.19 | 9.16E-01 |
| X - 21286 | 0.1±0.1 | 0 | 8.09E-01 | 7.36E-01 | 0.06±0.14 | 0 | 9.85E-01 | 9.78E-01 | 0.12±0.2 | 8.80E-01 | 0.06±0.21 | 9.40E-01 | 0.06±0.2 | 9.16E-01 | 0.06±0.2 | 9.19E-01 |
| gamma-glutamylglutamine | -0.1±0.12 | 0 | 5.82E-01 | 7.39E-01 | -0.04±0.15 | 0 | 5.04E-01 | 9.78E-01 | -0.16±0.2 | 8.55E-01 | -0.14±0.21 | 8.59E-01 | -0.02±0.18 | 9.73E-01 | 0.05±0.2 | 9.25E-01 |
| X - 23782 | 0.08±0.09 | 0 | 7.08E-01 | 7.36E-01 | 0.05±0.14 | 0 | 9.91E-01 | 9.78E-01 | 0.1±0.2 | 8.93E-01 | 0.04±0.2 | 9.60E-01 | 0.02±0.19 | 9.74E-01 | 0.05±0.2 | 9.31E-01 |
| 1-palmitoyl-2-docosahexaenoyl-GPE (16:0/22:6)* | 0.07±0.07 | 0 | 9.05E-01 | 7.36E-01 | 0.04±0.15 | 0 | 9.55E-01 | 9.78E-01 | 0.07±0.22 | 9.38E-01 | 0.03±0.22 | 9.73E-01 | 0.04±0.2 | 9.45E-01 | 0.05±0.2 | 9.38E-01 |
| 1-stearoyl-GPI (18:0) | -0.07±0.1 | 0 | 4.95E-01 | 7.49E-01 | -0.05±0.14 | 0 | 5.14E-01 | 9.78E-01 | -0.11±0.2 | 8.91E-01 | -0.15±0.21 | 8.59E-01 | 0.04±0.19 | 9.45E-01 | 0.04±0.2 | 9.46E-01 |
| X - 24970 | -0.03±0.03 | 0 | 8.45E-01 | 7.36E-01 | -0.04±0.14 | 0 | 9.98E-01 | 9.78E-01 | -0.03±0.2 | 9.70E-01 | -0.04±0.2 | 9.65E-01 | -0.07±0.19 | 9.15E-01 | -0.04±0.19 | 9.47E-01 |
| X - 16935 | -0.1±0.1 | 0 | 6.97E-01 | 7.36E-01 | -0.03±0.14 | 0 | 6.07E-01 | 9.78E-01 | -0.13±0.19 | 8.55E-01 | -0.1±0.19 | 8.86E-01 | -0.04±0.19 | 9.45E-01 | 0.04±0.2 | 9.47E-01 |
| X - 24544 | 0.03±0.03 | 0 | 9.90E-01 | 7.36E-01 | 0.04±0.14 | 0 | 9.54E-01 | 9.78E-01 | 0.03±0.2 | 9.70E-01 | 0.05±0.21 | 9.54E-01 | 0.03±0.19 | 9.63E-01 | 0.04±0.19 | 9.47E-01 |
| 5alpha-pregnan-3beta,20beta-diol monosulfate (1) | -0.09±0.12 | 0 | 4.92E-01 | 7.60E-01 | -0.04±0.14 | 0 | 5.72E-01 | 9.78E-01 | -0.15±0.2 | 8.55E-01 | -0.12±0.2 | 8.66E-01 | 0.01±0.19 | 9.82E-01 | 0.03±0.18 | 9.52E-01 |
| X - 11470 | 0.04±0.05 | 0 | 7.48E-01 | 7.36E-01 | 0.04±0.15 | 0 | 9.40E-01 | 9.78E-01 | 0.05±0.22 | 9.54E-01 | 0.05±0.23 | 9.58E-01 | -0.02±0.2 | 9.79E-01 | 0.03±0.2 | 9.63E-01 |
| tricosanoyl sphingomyelin (d18:1/23:0)* | -0.11±0.12 | 0 | 6.30E-01 | 7.36E-01 | -0.05±0.15 | 0 | 5.68E-01 | 9.78E-01 | -0.15±0.22 | 8.55E-01 | -0.15±0.23 | 8.63E-01 | -0.04±0.18 | 9.43E-01 | 0.03±0.2 | 9.69E-01 |
| caprate (10:0) | 0.07±0.08 | 0 | 6.36E-01 | 7.36E-01 | 0.05±0.14 | 0 | 8.49E-01 | 9.78E-01 | 0.09±0.2 | 9.06E-01 | 0.08±0.2 | 9.27E-01 | -0.01±0.19 | 9.82E-01 | 0.02±0.2 | 9.71E-01 |
| sphingomyelin (d18:0/18:0, d19:0/17:0)* | 0.12±0.11 | 0 | 9.38E-01 | 7.36E-01 | 0.04±0.14 | 0 | 8.66E-01 | 9.78E-01 | 0.12±0.2 | 8.72E-01 | 0.07±0.22 | 9.33E-01 | 0.11±0.2 | 8.27E-01 | 0.02±0.19 | 9.73E-01 |
| 4-hydroxycoumarin | -0.08±0.08 | 0 | 8.63E-01 | 7.36E-01 | -0.04±0.14 | 0 | 7.28E-01 | 9.78E-01 | -0.09±0.19 | 9.04E-01 | -0.08±0.19 | 9.17E-01 | -0.05±0.19 | 9.30E-01 | 0.01±0.2 | 9.88E-01 |
| sphingomyelin (d18:1/25:0, d19:0/24:1, d20:1/23:0, d19:1/24:0)* | -0.09±0.09 | 0 | 7.71E-01 | 7.36E-01 | -0.04±0.13 | 0 | 6.60E-01 | 9.78E-01 | -0.1±0.2 | 8.98E-01 | -0.11±0.2 | 8.84E-01 | -0.04±0.17 | 9.34E-01 | 0.01±0.18 | 9.88E-01 |
| hippurate | -0.09±0.09 | 0 | 7.58E-01 | 7.36E-01 | -0.05±0.13 | 0 | 7.60E-01 | 9.78E-01 | -0.1±0.18 | 8.81E-01 | -0.09±0.19 | 8.97E-01 | -0.04±0.19 | 9.50E-01 | -0.01±0.19 | 9.88E-01 |
| X - 23587 | 0.09±0.1 | 0 | 6.52E-01 | 7.36E-01 | 0.04±0.14 | 0 | 6.99E-01 | 9.78E-01 | 0.12±0.2 | 8.72E-01 | 0.1±0.2 | 9.07E-01 | 0.02±0.19 | 9.74E-01 | -0.01±0.19 | 9.88E-01 |
| X - 24812 | 0.02±0.02 | 0 | 7.42E-01 | 7.36E-01 | 0.04±0.16 | 0 | 7.46E-01 | 9.78E-01 | 0.02±0.19 | 9.84E-01 | 0.09±0.23 | 9.27E-01 | -0.05±0.2 | 9.38E-01 | -0.01±0.23 | 9.90E-01 |
| X - 21441 | -0.05±0.05 | 0 | 7.48E-01 | 7.36E-01 | -0.04±0.14 | 0 | 7.84E-01 | 9.78E-01 | -0.06±0.19 | 9.38E-01 | -0.08±0.2 | 9.27E-01 | 0±0.18 | 9.95E-01 | -0.01±0.19 | 9.93E-01 |
| beta-hydroxyisovaleroylcarnitine | 0.1±0.11 | 0 | 6.77E-01 | 7.36E-01 | 0.04±0.15 | 0 | 8.11E-01 | 9.78E-01 | 0.14±0.2 | 8.55E-01 | 0.08±0.21 | 9.33E-01 | 0.04±0.2 | 9.62E-01 | 0.01±0.2 | 9.94E-01 |
| sphingomyelin (d18:2/21:0, d16:2/23:0)* | -0.12±0.14 | 0 | 5.39E-01 | 7.44E-01 | -0.06±0.16 | 0 | 5.86E-01 | 9.78E-01 | -0.2±0.24 | 8.55E-01 | -0.17±0.25 | 8.59E-01 | -0.03±0.2 | 9.73E-01 | 0.01±0.2 | 9.94E-01 |
| taurodeoxycholate | 0.08±0.08 | 0 | 7.27E-01 | 7.36E-01 | 0.04±0.14 | 0 | 7.75E-01 | 9.78E-01 | 0.09±0.2 | 9.06E-01 | 0.09±0.21 | 9.27E-01 | 0.02±0.19 | 9.74E-01 | 0±0.19 | 9.94E-01 |
| isoleucylglycine | -0.07±0.06 | 0 | 9.82E-01 | 7.36E-01 | -0.05±0.14 | 0 | 7.68E-01 | 9.78E-01 | -0.07±0.19 | 9.32E-01 | -0.08±0.19 | 9.17E-01 | -0.06±0.19 | 9.15E-01 | 0±0.2 | 9.95E-01 |
| X - 21339 | 0.08±0.1 | 0 | 5.30E-01 | 7.44E-01 | 0.05±0.14 | 0 | 7.06E-01 | 9.78E-01 | 0.11±0.21 | 8.91E-01 | 0.11±0.21 | 8.87E-01 | -0.02±0.18 | 9.74E-01 | 0±0.19 | 9.96E-01 |
| 1-palmitoleoyl-2-linolenoyl-GPC (16:1/18:3)* | 0.07±0.09 | 0 | 5.08E-01 | 7.44E-01 | 0.05±0.14 | 0 | 7.04E-01 | 9.78E-01 | 0.1±0.2 | 8.99E-01 | 0.11±0.2 | 8.90E-01 | -0.04±0.19 | 9.43E-01 | 0±0.19 | 9.97E-01 |
| 11-ketoetiocholanolone glucuronide | -0.12±0.17 | 54.58 | 1.38E-01 | 7.60E-01 | 0.04±0.15 | 70.63 | 6.50E-02 | 9.79E-01 | 0.38±0.25 | 8.16E-01 | 0.41±0.25 | 8.44E-01 | -0.25±0.19 | 5.26E-01 | -0.18±0.2 | 6.88E-01 |
| 1-stearoyl-2-linoleoyl-GPI (18:0/18:2) | -0.1±0.12 | 0 | 5.39E-01 | 7.44E-01 | -0.04±0.15 | 0 | 4.95E-01 | 9.79E-01 | -0.15±0.2 | 8.55E-01 | -0.14±0.21 | 8.61E-01 | 0.01±0.21 | 9.95E-01 | 0.07±0.21 | 9.10E-01 |
| indolepropionate | -0.05±0.06 | 0 | 3.85E-01 | 7.44E-01 | 0.03±0.14 | 0 | 3.44E-01 | 9.80E-01 | -0.06±0.18 | 9.30E-01 | -0.08±0.19 | 9.21E-01 | 0.12±0.2 | 8.06E-01 | 0.19±0.22 | 7.06E-01 |
| p-cresol glucuronide* | 0.09±0.16 | 67.78 | 7.81E-02 | 8.02E-01 | 0.04±0.15 | 79.81 | 2.60E-02 | 9.82E-01 | -0.26±0.19 | 8.40E-01 | -0.26±0.2 | 8.44E-01 | 0.33±0.22 | 4.50E-01 | 0.41±0.22 | 4.09E-01 |
| palmitate (16:0) | -0.11±0.16 | 60.3 | 1.13E-01 | 7.62E-01 | -0.03±0.14 | 79.16 | 2.85E-02 | 9.82E-01 | 0.31±0.2 | 8.16E-01 | 0.31±0.21 | 8.44E-01 | -0.26±0.19 | 4.81E-01 | -0.3±0.18 | 4.67E-01 |
| oxalate (ethanedioate) | -0.12±0.14 | 52.58 | 1.46E-01 | 7.40E-01 | -0.03±0.14 | 47.36 | 1.68E-01 | 9.82E-01 | 0.36±0.27 | 8.40E-01 | 0.29±0.28 | 8.44E-01 | -0.21±0.16 | 5.17E-01 | -0.16±0.17 | 6.92E-01 |
| 4-ethylcatechol sulfate | 0.01±0.01 | 0 | 7.78E-01 | 7.36E-01 | 0.03±0.14 | 0 | 9.49E-01 | 9.82E-01 | 0.01±0.2 | 9.94E-01 | 0.04±0.2 | 9.61E-01 | -0.05±0.19 | 9.34E-01 | 0.02±0.19 | 9.69E-01 |
| X - 25790 | 0.04±0.2 | 45.61 | 1.75E-01 | 9.32E-01 | -0.04±0.16 | 71.5 | 6.10E-02 | 9.84E-01 | -0.35±0.2 | 8.16E-01 | -0.28±0.21 | 8.44E-01 | 0.22±0.24 | 6.68E-01 | 0.32±0.25 | 5.60E-01 |
| 1-palmitoyl-GPI (16:0) | -0.04±0.13 | 25.91 | 2.45E-01 | 8.77E-01 | -0.03±0.15 | 17.24 | 2.72E-01 | 9.84E-01 | -0.17±0.2 | 8.55E-01 | -0.19±0.21 | 8.45E-01 | 0.14±0.21 | 7.79E-01 | 0.13±0.21 | 8.13E-01 |
| docosahexaenoate (DHA; 22:6n3) | 0.05±0.09 | 4.92 | 3.05E-01 | 8.00E-01 | -0.03±0.14 | 0 | 5.05E-01 | 9.84E-01 | 0.11±0.19 | 8.81E-01 | 0.07±0.2 | 9.33E-01 | -0.11±0.19 | 8.02E-01 | -0.12±0.19 | 8.13E-01 |
| taurochenodeoxycholate | -0.05±0.07 | 0 | 3.56E-01 | 7.62E-01 | 0.03±0.13 | 0 | 5.32E-01 | 9.84E-01 | -0.08±0.19 | 9.12E-01 | -0.05±0.19 | 9.40E-01 | 0.11±0.18 | 8.13E-01 | 0.11±0.19 | 8.13E-01 |
| X - 24494 | -0.04±0.17 | 38.38 | 2.03E-01 | 9.25E-01 | 0.03±0.15 | 57.81 | 1.24E-01 | 9.85E-01 | 0.35±0.23 | 8.16E-01 | 0.32±0.24 | 8.44E-01 | -0.16±0.19 | 7.29E-01 | -0.16±0.19 | 7.36E-01 |
| quinate | -0.06±0.12 | 6.36 | 3.01E-01 | 8.27E-01 | -0.03±0.14 | 11.94 | 2.87E-01 | 9.85E-01 | -0.16±0.17 | 8.55E-01 | -0.15±0.17 | 8.45E-01 | 0.11±0.21 | 8.25E-01 | 0.15±0.22 | 7.81E-01 |
| docosapentaenoate (n3 DPA; 22:5n3) | 0.08±0.12 | 0 | 4.21E-01 | 7.80E-01 | 0.03±0.14 | 0 | 4.70E-01 | 9.85E-01 | 0.16±0.2 | 8.55E-01 | 0.14±0.2 | 8.59E-01 | -0.04±0.19 | 9.45E-01 | -0.06±0.19 | 8.98E-01 |
| octadecadienedioate (C18:2-DC)* | -0.09±0.12 | 0 | 4.92E-01 | 7.60E-01 | -0.03±0.15 | 0 | 4.96E-01 | 9.85E-01 | -0.16±0.23 | 8.55E-01 | -0.15±0.23 | 8.61E-01 | 0.01±0.19 | 9.82E-01 | 0.06±0.2 | 9.16E-01 |
| glutarylcarnitine (C5-DC) | 0.05±0.05 | 0 | 8.21E-01 | 7.36E-01 | 0.03±0.15 | 0 | 8.99E-01 | 9.86E-01 | 0.06±0.21 | 9.46E-01 | 0.05±0.22 | 9.55E-01 | 0.01±0.2 | 9.91E-01 | 0.01±0.2 | 9.85E-01 |
| 1-palmitoyl-2-linoleoyl-GPI (16:0/18:2) | 0±0.15 | 49.57 | 1.59E-01 | 9.91E-01 | -0.03±0.15 | 48.34 | 1.64E-01 | 9.86E-01 | -0.21±0.19 | 8.40E-01 | -0.22±0.2 | 8.44E-01 | 0.21±0.22 | 6.49E-01 | 0.19±0.22 | 7.03E-01 |
| 7-methylxanthine | 0±0.14 | 41.96 | 1.89E-01 | 9.91E-01 | -0.03±0.13 | 65.91 | 8.67E-02 | 9.92E-01 | -0.23±0.18 | 8.40E-01 | -0.24±0.18 | 8.44E-01 | 0.16±0.19 | 7.16E-01 | 0.21±0.19 | 6.16E-01 |
| 3-hydroxyphenylacetoylglutamine | -0.01±0.01 | 0 | 4.74E-01 | 7.36E-01 | 0.03±0.14 | 0 | 5.53E-01 | 9.92E-01 | -0.01±0.2 | 9.92E-01 | -0.06±0.2 | 9.40E-01 | 0.13±0.19 | 7.83E-01 | 0.11±0.19 | 8.27E-01 |
| X - 26119 | -0.06±0.12 | 8.84 | 2.95E-01 | 8.27E-01 | -0.03±0.14 | 0 | 3.19E-01 | 9.92E-01 | -0.15±0.21 | 8.55E-01 | -0.18±0.21 | 8.45E-01 | 0.11±0.2 | 8.20E-01 | 0.11±0.2 | 8.29E-01 |
| X - 24337 | 0.25±0.18 | 70.57 | 6.53E-02 | 7.36E-01 | 0.03±0.15 | 84.42 | 1.13E-02 | 9.92E-01 | -0.4±0.2 | 8.16E-01 | -0.35±0.21 | 8.44E-01 | 0.43±0.2 | 2.84E-01 | 0.41±0.21 | 3.75E-01 |
| 1,7-dimethylurate | 0.02±0.12 | 82.88 | 1.57E-02 | 9.51E-01 | 0.02±0.15 | 79.79 | 2.61E-02 | 9.92E-01 | -0.14±0.18 | 8.55E-01 | -0.2±0.18 | 8.44E-01 | 0.57±0.26 | 2.47E-01 | 0.49±0.26 | 3.78E-01 |
| 4-methylguaiacol sulfate | 0.16±0.17 | 66.18 | 8.55E-02 | 7.36E-01 | -0.01±0.13 | 84.74 | 1.05E-02 | 9.92E-01 | -0.33±0.19 | 8.16E-01 | -0.34±0.18 | 8.44E-01 | 0.33±0.19 | 4.07E-01 | 0.34±0.19 | 4.18E-01 |
| branched-chain, straight-chain, or cyclopropyl 12:1 fatty acid* | -0.21±0.16 | 54 | 1.40E-01 | 7.36E-01 | -0.02±0.14 | 87.43 | 4.79E-03 | 9.92E-01 | 0.52±0.23 | 8.16E-01 | 0.53±0.24 | 8.44E-01 | -0.28±0.17 | 4.07E-01 | -0.31±0.17 | 4.18E-01 |
| 7-methylguanine | 0.08±0.18 | 75.25 | 4.44E-02 | 8.32E-01 | 0.02±0.18 | 78.34 | 3.16E-02 | 9.92E-01 | -0.25±0.21 | 8.40E-01 | -0.28±0.23 | 8.44E-01 | 0.5±0.28 | 3.77E-01 | 0.51±0.29 | 4.28E-01 |
| X - 12680 | 0.1±0.19 | 58.59 | 1.20E-01 | 8.01E-01 | -0.03±0.15 | 83.24 | 1.46E-02 | 9.92E-01 | -0.34±0.19 | 8.16E-01 | -0.35±0.2 | 8.44E-01 | 0.29±0.22 | 5.27E-01 | 0.4±0.23 | 4.32E-01 |
| X - 17438 | -0.16±0.16 | 65.31 | 8.95E-02 | 7.36E-01 | 0.01±0.13 | 83.25 | 1.45E-02 | 9.92E-01 | 0.34±0.18 | 8.16E-01 | 0.33±0.18 | 8.44E-01 | -0.32±0.19 | 4.07E-01 | -0.32±0.19 | 4.40E-01 |
| tetradecadienoate (14:2)* | -0.16±0.17 | 58.78 | 1.19E-01 | 7.36E-01 | 0.02±0.14 | 83.92 | 1.26E-02 | 9.92E-01 | 0.39±0.2 | 8.16E-01 | 0.41±0.21 | 8.44E-01 | -0.28±0.18 | 4.50E-01 | -0.29±0.19 | 4.67E-01 |
| myristoleate (14:1n5) | -0.13±0.18 | 59.67 | 1.15E-01 | 7.62E-01 | -0.02±0.16 | 79.67 | 2.66E-02 | 9.92E-01 | 0.36±0.22 | 8.16E-01 | 0.36±0.23 | 8.44E-01 | -0.29±0.21 | 4.93E-01 | -0.33±0.21 | 4.67E-01 |
| X - 07765 | 0.14±0.13 | 56.07 | 1.31E-01 | 7.36E-01 | 0.01±0.12 | 83.74 | 1.31E-02 | 9.92E-01 | -0.35±0.19 | 8.16E-01 | -0.39±0.2 | 8.44E-01 | 0.22±0.14 | 4.51E-01 | 0.23±0.15 | 4.73E-01 |
| N-acetylproline | 0.01±0.14 | 68.83 | 7.33E-02 | 9.90E-01 | -0.01±0.13 | 71.22 | 6.23E-02 | 9.92E-01 | -0.17±0.15 | 8.40E-01 | -0.16±0.15 | 8.44E-01 | 0.35±0.24 | 4.71E-01 | 0.38±0.24 | 4.73E-01 |
| kynurenate | 0.17±0.17 | 69.68 | 6.93E-02 | 7.36E-01 | -0.03±0.15 | 82.85 | 1.57E-02 | 9.92E-01 | -0.32±0.21 | 8.16E-01 | -0.42±0.22 | 8.44E-01 | 0.37±0.21 | 3.77E-01 | 0.32±0.21 | 4.77E-01 |
| oleate/vaccenate (18:1) | -0.13±0.16 | 54.86 | 1.37E-01 | 7.44E-01 | -0.02±0.14 | 79.84 | 2.59E-02 | 9.92E-01 | 0.38±0.22 | 8.16E-01 | 0.37±0.23 | 8.44E-01 | -0.25±0.18 | 5.09E-01 | -0.27±0.18 | 4.84E-01 |
| 1-methylurate | 0.02±0.13 | 66.83 | 8.25E-02 | 9.47E-01 | 0.03±0.14 | 67.98 | 7.72E-02 | 9.92E-01 | -0.18±0.19 | 8.55E-01 | -0.19±0.19 | 8.44E-01 | 0.3±0.21 | 4.81E-01 | 0.31±0.21 | 5.19E-01 |
| sulfate of piperine metabolite C16H19NO3 (3)* | 0±0.11 | 74.11 | 4.94E-02 | 9.91E-01 | -0.02±0.15 | 73.47 | 5.22E-02 | 9.92E-01 | -0.13±0.19 | 8.55E-01 | -0.29±0.21 | 8.44E-01 | 0.38±0.23 | 4.08E-01 | 0.31±0.23 | 5.44E-01 |
| X - 23780 | 0.09±0.16 | 62.68 | 1.02E-01 | 7.91E-01 | -0.02±0.13 | 75.47 | 4.35E-02 | 9.92E-01 | -0.28±0.18 | 8.16E-01 | -0.27±0.18 | 8.44E-01 | 0.28±0.2 | 4.81E-01 | 0.27±0.2 | 5.44E-01 |
| N-acetylglucosaminylasparagine | 0.1±0.18 | 61.37 | 1.08E-01 | 8.02E-01 | -0.01±0.15 | 75.26 | 4.44E-02 | 9.92E-01 | -0.31±0.21 | 8.19E-01 | -0.32±0.22 | 8.44E-01 | 0.29±0.21 | 4.97E-01 | 0.3±0.22 | 5.44E-01 |
| 5-hydroxyhexanoate | -0.12±0.16 | 62.29 | 1.03E-01 | 7.60E-01 | -0.02±0.15 | 72.61 | 5.61E-02 | 9.92E-01 | 0.3±0.22 | 8.33E-01 | 0.31±0.22 | 8.44E-01 | -0.28±0.19 | 4.74E-01 | -0.26±0.19 | 5.46E-01 |
| 5-dodecenoate (12:1n7) | -0.09±0.17 | 56.26 | 1.31E-01 | 8.02E-01 | 0.02±0.15 | 76.92 | 3.74E-02 | 9.92E-01 | 0.32±0.2 | 8.16E-01 | 0.33±0.21 | 8.44E-01 | -0.25±0.2 | 5.53E-01 | -0.27±0.2 | 5.46E-01 |
| sulfate of piperine metabolite C16H19NO3 (2)* | -0.02±0.1 | 74.09 | 4.95E-02 | 9.45E-01 | -0.01±0.16 | 71.14 | 6.27E-02 | 9.92E-01 | -0.11±0.19 | 8.81E-01 | -0.27±0.21 | 8.44E-01 | 0.39±0.23 | 4.07E-01 | 0.31±0.24 | 5.46E-01 |
| tartarate | -0.1±0.16 | 62.48 | 1.03E-01 | 7.75E-01 | -0.02±0.15 | 67.71 | 7.84E-02 | 9.92E-01 | 0.28±0.22 | 8.40E-01 | 0.27±0.22 | 8.44E-01 | -0.27±0.19 | 4.80E-01 | -0.26±0.2 | 5.60E-01 |
| vanillic acid glycine | 0.09±0.16 | 57.67 | 1.24E-01 | 7.91E-01 | -0.01±0.13 | 73.3 | 5.29E-02 | 9.92E-01 | -0.3±0.19 | 8.16E-01 | -0.27±0.19 | 8.44E-01 | 0.24±0.18 | 5.28E-01 | 0.24±0.19 | 5.60E-01 |
| X - 12707 | 0.02±0.16 | 51.66 | 1.50E-01 | 9.56E-01 | -0.01±0.15 | 67.42 | 7.98E-02 | 9.92E-01 | -0.25±0.19 | 8.40E-01 | -0.23±0.19 | 8.44E-01 | 0.22±0.22 | 6.31E-01 | 0.29±0.23 | 5.62E-01 |
| phenylacetate | -0.01±0.14 | 49.76 | 1.58E-01 | 9.87E-01 | 0.02±0.14 | 58.7 | 1.20E-01 | 9.92E-01 | -0.19±0.19 | 8.55E-01 | -0.19±0.2 | 8.45E-01 | 0.2±0.2 | 6.48E-01 | 0.26±0.21 | 5.79E-01 |
| N,N,N-trimethyl-alanylproline betaine (TMAP) | -0.05±0.14 | 47.75 | 1.67E-01 | 8.55E-01 | 0.03±0.19 | 46.29 | 1.72E-01 | 9.92E-01 | -0.16±0.24 | 8.55E-01 | -0.18±0.25 | 8.59E-01 | 0.31±0.3 | 6.31E-01 | 0.36±0.31 | 6.04E-01 |
| X - 22834 | 0±0.12 | 62.42 | 1.03E-01 | 9.91E-01 | 0.03±0.14 | 50.63 | 1.55E-01 | 9.92E-01 | -0.16±0.2 | 8.55E-01 | -0.18±0.2 | 8.45E-01 | 0.25±0.2 | 5.37E-01 | 0.22±0.2 | 6.11E-01 |
| dimethylarginine (SDMA + ADMA) | -0.04±0.18 | 46.32 | 1.72E-01 | 9.21E-01 | -0.03±0.19 | 55.15 | 1.35E-01 | 9.92E-01 | -0.22±0.22 | 8.55E-01 | -0.23±0.23 | 8.44E-01 | 0.29±0.31 | 6.61E-01 | 0.36±0.32 | 6.11E-01 |
| 3-ureidopropionate | -0.02±0.14 | 41.99 | 1.89E-01 | 9.47E-01 | 0.02±0.15 | 55.85 | 1.32E-01 | 9.92E-01 | -0.18±0.22 | 8.55E-01 | -0.23±0.22 | 8.44E-01 | 0.17±0.2 | 7.13E-01 | 0.23±0.21 | 6.14E-01 |
| branched-chain, straight-chain, or cyclopropyl 10:1 fatty acid (1)* | -0.02±0.13 | 52.46 | 1.47E-01 | 9.47E-01 | -0.03±0.14 | 53.1 | 1.44E-01 | 9.92E-01 | 0.2±0.2 | 8.55E-01 | 0.2±0.21 | 8.45E-01 | -0.18±0.17 | 6.19E-01 | -0.2±0.18 | 6.15E-01 |
| alpha-hydroxyisocaproate | -0.02±0.13 | 57.79 | 1.24E-01 | 9.51E-01 | 0.01±0.15 | 49.24 | 1.60E-01 | 9.92E-01 | -0.16±0.2 | 8.55E-01 | -0.19±0.21 | 8.45E-01 | 0.27±0.23 | 5.73E-01 | 0.25±0.23 | 6.19E-01 |
| trans-4-hydroxyproline | -0.02±0.12 | 54.45 | 1.38E-01 | 9.47E-01 | 0.02±0.14 | 52.57 | 1.46E-01 | 9.92E-01 | -0.14±0.2 | 8.55E-01 | -0.2±0.21 | 8.45E-01 | 0.22±0.2 | 6.00E-01 | 0.21±0.2 | 6.19E-01 |
| 4-allylcatechol sulfate | -0.01±0.13 | 51.97 | 1.49E-01 | 9.91E-01 | -0.02±0.14 | 63.26 | 9.90E-02 | 9.92E-01 | -0.18±0.19 | 8.55E-01 | -0.24±0.2 | 8.44E-01 | 0.2±0.2 | 6.31E-01 | 0.22±0.21 | 6.19E-01 |
| X - 24546 | -0.02±0.15 | 52.27 | 1.48E-01 | 9.47E-01 | -0.01±0.15 | 53.71 | 1.42E-01 | 9.92E-01 | 0.24±0.21 | 8.40E-01 | 0.22±0.21 | 8.44E-01 | -0.21±0.2 | 6.22E-01 | -0.22±0.2 | 6.29E-01 |
| X - 11381 | -0.03±0.14 | 37.7 | 2.05E-01 | 9.35E-01 | 0.01±0.14 | 43.64 | 1.83E-01 | 9.92E-01 | -0.18±0.19 | 8.55E-01 | -0.16±0.2 | 8.45E-01 | 0.16±0.2 | 7.44E-01 | 0.22±0.21 | 6.42E-01 |
| 6-oxopiperidine-2-carboxylate | -0.02±0.12 | 57.13 | 1.27E-01 | 9.35E-01 | -0.03±0.14 | 55.58 | 1.34E-01 | 9.92E-01 | -0.14±0.18 | 8.55E-01 | -0.21±0.19 | 8.44E-01 | 0.26±0.22 | 5.78E-01 | 0.23±0.22 | 6.49E-01 |
| 1-palmitoyl-2-stearoyl-GPC (16:0/18:0) | -0.07±0.11 | 7.35 | 2.99E-01 | 7.83E-01 | 0.01±0.18 | 30.52 | 2.30E-01 | 9.92E-01 | -0.12±0.22 | 8.80E-01 | -0.14±0.22 | 8.61E-01 | 0.18±0.26 | 7.81E-01 | 0.3±0.3 | 6.58E-01 |
| 1-linoleoyl-GPG (18:2)* | -0.03±0.11 | 47.8 | 1.66E-01 | 9.21E-01 | 0.01±0.13 | 26.14 | 2.45E-01 | 9.92E-01 | -0.14±0.17 | 8.55E-01 | -0.12±0.17 | 8.59E-01 | 0.2±0.2 | 6.50E-01 | 0.19±0.2 | 6.82E-01 |
| 12-HETE | -0.01±0.14 | 47.27 | 1.68E-01 | 9.77E-01 | -0.01±0.14 | 47.26 | 1.69E-01 | 9.92E-01 | 0.21±0.21 | 8.55E-01 | 0.21±0.21 | 8.44E-01 | -0.17±0.18 | 6.66E-01 | -0.17±0.18 | 6.82E-01 |
| 16-hydroxypalmitate | 0.01±0.14 | 43.1 | 1.85E-01 | 9.76E-01 | 0.02±0.14 | 47.16 | 1.69E-01 | 9.92E-01 | 0.2±0.18 | 8.40E-01 | 0.19±0.19 | 8.44E-01 | -0.17±0.2 | 7.11E-01 | -0.19±0.2 | 6.88E-01 |
| 2-hydroxyoctanoate | 0.02±0.14 | 48.57 | 1.63E-01 | 9.47E-01 | -0.02±0.14 | 57.91 | 1.23E-01 | 9.92E-01 | -0.23±0.21 | 8.40E-01 | -0.26±0.21 | 8.44E-01 | 0.18±0.18 | 6.50E-01 | 0.17±0.19 | 6.91E-01 |
| dihomo-linoleate (20:2n6) | 0.01±0.15 | 32.43 | 2.24E-01 | 9.91E-01 | 0.02±0.14 | 56.43 | 1.30E-01 | 9.92E-01 | 0.25±0.2 | 8.40E-01 | 0.25±0.21 | 8.44E-01 | -0.13±0.19 | 7.79E-01 | -0.17±0.19 | 6.93E-01 |
| 17alpha-hydroxypregnanolone glucuronide | 0±0.16 | 50.99 | 1.53E-01 | 9.91E-01 | 0.01±0.17 | 46.29 | 1.72E-01 | 9.92E-01 | 0.23±0.24 | 8.55E-01 | 0.25±0.24 | 8.44E-01 | -0.23±0.23 | 6.36E-01 | -0.21±0.23 | 6.98E-01 |
| creatine | -0.05±0.18 | 37.64 | 2.05E-01 | 9.11E-01 | 0.02±0.16 | 63.61 | 9.74E-02 | 9.92E-01 | 0.4±0.26 | 8.16E-01 | 0.38±0.27 | 8.44E-01 | -0.17±0.2 | 7.29E-01 | -0.18±0.2 | 6.99E-01 |
| glycosyl ceramide (d18:1/20:0, d16:1/22:0)* | -0.06±0.09 | 0 | 3.81E-01 | 7.62E-01 | 0.02±0.17 | 17.04 | 2.72E-01 | 9.92E-01 | -0.1±0.22 | 9.04E-01 | -0.15±0.23 | 8.59E-01 | 0.11±0.22 | 8.37E-01 | 0.21±0.24 | 7.06E-01 |
| X - 16576 | -0.05±0.08 | 5.44 | 3.04E-01 | 7.80E-01 | 0.02±0.14 | 15.14 | 2.78E-01 | 9.92E-01 | -0.09±0.2 | 9.04E-01 | -0.13±0.19 | 8.59E-01 | 0.13±0.2 | 7.83E-01 | 0.18±0.2 | 7.09E-01 |
| 4-allylphenol sulfate | -0.06±0.1 | 0 | 3.95E-01 | 7.76E-01 | 0.02±0.14 | 10.3 | 2.91E-01 | 9.92E-01 | -0.12±0.19 | 8.72E-01 | -0.12±0.2 | 8.63E-01 | 0.07±0.18 | 9.09E-01 | 0.17±0.19 | 7.11E-01 |
| (14 or 15)-methylpalmitate (a17:0 or i17:0) | 0±0.16 | 32.18 | 2.25E-01 | 9.99E-01 | 0.03±0.14 | 53.13 | 1.44E-01 | 9.92E-01 | 0.28±0.21 | 8.40E-01 | 0.27±0.22 | 8.44E-01 | -0.13±0.19 | 7.79E-01 | -0.16±0.19 | 7.25E-01 |
| beta-hydroxyisovalerate | -0.03±0.04 | 30.69 | 2.30E-01 | 7.44E-01 | 0.03±0.15 | 0 | 3.37E-01 | 9.92E-01 | -0.04±0.2 | 9.57E-01 | -0.11±0.2 | 8.86E-01 | 0.21±0.21 | 6.31E-01 | 0.17±0.21 | 7.27E-01 |
| 4-methylhexanoylglutamine | 0.05±0.12 | 20.17 | 2.63E-01 | 8.61E-01 | -0.03±0.14 | 0 | 3.47E-01 | 9.92E-01 | 0.16±0.2 | 8.55E-01 | 0.11±0.2 | 8.84E-01 | -0.12±0.19 | 7.98E-01 | -0.15±0.19 | 7.49E-01 |
| uracil | -0.05±0.13 | 24.55 | 2.50E-01 | 8.42E-01 | -0.02±0.15 | 22.67 | 2.55E-01 | 9.92E-01 | -0.15±0.19 | 8.55E-01 | -0.16±0.19 | 8.45E-01 | 0.16±0.22 | 7.79E-01 | 0.18±0.23 | 7.56E-01 |
| N6-methyladenosine | -0.04±0.13 | 35.59 | 2.13E-01 | 9.02E-01 | -0.02±0.14 | 25.86 | 2.45E-01 | 9.92E-01 | -0.16±0.19 | 8.55E-01 | -0.18±0.2 | 8.45E-01 | 0.17±0.21 | 7.46E-01 | 0.16±0.21 | 7.63E-01 |
| 3-hydroxystachydrine* | -0.05±0.07 | 0 | 3.80E-01 | 7.60E-01 | 0.02±0.15 | 0 | 3.58E-01 | 9.92E-01 | -0.08±0.2 | 9.21E-01 | -0.11±0.21 | 8.84E-01 | 0.11±0.2 | 8.22E-01 | 0.16±0.21 | 7.66E-01 |
| indoleacetoylcarnitine* | 0±0.14 | 48.19 | 1.65E-01 | 9.91E-01 | -0.01±0.14 | 29.41 | 2.34E-01 | 9.92E-01 | -0.19±0.21 | 8.55E-01 | -0.19±0.21 | 8.45E-01 | 0.19±0.19 | 6.61E-01 | 0.14±0.19 | 7.72E-01 |
| 1-palmitoyl-GPE (16:0) | -0.07±0.11 | 0 | 3.94E-01 | 7.80E-01 | -0.02±0.15 | 24.65 | 2.49E-01 | 9.92E-01 | -0.14±0.2 | 8.55E-01 | -0.2±0.22 | 8.45E-01 | 0.07±0.21 | 9.14E-01 | 0.16±0.22 | 7.75E-01 |
| mannose | -0.05±0.07 | 37.63 | 2.05E-01 | 7.65E-01 | 0.02±0.15 | 0 | 4.22E-01 | 9.92E-01 | -0.07±0.19 | 9.30E-01 | -0.08±0.2 | 9.27E-01 | 0.26±0.25 | 6.31E-01 | 0.17±0.24 | 7.79E-01 |
| X - 12026 | -0.05±0.08 | 36.34 | 2.10E-01 | 7.86E-01 | 0.02±0.16 | 0 | 4.21E-01 | 9.92E-01 | -0.08±0.21 | 9.29E-01 | -0.1±0.22 | 9.13E-01 | 0.21±0.22 | 6.61E-01 | 0.15±0.22 | 7.89E-01 |
| sphingomyelin (d17:1/14:0, d16:1/15:0)* | -0.08±0.14 | 0 | 3.71E-01 | 8.02E-01 | -0.01±0.16 | 1.4 | 3.14E-01 | 9.92E-01 | -0.19±0.23 | 8.55E-01 | -0.18±0.23 | 8.54E-01 | 0.07±0.22 | 9.15E-01 | 0.15±0.23 | 8.02E-01 |
| cholesterol sulfate | -0.09±0.14 | 0 | 4.40E-01 | 7.72E-01 | -0.02±0.16 | 5.32 | 3.04E-01 | 9.92E-01 | -0.18±0.22 | 8.55E-01 | -0.18±0.23 | 8.50E-01 | 0.04±0.22 | 9.64E-01 | 0.15±0.23 | 8.02E-01 |
| maleate | 0.08±0.17 | 28.86 | 2.36E-01 | 8.32E-01 | -0.03±0.17 | 61.83 | 1.06E-01 | 9.92E-01 | -0.59±0.36 | 8.16E-01 | -0.56±0.37 | 8.44E-01 | 0.14±0.18 | 7.57E-01 | 0.11±0.19 | 8.13E-01 |
| 2'-O-methylcytidine | 0.04±0.11 | 31.22 | 2.28E-01 | 8.77E-01 | -0.01±0.14 | 0 | 4.26E-01 | 9.92E-01 | 0.13±0.2 | 8.55E-01 | 0.11±0.21 | 8.87E-01 | -0.13±0.18 | 7.57E-01 | -0.11±0.18 | 8.13E-01 |
| lithocholate sulfate (1) | -0.05±0.11 | 25.01 | 2.48E-01 | 8.32E-01 | -0.03±0.14 | 8.16 | 2.97E-01 | 9.92E-01 | -0.13±0.19 | 8.55E-01 | -0.17±0.2 | 8.45E-01 | 0.15±0.21 | 7.75E-01 | 0.13±0.21 | 8.13E-01 |
| cis-4-decenoate (10:1n6)* | 0.05±0.08 | 0 | 3.26E-01 | 7.76E-01 | -0.02±0.14 | 0 | 4.75E-01 | 9.92E-01 | 0.09±0.2 | 9.06E-01 | 0.08±0.2 | 9.27E-01 | -0.11±0.18 | 8.00E-01 | -0.12±0.19 | 8.13E-01 |
| lactosyl-N-behenoyl-sphingosine (d18:1/22:0)* | -0.07±0.11 | 0 | 4.35E-01 | 7.65E-01 | -0.02±0.15 | 0 | 3.37E-01 | 9.92E-01 | -0.13±0.2 | 8.55E-01 | -0.15±0.2 | 8.58E-01 | 0.05±0.2 | 9.30E-01 | 0.13±0.21 | 8.13E-01 |
| 3-hydroxydecanoylcarnitine | 0.02±0.14 | 32.81 | 2.22E-01 | 9.60E-01 | 0.03±0.14 | 13.52 | 2.82E-01 | 9.92E-01 | 0.22±0.2 | 8.40E-01 | 0.19±0.21 | 8.45E-01 | -0.13±0.19 | 7.79E-01 | -0.11±0.19 | 8.20E-01 |
| 1-(1-enyl-palmitoyl)-2-linoleoyl-GPE (P-16:0/18:2)* | -0.08±0.11 | 0 | 4.26E-01 | 7.65E-01 | -0.03±0.14 | 0 | 3.26E-01 | 9.92E-01 | -0.13±0.19 | 8.55E-01 | -0.16±0.2 | 8.47E-01 | 0.06±0.21 | 9.16E-01 | 0.12±0.21 | 8.22E-01 |
| succinate | -0.05±0.06 | 0 | 5.96E-01 | 7.36E-01 | 0.02±0.14 | 0 | 4.99E-01 | 9.92E-01 | -0.06±0.19 | 9.38E-01 | -0.08±0.2 | 9.27E-01 | 0.05±0.19 | 9.38E-01 | 0.11±0.2 | 8.27E-01 |
| N-palmitoylglycine | 0.06±0.1 | 0.85 | 3.15E-01 | 8.02E-01 | 0.01±0.14 | 0 | 3.73E-01 | 9.92E-01 | 0.13±0.19 | 8.62E-01 | 0.14±0.2 | 8.59E-01 | -0.1±0.19 | 8.25E-01 | -0.1±0.19 | 8.29E-01 |
| 5-(galactosylhydroxy)-L-lysine | -0.06±0.11 | 0.3 | 3.17E-01 | 7.91E-01 | -0.01±0.15 | 0 | 4.05E-01 | 9.92E-01 | -0.12±0.21 | 8.75E-01 | -0.14±0.21 | 8.62E-01 | 0.12±0.21 | 8.13E-01 | 0.11±0.21 | 8.41E-01 |
| sphingomyelin (d18:1/21:0, d17:1/22:0, d16:1/23:0)* | -0.06±0.07 | 0 | 6.06E-01 | 7.36E-01 | 0.02±0.16 | 0 | 5.79E-01 | 9.92E-01 | -0.08±0.23 | 9.32E-01 | -0.07±0.23 | 9.38E-01 | 0.04±0.21 | 9.61E-01 | 0.11±0.22 | 8.52E-01 |
| X - 16580 | 0.01±0.01 | 0 | 5.61E-01 | 7.36E-01 | 0.02±0.14 | 0 | 6.03E-01 | 9.92E-01 | 0.01±0.2 | 9.90E-01 | -0.05±0.21 | 9.46E-01 | 0.14±0.21 | 7.93E-01 | 0.09±0.2 | 8.57E-01 |
| glycolithocholate sulfate* | -0.06±0.11 | 1.82 | 3.13E-01 | 8.02E-01 | -0.02±0.14 | 0 | 4.04E-01 | 9.92E-01 | -0.13±0.19 | 8.55E-01 | -0.14±0.2 | 8.59E-01 | 0.11±0.2 | 8.22E-01 | 0.09±0.19 | 8.57E-01 |
| citrate | -0.07±0.08 | 0 | 5.90E-01 | 7.37E-01 | -0.02±0.15 | 0 | 4.36E-01 | 9.92E-01 | -0.08±0.2 | 9.12E-01 | -0.13±0.21 | 8.63E-01 | 0.03±0.19 | 9.67E-01 | 0.1±0.2 | 8.57E-01 |
| histidine | -0.06±0.07 | 0 | 6.09E-01 | 7.36E-01 | -0.01±0.15 | 0 | 4.21E-01 | 9.92E-01 | -0.08±0.21 | 9.30E-01 | -0.15±0.22 | 8.59E-01 | 0.03±0.19 | 9.67E-01 | 0.09±0.19 | 8.59E-01 |
| 1-(1-enyl-stearoyl)-2-linoleoyl-GPE (P-18:0/18:2)* | -0.07±0.08 | 0 | 6.75E-01 | 7.36E-01 | -0.02±0.15 | 0 | 4.68E-01 | 9.92E-01 | -0.09±0.2 | 9.06E-01 | -0.12±0.21 | 8.70E-01 | 0±0.19 | 9.97E-01 | 0.09±0.21 | 8.60E-01 |
| X - 25265 | 0.05±0.12 | 13.25 | 2.83E-01 | 8.32E-01 | 0.01±0.15 | 0 | 4.70E-01 | 9.92E-01 | 0.15±0.22 | 8.55E-01 | 0.14±0.23 | 8.70E-01 | -0.12±0.2 | 8.12E-01 | -0.09±0.21 | 8.65E-01 |
| X - 25936 | 0.03±0.1 | 26.69 | 2.43E-01 | 8.91E-01 | 0.02±0.13 | 0 | 4.39E-01 | 9.92E-01 | 0.14±0.2 | 8.55E-01 | 0.14±0.21 | 8.59E-01 | -0.11±0.16 | 7.79E-01 | -0.06±0.17 | 8.86E-01 |
| X - 23974 | 0.05±0.09 | 17.64 | 2.71E-01 | 8.14E-01 | -0.01±0.14 | 0 | 6.33E-01 | 9.92E-01 | 0.11±0.2 | 8.91E-01 | 0.06±0.2 | 9.38E-01 | -0.12±0.18 | 7.79E-01 | -0.07±0.18 | 8.86E-01 |
| alpha-tocopherol | -0.06±0.06 | 0 | 5.68E-01 | 7.36E-01 | -0.02±0.14 | 0 | 5.22E-01 | 9.92E-01 | -0.07±0.19 | 9.30E-01 | -0.1±0.19 | 8.87E-01 | 0.06±0.21 | 9.28E-01 | 0.08±0.21 | 8.86E-01 |
| linolenoylcarnitine (C18:3)* | -0.05±0.05 | 0 | 6.11E-01 | 7.36E-01 | 0.02±0.14 | 0 | 7.31E-01 | 9.92E-01 | -0.06±0.19 | 9.38E-01 | -0.03±0.2 | 9.73E-01 | 0.05±0.2 | 9.37E-01 | 0.07±0.21 | 8.98E-01 |
| corticosterone | 0.03±0.04 | 0 | 4.55E-01 | 7.36E-01 | -0.02±0.14 | 0 | 7.30E-01 | 9.92E-01 | 0.04±0.2 | 9.57E-01 | 0.03±0.2 | 9.71E-01 | -0.1±0.19 | 8.22E-01 | -0.07±0.2 | 8.98E-01 |
| dihomo-linolenate (20:3n3 or n6) | 0.05±0.06 | 0 | 6.63E-01 | 7.36E-01 | -0.01±0.14 | 0 | 7.16E-01 | 9.92E-01 | 0.06±0.2 | 9.38E-01 | 0.04±0.21 | 9.61E-01 | -0.03±0.19 | 9.69E-01 | -0.06±0.19 | 9.10E-01 |
| N-acetyl-1-methylhistidine* | -0.01±0.01 | 0 | 5.99E-01 | 7.36E-01 | 0.02±0.14 | 0 | 7.67E-01 | 9.92E-01 | -0.01±0.2 | 9.92E-01 | -0.03±0.2 | 9.73E-01 | 0.09±0.19 | 8.50E-01 | 0.06±0.19 | 9.16E-01 |
| 4-vinylcatechol sulfate | -0.09±0.11 | 0 | 5.91E-01 | 7.39E-01 | -0.02±0.14 | 0 | 5.87E-01 | 9.92E-01 | -0.13±0.18 | 8.55E-01 | -0.1±0.19 | 8.98E-01 | -0.01±0.18 | 9.82E-01 | 0.05±0.2 | 9.19E-01 |
| isovalerate (i5:0) | 0.03±0.03 | 0 | 7.93E-01 | 7.36E-01 | 0.02±0.14 | 0 | 7.89E-01 | 9.92E-01 | 0.03±0.2 | 9.65E-01 | -0.02±0.19 | 9.76E-01 | 0.08±0.19 | 8.75E-01 | 0.05±0.19 | 9.22E-01 |
| eicosenoylcarnitine (C20:1)* | 0±0 | 0 | 8.53E-01 | 7.36E-01 | 0.02±0.14 | 0 | 8.01E-01 | 9.92E-01 | 0±0.2 | 9.99E-01 | -0.02±0.2 | 9.84E-01 | 0.04±0.2 | 9.62E-01 | 0.06±0.21 | 9.22E-01 |
| behenoyl sphingomyelin (d18:1/22:0)* | -0.01±0.01 | 0 | 9.47E-01 | 7.36E-01 | 0.02±0.15 | 0 | 8.34E-01 | 9.92E-01 | -0.01±0.21 | 9.92E-01 | -0.01±0.22 | 9.97E-01 | 0±0.19 | 9.97E-01 | 0.05±0.2 | 9.24E-01 |
| X - 11880 | 0.03±0.03 | 0 | 6.15E-01 | 7.36E-01 | -0.02±0.15 | 0 | 8.83E-01 | 9.92E-01 | 0.03±0.22 | 9.65E-01 | 0.01±0.22 | 9.98E-01 | -0.06±0.19 | 9.15E-01 | -0.04±0.19 | 9.47E-01 |
| 3-bromo-5-chloro-2,6-dihydroxybenzoic acid* | -0.05±0.06 | 0 | 5.73E-01 | 7.36E-01 | -0.02±0.16 | 0 | 6.48E-01 | 9.92E-01 | -0.06±0.24 | 9.51E-01 | -0.11±0.25 | 9.17E-01 | 0.07±0.22 | 9.16E-01 | 0.04±0.21 | 9.47E-01 |
| 1-palmitoyl-2-docosahexaenoyl-GPC (16:0/22:6) | 0.06±0.07 | 0 | 5.38E-01 | 7.40E-01 | -0.02±0.14 | 0 | 8.84E-01 | 9.92E-01 | 0.07±0.2 | 9.30E-01 | 0.01±0.21 | 9.98E-01 | -0.05±0.19 | 9.29E-01 | -0.03±0.19 | 9.47E-01 |
| X - 24475 | -0.09±0.1 | 0 | 6.86E-01 | 7.36E-01 | -0.03±0.14 | 0 | 6.50E-01 | 9.92E-01 | -0.11±0.19 | 8.80E-01 | -0.09±0.2 | 9.13E-01 | -0.02±0.19 | 9.74E-01 | 0.04±0.2 | 9.47E-01 |
| pregnenediol sulfate (C21H34O5S)* | 0.06±0.07 | 0 | 5.52E-01 | 7.37E-01 | 0.03±0.15 | 0 | 6.71E-01 | 9.92E-01 | 0.07±0.22 | 9.38E-01 | 0.1±0.22 | 9.22E-01 | -0.06±0.21 | 9.17E-01 | -0.04±0.21 | 9.53E-01 |
| X - 21834 | 0±0 | 0 | 9.96E-01 | 7.36E-01 | 0.01±0.14 | 0 | 9.02E-01 | 9.92E-01 | 0±0.2 | 9.99E-01 | -0.01±0.21 | 9.98E-01 | 0±0.19 | 9.99E-01 | 0.03±0.19 | 9.59E-01 |
| isovalerylglycine | -0.01±0.01 | 0 | 8.00E-01 | 7.36E-01 | -0.03±0.14 | 0 | 9.81E-01 | 9.92E-01 | -0.01±0.2 | 9.92E-01 | -0.03±0.21 | 9.71E-01 | -0.06±0.18 | 9.16E-01 | -0.03±0.19 | 9.68E-01 |
| X - 15728 | -0.05±0.05 | 0 | 9.84E-01 | 7.36E-01 | -0.02±0.13 | 0 | 7.55E-01 | 9.92E-01 | -0.05±0.19 | 9.47E-01 | -0.06±0.19 | 9.38E-01 | -0.05±0.18 | 9.16E-01 | 0.02±0.18 | 9.70E-01 |
| carotene diol (2) | -0.05±0.04 | 0 | 9.46E-01 | 7.36E-01 | -0.02±0.15 | 0 | 7.47E-01 | 9.92E-01 | -0.04±0.21 | 9.54E-01 | -0.07±0.22 | 9.33E-01 | -0.06±0.18 | 9.15E-01 | 0.02±0.19 | 9.76E-01 |
| cis-3,4-methyleneheptanoate | -0.06±0.06 | 0 | 6.36E-01 | 7.36E-01 | -0.01±0.15 | 0 | 8.20E-01 | 9.92E-01 | -0.07±0.21 | 9.38E-01 | -0.05±0.21 | 9.60E-01 | 0.03±0.2 | 9.67E-01 | 0.02±0.2 | 9.76E-01 |
| 3-hydroxyoleoylcarnitine | 0.03±0.03 | 0 | 9.01E-01 | 7.36E-01 | 0.01±0.14 | 0 | 9.64E-01 | 9.92E-01 | 0.03±0.2 | 9.65E-01 | 0±0.2 | 9.98E-01 | 0.06±0.19 | 9.17E-01 | 0.02±0.2 | 9.80E-01 |
| X - 18901 | -0.02±0.02 | 0 | 8.27E-01 | 7.36E-01 | -0.02±0.15 | 0 | 8.97E-01 | 9.92E-01 | -0.02±0.2 | 9.84E-01 | -0.04±0.21 | 9.63E-01 | -0.06±0.19 | 9.15E-01 | 0±0.2 | 9.94E-01 |
| cerotoylcarnitine (C26)* | -0.05±0.14 | 31.93 | 2.25E-01 | 8.77E-01 | -0.01±0.15 | 48.77 | 1.62E-01 | 9.92E-01 | -0.17±0.2 | 8.55E-01 | -0.19±0.2 | 8.45E-01 | 0.18±0.23 | 7.57E-01 | 0.24±0.24 | 6.56E-01 |
| homostachydrine* | -0.08±0.11 | 0 | 4.84E-01 | 7.60E-01 | -0.01±0.14 | 0 | 4.73E-01 | 9.92E-01 | -0.14±0.19 | 8.55E-01 | -0.11±0.2 | 8.76E-01 | 0.02±0.19 | 9.74E-01 | 0.09±0.19 | 8.60E-01 |
| 2-hydroxydecanoate | 0.06±0.06 | 0 | 6.59E-01 | 7.36E-01 | 0.01±0.14 | 0 | 8.72E-01 | 9.92E-01 | 0.07±0.19 | 9.32E-01 | 0.03±0.2 | 9.71E-01 | -0.02±0.19 | 9.74E-01 | -0.01±0.19 | 9.88E-01 |
| (2 or 3)-decenoate (10:1n7 or n8) | -0.09±0.15 | 49.43 | 1.60E-01 | 7.83E-01 | -0.01±0.14 | 72.89 | 5.48E-02 | 9.93E-01 | 0.35±0.22 | 8.16E-01 | 0.34±0.23 | 8.44E-01 | -0.2±0.17 | 5.81E-01 | -0.21±0.18 | 5.91E-01 |
| C-glycosyltryptophan | -0.04±0.13 | 33.12 | 2.21E-01 | 8.91E-01 | 0.01±0.15 | 7.79 | 2.98E-01 | 9.93E-01 | -0.16±0.23 | 8.55E-01 | -0.16±0.22 | 8.59E-01 | 0.16±0.21 | 7.57E-01 | 0.16±0.21 | 7.60E-01 |
| X - 12830 | -0.02±0.02 | 0 | 5.40E-01 | 7.36E-01 | 0.01±0.14 | 0 | 5.71E-01 | 9.93E-01 | -0.02±0.2 | 9.90E-01 | -0.07±0.2 | 9.33E-01 | 0.11±0.2 | 8.27E-01 | 0.09±0.2 | 8.60E-01 |
| octadecanedioylcarnitine (C18-DC)* | -0.07±0.07 | 0 | 6.57E-01 | 7.36E-01 | -0.01±0.14 | 0 | 6.43E-01 | 9.93E-01 | -0.08±0.2 | 9.19E-01 | -0.08±0.2 | 9.27E-01 | 0.02±0.2 | 9.82E-01 | 0.06±0.21 | 9.16E-01 |
| pregnenolone sulfate | -0.01±0.01 | 0 | 8.08E-01 | 7.36E-01 | 0.01±0.14 | 0 | 9.16E-01 | 9.93E-01 | -0.01±0.2 | 9.92E-01 | 0.03±0.21 | 9.76E-01 | -0.06±0.19 | 9.16E-01 | -0.01±0.2 | 9.94E-01 |
| X - 21607 | 0.03±0.04 | 0 | 7.97E-01 | 7.36E-01 | 0.01±0.14 | 0 | 9.83E-01 | 9.93E-01 | 0.04±0.19 | 9.57E-01 | 0.01±0.2 | 9.88E-01 | -0.01±0.18 | 9.83E-01 | 0.01±0.19 | 9.94E-01 |
| X - 22162 | 0.05±0.17 | 68.83 | 7.32E-02 | 9.02E-01 | 0.01±0.16 | 72.75 | 5.54E-02 | 9.93E-01 | -0.23±0.22 | 8.55E-01 | -0.28±0.22 | 8.44E-01 | 0.37±0.25 | 4.58E-01 | 0.34±0.24 | 5.22E-01 |
| X - 25420 | 0.09±0.18 | 49.06 | 1.61E-01 | 8.14E-01 | -0.01±0.15 | 76.74 | 3.81E-02 | 9.93E-01 | -0.38±0.2 | 8.16E-01 | -0.32±0.21 | 8.44E-01 | 0.22±0.2 | 6.00E-01 | 0.29±0.2 | 5.31E-01 |
| tetradecadienedioate (C14:2-DC)* | -0.08±0.16 | 45.83 | 1.74E-01 | 8.26E-01 | 0.01±0.13 | 77.55 | 3.48E-02 | 9.93E-01 | 0.36±0.2 | 8.16E-01 | 0.32±0.2 | 8.44E-01 | -0.18±0.18 | 6.31E-01 | -0.24±0.18 | 5.44E-01 |
| X - 13728 | -0.05±0.1 | 21.53 | 2.59E-01 | 8.26E-01 | 0.01±0.13 | 6.2 | 3.02E-01 | 9.93E-01 | -0.12±0.19 | 8.72E-01 | -0.12±0.18 | 8.59E-01 | 0.14±0.19 | 7.79E-01 | 0.15±0.2 | 7.48E-01 |
| 1-(1-enyl-stearoyl)-2-arachidonoyl-GPE (P-18:0/20:4)* | -0.05±0.07 | 0 | 3.41E-01 | 7.51E-01 | 0.01±0.15 | 5.56 | 3.03E-01 | 9.93E-01 | -0.07±0.2 | 9.32E-01 | -0.14±0.21 | 8.59E-01 | 0.16±0.22 | 7.79E-01 | 0.18±0.22 | 7.48E-01 |
| N6,N6,N6-trimethyllysine | -0.06±0.13 | 27.2 | 2.41E-01 | 8.32E-01 | -0.01±0.15 | 4.62 | 3.06E-01 | 9.93E-01 | -0.15±0.2 | 8.55E-01 | -0.15±0.2 | 8.59E-01 | 0.18±0.24 | 7.57E-01 | 0.16±0.22 | 7.79E-01 |
| 3-(3-amino-3-carboxypropyl)uridine* | -0.04±0.12 | 33.79 | 2.19E-01 | 8.77E-01 | -0.01±0.15 | 13.79 | 2.81E-01 | 9.93E-01 | -0.15±0.22 | 8.55E-01 | -0.17±0.21 | 8.47E-01 | 0.17±0.21 | 7.49E-01 | 0.14±0.2 | 7.80E-01 |
| X - 14939 | 0.04±0.12 | 19.85 | 2.64E-01 | 8.71E-01 | -0.01±0.14 | 0 | 3.43E-01 | 9.93E-01 | 0.16±0.21 | 8.55E-01 | 0.15±0.22 | 8.59E-01 | -0.11±0.19 | 8.00E-01 | -0.12±0.19 | 7.94E-01 |
| X - 11849 | 0.05±0.15 | 2.12 | 3.12E-01 | 8.77E-01 | 0.01±0.15 | 0 | 3.84E-01 | 9.93E-01 | 0.23±0.22 | 8.40E-01 | 0.15±0.22 | 8.59E-01 | -0.07±0.19 | 9.06E-01 | -0.11±0.2 | 8.33E-01 |
| N-palmitoyl-heptadecasphingosine (d17:1/16:0)* | -0.07±0.07 | 0 | 7.88E-01 | 7.36E-01 | 0.01±0.14 | 0 | 8.31E-01 | 9.93E-01 | -0.07±0.19 | 9.21E-01 | -0.02±0.19 | 9.76E-01 | -0.02±0.19 | 9.80E-01 | 0.04±0.2 | 9.47E-01 |
| p-cresol sulfate | 0.13±0.18 | 77.75 | 3.40E-02 | 7.60E-01 | -0.01±0.14 | 87.07 | 5.42E-03 | 9.94E-01 | -0.25±0.17 | 8.16E-01 | -0.27±0.17 | 8.44E-01 | 0.5±0.25 | 3.07E-01 | 0.58±0.25 | 2.84E-01 |
| X - 11852 | 0.12±0.16 | 62.95 | 1.00E-01 | 7.44E-01 | 0.01±0.15 | 80.03 | 2.52E-02 | 9.94E-01 | -0.31±0.23 | 8.40E-01 | -0.4±0.23 | 8.44E-01 | 0.28±0.19 | 4.57E-01 | 0.28±0.19 | 5.19E-01 |
| N-acetylglucosamine/N-acetylgalactosamine | -0.06±0.08 | 0 | 3.67E-01 | 7.60E-01 | -0.01±0.15 | 0 | 4.75E-01 | 9.94E-01 | -0.08±0.2 | 9.13E-01 | -0.11±0.21 | 8.86E-01 | 0.12±0.21 | 8.13E-01 | 0.1±0.21 | 8.57E-01 |
| sphingomyelin (d17:2/16:0, d18:2/15:0)* | -0.07±0.09 | 0 | 4.69E-01 | 7.44E-01 | -0.01±0.16 | 0 | 5.17E-01 | 9.94E-01 | -0.1±0.23 | 9.06E-01 | -0.12±0.24 | 8.97E-01 | 0.08±0.23 | 9.15E-01 | 0.09±0.22 | 8.69E-01 |
| palmitoleate (16:1n7) | -0.17±0.19 | 55.85 | 1.32E-01 | 7.36E-01 | 0±0.16 | 85.07 | 9.64E-03 | 9.94E-01 | 0.46±0.23 | 8.16E-01 | 0.44±0.23 | 8.44E-01 | -0.3±0.21 | 4.81E-01 | -0.37±0.21 | 4.20E-01 |
| 10-heptadecenoate (17:1n7) | -0.11±0.17 | 53.25 | 1.44E-01 | 7.80E-01 | 0±0.14 | 78.28 | 3.19E-02 | 9.94E-01 | 0.36±0.21 | 8.16E-01 | 0.35±0.22 | 8.44E-01 | -0.24±0.19 | 5.56E-01 | -0.27±0.19 | 5.23E-01 |
| 1-oleoyl-GPI (18:1) | 0.03±0.15 | 60.23 | 1.13E-01 | 9.46E-01 | 0±0.15 | 65.61 | 8.81E-02 | 9.94E-01 | -0.21±0.2 | 8.55E-01 | -0.26±0.21 | 8.44E-01 | 0.26±0.21 | 5.56E-01 | 0.26±0.21 | 5.88E-01 |
| phenylpyruvate | -0.02±0.12 | 55.19 | 1.35E-01 | 9.51E-01 | 0±0.13 | 51.21 | 1.52E-01 | 9.94E-01 | -0.15±0.18 | 8.55E-01 | -0.16±0.17 | 8.45E-01 | 0.23±0.2 | 5.99E-01 | 0.22±0.2 | 6.14E-01 |
| 3-hydroxy-2-methylpyridine sulfate | -0.03±0.14 | 19.3 | 2.66E-01 | 9.23E-01 | 0±0.13 | 38.51 | 2.02E-01 | 9.94E-01 | -0.21±0.19 | 8.40E-01 | -0.18±0.2 | 8.45E-01 | 0.1±0.18 | 8.24E-01 | 0.16±0.18 | 7.02E-01 |
| cytidine | -0.02±0.12 | 45.04 | 1.77E-01 | 9.32E-01 | 0±0.14 | 20.19 | 2.63E-01 | 9.94E-01 | -0.15±0.19 | 8.55E-01 | -0.16±0.2 | 8.45E-01 | 0.18±0.19 | 6.76E-01 | 0.14±0.19 | 7.63E-01 |
| X - 18888 | 0.05±0.12 | 19.06 | 2.66E-01 | 8.49E-01 | 0±0.14 | 0 | 3.22E-01 | 9.94E-01 | 0.16±0.19 | 8.55E-01 | 0.13±0.19 | 8.59E-01 | -0.13±0.2 | 7.96E-01 | -0.15±0.21 | 7.79E-01 |
| X - 21442 | -0.06±0.08 | 0 | 4.08E-01 | 7.60E-01 | 0±0.14 | 0 | 3.80E-01 | 9.94E-01 | -0.08±0.18 | 9.03E-01 | -0.1±0.18 | 8.78E-01 | 0.09±0.19 | 8.56E-01 | 0.14±0.21 | 7.89E-01 |
| X - 11847 | 0.07±0.12 | 0 | 3.83E-01 | 7.91E-01 | -0.01±0.15 | 0 | 6.23E-01 | 9.94E-01 | 0.15±0.21 | 8.55E-01 | 0.07±0.22 | 9.33E-01 | -0.06±0.19 | 9.16E-01 | -0.07±0.2 | 8.94E-01 |
| 1-oleoyl-2-docosahexaenoyl-GPC (18:1/22:6)* | 0.04±0.06 | 0 | 4.05E-01 | 7.44E-01 | -0.01±0.14 | 0 | 8.03E-01 | 9.94E-01 | 0.06±0.2 | 9.38E-01 | 0.03±0.21 | 9.71E-01 | -0.1±0.18 | 8.23E-01 | -0.04±0.19 | 9.47E-01 |
| 16a-hydroxy DHEA 3-sulfate | 0±0 | 0 | 8.94E-01 | 7.36E-01 | 0±0.14 | 0 | 1.00E+00 | 9.95E-01 | 0±0.19 | 9.94E-01 | 0±0.2 | 9.99E-01 | 0.02±0.19 | 9.74E-01 | 0±0.19 | 9.95E-01 |
| 3-phenylpropionate (hydrocinnamate) | -0.04±0.05 | 0 | 7.60E-01 | 7.36E-01 | 0±0.14 | 0 | 7.39E-01 | 9.96E-01 | -0.05±0.19 | 9.53E-01 | -0.04±0.2 | 9.61E-01 | 0.01±0.19 | 9.82E-01 | 0.05±0.2 | 9.27E-01 |
| X - 12101 | 0.03±0.16 | 54.21 | 1.39E-01 | 9.42E-01 | 0±0.15 | 62.78 | 1.01E-01 | 9.97E-01 | -0.24±0.21 | 8.40E-01 | -0.26±0.22 | 8.44E-01 | 0.23±0.21 | 6.00E-01 | 0.24±0.21 | 6.06E-01 |
| 1-stearoyl-GPE (18:0) | -0.07±0.11 | 0 | 3.94E-01 | 7.72E-01 | 0±0.16 | 7.64 | 2.98E-01 | 9.97E-01 | -0.13±0.21 | 8.72E-01 | -0.15±0.22 | 8.59E-01 | 0.08±0.21 | 8.93E-01 | 0.18±0.23 | 7.56E-01 |
| behenoyl dihydrosphingomyelin (d18:0/22:0)* | -0.03±0.04 | 0 | 3.54E-01 | 7.40E-01 | 0±0.15 | 0 | 4.64E-01 | 9.97E-01 | -0.04±0.21 | 9.57E-01 | -0.11±0.21 | 8.87E-01 | 0.15±0.21 | 7.64E-01 | 0.1±0.2 | 8.44E-01 |
| margarate (17:0) | -0.02±0.14 | 49.5 | 1.59E-01 | 9.58E-01 | 0±0.14 | 57.74 | 1.24E-01 | 9.98E-01 | 0.22±0.2 | 8.40E-01 | 0.23±0.2 | 8.44E-01 | -0.19±0.19 | 6.48E-01 | -0.2±0.19 | 6.42E-01 |
| X - 21733 | -0.09±0.16 | 47.33 | 1.68E-01 | 7.91E-01 | 0±0.15 | 60.8 | 1.10E-01 | 9.98E-01 | 0.38±0.24 | 8.16E-01 | 0.32±0.25 | 8.44E-01 | -0.2±0.18 | 6.00E-01 | -0.17±0.19 | 6.88E-01 |
| valylglycine | -0.05±0.11 | 18.14 | 2.69E-01 | 8.32E-01 | 0±0.14 | 41.82 | 1.90E-01 | 9.98E-01 | -0.14±0.19 | 8.55E-01 | -0.18±0.19 | 8.45E-01 | 0.12±0.19 | 7.94E-01 | 0.19±0.2 | 6.88E-01 |
| guanidinoacetate | -0.08±0.1 | 0 | 5.38E-01 | 7.44E-01 | 0±0.15 | 0 | 4.42E-01 | 9.98E-01 | -0.12±0.2 | 8.72E-01 | -0.11±0.21 | 8.86E-01 | 0.02±0.2 | 9.74E-01 | 0.12±0.22 | 8.27E-01 |
| deoxycholate | -0.05±0.08 | 17.76 | 2.70E-01 | 7.80E-01 | 0±0.15 | 0 | 4.69E-01 | 9.98E-01 | -0.08±0.2 | 9.09E-01 | -0.11±0.21 | 8.87E-01 | 0.16±0.21 | 7.57E-01 | 0.1±0.21 | 8.47E-01 |
| 3,5-dichloro-2,6-dihydroxybenzoic acid | -0.05±0.06 | 0 | 5.64E-01 | 7.36E-01 | 0±0.16 | 0 | 6.84E-01 | 9.98E-01 | -0.06±0.24 | 9.54E-01 | -0.09±0.26 | 9.33E-01 | 0.06±0.21 | 9.16E-01 | 0.05±0.2 | 9.31E-01 |
| sphingomyelin (d18:1/19:0, d19:1/18:0)* | -0.08±0.09 | 0 | 5.95E-01 | 7.37E-01 | 0±0.16 | 0 | 7.05E-01 | 9.98E-01 | -0.1±0.24 | 9.12E-01 | -0.07±0.24 | 9.40E-01 | 0.02±0.2 | 9.78E-01 | 0.05±0.2 | 9.31E-01 |
| 4-hydroxyphenylacetate | 0.08±0.08 | 0 | 6.99E-01 | 7.36E-01 | 0±0.15 | 0 | 9.23E-01 | 9.98E-01 | 0.09±0.21 | 9.09E-01 | 0.02±0.22 | 9.84E-01 | 0.01±0.2 | 9.94E-01 | -0.01±0.2 | 9.88E-01 |
| methionine sulfone | -0.03±0.15 | 31.67 | 2.26E-01 | 9.35E-01 | 0±0.15 | 56.11 | 1.31E-01 | 1.00E+00 | -0.22±0.21 | 8.55E-01 | -0.23±0.22 | 8.44E-01 | 0.15±0.21 | 7.79E-01 | 0.23±0.22 | 6.29E-01 |
| X - 21821 | 0±0 | 0 | 8.86E-01 | 7.36E-01 | 0±0.14 | 0 | 8.84E-01 | 1.00E+00 | 0±0.2 | 9.99E-01 | -0.02±0.21 | 9.76E-01 | 0.03±0.19 | 9.69E-01 | 0.02±0.19 | 9.76E-01 |
| propionylglycine |  |  |  |  |  |  |  |  | -0.43±0.17 | 8.16E-01 | -0.48±0.17 | 8.44E-01 |  |  |  |  |
| adrenate (22:4n6) |  |  |  |  |  |  |  |  | 0.43±0.21 | 8.16E-01 | 0.42±0.21 | 8.44E-01 |  |  |  |  |
| N-acetyltaurine |  |  |  |  |  |  |  |  | -0.41±0.21 | 8.16E-01 | -0.42±0.22 | 8.44E-01 |  |  |  |  |
| 1,2,3-benzenetriol sulfate (2) |  |  |  |  |  |  |  |  | -0.36±0.21 | 8.16E-01 | -0.4±0.21 | 8.44E-01 |  |  |  |  |
| catechol glucuronide |  |  |  |  |  |  |  |  | -0.59±0.23 | 8.16E-01 | -0.57±0.23 | 8.44E-01 |  |  |  |  |
| docosatrienoate (22:3n6)* |  |  |  |  |  |  |  |  | 0.34±0.2 | 8.16E-01 | 0.34±0.21 | 8.44E-01 |  |  |  |  |
| nisinate (24:6n3) |  |  |  |  |  |  |  |  | 0.34±0.22 | 8.16E-01 | 0.31±0.23 | 8.44E-01 |  |  |  |  |
| docosahexaenoylcarnitine (C22:6)* |  |  |  |  |  |  |  |  | 0.32±0.21 | 8.16E-01 | 0.28±0.22 | 8.44E-01 |  |  |  |  |
| 3-hydroxyhippurate sulfate |  |  |  |  |  |  |  |  | -0.28±0.19 | 8.16E-01 | -0.32±0.19 | 8.44E-01 |  |  |  |  |
| (2,4 or 2,5)-dimethylphenol sulfate |  |  |  |  |  |  |  |  | -0.35±0.21 | 8.16E-01 | -0.39±0.25 | 8.44E-01 |  |  |  |  |
| 2-hydroxyfluorene sulfate |  |  |  |  |  |  |  |  | -0.6±0.27 | 8.16E-01 | -0.8±0.32 | 8.44E-01 |  |  |  |  |
| 3-hydroxypyridine glucuronide |  |  |  |  |  |  |  |  | -0.47±0.23 | 8.16E-01 | -0.44±0.24 | 8.44E-01 |  |  |  |  |
| dihydrocaffeate sulfate (2) |  |  |  |  |  |  |  |  | -0.42±0.21 | 8.16E-01 | -0.43±0.21 | 8.44E-01 |  |  |  |  |
| N,N-dimethyl-5-aminovalerate |  |  |  |  |  |  |  |  | -0.4±0.19 | 8.16E-01 | -0.45±0.19 | 8.44E-01 |  |  |  |  |
| 5-hydroxy-2-methylpyridine sulfate |  |  |  |  |  |  |  |  | -0.7±0.26 | 8.16E-01 | -0.69±0.26 | 8.44E-01 |  |  |  |  |
| oxindolylalanine |  |  |  |  |  |  |  |  | 0.42±0.21 | 8.16E-01 | 0.38±0.21 | 8.44E-01 |  |  |  |  |
| hypoxanthine |  |  |  |  |  |  |  |  | 0.33±0.21 | 8.16E-01 | 0.34±0.22 | 8.44E-01 |  |  |  |  |
| sucrose |  |  |  |  |  |  |  |  | -0.34±0.22 | 8.16E-01 | -0.31±0.23 | 8.44E-01 |  |  |  |  |
| X - 12261 |  |  |  |  |  |  |  |  | -0.37±0.21 | 8.16E-01 | -0.36±0.22 | 8.44E-01 |  |  |  |  |
| X - 12738 |  |  |  |  |  |  |  |  | -0.67±0.24 | 8.16E-01 | -0.64±0.24 | 8.44E-01 |  |  |  |  |
| X - 14056 |  |  |  |  |  |  |  |  | -0.47±0.2 | 8.16E-01 | -0.46±0.2 | 8.44E-01 |  |  |  |  |
| X - 14082 |  |  |  |  |  |  |  |  | -0.28±0.19 | 8.16E-01 | -0.3±0.19 | 8.44E-01 |  |  |  |  |
| X - 16124 |  |  |  |  |  |  |  |  | -0.34±0.22 | 8.16E-01 | -0.38±0.22 | 8.44E-01 |  |  |  |  |
| X - 17346 |  |  |  |  |  |  |  |  | -0.41±0.2 | 8.16E-01 | -0.4±0.2 | 8.44E-01 |  |  |  |  |
| X - 23648 |  |  |  |  |  |  |  |  | -0.28±0.18 | 8.16E-01 | -0.29±0.19 | 8.44E-01 |  |  |  |  |
| X - 23666 |  |  |  |  |  |  |  |  | -0.33±0.19 | 8.16E-01 | -0.31±0.19 | 8.44E-01 |  |  |  |  |
| X - 12015 |  |  |  |  |  |  |  |  | 0.3±0.2 | 8.19E-01 | 0.3±0.2 | 8.44E-01 |  |  |  |  |
| X - 23276 |  |  |  |  |  |  |  |  | 0.27±0.19 | 8.19E-01 | 0.32±0.2 | 8.44E-01 |  |  |  |  |
| X - 21312 |  |  |  |  |  |  |  |  | -0.27±0.2 | 8.35E-01 | -0.34±0.21 | 8.44E-01 |  |  |  |  |
| 1-stearoyl-2-oleoyl-GPS (18:0/18:1) |  |  |  |  |  |  |  |  | 0.28±0.21 | 8.40E-01 | 0.27±0.21 | 8.44E-01 |  |  |  |  |
| N-acetylaspartate (NAA) |  |  |  |  |  |  |  |  | 0.29±0.24 | 8.40E-01 | 0.26±0.24 | 8.44E-01 |  |  |  |  |
| 1-palmitoylglycerol (16:0) |  |  |  |  |  |  |  |  | -0.21±0.19 | 8.40E-01 | -0.2±0.19 | 8.44E-01 |  |  |  |  |
| gulonate* |  |  |  |  |  |  |  |  | -0.25±0.19 | 8.40E-01 | -0.21±0.2 | 8.44E-01 |  |  |  |  |
| cyclo(gly-pro) |  |  |  |  |  |  |  |  | -0.24±0.2 | 8.40E-01 | -0.23±0.2 | 8.44E-01 |  |  |  |  |
| dimethyl sulfone |  |  |  |  |  |  |  |  | -0.26±0.23 | 8.40E-01 | -0.26±0.22 | 8.44E-01 |  |  |  |  |
| 2,3-dihydroxy-2-methylbutyrate |  |  |  |  |  |  |  |  | -0.21±0.19 | 8.40E-01 | -0.21±0.19 | 8.44E-01 |  |  |  |  |
| stearoylcholine* |  |  |  |  |  |  |  |  | -0.21±0.17 | 8.40E-01 | -0.21±0.18 | 8.44E-01 |  |  |  |  |
| linoleoylcholine* |  |  |  |  |  |  |  |  | -0.19±0.18 | 8.40E-01 | -0.2±0.18 | 8.44E-01 |  |  |  |  |
| dihydroferulic acid sulfate |  |  |  |  |  |  |  |  | -0.25±0.21 | 8.40E-01 | -0.26±0.21 | 8.44E-01 |  |  |  |  |
| cholate |  |  |  |  |  |  |  |  | -0.3±0.23 | 8.40E-01 | -0.32±0.23 | 8.44E-01 |  |  |  |  |
| inosine |  |  |  |  |  |  |  |  | 0.3±0.22 | 8.40E-01 | 0.32±0.22 | 8.44E-01 |  |  |  |  |
| X - 23583 |  |  |  |  |  |  |  |  | -0.2±0.19 | 8.40E-01 | -0.23±0.19 | 8.44E-01 |  |  |  |  |
| X - 24411 |  |  |  |  |  |  |  |  | 0.25±0.22 | 8.40E-01 | 0.24±0.22 | 8.44E-01 |  |  |  |  |
| X - 24728 |  |  |  |  |  |  |  |  | -0.23±0.21 | 8.40E-01 | -0.27±0.22 | 8.44E-01 |  |  |  |  |
| X - 25948 |  |  |  |  |  |  |  |  | -0.23±0.2 | 8.40E-01 | -0.26±0.21 | 8.44E-01 |  |  |  |  |
| X - 18345 |  |  |  |  |  |  |  |  | -0.21±0.2 | 8.51E-01 | -0.26±0.21 | 8.44E-01 |  |  |  |  |
| succinoyltaurine |  |  |  |  |  |  |  |  | 0.21±0.21 | 8.55E-01 | 0.27±0.21 | 8.44E-01 |  |  |  |  |
| 1-arachidonylglycerol (20:4) |  |  |  |  |  |  |  |  | 0.19±0.2 | 8.55E-01 | 0.21±0.2 | 8.44E-01 |  |  |  |  |
| bilirubin degradation product, C17H20N2O5 (1)** |  |  |  |  |  |  |  |  | 0.27±0.22 | 8.40E-01 | 0.23±0.22 | 8.44E-01 |  |  |  |  |
| X - 12126 |  |  |  |  |  |  |  |  | 0.24±0.22 | 8.40E-01 | 0.23±0.23 | 8.44E-01 |  |  |  |  |
| X - 21327 |  |  |  |  |  |  |  |  | 0.2±0.23 | 8.55E-01 | 0.24±0.23 | 8.44E-01 |  |  |  |  |
| X - 23997 |  |  |  |  |  |  |  |  | -0.17±0.18 | 8.55E-01 | -0.19±0.18 | 8.44E-01 |  |  |  |  |
| bilirubin degradation product, C16H18N2O5 (2)** |  |  |  |  |  |  |  |  | 0.24±0.2 | 8.40E-01 | 0.18±0.21 | 8.45E-01 |  |  |  |  |
| bilirubin degradation product, C17H20N2O5 (2)** |  |  |  |  |  |  |  |  | 0.26±0.22 | 8.40E-01 | 0.21±0.22 | 8.45E-01 |  |  |  |  |
| bilirubin degradation product, C16H18N2O5 (3)** |  |  |  |  |  |  |  |  | 0.23±0.2 | 8.40E-01 | 0.17±0.21 | 8.45E-01 |  |  |  |  |
| S-methylmethionine |  |  |  |  |  |  |  |  | -0.17±0.21 | 8.55E-01 | -0.19±0.21 | 8.45E-01 |  |  |  |  |
| dihydroferulate |  |  |  |  |  |  |  |  | -0.18±0.2 | 8.55E-01 | -0.18±0.2 | 8.45E-01 |  |  |  |  |
| mannonate* |  |  |  |  |  |  |  |  | -0.21±0.21 | 8.55E-01 | -0.21±0.22 | 8.45E-01 |  |  |  |  |
| ferulic acid 4-sulfate |  |  |  |  |  |  |  |  | -0.17±0.2 | 8.55E-01 | -0.18±0.2 | 8.45E-01 |  |  |  |  |
| palmitoylcholine |  |  |  |  |  |  |  |  | -0.15±0.18 | 8.55E-01 | -0.16±0.19 | 8.45E-01 |  |  |  |  |
| docosahexaenoylcholine |  |  |  |  |  |  |  |  | -0.13±0.18 | 8.55E-01 | -0.16±0.19 | 8.45E-01 |  |  |  |  |
| 3-indoleglyoxylic acid |  |  |  |  |  |  |  |  | -0.17±0.2 | 8.55E-01 | -0.17±0.2 | 8.45E-01 |  |  |  |  |
| cystine |  |  |  |  |  |  |  |  | 0.2±0.21 | 8.55E-01 | 0.21±0.22 | 8.45E-01 |  |  |  |  |
| X - 24295 |  |  |  |  |  |  |  |  | -0.18±0.2 | 8.55E-01 | -0.2±0.21 | 8.45E-01 |  |  |  |  |
| X - 24531 |  |  |  |  |  |  |  |  | -0.17±0.2 | 8.55E-01 | -0.18±0.2 | 8.45E-01 |  |  |  |  |
| X - 24951 |  |  |  |  |  |  |  |  | -0.16±0.21 | 8.55E-01 | -0.18±0.21 | 8.45E-01 |  |  |  |  |
| X - 12713 |  |  |  |  |  |  |  |  | -0.12±0.19 | 8.72E-01 | -0.17±0.2 | 8.45E-01 |  |  |  |  |
| X - 13658 |  |  |  |  |  |  |  |  | -0.11±0.21 | 8.90E-01 | -0.2±0.22 | 8.45E-01 |  |  |  |  |
| glucuronide of piperine metabolite C17H21NO3 (5)* |  |  |  |  |  |  |  |  | -0.07±0.2 | 9.30E-01 | -0.18±0.2 | 8.45E-01 |  |  |  |  |
| bilirubin degradation product, C16H18N2O5 (1)** |  |  |  |  |  |  |  |  | 0.23±0.21 | 8.40E-01 | 0.17±0.22 | 8.52E-01 |  |  |  |  |
| tetrahydrocortisol sulfate (1) |  |  |  |  |  |  |  |  | 0.12±0.21 | 8.81E-01 | 0.17±0.22 | 8.52E-01 |  |  |  |  |
| (R)-3-hydroxybutyrylcarnitine |  |  |  |  |  |  |  |  | 0.19±0.19 | 8.55E-01 | 0.16±0.2 | 8.53E-01 |  |  |  |  |
| trans-3,4-methyleneheptanoate |  |  |  |  |  |  |  |  | 0.15±0.21 | 8.55E-01 | 0.17±0.21 | 8.53E-01 |  |  |  |  |
| 5-hydroxyindoleacetate |  |  |  |  |  |  |  |  | 0.14±0.21 | 8.55E-01 | 0.16±0.21 | 8.54E-01 |  |  |  |  |
| X - 12013 |  |  |  |  |  |  |  |  | -0.18±0.21 | 8.55E-01 | -0.16±0.21 | 8.54E-01 |  |  |  |  |
| dopamine 4-sulfate |  |  |  |  |  |  |  |  | -0.15±0.21 | 8.55E-01 | -0.16±0.21 | 8.58E-01 |  |  |  |  |
| cysteine s-sulfate |  |  |  |  |  |  |  |  | 0.17±0.2 | 8.55E-01 | 0.16±0.21 | 8.58E-01 |  |  |  |  |
| isoeugenol sulfate |  |  |  |  |  |  |  |  | -0.16±0.2 | 8.55E-01 | -0.16±0.21 | 8.58E-01 |  |  |  |  |
| N-formylanthranilic acid |  |  |  |  |  |  |  |  | -0.12±0.2 | 8.72E-01 | -0.16±0.21 | 8.58E-01 |  |  |  |  |
| lithocholate |  |  |  |  |  |  |  |  | -0.1±0.2 | 8.99E-01 | -0.15±0.2 | 8.58E-01 |  |  |  |  |
| histidine betaine (hercynine)* |  |  |  |  |  |  |  |  | 0.21±0.2 | 8.40E-01 | 0.15±0.2 | 8.59E-01 |  |  |  |  |
| ribonate |  |  |  |  |  |  |  |  | -0.17±0.21 | 8.55E-01 | -0.15±0.21 | 8.59E-01 |  |  |  |  |
| 5alpha-androstan-3alpha,17alpha-diol monosulfate |  |  |  |  |  |  |  |  | 0.14±0.2 | 8.55E-01 | 0.14±0.21 | 8.59E-01 |  |  |  |  |
| suberoylcarnitine (C8-DC) |  |  |  |  |  |  |  |  | 0.16±0.2 | 8.55E-01 | 0.14±0.2 | 8.59E-01 |  |  |  |  |
| nervonoylcarnitine (C24:1)* |  |  |  |  |  |  |  |  | 0.16±0.19 | 8.55E-01 | 0.14±0.2 | 8.59E-01 |  |  |  |  |
| bilirubin degradation product, C16H18N2O5 (4)** |  |  |  |  |  |  |  |  | 0.19±0.2 | 8.55E-01 | 0.14±0.21 | 8.59E-01 |  |  |  |  |
| gamma-glutamylalanine |  |  |  |  |  |  |  |  | 0.14±0.22 | 8.70E-01 | 0.15±0.22 | 8.59E-01 |  |  |  |  |
| X - 21959 |  |  |  |  |  |  |  |  | 0.11±0.19 | 8.75E-01 | 0.15±0.2 | 8.59E-01 |  |  |  |  |
| 5-acetylamino-6-formylamino-3-methyluracil |  |  |  |  |  |  |  |  | -0.09±0.2 | 9.04E-01 | -0.15±0.2 | 8.59E-01 |  |  |  |  |
| behenoylcarnitine (C22)* |  |  |  |  |  |  |  |  | -0.08±0.2 | 9.12E-01 | -0.14±0.21 | 8.59E-01 |  |  |  |  |
| 1-oleoyl-GPG (18:1)* |  |  |  |  |  |  |  |  | 0.15±0.21 | 8.55E-01 | 0.14±0.21 | 8.61E-01 |  |  |  |  |
| xanthosine |  |  |  |  |  |  |  |  | 0.14±0.2 | 8.55E-01 | 0.13±0.2 | 8.63E-01 |  |  |  |  |
| dihomo-linolenoyl-choline |  |  |  |  |  |  |  |  | -0.1±0.18 | 8.80E-01 | -0.12±0.18 | 8.63E-01 |  |  |  |  |
| gentisate |  |  |  |  |  |  |  |  | 0.16±0.21 | 8.55E-01 | 0.13±0.21 | 8.65E-01 |  |  |  |  |
| oleoylcholine |  |  |  |  |  |  |  |  | -0.1±0.19 | 8.81E-01 | -0.11±0.19 | 8.70E-01 |  |  |  |  |
| trans-2-hexenoylglycine |  |  |  |  |  |  |  |  | 0.1±0.2 | 8.99E-01 | 0.12±0.21 | 8.70E-01 |  |  |  |  |
| hexanoylglycine |  |  |  |  |  |  |  |  | 0.14±0.2 | 8.55E-01 | 0.12±0.2 | 8.75E-01 |  |  |  |  |
| prolylglycine |  |  |  |  |  |  |  |  | -0.07±0.21 | 9.32E-01 | -0.12±0.21 | 8.82E-01 |  |  |  |  |
| X - 26054 |  |  |  |  |  |  |  |  | 0.07±0.2 | 9.38E-01 | 0.11±0.21 | 8.84E-01 |  |  |  |  |
| glucuronide of piperine metabolite C17H21NO3 (3)* |  |  |  |  |  |  |  |  | -0.01±0.2 | 9.94E-01 | -0.11±0.2 | 8.84E-01 |  |  |  |  |
| X - 18935 |  |  |  |  |  |  |  |  | 0.12±0.2 | 8.75E-01 | 0.11±0.21 | 8.87E-01 |  |  |  |  |
| 4-hydroxy-2-oxoglutaric acid |  |  |  |  |  |  |  |  | 0.14±0.2 | 8.55E-01 | 0.11±0.21 | 8.90E-01 |  |  |  |  |
| suberate (C8-DC) |  |  |  |  |  |  |  |  | -0.09±0.2 | 9.06E-01 | -0.1±0.2 | 9.09E-01 |  |  |  |  |
| 2,2'-Methylenebis(6-tert-butyl-p-cresol) |  |  |  |  |  |  |  |  | 0.08±0.18 | 9.09E-01 | 0.09±0.19 | 9.11E-01 |  |  |  |  |
| delta-CEHC |  |  |  |  |  |  |  |  | -0.09±0.2 | 9.03E-01 | -0.09±0.2 | 9.13E-01 |  |  |  |  |
| X - 23641 |  |  |  |  |  |  |  |  | -0.09±0.19 | 9.03E-01 | -0.08±0.19 | 9.19E-01 |  |  |  |  |
| X - 12007 |  |  |  |  |  |  |  |  | -0.07±0.2 | 9.30E-01 | -0.08±0.2 | 9.27E-01 |  |  |  |  |
| X - 12726 |  |  |  |  |  |  |  |  | -0.06±0.19 | 9.38E-01 | -0.08±0.2 | 9.27E-01 |  |  |  |  |
| palmitoyl ethanolamide |  |  |  |  |  |  |  |  | 0.09±0.2 | 9.06E-01 | 0.08±0.2 | 9.27E-01 |  |  |  |  |
| 1-oleoylglycerol (18:1) |  |  |  |  |  |  |  |  | 0.09±0.2 | 9.06E-01 | 0.08±0.21 | 9.27E-01 |  |  |  |  |
| X - 24953 |  |  |  |  |  |  |  |  | -0.05±0.21 | 9.54E-01 | -0.09±0.21 | 9.27E-01 |  |  |  |  |
| X - 13007 |  |  |  |  |  |  |  |  | 0.1±0.21 | 8.99E-01 | 0.08±0.22 | 9.27E-01 |  |  |  |  |
| alpha-CEHC sulfate |  |  |  |  |  |  |  |  | -0.06±0.2 | 9.38E-01 | -0.08±0.2 | 9.27E-01 |  |  |  |  |
| iminodiacetate (IDA) |  |  |  |  |  |  |  |  | -0.06±0.2 | 9.38E-01 | -0.08±0.21 | 9.27E-01 |  |  |  |  |
| X - 21410 |  |  |  |  |  |  |  |  | -0.03±0.19 | 9.70E-01 | -0.07±0.19 | 9.27E-01 |  |  |  |  |
| 2,4-di-tert-butylphenol |  |  |  |  |  |  |  |  | -0.04±0.21 | 9.57E-01 | -0.08±0.21 | 9.28E-01 |  |  |  |  |
| 4-hydroxyphenylpyruvate |  |  |  |  |  |  |  |  | 0.04±0.21 | 9.57E-01 | 0.08±0.21 | 9.28E-01 |  |  |  |  |
| malonylcarnitine |  |  |  |  |  |  |  |  | 0.12±0.22 | 8.81E-01 | 0.07±0.22 | 9.33E-01 |  |  |  |  |
| 2-linoleoylglycerol (18:2) |  |  |  |  |  |  |  |  | 0.11±0.21 | 8.93E-01 | 0.07±0.21 | 9.33E-01 |  |  |  |  |
| X - 12127 |  |  |  |  |  |  |  |  | 0.07±0.2 | 9.30E-01 | 0.07±0.2 | 9.33E-01 |  |  |  |  |
| docosatrienoate (22:3n3) |  |  |  |  |  |  |  |  | -0.06±0.19 | 9.38E-01 | -0.06±0.19 | 9.33E-01 |  |  |  |  |
| arachidonoylcholine |  |  |  |  |  |  |  |  | -0.06±0.19 | 9.38E-01 | -0.07±0.19 | 9.33E-01 |  |  |  |  |
| gamma-glutamylhistidine |  |  |  |  |  |  |  |  | -0.06±0.2 | 9.44E-01 | -0.07±0.21 | 9.33E-01 |  |  |  |  |
| phenol glucuronide |  |  |  |  |  |  |  |  | -0.02±0.19 | 9.83E-01 | -0.06±0.19 | 9.33E-01 |  |  |  |  |
| X - 25519 |  |  |  |  |  |  |  |  | -0.08±0.21 | 9.25E-01 | -0.07±0.22 | 9.35E-01 |  |  |  |  |
| 4-hydroxyphenylacetoylcarnitine |  |  |  |  |  |  |  |  | 0.13±0.22 | 8.72E-01 | 0.07±0.22 | 9.38E-01 |  |  |  |  |
| 1-dihomo-linolenylglycerol (20:3) |  |  |  |  |  |  |  |  | 0.07±0.21 | 9.36E-01 | 0.07±0.21 | 9.38E-01 |  |  |  |  |
| X - 12839 |  |  |  |  |  |  |  |  | 0.04±0.2 | 9.54E-01 | 0.06±0.2 | 9.40E-01 |  |  |  |  |
| galactonate |  |  |  |  |  |  |  |  | 0.07±0.2 | 9.32E-01 | 0.05±0.2 | 9.46E-01 |  |  |  |  |
| X - 23890 |  |  |  |  |  |  |  |  | -0.05±0.21 | 9.54E-01 | -0.05±0.21 | 9.55E-01 |  |  |  |  |
| 2-oleoylglycerol (18:1) |  |  |  |  |  |  |  |  | 0.06±0.2 | 9.38E-01 | 0.04±0.2 | 9.60E-01 |  |  |  |  |
| 17alpha-hydroxypregnenolone 3-sulfate |  |  |  |  |  |  |  |  | -0.07±0.19 | 9.30E-01 | -0.04±0.2 | 9.64E-01 |  |  |  |  |
| X - 26058 |  |  |  |  |  |  |  |  | 0.05±0.18 | 9.38E-01 | 0.04±0.18 | 9.64E-01 |  |  |  |  |
| fructose |  |  |  |  |  |  |  |  | 0.02±0.19 | 9.77E-01 | 0.03±0.21 | 9.71E-01 |  |  |  |  |
| 1-linolenoylglycerol (18:3) |  |  |  |  |  |  |  |  | -0.06±0.19 | 9.38E-01 | -0.03±0.2 | 9.71E-01 |  |  |  |  |
| X - 13729 |  |  |  |  |  |  |  |  | -0.01±0.2 | 9.90E-01 | -0.03±0.21 | 9.71E-01 |  |  |  |  |
| gamma-CEHC glucuronide* |  |  |  |  |  |  |  |  | -0.02±0.21 | 9.86E-01 | 0.03±0.21 | 9.71E-01 |  |  |  |  |
| phosphate |  |  |  |  |  |  |  |  | 0.05±0.21 | 9.53E-01 | 0.03±0.22 | 9.73E-01 |  |  |  |  |
| X - 12096 |  |  |  |  |  |  |  |  | -0.05±0.21 | 9.54E-01 | -0.03±0.22 | 9.73E-01 |  |  |  |  |
| cholic acid glucuronide |  |  |  |  |  |  |  |  | -0.01±0.2 | 9.92E-01 | -0.03±0.2 | 9.73E-01 |  |  |  |  |
| X - 17351 |  |  |  |  |  |  |  |  | 0±0.2 | 9.96E-01 | -0.02±0.2 | 9.76E-01 |  |  |  |  |
| X - 11979 |  |  |  |  |  |  |  |  | 0±0.21 | 9.99E-01 | -0.02±0.21 | 9.76E-01 |  |  |  |  |
| GlcNAc sulfate conjugate of C21H34O2 steroid** |  |  |  |  |  |  |  |  | 0.03±0.21 | 9.73E-01 | 0.02±0.21 | 9.84E-01 |  |  |  |  |
| branched chain 14:0 dicarboxylic acid** |  |  |  |  |  |  |  |  | 0.03±0.19 | 9.70E-01 | 0.01±0.19 | 9.98E-01 |  |  |  |  |
| X - 12104 |  |  |  |  |  |  |  |  | -0.01±0.2 | 9.92E-01 | 0±0.2 | 9.99E-01 |  |  |  |  |
| methyl-4-hydroxybenzoate sulfate |  |  |  |  |  |  |  |  | 0.06±0.23 | 9.44E-01 | 0±0.24 | 1.00E+00 |  |  |  |  |
| 1-myristoylglycerol (14:0) |  |  |  |  |  |  |  |  | -0.01±0.19 | 9.90E-01 | 0±0.19 | 1.00E+00 |  |  |  |  |
| 1-carboxyethylphenylalanine |  |  |  |  |  |  |  |  |  |  |  |  | 0.85±0.19 | **2.83E-03** | 0.86±0.2 | **6.76E-03** |
| 1-carboxyethylleucine |  |  |  |  |  |  |  |  |  |  |  |  | 0.85±0.21 | **1.48E-02** | 0.86±0.22 | **3.40E-02** |
| 1-carboxyethyltyrosine |  |  |  |  |  |  |  |  |  |  |  |  | 0.82±0.21 | **1.84E-02** | 0.83±0.22 | **3.40E-02** |
| 1-carboxyethylisoleucine |  |  |  |  |  |  |  |  |  |  |  |  | 0.79±0.21 | **2.91E-02** | 0.8±0.22 | 6.49E-02 |
| 1-carboxyethylvaline |  |  |  |  |  |  |  |  |  |  |  |  | 0.71±0.2 | 5.06E-02 | 0.71±0.22 | 1.20E-01 |
| myristoyl-linoleoyl-glycerol (14:0/18:2) [2]* |  |  |  |  |  |  |  |  |  |  |  |  | 0.59±0.22 | 1.58E-01 | 0.57±0.23 | 2.38E-01 |
| X - 21829 |  |  |  |  |  |  |  |  |  |  |  |  | 0.51±0.19 | 1.73E-01 | 0.44±0.21 | 3.24E-01 |
| X - 25828 |  |  |  |  |  |  |  |  |  |  |  |  | -0.31±0.13 | 2.27E-01 | -0.27±0.14 | 3.86E-01 |
| palmitoyl-oleoyl-glycerol (16:0/18:1) [2]* |  |  |  |  |  |  |  |  |  |  |  |  | 0.49±0.22 | 2.35E-01 | 0.44±0.23 | 3.78E-01 |
| palmitoyl-docosahexaenoyl-glycerol (16:0/22:6) [1]* |  |  |  |  |  |  |  |  |  |  |  |  | 0.46±0.21 | 2.57E-01 | 0.42±0.22 | 3.70E-01 |
| 2-aminoadipate |  |  |  |  |  |  |  |  |  |  |  |  | 0.47±0.23 | 2.93E-01 | 0.44±0.23 | 3.86E-01 |
| palmitoyl-oleoyl-glycerol (16:0/18:1) [1]* |  |  |  |  |  |  |  |  |  |  |  |  | 0.44±0.22 | 3.03E-01 | 0.38±0.22 | 4.33E-01 |
| saccharin |  |  |  |  |  |  |  |  |  |  |  |  | 0.36±0.19 | 3.39E-01 | 0.39±0.19 | 3.38E-01 |
| 2-hydroxyglutarate |  |  |  |  |  |  |  |  |  |  |  |  | 0.42±0.23 | 3.68E-01 | 0.41±0.23 | 4.20E-01 |
| palmitoyl-arachidonoyl-glycerol (16:0/20:4) [2]* |  |  |  |  |  |  |  |  |  |  |  |  | 0.42±0.23 | 3.68E-01 | 0.37±0.23 | 4.67E-01 |
| dibutyl sulfosuccinate |  |  |  |  |  |  |  |  |  |  |  |  | 0.29±0.16 | 3.86E-01 | 0.28±0.16 | 4.32E-01 |
| palmitoleoyl-oleoyl-glycerol (16:1/18:1) [2]* |  |  |  |  |  |  |  |  |  |  |  |  | 0.39±0.23 | 4.10E-01 | 0.31±0.24 | 5.56E-01 |
| oleoyl-arachidonoyl-glycerol (18:1/20:4) [2]* |  |  |  |  |  |  |  |  |  |  |  |  | 0.36±0.23 | 4.50E-01 | 0.33±0.23 | 5.36E-01 |
| X - 24241 |  |  |  |  |  |  |  |  |  |  |  |  | 0.43±0.29 | 4.74E-01 | 0.49±0.31 | 4.67E-01 |
| diacylglycerol (16:1/18:2 [2], 16:0/18:3 [1])* |  |  |  |  |  |  |  |  |  |  |  |  | 0.3±0.21 | 4.81E-01 | 0.24±0.21 | 6.11E-01 |
| oleoyl-arachidonoyl-glycerol (18:1/20:4) [1]* |  |  |  |  |  |  |  |  |  |  |  |  | 0.33±0.24 | 4.96E-01 | 0.29±0.24 | 5.88E-01 |
| stearoyl-arachidonoyl-glycerol (18:0/20:4) [1]* |  |  |  |  |  |  |  |  |  |  |  |  | 0.28±0.22 | 5.52E-01 | 0.26±0.22 | 6.03E-01 |
| oleoyl-oleoyl-glycerol (18:1/18:1) [1]* |  |  |  |  |  |  |  |  |  |  |  |  | 0.3±0.24 | 5.56E-01 | 0.25±0.23 | 6.42E-01 |
| palmitoyl-linoleoyl-glycerol (16:0/18:2) [2]* |  |  |  |  |  |  |  |  |  |  |  |  | 0.25±0.21 | 5.71E-01 | 0.22±0.22 | 6.58E-01 |
| 1-methylhistidine |  |  |  |  |  |  |  |  |  |  |  |  | 0.23±0.2 | 5.92E-01 | 0.21±0.2 | 6.45E-01 |
| ceramide (d18:1/20:0, d16:1/22:0, d20:1/18:0)* |  |  |  |  |  |  |  |  |  |  |  |  | 0.32±0.28 | 5.96E-01 | 0.31±0.28 | 6.11E-01 |
| stearoyl-arachidonoyl-glycerol (18:0/20:4) [2]* |  |  |  |  |  |  |  |  |  |  |  |  | -0.2±0.18 | 6.02E-01 | -0.17±0.18 | 6.81E-01 |
| glycosyl ceramide (d18:1/23:1, d17:1/24:1)* |  |  |  |  |  |  |  |  |  |  |  |  | -0.17±0.18 | 6.66E-01 | -0.1±0.19 | 8.29E-01 |
| X - 16964 |  |  |  |  |  |  |  |  |  |  |  |  | 0.19±0.21 | 6.79E-01 | 0.21±0.21 | 6.58E-01 |
| 1-stearoyl-2-arachidonoyl-GPS (18:0/20:4) |  |  |  |  |  |  |  |  |  |  |  |  | 0.16±0.19 | 7.13E-01 | 0.17±0.19 | 6.92E-01 |
| linoleoyl-arachidonoyl-glycerol (18:2/20:4) [2]* |  |  |  |  |  |  |  |  |  |  |  |  | 0.18±0.21 | 7.15E-01 | 0.17±0.21 | 7.25E-01 |
| gamma-glutamyl-epsilon-lysine |  |  |  |  |  |  |  |  |  |  |  |  | 0.19±0.22 | 7.27E-01 | 0.15±0.22 | 7.81E-01 |
| linoleoyl-arachidonoyl-glycerol (18:2/20:4) [1]* |  |  |  |  |  |  |  |  |  |  |  |  | 0.16±0.21 | 7.57E-01 | 0.15±0.21 | 7.79E-01 |
| serotonin |  |  |  |  |  |  |  |  |  |  |  |  | -0.14±0.18 | 7.57E-01 | -0.1±0.18 | 8.27E-01 |
| X - 16938 |  |  |  |  |  |  |  |  |  |  |  |  | -0.14±0.19 | 7.75E-01 | -0.12±0.19 | 8.13E-01 |
| stearoyl ethanolamide |  |  |  |  |  |  |  |  |  |  |  |  | -0.12±0.18 | 7.79E-01 | -0.16±0.17 | 6.88E-01 |
| linoleoyl-linoleoyl-glycerol (18:2/18:2) [2]* |  |  |  |  |  |  |  |  |  |  |  |  | -0.12±0.17 | 7.79E-01 | -0.11±0.17 | 8.13E-01 |
| N1-Methyl-4-pyridone-3-carboxamide |  |  |  |  |  |  |  |  |  |  |  |  | 0.14±0.2 | 7.79E-01 | 0.1±0.2 | 8.47E-01 |
| 3-hydroxymyristate |  |  |  |  |  |  |  |  |  |  |  |  | 0.14±0.2 | 7.79E-01 | 0.09±0.2 | 8.59E-01 |
| X - 13684 |  |  |  |  |  |  |  |  |  |  |  |  | 0.14±0.2 | 7.85E-01 | 0.07±0.2 | 8.98E-01 |
| palmitoleoyl-linoleoyl-glycerol (16:1/18:2) [1]* |  |  |  |  |  |  |  |  |  |  |  |  | 0.12±0.2 | 8.01E-01 | 0.07±0.2 | 8.88E-01 |
| linoleoyl-linolenoyl-glycerol (18:2/18:3) [2]* |  |  |  |  |  |  |  |  |  |  |  |  | -0.09±0.18 | 8.32E-01 | -0.06±0.19 | 9.14E-01 |
| X - 22771 |  |  |  |  |  |  |  |  |  |  |  |  | 0.1±0.2 | 8.37E-01 | 0.11±0.2 | 8.27E-01 |
| X - 12849 |  |  |  |  |  |  |  |  |  |  |  |  | -0.08±0.18 | 8.85E-01 | -0.06±0.19 | 9.12E-01 |
| palmitoleoyl-arachidonoyl-glycerol (16:1/20:4) [2]* |  |  |  |  |  |  |  |  |  |  |  |  | 0.07±0.2 | 9.07E-01 | 0.07±0.2 | 8.98E-01 |
| thymidine |  |  |  |  |  |  |  |  |  |  |  |  | -0.06±0.18 | 9.16E-01 | -0.06±0.18 | 9.12E-01 |
| linoleoyl-linoleoyl-glycerol (18:2/18:2) [1]* |  |  |  |  |  |  |  |  |  |  |  |  | 0.01±0.18 | 9.82E-01 | 0.1±0.19 | 8.45E-01 |
| beta-sitosterol |  |  |  |  |  |  |  |  |  |  |  |  | -0.01±0.19 | 9.91E-01 | 0.11±0.21 | 8.44E-01 |
| 3-methylcrotonylglycine |  |  |  |  |  |  |  |  |  |  |  |  | 0.01±0.18 | 9.91E-01 | 0.04±0.19 | 9.38E-01 |
| N-behenoyl-sphingadienine (d18:2/22:0)* |  |  |  |  |  |  |  |  |  |  |  |  | 0±0.19 | 9.95E-01 | 0.04±0.2 | 9.47E-01 |
| (2-butoxyethoxy)acetic acid |  |  |  |  |  |  |  |  |  |  |  |  | 0±0.19 | 1.00E+00 | -0.02±0.19 | 9.77E-01 |

Note: The table shows associations from the logistic regression analysis where plasms metabolomics level from the Metabolon platform was main exposure and prevalent breast cancer as the main outcome in ERGO-4 and ERGO-5, respectively. ERGO-4 and ERGO-5 have 939 overlapping circulating metabolites, and ERGO-4&5 refers to the correlation between prevalent breast cancer and 939 metabolites derived from meta-analysis of ERGO-4 and ERGO-5. Model 1: adjusted for age, cohort, and lipid lowering medication. Model 2: adjusted for model1, smoking status, BMI, alcohol and education. The table is ranked based on the *FDR* values in model 2 within the E4&E5 cohort, using bold text indicates metabolites are surpassing *FDR* adjusted *P* value threshold (*P*<0.05). ERGO-4 and ERGO-5 refer to the fourth and fifth visits subcohort from the baseline cohort. Abbreviations: SE, standard error; I^2^: heterogeneity parameter, Het. *P*-value: significance of the heterogeneity; BMI, body mass index; FDR, false discovery rate.

**Supplementary Table 11. Serum metabolites associated with prevalent prostate cancer from the Nightingale platform at baseline.**

| **Metabolite** | **ERGO-4&5** | | | | | | **ERGO-4** | | | | | | **ERGO-5** | | | | | |
| --- | --- | --- | --- | --- | --- | --- | --- | --- | --- | --- | --- | --- | --- | --- | --- | --- | --- | --- |
|  | **Model 1** | | | **Model 2** | | | **Model 1** | | | **Model 2** | | | **Model 1** | | | **Model 2** | | |
|  | **Beta** | **SE** | ***FDR* value** | **Beta** | **SE** | ***FDR* value** | **Beta** | **SE** | ***FDR* value** | **Beta** | **SE** | ***FDR* value** | **Beta** | **SE** | ***FDR* value** | **Beta** | **SE** | ***FDR* value** |
| Citrate | -0.24 | 0.10 | 9.96E-01 | -0.25 | 0.10 | 9.99E-01 | -0.24 | 0.11 | 8.62E-01 | -0.25 | 0.11 | 8.83E-01 | -0.25 | 0.21 | 9.34E-01 | -0.24 | 0.30 | 9.86E-01 |
| Lactate | -0.23 | 0.10 | 9.96E-01 | -0.24 | 0.10 | 9.99E-01 | -0.30 | 0.11 | 8.62E-01 | -0.30 | 0.11 | 8.83E-01 | 0.08 | 0.24 | 9.34E-01 | 0.07 | 0.76 | 9.86E-01 |
| Ratio of monounsaturated fatty acids to total fatty acids | -0.22 | 0.10 | 9.96E-01 | -0.24 | 0.11 | 9.99E-01 | -0.25 | 0.11 | 8.62E-01 | -0.26 | 0.12 | 8.83E-01 | -0.09 | 0.24 | 9.34E-01 | -0.17 | 0.50 | 9.86E-01 |
| Ratio of docosahexaenoic acid to total fatty acids | 0.19 | 0.09 | 9.96E-01 | 0.21 | 0.09 | 9.99E-01 | 0.19 | 0.10 | 8.62E-01 | 0.20 | 0.11 | 8.83E-01 | 0.24 | 0.20 | 9.34E-01 | 0.27 | 0.20 | 9.86E-01 |
| Phenylalanine | -0.22 | 0.10 | 9.96E-01 | -0.22 | 0.11 | 9.99E-01 | -0.23 | 0.12 | 8.62E-01 | -0.22 | 0.12 | 8.83E-01 | -0.16 | 0.26 | 9.34E-01 | -0.33 | 0.24 | 9.86E-01 |
| Pyruvate | -0.17 | 0.10 | 9.96E-01 | -0.18 | 0.10 | 9.99E-01 | -0.26 | 0.11 | 8.62E-01 | -0.26 | 0.11 | 8.83E-01 | 0.14 | 0.22 | 9.34E-01 | 0.14 | 0.51 | 9.86E-01 |
| Ratio of omega-3 fatty acids to total fatty acids | 0.17 | 0.10 | 9.96E-01 | 0.19 | 0.10 | 9.99E-01 | 0.13 | 0.11 | 8.62E-01 | 0.15 | 0.11 | 8.83E-01 | 0.38 | 0.23 | 9.34E-01 | 0.39 | 0.09 | 9.86E-01 |
| Acetate | 0.19 | 0.10 | 9.96E-01 | 0.19 | 0.10 | 9.99E-01 | 0.20 | 0.11 | 8.62E-01 | 0.19 | 0.11 | 8.83E-01 | 0.12 | 0.27 | 9.34E-01 | 0.15 | 0.60 | 9.86E-01 |
| Ratio of omega-6 fatty acids to omega-3 fatty acids | -0.16 | 0.10 | 9.96E-01 | -0.17 | 0.10 | 9.99E-01 | -0.11 | 0.11 | 8.62E-01 | -0.13 | 0.11 | 8.83E-01 | -0.40 | 0.22 | 9.34E-01 | -0.40 | 0.09 | 9.86E-01 |
| Ratio of polyunsaturated fatty acids to monounsaturated fatty acids | 0.16 | 0.10 | 9.96E-01 | 0.19 | 0.11 | 9.99E-01 | 0.22 | 0.12 | 8.62E-01 | 0.23 | 0.12 | 8.83E-01 | -0.04 | 0.23 | 9.50E-01 | 0.05 | 0.84 | 9.89E-01 |
| Docosahexaenoic acid | 0.15 | 0.09 | 9.96E-01 | 0.16 | 0.10 | 9.99E-01 | 0.10 | 0.10 | 8.62E-01 | 0.12 | 0.11 | 8.83E-01 | 0.34 | 0.21 | 9.34E-01 | 0.34 | 0.11 | 9.86E-01 |
| Degree of unsaturation | 0.15 | 0.10 | 9.96E-01 | 0.16 | 0.10 | 9.99E-01 | 0.17 | 0.11 | 8.62E-01 | 0.17 | 0.11 | 8.83E-01 | 0.07 | 0.23 | 9.34E-01 | 0.15 | 0.53 | 9.86E-01 |
| Cholesteryl esters in chylomicrons and extremely large VLDL | -0.14 | 0.09 | 9.96E-01 | -0.16 | 0.10 | 9.99E-01 | -0.20 | 0.10 | 8.62E-01 | -0.20 | 0.11 | 8.83E-01 | 0.14 | 0.25 | 9.34E-01 | 0.10 | 0.71 | 9.86E-01 |
| Valine | -0.16 | 0.11 | 9.96E-01 | -0.18 | 0.11 | 9.99E-01 | -0.16 | 0.12 | 8.62E-01 | -0.14 | 0.13 | 8.83E-01 | -0.18 | 0.25 | 9.34E-01 | -0.39 | 0.16 | 9.86E-01 |
| Phospholipids to total lipids ratio in very large VLDL | -0.13 | 0.09 | 9.96E-01 | -0.14 | 0.10 | 9.99E-01 | -0.14 | 0.10 | 8.62E-01 | -0.15 | 0.10 | 8.83E-01 | -0.10 | 0.28 | 9.34E-01 | -0.02 | 0.94 | 9.89E-01 |
| Total concentration of branched-chain amino acids (leucine + isoleucine + valine) | -0.15 | 0.11 | 9.96E-01 | -0.17 | 0.12 | 9.99E-01 | -0.16 | 0.12 | 8.62E-01 | -0.14 | 0.13 | 8.83E-01 | -0.17 | 0.26 | 9.34E-01 | -0.39 | 0.16 | 9.86E-01 |
| Cholesterol in chylomicrons and extremely large VLDL | -0.13 | 0.10 | 9.96E-01 | -0.15 | 0.10 | 9.99E-01 | -0.20 | 0.10 | 8.62E-01 | -0.21 | 0.11 | 8.83E-01 | 0.19 | 0.26 | 9.34E-01 | 0.15 | 0.58 | 9.86E-01 |
| Monounsaturated fatty acids | -0.13 | 0.10 | 9.96E-01 | -0.15 | 0.10 | 9.99E-01 | -0.20 | 0.11 | 8.62E-01 | -0.20 | 0.12 | 8.83E-01 | 0.11 | 0.21 | 9.34E-01 | 0.05 | 0.81 | 9.86E-01 |
| Free cholesterol to total lipids ratio in medium LDL | 0.13 | 0.10 | 9.96E-01 | 0.15 | 0.11 | 9.99E-01 | 0.22 | 0.12 | 8.62E-01 | 0.22 | 0.13 | 8.83E-01 | -0.20 | 0.21 | 9.34E-01 | -0.14 | 0.54 | 9.86E-01 |
| Omega-3 fatty acids | 0.12 | 0.10 | 9.96E-01 | 0.13 | 0.10 | 9.99E-01 | 0.06 | 0.11 | 9.02E-01 | 0.08 | 0.11 | 8.83E-01 | 0.39 | 0.23 | 9.34E-01 | 0.38 | 0.11 | 9.86E-01 |
| Free cholesterol to total lipids ratio in small LDL | 0.13 | 0.10 | 9.96E-01 | 0.14 | 0.11 | 9.99E-01 | 0.24 | 0.12 | 8.62E-01 | 0.24 | 0.13 | 8.83E-01 | -0.24 | 0.20 | 9.34E-01 | -0.21 | 0.31 | 9.86E-01 |
| Phospholipids to total lipids ratio in large VLDL | -0.11 | 0.09 | 9.96E-01 | -0.13 | 0.10 | 9.99E-01 | -0.16 | 0.10 | 8.62E-01 | -0.17 | 0.10 | 8.83E-01 | 0.44 | 0.40 | 9.34E-01 | 0.45 | 0.30 | 9.86E-01 |
| Cholesteryl esters to total lipids ratio in medium LDL | -0.12 | 0.10 | 9.96E-01 | -0.13 | 0.11 | 9.99E-01 | -0.15 | 0.12 | 8.62E-01 | -0.14 | 0.12 | 8.83E-01 | 0.00 | 0.23 | 9.98E-01 | -0.08 | 0.73 | 9.86E-01 |
| Leucine | -0.14 | 0.11 | 9.96E-01 | -0.15 | 0.12 | 9.99E-01 | -0.14 | 0.13 | 8.62E-01 | -0.11 | 0.13 | 8.83E-01 | -0.21 | 0.26 | 9.34E-01 | -0.45 | 0.12 | 9.86E-01 |
| Free cholesterol to total lipids ratio in large LDL | 0.12 | 0.11 | 9.96E-01 | 0.14 | 0.11 | 9.99E-01 | 0.21 | 0.12 | 8.62E-01 | 0.20 | 0.13 | 8.83E-01 | -0.20 | 0.22 | 9.34E-01 | -0.10 | 0.66 | 9.86E-01 |
| Phospholipids to total lipids ratio in IDL | -0.11 | 0.10 | 9.96E-01 | -0.13 | 0.10 | 9.99E-01 | -0.16 | 0.12 | 8.62E-01 | -0.18 | 0.12 | 8.83E-01 | 0.00 | 0.21 | 9.98E-01 | 0.06 | 0.79 | 9.86E-01 |
| Phospholipids to total lipids ratio in medium LDL | 0.12 | 0.11 | 9.96E-01 | 0.13 | 0.11 | 9.99E-01 | 0.17 | 0.12 | 8.62E-01 | 0.16 | 0.13 | 8.83E-01 | -0.06 | 0.22 | 9.34E-01 | 0.02 | 0.93 | 9.89E-01 |
| Average diameter for LDL particles | 0.12 | 0.10 | 9.96E-01 | 0.13 | 0.11 | 9.99E-01 | 0.20 | 0.12 | 8.62E-01 | 0.19 | 0.12 | 8.83E-01 | -0.23 | 0.22 | 9.34E-01 | -0.12 | 0.60 | 9.86E-01 |
| Triglycerides in chylomicrons and extremely large VLDL | -0.11 | 0.10 | 9.96E-01 | -0.12 | 0.10 | 9.99E-01 | -0.16 | 0.11 | 8.62E-01 | -0.15 | 0.11 | 8.83E-01 | -0.01 | 0.21 | 9.98E-01 | -0.04 | 0.84 | 9.89E-01 |
| Total lipids in chylomicrons and extremely large VLDL | -0.11 | 0.10 | 9.96E-01 | -0.12 | 0.10 | 9.99E-01 | -0.19 | 0.11 | 8.62E-01 | -0.18 | 0.12 | 8.83E-01 | 0.16 | 0.24 | 9.34E-01 | 0.13 | 0.62 | 9.86E-01 |
| Isoleucine | -0.12 | 0.11 | 9.96E-01 | -0.13 | 0.11 | 9.99E-01 | -0.15 | 0.12 | 8.62E-01 | -0.13 | 0.13 | 8.83E-01 | -0.03 | 0.26 | 9.85E-01 | -0.21 | 0.46 | 9.86E-01 |
| Cholesterol to total lipids ratio in IDL | 0.11 | 0.11 | 9.96E-01 | 0.12 | 0.11 | 9.99E-01 | 0.23 | 0.13 | 8.62E-01 | 0.24 | 0.13 | 8.83E-01 | -0.32 | 0.20 | 9.34E-01 | -0.27 | 0.20 | 9.86E-01 |
| Triglycerides to total lipids ratio in small LDL | -0.11 | 0.10 | 9.96E-01 | -0.12 | 0.11 | 9.99E-01 | -0.23 | 0.12 | 8.62E-01 | -0.23 | 0.12 | 8.83E-01 | 0.36 | 0.22 | 9.34E-01 | 0.29 | 0.21 | 9.86E-01 |
| Linoleic acid | -0.11 | 0.10 | 9.96E-01 | -0.11 | 0.10 | 9.99E-01 | -0.14 | 0.11 | 8.62E-01 | -0.14 | 0.11 | 8.83E-01 | 0.02 | 0.22 | 9.98E-01 | 0.02 | 0.92 | 9.89E-01 |
| Phospholipids in very large VLDL | -0.10 | 0.10 | 9.96E-01 | -0.11 | 0.10 | 9.99E-01 | -0.16 | 0.11 | 8.62E-01 | -0.15 | 0.11 | 8.83E-01 | 0.17 | 0.27 | 9.34E-01 | 0.12 | 0.67 | 9.86E-01 |
| Cholesteryl esters to total lipids ratio in small LDL | -0.11 | 0.10 | 9.96E-01 | -0.11 | 0.10 | 9.99E-01 | -0.14 | 0.11 | 8.62E-01 | -0.13 | 0.12 | 8.83E-01 | 0.01 | 0.22 | 9.98E-01 | -0.02 | 0.95 | 9.89E-01 |
| Triglycerides in small LDL | -0.09 | 0.10 | 9.96E-01 | -0.11 | 0.10 | 9.99E-01 | -0.21 | 0.11 | 8.62E-01 | -0.21 | 0.12 | 8.83E-01 | 0.31 | 0.21 | 9.34E-01 | 0.27 | 0.22 | 9.86E-01 |
| Free cholesterol in very large VLDL | -0.09 | 0.10 | 9.96E-01 | -0.10 | 0.10 | 9.99E-01 | -0.14 | 0.11 | 8.62E-01 | -0.14 | 0.12 | 8.83E-01 | 0.14 | 0.25 | 9.34E-01 | 0.09 | 0.72 | 9.86E-01 |
| Ratio of saturated fatty acids to total fatty acids | 0.09 | 0.09 | 9.96E-01 | 0.10 | 0.10 | 9.99E-01 | 0.01 | 0.11 | 9.90E-01 | 0.03 | 0.11 | 9.77E-01 | 0.36 | 0.20 | 9.34E-01 | 0.32 | 0.14 | 9.86E-01 |
| Free cholesterol in chylomicrons and extremely large VLDL | -0.09 | 0.10 | 9.96E-01 | -0.10 | 0.10 | 9.99E-01 | -0.18 | 0.11 | 8.62E-01 | -0.18 | 0.11 | 8.83E-01 | 0.28 | 0.26 | 9.34E-01 | 0.24 | 0.35 | 9.86E-01 |
| Phospholipids to total lipids ratio in small LDL | 0.10 | 0.10 | 9.96E-01 | 0.10 | 0.11 | 9.99E-01 | 0.14 | 0.12 | 8.62E-01 | 0.12 | 0.12 | 8.83E-01 | -0.06 | 0.23 | 9.34E-01 | 0.00 | 0.99 | 9.95E-01 |
| Cholesterol in very large VLDL | -0.08 | 0.10 | 9.96E-01 | -0.10 | 0.10 | 9.99E-01 | -0.14 | 0.11 | 8.62E-01 | -0.13 | 0.11 | 8.83E-01 | 0.13 | 0.24 | 9.34E-01 | 0.09 | 0.72 | 9.86E-01 |
| Phospholipids in very large HDL | -0.08 | 0.10 | 9.96E-01 | -0.10 | 0.10 | 9.99E-01 | -0.07 | 0.11 | 8.62E-01 | -0.11 | 0.11 | 8.83E-01 | -0.13 | 0.22 | 9.34E-01 | -0.05 | 0.82 | 9.86E-01 |
| Triglycerides to total lipids ratio in chylomicrons and extremely large VLDL | -0.09 | 0.10 | 9.96E-01 | -0.09 | 0.10 | 9.99E-01 | -0.10 | 0.11 | 8.62E-01 | -0.09 | 0.11 | 8.83E-01 | -0.09 | 0.20 | 9.34E-01 | -0.12 | 0.56 | 9.86E-01 |
| Phospholipids to total lipids ratio in very small VLDL | -0.07 | 0.09 | 9.96E-01 | -0.09 | 0.09 | 9.99E-01 | -0.14 | 0.10 | 8.62E-01 | -0.15 | 0.11 | 8.83E-01 | 0.16 | 0.21 | 9.34E-01 | 0.17 | 0.42 | 9.86E-01 |
| Cholesteryl esters to total lipids ratio in very large HDL | 0.07 | 0.09 | 9.96E-01 | 0.08 | 0.09 | 9.99E-01 | 0.08 | 0.10 | 8.62E-01 | 0.09 | 0.11 | 8.83E-01 | 0.07 | 0.19 | 9.34E-01 | 0.06 | 0.77 | 9.86E-01 |
| Phospholipids to total lipids ratio in very large HDL | -0.07 | 0.08 | 9.96E-01 | -0.08 | 0.09 | 9.99E-01 | -0.05 | 0.10 | 9.20E-01 | -0.08 | 0.10 | 8.83E-01 | -0.13 | 0.17 | 9.34E-01 | -0.08 | 0.67 | 9.86E-01 |
| Total lipids in very large HDL | -0.09 | 0.11 | 9.96E-01 | -0.10 | 0.11 | 9.99E-01 | -0.09 | 0.12 | 8.62E-01 | -0.13 | 0.12 | 8.83E-01 | -0.07 | 0.26 | 9.34E-01 | 0.03 | 0.92 | 9.89E-01 |
| Cholesterol to total lipids ratio in very large HDL | 0.08 | 0.10 | 9.96E-01 | 0.09 | 0.10 | 9.99E-01 | 0.09 | 0.11 | 8.62E-01 | 0.12 | 0.11 | 8.83E-01 | 0.01 | 0.22 | 9.98E-01 | -0.03 | 0.89 | 9.89E-01 |
| Triglycerides to total lipids ratio in medium LDL | -0.09 | 0.10 | 9.96E-01 | -0.10 | 0.11 | 9.99E-01 | -0.23 | 0.12 | 8.62E-01 | -0.23 | 0.12 | 8.83E-01 | 0.53 | 0.23 | 9.34E-01 | 0.49 | 0.04 | 9.86E-01 |
| Ratio of polyunsaturated fatty acids to total fatty acids | 0.08 | 0.10 | 9.96E-01 | 0.10 | 0.11 | 9.99E-01 | 0.15 | 0.12 | 8.62E-01 | 0.15 | 0.12 | 8.83E-01 | -0.15 | 0.20 | 9.34E-01 | -0.08 | 0.73 | 9.86E-01 |
| Triglycerides to total lipids ratio in IDL | -0.08 | 0.10 | 9.96E-01 | -0.10 | 0.11 | 9.99E-01 | -0.21 | 0.12 | 8.62E-01 | -0.21 | 0.12 | 8.83E-01 | 0.45 | 0.22 | 9.34E-01 | 0.38 | 0.11 | 9.86E-01 |
| Cholesteryl esters in very large VLDL | -0.08 | 0.10 | 9.96E-01 | -0.09 | 0.10 | 9.99E-01 | -0.12 | 0.11 | 8.62E-01 | -0.12 | 0.11 | 8.83E-01 | 0.11 | 0.24 | 9.34E-01 | 0.08 | 0.75 | 9.86E-01 |
| Ratio of linoleic acid to total fatty acids | -0.08 | 0.10 | 9.96E-01 | -0.09 | 0.11 | 9.99E-01 | -0.01 | 0.11 | 9.90E-01 | -0.04 | 0.12 | 9.77E-01 | -0.34 | 0.22 | 9.34E-01 | -0.29 | 0.24 | 9.86E-01 |
| Cholesteryl esters to total lipids ratio in IDL | 0.08 | 0.10 | 9.96E-01 | 0.09 | 0.11 | 9.99E-01 | 0.18 | 0.12 | 8.62E-01 | 0.19 | 0.12 | 8.83E-01 | -0.25 | 0.20 | 9.34E-01 | -0.23 | 0.27 | 9.86E-01 |
| Alanine | -0.08 | 0.10 | 9.96E-01 | -0.09 | 0.10 | 9.99E-01 | -0.11 | 0.11 | 8.62E-01 | -0.10 | 0.11 | 8.83E-01 | 0.03 | 0.23 | 9.76E-01 | -0.04 | 0.87 | 9.89E-01 |
| Omega-6 fatty acids | -0.08 | 0.10 | 9.96E-01 | -0.09 | 0.10 | 9.99E-01 | -0.13 | 0.11 | 8.62E-01 | -0.13 | 0.11 | 8.83E-01 | 0.10 | 0.23 | 9.34E-01 | 0.09 | 0.69 | 9.86E-01 |
| Concentration of very large HDL particles | -0.09 | 0.11 | 9.96E-01 | -0.10 | 0.12 | 9.99E-01 | -0.10 | 0.12 | 8.62E-01 | -0.14 | 0.13 | 8.83E-01 | -0.01 | 0.27 | 9.98E-01 | 0.08 | 0.76 | 9.86E-01 |
| Cholesterol in very large HDL | -0.09 | 0.11 | 9.96E-01 | -0.10 | 0.11 | 9.99E-01 | -0.08 | 0.12 | 8.62E-01 | -0.13 | 0.13 | 8.83E-01 | -0.09 | 0.26 | 9.34E-01 | 0.02 | 0.95 | 9.89E-01 |
| Concentration of chylomicrons and extremely large VLDL particles | -0.07 | 0.10 | 9.96E-01 | -0.09 | 0.10 | 9.99E-01 | -0.15 | 0.11 | 8.62E-01 | -0.15 | 0.11 | 8.83E-01 | 0.22 | 0.26 | 9.34E-01 | 0.18 | 0.51 | 9.86E-01 |
| Cholesterol to total lipids ratio in very small VLDL | 0.08 | 0.10 | 9.96E-01 | 0.09 | 0.11 | 9.99E-01 | 0.18 | 0.12 | 8.62E-01 | 0.17 | 0.12 | 8.83E-01 | -0.38 | 0.23 | 9.34E-01 | -0.28 | 0.25 | 9.86E-01 |
| Cholesteryl esters to total lipids ratio in very small VLDL | 0.08 | 0.10 | 9.96E-01 | 0.09 | 0.11 | 9.99E-01 | 0.17 | 0.12 | 8.62E-01 | 0.17 | 0.12 | 8.83E-01 | -0.34 | 0.22 | 9.34E-01 | -0.25 | 0.28 | 9.86E-01 |
| Total lipids in very large VLDL | -0.07 | 0.10 | 9.96E-01 | -0.09 | 0.11 | 9.99E-01 | -0.14 | 0.11 | 8.62E-01 | -0.13 | 0.12 | 8.83E-01 | 0.20 | 0.25 | 9.34E-01 | 0.14 | 0.61 | 9.86E-01 |
| Phospholipids in chylomicrons and extremely large VLDL | -0.07 | 0.10 | 9.96E-01 | -0.09 | 0.11 | 9.99E-01 | -0.16 | 0.11 | 8.62E-01 | -0.16 | 0.11 | 8.83E-01 | 0.34 | 0.31 | 9.34E-01 | 0.31 | 0.32 | 9.86E-01 |
| Phospholipids to total lipids ratio in large HDL | 0.07 | 0.09 | 9.96E-01 | 0.08 | 0.10 | 9.99E-01 | 0.03 | 0.11 | 9.90E-01 | 0.06 | 0.11 | 9.49E-01 | 0.23 | 0.20 | 9.34E-01 | 0.15 | 0.49 | 9.86E-01 |
| 3-Hydroxybutyrate | -0.08 | 0.11 | 9.96E-01 | -0.09 | 0.11 | 9.99E-01 | -0.05 | 0.12 | 9.48E-01 | -0.06 | 0.12 | 9.49E-01 | -0.19 | 0.25 | 9.34E-01 | -0.18 | 0.50 | 9.86E-01 |
| Total fatty acids | -0.07 | 0.10 | 9.96E-01 | -0.08 | 0.10 | 9.99E-01 | -0.15 | 0.11 | 8.62E-01 | -0.14 | 0.12 | 8.83E-01 | 0.19 | 0.21 | 9.34E-01 | 0.14 | 0.51 | 9.86E-01 |
| Triglycerides to total lipids ratio in large LDL | -0.07 | 0.10 | 9.96E-01 | -0.08 | 0.10 | 9.99E-01 | -0.21 | 0.11 | 8.62E-01 | -0.21 | 0.12 | 8.83E-01 | 0.50 | 0.22 | 9.34E-01 | 0.45 | 0.05 | 9.86E-01 |
| Concentration of very large VLDL particles | -0.07 | 0.10 | 9.96E-01 | -0.08 | 0.10 | 9.99E-01 | -0.13 | 0.11 | 8.62E-01 | -0.12 | 0.11 | 8.83E-01 | 0.22 | 0.25 | 9.34E-01 | 0.16 | 0.55 | 9.86E-01 |
| Cholesteryl esters in very large HDL | -0.08 | 0.11 | 9.96E-01 | -0.09 | 0.11 | 9.99E-01 | -0.08 | 0.12 | 8.62E-01 | -0.13 | 0.13 | 8.83E-01 | -0.06 | 0.26 | 9.46E-01 | 0.05 | 0.86 | 9.89E-01 |
| Average diameter for VLDL particles | -0.07 | 0.10 | 9.96E-01 | -0.08 | 0.10 | 9.99E-01 | -0.10 | 0.11 | 8.62E-01 | -0.08 | 0.11 | 9.10E-01 | 0.02 | 0.23 | 9.86E-01 | -0.05 | 0.82 | 9.86E-01 |
| Phospholipids in VLDL | -0.06 | 0.10 | 9.96E-01 | -0.08 | 0.10 | 9.99E-01 | -0.13 | 0.11 | 8.62E-01 | -0.13 | 0.11 | 8.83E-01 | 0.16 | 0.22 | 9.34E-01 | 0.13 | 0.55 | 9.86E-01 |
| Triglycerides to total lipids ratio in large VLDL | 0.07 | 0.10 | 9.96E-01 | 0.08 | 0.10 | 9.99E-01 | 0.09 | 0.11 | 8.62E-01 | 0.10 | 0.11 | 8.83E-01 | 0.00 | 0.26 | 9.98E-01 | -0.09 | 0.72 | 9.86E-01 |
| Phospholipids in large VLDL | -0.06 | 0.10 | 9.96E-01 | -0.08 | 0.10 | 9.99E-01 | -0.12 | 0.11 | 8.62E-01 | -0.12 | 0.11 | 8.83E-01 | 0.21 | 0.25 | 9.34E-01 | 0.15 | 0.57 | 9.86E-01 |
| Triglycerides in medium LDL | -0.06 | 0.10 | 9.96E-01 | -0.07 | 0.10 | 9.99E-01 | -0.20 | 0.11 | 8.62E-01 | -0.21 | 0.11 | 8.83E-01 | 0.45 | 0.21 | 9.34E-01 | 0.41 | 0.06 | 9.86E-01 |
| Triglycerides to total lipids ratio in very small VLDL | -0.08 | 0.11 | 9.96E-01 | -0.08 | 0.11 | 9.99E-01 | -0.18 | 0.12 | 8.62E-01 | -0.17 | 0.12 | 8.83E-01 | 0.45 | 0.26 | 9.34E-01 | 0.33 | 0.24 | 9.86E-01 |
| Total lipids in VLDL | -0.06 | 0.10 | 9.96E-01 | -0.07 | 0.10 | 9.99E-01 | -0.13 | 0.11 | 8.62E-01 | -0.12 | 0.11 | 8.83E-01 | 0.16 | 0.21 | 9.34E-01 | 0.12 | 0.59 | 9.86E-01 |
| Free cholesterol in VLDL | -0.06 | 0.10 | 9.96E-01 | -0.07 | 0.10 | 9.99E-01 | -0.12 | 0.11 | 8.62E-01 | -0.12 | 0.11 | 8.83E-01 | 0.15 | 0.22 | 9.34E-01 | 0.13 | 0.57 | 9.86E-01 |
| Free cholesterol to total lipids ratio in chylomicrons and extremely large VLDL | 0.06 | 0.10 | 9.96E-01 | 0.07 | 0.10 | 9.99E-01 | 0.08 | 0.11 | 8.62E-01 | 0.07 | 0.11 | 9.30E-01 | 0.00 | 0.24 | 9.98E-01 | 0.07 | 0.78 | 9.86E-01 |
| Concentration of small LDL particles | -0.06 | 0.10 | 9.96E-01 | -0.07 | 0.10 | 9.99E-01 | -0.12 | 0.11 | 8.62E-01 | -0.12 | 0.12 | 8.83E-01 | 0.15 | 0.22 | 9.34E-01 | 0.13 | 0.55 | 9.86E-01 |
| Glutamine | -0.07 | 0.10 | 9.96E-01 | -0.07 | 0.10 | 9.99E-01 | 0.02 | 0.11 | 9.90E-01 | 0.01 | 0.11 | 9.87E-01 | -0.48 | 0.24 | 9.34E-01 | -0.48 | 0.06 | 9.86E-01 |
| VLDL cholesterol | -0.06 | 0.10 | 9.96E-01 | -0.07 | 0.10 | 9.99E-01 | -0.11 | 0.11 | 8.62E-01 | -0.12 | 0.11 | 8.83E-01 | 0.13 | 0.22 | 9.34E-01 | 0.12 | 0.59 | 9.86E-01 |
| Triglycerides in very large HDL | -0.06 | 0.10 | 9.96E-01 | -0.07 | 0.10 | 9.99E-01 | -0.15 | 0.11 | 8.62E-01 | -0.16 | 0.11 | 8.83E-01 | 0.34 | 0.22 | 9.34E-01 | 0.31 | 0.15 | 9.86E-01 |
| Cholesteryl esters in small HDL | 0.06 | 0.10 | 9.96E-01 | 0.07 | 0.10 | 9.99E-01 | 0.07 | 0.11 | 8.74E-01 | 0.09 | 0.12 | 8.83E-01 | 0.00 | 0.22 | 9.98E-01 | -0.06 | 0.80 | 9.86E-01 |
| Cholesterol to total lipids ratio in large LDL | 0.06 | 0.10 | 9.96E-01 | 0.07 | 0.10 | 9.99E-01 | 0.16 | 0.12 | 8.62E-01 | 0.17 | 0.12 | 8.83E-01 | -0.31 | 0.20 | 9.34E-01 | -0.27 | 0.21 | 9.86E-01 |
| Free cholesterol to total lipids ratio in very small VLDL | 0.07 | 0.10 | 9.96E-01 | 0.07 | 0.10 | 9.99E-01 | 0.12 | 0.12 | 8.62E-01 | 0.13 | 0.12 | 8.83E-01 | -0.19 | 0.22 | 9.34E-01 | -0.12 | 0.61 | 9.86E-01 |
| Total triglycerides | -0.05 | 0.10 | 9.96E-01 | -0.07 | 0.10 | 9.99E-01 | -0.13 | 0.11 | 8.62E-01 | -0.12 | 0.12 | 8.83E-01 | 0.22 | 0.21 | 9.34E-01 | 0.16 | 0.47 | 9.86E-01 |
| Phospholipids to total lipids ratio in chylomicrons and extremely large VLDL | -0.06 | 0.10 | 9.96E-01 | -0.06 | 0.10 | 9.99E-01 | -0.12 | 0.11 | 8.62E-01 | -0.12 | 0.11 | 8.83E-01 | 0.24 | 0.33 | 9.34E-01 | 0.25 | 0.46 | 9.86E-01 |
| Triglycerides in LDL | -0.05 | 0.10 | 9.96E-01 | -0.06 | 0.10 | 9.99E-01 | -0.20 | 0.11 | 8.62E-01 | -0.20 | 0.11 | 8.83E-01 | 0.47 | 0.20 | 9.34E-01 | 0.43 | 0.04 | 9.86E-01 |
| Cholesteryl esters in VLDL | -0.05 | 0.10 | 9.96E-01 | -0.06 | 0.10 | 9.99E-01 | -0.10 | 0.11 | 8.62E-01 | -0.11 | 0.12 | 8.83E-01 | 0.11 | 0.22 | 9.34E-01 | 0.12 | 0.60 | 9.86E-01 |
| Free cholesterol to total lipids ratio in large VLDL | -0.06 | 0.10 | 9.96E-01 | -0.06 | 0.10 | 9.99E-01 | -0.08 | 0.11 | 8.62E-01 | -0.09 | 0.11 | 8.83E-01 | 0.04 | 0.26 | 9.59E-01 | 0.11 | 0.68 | 9.86E-01 |
| Triglycerides in VLDL | -0.05 | 0.10 | 9.96E-01 | -0.06 | 0.10 | 9.99E-01 | -0.12 | 0.11 | 8.62E-01 | -0.11 | 0.12 | 8.83E-01 | 0.18 | 0.22 | 9.34E-01 | 0.12 | 0.60 | 9.86E-01 |
| Free cholesterol in very large HDL | -0.06 | 0.11 | 9.96E-01 | -0.07 | 0.11 | 9.99E-01 | -0.06 | 0.12 | 9.40E-01 | -0.10 | 0.13 | 8.83E-01 | -0.05 | 0.26 | 9.46E-01 | 0.06 | 0.84 | 9.89E-01 |
| Triglycerides in very large VLDL | -0.05 | 0.10 | 9.96E-01 | -0.06 | 0.11 | 9.99E-01 | -0.12 | 0.11 | 8.62E-01 | -0.11 | 0.12 | 8.83E-01 | 0.23 | 0.26 | 9.34E-01 | 0.16 | 0.56 | 9.86E-01 |
| Free cholesterol in large VLDL | -0.05 | 0.10 | 9.96E-01 | -0.06 | 0.10 | 9.99E-01 | -0.11 | 0.11 | 8.62E-01 | -0.10 | 0.11 | 8.83E-01 | 0.18 | 0.24 | 9.34E-01 | 0.12 | 0.62 | 9.86E-01 |
| Ratio of triglycerides to phosphoglycerides | -0.05 | 0.10 | 9.96E-01 | -0.06 | 0.11 | 9.99E-01 | -0.11 | 0.11 | 8.62E-01 | -0.10 | 0.12 | 8.83E-01 | 0.19 | 0.23 | 9.34E-01 | 0.13 | 0.59 | 9.86E-01 |
| Cholesterol in small HDL | 0.05 | 0.10 | 9.96E-01 | 0.06 | 0.10 | 9.99E-01 | 0.05 | 0.11 | 9.48E-01 | 0.06 | 0.12 | 9.49E-01 | 0.06 | 0.22 | 9.34E-01 | 0.01 | 0.98 | 9.92E-01 |
| Free cholesterol to total lipids ratio in IDL | 0.06 | 0.10 | 9.96E-01 | 0.06 | 0.10 | 9.99E-01 | 0.12 | 0.11 | 8.62E-01 | 0.11 | 0.11 | 8.83E-01 | -0.27 | 0.23 | 9.34E-01 | -0.19 | 0.44 | 9.86E-01 |
| Cholesterol in large VLDL | -0.05 | 0.10 | 9.96E-01 | -0.06 | 0.10 | 9.99E-01 | -0.10 | 0.11 | 8.62E-01 | -0.09 | 0.11 | 8.83E-01 | 0.15 | 0.23 | 9.34E-01 | 0.10 | 0.66 | 9.86E-01 |
| Concentration of large VLDL particles | -0.05 | 0.10 | 9.96E-01 | -0.06 | 0.10 | 9.99E-01 | -0.11 | 0.11 | 8.62E-01 | -0.10 | 0.11 | 8.83E-01 | 0.18 | 0.23 | 9.34E-01 | 0.13 | 0.60 | 9.86E-01 |
| Concentration of medium LDL particles | -0.05 | 0.10 | 9.96E-01 | -0.06 | 0.10 | 9.99E-01 | -0.11 | 0.11 | 8.62E-01 | -0.10 | 0.11 | 8.83E-01 | 0.16 | 0.22 | 9.34E-01 | 0.13 | 0.56 | 9.86E-01 |
| Histidine | -0.05 | 0.09 | 9.96E-01 | -0.05 | 0.10 | 9.99E-01 | -0.05 | 0.11 | 9.40E-01 | -0.05 | 0.11 | 9.49E-01 | -0.08 | 0.22 | 9.34E-01 | -0.06 | 0.78 | 9.86E-01 |
| Total lipids in medium VLDL | -0.04 | 0.09 | 9.96E-01 | -0.06 | 0.10 | 9.99E-01 | -0.09 | 0.11 | 8.62E-01 | -0.09 | 0.11 | 8.83E-01 | 0.12 | 0.21 | 9.34E-01 | 0.08 | 0.70 | 9.86E-01 |
| Cholesteryl esters in large VLDL | -0.04 | 0.10 | 9.96E-01 | -0.06 | 0.10 | 9.99E-01 | -0.09 | 0.11 | 8.62E-01 | -0.08 | 0.11 | 8.87E-01 | 0.11 | 0.23 | 9.34E-01 | 0.09 | 0.71 | 9.86E-01 |
| Phospholipids in medium VLDL | -0.05 | 0.09 | 9.96E-01 | -0.06 | 0.10 | 9.99E-01 | -0.09 | 0.11 | 8.62E-01 | -0.09 | 0.11 | 8.83E-01 | 0.11 | 0.21 | 9.34E-01 | 0.09 | 0.67 | 9.86E-01 |
| Concentration of VLDL particles | -0.04 | 0.10 | 9.96E-01 | -0.06 | 0.10 | 9.99E-01 | -0.11 | 0.11 | 8.62E-01 | -0.11 | 0.11 | 8.83E-01 | 0.20 | 0.21 | 9.34E-01 | 0.18 | 0.42 | 9.86E-01 |
| Concentration of small HDL particles | 0.05 | 0.10 | 9.96E-01 | 0.06 | 0.10 | 9.99E-01 | 0.04 | 0.11 | 9.80E-01 | 0.05 | 0.12 | 9.49E-01 | 0.09 | 0.22 | 9.34E-01 | 0.02 | 0.92 | 9.89E-01 |
| Free cholesterol to total lipids ratio in large HDL | -0.04 | 0.08 | 9.96E-01 | -0.05 | 0.08 | 9.99E-01 | -0.06 | 0.09 | 8.67E-01 | -0.08 | 0.10 | 8.83E-01 | 0.01 | 0.22 | 9.98E-01 | 0.08 | 0.74 | 9.86E-01 |
| Free cholesterol to total lipids ratio in very large HDL | 0.05 | 0.10 | 9.96E-01 | 0.06 | 0.11 | 9.99E-01 | 0.09 | 0.11 | 8.62E-01 | 0.12 | 0.12 | 8.83E-01 | -0.13 | 0.25 | 9.34E-01 | -0.21 | 0.42 | 9.86E-01 |
| Free cholesterol in large LDL | 0.06 | 0.11 | 9.96E-01 | 0.06 | 0.11 | 9.99E-01 | 0.05 | 0.12 | 9.48E-01 | 0.04 | 0.12 | 9.77E-01 | 0.08 | 0.24 | 9.34E-01 | 0.11 | 0.65 | 9.86E-01 |
| Triglycerides to total lipids ratio in large HDL | 0.05 | 0.09 | 9.96E-01 | 0.05 | 0.09 | 9.99E-01 | 0.02 | 0.10 | 9.90E-01 | 0.03 | 0.10 | 9.77E-01 | 0.19 | 0.20 | 9.34E-01 | 0.16 | 0.44 | 9.86E-01 |
| Concentration of medium VLDL particles | -0.04 | 0.09 | 9.96E-01 | -0.05 | 0.10 | 9.99E-01 | -0.09 | 0.11 | 8.62E-01 | -0.08 | 0.11 | 8.83E-01 | 0.11 | 0.21 | 9.34E-01 | 0.09 | 0.69 | 9.86E-01 |
| Total lipids in large VLDL | -0.04 | 0.10 | 9.96E-01 | -0.05 | 0.10 | 9.99E-01 | -0.10 | 0.11 | 8.62E-01 | -0.09 | 0.11 | 8.83E-01 | 0.17 | 0.23 | 9.34E-01 | 0.11 | 0.65 | 9.86E-01 |
| Cholesteryl esters to total lipids ratio in small HDL | 0.04 | 0.10 | 9.96E-01 | 0.05 | 0.10 | 9.99E-01 | 0.15 | 0.12 | 8.62E-01 | 0.16 | 0.12 | 8.83E-01 | -0.31 | 0.20 | 9.34E-01 | -0.28 | 0.17 | 9.86E-01 |
| Cholesterol in IDL | 0.06 | 0.11 | 9.96E-01 | 0.05 | 0.11 | 9.99E-01 | 0.05 | 0.12 | 9.74E-01 | 0.03 | 0.12 | 9.77E-01 | 0.08 | 0.26 | 9.34E-01 | 0.12 | 0.63 | 9.86E-01 |
| Cholesteryl esters in IDL | 0.06 | 0.11 | 9.96E-01 | 0.05 | 0.11 | 9.99E-01 | 0.04 | 0.12 | 9.75E-01 | 0.03 | 0.12 | 9.77E-01 | 0.08 | 0.26 | 9.34E-01 | 0.12 | 0.64 | 9.86E-01 |
| Free cholesterol to total lipids ratio in medium VLDL | 0.05 | 0.10 | 9.96E-01 | 0.05 | 0.11 | 9.99E-01 | 0.08 | 0.12 | 8.62E-01 | 0.05 | 0.12 | 9.49E-01 | -0.07 | 0.25 | 9.34E-01 | 0.07 | 0.79 | 9.86E-01 |
| Triglycerides in very small VLDL | -0.04 | 0.10 | 9.96E-01 | -0.05 | 0.10 | 9.99E-01 | -0.16 | 0.11 | 8.62E-01 | -0.17 | 0.11 | 8.83E-01 | 0.45 | 0.21 | 9.34E-01 | 0.41 | 0.07 | 9.86E-01 |
| Cholesteryl esters in small LDL | -0.04 | 0.10 | 9.96E-01 | -0.05 | 0.10 | 9.99E-01 | -0.09 | 0.11 | 8.62E-01 | -0.09 | 0.11 | 8.83E-01 | 0.12 | 0.23 | 9.34E-01 | 0.11 | 0.64 | 9.86E-01 |
| Cholesteryl esters to total lipids ratio in very large VLDL | 0.04 | 0.10 | 9.96E-01 | 0.05 | 0.11 | 9.99E-01 | 0.12 | 0.11 | 8.62E-01 | 0.11 | 0.12 | 8.83E-01 | -0.30 | 0.27 | 9.34E-01 | -0.20 | 0.51 | 9.86E-01 |
| Free cholesterol in LDL | 0.05 | 0.10 | 9.96E-01 | 0.05 | 0.10 | 9.99E-01 | 0.04 | 0.12 | 9.80E-01 | 0.03 | 0.12 | 9.77E-01 | 0.08 | 0.24 | 9.34E-01 | 0.10 | 0.68 | 9.86E-01 |
| Free cholesterol in IDL | 0.06 | 0.11 | 9.96E-01 | 0.05 | 0.11 | 9.99E-01 | 0.05 | 0.12 | 9.74E-01 | 0.03 | 0.12 | 9.77E-01 | 0.06 | 0.26 | 9.39E-01 | 0.12 | 0.63 | 9.86E-01 |
| Triglycerides in medium VLDL | -0.04 | 0.09 | 9.96E-01 | -0.05 | 0.10 | 9.99E-01 | -0.09 | 0.11 | 8.62E-01 | -0.08 | 0.11 | 9.03E-01 | 0.15 | 0.22 | 9.34E-01 | 0.09 | 0.68 | 9.86E-01 |
| Average diameter for HDL particles | -0.05 | 0.11 | 9.96E-01 | -0.05 | 0.11 | 9.99E-01 | -0.03 | 0.12 | 9.90E-01 | -0.07 | 0.13 | 9.49E-01 | -0.11 | 0.27 | 9.34E-01 | -0.04 | 0.89 | 9.89E-01 |
| Total lipids in small HDL | 0.04 | 0.10 | 9.96E-01 | 0.05 | 0.11 | 9.99E-01 | 0.01 | 0.12 | 9.90E-01 | 0.03 | 0.12 | 9.77E-01 | 0.16 | 0.22 | 9.34E-01 | 0.09 | 0.73 | 9.86E-01 |
| Cholesteryl esters to total lipids ratio in medium VLDL | 0.04 | 0.10 | 9.96E-01 | 0.05 | 0.10 | 9.99E-01 | 0.09 | 0.11 | 8.62E-01 | 0.06 | 0.12 | 9.49E-01 | -0.15 | 0.22 | 9.34E-01 | -0.03 | 0.89 | 9.89E-01 |
| Glycine | -0.04 | 0.11 | 9.96E-01 | -0.05 | 0.11 | 9.99E-01 | 0.02 | 0.12 | 9.90E-01 | -0.02 | 0.13 | 9.77E-01 | -0.39 | 0.27 | 9.34E-01 | -0.34 | 0.24 | 9.86E-01 |
| Tyrosine | -0.05 | 0.10 | 9.96E-01 | -0.05 | 0.10 | 9.99E-01 | -0.05 | 0.11 | 9.48E-01 | -0.01 | 0.12 | 9.77E-01 | -0.08 | 0.23 | 9.34E-01 | -0.18 | 0.46 | 9.86E-01 |
| Free cholesterol in medium VLDL | -0.04 | 0.10 | 9.96E-01 | -0.05 | 0.10 | 9.99E-01 | -0.08 | 0.11 | 8.62E-01 | -0.08 | 0.11 | 9.03E-01 | 0.11 | 0.22 | 9.34E-01 | 0.10 | 0.64 | 9.86E-01 |
| Triglycerides in large LDL | -0.04 | 0.10 | 9.96E-01 | -0.04 | 0.10 | 9.99E-01 | -0.19 | 0.11 | 8.62E-01 | -0.19 | 0.11 | 8.83E-01 | 0.51 | 0.21 | 9.34E-01 | 0.48 | 0.03 | 9.86E-01 |
| Free cholesterol to total lipids ratio in small VLDL | 0.05 | 0.11 | 9.96E-01 | 0.05 | 0.11 | 9.99E-01 | 0.11 | 0.12 | 8.62E-01 | 0.09 | 0.12 | 8.83E-01 | -0.27 | 0.26 | 9.34E-01 | -0.17 | 0.54 | 9.86E-01 |
| Saturated fatty acids | -0.04 | 0.10 | 9.96E-01 | -0.05 | 0.10 | 9.99E-01 | -0.13 | 0.11 | 8.62E-01 | -0.12 | 0.12 | 8.83E-01 | 0.27 | 0.21 | 9.34E-01 | 0.22 | 0.31 | 9.86E-01 |
| Phospholipids in small HDL | 0.04 | 0.10 | 9.96E-01 | 0.05 | 0.11 | 9.99E-01 | 0.00 | 0.12 | 9.90E-01 | 0.02 | 0.12 | 9.77E-01 | 0.16 | 0.23 | 9.34E-01 | 0.09 | 0.73 | 9.86E-01 |
| Total lipids in small VLDL | -0.03 | 0.09 | 9.96E-01 | -0.04 | 0.10 | 9.99E-01 | -0.10 | 0.11 | 8.62E-01 | -0.10 | 0.11 | 8.83E-01 | 0.23 | 0.22 | 9.34E-01 | 0.20 | 0.36 | 9.86E-01 |
| Free cholesterol in small HDL | 0.04 | 0.10 | 9.96E-01 | 0.05 | 0.11 | 9.99E-01 | -0.01 | 0.12 | 9.90E-01 | 0.00 | 0.12 | 9.92E-01 | 0.23 | 0.22 | 9.34E-01 | 0.19 | 0.43 | 9.86E-01 |
| Triglycerides in IDL | -0.03 | 0.10 | 9.96E-01 | -0.04 | 0.10 | 9.99E-01 | -0.17 | 0.11 | 8.62E-01 | -0.18 | 0.11 | 8.83E-01 | 0.49 | 0.21 | 9.34E-01 | 0.46 | 0.03 | 9.86E-01 |
| Total lipids in lipoprotein particles | -0.04 | 0.10 | 9.96E-01 | -0.04 | 0.10 | 9.99E-01 | -0.10 | 0.11 | 8.62E-01 | -0.09 | 0.12 | 8.83E-01 | 0.17 | 0.22 | 9.34E-01 | 0.14 | 0.55 | 9.86E-01 |
| Triglycerides in small VLDL | -0.03 | 0.10 | 9.96E-01 | -0.04 | 0.10 | 9.99E-01 | -0.11 | 0.11 | 8.62E-01 | -0.10 | 0.11 | 8.83E-01 | 0.26 | 0.22 | 9.34E-01 | 0.21 | 0.37 | 9.86E-01 |
| Triglycerides in large HDL | 0.04 | 0.10 | 9.96E-01 | 0.04 | 0.10 | 9.99E-01 | 0.00 | 0.11 | 9.90E-01 | -0.01 | 0.11 | 9.77E-01 | 0.28 | 0.25 | 9.34E-01 | 0.27 | 0.27 | 9.86E-01 |
| Cholesterol to total lipids ratio in medium VLDL | 0.04 | 0.10 | 9.96E-01 | 0.04 | 0.11 | 9.99E-01 | 0.08 | 0.11 | 8.62E-01 | 0.06 | 0.12 | 9.49E-01 | -0.15 | 0.24 | 9.34E-01 | -0.03 | 0.91 | 9.89E-01 |
| Concentration of small VLDL particles | -0.03 | 0.09 | 9.96E-01 | -0.04 | 0.10 | 9.99E-01 | -0.11 | 0.11 | 8.62E-01 | -0.10 | 0.11 | 8.83E-01 | 0.25 | 0.22 | 9.34E-01 | 0.22 | 0.32 | 9.86E-01 |
| Cholesterol to total lipids ratio in small HDL | 0.03 | 0.10 | 9.96E-01 | 0.04 | 0.10 | 9.99E-01 | 0.12 | 0.12 | 8.62E-01 | 0.12 | 0.12 | 8.83E-01 | -0.23 | 0.20 | 9.34E-01 | -0.20 | 0.34 | 9.86E-01 |
| Polyunsaturated fatty acids | -0.04 | 0.10 | 9.96E-01 | -0.04 | 0.10 | 9.99E-01 | -0.09 | 0.11 | 8.62E-01 | -0.08 | 0.11 | 8.83E-01 | 0.17 | 0.22 | 9.34E-01 | 0.16 | 0.50 | 9.86E-01 |
| Cholesterol to total lipids ratio in small VLDL | 0.04 | 0.10 | 9.96E-01 | 0.04 | 0.10 | 9.99E-01 | 0.08 | 0.11 | 8.62E-01 | 0.05 | 0.12 | 9.49E-01 | -0.14 | 0.23 | 9.34E-01 | -0.02 | 0.92 | 9.89E-01 |
| Cholesterol in medium VLDL | -0.03 | 0.10 | 9.96E-01 | -0.04 | 0.10 | 9.99E-01 | -0.06 | 0.11 | 9.20E-01 | -0.06 | 0.11 | 9.49E-01 | 0.05 | 0.23 | 9.46E-01 | 0.08 | 0.73 | 9.86E-01 |
| Triglycerides in large VLDL | -0.03 | 0.10 | 9.96E-01 | -0.04 | 0.10 | 9.99E-01 | -0.08 | 0.11 | 8.62E-01 | -0.07 | 0.12 | 9.44E-01 | 0.16 | 0.23 | 9.34E-01 | 0.09 | 0.70 | 9.86E-01 |
| Phospholipids in small VLDL | -0.03 | 0.10 | 9.96E-01 | -0.04 | 0.10 | 9.99E-01 | -0.09 | 0.11 | 8.62E-01 | -0.09 | 0.11 | 8.83E-01 | 0.19 | 0.22 | 9.34E-01 | 0.18 | 0.43 | 9.86E-01 |
| Cholesterol to total lipids ratio in large HDL | -0.04 | 0.09 | 9.96E-01 | -0.04 | 0.09 | 9.99E-01 | -0.03 | 0.10 | 9.80E-01 | -0.06 | 0.11 | 9.49E-01 | -0.07 | 0.18 | 9.34E-01 | -0.01 | 0.95 | 9.89E-01 |
| Free cholesterol in medium LDL | 0.04 | 0.10 | 9.96E-01 | 0.04 | 0.10 | 9.99E-01 | 0.03 | 0.11 | 9.90E-01 | 0.03 | 0.12 | 9.77E-01 | 0.08 | 0.23 | 9.34E-01 | 0.08 | 0.72 | 9.86E-01 |
| Phospholipids to total lipids ratio in small VLDL | 0.04 | 0.11 | 9.96E-01 | 0.04 | 0.11 | 9.99E-01 | 0.11 | 0.12 | 8.62E-01 | 0.09 | 0.12 | 8.83E-01 | -0.30 | 0.26 | 9.34E-01 | -0.20 | 0.47 | 9.86E-01 |
| Cholesteryl esters in medium LDL | -0.03 | 0.10 | 9.96E-01 | -0.04 | 0.10 | 9.99E-01 | -0.08 | 0.11 | 8.62E-01 | -0.07 | 0.11 | 9.21E-01 | 0.14 | 0.22 | 9.34E-01 | 0.10 | 0.66 | 9.86E-01 |
| Albumin | 0.03 | 0.10 | 9.96E-01 | 0.04 | 0.10 | 9.99E-01 | -0.01 | 0.11 | 9.90E-01 | -0.01 | 0.11 | 9.87E-01 | 0.24 | 0.23 | 9.34E-01 | 0.28 | 0.26 | 9.86E-01 |
| Total lipids in IDL | 0.04 | 0.11 | 9.96E-01 | 0.04 | 0.11 | 9.99E-01 | 0.01 | 0.12 | 9.90E-01 | 0.00 | 0.12 | 9.95E-01 | 0.14 | 0.26 | 9.34E-01 | 0.18 | 0.49 | 9.86E-01 |
| Total cholines | -0.03 | 0.11 | 9.96E-01 | -0.04 | 0.11 | 9.99E-01 | -0.09 | 0.12 | 8.62E-01 | -0.10 | 0.12 | 8.83E-01 | 0.19 | 0.25 | 9.34E-01 | 0.17 | 0.51 | 9.86E-01 |
| Glycoprotein acetyls | -0.02 | 0.10 | 9.96E-01 | -0.03 | 0.10 | 9.99E-01 | -0.08 | 0.11 | 8.62E-01 | -0.08 | 0.11 | 8.83E-01 | 0.36 | 0.23 | 9.34E-01 | 0.37 | 0.12 | 9.86E-01 |
| Concentration of large HDL particles | -0.03 | 0.10 | 9.96E-01 | -0.04 | 0.11 | 9.99E-01 | -0.02 | 0.11 | 9.90E-01 | -0.05 | 0.12 | 9.49E-01 | -0.09 | 0.23 | 9.34E-01 | -0.04 | 0.87 | 9.89E-01 |
| Free cholesterol to total lipids ratio in small HDL | -0.03 | 0.10 | 9.96E-01 | -0.03 | 0.10 | 9.99E-01 | -0.10 | 0.11 | 8.62E-01 | -0.12 | 0.11 | 8.83E-01 | 0.29 | 0.22 | 9.34E-01 | 0.30 | 0.17 | 9.86E-01 |
| Cholesteryl esters to total lipids ratio in large HDL | -0.03 | 0.09 | 9.96E-01 | -0.03 | 0.09 | 9.99E-01 | -0.02 | 0.10 | 9.90E-01 | -0.04 | 0.11 | 9.49E-01 | -0.10 | 0.18 | 9.34E-01 | -0.05 | 0.82 | 9.86E-01 |
| Total lipids in small LDL | -0.03 | 0.10 | 9.96E-01 | -0.03 | 0.10 | 9.99E-01 | -0.07 | 0.11 | 8.62E-01 | -0.07 | 0.11 | 9.30E-01 | 0.13 | 0.22 | 9.34E-01 | 0.12 | 0.61 | 9.86E-01 |
| Cholesteryl esters to total lipids ratio in small VLDL | 0.03 | 0.10 | 9.96E-01 | 0.03 | 0.10 | 9.99E-01 | 0.05 | 0.11 | 9.46E-01 | 0.03 | 0.11 | 9.77E-01 | -0.06 | 0.22 | 9.34E-01 | 0.04 | 0.86 | 9.89E-01 |
| Cholesterol in small VLDL | -0.02 | 0.10 | 9.96E-01 | -0.03 | 0.10 | 9.99E-01 | -0.09 | 0.11 | 8.62E-01 | -0.09 | 0.11 | 8.83E-01 | 0.20 | 0.23 | 9.34E-01 | 0.20 | 0.38 | 9.86E-01 |
| Total phospholipids in lipoprotein particles | -0.03 | 0.11 | 9.96E-01 | -0.03 | 0.11 | 9.99E-01 | -0.09 | 0.12 | 8.62E-01 | -0.09 | 0.12 | 8.83E-01 | 0.19 | 0.24 | 9.34E-01 | 0.17 | 0.50 | 9.86E-01 |
| Cholesterol in large LDL | 0.04 | 0.10 | 9.96E-01 | 0.03 | 0.10 | 9.99E-01 | 0.02 | 0.11 | 9.90E-01 | 0.01 | 0.12 | 9.77E-01 | 0.08 | 0.23 | 9.34E-01 | 0.10 | 0.68 | 9.86E-01 |
| Triglycerides to total lipids ratio in small HDL | -0.02 | 0.10 | 9.96E-01 | -0.03 | 0.11 | 9.99E-01 | -0.11 | 0.11 | 8.62E-01 | -0.10 | 0.12 | 8.83E-01 | 0.37 | 0.25 | 9.34E-01 | 0.32 | 0.21 | 9.86E-01 |
| Phospholipids in very small VLDL | -0.02 | 0.10 | 9.96E-01 | -0.03 | 0.10 | 9.99E-01 | -0.11 | 0.11 | 8.62E-01 | -0.12 | 0.12 | 8.83E-01 | 0.27 | 0.22 | 9.34E-01 | 0.27 | 0.22 | 9.86E-01 |
| Free cholesterol in small VLDL | -0.02 | 0.10 | 9.96E-01 | -0.03 | 0.10 | 9.99E-01 | -0.08 | 0.11 | 8.62E-01 | -0.08 | 0.11 | 9.10E-01 | 0.16 | 0.22 | 9.34E-01 | 0.16 | 0.47 | 9.86E-01 |
| Cholesteryl esters in small VLDL | -0.02 | 0.10 | 9.96E-01 | -0.03 | 0.10 | 9.99E-01 | -0.09 | 0.11 | 8.62E-01 | -0.09 | 0.11 | 8.83E-01 | 0.22 | 0.23 | 9.34E-01 | 0.22 | 0.34 | 9.86E-01 |
| Ratio of apolipoprotein B to apolipoprotein A1 | -0.02 | 0.10 | 9.96E-01 | -0.03 | 0.10 | 9.99E-01 | -0.05 | 0.11 | 9.40E-01 | -0.05 | 0.12 | 9.49E-01 | 0.08 | 0.23 | 9.34E-01 | 0.10 | 0.68 | 9.86E-01 |
| Apolipoprotein B | -0.02 | 0.10 | 9.96E-01 | -0.03 | 0.10 | 9.99E-01 | -0.07 | 0.11 | 8.88E-01 | -0.07 | 0.12 | 9.41E-01 | 0.13 | 0.23 | 9.34E-01 | 0.13 | 0.57 | 9.86E-01 |
| Cholesterol in small LDL | -0.02 | 0.10 | 9.96E-01 | -0.03 | 0.10 | 9.99E-01 | -0.06 | 0.11 | 9.04E-01 | -0.06 | 0.11 | 9.49E-01 | 0.11 | 0.23 | 9.34E-01 | 0.10 | 0.68 | 9.86E-01 |
| Free cholesterol in large HDL | -0.03 | 0.10 | 9.96E-01 | -0.03 | 0.10 | 9.99E-01 | -0.03 | 0.11 | 9.90E-01 | -0.06 | 0.11 | 9.49E-01 | -0.04 | 0.23 | 9.50E-01 | 0.02 | 0.94 | 9.89E-01 |
| Phospholipids in large LDL | 0.03 | 0.10 | 9.96E-01 | 0.03 | 0.10 | 9.99E-01 | 0.01 | 0.12 | 9.90E-01 | 0.00 | 0.12 | 9.92E-01 | 0.11 | 0.24 | 9.34E-01 | 0.13 | 0.61 | 9.86E-01 |
| Cholesterol in large HDL | -0.03 | 0.10 | 9.96E-01 | -0.03 | 0.10 | 9.99E-01 | -0.02 | 0.11 | 9.90E-01 | -0.05 | 0.12 | 9.49E-01 | -0.07 | 0.22 | 9.34E-01 | -0.02 | 0.94 | 9.89E-01 |
| Cholesterol to total lipids ratio in small LDL | 0.03 | 0.10 | 9.96E-01 | 0.03 | 0.10 | 9.99E-01 | 0.08 | 0.12 | 8.62E-01 | 0.08 | 0.12 | 9.17E-01 | -0.21 | 0.23 | 9.34E-01 | -0.21 | 0.40 | 9.86E-01 |
| Cholesteryl esters in large HDL | -0.03 | 0.10 | 9.96E-01 | -0.03 | 0.10 | 9.99E-01 | -0.02 | 0.11 | 9.90E-01 | -0.05 | 0.12 | 9.49E-01 | -0.08 | 0.22 | 9.34E-01 | -0.03 | 0.90 | 9.89E-01 |
| Phosphoglycerides | -0.03 | 0.11 | 9.96E-01 | -0.03 | 0.11 | 9.99E-01 | -0.08 | 0.12 | 8.62E-01 | -0.09 | 0.12 | 8.87E-01 | 0.20 | 0.24 | 9.34E-01 | 0.17 | 0.51 | 9.86E-01 |
| Cholesteryl esters in medium HDL | 0.02 | 0.10 | 9.96E-01 | 0.03 | 0.11 | 9.99E-01 | 0.04 | 0.11 | 9.80E-01 | 0.04 | 0.12 | 9.77E-01 | -0.06 | 0.23 | 9.34E-01 | -0.08 | 0.75 | 9.86E-01 |
| Total lipids in large LDL | 0.03 | 0.10 | 9.96E-01 | 0.03 | 0.10 | 9.99E-01 | 0.00 | 0.11 | 9.90E-01 | 0.00 | 0.12 | 9.99E-01 | 0.12 | 0.24 | 9.34E-01 | 0.13 | 0.59 | 9.86E-01 |
| Sphingomyelins | 0.03 | 0.11 | 9.96E-01 | 0.03 | 0.11 | 9.99E-01 | 0.00 | 0.12 | 9.90E-01 | -0.02 | 0.13 | 9.77E-01 | 0.18 | 0.24 | 9.34E-01 | 0.20 | 0.42 | 9.86E-01 |
| Cholesterol in medium HDL | 0.02 | 0.10 | 9.96E-01 | 0.03 | 0.11 | 9.99E-01 | 0.04 | 0.11 | 9.81E-01 | 0.03 | 0.12 | 9.77E-01 | -0.05 | 0.23 | 9.46E-01 | -0.07 | 0.79 | 9.86E-01 |
| Concentration of LDL particles | -0.02 | 0.10 | 9.96E-01 | -0.03 | 0.10 | 9.99E-01 | -0.06 | 0.11 | 9.20E-01 | -0.06 | 0.12 | 9.49E-01 | 0.11 | 0.23 | 9.34E-01 | 0.11 | 0.62 | 9.86E-01 |
| Phospholipids in medium HDL | 0.02 | 0.10 | 9.96E-01 | 0.03 | 0.11 | 9.99E-01 | 0.01 | 0.12 | 9.90E-01 | 0.01 | 0.13 | 9.77E-01 | 0.05 | 0.24 | 9.46E-01 | 0.01 | 0.96 | 9.89E-01 |
| Total lipids in medium HDL | 0.02 | 0.10 | 9.96E-01 | 0.03 | 0.11 | 9.99E-01 | 0.02 | 0.12 | 9.90E-01 | 0.02 | 0.12 | 9.77E-01 | 0.02 | 0.24 | 9.94E-01 | -0.01 | 0.97 | 9.92E-01 |
| Concentration of HDL particles | 0.02 | 0.10 | 9.96E-01 | 0.03 | 0.11 | 9.99E-01 | 0.02 | 0.12 | 9.90E-01 | 0.02 | 0.13 | 9.77E-01 | 0.03 | 0.24 | 9.76E-01 | 0.00 | 0.99 | 9.95E-01 |
| Phosphatidylcholines | -0.02 | 0.11 | 9.96E-01 | -0.03 | 0.11 | 9.99E-01 | -0.07 | 0.12 | 8.68E-01 | -0.08 | 0.12 | 9.21E-01 | 0.18 | 0.25 | 9.34E-01 | 0.14 | 0.57 | 9.86E-01 |
| Concentration of very small VLDL particles | -0.02 | 0.10 | 9.96E-01 | -0.03 | 0.10 | 9.99E-01 | -0.10 | 0.11 | 8.62E-01 | -0.11 | 0.12 | 8.83E-01 | 0.29 | 0.23 | 9.34E-01 | 0.29 | 0.21 | 9.86E-01 |
| Free cholesterol to total lipids ratio in very large VLDL | -0.02 | 0.10 | 9.96E-01 | -0.03 | 0.11 | 9.99E-01 | 0.01 | 0.12 | 9.90E-01 | -0.02 | 0.12 | 9.77E-01 | -0.13 | 0.23 | 9.34E-01 | -0.01 | 0.97 | 9.92E-01 |
| Cholesteryl esters in large LDL | 0.03 | 0.10 | 9.96E-01 | 0.02 | 0.10 | 9.99E-01 | 0.00 | 0.11 | 9.90E-01 | 0.01 | 0.11 | 9.92E-01 | 0.09 | 0.23 | 9.34E-01 | 0.09 | 0.69 | 9.86E-01 |
| Free cholesterol in small LDL | 0.03 | 0.10 | 9.96E-01 | 0.02 | 0.10 | 9.99E-01 | 0.01 | 0.12 | 9.90E-01 | 0.01 | 0.12 | 9.77E-01 | 0.06 | 0.23 | 9.34E-01 | 0.06 | 0.78 | 9.86E-01 |
| Remnant cholesterol (non-HDL | -0.02 | 0.10 | 9.96E-01 | -0.02 | 0.10 | 9.99E-01 | -0.06 | 0.12 | 9.20E-01 | -0.06 | 0.12 | 9.49E-01 | 0.13 | 0.24 | 9.34E-01 | 0.14 | 0.56 | 9.86E-01 |
| Clinical LDL cholesterol | 0.03 | 0.10 | 9.96E-01 | 0.02 | 0.10 | 9.99E-01 | 0.00 | 0.12 | 9.90E-01 | 0.00 | 0.12 | 9.95E-01 | 0.09 | 0.24 | 9.34E-01 | 0.10 | 0.67 | 9.86E-01 |
| Cholesteryl esters in medium VLDL | -0.02 | 0.10 | 9.96E-01 | -0.02 | 0.10 | 9.99E-01 | -0.03 | 0.12 | 9.90E-01 | -0.04 | 0.12 | 9.77E-01 | -0.01 | 0.24 | 9.98E-01 | 0.07 | 0.77 | 9.86E-01 |
| Triglycerides to total lipids ratio in small VLDL | -0.02 | 0.11 | 9.96E-01 | -0.02 | 0.11 | 9.99E-01 | -0.07 | 0.12 | 8.83E-01 | -0.05 | 0.12 | 9.49E-01 | 0.22 | 0.27 | 9.34E-01 | 0.10 | 0.72 | 9.86E-01 |
| Free cholesterol in medium HDL | 0.01 | 0.10 | 9.96E-01 | 0.02 | 0.11 | 9.99E-01 | 0.02 | 0.11 | 9.90E-01 | 0.01 | 0.12 | 9.77E-01 | 0.00 | 0.24 | 9.98E-01 | -0.02 | 0.94 | 9.89E-01 |
| Phospholipids in IDL | 0.03 | 0.11 | 9.96E-01 | 0.02 | 0.11 | 9.99E-01 | -0.01 | 0.12 | 9.90E-01 | -0.02 | 0.12 | 9.77E-01 | 0.14 | 0.26 | 9.34E-01 | 0.19 | 0.45 | 9.86E-01 |
| Concentration of medium HDL particles | 0.01 | 0.10 | 9.96E-01 | 0.02 | 0.11 | 9.99E-01 | 0.02 | 0.11 | 9.90E-01 | 0.02 | 0.12 | 9.77E-01 | -0.02 | 0.24 | 9.98E-01 | -0.04 | 0.87 | 9.89E-01 |
| Acetone | -0.01 | 0.09 | 9.96E-01 | -0.02 | 0.10 | 9.99E-01 | -0.11 | 0.11 | 8.62E-01 | -0.12 | 0.11 | 8.83E-01 | 0.31 | 0.19 | 9.34E-01 | 0.37 | 0.08 | 9.86E-01 |
| Triglycerides to total lipids ratio in very large HDL | 0.02 | 0.10 | 9.96E-01 | 0.02 | 0.10 | 9.99E-01 | -0.04 | 0.11 | 9.74E-01 | -0.02 | 0.11 | 9.77E-01 | 0.29 | 0.22 | 9.34E-01 | 0.22 | 0.35 | 9.86E-01 |
| Triglycerides in medium HDL | 0.02 | 0.10 | 9.96E-01 | 0.02 | 0.10 | 9.99E-01 | -0.05 | 0.11 | 9.40E-01 | -0.04 | 0.11 | 9.77E-01 | 0.32 | 0.23 | 9.34E-01 | 0.26 | 0.28 | 9.86E-01 |
| Total free cholesterol | -0.01 | 0.10 | 9.96E-01 | -0.02 | 0.10 | 9.99E-01 | -0.06 | 0.12 | 9.26E-01 | -0.06 | 0.12 | 9.49E-01 | 0.14 | 0.24 | 9.34E-01 | 0.15 | 0.55 | 9.86E-01 |
| Total lipids in very small VLDL | -0.01 | 0.10 | 9.96E-01 | -0.02 | 0.10 | 9.99E-01 | -0.09 | 0.11 | 8.62E-01 | -0.10 | 0.12 | 8.83E-01 | 0.29 | 0.23 | 9.34E-01 | 0.29 | 0.21 | 9.86E-01 |
| Cholesteryl esters to total lipids ratio in large VLDL | 0.02 | 0.10 | 9.96E-01 | 0.02 | 0.11 | 9.99E-01 | 0.09 | 0.11 | 8.62E-01 | 0.06 | 0.12 | 9.49E-01 | -0.22 | 0.24 | 9.34E-01 | -0.08 | 0.75 | 9.86E-01 |
| Total concentration of lipoprotein particles | 0.01 | 0.11 | 9.96E-01 | 0.02 | 0.11 | 9.99E-01 | 0.00 | 0.12 | 9.90E-01 | 0.00 | 0.12 | 9.95E-01 | 0.06 | 0.24 | 9.34E-01 | 0.03 | 0.90 | 9.89E-01 |
| Cholesterol in medium LDL | -0.01 | 0.10 | 9.96E-01 | -0.02 | 0.10 | 9.99E-01 | -0.05 | 0.11 | 9.40E-01 | -0.05 | 0.11 | 9.49E-01 | 0.12 | 0.23 | 9.34E-01 | 0.10 | 0.67 | 9.86E-01 |
| Cholesterol to total lipids ratio in very large VLDL | 0.02 | 0.11 | 9.96E-01 | 0.02 | 0.11 | 9.99E-01 | 0.10 | 0.11 | 8.62E-01 | 0.08 | 0.12 | 9.30E-01 | -0.35 | 0.28 | 9.34E-01 | -0.23 | 0.45 | 9.86E-01 |
| Total lipids in medium LDL | -0.01 | 0.10 | 9.96E-01 | -0.02 | 0.10 | 9.99E-01 | -0.06 | 0.11 | 9.20E-01 | -0.05 | 0.11 | 9.49E-01 | 0.15 | 0.23 | 9.34E-01 | 0.12 | 0.59 | 9.86E-01 |
| Free cholesterol in very small VLDL | -0.01 | 0.10 | 9.96E-01 | -0.02 | 0.10 | 9.99E-01 | -0.08 | 0.11 | 8.62E-01 | -0.09 | 0.12 | 8.83E-01 | 0.26 | 0.23 | 9.34E-01 | 0.26 | 0.25 | 9.86E-01 |
| Cholesterol to total lipids ratio in chylomicrons and extremely large VLDL | 0.01 | 0.10 | 9.96E-01 | 0.02 | 0.11 | 9.99E-01 | 0.08 | 0.11 | 8.62E-01 | 0.06 | 0.12 | 9.49E-01 | -0.19 | 0.25 | 9.34E-01 | -0.15 | 0.56 | 9.86E-01 |
| Phospholipids to total lipids ratio in medium VLDL | 0.02 | 0.10 | 9.96E-01 | 0.02 | 0.11 | 9.99E-01 | 0.04 | 0.12 | 9.80E-01 | 0.01 | 0.12 | 9.77E-01 | -0.07 | 0.24 | 9.34E-01 | 0.05 | 0.82 | 9.86E-01 |
| Total lipids in large HDL | -0.02 | 0.10 | 9.96E-01 | -0.02 | 0.11 | 9.99E-01 | -0.01 | 0.11 | 9.90E-01 | -0.03 | 0.12 | 9.77E-01 | -0.06 | 0.23 | 9.34E-01 | -0.01 | 0.97 | 9.92E-01 |
| Cholesteryl esters in very small VLDL | 0.02 | 0.11 | 9.96E-01 | 0.02 | 0.11 | 9.99E-01 | -0.01 | 0.12 | 9.90E-01 | -0.03 | 0.12 | 9.77E-01 | 0.12 | 0.26 | 9.34E-01 | 0.17 | 0.49 | 9.86E-01 |
| Phospholipids in LDL | 0.02 | 0.10 | 9.96E-01 | 0.01 | 0.10 | 9.99E-01 | -0.02 | 0.11 | 9.90E-01 | -0.02 | 0.12 | 9.77E-01 | 0.13 | 0.23 | 9.34E-01 | 0.13 | 0.58 | 9.86E-01 |
| Phospholipids to total lipids ratio in small HDL | -0.01 | 0.10 | 9.96E-01 | -0.01 | 0.10 | 9.99E-01 | -0.02 | 0.11 | 9.90E-01 | -0.03 | 0.11 | 9.77E-01 | 0.01 | 0.21 | 9.98E-01 | 0.00 | 1.00 | 9.95E-01 |
| Glucose | -0.02 | 0.10 | 9.96E-01 | -0.01 | 0.10 | 9.99E-01 | -0.11 | 0.12 | 8.62E-01 | -0.09 | 0.12 | 8.83E-01 | 0.26 | 0.19 | 9.34E-01 | 0.22 | 0.29 | 9.86E-01 |
| LDL cholesterol | 0.02 | 0.10 | 9.96E-01 | 0.01 | 0.10 | 9.99E-01 | -0.01 | 0.11 | 9.90E-01 | -0.01 | 0.11 | 9.78E-01 | 0.10 | 0.23 | 9.34E-01 | 0.10 | 0.68 | 9.86E-01 |
| Concentration of IDL particles | -0.01 | 0.11 | 9.96E-01 | -0.01 | 0.11 | 9.99E-01 | -0.04 | 0.12 | 9.80E-01 | -0.05 | 0.12 | 9.49E-01 | 0.11 | 0.25 | 9.34E-01 | 0.14 | 0.59 | 9.86E-01 |
| Phospholipids in small LDL | -0.01 | 0.10 | 9.96E-01 | -0.01 | 0.10 | 9.99E-01 | -0.05 | 0.11 | 9.48E-01 | -0.05 | 0.11 | 9.49E-01 | 0.13 | 0.22 | 9.34E-01 | 0.13 | 0.57 | 9.86E-01 |
| Cholesteryl esters to total lipids ratio in medium HDL | 0.00 | 0.09 | 9.96E-01 | 0.01 | 0.10 | 9.99E-01 | 0.08 | 0.11 | 8.62E-01 | 0.07 | 0.12 | 9.41E-01 | -0.23 | 0.18 | 9.34E-01 | -0.20 | 0.29 | 9.86E-01 |
| Cholesteryl esters to total lipids ratio in large LDL | -0.01 | 0.09 | 9.96E-01 | -0.01 | 0.09 | 9.99E-01 | 0.03 | 0.11 | 9.90E-01 | 0.05 | 0.11 | 9.49E-01 | -0.19 | 0.20 | 9.34E-01 | -0.21 | 0.32 | 9.86E-01 |
| Cholesterol to total lipids ratio in medium HDL | 0.00 | 0.09 | 9.96E-01 | 0.01 | 0.10 | 9.99E-01 | 0.07 | 0.11 | 8.62E-01 | 0.06 | 0.12 | 9.49E-01 | -0.22 | 0.19 | 9.34E-01 | -0.19 | 0.33 | 9.86E-01 |
| Triglycerides in small HDL | 0.00 | 0.10 | 9.96E-01 | -0.01 | 0.11 | 9.99E-01 | -0.10 | 0.11 | 8.62E-01 | -0.08 | 0.12 | 8.87E-01 | 0.40 | 0.24 | 9.34E-01 | 0.34 | 0.19 | 9.86E-01 |
| Cholesterol to total lipids ratio in medium LDL | 0.00 | 0.10 | 9.96E-01 | -0.01 | 0.10 | 9.99E-01 | 0.09 | 0.12 | 8.62E-01 | 0.09 | 0.12 | 8.83E-01 | -0.37 | 0.21 | 9.34E-01 | -0.39 | 0.08 | 9.86E-01 |
| Apolipoprotein A1 | 0.00 | 0.11 | 9.96E-01 | 0.01 | 0.11 | 9.99E-01 | 0.00 | 0.12 | 9.90E-01 | -0.01 | 0.13 | 9.78E-01 | 0.04 | 0.25 | 9.62E-01 | 0.02 | 0.94 | 9.89E-01 |
| Creatinine | -0.01 | 0.11 | 9.96E-01 | -0.01 | 0.12 | 9.99E-01 | -0.03 | 0.12 | 9.90E-01 | -0.02 | 0.12 | 9.77E-01 | 1.26 | 1.66 | 9.34E-01 | 1.31 | 0.45 | 9.86E-01 |
| Total lipids in LDL | 0.01 | 0.10 | 9.96E-01 | 0.01 | 0.10 | 9.99E-01 | -0.03 | 0.11 | 9.90E-01 | -0.02 | 0.11 | 9.77E-01 | 0.13 | 0.23 | 9.34E-01 | 0.12 | 0.59 | 9.86E-01 |
| Phospholipids to total lipids ratio in medium HDL | 0.00 | 0.09 | 9.96E-01 | -0.01 | 0.10 | 9.99E-01 | -0.05 | 0.11 | 9.40E-01 | -0.04 | 0.12 | 9.77E-01 | 0.16 | 0.20 | 9.34E-01 | 0.14 | 0.52 | 9.86E-01 |
| Total cholesterol | 0.00 | 0.11 | 9.96E-01 | -0.01 | 0.11 | 9.99E-01 | -0.03 | 0.12 | 9.90E-01 | -0.04 | 0.12 | 9.77E-01 | 0.10 | 0.24 | 9.34E-01 | 0.11 | 0.65 | 9.86E-01 |
| Triglycerides to total lipids ratio in very large VLDL | 0.01 | 0.11 | 9.96E-01 | 0.01 | 0.12 | 9.99E-01 | -0.06 | 0.12 | 9.20E-01 | -0.04 | 0.12 | 9.77E-01 | 0.39 | 0.32 | 9.34E-01 | 0.23 | 0.48 | 9.86E-01 |
| Triglycerides to total lipids ratio in medium VLDL | -0.01 | 0.11 | 9.96E-01 | -0.01 | 0.11 | 9.99E-01 | -0.05 | 0.12 | 9.46E-01 | -0.02 | 0.12 | 9.77E-01 | 0.20 | 0.26 | 9.34E-01 | 0.07 | 0.82 | 9.86E-01 |
| Phospholipids in large HDL | -0.01 | 0.10 | 9.96E-01 | -0.01 | 0.10 | 9.99E-01 | 0.00 | 0.11 | 9.90E-01 | -0.03 | 0.12 | 9.77E-01 | -0.03 | 0.23 | 9.65E-01 | 0.01 | 0.98 | 9.92E-01 |
| Free cholesterol in HDL | -0.01 | 0.11 | 9.96E-01 | -0.01 | 0.11 | 9.99E-01 | -0.02 | 0.12 | 9.90E-01 | -0.04 | 0.13 | 9.77E-01 | 0.05 | 0.26 | 9.50E-01 | 0.06 | 0.81 | 9.86E-01 |
| Cholesterol in very small VLDL | 0.01 | 0.11 | 9.96E-01 | 0.01 | 0.11 | 9.99E-01 | -0.03 | 0.12 | 9.90E-01 | -0.05 | 0.12 | 9.51E-01 | 0.17 | 0.25 | 9.34E-01 | 0.21 | 0.39 | 9.86E-01 |
| Total cholesterol minus HDL-C | 0.00 | 0.10 | 9.96E-01 | 0.00 | 0.10 | 9.99E-01 | -0.04 | 0.11 | 9.80E-01 | -0.04 | 0.12 | 9.77E-01 | 0.12 | 0.23 | 9.34E-01 | 0.12 | 0.61 | 9.86E-01 |
| Triglycerides to total lipids ratio in medium HDL | 0.01 | 0.09 | 9.96E-01 | 0.00 | 0.10 | 9.99E-01 | -0.05 | 0.10 | 9.40E-01 | -0.04 | 0.11 | 9.69E-01 | 0.27 | 0.23 | 9.34E-01 | 0.23 | 0.33 | 9.86E-01 |
| Cholesteryl esters to total lipids ratio in chylomicrons and extremely large VLDL | -0.01 | 0.10 | 9.96E-01 | 0.00 | 0.11 | 9.99E-01 | 0.07 | 0.12 | 9.12E-01 | 0.05 | 0.12 | 9.51E-01 | -0.21 | 0.24 | 9.34E-01 | -0.17 | 0.49 | 9.86E-01 |
| Phospholipids in HDL | 0.00 | 0.11 | 9.96E-01 | 0.00 | 0.11 | 9.99E-01 | -0.01 | 0.12 | 9.90E-01 | -0.02 | 0.13 | 9.77E-01 | 0.04 | 0.25 | 9.59E-01 | 0.03 | 0.90 | 9.89E-01 |
| Free cholesterol to total lipids ratio in medium HDL | -0.01 | 0.10 | 9.96E-01 | 0.00 | 0.10 | 9.99E-01 | 0.01 | 0.11 | 9.90E-01 | 0.00 | 0.11 | 9.92E-01 | -0.06 | 0.22 | 9.34E-01 | -0.04 | 0.85 | 9.89E-01 |
| Concentration of large LDL particles | 0.01 | 0.10 | 9.96E-01 | 0.00 | 0.10 | 9.99E-01 | -0.02 | 0.11 | 9.90E-01 | -0.02 | 0.12 | 9.77E-01 | 0.07 | 0.23 | 9.34E-01 | 0.10 | 0.67 | 9.86E-01 |
| Cholesterol to total lipids ratio in large VLDL | 0.01 | 0.10 | 9.96E-01 | 0.00 | 0.11 | 9.99E-01 | 0.05 | 0.11 | 9.48E-01 | 0.02 | 0.12 | 9.77E-01 | -0.16 | 0.25 | 9.34E-01 | -0.03 | 0.90 | 9.89E-01 |
| Phospholipids to total lipids ratio in large LDL | 0.00 | 0.10 | 9.96E-01 | 0.00 | 0.10 | 9.99E-01 | 0.04 | 0.11 | 9.80E-01 | 0.03 | 0.11 | 9.77E-01 | -0.11 | 0.22 | 9.34E-01 | -0.10 | 0.66 | 9.86E-01 |
| Ratio of omega-6 fatty acids to total fatty acids | 0.00 | 0.10 | 9.96E-01 | 0.00 | 0.10 | 9.99E-01 | 0.07 | 0.11 | 8.68E-01 | 0.06 | 0.12 | 9.49E-01 | -0.25 | 0.20 | 9.34E-01 | -0.19 | 0.39 | 9.86E-01 |
| Phospholipids in medium LDL | 0.01 | 0.10 | 9.96E-01 | 0.00 | 0.10 | 9.99E-01 | -0.04 | 0.11 | 9.80E-01 | -0.03 | 0.11 | 9.77E-01 | 0.15 | 0.23 | 9.34E-01 | 0.14 | 0.56 | 9.86E-01 |
| Acetoacetate | 0.00 | 0.10 | 9.96E-01 | 0.00 | 0.10 | 9.99E-01 | -0.01 | 0.11 | 9.90E-01 | -0.01 | 0.11 | 9.77E-01 | 0.05 | 0.21 | 9.34E-01 | 0.05 | 0.81 | 9.86E-01 |
| Triglycerides in HDL | 0.01 | 0.10 | 9.96E-01 | 0.00 | 0.10 | 9.99E-01 | -0.08 | 0.11 | 8.62E-01 | -0.08 | 0.11 | 9.10E-01 | 0.37 | 0.22 | 9.34E-01 | 0.31 | 0.18 | 9.86E-01 |
| HDL cholesterol | -0.01 | 0.10 | 9.96E-01 | 0.00 | 0.11 | 9.99E-01 | 0.01 | 0.12 | 9.90E-01 | -0.01 | 0.13 | 9.87E-01 | -0.06 | 0.25 | 9.34E-01 | -0.05 | 0.85 | 9.89E-01 |
| Cholesteryl esters in HDL | -0.01 | 0.10 | 9.96E-01 | 0.00 | 0.11 | 9.99E-01 | 0.02 | 0.12 | 9.90E-01 | 0.00 | 0.13 | 9.95E-01 | -0.09 | 0.24 | 9.34E-01 | -0.08 | 0.76 | 9.86E-01 |
| Total lipids in HDL | -0.01 | 0.11 | 9.96E-01 | 0.00 | 0.11 | 9.99E-01 | -0.01 | 0.12 | 9.90E-01 | -0.02 | 0.13 | 9.77E-01 | 0.02 | 0.25 | 9.98E-01 | 0.02 | 0.95 | 9.89E-01 |
| Total esterified cholesterol | 0.00 | 0.11 | 9.96E-01 | 0.00 | 0.11 | 9.99E-01 | -0.02 | 0.12 | 9.90E-01 | -0.03 | 0.12 | 9.77E-01 | 0.08 | 0.25 | 9.34E-01 | 0.10 | 0.70 | 9.86E-01 |
| Cholesteryl esters in LDL | 0.00 | 0.10 | 9.96E-01 | 0.00 | 0.10 | 9.99E-01 | -0.03 | 0.11 | 9.90E-01 | -0.03 | 0.11 | 9.77E-01 | 0.10 | 0.23 | 9.34E-01 | 0.10 | 0.68 | 9.86E-01 |

Note: The table shows associations from the logistic regression analysis where plasms metabolomics level from the Nightingale platform was main exposure and prevalent prostate cancer as the main outcome. Model 1 : adjusted for age, cohort, time differences (ERGO), and lipid lowering medication. Model 2: adjusted for model1, smoking status, BMI, alcohol and education. No adjustment for time differences was made in the analyses of the subcohorts (ERGO-4 and ERGO-5). The table is ranked based on the *FDR* values in model 2 within the E4&E5 cohort, using bold text indicates metabolites are surpassing *FDR* adjusted *P* value threshold (*P*<0.05). ERGO-4 and ERGO-5 refer to the fourth and fifth visits subcohort from the baseline cohort. ERGO-4&5 refers to the combined data from the fourth and fifth visits of the baseline cohort. Abbreviations: SE, standard error; IDL, intermediate-density lipoprotein; LDL, low-density lipoprotein; HDL, high-density lipoprotein; VLDL, very-low-density lipoprotein; BMI, body mass index; FDR, false discovery rate.

**Supplementary Table 12. Serum metabolites associated with prevalent prostate cancer from the Metabolon platform at baseline.**

| **Metabolite** | **ERGO-4&5** | | | | | | | | **ERGO-4** | | | | **ERGO-5** | | | |
| --- | --- | --- | --- | --- | --- | --- | --- | --- | --- | --- | --- | --- | --- | --- | --- | --- |
|  | **Model 1** | | | | **Model 2** | | | | **Model 1** | | **Model 2** | | **Model 1** | | **Model 2** | |
|  | **Beta±SE** | **I^2^** | **Het.*P-*value** | ***FDR* value** | **Beta±SE** | **I^2^** | **Het.*P-*value** | ***FDR* value** | **Beta±SE** | ***FDR* value** | **Beta±SE** | ***FDR* value** | **Beta±SE** | ***FDR* value** | **Beta±SE** | ***FDR* value** |
| sphingomyelin (d18:1/18:1, d18:2/18:0) | 0.67±0.39 | 0 | 7.71E-01 | 5.24E-01 | 0.63±0.17 | 0 | 5.48E-01 | 1.23E-01 | 0.55±0.17 | 4.25E-01 | 0.6±0.18 | 3.91E-01 | 0.78±0.56 | 9.99E-01 | 0.99±0.63 | 1.00E+00 |
| stearoyl sphingomyelin (d18:1/18:0) | 0.68±0.4 | 0 | 7.10E-01 | 5.24E-01 | 0.59±0.16 | 0 | 6.18E-01 | 1.23E-01 | 0.54±0.16 | 4.25E-01 | 0.57±0.16 | 3.91E-01 | 0.84±0.59 | 9.99E-01 | 0.89±0.64 | 1.00E+00 |
| sphingomyelin (d18:0/18:0, d19:0/17:0)* | 0.92±0.38 | 67.07 | 8.14E-02 | 5.24E-01 | 0.57±0.16 | 81.39 | 2.05E-02 | 1.52E-01 | 0.46±0.16 | 4.41E-01 | 0.52±0.17 | 3.91E-01 | 1.86±0.66 | 9.99E-01 | 3.22±1.15 | 1.00E+00 |
| 7-alpha-hydroxy-3-oxo-4-cholestenoate (7-Hoca) | -0.37±0.26 | 0 | 7.33E-01 | 5.24E-01 | -0.54±0.16 | 0 | 8.40E-01 | 1.56E-01 | -0.52±0.18 | 4.41E-01 | -0.55±0.18 | 3.91E-01 | -0.31±0.31 | 9.99E-01 | -0.48±0.34 | 1.00E+00 |
| 5,6-dihydrothymine | -0.29±0.26 | 0 | 6.48E-01 | 5.24E-01 | -0.46±0.14 | 0 | 9.33E-01 | 1.56E-01 | -0.46±0.15 | 4.25E-01 | -0.45±0.15 | 3.91E-01 | -0.21±0.31 | 9.99E-01 | -0.49±0.36 | 1.00E+00 |
| androsterone glucuronide | -0.43±0.28 | 0 | 9.14E-01 | 5.24E-01 | -0.51±0.15 | 0 | 9.54E-01 | 1.63E-01 | -0.48±0.17 | 4.41E-01 | -0.51±0.17 | 3.91E-01 | -0.41±0.34 | 9.99E-01 | -0.49±0.38 | 1.00E+00 |
| ribitol | -0.24±0.28 | 0 | 5.76E-01 | 5.24E-01 | -0.41±0.14 | 0 | 6.79E-01 | 2.23E-01 | -0.38±0.14 | 4.41E-01 | -0.43±0.15 | 3.91E-01 | -0.06±0.43 | 9.99E-01 | -0.23±0.46 | 1.00E+00 |
| sphingomyelin (d18:1/19:0, d19:1/18:0)* | 0.42±0.35 | 0 | 9.90E-01 | 5.24E-01 | 0.48±0.16 | 0 | 9.04E-01 | 2.23E-01 | 0.42±0.16 | 4.41E-01 | 0.47±0.17 | 3.91E-01 | 0.41±0.62 | 9.99E-01 | 0.56±0.7 | 1.00E+00 |
| N-stearoyl-sphingosine (d18:1/18:0)* | 0.52±0.35 | 0 | 7.70E-01 | 5.24E-01 | 0.48±0.16 | 0 | 6.58E-01 | 2.23E-01 | 0.44±0.16 | 4.41E-01 | 0.46±0.16 | 3.91E-01 | 0.65±0.59 | 9.99E-01 | 0.75±0.63 | 1.00E+00 |
| orotate | -0.32±0.26 | 0 | 6.34E-01 | 5.24E-01 | -0.52±0.17 | 0 | 8.22E-01 | 2.23E-01 | -0.55±0.19 | 4.41E-01 | -0.54±0.19 | 3.91E-01 | -0.25±0.29 | 9.99E-01 | -0.45±0.37 | 1.00E+00 |
| pristanate | -0.28±0.3 | 0 | 6.32E-01 | 5.24E-01 | -0.41±0.14 | 0 | 8.08E-01 | 2.23E-01 | -0.44±0.14 | 4.25E-01 | -0.42±0.14 | 3.91E-01 | -0.15±0.41 | 9.99E-01 | -0.3±0.47 | 1.00E+00 |
| X - 18921 | -0.47±0.29 | 0 | 8.00E-01 | 5.24E-01 | -0.45±0.14 | 39.08 | 2.00E-01 | 2.23E-01 | -0.4±0.15 | 4.41E-01 | -0.4±0.15 | 4.32E-01 | -0.55±0.41 | 9.99E-01 | -1.25±0.64 | 1.00E+00 |
| linolenoylcarnitine (C18:3)* | -0.48±0.26 | 0 | 9.12E-01 | 5.24E-01 | -0.48±0.15 | 0 | 3.39E-01 | 2.23E-01 | -0.44±0.16 | 4.41E-01 | -0.42±0.16 | 5.29E-01 | -0.5±0.33 | 9.99E-01 | -0.88±0.45 | 1.00E+00 |
| 5alpha-androstan-3alpha,17beta-diol monosulfate (2) | -0.76±0.33 | 2.87 | 3.10E-01 | 5.24E-01 | -0.54±0.19 | 68.78 | 7.35E-02 | 2.32E-01 | -0.45±0.19 | 5.66E-01 | -0.44±0.19 | 5.97E-01 | -1.12±0.49 | 9.99E-01 | -1.58±0.61 | 1.00E+00 |
| 5alpha-androstan-3beta,17beta-diol disulfate | -0.44±0.31 | 0 | 9.57E-01 | 5.24E-01 | -0.49±0.17 | 0 | 7.64E-01 | 2.36E-01 | -0.46±0.18 | 5.66E-01 | -0.47±0.18 | 5.29E-01 | -0.42±0.41 | 9.99E-01 | -0.62±0.46 | 1.00E+00 |
| glycochenodeoxycholate 3-sulfate | -0.3±0.28 | 0 | 7.03E-01 | 5.24E-01 | -0.41±0.14 | 0 | 4.98E-01 | 2.50E-01 | -0.42±0.15 | 4.41E-01 | -0.45±0.15 | 3.91E-01 | -0.21±0.37 | 9.99E-01 | -0.16±0.38 | 1.00E+00 |
| glycine conjugate of C10H12O2* | -0.46±0.25 | 0 | 4.49E-01 | 5.24E-01 | -0.39±0.14 | 43.94 | 1.82E-01 | 2.58E-01 | -0.32±0.14 | 5.87E-01 | -0.34±0.14 | 5.80E-01 | -0.72±0.43 | 9.99E-01 | -1.07±0.53 | 1.00E+00 |
| phytanate | -0.3±0.31 | 0 | 6.64E-01 | 5.24E-01 | -0.4±0.14 | 0 | 8.53E-01 | 2.69E-01 | -0.43±0.14 | 4.41E-01 | -0.4±0.15 | 4.24E-01 | -0.16±0.45 | 9.99E-01 | -0.31±0.5 | 1.00E+00 |
| sphingomyelin (d18:1/20:1, d18:2/20:0)* | 0.23±0.32 | 0 | 4.95E-01 | 5.37E-01 | 0.47±0.17 | 0 | 4.86E-01 | 2.73E-01 | 0.47±0.17 | 4.41E-01 | 0.5±0.18 | 3.91E-01 | 0.04±0.42 | 9.99E-01 | 0.07±0.59 | 1.00E+00 |
| N-stearoyl-sphingadienine (d18:2/18:0)* | 0.57±0.32 | 0 | 3.77E-01 | 5.24E-01 | 0.44±0.16 | 0 | 3.33E-01 | 2.73E-01 | 0.38±0.16 | 5.66E-01 | 0.4±0.17 | 5.80E-01 | 1.01±0.6 | 9.99E-01 | 1.06±0.66 | 1.00E+00 |
| 10-undecenoate (11:1n1) | -0.3±0.3 | 0 | 7.10E-01 | 5.24E-01 | -0.4±0.15 | 0 | 7.93E-01 | 3.10E-01 | -0.4±0.15 | 4.41E-01 | -0.39±0.15 | 5.58E-01 | -0.18±0.45 | 9.99E-01 | -0.53±0.54 | 1.00E+00 |
| X - 18888 | -0.25±0.29 | 0 | 5.87E-01 | 5.24E-01 | -0.4±0.15 | 0 | 5.02E-01 | 3.10E-01 | -0.41±0.16 | 4.78E-01 | -0.44±0.16 | 4.24E-01 | -0.1±0.41 | 9.99E-01 | -0.12±0.44 | 1.00E+00 |
| sphingomyelin (d18:1/17:0, d17:1/18:0, d19:1/16:0) | 0.41±0.32 | 0 | 7.60E-01 | 5.24E-01 | 0.44±0.16 | 0 | 5.51E-01 | 3.17E-01 | 0.36±0.16 | 5.73E-01 | 0.42±0.17 | 5.58E-01 | 0.61±0.75 | 9.99E-01 | 0.95±0.87 | 1.00E+00 |
| 3-ureidopropionate | -0.28±0.28 | 0 | 6.95E-01 | 5.24E-01 | -0.4±0.15 | 0 | 9.96E-01 | 3.17E-01 | -0.38±0.16 | 5.66E-01 | -0.4±0.16 | 5.58E-01 | -0.16±0.42 | 9.99E-01 | -0.4±0.48 | 1.00E+00 |
| arachidonoylcarnitine (C20:4) | -0.33±0.27 | 0 | 8.48E-01 | 5.24E-01 | -0.4±0.15 | 0 | 6.91E-01 | 3.17E-01 | -0.39±0.16 | 5.66E-01 | -0.38±0.16 | 5.89E-01 | -0.28±0.37 | 9.99E-01 | -0.56±0.44 | 1.00E+00 |
| taurochenodeoxycholic acid 3-sulfate | -0.41±0.28 | 0 | 8.16E-01 | 5.24E-01 | -0.39±0.15 | 0 | 8.13E-01 | 3.31E-01 | -0.36±0.15 | 5.66E-01 | -0.38±0.16 | 5.80E-01 | -0.49±0.44 | 9.99E-01 | -0.5±0.49 | 1.00E+00 |
| sphingomyelin (d18:2/21:0, d16:2/23:0)* | 0.22±0.3 | 0 | 4.93E-01 | 5.39E-01 | 0.46±0.18 | 0 | 4.52E-01 | 3.53E-01 | 0.43±0.18 | 5.66E-01 | 0.51±0.19 | 4.49E-01 | 0.02±0.41 | 9.99E-01 | 0.04±0.59 | 1.00E+00 |
| X - 21353 | -0.34±0.28 | 0 | 9.49E-01 | 5.24E-01 | -0.37±0.14 | 0 | 7.73E-01 | 3.53E-01 | -0.36±0.15 | 5.66E-01 | -0.36±0.15 | 5.80E-01 | -0.32±0.45 | 9.99E-01 | -0.52±0.55 | 1.00E+00 |
| X - 12812 | -0.47±0.25 | 0 | 4.69E-01 | 5.24E-01 | -0.36±0.14 | 21.14 | 2.60E-01 | 4.12E-01 | -0.32±0.15 | 6.11E-01 | -0.3±0.15 | 6.30E-01 | -0.7±0.41 | 9.99E-01 | -0.85±0.46 | 1.00E+00 |
| sphingomyelin (d18:0/20:0, d16:0/22:0)* | 0.52±0.28 | 62.16 | 1.04E-01 | 5.24E-01 | 0.4±0.16 | 72.37 | 5.71E-02 | 4.13E-01 | 0.31±0.16 | 6.58E-01 | 0.35±0.16 | 6.30E-01 | 1.55±0.7 | 9.99E-01 | 2.33±1.03 | 1.00E+00 |
| 3-carboxy-4-methyl-5-propyl-2-furanpropanoate (CMPF) | 0.25±0.3 | 0 | 5.76E-01 | 5.24E-01 | 0.38±0.16 | 0 | 4.05E-01 | 4.35E-01 | 0.4±0.16 | 5.66E-01 | 0.43±0.17 | 5.36E-01 | 0.06±0.46 | 9.99E-01 | 0.03±0.44 | 1.00E+00 |
| sphingomyelin (d18:1/20:2, d18:2/20:1, d16:1/22:2)* | 0.29±0.3 | 0 | 6.99E-01 | 5.24E-01 | 0.4±0.17 | 0 | 8.33E-01 | 4.35E-01 | 0.38±0.16 | 5.73E-01 | 0.41±0.17 | 5.80E-01 | 0.14±0.48 | 9.99E-01 | 0.28±0.62 | 1.00E+00 |
| glycohyocholate | -0.52±0.27 | 55.11 | 1.36E-01 | 5.24E-01 | -0.37±0.15 | 46.54 | 1.71E-01 | 4.35E-01 | -0.3±0.16 | 6.58E-01 | -0.31±0.16 | 6.30E-01 | -1.27±0.57 | 9.99E-01 | -1.14±0.59 | 1.00E+00 |
| branched-chain, straight-chain, or cyclopropyl 10:1 fatty acid (1)* | -0.16±0.32 | 5.69 | 3.03E-01 | 6.42E-01 | -0.36±0.15 | 0 | 5.37E-01 | 4.43E-01 | -0.39±0.16 | 5.66E-01 | -0.39±0.16 | 5.80E-01 | 0.32±0.56 | 9.99E-01 | -0.03±0.56 | 1.00E+00 |
| tetrahydrocortisone glucuronide (5) | -0.22±0.25 | 0 | 6.50E-01 | 5.24E-01 | -0.3±0.13 | 0 | 7.54E-01 | 4.43E-01 | -0.29±0.13 | 5.73E-01 | -0.31±0.13 | 5.80E-01 | -0.04±0.47 | 9.99E-01 | -0.16±0.48 | 1.00E+00 |
| indolin-2-one | 0.42±0.29 | 0 | 6.86E-01 | 5.24E-01 | 0.38±0.16 | 0 | 7.26E-01 | 4.43E-01 | 0.35±0.17 | 6.13E-01 | 0.37±0.17 | 6.22E-01 | 0.61±0.56 | 9.99E-01 | 0.59±0.62 | 1.00E+00 |
| octadecanedioate (C18-DC) | -0.37±0.26 | 0 | 8.57E-01 | 5.24E-01 | -0.35±0.15 | 0 | 5.01E-01 | 4.43E-01 | -0.33±0.15 | 6.11E-01 | -0.32±0.16 | 6.30E-01 | -0.42±0.4 | 9.99E-01 | -0.66±0.48 | 1.00E+00 |
| undecenoylcarnitine (C11:1) | -0.35±0.25 | 0 | 1.00E+00 | 5.24E-01 | -0.35±0.15 | 0 | 3.57E-01 | 4.43E-01 | -0.35±0.15 | 5.66E-01 | -0.31±0.15 | 6.30E-01 | -0.35±0.36 | 9.99E-01 | -0.84±0.55 | 1.00E+00 |
| behenoyl dihydrosphingomyelin (d18:0/22:0)* | 0.45±0.26 | 37.23 | 2.07E-01 | 5.24E-01 | 0.36±0.15 | 51.93 | 1.49E-01 | 4.47E-01 | 0.29±0.15 | 6.58E-01 | 0.32±0.16 | 6.30E-01 | 1.13±0.6 | 9.99E-01 | 1.42±0.75 | 1.00E+00 |
| docosapentaenoate (n6 DPA; 22:5n6) | -0.16±0.34 | 27.26 | 2.41E-01 | 6.56E-01 | -0.38±0.16 | 0 | 3.99E-01 | 4.48E-01 | -0.39±0.16 | 5.66E-01 | -0.41±0.17 | 5.72E-01 | 0.54±0.69 | 9.99E-01 | 0.16±0.66 | 1.00E+00 |
| cinnamoylglycine | -0.39±0.18 | 67.94 | 7.74E-02 | 5.24E-01 | -0.32±0.14 | 67.12 | 8.12E-02 | 4.52E-01 | -0.2±0.14 | 7.58E-01 | -0.23±0.15 | 7.63E-01 | -0.91±0.35 | 9.99E-01 | -0.94±0.38 | 1.00E+00 |
| 1-oleoyl-2-linoleoyl-GPE (18:1/18:2)* | -0.27±0.22 | 0 | 9.89E-01 | 5.24E-01 | -0.29±0.13 | 0 | 7.89E-01 | 4.87E-01 | -0.27±0.13 | 6.13E-01 | -0.3±0.14 | 5.97E-01 | -0.27±0.36 | 9.99E-01 | -0.2±0.39 | 1.00E+00 |
| N-acetylhistidine | -0.27±0.26 | 0 | 7.66E-01 | 5.24E-01 | -0.33±0.15 | 0 | 5.87E-01 | 4.88E-01 | -0.33±0.15 | 5.87E-01 | -0.36±0.15 | 5.89E-01 | -0.17±0.41 | 9.99E-01 | -0.1±0.45 | 1.00E+00 |
| 3-hydroxyoctanoate | -0.21±0.28 | 0 | 5.10E-01 | 5.33E-01 | -0.38±0.16 | 0 | 6.48E-01 | 4.88E-01 | -0.41±0.17 | 5.66E-01 | -0.41±0.18 | 5.97E-01 | -0.04±0.38 | 9.99E-01 | -0.18±0.46 | 1.00E+00 |
| octadecenedioate (C18:1-DC) | -0.29±0.23 | 0 | 8.98E-01 | 5.24E-01 | -0.33±0.15 | 0 | 6.39E-01 | 4.88E-01 | -0.32±0.15 | 6.13E-01 | -0.31±0.16 | 6.39E-01 | -0.26±0.33 | 9.99E-01 | -0.52±0.42 | 1.00E+00 |
| phenyllactate (PLA) | -0.37±0.21 | 0 | 4.75E-01 | 5.24E-01 | -0.38±0.17 | 36.05 | 2.11E-01 | 4.98E-01 | -0.26±0.18 | 7.45E-01 | -0.29±0.18 | 7.56E-01 | -0.58±0.36 | 9.99E-01 | -0.87±0.42 | 1.00E+00 |
| dihomo-linolenoylcarnitine (C20:3n3 or 6)* | -0.23±0.27 | 0 | 5.77E-01 | 5.24E-01 | -0.32±0.14 | 0 | 8.70E-01 | 5.03E-01 | -0.35±0.15 | 5.66E-01 | -0.33±0.15 | 6.24E-01 | -0.04±0.43 | 9.99E-01 | -0.24±0.51 | 1.00E+00 |
| glycine conjugate of C10H14O2 (1)* | -0.36±0.21 | 0 | 3.42E-01 | 5.24E-01 | -0.34±0.15 | 59.52 | 1.16E-01 | 5.10E-01 | -0.24±0.15 | 7.35E-01 | -0.27±0.16 | 7.30E-01 | -0.73±0.44 | 9.99E-01 | -1.23±0.59 | 1.00E+00 |
| X - 23665 | -0.33±0.25 | 0 | 9.73E-01 | 5.24E-01 | -0.33±0.15 | 0 | 7.01E-01 | 5.18E-01 | -0.33±0.16 | 6.13E-01 | -0.31±0.16 | 6.30E-01 | -0.34±0.37 | 9.99E-01 | -0.5±0.46 | 1.00E+00 |
| X - 15486 | -0.31±0.22 | 0 | 7.09E-01 | 5.24E-01 | -0.33±0.15 | 25.5 | 2.47E-01 | 5.18E-01 | -0.26±0.15 | 6.62E-01 | -0.28±0.15 | 7.00E-01 | -0.43±0.4 | 9.99E-01 | -0.91±0.53 | 1.00E+00 |
| lactate | -0.21±0.26 | 0 | 5.58E-01 | 5.25E-01 | -0.33±0.15 | 0 | 5.93E-01 | 5.18E-01 | -0.33±0.15 | 6.11E-01 | -0.36±0.16 | 5.97E-01 | -0.02±0.43 | 9.99E-01 | -0.09±0.48 | 1.00E+00 |
| X - 13431 | -0.26±0.27 | 0 | 6.75E-01 | 5.24E-01 | -0.33±0.15 | 0 | 9.24E-01 | 5.18E-01 | -0.36±0.15 | 5.66E-01 | -0.33±0.16 | 6.30E-01 | -0.13±0.42 | 9.99E-01 | -0.39±0.61 | 1.00E+00 |
| 3-hydroxydecanoate | -0.18±0.29 | 0 | 4.17E-01 | 5.83E-01 | -0.35±0.16 | 0 | 4.41E-01 | 5.51E-01 | -0.4±0.17 | 5.66E-01 | -0.39±0.17 | 5.97E-01 | 0.07±0.42 | 9.99E-01 | 0.01±0.49 | 1.00E+00 |
| gamma-glutamylthreonine | 0.24±0.29 | 0 | 5.76E-01 | 5.24E-01 | 0.31±0.15 | 6.87 | 3.00E-01 | 5.52E-01 | 0.39±0.16 | 5.66E-01 | 0.37±0.16 | 5.89E-01 | 0.06±0.43 | 9.99E-01 | -0.08±0.41 | 1.00E+00 |
| linolenate [alpha or gamma; (18:3n3 or 6)] | -0.24±0.28 | 0 | 5.98E-01 | 5.24E-01 | -0.32±0.15 | 0 | 8.58E-01 | 5.52E-01 | -0.34±0.16 | 6.13E-01 | -0.33±0.16 | 6.30E-01 | -0.02±0.5 | 9.99E-01 | -0.23±0.52 | 1.00E+00 |
| glycosyl-N-stearoyl-sphingosine (d18:1/18:0) | 0.32±0.28 | 0 | 8.88E-01 | 5.24E-01 | 0.34±0.16 | 0 | 9.60E-01 | 5.52E-01 | 0.35±0.16 | 6.13E-01 | 0.34±0.17 | 6.30E-01 | 0.26±0.49 | 9.99E-01 | 0.31±0.64 | 1.00E+00 |
| 13-HODE + 9-HODE | -0.19±0.28 | 0 | 4.28E-01 | 5.58E-01 | -0.29±0.14 | 0 | 4.67E-01 | 5.52E-01 | -0.34±0.15 | 5.87E-01 | -0.33±0.15 | 6.30E-01 | 0.14±0.5 | 9.99E-01 | 0.05±0.49 | 1.00E+00 |
| 1-stearoyl-2-docosahexaenoyl-GPC (18:0/22:6) | 0.28±0.26 | 0 | 8.27E-01 | 5.24E-01 | 0.32±0.15 | 0 | 9.48E-01 | 5.52E-01 | 0.32±0.16 | 6.58E-01 | 0.32±0.16 | 6.30E-01 | 0.19±0.47 | 9.99E-01 | 0.29±0.54 | 1.00E+00 |
| hydroxy-CMPF* | 0.24±0.26 | 0 | 6.78E-01 | 5.24E-01 | 0.32±0.15 | 0 | 7.53E-01 | 5.52E-01 | 0.31±0.16 | 6.58E-01 | 0.34±0.16 | 6.30E-01 | 0.08±0.46 | 9.99E-01 | 0.18±0.47 | 1.00E+00 |
| X - 12798 | -0.17±0.22 | 0 | 4.84E-01 | 5.33E-01 | -0.24±0.11 | 0 | 7.37E-01 | 5.52E-01 | -0.25±0.11 | 6.11E-01 | -0.25±0.12 | 6.30E-01 | 0.12±0.46 | 9.99E-01 | -0.06±0.55 | 1.00E+00 |
| X - 11444 | -0.24±0.19 | 0 | 9.78E-01 | 5.24E-01 | -0.26±0.12 | 0 | 8.18E-01 | 5.52E-01 | -0.24±0.13 | 6.58E-01 | -0.25±0.13 | 6.48E-01 | -0.25±0.32 | 9.99E-01 | -0.34±0.37 | 1.00E+00 |
| 2S,3R-dihydroxybutyrate | -0.33±0.26 | 0 | 9.74E-01 | 5.24E-01 | -0.35±0.16 | 0 | 7.43E-01 | 5.52E-01 | -0.34±0.17 | 6.58E-01 | -0.32±0.18 | 6.72E-01 | -0.32±0.4 | 9.99E-01 | -0.48±0.44 | 1.00E+00 |
| X - 21286 | 0.36±0.24 | 0 | 4.34E-01 | 5.24E-01 | 0.32±0.16 | 0 | 5.76E-01 | 5.52E-01 | 0.26±0.16 | 7.05E-01 | 0.29±0.16 | 6.79E-01 | 0.73±0.53 | 9.99E-01 | 0.65±0.62 | 1.00E+00 |
| glutamine conjugate of C6H10O2 (1)* | -0.28±0.23 | 0 | 9.23E-01 | 5.24E-01 | -0.33±0.16 | 0 | 7.24E-01 | 5.52E-01 | -0.29±0.17 | 6.58E-01 | -0.31±0.17 | 6.89E-01 | -0.25±0.38 | 9.99E-01 | -0.49±0.49 | 1.00E+00 |
| cis-3,4-methyleneheptanoate | -0.22±0.2 | 0 | 9.04E-01 | 5.24E-01 | -0.27±0.13 | 0 | 4.62E-01 | 5.52E-01 | -0.24±0.13 | 6.58E-01 | -0.24±0.13 | 7.30E-01 | -0.19±0.34 | 9.99E-01 | -0.55±0.4 | 1.00E+00 |
| citrate | -0.21±0.18 | 0 | 8.25E-01 | 5.24E-01 | -0.27±0.13 | 0 | 7.86E-01 | 5.52E-01 | -0.25±0.14 | 6.58E-01 | -0.25±0.15 | 7.32E-01 | -0.17±0.26 | 9.99E-01 | -0.34±0.29 | 1.00E+00 |
| biliverdin | -0.21±0.23 | 0 | 6.82E-01 | 5.24E-01 | -0.29±0.14 | 0 | 5.04E-01 | 5.52E-01 | -0.27±0.14 | 6.58E-01 | -0.25±0.15 | 7.32E-01 | -0.07±0.41 | 9.99E-01 | -0.55±0.42 | 1.00E+00 |
| X - 11478 | -0.33±0.21 | 18.08 | 2.69E-01 | 5.24E-01 | -0.29±0.14 | 67.57 | 7.91E-02 | 5.52E-01 | -0.23±0.14 | 7.44E-01 | -0.24±0.14 | 7.32E-01 | -0.82±0.49 | 9.99E-01 | -1.41±0.65 | 1.00E+00 |
| eicosanedioate (C20-DC) | -0.33±0.21 | 0 | 5.89E-01 | 5.24E-01 | -0.29±0.14 | 30.31 | 2.31E-01 | 5.52E-01 | -0.25±0.15 | 6.69E-01 | -0.23±0.15 | 7.61E-01 | -0.5±0.38 | 9.99E-01 | -0.8±0.45 | 1.00E+00 |
| N-acetyl-2-aminooctanoate* | -0.31±0.23 | 0 | 8.68E-01 | 5.24E-01 | -0.31±0.15 | 4.7 | 3.06E-01 | 5.52E-01 | -0.29±0.16 | 6.58E-01 | -0.26±0.16 | 7.61E-01 | -0.37±0.4 | 9.99E-01 | -0.74±0.44 | 1.00E+00 |
| lactosyl-N-palmitoyl-sphingosine (d18:1/16:0) | -0.23±0.14 | 0 | 7.05E-01 | 5.24E-01 | -0.3±0.14 | 31.34 | 2.27E-01 | 5.52E-01 | -0.19±0.16 | 7.99E-01 | -0.19±0.16 | 8.25E-01 | -0.29±0.21 | 9.99E-01 | -0.58±0.28 | 1.00E+00 |
| creatine | 0.05±0.27 | 29.6 | 2.33E-01 | 8.65E-01 | 0.3±0.16 | 54.72 | 1.37E-01 | 5.54E-01 | 0.39±0.16 | 5.66E-01 | 0.39±0.17 | 5.91E-01 | -0.25±0.36 | 9.99E-01 | -0.34±0.46 | 1.00E+00 |
| glycosyl-N-palmitoyl-sphingosine (d18:1/16:0) | 0.13±0.23 | 0 | 3.98E-01 | 6.07E-01 | 0.29±0.15 | 43.34 | 1.84E-01 | 5.54E-01 | 0.34±0.16 | 6.11E-01 | 0.37±0.16 | 5.97E-01 | -0.06±0.33 | 9.99E-01 | -0.22±0.41 | 1.00E+00 |
| 3-hydroxylaurate | -0.21±0.27 | 0 | 5.39E-01 | 5.29E-01 | -0.29±0.15 | 0 | 5.76E-01 | 5.54E-01 | -0.33±0.16 | 6.13E-01 | -0.32±0.16 | 6.30E-01 | 0.02±0.47 | 9.99E-01 | -0.04±0.48 | 1.00E+00 |
| indolebutyrate | -0.19±0.26 | 0 | 4.92E-01 | 5.35E-01 | -0.28±0.15 | 0 | 4.62E-01 | 5.54E-01 | -0.31±0.15 | 6.58E-01 | -0.31±0.15 | 6.30E-01 | 0.08±0.47 | 9.99E-01 | 0.07±0.5 | 1.00E+00 |
| androstenediol (3beta,17beta) disulfate (2) | -0.21±0.28 | 0 | 4.53E-01 | 5.38E-01 | -0.32±0.17 | 0 | 5.93E-01 | 5.54E-01 | -0.32±0.17 | 6.58E-01 | -0.35±0.17 | 6.30E-01 | 0.19±0.59 | 9.99E-01 | -0.01±0.61 | 1.00E+00 |
| 16-hydroxypalmitate | -0.24±0.29 | 0 | 5.77E-01 | 5.24E-01 | -0.34±0.17 | 0 | 7.41E-01 | 5.54E-01 | -0.37±0.18 | 6.13E-01 | -0.36±0.18 | 6.30E-01 | -0.04±0.48 | 9.99E-01 | -0.19±0.48 | 1.00E+00 |
| octadecenedioylcarnitine (C18:1-DC)* | -0.22±0.24 | 0 | 7.03E-01 | 5.24E-01 | -0.28±0.14 | 0 | 8.17E-01 | 5.54E-01 | -0.28±0.14 | 6.58E-01 | -0.29±0.15 | 6.30E-01 | -0.08±0.44 | 9.99E-01 | -0.17±0.5 | 1.00E+00 |
| sphingomyelin (d18:1/22:2, d18:2/22:1, d16:1/24:2)* | 0.2±0.25 | 0 | 5.43E-01 | 5.29E-01 | 0.33±0.17 | 0 | 5.07E-01 | 5.54E-01 | 0.32±0.17 | 6.58E-01 | 0.36±0.17 | 6.30E-01 | 0.01±0.4 | 9.99E-01 | -0.04±0.58 | 1.00E+00 |
| tetrahydrocortisol glucuronide | -0.17±0.22 | 0 | 5.04E-01 | 5.29E-01 | -0.24±0.12 | 0 | 5.15E-01 | 5.54E-01 | -0.24±0.13 | 6.58E-01 | -0.26±0.13 | 6.30E-01 | 0.14±0.52 | 9.99E-01 | 0.09±0.52 | 1.00E+00 |
| alpha-ketoglutarate | -0.17±0.27 | 0 | 3.44E-01 | 5.86E-01 | -0.31±0.16 | 0 | 5.28E-01 | 5.54E-01 | -0.32±0.16 | 6.58E-01 | -0.34±0.16 | 6.30E-01 | 0.28±0.54 | 9.99E-01 | 0.01±0.52 | 1.00E+00 |
| X - 23782 | -0.21±0.27 | 0 | 5.15E-01 | 5.33E-01 | -0.3±0.15 | 0 | 5.84E-01 | 5.54E-01 | -0.33±0.16 | 6.13E-01 | -0.32±0.16 | 6.30E-01 | 0.03±0.46 | 9.99E-01 | -0.01±0.54 | 1.00E+00 |
| octadecanedioylcarnitine (C18-DC)* | -0.23±0.27 | 0 | 5.94E-01 | 5.24E-01 | -0.3±0.16 | 0 | 7.07E-01 | 5.54E-01 | -0.34±0.16 | 6.13E-01 | -0.32±0.16 | 6.46E-01 | -0.05±0.42 | 9.99E-01 | -0.12±0.5 | 1.00E+00 |
| xanthurenate | -0.2±0.22 | 0 | 6.35E-01 | 5.24E-01 | -0.31±0.16 | 0 | 8.72E-01 | 5.54E-01 | -0.26±0.15 | 6.58E-01 | -0.3±0.16 | 6.72E-01 | -0.02±0.43 | 9.99E-01 | -0.39±0.54 | 1.00E+00 |
| tricosanoyl sphingomyelin (d18:1/23:0)* | 0.29±0.26 | 0 | 9.96E-01 | 5.24E-01 | 0.32±0.16 | 0 | 6.76E-01 | 5.54E-01 | 0.29±0.16 | 6.58E-01 | 0.31±0.16 | 6.72E-01 | 0.29±0.6 | 9.99E-01 | 0.69±0.89 | 1.00E+00 |
| glutamine conjugate of C7H12O2* | -0.25±0.24 | 0 | 7.86E-01 | 5.24E-01 | -0.31±0.16 | 0 | 9.99E-01 | 5.54E-01 | -0.3±0.16 | 6.58E-01 | -0.31±0.17 | 6.72E-01 | -0.16±0.41 | 9.99E-01 | -0.31±0.48 | 1.00E+00 |
| palmitoyl dihydrosphingomyelin (d18:0/16:0)* | 0.33±0.27 | 0 | 6.92E-01 | 5.24E-01 | 0.32±0.16 | 0 | 4.93E-01 | 5.54E-01 | 0.29±0.16 | 6.58E-01 | 0.3±0.16 | 6.93E-01 | 0.62±0.78 | 9.99E-01 | 0.96±0.96 | 1.00E+00 |
| linoleoylcarnitine (C18:2)* | -0.26±0.21 | 0 | 8.62E-01 | 5.24E-01 | -0.29±0.15 | 0 | 6.43E-01 | 5.54E-01 | -0.29±0.16 | 6.58E-01 | -0.26±0.16 | 7.32E-01 | -0.22±0.31 | 9.99E-01 | -0.45±0.36 | 1.00E+00 |
| 4-methoxyphenol sulfate | 0.33±0.23 | 0 | 6.54E-01 | 5.24E-01 | 0.31±0.16 | 0 | 5.82E-01 | 5.54E-01 | 0.27±0.17 | 7.45E-01 | 0.28±0.17 | 7.32E-01 | 0.5±0.44 | 9.99E-01 | 0.57±0.5 | 1.00E+00 |
| 2-hydroxyoctanoate | -0.32±0.22 | 0 | 7.31E-01 | 5.24E-01 | -0.31±0.15 | 31.22 | 2.28E-01 | 5.54E-01 | -0.27±0.16 | 6.90E-01 | -0.25±0.16 | 7.61E-01 | -0.43±0.37 | 9.99E-01 | -0.9±0.52 | 1.00E+00 |
| N-stearoyl-sphinganine (d18:0/18:0)* | 0.32±0.21 | 57.69 | 1.24E-01 | 5.24E-01 | 0.31±0.16 | 70.77 | 6.44E-02 | 5.54E-01 | 0.22±0.15 | 7.58E-01 | 0.25±0.16 | 7.74E-01 | 1.27±0.65 | 9.99E-01 | 1.75±0.8 | 1.00E+00 |
| 1-methylnicotinamide | 0.34±0.18 | 70.93 | 6.37E-02 | 5.24E-01 | 0.3±0.15 | 63.82 | 9.64E-02 | 5.54E-01 | 0.2±0.15 | 7.86E-01 | 0.24±0.16 | 7.81E-01 | 1.14±0.47 | 9.99E-01 | 1.31±0.63 | 1.00E+00 |
| androsterone sulfate | -0.35±0.18 | 10.29 | 2.91E-01 | 5.24E-01 | -0.28±0.14 | 0 | 3.28E-01 | 5.54E-01 | -0.22±0.15 | 7.58E-01 | -0.21±0.16 | 7.84E-01 | -0.61±0.3 | 9.99E-01 | -0.58±0.34 | 1.00E+00 |
| 1-linoleoyl-2-linolenoyl-GPC (18:2/18:3)* | -0.33±0.16 | 34.72 | 2.16E-01 | 5.24E-01 | -0.3±0.15 | 55.57 | 1.34E-01 | 5.54E-01 | -0.19±0.16 | 8.05E-01 | -0.19±0.17 | 8.53E-01 | -0.62±0.28 | 9.99E-01 | -0.76±0.34 | 1.00E+00 |
| trans-4-hydroxyproline | 0.19±0.27 | 0 | 4.67E-01 | 5.48E-01 | 0.28±0.15 | 34.83 | 2.15E-01 | 5.58E-01 | 0.34±0.15 | 5.73E-01 | 0.33±0.15 | 6.24E-01 | -0.05±0.42 | 9.99E-01 | -0.28±0.47 | 1.00E+00 |
| caprate (10:0) | -0.07±0.3 | 37.13 | 2.07E-01 | 8.26E-01 | -0.31±0.16 | 26.17 | 2.45E-01 | 5.58E-01 | -0.39±0.17 | 5.73E-01 | -0.38±0.17 | 6.26E-01 | 0.37±0.45 | 9.99E-01 | 0.2±0.47 | 1.00E+00 |
| 1-palmitoyl-2-dihomo-linolenoyl-GPC (16:0/20:3n3 or 6)* | -0.19±0.26 | 0 | 4.09E-01 | 5.39E-01 | -0.28±0.15 | 0 | 4.98E-01 | 5.58E-01 | -0.29±0.15 | 6.58E-01 | -0.3±0.15 | 6.30E-01 | 0.29±0.64 | 9.99E-01 | 0.2±0.73 | 1.00E+00 |
| dodecadienoate (12:2)* | -0.24±0.23 | 0 | 8.64E-01 | 5.24E-01 | -0.27±0.14 | 0 | 6.42E-01 | 5.58E-01 | -0.26±0.14 | 6.58E-01 | -0.25±0.15 | 7.32E-01 | -0.17±0.44 | 9.99E-01 | -0.5±0.52 | 1.00E+00 |
| N,N,N-trimethyl-5-aminovalerate | -0.21±0.15 | 0 | 7.39E-01 | 5.24E-01 | -0.25±0.13 | 8.55 | 2.96E-01 | 5.58E-01 | -0.17±0.14 | 7.92E-01 | -0.2±0.15 | 7.84E-01 | -0.28±0.26 | 9.99E-01 | -0.6±0.36 | 1.00E+00 |
| 5-hydroxylysine | 0.21±0.2 | 0 | 8.05E-01 | 5.24E-01 | 0.23±0.12 | 0 | 8.94E-01 | 5.63E-01 | 0.23±0.12 | 6.58E-01 | 0.23±0.13 | 6.72E-01 | 0.11±0.43 | 9.99E-01 | 0.16±0.53 | 1.00E+00 |
| N6-methyladenosine | 0.35±0.23 | 29.59 | 2.33E-01 | 5.24E-01 | 0.28±0.15 | 9.33 | 2.94E-01 | 5.63E-01 | 0.24±0.15 | 7.45E-01 | 0.25±0.16 | 7.51E-01 | 1.03±0.62 | 9.99E-01 | 0.96±0.66 | 1.00E+00 |
| cis-3,4-methyleneheptanoylglycine | -0.29±0.16 | 59.65 | 1.15E-01 | 5.24E-01 | -0.27±0.15 | 71.53 | 6.09E-02 | 5.70E-01 | -0.18±0.15 | 7.99E-01 | -0.19±0.15 | 8.11E-01 | -0.86±0.4 | 9.99E-01 | -1.2±0.51 | 1.00E+00 |
| 4-vinylcatechol sulfate | 0.1±0.29 | 16.32 | 2.74E-01 | 7.51E-01 | 0.32±0.17 | 0 | 3.98E-01 | 5.76E-01 | 0.39±0.18 | 6.11E-01 | 0.37±0.18 | 6.30E-01 | -0.23±0.41 | 9.99E-01 | -0.05±0.46 | 1.00E+00 |
| 3-methylcytidine | 0.32±0.21 | 0 | 3.22E-01 | 5.24E-01 | 0.31±0.17 | 6.29 | 3.02E-01 | 5.76E-01 | 0.23±0.17 | 7.66E-01 | 0.26±0.17 | 7.70E-01 | 0.83±0.56 | 9.99E-01 | 0.96±0.65 | 1.00E+00 |
| cysteinylglycine | 0.32±0.21 | 0 | 4.45E-01 | 5.24E-01 | 0.3±0.16 | 0 | 4.12E-01 | 5.94E-01 | 0.24±0.17 | 7.58E-01 | 0.26±0.17 | 7.81E-01 | 0.65±0.48 | 9.99E-01 | 0.71±0.53 | 1.00E+00 |
| taurochenodeoxycholate | -0.36±0.21 | 0 | 3.29E-01 | 5.24E-01 | -0.28±0.15 | 15.69 | 2.76E-01 | 5.94E-01 | -0.24±0.16 | 7.45E-01 | -0.22±0.16 | 7.84E-01 | -0.72±0.42 | 9.99E-01 | -0.75±0.46 | 1.00E+00 |
| lignoceroylcarnitine (C24)* | -0.22±0.21 | 0 | 7.87E-01 | 5.24E-01 | -0.25±0.14 | 0 | 7.47E-01 | 5.94E-01 | -0.26±0.15 | 6.58E-01 | -0.24±0.15 | 7.34E-01 | -0.14±0.36 | 9.99E-01 | -0.38±0.42 | 1.00E+00 |
| adenosine 3',5'-cyclic monophosphate (cAMP) | -0.18±0.29 | 11.27 | 2.88E-01 | 5.84E-01 | -0.29±0.16 | 0 | 3.26E-01 | 6.00E-01 | -0.32±0.16 | 6.58E-01 | -0.32±0.16 | 6.30E-01 | 0.48±0.69 | 9.99E-01 | 0.53±0.85 | 1.00E+00 |
| androstenediol (3alpha, 17alpha) monosulfate (2) | -0.25±0.21 | 0 | 9.41E-01 | 5.24E-01 | -0.27±0.15 | 0 | 7.33E-01 | 6.00E-01 | -0.24±0.16 | 7.45E-01 | -0.25±0.16 | 7.33E-01 | -0.28±0.46 | 9.99E-01 | -0.43±0.49 | 1.00E+00 |
| 5alpha-androstan-3alpha,17beta-diol monosulfate (1) | -0.27±0.21 | 0 | 9.54E-01 | 5.24E-01 | -0.25±0.14 | 0 | 9.52E-01 | 6.06E-01 | -0.26±0.15 | 6.62E-01 | -0.25±0.15 | 7.32E-01 | -0.29±0.35 | 9.99E-01 | -0.28±0.37 | 1.00E+00 |
| X - 26111 | -0.02±0.29 | 60.77 | 1.10E-01 | 9.55E-01 | 0.3±0.17 | 72.06 | 5.85E-02 | 6.15E-01 | 0.38±0.18 | 6.11E-01 | 0.42±0.18 | 5.89E-01 | -0.55±0.44 | 9.99E-01 | -0.53±0.47 | 1.00E+00 |
| hydantoin-5-propionate | -0.2±0.23 | 0 | 6.30E-01 | 5.24E-01 | -0.27±0.15 | 0 | 5.34E-01 | 6.15E-01 | -0.28±0.15 | 6.58E-01 | -0.31±0.16 | 6.72E-01 | -0.03±0.42 | 9.99E-01 | 0±0.46 | 1.00E+00 |
| branched-chain, straight-chain, or cyclopropyl 12:1 fatty acid* | -0.21±0.26 | 0 | 5.56E-01 | 5.25E-01 | -0.27±0.15 | 0 | 8.27E-01 | 6.19E-01 | -0.31±0.16 | 6.58E-01 | -0.28±0.16 | 7.23E-01 | 0.04±0.51 | 9.99E-01 | -0.16±0.53 | 1.00E+00 |
| betaine | -0.2±0.22 | 0 | 6.60E-01 | 5.24E-01 | -0.27±0.15 | 0 | 9.76E-01 | 6.19E-01 | -0.27±0.16 | 6.62E-01 | -0.27±0.16 | 7.32E-01 | -0.07±0.37 | 9.99E-01 | -0.26±0.48 | 1.00E+00 |
| X - 25419 | 0.13±0.22 | 0 | 3.45E-01 | 5.98E-01 | 0.24±0.14 | 10.44 | 2.91E-01 | 6.19E-01 | 0.26±0.14 | 6.58E-01 | 0.29±0.14 | 6.30E-01 | -0.2±0.41 | 9.99E-01 | -0.23±0.47 | 1.00E+00 |
| hexadecanedioate (C16-DC) | -0.1±0.25 | 2.13 | 3.12E-01 | 6.99E-01 | -0.28±0.16 | 0 | 3.91E-01 | 6.19E-01 | -0.34±0.17 | 6.45E-01 | -0.33±0.17 | 6.40E-01 | 0.16±0.36 | 9.99E-01 | 0.06±0.43 | 1.00E+00 |
| sphingomyelin (d18:2/23:1)* | 0.22±0.24 | 0 | 7.00E-01 | 5.24E-01 | 0.31±0.18 | 0 | 8.32E-01 | 6.19E-01 | 0.28±0.17 | 7.35E-01 | 0.32±0.18 | 7.23E-01 | 0.08±0.46 | 9.99E-01 | 0.18±0.67 | 1.00E+00 |
| docosadienoate (22:2n6) | -0.19±0.23 | 0 | 6.00E-01 | 5.24E-01 | -0.27±0.15 | 0 | 8.10E-01 | 6.19E-01 | -0.27±0.15 | 6.58E-01 | -0.28±0.16 | 7.30E-01 | 0±0.43 | 9.99E-01 | -0.16±0.47 | 1.00E+00 |
| N-palmitoyl-sphingosine (d18:1/16:0) | 0.27±0.24 | 0 | 9.73E-01 | 5.24E-01 | 0.25±0.15 | 0 | 9.72E-01 | 6.19E-01 | 0.26±0.15 | 6.58E-01 | 0.26±0.15 | 7.32E-01 | 0.28±0.55 | 9.99E-01 | 0.23±0.64 | 1.00E+00 |
| bilirubin (E,E)* | -0.22±0.2 | 0 | 9.86E-01 | 5.24E-01 | -0.25±0.14 | 0 | 3.18E-01 | 6.19E-01 | -0.22±0.14 | 7.45E-01 | -0.21±0.15 | 7.84E-01 | -0.21±0.43 | 9.99E-01 | -0.7±0.47 | 1.00E+00 |
| allantoin | -0.25±0.18 | 0 | 7.19E-01 | 5.24E-01 | -0.26±0.15 | 0 | 3.51E-01 | 6.19E-01 | -0.21±0.16 | 7.66E-01 | -0.2±0.16 | 8.05E-01 | -0.35±0.33 | 9.99E-01 | -0.59±0.38 | 1.00E+00 |
| X - 21310 | 0.26±0.17 | 62.66 | 1.02E-01 | 5.24E-01 | 0.29±0.16 | 47.72 | 1.67E-01 | 6.19E-01 | 0.18±0.17 | 8.44E-01 | 0.22±0.17 | 8.05E-01 | 1.18±0.59 | 9.99E-01 | 1.04±0.57 | 1.00E+00 |
| thyroxine | 0.16±0.21 | 0 | 5.29E-01 | 5.30E-01 | 0.26±0.15 | 0 | 4.05E-01 | 6.25E-01 | 0.26±0.15 | 6.66E-01 | 0.31±0.16 | 6.58E-01 | -0.02±0.36 | 9.99E-01 | -0.09±0.45 | 1.00E+00 |
| cis-4-decenoate (10:1n6)* | -0.28±0.21 | 0 | 7.24E-01 | 5.24E-01 | -0.27±0.16 | 0 | 3.66E-01 | 6.29E-01 | -0.24±0.16 | 7.58E-01 | -0.22±0.16 | 7.84E-01 | -0.41±0.43 | 9.99E-01 | -0.67±0.47 | 1.00E+00 |
| glucuronide of C10H18O2 (7)* | -0.16±0.22 | 0 | 4.76E-01 | 5.39E-01 | -0.24±0.14 | 0 | 3.61E-01 | 6.32E-01 | -0.27±0.15 | 6.58E-01 | -0.29±0.15 | 6.58E-01 | 0.07±0.39 | 9.99E-01 | 0.12±0.42 | 1.00E+00 |
| cortolone glucuronide (1) | -0.17±0.18 | 0 | 6.77E-01 | 5.24E-01 | -0.23±0.13 | 0 | 9.98E-01 | 6.32E-01 | -0.2±0.14 | 7.45E-01 | -0.23±0.14 | 7.32E-01 | -0.02±0.4 | 9.99E-01 | -0.23±0.44 | 1.00E+00 |
| dihomo-linoleoylcarnitine (C20:2)* | -0.23±0.23 | 0 | 7.82E-01 | 5.24E-01 | -0.27±0.15 | 0 | 8.72E-01 | 6.32E-01 | -0.28±0.16 | 6.58E-01 | -0.26±0.16 | 7.55E-01 | -0.14±0.42 | 9.99E-01 | -0.34±0.49 | 1.00E+00 |
| 5alpha-androstan-3beta,17alpha-diol disulfate | -0.33±0.23 | 0 | 6.35E-01 | 5.24E-01 | -0.29±0.17 | 0 | 6.34E-01 | 6.32E-01 | -0.27±0.18 | 7.58E-01 | -0.25±0.18 | 7.84E-01 | -0.5±0.43 | 9.99E-01 | -0.48±0.44 | 1.00E+00 |
| epiandrosterone sulfate | -0.3±0.16 | 51.57 | 1.51E-01 | 5.24E-01 | -0.26±0.15 | 34.18 | 2.18E-01 | 6.32E-01 | -0.18±0.16 | 8.19E-01 | -0.18±0.17 | 8.53E-01 | -0.77±0.36 | 9.99E-01 | -0.72±0.4 | 1.00E+00 |
| sphingomyelin (d18:1/24:1, d18:2/24:0)* | 0.17±0.23 | 0 | 4.95E-01 | 5.38E-01 | 0.26±0.15 | 0 | 4.16E-01 | 6.34E-01 | 0.31±0.16 | 6.58E-01 | 0.3±0.16 | 6.72E-01 | -0.01±0.36 | 9.99E-01 | -0.12±0.49 | 1.00E+00 |
| X - 11483 | -0.01±0.27 | 63.28 | 9.89E-02 | 9.67E-01 | 0.28±0.16 | 72.23 | 5.77E-02 | 6.42E-01 | 0.35±0.17 | 6.56E-01 | 0.39±0.17 | 5.97E-01 | -0.57±0.44 | 9.99E-01 | -0.57±0.47 | 1.00E+00 |
| succinate | -0.22±0.14 | 0 | 5.67E-01 | 5.24E-01 | -0.23±0.14 | 51.97 | 1.49E-01 | 6.45E-01 | -0.17±0.15 | 8.05E-01 | -0.15±0.15 | 8.97E-01 | -0.35±0.26 | 9.99E-01 | -0.69±0.35 | 1.00E+00 |
| 5-hydroxyindole sulfate | 0.27±0.2 | 0 | 6.00E-01 | 5.24E-01 | 0.28±0.16 | 0 | 6.23E-01 | 6.45E-01 | 0.22±0.17 | 7.66E-01 | 0.25±0.17 | 7.84E-01 | 0.49±0.45 | 9.99E-01 | 0.51±0.51 | 1.00E+00 |
| tetradecadienoate (14:2)* | -0.21±0.23 | 0 | 6.75E-01 | 5.24E-01 | -0.26±0.15 | 0 | 9.91E-01 | 6.49E-01 | -0.26±0.15 | 6.65E-01 | -0.26±0.16 | 7.34E-01 | -0.03±0.49 | 9.99E-01 | -0.25±0.56 | 1.00E+00 |
| 4-allylcatechol sulfate | -0.18±0.24 | 0 | 5.05E-01 | 5.33E-01 | -0.25±0.15 | 0 | 6.26E-01 | 6.50E-01 | -0.29±0.16 | 6.58E-01 | -0.28±0.16 | 7.30E-01 | 0.06±0.44 | 9.99E-01 | -0.05±0.45 | 1.00E+00 |
| sphingomyelin (d18:2/23:0, d18:1/23:1, d17:1/24:1)* | 0.19±0.21 | 0 | 6.38E-01 | 5.24E-01 | 0.27±0.16 | 0 | 7.45E-01 | 6.50E-01 | 0.24±0.16 | 7.45E-01 | 0.28±0.17 | 7.32E-01 | 0.02±0.42 | 9.99E-01 | 0.07±0.65 | 1.00E+00 |
| 2-ketocaprylate | -0.27±0.2 | 0 | 7.21E-01 | 5.24E-01 | -0.25±0.15 | 0 | 4.30E-01 | 6.50E-01 | -0.23±0.16 | 7.58E-01 | -0.21±0.16 | 7.84E-01 | -0.4±0.43 | 9.99E-01 | -0.61±0.48 | 1.00E+00 |
| X - 23680 | -0.22±0.16 | 0 | 5.80E-01 | 5.24E-01 | -0.24±0.14 | 57.12 | 1.27E-01 | 6.50E-01 | -0.18±0.14 | 7.92E-01 | -0.18±0.15 | 8.25E-01 | -0.4±0.35 | 9.99E-01 | -1.02±0.53 | 1.00E+00 |
| bilirubin (Z,Z) | -0.19±0.21 | 0 | 6.74E-01 | 5.24E-01 | -0.24±0.15 | 0 | 5.92E-01 | 6.53E-01 | -0.24±0.15 | 7.45E-01 | -0.21±0.16 | 7.84E-01 | -0.03±0.43 | 9.99E-01 | -0.46±0.42 | 1.00E+00 |
| ethyl beta-glucopyranoside | -0.22±0.24 | 0 | 6.65E-01 | 5.24E-01 | -0.24±0.15 | 0 | 6.83E-01 | 6.56E-01 | -0.28±0.15 | 6.58E-01 | -0.26±0.16 | 7.32E-01 | -0.06±0.43 | 9.99E-01 | -0.06±0.47 | 1.00E+00 |
| sphingomyelin (d18:2/18:1)* | 0.26±0.23 | 0 | 8.85E-01 | 5.24E-01 | 0.29±0.18 | 0 | 6.69E-01 | 6.56E-01 | 0.24±0.17 | 7.58E-01 | 0.27±0.18 | 7.74E-01 | 0.34±0.62 | 9.99E-01 | 0.64±0.83 | 1.00E+00 |
| ceramide (d18:1/17:0, d17:1/18:0)* | 0.26±0.2 | 0 | 3.80E-01 | 5.24E-01 | 0.25±0.15 | 9.8 | 2.92E-01 | 6.56E-01 | 0.21±0.15 | 7.66E-01 | 0.22±0.16 | 7.84E-01 | 0.79±0.63 | 9.99E-01 | 0.99±0.72 | 1.00E+00 |
| glycosyl ceramide (d18:1/20:0, d16:1/22:0)* | 0.08±0.22 | 8.15 | 2.97E-01 | 7.31E-01 | 0.23±0.14 | 57.27 | 1.26E-01 | 6.60E-01 | 0.31±0.15 | 6.58E-01 | 0.32±0.16 | 6.30E-01 | -0.15±0.31 | 9.99E-01 | -0.31±0.38 | 1.00E+00 |
| laurate (12:0) | -0.03±0.28 | 62.68 | 1.02E-01 | 9.09E-01 | -0.25±0.16 | 56.32 | 1.30E-01 | 6.60E-01 | -0.34±0.16 | 6.13E-01 | -0.33±0.17 | 6.30E-01 | 0.67±0.52 | 9.99E-01 | 0.45±0.49 | 1.00E+00 |
| pro-hydroxy-pro | 0.16±0.22 | 0 | 4.80E-01 | 5.42E-01 | 0.23±0.14 | 4.54 | 3.06E-01 | 6.60E-01 | 0.29±0.15 | 6.58E-01 | 0.28±0.15 | 6.72E-01 | -0.03±0.35 | 9.99E-01 | -0.18±0.43 | 1.00E+00 |
| N-methylproline | -0.14±0.24 | 0 | 3.47E-01 | 5.86E-01 | -0.23±0.14 | 4.92 | 3.05E-01 | 6.60E-01 | -0.28±0.14 | 6.58E-01 | -0.27±0.15 | 6.72E-01 | 0.23±0.46 | 9.99E-01 | 0.25±0.49 | 1.00E+00 |
| glycosyl ceramide (d18:2/24:1, d18:1/24:2)* | 0.11±0.21 | 0 | 3.48E-01 | 6.38E-01 | 0.24±0.16 | 28.49 | 2.37E-01 | 6.60E-01 | 0.28±0.16 | 6.58E-01 | 0.31±0.17 | 6.72E-01 | -0.12±0.33 | 9.99E-01 | -0.23±0.43 | 1.00E+00 |
| sphingomyelin (d18:2/24:1, d18:1/24:2)* | 0.13±0.22 | 0 | 4.12E-01 | 5.86E-01 | 0.25±0.16 | 0 | 3.32E-01 | 6.60E-01 | 0.3±0.16 | 6.58E-01 | 0.3±0.17 | 6.89E-01 | -0.06±0.32 | 9.99E-01 | -0.17±0.46 | 1.00E+00 |
| 1-palmitoyl-2-docosahexaenoyl-GPC (16:0/22:6) | 0.15±0.22 | 0 | 4.49E-01 | 5.53E-01 | 0.24±0.15 | 0 | 3.71E-01 | 6.60E-01 | 0.28±0.16 | 6.58E-01 | 0.29±0.16 | 6.93E-01 | -0.07±0.37 | 9.99E-01 | -0.14±0.46 | 1.00E+00 |
| nonadecanoate (19:0) | -0.16±0.26 | 0 | 3.91E-01 | 5.74E-01 | -0.25±0.16 | 0 | 4.49E-01 | 6.60E-01 | -0.31±0.16 | 6.58E-01 | -0.3±0.17 | 7.30E-01 | 0.17±0.47 | 9.99E-01 | 0.09±0.49 | 1.00E+00 |
| sphingosine 1-phosphate | 0.18±0.2 | 0 | 6.18E-01 | 5.24E-01 | 0.22±0.14 | 0 | 6.65E-01 | 6.60E-01 | 0.24±0.14 | 6.62E-01 | 0.23±0.14 | 7.32E-01 | 0.02±0.37 | 9.99E-01 | 0±0.51 | 1.00E+00 |
| palmitoyl sphingomyelin (d18:1/16:0) | 0.17±0.21 | 0 | 5.52E-01 | 5.26E-01 | 0.24±0.16 | 0 | 4.29E-01 | 6.60E-01 | 0.26±0.16 | 7.30E-01 | 0.28±0.17 | 7.32E-01 | -0.01±0.37 | 9.99E-01 | -0.14±0.51 | 1.00E+00 |
| phenylacetylcarnitine | 0.21±0.24 | 0 | 6.26E-01 | 5.24E-01 | 0.25±0.16 | 0 | 6.34E-01 | 6.60E-01 | 0.3±0.17 | 6.58E-01 | 0.28±0.17 | 7.32E-01 | 0.04±0.43 | 9.99E-01 | 0.04±0.47 | 1.00E+00 |
| taurodeoxycholic acid 3-sulfate | -0.17±0.19 | 0 | 6.42E-01 | 5.24E-01 | -0.22±0.14 | 0 | 5.64E-01 | 6.60E-01 | -0.22±0.15 | 7.45E-01 | -0.25±0.15 | 7.32E-01 | 0±0.43 | 9.99E-01 | 0.03±0.46 | 1.00E+00 |
| thioproline | 0.27±0.25 | 0 | 8.93E-01 | 5.24E-01 | 0.24±0.15 | 0 | 6.82E-01 | 6.60E-01 | 0.29±0.16 | 6.58E-01 | 0.26±0.16 | 7.32E-01 | 0.21±0.48 | 9.99E-01 | 0.01±0.59 | 1.00E+00 |
| N-palmitoyl-heptadecasphingosine (d17:1/16:0)* | -0.2±0.22 | 0 | 6.43E-01 | 5.24E-01 | -0.23±0.15 | 0 | 5.14E-01 | 6.60E-01 | -0.25±0.15 | 6.90E-01 | -0.26±0.15 | 7.32E-01 | -0.02±0.45 | 9.99E-01 | 0.11±0.54 | 1.00E+00 |
| homovanillate (HVA) | -0.2±0.21 | 0 | 7.36E-01 | 5.24E-01 | -0.22±0.14 | 0 | 6.00E-01 | 6.60E-01 | -0.24±0.15 | 7.45E-01 | -0.25±0.15 | 7.32E-01 | -0.07±0.44 | 9.99E-01 | -0.01±0.42 | 1.00E+00 |
| X - 17335 | -0.21±0.23 | 0 | 6.52E-01 | 5.24E-01 | -0.25±0.15 | 0 | 8.48E-01 | 6.60E-01 | -0.27±0.15 | 6.58E-01 | -0.26±0.16 | 7.35E-01 | -0.03±0.45 | 9.99E-01 | -0.15±0.51 | 1.00E+00 |
| myristoleate (14:1n5) | -0.18±0.24 | 0 | 4.81E-01 | 5.33E-01 | -0.26±0.17 | 0 | 7.77E-01 | 6.60E-01 | -0.27±0.17 | 7.28E-01 | -0.27±0.17 | 7.55E-01 | 0.14±0.53 | 9.99E-01 | -0.1±0.57 | 1.00E+00 |
| threonine | 0.27±0.24 | 0 | 9.52E-01 | 5.24E-01 | 0.24±0.15 | 0 | 9.02E-01 | 6.60E-01 | 0.26±0.16 | 6.92E-01 | 0.25±0.16 | 7.56E-01 | 0.3±0.59 | 9.99E-01 | 0.16±0.67 | 1.00E+00 |
| X - 25343 | -0.17±0.2 | 0 | 6.02E-01 | 5.24E-01 | -0.23±0.15 | 0 | 7.91E-01 | 6.60E-01 | -0.22±0.15 | 7.58E-01 | -0.24±0.15 | 7.60E-01 | 0.05±0.47 | 9.99E-01 | -0.1±0.52 | 1.00E+00 |
| glycerophosphorylcholine (GPC) | 0.24±0.22 | 0 | 9.98E-01 | 5.24E-01 | 0.26±0.17 | 0 | 7.84E-01 | 6.60E-01 | 0.24±0.17 | 7.59E-01 | 0.28±0.18 | 7.70E-01 | 0.23±0.53 | 9.99E-01 | 0.1±0.61 | 1.00E+00 |
| sphingomyelin (d18:1/20:0, d16:1/22:0)* | 0.22±0.2 | 0 | 9.91E-01 | 5.24E-01 | 0.22±0.14 | 0 | 9.69E-01 | 6.60E-01 | 0.22±0.14 | 7.45E-01 | 0.22±0.15 | 7.74E-01 | 0.21±0.56 | 9.99E-01 | 0.19±0.68 | 1.00E+00 |
| cholesterol | 0.22±0.22 | 0 | 8.17E-01 | 5.24E-01 | 0.23±0.15 | 0 | 8.79E-01 | 6.60E-01 | 0.25±0.16 | 7.45E-01 | 0.24±0.16 | 7.74E-01 | 0.13±0.48 | 9.99E-01 | 0.14±0.64 | 1.00E+00 |
| X - 11470 | -0.17±0.17 | 0 | 8.04E-01 | 5.24E-01 | -0.2±0.13 | 0 | 9.52E-01 | 6.60E-01 | -0.2±0.13 | 7.50E-01 | -0.2±0.13 | 7.74E-01 | -0.09±0.37 | 9.99E-01 | -0.18±0.41 | 1.00E+00 |
| cysteine | 0.23±0.2 | 0 | 8.50E-01 | 5.24E-01 | 0.23±0.15 | 0 | 9.13E-01 | 6.60E-01 | 0.21±0.15 | 7.58E-01 | 0.23±0.16 | 7.81E-01 | 0.33±0.57 | 9.99E-01 | 0.31±0.66 | 1.00E+00 |
| 4-methyl-2-oxopentanoate | -0.19±0.2 | 0 | 7.33E-01 | 5.24E-01 | -0.23±0.15 | 0 | 8.42E-01 | 6.60E-01 | -0.22±0.15 | 7.58E-01 | -0.21±0.16 | 7.84E-01 | -0.05±0.43 | 9.99E-01 | -0.3±0.42 | 1.00E+00 |
| 3-methyl-2-oxobutyrate | -0.18±0.18 | 0 | 8.09E-01 | 5.24E-01 | -0.21±0.14 | 0 | 9.34E-01 | 6.60E-01 | -0.21±0.15 | 7.58E-01 | -0.21±0.15 | 7.84E-01 | -0.11±0.34 | 9.99E-01 | -0.24±0.34 | 1.00E+00 |
| glutamine conjugate of C6H10O2 (2)* | -0.23±0.2 | 0 | 9.57E-01 | 5.24E-01 | -0.26±0.16 | 0 | 6.84E-01 | 6.60E-01 | -0.22±0.17 | 7.66E-01 | -0.24±0.17 | 7.84E-01 | -0.25±0.42 | 9.99E-01 | -0.46±0.51 | 1.00E+00 |
| bilirubin degradation product, C17H18N2O4 (2)** | -0.17±0.2 | 0 | 6.05E-01 | 5.24E-01 | -0.21±0.14 | 0 | 6.62E-01 | 6.60E-01 | -0.22±0.14 | 7.45E-01 | -0.19±0.14 | 7.84E-01 | 0.04±0.45 | 9.99E-01 | -0.4±0.46 | 1.00E+00 |
| bilirubin degradation product, C17H18N2O4 (3)** | -0.17±0.21 | 0 | 5.54E-01 | 5.24E-01 | -0.22±0.14 | 0 | 7.41E-01 | 6.60E-01 | -0.23±0.14 | 6.92E-01 | -0.21±0.14 | 7.84E-01 | 0.07±0.46 | 9.99E-01 | -0.37±0.46 | 1.00E+00 |
| X - 12544 | -0.25±0.18 | 42.2 | 1.88E-01 | 5.24E-01 | -0.23±0.15 | 49.07 | 1.61E-01 | 6.60E-01 | -0.19±0.15 | 7.86E-01 | -0.19±0.15 | 7.93E-01 | -1.13±0.69 | 9.99E-01 | -1.61±1 | 1.00E+00 |
| 2,3-dihydroxypyridine | 0.29±0.23 | 0 | 7.70E-01 | 5.24E-01 | 0.28±0.18 | 0 | 4.64E-01 | 6.60E-01 | 0.26±0.18 | 7.58E-01 | 0.24±0.18 | 8.05E-01 | 0.44±0.58 | 9.99E-01 | 0.68±0.57 | 1.00E+00 |
| N-acetylthreonine | -0.18±0.16 | 0 | 9.08E-01 | 5.24E-01 | -0.22±0.15 | 0 | 8.45E-01 | 6.60E-01 | -0.19±0.16 | 7.92E-01 | -0.21±0.16 | 8.05E-01 | -0.15±0.28 | 9.99E-01 | -0.28±0.33 | 1.00E+00 |
| 5alpha-androstan-3beta,17beta-diol monosulfate (2) | -0.24±0.18 | 0 | 7.00E-01 | 5.24E-01 | -0.23±0.15 | 0 | 6.32E-01 | 6.60E-01 | -0.2±0.16 | 7.86E-01 | -0.2±0.16 | 8.12E-01 | -0.37±0.38 | 9.99E-01 | -0.42±0.42 | 1.00E+00 |
| X - 10458 | 0.28±0.19 | 62.52 | 1.02E-01 | 5.24E-01 | 0.23±0.15 | 66.83 | 8.25E-02 | 6.60E-01 | 0.2±0.15 | 7.66E-01 | 0.19±0.15 | 8.12E-01 | 1.53±0.79 | 9.99E-01 | 1.72±0.87 | 1.00E+00 |
| X - 17676 | -0.24±0.16 | 0 | 3.80E-01 | 5.24E-01 | -0.24±0.15 | 0 | 3.75E-01 | 6.60E-01 | -0.18±0.15 | 8.05E-01 | -0.2±0.16 | 8.14E-01 | -0.56±0.4 | 9.99E-01 | -0.63±0.46 | 1.00E+00 |
| X - 21319 | -0.2±0.15 | 0 | 5.90E-01 | 5.24E-01 | -0.22±0.14 | 35.83 | 2.12E-01 | 6.60E-01 | -0.16±0.14 | 8.05E-01 | -0.17±0.14 | 8.25E-01 | -0.4±0.41 | 9.99E-01 | -0.81±0.49 | 1.00E+00 |
| X - 24812 | -0.05±0.04 | 56.89 | 1.28E-01 | 5.24E-01 | -0.27±0.18 | 0 | 4.92E-01 | 6.60E-01 | -0.04±0.15 | 9.80E-01 | -0.23±0.19 | 8.25E-01 | -0.71±0.43 | 9.99E-01 | -0.59±0.49 | 1.00E+00 |
| 2,6-dihydroxybenzoic acid | -0.3±0.18 | 33.1 | 2.21E-01 | 5.24E-01 | -0.23±0.14 | 38.23 | 2.03E-01 | 6.60E-01 | -0.2±0.15 | 7.66E-01 | -0.17±0.15 | 8.53E-01 | -0.74±0.4 | 9.99E-01 | -0.74±0.42 | 1.00E+00 |
| X - 24949 | -0.23±0.16 | 0 | 3.73E-01 | 5.24E-01 | -0.23±0.14 | 26.65 | 2.43E-01 | 6.60E-01 | -0.17±0.15 | 8.11E-01 | -0.17±0.15 | 8.53E-01 | -0.56±0.4 | 9.99E-01 | -0.71±0.44 | 1.00E+00 |
| X - 12410 | -0.17±0.1 | 80.72 | 2.28E-02 | 5.24E-01 | -0.22±0.14 | 74.34 | 4.84E-02 | 6.60E-01 | -0.11±0.15 | 9.32E-01 | -0.12±0.15 | 9.15E-01 | -1.1±0.42 | 9.99E-01 | -1.04±0.44 | 1.00E+00 |
| imidazole propionate | 0.24±0.13 | 73.2 | 5.34E-02 | 5.24E-01 | 0.25±0.16 | 78.59 | 3.07E-02 | 6.60E-01 | 0.14±0.17 | 9.12E-01 | 0.12±0.17 | 9.23E-01 | 0.9±0.37 | 9.99E-01 | 1.14±0.44 | 1.00E+00 |
| lactosyl-N-nervonoyl-sphingosine (d18:1/24:1)* | -0.14±0.09 | 29.42 | 2.34E-01 | 5.24E-01 | -0.23±0.15 | 57.2 | 1.26E-01 | 6.60E-01 | -0.1±0.16 | 9.32E-01 | -0.11±0.17 | 9.23E-01 | -0.4±0.24 | 9.99E-01 | -0.65±0.31 | 1.00E+00 |
| 4-hydroxycoumarin | -0.22±0.14 | 54.01 | 1.40E-01 | 5.24E-01 | -0.22±0.14 | 52.3 | 1.48E-01 | 6.67E-01 | -0.15±0.15 | 8.52E-01 | -0.15±0.15 | 8.97E-01 | -0.85±0.45 | 9.99E-01 | -0.86±0.47 | 1.00E+00 |
| 2-methylserine | 0.15±0.2 | 0 | 5.08E-01 | 5.30E-01 | 0.2±0.14 | 0 | 5.51E-01 | 6.74E-01 | 0.23±0.14 | 7.30E-01 | 0.23±0.14 | 7.35E-01 | -0.08±0.41 | 9.99E-01 | -0.04±0.43 | 1.00E+00 |
| sphingomyelin (d18:1/21:0, d17:1/22:0, d16:1/23:0)* | 0.19±0.19 | 0 | 8.50E-01 | 5.24E-01 | 0.24±0.16 | 0 | 8.10E-01 | 6.74E-01 | 0.21±0.16 | 7.66E-01 | 0.25±0.17 | 7.74E-01 | 0.11±0.47 | 9.99E-01 | 0.1±0.59 | 1.00E+00 |
| uracil | -0.17±0.16 | 0 | 8.86E-01 | 5.24E-01 | -0.2±0.13 | 0 | 8.65E-01 | 6.83E-01 | -0.18±0.14 | 7.66E-01 | -0.2±0.14 | 7.81E-01 | -0.12±0.39 | 9.99E-01 | -0.13±0.43 | 1.00E+00 |
| isoleucylglycine | 0.25±0.17 | 0 | 3.41E-01 | 5.24E-01 | 0.25±0.17 | 0 | 3.63E-01 | 6.83E-01 | 0.19±0.17 | 8.24E-01 | 0.2±0.17 | 8.43E-01 | 0.67±0.47 | 9.99E-01 | 0.74±0.57 | 1.00E+00 |
| X - 11299 | -0.04±0.26 | 72.18 | 5.80E-02 | 8.75E-01 | 0.24±0.16 | 74.69 | 4.68E-02 | 6.87E-01 | 0.33±0.17 | 6.58E-01 | 0.36±0.17 | 6.30E-01 | -0.71±0.44 | 9.99E-01 | -0.66±0.48 | 1.00E+00 |
| X - 12216 | 0.1±0.24 | 28.25 | 2.38E-01 | 6.84E-01 | 0.23±0.16 | 49.27 | 1.60E-01 | 6.87E-01 | 0.28±0.16 | 6.62E-01 | 0.31±0.17 | 6.72E-01 | -0.34±0.45 | 9.99E-01 | -0.42±0.5 | 1.00E+00 |
| 5-dodecenoate (12:1n7) | -0.19±0.23 | 0 | 5.33E-01 | 5.25E-01 | -0.25±0.17 | 0 | 8.65E-01 | 6.87E-01 | -0.26±0.17 | 7.53E-01 | -0.26±0.18 | 7.81E-01 | 0.12±0.54 | 9.99E-01 | -0.15±0.59 | 1.00E+00 |
| linoleate (18:2n6) | -0.19±0.2 | 0 | 7.36E-01 | 5.24E-01 | -0.21±0.14 | 0 | 9.32E-01 | 6.87E-01 | -0.23±0.15 | 7.45E-01 | -0.21±0.15 | 7.84E-01 | -0.05±0.48 | 9.99E-01 | -0.25±0.5 | 1.00E+00 |
| X - 25371 | 0.18±0.16 | 0 | 8.92E-01 | 5.24E-01 | 0.2±0.14 | 0 | 8.47E-01 | 6.87E-01 | 0.17±0.14 | 7.87E-01 | 0.21±0.15 | 7.84E-01 | 0.23±0.37 | 9.99E-01 | 0.13±0.43 | 1.00E+00 |
| bilirubin degradation product, C17H18N2O4 (1)** | -0.17±0.2 | 0 | 5.72E-01 | 5.24E-01 | -0.21±0.14 | 0 | 7.68E-01 | 6.87E-01 | -0.22±0.14 | 7.45E-01 | -0.19±0.15 | 7.91E-01 | 0.07±0.47 | 9.99E-01 | -0.33±0.46 | 1.00E+00 |
| X - 13553 | -0.21±0.15 | 0 | 5.24E-01 | 5.24E-01 | -0.23±0.16 | 0 | 3.21E-01 | 6.87E-01 | -0.17±0.17 | 8.55E-01 | -0.17±0.17 | 8.97E-01 | -0.43±0.38 | 9.99E-01 | -0.61±0.42 | 1.00E+00 |
| taurine | 0.08±0.22 | 6.98 | 3.00E-01 | 7.25E-01 | 0.22±0.16 | 64.41 | 9.37E-02 | 6.95E-01 | 0.31±0.17 | 6.58E-01 | 0.34±0.17 | 6.30E-01 | -0.14±0.31 | 9.99E-01 | -0.37±0.38 | 1.00E+00 |
| X - 13507 | 0.03±0.2 | 66.83 | 8.25E-02 | 8.74E-01 | 0.18±0.13 | 64.43 | 9.36E-02 | 6.95E-01 | 0.24±0.13 | 6.58E-01 | 0.24±0.13 | 6.97E-01 | -0.6±0.42 | 9.99E-01 | -0.67±0.52 | 1.00E+00 |
| stachydrine | -0.15±0.21 | 0 | 3.97E-01 | 5.45E-01 | -0.18±0.13 | 8.98 | 2.95E-01 | 6.95E-01 | -0.23±0.13 | 6.58E-01 | -0.22±0.13 | 7.32E-01 | 0.23±0.49 | 9.99E-01 | 0.31±0.48 | 1.00E+00 |
| 12,13-DiHOME | -0.14±0.23 | 0 | 3.43E-01 | 5.75E-01 | -0.2±0.14 | 0 | 4.22E-01 | 6.95E-01 | -0.26±0.14 | 6.58E-01 | -0.23±0.14 | 7.35E-01 | 0.26±0.48 | 9.99E-01 | 0.22±0.54 | 1.00E+00 |
| pyruvate | -0.17±0.19 | 0 | 6.18E-01 | 5.24E-01 | -0.21±0.15 | 0 | 7.56E-01 | 6.95E-01 | -0.21±0.15 | 7.58E-01 | -0.23±0.15 | 7.81E-01 | 0.04±0.45 | 9.99E-01 | -0.07±0.49 | 1.00E+00 |
| N-acetylglutamate | 0.26±0.23 | 0 | 9.76E-01 | 5.24E-01 | 0.27±0.19 | 0 | 9.45E-01 | 6.95E-01 | 0.27±0.2 | 7.66E-01 | 0.27±0.2 | 7.84E-01 | 0.25±0.49 | 9.99E-01 | 0.24±0.5 | 1.00E+00 |
| dihomo-linoleate (20:2n6) | -0.2±0.2 | 0 | 7.68E-01 | 5.24E-01 | -0.22±0.15 | 0 | 9.99E-01 | 6.95E-01 | -0.23±0.16 | 7.58E-01 | -0.22±0.16 | 7.84E-01 | -0.08±0.44 | 9.99E-01 | -0.22±0.48 | 1.00E+00 |
| mannose | -0.15±0.16 | 0 | 6.44E-01 | 5.24E-01 | -0.22±0.15 | 0 | 9.69E-01 | 6.95E-01 | -0.18±0.16 | 8.05E-01 | -0.22±0.16 | 7.84E-01 | 0±0.35 | 9.99E-01 | -0.24±0.43 | 1.00E+00 |
| caprylate (8:0) | -0.2±0.2 | 0 | 7.91E-01 | 5.24E-01 | -0.22±0.15 | 0 | 9.23E-01 | 6.95E-01 | -0.23±0.16 | 7.58E-01 | -0.22±0.16 | 7.84E-01 | -0.1±0.42 | 9.99E-01 | -0.17±0.5 | 1.00E+00 |
| erythronate* | -0.17±0.17 | 0 | 8.01E-01 | 5.24E-01 | -0.21±0.15 | 0 | 9.34E-01 | 6.95E-01 | -0.2±0.16 | 7.87E-01 | -0.21±0.16 | 8.08E-01 | -0.11±0.3 | 9.99E-01 | -0.24±0.35 | 1.00E+00 |
| glycerol | -0.22±0.2 | 0 | 9.53E-01 | 5.24E-01 | -0.23±0.16 | 0 | 5.31E-01 | 6.95E-01 | -0.22±0.17 | 7.66E-01 | -0.2±0.17 | 8.35E-01 | -0.25±0.46 | 9.99E-01 | -0.54±0.51 | 1.00E+00 |
| beta-hydroxyisovaleroylcarnitine | -0.23±0.17 | 0 | 7.92E-01 | 5.24E-01 | -0.22±0.15 | 0 | 6.72E-01 | 6.95E-01 | -0.2±0.17 | 7.99E-01 | -0.19±0.17 | 8.44E-01 | -0.3±0.33 | 9.99E-01 | -0.37±0.38 | 1.00E+00 |
| 1,3,7-trimethylurate | 0.21±0.18 | 0 | 7.12E-01 | 5.24E-01 | 0.25±0.17 | 0 | 9.97E-01 | 6.96E-01 | 0.19±0.17 | 8.11E-01 | 0.25±0.18 | 7.84E-01 | 0.4±0.54 | 9.99E-01 | 0.24±0.64 | 1.00E+00 |
| corticosterone | -0.18±0.16 | 0 | 9.73E-01 | 5.24E-01 | -0.19±0.13 | 0 | 9.22E-01 | 6.97E-01 | -0.18±0.13 | 7.66E-01 | -0.19±0.14 | 7.84E-01 | -0.16±0.44 | 9.99E-01 | -0.14±0.5 | 1.00E+00 |
| 4-ethylcatechol sulfate | 0.03±0.23 | 61.87 | 1.05E-01 | 9.09E-01 | 0.2±0.14 | 52.43 | 1.47E-01 | 7.13E-01 | 0.28±0.15 | 6.58E-01 | 0.26±0.15 | 7.23E-01 | -0.53±0.41 | 9.99E-01 | -0.48±0.49 | 1.00E+00 |
| alpha-hydroxyisocaproate | -0.2±0.2 | 0 | 7.86E-01 | 5.24E-01 | -0.22±0.16 | 0 | 7.48E-01 | 7.13E-01 | -0.23±0.16 | 7.58E-01 | -0.24±0.17 | 7.84E-01 | -0.09±0.44 | 9.99E-01 | -0.09±0.44 | 1.00E+00 |
| xanthine | -0.15±0.18 | 0 | 5.28E-01 | 5.29E-01 | -0.2±0.14 | 0 | 9.05E-01 | 7.17E-01 | -0.21±0.15 | 7.64E-01 | -0.21±0.15 | 7.84E-01 | 0.07±0.38 | 9.99E-01 | -0.15±0.42 | 1.00E+00 |
| N-acetylisoleucine | -0.16±0.13 | 0 | 7.54E-01 | 5.24E-01 | -0.21±0.15 | 0 | 4.20E-01 | 7.22E-01 | -0.14±0.15 | 8.75E-01 | -0.17±0.16 | 8.61E-01 | -0.28±0.41 | 9.99E-01 | -0.57±0.48 | 1.00E+00 |
| 3beta-hydroxy-5-cholestenoate | -0.15±0.11 | 0 | 5.23E-01 | 5.24E-01 | -0.2±0.15 | 0 | 4.05E-01 | 7.22E-01 | -0.12±0.15 | 9.22E-01 | -0.14±0.16 | 9.15E-01 | -0.33±0.3 | 9.99E-01 | -0.45±0.33 | 1.00E+00 |
| ximenoylcarnitine (C26:1)* | -0.17±0.2 | 0 | 6.25E-01 | 5.24E-01 | -0.21±0.15 | 0 | 9.48E-01 | 7.25E-01 | -0.23±0.16 | 7.58E-01 | -0.21±0.16 | 7.87E-01 | 0±0.41 | 9.99E-01 | -0.18±0.46 | 1.00E+00 |
| 3,7-dimethylurate | 0.19±0.22 | 0 | 5.88E-01 | 5.24E-01 | 0.22±0.16 | 23.53 | 2.53E-01 | 7.32E-01 | 0.25±0.16 | 7.45E-01 | 0.28±0.17 | 7.32E-01 | -0.03±0.46 | 9.99E-01 | -0.28±0.46 | 1.00E+00 |
| retinol (Vitamin A) | 0.13±0.19 | 0 | 4.28E-01 | 5.59E-01 | 0.2±0.15 | 18.25 | 2.69E-01 | 7.32E-01 | 0.23±0.15 | 7.45E-01 | 0.26±0.15 | 7.32E-01 | -0.09±0.33 | 9.99E-01 | -0.24±0.42 | 1.00E+00 |
| pregnenediol disulfate (C21H34O8S2)* | -0.17±0.21 | 0 | 5.11E-01 | 5.25E-01 | -0.24±0.18 | 0 | 5.80E-01 | 7.32E-01 | -0.23±0.18 | 7.86E-01 | -0.27±0.19 | 7.81E-01 | 0.17±0.56 | 9.99E-01 | 0.08±0.61 | 1.00E+00 |
| dimethylglycine | 0.21±0.21 | 0 | 8.59E-01 | 5.24E-01 | 0.18±0.13 | 0 | 6.56E-01 | 7.32E-01 | 0.23±0.13 | 6.58E-01 | 0.19±0.14 | 7.84E-01 | 0.14±0.47 | 9.99E-01 | -0.04±0.5 | 1.00E+00 |
| X - 24334 | -0.16±0.21 | 0 | 4.52E-01 | 5.37E-01 | -0.22±0.16 | 0 | 8.93E-01 | 7.32E-01 | -0.23±0.17 | 7.58E-01 | -0.23±0.17 | 7.84E-01 | 0.16±0.47 | 9.99E-01 | -0.16±0.51 | 1.00E+00 |
| methionine sulfoxide | -0.18±0.19 | 0 | 6.96E-01 | 5.24E-01 | -0.21±0.15 | 0 | 9.54E-01 | 7.32E-01 | -0.21±0.16 | 7.66E-01 | -0.21±0.16 | 7.91E-01 | -0.03±0.42 | 9.99E-01 | -0.19±0.48 | 1.00E+00 |
| X - 12707 | -0.19±0.17 | 0 | 9.87E-01 | 5.24E-01 | -0.2±0.15 | 0 | 7.91E-01 | 7.32E-01 | -0.19±0.15 | 7.87E-01 | -0.19±0.16 | 8.25E-01 | -0.18±0.41 | 9.99E-01 | -0.31±0.44 | 1.00E+00 |
| X - 21842 | -0.21±0.16 | 0 | 4.93E-01 | 5.24E-01 | -0.19±0.14 | 0 | 4.33E-01 | 7.32E-01 | -0.17±0.15 | 8.05E-01 | -0.16±0.15 | 8.53E-01 | -0.54±0.51 | 9.99E-01 | -0.59±0.52 | 1.00E+00 |
| hypotaurine | -0.21±0.15 | 0 | 4.28E-01 | 5.24E-01 | -0.21±0.15 | 13.36 | 2.83E-01 | 7.32E-01 | -0.16±0.16 | 8.55E-01 | -0.14±0.16 | 9.15E-01 | -0.48±0.37 | 9.99E-01 | -0.63±0.42 | 1.00E+00 |
| 3-hydroxybutyrate (BHBA) | -0.17±0.21 | 0 | 5.20E-01 | 5.29E-01 | -0.22±0.16 | 0 | 4.66E-01 | 7.45E-01 | -0.25±0.17 | 7.57E-01 | -0.26±0.17 | 7.74E-01 | 0.07±0.43 | 9.99E-01 | 0.11±0.47 | 1.00E+00 |
| homocitrulline | -0.14±0.1 | 76.13 | 4.07E-02 | 5.24E-01 | -0.2±0.15 | 77.17 | 3.64E-02 | 7.45E-01 | -0.1±0.15 | 9.32E-01 | -0.12±0.15 | 9.15E-01 | -1.03±0.44 | 9.99E-01 | -1.35±0.57 | 1.00E+00 |
| 3-phenylpropionate (hydrocinnamate) | -0.14±0.09 | 63.25 | 9.90E-02 | 5.24E-01 | -0.18±0.13 | 62.87 | 1.01E-01 | 7.45E-01 | -0.1±0.14 | 9.32E-01 | -0.1±0.14 | 9.23E-01 | -0.68±0.34 | 9.99E-01 | -0.79±0.4 | 1.00E+00 |
| X - 26106 | -0.2±0.19 | 0 | 8.98E-01 | 5.24E-01 | -0.2±0.15 | 0 | 8.02E-01 | 7.46E-01 | -0.22±0.16 | 7.64E-01 | -0.19±0.16 | 8.35E-01 | -0.16±0.38 | 9.99E-01 | -0.3±0.43 | 1.00E+00 |
| guanidinoacetate | -0.13±0.1 | 0 | 5.07E-01 | 5.24E-01 | -0.19±0.14 | 12 | 2.86E-01 | 7.46E-01 | -0.11±0.15 | 9.32E-01 | -0.12±0.15 | 9.15E-01 | -0.3±0.27 | 9.99E-01 | -0.52±0.34 | 1.00E+00 |
| ergothioneine | 0.16±0.21 | 0 | 5.27E-01 | 5.29E-01 | 0.21±0.16 | 10.07 | 2.92E-01 | 7.47E-01 | 0.24±0.16 | 7.58E-01 | 0.27±0.17 | 7.35E-01 | -0.06±0.41 | 9.99E-01 | -0.24±0.45 | 1.00E+00 |
| 17alpha-hydroxypregnanolone glucuronide | -0.17±0.13 | 0 | 3.99E-01 | 5.24E-01 | -0.26±0.2 | 1.49 | 3.14E-01 | 7.50E-01 | -0.14±0.21 | 9.32E-01 | -0.18±0.21 | 9.15E-01 | -0.55±0.46 | 9.99E-01 | -0.73±0.51 | 1.00E+00 |
| phenylacetylglutamine | 0.23±0.2 | 0 | 9.32E-01 | 5.24E-01 | 0.21±0.16 | 0 | 9.55E-01 | 7.50E-01 | 0.22±0.17 | 7.66E-01 | 0.22±0.17 | 8.05E-01 | 0.26±0.49 | 9.99E-01 | 0.18±0.56 | 1.00E+00 |
| N-methylhydroxyproline** | -0.14±0.2 | 0 | 3.77E-01 | 5.47E-01 | -0.18±0.14 | 0 | 3.20E-01 | 7.52E-01 | -0.22±0.14 | 7.45E-01 | -0.22±0.14 | 7.63E-01 | 0.25±0.49 | 9.99E-01 | 0.29±0.5 | 1.00E+00 |
| glutamine | 0.2±0.2 | 0 | 8.82E-01 | 5.24E-01 | 0.2±0.15 | 0 | 6.59E-01 | 7.55E-01 | 0.21±0.15 | 7.58E-01 | 0.21±0.15 | 7.84E-01 | 0.13±0.52 | 9.99E-01 | -0.04±0.56 | 1.00E+00 |
| 4-cholesten-3-one | -0.12±0.15 | 0 | 5.28E-01 | 5.29E-01 | -0.18±0.13 | 0 | 9.64E-01 | 7.55E-01 | -0.17±0.14 | 7.92E-01 | -0.18±0.14 | 8.12E-01 | 0.06±0.32 | 9.99E-01 | -0.16±0.38 | 1.00E+00 |
| cis-4-decenoylcarnitine (C10:1) | -0.2±0.14 | 0 | 4.70E-01 | 5.24E-01 | -0.19±0.15 | 48.11 | 1.65E-01 | 7.55E-01 | -0.16±0.15 | 8.52E-01 | -0.13±0.16 | 9.15E-01 | -0.43±0.35 | 9.99E-01 | -0.88±0.52 | 1.00E+00 |
| 2-hydroxy-4-(methylthio)butanoic acid | -0.08±0.06 | 56.64 | 1.29E-01 | 5.24E-01 | -0.21±0.16 | 45.56 | 1.75E-01 | 7.59E-01 | -0.06±0.16 | 9.80E-01 | -0.12±0.17 | 9.23E-01 | -0.62±0.36 | 9.99E-01 | -0.74±0.42 | 1.00E+00 |
| 3-(4-hydroxyphenyl)lactate | -0.14±0.1 | 0 | 3.83E-01 | 5.24E-01 | -0.2±0.16 | 48.11 | 1.65E-01 | 7.59E-01 | -0.11±0.16 | 9.32E-01 | -0.11±0.17 | 9.23E-01 | -0.43±0.35 | 9.99E-01 | -0.72±0.41 | 1.00E+00 |
| X - 12111 | -0.13±0.23 | 5.58 | 3.03E-01 | 6.15E-01 | -0.19±0.15 | 30.08 | 2.32E-01 | 7.73E-01 | -0.27±0.15 | 6.58E-01 | -0.25±0.15 | 7.35E-01 | 0.27±0.44 | 9.99E-01 | 0.33±0.46 | 1.00E+00 |
| 1-methylxanthine | 0.22±0.18 | 0 | 7.08E-01 | 5.24E-01 | 0.21±0.16 | 0 | 7.29E-01 | 7.73E-01 | 0.2±0.17 | 8.05E-01 | 0.19±0.17 | 8.53E-01 | 0.4±0.52 | 9.99E-01 | 0.38±0.52 | 1.00E+00 |
| 3-hydroxyisobutyrate | -0.13±0.22 | 1.05 | 3.15E-01 | 5.89E-01 | -0.2±0.15 | 0 | 3.78E-01 | 7.86E-01 | -0.25±0.16 | 7.45E-01 | -0.24±0.16 | 7.81E-01 | 0.28±0.46 | 9.99E-01 | 0.24±0.52 | 1.00E+00 |
| alpha-tocopherol | 0.17±0.14 | 0 | 4.79E-01 | 5.24E-01 | 0.19±0.15 | 0 | 5.16E-01 | 7.88E-01 | 0.14±0.16 | 8.83E-01 | 0.17±0.16 | 8.53E-01 | 0.65±0.69 | 9.99E-01 | 0.65±0.72 | 1.00E+00 |
| urea | 0.09±0.19 | 15.53 | 2.77E-01 | 6.69E-01 | 0.18±0.14 | 33.57 | 2.20E-01 | 7.99E-01 | 0.23±0.15 | 7.45E-01 | 0.23±0.15 | 7.60E-01 | -0.21±0.33 | 9.99E-01 | -0.37±0.47 | 1.00E+00 |
| 1-palmitoyl-GPE (16:0) | 0.17±0.19 | 0 | 5.98E-01 | 5.24E-01 | 0.19±0.15 | 0 | 4.09E-01 | 7.99E-01 | 0.22±0.16 | 7.58E-01 | 0.24±0.16 | 7.84E-01 | -0.03±0.41 | 9.99E-01 | -0.17±0.47 | 1.00E+00 |
| 4-methylhexanoylglutamine | -0.15±0.22 | 0 | 4.09E-01 | 5.53E-01 | -0.2±0.16 | 0 | 5.50E-01 | 7.99E-01 | -0.26±0.16 | 7.45E-01 | -0.23±0.17 | 7.84E-01 | 0.17±0.45 | 9.99E-01 | 0.08±0.5 | 1.00E+00 |
| 5-oxoproline | 0.19±0.2 | 0 | 7.79E-01 | 5.24E-01 | 0.19±0.15 | 0 | 4.48E-01 | 7.99E-01 | 0.22±0.16 | 7.58E-01 | 0.23±0.16 | 7.84E-01 | 0.09±0.42 | 9.99E-01 | -0.14±0.46 | 1.00E+00 |
| dihydroorotate | -0.12±0.2 | 22.57 | 2.56E-01 | 6.05E-01 | -0.18±0.14 | 0 | 4.09E-01 | 7.99E-01 | -0.23±0.15 | 7.45E-01 | -0.22±0.15 | 7.84E-01 | 0.38±0.48 | 9.99E-01 | 0.2±0.48 | 1.00E+00 |
| 2-hydroxydecanoate | -0.15±0.19 | 0 | 5.33E-01 | 5.25E-01 | -0.18±0.15 | 0 | 6.59E-01 | 7.99E-01 | -0.21±0.16 | 7.66E-01 | -0.21±0.16 | 7.91E-01 | 0.1±0.44 | 9.99E-01 | 0.02±0.49 | 1.00E+00 |
| myristoylcarnitine (C14) | -0.16±0.21 | 0 | 4.98E-01 | 5.30E-01 | -0.19±0.15 | 0 | 7.45E-01 | 7.99E-01 | -0.24±0.16 | 7.45E-01 | -0.21±0.16 | 8.03E-01 | 0.12±0.46 | 9.99E-01 | -0.02±0.55 | 1.00E+00 |
| X - 13728 | 0.21±0.18 | 0 | 8.13E-01 | 5.24E-01 | 0.21±0.16 | 0 | 9.04E-01 | 7.99E-01 | 0.2±0.17 | 8.05E-01 | 0.21±0.17 | 8.14E-01 | 0.33±0.55 | 9.99E-01 | 0.14±0.59 | 1.00E+00 |
| 3-hydroxyoctanoylcarnitine (2) | -0.19±0.21 | 0 | 7.25E-01 | 5.24E-01 | -0.19±0.15 | 0 | 8.64E-01 | 7.99E-01 | -0.23±0.16 | 7.58E-01 | -0.2±0.16 | 8.16E-01 | -0.05±0.45 | 9.99E-01 | -0.1±0.54 | 1.00E+00 |
| pentadecanoate (15:0) | -0.19±0.21 | 0 | 6.52E-01 | 5.24E-01 | -0.2±0.16 | 0 | 9.33E-01 | 7.99E-01 | -0.24±0.17 | 7.58E-01 | -0.21±0.17 | 8.25E-01 | 0.01±0.49 | 9.99E-01 | -0.16±0.53 | 1.00E+00 |
| X - 18901 | 0.14±0.14 | 0 | 8.96E-01 | 5.24E-01 | 0.18±0.15 | 0 | 9.73E-01 | 7.99E-01 | 0.14±0.15 | 8.75E-01 | 0.18±0.15 | 8.32E-01 | 0.08±0.45 | 9.99E-01 | 0.2±0.55 | 1.00E+00 |
| X - 21607 | -0.17±0.15 | 0 | 8.33E-01 | 5.24E-01 | -0.18±0.14 | 0 | 5.75E-01 | 7.99E-01 | -0.16±0.15 | 8.17E-01 | -0.15±0.15 | 8.75E-01 | -0.27±0.46 | 9.99E-01 | -0.48±0.56 | 1.00E+00 |
| 12-HETE | -0.16±0.13 | 0 | 4.95E-01 | 5.24E-01 | -0.18±0.14 | 0 | 5.48E-01 | 7.99E-01 | -0.13±0.14 | 8.83E-01 | -0.15±0.15 | 8.95E-01 | -0.42±0.4 | 9.99E-01 | -0.42±0.44 | 1.00E+00 |
| oleoyl-linoleoyl-glycerol (18:1/18:2) [2] | -0.12±0.09 | 0 | 5.10E-01 | 5.24E-01 | -0.16±0.13 | 0 | 4.86E-01 | 7.99E-01 | -0.1±0.14 | 9.32E-01 | -0.13±0.14 | 9.10E-01 | -0.33±0.34 | 9.99E-01 | -0.42±0.4 | 1.00E+00 |
| 2-piperidinone | -0.12±0.1 | 33.9 | 2.19E-01 | 5.24E-01 | -0.17±0.14 | 64.6 | 9.28E-02 | 7.99E-01 | -0.1±0.14 | 9.32E-01 | -0.12±0.14 | 9.15E-01 | -0.77±0.54 | 9.99E-01 | -1.25±0.66 | 1.00E+00 |
| kynurenate | -0.16±0.14 | 0 | 8.80E-01 | 5.24E-01 | -0.19±0.15 | 0 | 3.98E-01 | 7.99E-01 | -0.15±0.15 | 8.55E-01 | -0.14±0.16 | 9.15E-01 | -0.22±0.39 | 9.99E-01 | -0.57±0.47 | 1.00E+00 |
| 1-palmitoyl-GPC (16:0) | 0.17±0.18 | 0 | 7.32E-01 | 5.24E-01 | 0.18±0.15 | 0 | 3.97E-01 | 7.99E-01 | 0.19±0.15 | 7.87E-01 | 0.23±0.16 | 7.84E-01 | 0.03±0.43 | 9.99E-01 | -0.17±0.44 | 1.00E+00 |
| (16 or 17)-methylstearate (a19:0 or i19:0) | -0.16±0.22 | 0 | 4.32E-01 | 5.39E-01 | -0.19±0.16 | 0 | 5.81E-01 | 8.00E-01 | -0.24±0.16 | 7.45E-01 | -0.22±0.16 | 7.84E-01 | 0.17±0.47 | 9.99E-01 | 0.06±0.48 | 1.00E+00 |
| X - 23639 | 0.16±0.19 | 0 | 5.88E-01 | 5.24E-01 | 0.19±0.16 | 0 | 7.04E-01 | 8.00E-01 | 0.21±0.16 | 7.66E-01 | 0.21±0.17 | 8.05E-01 | -0.05±0.42 | 9.99E-01 | -0.02±0.58 | 1.00E+00 |
| 6-hydroxyindole sulfate | 0.16±0.15 | 0 | 9.06E-01 | 5.24E-01 | 0.19±0.16 | 0 | 9.90E-01 | 8.03E-01 | 0.16±0.16 | 8.75E-01 | 0.19±0.17 | 8.52E-01 | 0.21±0.41 | 9.99E-01 | 0.18±0.43 | 1.00E+00 |
| deoxycholate | 0.13±0.2 | 0 | 3.82E-01 | 5.62E-01 | 0.18±0.15 | 16.25 | 2.75E-01 | 8.03E-01 | 0.23±0.15 | 7.50E-01 | 0.23±0.16 | 7.81E-01 | -0.18±0.41 | 9.99E-01 | -0.3±0.46 | 1.00E+00 |
| S-carboxyethylcysteine | 0.11±0.21 | 33.97 | 2.18E-01 | 6.34E-01 | 0.18±0.15 | 0 | 4.07E-01 | 8.03E-01 | 0.23±0.14 | 6.90E-01 | 0.21±0.15 | 7.84E-01 | -0.43±0.48 | 9.99E-01 | -0.3±0.59 | 1.00E+00 |
| myristate (14:0) | -0.13±0.23 | 10.52 | 2.90E-01 | 5.93E-01 | -0.19±0.16 | 0 | 4.98E-01 | 8.03E-01 | -0.25±0.17 | 7.45E-01 | -0.23±0.17 | 7.84E-01 | 0.35±0.51 | 9.99E-01 | 0.15±0.54 | 1.00E+00 |
| arachidate (20:0) | -0.15±0.2 | 0 | 4.90E-01 | 5.29E-01 | -0.19±0.16 | 0 | 6.21E-01 | 8.03E-01 | -0.21±0.16 | 7.66E-01 | -0.22±0.17 | 8.05E-01 | 0.15±0.48 | 9.99E-01 | 0.04±0.49 | 1.00E+00 |
| p-cresol sulfate | 0.21±0.22 | 0 | 8.01E-01 | 5.24E-01 | 0.2±0.17 | 0 | 6.76E-01 | 8.03E-01 | 0.24±0.17 | 7.58E-01 | 0.22±0.18 | 8.11E-01 | 0.11±0.48 | 9.99E-01 | -0.02±0.55 | 1.00E+00 |
| androstenediol (3alpha, 17alpha) monosulfate (3) | -0.18±0.18 | 0 | 8.46E-01 | 5.24E-01 | -0.18±0.15 | 0 | 7.70E-01 | 8.03E-01 | -0.19±0.16 | 7.92E-01 | -0.2±0.16 | 8.14E-01 | -0.09±0.48 | 9.99E-01 | -0.05±0.48 | 1.00E+00 |
| hexadecenedioate (C16:1-DC)* | -0.13±0.17 | 0 | 5.04E-01 | 5.29E-01 | -0.18±0.15 | 0 | 9.04E-01 | 8.03E-01 | -0.19±0.15 | 7.87E-01 | -0.19±0.16 | 8.25E-01 | 0.09±0.38 | 9.99E-01 | -0.12±0.48 | 1.00E+00 |
| X - 13866 | -0.19±0.2 | 0 | 7.77E-01 | 5.24E-01 | -0.18±0.15 | 0 | 8.83E-01 | 8.03E-01 | -0.22±0.15 | 7.58E-01 | -0.18±0.16 | 8.33E-01 | -0.07±0.46 | 9.99E-01 | -0.1±0.52 | 1.00E+00 |
| 1-ribosyl-imidazoleacetate* | 0.16±0.15 | 0 | 9.05E-01 | 5.24E-01 | 0.16±0.13 | 0 | 9.69E-01 | 8.03E-01 | 0.17±0.13 | 7.87E-01 | 0.16±0.14 | 8.35E-01 | 0.12±0.37 | 9.99E-01 | 0.14±0.49 | 1.00E+00 |
| myristoleoylcarnitine (C14:1)* | -0.18±0.18 | 0 | 7.80E-01 | 5.24E-01 | -0.19±0.16 | 0 | 9.43E-01 | 8.03E-01 | -0.2±0.16 | 7.87E-01 | -0.18±0.17 | 8.53E-01 | -0.06±0.44 | 9.99E-01 | -0.23±0.55 | 1.00E+00 |
| gamma-glutamylphenylalanine | -0.15±0.15 | 0 | 7.73E-01 | 5.24E-01 | -0.18±0.15 | 0 | 8.50E-01 | 8.03E-01 | -0.17±0.16 | 8.48E-01 | -0.17±0.16 | 8.77E-01 | -0.04±0.39 | 9.99E-01 | -0.25±0.39 | 1.00E+00 |
| kynurenine | 0.14±0.12 | 0 | 3.50E-01 | 5.24E-01 | 0.19±0.16 | 0 | 4.55E-01 | 8.03E-01 | 0.12±0.15 | 9.32E-01 | 0.16±0.17 | 9.08E-01 | 0.63±0.53 | 9.99E-01 | 0.64±0.62 | 1.00E+00 |
| carotene diol (1) | 0.1±0.09 | 0 | 6.38E-01 | 5.24E-01 | 0.18±0.15 | 0 | 4.25E-01 | 8.03E-01 | 0.09±0.15 | 9.32E-01 | 0.15±0.16 | 9.08E-01 | 0.34±0.52 | 9.99E-01 | 0.65±0.61 | 1.00E+00 |
| glycolithocholate sulfate* | -0.13±0.1 | 0 | 5.11E-01 | 5.24E-01 | -0.16±0.13 | 0 | 4.57E-01 | 8.03E-01 | -0.11±0.14 | 9.22E-01 | -0.13±0.14 | 9.09E-01 | -0.36±0.37 | 9.99E-01 | -0.49±0.47 | 1.00E+00 |
| X - 14939 | -0.13±0.11 | 0 | 6.61E-01 | 5.24E-01 | -0.16±0.14 | 0 | 3.76E-01 | 8.03E-01 | -0.11±0.14 | 9.29E-01 | -0.12±0.15 | 9.15E-01 | -0.3±0.41 | 9.99E-01 | -0.53±0.44 | 1.00E+00 |
| dopamine 3-O-sulfate | -0.14±0.09 | 82.5 | 1.68E-02 | 5.24E-01 | -0.17±0.14 | 80.72 | 2.28E-02 | 8.03E-01 | -0.09±0.15 | 9.32E-01 | -0.09±0.15 | 9.26E-01 | -1.18±0.45 | 9.99E-01 | -1.52±0.61 | 1.00E+00 |
| 1-palmitoyl-2-oleoyl-GPC (16:0/18:1) | 0.17±0.18 | 0 | 7.48E-01 | 5.24E-01 | 0.18±0.16 | 0 | 5.33E-01 | 8.07E-01 | 0.2±0.16 | 7.86E-01 | 0.22±0.17 | 7.91E-01 | 0.05±0.42 | 9.99E-01 | -0.11±0.5 | 1.00E+00 |
| arachidonate (20:4n6) | -0.13±0.19 | 0 | 3.64E-01 | 5.43E-01 | -0.17±0.15 | 0 | 7.01E-01 | 8.07E-01 | -0.2±0.15 | 7.66E-01 | -0.19±0.15 | 8.14E-01 | 0.29±0.5 | 9.99E-01 | 0.02±0.52 | 1.00E+00 |
| EDTA | -0.13±0.15 | 0 | 5.93E-01 | 5.24E-01 | -0.14±0.12 | 0 | 7.38E-01 | 8.07E-01 | -0.16±0.13 | 7.92E-01 | -0.16±0.13 | 8.19E-01 | 0.12±0.49 | 9.99E-01 | -0.01±0.41 | 1.00E+00 |
| X - 11632 | 0.19±0.15 | 2.65 | 3.11E-01 | 5.24E-01 | 0.18±0.15 | 4.91 | 3.05E-01 | 8.07E-01 | 0.16±0.15 | 8.48E-01 | 0.14±0.16 | 9.12E-01 | 0.8±0.62 | 9.99E-01 | 0.92±0.74 | 1.00E+00 |
| androstenediol (3beta,17beta) disulfate (1) | -0.08±0.21 | 58.95 | 1.19E-01 | 7.33E-01 | -0.2±0.17 | 64.29 | 9.43E-02 | 8.08E-01 | -0.23±0.17 | 7.66E-01 | -0.28±0.18 | 7.56E-01 | 0.6±0.48 | 9.99E-01 | 0.81±0.63 | 1.00E+00 |
| androstenediol (3beta,17beta) monosulfate (2) | -0.12±0.19 | 39.79 | 1.97E-01 | 5.79E-01 | -0.16±0.14 | 16.82 | 2.73E-01 | 8.08E-01 | -0.2±0.14 | 7.58E-01 | -0.19±0.14 | 7.84E-01 | 0.59±0.58 | 9.99E-01 | 0.53±0.65 | 1.00E+00 |
| N-acetylglutamine | -0.13±0.18 | 0 | 3.76E-01 | 5.39E-01 | -0.16±0.14 | 0 | 4.06E-01 | 8.08E-01 | -0.19±0.15 | 7.66E-01 | -0.2±0.15 | 7.87E-01 | 0.25±0.47 | 9.99E-01 | 0.27±0.54 | 1.00E+00 |
| phenylacetate | 0.2±0.18 | 0 | 8.82E-01 | 5.24E-01 | 0.18±0.16 | 0 | 9.78E-01 | 8.08E-01 | 0.19±0.16 | 8.05E-01 | 0.18±0.17 | 8.61E-01 | 0.27±0.49 | 9.99E-01 | 0.2±0.52 | 1.00E+00 |
| glutarylcarnitine (C5-DC) | -0.14±0.14 | 0 | 7.49E-01 | 5.24E-01 | -0.17±0.15 | 0 | 9.86E-01 | 8.08E-01 | -0.15±0.15 | 8.55E-01 | -0.17±0.16 | 8.61E-01 | -0.02±0.39 | 9.99E-01 | -0.18±0.44 | 1.00E+00 |
| 1-linoleoyl-2-arachidonoyl-GPC (18:2/20:4n6)* | -0.14±0.15 | 0 | 7.29E-01 | 5.24E-01 | -0.17±0.14 | 0 | 9.53E-01 | 8.08E-01 | -0.16±0.15 | 8.27E-01 | -0.16±0.15 | 8.61E-01 | -0.02±0.37 | 9.99E-01 | -0.19±0.43 | 1.00E+00 |
| X - 21736 | -0.17±0.16 | 0 | 9.44E-01 | 5.24E-01 | -0.17±0.15 | 0 | 7.61E-01 | 8.08E-01 | -0.17±0.15 | 8.11E-01 | -0.16±0.16 | 8.92E-01 | -0.14±0.45 | 9.99E-01 | -0.32±0.5 | 1.00E+00 |
| 6-oxopiperidine-2-carboxylate | -0.15±0.11 | 0 | 4.43E-01 | 5.24E-01 | -0.17±0.15 | 0 | 4.69E-01 | 8.08E-01 | -0.12±0.15 | 9.32E-01 | -0.12±0.16 | 9.15E-01 | -0.37±0.31 | 9.99E-01 | -0.4±0.36 | 1.00E+00 |
| N1-methylinosine | 0.11±0.23 | 0.97 | 3.15E-01 | 6.49E-01 | 0.19±0.16 | 60.86 | 1.10E-01 | 8.09E-01 | 0.29±0.17 | 6.62E-01 | 0.29±0.18 | 7.33E-01 | -0.19±0.38 | 9.99E-01 | -0.51±0.46 | 1.00E+00 |
| X - 21471 | -0.14±0.17 | 0 | 4.72E-01 | 5.26E-01 | -0.18±0.15 | 0 | 5.37E-01 | 8.09E-01 | -0.18±0.16 | 8.05E-01 | -0.2±0.16 | 8.05E-01 | 0.21±0.51 | 9.99E-01 | 0.16±0.57 | 1.00E+00 |
| lyxonate | -0.16±0.19 | 0 | 5.77E-01 | 5.24E-01 | -0.17±0.15 | 0 | 5.67E-01 | 8.09E-01 | -0.21±0.15 | 7.58E-01 | -0.19±0.15 | 8.12E-01 | 0.07±0.47 | 9.99E-01 | 0.12±0.52 | 1.00E+00 |
| N2,N2-dimethylguanosine | 0.17±0.16 | 0 | 9.93E-01 | 5.24E-01 | 0.19±0.16 | 0 | 6.62E-01 | 8.09E-01 | 0.17±0.16 | 8.48E-01 | 0.21±0.17 | 8.16E-01 | 0.16±0.48 | 9.99E-01 | -0.04±0.54 | 1.00E+00 |
| pregnenetriol disulfate* | -0.15±0.17 | 0 | 6.13E-01 | 5.24E-01 | -0.19±0.17 | 0 | 6.79E-01 | 8.09E-01 | -0.18±0.17 | 8.48E-01 | -0.21±0.18 | 8.25E-01 | 0.11±0.55 | 9.99E-01 | 0.05±0.62 | 1.00E+00 |
| cyclo(leu-pro) | 0.14±0.11 | 0 | 3.58E-01 | 5.24E-01 | 0.2±0.18 | 0 | 7.76E-01 | 8.09E-01 | 0.12±0.17 | 9.32E-01 | 0.19±0.19 | 8.97E-01 | 0.58±0.5 | 9.99E-01 | 0.36±0.59 | 1.00E+00 |
| 1-stearoyl-2-linoleoyl-GPC (18:0/18:2)* | -0.12±0.11 | 0 | 8.96E-01 | 5.24E-01 | -0.17±0.15 | 0 | 5.99E-01 | 8.09E-01 | -0.12±0.16 | 9.32E-01 | -0.13±0.16 | 9.15E-01 | -0.16±0.29 | 9.99E-01 | -0.33±0.35 | 1.00E+00 |
| 1-(1-enyl-palmitoyl)-2-linoleoyl-GPE (P-16:0/18:2)* | -0.12±0.09 | 0 | 4.82E-01 | 5.24E-01 | -0.16±0.14 | 19.35 | 2.65E-01 | 8.09E-01 | -0.1±0.15 | 9.32E-01 | -0.09±0.15 | 9.26E-01 | -0.32±0.3 | 9.99E-01 | -0.53±0.36 | 1.00E+00 |
| citrulline | -0.03±0.02 | 46.92 | 1.70E-01 | 5.24E-01 | -0.15±0.13 | 61.97 | 1.05E-01 | 8.11E-01 | -0.02±0.15 | 9.94E-01 | -0.04±0.15 | 9.69E-01 | -0.36±0.24 | 9.99E-01 | -0.56±0.29 | 1.00E+00 |
| palmitoleoylcarnitine (C16:1)* | -0.15±0.18 | 0 | 5.47E-01 | 5.24E-01 | -0.18±0.16 | 0 | 9.06E-01 | 8.15E-01 | -0.2±0.16 | 7.86E-01 | -0.18±0.16 | 8.53E-01 | 0.1±0.45 | 9.99E-01 | -0.11±0.54 | 1.00E+00 |
| X - 23659 | -0.2±0.17 | 0 | 9.49E-01 | 5.24E-01 | -0.17±0.15 | 0 | 9.23E-01 | 8.15E-01 | -0.19±0.16 | 7.92E-01 | -0.16±0.16 | 8.77E-01 | -0.22±0.44 | 9.99E-01 | -0.21±0.49 | 1.00E+00 |
| cysteine sulfinic acid | 0.19±0.16 | 0 | 7.84E-01 | 5.24E-01 | 0.17±0.15 | 0 | 3.99E-01 | 8.15E-01 | 0.18±0.15 | 8.05E-01 | 0.13±0.16 | 9.15E-01 | 0.31±0.45 | 9.99E-01 | 0.59±0.52 | 1.00E+00 |
| X - 24947 | -0.14±0.11 | 0 | 4.77E-01 | 5.24E-01 | -0.15±0.14 | 0 | 4.60E-01 | 8.15E-01 | -0.12±0.14 | 9.17E-01 | -0.12±0.15 | 9.15E-01 | -0.39±0.37 | 9.99E-01 | -0.43±0.39 | 1.00E+00 |
| theobromine | 0.16±0.13 | 0 | 5.80E-01 | 5.24E-01 | 0.17±0.16 | 0 | 9.09E-01 | 8.20E-01 | 0.14±0.16 | 8.89E-01 | 0.18±0.16 | 8.53E-01 | 0.43±0.5 | 9.99E-01 | 0.12±0.54 | 1.00E+00 |
| gamma-carboxyglutamate | 0.16±0.16 | 0 | 9.50E-01 | 5.24E-01 | 0.15±0.14 | 0 | 8.44E-01 | 8.20E-01 | 0.17±0.14 | 8.05E-01 | 0.16±0.14 | 8.53E-01 | 0.14±0.46 | 9.99E-01 | 0.05±0.52 | 1.00E+00 |
| 1,2-dilinoleoyl-GPC (18:2/18:2) | -0.12±0.1 | 0 | 4.66E-01 | 5.24E-01 | -0.16±0.15 | 10.49 | 2.91E-01 | 8.20E-01 | -0.1±0.15 | 9.32E-01 | -0.09±0.16 | 9.39E-01 | -0.33±0.3 | 9.99E-01 | -0.5±0.35 | 1.00E+00 |
| cis-3,4-methyleneheptanoylcarnitine | -0.07±0.05 | 70.96 | 6.35E-02 | 5.24E-01 | -0.16±0.14 | 81.26 | 2.09E-02 | 8.20E-01 | -0.05±0.15 | 9.80E-01 | -0.07±0.15 | 9.45E-01 | -0.71±0.35 | 9.99E-01 | -1.37±0.54 | 1.00E+00 |
| 1-stearoyl-GPC (18:0) | 0.14±0.18 | 0 | 5.21E-01 | 5.29E-01 | 0.17±0.16 | 13.17 | 2.83E-01 | 8.21E-01 | 0.21±0.16 | 7.86E-01 | 0.24±0.17 | 7.84E-01 | -0.06±0.37 | 9.99E-01 | -0.24±0.42 | 1.00E+00 |
| glycerophosphoethanolamine | 0.13±0.19 | 0 | 4.14E-01 | 5.61E-01 | 0.17±0.16 | 11.77 | 2.87E-01 | 8.21E-01 | 0.23±0.17 | 7.58E-01 | 0.24±0.17 | 7.84E-01 | -0.11±0.35 | 9.99E-01 | -0.24±0.42 | 1.00E+00 |
| X - 24546 | -0.13±0.19 | 0 | 3.18E-01 | 5.59E-01 | -0.17±0.16 | 0 | 3.53E-01 | 8.21E-01 | -0.21±0.17 | 7.86E-01 | -0.22±0.17 | 7.91E-01 | 0.33±0.5 | 9.99E-01 | 0.3±0.53 | 1.00E+00 |
| gamma-glutamylvaline | 0.16±0.17 | 0 | 7.73E-01 | 5.24E-01 | 0.17±0.16 | 0 | 6.19E-01 | 8.21E-01 | 0.18±0.16 | 8.05E-01 | 0.19±0.16 | 8.25E-01 | 0.05±0.42 | 9.99E-01 | -0.07±0.5 | 1.00E+00 |
| 10-nonadecenoate (19:1n9) | -0.15±0.2 | 0 | 4.30E-01 | 5.37E-01 | -0.17±0.16 | 0 | 6.22E-01 | 8.21E-01 | -0.22±0.16 | 7.66E-01 | -0.2±0.17 | 8.25E-01 | 0.2±0.48 | 9.99E-01 | 0.06±0.5 | 1.00E+00 |
| sphinganine | -0.1±0.09 | 0 | 7.33E-01 | 5.24E-01 | -0.15±0.14 | 0 | 9.23E-01 | 8.21E-01 | -0.09±0.14 | 9.32E-01 | -0.14±0.14 | 8.95E-01 | -0.23±0.41 | 9.99E-01 | -0.19±0.46 | 1.00E+00 |
| hexadecasphingosine (d16:1)* | 0.15±0.14 | 0 | 8.98E-01 | 5.24E-01 | 0.16±0.15 | 0 | 8.03E-01 | 8.21E-01 | 0.14±0.15 | 8.75E-01 | 0.15±0.15 | 8.97E-01 | 0.2±0.47 | 9.99E-01 | 0.28±0.5 | 1.00E+00 |
| gamma-glutamyltryptophan | -0.15±0.14 | 0 | 9.63E-01 | 5.24E-01 | -0.16±0.15 | 0 | 6.79E-01 | 8.21E-01 | -0.15±0.16 | 8.75E-01 | -0.14±0.16 | 9.15E-01 | -0.13±0.43 | 9.99E-01 | -0.35±0.48 | 1.00E+00 |
| thymol sulfate | -0.19±0.15 | 23.92 | 2.52E-01 | 5.24E-01 | -0.18±0.17 | 10.76 | 2.90E-01 | 8.21E-01 | -0.15±0.17 | 8.85E-01 | -0.15±0.17 | 9.15E-01 | -1.01±0.73 | 9.99E-01 | -1.08±0.87 | 1.00E+00 |
| myristoyl dihydrosphingomyelin (d18:0/14:0)* | 0.14±0.12 | 35.63 | 2.13E-01 | 5.24E-01 | 0.17±0.16 | 48.32 | 1.64E-01 | 8.21E-01 | 0.12±0.16 | 9.32E-01 | 0.13±0.16 | 9.15E-01 | 1.01±0.71 | 9.99E-01 | 1.34±0.85 | 1.00E+00 |
| N,N,N-trimethyl-alanylproline betaine (TMAP) | -0.13±0.11 | 0 | 9.24E-01 | 5.24E-01 | -0.16±0.15 | 0 | 5.38E-01 | 8.21E-01 | -0.12±0.16 | 9.32E-01 | -0.12±0.17 | 9.18E-01 | -0.15±0.3 | 9.99E-01 | -0.36±0.36 | 1.00E+00 |
| malate | -0.14±0.12 | 0 | 8.59E-01 | 5.24E-01 | -0.15±0.14 | 0 | 3.57E-01 | 8.21E-01 | -0.13±0.14 | 8.85E-01 | -0.11±0.14 | 9.18E-01 | -0.2±0.36 | 9.99E-01 | -0.53±0.43 | 1.00E+00 |
| N-acetylleucine | -0.1±0.08 | 0 | 5.76E-01 | 5.24E-01 | -0.16±0.15 | 18.81 | 2.67E-01 | 8.21E-01 | -0.09±0.15 | 9.32E-01 | -0.1±0.16 | 9.26E-01 | -0.3±0.36 | 9.99E-01 | -0.59±0.42 | 1.00E+00 |
| pyridoxate | -0.02±0.04 | 89.16 | 2.38E-03 | 6.27E-01 | 0.16±0.15 | 75.67 | 4.26E-02 | 8.21E-01 | -0.04±0.17 | 9.80E-01 | 0±0.17 | 9.99E-01 | 0.78±0.27 | 9.99E-01 | 0.7±0.3 | 1.00E+00 |
| laurylcarnitine (C12) | -0.17±0.18 | 0 | 7.17E-01 | 5.24E-01 | -0.17±0.16 | 0 | 9.96E-01 | 8.24E-01 | -0.2±0.16 | 7.87E-01 | -0.17±0.16 | 8.77E-01 | -0.02±0.45 | 9.99E-01 | -0.17±0.57 | 1.00E+00 |
| gamma-tocopherol/beta-tocopherol | 0.01±0.24 | 53.92 | 1.41E-01 | 9.67E-01 | 0.17±0.16 | 79.5 | 2.72E-02 | 8.28E-01 | 0.32±0.17 | 6.58E-01 | 0.33±0.17 | 6.72E-01 | -0.39±0.36 | 9.99E-01 | -0.59±0.38 | 1.00E+00 |
| glucuronate | -0.12±0.14 | 0 | 6.25E-01 | 5.24E-01 | -0.15±0.14 | 0 | 5.07E-01 | 8.28E-01 | -0.15±0.15 | 8.55E-01 | -0.18±0.15 | 8.16E-01 | 0.04±0.35 | 9.99E-01 | 0.09±0.39 | 1.00E+00 |
| phenylalanine | -0.14±0.16 | 0 | 5.25E-01 | 5.24E-01 | -0.16±0.15 | 0 | 7.79E-01 | 8.28E-01 | -0.17±0.15 | 8.27E-01 | -0.17±0.16 | 8.53E-01 | 0.25±0.64 | 9.99E-01 | -0.01±0.58 | 1.00E+00 |
| citraconate/glutaconate | 0.14±0.17 | 0 | 5.42E-01 | 5.24E-01 | 0.16±0.15 | 0 | 8.33E-01 | 8.28E-01 | 0.18±0.16 | 8.05E-01 | 0.17±0.16 | 8.61E-01 | -0.12±0.46 | 9.99E-01 | 0.07±0.44 | 1.00E+00 |
| pregnenediol sulfate (C21H34O5S)* | -0.14±0.13 | 0 | 8.72E-01 | 5.24E-01 | -0.18±0.16 | 0 | 7.54E-01 | 8.28E-01 | -0.14±0.17 | 9.16E-01 | -0.16±0.17 | 9.08E-01 | -0.23±0.57 | 9.99E-01 | -0.36±0.62 | 1.00E+00 |
| carboxyethyl-GABA | 0.13±0.12 | 0 | 8.56E-01 | 5.24E-01 | 0.16±0.15 | 0 | 7.54E-01 | 8.28E-01 | 0.12±0.16 | 9.27E-01 | 0.15±0.16 | 9.12E-01 | 0.21±0.45 | 9.99E-01 | 0.33±0.54 | 1.00E+00 |
| aconitate [cis or trans] | -0.11±0.1 | 0 | 9.72E-01 | 5.24E-01 | -0.15±0.14 | 0 | 5.97E-01 | 8.28E-01 | -0.11±0.15 | 9.32E-01 | -0.12±0.16 | 9.15E-01 | -0.12±0.31 | 9.99E-01 | -0.33±0.37 | 1.00E+00 |
| 4-ethylphenylsulfate | -0.12±0.09 | 42.82 | 1.86E-01 | 5.24E-01 | -0.16±0.15 | 0 | 3.70E-01 | 8.28E-01 | -0.1±0.15 | 9.32E-01 | -0.13±0.16 | 9.15E-01 | -0.86±0.57 | 9.99E-01 | -0.71±0.63 | 1.00E+00 |
| 8-methoxykynurenate | -0.14±0.11 | 13.86 | 2.81E-01 | 5.24E-01 | -0.17±0.16 | 61.91 | 1.05E-01 | 8.28E-01 | -0.11±0.16 | 9.32E-01 | -0.08±0.16 | 9.44E-01 | -0.58±0.42 | 9.99E-01 | -0.97±0.52 | 1.00E+00 |
| imidazole lactate | 0.1±0.18 | 0 | 3.32E-01 | 6.05E-01 | 0.15±0.15 | 45.03 | 1.77E-01 | 8.30E-01 | 0.21±0.15 | 7.58E-01 | 0.22±0.16 | 7.84E-01 | -0.17±0.34 | 9.99E-01 | -0.45±0.47 | 1.00E+00 |
| 2-hydroxysebacate | -0.12±0.16 | 0 | 4.41E-01 | 5.34E-01 | -0.15±0.15 | 0 | 4.14E-01 | 8.30E-01 | -0.18±0.15 | 8.05E-01 | -0.2±0.16 | 8.11E-01 | 0.16±0.41 | 9.99E-01 | 0.19±0.45 | 1.00E+00 |
| eicosenoate (20:1) | -0.16±0.16 | 0 | 8.42E-01 | 5.24E-01 | -0.16±0.16 | 0 | 9.53E-01 | 8.30E-01 | -0.17±0.16 | 8.44E-01 | -0.16±0.16 | 8.97E-01 | -0.07±0.46 | 9.99E-01 | -0.19±0.51 | 1.00E+00 |
| sebacate (C10-DC) | -0.12±0.17 | 0 | 4.41E-01 | 5.39E-01 | -0.16±0.15 | 0 | 5.52E-01 | 8.30E-01 | -0.19±0.16 | 8.05E-01 | -0.19±0.16 | 8.25E-01 | 0.12±0.36 | 9.99E-01 | 0.08±0.43 | 1.00E+00 |
| X - 17357 | -0.09±0.08 | 0 | 5.04E-01 | 5.24E-01 | -0.13±0.13 | 22.79 | 2.55E-01 | 8.30E-01 | -0.08±0.13 | 9.32E-01 | -0.08±0.13 | 9.26E-01 | -0.32±0.35 | 9.99E-01 | -0.56±0.4 | 1.00E+00 |
| glycosyl-N-behenoyl-sphingadienine (d18:2/22:0)* | 0.05±0.18 | 33.31 | 2.21E-01 | 7.90E-01 | 0.15±0.15 | 66.36 | 8.47E-02 | 8.31E-01 | 0.24±0.16 | 7.45E-01 | 0.27±0.16 | 7.32E-01 | -0.22±0.29 | 9.99E-01 | -0.42±0.36 | 1.00E+00 |
| 1-stearoyl-2-oleoyl-GPI (18:0/18:1)* | 0.11±0.17 | 0 | 3.95E-01 | 5.72E-01 | 0.15±0.14 | 7.21 | 2.99E-01 | 8.31E-01 | 0.21±0.15 | 7.64E-01 | 0.21±0.15 | 7.84E-01 | -0.11±0.32 | 9.99E-01 | -0.21±0.38 | 1.00E+00 |
| erythritol | 0.15±0.16 | 0 | 7.16E-01 | 5.24E-01 | 0.14±0.14 | 0 | 4.88E-01 | 8.31E-01 | 0.17±0.15 | 8.05E-01 | 0.18±0.15 | 8.16E-01 | 0.02±0.39 | 9.99E-01 | -0.09±0.37 | 1.00E+00 |
| tartarate | 0.13±0.13 | 0 | 9.38E-01 | 5.24E-01 | 0.15±0.14 | 0 | 9.46E-01 | 8.31E-01 | 0.13±0.15 | 8.85E-01 | 0.15±0.15 | 8.97E-01 | 0.1±0.38 | 9.99E-01 | 0.12±0.41 | 1.00E+00 |
| caffeine | 0.11±0.1 | 0 | 4.99E-01 | 5.24E-01 | 0.17±0.17 | 0 | 8.69E-01 | 8.31E-01 | 0.1±0.16 | 9.32E-01 | 0.17±0.17 | 9.08E-01 | 0.49±0.58 | 9.99E-01 | 0.28±0.69 | 1.00E+00 |
| 1-stearoyl-GPG (18:0) | 0.14±0.12 | 0 | 4.88E-01 | 5.24E-01 | 0.16±0.16 | 0 | 6.51E-01 | 8.31E-01 | 0.12±0.16 | 9.32E-01 | 0.14±0.16 | 9.15E-01 | 0.51±0.55 | 9.99E-01 | 0.4±0.56 | 1.00E+00 |
| 3-ethylcatechol sulfate (1) | -0.08±0.07 | 17.13 | 2.72E-01 | 5.24E-01 | -0.16±0.16 | 0 | 6.58E-01 | 8.31E-01 | -0.07±0.17 | 9.77E-01 | -0.14±0.17 | 9.15E-01 | -0.52±0.41 | 9.99E-01 | -0.34±0.43 | 1.00E+00 |
| X - 17653 | -0.12±0.12 | 0 | 9.22E-01 | 5.24E-01 | -0.14±0.13 | 0 | 7.26E-01 | 8.31E-01 | -0.13±0.14 | 8.83E-01 | -0.12±0.14 | 9.15E-01 | -0.09±0.38 | 9.99E-01 | -0.28±0.41 | 1.00E+00 |
| (N(1) + N(8))-acetylspermidine | 0.06±0.25 | 82.55 | 1.67E-02 | 8.28E-01 | -0.16±0.16 | 84.19 | 1.19E-02 | 8.33E-01 | -0.29±0.16 | 6.58E-01 | -0.27±0.17 | 7.32E-01 | 1.1±0.5 | 9.99E-01 | 1.3±0.6 | 1.00E+00 |
| stearate (18:0) | -0.14±0.2 | 0 | 3.73E-01 | 5.48E-01 | -0.16±0.16 | 0 | 5.52E-01 | 8.33E-01 | -0.21±0.17 | 7.86E-01 | -0.2±0.17 | 8.35E-01 | 0.25±0.48 | 9.99E-01 | 0.1±0.47 | 1.00E+00 |
| X - 15245 | -0.1±0.19 | 54.45 | 1.38E-01 | 6.34E-01 | -0.14±0.14 | 49.46 | 1.60E-01 | 8.35E-01 | -0.2±0.15 | 7.58E-01 | -0.2±0.15 | 7.84E-01 | 0.66±0.54 | 9.99E-01 | 0.54±0.51 | 1.00E+00 |
| X - 16576 | -0.03±0.2 | 82.64 | 1.64E-02 | 8.78E-01 | -0.15±0.15 | 82.28 | 1.75E-02 | 8.37E-01 | -0.21±0.15 | 7.58E-01 | -0.22±0.15 | 7.81E-01 | 1.21±0.55 | 9.99E-01 | 1.58±0.74 | 1.00E+00 |
| X - 12411 | 0.11±0.16 | 0 | 3.33E-01 | 5.59E-01 | 0.15±0.15 | 0 | 4.52E-01 | 8.37E-01 | 0.18±0.15 | 8.05E-01 | 0.19±0.16 | 8.25E-01 | -0.25±0.4 | 9.99E-01 | -0.19±0.48 | 1.00E+00 |
| glutamine_degradant* | -0.15±0.19 | 0 | 5.24E-01 | 5.24E-01 | -0.19±0.19 | 0 | 7.44E-01 | 8.39E-01 | -0.2±0.19 | 8.48E-01 | -0.21±0.2 | 8.61E-01 | 0.15±0.51 | 9.99E-01 | 0±0.6 | 1.00E+00 |
| 3-methoxytyrosine | 0.11±0.21 | 0 | 3.63E-01 | 6.24E-01 | 0.18±0.18 | 42.09 | 1.89E-01 | 8.40E-01 | 0.27±0.19 | 7.58E-01 | 0.29±0.2 | 7.81E-01 | -0.1±0.31 | 9.99E-01 | -0.31±0.41 | 1.00E+00 |
| X - 17438 | -0.02±0.21 | 77.48 | 3.51E-02 | 9.19E-01 | -0.14±0.15 | 79.77 | 2.62E-02 | 8.49E-01 | -0.23±0.16 | 7.50E-01 | -0.24±0.16 | 7.74E-01 | 0.92±0.49 | 9.99E-01 | 1.06±0.56 | 1.00E+00 |
| glycolithocholate | 0.06±0.21 | 63.24 | 9.91E-02 | 8.05E-01 | 0.14±0.15 | 75.56 | 4.31E-02 | 8.49E-01 | 0.24±0.16 | 7.45E-01 | 0.24±0.16 | 7.81E-01 | -0.62±0.46 | 9.99E-01 | -0.83±0.5 | 1.00E+00 |
| indolelactate | 0.1±0.17 | 0 | 3.32E-01 | 5.82E-01 | 0.14±0.15 | 50.06 | 1.57E-01 | 8.49E-01 | 0.19±0.15 | 7.87E-01 | 0.23±0.16 | 7.84E-01 | -0.2±0.35 | 9.99E-01 | -0.35±0.38 | 1.00E+00 |
| 1-(1-enyl-palmitoyl)-GPE (P-16:0)* | 0.09±0.17 | 7.13 | 2.99E-01 | 6.15E-01 | 0.12±0.14 | 50.74 | 1.54E-01 | 8.49E-01 | 0.19±0.15 | 7.80E-01 | 0.2±0.15 | 7.84E-01 | -0.2±0.33 | 9.99E-01 | -0.39±0.39 | 1.00E+00 |
| 1-palmitoyl-2-arachidonoyl-GPE (16:0/20:4)* | -0.08±0.15 | 64.98 | 9.11E-02 | 6.20E-01 | -0.12±0.14 | 66.39 | 8.46E-02 | 8.49E-01 | -0.16±0.14 | 8.05E-01 | -0.19±0.14 | 7.91E-01 | 0.79±0.54 | 9.99E-01 | 0.76±0.53 | 1.00E+00 |
| 2-oxoarginine* | 0.11±0.16 | 0 | 3.26E-01 | 5.55E-01 | 0.13±0.14 | 32 | 2.25E-01 | 8.49E-01 | 0.17±0.14 | 7.92E-01 | 0.19±0.15 | 8.08E-01 | -0.27±0.41 | 9.99E-01 | -0.39±0.45 | 1.00E+00 |
| 1-(1-enyl-palmitoyl)-2-oleoyl-GPC (P-16:0/18:1)* | 0.13±0.16 | 0 | 4.98E-01 | 5.30E-01 | 0.14±0.16 | 19.29 | 2.66E-01 | 8.49E-01 | 0.18±0.17 | 8.27E-01 | 0.22±0.18 | 8.12E-01 | -0.09±0.35 | 9.99E-01 | -0.29±0.42 | 1.00E+00 |
| 3-hydroxydodecanedioate* | -0.1±0.19 | 22.85 | 2.55E-01 | 6.27E-01 | -0.16±0.17 | 0 | 3.21E-01 | 8.49E-01 | -0.22±0.17 | 7.86E-01 | -0.22±0.18 | 8.13E-01 | 0.32±0.42 | 9.99E-01 | 0.28±0.47 | 1.00E+00 |
| p-cresol glucuronide* | 0.16±0.19 | 0 | 6.11E-01 | 5.24E-01 | 0.14±0.15 | 0 | 4.67E-01 | 8.49E-01 | 0.21±0.16 | 7.67E-01 | 0.18±0.16 | 8.53E-01 | -0.05±0.45 | 9.99E-01 | -0.21±0.51 | 1.00E+00 |
| 1-palmitoyl-2-linoleoyl-GPI (16:0/18:2) | 0.12±0.16 | 0 | 4.81E-01 | 5.29E-01 | 0.14±0.15 | 0 | 4.45E-01 | 8.49E-01 | 0.17±0.16 | 8.35E-01 | 0.18±0.16 | 8.53E-01 | -0.13±0.4 | 9.99E-01 | -0.2±0.46 | 1.00E+00 |
| X - 21470 | -0.12±0.15 | 0 | 3.77E-01 | 5.34E-01 | -0.14±0.16 | 0 | 3.93E-01 | 8.49E-01 | -0.16±0.17 | 8.57E-01 | -0.19±0.17 | 8.53E-01 | 0.28±0.47 | 9.99E-01 | 0.29±0.53 | 1.00E+00 |
| X - 25520 | -0.13±0.17 | 0 | 5.12E-01 | 5.26E-01 | -0.14±0.15 | 0 | 5.34E-01 | 8.49E-01 | -0.18±0.15 | 7.92E-01 | -0.17±0.15 | 8.53E-01 | 0.12±0.42 | 9.99E-01 | 0.15±0.5 | 1.00E+00 |
| stearidonate (18:4n3) | -0.14±0.17 | 0 | 4.91E-01 | 5.25E-01 | -0.15±0.15 | 0 | 5.72E-01 | 8.49E-01 | -0.18±0.15 | 8.05E-01 | -0.17±0.16 | 8.58E-01 | 0.18±0.5 | 9.99E-01 | 0.14±0.54 | 1.00E+00 |
| dihomo-linolenate (20:3n3 or n6) | -0.12±0.18 | 1.48 | 3.14E-01 | 5.55E-01 | -0.14±0.16 | 0 | 5.20E-01 | 8.49E-01 | -0.2±0.16 | 7.87E-01 | -0.18±0.16 | 8.61E-01 | 0.34±0.5 | 9.99E-01 | 0.19±0.54 | 1.00E+00 |
| ceramide (d18:2/24:1, d18:1/24:2)* | 0.12±0.15 | 0 | 5.54E-01 | 5.24E-01 | 0.13±0.15 | 0 | 4.81E-01 | 8.49E-01 | 0.16±0.15 | 8.44E-01 | 0.17±0.16 | 8.61E-01 | -0.07±0.36 | 9.99E-01 | -0.18±0.47 | 1.00E+00 |
| glycerate | -0.15±0.19 | 0 | 4.31E-01 | 5.30E-01 | -0.14±0.15 | 0 | 5.40E-01 | 8.49E-01 | -0.2±0.14 | 7.58E-01 | -0.16±0.15 | 8.61E-01 | 0.26±0.55 | 9.99E-01 | 0.24±0.65 | 1.00E+00 |
| hydroxypalmitoyl sphingomyelin (d18:1/16:0(OH))** | 0.1±0.13 | 0 | 5.06E-01 | 5.29E-01 | 0.14±0.15 | 0 | 5.13E-01 | 8.49E-01 | 0.14±0.16 | 8.85E-01 | 0.17±0.16 | 8.75E-01 | -0.08±0.31 | 9.99E-01 | -0.15±0.46 | 1.00E+00 |
| hexanoylglutamine | -0.13±0.17 | 0 | 4.39E-01 | 5.33E-01 | -0.15±0.16 | 0 | 6.56E-01 | 8.49E-01 | -0.19±0.17 | 8.11E-01 | -0.18±0.17 | 8.77E-01 | 0.19±0.45 | 9.99E-01 | 0.06±0.5 | 1.00E+00 |
| isoursodeoxycholate | 0.16±0.15 | 0 | 9.45E-01 | 5.24E-01 | 0.15±0.15 | 0 | 7.83E-01 | 8.49E-01 | 0.16±0.15 | 8.44E-01 | 0.16±0.16 | 8.77E-01 | 0.13±0.45 | 9.99E-01 | 0.03±0.45 | 1.00E+00 |
| 9,10-DiHOME | -0.12±0.16 | 0 | 4.27E-01 | 5.29E-01 | -0.13±0.14 | 0 | 5.26E-01 | 8.49E-01 | -0.16±0.15 | 8.17E-01 | -0.15±0.15 | 8.79E-01 | 0.26±0.51 | 9.99E-01 | 0.2±0.54 | 1.00E+00 |
| cys-gly, oxidized | -0.13±0.16 | 0 | 4.73E-01 | 5.26E-01 | -0.13±0.15 | 0 | 5.43E-01 | 8.49E-01 | -0.17±0.15 | 8.11E-01 | -0.15±0.15 | 8.94E-01 | 0.2±0.48 | 9.99E-01 | 0.24±0.63 | 1.00E+00 |
| X - 25271 | 0.12±0.13 | 0 | 6.48E-01 | 5.24E-01 | 0.14±0.15 | 0 | 7.54E-01 | 8.49E-01 | 0.13±0.15 | 8.89E-01 | 0.15±0.16 | 8.97E-01 | -0.08±0.44 | 9.99E-01 | 0±0.47 | 1.00E+00 |
| etiocholanolone glucuronide | -0.13±0.13 | 0 | 8.24E-01 | 5.24E-01 | -0.12±0.14 | 0 | 6.81E-01 | 8.49E-01 | -0.14±0.14 | 8.55E-01 | -0.14±0.14 | 9.01E-01 | -0.04±0.45 | 9.99E-01 | 0.04±0.42 | 1.00E+00 |
| 1,5-anhydroglucitol (1,5-AG) | -0.1±0.12 | 0 | 5.88E-01 | 5.24E-01 | -0.13±0.13 | 0 | 9.26E-01 | 8.49E-01 | -0.13±0.14 | 8.83E-01 | -0.13±0.14 | 9.08E-01 | 0.08±0.36 | 9.99E-01 | -0.09±0.39 | 1.00E+00 |
| 7-methylguanine | 0.14±0.13 | 0 | 8.37E-01 | 5.24E-01 | 0.15±0.15 | 0 | 9.34E-01 | 8.49E-01 | 0.14±0.15 | 8.85E-01 | 0.15±0.16 | 9.09E-01 | 0.26±0.57 | 9.99E-01 | 0.09±0.65 | 1.00E+00 |
| glucose | -0.12±0.14 | 0 | 5.13E-01 | 5.24E-01 | -0.16±0.17 | 0 | 7.96E-01 | 8.49E-01 | -0.15±0.18 | 8.93E-01 | -0.17±0.18 | 9.09E-01 | 0.2±0.51 | 9.99E-01 | -0.03±0.52 | 1.00E+00 |
| 3-methyl-2-oxovalerate | -0.12±0.13 | 0 | 7.00E-01 | 5.24E-01 | -0.15±0.15 | 0 | 9.42E-01 | 8.49E-01 | -0.14±0.16 | 8.85E-01 | -0.14±0.16 | 9.15E-01 | 0.05±0.47 | 9.99E-01 | -0.18±0.45 | 1.00E+00 |
| 3-indoxyl sulfate | 0.12±0.11 | 0 | 7.41E-01 | 5.24E-01 | 0.15±0.16 | 0 | 9.23E-01 | 8.49E-01 | 0.11±0.16 | 9.32E-01 | 0.15±0.17 | 9.15E-01 | 0.25±0.41 | 9.99E-01 | 0.19±0.43 | 1.00E+00 |
| threonate | -0.16±0.14 | 0 | 9.55E-01 | 5.24E-01 | -0.13±0.14 | 0 | 7.49E-01 | 8.49E-01 | -0.16±0.14 | 8.11E-01 | -0.12±0.15 | 9.15E-01 | -0.18±0.35 | 9.99E-01 | -0.26±0.44 | 1.00E+00 |
| 1-methyl-4-imidazoleacetate | 0.14±0.13 | 0 | 8.41E-01 | 5.24E-01 | 0.13±0.13 | 0 | 8.83E-01 | 8.49E-01 | 0.13±0.13 | 8.48E-01 | 0.12±0.14 | 9.15E-01 | 0.23±0.45 | 9.99E-01 | 0.19±0.45 | 1.00E+00 |
| N4-acetylcytidine | 0.12±0.11 | 0 | 7.45E-01 | 5.24E-01 | 0.14±0.15 | 0 | 6.65E-01 | 8.49E-01 | 0.11±0.16 | 9.32E-01 | 0.12±0.16 | 9.15E-01 | 0.28±0.51 | 9.99E-01 | 0.39±0.6 | 1.00E+00 |
| bilirubin (E,Z or Z,E)* | -0.12±0.14 | 0 | 5.45E-01 | 5.24E-01 | -0.13±0.14 | 0 | 7.30E-01 | 8.49E-01 | -0.15±0.14 | 8.48E-01 | -0.12±0.15 | 9.15E-01 | 0.15±0.47 | 9.99E-01 | -0.3±0.51 | 1.00E+00 |
| pregnenolone sulfate | -0.1±0.09 | 0 | 7.56E-01 | 5.24E-01 | -0.14±0.15 | 0 | 9.49E-01 | 8.49E-01 | -0.09±0.16 | 9.32E-01 | -0.14±0.16 | 9.15E-01 | -0.27±0.55 | 9.99E-01 | -0.17±0.55 | 1.00E+00 |
| tridecenedioate (C13:1-DC)* | -0.14±0.15 | 0 | 7.37E-01 | 5.24E-01 | -0.14±0.15 | 0 | 7.78E-01 | 8.49E-01 | -0.16±0.15 | 8.44E-01 | -0.12±0.15 | 9.15E-01 | -0.02±0.39 | 9.99E-01 | -0.27±0.51 | 1.00E+00 |
| 1H-indole-7-acetic acid | 0.16±0.13 | 0 | 6.57E-01 | 5.24E-01 | 0.15±0.16 | 0 | 6.57E-01 | 8.49E-01 | 0.14±0.16 | 8.93E-01 | 0.13±0.17 | 9.15E-01 | 0.36±0.48 | 9.99E-01 | 0.39±0.56 | 1.00E+00 |
| methyl glucopyranoside (alpha + beta) | -0.17±0.16 | 0 | 9.16E-01 | 5.24E-01 | -0.14±0.15 | 0 | 9.25E-01 | 8.49E-01 | -0.17±0.15 | 8.11E-01 | -0.13±0.16 | 9.15E-01 | -0.12±0.44 | 9.99E-01 | -0.18±0.5 | 1.00E+00 |
| lignoceroyl sphingomyelin (d18:1/24:0) | 0.11±0.11 | 0 | 9.12E-01 | 5.24E-01 | 0.13±0.15 | 0 | 8.14E-01 | 8.49E-01 | 0.11±0.15 | 9.32E-01 | 0.12±0.15 | 9.15E-01 | 0.17±0.5 | 9.99E-01 | 0.28±0.67 | 1.00E+00 |
| arabitol/xylitol | -0.12±0.12 | 0 | 8.51E-01 | 5.24E-01 | -0.12±0.14 | 0 | 9.67E-01 | 8.49E-01 | -0.12±0.15 | 9.00E-01 | -0.12±0.14 | 9.15E-01 | -0.05±0.38 | 9.99E-01 | -0.14±0.44 | 1.00E+00 |
| 1-(1-enyl-palmitoyl)-2-oleoyl-GPE (P-16:0/18:1)* | -0.1±0.1 | 0 | 8.36E-01 | 5.24E-01 | -0.12±0.14 | 0 | 8.55E-01 | 8.49E-01 | -0.11±0.15 | 9.32E-01 | -0.12±0.15 | 9.15E-01 | -0.03±0.39 | 9.99E-01 | -0.2±0.42 | 1.00E+00 |
| N-palmitoyl-sphingadienine (d18:2/16:0)* | -0.12±0.12 | 0 | 8.38E-01 | 5.24E-01 | -0.13±0.15 | 0 | 9.01E-01 | 8.49E-01 | -0.13±0.15 | 8.93E-01 | -0.13±0.16 | 9.15E-01 | -0.05±0.35 | 9.99E-01 | -0.19±0.47 | 1.00E+00 |
| 3-hydroxydecanoylcarnitine | -0.16±0.16 | 0 | 8.44E-01 | 5.24E-01 | -0.14±0.16 | 0 | 9.00E-01 | 8.49E-01 | -0.17±0.16 | 8.27E-01 | -0.13±0.16 | 9.15E-01 | -0.08±0.43 | 9.99E-01 | -0.21±0.56 | 1.00E+00 |
| 3-hydroxyoctanoylcarnitine (1) | -0.16±0.17 | 0 | 7.51E-01 | 5.24E-01 | -0.15±0.16 | 0 | 9.45E-01 | 8.49E-01 | -0.19±0.16 | 8.05E-01 | -0.15±0.16 | 9.15E-01 | -0.03±0.44 | 9.99E-01 | -0.11±0.55 | 1.00E+00 |
| X - 22776 | -0.11±0.12 | 0 | 6.15E-01 | 5.24E-01 | -0.13±0.15 | 0 | 9.46E-01 | 8.49E-01 | -0.13±0.15 | 9.17E-01 | -0.14±0.16 | 9.15E-01 | 0.08±0.39 | 9.99E-01 | -0.11±0.38 | 1.00E+00 |
| anthranilate | 0.1±0.08 | 4.79 | 3.05E-01 | 5.24E-01 | 0.16±0.16 | 0 | 3.52E-01 | 8.49E-01 | 0.08±0.16 | 9.58E-01 | 0.12±0.17 | 9.18E-01 | 0.73±0.62 | 9.99E-01 | 0.85±0.76 | 1.00E+00 |
| nicotinamide | 0.11±0.1 | 0 | 7.20E-01 | 5.24E-01 | 0.14±0.15 | 0 | 5.51E-01 | 8.49E-01 | 0.1±0.15 | 9.32E-01 | 0.11±0.16 | 9.18E-01 | 0.26±0.42 | 9.99E-01 | 0.43±0.5 | 1.00E+00 |
| N1-Methyl-2-pyridone-5-carboxamide | 0.08±0.07 | 0 | 4.88E-01 | 5.24E-01 | 0.15±0.16 | 0 | 5.16E-01 | 8.49E-01 | 0.07±0.16 | 9.74E-01 | 0.11±0.17 | 9.23E-01 | 0.4±0.46 | 9.99E-01 | 0.46±0.5 | 1.00E+00 |
| tyramine O-sulfate | 0.16±0.12 | 0 | 5.19E-01 | 5.24E-01 | 0.13±0.14 | 0 | 6.20E-01 | 8.49E-01 | 0.13±0.15 | 8.85E-01 | 0.1±0.15 | 9.23E-01 | 0.38±0.37 | 9.99E-01 | 0.3±0.36 | 1.00E+00 |
| 11beta-hydroxyandrosterone glucuronide | -0.08±0.07 | 0 | 6.68E-01 | 5.24E-01 | -0.13±0.13 | 0 | 6.63E-01 | 8.49E-01 | -0.07±0.14 | 9.58E-01 | -0.1±0.14 | 9.23E-01 | -0.2±0.29 | 9.99E-01 | -0.25±0.32 | 1.00E+00 |
| X - 18913 | -0.11±0.1 | 0 | 7.46E-01 | 5.24E-01 | -0.14±0.15 | 0 | 4.44E-01 | 8.49E-01 | -0.11±0.15 | 9.32E-01 | -0.1±0.15 | 9.25E-01 | -0.24±0.42 | 9.99E-01 | -0.48±0.47 | 1.00E+00 |
| acetylcarnitine (C2) | -0.11±0.1 | 0 | 9.74E-01 | 5.24E-01 | -0.14±0.15 | 0 | 5.14E-01 | 8.49E-01 | -0.11±0.16 | 9.32E-01 | -0.1±0.16 | 9.26E-01 | -0.12±0.29 | 9.99E-01 | -0.39±0.41 | 1.00E+00 |
| N-acetylphenylalanine | -0.1±0.09 | 0 | 9.11E-01 | 5.24E-01 | -0.12±0.14 | 0 | 3.67E-01 | 8.49E-01 | -0.09±0.15 | 9.32E-01 | -0.09±0.15 | 9.26E-01 | -0.14±0.39 | 9.99E-01 | -0.57±0.51 | 1.00E+00 |
| taurolithocholate 3-sulfate | -0.08±0.07 | 0 | 4.39E-01 | 5.24E-01 | -0.13±0.14 | 0 | 3.78E-01 | 8.49E-01 | -0.07±0.15 | 9.72E-01 | -0.09±0.15 | 9.26E-01 | -0.37±0.38 | 9.99E-01 | -0.51±0.46 | 1.00E+00 |
| taurocholate | -0.12±0.1 | 0 | 3.43E-01 | 5.24E-01 | -0.13±0.15 | 0 | 5.22E-01 | 8.49E-01 | -0.1±0.16 | 9.32E-01 | -0.1±0.16 | 9.26E-01 | -0.56±0.47 | 9.99E-01 | -0.42±0.47 | 1.00E+00 |
| X - 23644 | -0.11±0.09 | 49.64 | 1.59E-01 | 5.24E-01 | -0.14±0.14 | 28.32 | 2.38E-01 | 8.49E-01 | -0.09±0.15 | 9.32E-01 | -0.09±0.15 | 9.26E-01 | -0.77±0.48 | 9.99E-01 | -0.71±0.5 | 1.00E+00 |
| 5alpha-pregnan-3beta,20alpha-diol disulfate | -0.07±0.06 | 0 | 4.20E-01 | 5.24E-01 | -0.15±0.16 | 0 | 3.88E-01 | 8.49E-01 | -0.06±0.17 | 9.80E-01 | -0.09±0.17 | 9.39E-01 | -0.37±0.38 | 9.99E-01 | -0.49±0.43 | 1.00E+00 |
| 1-stearoyl-2-linoleoyl-GPI (18:0/18:2) | -0.1±0.08 | 0 | 5.98E-01 | 5.24E-01 | -0.12±0.13 | 0 | 4.67E-01 | 8.49E-01 | -0.09±0.14 | 9.32E-01 | -0.08±0.14 | 9.39E-01 | -0.25±0.3 | 9.99E-01 | -0.35±0.35 | 1.00E+00 |
| X - 23587 | -0.08±0.07 | 0 | 3.81E-01 | 5.24E-01 | -0.13±0.14 | 16.74 | 2.73E-01 | 8.49E-01 | -0.07±0.15 | 9.72E-01 | -0.08±0.15 | 9.39E-01 | -0.41±0.38 | 9.99E-01 | -0.65±0.5 | 1.00E+00 |
| carotene diol (3) | 0.06±0.05 | 72.69 | 5.57E-02 | 5.24E-01 | 0.15±0.15 | 70.3 | 6.65E-02 | 8.49E-01 | 0.06±0.15 | 9.80E-01 | 0.08±0.16 | 9.39E-01 | 1.24±0.62 | 9.99E-01 | 1.44±0.72 | 1.00E+00 |
| (S)-3-hydroxybutyrylcarnitine | -0.08±0.06 | 0 | 3.27E-01 | 5.24E-01 | -0.15±0.16 | 41.02 | 1.93E-01 | 8.49E-01 | -0.07±0.16 | 9.78E-01 | -0.09±0.16 | 9.43E-01 | -0.42±0.36 | 9.99E-01 | -0.83±0.55 | 1.00E+00 |
| X - 25957 | -0.05±0.05 | 0 | 3.93E-01 | 5.24E-01 | -0.14±0.14 | 45.19 | 1.77E-01 | 8.49E-01 | -0.05±0.14 | 9.80E-01 | -0.08±0.14 | 9.44E-01 | -0.36±0.36 | 9.99E-01 | -0.7±0.44 | 1.00E+00 |
| X - 24757 | 0.07±0.06 | 22.03 | 2.57E-01 | 5.24E-01 | 0.15±0.17 | 24.15 | 2.51E-01 | 8.49E-01 | 0.06±0.17 | 9.80E-01 | 0.09±0.17 | 9.44E-01 | 0.74±0.6 | 9.99E-01 | 0.78±0.58 | 1.00E+00 |
| propionylcarnitine (C3) | -0.06±0.05 | 0 | 6.11E-01 | 5.24E-01 | -0.13±0.14 | 9.06 | 2.94E-01 | 8.49E-01 | -0.06±0.15 | 9.80E-01 | -0.07±0.15 | 9.45E-01 | -0.2±0.28 | 9.99E-01 | -0.5±0.37 | 1.00E+00 |
| alpha-hydroxyisovalerate | -0.05±0.05 | 0 | 4.91E-01 | 5.24E-01 | -0.16±0.17 | 0 | 3.50E-01 | 8.49E-01 | -0.05±0.17 | 9.80E-01 | -0.09±0.18 | 9.45E-01 | -0.31±0.38 | 9.99E-01 | -0.5±0.41 | 1.00E+00 |
| 3-acetylphenol sulfate | 0.14±0.11 | 0 | 3.52E-01 | 5.24E-01 | 0.15±0.16 | 4.68 | 3.06E-01 | 8.49E-01 | 0.11±0.17 | 9.32E-01 | 0.09±0.17 | 9.45E-01 | 0.59±0.49 | 9.99E-01 | 0.62±0.49 | 1.00E+00 |
| sulfate of piperine metabolite C16H19NO3 (3)* | -0.08±0.07 | 0 | 5.01E-01 | 5.24E-01 | -0.14±0.16 | 37.85 | 2.05E-01 | 8.49E-01 | -0.07±0.16 | 9.72E-01 | -0.08±0.16 | 9.45E-01 | -0.35±0.41 | 9.99E-01 | -0.78±0.53 | 1.00E+00 |
| N-acetylserine | -0.06±0.05 | 0 | 5.99E-01 | 5.24E-01 | -0.13±0.14 | 0 | 3.75E-01 | 8.49E-01 | -0.06±0.16 | 9.80E-01 | -0.07±0.16 | 9.56E-01 | -0.19±0.24 | 9.99E-01 | -0.39±0.32 | 1.00E+00 |
| 2-aminooctanoate | -0.09±0.07 | 61.29 | 1.08E-01 | 5.24E-01 | -0.13±0.15 | 82.21 | 1.77E-02 | 8.49E-01 | -0.07±0.15 | 9.72E-01 | -0.05±0.15 | 9.65E-01 | -0.72±0.4 | 9.99E-01 | -1.53±0.61 | 1.00E+00 |
| glycochenodeoxycholate | -0.06±0.05 | 70.9 | 6.38E-02 | 5.24E-01 | -0.15±0.16 | 61.05 | 1.09E-01 | 8.49E-01 | -0.05±0.17 | 9.80E-01 | -0.04±0.17 | 9.71E-01 | -0.75±0.38 | 9.99E-01 | -0.71±0.38 | 1.00E+00 |
| X - 24328 | -0.02±0.02 | 67.04 | 8.15E-02 | 5.24E-01 | -0.16±0.19 | 66.34 | 8.48E-02 | 8.49E-01 | -0.02±0.2 | 9.94E-01 | -0.03±0.2 | 9.84E-01 | -0.73±0.41 | 9.99E-01 | -0.95±0.49 | 1.00E+00 |
| pantothenate | -0.02±0.02 | 81.72 | 1.93E-02 | 5.24E-01 | 0.16±0.16 | 71.88 | 5.93E-02 | 8.49E-01 | -0.02±0.18 | 9.94E-01 | 0.02±0.18 | 9.92E-01 | 0.85±0.37 | 9.99E-01 | 0.82±0.39 | 1.00E+00 |
| hydroxy-N6,N6,N6-trimethyllysine* | 0.06±0.17 | 35.38 | 2.13E-01 | 7.65E-01 | 0.12±0.14 | 51.25 | 1.52E-01 | 8.50E-01 | 0.22±0.15 | 7.58E-01 | 0.21±0.16 | 7.84E-01 | -0.23±0.29 | 9.99E-01 | -0.36±0.37 | 1.00E+00 |
| glycoursodeoxycholate | 0.12±0.17 | 0 | 3.77E-01 | 5.42E-01 | 0.13±0.15 | 0 | 3.54E-01 | 8.50E-01 | 0.18±0.16 | 8.05E-01 | 0.18±0.16 | 8.53E-01 | -0.23±0.43 | 9.99E-01 | -0.26±0.45 | 1.00E+00 |
| 1-stearoyl-GPE (18:0) | 0.13±0.15 | 0 | 6.47E-01 | 5.24E-01 | 0.13±0.16 | 0 | 4.92E-01 | 8.50E-01 | 0.16±0.16 | 8.55E-01 | 0.17±0.16 | 8.77E-01 | -0.03±0.39 | 9.99E-01 | -0.17±0.47 | 1.00E+00 |
| X - 23739 | 0.13±0.14 | 0 | 6.60E-01 | 5.24E-01 | 0.12±0.14 | 0 | 6.93E-01 | 8.50E-01 | 0.15±0.14 | 8.44E-01 | 0.14±0.14 | 9.08E-01 | -0.03±0.38 | 9.99E-01 | -0.06±0.49 | 1.00E+00 |
| 3-hydroxyhexanoate | -0.13±0.13 | 0 | 7.81E-01 | 5.24E-01 | -0.13±0.15 | 0 | 7.64E-01 | 8.50E-01 | -0.14±0.16 | 8.85E-01 | -0.15±0.16 | 9.15E-01 | 0±0.47 | 9.99E-01 | 0.01±0.49 | 1.00E+00 |
| oleate/vaccenate (18:1) | -0.13±0.16 | 0 | 4.62E-01 | 5.25E-01 | -0.13±0.15 | 0 | 7.39E-01 | 8.50E-01 | -0.17±0.16 | 8.44E-01 | -0.15±0.16 | 9.15E-01 | 0.23±0.51 | 9.99E-01 | 0.03±0.51 | 1.00E+00 |
| (2 or 3)-decenoate (10:1n7 or n8) | -0.14±0.15 | 0 | 7.33E-01 | 5.24E-01 | -0.13±0.15 | 0 | 9.20E-01 | 8.50E-01 | -0.15±0.15 | 8.55E-01 | -0.13±0.16 | 9.15E-01 | 0.02±0.49 | 9.99E-01 | -0.18±0.54 | 1.00E+00 |
| N1-methyladenosine | 0.13±0.12 | 0 | 5.05E-01 | 5.24E-01 | 0.14±0.16 | 0 | 6.49E-01 | 8.50E-01 | 0.12±0.16 | 9.32E-01 | 0.13±0.17 | 9.15E-01 | 0.68±0.84 | 9.99E-01 | 0.57±0.95 | 1.00E+00 |
| X - 12844 | -0.09±0.1 | 0 | 7.87E-01 | 5.24E-01 | -0.11±0.13 | 0 | 9.72E-01 | 8.50E-01 | -0.1±0.14 | 9.32E-01 | -0.11±0.14 | 9.15E-01 | 0.01±0.39 | 9.99E-01 | -0.13±0.48 | 1.00E+00 |
| X - 25172 | 0.15±0.14 | 0 | 8.19E-01 | 5.24E-01 | 0.13±0.15 | 0 | 8.82E-01 | 8.50E-01 | 0.14±0.15 | 8.83E-01 | 0.12±0.15 | 9.15E-01 | 0.26±0.51 | 9.99E-01 | 0.21±0.58 | 1.00E+00 |
| picolinoylglycine | -0.1±0.1 | 0 | 9.35E-01 | 5.24E-01 | -0.13±0.15 | 0 | 6.46E-01 | 8.50E-01 | -0.1±0.14 | 9.32E-01 | -0.11±0.15 | 9.23E-01 | -0.14±0.45 | 9.99E-01 | -0.38±0.57 | 1.00E+00 |
| X - 23655 | 0.14±0.13 | 0 | 8.98E-01 | 5.24E-01 | 0.14±0.16 | 0 | 7.09E-01 | 8.50E-01 | 0.14±0.17 | 9.15E-01 | 0.12±0.17 | 9.23E-01 | 0.2±0.5 | 9.99E-01 | 0.3±0.48 | 1.00E+00 |
| citramalate | 0.12±0.1 | 0 | 5.40E-01 | 5.24E-01 | 0.13±0.15 | 0 | 3.87E-01 | 8.50E-01 | 0.11±0.15 | 9.32E-01 | 0.09±0.15 | 9.26E-01 | 0.42±0.5 | 9.99E-01 | 0.6±0.57 | 1.00E+00 |
| N2,N5-diacetylornithine | -0.13±0.11 | 0 | 7.98E-01 | 5.24E-01 | -0.12±0.14 | 0 | 6.75E-01 | 8.50E-01 | -0.12±0.15 | 9.17E-01 | -0.1±0.15 | 9.23E-01 | -0.23±0.39 | 9.99E-01 | -0.28±0.41 | 1.00E+00 |
| sphingomyelin (d18:1/14:0, d16:1/16:0)* | 0.12±0.12 | 0 | 8.79E-01 | 5.24E-01 | 0.13±0.15 | 0 | 6.48E-01 | 8.50E-01 | 0.13±0.15 | 9.14E-01 | 0.15±0.16 | 9.08E-01 | 0.06±0.42 | 9.99E-01 | -0.09±0.49 | 1.00E+00 |
| X - 21339 | -0.11±0.13 | 0 | 4.60E-01 | 5.24E-01 | -0.12±0.14 | 0 | 8.61E-01 | 8.50E-01 | -0.13±0.15 | 8.85E-01 | -0.13±0.15 | 9.15E-01 | 0.23±0.48 | 9.99E-01 | -0.03±0.52 | 1.00E+00 |
| glycocholate | -0.09±0.07 | 65.48 | 8.87E-02 | 5.24E-01 | -0.13±0.16 | 50.31 | 1.56E-01 | 8.50E-01 | -0.07±0.16 | 9.74E-01 | -0.06±0.16 | 9.61E-01 | -0.96±0.52 | 9.99E-01 | -0.86±0.54 | 1.00E+00 |
| 4-guanidinobutanoate | -0.07±0.06 | 40.43 | 1.95E-01 | 5.24E-01 | -0.13±0.15 | 37.09 | 2.07E-01 | 8.50E-01 | -0.06±0.16 | 9.80E-01 | -0.06±0.16 | 9.62E-01 | -0.55±0.37 | 9.99E-01 | -0.63±0.43 | 1.00E+00 |
| androstenediol (3beta,17beta) monosulfate (1) | -0.1±0.16 | 43.94 | 1.82E-01 | 5.74E-01 | -0.13±0.16 | 56.31 | 1.30E-01 | 8.54E-01 | -0.16±0.16 | 8.48E-01 | -0.2±0.16 | 8.20E-01 | 0.57±0.52 | 9.99E-01 | 0.72±0.58 | 1.00E+00 |
| X - 16935 | -0.12±0.14 | 0 | 5.70E-01 | 5.24E-01 | -0.12±0.15 | 0 | 7.38E-01 | 8.54E-01 | -0.15±0.15 | 8.75E-01 | -0.14±0.15 | 9.15E-01 | 0.13±0.46 | 9.99E-01 | 0.05±0.54 | 1.00E+00 |
| X - 12306 | -0.11±0.18 | 19.97 | 2.64E-01 | 5.80E-01 | -0.12±0.15 | 35.01 | 2.15E-01 | 8.57E-01 | -0.2±0.15 | 7.66E-01 | -0.18±0.16 | 8.38E-01 | 0.38±0.48 | 9.99E-01 | 0.45±0.48 | 1.00E+00 |
| vanillic acid glycine | 0.09±0.13 | 24.93 | 2.48E-01 | 5.59E-01 | 0.12±0.15 | 8.3 | 2.96E-01 | 8.57E-01 | 0.14±0.15 | 8.85E-01 | 0.18±0.16 | 8.53E-01 | -0.36±0.41 | 9.99E-01 | -0.29±0.42 | 1.00E+00 |
| N6-acetyllysine | 0.14±0.15 | 0 | 7.40E-01 | 5.24E-01 | 0.12±0.15 | 0 | 4.82E-01 | 8.57E-01 | 0.16±0.15 | 8.48E-01 | 0.16±0.16 | 8.92E-01 | 0.02±0.4 | 9.99E-01 | -0.18±0.45 | 1.00E+00 |
| butyrylcarnitine (C4) | -0.11±0.13 | 0 | 5.70E-01 | 5.24E-01 | -0.13±0.16 | 0 | 9.06E-01 | 8.57E-01 | -0.14±0.17 | 9.13E-01 | -0.14±0.17 | 9.15E-01 | 0.11±0.41 | 9.99E-01 | -0.08±0.45 | 1.00E+00 |
| 4-hydroxychlorothalonil | 0.08±0.08 | 0 | 8.55E-01 | 5.24E-01 | 0.12±0.14 | 0 | 7.29E-01 | 8.57E-01 | 0.08±0.14 | 9.32E-01 | 0.14±0.15 | 9.15E-01 | 0.02±0.36 | 9.99E-01 | -0.02±0.42 | 1.00E+00 |
| 1-stearoyl-2-linoleoyl-GPE (18:0/18:2)* | -0.1±0.1 | 0 | 7.37E-01 | 5.24E-01 | -0.11±0.14 | 0 | 7.05E-01 | 8.57E-01 | -0.11±0.14 | 9.32E-01 | -0.13±0.14 | 9.15E-01 | 0.04±0.42 | 9.99E-01 | 0.05±0.44 | 1.00E+00 |
| fumarate | -0.1±0.11 | 0 | 7.01E-01 | 5.24E-01 | -0.11±0.13 | 0 | 9.30E-01 | 8.57E-01 | -0.11±0.13 | 9.15E-01 | -0.1±0.14 | 9.15E-01 | 0.06±0.44 | 9.99E-01 | -0.15±0.52 | 1.00E+00 |
| X - 19438 | -0.12±0.13 | 0 | 7.71E-01 | 5.24E-01 | -0.11±0.13 | 0 | 9.78E-01 | 8.57E-01 | -0.14±0.13 | 8.48E-01 | -0.11±0.14 | 9.15E-01 | -0.01±0.42 | 9.99E-01 | -0.12±0.46 | 1.00E+00 |
| X - 21733 | 0.06±0.06 | 0 | 6.38E-01 | 5.24E-01 | 0.13±0.16 | 0 | 9.80E-01 | 8.57E-01 | 0.06±0.16 | 9.80E-01 | 0.13±0.17 | 9.15E-01 | 0.29±0.49 | 9.99E-01 | 0.14±0.49 | 1.00E+00 |
| 3-carboxy-4-methyl-5-pentyl-2-furanpropionate (3-CMPFP)** | 0.08±0.07 | 0 | 7.11E-01 | 5.24E-01 | 0.12±0.15 | 0 | 9.11E-01 | 8.57E-01 | 0.08±0.15 | 9.58E-01 | 0.11±0.15 | 9.18E-01 | 0.25±0.47 | 9.99E-01 | 0.18±0.52 | 1.00E+00 |
| 1-palmitoleoyl-GPC (16:1)* | 0.12±0.1 | 0 | 5.04E-01 | 5.24E-01 | 0.13±0.16 | 0 | 8.42E-01 | 8.57E-01 | 0.1±0.16 | 9.32E-01 | 0.12±0.17 | 9.18E-01 | 0.48±0.55 | 9.99E-01 | 0.25±0.63 | 1.00E+00 |
| X - 26109 | -0.11±0.1 | 0 | 8.09E-01 | 5.24E-01 | -0.13±0.15 | 0 | 7.00E-01 | 8.57E-01 | -0.1±0.16 | 9.32E-01 | -0.1±0.16 | 9.26E-01 | -0.21±0.42 | 9.99E-01 | -0.28±0.44 | 1.00E+00 |
| 3-hydroxyhexanoylcarnitine (1) | -0.09±0.08 | 0 | 6.05E-01 | 5.24E-01 | -0.13±0.16 | 0 | 4.27E-01 | 8.57E-01 | -0.08±0.17 | 9.69E-01 | -0.09±0.17 | 9.43E-01 | -0.27±0.37 | 9.99E-01 | -0.51±0.51 | 1.00E+00 |
| N-palmitoyl-sphinganine (d18:0/16:0) | 0.09±0.07 | 0 | 3.41E-01 | 5.24E-01 | 0.12±0.15 | 16.4 | 2.74E-01 | 8.57E-01 | 0.08±0.15 | 9.58E-01 | 0.08±0.15 | 9.45E-01 | 0.52±0.46 | 9.99E-01 | 0.68±0.53 | 1.00E+00 |
| sulfate of piperine metabolite C16H19NO3 (2)* | -0.06±0.05 | 0 | 4.25E-01 | 5.24E-01 | -0.12±0.15 | 36 | 2.11E-01 | 8.57E-01 | -0.05±0.16 | 9.80E-01 | -0.06±0.16 | 9.62E-01 | -0.39±0.41 | 9.99E-01 | -0.68±0.47 | 1.00E+00 |
| X - 24494 | 0.05±0.04 | 57.59 | 1.25E-01 | 5.24E-01 | 0.12±0.14 | 46.34 | 1.72E-01 | 8.57E-01 | 0.04±0.15 | 9.80E-01 | 0.05±0.15 | 9.62E-01 | 0.69±0.42 | 9.99E-01 | 0.7±0.45 | 1.00E+00 |
| 3-(3-amino-3-carboxypropyl)uridine* | 0±0 | 77.24 | 3.61E-02 | 5.24E-01 | -0.12±0.15 | 73.96 | 5.00E-02 | 8.57E-01 | 0±0.16 | 9.99E-01 | 0±0.16 | 9.99E-01 | -0.68±0.32 | 9.99E-01 | -0.84±0.4 | 1.00E+00 |
| dodecenedioate (C12:1-DC)* | -0.08±0.19 | 52.08 | 1.49E-01 | 7.05E-01 | -0.13±0.16 | 35.51 | 2.13E-01 | 8.57E-01 | -0.21±0.17 | 7.87E-01 | -0.2±0.17 | 8.26E-01 | 0.48±0.43 | 9.99E-01 | 0.41±0.46 | 1.00E+00 |
| cortisone | -0.08±0.12 | 70.93 | 6.36E-02 | 5.63E-01 | -0.1±0.13 | 63.68 | 9.70E-02 | 8.57E-01 | -0.12±0.13 | 8.75E-01 | -0.14±0.13 | 8.61E-01 | 1.07±0.63 | 9.99E-01 | 1.12±0.75 | 1.00E+00 |
| 3-hydroxysebacate | -0.11±0.17 | 10.51 | 2.90E-01 | 5.71E-01 | -0.13±0.16 | 0 | 3.48E-01 | 8.57E-01 | -0.18±0.17 | 8.44E-01 | -0.18±0.17 | 8.66E-01 | 0.31±0.43 | 9.99E-01 | 0.31±0.5 | 1.00E+00 |
| X - 12456 | -0.11±0.14 | 0 | 4.00E-01 | 5.25E-01 | -0.13±0.16 | 0 | 3.67E-01 | 8.57E-01 | -0.14±0.15 | 8.85E-01 | -0.16±0.16 | 8.95E-01 | 0.33±0.54 | 9.99E-01 | 0.43±0.63 | 1.00E+00 |
| valine | 0.18±0.18 | 0 | 8.84E-01 | 5.24E-01 | 0.16±0.2 | 0 | 6.05E-01 | 8.57E-01 | 0.19±0.2 | 8.83E-01 | 0.21±0.22 | 9.09E-01 | 0.12±0.48 | 9.99E-01 | -0.08±0.5 | 1.00E+00 |
| valylglycine | 0.13±0.14 | 0 | 7.19E-01 | 5.24E-01 | 0.12±0.15 | 0 | 7.57E-01 | 8.57E-01 | 0.14±0.16 | 8.83E-01 | 0.14±0.16 | 9.15E-01 | -0.03±0.47 | 9.99E-01 | -0.02±0.49 | 1.00E+00 |
| hexadecadienoate (16:2n6) | -0.13±0.16 | 0 | 4.91E-01 | 5.24E-01 | -0.13±0.16 | 0 | 6.59E-01 | 8.57E-01 | -0.16±0.16 | 8.55E-01 | -0.15±0.17 | 9.15E-01 | 0.22±0.54 | 9.99E-01 | 0.12±0.58 | 1.00E+00 |
| cerotoylcarnitine (C26)* | -0.12±0.15 | 0 | 5.16E-01 | 5.24E-01 | -0.11±0.14 | 0 | 6.13E-01 | 8.57E-01 | -0.16±0.15 | 8.44E-01 | -0.14±0.15 | 9.15E-01 | 0.16±0.46 | 9.99E-01 | 0.11±0.47 | 1.00E+00 |
| uridine | -0.12±0.12 | 0 | 7.97E-01 | 5.24E-01 | -0.12±0.15 | 0 | 9.16E-01 | 8.57E-01 | -0.13±0.15 | 8.92E-01 | -0.11±0.16 | 9.23E-01 | -0.03±0.37 | 9.99E-01 | -0.16±0.42 | 1.00E+00 |
| glycerol 3-phosphate | -0.1±0.09 | 0 | 9.18E-01 | 5.24E-01 | -0.11±0.14 | 0 | 5.35E-01 | 8.57E-01 | -0.09±0.14 | 9.32E-01 | -0.08±0.14 | 9.35E-01 | -0.14±0.41 | 9.99E-01 | -0.37±0.44 | 1.00E+00 |
| pipecolate | -0.1±0.09 | 0 | 6.02E-01 | 5.24E-01 | -0.11±0.14 | 0 | 5.41E-01 | 8.58E-01 | -0.09±0.14 | 9.32E-01 | -0.09±0.14 | 9.26E-01 | -0.31±0.41 | 9.99E-01 | -0.43±0.54 | 1.00E+00 |
| sulfate* | 0.12±0.14 | 0 | 5.47E-01 | 5.24E-01 | 0.11±0.14 | 0 | 3.52E-01 | 8.60E-01 | 0.15±0.14 | 8.48E-01 | 0.15±0.15 | 8.77E-01 | -0.08±0.36 | 9.99E-01 | -0.3±0.46 | 1.00E+00 |
| 1,3-dimethylurate | 0.1±0.1 | 0 | 9.25E-01 | 5.24E-01 | 0.12±0.15 | 0 | 9.37E-01 | 8.60E-01 | 0.1±0.15 | 9.32E-01 | 0.12±0.16 | 9.15E-01 | 0.05±0.48 | 9.99E-01 | 0.08±0.51 | 1.00E+00 |
| paraxanthine | 0.09±0.08 | 0 | 6.01E-01 | 5.24E-01 | 0.13±0.16 | 0 | 8.09E-01 | 8.60E-01 | 0.08±0.16 | 9.58E-01 | 0.12±0.17 | 9.23E-01 | 0.38±0.56 | 9.99E-01 | 0.28±0.66 | 1.00E+00 |
| glycochenodeoxycholate glucuronide (1) | -0.07±0.06 | 0 | 9.21E-01 | 5.24E-01 | -0.13±0.17 | 0 | 8.10E-01 | 8.60E-01 | -0.06±0.17 | 9.80E-01 | -0.12±0.17 | 9.23E-01 | -0.11±0.46 | 9.99E-01 | -0.25±0.53 | 1.00E+00 |
| 1-(1-enyl-stearoyl)-2-linoleoyl-GPE (P-18:0/18:2)* | -0.08±0.08 | 0 | 7.14E-01 | 5.24E-01 | -0.11±0.15 | 0 | 4.80E-01 | 8.60E-01 | -0.08±0.15 | 9.58E-01 | -0.07±0.16 | 9.45E-01 | -0.2±0.32 | 9.99E-01 | -0.36±0.38 | 1.00E+00 |
| X - 18922 | -0.04±0.04 | 1.9 | 3.13E-01 | 5.24E-01 | -0.11±0.14 | 46.47 | 1.72E-01 | 8.62E-01 | -0.04±0.15 | 9.80E-01 | -0.04±0.15 | 9.67E-01 | -0.4±0.35 | 9.99E-01 | -0.63±0.41 | 1.00E+00 |
| ascorbic acid 2-sulfate | -0.08±0.07 | 0 | 6.65E-01 | 5.24E-01 | -0.11±0.14 | 0 | 4.40E-01 | 8.63E-01 | -0.07±0.14 | 9.58E-01 | -0.07±0.15 | 9.45E-01 | -0.21±0.31 | 9.99E-01 | -0.38±0.39 | 1.00E+00 |
| tetradecadienedioate (C14:2-DC)* | 0.09±0.08 | 30.52 | 2.30E-01 | 5.24E-01 | 0.12±0.15 | 0 | 3.49E-01 | 8.65E-01 | 0.08±0.15 | 9.58E-01 | 0.09±0.16 | 9.39E-01 | 0.86±0.65 | 9.99E-01 | 0.78±0.72 | 1.00E+00 |
| sphingomyelin (d18:1/25:0, d19:0/24:1, d20:1/23:0, d19:1/24:0)* | 0.04±0.04 | 19.45 | 2.65E-01 | 5.24E-01 | 0.11±0.15 | 21.66 | 2.59E-01 | 8.65E-01 | 0.04±0.15 | 9.80E-01 | 0.08±0.15 | 9.45E-01 | 0.73±0.62 | 9.99E-01 | 0.84±0.66 | 1.00E+00 |
| margarate (17:0) | -0.13±0.16 | 0 | 5.07E-01 | 5.24E-01 | -0.12±0.16 | 0 | 7.29E-01 | 8.66E-01 | -0.17±0.17 | 8.55E-01 | -0.14±0.17 | 9.15E-01 | 0.16±0.46 | 9.99E-01 | 0.04±0.49 | 1.00E+00 |
| N-acetyl-2-aminoadipate | 0.12±0.13 | 0 | 7.09E-01 | 5.24E-01 | 0.12±0.15 | 0 | 4.11E-01 | 8.66E-01 | 0.14±0.15 | 8.85E-01 | 0.16±0.16 | 8.97E-01 | -0.03±0.41 | 9.99E-01 | -0.25±0.47 | 1.00E+00 |
| X - 21364 | -0.1±0.12 | 0 | 4.92E-01 | 5.24E-01 | -0.13±0.17 | 0 | 5.94E-01 | 8.67E-01 | -0.12±0.18 | 9.32E-01 | -0.16±0.18 | 9.15E-01 | 0.28±0.57 | 9.99E-01 | 0.19±0.63 | 1.00E+00 |
| theophylline | 0.06±0.06 | 0 | 5.14E-01 | 5.24E-01 | 0.12±0.15 | 0 | 7.11E-01 | 8.69E-01 | 0.06±0.15 | 9.80E-01 | 0.1±0.16 | 9.23E-01 | 0.45±0.6 | 9.99E-01 | 0.37±0.69 | 1.00E+00 |
| 2-palmitoleoyl-GPC (16:1)* | -0.1±0.16 | 71.67 | 6.03E-02 | 5.77E-01 | -0.12±0.16 | 61 | 1.09E-01 | 8.73E-01 | -0.16±0.15 | 8.44E-01 | -0.16±0.16 | 8.79E-01 | 1.33±0.78 | 9.99E-01 | 1.27±0.88 | 1.00E+00 |
| tyrosine | -0.1±0.13 | 8.6 | 2.96E-01 | 5.32E-01 | -0.11±0.15 | 0 | 5.53E-01 | 8.73E-01 | -0.14±0.15 | 8.83E-01 | -0.13±0.15 | 9.15E-01 | 0.45±0.55 | 9.99E-01 | 0.26±0.64 | 1.00E+00 |
| orotidine | -0.08±0.07 | 0 | 7.05E-01 | 5.24E-01 | -0.13±0.17 | 0 | 6.99E-01 | 8.73E-01 | -0.07±0.18 | 9.80E-01 | -0.09±0.19 | 9.44E-01 | -0.21±0.35 | 9.99E-01 | -0.26±0.38 | 1.00E+00 |
| sphingosine | 0.07±0.17 | 42.04 | 1.89E-01 | 6.84E-01 | 0.11±0.15 | 30.14 | 2.32E-01 | 8.73E-01 | 0.19±0.15 | 7.87E-01 | 0.17±0.16 | 8.53E-01 | -0.35±0.36 | 9.99E-01 | -0.36±0.42 | 1.00E+00 |
| X - 07765 | 0.11±0.15 | 45.86 | 1.74E-01 | 5.37E-01 | 0.12±0.16 | 43.78 | 1.82E-01 | 8.73E-01 | 0.16±0.16 | 8.75E-01 | 0.16±0.16 | 8.97E-01 | -0.9±0.76 | 9.99E-01 | -1.04±0.88 | 1.00E+00 |
| sphingomyelin (d18:2/16:0, d18:1/16:1)* | 0.12±0.12 | 0 | 8.57E-01 | 5.24E-01 | 0.12±0.16 | 0 | 7.24E-01 | 8.73E-01 | 0.13±0.17 | 9.32E-01 | 0.14±0.17 | 9.15E-01 | 0.05±0.42 | 9.99E-01 | -0.06±0.54 | 1.00E+00 |
| glycodeoxycholate 3-sulfate | -0.09±0.1 | 0 | 6.41E-01 | 5.24E-01 | -0.11±0.14 | 0 | 6.18E-01 | 8.73E-01 | -0.1±0.14 | 9.32E-01 | -0.13±0.15 | 9.15E-01 | 0.11±0.44 | 9.99E-01 | 0.11±0.46 | 1.00E+00 |
| linoleoyl ethanolamide | -0.11±0.13 | 0 | 4.85E-01 | 5.24E-01 | -0.11±0.15 | 0 | 7.45E-01 | 8.73E-01 | -0.14±0.15 | 8.83E-01 | -0.12±0.15 | 9.15E-01 | 0.24±0.52 | 9.99E-01 | 0.07±0.57 | 1.00E+00 |
| methylsuccinoylcarnitine | 0.11±0.12 | 0 | 8.01E-01 | 5.24E-01 | 0.1±0.13 | 0 | 8.19E-01 | 8.73E-01 | 0.12±0.14 | 8.85E-01 | 0.11±0.14 | 9.15E-01 | 0.02±0.37 | 9.99E-01 | 0±0.44 | 1.00E+00 |
| gamma-glutamylglutamine | 0.11±0.11 | 0 | 9.89E-01 | 5.24E-01 | 0.11±0.15 | 0 | 7.67E-01 | 8.73E-01 | 0.11±0.15 | 9.32E-01 | 0.12±0.16 | 9.15E-01 | 0.11±0.49 | 9.99E-01 | -0.05±0.57 | 1.00E+00 |
| taurocholenate sulfate* | -0.07±0.08 | 0 | 7.43E-01 | 5.24E-01 | -0.11±0.15 | 0 | 8.60E-01 | 8.73E-01 | -0.08±0.15 | 9.58E-01 | -0.12±0.16 | 9.18E-01 | 0.06±0.43 | 9.99E-01 | -0.03±0.5 | 1.00E+00 |
| X - 11880 | -0.11±0.13 | 0 | 4.74E-01 | 5.24E-01 | -0.1±0.13 | 0 | 9.46E-01 | 8.73E-01 | -0.13±0.14 | 8.67E-01 | -0.1±0.14 | 9.18E-01 | 0.22±0.48 | 9.99E-01 | -0.07±0.49 | 1.00E+00 |
| picolinate | -0.1±0.11 | 0 | 7.02E-01 | 5.24E-01 | -0.12±0.16 | 0 | 9.16E-01 | 8.73E-01 | -0.11±0.17 | 9.32E-01 | -0.12±0.17 | 9.19E-01 | 0.06±0.43 | 9.99E-01 | -0.07±0.49 | 1.00E+00 |
| cytidine | 0.11±0.11 | 0 | 9.12E-01 | 5.24E-01 | 0.11±0.16 | 0 | 9.42E-01 | 8.73E-01 | 0.11±0.16 | 9.32E-01 | 0.11±0.17 | 9.23E-01 | 0.16±0.41 | 9.99E-01 | 0.15±0.46 | 1.00E+00 |
| 3-methoxycatechol sulfate (1) | -0.11±0.1 | 0 | 7.76E-01 | 5.24E-01 | -0.11±0.15 | 0 | 7.72E-01 | 8.73E-01 | -0.1±0.16 | 9.32E-01 | -0.1±0.17 | 9.27E-01 | -0.24±0.47 | 9.99E-01 | -0.23±0.43 | 1.00E+00 |
| erucate (22:1n9) | -0.11±0.1 | 0 | 5.69E-01 | 5.24E-01 | -0.11±0.15 | 0 | 5.73E-01 | 8.73E-01 | -0.1±0.15 | 9.32E-01 | -0.09±0.15 | 9.27E-01 | -0.41±0.54 | 9.99E-01 | -0.44±0.61 | 1.00E+00 |
| 2-hydroxyhippurate (salicylurate) | -0.05±0.04 | 85.12 | 9.52E-03 | 5.24E-01 | -0.11±0.15 | 77.58 | 3.47E-02 | 8.73E-01 | -0.04±0.15 | 9.80E-01 | -0.08±0.15 | 9.44E-01 | -2.86±1.09 | 9.99E-01 | -3.08±1.42 | 1.00E+00 |
| 1-palmitoyl-2-palmitoleoyl-GPC (16:0/16:1)* | 0.08±0.07 | 0 | 3.25E-01 | 5.24E-01 | 0.11±0.16 | 0 | 4.18E-01 | 8.73E-01 | 0.07±0.15 | 9.72E-01 | 0.08±0.17 | 9.45E-01 | 0.52±0.45 | 9.99E-01 | 0.53±0.54 | 1.00E+00 |
| sulfate of piperine metabolite C18H21NO3 (3)* | -0.06±0.05 | 0 | 4.67E-01 | 5.24E-01 | -0.11±0.15 | 0 | 4.73E-01 | 8.73E-01 | -0.05±0.15 | 9.80E-01 | -0.07±0.16 | 9.45E-01 | -0.35±0.41 | 9.99E-01 | -0.42±0.45 | 1.00E+00 |
| X - 12847 | -0.04±0.04 | 15.04 | 2.78E-01 | 5.24E-01 | -0.11±0.15 | 0 | 3.84E-01 | 8.73E-01 | -0.04±0.15 | 9.80E-01 | -0.08±0.16 | 9.45E-01 | -0.59±0.51 | 9.99E-01 | -0.63±0.61 | 1.00E+00 |
| X - 17325 | 0.08±0.07 | 0 | 4.78E-01 | 5.24E-01 | 0.12±0.16 | 0 | 4.67E-01 | 8.73E-01 | 0.07±0.17 | 9.78E-01 | 0.08±0.17 | 9.45E-01 | 0.48±0.57 | 9.99E-01 | 0.52±0.57 | 1.00E+00 |
| myo-inositol | -0.04±0.03 | 28.51 | 2.37E-01 | 5.24E-01 | -0.1±0.14 | 54.72 | 1.37E-01 | 8.73E-01 | -0.03±0.15 | 9.91E-01 | -0.01±0.15 | 9.93E-01 | -0.35±0.27 | 9.99E-01 | -0.56±0.34 | 1.00E+00 |
| 1-methylurate | 0.11±0.1 | 0 | 9.25E-01 | 5.24E-01 | 0.12±0.16 | 0 | 7.83E-01 | 8.75E-01 | 0.11±0.17 | 9.32E-01 | 0.1±0.17 | 9.26E-01 | 0.16±0.53 | 9.99E-01 | 0.27±0.59 | 1.00E+00 |
| X - 25267 | 0.1±0.15 | 0 | 4.05E-01 | 5.50E-01 | 0.1±0.14 | 0 | 3.87E-01 | 8.75E-01 | 0.16±0.15 | 8.27E-01 | 0.15±0.15 | 8.97E-01 | -0.13±0.31 | 9.99E-01 | -0.21±0.38 | 1.00E+00 |
| isovalerate (i5:0) | -0.11±0.12 | 0 | 5.60E-01 | 5.24E-01 | -0.11±0.16 | 0 | 6.67E-01 | 8.75E-01 | -0.13±0.16 | 9.22E-01 | -0.13±0.16 | 9.15E-01 | 0.23±0.59 | 9.99E-01 | 0.17±0.66 | 1.00E+00 |
| indoleacetate | -0.09±0.1 | 0 | 6.16E-01 | 5.24E-01 | -0.1±0.14 | 0 | 6.15E-01 | 8.75E-01 | -0.1±0.15 | 9.32E-01 | -0.12±0.15 | 9.15E-01 | 0.08±0.35 | 9.99E-01 | 0.1±0.42 | 1.00E+00 |
| 4-hydroxyglutamate | 0.13±0.13 | 0 | 8.55E-01 | 5.24E-01 | 0.11±0.16 | 0 | 6.94E-01 | 8.75E-01 | 0.14±0.16 | 8.92E-01 | 0.13±0.17 | 9.15E-01 | 0.05±0.5 | 9.99E-01 | -0.09±0.55 | 1.00E+00 |
| (14 or 15)-methylpalmitate (a17:0 or i17:0) | -0.11±0.15 | 0 | 3.63E-01 | 5.30E-01 | -0.11±0.16 | 0 | 6.46E-01 | 8.75E-01 | -0.15±0.16 | 8.75E-01 | -0.13±0.17 | 9.15E-01 | 0.34±0.52 | 9.99E-01 | 0.13±0.55 | 1.00E+00 |
| 2R,3R-dihydroxybutyrate | 0.1±0.1 | 0 | 8.65E-01 | 5.24E-01 | 0.1±0.15 | 0 | 8.36E-01 | 8.75E-01 | 0.11±0.15 | 9.32E-01 | 0.11±0.15 | 9.18E-01 | 0.04±0.38 | 9.99E-01 | 0.02±0.43 | 1.00E+00 |
| phosphoethanolamine | -0.05±0.04 | 0 | 5.37E-01 | 5.24E-01 | -0.1±0.14 | 15.2 | 2.78E-01 | 8.75E-01 | -0.04±0.15 | 9.80E-01 | -0.03±0.15 | 9.84E-01 | -0.2±0.26 | 9.99E-01 | -0.41±0.32 | 1.00E+00 |
| 1-stearoyl-2-arachidonoyl-GPE (18:0/20:4) | -0.08±0.11 | 26.59 | 2.43E-01 | 5.30E-01 | -0.1±0.14 | 30.34 | 2.31E-01 | 8.75E-01 | -0.11±0.14 | 9.32E-01 | -0.14±0.15 | 8.99E-01 | 0.51±0.52 | 9.99E-01 | 0.51±0.53 | 1.00E+00 |
| N6-methyllysine | -0.11±0.15 | 0 | 3.30E-01 | 5.39E-01 | -0.1±0.15 | 8.04 | 2.97E-01 | 8.75E-01 | -0.15±0.15 | 8.52E-01 | -0.14±0.15 | 9.08E-01 | 0.32±0.46 | 9.99E-01 | 0.41±0.51 | 1.00E+00 |
| 4-methylguaiacol sulfate | -0.07±0.09 | 0 | 3.87E-01 | 5.24E-01 | -0.1±0.14 | 0 | 5.20E-01 | 8.75E-01 | -0.09±0.15 | 9.32E-01 | -0.12±0.15 | 9.15E-01 | 0.34±0.48 | 9.99E-01 | 0.21±0.5 | 1.00E+00 |
| pregnenetriol sulfate* | -0.11±0.11 | 0 | 6.63E-01 | 5.24E-01 | -0.12±0.17 | 0 | 6.78E-01 | 8.75E-01 | -0.11±0.18 | 9.32E-01 | -0.14±0.18 | 9.15E-01 | 0.15±0.61 | 9.99E-01 | 0.14±0.65 | 1.00E+00 |
| X - 12462 | 0.12±0.13 | 0 | 7.70E-01 | 5.24E-01 | 0.1±0.14 | 0 | 5.70E-01 | 8.75E-01 | 0.14±0.15 | 8.83E-01 | 0.12±0.15 | 9.15E-01 | 0.01±0.39 | 9.99E-01 | -0.13±0.43 | 1.00E+00 |
| X - 12740 | 0.1±0.11 | 0 | 8.14E-01 | 5.24E-01 | 0.1±0.15 | 0 | 6.35E-01 | 8.75E-01 | 0.11±0.15 | 9.32E-01 | 0.12±0.15 | 9.15E-01 | 0±0.44 | 9.99E-01 | -0.11±0.46 | 1.00E+00 |
| X - 26107 | -0.09±0.1 | 0 | 5.86E-01 | 5.24E-01 | -0.09±0.13 | 0 | 7.16E-01 | 8.75E-01 | -0.1±0.13 | 9.32E-01 | -0.1±0.14 | 9.18E-01 | 0.17±0.48 | 9.99E-01 | 0.11±0.56 | 1.00E+00 |
| gamma-glutamyl-alpha-lysine | 0.09±0.09 | 0 | 9.25E-01 | 5.24E-01 | 0.1±0.14 | 0 | 9.08E-01 | 8.75E-01 | 0.09±0.14 | 9.32E-01 | 0.1±0.15 | 9.23E-01 | 0.05±0.41 | 9.99E-01 | 0.02±0.64 | 1.00E+00 |
| ceramide (d18:1/14:0, d16:1/16:0)* | -0.11±0.11 | 0 | 7.93E-01 | 5.24E-01 | -0.1±0.14 | 0 | 9.85E-01 | 8.75E-01 | -0.11±0.15 | 9.30E-01 | -0.1±0.15 | 9.23E-01 | -0.01±0.38 | 9.99E-01 | -0.09±0.45 | 1.00E+00 |
| 2-hydroxyphenylacetate | 0.1±0.11 | 0 | 8.24E-01 | 5.24E-01 | 0.12±0.17 | 0 | 9.07E-01 | 8.75E-01 | 0.11±0.17 | 9.32E-01 | 0.12±0.17 | 9.23E-01 | 0±0.49 | 9.99E-01 | 0.05±0.59 | 1.00E+00 |
| heptenedioate (C7:1-DC)* | -0.11±0.12 | 0 | 7.60E-01 | 5.24E-01 | -0.1±0.14 | 0 | 9.03E-01 | 8.75E-01 | -0.13±0.15 | 9.10E-01 | -0.1±0.15 | 9.26E-01 | 0.01±0.42 | 9.99E-01 | -0.16±0.52 | 1.00E+00 |
| alpha-ketobutyrate | 0.1±0.1 | 0 | 7.28E-01 | 5.24E-01 | 0.11±0.16 | 0 | 8.81E-01 | 8.75E-01 | 0.1±0.16 | 9.32E-01 | 0.1±0.17 | 9.26E-01 | 0.27±0.5 | 9.99E-01 | 0.19±0.54 | 1.00E+00 |
| X - 17654 | -0.1±0.1 | 0 | 8.54E-01 | 5.24E-01 | -0.09±0.13 | 0 | 8.50E-01 | 8.75E-01 | -0.1±0.14 | 9.32E-01 | -0.08±0.14 | 9.27E-01 | -0.03±0.4 | 9.99E-01 | -0.17±0.43 | 1.00E+00 |
| eicosenoylcarnitine (C20:1)* | -0.12±0.11 | 0 | 8.72E-01 | 5.24E-01 | -0.1±0.15 | 0 | 7.95E-01 | 8.75E-01 | -0.11±0.15 | 9.32E-01 | -0.09±0.16 | 9.34E-01 | -0.18±0.4 | 9.99E-01 | -0.21±0.45 | 1.00E+00 |
| eicosenedioate (C20:1-DC)* | -0.09±0.09 | 0 | 9.10E-01 | 5.24E-01 | -0.1±0.15 | 0 | 6.85E-01 | 8.75E-01 | -0.09±0.15 | 9.32E-01 | -0.08±0.15 | 9.39E-01 | -0.04±0.43 | 9.99E-01 | -0.29±0.49 | 1.00E+00 |
| oleoyl-linoleoyl-glycerol (18:1/18:2) [1] | -0.04±0.04 | 0 | 6.13E-01 | 5.24E-01 | -0.1±0.14 | 0 | 5.89E-01 | 8.75E-01 | -0.04±0.15 | 9.80E-01 | -0.07±0.15 | 9.45E-01 | -0.22±0.36 | 9.99E-01 | -0.32±0.43 | 1.00E+00 |
| fructosyllysine | -0.05±0.05 | 0 | 6.76E-01 | 5.24E-01 | -0.1±0.15 | 0 | 5.08E-01 | 8.75E-01 | -0.05±0.16 | 9.80E-01 | -0.06±0.16 | 9.61E-01 | -0.19±0.34 | 9.99E-01 | -0.35±0.4 | 1.00E+00 |
| X - 21258 | -0.07±0.06 | 21.57 | 2.59E-01 | 5.24E-01 | -0.11±0.15 | 0 | 3.52E-01 | 8.75E-01 | -0.06±0.16 | 9.80E-01 | -0.06±0.16 | 9.62E-01 | -0.59±0.47 | 9.99E-01 | -0.52±0.47 | 1.00E+00 |
| X - 24306 | -0.05±0.04 | 0 | 6.44E-01 | 5.24E-01 | -0.09±0.14 | 0 | 3.85E-01 | 8.75E-01 | -0.05±0.15 | 9.80E-01 | -0.05±0.15 | 9.62E-01 | -0.21±0.36 | 9.99E-01 | -0.42±0.4 | 1.00E+00 |
| X - 25790 | -0.06±0.05 | 0 | 6.87E-01 | 5.24E-01 | -0.1±0.15 | 0 | 5.29E-01 | 8.75E-01 | -0.05±0.16 | 9.80E-01 | -0.06±0.16 | 9.62E-01 | -0.17±0.28 | 9.99E-01 | -0.31±0.36 | 1.00E+00 |
| indoleacetoylcarnitine* | 0.07±0.06 | 32.01 | 2.25E-01 | 5.24E-01 | 0.1±0.14 | 44.4 | 1.80E-01 | 8.75E-01 | 0.06±0.15 | 9.78E-01 | 0.05±0.15 | 9.65E-01 | 0.57±0.42 | 9.99E-01 | 0.8±0.54 | 1.00E+00 |
| glyco-beta-muricholate** | -0.02±0.01 | 69.15 | 7.18E-02 | 5.24E-01 | -0.1±0.15 | 57.31 | 1.26E-01 | 8.75E-01 | -0.01±0.15 | 9.94E-01 | -0.03±0.15 | 9.89E-01 | -0.82±0.44 | 9.99E-01 | -0.77±0.46 | 1.00E+00 |
| X - 21834 | 0.02±0.02 | 62.05 | 1.05E-01 | 5.24E-01 | 0.1±0.15 | 69.83 | 6.87E-02 | 8.75E-01 | 0.02±0.15 | 9.94E-01 | 0.02±0.16 | 9.92E-01 | 0.77±0.46 | 9.99E-01 | 0.99±0.51 | 1.00E+00 |
| X - 12680 | -0.01±0.01 | 52.04 | 1.49E-01 | 5.24E-01 | -0.09±0.14 | 60.2 | 1.13E-01 | 8.75E-01 | -0.01±0.15 | 9.99E-01 | 0±0.15 | 9.98E-01 | -0.43±0.29 | 9.99E-01 | -0.63±0.37 | 1.00E+00 |
| 1-(1-enyl-palmitoyl)-GPC (P-16:0)* | 0.11±0.14 | 0 | 4.52E-01 | 5.33E-01 | 0.1±0.15 | 39.02 | 2.00E-01 | 8.78E-01 | 0.15±0.16 | 8.75E-01 | 0.19±0.17 | 8.53E-01 | -0.13±0.34 | 9.99E-01 | -0.35±0.38 | 1.00E+00 |
| 1-arachidonoyl-GPI (20:4)* | -0.1±0.11 | 0 | 5.65E-01 | 5.24E-01 | -0.1±0.14 | 0 | 8.51E-01 | 8.78E-01 | -0.11±0.15 | 9.32E-01 | -0.1±0.15 | 9.23E-01 | 0.18±0.49 | 9.99E-01 | 0±0.53 | 1.00E+00 |
| 4-vinylguaiacol sulfate | 0.12±0.17 | 0 | 3.45E-01 | 5.39E-01 | 0.11±0.17 | 0 | 6.10E-01 | 8.83E-01 | 0.18±0.18 | 8.55E-01 | 0.14±0.18 | 9.15E-01 | -0.32±0.5 | 9.99E-01 | -0.13±0.51 | 1.00E+00 |
| X - 21383 | 0.09±0.08 | 0 | 7.50E-01 | 5.24E-01 | 0.09±0.14 | 0 | 8.82E-01 | 8.83E-01 | 0.09±0.15 | 9.32E-01 | 0.1±0.15 | 9.23E-01 | 0.21±0.38 | 9.99E-01 | 0.03±0.44 | 1.00E+00 |
| 2-hydroxy-3-methylvalerate | -0.08±0.07 | 0 | 9.83E-01 | 5.24E-01 | -0.11±0.17 | 0 | 9.87E-01 | 8.83E-01 | -0.07±0.17 | 9.74E-01 | -0.11±0.18 | 9.26E-01 | -0.08±0.45 | 9.99E-01 | -0.12±0.45 | 1.00E+00 |
| X - 12101 | -0.02±0.02 | 0 | 3.62E-01 | 5.24E-01 | -0.1±0.15 | 34.36 | 2.17E-01 | 8.83E-01 | -0.02±0.16 | 9.94E-01 | -0.04±0.16 | 9.69E-01 | -0.36±0.37 | 9.99E-01 | -0.66±0.48 | 1.00E+00 |
| N-acetyl-1-methylhistidine* | 0.04±0.04 | 12.2 | 2.86E-01 | 5.24E-01 | 0.09±0.14 | 0 | 3.96E-01 | 8.85E-01 | 0.04±0.15 | 9.86E-01 | 0.05±0.15 | 9.65E-01 | 0.47±0.41 | 9.99E-01 | 0.44±0.43 | 1.00E+00 |
| X - 24462 | -0.04±0.03 | 32.01 | 2.25E-01 | 5.24E-01 | -0.09±0.14 | 33.91 | 2.19E-01 | 8.85E-01 | -0.03±0.14 | 9.86E-01 | -0.03±0.15 | 9.69E-01 | -0.49±0.37 | 9.99E-01 | -0.61±0.44 | 1.00E+00 |
| N,N-dimethyl-pro-pro | -0.06±0.06 | 0 | 8.42E-01 | 5.24E-01 | -0.1±0.15 | 0 | 4.91E-01 | 9.05E-01 | -0.06±0.16 | 9.80E-01 | -0.05±0.16 | 9.65E-01 | -0.12±0.33 | 9.99E-01 | -0.35±0.4 | 1.00E+00 |
| argininate* | 0.07±0.14 | 37.37 | 2.06E-01 | 6.20E-01 | 0.09±0.15 | 53.98 | 1.40E-01 | 9.10E-01 | 0.15±0.15 | 8.55E-01 | 0.17±0.16 | 8.53E-01 | -0.32±0.34 | 9.99E-01 | -0.48±0.41 | 1.00E+00 |
| 10-heptadecenoate (17:1n7) | -0.11±0.14 | 0 | 4.11E-01 | 5.26E-01 | -0.1±0.16 | 0 | 7.00E-01 | 9.10E-01 | -0.15±0.17 | 8.89E-01 | -0.12±0.17 | 9.23E-01 | 0.29±0.51 | 9.99E-01 | 0.1±0.55 | 1.00E+00 |
| docosapentaenoate (n3 DPA; 22:5n3) | -0.12±0.14 | 0 | 5.94E-01 | 5.24E-01 | -0.1±0.17 | 0 | 9.31E-01 | 9.10E-01 | -0.14±0.17 | 9.00E-01 | -0.11±0.18 | 9.26E-01 | 0.12±0.47 | 9.99E-01 | -0.06±0.51 | 1.00E+00 |
| 1-linoleoyl-GPG (18:2)* | -0.1±0.12 | 0 | 5.48E-01 | 5.24E-01 | -0.1±0.16 | 0 | 9.23E-01 | 9.10E-01 | -0.12±0.16 | 9.32E-01 | -0.1±0.16 | 9.26E-01 | 0.23±0.58 | 9.99E-01 | -0.04±0.57 | 1.00E+00 |
| oleoylcarnitine (C18:1) | -0.1±0.1 | 0 | 9.34E-01 | 5.24E-01 | -0.1±0.15 | 0 | 5.40E-01 | 9.10E-01 | -0.1±0.16 | 9.32E-01 | -0.06±0.16 | 9.62E-01 | -0.07±0.38 | 9.99E-01 | -0.34±0.43 | 1.00E+00 |
| decanoylcarnitine (C10) | -0.1±0.09 | 0 | 6.85E-01 | 5.24E-01 | -0.1±0.15 | 0 | 3.53E-01 | 9.10E-01 | -0.09±0.16 | 9.32E-01 | -0.05±0.16 | 9.63E-01 | -0.26±0.42 | 9.99E-01 | -0.57±0.53 | 1.00E+00 |
| formiminoglutamate | -0.03±0.03 | 0 | 6.67E-01 | 5.24E-01 | -0.1±0.16 | 0 | 4.70E-01 | 9.11E-01 | -0.03±0.16 | 9.94E-01 | -0.06±0.17 | 9.62E-01 | -0.21±0.42 | 9.99E-01 | -0.48±0.54 | 1.00E+00 |
| X - 12816 | -0.05±0.05 | 0 | 3.52E-01 | 5.24E-01 | -0.09±0.14 | 0 | 4.84E-01 | 9.11E-01 | -0.05±0.15 | 9.80E-01 | -0.06±0.15 | 9.62E-01 | -0.45±0.44 | 9.99E-01 | -0.4±0.47 | 1.00E+00 |
| 1-oleoyl-GPE (18:1) | -0.01±0.01 | 0 | 4.41E-01 | 5.24E-01 | -0.08±0.14 | 25.18 | 2.48E-01 | 9.11E-01 | -0.01±0.15 | 9.94E-01 | -0.02±0.15 | 9.92E-01 | -0.29±0.36 | 9.99E-01 | -0.48±0.37 | 1.00E+00 |
| 1-palmitoyl-2-linoleoyl-GPE (16:0/18:2) | -0.09±0.11 | 0 | 4.87E-01 | 5.24E-01 | -0.08±0.14 | 0 | 4.04E-01 | 9.12E-01 | -0.11±0.14 | 9.22E-01 | -0.12±0.15 | 9.15E-01 | 0.2±0.44 | 9.99E-01 | 0.26±0.43 | 1.00E+00 |
| 2-hydroxyarachidate* | -0.05±0.05 | 0 | 6.79E-01 | 5.24E-01 | -0.08±0.14 | 0 | 6.67E-01 | 9.12E-01 | -0.05±0.14 | 9.78E-01 | -0.1±0.14 | 9.23E-01 | 0.13±0.45 | 9.99E-01 | 0.1±0.46 | 1.00E+00 |
| 4-hydroxyphenylacetate | 0.11±0.1 | 0 | 9.14E-01 | 5.24E-01 | 0.09±0.14 | 0 | 9.50E-01 | 9.12E-01 | 0.1±0.14 | 9.32E-01 | 0.09±0.15 | 9.26E-01 | 0.15±0.4 | 9.99E-01 | 0.06±0.48 | 1.00E+00 |
| sulfate of piperine metabolite C18H21NO3 (1)* | -0.06±0.05 | 0 | 6.57E-01 | 5.24E-01 | -0.09±0.16 | 0 | 6.79E-01 | 9.12E-01 | -0.05±0.16 | 9.80E-01 | -0.07±0.16 | 9.47E-01 | -0.25±0.44 | 9.99E-01 | -0.28±0.47 | 1.00E+00 |
| N-acetylglucosaminylasparagine | 0.1±0.13 | 0 | 4.40E-01 | 5.29E-01 | 0.09±0.14 | 0 | 4.03E-01 | 9.12E-01 | 0.14±0.15 | 8.83E-01 | 0.13±0.15 | 9.15E-01 | -0.17±0.38 | 9.99E-01 | -0.27±0.45 | 1.00E+00 |
| 1-myristoyl-2-palmitoyl-GPC (14:0/16:0) | 0.05±0.05 | 0 | 3.54E-01 | 5.24E-01 | 0.09±0.15 | 0 | 4.53E-01 | 9.12E-01 | 0.05±0.15 | 9.80E-01 | 0.06±0.15 | 9.62E-01 | 0.51±0.5 | 9.99E-01 | 0.49±0.55 | 1.00E+00 |
| X - 18886 | -0.07±0.07 | 0 | 8.16E-01 | 5.24E-01 | -0.1±0.17 | 0 | 4.80E-01 | 9.12E-01 | -0.07±0.18 | 9.80E-01 | -0.06±0.18 | 9.62E-01 | -0.17±0.43 | 9.99E-01 | -0.45±0.52 | 1.00E+00 |
| adipoylcarnitine (C6-DC) | -0.04±0.04 | 0 | 3.40E-01 | 5.24E-01 | -0.09±0.15 | 0 | 3.41E-01 | 9.12E-01 | -0.04±0.16 | 9.86E-01 | -0.04±0.16 | 9.69E-01 | -0.36±0.33 | 9.99E-01 | -0.47±0.43 | 1.00E+00 |
| X - 25810 | 0.04±0.14 | 76.72 | 3.82E-02 | 7.77E-01 | 0.07±0.14 | 78.98 | 2.92E-02 | 9.14E-01 | 0.15±0.14 | 8.46E-01 | 0.15±0.14 | 8.74E-01 | -0.73±0.4 | 9.99E-01 | -1.06±0.54 | 1.00E+00 |
| N-acetyltyrosine | -0.08±0.12 | 53.35 | 1.43E-01 | 5.73E-01 | -0.08±0.15 | 0 | 3.64E-01 | 9.14E-01 | -0.13±0.15 | 8.85E-01 | -0.12±0.15 | 9.15E-01 | 0.59±0.47 | 9.99E-01 | 0.38±0.53 | 1.00E+00 |
| isovalerylcarnitine (C5) | 0.1±0.13 | 0 | 4.35E-01 | 5.33E-01 | 0.09±0.16 | 9.63 | 2.93E-01 | 9.14E-01 | 0.14±0.16 | 8.85E-01 | 0.15±0.17 | 9.15E-01 | -0.15±0.35 | 9.99E-01 | -0.35±0.44 | 1.00E+00 |
| N-delta-acetylornithine | 0.07±0.11 | 23.76 | 2.52E-01 | 5.52E-01 | 0.09±0.15 | 0 | 3.82E-01 | 9.14E-01 | 0.11±0.15 | 9.32E-01 | 0.14±0.16 | 9.15E-01 | -0.31±0.35 | 9.99E-01 | -0.26±0.43 | 1.00E+00 |
| 2-aminoheptanoate | 0.11±0.14 | 0 | 5.12E-01 | 5.24E-01 | 0.09±0.15 | 0 | 4.01E-01 | 9.14E-01 | 0.15±0.16 | 8.83E-01 | 0.13±0.16 | 9.15E-01 | -0.13±0.39 | 9.99E-01 | -0.32±0.51 | 1.00E+00 |
| 1-palmitoyl-GPG (16:0)* | 0.09±0.12 | 0 | 3.22E-01 | 5.34E-01 | 0.08±0.15 | 21.07 | 2.60E-01 | 9.14E-01 | 0.12±0.16 | 9.32E-01 | 0.15±0.17 | 9.15E-01 | -0.31±0.42 | 9.99E-01 | -0.35±0.41 | 1.00E+00 |
| 2-acetamidophenol sulfate | 0.09±0.1 | 0 | 6.04E-01 | 5.24E-01 | 0.08±0.15 | 0 | 4.33E-01 | 9.14E-01 | 0.1±0.16 | 9.32E-01 | 0.12±0.16 | 9.15E-01 | -0.11±0.4 | 9.99E-01 | -0.28±0.48 | 1.00E+00 |
| arachidoylcarnitine (C20)* | -0.11±0.14 | 18.3 | 2.69E-01 | 5.33E-01 | -0.09±0.15 | 0 | 3.54E-01 | 9.14E-01 | -0.14±0.15 | 8.83E-01 | -0.12±0.16 | 9.15E-01 | 0.52±0.58 | 9.99E-01 | 0.54±0.69 | 1.00E+00 |
| aspartate | -0.06±0.07 | 4.92 | 3.05E-01 | 5.24E-01 | -0.09±0.17 | 0 | 3.40E-01 | 9.14E-01 | -0.07±0.16 | 9.74E-01 | -0.13±0.17 | 9.18E-01 | 0.63±0.67 | 9.99E-01 | 0.57±0.71 | 1.00E+00 |
| X - 25936 | 0.11±0.12 | 0 | 7.48E-01 | 5.24E-01 | 0.08±0.14 | 0 | 5.46E-01 | 9.14E-01 | 0.13±0.15 | 8.92E-01 | 0.11±0.15 | 9.18E-01 | -0.01±0.4 | 9.99E-01 | -0.2±0.49 | 1.00E+00 |
| dehydroepiandrosterone sulfate (DHEA-S) | -0.08±0.08 | 0 | 5.70E-01 | 5.24E-01 | -0.08±0.16 | 0 | 4.93E-01 | 9.14E-01 | -0.08±0.16 | 9.58E-01 | -0.11±0.16 | 9.23E-01 | 0.2±0.5 | 9.99E-01 | 0.26±0.53 | 1.00E+00 |
| 1,7-dimethylurate | 0.07±0.07 | 0 | 8.10E-01 | 5.24E-01 | 0.09±0.16 | 0 | 6.20E-01 | 9.14E-01 | 0.07±0.16 | 9.71E-01 | 0.11±0.17 | 9.23E-01 | -0.07±0.57 | 9.99E-01 | -0.23±0.66 | 1.00E+00 |
| ectoine | 0.09±0.09 | 0 | 8.19E-01 | 5.24E-01 | 0.08±0.15 | 0 | 6.06E-01 | 9.14E-01 | 0.1±0.16 | 9.32E-01 | 0.11±0.16 | 9.23E-01 | 0±0.42 | 9.99E-01 | -0.15±0.47 | 1.00E+00 |
| andro steroid monosulfate C19H28O6S (1)* | -0.07±0.08 | 0 | 5.12E-01 | 5.24E-01 | -0.09±0.16 | 0 | 6.49E-01 | 9.14E-01 | -0.08±0.17 | 9.59E-01 | -0.11±0.17 | 9.23E-01 | 0.22±0.45 | 9.99E-01 | 0.13±0.51 | 1.00E+00 |
| 4-hydroxyphenylacetylglutamine | 0.1±0.1 | 0 | 9.76E-01 | 5.24E-01 | 0.08±0.14 | 0 | 7.62E-01 | 9.14E-01 | 0.1±0.14 | 9.32E-01 | 0.1±0.15 | 9.23E-01 | 0.11±0.36 | 9.99E-01 | -0.04±0.43 | 1.00E+00 |
| sphingomyelin (d18:2/24:2)* | 0.09±0.1 | 0 | 6.08E-01 | 5.24E-01 | 0.09±0.16 | 0 | 6.17E-01 | 9.14E-01 | 0.1±0.17 | 9.32E-01 | 0.11±0.17 | 9.23E-01 | -0.09±0.36 | 9.99E-01 | -0.17±0.54 | 1.00E+00 |
| N6,N6-dimethyllysine | -0.09±0.1 | 0 | 5.52E-01 | 5.24E-01 | -0.08±0.15 | 0 | 5.55E-01 | 9.14E-01 | -0.1±0.15 | 9.32E-01 | -0.1±0.15 | 9.23E-01 | 0.22±0.53 | 9.99E-01 | 0.31±0.68 | 1.00E+00 |
| 3-hydroxy-3-methylglutarate | -0.08±0.11 | 8.58 | 2.96E-01 | 5.37E-01 | -0.08±0.15 | 0 | 5.45E-01 | 9.14E-01 | -0.11±0.16 | 9.32E-01 | -0.11±0.16 | 9.23E-01 | 0.31±0.39 | 9.99E-01 | 0.19±0.48 | 1.00E+00 |
| arginine | 0.12±0.13 | 0 | 7.96E-01 | 5.24E-01 | 0.09±0.15 | 0 | 6.62E-01 | 9.14E-01 | 0.13±0.16 | 8.99E-01 | 0.11±0.16 | 9.23E-01 | 0.02±0.4 | 9.99E-01 | -0.11±0.47 | 1.00E+00 |
| 7-methylxanthine | 0.1±0.1 | 0 | 6.61E-01 | 5.24E-01 | 0.08±0.16 | 0 | 6.14E-01 | 9.14E-01 | 0.11±0.16 | 9.32E-01 | 0.11±0.17 | 9.25E-01 | -0.1±0.47 | 9.99E-01 | -0.16±0.51 | 1.00E+00 |
| 3-methylxanthine | 0.08±0.09 | 0 | 7.46E-01 | 5.24E-01 | 0.08±0.16 | 0 | 6.26E-01 | 9.14E-01 | 0.09±0.16 | 9.55E-01 | 0.11±0.17 | 9.25E-01 | -0.07±0.48 | 9.99E-01 | -0.16±0.52 | 1.00E+00 |
| 5-(galactosylhydroxy)-L-lysine | 0.1±0.11 | 0 | 6.89E-01 | 5.24E-01 | 0.08±0.15 | 0 | 6.70E-01 | 9.14E-01 | 0.11±0.15 | 9.32E-01 | 0.1±0.16 | 9.25E-01 | -0.06±0.41 | 9.99E-01 | -0.12±0.5 | 1.00E+00 |
| ascorbic acid 3-sulfate* | -0.1±0.11 | 0 | 6.48E-01 | 5.24E-01 | -0.08±0.14 | 0 | 7.64E-01 | 9.14E-01 | -0.11±0.15 | 9.32E-01 | -0.1±0.15 | 9.26E-01 | 0.08±0.41 | 9.99E-01 | 0.05±0.47 | 1.00E+00 |
| 5-methyluridine (ribothymidine) | -0.1±0.11 | 0 | 5.88E-01 | 5.24E-01 | -0.09±0.16 | 0 | 8.07E-01 | 9.14E-01 | -0.12±0.16 | 9.32E-01 | -0.1±0.17 | 9.26E-01 | 0.15±0.48 | 9.99E-01 | 0.02±0.49 | 1.00E+00 |
| 2-hydroxybutyrate/2-hydroxyisobutyrate | -0.09±0.1 | 0 | 5.19E-01 | 5.24E-01 | -0.08±0.15 | 0 | 7.18E-01 | 9.14E-01 | -0.1±0.15 | 9.32E-01 | -0.1±0.16 | 9.26E-01 | 0.19±0.44 | 9.99E-01 | 0.08±0.48 | 1.00E+00 |
| 3-bromo-5-chloro-2,6-dihydroxybenzoic acid* | -0.08±0.09 | 0 | 5.81E-01 | 5.24E-01 | -0.07±0.13 | 0 | 9.45E-01 | 9.14E-01 | -0.09±0.13 | 9.32E-01 | -0.08±0.13 | 9.27E-01 | 0.16±0.44 | 9.99E-01 | -0.05±0.44 | 1.00E+00 |
| vanillylmandelate (VMA) | -0.1±0.11 | 0 | 5.07E-01 | 5.24E-01 | -0.08±0.16 | 0 | 7.47E-01 | 9.14E-01 | -0.12±0.16 | 9.32E-01 | -0.1±0.17 | 9.27E-01 | 0.17±0.41 | 9.99E-01 | 0.06±0.46 | 1.00E+00 |
| methyl indole-3-acetate | -0.07±0.06 | 0 | 9.22E-01 | 5.24E-01 | -0.08±0.14 | 0 | 8.89E-01 | 9.14E-01 | -0.07±0.14 | 9.72E-01 | -0.08±0.14 | 9.34E-01 | -0.11±0.4 | 9.99E-01 | -0.02±0.44 | 1.00E+00 |
| lysine | 0.07±0.07 | 0 | 9.59E-01 | 5.24E-01 | 0.07±0.14 | 0 | 8.14E-01 | 9.14E-01 | 0.07±0.14 | 9.66E-01 | 0.08±0.14 | 9.35E-01 | 0.05±0.4 | 9.99E-01 | -0.05±0.53 | 1.00E+00 |
| N-acetylglucosamine/N-acetylgalactosamine | 0.12±0.12 | 0 | 8.44E-01 | 5.24E-01 | 0.09±0.16 | 0 | 9.74E-01 | 9.14E-01 | 0.12±0.16 | 9.32E-01 | 0.09±0.17 | 9.39E-01 | 0.03±0.44 | 9.99E-01 | 0.11±0.61 | 1.00E+00 |
| N-acetyl-isoputreanine | 0.09±0.09 | 0 | 8.04E-01 | 5.24E-01 | 0.09±0.15 | 0 | 9.74E-01 | 9.14E-01 | 0.09±0.15 | 9.32E-01 | 0.08±0.16 | 9.39E-01 | 0.22±0.5 | 9.99E-01 | 0.1±0.6 | 1.00E+00 |
| sphinganine-1-phosphate | 0.09±0.08 | 0 | 9.63E-01 | 5.24E-01 | 0.08±0.14 | 0 | 9.04E-01 | 9.14E-01 | 0.09±0.15 | 9.32E-01 | 0.07±0.15 | 9.44E-01 | 0.11±0.45 | 9.99E-01 | 0.14±0.53 | 1.00E+00 |
| N-carbamoylalanine | 0.08±0.08 | 0 | 9.06E-01 | 5.24E-01 | 0.09±0.15 | 0 | 8.74E-01 | 9.14E-01 | 0.09±0.15 | 9.48E-01 | 0.08±0.16 | 9.44E-01 | 0.03±0.45 | 9.99E-01 | 0.17±0.55 | 1.00E+00 |
| 3,5-dichloro-2,6-dihydroxybenzoic acid | -0.07±0.07 | 0 | 7.31E-01 | 5.24E-01 | -0.07±0.12 | 0 | 9.55E-01 | 9.14E-01 | -0.08±0.13 | 9.32E-01 | -0.07±0.13 | 9.44E-01 | 0.06±0.38 | 9.99E-01 | -0.09±0.42 | 1.00E+00 |
| 4-acetylphenol sulfate | -0.08±0.08 | 0 | 9.28E-01 | 5.24E-01 | -0.09±0.16 | 0 | 8.18E-01 | 9.14E-01 | -0.08±0.16 | 9.66E-01 | -0.07±0.17 | 9.45E-01 | -0.12±0.49 | 9.99E-01 | -0.2±0.5 | 1.00E+00 |
| N6-carbamoylthreonyladenosine | -0.08±0.08 | 0 | 7.63E-01 | 5.24E-01 | -0.09±0.15 | 0 | 8.69E-01 | 9.14E-01 | -0.08±0.16 | 9.58E-01 | -0.08±0.16 | 9.45E-01 | 0.04±0.4 | 9.99E-01 | -0.16±0.47 | 1.00E+00 |
| beta-citrylglutamate | -0.07±0.07 | 0 | 7.58E-01 | 5.24E-01 | -0.07±0.14 | 0 | 8.75E-01 | 9.14E-01 | -0.07±0.14 | 9.58E-01 | -0.07±0.15 | 9.45E-01 | 0.06±0.42 | 9.99E-01 | -0.14±0.44 | 1.00E+00 |
| octadecadienedioate (C18:2-DC)* | -0.1±0.09 | 0 | 9.80E-01 | 5.24E-01 | -0.08±0.14 | 0 | 8.25E-01 | 9.14E-01 | -0.1±0.14 | 9.32E-01 | -0.07±0.14 | 9.45E-01 | -0.09±0.45 | 9.99E-01 | -0.19±0.52 | 1.00E+00 |
| X - 12193 | -0.05±0.05 | 0 | 9.77E-01 | 5.24E-01 | -0.07±0.14 | 0 | 9.33E-01 | 9.14E-01 | -0.05±0.15 | 9.80E-01 | -0.07±0.15 | 9.45E-01 | -0.06±0.43 | 9.99E-01 | -0.11±0.48 | 1.00E+00 |
| butyrate/isobutyrate (4:0) | 0.06±0.06 | 0 | 5.13E-01 | 5.24E-01 | 0.09±0.15 | 0 | 5.64E-01 | 9.14E-01 | 0.06±0.15 | 9.80E-01 | 0.07±0.16 | 9.50E-01 | 0.42±0.55 | 9.99E-01 | 0.46±0.66 | 1.00E+00 |
| N-acetylproline | -0.04±0.04 | 0 | 7.09E-01 | 5.24E-01 | -0.07±0.14 | 0 | 8.19E-01 | 9.14E-01 | -0.04±0.15 | 9.80E-01 | -0.06±0.15 | 9.58E-01 | -0.18±0.37 | 9.99E-01 | -0.16±0.4 | 1.00E+00 |
| leucine | -0.05±0.05 | 0 | 8.28E-01 | 5.24E-01 | -0.09±0.17 | 0 | 8.38E-01 | 9.14E-01 | -0.05±0.17 | 9.80E-01 | -0.07±0.18 | 9.58E-01 | 0.04±0.43 | 9.99E-01 | -0.17±0.42 | 1.00E+00 |
| X - 25420 | -0.04±0.04 | 0 | 6.71E-01 | 5.24E-01 | -0.1±0.18 | 0 | 6.81E-01 | 9.14E-01 | -0.04±0.18 | 9.87E-01 | -0.08±0.19 | 9.61E-01 | -0.22±0.42 | 9.99E-01 | -0.32±0.57 | 1.00E+00 |
| glycocholenate sulfate* | -0.03±0.03 | 0 | 5.70E-01 | 5.24E-01 | -0.09±0.15 | 0 | 5.55E-01 | 9.14E-01 | -0.03±0.16 | 9.94E-01 | -0.05±0.16 | 9.65E-01 | -0.21±0.32 | 9.99E-01 | -0.28±0.36 | 1.00E+00 |
| 1-stearoyl-2-docosahexaenoyl-GPE (18:0/22:6)* | 0.05±0.05 | 0 | 6.35E-01 | 5.24E-01 | 0.08±0.15 | 0 | 5.46E-01 | 9.14E-01 | 0.05±0.15 | 9.80E-01 | 0.05±0.16 | 9.65E-01 | 0.26±0.45 | 9.99E-01 | 0.33±0.44 | 1.00E+00 |
| 2'-O-methylcytidine | -0.06±0.06 | 0 | 6.21E-01 | 5.24E-01 | -0.07±0.14 | 0 | 5.41E-01 | 9.14E-01 | -0.06±0.15 | 9.80E-01 | -0.04±0.15 | 9.67E-01 | -0.24±0.37 | 9.99E-01 | -0.31±0.42 | 1.00E+00 |
| N-acetylmethionine | 0.03±0.05 | 73.79 | 5.08E-02 | 5.61E-01 | -0.09±0.15 | 66.89 | 8.22E-02 | 9.14E-01 | 0.05±0.16 | 9.80E-01 | 0.04±0.17 | 9.67E-01 | -0.48±0.26 | 9.99E-01 | -0.6±0.33 | 1.00E+00 |
| piperine | -0.04±0.04 | 0 | 5.37E-01 | 5.24E-01 | -0.09±0.16 | 11.24 | 2.88E-01 | 9.14E-01 | -0.04±0.16 | 9.80E-01 | -0.04±0.17 | 9.69E-01 | -0.31±0.43 | 9.99E-01 | -0.65±0.55 | 1.00E+00 |
| X - 17367 | 0.03±0.03 | 37.55 | 2.06E-01 | 5.24E-01 | 0.09±0.16 | 34.78 | 2.16E-01 | 9.14E-01 | 0.03±0.16 | 9.94E-01 | 0.04±0.16 | 9.69E-01 | 0.78±0.59 | 9.99E-01 | 0.79±0.59 | 1.00E+00 |
| 5alpha-pregnan-3beta,20alpha-diol monosulfate (2) | 0.03±0.05 | 70.85 | 6.40E-02 | 5.37E-01 | -0.07±0.14 | 73.9 | 5.03E-02 | 9.14E-01 | 0.05±0.15 | 9.80E-01 | 0.03±0.15 | 9.69E-01 | -0.55±0.32 | 9.99E-01 | -0.77±0.38 | 1.00E+00 |
| 1-linolenoyl-GPC (18:3)* | -0.06±0.05 | 0 | 7.60E-01 | 5.24E-01 | -0.08±0.15 | 0 | 4.27E-01 | 9.14E-01 | -0.05±0.15 | 9.80E-01 | -0.03±0.16 | 9.79E-01 | -0.18±0.42 | 9.99E-01 | -0.38±0.41 | 1.00E+00 |
| C-glycosyltryptophan | 0.03±0.03 | 57.06 | 1.27E-01 | 5.24E-01 | -0.08±0.14 | 55.57 | 1.34E-01 | 9.14E-01 | 0.03±0.16 | 9.90E-01 | 0.03±0.16 | 9.84E-01 | -0.4±0.28 | 9.99E-01 | -0.51±0.32 | 1.00E+00 |
| 1-palmitoleoylglycerol (16:1)* | 0.03±0.02 | 74.18 | 4.91E-02 | 5.24E-01 | 0.08±0.15 | 58.27 | 1.22E-01 | 9.14E-01 | 0.02±0.15 | 9.94E-01 | 0.03±0.15 | 9.88E-01 | 1.14±0.57 | 9.99E-01 | 1.06±0.65 | 1.00E+00 |
| 3-(3-hydroxyphenyl)propionate | 0.01±0.01 | 76.83 | 3.77E-02 | 5.24E-01 | 0.09±0.16 | 80.43 | 2.38E-02 | 9.14E-01 | 0.01±0.16 | 9.94E-01 | 0.01±0.16 | 9.92E-01 | 1.44±0.69 | 9.99E-01 | 1.8±0.77 | 1.00E+00 |
| succinylcarnitine (C4-DC) | 0±0 | 0 | 4.35E-01 | 5.24E-01 | -0.09±0.15 | 20.42 | 2.62E-01 | 9.14E-01 | 0±0.15 | 9.99E-01 | -0.02±0.16 | 9.92E-01 | -0.27±0.35 | 9.99E-01 | -0.55±0.45 | 1.00E+00 |
| guaiacol sulfate | -0.02±0.02 | 59.34 | 1.17E-01 | 5.24E-01 | -0.08±0.15 | 15.13 | 2.78E-01 | 9.14E-01 | -0.02±0.16 | 9.94E-01 | -0.02±0.16 | 9.92E-01 | -0.68±0.42 | 9.99E-01 | -0.52±0.43 | 1.00E+00 |
| glycoursodeoxycholic acid sulfate (1) | 0.06±0.05 | 52.5 | 1.47E-01 | 5.24E-01 | 0.09±0.16 | 47.68 | 1.67E-01 | 9.14E-01 | 0.05±0.16 | 9.80E-01 | 0.03±0.17 | 9.92E-01 | 0.8±0.51 | 9.99E-01 | 0.82±0.55 | 1.00E+00 |
| cytosine | 0.02±0.02 | 57.15 | 1.27E-01 | 5.24E-01 | 0.07±0.14 | 57.49 | 1.25E-01 | 9.14E-01 | 0.02±0.14 | 9.94E-01 | 0.01±0.15 | 9.92E-01 | 0.74±0.47 | 9.99E-01 | 0.84±0.52 | 1.00E+00 |
| X - 11852 | -0.04±0.03 | 75.3 | 4.42E-02 | 5.24E-01 | -0.08±0.14 | 79.12 | 2.86E-02 | 9.14E-01 | -0.03±0.15 | 9.86E-01 | -0.01±0.15 | 9.92E-01 | -1.25±0.6 | 9.99E-01 | -1.6±0.71 | 1.00E+00 |
| X - 12906 | -0.02±0.02 | 16.71 | 2.73E-01 | 5.24E-01 | -0.07±0.14 | 8.99 | 2.95E-01 | 9.14E-01 | -0.02±0.15 | 9.94E-01 | -0.02±0.15 | 9.92E-01 | -0.39±0.34 | 9.99E-01 | -0.46±0.4 | 1.00E+00 |
| X - 24811 | 0.05±0.04 | 0 | 3.20E-01 | 5.24E-01 | 0.09±0.17 | 32.19 | 2.25E-01 | 9.14E-01 | 0.04±0.18 | 9.80E-01 | 0.01±0.18 | 9.94E-01 | 0.53±0.49 | 9.99E-01 | 0.64±0.48 | 1.00E+00 |
| 2-pyrrolidinone | -0.02±0.02 | 62.56 | 1.02E-01 | 5.24E-01 | -0.08±0.13 | 22.51 | 2.56E-01 | 9.14E-01 | -0.02±0.15 | 9.94E-01 | 0±0.15 | 9.99E-01 | -0.4±0.23 | 9.99E-01 | -0.35±0.28 | 1.00E+00 |
| X - 17690 | -0.04±0.04 | 33.65 | 2.20E-01 | 5.24E-01 | -0.07±0.14 | 0 | 3.69E-01 | 9.14E-01 | -0.04±0.15 | 9.80E-01 | -0.04±0.15 | 9.67E-01 | -0.65±0.49 | 9.99E-01 | -0.55±0.55 | 1.00E+00 |
| N-oleoylserine | 0.08±0.07 | 0 | 6.19E-01 | 5.24E-01 | 0.08±0.15 | 0 | 8.87E-01 | 9.16E-01 | 0.07±0.15 | 9.72E-01 | 0.07±0.16 | 9.45E-01 | 0.36±0.57 | 9.99E-01 | 0.16±0.58 | 1.00E+00 |
| 1-(1-enyl-stearoyl)-2-oleoyl-GPE (P-18:0/18:1) | -0.05±0.04 | 0 | 9.91E-01 | 5.24E-01 | -0.07±0.14 | 0 | 7.00E-01 | 9.16E-01 | -0.05±0.15 | 9.80E-01 | -0.05±0.15 | 9.62E-01 | -0.04±0.36 | 9.99E-01 | -0.23±0.43 | 1.00E+00 |
| salicylate | -0.01±0.01 | 79.35 | 2.78E-02 | 5.24E-01 | -0.07±0.15 | 72.43 | 5.68E-02 | 9.16E-01 | -0.01±0.15 | 9.94E-01 | -0.04±0.15 | 9.67E-01 | -2.69±1.22 | 9.99E-01 | -2.49±1.28 | 1.00E+00 |
| X - 17612 | 0.03±0.21 | 56.8 | 1.28E-01 | 8.85E-01 | 0.09±0.18 | 66.34 | 8.48E-02 | 9.16E-01 | 0.26±0.2 | 7.70E-01 | 0.24±0.2 | 8.16E-01 | -0.42±0.36 | 9.99E-01 | -0.51±0.39 | 1.00E+00 |
| X - 11381 | 0.07±0.07 | 0 | 9.53E-01 | 5.24E-01 | 0.08±0.15 | 0 | 8.64E-01 | 9.16E-01 | 0.07±0.16 | 9.70E-01 | 0.08±0.16 | 9.43E-01 | 0.05±0.44 | 9.99E-01 | 0±0.49 | 1.00E+00 |
| X - 11372 | -0.08±0.09 | 0 | 5.58E-01 | 5.24E-01 | -0.07±0.14 | 0 | 9.96E-01 | 9.16E-01 | -0.09±0.14 | 9.32E-01 | -0.07±0.14 | 9.45E-01 | 0.19±0.47 | 9.99E-01 | -0.07±0.51 | 1.00E+00 |
| X - 11849 | 0.01±0.01 | 0 | 4.29E-01 | 5.24E-01 | 0.07±0.14 | 0 | 6.49E-01 | 9.16E-01 | 0.01±0.15 | 9.94E-01 | 0.05±0.15 | 9.62E-01 | 0.4±0.49 | 9.99E-01 | 0.29±0.51 | 1.00E+00 |
| carnitine | 0.09±0.15 | 0 | 3.52E-01 | 5.95E-01 | 0.07±0.14 | 52.38 | 1.47E-01 | 9.19E-01 | 0.18±0.16 | 8.11E-01 | 0.17±0.16 | 8.61E-01 | -0.12±0.27 | 9.99E-01 | -0.37±0.34 | 1.00E+00 |
| 1-palmitoyl-2-oleoyl-GPI (16:0/18:1)* | 0.09±0.09 | 0 | 9.05E-01 | 5.24E-01 | 0.07±0.15 | 0 | 8.69E-01 | 9.19E-01 | 0.1±0.15 | 9.32E-01 | 0.08±0.16 | 9.43E-01 | 0.05±0.4 | 9.99E-01 | 0±0.46 | 1.00E+00 |
| 11beta-hydroxyetiocholanolone glucuronide* | 0.05±0.05 | 0 | 5.73E-01 | 5.24E-01 | 0.07±0.15 | 0 | 6.56E-01 | 9.19E-01 | 0.05±0.15 | 9.80E-01 | 0.05±0.16 | 9.65E-01 | 0.3±0.45 | 9.99E-01 | 0.27±0.47 | 1.00E+00 |
| 3-hydroxypyridine sulfate | 0.08±0.07 | 0 | 7.17E-01 | 5.24E-01 | 0.08±0.17 | 0 | 5.35E-01 | 9.19E-01 | 0.07±0.17 | 9.74E-01 | 0.05±0.18 | 9.67E-01 | 0.27±0.55 | 9.99E-01 | 0.38±0.51 | 1.00E+00 |
| gamma-glutamylleucine | -0.02±0.02 | 0 | 7.05E-01 | 5.24E-01 | -0.07±0.15 | 0 | 4.32E-01 | 9.19E-01 | -0.02±0.16 | 9.94E-01 | -0.02±0.16 | 9.92E-01 | -0.16±0.36 | 9.99E-01 | -0.35±0.38 | 1.00E+00 |
| X - 19141 | -0.08±0.09 | 0 | 7.23E-01 | 5.24E-01 | -0.07±0.14 | 0 | 7.38E-01 | 9.21E-01 | -0.09±0.15 | 9.32E-01 | -0.08±0.15 | 9.39E-01 | 0.07±0.44 | 9.99E-01 | 0.09±0.5 | 1.00E+00 |
| 3-methylhistidine | 0.07±0.12 | 61.93 | 1.05E-01 | 5.89E-01 | 0.07±0.15 | 54.07 | 1.40E-01 | 9.23E-01 | 0.12±0.15 | 9.22E-01 | 0.14±0.15 | 9.15E-01 | -0.64±0.45 | 9.99E-01 | -0.59±0.47 | 1.00E+00 |
| 2-methylbutyrylcarnitine (C5) | 0.09±0.12 | 0 | 4.49E-01 | 5.27E-01 | 0.07±0.15 | 25.03 | 2.48E-01 | 9.23E-01 | 0.12±0.15 | 9.23E-01 | 0.13±0.16 | 9.15E-01 | -0.17±0.36 | 9.99E-01 | -0.4±0.44 | 1.00E+00 |
| ceramide (d16:1/24:1, d18:1/22:1)* | 0.08±0.08 | 0 | 9.20E-01 | 5.24E-01 | 0.07±0.15 | 0 | 7.81E-01 | 9.23E-01 | 0.08±0.15 | 9.58E-01 | 0.09±0.16 | 9.39E-01 | 0.04±0.39 | 9.99E-01 | -0.05±0.46 | 1.00E+00 |
| 2,3-dihydroxy-5-methylthio-4-pentenoate (DMTPA)* | 0±0 | 0 | 5.00E-01 | 5.24E-01 | -0.07±0.14 | 0 | 3.24E-01 | 9.23E-01 | 0±0.15 | 9.99E-01 | 0±0.16 | 9.99E-01 | -0.2±0.3 | 9.99E-01 | -0.37±0.34 | 1.00E+00 |
| 3-hydroxy-2-methylpyridine sulfate | 0.08±0.08 | 0 | 8.65E-01 | 5.24E-01 | 0.07±0.15 | 0 | 6.91E-01 | 9.29E-01 | 0.08±0.16 | 9.58E-01 | 0.05±0.16 | 9.65E-01 | 0.16±0.48 | 9.99E-01 | 0.24±0.46 | 1.00E+00 |
| 1-palmitoleoyl-2-linolenoyl-GPC (16:1/18:3)* | -0.08±0.09 | 0 | 4.76E-01 | 5.24E-01 | -0.08±0.16 | 0 | 5.63E-01 | 9.30E-01 | -0.1±0.17 | 9.32E-01 | -0.11±0.17 | 9.26E-01 | 0.27±0.5 | 9.99E-01 | 0.21±0.51 | 1.00E+00 |
| 1-(1-enyl-stearoyl)-GPE (P-18:0)* | 0.08±0.12 | 0 | 3.55E-01 | 5.39E-01 | 0.07±0.15 | 36.52 | 2.09E-01 | 9.30E-01 | 0.12±0.15 | 9.17E-01 | 0.14±0.16 | 9.15E-01 | -0.2±0.32 | 9.99E-01 | -0.38±0.39 | 1.00E+00 |
| sphingomyelin (d18:2/14:0, d18:1/14:1)* | 0.09±0.09 | 0 | 9.80E-01 | 5.24E-01 | 0.09±0.19 | 0 | 7.64E-01 | 9.30E-01 | 0.09±0.2 | 9.70E-01 | 0.11±0.21 | 9.39E-01 | 0.08±0.51 | 9.99E-01 | -0.08±0.59 | 1.00E+00 |
| 3-methyl catechol sulfate (2) | 0.07±0.07 | 0 | 9.96E-01 | 5.24E-01 | 0.07±0.16 | 0 | 7.10E-01 | 9.30E-01 | 0.07±0.16 | 9.74E-01 | 0.05±0.17 | 9.65E-01 | 0.07±0.49 | 9.99E-01 | 0.23±0.46 | 1.00E+00 |
| 1-palmitoyl-GPI (16:0) | 0.06±0.06 | 0 | 8.51E-01 | 5.24E-01 | 0.07±0.16 | 0 | 9.88E-01 | 9.31E-01 | 0.06±0.17 | 9.80E-01 | 0.08±0.17 | 9.45E-01 | 0.15±0.48 | 9.99E-01 | 0.07±0.53 | 1.00E+00 |
| trans-urocanate | 0.03±0.03 | 57.7 | 1.24E-01 | 5.24E-01 | -0.07±0.15 | 49.9 | 1.58E-01 | 9.31E-01 | 0.03±0.16 | 9.94E-01 | 0±0.16 | 9.99E-01 | -0.53±0.36 | 9.99E-01 | -0.74±0.49 | 1.00E+00 |
| O-sulfo-L-tyrosine | -0.03±0.16 | 72.29 | 5.75E-02 | 8.60E-01 | 0.06±0.14 | 81.44 | 2.03E-02 | 9.32E-01 | 0.2±0.15 | 7.66E-01 | 0.21±0.16 | 7.84E-01 | -0.44±0.27 | 9.99E-01 | -0.64±0.33 | 1.00E+00 |
| X - 25422 | 0±0.18 | 61.34 | 1.08E-01 | 9.95E-01 | 0.06±0.16 | 74.99 | 4.56E-02 | 9.32E-01 | 0.23±0.17 | 7.66E-01 | 0.2±0.17 | 8.32E-01 | -0.36±0.29 | 9.99E-01 | -0.65±0.39 | 1.00E+00 |
| N-carbamoylvaline | 0.05±0.13 | 61.36 | 1.08E-01 | 7.09E-01 | 0.05±0.15 | 61.06 | 1.09E-01 | 9.32E-01 | 0.15±0.16 | 8.85E-01 | 0.15±0.16 | 9.08E-01 | -0.46±0.34 | 9.99E-01 | -0.53±0.4 | 1.00E+00 |
| serine | 0.1±0.16 | 0 | 3.81E-01 | 5.74E-01 | 0.06±0.14 | 44.12 | 1.81E-01 | 9.32E-01 | 0.19±0.15 | 7.92E-01 | 0.15±0.16 | 9.08E-01 | -0.12±0.3 | 9.99E-01 | -0.35±0.34 | 1.00E+00 |
| isobutyrylcarnitine (C4) | 0.08±0.1 | 0 | 3.64E-01 | 5.33E-01 | 0.06±0.14 | 27.22 | 2.41E-01 | 9.32E-01 | 0.11±0.15 | 9.32E-01 | 0.11±0.15 | 9.15E-01 | -0.21±0.33 | 9.99E-01 | -0.45±0.46 | 1.00E+00 |
| gamma-glutamylglycine | 0.08±0.15 | 3.76 | 3.08E-01 | 6.24E-01 | 0.07±0.17 | 19.91 | 2.64E-01 | 9.32E-01 | 0.18±0.18 | 8.55E-01 | 0.17±0.19 | 9.15E-01 | -0.16±0.29 | 9.99E-01 | -0.29±0.37 | 1.00E+00 |
| tetradecanedioate (C14-DC) | -0.05±0.14 | 61.47 | 1.07E-01 | 7.19E-01 | -0.06±0.15 | 34.73 | 2.16E-01 | 9.32E-01 | -0.15±0.16 | 8.83E-01 | -0.13±0.16 | 9.15E-01 | 0.45±0.34 | 9.99E-01 | 0.39±0.39 | 1.00E+00 |
| pyrraline | 0.07±0.1 | 42.93 | 1.86E-01 | 5.59E-01 | 0.05±0.14 | 66.43 | 8.44E-02 | 9.32E-01 | 0.11±0.15 | 9.32E-01 | 0.13±0.15 | 9.15E-01 | -0.41±0.37 | 9.99E-01 | -0.71±0.46 | 1.00E+00 |
| methionine sulfone | 0.07±0.11 | 32.89 | 2.22E-01 | 5.75E-01 | 0.07±0.15 | 20.15 | 2.63E-01 | 9.32E-01 | 0.12±0.15 | 9.32E-01 | 0.13±0.16 | 9.15E-01 | -0.31±0.33 | 9.99E-01 | -0.33±0.38 | 1.00E+00 |
| 2-aminobutyrate | 0.09±0.1 | 0 | 5.44E-01 | 5.24E-01 | 0.07±0.15 | 22.61 | 2.56E-01 | 9.32E-01 | 0.11±0.15 | 9.32E-01 | 0.13±0.16 | 9.15E-01 | -0.13±0.38 | 9.99E-01 | -0.4±0.44 | 1.00E+00 |
| glycine | 0.09±0.16 | 0 | 3.22E-01 | 6.10E-01 | 0.07±0.18 | 32.36 | 2.24E-01 | 9.32E-01 | 0.19±0.19 | 8.57E-01 | 0.18±0.2 | 9.15E-01 | -0.17±0.31 | 9.99E-01 | -0.35±0.39 | 1.00E+00 |
| carotene diol (2) | -0.11±0.14 | 6.46 | 3.01E-01 | 5.33E-01 | -0.07±0.15 | 47.18 | 1.69E-01 | 9.32E-01 | -0.15±0.14 | 8.48E-01 | -0.11±0.16 | 9.18E-01 | 0.47±0.58 | 9.99E-01 | 0.86±0.69 | 1.00E+00 |
| N-acetylneuraminate | 0.09±0.12 | 0 | 4.31E-01 | 5.38E-01 | 0.06±0.14 | 0 | 3.88E-01 | 9.32E-01 | 0.13±0.14 | 8.83E-01 | 0.11±0.15 | 9.18E-01 | -0.11±0.28 | 9.99E-01 | -0.25±0.39 | 1.00E+00 |
| gamma-glutamylisoleucine* | 0.1±0.11 | 0 | 6.33E-01 | 5.24E-01 | 0.07±0.15 | 0 | 3.82E-01 | 9.32E-01 | 0.12±0.15 | 9.22E-01 | 0.12±0.16 | 9.19E-01 | -0.06±0.36 | 9.99E-01 | -0.28±0.43 | 1.00E+00 |
| X - 21796 | -0.05±0.14 | 73.09 | 5.39E-02 | 7.47E-01 | -0.06±0.16 | 20.13 | 2.63E-01 | 9.32E-01 | -0.14±0.16 | 8.85E-01 | -0.12±0.16 | 9.19E-01 | 0.65±0.39 | 9.99E-01 | 0.5±0.53 | 1.00E+00 |
| 1-oleoyl-GPC (18:1) | 0.07±0.07 | 0 | 8.60E-01 | 5.24E-01 | 0.07±0.15 | 0 | 4.65E-01 | 9.32E-01 | 0.08±0.15 | 9.58E-01 | 0.11±0.16 | 9.23E-01 | 0±0.43 | 1.00E+00 | -0.22±0.43 | 1.00E+00 |
| gamma-glutamylcitrulline* | 0.03±0.09 | 70.96 | 6.35E-02 | 7.87E-01 | -0.06±0.14 | 76.42 | 3.95E-02 | 9.32E-01 | 0.1±0.16 | 9.32E-01 | 0.11±0.16 | 9.23E-01 | -0.36±0.22 | 9.99E-01 | -0.56±0.28 | 1.00E+00 |
| X - 24422 | 0.09±0.12 | 0 | 3.45E-01 | 5.39E-01 | 0.06±0.14 | 0 | 3.36E-01 | 9.32E-01 | 0.13±0.15 | 8.93E-01 | 0.1±0.15 | 9.23E-01 | -0.22±0.35 | 9.99E-01 | -0.35±0.45 | 1.00E+00 |
| dodecanedioate (C12-DC) | -0.07±0.11 | 36.56 | 2.09E-01 | 5.59E-01 | -0.06±0.15 | 0 | 3.94E-01 | 9.32E-01 | -0.11±0.16 | 9.32E-01 | -0.11±0.16 | 9.25E-01 | 0.37±0.37 | 9.99E-01 | 0.29±0.44 | 1.00E+00 |
| quinolinate | -0.08±0.12 | 21.29 | 2.60E-01 | 5.54E-01 | -0.07±0.16 | 0 | 5.14E-01 | 9.32E-01 | -0.13±0.16 | 9.17E-01 | -0.11±0.17 | 9.25E-01 | 0.33±0.39 | 9.99E-01 | 0.2±0.44 | 1.00E+00 |
| cholesterol sulfate | -0.05±0.05 | 0 | 6.81E-01 | 5.24E-01 | -0.06±0.15 | 0 | 5.35E-01 | 9.32E-01 | -0.05±0.16 | 9.80E-01 | -0.1±0.17 | 9.26E-01 | 0.12±0.43 | 9.99E-01 | 0.16±0.4 | 1.00E+00 |
| palmitoyl-sphingosine-phosphoethanolamine (d18:1/16:0) | 0.07±0.08 | 0 | 5.35E-01 | 5.24E-01 | 0.06±0.15 | 0 | 4.27E-01 | 9.32E-01 | 0.09±0.16 | 9.41E-01 | 0.1±0.16 | 9.26E-01 | -0.14±0.36 | 9.99E-01 | -0.28±0.45 | 1.00E+00 |
| X - 12729 | 0.07±0.08 | 21.34 | 2.60E-01 | 5.24E-01 | 0.05±0.13 | 46.07 | 1.73E-01 | 9.32E-01 | 0.08±0.14 | 9.32E-01 | 0.09±0.14 | 9.26E-01 | -0.56±0.56 | 9.99E-01 | -0.82±0.65 | 1.00E+00 |
| X - 21821 | -0.09±0.11 | 17.79 | 2.70E-01 | 5.32E-01 | -0.05±0.15 | 13.69 | 2.82E-01 | 9.32E-01 | -0.12±0.15 | 9.23E-01 | -0.1±0.15 | 9.26E-01 | 0.46±0.51 | 9.99E-01 | 0.55±0.58 | 1.00E+00 |
| campesterol | 0.08±0.08 | 0 | 7.12E-01 | 5.24E-01 | 0.06±0.15 | 0 | 5.31E-01 | 9.32E-01 | 0.08±0.16 | 9.58E-01 | 0.1±0.16 | 9.27E-01 | -0.08±0.43 | 9.99E-01 | -0.22±0.47 | 1.00E+00 |
| 2-methoxyhydroquinone sulfate (1) | -0.07±0.08 | 0 | 5.47E-01 | 5.24E-01 | -0.07±0.15 | 0 | 6.68E-01 | 9.32E-01 | -0.08±0.15 | 9.58E-01 | -0.09±0.16 | 9.38E-01 | 0.19±0.44 | 9.99E-01 | 0.1±0.42 | 1.00E+00 |
| indolepropionate | -0.1±0.12 | 0 | 4.86E-01 | 5.24E-01 | -0.06±0.16 | 0 | 4.74E-01 | 9.32E-01 | -0.12±0.16 | 9.32E-01 | -0.09±0.17 | 9.39E-01 | 0.22±0.48 | 9.99E-01 | 0.33±0.57 | 1.00E+00 |
| pimeloylcarnitine/3-methyladipoylcarnitine (C7-DC) | 0.06±0.07 | 0 | 6.51E-01 | 5.24E-01 | 0.07±0.15 | 0 | 7.63E-01 | 9.32E-01 | 0.07±0.16 | 9.74E-01 | 0.09±0.16 | 9.43E-01 | -0.1±0.37 | 9.99E-01 | -0.06±0.46 | 1.00E+00 |
| palmitate (16:0) | -0.09±0.11 | 0 | 3.24E-01 | 5.25E-01 | -0.06±0.16 | 0 | 6.18E-01 | 9.32E-01 | -0.11±0.16 | 9.32E-01 | -0.08±0.17 | 9.44E-01 | 0.41±0.51 | 9.99E-01 | 0.19±0.53 | 1.00E+00 |
| palmitoleate (16:1n7) | -0.08±0.1 | 0 | 3.53E-01 | 5.24E-01 | -0.06±0.16 | 0 | 6.27E-01 | 9.32E-01 | -0.1±0.16 | 9.32E-01 | -0.09±0.17 | 9.44E-01 | 0.38±0.51 | 9.99E-01 | 0.2±0.57 | 1.00E+00 |
| homoarginine | -0.06±0.06 | 0 | 6.98E-01 | 5.24E-01 | -0.06±0.16 | 0 | 7.68E-01 | 9.32E-01 | -0.06±0.16 | 9.80E-01 | -0.07±0.17 | 9.45E-01 | 0.13±0.49 | 9.99E-01 | 0.13±0.67 | 1.00E+00 |
| N-acetyl-aspartyl-glutamate (NAAG) | 0.08±0.09 | 0 | 7.33E-01 | 5.24E-01 | 0.06±0.14 | 0 | 7.61E-01 | 9.32E-01 | 0.09±0.15 | 9.32E-01 | 0.07±0.15 | 9.45E-01 | -0.05±0.41 | 9.99E-01 | -0.06±0.42 | 1.00E+00 |
| N-stearoyltaurine | 0.07±0.06 | 0 | 9.05E-01 | 5.24E-01 | 0.07±0.15 | 0 | 8.75E-01 | 9.32E-01 | 0.06±0.16 | 9.78E-01 | 0.07±0.16 | 9.45E-01 | 0.12±0.44 | 9.99E-01 | 0±0.44 | 1.00E+00 |
| ursodeoxycholate | 0.06±0.06 | 0 | 9.35E-01 | 5.24E-01 | 0.06±0.15 | 0 | 8.98E-01 | 9.32E-01 | 0.06±0.15 | 9.80E-01 | 0.07±0.15 | 9.45E-01 | 0.02±0.44 | 9.99E-01 | 0.01±0.48 | 1.00E+00 |
| choline phosphate | 0.05±0.05 | 0 | 8.74E-01 | 5.24E-01 | 0.06±0.15 | 0 | 8.05E-01 | 9.32E-01 | 0.05±0.15 | 9.80E-01 | 0.07±0.15 | 9.45E-01 | -0.02±0.44 | 9.99E-01 | -0.06±0.48 | 1.00E+00 |
| X - 12112 | 0.07±0.07 | 0 | 6.11E-01 | 5.24E-01 | 0.05±0.14 | 0 | 7.42E-01 | 9.32E-01 | 0.07±0.15 | 9.62E-01 | 0.07±0.15 | 9.45E-01 | -0.12±0.38 | 9.99E-01 | -0.08±0.41 | 1.00E+00 |
| lithocholate sulfate (1) | 0.08±0.09 | 0 | 7.01E-01 | 5.24E-01 | 0.05±0.15 | 0 | 7.41E-01 | 9.32E-01 | 0.09±0.16 | 9.32E-01 | 0.07±0.16 | 9.50E-01 | -0.07±0.42 | 9.99E-01 | -0.1±0.49 | 1.00E+00 |
| hippurate | -0.07±0.07 | 0 | 9.05E-01 | 5.24E-01 | -0.06±0.16 | 0 | 8.10E-01 | 9.32E-01 | -0.08±0.16 | 9.70E-01 | -0.07±0.17 | 9.57E-01 | -0.02±0.48 | 9.99E-01 | 0.06±0.49 | 1.00E+00 |
| o-cresol sulfate | 0.05±0.05 | 0 | 3.87E-01 | 5.24E-01 | -0.06±0.17 | 0 | 8.87E-01 | 9.32E-01 | 0.05±0.15 | 9.80E-01 | -0.07±0.18 | 9.61E-01 | -0.38±0.49 | 9.99E-01 | 0.01±0.54 | 1.00E+00 |
| N-palmitoylserine | 0.08±0.08 | 0 | 9.68E-01 | 5.24E-01 | 0.06±0.16 | 0 | 8.91E-01 | 9.32E-01 | 0.08±0.16 | 9.58E-01 | 0.07±0.16 | 9.61E-01 | 0.1±0.47 | 9.99E-01 | -0.01±0.53 | 1.00E+00 |
| X - 11315 | 0.03±0.03 | 0 | 9.95E-01 | 5.24E-01 | 0.05±0.14 | 0 | 8.66E-01 | 9.32E-01 | 0.03±0.14 | 9.94E-01 | 0.06±0.15 | 9.61E-01 | 0.02±0.45 | 9.99E-01 | -0.02±0.45 | 1.00E+00 |
| X - 12117 | 0.07±0.07 | 0 | 8.41E-01 | 5.24E-01 | 0.05±0.16 | 0 | 8.29E-01 | 9.32E-01 | 0.08±0.15 | 9.58E-01 | 0.06±0.16 | 9.61E-01 | -0.01±0.43 | 9.99E-01 | -0.05±0.52 | 1.00E+00 |
| N-acetylcitrulline | -0.05±0.05 | 0 | 6.65E-01 | 5.24E-01 | -0.05±0.14 | 0 | 9.44E-01 | 9.32E-01 | -0.05±0.15 | 9.80E-01 | -0.05±0.15 | 9.62E-01 | 0.15±0.46 | 9.99E-01 | -0.02±0.49 | 1.00E+00 |
| 3,4-dihydroxybutyrate | 0.07±0.07 | 0 | 8.86E-01 | 5.24E-01 | 0.05±0.14 | 0 | 8.66E-01 | 9.32E-01 | 0.07±0.14 | 9.62E-01 | 0.06±0.15 | 9.62E-01 | 0.02±0.37 | 9.99E-01 | -0.02±0.47 | 1.00E+00 |
| 2-stearoyl-GPE (18:0)* | 0.03±0.03 | 0 | 9.60E-01 | 5.24E-01 | 0.05±0.14 | 0 | 9.01E-01 | 9.32E-01 | 0.03±0.15 | 9.92E-01 | 0.06±0.15 | 9.62E-01 | 0.05±0.43 | 9.99E-01 | 0±0.45 | 1.00E+00 |
| 1-(1-enyl-palmitoyl)-2-palmitoleoyl-GPC (P-16:0/16:1)* | 0.06±0.06 | 0 | 8.47E-01 | 5.24E-01 | 0.06±0.16 | 0 | 9.35E-01 | 9.32E-01 | 0.06±0.16 | 9.80E-01 | 0.06±0.17 | 9.62E-01 | 0.15±0.48 | 9.99E-01 | 0.02±0.53 | 1.00E+00 |
| N-acetylputrescine | -0.09±0.1 | 0 | 7.19E-01 | 5.24E-01 | -0.05±0.15 | 0 | 9.51E-01 | 9.32E-01 | -0.1±0.15 | 9.32E-01 | -0.06±0.16 | 9.62E-01 | 0.04±0.39 | 9.99E-01 | -0.02±0.52 | 1.00E+00 |
| gamma-glutamylglutamate | -0.03±0.03 | 0 | 9.77E-01 | 5.24E-01 | -0.07±0.15 | 0 | 7.91E-01 | 9.32E-01 | -0.03±0.15 | 9.94E-01 | -0.05±0.16 | 9.62E-01 | -0.04±0.41 | 9.99E-01 | -0.19±0.47 | 1.00E+00 |
| X - 23974 | 0.05±0.05 | 0 | 9.73E-01 | 5.24E-01 | 0.06±0.15 | 0 | 9.60E-01 | 9.32E-01 | 0.05±0.15 | 9.80E-01 | 0.06±0.15 | 9.62E-01 | 0.06±0.46 | 9.99E-01 | 0.08±0.48 | 1.00E+00 |
| X - 24418 | -0.05±0.05 | 0 | 8.74E-01 | 5.24E-01 | -0.06±0.14 | 0 | 8.87E-01 | 9.32E-01 | -0.05±0.15 | 9.80E-01 | -0.05±0.15 | 9.62E-01 | -0.11±0.4 | 9.99E-01 | -0.12±0.42 | 1.00E+00 |
| X - 24475 | 0.03±0.03 | 0 | 9.45E-01 | 5.24E-01 | 0.05±0.14 | 0 | 9.42E-01 | 9.32E-01 | 0.03±0.15 | 9.90E-01 | 0.05±0.15 | 9.62E-01 | 0±0.47 | 9.99E-01 | 0.02±0.44 | 1.00E+00 |
| asparagine | 0.05±0.05 | 0 | 5.74E-01 | 5.24E-01 | 0.06±0.16 | 0 | 7.69E-01 | 9.32E-01 | 0.05±0.16 | 9.80E-01 | 0.05±0.16 | 9.63E-01 | 0.42±0.65 | 9.99E-01 | 0.26±0.69 | 1.00E+00 |
| X - 12100 | -0.08±0.08 | 0 | 6.92E-01 | 5.24E-01 | -0.06±0.15 | 0 | 9.83E-01 | 9.32E-01 | -0.08±0.16 | 9.58E-01 | -0.05±0.16 | 9.63E-01 | 0.08±0.41 | 9.99E-01 | -0.07±0.51 | 1.00E+00 |
| 2'-deoxyuridine | -0.07±0.07 | 0 | 7.85E-01 | 5.24E-01 | -0.06±0.15 | 0 | 9.30E-01 | 9.32E-01 | -0.07±0.15 | 9.58E-01 | -0.05±0.16 | 9.65E-01 | 0.03±0.39 | 9.99E-01 | -0.09±0.45 | 1.00E+00 |
| 3b-hydroxy-5-cholenoic acid | 0.04±0.05 | 71.28 | 6.20E-02 | 5.30E-01 | -0.06±0.16 | 69.44 | 7.05E-02 | 9.32E-01 | 0.05±0.16 | 9.80E-01 | 0.05±0.17 | 9.65E-01 | -0.73±0.42 | 9.99E-01 | -0.81±0.44 | 1.00E+00 |
| 1-palmitoyl-2-stearoyl-GPC (16:0/18:0) | 0.04±0.04 | 0 | 6.76E-01 | 5.24E-01 | 0.06±0.15 | 0 | 7.24E-01 | 9.32E-01 | 0.04±0.15 | 9.80E-01 | 0.05±0.15 | 9.65E-01 | 0.28±0.58 | 9.99E-01 | 0.31±0.71 | 1.00E+00 |
| beta-hydroxyisovalerate | -0.04±0.04 | 0 | 9.69E-01 | 5.24E-01 | -0.05±0.14 | 0 | 9.58E-01 | 9.32E-01 | -0.04±0.15 | 9.80E-01 | -0.05±0.15 | 9.65E-01 | -0.02±0.45 | 9.99E-01 | -0.07±0.46 | 1.00E+00 |
| methylsuccinate | -0.04±0.04 | 0 | 9.37E-01 | 5.24E-01 | -0.05±0.14 | 0 | 8.76E-01 | 9.32E-01 | -0.04±0.14 | 9.80E-01 | -0.04±0.14 | 9.65E-01 | -0.01±0.42 | 9.99E-01 | -0.11±0.44 | 1.00E+00 |
| palmitoylcarnitine (C16) | 0±0.01 | 0 | 3.54E-01 | 5.24E-01 | 0.06±0.16 | 0 | 6.79E-01 | 9.32E-01 | -0.01±0.16 | 9.99E-01 | 0.05±0.16 | 9.67E-01 | 0.54±0.59 | 9.99E-01 | 0.3±0.6 | 1.00E+00 |
| oleoyl ethanolamide | -0.05±0.05 | 0 | 9.15E-01 | 5.24E-01 | -0.07±0.16 | 0 | 7.12E-01 | 9.32E-01 | -0.05±0.16 | 9.80E-01 | -0.05±0.17 | 9.67E-01 | -0.1±0.44 | 9.99E-01 | -0.23±0.47 | 1.00E+00 |
| eugenol sulfate | -0.03±0.02 | 0 | 5.54E-01 | 5.24E-01 | -0.06±0.15 | 0 | 6.17E-01 | 9.32E-01 | -0.02±0.15 | 9.94E-01 | -0.04±0.15 | 9.67E-01 | -0.3±0.46 | 9.99E-01 | -0.29±0.48 | 1.00E+00 |
| metabolonic lactone sulfate | -0.05±0.05 | 0 | 9.61E-01 | 5.24E-01 | -0.05±0.15 | 0 | 8.71E-01 | 9.32E-01 | -0.05±0.15 | 9.80E-01 | -0.04±0.16 | 9.67E-01 | -0.08±0.44 | 9.99E-01 | -0.13±0.47 | 1.00E+00 |
| S-adenosylhomocysteine (SAH) | -0.02±0.02 | 0 | 8.67E-01 | 5.24E-01 | -0.06±0.16 | 0 | 7.09E-01 | 9.32E-01 | -0.02±0.16 | 9.94E-01 | -0.04±0.17 | 9.67E-01 | -0.09±0.43 | 9.99E-01 | -0.23±0.46 | 1.00E+00 |
| caproate (6:0) | 0.02±0.02 | 0 | 3.90E-01 | 5.24E-01 | 0.05±0.15 | 0 | 5.85E-01 | 9.32E-01 | 0.02±0.15 | 9.94E-01 | 0.04±0.15 | 9.67E-01 | 0.71±0.8 | 9.99E-01 | 0.51±0.85 | 1.00E+00 |
| quinate | 0.07±0.07 | 0 | 6.71E-01 | 5.24E-01 | 0.08±0.18 | 0 | 4.22E-01 | 9.32E-01 | 0.07±0.19 | 9.80E-01 | 0.04±0.19 | 9.69E-01 | 0.35±0.67 | 9.99E-01 | 0.68±0.77 | 1.00E+00 |
| homostachydrine* | -0.01±0.01 | 0 | 5.86E-01 | 5.24E-01 | -0.06±0.15 | 0 | 7.20E-01 | 9.32E-01 | -0.01±0.16 | 9.94E-01 | -0.04±0.16 | 9.69E-01 | -0.21±0.38 | 9.99E-01 | -0.21±0.46 | 1.00E+00 |
| margaroylcarnitine (C17)* | -0.01±0.01 | 9.92 | 2.92E-01 | 5.24E-01 | 0.06±0.15 | 0 | 4.85E-01 | 9.32E-01 | -0.01±0.15 | 9.94E-01 | 0.04±0.16 | 9.69E-01 | 0.61±0.6 | 9.99E-01 | 0.48±0.62 | 1.00E+00 |
| X - 15461 | 0.04±0.05 | 0 | 3.25E-01 | 5.24E-01 | -0.06±0.14 | 58.71 | 1.20E-01 | 9.32E-01 | 0.05±0.15 | 9.80E-01 | 0.04±0.15 | 9.69E-01 | -0.21±0.26 | 9.99E-01 | -0.56±0.35 | 1.00E+00 |
| perfluorooctanoate (PFOA) | -0.05±0.05 | 0 | 9.74E-01 | 5.24E-01 | -0.05±0.13 | 0 | 7.45E-01 | 9.32E-01 | -0.05±0.14 | 9.80E-01 | -0.03±0.14 | 9.74E-01 | -0.06±0.37 | 9.99E-01 | -0.17±0.39 | 1.00E+00 |
| N-oleoyltaurine | -0.04±0.04 | 0 | 9.42E-01 | 5.24E-01 | -0.06±0.15 | 0 | 6.34E-01 | 9.32E-01 | -0.04±0.16 | 9.80E-01 | -0.03±0.16 | 9.79E-01 | -0.07±0.43 | 9.99E-01 | -0.26±0.45 | 1.00E+00 |
| taurodeoxycholate | -0.02±0.02 | 0 | 6.55E-01 | 5.24E-01 | -0.06±0.14 | 0 | 5.09E-01 | 9.32E-01 | -0.02±0.15 | 9.94E-01 | -0.03±0.15 | 9.79E-01 | -0.21±0.43 | 9.99E-01 | -0.37±0.49 | 1.00E+00 |
| ornithine | 0.04±0.06 | 14.97 | 2.78E-01 | 5.32E-01 | -0.06±0.13 | 40.02 | 1.97E-01 | 9.32E-01 | 0.06±0.15 | 9.78E-01 | 0.03±0.15 | 9.79E-01 | -0.22±0.25 | 9.99E-01 | -0.4±0.3 | 1.00E+00 |
| 3-hydroxyhippurate | -0.03±0.04 | 78.79 | 2.99E-02 | 5.24E-01 | 0.06±0.15 | 79.62 | 2.67E-02 | 9.32E-01 | -0.04±0.15 | 9.86E-01 | -0.03±0.16 | 9.84E-01 | 1.26±0.6 | 9.99E-01 | 1.38±0.61 | 1.00E+00 |
| 5,6-dihydrouridine | 0.02±0.02 | 28.19 | 2.38E-01 | 5.24E-01 | -0.06±0.14 | 0 | 3.45E-01 | 9.32E-01 | 0.02±0.16 | 9.94E-01 | 0.01±0.16 | 9.92E-01 | -0.3±0.27 | 9.99E-01 | -0.31±0.31 | 1.00E+00 |
| phenol sulfate | -0.04±0.04 | 0 | 4.05E-01 | 5.24E-01 | -0.06±0.15 | 14.6 | 2.79E-01 | 9.32E-01 | -0.04±0.15 | 9.80E-01 | -0.01±0.15 | 9.92E-01 | -0.45±0.49 | 9.99E-01 | -0.68±0.59 | 1.00E+00 |
| 1-oleoyl-GPI (18:1) | 0.02±0.02 | 28.74 | 2.36E-01 | 5.24E-01 | -0.05±0.14 | 41.66 | 1.90E-01 | 9.32E-01 | 0.02±0.15 | 9.94E-01 | 0.02±0.15 | 9.92E-01 | -0.37±0.33 | 9.99E-01 | -0.49±0.36 | 1.00E+00 |
| 1-linoleoyl-GPI (18:2)* | -0.04±0.04 | 0 | 6.21E-01 | 5.24E-01 | -0.05±0.14 | 0 | 5.02E-01 | 9.32E-01 | -0.04±0.15 | 9.80E-01 | -0.02±0.15 | 9.92E-01 | -0.22±0.37 | 9.99E-01 | -0.31±0.41 | 1.00E+00 |
| 1-palmitoyl-2-docosahexaenoyl-GPE (16:0/22:6)* | 0.01±0.01 | 0 | 4.79E-01 | 5.24E-01 | 0.05±0.14 | 0 | 4.00E-01 | 9.32E-01 | 0.01±0.15 | 9.94E-01 | 0.01±0.15 | 9.92E-01 | 0.34±0.47 | 9.99E-01 | 0.42±0.47 | 1.00E+00 |
| N-acetylvaline | 0.02±0.02 | 34.47 | 2.17E-01 | 5.24E-01 | -0.06±0.14 | 37.29 | 2.07E-01 | 9.32E-01 | 0.02±0.15 | 9.94E-01 | 0.02±0.15 | 9.92E-01 | -0.32±0.28 | 9.99E-01 | -0.45±0.33 | 1.00E+00 |
| N-acetylalanine | -0.01±0.01 | 0 | 7.55E-01 | 5.24E-01 | -0.06±0.15 | 0 | 5.33E-01 | 9.32E-01 | -0.01±0.16 | 9.99E-01 | -0.01±0.17 | 9.92E-01 | -0.1±0.3 | 9.99E-01 | -0.26±0.36 | 1.00E+00 |
| 4-acetamidobutanoate | 0±0 | 5.06 | 3.05E-01 | 5.24E-01 | -0.07±0.15 | 22.26 | 2.57E-01 | 9.32E-01 | 0±0.16 | 9.99E-01 | 0.02±0.16 | 9.92E-01 | -0.29±0.28 | 9.99E-01 | -0.39±0.32 | 1.00E+00 |
| X - 24337 | 0.01±0.01 | 0 | 7.13E-01 | 5.24E-01 | -0.06±0.16 | 0 | 4.89E-01 | 9.32E-01 | 0.01±0.16 | 9.94E-01 | -0.02±0.17 | 9.92E-01 | -0.14±0.42 | 9.99E-01 | -0.38±0.48 | 1.00E+00 |
| 1-(1-enyl-palmitoyl)-2-linoleoyl-GPC (P-16:0/18:2)* | -0.02±0.02 | 0 | 5.39E-01 | 5.24E-01 | -0.06±0.15 | 2.23 | 3.12E-01 | 9.32E-01 | -0.02±0.15 | 9.94E-01 | 0.01±0.16 | 9.94E-01 | -0.22±0.32 | 9.99E-01 | -0.41±0.38 | 1.00E+00 |
| isobutyrylglycine | 0±0 | 21.25 | 2.60E-01 | 5.24E-01 | -0.05±0.14 | 0 | 4.04E-01 | 9.32E-01 | 0±0.15 | 9.99E-01 | 0±0.15 | 9.97E-01 | -0.4±0.35 | 9.99E-01 | -0.34±0.38 | 1.00E+00 |
| alanine | 0.08±0.08 | 0 | 9.27E-01 | 5.24E-01 | 0.05±0.15 | 0 | 7.66E-01 | 9.33E-01 | 0.08±0.15 | 9.58E-01 | 0.06±0.16 | 9.57E-01 | 0.12±0.47 | 9.99E-01 | -0.09±0.5 | 1.00E+00 |
| 3-amino-2-piperidone | 0.02±0.02 | 0 | 6.01E-01 | 5.24E-01 | -0.05±0.14 | 0 | 5.19E-01 | 9.33E-01 | 0.02±0.15 | 9.94E-01 | -0.01±0.15 | 9.94E-01 | -0.13±0.29 | 9.99E-01 | -0.25±0.35 | 1.00E+00 |
| 1-palmitoyl-2-arachidonoyl-GPI (16:0/20:4)* | 0.06±0.05 | 0 | 8.19E-01 | 5.24E-01 | 0.05±0.16 | 0 | 8.32E-01 | 9.34E-01 | 0.05±0.16 | 9.80E-01 | 0.04±0.17 | 9.67E-01 | 0.16±0.46 | 9.99E-01 | 0.17±0.55 | 1.00E+00 |
| X - 24307 | 0.02±0.02 | 54.4 | 1.39E-01 | 5.24E-01 | -0.05±0.14 | 51.98 | 1.49E-01 | 9.34E-01 | 0.02±0.14 | 9.94E-01 | 0.01±0.14 | 9.92E-01 | -0.49±0.34 | 9.99E-01 | -0.72±0.49 | 1.00E+00 |
| X - 18899 | 0.03±0.03 | 0 | 9.83E-01 | 5.24E-01 | 0.05±0.15 | 0 | 7.34E-01 | 9.34E-01 | 0.03±0.15 | 9.90E-01 | 0.07±0.16 | 9.50E-01 | 0.04±0.42 | 9.99E-01 | -0.09±0.43 | 1.00E+00 |
| S-methylcysteine | -0.03±0.03 | 0 | 4.66E-01 | 5.24E-01 | -0.05±0.15 | 0 | 4.71E-01 | 9.34E-01 | -0.03±0.15 | 9.90E-01 | -0.02±0.16 | 9.92E-01 | -0.39±0.5 | 9.99E-01 | -0.42±0.53 | 1.00E+00 |
| retinal | -0.04±0.04 | 0 | 5.18E-01 | 5.24E-01 | -0.05±0.14 | 0 | 5.37E-01 | 9.34E-01 | -0.04±0.14 | 9.80E-01 | -0.02±0.15 | 9.92E-01 | -0.31±0.42 | 9.99E-01 | -0.31±0.45 | 1.00E+00 |
| 1-linoleoyl-GPC (18:2) | 0.06±0.07 | 0 | 6.16E-01 | 5.24E-01 | 0.05±0.15 | 0 | 3.28E-01 | 9.35E-01 | 0.07±0.15 | 9.72E-01 | 0.11±0.16 | 9.23E-01 | -0.12±0.36 | 9.99E-01 | -0.29±0.38 | 1.00E+00 |
| gamma-glutamyl-2-aminobutyrate | 0.07±0.08 | 0 | 5.62E-01 | 5.24E-01 | 0.05±0.15 | 0 | 3.62E-01 | 9.35E-01 | 0.08±0.16 | 9.58E-01 | 0.1±0.16 | 9.26E-01 | -0.15±0.39 | 9.99E-01 | -0.32±0.43 | 1.00E+00 |
| X - 11787 | 0.07±0.08 | 0 | 3.94E-01 | 5.29E-01 | 0.05±0.15 | 0 | 4.30E-01 | 9.35E-01 | 0.09±0.16 | 9.41E-01 | 0.1±0.16 | 9.26E-01 | -0.17±0.29 | 9.99E-01 | -0.21±0.36 | 1.00E+00 |
| X - 22834 | 0.08±0.13 | 53.97 | 1.40E-01 | 5.86E-01 | 0.05±0.15 | 62.55 | 1.02E-01 | 9.36E-01 | 0.14±0.15 | 8.85E-01 | 0.13±0.16 | 9.15E-01 | -0.57±0.46 | 9.99E-01 | -0.72±0.5 | 1.00E+00 |
| X - 17685 | -0.04±0.04 | 0 | 8.00E-01 | 5.24E-01 | -0.05±0.16 | 0 | 7.66E-01 | 9.36E-01 | -0.04±0.16 | 9.80E-01 | -0.03±0.17 | 9.79E-01 | -0.16±0.47 | 9.99E-01 | -0.17±0.44 | 1.00E+00 |
| 3beta,7alpha-dihydroxy-5-cholestenoate | 0.1±0.11 | 0 | 6.41E-01 | 5.24E-01 | 0.05±0.16 | 0 | 5.29E-01 | 9.37E-01 | 0.11±0.16 | 9.32E-01 | 0.09±0.17 | 9.43E-01 | -0.08±0.38 | 9.99E-01 | -0.21±0.44 | 1.00E+00 |
| 1-myristoyl-2-arachidonoyl-GPC (14:0/20:4)* | -0.05±0.05 | 0 | 7.15E-01 | 5.24E-01 | -0.05±0.16 | 0 | 9.59E-01 | 9.37E-01 | -0.05±0.16 | 9.80E-01 | -0.05±0.16 | 9.67E-01 | 0.12±0.47 | 9.99E-01 | -0.07±0.49 | 1.00E+00 |
| 1-linoleoylglycerol (18:2) | -0.03±0.04 | 73.37 | 5.27E-02 | 5.24E-01 | 0.05±0.15 | 66.77 | 8.28E-02 | 9.37E-01 | -0.04±0.15 | 9.80E-01 | -0.03±0.15 | 9.79E-01 | 0.83±0.44 | 9.99E-01 | 0.89±0.51 | 1.00E+00 |
| X - 13695 | -0.02±0.02 | 0 | 4.92E-01 | 5.24E-01 | -0.05±0.16 | 0 | 6.94E-01 | 9.37E-01 | -0.02±0.16 | 9.94E-01 | -0.03±0.17 | 9.89E-01 | -0.32±0.44 | 9.99E-01 | -0.22±0.45 | 1.00E+00 |
| tryptophan betaine | 0.01±0.01 | 35.41 | 2.13E-01 | 5.24E-01 | -0.05±0.15 | 34.53 | 2.17E-01 | 9.37E-01 | 0.01±0.16 | 9.94E-01 | 0.01±0.16 | 9.92E-01 | -0.51±0.42 | 9.99E-01 | -0.61±0.48 | 1.00E+00 |
| 11-ketoetiocholanolone glucuronide | -0.04±0.04 | 0 | 6.65E-01 | 5.24E-01 | -0.05±0.15 | 0 | 8.82E-01 | 9.37E-01 | -0.04±0.15 | 9.82E-01 | -0.05±0.15 | 9.62E-01 | 0.15±0.42 | 9.99E-01 | 0.02±0.45 | 1.00E+00 |
| X - 12026 | 0.06±0.09 | 34.33 | 2.17E-01 | 5.52E-01 | 0.05±0.16 | 54.98 | 1.36E-01 | 9.39E-01 | 0.1±0.16 | 9.32E-01 | 0.15±0.17 | 9.15E-01 | -0.34±0.34 | 9.99E-01 | -0.48±0.39 | 1.00E+00 |
| X - 23787 | -0.05±0.05 | 0 | 9.32E-01 | 5.24E-01 | -0.05±0.16 | 0 | 8.83E-01 | 9.39E-01 | -0.05±0.17 | 9.80E-01 | -0.06±0.17 | 9.62E-01 | -0.01±0.43 | 9.99E-01 | 0.02±0.48 | 1.00E+00 |
| 2-O-methylascorbic acid | -0.07±0.08 | 0 | 3.94E-01 | 5.24E-01 | -0.04±0.15 | 0 | 6.17E-01 | 9.41E-01 | -0.09±0.15 | 9.32E-01 | -0.07±0.15 | 9.45E-01 | 0.25±0.38 | 9.99E-01 | 0.17±0.45 | 1.00E+00 |
| 2-naphthol sulfate | 0.09±0.1 | 0 | 4.36E-01 | 5.24E-01 | -0.06±0.2 | 0 | 7.07E-01 | 9.41E-01 | 0.11±0.15 | 9.32E-01 | -0.09±0.22 | 9.58E-01 | -0.28±0.48 | 9.99E-01 | 0.16±0.63 | 1.00E+00 |
| indole-3-carboxylate | 0.07±0.07 | 0 | 9.39E-01 | 5.24E-01 | 0.05±0.15 | 0 | 8.14E-01 | 9.41E-01 | 0.07±0.15 | 9.72E-01 | 0.06±0.16 | 9.62E-01 | 0.04±0.43 | 9.99E-01 | -0.06±0.46 | 1.00E+00 |
| X - 12851 | 0.05±0.05 | 0 | 6.45E-01 | 5.24E-01 | 0.04±0.15 | 0 | 7.59E-01 | 9.41E-01 | 0.05±0.15 | 9.80E-01 | 0.06±0.15 | 9.62E-01 | -0.16±0.44 | 9.99E-01 | -0.1±0.49 | 1.00E+00 |
| X - 17010 | -0.05±0.05 | 0 | 4.76E-01 | 5.24E-01 | -0.04±0.14 | 0 | 9.83E-01 | 9.41E-01 | -0.05±0.15 | 9.80E-01 | -0.04±0.15 | 9.65E-01 | 0.29±0.48 | 9.99E-01 | -0.03±0.55 | 1.00E+00 |
| ethylmalonate | 0.03±0.03 | 0 | 6.76E-01 | 5.24E-01 | 0.04±0.15 | 0 | 9.68E-01 | 9.41E-01 | 0.03±0.15 | 9.94E-01 | 0.04±0.15 | 9.67E-01 | 0.21±0.45 | 9.99E-01 | 0.06±0.45 | 1.00E+00 |
| 2-aminophenol sulfate | 0.06±0.06 | 0 | 4.97E-01 | 5.24E-01 | 0.04±0.15 | 0 | 4.82E-01 | 9.41E-01 | 0.06±0.16 | 9.78E-01 | 0.08±0.16 | 9.43E-01 | -0.2±0.38 | 9.99E-01 | -0.24±0.42 | 1.00E+00 |
| sphingomyelin (d17:1/14:0, d16:1/15:0)* | 0.05±0.05 | 0 | 7.97E-01 | 5.24E-01 | 0.05±0.17 | 0 | 6.72E-01 | 9.41E-01 | 0.05±0.17 | 9.80E-01 | 0.07±0.18 | 9.53E-01 | -0.06±0.43 | 9.99E-01 | -0.15±0.5 | 1.00E+00 |
| 1-palmitoyl-2-linoleoyl-GPC (16:0/18:2) | -0.01±0.01 | 0 | 7.60E-01 | 5.24E-01 | -0.04±0.15 | 0 | 4.88E-01 | 9.41E-01 | -0.01±0.16 | 9.94E-01 | 0±0.16 | 9.99E-01 | -0.11±0.33 | 9.99E-01 | -0.28±0.38 | 1.00E+00 |
| hydroxyasparagine** | 0.08±0.12 | 0 | 4.00E-01 | 5.55E-01 | 0.04±0.14 | 40.74 | 1.94E-01 | 9.43E-01 | 0.14±0.15 | 8.83E-01 | 0.13±0.16 | 9.15E-01 | -0.1±0.26 | 9.99E-01 | -0.33±0.32 | 1.00E+00 |
| 3-hydroxystachydrine* | -0.07±0.09 | 15.6 | 2.76E-01 | 5.24E-01 | -0.04±0.13 | 26.21 | 2.44E-01 | 9.43E-01 | -0.09±0.13 | 9.32E-01 | -0.08±0.14 | 9.38E-01 | 0.49±0.52 | 9.99E-01 | 0.57±0.54 | 1.00E+00 |
| 5-hydroxyhexanoate | -0.01±0.01 | 0 | 7.68E-01 | 5.24E-01 | -0.04±0.14 | 0 | 8.87E-01 | 9.43E-01 | -0.01±0.15 | 9.94E-01 | -0.04±0.15 | 9.69E-01 | -0.15±0.46 | 9.99E-01 | -0.11±0.5 | 1.00E+00 |
| X - 16087 | 0±0 | 0 | 4.67E-01 | 5.24E-01 | -0.04±0.15 | 0 | 3.51E-01 | 9.43E-01 | 0±0.16 | 9.99E-01 | 0±0.16 | 9.98E-01 | -0.29±0.4 | 9.99E-01 | -0.46±0.47 | 1.00E+00 |
| urate | 0.05±0.07 | 58.95 | 1.19E-01 | 5.51E-01 | -0.05±0.16 | 66.73 | 8.30E-02 | 9.43E-01 | 0.07±0.17 | 9.78E-01 | 0.08±0.17 | 9.45E-01 | -0.45±0.33 | 9.99E-01 | -0.62±0.37 | 1.00E+00 |
| phenylacetylglutamate | 0.06±0.06 | 0 | 9.31E-01 | 5.24E-01 | 0.04±0.15 | 0 | 9.37E-01 | 9.45E-01 | 0.06±0.15 | 9.78E-01 | 0.05±0.15 | 9.65E-01 | 0.1±0.44 | 9.99E-01 | 0±0.51 | 1.00E+00 |
| tiglylcarnitine (C5:1-DC) | -0.02±0.02 | 0 | 5.80E-01 | 5.24E-01 | -0.04±0.14 | 7.55 | 2.98E-01 | 9.47E-01 | -0.02±0.15 | 9.94E-01 | 0±0.15 | 9.99E-01 | -0.24±0.38 | 9.99E-01 | -0.56±0.52 | 1.00E+00 |
| N-acetyl-beta-alanine | -0.07±0.11 | 72.52 | 5.65E-02 | 5.53E-01 | -0.04±0.15 | 58.23 | 1.22E-01 | 9.49E-01 | -0.11±0.15 | 9.32E-01 | -0.09±0.16 | 9.26E-01 | 1.11±0.63 | 9.99E-01 | 1.05±0.72 | 1.00E+00 |
| 1-stearoyl-2-oleoyl-GPE (18:0/18:1) | -0.01±0.01 | 0 | 9.74E-01 | 5.24E-01 | -0.04±0.14 | 0 | 9.77E-01 | 9.50E-01 | -0.01±0.14 | 9.94E-01 | -0.04±0.15 | 9.67E-01 | -0.02±0.4 | 9.99E-01 | -0.05±0.44 | 1.00E+00 |
| creatinine | 0.06±0.14 | 31.15 | 2.28E-01 | 6.70E-01 | 0.04±0.15 | 56.71 | 1.29E-01 | 9.51E-01 | 0.16±0.17 | 8.83E-01 | 0.16±0.17 | 9.12E-01 | -0.22±0.27 | 9.99E-01 | -0.42±0.34 | 1.00E+00 |
| N2-acetyl,N6-methyllysine | -0.07±0.1 | 52.74 | 1.46E-01 | 5.39E-01 | -0.04±0.14 | 39.2 | 2.00E-01 | 9.51E-01 | -0.11±0.15 | 9.32E-01 | -0.09±0.15 | 9.27E-01 | 0.66±0.51 | 9.99E-01 | 0.64±0.55 | 1.00E+00 |
| X - 11308 | -0.05±0.05 | 0 | 7.26E-01 | 5.24E-01 | -0.04±0.14 | 0 | 9.94E-01 | 9.51E-01 | -0.05±0.14 | 9.80E-01 | -0.04±0.15 | 9.67E-01 | 0.11±0.45 | 9.99E-01 | -0.04±0.5 | 1.00E+00 |
| arabonate/xylonate | -0.04±0.04 | 0 | 7.71E-01 | 5.24E-01 | -0.04±0.13 | 0 | 9.72E-01 | 9.60E-01 | -0.04±0.14 | 9.80E-01 | -0.04±0.14 | 9.67E-01 | 0.07±0.38 | 9.99E-01 | -0.02±0.43 | 1.00E+00 |
| choline | 0.03±0.03 | 0 | 6.31E-01 | 5.24E-01 | -0.04±0.14 | 0 | 3.30E-01 | 9.60E-01 | 0.03±0.15 | 9.86E-01 | 0.02±0.15 | 9.92E-01 | -0.12±0.31 | 9.99E-01 | -0.39±0.39 | 1.00E+00 |
| 3-hydroxybutyroylglycine** | 0.05±0.14 | 69.97 | 6.80E-02 | 7.24E-01 | 0.04±0.16 | 72.62 | 5.60E-02 | 9.60E-01 | 0.15±0.17 | 8.92E-01 | 0.14±0.17 | 9.15E-01 | -0.62±0.39 | 9.99E-01 | -0.99±0.56 | 1.00E+00 |
| X - 23593 | 0.08±0.14 | 0 | 3.67E-01 | 5.79E-01 | 0.04±0.15 | 40.63 | 1.94E-01 | 9.60E-01 | 0.16±0.17 | 8.83E-01 | 0.14±0.17 | 9.15E-01 | -0.11±0.26 | 9.99E-01 | -0.33±0.32 | 1.00E+00 |
| 4-methylcatechol sulfate | -0.05±0.06 | 58.72 | 1.20E-01 | 5.24E-01 | -0.03±0.14 | 28.38 | 2.37E-01 | 9.60E-01 | -0.06±0.14 | 9.78E-01 | -0.08±0.14 | 9.39E-01 | 0.73±0.51 | 9.99E-01 | 0.56±0.52 | 1.00E+00 |
| X - 26108 | -0.07±0.08 | 28.39 | 2.37E-01 | 5.24E-01 | -0.04±0.14 | 15.01 | 2.78E-01 | 9.60E-01 | -0.09±0.15 | 9.32E-01 | -0.07±0.15 | 9.45E-01 | 0.72±0.68 | 9.99E-01 | 0.69±0.69 | 1.00E+00 |
| tartronate (hydroxymalonate) | -0.08±0.1 | 6.81 | 3.00E-01 | 5.24E-01 | -0.04±0.14 | 0 | 4.10E-01 | 9.60E-01 | -0.1±0.14 | 9.32E-01 | -0.06±0.15 | 9.50E-01 | 0.51±0.58 | 9.99E-01 | 0.46±0.62 | 1.00E+00 |
| pentose acid* | -0.08±0.08 | 0 | 6.62E-01 | 5.24E-01 | -0.04±0.14 | 0 | 6.34E-01 | 9.60E-01 | -0.08±0.15 | 9.32E-01 | -0.06±0.15 | 9.61E-01 | 0.1±0.42 | 9.99E-01 | 0.17±0.44 | 1.00E+00 |
| 1-stearoyl-2-arachidonoyl-GPI (18:0/20:4) | 0.07±0.08 | 0 | 8.41E-01 | 5.24E-01 | 0.04±0.15 | 0 | 6.84E-01 | 9.60E-01 | 0.08±0.16 | 9.58E-01 | 0.06±0.16 | 9.62E-01 | 0±0.39 | 9.99E-01 | -0.15±0.48 | 1.00E+00 |
| gamma-glutamyltyrosine | 0.01±0.01 | 23.21 | 2.54E-01 | 5.24E-01 | 0.04±0.15 | 0 | 4.09E-01 | 9.60E-01 | 0.01±0.14 | 9.94E-01 | 0.02±0.15 | 9.92E-01 | 0.85±0.74 | 9.99E-01 | 0.72±0.84 | 1.00E+00 |
| catechol sulfate | -0.02±0.02 | 0 | 5.03E-01 | 5.24E-01 | 0.04±0.16 | 0 | 3.49E-01 | 9.60E-01 | -0.02±0.16 | 9.94E-01 | 0±0.17 | 9.98E-01 | 0.33±0.53 | 9.99E-01 | 0.54±0.56 | 1.00E+00 |
| sarcosine | 0.07±0.08 | 0 | 6.88E-01 | 5.24E-01 | 0.03±0.13 | 0 | 5.32E-01 | 9.62E-01 | 0.08±0.14 | 9.32E-01 | 0.06±0.14 | 9.47E-01 | -0.06±0.33 | 9.99E-01 | -0.19±0.39 | 1.00E+00 |
| 2-hydroxynervonate* | 0.06±0.06 | 0 | 9.75E-01 | 5.24E-01 | 0.04±0.15 | 0 | 9.45E-01 | 9.62E-01 | 0.06±0.16 | 9.80E-01 | 0.03±0.16 | 9.79E-01 | 0.07±0.46 | 9.99E-01 | 0.06±0.44 | 1.00E+00 |
| glycosyl-N-(2-hydroxynervonoyl)-sphingosine (d18:1/24:1(2OH))* | 0.01±0.01 | 0 | 4.95E-01 | 5.24E-01 | -0.04±0.14 | 0 | 4.97E-01 | 9.62E-01 | 0.01±0.15 | 9.99E-01 | 0±0.15 | 9.99E-01 | -0.27±0.4 | 9.99E-01 | -0.31±0.43 | 1.00E+00 |
| 3-hydroxy-2-ethylpropionate | -0.01±0.01 | 0 | 7.53E-01 | 5.24E-01 | -0.04±0.16 | 0 | 6.96E-01 | 9.63E-01 | -0.01±0.17 | 9.94E-01 | -0.01±0.17 | 9.93E-01 | -0.14±0.39 | 9.99E-01 | -0.19±0.41 | 1.00E+00 |
| 1-(1-enyl-palmitoyl)-2-arachidonoyl-GPE (P-16:0/20:4)* | 0±0 | 0 | 8.23E-01 | 5.24E-01 | -0.03±0.14 | 0 | 6.13E-01 | 9.65E-01 | 0±0.15 | 9.99E-01 | -0.01±0.15 | 9.94E-01 | -0.09±0.38 | 9.99E-01 | -0.23±0.41 | 1.00E+00 |
| X - 24344 | 0.03±0.03 | 0 | 5.82E-01 | 5.24E-01 | -0.04±0.16 | 0 | 8.46E-01 | 9.66E-01 | 0.03±0.16 | 9.91E-01 | -0.05±0.17 | 9.67E-01 | -0.21±0.45 | 9.99E-01 | 0.05±0.48 | 1.00E+00 |
| 3-formylindole | 0.06±0.07 | 0 | 5.49E-01 | 5.24E-01 | 0.04±0.15 | 0 | 4.63E-01 | 9.66E-01 | 0.07±0.16 | 9.74E-01 | 0.08±0.16 | 9.45E-01 | -0.15±0.36 | 9.99E-01 | -0.28±0.45 | 1.00E+00 |
| gamma-glutamylmethionine | -0.04±0.04 | 0 | 5.51E-01 | 5.24E-01 | -0.03±0.15 | 0 | 6.88E-01 | 9.66E-01 | -0.04±0.15 | 9.80E-01 | -0.05±0.15 | 9.65E-01 | 0.33±0.61 | 9.99E-01 | 0.24±0.7 | 1.00E+00 |
| glutarate (C5-DC) | -0.01±0.01 | 0 | 7.59E-01 | 5.24E-01 | -0.03±0.15 | 0 | 6.74E-01 | 9.66E-01 | -0.01±0.15 | 9.94E-01 | -0.01±0.15 | 9.92E-01 | -0.14±0.43 | 9.99E-01 | -0.21±0.44 | 1.00E+00 |
| X - 24473 | -0.06±0.06 | 0 | 9.41E-01 | 5.24E-01 | -0.03±0.14 | 0 | 9.03E-01 | 9.67E-01 | -0.06±0.15 | 9.80E-01 | -0.03±0.15 | 9.84E-01 | -0.09±0.42 | 9.99E-01 | -0.08±0.44 | 1.00E+00 |
| docosahexaenoate (DHA; 22:6n3) | 0.02±0.02 | 0 | 8.64E-01 | 5.24E-01 | 0.03±0.15 | 0 | 6.38E-01 | 9.69E-01 | 0.02±0.16 | 9.94E-01 | 0.06±0.16 | 9.62E-01 | -0.06±0.44 | 9.99E-01 | -0.18±0.47 | 1.00E+00 |
| 1-stearoyl-2-oleoyl-GPC (18:0/18:1) | 0.04±0.04 | 0 | 6.81E-01 | 5.24E-01 | 0.04±0.16 | 0 | 7.32E-01 | 9.69E-01 | 0.04±0.16 | 9.87E-01 | 0.02±0.17 | 9.92E-01 | 0.26±0.54 | 9.99E-01 | 0.24±0.63 | 1.00E+00 |
| X - 24588 | -0.03±0.03 | 0 | 9.42E-01 | 5.24E-01 | -0.03±0.14 | 0 | 7.23E-01 | 9.70E-01 | -0.03±0.15 | 9.91E-01 | -0.01±0.15 | 9.92E-01 | -0.06±0.36 | 9.99E-01 | -0.18±0.45 | 1.00E+00 |
| X - 23780 | 0.02±0.02 | 0 | 4.45E-01 | 5.24E-01 | -0.03±0.14 | 0 | 6.07E-01 | 9.70E-01 | 0.02±0.14 | 9.94E-01 | -0.01±0.15 | 9.94E-01 | -0.3±0.41 | 9.99E-01 | -0.25±0.44 | 1.00E+00 |
| trigonelline (N'-methylnicotinate) | -0.02±0.02 | 15.36 | 2.77E-01 | 5.24E-01 | 0.04±0.17 | 46.9 | 1.70E-01 | 9.71E-01 | -0.02±0.17 | 9.94E-01 | -0.03±0.17 | 9.84E-01 | 0.63±0.59 | 9.99E-01 | 0.82±0.59 | 1.00E+00 |
| 5alpha-pregnan-3beta,20beta-diol monosulfate (1) | 0.07±0.08 | 0 | 3.79E-01 | 5.24E-01 | 0.03±0.15 | 0 | 3.48E-01 | 9.73E-01 | 0.08±0.16 | 9.58E-01 | 0.08±0.16 | 9.44E-01 | -0.3±0.43 | 9.99E-01 | -0.36±0.45 | 1.00E+00 |
| N6,N6,N6-trimethyllysine | 0.02±0.03 | 0 | 5.26E-01 | 5.24E-01 | -0.03±0.15 | 0 | 4.09E-01 | 9.75E-01 | 0.03±0.16 | 9.94E-01 | 0.02±0.16 | 9.92E-01 | -0.16±0.29 | 9.99E-01 | -0.31±0.37 | 1.00E+00 |
| ethyl alpha-glucopyranoside | -0.01±0.01 | 0 | 5.58E-01 | 5.24E-01 | 0.03±0.16 | 0 | 8.32E-01 | 9.76E-01 | -0.01±0.14 | 9.94E-01 | 0.02±0.17 | 9.92E-01 | 0.24±0.43 | 9.99E-01 | 0.14±0.51 | 1.00E+00 |
| pregnanediol-3-glucuronide | 0.02±0.02 | 0 | 5.30E-01 | 5.24E-01 | -0.03±0.15 | 0 | 4.28E-01 | 9.76E-01 | 0.02±0.15 | 9.94E-01 | 0±0.15 | 9.99E-01 | -0.26±0.45 | 9.99E-01 | -0.44±0.54 | 1.00E+00 |
| X - 16397 | 0.03±0.03 | 0 | 8.08E-01 | 5.24E-01 | 0.03±0.15 | 0 | 6.52E-01 | 9.78E-01 | 0.03±0.16 | 9.94E-01 | 0.06±0.16 | 9.62E-01 | -0.07±0.4 | 9.99E-01 | -0.16±0.44 | 1.00E+00 |
| adenosine 5'-monophosphate (AMP) | 0.06±0.1 | 66.62 | 8.35E-02 | 5.54E-01 | 0.03±0.15 | 50.27 | 1.56E-01 | 9.79E-01 | 0.1±0.15 | 9.32E-01 | 0.09±0.16 | 9.27E-01 | -0.78±0.5 | 9.99E-01 | -0.7±0.54 | 1.00E+00 |
| perfluorooctanesulfonate (PFOS) | 0.07±0.07 | 0 | 5.29E-01 | 5.24E-01 | 0.03±0.15 | 0 | 4.53E-01 | 9.79E-01 | 0.08±0.16 | 9.62E-01 | 0.07±0.16 | 9.45E-01 | -0.15±0.35 | 9.99E-01 | -0.26±0.42 | 1.00E+00 |
| 3-methoxycatechol sulfate (2) | 0.04±0.04 | 0 | 4.00E-01 | 5.24E-01 | 0.03±0.15 | 0 | 6.14E-01 | 9.79E-01 | 0.04±0.16 | 9.80E-01 | 0.06±0.16 | 9.62E-01 | -0.3±0.41 | 9.99E-01 | -0.17±0.42 | 1.00E+00 |
| 16a-hydroxy DHEA 3-sulfate | -0.01±0.01 | 0 | 6.20E-01 | 5.24E-01 | -0.03±0.16 | 0 | 6.84E-01 | 9.79E-01 | -0.01±0.16 | 9.94E-01 | -0.05±0.16 | 9.65E-01 | 0.23±0.49 | 9.99E-01 | 0.17±0.52 | 1.00E+00 |
| 5-dodecenoylcarnitine (C12:1) | 0.05±0.06 | 26.48 | 2.43E-01 | 5.25E-01 | 0.03±0.14 | 25.15 | 2.48E-01 | 9.82E-01 | 0.06±0.14 | 9.78E-01 | 0.09±0.15 | 9.26E-01 | -0.35±0.35 | 9.99E-01 | -0.37±0.37 | 1.00E+00 |
| X - 24243 | 0.05±0.06 | 47.96 | 1.66E-01 | 5.35E-01 | -0.03±0.14 | 59.98 | 1.14E-01 | 9.82E-01 | 0.06±0.16 | 9.78E-01 | 0.07±0.16 | 9.45E-01 | -0.4±0.33 | 9.99E-01 | -0.55±0.36 | 1.00E+00 |
| nonanoylcarnitine (C9) | 0.02±0.03 | 0 | 6.38E-01 | 5.24E-01 | 0.03±0.16 | 0 | 4.27E-01 | 9.82E-01 | 0.03±0.17 | 9.94E-01 | 0.07±0.17 | 9.57E-01 | -0.18±0.44 | 9.99E-01 | -0.37±0.53 | 1.00E+00 |
| X - 12830 | 0.05±0.06 | 0 | 5.54E-01 | 5.24E-01 | 0.03±0.14 | 0 | 6.89E-01 | 9.82E-01 | 0.06±0.15 | 9.80E-01 | 0.05±0.15 | 9.65E-01 | -0.18±0.4 | 9.99E-01 | -0.14±0.44 | 1.00E+00 |
| dimethylarginine (SDMA + ADMA) | 0.03±0.04 | 0 | 5.81E-01 | 5.24E-01 | -0.03±0.14 | 0 | 3.41E-01 | 9.82E-01 | 0.04±0.16 | 9.82E-01 | 0.04±0.16 | 9.67E-01 | -0.11±0.27 | 9.99E-01 | -0.3±0.32 | 1.00E+00 |
| cystathionine | 0.05±0.05 | 0 | 9.07E-01 | 5.24E-01 | 0.03±0.15 | 0 | 9.83E-01 | 9.82E-01 | 0.05±0.14 | 9.80E-01 | 0.03±0.15 | 9.84E-01 | 0±0.44 | 9.99E-01 | 0.02±0.54 | 1.00E+00 |
| octanoylcarnitine (C8) | -0.01±0.01 | 0 | 6.14E-01 | 5.24E-01 | -0.03±0.16 | 0 | 3.63E-01 | 9.82E-01 | -0.01±0.17 | 9.94E-01 | 0.02±0.17 | 9.92E-01 | -0.21±0.4 | 9.99E-01 | -0.49±0.53 | 1.00E+00 |
| 3-methyl catechol sulfate (1) | 0.01±0.01 | 0 | 4.78E-01 | 5.24E-01 | -0.03±0.16 | 0 | 8.93E-01 | 9.82E-01 | 0.01±0.17 | 9.94E-01 | -0.02±0.17 | 9.92E-01 | -0.33±0.48 | 9.99E-01 | -0.09±0.47 | 1.00E+00 |
| heneicosapentaenoate (21:5n3) | 0±0 | 0 | 8.77E-01 | 5.24E-01 | 0.03±0.15 | 0 | 9.04E-01 | 9.82E-01 | 0±0.16 | 9.99E-01 | 0.02±0.16 | 9.92E-01 | 0.07±0.44 | 9.99E-01 | 0.09±0.5 | 1.00E+00 |
| glucuronide of piperine metabolite C17H21NO3 (4)* | 0±0 | 0 | 7.93E-01 | 5.24E-01 | -0.03±0.17 | 0 | 5.99E-01 | 9.82E-01 | 0±0.17 | 9.99E-01 | 0±0.17 | 9.99E-01 | -0.13±0.49 | 9.99E-01 | -0.3±0.55 | 1.00E+00 |
| X - 26097 | -0.03±0.03 | 0 | 9.06E-01 | 5.24E-01 | -0.03±0.15 | 0 | 5.13E-01 | 9.82E-01 | -0.03±0.15 | 9.94E-01 | 0±0.16 | 9.99E-01 | -0.08±0.42 | 9.99E-01 | -0.36±0.52 | 1.00E+00 |
| X - 24544 | 0.01±0.01 | 0 | 7.54E-01 | 5.24E-01 | -0.03±0.15 | 0 | 5.32E-01 | 9.82E-01 | 0.01±0.15 | 9.94E-01 | 0±0.15 | 1.00E+00 | -0.13±0.45 | 9.99E-01 | -0.33±0.51 | 1.00E+00 |
| X - 25265 | -0.04±0.04 | 0 | 5.44E-01 | 5.24E-01 | -0.03±0.15 | 0 | 7.93E-01 | 9.83E-01 | -0.04±0.16 | 9.80E-01 | -0.04±0.16 | 9.67E-01 | 0.21±0.41 | 9.99E-01 | 0.07±0.39 | 1.00E+00 |
| 5-methylthioadenosine (MTA) | 0.06±0.06 | 0 | 9.63E-01 | 5.24E-01 | 0.03±0.15 | 0 | 7.91E-01 | 9.83E-01 | 0.06±0.15 | 9.80E-01 | 0.04±0.16 | 9.69E-01 | 0.04±0.46 | 9.99E-01 | -0.11±0.55 | 1.00E+00 |
| 3-aminoisobutyrate | -0.01±0.01 | 0 | 5.71E-01 | 5.24E-01 | -0.02±0.14 | 0 | 4.77E-01 | 9.83E-01 | -0.01±0.15 | 9.95E-01 | 0.01±0.15 | 9.93E-01 | -0.2±0.35 | 9.99E-01 | -0.33±0.45 | 1.00E+00 |
| 1-lignoceroyl-GPC (24:0) | 0.01±0.01 | 0 | 5.48E-01 | 5.24E-01 | -0.03±0.15 | 0 | 4.94E-01 | 9.83E-01 | 0.01±0.15 | 9.94E-01 | 0.01±0.16 | 9.94E-01 | -0.25±0.43 | 9.99E-01 | -0.34±0.49 | 1.00E+00 |
| butyrylglycine | 0.05±0.05 | 0 | 4.23E-01 | 5.24E-01 | 0.02±0.14 | 0 | 5.77E-01 | 9.85E-01 | 0.05±0.15 | 9.80E-01 | 0.05±0.15 | 9.62E-01 | -0.28±0.41 | 9.99E-01 | -0.2±0.42 | 1.00E+00 |
| umbelliferone sulfate | -0.05±0.06 | 20.69 | 2.61E-01 | 5.24E-01 | -0.02±0.14 | 0 | 4.69E-01 | 9.87E-01 | -0.06±0.15 | 9.78E-01 | -0.06±0.15 | 9.61E-01 | 0.41±0.42 | 9.99E-01 | 0.26±0.42 | 1.00E+00 |
| hexanoylcarnitine (C6) | 0±0 | 0 | 6.83E-01 | 5.24E-01 | -0.03±0.17 | 0 | 3.86E-01 | 9.87E-01 | 0±0.17 | 9.99E-01 | 0.02±0.18 | 9.92E-01 | -0.16±0.38 | 9.99E-01 | -0.46±0.52 | 1.00E+00 |
| 1-linoleoyl-GPE (18:2)* | 0.03±0.04 | 0 | 3.86E-01 | 5.24E-01 | -0.02±0.14 | 28.17 | 2.38E-01 | 9.91E-01 | 0.04±0.15 | 9.80E-01 | 0.05±0.15 | 9.65E-01 | -0.26±0.35 | 9.99E-01 | -0.42±0.37 | 1.00E+00 |
| X - 21441 | -0.03±0.03 | 0 | 6.32E-01 | 5.24E-01 | -0.03±0.17 | 0 | 5.31E-01 | 9.91E-01 | -0.03±0.17 | 9.94E-01 | -0.05±0.17 | 9.65E-01 | 0.22±0.52 | 9.99E-01 | 0.4±0.69 | 1.00E+00 |
| 9-hydroxystearate | -0.04±0.04 | 17.34 | 2.71E-01 | 5.24E-01 | -0.02±0.14 | 1.09 | 3.15E-01 | 9.92E-01 | -0.04±0.14 | 9.80E-01 | -0.05±0.15 | 9.62E-01 | 0.71±0.68 | 9.99E-01 | 0.65±0.68 | 1.00E+00 |
| deoxycholic acid glucuronide | 0.02±0.02 | 0 | 9.44E-01 | 5.24E-01 | 0.02±0.15 | 0 | 9.44E-01 | 9.92E-01 | 0.02±0.16 | 9.94E-01 | 0.02±0.16 | 9.92E-01 | 0.05±0.47 | 9.99E-01 | 0.06±0.51 | 1.00E+00 |
| sphingomyelin (d17:1/16:0, d18:1/15:0, d16:1/17:0)* | -0.01±0.01 | 0 | 8.74E-01 | 5.24E-01 | -0.02±0.16 | 0 | 6.14E-01 | 9.92E-01 | -0.01±0.16 | 9.94E-01 | 0.01±0.17 | 9.94E-01 | -0.07±0.33 | 9.99E-01 | -0.23±0.43 | 1.00E+00 |
| 3-hydroxyphenylacetoylglutamine | 0.06±0.09 | 79.98 | 2.54E-02 | 5.79E-01 | 0.02±0.14 | 79.45 | 2.74E-02 | 9.92E-01 | 0.09±0.15 | 9.32E-01 | 0.1±0.15 | 9.23E-01 | -1.08±0.52 | 9.99E-01 | -1.18±0.56 | 1.00E+00 |
| spermidine | -0.05±0.13 | 78.73 | 3.01E-02 | 7.12E-01 | -0.02±0.15 | 68.35 | 7.55E-02 | 9.92E-01 | -0.14±0.15 | 8.85E-01 | -0.1±0.16 | 9.23E-01 | 0.91±0.46 | 9.99E-01 | 0.84±0.51 | 1.00E+00 |
| 3-phosphoglycerate | 0.05±0.08 | 54.28 | 1.39E-01 | 5.39E-01 | 0.02±0.14 | 53.35 | 1.43E-01 | 9.92E-01 | 0.08±0.15 | 9.58E-01 | 0.09±0.15 | 9.27E-01 | -0.5±0.38 | 9.99E-01 | -0.61±0.46 | 1.00E+00 |
| N-formylmethionine | 0.05±0.08 | 28.43 | 2.37E-01 | 5.53E-01 | -0.02±0.15 | 57.46 | 1.25E-01 | 9.92E-01 | 0.08±0.16 | 9.58E-01 | 0.1±0.16 | 9.27E-01 | -0.25±0.27 | 9.99E-01 | -0.46±0.32 | 1.00E+00 |
| beta-cryptoxanthin | -0.06±0.13 | 80.37 | 2.40E-02 | 6.51E-01 | -0.02±0.15 | 79.25 | 2.81E-02 | 9.92E-01 | -0.13±0.14 | 8.83E-01 | -0.08±0.15 | 9.39E-01 | 1.16±0.55 | 9.99E-01 | 1.67±0.78 | 1.00E+00 |
| gamma-CEHC | 0.03±0.04 | 60.8 | 1.10E-01 | 5.24E-01 | -0.02±0.14 | 69.99 | 6.79E-02 | 9.92E-01 | 0.04±0.15 | 9.80E-01 | 0.07±0.15 | 9.45E-01 | -0.51±0.34 | 9.99E-01 | -0.77±0.44 | 1.00E+00 |
| histidine | 0.07±0.08 | 0 | 5.56E-01 | 5.24E-01 | 0.02±0.15 | 11.43 | 2.88E-01 | 9.92E-01 | 0.08±0.16 | 9.54E-01 | 0.07±0.16 | 9.45E-01 | -0.14±0.37 | 9.99E-01 | -0.41±0.42 | 1.00E+00 |
| pseudouridine | 0.05±0.07 | 28.88 | 2.36E-01 | 5.44E-01 | -0.02±0.14 | 41.72 | 1.90E-01 | 9.92E-01 | 0.08±0.16 | 9.58E-01 | 0.08±0.16 | 9.45E-01 | -0.26±0.28 | 9.99E-01 | -0.39±0.32 | 1.00E+00 |
| lactosyl-N-behenoyl-sphingosine (d18:1/22:0)* | 0.06±0.07 | 0 | 3.63E-01 | 5.24E-01 | 0.02±0.16 | 0 | 3.83E-01 | 9.92E-01 | 0.07±0.17 | 9.78E-01 | 0.07±0.17 | 9.57E-01 | -0.31±0.41 | 9.99E-01 | -0.36±0.46 | 1.00E+00 |
| N-palmitoylglycine | 0.04±0.04 | 0 | 7.18E-01 | 5.24E-01 | 0.02±0.16 | 0 | 4.97E-01 | 9.92E-01 | 0.04±0.17 | 9.90E-01 | 0.06±0.18 | 9.62E-01 | -0.1±0.39 | 9.99E-01 | -0.24±0.41 | 1.00E+00 |
| N-acetylarginine | 0.04±0.04 | 0 | 8.80E-01 | 5.24E-01 | 0.02±0.14 | 0 | 5.05E-01 | 9.92E-01 | 0.04±0.15 | 9.80E-01 | 0.05±0.15 | 9.65E-01 | -0.03±0.42 | 9.99E-01 | -0.27±0.45 | 1.00E+00 |
| 2-hydroxystearate | 0.04±0.04 | 0 | 7.94E-01 | 5.24E-01 | 0.02±0.15 | 0 | 5.22E-01 | 9.92E-01 | 0.04±0.15 | 9.80E-01 | 0.05±0.16 | 9.65E-01 | -0.07±0.42 | 9.99E-01 | -0.27±0.47 | 1.00E+00 |
| deoxycarnitine | 0.02±0.02 | 0 | 5.94E-01 | 5.24E-01 | -0.02±0.13 | 13.98 | 2.81E-01 | 9.92E-01 | 0.02±0.14 | 9.94E-01 | 0.04±0.14 | 9.67E-01 | -0.14±0.29 | 9.99E-01 | -0.37±0.35 | 1.00E+00 |
| 1-arachidonoyl-GPE (20:4n6)* | -0.02±0.02 | 0 | 4.42E-01 | 5.24E-01 | -0.02±0.15 | 0 | 7.30E-01 | 9.92E-01 | -0.02±0.15 | 9.94E-01 | -0.03±0.15 | 9.69E-01 | 0.4±0.55 | 9.99E-01 | 0.17±0.58 | 1.00E+00 |
| 4-allylphenol sulfate | 0.03±0.03 | 0 | 9.47E-01 | 5.24E-01 | 0.02±0.15 | 0 | 7.22E-01 | 9.92E-01 | 0.03±0.15 | 9.94E-01 | 0.03±0.15 | 9.69E-01 | 0±0.46 | 9.99E-01 | -0.14±0.46 | 1.00E+00 |
| lanthionine | -0.02±0.02 | 0 | 6.02E-01 | 5.24E-01 | -0.02±0.14 | 0 | 6.51E-01 | 9.92E-01 | -0.02±0.14 | 9.94E-01 | -0.04±0.14 | 9.69E-01 | 0.21±0.45 | 9.99E-01 | 0.21±0.51 | 1.00E+00 |
| X - 15503 | 0±0 | 0 | 5.53E-01 | 5.24E-01 | -0.02±0.16 | 20.34 | 2.63E-01 | 9.92E-01 | 0±0.16 | 9.99E-01 | 0.04±0.17 | 9.69E-01 | -0.23±0.39 | 9.99E-01 | -0.53±0.48 | 1.00E+00 |
| 3-hydroxyoleoylcarnitine | -0.04±0.04 | 0 | 6.81E-01 | 5.24E-01 | -0.02±0.14 | 0 | 7.71E-01 | 9.92E-01 | -0.04±0.14 | 9.80E-01 | -0.03±0.15 | 9.79E-01 | 0.16±0.5 | 9.99E-01 | 0.14±0.55 | 1.00E+00 |
| isoleucine | 0.07±0.07 | 0 | 9.28E-01 | 5.24E-01 | 0.02±0.17 | 0 | 8.24E-01 | 9.92E-01 | 0.07±0.17 | 9.78E-01 | 0.04±0.18 | 9.79E-01 | 0.11±0.48 | 9.99E-01 | -0.08±0.5 | 1.00E+00 |
| glutamate | 0.02±0.02 | 0 | 8.92E-01 | 5.24E-01 | -0.02±0.16 | 0 | 9.23E-01 | 9.92E-01 | 0.02±0.15 | 9.94E-01 | -0.03±0.17 | 9.91E-01 | 0.08±0.47 | 9.99E-01 | 0.03±0.54 | 1.00E+00 |
| isovalerylglycine | 0.02±0.02 | 0 | 4.33E-01 | 5.24E-01 | -0.02±0.13 | 0 | 4.43E-01 | 9.92E-01 | 0.02±0.14 | 9.94E-01 | 0.02±0.14 | 9.92E-01 | -0.29±0.39 | 9.99E-01 | -0.31±0.41 | 1.00E+00 |
| mannitol/sorbitol | 0.03±0.03 | 0 | 8.54E-01 | 5.24E-01 | 0.02±0.14 | 0 | 9.40E-01 | 9.92E-01 | 0.03±0.14 | 9.94E-01 | 0.02±0.15 | 9.92E-01 | -0.06±0.45 | 9.99E-01 | -0.02±0.49 | 1.00E+00 |
| pantoate | -0.03±0.03 | 27.77 | 2.39E-01 | 5.24E-01 | 0.02±0.16 | 0 | 3.39E-01 | 9.92E-01 | -0.03±0.16 | 9.94E-01 | -0.03±0.16 | 9.92E-01 | 0.59±0.52 | 9.99E-01 | 0.54±0.56 | 1.00E+00 |
| 3-methoxytyramine sulfate | 0.03±0.03 | 0 | 3.47E-01 | 5.24E-01 | -0.02±0.14 | 0 | 4.00E-01 | 9.92E-01 | 0.03±0.14 | 9.90E-01 | 0.02±0.14 | 9.92E-01 | -0.39±0.45 | 9.99E-01 | -0.42±0.5 | 1.00E+00 |
| behenoyl sphingomyelin (d18:1/22:0)* | -0.02±0.02 | 0 | 9.04E-01 | 5.24E-01 | -0.02±0.14 | 0 | 9.81E-01 | 9.92E-01 | -0.02±0.15 | 9.94E-01 | -0.01±0.15 | 9.92E-01 | 0.02±0.4 | 9.99E-01 | -0.03±0.57 | 1.00E+00 |
| proline | 0.02±0.02 | 0 | 7.71E-01 | 5.24E-01 | -0.02±0.15 | 0 | 5.26E-01 | 9.92E-01 | 0.02±0.15 | 9.94E-01 | 0.02±0.16 | 9.92E-01 | -0.09±0.37 | 9.99E-01 | -0.28±0.44 | 1.00E+00 |
| X - 21442 | 0±0 | 0 | 8.63E-01 | 5.24E-01 | -0.02±0.17 | 0 | 9.59E-01 | 9.92E-01 | 0±0.18 | 9.99E-01 | -0.02±0.19 | 9.92E-01 | -0.09±0.47 | 9.99E-01 | 0±0.51 | 1.00E+00 |
| X - 15728 | 0.03±0.03 | 0 | 4.18E-01 | 5.24E-01 | -0.02±0.15 | 0 | 5.46E-01 | 9.92E-01 | 0.03±0.16 | 9.94E-01 | 0.01±0.16 | 9.93E-01 | -0.34±0.45 | 9.99E-01 | -0.31±0.5 | 1.00E+00 |
| X - 11847 | -0.02±0.02 | 0 | 6.27E-01 | 5.24E-01 | 0.02±0.14 | 0 | 8.27E-01 | 9.92E-01 | -0.02±0.14 | 9.94E-01 | 0.01±0.15 | 9.94E-01 | 0.21±0.48 | 9.99E-01 | 0.12±0.49 | 1.00E+00 |
| X - 24518 | 0±0 | 0 | 8.98E-01 | 5.24E-01 | -0.02±0.15 | 0 | 8.65E-01 | 9.92E-01 | 0±0.16 | 9.99E-01 | -0.01±0.16 | 9.94E-01 | -0.05±0.38 | 9.99E-01 | -0.09±0.45 | 1.00E+00 |
| 5-acetylamino-6-amino-3-methyluracil | 0.01±0.01 | 0 | 3.82E-01 | 5.24E-01 | -0.02±0.15 | 0 | 5.81E-01 | 9.92E-01 | 0.01±0.16 | 9.94E-01 | 0±0.16 | 9.98E-01 | -0.39±0.46 | 9.99E-01 | -0.34±0.61 | 1.00E+00 |
| 1-arachidonoyl-GPC (20:4n6)* | 0±0 | 0 | 4.44E-01 | 5.24E-01 | 0.02±0.15 | 0 | 7.20E-01 | 9.92E-01 | 0±0.15 | 9.99E-01 | 0±0.15 | 9.98E-01 | 0.43±0.56 | 9.99E-01 | 0.22±0.58 | 1.00E+00 |
| N,N-dimethylalanine | 0.04±0.05 | 15.95 | 2.75E-01 | 5.24E-01 | -0.01±0.14 | 31.67 | 2.26E-01 | 9.93E-01 | 0.05±0.14 | 9.80E-01 | 0.03±0.14 | 9.69E-01 | -0.38±0.4 | 9.99E-01 | -0.6±0.5 | 1.00E+00 |
| 3-methylglutarylcarnitine (2) | 0.07±0.09 | 16.16 | 2.75E-01 | 5.34E-01 | 0.01±0.15 | 56.96 | 1.27E-01 | 9.95E-01 | 0.09±0.16 | 9.32E-01 | 0.1±0.16 | 9.26E-01 | -0.31±0.36 | 9.99E-01 | -0.64±0.45 | 1.00E+00 |
| indoleacetylglutamine | -0.02±0.03 | 0 | 3.81E-01 | 5.24E-01 | -0.01±0.14 | 0 | 4.89E-01 | 9.95E-01 | -0.03±0.15 | 9.94E-01 | -0.05±0.15 | 9.65E-01 | 0.29±0.36 | 9.99E-01 | 0.28±0.45 | 1.00E+00 |
| beta-alanine | -0.02±0.02 | 0 | 4.63E-01 | 5.24E-01 | -0.02±0.15 | 0 | 6.22E-01 | 9.95E-01 | -0.02±0.15 | 9.94E-01 | -0.03±0.16 | 9.79E-01 | 0.45±0.64 | 9.99E-01 | 0.32±0.69 | 1.00E+00 |
| oxalate (ethanedioate) | -0.07±0.08 | 0 | 6.12E-01 | 5.24E-01 | -0.01±0.15 | 0 | 7.35E-01 | 9.95E-01 | -0.08±0.15 | 9.48E-01 | -0.03±0.15 | 9.84E-01 | 0.18±0.5 | 9.99E-01 | 0.17±0.56 | 1.00E+00 |
| N-(2-furoyl)glycine | 0.01±0.01 | 0 | 8.54E-01 | 5.24E-01 | -0.01±0.16 | 0 | 9.89E-01 | 9.95E-01 | 0.01±0.16 | 9.94E-01 | -0.02±0.16 | 9.92E-01 | -0.09±0.55 | 9.99E-01 | -0.01±0.48 | 1.00E+00 |
| vanillactate | 0.02±0.02 | 0 | 5.49E-01 | 5.24E-01 | -0.01±0.15 | 0 | 6.84E-01 | 9.95E-01 | 0.02±0.16 | 9.94E-01 | 0.01±0.16 | 9.92E-01 | -0.21±0.38 | 9.99E-01 | -0.15±0.38 | 1.00E+00 |
| X - 24556 | -0.01±0.01 | 0 | 9.34E-01 | 5.24E-01 | 0.01±0.14 | 0 | 9.36E-01 | 9.95E-01 | -0.01±0.14 | 9.94E-01 | 0.02±0.15 | 9.92E-01 | 0.02±0.39 | 9.99E-01 | -0.02±0.44 | 1.00E+00 |
| cortisol | 0±0 | 0 | 9.11E-01 | 5.24E-01 | 0.01±0.15 | 0 | 8.79E-01 | 9.95E-01 | 0±0.15 | 9.99E-01 | 0.01±0.16 | 9.94E-01 | 0.05±0.46 | 9.99E-01 | 0.09±0.49 | 1.00E+00 |
| X - 21467 | 0.01±0.01 | 0 | 7.74E-01 | 5.24E-01 | -0.01±0.16 | 0 | 5.21E-01 | 9.96E-01 | 0.01±0.16 | 9.95E-01 | 0.02±0.17 | 9.92E-01 | -0.11±0.41 | 9.99E-01 | -0.29±0.46 | 1.00E+00 |
| 4-vinylphenol sulfate | 0.04±0.13 | 81.46 | 2.02E-02 | 7.91E-01 | 0.01±0.15 | 72.63 | 5.60E-02 | 9.96E-01 | 0.14±0.15 | 8.83E-01 | 0.12±0.16 | 9.19E-01 | -0.9±0.43 | 9.99E-01 | -0.79±0.44 | 1.00E+00 |
| 1-(1-enyl-palmitoyl)-2-arachidonoyl-GPC (P-16:0/20:4)* | 0.01±0.01 | 0 | 8.70E-01 | 5.24E-01 | -0.01±0.14 | 0 | 8.81E-01 | 9.96E-01 | 0.01±0.15 | 9.94E-01 | -0.01±0.15 | 9.96E-01 | 0.08±0.44 | 9.99E-01 | -0.08±0.47 | 1.00E+00 |
| 1-palmitoyl-2-arachidonoyl-GPC (16:0/20:4n6) | -0.01±0.01 | 0 | 4.81E-01 | 5.24E-01 | -0.01±0.15 | 0 | 6.62E-01 | 9.96E-01 | -0.01±0.15 | 9.95E-01 | -0.03±0.16 | 9.82E-01 | 0.4±0.57 | 9.99E-01 | 0.24±0.61 | 1.00E+00 |
| 4-hydroxyhippurate | -0.04±0.11 | 75.58 | 4.30E-02 | 7.25E-01 | 0±0.14 | 69.99 | 6.79E-02 | 9.97E-01 | -0.12±0.15 | 9.22E-01 | -0.1±0.15 | 9.23E-01 | 0.63±0.35 | 9.99E-01 | 0.64±0.38 | 1.00E+00 |
| 1-(1-enyl-oleoyl)-GPE (P-18:1)* | 0.05±0.1 | 66.56 | 8.38E-02 | 6.12E-01 | 0.01±0.15 | 71.97 | 5.89E-02 | 9.97E-01 | 0.1±0.15 | 9.32E-01 | 0.1±0.15 | 9.23E-01 | -0.54±0.36 | 9.99E-01 | -0.81±0.45 | 1.00E+00 |
| sphingomyelin (d18:1/22:1, d18:2/22:0, d16:1/24:1)* | 0.06±0.09 | 0 | 3.67E-01 | 5.34E-01 | 0.01±0.14 | 36.12 | 2.11E-01 | 9.97E-01 | 0.09±0.16 | 9.32E-01 | 0.1±0.16 | 9.26E-01 | -0.16±0.27 | 9.99E-01 | -0.36±0.33 | 1.00E+00 |
| 6-bromotryptophan | 0.04±0.05 | 9.8 | 2.92E-01 | 5.24E-01 | -0.01±0.14 | 59.08 | 1.18E-01 | 9.97E-01 | 0.05±0.15 | 9.80E-01 | 0.09±0.15 | 9.27E-01 | -0.28±0.31 | 9.99E-01 | -0.49±0.34 | 1.00E+00 |
| hyocholate | 0.06±0.08 | 53.57 | 1.42E-01 | 5.38E-01 | 0±0.14 | 64.74 | 9.21E-02 | 9.97E-01 | 0.08±0.15 | 9.58E-01 | 0.09±0.15 | 9.38E-01 | -0.51±0.4 | 9.99E-01 | -0.71±0.45 | 1.00E+00 |
| 1-methyl-5-imidazoleacetate | 0.05±0.08 | 65.92 | 8.67E-02 | 5.46E-01 | 0.01±0.15 | 64.29 | 9.42E-02 | 9.97E-01 | 0.08±0.16 | 9.58E-01 | 0.09±0.16 | 9.38E-01 | -0.67±0.43 | 9.99E-01 | -0.78±0.49 | 1.00E+00 |
| 1-methyl-5-imidazolelactate | 0.05±0.08 | 73.61 | 5.16E-02 | 5.71E-01 | -0.01±0.16 | 70.04 | 6.77E-02 | 9.97E-01 | 0.08±0.17 | 9.58E-01 | 0.1±0.17 | 9.39E-01 | -0.78±0.44 | 9.99E-01 | -0.88±0.5 | 1.00E+00 |
| 3-(3-hydroxyphenyl)propionate sulfate | -0.06±0.09 | 62.64 | 1.02E-01 | 5.39E-01 | 0±0.15 | 69.15 | 7.18E-02 | 9.97E-01 | -0.09±0.15 | 9.32E-01 | -0.08±0.16 | 9.43E-01 | 0.74±0.5 | 9.99E-01 | 0.9±0.52 | 1.00E+00 |
| 1-oleoyl-2-docosahexaenoyl-GPC (18:1/22:6)* | 0.05±0.07 | 18.6 | 2.68E-01 | 5.34E-01 | -0.01±0.14 | 40.1 | 1.96E-01 | 9.97E-01 | 0.07±0.15 | 9.72E-01 | 0.07±0.16 | 9.45E-01 | -0.24±0.28 | 9.99E-01 | -0.43±0.36 | 1.00E+00 |
| 4-chlorobenzoic acid | 0.05±0.07 | 39.23 | 2.00E-01 | 5.24E-01 | 0.01±0.14 | 47.09 | 1.69E-01 | 9.97E-01 | 0.07±0.15 | 9.74E-01 | 0.07±0.15 | 9.45E-01 | -0.53±0.46 | 9.99E-01 | -0.57±0.45 | 1.00E+00 |
| X - 12822 | 0.04±0.04 | 5.4 | 3.04E-01 | 5.24E-01 | 0±0.15 | 33.3 | 2.21E-01 | 9.97E-01 | 0.04±0.16 | 9.80E-01 | 0.08±0.16 | 9.45E-01 | -0.32±0.35 | 9.99E-01 | -0.44±0.39 | 1.00E+00 |
| X - 17354 | 0.03±0.04 | 55.52 | 1.34E-01 | 5.24E-01 | 0±0.15 | 52.67 | 1.46E-01 | 9.97E-01 | 0.04±0.15 | 9.80E-01 | 0.07±0.16 | 9.45E-01 | -0.68±0.48 | 9.99E-01 | -0.7±0.51 | 1.00E+00 |
| X - 11843 | 0.07±0.09 | 48.91 | 1.62E-01 | 5.34E-01 | 0±0.15 | 48.43 | 1.64E-01 | 9.97E-01 | 0.09±0.16 | 9.32E-01 | 0.07±0.16 | 9.50E-01 | -0.59±0.48 | 9.99E-01 | -0.67±0.5 | 1.00E+00 |
| X - 12221 | -0.06±0.07 | 12.72 | 2.84E-01 | 5.24E-01 | -0.01±0.17 | 32.72 | 2.23E-01 | 9.97E-01 | -0.07±0.17 | 9.80E-01 | -0.08±0.18 | 9.50E-01 | 0.48±0.5 | 9.99E-01 | 0.59±0.51 | 1.00E+00 |
| N2-acetyl,N6,N6-dimethyllysine | -0.06±0.07 | 31.01 | 2.29E-01 | 5.25E-01 | -0.01±0.14 | 29.58 | 2.33E-01 | 9.97E-01 | -0.07±0.14 | 9.58E-01 | -0.06±0.14 | 9.58E-01 | 0.47±0.44 | 9.99E-01 | 0.56±0.5 | 1.00E+00 |
| N-acetyltryptophan | 0.04±0.04 | 0 | 4.36E-01 | 5.24E-01 | 0±0.15 | 0 | 3.32E-01 | 9.97E-01 | 0.04±0.16 | 9.80E-01 | 0.06±0.16 | 9.62E-01 | -0.29±0.43 | 9.99E-01 | -0.42±0.47 | 1.00E+00 |
| 1-(1-enyl-palmitoyl)-2-palmitoyl-GPC (P-16:0/16:0)* | 0.04±0.04 | 0 | 6.61E-01 | 5.24E-01 | 0.01±0.15 | 0 | 4.25E-01 | 9.97E-01 | 0.04±0.16 | 9.80E-01 | 0.06±0.16 | 9.62E-01 | -0.09±0.31 | 9.99E-01 | -0.27±0.37 | 1.00E+00 |
| X - 11850 | 0.06±0.08 | 46.35 | 1.72E-01 | 5.28E-01 | -0.01±0.15 | 40.23 | 1.96E-01 | 9.97E-01 | 0.08±0.15 | 9.58E-01 | 0.05±0.16 | 9.65E-01 | -0.6±0.49 | 9.99E-01 | -0.64±0.51 | 1.00E+00 |
| X - 13844 | -0.05±0.05 | 0 | 3.95E-01 | 5.24E-01 | 0±0.15 | 34.4 | 2.17E-01 | 9.97E-01 | -0.05±0.16 | 9.80E-01 | -0.05±0.16 | 9.65E-01 | 0.38±0.51 | 9.99E-01 | 0.7±0.58 | 1.00E+00 |
| X - 22509 | 0.02±0.02 | 32.83 | 2.22E-01 | 5.24E-01 | 0±0.15 | 0 | 3.31E-01 | 9.97E-01 | 0.02±0.15 | 9.94E-01 | 0.05±0.15 | 9.65E-01 | -0.55±0.47 | 9.99E-01 | -0.45±0.49 | 1.00E+00 |
| X - 24456 | 0.03±0.04 | 0 | 4.18E-01 | 5.24E-01 | 0±0.16 | 0 | 4.10E-01 | 9.97E-01 | 0.04±0.17 | 9.90E-01 | 0.06±0.18 | 9.65E-01 | -0.32±0.44 | 9.99E-01 | -0.35±0.45 | 1.00E+00 |
| 1-palmitoyl-2-oleoyl-GPE (16:0/18:1) | -0.02±0.03 | 0 | 3.29E-01 | 5.24E-01 | 0±0.15 | 0 | 3.23E-01 | 9.97E-01 | -0.03±0.15 | 9.94E-01 | -0.04±0.15 | 9.67E-01 | 0.47±0.51 | 9.99E-01 | 0.49±0.52 | 1.00E+00 |
| X - 12283 | -0.06±0.06 | 0 | 3.23E-01 | 5.24E-01 | 0±0.15 | 0 | 3.34E-01 | 9.97E-01 | -0.06±0.15 | 9.78E-01 | -0.04±0.16 | 9.67E-01 | 0.43±0.49 | 9.99E-01 | 0.51±0.55 | 1.00E+00 |
| N-acetylglycine | 0.02±0.03 | 0 | 3.85E-01 | 5.24E-01 | 0±0.16 | 0 | 4.06E-01 | 9.97E-01 | 0.03±0.16 | 9.94E-01 | 0.04±0.17 | 9.67E-01 | -0.31±0.39 | 9.99E-01 | -0.38±0.48 | 1.00E+00 |
| 2-palmitoyl-GPC (16:0)* | 0.01±0.01 | 0 | 6.91E-01 | 5.24E-01 | -0.01±0.15 | 0 | 3.54E-01 | 9.97E-01 | 0.01±0.15 | 9.94E-01 | 0.04±0.16 | 9.67E-01 | -0.15±0.4 | 9.99E-01 | -0.36±0.41 | 1.00E+00 |
| N-acetylcarnosine | 0.04±0.04 | 0 | 5.65E-01 | 5.24E-01 | -0.01±0.18 | 0 | 3.60E-01 | 9.97E-01 | 0.04±0.18 | 9.86E-01 | 0.05±0.19 | 9.67E-01 | -0.19±0.4 | 9.99E-01 | -0.42±0.48 | 1.00E+00 |
| X - 16580 | 0.04±0.04 | 0 | 6.42E-01 | 5.24E-01 | 0±0.15 | 0 | 4.11E-01 | 9.97E-01 | 0.04±0.16 | 9.82E-01 | 0.04±0.16 | 9.67E-01 | -0.12±0.34 | 9.99E-01 | -0.34±0.44 | 1.00E+00 |
| N-acetylasparagine | -0.04±0.05 | 40.45 | 1.95E-01 | 5.24E-01 | 0.01±0.14 | 0.91 | 3.15E-01 | 9.97E-01 | -0.05±0.14 | 9.80E-01 | -0.04±0.15 | 9.69E-01 | 0.53±0.44 | 9.99E-01 | 0.46±0.47 | 1.00E+00 |
| 3-methylglutaconate | 0.02±0.02 | 0 | 8.64E-01 | 5.24E-01 | 0±0.16 | 0 | 4.94E-01 | 9.97E-01 | 0.02±0.17 | 9.94E-01 | 0.04±0.17 | 9.69E-01 | -0.04±0.37 | 9.99E-01 | -0.28±0.43 | 1.00E+00 |
| sphingomyelin (d17:2/16:0, d18:2/15:0)* | 0.01±0.01 | 0 | 6.67E-01 | 5.24E-01 | 0.01±0.17 | 0 | 6.01E-01 | 9.97E-01 | 0.01±0.18 | 9.94E-01 | 0.04±0.18 | 9.69E-01 | -0.17±0.41 | 9.99E-01 | -0.24±0.51 | 1.00E+00 |
| gluconate | 0.03±0.03 | 0 | 7.44E-01 | 5.24E-01 | 0±0.15 | 0 | 5.23E-01 | 9.97E-01 | 0.03±0.15 | 9.93E-01 | 0.03±0.16 | 9.75E-01 | -0.08±0.34 | 9.99E-01 | -0.24±0.39 | 1.00E+00 |
| 1-stearoyl-2-arachidonoyl-GPC (18:0/20:4) | 0±0 | 0 | 4.55E-01 | 5.24E-01 | -0.01±0.15 | 0 | 5.52E-01 | 9.97E-01 | 0±0.15 | 9.99E-01 | -0.03±0.16 | 9.79E-01 | 0.48±0.64 | 9.99E-01 | 0.38±0.68 | 1.00E+00 |
| tryptophan | 0.02±0.02 | 0 | 9.28E-01 | 5.24E-01 | 0.01±0.15 | 0 | 7.19E-01 | 9.97E-01 | 0.02±0.16 | 9.94E-01 | 0.03±0.16 | 9.83E-01 | 0.06±0.44 | 9.99E-01 | -0.15±0.47 | 1.00E+00 |
| decadienedioic acid (C10:2-DC)** | -0.01±0.01 | 14.71 | 2.79E-01 | 5.24E-01 | 0.01±0.15 | 0 | 3.74E-01 | 9.97E-01 | -0.01±0.15 | 9.94E-01 | -0.03±0.15 | 9.84E-01 | 0.52±0.5 | 9.99E-01 | 0.49±0.56 | 1.00E+00 |
| trimethylamine N-oxide | 0.03±0.03 | 0 | 5.40E-01 | 5.24E-01 | -0.01±0.14 | 0 | 5.46E-01 | 9.97E-01 | 0.03±0.15 | 9.92E-01 | 0.02±0.15 | 9.89E-01 | -0.18±0.34 | 9.99E-01 | -0.24±0.41 | 1.00E+00 |
| maleate | 0.01±0.01 | 0 | 7.80E-01 | 5.24E-01 | 0±0.13 | 0 | 6.43E-01 | 9.97E-01 | 0.01±0.13 | 9.94E-01 | 0.02±0.14 | 9.92E-01 | -0.13±0.5 | 9.99E-01 | -0.25±0.56 | 1.00E+00 |
| 2-hydroxypalmitate | 0±0 | 0 | 9.98E-01 | 5.24E-01 | 0±0.15 | 0 | 6.80E-01 | 9.97E-01 | 0±0.15 | 9.99E-01 | 0.02±0.16 | 9.92E-01 | 0±0.43 | 9.99E-01 | -0.19±0.47 | 1.00E+00 |
| hydroquinone sulfate | 0.01±0.01 | 0 | 6.28E-01 | 5.24E-01 | 0±0.14 | 0 | 6.54E-01 | 9.97E-01 | 0.01±0.15 | 9.94E-01 | 0.02±0.15 | 9.92E-01 | -0.19±0.42 | 9.99E-01 | -0.19±0.44 | 1.00E+00 |
| S-methylcysteine sulfoxide | -0.01±0.01 | 0 | 6.25E-01 | 5.24E-01 | 0±0.15 | 0 | 6.67E-01 | 9.97E-01 | -0.01±0.15 | 9.94E-01 | 0.02±0.15 | 9.92E-01 | -0.24±0.48 | 9.99E-01 | -0.22±0.53 | 1.00E+00 |
| 1-oleoyl-2-arachidonoyl-GPE (18:1/20:4)* | -0.02±0.02 | 0 | 4.81E-01 | 5.24E-01 | 0±0.13 | 0 | 5.39E-01 | 9.97E-01 | -0.02±0.13 | 9.94E-01 | -0.02±0.13 | 9.92E-01 | 0.35±0.53 | 9.99E-01 | 0.34±0.57 | 1.00E+00 |
| 2-hydroxybehenate | 0.04±0.04 | 0 | 6.47E-01 | 5.24E-01 | -0.01±0.14 | 0 | 5.03E-01 | 9.97E-01 | 0.04±0.15 | 9.80E-01 | 0.02±0.15 | 9.92E-01 | -0.15±0.41 | 9.99E-01 | -0.3±0.46 | 1.00E+00 |
| phenylpyruvate | -0.01±0.01 | 0 | 6.99E-01 | 5.24E-01 | -0.01±0.16 | 0 | 8.79E-01 | 9.97E-01 | -0.01±0.16 | 9.94E-01 | -0.02±0.17 | 9.92E-01 | 0.17±0.47 | 9.99E-01 | 0.06±0.5 | 1.00E+00 |
| methionine | 0±0 | 0 | 5.68E-01 | 5.24E-01 | -0.01±0.14 | 0 | 8.06E-01 | 9.97E-01 | 0±0.15 | 9.99E-01 | -0.01±0.15 | 9.92E-01 | 0.3±0.53 | 9.99E-01 | 0.13±0.57 | 1.00E+00 |
| X - 12407 | -0.01±0.01 | 0 | 5.30E-01 | 5.24E-01 | 0±0.14 | 0 | 7.14E-01 | 9.97E-01 | -0.01±0.15 | 9.94E-01 | -0.01±0.15 | 9.92E-01 | 0.28±0.46 | 9.99E-01 | 0.16±0.46 | 1.00E+00 |
| X - 18887 | 0.01±0.01 | 0 | 9.92E-01 | 5.24E-01 | 0.01±0.15 | 0 | 8.94E-01 | 9.97E-01 | 0.01±0.16 | 9.94E-01 | 0.01±0.16 | 9.92E-01 | 0.02±0.42 | 9.99E-01 | -0.05±0.47 | 1.00E+00 |
| X - 24970 | 0.02±0.01 | 0 | 7.98E-01 | 5.24E-01 | 0.01±0.14 | 0 | 9.65E-01 | 9.97E-01 | 0.01±0.14 | 9.94E-01 | 0.01±0.15 | 9.92E-01 | 0.13±0.44 | 9.99E-01 | -0.01±0.52 | 1.00E+00 |
| N-methylpipecolate | -0.01±0.01 | 0 | 8.00E-01 | 5.24E-01 | 0.01±0.14 | 0 | 7.07E-01 | 9.97E-01 | -0.01±0.15 | 9.94E-01 | -0.01±0.15 | 9.93E-01 | 0.1±0.43 | 9.99E-01 | 0.19±0.52 | 1.00E+00 |
| X - 23636 | 0.01±0.01 | 0 | 7.45E-01 | 5.24E-01 | 0±0.14 | 0 | 8.64E-01 | 9.97E-01 | 0.01±0.14 | 9.94E-01 | 0.01±0.15 | 9.93E-01 | 0.19±0.54 | 9.99E-01 | -0.08±0.52 | 1.00E+00 |
| cysteinylglycine disulfide* | 0±0 | 0 | 7.97E-01 | 5.24E-01 | -0.01±0.15 | 0 | 9.71E-01 | 9.97E-01 | 0±0.16 | 9.99E-01 | -0.01±0.16 | 9.94E-01 | 0.12±0.48 | 9.99E-01 | 0.02±0.61 | 1.00E+00 |
| 1-(1-enyl-stearoyl)-2-arachidonoyl-GPE (P-18:0/20:4)* | 0.02±0.02 | 0 | 9.04E-01 | 5.24E-01 | 0±0.15 | 0 | 8.76E-01 | 9.97E-01 | 0.02±0.15 | 9.94E-01 | 0.01±0.15 | 9.94E-01 | 0.07±0.42 | 9.99E-01 | -0.07±0.47 | 1.00E+00 |
| X - 11795 | -0.01±0.01 | 0 | 7.75E-01 | 5.24E-01 | 0.01±0.15 | 0 | 9.60E-01 | 9.97E-01 | -0.01±0.15 | 9.94E-01 | 0.01±0.16 | 9.94E-01 | 0.12±0.46 | 9.99E-01 | -0.02±0.56 | 1.00E+00 |
| X - 26119 | 0.01±0.01 | 0 | 8.71E-01 | 5.24E-01 | -0.01±0.15 | 0 | 9.55E-01 | 9.97E-01 | 0.01±0.16 | 9.99E-01 | -0.01±0.16 | 9.94E-01 | 0.09±0.5 | 9.99E-01 | 0.03±0.57 | 1.00E+00 |
| 1,2-dipalmitoyl-GPC (16:0/16:0) | -0.01±0.01 | 0 | 9.08E-01 | 5.24E-01 | 0±0.16 | 0 | 8.18E-01 | 9.97E-01 | -0.01±0.16 | 9.94E-01 | 0.01±0.16 | 9.94E-01 | 0.03±0.4 | 9.99E-01 | -0.11±0.5 | 1.00E+00 |
| eicosapentaenoate (EPA; 20:5n3) | -0.04±0.04 | 0 | 7.86E-01 | 5.24E-01 | -0.01±0.15 | 0 | 9.86E-01 | 9.97E-01 | -0.04±0.16 | 9.80E-01 | -0.01±0.16 | 9.96E-01 | 0.09±0.46 | 9.99E-01 | -0.02±0.52 | 1.00E+00 |
| 2,3-dihydroxyisovalerate | -0.03±0.04 | 0 | 6.60E-01 | 5.24E-01 | 0.01±0.15 | 0 | 7.44E-01 | 9.97E-01 | -0.04±0.15 | 9.86E-01 | -0.01±0.16 | 9.96E-01 | 0.16±0.44 | 9.99E-01 | 0.15±0.47 | 1.00E+00 |
| N-acetylkynurenine (2) | -0.02±0.02 | 0 | 5.40E-01 | 5.24E-01 | 0±0.15 | 0 | 8.90E-01 | 9.97E-01 | -0.02±0.16 | 9.94E-01 | 0±0.16 | 9.99E-01 | 0.25±0.44 | 9.99E-01 | 0.07±0.53 | 1.00E+00 |
| 1-stearoyl-GPI (18:0) | -0.01±0.01 | 0 | 8.99E-01 | 5.24E-01 | 0±0.15 | 0 | 9.30E-01 | 9.97E-01 | -0.01±0.16 | 9.94E-01 | 0±0.16 | 9.99E-01 | 0.05±0.46 | 9.99E-01 | -0.05±0.54 | 1.00E+00 |
| stearoylcarnitine (C18) | -0.04±0.04 | 0 | 6.99E-01 | 5.24E-01 | 0±0.16 | 0 | 9.88E-01 | 9.97E-01 | -0.04±0.16 | 9.80E-01 | 0±0.16 | 9.99E-01 | 0.15±0.48 | 9.99E-01 | 0.01±0.58 | 1.00E+00 |
| X - 22162 | -0.01±0.01 | 0 | 9.14E-01 | 5.24E-01 | -0.01±0.14 | 0 | 8.61E-01 | 9.97E-01 | -0.01±0.14 | 9.94E-01 | 0±0.14 | 9.99E-01 | 0.03±0.39 | 9.99E-01 | -0.09±0.48 | 1.00E+00 |
| acisoga | 0.04±0.05 | 0 | 4.93E-01 | 5.24E-01 | 0±0.15 | 0 | 5.17E-01 | 9.98E-01 | 0.05±0.15 | 9.80E-01 | 0.04±0.16 | 9.69E-01 | -0.22±0.39 | 9.99E-01 | -0.24±0.4 | 1.00E+00 |
| trans-3,4-methyleneheptanoate |  |  |  |  |  |  |  |  | -0.44±0.14 | 4.33E-01 | -0.44±0.14 | 3.91E-01 |  |  |  |  |
| suberate (C8-DC) |  |  |  |  |  |  |  |  | -0.32±0.14 | 6.11E-01 | -0.35±0.15 | 5.80E-01 |  |  |  |  |
| hypoxanthine |  |  |  |  |  |  |  |  | -0.32±0.14 | 5.66E-01 | -0.31±0.14 | 5.97E-01 |  |  |  |  |
| X - 12839 |  |  |  |  |  |  |  |  | -0.35±0.16 | 6.11E-01 | -0.35±0.16 | 5.97E-01 |  |  |  |  |
| 1-linolenoylglycerol (18:3) |  |  |  |  |  |  |  |  | -0.3±0.15 | 6.58E-01 | -0.3±0.15 | 6.30E-01 |  |  |  |  |
| GlcNAc sulfate conjugate of C21H34O2 steroid** | |  |  |  |  |  |  |  | -0.27±0.15 | 6.58E-01 | -0.29±0.15 | 6.30E-01 |  |  |  |  |
| X - 12126 |  |  |  |  |  |  |  |  | 0.33±0.17 | 6.58E-01 | 0.35±0.17 | 6.30E-01 |  |  |  |  |
| xanthosine |  |  |  |  |  |  |  |  | -0.25±0.14 | 6.58E-01 | -0.28±0.15 | 6.72E-01 |  |  |  |  |
| X - 21327 |  |  |  |  |  |  |  |  | -0.23±0.12 | 6.58E-01 | -0.23±0.12 | 6.72E-01 |  |  |  |  |
| X - 24295 |  |  |  |  |  |  |  |  | -0.27±0.15 | 6.58E-01 | -0.29±0.16 | 6.72E-01 |  |  |  |  |
| X - 26058 |  |  |  |  |  |  |  |  | 0.25±0.15 | 6.92E-01 | 0.29±0.16 | 6.79E-01 |  |  |  |  |
| trans-2-hexenoylglycine |  |  |  |  |  |  |  |  | -0.25±0.16 | 7.45E-01 | -0.28±0.16 | 7.30E-01 |  |  |  |  |
| inosine |  |  |  |  |  |  |  |  | -0.23±0.13 | 6.62E-01 | -0.24±0.14 | 7.30E-01 |  |  |  |  |
| adrenate (22:4n6) |  |  |  |  |  |  |  |  | -0.28±0.15 | 6.58E-01 | -0.26±0.15 | 7.32E-01 |  |  |  |  |
| 4-hydroxyphenylacetoylcarnitine |  |  |  |  |  |  |  |  | 0.24±0.14 | 6.69E-01 | 0.25±0.14 | 7.32E-01 |  |  |  |  |
| behenoylcarnitine (C22)* |  |  |  |  |  |  |  |  | -0.24±0.14 | 6.58E-01 | -0.23±0.14 | 7.32E-01 |  |  |  |  |
| X - 21312 |  |  |  |  |  |  |  |  | -0.27±0.15 | 6.58E-01 | -0.25±0.15 | 7.32E-01 |  |  |  |  |
| gentisate |  |  |  |  |  |  |  |  | 0.27±0.18 | 7.45E-01 | 0.29±0.18 | 7.35E-01 |  |  |  |  |
| galactonate |  |  |  |  |  |  |  |  | -0.19±0.14 | 7.66E-01 | -0.21±0.14 | 7.81E-01 |  |  |  |  |
| cyclo(gly-pro) |  |  |  |  |  |  |  |  | 0.25±0.16 | 7.45E-01 | 0.24±0.16 | 7.81E-01 |  |  |  |  |
| 2,3-dihydroxy-2-methylbutyrate |  |  |  |  |  |  |  |  | 0.24±0.16 | 7.45E-01 | 0.24±0.16 | 7.81E-01 |  |  |  |  |
| tetrahydrocortisol sulfate (1) |  |  |  |  |  |  |  |  | -0.2±0.14 | 7.58E-01 | -0.22±0.15 | 7.81E-01 |  |  |  |  |
| X - 18935 |  |  |  |  |  |  |  |  | 0.19±0.15 | 7.86E-01 | 0.22±0.15 | 7.81E-01 |  |  |  |  |
| N-acetylaspartate (NAA) |  |  |  |  |  |  |  |  | -0.2±0.14 | 7.58E-01 | -0.21±0.14 | 7.84E-01 |  |  |  |  |
| dimethyl sulfone |  |  |  |  |  |  |  |  | 0.19±0.14 | 7.58E-01 | 0.19±0.14 | 7.84E-01 |  |  |  |  |
| glucuronide of piperine metabolite C17H21NO3 (3)* | |  |  |  |  |  |  |  | -0.2±0.15 | 7.66E-01 | -0.21±0.16 | 7.84E-01 |  |  |  |  |
| cholate |  |  |  |  |  |  |  |  | 0.18±0.15 | 7.92E-01 | 0.2±0.15 | 7.84E-01 |  |  |  |  |
| X - 24728 |  |  |  |  |  |  |  |  | 0.21±0.15 | 7.66E-01 | 0.21±0.16 | 7.84E-01 |  |  |  |  |
| malonylcarnitine |  |  |  |  |  |  |  |  | 0.18±0.14 | 7.86E-01 | 0.19±0.14 | 7.91E-01 |  |  |  |  |
| 5alpha-androstan-3alpha,17alpha-diol monosulfate | |  |  |  |  |  |  |  | -0.25±0.16 | 7.45E-01 | -0.22±0.17 | 7.91E-01 |  |  |  |  |
| 2,2'-Methylenebis(6-tert-butyl-p-cresol) |  |  |  |  |  |  |  |  | -0.22±0.16 | 7.58E-01 | -0.21±0.16 | 7.91E-01 |  |  |  |  |
| docosatrienoate (22:3n6)* |  |  |  |  |  |  |  |  | -0.21±0.16 | 7.66E-01 | -0.21±0.16 | 7.97E-01 |  |  |  |  |
| methyl-4-hydroxybenzoate sulfate |  |  |  |  |  |  |  |  | -0.22±0.15 | 7.45E-01 | -0.19±0.15 | 8.05E-01 |  |  |  |  |
| sucrose |  |  |  |  |  |  |  |  | -0.15±0.15 | 8.75E-01 | -0.19±0.16 | 8.25E-01 |  |  |  |  |
| X - 18345 |  |  |  |  |  |  |  |  | 0.17±0.14 | 7.87E-01 | 0.17±0.14 | 8.35E-01 |  |  |  |  |
| prolylglycine |  |  |  |  |  |  |  |  | 0.15±0.14 | 8.44E-01 | 0.17±0.15 | 8.44E-01 |  |  |  |  |
| succinoyltaurine |  |  |  |  |  |  |  |  | -0.16±0.14 | 8.11E-01 | -0.17±0.14 | 8.44E-01 |  |  |  |  |
| fructose |  |  |  |  |  |  |  |  | -0.17±0.15 | 8.24E-01 | -0.17±0.15 | 8.53E-01 |  |  |  |  |
| 1-oleoyl-GPG (18:1)* |  |  |  |  |  |  |  |  | 0.17±0.17 | 8.48E-01 | 0.18±0.17 | 8.61E-01 |  |  |  |  |
| docosahexaenoylcholine |  |  |  |  |  |  |  |  | 0.16±0.15 | 8.48E-01 | 0.17±0.16 | 8.61E-01 |  |  |  |  |
| X - 17351 |  |  |  |  |  |  |  |  | -0.18±0.15 | 7.93E-01 | -0.17±0.16 | 8.61E-01 |  |  |  |  |
| X - 23641 |  |  |  |  |  |  |  |  | 0.23±0.2 | 8.05E-01 | 0.22±0.2 | 8.61E-01 |  |  |  |  |
| X - 24531 |  |  |  |  |  |  |  |  | 0.2±0.15 | 7.66E-01 | 0.17±0.15 | 8.61E-01 |  |  |  |  |
| bilirubin degradation product, C16H18N2O5 (3)** | |  |  |  |  |  |  |  | -0.18±0.14 | 7.86E-01 | -0.15±0.15 | 8.77E-01 |  |  |  |  |
| iminodiacetate (IDA) |  |  |  |  |  |  |  |  | -0.12±0.14 | 8.85E-01 | -0.14±0.14 | 8.92E-01 |  |  |  |  |
| bilirubin degradation product, C16H18N2O5 (2)** | |  |  |  |  |  |  |  | -0.17±0.14 | 8.02E-01 | -0.14±0.14 | 9.01E-01 |  |  |  |  |
| bilirubin degradation product, C16H18N2O5 (4)** | |  |  |  |  |  |  |  | -0.17±0.14 | 8.02E-01 | -0.14±0.15 | 9.01E-01 |  |  |  |  |
| nervonoylcarnitine (C24:1)* |  |  |  |  |  |  |  |  | -0.17±0.15 | 8.05E-01 | -0.14±0.15 | 9.08E-01 |  |  |  |  |
| lithocholate |  |  |  |  |  |  |  |  | 0.16±0.16 | 8.55E-01 | 0.16±0.16 | 9.08E-01 |  |  |  |  |
| 1-stearoyl-2-oleoyl-GPS (18:0/18:1) |  |  |  |  |  |  |  |  | -0.09±0.14 | 9.32E-01 | -0.13±0.15 | 9.15E-01 |  |  |  |  |
| hexanoylglycine |  |  |  |  |  |  |  |  | -0.15±0.16 | 8.57E-01 | -0.15±0.16 | 9.15E-01 |  |  |  |  |
| gamma-glutamylalanine |  |  |  |  |  |  |  |  | 0.13±0.16 | 9.22E-01 | 0.12±0.16 | 9.15E-01 |  |  |  |  |
| 17alpha-hydroxypregnenolone 3-sulfate |  |  |  |  |  |  |  |  | -0.07±0.15 | 9.62E-01 | -0.12±0.16 | 9.15E-01 |  |  |  |  |
| N-formylanthranilic acid |  |  |  |  |  |  |  |  | 0.1±0.16 | 9.32E-01 | 0.13±0.16 | 9.15E-01 |  |  |  |  |
| alpha-CEHC sulfate |  |  |  |  |  |  |  |  | -0.15±0.15 | 8.48E-01 | -0.11±0.15 | 9.15E-01 |  |  |  |  |
| docosahexaenoylcarnitine (C22:6)* |  |  |  |  |  |  |  |  | 0.1±0.16 | 9.32E-01 | 0.14±0.17 | 9.15E-01 |  |  |  |  |
| 3-hydroxypyridine glucuronide |  |  |  |  |  |  |  |  | 0.14±0.15 | 8.85E-01 | 0.12±0.16 | 9.15E-01 |  |  |  |  |
| bilirubin degradation product, C16H18N2O5 (1)** | |  |  |  |  |  |  |  | -0.15±0.14 | 8.29E-01 | -0.12±0.14 | 9.15E-01 |  |  |  |  |
| cystine |  |  |  |  |  |  |  |  | 0.14±0.17 | 9.17E-01 | 0.13±0.17 | 9.15E-01 |  |  |  |  |
| 5-hydroxyindoleacetate |  |  |  |  |  |  |  |  | -0.13±0.15 | 8.89E-01 | -0.12±0.15 | 9.15E-01 |  |  |  |  |
| X - 11979 |  |  |  |  |  |  |  |  | -0.09±0.15 | 9.32E-01 | -0.12±0.15 | 9.15E-01 |  |  |  |  |
| X - 21959 |  |  |  |  |  |  |  |  | 0.1±0.14 | 9.32E-01 | 0.12±0.15 | 9.15E-01 |  |  |  |  |
| X - 23997 |  |  |  |  |  |  |  |  | 0.15±0.17 | 8.89E-01 | 0.15±0.17 | 9.15E-01 |  |  |  |  |
| 4-hydroxy-2-oxoglutaric acid |  |  |  |  |  |  |  |  | -0.12±0.15 | 9.17E-01 | -0.11±0.15 | 9.18E-01 |  |  |  |  |
| (2,4 or 2,5)-dimethylphenol sulfate |  |  |  |  |  |  |  |  | 0.02±0.15 | 9.94E-01 | -0.14±0.19 | 9.18E-01 |  |  |  |  |
| 4-hydroxyphenylpyruvate |  |  |  |  |  |  |  |  | 0.09±0.15 | 9.32E-01 | 0.11±0.15 | 9.19E-01 |  |  |  |  |
| propionylglycine |  |  |  |  |  |  |  |  | -0.1±0.15 | 9.32E-01 | -0.1±0.15 | 9.23E-01 |  |  |  |  |
| gulonate* |  |  |  |  |  |  |  |  | -0.09±0.13 | 9.32E-01 | -0.09±0.13 | 9.23E-01 |  |  |  |  |
| S-methylmethionine |  |  |  |  |  |  |  |  | -0.12±0.14 | 9.00E-01 | -0.1±0.15 | 9.23E-01 |  |  |  |  |
| mannonate* |  |  |  |  |  |  |  |  | -0.09±0.15 | 9.41E-01 | -0.11±0.16 | 9.23E-01 |  |  |  |  |
| delta-CEHC |  |  |  |  |  |  |  |  | 0.09±0.15 | 9.32E-01 | 0.11±0.15 | 9.23E-01 |  |  |  |  |
| X - 12015 |  |  |  |  |  |  |  |  | 0.07±0.15 | 9.72E-01 | 0.11±0.16 | 9.23E-01 |  |  |  |  |
| X - 12738 |  |  |  |  |  |  |  |  | 0.13±0.16 | 9.12E-01 | 0.11±0.16 | 9.23E-01 |  |  |  |  |
| X - 21410 |  |  |  |  |  |  |  |  | -0.11±0.18 | 9.32E-01 | -0.12±0.18 | 9.23E-01 |  |  |  |  |
| 3-hydroxyhippurate sulfate |  |  |  |  |  |  |  |  | -0.09±0.15 | 9.32E-01 | -0.09±0.16 | 9.26E-01 |  |  |  |  |
| X - 12096 |  |  |  |  |  |  |  |  | 0.11±0.14 | 9.32E-01 | 0.1±0.16 | 9.26E-01 |  |  |  |  |
| X - 23890 |  |  |  |  |  |  |  |  | 0.13±0.16 | 9.22E-01 | 0.1±0.16 | 9.26E-01 |  |  |  |  |
| dihydroferulate |  |  |  |  |  |  |  |  | 0.07±0.16 | 9.70E-01 | 0.09±0.16 | 9.39E-01 |  |  |  |  |
| 1-dihomo-linolenylglycerol (20:3) |  |  |  |  |  |  |  |  | -0.1±0.15 | 9.32E-01 | -0.09±0.16 | 9.39E-01 |  |  |  |  |
| dopamine 4-sulfate |  |  |  |  |  |  |  |  | 0.08±0.14 | 9.52E-01 | 0.08±0.14 | 9.39E-01 |  |  |  |  |
| 1-palmitoylglycerol (16:0) |  |  |  |  |  |  |  |  | 0.09±0.15 | 9.32E-01 | 0.08±0.16 | 9.39E-01 |  |  |  |  |
| oxindolylalanine |  |  |  |  |  |  |  |  | 0.05±0.14 | 9.80E-01 | 0.08±0.15 | 9.39E-01 |  |  |  |  |
| X - 13007 |  |  |  |  |  |  |  |  | 0.08±0.14 | 9.32E-01 | 0.08±0.15 | 9.39E-01 |  |  |  |  |
| ferulic acid 4-sulfate |  |  |  |  |  |  |  |  | -0.08±0.16 | 9.58E-01 | -0.08±0.16 | 9.44E-01 |  |  |  |  |
| stearoylcholine* |  |  |  |  |  |  |  |  | 0.06±0.15 | 9.80E-01 | 0.08±0.16 | 9.44E-01 |  |  |  |  |
| oleoylcholine |  |  |  |  |  |  |  |  | 0.05±0.15 | 9.80E-01 | 0.08±0.16 | 9.45E-01 |  |  |  |  |
| docosatrienoate (22:3n3) |  |  |  |  |  |  |  |  | -0.1±0.16 | 9.32E-01 | -0.08±0.16 | 9.45E-01 |  |  |  |  |
| phenol glucuronide |  |  |  |  |  |  |  |  | 0.07±0.15 | 9.74E-01 | 0.08±0.16 | 9.45E-01 |  |  |  |  |
| catechol glucuronide |  |  |  |  |  |  |  |  | 0.07±0.15 | 9.72E-01 | 0.07±0.16 | 9.45E-01 |  |  |  |  |
| dihydrocaffeate sulfate (2) |  |  |  |  |  |  |  |  | 0.05±0.16 | 9.80E-01 | 0.08±0.16 | 9.45E-01 |  |  |  |  |
| N,N-dimethyl-5-aminovalerate |  |  |  |  |  |  |  |  | -0.06±0.15 | 9.78E-01 | -0.07±0.15 | 9.45E-01 |  |  |  |  |
| X - 12127 |  |  |  |  |  |  |  |  | 0.06±0.16 | 9.78E-01 | 0.07±0.16 | 9.45E-01 |  |  |  |  |
| X - 12013 |  |  |  |  |  |  |  |  | 0.09±0.15 | 9.32E-01 | 0.07±0.15 | 9.52E-01 |  |  |  |  |
| X - 24411 |  |  |  |  |  |  |  |  | 0.1±0.15 | 9.32E-01 | 0.06±0.15 | 9.58E-01 |  |  |  |  |
| X - 24951 |  |  |  |  |  |  |  |  | -0.06±0.13 | 9.74E-01 | -0.05±0.13 | 9.58E-01 |  |  |  |  |
| nisinate (24:6n3) |  |  |  |  |  |  |  |  | -0.08±0.15 | 9.46E-01 | -0.06±0.15 | 9.61E-01 |  |  |  |  |
| X - 12726 |  |  |  |  |  |  |  |  | 0.05±0.16 | 9.80E-01 | 0.06±0.16 | 9.61E-01 |  |  |  |  |
| isoeugenol sulfate |  |  |  |  |  |  |  |  | -0.01±0.14 | 9.94E-01 | -0.06±0.15 | 9.62E-01 |  |  |  |  |
| palmitoylcholine |  |  |  |  |  |  |  |  | 0.03±0.15 | 9.87E-01 | 0.06±0.16 | 9.62E-01 |  |  |  |  |
| cholic acid glucuronide |  |  |  |  |  |  |  |  | 0.04±0.15 | 9.80E-01 | 0.06±0.15 | 9.62E-01 |  |  |  |  |
| 2-hydroxyfluorene sulfate |  |  |  |  |  |  |  |  | 0.16±0.14 | 8.24E-01 | 0.07±0.19 | 9.62E-01 |  |  |  |  |
| branched chain 14:0 dicarboxylic acid** |  |  |  |  |  |  |  |  | 0.02±0.16 | 9.94E-01 | 0.06±0.17 | 9.62E-01 |  |  |  |  |
| X - 23276 |  |  |  |  |  |  |  |  | 0.03±0.14 | 9.87E-01 | 0.06±0.15 | 9.62E-01 |  |  |  |  |
| X - 23648 |  |  |  |  |  |  |  |  | 0.07±0.16 | 9.74E-01 | 0.06±0.16 | 9.62E-01 |  |  |  |  |
| X - 26054 |  |  |  |  |  |  |  |  | -0.06±0.15 | 9.80E-01 | -0.05±0.15 | 9.63E-01 |  |  |  |  |
| suberoylcarnitine (C8-DC) |  |  |  |  |  |  |  |  | -0.05±0.16 | 9.80E-01 | -0.05±0.17 | 9.65E-01 |  |  |  |  |
| linoleoylcholine* |  |  |  |  |  |  |  |  | 0.02±0.15 | 9.94E-01 | 0.05±0.16 | 9.65E-01 |  |  |  |  |
| phosphate |  |  |  |  |  |  |  |  | 0.07±0.16 | 9.74E-01 | 0.05±0.16 | 9.65E-01 |  |  |  |  |
| X - 25948 |  |  |  |  |  |  |  |  | 0.07±0.14 | 9.70E-01 | 0.05±0.15 | 9.65E-01 |  |  |  |  |
| X - 13729 |  |  |  |  |  |  |  |  | 0.04±0.16 | 9.80E-01 | 0.05±0.16 | 9.65E-01 |  |  |  |  |
| 5-acetylamino-6-formylamino-3-methyluracil | |  |  |  |  |  |  |  | 0.02±0.15 | 9.94E-01 | 0.04±0.16 | 9.67E-01 |  |  |  |  |
| 5-hydroxy-2-methylpyridine sulfate |  |  |  |  |  |  |  |  | -0.01±0.15 | 9.94E-01 | -0.04±0.15 | 9.67E-01 |  |  |  |  |
| (R)-3-hydroxybutyrylcarnitine |  |  |  |  |  |  |  |  | -0.02±0.16 | 9.94E-01 | -0.04±0.16 | 9.67E-01 |  |  |  |  |
| gamma-CEHC glucuronide* |  |  |  |  |  |  |  |  | -0.05±0.14 | 9.80E-01 | -0.04±0.15 | 9.67E-01 |  |  |  |  |
| gamma-glutamylhistidine |  |  |  |  |  |  |  |  | 0.05±0.15 | 9.80E-01 | 0.04±0.15 | 9.67E-01 |  |  |  |  |
| N-acetyltaurine |  |  |  |  |  |  |  |  | 0.05±0.14 | 9.80E-01 | 0.04±0.15 | 9.69E-01 |  |  |  |  |
| dihomo-linolenoyl-choline |  |  |  |  |  |  |  |  | 0.02±0.15 | 9.94E-01 | 0.04±0.16 | 9.69E-01 |  |  |  |  |
| ribonate |  |  |  |  |  |  |  |  | -0.02±0.15 | 9.94E-01 | -0.03±0.15 | 9.69E-01 |  |  |  |  |
| 1-arachidonylglycerol (20:4) |  |  |  |  |  |  |  |  | 0±0.16 | 9.99E-01 | 0.03±0.16 | 9.84E-01 |  |  |  |  |
| 1-myristoylglycerol (14:0) |  |  |  |  |  |  |  |  | -0.03±0.15 | 9.94E-01 | -0.03±0.15 | 9.84E-01 |  |  |  |  |
| X - 13658 |  |  |  |  |  |  |  |  | 0.02±0.14 | 9.94E-01 | 0.03±0.15 | 9.84E-01 |  |  |  |  |
| 3-indoleglyoxylic acid |  |  |  |  |  |  |  |  | 0.02±0.14 | 9.94E-01 | 0.02±0.15 | 9.88E-01 |  |  |  |  |
| 2-oleoylglycerol (18:1) |  |  |  |  |  |  |  |  | -0.01±0.16 | 9.94E-01 | -0.01±0.16 | 9.92E-01 |  |  |  |  |
| 2-linoleoylglycerol (18:2) |  |  |  |  |  |  |  |  | 0±0.16 | 9.99E-01 | 0.02±0.16 | 9.92E-01 |  |  |  |  |
| glucuronide of piperine metabolite C17H21NO3 (5)* | |  |  |  |  |  |  |  | 0±0.16 | 9.99E-01 | -0.02±0.16 | 9.92E-01 |  |  |  |  |
| 2,4-di-tert-butylphenol |  |  |  |  |  |  |  |  | 0±0.15 | 9.99E-01 | -0.01±0.16 | 9.92E-01 |  |  |  |  |
| palmitoyl ethanolamide |  |  |  |  |  |  |  |  | -0.04±0.16 | 9.80E-01 | -0.02±0.16 | 9.92E-01 |  |  |  |  |
| X - 12007 |  |  |  |  |  |  |  |  | 0.02±0.16 | 9.94E-01 | 0.02±0.16 | 9.92E-01 |  |  |  |  |
| X - 12713 |  |  |  |  |  |  |  |  | 0±0.16 | 9.99E-01 | 0.01±0.16 | 9.92E-01 |  |  |  |  |
| X - 14056 |  |  |  |  |  |  |  |  | 0±0.15 | 9.99E-01 | -0.01±0.15 | 9.92E-01 |  |  |  |  |
| X - 14082 |  |  |  |  |  |  |  |  | 0±0.16 | 9.99E-01 | -0.01±0.17 | 9.92E-01 |  |  |  |  |
| X - 17346 |  |  |  |  |  |  |  |  | 0.03±0.16 | 9.92E-01 | 0.02±0.16 | 9.92E-01 |  |  |  |  |
| X - 23583 |  |  |  |  |  |  |  |  | -0.04±0.15 | 9.80E-01 | -0.02±0.15 | 9.92E-01 |  |  |  |  |
| X - 24953 |  |  |  |  |  |  |  |  | 0±0.14 | 9.99E-01 | 0.02±0.15 | 9.92E-01 |  |  |  |  |
| histidine betaine (hercynine)* |  |  |  |  |  |  |  |  | -0.02±0.15 | 9.94E-01 | -0.01±0.15 | 9.93E-01 |  |  |  |  |
| dihydroferulic acid sulfate |  |  |  |  |  |  |  |  | 0±0.15 | 9.99E-01 | 0.01±0.16 | 9.93E-01 |  |  |  |  |
| bilirubin degradation product, C17H20N2O5 (2)** | |  |  |  |  |  |  |  | -0.03±0.14 | 9.90E-01 | -0.01±0.14 | 9.93E-01 |  |  |  |  |
| X - 16124 |  |  |  |  |  |  |  |  | -0.02±0.15 | 9.94E-01 | -0.01±0.15 | 9.93E-01 |  |  |  |  |
| X - 23666 |  |  |  |  |  |  |  |  | -0.03±0.15 | 9.94E-01 | -0.01±0.15 | 9.93E-01 |  |  |  |  |
| arachidonoylcholine |  |  |  |  |  |  |  |  | 0±0.15 | 9.99E-01 | 0.01±0.15 | 9.94E-01 |  |  |  |  |
| X - 12104 |  |  |  |  |  |  |  |  | -0.01±0.15 | 9.99E-01 | -0.01±0.15 | 9.94E-01 |  |  |  |  |
| X - 25519 |  |  |  |  |  |  |  |  | 0.04±0.15 | 9.82E-01 | 0.01±0.15 | 9.94E-01 |  |  |  |  |
| 1,2,3-benzenetriol sulfate (2) |  |  |  |  |  |  |  |  | -0.03±0.15 | 9.94E-01 | 0±0.15 | 9.98E-01 |  |  |  |  |
| 1-oleoylglycerol (18:1) |  |  |  |  |  |  |  |  | 0.01±0.15 | 9.94E-01 | 0±0.15 | 9.98E-01 |  |  |  |  |
| cysteine s-sulfate |  |  |  |  |  |  |  |  | 0.02±0.15 | 9.94E-01 | 0±0.15 | 9.99E-01 |  |  |  |  |
| bilirubin degradation product, C17H20N2O5 (1)** | |  |  |  |  |  |  |  | -0.02±0.14 | 9.94E-01 | 0±0.14 | 9.99E-01 |  |  |  |  |
| X - 12261 |  |  |  |  |  |  |  |  | 0.04±0.15 | 9.80E-01 | 0±0.15 | 9.99E-01 |  |  |  |  |
| saccharin |  |  |  |  |  |  |  |  |  |  |  |  | -0.56±0.47 | 9.99E-01 | -0.9±0.56 | 1.00E+00 |
| 3-hydroxymyristate |  |  |  |  |  |  |  |  |  |  |  |  | -0.23±0.47 | 9.99E-01 | -0.3±0.47 | 1.00E+00 |
| beta-sitosterol |  |  |  |  |  |  |  |  |  |  |  |  | -1.06±0.58 | 9.99E-01 | -1.19±0.61 | 1.00E+00 |
| 1-methylhistidine |  |  |  |  |  |  |  |  |  |  |  |  | -0.49±0.32 | 9.99E-01 | -0.68±0.39 | 1.00E+00 |
| 3-methylcrotonylglycine |  |  |  |  |  |  |  |  |  |  |  |  | 0.11±0.48 | 9.99E-01 | 0.31±0.54 | 1.00E+00 |
| gamma-glutamyl-epsilon-lysine |  |  |  |  |  |  |  |  |  |  |  |  | -1±0.35 | 9.99E-01 | -1.19±0.41 | 1.00E+00 |
| N1-Methyl-4-pyridone-3-carboxamide |  |  |  |  |  |  |  |  |  |  |  |  | 1.3±0.73 | 9.99E-01 | 1.38±0.76 | 1.00E+00 |
| 1-stearoyl-2-arachidonoyl-GPS (18:0/20:4) |  |  |  |  |  |  |  |  |  |  |  |  | -0.46±0.44 | 9.99E-01 | -0.39±0.48 | 1.00E+00 |
| 2-hydroxyglutarate |  |  |  |  |  |  |  |  |  |  |  |  | -0.03±0.4 | 9.99E-01 | -0.23±0.43 | 1.00E+00 |
| stearoyl ethanolamide |  |  |  |  |  |  |  |  |  |  |  |  | 0.07±0.44 | 9.99E-01 | 0.06±0.47 | 1.00E+00 |
| palmitoleoyl-oleoyl-glycerol (16:1/18:1) [2]* |  |  |  |  |  |  |  |  |  |  |  |  | 0.25±0.47 | 9.99E-01 | 0.18±0.49 | 1.00E+00 |
| palmitoyl-linoleoyl-glycerol (16:0/18:2) [2]* |  |  |  |  |  |  |  |  |  |  |  |  | 0.08±0.45 | 9.99E-01 | 0.06±0.5 | 1.00E+00 |
| palmitoyl-oleoyl-glycerol (16:0/18:1) [1]* |  |  |  |  |  |  |  |  |  |  |  |  | 0.78±0.54 | 9.99E-01 | 0.91±0.58 | 1.00E+00 |
| palmitoyl-oleoyl-glycerol (16:0/18:1) [2]* |  |  |  |  |  |  |  |  |  |  |  |  | 0.33±0.47 | 9.99E-01 | 0.32±0.5 | 1.00E+00 |
| oleoyl-oleoyl-glycerol (18:1/18:1) [1]* |  |  |  |  |  |  |  |  |  |  |  |  | 0.04±0.45 | 9.99E-01 | -0.06±0.52 | 1.00E+00 |
| linoleoyl-arachidonoyl-glycerol (18:2/20:4) [1]* | |  |  |  |  |  |  |  |  |  |  |  | 0.23±0.43 | 9.99E-01 | 0.25±0.47 | 1.00E+00 |
| linoleoyl-arachidonoyl-glycerol (18:2/20:4) [2]* | |  |  |  |  |  |  |  |  |  |  |  | 0.15±0.43 | 9.99E-01 | 0.2±0.48 | 1.00E+00 |
| palmitoyl-arachidonoyl-glycerol (16:0/20:4) [2]* | |  |  |  |  |  |  |  |  |  |  |  | 0.81±0.57 | 9.99E-01 | 0.99±0.71 | 1.00E+00 |
| linoleoyl-linolenoyl-glycerol (18:2/18:3) [2]* |  |  |  |  |  |  |  |  |  |  |  |  | -0.44±0.34 | 9.99E-01 | -0.49±0.4 | 1.00E+00 |
| palmitoleoyl-linoleoyl-glycerol (16:1/18:2) [1]* | |  |  |  |  |  |  |  |  |  |  |  | 0.4±0.51 | 9.99E-01 | 0.29±0.57 | 1.00E+00 |
| oleoyl-arachidonoyl-glycerol (18:1/20:4) [1]* | |  |  |  |  |  |  |  |  |  |  |  | 0.32±0.48 | 9.99E-01 | 0.3±0.56 | 1.00E+00 |
| oleoyl-arachidonoyl-glycerol (18:1/20:4) [2]* | |  |  |  |  |  |  |  |  |  |  |  | 0.24±0.45 | 9.99E-01 | 0.25±0.53 | 1.00E+00 |
| diacylglycerol (16:1/18:2 [2], 16:0/18:3 [1])* |  |  |  |  |  |  |  |  |  |  |  |  | 0.02±0.44 | 9.99E-01 | -0.04±0.48 | 1.00E+00 |
| linoleoyl-linoleoyl-glycerol (18:2/18:2) [1]* |  |  |  |  |  |  |  |  |  |  |  |  | -0.1±0.43 | 9.99E-01 | -0.14±0.5 | 1.00E+00 |
| linoleoyl-linoleoyl-glycerol (18:2/18:2) [2]* |  |  |  |  |  |  |  |  |  |  |  |  | -0.09±0.42 | 9.99E-01 | -0.16±0.49 | 1.00E+00 |
| stearoyl-arachidonoyl-glycerol (18:0/20:4) [1]* | |  |  |  |  |  |  |  |  |  |  |  | 0.32±0.43 | 9.99E-01 | 0.33±0.49 | 1.00E+00 |
| stearoyl-arachidonoyl-glycerol (18:0/20:4) [2]* | |  |  |  |  |  |  |  |  |  |  |  | -0.01±0.43 | 9.99E-01 | 0.01±0.51 | 1.00E+00 |
| palmitoyl-docosahexaenoyl-glycerol (16:0/22:6) [1]* | |  |  |  |  |  |  |  |  |  |  |  | -0.28±0.43 | 9.99E-01 | -0.23±0.46 | 1.00E+00 |
| N-behenoyl-sphingadienine (d18:2/22:0)* |  |  |  |  |  |  |  |  |  |  |  |  | -0.04±0.36 | 9.99E-01 | -0.16±0.42 | 1.00E+00 |
| palmitoleoyl-arachidonoyl-glycerol (16:1/20:4) [2]* | |  |  |  |  |  |  |  |  |  |  |  | 0.75±0.6 | 9.99E-01 | 0.73±0.68 | 1.00E+00 |
| myristoyl-linoleoyl-glycerol (14:0/18:2) [2]* |  |  |  |  |  |  |  |  |  |  |  |  | 0±0.42 | 9.99E-01 | 0.03±0.45 | 1.00E+00 |
| glycosyl ceramide (d18:1/23:1, d17:1/24:1)* |  |  |  |  |  |  |  |  |  |  |  |  | -0.13±0.41 | 9.99E-01 | -0.13±0.48 | 1.00E+00 |
| ceramide (d18:1/20:0, d16:1/22:0, d20:1/18:0)* | |  |  |  |  |  |  |  |  |  |  |  | 0.01±0.38 | 9.99E-01 | -0.12±0.45 | 1.00E+00 |
| 1-carboxyethylphenylalanine |  |  |  |  |  |  |  |  |  |  |  |  | -0.2±0.45 | 9.99E-01 | -0.46±0.54 | 1.00E+00 |
| 1-carboxyethyltyrosine |  |  |  |  |  |  |  |  |  |  |  |  | 0.06±0.44 | 9.99E-01 | -0.14±0.55 | 1.00E+00 |
| 1-carboxyethylvaline |  |  |  |  |  |  |  |  |  |  |  |  | -0.19±0.45 | 9.99E-01 | -0.36±0.5 | 1.00E+00 |
| 1-carboxyethylleucine |  |  |  |  |  |  |  |  |  |  |  |  | -0.27±0.41 | 9.99E-01 | -0.51±0.49 | 1.00E+00 |
| 1-carboxyethylisoleucine |  |  |  |  |  |  |  |  |  |  |  |  | -0.02±0.42 | 9.99E-01 | -0.23±0.55 | 1.00E+00 |
| (2-butoxyethoxy)acetic acid |  |  |  |  |  |  |  |  |  |  |  |  | -0.15±0.44 | 9.99E-01 | -0.03±0.45 | 1.00E+00 |
| dibutyl sulfosuccinate |  |  |  |  |  |  |  |  |  |  |  |  | 0.1±0.35 | 9.99E-01 | 0.06±0.41 | 1.00E+00 |
| 2-aminoadipate |  |  |  |  |  |  |  |  |  |  |  |  | 0.33±0.49 | 9.99E-01 | 0.33±0.58 | 1.00E+00 |
| serotonin |  |  |  |  |  |  |  |  |  |  |  |  | 0.17±0.46 | 9.99E-01 | 0.43±0.49 | 1.00E+00 |
| thymidine |  |  |  |  |  |  |  |  |  |  |  |  | -0.32±0.4 | 9.99E-01 | -0.36±0.43 | 1.00E+00 |
| X - 12849 |  |  |  |  |  |  |  |  |  |  |  |  | -0.5±0.33 | 9.99E-01 | -0.58±0.38 | 1.00E+00 |
| X - 13684 |  |  |  |  |  |  |  |  |  |  |  |  | -0.27±0.37 | 9.99E-01 | -0.59±0.47 | 1.00E+00 |
| X - 16938 |  |  |  |  |  |  |  |  |  |  |  |  | 0.57±0.47 | 9.99E-01 | 0.51±0.49 | 1.00E+00 |
| X - 16964 |  |  |  |  |  |  |  |  |  |  |  |  | 0.15±0.46 | 9.99E-01 | 0.21±0.49 | 1.00E+00 |
| X - 21829 |  |  |  |  |  |  |  |  |  |  |  |  | -0.29±0.43 | 9.99E-01 | -0.67±0.58 | 1.00E+00 |
| X - 22771 |  |  |  |  |  |  |  |  |  |  |  |  | 0.31±0.58 | 9.99E-01 | 0.44±0.67 | 1.00E+00 |
| X - 24241 |  |  |  |  |  |  |  |  |  |  |  |  | 0.19±0.57 | 9.99E-01 | 0.25±0.62 | 1.00E+00 |
| X - 25828 |  |  |  |  |  |  |  |  |  |  |  |  | 0.14±0.47 | 9.99E-01 | 0.22±0.45 | 1.00E+00 |

Note: The table shows associations from the logistic regression analysis where plasms metabolomics level from the Metabolon platform was main exposure and prevalent prostate cancer as the main outcome in ERGO-4 and ERGO-5, respectively. ERGO-4 and ERGO-5 have 939 overlapping circulating metabolites, and ERGO-4&5 refers to the correlation between prevalent prostate cancer and 939 metabolites derived from meta-analysis of ERGO-4 and ERGO-5. Model 1: adjusted for age, cohort, and lipid lowering medication. Model 2: adjusted for model1, smoking status, BMI, alcohol and education. The table is ranked based on the *FDR* values in model 2 within the E4&E5 cohort, using bold text indicates metabolites are surpassing *FDR* adjusted *P* value threshold (*P*<0.05). ERGO-4 and ERGO-5 refer to the fourth and fifth visits subcohort from the baseline cohort. Abbreviations: SE, standard error; I^2^: heterogeneity parameter, Het. *P*-value: significance of the heterogeneity; BMI, body mass index; FDR, false discovery rate.

**Supplementary Table 13. Serum metabolites associated with incident hematological malignancy from the Nightingale platform.**

| **Metabolite** | **Model 1** | | | **Model 2** | | |
| --- | --- | --- | --- | --- | --- | --- |
|  | **HR** | **95% CI** | ***FDR* value** | **HR** | **95% CI** | ***FDR* value** |
| Cholesteryl esters to total lipids ratio in very large HDL | 0.49 | 0.37-0.64 | **9.67E-05** | 0.48 | 0.36-0.64 | **1.20E-04** |
| Cholesteryl esters to total lipids ratio in small HDL | 0.64 | 0.52-0.78 | **2.10E-03** | 0.66 | 0.54-0.81 | **7.70E-03** |
| Cholesteryl esters to total lipids ratio in large HDL | 0.69 | 0.57-0.85 | **1.65E-02** | 0.68 | 0.56-0.83 | **7.70E-03** |
| Cholesterol to total lipids ratio in large LDL | 0.61 | 0.46-0.79 | **1.65E-02** | 0.61 | 0.47-0.8 | **1.05E-02** |
| Cholesteryl esters to total lipids ratio in medium HDL | 0.67 | 0.54-0.84 | **2.14E-02** | 0.66 | 0.53-0.83 | **1.05E-02** |
| Cholesterol to total lipids ratio in medium HDL | 0.66 | 0.51-0.85 | **2.68E-02** | 0.63 | 0.49-0.81 | **1.05E-02** |
| Cholesteryl esters in small HDL | 0.57 | 0.41-0.81 | **3.30E-02** | 0.53 | 0.38-0.74 | **1.05E-02** |
| Cholesterol to total lipids ratio in small HDL | 0.63 | 0.49-0.8 | **1.39E-02** | 0.64 | 0.51-0.82 | **1.20E-02** |
| Cholesterol to total lipids ratio in IDL | 0.68 | 0.54-0.86 | **2.68E-02** | 0.68 | 0.54-0.84 | **1.51E-02** |
| Cholesterol in small HDL | 0.6 | 0.42-0.86 | 7.22E-02 | 0.55 | 0.39-0.78 | **1.51E-02** |
| Triglycerides to total lipids ratio in large LDL | 1.61 | 1.23-2.12 | **2.14E-02** | 1.6 | 1.21-2.11 | **1.91E-02** |
| Cholesteryl esters to total lipids ratio in large LDL | 0.64 | 0.49-0.84 | **2.68E-02** | 0.66 | 0.52-0.85 | **2.07E-02** |
| Concentration of small HDL particles | 0.62 | 0.44-0.88 | 9.70E-02 | 0.57 | 0.41-0.8 | **2.07E-02** |
| Triglycerides to total lipids ratio in medium LDL | 1.53 | 1.19-1.97 | **2.22E-02** | 1.52 | 1.18-1.96 | **2.14E-02** |
| Cholesterol to total lipids ratio in large HDL | 0.7 | 0.55-0.89 | 5.45E-02 | 0.68 | 0.54-0.86 | **2.26E-02** |
| Cholesteryl esters to total lipids ratio in IDL | 0.68 | 0.53-0.89 | 6.50E-02 | 0.67 | 0.52-0.86 | **2.59E-02** |
| Triglycerides to total lipids ratio in large HDL | 1.53 | 1.14-2.05 | 6.79E-02 | 1.59 | 1.19-2.12 | **2.59E-02** |
| Phospholipids to total lipids ratio in medium HDL | 1.48 | 1.12-1.95 | 7.99E-02 | 1.57 | 1.18-2.09 | **2.83E-02** |
| Triglycerides to total lipids ratio in IDL | 1.52 | 1.13-2.03 | 7.22E-02 | 1.53 | 1.14-2.05 | **6.26E-02** |
| Concentration of HDL particles | 0.68 | 0.46-1 | 3.50E-01 | 0.61 | 0.42-0.88 | 1.05E-01 |
| Total concentration of lipoprotein particles | 0.68 | 0.47-1 | 3.50E-01 | 0.62 | 0.43-0.89 | 1.10E-01 |
| Total lipids in small HDL | 0.71 | 0.49-1.02 | 3.67E-01 | 0.63 | 0.45-0.9 | 1.13E-01 |
| Free cholesterol to total lipids ratio in small LDL | 0.72 | 0.56-0.93 | 1.13E-01 | 0.72 | 0.56-0.93 | 1.33E-01 |
| Cholesteryl esters in medium HDL | 0.72 | 0.5-1.03 | 3.74E-01 | 0.64 | 0.46-0.91 | 1.33E-01 |
| Triglycerides to total lipids ratio in large VLDL | 0.69 | 0.51-0.93 | 1.68E-01 | 0.68 | 0.51-0.93 | 1.37E-01 |
| Cholesterol in medium HDL | 0.73 | 0.51-1.05 | 3.74E-01 | 0.65 | 0.46-0.92 | 1.46E-01 |
| Triglycerides in large HDL | 1.67 | 1.24-2.25 | **2.22E-02** | 1.42 | 1.05-1.91 | 1.98E-01 |
| Triglycerides to total lipids ratio in medium HDL | 1.55 | 1.04-2.3 | 2.63E-01 | 1.59 | 1.07-2.36 | 1.99E-01 |
| Free cholesterol in small HDL | 0.76 | 0.53-1.08 | 4.81E-01 | 0.67 | 0.48-0.95 | 1.99E-01 |
| Phospholipids in small HDL | 0.76 | 0.53-1.1 | 4.99E-01 | 0.67 | 0.47-0.95 | 2.07E-01 |
| Cholesteryl esters in HDL | 0.72 | 0.49-1.07 | 4.33E-01 | 0.65 | 0.44-0.95 | 2.12E-01 |
| Triglycerides to total lipids ratio in small LDL | 1.4 | 1.04-1.88 | 2.31E-01 | 1.4 | 1.03-1.89 | 2.19E-01 |
| Free cholesterol in medium LDL | 0.75 | 0.56-1.01 | 3.50E-01 | 0.72 | 0.54-0.97 | 2.19E-01 |
| Free cholesterol in small LDL | 0.75 | 0.56-1 | 3.50E-01 | 0.73 | 0.54-0.97 | 2.19E-01 |
| Concentration of medium HDL particles | 0.78 | 0.54-1.11 | 5.21E-01 | 0.69 | 0.49-0.97 | 2.19E-01 |
| Free cholesterol in medium HDL | 0.78 | 0.54-1.12 | 5.21E-01 | 0.69 | 0.49-0.97 | 2.19E-01 |
| Phospholipids to total lipids ratio in large LDL | 1.36 | 1.02-1.81 | 2.99E-01 | 1.34 | 1.02-1.75 | 2.26E-01 |
| Free cholesterol to total lipids ratio in large VLDL | 1.4 | 1-1.96 | 3.50E-01 | 1.4 | 1.01-1.95 | 2.39E-01 |
| Triglycerides to total lipids ratio in small HDL | 1.45 | 0.98-2.13 | 3.67E-01 | 1.49 | 1.01-2.19 | 2.39E-01 |
| Cholesteryl esters in large LDL | 0.77 | 0.57-1.03 | 3.74E-01 | 0.73 | 0.54-0.98 | 2.39E-01 |
| Cholesterol in large LDL | 0.76 | 0.56-1.04 | 3.74E-01 | 0.73 | 0.53-0.99 | 2.39E-01 |
| Free cholesterol in LDL | 0.76 | 0.55-1.03 | 3.74E-01 | 0.72 | 0.53-0.99 | 2.39E-01 |
| HDL cholesterol | 0.74 | 0.5-1.11 | 4.99E-01 | 0.66 | 0.45-0.98 | 2.39E-01 |
| Free cholesterol to total lipids ratio in medium HDL | 0.77 | 0.53-1.1 | 5.05E-01 | 0.69 | 0.49-0.99 | 2.39E-01 |
| Total lipids in medium HDL | 0.79 | 0.56-1.14 | 5.73E-01 | 0.7 | 0.5-0.98 | 2.39E-01 |
| Phospholipids to total lipids ratio in small HDL | 1.41 | 1.09-1.83 | 9.84E-02 | 1.28 | 1-1.63 | 2.41E-01 |
| Cholesterol to total lipids ratio in very large HDL | 0.63 | 0.42-0.93 | 2.05E-01 | 0.68 | 0.46-0.99 | 2.41E-01 |
| Phospholipids to total lipids ratio in very large VLDL | 1.51 | 0.99-2.29 | 3.50E-01 | 1.55 | 1.01-2.38 | 2.41E-01 |
| Phospholipids to total lipids ratio in IDL | 1.32 | 0.91-1.93 | 4.99E-01 | 1.41 | 02-01-2024 | 2.41E-01 |
| Apolipoprotein A1 | 0.78 | 0.53-1.15 | 5.76E-01 | 0.69 | 0.48-1 | 2.41E-01 |
| Triglycerides in very large HDL | 1.45 | 1.09-1.94 | 1.18E-01 | 1.33 | 1-1.78 | 2.46E-01 |
| Free cholesterol in large LDL | 0.76 | 0.55-1.06 | 4.33E-01 | 0.73 | 0.52-1 | 2.51E-01 |
| Phospholipids in medium HDL | 0.83 | 0.59-1.18 | 6.84E-01 | 0.72 | 0.52-1 | 2.51E-01 |
| Free cholesterol to total lipids ratio in medium LDL | 0.74 | 0.55-0.99 | 3.50E-01 | 0.74 | 0.55-1.01 | 2.53E-01 |
| Total esterified cholesterol | 0.78 | 0.54-1.11 | 5.21E-01 | 0.72 | 0.51-1.02 | 2.81E-01 |
| Free cholesterol to total lipids ratio in very large VLDL | 1.33 | 0.97-1.82 | 3.74E-01 | 1.35 | 0.98-1.86 | 2.88E-01 |
| LDL cholesterol | 0.79 | 0.58-1.07 | 4.99E-01 | 0.75 | 0.55-1.02 | 2.93E-01 |
| Total lipids in large LDL | 0.79 | 0.58-1.08 | 4.99E-01 | 0.75 | 0.55-1.03 | 3.06E-01 |
| Triglycerides in HDL | 1.46 | 1.06-2.02 | 2.15E-01 | 1.31 | 0.97-1.79 | 3.42E-01 |
| Cholesteryl esters in LDL | 0.81 | 0.59-1.09 | 5.21E-01 | 0.76 | 0.56-1.04 | 3.42E-01 |
| Total lipids in HDL | 0.83 | 0.57-1.21 | 6.95E-01 | 0.72 | 0.5-1.04 | 3.44E-01 |
| Clinical LDL cholesterol | 0.81 | 0.59-1.1 | 5.21E-01 | 0.76 | 0.56-1.04 | 3.48E-01 |
| Phospholipids in medium LDL | 0.81 | 0.6-1.09 | 5.21E-01 | 0.77 | 0.57-1.04 | 3.48E-01 |
| Phospholipids to total lipids ratio in very small VLDL | 1.28 | 0.91-1.81 | 5.05E-01 | 1.36 | 0.95-1.93 | 3.48E-01 |
| Histidine | 0.77 | 0.6-1 | 3.50E-01 | 0.8 | 0.62-1.04 | 3.52E-01 |
| Average diameter for LDL particles | 0.75 | 0.55-1.02 | 3.74E-01 | 0.77 | 0.57-1.05 | 3.52E-01 |
| Phospholipids in LDL | 0.81 | 0.59-1.11 | 5.59E-01 | 0.77 | 0.56-1.05 | 3.52E-01 |
| Total lipids in LDL | 0.81 | 0.6-1.11 | 5.63E-01 | 0.77 | 0.56-1.05 | 3.52E-01 |
| Total cholesterol | 0.81 | 0.56-1.16 | 6.31E-01 | 0.74 | 0.52-1.06 | 3.52E-01 |
| Cholesteryl esters in large HDL | 0.82 | 0.57-1.17 | 6.57E-01 | 0.75 | 0.53-1.05 | 3.52E-01 |
| Sphingomyelins | 0.82 | 0.56-1.2 | 6.84E-01 | 0.74 | 0.51-1.06 | 3.52E-01 |
| Phospholipids to total lipids ratio in medium LDL | 0.75 | 0.55-1.02 | 3.67E-01 | 0.78 | 0.58-1.05 | 3.54E-01 |
| Phospholipids in large LDL | 0.82 | 0.59-1.13 | 5.79E-01 | 0.77 | 0.55-1.06 | 3.54E-01 |
| Triglycerides in IDL | 1.39 | 1.03-1.86 | 2.62E-01 | 1.27 | 0.94-1.7 | 3.63E-01 |
| Triglycerides to total lipids ratio in very large VLDL | 0.8 | 0.6-1.08 | 4.99E-01 | 0.79 | 0.59-1.06 | 3.63E-01 |
| Cholesteryl esters to total lipids ratio in very small VLDL | 0.78 | 0.56-1.08 | 4.99E-01 | 0.77 | 0.55-1.06 | 3.63E-01 |
| Free cholesterol to total lipids ratio in small VLDL | 0.76 | 0.52-1.1 | 4.99E-01 | 0.74 | 0.51-1.08 | 3.63E-01 |
| Phospholipids to total lipids ratio in small VLDL | 0.75 | 0.51-1.09 | 4.99E-01 | 0.74 | 0.5-1.08 | 3.63E-01 |
| Cholesterol in large HDL | 0.83 | 0.57-1.2 | 6.84E-01 | 0.75 | 0.53-1.07 | 3.63E-01 |
| Free cholesterol in HDL | 0.84 | 0.57-1.25 | 7.57E-01 | 0.74 | 0.5-1.08 | 3.63E-01 |
| Phospholipids in HDL | 0.87 | 0.61-1.25 | 7.93E-01 | 0.76 | 0.54-1.07 | 3.63E-01 |
| Phospholipids to total lipids ratio in large HDL | 1.35 | 0.95-1.91 | 3.97E-01 | 1.34 | 0.93-1.95 | 3.65E-01 |
| Triglycerides in very small VLDL | 1.36 | 0.99-1.87 | 3.67E-01 | 1.28 | 0.93-1.74 | 3.81E-01 |
| Phospholipids in small LDL | 0.82 | 0.6-1.11 | 5.66E-01 | 0.79 | 0.58-1.07 | 3.85E-01 |
| Cholesterol to total lipids ratio in large VLDL | 1.32 | 0.91-1.93 | 4.99E-01 | 1.33 | 0.92-1.94 | 3.92E-01 |
| Cholesterol to total lipids ratio in very small VLDL | 0.79 | 0.56-1.1 | 5.15E-01 | 0.78 | 0.55-1.09 | 4.14E-01 |
| Triglycerides in medium HDL | 1.45 | 1-2.08 | 3.50E-01 | 1.28 | 0.92-1.79 | 4.20E-01 |
| Glucose | 0.77 | 0.53-1.13 | 5.36E-01 | 0.74 | 0.49-1.12 | 4.32E-01 |
| Cholesteryl esters in IDL | 0.85 | 0.6-1.21 | 7.37E-01 | 0.79 | 0.56-1.1 | 4.52E-01 |
| Cholesterol in medium LDL | 0.85 | 0.63-1.16 | 6.84E-01 | 0.8 | 0.59-1.09 | 4.53E-01 |
| Total lipids in medium LDL | 0.86 | 0.63-1.17 | 6.85E-01 | 0.8 | 0.59-1.1 | 4.64E-01 |
| Total cholines | 0.9 | 0.61-1.31 | 8.17E-01 | 0.78 | 0.55-1.11 | 4.64E-01 |
| Phospholipids in very small VLDL | 1.36 | 0.98-1.88 | 3.67E-01 | 1.24 | 0.91-1.71 | 4.75E-01 |
| Cholesterol in IDL | 0.86 | 0.6-1.23 | 7.62E-01 | 0.79 | 0.56-1.12 | 4.89E-01 |
| Concentration of large HDL particles | 0.88 | 0.6-1.27 | 8.02E-01 | 0.79 | 0.55-1.13 | 4.98E-01 |
| Cholesterol in small LDL | 0.85 | 0.62-1.17 | 6.84E-01 | 0.81 | 0.59-1.11 | 5.00E-01 |
| Total phospholipids in lipoprotein particles | 0.9 | 0.62-1.31 | 8.35E-01 | 0.79 | 0.56-1.13 | 5.00E-01 |
| Phosphatidylcholines | 0.92 | 0.64-1.33 | 8.49E-01 | 0.8 | 0.57-1.12 | 5.00E-01 |
| Phosphoglycerides | 0.93 | 0.64-1.34 | 8.59E-01 | 0.8 | 0.57-1.13 | 5.03E-01 |
| Total lipids in small LDL | 0.86 | 0.62-1.18 | 6.97E-01 | 0.82 | 0.59-1.12 | 5.36E-01 |
| Ratio of saturated fatty acids to total fatty acids | 1.27 | 0.99-1.63 | 3.67E-01 | 1.16 | 0.9-1.49 | 5.42E-01 |
| Free cholesterol in very small VLDL | 1.35 | 0.97-1.88 | 3.74E-01 | 1.22 | 0.88-1.69 | 5.42E-01 |
| Concentration of very small VLDL particles | 1.36 | 0.97-1.9 | 3.74E-01 | 1.22 | 0.88-1.7 | 5.42E-01 |
| Total lipids in very small VLDL | 1.34 | 0.97-1.86 | 3.74E-01 | 1.21 | 0.88-1.67 | 5.42E-01 |
| Ratio of omega-6 fatty acids to total fatty acids | 0.81 | 0.64-1.02 | 3.74E-01 | 0.87 | 0.7-1.09 | 5.42E-01 |
| Ratio of polyunsaturated fatty acids to total fatty acids | 0.82 | 0.64-1.04 | 4.33E-01 | 0.87 | 0.68-1.1 | 5.42E-01 |
| Free cholesterol to total lipids ratio in large LDL | 0.83 | 0.61-1.14 | 6.31E-01 | 0.83 | 0.61-1.13 | 5.42E-01 |
| Cholesteryl esters to total lipids ratio in large VLDL | 1.26 | 0.85-1.88 | 6.40E-01 | 1.28 | 0.85-1.91 | 5.42E-01 |
| Phospholipids to total lipids ratio in large VLDL | 1.34 | 0.8-2.25 | 6.57E-01 | 1.36 | 0.81-2.27 | 5.42E-01 |
| Total lipids in large HDL | 0.9 | 0.62-1.3 | 8.17E-01 | 0.81 | 0.57-1.14 | 5.42E-01 |
| Creatinine | 1.03 | 0.76-1.38 | 9.31E-01 | 1.18 | 0.9-1.56 | 5.42E-01 |
| Concentration of large LDL particles | 0.85 | 0.62-1.17 | 6.84E-01 | 0.83 | 0.61-1.13 | 5.50E-01 |
| Alanine | 0.85 | 0.61-1.17 | 6.84E-01 | 0.82 | 0.58-1.16 | 5.56E-01 |
| Pyruvate | 0.87 | 0.66-1.14 | 6.84E-01 | 0.85 | 0.63-1.13 | 5.56E-01 |
| Phospholipids in large HDL | 0.93 | 0.65-1.31 | 8.49E-01 | 0.83 | 0.6-1.15 | 5.64E-01 |
| Triglycerides in large LDL | 1.3 | 0.96-1.76 | 3.74E-01 | 1.19 | 0.87-1.61 | 5.64E-01 |
| Ratio of linoleic acid to total fatty acids | 0.8 | 0.61-1.05 | 4.33E-01 | 0.87 | 0.67-1.12 | 5.64E-01 |
| Triglycerides to total lipids ratio in very large HDL | 1.21 | 0.86-1.69 | 6.57E-01 | 1.2 | 0.87-1.66 | 5.64E-01 |
| Total cholesterol minus HDL-C | 0.89 | 0.65-1.23 | 8.02E-01 | 0.83 | 0.6-1.15 | 5.64E-01 |
| Free cholesterol in IDL | 0.89 | 0.6-1.31 | 8.17E-01 | 0.81 | 0.56-1.18 | 5.64E-01 |
| Total free cholesterol | 0.9 | 0.63-1.28 | 8.17E-01 | 0.82 | 0.58-1.17 | 5.64E-01 |
| Ratio of triglycerides to phosphoglycerides | 1.16 | 0.82-1.63 | 7.57E-01 | 1.21 | 0.86-1.7 | 5.65E-01 |
| Free cholesterol in large HDL | 0.91 | 0.63-1.33 | 8.41E-01 | 0.82 | 0.58-1.17 | 5.65E-01 |
| Acetone | 1.15 | 0.81-1.63 | 7.89E-01 | 1.19 | 0.86-1.66 | 5.73E-01 |
| Ratio of apolipoprotein B to apolipoprotein A1 | 1.11 | 0.82-1.52 | 8.07E-01 | 1.18 | 0.87-1.59 | 5.73E-01 |
| Cholesteryl esters in medium LDL | 0.9 | 0.65-1.24 | 8.17E-01 | 0.84 | 0.61-1.16 | 5.73E-01 |
| Total lipids in lipoprotein particles | 0.93 | 0.65-1.31 | 8.49E-01 | 0.83 | 0.59-1.17 | 5.77E-01 |
| Triglycerides in LDL | 1.27 | 0.94-1.72 | 4.48E-01 | 1.17 | 0.87-1.59 | 5.80E-01 |
| Triglycerides in small HDL | 1.26 | 0.87-1.82 | 5.98E-01 | 1.2 | 0.84-1.71 | 5.94E-01 |
| Albumin | 0.85 | 0.62-1.15 | 6.84E-01 | 0.85 | 0.62-1.16 | 5.94E-01 |
| Glycine | 0.9 | 0.68-1.2 | 8.02E-01 | 0.86 | 0.65-1.15 | 5.94E-01 |
| Cholesteryl esters to total lipids ratio in small LDL | 1.25 | 0.9-1.73 | 5.30E-01 | 1.19 | 0.85-1.68 | 5.97E-01 |
| Omega-6 fatty acids | 0.89 | 0.61-1.29 | 8.17E-01 | 0.83 | 0.57-1.2 | 5.97E-01 |
| Total lipids in IDL | 0.92 | 0.64-1.33 | 8.49E-01 | 0.84 | 0.59-1.19 | 6.13E-01 |
| Cholesteryl esters in small LDL | 0.9 | 0.65-1.25 | 8.17E-01 | 0.85 | 0.61-1.18 | 6.16E-01 |
| Cholesteryl esters to total lipids ratio in medium LDL | 1.23 | 0.85-1.79 | 6.57E-01 | 1.19 | 0.83-1.72 | 6.17E-01 |
| Triglycerides to total lipids ratio in very small VLDL | 1.21 | 0.83-1.76 | 6.84E-01 | 1.21 | 0.82-1.77 | 6.18E-01 |
| Cholesterol to total lipids ratio in small LDL | 0.88 | 0.64-1.2 | 7.67E-01 | 0.85 | 0.62-1.18 | 6.21E-01 |
| Linoleic acid | 0.88 | 0.63-1.25 | 8.02E-01 | 0.85 | 0.6-1.2 | 6.33E-01 |
| Phospholipids to total lipids ratio in small LDL | 0.81 | 0.59-1.12 | 5.70E-01 | 0.85 | 0.61-1.2 | 6.43E-01 |
| Ratio of polyunsaturated fatty acids to monounsaturated fatty acids | 0.84 | 0.63-1.12 | 6.31E-01 | 0.88 | 0.66-1.17 | 6.43E-01 |
| Degree of unsaturation | 0.87 | 0.63-1.2 | 7.57E-01 | 0.86 | 0.63-1.19 | 6.44E-01 |
| Polyunsaturated fatty acids | 0.91 | 0.62-1.34 | 8.41E-01 | 0.84 | 0.57-1.23 | 6.44E-01 |
| Citrate | 1.14 | 0.79-1.65 | 8.02E-01 | 1.18 | 0.82-1.69 | 6.67E-01 |
| Triglycerides in small LDL | 1.19 | 0.87-1.61 | 6.57E-01 | 1.14 | 0.84-1.54 | 6.88E-01 |
| Free cholesterol in very large HDL | 1.19 | 0.81-1.74 | 7.45E-01 | 1.17 | 0.81-1.71 | 6.88E-01 |
| Triglycerides in medium LDL | 1.22 | 0.9-1.66 | 5.73E-01 | 1.14 | 0.84-1.55 | 6.93E-01 |
| Lactate | 0.85 | 0.61-1.19 | 6.97E-01 | 0.86 | 0.61-1.22 | 6.93E-01 |
| Concentration of LDL particles | 0.91 | 0.66-1.25 | 8.17E-01 | 0.88 | 0.64-1.2 | 6.93E-01 |
| Concentration of small VLDL particles | 1.17 | 0.85-1.62 | 6.97E-01 | 1.14 | 0.83-1.56 | 7.10E-01 |
| Cholesteryl esters in small VLDL | 1.18 | 0.86-1.62 | 6.84E-01 | 1.13 | 0.83-1.53 | 7.14E-01 |
| Concentration of VLDL particles | 1.18 | 0.86-1.63 | 6.84E-01 | 1.13 | 0.83-1.54 | 7.20E-01 |
| Isoleucine | 1.01 | 0.75-1.35 | 9.71E-01 | 1.11 | 0.86-1.43 | 7.20E-01 |
| Cholesteryl esters in large VLDL | 1.14 | 0.82-1.59 | 7.76E-01 | 1.13 | 0.82-1.56 | 7.20E-01 |
| Cholesterol in very large VLDL | 1.13 | 0.82-1.57 | 7.93E-01 | 1.12 | 0.82-1.54 | 7.43E-01 |
| Cholesteryl esters in medium VLDL | 0.92 | 0.65-1.29 | 8.41E-01 | 0.88 | 0.62-1.24 | 7.43E-01 |
| Glutamine | 0.85 | 0.63-1.15 | 6.84E-01 | 0.89 | 0.64-1.23 | 7.46E-01 |
| Cholesteryl esters in very large VLDL | 1.13 | 0.82-1.55 | 7.97E-01 | 1.12 | 0.82-1.52 | 7.50E-01 |
| Phospholipids in VLDL | 1.15 | 0.84-1.58 | 7.54E-01 | 1.12 | 0.82-1.52 | 7.57E-01 |
| Cholesterol in very small VLDL | 1.27 | 0.87-1.83 | 5.74E-01 | 1.13 | 0.79-1.62 | 7.72E-01 |
| Phospholipids to total lipids ratio in very large HDL | 1.29 | 0.69-2.42 | 7.67E-01 | 1.22 | 0.68-2.18 | 7.72E-01 |
| Cholesteryl esters to total lipids ratio in medium VLDL | 0.89 | 0.61-1.31 | 8.17E-01 | 0.88 | 0.6-1.28 | 7.72E-01 |
| Phospholipids in IDL | 0.96 | 0.66-1.4 | 9.29E-01 | 0.88 | 0.61-1.27 | 7.72E-01 |
| VLDL cholesterol | 1.16 | 0.84-1.59 | 7.37E-01 | 1.11 | 0.81-1.51 | 7.74E-01 |
| Triglycerides in small VLDL | 1.15 | 0.81-1.63 | 7.89E-01 | 1.12 | 0.8-1.58 | 7.74E-01 |
| Ratio of monounsaturated fatty acids to total fatty acids | 1.13 | 0.81-1.57 | 7.96E-01 | 1.11 | 0.8-1.55 | 7.74E-01 |
| Free cholesterol to total lipids ratio in IDL | 0.86 | 0.58-1.29 | 8.02E-01 | 0.87 | 0.58-1.32 | 7.74E-01 |
| Free cholesterol in VLDL | 1.14 | 0.83-1.57 | 7.62E-01 | 1.1 | 0.81-1.5 | 7.80E-01 |
| Total lipids in small VLDL | 1.14 | 0.83-1.58 | 7.62E-01 | 1.1 | 0.81-1.51 | 7.85E-01 |
| Cholesterol in large VLDL | 1.11 | 0.8-1.55 | 8.17E-01 | 1.11 | 0.8-1.52 | 7.85E-01 |
| Free cholesterol in very large VLDL | 1.12 | 0.79-1.58 | 8.17E-01 | 1.11 | 0.79-1.56 | 7.85E-01 |
| Cholesterol to total lipids ratio in medium LDL | 0.9 | 0.61-1.34 | 8.41E-01 | 0.89 | 0.61-1.29 | 7.85E-01 |
| Cholesteryl esters in VLDL | 1.16 | 0.84-1.6 | 7.37E-01 | 1.1 | 0.8-1.51 | 7.89E-01 |
| Cholesteryl esters in chylomicrons and extremely large VLDL | 1.13 | 0.76-1.67 | 8.17E-01 | 1.13 | 0.76-1.67 | 7.90E-01 |
| Phospholipids in very large HDL | 1.18 | 0.79-1.76 | 7.62E-01 | 1.11 | 0.76-1.63 | 8.07E-01 |
| Total lipids in VLDL | 1.12 | 0.81-1.54 | 8.02E-01 | 1.09 | 0.8-1.5 | 8.07E-01 |
| Cholesterol in chylomicrons and extremely large VLDL | 1.12 | 0.76-1.66 | 8.17E-01 | 1.12 | 0.75-1.67 | 8.07E-01 |
| Cholesterol to total lipids ratio in very large VLDL | 1.1 | 0.75-1.61 | 8.41E-01 | 1.11 | 0.76-1.64 | 8.07E-01 |
| Free cholesterol to total lipids ratio in very small VLDL | 1.05 | 0.73-1.52 | 9.10E-01 | 1.11 | 0.76-1.61 | 8.07E-01 |
| Cholesteryl esters in very large HDL | 0.95 | 0.64-1.43 | 9.28E-01 | 0.89 | 0.6-1.34 | 8.07E-01 |
| Free cholesterol to total lipids ratio in very large HDL | 0.99 | 0.69-1.42 | 9.71E-01 | 1.1 | 0.78-1.54 | 8.07E-01 |
| Total triglycerides | 1.12 | 0.81-1.56 | 8.02E-01 | 1.09 | 0.79-1.51 | 8.07E-01 |
| Total fatty acids | 1.02 | 0.72-1.44 | 9.57E-01 | 0.91 | 0.64-1.29 | 8.07E-01 |
| Free cholesterol to total lipids ratio in chylomicrons and extremely large VLDL | 1.11 | 0.79-1.56 | 8.17E-01 | 1.1 | 0.78-1.54 | 8.07E-01 |
| Apolipoprotein B | 0.97 | 0.7-1.34 | 9.29E-01 | 0.92 | 0.66-1.27 | 8.07E-01 |
| Docosahexaenoic acid | 1.01 | 0.73-1.42 | 9.60E-01 | 0.91 | 0.64-1.3 | 8.07E-01 |
| Free cholesterol in chylomicrons and extremely large VLDL | 1.11 | 0.75-1.65 | 8.35E-01 | 1.11 | 0.74-1.65 | 8.21E-01 |
| Cholesterol in small VLDL | 1.13 | 0.82-1.54 | 7.93E-01 | 1.08 | 0.79-1.46 | 8.38E-01 |
| Phospholipids in very large VLDL | 1.09 | 0.77-1.55 | 8.41E-01 | 1.09 | 0.77-1.54 | 8.38E-01 |
| Triglycerides to total lipids ratio in small VLDL | 1.09 | 0.73-1.64 | 8.49E-01 | 1.1 | 0.73-1.66 | 8.38E-01 |
| Free cholesterol in large VLDL | 1.08 | 0.78-1.51 | 8.41E-01 | 1.08 | 0.78-1.49 | 8.61E-01 |
| Cholesteryl esters in very small VLDL | 1.21 | 0.82-1.79 | 6.97E-01 | 1.08 | 0.74-1.58 | 8.73E-01 |
| Phospholipids in large VLDL | 1.08 | 0.77-1.52 | 8.49E-01 | 1.07 | 0.77-1.49 | 8.73E-01 |
| Cholesterol to total lipids ratio in small VLDL | 0.93 | 0.63-1.38 | 8.81E-01 | 0.92 | 0.62-1.36 | 8.73E-01 |
| Valine | 0.91 | 0.68-1.21 | 8.15E-01 | 0.95 | 0.72-1.25 | 8.86E-01 |
| Phospholipids to total lipids ratio in medium VLDL | 1.12 | 0.73-1.72 | 8.35E-01 | 1.09 | 0.72-1.65 | 8.86E-01 |
| Cholesterol to total lipids ratio in medium VLDL | 0.94 | 0.63-1.42 | 9.10E-01 | 0.92 | 0.62-1.39 | 8.86E-01 |
| 3-Hydroxybutyrate | 1.04 | 0.76-1.44 | 9.13E-01 | 1.06 | 0.78-1.44 | 8.86E-01 |
| Cholesterol in medium VLDL | 0.98 | 0.71-1.36 | 9.58E-01 | 0.94 | 0.68-1.3 | 8.94E-01 |
| Cholesteryl esters to total lipids ratio in small VLDL | 1.08 | 0.71-1.65 | 8.85E-01 | 1.08 | 0.71-1.64 | 9.05E-01 |
| Concentration of very large VLDL particles | 1.07 | 0.76-1.51 | 8.79E-01 | 1.06 | 0.75-1.49 | 9.09E-01 |
| Cholesteryl esters to total lipids ratio in very large VLDL | 1.06 | 0.7-1.6 | 9.10E-01 | 1.07 | 0.71-1.63 | 9.10E-01 |
| Total lipids in chylomicrons and extremely large VLDL | 1.06 | 0.73-1.55 | 9.03E-01 | 1.07 | 0.73-1.56 | 9.17E-01 |
| Ratio of omega-6 fatty acids to omega-3 fatty acids | 0.87 | 0.64-1.19 | 7.54E-01 | 0.95 | 0.69-1.32 | 9.18E-01 |
| Total lipids in very large HDL | 1.11 | 0.76-1.62 | 8.17E-01 | 1.05 | 0.73-1.52 | 9.18E-01 |
| Saturated fatty acids | 1.09 | 0.8-1.49 | 8.17E-01 | 0.96 | 0.7-1.31 | 9.18E-01 |
| Free cholesterol to total lipids ratio in small HDL | 1.1 | 0.76-1.59 | 8.36E-01 | 1.06 | 0.72-1.55 | 9.18E-01 |
| Phenylalanine | 0.93 | 0.71-1.23 | 8.41E-01 | 0.97 | 0.73-1.28 | 9.18E-01 |
| Free cholesterol to total lipids ratio in medium VLDL | 1.09 | 0.7-1.69 | 8.81E-01 | 1.06 | 0.69-1.63 | 9.18E-01 |
| Triglycerides to total lipids ratio in medium VLDL | 0.94 | 0.61-1.42 | 9.03E-01 | 0.95 | 0.63-1.44 | 9.18E-01 |
| Concentration of large VLDL particles | 1.06 | 0.75-1.49 | 9.03E-01 | 1.05 | 0.75-1.46 | 9.18E-01 |
| Triglycerides in VLDL | 1.06 | 0.75-1.5 | 9.03E-01 | 1.04 | 0.74-1.47 | 9.18E-01 |
| Concentration of chylomicrons and extremely large VLDL particles | 1.06 | 0.73-1.54 | 9.03E-01 | 1.06 | 0.72-1.55 | 9.18E-01 |
| Total lipids in very large VLDL | 1.05 | 0.74-1.5 | 9.10E-01 | 1.05 | 0.74-1.47 | 9.18E-01 |
| Phospholipids in chylomicrons and extremely large VLDL | 1.05 | 0.72-1.54 | 9.13E-01 | 1.06 | 0.72-1.56 | 9.18E-01 |
| Remnant cholesterol (non-HDL | 1.03 | 0.74-1.44 | 9.29E-01 | 0.95 | 0.68-1.33 | 9.18E-01 |
| Triglycerides in large VLDL | 0.97 | 0.68-1.39 | 9.31E-01 | 0.96 | 0.67-1.36 | 9.18E-01 |
| Concentration of medium LDL particles | 1.03 | 0.73-1.43 | 9.34E-01 | 0.96 | 0.69-1.34 | 9.18E-01 |
| Ratio of docosahexaenoic acid to total fatty acids | 1.02 | 0.74-1.4 | 9.57E-01 | 0.96 | 0.68-1.34 | 9.18E-01 |
| Free cholesterol to total lipids ratio in large HDL | 0.99 | 0.65-1.5 | 9.71E-01 | 0.94 | 0.62-1.42 | 9.18E-01 |
| Cholesterol in very large HDL | 1.01 | 0.67-1.5 | 9.83E-01 | 0.95 | 0.64-1.42 | 9.18E-01 |
| Concentration of small LDL particles | 1 | 0.71-1.39 | 9.84E-01 | 0.95 | 0.68-1.33 | 9.18E-01 |
| Glycoprotein acetyls | 1.03 | 0.78-1.35 | 9.31E-01 | 0.97 | 0.71-1.31 | 9.21E-01 |
| Phospholipids in small VLDL | 1.08 | 0.79-1.48 | 8.41E-01 | 1.03 | 0.76-1.41 | 9.29E-01 |
| Monounsaturated fatty acids | 1.06 | 0.77-1.47 | 8.85E-01 | 0.97 | 0.7-1.34 | 9.29E-01 |
| Acetoacetate | 1.03 | 0.76-1.42 | 9.29E-01 | 1.03 | 0.76-1.39 | 9.29E-01 |
| Average diameter for VLDL particles | 0.94 | 0.65-1.37 | 9.03E-01 | 0.97 | 0.67-1.39 | 9.31E-01 |
| Average diameter for HDL particles | 1.05 | 0.75-1.48 | 9.10E-01 | 0.97 | 0.69-1.36 | 9.31E-01 |
| Cholesterol to total lipids ratio in chylomicrons and extremely large VLDL | 1.04 | 0.75-1.44 | 9.29E-01 | 1.03 | 0.74-1.44 | 9.31E-01 |
| Phospholipids to total lipids ratio in chylomicrons and extremely large VLDL | 0.97 | 0.7-1.33 | 9.29E-01 | 0.97 | 0.7-1.36 | 9.35E-01 |
| Triglycerides to total lipids ratio in chylomicrons and extremely large VLDL | 0.97 | 0.74-1.27 | 9.29E-01 | 0.98 | 0.75-1.28 | 9.35E-01 |
| Triglycerides in medium VLDL | 0.99 | 0.7-1.41 | 9.71E-01 | 0.97 | 0.69-1.37 | 9.35E-01 |
| Acetate | 1 | 0.73-1.39 | 9.83E-01 | 1.03 | 0.74-1.43 | 9.35E-01 |
| Total lipids in large VLDL | 1.03 | 0.73-1.46 | 9.29E-01 | 1.02 | 0.73-1.43 | 9.43E-01 |
| Cholesteryl esters to total lipids ratio in chylomicrons and extremely large VLDL | 1.03 | 0.74-1.43 | 9.34E-01 | 1.02 | 0.73-1.44 | 9.43E-01 |
| Ratio of omega-3 fatty acids to total fatty acids | 1.1 | 0.81-1.5 | 8.17E-01 | 1.02 | 0.74-1.42 | 9.49E-01 |
| Concentration of IDL particles | 1.08 | 0.75-1.55 | 8.49E-01 | 0.98 | 0.69-1.39 | 9.49E-01 |
| Leucine | 0.92 | 0.69-1.22 | 8.17E-01 | 1.01 | 0.79-1.31 | 9.57E-01 |
| Concentration of very large HDL particles | 1.1 | 0.76-1.58 | 8.41E-01 | 1.02 | 0.7-1.48 | 9.59E-01 |
| Tyrosine | 1.02 | 0.75-1.4 | 9.34E-01 | 0.99 | 0.72-1.34 | 9.60E-01 |
| Total lipids in medium VLDL | 1.02 | 0.74-1.41 | 9.57E-01 | 0.99 | 0.72-1.35 | 9.60E-01 |
| Free cholesterol in small VLDL | 1.04 | 0.76-1.42 | 9.29E-01 | 0.99 | 0.72-1.35 | 9.66E-01 |
| Triglycerides in chylomicrons and extremely large VLDL | 0.98 | 0.7-1.39 | 9.57E-01 | 0.99 | 0.7-1.39 | 9.67E-01 |
| Total concentration of branched-chain amino acids (leucine + isoleucine + valine) | 0.92 | 0.69-1.24 | 8.29E-01 | 1 | 0.76-1.3 | 9.88E-01 |
| Concentration of medium VLDL particles | 1.03 | 0.75-1.42 | 9.31E-01 | 0.99 | 0.72-1.36 | 9.88E-01 |
| Triglycerides in very large VLDL | 1.02 | 0.71-1.45 | 9.60E-01 | 1.01 | 0.71-1.42 | 9.88E-01 |
| Omega-3 fatty acids | 1.11 | 0.8-1.54 | 8.17E-01 | 1 | 0.71-1.4 | 1.00E+00 |
| Free cholesterol in medium VLDL | 1.04 | 0.76-1.43 | 9.16E-01 | 1 | 0.73-1.37 | 1.00E+00 |
| Phospholipids in medium VLDL | 1.04 | 0.76-1.43 | 9.23E-01 | 1 | 0.73-1.37 | 1.00E+00 |

Note: the table shows associations from The competing risk Cox proportional hazards regression analysis where plasms metabolomics level from the Nightingale platform was main exposure and incident blood tumors as the main outcome in ERGO-4 cohort. Model 1 : adjusted for age, sex, cohort, and lipid lowering medication. Model 2: adjusted for model1, smoking status, BMI, alcohol and education. The table is ranked based on the *FDR* values in model 2, using bold text indicates metabolites are surpassing *FDR* adjusted P value treshold (*P*<0.05). ERGO-4 refer to the fourth and fifth visits from the baseline cohort. Abbreviations: IDL, intermediate-density lipoprotein; LDL, low-density lipoprotein; HDL, high-density lipoprotein; VLDL, very-low-density lipoprotein; BMI, body mass index; HR, hazard ratio; CI, confidence interval; FDR, false discovery rate.

**Supplementary Table 14. Serum metabolites associated with incident hematological malignancy from the Metabolon platform.**

| **Metabolite** | **Model 1** | | | **Model 2** | | |
| --- | --- | --- | --- | --- | --- | --- |
|  | **HR** | **95% Ci** | ***FDR* value** | **HR** | **95% Ci** | ***FDR* value** |
| Carnitine | 1.40 | 0.76-2.56 | 9.27E-01 | 1.47 | 0.78-2.76 | 9.51E-01 |
| 3-methyl-2-oxovalerate | 0.66 | 0.4-1.09 | 9.27E-01 | 0.62 | 0.39-0.98 | 9.51E-01 |
| glycerol 3-phosphate | 0.81 | 0.58-1.13 | 9.27E-01 | 0.82 | 0.59-1.15 | 9.51E-01 |
| imidazole lactate | 1.23 | 0.84-1.8 | 9.27E-01 | 1.28 | 0.87-1.86 | 9.51E-01 |
| Xanthosine | 0.82 | 0.59-1.14 | 9.27E-01 | 0.77 | 0.56-1.07 | 9.51E-01 |
| Theophylline | 0.80 | 0.58-1.11 | 9.27E-01 | 0.82 | 0.6-1.11 | 9.51E-01 |
| Gentisate | 0.75 | 0.53-1.07 | 9.27E-01 | 0.78 | 0.56-1.1 | 9.51E-01 |
| 4-methyl-2-oxopentanoate | 0.66 | 0.4-1.09 | 9.27E-01 | 0.65 | 0.41-1.04 | 9.51E-01 |
| 1,5-anhydroglucitol (1,5-AG) | 1.49 | 0.88-2.53 | 9.27E-01 | 1.55 | 0.91-2.63 | 9.51E-01 |
| 1-myristoyl-2-palmitoyl-GPC (14:0/16:0) | 1.32 | 0.95-1.82 | 9.27E-01 | 1.31 | 0.94-1.84 | 9.51E-01 |
| alpha-hydroxyisocaproate | 0.68 | 0.46-0.99 | 9.27E-01 | 0.67 | 0.45-1.01 | 9.51E-01 |
| 2-hydroxyoctanoate | 0.62 | 0.32-1.21 | 9.27E-01 | 0.63 | 0.33-1.19 | 9.51E-01 |
| cysteine s-sulfate | 1.25 | 0.9-1.74 | 9.27E-01 | 1.27 | 0.92-1.75 | 9.51E-01 |
| 3-methyl-2-oxobutyrate | 0.70 | 0.44-1.13 | 9.27E-01 | 0.68 | 0.45-1.04 | 9.51E-01 |
| 1-linoleoylglycerol (18:2) | 0.63 | 0.39-0.99 | 9.27E-01 | 0.65 | 0.4-1.06 | 9.51E-01 |
| androsterone sulfate | 1.38 | 0.78-2.43 | 9.27E-01 | 1.40 | 0.81-2.41 | 9.51E-01 |
| Indolepropionate | 0.81 | 0.61-1.07 | 9.27E-01 | 0.81 | 0.6-1.09 | 9.51E-01 |
| gamma-glutamylvaline | 0.73 | 0.41-1.32 | 9.28E-01 | 0.66 | 0.37-1.2 | 9.51E-01 |
| 5-hydroxyhexanoate | 0.83 | 0.58-1.17 | 9.27E-01 | 0.83 | 0.58-1.18 | 9.51E-01 |
| Butyrylglycine | 1.36 | 0.79-2.32 | 9.27E-01 | 1.45 | 0.81-2.61 | 9.51E-01 |
| propionylcarnitine (C3) | 1.26 | 0.94-1.69 | 9.27E-01 | 1.23 | 0.94-1.62 | 9.51E-01 |
| pro-hydroxy-pro | 1.20 | 0.82-1.77 | 9.58E-01 | 1.23 | 0.83-1.81 | 9.51E-01 |
| docosapentaenoate (n3 DPA; 22:5n3) | 0.67 | 0.42-1.05 | 9.27E-01 | 0.69 | 0.42-1.13 | 9.51E-01 |
| docosadienoate (22:2n6) | 0.71 | 0.45-1.13 | 9.27E-01 | 0.75 | 0.47-1.22 | 9.51E-01 |
| adrenate (22:4n6) | 0.73 | 0.48-1.11 | 9.27E-01 | 0.75 | 0.45-1.24 | 9.51E-01 |
| 10-undecenoate (11:1n1) | 0.72 | 0.49-1.05 | 9.27E-01 | 0.74 | 0.49-1.11 | 9.51E-01 |
| 1-methyl-4-imidazoleacetate | 1.41 | 1.04-1.91 | 9.27E-01 | 1.39 | 1.02-1.89 | 9.51E-01 |
| 5-dodecenoate (12:1n7) | 0.74 | 0.43-1.26 | 9.27E-01 | 0.76 | 0.45-1.28 | 9.51E-01 |
| octanoylcarnitine (C8) | 0.69 | 0.42-1.14 | 9.27E-01 | 0.70 | 0.41-1.18 | 9.51E-01 |
| N-acetyltryptophan | 0.80 | 0.51-1.26 | 9.44E-01 | 0.76 | 0.47-1.23 | 9.51E-01 |
| N-acetylphenylalanine | 0.82 | 0.57-1.18 | 9.27E-01 | 0.81 | 0.58-1.13 | 9.51E-01 |
| N-acetylarginine | 1.33 | 0.79-2.23 | 9.27E-01 | 1.33 | 0.8-2.22 | 9.51E-01 |
| Piperine | 0.76 | 0.55-1.06 | 9.27E-01 | 0.74 | 0.5-1.1 | 9.51E-01 |
| 1-stearoyl-GPC (18:0) | 0.73 | 0.47-1.13 | 9.27E-01 | 0.78 | 0.48-1.26 | 9.51E-01 |
| N-acetylhistidine | 1.23 | 0.88-1.72 | 9.27E-01 | 1.28 | 0.91-1.81 | 9.51E-01 |
| alpha-hydroxyisovalerate | 0.72 | 0.47-1.12 | 9.27E-01 | 0.72 | 0.44-1.15 | 9.51E-01 |
| N-acetylproline | 0.92 | 0.75-1.12 | 9.62E-01 | 0.89 | 0.72-1.09 | 9.51E-01 |
| linolenate [alpha or gamma; (18:3n3 or 6)] | 0.65 | 0.45-0.94 | 9.27E-01 | 0.67 | 0.46-0.98 | 9.51E-01 |
| laurylcarnitine (C12) | 0.73 | 0.46-1.15 | 9.27E-01 | 0.74 | 0.43-1.26 | 9.51E-01 |
| 1-linoleoyl-GPC (18:2) | 0.72 | 0.43-1.19 | 9.27E-01 | 0.78 | 0.49-1.24 | 9.51E-01 |
| 1,3,7-trimethylurate | 0.80 | 0.54-1.18 | 9.27E-01 | 0.78 | 0.53-1.16 | 9.51E-01 |
| N1-methylinosine | 0.75 | 0.47-1.18 | 9.27E-01 | 0.71 | 0.43-1.18 | 9.51E-01 |
| Orotidine | 1.24 | 0.96-1.61 | 9.27E-01 | 1.25 | 0.93-1.68 | 9.51E-01 |
| 4-hydroxyhippurate | 1.48 | 1.06-2.07 | 9.27E-01 | 1.47 | 1.03-2.1 | 9.51E-01 |
| 1-arachidonylglycerol (20:4) | 0.66 | 0.41-1.08 | 9.27E-01 | 0.70 | 0.4-1.22 | 9.51E-01 |
| 1-linolenoylglycerol (18:3) | 0.75 | 0.53-1.06 | 9.27E-01 | 0.76 | 0.53-1.09 | 9.51E-01 |
| 5-methyluridine (ribothymidine) | 1.33 | 0.75-2.34 | 9.32E-01 | 1.37 | 0.75-2.5 | 9.51E-01 |
| 3-methylcytidine | 1.33 | 0.8-2.2 | 9.27E-01 | 1.35 | 0.79-2.29 | 9.51E-01 |
| 2-hydroxy-3-methylvalerate | 0.65 | 0.39-1.08 | 9.27E-01 | 0.62 | 0.36-1.06 | 9.51E-01 |
| 1-arachidonoyl-GPC (20:4n6)* | 0.63 | 0.41-0.96 | 9.27E-01 | 0.66 | 0.41-1.06 | 9.51E-01 |
| catechol sulfate | 1.54 | 0.94-2.53 | 9.27E-01 | 1.58 | 0.95-2.63 | 9.51E-01 |
| 7-alpha-hydroxy-3-oxo-4-cholestenoate (7-Hoca) | 0.72 | 0.42-1.23 | 9.27E-01 | 0.73 | 0.43-1.23 | 9.51E-01 |
| hexadecanedioate (C16-DC) | 0.73 | 0.49-1.11 | 9.27E-01 | 0.73 | 0.48-1.13 | 9.51E-01 |
| glycerophosphoethanolamine | 1.18 | 0.83-1.68 | 9.62E-01 | 1.20 | 0.87-1.66 | 9.51E-01 |
| 3-(3-hydroxyphenyl)propionate | 1.52 | 0.92-2.53 | 9.27E-01 | 1.56 | 0.94-2.58 | 9.51E-01 |
| glycolithocholate sulfate* | 0.84 | 0.59-1.18 | 9.28E-01 | 0.82 | 0.57-1.18 | 9.51E-01 |
| taurolithocholate 3-sulfate | 0.77 | 0.54-1.11 | 9.27E-01 | 0.76 | 0.52-1.12 | 9.51E-01 |
| 3,4-dihydroxybutyrate | 1.33 | 1.04-1.72 | 9.27E-01 | 1.25 | 0.97-1.62 | 9.51E-01 |
| 4-ethylphenylsulfate | 0.71 | 0.53-0.95 | 9.27E-01 | 0.72 | 0.53-0.97 | 9.51E-01 |
| 1-oleoyl-GPI (18:1) | 1.27 | 0.73-2.2 | 9.62E-01 | 1.33 | 0.77-2.29 | 9.51E-01 |
| dimethylarginine (SDMA + ADMA) | 1.65 | 1.12-2.43 | 8.56E-01 | 1.64 | 1.11-2.42 | 9.51E-01 |
| 1-stearoyl-2-arachidonoyl-GPC (18:0/20:4) | 0.58 | 0.34-0.98 | 9.27E-01 | 0.60 | 0.35-1.03 | 9.51E-01 |
| 1-palmitoyl-2-linoleoyl-GPE (16:0/18:2) | 1.26 | 0.82-1.93 | 9.27E-01 | 1.24 | 0.83-1.86 | 9.51E-01 |
| cyclo(leu-pro) | 0.71 | 0.44-1.14 | 9.27E-01 | 0.69 | 0.42-1.14 | 9.51E-01 |
| succinylcarnitine (C4-DC) | 0.66 | 0.42-1.03 | 9.27E-01 | 0.62 | 0.39-1 | 9.51E-01 |
| taurocholenate sulfate* | 0.75 | 0.49-1.14 | 9.27E-01 | 0.73 | 0.47-1.13 | 9.51E-01 |
| 5alpha-androstan-3alpha,17alpha-diol monosulfate | 1.50 | 0.96-2.36 | 9.27E-01 | 1.47 | 0.97-2.24 | 9.51E-01 |
| 5alpha-androstan-3alpha,17beta-diol monosulfate (1) | 1.42 | 0.77-2.59 | 9.27E-01 | 1.41 | 0.77-2.57 | 9.51E-01 |
| 5alpha-androstan-3beta,17beta-diol monosulfate (2) | 1.30 | 0.78-2.15 | 9.28E-01 | 1.33 | 0.79-2.22 | 9.51E-01 |
| androstenediol (3alpha, 17alpha) monosulfate (3) | 1.26 | 0.82-1.96 | 9.27E-01 | 1.30 | 0.85-1.99 | 9.51E-01 |
| androstenediol (3beta,17beta) monosulfate (2) | 0.70 | 0.47-1.04 | 9.27E-01 | 0.73 | 0.49-1.08 | 9.51E-01 |
| gamma-CEHC | 1.26 | 0.88-1.81 | 9.27E-01 | 1.27 | 0.87-1.85 | 9.51E-01 |
| sphingomyelin (d18:1/18:1, d18:2/18:0) | 0.61 | 0.37-1 | 9.27E-01 | 0.58 | 0.35-0.96 | 9.51E-01 |
| 3-hydroxyhippurate | 1.42 | 0.87-2.33 | 9.27E-01 | 1.43 | 0.9-2.28 | 9.51E-01 |
| 16a-hydroxy DHEA 3-sulfate | 1.21 | 0.92-1.59 | 9.27E-01 | 1.23 | 0.89-1.71 | 9.51E-01 |
| 12-HETE | 0.76 | 0.48-1.19 | 9.27E-01 | 0.75 | 0.48-1.18 | 9.51E-01 |
| 4-cholesten-3-one | 0.63 | 0.43-0.92 | 9.27E-01 | 0.61 | 0.41-0.9 | 9.51E-01 |
| cinnamoylglycine | 0.77 | 0.53-1.11 | 9.27E-01 | 0.75 | 0.51-1.11 | 9.51E-01 |
| cis-4-decenoylcarnitine (C10:1) | 0.62 | 0.42-0.91 | 9.27E-01 | 0.63 | 0.43-0.94 | 9.51E-01 |
| androsterone glucuronide | 1.36 | 0.81-2.26 | 9.27E-01 | 1.40 | 0.83-2.37 | 9.51E-01 |
| cis-4-decenoate (10:1n6)* | 0.74 | 0.5-1.1 | 9.27E-01 | 0.76 | 0.51-1.13 | 9.51E-01 |
| 1-methyl-5-imidazoleacetate | 0.68 | 0.38-1.22 | 9.27E-01 | 0.67 | 0.38-1.18 | 9.51E-01 |
| S-methylcysteine sulfoxide | 1.23 | 0.83-1.81 | 9.28E-01 | 1.26 | 0.84-1.88 | 9.51E-01 |
| eicosanedioate (C20-DC) | 0.71 | 0.46-1.09 | 9.27E-01 | 0.74 | 0.48-1.12 | 9.51E-01 |
| oleoyl-linoleoyl-glycerol (18:1/18:2) [1] | 0.71 | 0.49-1.02 | 9.27E-01 | 0.69 | 0.48-0.98 | 9.51E-01 |
| oleoyl-linoleoyl-glycerol (18:1/18:2) [2] | 0.71 | 0.48-1.06 | 9.27E-01 | 0.69 | 0.47-1 | 9.51E-01 |
| N-oleoyltaurine | 0.65 | 0.37-1.14 | 9.27E-01 | 0.67 | 0.39-1.16 | 9.51E-01 |
| linoleoylcarnitine (C18:2)* | 0.70 | 0.45-1.08 | 9.27E-01 | 0.73 | 0.47-1.13 | 9.51E-01 |
| trimethylamine N-oxide | 0.77 | 0.53-1.13 | 9.27E-01 | 0.75 | 0.52-1.1 | 9.51E-01 |
| N6-methyllysine | 0.60 | 0.37-0.99 | 9.27E-01 | 0.59 | 0.36-0.97 | 9.51E-01 |
| dihydroferulate | 1.42 | 0.89-2.25 | 9.27E-01 | 1.44 | 0.89-2.32 | 9.51E-01 |
| imidazole propionate | 1.27 | 0.81-1.98 | 9.27E-01 | 1.30 | 0.82-2.06 | 9.51E-01 |
| prolylglycine | 0.75 | 0.47-1.2 | 9.27E-01 | 0.76 | 0.47-1.21 | 9.51E-01 |
| histidine betaine (hercynine)* | 1.51 | 1.02-2.23 | 9.27E-01 | 1.56 | 1.05-2.32 | 9.51E-01 |
| guaiacol sulfate | 1.37 | 0.91-2.07 | 9.27E-01 | 1.36 | 0.9-2.06 | 9.51E-01 |
| 3-acetylphenol sulfate | 1.25 | 0.8-1.93 | 9.31E-01 | 1.26 | 0.81-1.96 | 9.51E-01 |
| sphingomyelin (d18:2/16:0, d18:1/16:1)* | 0.61 | 0.37-1 | 9.27E-01 | 0.65 | 0.39-1.06 | 9.51E-01 |
| 6-oxopiperidine-2-carboxylate | 1.36 | 0.93-2 | 9.27E-01 | 1.31 | 0.89-1.93 | 9.51E-01 |
| N-delta-acetylornithine | 0.78 | 0.54-1.13 | 9.27E-01 | 0.79 | 0.51-1.22 | 9.51E-01 |
| acisoga | 0.75 | 0.49-1.14 | 9.27E-01 | 0.75 | 0.47-1.2 | 9.51E-01 |
| 1-linolenoyl-GPC (18:3)* | 0.75 | 0.52-1.08 | 9.27E-01 | 0.81 | 0.57-1.14 | 9.51E-01 |
| N-methylpipecolate | 0.68 | 0.46-1.01 | 9.27E-01 | 0.65 | 0.43-0.98 | 9.51E-01 |
| etiocholanolone glucuronide | 0.65 | 0.45-0.93 | 9.27E-01 | 0.66 | 0.45-0.95 | 9.51E-01 |
| methyl glucopyranoside (alpha + beta) | 1.44 | 0.95-2.2 | 9.27E-01 | 1.44 | 0.97-2.14 | 9.51E-01 |
| sphingomyelin (d18:1/24:1, d18:2/24:0)* | 0.73 | 0.47-1.14 | 9.27E-01 | 0.77 | 0.51-1.17 | 9.51E-01 |
| myristoleoylcarnitine (C14:1)* | 0.74 | 0.5-1.1 | 9.27E-01 | 0.75 | 0.49-1.15 | 9.51E-01 |
| 4-hydroxychlorothalonil | 1.45 | 0.93-2.26 | 9.27E-01 | 1.38 | 0.85-2.25 | 9.51E-01 |
| 4-methylguaiacol sulfate | 1.32 | 0.81-2.15 | 9.27E-01 | 1.33 | 0.83-2.14 | 9.51E-01 |
| 6-hydroxyindole sulfate | 0.82 | 0.56-1.2 | 9.28E-01 | 0.80 | 0.55-1.17 | 9.51E-01 |
| umbelliferone sulfate | 1.30 | 0.86-1.95 | 9.27E-01 | 1.30 | 0.88-1.92 | 9.51E-01 |
| sphingomyelin (d18:1/20:1, d18:2/20:0)* | 0.83 | 0.57-1.21 | 9.32E-01 | 0.82 | 0.57-1.17 | 9.51E-01 |
| sphingomyelin (d17:1/16:0, d18:1/15:0, d16:1/17:0)* | 1.34 | 0.79-2.26 | 9.27E-01 | 1.37 | 0.77-2.42 | 9.51E-01 |
| dopamine 4-sulfate | 1.19 | 0.87-1.64 | 9.27E-01 | 1.20 | 0.86-1.67 | 9.51E-01 |
| 3-methoxycatechol sulfate (2) | 0.77 | 0.5-1.18 | 9.27E-01 | 0.75 | 0.49-1.14 | 9.51E-01 |
| N-acetylkynurenine (2) | 0.87 | 0.67-1.14 | 9.28E-01 | 0.86 | 0.65-1.13 | 9.51E-01 |
| citraconate/glutaconate | 1.29 | 0.83-2 | 9.27E-01 | 1.29 | 0.82-2.03 | 9.51E-01 |
| adipoylcarnitine (C6-DC) | 1.29 | 0.85-1.94 | 9.27E-01 | 1.26 | 0.83-1.92 | 9.51E-01 |
| 4-hydroxyphenylacetoylcarnitine | 0.69 | 0.44-1.09 | 9.27E-01 | 0.69 | 0.44-1.07 | 9.51E-01 |
| nonanoylcarnitine (C9) | 0.66 | 0.36-1.2 | 9.27E-01 | 0.66 | 0.35-1.22 | 9.51E-01 |
| linoleoyl ethanolamide | 0.77 | 0.53-1.12 | 9.27E-01 | 0.82 | 0.56-1.19 | 9.51E-01 |
| 1-palmitoyl-2-arachidonoyl-GPC (16:0/20:4n6) | 0.70 | 0.43-1.13 | 9.27E-01 | 0.72 | 0.44-1.2 | 9.51E-01 |
| 1-(1-enyl-stearoyl)-2-oleoyl-GPE (P-18:0/18:1) | 1.37 | 1.01-1.85 | 9.27E-01 | 1.44 | 1.05-1.98 | 9.51E-01 |
| 2-hydroxybutyrate/2-hydroxyisobutyrate | 0.78 | 0.5-1.23 | 9.27E-01 | 0.72 | 0.41-1.25 | 9.51E-01 |
| sphingomyelin (d18:2/24:1, d18:1/24:2)* | 0.69 | 0.44-1.09 | 9.27E-01 | 0.71 | 0.45-1.13 | 9.51E-01 |
| 1-palmitoyl-2-palmitoleoyl-GPC (16:0/16:1)* | 1.23 | 0.91-1.67 | 9.27E-01 | 1.25 | 0.91-1.72 | 9.51E-01 |
| 1-palmitoyl-2-dihomo-linolenoyl-GPC (16:0/20:3n3 or 6)* | 1.30 | 0.85-1.99 | 9.27E-01 | 1.26 | 0.85-1.85 | 9.51E-01 |
| 1-stearoyl-2-linoleoyl-GPI (18:0/18:2) | 0.75 | 0.46-1.24 | 9.27E-01 | 0.75 | 0.45-1.25 | 9.51E-01 |
| gamma-tocopherol/beta-tocopherol | 0.77 | 0.6-1 | 9.27E-01 | 0.78 | 0.59-1.03 | 9.51E-01 |
| 1-(1-enyl-palmitoyl)-2-oleoyl-GPE (P-16:0/18:1)* | 1.31 | 0.93-1.84 | 9.27E-01 | 1.38 | 0.99-1.9 | 9.51E-01 |
| 1-(1-enyl-palmitoyl)-2-oleoyl-GPC (P-16:0/18:1)* | 1.21 | 0.79-1.84 | 9.62E-01 | 1.40 | 0.95-2.06 | 9.51E-01 |
| sphingomyelin (d18:1/21:0, d17:1/22:0, d16:1/23:0)* | 1.50 | 0.81-2.76 | 9.27E-01 | 1.51 | 0.78-2.92 | 9.51E-01 |
| behenoyl dihydrosphingomyelin (d18:0/22:0)* | 1.29 | 0.83-2.01 | 9.27E-01 | 1.31 | 0.83-2.06 | 9.51E-01 |
| myristoyl dihydrosphingomyelin (d18:0/14:0)* | 1.41 | 0.86-2.32 | 9.27E-01 | 1.47 | 0.89-2.42 | 9.51E-01 |
| 1-palmitoyl-2-oleoyl-GPI (16:0/18:1)* | 1.34 | 0.89-2.02 | 9.27E-01 | 1.35 | 0.92-2 | 9.51E-01 |
| 1-(1-enyl-palmitoyl)-2-linoleoyl-GPE (P-16:0/18:2)* | 1.41 | 0.87-2.3 | 9.27E-01 | 1.45 | 0.88-2.41 | 9.51E-01 |
| 1-oleoyl-2-linoleoyl-GPE (18:1/18:2)* | 0.79 | 0.51-1.22 | 9.27E-01 | 0.80 | 0.51-1.23 | 9.51E-01 |
| 1-linoleoyl-2-arachidonoyl-GPC (18:2/20:4n6)* | 0.67 | 0.5-0.88 | 8.16E-01 | 0.72 | 0.52-0.99 | 9.51E-01 |
| 1-(1-enyl-palmitoyl)-2-palmitoleoyl-GPC (P-16:0/16:1)* | 1.35 | 0.88-2.08 | 9.27E-01 | 1.46 | 0.95-2.25 | 9.51E-01 |
| 1-(1-enyl-palmitoyl)-2-palmitoyl-GPC (P-16:0/16:0)* | 1.31 | 0.84-2.03 | 9.27E-01 | 1.44 | 0.93-2.22 | 9.51E-01 |
| 1-stearoyl-2-oleoyl-GPI (18:0/18:1)* | 1.30 | 0.82-2.06 | 9.27E-01 | 1.35 | 0.85-2.15 | 9.51E-01 |
| 1-oleoyl-2-arachidonoyl-GPE (18:1/20:4)* | 0.78 | 0.54-1.14 | 9.27E-01 | 0.79 | 0.54-1.15 | 9.51E-01 |
| 1-(1-enyl-stearoyl)-2-linoleoyl-GPE (P-18:0/18:2)* | 1.17 | 0.78-1.76 | 9.62E-01 | 1.26 | 0.84-1.89 | 9.51E-01 |
| 1-linoleoyl-GPG (18:2)* | 0.69 | 0.54-0.9 | 8.16E-01 | 0.70 | 0.54-0.91 | 9.51E-01 |
| (S)-3-hydroxybutyrylcarnitine | 0.72 | 0.46-1.12 | 9.27E-01 | 0.69 | 0.44-1.07 | 9.51E-01 |
| methylsuccinoylcarnitine | 1.37 | 0.88-2.14 | 9.27E-01 | 1.37 | 0.85-2.2 | 9.51E-01 |
| catechol glucuronide | 1.48 | 0.95-2.32 | 9.27E-01 | 1.49 | 0.95-2.35 | 9.51E-01 |
| ascorbic acid 2-sulfate | 0.81 | 0.6-1.09 | 9.27E-01 | 0.79 | 0.58-1.07 | 9.51E-01 |
| 1-linoleoyl-2-linolenoyl-GPC (18:2/18:3)* | 0.79 | 0.57-1.11 | 9.27E-01 | 0.83 | 0.6-1.14 | 9.51E-01 |
| hexadecadienoate (16:2n6) | 0.77 | 0.51-1.17 | 9.27E-01 | 0.78 | 0.52-1.19 | 9.51E-01 |
| lactosyl-N-nervonoyl-sphingosine (d18:1/24:1)* | 1.35 | 0.86-2.1 | 9.27E-01 | 1.43 | 0.91-2.25 | 9.51E-01 |
| N-stearoyl-sphingadienine (d18:2/18:0)* | 0.64 | 0.43-0.96 | 9.27E-01 | 0.61 | 0.4-0.93 | 9.51E-01 |
| 2-hydroxybehenate | 1.25 | 0.83-1.9 | 9.27E-01 | 1.30 | 0.8-2.11 | 9.51E-01 |
| 2-hydroxynervonate* | 1.29 | 0.82-2.01 | 9.27E-01 | 1.31 | 0.78-2.19 | 9.51E-01 |
| hexadecasphingosine (d16:1)* | 1.70 | 0.96-3.03 | 9.27E-01 | 1.75 | 0.98-3.13 | 9.51E-01 |
| ceramide (d16:1/24:1, d18:1/22:1)* | 1.39 | 0.83-2.32 | 9.27E-01 | 1.34 | 0.82-2.17 | 9.51E-01 |
| ceramide (d18:1/14:0, d16:1/16:0)* | 1.51 | 0.99-2.32 | 9.27E-01 | 1.51 | 0.97-2.34 | 9.51E-01 |
| ceramide (d18:2/24:1, d18:1/24:2)* | 0.76 | 0.45-1.29 | 9.29E-01 | 0.76 | 0.46-1.25 | 9.51E-01 |
| nisinate (24:6n3) | 0.79 | 0.51-1.22 | 9.27E-01 | 0.79 | 0.5-1.25 | 9.51E-01 |
| sphingomyelin (d18:0/20:0, d16:0/22:0)* | 1.60 | 1-2.56 | 9.27E-01 | 1.66 | 1.02-2.71 | 9.51E-01 |
| sphingomyelin (d18:2/18:1)* | 0.71 | 0.43-1.18 | 9.27E-01 | 0.69 | 0.42-1.13 | 9.51E-01 |
| sphingomyelin (d18:2/24:2)* | 0.70 | 0.47-1.03 | 9.27E-01 | 0.70 | 0.47-1.04 | 9.51E-01 |
| sphingomyelin (d18:1/25:0, d19:0/24:1, d20:1/23:0, d19:1/24:0)* | 1.41 | 0.91-2.19 | 9.27E-01 | 1.45 | 0.9-2.34 | 9.51E-01 |
| linolenoylcarnitine (C18:3)* | 0.73 | 0.51-1.03 | 9.27E-01 | 0.75 | 0.53-1.07 | 9.51E-01 |
| arachidoylcarnitine (C20)* | 1.29 | 0.74-2.27 | 9.62E-01 | 1.42 | 0.84-2.38 | 9.51E-01 |
| lignoceroylcarnitine (C24)* | 1.34 | 0.79-2.28 | 9.27E-01 | 1.41 | 0.83-2.4 | 9.51E-01 |
| cerotoylcarnitine (C26)* | 1.32 | 0.79-2.2 | 9.27E-01 | 1.33 | 0.78-2.27 | 9.51E-01 |
| ximenoylcarnitine (C26:1)* | 1.30 | 0.86-1.98 | 9.27E-01 | 1.33 | 0.85-2.08 | 9.51E-01 |
| N,N,N-trimethyl-5-aminovalerate | 1.30 | 0.94-1.79 | 9.27E-01 | 1.22 | 0.86-1.73 | 9.51E-01 |
| ethyl alpha-glucopyranoside | 0.59 | 0.38-0.91 | 9.27E-01 | 0.56 | 0.32-0.96 | 9.51E-01 |
| carotene diol (1) | 1.32 | 0.84-2.06 | 9.27E-01 | 1.36 | 0.87-2.1 | 9.51E-01 |
| carotene diol (2) | 1.35 | 0.87-2.07 | 9.27E-01 | 1.45 | 0.95-2.21 | 9.51E-01 |
| carotene diol (3) | 1.45 | 0.86-2.45 | 9.27E-01 | 1.57 | 0.91-2.71 | 9.51E-01 |
| (N(1) + N(8))-acetylspermidine | 1.28 | 0.88-1.87 | 9.27E-01 | 1.30 | 0.86-1.97 | 9.51E-01 |
| 5-dodecenoylcarnitine (C12:1) | 0.65 | 0.44-0.97 | 9.27E-01 | 0.65 | 0.43-0.99 | 9.51E-01 |
| 3-hydroxyphenylacetoylglutamine | 0.76 | 0.51-1.13 | 9.27E-01 | 0.75 | 0.52-1.08 | 9.51E-01 |
| trans-2-hexenoylglycine | 0.59 | 0.4-0.87 | 8.16E-01 | 0.58 | 0.39-0.88 | 9.51E-01 |
| dodecenedioate (C12:1-DC)* | 0.71 | 0.41-1.25 | 9.27E-01 | 0.73 | 0.42-1.28 | 9.51E-01 |
| octadecenedioate (C18:1-DC) | 0.79 | 0.5-1.24 | 9.28E-01 | 0.78 | 0.49-1.24 | 9.51E-01 |
| glucuronide of C10H18O2 (7)* | 1.58 | 0.97-2.56 | 9.27E-01 | 1.56 | 0.96-2.54 | 9.51E-01 |
| 3-hydroxybutyroylglycine** | 0.70 | 0.54-0.91 | 8.16E-01 | 0.68 | 0.51-0.91 | 9.51E-01 |
| glyco-beta-muricholate** | 1.33 | 0.82-2.15 | 9.27E-01 | 1.32 | 0.81-2.16 | 9.51E-01 |
| N-methylhydroxyproline** | 1.51 | 0.87-2.63 | 9.27E-01 | 1.48 | 0.84-2.62 | 9.51E-01 |
| glycine conjugate of C10H14O2 (1)* | 0.63 | 0.49-0.82 | 4.40E-01 | 0.63 | 0.47-0.85 | 9.51E-01 |
| glutamine conjugate of C6H10O2 (1)* | 0.72 | 0.46-1.12 | 9.27E-01 | 0.69 | 0.45-1.07 | 9.51E-01 |
| glutamine conjugate of C6H10O2 (2)* | 0.75 | 0.5-1.13 | 9.27E-01 | 0.72 | 0.46-1.12 | 9.51E-01 |
| dihydroferulic acid sulfate | 1.32 | 0.86-2.03 | 9.27E-01 | 1.32 | 0.85-2.06 | 9.51E-01 |
| sphingomyelin (d17:1/14:0, d16:1/15:0)* | 1.49 | 0.9-2.47 | 9.27E-01 | 1.50 | 0.86-2.62 | 9.51E-01 |
| tetradecadienoate (14:2)* | 0.57 | 0.37-0.86 | 8.16E-01 | 0.59 | 0.39-0.9 | 9.51E-01 |
| 2,2'-Methylenebis(6-tert-butyl-p-cresol) | 0.71 | 0.46-1.11 | 9.27E-01 | 0.75 | 0.49-1.15 | 9.51E-01 |
| ethyl beta-glucopyranoside | 1.20 | 0.79-1.82 | 9.62E-01 | 1.25 | 0.82-1.89 | 9.51E-01 |
| 3-hydroxyhippurate sulfate | 1.36 | 0.81-2.26 | 9.27E-01 | 1.35 | 0.82-2.21 | 9.51E-01 |
| N6,N6-dimethyllysine | 0.57 | 0.34-0.96 | 9.27E-01 | 0.56 | 0.34-0.92 | 9.51E-01 |
| dodecadienoate (12:2)* | 0.48 | 0.31-0.74 | 4.40E-01 | 0.49 | 0.31-0.78 | 9.51E-01 |
| glucuronide of piperine metabolite C17H21NO3 (3)* | 0.69 | 0.48-1.01 | 9.27E-01 | 0.66 | 0.45-0.97 | 9.51E-01 |
| glucuronide of piperine metabolite C17H21NO3 (4)* | 0.79 | 0.54-1.15 | 9.27E-01 | 0.77 | 0.5-1.18 | 9.51E-01 |
| glucuronide of piperine metabolite C17H21NO3 (5)* | 0.75 | 0.53-1.06 | 9.27E-01 | 0.72 | 0.49-1.07 | 9.51E-01 |
| sulfate of piperine metabolite C16H19NO3 (3)* | 0.78 | 0.54-1.11 | 9.27E-01 | 0.76 | 0.5-1.16 | 9.51E-01 |
| 2-naphthol sulfate | 0.77 | 0.46-1.29 | 9.29E-01 | 0.71 | 0.39-1.28 | 9.51E-01 |
| N2-acetyl,N6,N6-dimethyllysine | 0.77 | 0.51-1.16 | 9.27E-01 | 0.77 | 0.52-1.16 | 9.51E-01 |
| N2-acetyl,N6-methyllysine | 0.79 | 0.51-1.21 | 9.27E-01 | 0.79 | 0.51-1.22 | 9.51E-01 |
| dihydrocaffeate sulfate (2) | 1.38 | 0.89-2.14 | 9.27E-01 | 1.38 | 0.87-2.2 | 9.51E-01 |
| 3-hydroxyhexanoylcarnitine (1) | 0.76 | 0.51-1.12 | 9.27E-01 | 0.74 | 0.5-1.1 | 9.51E-01 |
| 2-ketocaprylate | 0.68 | 0.41-1.13 | 9.27E-01 | 0.73 | 0.47-1.13 | 9.51E-01 |
| eicosenedioate (C20:1-DC)* | 0.79 | 0.58-1.08 | 9.27E-01 | 0.83 | 0.61-1.14 | 9.51E-01 |
| 3,5-dichloro-2,6-dihydroxybenzoic acid | 0.80 | 0.56-1.13 | 9.27E-01 | 0.77 | 0.53-1.11 | 9.51E-01 |
| vanillic acid glycine | 1.48 | 0.77-2.81 | 9.27E-01 | 1.46 | 0.72-2.96 | 9.51E-01 |
| 1-methyl-5-imidazolelactate | 0.67 | 0.4-1.14 | 9.27E-01 | 0.65 | 0.37-1.14 | 9.51E-01 |
| (2 or 3)-decenoate (10:1n7 or n8) | 0.80 | 0.56-1.13 | 9.27E-01 | 0.80 | 0.55-1.16 | 9.51E-01 |
| branched-chain, straight-chain, or cyclopropyl 10:1 fatty acid (1)* | 0.67 | 0.45-1.01 | 9.27E-01 | 0.70 | 0.47-1.03 | 9.51E-01 |
| branched-chain, straight-chain, or cyclopropyl 12:1 fatty acid* | 0.57 | 0.37-0.88 | 8.56E-01 | 0.57 | 0.36-0.92 | 9.51E-01 |
| cis-3,4-methyleneheptanoate | 0.69 | 0.51-0.94 | 9.27E-01 | 0.68 | 0.49-0.94 | 9.51E-01 |
| cis-3,4-methyleneheptanoylcarnitine | 0.81 | 0.59-1.13 | 9.27E-01 | 0.81 | 0.57-1.14 | 9.51E-01 |
| 3-hydroxyoctanoylcarnitine (1) | 0.71 | 0.45-1.14 | 9.27E-01 | 0.73 | 0.44-1.2 | 9.51E-01 |
| 3-hydroxyoctanoylcarnitine (2) | 0.69 | 0.41-1.17 | 9.27E-01 | 0.69 | 0.4-1.21 | 9.51E-01 |
| cis-3,4-methyleneheptanoylglycine | 0.73 | 0.53-0.99 | 9.27E-01 | 0.75 | 0.54-1.03 | 9.51E-01 |
| bilirubin degradation product, C16H18N2O5 (2)** | 0.80 | 0.56-1.13 | 9.27E-01 | 0.82 | 0.57-1.18 | 9.51E-01 |
| bilirubin degradation product, C17H18N2O4 (1)** | 0.75 | 0.51-1.11 | 9.27E-01 | 0.75 | 0.5-1.12 | 9.51E-01 |
| bilirubin degradation product, C17H18N2O4 (2)** | 0.72 | 0.49-1.05 | 9.27E-01 | 0.70 | 0.47-1.04 | 9.51E-01 |
| bilirubin degradation product, C17H18N2O4 (3)** | 0.74 | 0.5-1.12 | 9.27E-01 | 0.76 | 0.5-1.15 | 9.51E-01 |
| tetrahydrocortisol glucuronide | 0.84 | 0.59-1.2 | 9.44E-01 | 0.78 | 0.54-1.13 | 9.51E-01 |
| bilirubin degradation product, C16H18N2O5 (3)** | 0.80 | 0.56-1.16 | 9.27E-01 | 0.82 | 0.57-1.18 | 9.51E-01 |
| bilirubin degradation product, C16H18N2O5 (4)** | 0.78 | 0.53-1.16 | 9.27E-01 | 0.81 | 0.55-1.18 | 9.51E-01 |
| tetrahydrocortisone glucuronide (5) | 0.75 | 0.53-1.06 | 9.27E-01 | 0.68 | 0.47-0.98 | 9.51E-01 |
| picolinate | 1.54 | 0.81-2.93 | 9.27E-01 | 1.52 | 0.78-2.98 | 9.51E-01 |
| N-acetylmethionine | 0.78 | 0.52-1.16 | 9.27E-01 | 0.74 | 0.48-1.13 | 9.51E-01 |
| bilirubin (Z,Z) | 0.69 | 0.43-1.11 | 9.27E-01 | 0.70 | 0.42-1.17 | 9.51E-01 |
| alpha-tocopherol | 0.69 | 0.52-0.92 | 8.56E-01 | 0.69 | 0.52-0.92 | 9.51E-01 |
| 3-aminoisobutyrate | 1.37 | 0.88-2.13 | 9.27E-01 | 1.41 | 0.91-2.19 | 9.51E-01 |
| citrate | 0.76 | 0.47-1.23 | 9.27E-01 | 0.79 | 0.5-1.24 | 9.51E-01 |
| ursodeoxycholate | 1.35 | 0.79-2.31 | 9.27E-01 | 1.41 | 0.8-2.49 | 9.51E-01 |
| N-acetylneuraminate | 1.33 | 0.99-1.77 | 9.27E-01 | 1.26 | 0.99-1.6 | 9.51E-01 |
| N-acetylglucosaminylasparagine | 0.77 | 0.52-1.15 | 9.27E-01 | 0.76 | 0.53-1.1 | 9.51E-01 |
| creatine | 1.58 | 1.04-2.4 | 9.27E-01 | 1.61 | 1.1-2.34 | 9.51E-01 |
| cys-gly, oxidized | 0.69 | 0.48-1.01 | 9.27E-01 | 0.67 | 0.44-1.02 | 9.51E-01 |
| dihomo-linoleate (20:2n6) | 0.71 | 0.48-1.05 | 9.27E-01 | 0.74 | 0.5-1.1 | 9.51E-01 |
| glycerol | 0.57 | 0.33-1 | 9.27E-01 | 0.57 | 0.33-1 | 9.51E-01 |
| cholate | 1.48 | 0.93-2.36 | 9.27E-01 | 1.48 | 0.9-2.43 | 9.51E-01 |
| beta-hydroxyisovalerate | 0.77 | 0.54-1.1 | 9.27E-01 | 0.77 | 0.5-1.17 | 9.51E-01 |
| N-palmitoyl-sphingosine (d18:1/16:0) | 0.77 | 0.51-1.16 | 9.27E-01 | 0.77 | 0.51-1.16 | 9.51E-01 |
| stearoyl sphingomyelin (d18:1/18:0) | 0.84 | 0.56-1.27 | 9.62E-01 | 0.81 | 0.55-1.2 | 9.51E-01 |
| 1-palmitoyl-2-oleoyl-GPC (16:0/18:1) | 1.25 | 0.86-1.8 | 9.27E-01 | 1.30 | 0.91-1.85 | 9.51E-01 |
| 5,6-dihydrothymine | 0.77 | 0.5-1.19 | 9.27E-01 | 0.75 | 0.48-1.16 | 9.51E-01 |
| glycochenodeoxycholate | 0.73 | 0.41-1.3 | 9.27E-01 | 0.74 | 0.41-1.31 | 9.51E-01 |
| taurocholate | 1.29 | 0.89-1.88 | 9.27E-01 | 1.27 | 0.86-1.87 | 9.51E-01 |
| linoleate (18:2n6) | 0.70 | 0.5-0.97 | 9.27E-01 | 0.72 | 0.52-1 | 9.51E-01 |
| quinolinate | 1.26 | 0.95-1.66 | 9.27E-01 | 1.22 | 0.93-1.62 | 9.51E-01 |
| N-formylmethionine | 0.71 | 0.41-1.24 | 9.27E-01 | 0.68 | 0.39-1.19 | 9.51E-01 |
| methylsuccinate | 0.79 | 0.56-1.12 | 9.27E-01 | 0.78 | 0.55-1.1 | 9.51E-01 |
| ethylmalonate | 1.28 | 0.92-1.78 | 9.27E-01 | 1.29 | 0.94-1.75 | 9.51E-01 |
| adenosine 3',5'-cyclic monophosphate (cAMP) | 0.76 | 0.47-1.21 | 9.27E-01 | 0.76 | 0.49-1.19 | 9.51E-01 |
| arachidonate (20:4n6) | 0.70 | 0.43-1.14 | 9.27E-01 | 0.74 | 0.42-1.29 | 9.51E-01 |
| aspartate | 1.37 | 0.91-2.07 | 9.27E-01 | 1.29 | 0.85-1.96 | 9.51E-01 |
| phenylpyruvate | 0.73 | 0.5-1.06 | 9.27E-01 | 0.70 | 0.48-1.03 | 9.51E-01 |
| beta-alanine | 0.73 | 0.51-1.06 | 9.27E-01 | 0.72 | 0.48-1.09 | 9.51E-01 |
| biliverdin | 0.76 | 0.53-1.1 | 9.27E-01 | 0.78 | 0.56-1.09 | 9.51E-01 |
| succinate | 0.55 | 0.36-0.85 | 8.16E-01 | 0.57 | 0.38-0.85 | 9.51E-01 |
| cortisone | 0.83 | 0.65-1.06 | 9.27E-01 | 0.82 | 0.62-1.09 | 9.51E-01 |
| cystathionine | 0.82 | 0.5-1.34 | 9.62E-01 | 0.77 | 0.47-1.26 | 9.51E-01 |
| fumarate | 0.77 | 0.59-1 | 9.27E-01 | 0.75 | 0.56-1 | 9.51E-01 |
| gamma-glutamylglutamate | 1.31 | 0.9-1.93 | 9.27E-01 | 1.28 | 0.85-1.93 | 9.51E-01 |
| glycocholate | 1.48 | 0.98-2.23 | 9.27E-01 | 1.46 | 0.97-2.21 | 9.51E-01 |
| histidine | 1.25 | 0.85-1.82 | 9.27E-01 | 1.27 | 0.84-1.92 | 9.51E-01 |
| cortisol | 0.83 | 0.61-1.12 | 9.27E-01 | 0.83 | 0.59-1.16 | 9.51E-01 |
| hypotaurine | 0.75 | 0.47-1.19 | 9.27E-01 | 0.75 | 0.48-1.16 | 9.51E-01 |
| lysine | 1.26 | 0.82-1.92 | 9.27E-01 | 1.27 | 0.83-1.94 | 9.51E-01 |
| malate | 0.77 | 0.5-1.2 | 9.27E-01 | 0.79 | 0.51-1.21 | 9.51E-01 |
| phenylalanine | 1.34 | 0.86-2.09 | 9.27E-01 | 1.32 | 0.83-2.09 | 9.51E-01 |
| spermidine | 1.27 | 1.07-1.51 | 8.16E-01 | 1.25 | 1.06-1.48 | 9.51E-01 |
| serine | 1.46 | 0.88-2.43 | 9.27E-01 | 1.50 | 0.86-2.62 | 9.51E-01 |
| 2'-deoxyuridine | 1.44 | 0.97-2.14 | 9.27E-01 | 1.46 | 0.99-2.15 | 9.51E-01 |
| trans-urocanate | 0.61 | 0.42-0.88 | 8.16E-01 | 0.65 | 0.45-0.95 | 9.51E-01 |
| glutamate | 1.42 | 1-2 | 9.27E-01 | 1.33 | 0.91-1.93 | 9.51E-01 |
| threonine | 1.24 | 0.78-1.96 | 9.62E-01 | 1.27 | 0.8-1.99 | 9.51E-01 |
| tryptophan | 0.80 | 0.48-1.33 | 9.62E-01 | 0.76 | 0.49-1.19 | 9.51E-01 |
| glucose | 0.65 | 0.38-1.1 | 9.27E-01 | 0.60 | 0.34-1.04 | 9.51E-01 |
| 12,13-DiHOME | 0.78 | 0.52-1.17 | 9.27E-01 | 0.77 | 0.52-1.15 | 9.51E-01 |
| alpha-ketobutyrate | 0.73 | 0.5-1.08 | 9.27E-01 | 0.68 | 0.44-1.03 | 9.51E-01 |
| betaine | 0.66 | 0.43-1.02 | 9.27E-01 | 0.67 | 0.43-1.07 | 9.51E-01 |
| pseudouridine | 1.34 | 0.95-1.88 | 9.27E-01 | 1.31 | 0.96-1.79 | 9.51E-01 |
| cytidine | 1.28 | 0.93-1.77 | 9.27E-01 | 1.25 | 0.89-1.75 | 9.51E-01 |
| caffeine | 0.77 | 0.52-1.15 | 9.27E-01 | 0.76 | 0.52-1.12 | 9.51E-01 |
| dihydroorotate | 1.29 | 0.91-1.84 | 9.27E-01 | 1.28 | 0.88-1.85 | 9.51E-01 |
| sucrose | 1.59 | 1.04-2.44 | 9.27E-01 | 1.55 | 1-2.43 | 9.51E-01 |
| X - 10458 | 1.32 | 0.85-2.04 | 9.27E-01 | 1.33 | 0.87-2.04 | 9.51E-01 |
| X - 11308 | 0.71 | 0.47-1.05 | 9.27E-01 | 0.73 | 0.48-1.09 | 9.51E-01 |
| X - 11315 | 0.73 | 0.59-0.91 | 8.16E-01 | 0.76 | 0.6-0.96 | 9.51E-01 |
| X - 11372 | 0.82 | 0.58-1.17 | 9.27E-01 | 0.83 | 0.59-1.17 | 9.51E-01 |
| X - 11478 | 0.79 | 0.6-1.04 | 9.27E-01 | 0.80 | 0.59-1.07 | 9.51E-01 |
| X - 11852 | 0.69 | 0.41-1.15 | 9.27E-01 | 0.71 | 0.42-1.2 | 9.51E-01 |
| X - 12013 | 1.31 | 0.76-2.27 | 9.43E-01 | 1.34 | 0.76-2.37 | 9.51E-01 |
| X - 12101 | 0.77 | 0.47-1.25 | 9.27E-01 | 0.76 | 0.47-1.24 | 9.51E-01 |
| X - 12112 | 0.75 | 0.6-0.94 | 8.71E-01 | 0.76 | 0.61-0.94 | 9.51E-01 |
| X - 12117 | 0.74 | 0.41-1.33 | 9.28E-01 | 0.66 | 0.38-1.18 | 9.51E-01 |
| X - 12283 | 0.69 | 0.46-1.02 | 9.27E-01 | 0.69 | 0.46-1.03 | 9.51E-01 |
| X - 12410 | 0.78 | 0.53-1.14 | 9.27E-01 | 0.75 | 0.52-1.07 | 9.51E-01 |
| X - 12707 | 0.68 | 0.42-1.09 | 9.27E-01 | 0.68 | 0.44-1.06 | 9.51E-01 |
| X - 12713 | 0.83 | 0.57-1.22 | 9.56E-01 | 0.81 | 0.55-1.2 | 9.51E-01 |
| X - 12726 | 0.87 | 0.61-1.23 | 9.62E-01 | 0.83 | 0.58-1.19 | 9.51E-01 |
| X - 12729 | 0.76 | 0.6-0.96 | 9.27E-01 | 0.78 | 0.62-0.98 | 9.51E-01 |
| X - 12812 | 1.29 | 0.82-2.02 | 9.27E-01 | 1.24 | 0.82-1.88 | 9.51E-01 |
| X - 12830 | 0.65 | 0.41-1.01 | 9.27E-01 | 0.65 | 0.41-1.05 | 9.51E-01 |
| X - 12839 | 0.67 | 0.43-1.05 | 9.27E-01 | 0.65 | 0.41-1.05 | 9.51E-01 |
| X - 12844 | 0.83 | 0.58-1.19 | 9.28E-01 | 0.78 | 0.53-1.15 | 9.51E-01 |
| X - 12906 | 1.37 | 0.92-2.02 | 9.27E-01 | 1.37 | 0.97-1.93 | 9.51E-01 |
| X - 13658 | 0.67 | 0.42-1.07 | 9.27E-01 | 0.72 | 0.45-1.15 | 9.51E-01 |
| X - 15486 | 0.78 | 0.61-1 | 9.27E-01 | 0.78 | 0.58-1.04 | 9.51E-01 |
| X - 15503 | 0.70 | 0.41-1.2 | 9.27E-01 | 0.65 | 0.4-1.06 | 9.51E-01 |
| X - 15728 | 0.83 | 0.58-1.19 | 9.28E-01 | 0.81 | 0.55-1.2 | 9.51E-01 |
| X - 17351 | 0.76 | 0.52-1.1 | 9.27E-01 | 0.74 | 0.5-1.1 | 9.51E-01 |
| X - 17354 | 0.74 | 0.47-1.17 | 9.27E-01 | 0.74 | 0.47-1.18 | 9.51E-01 |
| X - 17357 | 0.83 | 0.6-1.15 | 9.27E-01 | 0.80 | 0.58-1.11 | 9.51E-01 |
| X - 17438 | 1.21 | 0.89-1.65 | 9.27E-01 | 1.24 | 0.9-1.71 | 9.51E-01 |
| X - 17654 | 0.71 | 0.5-1.01 | 9.27E-01 | 0.70 | 0.49-1.01 | 9.51E-01 |
| X - 17676 | 0.72 | 0.38-1.39 | 9.34E-01 | 0.71 | 0.37-1.36 | 9.51E-01 |
| X - 17685 | 1.28 | 0.84-1.93 | 9.27E-01 | 1.29 | 0.84-1.97 | 9.51E-01 |
| X - 18913 | 0.66 | 0.32-1.37 | 9.27E-01 | 0.70 | 0.37-1.35 | 9.51E-01 |
| X - 18921 | 0.73 | 0.51-1.04 | 9.27E-01 | 0.76 | 0.53-1.09 | 9.51E-01 |
| X - 21286 | 0.72 | 0.44-1.17 | 9.27E-01 | 0.70 | 0.43-1.14 | 9.51E-01 |
| X - 21312 | 1.27 | 0.8-2.02 | 9.28E-01 | 1.31 | 0.81-2.11 | 9.51E-01 |
| X - 21319 | 0.77 | 0.57-1.03 | 9.27E-01 | 0.78 | 0.57-1.06 | 9.51E-01 |
| X - 21327 | 1.34 | 0.89-2 | 9.27E-01 | 1.27 | 0.84-1.92 | 9.51E-01 |
| X - 21339 | 0.73 | 0.51-1.05 | 9.27E-01 | 0.72 | 0.49-1.06 | 9.51E-01 |
| X - 21353 | 0.68 | 0.43-1.07 | 9.27E-01 | 0.71 | 0.44-1.13 | 9.51E-01 |
| X - 21383 | 0.68 | 0.41-1.14 | 9.27E-01 | 0.68 | 0.4-1.16 | 9.51E-01 |
| X - 21607 | 0.82 | 0.6-1.13 | 9.27E-01 | 0.84 | 0.6-1.17 | 9.51E-01 |
| X - 21736 | 0.72 | 0.49-1.04 | 9.27E-01 | 0.70 | 0.47-1.05 | 9.51E-01 |
| X - 21796 | 0.75 | 0.53-1.06 | 9.27E-01 | 0.75 | 0.52-1.06 | 9.51E-01 |
| X - 21821 | 0.78 | 0.52-1.18 | 9.27E-01 | 0.78 | 0.51-1.2 | 9.51E-01 |
| X - 23587 | 0.76 | 0.51-1.12 | 9.27E-01 | 0.76 | 0.51-1.15 | 9.51E-01 |
| X - 23639 | 1.37 | 0.81-2.34 | 9.27E-01 | 1.48 | 0.83-2.64 | 9.51E-01 |
| X - 23666 | 0.74 | 0.54-1.01 | 9.27E-01 | 0.73 | 0.53-1 | 9.51E-01 |
| X - 23680 | 0.81 | 0.65-1.02 | 9.27E-01 | 0.80 | 0.62-1.03 | 9.51E-01 |
| X - 23780 | 1.23 | 0.93-1.64 | 9.27E-01 | 1.23 | 0.93-1.62 | 9.51E-01 |
| X - 24295 | 0.74 | 0.49-1.12 | 9.27E-01 | 0.68 | 0.44-1.05 | 9.51E-01 |
| X - 24306 | 0.77 | 0.46-1.26 | 9.27E-01 | 0.75 | 0.47-1.18 | 9.51E-01 |
| X - 24418 | 0.78 | 0.52-1.17 | 9.27E-01 | 0.78 | 0.52-1.16 | 9.51E-01 |
| X - 24462 | 0.69 | 0.47-1.03 | 9.27E-01 | 0.67 | 0.44-1.02 | 9.51E-01 |
| X - 24473 | 1.30 | 0.97-1.74 | 9.27E-01 | 1.35 | 1.01-1.81 | 9.51E-01 |
| X - 24475 | 1.30 | 0.85-1.99 | 9.27E-01 | 1.29 | 0.81-2.06 | 9.51E-01 |
| X - 24494 | 0.80 | 0.54-1.19 | 9.27E-01 | 0.81 | 0.56-1.19 | 9.51E-01 |
| X - 24518 | 0.83 | 0.49-1.43 | 9.65E-01 | 0.76 | 0.45-1.27 | 9.51E-01 |
| X - 24546 | 1.43 | 1.05-1.95 | 9.27E-01 | 1.35 | 0.99-1.84 | 9.51E-01 |
| X - 24947 | 1.42 | 0.96-2.1 | 9.27E-01 | 1.41 | 0.97-2.05 | 9.51E-01 |
| X - 24949 | 0.77 | 0.57-1.04 | 9.27E-01 | 0.78 | 0.59-1.04 | 9.51E-01 |
| X - 24951 | 0.85 | 0.62-1.17 | 9.28E-01 | 0.85 | 0.64-1.13 | 9.51E-01 |
| X - 24953 | 0.81 | 0.58-1.12 | 9.27E-01 | 0.84 | 0.61-1.15 | 9.51E-01 |
| X - 24970 | 1.38 | 0.93-2.05 | 9.27E-01 | 1.37 | 0.9-2.1 | 9.51E-01 |
| X - 25172 | 0.76 | 0.47-1.23 | 9.27E-01 | 0.74 | 0.44-1.25 | 9.51E-01 |
| X - 25271 | 0.76 | 0.48-1.2 | 9.27E-01 | 0.78 | 0.5-1.22 | 9.51E-01 |
| X - 25420 | 1.40 | 0.85-2.31 | 9.27E-01 | 1.46 | 0.87-2.45 | 9.51E-01 |
| X - 25519 | 0.74 | 0.44-1.25 | 9.27E-01 | 0.73 | 0.43-1.24 | 9.51E-01 |
| X - 25810 | 0.71 | 0.43-1.17 | 9.27E-01 | 0.70 | 0.41-1.2 | 9.51E-01 |
| X - 25957 | 1.39 | 0.9-2.16 | 9.27E-01 | 1.36 | 0.89-2.08 | 9.51E-01 |
| threonate | 1.54 | 0.69-3.46 | 9.27E-01 | 1.49 | 0.69-3.23 | 9.54E-01 |
| 2-acetamidophenol sulfate | 1.22 | 0.82-1.82 | 9.29E-01 | 1.25 | 0.81-1.91 | 9.54E-01 |
| 3-methylhistidine | 0.76 | 0.43-1.33 | 9.37E-01 | 0.76 | 0.42-1.35 | 9.55E-01 |
| paraxanthine | 0.84 | 0.59-1.18 | 9.28E-01 | 0.85 | 0.6-1.19 | 9.55E-01 |
| gamma-glutamylphenylalanine | 1.26 | 0.83-1.92 | 9.27E-01 | 1.24 | 0.8-1.93 | 9.55E-01 |
| 2-pyrrolidinone | 0.84 | 0.57-1.25 | 9.62E-01 | 0.83 | 0.57-1.21 | 9.55E-01 |
| propionylglycine | 1.28 | 0.75-2.17 | 9.62E-01 | 1.29 | 0.77-2.17 | 9.55E-01 |
| 4-hydroxycoumarin | 0.83 | 0.55-1.25 | 9.62E-01 | 0.81 | 0.54-1.23 | 9.55E-01 |
| andro steroid monosulfate C19H28O6S (1)* | 1.18 | 0.85-1.64 | 9.32E-01 | 1.19 | 0.83-1.7 | 9.55E-01 |
| 2R,3R-dihydroxybutyrate | 1.30 | 0.78-2.17 | 9.28E-01 | 1.30 | 0.77-2.2 | 9.55E-01 |
| glycoursodeoxycholate | 1.19 | 0.8-1.77 | 9.62E-01 | 1.24 | 0.81-1.89 | 9.55E-01 |
| 2-O-methylascorbic acid | 0.90 | 0.67-1.22 | 9.65E-01 | 0.86 | 0.63-1.17 | 9.55E-01 |
| 2-hydroxydecanoate | 0.72 | 0.39-1.35 | 9.28E-01 | 0.78 | 0.46-1.31 | 9.55E-01 |
| dimethyl sulfone | 0.80 | 0.54-1.2 | 9.27E-01 | 0.82 | 0.55-1.22 | 9.55E-01 |
| 3-(3-hydroxyphenyl)propionate sulfate | 1.30 | 0.73-2.3 | 9.62E-01 | 1.31 | 0.77-2.24 | 9.55E-01 |
| isoeugenol sulfate | 0.84 | 0.59-1.19 | 9.32E-01 | 0.83 | 0.56-1.21 | 9.55E-01 |
| methyl-4-hydroxybenzoate sulfate | 0.83 | 0.56-1.24 | 9.62E-01 | 0.82 | 0.55-1.23 | 9.55E-01 |
| 1-palmitoyl-2-docosahexaenoyl-GPE (16:0/22:6)* | 1.31 | 0.78-2.2 | 9.28E-01 | 1.29 | 0.77-2.15 | 9.55E-01 |
| lactosyl-N-palmitoyl-sphingosine (d18:1/16:0) | 1.23 | 0.77-1.95 | 9.62E-01 | 1.25 | 0.79-1.97 | 9.55E-01 |
| oleoylcholine | 1.15 | 0.75-1.75 | 9.65E-01 | 1.24 | 0.8-1.92 | 9.55E-01 |
| glycosyl ceramide (d18:1/20:0, d16:1/22:0)* | 1.20 | 0.75-1.93 | 9.62E-01 | 1.26 | 0.79-2.03 | 9.55E-01 |
| N-acetyl-2-aminooctanoate* | 0.78 | 0.51-1.17 | 9.27E-01 | 0.83 | 0.57-1.21 | 9.55E-01 |
| 8-methoxykynurenate | 0.84 | 0.53-1.32 | 9.62E-01 | 0.79 | 0.5-1.26 | 9.55E-01 |
| sulfate of piperine metabolite C16H19NO3 (2)* | 0.81 | 0.55-1.18 | 9.27E-01 | 0.80 | 0.51-1.25 | 9.55E-01 |
| sulfate of piperine metabolite C18H21NO3 (1)* | 0.77 | 0.52-1.15 | 9.27E-01 | 0.80 | 0.5-1.27 | 9.55E-01 |
| 11beta-hydroxyetiocholanolone glucuronide* | 0.80 | 0.51-1.24 | 9.29E-01 | 0.80 | 0.51-1.26 | 9.55E-01 |
| undecenoylcarnitine (C11:1) | 1.27 | 0.84-1.92 | 9.27E-01 | 1.27 | 0.8-2 | 9.55E-01 |
| decadienedioic acid (C10:2-DC)** | 0.80 | 0.52-1.23 | 9.28E-01 | 0.81 | 0.52-1.25 | 9.55E-01 |
| homovanillate (HVA) | 0.80 | 0.54-1.18 | 9.27E-01 | 0.83 | 0.56-1.22 | 9.55E-01 |
| N-stearoyl-sphingosine (d18:1/18:0)* | 0.88 | 0.63-1.23 | 9.65E-01 | 0.84 | 0.59-1.19 | 9.55E-01 |
| hypoxanthine | 0.85 | 0.58-1.26 | 9.62E-01 | 0.82 | 0.55-1.22 | 9.55E-01 |
| N-acetylputrescine | 1.25 | 0.79-1.97 | 9.43E-01 | 1.26 | 0.79-2.02 | 9.55E-01 |
| alpha-ketoglutarate | 1.23 | 0.85-1.78 | 9.27E-01 | 1.20 | 0.84-1.73 | 9.55E-01 |
| X - 11850 | 1.23 | 0.76-2 | 9.62E-01 | 1.27 | 0.78-2.09 | 9.55E-01 |
| X - 12126 | 0.79 | 0.52-1.21 | 9.27E-01 | 0.82 | 0.54-1.23 | 9.55E-01 |
| X - 12261 | 1.23 | 0.77-1.95 | 9.62E-01 | 1.25 | 0.79-2 | 9.55E-01 |
| X - 23655 | 1.27 | 0.8-2.02 | 9.28E-01 | 1.27 | 0.77-2.1 | 9.58E-01 |
| 2-piperidinone | 1.19 | 0.77-1.83 | 9.62E-01 | 1.22 | 0.8-1.86 | 9.59E-01 |
| X - 12096 | 0.85 | 0.49-1.48 | 9.69E-01 | 0.78 | 0.46-1.31 | 9.59E-01 |
| 11-ketoetiocholanolone glucuronide | 0.82 | 0.53-1.27 | 9.62E-01 | 0.81 | 0.51-1.27 | 9.65E-01 |
| 1-arachidonoyl-GPE (20:4n6)* | 0.71 | 0.42-1.21 | 9.27E-01 | 0.75 | 0.41-1.37 | 9.67E-01 |
| 1-stearoyl-2-linoleoyl-GPC (18:0/18:2)* | 0.79 | 0.53-1.17 | 9.27E-01 | 0.83 | 0.55-1.24 | 9.67E-01 |
| ceramide (d18:1/17:0, d17:1/18:0)* | 0.88 | 0.61-1.25 | 9.65E-01 | 0.84 | 0.59-1.21 | 9.67E-01 |
| phosphate | 1.12 | 0.79-1.61 | 9.65E-01 | 1.17 | 0.84-1.65 | 9.67E-01 |
| dimethylglycine | 1.21 | 0.81-1.81 | 9.62E-01 | 1.21 | 0.81-1.82 | 9.67E-01 |
| X - 17335 | 0.78 | 0.52-1.18 | 9.27E-01 | 0.80 | 0.5-1.28 | 9.67E-01 |
| phenylacetate | 1.26 | 0.58-2.75 | 9.69E-01 | 1.26 | 0.55-2.91 | 9.69E-01 |
| hippurate | 1.21 | 0.67-2.2 | 9.65E-01 | 1.24 | 0.69-2.22 | 9.69E-01 |
| xanthurenate | 0.89 | 0.59-1.33 | 9.69E-01 | 0.84 | 0.57-1.24 | 9.69E-01 |
| methionine sulfoxide | 1.18 | 0.72-1.95 | 9.65E-01 | 1.16 | 0.71-1.89 | 9.69E-01 |
| kynurenine | 0.88 | 0.54-1.42 | 9.69E-01 | 0.82 | 0.5-1.33 | 9.69E-01 |
| quinate | 1.23 | 0.61-2.45 | 9.69E-01 | 1.25 | 0.6-2.6 | 9.69E-01 |
| theobromine | 0.87 | 0.61-1.25 | 9.62E-01 | 0.86 | 0.6-1.21 | 9.69E-01 |
| indolelactate | 0.83 | 0.47-1.45 | 9.65E-01 | 0.80 | 0.47-1.36 | 9.69E-01 |
| 3-indoxyl sulfate | 0.86 | 0.58-1.28 | 9.65E-01 | 0.86 | 0.59-1.25 | 9.69E-01 |
| 1-stearoyl-2-arachidonoyl-GPI (18:0/20:4) | 0.79 | 0.45-1.37 | 9.62E-01 | 0.81 | 0.45-1.46 | 9.69E-01 |
| sphingosine 1-phosphate | 0.88 | 0.55-1.41 | 9.69E-01 | 0.89 | 0.57-1.41 | 9.69E-01 |
| 1-stearoyl-2-oleoyl-GPS (18:0/18:1) | 1.16 | 0.78-1.72 | 9.65E-01 | 1.13 | 0.77-1.64 | 9.69E-01 |
| maleate | 0.91 | 0.67-1.23 | 9.65E-01 | 0.88 | 0.63-1.23 | 9.69E-01 |
| isovalerate (i5:0) | 1.21 | 0.76-1.92 | 9.62E-01 | 1.16 | 0.74-1.81 | 9.69E-01 |
| 4-acetylphenol sulfate | 0.93 | 0.66-1.31 | 9.80E-01 | 0.91 | 0.65-1.28 | 9.69E-01 |
| 3-hydroxyoctanoate | 0.79 | 0.38-1.67 | 9.69E-01 | 0.84 | 0.42-1.68 | 9.69E-01 |
| palmitoylcarnitine (C16) | 1.06 | 0.75-1.5 | 9.84E-01 | 1.10 | 0.73-1.66 | 9.69E-01 |
| hexanoylcarnitine (C6) | 0.83 | 0.54-1.27 | 9.62E-01 | 0.83 | 0.53-1.3 | 9.69E-01 |
| N-acetylaspartate (NAA) | 0.86 | 0.57-1.3 | 9.65E-01 | 0.88 | 0.58-1.34 | 9.69E-01 |
| dehydroepiandrosterone sulfate (DHEA-S) | 1.08 | 0.67-1.73 | 9.84E-01 | 1.15 | 0.71-1.86 | 9.69E-01 |
| acetylcarnitine (C2) | 0.84 | 0.58-1.22 | 9.62E-01 | 0.82 | 0.55-1.25 | 9.69E-01 |
| iminodiacetate (IDA) | 0.90 | 0.55-1.48 | 9.80E-01 | 0.88 | 0.54-1.43 | 9.69E-01 |
| 1-oleoylglycerol (18:1) | 0.89 | 0.57-1.39 | 9.69E-01 | 0.88 | 0.56-1.38 | 9.69E-01 |
| homoarginine | 1.21 | 0.72-2.03 | 9.65E-01 | 1.16 | 0.69-1.96 | 9.69E-01 |
| homocitrulline | 1.15 | 0.71-1.87 | 9.69E-01 | 1.14 | 0.72-1.81 | 9.69E-01 |
| 3-hydroxydecanoate | 0.77 | 0.41-1.44 | 9.62E-01 | 0.80 | 0.43-1.5 | 9.69E-01 |
| citramalate | 0.91 | 0.56-1.47 | 9.80E-01 | 0.88 | 0.55-1.39 | 9.69E-01 |
| EDTA | 0.92 | 0.6-1.41 | 9.80E-01 | 0.91 | 0.62-1.34 | 9.69E-01 |
| ribonate | 1.15 | 0.79-1.66 | 9.65E-01 | 1.12 | 0.79-1.59 | 9.69E-01 |
| butyrylcarnitine (C4) | 1.17 | 0.74-1.83 | 9.65E-01 | 1.16 | 0.77-1.76 | 9.69E-01 |
| isobutyrylcarnitine (C4) | 1.09 | 0.77-1.54 | 9.69E-01 | 1.09 | 0.78-1.54 | 9.69E-01 |
| N-(2-furoyl)glycine | 1.17 | 0.8-1.71 | 9.62E-01 | 1.16 | 0.77-1.75 | 9.69E-01 |
| trigonelline (N'-methylnicotinate) | 0.87 | 0.54-1.41 | 9.69E-01 | 0.88 | 0.54-1.44 | 9.69E-01 |
| 1,3-dimethylurate | 0.89 | 0.66-1.21 | 9.62E-01 | 0.89 | 0.66-1.21 | 9.69E-01 |
| pyridoxate | 1.15 | 0.82-1.62 | 9.62E-01 | 1.13 | 0.77-1.65 | 9.69E-01 |
| 3-hydroxy-2-ethylpropionate | 0.88 | 0.56-1.36 | 9.69E-01 | 0.86 | 0.52-1.4 | 9.69E-01 |
| stearidonate (18:4n3) | 0.81 | 0.53-1.24 | 9.32E-01 | 0.84 | 0.56-1.28 | 9.69E-01 |
| decanoylcarnitine (C10) | 0.82 | 0.51-1.34 | 9.62E-01 | 0.82 | 0.48-1.39 | 9.69E-01 |
| N-acetylasparagine | 1.12 | 0.71-1.75 | 9.69E-01 | 1.13 | 0.71-1.79 | 9.69E-01 |
| 1-palmitoyl-GPC (16:0) | 0.80 | 0.54-1.18 | 9.27E-01 | 0.85 | 0.55-1.31 | 9.69E-01 |
| myristoylcarnitine (C14) | 0.90 | 0.64-1.28 | 9.69E-01 | 0.90 | 0.6-1.34 | 9.69E-01 |
| N-acetylthreonine | 0.85 | 0.54-1.35 | 9.65E-01 | 0.84 | 0.54-1.3 | 9.69E-01 |
| N-acetylisoleucine | 0.92 | 0.6-1.42 | 9.80E-01 | 0.85 | 0.56-1.29 | 9.69E-01 |
| hyocholate | 1.21 | 0.69-2.13 | 9.65E-01 | 1.21 | 0.67-2.17 | 9.69E-01 |
| epiandrosterone sulfate | 1.16 | 0.65-2.07 | 9.69E-01 | 1.21 | 0.68-2.15 | 9.69E-01 |
| gamma-glutamylglycine | 1.03 | 0.76-1.4 | 9.93E-01 | 1.14 | 0.79-1.65 | 9.69E-01 |
| gamma-glutamyltryptophan | 0.85 | 0.54-1.36 | 9.65E-01 | 0.82 | 0.52-1.28 | 9.69E-01 |
| gamma-glutamylthreonine | 1.20 | 0.7-2.05 | 9.65E-01 | 1.21 | 0.7-2.07 | 9.69E-01 |
| 3,7-dimethylurate | 1.08 | 0.76-1.53 | 9.79E-01 | 1.10 | 0.79-1.54 | 9.69E-01 |
| 1,7-dimethylurate | 0.86 | 0.62-1.2 | 9.62E-01 | 0.87 | 0.63-1.19 | 9.69E-01 |
| indolebutyrate | 1.13 | 0.78-1.64 | 9.65E-01 | 1.18 | 0.81-1.71 | 9.69E-01 |
| 3-(3-amino-3-carboxypropyl)uridine* | 1.20 | 0.81-1.77 | 9.62E-01 | 1.19 | 0.81-1.74 | 9.69E-01 |
| 1-stearoyl-GPE (18:0) | 0.82 | 0.46-1.45 | 9.65E-01 | 0.86 | 0.47-1.57 | 9.69E-01 |
| 1-stearoyl-GPG (18:0) | 1.11 | 0.68-1.8 | 9.79E-01 | 1.13 | 0.69-1.83 | 9.69E-01 |
| oleoylcarnitine (C18:1) | 0.90 | 0.66-1.23 | 9.65E-01 | 0.92 | 0.67-1.28 | 9.69E-01 |
| malonylcarnitine | 1.17 | 0.72-1.89 | 9.66E-01 | 1.15 | 0.71-1.85 | 9.69E-01 |
| 1-palmitoyl-GPE (16:0) | 1.12 | 0.68-1.83 | 9.79E-01 | 1.15 | 0.69-1.93 | 9.69E-01 |
| 1-linoleoyl-GPE (18:2)* | 0.83 | 0.49-1.39 | 9.65E-01 | 0.89 | 0.53-1.48 | 9.69E-01 |
| N-acetylcitrulline | 1.17 | 0.82-1.68 | 9.62E-01 | 1.15 | 0.77-1.7 | 9.69E-01 |
| glutarylcarnitine (C5-DC) | 0.87 | 0.51-1.46 | 9.69E-01 | 0.81 | 0.47-1.39 | 9.69E-01 |
| beta-hydroxyisovaleroylcarnitine | 0.91 | 0.6-1.39 | 9.79E-01 | 0.88 | 0.57-1.38 | 9.69E-01 |
| tiglylcarnitine (C5:1-DC) | 0.92 | 0.67-1.26 | 9.69E-01 | 0.89 | 0.63-1.28 | 9.69E-01 |
| hydroquinone sulfate | 1.17 | 0.76-1.8 | 9.65E-01 | 1.14 | 0.71-1.81 | 9.69E-01 |
| tetradecanedioate (C14-DC) | 0.87 | 0.57-1.33 | 9.65E-01 | 0.89 | 0.59-1.36 | 9.69E-01 |
| octadecanedioate (C18-DC) | 0.84 | 0.55-1.29 | 9.62E-01 | 0.85 | 0.57-1.27 | 9.69E-01 |
| 1-myristoylglycerol (14:0) | 1.12 | 0.74-1.68 | 9.69E-01 | 1.14 | 0.76-1.73 | 9.69E-01 |
| ectoine | 0.73 | 0.37-1.46 | 9.62E-01 | 0.76 | 0.39-1.46 | 9.69E-01 |
| 1-arachidonoyl-GPI (20:4)* | 0.81 | 0.48-1.37 | 9.62E-01 | 0.86 | 0.51-1.45 | 9.69E-01 |
| 1-palmitoyl-GPI (16:0) | 1.21 | 0.63-2.31 | 9.69E-01 | 1.23 | 0.65-2.32 | 9.69E-01 |
| hexanoylglutamine | 0.86 | 0.55-1.35 | 9.65E-01 | 0.87 | 0.56-1.35 | 9.69E-01 |
| N6-acetyllysine | 1.20 | 0.85-1.7 | 9.28E-01 | 1.18 | 0.82-1.7 | 9.69E-01 |
| mannitol/sorbitol | 0.89 | 0.51-1.56 | 9.80E-01 | 0.86 | 0.48-1.56 | 9.69E-01 |
| tryptophan betaine | 1.17 | 0.61-2.25 | 9.69E-01 | 1.21 | 0.63-2.32 | 9.69E-01 |
| 1-linoleoyl-GPI (18:2)* | 0.77 | 0.42-1.42 | 9.62E-01 | 0.80 | 0.45-1.44 | 9.69E-01 |
| gamma-glutamylalanine | 0.89 | 0.56-1.43 | 9.70E-01 | 0.89 | 0.56-1.43 | 9.69E-01 |
| sphinganine-1-phosphate | 0.86 | 0.55-1.34 | 9.65E-01 | 0.83 | 0.52-1.31 | 9.69E-01 |
| cyclo(gly-pro) | 0.84 | 0.53-1.33 | 9.65E-01 | 0.87 | 0.54-1.38 | 9.69E-01 |
| bilirubin (E,E)* | 0.85 | 0.54-1.33 | 9.65E-01 | 0.86 | 0.56-1.33 | 9.69E-01 |
| N-methylproline | 1.30 | 0.75-2.25 | 9.56E-01 | 1.29 | 0.74-2.26 | 9.69E-01 |
| beta-cryptoxanthin | 1.25 | 0.75-2.08 | 9.62E-01 | 1.28 | 0.72-2.29 | 9.69E-01 |
| 5alpha-androstan-3beta,17beta-diol disulfate | 1.15 | 0.72-1.84 | 9.69E-01 | 1.18 | 0.74-1.9 | 9.69E-01 |
| 5alpha-pregnan-3beta,20beta-diol monosulfate (1) | 0.91 | 0.63-1.31 | 9.69E-01 | 0.92 | 0.63-1.34 | 9.69E-01 |
| 5alpha-androstan-3beta,17alpha-diol disulfate | 0.88 | 0.55-1.41 | 9.69E-01 | 0.86 | 0.52-1.42 | 9.69E-01 |
| androstenediol (3beta,17beta) monosulfate (1) | 1.06 | 0.68-1.66 | 9.84E-01 | 1.11 | 0.71-1.74 | 9.69E-01 |
| pregnenediol sulfate (C21H34O5S)* | 0.90 | 0.65-1.25 | 9.65E-01 | 0.92 | 0.65-1.29 | 9.69E-01 |
| pregnenolone sulfate | 0.89 | 0.68-1.18 | 9.62E-01 | 0.93 | 0.67-1.28 | 9.69E-01 |
| indole-3-carboxylate | 1.15 | 0.73-1.83 | 9.69E-01 | 1.16 | 0.72-1.87 | 9.69E-01 |
| 2,3-dihydroxyisovalerate | 0.85 | 0.58-1.25 | 9.62E-01 | 0.88 | 0.6-1.3 | 9.69E-01 |
| cysteinylglycine disulfide* | 0.86 | 0.59-1.25 | 9.62E-01 | 0.84 | 0.56-1.27 | 9.69E-01 |
| hydantoin-5-propionate | 1.26 | 0.88-1.81 | 9.27E-01 | 1.14 | 0.78-1.68 | 9.69E-01 |
| gamma-carboxyglutamate | 1.15 | 0.81-1.61 | 9.62E-01 | 1.10 | 0.8-1.51 | 9.69E-01 |
| argininate* | 1.25 | 0.82-1.9 | 9.27E-01 | 1.20 | 0.81-1.78 | 9.69E-01 |
| 2-oxoarginine* | 1.25 | 0.7-2.21 | 9.62E-01 | 1.18 | 0.66-2.12 | 9.69E-01 |
| 1-lignoceroyl-GPC (24:0) | 1.02 | 0.59-1.77 | 9.93E-01 | 1.14 | 0.64-2.05 | 9.69E-01 |
| 1-(1-enyl-palmitoyl)-GPC (P-16:0)* | 1.05 | 0.75-1.47 | 9.84E-01 | 1.16 | 0.78-1.73 | 9.69E-01 |
| isoleucylglycine | 0.85 | 0.54-1.35 | 9.65E-01 | 0.89 | 0.54-1.46 | 9.69E-01 |
| pregnanediol-3-glucuronide | 0.79 | 0.47-1.31 | 9.62E-01 | 0.81 | 0.48-1.37 | 9.69E-01 |
| valylglycine | 0.80 | 0.54-1.2 | 9.27E-01 | 0.86 | 0.56-1.31 | 9.69E-01 |
| mannonate* | 1.19 | 0.8-1.76 | 9.62E-01 | 1.11 | 0.75-1.65 | 9.69E-01 |
| lanthionine | 1.16 | 0.72-1.87 | 9.66E-01 | 1.18 | 0.73-1.9 | 9.69E-01 |
| 2-stearoyl-GPE (18:0)* | 0.74 | 0.42-1.28 | 9.27E-01 | 0.79 | 0.45-1.39 | 9.69E-01 |
| (R)-3-hydroxybutyrylcarnitine | 0.86 | 0.54-1.37 | 9.65E-01 | 0.82 | 0.49-1.36 | 9.69E-01 |
| margaroylcarnitine (C17)* | 1.08 | 0.74-1.56 | 9.80E-01 | 1.11 | 0.72-1.71 | 9.69E-01 |
| 4-methylcatechol sulfate | 1.19 | 0.74-1.92 | 9.65E-01 | 1.21 | 0.74-1.98 | 9.69E-01 |
| 2-aminooctanoate | 0.82 | 0.58-1.15 | 9.27E-01 | 0.86 | 0.61-1.2 | 9.69E-01 |
| gamma-CEHC glucuronide* | 1.18 | 0.75-1.85 | 9.65E-01 | 1.15 | 0.71-1.84 | 9.69E-01 |
| indolin-2-one | 0.91 | 0.63-1.32 | 9.69E-01 | 0.91 | 0.63-1.3 | 9.69E-01 |
| 2-aminophenol sulfate | 1.11 | 0.73-1.7 | 9.69E-01 | 1.11 | 0.72-1.72 | 9.69E-01 |
| sphingomyelin (d18:1/14:0, d16:1/16:0)* | 1.18 | 0.71-1.95 | 9.65E-01 | 1.20 | 0.73-1.98 | 9.69E-01 |
| N-formylanthranilic acid | 0.82 | 0.51-1.3 | 9.62E-01 | 0.84 | 0.54-1.32 | 9.69E-01 |
| 1H-indole-7-acetic acid | 0.85 | 0.56-1.28 | 9.62E-01 | 0.84 | 0.56-1.27 | 9.69E-01 |
| 3-methoxytyramine sulfate | 0.87 | 0.62-1.21 | 9.62E-01 | 0.89 | 0.63-1.27 | 9.69E-01 |
| methionine sulfone | 0.81 | 0.51-1.3 | 9.62E-01 | 0.83 | 0.51-1.34 | 9.69E-01 |
| 1-(1-enyl-oleoyl)-GPE (P-18:1)* | 1.13 | 0.69-1.87 | 9.69E-01 | 1.14 | 0.72-1.8 | 9.69E-01 |
| O-sulfo-L-tyrosine | 0.78 | 0.49-1.23 | 9.27E-01 | 0.81 | 0.5-1.31 | 9.69E-01 |
| ferulic acid 4-sulfate | 1.27 | 0.81-1.99 | 9.28E-01 | 1.23 | 0.79-1.94 | 9.69E-01 |
| N-acetyltaurine | 1.12 | 0.79-1.59 | 9.65E-01 | 1.11 | 0.78-1.57 | 9.69E-01 |
| 9-hydroxystearate | 1.12 | 0.79-1.61 | 9.65E-01 | 1.17 | 0.79-1.72 | 9.69E-01 |
| 3-methylglutarylcarnitine (2) | 0.89 | 0.59-1.34 | 9.69E-01 | 0.85 | 0.55-1.31 | 9.69E-01 |
| octadecenedioylcarnitine (C18:1-DC)* | 0.87 | 0.57-1.32 | 9.65E-01 | 0.85 | 0.54-1.33 | 9.69E-01 |
| octadecanedioylcarnitine (C18-DC)* | 1.14 | 0.79-1.65 | 9.65E-01 | 1.12 | 0.74-1.68 | 9.69E-01 |
| 5alpha-androstan-3alpha,17beta-diol monosulfate (2) | 1.27 | 0.73-2.22 | 9.62E-01 | 1.20 | 0.7-2.05 | 9.69E-01 |
| tyramine O-sulfate | 1.11 | 0.78-1.6 | 9.69E-01 | 1.13 | 0.81-1.58 | 9.69E-01 |
| 1-dihomo-linolenylglycerol (20:3) | 0.81 | 0.5-1.32 | 9.62E-01 | 0.84 | 0.52-1.37 | 9.69E-01 |
| 4-vinylguaiacol sulfate | 1.15 | 0.78-1.69 | 9.65E-01 | 1.17 | 0.73-1.88 | 9.69E-01 |
| vanillactate | 0.83 | 0.51-1.37 | 9.65E-01 | 0.84 | 0.52-1.37 | 9.69E-01 |
| eugenol sulfate | 0.85 | 0.51-1.41 | 9.65E-01 | 0.86 | 0.54-1.38 | 9.69E-01 |
| sphingomyelin (d18:1/20:0, d16:1/22:0)* | 1.16 | 0.73-1.86 | 9.65E-01 | 1.16 | 0.71-1.88 | 9.69E-01 |
| behenoyl sphingomyelin (d18:1/22:0)* | 1.08 | 0.72-1.63 | 9.80E-01 | 1.12 | 0.75-1.67 | 9.69E-01 |
| sphingomyelin (d18:1/22:2, d18:2/22:1, d16:1/24:2)* | 0.92 | 0.61-1.38 | 9.79E-01 | 0.88 | 0.56-1.38 | 9.69E-01 |
| lignoceroyl sphingomyelin (d18:1/24:0) | 0.81 | 0.47-1.41 | 9.65E-01 | 0.89 | 0.53-1.48 | 9.69E-01 |
| dopamine 3-O-sulfate | 0.88 | 0.58-1.33 | 9.69E-01 | 0.91 | 0.61-1.35 | 9.69E-01 |
| 3-hydroxyhexanoate | 0.78 | 0.44-1.41 | 9.62E-01 | 0.79 | 0.44-1.43 | 9.69E-01 |
| N-carbamoylalanine | 1.17 | 0.84-1.62 | 9.56E-01 | 1.14 | 0.81-1.59 | 9.69E-01 |
| 3beta-hydroxy-5-cholestenoate | 0.76 | 0.49-1.17 | 9.27E-01 | 0.83 | 0.52-1.33 | 9.69E-01 |
| 1,2,3-benzenetriol sulfate (2) | 1.15 | 0.75-1.76 | 9.65E-01 | 1.16 | 0.76-1.77 | 9.69E-01 |
| 3-methoxycatechol sulfate (1) | 1.16 | 0.73-1.87 | 9.65E-01 | 1.17 | 0.72-1.89 | 9.69E-01 |
| C-glycosyltryptophan | 1.18 | 0.77-1.81 | 9.62E-01 | 1.15 | 0.74-1.8 | 9.69E-01 |
| arabitol/xylitol | 0.91 | 0.63-1.33 | 9.69E-01 | 0.90 | 0.61-1.33 | 9.69E-01 |
| suberoylcarnitine (C8-DC) | 1.17 | 0.81-1.69 | 9.62E-01 | 1.13 | 0.78-1.62 | 9.69E-01 |
| glycochenodeoxycholate 3-sulfate | 0.89 | 0.58-1.36 | 9.69E-01 | 0.85 | 0.52-1.38 | 9.69E-01 |
| 1,2-dilinoleoyl-GPC (18:2/18:2) | 0.77 | 0.5-1.19 | 9.27E-01 | 0.83 | 0.55-1.24 | 9.69E-01 |
| 1-stearoyl-2-oleoyl-GPC (18:0/18:1) | 1.14 | 0.82-1.58 | 9.62E-01 | 1.16 | 0.84-1.6 | 9.69E-01 |
| oleate/vaccenate (18:1) | 0.87 | 0.58-1.3 | 9.65E-01 | 0.89 | 0.58-1.37 | 9.69E-01 |
| 1-palmitoleoylglycerol (16:1)* | 0.82 | 0.51-1.33 | 9.62E-01 | 0.83 | 0.49-1.39 | 9.69E-01 |
| 1-stearoyl-2-arachidonoyl-GPE (18:0/20:4) | 0.84 | 0.44-1.58 | 9.69E-01 | 0.86 | 0.48-1.54 | 9.69E-01 |
| 1-palmitoyl-2-arachidonoyl-GPE (16:0/20:4)* | 1.16 | 0.65-2.07 | 9.69E-01 | 1.18 | 0.71-1.96 | 9.69E-01 |
| 1-(1-enyl-palmitoyl)-2-arachidonoyl-GPE (P-16:0/20:4)* | 1.14 | 0.71-1.83 | 9.69E-01 | 1.18 | 0.78-1.8 | 9.69E-01 |
| 1-(1-enyl-palmitoyl)-2-linoleoyl-GPC (P-16:0/18:2)* | 1.04 | 0.67-1.63 | 9.93E-01 | 1.19 | 0.79-1.78 | 9.69E-01 |
| 1-(1-enyl-palmitoyl)-2-arachidonoyl-GPC (P-16:0/20:4)* | 1.10 | 0.71-1.71 | 9.79E-01 | 1.18 | 0.76-1.84 | 9.69E-01 |
| sphingomyelin (d18:0/18:0, d19:0/17:0)* | 1.20 | 0.79-1.82 | 9.62E-01 | 1.18 | 0.76-1.83 | 9.69E-01 |
| 1-oleoyl-2-docosahexaenoyl-GPC (18:1/22:6)* | 1.21 | 0.84-1.74 | 9.29E-01 | 1.20 | 0.79-1.82 | 9.69E-01 |
| 1-myristoyl-2-arachidonoyl-GPC (14:0/20:4)* | 0.84 | 0.54-1.3 | 9.62E-01 | 0.86 | 0.55-1.35 | 9.69E-01 |
| palmitoylcholine | 1.09 | 0.7-1.7 | 9.80E-01 | 1.19 | 0.76-1.86 | 9.69E-01 |
| glycochenodeoxycholate glucuronide (1) | 1.25 | 0.69-2.27 | 9.65E-01 | 1.23 | 0.7-2.16 | 9.69E-01 |
| glycosyl-N-palmitoyl-sphingosine (d18:1/16:0) | 1.07 | 0.68-1.68 | 9.84E-01 | 1.12 | 0.73-1.72 | 9.69E-01 |
| dihomo-linolenoyl-choline | 1.13 | 0.73-1.75 | 9.69E-01 | 1.18 | 0.77-1.8 | 9.69E-01 |
| palmitoleoylcarnitine (C16:1)* | 0.84 | 0.58-1.23 | 9.62E-01 | 0.84 | 0.56-1.25 | 9.69E-01 |
| 4-hydroxyphenylacetylglutamine | 0.88 | 0.52-1.49 | 9.69E-01 | 0.83 | 0.47-1.45 | 9.69E-01 |
| 2,3-dihydroxy-2-methylbutyrate | 1.20 | 0.71-2.02 | 9.65E-01 | 1.20 | 0.71-2.02 | 9.69E-01 |
| 2'-O-methylcytidine | 1.18 | 0.7-1.99 | 9.69E-01 | 1.19 | 0.7-2.04 | 9.69E-01 |
| gamma-glutamyl-alpha-lysine | 1.17 | 0.79-1.72 | 9.62E-01 | 1.17 | 0.79-1.74 | 9.69E-01 |
| N-palmitoyl-sphingadienine (d18:2/16:0)* | 1.23 | 0.75-2.02 | 9.62E-01 | 1.22 | 0.77-1.94 | 9.69E-01 |
| sphingomyelin (d18:1/19:0, d19:1/18:0)* | 1.24 | 0.78-1.99 | 9.62E-01 | 1.18 | 0.7-1.97 | 9.69E-01 |
| eicosenoylcarnitine (C20:1)* | 0.89 | 0.64-1.23 | 9.65E-01 | 0.93 | 0.66-1.3 | 9.69E-01 |
| dihomo-linoleoylcarnitine (C20:2)* | 0.74 | 0.43-1.28 | 9.27E-01 | 0.79 | 0.45-1.37 | 9.69E-01 |
| docosahexaenoylcarnitine (C22:6)* | 1.18 | 0.77-1.8 | 9.62E-01 | 1.19 | 0.73-1.93 | 9.69E-01 |
| nervonoylcarnitine (C24:1)* | 1.15 | 0.76-1.75 | 9.65E-01 | 1.17 | 0.76-1.79 | 9.69E-01 |
| cortolone glucuronide (1) | 0.91 | 0.56-1.47 | 9.80E-01 | 0.83 | 0.49-1.42 | 9.69E-01 |
| hydroxy-CMPF* | 1.14 | 0.73-1.8 | 9.69E-01 | 1.16 | 0.74-1.83 | 9.69E-01 |
| 3-hydroxyoleoylcarnitine | 1.20 | 0.75-1.9 | 9.62E-01 | 1.20 | 0.74-1.94 | 9.69E-01 |
| 2-hydroxyarachidate* | 0.85 | 0.56-1.27 | 9.62E-01 | 0.87 | 0.56-1.36 | 9.69E-01 |
| lyxonate | 0.90 | 0.63-1.29 | 9.69E-01 | 0.91 | 0.64-1.29 | 9.69E-01 |
| hexadecenedioate (C16:1-DC)* | 0.81 | 0.53-1.24 | 9.34E-01 | 0.81 | 0.51-1.28 | 9.69E-01 |
| 3-hydroxystachydrine* | 1.17 | 0.65-2.11 | 9.69E-01 | 1.15 | 0.65-2.05 | 9.69E-01 |
| perfluorooctanoate (PFOA) | 0.76 | 0.46-1.27 | 9.27E-01 | 0.79 | 0.46-1.33 | 9.69E-01 |
| glycine conjugate of C10H12O2* | 0.96 | 0.78-1.18 | 9.79E-01 | 0.94 | 0.74-1.2 | 9.69E-01 |
| glutamine conjugate of C7H12O2* | 0.86 | 0.55-1.32 | 9.65E-01 | 0.82 | 0.53-1.27 | 9.69E-01 |
| N-carbamoylvaline | 1.27 | 0.69-2.31 | 9.62E-01 | 1.26 | 0.69-2.32 | 9.69E-01 |
| N,N-dimethylalanine | 0.87 | 0.54-1.39 | 9.69E-01 | 0.89 | 0.55-1.44 | 9.69E-01 |
| 3-indoleglyoxylic acid | 0.88 | 0.57-1.34 | 9.69E-01 | 0.87 | 0.58-1.31 | 9.69E-01 |
| 6-bromotryptophan | 0.82 | 0.47-1.41 | 9.65E-01 | 0.81 | 0.48-1.36 | 9.69E-01 |
| delta-CEHC | 1.16 | 0.76-1.78 | 9.65E-01 | 1.17 | 0.74-1.82 | 9.69E-01 |
| indoleacetoylcarnitine* | 1.11 | 0.73-1.69 | 9.69E-01 | 1.12 | 0.73-1.74 | 9.69E-01 |
| sulfate of piperine metabolite C18H21NO3 (3)* | 0.77 | 0.5-1.19 | 9.27E-01 | 0.80 | 0.49-1.32 | 9.69E-01 |
| 11beta-hydroxyandrosterone glucuronide | 1.18 | 0.77-1.82 | 9.62E-01 | 1.19 | 0.75-1.87 | 9.69E-01 |
| cholic acid glucuronide | 1.32 | 0.72-2.43 | 9.62E-01 | 1.30 | 0.71-2.39 | 9.69E-01 |
| deoxycholic acid glucuronide | 1.29 | 0.72-2.3 | 9.62E-01 | 1.27 | 0.69-2.33 | 9.69E-01 |
| 3-hydroxypyridine glucuronide | 1.22 | 0.8-1.85 | 9.62E-01 | 1.21 | 0.76-1.92 | 9.69E-01 |
| glycoursodeoxycholic acid sulfate (1) | 1.22 | 0.76-1.96 | 9.62E-01 | 1.25 | 0.73-2.14 | 9.69E-01 |
| lithocholate sulfate (1) | 0.88 | 0.56-1.4 | 9.69E-01 | 0.86 | 0.52-1.42 | 9.69E-01 |
| tetrahydrocortisol sulfate (1) | 1.20 | 0.76-1.91 | 9.62E-01 | 1.17 | 0.72-1.9 | 9.69E-01 |
| 2,3-dihydroxy-5-methylthio-4-pentenoate (DMTPA)* | 0.93 | 0.61-1.41 | 9.83E-01 | 0.88 | 0.58-1.34 | 9.69E-01 |
| 5-hydroxy-2-methylpyridine sulfate | 1.20 | 0.8-1.81 | 9.62E-01 | 1.19 | 0.76-1.86 | 9.69E-01 |
| 3-hydroxy-2-methylpyridine sulfate | 1.22 | 0.82-1.81 | 9.31E-01 | 1.20 | 0.77-1.86 | 9.69E-01 |
| pregnenetriol sulfate* | 1.09 | 0.77-1.55 | 9.69E-01 | 1.10 | 0.77-1.58 | 9.69E-01 |
| hydroxy-N6,N6,N6-trimethyllysine* | 0.96 | 0.64-1.45 | 9.93E-01 | 0.90 | 0.61-1.34 | 9.69E-01 |
| 3-hydroxydecanoylcarnitine | 0.75 | 0.44-1.27 | 9.27E-01 | 0.78 | 0.45-1.35 | 9.69E-01 |
| palmitoyl-sphingosine-phosphoethanolamine (d18:1/16:0) | 0.88 | 0.6-1.27 | 9.65E-01 | 0.90 | 0.61-1.33 | 9.69E-01 |
| picolinoylglycine | 0.93 | 0.58-1.52 | 9.84E-01 | 0.87 | 0.54-1.4 | 9.69E-01 |
| 4-vinylcatechol sulfate | 0.87 | 0.59-1.27 | 9.65E-01 | 0.87 | 0.56-1.37 | 9.69E-01 |
| 2-hydroxy-4-(methylthio)butanoic acid | 0.83 | 0.53-1.32 | 9.62E-01 | 0.81 | 0.49-1.32 | 9.69E-01 |
| N-acetyl-2-aminoadipate | 1.23 | 0.82-1.85 | 9.29E-01 | 1.17 | 0.76-1.81 | 9.69E-01 |
| bilirubin degradation product, C16H18N2O5 (1)** | 0.83 | 0.61-1.15 | 9.27E-01 | 0.86 | 0.62-1.2 | 9.69E-01 |
| bilirubin degradation product, C17H20N2O5 (1)** | 0.85 | 0.58-1.27 | 9.62E-01 | 0.88 | 0.57-1.34 | 9.69E-01 |
| bilirubin degradation product, C17H20N2O5 (2)** | 0.88 | 0.59-1.31 | 9.65E-01 | 0.90 | 0.58-1.39 | 9.69E-01 |
| allantoin | 1.10 | 0.75-1.59 | 9.69E-01 | 1.12 | 0.76-1.66 | 9.69E-01 |
| 5-oxoproline | 0.86 | 0.61-1.22 | 9.62E-01 | 0.87 | 0.6-1.26 | 9.69E-01 |
| 3-ureidopropionate | 1.16 | 0.75-1.77 | 9.65E-01 | 1.14 | 0.73-1.8 | 9.69E-01 |
| N-acetylvaline | 0.90 | 0.59-1.36 | 9.69E-01 | 0.84 | 0.56-1.26 | 9.69E-01 |
| erucate (22:1n9) | 1.05 | 0.73-1.53 | 9.84E-01 | 1.12 | 0.8-1.57 | 9.69E-01 |
| thyroxine | 1.15 | 0.75-1.75 | 9.65E-01 | 1.11 | 0.73-1.71 | 9.69E-01 |
| methyl indole-3-acetate | 1.10 | 0.84-1.45 | 9.65E-01 | 1.09 | 0.81-1.47 | 9.69E-01 |
| 3-hydroxyisobutyrate | 0.88 | 0.56-1.37 | 9.69E-01 | 0.81 | 0.49-1.35 | 9.69E-01 |
| N-acetylalanine | 0.84 | 0.5-1.4 | 9.65E-01 | 0.80 | 0.47-1.36 | 9.69E-01 |
| vanillylmandelate (VMA) | 0.87 | 0.55-1.39 | 9.69E-01 | 0.86 | 0.56-1.3 | 9.69E-01 |
| 4-acetamidobutanoate | 0.91 | 0.61-1.37 | 9.79E-01 | 0.89 | 0.59-1.32 | 9.69E-01 |
| urate | 0.89 | 0.63-1.26 | 9.65E-01 | 0.88 | 0.62-1.26 | 9.69E-01 |
| oleoyl ethanolamide | 1.14 | 0.65-1.98 | 9.75E-01 | 1.20 | 0.71-2.03 | 9.69E-01 |
| gamma-glutamylglutamine | 1.07 | 0.73-1.56 | 9.84E-01 | 1.14 | 0.74-1.76 | 9.69E-01 |
| gamma-glutamylhistidine | 1.13 | 0.79-1.6 | 9.65E-01 | 1.11 | 0.79-1.58 | 9.69E-01 |
| 2-hydroxystearate | 1.09 | 0.68-1.73 | 9.84E-01 | 1.14 | 0.69-1.87 | 9.69E-01 |
| N1-methyladenosine | 1.25 | 0.77-2.02 | 9.62E-01 | 1.22 | 0.76-1.96 | 9.69E-01 |
| choline | 1.10 | 0.68-1.79 | 9.80E-01 | 1.11 | 0.7-1.77 | 9.69E-01 |
| anthranilate | 1.22 | 0.73-2.06 | 9.62E-01 | 1.22 | 0.75-1.98 | 9.69E-01 |
| 4-hydroxyphenylacetate | 0.87 | 0.5-1.52 | 9.69E-01 | 0.85 | 0.49-1.47 | 9.69E-01 |
| 1-palmitoyl-2-linoleoyl-GPC (16:0/18:2) | 0.83 | 0.51-1.37 | 9.65E-01 | 0.88 | 0.54-1.44 | 9.69E-01 |
| 9,10-DiHOME | 0.89 | 0.54-1.47 | 9.70E-01 | 0.90 | 0.56-1.44 | 9.69E-01 |
| laurate (12:0) | 0.86 | 0.54-1.37 | 9.65E-01 | 0.90 | 0.56-1.43 | 9.69E-01 |
| S-adenosylhomocysteine (SAH) | 0.93 | 0.59-1.46 | 9.84E-01 | 0.88 | 0.55-1.4 | 9.69E-01 |
| eicosapentaenoate (EPA; 20:5n3) | 0.81 | 0.46-1.42 | 9.65E-01 | 0.86 | 0.48-1.54 | 9.69E-01 |
| 5-methylthioadenosine (MTA) | 1.22 | 0.83-1.81 | 9.28E-01 | 1.16 | 0.81-1.66 | 9.69E-01 |
| N6-methyladenosine | 1.15 | 0.77-1.71 | 9.65E-01 | 1.13 | 0.77-1.67 | 9.69E-01 |
| arginine | 1.16 | 0.71-1.88 | 9.69E-01 | 1.21 | 0.73-2.01 | 9.69E-01 |
| 2-hydroxyphenylacetate | 0.93 | 0.59-1.45 | 9.84E-01 | 0.90 | 0.58-1.41 | 9.69E-01 |
| cholesterol | 0.81 | 0.46-1.42 | 9.65E-01 | 0.86 | 0.5-1.49 | 9.69E-01 |
| corticosterone | 0.85 | 0.68-1.07 | 9.27E-01 | 0.90 | 0.72-1.13 | 9.69E-01 |
| creatinine | 1.13 | 0.77-1.68 | 9.65E-01 | 1.15 | 0.8-1.64 | 9.69E-01 |
| cysteinylglycine | 0.83 | 0.54-1.29 | 9.62E-01 | 0.84 | 0.56-1.25 | 9.69E-01 |
| sphingosine | 1.23 | 0.8-1.91 | 9.44E-01 | 1.21 | 0.78-1.88 | 9.69E-01 |
| deoxycholate | 1.15 | 0.7-1.89 | 9.69E-01 | 1.17 | 0.71-1.92 | 9.69E-01 |
| isoleucine | 0.92 | 0.6-1.41 | 9.80E-01 | 0.80 | 0.5-1.28 | 9.69E-01 |
| citrulline | 1.17 | 0.69-1.99 | 9.69E-01 | 1.21 | 0.71-2.07 | 9.69E-01 |
| leucine | 1.00 | 0.68-1.48 | 9.93E-01 | 0.92 | 0.64-1.31 | 9.69E-01 |
| lithocholate | 0.86 | 0.52-1.43 | 9.69E-01 | 0.87 | 0.5-1.51 | 9.69E-01 |
| palmitate (16:0) | 0.85 | 0.55-1.3 | 9.62E-01 | 0.87 | 0.54-1.39 | 9.69E-01 |
| stearate (18:0) | 0.83 | 0.52-1.32 | 9.62E-01 | 0.88 | 0.55-1.4 | 9.69E-01 |
| orotate | 1.16 | 0.79-1.71 | 9.62E-01 | 1.19 | 0.79-1.78 | 9.69E-01 |
| palmitoleate (16:1n7) | 0.83 | 0.53-1.31 | 9.62E-01 | 0.85 | 0.53-1.38 | 9.69E-01 |
| phytanate | 1.10 | 0.73-1.65 | 9.70E-01 | 1.18 | 0.8-1.74 | 9.69E-01 |
| proline | 0.82 | 0.49-1.37 | 9.62E-01 | 0.77 | 0.43-1.37 | 9.69E-01 |
| lactate | 0.92 | 0.58-1.47 | 9.84E-01 | 0.88 | 0.57-1.36 | 9.69E-01 |
| taurine | 1.18 | 0.66-2.1 | 9.69E-01 | 1.22 | 0.71-2.1 | 9.69E-01 |
| myristate (14:0) | 0.85 | 0.54-1.36 | 9.65E-01 | 0.88 | 0.54-1.44 | 9.69E-01 |
| urea | 1.17 | 0.79-1.73 | 9.62E-01 | 1.13 | 0.77-1.67 | 9.69E-01 |
| uridine | 1.22 | 0.76-1.97 | 9.62E-01 | 1.24 | 0.76-2.01 | 9.69E-01 |
| cysteine | 0.91 | 0.56-1.47 | 9.80E-01 | 0.89 | 0.55-1.44 | 9.69E-01 |
| pyruvate | 0.94 | 0.59-1.48 | 9.84E-01 | 0.88 | 0.56-1.38 | 9.69E-01 |
| fructose | 0.84 | 0.51-1.39 | 9.65E-01 | 0.84 | 0.49-1.45 | 9.69E-01 |
| cytosine | 1.20 | 0.79-1.84 | 9.62E-01 | 1.20 | 0.79-1.82 | 9.69E-01 |
| margarate (17:0) | 0.73 | 0.37-1.41 | 9.54E-01 | 0.74 | 0.36-1.51 | 9.69E-01 |
| arachidate (20:0) | 1.02 | 0.64-1.62 | 9.93E-01 | 1.12 | 0.73-1.71 | 9.69E-01 |
| asparagine | 1.10 | 0.77-1.57 | 9.69E-01 | 1.18 | 0.79-1.76 | 9.69E-01 |
| caproate (6:0) | 0.80 | 0.48-1.31 | 9.62E-01 | 0.80 | 0.48-1.34 | 9.69E-01 |
| caprylate (8:0) | 0.88 | 0.54-1.42 | 9.69E-01 | 0.89 | 0.54-1.48 | 9.69E-01 |
| X - 11299 | 1.13 | 0.74-1.72 | 9.69E-01 | 1.14 | 0.73-1.76 | 9.69E-01 |
| X - 11381 | 1.10 | 0.73-1.64 | 9.79E-01 | 1.10 | 0.74-1.63 | 9.69E-01 |
| X - 11470 | 0.91 | 0.66-1.26 | 9.69E-01 | 0.91 | 0.64-1.28 | 9.69E-01 |
| X - 11795 | 0.81 | 0.47-1.39 | 9.62E-01 | 0.87 | 0.5-1.51 | 9.69E-01 |
| X - 11843 | 1.23 | 0.74-2.05 | 9.62E-01 | 1.25 | 0.75-2.08 | 9.69E-01 |
| X - 12104 | 0.91 | 0.6-1.39 | 9.79E-01 | 0.90 | 0.58-1.38 | 9.69E-01 |
| X - 12111 | 1.16 | 0.7-1.93 | 9.69E-01 | 1.14 | 0.68-1.92 | 9.69E-01 |
| X - 12221 | 1.18 | 0.77-1.82 | 9.62E-01 | 1.18 | 0.78-1.77 | 9.69E-01 |
| X - 12306 | 1.26 | 0.78-2.05 | 9.49E-01 | 1.24 | 0.74-2.07 | 9.69E-01 |
| X - 12407 | 0.93 | 0.64-1.35 | 9.80E-01 | 0.89 | 0.62-1.27 | 9.69E-01 |
| X - 12411 | 1.28 | 0.78-2.08 | 9.32E-01 | 1.28 | 0.73-2.24 | 9.69E-01 |
| X - 12456 | 1.21 | 0.75-1.95 | 9.62E-01 | 1.14 | 0.66-1.99 | 9.69E-01 |
| X - 12544 | 0.93 | 0.65-1.34 | 9.80E-01 | 0.91 | 0.64-1.29 | 9.69E-01 |
| X - 12680 | 1.21 | 0.76-1.94 | 9.62E-01 | 1.19 | 0.73-1.92 | 9.69E-01 |
| X - 12740 | 0.85 | 0.57-1.29 | 9.62E-01 | 0.86 | 0.57-1.29 | 9.69E-01 |
| X - 12798 | 1.24 | 0.81-1.9 | 9.29E-01 | 1.21 | 0.8-1.83 | 9.69E-01 |
| X - 12847 | 1.12 | 0.79-1.59 | 9.65E-01 | 1.16 | 0.77-1.74 | 9.69E-01 |
| X - 12851 | 0.84 | 0.51-1.37 | 9.65E-01 | 0.82 | 0.49-1.37 | 9.69E-01 |
| X - 13553 | 0.92 | 0.61-1.41 | 9.80E-01 | 0.90 | 0.59-1.38 | 9.69E-01 |
| X - 13695 | 1.21 | 0.69-2.14 | 9.65E-01 | 1.19 | 0.67-2.11 | 9.69E-01 |
| X - 13729 | 0.88 | 0.55-1.39 | 9.69E-01 | 0.88 | 0.57-1.36 | 9.69E-01 |
| X - 13844 | 0.81 | 0.49-1.35 | 9.62E-01 | 0.79 | 0.46-1.34 | 9.69E-01 |
| X - 13866 | 0.89 | 0.59-1.35 | 9.69E-01 | 0.89 | 0.56-1.4 | 9.69E-01 |
| X - 14082 | 0.87 | 0.59-1.3 | 9.65E-01 | 0.88 | 0.59-1.31 | 9.69E-01 |
| X - 14939 | 0.88 | 0.65-1.18 | 9.62E-01 | 0.88 | 0.64-1.22 | 9.69E-01 |
| X - 15245 | 0.86 | 0.54-1.39 | 9.69E-01 | 0.86 | 0.55-1.34 | 9.69E-01 |
| X - 15461 | 1.14 | 0.76-1.7 | 9.69E-01 | 1.16 | 0.8-1.71 | 9.69E-01 |
| X - 16087 | 0.89 | 0.62-1.27 | 9.65E-01 | 0.88 | 0.59-1.3 | 9.69E-01 |
| X - 16124 | 0.84 | 0.55-1.28 | 9.62E-01 | 0.85 | 0.55-1.33 | 9.69E-01 |
| X - 17325 | 1.13 | 0.65-1.95 | 9.79E-01 | 1.17 | 0.69-1.98 | 9.69E-01 |
| X - 17367 | 1.19 | 0.69-2.04 | 9.66E-01 | 1.24 | 0.73-2.11 | 9.69E-01 |
| X - 17612 | 0.88 | 0.59-1.33 | 9.69E-01 | 0.88 | 0.58-1.33 | 9.69E-01 |
| X - 18886 | 0.86 | 0.47-1.55 | 9.69E-01 | 0.83 | 0.46-1.48 | 9.69E-01 |
| X - 18935 | 1.11 | 0.7-1.76 | 9.79E-01 | 1.13 | 0.72-1.78 | 9.69E-01 |
| X - 19141 | 0.83 | 0.5-1.37 | 9.65E-01 | 0.84 | 0.5-1.42 | 9.69E-01 |
| X - 21258 | 1.13 | 0.67-1.89 | 9.72E-01 | 1.16 | 0.69-1.96 | 9.69E-01 |
| X - 21410 | 1.14 | 0.75-1.75 | 9.65E-01 | 1.12 | 0.71-1.75 | 9.69E-01 |
| X - 21733 | 0.88 | 0.53-1.45 | 9.69E-01 | 0.83 | 0.47-1.48 | 9.69E-01 |
| X - 21834 | 0.73 | 0.41-1.31 | 9.27E-01 | 0.78 | 0.42-1.43 | 9.69E-01 |
| X - 21959 | 1.20 | 0.85-1.68 | 9.28E-01 | 1.17 | 0.81-1.68 | 9.69E-01 |
| X - 22834 | 1.10 | 0.71-1.71 | 9.79E-01 | 1.13 | 0.72-1.78 | 9.69E-01 |
| X - 23644 | 1.11 | 0.69-1.78 | 9.79E-01 | 1.12 | 0.69-1.82 | 9.69E-01 |
| X - 23648 | 1.20 | 0.82-1.75 | 9.44E-01 | 1.20 | 0.81-1.77 | 9.69E-01 |
| X - 23659 | 0.86 | 0.61-1.22 | 9.62E-01 | 0.85 | 0.6-1.22 | 9.69E-01 |
| X - 23739 | 1.10 | 0.78-1.54 | 9.69E-01 | 1.13 | 0.82-1.55 | 9.69E-01 |
| X - 23782 | 0.86 | 0.56-1.31 | 9.65E-01 | 0.89 | 0.58-1.36 | 9.69E-01 |
| X - 23787 | 1.12 | 0.75-1.67 | 9.69E-01 | 1.16 | 0.78-1.7 | 9.69E-01 |
| X - 23890 | 1.22 | 0.76-1.98 | 9.62E-01 | 1.23 | 0.73-2.08 | 9.69E-01 |
| X - 23974 | 0.90 | 0.57-1.41 | 9.70E-01 | 0.86 | 0.54-1.38 | 9.69E-01 |
| X - 23997 | 1.15 | 0.7-1.88 | 9.69E-01 | 1.15 | 0.68-1.95 | 9.69E-01 |
| X - 24328 | 0.86 | 0.47-1.57 | 9.69E-01 | 0.85 | 0.49-1.49 | 9.69E-01 |
| X - 24337 | 1.17 | 0.75-1.84 | 9.65E-01 | 1.17 | 0.71-1.94 | 9.69E-01 |
| X - 24456 | 0.84 | 0.54-1.31 | 9.62E-01 | 0.83 | 0.55-1.24 | 9.69E-01 |
| X - 24588 | 0.93 | 0.61-1.4 | 9.81E-01 | 0.90 | 0.58-1.4 | 9.69E-01 |
| X - 24728 | 0.81 | 0.5-1.31 | 9.62E-01 | 0.80 | 0.49-1.32 | 9.69E-01 |
| X - 24757 | 1.12 | 0.62-2.04 | 9.80E-01 | 1.16 | 0.65-2.05 | 9.69E-01 |
| X - 24811 | 1.18 | 0.72-1.93 | 9.65E-01 | 1.18 | 0.69-2.01 | 9.69E-01 |
| X - 24812 | 0.90 | 0.61-1.31 | 9.69E-01 | 0.87 | 0.6-1.25 | 9.69E-01 |
| X - 25265 | 1.09 | 0.7-1.69 | 9.80E-01 | 1.12 | 0.71-1.76 | 9.69E-01 |
| X - 25267 | 0.91 | 0.59-1.4 | 9.79E-01 | 0.91 | 0.59-1.39 | 9.69E-01 |
| X - 25371 | 0.97 | 0.59-1.62 | 9.93E-01 | 0.88 | 0.53-1.47 | 9.69E-01 |
| X - 25419 | 0.84 | 0.58-1.22 | 9.62E-01 | 0.86 | 0.59-1.24 | 9.69E-01 |
| X - 25422 | 1.15 | 0.71-1.85 | 9.69E-01 | 1.12 | 0.69-1.81 | 9.69E-01 |
| X - 25520 | 0.90 | 0.6-1.35 | 9.69E-01 | 0.88 | 0.57-1.36 | 9.69E-01 |
| X - 25790 | 0.89 | 0.56-1.4 | 9.69E-01 | 0.91 | 0.61-1.36 | 9.69E-01 |
| X - 25936 | 0.83 | 0.55-1.24 | 9.62E-01 | 0.84 | 0.56-1.26 | 9.69E-01 |
| X - 26097 | 1.20 | 0.79-1.84 | 9.62E-01 | 1.16 | 0.76-1.76 | 9.69E-01 |
| X - 26106 | 0.85 | 0.53-1.35 | 9.65E-01 | 0.83 | 0.49-1.4 | 9.69E-01 |
| X - 26111 | 1.12 | 0.74-1.69 | 9.69E-01 | 1.12 | 0.72-1.74 | 9.69E-01 |
| X - 12216 | 0.88 | 0.53-1.45 | 9.69E-01 | 0.90 | 0.56-1.45 | 9.70E-01 |
| suberate (C8-DC) | 0.95 | 0.62-1.44 | 9.84E-01 | 0.90 | 0.58-1.42 | 9.71E-01 |
| pantoate | 0.90 | 0.63-1.28 | 9.69E-01 | 0.93 | 0.66-1.3 | 9.71E-01 |
| N-stearoyl-sphinganine (d18:0/18:0)* | 1.14 | 0.79-1.64 | 9.65E-01 | 1.09 | 0.74-1.62 | 9.73E-01 |
| cholesterol sulfate | 0.83 | 0.52-1.32 | 9.62E-01 | 0.90 | 0.55-1.47 | 9.76E-01 |
| palmitoyl dihydrosphingomyelin (d18:0/16:0)* | 1.05 | 0.59-1.9 | 9.93E-01 | 1.13 | 0.65-1.98 | 9.76E-01 |
| 1-stearoyl-2-docosahexaenoyl-GPE (18:0/22:6)* | 1.16 | 0.7-1.94 | 9.69E-01 | 1.11 | 0.68-1.83 | 9.76E-01 |
| N-palmitoyl-sphinganine (d18:0/16:0) | 0.93 | 0.63-1.38 | 9.81E-01 | 0.92 | 0.61-1.37 | 9.76E-01 |
| linoleoylcholine* | 1.00 | 0.64-1.57 | 9.93E-01 | 1.10 | 0.71-1.71 | 9.76E-01 |
| 3-hydroxy-3-methylglutarate | 1.15 | 0.76-1.74 | 9.65E-01 | 1.09 | 0.74-1.61 | 9.76E-01 |
| palmitoyl ethanolamide | 1.05 | 0.64-1.71 | 9.93E-01 | 1.11 | 0.69-1.8 | 9.76E-01 |
| X - 12822 | 1.12 | 0.71-1.74 | 9.69E-01 | 1.10 | 0.7-1.75 | 9.76E-01 |
| X - 23593 | 1.11 | 0.7-1.74 | 9.79E-01 | 1.10 | 0.72-1.67 | 9.76E-01 |
| X - 24411 | 0.92 | 0.62-1.35 | 9.79E-01 | 0.92 | 0.62-1.37 | 9.76E-01 |
| X - 24544 | 0.93 | 0.68-1.29 | 9.79E-01 | 0.93 | 0.67-1.29 | 9.76E-01 |
| 3-phosphoglycerate | 0.96 | 0.68-1.35 | 9.87E-01 | 0.93 | 0.66-1.3 | 9.76E-01 |
| X - 26109 | 1.20 | 0.68-2.11 | 9.65E-01 | 1.14 | 0.63-2.06 | 9.76E-01 |
| 1-palmitoyl-2-stearoyl-GPC (16:0/18:0) | 0.83 | 0.41-1.68 | 9.69E-01 | 0.87 | 0.45-1.67 | 9.78E-01 |
| 5-hydroxylysine | 1.08 | 0.8-1.45 | 9.69E-01 | 1.06 | 0.8-1.42 | 9.80E-01 |
| tartarate | 0.94 | 0.68-1.29 | 9.80E-01 | 0.93 | 0.66-1.31 | 9.80E-01 |
| eicosenoate (20:1) | 0.86 | 0.55-1.35 | 9.65E-01 | 0.91 | 0.58-1.43 | 9.80E-01 |
| fructosyllysine | 0.95 | 0.59-1.52 | 9.88E-01 | 0.91 | 0.57-1.45 | 9.80E-01 |
| taurodeoxycholic acid 3-sulfate | 0.94 | 0.6-1.46 | 9.84E-01 | 0.91 | 0.58-1.43 | 9.80E-01 |
| hydroxyasparagine** | 1.15 | 0.73-1.82 | 9.69E-01 | 1.10 | 0.7-1.74 | 9.80E-01 |
| X - 26107 | 1.07 | 0.81-1.41 | 9.70E-01 | 1.07 | 0.78-1.46 | 9.80E-01 |
| bilirubin (E,Z or Z,E)* | 0.90 | 0.61-1.33 | 9.69E-01 | 0.92 | 0.61-1.39 | 9.80E-01 |
| X - 18888 | 0.90 | 0.56-1.42 | 9.70E-01 | 0.91 | 0.56-1.47 | 9.80E-01 |
| dihomo-linolenate (20:3n3 or n6) | 0.87 | 0.53-1.43 | 9.69E-01 | 0.90 | 0.52-1.55 | 9.82E-01 |
| (16 or 17)-methylstearate (a19:0 or i19:0) | 1.03 | 0.7-1.53 | 9.93E-01 | 1.09 | 0.73-1.63 | 9.82E-01 |
| 5-(galactosylhydroxy)-L-lysine | 0.94 | 0.57-1.54 | 9.84E-01 | 0.90 | 0.53-1.52 | 9.82E-01 |
| 2-aminoheptanoate | 0.90 | 0.52-1.56 | 9.80E-01 | 0.90 | 0.53-1.53 | 9.82E-01 |
| p-cresol glucuronide* | 1.09 | 0.69-1.72 | 9.81E-01 | 1.10 | 0.68-1.77 | 9.82E-01 |
| glycerate | 1.15 | 0.61-2.15 | 9.79E-01 | 1.15 | 0.59-2.24 | 9.82E-01 |
| taurodeoxycholate | 1.09 | 0.71-1.66 | 9.80E-01 | 1.09 | 0.71-1.68 | 9.82E-01 |
| methionine | 1.11 | 0.64-1.92 | 9.80E-01 | 1.12 | 0.63-2 | 9.82E-01 |
| retinol (Vitamin A) | 1.06 | 0.74-1.51 | 9.84E-01 | 1.08 | 0.74-1.59 | 9.82E-01 |
| salicylate | 1.06 | 0.69-1.62 | 9.84E-01 | 1.08 | 0.73-1.62 | 9.82E-01 |
| alanine | 0.94 | 0.58-1.51 | 9.84E-01 | 0.91 | 0.56-1.47 | 9.82E-01 |
| X - 21441 | 1.09 | 0.69-1.73 | 9.80E-01 | 1.10 | 0.68-1.79 | 9.82E-01 |
| X - 23665 | 1.15 | 0.73-1.83 | 9.69E-01 | 1.10 | 0.67-1.81 | 9.83E-01 |
| glycohyocholate | 1.08 | 0.66-1.76 | 9.84E-01 | 1.10 | 0.68-1.78 | 9.83E-01 |
| X - 23276 | 1.09 | 0.76-1.55 | 9.70E-01 | 1.08 | 0.74-1.56 | 9.83E-01 |
| myristoleate (14:1n5) | 0.90 | 0.56-1.42 | 9.70E-01 | 0.91 | 0.57-1.47 | 9.84E-01 |
| gamma-glutamylmethionine | 1.07 | 0.63-1.83 | 9.84E-01 | 1.11 | 0.64-1.93 | 9.84E-01 |
| 7-methylguanine | 1.07 | 0.68-1.68 | 9.84E-01 | 1.09 | 0.68-1.76 | 9.84E-01 |
| 2-palmitoleoyl-GPC (16:1)* | 1.02 | 0.68-1.52 | 9.93E-01 | 1.08 | 0.71-1.65 | 9.84E-01 |
| beta-citrylglutamate | 1.08 | 0.73-1.59 | 9.80E-01 | 1.08 | 0.72-1.62 | 9.84E-01 |
| stearoylcholine* | 1.00 | 0.66-1.52 | 9.95E-01 | 1.08 | 0.71-1.65 | 9.84E-01 |
| 4-ethylcatechol sulfate | 0.91 | 0.65-1.27 | 9.69E-01 | 0.93 | 0.64-1.35 | 9.84E-01 |
| phosphoethanolamine | 0.90 | 0.49-1.64 | 9.84E-01 | 0.89 | 0.48-1.65 | 9.84E-01 |
| 3-methoxytyrosine | 0.93 | 0.62-1.38 | 9.80E-01 | 0.94 | 0.66-1.33 | 9.84E-01 |
| choline phosphate | 0.88 | 0.46-1.67 | 9.80E-01 | 0.87 | 0.43-1.76 | 9.84E-01 |
| gluconate | 0.98 | 0.68-1.42 | 9.93E-01 | 0.94 | 0.68-1.3 | 9.84E-01 |
| X - 23583 | 1.06 | 0.75-1.51 | 9.84E-01 | 1.07 | 0.75-1.53 | 9.84E-01 |
| glutamine_degradant* | 0.89 | 0.58-1.37 | 9.69E-01 | 0.92 | 0.6-1.42 | 9.88E-01 |
| 3-methyl catechol sulfate (2) | 0.92 | 0.6-1.42 | 9.80E-01 | 0.92 | 0.58-1.45 | 9.93E-01 |
| isovalerylcarnitine (C5) | 1.15 | 0.69-1.91 | 9.69E-01 | 1.09 | 0.67-1.79 | 9.95E-01 |
| 3-methylglutaconate | 0.88 | 0.54-1.43 | 9.69E-01 | 0.91 | 0.54-1.53 | 9.95E-01 |
| succinoyltaurine | 1.14 | 0.77-1.69 | 9.65E-01 | 1.07 | 0.75-1.52 | 9.95E-01 |
| tyrosine | 1.13 | 0.73-1.76 | 9.69E-01 | 1.09 | 0.68-1.73 | 9.95E-01 |
| 3-carboxy-4-methyl-5-propyl-2-furanpropanoate (CMPF) | 0.93 | 0.65-1.34 | 9.80E-01 | 0.94 | 0.66-1.34 | 9.96E-01 |
| N-acetyl-beta-alanine | 0.93 | 0.59-1.45 | 9.84E-01 | 0.93 | 0.62-1.4 | 9.96E-01 |
| taurochenodeoxycholic acid 3-sulfate | 0.94 | 0.61-1.47 | 9.84E-01 | 0.92 | 0.57-1.49 | 9.96E-01 |
| pregnenediol disulfate (C21H34O8S2)* | 0.91 | 0.59-1.4 | 9.79E-01 | 0.92 | 0.58-1.47 | 9.97E-01 |
| X - 26119 | 0.88 | 0.48-1.62 | 9.80E-01 | 0.90 | 0.48-1.68 | 9.97E-01 |
| 3-phenylpropionate (hydrocinnamate) | 0.93 | 0.59-1.46 | 9.84E-01 | 0.93 | 0.58-1.5 | 9.99E-01 |
| 4-guanidinobutanoate | 0.97 | 0.57-1.66 | 9.93E-01 | 1.00 | 0.56-1.77 | 9.99E-01 |
| glucuronate | 0.96 | 0.65-1.41 | 9.88E-01 | 0.99 | 0.68-1.44 | 9.99E-01 |
| glycerophosphorylcholine (GPC) | 0.88 | 0.55-1.39 | 9.69E-01 | 0.94 | 0.56-1.57 | 9.99E-01 |
| N-acetylglutamate | 0.93 | 0.62-1.39 | 9.80E-01 | 0.98 | 0.64-1.48 | 9.99E-01 |
| ribitol | 1.04 | 0.68-1.6 | 9.93E-01 | 1.03 | 0.66-1.59 | 9.99E-01 |
| 1-stearoyl-GPI (18:0) | 0.95 | 0.52-1.73 | 9.93E-01 | 0.98 | 0.54-1.79 | 9.99E-01 |
| 1,2-dipalmitoyl-GPC (16:0/16:0) | 0.95 | 0.56-1.62 | 9.93E-01 | 0.99 | 0.56-1.76 | 9.99E-01 |
| docosahexaenoate (DHA; 22:6n3) | 0.95 | 0.61-1.49 | 9.88E-01 | 1.00 | 0.62-1.61 | 9.99E-01 |
| phenyllactate (PLA) | 1.01 | 0.66-1.54 | 9.93E-01 | 0.98 | 0.63-1.53 | 9.99E-01 |
| 1-palmitoylglycerol (16:0) | 1.05 | 0.67-1.63 | 9.93E-01 | 1.06 | 0.69-1.63 | 9.99E-01 |
| tartronate (hydroxymalonate) | 1.01 | 0.58-1.77 | 9.93E-01 | 1.02 | 0.56-1.88 | 9.99E-01 |
| oxalate (ethanedioate) | 1.08 | 0.57-2.04 | 9.84E-01 | 1.08 | 0.56-2.1 | 9.99E-01 |
| erythritol | 0.97 | 0.59-1.58 | 9.93E-01 | 0.93 | 0.56-1.54 | 9.99E-01 |
| 2,3-dihydroxypyridine | 1.05 | 0.62-1.77 | 9.93E-01 | 1.02 | 0.59-1.77 | 9.99E-01 |
| 2-oleoylglycerol (18:1) | 1.06 | 0.7-1.62 | 9.84E-01 | 1.03 | 0.67-1.59 | 9.99E-01 |
| 2-linoleoylglycerol (18:2) | 0.98 | 0.71-1.35 | 9.93E-01 | 0.99 | 0.71-1.39 | 9.99E-01 |
| N-acetylglycine | 0.94 | 0.59-1.48 | 9.84E-01 | 1.01 | 0.63-1.63 | 9.99E-01 |
| galactonate | 1.03 | 0.72-1.47 | 9.93E-01 | 0.96 | 0.68-1.34 | 9.99E-01 |
| indoleacetate | 1.03 | 0.71-1.49 | 9.93E-01 | 1.06 | 0.73-1.53 | 9.99E-01 |
| glycolithocholate | 0.93 | 0.57-1.51 | 9.84E-01 | 0.96 | 0.58-1.59 | 9.99E-01 |
| dodecanedioate (C12-DC) | 0.95 | 0.62-1.44 | 9.84E-01 | 0.98 | 0.64-1.48 | 9.99E-01 |
| N-acetyltyrosine | 1.04 | 0.78-1.38 | 9.87E-01 | 1.03 | 0.77-1.37 | 9.99E-01 |
| 3-methylxanthine | 0.94 | 0.64-1.37 | 9.84E-01 | 0.96 | 0.66-1.39 | 9.99E-01 |
| 3-hydroxylaurate | 0.88 | 0.54-1.46 | 9.69E-01 | 0.93 | 0.54-1.57 | 9.99E-01 |
| 3-hydroxysebacate | 1.03 | 0.64-1.66 | 9.93E-01 | 1.04 | 0.65-1.65 | 9.99E-01 |
| docosatrienoate (22:3n3) | 1.01 | 0.7-1.47 | 9.93E-01 | 1.00 | 0.67-1.49 | 9.99E-01 |
| sebacate (C10-DC) | 1.04 | 0.72-1.51 | 9.88E-01 | 1.06 | 0.7-1.61 | 9.99E-01 |
| N-acetylglutamine | 0.98 | 0.74-1.29 | 9.93E-01 | 1.01 | 0.75-1.36 | 9.99E-01 |
| campesterol | 0.92 | 0.62-1.36 | 9.79E-01 | 1.03 | 0.67-1.6 | 9.99E-01 |
| 1-oleoyl-GPC (18:1) | 0.96 | 0.67-1.35 | 9.84E-01 | 1.04 | 0.71-1.54 | 9.99E-01 |
| 10-nonadecenoate (19:1n9) | 0.97 | 0.64-1.48 | 9.93E-01 | 1.02 | 0.66-1.58 | 9.99E-01 |
| 10-heptadecenoate (17:1n7) | 0.90 | 0.56-1.42 | 9.70E-01 | 0.92 | 0.56-1.51 | 9.99E-01 |
| stachydrine | 1.08 | 0.6-1.94 | 9.84E-01 | 1.07 | 0.6-1.91 | 9.99E-01 |
| p-cresol sulfate | 0.93 | 0.6-1.43 | 9.84E-01 | 0.94 | 0.61-1.45 | 9.99E-01 |
| erythronate* | 1.10 | 0.74-1.64 | 9.69E-01 | 1.03 | 0.69-1.54 | 9.99E-01 |
| aconitate [cis or trans] | 0.95 | 0.54-1.67 | 9.93E-01 | 0.92 | 0.53-1.61 | 9.99E-01 |
| stearoylcarnitine (C18) | 0.98 | 0.62-1.56 | 9.93E-01 | 1.05 | 0.66-1.66 | 9.99E-01 |
| 7-methylxanthine | 0.98 | 0.61-1.57 | 9.93E-01 | 0.99 | 0.61-1.61 | 9.99E-01 |
| 1-methylurate | 0.98 | 0.62-1.53 | 9.93E-01 | 0.98 | 0.61-1.56 | 9.99E-01 |
| 5-acetylamino-6-formylamino-3-methyluracil | 1.06 | 0.72-1.56 | 9.84E-01 | 1.05 | 0.71-1.57 | 9.99E-01 |
| 5-acetylamino-6-amino-3-methyluracil | 0.99 | 0.76-1.27 | 9.93E-01 | 1.00 | 0.76-1.32 | 9.99E-01 |
| 1-methylxanthine | 0.92 | 0.66-1.27 | 9.69E-01 | 0.96 | 0.68-1.35 | 9.99E-01 |
| N2,N2-dimethylguanosine | 1.07 | 0.69-1.67 | 9.84E-01 | 1.02 | 0.66-1.57 | 9.99E-01 |
| N4-acetylcytidine | 1.08 | 0.64-1.85 | 9.84E-01 | 1.05 | 0.63-1.74 | 9.99E-01 |
| N6-carbamoylthreonyladenosine | 1.04 | 0.72-1.51 | 9.88E-01 | 1.00 | 0.69-1.46 | 9.99E-01 |
| phenylacetylglutamine | 0.97 | 0.64-1.46 | 9.93E-01 | 0.96 | 0.64-1.44 | 9.99E-01 |
| 5,6-dihydrouridine | 0.92 | 0.51-1.63 | 9.84E-01 | 0.91 | 0.51-1.62 | 9.99E-01 |
| isovalerylglycine | 1.01 | 0.62-1.65 | 9.93E-01 | 0.99 | 0.61-1.61 | 9.99E-01 |
| 3-hydroxydodecanedioate* | 1.00 | 0.61-1.64 | 9.93E-01 | 1.02 | 0.63-1.67 | 9.99E-01 |
| N1-Methyl-2-pyridone-5-carboxamide | 1.01 | 0.57-1.8 | 9.93E-01 | 0.95 | 0.53-1.69 | 9.99E-01 |
| gamma-glutamylisoleucine* | 1.12 | 0.75-1.68 | 9.69E-01 | 1.02 | 0.71-1.47 | 9.99E-01 |
| gamma-glutamyl-2-aminobutyrate | 1.01 | 0.67-1.52 | 9.93E-01 | 1.01 | 0.64-1.6 | 9.99E-01 |
| 2-methylbutyrylcarnitine (C5) | 1.03 | 0.73-1.44 | 9.93E-01 | 0.99 | 0.71-1.36 | 9.99E-01 |
| phenol sulfate | 1.02 | 0.65-1.6 | 9.93E-01 | 1.02 | 0.66-1.58 | 9.99E-01 |
| 1-palmitoleoyl-GPC (16:1)* | 1.03 | 0.71-1.49 | 9.93E-01 | 1.05 | 0.7-1.58 | 9.99E-01 |
| hexanoylglycine | 0.94 | 0.65-1.37 | 9.84E-01 | 0.98 | 0.67-1.44 | 9.99E-01 |
| homostachydrine* | 1.00 | 0.62-1.64 | 9.93E-01 | 1.02 | 0.61-1.7 | 9.99E-01 |
| 2-palmitoyl-GPC (16:0)* | 0.87 | 0.53-1.45 | 9.69E-01 | 0.93 | 0.54-1.59 | 9.99E-01 |
| 1-oleoyl-GPE (18:1) | 0.91 | 0.58-1.43 | 9.79E-01 | 0.97 | 0.61-1.55 | 9.99E-01 |
| 2-hydroxypalmitate | 0.88 | 0.52-1.49 | 9.69E-01 | 0.93 | 0.55-1.58 | 9.99E-01 |
| docosapentaenoate (n6 DPA; 22:5n6) | 1.04 | 0.62-1.75 | 9.93E-01 | 1.06 | 0.6-1.87 | 9.99E-01 |
| gulonate* | 1.10 | 0.6-2.04 | 9.84E-01 | 1.06 | 0.59-1.89 | 9.99E-01 |
| isobutyrylglycine | 1.09 | 0.64-1.86 | 9.84E-01 | 1.06 | 0.66-1.73 | 9.99E-01 |
| 3beta,7alpha-dihydroxy-5-cholestenoate | 0.93 | 0.62-1.39 | 9.80E-01 | 0.96 | 0.59-1.54 | 9.99E-01 |
| N-acetyl-aspartyl-glutamate (NAAG) | 1.01 | 0.69-1.48 | 9.93E-01 | 1.02 | 0.7-1.49 | 9.99E-01 |
| deoxycarnitine | 1.01 | 0.82-1.25 | 9.93E-01 | 1.02 | 0.79-1.32 | 9.99E-01 |
| 1-ribosyl-imidazoleacetate* | 1.03 | 0.72-1.5 | 9.93E-01 | 1.01 | 0.66-1.57 | 9.99E-01 |
| indoleacetylglutamine | 1.02 | 0.64-1.63 | 9.93E-01 | 1.02 | 0.63-1.67 | 9.99E-01 |
| 4-vinylphenol sulfate | 0.97 | 0.6-1.56 | 9.93E-01 | 1.01 | 0.63-1.61 | 9.99E-01 |
| thymol sulfate | 0.91 | 0.59-1.39 | 9.79E-01 | 0.94 | 0.61-1.42 | 9.99E-01 |
| pyrraline | 1.03 | 0.68-1.57 | 9.93E-01 | 1.03 | 0.67-1.58 | 9.99E-01 |
| o-cresol sulfate | 0.97 | 0.64-1.46 | 9.93E-01 | 0.97 | 0.54-1.75 | 9.99E-01 |
| 1-stearoyl-2-oleoyl-GPE (18:0/18:1) | 1.01 | 0.59-1.74 | 9.93E-01 | 0.99 | 0.6-1.64 | 9.99E-01 |
| 4-allylphenol sulfate | 0.97 | 0.67-1.4 | 9.93E-01 | 0.96 | 0.68-1.34 | 9.99E-01 |
| glycosyl-N-stearoyl-sphingosine (d18:1/18:0) | 0.94 | 0.54-1.63 | 9.88E-01 | 0.95 | 0.55-1.65 | 9.99E-01 |
| 5alpha-pregnan-3beta,20alpha-diol disulfate | 0.93 | 0.6-1.43 | 9.84E-01 | 0.94 | 0.58-1.53 | 9.99E-01 |
| glycocholenate sulfate* | 1.08 | 0.65-1.78 | 9.84E-01 | 1.06 | 0.63-1.8 | 9.99E-01 |
| androstenediol (3beta,17beta) disulfate (1) | 0.98 | 0.68-1.43 | 9.93E-01 | 1.06 | 0.72-1.57 | 9.99E-01 |
| androstenediol (3beta,17beta) disulfate (2) | 0.98 | 0.71-1.33 | 9.93E-01 | 0.99 | 0.72-1.37 | 9.99E-01 |
| 5alpha-pregnan-3beta,20alpha-diol monosulfate (2) | 0.94 | 0.61-1.44 | 9.84E-01 | 0.96 | 0.61-1.51 | 9.99E-01 |
| androstenediol (3alpha, 17alpha) monosulfate (2) | 0.98 | 0.69-1.38 | 9.93E-01 | 0.98 | 0.69-1.37 | 9.99E-01 |
| palmitoyl sphingomyelin (d18:1/16:0) | 0.89 | 0.53-1.51 | 9.79E-01 | 0.95 | 0.57-1.6 | 9.99E-01 |
| cysteine sulfinic acid | 1.03 | 0.64-1.67 | 9.93E-01 | 1.01 | 0.59-1.7 | 9.99E-01 |
| 17alpha-hydroxypregnenolone 3-sulfate | 0.97 | 0.69-1.37 | 9.93E-01 | 1.00 | 0.7-1.44 | 9.99E-01 |
| ergothioneine | 0.95 | 0.72-1.25 | 9.80E-01 | 1.01 | 0.75-1.36 | 9.99E-01 |
| S-methylmethionine | 0.99 | 0.63-1.55 | 9.93E-01 | 0.99 | 0.61-1.62 | 9.99E-01 |
| 13-HODE + 9-HODE | 0.92 | 0.51-1.66 | 9.84E-01 | 0.94 | 0.5-1.76 | 9.99E-01 |
| tridecenedioate (C13:1-DC)* | 0.94 | 0.64-1.39 | 9.84E-01 | 0.96 | 0.63-1.46 | 9.99E-01 |
| 2S,3R-dihydroxybutyrate | 0.92 | 0.53-1.6 | 9.84E-01 | 0.90 | 0.45-1.77 | 9.99E-01 |
| isoursodeoxycholate | 0.99 | 0.69-1.41 | 9.93E-01 | 1.01 | 0.68-1.51 | 9.99E-01 |
| formiminoglutamate | 1.08 | 0.64-1.83 | 9.84E-01 | 0.96 | 0.55-1.68 | 9.99E-01 |
| sulfate* | 0.97 | 0.7-1.34 | 9.93E-01 | 0.96 | 0.7-1.33 | 9.99E-01 |
| 4-hydroxy-2-oxoglutaric acid | 0.99 | 0.59-1.67 | 9.93E-01 | 0.99 | 0.59-1.66 | 9.99E-01 |
| 4-hydroxyglutamate | 1.00 | 0.6-1.66 | 9.97E-01 | 0.92 | 0.55-1.54 | 9.99E-01 |
| S-methylcysteine | 0.99 | 0.73-1.34 | 9.93E-01 | 1.00 | 0.75-1.33 | 9.99E-01 |
| (14 or 15)-methylpalmitate (a17:0 or i17:0) | 0.92 | 0.51-1.66 | 9.84E-01 | 0.93 | 0.49-1.78 | 9.99E-01 |
| 16-hydroxypalmitate | 0.94 | 0.63-1.41 | 9.84E-01 | 0.96 | 0.62-1.48 | 9.99E-01 |
| 1-(1-enyl-palmitoyl)-GPE (P-16:0)* | 1.03 | 0.62-1.71 | 9.93E-01 | 1.08 | 0.69-1.68 | 9.99E-01 |
| 1-(1-enyl-stearoyl)-GPE (P-18:0)* | 0.87 | 0.52-1.45 | 9.69E-01 | 0.93 | 0.58-1.49 | 9.99E-01 |
| N-stearoyltaurine | 0.92 | 0.63-1.36 | 9.80E-01 | 0.99 | 0.67-1.47 | 9.99E-01 |
| carboxyethyl-GABA | 1.03 | 0.74-1.42 | 9.93E-01 | 1.01 | 0.74-1.4 | 9.99E-01 |
| N-palmitoylglycine | 0.95 | 0.52-1.76 | 9.93E-01 | 1.02 | 0.55-1.89 | 9.99E-01 |
| N-acetylcarnosine | 1.03 | 0.57-1.86 | 9.93E-01 | 0.98 | 0.55-1.76 | 9.99E-01 |
| retinal | 0.94 | 0.58-1.52 | 9.84E-01 | 0.94 | 0.56-1.57 | 9.99E-01 |
| 3-methyl catechol sulfate (1) | 1.04 | 0.67-1.59 | 9.93E-01 | 1.06 | 0.64-1.75 | 9.99E-01 |
| 3b-hydroxy-5-cholenoic acid | 0.99 | 0.57-1.72 | 9.93E-01 | 1.05 | 0.6-1.83 | 9.99E-01 |
| N-acetyl-1-methylhistidine* | 0.98 | 0.67-1.42 | 9.93E-01 | 0.99 | 0.66-1.48 | 9.99E-01 |
| N2,N5-diacetylornithine | 0.90 | 0.64-1.25 | 9.65E-01 | 0.96 | 0.67-1.4 | 9.99E-01 |
| 17alpha-hydroxypregnanolone glucuronide | 0.96 | 0.62-1.51 | 9.93E-01 | 0.98 | 0.62-1.56 | 9.99E-01 |
| 1-palmitoyl-GPG (16:0)* | 1.00 | 0.63-1.6 | 9.93E-01 | 1.01 | 0.62-1.64 | 9.99E-01 |
| alpha-CEHC sulfate | 0.96 | 0.73-1.27 | 9.84E-01 | 0.97 | 0.75-1.25 | 9.99E-01 |
| sphingomyelin (d18:2/14:0, d18:1/14:1)* | 1.08 | 0.62-1.88 | 9.84E-01 | 1.06 | 0.61-1.83 | 9.99E-01 |
| 3-hydroxypyridine sulfate | 1.09 | 0.64-1.86 | 9.84E-01 | 1.09 | 0.62-1.92 | 9.99E-01 |
| phenylacetylcarnitine | 1.07 | 0.58-1.96 | 9.93E-01 | 1.06 | 0.57-1.94 | 9.99E-01 |
| arabonate/xylonate | 0.99 | 0.75-1.31 | 9.93E-01 | 0.96 | 0.73-1.26 | 9.99E-01 |
| 4-methoxyphenol sulfate | 1.03 | 0.65-1.63 | 9.93E-01 | 1.03 | 0.66-1.63 | 9.99E-01 |
| sphingomyelin (d18:1/20:2, d18:2/20:1, d16:1/22:2)* | 1.04 | 0.72-1.5 | 9.88E-01 | 0.98 | 0.66-1.45 | 9.99E-01 |
| sphingomyelin (d18:1/22:1, d18:2/22:0, d16:1/24:1)* | 1.06 | 0.59-1.9 | 9.93E-01 | 1.08 | 0.6-1.93 | 9.99E-01 |
| N-acetylglucosamine/N-acetylgalactosamine | 1.07 | 0.71-1.63 | 9.84E-01 | 1.02 | 0.69-1.52 | 9.99E-01 |
| glycodeoxycholate 3-sulfate | 0.95 | 0.62-1.46 | 9.88E-01 | 0.93 | 0.61-1.43 | 9.99E-01 |
| trans-3,4-methyleneheptanoate | 0.95 | 0.66-1.37 | 9.84E-01 | 0.96 | 0.66-1.41 | 9.99E-01 |
| phenol glucuronide | 1.05 | 0.7-1.55 | 9.88E-01 | 1.06 | 0.72-1.57 | 9.99E-01 |
| 1-palmitoyl-2-docosahexaenoyl-GPC (16:0/22:6) | 0.98 | 0.63-1.52 | 9.93E-01 | 0.99 | 0.64-1.53 | 9.99E-01 |
| 1-stearoyl-2-docosahexaenoyl-GPC (18:0/22:6) | 0.99 | 0.66-1.49 | 9.93E-01 | 0.96 | 0.64-1.45 | 9.99E-01 |
| sphingomyelin (d18:1/17:0, d17:1/18:0, d19:1/16:0) | 1.07 | 0.65-1.75 | 9.84E-01 | 1.03 | 0.61-1.75 | 9.99E-01 |
| tricosanoyl sphingomyelin (d18:1/23:0)* | 0.91 | 0.53-1.55 | 9.81E-01 | 0.93 | 0.56-1.54 | 9.99E-01 |
| sphingomyelin (d18:2/23:0, d18:1/23:1, d17:1/24:1)* | 1.02 | 0.58-1.76 | 9.93E-01 | 1.03 | 0.59-1.79 | 9.99E-01 |
| 1-stearoyl-2-linoleoyl-GPE (18:0/18:2)* | 1.00 | 0.61-1.63 | 9.97E-01 | 0.99 | 0.62-1.58 | 9.99E-01 |
| 1-palmitoyl-2-arachidonoyl-GPI (16:0/20:4)* | 1.02 | 0.57-1.85 | 9.93E-01 | 1.04 | 0.58-1.86 | 9.99E-01 |
| 1-(1-enyl-stearoyl)-2-arachidonoyl-GPE (P-18:0/20:4)* | 0.98 | 0.61-1.58 | 9.93E-01 | 1.05 | 0.68-1.62 | 9.99E-01 |
| 5-hydroxyindole sulfate | 1.04 | 0.65-1.66 | 9.93E-01 | 1.01 | 0.63-1.61 | 9.99E-01 |
| phenylacetylglutamate | 0.95 | 0.63-1.44 | 9.88E-01 | 0.93 | 0.61-1.44 | 9.99E-01 |
| thioproline | 1.06 | 0.7-1.62 | 9.84E-01 | 1.07 | 0.69-1.67 | 9.99E-01 |
| 2-methylserine | 1.02 | 0.67-1.54 | 9.93E-01 | 1.02 | 0.68-1.54 | 9.99E-01 |
| arachidonoylcholine | 0.91 | 0.61-1.35 | 9.72E-01 | 1.00 | 0.66-1.5 | 9.99E-01 |
| docosahexaenoylcholine | 0.95 | 0.61-1.46 | 9.84E-01 | 1.01 | 0.67-1.51 | 9.99E-01 |
| 1-palmitoleoyl-2-linolenoyl-GPC (16:1/18:3)* | 1.03 | 0.65-1.63 | 9.93E-01 | 1.04 | 0.66-1.66 | 9.99E-01 |
| pimeloylcarnitine/3-methyladipoylcarnitine (C7-DC) | 1.06 | 0.72-1.57 | 9.84E-01 | 1.03 | 0.68-1.56 | 9.99E-01 |
| perfluorooctanesulfonate (PFOS) | 1.01 | 0.64-1.6 | 9.93E-01 | 1.05 | 0.67-1.66 | 9.99E-01 |
| lactosyl-N-behenoyl-sphingosine (d18:1/22:0)* | 0.87 | 0.5-1.53 | 9.69E-01 | 0.93 | 0.52-1.64 | 9.99E-01 |
| glycosyl-N-behenoyl-sphingadienine (d18:2/22:0)* | 1.01 | 0.64-1.61 | 9.93E-01 | 1.07 | 0.7-1.63 | 9.99E-01 |
| N-palmitoylserine | 1.05 | 0.59-1.87 | 9.93E-01 | 1.10 | 0.61-2.01 | 9.99E-01 |
| N-oleoylserine | 0.94 | 0.53-1.66 | 9.88E-01 | 0.98 | 0.54-1.77 | 9.99E-01 |
| N-palmitoyl-heptadecasphingosine (d17:1/16:0)* | 0.93 | 0.6-1.45 | 9.84E-01 | 0.93 | 0.6-1.45 | 9.99E-01 |
| glycosyl ceramide (d18:2/24:1, d18:1/24:2)* | 0.95 | 0.6-1.48 | 9.84E-01 | 0.99 | 0.63-1.54 | 9.99E-01 |
| glycosyl-N-(2-hydroxynervonoyl)-sphingosine (d18:1/24:1(2OH))* | 1.09 | 0.56-2.13 | 9.84E-01 | 1.04 | 0.55-1.97 | 9.99E-01 |
| docosatrienoate (22:3n6)* | 0.97 | 0.68-1.4 | 9.93E-01 | 1.02 | 0.66-1.58 | 9.99E-01 |
| sphingomyelin (d18:2/21:0, d16:2/23:0)* | 1.16 | 0.68-1.99 | 9.69E-01 | 1.09 | 0.6-1.95 | 9.99E-01 |
| sphingomyelin (d18:2/23:1)* | 1.07 | 0.59-1.94 | 9.88E-01 | 1.02 | 0.53-1.96 | 9.99E-01 |
| sphingomyelin (d17:2/16:0, d18:2/15:0)* | 1.10 | 0.62-1.94 | 9.84E-01 | 1.05 | 0.57-1.91 | 9.99E-01 |
| heneicosapentaenoate (21:5n3) | 1.01 | 0.65-1.55 | 9.93E-01 | 1.05 | 0.67-1.66 | 9.99E-01 |
| behenoylcarnitine (C22)* | 0.97 | 0.59-1.61 | 9.93E-01 | 0.99 | 0.61-1.61 | 9.99E-01 |
| arachidonoylcarnitine (C20:4) | 0.91 | 0.59-1.39 | 9.79E-01 | 0.94 | 0.59-1.5 | 9.99E-01 |
| dihomo-linolenoylcarnitine (C20:3n3 or 6)* | 0.90 | 0.52-1.55 | 9.80E-01 | 0.93 | 0.53-1.62 | 9.99E-01 |
| heptenedioate (C7:1-DC)* | 0.93 | 0.54-1.6 | 9.84E-01 | 0.93 | 0.52-1.66 | 9.99E-01 |
| octadecadienedioate (C18:2-DC)* | 1.01 | 0.63-1.63 | 9.93E-01 | 1.01 | 0.64-1.6 | 9.99E-01 |
| 3-carboxy-4-methyl-5-pentyl-2-furanpropionate (3-CMPFP)** | 0.95 | 0.62-1.47 | 9.88E-01 | 0.96 | 0.6-1.52 | 9.99E-01 |
| N,N,N-trimethyl-alanylproline betaine (TMAP) | 1.04 | 0.71-1.53 | 9.93E-01 | 1.03 | 0.7-1.51 | 9.99E-01 |
| 3-formylindole | 1.01 | 0.58-1.75 | 9.93E-01 | 0.96 | 0.55-1.66 | 9.99E-01 |
| gamma-glutamylcitrulline* | 1.06 | 0.66-1.69 | 9.88E-01 | 1.08 | 0.66-1.77 | 9.99E-01 |
| 3-amino-2-piperidone | 1.03 | 0.62-1.7 | 9.93E-01 | 1.03 | 0.62-1.7 | 9.99E-01 |
| 2-hydroxysebacate | 1.01 | 0.65-1.56 | 9.93E-01 | 1.01 | 0.63-1.62 | 9.99E-01 |
| ascorbic acid 3-sulfate* | 1.07 | 0.67-1.72 | 9.84E-01 | 1.02 | 0.66-1.57 | 9.99E-01 |
| N-acetyl-isoputreanine | 0.97 | 0.6-1.56 | 9.93E-01 | 1.01 | 0.62-1.64 | 9.99E-01 |
| (2,4 or 2,5)-dimethylphenol sulfate | 0.99 | 0.73-1.34 | 9.93E-01 | 1.04 | 0.69-1.58 | 9.99E-01 |
| 4-allylcatechol sulfate | 0.94 | 0.64-1.39 | 9.84E-01 | 0.96 | 0.66-1.39 | 9.99E-01 |
| 2-hydroxyfluorene sulfate | 0.96 | 0.67-1.37 | 9.88E-01 | 0.98 | 0.63-1.52 | 9.99E-01 |
| 4-methylhexanoylglutamine | 1.09 | 0.7-1.72 | 9.80E-01 | 1.08 | 0.69-1.68 | 9.99E-01 |
| 2,6-dihydroxybenzoic acid | 0.98 | 0.72-1.33 | 9.93E-01 | 1.01 | 0.74-1.39 | 9.99E-01 |
| N,N-dimethyl-5-aminovalerate | 0.98 | 0.61-1.58 | 9.93E-01 | 0.96 | 0.6-1.53 | 9.99E-01 |
| 3-ethylcatechol sulfate (1) | 0.96 | 0.74-1.26 | 9.84E-01 | 0.97 | 0.69-1.35 | 9.99E-01 |
| hydroxypalmitoyl sphingomyelin (d18:1/16:0(OH))** | 0.99 | 0.57-1.72 | 9.93E-01 | 0.98 | 0.58-1.67 | 9.99E-01 |
| tetradecadienedioate (C14:2-DC)* | 0.94 | 0.59-1.5 | 9.84E-01 | 0.97 | 0.6-1.58 | 9.99E-01 |
| pregnenetriol disulfate* | 1.01 | 0.68-1.48 | 9.93E-01 | 1.03 | 0.68-1.55 | 9.99E-01 |
| metabolonic lactone sulfate | 1.05 | 0.67-1.66 | 9.88E-01 | 0.99 | 0.61-1.6 | 9.99E-01 |
| branched chain 14:0 dicarboxylic acid** | 1.10 | 0.74-1.63 | 9.69E-01 | 1.06 | 0.71-1.59 | 9.99E-01 |
| pentose acid* | 0.98 | 0.67-1.44 | 9.93E-01 | 0.98 | 0.67-1.43 | 9.99E-01 |
| S-carboxyethylcysteine | 1.07 | 0.69-1.66 | 9.84E-01 | 1.05 | 0.65-1.72 | 9.99E-01 |
| 3-bromo-5-chloro-2,6-dihydroxybenzoic acid* | 1.03 | 0.6-1.76 | 9.93E-01 | 1.02 | 0.6-1.73 | 9.99E-01 |
| GlcNAc sulfate conjugate of C21H34O2 steroid** | 1.01 | 0.66-1.56 | 9.93E-01 | 1.03 | 0.68-1.58 | 9.99E-01 |
| 4-chlorobenzoic acid | 0.98 | 0.67-1.43 | 9.93E-01 | 0.99 | 0.68-1.44 | 9.99E-01 |
| 2-methoxyhydroquinone sulfate (1) | 1.00 | 0.63-1.6 | 9.98E-01 | 0.98 | 0.6-1.6 | 9.99E-01 |
| 2,4-di-tert-butylphenol | 1.05 | 0.69-1.61 | 9.84E-01 | 1.08 | 0.7-1.67 | 9.99E-01 |
| N,N-dimethyl-pro-pro | 0.99 | 0.71-1.38 | 9.93E-01 | 1.02 | 0.71-1.45 | 9.99E-01 |
| oxindolylalanine | 0.96 | 0.58-1.58 | 9.93E-01 | 0.94 | 0.57-1.54 | 9.99E-01 |
| trans-4-hydroxyproline | 0.91 | 0.54-1.51 | 9.80E-01 | 0.95 | 0.55-1.61 | 9.99E-01 |
| xanthine | 1.05 | 0.65-1.68 | 9.93E-01 | 0.99 | 0.63-1.57 | 9.99E-01 |
| sarcosine | 0.95 | 0.56-1.62 | 9.93E-01 | 0.99 | 0.58-1.68 | 9.99E-01 |
| pantothenate | 1.07 | 0.61-1.87 | 9.88E-01 | 1.01 | 0.56-1.83 | 9.99E-01 |
| pipecolate | 0.99 | 0.66-1.48 | 9.93E-01 | 1.02 | 0.68-1.51 | 9.99E-01 |
| N-acetylleucine | 1.02 | 0.71-1.45 | 9.93E-01 | 0.97 | 0.67-1.4 | 9.99E-01 |
| gamma-glutamyltyrosine | 1.10 | 0.73-1.64 | 9.79E-01 | 1.07 | 0.71-1.62 | 9.99E-01 |
| 2-aminobutyrate | 0.98 | 0.67-1.43 | 9.93E-01 | 0.99 | 0.64-1.53 | 9.99E-01 |
| 4-hydroxyphenylpyruvate | 1.01 | 0.66-1.53 | 9.93E-01 | 0.98 | 0.66-1.46 | 9.99E-01 |
| butyrate/isobutyrate (4:0) | 1.08 | 0.69-1.69 | 9.84E-01 | 1.03 | 0.65-1.63 | 9.99E-01 |
| gamma-glutamylleucine | 1.04 | 0.69-1.56 | 9.93E-01 | 0.97 | 0.63-1.5 | 9.99E-01 |
| 1-palmitoyl-2-oleoyl-GPE (16:0/18:1) | 1.00 | 0.53-1.91 | 9.93E-01 | 0.99 | 0.55-1.79 | 9.99E-01 |
| 1-palmitoyl-2-linoleoyl-GPI (16:0/18:2) | 1.03 | 0.73-1.45 | 9.93E-01 | 1.04 | 0.74-1.47 | 9.99E-01 |
| taurochenodeoxycholate | 1.04 | 0.66-1.64 | 9.93E-01 | 1.04 | 0.64-1.68 | 9.99E-01 |
| 2-hydroxyhippurate (salicylurate) | 0.93 | 0.62-1.41 | 9.84E-01 | 0.96 | 0.66-1.4 | 9.99E-01 |
| N6,N6,N6-trimethyllysine | 0.97 | 0.55-1.73 | 9.93E-01 | 0.95 | 0.57-1.57 | 9.99E-01 |
| adenosine 5'-monophosphate (AMP) | 0.99 | 0.66-1.49 | 9.93E-01 | 0.96 | 0.65-1.42 | 9.99E-01 |
| 3-(4-hydroxyphenyl)lactate | 1.08 | 0.66-1.77 | 9.84E-01 | 1.07 | 0.65-1.75 | 9.99E-01 |
| 3-hydroxybutyrate (BHBA) | 0.94 | 0.57-1.54 | 9.84E-01 | 0.93 | 0.56-1.56 | 9.99E-01 |
| cystine | 0.92 | 0.55-1.56 | 9.84E-01 | 0.97 | 0.58-1.63 | 9.99E-01 |
| sphinganine | 1.10 | 0.66-1.84 | 9.81E-01 | 1.07 | 0.63-1.81 | 9.99E-01 |
| glutarate (C5-DC) | 1.00 | 0.6-1.66 | 9.93E-01 | 1.04 | 0.63-1.7 | 9.99E-01 |
| glycine | 0.97 | 0.61-1.54 | 9.93E-01 | 1.07 | 0.65-1.74 | 9.99E-01 |
| guanidinoacetate | 0.95 | 0.61-1.49 | 9.92E-01 | 1.05 | 0.66-1.69 | 9.99E-01 |
| inosine | 0.98 | 0.54-1.76 | 9.93E-01 | 0.97 | 0.55-1.72 | 9.99E-01 |
| myo-inositol | 1.06 | 0.61-1.84 | 9.92E-01 | 1.03 | 0.61-1.72 | 9.99E-01 |
| nicotinamide | 1.08 | 0.68-1.72 | 9.84E-01 | 1.04 | 0.68-1.61 | 9.99E-01 |
| ornithine | 1.00 | 0.6-1.68 | 9.96E-01 | 0.98 | 0.59-1.63 | 9.99E-01 |
| pristanate | 0.88 | 0.53-1.46 | 9.69E-01 | 0.96 | 0.59-1.55 | 9.99E-01 |
| 1-methylnicotinamide | 0.98 | 0.65-1.48 | 9.93E-01 | 0.97 | 0.63-1.49 | 9.99E-01 |
| glutamine | 0.99 | 0.67-1.47 | 9.93E-01 | 1.05 | 0.66-1.69 | 9.99E-01 |
| valine | 1.07 | 0.72-1.6 | 9.84E-01 | 1.01 | 0.76-1.35 | 9.99E-01 |
| 5-hydroxyindoleacetate | 1.12 | 0.65-1.92 | 9.80E-01 | 1.08 | 0.65-1.8 | 9.99E-01 |
| mannose | 1.10 | 0.8-1.51 | 9.69E-01 | 1.02 | 0.7-1.49 | 9.99E-01 |
| uracil | 1.03 | 0.65-1.64 | 9.93E-01 | 1.02 | 0.65-1.61 | 9.99E-01 |
| caprate (10:0) | 0.88 | 0.52-1.49 | 9.69E-01 | 0.93 | 0.58-1.5 | 9.99E-01 |
| nonadecanoate (19:0) | 0.99 | 0.57-1.71 | 9.93E-01 | 1.07 | 0.62-1.84 | 9.99E-01 |
| kynurenate | 0.98 | 0.66-1.45 | 9.93E-01 | 0.95 | 0.62-1.44 | 9.99E-01 |
| pentadecanoate (15:0) | 1.05 | 0.66-1.68 | 9.88E-01 | 1.07 | 0.65-1.77 | 9.99E-01 |
| X - 11444 | 1.03 | 0.63-1.68 | 9.93E-01 | 1.00 | 0.6-1.64 | 9.99E-01 |
| X - 11483 | 0.97 | 0.62-1.5 | 9.93E-01 | 0.99 | 0.62-1.57 | 9.99E-01 |
| X - 11632 | 1.02 | 0.63-1.63 | 9.93E-01 | 1.04 | 0.64-1.7 | 9.99E-01 |
| X - 11847 | 1.03 | 0.72-1.49 | 9.93E-01 | 1.06 | 0.72-1.56 | 9.99E-01 |
| X - 11849 | 1.00 | 0.72-1.37 | 9.93E-01 | 1.02 | 0.72-1.44 | 9.99E-01 |
| X - 11880 | 0.93 | 0.65-1.33 | 9.80E-01 | 0.95 | 0.67-1.35 | 9.99E-01 |
| X - 11979 | 1.03 | 0.65-1.64 | 9.93E-01 | 1.03 | 0.63-1.68 | 9.99E-01 |
| X - 12007 | 1.05 | 0.71-1.56 | 9.84E-01 | 1.03 | 0.69-1.54 | 9.99E-01 |
| X - 12015 | 1.07 | 0.69-1.65 | 9.84E-01 | 1.04 | 0.67-1.61 | 9.99E-01 |
| X - 12026 | 1.13 | 0.72-1.78 | 9.69E-01 | 1.08 | 0.69-1.68 | 9.99E-01 |
| X - 12100 | 1.07 | 0.76-1.49 | 9.80E-01 | 1.03 | 0.74-1.42 | 9.99E-01 |
| X - 12127 | 1.07 | 0.7-1.64 | 9.84E-01 | 1.01 | 0.65-1.56 | 9.99E-01 |
| X - 12193 | 1.03 | 0.72-1.49 | 9.93E-01 | 1.02 | 0.68-1.54 | 9.99E-01 |
| X - 12462 | 0.98 | 0.6-1.58 | 9.93E-01 | 1.03 | 0.63-1.68 | 9.99E-01 |
| X - 12738 | 1.02 | 0.65-1.62 | 9.93E-01 | 1.03 | 0.62-1.7 | 9.99E-01 |
| X - 12816 | 0.96 | 0.66-1.38 | 9.88E-01 | 0.97 | 0.66-1.42 | 9.99E-01 |
| X - 13007 | 1.04 | 0.7-1.55 | 9.93E-01 | 1.02 | 0.68-1.53 | 9.99E-01 |
| X - 13431 | 0.99 | 0.71-1.38 | 9.93E-01 | 1.00 | 0.71-1.41 | 9.99E-01 |
| X - 13507 | 1.02 | 0.69-1.52 | 9.93E-01 | 1.00 | 0.69-1.45 | 9.99E-01 |
| X - 13728 | 0.96 | 0.69-1.33 | 9.84E-01 | 0.97 | 0.7-1.35 | 9.99E-01 |
| X - 14056 | 1.08 | 0.67-1.76 | 9.84E-01 | 1.04 | 0.65-1.66 | 9.99E-01 |
| X - 16397 | 0.92 | 0.59-1.41 | 9.80E-01 | 0.93 | 0.59-1.47 | 9.99E-01 |
| X - 16576 | 1.02 | 0.67-1.57 | 9.93E-01 | 1.05 | 0.68-1.62 | 9.99E-01 |
| X - 16580 | 0.96 | 0.59-1.55 | 9.93E-01 | 0.96 | 0.56-1.62 | 9.99E-01 |
| X - 16935 | 1.00 | 0.68-1.48 | 9.93E-01 | 1.02 | 0.67-1.56 | 9.99E-01 |
| X - 17010 | 1.01 | 0.63-1.62 | 9.93E-01 | 1.00 | 0.62-1.61 | 9.99E-01 |
| X - 17346 | 1.02 | 0.73-1.41 | 9.93E-01 | 1.03 | 0.73-1.43 | 9.99E-01 |
| X - 17653 | 0.94 | 0.65-1.36 | 9.84E-01 | 1.00 | 0.69-1.43 | 9.99E-01 |
| X - 17690 | 0.98 | 0.62-1.55 | 9.93E-01 | 0.98 | 0.62-1.56 | 9.99E-01 |
| X - 18345 | 0.94 | 0.64-1.39 | 9.84E-01 | 0.95 | 0.64-1.39 | 9.99E-01 |
| X - 18887 | 0.99 | 0.69-1.43 | 9.93E-01 | 1.02 | 0.7-1.51 | 9.99E-01 |
| X - 18899 | 0.93 | 0.62-1.39 | 9.80E-01 | 0.94 | 0.61-1.44 | 9.99E-01 |
| X - 18901 | 1.03 | 0.68-1.58 | 9.93E-01 | 1.02 | 0.67-1.54 | 9.99E-01 |
| X - 18922 | 1.00 | 0.75-1.34 | 9.93E-01 | 1.02 | 0.74-1.41 | 9.99E-01 |
| X - 19438 | 1.00 | 0.67-1.48 | 9.93E-01 | 0.98 | 0.68-1.41 | 9.99E-01 |
| X - 21310 | 0.92 | 0.6-1.4 | 9.80E-01 | 0.93 | 0.59-1.48 | 9.99E-01 |
| X - 21364 | 1.02 | 0.66-1.59 | 9.93E-01 | 1.02 | 0.62-1.68 | 9.99E-01 |
| X - 21442 | 1.00 | 0.62-1.64 | 9.93E-01 | 1.02 | 0.63-1.64 | 9.99E-01 |
| X - 21467 | 1.14 | 0.65-2 | 9.70E-01 | 1.09 | 0.63-1.88 | 9.99E-01 |
| X - 21470 | 0.99 | 0.71-1.37 | 9.93E-01 | 1.02 | 0.71-1.45 | 9.99E-01 |
| X - 21471 | 0.98 | 0.54-1.78 | 9.93E-01 | 0.95 | 0.52-1.73 | 9.99E-01 |
| X - 21842 | 1.02 | 0.71-1.46 | 9.93E-01 | 1.01 | 0.7-1.46 | 9.99E-01 |
| X - 22162 | 0.96 | 0.65-1.41 | 9.88E-01 | 0.96 | 0.66-1.38 | 9.99E-01 |
| X - 22509 | 0.97 | 0.66-1.41 | 9.93E-01 | 1.00 | 0.68-1.47 | 9.99E-01 |
| X - 22776 | 1.08 | 0.62-1.87 | 9.84E-01 | 1.08 | 0.63-1.86 | 9.99E-01 |
| X - 23636 | 1.05 | 0.74-1.48 | 9.84E-01 | 0.99 | 0.67-1.44 | 9.99E-01 |
| X - 24243 | 0.99 | 0.64-1.54 | 9.93E-01 | 0.95 | 0.62-1.47 | 9.99E-01 |
| X - 24307 | 0.96 | 0.6-1.55 | 9.93E-01 | 0.92 | 0.55-1.54 | 9.99E-01 |
| X - 24334 | 1.13 | 0.64-2 | 9.79E-01 | 1.06 | 0.62-1.82 | 9.99E-01 |
| X - 24344 | 0.96 | 0.67-1.37 | 9.88E-01 | 0.95 | 0.59-1.53 | 9.99E-01 |
| X - 24422 | 0.98 | 0.72-1.32 | 9.93E-01 | 1.03 | 0.72-1.47 | 9.99E-01 |
| X - 24531 | 0.97 | 0.6-1.56 | 9.93E-01 | 0.97 | 0.59-1.59 | 9.99E-01 |
| X - 24556 | 1.08 | 0.71-1.63 | 9.80E-01 | 1.07 | 0.7-1.62 | 9.99E-01 |
| X - 25343 | 1.11 | 0.7-1.75 | 9.79E-01 | 1.06 | 0.68-1.65 | 9.99E-01 |
| X - 25948 | 1.11 | 0.68-1.8 | 9.80E-01 | 1.07 | 0.66-1.74 | 9.99E-01 |
| X - 26054 | 1.02 | 0.62-1.66 | 9.93E-01 | 1.01 | 0.63-1.61 | 9.99E-01 |
| X - 26058 | 1.03 | 0.63-1.66 | 9.93E-01 | 1.00 | 0.64-1.56 | 9.99E-01 |
| X - 26108 | 0.98 | 0.66-1.46 | 9.93E-01 | 0.98 | 0.64-1.5 | 9.99E-01 |
| X - 11787 | 1.01 | 0.59-1.74 | 9.93E-01 | 1.00 | 0.57-1.74 | 9.99E-01 |
| X - 23641 | 1.01 | 0.58-1.75 | 9.93E-01 | 1.00 | 0.58-1.73 | 9.99E-01 |
| N-acetylserine | 1.05 | 0.69-1.61 | 9.84E-01 | 1.00 | 0.66-1.52 | 1.00E+00 |
| 1-oleoyl-GPG (18:1)* | 1.00 | 0.68-1.48 | 9.93E-01 | 1.00 | 0.66-1.52 | 1.00E+00 |

Note: the table shows associations from The competing risk Cox proportional hazards regression analysis where plasms metabolomics level from the Metabolon platform was main exposure and incident blood tumors as the main outcome in ERGO-4 cohort. Model 1 : adjusted for age, sex, cohort, and lipid lowering medication. Model 2: adjusted for model1, smoking status, BMI, alcohol and education. The table is ranked based on the *FDR* values in model 2, using bold text indicates metabolites are surpassing *FDR* adjusted P value threshold (*P*<0.05). ERGO-4 refer to the fourth and fifth visits from the baseline cohort. Abbreviations: BMI, body mass index; HR, hazard ratio; CI, confidence interval; FDR, false discovery rate.

**Supplementary Table 15. Serum metabolites associated with incident colorectal cancer from the Nightingale platform.**

| **Metabolite** | **Model 1** | | | **Model 2** | | |
| --- | --- | --- | --- | --- | --- | --- |
|  | **HR** | **95% CI** | ***FDR* value** | **HR** | **95% CI** | ***FDR* value** |
| Free cholesterol to total lipids ratio in IDL | 0.77 | 0.61-0.96 | 9.99E-01 | 0.76 | 0.6-0.96 | 9.94E-01 |
| Histidine | 0.86 | 0.72-1.03 | 9.99E-01 | 0.85 | 0.71-1.02 | 9.94E-01 |
| Phospholipids to total lipids ratio in IDL | 0.75 | 0.57-0.98 | 9.99E-01 | 0.76 | 0.58-0.99 | 9.94E-01 |
| Glutamine | 0.86 | 0.69-1.07 | 9.99E-01 | 0.85 | 0.68-1.06 | 9.94E-01 |
| Albumin | 0.88 | 0.73-1.06 | 9.99E-01 | 0.86 | 0.71-1.04 | 9.94E-01 |
| Cholesterol to total lipids ratio in medium LDL | 0.86 | 0.68-1.08 | 9.99E-01 | 0.85 | 0.67-1.08 | 9.94E-01 |
| Phospholipids to total lipids ratio in chylomicrons and extremely large VLDL | 0.86 | 0.71-1.04 | 9.99E-01 | 0.86 | 0.71-1.05 | 9.94E-01 |
| Leucine | 0.93 | 0.76-1.12 | 9.99E-01 | 0.94 | 0.77-1.14 | 9.94E-01 |
| Cholesteryl esters to total lipids ratio in medium VLDL | 0.85 | 0.68-1.07 | 9.99E-01 | 0.83 | 0.66-1.05 | 9.94E-01 |
| Cholesterol to total lipids ratio in small LDL | 0.86 | 0.67-1.1 | 9.99E-01 | 0.83 | 0.65-1.06 | 9.94E-01 |
| Average diameter for HDL particles | 0.89 | 0.7-1.14 | 9.99E-01 | 0.86 | 0.66-1.11 | 9.94E-01 |
| Free cholesterol to total lipids ratio in large HDL | 0.91 | 0.72-1.16 | 9.99E-01 | 0.92 | 0.73-1.16 | 9.94E-01 |
| Glucose | 0.88 | 0.67-1.16 | 9.99E-01 | 0.89 | 0.67-1.17 | 9.94E-01 |
| Glycoprotein acetyls | 1.04 | 0.82-1.32 | 9.99E-01 | 1.08 | 0.86-1.36 | 9.94E-01 |
| Free cholesterol to total lipids ratio in very large HDL | 1.11 | 0.89-1.38 | 9.99E-01 | 1.15 | 0.91-1.44 | 9.94E-01 |
| Cholesterol to total lipids ratio in medium VLDL | 0.85 | 0.67-1.09 | 9.99E-01 | 0.83 | 0.65-1.06 | 9.94E-01 |
| Free cholesterol in large HDL | 0.91 | 0.73-1.15 | 9.99E-01 | 0.88 | 0.7-1.11 | 9.94E-01 |
| Phospholipids to total lipids ratio in small HDL | 0.9 | 0.69-1.17 | 9.99E-01 | 0.89 | 0.69-1.15 | 9.94E-01 |
| Free cholesterol to total lipids ratio in large LDL | 0.89 | 0.7-1.12 | 9.99E-01 | 0.86 | 0.68-1.09 | 9.94E-01 |
| Phospholipids in HDL | 0.92 | 0.71-1.19 | 9.99E-01 | 0.88 | 0.67-1.14 | 9.94E-01 |
| Cholesteryl esters to total lipids ratio in small VLDL | 0.86 | 0.67-1.1 | 9.99E-01 | 0.85 | 0.66-1.09 | 9.94E-01 |
| Free cholesterol to total lipids ratio in medium VLDL | 0.85 | 0.66-1.11 | 9.99E-01 | 0.83 | 0.64-1.09 | 9.94E-01 |
| Concentration of large HDL particles | 0.92 | 0.74-1.16 | 9.99E-01 | 0.89 | 0.7-1.12 | 9.94E-01 |
| Phospholipids in very large HDL | 0.91 | 0.73-1.14 | 9.99E-01 | 0.91 | 0.73-1.13 | 9.94E-01 |
| Total lipids in HDL | 0.92 | 0.71-1.19 | 9.99E-01 | 0.87 | 0.67-1.14 | 9.94E-01 |
| Cholesterol to total lipids ratio in small VLDL | 0.86 | 0.67-1.12 | 9.99E-01 | 0.85 | 0.65-1.1 | 9.94E-01 |
| Cholesterol to total lipids ratio in very large HDL | 1.09 | 0.87-1.37 | 9.99E-01 | 1.09 | 0.87-1.36 | 9.94E-01 |
| Phospholipids in large HDL | 0.93 | 0.75-1.16 | 9.99E-01 | 0.9 | 0.72-1.12 | 9.94E-01 |
| Total lipids in very large HDL | 0.91 | 0.72-1.16 | 9.99E-01 | 0.9 | 0.7-1.14 | 9.94E-01 |
| Total lipids in large HDL | 0.93 | 0.74-1.16 | 9.99E-01 | 0.89 | 0.71-1.12 | 9.94E-01 |
| Concentration of medium HDL particles | 0.92 | 0.71-1.19 | 9.99E-01 | 0.87 | 0.67-1.14 | 9.94E-01 |
| Phospholipids to total lipids ratio in very large HDL | 0.92 | 0.76-1.12 | 9.99E-01 | 0.93 | 0.77-1.12 | 9.94E-01 |
| Concentration of very large HDL particles | 0.92 | 0.72-1.18 | 9.99E-01 | 0.9 | 0.69-1.16 | 9.94E-01 |
| Cholesteryl esters to total lipids ratio in IDL | 1.21 | 0.89-1.66 | 9.99E-01 | 1.19 | 0.88-1.61 | 9.94E-01 |
| Citrate | 0.93 | 0.74-1.18 | 9.99E-01 | 0.92 | 0.73-1.17 | 9.94E-01 |
| Phospholipids in medium HDL | 0.93 | 0.72-1.2 | 9.99E-01 | 0.88 | 0.68-1.15 | 9.94E-01 |
| HDL cholesterol | 0.92 | 0.72-1.18 | 9.99E-01 | 0.87 | 0.67-1.13 | 9.94E-01 |
| Total concentration of branched-chain amino acids (leucine + isoleucine + valine) | 0.94 | 0.77-1.15 | 9.99E-01 | 0.95 | 0.78-1.17 | 9.94E-01 |
| Cholesteryl esters in HDL | 0.92 | 0.72-1.18 | 9.99E-01 | 0.87 | 0.67-1.13 | 9.94E-01 |
| Total lipids in medium HDL | 0.93 | 0.72-1.2 | 9.99E-01 | 0.88 | 0.68-1.15 | 9.94E-01 |
| Apolipoprotein A1 | 0.93 | 0.72-1.21 | 9.99E-01 | 0.88 | 0.67-1.15 | 9.94E-01 |
| Cholesteryl esters in very large HDL | 0.91 | 0.72-1.16 | 9.99E-01 | 0.89 | 0.69-1.14 | 9.94E-01 |
| Cholesterol in large HDL | 0.92 | 0.74-1.15 | 9.99E-01 | 0.89 | 0.71-1.11 | 9.94E-01 |
| Free cholesterol in HDL | 0.94 | 0.73-1.21 | 9.99E-01 | 0.89 | 0.69-1.16 | 9.94E-01 |
| Cholesteryl esters in medium HDL | 0.92 | 0.72-1.18 | 9.99E-01 | 0.87 | 0.67-1.13 | 9.94E-01 |
| Cholesterol in medium HDL | 0.92 | 0.72-1.19 | 9.99E-01 | 0.88 | 0.67-1.13 | 9.94E-01 |
| Phospholipids to total lipids ratio in medium VLDL | 0.88 | 0.68-1.14 | 9.99E-01 | 0.86 | 0.66-1.13 | 9.94E-01 |
| Cholesterol in very large HDL | 0.92 | 0.72-1.17 | 9.99E-01 | 0.9 | 0.7-1.16 | 9.94E-01 |
| Cholesteryl esters in large HDL | 0.92 | 0.74-1.15 | 9.99E-01 | 0.89 | 0.71-1.11 | 9.94E-01 |
| Triglycerides in large HDL | 0.94 | 0.73-1.2 | 9.99E-01 | 0.93 | 0.72-1.2 | 9.94E-01 |
| Phospholipids to total lipids ratio in medium LDL | 1.07 | 0.86-1.34 | 9.99E-01 | 1.06 | 0.84-1.33 | 9.94E-01 |
| Cholesteryl esters to total lipids ratio in very small VLDL | 0.9 | 0.7-1.16 | 9.99E-01 | 0.88 | 0.68-1.13 | 9.94E-01 |
| Free cholesterol in medium HDL | 0.95 | 0.74-1.22 | 9.99E-01 | 0.9 | 0.7-1.17 | 9.94E-01 |
| Cholesterol to total lipids ratio in very small VLDL | 0.91 | 0.7-1.17 | 9.99E-01 | 0.88 | 0.68-1.14 | 9.94E-01 |
| Phospholipids to total lipids ratio in small LDL | 1.08 | 0.85-1.39 | 9.99E-01 | 1.1 | 0.86-1.41 | 9.94E-01 |
| Phospholipids to total lipids ratio in very large VLDL | 0.9 | 0.71-1.12 | 9.99E-01 | 0.9 | 0.71-1.14 | 9.94E-01 |
| Valine | 0.95 | 0.78-1.16 | 9.99E-01 | 0.96 | 0.78-1.19 | 9.94E-01 |
| Free cholesterol in IDL | 0.9 | 0.67-1.22 | 9.99E-01 | 0.9 | 0.66-1.21 | 9.94E-01 |
| Average diameter for VLDL particles | 1.11 | 0.88-1.39 | 9.99E-01 | 1.14 | 0.89-1.44 | 9.94E-01 |
| Free cholesterol to total lipids ratio in small VLDL | 0.9 | 0.69-1.17 | 9.99E-01 | 0.87 | 0.67-1.14 | 9.94E-01 |
| Triglycerides to total lipids ratio in medium VLDL | 1.12 | 0.86-1.48 | 9.99E-01 | 1.16 | 0.87-1.53 | 9.94E-01 |
| Acetate | 1.08 | 0.85-1.38 | 9.99E-01 | 1.08 | 0.84-1.38 | 9.94E-01 |
| Tyrosine | 0.96 | 0.78-1.18 | 9.99E-01 | 0.96 | 0.78-1.18 | 9.94E-01 |
| Triglycerides in medium VLDL | 1.1 | 0.87-1.41 | 9.99E-01 | 1.13 | 0.88-1.46 | 9.94E-01 |
| Ratio of triglycerides to phosphoglycerides | 1.09 | 0.86-1.38 | 9.99E-01 | 1.13 | 0.89-1.45 | 9.94E-01 |
| Triglycerides to total lipids ratio in very small VLDL | 1.1 | 0.84-1.45 | 9.99E-01 | 1.13 | 0.85-1.5 | 9.94E-01 |
| Free cholesterol in very large HDL | 0.93 | 0.73-1.19 | 9.99E-01 | 0.93 | 0.72-1.2 | 9.94E-01 |
| Ratio of monounsaturated fatty acids to total fatty acids | 0.91 | 0.68-1.21 | 9.99E-01 | 0.94 | 0.69-1.27 | 9.94E-01 |
| Concentration of HDL particles | 0.96 | 0.74-1.24 | 9.99E-01 | 0.91 | 0.7-1.19 | 9.94E-01 |
| Cholesteryl esters in very small VLDL | 0.92 | 0.68-1.23 | 9.99E-01 | 0.92 | 0.69-1.24 | 9.94E-01 |
| Cholesterol in very small VLDL | 0.92 | 0.68-1.23 | 9.99E-01 | 0.92 | 0.68-1.24 | 9.94E-01 |
| Average diameter for LDL particles | 0.92 | 0.74-1.14 | 9.99E-01 | 0.91 | 0.73-1.13 | 9.94E-01 |
| Ratio of apolipoprotein B to apolipoprotein A1 | 1.06 | 0.82-1.38 | 9.99E-01 | 1.1 | 0.84-1.43 | 9.94E-01 |
| 3-Hydroxybutyrate | 1.03 | 0.85-1.25 | 9.99E-01 | 1.04 | 0.86-1.26 | 9.94E-01 |
| Phospholipids in IDL | 0.93 | 0.69-1.26 | 9.99E-01 | 0.93 | 0.69-1.25 | 9.94E-01 |
| Total concentration of lipoprotein particles | 0.97 | 0.74-1.26 | 9.99E-01 | 0.93 | 0.71-1.21 | 9.94E-01 |
| Cholesterol to total lipids ratio in large HDL | 0.92 | 0.73-1.15 | 9.99E-01 | 0.89 | 0.7-1.13 | 9.94E-01 |
| Cholesteryl esters in medium VLDL | 0.91 | 0.69-1.2 | 9.99E-01 | 0.91 | 0.69-1.2 | 9.94E-01 |
| Ratio of polyunsaturated fatty acids to monounsaturated fatty acids | 1.07 | 0.81-1.43 | 9.99E-01 | 1.04 | 0.77-1.41 | 9.94E-01 |
| Cholesteryl esters to total lipids ratio in very large HDL | 1.06 | 0.82-1.36 | 9.99E-01 | 1.04 | 0.81-1.33 | 9.94E-01 |
| Phospholipids in chylomicrons and extremely large VLDL | 0.92 | 0.73-1.16 | 9.99E-01 | 0.93 | 0.73-1.19 | 9.94E-01 |
| Triglycerides in small VLDL | 1.08 | 0.85-1.37 | 9.99E-01 | 1.11 | 0.86-1.41 | 9.94E-01 |
| Triglycerides in large VLDL | 1.09 | 0.86-1.39 | 9.99E-01 | 1.12 | 0.87-1.45 | 9.94E-01 |
| Pyruvate | 1.09 | 0.89-1.34 | 9.99E-01 | 1.1 | 0.89-1.35 | 9.94E-01 |
| Cholesterol to total lipids ratio in large LDL | 0.94 | 0.74-1.2 | 9.99E-01 | 0.92 | 0.72-1.17 | 9.94E-01 |
| Phosphoglycerides | 0.99 | 0.75-1.29 | 9.99E-01 | 0.96 | 0.73-1.27 | 9.94E-01 |
| Ratio of linoleic acid to total fatty acids | 1.07 | 0.82-1.38 | 9.99E-01 | 1.05 | 0.8-1.37 | 9.94E-01 |
| Lactate | 1.09 | 0.88-1.35 | 9.99E-01 | 1.09 | 0.88-1.35 | 9.94E-01 |
| Triglycerides to total lipids ratio in very large VLDL | 0.96 | 0.74-1.25 | 9.99E-01 | 0.97 | 0.73-1.28 | 9.94E-01 |
| Total lipids in medium VLDL | 1.08 | 0.84-1.39 | 9.99E-01 | 1.1 | 0.85-1.42 | 9.94E-01 |
| Total lipids in large VLDL | 1.08 | 0.84-1.39 | 9.99E-01 | 1.11 | 0.86-1.44 | 9.94E-01 |
| Total cholines | 0.98 | 0.75-1.29 | 9.99E-01 | 0.96 | 0.72-1.27 | 9.94E-01 |
| Phosphatidylcholines | 1.01 | 0.77-1.31 | 9.99E-01 | 0.98 | 0.74-1.3 | 9.94E-01 |
| Concentration of large VLDL particles | 1.08 | 0.84-1.38 | 9.99E-01 | 1.11 | 0.85-1.43 | 9.94E-01 |
| Triglycerides to total lipids ratio in very large HDL | 1.06 | 0.85-1.33 | 9.99E-01 | 1.08 | 0.86-1.37 | 9.94E-01 |
| Free cholesterol in large VLDL | 1.07 | 0.83-1.38 | 9.99E-01 | 1.1 | 0.84-1.44 | 9.94E-01 |
| Triglycerides in VLDL | 1.08 | 0.85-1.36 | 9.99E-01 | 1.11 | 0.87-1.42 | 9.94E-01 |
| Phospholipids in small LDL | 1.09 | 0.83-1.42 | 9.99E-01 | 1.1 | 0.84-1.42 | 9.94E-01 |
| Cholesteryl esters to total lipids ratio in large VLDL | 0.9 | 0.71-1.13 | 9.99E-01 | 0.88 | 0.68-1.13 | 9.94E-01 |
| Triglycerides to total lipids ratio in small VLDL | 1.08 | 0.81-1.44 | 9.99E-01 | 1.11 | 0.83-1.49 | 9.94E-01 |
| Triglycerides to total lipids ratio in chylomicrons and extremely large VLDL | 0.94 | 0.77-1.15 | 9.99E-01 | 0.95 | 0.77-1.17 | 9.94E-01 |
| Acetone | 0.93 | 0.77-1.14 | 9.99E-01 | 0.94 | 0.77-1.15 | 9.94E-01 |
| Total triglycerides | 1.08 | 0.85-1.36 | 9.99E-01 | 1.11 | 0.87-1.41 | 9.94E-01 |
| Cholesterol in large VLDL | 1.06 | 0.82-1.37 | 9.99E-01 | 1.09 | 0.84-1.42 | 9.94E-01 |
| Linoleic acid | 1.1 | 0.84-1.44 | 9.99E-01 | 1.08 | 0.83-1.41 | 9.94E-01 |
| Phospholipids to total lipids ratio in very small VLDL | 0.92 | 0.71-1.19 | 9.99E-01 | 0.94 | 0.73-1.22 | 9.94E-01 |
| Cholesteryl esters to total lipids ratio in small LDL | 0.96 | 0.76-1.21 | 9.99E-01 | 0.95 | 0.74-1.2 | 9.94E-01 |
| Creatinine | 0.89 | 0.67-1.2 | 9.99E-01 | 0.91 | 0.68-1.21 | 9.94E-01 |
| Ratio of omega-6 fatty acids to omega-3 fatty acids | 1.06 | 0.83-1.34 | 9.99E-01 | 1.05 | 0.83-1.35 | 9.94E-01 |
| Omega-6 fatty acids | 1.1 | 0.84-1.44 | 9.99E-01 | 1.09 | 0.83-1.42 | 9.94E-01 |
| Free cholesterol to total lipids ratio in small LDL | 0.94 | 0.74-1.18 | 9.99E-01 | 0.92 | 0.73-1.16 | 9.94E-01 |
| Total lipids in small VLDL | 1.06 | 0.83-1.35 | 9.99E-01 | 1.08 | 0.84-1.39 | 9.94E-01 |
| Concentration of small VLDL particles | 1.05 | 0.82-1.34 | 9.99E-01 | 1.08 | 0.84-1.38 | 9.94E-01 |
| Phospholipids to total lipids ratio in small VLDL | 0.94 | 0.72-1.22 | 9.99E-01 | 0.91 | 0.7-1.19 | 9.94E-01 |
| Ratio of polyunsaturated fatty acids to total fatty acids | 1.04 | 0.8-1.35 | 9.99E-01 | 1.01 | 0.77-1.33 | 9.94E-01 |
| Triglycerides in medium HDL | 0.97 | 0.76-1.25 | 9.99E-01 | 0.98 | 0.75-1.27 | 9.94E-01 |
| Total lipids in VLDL | 1.07 | 0.84-1.36 | 9.99E-01 | 1.09 | 0.85-1.4 | 9.94E-01 |
| Free cholesterol to total lipids ratio in medium LDL | 0.93 | 0.73-1.18 | 9.99E-01 | 0.91 | 0.71-1.16 | 9.94E-01 |
| Cholesterol to total lipids ratio in large VLDL | 0.9 | 0.7-1.14 | 9.99E-01 | 0.88 | 0.69-1.13 | 9.94E-01 |
| Ratio of omega-6 fatty acids to total fatty acids | 1.04 | 0.8-1.36 | 9.99E-01 | 1.02 | 0.77-1.34 | 9.94E-01 |
| Polyunsaturated fatty acids | 1.09 | 0.84-1.42 | 9.99E-01 | 1.08 | 0.83-1.4 | 9.94E-01 |
| Cholesteryl esters in large VLDL | 1.05 | 0.81-1.36 | 9.99E-01 | 1.08 | 0.83-1.4 | 9.94E-01 |
| Concentration of chylomicrons and extremely large VLDL particles | 0.95 | 0.74-1.2 | 9.99E-01 | 0.96 | 0.75-1.24 | 9.94E-01 |
| Free cholesterol to total lipids ratio in chylomicrons and extremely large VLDL | 0.91 | 0.73-1.13 | 9.99E-01 | 0.89 | 0.72-1.11 | 9.94E-01 |
| Phospholipids to total lipids ratio in large HDL | 1.06 | 0.84-1.35 | 9.99E-01 | 1.09 | 0.85-1.39 | 9.94E-01 |
| Cholesterol to total lipids ratio in medium HDL | 0.95 | 0.75-1.2 | 9.99E-01 | 0.91 | 0.71-1.17 | 9.94E-01 |
| Triglycerides to total lipids ratio in IDL | 1.05 | 0.81-1.35 | 9.99E-01 | 1.08 | 0.83-1.39 | 9.94E-01 |
| Isoleucine | 0.96 | 0.79-1.18 | 9.99E-01 | 0.99 | 0.81-1.21 | 9.94E-01 |
| Total phospholipids in lipoprotein particles | 1 | 0.76-1.32 | 9.99E-01 | 0.98 | 0.74-1.3 | 9.94E-01 |
| Concentration of medium VLDL particles | 1.06 | 0.82-1.36 | 9.99E-01 | 1.07 | 0.83-1.39 | 9.94E-01 |
| Ratio of omega-3 fatty acids to total fatty acids | 0.95 | 0.75-1.2 | 9.99E-01 | 0.95 | 0.75-1.2 | 9.94E-01 |
| Cholesteryl esters to total lipids ratio in medium HDL | 0.94 | 0.74-1.18 | 9.99E-01 | 0.91 | 0.71-1.15 | 9.94E-01 |
| Phospholipids to total lipids ratio in medium HDL | 1.05 | 0.83-1.34 | 9.99E-01 | 1.1 | 0.85-1.41 | 9.94E-01 |
| Cholesteryl esters in very large VLDL | 1.05 | 0.81-1.35 | 9.99E-01 | 1.07 | 0.82-1.4 | 9.94E-01 |
| Phospholipids in VLDL | 1.06 | 0.83-1.35 | 9.99E-01 | 1.08 | 0.84-1.39 | 9.94E-01 |
| Free cholesterol to total lipids ratio in very small VLDL | 0.91 | 0.72-1.16 | 9.99E-01 | 0.93 | 0.73-1.18 | 9.94E-01 |
| Triglycerides in very large HDL | 0.98 | 0.76-1.26 | 9.99E-01 | 0.99 | 0.77-1.28 | 9.94E-01 |
| Phospholipids in very small VLDL | 0.95 | 0.72-1.25 | 9.99E-01 | 0.97 | 0.73-1.28 | 9.94E-01 |
| Phospholipids in medium VLDL | 1.06 | 0.82-1.37 | 9.99E-01 | 1.07 | 0.83-1.39 | 9.94E-01 |
| Phospholipids in small HDL | 1 | 0.78-1.29 | 9.99E-01 | 0.97 | 0.75-1.26 | 9.94E-01 |
| Triglycerides to total lipids ratio in large LDL | 1.06 | 0.82-1.36 | 9.99E-01 | 1.09 | 0.84-1.4 | 9.94E-01 |
| Free cholesterol in very small VLDL | 0.95 | 0.72-1.26 | 9.99E-01 | 0.97 | 0.73-1.28 | 9.94E-01 |
| Cholesterol in very large VLDL | 1.05 | 0.81-1.35 | 9.99E-01 | 1.07 | 0.82-1.4 | 9.94E-01 |
| Phospholipids in small VLDL | 1.05 | 0.81-1.35 | 9.99E-01 | 1.06 | 0.82-1.37 | 9.94E-01 |
| Triglycerides in medium LDL | 1.07 | 0.85-1.35 | 9.99E-01 | 1.09 | 0.86-1.39 | 9.94E-01 |
| Ratio of saturated fatty acids to total fatty acids | 0.98 | 0.78-1.24 | 9.99E-01 | 1 | 0.79-1.26 | 9.94E-01 |
| Phospholipids in medium LDL | 1.07 | 0.81-1.4 | 9.99E-01 | 1.07 | 0.82-1.39 | 9.94E-01 |
| Total lipids in very small VLDL | 0.96 | 0.73-1.26 | 9.99E-01 | 0.97 | 0.74-1.28 | 9.94E-01 |
| Phenylalanine | 1.04 | 0.84-1.28 | 9.99E-01 | 1.04 | 0.84-1.3 | 9.94E-01 |
| Alanine | 1.1 | 0.89-1.36 | 9.99E-01 | 1.11 | 0.9-1.38 | 9.94E-01 |
| Phospholipids in large VLDL | 1.05 | 0.81-1.37 | 9.99E-01 | 1.08 | 0.82-1.43 | 9.94E-01 |
| Concentration of small LDL particles | 1.06 | 0.82-1.37 | 9.99E-01 | 1.07 | 0.83-1.38 | 9.94E-01 |
| Free cholesterol in VLDL | 1.05 | 0.82-1.34 | 9.99E-01 | 1.07 | 0.83-1.38 | 9.94E-01 |
| Cholesterol to total lipids ratio in small HDL | 1.02 | 0.78-1.32 | 9.99E-01 | 1 | 0.76-1.31 | 9.94E-01 |
| Glycine | 0.92 | 0.76-1.12 | 9.99E-01 | 0.91 | 0.75-1.11 | 9.94E-01 |
| Cholesteryl esters to total lipids ratio in large HDL | 0.93 | 0.75-1.16 | 9.99E-01 | 0.9 | 0.72-1.14 | 9.94E-01 |
| Concentration of very large VLDL particles | 1.05 | 0.82-1.36 | 9.99E-01 | 1.08 | 0.82-1.42 | 9.94E-01 |
| Total lipids in small LDL | 1.06 | 0.81-1.39 | 9.99E-01 | 1.07 | 0.82-1.39 | 9.94E-01 |
| Free cholesterol to total lipids ratio in medium HDL | 1.02 | 0.79-1.31 | 9.99E-01 | 0.99 | 0.77-1.29 | 9.94E-01 |
| Triglycerides in HDL | 0.99 | 0.77-1.26 | 9.99E-01 | 1 | 0.77-1.3 | 9.94E-01 |
| Triglycerides in small LDL | 1.06 | 0.84-1.34 | 9.99E-01 | 1.08 | 0.85-1.38 | 9.94E-01 |
| Triglycerides to total lipids ratio in large VLDL | 1.1 | 0.85-1.44 | 9.99E-01 | 1.11 | 0.85-1.44 | 9.94E-01 |
| Omega-3 fatty acids | 0.97 | 0.77-1.23 | 9.99E-01 | 0.97 | 0.76-1.24 | 9.94E-01 |
| Free cholesterol to total lipids ratio in large VLDL | 0.91 | 0.71-1.17 | 9.99E-01 | 0.91 | 0.71-1.17 | 9.94E-01 |
| Free cholesterol to total lipids ratio in small HDL | 1.06 | 0.83-1.37 | 9.99E-01 | 1.07 | 0.83-1.39 | 9.94E-01 |
| Triglycerides in LDL | 1.07 | 0.84-1.35 | 9.99E-01 | 1.09 | 0.85-1.39 | 9.94E-01 |
| Concentration of VLDL particles | 1.04 | 0.81-1.34 | 9.99E-01 | 1.07 | 0.82-1.38 | 9.94E-01 |
| Free cholesterol in large LDL | 0.98 | 0.73-1.3 | 9.99E-01 | 0.96 | 0.73-1.28 | 9.94E-01 |
| Triglycerides to total lipids ratio in small HDL | 1.04 | 0.81-1.32 | 9.99E-01 | 1.07 | 0.83-1.39 | 9.94E-01 |
| Total esterified cholesterol | 0.99 | 0.75-1.33 | 9.99E-01 | 0.98 | 0.73-1.3 | 9.94E-01 |
| Total lipids in IDL | 0.99 | 0.73-1.33 | 9.99E-01 | 0.98 | 0.73-1.31 | 9.94E-01 |
| Concentration of very small VLDL particles | 0.97 | 0.73-1.27 | 9.99E-01 | 0.98 | 0.74-1.29 | 9.94E-01 |
| Free cholesterol in medium VLDL | 1.04 | 0.8-1.35 | 9.99E-01 | 1.05 | 0.81-1.37 | 9.94E-01 |
| Free cholesterol in very large VLDL | 1.04 | 0.8-1.34 | 9.99E-01 | 1.07 | 0.81-1.4 | 9.94E-01 |
| Triglycerides in large LDL | 1.06 | 0.84-1.35 | 9.99E-01 | 1.08 | 0.85-1.38 | 9.94E-01 |
| Cholesteryl esters to total lipids ratio in small HDL | 1 | 0.77-1.29 | 9.99E-01 | 0.98 | 0.76-1.27 | 9.94E-01 |
| Cholesteryl esters to total lipids ratio in medium LDL | 0.99 | 0.79-1.24 | 9.99E-01 | 1 | 0.79-1.27 | 9.94E-01 |
| Cholesterol in IDL | 0.99 | 0.74-1.33 | 9.99E-01 | 0.98 | 0.73-1.32 | 9.94E-01 |
| Total cholesterol | 1 | 0.75-1.33 | 9.99E-01 | 0.98 | 0.74-1.31 | 9.94E-01 |
| Total lipids in medium LDL | 1.05 | 0.81-1.37 | 9.99E-01 | 1.06 | 0.81-1.38 | 9.94E-01 |
| Cholesteryl esters in small LDL | 1.05 | 0.81-1.37 | 9.99E-01 | 1.06 | 0.81-1.38 | 9.94E-01 |
| Phospholipids to total lipids ratio in large VLDL | 0.96 | 0.75-1.22 | 9.99E-01 | 0.97 | 0.75-1.25 | 9.94E-01 |
| Total lipids in very large VLDL | 1.04 | 0.8-1.35 | 9.99E-01 | 1.07 | 0.81-1.41 | 9.94E-01 |
| Free cholesterol in chylomicrons and extremely large VLDL | 0.97 | 0.76-1.23 | 9.99E-01 | 0.99 | 0.76-1.27 | 9.94E-01 |
| Triglycerides to total lipids ratio in medium LDL | 1.04 | 0.81-1.35 | 9.99E-01 | 1.07 | 0.82-1.38 | 9.94E-01 |
| Triglycerides in chylomicrons and extremely large VLDL | 0.97 | 0.77-1.21 | 9.99E-01 | 0.98 | 0.78-1.24 | 9.94E-01 |
| Triglycerides in small HDL | 1.04 | 0.82-1.32 | 9.99E-01 | 1.07 | 0.82-1.38 | 9.94E-01 |
| Cholesterol in small LDL | 1.05 | 0.8-1.37 | 9.99E-01 | 1.05 | 0.8-1.37 | 9.94E-01 |
| Cholesteryl esters to total lipids ratio in chylomicrons and extremely large VLDL | 1.01 | 0.79-1.31 | 9.99E-01 | 0.99 | 0.76-1.3 | 9.94E-01 |
| Degree of unsaturation | 1.01 | 0.79-1.29 | 9.99E-01 | 0.99 | 0.77-1.27 | 9.94E-01 |
| Concentration of IDL particles | 1.05 | 0.79-1.39 | 9.99E-01 | 1.05 | 0.79-1.4 | 9.94E-01 |
| Cholesteryl esters in medium LDL | 1.05 | 0.81-1.36 | 9.99E-01 | 1.05 | 0.81-1.37 | 9.94E-01 |
| Ratio of docosahexaenoic acid to total fatty acids | 0.95 | 0.76-1.18 | 9.99E-01 | 0.93 | 0.73-1.18 | 9.94E-01 |
| Cholesterol to total lipids ratio in IDL | 1.04 | 0.8-1.37 | 9.99E-01 | 1.02 | 0.79-1.33 | 9.94E-01 |
| Total lipids in small HDL | 1.02 | 0.8-1.31 | 9.99E-01 | 0.99 | 0.77-1.28 | 9.94E-01 |
| Cholesterol in medium LDL | 1.04 | 0.8-1.36 | 9.99E-01 | 1.04 | 0.8-1.36 | 9.94E-01 |
| Concentration of medium LDL particles | 1.03 | 0.81-1.32 | 9.99E-01 | 1.04 | 0.82-1.33 | 9.94E-01 |
| Monounsaturated fatty acids | 1 | 0.78-1.3 | 9.99E-01 | 1.02 | 0.78-1.33 | 9.94E-01 |
| Cholesteryl esters to total lipids ratio in large LDL | 1.03 | 0.81-1.3 | 9.99E-01 | 1.02 | 0.81-1.28 | 9.94E-01 |
| Cholesterol to total lipids ratio in chylomicrons and extremely large VLDL | 1 | 0.78-1.28 | 9.99E-01 | 0.98 | 0.76-1.27 | 9.94E-01 |
| Total free cholesterol | 1.01 | 0.76-1.34 | 9.99E-01 | 1 | 0.75-1.33 | 9.94E-01 |
| Free cholesterol to total lipids ratio in very large VLDL | 0.98 | 0.76-1.27 | 9.99E-01 | 0.97 | 0.73-1.27 | 9.94E-01 |
| Cholesterol to total lipids ratio in very large VLDL | 0.99 | 0.76-1.3 | 9.99E-01 | 0.97 | 0.73-1.3 | 9.94E-01 |
| Triglycerides in very small VLDL | 1.03 | 0.81-1.31 | 9.99E-01 | 1.05 | 0.82-1.35 | 9.94E-01 |
| Triglycerides to total lipids ratio in large HDL | 1.02 | 0.81-1.29 | 9.99E-01 | 1.05 | 0.82-1.33 | 9.94E-01 |
| Free cholesterol in small VLDL | 1.02 | 0.78-1.32 | 9.99E-01 | 1.03 | 0.79-1.34 | 9.94E-01 |
| Triglycerides to total lipids ratio in small LDL | 1.03 | 0.8-1.33 | 9.99E-01 | 1.06 | 0.82-1.36 | 9.94E-01 |
| Free cholesterol in LDL | 0.99 | 0.75-1.32 | 9.99E-01 | 0.99 | 0.75-1.3 | 9.94E-01 |
| VLDL cholesterol | 1.02 | 0.79-1.32 | 9.99E-01 | 1.04 | 0.8-1.35 | 9.94E-01 |
| Phospholipids in very large VLDL | 1.02 | 0.78-1.34 | 9.99E-01 | 1.05 | 0.79-1.4 | 9.94E-01 |
| Apolipoprotein B | 1.03 | 0.78-1.36 | 9.99E-01 | 1.04 | 0.79-1.36 | 9.94E-01 |
| Total fatty acids | 1.06 | 0.82-1.36 | 9.99E-01 | 1.06 | 0.82-1.37 | 9.94E-01 |
| Docosahexaenoic acid | 0.98 | 0.78-1.23 | 9.99E-01 | 0.96 | 0.76-1.23 | 9.94E-01 |
| Phospholipids in LDL | 1.04 | 0.79-1.37 | 9.99E-01 | 1.03 | 0.79-1.36 | 9.94E-01 |
| Cholesteryl esters in chylomicrons and extremely large VLDL | 1.01 | 0.8-1.27 | 9.99E-01 | 1.03 | 0.81-1.31 | 9.94E-01 |
| Sphingomyelins | 0.98 | 0.75-1.3 | 9.99E-01 | 0.97 | 0.73-1.28 | 9.94E-01 |
| Triglycerides in very large VLDL | 1.03 | 0.79-1.34 | 9.99E-01 | 1.06 | 0.8-1.4 | 9.94E-01 |
| Triglycerides in IDL | 1.04 | 0.81-1.32 | 9.99E-01 | 1.05 | 0.82-1.35 | 9.94E-01 |
| Cholesterol in large LDL | 1 | 0.76-1.33 | 9.99E-01 | 1 | 0.76-1.31 | 9.94E-01 |
| Cholesterol in medium VLDL | 0.98 | 0.75-1.29 | 9.99E-01 | 0.99 | 0.76-1.29 | 9.94E-01 |
| Cholesteryl esters in IDL | 1.02 | 0.76-1.37 | 9.99E-01 | 1.01 | 0.75-1.35 | 9.94E-01 |
| Phospholipids in large LDL | 1.01 | 0.76-1.34 | 9.99E-01 | 1 | 0.76-1.32 | 9.94E-01 |
| Concentration of LDL particles | 1.02 | 0.78-1.34 | 9.99E-01 | 1.03 | 0.78-1.34 | 9.94E-01 |
| Cholesterol in small VLDL | 1 | 0.77-1.29 | 9.99E-01 | 1.01 | 0.78-1.32 | 9.94E-01 |
| Free cholesterol in small LDL | 1.02 | 0.77-1.35 | 9.99E-01 | 1.02 | 0.78-1.34 | 9.94E-01 |
| Free cholesterol in medium LDL | 1.02 | 0.77-1.35 | 9.99E-01 | 1.01 | 0.77-1.33 | 9.94E-01 |
| Triglycerides to total lipids ratio in medium HDL | 1.01 | 0.79-1.29 | 9.99E-01 | 1.04 | 0.8-1.35 | 9.94E-01 |
| Phospholipids to total lipids ratio in large LDL | 0.96 | 0.75-1.21 | 9.99E-01 | 0.96 | 0.76-1.22 | 9.94E-01 |
| Total lipids in large LDL | 1.01 | 0.77-1.34 | 9.99E-01 | 1 | 0.76-1.32 | 9.94E-01 |
| Clinical LDL cholesterol | 1.01 | 0.76-1.34 | 9.99E-01 | 1 | 0.76-1.32 | 9.94E-01 |
| Cholesteryl esters to total lipids ratio in very large VLDL | 0.98 | 0.75-1.28 | 9.99E-01 | 0.96 | 0.72-1.27 | 9.94E-01 |
| Total lipids in LDL | 1.03 | 0.78-1.36 | 9.99E-01 | 1.03 | 0.78-1.35 | 9.94E-01 |
| Cholesteryl esters in large LDL | 1.01 | 0.77-1.34 | 9.99E-01 | 1.01 | 0.77-1.32 | 9.94E-01 |
| Cholesteryl esters in LDL | 1.03 | 0.78-1.35 | 9.99E-01 | 1.02 | 0.78-1.34 | 9.94E-01 |
| Acetoacetate | 0.99 | 0.8-1.23 | 9.99E-01 | 1 | 0.8-1.24 | 9.94E-01 |
| Total lipids in chylomicrons and extremely large VLDL | 1 | 0.78-1.27 | 9.99E-01 | 1.02 | 0.79-1.31 | 9.94E-01 |
| Saturated fatty acids | 1.05 | 0.82-1.33 | 9.99E-01 | 1.06 | 0.82-1.36 | 9.94E-01 |
| Cholesteryl esters in small HDL | 1.02 | 0.8-1.3 | 9.99E-01 | 0.98 | 0.77-1.26 | 9.94E-01 |
| Free cholesterol in small HDL | 1.05 | 0.81-1.35 | 9.99E-01 | 1.02 | 0.79-1.33 | 9.94E-01 |
| Cholesteryl esters in small VLDL | 0.99 | 0.77-1.28 | 9.99E-01 | 1.01 | 0.78-1.3 | 9.94E-01 |
| Cholesteryl esters in VLDL | 1 | 0.77-1.29 | 9.99E-01 | 1.01 | 0.77-1.31 | 9.94E-01 |
| Cholesterol in chylomicrons and extremely large VLDL | 1 | 0.79-1.26 | 9.99E-01 | 1.02 | 0.79-1.3 | 9.94E-01 |
| Cholesterol in small HDL | 1.03 | 0.8-1.31 | 9.99E-01 | 0.99 | 0.77-1.28 | 9.94E-01 |
| Concentration of small HDL particles | 1.03 | 0.81-1.32 | 9.99E-01 | 1 | 0.78-1.29 | 9.94E-01 |
| Total cholesterol minus HDL-C | 1.02 | 0.77-1.35 | 9.99E-01 | 1.02 | 0.77-1.35 | 9.94E-01 |
| Remnant cholesterol (non-HDL | 1.02 | 0.77-1.35 | 9.99E-01 | 1.03 | 0.78-1.36 | 9.94E-01 |
| Total lipids in lipoprotein particles | 1.05 | 0.8-1.37 | 9.99E-01 | 1.04 | 0.8-1.37 | 9.94E-01 |
| LDL cholesterol | 1.02 | 0.77-1.35 | 9.99E-01 | 1.01 | 0.77-1.33 | 9.94E-01 |
| Concentration of large LDL particles | 1.01 | 0.76-1.33 | 9.99E-01 | 1.01 | 0.77-1.33 | 9.94E-01 |

Note: the table shows associations from The competing risk Cox proportional hazards regression analysis where plasms metabolomics level from the Nightingale platform was main exposure and incident colorectal cancer as the main outcome in ERGO-4 cohort. Model 1 : adjusted for age, sex, cohort, and lipid lowering medication. Model 2: adjusted for model1, smoking status, BMI, alcohol and education. The table is ranked based on the *FDR* values in model 2, using bold text indicates metabolites are surpassing *FDR* adjusted P value treshold (*P*<0.05). ERGO-4 refer to the fourth and fifth visits from the baseline cohort. Abbreviations: IDL, intermediate-density lipoprotein; LDL, low-density lipoprotein; HDL, high-density lipoprotein; VLDL, very-low-density lipoprotein; BMI, body mass index; HR, hazard ratio; CI, confidence interval; FDR, false discovery rate.

**Supplementary Table 16. Serum metabolites associated with incident colorectal cancer from the Metabolon platform.**

| **Metabolite** | **Model 1** | | | **Model 2** | | |
| --- | --- | --- | --- | --- | --- | --- |
|  | **HR** | **95% Ci** | ***FDR* value** | **HR** | **95% Ci** | ***FDR* value** |
| perfluorooctanesulfonate (PFOS) | 0.51 | 0.4-0.66 | **7.48E-05** | 0.47 | 0.37-0.59 | **1.50E-07** |
| X - 24951 | 0.58 | 0.47-0.72 | **1.07E-04** | 0.52 | 0.42-0.65 | **5.39E-06** |
| perfluorooctanoate (PFOA) | 0.55 | 0.44-0.68 | **6.09E-05** | 0.52 | 0.4-0.66 | **7.20E-05** |
| 3-bromo-5-chloro-2,6-dihydroxybenzoic acid* | 0.58 | 0.47-0.73 | **6.04E-04** | 0.53 | 0.41-0.69 | **7.73E-04** |
| X - 11372 | 0.54 | 0.41-0.71 | **2.08E-03** | 0.52 | 0.39-0.69 | **1.33E-03** |
| 3,5-dichloro-2,6-dihydroxybenzoic acid | 0.63 | 0.51-0.78 | **2.30E-03** | 0.60 | 0.48-0.75 | **1.33E-03** |
| X - 24953 | 0.64 | 0.52-0.78 | **2.30E-03** | 0.64 | 0.52-0.79 | **4.32E-03** |
| 1-palmitoyl-2-arachidonoyl-GPC (16:0/20:4n6) | 1.68 | 1.21-2.35 | 1.47E-01 | 1.77 | 1.32-2.38 | **1.97E-02** |
| X - 13658 | 0.61 | 0.46-0.81 | 6.34E-02 | 0.59 | 0.44-0.78 | **3.08E-02** |
| X - 11880 | 0.62 | 0.47-0.81 | **4.20E-02** | 0.60 | 0.45-0.79 | **3.69E-02** |
| pristanate | 0.65 | 0.5-0.85 | 9.90E-02 | 0.64 | 0.5-0.82 | **3.69E-02** |
| cortisone | 0.77 | 0.67-0.89 | **3.26E-02** | 0.78 | 0.67-0.9 | 5.50E-02 |
| 4-hydroxychlorothalonil | 0.67 | 0.55-0.83 | **2.12E-02** | 0.65 | 0.51-0.83 | 5.53E-02 |
| 1-(1-enyl-palmitoyl)-2-linoleoyl-GPE (P-16:0/18:2)* | 0.65 | 0.5-0.84 | 7.46E-02 | 0.61 | 0.46-0.82 | 5.86E-02 |
| thymol sulfate | 1.52 | 1.18-1.96 | 8.99E-02 | 1.55 | 1.19-2.01 | 8.67E-02 |
| X - 18345 | 0.62 | 0.46-0.83 | 1.27E-01 | 0.61 | 0.45-0.83 | 9.86E-02 |
| 1-(1-enyl-oleoyl)-GPE (P-18:1)* | 0.67 | 0.51-0.87 | 1.59E-01 | 0.65 | 0.5-0.85 | 1.15E-01 |
| X - 17690 | 1.62 | 1.18-2.21 | 1.59E-01 | 1.60 | 1.19-2.17 | 1.34E-01 |
| bilirubin degradation product, C17H18N2O4 (3)** | 0.73 | 0.58-0.91 | 2.28E-01 | 0.72 | 0.58-0.89 | 1.45E-01 |
| bilirubin degradation product, C17H18N2O4 (1)** | 0.73 | 0.59-0.91 | 2.26E-01 | 0.74 | 0.6-0.9 | 1.69E-01 |
| X - 21339 | 0.62 | 0.46-0.85 | 1.60E-01 | 0.61 | 0.44-0.85 | 1.72E-01 |
| N-acetyl-isoputreanine | 1.41 | 1.1-1.79 | 2.43E-01 | 1.40 | 1.11-1.77 | 2.56E-01 |
| 1-stearoyl-2-arachidonoyl-GPE (18:0/20:4) | 1.65 | 1.13-2.41 | 3.60E-01 | 1.74 | 1.17-2.58 | 2.87E-01 |
| X - 11308 | 0.67 | 0.48-0.92 | 4.17E-01 | 0.63 | 0.46-0.88 | 3.08E-01 |
| picolinate | 0.69 | 0.53-0.91 | 3.60E-01 | 0.69 | 0.52-0.9 | 3.13E-01 |
| 10-undecenoate (11:1n1) | 0.71 | 0.56-0.9 | 2.10E-01 | 0.73 | 0.57-0.92 | 3.26E-01 |
| X - 24947 | 0.70 | 0.56-0.9 | 2.10E-01 | 0.72 | 0.56-0.92 | 3.26E-01 |
| 1-(1-enyl-palmitoyl)-GPE (P-16:0)* | 0.67 | 0.49-0.91 | 3.93E-01 | 0.63 | 0.45-0.89 | 3.26E-01 |
| X - 24544 | 0.73 | 0.57-0.93 | 3.96E-01 | 0.71 | 0.55-0.92 | 3.26E-01 |
| 1-(1-enyl-stearoyl)-2-linoleoyl-GPE (P-18:0/18:2)* | 0.70 | 0.52-0.93 | 4.17E-01 | 0.66 | 0.49-0.9 | 3.26E-01 |
| N-acetylglucosaminylasparagine | 0.66 | 0.49-0.89 | 2.65E-01 | 0.68 | 0.5-0.91 | 3.27E-01 |
| 1-stearoyl-2-arachidonoyl-GPC (18:0/20:4) | 1.63 | 1.1-2.41 | 4.17E-01 | 1.67 | 1.13-2.46 | 3.27E-01 |
| phytanate | 0.70 | 0.53-0.93 | 4.17E-01 | 0.68 | 0.51-0.91 | 3.27E-01 |
| androstenediol (3beta,17beta) disulfate (2) | 0.70 | 0.55-0.89 | 1.60E-01 | 0.71 | 0.54-0.92 | 3.41E-01 |
| 3-hydroxylaurate | 0.69 | 0.51-0.93 | 4.17E-01 | 0.69 | 0.52-0.92 | 3.44E-01 |
| 1-palmitoyl-2-arachidonoyl-GPE (16:0/20:4)* | 1.63 | 1.1-2.41 | 4.17E-01 | 1.67 | 1.12-2.5 | 3.62E-01 |
| X - 12844 | 0.73 | 0.57-0.93 | 3.93E-01 | 0.73 | 0.57-0.94 | 3.76E-01 |
| 5alpha-pregnan-3beta,20beta-diol monosulfate (1) | 0.75 | 0.58-0.97 | 5.71E-01 | 0.72 | 0.56-0.94 | 3.92E-01 |
| 1-(1-enyl-palmitoyl)-2-oleoyl-GPE (P-16:0/18:1)* | 0.67 | 0.47-0.95 | 5.43E-01 | 0.66 | 0.47-0.93 | 4.74E-01 |
| hexadecanedioate (C16-DC) | 0.67 | 0.49-0.92 | 4.12E-01 | 0.70 | 0.52-0.94 | 4.75E-01 |
| hexadecenedioate (C16:1-DC)* | 0.71 | 0.54-0.94 | 4.17E-01 | 0.73 | 0.56-0.95 | 5.24E-01 |
| (14 or 15)-methylpalmitate (a17:0 or i17:0) | 0.69 | 0.49-0.96 | 5.70E-01 | 0.68 | 0.49-0.94 | 5.45E-01 |
| gamma-glutamylleucine | 0.65 | 0.47-0.9 | 3.93E-01 | 0.67 | 0.47-0.95 | 5.62E-01 |
| bilirubin degradation product, C17H18N2O4 (2)** | 0.78 | 0.63-0.97 | 5.43E-01 | 0.79 | 0.65-0.97 | 5.62E-01 |
| tetrahydrocortisone glucuronide (5) | 0.76 | 0.6-0.97 | 5.44E-01 | 0.76 | 0.6-0.96 | 5.62E-01 |
| 1-oleoyl-GPI (18:1) | 0.71 | 0.52-0.97 | 5.77E-01 | 0.69 | 0.51-0.95 | 5.62E-01 |
| 5alpha-androstan-3alpha,17alpha-diol monosulfate | 0.68 | 0.48-0.97 | 5.88E-01 | 0.66 | 0.46-0.95 | 5.62E-01 |
| bilirubin (E,Z or Z,E)* | 0.75 | 0.57-0.98 | 6.61E-01 | 0.72 | 0.55-0.96 | 5.62E-01 |
| cysteine s-sulfate | 1.39 | 1.01-1.92 | 6.85E-01 | 1.42 | 1.04-1.95 | 6.15E-01 |
| X - 21470 | 0.72 | 0.55-0.95 | 4.90E-01 | 0.74 | 0.56-0.99 | 6.47E-01 |
| X - 25419 | 0.69 | 0.5-0.94 | 4.90E-01 | 0.69 | 0.48-0.97 | 6.47E-01 |
| catechol glucuronide | 1.55 | 1.06-2.26 | 5.43E-01 | 1.51 | 1.03-2.23 | 6.47E-01 |
| tetrahydrocortisol glucuronide | 0.76 | 0.6-0.96 | 5.43E-01 | 0.77 | 0.61-0.98 | 6.47E-01 |
| cytidine | 1.37 | 1.04-1.79 | 5.43E-01 | 1.32 | 1.02-1.69 | 6.47E-01 |
| 5alpha-pregnan-3beta,20alpha-diol disulfate | 0.72 | 0.54-0.96 | 5.70E-01 | 0.73 | 0.55-0.97 | 6.47E-01 |
| 5alpha-androstan-3beta,17alpha-diol disulfate | 0.67 | 0.46-0.96 | 5.70E-01 | 0.68 | 0.48-0.97 | 6.47E-01 |
| imidazole propionate | 1.33 | 1.03-1.72 | 5.70E-01 | 1.32 | 1.01-1.72 | 6.47E-01 |
| methylsuccinoylcarnitine | 1.45 | 1.04-2.03 | 5.70E-01 | 1.44 | 1-2.08 | 6.47E-01 |
| X - 12830 | 0.67 | 0.48-0.96 | 5.70E-01 | 0.70 | 0.49-0.98 | 6.47E-01 |
| 1-(1-enyl-palmitoyl)-2-arachidonoyl-GPE (P-16:0/20:4)* | 0.72 | 0.53-0.97 | 5.99E-01 | 0.72 | 0.52-0.99 | 6.47E-01 |
| X - 25957 | 1.47 | 1.02-2.11 | 6.58E-01 | 1.46 | 1.01-2.11 | 6.47E-01 |
| orotidine | 1.30 | 1.01-1.66 | 6.70E-01 | 1.34 | 1.02-1.78 | 6.47E-01 |
| hydroquinone sulfate | 1.41 | 1.02-1.96 | 6.75E-01 | 1.38 | 1-1.92 | 6.47E-01 |
| succinylcarnitine (C4-DC) | 1.48 | 1.01-2.17 | 6.85E-01 | 1.52 | 1-2.32 | 6.47E-01 |
| 1-(1-enyl-stearoyl)-2-oleoyl-GPE (P-18:0/18:1) | 0.68 | 0.47-0.99 | 6.85E-01 | 0.68 | 0.47-0.97 | 6.47E-01 |
| gamma-glutamyl-alpha-lysine | 0.76 | 0.58-0.99 | 6.85E-01 | 0.75 | 0.56-0.99 | 6.47E-01 |
| X - 13728 | 1.33 | 1.01-1.75 | 6.85E-01 | 1.33 | 1.01-1.76 | 6.47E-01 |
| X - 23739 | 0.71 | 0.51-0.99 | 6.85E-01 | 0.69 | 0.48-0.97 | 6.47E-01 |
| 1-stearoyl-2-oleoyl-GPS (18:0/18:1) | 0.74 | 0.54-0.99 | 6.87E-01 | 0.73 | 0.53-0.99 | 6.47E-01 |
| glucuronate | 1.35 | 0.99-1.83 | 6.94E-01 | 1.36 | 1.01-1.84 | 6.47E-01 |
| citramalate | 1.31 | 0.99-1.72 | 6.94E-01 | 1.34 | 1.01-1.78 | 6.47E-01 |
| sebacate (C10-DC) | 0.74 | 0.55-1 | 6.94E-01 | 0.75 | 0.56-1 | 6.47E-01 |
| 5-dodecenoate (12:1n7) | 0.71 | 0.51-1 | 6.94E-01 | 0.72 | 0.51-1 | 6.47E-01 |
| N4-acetylcytidine | 1.40 | 1-1.96 | 6.94E-01 | 1.42 | 1.01-2 | 6.47E-01 |
| 5alpha-pregnan-3beta,20alpha-diol monosulfate (2) | 0.75 | 0.55-1.01 | 6.94E-01 | 0.72 | 0.54-0.97 | 6.47E-01 |
| pregnenediol sulfate (C21H34O5S)* | 0.76 | 0.57-1.01 | 6.94E-01 | 0.75 | 0.56-0.99 | 6.47E-01 |
| pregnenolone sulfate | 0.75 | 0.55-1.02 | 6.94E-01 | 0.74 | 0.55-1 | 6.47E-01 |
| eicosanedioate (C20-DC) | 0.78 | 0.6-1.01 | 6.94E-01 | 0.76 | 0.59-0.99 | 6.47E-01 |
| 1-(1-enyl-stearoyl)-GPE (P-18:0)* | 0.74 | 0.53-1.02 | 6.94E-01 | 0.68 | 0.47-0.97 | 6.47E-01 |
| 2-hydroxydecanoate | 0.73 | 0.53-1 | 6.94E-01 | 0.71 | 0.52-0.98 | 6.47E-01 |
| N-acetylneuraminate | 1.41 | 0.98-2.03 | 6.94E-01 | 1.43 | 1-2.06 | 6.47E-01 |
| phosphate | 1.39 | 1-1.92 | 6.94E-01 | 1.38 | 1-1.92 | 6.47E-01 |
| alanine | 1.36 | 0.98-1.89 | 6.94E-01 | 1.38 | 1-1.91 | 6.47E-01 |
| fructose | 1.35 | 0.98-1.85 | 6.94E-01 | 1.38 | 1-1.9 | 6.47E-01 |
| X - 15245 | 1.32 | 0.98-1.78 | 6.94E-01 | 1.38 | 1-1.9 | 6.47E-01 |
| X - 16935 | 0.74 | 0.54-1 | 6.94E-01 | 0.73 | 0.53-1 | 6.47E-01 |
| linolenate [alpha or gamma; (18:3n3 or 6)] | 0.75 | 0.55-1.03 | 7.00E-01 | 0.73 | 0.54-1 | 6.47E-01 |
| 1-myristoyl-2-arachidonoyl-GPC (14:0/20:4)* | 1.43 | 0.96-2.13 | 7.15E-01 | 1.48 | 1-2.2 | 6.47E-01 |
| N-acetyltryptophan | 1.32 | 0.92-1.9 | 7.96E-01 | 1.47 | 1-2.16 | 6.47E-01 |
| X - 17357 | 0.78 | 0.61-0.99 | 6.75E-01 | 0.77 | 0.59-1.01 | 6.73E-01 |
| 10-nonadecenoate (19:1n9) | 0.70 | 0.49-1.01 | 6.94E-01 | 0.71 | 0.49-1.01 | 6.78E-01 |
| X - 11381 | 0.73 | 0.51-1.05 | 7.30E-01 | 0.71 | 0.5-1.01 | 7.02E-01 |
| lanthionine | 0.76 | 0.56-1.02 | 6.94E-01 | 0.75 | 0.56-1.01 | 7.18E-01 |
| X - 11470 | 0.80 | 0.62-1.04 | 7.67E-01 | 0.78 | 0.6-1.01 | 7.18E-01 |
| 3-hydroxydecanoate | 0.73 | 0.52-1.02 | 6.94E-01 | 0.74 | 0.53-1.02 | 7.22E-01 |
| androstenediol (3alpha, 17alpha) monosulfate (2) | 0.78 | 0.61-1.01 | 6.94E-01 | 0.78 | 0.61-1.01 | 7.22E-01 |
| myristate (14:0) | 0.72 | 0.5-1.03 | 7.04E-01 | 0.72 | 0.51-1.02 | 7.22E-01 |
| 2-hydroxyfluorene sulfate | 0.70 | 0.46-1.05 | 7.23E-01 | 0.65 | 0.41-1.03 | 7.22E-01 |
| laurate (12:0) | 0.76 | 0.55-1.07 | 7.96E-01 | 0.72 | 0.51-1.02 | 7.22E-01 |
| X - 07765 | 1.38 | 1.01-1.87 | 6.75E-01 | 1.34 | 0.98-1.83 | 7.22E-01 |
| X - 12812 | 1.33 | 0.98-1.79 | 6.94E-01 | 1.34 | 0.98-1.82 | 7.23E-01 |
| deoxycarnitine | 0.87 | 0.78-0.98 | 4.90E-01 | 0.89 | 0.78-1.01 | 7.37E-01 |
| docosadienoate (22:2n6) | 0.73 | 0.53-1 | 6.94E-01 | 0.74 | 0.53-1.02 | 7.37E-01 |
| gamma-glutamyltryptophan | 0.70 | 0.48-1.02 | 6.94E-01 | 0.70 | 0.48-1.04 | 7.37E-01 |
| octadecanedioate (C18-DC) | 0.77 | 0.59-1.01 | 6.94E-01 | 0.79 | 0.61-1.02 | 7.37E-01 |
| glycocholenate sulfate* | 0.72 | 0.51-1.02 | 6.94E-01 | 0.73 | 0.52-1.03 | 7.37E-01 |
| guaiacol sulfate | 1.46 | 0.99-2.16 | 6.94E-01 | 1.45 | 0.96-2.19 | 7.37E-01 |
| N,N-dimethylalanine | 1.28 | 0.98-1.66 | 7.00E-01 | 1.28 | 0.98-1.68 | 7.37E-01 |
| oleate/vaccenate (18:1) | 0.76 | 0.56-1.03 | 7.15E-01 | 0.76 | 0.57-1.03 | 7.37E-01 |
| 1-palmitoyl-2-arachidonoyl-GPI (16:0/20:4)* | 1.30 | 0.96-1.75 | 7.27E-01 | 1.32 | 0.97-1.81 | 7.37E-01 |
| lysine | 0.77 | 0.56-1.05 | 7.64E-01 | 0.75 | 0.54-1.03 | 7.37E-01 |
| X - 12117 | 1.41 | 0.94-2.13 | 7.64E-01 | 1.47 | 0.96-2.26 | 7.37E-01 |
| X - 21353 | 0.79 | 0.6-1.04 | 7.64E-01 | 0.78 | 0.59-1.02 | 7.37E-01 |
| 1-palmitoyl-2-oleoyl-GPE (16:0/18:1) | 1.40 | 0.92-2.11 | 7.96E-01 | 1.47 | 0.96-2.25 | 7.37E-01 |
| spermidine | 0.83 | 0.63-1.08 | 8.17E-01 | 0.80 | 0.62-1.03 | 7.37E-01 |
| 3-methylxanthine | 1.30 | 0.97-1.73 | 7.15E-01 | 1.30 | 0.97-1.74 | 7.38E-01 |
| X - 21834 | 1.41 | 0.99-2 | 6.94E-01 | 1.37 | 0.96-1.96 | 7.40E-01 |
| ribonate | 1.40 | 0.96-2.03 | 7.23E-01 | 1.40 | 0.96-2.03 | 7.40E-01 |
| docosatrienoate (22:3n3) | 0.77 | 0.58-1.02 | 7.00E-01 | 0.77 | 0.57-1.03 | 7.41E-01 |
| bilirubin degradation product, C16H18N2O5 (3)** | 0.80 | 0.62-1.04 | 7.64E-01 | 0.79 | 0.6-1.03 | 7.48E-01 |
| myristoylcarnitine (C14) | 0.76 | 0.58-0.99 | 6.85E-01 | 0.79 | 0.6-1.03 | 7.54E-01 |
| alpha-hydroxyisocaproate | 0.72 | 0.51-1 | 6.94E-01 | 0.74 | 0.52-1.04 | 7.55E-01 |
| X - 23787 | 0.77 | 0.59-1.02 | 6.94E-01 | 0.77 | 0.57-1.04 | 7.55E-01 |
| androstenediol (3alpha, 17alpha) monosulfate (3) | 0.80 | 0.61-1.04 | 7.64E-01 | 0.79 | 0.6-1.04 | 7.55E-01 |
| octadecenedioate (C18:1-DC) | 0.78 | 0.58-1.05 | 7.67E-01 | 0.77 | 0.57-1.04 | 7.55E-01 |
| tetradecanedioate (C14-DC) | 0.82 | 0.64-1.04 | 7.94E-01 | 0.81 | 0.64-1.03 | 7.55E-01 |
| caprate (10:0) | 0.78 | 0.57-1.06 | 7.96E-01 | 0.77 | 0.57-1.04 | 7.55E-01 |
| X - 12261 | 0.78 | 0.56-1.09 | 8.17E-01 | 0.75 | 0.54-1.04 | 7.55E-01 |
| 1-arachidonoyl-GPC (20:4n6)* | 1.34 | 0.97-1.86 | 7.00E-01 | 1.32 | 0.95-1.83 | 7.61E-01 |
| nicotinamide | 1.25 | 0.97-1.6 | 7.23E-01 | 1.23 | 0.97-1.57 | 7.61E-01 |
| 16-hydroxypalmitate | 0.73 | 0.51-1.05 | 7.30E-01 | 0.74 | 0.52-1.05 | 7.61E-01 |
| N-stearoyltaurine | 0.80 | 0.6-1.06 | 7.96E-01 | 0.76 | 0.56-1.04 | 7.61E-01 |
| nonadecanoate (19:0) | 0.76 | 0.54-1.08 | 7.96E-01 | 0.74 | 0.52-1.05 | 7.67E-01 |
| gamma-glutamylvaline | 0.75 | 0.55-1.02 | 6.94E-01 | 0.76 | 0.56-1.05 | 7.70E-01 |
| beta-cryptoxanthin | 1.34 | 0.98-1.85 | 6.94E-01 | 1.32 | 0.93-1.88 | 7.70E-01 |
| eicosenoate (20:1) | 0.75 | 0.55-1.04 | 7.23E-01 | 0.76 | 0.55-1.06 | 7.70E-01 |
| X - 25265 | 1.28 | 0.97-1.7 | 7.23E-01 | 1.29 | 0.95-1.74 | 7.70E-01 |
| 1-arachidonoyl-GPE (20:4n6)* | 1.32 | 0.96-1.82 | 7.30E-01 | 1.29 | 0.95-1.77 | 7.70E-01 |
| tyrosine | 0.77 | 0.57-1.04 | 7.51E-01 | 0.77 | 0.56-1.07 | 7.70E-01 |
| androsterone sulfate | 0.84 | 0.68-1.03 | 7.64E-01 | 0.83 | 0.67-1.04 | 7.70E-01 |
| tyramine O-sulfate | 0.79 | 0.6-1.04 | 7.64E-01 | 0.80 | 0.61-1.05 | 7.70E-01 |
| deoxycholate | 0.79 | 0.6-1.05 | 7.67E-01 | 0.80 | 0.6-1.06 | 7.70E-01 |
| (16 or 17)-methylstearate (a19:0 or i19:0) | 0.73 | 0.49-1.07 | 7.72E-01 | 0.73 | 0.5-1.06 | 7.70E-01 |
| bilirubin (E,E)* | 0.76 | 0.54-1.07 | 7.96E-01 | 0.76 | 0.54-1.05 | 7.70E-01 |
| 1-stearoyl-2-oleoyl-GPI (18:0/18:1)* | 0.79 | 0.58-1.07 | 7.96E-01 | 0.78 | 0.58-1.06 | 7.70E-01 |
| tetradecadienoate (14:2)* | 0.78 | 0.57-1.07 | 7.96E-01 | 0.78 | 0.57-1.05 | 7.70E-01 |
| 3-hydroxypyridine glucuronide | 1.34 | 0.92-1.96 | 7.96E-01 | 1.38 | 0.92-2.08 | 7.70E-01 |
| 4-acetamidobutanoate | 1.39 | 0.91-2.11 | 7.96E-01 | 1.40 | 0.92-2.12 | 7.70E-01 |
| 3-hydroxy-3-methylglutarate | 1.39 | 0.91-2.11 | 7.96E-01 | 1.43 | 0.92-2.23 | 7.70E-01 |
| biliverdin | 0.76 | 0.54-1.08 | 7.96E-01 | 0.74 | 0.52-1.06 | 7.70E-01 |
| cortisol | 0.81 | 0.62-1.06 | 7.96E-01 | 0.81 | 0.62-1.05 | 7.70E-01 |
| N-(2-furoyl)glycine | 1.29 | 0.92-1.81 | 8.05E-01 | 1.33 | 0.93-1.91 | 7.70E-01 |
| bilirubin (Z,Z) | 0.79 | 0.58-1.08 | 8.14E-01 | 0.78 | 0.57-1.06 | 7.70E-01 |
| X - 22776 | 1.27 | 0.93-1.73 | 8.14E-01 | 1.31 | 0.95-1.8 | 7.70E-01 |
| 2-palmitoyl-GPC (16:0)* | 0.77 | 0.53-1.1 | 8.17E-01 | 0.74 | 0.5-1.08 | 7.70E-01 |
| 4-vinylphenol sulfate | 1.26 | 0.92-1.73 | 8.17E-01 | 1.29 | 0.95-1.75 | 7.70E-01 |
| 1-linoleoyl-GPI (18:2)* | 0.78 | 0.55-1.11 | 8.17E-01 | 0.75 | 0.53-1.07 | 7.70E-01 |
| trimethylamine N-oxide | 1.22 | 0.93-1.6 | 8.17E-01 | 1.24 | 0.95-1.61 | 7.70E-01 |
| alpha-CEHC sulfate | 0.80 | 0.58-1.1 | 8.17E-01 | 0.78 | 0.57-1.06 | 7.70E-01 |
| linolenoylcarnitine (C18:3)* | 0.83 | 0.64-1.08 | 8.17E-01 | 0.80 | 0.62-1.05 | 7.70E-01 |
| octadecadienedioate (C18:2-DC)* | 0.82 | 0.63-1.07 | 8.17E-01 | 0.80 | 0.61-1.04 | 7.70E-01 |
| dodecadienoate (12:2)* | 0.81 | 0.61-1.09 | 8.17E-01 | 0.80 | 0.61-1.06 | 7.70E-01 |
| anthranilate | 0.81 | 0.61-1.09 | 8.17E-01 | 0.78 | 0.58-1.05 | 7.70E-01 |
| margarate (17:0) | 0.74 | 0.5-1.1 | 8.17E-01 | 0.73 | 0.49-1.07 | 7.70E-01 |
| X - 21842 | 0.83 | 0.64-1.07 | 8.17E-01 | 0.81 | 0.62-1.05 | 7.70E-01 |
| choline phosphate | 0.81 | 0.6-1.11 | 8.24E-01 | 0.76 | 0.55-1.06 | 7.70E-01 |
| X - 21410 | 1.23 | 0.9-1.68 | 8.24E-01 | 1.31 | 0.95-1.81 | 7.70E-01 |
| hypotaurine | 0.81 | 0.58-1.12 | 8.33E-01 | 0.76 | 0.54-1.07 | 7.70E-01 |
| laurylcarnitine (C12) | 0.78 | 0.6-1.02 | 6.94E-01 | 0.81 | 0.61-1.06 | 7.70E-01 |
| X - 12015 | 1.35 | 0.97-1.89 | 7.11E-01 | 1.31 | 0.93-1.85 | 7.70E-01 |
| pregnenediol disulfate (C21H34O8S2)* | 0.76 | 0.55-1.04 | 7.23E-01 | 0.77 | 0.56-1.07 | 7.70E-01 |
| X - 24475 | 0.79 | 0.57-1.11 | 8.17E-01 | 0.77 | 0.55-1.07 | 7.70E-01 |
| glycosyl ceramide (d18:1/20:0, d16:1/22:0)* | 0.80 | 0.58-1.11 | 8.24E-01 | 0.77 | 0.55-1.07 | 7.70E-01 |
| X - 11787 | 0.79 | 0.55-1.13 | 8.30E-01 | 0.74 | 0.51-1.08 | 7.70E-01 |
| X - 11843 | 0.83 | 0.62-1.11 | 8.47E-01 | 0.80 | 0.6-1.06 | 7.70E-01 |
| myristoleoylcarnitine (C14:1)* | 0.80 | 0.64-1.01 | 6.94E-01 | 0.83 | 0.66-1.06 | 7.71E-01 |
| X - 21364 | 0.79 | 0.61-1.02 | 7.00E-01 | 0.80 | 0.6-1.07 | 7.71E-01 |
| gamma-glutamyltyrosine | 0.79 | 0.6-1.03 | 7.23E-01 | 0.80 | 0.6-1.07 | 7.71E-01 |
| 1-stearoyl-2-arachidonoyl-GPI (18:0/20:4) | 1.33 | 0.92-1.92 | 7.96E-01 | 1.33 | 0.92-1.91 | 7.71E-01 |
| 2,2'-Methylenebis(6-tert-butyl-p-cresol) | 0.74 | 0.5-1.09 | 7.96E-01 | 0.74 | 0.5-1.1 | 7.71E-01 |
| adenosine 5'-monophosphate (AMP) | 0.79 | 0.58-1.07 | 7.96E-01 | 0.79 | 0.58-1.08 | 7.71E-01 |
| X - 23974 | 0.79 | 0.59-1.05 | 7.96E-01 | 0.81 | 0.61-1.07 | 7.71E-01 |
| pregnenetriol sulfate* | 0.80 | 0.6-1.07 | 8.17E-01 | 0.80 | 0.6-1.07 | 7.71E-01 |
| bilirubin degradation product, C16H18N2O5 (4)** | 0.82 | 0.63-1.07 | 8.17E-01 | 0.81 | 0.62-1.07 | 7.71E-01 |
| pipecolate | 0.79 | 0.58-1.09 | 8.17E-01 | 0.78 | 0.57-1.08 | 7.71E-01 |
| adenosine 3',5'-cyclic monophosphate (cAMP) | 0.83 | 0.64-1.08 | 8.17E-01 | 0.82 | 0.63-1.06 | 7.71E-01 |
| cysteinylglycine | 1.25 | 0.92-1.7 | 8.17E-01 | 1.29 | 0.93-1.78 | 7.71E-01 |
| X - 23780 | 1.24 | 0.93-1.64 | 8.17E-01 | 1.24 | 0.94-1.65 | 7.71E-01 |
| stearate (18:0) | 0.77 | 0.53-1.12 | 8.23E-01 | 0.75 | 0.52-1.08 | 7.71E-01 |
| 12,13-DiHOME | 0.85 | 0.66-1.08 | 8.24E-01 | 0.82 | 0.64-1.06 | 7.71E-01 |
| 1-palmitoylglycerol (16:0) | 0.82 | 0.61-1.11 | 8.29E-01 | 0.79 | 0.59-1.07 | 7.71E-01 |
| 9,10-DiHOME | 0.86 | 0.69-1.08 | 8.29E-01 | 0.84 | 0.67-1.05 | 7.71E-01 |
| X - 17653 | 0.86 | 0.64-1.17 | 9.20E-01 | 0.79 | 0.58-1.08 | 7.71E-01 |
| (R)-3-hydroxybutyrylcarnitine | 1.09 | 0.88-1.35 | 9.39E-01 | 1.19 | 0.95-1.5 | 7.71E-01 |
| X - 12726 | 0.83 | 0.62-1.11 | 8.37E-01 | 0.80 | 0.6-1.08 | 7.76E-01 |
| branched-chain, straight-chain, or cyclopropyl 10:1 fatty acid (1)* | 0.82 | 0.62-1.1 | 8.24E-01 | 0.81 | 0.61-1.07 | 7.78E-01 |
| 3-hydroxybutyrate (BHBA) | 0.76 | 0.54-1.07 | 7.96E-01 | 0.78 | 0.55-1.09 | 7.78E-01 |
| 2-hydroxybutyrate/2-hydroxyisobutyrate | 0.78 | 0.6-1.02 | 7.00E-01 | 0.81 | 0.61-1.08 | 7.79E-01 |
| X - 21441 | 0.78 | 0.59-1.03 | 7.15E-01 | 0.80 | 0.6-1.08 | 7.79E-01 |
| alpha-ketobutyrate | 0.79 | 0.61-1.03 | 7.23E-01 | 0.81 | 0.6-1.08 | 7.79E-01 |
| X - 24531 | 1.33 | 0.96-1.84 | 7.30E-01 | 1.27 | 0.92-1.77 | 7.79E-01 |
| X - 23587 | 0.78 | 0.57-1.05 | 7.67E-01 | 0.80 | 0.59-1.08 | 7.79E-01 |
| dehydroepiandrosterone sulfate (DHEA-S) | 0.82 | 0.64-1.06 | 7.96E-01 | 0.82 | 0.63-1.07 | 7.79E-01 |
| 10-heptadecenoate (17:1n7) | 0.75 | 0.51-1.09 | 7.96E-01 | 0.76 | 0.52-1.1 | 7.79E-01 |
| epiandrosterone sulfate | 0.84 | 0.68-1.04 | 7.96E-01 | 0.85 | 0.67-1.06 | 7.79E-01 |
| 3-methylglutarylcarnitine (2) | 1.29 | 0.94-1.77 | 7.96E-01 | 1.28 | 0.91-1.81 | 7.79E-01 |
| X - 12456 | 0.80 | 0.61-1.06 | 7.96E-01 | 0.81 | 0.61-1.08 | 7.79E-01 |
| 1-stearoyl-GPG (18:0) | 0.81 | 0.6-1.09 | 8.17E-01 | 0.80 | 0.59-1.08 | 7.79E-01 |
| N-oleoyltaurine | 0.76 | 0.51-1.12 | 8.17E-01 | 0.75 | 0.5-1.11 | 7.79E-01 |
| 1-(1-enyl-palmitoyl)-2-arachidonoyl-GPC (P-16:0/20:4)* | 1.27 | 0.91-1.77 | 8.17E-01 | 1.28 | 0.91-1.81 | 7.79E-01 |
| branched-chain, straight-chain, or cyclopropyl 12:1 fatty acid* | 0.85 | 0.66-1.08 | 8.17E-01 | 0.84 | 0.66-1.07 | 7.79E-01 |
| dihomo-linoleate (20:2n6) | 0.79 | 0.57-1.1 | 8.17E-01 | 0.79 | 0.56-1.1 | 7.79E-01 |
| uridine | 0.82 | 0.61-1.1 | 8.24E-01 | 0.81 | 0.6-1.08 | 7.79E-01 |
| 2,4-di-tert-butylphenol | 0.80 | 0.57-1.12 | 8.26E-01 | 0.78 | 0.55-1.1 | 7.79E-01 |
| hexadecasphingosine (d16:1)* | 0.82 | 0.61-1.11 | 8.29E-01 | 0.79 | 0.58-1.09 | 7.79E-01 |
| glycosyl-N-behenoyl-sphingadienine (d18:2/22:0)* | 0.83 | 0.61-1.13 | 8.65E-01 | 0.79 | 0.58-1.09 | 7.79E-01 |
| glycerophosphoethanolamine | 0.81 | 0.57-1.16 | 8.83E-01 | 0.76 | 0.52-1.11 | 7.79E-01 |
| valylglycine | 0.85 | 0.64-1.13 | 8.96E-01 | 0.81 | 0.61-1.08 | 7.79E-01 |
| 1-palmitoyl-2-palmitoleoyl-GPC (16:0/16:1)* | 1.17 | 0.84-1.62 | 9.20E-01 | 1.28 | 0.91-1.79 | 7.79E-01 |
| 1-linoleoyl-2-linolenoyl-GPC (18:2/18:3)* | 0.90 | 0.69-1.18 | 9.39E-01 | 0.82 | 0.63-1.08 | 7.79E-01 |
| X - 24812 | 1.12 | 0.8-1.56 | 9.50E-01 | 1.32 | 0.91-1.93 | 7.79E-01 |
| glycerol 3-phosphate | 1.30 | 0.94-1.78 | 7.96E-01 | 1.26 | 0.91-1.75 | 7.92E-01 |
| bilirubin degradation product, C16H18N2O5 (2)** | 0.85 | 0.65-1.1 | 8.42E-01 | 0.82 | 0.63-1.08 | 7.92E-01 |
| tridecenedioate (C13:1-DC)* | 0.81 | 0.6-1.1 | 8.24E-01 | 0.81 | 0.6-1.09 | 7.96E-01 |
| 4-hydroxyhippurate | 1.27 | 0.95-1.68 | 7.72E-01 | 1.23 | 0.91-1.65 | 8.01E-01 |
| p-cresol glucuronide* | 1.42 | 0.9-2.25 | 7.96E-01 | 1.38 | 0.87-2.19 | 8.01E-01 |
| 2,3-dihydroxy-2-methylbutyrate | 1.33 | 0.92-1.93 | 7.96E-01 | 1.31 | 0.88-1.93 | 8.01E-01 |
| 1-palmitoyl-2-linoleoyl-GPE (16:0/18:2) | 1.28 | 0.91-1.81 | 8.17E-01 | 1.28 | 0.9-1.82 | 8.01E-01 |
| isoeugenol sulfate | 0.82 | 0.63-1.08 | 8.17E-01 | 0.83 | 0.63-1.09 | 8.01E-01 |
| 1-palmitoyl-2-oleoyl-GPI (16:0/18:1)* | 0.79 | 0.57-1.1 | 8.17E-01 | 0.80 | 0.58-1.1 | 8.01E-01 |
| X - 13507 | 1.24 | 0.93-1.66 | 8.17E-01 | 1.23 | 0.91-1.66 | 8.01E-01 |
| X - 15728 | 0.83 | 0.65-1.07 | 8.17E-01 | 0.84 | 0.65-1.08 | 8.01E-01 |
| sulfate* | 1.29 | 0.89-1.87 | 8.24E-01 | 1.30 | 0.9-1.87 | 8.01E-01 |
| oxindolylalanine | 0.82 | 0.6-1.1 | 8.24E-01 | 0.81 | 0.59-1.1 | 8.01E-01 |
| (2 or 3)-decenoate (10:1n7 or n8) | 0.84 | 0.65-1.09 | 8.29E-01 | 0.84 | 0.65-1.08 | 8.01E-01 |
| X - 11852 | 0.80 | 0.58-1.12 | 8.29E-01 | 0.80 | 0.57-1.11 | 8.01E-01 |
| myristoyl dihydrosphingomyelin (d18:0/14:0)* | 0.79 | 0.56-1.13 | 8.34E-01 | 0.78 | 0.54-1.12 | 8.01E-01 |
| X - 25810 | 1.22 | 0.9-1.64 | 8.34E-01 | 1.22 | 0.92-1.62 | 8.01E-01 |
| cholate | 0.83 | 0.62-1.11 | 8.42E-01 | 0.81 | 0.6-1.1 | 8.01E-01 |
| taurine | 0.77 | 0.51-1.16 | 8.52E-01 | 0.74 | 0.49-1.14 | 8.01E-01 |
| X - 11795 | 1.22 | 0.88-1.68 | 8.52E-01 | 1.25 | 0.9-1.72 | 8.01E-01 |
| 1-palmitoyl-2-dihomo-linolenoyl-GPC (16:0/20:3n3 or 6)* | 1.24 | 0.87-1.76 | 8.68E-01 | 1.25 | 0.9-1.75 | 8.01E-01 |
| 1-palmitoyl-2-docosahexaenoyl-GPE (16:0/22:6)* | 1.29 | 0.84-2 | 8.78E-01 | 1.37 | 0.87-2.14 | 8.01E-01 |
| X - 12013 | 0.83 | 0.6-1.14 | 8.79E-01 | 0.80 | 0.59-1.1 | 8.01E-01 |
| 3beta-hydroxy-5-cholestenoate | 0.81 | 0.57-1.16 | 8.83E-01 | 0.78 | 0.54-1.12 | 8.01E-01 |
| gamma-carboxyglutamate | 1.25 | 0.85-1.85 | 8.94E-01 | 1.32 | 0.88-1.99 | 8.01E-01 |
| glucuronide of piperine metabolite C17H21NO3 (4)* | 1.17 | 0.88-1.55 | 9.01E-01 | 1.22 | 0.91-1.65 | 8.01E-01 |
| X - 12112 | 1.16 | 0.89-1.51 | 9.01E-01 | 1.21 | 0.92-1.59 | 8.01E-01 |
| cis-4-decenoate (10:1n6)* | 0.78 | 0.54-1.12 | 8.17E-01 | 0.77 | 0.53-1.13 | 8.12E-01 |
| glucuronide of piperine metabolite C17H21NO3 (5)* | 1.18 | 0.88-1.58 | 8.96E-01 | 1.22 | 0.91-1.63 | 8.12E-01 |
| linoleate (18:2n6) | 0.84 | 0.61-1.15 | 8.96E-01 | 0.81 | 0.6-1.1 | 8.12E-01 |
| X - 21383 | 0.80 | 0.6-1.08 | 8.17E-01 | 0.82 | 0.6-1.11 | 8.24E-01 |
| N2,N2-dimethylguanosine | 1.31 | 0.89-1.94 | 8.24E-01 | 1.31 | 0.87-1.97 | 8.24E-01 |
| X - 25343 | 1.18 | 0.88-1.58 | 8.96E-01 | 1.23 | 0.9-1.67 | 8.24E-01 |
| X - 16580 | 1.14 | 0.82-1.57 | 9.39E-01 | 1.24 | 0.9-1.71 | 8.24E-01 |
| phenylacetylglutamine | 1.42 | 0.91-2.21 | 7.96E-01 | 1.34 | 0.86-2.08 | 8.30E-01 |
| 4-methylguaiacol sulfate | 1.25 | 0.94-1.66 | 7.96E-01 | 1.22 | 0.9-1.64 | 8.30E-01 |
| 2-aminobutyrate | 0.78 | 0.57-1.07 | 7.96E-01 | 0.81 | 0.58-1.13 | 8.30E-01 |
| carboxyethyl-GABA | 1.30 | 0.91-1.86 | 8.17E-01 | 1.26 | 0.88-1.8 | 8.30E-01 |
| sphingomyelin (d18:2/16:0, d18:1/16:1)* | 1.25 | 0.93-1.68 | 8.17E-01 | 1.21 | 0.9-1.64 | 8.30E-01 |
| X - 24328 | 0.78 | 0.54-1.11 | 8.17E-01 | 0.79 | 0.56-1.13 | 8.30E-01 |
| X - 24334 | 0.74 | 0.49-1.12 | 8.17E-01 | 0.77 | 0.51-1.15 | 8.30E-01 |
| X - 23782 | 0.77 | 0.53-1.12 | 8.24E-01 | 0.79 | 0.54-1.14 | 8.30E-01 |
| sphingosine 1-phosphate | 1.19 | 0.9-1.59 | 8.52E-01 | 1.20 | 0.91-1.58 | 8.30E-01 |
| X - 11444 | 0.83 | 0.61-1.12 | 8.52E-01 | 0.82 | 0.6-1.11 | 8.30E-01 |
| X - 18899 | 0.84 | 0.63-1.11 | 8.52E-01 | 0.84 | 0.63-1.1 | 8.30E-01 |
| 1-myristoylglycerol (14:0) | 0.83 | 0.6-1.14 | 8.83E-01 | 0.81 | 0.59-1.12 | 8.30E-01 |
| glycosyl-N-stearoyl-sphingosine (d18:1/18:0) | 0.84 | 0.62-1.13 | 8.84E-01 | 0.82 | 0.61-1.11 | 8.30E-01 |
| methionine sulfoxide | 0.82 | 0.58-1.18 | 9.01E-01 | 0.79 | 0.54-1.14 | 8.30E-01 |
| lactate | 1.15 | 0.89-1.49 | 9.01E-01 | 1.19 | 0.91-1.54 | 8.30E-01 |
| adipoylcarnitine (C6-DC) | 1.18 | 0.87-1.61 | 9.12E-01 | 1.23 | 0.9-1.69 | 8.30E-01 |
| 1-stearoyl-2-docosahexaenoyl-GPE (18:0/22:6)* | 1.22 | 0.82-1.83 | 9.20E-01 | 1.32 | 0.86-2.01 | 8.30E-01 |
| cortolone glucuronide (1) | 0.79 | 0.59-1.06 | 7.96E-01 | 0.81 | 0.59-1.12 | 8.40E-01 |
| gamma-glutamylphenylalanine | 0.75 | 0.5-1.12 | 8.17E-01 | 0.77 | 0.51-1.17 | 8.41E-01 |
| pregnenetriol disulfate* | 0.79 | 0.58-1.09 | 8.17E-01 | 0.81 | 0.58-1.13 | 8.41E-01 |
| 3-hydroxyoctanoate | 0.79 | 0.56-1.12 | 8.24E-01 | 0.81 | 0.57-1.14 | 8.41E-01 |
| 1-stearoyl-2-linoleoyl-GPE (18:0/18:2)* | 1.25 | 0.89-1.75 | 8.29E-01 | 1.25 | 0.88-1.79 | 8.41E-01 |
| 1-arachidonylglycerol (20:4) | 1.20 | 0.9-1.6 | 8.42E-01 | 1.20 | 0.9-1.62 | 8.41E-01 |
| homostachydrine* | 1.25 | 0.87-1.82 | 8.52E-01 | 1.27 | 0.86-1.87 | 8.41E-01 |
| umbelliferone sulfate | 0.79 | 0.53-1.16 | 8.52E-01 | 0.79 | 0.54-1.15 | 8.41E-01 |
| 3-amino-2-piperidone | 1.23 | 0.88-1.7 | 8.52E-01 | 1.21 | 0.89-1.65 | 8.41E-01 |
| X - 26119 | 0.84 | 0.63-1.12 | 8.52E-01 | 0.83 | 0.63-1.11 | 8.41E-01 |
| 4-ethylphenylsulfate | 1.19 | 0.88-1.6 | 8.83E-01 | 1.20 | 0.9-1.6 | 8.41E-01 |
| hexadecadienoate (16:2n6) | 0.83 | 0.6-1.15 | 8.96E-01 | 0.81 | 0.58-1.13 | 8.41E-01 |
| X - 12816 | 1.27 | 0.84-1.91 | 8.96E-01 | 1.28 | 0.86-1.91 | 8.41E-01 |
| N-palmitoylserine | 0.84 | 0.62-1.15 | 9.01E-01 | 0.82 | 0.59-1.13 | 8.41E-01 |
| ceramide (d18:2/24:1, d18:1/24:2)* | 1.15 | 0.88-1.51 | 9.20E-01 | 1.18 | 0.91-1.55 | 8.41E-01 |
| betaine | 0.84 | 0.59-1.2 | 9.20E-01 | 0.80 | 0.56-1.14 | 8.41E-01 |
| X - 11850 | 0.87 | 0.64-1.18 | 9.23E-01 | 0.83 | 0.62-1.12 | 8.41E-01 |
| X - 17325 | 1.26 | 0.91-1.75 | 8.17E-01 | 1.23 | 0.88-1.73 | 8.49E-01 |
| bilirubin degradation product, C16H18N2O5 (1)** | 0.87 | 0.67-1.12 | 9.01E-01 | 0.85 | 0.65-1.11 | 8.58E-01 |
| 1-oleoyl-2-arachidonoyl-GPE (18:1/20:4)* | 1.42 | 0.82-2.48 | 8.48E-01 | 1.40 | 0.81-2.41 | 8.61E-01 |
| 1-stearoyl-2-oleoyl-GPE (18:0/18:1) | 1.23 | 0.84-1.78 | 9.01E-01 | 1.27 | 0.86-1.88 | 8.71E-01 |
| palmitate (16:0) | 0.81 | 0.57-1.15 | 8.67E-01 | 0.81 | 0.57-1.15 | 8.72E-01 |
| stachydrine | 1.22 | 0.91-1.65 | 8.24E-01 | 1.20 | 0.89-1.62 | 8.74E-01 |
| X - 25420 | 0.83 | 0.58-1.18 | 9.12E-01 | 0.79 | 0.54-1.17 | 8.74E-01 |
| 1H-indole-7-acetic acid | 0.84 | 0.64-1.1 | 8.42E-01 | 0.85 | 0.65-1.12 | 8.76E-01 |
| histidine | 0.84 | 0.58-1.21 | 9.20E-01 | 0.80 | 0.56-1.16 | 8.76E-01 |
| dodecanedioate (C12-DC) | 0.89 | 0.66-1.19 | 9.39E-01 | 0.83 | 0.6-1.14 | 8.76E-01 |
| N-palmitoyl-sphinganine (d18:0/16:0) | 0.81 | 0.62-1.06 | 7.96E-01 | 0.85 | 0.64-1.12 | 8.78E-01 |
| X - 17685 | 1.25 | 0.9-1.74 | 8.24E-01 | 1.23 | 0.87-1.73 | 8.78E-01 |
| myristoleate (14:1n5) | 0.79 | 0.55-1.13 | 8.29E-01 | 0.81 | 0.56-1.16 | 8.78E-01 |
| X - 17354 | 1.23 | 0.9-1.69 | 8.29E-01 | 1.21 | 0.88-1.66 | 8.78E-01 |
| 2,3-dihydroxypyridine | 1.28 | 0.82-1.99 | 9.01E-01 | 1.30 | 0.83-2.03 | 8.78E-01 |
| 3-hydroxy-2-ethylpropionate | 0.85 | 0.67-1.07 | 8.17E-01 | 0.86 | 0.68-1.11 | 8.79E-01 |
| 13-HODE + 9-HODE | 0.84 | 0.63-1.11 | 8.52E-01 | 0.85 | 0.64-1.13 | 8.88E-01 |
| 1-palmitoyl-GPG (16:0)* | 0.84 | 0.62-1.13 | 8.83E-01 | 0.84 | 0.62-1.13 | 8.88E-01 |
| thioproline | 1.25 | 0.85-1.84 | 8.83E-01 | 1.24 | 0.85-1.81 | 8.88E-01 |
| tetrahydrocortisol sulfate (1) | 0.85 | 0.63-1.14 | 8.96E-01 | 0.84 | 0.63-1.13 | 8.88E-01 |
| glycosyl ceramide (d18:2/24:1, d18:1/24:2)* | 0.87 | 0.67-1.14 | 9.20E-01 | 0.85 | 0.63-1.13 | 8.88E-01 |
| hydroxyasparagine** | 1.23 | 0.81-1.87 | 9.20E-01 | 1.27 | 0.84-1.91 | 8.88E-01 |
| X - 18921 | 0.87 | 0.66-1.15 | 9.20E-01 | 0.85 | 0.65-1.12 | 8.88E-01 |
| 4-hydroxyglutamate | 1.17 | 0.83-1.63 | 9.23E-01 | 1.22 | 0.86-1.73 | 8.88E-01 |
| X - 12707 | 0.86 | 0.61-1.22 | 9.24E-01 | 0.81 | 0.56-1.17 | 8.88E-01 |
| gamma-glutamylcitrulline* | 0.89 | 0.66-1.2 | 9.39E-01 | 0.84 | 0.63-1.13 | 8.88E-01 |
| X - 17654 | 0.89 | 0.67-1.18 | 9.39E-01 | 0.86 | 0.67-1.11 | 8.88E-01 |
| 12-HETE | 0.86 | 0.65-1.13 | 8.96E-01 | 0.85 | 0.65-1.13 | 8.88E-01 |
| X - 23276 | 0.82 | 0.62-1.08 | 8.17E-01 | 0.85 | 0.65-1.13 | 8.90E-01 |
| 3-methoxycatechol sulfate (2) | 1.29 | 0.85-1.97 | 8.52E-01 | 1.28 | 0.83-1.96 | 8.90E-01 |
| docosapentaenoate (n3 DPA; 22:5n3) | 0.80 | 0.55-1.17 | 8.83E-01 | 0.80 | 0.55-1.18 | 8.90E-01 |
| pentadecanoate (15:0) | 0.82 | 0.55-1.21 | 9.20E-01 | 0.80 | 0.55-1.18 | 8.92E-01 |
| 3-indoleglyoxylic acid | 1.24 | 0.91-1.68 | 8.17E-01 | 1.18 | 0.86-1.6 | 8.93E-01 |
| N-stearoyl-sphinganine (d18:0/18:0)* | 0.81 | 0.6-1.08 | 8.17E-01 | 0.85 | 0.63-1.15 | 8.93E-01 |
| X - 21959 | 0.84 | 0.65-1.08 | 8.17E-01 | 0.86 | 0.66-1.13 | 8.93E-01 |
| hippurate | 1.20 | 0.92-1.56 | 8.24E-01 | 1.16 | 0.88-1.54 | 8.93E-01 |
| 1-stearoyl-GPE (18:0) | 1.29 | 0.88-1.87 | 8.24E-01 | 1.23 | 0.83-1.81 | 8.93E-01 |
| hydroxy-CMPF* | 0.84 | 0.65-1.09 | 8.24E-01 | 0.87 | 0.67-1.13 | 8.93E-01 |
| X - 24546 | 0.79 | 0.57-1.11 | 8.24E-01 | 0.83 | 0.58-1.17 | 8.93E-01 |
| 4-methylcatechol sulfate | 1.23 | 0.9-1.68 | 8.29E-01 | 1.19 | 0.85-1.65 | 8.93E-01 |
| 4-hydroxyphenylacetoylcarnitine | 1.20 | 0.91-1.59 | 8.29E-01 | 1.17 | 0.87-1.58 | 8.93E-01 |
| trans-3,4-methyleneheptanoate | 1.25 | 0.89-1.74 | 8.29E-01 | 1.20 | 0.86-1.68 | 8.93E-01 |
| trans-urocanate | 1.21 | 0.9-1.63 | 8.42E-01 | 1.17 | 0.88-1.56 | 8.93E-01 |
| andro steroid monosulfate C19H28O6S (1)* | 0.84 | 0.63-1.11 | 8.48E-01 | 0.86 | 0.65-1.14 | 8.93E-01 |
| glycosyl-N-(2-hydroxynervonoyl)-sphingosine (d18:1/24:1(2OH))* | 0.83 | 0.63-1.11 | 8.48E-01 | 0.86 | 0.65-1.15 | 8.93E-01 |
| X - 17367 | 1.24 | 0.88-1.76 | 8.49E-01 | 1.21 | 0.85-1.74 | 8.93E-01 |
| catechol sulfate | 1.28 | 0.86-1.9 | 8.52E-01 | 1.24 | 0.82-1.87 | 8.93E-01 |
| X - 23644 | 1.24 | 0.88-1.74 | 8.52E-01 | 1.20 | 0.85-1.7 | 8.93E-01 |
| X - 26111 | 0.86 | 0.66-1.1 | 8.52E-01 | 0.87 | 0.68-1.13 | 8.93E-01 |
| 2-hydroxynervonate* | 0.85 | 0.66-1.11 | 8.60E-01 | 0.86 | 0.66-1.12 | 8.93E-01 |
| 1-linoleoyl-2-arachidonoyl-GPC (18:2/20:4n6)* | 1.27 | 0.85-1.89 | 8.78E-01 | 1.26 | 0.83-1.9 | 8.93E-01 |
| X - 17676 | 1.28 | 0.85-1.94 | 8.78E-01 | 1.26 | 0.82-1.95 | 8.93E-01 |
| N-acetylcitrulline | 0.82 | 0.58-1.16 | 8.83E-01 | 0.82 | 0.58-1.16 | 8.93E-01 |
| tiglylcarnitine (C5:1-DC) | 0.78 | 0.52-1.19 | 8.83E-01 | 0.79 | 0.51-1.21 | 8.93E-01 |
| creatinine | 1.33 | 0.81-2.18 | 8.83E-01 | 1.30 | 0.79-2.16 | 8.93E-01 |
| X - 12096 | 1.23 | 0.86-1.74 | 8.83E-01 | 1.22 | 0.83-1.79 | 8.93E-01 |
| X - 21607 | 0.87 | 0.68-1.11 | 8.83E-01 | 0.87 | 0.67-1.12 | 8.93E-01 |
| X - 23680 | 1.19 | 0.89-1.58 | 8.83E-01 | 1.17 | 0.87-1.56 | 8.93E-01 |
| X - 11632 | 1.21 | 0.86-1.69 | 8.96E-01 | 1.21 | 0.85-1.71 | 8.93E-01 |
| docosahexaenoate (DHA; 22:6n3) | 0.82 | 0.58-1.18 | 9.01E-01 | 0.83 | 0.57-1.2 | 8.93E-01 |
| 1-methylxanthine | 1.18 | 0.87-1.59 | 9.01E-01 | 1.17 | 0.87-1.57 | 8.93E-01 |
| 7-alpha-hydroxy-3-oxo-4-cholestenoate (7-Hoca) | 0.81 | 0.55-1.19 | 9.01E-01 | 0.82 | 0.56-1.2 | 8.93E-01 |
| 17alpha-hydroxypregnenolone 3-sulfate | 0.83 | 0.6-1.16 | 9.01E-01 | 0.84 | 0.61-1.16 | 8.93E-01 |
| arachidonoylcarnitine (C20:4) | 1.33 | 0.8-2.22 | 9.01E-01 | 1.33 | 0.77-2.29 | 8.93E-01 |
| glucuronide of piperine metabolite C17H21NO3 (3)* | 0.84 | 0.61-1.15 | 9.01E-01 | 0.84 | 0.6-1.18 | 8.93E-01 |
| stearoylcarnitine (C18) | 0.84 | 0.6-1.17 | 9.09E-01 | 0.82 | 0.58-1.16 | 8.93E-01 |
| X - 15486 | 1.17 | 0.87-1.58 | 9.12E-01 | 1.16 | 0.87-1.56 | 8.93E-01 |
| oleoylcarnitine (C18:1) | 0.86 | 0.65-1.14 | 9.16E-01 | 0.86 | 0.64-1.15 | 8.93E-01 |
| 5-acetylamino-6-amino-3-methyluracil | 1.22 | 0.82-1.82 | 9.20E-01 | 1.23 | 0.83-1.84 | 8.93E-01 |
| glutarylcarnitine (C5-DC) | 1.21 | 0.82-1.77 | 9.20E-01 | 1.24 | 0.83-1.84 | 8.93E-01 |
| cyclo(leu-pro) | 1.13 | 0.87-1.47 | 9.20E-01 | 1.17 | 0.87-1.58 | 8.93E-01 |
| N-methylpipecolate | 1.16 | 0.85-1.57 | 9.20E-01 | 1.17 | 0.86-1.59 | 8.93E-01 |
| eugenol sulfate | 0.86 | 0.64-1.16 | 9.20E-01 | 0.86 | 0.65-1.15 | 8.93E-01 |
| pentose acid* | 1.18 | 0.85-1.63 | 9.20E-01 | 1.20 | 0.86-1.66 | 8.93E-01 |
| trans-4-hydroxyproline | 1.17 | 0.85-1.62 | 9.20E-01 | 1.18 | 0.86-1.62 | 8.93E-01 |
| N-acetylalanine | 1.24 | 0.82-1.89 | 9.20E-01 | 1.24 | 0.82-1.88 | 8.93E-01 |
| hypoxanthine | 1.16 | 0.85-1.59 | 9.20E-01 | 1.19 | 0.87-1.63 | 8.93E-01 |
| X - 18901 | 0.86 | 0.62-1.18 | 9.20E-01 | 0.84 | 0.61-1.16 | 8.93E-01 |
| X - 19141 | 1.19 | 0.85-1.66 | 9.20E-01 | 1.19 | 0.86-1.66 | 8.93E-01 |
| X - 25267 | 1.19 | 0.84-1.68 | 9.20E-01 | 1.21 | 0.85-1.74 | 8.93E-01 |
| N-acetylglucosamine/N-acetylgalactosamine | 1.20 | 0.81-1.78 | 9.23E-01 | 1.23 | 0.82-1.86 | 8.93E-01 |
| X - 24556 | 1.18 | 0.82-1.69 | 9.23E-01 | 1.22 | 0.85-1.75 | 8.93E-01 |
| methyl-4-hydroxybenzoate sulfate | 0.87 | 0.63-1.19 | 9.24E-01 | 0.84 | 0.61-1.17 | 8.93E-01 |
| suberoylcarnitine (C8-DC) | 1.16 | 0.82-1.63 | 9.24E-01 | 1.19 | 0.85-1.66 | 8.93E-01 |
| docosahexaenoylcholine | 0.89 | 0.67-1.17 | 9.36E-01 | 0.86 | 0.65-1.15 | 8.93E-01 |
| 2-oxoarginine* | 0.89 | 0.68-1.18 | 9.39E-01 | 0.85 | 0.63-1.15 | 8.93E-01 |
| 1-palmitoleoylglycerol (16:1)* | 1.14 | 0.82-1.58 | 9.39E-01 | 1.20 | 0.86-1.67 | 8.93E-01 |
| lactosyl-N-behenoyl-sphingosine (d18:1/22:0)* | 0.87 | 0.62-1.23 | 9.39E-01 | 0.83 | 0.59-1.17 | 8.93E-01 |
| pyruvate | 1.12 | 0.85-1.46 | 9.39E-01 | 1.18 | 0.87-1.6 | 8.93E-01 |
| X - 12847 | 1.20 | 0.77-1.88 | 9.39E-01 | 1.24 | 0.81-1.89 | 8.93E-01 |
| X - 17438 | 0.89 | 0.66-1.18 | 9.39E-01 | 0.86 | 0.65-1.14 | 8.93E-01 |
| X - 26058 | 0.87 | 0.61-1.24 | 9.39E-01 | 0.83 | 0.59-1.18 | 8.93E-01 |
| dodecenedioate (C12:1-DC)* | 0.89 | 0.67-1.2 | 9.42E-01 | 0.86 | 0.64-1.15 | 8.93E-01 |
| 1-(1-enyl-palmitoyl)-2-linoleoyl-GPC (P-16:0/18:2)* | 0.92 | 0.69-1.22 | 9.55E-01 | 0.84 | 0.61-1.16 | 8.93E-01 |
| o-cresol sulfate | 1.09 | 0.77-1.53 | 9.70E-01 | 1.24 | 0.83-1.85 | 8.93E-01 |
| X - 24757 | 1.21 | 0.89-1.66 | 8.52E-01 | 1.18 | 0.85-1.64 | 9.04E-01 |
| 3,7-dimethylurate | 1.17 | 0.85-1.62 | 9.20E-01 | 1.18 | 0.85-1.63 | 9.04E-01 |
| carnitine | 1.19 | 0.81-1.73 | 9.24E-01 | 1.22 | 0.83-1.8 | 9.04E-01 |
| corticosterone | 0.89 | 0.66-1.2 | 9.39E-01 | 0.86 | 0.63-1.17 | 9.08E-01 |
| 1-palmitoleoyl-GPC (16:1)* | 1.15 | 0.84-1.58 | 9.24E-01 | 1.18 | 0.85-1.63 | 9.10E-01 |
| palmitoyl-sphingosine-phosphoethanolamine (d18:1/16:0) | 1.21 | 0.82-1.78 | 9.20E-01 | 1.20 | 0.83-1.74 | 9.13E-01 |
| sphingomyelin (d18:1/18:1, d18:2/18:0) | 1.19 | 0.8-1.75 | 9.24E-01 | 1.21 | 0.83-1.77 | 9.13E-01 |
| eicosapentaenoate (EPA; 20:5n3) | 0.82 | 0.58-1.16 | 8.96E-01 | 0.84 | 0.59-1.19 | 9.15E-01 |
| 3-methylglutaconate | 1.24 | 0.83-1.84 | 9.01E-01 | 1.22 | 0.82-1.82 | 9.15E-01 |
| cholesterol sulfate | 1.20 | 0.84-1.72 | 9.20E-01 | 1.20 | 0.83-1.72 | 9.15E-01 |
| X - 14056 | 1.14 | 0.84-1.56 | 9.24E-01 | 1.19 | 0.84-1.68 | 9.15E-01 |
| X - 12462 | 0.92 | 0.68-1.24 | 9.55E-01 | 0.86 | 0.64-1.16 | 9.15E-01 |
| mannose | 0.76 | 0.54-1.09 | 8.14E-01 | 0.82 | 0.55-1.22 | 9.15E-01 |
| 2-oleoylglycerol (18:1) | 0.85 | 0.62-1.16 | 9.12E-01 | 0.86 | 0.64-1.16 | 9.15E-01 |
| 3-hydroxybutyroylglycine** | 1.17 | 0.87-1.56 | 9.12E-01 | 1.16 | 0.86-1.56 | 9.18E-01 |
| stearidonate (18:4n3) | 0.84 | 0.6-1.18 | 9.20E-01 | 0.84 | 0.6-1.19 | 9.18E-01 |
| theobromine | 1.16 | 0.88-1.52 | 9.01E-01 | 1.14 | 0.87-1.51 | 9.19E-01 |
| xanthosine | 1.19 | 0.83-1.71 | 9.20E-01 | 1.19 | 0.83-1.7 | 9.19E-01 |
| propionylcarnitine (C3) | 0.88 | 0.67-1.14 | 9.20E-01 | 0.87 | 0.66-1.16 | 9.19E-01 |
| isoursodeoxycholate | 1.15 | 0.86-1.53 | 9.20E-01 | 1.15 | 0.86-1.53 | 9.19E-01 |
| gamma-tocopherol/beta-tocopherol | 1.19 | 0.82-1.72 | 9.20E-01 | 1.19 | 0.83-1.69 | 9.19E-01 |
| 3-hydroxy-2-methylpyridine sulfate | 1.20 | 0.82-1.76 | 9.20E-01 | 1.22 | 0.8-1.86 | 9.19E-01 |
| erucate (22:1n9) | 0.82 | 0.56-1.21 | 9.20E-01 | 0.82 | 0.54-1.25 | 9.19E-01 |
| X - 12100 | 0.79 | 0.49-1.28 | 9.20E-01 | 0.79 | 0.49-1.28 | 9.19E-01 |
| X - 21442 | 0.86 | 0.64-1.16 | 9.20E-01 | 0.87 | 0.64-1.17 | 9.19E-01 |
| X - 21467 | 0.87 | 0.66-1.16 | 9.20E-01 | 0.87 | 0.65-1.16 | 9.19E-01 |
| 3-acetylphenol sulfate | 1.22 | 0.79-1.88 | 9.23E-01 | 1.24 | 0.79-1.95 | 9.19E-01 |
| X - 23655 | 1.22 | 0.79-1.89 | 9.24E-01 | 1.25 | 0.79-1.97 | 9.19E-01 |
| X - 11849 | 1.15 | 0.84-1.57 | 9.24E-01 | 1.15 | 0.85-1.57 | 9.19E-01 |
| 7-methylguanine | 1.13 | 0.83-1.53 | 9.39E-01 | 1.16 | 0.85-1.58 | 9.19E-01 |
| 1-linoleoyl-GPG (18:2)* | 0.88 | 0.63-1.22 | 9.39E-01 | 0.85 | 0.61-1.19 | 9.19E-01 |
| (S)-3-hydroxybutyrylcarnitine | 1.12 | 0.84-1.49 | 9.39E-01 | 1.15 | 0.86-1.53 | 9.19E-01 |
| 1-stearoyl-GPI (18:0) | 0.88 | 0.62-1.25 | 9.44E-01 | 0.85 | 0.6-1.2 | 9.19E-01 |
| 1,2-dilinoleoyl-GPC (18:2/18:2) | 0.98 | 0.75-1.27 | 9.93E-01 | 0.89 | 0.69-1.14 | 9.19E-01 |
| X - 10458 | 1.18 | 0.84-1.66 | 9.20E-01 | 1.18 | 0.83-1.69 | 9.19E-01 |
| X - 24473 | 1.15 | 0.83-1.6 | 9.24E-01 | 1.16 | 0.84-1.61 | 9.19E-01 |
| 5-methyluridine (ribothymidine) | 0.89 | 0.66-1.21 | 9.39E-01 | 0.87 | 0.64-1.17 | 9.19E-01 |
| X - 21796 | 0.89 | 0.65-1.2 | 9.39E-01 | 0.86 | 0.63-1.18 | 9.19E-01 |
| 3-hydroxyisobutyrate | 0.83 | 0.62-1.12 | 8.62E-01 | 0.87 | 0.64-1.17 | 9.20E-01 |
| X - 23665 | 0.83 | 0.57-1.2 | 9.20E-01 | 0.84 | 0.57-1.23 | 9.20E-01 |
| mannonate* | 1.10 | 0.78-1.56 | 9.68E-01 | 1.19 | 0.82-1.72 | 9.20E-01 |
| arachidate (20:0) | 0.89 | 0.61-1.29 | 9.55E-01 | 0.84 | 0.57-1.23 | 9.21E-01 |
| palmitoleoylcarnitine (C16:1)* | 0.85 | 0.66-1.11 | 8.52E-01 | 0.89 | 0.67-1.17 | 9.26E-01 |
| indolin-2-one | 1.21 | 0.87-1.7 | 8.94E-01 | 1.18 | 0.83-1.68 | 9.26E-01 |
| palmitoleate (16:1n7) | 0.82 | 0.57-1.17 | 8.96E-01 | 0.85 | 0.58-1.22 | 9.26E-01 |
| X - 12283 | 1.22 | 0.85-1.75 | 9.01E-01 | 1.18 | 0.8-1.75 | 9.26E-01 |
| 5-acetylamino-6-formylamino-3-methyluracil | 0.84 | 0.61-1.16 | 9.09E-01 | 0.86 | 0.63-1.19 | 9.26E-01 |
| undecenoylcarnitine (C11:1) | 0.87 | 0.67-1.13 | 9.09E-01 | 0.90 | 0.7-1.15 | 9.26E-01 |
| decanoylcarnitine (C10) | 0.89 | 0.71-1.11 | 9.12E-01 | 0.90 | 0.71-1.14 | 9.26E-01 |
| tryptophan betaine | 1.24 | 0.79-1.93 | 9.20E-01 | 1.23 | 0.79-1.91 | 9.26E-01 |
| dihomo-linolenoylcarnitine (C20:3n3 or 6)* | 1.17 | 0.85-1.62 | 9.20E-01 | 1.17 | 0.84-1.63 | 9.26E-01 |
| 2-methoxyhydroquinone sulfate (1) | 1.18 | 0.84-1.64 | 9.20E-01 | 1.16 | 0.83-1.62 | 9.26E-01 |
| sphingosine | 0.87 | 0.67-1.15 | 9.20E-01 | 0.88 | 0.67-1.16 | 9.26E-01 |
| X - 25790 | 1.20 | 0.83-1.75 | 9.20E-01 | 1.18 | 0.82-1.7 | 9.26E-01 |
| N-acetylputrescine | 1.12 | 0.87-1.45 | 9.23E-01 | 1.12 | 0.87-1.46 | 9.26E-01 |
| orotate | 0.77 | 0.44-1.35 | 9.23E-01 | 0.77 | 0.44-1.37 | 9.26E-01 |
| X - 25371 | 1.23 | 0.78-1.94 | 9.23E-01 | 1.24 | 0.76-2.03 | 9.26E-01 |
| N-acetylglutamate | 1.21 | 0.8-1.83 | 9.24E-01 | 1.21 | 0.79-1.86 | 9.26E-01 |
| quinate | 1.24 | 0.76-2 | 9.24E-01 | 1.24 | 0.77-2 | 9.26E-01 |
| N-acetylglycine | 0.88 | 0.66-1.17 | 9.24E-01 | 0.88 | 0.65-1.18 | 9.26E-01 |
| erythronate* | 1.22 | 0.79-1.88 | 9.24E-01 | 1.22 | 0.78-1.92 | 9.26E-01 |
| 1-(1-enyl-stearoyl)-2-arachidonoyl-GPE (P-18:0/20:4)* | 0.87 | 0.63-1.2 | 9.24E-01 | 0.86 | 0.62-1.21 | 9.26E-01 |
| sphingomyelin (d18:1/25:0, d19:0/24:1, d20:1/23:0, d19:1/24:0)* | 1.19 | 0.8-1.78 | 9.24E-01 | 1.20 | 0.8-1.81 | 9.26E-01 |
| sulfate of piperine metabolite C16H19NO3 (3)* | 0.87 | 0.63-1.2 | 9.24E-01 | 0.86 | 0.62-1.21 | 9.26E-01 |
| X - 21310 | 0.87 | 0.64-1.19 | 9.24E-01 | 0.87 | 0.64-1.19 | 9.26E-01 |
| 4-allylphenol sulfate | 0.87 | 0.6-1.25 | 9.39E-01 | 0.85 | 0.6-1.21 | 9.26E-01 |
| 6-bromotryptophan | 0.88 | 0.64-1.21 | 9.39E-01 | 0.86 | 0.62-1.19 | 9.26E-01 |
| 1-palmitoyl-GPI (16:0) | 0.89 | 0.64-1.24 | 9.45E-01 | 0.86 | 0.62-1.21 | 9.26E-01 |
| 2,3-dihydroxyisovalerate | 1.10 | 0.84-1.46 | 9.45E-01 | 1.14 | 0.86-1.52 | 9.26E-01 |
| S-methylmethionine | 0.90 | 0.64-1.27 | 9.55E-01 | 0.86 | 0.62-1.2 | 9.26E-01 |
| N-acetyl-2-aminoadipate | 1.11 | 0.79-1.57 | 9.55E-01 | 1.18 | 0.82-1.69 | 9.26E-01 |
| X - 12544 | 0.91 | 0.65-1.26 | 9.55E-01 | 0.86 | 0.61-1.22 | 9.26E-01 |
| citrulline | 0.92 | 0.67-1.26 | 9.68E-01 | 0.86 | 0.62-1.2 | 9.26E-01 |
| metabolonic lactone sulfate | 1.06 | 0.82-1.38 | 9.71E-01 | 1.14 | 0.85-1.51 | 9.26E-01 |
| linoleoylcholine* | 0.96 | 0.73-1.26 | 9.86E-01 | 0.88 | 0.67-1.17 | 9.26E-01 |
| N-stearoyl-sphingadienine (d18:2/18:0)* | 1.12 | 0.76-1.65 | 9.55E-01 | 1.18 | 0.81-1.7 | 9.29E-01 |
| 4-chlorobenzoic acid | 1.19 | 0.84-1.68 | 9.20E-01 | 1.15 | 0.83-1.59 | 9.30E-01 |
| 2-pyrrolidinone | 0.87 | 0.63-1.18 | 9.23E-01 | 0.87 | 0.63-1.2 | 9.30E-01 |
| X - 23641 | 1.20 | 0.79-1.81 | 9.24E-01 | 1.21 | 0.78-1.89 | 9.30E-01 |
| X - 17335 | 0.86 | 0.67-1.11 | 8.78E-01 | 0.90 | 0.69-1.16 | 9.32E-01 |
| 3-hydroxystachydrine* | 1.18 | 0.83-1.68 | 9.23E-01 | 1.17 | 0.82-1.67 | 9.32E-01 |
| X - 12851 | 1.15 | 0.85-1.57 | 9.23E-01 | 1.14 | 0.84-1.56 | 9.32E-01 |
| citraconate/glutaconate | 1.19 | 0.79-1.79 | 9.39E-01 | 1.20 | 0.79-1.82 | 9.32E-01 |
| paraxanthine | 1.14 | 0.86-1.52 | 9.20E-01 | 1.14 | 0.83-1.57 | 9.34E-01 |
| palmitoylcarnitine (C16) | 0.86 | 0.64-1.16 | 9.20E-01 | 0.88 | 0.64-1.2 | 9.34E-01 |
| methionine sulfone | 1.14 | 0.87-1.49 | 9.20E-01 | 1.12 | 0.86-1.47 | 9.34E-01 |
| indolelactate | 1.17 | 0.82-1.67 | 9.24E-01 | 1.17 | 0.8-1.71 | 9.34E-01 |
| lyxonate | 1.12 | 0.87-1.45 | 9.24E-01 | 1.13 | 0.85-1.5 | 9.34E-01 |
| palmitoyl dihydrosphingomyelin (d18:0/16:0)* | 0.87 | 0.62-1.22 | 9.39E-01 | 0.85 | 0.58-1.24 | 9.34E-01 |
| butyrylcarnitine (C4) | 1.11 | 0.81-1.53 | 9.50E-01 | 1.14 | 0.84-1.54 | 9.34E-01 |
| lactosyl-N-nervonoyl-sphingosine (d18:1/24:1)* | 0.88 | 0.58-1.34 | 9.55E-01 | 0.82 | 0.52-1.3 | 9.34E-01 |
| X - 18887 | 0.89 | 0.62-1.28 | 9.55E-01 | 0.86 | 0.6-1.23 | 9.34E-01 |
| 6-oxopiperidine-2-carboxylate | 0.93 | 0.69-1.25 | 9.70E-01 | 0.88 | 0.65-1.2 | 9.34E-01 |
| eicosenedioate (C20:1-DC)* | 0.94 | 0.72-1.23 | 9.72E-01 | 0.89 | 0.69-1.17 | 9.34E-01 |
| X - 21736 | 1.05 | 0.78-1.41 | 9.86E-01 | 1.14 | 0.84-1.54 | 9.34E-01 |
| threonate | 0.90 | 0.65-1.25 | 9.55E-01 | 0.86 | 0.61-1.23 | 9.36E-01 |
| N-acetylthreonine | 1.18 | 0.77-1.81 | 9.39E-01 | 1.19 | 0.79-1.79 | 9.39E-01 |
| beta-hydroxyisovalerate | 0.88 | 0.75-1.05 | 8.17E-01 | 0.93 | 0.78-1.12 | 9.42E-01 |
| carotene diol (1) | 1.21 | 0.89-1.63 | 8.52E-01 | 1.15 | 0.82-1.6 | 9.42E-01 |
| branched chain 14:0 dicarboxylic acid** | 1.15 | 0.9-1.46 | 8.94E-01 | 1.10 | 0.86-1.4 | 9.42E-01 |
| 5alpha-androstan-3beta,17beta-diol disulfate | 0.86 | 0.66-1.13 | 9.01E-01 | 0.92 | 0.68-1.24 | 9.42E-01 |
| X - 26106 | 0.86 | 0.64-1.14 | 9.01E-01 | 0.90 | 0.67-1.2 | 9.42E-01 |
| docosahexaenoylcarnitine (C22:6)* | 0.85 | 0.62-1.16 | 9.14E-01 | 0.89 | 0.64-1.24 | 9.42E-01 |
| sphingomyelin (d18:0/18:0, d19:0/17:0)* | 0.83 | 0.58-1.18 | 9.16E-01 | 0.88 | 0.61-1.28 | 9.42E-01 |
| 4-acetylphenol sulfate | 1.18 | 0.84-1.66 | 9.20E-01 | 1.14 | 0.81-1.61 | 9.42E-01 |
| 1-palmitoyl-GPE (16:0) | 1.21 | 0.82-1.79 | 9.20E-01 | 1.17 | 0.78-1.75 | 9.42E-01 |
| taurocholenate sulfate* | 0.86 | 0.63-1.17 | 9.20E-01 | 0.90 | 0.66-1.22 | 9.42E-01 |
| 16a-hydroxy DHEA 3-sulfate | 0.87 | 0.66-1.15 | 9.20E-01 | 0.89 | 0.66-1.19 | 9.42E-01 |
| sphingomyelin (d18:1/20:1, d18:2/20:0)* | 1.19 | 0.83-1.7 | 9.20E-01 | 1.14 | 0.81-1.61 | 9.42E-01 |
| 5-dodecenoylcarnitine (C12:1) | 0.89 | 0.69-1.13 | 9.20E-01 | 0.93 | 0.71-1.21 | 9.42E-01 |
| 3-hydroxyoleoylcarnitine | 0.87 | 0.65-1.17 | 9.20E-01 | 0.90 | 0.67-1.2 | 9.42E-01 |
| GlcNAc sulfate conjugate of C21H34O2 steroid** | 0.85 | 0.61-1.18 | 9.20E-01 | 0.88 | 0.63-1.23 | 9.42E-01 |
| valine | 0.93 | 0.8-1.08 | 9.20E-01 | 0.93 | 0.77-1.12 | 9.42E-01 |
| X - 12680 | 1.17 | 0.86-1.58 | 9.20E-01 | 1.12 | 0.81-1.55 | 9.42E-01 |
| X - 21471 | 0.88 | 0.67-1.15 | 9.20E-01 | 0.90 | 0.68-1.18 | 9.42E-01 |
| X - 24588 | 0.84 | 0.59-1.21 | 9.20E-01 | 0.87 | 0.58-1.3 | 9.42E-01 |
| 3-methyl-2-oxobutyrate | 0.86 | 0.63-1.19 | 9.23E-01 | 0.90 | 0.63-1.29 | 9.42E-01 |
| 3-hydroxyhippurate | 1.18 | 0.82-1.7 | 9.23E-01 | 1.16 | 0.79-1.7 | 9.42E-01 |
| arachidonoylcholine | 1.17 | 0.83-1.65 | 9.23E-01 | 1.11 | 0.78-1.59 | 9.42E-01 |
| X - 15461 | 1.21 | 0.8-1.83 | 9.23E-01 | 1.18 | 0.78-1.79 | 9.42E-01 |
| 4-vinylcatechol sulfate | 0.84 | 0.58-1.23 | 9.24E-01 | 0.87 | 0.59-1.27 | 9.42E-01 |
| hexanoylcarnitine (C6) | 0.87 | 0.63-1.19 | 9.24E-01 | 0.90 | 0.66-1.23 | 9.42E-01 |
| 1,7-dimethylurate | 1.17 | 0.81-1.7 | 9.24E-01 | 1.18 | 0.79-1.75 | 9.42E-01 |
| gulonate* | 1.18 | 0.81-1.71 | 9.24E-01 | 1.15 | 0.76-1.72 | 9.42E-01 |
| 5alpha-androstan-3beta,17beta-diol monosulfate (2) | 0.90 | 0.7-1.15 | 9.24E-01 | 0.91 | 0.7-1.19 | 9.42E-01 |
| 2'-O-methylcytidine | 1.14 | 0.84-1.55 | 9.24E-01 | 1.11 | 0.82-1.52 | 9.42E-01 |
| ceramide (d16:1/24:1, d18:1/22:1)* | 0.89 | 0.68-1.17 | 9.24E-01 | 0.92 | 0.7-1.22 | 9.42E-01 |
| 3-hydroxyhippurate sulfate | 1.17 | 0.83-1.65 | 9.24E-01 | 1.15 | 0.81-1.65 | 9.42E-01 |
| 2,6-dihydroxybenzoic acid | 1.16 | 0.82-1.64 | 9.24E-01 | 1.13 | 0.8-1.62 | 9.42E-01 |
| picolinoylglycine | 0.84 | 0.56-1.25 | 9.24E-01 | 0.85 | 0.55-1.31 | 9.42E-01 |
| N6-methyladenosine | 1.15 | 0.83-1.6 | 9.24E-01 | 1.13 | 0.82-1.57 | 9.42E-01 |
| proline | 1.19 | 0.8-1.77 | 9.24E-01 | 1.16 | 0.78-1.71 | 9.42E-01 |
| X - 14082 | 1.17 | 0.81-1.68 | 9.24E-01 | 1.15 | 0.8-1.65 | 9.42E-01 |
| 3-methylhistidine | 0.89 | 0.66-1.2 | 9.39E-01 | 0.91 | 0.68-1.22 | 9.42E-01 |
| 5,6-dihydrouridine | 1.19 | 0.77-1.82 | 9.39E-01 | 1.17 | 0.76-1.81 | 9.42E-01 |
| 2-palmitoleoyl-GPC (16:1)* | 1.13 | 0.82-1.55 | 9.39E-01 | 1.14 | 0.82-1.59 | 9.42E-01 |
| N-acetylserine | 1.19 | 0.76-1.88 | 9.39E-01 | 1.19 | 0.76-1.87 | 9.42E-01 |
| 5alpha-androstan-3alpha,17beta-diol monosulfate (1) | 0.91 | 0.73-1.14 | 9.39E-01 | 0.92 | 0.73-1.16 | 9.42E-01 |
| hydantoin-5-propionate | 0.86 | 0.59-1.27 | 9.39E-01 | 0.86 | 0.57-1.29 | 9.42E-01 |
| retinal | 0.88 | 0.63-1.22 | 9.39E-01 | 0.89 | 0.63-1.24 | 9.42E-01 |
| gamma-CEHC glucuronide* | 1.18 | 0.78-1.77 | 9.39E-01 | 1.15 | 0.76-1.73 | 9.42E-01 |
| 9-hydroxystearate | 0.91 | 0.72-1.15 | 9.39E-01 | 0.92 | 0.73-1.16 | 9.42E-01 |
| phenylacetylcarnitine | 1.14 | 0.82-1.58 | 9.39E-01 | 1.10 | 0.79-1.53 | 9.42E-01 |
| lignoceroyl sphingomyelin (d18:1/24:0) | 1.18 | 0.79-1.75 | 9.39E-01 | 1.18 | 0.78-1.77 | 9.42E-01 |
| glycodeoxycholate 3-sulfate | 0.88 | 0.64-1.2 | 9.39E-01 | 0.90 | 0.67-1.22 | 9.42E-01 |
| taurodeoxycholic acid 3-sulfate | 0.89 | 0.65-1.21 | 9.39E-01 | 0.92 | 0.68-1.24 | 9.42E-01 |
| sphingomyelin (d18:2/23:0, d18:1/23:1, d17:1/24:1)* | 1.18 | 0.79-1.77 | 9.39E-01 | 1.14 | 0.74-1.73 | 9.42E-01 |
| N,N,N-trimethyl-alanylproline betaine (TMAP) | 1.19 | 0.78-1.83 | 9.39E-01 | 1.17 | 0.76-1.8 | 9.42E-01 |
| glycine conjugate of C10H14O2 (1)* | 1.13 | 0.82-1.56 | 9.39E-01 | 1.12 | 0.81-1.54 | 9.42E-01 |
| 3-hydroxyoctanoylcarnitine (2) | 0.88 | 0.64-1.21 | 9.39E-01 | 0.90 | 0.66-1.23 | 9.42E-01 |
| butyrate/isobutyrate (4:0) | 0.88 | 0.63-1.23 | 9.39E-01 | 0.87 | 0.62-1.22 | 9.42E-01 |
| 3-phosphoglycerate | 1.14 | 0.81-1.61 | 9.39E-01 | 1.14 | 0.82-1.59 | 9.42E-01 |
| ethylmalonate | 1.14 | 0.81-1.62 | 9.39E-01 | 1.13 | 0.82-1.57 | 9.42E-01 |
| gluconate | 1.19 | 0.78-1.82 | 9.39E-01 | 1.18 | 0.76-1.84 | 9.42E-01 |
| glutarate (C5-DC) | 1.14 | 0.83-1.57 | 9.39E-01 | 1.12 | 0.81-1.55 | 9.42E-01 |
| caffeine | 1.11 | 0.85-1.44 | 9.39E-01 | 1.12 | 0.81-1.53 | 9.42E-01 |
| X - 18913 | 1.10 | 0.87-1.41 | 9.39E-01 | 1.09 | 0.84-1.41 | 9.42E-01 |
| X - 21312 | 1.14 | 0.82-1.59 | 9.39E-01 | 1.15 | 0.82-1.61 | 9.42E-01 |
| X - 21821 | 1.16 | 0.81-1.68 | 9.39E-01 | 1.13 | 0.76-1.66 | 9.42E-01 |
| 1,3,7-trimethylurate | 1.11 | 0.84-1.48 | 9.39E-01 | 1.14 | 0.83-1.56 | 9.42E-01 |
| X - 19438 | 1.14 | 0.81-1.62 | 9.41E-01 | 1.16 | 0.79-1.7 | 9.42E-01 |
| N-methylproline | 1.12 | 0.83-1.49 | 9.42E-01 | 1.09 | 0.81-1.45 | 9.42E-01 |
| 3-(3-hydroxyphenyl)propionate | 1.14 | 0.8-1.63 | 9.42E-01 | 1.12 | 0.77-1.61 | 9.42E-01 |
| gamma-glutamylhistidine | 0.87 | 0.59-1.27 | 9.42E-01 | 0.87 | 0.59-1.27 | 9.42E-01 |
| N-acetyl-aspartyl-glutamate (NAAG) | 1.13 | 0.8-1.61 | 9.45E-01 | 1.14 | 0.81-1.6 | 9.42E-01 |
| glycohyocholate | 1.12 | 0.82-1.52 | 9.45E-01 | 1.11 | 0.8-1.54 | 9.42E-01 |
| sphingomyelin (d18:1/14:0, d16:1/16:0)* | 0.89 | 0.63-1.25 | 9.45E-01 | 0.89 | 0.64-1.23 | 9.42E-01 |
| vanillactate | 1.13 | 0.81-1.56 | 9.45E-01 | 1.12 | 0.82-1.55 | 9.42E-01 |
| 1-oleoyl-2-linoleoyl-GPE (18:1/18:2)* | 1.14 | 0.79-1.64 | 9.45E-01 | 1.12 | 0.78-1.59 | 9.42E-01 |
| 4-hydroxyphenylacetylglutamine | 1.13 | 0.79-1.62 | 9.45E-01 | 1.12 | 0.77-1.63 | 9.42E-01 |
| N-palmitoyl-heptadecasphingosine (d17:1/16:0)* | 1.14 | 0.79-1.67 | 9.45E-01 | 1.12 | 0.77-1.62 | 9.42E-01 |
| glyco-beta-muricholate** | 0.91 | 0.71-1.18 | 9.45E-01 | 0.91 | 0.7-1.17 | 9.42E-01 |
| delta-CEHC | 1.14 | 0.79-1.64 | 9.45E-01 | 1.12 | 0.77-1.64 | 9.42E-01 |
| indoleacetoylcarnitine* | 1.12 | 0.81-1.54 | 9.45E-01 | 1.10 | 0.8-1.51 | 9.42E-01 |
| 2-hydroxy-4-(methylthio)butanoic acid | 0.87 | 0.6-1.28 | 9.45E-01 | 0.87 | 0.59-1.29 | 9.42E-01 |
| oleoyl ethanolamide | 0.88 | 0.62-1.26 | 9.45E-01 | 0.88 | 0.61-1.26 | 9.42E-01 |
| 1-palmitoyl-2-linoleoyl-GPC (16:0/18:2) | 1.15 | 0.77-1.72 | 9.45E-01 | 1.13 | 0.77-1.65 | 9.42E-01 |
| methylsuccinate | 1.19 | 0.73-1.93 | 9.45E-01 | 1.18 | 0.74-1.89 | 9.42E-01 |
| succinate | 0.92 | 0.71-1.18 | 9.45E-01 | 0.91 | 0.71-1.18 | 9.42E-01 |
| phenylalanine | 0.89 | 0.64-1.23 | 9.45E-01 | 0.89 | 0.64-1.25 | 9.42E-01 |
| 5-hydroxyindoleacetate | 0.89 | 0.63-1.25 | 9.45E-01 | 0.88 | 0.62-1.24 | 9.42E-01 |
| cysteine | 1.10 | 0.83-1.47 | 9.45E-01 | 1.10 | 0.83-1.48 | 9.42E-01 |
| dimethylglycine | 1.13 | 0.8-1.6 | 9.45E-01 | 1.14 | 0.79-1.63 | 9.42E-01 |
| X - 11299 | 0.90 | 0.66-1.22 | 9.45E-01 | 0.90 | 0.66-1.23 | 9.42E-01 |
| X - 17612 | 0.91 | 0.69-1.19 | 9.45E-01 | 0.90 | 0.68-1.19 | 9.42E-01 |
| X - 21327 | 0.91 | 0.7-1.19 | 9.45E-01 | 0.92 | 0.69-1.22 | 9.42E-01 |
| X - 24411 | 0.90 | 0.69-1.19 | 9.45E-01 | 0.90 | 0.68-1.19 | 9.42E-01 |
| X - 24811 | 1.17 | 0.74-1.85 | 9.45E-01 | 1.20 | 0.76-1.89 | 9.42E-01 |
| X - 24970 | 0.89 | 0.63-1.25 | 9.45E-01 | 0.89 | 0.63-1.25 | 9.42E-01 |
| X - 25520 | 1.13 | 0.8-1.59 | 9.45E-01 | 1.12 | 0.79-1.58 | 9.42E-01 |
| X - 25936 | 1.12 | 0.81-1.54 | 9.45E-01 | 1.11 | 0.8-1.55 | 9.42E-01 |
| N-acetylaspartate (NAA) | 1.12 | 0.8-1.59 | 9.50E-01 | 1.13 | 0.8-1.61 | 9.42E-01 |
| iminodiacetate (IDA) | 1.11 | 0.81-1.51 | 9.50E-01 | 1.09 | 0.81-1.47 | 9.42E-01 |
| glycolithocholate | 0.91 | 0.68-1.22 | 9.50E-01 | 0.90 | 0.66-1.22 | 9.42E-01 |
| 7-methylxanthine | 1.14 | 0.77-1.69 | 9.50E-01 | 1.14 | 0.77-1.69 | 9.42E-01 |
| beta-hydroxyisovaleroylcarnitine | 0.88 | 0.6-1.29 | 9.50E-01 | 0.88 | 0.59-1.32 | 9.42E-01 |
| pregnanediol-3-glucuronide | 0.90 | 0.66-1.24 | 9.50E-01 | 0.90 | 0.65-1.25 | 9.42E-01 |
| C-glycosyltryptophan | 0.86 | 0.54-1.35 | 9.50E-01 | 0.87 | 0.56-1.36 | 9.42E-01 |
| N,N,N-trimethyl-5-aminovalerate | 1.14 | 0.77-1.68 | 9.50E-01 | 1.13 | 0.76-1.69 | 9.42E-01 |
| 5-hydroxy-2-methylpyridine sulfate | 1.12 | 0.8-1.58 | 9.50E-01 | 1.12 | 0.78-1.61 | 9.42E-01 |
| fumarate | 1.10 | 0.83-1.47 | 9.50E-01 | 1.12 | 0.83-1.52 | 9.42E-01 |
| X - 13007 | 0.90 | 0.67-1.23 | 9.50E-01 | 0.89 | 0.65-1.23 | 9.42E-01 |
| 1,2,3-benzenetriol sulfate (2) | 0.89 | 0.63-1.26 | 9.51E-01 | 0.88 | 0.62-1.25 | 9.42E-01 |
| cystine | 1.11 | 0.81-1.51 | 9.51E-01 | 1.11 | 0.82-1.52 | 9.42E-01 |
| 3-hydroxydodecanedioate* | 0.90 | 0.66-1.23 | 9.53E-01 | 0.89 | 0.65-1.21 | 9.42E-01 |
| sphinganine-1-phosphate | 1.11 | 0.8-1.55 | 9.53E-01 | 1.14 | 0.81-1.6 | 9.42E-01 |
| oleoyl-linoleoyl-glycerol (18:1/18:2) [2] | 1.14 | 0.77-1.68 | 9.53E-01 | 1.14 | 0.74-1.75 | 9.42E-01 |
| 1-stearoyl-2-oleoyl-GPC (18:0/18:1) | 0.90 | 0.64-1.25 | 9.53E-01 | 0.91 | 0.66-1.26 | 9.42E-01 |
| 1-palmitoyl-2-stearoyl-GPC (16:0/18:0) | 0.89 | 0.62-1.27 | 9.53E-01 | 0.86 | 0.59-1.24 | 9.42E-01 |
| N-acetylvaline | 1.14 | 0.76-1.72 | 9.53E-01 | 1.19 | 0.77-1.83 | 9.42E-01 |
| 4-hydroxyphenylpyruvate | 0.91 | 0.69-1.21 | 9.53E-01 | 0.91 | 0.68-1.22 | 9.42E-01 |
| urea | 0.88 | 0.59-1.31 | 9.53E-01 | 0.88 | 0.58-1.33 | 9.42E-01 |
| X - 12104 | 1.11 | 0.81-1.51 | 9.53E-01 | 1.13 | 0.82-1.54 | 9.42E-01 |
| theophylline | 1.08 | 0.84-1.4 | 9.55E-01 | 1.09 | 0.82-1.45 | 9.42E-01 |
| maleate | 0.86 | 0.54-1.39 | 9.55E-01 | 0.88 | 0.56-1.38 | 9.42E-01 |
| galactonate | 1.12 | 0.77-1.61 | 9.55E-01 | 1.11 | 0.77-1.6 | 9.42E-01 |
| isobutyrylglycine | 1.13 | 0.76-1.67 | 9.55E-01 | 1.12 | 0.76-1.66 | 9.42E-01 |
| 4-hydroxycoumarin | 0.90 | 0.63-1.27 | 9.55E-01 | 0.88 | 0.62-1.24 | 9.42E-01 |
| N-palmitoylglycine | 0.87 | 0.57-1.35 | 9.55E-01 | 0.84 | 0.54-1.32 | 9.42E-01 |
| 3-methyl catechol sulfate (1) | 1.14 | 0.74-1.76 | 9.55E-01 | 1.18 | 0.76-1.84 | 9.42E-01 |
| 2-piperidinone | 0.91 | 0.67-1.24 | 9.55E-01 | 0.90 | 0.66-1.24 | 9.42E-01 |
| 6-hydroxyindole sulfate | 0.90 | 0.62-1.29 | 9.55E-01 | 0.88 | 0.63-1.24 | 9.42E-01 |
| ascorbic acid 2-sulfate | 1.08 | 0.82-1.43 | 9.55E-01 | 1.09 | 0.82-1.44 | 9.42E-01 |
| sulfate of piperine metabolite C16H19NO3 (2)* | 0.91 | 0.66-1.25 | 9.55E-01 | 0.90 | 0.65-1.25 | 9.42E-01 |
| 3-hydroxyhexanoylcarnitine (1) | 1.10 | 0.8-1.51 | 9.55E-01 | 1.12 | 0.82-1.53 | 9.42E-01 |
| succinoyltaurine | 1.11 | 0.79-1.56 | 9.55E-01 | 1.12 | 0.79-1.58 | 9.42E-01 |
| myo-inositol | 0.92 | 0.7-1.21 | 9.55E-01 | 0.91 | 0.69-1.2 | 9.42E-01 |
| lithocholate | 0.91 | 0.67-1.23 | 9.55E-01 | 0.91 | 0.65-1.26 | 9.42E-01 |
| ornithine | 1.09 | 0.81-1.47 | 9.55E-01 | 1.10 | 0.82-1.47 | 9.42E-01 |
| X - 22834 | 0.92 | 0.69-1.22 | 9.55E-01 | 0.92 | 0.69-1.22 | 9.42E-01 |
| X - 23636 | 1.11 | 0.79-1.56 | 9.55E-01 | 1.11 | 0.8-1.54 | 9.42E-01 |
| ectoine | 0.91 | 0.67-1.25 | 9.57E-01 | 0.90 | 0.65-1.24 | 9.42E-01 |
| 3-phenylpropionate (hydrocinnamate) | 0.92 | 0.69-1.24 | 9.68E-01 | 0.91 | 0.68-1.22 | 9.42E-01 |
| gamma-glutamylmethionine | 0.92 | 0.66-1.28 | 9.68E-01 | 0.91 | 0.64-1.28 | 9.42E-01 |
| N1-methylinosine | 1.12 | 0.73-1.71 | 9.68E-01 | 1.17 | 0.76-1.8 | 9.42E-01 |
| 3,4-dihydroxybutyrate | 1.08 | 0.81-1.44 | 9.68E-01 | 1.09 | 0.82-1.46 | 9.42E-01 |
| dihomo-linolenate (20:3n3 or n6) | 0.91 | 0.65-1.28 | 9.68E-01 | 0.90 | 0.64-1.27 | 9.42E-01 |
| 11-ketoetiocholanolone glucuronide | 0.89 | 0.59-1.34 | 9.68E-01 | 0.88 | 0.58-1.36 | 9.42E-01 |
| 1-(1-enyl-palmitoyl)-2-palmitoleoyl-GPC (P-16:0/16:1)* | 1.09 | 0.79-1.5 | 9.68E-01 | 1.11 | 0.78-1.59 | 9.42E-01 |
| eicosenoylcarnitine (C20:1)* | 0.92 | 0.68-1.25 | 9.68E-01 | 0.89 | 0.65-1.24 | 9.42E-01 |
| cis-3,4-methyleneheptanoate | 0.93 | 0.71-1.22 | 9.68E-01 | 0.91 | 0.7-1.2 | 9.42E-01 |
| N-palmitoyl-sphingosine (d18:1/16:0) | 1.10 | 0.77-1.57 | 9.68E-01 | 1.11 | 0.79-1.56 | 9.42E-01 |
| serine | 0.91 | 0.66-1.27 | 9.68E-01 | 0.89 | 0.64-1.24 | 9.42E-01 |
| X - 13553 | 0.90 | 0.61-1.32 | 9.68E-01 | 0.88 | 0.59-1.3 | 9.42E-01 |
| X - 26109 | 1.09 | 0.8-1.49 | 9.68E-01 | 1.14 | 0.81-1.6 | 9.42E-01 |
| 3-hydroxypyridine sulfate | 1.12 | 0.72-1.73 | 9.70E-01 | 1.14 | 0.73-1.78 | 9.42E-01 |
| arabitol/xylitol | 1.10 | 0.75-1.62 | 9.70E-01 | 1.12 | 0.76-1.65 | 9.42E-01 |
| X - 13729 | 0.92 | 0.66-1.28 | 9.70E-01 | 0.89 | 0.63-1.26 | 9.42E-01 |
| homocitrulline | 0.92 | 0.65-1.29 | 9.70E-01 | 0.91 | 0.65-1.26 | 9.42E-01 |
| sphingomyelin (d18:1/20:2, d18:2/20:1, d16:1/22:2)* | 0.91 | 0.63-1.33 | 9.70E-01 | 0.89 | 0.61-1.29 | 9.42E-01 |
| bilirubin degradation product, C17H20N2O5 (2)** | 0.94 | 0.71-1.23 | 9.70E-01 | 0.91 | 0.69-1.22 | 9.42E-01 |
| 1-palmitoyl-2-oleoyl-GPC (16:0/18:1) | 1.10 | 0.76-1.6 | 9.70E-01 | 1.16 | 0.8-1.68 | 9.42E-01 |
| 2-hydroxypalmitate | 0.93 | 0.67-1.29 | 9.71E-01 | 0.89 | 0.62-1.26 | 9.42E-01 |
| hexanoylglutamine | 1.06 | 0.81-1.39 | 9.71E-01 | 1.09 | 0.84-1.42 | 9.42E-01 |
| decadienedioic acid (C10:2-DC)** | 1.07 | 0.81-1.41 | 9.71E-01 | 1.13 | 0.84-1.52 | 9.42E-01 |
| X - 21258 | 0.91 | 0.63-1.34 | 9.71E-01 | 0.90 | 0.63-1.29 | 9.42E-01 |
| isoleucine | 1.08 | 0.77-1.53 | 9.71E-01 | 1.13 | 0.76-1.68 | 9.42E-01 |
| bilirubin degradation product, C17H20N2O5 (1)** | 0.94 | 0.72-1.23 | 9.72E-01 | 0.92 | 0.7-1.22 | 9.42E-01 |
| 2-aminooctanoate | 0.94 | 0.72-1.24 | 9.75E-01 | 0.92 | 0.71-1.18 | 9.42E-01 |
| N6-methyllysine | 1.06 | 0.79-1.42 | 9.80E-01 | 1.09 | 0.82-1.46 | 9.42E-01 |
| 4-allylcatechol sulfate | 0.94 | 0.68-1.3 | 9.84E-01 | 0.91 | 0.65-1.26 | 9.42E-01 |
| tartronate (hydroxymalonate) | 0.95 | 0.7-1.28 | 9.86E-01 | 0.89 | 0.66-1.2 | 9.42E-01 |
| oxalate (ethanedioate) | 0.95 | 0.71-1.27 | 9.86E-01 | 0.90 | 0.67-1.22 | 9.42E-01 |
| isobutyrylcarnitine (C4) | 0.94 | 0.66-1.34 | 9.86E-01 | 0.90 | 0.63-1.29 | 9.42E-01 |
| N-acetylphenylalanine | 1.06 | 0.74-1.52 | 9.86E-01 | 1.12 | 0.77-1.62 | 9.42E-01 |
| indole-3-carboxylate | 0.96 | 0.7-1.31 | 9.86E-01 | 0.91 | 0.66-1.26 | 9.42E-01 |
| N-acetyl-1-methylhistidine* | 1.06 | 0.76-1.48 | 9.86E-01 | 1.11 | 0.8-1.54 | 9.42E-01 |
| glycochenodeoxycholate 3-sulfate | 1.07 | 0.76-1.5 | 9.86E-01 | 1.11 | 0.78-1.57 | 9.42E-01 |
| 2-ketocaprylate | 0.97 | 0.76-1.23 | 9.86E-01 | 0.93 | 0.75-1.16 | 9.42E-01 |
| 2-hydroxystearate | 0.95 | 0.66-1.35 | 9.86E-01 | 0.90 | 0.62-1.28 | 9.42E-01 |
| methionine | 0.96 | 0.71-1.29 | 9.86E-01 | 0.91 | 0.67-1.24 | 9.42E-01 |
| asparagine | 0.95 | 0.68-1.32 | 9.86E-01 | 0.91 | 0.65-1.26 | 9.42E-01 |
| alpha-ketoglutarate | 1.06 | 0.76-1.46 | 9.86E-01 | 1.14 | 0.78-1.67 | 9.42E-01 |
| X - 17010 | 1.05 | 0.78-1.42 | 9.86E-01 | 1.10 | 0.81-1.49 | 9.42E-01 |
| X - 18886 | 1.06 | 0.72-1.55 | 9.86E-01 | 1.13 | 0.76-1.67 | 9.42E-01 |
| X - 21733 | 0.95 | 0.72-1.27 | 9.86E-01 | 0.88 | 0.64-1.2 | 9.42E-01 |
| X - 24728 | 1.06 | 0.73-1.56 | 9.86E-01 | 1.13 | 0.75-1.69 | 9.42E-01 |
| X - 26107 | 1.05 | 0.79-1.39 | 9.86E-01 | 1.09 | 0.81-1.46 | 9.42E-01 |
| glutamine | 0.97 | 0.71-1.33 | 9.92E-01 | 0.91 | 0.66-1.25 | 9.42E-01 |
| glycerophosphorylcholine (GPC) | 0.97 | 0.7-1.34 | 9.93E-01 | 0.90 | 0.64-1.25 | 9.42E-01 |
| 1-stearoyl-GPC (18:0) | 0.97 | 0.66-1.44 | 9.94E-01 | 0.89 | 0.6-1.33 | 9.42E-01 |
| 1-linoleoyl-GPC (18:2) | 0.99 | 0.75-1.32 | 9.94E-01 | 0.89 | 0.66-1.2 | 9.42E-01 |
| stearoylcholine* | 0.99 | 0.74-1.33 | 9.94E-01 | 0.92 | 0.68-1.24 | 9.42E-01 |
| isoleucylglycine | 1.14 | 0.89-1.46 | 9.14E-01 | 1.07 | 0.83-1.37 | 9.42E-01 |
| 3-hydroxyhexanoate | 0.90 | 0.71-1.14 | 9.24E-01 | 0.94 | 0.75-1.19 | 9.42E-01 |
| 1-oleoyl-GPE (18:1) | 1.15 | 0.8-1.65 | 9.39E-01 | 1.10 | 0.78-1.56 | 9.42E-01 |
| vanillic acid glycine | 1.18 | 0.78-1.8 | 9.39E-01 | 1.13 | 0.73-1.77 | 9.42E-01 |
| glucose | 0.85 | 0.55-1.3 | 9.39E-01 | 0.89 | 0.57-1.38 | 9.42E-01 |
| leucine | 0.89 | 0.65-1.22 | 9.41E-01 | 0.91 | 0.64-1.31 | 9.42E-01 |
| arginine | 1.15 | 0.79-1.68 | 9.42E-01 | 1.10 | 0.76-1.59 | 9.42E-01 |
| androstenediol (3beta,17beta) monosulfate (1) | 0.89 | 0.66-1.21 | 9.44E-01 | 0.91 | 0.67-1.25 | 9.42E-01 |
| sphingomyelin (d18:0/20:0, d16:0/22:0)* | 0.88 | 0.61-1.25 | 9.44E-01 | 0.91 | 0.62-1.32 | 9.42E-01 |
| glycine conjugate of C10H12O2* | 1.13 | 0.8-1.59 | 9.45E-01 | 1.10 | 0.77-1.59 | 9.42E-01 |
| X - 12740 | 1.12 | 0.81-1.53 | 9.45E-01 | 1.10 | 0.79-1.53 | 9.42E-01 |
| palmitoyl sphingomyelin (d18:1/16:0) | 1.14 | 0.77-1.7 | 9.50E-01 | 1.11 | 0.73-1.7 | 9.42E-01 |
| N6-carbamoylthreonyladenosine | 0.88 | 0.59-1.31 | 9.53E-01 | 0.91 | 0.62-1.32 | 9.42E-01 |
| 1-linolenoylglycerol (18:3) | 1.10 | 0.8-1.5 | 9.55E-01 | 1.08 | 0.79-1.49 | 9.42E-01 |
| docosapentaenoate (n6 DPA; 22:5n6) | 0.91 | 0.67-1.23 | 9.55E-01 | 0.92 | 0.67-1.25 | 9.42E-01 |
| glycolithocholate sulfate* | 0.93 | 0.72-1.2 | 9.55E-01 | 0.94 | 0.73-1.21 | 9.42E-01 |
| 1-dihomo-linolenylglycerol (20:3) | 1.10 | 0.81-1.49 | 9.55E-01 | 1.09 | 0.8-1.48 | 9.42E-01 |
| tetradecadienedioate (C14:2-DC)* | 0.90 | 0.64-1.27 | 9.55E-01 | 0.92 | 0.65-1.29 | 9.42E-01 |
| X - 12007 | 1.11 | 0.79-1.57 | 9.55E-01 | 1.10 | 0.78-1.54 | 9.42E-01 |
| 4-hydroxy-2-oxoglutaric acid | 1.10 | 0.77-1.57 | 9.68E-01 | 1.10 | 0.78-1.56 | 9.42E-01 |
| sphingomyelin (d18:2/24:1, d18:1/24:2)* | 1.10 | 0.78-1.54 | 9.68E-01 | 1.10 | 0.76-1.59 | 9.42E-01 |
| kynurenate | 0.90 | 0.62-1.32 | 9.68E-01 | 0.90 | 0.59-1.35 | 9.42E-01 |
| dimethyl sulfone | 0.92 | 0.65-1.29 | 9.70E-01 | 0.91 | 0.64-1.3 | 9.42E-01 |
| ximenoylcarnitine (C26:1)* | 0.92 | 0.65-1.29 | 9.70E-01 | 0.91 | 0.64-1.3 | 9.42E-01 |
| phenylpyruvate | 1.08 | 0.8-1.47 | 9.70E-01 | 1.09 | 0.79-1.5 | 9.42E-01 |
| X - 13431 | 0.93 | 0.69-1.25 | 9.70E-01 | 0.92 | 0.69-1.24 | 9.42E-01 |
| tartarate | 1.06 | 0.83-1.36 | 9.70E-01 | 1.07 | 0.83-1.38 | 9.42E-01 |
| 1-myristoyl-2-palmitoyl-GPC (14:0/16:0) | 1.09 | 0.76-1.57 | 9.70E-01 | 1.10 | 0.78-1.57 | 9.42E-01 |
| N-acetylhistidine | 1.09 | 0.76-1.59 | 9.70E-01 | 1.11 | 0.76-1.61 | 9.42E-01 |
| octadecenedioylcarnitine (C18:1-DC)* | 1.05 | 0.85-1.3 | 9.70E-01 | 1.06 | 0.86-1.29 | 9.42E-01 |
| retinol (Vitamin A) | 1.09 | 0.75-1.57 | 9.71E-01 | 1.11 | 0.75-1.64 | 9.42E-01 |
| X - 13695 | 0.91 | 0.61-1.36 | 9.71E-01 | 0.90 | 0.6-1.34 | 9.42E-01 |
| 2-hydroxyhippurate (salicylurate) | 1.06 | 0.8-1.42 | 9.76E-01 | 1.08 | 0.8-1.45 | 9.42E-01 |
| X - 12411 | 0.93 | 0.66-1.32 | 9.80E-01 | 0.92 | 0.65-1.29 | 9.42E-01 |
| 1-oleoyl-GPG (18:1)* | 1.06 | 0.79-1.43 | 9.82E-01 | 1.08 | 0.81-1.45 | 9.42E-01 |
| X - 12216 | 0.93 | 0.65-1.33 | 9.82E-01 | 0.90 | 0.63-1.3 | 9.42E-01 |
| gentisate | 0.94 | 0.7-1.27 | 9.84E-01 | 0.92 | 0.67-1.25 | 9.42E-01 |
| ferulic acid 4-sulfate | 0.93 | 0.64-1.35 | 9.84E-01 | 0.91 | 0.62-1.33 | 9.42E-01 |
| indoleacetate | 0.94 | 0.65-1.35 | 9.86E-01 | 0.91 | 0.64-1.3 | 9.42E-01 |
| 1-stearoyl-2-linoleoyl-GPI (18:0/18:2) | 0.96 | 0.68-1.35 | 9.86E-01 | 0.92 | 0.65-1.3 | 9.42E-01 |
| dihomo-linolenoyl-choline | 0.96 | 0.7-1.32 | 9.86E-01 | 0.92 | 0.66-1.27 | 9.42E-01 |
| thyroxine | 0.97 | 0.78-1.21 | 9.86E-01 | 0.94 | 0.75-1.19 | 9.42E-01 |
| 1-oleoyl-GPC (18:1) | 0.96 | 0.69-1.34 | 9.89E-01 | 0.91 | 0.65-1.28 | 9.42E-01 |
| X - 12306 | 0.98 | 0.79-1.21 | 9.89E-01 | 0.94 | 0.76-1.17 | 9.42E-01 |
| 2-hydroxy-3-methylvalerate | 1.03 | 0.73-1.44 | 9.93E-01 | 1.10 | 0.76-1.57 | 9.42E-01 |
| glycerate | 0.97 | 0.69-1.38 | 9.93E-01 | 0.91 | 0.64-1.3 | 9.42E-01 |
| palmitoylcholine | 0.99 | 0.75-1.32 | 9.94E-01 | 0.93 | 0.69-1.25 | 9.42E-01 |
| oleoylcholine | 0.98 | 0.72-1.33 | 9.94E-01 | 0.92 | 0.67-1.27 | 9.42E-01 |
| homovanillate (HVA) | 0.92 | 0.66-1.29 | 9.70E-01 | 0.92 | 0.66-1.29 | 9.46E-01 |
| gamma-glutamylthreonine | 0.93 | 0.68-1.28 | 9.76E-01 | 0.92 | 0.67-1.27 | 9.46E-01 |
| 2'-deoxyuridine | 0.93 | 0.66-1.32 | 9.82E-01 | 0.91 | 0.64-1.31 | 9.46E-01 |
| X - 23890 | 0.94 | 0.7-1.28 | 9.86E-01 | 0.93 | 0.68-1.26 | 9.49E-01 |
| N-formylanthranilic acid | 0.95 | 0.64-1.39 | 9.86E-01 | 0.91 | 0.61-1.36 | 9.50E-01 |
| gamma-glutamyl-2-aminobutyrate | 0.92 | 0.68-1.24 | 9.58E-01 | 0.92 | 0.67-1.28 | 9.52E-01 |
| X - 26097 | 1.08 | 0.81-1.42 | 9.68E-01 | 1.07 | 0.8-1.44 | 9.52E-01 |
| dopamine 4-sulfate | 0.96 | 0.71-1.3 | 9.86E-01 | 0.93 | 0.67-1.27 | 9.52E-01 |
| dihydrocaffeate sulfate (2) | 0.93 | 0.61-1.41 | 9.86E-01 | 0.90 | 0.59-1.38 | 9.52E-01 |
| 1-palmitoyl-2-linoleoyl-GPI (16:0/18:2) | 0.95 | 0.69-1.31 | 9.86E-01 | 0.92 | 0.66-1.28 | 9.52E-01 |
| X - 14939 | 0.93 | 0.68-1.27 | 9.70E-01 | 0.93 | 0.68-1.27 | 9.52E-01 |
| N-acetylarginine | 0.95 | 0.69-1.29 | 9.86E-01 | 0.93 | 0.68-1.27 | 9.52E-01 |
| X - 25422 | 0.91 | 0.62-1.33 | 9.70E-01 | 0.91 | 0.62-1.34 | 9.55E-01 |
| X - 23666 | 0.92 | 0.63-1.34 | 9.71E-01 | 0.92 | 0.64-1.32 | 9.55E-01 |
| sphingomyelin (d17:1/14:0, d16:1/15:0)* | 0.94 | 0.65-1.36 | 9.86E-01 | 0.91 | 0.62-1.34 | 9.55E-01 |
| 3-methoxytyramine sulfate | 1.08 | 0.81-1.45 | 9.68E-01 | 1.08 | 0.78-1.49 | 9.56E-01 |
| 3-(3-hydroxyphenyl)propionate sulfate | 0.94 | 0.65-1.36 | 9.86E-01 | 0.91 | 0.62-1.34 | 9.56E-01 |
| dimethylarginine (SDMA + ADMA) | 1.11 | 0.75-1.63 | 9.68E-01 | 1.10 | 0.74-1.62 | 9.57E-01 |
| cysteine sulfinic acid | 0.91 | 0.69-1.19 | 9.45E-01 | 0.93 | 0.7-1.25 | 9.58E-01 |
| X - 17351 | 1.13 | 0.77-1.66 | 9.53E-01 | 1.10 | 0.73-1.64 | 9.58E-01 |
| quinolinate | 0.91 | 0.6-1.39 | 9.75E-01 | 0.90 | 0.58-1.4 | 9.58E-01 |
| 2,3-dihydroxy-5-methylthio-4-pentenoate (DMTPA)* | 1.10 | 0.71-1.71 | 9.80E-01 | 1.11 | 0.7-1.75 | 9.58E-01 |
| X - 24243 | 0.94 | 0.68-1.3 | 9.82E-01 | 0.93 | 0.66-1.29 | 9.58E-01 |
| threonine | 0.95 | 0.68-1.33 | 9.86E-01 | 0.93 | 0.66-1.3 | 9.58E-01 |
| X - 11847 | 1.07 | 0.8-1.44 | 9.70E-01 | 1.07 | 0.8-1.42 | 9.61E-01 |
| glycoursodeoxycholate | 1.06 | 0.77-1.46 | 9.86E-01 | 1.08 | 0.77-1.51 | 9.61E-01 |
| S-carboxyethylcysteine | 1.03 | 0.7-1.51 | 9.93E-01 | 1.09 | 0.75-1.59 | 9.61E-01 |
| EDTA | 0.90 | 0.7-1.17 | 9.39E-01 | 0.94 | 0.71-1.25 | 9.64E-01 |
| methyl glucopyranoside (alpha + beta) | 0.97 | 0.75-1.24 | 9.86E-01 | 0.94 | 0.71-1.24 | 9.64E-01 |
| N-acetylasparagine | 1.13 | 0.81-1.59 | 9.45E-01 | 1.08 | 0.76-1.52 | 9.67E-01 |
| inosine | 0.91 | 0.65-1.27 | 9.59E-01 | 0.93 | 0.66-1.3 | 9.67E-01 |
| N,N-dimethyl-pro-pro | 1.14 | 0.73-1.77 | 9.60E-01 | 1.10 | 0.7-1.73 | 9.67E-01 |
| taurolithocholate 3-sulfate | 0.93 | 0.72-1.21 | 9.68E-01 | 0.94 | 0.73-1.22 | 9.67E-01 |
| cytosine | 0.92 | 0.65-1.29 | 9.70E-01 | 0.93 | 0.67-1.3 | 9.67E-01 |
| X - 11483 | 0.94 | 0.71-1.24 | 9.71E-01 | 0.94 | 0.72-1.24 | 9.67E-01 |
| 5-(galactosylhydroxy)-L-lysine | 0.92 | 0.62-1.38 | 9.80E-01 | 0.92 | 0.62-1.36 | 9.67E-01 |
| linoleoyl ethanolamide | 0.95 | 0.67-1.36 | 9.86E-01 | 0.92 | 0.64-1.33 | 9.67E-01 |
| lactosyl-N-palmitoyl-sphingosine (d18:1/16:0) | 0.95 | 0.68-1.34 | 9.86E-01 | 0.92 | 0.64-1.33 | 9.67E-01 |
| 5,6-dihydrothymine | 1.05 | 0.77-1.43 | 9.86E-01 | 1.07 | 0.79-1.45 | 9.67E-01 |
| malate | 1.07 | 0.76-1.49 | 9.86E-01 | 1.08 | 0.77-1.51 | 9.67E-01 |
| phenyllactate (PLA) | 0.92 | 0.64-1.31 | 9.70E-01 | 0.92 | 0.63-1.35 | 9.67E-01 |
| X - 12126 | 1.10 | 0.78-1.55 | 9.68E-01 | 1.08 | 0.76-1.54 | 9.67E-01 |
| androstenediol (3beta,17beta) disulfate (1) | 0.87 | 0.64-1.19 | 9.24E-01 | 0.93 | 0.65-1.34 | 9.68E-01 |
| formiminoglutamate | 0.90 | 0.68-1.19 | 9.41E-01 | 0.95 | 0.7-1.28 | 9.68E-01 |
| N-acetyltyrosine | 0.89 | 0.66-1.21 | 9.44E-01 | 0.95 | 0.7-1.29 | 9.68E-01 |
| glycine | 1.12 | 0.82-1.53 | 9.45E-01 | 1.06 | 0.77-1.47 | 9.68E-01 |
| phenylacetylglutamate | 1.13 | 0.78-1.64 | 9.51E-01 | 1.08 | 0.74-1.58 | 9.68E-01 |
| 2-stearoyl-GPE (18:0)* | 1.12 | 0.77-1.63 | 9.55E-01 | 1.08 | 0.73-1.6 | 9.68E-01 |
| behenoyl dihydrosphingomyelin (d18:0/22:0)* | 0.91 | 0.67-1.24 | 9.55E-01 | 0.94 | 0.69-1.29 | 9.68E-01 |
| glycochenodeoxycholate glucuronide (1) | 0.92 | 0.7-1.22 | 9.55E-01 | 0.95 | 0.71-1.26 | 9.68E-01 |
| 5-oxoproline | 1.11 | 0.79-1.55 | 9.55E-01 | 1.07 | 0.77-1.49 | 9.68E-01 |
| indoleacetylglutamine | 1.10 | 0.77-1.58 | 9.68E-01 | 1.07 | 0.74-1.55 | 9.68E-01 |
| fructosyllysine | 0.89 | 0.58-1.37 | 9.68E-01 | 0.91 | 0.59-1.42 | 9.68E-01 |
| 11beta-hydroxyandrosterone glucuronide | 0.92 | 0.68-1.24 | 9.68E-01 | 0.94 | 0.68-1.29 | 9.68E-01 |
| taurochenodeoxycholate | 0.92 | 0.68-1.25 | 9.68E-01 | 0.94 | 0.69-1.28 | 9.68E-01 |
| taurocholate | 0.92 | 0.67-1.27 | 9.68E-01 | 0.93 | 0.67-1.3 | 9.68E-01 |
| taurodeoxycholate | 0.93 | 0.69-1.24 | 9.68E-01 | 0.94 | 0.71-1.26 | 9.68E-01 |
| N-carbamoylalanine | 1.09 | 0.76-1.56 | 9.70E-01 | 1.07 | 0.76-1.51 | 9.68E-01 |
| tricosanoyl sphingomyelin (d18:1/23:0)* | 1.11 | 0.72-1.7 | 9.70E-01 | 1.08 | 0.7-1.67 | 9.68E-01 |
| xanthine | 0.91 | 0.62-1.33 | 9.70E-01 | 0.93 | 0.62-1.39 | 9.68E-01 |
| X - 11979 | 1.09 | 0.77-1.54 | 9.70E-01 | 1.08 | 0.75-1.54 | 9.68E-01 |
| X - 18935 | 1.08 | 0.78-1.5 | 9.70E-01 | 1.06 | 0.76-1.48 | 9.68E-01 |
| 2-acetamidophenol sulfate | 1.09 | 0.75-1.57 | 9.71E-01 | 1.08 | 0.74-1.56 | 9.68E-01 |
| malonylcarnitine | 1.08 | 0.76-1.52 | 9.75E-01 | 1.06 | 0.75-1.51 | 9.68E-01 |
| arabonate/xylonate | 1.09 | 0.74-1.6 | 9.76E-01 | 1.09 | 0.72-1.63 | 9.68E-01 |
| X - 12798 | 0.93 | 0.65-1.32 | 9.76E-01 | 0.93 | 0.67-1.31 | 9.68E-01 |
| glutamine conjugate of C7H12O2* | 0.94 | 0.69-1.27 | 9.77E-01 | 0.94 | 0.7-1.26 | 9.68E-01 |
| 3-hydroxyphenylacetoylglutamine | 0.94 | 0.71-1.25 | 9.80E-01 | 0.95 | 0.7-1.28 | 9.68E-01 |
| X - 12738 | 1.09 | 0.71-1.69 | 9.80E-01 | 1.09 | 0.7-1.69 | 9.68E-01 |
| 3-(3-amino-3-carboxypropyl)uridine* | 0.92 | 0.6-1.4 | 9.84E-01 | 0.93 | 0.62-1.39 | 9.68E-01 |
| ursodeoxycholate | 1.06 | 0.79-1.43 | 9.85E-01 | 1.06 | 0.78-1.45 | 9.68E-01 |
| imidazole lactate | 1.05 | 0.79-1.41 | 9.86E-01 | 1.07 | 0.78-1.47 | 9.68E-01 |
| 3-indoxyl sulfate | 0.94 | 0.64-1.4 | 9.86E-01 | 0.94 | 0.65-1.34 | 9.68E-01 |
| butyrylglycine | 0.95 | 0.67-1.34 | 9.86E-01 | 0.93 | 0.66-1.31 | 9.68E-01 |
| dihydroferulate | 0.96 | 0.67-1.37 | 9.86E-01 | 0.94 | 0.65-1.34 | 9.68E-01 |
| 3-methyl catechol sulfate (2) | 1.06 | 0.71-1.57 | 9.86E-01 | 1.07 | 0.72-1.61 | 9.68E-01 |
| sphingomyelin (d18:2/14:0, d18:1/14:1)* | 1.06 | 0.74-1.52 | 9.86E-01 | 1.07 | 0.74-1.55 | 9.68E-01 |
| sphingomyelin (d18:1/22:2, d18:2/22:1, d16:1/24:2)* | 0.93 | 0.59-1.45 | 9.86E-01 | 0.92 | 0.59-1.43 | 9.68E-01 |
| 5-hydroxyindole sulfate | 0.96 | 0.69-1.33 | 9.86E-01 | 0.94 | 0.68-1.31 | 9.68E-01 |
| N-palmitoyl-sphingadienine (d18:2/16:0)* | 1.04 | 0.76-1.44 | 9.86E-01 | 1.07 | 0.77-1.48 | 9.68E-01 |
| N-oleoylserine | 0.96 | 0.69-1.34 | 9.86E-01 | 0.94 | 0.66-1.32 | 9.68E-01 |
| sphingomyelin (d18:2/23:1)* | 0.93 | 0.61-1.42 | 9.86E-01 | 0.92 | 0.6-1.42 | 9.68E-01 |
| dihomo-linoleoylcarnitine (C20:2)* | 0.96 | 0.69-1.34 | 9.86E-01 | 0.93 | 0.66-1.32 | 9.68E-01 |
| trans-2-hexenoylglycine | 0.97 | 0.72-1.29 | 9.86E-01 | 0.94 | 0.7-1.27 | 9.68E-01 |
| cholesterol | 1.06 | 0.76-1.48 | 9.86E-01 | 1.07 | 0.76-1.51 | 9.68E-01 |
| X - 15503 | 0.95 | 0.64-1.41 | 9.86E-01 | 0.93 | 0.61-1.41 | 9.68E-01 |
| X - 23659 | 0.92 | 0.61-1.4 | 9.86E-01 | 0.92 | 0.6-1.39 | 9.68E-01 |
| X - 24418 | 0.95 | 0.65-1.37 | 9.86E-01 | 0.93 | 0.63-1.37 | 9.68E-01 |
| X - 24949 | 1.05 | 0.8-1.37 | 9.86E-01 | 1.05 | 0.8-1.39 | 9.68E-01 |
| X - 26108 | 1.05 | 0.78-1.41 | 9.86E-01 | 1.06 | 0.78-1.44 | 9.68E-01 |
| 3beta,7alpha-dihydroxy-5-cholestenoate | 1.03 | 0.8-1.32 | 9.89E-01 | 1.05 | 0.82-1.34 | 9.68E-01 |
| argininate* | 0.97 | 0.71-1.31 | 9.89E-01 | 0.94 | 0.68-1.31 | 9.68E-01 |
| docosatrienoate (22:3n6)* | 1.03 | 0.76-1.42 | 9.89E-01 | 1.07 | 0.75-1.51 | 9.68E-01 |
| tryptophan | 1.03 | 0.77-1.37 | 9.92E-01 | 1.07 | 0.77-1.48 | 9.68E-01 |
| (N(1) + N(8))-acetylspermidine | 0.97 | 0.71-1.32 | 9.93E-01 | 0.94 | 0.69-1.29 | 9.68E-01 |
| heptenedioate (C7:1-DC)* | 1.03 | 0.73-1.44 | 9.93E-01 | 1.07 | 0.76-1.5 | 9.68E-01 |
| 1-methylnicotinamide | 1.02 | 0.74-1.42 | 9.93E-01 | 1.06 | 0.77-1.47 | 9.68E-01 |
| X - 11315 | 0.97 | 0.62-1.5 | 9.93E-01 | 0.92 | 0.58-1.45 | 9.68E-01 |
| 1-palmitoyl-GPC (16:0) | 0.98 | 0.68-1.4 | 9.94E-01 | 0.93 | 0.65-1.34 | 9.68E-01 |
| (2,4 or 2,5)-dimethylphenol sulfate | 0.99 | 0.74-1.33 | 9.94E-01 | 1.06 | 0.78-1.45 | 9.68E-01 |
| 3-ethylcatechol sulfate (1) | 1.01 | 0.72-1.41 | 9.94E-01 | 1.07 | 0.75-1.55 | 9.68E-01 |
| androstenediol (3beta,17beta) monosulfate (2) | 0.92 | 0.74-1.15 | 9.42E-01 | 0.96 | 0.75-1.22 | 9.68E-01 |
| N-methylhydroxyproline** | 1.07 | 0.83-1.39 | 9.68E-01 | 1.05 | 0.8-1.37 | 9.68E-01 |
| kynurenine | 0.92 | 0.64-1.32 | 9.71E-01 | 0.93 | 0.63-1.39 | 9.68E-01 |
| N,N-dimethyl-5-aminovalerate | 1.08 | 0.78-1.49 | 9.71E-01 | 1.06 | 0.76-1.47 | 9.68E-01 |
| X - 24306 | 0.94 | 0.7-1.26 | 9.74E-01 | 0.95 | 0.71-1.28 | 9.68E-01 |
| homoarginine | 0.94 | 0.62-1.4 | 9.86E-01 | 0.93 | 0.61-1.41 | 9.68E-01 |
| 1-methyl-5-imidazoleacetate | 1.05 | 0.72-1.51 | 9.86E-01 | 1.06 | 0.74-1.53 | 9.68E-01 |
| arachidoylcarnitine (C20)* | 0.96 | 0.69-1.34 | 9.86E-01 | 0.94 | 0.68-1.31 | 9.68E-01 |
| X - 24518 | 1.05 | 0.71-1.55 | 9.86E-01 | 1.07 | 0.72-1.59 | 9.68E-01 |
| gamma-glutamylglycine | 0.99 | 0.68-1.44 | 9.94E-01 | 0.93 | 0.63-1.39 | 9.68E-01 |
| gamma-glutamylglutamate | 0.89 | 0.64-1.24 | 9.45E-01 | 0.94 | 0.64-1.38 | 9.69E-01 |
| 1-lignoceroyl-GPC (24:0) | 1.12 | 0.78-1.61 | 9.53E-01 | 1.06 | 0.74-1.54 | 9.69E-01 |
| 3-carboxy-4-methyl-5-propyl-2-furanpropanoate (CMPF) | 0.90 | 0.63-1.28 | 9.55E-01 | 0.94 | 0.65-1.37 | 9.69E-01 |
| 1-ribosyl-imidazoleacetate* | 1.09 | 0.82-1.46 | 9.55E-01 | 1.05 | 0.78-1.41 | 9.69E-01 |
| p-cresol sulfate | 1.12 | 0.76-1.64 | 9.59E-01 | 1.06 | 0.72-1.55 | 9.69E-01 |
| heneicosapentaenoate (21:5n3) | 0.92 | 0.68-1.25 | 9.68E-01 | 0.95 | 0.7-1.29 | 9.69E-01 |
| nisinate (24:6n3) | 0.92 | 0.67-1.27 | 9.69E-01 | 0.95 | 0.68-1.33 | 9.69E-01 |
| sphingomyelin (d18:2/24:2)* | 1.10 | 0.76-1.6 | 9.70E-01 | 1.06 | 0.73-1.54 | 9.69E-01 |
| octanoylcarnitine (C8) | 0.93 | 0.69-1.25 | 9.70E-01 | 0.95 | 0.71-1.29 | 9.69E-01 |
| cis-3,4-methyleneheptanoylglycine | 1.09 | 0.77-1.55 | 9.70E-01 | 1.06 | 0.75-1.51 | 9.69E-01 |
| dihydroorotate | 0.94 | 0.72-1.22 | 9.70E-01 | 0.96 | 0.73-1.26 | 9.69E-01 |
| 3-methoxycatechol sulfate (1) | 1.08 | 0.77-1.52 | 9.71E-01 | 1.05 | 0.75-1.48 | 9.69E-01 |
| X - 23583 | 1.07 | 0.78-1.48 | 9.71E-01 | 1.05 | 0.77-1.43 | 9.69E-01 |
| X - 13844 | 1.10 | 0.72-1.68 | 9.74E-01 | 1.07 | 0.7-1.64 | 9.69E-01 |
| X - 22509 | 1.07 | 0.78-1.46 | 9.76E-01 | 1.05 | 0.76-1.45 | 9.69E-01 |
| isovalerylglycine | 1.07 | 0.76-1.51 | 9.80E-01 | 1.05 | 0.74-1.51 | 9.69E-01 |
| oleoyl-linoleoyl-glycerol (18:1/18:2) [1] | 1.08 | 0.73-1.6 | 9.80E-01 | 1.07 | 0.71-1.62 | 9.69E-01 |
| glutamine conjugate of C6H10O2 (1)* | 1.07 | 0.78-1.47 | 9.80E-01 | 1.05 | 0.78-1.43 | 9.69E-01 |
| choline | 1.07 | 0.78-1.45 | 9.80E-01 | 1.05 | 0.76-1.46 | 9.69E-01 |
| sulfate of piperine metabolite C18H21NO3 (1)* | 0.94 | 0.69-1.28 | 9.84E-01 | 0.95 | 0.69-1.32 | 9.69E-01 |
| glycocholate | 0.94 | 0.67-1.31 | 9.84E-01 | 0.94 | 0.67-1.33 | 9.69E-01 |
| X - 24456 | 1.09 | 0.7-1.71 | 9.84E-01 | 1.08 | 0.68-1.71 | 9.69E-01 |
| xanthurenate | 0.94 | 0.66-1.35 | 9.86E-01 | 0.94 | 0.65-1.37 | 9.69E-01 |
| 1-methylurate | 1.07 | 0.72-1.59 | 9.86E-01 | 1.06 | 0.71-1.58 | 9.69E-01 |
| N2,N5-diacetylornithine | 0.93 | 0.63-1.39 | 9.86E-01 | 0.94 | 0.63-1.41 | 9.69E-01 |
| O-sulfo-L-tyrosine | 1.08 | 0.71-1.63 | 9.86E-01 | 1.06 | 0.7-1.61 | 9.69E-01 |
| etiocholanolone glucuronide | 1.04 | 0.76-1.43 | 9.86E-01 | 1.05 | 0.76-1.45 | 9.69E-01 |
| 2-hydroxyarachidate* | 0.96 | 0.76-1.22 | 9.86E-01 | 0.97 | 0.76-1.23 | 9.69E-01 |
| glucuronide of C10H18O2 (7)* | 0.94 | 0.68-1.31 | 9.86E-01 | 0.95 | 0.67-1.35 | 9.69E-01 |
| creatine | 1.05 | 0.75-1.49 | 9.86E-01 | 1.06 | 0.73-1.54 | 9.69E-01 |
| 4-hydroxyphenylacetate | 1.05 | 0.74-1.47 | 9.86E-01 | 1.05 | 0.75-1.48 | 9.69E-01 |
| X - 23593 | 1.07 | 0.7-1.62 | 9.86E-01 | 1.07 | 0.69-1.65 | 9.69E-01 |
| X - 25519 | 0.95 | 0.66-1.36 | 9.86E-01 | 0.94 | 0.65-1.37 | 9.69E-01 |
| X - 26054 | 1.06 | 0.73-1.55 | 9.86E-01 | 1.06 | 0.73-1.55 | 9.69E-01 |
| S-methylcysteine | 0.97 | 0.71-1.32 | 9.92E-01 | 0.95 | 0.71-1.28 | 9.69E-01 |
| 1-oleoylglycerol (18:1) | 1.03 | 0.72-1.46 | 9.93E-01 | 1.06 | 0.73-1.53 | 9.69E-01 |
| histidine betaine (hercynine)* | 1.03 | 0.76-1.39 | 9.93E-01 | 1.05 | 0.78-1.41 | 9.69E-01 |
| citrate | 0.97 | 0.68-1.4 | 9.93E-01 | 0.94 | 0.66-1.35 | 9.69E-01 |
| N-acetylisoleucine | 1.03 | 0.66-1.63 | 9.94E-01 | 1.07 | 0.67-1.72 | 9.69E-01 |
| 4-methoxyphenol sulfate | 0.98 | 0.7-1.36 | 9.94E-01 | 0.95 | 0.68-1.32 | 9.69E-01 |
| X - 24295 | 0.99 | 0.76-1.29 | 9.94E-01 | 1.05 | 0.79-1.39 | 9.69E-01 |
| hydroxypalmitoyl sphingomyelin (d18:1/16:0(OH))** | 1.00 | 0.7-1.43 | 9.96E-01 | 0.94 | 0.65-1.37 | 9.69E-01 |
| X - 24462 | 0.95 | 0.68-1.35 | 9.86E-01 | 0.95 | 0.66-1.36 | 9.69E-01 |
| 4-guanidinobutanoate | 0.96 | 0.7-1.31 | 9.86E-01 | 0.96 | 0.7-1.32 | 9.74E-01 |
| cis-4-decenoylcarnitine (C10:1) | 1.04 | 0.78-1.38 | 9.86E-01 | 1.04 | 0.78-1.38 | 9.74E-01 |
| prolylglycine | 0.96 | 0.68-1.35 | 9.86E-01 | 0.95 | 0.67-1.35 | 9.74E-01 |
| sphingomyelin (d18:1/24:1, d18:2/24:0)* | 0.94 | 0.65-1.34 | 9.86E-01 | 0.95 | 0.64-1.4 | 9.74E-01 |
| ethyl beta-glucopyranoside | 0.96 | 0.71-1.3 | 9.86E-01 | 0.96 | 0.7-1.31 | 9.74E-01 |
| 1-arachidonoyl-GPI (20:4)* | 0.96 | 0.68-1.36 | 9.89E-01 | 0.95 | 0.67-1.35 | 9.74E-01 |
| N6,N6,N6-trimethyllysine | 0.95 | 0.58-1.55 | 9.89E-01 | 0.94 | 0.59-1.49 | 9.74E-01 |
| X - 12127 | 1.04 | 0.75-1.43 | 9.89E-01 | 1.04 | 0.76-1.43 | 9.74E-01 |
| indolebutyrate | 0.96 | 0.66-1.4 | 9.89E-01 | 0.95 | 0.65-1.39 | 9.74E-01 |
| 3-ureidopropionate | 1.03 | 0.75-1.4 | 9.93E-01 | 1.05 | 0.76-1.43 | 9.74E-01 |
| campesterol | 0.99 | 0.7-1.39 | 9.94E-01 | 0.95 | 0.66-1.37 | 9.74E-01 |
| pyrraline | 0.98 | 0.71-1.36 | 9.94E-01 | 0.96 | 0.69-1.33 | 9.74E-01 |
| N2-acetyl,N6,N6-dimethyllysine | 1.02 | 0.76-1.37 | 9.94E-01 | 1.04 | 0.77-1.41 | 9.74E-01 |
| 2S,3R-dihydroxybutyrate | 0.90 | 0.6-1.35 | 9.68E-01 | 0.94 | 0.61-1.45 | 9.74E-01 |
| mannitol/sorbitol | 1.05 | 0.72-1.53 | 9.86E-01 | 1.05 | 0.72-1.54 | 9.74E-01 |
| N-formylmethionine | 1.06 | 0.7-1.6 | 9.86E-01 | 1.06 | 0.7-1.59 | 9.74E-01 |
| 1-stearoyl-2-linoleoyl-GPC (18:0/18:2)* | 1.02 | 0.71-1.46 | 9.94E-01 | 0.95 | 0.67-1.36 | 9.74E-01 |
| gamma-CEHC | 1.00 | 0.72-1.39 | 9.96E-01 | 0.96 | 0.69-1.33 | 9.74E-01 |
| sulfate of piperine metabolite C18H21NO3 (3)* | 0.95 | 0.7-1.3 | 9.86E-01 | 0.96 | 0.7-1.31 | 9.75E-01 |
| 2-hydroxysebacate | 1.05 | 0.73-1.52 | 9.86E-01 | 1.05 | 0.74-1.48 | 9.75E-01 |
| X - 24307 | 0.96 | 0.7-1.31 | 9.86E-01 | 0.96 | 0.71-1.31 | 9.75E-01 |
| uracil | 1.05 | 0.77-1.42 | 9.86E-01 | 1.04 | 0.77-1.41 | 9.79E-01 |
| X - 12407 | 1.06 | 0.74-1.52 | 9.86E-01 | 1.05 | 0.73-1.5 | 9.79E-01 |
| phenylacetate | 0.99 | 0.69-1.44 | 9.94E-01 | 0.95 | 0.66-1.38 | 9.79E-01 |
| 4-methyl-2-oxopentanoate | 0.92 | 0.66-1.28 | 9.68E-01 | 0.96 | 0.67-1.37 | 9.82E-01 |
| 3-methylcytidine | 0.94 | 0.75-1.18 | 9.68E-01 | 0.98 | 0.78-1.23 | 9.82E-01 |
| sphingomyelin (d18:1/22:1, d18:2/22:0, d16:1/24:1)* | 1.09 | 0.78-1.52 | 9.68E-01 | 1.04 | 0.74-1.45 | 9.82E-01 |
| glycoursodeoxycholic acid sulfate (1) | 0.92 | 0.64-1.32 | 9.70E-01 | 0.95 | 0.65-1.41 | 9.82E-01 |
| X - 12193 | 0.93 | 0.66-1.31 | 9.80E-01 | 0.96 | 0.69-1.34 | 9.82E-01 |
| X - 18922 | 0.94 | 0.68-1.29 | 9.82E-01 | 0.96 | 0.7-1.33 | 9.82E-01 |
| X - 13866 | 0.92 | 0.62-1.37 | 9.84E-01 | 0.96 | 0.65-1.41 | 9.82E-01 |
| suberate (C8-DC) | 0.94 | 0.67-1.31 | 9.86E-01 | 0.97 | 0.69-1.36 | 9.82E-01 |
| 1,3-dimethylurate | 0.95 | 0.68-1.31 | 9.86E-01 | 0.97 | 0.7-1.34 | 9.82E-01 |
| propionylglycine | 1.06 | 0.74-1.5 | 9.86E-01 | 1.04 | 0.73-1.48 | 9.82E-01 |
| adrenate (22:4n6) | 0.95 | 0.66-1.35 | 9.86E-01 | 0.96 | 0.66-1.39 | 9.82E-01 |
| 1-linoleoyl-GPE (18:2)* | 1.04 | 0.77-1.42 | 9.86E-01 | 0.96 | 0.71-1.3 | 9.82E-01 |
| 1-(1-enyl-palmitoyl)-GPC (P-16:0)* | 1.05 | 0.76-1.44 | 9.86E-01 | 0.97 | 0.68-1.38 | 9.82E-01 |
| 2-O-methylascorbic acid | 0.95 | 0.67-1.35 | 9.86E-01 | 0.95 | 0.65-1.41 | 9.82E-01 |
| 5alpha-androstan-3alpha,17beta-diol monosulfate (2) | 0.94 | 0.6-1.47 | 9.86E-01 | 0.96 | 0.61-1.51 | 9.82E-01 |
| behenoyl sphingomyelin (d18:1/22:0)* | 0.96 | 0.69-1.32 | 9.86E-01 | 0.96 | 0.7-1.32 | 9.82E-01 |
| pimeloylcarnitine/3-methyladipoylcarnitine (C7-DC) | 0.94 | 0.62-1.43 | 9.86E-01 | 0.96 | 0.65-1.42 | 9.82E-01 |
| 2-hydroxybehenate | 1.04 | 0.74-1.47 | 9.86E-01 | 1.03 | 0.73-1.46 | 9.82E-01 |
| ceramide (d18:1/17:0, d17:1/18:0)* | 0.95 | 0.66-1.36 | 9.86E-01 | 0.96 | 0.67-1.36 | 9.82E-01 |
| 3-hydroxydecanoylcarnitine | 0.95 | 0.7-1.29 | 9.86E-01 | 0.97 | 0.71-1.32 | 9.82E-01 |
| pantothenate | 0.95 | 0.6-1.48 | 9.86E-01 | 0.96 | 0.61-1.52 | 9.82E-01 |
| palmitoyl ethanolamide | 0.95 | 0.62-1.44 | 9.86E-01 | 0.95 | 0.61-1.47 | 9.82E-01 |
| S-adenosylhomocysteine (SAH) | 0.95 | 0.6-1.49 | 9.86E-01 | 0.95 | 0.6-1.51 | 9.82E-01 |
| sphinganine | 1.05 | 0.76-1.45 | 9.86E-01 | 1.03 | 0.74-1.42 | 9.82E-01 |
| X - 12221 | 1.05 | 0.78-1.42 | 9.86E-01 | 1.03 | 0.76-1.39 | 9.82E-01 |
| X - 12839 | 0.97 | 0.74-1.26 | 9.86E-01 | 0.98 | 0.75-1.27 | 9.82E-01 |
| X - 22162 | 1.05 | 0.71-1.57 | 9.86E-01 | 1.05 | 0.69-1.59 | 9.82E-01 |
| X - 25172 | 1.06 | 0.76-1.47 | 9.86E-01 | 1.03 | 0.74-1.43 | 9.82E-01 |
| N-acetylcarnosine | 0.95 | 0.62-1.46 | 9.89E-01 | 0.96 | 0.61-1.5 | 9.82E-01 |
| 1-(1-enyl-palmitoyl)-2-palmitoyl-GPC (P-16:0/16:0)* | 0.97 | 0.72-1.3 | 9.89E-01 | 0.96 | 0.69-1.34 | 9.82E-01 |
| hydroxy-N6,N6,N6-trimethyllysine* | 0.95 | 0.61-1.5 | 9.89E-01 | 0.96 | 0.62-1.49 | 9.82E-01 |
| 3-hydroxyoctanoylcarnitine (1) | 0.97 | 0.72-1.3 | 9.89E-01 | 0.97 | 0.72-1.31 | 9.82E-01 |
| 5-hydroxyhexanoate | 1.04 | 0.74-1.45 | 9.89E-01 | 1.04 | 0.75-1.45 | 9.82E-01 |
| X - 24337 | 0.96 | 0.64-1.43 | 9.92E-01 | 0.97 | 0.64-1.45 | 9.82E-01 |
| pyridoxate | 0.97 | 0.64-1.47 | 9.93E-01 | 0.96 | 0.62-1.49 | 9.82E-01 |
| 2R,3R-dihydroxybutyrate | 1.03 | 0.72-1.49 | 9.93E-01 | 1.04 | 0.72-1.51 | 9.82E-01 |
| acisoga | 1.03 | 0.74-1.42 | 9.93E-01 | 1.03 | 0.76-1.4 | 9.82E-01 |
| 4-ethylcatechol sulfate | 1.03 | 0.74-1.42 | 9.93E-01 | 1.04 | 0.73-1.46 | 9.82E-01 |
| cholic acid glucuronide | 1.02 | 0.75-1.41 | 9.93E-01 | 1.03 | 0.75-1.4 | 9.82E-01 |
| glycerol | 0.98 | 0.73-1.31 | 9.93E-01 | 1.03 | 0.77-1.38 | 9.82E-01 |
| X - 16576 | 1.02 | 0.78-1.34 | 9.93E-01 | 1.03 | 0.78-1.35 | 9.82E-01 |
| X - 24344 | 0.97 | 0.68-1.39 | 9.93E-01 | 1.03 | 0.71-1.51 | 9.82E-01 |
| X - 25948 | 1.03 | 0.72-1.46 | 9.93E-01 | 1.04 | 0.73-1.48 | 9.82E-01 |
| indolepropionate | 1.01 | 0.69-1.48 | 9.94E-01 | 0.97 | 0.66-1.43 | 9.82E-01 |
| hyocholate | 0.99 | 0.74-1.32 | 9.94E-01 | 0.98 | 0.74-1.3 | 9.82E-01 |
| N1-Methyl-2-pyridone-5-carboxamide | 1.01 | 0.69-1.48 | 9.94E-01 | 1.04 | 0.7-1.53 | 9.82E-01 |
| ergothioneine | 0.98 | 0.69-1.41 | 9.94E-01 | 1.03 | 0.72-1.48 | 9.82E-01 |
| margaroylcarnitine (C17)* | 0.98 | 0.69-1.4 | 9.94E-01 | 0.96 | 0.67-1.39 | 9.82E-01 |
| 1-linolenoyl-GPC (18:3)* | 1.02 | 0.72-1.44 | 9.94E-01 | 0.96 | 0.68-1.37 | 9.82E-01 |
| octadecanedioylcarnitine (C18-DC)* | 0.99 | 0.78-1.25 | 9.94E-01 | 0.98 | 0.79-1.21 | 9.82E-01 |
| phenol glucuronide | 0.99 | 0.69-1.43 | 9.94E-01 | 0.96 | 0.66-1.39 | 9.82E-01 |
| 1-stearoyl-2-docosahexaenoyl-GPC (18:0/22:6) | 0.99 | 0.74-1.32 | 9.94E-01 | 1.02 | 0.76-1.37 | 9.82E-01 |
| lignoceroylcarnitine (C24)* | 0.99 | 0.73-1.34 | 9.94E-01 | 0.97 | 0.71-1.32 | 9.82E-01 |
| 8-methoxykynurenate | 1.02 | 0.73-1.42 | 9.94E-01 | 1.04 | 0.72-1.49 | 9.82E-01 |
| salicylate | 1.02 | 0.76-1.36 | 9.94E-01 | 1.03 | 0.76-1.39 | 9.82E-01 |
| X - 12026 | 1.01 | 0.7-1.47 | 9.94E-01 | 0.96 | 0.64-1.44 | 9.82E-01 |
| X - 12729 | 0.99 | 0.72-1.37 | 9.94E-01 | 0.96 | 0.68-1.36 | 9.82E-01 |
| X - 12906 | 0.99 | 0.7-1.41 | 9.94E-01 | 0.96 | 0.68-1.37 | 9.82E-01 |
| X - 18888 | 0.99 | 0.74-1.33 | 9.94E-01 | 0.97 | 0.73-1.3 | 9.82E-01 |
| X - 21286 | 1.01 | 0.72-1.42 | 9.94E-01 | 0.96 | 0.67-1.37 | 9.82E-01 |
| X - 21319 | 0.98 | 0.74-1.31 | 9.94E-01 | 0.97 | 0.73-1.29 | 9.82E-01 |
| X - 23648 | 0.98 | 0.7-1.37 | 9.94E-01 | 0.97 | 0.69-1.36 | 9.82E-01 |
| X - 23997 | 1.02 | 0.74-1.39 | 9.94E-01 | 0.97 | 0.71-1.34 | 9.82E-01 |
| 1-(1-enyl-palmitoyl)-2-oleoyl-GPC (P-16:0/18:1)* | 1.00 | 0.74-1.35 | 9.94E-01 | 0.97 | 0.67-1.4 | 9.82E-01 |
| N-acetyl-2-aminooctanoate* | 1.00 | 0.73-1.38 | 9.94E-01 | 0.98 | 0.72-1.32 | 9.82E-01 |
| 4-methylhexanoylglutamine | 1.00 | 0.74-1.37 | 9.94E-01 | 1.03 | 0.76-1.39 | 9.82E-01 |
| alpha-tocopherol | 0.99 | 0.73-1.36 | 9.94E-01 | 0.97 | 0.7-1.33 | 9.82E-01 |
| N6-acetyllysine | 1.00 | 0.76-1.32 | 9.94E-01 | 0.97 | 0.74-1.28 | 9.82E-01 |
| beta-alanine | 1.00 | 0.71-1.43 | 9.94E-01 | 1.03 | 0.72-1.48 | 9.82E-01 |
| ceramide (d18:1/14:0, d16:1/16:0)* | 1.00 | 0.75-1.33 | 9.95E-01 | 1.03 | 0.78-1.38 | 9.82E-01 |
| linoleoylcarnitine (C18:2)* | 1.00 | 0.72-1.4 | 9.95E-01 | 0.96 | 0.69-1.35 | 9.82E-01 |
| 17alpha-hydroxypregnanolone glucuronide | 1.00 | 0.7-1.43 | 9.96E-01 | 1.03 | 0.72-1.48 | 9.82E-01 |
| N2-acetyl,N6-methyllysine | 1.00 | 0.75-1.33 | 9.96E-01 | 1.03 | 0.77-1.38 | 9.82E-01 |
| pro-hydroxy-pro | 1.00 | 0.66-1.53 | 9.96E-01 | 0.96 | 0.63-1.46 | 9.82E-01 |
| N-acetylmethionine | 0.99 | 0.69-1.41 | 9.94E-01 | 0.97 | 0.67-1.4 | 9.86E-01 |
| urate | 0.94 | 0.67-1.33 | 9.86E-01 | 0.97 | 0.69-1.38 | 9.88E-01 |
| isovalerate (i5:0) | 1.01 | 0.71-1.42 | 9.94E-01 | 1.03 | 0.73-1.45 | 9.88E-01 |
| trigonelline (N'-methylnicotinate) | 1.04 | 0.72-1.49 | 9.92E-01 | 1.03 | 0.72-1.45 | 9.90E-01 |
| nervonoylcarnitine (C24:1)* | 0.99 | 0.75-1.31 | 9.94E-01 | 1.02 | 0.76-1.37 | 9.91E-01 |
| carotene diol (2) | 1.09 | 0.81-1.46 | 9.55E-01 | 1.02 | 0.75-1.37 | 9.91E-01 |
| pantoate | 1.05 | 0.73-1.52 | 9.86E-01 | 1.03 | 0.69-1.53 | 9.91E-01 |
| beta-citrylglutamate | 0.94 | 0.68-1.31 | 9.86E-01 | 0.98 | 0.69-1.38 | 9.91E-01 |
| phosphoethanolamine | 1.04 | 0.81-1.34 | 9.86E-01 | 0.99 | 0.76-1.28 | 9.91E-01 |
| N-stearoyl-sphingosine (d18:1/18:0)* | 0.96 | 0.69-1.34 | 9.86E-01 | 1.02 | 0.74-1.4 | 9.91E-01 |
| 5-methylthioadenosine (MTA) | 0.96 | 0.65-1.4 | 9.86E-01 | 0.97 | 0.66-1.44 | 9.91E-01 |
| X - 12111 | 1.05 | 0.75-1.47 | 9.86E-01 | 1.02 | 0.72-1.43 | 9.91E-01 |
| 1-palmitoyl-2-docosahexaenoyl-GPC (16:0/22:6) | 0.97 | 0.73-1.28 | 9.89E-01 | 1.02 | 0.76-1.37 | 9.91E-01 |
| 3-(4-hydroxyphenyl)lactate | 0.95 | 0.6-1.51 | 9.89E-01 | 0.97 | 0.58-1.6 | 9.91E-01 |
| alpha-hydroxyisovalerate | 0.96 | 0.62-1.48 | 9.92E-01 | 1.03 | 0.63-1.68 | 9.91E-01 |
| 5-hydroxylysine | 0.97 | 0.68-1.37 | 9.93E-01 | 1.02 | 0.72-1.44 | 9.91E-01 |
| 1,2-dipalmitoyl-GPC (16:0/16:0) | 1.03 | 0.69-1.55 | 9.93E-01 | 1.02 | 0.67-1.54 | 9.91E-01 |
| erythritol | 1.03 | 0.74-1.44 | 9.93E-01 | 1.02 | 0.73-1.42 | 9.91E-01 |
| 1-linoleoylglycerol (18:2) | 1.03 | 0.72-1.47 | 9.93E-01 | 1.02 | 0.71-1.47 | 9.91E-01 |
| S-methylcysteine sulfoxide | 1.03 | 0.76-1.39 | 9.93E-01 | 1.01 | 0.76-1.36 | 9.91E-01 |
| 2-aminophenol sulfate | 1.04 | 0.67-1.61 | 9.93E-01 | 1.02 | 0.66-1.59 | 9.91E-01 |
| N-acetylkynurenine (2) | 0.97 | 0.7-1.36 | 9.93E-01 | 1.02 | 0.73-1.43 | 9.91E-01 |
| carotene diol (3) | 1.02 | 0.78-1.35 | 9.93E-01 | 0.98 | 0.75-1.3 | 9.91E-01 |
| 3-methoxytyrosine | 1.02 | 0.78-1.34 | 9.93E-01 | 1.01 | 0.77-1.34 | 9.91E-01 |
| 2-hydroxyphenylacetate | 0.96 | 0.62-1.49 | 9.93E-01 | 0.98 | 0.63-1.51 | 9.91E-01 |
| pseudouridine | 1.03 | 0.68-1.58 | 9.93E-01 | 1.02 | 0.68-1.54 | 9.91E-01 |
| X - 23639 | 1.02 | 0.75-1.4 | 9.93E-01 | 0.98 | 0.69-1.38 | 9.91E-01 |
| ribitol | 1.01 | 0.71-1.43 | 9.94E-01 | 1.02 | 0.7-1.47 | 9.91E-01 |
| acetylcarnitine (C2) | 0.99 | 0.74-1.32 | 9.94E-01 | 1.02 | 0.75-1.39 | 9.91E-01 |
| 1-methyl-4-imidazoleacetate | 0.98 | 0.63-1.51 | 9.94E-01 | 0.98 | 0.62-1.54 | 9.91E-01 |
| cyclo(gly-pro) | 1.02 | 0.73-1.42 | 9.94E-01 | 1.02 | 0.73-1.42 | 9.91E-01 |
| cinnamoylglycine | 1.01 | 0.72-1.41 | 9.94E-01 | 0.98 | 0.71-1.36 | 9.91E-01 |
| cysteinylglycine disulfide* | 0.99 | 0.73-1.35 | 9.94E-01 | 1.02 | 0.75-1.38 | 9.91E-01 |
| androsterone glucuronide | 1.02 | 0.71-1.45 | 9.94E-01 | 1.02 | 0.71-1.47 | 9.91E-01 |
| sphingomyelin (d18:1/19:0, d19:1/18:0)* | 0.99 | 0.63-1.55 | 9.94E-01 | 0.98 | 0.61-1.57 | 9.91E-01 |
| 3-carboxy-4-methyl-5-pentyl-2-furanpropionate (3-CMPFP)** | 1.02 | 0.69-1.51 | 9.94E-01 | 1.02 | 0.68-1.53 | 9.91E-01 |
| glutamine conjugate of C6H10O2 (2)* | 1.01 | 0.74-1.38 | 9.94E-01 | 1.02 | 0.76-1.36 | 9.91E-01 |
| N-carbamoylvaline | 1.01 | 0.7-1.45 | 9.94E-01 | 1.02 | 0.72-1.45 | 9.91E-01 |
| deoxycholic acid glucuronide | 0.99 | 0.73-1.35 | 9.94E-01 | 1.01 | 0.75-1.37 | 9.91E-01 |
| allantoin | 1.02 | 0.74-1.39 | 9.94E-01 | 0.98 | 0.73-1.33 | 9.91E-01 |
| N-acetylleucine | 0.99 | 0.66-1.48 | 9.94E-01 | 1.02 | 0.68-1.53 | 9.91E-01 |
| guanidinoacetate | 1.01 | 0.74-1.39 | 9.94E-01 | 0.98 | 0.72-1.33 | 9.91E-01 |
| X - 12101 | 1.02 | 0.72-1.44 | 9.94E-01 | 1.02 | 0.74-1.41 | 9.91E-01 |
| X - 12822 | 1.02 | 0.7-1.49 | 9.94E-01 | 1.02 | 0.68-1.52 | 9.91E-01 |
| X - 24422 | 0.98 | 0.74-1.31 | 9.94E-01 | 0.99 | 0.72-1.35 | 9.91E-01 |
| X - 24494 | 0.98 | 0.68-1.43 | 9.94E-01 | 0.98 | 0.66-1.45 | 9.91E-01 |
| N-acetylproline | 1.00 | 0.68-1.48 | 9.94E-01 | 0.98 | 0.66-1.46 | 9.91E-01 |
| 1-oleoyl-2-docosahexaenoyl-GPC (18:1/22:6)* | 1.00 | 0.73-1.36 | 9.94E-01 | 0.98 | 0.72-1.33 | 9.91E-01 |
| 4-vinylguaiacol sulfate | 1.00 | 0.69-1.46 | 9.96E-01 | 1.02 | 0.68-1.53 | 9.91E-01 |
| N-acetylglutamine | 1.00 | 0.74-1.36 | 9.97E-01 | 1.02 | 0.75-1.37 | 9.91E-01 |
| N6,N6-dimethyllysine | 1.00 | 0.72-1.4 | 9.97E-01 | 1.02 | 0.73-1.43 | 9.91E-01 |
| ethyl alpha-glucopyranoside | 0.94 | 0.73-1.21 | 9.70E-01 | 1.01 | 0.74-1.4 | 9.92E-01 |
| 2-hydroxyoctanoate | 1.04 | 0.82-1.32 | 9.86E-01 | 1.01 | 0.8-1.27 | 9.92E-01 |
| aspartate | 0.96 | 0.73-1.25 | 9.86E-01 | 1.01 | 0.75-1.37 | 9.92E-01 |
| arachidonate (20:4n6) | 1.03 | 0.74-1.45 | 9.93E-01 | 1.01 | 0.71-1.45 | 9.92E-01 |
| dihydroferulic acid sulfate | 1.03 | 0.7-1.52 | 9.93E-01 | 1.02 | 0.68-1.52 | 9.92E-01 |
| N-acetyl-beta-alanine | 0.98 | 0.73-1.33 | 9.94E-01 | 1.01 | 0.74-1.39 | 9.92E-01 |
| glycosyl-N-palmitoyl-sphingosine (d18:1/16:0) | 0.98 | 0.69-1.39 | 9.94E-01 | 0.98 | 0.67-1.45 | 9.92E-01 |
| ascorbic acid 3-sulfate* | 0.99 | 0.71-1.39 | 9.94E-01 | 0.99 | 0.69-1.41 | 9.92E-01 |
| cystathionine | 0.98 | 0.64-1.51 | 9.94E-01 | 0.98 | 0.63-1.54 | 9.92E-01 |
| taurochenodeoxycholic acid 3-sulfate | 0.96 | 0.7-1.32 | 9.86E-01 | 0.99 | 0.72-1.36 | 9.93E-01 |
| N-delta-acetylornithine | 0.99 | 0.7-1.4 | 9.94E-01 | 0.99 | 0.7-1.39 | 9.93E-01 |
| methyl indole-3-acetate | 1.00 | 0.66-1.52 | 9.95E-01 | 0.99 | 0.67-1.45 | 9.93E-01 |
| cys-gly, oxidized | 1.00 | 0.73-1.37 | 9.95E-01 | 1.01 | 0.74-1.39 | 9.93E-01 |
| X - 16124 | 1.04 | 0.78-1.4 | 9.86E-01 | 1.01 | 0.75-1.36 | 9.94E-01 |
| cis-3,4-methyleneheptanoylcarnitine | 1.02 | 0.76-1.37 | 9.93E-01 | 1.01 | 0.75-1.37 | 9.94E-01 |
| 2-methylserine | 1.05 | 0.79-1.41 | 9.86E-01 | 0.99 | 0.72-1.35 | 9.95E-01 |
| 2-naphthol sulfate | 0.91 | 0.64-1.3 | 9.70E-01 | 1.01 | 0.64-1.62 | 9.96E-01 |
| sphingomyelin (d18:2/18:1)* | 1.07 | 0.77-1.5 | 9.80E-01 | 1.01 | 0.71-1.43 | 9.96E-01 |
| gamma-glutamylisoleucine* | 0.96 | 0.71-1.31 | 9.86E-01 | 0.99 | 0.7-1.4 | 9.96E-01 |
| sphingomyelin (d17:1/16:0, d18:1/15:0, d16:1/17:0)* | 1.05 | 0.72-1.51 | 9.86E-01 | 0.99 | 0.67-1.45 | 9.96E-01 |
| 1-methyl-5-imidazolelactate | 0.96 | 0.69-1.33 | 9.86E-01 | 1.01 | 0.73-1.39 | 9.96E-01 |
| sphingomyelin (d18:1/20:0, d16:1/22:0)* | 1.02 | 0.74-1.43 | 9.93E-01 | 0.99 | 0.71-1.38 | 9.96E-01 |
| 1-palmitoleoyl-2-linolenoyl-GPC (16:1/18:3)* | 1.03 | 0.74-1.44 | 9.93E-01 | 1.01 | 0.72-1.41 | 9.96E-01 |
| 2-methylbutyrylcarnitine (C5) | 0.99 | 0.73-1.35 | 9.94E-01 | 0.99 | 0.72-1.37 | 9.96E-01 |
| sphingomyelin (d18:1/17:0, d17:1/18:0, d19:1/16:0) | 1.02 | 0.66-1.57 | 9.94E-01 | 0.99 | 0.63-1.56 | 9.96E-01 |
| sphingomyelin (d18:1/21:0, d17:1/22:0, d16:1/23:0)* | 1.02 | 0.69-1.51 | 9.94E-01 | 0.99 | 0.66-1.48 | 9.96E-01 |
| cerotoylcarnitine (C26)* | 1.01 | 0.69-1.49 | 9.94E-01 | 0.99 | 0.68-1.44 | 9.96E-01 |
| vanillylmandelate (VMA) | 1.02 | 0.63-1.66 | 9.94E-01 | 0.99 | 0.6-1.63 | 9.96E-01 |
| stearoyl sphingomyelin (d18:1/18:0) | 0.98 | 0.65-1.48 | 9.94E-01 | 0.99 | 0.66-1.49 | 9.96E-01 |
| X - 25271 | 1.01 | 0.74-1.39 | 9.94E-01 | 1.01 | 0.72-1.41 | 9.96E-01 |
| glutamate | 0.93 | 0.68-1.27 | 9.71E-01 | 1.00 | 0.71-1.42 | 9.98E-01 |
| X - 16397 | 0.95 | 0.72-1.25 | 9.85E-01 | 1.00 | 0.75-1.34 | 9.98E-01 |
| 1,5-anhydroglucitol (1,5-AG) | 1.04 | 0.76-1.44 | 9.86E-01 | 0.99 | 0.72-1.37 | 9.98E-01 |
| gamma-glutamylglutamine | 1.05 | 0.79-1.39 | 9.86E-01 | 1.00 | 0.75-1.33 | 9.98E-01 |
| caproate (6:0) | 0.95 | 0.69-1.32 | 9.86E-01 | 1.00 | 0.71-1.4 | 9.98E-01 |
| 3-methyl-2-oxovalerate | 0.96 | 0.68-1.37 | 9.89E-01 | 1.00 | 0.68-1.47 | 9.98E-01 |
| isovalerylcarnitine (C5) | 0.97 | 0.71-1.32 | 9.89E-01 | 1.00 | 0.72-1.39 | 9.98E-01 |
| glutamine_degradant* | 1.03 | 0.69-1.55 | 9.93E-01 | 1.00 | 0.67-1.49 | 9.98E-01 |
| sphingomyelin (d18:2/21:0, d16:2/23:0)* | 1.03 | 0.68-1.56 | 9.93E-01 | 1.00 | 0.65-1.55 | 9.98E-01 |
| 3-formylindole | 1.03 | 0.74-1.44 | 9.93E-01 | 1.00 | 0.7-1.44 | 9.98E-01 |
| sarcosine | 1.03 | 0.74-1.43 | 9.93E-01 | 1.00 | 0.71-1.39 | 9.98E-01 |
| caprylate (8:0) | 0.97 | 0.65-1.44 | 9.93E-01 | 0.99 | 0.67-1.49 | 9.98E-01 |
| X - 16087 | 0.98 | 0.72-1.32 | 9.93E-01 | 1.01 | 0.74-1.37 | 9.98E-01 |
| N1-methyladenosine | 0.98 | 0.73-1.31 | 9.94E-01 | 1.00 | 0.75-1.34 | 9.98E-01 |
| 2-linoleoylglycerol (18:2) | 1.01 | 0.71-1.45 | 9.94E-01 | 1.00 | 0.7-1.43 | 9.98E-01 |
| piperine | 0.99 | 0.75-1.31 | 9.94E-01 | 0.99 | 0.74-1.34 | 9.98E-01 |
| aconitate [cis or trans] | 0.98 | 0.66-1.46 | 9.94E-01 | 1.00 | 0.65-1.53 | 9.98E-01 |
| phenol sulfate | 1.02 | 0.68-1.54 | 9.94E-01 | 1.01 | 0.68-1.5 | 9.98E-01 |
| hexanoylglycine | 0.98 | 0.72-1.35 | 9.94E-01 | 0.99 | 0.72-1.37 | 9.98E-01 |
| gamma-glutamylalanine | 0.99 | 0.65-1.5 | 9.94E-01 | 1.00 | 0.67-1.49 | 9.98E-01 |
| 4-cholesten-3-one | 1.01 | 0.73-1.41 | 9.94E-01 | 1.00 | 0.73-1.39 | 9.98E-01 |
| 3b-hydroxy-5-cholenoic acid | 1.01 | 0.75-1.37 | 9.94E-01 | 1.00 | 0.74-1.35 | 9.98E-01 |
| 2-aminoheptanoate | 1.01 | 0.78-1.32 | 9.94E-01 | 1.01 | 0.77-1.32 | 9.98E-01 |
| N-acetyltaurine | 0.98 | 0.67-1.42 | 9.94E-01 | 1.01 | 0.68-1.48 | 9.98E-01 |
| dopamine 3-O-sulfate | 1.01 | 0.76-1.35 | 9.94E-01 | 1.00 | 0.74-1.35 | 9.98E-01 |
| nonanoylcarnitine (C9) | 0.99 | 0.73-1.33 | 9.94E-01 | 1.00 | 0.74-1.37 | 9.98E-01 |
| sphingomyelin (d17:2/16:0, d18:2/15:0)* | 1.01 | 0.72-1.42 | 9.94E-01 | 1.00 | 0.7-1.42 | 9.98E-01 |
| 11beta-hydroxyetiocholanolone glucuronide* | 1.01 | 0.66-1.55 | 9.94E-01 | 1.00 | 0.64-1.58 | 9.98E-01 |
| lithocholate sulfate (1) | 0.99 | 0.7-1.4 | 9.94E-01 | 0.99 | 0.69-1.42 | 9.98E-01 |
| 3-aminoisobutyrate | 1.01 | 0.74-1.37 | 9.94E-01 | 1.00 | 0.73-1.38 | 9.98E-01 |
| glycochenodeoxycholate | 0.99 | 0.72-1.35 | 9.94E-01 | 1.00 | 0.72-1.38 | 9.98E-01 |
| sucrose | 1.03 | 0.68-1.55 | 9.94E-01 | 1.00 | 0.65-1.56 | 9.98E-01 |
| X - 11478 | 1.02 | 0.76-1.37 | 9.94E-01 | 1.00 | 0.75-1.33 | 9.98E-01 |
| X - 12410 | 1.01 | 0.73-1.38 | 9.94E-01 | 1.00 | 0.72-1.39 | 9.98E-01 |
| X - 17346 | 1.01 | 0.67-1.52 | 9.94E-01 | 1.00 | 0.66-1.52 | 9.98E-01 |
| X - 12713 | 1.00 | 0.69-1.46 | 9.96E-01 | 1.01 | 0.7-1.45 | 9.98E-01 |
| 3-hydroxysebacate | 1.00 | 0.72-1.4 | 9.98E-01 | 1.00 | 0.72-1.4 | 9.98E-01 |
| behenoylcarnitine (C22)* | 1.01 | 0.75-1.36 | 9.94E-01 | 1.00 | 0.74-1.34 | 9.99E-01 |

Note: the table shows associations from the competing risk Cox proportional hazards regression analysis where plasms metabolomics level from the Metabolon platform was main exposure and incident colorectal cancer as the main outcome in ERGO-4 cohort. Model 1: adjusted for age, sex, cohort, and lipid lowering medication. Model 2: adjusted for model1, smoking status, BMI, alcohol and education. The table is ranked based on the *FDR* values in model 2, using bold text indicates metabolites are surpassing *FDR* adjusted P value treshold (*P*<0.05). ERGO-4 refers to the fourth and fifth visits from the baseline cohort. Abbreviations: BMI, body mass index; HR, hazard ratio; CI, confidence interval; FDR, false discovery rate.

**Supplementary Table 17. Serum metabolites associated with incident all cancer from the Nightingale platform.**

| **Metabolite** | **Model 1** | | | **Model 2** | | |
| --- | --- | --- | --- | --- | --- | --- |
|  | **HR** | **95% CI** | ***FDR* value** | **HR** | **95% CI** | ***FDR* value** |
| Glycoprotein acetyls | 1.25 | 1.13-1.39 | **6.25E-03** | 1.24 | 1.11-1.37 | **2.22E-02** |
| Cholesterol to total lipids ratio in large HDL | 0.86 | 0.78-0.94 | 5.32E-02 | 0.86 | 0.78-0.95 | 1.36E-01 |
| Cholesteryl esters to total lipids ratio in large HDL | 0.86 | 0.79-0.94 | 5.32E-02 | 0.87 | 0.79-0.95 | 1.36E-01 |
| Free cholesterol to total lipids ratio in small LDL | 0.86 | 0.78-0.94 | 5.32E-02 | 0.86 | 0.78-0.95 | 1.36E-01 |
| Free cholesterol to total lipids ratio in large LDL | 0.86 | 0.78-0.94 | 5.76E-02 | 0.86 | 0.78-0.95 | 1.36E-01 |
| Free cholesterol to total lipids ratio in medium LDL | 0.85 | 0.78-0.94 | 5.76E-02 | 0.86 | 0.78-0.95 | 1.36E-01 |
| Cholesteryl esters to total lipids ratio in medium HDL | 0.86 | 0.79-0.95 | 7.82E-02 | 0.87 | 0.79-0.96 | 1.58E-01 |
| Phospholipids to total lipids ratio in medium HDL | 1.16 | 1.05-1.28 | 7.82E-02 | 1.15 | 1.04-1.28 | 1.58E-01 |
| Cholesterol to total lipids ratio in medium HDL | 0.86 | 0.78-0.95 | 7.82E-02 | 0.87 | 0.78-0.96 | 1.58E-01 |
| Triglycerides to total lipids ratio in small LDL | 1.17 | 1.05-1.29 | 7.82E-02 | 1.16 | 1.04-1.29 | 1.58E-01 |
| Cholesteryl esters to total lipids ratio in very small VLDL | 0.86 | 0.78-0.95 | 8.27E-02 | 0.86 | 0.78-0.96 | 1.58E-01 |
| Cholesteryl esters to total lipids ratio in medium VLDL | 0.87 | 0.78-0.96 | 8.27E-02 | 0.87 | 0.78-0.97 | 1.58E-01 |
| Triglycerides to total lipids ratio in IDL | 1.16 | 1.05-1.29 | 8.27E-02 | 1.15 | 1.04-1.28 | 1.58E-01 |
| Cholesterol to total lipids ratio in very small VLDL | 0.86 | 0.78-0.96 | 8.27E-02 | 0.87 | 0.78-0.97 | 1.58E-01 |
| Free cholesterol to total lipids ratio in small VLDL | 0.86 | 0.77-0.96 | 8.27E-02 | 0.86 | 0.77-0.96 | 1.58E-01 |
| Average diameter for LDL particles | 0.88 | 0.8-0.96 | 8.27E-02 | 0.88 | 0.8-0.97 | 1.58E-01 |
| Triglycerides to total lipids ratio in medium LDL | 1.16 | 1.04-1.28 | 8.27E-02 | 1.14 | 1.03-1.27 | 1.66E-01 |
| Cholesterol to total lipids ratio in small VLDL | 0.86 | 0.78-0.96 | 8.56E-02 | 0.87 | 0.78-0.97 | 1.66E-01 |
| Triglycerides to total lipids ratio in large LDL | 1.16 | 1.04-1.28 | 8.27E-02 | 1.14 | 1.03-1.27 | 1.74E-01 |
| Cholesteryl esters to total lipids ratio in small HDL | 0.87 | 0.79-0.96 | 8.29E-02 | 0.88 | 0.8-0.98 | 1.74E-01 |
| Cholesterol to total lipids ratio in IDL | 0.88 | 0.8-0.97 | 8.56E-02 | 0.89 | 0.8-0.98 | 1.74E-01 |
| Triglycerides in small LDL | 1.14 | 1.04-1.26 | 8.56E-02 | 1.13 | 1.02-1.25 | 1.74E-01 |
| Cholesterol to total lipids ratio in small HDL | 0.87 | 0.79-0.97 | 8.58E-02 | 0.88 | 0.8-0.98 | 1.74E-01 |
| Triglycerides to total lipids ratio in very small VLDL | 1.16 | 1.04-1.3 | 9.43E-02 | 1.15 | 1.03-1.3 | 1.74E-01 |
| Free cholesterol to total lipids ratio in IDL | 0.87 | 0.78-0.97 | 9.53E-02 | 0.88 | 0.79-0.98 | 1.74E-01 |
| Phospholipids to total lipids ratio in medium LDL | 0.88 | 0.8-0.97 | 9.43E-02 | 0.89 | 0.81-0.98 | 1.75E-01 |
| Phospholipids to total lipids ratio in small VLDL | 0.87 | 0.78-0.97 | 9.53E-02 | 0.87 | 0.78-0.98 | 1.75E-01 |
| Triglycerides in medium LDL | 1.14 | 1.03-1.26 | 9.28E-02 | 1.12 | 1.02-1.24 | 1.81E-01 |
| Phospholipids to total lipids ratio in large HDL | 1.14 | 1.03-1.26 | 9.43E-02 | 1.13 | 1.02-1.26 | 1.81E-01 |
| Cholesterol to total lipids ratio in medium VLDL | 0.87 | 0.79-0.97 | 9.53E-02 | 0.88 | 0.79-0.98 | 1.81E-01 |
| Triglycerides in LDL | 1.14 | 1.03-1.25 | 9.53E-02 | 1.12 | 1.01-1.24 | 2.04E-01 |
| Monounsaturated fatty acids | 1.14 | 1.03-1.26 | 9.55E-02 | 1.12 | 1.01-1.25 | 2.04E-01 |
| Cholesteryl esters to total lipids ratio in medium LDL | 1.13 | 1.02-1.25 | 1.13E-01 | 1.12 | 1.01-1.24 | 2.04E-01 |
| Triglycerides in small VLDL | 1.13 | 1.02-1.26 | 1.17E-01 | 1.12 | 1.01-1.25 | 2.04E-01 |
| Triglycerides to total lipids ratio in very large HDL | 1.13 | 1.02-1.25 | 1.25E-01 | 1.13 | 1.01-1.25 | 2.04E-01 |
| Cholesteryl esters to total lipids ratio in small VLDL | 0.88 | 0.79-0.98 | 1.25E-01 | 0.89 | 0.8-0.99 | 2.04E-01 |
| Total triglycerides | 1.13 | 1.02-1.25 | 1.13E-01 | 1.12 | 1.01-1.24 | 2.07E-01 |
| Triglycerides in very small VLDL | 1.13 | 1.02-1.25 | 1.15E-01 | 1.12 | 1.01-1.24 | 2.16E-01 |
| Triglycerides in small HDL | 1.13 | 1.02-1.26 | 1.25E-01 | 1.13 | 1.01-1.26 | 2.28E-01 |
| Cholesterol to total lipids ratio in large LDL | 0.89 | 0.8-0.98 | 1.26E-01 | 0.9 | 0.81-0.99 | 2.28E-01 |
| Triglycerides in large LDL | 1.13 | 1.02-1.24 | 1.25E-01 | 1.11 | 1-1.23 | 2.45E-01 |
| Ratio of triglycerides to phosphoglycerides | 1.13 | 1.02-1.25 | 1.26E-01 | 1.12 | 1-1.25 | 2.45E-01 |
| Triglycerides to total lipids ratio in small HDL | 1.13 | 1.02-1.26 | 1.31E-01 | 1.12 | 1-1.25 | 2.45E-01 |
| Free cholesterol to total lipids ratio in large HDL | 0.91 | 0.82-1 | 1.96E-01 | 0.9 | 0.81-1 | 2.45E-01 |
| Total fatty acids | 1.13 | 1.01-1.25 | 1.36E-01 | 1.11 | 1-1.24 | 2.49E-01 |
| Triglycerides to total lipids ratio in large HDL | 1.12 | 1.01-1.24 | 1.51E-01 | 1.11 | 1-1.24 | 2.49E-01 |
| Triglycerides to total lipids ratio in small VLDL | 1.14 | 1.01-1.27 | 1.51E-01 | 1.13 | 1-1.27 | 2.49E-01 |
| Triglycerides in IDL | 1.12 | 1.02-1.24 | 1.31E-01 | 1.11 | 1-1.23 | 2.52E-01 |
| Saturated fatty acids | 1.12 | 1.02-1.24 | 1.31E-01 | 1.11 | 1-1.23 | 2.58E-01 |
| Triglycerides in VLDL | 1.12 | 1.01-1.24 | 1.43E-01 | 1.11 | 1-1.23 | 2.58E-01 |
| Cholesteryl esters in large HDL | 0.89 | 0.8-0.99 | 1.58E-01 | 0.9 | 0.8-1 | 2.58E-01 |
| Triglycerides in HDL | 1.12 | 1.01-1.25 | 1.59E-01 | 1.11 | 1-1.24 | 2.58E-01 |
| Histidine | 0.91 | 0.82-1 | 1.88E-01 | 0.91 | 0.82-1 | 2.58E-01 |
| Total lipids in VLDL | 1.12 | 1.01-1.23 | 1.58E-01 | 1.1 | 1-1.23 | 2.75E-01 |
| Cholesterol in large HDL | 0.89 | 0.8-1 | 1.79E-01 | 0.9 | 0.8-1.01 | 2.80E-01 |
| Ratio of polyunsaturated fatty acids to monounsaturated fatty acids | 0.89 | 0.81-0.98 | 1.26E-01 | 0.91 | 0.82-1.01 | 2.85E-01 |
| Ratio of monounsaturated fatty acids to total fatty acids | 1.12 | 1.01-1.24 | 1.36E-01 | 1.1 | 0.99-1.23 | 2.85E-01 |
| Triglycerides to total lipids ratio in medium HDL | 1.12 | 1-1.24 | 1.79E-01 | 1.11 | 0.99-1.24 | 2.85E-01 |
| Cholesteryl esters in chylomicrons and extremely large VLDL | 1.11 | 1-1.24 | 1.79E-01 | 1.11 | 0.99-1.23 | 2.85E-01 |
| Triglycerides in very large HDL | 1.11 | 1-1.23 | 1.88E-01 | 1.1 | 0.99-1.22 | 2.99E-01 |
| Concentration of small VLDL particles | 1.11 | 1-1.23 | 1.88E-01 | 1.1 | 0.99-1.22 | 3.00E-01 |
| Cholesterol in chylomicrons and extremely large VLDL | 1.11 | 1-1.23 | 1.88E-01 | 1.1 | 0.99-1.23 | 3.02E-01 |
| Free cholesterol to total lipids ratio in medium VLDL | 0.9 | 0.8-1 | 1.94E-01 | 0.9 | 0.8-1.01 | 3.02E-01 |
| Triglycerides in medium HDL | 1.11 | 1-1.24 | 1.94E-01 | 1.11 | 0.99-1.24 | 3.02E-01 |
| Cholesteryl esters to total lipids ratio in small LDL | 1.09 | 0.98-1.2 | 2.68E-01 | 1.1 | 0.99-1.21 | 3.02E-01 |
| Phospholipids in VLDL | 1.11 | 1-1.23 | 1.88E-01 | 1.1 | 0.99-1.22 | 3.02E-01 |
| Triglycerides in medium VLDL | 1.11 | 1-1.23 | 1.94E-01 | 1.1 | 0.99-1.22 | 3.02E-01 |
| Total lipids in small VLDL | 1.1 | 1-1.22 | 1.94E-01 | 1.09 | 0.99-1.21 | 3.09E-01 |
| Average diameter for VLDL particles | 1.11 | 1-1.22 | 1.94E-01 | 1.1 | 0.99-1.22 | 3.09E-01 |
| Free cholesterol in large HDL | 0.9 | 0.81-1.01 | 2.19E-01 | 0.91 | 0.81-1.01 | 3.11E-01 |
| Concentration of large VLDL particles | 1.11 | 1-1.23 | 1.94E-01 | 1.1 | 0.99-1.22 | 3.12E-01 |
| Free cholesterol in large VLDL | 1.11 | 1-1.23 | 1.94E-01 | 1.1 | 0.99-1.22 | 3.12E-01 |
| Triglycerides in large VLDL | 1.11 | 1-1.23 | 1.94E-01 | 1.1 | 0.98-1.22 | 3.12E-01 |
| Total lipids in large VLDL | 1.11 | 1-1.23 | 1.96E-01 | 1.1 | 0.98-1.22 | 3.12E-01 |
| Free cholesterol in VLDL | 1.1 | 1-1.22 | 1.96E-01 | 1.09 | 0.98-1.21 | 3.12E-01 |
| Glutamine | 0.91 | 0.82-1.01 | 2.30E-01 | 0.92 | 0.82-1.02 | 3.12E-01 |
| Phospholipids to total lipids ratio in small LDL | 0.92 | 0.83-1.02 | 3.07E-01 | 0.91 | 0.82-1.01 | 3.12E-01 |
| Concentration of VLDL particles | 1.1 | 1-1.22 | 1.99E-01 | 1.09 | 0.98-1.21 | 3.17E-01 |
| Ratio of polyunsaturated fatty acids to total fatty acids | 0.9 | 0.82-0.99 | 1.58E-01 | 0.92 | 0.83-1.02 | 3.30E-01 |
| Cholesterol in large VLDL | 1.1 | 0.99-1.22 | 2.22E-01 | 1.09 | 0.98-1.22 | 3.30E-01 |
| Triglycerides to total lipids ratio in medium VLDL | 1.11 | 0.99-1.24 | 2.23E-01 | 1.1 | 0.98-1.24 | 3.30E-01 |
| Phospholipids to total lipids ratio in medium VLDL | 0.91 | 0.81-1.01 | 2.37E-01 | 0.91 | 0.81-1.02 | 3.30E-01 |
| Concentration of very large VLDL particles | 1.11 | 0.99-1.23 | 2.14E-01 | 1.09 | 0.98-1.22 | 3.37E-01 |
| Cholesteryl esters to total lipids ratio in IDL | 0.91 | 0.83-1.01 | 2.30E-01 | 0.92 | 0.83-1.02 | 3.37E-01 |
| Ratio of omega-6 fatty acids to total fatty acids | 0.91 | 0.83-1 | 1.88E-01 | 0.92 | 0.84-1.02 | 3.38E-01 |
| Free cholesterol in very large VLDL | 1.1 | 0.99-1.23 | 2.23E-01 | 1.09 | 0.98-1.22 | 3.47E-01 |
| Cholesterol in very large VLDL | 1.1 | 0.99-1.22 | 2.37E-01 | 1.09 | 0.98-1.21 | 3.56E-01 |
| Concentration of large HDL particles | 0.91 | 0.82-1.01 | 2.47E-01 | 0.91 | 0.82-1.03 | 3.56E-01 |
| Polyunsaturated fatty acids | 1.09 | 0.98-1.21 | 3.07E-01 | 1.09 | 0.98-1.21 | 3.64E-01 |
| Omega-6 fatty acids | 1.09 | 0.98-1.21 | 3.14E-01 | 1.09 | 0.98-1.21 | 3.64E-01 |
| Total lipids in very large VLDL | 1.1 | 0.99-1.22 | 2.37E-01 | 1.09 | 0.97-1.22 | 3.73E-01 |
| Phospholipids in large VLDL | 1.1 | 0.99-1.22 | 2.53E-01 | 1.09 | 0.97-1.22 | 3.74E-01 |
| Total lipids in large HDL | 0.91 | 0.82-1.02 | 2.67E-01 | 0.92 | 0.82-1.03 | 3.74E-01 |
| Cholesteryl esters in large VLDL | 1.09 | 0.98-1.21 | 2.67E-01 | 1.08 | 0.97-1.21 | 3.74E-01 |
| Total lipids in medium VLDL | 1.09 | 0.98-1.21 | 2.67E-01 | 1.08 | 0.97-1.2 | 3.74E-01 |
| Cholesteryl esters in very large HDL | 0.92 | 0.82-1.03 | 3.07E-01 | 0.92 | 0.81-1.03 | 3.95E-01 |
| Triglycerides in very large VLDL | 1.1 | 0.98-1.22 | 2.61E-01 | 1.09 | 0.97-1.22 | 4.08E-01 |
| Phospholipids in very large VLDL | 1.09 | 0.98-1.22 | 2.79E-01 | 1.08 | 0.97-1.21 | 4.27E-01 |
| Cholesteryl esters in HDL | 0.91 | 0.81-1.02 | 2.87E-01 | 0.92 | 0.81-1.04 | 4.27E-01 |
| Cholesterol in very large HDL | 0.92 | 0.82-1.03 | 3.44E-01 | 0.92 | 0.81-1.04 | 4.27E-01 |
| Linoleic acid | 1.07 | 0.96-1.19 | 4.00E-01 | 1.08 | 0.97-1.2 | 4.27E-01 |
| Cholesteryl esters in very large VLDL | 1.09 | 0.98-1.21 | 3.07E-01 | 1.08 | 0.97-1.2 | 4.34E-01 |
| Free cholesterol in chylomicrons and extremely large VLDL | 1.09 | 0.98-1.22 | 2.84E-01 | 1.08 | 0.96-1.21 | 4.38E-01 |
| Total lipids in chylomicrons and extremely large VLDL | 1.09 | 0.98-1.21 | 2.86E-01 | 1.08 | 0.97-1.2 | 4.42E-01 |
| Phospholipids in large HDL | 0.92 | 0.83-1.03 | 3.26E-01 | 0.93 | 0.83-1.04 | 4.44E-01 |
| Free cholesterol in IDL | 0.93 | 0.83-1.04 | 4.00E-01 | 0.93 | 0.82-1.04 | 4.47E-01 |
| Albumin | 0.92 | 0.83-1.01 | 2.61E-01 | 0.93 | 0.84-1.04 | 4.53E-01 |
| Cholesteryl esters to total lipids ratio in large VLDL | 0.92 | 0.83-1.02 | 2.79E-01 | 0.93 | 0.83-1.04 | 4.53E-01 |
| VLDL cholesterol | 1.08 | 0.98-1.2 | 3.24E-01 | 1.07 | 0.96-1.19 | 4.53E-01 |
| Cholesteryl esters to total lipids ratio in very large VLDL | 0.91 | 0.82-1.02 | 2.83E-01 | 0.93 | 0.82-1.04 | 4.57E-01 |
| Tyrosine | 0.95 | 0.86-1.04 | 4.80E-01 | 0.94 | 0.85-1.03 | 4.57E-01 |
| HDL cholesterol | 0.92 | 0.82-1.03 | 3.44E-01 | 0.92 | 0.82-1.05 | 4.66E-01 |
| Concentration of medium VLDL particles | 1.08 | 0.97-1.2 | 3.44E-01 | 1.07 | 0.96-1.19 | 4.66E-01 |
| Phospholipids in small VLDL | 1.08 | 0.97-1.19 | 3.51E-01 | 1.07 | 0.96-1.18 | 4.73E-01 |
| Cholesteryl esters in medium HDL | 0.92 | 0.82-1.03 | 3.51E-01 | 0.93 | 0.82-1.05 | 4.95E-01 |
| Phospholipids in medium VLDL | 1.08 | 0.97-1.19 | 3.74E-01 | 1.07 | 0.96-1.18 | 5.00E-01 |
| Free cholesterol in very large HDL | 0.93 | 0.83-1.05 | 4.61E-01 | 0.93 | 0.82-1.05 | 5.00E-01 |
| Creatinine | 1.07 | 0.96-1.2 | 4.60E-01 | 1.07 | 0.96-1.2 | 5.06E-01 |
| Free cholesterol to total lipids ratio in medium HDL | 0.93 | 0.84-1.04 | 4.25E-01 | 0.93 | 0.83-1.05 | 5.14E-01 |
| Phospholipids in very large HDL | 0.94 | 0.85-1.04 | 4.53E-01 | 0.94 | 0.84-1.05 | 5.32E-01 |
| Cholesterol in medium HDL | 0.93 | 0.83-1.04 | 4.00E-01 | 0.93 | 0.83-1.05 | 5.44E-01 |
| Total lipids in very large HDL | 0.94 | 0.84-1.05 | 4.58E-01 | 0.94 | 0.83-1.05 | 5.44E-01 |
| Phospholipids to total lipids ratio in very large HDL | 0.95 | 0.86-1.04 | 4.61E-01 | 0.95 | 0.86-1.04 | 5.48E-01 |
| Concentration of medium LDL particles | 1.07 | 0.97-1.19 | 4.00E-01 | 1.06 | 0.95-1.18 | 5.51E-01 |
| Free cholesterol in large LDL | 0.94 | 0.84-1.05 | 4.77E-01 | 0.94 | 0.84-1.05 | 5.51E-01 |
| Omega-3 fatty acids | 1.06 | 0.96-1.17 | 4.80E-01 | 1.06 | 0.95-1.17 | 5.63E-01 |
| Cholesterol to total lipids ratio in very large VLDL | 0.92 | 0.83-1.03 | 3.68E-01 | 0.94 | 0.83-1.06 | 5.69E-01 |
| Average diameter for HDL particles | 0.93 | 0.83-1.04 | 4.47E-01 | 0.94 | 0.83-1.06 | 5.69E-01 |
| Total lipids in lipoprotein particles | 1.07 | 0.96-1.19 | 4.53E-01 | 1.06 | 0.95-1.18 | 5.69E-01 |
| Cholesterol to total lipids ratio in small LDL | 0.93 | 0.83-1.03 | 3.42E-01 | 0.95 | 0.85-1.05 | 5.74E-01 |
| Cholesterol to total lipids ratio in large VLDL | 0.93 | 0.84-1.04 | 4.00E-01 | 0.95 | 0.85-1.05 | 5.74E-01 |
| Concentration of chylomicrons and extremely large VLDL particles | 1.07 | 0.96-1.19 | 4.07E-01 | 1.06 | 0.95-1.18 | 5.74E-01 |
| Free cholesterol in medium VLDL | 1.07 | 0.96-1.18 | 4.53E-01 | 1.06 | 0.95-1.17 | 5.74E-01 |
| Concentration of small LDL particles | 1.07 | 0.96-1.18 | 4.54E-01 | 1.06 | 0.95-1.17 | 5.74E-01 |
| Cholesterol in IDL | 0.94 | 0.85-1.06 | 5.30E-01 | 0.94 | 0.84-1.06 | 5.74E-01 |
| Triglycerides in large HDL | 1.06 | 0.95-1.18 | 5.34E-01 | 1.06 | 0.95-1.18 | 5.74E-01 |
| Ratio of linoleic acid to total fatty acids | 0.94 | 0.85-1.03 | 3.92E-01 | 0.95 | 0.86-1.06 | 6.40E-01 |
| Cholesteryl esters in medium LDL | 1.06 | 0.95-1.18 | 4.87E-01 | 1.05 | 0.95-1.17 | 6.40E-01 |
| Free cholesterol in LDL | 0.95 | 0.85-1.06 | 5.59E-01 | 0.95 | 0.85-1.06 | 6.40E-01 |
| Cholesteryl esters in IDL | 0.95 | 0.85-1.06 | 5.82E-01 | 0.95 | 0.85-1.06 | 6.42E-01 |
| Cholesteryl esters in VLDL | 1.06 | 0.95-1.18 | 5.06E-01 | 1.05 | 0.94-1.17 | 6.54E-01 |
| Glycine | 0.95 | 0.85-1.05 | 5.04E-01 | 0.95 | 0.86-1.06 | 6.57E-01 |
| Phospholipids in very small VLDL | 1.06 | 0.96-1.18 | 4.87E-01 | 1.05 | 0.94-1.17 | 6.74E-01 |
| Concentration of very small VLDL particles | 1.06 | 0.95-1.18 | 5.06E-01 | 1.05 | 0.94-1.17 | 6.74E-01 |
| Total lipids in very small VLDL | 1.06 | 0.95-1.18 | 5.06E-01 | 1.05 | 0.94-1.16 | 6.74E-01 |
| Cholesteryl esters in small VLDL | 1.06 | 0.95-1.17 | 5.22E-01 | 1.05 | 0.94-1.16 | 6.74E-01 |
| Cholesteryl esters to total lipids ratio in very large HDL | 0.95 | 0.85-1.05 | 5.22E-01 | 0.96 | 0.86-1.06 | 6.74E-01 |
| Cholesterol in small VLDL | 1.05 | 0.95-1.17 | 5.32E-01 | 1.05 | 0.94-1.16 | 6.74E-01 |
| Phospholipids to total lipids ratio in small HDL | 1.05 | 0.95-1.16 | 5.82E-01 | 1.05 | 0.94-1.16 | 6.74E-01 |
| Acetate | 0.95 | 0.86-1.05 | 5.21E-01 | 0.96 | 0.87-1.06 | 6.80E-01 |
| Degree of unsaturation | 0.94 | 0.85-1.04 | 4.54E-01 | 0.96 | 0.87-1.06 | 6.89E-01 |
| Concentration of very large HDL particles | 0.95 | 0.85-1.07 | 6.07E-01 | 0.95 | 0.85-1.07 | 7.06E-01 |
| Free cholesterol in very small VLDL | 1.06 | 0.95-1.17 | 5.32E-01 | 1.04 | 0.94-1.16 | 7.09E-01 |
| Concentration of medium HDL particles | 0.95 | 0.84-1.06 | 5.56E-01 | 0.95 | 0.84-1.08 | 7.09E-01 |
| Free cholesterol to total lipids ratio in very large HDL | 1.05 | 0.95-1.17 | 5.56E-01 | 1.04 | 0.93-1.17 | 7.09E-01 |
| Free cholesterol in small VLDL | 1.05 | 0.95-1.16 | 5.82E-01 | 1.04 | 0.94-1.16 | 7.09E-01 |
| Cholesteryl esters in medium VLDL | 0.96 | 0.86-1.07 | 6.61E-01 | 0.96 | 0.86-1.07 | 7.09E-01 |
| Free cholesterol in small LDL | 0.96 | 0.87-1.07 | 6.87E-01 | 0.96 | 0.86-1.07 | 7.09E-01 |
| Free cholesterol in HDL | 0.95 | 0.84-1.07 | 5.93E-01 | 0.95 | 0.84-1.08 | 7.11E-01 |
| Phospholipids to total lipids ratio in very small VLDL | 1.06 | 0.95-1.18 | 5.25E-01 | 1.04 | 0.93-1.16 | 7.13E-01 |
| Docosahexaenoic acid | 1.04 | 0.94-1.14 | 6.88E-01 | 1.04 | 0.94-1.15 | 7.17E-01 |
| Free cholesterol in medium HDL | 0.95 | 0.85-1.07 | 5.93E-01 | 0.96 | 0.85-1.08 | 7.26E-01 |
| Phospholipids in chylomicrons and extremely large VLDL | 1.05 | 0.95-1.18 | 5.56E-01 | 1.04 | 0.93-1.16 | 7.34E-01 |
| Total lipids in HDL | 0.95 | 0.85-1.07 | 6.07E-01 | 0.96 | 0.85-1.09 | 7.34E-01 |
| Cholesteryl esters in small LDL | 1.05 | 0.94-1.16 | 6.07E-01 | 1.04 | 0.93-1.16 | 7.34E-01 |
| Phospholipids to total lipids ratio in large VLDL | 1.05 | 0.94-1.16 | 6.07E-01 | 1.04 | 0.93-1.16 | 7.34E-01 |
| Ratio of omega-6 fatty acids to omega-3 fatty acids | 0.96 | 0.87-1.06 | 6.61E-01 | 0.96 | 0.87-1.07 | 7.34E-01 |
| Phosphoglycerides | 1.04 | 0.93-1.17 | 6.87E-01 | 1.04 | 0.92-1.18 | 7.34E-01 |
| Total lipids in IDL | 0.96 | 0.86-1.08 | 7.16E-01 | 0.96 | 0.86-1.08 | 7.34E-01 |
| Free cholesterol to total lipids ratio in very small VLDL | 0.97 | 0.87-1.08 | 7.38E-01 | 0.96 | 0.87-1.07 | 7.34E-01 |
| Phospholipids in IDL | 0.97 | 0.86-1.08 | 7.24E-01 | 0.96 | 0.86-1.08 | 7.35E-01 |
| Phenylalanine | 1.03 | 0.93-1.14 | 7.24E-01 | 1.03 | 0.93-1.15 | 7.35E-01 |
| Triglycerides to total lipids ratio in chylomicrons and extremely large VLDL | 0.98 | 0.89-1.07 | 7.60E-01 | 0.97 | 0.88-1.06 | 7.35E-01 |
| Phospholipids in small HDL | 1.04 | 0.92-1.17 | 7.24E-01 | 1.04 | 0.92-1.18 | 7.56E-01 |
| Ratio of apolipoprotein B to apolipoprotein A1 | 1.05 | 0.94-1.16 | 6.07E-01 | 1.04 | 0.93-1.15 | 7.57E-01 |
| Total phospholipids in lipoprotein particles | 1.04 | 0.93-1.17 | 7.03E-01 | 1.04 | 0.92-1.17 | 7.58E-01 |
| Free cholesterol in medium LDL | 0.97 | 0.87-1.08 | 7.24E-01 | 0.97 | 0.87-1.08 | 7.58E-01 |
| Cholesteryl esters in small HDL | 0.96 | 0.86-1.07 | 6.68E-01 | 0.96 | 0.86-1.09 | 7.66E-01 |
| Phosphatidylcholines | 1.04 | 0.92-1.16 | 7.24E-01 | 1.04 | 0.92-1.17 | 7.66E-01 |
| Leucine | 0.98 | 0.88-1.09 | 8.25E-01 | 0.97 | 0.87-1.08 | 7.75E-01 |
| Concentration of HDL particles | 0.96 | 0.85-1.08 | 6.61E-01 | 0.96 | 0.85-1.09 | 7.77E-01 |
| Total lipids in medium HDL | 0.96 | 0.85-1.08 | 6.68E-01 | 0.97 | 0.85-1.09 | 7.78E-01 |
| Total lipids in medium LDL | 1.04 | 0.93-1.15 | 6.81E-01 | 1.03 | 0.93-1.15 | 7.78E-01 |
| Free cholesterol in small HDL | 1.03 | 0.92-1.16 | 7.47E-01 | 1.04 | 0.92-1.17 | 7.78E-01 |
| Apolipoprotein A1 | 0.96 | 0.85-1.08 | 6.70E-01 | 0.97 | 0.85-1.1 | 7.82E-01 |
| Cholesterol in medium LDL | 1.04 | 0.93-1.15 | 6.88E-01 | 1.03 | 0.93-1.15 | 7.82E-01 |
| Cholesterol in large LDL | 0.97 | 0.87-1.08 | 7.48E-01 | 0.97 | 0.87-1.08 | 7.82E-01 |
| 3-Hydroxybutyrate | 1.03 | 0.94-1.14 | 7.12E-01 | 1.03 | 0.93-1.13 | 7.87E-01 |
| Total lipids in small HDL | 1.03 | 0.92-1.16 | 7.76E-01 | 1.03 | 0.91-1.17 | 8.00E-01 |
| Clinical LDL cholesterol | 0.98 | 0.88-1.09 | 7.88E-01 | 0.97 | 0.87-1.08 | 8.12E-01 |
| Isoleucine | 1.04 | 0.94-1.15 | 6.41E-01 | 1.03 | 0.92-1.14 | 8.17E-01 |
| Cholesteryl esters to total lipids ratio in chylomicrons and extremely large VLDL | 0.96 | 0.86-1.07 | 6.61E-01 | 0.97 | 0.87-1.09 | 8.17E-01 |
| Phospholipids in HDL | 0.97 | 0.86-1.08 | 7.24E-01 | 0.97 | 0.86-1.1 | 8.17E-01 |
| Total concentration of lipoprotein particles | 0.96 | 0.86-1.09 | 7.24E-01 | 0.97 | 0.86-1.1 | 8.17E-01 |
| Total lipids in small LDL | 1.03 | 0.93-1.15 | 7.24E-01 | 1.02 | 0.92-1.14 | 8.17E-01 |
| Lactate | 1.03 | 0.93-1.14 | 7.47E-01 | 1.03 | 0.92-1.14 | 8.17E-01 |
| Pyruvate | 1.03 | 0.93-1.13 | 7.54E-01 | 1.02 | 0.93-1.13 | 8.17E-01 |
| Phospholipids to total lipids ratio in large LDL | 0.98 | 0.88-1.08 | 7.87E-01 | 0.98 | 0.88-1.08 | 8.17E-01 |
| Phospholipids in large LDL | 0.98 | 0.88-1.09 | 7.95E-01 | 0.97 | 0.87-1.09 | 8.17E-01 |
| Cholesteryl esters in very small VLDL | 0.98 | 0.87-1.1 | 8.20E-01 | 0.97 | 0.87-1.09 | 8.17E-01 |
| Remnant cholesterol (non-HDL | 1.03 | 0.93-1.15 | 7.36E-01 | 1.02 | 0.92-1.14 | 8.22E-01 |
| Ratio of omega-3 fatty acids to total fatty acids | 1.02 | 0.93-1.13 | 8.04E-01 | 1.02 | 0.92-1.13 | 8.22E-01 |
| Total cholines | 1.02 | 0.91-1.15 | 8.04E-01 | 1.03 | 0.91-1.16 | 8.22E-01 |
| Triglycerides to total lipids ratio in large VLDL | 1.03 | 0.93-1.14 | 7.46E-01 | 1.02 | 0.92-1.14 | 8.47E-01 |
| Apolipoprotein B | 1.03 | 0.93-1.14 | 7.49E-01 | 1.02 | 0.92-1.13 | 8.47E-01 |
| Total esterified cholesterol | 0.98 | 0.87-1.09 | 8.04E-01 | 0.98 | 0.87-1.1 | 8.47E-01 |
| Alanine | 0.98 | 0.89-1.08 | 8.25E-01 | 0.98 | 0.89-1.08 | 8.49E-01 |
| Total lipids in large LDL | 0.98 | 0.88-1.09 | 8.34E-01 | 0.98 | 0.88-1.09 | 8.53E-01 |
| Cholesteryl esters in large LDL | 0.98 | 0.88-1.09 | 8.34E-01 | 0.98 | 0.88-1.09 | 8.64E-01 |
| Ratio of saturated fatty acids to total fatty acids | 1.04 | 0.94-1.14 | 6.70E-01 | 1.02 | 0.92-1.12 | 8.72E-01 |
| Cholesterol in small LDL | 1.03 | 0.92-1.14 | 7.88E-01 | 1.02 | 0.92-1.13 | 8.78E-01 |
| Cholesterol in small HDL | 0.98 | 0.87-1.09 | 7.97E-01 | 0.98 | 0.87-1.11 | 8.89E-01 |
| Phospholipids in medium HDL | 0.97 | 0.87-1.09 | 7.95E-01 | 0.98 | 0.87-1.11 | 9.03E-01 |
| Concentration of large LDL particles | 0.99 | 0.89-1.1 | 8.88E-01 | 0.98 | 0.89-1.09 | 9.03E-01 |
| Cholesterol to total lipids ratio in chylomicrons and extremely large VLDL | 0.97 | 0.87-1.08 | 7.40E-01 | 0.99 | 0.89-1.1 | 9.18E-01 |
| Ratio of docosahexaenoic acid to total fatty acids | 0.98 | 0.89-1.08 | 7.95E-01 | 0.99 | 0.89-1.09 | 9.18E-01 |
| Phospholipids to total lipids ratio in very large VLDL | 1.02 | 0.92-1.13 | 8.34E-01 | 1.01 | 0.91-1.13 | 9.18E-01 |
| Concentration of LDL particles | 1.02 | 0.92-1.13 | 8.23E-01 | 1.01 | 0.91-1.13 | 9.18E-01 |
| Acetoacetate | 1.02 | 0.92-1.12 | 8.25E-01 | 1.01 | 0.92-1.12 | 9.29E-01 |
| Cholesterol in medium VLDL | 1.02 | 0.91-1.13 | 8.37E-01 | 1.01 | 0.91-1.13 | 9.29E-01 |
| Triglycerides to total lipids ratio in very large VLDL | 1.03 | 0.92-1.16 | 7.70E-01 | 1.01 | 0.9-1.14 | 9.32E-01 |
| Free cholesterol to total lipids ratio in very large VLDL | 0.97 | 0.87-1.09 | 7.79E-01 | 0.99 | 0.88-1.11 | 9.32E-01 |
| Phospholipids in medium LDL | 1.02 | 0.91-1.13 | 8.55E-01 | 1.01 | 0.91-1.12 | 9.32E-01 |
| Total free cholesterol | 1.02 | 0.91-1.13 | 8.55E-01 | 1.01 | 0.91-1.13 | 9.32E-01 |
| Cholesterol to total lipids ratio in medium LDL | 0.99 | 0.89-1.1 | 8.73E-01 | 0.99 | 0.89-1.1 | 9.32E-01 |
| Total cholesterol | 0.99 | 0.88-1.1 | 8.88E-01 | 0.99 | 0.88-1.11 | 9.32E-01 |
| Sphingomyelins | 0.99 | 0.88-1.11 | 8.97E-01 | 0.99 | 0.88-1.11 | 9.32E-01 |
| Phospholipids in LDL | 0.99 | 0.89-1.11 | 9.41E-01 | 0.99 | 0.89-1.1 | 9.32E-01 |
| Total concentration of branched-chain amino acids (leucine + isoleucine + valine) | 1 | 0.91-1.11 | 9.56E-01 | 0.99 | 0.89-1.1 | 9.32E-01 |
| Phospholipids to total lipids ratio in IDL | 1.01 | 0.91-1.13 | 8.88E-01 | 1.01 | 0.91-1.13 | 9.36E-01 |
| LDL cholesterol | 0.99 | 0.89-1.11 | 9.40E-01 | 0.99 | 0.89-1.1 | 9.40E-01 |
| Triglycerides in chylomicrons and extremely large VLDL | 1.02 | 0.92-1.13 | 8.25E-01 | 1.01 | 0.91-1.12 | 9.43E-01 |
| Free cholesterol to total lipids ratio in large VLDL | 0.99 | 0.89-1.09 | 8.55E-01 | 0.99 | 0.89-1.1 | 9.43E-01 |
| Free cholesterol to total lipids ratio in small HDL | 1.01 | 0.91-1.12 | 8.97E-01 | 1.01 | 0.91-1.12 | 9.43E-01 |
| Acetone | 1.01 | 0.91-1.12 | 8.90E-01 | 1.01 | 0.91-1.11 | 9.45E-01 |
| Total cholesterol minus HDL-C | 1.01 | 0.91-1.13 | 8.88E-01 | 1.01 | 0.9-1.12 | 9.50E-01 |
| Phospholipids to total lipids ratio in chylomicrons and extremely large VLDL | 1 | 0.91-1.11 | 9.63E-01 | 0.99 | 0.9-1.1 | 9.50E-01 |
| Glucose | 1.02 | 0.92-1.12 | 8.35E-01 | 1.01 | 0.91-1.11 | 9.56E-01 |
| Cholesteryl esters in LDL | 1.01 | 0.91-1.12 | 8.97E-01 | 1.01 | 0.9-1.12 | 9.58E-01 |
| Cholesterol in very small VLDL | 1 | 0.9-1.12 | 9.69E-01 | 0.99 | 0.89-1.11 | 9.58E-01 |
| Concentration of IDL particles | 1 | 0.9-1.12 | 9.83E-01 | 0.99 | 0.89-1.11 | 9.58E-01 |
| Phospholipids in small LDL | 1.02 | 0.92-1.13 | 8.48E-01 | 1 | 0.91-1.11 | 9.59E-01 |
| Cholesteryl esters to total lipids ratio in large LDL | 0.99 | 0.9-1.1 | 9.24E-01 | 1 | 0.9-1.1 | 9.67E-01 |
| Concentration of small HDL particles | 0.99 | 0.88-1.11 | 9.13E-01 | 1 | 0.88-1.12 | 9.67E-01 |
| Citrate | 0.99 | 0.89-1.1 | 8.65E-01 | 1 | 0.9-1.12 | 9.81E-01 |
| Valine | 1.01 | 0.91-1.12 | 8.97E-01 | 1 | 0.9-1.11 | 9.81E-01 |
| Free cholesterol to total lipids ratio in chylomicrons and extremely large VLDL | 0.99 | 0.9-1.09 | 8.97E-01 | 1 | 0.91-1.11 | 9.88E-01 |
| Total lipids in LDL | 1 | 0.9-1.12 | 9.63E-01 | 1 | 0.9-1.11 | 9.90E-01 |
| Cholesterol to total lipids ratio in very large HDL | 1 | 0.9-1.11 | 9.83E-01 | 1 | 0.9-1.11 | 1.00E+00 |

Note: the table shows associations from the competing risk Cox proportional hazards regression analysis where plasms metabolomics level from the Nightingale platform was main exposure and incident all cancer as the main outcome in ERGO-4 cohort. Model 1: adjusted for age, sex, cohort, and lipid lowering medication. Model 2: adjusted for model1, smoking status, BMI, alcohol and education. The table is ranked based on the *FDR* values in model 2, using bold text indicates metabolites are surpassing *FDR* adjusted P value treshold (*P*<0.05). ERGO-4 refer to the fourth and fifth visits from the baseline cohort. Abbreviations: IDL, intermediate-density lipoprotein; LDL, low-density lipoprotein; HDL, high-density lipoprotein; VLDL, very-low-density lipoprotein; BMI, body mass index; HR, hazard ratio; CI, confidence interval; FDR, false discovery rate.

**Supplementary Table 18. Serum metabolites associated with incident all cancer from the Metabolon platform.**

| **Metabolite** | **Model 1** | | | **Model 2** | | |
| --- | --- | --- | --- | --- | --- | --- |
|  | **HR** | **95% Ci** | ***FDR* value** | **HR** | **95% Ci** | ***FDR* value** |
| 3-methylxanthine | 1.25 | 1.09-1.44 | 2.79E-01 | 1.26 | 1.09-1.45 | 3.02E-01 |
| hexadecanedioate (C16-DC) | 0.79 | 0.68-0.91 | 2.79E-01 | 0.79 | 0.68-0.92 | 3.02E-01 |
| trimethylamine N-oxide | 1.22 | 1.08-1.37 | 2.79E-01 | 1.23 | 1.09-1.38 | 3.02E-01 |
| 1-palmitoyl-2-palmitoleoyl-GPC (16:0/16:1)* | 1.24 | 1.07-1.44 | 2.79E-01 | 1.27 | 1.09-1.47 | 3.02E-01 |
| methylsuccinoylcarnitine | 1.26 | 1.1-1.44 | 2.79E-01 | 1.25 | 1.09-1.44 | 3.02E-01 |
| hexadecenedioate (C16:1-DC)* | 0.80 | 0.69-0.92 | 2.79E-01 | 0.80 | 0.69-0.93 | 3.02E-01 |
| octadecenedioate (C18:1-DC) | 0.80 | 0.69-0.92 | 2.79E-01 | 0.80 | 0.7-0.93 | 3.02E-01 |
| bilirubin degradation product, C17H18N2O4 (1)** | 0.82 | 0.72-0.93 | 2.79E-01 | 0.83 | 0.73-0.94 | 3.02E-01 |
| bilirubin degradation product, C17H18N2O4 (3)** | 0.81 | 0.71-0.92 | 2.79E-01 | 0.82 | 0.72-0.93 | 3.02E-01 |
| 1-palmitoyl-2-oleoyl-GPC (16:0/18:1) | 1.25 | 1.08-1.45 | 2.79E-01 | 1.26 | 1.09-1.46 | 3.02E-01 |
| X - 23780 | 1.28 | 1.11-1.48 | 2.79E-01 | 1.28 | 1.1-1.48 | 3.02E-01 |
| X - 24951 | 0.82 | 0.72-0.93 | 2.79E-01 | 0.81 | 0.7-0.92 | 3.02E-01 |
| N-acetylglucosamine/N-acetylgalactosamine | 1.24 | 1.07-1.44 | 2.79E-01 | 1.23 | 1.06-1.43 | 3.76E-01 |
| bilirubin degradation product, C17H18N2O4 (2)** | 0.83 | 0.73-0.94 | 2.79E-01 | 0.84 | 0.74-0.95 | 3.76E-01 |
| dodecadienoate (12:2)* | 0.82 | 0.72-0.94 | 3.05E-01 | 0.83 | 0.72-0.95 | 3.76E-01 |
| androstenediol (3alpha, 17alpha) monosulfate (2) | 0.84 | 0.74-0.95 | 3.80E-01 | 0.84 | 0.74-0.95 | 3.76E-01 |
| phosphate | 1.24 | 1.06-1.44 | 3.80E-01 | 1.23 | 1.06-1.43 | 3.99E-01 |
| glucuronate | 1.20 | 1.05-1.38 | 3.80E-01 | 1.20 | 1.05-1.38 | 4.30E-01 |
| butyrylcarnitine (C4) | 1.16 | 1.03-1.31 | 3.80E-01 | 1.17 | 1.03-1.32 | 4.30E-01 |
| N4-acetylcytidine | 1.21 | 1.04-1.4 | 3.80E-01 | 1.21 | 1.04-1.41 | 4.30E-01 |
| 5alpha-pregnan-3beta,20alpha-diol monosulfate (2) | 0.84 | 0.74-0.96 | 3.80E-01 | 0.84 | 0.73-0.96 | 4.30E-01 |
| beta-citrylglutamate | 1.19 | 1.04-1.37 | 3.80E-01 | 1.21 | 1.05-1.4 | 4.30E-01 |
| sphingomyelin (d18:0/20:0, d16:0/22:0)* | 1.20 | 1.03-1.38 | 3.80E-01 | 1.21 | 1.04-1.41 | 4.30E-01 |
| tetradecadienoate (14:2)* | 0.83 | 0.72-0.95 | 3.80E-01 | 0.84 | 0.73-0.96 | 4.30E-01 |
| branched-chain, straight-chain, or cyclopropyl 12:1 fatty acid* | 0.83 | 0.73-0.95 | 3.80E-01 | 0.84 | 0.73-0.96 | 4.30E-01 |
| bilirubin degradation product, C16H18N2O5 (4)** | 0.83 | 0.73-0.95 | 3.80E-01 | 0.84 | 0.74-0.97 | 4.30E-01 |
| N-acetylneuraminate | 1.22 | 1.05-1.41 | 3.80E-01 | 1.20 | 1.04-1.39 | 4.30E-01 |
| 9,10-DiHOME | 0.85 | 0.74-0.96 | 3.80E-01 | 0.85 | 0.74-0.96 | 4.30E-01 |
| biliverdin | 0.82 | 0.71-0.95 | 3.80E-01 | 0.83 | 0.72-0.96 | 4.30E-01 |
| retinol (Vitamin A) | 1.19 | 1.03-1.37 | 3.80E-01 | 1.21 | 1.05-1.4 | 4.30E-01 |
| fructose | 1.17 | 1.03-1.33 | 3.80E-01 | 1.18 | 1.04-1.35 | 4.30E-01 |
| X - 18345 | 0.84 | 0.73-0.96 | 3.80E-01 | 0.84 | 0.73-0.96 | 4.30E-01 |
| X - 21353 | 0.84 | 0.74-0.96 | 3.80E-01 | 0.85 | 0.74-0.97 | 4.30E-01 |
| X - 24531 | 1.21 | 1.05-1.4 | 3.80E-01 | 1.20 | 1.04-1.39 | 4.30E-01 |
| citramalate | 1.20 | 1.04-1.39 | 3.80E-01 | 1.20 | 1.03-1.39 | 4.31E-01 |
| 1-methylxanthine | 1.19 | 1.03-1.37 | 3.80E-01 | 1.19 | 1.03-1.38 | 4.31E-01 |
| octadecanedioate (C18-DC) | 0.84 | 0.74-0.96 | 3.80E-01 | 0.85 | 0.74-0.97 | 4.31E-01 |
| 5alpha-pregnan-3beta,20beta-diol monosulfate (1) | 0.86 | 0.75-0.97 | 3.80E-01 | 0.85 | 0.75-0.97 | 4.31E-01 |
| 3-methylglutarylcarnitine (2) | 1.20 | 1.04-1.38 | 3.80E-01 | 1.19 | 1.03-1.37 | 4.31E-01 |
| 4-methylguaiacol sulfate | 1.20 | 1.05-1.38 | 3.80E-01 | 1.18 | 1.03-1.36 | 4.31E-01 |
| 4-hydroxyphenylacetoylcarnitine | 1.20 | 1.04-1.38 | 3.80E-01 | 1.19 | 1.03-1.38 | 4.31E-01 |
| 3,5-dichloro-2,6-dihydroxybenzoic acid | 0.84 | 0.73-0.97 | 3.80E-01 | 0.83 | 0.72-0.97 | 4.31E-01 |
| 3-bromo-5-chloro-2,6-dihydroxybenzoic acid* | 0.83 | 0.72-0.96 | 3.80E-01 | 0.83 | 0.71-0.97 | 4.31E-01 |
| X - 17654 | 0.85 | 0.75-0.96 | 3.80E-01 | 0.86 | 0.76-0.97 | 4.31E-01 |
| X - 17690 | 1.18 | 1.03-1.36 | 3.80E-01 | 1.19 | 1.03-1.37 | 4.31E-01 |
| ethylmalonate | 1.16 | 1.02-1.31 | 4.26E-01 | 1.16 | 1.03-1.32 | 4.31E-01 |
| alpha-ketobutyrate | 0.85 | 0.74-0.98 | 4.59E-01 | 0.84 | 0.73-0.97 | 4.31E-01 |
| carnitine | 1.21 | 1.03-1.41 | 3.87E-01 | 1.21 | 1.03-1.41 | 4.75E-01 |
| tetradecanedioate (C14-DC) | 0.84 | 0.72-0.97 | 3.80E-01 | 0.85 | 0.73-0.98 | 4.87E-01 |
| 5alpha-androstan-3beta,17alpha-diol disulfate | 0.84 | 0.72-0.97 | 3.80E-01 | 0.85 | 0.73-0.98 | 4.87E-01 |
| bilirubin degradation product, C16H18N2O5 (3)** | 0.85 | 0.74-0.97 | 3.80E-01 | 0.86 | 0.75-0.98 | 4.87E-01 |
| bilirubin (Z,Z) | 0.84 | 0.73-0.97 | 3.80E-01 | 0.85 | 0.73-0.98 | 4.87E-01 |
| 12,13-DiHOME | 0.85 | 0.75-0.97 | 3.80E-01 | 0.86 | 0.76-0.98 | 4.87E-01 |
| X - 12830 | 0.84 | 0.73-0.97 | 3.87E-01 | 0.85 | 0.73-0.98 | 4.87E-01 |
| androstenediol (3beta,17beta) disulfate (2) | 0.85 | 0.74-0.98 | 4.59E-01 | 0.84 | 0.73-0.98 | 4.87E-01 |
| 1-stearoyl-2-docosahexaenoyl-GPE (18:0/22:6)* | 1.20 | 1.02-1.4 | 4.72E-01 | 1.20 | 1.02-1.42 | 4.87E-01 |
| alpha-hydroxyisocaproate | 0.85 | 0.74-0.98 | 4.75E-01 | 0.84 | 0.73-0.98 | 4.87E-01 |
| theobromine | 1.16 | 1.01-1.33 | 5.11E-01 | 1.18 | 1.02-1.36 | 4.87E-01 |
| 1,7-dimethylurate | 1.16 | 1.01-1.33 | 5.38E-01 | 1.19 | 1.02-1.38 | 4.87E-01 |
| X - 11372 | 0.86 | 0.75-0.98 | 4.59E-01 | 0.86 | 0.75-0.98 | 4.90E-01 |
| phenylacetylglutamine | 1.20 | 1.02-1.4 | 4.75E-01 | 1.20 | 1.02-1.41 | 4.90E-01 |
| X - 25420 | 1.19 | 1.02-1.39 | 4.53E-01 | 1.19 | 1.02-1.39 | 5.21E-01 |
| p-cresol glucuronide* | 1.19 | 1.02-1.38 | 4.51E-01 | 1.18 | 1.01-1.37 | 5.24E-01 |
| isobutyrylglycine | 1.18 | 1.02-1.37 | 4.75E-01 | 1.18 | 1.01-1.36 | 5.24E-01 |
| guaiacol sulfate | 1.19 | 1.02-1.39 | 4.75E-01 | 1.19 | 1.02-1.39 | 5.24E-01 |
| 1-palmitoyl-2-oleoyl-GPI (16:0/18:1)* | 1.18 | 1.02-1.38 | 4.78E-01 | 1.18 | 1.01-1.38 | 5.24E-01 |
| branched-chain, straight-chain, or cyclopropyl 10:1 fatty acid (1)* | 0.86 | 0.74-0.99 | 4.85E-01 | 0.86 | 0.74-0.99 | 5.24E-01 |
| octadecadienedioate (C18:2-DC)* | 0.84 | 0.71-0.99 | 5.10E-01 | 0.83 | 0.71-0.98 | 5.24E-01 |
| alanine | 1.16 | 1.01-1.34 | 5.11E-01 | 1.17 | 1.01-1.36 | 5.24E-01 |
| 1-palmitoleoyl-GPC (16:1)* | 1.16 | 1-1.34 | 5.41E-01 | 1.17 | 1.01-1.36 | 5.24E-01 |
| 1-palmitoyl-2-docosahexaenoyl-GPC (16:0/22:6) | 1.14 | 1-1.31 | 5.41E-01 | 1.16 | 1.01-1.33 | 5.24E-01 |
| sphingomyelin (d18:0/18:0, d19:0/17:0)* | 1.15 | 1-1.33 | 5.41E-01 | 1.18 | 1.01-1.37 | 5.24E-01 |
| N-acetylputrescine | 1.15 | 1-1.31 | 5.41E-01 | 1.17 | 1.01-1.34 | 5.24E-01 |
| (2,4 or 2,5)-dimethylphenol sulfate | 1.18 | 1.04-1.34 | 3.80E-01 | 1.17 | 1.01-1.35 | 5.35E-01 |
| 1-palmitoyl-2-docosahexaenoyl-GPE (16:0/22:6)* | 1.17 | 1-1.37 | 5.41E-01 | 1.18 | 1.01-1.39 | 5.35E-01 |
| X - 12015 | 1.15 | 1-1.33 | 5.41E-01 | 1.16 | 1.01-1.34 | 5.35E-01 |
| sphingosine 1-phosphate | 1.15 | 1.01-1.31 | 4.75E-01 | 1.15 | 1-1.31 | 5.39E-01 |
| 4-hydroxyglutamate | 1.18 | 1.02-1.38 | 4.75E-01 | 1.18 | 1.01-1.39 | 5.39E-01 |
| bilirubin degradation product, C16H18N2O5 (1)** | 0.87 | 0.76-0.98 | 4.75E-01 | 0.87 | 0.77-0.99 | 5.39E-01 |
| bilirubin degradation product, C16H18N2O5 (2)** | 0.87 | 0.76-0.99 | 4.75E-01 | 0.87 | 0.76-1 | 5.39E-01 |
| 4-hydroxyphenylacetate | 1.17 | 1.01-1.35 | 4.85E-01 | 1.16 | 1-1.34 | 5.39E-01 |
| 3-hydroxydecanoate | 0.86 | 0.74-0.99 | 5.11E-01 | 0.86 | 0.74-1 | 5.39E-01 |
| 1-linolenoylglycerol (18:3) | 1.16 | 1.01-1.34 | 5.30E-01 | 1.17 | 1.01-1.35 | 5.39E-01 |
| 3,7-dimethylurate | 1.15 | 1-1.33 | 5.41E-01 | 1.16 | 1-1.34 | 5.39E-01 |
| 1-stearoyl-2-docosahexaenoyl-GPC (18:0/22:6) | 1.15 | 1-1.32 | 5.41E-01 | 1.15 | 1-1.32 | 5.39E-01 |
| behenoyl dihydrosphingomyelin (d18:0/22:0)* | 1.16 | 1-1.34 | 5.41E-01 | 1.17 | 1.01-1.35 | 5.39E-01 |
| 5alpha-pregnan-3beta,20alpha-diol disulfate | 0.87 | 0.75-1 | 5.43E-01 | 0.86 | 0.74-1 | 5.39E-01 |
| 2-palmitoleoyl-GPC (16:1)* | 1.15 | 0.99-1.33 | 5.51E-01 | 1.17 | 1-1.35 | 5.39E-01 |
| X - 24556 | 1.13 | 0.99-1.3 | 5.73E-01 | 1.15 | 1-1.32 | 5.39E-01 |
| cortisone | 0.90 | 0.81-1.01 | 5.83E-01 | 0.89 | 0.8-1 | 5.39E-01 |
| 4-hydroxyphenylacetylglutamine | 1.17 | 1.01-1.35 | 5.11E-01 | 1.16 | 1-1.34 | 5.50E-01 |
| butyrylglycine | 1.18 | 1.01-1.37 | 5.14E-01 | 1.17 | 1-1.37 | 5.59E-01 |
| X - 21364 | 0.87 | 0.75-1 | 5.41E-01 | 0.86 | 0.75-1 | 5.60E-01 |
| N-acetylhistidine | 1.15 | 1-1.32 | 5.41E-01 | 1.15 | 1-1.33 | 5.66E-01 |
| S-methylcysteine | 0.87 | 0.75-1 | 5.43E-01 | 0.86 | 0.74-1 | 5.68E-01 |
| orotidine | 1.12 | 1-1.26 | 5.41E-01 | 1.13 | 1-1.27 | 5.73E-01 |
| 1-palmitoyl-2-dihomo-linolenoyl-GPC (16:0/20:3n3 or 6)* | 1.16 | 1-1.35 | 5.41E-01 | 1.16 | 1-1.35 | 5.74E-01 |
| bilirubin (E,Z or Z,E)* | 0.86 | 0.75-0.99 | 4.76E-01 | 0.87 | 0.76-1 | 5.74E-01 |
| laurate (12:0) | 0.85 | 0.73-0.99 | 5.11E-01 | 0.86 | 0.74-1 | 5.74E-01 |
| 5-dodecenoate (12:1n7) | 0.85 | 0.72-1 | 5.41E-01 | 0.85 | 0.72-1 | 5.74E-01 |
| betaine | 0.86 | 0.73-1 | 5.41E-01 | 0.86 | 0.73-1 | 5.74E-01 |
| X - 10458 | 1.15 | 1-1.32 | 5.41E-01 | 1.14 | 1-1.31 | 5.74E-01 |
| X - 24456 | 0.87 | 0.76-1 | 5.41E-01 | 0.87 | 0.76-1 | 5.74E-01 |
| heneicosapentaenoate (21:5n3) | 1.12 | 0.98-1.27 | 6.49E-01 | 1.14 | 1-1.3 | 5.74E-01 |
| perfluorooctanoate (PFOA) | 0.85 | 0.73-1 | 5.41E-01 | 0.85 | 0.72-1 | 5.86E-01 |
| methyl indole-3-acetate | 1.15 | 1.01-1.32 | 5.26E-01 | 1.14 | 1-1.3 | 5.87E-01 |
| dodecanedioate (C12-DC) | 0.86 | 0.73-1 | 5.41E-01 | 0.86 | 0.74-1.01 | 5.90E-01 |
| dodecenedioate (C12:1-DC)* | 0.86 | 0.74-1 | 5.43E-01 | 0.86 | 0.74-1.01 | 5.90E-01 |
| 7-methylxanthine | 1.17 | 0.99-1.37 | 5.43E-01 | 1.17 | 0.99-1.37 | 5.90E-01 |
| catechol glucuronide | 1.15 | 1-1.32 | 5.43E-01 | 1.15 | 0.99-1.32 | 5.90E-01 |
| 1-myristoyl-2-arachidonoyl-GPC (14:0/20:4)* | 1.16 | 0.98-1.36 | 6.23E-01 | 1.17 | 0.99-1.38 | 5.90E-01 |
| 1-stearoyl-2-oleoyl-GPC (18:0/18:1) | 1.14 | 1-1.3 | 5.41E-01 | 1.13 | 0.99-1.29 | 6.08E-01 |
| 2,2'-Methylenebis(6-tert-butyl-p-cresol) | 0.86 | 0.74-1 | 5.41E-01 | 0.87 | 0.75-1.01 | 6.12E-01 |
| 1-palmitoyl-2-oleoyl-GPE (16:0/18:1) | 1.17 | 0.99-1.37 | 5.43E-01 | 1.16 | 0.99-1.37 | 6.12E-01 |
| paraxanthine | 1.12 | 0.98-1.28 | 6.49E-01 | 1.14 | 0.99-1.31 | 6.23E-01 |
| o-cresol sulfate | 1.17 | 1.02-1.34 | 4.26E-01 | 1.16 | 0.99-1.36 | 6.29E-01 |
| linolenoylcarnitine (C18:3)* | 0.88 | 0.77-1 | 5.43E-01 | 0.88 | 0.77-1.01 | 6.29E-01 |
| X - 13658 | 0.88 | 0.77-1 | 5.43E-01 | 0.88 | 0.77-1.01 | 6.29E-01 |
| 4-methylcatechol sulfate | 1.14 | 0.99-1.31 | 5.43E-01 | 1.13 | 0.99-1.3 | 6.29E-01 |
| X - 11308 | 0.87 | 0.75-1.01 | 5.61E-01 | 0.87 | 0.75-1.01 | 6.29E-01 |
| 1-methylurate | 1.15 | 0.99-1.35 | 5.73E-01 | 1.15 | 0.99-1.34 | 6.29E-01 |
| pantoate | 1.15 | 0.99-1.33 | 5.73E-01 | 1.15 | 0.99-1.33 | 6.29E-01 |
| imidazole propionate | 1.13 | 0.99-1.3 | 6.07E-01 | 1.13 | 0.99-1.3 | 6.29E-01 |
| ribonate | 1.14 | 0.99-1.31 | 6.23E-01 | 1.14 | 0.99-1.31 | 6.29E-01 |
| X - 24462 | 0.88 | 0.77-1.01 | 6.23E-01 | 0.88 | 0.76-1.01 | 6.29E-01 |
| 1-myristoyl-2-palmitoyl-GPC (14:0/16:0) | 1.13 | 0.98-1.31 | 6.49E-01 | 1.14 | 0.99-1.32 | 6.29E-01 |
| 1-palmitoyl-GPE (16:0) | 1.14 | 0.98-1.33 | 6.49E-01 | 1.15 | 0.99-1.34 | 6.29E-01 |
| thymol sulfate | 1.12 | 0.98-1.29 | 6.87E-01 | 1.14 | 0.99-1.31 | 6.29E-01 |
| 2-hydroxy-4-(methylthio)butanoic acid | 0.90 | 0.78-1.04 | 7.57E-01 | 0.87 | 0.75-1.01 | 6.29E-01 |
| cis-4-decenoate (10:1n6)* | 0.87 | 0.75-1.01 | 5.58E-01 | 0.88 | 0.76-1.01 | 6.38E-01 |
| 5-acetylamino-6-amino-3-methyluracil | 1.13 | 0.99-1.29 | 6.23E-01 | 1.13 | 0.99-1.29 | 6.38E-01 |
| linoleate (18:2n6) | 0.88 | 0.77-1 | 5.41E-01 | 0.89 | 0.78-1.01 | 6.44E-01 |
| linolenate [alpha or gamma; (18:3n3 or 6)] | 0.87 | 0.76-1 | 5.43E-01 | 0.88 | 0.77-1.02 | 6.44E-01 |
| sphinganine-1-phosphate | 1.15 | 1-1.32 | 5.43E-01 | 1.14 | 0.99-1.31 | 6.44E-01 |
| propionylglycine | 1.14 | 0.99-1.31 | 6.01E-01 | 1.14 | 0.98-1.31 | 6.44E-01 |
| theophylline | 1.11 | 0.97-1.26 | 7.28E-01 | 1.13 | 0.99-1.29 | 6.44E-01 |
| 2,6-dihydroxybenzoic acid | 1.11 | 0.97-1.27 | 7.39E-01 | 1.13 | 0.99-1.3 | 6.44E-01 |
| lactosyl-N-behenoyl-sphingosine (d18:1/22:0)* | 0.88 | 0.76-1.02 | 6.23E-01 | 0.87 | 0.75-1.02 | 6.47E-01 |
| 3-amino-2-piperidone | 0.90 | 0.77-1.04 | 7.58E-01 | 0.88 | 0.75-1.02 | 6.47E-01 |
| 1-stearoyl-2-oleoyl-GPE (18:0/18:1) | 1.15 | 0.99-1.35 | 5.73E-01 | 1.15 | 0.98-1.34 | 6.47E-01 |
| ceramide (d18:1/14:0, d16:1/16:0)* | 1.13 | 0.98-1.29 | 6.49E-01 | 1.13 | 0.98-1.3 | 6.47E-01 |
| X - 24812 | 1.16 | 1.01-1.33 | 5.11E-01 | 1.16 | 0.98-1.36 | 6.54E-01 |
| trans-4-hydroxyproline | 1.14 | 1-1.31 | 5.43E-01 | 1.13 | 0.98-1.3 | 6.54E-01 |
| 1-palmitoyl-2-linoleoyl-GPC (16:0/18:2) | 1.14 | 0.98-1.33 | 6.49E-01 | 1.14 | 0.98-1.33 | 6.54E-01 |
| 5alpha-androstan-3alpha,17beta-diol monosulfate (2) | 0.84 | 0.69-1.03 | 6.49E-01 | 0.84 | 0.69-1.03 | 6.59E-01 |
| 3-hydroxydodecanedioate* | 0.88 | 0.75-1.02 | 6.49E-01 | 0.88 | 0.75-1.02 | 6.71E-01 |
| X - 24544 | 0.90 | 0.79-1.03 | 7.30E-01 | 0.89 | 0.78-1.02 | 6.71E-01 |
| nisinate (24:6n3) | 1.12 | 0.97-1.29 | 7.35E-01 | 1.13 | 0.98-1.31 | 6.71E-01 |
| X - 12462 | 1.14 | 0.99-1.31 | 5.78E-01 | 1.13 | 0.98-1.3 | 6.78E-01 |
| dihomo-linoleate (20:2n6) | 0.88 | 0.76-1.01 | 5.90E-01 | 0.89 | 0.77-1.02 | 6.78E-01 |
| 3-hydroxylaurate | 0.88 | 0.77-1.01 | 6.23E-01 | 0.89 | 0.77-1.02 | 6.78E-01 |
| oxindolylalanine | 0.89 | 0.77-1.01 | 6.23E-01 | 0.89 | 0.78-1.02 | 6.78E-01 |
| X - 12847 | 1.15 | 0.98-1.34 | 6.23E-01 | 1.14 | 0.97-1.33 | 6.78E-01 |
| phenylacetylcarnitine | 1.14 | 0.98-1.34 | 6.49E-01 | 1.14 | 0.98-1.33 | 6.78E-01 |
| perfluorooctanesulfonate (PFOS) | 0.87 | 0.74-1.02 | 6.49E-01 | 0.87 | 0.73-1.03 | 6.78E-01 |
| N-stearoyl-sphingosine (d18:1/18:0)* | 1.13 | 0.98-1.29 | 6.49E-01 | 1.13 | 0.98-1.29 | 6.78E-01 |
| X - 24953 | 0.90 | 0.8-1.02 | 6.49E-01 | 0.90 | 0.8-1.02 | 6.78E-01 |
| creatine | 1.15 | 0.98-1.35 | 6.69E-01 | 1.15 | 0.97-1.35 | 6.78E-01 |
| 1-palmitoleoylglycerol (16:1)* | 1.13 | 0.97-1.31 | 6.93E-01 | 1.14 | 0.98-1.32 | 6.78E-01 |
| X - 21319 | 0.90 | 0.8-1.02 | 7.09E-01 | 0.90 | 0.79-1.02 | 6.78E-01 |
| arabitol/xylitol | 1.10 | 0.97-1.25 | 7.26E-01 | 1.11 | 0.98-1.26 | 6.78E-01 |
| uracil | 0.91 | 0.8-1.03 | 7.35E-01 | 0.90 | 0.79-1.02 | 6.78E-01 |
| X - 12812 | 1.11 | 0.96-1.27 | 7.57E-01 | 1.12 | 0.98-1.29 | 6.78E-01 |
| glycocholenate sulfate* | 0.89 | 0.76-1.04 | 7.58E-01 | 0.88 | 0.75-1.03 | 6.78E-01 |
| taurocholenate sulfate* | 0.91 | 0.79-1.04 | 7.77E-01 | 0.88 | 0.77-1.02 | 6.78E-01 |
| trans-urocanate | 0.89 | 0.77-1.02 | 6.51E-01 | 0.89 | 0.78-1.02 | 6.86E-01 |
| erythronate* | 1.12 | 0.98-1.29 | 6.87E-01 | 1.13 | 0.98-1.3 | 6.88E-01 |
| X - 23665 | 0.89 | 0.76-1.03 | 6.93E-01 | 0.88 | 0.76-1.03 | 6.93E-01 |
| phenylacetylglutamate | 1.13 | 0.98-1.3 | 6.77E-01 | 1.13 | 0.97-1.31 | 6.94E-01 |
| indoleacetate | 1.12 | 0.99-1.27 | 6.39E-01 | 1.11 | 0.97-1.26 | 6.99E-01 |
| 2-hydroxyphenylacetate | 0.88 | 0.75-1.02 | 6.49E-01 | 0.89 | 0.76-1.03 | 6.99E-01 |
| X - 17653 | 0.90 | 0.79-1.02 | 6.49E-01 | 0.90 | 0.79-1.03 | 6.99E-01 |
| 3-hydroxyoctanoate | 0.88 | 0.76-1.02 | 6.52E-01 | 0.89 | 0.77-1.03 | 6.99E-01 |
| trans-3,4-methyleneheptanoate | 1.12 | 0.98-1.28 | 6.69E-01 | 1.11 | 0.97-1.28 | 6.99E-01 |
| thyroxine | 1.12 | 0.98-1.29 | 6.71E-01 | 1.12 | 0.97-1.29 | 6.99E-01 |
| 2,3-dihydroxy-2-methylbutyrate | 1.13 | 0.98-1.31 | 6.77E-01 | 1.13 | 0.97-1.31 | 6.99E-01 |
| lactosyl-N-nervonoyl-sphingosine (d18:1/24:1)* | 0.88 | 0.75-1.03 | 6.77E-01 | 0.88 | 0.74-1.04 | 6.99E-01 |
| tetradecadienedioate (C14:2-DC)* | 0.89 | 0.77-1.02 | 6.77E-01 | 0.89 | 0.78-1.03 | 6.99E-01 |
| 3-hydroxybutyrate (BHBA) | 0.88 | 0.76-1.03 | 6.77E-01 | 0.88 | 0.75-1.03 | 6.99E-01 |
| octadecenedioylcarnitine (C18:1-DC)* | 0.90 | 0.79-1.02 | 6.88E-01 | 0.90 | 0.79-1.02 | 6.99E-01 |
| octadecanedioylcarnitine (C18-DC)* | 0.89 | 0.78-1.03 | 7.12E-01 | 0.89 | 0.78-1.03 | 6.99E-01 |
| eugenol sulfate | 0.90 | 0.78-1.03 | 7.24E-01 | 0.89 | 0.78-1.03 | 6.99E-01 |
| X - 12007 | 1.11 | 0.97-1.27 | 7.26E-01 | 1.11 | 0.97-1.28 | 6.99E-01 |
| methylsuccinate | 1.16 | 0.96-1.4 | 7.32E-01 | 1.16 | 0.96-1.4 | 6.99E-01 |
| N-acetyltryptophan | 1.12 | 0.97-1.3 | 7.35E-01 | 1.14 | 0.97-1.33 | 6.99E-01 |
| X - 23583 | 0.90 | 0.78-1.03 | 7.35E-01 | 0.90 | 0.78-1.03 | 6.99E-01 |
| X - 26107 | 1.12 | 0.97-1.29 | 7.35E-01 | 1.13 | 0.97-1.31 | 6.99E-01 |
| 1-stearoyl-GPE (18:0) | 1.13 | 0.96-1.32 | 7.57E-01 | 1.14 | 0.97-1.34 | 6.99E-01 |
| pregnenediol sulfate (C21H34O5S)* | 0.90 | 0.78-1.04 | 7.57E-01 | 0.89 | 0.77-1.03 | 6.99E-01 |
| pregnenolone sulfate | 0.91 | 0.79-1.04 | 7.66E-01 | 0.90 | 0.78-1.03 | 6.99E-01 |
| 1-palmitoyl-2-arachidonoyl-GPC (16:0/20:4n6) | 1.13 | 0.95-1.33 | 7.67E-01 | 1.14 | 0.96-1.36 | 6.99E-01 |
| 1,3,7-trimethylurate | 1.10 | 0.96-1.25 | 7.76E-01 | 1.12 | 0.97-1.29 | 6.99E-01 |
| hexadecadienoate (16:2n6) | 0.88 | 0.77-1.01 | 6.23E-01 | 0.90 | 0.78-1.03 | 7.07E-01 |
| sebacate (C10-DC) | 0.90 | 0.78-1.03 | 7.12E-01 | 0.90 | 0.78-1.03 | 7.07E-01 |
| hypotaurine | 0.89 | 0.77-1.03 | 6.93E-01 | 0.89 | 0.77-1.03 | 7.08E-01 |
| caffeine | 1.08 | 0.95-1.23 | 8.26E-01 | 1.12 | 0.97-1.29 | 7.08E-01 |
| bilirubin (E,E)* | 0.88 | 0.77-1.02 | 6.49E-01 | 0.90 | 0.78-1.03 | 7.09E-01 |
| N2,N2-dimethylguanosine | 1.12 | 0.96-1.31 | 7.57E-01 | 1.13 | 0.96-1.33 | 7.09E-01 |
| N-stearoyl-sphinganine (d18:0/18:0)* | 1.11 | 0.96-1.28 | 7.65E-01 | 1.12 | 0.97-1.31 | 7.18E-01 |
| cis-3,4-methyleneheptanoate | 0.91 | 0.8-1.03 | 7.39E-01 | 0.91 | 0.8-1.03 | 7.25E-01 |
| X - 11849 | 1.09 | 0.95-1.24 | 8.06E-01 | 1.11 | 0.97-1.26 | 7.25E-01 |
| X - 17685 | 1.13 | 0.97-1.32 | 7.26E-01 | 1.13 | 0.96-1.31 | 7.28E-01 |
| 2-hydroxybehenate | 1.13 | 0.98-1.3 | 6.49E-01 | 1.11 | 0.96-1.29 | 7.29E-01 |
| 10-undecenoate (11:1n1) | 0.89 | 0.78-1.02 | 6.71E-01 | 0.90 | 0.79-1.03 | 7.29E-01 |
| X - 13728 | 1.13 | 0.97-1.32 | 7.32E-01 | 1.12 | 0.96-1.31 | 7.29E-01 |
| hexadecasphingosine (d16:1)* | 1.12 | 0.97-1.29 | 7.35E-01 | 1.12 | 0.96-1.3 | 7.29E-01 |
| hydroxyasparagine** | 1.12 | 0.97-1.31 | 7.35E-01 | 1.12 | 0.96-1.32 | 7.29E-01 |
| cytidine | 1.12 | 0.97-1.3 | 7.35E-01 | 1.12 | 0.96-1.31 | 7.29E-01 |
| docosapentaenoate (n6 DPA; 22:5n6) | 0.90 | 0.79-1.03 | 7.45E-01 | 0.90 | 0.78-1.04 | 7.29E-01 |
| mannitol/sorbitol | 1.11 | 0.97-1.27 | 7.45E-01 | 1.11 | 0.97-1.27 | 7.29E-01 |
| 1-palmitoyl-2-arachidonoyl-GPE (16:0/20:4)* | 1.13 | 0.96-1.33 | 7.45E-01 | 1.13 | 0.96-1.33 | 7.29E-01 |
| N-acetylalanine | 1.12 | 0.96-1.3 | 7.62E-01 | 1.12 | 0.96-1.31 | 7.29E-01 |
| EDTA | 0.92 | 0.81-1.03 | 7.66E-01 | 0.91 | 0.81-1.03 | 7.29E-01 |
| 1-palmitoyl-2-arachidonoyl-GPI (16:0/20:4)* | 1.12 | 0.96-1.31 | 7.66E-01 | 1.13 | 0.96-1.32 | 7.29E-01 |
| 5alpha-androstan-3beta,17beta-diol disulfate | 0.89 | 0.75-1.05 | 7.77E-01 | 0.88 | 0.74-1.05 | 7.29E-01 |
| 3-methylglutaconate | 1.12 | 0.95-1.32 | 7.77E-01 | 1.13 | 0.96-1.33 | 7.29E-01 |
| N-acetylglucosaminylasparagine | 0.91 | 0.79-1.05 | 8.06E-01 | 0.90 | 0.78-1.04 | 7.29E-01 |
| X - 11483 | 1.10 | 0.95-1.26 | 8.06E-01 | 1.11 | 0.96-1.29 | 7.29E-01 |
| sphingomyelin (d18:1/25:0, d19:0/24:1, d20:1/23:0, d19:1/24:0)* | 1.09 | 0.95-1.26 | 8.18E-01 | 1.12 | 0.97-1.29 | 7.29E-01 |
| 5alpha-androstan-3alpha,17alpha-diol monosulfate | 0.89 | 0.77-1.04 | 7.39E-01 | 0.89 | 0.77-1.04 | 7.37E-01 |
| phenyllactate (PLA) | 0.91 | 0.78-1.06 | 8.06E-01 | 0.89 | 0.76-1.04 | 7.37E-01 |
| trans-2-hexenoylglycine | 0.91 | 0.8-1.05 | 8.06E-01 | 0.90 | 0.78-1.04 | 7.37E-01 |
| 4-cholesten-3-one | 0.91 | 0.8-1.04 | 7.77E-01 | 0.91 | 0.79-1.04 | 7.50E-01 |
| X - 21607 | 0.92 | 0.81-1.04 | 7.97E-01 | 0.92 | 0.81-1.03 | 7.50E-01 |
| N-methylhydroxyproline** | 1.09 | 0.95-1.25 | 8.06E-01 | 1.11 | 0.96-1.27 | 7.50E-01 |
| sphingomyelin (d18:2/24:2)* | 0.89 | 0.77-1.04 | 7.35E-01 | 0.90 | 0.77-1.04 | 7.50E-01 |
| threonine | 0.91 | 0.79-1.05 | 8.02E-01 | 0.90 | 0.79-1.04 | 7.50E-01 |
| gamma-glutamylleucine | 0.91 | 0.79-1.05 | 8.06E-01 | 0.90 | 0.78-1.04 | 7.54E-01 |
| (2 or 3)-decenoate (10:1n7 or n8) | 0.91 | 0.8-1.03 | 7.35E-01 | 0.91 | 0.8-1.04 | 7.78E-01 |
| X - 25936 | 1.10 | 0.96-1.25 | 7.77E-01 | 1.10 | 0.96-1.25 | 7.80E-01 |
| indolebutyrate | 1.11 | 0.96-1.28 | 7.65E-01 | 1.11 | 0.96-1.28 | 7.81E-01 |
| 5-methylthioadenosine (MTA) | 1.12 | 0.97-1.28 | 7.26E-01 | 1.11 | 0.96-1.28 | 7.84E-01 |
| 1-oleoyl-2-docosahexaenoyl-GPC (18:1/22:6)* | 1.09 | 0.95-1.25 | 8.06E-01 | 1.10 | 0.96-1.27 | 7.86E-01 |
| lignoceroylcarnitine (C24)* | 1.10 | 0.96-1.26 | 8.02E-01 | 1.10 | 0.96-1.27 | 7.90E-01 |
| 3-acetylphenol sulfate | 1.13 | 0.96-1.34 | 7.35E-01 | 1.12 | 0.95-1.33 | 7.93E-01 |
| sucrose | 1.12 | 0.97-1.29 | 7.12E-01 | 1.10 | 0.96-1.28 | 7.99E-01 |
| taurocholate | 1.11 | 0.96-1.28 | 7.76E-01 | 1.11 | 0.96-1.28 | 7.99E-01 |
| X - 21383 | 0.90 | 0.78-1.05 | 7.77E-01 | 0.90 | 0.77-1.05 | 7.99E-01 |
| 1-palmitoyl-2-linoleoyl-GPE (16:0/18:2) | 1.11 | 0.96-1.29 | 7.57E-01 | 1.11 | 0.95-1.29 | 8.02E-01 |
| glycosyl-N-stearoyl-sphingosine (d18:1/18:0) | 1.12 | 0.96-1.3 | 7.66E-01 | 1.11 | 0.95-1.3 | 8.03E-01 |
| 4-acetamidobutanoate | 1.12 | 0.95-1.31 | 7.72E-01 | 1.12 | 0.95-1.31 | 8.03E-01 |
| cytosine | 1.10 | 0.96-1.27 | 7.77E-01 | 1.10 | 0.96-1.27 | 8.03E-01 |
| X - 26054 | 1.10 | 0.95-1.29 | 8.06E-01 | 1.11 | 0.95-1.29 | 8.04E-01 |
| glutamine conjugate of C6H10O2 (2)* | 0.92 | 0.8-1.06 | 8.26E-01 | 0.91 | 0.79-1.05 | 8.31E-01 |
| X - 11880 | 0.91 | 0.79-1.04 | 7.77E-01 | 0.91 | 0.79-1.05 | 8.33E-01 |
| 3-ethylcatechol sulfate (1) | 1.12 | 0.97-1.29 | 7.35E-01 | 1.10 | 0.95-1.27 | 8.34E-01 |
| X - 12096 | 1.12 | 0.96-1.31 | 7.57E-01 | 1.11 | 0.95-1.31 | 8.34E-01 |
| N,N,N-trimethyl-5-aminovalerate | 1.11 | 0.96-1.27 | 7.65E-01 | 1.10 | 0.95-1.26 | 8.34E-01 |
| X - 11632 | 1.10 | 0.96-1.27 | 7.77E-01 | 1.10 | 0.95-1.27 | 8.34E-01 |
| X - 13507 | 1.09 | 0.96-1.24 | 8.04E-01 | 1.09 | 0.96-1.24 | 8.34E-01 |
| acetylcarnitine (C2) | 1.10 | 0.95-1.28 | 8.06E-01 | 1.10 | 0.95-1.28 | 8.34E-01 |
| cholesterol | 1.11 | 0.95-1.29 | 8.06E-01 | 1.11 | 0.95-1.3 | 8.34E-01 |
| caprylate (8:0) | 0.90 | 0.77-1.05 | 8.06E-01 | 0.90 | 0.77-1.05 | 8.34E-01 |
| X - 18921 | 0.92 | 0.81-1.05 | 8.06E-01 | 0.92 | 0.81-1.04 | 8.34E-01 |
| X - 25343 | 1.09 | 0.95-1.25 | 8.06E-01 | 1.10 | 0.95-1.27 | 8.34E-01 |
| X - 11470 | 0.92 | 0.82-1.05 | 8.18E-01 | 0.92 | 0.81-1.04 | 8.34E-01 |
| serine | 0.92 | 0.79-1.06 | 8.26E-01 | 0.91 | 0.78-1.05 | 8.34E-01 |
| sphinganine | 1.14 | 0.98-1.32 | 6.49E-01 | 1.11 | 0.95-1.29 | 8.37E-01 |
| oleate/vaccenate (18:1) | 0.90 | 0.78-1.04 | 7.57E-01 | 0.91 | 0.79-1.05 | 8.37E-01 |
| docosadienoate (22:2n6) | 0.90 | 0.78-1.04 | 7.66E-01 | 0.91 | 0.78-1.05 | 8.37E-01 |
| eicosanedioate (C20-DC) | 0.91 | 0.8-1.04 | 7.89E-01 | 0.92 | 0.8-1.05 | 8.37E-01 |
| (R)-3-hydroxybutyrylcarnitine | 1.09 | 0.95-1.24 | 8.06E-01 | 1.09 | 0.95-1.25 | 8.37E-01 |
| pyridoxate | 1.07 | 0.93-1.24 | 8.42E-01 | 1.10 | 0.95-1.27 | 8.37E-01 |
| ceramide (d16:1/24:1, d18:1/22:1)* | 1.09 | 0.95-1.26 | 8.06E-01 | 1.10 | 0.95-1.27 | 8.44E-01 |
| X - 24411 | 1.12 | 0.97-1.3 | 7.35E-01 | 1.10 | 0.95-1.27 | 8.47E-01 |
| p-cresol sulfate | 1.11 | 0.95-1.28 | 8.06E-01 | 1.10 | 0.95-1.28 | 8.47E-01 |
| N-acetyl-aspartyl-glutamate (NAAG) | 1.10 | 0.95-1.27 | 8.06E-01 | 1.10 | 0.95-1.27 | 8.47E-01 |
| androstenediol (3beta,17beta) monosulfate (2) | 0.92 | 0.8-1.05 | 8.06E-01 | 0.92 | 0.8-1.05 | 8.47E-01 |
| gamma-carboxyglutamate | 1.10 | 0.95-1.27 | 8.06E-01 | 1.10 | 0.95-1.27 | 8.50E-01 |
| X - 23890 | 1.10 | 0.95-1.27 | 8.06E-01 | 1.10 | 0.95-1.27 | 8.50E-01 |
| 5-acetylamino-6-formylamino-3-methyluracil | 0.90 | 0.78-1.04 | 7.55E-01 | 0.92 | 0.8-1.06 | 8.53E-01 |
| dihomo-linoleoylcarnitine (C20:2)* | 0.90 | 0.78-1.04 | 7.65E-01 | 0.91 | 0.78-1.06 | 8.53E-01 |
| X - 21842 | 0.91 | 0.8-1.04 | 7.65E-01 | 0.92 | 0.81-1.06 | 8.53E-01 |
| glutamate | 1.10 | 0.96-1.25 | 7.77E-01 | 1.09 | 0.94-1.27 | 8.53E-01 |
| indoleacetylglutamine | 1.11 | 0.95-1.28 | 7.97E-01 | 1.09 | 0.94-1.27 | 8.53E-01 |
| tridecenedioate (C13:1-DC)* | 0.91 | 0.79-1.05 | 7.99E-01 | 0.92 | 0.8-1.06 | 8.53E-01 |
| 5-hydroxyhexanoate | 1.10 | 0.96-1.25 | 8.02E-01 | 1.09 | 0.95-1.25 | 8.53E-01 |
| pseudouridine | 1.11 | 0.95-1.29 | 8.02E-01 | 1.10 | 0.95-1.28 | 8.53E-01 |
| 4-ethylphenylsulfate | 1.09 | 0.96-1.24 | 8.04E-01 | 1.08 | 0.95-1.23 | 8.53E-01 |
| glycine conjugate of C10H12O2* | 1.10 | 0.95-1.26 | 8.04E-01 | 1.08 | 0.94-1.25 | 8.53E-01 |
| adrenate (22:4n6) | 0.91 | 0.79-1.05 | 8.06E-01 | 0.92 | 0.8-1.07 | 8.53E-01 |
| myristoleate (14:1n5) | 0.90 | 0.77-1.06 | 8.06E-01 | 0.91 | 0.78-1.07 | 8.53E-01 |
| 1-ribosyl-imidazoleacetate* | 1.10 | 0.95-1.26 | 8.06E-01 | 1.08 | 0.95-1.24 | 8.53E-01 |
| indole-3-carboxylate | 1.09 | 0.95-1.25 | 8.06E-01 | 1.08 | 0.94-1.25 | 8.53E-01 |
| 3-methyl catechol sulfate (2) | 1.10 | 0.95-1.28 | 8.06E-01 | 1.09 | 0.93-1.26 | 8.53E-01 |
| 3-methyl catechol sulfate (1) | 1.10 | 0.95-1.28 | 8.06E-01 | 1.09 | 0.93-1.27 | 8.53E-01 |
| 2-aminoheptanoate | 1.09 | 0.95-1.26 | 8.06E-01 | 1.08 | 0.94-1.25 | 8.53E-01 |
| 3-methoxytyramine sulfate | 1.09 | 0.96-1.23 | 8.06E-01 | 1.08 | 0.95-1.23 | 8.53E-01 |
| myristoleoylcarnitine (C14:1)* | 0.92 | 0.81-1.05 | 8.06E-01 | 0.93 | 0.81-1.06 | 8.53E-01 |
| indoleacetoylcarnitine* | 1.10 | 0.95-1.26 | 8.06E-01 | 1.08 | 0.94-1.25 | 8.53E-01 |
| bilirubin degradation product, C17H20N2O5 (1)** | 0.92 | 0.81-1.05 | 8.06E-01 | 0.92 | 0.81-1.05 | 8.53E-01 |
| N6-methyladenosine | 1.09 | 0.95-1.25 | 8.06E-01 | 1.08 | 0.94-1.24 | 8.53E-01 |
| X - 07765 | 1.09 | 0.96-1.25 | 8.06E-01 | 1.08 | 0.94-1.24 | 8.53E-01 |
| X - 11381 | 0.92 | 0.8-1.05 | 8.06E-01 | 0.92 | 0.8-1.05 | 8.53E-01 |
| X - 21339 | 0.92 | 0.8-1.05 | 8.06E-01 | 0.92 | 0.79-1.06 | 8.53E-01 |
| X - 24811 | 1.10 | 0.95-1.28 | 8.06E-01 | 1.10 | 0.94-1.27 | 8.53E-01 |
| cysteinylglycine disulfide* | 1.09 | 0.95-1.24 | 8.18E-01 | 1.08 | 0.95-1.24 | 8.53E-01 |
| 1-stearoyl-2-linoleoyl-GPE (18:0/18:2)* | 1.10 | 0.94-1.28 | 8.18E-01 | 1.09 | 0.94-1.27 | 8.53E-01 |
| bilirubin degradation product, C17H20N2O5 (2)** | 0.92 | 0.81-1.05 | 8.18E-01 | 0.92 | 0.81-1.05 | 8.53E-01 |
| X - 23636 | 1.09 | 0.95-1.26 | 8.18E-01 | 1.08 | 0.94-1.24 | 8.53E-01 |
| gamma-glutamylhistidine | 1.09 | 0.95-1.26 | 8.26E-01 | 1.09 | 0.94-1.26 | 8.53E-01 |
| X - 17346 | 1.09 | 0.95-1.26 | 8.26E-01 | 1.09 | 0.94-1.26 | 8.53E-01 |
| X - 23644 | 1.09 | 0.95-1.26 | 8.26E-01 | 1.09 | 0.94-1.26 | 8.53E-01 |
| ascorbic acid 3-sulfate* | 1.09 | 0.94-1.27 | 8.27E-01 | 1.09 | 0.95-1.27 | 8.53E-01 |
| pregnanediol-3-glucuronide | 0.92 | 0.81-1.06 | 8.31E-01 | 0.93 | 0.81-1.06 | 8.53E-01 |
| X - 21959 | 0.92 | 0.81-1.05 | 8.31E-01 | 0.93 | 0.81-1.06 | 8.53E-01 |
| X - 12713 | 1.10 | 0.94-1.28 | 8.40E-01 | 1.10 | 0.94-1.28 | 8.53E-01 |
| quinate | 1.09 | 0.93-1.27 | 8.42E-01 | 1.09 | 0.93-1.27 | 8.53E-01 |
| 1,5-anhydroglucitol (1,5-AG) | 0.93 | 0.8-1.07 | 8.42E-01 | 0.92 | 0.79-1.06 | 8.53E-01 |
| N-acetylaspartate (NAA) | 1.08 | 0.93-1.26 | 8.42E-01 | 1.09 | 0.94-1.27 | 8.53E-01 |
| dehydroepiandrosterone sulfate (DHEA-S) | 0.92 | 0.8-1.06 | 8.42E-01 | 0.92 | 0.8-1.06 | 8.53E-01 |
| 1-oleoylglycerol (18:1) | 1.09 | 0.94-1.26 | 8.42E-01 | 1.08 | 0.93-1.26 | 8.53E-01 |
| trigonelline (N'-methylnicotinate) | 1.09 | 0.93-1.26 | 8.42E-01 | 1.09 | 0.93-1.26 | 8.53E-01 |
| hyocholate | 0.93 | 0.81-1.06 | 8.42E-01 | 0.93 | 0.81-1.06 | 8.53E-01 |
| 4-hydroxyhippurate | 1.07 | 0.94-1.22 | 8.42E-01 | 1.08 | 0.95-1.23 | 8.53E-01 |
| malonylcarnitine | 1.09 | 0.94-1.26 | 8.42E-01 | 1.09 | 0.94-1.26 | 8.53E-01 |
| 2-hydroxy-3-methylvalerate | 0.92 | 0.79-1.08 | 8.42E-01 | 0.91 | 0.77-1.07 | 8.53E-01 |
| hydroquinone sulfate | 1.09 | 0.94-1.25 | 8.42E-01 | 1.08 | 0.94-1.25 | 8.53E-01 |
| catechol sulfate | 1.09 | 0.93-1.28 | 8.42E-01 | 1.10 | 0.94-1.29 | 8.53E-01 |
| taurolithocholate 3-sulfate | 0.94 | 0.83-1.06 | 8.42E-01 | 0.93 | 0.83-1.06 | 8.53E-01 |
| gamma-glutamylalanine | 1.10 | 0.93-1.3 | 8.42E-01 | 1.10 | 0.93-1.3 | 8.53E-01 |
| N-acetylserine | 1.09 | 0.93-1.27 | 8.42E-01 | 1.09 | 0.93-1.27 | 8.53E-01 |
| sulfate* | 1.09 | 0.93-1.27 | 8.42E-01 | 1.09 | 0.93-1.27 | 8.53E-01 |
| 1-methyl-5-imidazoleacetate | 1.08 | 0.93-1.25 | 8.42E-01 | 1.09 | 0.94-1.26 | 8.53E-01 |
| valylglycine | 0.93 | 0.81-1.06 | 8.42E-01 | 0.92 | 0.8-1.05 | 8.53E-01 |
| histidine betaine (hercynine)* | 1.07 | 0.93-1.23 | 8.42E-01 | 1.08 | 0.94-1.25 | 8.53E-01 |
| glycohyocholate | 0.93 | 0.8-1.08 | 8.42E-01 | 0.92 | 0.79-1.07 | 8.53E-01 |
| glycodeoxycholate 3-sulfate | 0.93 | 0.81-1.06 | 8.42E-01 | 0.92 | 0.8-1.06 | 8.53E-01 |
| palmitoyl dihydrosphingomyelin (d18:0/16:0)* | 1.10 | 0.94-1.28 | 8.42E-01 | 1.10 | 0.93-1.3 | 8.53E-01 |
| 1-stearoyl-2-arachidonoyl-GPE (18:0/20:4) | 1.10 | 0.93-1.31 | 8.42E-01 | 1.10 | 0.92-1.31 | 8.53E-01 |
| 1-linoleoyl-2-linolenoyl-GPC (18:2/18:3)* | 0.92 | 0.79-1.07 | 8.42E-01 | 0.92 | 0.78-1.08 | 8.53E-01 |
| glycosyl ceramide (d18:2/24:1, d18:1/24:2)* | 0.92 | 0.8-1.06 | 8.42E-01 | 0.92 | 0.79-1.07 | 8.53E-01 |
| sphingomyelin (d18:2/18:1)* | 0.92 | 0.78-1.08 | 8.42E-01 | 0.91 | 0.78-1.08 | 8.53E-01 |
| ximenoylcarnitine (C26:1)* | 1.08 | 0.94-1.23 | 8.42E-01 | 1.08 | 0.95-1.23 | 8.53E-01 |
| docosahexaenoylcarnitine (C22:6)* | 1.08 | 0.94-1.24 | 8.42E-01 | 1.09 | 0.95-1.26 | 8.53E-01 |
| nervonoylcarnitine (C24:1)* | 1.06 | 0.94-1.2 | 8.42E-01 | 1.07 | 0.95-1.21 | 8.53E-01 |
| glutamine conjugate of C7H12O2* | 0.93 | 0.81-1.06 | 8.42E-01 | 0.92 | 0.8-1.06 | 8.53E-01 |
| 3-hydroxyhippurate sulfate | 1.08 | 0.94-1.25 | 8.42E-01 | 1.09 | 0.94-1.26 | 8.53E-01 |
| N6,N6-dimethyllysine | 0.92 | 0.8-1.06 | 8.42E-01 | 0.92 | 0.8-1.07 | 8.53E-01 |
| glucuronide of piperine metabolite C17H21NO3 (4)* | 1.08 | 0.93-1.26 | 8.42E-01 | 1.09 | 0.93-1.27 | 8.53E-01 |
| glucuronide of piperine metabolite C17H21NO3 (5)* | 1.08 | 0.94-1.25 | 8.42E-01 | 1.08 | 0.93-1.26 | 8.53E-01 |
| 3-hydroxy-3-methylglutarate | 1.10 | 0.93-1.3 | 8.42E-01 | 1.10 | 0.93-1.3 | 8.53E-01 |
| gamma-glutamylglutamine | 1.08 | 0.94-1.25 | 8.42E-01 | 1.09 | 0.94-1.26 | 8.53E-01 |
| cys-gly, oxidized | 1.08 | 0.94-1.24 | 8.42E-01 | 1.08 | 0.94-1.25 | 8.53E-01 |
| quinolinate | 1.08 | 0.93-1.25 | 8.42E-01 | 1.09 | 0.94-1.27 | 8.53E-01 |
| phenylpyruvate | 0.93 | 0.82-1.06 | 8.42E-01 | 0.92 | 0.81-1.05 | 8.53E-01 |
| cortisol | 0.93 | 0.81-1.06 | 8.42E-01 | 0.93 | 0.81-1.07 | 8.53E-01 |
| X - 12456 | 0.93 | 0.8-1.07 | 8.42E-01 | 0.91 | 0.78-1.06 | 8.53E-01 |
| X - 12544 | 0.94 | 0.82-1.07 | 8.42E-01 | 0.93 | 0.8-1.06 | 8.53E-01 |
| X - 12726 | 0.93 | 0.81-1.06 | 8.42E-01 | 0.93 | 0.81-1.07 | 8.53E-01 |
| X - 14082 | 1.08 | 0.94-1.25 | 8.42E-01 | 1.08 | 0.94-1.25 | 8.53E-01 |
| X - 14939 | 0.93 | 0.82-1.06 | 8.42E-01 | 0.92 | 0.81-1.05 | 8.53E-01 |
| X - 15728 | 0.94 | 0.83-1.06 | 8.42E-01 | 0.93 | 0.82-1.06 | 8.53E-01 |
| X - 16580 | 1.07 | 0.93-1.24 | 8.42E-01 | 1.08 | 0.94-1.25 | 8.53E-01 |
| X - 19438 | 1.09 | 0.93-1.27 | 8.42E-01 | 1.09 | 0.93-1.29 | 8.53E-01 |
| X - 21467 | 0.93 | 0.8-1.07 | 8.42E-01 | 0.92 | 0.8-1.06 | 8.53E-01 |
| X - 21471 | 0.93 | 0.8-1.07 | 8.42E-01 | 0.92 | 0.79-1.06 | 8.53E-01 |
| X - 23276 | 0.93 | 0.81-1.05 | 8.42E-01 | 0.93 | 0.81-1.06 | 8.53E-01 |
| X - 24306 | 0.94 | 0.83-1.06 | 8.42E-01 | 0.93 | 0.82-1.06 | 8.53E-01 |
| sphingomyelin (d18:1/21:0, d17:1/22:0, d16:1/23:0)* | 1.08 | 0.92-1.28 | 8.43E-01 | 1.10 | 0.93-1.3 | 8.53E-01 |
| pregnenetriol sulfate* | 0.93 | 0.8-1.08 | 8.43E-01 | 0.92 | 0.79-1.07 | 8.53E-01 |
| palmitoyl-sphingosine-phosphoethanolamine (d18:1/16:0) | 1.07 | 0.93-1.24 | 8.43E-01 | 1.09 | 0.94-1.26 | 8.53E-01 |
| androsterone sulfate | 0.94 | 0.82-1.07 | 8.44E-01 | 0.93 | 0.81-1.06 | 8.53E-01 |
| X - 26111 | 1.07 | 0.93-1.22 | 8.44E-01 | 1.08 | 0.94-1.24 | 8.53E-01 |
| X - 16397 | 1.07 | 0.93-1.23 | 8.50E-01 | 1.09 | 0.94-1.26 | 8.53E-01 |
| cyclo(leu-pro) | 1.08 | 0.92-1.26 | 8.54E-01 | 1.10 | 0.92-1.32 | 8.53E-01 |
| isoeugenol sulfate | 0.94 | 0.83-1.07 | 8.54E-01 | 0.92 | 0.81-1.05 | 8.53E-01 |
| pantothenate | 1.07 | 0.92-1.25 | 8.54E-01 | 1.09 | 0.94-1.28 | 8.53E-01 |
| 1-arachidonylglycerol (20:4) | 1.07 | 0.92-1.23 | 8.55E-01 | 1.09 | 0.94-1.25 | 8.53E-01 |
| ornithine | 0.94 | 0.82-1.09 | 8.71E-01 | 0.92 | 0.8-1.06 | 8.53E-01 |
| 1-stearoyl-2-oleoyl-GPI (18:0/18:1)* | 1.08 | 0.94-1.24 | 8.42E-01 | 1.08 | 0.94-1.24 | 8.59E-01 |
| lactosyl-N-palmitoyl-sphingosine (d18:1/16:0) | 0.91 | 0.78-1.06 | 8.40E-01 | 0.92 | 0.78-1.08 | 8.61E-01 |
| 5alpha-androstan-3alpha,17beta-diol monosulfate (1) | 0.94 | 0.82-1.08 | 8.54E-01 | 0.93 | 0.81-1.07 | 8.62E-01 |
| glycerol | 0.90 | 0.77-1.05 | 7.99E-01 | 0.92 | 0.79-1.08 | 8.63E-01 |
| myristate (14:0) | 0.91 | 0.78-1.05 | 8.06E-01 | 0.92 | 0.79-1.08 | 8.63E-01 |
| sphingosine | 1.08 | 0.95-1.24 | 8.18E-01 | 1.07 | 0.94-1.22 | 8.63E-01 |
| linoleoylcarnitine (C18:2)* | 0.92 | 0.79-1.06 | 8.26E-01 | 0.93 | 0.8-1.07 | 8.63E-01 |
| N2-acetyl,N6-methyllysine | 0.92 | 0.81-1.05 | 8.26E-01 | 0.94 | 0.82-1.07 | 8.63E-01 |
| 5-hydroxylysine | 1.07 | 0.94-1.2 | 8.42E-01 | 1.07 | 0.94-1.22 | 8.63E-01 |
| N-(2-furoyl)glycine | 1.08 | 0.94-1.24 | 8.42E-01 | 1.07 | 0.93-1.23 | 8.63E-01 |
| 7-methylguanine | 1.07 | 0.93-1.24 | 8.42E-01 | 1.07 | 0.93-1.24 | 8.63E-01 |
| glycerophosphoethanolamine | 1.09 | 0.92-1.28 | 8.42E-01 | 1.09 | 0.93-1.27 | 8.63E-01 |
| 1-palmitoyl-GPI (16:0) | 1.08 | 0.93-1.26 | 8.42E-01 | 1.08 | 0.93-1.26 | 8.63E-01 |
| glycolithocholate sulfate* | 0.94 | 0.83-1.06 | 8.42E-01 | 0.94 | 0.83-1.06 | 8.63E-01 |
| S-methylmethionine | 0.92 | 0.8-1.06 | 8.42E-01 | 0.93 | 0.81-1.07 | 8.63E-01 |
| hydantoin-5-propionate | 0.93 | 0.82-1.06 | 8.42E-01 | 0.93 | 0.82-1.06 | 8.63E-01 |
| (14 or 15)-methylpalmitate (a17:0 or i17:0) | 0.91 | 0.77-1.07 | 8.42E-01 | 0.92 | 0.78-1.09 | 8.63E-01 |
| N6-methyllysine | 0.93 | 0.81-1.06 | 8.42E-01 | 0.93 | 0.81-1.07 | 8.63E-01 |
| 3b-hydroxy-5-cholenoic acid | 0.93 | 0.81-1.06 | 8.42E-01 | 0.93 | 0.81-1.07 | 8.63E-01 |
| 4-methoxyphenol sulfate | 1.08 | 0.93-1.26 | 8.42E-01 | 1.08 | 0.93-1.26 | 8.63E-01 |
| sphingomyelin (d18:1/20:2, d18:2/20:1, d16:1/22:2)* | 0.93 | 0.8-1.08 | 8.42E-01 | 0.93 | 0.79-1.08 | 8.63E-01 |
| N-carbamoylalanine | 1.07 | 0.94-1.22 | 8.42E-01 | 1.07 | 0.94-1.22 | 8.63E-01 |
| C-glycosyltryptophan | 0.92 | 0.79-1.09 | 8.42E-01 | 0.92 | 0.78-1.08 | 8.63E-01 |
| 1,2-dilinoleoyl-GPC (18:2/18:2) | 0.93 | 0.81-1.06 | 8.42E-01 | 0.93 | 0.81-1.07 | 8.63E-01 |
| 2'-O-methylcytidine | 1.08 | 0.93-1.24 | 8.42E-01 | 1.08 | 0.93-1.25 | 8.63E-01 |
| N-palmitoylserine | 1.08 | 0.94-1.25 | 8.42E-01 | 1.08 | 0.93-1.24 | 8.63E-01 |
| butyrate/isobutyrate (4:0) | 0.93 | 0.81-1.07 | 8.42E-01 | 0.93 | 0.81-1.07 | 8.63E-01 |
| N-palmitoyl-sphingosine (d18:1/16:0) | 1.07 | 0.94-1.23 | 8.42E-01 | 1.07 | 0.93-1.22 | 8.63E-01 |
| corticosterone | 0.94 | 0.83-1.06 | 8.42E-01 | 0.94 | 0.83-1.06 | 8.63E-01 |
| caprate (10:0) | 0.91 | 0.78-1.07 | 8.42E-01 | 0.92 | 0.79-1.08 | 8.63E-01 |
| kynurenate | 0.93 | 0.81-1.07 | 8.42E-01 | 0.93 | 0.8-1.07 | 8.63E-01 |
| X - 12193 | 1.08 | 0.95-1.23 | 8.42E-01 | 1.07 | 0.94-1.23 | 8.63E-01 |
| X - 12822 | 0.92 | 0.8-1.07 | 8.42E-01 | 0.93 | 0.8-1.08 | 8.63E-01 |
| X - 23587 | 1.07 | 0.93-1.23 | 8.42E-01 | 1.07 | 0.93-1.23 | 8.63E-01 |
| X - 23655 | 1.08 | 0.93-1.25 | 8.42E-01 | 1.08 | 0.93-1.24 | 8.63E-01 |
| X - 23739 | 0.93 | 0.81-1.07 | 8.42E-01 | 0.93 | 0.81-1.07 | 8.63E-01 |
| X - 23997 | 1.08 | 0.94-1.25 | 8.42E-01 | 1.08 | 0.93-1.25 | 8.63E-01 |
| gamma-glutamyltryptophan | 0.93 | 0.81-1.08 | 8.43E-01 | 0.93 | 0.8-1.07 | 8.63E-01 |
| gluconate | 1.08 | 0.92-1.25 | 8.43E-01 | 1.08 | 0.92-1.26 | 8.63E-01 |
| 1,3-dimethylurate | 1.07 | 0.93-1.24 | 8.44E-01 | 1.08 | 0.93-1.25 | 8.63E-01 |
| 3-carboxy-4-methyl-5-propyl-2-furanpropanoate (CMPF) | 1.07 | 0.93-1.23 | 8.52E-01 | 1.08 | 0.93-1.24 | 8.63E-01 |
| epiandrosterone sulfate | 0.94 | 0.82-1.08 | 8.54E-01 | 0.93 | 0.81-1.07 | 8.63E-01 |
| alpha-ketoglutarate | 1.08 | 0.91-1.27 | 8.54E-01 | 1.09 | 0.92-1.29 | 8.63E-01 |
| X - 18888 | 0.94 | 0.82-1.08 | 8.54E-01 | 0.93 | 0.81-1.07 | 8.63E-01 |
| X - 23639 | 0.94 | 0.82-1.08 | 8.57E-01 | 0.93 | 0.8-1.08 | 8.63E-01 |
| deoxycholate | 1.06 | 0.93-1.22 | 8.59E-01 | 1.08 | 0.93-1.24 | 8.63E-01 |
| pregnenediol disulfate (C21H34O8S2)* | 0.93 | 0.79-1.1 | 8.68E-01 | 0.92 | 0.78-1.09 | 8.63E-01 |
| androstenediol (3alpha, 17alpha) monosulfate (3) | 0.94 | 0.81-1.09 | 8.70E-01 | 0.92 | 0.79-1.07 | 8.63E-01 |
| indolin-2-one | 1.06 | 0.92-1.21 | 8.70E-01 | 1.07 | 0.94-1.23 | 8.63E-01 |
| X - 22509 | 1.06 | 0.92-1.21 | 8.71E-01 | 1.07 | 0.93-1.23 | 8.63E-01 |
| X - 11847 | 1.05 | 0.93-1.2 | 8.72E-01 | 1.07 | 0.94-1.22 | 8.63E-01 |
| N-acetyl-isoputreanine | 1.08 | 0.93-1.24 | 8.42E-01 | 1.07 | 0.93-1.23 | 8.65E-01 |
| X - 18935 | 1.05 | 0.92-1.21 | 8.77E-01 | 1.07 | 0.93-1.23 | 8.66E-01 |
| 1-(1-enyl-palmitoyl)-2-linoleoyl-GPC (P-16:0/18:2)* | 0.92 | 0.8-1.06 | 8.42E-01 | 0.93 | 0.8-1.08 | 8.67E-01 |
| phenylacetate | 1.08 | 0.92-1.27 | 8.42E-01 | 1.08 | 0.92-1.27 | 8.69E-01 |
| propionylcarnitine (C3) | 1.07 | 0.94-1.22 | 8.42E-01 | 1.07 | 0.93-1.22 | 8.69E-01 |
| 10-nonadecenoate (19:1n9) | 0.91 | 0.78-1.07 | 8.42E-01 | 0.93 | 0.79-1.09 | 8.69E-01 |
| 5-methyluridine (ribothymidine) | 0.94 | 0.82-1.07 | 8.42E-01 | 0.94 | 0.82-1.08 | 8.69E-01 |
| retinal | 0.93 | 0.81-1.06 | 8.42E-01 | 0.94 | 0.81-1.08 | 8.69E-01 |
| 1-palmitoyl-2-stearoyl-GPC (16:0/18:0) | 0.93 | 0.81-1.07 | 8.42E-01 | 0.93 | 0.81-1.08 | 8.69E-01 |
| glucuronide of C10H18O2 (7)* | 1.07 | 0.93-1.24 | 8.42E-01 | 1.07 | 0.93-1.24 | 8.69E-01 |
| N2-acetyl,N6,N6-dimethyllysine | 0.93 | 0.81-1.06 | 8.42E-01 | 0.94 | 0.82-1.07 | 8.69E-01 |
| 5-hydroxyindoleacetate | 1.08 | 0.93-1.25 | 8.42E-01 | 1.07 | 0.93-1.24 | 8.69E-01 |
| X - 11315 | 0.92 | 0.8-1.06 | 8.42E-01 | 0.93 | 0.8-1.08 | 8.69E-01 |
| X - 15461 | 1.08 | 0.93-1.25 | 8.42E-01 | 1.07 | 0.93-1.25 | 8.69E-01 |
| X - 25810 | 1.07 | 0.94-1.21 | 8.42E-01 | 1.06 | 0.94-1.21 | 8.69E-01 |
| X - 12816 | 1.08 | 0.92-1.26 | 8.43E-01 | 1.08 | 0.92-1.25 | 8.69E-01 |
| N-acetyl-2-aminoadipate | 1.07 | 0.92-1.24 | 8.54E-01 | 1.07 | 0.92-1.25 | 8.69E-01 |
| X - 21470 | 0.94 | 0.81-1.08 | 8.54E-01 | 0.93 | 0.8-1.08 | 8.69E-01 |
| X - 24328 | 0.92 | 0.77-1.11 | 8.54E-01 | 0.92 | 0.77-1.1 | 8.69E-01 |
| oleoylcholine | 1.06 | 0.92-1.21 | 8.68E-01 | 1.07 | 0.93-1.23 | 8.69E-01 |
| 3-hydroxyoleoylcarnitine | 1.07 | 0.91-1.24 | 8.69E-01 | 1.08 | 0.92-1.26 | 8.69E-01 |
| X - 22162 | 1.06 | 0.92-1.22 | 8.69E-01 | 1.07 | 0.93-1.23 | 8.69E-01 |
| stachydrine | 1.05 | 0.91-1.2 | 9.27E-01 | 1.07 | 0.93-1.23 | 8.69E-01 |
| beta-cryptoxanthin | 1.03 | 0.9-1.19 | 9.58E-01 | 1.07 | 0.92-1.25 | 8.69E-01 |
| 13-HODE + 9-HODE | 0.92 | 0.8-1.07 | 8.42E-01 | 0.93 | 0.8-1.08 | 8.72E-01 |
| N-formylanthranilic acid | 1.07 | 0.92-1.23 | 8.68E-01 | 1.07 | 0.92-1.24 | 8.72E-01 |
| creatinine | 1.09 | 0.91-1.29 | 8.43E-01 | 1.08 | 0.91-1.29 | 8.73E-01 |
| 2-linoleoylglycerol (18:2) | 1.07 | 0.92-1.25 | 8.52E-01 | 1.07 | 0.92-1.25 | 8.74E-01 |
| 3beta-hydroxy-5-cholestenoate | 0.94 | 0.82-1.09 | 8.69E-01 | 0.93 | 0.8-1.08 | 8.74E-01 |
| indolepropionate | 0.93 | 0.82-1.06 | 8.42E-01 | 0.94 | 0.82-1.07 | 8.74E-01 |
| gamma-CEHC glucuronide* | 1.08 | 0.92-1.26 | 8.42E-01 | 1.07 | 0.92-1.26 | 8.74E-01 |
| 2-hydroxynervonate* | 1.09 | 0.94-1.27 | 8.42E-01 | 1.07 | 0.92-1.25 | 8.74E-01 |
| 3-hydroxyphenylacetoylglutamine | 0.93 | 0.81-1.07 | 8.42E-01 | 0.94 | 0.82-1.08 | 8.74E-01 |
| 3-hydroxy-2-methylpyridine sulfate | 1.08 | 0.94-1.24 | 8.42E-01 | 1.07 | 0.93-1.23 | 8.74E-01 |
| succinate | 0.92 | 0.81-1.05 | 8.42E-01 | 0.94 | 0.82-1.08 | 8.74E-01 |
| palmitoleate (16:1n7) | 0.92 | 0.79-1.07 | 8.42E-01 | 0.93 | 0.8-1.09 | 8.74E-01 |
| X - 21796 | 0.93 | 0.81-1.07 | 8.42E-01 | 0.94 | 0.82-1.08 | 8.74E-01 |
| 2-aminooctanoate | 0.94 | 0.82-1.07 | 8.43E-01 | 0.94 | 0.83-1.07 | 8.74E-01 |
| X - 24728 | 1.07 | 0.93-1.24 | 8.44E-01 | 1.07 | 0.92-1.24 | 8.74E-01 |
| citrate | 0.93 | 0.81-1.08 | 8.54E-01 | 0.94 | 0.81-1.08 | 8.74E-01 |
| 1-palmitoyl-2-linoleoyl-GPI (16:0/18:2) | 1.07 | 0.92-1.24 | 8.54E-01 | 1.07 | 0.92-1.24 | 8.74E-01 |
| X - 25265 | 1.07 | 0.92-1.25 | 8.54E-01 | 1.07 | 0.92-1.25 | 8.74E-01 |
| X - 25371 | 1.07 | 0.91-1.26 | 8.68E-01 | 1.08 | 0.91-1.27 | 8.74E-01 |
| gamma-glutamylthreonine | 0.94 | 0.81-1.09 | 8.72E-01 | 0.93 | 0.81-1.08 | 8.74E-01 |
| X - 21312 | 1.06 | 0.92-1.21 | 8.72E-01 | 1.07 | 0.93-1.22 | 8.74E-01 |
| 1-oleoyl-GPC (18:1) | 1.05 | 0.91-1.21 | 9.06E-01 | 1.07 | 0.92-1.24 | 8.74E-01 |
| S-carboxyethylcysteine | 1.09 | 0.95-1.25 | 8.06E-01 | 1.07 | 0.92-1.23 | 8.77E-01 |
| eicosenoate (20:1) | 0.93 | 0.81-1.07 | 8.42E-01 | 0.94 | 0.81-1.08 | 8.77E-01 |
| prolylglycine | 0.93 | 0.82-1.07 | 8.42E-01 | 0.94 | 0.82-1.08 | 8.77E-01 |
| tyramine O-sulfate | 1.07 | 0.94-1.22 | 8.42E-01 | 1.06 | 0.93-1.22 | 8.77E-01 |
| stearate (18:0) | 0.93 | 0.8-1.08 | 8.42E-01 | 0.94 | 0.81-1.09 | 8.77E-01 |
| uridine | 0.93 | 0.81-1.06 | 8.42E-01 | 0.94 | 0.82-1.08 | 8.77E-01 |
| nonadecanoate (19:0) | 0.92 | 0.8-1.07 | 8.42E-01 | 0.94 | 0.8-1.09 | 8.77E-01 |
| 3-hydroxysebacate | 0.93 | 0.8-1.09 | 8.54E-01 | 0.94 | 0.81-1.09 | 8.77E-01 |
| nicotinamide | 1.06 | 0.93-1.22 | 8.54E-01 | 1.06 | 0.93-1.22 | 8.77E-01 |
| isovalerylglycine | 1.07 | 0.92-1.23 | 8.57E-01 | 1.07 | 0.92-1.24 | 8.77E-01 |
| 4-guanidinobutanoate | 1.06 | 0.93-1.22 | 8.63E-01 | 1.06 | 0.93-1.22 | 8.77E-01 |
| glycochenodeoxycholate | 0.93 | 0.8-1.09 | 8.68E-01 | 0.93 | 0.8-1.09 | 8.77E-01 |
| 1-stearoyl-2-arachidonoyl-GPC (18:0/20:4) | 1.08 | 0.9-1.28 | 8.68E-01 | 1.08 | 0.9-1.29 | 8.77E-01 |
| taurodeoxycholic acid 3-sulfate | 0.95 | 0.83-1.08 | 8.68E-01 | 0.94 | 0.83-1.08 | 8.77E-01 |
| X - 21410 | 1.07 | 0.91-1.25 | 8.68E-01 | 1.07 | 0.92-1.25 | 8.77E-01 |
| 1-oleoyl-GPE (18:1) | 1.07 | 0.92-1.24 | 8.69E-01 | 1.07 | 0.92-1.25 | 8.77E-01 |
| X - 25790 | 0.94 | 0.8-1.09 | 8.69E-01 | 0.93 | 0.8-1.09 | 8.77E-01 |
| lactate | 1.06 | 0.92-1.22 | 8.70E-01 | 1.07 | 0.92-1.23 | 8.77E-01 |
| N-oleoyltaurine | 0.94 | 0.81-1.1 | 8.72E-01 | 0.94 | 0.8-1.09 | 8.77E-01 |
| 1-palmitoyl-GPC (16:0) | 1.06 | 0.92-1.22 | 8.77E-01 | 1.07 | 0.92-1.25 | 8.77E-01 |
| sphingomyelin (d18:1/19:0, d19:1/18:0)* | 1.06 | 0.9-1.26 | 8.83E-01 | 1.08 | 0.91-1.27 | 8.77E-01 |
| 1-linolenoyl-GPC (18:3)* | 1.06 | 0.91-1.23 | 8.93E-01 | 1.07 | 0.92-1.25 | 8.77E-01 |
| 4-allylcatechol sulfate | 1.05 | 0.92-1.2 | 9.00E-01 | 1.06 | 0.93-1.21 | 8.77E-01 |
| 2'-deoxyuridine | 0.93 | 0.81-1.08 | 8.50E-01 | 0.94 | 0.81-1.09 | 8.80E-01 |
| 3-methoxycatechol sulfate (1) | 1.07 | 0.92-1.24 | 8.54E-01 | 1.07 | 0.92-1.23 | 8.80E-01 |
| mannonate* | 1.06 | 0.92-1.22 | 8.68E-01 | 1.06 | 0.92-1.23 | 8.80E-01 |
| N-palmitoyl-sphingadienine (d18:2/16:0)* | 1.06 | 0.92-1.22 | 8.68E-01 | 1.06 | 0.92-1.22 | 8.80E-01 |
| X - 16935 | 0.94 | 0.82-1.09 | 8.72E-01 | 0.94 | 0.82-1.09 | 8.80E-01 |
| hydroxy-CMPF* | 1.05 | 0.92-1.19 | 9.20E-01 | 1.06 | 0.93-1.21 | 8.80E-01 |
| isovalerate (i5:0) | 0.95 | 0.84-1.08 | 8.70E-01 | 0.95 | 0.84-1.08 | 8.83E-01 |
| X - 15245 | 1.05 | 0.91-1.21 | 9.28E-01 | 1.06 | 0.92-1.23 | 8.83E-01 |
| X - 22776 | 1.06 | 0.92-1.23 | 8.71E-01 | 1.06 | 0.92-1.23 | 8.84E-01 |
| N-acetylcarnosine | 0.93 | 0.78-1.12 | 8.79E-01 | 0.92 | 0.77-1.11 | 8.84E-01 |
| sphingomyelin (d18:1/17:0, d17:1/18:0, d19:1/16:0) | 1.06 | 0.9-1.24 | 9.05E-01 | 1.07 | 0.91-1.27 | 8.85E-01 |
| myristoylcarnitine (C14) | 0.93 | 0.81-1.07 | 8.42E-01 | 0.95 | 0.82-1.09 | 8.88E-01 |
| palmitoleoylcarnitine (C16:1)* | 0.93 | 0.81-1.07 | 8.42E-01 | 0.94 | 0.82-1.09 | 8.88E-01 |
| eicosenoylcarnitine (C20:1)* | 0.94 | 0.83-1.07 | 8.42E-01 | 0.95 | 0.83-1.08 | 8.88E-01 |
| carotene diol (3) | 0.94 | 0.82-1.07 | 8.42E-01 | 0.95 | 0.83-1.08 | 8.88E-01 |
| palmitate (16:0) | 0.93 | 0.8-1.07 | 8.42E-01 | 0.94 | 0.81-1.09 | 8.88E-01 |
| ribitol | 1.07 | 0.93-1.23 | 8.43E-01 | 1.06 | 0.92-1.22 | 8.88E-01 |
| proline | 1.07 | 0.93-1.24 | 8.43E-01 | 1.06 | 0.92-1.23 | 8.88E-01 |
| suberoylcarnitine (C8-DC) | 1.07 | 0.93-1.22 | 8.44E-01 | 1.06 | 0.92-1.22 | 8.88E-01 |
| 5-dodecenoylcarnitine (C12:1) | 0.94 | 0.83-1.07 | 8.44E-01 | 0.95 | 0.83-1.08 | 8.88E-01 |
| X - 11850 | 1.07 | 0.93-1.24 | 8.44E-01 | 1.06 | 0.91-1.24 | 8.88E-01 |
| X - 23680 | 1.06 | 0.94-1.21 | 8.44E-01 | 1.05 | 0.93-1.2 | 8.88E-01 |
| 3-indoleglyoxylic acid | 1.07 | 0.93-1.23 | 8.44E-01 | 1.06 | 0.92-1.22 | 8.88E-01 |
| gamma-glutamylglutamate | 1.06 | 0.93-1.22 | 8.44E-01 | 1.06 | 0.92-1.22 | 8.88E-01 |
| glycosyl-N-behenoyl-sphingadienine (d18:2/22:0)* | 0.94 | 0.82-1.08 | 8.54E-01 | 0.94 | 0.82-1.09 | 8.88E-01 |
| arachidate (20:0) | 0.94 | 0.81-1.09 | 8.68E-01 | 0.94 | 0.81-1.09 | 8.88E-01 |
| S-methylcysteine sulfoxide | 0.94 | 0.82-1.08 | 8.68E-01 | 0.94 | 0.82-1.09 | 8.88E-01 |
| 1-oleoyl-GPG (18:1)* | 1.06 | 0.92-1.22 | 8.68E-01 | 1.06 | 0.92-1.22 | 8.88E-01 |
| X - 12104 | 0.95 | 0.83-1.08 | 8.68E-01 | 0.95 | 0.83-1.08 | 8.88E-01 |
| 1-palmitoyl-GPG (16:0)* | 1.06 | 0.91-1.23 | 8.72E-01 | 1.06 | 0.91-1.23 | 8.88E-01 |
| urate | 1.06 | 0.92-1.22 | 8.76E-01 | 1.06 | 0.92-1.23 | 8.88E-01 |
| 2-hydroxydecanoate | 0.95 | 0.82-1.09 | 8.77E-01 | 0.94 | 0.82-1.09 | 8.88E-01 |
| myristoyl dihydrosphingomyelin (d18:0/14:0)* | 1.06 | 0.91-1.23 | 8.99E-01 | 1.06 | 0.91-1.24 | 8.88E-01 |
| glycerol 3-phosphate | 1.05 | 0.91-1.21 | 9.05E-01 | 1.06 | 0.92-1.22 | 8.88E-01 |
| 5alpha-androstan-3beta,17beta-diol monosulfate (2) | 0.95 | 0.83-1.1 | 9.28E-01 | 0.94 | 0.81-1.09 | 8.88E-01 |
| lyxonate | 1.04 | 0.91-1.19 | 9.29E-01 | 1.05 | 0.92-1.2 | 8.88E-01 |
| X - 11299 | 1.05 | 0.91-1.21 | 9.29E-01 | 1.06 | 0.92-1.23 | 8.88E-01 |
| X - 12117 | 1.08 | 0.92-1.28 | 8.42E-01 | 1.07 | 0.9-1.27 | 8.88E-01 |
| 3-hydroxypyridine sulfate | 1.07 | 0.92-1.24 | 8.68E-01 | 1.06 | 0.91-1.23 | 8.90E-01 |
| 4-hydroxyphenylpyruvate | 1.06 | 0.92-1.22 | 8.70E-01 | 1.06 | 0.92-1.22 | 8.90E-01 |
| X - 12906 | 1.06 | 0.92-1.22 | 8.70E-01 | 1.06 | 0.92-1.23 | 8.90E-01 |
| glucuronide of piperine metabolite C17H21NO3 (3)* | 0.95 | 0.82-1.1 | 8.99E-01 | 0.94 | 0.81-1.09 | 8.90E-01 |
| X - 24970 | 0.95 | 0.83-1.09 | 9.00E-01 | 0.95 | 0.83-1.09 | 8.90E-01 |
| androsterone glucuronide | 0.94 | 0.81-1.11 | 9.00E-01 | 0.94 | 0.8-1.1 | 8.90E-01 |
| arabonate/xylonate | 1.04 | 0.92-1.19 | 9.28E-01 | 1.05 | 0.93-1.2 | 8.90E-01 |
| 3-hydroxystachydrine* | 1.04 | 0.9-1.19 | 9.48E-01 | 1.06 | 0.92-1.22 | 8.90E-01 |
| gamma-glutamylisoleucine* | 1.07 | 0.94-1.22 | 8.42E-01 | 1.05 | 0.92-1.21 | 8.91E-01 |
| isoleucine | 1.09 | 0.93-1.26 | 8.42E-01 | 1.07 | 0.91-1.26 | 8.91E-01 |
| palmitoyl ethanolamide | 1.05 | 0.9-1.23 | 9.29E-01 | 1.06 | 0.91-1.24 | 8.93E-01 |
| aspartate | 1.07 | 0.94-1.22 | 8.42E-01 | 1.06 | 0.92-1.22 | 8.94E-01 |
| X - 24422 | 1.06 | 0.92-1.22 | 8.68E-01 | 1.06 | 0.91-1.23 | 8.94E-01 |
| 1-linoleoyl-GPG (18:2)* | 0.94 | 0.82-1.09 | 8.71E-01 | 0.95 | 0.82-1.09 | 8.94E-01 |
| pyruvate | 1.06 | 0.91-1.22 | 8.83E-01 | 1.06 | 0.91-1.23 | 8.94E-01 |
| palmitoylcholine | 1.04 | 0.91-1.19 | 9.29E-01 | 1.06 | 0.92-1.21 | 8.94E-01 |
| X - 24473 | 1.04 | 0.91-1.2 | 9.31E-01 | 1.06 | 0.92-1.22 | 8.94E-01 |
| 3-hydroxybutyroylglycine** | 0.97 | 0.85-1.1 | 9.50E-01 | 0.95 | 0.83-1.09 | 8.94E-01 |
| X - 23659 | 1.04 | 0.9-1.2 | 9.50E-01 | 1.06 | 0.92-1.21 | 8.94E-01 |
| 10-heptadecenoate (17:1n7) | 0.92 | 0.79-1.08 | 8.42E-01 | 0.94 | 0.8-1.1 | 8.95E-01 |
| X - 24307 | 0.96 | 0.85-1.09 | 9.28E-01 | 0.95 | 0.84-1.08 | 8.95E-01 |
| 6-bromotryptophan | 0.94 | 0.81-1.09 | 8.69E-01 | 0.95 | 0.82-1.1 | 8.98E-01 |
| cholate | 1.06 | 0.92-1.22 | 8.69E-01 | 1.05 | 0.92-1.21 | 8.98E-01 |
| 3-hydroxypyridine glucuronide | 1.06 | 0.92-1.23 | 8.70E-01 | 1.06 | 0.91-1.22 | 8.98E-01 |
| N-palmitoyl-sphinganine (d18:0/16:0) | 1.05 | 0.92-1.2 | 8.77E-01 | 1.05 | 0.92-1.21 | 8.98E-01 |
| 2,3-dihydroxy-5-methylthio-4-pentenoate (DMTPA)* | 1.06 | 0.91-1.24 | 8.77E-01 | 1.06 | 0.91-1.24 | 8.98E-01 |
| X - 25520 | 1.05 | 0.92-1.2 | 9.06E-01 | 1.05 | 0.92-1.21 | 8.98E-01 |
| arachidonoylcholine | 1.04 | 0.91-1.2 | 9.29E-01 | 1.05 | 0.92-1.21 | 8.98E-01 |
| glutamine conjugate of C6H10O2 (1)* | 0.96 | 0.83-1.1 | 9.29E-01 | 0.95 | 0.82-1.09 | 8.98E-01 |
| X - 17354 | 1.04 | 0.91-1.2 | 9.35E-01 | 1.06 | 0.91-1.22 | 8.98E-01 |
| 2-hydroxybutyrate/2-hydroxyisobutyrate | 0.95 | 0.81-1.1 | 9.00E-01 | 0.94 | 0.8-1.11 | 8.99E-01 |
| arachidoylcarnitine (C20)* | 1.04 | 0.9-1.2 | 9.50E-01 | 1.06 | 0.91-1.23 | 9.01E-01 |
| 2-hydroxyfluorene sulfate | 1.02 | 0.88-1.18 | 9.80E-01 | 0.94 | 0.79-1.12 | 9.09E-01 |
| hippurate | 1.04 | 0.91-1.18 | 9.50E-01 | 1.05 | 0.92-1.2 | 9.10E-01 |
| arachidonate (20:4n6) | 0.94 | 0.82-1.08 | 8.63E-01 | 0.95 | 0.82-1.1 | 9.11E-01 |
| 2S,3R-dihydroxybutyrate | 0.94 | 0.8-1.11 | 8.99E-01 | 0.94 | 0.8-1.11 | 9.11E-01 |
| choline phosphate | 0.94 | 0.8-1.1 | 8.69E-01 | 0.94 | 0.8-1.11 | 9.13E-01 |
| X - 12013 | 1.06 | 0.92-1.23 | 8.70E-01 | 1.05 | 0.91-1.22 | 9.13E-01 |
| xanthine | 0.94 | 0.81-1.1 | 8.91E-01 | 0.94 | 0.81-1.11 | 9.13E-01 |
| androstenediol (3beta,17beta) monosulfate (1) | 0.95 | 0.82-1.1 | 9.28E-01 | 0.95 | 0.81-1.1 | 9.13E-01 |
| N-acetylglutamate | 1.04 | 0.9-1.21 | 9.41E-01 | 1.05 | 0.91-1.22 | 9.13E-01 |
| undecenoylcarnitine (C11:1) | 1.03 | 0.9-1.18 | 9.50E-01 | 1.05 | 0.92-1.2 | 9.13E-01 |
| glycochenodeoxycholate 3-sulfate | 0.96 | 0.82-1.13 | 9.55E-01 | 0.94 | 0.8-1.11 | 9.13E-01 |
| 1-dihomo-linolenylglycerol (20:3) | 1.04 | 0.9-1.21 | 9.41E-01 | 1.05 | 0.91-1.22 | 9.16E-01 |
| docosapentaenoate (n3 DPA; 22:5n3) | 0.93 | 0.8-1.08 | 8.42E-01 | 0.95 | 0.81-1.1 | 9.18E-01 |
| 2-methylbutyrylcarnitine (C5) | 1.05 | 0.93-1.19 | 8.77E-01 | 1.05 | 0.92-1.19 | 9.18E-01 |
| hypoxanthine | 0.94 | 0.82-1.08 | 8.54E-01 | 0.95 | 0.83-1.09 | 9.20E-01 |
| margarate (17:0) | 0.93 | 0.79-1.09 | 8.54E-01 | 0.94 | 0.8-1.12 | 9.20E-01 |
| 1-stearoyl-2-oleoyl-GPS (18:0/18:1) | 1.06 | 0.93-1.22 | 8.57E-01 | 1.05 | 0.91-1.21 | 9.20E-01 |
| 3,4-dihydroxybutyrate | 1.06 | 0.93-1.21 | 8.68E-01 | 1.05 | 0.91-1.2 | 9.20E-01 |
| 2-aminobutyrate | 0.94 | 0.82-1.08 | 8.68E-01 | 0.95 | 0.83-1.1 | 9.20E-01 |
| X - 12261 | 1.06 | 0.92-1.23 | 8.68E-01 | 1.05 | 0.9-1.22 | 9.20E-01 |
| laurylcarnitine (C12) | 0.94 | 0.82-1.08 | 8.69E-01 | 0.96 | 0.83-1.1 | 9.20E-01 |
| 2-oleoylglycerol (18:1) | 1.07 | 0.91-1.26 | 8.70E-01 | 1.06 | 0.9-1.24 | 9.20E-01 |
| 3-methoxytyrosine | 0.94 | 0.81-1.1 | 8.72E-01 | 0.95 | 0.82-1.1 | 9.20E-01 |
| 4-methyl-2-oxopentanoate | 0.94 | 0.8-1.1 | 8.77E-01 | 0.95 | 0.8-1.12 | 9.20E-01 |
| indolelactate | 1.06 | 0.91-1.24 | 8.77E-01 | 1.05 | 0.9-1.23 | 9.20E-01 |
| 5-(galactosylhydroxy)-L-lysine | 1.06 | 0.91-1.24 | 8.77E-01 | 1.05 | 0.9-1.23 | 9.20E-01 |
| sphingomyelin (d18:1/22:2, d18:2/22:1, d16:1/24:2)* | 0.94 | 0.8-1.1 | 8.77E-01 | 0.95 | 0.81-1.11 | 9.20E-01 |
| 1-(1-enyl-stearoyl)-2-oleoyl-GPE (P-18:0/18:1) | 1.06 | 0.91-1.24 | 8.77E-01 | 1.05 | 0.9-1.23 | 9.20E-01 |
| beta-hydroxyisovalerate | 0.95 | 0.82-1.09 | 8.77E-01 | 0.95 | 0.82-1.1 | 9.20E-01 |
| 3-methyl-2-oxobutyrate | 0.95 | 0.82-1.1 | 9.00E-01 | 0.95 | 0.81-1.11 | 9.20E-01 |
| docosatrienoate (22:3n3) | 0.95 | 0.83-1.09 | 9.00E-01 | 0.95 | 0.83-1.09 | 9.20E-01 |
| gamma-glutamylphenylalanine | 0.95 | 0.82-1.1 | 9.09E-01 | 0.95 | 0.82-1.1 | 9.20E-01 |
| N-acetylthreonine | 1.06 | 0.9-1.24 | 9.09E-01 | 1.05 | 0.9-1.23 | 9.20E-01 |
| aconitate [cis or trans] | 1.06 | 0.9-1.24 | 9.20E-01 | 1.05 | 0.89-1.24 | 9.20E-01 |
| 4-hydroxycoumarin | 1.05 | 0.91-1.22 | 9.20E-01 | 1.05 | 0.9-1.23 | 9.20E-01 |
| (N(1) + N(8))-acetylspermidine | 0.95 | 0.83-1.1 | 9.21E-01 | 0.95 | 0.83-1.1 | 9.20E-01 |
| adenosine 3',5'-cyclic monophosphate (cAMP) | 0.95 | 0.83-1.1 | 9.27E-01 | 0.95 | 0.83-1.1 | 9.20E-01 |
| tyrosine | 0.96 | 0.83-1.09 | 9.27E-01 | 0.95 | 0.83-1.1 | 9.20E-01 |
| dihomo-linolenoyl-choline | 1.04 | 0.91-1.19 | 9.28E-01 | 1.05 | 0.91-1.2 | 9.20E-01 |
| isobutyrylcarnitine (C4) | 1.05 | 0.91-1.2 | 9.29E-01 | 1.05 | 0.91-1.21 | 9.20E-01 |
| gamma-glutamyl-2-aminobutyrate | 1.05 | 0.9-1.22 | 9.29E-01 | 1.05 | 0.9-1.23 | 9.20E-01 |
| N-acetylvaline | 1.05 | 0.9-1.22 | 9.29E-01 | 1.05 | 0.9-1.23 | 9.20E-01 |
| X - 12407 | 0.96 | 0.84-1.09 | 9.29E-01 | 0.96 | 0.84-1.09 | 9.20E-01 |
| N6-acetyllysine | 0.96 | 0.83-1.1 | 9.31E-01 | 0.95 | 0.82-1.1 | 9.20E-01 |
| sphingomyelin (d18:2/21:0, d16:2/23:0)* | 1.06 | 0.89-1.26 | 9.31E-01 | 1.06 | 0.89-1.27 | 9.20E-01 |
| behenoylcarnitine (C22)* | 1.05 | 0.91-1.21 | 9.31E-01 | 1.05 | 0.91-1.21 | 9.20E-01 |
| 2-methoxyhydroquinone sulfate (1) | 1.05 | 0.91-1.21 | 9.31E-01 | 1.05 | 0.91-1.21 | 9.20E-01 |
| glucose | 1.05 | 0.9-1.21 | 9.31E-01 | 1.05 | 0.9-1.23 | 9.20E-01 |
| X - 24546 | 0.95 | 0.82-1.11 | 9.31E-01 | 0.95 | 0.82-1.11 | 9.20E-01 |
| 7-alpha-hydroxy-3-oxo-4-cholestenoate (7-Hoca) | 0.95 | 0.82-1.11 | 9.35E-01 | 0.95 | 0.81-1.11 | 9.20E-01 |
| cysteinylglycine | 1.04 | 0.9-1.21 | 9.41E-01 | 1.05 | 0.9-1.22 | 9.20E-01 |
| X - 12839 | 0.96 | 0.84-1.1 | 9.41E-01 | 0.96 | 0.83-1.1 | 9.20E-01 |
| 17alpha-hydroxypregnenolone 3-sulfate | 0.96 | 0.84-1.1 | 9.41E-01 | 0.96 | 0.83-1.1 | 9.20E-01 |
| X - 12101 | 0.96 | 0.84-1.1 | 9.41E-01 | 0.96 | 0.84-1.09 | 9.20E-01 |
| X - 21736 | 1.04 | 0.91-1.18 | 9.41E-01 | 1.04 | 0.91-1.19 | 9.20E-01 |
| isovalerylcarnitine (C5) | 0.96 | 0.83-1.11 | 9.50E-01 | 0.95 | 0.82-1.11 | 9.20E-01 |
| phenol sulfate | 1.04 | 0.9-1.19 | 9.50E-01 | 1.05 | 0.91-1.2 | 9.20E-01 |
| X - 11787 | 0.96 | 0.82-1.12 | 9.50E-01 | 0.95 | 0.82-1.11 | 9.20E-01 |
| X - 12844 | 0.97 | 0.84-1.12 | 9.56E-01 | 0.95 | 0.82-1.1 | 9.20E-01 |
| ergothioneine | 1.04 | 0.88-1.23 | 9.58E-01 | 1.06 | 0.89-1.26 | 9.20E-01 |
| 2-acetamidophenol sulfate | 1.03 | 0.9-1.18 | 9.58E-01 | 1.05 | 0.91-1.2 | 9.20E-01 |
| 1-linoleoyl-2-arachidonoyl-GPC (18:2/20:4n6)* | 1.03 | 0.89-1.2 | 9.59E-01 | 1.06 | 0.9-1.24 | 9.20E-01 |
| X - 12111 | 1.03 | 0.89-1.18 | 9.70E-01 | 1.05 | 0.91-1.21 | 9.20E-01 |
| X - 18899 | 0.94 | 0.82-1.08 | 8.54E-01 | 0.96 | 0.84-1.1 | 9.24E-01 |
| N1-methylinosine | 1.07 | 0.91-1.26 | 8.68E-01 | 1.05 | 0.89-1.24 | 9.24E-01 |
| 17alpha-hydroxypregnanolone glucuronide | 1.08 | 0.91-1.28 | 8.68E-01 | 1.06 | 0.89-1.26 | 9.24E-01 |
| 4-vinylphenol sulfate | 1.06 | 0.92-1.23 | 8.71E-01 | 1.05 | 0.9-1.22 | 9.24E-01 |
| succinylcarnitine (C4-DC) | 1.06 | 0.91-1.24 | 8.72E-01 | 1.05 | 0.9-1.23 | 9.24E-01 |
| 16-hydroxypalmitate | 0.94 | 0.81-1.1 | 8.83E-01 | 0.95 | 0.82-1.11 | 9.24E-01 |
| methyl-4-hydroxybenzoate sulfate | 0.95 | 0.82-1.1 | 9.00E-01 | 0.96 | 0.82-1.11 | 9.24E-01 |
| phosphoethanolamine | 0.95 | 0.82-1.1 | 9.09E-01 | 0.96 | 0.82-1.11 | 9.24E-01 |
| 4-acetylphenol sulfate | 1.05 | 0.91-1.2 | 9.29E-01 | 1.05 | 0.91-1.2 | 9.24E-01 |
| sphingomyelin (d18:2/16:0, d18:1/16:1)* | 0.95 | 0.81-1.11 | 9.31E-01 | 0.95 | 0.81-1.12 | 9.24E-01 |
| homovanillate (HVA) | 0.96 | 0.83-1.1 | 9.32E-01 | 0.96 | 0.83-1.1 | 9.24E-01 |
| X - 26108 | 1.05 | 0.9-1.22 | 9.35E-01 | 1.05 | 0.9-1.23 | 9.24E-01 |
| X - 17438 | 0.96 | 0.84-1.1 | 9.41E-01 | 0.96 | 0.84-1.09 | 9.24E-01 |
| homoarginine | 0.96 | 0.83-1.12 | 9.50E-01 | 0.95 | 0.81-1.11 | 9.24E-01 |
| N-acetylphenylalanine | 1.04 | 0.9-1.2 | 9.50E-01 | 1.05 | 0.9-1.21 | 9.24E-01 |
| sphingomyelin (d18:2/14:0, d18:1/14:1)* | 1.04 | 0.87-1.25 | 9.50E-01 | 1.06 | 0.88-1.27 | 9.24E-01 |
| 3-methoxycatechol sulfate (2) | 1.04 | 0.9-1.2 | 9.50E-01 | 1.05 | 0.9-1.21 | 9.24E-01 |
| stearoylcholine* | 1.04 | 0.91-1.19 | 9.50E-01 | 1.04 | 0.91-1.2 | 9.24E-01 |
| 4-chlorobenzoic acid | 1.04 | 0.9-1.2 | 9.50E-01 | 1.05 | 0.91-1.2 | 9.24E-01 |
| adenosine 5'-monophosphate (AMP) | 0.97 | 0.84-1.1 | 9.50E-01 | 0.96 | 0.84-1.1 | 9.24E-01 |
| histidine | 0.96 | 0.83-1.12 | 9.50E-01 | 0.96 | 0.82-1.11 | 9.24E-01 |
| spermidine | 1.04 | 0.9-1.19 | 9.50E-01 | 1.04 | 0.91-1.2 | 9.24E-01 |
| X - 23787 | 0.96 | 0.83-1.11 | 9.50E-01 | 0.96 | 0.82-1.11 | 9.24E-01 |
| X - 24475 | 1.04 | 0.9-1.2 | 9.52E-01 | 1.05 | 0.9-1.22 | 9.24E-01 |
| cis-3,4-methyleneheptanoylcarnitine | 0.97 | 0.85-1.11 | 9.56E-01 | 0.96 | 0.84-1.1 | 9.24E-01 |
| X - 25172 | 0.97 | 0.85-1.12 | 9.70E-01 | 0.96 | 0.83-1.1 | 9.24E-01 |
| threonate | 1.03 | 0.88-1.2 | 9.70E-01 | 1.05 | 0.9-1.23 | 9.24E-01 |
| margaroylcarnitine (C17)* | 1.02 | 0.89-1.17 | 9.75E-01 | 1.04 | 0.91-1.21 | 9.24E-01 |
| pristanate | 0.94 | 0.82-1.09 | 8.70E-01 | 0.96 | 0.83-1.11 | 9.29E-01 |
| taurine | 0.95 | 0.81-1.11 | 9.00E-01 | 0.95 | 0.81-1.12 | 9.29E-01 |
| etiocholanolone glucuronide | 0.95 | 0.82-1.1 | 9.05E-01 | 0.96 | 0.83-1.1 | 9.29E-01 |
| glycocholate | 1.05 | 0.9-1.22 | 9.35E-01 | 1.05 | 0.9-1.22 | 9.29E-01 |
| dimethyl sulfone | 0.97 | 0.85-1.1 | 9.50E-01 | 0.96 | 0.84-1.1 | 9.29E-01 |
| taurodeoxycholate | 1.03 | 0.9-1.19 | 9.50E-01 | 1.04 | 0.91-1.2 | 9.29E-01 |
| N-acetyltaurine | 1.05 | 0.92-1.2 | 8.78E-01 | 1.04 | 0.91-1.19 | 9.31E-01 |
| 2-palmitoyl-GPC (16:0)* | 0.95 | 0.82-1.09 | 8.91E-01 | 0.96 | 0.83-1.11 | 9.31E-01 |
| delta-CEHC | 1.05 | 0.9-1.22 | 9.29E-01 | 1.04 | 0.9-1.21 | 9.31E-01 |
| cis-4-decenoylcarnitine (C10:1) | 1.03 | 0.91-1.18 | 9.50E-01 | 1.04 | 0.91-1.18 | 9.31E-01 |
| umbelliferone sulfate | 1.04 | 0.9-1.2 | 9.50E-01 | 1.04 | 0.9-1.21 | 9.32E-01 |
| X - 21442 | 1.04 | 0.9-1.19 | 9.50E-01 | 1.04 | 0.91-1.19 | 9.32E-01 |
| 1-methyl-5-imidazolelactate | 1.04 | 0.9-1.19 | 9.50E-01 | 1.04 | 0.9-1.2 | 9.32E-01 |
| X - 12126 | 1.03 | 0.9-1.18 | 9.67E-01 | 1.04 | 0.91-1.2 | 9.32E-01 |
| dihomo-linolenate (20:3n3 or n6) | 0.95 | 0.83-1.09 | 8.93E-01 | 0.96 | 0.83-1.11 | 9.33E-01 |
| heptenedioate (C7:1-DC)* | 0.94 | 0.81-1.1 | 8.71E-01 | 0.96 | 0.82-1.12 | 9.33E-01 |
| X - 23593 | 1.05 | 0.91-1.22 | 9.09E-01 | 1.04 | 0.9-1.21 | 9.33E-01 |
| X - 21310 | 1.03 | 0.88-1.21 | 9.70E-01 | 1.05 | 0.89-1.23 | 9.33E-01 |
| N-acetylisoleucine | 1.05 | 0.9-1.22 | 9.29E-01 | 1.04 | 0.9-1.22 | 9.36E-01 |
| 9-hydroxystearate | 1.04 | 0.91-1.19 | 9.38E-01 | 1.04 | 0.91-1.19 | 9.40E-01 |
| N6,N6,N6-trimethyllysine | 0.97 | 0.83-1.13 | 9.58E-01 | 0.96 | 0.83-1.11 | 9.40E-01 |
| X - 13431 | 0.94 | 0.83-1.08 | 8.57E-01 | 0.96 | 0.84-1.1 | 9.42E-01 |
| oleoylcarnitine (C18:1) | 0.95 | 0.83-1.09 | 8.86E-01 | 0.96 | 0.83-1.11 | 9.42E-01 |
| eicosenedioate (C20:1-DC)* | 0.95 | 0.83-1.09 | 8.93E-01 | 0.96 | 0.84-1.11 | 9.42E-01 |
| caproate (6:0) | 0.95 | 0.83-1.1 | 9.28E-01 | 0.96 | 0.83-1.11 | 9.42E-01 |
| adipoylcarnitine (C6-DC) | 1.04 | 0.9-1.21 | 9.38E-01 | 1.04 | 0.9-1.2 | 9.42E-01 |
| 3-methylcytidine | 1.04 | 0.9-1.2 | 9.41E-01 | 1.04 | 0.9-1.2 | 9.42E-01 |
| hexanoylcarnitine (C6) | 1.04 | 0.9-1.19 | 9.50E-01 | 1.04 | 0.91-1.19 | 9.42E-01 |
| X - 18887 | 0.97 | 0.84-1.11 | 9.50E-01 | 0.96 | 0.84-1.11 | 9.42E-01 |
| 3-hydroxyoctanoylcarnitine (1) | 1.03 | 0.89-1.2 | 9.58E-01 | 1.04 | 0.9-1.21 | 9.42E-01 |
| X - 17367 | 1.03 | 0.89-1.19 | 9.58E-01 | 1.04 | 0.9-1.21 | 9.42E-01 |
| eicosapentaenoate (EPA; 20:5n3) | 1.02 | 0.89-1.17 | 9.70E-01 | 1.04 | 0.91-1.19 | 9.42E-01 |
| tartronate (hydroxymalonate) | 1.02 | 0.88-1.18 | 9.80E-01 | 1.04 | 0.89-1.22 | 9.42E-01 |
| 2-naphthol sulfate | 1.10 | 0.95-1.26 | 8.06E-01 | 1.04 | 0.87-1.25 | 9.43E-01 |
| X - 24344 | 1.07 | 0.92-1.24 | 8.52E-01 | 1.04 | 0.89-1.21 | 9.43E-01 |
| 4-hydroxychlorothalonil | 0.94 | 0.81-1.1 | 8.72E-01 | 0.96 | 0.82-1.13 | 9.43E-01 |
| 1-methyl-4-imidazoleacetate | 1.06 | 0.91-1.24 | 8.77E-01 | 1.04 | 0.89-1.22 | 9.43E-01 |
| phytanate | 0.95 | 0.83-1.09 | 9.00E-01 | 0.96 | 0.84-1.11 | 9.43E-01 |
| 1-linoleoyl-GPC (18:2) | 0.95 | 0.83-1.1 | 9.05E-01 | 0.96 | 0.82-1.12 | 9.43E-01 |
| tetrahydrocortisol sulfate (1) | 1.05 | 0.91-1.2 | 9.27E-01 | 1.04 | 0.9-1.2 | 9.43E-01 |
| X - 12851 | 0.96 | 0.83-1.1 | 9.27E-01 | 0.97 | 0.84-1.11 | 9.43E-01 |
| X - 23782 | 0.96 | 0.83-1.1 | 9.27E-01 | 0.97 | 0.84-1.11 | 9.43E-01 |
| X - 17335 | 0.96 | 0.83-1.1 | 9.28E-01 | 0.97 | 0.84-1.11 | 9.43E-01 |
| ectoine | 0.95 | 0.82-1.1 | 9.29E-01 | 0.97 | 0.83-1.12 | 9.43E-01 |
| dihydroferulate | 0.96 | 0.83-1.1 | 9.29E-01 | 0.96 | 0.83-1.11 | 9.43E-01 |
| X - 19141 | 0.95 | 0.82-1.11 | 9.29E-01 | 0.97 | 0.84-1.12 | 9.43E-01 |
| 3-phosphoglycerate | 1.04 | 0.91-1.2 | 9.30E-01 | 1.04 | 0.9-1.19 | 9.43E-01 |
| arginine | 1.05 | 0.91-1.21 | 9.31E-01 | 1.03 | 0.89-1.2 | 9.43E-01 |
| arachidonoylcarnitine (C20:4) | 0.95 | 0.81-1.12 | 9.35E-01 | 0.96 | 0.82-1.13 | 9.43E-01 |
| gamma-glutamylglycine | 1.05 | 0.9-1.22 | 9.35E-01 | 1.04 | 0.89-1.23 | 9.43E-01 |
| 1-myristoylglycerol (14:0) | 0.96 | 0.84-1.1 | 9.41E-01 | 0.97 | 0.84-1.11 | 9.43E-01 |
| gulonate* | 1.04 | 0.91-1.19 | 9.41E-01 | 1.04 | 0.9-1.19 | 9.43E-01 |
| (S)-3-hydroxybutyrylcarnitine | 1.04 | 0.9-1.21 | 9.41E-01 | 1.04 | 0.89-1.2 | 9.43E-01 |
| GlcNAc sulfate conjugate of C21H34O2 steroid** | 0.96 | 0.84-1.1 | 9.41E-01 | 0.97 | 0.84-1.11 | 9.43E-01 |
| X - 17676 | 1.04 | 0.9-1.21 | 9.41E-01 | 1.04 | 0.89-1.21 | 9.43E-01 |
| 1-palmitoleoyl-2-linolenoyl-GPC (16:1/18:3)* | 0.96 | 0.83-1.11 | 9.41E-01 | 0.96 | 0.83-1.12 | 9.43E-01 |
| gamma-glutamylmethionine | 1.04 | 0.9-1.2 | 9.44E-01 | 1.04 | 0.9-1.2 | 9.43E-01 |
| gamma-CEHC | 0.96 | 0.83-1.11 | 9.45E-01 | 0.96 | 0.83-1.11 | 9.43E-01 |
| X - 21327 | 0.97 | 0.85-1.09 | 9.45E-01 | 0.97 | 0.85-1.1 | 9.43E-01 |
| behenoyl sphingomyelin (d18:1/22:0)* | 1.04 | 0.9-1.2 | 9.50E-01 | 1.04 | 0.9-1.2 | 9.43E-01 |
| dimethylarginine (SDMA + ADMA) | 1.04 | 0.89-1.21 | 9.50E-01 | 1.04 | 0.89-1.21 | 9.43E-01 |
| lanthionine | 0.97 | 0.85-1.1 | 9.50E-01 | 0.97 | 0.86-1.1 | 9.43E-01 |
| fructosyllysine | 1.04 | 0.89-1.21 | 9.50E-01 | 1.04 | 0.89-1.22 | 9.43E-01 |
| ceramide (d18:2/24:1, d18:1/24:2)* | 1.04 | 0.9-1.2 | 9.50E-01 | 1.04 | 0.9-1.2 | 9.43E-01 |
| succinoyltaurine | 1.04 | 0.9-1.19 | 9.50E-01 | 1.03 | 0.9-1.18 | 9.43E-01 |
| cystine | 1.04 | 0.89-1.21 | 9.50E-01 | 1.04 | 0.89-1.21 | 9.43E-01 |
| X - 12410 | 1.03 | 0.91-1.18 | 9.50E-01 | 1.03 | 0.9-1.18 | 9.43E-01 |
| X - 24334 | 0.96 | 0.81-1.14 | 9.50E-01 | 0.96 | 0.81-1.14 | 9.43E-01 |
| X - 24494 | 1.04 | 0.9-1.2 | 9.50E-01 | 1.04 | 0.9-1.2 | 9.43E-01 |
| X - 26119 | 1.04 | 0.89-1.2 | 9.50E-01 | 1.04 | 0.89-1.21 | 9.43E-01 |
| X - 12798 | 1.04 | 0.89-1.2 | 9.52E-01 | 1.04 | 0.9-1.2 | 9.43E-01 |
| hydroxypalmitoyl sphingomyelin (d18:1/16:0(OH))** | 0.96 | 0.83-1.12 | 9.52E-01 | 0.96 | 0.82-1.12 | 9.43E-01 |
| phenol glucuronide | 1.03 | 0.9-1.19 | 9.56E-01 | 1.04 | 0.9-1.19 | 9.43E-01 |
| 3-methylhistidine | 1.03 | 0.9-1.18 | 9.58E-01 | 1.03 | 0.9-1.18 | 9.43E-01 |
| 2-hydroxyoctanoate | 0.97 | 0.85-1.11 | 9.58E-01 | 0.97 | 0.84-1.11 | 9.43E-01 |
| glutamine_degradant* | 1.04 | 0.88-1.23 | 9.58E-01 | 1.04 | 0.88-1.24 | 9.43E-01 |
| pyrraline | 1.03 | 0.9-1.17 | 9.58E-01 | 1.04 | 0.91-1.19 | 9.43E-01 |
| 4-hydroxy-2-oxoglutaric acid | 1.03 | 0.89-1.19 | 9.58E-01 | 1.04 | 0.9-1.2 | 9.43E-01 |
| sphingomyelin (d18:1/20:1, d18:2/20:0)* | 0.97 | 0.82-1.14 | 9.58E-01 | 0.96 | 0.82-1.13 | 9.43E-01 |
| valine | 1.03 | 0.91-1.15 | 9.58E-01 | 1.03 | 0.91-1.16 | 9.43E-01 |
| X - 23648 | 0.97 | 0.85-1.11 | 9.59E-01 | 0.97 | 0.84-1.11 | 9.43E-01 |
| X - 13844 | 1.03 | 0.89-1.2 | 9.64E-01 | 1.04 | 0.89-1.21 | 9.43E-01 |
| erythritol | 1.03 | 0.89-1.19 | 9.67E-01 | 1.04 | 0.9-1.19 | 9.43E-01 |
| nonanoylcarnitine (C9) | 1.03 | 0.88-1.21 | 9.70E-01 | 1.04 | 0.89-1.22 | 9.43E-01 |
| tricosanoyl sphingomyelin (d18:1/23:0)* | 1.03 | 0.88-1.21 | 9.70E-01 | 1.04 | 0.88-1.22 | 9.43E-01 |
| 4-methylhexanoylglutamine | 1.03 | 0.89-1.18 | 9.70E-01 | 1.03 | 0.9-1.19 | 9.43E-01 |
| N-acetylleucine | 1.03 | 0.88-1.2 | 9.70E-01 | 1.04 | 0.89-1.21 | 9.43E-01 |
| X - 11478 | 0.98 | 0.86-1.11 | 9.70E-01 | 0.97 | 0.85-1.1 | 9.43E-01 |
| methionine sulfone | 1.02 | 0.89-1.18 | 9.70E-01 | 1.04 | 0.9-1.2 | 9.43E-01 |
| glycochenodeoxycholate glucuronide (1) | 0.97 | 0.84-1.14 | 9.70E-01 | 0.96 | 0.82-1.13 | 9.43E-01 |
| glycosyl ceramide (d18:1/20:0, d16:1/22:0)* | 1.03 | 0.89-1.18 | 9.70E-01 | 1.04 | 0.89-1.2 | 9.43E-01 |
| gamma-glutamylcitrulline* | 0.98 | 0.85-1.12 | 9.70E-01 | 0.97 | 0.84-1.11 | 9.43E-01 |
| dihydroorotate | 1.02 | 0.9-1.16 | 9.70E-01 | 1.03 | 0.91-1.17 | 9.43E-01 |
| X - 16087 | 1.02 | 0.9-1.17 | 9.70E-01 | 1.04 | 0.9-1.19 | 9.43E-01 |
| X - 24757 | 1.03 | 0.89-1.18 | 9.70E-01 | 1.04 | 0.9-1.2 | 9.43E-01 |
| X - 24949 | 0.98 | 0.85-1.11 | 9.70E-01 | 0.97 | 0.85-1.11 | 9.43E-01 |
| N-acetyl-2-aminooctanoate* | 1.02 | 0.89-1.18 | 9.77E-01 | 1.04 | 0.9-1.2 | 9.43E-01 |
| palmitoylcarnitine (C16) | 1.02 | 0.89-1.17 | 9.80E-01 | 1.04 | 0.9-1.2 | 9.43E-01 |
| 1-palmitoylglycerol (16:0) | 0.97 | 0.84-1.11 | 9.50E-01 | 0.97 | 0.84-1.11 | 9.45E-01 |
| 3-hydroxyhexanoate | 0.97 | 0.85-1.1 | 9.50E-01 | 0.97 | 0.85-1.11 | 9.45E-01 |
| 5,6-dihydrouridine | 1.04 | 0.89-1.22 | 9.50E-01 | 1.04 | 0.88-1.22 | 9.48E-01 |
| X - 21834 | 1.03 | 0.9-1.19 | 9.52E-01 | 1.03 | 0.89-1.19 | 9.48E-01 |
| glycoursodeoxycholate | 0.97 | 0.83-1.13 | 9.58E-01 | 0.97 | 0.83-1.13 | 9.48E-01 |
| androstenediol (3beta,17beta) disulfate (1) | 0.97 | 0.84-1.13 | 9.70E-01 | 0.96 | 0.82-1.13 | 9.48E-01 |
| lignoceroyl sphingomyelin (d18:1/24:0) | 1.03 | 0.88-1.2 | 9.70E-01 | 1.04 | 0.88-1.21 | 9.48E-01 |
| X - 18922 | 0.98 | 0.85-1.12 | 9.70E-01 | 0.97 | 0.85-1.11 | 9.48E-01 |
| alpha-CEHC sulfate | 1.02 | 0.89-1.16 | 9.81E-01 | 1.03 | 0.9-1.19 | 9.48E-01 |
| sphingomyelin (d17:1/14:0, d16:1/15:0)* | 1.02 | 0.87-1.2 | 9.83E-01 | 1.04 | 0.88-1.23 | 9.48E-01 |
| 11beta-hydroxyandrosterone glucuronide | 1.05 | 0.91-1.22 | 9.00E-01 | 1.03 | 0.89-1.2 | 9.50E-01 |
| X - 12026 | 0.96 | 0.82-1.11 | 9.41E-01 | 0.97 | 0.83-1.13 | 9.50E-01 |
| 2,3-dihydroxyisovalerate | 1.02 | 0.9-1.16 | 9.70E-01 | 1.03 | 0.9-1.17 | 9.50E-01 |
| andro steroid monosulfate C19H28O6S (1)* | 0.97 | 0.84-1.13 | 9.70E-01 | 0.97 | 0.83-1.13 | 9.50E-01 |
| metabolonic lactone sulfate | 0.98 | 0.85-1.12 | 9.70E-01 | 0.97 | 0.84-1.12 | 9.50E-01 |
| X - 24518 | 1.05 | 0.89-1.22 | 9.41E-01 | 1.03 | 0.88-1.21 | 9.52E-01 |
| xanthosine | 1.04 | 0.89-1.21 | 9.56E-01 | 1.03 | 0.89-1.2 | 9.52E-01 |
| N-acetylkynurenine (2) | 1.03 | 0.89-1.19 | 9.70E-01 | 1.03 | 0.88-1.21 | 9.52E-01 |
| (16 or 17)-methylstearate (a19:0 or i19:0) | 0.96 | 0.82-1.11 | 9.35E-01 | 0.97 | 0.83-1.13 | 9.54E-01 |
| X - 17351 | 0.96 | 0.84-1.1 | 9.37E-01 | 0.97 | 0.85-1.12 | 9.54E-01 |
| 3-formylindole | 1.05 | 0.9-1.21 | 9.41E-01 | 1.03 | 0.89-1.2 | 9.54E-01 |
| N-delta-acetylornithine | 0.96 | 0.84-1.1 | 9.41E-01 | 0.97 | 0.85-1.12 | 9.54E-01 |
| deoxycarnitine | 0.98 | 0.89-1.07 | 9.50E-01 | 0.98 | 0.89-1.08 | 9.54E-01 |
| isoleucylglycine | 1.03 | 0.9-1.18 | 9.50E-01 | 1.03 | 0.9-1.18 | 9.54E-01 |
| 1-(1-enyl-palmitoyl)-2-oleoyl-GPE (P-16:0/18:1)* | 1.04 | 0.9-1.21 | 9.50E-01 | 1.03 | 0.89-1.2 | 9.54E-01 |
| X - 26097 | 0.96 | 0.84-1.11 | 9.50E-01 | 0.97 | 0.84-1.12 | 9.54E-01 |
| maleate | 1.03 | 0.9-1.18 | 9.56E-01 | 1.03 | 0.9-1.18 | 9.54E-01 |
| lysine | 0.97 | 0.84-1.11 | 9.56E-01 | 0.97 | 0.85-1.11 | 9.54E-01 |
| 2-O-methylascorbic acid | 1.03 | 0.89-1.19 | 9.58E-01 | 1.03 | 0.89-1.2 | 9.54E-01 |
| N-methylpipecolate | 0.97 | 0.85-1.11 | 9.58E-01 | 0.97 | 0.85-1.12 | 9.54E-01 |
| 1-stearoyl-2-linoleoyl-GPC (18:0/18:2)* | 1.04 | 0.88-1.22 | 9.58E-01 | 1.03 | 0.87-1.22 | 9.54E-01 |
| 5,6-dihydrothymine | 0.97 | 0.84-1.12 | 9.58E-01 | 0.97 | 0.84-1.13 | 9.54E-01 |
| asparagine | 0.97 | 0.83-1.13 | 9.58E-01 | 0.97 | 0.83-1.14 | 9.54E-01 |
| X - 12740 | 0.97 | 0.84-1.11 | 9.58E-01 | 0.97 | 0.84-1.12 | 9.54E-01 |
| vanillactate | 1.03 | 0.9-1.17 | 9.64E-01 | 1.03 | 0.9-1.17 | 9.54E-01 |
| lithocholate sulfate (1) | 1.03 | 0.89-1.19 | 9.64E-01 | 1.03 | 0.89-1.19 | 9.54E-01 |
| X - 12112 | 1.03 | 0.91-1.16 | 9.64E-01 | 1.03 | 0.91-1.16 | 9.54E-01 |
| X - 13553 | 1.03 | 0.88-1.21 | 9.64E-01 | 1.03 | 0.88-1.21 | 9.54E-01 |
| N,N-dimethyl-5-aminovalerate | 0.97 | 0.85-1.12 | 9.67E-01 | 0.97 | 0.84-1.12 | 9.54E-01 |
| X - 14056 | 1.03 | 0.89-1.19 | 9.70E-01 | 1.03 | 0.88-1.2 | 9.54E-01 |
| X - 18886 | 1.03 | 0.88-1.21 | 9.70E-01 | 1.03 | 0.88-1.21 | 9.54E-01 |
| sphingomyelin (d18:1/18:1, d18:2/18:0) | 1.03 | 0.87-1.22 | 9.70E-01 | 1.03 | 0.87-1.22 | 9.54E-01 |
| 16a-hydroxy DHEA 3-sulfate | 0.98 | 0.85-1.13 | 9.70E-01 | 0.97 | 0.84-1.13 | 9.54E-01 |
| isoursodeoxycholate | 1.02 | 0.89-1.18 | 9.70E-01 | 1.03 | 0.89-1.19 | 9.54E-01 |
| argininate* | 1.02 | 0.9-1.17 | 9.70E-01 | 1.03 | 0.9-1.17 | 9.54E-01 |
| 1-(1-enyl-stearoyl)-GPE (P-18:0)* | 1.03 | 0.89-1.19 | 9.70E-01 | 1.03 | 0.89-1.2 | 9.54E-01 |
| dopamine 4-sulfate | 1.03 | 0.9-1.17 | 9.70E-01 | 1.03 | 0.9-1.18 | 9.54E-01 |
| sphingomyelin (d18:2/23:0, d18:1/23:1, d17:1/24:1)* | 1.03 | 0.88-1.2 | 9.70E-01 | 1.03 | 0.88-1.21 | 9.54E-01 |
| gamma-tocopherol/beta-tocopherol | 0.98 | 0.85-1.12 | 9.70E-01 | 0.97 | 0.85-1.12 | 9.54E-01 |
| cholic acid glucuronide | 1.03 | 0.89-1.19 | 9.70E-01 | 1.03 | 0.89-1.19 | 9.54E-01 |
| decadienedioic acid (C10:2-DC)** | 0.98 | 0.85-1.12 | 9.70E-01 | 0.97 | 0.85-1.12 | 9.54E-01 |
| allantoin | 1.02 | 0.9-1.16 | 9.70E-01 | 1.03 | 0.9-1.17 | 9.54E-01 |
| N-acetylmethionine | 0.97 | 0.84-1.13 | 9.70E-01 | 0.97 | 0.83-1.13 | 9.54E-01 |
| N-formylmethionine | 1.03 | 0.88-1.2 | 9.70E-01 | 1.03 | 0.88-1.2 | 9.54E-01 |
| X - 12729 | 1.02 | 0.9-1.16 | 9.70E-01 | 1.03 | 0.9-1.17 | 9.54E-01 |
| imidazole lactate | 1.02 | 0.89-1.17 | 9.80E-01 | 1.03 | 0.89-1.18 | 9.54E-01 |
| X - 12738 | 0.98 | 0.84-1.14 | 9.80E-01 | 0.97 | 0.83-1.13 | 9.54E-01 |
| X - 26109 | 0.98 | 0.84-1.14 | 9.80E-01 | 0.97 | 0.83-1.13 | 9.54E-01 |
| piperine | 1.02 | 0.88-1.18 | 9.83E-01 | 1.03 | 0.89-1.19 | 9.54E-01 |
| campesterol | 1.02 | 0.88-1.17 | 9.83E-01 | 1.03 | 0.88-1.2 | 9.54E-01 |
| cortolone glucuronide (1) | 0.98 | 0.84-1.15 | 9.86E-01 | 0.97 | 0.82-1.14 | 9.54E-01 |
| malate | 1.01 | 0.88-1.17 | 9.86E-01 | 1.03 | 0.89-1.19 | 9.54E-01 |
| ethyl beta-glucopyranoside | 1.00 | 0.87-1.15 | 9.99E-01 | 1.03 | 0.9-1.18 | 9.54E-01 |
| 2,3-dihydroxypyridine | 1.04 | 0.9-1.2 | 9.50E-01 | 1.03 | 0.89-1.19 | 9.55E-01 |
| 6-hydroxyindole sulfate | 0.96 | 0.83-1.12 | 9.50E-01 | 0.97 | 0.84-1.13 | 9.55E-01 |
| X - 12100 | 0.96 | 0.81-1.14 | 9.58E-01 | 0.97 | 0.82-1.15 | 9.55E-01 |
| cyclo(gly-pro) | 1.03 | 0.89-1.19 | 9.70E-01 | 1.03 | 0.89-1.19 | 9.55E-01 |
| gamma-glutamylvaline | 0.98 | 0.84-1.13 | 9.70E-01 | 0.97 | 0.84-1.13 | 9.55E-01 |
| stearoyl sphingomyelin (d18:1/18:0) | 1.03 | 0.88-1.2 | 9.70E-01 | 1.03 | 0.88-1.2 | 9.55E-01 |
| X - 13695 | 1.03 | 0.89-1.19 | 9.70E-01 | 1.03 | 0.88-1.19 | 9.55E-01 |
| X - 17612 | 0.98 | 0.85-1.12 | 9.70E-01 | 0.98 | 0.85-1.12 | 9.55E-01 |
| erucate (22:1n9) | 1.02 | 0.89-1.16 | 9.77E-01 | 1.02 | 0.9-1.17 | 9.55E-01 |
| alpha-hydroxyisovalerate | 0.98 | 0.83-1.15 | 9.80E-01 | 0.97 | 0.81-1.15 | 9.55E-01 |
| cysteine | 1.02 | 0.88-1.18 | 9.80E-01 | 1.03 | 0.88-1.2 | 9.55E-01 |
| 1-(1-enyl-palmitoyl)-2-linoleoyl-GPE (P-16:0/18:2)* | 0.98 | 0.85-1.14 | 9.83E-01 | 0.97 | 0.84-1.13 | 9.55E-01 |
| branched chain 14:0 dicarboxylic acid** | 1.01 | 0.89-1.15 | 9.86E-01 | 1.03 | 0.9-1.17 | 9.55E-01 |
| 3-carboxy-4-methyl-5-pentyl-2-furanpropionate (3-CMPFP)** | 1.01 | 0.87-1.17 | 9.87E-01 | 1.03 | 0.88-1.19 | 9.55E-01 |
| X - 21286 | 1.01 | 0.88-1.16 | 9.87E-01 | 1.03 | 0.89-1.19 | 9.55E-01 |
| oleoyl-linoleoyl-glycerol (18:1/18:2) [2] | 0.99 | 0.85-1.15 | 9.91E-01 | 0.97 | 0.84-1.13 | 9.55E-01 |
| oxalate (ethanedioate) | 1.00 | 0.86-1.16 | 9.98E-01 | 1.03 | 0.88-1.2 | 9.55E-01 |
| methyl glucopyranoside (alpha + beta) | 1.00 | 0.87-1.15 | 9.98E-01 | 1.03 | 0.89-1.18 | 9.55E-01 |
| pentadecanoate (15:0) | 0.95 | 0.82-1.11 | 9.35E-01 | 0.97 | 0.83-1.14 | 9.56E-01 |
| tetrahydrocortisol glucuronide | 1.04 | 0.89-1.23 | 9.50E-01 | 1.03 | 0.87-1.22 | 9.56E-01 |
| glutarylcarnitine (C5-DC) | 1.03 | 0.89-1.2 | 9.58E-01 | 1.03 | 0.88-1.19 | 9.56E-01 |
| X - 16124 | 1.03 | 0.9-1.18 | 9.58E-01 | 1.02 | 0.89-1.18 | 9.56E-01 |
| X - 23666 | 0.98 | 0.86-1.11 | 9.70E-01 | 0.98 | 0.86-1.11 | 9.56E-01 |
| pipecolate | 0.98 | 0.85-1.12 | 9.70E-01 | 0.98 | 0.85-1.12 | 9.56E-01 |
| X - 12221 | 1.02 | 0.89-1.17 | 9.70E-01 | 1.02 | 0.89-1.17 | 9.56E-01 |
| glutamine | 0.98 | 0.85-1.13 | 9.80E-01 | 0.97 | 0.84-1.13 | 9.56E-01 |
| glycoursodeoxycholic acid sulfate (1) | 0.98 | 0.84-1.15 | 9.83E-01 | 0.97 | 0.82-1.15 | 9.56E-01 |
| pregnenetriol disulfate* | 0.98 | 0.84-1.15 | 9.83E-01 | 0.97 | 0.83-1.14 | 9.56E-01 |
| glycosyl-N-palmitoyl-sphingosine (d18:1/16:0) | 1.01 | 0.87-1.18 | 9.88E-01 | 1.03 | 0.88-1.2 | 9.56E-01 |
| suberate (C8-DC) | 0.99 | 0.86-1.14 | 9.91E-01 | 0.98 | 0.85-1.12 | 9.56E-01 |
| formiminoglutamate | 1.00 | 0.86-1.16 | 9.98E-01 | 0.97 | 0.83-1.14 | 9.56E-01 |
| sphingomyelin (d17:2/16:0, d18:2/15:0)* | 1.01 | 0.85-1.19 | 9.98E-01 | 1.03 | 0.87-1.22 | 9.56E-01 |
| 3-hydroxydecanoylcarnitine | 0.96 | 0.83-1.11 | 9.41E-01 | 0.98 | 0.84-1.13 | 9.59E-01 |
| X - 25271 | 0.96 | 0.84-1.1 | 9.41E-01 | 0.98 | 0.85-1.13 | 9.59E-01 |
| 2-oxoarginine* | 1.03 | 0.9-1.18 | 9.58E-01 | 1.02 | 0.89-1.18 | 9.59E-01 |
| homostachydrine* | 1.03 | 0.9-1.18 | 9.70E-01 | 1.02 | 0.89-1.17 | 9.59E-01 |
| 3-hydroxyoctanoylcarnitine (2) | 1.02 | 0.87-1.18 | 9.84E-01 | 1.02 | 0.88-1.19 | 9.59E-01 |
| N-acetyl-beta-alanine | 1.01 | 0.88-1.16 | 9.86E-01 | 1.02 | 0.89-1.17 | 9.59E-01 |
| X - 25419 | 1.02 | 0.89-1.17 | 9.80E-01 | 1.02 | 0.89-1.18 | 9.59E-01 |
| X - 23974 | 0.98 | 0.86-1.12 | 9.80E-01 | 0.98 | 0.86-1.12 | 9.63E-01 |
| X - 17325 | 1.01 | 0.88-1.17 | 9.86E-01 | 1.02 | 0.88-1.19 | 9.63E-01 |
| N-methylproline | 1.01 | 0.88-1.15 | 9.98E-01 | 1.02 | 0.89-1.17 | 9.63E-01 |
| 5-hydroxy-2-methylpyridine sulfate | 1.04 | 0.9-1.19 | 9.50E-01 | 1.02 | 0.89-1.17 | 9.64E-01 |
| X - 11843 | 1.03 | 0.89-1.2 | 9.58E-01 | 1.02 | 0.88-1.19 | 9.64E-01 |
| tiglylcarnitine (C5:1-DC) | 0.97 | 0.85-1.11 | 9.64E-01 | 0.98 | 0.85-1.12 | 9.64E-01 |
| urea | 0.98 | 0.85-1.12 | 9.70E-01 | 0.98 | 0.85-1.13 | 9.64E-01 |
| docosahexaenoylcholine | 1.02 | 0.88-1.18 | 9.80E-01 | 1.02 | 0.88-1.18 | 9.64E-01 |
| N-stearoyl-sphingadienine (d18:2/18:0)* | 1.02 | 0.88-1.18 | 9.80E-01 | 1.02 | 0.88-1.18 | 9.64E-01 |
| octanoylcarnitine (C8) | 1.02 | 0.88-1.17 | 9.83E-01 | 1.02 | 0.89-1.17 | 9.64E-01 |
| 5-hydroxyindole sulfate | 1.01 | 0.88-1.17 | 9.86E-01 | 1.02 | 0.89-1.18 | 9.64E-01 |
| citrulline | 0.99 | 0.86-1.14 | 9.91E-01 | 0.98 | 0.84-1.13 | 9.64E-01 |
| glycosyl-N-(2-hydroxynervonoyl)-sphingosine (d18:1/24:1(2OH))* | 0.99 | 0.85-1.15 | 9.98E-01 | 0.98 | 0.84-1.13 | 9.64E-01 |
| X - 13007 | 0.99 | 0.87-1.14 | 9.98E-01 | 0.98 | 0.85-1.12 | 9.64E-01 |
| 8-methoxykynurenate | 0.97 | 0.84-1.12 | 9.67E-01 | 0.98 | 0.84-1.13 | 9.65E-01 |
| cysteine s-sulfate | 0.98 | 0.85-1.12 | 9.70E-01 | 0.98 | 0.85-1.13 | 9.65E-01 |
| ferulic acid 4-sulfate | 0.98 | 0.85-1.13 | 9.70E-01 | 0.98 | 0.85-1.13 | 9.65E-01 |
| 3-phenylpropionate (hydrocinnamate) | 1.02 | 0.89-1.17 | 9.83E-01 | 1.02 | 0.89-1.17 | 9.65E-01 |
| X - 24947 | 0.99 | 0.86-1.13 | 9.86E-01 | 0.98 | 0.85-1.13 | 9.65E-01 |
| N1-Methyl-2-pyridone-5-carboxamide | 1.01 | 0.87-1.18 | 9.91E-01 | 1.02 | 0.88-1.19 | 9.65E-01 |
| sphingomyelin (d18:1/14:0, d16:1/16:0)* | 1.01 | 0.88-1.17 | 9.91E-01 | 1.02 | 0.88-1.18 | 9.65E-01 |
| X - 26106 | 0.96 | 0.83-1.12 | 9.50E-01 | 0.98 | 0.84-1.14 | 9.70E-01 |
| tryptophan betaine | 1.02 | 0.88-1.19 | 9.70E-01 | 1.02 | 0.88-1.19 | 9.70E-01 |
| docosatrienoate (22:3n6)* | 1.01 | 0.88-1.15 | 9.98E-01 | 1.02 | 0.89-1.17 | 9.70E-01 |
| 1-lignoceroyl-GPC (24:0) | 0.97 | 0.84-1.13 | 9.70E-01 | 0.98 | 0.84-1.15 | 9.70E-01 |
| beta-hydroxyisovaleroylcarnitine | 1.02 | 0.87-1.19 | 9.83E-01 | 1.02 | 0.87-1.19 | 9.70E-01 |
| dimethylglycine | 1.03 | 0.9-1.19 | 9.50E-01 | 1.02 | 0.89-1.17 | 9.71E-01 |
| 1-arachidonoyl-GPC (20:4n6)* | 1.00 | 0.87-1.16 | 9.98E-01 | 1.02 | 0.88-1.18 | 9.71E-01 |
| 1,2,3-benzenetriol sulfate (2) | 0.97 | 0.84-1.12 | 9.58E-01 | 0.98 | 0.85-1.14 | 9.72E-01 |
| glycine conjugate of C10H14O2 (1)* | 1.03 | 0.9-1.18 | 9.58E-01 | 1.02 | 0.89-1.17 | 9.72E-01 |
| X - 21821 | 0.97 | 0.85-1.11 | 9.58E-01 | 0.98 | 0.85-1.13 | 9.72E-01 |
| kynurenine | 1.03 | 0.88-1.19 | 9.70E-01 | 1.02 | 0.87-1.2 | 9.72E-01 |
| N-acetylglutamine | 0.98 | 0.85-1.12 | 9.70E-01 | 0.98 | 0.86-1.13 | 9.72E-01 |
| 2-methylserine | 1.02 | 0.9-1.16 | 9.70E-01 | 1.02 | 0.89-1.16 | 9.72E-01 |
| N,N-dimethylalanine | 1.02 | 0.89-1.17 | 9.70E-01 | 1.02 | 0.88-1.17 | 9.72E-01 |
| sulfate of piperine metabolite C16H19NO3 (3)* | 0.98 | 0.84-1.13 | 9.70E-01 | 0.98 | 0.85-1.14 | 9.72E-01 |
| X - 17010 | 0.98 | 0.86-1.12 | 9.70E-01 | 0.98 | 0.86-1.12 | 9.72E-01 |
| 6-oxopiperidine-2-carboxylate | 1.02 | 0.88-1.18 | 9.78E-01 | 1.02 | 0.88-1.18 | 9.72E-01 |
| 1-linoleoyl-GPE (18:2)* | 0.98 | 0.84-1.14 | 9.80E-01 | 0.98 | 0.84-1.15 | 9.72E-01 |
| cinnamoylglycine | 1.02 | 0.89-1.17 | 9.80E-01 | 1.02 | 0.88-1.18 | 9.72E-01 |
| citraconate/glutaconate | 1.02 | 0.88-1.18 | 9.80E-01 | 1.02 | 0.88-1.18 | 9.72E-01 |
| glyco-beta-muricholate** | 0.98 | 0.86-1.12 | 9.80E-01 | 0.98 | 0.86-1.13 | 9.72E-01 |
| beta-alanine | 0.98 | 0.85-1.13 | 9.80E-01 | 0.98 | 0.85-1.13 | 9.72E-01 |
| 3-methyl-2-oxovalerate | 0.98 | 0.84-1.15 | 9.83E-01 | 0.98 | 0.83-1.16 | 9.72E-01 |
| 3-(3-amino-3-carboxypropyl)uridine* | 0.98 | 0.84-1.15 | 9.83E-01 | 0.98 | 0.84-1.15 | 9.72E-01 |
| gamma-glutamyl-alpha-lysine | 0.99 | 0.86-1.12 | 9.83E-01 | 0.98 | 0.86-1.12 | 9.72E-01 |
| N-oleoylserine | 0.98 | 0.85-1.14 | 9.83E-01 | 0.98 | 0.85-1.13 | 9.72E-01 |
| 1-(1-enyl-palmitoyl)-GPE (P-16:0)* | 1.02 | 0.88-1.17 | 9.83E-01 | 1.02 | 0.88-1.17 | 9.72E-01 |
| 1-(1-enyl-palmitoyl)-2-arachidonoyl-GPC (P-16:0/20:4)* | 1.02 | 0.87-1.19 | 9.83E-01 | 1.02 | 0.87-1.19 | 9.72E-01 |
| 1-oleoyl-2-arachidonoyl-GPE (18:1/20:4)* | 1.02 | 0.88-1.18 | 9.86E-01 | 1.02 | 0.88-1.18 | 9.72E-01 |
| 1-linoleoylglycerol (18:2) | 1.01 | 0.88-1.17 | 9.86E-01 | 1.02 | 0.88-1.18 | 9.72E-01 |
| N6-carbamoylthreonyladenosine | 0.99 | 0.84-1.15 | 9.86E-01 | 0.98 | 0.84-1.15 | 9.72E-01 |
| hexanoylglycine | 1.01 | 0.88-1.17 | 9.86E-01 | 1.02 | 0.88-1.18 | 9.72E-01 |
| linoleoylcholine* | 1.01 | 0.88-1.15 | 9.98E-01 | 1.02 | 0.88-1.17 | 9.72E-01 |
| sarcosine | 0.99 | 0.87-1.13 | 9.98E-01 | 0.98 | 0.86-1.13 | 9.72E-01 |
| docosahexaenoate (DHA; 22:6n3) | 1.00 | 0.88-1.15 | 9.98E-01 | 1.02 | 0.89-1.17 | 9.72E-01 |
| N2,N5-diacetylornithine | 1.00 | 0.86-1.16 | 9.98E-01 | 1.02 | 0.88-1.19 | 9.72E-01 |
| 4-vinylcatechol sulfate | 0.99 | 0.86-1.15 | 9.98E-01 | 0.98 | 0.85-1.14 | 9.72E-01 |
| 1-methylnicotinamide | 1.01 | 0.88-1.15 | 9.98E-01 | 1.02 | 0.89-1.16 | 9.72E-01 |
| X - 12306 | 1.01 | 0.88-1.15 | 9.98E-01 | 1.02 | 0.89-1.17 | 9.72E-01 |
| X - 13729 | 1.01 | 0.87-1.16 | 9.98E-01 | 1.02 | 0.88-1.17 | 9.72E-01 |
| X - 21733 | 0.99 | 0.86-1.14 | 9.98E-01 | 1.02 | 0.88-1.18 | 9.72E-01 |
| stearidonate (18:4n3) | 0.98 | 0.85-1.12 | 9.70E-01 | 0.99 | 0.86-1.13 | 9.75E-01 |
| N-acetylasparagine | 0.98 | 0.86-1.11 | 9.70E-01 | 0.98 | 0.86-1.12 | 9.75E-01 |
| 3-ureidopropionate | 1.02 | 0.88-1.19 | 9.70E-01 | 1.02 | 0.88-1.18 | 9.75E-01 |
| S-adenosylhomocysteine (SAH) | 1.03 | 0.89-1.19 | 9.70E-01 | 1.02 | 0.87-1.18 | 9.75E-01 |
| 1-arachidonoyl-GPI (20:4)* | 0.98 | 0.85-1.13 | 9.79E-01 | 0.99 | 0.86-1.13 | 9.75E-01 |
| N-acetylproline | 1.02 | 0.89-1.17 | 9.80E-01 | 1.01 | 0.89-1.16 | 9.75E-01 |
| 2-ketocaprylate | 0.98 | 0.86-1.12 | 9.80E-01 | 0.99 | 0.87-1.12 | 9.75E-01 |
| gamma-glutamyltyrosine | 0.98 | 0.86-1.12 | 9.80E-01 | 0.99 | 0.86-1.13 | 9.75E-01 |
| 3-hydroxyisobutyrate | 0.98 | 0.85-1.13 | 9.80E-01 | 0.98 | 0.85-1.14 | 9.75E-01 |
| 1-stearoyl-GPG (18:0) | 1.02 | 0.88-1.18 | 9.83E-01 | 1.02 | 0.88-1.18 | 9.75E-01 |
| X - 11795 | 0.98 | 0.85-1.13 | 9.83E-01 | 0.99 | 0.85-1.14 | 9.75E-01 |
| 2R,3R-dihydroxybutyrate | 0.99 | 0.85-1.14 | 9.86E-01 | 0.98 | 0.85-1.14 | 9.75E-01 |
| N-palmitoyl-heptadecasphingosine (d17:1/16:0)* | 1.01 | 0.88-1.17 | 9.86E-01 | 1.02 | 0.88-1.17 | 9.75E-01 |
| N-carbamoylvaline | 0.99 | 0.85-1.14 | 9.86E-01 | 0.98 | 0.85-1.14 | 9.75E-01 |
| X - 24588 | 0.98 | 0.84-1.15 | 9.86E-01 | 0.98 | 0.84-1.15 | 9.75E-01 |
| ascorbic acid 2-sulfate | 1.01 | 0.88-1.17 | 9.87E-01 | 1.01 | 0.88-1.17 | 9.75E-01 |
| acisoga | 0.99 | 0.87-1.13 | 9.97E-01 | 0.99 | 0.86-1.13 | 9.75E-01 |
| N-acetylarginine | 1.01 | 0.88-1.16 | 9.98E-01 | 1.01 | 0.88-1.16 | 9.75E-01 |
| 4-allylphenol sulfate | 1.00 | 0.87-1.15 | 9.98E-01 | 1.01 | 0.88-1.16 | 9.75E-01 |
| dihydroferulic acid sulfate | 1.01 | 0.87-1.17 | 9.98E-01 | 1.02 | 0.87-1.18 | 9.75E-01 |
| 3-hydroxyhexanoylcarnitine (1) | 0.99 | 0.86-1.15 | 9.98E-01 | 0.98 | 0.85-1.14 | 9.75E-01 |
| tetrahydrocortisone glucuronide (5) | 1.00 | 0.86-1.16 | 9.98E-01 | 0.98 | 0.84-1.15 | 9.75E-01 |
| ethyl alpha-glucopyranoside | 1.00 | 0.87-1.14 | 1.00E+00 | 1.02 | 0.87-1.19 | 9.75E-01 |
| myo-inositol | 0.98 | 0.86-1.12 | 9.80E-01 | 0.99 | 0.87-1.12 | 9.75E-01 |
| glycine | 1.02 | 0.88-1.19 | 9.80E-01 | 1.02 | 0.86-1.2 | 9.76E-01 |
| sphingomyelin (d18:1/20:0, d16:1/22:0)* | 1.02 | 0.89-1.17 | 9.70E-01 | 1.01 | 0.88-1.16 | 9.76E-01 |
| X - 21441 | 0.99 | 0.86-1.14 | 9.98E-01 | 0.99 | 0.85-1.14 | 9.76E-01 |
| 3-hydroxy-2-ethylpropionate | 0.98 | 0.84-1.14 | 9.80E-01 | 0.98 | 0.84-1.15 | 9.77E-01 |
| anthranilate | 0.98 | 0.85-1.12 | 9.70E-01 | 0.99 | 0.85-1.14 | 9.79E-01 |
| 3-(3-hydroxyphenyl)propionate sulfate | 1.01 | 0.87-1.17 | 9.98E-01 | 1.01 | 0.88-1.18 | 9.79E-01 |
| cholesterol sulfate | 1.02 | 0.88-1.18 | 9.80E-01 | 1.01 | 0.87-1.18 | 9.81E-01 |
| 2-hydroxystearate | 0.99 | 0.86-1.14 | 9.97E-01 | 0.99 | 0.86-1.14 | 9.81E-01 |
| N1-methyladenosine | 0.99 | 0.86-1.14 | 9.98E-01 | 0.99 | 0.86-1.13 | 9.81E-01 |
| 2,4-di-tert-butylphenol | 0.99 | 0.87-1.14 | 9.98E-01 | 0.99 | 0.86-1.13 | 9.82E-01 |
| pentose acid* | 1.00 | 0.88-1.14 | 9.99E-01 | 1.01 | 0.88-1.16 | 9.83E-01 |
| 11beta-hydroxyetiocholanolone glucuronide* | 0.98 | 0.85-1.14 | 9.80E-01 | 0.99 | 0.85-1.15 | 9.84E-01 |
| 3-hydroxyhippurate | 1.01 | 0.87-1.16 | 9.98E-01 | 1.01 | 0.88-1.17 | 9.84E-01 |
| iminodiacetate (IDA) | 1.02 | 0.89-1.17 | 9.70E-01 | 1.01 | 0.88-1.15 | 9.85E-01 |
| N-acetyltyrosine | 0.98 | 0.85-1.12 | 9.70E-01 | 0.99 | 0.86-1.15 | 9.85E-01 |
| 1-(1-enyl-palmitoyl)-GPC (P-16:0)* | 0.98 | 0.85-1.12 | 9.70E-01 | 0.99 | 0.85-1.15 | 9.85E-01 |
| 2-stearoyl-GPE (18:0)* | 0.98 | 0.85-1.13 | 9.70E-01 | 0.99 | 0.86-1.15 | 9.85E-01 |
| carotene diol (2) | 0.97 | 0.85-1.12 | 9.70E-01 | 0.99 | 0.86-1.14 | 9.85E-01 |
| X - 25267 | 1.02 | 0.9-1.17 | 9.70E-01 | 1.01 | 0.88-1.15 | 9.85E-01 |
| oleoyl-linoleoyl-glycerol (18:1/18:2) [1] | 1.02 | 0.88-1.19 | 9.75E-01 | 1.01 | 0.87-1.17 | 9.85E-01 |
| gentisate | 0.98 | 0.86-1.13 | 9.80E-01 | 0.99 | 0.86-1.14 | 9.85E-01 |
| N-acetylglycine | 0.98 | 0.85-1.13 | 9.80E-01 | 0.99 | 0.85-1.16 | 9.85E-01 |
| pro-hydroxy-pro | 1.02 | 0.88-1.19 | 9.80E-01 | 1.01 | 0.87-1.17 | 9.85E-01 |
| decanoylcarnitine (C10) | 0.98 | 0.86-1.13 | 9.80E-01 | 0.99 | 0.86-1.14 | 9.85E-01 |
| stearoylcarnitine (C18) | 0.98 | 0.85-1.13 | 9.80E-01 | 0.99 | 0.86-1.15 | 9.85E-01 |
| 2-hydroxypalmitate | 0.98 | 0.85-1.13 | 9.80E-01 | 0.99 | 0.85-1.14 | 9.85E-01 |
| taurochenodeoxycholic acid 3-sulfate | 1.02 | 0.89-1.17 | 9.80E-01 | 1.01 | 0.87-1.16 | 9.85E-01 |
| leucine | 1.02 | 0.87-1.19 | 9.80E-01 | 1.01 | 0.86-1.19 | 9.85E-01 |
| X - 25422 | 1.02 | 0.88-1.19 | 9.80E-01 | 1.01 | 0.86-1.18 | 9.85E-01 |
| 1-arachidonoyl-GPE (20:4n6)* | 0.98 | 0.84-1.14 | 9.83E-01 | 0.99 | 0.85-1.15 | 9.85E-01 |
| cysteine sulfinic acid | 1.02 | 0.87-1.19 | 9.83E-01 | 0.99 | 0.85-1.15 | 9.85E-01 |
| 2-aminophenol sulfate | 0.98 | 0.85-1.14 | 9.83E-01 | 0.99 | 0.86-1.15 | 9.85E-01 |
| linoleoyl ethanolamide | 0.98 | 0.86-1.13 | 9.83E-01 | 0.99 | 0.86-1.14 | 9.85E-01 |
| pimeloylcarnitine/3-methyladipoylcarnitine (C7-DC) | 0.98 | 0.85-1.14 | 9.83E-01 | 0.99 | 0.85-1.15 | 9.85E-01 |
| dihydrocaffeate sulfate (2) | 0.98 | 0.85-1.14 | 9.83E-01 | 0.99 | 0.86-1.15 | 9.85E-01 |
| X - 12127 | 1.02 | 0.89-1.16 | 9.83E-01 | 1.01 | 0.88-1.16 | 9.85E-01 |
| X - 24418 | 1.02 | 0.88-1.18 | 9.84E-01 | 1.01 | 0.87-1.17 | 9.85E-01 |
| methionine sulfoxide | 0.98 | 0.85-1.14 | 9.85E-01 | 0.99 | 0.86-1.15 | 9.85E-01 |
| 4-vinylguaiacol sulfate | 1.02 | 0.88-1.18 | 9.85E-01 | 0.99 | 0.86-1.15 | 9.85E-01 |
| 1-oleoyl-GPI (18:1) | 1.01 | 0.88-1.16 | 9.86E-01 | 1.01 | 0.88-1.16 | 9.85E-01 |
| 1-linoleoyl-GPI (18:2)* | 0.99 | 0.86-1.13 | 9.86E-01 | 0.99 | 0.86-1.14 | 9.85E-01 |
| 12-HETE | 1.01 | 0.88-1.17 | 9.86E-01 | 1.01 | 0.88-1.16 | 9.85E-01 |
| 1H-indole-7-acetic acid | 1.01 | 0.88-1.17 | 9.86E-01 | 1.01 | 0.87-1.17 | 9.85E-01 |
| 11-ketoetiocholanolone glucuronide | 1.01 | 0.87-1.18 | 9.86E-01 | 1.01 | 0.86-1.18 | 9.85E-01 |
| ceramide (d18:1/17:0, d17:1/18:0)* | 0.99 | 0.86-1.14 | 9.86E-01 | 0.99 | 0.86-1.15 | 9.85E-01 |
| ursodeoxycholate | 1.01 | 0.87-1.18 | 9.86E-01 | 1.01 | 0.87-1.18 | 9.85E-01 |
| guanidinoacetate | 1.02 | 0.88-1.17 | 9.86E-01 | 1.01 | 0.87-1.17 | 9.85E-01 |
| phenylalanine | 1.01 | 0.88-1.17 | 9.86E-01 | 1.01 | 0.87-1.16 | 9.85E-01 |
| X - 12707 | 0.99 | 0.84-1.15 | 9.86E-01 | 0.99 | 0.84-1.16 | 9.85E-01 |
| X - 24243 | 1.01 | 0.88-1.17 | 9.86E-01 | 1.01 | 0.87-1.16 | 9.85E-01 |
| carotene diol (1) | 0.99 | 0.86-1.14 | 9.91E-01 | 1.01 | 0.86-1.17 | 9.85E-01 |
| 2-hydroxyhippurate (salicylurate) | 1.01 | 0.88-1.16 | 9.91E-01 | 1.01 | 0.88-1.16 | 9.85E-01 |
| X - 24295 | 0.99 | 0.86-1.14 | 9.91E-01 | 0.99 | 0.86-1.15 | 9.85E-01 |
| taurochenodeoxycholate | 0.99 | 0.84-1.16 | 9.93E-01 | 0.99 | 0.85-1.16 | 9.85E-01 |
| sphingomyelin (d18:2/24:1, d18:1/24:2)* | 0.99 | 0.84-1.16 | 9.93E-01 | 0.99 | 0.84-1.17 | 9.85E-01 |
| dopamine 3-O-sulfate | 0.99 | 0.87-1.13 | 9.98E-01 | 1.00 | 0.87-1.13 | 9.85E-01 |
| 1-stearoyl-2-linoleoyl-GPI (18:0/18:2) | 1.01 | 0.87-1.16 | 9.98E-01 | 1.00 | 0.87-1.16 | 9.85E-01 |
| glycerate | 0.99 | 0.86-1.15 | 9.98E-01 | 1.01 | 0.87-1.18 | 9.85E-01 |
| alpha-tocopherol | 1.01 | 0.87-1.17 | 9.98E-01 | 1.01 | 0.87-1.17 | 9.85E-01 |
| X - 25957 | 1.01 | 0.87-1.18 | 9.98E-01 | 1.01 | 0.86-1.17 | 9.85E-01 |
| xanthurenate | 1.00 | 0.87-1.16 | 9.98E-01 | 0.99 | 0.85-1.15 | 9.85E-01 |
| glycerophosphorylcholine (GPC) | 1.00 | 0.86-1.15 | 9.98E-01 | 1.01 | 0.87-1.17 | 9.85E-01 |
| tartarate | 0.99 | 0.88-1.12 | 9.98E-01 | 0.99 | 0.88-1.13 | 9.85E-01 |
| 3-indoxyl sulfate | 1.00 | 0.86-1.17 | 9.98E-01 | 1.01 | 0.87-1.18 | 9.85E-01 |
| 1-stearoyl-2-arachidonoyl-GPI (18:0/20:4) | 1.00 | 0.86-1.17 | 9.98E-01 | 1.01 | 0.86-1.18 | 9.85E-01 |
| 1,2-dipalmitoyl-GPC (16:0/16:0) | 1.00 | 0.85-1.17 | 9.98E-01 | 1.01 | 0.86-1.18 | 9.85E-01 |
| 2-pyrrolidinone | 1.01 | 0.87-1.16 | 9.98E-01 | 1.01 | 0.87-1.17 | 9.85E-01 |
| 3beta,7alpha-dihydroxy-5-cholestenoate | 1.01 | 0.88-1.14 | 9.98E-01 | 0.99 | 0.87-1.13 | 9.85E-01 |
| hexanoylglutamine | 1.00 | 0.87-1.15 | 9.98E-01 | 1.00 | 0.87-1.16 | 9.85E-01 |
| N-stearoyltaurine | 1.00 | 0.86-1.15 | 9.98E-01 | 0.99 | 0.86-1.15 | 9.85E-01 |
| 2-piperidinone | 1.00 | 0.86-1.15 | 9.98E-01 | 0.99 | 0.86-1.15 | 9.85E-01 |
| N-acetyl-1-methylhistidine* | 1.00 | 0.87-1.16 | 9.98E-01 | 1.01 | 0.87-1.17 | 9.85E-01 |
| sphingomyelin (d17:1/16:0, d18:1/15:0, d16:1/17:0)* | 1.00 | 0.85-1.17 | 9.98E-01 | 1.01 | 0.85-1.19 | 9.85E-01 |
| 1-(1-enyl-stearoyl)-2-arachidonoyl-GPE (P-18:0/20:4)* | 1.00 | 0.86-1.17 | 9.98E-01 | 1.01 | 0.87-1.17 | 9.85E-01 |
| 1-oleoyl-2-linoleoyl-GPE (18:1/18:2)* | 0.99 | 0.86-1.14 | 9.98E-01 | 0.99 | 0.86-1.15 | 9.85E-01 |
| 1-(1-enyl-stearoyl)-2-linoleoyl-GPE (P-18:0/18:2)* | 1.00 | 0.86-1.16 | 9.98E-01 | 0.99 | 0.85-1.15 | 9.85E-01 |
| cerotoylcarnitine (C26)* | 1.00 | 0.87-1.16 | 9.98E-01 | 1.01 | 0.87-1.17 | 9.85E-01 |
| dihomo-linolenoylcarnitine (C20:3n3 or 6)* | 0.99 | 0.85-1.16 | 9.98E-01 | 1.01 | 0.86-1.17 | 9.85E-01 |
| sulfate of piperine metabolite C18H21NO3 (1)* | 0.99 | 0.86-1.15 | 9.98E-01 | 0.99 | 0.86-1.15 | 9.85E-01 |
| 4-ethylcatechol sulfate | 1.00 | 0.87-1.15 | 9.98E-01 | 1.01 | 0.88-1.16 | 9.85E-01 |
| hydroxy-N6,N6,N6-trimethyllysine* | 1.00 | 0.87-1.16 | 9.98E-01 | 0.99 | 0.86-1.14 | 9.85E-01 |
| vanillic acid glycine | 1.00 | 0.86-1.17 | 9.98E-01 | 1.01 | 0.86-1.19 | 9.85E-01 |
| cis-3,4-methyleneheptanoylglycine | 1.00 | 0.87-1.15 | 9.98E-01 | 0.99 | 0.86-1.14 | 9.85E-01 |
| N,N-dimethyl-pro-pro | 1.00 | 0.86-1.17 | 9.98E-01 | 1.01 | 0.86-1.18 | 9.85E-01 |
| 5-oxoproline | 0.99 | 0.86-1.15 | 9.98E-01 | 0.99 | 0.86-1.15 | 9.85E-01 |
| picolinate | 0.99 | 0.86-1.15 | 9.98E-01 | 0.99 | 0.86-1.15 | 9.85E-01 |
| vanillylmandelate (VMA) | 1.00 | 0.85-1.18 | 9.98E-01 | 1.01 | 0.85-1.19 | 9.85E-01 |
| oleoyl ethanolamide | 1.00 | 0.85-1.16 | 9.98E-01 | 1.01 | 0.86-1.17 | 9.85E-01 |
| choline | 0.99 | 0.85-1.16 | 9.98E-01 | 0.99 | 0.85-1.15 | 9.85E-01 |
| 3-(4-hydroxyphenyl)lactate | 0.99 | 0.85-1.17 | 9.98E-01 | 0.99 | 0.84-1.17 | 9.85E-01 |
| fumarate | 1.00 | 0.87-1.15 | 9.98E-01 | 1.01 | 0.88-1.16 | 9.85E-01 |
| glutarate (C5-DC) | 1.01 | 0.87-1.16 | 9.98E-01 | 1.01 | 0.87-1.16 | 9.85E-01 |
| lithocholate | 1.01 | 0.88-1.16 | 9.98E-01 | 1.01 | 0.87-1.17 | 9.85E-01 |
| methionine | 1.01 | 0.87-1.16 | 9.98E-01 | 0.99 | 0.86-1.15 | 9.85E-01 |
| orotate | 0.99 | 0.85-1.16 | 9.98E-01 | 0.99 | 0.84-1.16 | 9.85E-01 |
| tryptophan | 1.00 | 0.87-1.15 | 9.98E-01 | 0.99 | 0.86-1.15 | 9.85E-01 |
| mannose | 1.00 | 0.87-1.14 | 9.98E-01 | 0.99 | 0.85-1.15 | 9.85E-01 |
| X - 11444 | 1.00 | 0.86-1.17 | 9.98E-01 | 0.99 | 0.85-1.16 | 9.85E-01 |
| X - 11852 | 1.01 | 0.88-1.16 | 9.98E-01 | 1.01 | 0.88-1.16 | 9.85E-01 |
| X - 12411 | 1.00 | 0.87-1.15 | 9.98E-01 | 0.99 | 0.86-1.14 | 9.85E-01 |
| X - 13866 | 1.01 | 0.88-1.15 | 9.98E-01 | 1.01 | 0.88-1.15 | 9.85E-01 |
| X - 17357 | 1.00 | 0.87-1.15 | 9.98E-01 | 0.99 | 0.86-1.15 | 9.85E-01 |
| X - 18901 | 1.00 | 0.87-1.14 | 9.98E-01 | 1.01 | 0.88-1.15 | 9.85E-01 |
| X - 24337 | 1.01 | 0.86-1.18 | 9.98E-01 | 0.99 | 0.85-1.16 | 9.85E-01 |
| X - 25519 | 1.01 | 0.86-1.18 | 9.98E-01 | 0.99 | 0.85-1.16 | 9.85E-01 |
| 1-(1-enyl-palmitoyl)-2-palmitoleoyl-GPC (P-16:0/16:1)* | 1.00 | 0.86-1.16 | 9.99E-01 | 1.01 | 0.86-1.18 | 9.85E-01 |
| picolinoylglycine | 1.00 | 0.86-1.16 | 1.00E+00 | 0.99 | 0.85-1.16 | 9.85E-01 |
| 1-(1-enyl-palmitoyl)-2-oleoyl-GPC (P-16:0/18:1)* | 1.00 | 0.87-1.15 | 1.00E+00 | 1.01 | 0.87-1.18 | 9.85E-01 |
| sulfate of piperine metabolite C16H19NO3 (2)* | 1.00 | 0.86-1.16 | 1.00E+00 | 1.01 | 0.87-1.17 | 9.85E-01 |
| cystathionine | 1.01 | 0.87-1.17 | 9.98E-01 | 1.00 | 0.85-1.16 | 9.86E-01 |
| X - 12216 | 0.99 | 0.86-1.15 | 9.98E-01 | 1.00 | 0.87-1.16 | 9.86E-01 |
| X - 15486 | 1.01 | 0.89-1.15 | 9.98E-01 | 1.00 | 0.88-1.13 | 9.87E-01 |
| sulfate of piperine metabolite C18H21NO3 (3)* | 1.01 | 0.87-1.16 | 9.98E-01 | 1.00 | 0.87-1.16 | 9.87E-01 |
| deoxycholic acid glucuronide | 1.00 | 0.86-1.16 | 9.98E-01 | 1.00 | 0.86-1.15 | 9.87E-01 |
| thioproline | 1.01 | 0.87-1.18 | 9.86E-01 | 1.00 | 0.86-1.17 | 9.88E-01 |
| sphingomyelin (d18:2/23:1)* | 0.98 | 0.83-1.17 | 9.86E-01 | 1.00 | 0.83-1.19 | 9.88E-01 |
| 1-(1-enyl-palmitoyl)-2-palmitoyl-GPC (P-16:0/16:0)* | 0.99 | 0.85-1.14 | 9.91E-01 | 1.00 | 0.86-1.16 | 9.88E-01 |
| X - 11979 | 1.01 | 0.87-1.17 | 9.91E-01 | 1.00 | 0.87-1.16 | 9.88E-01 |
| X - 12283 | 0.99 | 0.86-1.14 | 9.91E-01 | 1.00 | 0.87-1.16 | 9.88E-01 |
| X - 23641 | 1.01 | 0.86-1.19 | 9.91E-01 | 1.00 | 0.85-1.19 | 9.88E-01 |
| carboxyethyl-GABA | 1.00 | 0.88-1.15 | 9.98E-01 | 1.00 | 0.87-1.15 | 9.88E-01 |
| 1-(1-enyl-oleoyl)-GPE (P-18:1)* | 1.00 | 0.86-1.16 | 9.98E-01 | 1.00 | 0.86-1.16 | 9.88E-01 |
| sphingomyelin (d18:1/22:1, d18:2/22:0, d16:1/24:1)* | 1.00 | 0.85-1.17 | 9.98E-01 | 1.00 | 0.85-1.17 | 9.88E-01 |
| X - 16576 | 0.99 | 0.87-1.13 | 9.87E-01 | 1.00 | 0.87-1.14 | 9.88E-01 |
| 1-stearoyl-GPI (18:0) | 0.99 | 0.86-1.15 | 9.98E-01 | 1.00 | 0.86-1.15 | 9.88E-01 |
| homocitrulline | 1.01 | 0.88-1.16 | 9.98E-01 | 1.00 | 0.87-1.15 | 9.88E-01 |
| 3-(3-hydroxyphenyl)propionate | 1.00 | 0.86-1.16 | 9.98E-01 | 1.00 | 0.87-1.16 | 9.88E-01 |
| 2-hydroxysebacate | 1.00 | 0.87-1.16 | 9.98E-01 | 1.00 | 0.86-1.15 | 9.88E-01 |
| inosine | 0.99 | 0.87-1.14 | 9.98E-01 | 1.00 | 0.87-1.14 | 9.88E-01 |
| X - 26058 | 1.00 | 0.87-1.15 | 9.98E-01 | 1.00 | 0.87-1.15 | 9.88E-01 |
| X - 15503 | 0.99 | 0.84-1.17 | 9.98E-01 | 1.00 | 0.84-1.2 | 9.89E-01 |
| O-sulfo-L-tyrosine | 1.00 | 0.85-1.18 | 9.98E-01 | 1.00 | 0.85-1.18 | 9.91E-01 |
| N,N,N-trimethyl-alanylproline betaine (TMAP) | 1.00 | 0.85-1.16 | 9.98E-01 | 1.00 | 0.86-1.16 | 9.91E-01 |
| X - 18913 | 1.01 | 0.88-1.15 | 9.98E-01 | 1.00 | 0.87-1.15 | 9.91E-01 |
| galactonate | 1.01 | 0.89-1.16 | 9.84E-01 | 1.00 | 0.88-1.14 | 9.93E-01 |
| N-palmitoylglycine | 1.00 | 0.85-1.17 | 9.98E-01 | 1.00 | 0.85-1.18 | 9.94E-01 |
| palmitoyl sphingomyelin (d18:1/16:0) | 1.00 | 0.85-1.17 | 9.98E-01 | 1.00 | 0.85-1.18 | 9.95E-01 |
| salicylate | 1.00 | 0.87-1.15 | 9.98E-01 | 1.00 | 0.87-1.15 | 9.95E-01 |
| 1-stearoyl-GPC (18:0) | 0.99 | 0.85-1.16 | 9.98E-01 | 1.00 | 0.85-1.18 | 9.96E-01 |
| X - 22834 | 0.99 | 0.87-1.14 | 9.98E-01 | 1.00 | 0.87-1.15 | 9.96E-01 |
| X - 25948 | 1.00 | 0.87-1.15 | 9.98E-01 | 1.00 | 0.87-1.15 | 9.96E-01 |
| 1-(1-enyl-palmitoyl)-2-arachidonoyl-GPE (P-16:0/20:4)* | 1.00 | 0.86-1.16 | 9.99E-01 | 1.00 | 0.86-1.16 | 9.96E-01 |
| N-acetylcitrulline | 1.00 | 0.86-1.16 | 9.98E-01 | 1.00 | 0.86-1.16 | 9.96E-01 |
| 2-hydroxyarachidate* | 1.02 | 0.9-1.16 | 9.70E-01 | 1.00 | 0.88-1.14 | 9.97E-01 |
| glycolithocholate | 0.99 | 0.86-1.14 | 9.98E-01 | 1.00 | 0.87-1.15 | 9.97E-01 |
| sphingomyelin (d18:1/24:1, d18:2/24:0)* | 1.00 | 0.86-1.17 | 9.98E-01 | 1.00 | 0.85-1.18 | 9.97E-01 |
| X - 12680 | 1.00 | 0.88-1.14 | 9.99E-01 | 1.00 | 0.88-1.14 | 9.97E-01 |
| 3-aminoisobutyrate | 1.00 | 0.87-1.16 | 9.98E-01 | 1.00 | 0.86-1.16 | 9.98E-01 |
| X - 21258 | 1.00 | 0.87-1.16 | 9.98E-01 | 1.00 | 0.86-1.16 | 9.98E-01 |

Note: the table shows associations from the competing risk Cox proportional hazards regression analysis where plasms metabolomics level from the Metabolon platform was main exposure and incident all cancer as the main outcome in ERGO-4 cohort. Model 1 : adjusted for age, sex, cohort, and lipid lowering medication. Model 2: adjusted for model1, smoking status, BMI, alcohol and education. The table is ranked based on the *FDR* values in model 2, using bold text indicates metabolites are surpassing *FDR* adjusted P value treshold (*P*<0.05). ERGO-4 refer to the fourth and fifth visits from the baseline cohort. Abbreviations: BMI, body mass index; HR, hazard ratio; CI, confidence interval; FDR, false discovery rate.

**Supplementary Table 19. Serum metabolites associated with incident lung cancer from the Nightingale platform.**

| **Metabolite** | **Model 1** | | | **Model 2** | | |
| --- | --- | --- | --- | --- | --- | --- |
|  | **HR** | **95% CI** | ***FDR* value** | **HR** | **95% CI** | ***FDR* value** |
| Glycoprotein acetyls | 1.53 | 1.12-2.09 | 9.26E-01 | 1.47 | 1.04-2.07 | 9.45E-01 |
| Average diameter for LDL particles | 0.79 | 0.62-1 | 9.26E-01 | 0.76 | 0.59-1 | 9.45E-01 |
| Phospholipids to total lipids ratio in medium LDL | 0.79 | 0.63-1 | 9.26E-01 | 0.78 | 0.61-1 | 9.45E-01 |
| Cholesterol to total lipids ratio in IDL | 0.8 | 0.63-1.01 | 9.26E-01 | 0.78 | 0.59-1.03 | 9.45E-01 |
| Triglycerides to total lipids ratio in medium LDL | 1.31 | 1-1.72 | 9.26E-01 | 1.29 | 0.97-1.71 | 9.45E-01 |
| Triglycerides to total lipids ratio in large LDL | 1.3 | 0.98-1.72 | 9.26E-01 | 1.28 | 0.95-1.73 | 9.45E-01 |
| Phospholipids to total lipids ratio in small HDL | 1.4 | 1.03-1.89 | 9.26E-01 | 1.35 | 1.01-1.82 | 9.45E-01 |
| Cholesterol to total lipids ratio in large LDL | 0.77 | 0.58-1.02 | 9.26E-01 | 0.76 | 0.56-1.02 | 9.45E-01 |
| Triglycerides to total lipids ratio in IDL | 1.25 | 0.94-1.66 | 9.26E-01 | 1.28 | 0.93-1.77 | 9.45E-01 |
| Cholesteryl esters to total lipids ratio in very large HDL | 0.72 | 0.56-0.93 | 9.26E-01 | 0.76 | 0.57-1 | 9.45E-01 |
| Free cholesterol in large LDL | 0.79 | 0.59-1.07 | 9.26E-01 | 0.76 | 0.55-1.05 | 9.45E-01 |
| Free cholesterol in LDL | 0.79 | 0.59-1.05 | 9.26E-01 | 0.76 | 0.56-1.04 | 9.45E-01 |
| Cholesterol to total lipids ratio in small HDL | 0.79 | 0.61-1.02 | 9.26E-01 | 0.79 | 0.6-1.04 | 9.45E-01 |
| Free cholesterol to total lipids ratio in medium LDL | 0.83 | 0.64-1.07 | 9.26E-01 | 0.79 | 0.59-1.07 | 9.45E-01 |
| Free cholesterol in medium LDL | 0.78 | 0.59-1.03 | 9.26E-01 | 0.77 | 0.57-1.03 | 9.45E-01 |
| Cholesteryl esters to total lipids ratio in small VLDL | 0.83 | 0.62-1.11 | 9.26E-01 | 0.8 | 0.58-1.1 | 9.45E-01 |
| Cholesteryl esters to total lipids ratio in IDL | 0.81 | 0.64-1.03 | 9.26E-01 | 0.8 | 0.61-1.04 | 9.45E-01 |
| Cholesterol to total lipids ratio in small VLDL | 0.82 | 0.6-1.12 | 9.26E-01 | 0.77 | 0.54-1.11 | 9.45E-01 |
| Free cholesterol to total lipids ratio in large LDL | 0.85 | 0.66-1.1 | 9.26E-01 | 0.8 | 0.6-1.08 | 9.45E-01 |
| Isoleucine | 1.22 | 0.93-1.61 | 9.26E-01 | 1.28 | 0.96-1.71 | 9.45E-01 |
| Triglycerides to total lipids ratio in small LDL | 1.25 | 0.95-1.66 | 9.26E-01 | 1.31 | 0.95-1.81 | 9.45E-01 |
| Free cholesterol to total lipids ratio in small LDL | 0.82 | 0.64-1.04 | 9.26E-01 | 0.78 | 0.59-1.03 | 9.45E-01 |
| Citrate | 0.74 | 0.55-0.98 | 9.26E-01 | 0.78 | 0.59-1.04 | 9.45E-01 |
| Free cholesterol in small LDL | 0.79 | 0.59-1.05 | 9.26E-01 | 0.76 | 0.56-1.03 | 9.45E-01 |
| Ratio of omega-6 fatty acids to omega-3 fatty acids | 0.85 | 0.67-1.07 | 9.26E-01 | 0.81 | 0.62-1.06 | 9.45E-01 |
| Cholesteryl esters to total lipids ratio in small HDL | 0.81 | 0.64-1.02 | 9.26E-01 | 0.83 | 0.64-1.06 | 9.45E-01 |
| Clinical LDL cholesterol | 0.8 | 0.6-1.08 | 9.26E-01 | 0.78 | 0.57-1.07 | 9.45E-01 |
| Cholesterol to total lipids ratio in very small VLDL | 0.85 | 0.63-1.15 | 9.26E-01 | 0.8 | 0.56-1.15 | 9.45E-01 |
| Cholesteryl esters to total lipids ratio in very small VLDL | 0.86 | 0.64-1.16 | 9.26E-01 | 0.81 | 0.57-1.16 | 9.45E-01 |
| Ratio of omega-3 fatty acids to total fatty acids | 1.17 | 0.92-1.5 | 9.26E-01 | 1.24 | 0.94-1.64 | 9.45E-01 |
| Concentration of large LDL particles | 0.8 | 0.6-1.07 | 9.26E-01 | 0.79 | 0.59-1.06 | 9.45E-01 |
| Free cholesterol to total lipids ratio in small VLDL | 0.83 | 0.59-1.16 | 9.26E-01 | 0.78 | 0.53-1.15 | 9.45E-01 |
| Cholesteryl esters in medium VLDL | 0.83 | 0.62-1.12 | 9.26E-01 | 0.81 | 0.6-1.1 | 9.45E-01 |
| Cholesterol in large LDL | 0.81 | 0.6-1.09 | 9.26E-01 | 0.79 | 0.58-1.08 | 9.45E-01 |
| Free cholesterol in IDL | 0.85 | 0.61-1.18 | 9.26E-01 | 0.79 | 0.55-1.14 | 9.45E-01 |
| Phospholipids in large LDL | 0.82 | 0.61-1.1 | 9.26E-01 | 0.8 | 0.59-1.09 | 9.45E-01 |
| Cholesterol in IDL | 0.84 | 0.62-1.16 | 9.26E-01 | 0.8 | 0.57-1.12 | 9.45E-01 |
| Cholesteryl esters in IDL | 0.84 | 0.62-1.15 | 9.26E-01 | 0.8 | 0.57-1.12 | 9.45E-01 |
| Omega-3 fatty acids | 1.19 | 0.92-1.53 | 9.26E-01 | 1.25 | 0.93-1.68 | 9.45E-01 |
| Cholesteryl esters to total lipids ratio in medium LDL | 1.16 | 0.9-1.51 | 9.26E-01 | 1.2 | 0.9-1.59 | 9.45E-01 |
| Phospholipids in LDL | 0.82 | 0.61-1.09 | 9.26E-01 | 0.8 | 0.59-1.08 | 9.45E-01 |
| Phospholipids to total lipids ratio in medium HDL | 1.19 | 0.92-1.53 | 9.26E-01 | 1.21 | 0.9-1.62 | 9.45E-01 |
| Total lipids in large LDL | 0.82 | 0.61-1.1 | 9.26E-01 | 0.8 | 0.59-1.09 | 9.45E-01 |
| 3-Hydroxybutyrate | 0.91 | 0.68-1.22 | 9.26E-01 | 0.84 | 0.62-1.14 | 9.45E-01 |
| Cholesteryl esters to total lipids ratio in medium VLDL | 0.88 | 0.64-1.2 | 9.26E-01 | 0.82 | 0.57-1.2 | 9.45E-01 |
| Cholesteryl esters in large LDL | 0.82 | 0.61-1.1 | 9.26E-01 | 0.8 | 0.59-1.09 | 9.45E-01 |
| Albumin | 0.76 | 0.57-1.01 | 9.26E-01 | 0.84 | 0.61-1.15 | 9.45E-01 |
| Glucose | 1.12 | 0.89-1.4 | 9.26E-01 | 1.19 | 0.92-1.52 | 9.45E-01 |
| LDL cholesterol | 0.82 | 0.61-1.09 | 9.26E-01 | 0.8 | 0.59-1.09 | 9.45E-01 |
| Cholesterol to total lipids ratio in very large HDL | 0.74 | 0.53-1.03 | 9.26E-01 | 0.78 | 0.54-1.11 | 9.45E-01 |
| Phospholipids in medium LDL | 0.81 | 0.61-1.07 | 9.26E-01 | 0.8 | 0.6-1.08 | 9.45E-01 |
| Total lipids in IDL | 0.87 | 0.64-1.19 | 9.26E-01 | 0.82 | 0.59-1.15 | 9.45E-01 |
| Concentration of IDL particles | 0.88 | 0.65-1.17 | 9.26E-01 | 0.84 | 0.62-1.14 | 9.45E-01 |
| Ratio of apolipoprotein B to apolipoprotein A1 | 0.82 | 0.61-1.1 | 9.26E-01 | 0.81 | 0.6-1.08 | 9.45E-01 |
| Phospholipids to total lipids ratio in small VLDL | 0.86 | 0.61-1.2 | 9.26E-01 | 0.82 | 0.56-1.19 | 9.45E-01 |
| Total lipids in LDL | 0.83 | 0.62-1.11 | 9.26E-01 | 0.81 | 0.6-1.1 | 9.45E-01 |
| Phospholipids to total lipids ratio in very small VLDL | 1.21 | 0.92-1.58 | 9.26E-01 | 1.22 | 0.88-1.68 | 9.45E-01 |
| Triglycerides to total lipids ratio in small VLDL | 1.17 | 0.84-1.64 | 9.26E-01 | 1.22 | 0.84-1.77 | 9.45E-01 |
| Phospholipids in IDL | 0.89 | 0.65-1.21 | 9.26E-01 | 0.84 | 0.6-1.17 | 9.45E-01 |
| Triglycerides to total lipids ratio in very small VLDL | 1.15 | 0.83-1.59 | 9.26E-01 | 1.21 | 0.83-1.74 | 9.45E-01 |
| Phospholipids in small LDL | 0.84 | 0.63-1.13 | 9.26E-01 | 0.8 | 0.58-1.09 | 9.45E-01 |
| Cholesterol to total lipids ratio in medium VLDL | 0.89 | 0.64-1.25 | 9.26E-01 | 0.84 | 0.57-1.24 | 9.45E-01 |
| Total cholesterol minus HDL-C | 0.84 | 0.63-1.13 | 9.26E-01 | 0.83 | 0.61-1.13 | 9.45E-01 |
| Cholesteryl esters in LDL | 0.83 | 0.62-1.11 | 9.26E-01 | 0.82 | 0.61-1.11 | 9.45E-01 |
| Concentration of LDL particles | 0.84 | 0.63-1.12 | 9.26E-01 | 0.82 | 0.61-1.11 | 9.45E-01 |
| Cholesterol in small LDL | 0.83 | 0.62-1.12 | 9.26E-01 | 0.82 | 0.6-1.12 | 9.45E-01 |
| Acetate | 0.85 | 0.67-1.07 | 9.26E-01 | 0.87 | 0.7-1.1 | 9.45E-01 |
| Phospholipids to total lipids ratio in IDL | 1.14 | 0.88-1.48 | 9.26E-01 | 1.13 | 0.84-1.51 | 9.45E-01 |
| Apolipoprotein B | 0.85 | 0.64-1.14 | 9.26E-01 | 0.84 | 0.62-1.13 | 9.45E-01 |
| Phospholipids in medium HDL | 1.23 | 0.87-1.72 | 9.26E-01 | 1.25 | 0.88-1.77 | 9.45E-01 |
| Free cholesterol to total lipids ratio in very small VLDL | 0.87 | 0.66-1.15 | 9.26E-01 | 0.86 | 0.65-1.13 | 9.45E-01 |
| Total lipids in small LDL | 0.85 | 0.63-1.14 | 9.26E-01 | 0.83 | 0.61-1.13 | 9.45E-01 |
| Cholesterol in medium VLDL | 0.86 | 0.65-1.15 | 9.26E-01 | 0.85 | 0.64-1.14 | 9.45E-01 |
| Free cholesterol to total lipids ratio in medium VLDL | 0.92 | 0.64-1.31 | 9.26E-01 | 0.86 | 0.57-1.29 | 9.45E-01 |
| Docosahexaenoic acid | 1.14 | 0.88-1.48 | 9.26E-01 | 1.19 | 0.88-1.61 | 9.45E-01 |
| Phospholipids to total lipids ratio in medium VLDL | 0.91 | 0.63-1.3 | 9.26E-01 | 0.86 | 0.57-1.29 | 9.45E-01 |
| Total lipids in medium LDL | 0.84 | 0.63-1.12 | 9.26E-01 | 0.84 | 0.62-1.13 | 9.45E-01 |
| Cholesterol in medium LDL | 0.84 | 0.63-1.12 | 9.26E-01 | 0.84 | 0.62-1.13 | 9.45E-01 |
| Cholesteryl esters in very small VLDL | 0.94 | 0.69-1.28 | 9.34E-01 | 0.88 | 0.64-1.23 | 9.45E-01 |
| Acetoacetate | 0.99 | 0.76-1.28 | 9.88E-01 | 0.94 | 0.73-1.22 | 9.45E-01 |
| Phospholipids to total lipids ratio in large HDL | 1.1 | 0.85-1.44 | 9.26E-01 | 1.13 | 0.83-1.55 | 9.45E-01 |
| Remnant cholesterol (non-HDL | 0.88 | 0.66-1.18 | 9.26E-01 | 0.87 | 0.64-1.17 | 9.45E-01 |
| Cholesteryl esters in small LDL | 0.85 | 0.63-1.15 | 9.26E-01 | 0.85 | 0.62-1.16 | 9.45E-01 |
| Phospholipids in HDL | 1.23 | 0.88-1.73 | 9.26E-01 | 1.22 | 0.86-1.73 | 9.45E-01 |
| Total esterified cholesterol | 0.89 | 0.65-1.22 | 9.26E-01 | 0.87 | 0.62-1.22 | 9.45E-01 |
| Free cholesterol to total lipids ratio in IDL | 0.9 | 0.65-1.25 | 9.26E-01 | 0.9 | 0.64-1.25 | 9.45E-01 |
| Total cholesterol | 0.9 | 0.66-1.22 | 9.26E-01 | 0.87 | 0.62-1.21 | 9.45E-01 |
| Total concentration of branched-chain amino acids (leucine + isoleucine + valine) | 1.09 | 0.81-1.45 | 9.26E-01 | 1.2 | 0.87-1.66 | 9.45E-01 |
| Cholesterol to total lipids ratio in small LDL | 0.81 | 0.62-1.06 | 9.26E-01 | 0.89 | 0.65-1.22 | 9.45E-01 |
| Valine | 1.08 | 0.81-1.44 | 9.26E-01 | 1.22 | 0.88-1.7 | 9.45E-01 |
| Total lipids in medium HDL | 1.18 | 0.85-1.66 | 9.26E-01 | 1.21 | 0.85-1.7 | 9.45E-01 |
| Cholesteryl esters to total lipids ratio in medium HDL | 0.92 | 0.72-1.17 | 9.26E-01 | 0.91 | 0.69-1.2 | 9.45E-01 |
| Cholesterol in small VLDL | 0.88 | 0.67-1.15 | 9.26E-01 | 0.88 | 0.68-1.15 | 9.45E-01 |
| Cholesteryl esters in small VLDL | 0.89 | 0.68-1.15 | 9.26E-01 | 0.89 | 0.69-1.16 | 9.45E-01 |
| Free cholesterol in small VLDL | 0.87 | 0.66-1.15 | 9.26E-01 | 0.87 | 0.66-1.15 | 9.45E-01 |
| Tyrosine | 1.08 | 0.82-1.42 | 9.26E-01 | 1.19 | 0.9-1.58 | 9.45E-01 |
| Total free cholesterol | 0.9 | 0.67-1.22 | 9.26E-01 | 0.88 | 0.64-1.21 | 9.45E-01 |
| Free cholesterol to total lipids ratio in chylomicrons and extremely large VLDL | 1.16 | 0.88-1.51 | 9.26E-01 | 1.16 | 0.87-1.54 | 9.45E-01 |
| Triglycerides in very small VLDL | 1.1 | 0.86-1.42 | 9.26E-01 | 1.1 | 0.84-1.44 | 9.45E-01 |
| Free cholesterol to total lipids ratio in very large HDL | 0.83 | 0.59-1.18 | 9.26E-01 | 0.85 | 0.58-1.25 | 9.45E-01 |
| Triglycerides in very large HDL | 1.17 | 0.89-1.52 | 9.26E-01 | 1.13 | 0.86-1.49 | 9.45E-01 |
| Triglycerides to total lipids ratio in chylomicrons and extremely large VLDL | 0.91 | 0.73-1.13 | 9.26E-01 | 0.92 | 0.73-1.15 | 9.45E-01 |
| Ratio of docosahexaenoic acid to total fatty acids | 1.1 | 0.84-1.44 | 9.26E-01 | 1.14 | 0.84-1.54 | 9.45E-01 |
| Cholesterol in very small VLDL | 0.97 | 0.72-1.29 | 9.48E-01 | 0.92 | 0.68-1.24 | 9.45E-01 |
| Cholesterol to total lipids ratio in medium HDL | 0.93 | 0.71-1.21 | 9.26E-01 | 0.91 | 0.68-1.22 | 9.45E-01 |
| Triglycerides in medium HDL | 1.09 | 0.8-1.47 | 9.26E-01 | 1.12 | 0.81-1.54 | 9.45E-01 |
| Cholesteryl esters to total lipids ratio in large LDL | 0.85 | 0.65-1.1 | 9.26E-01 | 0.87 | 0.66-1.14 | 9.45E-01 |
| Total lipids in HDL | 1.2 | 0.85-1.68 | 9.26E-01 | 1.18 | 0.84-1.68 | 9.45E-01 |
| Phospholipids to total lipids ratio in very large HDL | 1.26 | 0.84-1.9 | 9.26E-01 | 1.18 | 0.78-1.78 | 9.45E-01 |
| Cholesteryl esters in medium LDL | 0.87 | 0.66-1.16 | 9.26E-01 | 0.87 | 0.65-1.18 | 9.45E-01 |
| Triglycerides to total lipids ratio in medium VLDL | 1.05 | 0.74-1.48 | 9.36E-01 | 1.09 | 0.74-1.6 | 9.45E-01 |
| Concentration of medium HDL particles | 1.15 | 0.82-1.62 | 9.26E-01 | 1.18 | 0.83-1.66 | 9.45E-01 |
| Average diameter for HDL particles | 1.21 | 0.9-1.63 | 9.26E-01 | 1.16 | 0.85-1.58 | 9.45E-01 |
| Triglycerides in HDL | 1.09 | 0.81-1.45 | 9.26E-01 | 1.11 | 0.81-1.5 | 9.45E-01 |
| Free cholesterol in medium HDL | 1.16 | 0.82-1.63 | 9.26E-01 | 1.16 | 0.81-1.65 | 9.45E-01 |
| Phospholipids in small HDL | 1.08 | 0.8-1.46 | 9.26E-01 | 1.14 | 0.82-1.59 | 9.45E-01 |
| Pyruvate | 1.06 | 0.83-1.37 | 9.26E-01 | 1.11 | 0.85-1.43 | 9.45E-01 |
| Free cholesterol to total lipids ratio in small HDL | 0.95 | 0.7-1.29 | 9.34E-01 | 0.86 | 0.62-1.19 | 9.45E-01 |
| Concentration of small LDL particles | 0.9 | 0.67-1.21 | 9.26E-01 | 0.89 | 0.66-1.21 | 9.45E-01 |
| Cholesteryl esters in VLDL | 0.91 | 0.69-1.2 | 9.26E-01 | 0.91 | 0.69-1.2 | 9.45E-01 |
| Triglycerides in IDL | 1.12 | 0.87-1.43 | 9.26E-01 | 1.08 | 0.83-1.4 | 9.45E-01 |
| Apolipoprotein A1 | 1.16 | 0.82-1.63 | 9.26E-01 | 1.17 | 0.82-1.66 | 9.45E-01 |
| Phospholipids in very large HDL | 1.26 | 0.89-1.77 | 9.26E-01 | 1.16 | 0.8-1.67 | 9.45E-01 |
| Phospholipids in large HDL | 1.18 | 0.85-1.66 | 9.26E-01 | 1.14 | 0.8-1.63 | 9.45E-01 |
| Polyunsaturated fatty acids | 1.1 | 0.81-1.48 | 9.26E-01 | 1.13 | 0.83-1.54 | 9.45E-01 |
| Phospholipids to total lipids ratio in small LDL | 1 | 0.73-1.36 | 9.95E-01 | 0.88 | 0.64-1.2 | 9.45E-01 |
| Monounsaturated fatty acids | 1.11 | 0.85-1.45 | 9.26E-01 | 1.1 | 0.82-1.47 | 9.45E-01 |
| Free cholesterol to total lipids ratio in large VLDL | 0.92 | 0.68-1.23 | 9.26E-01 | 0.93 | 0.69-1.26 | 9.45E-01 |
| Free cholesterol in medium VLDL | 0.89 | 0.67-1.18 | 9.26E-01 | 0.89 | 0.67-1.19 | 9.45E-01 |
| Total fatty acids | 1.11 | 0.84-1.48 | 9.26E-01 | 1.11 | 0.82-1.51 | 9.45E-01 |
| Cholesterol in medium HDL | 1.13 | 0.82-1.57 | 9.26E-01 | 1.15 | 0.82-1.61 | 9.45E-01 |
| Triglycerides in small LDL | 1.08 | 0.82-1.41 | 9.26E-01 | 1.08 | 0.8-1.46 | 9.45E-01 |
| Cholesteryl esters to total lipids ratio in very large VLDL | 0.93 | 0.66-1.33 | 9.34E-01 | 0.92 | 0.62-1.36 | 9.45E-01 |
| Cholesteryl esters in medium HDL | 1.13 | 0.82-1.56 | 9.26E-01 | 1.15 | 0.82-1.6 | 9.45E-01 |
| Ratio of omega-6 fatty acids to total fatty acids | 0.91 | 0.71-1.15 | 9.26E-01 | 0.93 | 0.71-1.23 | 9.45E-01 |
| Degree of unsaturation | 1.06 | 0.8-1.41 | 9.26E-01 | 1.14 | 0.83-1.56 | 9.45E-01 |
| Creatinine | 1.04 | 0.77-1.42 | 9.36E-01 | 1.07 | 0.79-1.47 | 9.45E-01 |
| Sphingomyelins | 0.99 | 0.72-1.38 | 9.94E-01 | 0.93 | 0.65-1.31 | 9.45E-01 |
| Triglycerides in large HDL | 1.12 | 0.81-1.54 | 9.26E-01 | 1.1 | 0.8-1.51 | 9.45E-01 |
| Total lipids in large HDL | 1.17 | 0.83-1.63 | 9.26E-01 | 1.13 | 0.79-1.62 | 9.45E-01 |
| Cholesterol to total lipids ratio in very large VLDL | 0.95 | 0.67-1.34 | 9.34E-01 | 0.94 | 0.64-1.37 | 9.45E-01 |
| Total lipids in very large HDL | 1.24 | 0.9-1.72 | 9.26E-01 | 1.14 | 0.8-1.62 | 9.45E-01 |
| Free cholesterol in HDL | 1.18 | 0.83-1.66 | 9.26E-01 | 1.14 | 0.79-1.63 | 9.45E-01 |
| Triglycerides in small VLDL | 1.02 | 0.77-1.35 | 9.59E-01 | 1.04 | 0.77-1.4 | 9.45E-01 |
| Cholesterol to total lipids ratio in large HDL | 0.96 | 0.72-1.28 | 9.36E-01 | 0.94 | 0.67-1.3 | 9.45E-01 |
| Cholesteryl esters to total lipids ratio in large HDL | 0.94 | 0.73-1.22 | 9.26E-01 | 0.94 | 0.7-1.28 | 9.45E-01 |
| Phospholipids to total lipids ratio in chylomicrons and extremely large VLDL | 1.06 | 0.79-1.41 | 9.34E-01 | 1.09 | 0.8-1.47 | 9.45E-01 |
| Free cholesterol in chylomicrons and extremely large VLDL | 1.07 | 0.81-1.42 | 9.26E-01 | 1.11 | 0.81-1.51 | 9.45E-01 |
| Phospholipids in medium VLDL | 0.9 | 0.68-1.19 | 9.26E-01 | 0.91 | 0.68-1.21 | 9.45E-01 |
| Cholesterol in chylomicrons and extremely large VLDL | 1.04 | 0.79-1.36 | 9.36E-01 | 1.08 | 0.81-1.45 | 9.45E-01 |
| Concentration of medium LDL particles | 0.92 | 0.7-1.23 | 9.26E-01 | 0.91 | 0.68-1.23 | 9.45E-01 |
| Phospholipids in small VLDL | 0.91 | 0.69-1.19 | 9.26E-01 | 0.91 | 0.69-1.2 | 9.45E-01 |
| Concentration of large HDL particles | 1.14 | 0.82-1.61 | 9.26E-01 | 1.11 | 0.77-1.59 | 9.45E-01 |
| Saturated fatty acids | 1.11 | 0.84-1.46 | 9.26E-01 | 1.09 | 0.8-1.49 | 9.45E-01 |
| Total triglycerides | 1.03 | 0.78-1.37 | 9.48E-01 | 1.05 | 0.76-1.44 | 9.45E-01 |
| Omega-6 fatty acids | 1.07 | 0.79-1.45 | 9.26E-01 | 1.09 | 0.81-1.47 | 9.45E-01 |
| Triglycerides in small HDL | 1.01 | 0.76-1.34 | 9.90E-01 | 1.04 | 0.76-1.43 | 9.45E-01 |
| Ratio of monounsaturated fatty acids to total fatty acids | 1.08 | 0.83-1.42 | 9.26E-01 | 1.04 | 0.76-1.42 | 9.45E-01 |
| Linoleic acid | 1.05 | 0.77-1.42 | 9.34E-01 | 1.08 | 0.81-1.44 | 9.45E-01 |
| HDL cholesterol | 1.14 | 0.81-1.59 | 9.26E-01 | 1.12 | 0.79-1.59 | 9.45E-01 |
| Triglycerides in LDL | 1.08 | 0.83-1.4 | 9.26E-01 | 1.05 | 0.79-1.39 | 9.45E-01 |
| Concentration of very large HDL particles | 1.22 | 0.89-1.67 | 9.26E-01 | 1.12 | 0.79-1.57 | 9.45E-01 |
| Free cholesterol in large HDL | 1.17 | 0.82-1.66 | 9.26E-01 | 1.11 | 0.76-1.62 | 9.45E-01 |
| Cholesteryl esters in chylomicrons and extremely large VLDL | 1.02 | 0.78-1.32 | 9.60E-01 | 1.06 | 0.8-1.41 | 9.45E-01 |
| Phosphoglycerides | 1.12 | 0.8-1.58 | 9.26E-01 | 1.11 | 0.78-1.59 | 9.45E-01 |
| VLDL cholesterol | 0.93 | 0.71-1.23 | 9.26E-01 | 0.93 | 0.7-1.24 | 9.45E-01 |
| Lactate | 1 | 0.76-1.33 | 9.95E-01 | 1.02 | 0.78-1.35 | 9.45E-01 |
| Cholesteryl esters in HDL | 1.12 | 0.81-1.56 | 9.26E-01 | 1.12 | 0.79-1.58 | 9.45E-01 |
| Ratio of saturated fatty acids to total fatty acids | 1.02 | 0.78-1.34 | 9.59E-01 | 0.97 | 0.74-1.26 | 9.45E-01 |
| Total lipids in small HDL | 1.01 | 0.76-1.35 | 9.75E-01 | 1.07 | 0.78-1.48 | 9.45E-01 |
| Cholesteryl esters in very large VLDL | 0.89 | 0.67-1.18 | 9.26E-01 | 0.91 | 0.68-1.23 | 9.45E-01 |
| Concentration of medium VLDL particles | 0.91 | 0.68-1.21 | 9.26E-01 | 0.92 | 0.68-1.22 | 9.45E-01 |
| Triglycerides in large LDL | 1.08 | 0.84-1.4 | 9.26E-01 | 1.04 | 0.79-1.37 | 9.45E-01 |
| Triglycerides in chylomicrons and extremely large VLDL | 0.93 | 0.72-1.21 | 9.26E-01 | 0.95 | 0.73-1.24 | 9.45E-01 |
| Cholesterol in large HDL | 1.14 | 0.81-1.6 | 9.26E-01 | 1.1 | 0.76-1.59 | 9.45E-01 |
| Cholesterol to total lipids ratio in large VLDL | 0.97 | 0.7-1.34 | 9.48E-01 | 0.97 | 0.68-1.37 | 9.45E-01 |
| Triglycerides in medium LDL | 1.06 | 0.81-1.38 | 9.26E-01 | 1.04 | 0.78-1.38 | 9.45E-01 |
| Cholesteryl esters in small HDL | 0.91 | 0.7-1.17 | 9.26E-01 | 0.96 | 0.73-1.27 | 9.45E-01 |
| Cholesteryl esters in large HDL | 1.13 | 0.8-1.59 | 9.26E-01 | 1.1 | 0.76-1.58 | 9.45E-01 |
| Concentration of HDL particles | 1.08 | 0.78-1.49 | 9.26E-01 | 1.1 | 0.78-1.55 | 9.45E-01 |
| Cholesterol to total lipids ratio in chylomicrons and extremely large VLDL | 1.04 | 0.77-1.4 | 9.48E-01 | 1.03 | 0.75-1.41 | 9.45E-01 |
| Cholesteryl esters to total lipids ratio in small LDL | 0.99 | 0.74-1.33 | 9.90E-01 | 1.1 | 0.81-1.48 | 9.45E-01 |
| Ratio of polyunsaturated fatty acids to monounsaturated fatty acids | 0.93 | 0.71-1.21 | 9.26E-01 | 0.97 | 0.71-1.33 | 9.45E-01 |
| Phospholipids to total lipids ratio in large LDL | 1.09 | 0.81-1.47 | 9.26E-01 | 1.13 | 0.83-1.54 | 9.45E-01 |
| Cholesteryl esters to total lipids ratio in large VLDL | 0.97 | 0.7-1.35 | 9.59E-01 | 0.97 | 0.68-1.39 | 9.45E-01 |
| Leucine | 1 | 0.74-1.36 | 9.99E-01 | 1.08 | 0.78-1.49 | 9.45E-01 |
| Phosphatidylcholines | 1.09 | 0.77-1.54 | 9.26E-01 | 1.08 | 0.75-1.55 | 9.45E-01 |
| Ratio of linoleic acid to total fatty acids | 0.92 | 0.7-1.19 | 9.26E-01 | 0.96 | 0.73-1.26 | 9.45E-01 |
| Triglycerides to total lipids ratio in large VLDL | 1.04 | 0.77-1.42 | 9.36E-01 | 1.02 | 0.76-1.39 | 9.45E-01 |
| Free cholesterol to total lipids ratio in large HDL | 1.09 | 0.76-1.56 | 9.26E-01 | 0.96 | 0.67-1.36 | 9.45E-01 |
| Phenylalanine | 0.98 | 0.72-1.32 | 9.59E-01 | 1.07 | 0.8-1.42 | 9.45E-01 |
| Phospholipids in chylomicrons and extremely large VLDL | 1.02 | 0.77-1.35 | 9.59E-01 | 1.05 | 0.78-1.42 | 9.45E-01 |
| Concentration of chylomicrons and extremely large VLDL particles | 1.03 | 0.78-1.36 | 9.48E-01 | 1.05 | 0.78-1.42 | 9.45E-01 |
| Cholesterol in very large HDL | 1.2 | 0.86-1.66 | 9.26E-01 | 1.09 | 0.76-1.57 | 9.45E-01 |
| Cholesteryl esters in very large HDL | 1.17 | 0.84-1.65 | 9.26E-01 | 1.08 | 0.74-1.57 | 9.45E-01 |
| Cholesterol in small HDL | 0.93 | 0.71-1.21 | 9.26E-01 | 0.97 | 0.73-1.3 | 9.45E-01 |
| Concentration of small HDL particles | 0.92 | 0.71-1.2 | 9.26E-01 | 0.97 | 0.73-1.3 | 9.45E-01 |
| Total lipids in medium VLDL | 0.92 | 0.69-1.22 | 9.26E-01 | 0.93 | 0.69-1.25 | 9.45E-01 |
| Total cholines | 1.09 | 0.78-1.53 | 9.26E-01 | 1.07 | 0.75-1.53 | 9.45E-01 |
| Total lipids in chylomicrons and extremely large VLDL | 1.02 | 0.77-1.35 | 9.73E-01 | 1.05 | 0.77-1.42 | 9.45E-01 |
| Free cholesterol in very large HDL | 1.24 | 0.91-1.67 | 9.26E-01 | 1.09 | 0.78-1.52 | 9.45E-01 |
| Cholesterol in very large VLDL | 0.92 | 0.69-1.23 | 9.26E-01 | 0.95 | 0.7-1.28 | 9.45E-01 |
| Total concentration of lipoprotein particles | 1.05 | 0.75-1.45 | 9.36E-01 | 1.06 | 0.75-1.51 | 9.45E-01 |
| Histidine | 1.05 | 0.78-1.42 | 9.34E-01 | 1.11 | 0.81-1.52 | 9.45E-01 |
| Total lipids in lipoprotein particles | 0.99 | 0.74-1.33 | 9.88E-01 | 0.98 | 0.71-1.34 | 9.45E-01 |
| Cholesteryl esters in large VLDL | 0.92 | 0.7-1.22 | 9.26E-01 | 0.94 | 0.71-1.25 | 9.45E-01 |
| Free cholesterol in VLDL | 0.96 | 0.72-1.26 | 9.34E-01 | 0.97 | 0.72-1.29 | 9.45E-01 |
| Total phospholipids in lipoprotein particles | 1.07 | 0.78-1.49 | 9.26E-01 | 1.05 | 0.74-1.48 | 9.45E-01 |
| Phospholipids to total lipids ratio in large VLDL | 0.94 | 0.74-1.19 | 9.26E-01 | 0.97 | 0.75-1.26 | 9.45E-01 |
| Phospholipids to total lipids ratio in very large VLDL | 1 | 0.78-1.28 | 9.94E-01 | 1.02 | 0.81-1.28 | 9.45E-01 |
| Triglycerides in medium VLDL | 0.95 | 0.71-1.27 | 9.34E-01 | 0.97 | 0.71-1.32 | 9.45E-01 |
| Triglycerides to total lipids ratio in very large VLDL | 0.98 | 0.7-1.37 | 9.73E-01 | 0.97 | 0.7-1.36 | 9.45E-01 |
| Phospholipids in very small VLDL | 1.06 | 0.83-1.34 | 9.26E-01 | 1.02 | 0.8-1.31 | 9.45E-01 |
| Free cholesterol to total lipids ratio in very large VLDL | 1.05 | 0.76-1.45 | 9.34E-01 | 1.05 | 0.75-1.47 | 9.45E-01 |
| Total lipids in small VLDL | 0.95 | 0.73-1.24 | 9.34E-01 | 0.96 | 0.73-1.26 | 9.45E-01 |
| Cholesterol in large VLDL | 0.93 | 0.7-1.23 | 9.26E-01 | 0.95 | 0.71-1.28 | 9.45E-01 |
| Triglycerides to total lipids ratio in large HDL | 0.95 | 0.73-1.24 | 9.34E-01 | 0.97 | 0.73-1.3 | 9.45E-01 |
| Concentration of VLDL particles | 0.97 | 0.74-1.27 | 9.48E-01 | 0.97 | 0.73-1.28 | 9.45E-01 |
| Glycine | 1.07 | 0.79-1.45 | 9.26E-01 | 1.03 | 0.74-1.44 | 9.45E-01 |
| Acetone | 1.04 | 0.79-1.35 | 9.38E-01 | 0.97 | 0.75-1.26 | 9.45E-01 |
| Concentration of small VLDL particles | 0.95 | 0.73-1.24 | 9.34E-01 | 0.96 | 0.73-1.27 | 9.45E-01 |
| Glutamine | 0.95 | 0.75-1.21 | 9.34E-01 | 0.91 | 0.71-1.16 | 9.45E-01 |
| Concentration of large VLDL particles | 0.95 | 0.71-1.27 | 9.34E-01 | 0.97 | 0.71-1.33 | 9.45E-01 |
| Triglycerides in large VLDL | 0.95 | 0.7-1.29 | 9.34E-01 | 0.96 | 0.69-1.34 | 9.45E-01 |
| Free cholesterol in large VLDL | 0.93 | 0.7-1.24 | 9.26E-01 | 0.96 | 0.71-1.29 | 9.45E-01 |
| Cholesteryl esters to total lipids ratio in chylomicrons and extremely large VLDL | 0.98 | 0.72-1.33 | 9.59E-01 | 0.96 | 0.7-1.33 | 9.45E-01 |
| Total lipids in very large VLDL | 0.95 | 0.71-1.29 | 9.34E-01 | 0.97 | 0.71-1.34 | 9.45E-01 |
| Concentration of very large VLDL particles | 0.95 | 0.71-1.28 | 9.34E-01 | 0.97 | 0.7-1.34 | 9.45E-01 |
| Phospholipids in large VLDL | 0.94 | 0.7-1.25 | 9.26E-01 | 0.96 | 0.71-1.3 | 9.45E-01 |
| Total lipids in large VLDL | 0.94 | 0.7-1.27 | 9.34E-01 | 0.96 | 0.7-1.32 | 9.45E-01 |
| Phospholipids in very large VLDL | 0.95 | 0.71-1.28 | 9.34E-01 | 0.97 | 0.71-1.33 | 9.45E-01 |
| Free cholesterol in very large VLDL | 0.95 | 0.71-1.28 | 9.34E-01 | 0.97 | 0.71-1.33 | 9.45E-01 |
| Triglycerides in VLDL | 1 | 0.75-1.35 | 9.95E-01 | 1.02 | 0.74-1.41 | 9.48E-01 |
| Free cholesterol in very small VLDL | 1.02 | 0.79-1.31 | 9.59E-01 | 0.98 | 0.76-1.27 | 9.48E-01 |
| Free cholesterol to total lipids ratio in medium HDL | 1.04 | 0.74-1.45 | 9.48E-01 | 0.98 | 0.68-1.4 | 9.48E-01 |
| Triglycerides to total lipids ratio in small HDL | 1 | 0.75-1.34 | 9.94E-01 | 1.02 | 0.74-1.4 | 9.52E-01 |
| Phospholipids in VLDL | 0.97 | 0.74-1.28 | 9.50E-01 | 0.98 | 0.73-1.32 | 9.55E-01 |
| Ratio of triglycerides to phosphoglycerides | 1 | 0.73-1.35 | 9.94E-01 | 1.01 | 0.72-1.43 | 9.63E-01 |
| Average diameter for VLDL particles | 0.97 | 0.7-1.35 | 9.59E-01 | 1.02 | 0.71-1.45 | 9.63E-01 |
| Triglycerides to total lipids ratio in medium HDL | 0.99 | 0.76-1.3 | 9.90E-01 | 1.01 | 0.76-1.35 | 9.63E-01 |
| Concentration of very small VLDL particles | 1.02 | 0.79-1.33 | 9.59E-01 | 0.99 | 0.75-1.29 | 9.63E-01 |
| Triglycerides in very large VLDL | 0.97 | 0.72-1.3 | 9.48E-01 | 0.99 | 0.72-1.36 | 9.63E-01 |
| Ratio of polyunsaturated fatty acids to total fatty acids | 0.94 | 0.72-1.22 | 9.26E-01 | 0.99 | 0.73-1.35 | 9.79E-01 |
| Free cholesterol in small HDL | 0.99 | 0.74-1.34 | 9.94E-01 | 1.01 | 0.73-1.4 | 9.79E-01 |
| Total lipids in very small VLDL | 1.03 | 0.8-1.33 | 9.38E-01 | 1 | 0.77-1.29 | 9.87E-01 |
| Triglycerides to total lipids ratio in very large HDL | 0.94 | 0.7-1.27 | 9.34E-01 | 0.99 | 0.71-1.39 | 9.87E-01 |
| Alanine | 0.96 | 0.73-1.25 | 9.34E-01 | 1 | 0.77-1.3 | 9.93E-01 |
| Total lipids in VLDL | 0.98 | 0.74-1.31 | 9.73E-01 | 1 | 0.74-1.36 | 9.99E-01 |
| Cholesterol to total lipids ratio in medium LDL | 0.98 | 0.71-1.35 | 9.60E-01 | 1 | 0.74-1.35 | 9.99E-01 |

Note: the table shows associations from the competing risk Cox proportional hazards regression analysis where plasms metabolomics level from the Nightingale platform was main exposure and incident lung cancer as the main outcome in ERGO-4 cohort. Model 1 : adjusted for age, sex, cohort, and lipid lowering medication. Model 2: adjusted for model1, smoking status, BMI, alcohol and education. The table is ranked based on the *FDR* values in model 2, using bold text indicates metabolites are surpassing *FDR* adjusted P value treshold (*P*<0.05). ERGO-4 refer to the fourth and fifth visits from the baseline cohort. Abbreviations: IDL, intermediate-density lipoprotein; LDL, low-density lipoprotein; HDL, high-density lipoprotein; VLDL, very-low-density lipoprotein; BMI, body mass index; HR, hazard ratio; CI, confidence interval; FDR, false discovery rate.

**Supplementary Table 20. Serum metabolites associated with incident lung cancer from the Metabolon platform.**

| **Metabolite** | **Model 1** | | | **Model 2** | | |
| --- | --- | --- | --- | --- | --- | --- |
|  | **HR** | **95% Ci** | ***FDR* value** | **HR** | **95% Ci** | ***FDR* value** |
| X - 12462 | 1.69 | 1.35-2.11 | **5.20E-03** | 1.68 | 1.34-2.12 | **9.36E-03** |
| X - 23780 | 2.37 | 1.56-3.59 | **2.76E-02** | 2.33 | 1.53-3.57 | **4.85E-02** |
| 1-methylxanthine | 2.47 | 1.51-4.04 | 5.88E-02 | 2.58 | 1.47-4.52 | 1.77E-01 |
| bilirubin (Z,Z) | 0.49 | 0.33-0.73 | 5.88E-02 | 0.53 | 0.37-0.77 | 1.77E-01 |
| carnitine | 2.04 | 1.36-3.05 | 7.27E-02 | 1.99 | 1.32-3.02 | 1.77E-01 |
| 1,3-dimethylurate | 1.75 | 1.27-2.42 | 7.81E-02 | 1.91 | 1.28-2.84 | 1.77E-01 |
| trimethylamine N-oxide | 1.61 | 1.21-2.15 | 1.10E-01 | 1.58 | 1.21-2.07 | 1.77E-01 |
| dodecenedioate (C12:1-DC)* | 0.52 | 0.35-0.79 | 1.28E-01 | 0.57 | 0.41-0.81 | 1.77E-01 |
| fructose | 1.54 | 1.17-2.03 | 1.28E-01 | 1.56 | 1.18-2.05 | 1.77E-01 |
| tetradecadienedioate (C14:2-DC)* | 0.67 | 0.51-0.88 | 1.44E-01 | 0.64 | 0.48-0.84 | 1.77E-01 |
| biliverdin | 0.49 | 0.32-0.75 | 9.82E-02 | 0.53 | 0.36-0.8 | 2.04E-01 |
| 1-methyl-5-imidazolelactate | 1.49 | 1.14-1.95 | 1.44E-01 | 1.55 | 1.16-2.05 | 2.04E-01 |
| glycosyl-N-behenoyl-sphingadienine (d18:2/22:0)* | 0.61 | 0.43-0.86 | 1.74E-01 | 0.60 | 0.42-0.84 | 2.04E-01 |
| uracil | 0.64 | 0.46-0.89 | 2.25E-01 | 0.60 | 0.43-0.85 | 2.04E-01 |
| X - 11483 | 1.81 | 1.16-2.84 | 2.51E-01 | 2.02 | 1.26-3.24 | 2.04E-01 |
| beta-citrylglutamate | 1.50 | 1.09-2.06 | 3.10E-01 | 1.57 | 1.17-2.1 | 2.04E-01 |
| picolinate | 1.56 | 1.09-2.22 | 3.22E-01 | 1.67 | 1.19-2.34 | 2.04E-01 |
| theophylline | 1.38 | 1.04-1.83 | 3.97E-01 | 1.70 | 1.2-2.39 | 2.04E-01 |
| isobutyrylcarnitine (C4) | 1.43 | 1.04-1.97 | 4.01E-01 | 1.59 | 1.16-2.16 | 2.04E-01 |
| cysteine sulfinic acid | 2.32 | 1.48-3.64 | 5.88E-02 | 1.87 | 1.22-2.88 | 2.17E-01 |
| isobutyrylglycine | 1.71 | 1.21-2.42 | 1.28E-01 | 1.69 | 1.18-2.42 | 2.17E-01 |
| N,N,N-trimethyl-5-aminovalerate | 1.54 | 1.15-2.07 | 1.62E-01 | 1.50 | 1.14-1.97 | 2.17E-01 |
| 1-palmitoyl-2-linoleoyl-GPC (16:0/18:2) | 1.57 | 1.15-2.14 | 1.62E-01 | 1.53 | 1.14-2.06 | 2.30E-01 |
| 1,3,7-trimethylurate | 1.55 | 1-2.39 | 5.47E-01 | 2.04 | 1.24-3.36 | 2.30E-01 |
| 3-hydroxy-2-methylpyridine sulfate | 1.57 | 1.16-2.14 | 1.53E-01 | 1.46 | 1.12-1.9 | 2.30E-01 |
| X - 23644 | 1.45 | 1.07-1.95 | 3.35E-01 | 1.60 | 1.14-2.24 | 2.52E-01 |
| hexadecanedioate (C16-DC) | 0.67 | 0.47-0.94 | 3.78E-01 | 0.62 | 0.44-0.87 | 2.53E-01 |
| bilirubin degradation product, C17H18N2O4 (3)** | 0.58 | 0.41-0.83 | 1.28E-01 | 0.62 | 0.44-0.88 | 2.63E-01 |
| 2'-O-methylcytidine | 1.67 | 1.2-2.33 | 1.28E-01 | 1.55 | 1.12-2.13 | 2.78E-01 |
| bilirubin degradation product, C17H18N2O4 (1)** | 0.63 | 0.46-0.85 | 1.28E-01 | 0.67 | 0.5-0.9 | 2.78E-01 |
| trans-urocanate | 0.63 | 0.44-0.92 | 3.35E-01 | 0.61 | 0.43-0.88 | 2.78E-01 |
| bilirubin (E,Z or Z,E)* | 0.57 | 0.4-0.81 | 1.28E-01 | 0.63 | 0.44-0.89 | 2.82E-01 |
| bilirubin degradation product, C17H18N2O4 (2)** | 0.66 | 0.5-0.86 | 1.28E-01 | 0.68 | 0.51-0.91 | 2.82E-01 |
| X - 12740 | 0.57 | 0.4-0.82 | 1.28E-01 | 0.60 | 0.41-0.89 | 2.82E-01 |
| 1-methyl-5-imidazoleacetate | 1.39 | 1.07-1.81 | 3.22E-01 | 1.47 | 1.1-1.97 | 2.82E-01 |
| sebacate (C10-DC) | 0.74 | 0.58-0.94 | 3.35E-01 | 0.71 | 0.55-0.92 | 2.82E-01 |
| 3-hydroxysebacate | 0.66 | 0.46-0.94 | 3.78E-01 | 0.65 | 0.47-0.9 | 2.82E-01 |
| paraxanthine | 1.67 | 1.06-2.63 | 4.00E-01 | 2.12 | 1.21-3.72 | 2.82E-01 |
| X - 11299 | 1.52 | 1.05-2.21 | 4.00E-01 | 1.63 | 1.12-2.37 | 2.82E-01 |
| 2-methylbutyrylcarnitine (C5) | 1.23 | 0.97-1.56 | 6.72E-01 | 1.35 | 1.07-1.7 | 2.82E-01 |
| caffeine | 1.33 | 0.9-1.98 | 7.52E-01 | 1.85 | 1.15-2.97 | 2.82E-01 |
| bilirubin degradation product, C16H18N2O5 (4)** | 0.57 | 0.39-0.84 | 1.62E-01 | 0.62 | 0.43-0.9 | 2.83E-01 |
| trans-4-hydroxyproline | 1.59 | 1.13-2.22 | 2.01E-01 | 1.55 | 1.1-2.17 | 3.00E-01 |
| bilirubin degradation product, C16H18N2O5 (1)** | 0.63 | 0.47-0.86 | 1.42E-01 | 0.70 | 0.54-0.93 | 3.11E-01 |
| 3-methylhistidine | 1.37 | 1.06-1.76 | 3.35E-01 | 1.41 | 1.07-1.84 | 3.16E-01 |
| bilirubin degradation product, C16H18N2O5 (3)** | 0.57 | 0.38-0.84 | 1.65E-01 | 0.62 | 0.43-0.91 | 3.25E-01 |
| X - 24456 | 0.69 | 0.52-0.9 | 1.80E-01 | 0.69 | 0.52-0.93 | 3.25E-01 |
| X - 24953 | 0.74 | 0.59-0.92 | 1.87E-01 | 0.73 | 0.57-0.94 | 3.25E-01 |
| glutamine conjugate of C7H12O2* | 0.67 | 0.48-0.95 | 3.87E-01 | 0.66 | 0.48-0.92 | 3.25E-01 |
| bilirubin degradation product, C16H18N2O5 (2)** | 0.61 | 0.44-0.86 | 1.62E-01 | 0.65 | 0.46-0.92 | 3.34E-01 |
| 5-hydroxy-2-methylpyridine sulfate | 1.49 | 1.09-2.04 | 3.04E-01 | 1.40 | 1.07-1.85 | 3.34E-01 |
| 3-hydroxydodecanedioate* | 0.60 | 0.4-0.91 | 3.35E-01 | 0.59 | 0.39-0.91 | 3.34E-01 |
| N-carbamoylvaline | 0.66 | 0.46-0.94 | 3.78E-01 | 0.66 | 0.47-0.93 | 3.71E-01 |
| gamma-glutamylhistidine | 1.50 | 1.02-2.2 | 4.92E-01 | 1.53 | 1.07-2.19 | 3.71E-01 |
| cytosine | 1.45 | 1.06-1.98 | 3.78E-01 | 1.46 | 1.06-2 | 3.85E-01 |
| pyrraline | 1.30 | 0.95-1.78 | 6.90E-01 | 1.44 | 1.06-1.95 | 3.94E-01 |
| tetradecanedioate (C14-DC) | 0.62 | 0.41-0.94 | 4.00E-01 | 0.60 | 0.39-0.93 | 4.08E-01 |
| X - 23587 | 1.62 | 1.13-2.34 | 2.53E-01 | 1.58 | 1.06-2.35 | 4.40E-01 |
| beta-hydroxyisovaleroylcarnitine | 1.27 | 0.9-1.79 | 7.52E-01 | 1.42 | 1.04-1.92 | 4.78E-01 |
| 2,3-dihydroxypyridine | 1.80 | 1.05-3.07 | 4.39E-01 | 1.74 | 1.06-2.86 | 5.24E-01 |
| glutamine conjugate of C6H10O2 (2)* | 0.69 | 0.47-1.01 | 5.86E-01 | 0.62 | 0.41-0.95 | 5.24E-01 |
| N-methylproline | 0.60 | 0.44-0.81 | 1.10E-01 | 0.68 | 0.47-0.97 | 5.25E-01 |
| bilirubin (E,E)* | 0.62 | 0.43-0.91 | 3.22E-01 | 0.67 | 0.46-0.96 | 5.25E-01 |
| 5-acetylamino-6-amino-3-methyluracil | 1.74 | 1.09-2.8 | 3.78E-01 | 1.62 | 1.04-2.51 | 5.25E-01 |
| 3-hydroxypyridine glucuronide | 1.45 | 1.03-2.04 | 4.79E-01 | 1.37 | 1.03-1.83 | 5.25E-01 |
| octadecadienedioate (C18:2-DC)* | 0.62 | 0.4-0.97 | 4.92E-01 | 0.64 | 0.42-0.97 | 5.25E-01 |
| 1-myristoylglycerol (14:0) | 1.37 | 0.97-1.95 | 6.31E-01 | 1.44 | 1.03-2.02 | 5.25E-01 |
| ergothioneine | 1.49 | 0.97-2.29 | 6.31E-01 | 1.53 | 1.03-2.27 | 5.25E-01 |
| hexadecenedioate (C16:1-DC)* | 0.69 | 0.45-1.04 | 6.38E-01 | 0.63 | 0.41-0.96 | 5.25E-01 |
| 1,7-dimethylurate | 1.42 | 0.95-2.13 | 6.72E-01 | 1.84 | 1.05-3.22 | 5.25E-01 |
| 1-stearoyl-2-docosahexaenoyl-GPE (18:0/22:6)* | 1.41 | 0.95-2.09 | 6.72E-01 | 1.55 | 1.04-2.32 | 5.25E-01 |
| glycosyl ceramide (d18:2/24:1, d18:1/24:2)* | 0.72 | 0.49-1.07 | 6.90E-01 | 0.66 | 0.45-0.97 | 5.25E-01 |
| 3-amino-2-piperidone | 0.69 | 0.44-1.08 | 6.90E-01 | 0.61 | 0.39-0.96 | 5.25E-01 |
| carboxyethyl-GABA | 0.72 | 0.53-0.96 | 4.00E-01 | 0.72 | 0.53-0.98 | 5.73E-01 |
| EDTA | 0.82 | 0.66-1.02 | 6.31E-01 | 0.78 | 0.62-0.99 | 5.73E-01 |
| X - 26058 | 1.16 | 0.91-1.47 | 8.13E-01 | 1.28 | 1.01-1.61 | 5.73E-01 |
| hydantoin-5-propionate | 0.67 | 0.5-0.89 | 1.86E-01 | 0.76 | 0.58-0.99 | 5.79E-01 |
| choline | 0.67 | 0.48-0.95 | 3.87E-01 | 0.68 | 0.47-0.99 | 5.79E-01 |
| N1-methyladenosine | 0.70 | 0.5-0.99 | 5.26E-01 | 0.71 | 0.51-0.99 | 5.79E-01 |
| X - 26111 | 1.32 | 0.97-1.82 | 6.67E-01 | 1.39 | 1.01-1.9 | 5.79E-01 |
| octadecenedioate (C18:1-DC) | 0.73 | 0.52-1.04 | 6.45E-01 | 0.71 | 0.51-0.99 | 5.85E-01 |
| X - 25419 | 1.35 | 1.01-1.82 | 5.14E-01 | 1.37 | 1.01-1.86 | 6.02E-01 |
| N-(2-furoyl)glycine | 1.35 | 1-1.84 | 5.65E-01 | 1.31 | 1-1.71 | 6.02E-01 |
| 1-methylurate | 1.71 | 0.96-3.05 | 6.31E-01 | 1.76 | 1.01-3.08 | 6.02E-01 |
| o-cresol sulfate | 1.78 | 1.33-2.38 | **3.53E-02** | 1.38 | 1-1.92 | 6.05E-01 |
| X - 11470 | 0.75 | 0.57-0.98 | 4.97E-01 | 0.73 | 0.53-1 | 6.05E-01 |
| X - 23655 | 1.51 | 1.02-2.24 | 5.00E-01 | 1.44 | 1-2.07 | 6.05E-01 |
| methylsuccinoylcarnitine | 1.31 | 1-1.74 | 5.72E-01 | 1.32 | 1-1.74 | 6.05E-01 |
| dodecanedioate (C12-DC) | 0.62 | 0.37-1.02 | 5.86E-01 | 0.64 | 0.4-1.01 | 6.05E-01 |
| S-methylcysteine | 0.67 | 0.45-1.01 | 5.86E-01 | 0.62 | 0.38-1.02 | 6.05E-01 |
| 1-stearoyl-2-linoleoyl-GPI (18:0/18:2) | 1.38 | 0.99-1.92 | 5.86E-01 | 1.41 | 0.99-2 | 6.05E-01 |
| X - 21364 | 0.67 | 0.45-1.01 | 5.86E-01 | 0.64 | 0.4-1.01 | 6.05E-01 |
| 1-stearoyl-2-oleoyl-GPE (18:0/18:1) | 1.55 | 0.97-2.47 | 6.14E-01 | 1.53 | 0.99-2.37 | 6.05E-01 |
| X - 22162 | 1.29 | 0.98-1.71 | 6.31E-01 | 1.32 | 1-1.76 | 6.05E-01 |
| docosapentaenoate (n6 DPA; 22:5n6) | 0.72 | 0.5-1.03 | 6.38E-01 | 0.69 | 0.47-1.01 | 6.05E-01 |
| androstenediol (3beta,17beta) disulfate (2) | 0.72 | 0.49-1.05 | 6.72E-01 | 0.65 | 0.43-1 | 6.05E-01 |
| phenylacetylcarnitine | 1.62 | 0.93-2.82 | 6.72E-01 | 1.78 | 1-3.16 | 6.05E-01 |
| lactosyl-N-behenoyl-sphingosine (d18:1/22:0)* | 0.71 | 0.48-1.05 | 6.72E-01 | 0.68 | 0.46-1.01 | 6.05E-01 |
| glucose | 1.35 | 0.96-1.89 | 6.72E-01 | 1.42 | 1-2.01 | 6.05E-01 |
| 3-hydroxybutyrate (BHBA) | 0.68 | 0.43-1.08 | 6.90E-01 | 0.64 | 0.41-1.01 | 6.05E-01 |
| 1-palmitoyl-2-docosahexaenoyl-GPE (16:0/22:6)* | 1.38 | 0.93-2.03 | 6.91E-01 | 1.46 | 0.98-2.16 | 6.05E-01 |
| 1-(1-enyl-palmitoyl)-2-linoleoyl-GPE (P-16:0/18:2)* | 1.37 | 0.94-2.01 | 6.91E-01 | 1.41 | 0.99-2.01 | 6.05E-01 |
| lactosyl-N-nervonoyl-sphingosine (d18:1/24:1)* | 0.69 | 0.44-1.09 | 6.91E-01 | 0.61 | 0.37-1.01 | 6.05E-01 |
| hexadecasphingosine (d16:1)* | 1.40 | 0.92-2.12 | 6.91E-01 | 1.57 | 0.99-2.51 | 6.05E-01 |
| pregnenetriol sulfate* | 0.74 | 0.51-1.07 | 6.91E-01 | 0.70 | 0.48-1.01 | 6.05E-01 |
| isovalerylglycine | 1.37 | 0.9-2.08 | 7.45E-01 | 1.46 | 0.99-2.15 | 6.05E-01 |
| gamma-glutamylalanine | 1.67 | 0.82-3.4 | 7.52E-01 | 1.74 | 0.98-3.08 | 6.05E-01 |
| 2-aminoheptanoate | 1.42 | 0.98-2.06 | 6.11E-01 | 1.44 | 0.98-2.1 | 6.15E-01 |
| phosphate | 1.67 | 1.09-2.56 | 3.78E-01 | 1.51 | 0.98-2.33 | 6.19E-01 |
| 3-carboxy-4-methyl-5-propyl-2-furanpropanoate (CMPF) | 1.26 | 0.92-1.72 | 7.52E-01 | 1.37 | 0.98-1.9 | 6.19E-01 |
| trigonelline (N'-methylnicotinate) | 1.60 | 0.92-2.78 | 6.79E-01 | 1.62 | 0.97-2.69 | 6.36E-01 |
| 6-bromotryptophan | 0.64 | 0.44-0.94 | 4.00E-01 | 0.73 | 0.51-1.03 | 6.38E-01 |
| tridecenedioate (C13:1-DC)* | 0.67 | 0.47-0.95 | 4.00E-01 | 0.74 | 0.53-1.02 | 6.38E-01 |
| bilirubin degradation product, C17H20N2O5 (2)** | 0.73 | 0.54-0.98 | 5.00E-01 | 0.74 | 0.54-1.02 | 6.38E-01 |
| salicylate | 1.40 | 1.01-1.93 | 5.08E-01 | 1.36 | 0.98-1.88 | 6.38E-01 |
| bilirubin degradation product, C17H20N2O5 (1)** | 0.74 | 0.55-0.99 | 5.14E-01 | 0.75 | 0.55-1.02 | 6.38E-01 |
| X - 17010 | 1.23 | 0.96-1.58 | 6.90E-01 | 1.28 | 0.98-1.67 | 6.38E-01 |
| propionylcarnitine (C3) | 1.18 | 0.9-1.55 | 8.14E-01 | 1.30 | 0.98-1.73 | 6.38E-01 |
| octadecenedioylcarnitine (C18:1-DC)* | 0.77 | 0.55-1.08 | 7.01E-01 | 0.72 | 0.5-1.03 | 6.43E-01 |
| X - 12126 | 1.30 | 0.91-1.85 | 7.52E-01 | 1.38 | 0.97-1.94 | 6.43E-01 |
| X - 17346 | 1.36 | 0.93-2.01 | 6.91E-01 | 1.44 | 0.97-2.13 | 6.44E-01 |
| X - 21327 | 1.22 | 0.92-1.61 | 7.52E-01 | 1.30 | 0.98-1.72 | 6.44E-01 |
| X - 12839 | 0.76 | 0.54-1.07 | 6.91E-01 | 0.73 | 0.52-1.03 | 6.46E-01 |
| 1-oleoyl-GPG (18:1)* | 0.77 | 0.56-1.05 | 6.81E-01 | 0.75 | 0.55-1.03 | 6.56E-01 |
| uridine | 1.19 | 0.82-1.72 | 8.62E-01 | 1.48 | 0.96-2.27 | 6.56E-01 |
| gamma-glutamylmethionine | 1.36 | 1.03-1.81 | 4.55E-01 | 1.30 | 0.97-1.74 | 6.74E-01 |
| N1-methylinosine | 1.55 | 1.07-2.25 | 3.78E-01 | 1.42 | 0.96-2.11 | 6.78E-01 |
| retinol (Vitamin A) | 1.31 | 0.89-1.93 | 7.52E-01 | 1.41 | 0.96-2.08 | 6.80E-01 |
| X - 21467 | 0.65 | 0.44-0.95 | 4.00E-01 | 0.70 | 0.46-1.05 | 6.92E-01 |
| X - 24411 | 1.40 | 1.01-1.93 | 5.21E-01 | 1.35 | 0.96-1.91 | 6.92E-01 |
| umbelliferone sulfate | 0.68 | 0.47-0.99 | 5.34E-01 | 0.72 | 0.49-1.05 | 6.92E-01 |
| S-methylcysteine sulfoxide | 0.73 | 0.51-1.03 | 6.31E-01 | 0.73 | 0.51-1.05 | 6.92E-01 |
| 2-piperidinone | 0.77 | 0.55-1.06 | 6.91E-01 | 0.76 | 0.55-1.04 | 6.92E-01 |
| X - 18913 | 1.28 | 0.94-1.75 | 6.91E-01 | 1.32 | 0.96-1.81 | 6.92E-01 |
| N-acetylisoleucine | 1.30 | 0.9-1.88 | 7.52E-01 | 1.37 | 0.96-1.96 | 6.92E-01 |
| 4-hydroxycoumarin | 1.36 | 0.88-2.11 | 7.52E-01 | 1.51 | 0.95-2.41 | 6.92E-01 |
| glycoursodeoxycholic acid sulfate (1) | 0.79 | 0.52-1.19 | 8.15E-01 | 0.70 | 0.47-1.05 | 6.92E-01 |
| X - 17354 | 1.27 | 0.83-1.94 | 8.15E-01 | 1.46 | 0.95-2.24 | 6.92E-01 |
| 2-hydroxyhippurate (salicylurate) | 1.40 | 1.01-1.94 | 5.00E-01 | 1.33 | 0.96-1.86 | 6.95E-01 |
| 1-stearoyl-2-oleoyl-GPI (18:0/18:1)* | 1.50 | 1-2.26 | 5.63E-01 | 1.42 | 0.95-2.13 | 6.95E-01 |
| X - 17690 | 1.33 | 0.94-1.89 | 6.90E-01 | 1.40 | 0.95-2.07 | 6.96E-01 |
| palmitoleoylcarnitine (C16:1)* | 0.74 | 0.5-1.09 | 6.97E-01 | 0.75 | 0.54-1.05 | 6.96E-01 |
| N-acetyltyrosine | 1.34 | 0.88-2.03 | 7.52E-01 | 1.42 | 0.94-2.13 | 7.02E-01 |
| hydroxy-CMPF* | 1.24 | 0.89-1.72 | 7.97E-01 | 1.33 | 0.95-1.86 | 7.02E-01 |
| X - 24811 | 1.67 | 0.98-2.86 | 6.06E-01 | 1.49 | 0.93-2.38 | 7.06E-01 |
| 3-hydroxypyridine sulfate | 1.46 | 0.97-2.21 | 6.31E-01 | 1.37 | 0.94-1.99 | 7.06E-01 |
| 1-(1-enyl-stearoyl)-2-linoleoyl-GPE (P-18:0/18:2)* | 1.51 | 0.94-2.43 | 6.72E-01 | 1.51 | 0.93-2.47 | 7.06E-01 |
| N2-acetyl,N6-methyllysine | 0.77 | 0.57-1.04 | 6.72E-01 | 0.78 | 0.59-1.05 | 7.06E-01 |
| X - 23890 | 1.32 | 0.89-1.94 | 7.52E-01 | 1.38 | 0.94-2.03 | 7.06E-01 |
| gluconate | 1.21 | 0.85-1.7 | 8.33E-01 | 1.35 | 0.95-1.92 | 7.06E-01 |
| cyclo(leu-pro) | 1.33 | 0.74-2.41 | 8.56E-01 | 1.67 | 0.91-3.05 | 7.06E-01 |
| glycochenodeoxycholate 3-sulfate | 0.84 | 0.53-1.33 | 8.81E-01 | 0.75 | 0.53-1.06 | 7.06E-01 |
| X - 12111 | 0.63 | 0.45-0.87 | 1.74E-01 | 0.75 | 0.52-1.06 | 7.21E-01 |
| eicosenoylcarnitine (C20:1)* | 0.75 | 0.55-1.02 | 6.14E-01 | 0.76 | 0.55-1.06 | 7.21E-01 |
| gamma-glutamylglutamine | 1.55 | 0.95-2.54 | 6.68E-01 | 1.52 | 0.92-2.52 | 7.21E-01 |
| X - 24473 | 1.38 | 0.92-2.08 | 6.97E-01 | 1.40 | 0.93-2.11 | 7.21E-01 |
| X - 13431 | 1.10 | 0.84-1.43 | 9.10E-01 | 1.26 | 0.95-1.66 | 7.21E-01 |
| X - 22509 | 1.27 | 0.84-1.92 | 8.14E-01 | 1.39 | 0.93-2.08 | 7.23E-01 |
| (2,4 or 2,5)-dimethylphenol sulfate | 1.79 | 1.3-2.47 | 5.88E-02 | 1.36 | 0.93-1.97 | 7.25E-01 |
| linolenoylcarnitine (C18:3)* | 0.74 | 0.54-1.03 | 6.36E-01 | 0.78 | 0.58-1.06 | 7.25E-01 |
| 1-stearoyl-2-linoleoyl-GPC (18:0/18:2)* | 1.47 | 0.91-2.39 | 6.91E-01 | 1.51 | 0.91-2.51 | 7.25E-01 |
| N-stearoyl-sphingosine (d18:1/18:0)* | 1.28 | 0.95-1.74 | 6.91E-01 | 1.31 | 0.94-1.84 | 7.25E-01 |
| 1-linoleoylglycerol (18:2) | 1.36 | 0.9-2.06 | 7.52E-01 | 1.39 | 0.93-2.07 | 7.25E-01 |
| 2,3-dihydroxy-2-methylbutyrate | 1.33 | 0.88-2.01 | 7.52E-01 | 1.41 | 0.93-2.14 | 7.25E-01 |
| fructosyllysine | 1.32 | 0.87-2.01 | 7.74E-01 | 1.38 | 0.93-2.04 | 7.25E-01 |
| catechol glucuronide | 1.22 | 0.84-1.77 | 8.33E-01 | 1.31 | 0.94-1.83 | 7.25E-01 |
| X - 24462 | 0.69 | 0.48-1 | 5.63E-01 | 0.72 | 0.48-1.08 | 7.29E-01 |
| alpha-hydroxyisocaproate | 0.76 | 0.53-1.09 | 7.40E-01 | 0.74 | 0.52-1.07 | 7.29E-01 |
| X - 21733 | 0.94 | 0.67-1.32 | 9.59E-01 | 1.28 | 0.94-1.73 | 7.29E-01 |
| ceramide (d16:1/24:1, d18:1/22:1)* | 1.51 | 0.97-2.35 | 6.16E-01 | 1.47 | 0.91-2.37 | 7.29E-01 |
| N6-methyllysine | 0.76 | 0.55-1.07 | 6.91E-01 | 0.76 | 0.54-1.07 | 7.29E-01 |
| X - 23787 | 0.78 | 0.54-1.12 | 7.57E-01 | 0.76 | 0.54-1.07 | 7.29E-01 |
| N-acetyltryptophan | 1.41 | 0.9-2.21 | 7.01E-01 | 1.43 | 0.91-2.23 | 7.35E-01 |
| N-acetylleucine | 1.29 | 0.84-2 | 8.14E-01 | 1.41 | 0.92-2.17 | 7.35E-01 |
| X - 21441 | 0.83 | 0.55-1.24 | 8.62E-01 | 0.72 | 0.48-1.09 | 7.44E-01 |
| caprylate (8:0) | 0.76 | 0.48-1.19 | 8.14E-01 | 0.71 | 0.46-1.09 | 7.49E-01 |
| X - 12847 | 1.52 | 1.07-2.17 | 3.78E-01 | 1.37 | 0.91-2.08 | 7.53E-01 |
| arginine | 1.46 | 1.04-2.06 | 4.06E-01 | 1.35 | 0.92-1.99 | 7.53E-01 |
| homostachydrine* | 1.54 | 1-2.37 | 5.39E-01 | 1.37 | 0.91-2.08 | 7.53E-01 |
| 1-palmitoyl-2-oleoyl-GPI (16:0/18:1)* | 1.41 | 1-2 | 5.60E-01 | 1.30 | 0.92-1.84 | 7.53E-01 |
| perfluorooctanoate (PFOA) | 0.78 | 0.59-1.04 | 6.72E-01 | 0.79 | 0.59-1.07 | 7.53E-01 |
| 5alpha-pregnan-3beta,20beta-diol monosulfate (1) | 0.73 | 0.5-1.07 | 6.90E-01 | 0.72 | 0.47-1.1 | 7.53E-01 |
| 2-methylserine | 0.82 | 0.65-1.05 | 6.91E-01 | 0.82 | 0.62-1.06 | 7.53E-01 |
| isoursodeoxycholate | 0.72 | 0.48-1.1 | 7.01E-01 | 0.73 | 0.48-1.1 | 7.53E-01 |
| X - 21442 | 1.36 | 0.9-2.06 | 7.40E-01 | 1.39 | 0.91-2.13 | 7.53E-01 |
| hyocholate | 0.80 | 0.59-1.08 | 7.44E-01 | 0.78 | 0.57-1.07 | 7.53E-01 |
| isoleucine | 1.38 | 0.89-2.15 | 7.52E-01 | 1.43 | 0.9-2.28 | 7.53E-01 |
| X - 11632 | 1.31 | 0.9-1.9 | 7.52E-01 | 1.30 | 0.92-1.84 | 7.53E-01 |
| methionine | 1.23 | 0.91-1.65 | 7.66E-01 | 1.26 | 0.93-1.69 | 7.53E-01 |
| X - 12104 | 0.80 | 0.56-1.12 | 7.74E-01 | 0.77 | 0.55-1.08 | 7.53E-01 |
| urate | 1.26 | 0.89-1.8 | 7.79E-01 | 1.38 | 0.91-2.09 | 7.53E-01 |
| 1-arachidonylglycerol (20:4) | 1.27 | 0.87-1.86 | 7.97E-01 | 1.33 | 0.92-1.9 | 7.53E-01 |
| 4-hydroxyphenylacetoylcarnitine | 1.24 | 0.88-1.75 | 8.07E-01 | 1.31 | 0.93-1.86 | 7.53E-01 |
| gentisate | 1.29 | 0.85-1.96 | 8.14E-01 | 1.37 | 0.91-2.04 | 7.53E-01 |
| decadienedioic acid (C10:2-DC)** | 0.87 | 0.65-1.16 | 8.56E-01 | 0.78 | 0.58-1.07 | 7.53E-01 |
| X - 12726 | 1.20 | 0.81-1.77 | 8.62E-01 | 1.37 | 0.91-2.08 | 7.53E-01 |
| prolylglycine | 0.73 | 0.55-0.97 | 4.32E-01 | 0.79 | 0.57-1.08 | 7.53E-01 |
| butyrylcarnitine (C4) | 1.22 | 0.93-1.6 | 7.52E-01 | 1.25 | 0.93-1.67 | 7.53E-01 |
| X - 12798 | 1.34 | 0.86-2.11 | 7.79E-01 | 1.41 | 0.89-2.23 | 7.53E-01 |
| X - 12007 | 1.23 | 0.88-1.71 | 7.98E-01 | 1.28 | 0.92-1.77 | 7.53E-01 |
| X - 19438 | 1.39 | 0.79-2.45 | 8.14E-01 | 1.59 | 0.86-2.95 | 7.53E-01 |
| X - 24337 | 0.78 | 0.49-1.24 | 8.33E-01 | 0.71 | 0.45-1.12 | 7.53E-01 |
| glycoursodeoxycholate | 0.83 | 0.6-1.16 | 8.15E-01 | 0.78 | 0.56-1.09 | 7.53E-01 |
| cis-4-decenoate (10:1n6)* | 0.71 | 0.47-1.07 | 6.90E-01 | 0.77 | 0.55-1.09 | 7.55E-01 |
| X - 10458 | 1.27 | 0.95-1.69 | 6.90E-01 | 1.25 | 0.93-1.69 | 7.55E-01 |
| 1-stearoyl-2-linoleoyl-GPE (18:0/18:2)* | 1.40 | 0.87-2.24 | 7.52E-01 | 1.41 | 0.89-2.22 | 7.55E-01 |
| glutarate (C5-DC) | 0.77 | 0.56-1.07 | 6.97E-01 | 0.79 | 0.57-1.09 | 7.61E-01 |
| sphingosine | 1.32 | 0.99-1.76 | 5.99E-01 | 1.29 | 0.91-1.83 | 7.63E-01 |
| 1-(1-enyl-palmitoyl)-2-arachidonoyl-GPE (P-16:0/20:4)* | 1.43 | 0.91-2.26 | 6.97E-01 | 1.42 | 0.88-2.29 | 7.73E-01 |
| 1-linolenoylglycerol (18:3) | 1.42 | 0.86-2.35 | 7.52E-01 | 1.43 | 0.88-2.31 | 7.73E-01 |
| X - 21471 | 0.76 | 0.5-1.14 | 7.66E-01 | 0.74 | 0.49-1.12 | 7.73E-01 |
| ceramide (d18:1/14:0, d16:1/16:0)* | 1.26 | 0.87-1.83 | 7.98E-01 | 1.36 | 0.89-2.07 | 7.80E-01 |
| glutamine conjugate of C6H10O2 (1)* | 0.75 | 0.5-1.12 | 7.52E-01 | 0.73 | 0.47-1.12 | 7.80E-01 |
| 4-ethylcatechol sulfate | 1.37 | 0.93-2.02 | 6.91E-01 | 1.34 | 0.9-2 | 7.83E-01 |
| 1-(1-enyl-palmitoyl)-GPE (P-16:0)* | 1.29 | 0.89-1.87 | 7.52E-01 | 1.29 | 0.91-1.85 | 7.83E-01 |
| N-palmitoyl-sphinganine (d18:0/16:0) | 1.43 | 0.92-2.22 | 6.91E-01 | 1.35 | 0.89-2.06 | 7.88E-01 |
| N6,N6-dimethyllysine | 0.74 | 0.5-1.1 | 7.44E-01 | 0.75 | 0.5-1.12 | 7.88E-01 |
| X - 12822 | 0.71 | 0.53-0.96 | 4.00E-01 | 0.82 | 0.58-1.14 | 7.95E-01 |
| 3beta,7alpha-dihydroxy-5-cholestenoate | 1.55 | 1.05-2.29 | 4.12E-01 | 1.26 | 0.88-1.82 | 7.95E-01 |
| isoeugenol sulfate | 1.41 | 1.02-1.94 | 4.92E-01 | 1.25 | 0.87-1.78 | 7.95E-01 |
| X - 25172 | 1.30 | 0.99-1.72 | 6.11E-01 | 1.19 | 0.88-1.61 | 7.95E-01 |
| gamma-CEHC | 0.72 | 0.51-1.03 | 6.31E-01 | 0.81 | 0.57-1.17 | 7.95E-01 |
| sphingomyelin (d18:2/18:1)* | 0.65 | 0.4-1.05 | 6.43E-01 | 0.73 | 0.45-1.19 | 7.95E-01 |
| 3-methyl catechol sulfate (1) | 1.50 | 0.95-2.37 | 6.70E-01 | 1.31 | 0.86-2.01 | 7.95E-01 |
| 1-(1-enyl-stearoyl)-2-oleoyl-GPE (P-18:0/18:1) | 1.37 | 0.95-1.98 | 6.72E-01 | 1.28 | 0.89-1.83 | 7.95E-01 |
| N,N,N-trimethyl-alanylproline betaine (TMAP) | 0.75 | 0.54-1.05 | 6.72E-01 | 0.81 | 0.57-1.13 | 7.95E-01 |
| 3-methyl catechol sulfate (2) | 1.56 | 0.93-2.64 | 6.84E-01 | 1.40 | 0.87-2.24 | 7.95E-01 |
| N6-acetyllysine | 0.73 | 0.51-1.06 | 6.90E-01 | 0.80 | 0.57-1.1 | 7.95E-01 |
| 3,5-dichloro-2,6-dihydroxybenzoic acid | 0.78 | 0.58-1.05 | 6.90E-01 | 0.83 | 0.6-1.15 | 7.95E-01 |
| cortisone | 1.57 | 0.91-2.71 | 6.90E-01 | 1.39 | 0.83-2.34 | 7.95E-01 |
| N,N-dimethyl-pro-pro | 0.76 | 0.54-1.07 | 6.91E-01 | 0.80 | 0.55-1.16 | 7.95E-01 |
| sucrose | 1.33 | 0.93-1.9 | 6.91E-01 | 1.23 | 0.89-1.7 | 7.95E-01 |
| butyrylglycine | 1.35 | 0.92-1.98 | 6.98E-01 | 1.27 | 0.86-1.89 | 7.95E-01 |
| cyclo(gly-pro) | 1.44 | 0.9-2.29 | 7.01E-01 | 1.38 | 0.87-2.19 | 7.95E-01 |
| ursodeoxycholate | 0.75 | 0.52-1.09 | 7.01E-01 | 0.78 | 0.54-1.14 | 7.95E-01 |
| N-palmitoyl-sphingosine (d18:1/16:0) | 1.31 | 0.92-1.87 | 7.01E-01 | 1.21 | 0.87-1.69 | 7.95E-01 |
| X - 19141 | 0.75 | 0.53-1.08 | 7.01E-01 | 0.79 | 0.55-1.13 | 7.95E-01 |
| X - 12816 | 1.59 | 0.86-2.93 | 7.33E-01 | 1.52 | 0.8-2.89 | 7.95E-01 |
| p-cresol glucuronide* | 1.42 | 0.89-2.27 | 7.40E-01 | 1.32 | 0.86-2.03 | 7.95E-01 |
| branched-chain, straight-chain, or cyclopropyl 12:1 fatty acid* | 0.74 | 0.5-1.1 | 7.40E-01 | 0.80 | 0.56-1.14 | 7.95E-01 |
| adrenate (22:4n6) | 0.76 | 0.52-1.13 | 7.52E-01 | 0.80 | 0.56-1.14 | 7.95E-01 |
| oleoylcarnitine (C18:1) | 0.75 | 0.5-1.13 | 7.52E-01 | 0.80 | 0.54-1.18 | 7.95E-01 |
| 5alpha-androstan-3beta,17alpha-diol disulfate | 0.75 | 0.51-1.12 | 7.52E-01 | 0.78 | 0.52-1.17 | 7.95E-01 |
| androstenediol (3alpha, 17alpha) monosulfate (2) | 0.80 | 0.58-1.1 | 7.52E-01 | 0.77 | 0.53-1.11 | 7.95E-01 |
| octadecanedioylcarnitine (C18-DC)* | 0.73 | 0.48-1.13 | 7.52E-01 | 0.76 | 0.5-1.16 | 7.95E-01 |
| 1-palmitoyl-2-dihomo-linolenoyl-GPC (16:0/20:3n3 or 6)* | 1.41 | 0.87-2.29 | 7.52E-01 | 1.41 | 0.84-2.35 | 7.95E-01 |
| 1-oleoyl-2-linoleoyl-GPE (18:1/18:2)* | 1.40 | 0.86-2.27 | 7.52E-01 | 1.40 | 0.85-2.31 | 7.95E-01 |
| tetradecadienoate (14:2)* | 0.78 | 0.55-1.11 | 7.52E-01 | 0.81 | 0.58-1.13 | 7.95E-01 |
| 2-hydroxysebacate | 1.29 | 0.9-1.87 | 7.52E-01 | 1.25 | 0.89-1.74 | 7.95E-01 |
| 1-palmitoyl-2-oleoyl-GPE (16:0/18:1) | 1.38 | 0.88-2.16 | 7.52E-01 | 1.34 | 0.86-2.09 | 7.95E-01 |
| X - 12117 | 0.80 | 0.58-1.1 | 7.52E-01 | 0.81 | 0.57-1.15 | 7.95E-01 |
| X - 24531 | 1.36 | 0.88-2.11 | 7.52E-01 | 1.32 | 0.85-2.06 | 7.95E-01 |
| creatine | 1.39 | 0.86-2.25 | 7.57E-01 | 1.41 | 0.85-2.31 | 7.95E-01 |
| glucuronate | 1.28 | 0.89-1.83 | 7.66E-01 | 1.27 | 0.86-1.87 | 7.95E-01 |
| 1-palmitoyl-2-linoleoyl-GPE (16:0/18:2) | 1.30 | 0.88-1.93 | 7.67E-01 | 1.32 | 0.88-1.98 | 7.95E-01 |
| androsterone glucuronide | 0.74 | 0.48-1.15 | 7.67E-01 | 0.74 | 0.48-1.16 | 7.95E-01 |
| gamma-glutamyl-2-aminobutyrate | 1.48 | 0.82-2.68 | 7.74E-01 | 1.43 | 0.84-2.44 | 7.95E-01 |
| 1-linoleoyl-GPI (18:2)* | 1.23 | 0.9-1.69 | 7.74E-01 | 1.25 | 0.9-1.72 | 7.95E-01 |
| 1-(1-enyl-stearoyl)-2-arachidonoyl-GPE (P-18:0/20:4)* | 1.38 | 0.85-2.23 | 7.74E-01 | 1.32 | 0.84-2.07 | 7.95E-01 |
| N-acetylphenylalanine | 1.30 | 0.88-1.93 | 7.78E-01 | 1.32 | 0.87-1.99 | 7.95E-01 |
| glycine conjugate of C10H12O2* | 1.34 | 0.86-2.09 | 7.78E-01 | 1.33 | 0.86-2.06 | 7.95E-01 |
| quinate | 1.49 | 0.81-2.72 | 7.79E-01 | 1.43 | 0.81-2.5 | 7.95E-01 |
| glycosyl ceramide (d18:1/20:0, d16:1/22:0)* | 0.77 | 0.52-1.15 | 7.79E-01 | 0.78 | 0.54-1.13 | 7.95E-01 |
| X - 12738 | 1.32 | 0.86-2 | 7.89E-01 | 1.30 | 0.87-1.95 | 7.95E-01 |
| 2-linoleoylglycerol (18:2) | 1.52 | 0.78-2.96 | 7.97E-01 | 1.53 | 0.84-2.8 | 7.95E-01 |
| 4-methylhexanoylglutamine | 0.82 | 0.6-1.12 | 7.97E-01 | 0.82 | 0.6-1.12 | 7.95E-01 |
| 3-phosphoglycerate | 1.21 | 0.9-1.63 | 7.97E-01 | 1.25 | 0.91-1.74 | 7.95E-01 |
| alanine | 1.31 | 0.85-2.02 | 7.97E-01 | 1.33 | 0.87-2.04 | 7.95E-01 |
| X - 12844 | 0.82 | 0.61-1.12 | 7.97E-01 | 0.80 | 0.58-1.1 | 7.95E-01 |
| X - 12906 | 1.24 | 0.88-1.74 | 7.97E-01 | 1.24 | 0.85-1.81 | 7.95E-01 |
| histidine betaine (hercynine)* | 1.26 | 0.87-1.82 | 7.98E-01 | 1.28 | 0.87-1.88 | 7.95E-01 |
| 3-methylxanthine | 1.24 | 0.88-1.76 | 8.06E-01 | 1.27 | 0.88-1.84 | 7.95E-01 |
| 5-dodecenoate (12:1n7) | 0.78 | 0.52-1.18 | 8.14E-01 | 0.75 | 0.51-1.12 | 7.95E-01 |
| 11-ketoetiocholanolone glucuronide | 1.32 | 0.83-2.12 | 8.14E-01 | 1.33 | 0.81-2.18 | 7.95E-01 |
| 1-linoleoyl-2-arachidonoyl-GPC (18:2/20:4n6)* | 1.40 | 0.79-2.46 | 8.14E-01 | 1.41 | 0.77-2.59 | 7.95E-01 |
| N-oleoylserine | 0.82 | 0.58-1.15 | 8.14E-01 | 0.78 | 0.55-1.12 | 7.95E-01 |
| N2-acetyl,N6,N6-dimethyllysine | 0.81 | 0.57-1.16 | 8.14E-01 | 0.81 | 0.56-1.16 | 7.95E-01 |
| 2-aminobutyrate | 1.30 | 0.85-1.99 | 8.14E-01 | 1.28 | 0.89-1.86 | 7.95E-01 |
| histidine | 1.23 | 0.87-1.74 | 8.14E-01 | 1.25 | 0.87-1.78 | 7.95E-01 |
| X - 14082 | 1.35 | 0.81-2.27 | 8.14E-01 | 1.41 | 0.85-2.35 | 7.95E-01 |
| X - 15461 | 1.22 | 0.87-1.7 | 8.14E-01 | 1.21 | 0.88-1.65 | 7.95E-01 |
| X - 24518 | 0.80 | 0.56-1.15 | 8.14E-01 | 0.80 | 0.58-1.12 | 7.95E-01 |
| myristoleate (14:1n5) | 0.80 | 0.55-1.18 | 8.15E-01 | 0.80 | 0.55-1.16 | 7.95E-01 |
| gamma-glutamylisoleucine* | 1.19 | 0.88-1.62 | 8.15E-01 | 1.23 | 0.89-1.69 | 7.95E-01 |
| pregnenediol sulfate (C21H34O5S)* | 0.79 | 0.51-1.21 | 8.15E-01 | 0.71 | 0.45-1.15 | 7.95E-01 |
| dopamine 4-sulfate | 1.22 | 0.86-1.73 | 8.15E-01 | 1.27 | 0.9-1.79 | 7.95E-01 |
| 1-palmitoyl-2-docosahexaenoyl-GPC (16:0/22:6) | 1.24 | 0.85-1.82 | 8.15E-01 | 1.29 | 0.87-1.93 | 7.95E-01 |
| 3-hydroxyoctanoylcarnitine (2) | 1.23 | 0.85-1.76 | 8.15E-01 | 1.21 | 0.87-1.69 | 7.95E-01 |
| cytidine | 1.36 | 0.8-2.3 | 8.15E-01 | 1.40 | 0.81-2.42 | 7.95E-01 |
| X - 18345 | 0.83 | 0.61-1.15 | 8.15E-01 | 0.83 | 0.6-1.14 | 7.95E-01 |
| X - 21319 | 0.82 | 0.57-1.17 | 8.15E-01 | 0.78 | 0.55-1.1 | 7.95E-01 |
| X - 23276 | 1.14 | 0.9-1.45 | 8.15E-01 | 1.22 | 0.92-1.63 | 7.95E-01 |
| methylsuccinate | 1.34 | 0.79-2.25 | 8.19E-01 | 1.39 | 0.83-2.33 | 7.95E-01 |
| N-acetylproline | 1.26 | 0.83-1.89 | 8.20E-01 | 1.31 | 0.88-1.93 | 7.95E-01 |
| maleate | 1.15 | 0.89-1.49 | 8.33E-01 | 1.13 | 0.92-1.38 | 7.95E-01 |
| p-cresol sulfate | 1.32 | 0.79-2.18 | 8.33E-01 | 1.33 | 0.81-2.2 | 7.95E-01 |
| 1-linoleoyl-GPC (18:2) | 1.27 | 0.81-1.98 | 8.33E-01 | 1.40 | 0.84-2.32 | 7.95E-01 |
| tryptophan betaine | 1.23 | 0.83-1.83 | 8.33E-01 | 1.25 | 0.88-1.8 | 7.95E-01 |
| nisinate (24:6n3) | 1.23 | 0.83-1.81 | 8.33E-01 | 1.26 | 0.89-1.8 | 7.95E-01 |
| heneicosapentaenoate (21:5n3) | 1.21 | 0.84-1.74 | 8.33E-01 | 1.27 | 0.9-1.8 | 7.95E-01 |
| glucuronide of piperine metabolite C17H21NO3 (5)* | 1.25 | 0.81-1.94 | 8.33E-01 | 1.28 | 0.88-1.84 | 7.95E-01 |
| 2-ketocaprylate | 1.19 | 0.86-1.65 | 8.33E-01 | 1.24 | 0.87-1.75 | 7.95E-01 |
| 2-hydroxy-4-(methylthio)butanoic acid | 0.81 | 0.55-1.21 | 8.33E-01 | 0.78 | 0.55-1.11 | 7.95E-01 |
| leucine | 1.27 | 0.81-1.99 | 8.33E-01 | 1.39 | 0.82-2.36 | 7.95E-01 |
| orotate | 0.76 | 0.46-1.27 | 8.33E-01 | 0.72 | 0.44-1.19 | 7.95E-01 |
| X - 24544 | 0.82 | 0.55-1.2 | 8.34E-01 | 0.81 | 0.57-1.16 | 7.95E-01 |
| X - 23997 | 1.26 | 0.8-2 | 8.42E-01 | 1.42 | 0.85-2.36 | 7.95E-01 |
| taurodeoxycholate | 1.25 | 0.8-1.94 | 8.53E-01 | 1.30 | 0.83-2.05 | 7.95E-01 |
| 1-myristoyl-2-palmitoyl-GPC (14:0/16:0) | 1.24 | 0.8-1.9 | 8.56E-01 | 1.31 | 0.83-2.05 | 7.95E-01 |
| C-glycosyltryptophan | 0.84 | 0.59-1.2 | 8.56E-01 | 0.80 | 0.55-1.17 | 7.95E-01 |
| 1-palmitoyl-GPE (16:0) | 1.21 | 0.82-1.8 | 8.56E-01 | 1.29 | 0.87-1.9 | 7.95E-01 |
| 3-phenylpropionate (hydrocinnamate) | 1.17 | 0.84-1.63 | 8.62E-01 | 1.25 | 0.85-1.84 | 7.95E-01 |
| 1-stearoyl-GPE (18:0) | 1.26 | 0.76-2.07 | 8.62E-01 | 1.33 | 0.82-2.14 | 7.95E-01 |
| hexanoylglutamine | 0.86 | 0.62-1.21 | 8.62E-01 | 0.82 | 0.6-1.13 | 7.95E-01 |
| 5alpha-pregnan-3beta,20alpha-diol disulfate | 0.80 | 0.49-1.29 | 8.62E-01 | 0.72 | 0.42-1.24 | 7.95E-01 |
| glycocholenate sulfate* | 0.80 | 0.49-1.32 | 8.62E-01 | 0.72 | 0.42-1.21 | 7.95E-01 |
| formiminoglutamate | 0.82 | 0.51-1.31 | 8.62E-01 | 0.76 | 0.49-1.18 | 7.95E-01 |
| guaiacol sulfate | 1.21 | 0.8-1.83 | 8.62E-01 | 1.32 | 0.89-1.95 | 7.95E-01 |
| N-acetyl-1-methylhistidine* | 0.83 | 0.56-1.23 | 8.62E-01 | 0.78 | 0.51-1.19 | 7.95E-01 |
| N-palmitoyl-sphingadienine (d18:2/16:0)* | 1.19 | 0.82-1.73 | 8.62E-01 | 1.25 | 0.85-1.82 | 7.95E-01 |
| trans-2-hexenoylglycine | 0.87 | 0.63-1.2 | 8.62E-01 | 0.79 | 0.54-1.15 | 7.95E-01 |
| N-acetyl-2-aminooctanoate* | 1.21 | 0.81-1.8 | 8.62E-01 | 1.30 | 0.83-2.02 | 7.95E-01 |
| alpha-tocopherol | 1.18 | 0.81-1.72 | 8.62E-01 | 1.29 | 0.88-1.89 | 7.95E-01 |
| X - 12851 | 1.15 | 0.85-1.56 | 8.62E-01 | 1.19 | 0.88-1.61 | 7.95E-01 |
| X - 18888 | 0.87 | 0.63-1.2 | 8.62E-01 | 0.83 | 0.6-1.14 | 7.95E-01 |
| X - 21258 | 0.84 | 0.57-1.25 | 8.62E-01 | 0.79 | 0.53-1.17 | 7.95E-01 |
| X - 12216 | 1.11 | 0.86-1.43 | 8.63E-01 | 1.19 | 0.92-1.53 | 7.95E-01 |
| N-acetyl-2-aminoadipate | 1.16 | 0.8-1.69 | 8.72E-01 | 1.27 | 0.85-1.89 | 7.95E-01 |
| pristanate | 1.16 | 0.8-1.68 | 8.72E-01 | 1.31 | 0.9-1.91 | 7.95E-01 |
| 16a-hydroxy DHEA 3-sulfate | 0.84 | 0.54-1.29 | 8.74E-01 | 0.75 | 0.47-1.19 | 7.95E-01 |
| lysine | 1.12 | 0.84-1.5 | 8.75E-01 | 1.17 | 0.9-1.53 | 7.95E-01 |
| pregnanediol-3-glucuronide | 0.86 | 0.57-1.27 | 8.76E-01 | 0.80 | 0.54-1.17 | 7.95E-01 |
| pregnenetriol disulfate* | 0.84 | 0.54-1.31 | 8.76E-01 | 0.74 | 0.48-1.14 | 7.95E-01 |
| X - 24546 | 0.84 | 0.54-1.31 | 8.76E-01 | 0.75 | 0.48-1.19 | 7.95E-01 |
| tyrosine | 1.13 | 0.8-1.62 | 9.09E-01 | 1.26 | 0.89-1.78 | 7.95E-01 |
| pregnenediol disulfate (C21H34O8S2)* | 0.84 | 0.5-1.41 | 9.10E-01 | 0.71 | 0.41-1.23 | 7.95E-01 |
| etiocholanolone glucuronide | 0.88 | 0.6-1.27 | 9.10E-01 | 0.81 | 0.58-1.13 | 7.95E-01 |
| X - 24494 | 0.90 | 0.67-1.21 | 9.10E-01 | 0.84 | 0.63-1.13 | 7.95E-01 |
| androstenediol (3beta,17beta) monosulfate (2) | 0.89 | 0.6-1.3 | 9.23E-01 | 0.81 | 0.58-1.14 | 7.95E-01 |
| picolinoylglycine | 1.11 | 0.8-1.54 | 9.27E-01 | 1.28 | 0.89-1.84 | 7.95E-01 |
| X - 21470 | 0.87 | 0.54-1.4 | 9.30E-01 | 0.74 | 0.46-1.2 | 7.95E-01 |
| docosatrienoate (22:3n6)* | 0.90 | 0.62-1.3 | 9.30E-01 | 0.81 | 0.56-1.16 | 7.95E-01 |
| theobromine | 1.08 | 0.8-1.46 | 9.42E-01 | 1.27 | 0.88-1.83 | 7.95E-01 |
| X - 13844 | 1.10 | 0.76-1.6 | 9.42E-01 | 1.25 | 0.88-1.77 | 7.95E-01 |
| X - 23666 | 1.09 | 0.79-1.51 | 9.42E-01 | 1.21 | 0.88-1.67 | 7.95E-01 |
| ectoine | 1.07 | 0.79-1.46 | 9.48E-01 | 1.20 | 0.88-1.63 | 7.95E-01 |
| X - 11787 | 1.09 | 0.75-1.57 | 9.48E-01 | 1.26 | 0.87-1.83 | 7.95E-01 |
| piperine | 1.09 | 0.73-1.65 | 9.52E-01 | 1.25 | 0.9-1.74 | 7.95E-01 |
| serine | 0.93 | 0.67-1.31 | 9.56E-01 | 0.83 | 0.59-1.15 | 7.95E-01 |
| X - 12015 | 1.07 | 0.75-1.53 | 9.58E-01 | 1.22 | 0.88-1.71 | 7.95E-01 |
| xanthurenate | 1.07 | 0.74-1.54 | 9.59E-01 | 1.27 | 0.86-1.88 | 7.95E-01 |
| tiglylcarnitine (C5:1-DC) | 1.06 | 0.76-1.46 | 9.59E-01 | 1.19 | 0.89-1.59 | 7.95E-01 |
| argininate* | 1.06 | 0.75-1.49 | 9.59E-01 | 1.25 | 0.87-1.78 | 7.95E-01 |
| anthranilate | 1.07 | 0.7-1.63 | 9.61E-01 | 1.34 | 0.84-2.12 | 7.95E-01 |
| 3-carboxy-4-methyl-5-pentyl-2-furanpropionate (3-CMPFP)** | 1.02 | 0.72-1.45 | 9.89E-01 | 1.24 | 0.87-1.76 | 7.95E-01 |
| tartronate (hydroxymalonate) | 1.01 | 0.66-1.55 | 9.96E-01 | 1.26 | 0.84-1.88 | 7.95E-01 |
| X - 21286 | 1.00 | 0.7-1.42 | 9.96E-01 | 1.25 | 0.85-1.82 | 7.95E-01 |
| N-methylpipecolate | 0.84 | 0.61-1.16 | 8.33E-01 | 0.83 | 0.61-1.15 | 7.95E-01 |
| ethyl beta-glucopyranoside | 0.76 | 0.58-0.99 | 5.00E-01 | 0.86 | 0.65-1.12 | 7.96E-01 |
| 5alpha-pregnan-3beta,20alpha-diol monosulfate (2) | 0.78 | 0.52-1.16 | 7.98E-01 | 0.80 | 0.53-1.19 | 7.96E-01 |
| 5-(galactosylhydroxy)-L-lysine | 1.17 | 0.81-1.7 | 8.62E-01 | 1.27 | 0.84-1.91 | 7.96E-01 |
| 1-stearoyl-2-docosahexaenoyl-GPC (18:0/22:6) | 1.15 | 0.79-1.67 | 8.94E-01 | 1.23 | 0.85-1.79 | 7.96E-01 |
| cortisol | 1.30 | 0.82-2.05 | 8.15E-01 | 1.35 | 0.8-2.28 | 7.96E-01 |
| sarcosine | 0.92 | 0.69-1.24 | 9.42E-01 | 0.86 | 0.66-1.12 | 7.96E-01 |
| GlcNAc sulfate conjugate of C21H34O2 steroid** | 0.86 | 0.6-1.25 | 8.76E-01 | 0.81 | 0.55-1.18 | 7.97E-01 |
| X - 23680 | 1.22 | 0.89-1.68 | 7.97E-01 | 1.20 | 0.87-1.67 | 7.98E-01 |
| 5-hydroxylysine | 1.18 | 0.89-1.58 | 8.14E-01 | 1.22 | 0.86-1.72 | 7.98E-01 |
| citraconate/glutaconate | 1.21 | 0.82-1.8 | 8.56E-01 | 1.24 | 0.85-1.8 | 7.98E-01 |
| tartarate | 1.20 | 0.75-1.91 | 8.76E-01 | 1.30 | 0.81-2.09 | 7.98E-01 |
| glycerol 3-phosphate | 1.08 | 0.77-1.5 | 9.52E-01 | 1.21 | 0.86-1.71 | 7.98E-01 |
| dehydroepiandrosterone sulfate (DHEA-S) | 0.93 | 0.64-1.34 | 9.56E-01 | 0.81 | 0.56-1.17 | 7.98E-01 |
| 3-methoxytyramine sulfate | 1.28 | 0.9-1.82 | 7.52E-01 | 1.22 | 0.85-1.75 | 7.98E-01 |
| 1-myristoyl-2-arachidonoyl-GPC (14:0/20:4)* | 1.30 | 0.78-2.18 | 8.42E-01 | 1.36 | 0.78-2.39 | 7.98E-01 |
| sulfate of piperine metabolite C18H21NO3 (3)* | 1.21 | 0.82-1.79 | 8.56E-01 | 1.21 | 0.86-1.72 | 7.98E-01 |
| phenyllactate (PLA) | 0.82 | 0.52-1.29 | 8.62E-01 | 0.79 | 0.52-1.21 | 7.98E-01 |
| N4-acetylcytidine | 1.28 | 0.76-2.15 | 8.62E-01 | 1.34 | 0.79-2.29 | 7.98E-01 |
| X - 24307 | 0.89 | 0.67-1.18 | 8.62E-01 | 0.85 | 0.64-1.14 | 7.98E-01 |
| stachydrine | 0.73 | 0.56-0.94 | 3.35E-01 | 0.85 | 0.63-1.14 | 7.99E-01 |
| dihydroorotate | 0.79 | 0.6-1.03 | 6.45E-01 | 0.85 | 0.62-1.15 | 7.99E-01 |
| dihomo-linoleoylcarnitine (C20:2)* | 0.77 | 0.5-1.19 | 8.14E-01 | 0.79 | 0.52-1.21 | 7.99E-01 |
| citramalate | 1.24 | 0.84-1.82 | 8.33E-01 | 1.26 | 0.83-1.9 | 7.99E-01 |
| succinylcarnitine (C4-DC) | 0.84 | 0.61-1.16 | 8.33E-01 | 0.84 | 0.61-1.16 | 7.99E-01 |
| N2,N5-diacetylornithine | 1.21 | 0.85-1.72 | 8.33E-01 | 1.20 | 0.86-1.65 | 7.99E-01 |
| 1-(1-enyl-oleoyl)-GPE (P-18:1)* | 1.23 | 0.82-1.86 | 8.42E-01 | 1.26 | 0.83-1.91 | 7.99E-01 |
| 2-hydroxyoctanoate | 1.18 | 0.84-1.65 | 8.56E-01 | 1.21 | 0.86-1.71 | 7.99E-01 |
| O-sulfo-L-tyrosine | 0.82 | 0.54-1.23 | 8.56E-01 | 0.80 | 0.53-1.21 | 7.99E-01 |
| octadecanedioate (C18-DC) | 0.87 | 0.63-1.2 | 8.62E-01 | 0.84 | 0.61-1.16 | 7.99E-01 |
| sulfate* | 1.19 | 0.81-1.73 | 8.62E-01 | 1.22 | 0.85-1.74 | 7.99E-01 |
| cystathionine | 1.17 | 0.81-1.7 | 8.62E-01 | 1.26 | 0.82-1.93 | 7.99E-01 |
| myristoleoylcarnitine (C14:1)* | 0.89 | 0.65-1.22 | 9.01E-01 | 0.85 | 0.63-1.15 | 7.99E-01 |
| sphinganine-1-phosphate | 0.91 | 0.66-1.25 | 9.30E-01 | 0.83 | 0.6-1.16 | 7.99E-01 |
| 3-indoleglyoxylic acid | 1.10 | 0.75-1.61 | 9.42E-01 | 1.22 | 0.84-1.77 | 7.99E-01 |
| 2,6-dihydroxybenzoic acid | 1.09 | 0.78-1.53 | 9.42E-01 | 1.19 | 0.87-1.63 | 7.99E-01 |
| (2 or 3)-decenoate (10:1n7 or n8) | 1.11 | 0.85-1.45 | 8.90E-01 | 1.16 | 0.88-1.54 | 8.00E-01 |
| 1-palmitoyl-2-arachidonoyl-GPC (16:0/20:4n6) | 1.41 | 0.84-2.38 | 7.79E-01 | 1.31 | 0.79-2.18 | 8.00E-01 |
| 4-hydroxyglutamate | 1.23 | 0.83-1.84 | 8.33E-01 | 1.29 | 0.8-2.06 | 8.00E-01 |
| phenylacetylglutamine | 1.25 | 0.74-2.11 | 8.63E-01 | 1.32 | 0.79-2.21 | 8.00E-01 |
| phenol sulfate | 1.05 | 0.73-1.51 | 9.69E-01 | 1.22 | 0.84-1.76 | 8.00E-01 |
| taurocholenate sulfate* | 1.02 | 0.74-1.4 | 9.89E-01 | 0.83 | 0.59-1.17 | 8.00E-01 |
| phenylacetate | 1.25 | 0.74-2.11 | 8.63E-01 | 1.34 | 0.77-2.32 | 8.04E-01 |
| decanoylcarnitine (C10) | 0.85 | 0.65-1.11 | 8.14E-01 | 0.87 | 0.67-1.13 | 8.06E-01 |
| methyl-4-hydroxybenzoate sulfate | 0.72 | 0.49-1.06 | 6.90E-01 | 0.82 | 0.55-1.21 | 8.09E-01 |
| metabolonic lactone sulfate | 0.80 | 0.59-1.07 | 7.35E-01 | 0.83 | 0.57-1.19 | 8.09E-01 |
| 1-(1-enyl-palmitoyl)-2-oleoyl-GPE (P-16:0/18:1)* | 1.30 | 0.91-1.84 | 7.45E-01 | 1.19 | 0.85-1.68 | 8.09E-01 |
| myristoylcarnitine (C14) | 0.82 | 0.6-1.14 | 8.14E-01 | 0.86 | 0.64-1.15 | 8.09E-01 |
| 3-hydroxydecanoate | 0.81 | 0.55-1.18 | 8.15E-01 | 0.82 | 0.57-1.19 | 8.09E-01 |
| 13-HODE + 9-HODE | 1.28 | 0.83-1.99 | 8.15E-01 | 1.25 | 0.81-1.93 | 8.09E-01 |
| deoxycholic acid glucuronide | 0.82 | 0.57-1.18 | 8.33E-01 | 0.82 | 0.56-1.2 | 8.09E-01 |
| imidazole propionate | 0.78 | 0.49-1.27 | 8.42E-01 | 0.77 | 0.46-1.27 | 8.09E-01 |
| X - 26107 | 1.16 | 0.87-1.54 | 8.42E-01 | 1.18 | 0.85-1.63 | 8.09E-01 |
| 3-hydroxyoctanoate | 0.85 | 0.62-1.18 | 8.56E-01 | 0.85 | 0.62-1.16 | 8.09E-01 |
| X - 22776 | 1.22 | 0.81-1.84 | 8.62E-01 | 1.22 | 0.83-1.8 | 8.09E-01 |
| 3-methylcytidine | 0.85 | 0.59-1.23 | 8.62E-01 | 0.84 | 0.59-1.18 | 8.09E-01 |
| taurocholate | 1.21 | 0.79-1.84 | 8.62E-01 | 1.25 | 0.81-1.92 | 8.09E-01 |
| eicosapentaenoate (EPA; 20:5n3) | 1.17 | 0.82-1.67 | 8.62E-01 | 1.21 | 0.83-1.76 | 8.09E-01 |
| phenylacetylglutamate | 1.21 | 0.75-1.96 | 8.76E-01 | 1.30 | 0.79-2.12 | 8.09E-01 |
| 3b-hydroxy-5-cholenoic acid | 0.91 | 0.7-1.19 | 9.10E-01 | 0.86 | 0.64-1.15 | 8.09E-01 |
| arabonate/xylonate | 1.12 | 0.78-1.61 | 9.28E-01 | 1.19 | 0.84-1.69 | 8.09E-01 |
| 5alpha-androstan-3beta,17beta-diol disulfate | 0.88 | 0.52-1.49 | 9.42E-01 | 0.77 | 0.46-1.28 | 8.09E-01 |
| phenol glucuronide | 1.10 | 0.77-1.56 | 9.42E-01 | 1.21 | 0.84-1.73 | 8.09E-01 |
| N-acetylputrescine | 1.10 | 0.76-1.59 | 9.42E-01 | 1.22 | 0.83-1.79 | 8.09E-01 |
| phytanate | 1.08 | 0.75-1.53 | 9.56E-01 | 1.20 | 0.85-1.7 | 8.09E-01 |
| N-acetylglycine | 0.94 | 0.63-1.4 | 9.59E-01 | 0.80 | 0.52-1.23 | 8.09E-01 |
| oxalate (ethanedioate) | 0.95 | 0.64-1.42 | 9.69E-01 | 1.22 | 0.83-1.81 | 8.09E-01 |
| indole-3-carboxylate | 1.22 | 0.79-1.88 | 8.62E-01 | 1.23 | 0.82-1.84 | 8.15E-01 |
| 3-methylglutaconate | 1.21 | 0.79-1.86 | 8.62E-01 | 1.21 | 0.83-1.78 | 8.15E-01 |
| behenoylcarnitine (C22)* | 1.20 | 0.79-1.81 | 8.62E-01 | 1.22 | 0.83-1.8 | 8.15E-01 |
| X - 25790 | 0.91 | 0.6-1.37 | 9.42E-01 | 0.84 | 0.59-1.19 | 8.15E-01 |
| branched-chain, straight-chain, or cyclopropyl 10:1 fatty acid (1)* | 0.89 | 0.62-1.27 | 9.12E-01 | 0.82 | 0.56-1.21 | 8.19E-01 |
| X - 12411 | 0.85 | 0.61-1.18 | 8.56E-01 | 0.86 | 0.64-1.16 | 8.22E-01 |
| X - 11847 | 1.11 | 0.77-1.58 | 9.41E-01 | 1.19 | 0.84-1.67 | 8.27E-01 |
| ferulic acid 4-sulfate | 1.06 | 0.76-1.48 | 9.59E-01 | 1.19 | 0.84-1.68 | 8.27E-01 |
| 3-ethylcatechol sulfate (1) | 1.72 | 0.96-3.1 | 6.31E-01 | 1.26 | 0.79-2 | 8.35E-01 |
| 4-ethylphenylsulfate | 1.28 | 0.94-1.76 | 6.91E-01 | 1.19 | 0.84-1.69 | 8.35E-01 |
| X - 24306 | 0.89 | 0.69-1.15 | 8.62E-01 | 0.87 | 0.66-1.15 | 8.35E-01 |
| cortolone glucuronide (1) | 0.84 | 0.64-1.11 | 7.98E-01 | 0.86 | 0.62-1.18 | 8.41E-01 |
| 1-(1-enyl-palmitoyl)-2-linoleoyl-GPC (P-16:0/18:2)* | 1.16 | 0.76-1.79 | 9.09E-01 | 1.23 | 0.81-1.87 | 8.42E-01 |
| X - 24812 | 1.69 | 1.21-2.34 | 1.28E-01 | 1.20 | 0.83-1.73 | 8.43E-01 |
| X - 13007 | 1.17 | 0.86-1.6 | 8.42E-01 | 1.18 | 0.84-1.66 | 8.43E-01 |
| succinoyltaurine | 1.11 | 0.84-1.47 | 8.76E-01 | 1.16 | 0.86-1.56 | 8.43E-01 |
| quinolinate | 1.01 | 0.69-1.48 | 9.96E-01 | 1.22 | 0.81-1.84 | 8.43E-01 |
| hexadecadienoate (16:2n6) | 0.75 | 0.52-1.08 | 6.97E-01 | 0.85 | 0.6-1.2 | 8.44E-01 |
| X - 11849 | 1.06 | 0.78-1.45 | 9.56E-01 | 1.16 | 0.85-1.58 | 8.44E-01 |
| N-acetylhistidine | 1.32 | 0.94-1.85 | 6.91E-01 | 1.20 | 0.82-1.75 | 8.48E-01 |
| arabitol/xylitol | 1.13 | 0.89-1.44 | 8.42E-01 | 1.12 | 0.88-1.43 | 8.48E-01 |
| hydroxy-N6,N6,N6-trimethyllysine* | 1.11 | 0.83-1.47 | 9.10E-01 | 1.15 | 0.86-1.52 | 8.48E-01 |
| pregnenolone sulfate | 0.94 | 0.61-1.44 | 9.61E-01 | 0.80 | 0.51-1.27 | 8.48E-01 |
| asparagine | 1.23 | 0.8-1.88 | 8.56E-01 | 1.20 | 0.81-1.78 | 8.50E-01 |
| N-acetylglutamate | 1.17 | 0.81-1.7 | 8.62E-01 | 1.19 | 0.82-1.74 | 8.50E-01 |
| malonylcarnitine | 1.15 | 0.8-1.64 | 8.76E-01 | 1.19 | 0.82-1.73 | 8.50E-01 |
| X - 12306 | 1.03 | 0.76-1.41 | 9.85E-01 | 1.19 | 0.82-1.72 | 8.50E-01 |
| N-acetylkynurenine (2) | 1.18 | 0.73-1.92 | 9.10E-01 | 1.25 | 0.78-2.02 | 8.52E-01 |
| 1-arachidonoyl-GPE (20:4n6)* | 0.88 | 0.57-1.35 | 9.27E-01 | 0.82 | 0.54-1.25 | 8.52E-01 |
| sphingosine 1-phosphate | 0.92 | 0.63-1.32 | 9.42E-01 | 0.83 | 0.57-1.23 | 8.52E-01 |
| 3-methyl-2-oxovalerate | 1.18 | 0.78-1.78 | 8.76E-01 | 1.23 | 0.79-1.91 | 8.55E-01 |
| glucuronide of piperine metabolite C17H21NO3 (4)* | 1.14 | 0.73-1.77 | 9.33E-01 | 1.19 | 0.82-1.71 | 8.57E-01 |
| 1-palmitoyl-2-oleoyl-GPC (16:0/18:1) | 1.41 | 0.87-2.28 | 7.52E-01 | 1.22 | 0.79-1.88 | 8.57E-01 |
| 3-(3-hydroxyphenyl)propionate | 0.76 | 0.52-1.12 | 7.52E-01 | 0.82 | 0.53-1.26 | 8.59E-01 |
| 3-hydroxyoctanoylcarnitine (1) | 1.21 | 0.8-1.82 | 8.62E-01 | 1.20 | 0.81-1.76 | 8.59E-01 |
| 2-hydroxypalmitate | 1.15 | 0.81-1.64 | 8.71E-01 | 1.17 | 0.83-1.64 | 8.59E-01 |
| pro-hydroxy-pro | 0.85 | 0.55-1.3 | 8.76E-01 | 0.83 | 0.55-1.24 | 8.59E-01 |
| sulfate of piperine metabolite C18H21NO3 (1)* | 1.18 | 0.78-1.78 | 8.76E-01 | 1.18 | 0.82-1.69 | 8.59E-01 |
| 2-hydroxystearate | 1.13 | 0.82-1.55 | 8.81E-01 | 1.15 | 0.85-1.57 | 8.59E-01 |
| X - 21410 | 0.87 | 0.57-1.32 | 9.18E-01 | 0.83 | 0.55-1.24 | 8.59E-01 |
| sphinganine | 1.33 | 0.88-1.99 | 7.52E-01 | 1.23 | 0.78-1.92 | 8.62E-01 |
| X - 12456 | 0.80 | 0.59-1.09 | 7.52E-01 | 0.86 | 0.6-1.21 | 8.62E-01 |
| 2,3-dihydroxy-5-methylthio-4-pentenoate (DMTPA)* | 0.78 | 0.54-1.14 | 7.89E-01 | 0.83 | 0.56-1.25 | 8.62E-01 |
| arachidonoylcarnitine (C20:4) | 0.78 | 0.52-1.18 | 8.14E-01 | 0.84 | 0.57-1.24 | 8.62E-01 |
| citrate | 0.80 | 0.55-1.16 | 8.14E-01 | 0.84 | 0.57-1.23 | 8.62E-01 |
| eugenol sulfate | 0.83 | 0.6-1.15 | 8.15E-01 | 0.85 | 0.58-1.23 | 8.62E-01 |
| delta-CEHC | 0.78 | 0.51-1.2 | 8.15E-01 | 0.83 | 0.53-1.28 | 8.62E-01 |
| laurate (12:0) | 0.77 | 0.5-1.21 | 8.15E-01 | 0.82 | 0.53-1.28 | 8.62E-01 |
| glutamine | 1.28 | 0.83-1.98 | 8.15E-01 | 1.23 | 0.78-1.92 | 8.62E-01 |
| mannitol/sorbitol | 0.83 | 0.59-1.18 | 8.33E-01 | 0.86 | 0.62-1.2 | 8.62E-01 |
| 1-palmitoleoylglycerol (16:1)* | 1.28 | 0.78-2.12 | 8.56E-01 | 1.23 | 0.77-1.97 | 8.62E-01 |
| N6-carbamoylthreonyladenosine | 0.86 | 0.63-1.18 | 8.62E-01 | 0.85 | 0.59-1.22 | 8.62E-01 |
| 5alpha-androstan-3alpha,17beta-diol monosulfate (1) | 0.88 | 0.64-1.2 | 8.62E-01 | 0.87 | 0.63-1.2 | 8.62E-01 |
| lactosyl-N-palmitoyl-sphingosine (d18:1/16:0) | 0.80 | 0.49-1.31 | 8.62E-01 | 0.79 | 0.47-1.35 | 8.62E-01 |
| 4-hydroxyphenylacetate | 1.16 | 0.82-1.64 | 8.62E-01 | 1.14 | 0.85-1.54 | 8.62E-01 |
| X - 07765 | 1.14 | 0.84-1.56 | 8.62E-01 | 1.14 | 0.84-1.54 | 8.62E-01 |
| X - 12261 | 1.22 | 0.79-1.87 | 8.62E-01 | 1.22 | 0.78-1.9 | 8.62E-01 |
| 1,2-dipalmitoyl-GPC (16:0/16:0) | 1.24 | 0.75-2.05 | 8.63E-01 | 1.24 | 0.76-2.01 | 8.62E-01 |
| X - 12013 | 1.16 | 0.77-1.73 | 9.05E-01 | 1.20 | 0.8-1.8 | 8.62E-01 |
| 1-(1-enyl-stearoyl)-GPE (P-18:0)* | 1.16 | 0.75-1.79 | 9.10E-01 | 1.19 | 0.8-1.78 | 8.62E-01 |
| 16-hydroxypalmitate | 0.88 | 0.61-1.28 | 9.10E-01 | 0.83 | 0.55-1.26 | 8.62E-01 |
| androsterone sulfate | 0.90 | 0.66-1.24 | 9.20E-01 | 0.87 | 0.63-1.19 | 8.62E-01 |
| 4-acetylphenol sulfate | 1.11 | 0.74-1.66 | 9.42E-01 | 1.19 | 0.81-1.76 | 8.62E-01 |
| glycosyl-N-palmitoyl-sphingosine (d18:1/16:0) | 0.89 | 0.55-1.44 | 9.42E-01 | 0.83 | 0.54-1.27 | 8.62E-01 |
| docosahexaenoate (DHA; 22:6n3) | 1.07 | 0.76-1.53 | 9.56E-01 | 1.17 | 0.82-1.66 | 8.62E-01 |
| eicosenedioate (C20:1-DC)* | 1.10 | 0.69-1.74 | 9.56E-01 | 1.26 | 0.75-2.14 | 8.62E-01 |
| deoxycholate | 1.07 | 0.72-1.58 | 9.59E-01 | 1.20 | 0.8-1.81 | 8.62E-01 |
| 4-allylphenol sulfate | 1.01 | 0.72-1.42 | 9.91E-01 | 1.17 | 0.82-1.67 | 8.62E-01 |
| 2-palmitoleoyl-GPC (16:1)* | 1.30 | 0.88-1.92 | 7.78E-01 | 1.18 | 0.8-1.74 | 8.70E-01 |
| perfluorooctanesulfonate (PFOS) | 0.84 | 0.63-1.13 | 8.14E-01 | 0.87 | 0.63-1.2 | 8.70E-01 |
| cysteinylglycine disulfide* | 1.17 | 0.83-1.65 | 8.62E-01 | 1.17 | 0.81-1.68 | 8.70E-01 |
| X - 18887 | 0.85 | 0.59-1.24 | 8.62E-01 | 0.86 | 0.61-1.22 | 8.70E-01 |
| X - 17676 | 1.16 | 0.77-1.75 | 9.01E-01 | 1.17 | 0.81-1.7 | 8.70E-01 |
| 1-palmitoylglycerol (16:0) | 1.14 | 0.78-1.66 | 9.10E-01 | 1.18 | 0.81-1.72 | 8.70E-01 |
| 2-hydroxydecanoate | 1.14 | 0.78-1.67 | 9.10E-01 | 1.18 | 0.8-1.73 | 8.70E-01 |
| lignoceroylcarnitine (C24)* | 1.13 | 0.8-1.6 | 9.10E-01 | 1.16 | 0.82-1.63 | 8.70E-01 |
| glycochenodeoxycholate | 0.87 | 0.53-1.43 | 9.42E-01 | 0.81 | 0.5-1.32 | 8.70E-01 |
| gamma-CEHC glucuronide* | 0.82 | 0.57-1.17 | 8.15E-01 | 0.86 | 0.6-1.22 | 8.71E-01 |
| 3-bromo-5-chloro-2,6-dihydroxybenzoic acid* | 0.79 | 0.55-1.12 | 7.67E-01 | 0.85 | 0.58-1.25 | 8.73E-01 |
| X - 21383 | 0.84 | 0.57-1.25 | 8.62E-01 | 0.84 | 0.55-1.27 | 8.73E-01 |
| vanillic acid glycine | 0.71 | 0.51-1 | 5.39E-01 | 0.85 | 0.58-1.25 | 8.76E-01 |
| palmitoyl dihydrosphingomyelin (d18:0/16:0)* | 1.31 | 0.89-1.93 | 7.52E-01 | 1.20 | 0.78-1.85 | 8.76E-01 |
| 2-methoxyhydroquinone sulfate (1) | 0.79 | 0.54-1.15 | 7.98E-01 | 0.84 | 0.56-1.26 | 8.76E-01 |
| 7-methylxanthine | 1.21 | 0.79-1.86 | 8.62E-01 | 1.19 | 0.79-1.78 | 8.76E-01 |
| cinnamoylglycine | 1.14 | 0.84-1.55 | 8.63E-01 | 1.14 | 0.84-1.55 | 8.76E-01 |
| N-palmitoyl-heptadecasphingosine (d17:1/16:0)* | 1.14 | 0.78-1.66 | 9.10E-01 | 1.19 | 0.79-1.8 | 8.76E-01 |
| cys-gly, oxidized | 1.14 | 0.78-1.66 | 9.13E-01 | 1.17 | 0.8-1.7 | 8.76E-01 |
| arachidate (20:0) | 0.88 | 0.57-1.34 | 9.23E-01 | 0.84 | 0.55-1.28 | 8.76E-01 |
| 4-methyl-2-oxopentanoate | 1.14 | 0.74-1.74 | 9.27E-01 | 1.21 | 0.77-1.89 | 8.76E-01 |
| 3-aminoisobutyrate | 0.88 | 0.59-1.32 | 9.27E-01 | 0.84 | 0.56-1.27 | 8.76E-01 |
| X - 21959 | 1.08 | 0.83-1.39 | 9.40E-01 | 1.13 | 0.84-1.52 | 8.76E-01 |
| gamma-glutamyltyrosine | 1.10 | 0.76-1.59 | 9.42E-01 | 1.16 | 0.81-1.67 | 8.76E-01 |
| malate | 1.08 | 0.78-1.5 | 9.42E-01 | 1.14 | 0.83-1.56 | 8.76E-01 |
| palmitoleate (16:1n7) | 0.89 | 0.58-1.36 | 9.42E-01 | 0.85 | 0.57-1.26 | 8.76E-01 |
| X - 11381 | 1.09 | 0.79-1.51 | 9.42E-01 | 1.15 | 0.82-1.61 | 8.76E-01 |
| X - 25936 | 0.93 | 0.69-1.25 | 9.42E-01 | 0.88 | 0.64-1.2 | 8.76E-01 |
| 1-methyl-4-imidazoleacetate | 0.90 | 0.57-1.42 | 9.48E-01 | 0.79 | 0.45-1.39 | 8.76E-01 |
| andro steroid monosulfate C19H28O6S (1)* | 0.93 | 0.58-1.48 | 9.59E-01 | 0.82 | 0.5-1.33 | 8.76E-01 |
| catechol sulfate | 1.04 | 0.69-1.57 | 9.85E-01 | 1.20 | 0.77-1.85 | 8.76E-01 |
| X - 21796 | 0.78 | 0.54-1.15 | 7.97E-01 | 0.87 | 0.63-1.22 | 8.78E-01 |
| 1,2-dilinoleoyl-GPC (18:2/18:2) | 1.20 | 0.72-2 | 9.10E-01 | 1.28 | 0.7-2.33 | 8.79E-01 |
| vanillylmandelate (VMA) | 0.77 | 0.52-1.13 | 7.61E-01 | 0.86 | 0.6-1.25 | 8.83E-01 |
| X - 11444 | 0.83 | 0.59-1.18 | 8.33E-01 | 0.85 | 0.57-1.27 | 8.84E-01 |
| X - 23636 | 1.22 | 0.81-1.83 | 8.56E-01 | 1.17 | 0.79-1.75 | 8.84E-01 |
| S-adenosylhomocysteine (SAH) | 0.86 | 0.61-1.22 | 8.62E-01 | 0.86 | 0.58-1.26 | 8.84E-01 |
| nervonoylcarnitine (C24:1)* | 0.90 | 0.67-1.21 | 8.96E-01 | 0.89 | 0.67-1.19 | 8.84E-01 |
| sphingomyelin (d18:1/14:0, d16:1/16:0)* | 1.12 | 0.72-1.74 | 9.42E-01 | 1.22 | 0.74-2 | 8.84E-01 |
| 3-hydroxy-3-methylglutarate | 1.09 | 0.78-1.51 | 9.42E-01 | 1.14 | 0.82-1.59 | 8.84E-01 |
| dimethylglycine | 0.92 | 0.56-1.5 | 9.59E-01 | 0.82 | 0.5-1.34 | 8.84E-01 |
| N-methylhydroxyproline** | 0.75 | 0.57-0.98 | 4.92E-01 | 0.88 | 0.63-1.22 | 8.84E-01 |
| 2-aminophenol sulfate | 0.82 | 0.57-1.18 | 8.33E-01 | 0.87 | 0.61-1.24 | 8.84E-01 |
| X - 25810 | 1.15 | 0.87-1.52 | 8.42E-01 | 1.13 | 0.83-1.52 | 8.84E-01 |
| glycerol | 0.84 | 0.58-1.2 | 8.48E-01 | 0.86 | 0.59-1.26 | 8.84E-01 |
| 1-oleoyl-GPI (18:1) | 1.19 | 0.83-1.7 | 8.62E-01 | 1.15 | 0.8-1.64 | 8.84E-01 |
| acetylcarnitine (C2) | 1.20 | 0.77-1.86 | 8.71E-01 | 1.16 | 0.79-1.71 | 8.84E-01 |
| X - 12713 | 0.86 | 0.59-1.25 | 8.72E-01 | 0.85 | 0.57-1.28 | 8.84E-01 |
| X - 21607 | 0.89 | 0.6-1.33 | 9.40E-01 | 0.88 | 0.65-1.21 | 8.84E-01 |
| 3-hydroxyhexanoylcarnitine (1) | 0.90 | 0.62-1.32 | 9.42E-01 | 0.86 | 0.58-1.27 | 8.84E-01 |
| X - 13695 | 1.11 | 0.7-1.75 | 9.48E-01 | 1.21 | 0.74-1.97 | 8.84E-01 |
| ceramide (d18:1/17:0, d17:1/18:0)* | 1.08 | 0.75-1.54 | 9.56E-01 | 1.16 | 0.79-1.71 | 8.84E-01 |
| citrulline | 0.93 | 0.65-1.33 | 9.58E-01 | 0.87 | 0.61-1.25 | 8.84E-01 |
| 2-pyrrolidinone | 1.06 | 0.75-1.5 | 9.59E-01 | 1.15 | 0.8-1.66 | 8.84E-01 |
| hydroquinone sulfate | 1.06 | 0.74-1.53 | 9.59E-01 | 1.15 | 0.8-1.65 | 8.84E-01 |
| androstenediol (3beta,17beta) disulfate (1) | 1.05 | 0.66-1.66 | 9.85E-01 | 0.84 | 0.54-1.3 | 8.84E-01 |
| glycohyocholate | 0.97 | 0.65-1.44 | 9.89E-01 | 0.85 | 0.55-1.3 | 8.84E-01 |
| 2-oxoarginine* | 1.00 | 0.68-1.48 | 9.96E-01 | 1.16 | 0.8-1.68 | 8.84E-01 |
| X - 24344 | 1.61 | 0.96-2.7 | 6.31E-01 | 1.18 | 0.77-1.83 | 8.84E-01 |
| adenosine 5'-monophosphate (AMP) | 0.92 | 0.69-1.22 | 9.23E-01 | 0.87 | 0.62-1.24 | 8.84E-01 |
| X - 12221 | 1.08 | 0.72-1.62 | 9.58E-01 | 1.18 | 0.77-1.79 | 8.85E-01 |
| palmitoylcarnitine (C16) | 0.80 | 0.53-1.2 | 8.33E-01 | 0.87 | 0.6-1.26 | 8.85E-01 |
| oleate/vaccenate (18:1) | 0.85 | 0.58-1.23 | 8.62E-01 | 0.87 | 0.6-1.26 | 8.88E-01 |
| ribonate | 1.13 | 0.72-1.77 | 9.42E-01 | 1.17 | 0.77-1.79 | 8.88E-01 |
| cysteine | 1.02 | 0.67-1.54 | 9.91E-01 | 1.18 | 0.77-1.8 | 8.88E-01 |
| thioproline | 0.85 | 0.51-1.41 | 9.19E-01 | 0.84 | 0.53-1.34 | 8.93E-01 |
| X - 13658 | 0.82 | 0.55-1.24 | 8.62E-01 | 0.86 | 0.58-1.28 | 8.98E-01 |
| 5-hydroxyindoleacetate | 0.86 | 0.59-1.26 | 8.76E-01 | 0.87 | 0.6-1.27 | 8.98E-01 |
| hypoxanthine | 1.06 | 0.72-1.57 | 9.59E-01 | 1.15 | 0.79-1.69 | 8.98E-01 |
| N-acetylaspartate (NAA) | 1.19 | 0.79-1.8 | 8.62E-01 | 1.16 | 0.77-1.75 | 8.99E-01 |
| 2'-deoxyuridine | 0.79 | 0.53-1.19 | 8.15E-01 | 0.86 | 0.57-1.3 | 8.99E-01 |
| 1-oleoyl-GPE (18:1) | 1.17 | 0.82-1.67 | 8.62E-01 | 1.15 | 0.78-1.7 | 8.99E-01 |
| 1,5-anhydroglucitol (1,5-AG) | 0.88 | 0.57-1.37 | 9.40E-01 | 0.86 | 0.57-1.3 | 8.99E-01 |
| 3,7-dimethylurate | 1.11 | 0.76-1.63 | 9.41E-01 | 1.17 | 0.77-1.79 | 8.99E-01 |
| 1-palmitoyl-GPI (16:0) | 1.11 | 0.77-1.6 | 9.42E-01 | 1.15 | 0.79-1.67 | 8.99E-01 |
| dimethylarginine (SDMA + ADMA) | 0.90 | 0.58-1.39 | 9.42E-01 | 0.85 | 0.54-1.33 | 8.99E-01 |
| linoleoyl ethanolamide | 1.13 | 0.77-1.65 | 9.20E-01 | 1.15 | 0.79-1.67 | 8.99E-01 |
| glycerophosphoethanolamine | 1.13 | 0.72-1.78 | 9.42E-01 | 1.17 | 0.76-1.81 | 8.99E-01 |
| erythritol | 1.03 | 0.75-1.42 | 9.89E-01 | 1.14 | 0.79-1.64 | 8.99E-01 |
| ornithine | 0.98 | 0.66-1.46 | 9.89E-01 | 0.87 | 0.58-1.28 | 8.99E-01 |
| X - 21842 | 0.77 | 0.56-1.07 | 6.91E-01 | 0.88 | 0.62-1.25 | 8.99E-01 |
| 4-hydroxychlorothalonil | 0.80 | 0.59-1.08 | 7.52E-01 | 0.88 | 0.63-1.24 | 8.99E-01 |
| sphingomyelin (d18:2/23:0, d18:1/23:1, d17:1/24:1)* | 0.77 | 0.49-1.18 | 8.14E-01 | 0.85 | 0.54-1.35 | 8.99E-01 |
| cholic acid glucuronide | 0.83 | 0.59-1.17 | 8.33E-01 | 0.89 | 0.64-1.23 | 8.99E-01 |
| taurolithocholate 3-sulfate | 1.24 | 0.81-1.89 | 8.42E-01 | 1.16 | 0.77-1.73 | 8.99E-01 |
| 1-stearoyl-2-arachidonoyl-GPC (18:0/20:4) | 1.24 | 0.78-1.97 | 8.62E-01 | 1.19 | 0.74-1.91 | 8.99E-01 |
| cis-3,4-methyleneheptanoate | 0.85 | 0.58-1.24 | 8.62E-01 | 0.88 | 0.61-1.26 | 8.99E-01 |
| X - 16576 | 1.16 | 0.79-1.7 | 8.76E-01 | 1.15 | 0.78-1.7 | 8.99E-01 |
| phenylpyruvate | 0.89 | 0.64-1.24 | 9.07E-01 | 0.88 | 0.6-1.27 | 8.99E-01 |
| X - 16124 | 1.12 | 0.8-1.56 | 9.12E-01 | 1.14 | 0.79-1.65 | 8.99E-01 |
| hypotaurine | 0.85 | 0.51-1.42 | 9.20E-01 | 0.83 | 0.49-1.4 | 8.99E-01 |
| X - 11850 | 1.16 | 0.73-1.86 | 9.20E-01 | 1.19 | 0.73-1.92 | 8.99E-01 |
| gamma-glutamylleucine | 1.08 | 0.81-1.45 | 9.42E-01 | 1.12 | 0.81-1.54 | 8.99E-01 |
| 3-methyl-2-oxobutyrate | 1.07 | 0.75-1.52 | 9.59E-01 | 1.16 | 0.77-1.72 | 8.99E-01 |
| 3-hydroxyhexanoate | 0.94 | 0.67-1.33 | 9.59E-01 | 0.88 | 0.61-1.27 | 8.99E-01 |
| X - 18901 | 1.01 | 0.7-1.46 | 9.94E-01 | 1.14 | 0.79-1.64 | 8.99E-01 |
| X - 24728 | 1.21 | 0.87-1.68 | 8.15E-01 | 1.12 | 0.81-1.54 | 9.00E-01 |
| N-formylmethionine | 1.10 | 0.74-1.63 | 9.42E-01 | 1.16 | 0.76-1.76 | 9.00E-01 |
| sulfate of piperine metabolite C16H19NO3 (3)* | 1.01 | 0.7-1.48 | 9.91E-01 | 1.12 | 0.81-1.55 | 9.00E-01 |
| X - 12680 | 0.85 | 0.67-1.07 | 7.52E-01 | 0.92 | 0.72-1.17 | 9.02E-01 |
| kynurenate | 0.78 | 0.52-1.18 | 8.14E-01 | 0.87 | 0.59-1.29 | 9.02E-01 |
| 1-palmitoyl-2-palmitoleoyl-GPC (16:0/16:1)* | 1.28 | 0.82-2 | 8.25E-01 | 1.16 | 0.75-1.8 | 9.02E-01 |
| tetrahydrocortisol sulfate (1) | 0.85 | 0.6-1.22 | 8.62E-01 | 0.89 | 0.62-1.26 | 9.02E-01 |
| X - 21834 | 0.88 | 0.59-1.31 | 9.21E-01 | 0.87 | 0.57-1.31 | 9.03E-01 |
| mannose | 1.17 | 0.83-1.64 | 8.62E-01 | 1.13 | 0.79-1.62 | 9.03E-01 |
| 1-stearoyl-2-arachidonoyl-GPE (18:0/20:4) | 1.23 | 0.73-2.09 | 8.76E-01 | 1.21 | 0.7-2.07 | 9.04E-01 |
| 5-acetylamino-6-formylamino-3-methyluracil | 0.84 | 0.55-1.27 | 8.62E-01 | 0.87 | 0.58-1.31 | 9.04E-01 |
| gamma-tocopherol/beta-tocopherol | 1.17 | 0.79-1.73 | 8.71E-01 | 1.15 | 0.77-1.72 | 9.09E-01 |
| N-stearoyl-sphinganine (d18:0/18:0)* | 1.15 | 0.78-1.71 | 9.06E-01 | 1.15 | 0.76-1.73 | 9.09E-01 |
| 4-chlorobenzoic acid | 1.10 | 0.73-1.66 | 9.42E-01 | 1.16 | 0.75-1.79 | 9.09E-01 |
| 2-acetamidophenol sulfate | 0.86 | 0.6-1.24 | 8.63E-01 | 0.89 | 0.62-1.26 | 9.10E-01 |
| caproate (6:0) | 1.06 | 0.82-1.39 | 9.42E-01 | 1.10 | 0.84-1.44 | 9.10E-01 |
| glycerate | 0.89 | 0.61-1.31 | 9.30E-01 | 1.12 | 0.79-1.59 | 9.11E-01 |
| X - 26109 | 0.84 | 0.62-1.14 | 8.15E-01 | 0.88 | 0.62-1.27 | 9.11E-01 |
| 3-hydroxyoleoylcarnitine | 0.87 | 0.6-1.27 | 9.01E-01 | 0.88 | 0.61-1.28 | 9.11E-01 |
| isoleucylglycine | 0.92 | 0.64-1.32 | 9.42E-01 | 0.88 | 0.59-1.3 | 9.12E-01 |
| sphingomyelin (d18:1/24:1, d18:2/24:0)* | 1.04 | 0.7-1.56 | 9.85E-01 | 0.87 | 0.56-1.33 | 9.12E-01 |
| retinal | 0.85 | 0.59-1.23 | 8.62E-01 | 0.88 | 0.6-1.29 | 9.12E-01 |
| undecenoylcarnitine (C11:1) | 1.01 | 0.75-1.37 | 9.91E-01 | 1.12 | 0.79-1.59 | 9.12E-01 |
| glycosyl-N-(2-hydroxynervonoyl)-sphingosine (d18:1/24:1(2OH))* | 0.87 | 0.55-1.37 | 9.27E-01 | 0.89 | 0.64-1.25 | 9.16E-01 |
| sphingomyelin (d18:0/20:0, d16:0/22:0)* | 1.12 | 0.75-1.67 | 9.42E-01 | 1.16 | 0.74-1.83 | 9.18E-01 |
| 1-palmitoyl-2-arachidonoyl-GPE (16:0/20:4)* | 1.22 | 0.77-1.93 | 8.62E-01 | 1.17 | 0.73-1.87 | 9.20E-01 |
| X - 12707 | 0.87 | 0.58-1.29 | 9.01E-01 | 0.88 | 0.6-1.29 | 9.22E-01 |
| caprate (10:0) | 0.89 | 0.61-1.3 | 9.20E-01 | 0.88 | 0.59-1.31 | 9.22E-01 |
| sphingomyelin (d18:2/23:1)* | 0.73 | 0.43-1.25 | 8.15E-01 | 0.84 | 0.5-1.43 | 9.24E-01 |
| X - 13728 | 0.90 | 0.63-1.3 | 9.40E-01 | 0.89 | 0.62-1.28 | 9.24E-01 |
| aconitate [cis or trans] | 1.12 | 0.73-1.71 | 9.42E-01 | 1.16 | 0.74-1.82 | 9.24E-01 |
| creatinine | 1.09 | 0.74-1.61 | 9.50E-01 | 1.14 | 0.76-1.72 | 9.24E-01 |
| X - 24475 | 1.02 | 0.67-1.55 | 9.89E-01 | 1.16 | 0.73-1.86 | 9.24E-01 |
| 3-indoxyl sulfate | 1.00 | 0.67-1.49 | 9.96E-01 | 1.16 | 0.74-1.81 | 9.24E-01 |
| X - 24757 | 0.80 | 0.55-1.16 | 8.14E-01 | 0.88 | 0.6-1.3 | 9.25E-01 |
| tyramine O-sulfate | 1.16 | 0.89-1.51 | 8.28E-01 | 1.10 | 0.81-1.48 | 9.27E-01 |
| sphingomyelin (d18:1/20:1, d18:2/20:0)* | 0.75 | 0.44-1.29 | 8.33E-01 | 0.85 | 0.49-1.45 | 9.27E-01 |
| heptenedioate (C7:1-DC)* | 0.82 | 0.56-1.19 | 8.33E-01 | 0.89 | 0.6-1.31 | 9.27E-01 |
| X - 25422 | 1.28 | 0.79-2.08 | 8.42E-01 | 1.16 | 0.71-1.9 | 9.27E-01 |
| 4-methylguaiacol sulfate | 1.16 | 0.82-1.64 | 8.62E-01 | 1.10 | 0.81-1.51 | 9.27E-01 |
| 1-lignoceroyl-GPC (24:0) | 1.19 | 0.79-1.79 | 8.63E-01 | 1.15 | 0.73-1.82 | 9.27E-01 |
| 3-methoxytyrosine | 0.79 | 0.44-1.44 | 8.76E-01 | 0.83 | 0.45-1.53 | 9.27E-01 |
| betaine | 0.85 | 0.54-1.32 | 8.89E-01 | 0.88 | 0.57-1.35 | 9.27E-01 |
| gamma-glutamylphenylalanine | 0.89 | 0.61-1.28 | 9.18E-01 | 0.89 | 0.62-1.28 | 9.27E-01 |
| X - 23641 | 1.19 | 0.69-2.07 | 9.20E-01 | 1.19 | 0.67-2.1 | 9.27E-01 |
| N-acetylthreonine | 0.90 | 0.61-1.34 | 9.42E-01 | 0.88 | 0.59-1.32 | 9.27E-01 |
| 1-palmitoyl-GPG (16:0)* | 1.10 | 0.75-1.61 | 9.42E-01 | 1.12 | 0.77-1.62 | 9.27E-01 |
| X - 11843 | 1.12 | 0.71-1.76 | 9.42E-01 | 1.17 | 0.72-1.89 | 9.27E-01 |
| X - 24328 | 0.88 | 0.54-1.42 | 9.42E-01 | 0.84 | 0.48-1.46 | 9.27E-01 |
| X - 25948 | 1.09 | 0.74-1.62 | 9.48E-01 | 1.13 | 0.77-1.66 | 9.27E-01 |
| gamma-glutamylglutamate | 0.94 | 0.71-1.24 | 9.49E-01 | 0.92 | 0.71-1.2 | 9.27E-01 |
| gamma-glutamyltryptophan | 1.07 | 0.74-1.53 | 9.59E-01 | 1.11 | 0.79-1.55 | 9.27E-01 |
| N,N-dimethylalanine | 0.93 | 0.61-1.43 | 9.59E-01 | 0.88 | 0.6-1.31 | 9.27E-01 |
| 3-(4-hydroxyphenyl)lactate | 1.07 | 0.71-1.61 | 9.60E-01 | 1.13 | 0.75-1.7 | 9.27E-01 |
| urea | 1.03 | 0.73-1.46 | 9.88E-01 | 1.11 | 0.79-1.56 | 9.27E-01 |
| carotene diol (2) | 0.99 | 0.63-1.54 | 9.95E-01 | 1.15 | 0.74-1.78 | 9.27E-01 |
| cerotoylcarnitine (C26)* | 1.00 | 0.69-1.46 | 9.96E-01 | 1.13 | 0.76-1.67 | 9.27E-01 |
| sulfate of piperine metabolite C16H19NO3 (2)* | 1.00 | 0.69-1.45 | 9.96E-01 | 1.11 | 0.8-1.53 | 9.27E-01 |
| 4-vinylcatechol sulfate | 1.38 | 0.87-2.19 | 7.57E-01 | 1.14 | 0.73-1.79 | 9.29E-01 |
| 1-oleoyl-GPC (18:1) | 1.14 | 0.79-1.65 | 9.10E-01 | 1.13 | 0.76-1.68 | 9.29E-01 |
| myristoyl dihydrosphingomyelin (d18:0/14:0)* | 1.14 | 0.74-1.75 | 9.28E-01 | 1.16 | 0.71-1.89 | 9.29E-01 |
| X - 22834 | 1.07 | 0.68-1.69 | 9.67E-01 | 1.15 | 0.71-1.86 | 9.29E-01 |
| thyroxine | 1.04 | 0.71-1.55 | 9.81E-01 | 1.12 | 0.77-1.63 | 9.29E-01 |
| X - 15245 | 1.02 | 0.7-1.5 | 9.89E-01 | 1.12 | 0.76-1.66 | 9.29E-01 |
| 2-oleoylglycerol (18:1) | 1.17 | 0.74-1.83 | 9.10E-01 | 1.15 | 0.72-1.85 | 9.34E-01 |
| X - 24951 | 0.82 | 0.6-1.12 | 7.89E-01 | 0.90 | 0.63-1.29 | 9.35E-01 |
| dihydroferulic acid sulfate | 0.77 | 0.51-1.17 | 7.97E-01 | 0.87 | 0.54-1.41 | 9.35E-01 |
| stearoylcarnitine (C18) | 0.87 | 0.61-1.25 | 8.76E-01 | 0.91 | 0.66-1.26 | 9.35E-01 |
| N-palmitoylglycine | 0.86 | 0.53-1.41 | 9.28E-01 | 0.88 | 0.56-1.38 | 9.35E-01 |
| 1-oleoyl-2-arachidonoyl-GPE (18:1/20:4)* | 1.09 | 0.8-1.49 | 9.42E-01 | 1.09 | 0.81-1.47 | 9.35E-01 |
| pimeloylcarnitine/3-methyladipoylcarnitine (C7-DC) | 0.90 | 0.59-1.36 | 9.42E-01 | 0.89 | 0.59-1.34 | 9.35E-01 |
| docosahexaenoylcholine | 1.09 | 0.75-1.58 | 9.48E-01 | 1.15 | 0.72-1.83 | 9.35E-01 |
| oleoyl ethanolamide | 0.90 | 0.57-1.43 | 9.52E-01 | 0.88 | 0.58-1.35 | 9.35E-01 |
| N1-Methyl-2-pyridone-5-carboxamide | 0.93 | 0.66-1.31 | 9.56E-01 | 1.09 | 0.81-1.48 | 9.35E-01 |
| propionylglycine | 0.94 | 0.66-1.34 | 9.59E-01 | 0.91 | 0.65-1.26 | 9.35E-01 |
| N-acetylalanine | 1.06 | 0.74-1.53 | 9.59E-01 | 1.12 | 0.76-1.63 | 9.35E-01 |
| oleoyl-linoleoyl-glycerol (18:1/18:2) [1] | 1.05 | 0.7-1.6 | 9.69E-01 | 1.12 | 0.75-1.66 | 9.35E-01 |
| valine | 1.06 | 0.69-1.63 | 9.69E-01 | 1.20 | 0.64-2.24 | 9.35E-01 |
| X - 18935 | 1.02 | 0.74-1.41 | 9.89E-01 | 1.10 | 0.78-1.56 | 9.35E-01 |
| 6-oxopiperidine-2-carboxylate | 0.98 | 0.63-1.52 | 9.91E-01 | 1.13 | 0.73-1.76 | 9.35E-01 |
| guanidinoacetate | 0.99 | 0.66-1.46 | 9.91E-01 | 0.88 | 0.58-1.35 | 9.35E-01 |
| S-carboxyethylcysteine | 1.35 | 1.01-1.81 | 5.20E-01 | 1.10 | 0.77-1.56 | 9.35E-01 |
| alpha-CEHC sulfate | 0.79 | 0.58-1.06 | 6.91E-01 | 0.92 | 0.67-1.26 | 9.35E-01 |
| X - 11372 | 0.80 | 0.59-1.09 | 7.52E-01 | 0.92 | 0.67-1.26 | 9.35E-01 |
| gamma-glutamylglycine | 1.31 | 0.83-2.05 | 8.14E-01 | 1.14 | 0.71-1.8 | 9.35E-01 |
| 3-hydroxyhippurate | 0.79 | 0.53-1.19 | 8.15E-01 | 0.88 | 0.57-1.36 | 9.35E-01 |
| sphingomyelin (d18:1/20:2, d18:2/20:1, d16:1/22:2)* | 0.81 | 0.55-1.18 | 8.20E-01 | 0.90 | 0.6-1.34 | 9.35E-01 |
| 2-hydroxyphenylacetate | 0.79 | 0.51-1.21 | 8.33E-01 | 0.88 | 0.56-1.39 | 9.35E-01 |
| 5alpha-androstan-3alpha,17beta-diol monosulfate (2) | 0.79 | 0.47-1.31 | 8.62E-01 | 0.85 | 0.49-1.5 | 9.35E-01 |
| erucate (22:1n9) | 1.15 | 0.85-1.56 | 8.62E-01 | 1.10 | 0.79-1.52 | 9.35E-01 |
| X - 26097 | 0.83 | 0.54-1.28 | 8.62E-01 | 0.88 | 0.54-1.42 | 9.35E-01 |
| 3-hydroxylaurate | 0.87 | 0.62-1.23 | 8.76E-01 | 0.91 | 0.66-1.27 | 9.35E-01 |
| sphingomyelin (d18:2/24:2)* | 0.83 | 0.52-1.34 | 8.80E-01 | 0.88 | 0.57-1.38 | 9.35E-01 |
| ximenoylcarnitine (C26:1)* | 0.90 | 0.66-1.22 | 9.10E-01 | 0.92 | 0.7-1.23 | 9.35E-01 |
| 5-hydroxyhexanoate | 1.15 | 0.76-1.73 | 9.11E-01 | 1.12 | 0.73-1.74 | 9.35E-01 |
| gamma-glutamyl-alpha-lysine | 1.13 | 0.75-1.69 | 9.30E-01 | 1.12 | 0.75-1.69 | 9.35E-01 |
| 2,4-di-tert-butylphenol | 1.11 | 0.78-1.56 | 9.37E-01 | 1.11 | 0.77-1.6 | 9.35E-01 |
| X - 17438 | 0.90 | 0.61-1.32 | 9.40E-01 | 0.90 | 0.59-1.35 | 9.35E-01 |
| N-oleoyltaurine | 0.90 | 0.57-1.41 | 9.42E-01 | 0.89 | 0.59-1.35 | 9.35E-01 |
| 3-methylglutarylcarnitine (2) | 1.08 | 0.75-1.53 | 9.56E-01 | 1.10 | 0.78-1.54 | 9.35E-01 |
| 1-palmitoyl-GPC (16:0) | 1.07 | 0.7-1.64 | 9.59E-01 | 1.12 | 0.73-1.72 | 9.35E-01 |
| indolebutyrate | 0.94 | 0.63-1.41 | 9.68E-01 | 0.89 | 0.59-1.35 | 9.35E-01 |
| indolelactate | 1.05 | 0.67-1.65 | 9.85E-01 | 1.13 | 0.72-1.76 | 9.35E-01 |
| N-acetylasparagine | 1.02 | 0.72-1.45 | 9.89E-01 | 1.11 | 0.75-1.63 | 9.35E-01 |
| 3-hydroxyisobutyrate | 1.02 | 0.67-1.56 | 9.89E-01 | 1.10 | 0.77-1.59 | 9.35E-01 |
| cysteinylglycine | 1.03 | 0.72-1.49 | 9.89E-01 | 1.12 | 0.74-1.68 | 9.35E-01 |
| epiandrosterone sulfate | 0.99 | 0.69-1.42 | 9.95E-01 | 0.90 | 0.63-1.3 | 9.35E-01 |
| X - 12026 | 0.71 | 0.47-1.07 | 6.90E-01 | 0.89 | 0.57-1.39 | 9.36E-01 |
| methyl glucopyranoside (alpha + beta) | 0.77 | 0.56-1.07 | 6.91E-01 | 0.91 | 0.64-1.29 | 9.36E-01 |
| X - 25420 | 1.36 | 0.84-2.21 | 7.90E-01 | 1.14 | 0.69-1.88 | 9.36E-01 |
| S-methylmethionine | 0.85 | 0.64-1.15 | 8.33E-01 | 0.92 | 0.68-1.25 | 9.36E-01 |
| glycolithocholate sulfate* | 1.13 | 0.75-1.7 | 9.30E-01 | 1.10 | 0.76-1.6 | 9.36E-01 |
| X - 21736 | 1.09 | 0.75-1.58 | 9.42E-01 | 1.12 | 0.73-1.7 | 9.36E-01 |
| X - 16087 | 0.79 | 0.56-1.12 | 7.67E-01 | 0.91 | 0.65-1.29 | 9.36E-01 |
| X - 23593 | 0.99 | 0.61-1.61 | 9.96E-01 | 0.89 | 0.57-1.38 | 9.36E-01 |
| stearoylcholine* | 1.04 | 0.72-1.5 | 9.88E-01 | 1.12 | 0.73-1.72 | 9.36E-01 |
| gamma-carboxyglutamate | 0.94 | 0.66-1.33 | 9.59E-01 | 0.91 | 0.64-1.3 | 9.37E-01 |
| spermidine | 0.97 | 0.7-1.35 | 9.89E-01 | 1.09 | 0.78-1.54 | 9.37E-01 |
| N-acetylglucosaminylasparagine | 0.93 | 0.61-1.42 | 9.59E-01 | 0.89 | 0.57-1.4 | 9.38E-01 |
| oleoyl-linoleoyl-glycerol (18:1/18:2) [2] | 1.04 | 0.69-1.56 | 9.88E-01 | 1.10 | 0.76-1.6 | 9.38E-01 |
| xanthosine | 0.85 | 0.59-1.22 | 8.62E-01 | 0.89 | 0.58-1.38 | 9.40E-01 |
| 1-arachidonoyl-GPC (20:4n6)* | 1.11 | 0.79-1.57 | 9.27E-01 | 1.10 | 0.77-1.57 | 9.40E-01 |
| sphingomyelin (d18:1/20:0, d16:1/22:0)* | 0.86 | 0.57-1.29 | 8.88E-01 | 0.90 | 0.59-1.37 | 9.42E-01 |
| 3-hydroxyphenylacetoylglutamine | 0.85 | 0.59-1.21 | 8.62E-01 | 0.92 | 0.66-1.29 | 9.44E-01 |
| sphingomyelin (d18:2/16:0, d18:1/16:1)* | 0.86 | 0.54-1.35 | 9.10E-01 | 0.89 | 0.55-1.42 | 9.44E-01 |
| X - 25520 | 1.06 | 0.69-1.62 | 9.69E-01 | 1.11 | 0.73-1.69 | 9.44E-01 |
| indolin-2-one | 0.96 | 0.67-1.38 | 9.81E-01 | 1.10 | 0.75-1.64 | 9.44E-01 |
| X - 24556 | 1.01 | 0.76-1.32 | 9.96E-01 | 1.08 | 0.78-1.5 | 9.44E-01 |
| 1-stearoyl-2-oleoyl-GPC (18:0/18:1) | 1.18 | 0.84-1.64 | 8.56E-01 | 1.09 | 0.77-1.55 | 9.45E-01 |
| glucuronide of piperine metabolite C17H21NO3 (3)* | 0.88 | 0.58-1.35 | 9.37E-01 | 0.91 | 0.63-1.32 | 9.45E-01 |
| acisoga | 1.14 | 0.83-1.57 | 8.62E-01 | 1.09 | 0.77-1.53 | 9.46E-01 |
| glycocholate | 1.09 | 0.71-1.68 | 9.58E-01 | 1.11 | 0.72-1.71 | 9.46E-01 |
| linoleoylcholine* | 1.04 | 0.7-1.55 | 9.85E-01 | 1.12 | 0.71-1.75 | 9.46E-01 |
| androstenediol (3beta,17beta) monosulfate (1) | 1.04 | 0.69-1.54 | 9.89E-01 | 0.91 | 0.61-1.35 | 9.46E-01 |
| 4-vinylphenol sulfate | 1.31 | 0.86-1.99 | 7.97E-01 | 1.12 | 0.7-1.8 | 9.46E-01 |
| sphingomyelin (d18:1/22:1, d18:2/22:0, d16:1/24:1)* | 0.81 | 0.5-1.32 | 8.62E-01 | 0.88 | 0.52-1.48 | 9.46E-01 |
| palmitoylcholine | 1.05 | 0.73-1.51 | 9.69E-01 | 1.11 | 0.74-1.66 | 9.46E-01 |
| androstenediol (3alpha, 17alpha) monosulfate (3) | 1.01 | 0.67-1.52 | 9.96E-01 | 0.91 | 0.62-1.34 | 9.46E-01 |
| 4-vinylguaiacol sulfate | 1.29 | 0.75-2.21 | 8.62E-01 | 1.12 | 0.7-1.8 | 9.49E-01 |
| cholesterol | 1.19 | 0.78-1.8 | 8.71E-01 | 1.11 | 0.73-1.67 | 9.49E-01 |
| trans-3,4-methyleneheptanoate | 1.15 | 0.76-1.76 | 9.11E-01 | 1.11 | 0.72-1.69 | 9.51E-01 |
| dihomo-linoleate (20:2n6) | 0.89 | 0.59-1.34 | 9.40E-01 | 0.91 | 0.63-1.33 | 9.51E-01 |
| X - 17357 | 0.92 | 0.67-1.24 | 9.40E-01 | 0.92 | 0.66-1.29 | 9.51E-01 |
| 1-linolenoyl-GPC (18:3)* | 1.09 | 0.74-1.61 | 9.52E-01 | 1.10 | 0.74-1.65 | 9.51E-01 |
| 2S,3R-dihydroxybutyrate | 1.09 | 0.7-1.68 | 9.58E-01 | 1.09 | 0.76-1.55 | 9.51E-01 |
| 3-hydroxybutyroylglycine** | 1.03 | 0.7-1.52 | 9.89E-01 | 0.93 | 0.67-1.28 | 9.51E-01 |
| N-acetylarginine | 0.88 | 0.65-1.18 | 8.62E-01 | 0.93 | 0.7-1.25 | 9.54E-01 |
| 1-stearoyl-GPC (18:0) | 1.03 | 0.62-1.69 | 9.89E-01 | 1.13 | 0.66-1.93 | 9.54E-01 |
| glycerophosphorylcholine (GPC) | 1.05 | 0.68-1.62 | 9.81E-01 | 1.10 | 0.74-1.63 | 9.54E-01 |
| dihydrocaffeate sulfate (2) | 0.79 | 0.54-1.14 | 7.97E-01 | 0.90 | 0.59-1.4 | 9.54E-01 |
| 3-(3-hydroxyphenyl)propionate sulfate | 0.81 | 0.54-1.22 | 8.42E-01 | 0.91 | 0.59-1.39 | 9.54E-01 |
| phosphoethanolamine | 0.86 | 0.61-1.21 | 8.62E-01 | 0.92 | 0.65-1.31 | 9.54E-01 |
| dodecadienoate (12:2)* | 0.85 | 0.56-1.29 | 8.76E-01 | 0.91 | 0.61-1.36 | 9.54E-01 |
| glycine conjugate of C10H14O2 (1)* | 1.14 | 0.77-1.7 | 9.10E-01 | 1.10 | 0.73-1.64 | 9.54E-01 |
| laurylcarnitine (C12) | 0.96 | 0.73-1.27 | 9.69E-01 | 0.94 | 0.72-1.22 | 9.54E-01 |
| ethylmalonate | 1.04 | 0.74-1.46 | 9.80E-01 | 1.09 | 0.76-1.56 | 9.54E-01 |
| glycine | 1.26 | 0.81-1.94 | 8.33E-01 | 1.11 | 0.69-1.79 | 9.56E-01 |
| lyxonate | 0.83 | 0.57-1.22 | 8.56E-01 | 0.92 | 0.65-1.31 | 9.56E-01 |
| valylglycine | 0.94 | 0.62-1.45 | 9.69E-01 | 0.90 | 0.58-1.41 | 9.56E-01 |
| N-acetylcarnosine | 0.79 | 0.51-1.23 | 8.33E-01 | 0.90 | 0.57-1.43 | 9.58E-01 |
| X - 12407 | 0.87 | 0.64-1.18 | 8.62E-01 | 0.93 | 0.67-1.29 | 9.58E-01 |
| behenoyl dihydrosphingomyelin (d18:0/22:0)* | 1.10 | 0.73-1.67 | 9.42E-01 | 1.09 | 0.73-1.63 | 9.58E-01 |
| indoleacetate | 1.09 | 0.75-1.58 | 9.52E-01 | 1.08 | 0.77-1.5 | 9.58E-01 |
| 1-palmitoyl-2-linoleoyl-GPI (16:0/18:2) | 1.09 | 0.73-1.62 | 9.56E-01 | 1.10 | 0.73-1.66 | 9.58E-01 |
| pyruvate | 1.08 | 0.72-1.64 | 9.58E-01 | 1.09 | 0.73-1.64 | 9.58E-01 |
| sphingomyelin (d18:1/17:0, d17:1/18:0, d19:1/16:0) | 0.96 | 0.59-1.57 | 9.89E-01 | 1.12 | 0.68-1.84 | 9.58E-01 |
| 1-(1-enyl-palmitoyl)-2-palmitoyl-GPC (P-16:0/16:0)* | 0.98 | 0.63-1.51 | 9.89E-01 | 0.91 | 0.59-1.4 | 9.58E-01 |
| kynurenine | 0.98 | 0.64-1.51 | 9.91E-01 | 1.12 | 0.67-1.87 | 9.58E-01 |
| pentose acid* | 0.99 | 0.73-1.35 | 9.91E-01 | 1.07 | 0.79-1.45 | 9.58E-01 |
| 4-hydroxyhippurate | 0.80 | 0.59-1.08 | 7.52E-01 | 0.93 | 0.68-1.28 | 9.61E-01 |
| homovanillate (HVA) | 0.92 | 0.65-1.3 | 9.42E-01 | 0.93 | 0.65-1.32 | 9.61E-01 |
| gamma-glutamylthreonine | 1.01 | 0.67-1.54 | 9.94E-01 | 0.92 | 0.62-1.36 | 9.62E-01 |
| 1-(1-enyl-palmitoyl)-2-oleoyl-GPC (P-16:0/18:1)* | 1.18 | 0.75-1.87 | 8.96E-01 | 1.10 | 0.71-1.7 | 9.63E-01 |
| X - 13729 | 0.99 | 0.68-1.43 | 9.91E-01 | 1.08 | 0.76-1.54 | 9.63E-01 |
| 2-naphthol sulfate | 1.72 | 1.2-2.47 | 1.44E-01 | 1.10 | 0.69-1.74 | 9.63E-01 |
| 3-hydroxystachydrine* | 0.81 | 0.64-1.03 | 6.72E-01 | 0.95 | 0.72-1.24 | 9.63E-01 |
| dihydroferulate | 0.81 | 0.57-1.16 | 8.15E-01 | 0.92 | 0.61-1.38 | 9.63E-01 |
| X - 24422 | 1.25 | 0.8-1.93 | 8.48E-01 | 1.08 | 0.72-1.62 | 9.63E-01 |
| sphingomyelin (d18:1/25:0, d19:0/24:1, d20:1/23:0, d19:1/24:0)* | 0.86 | 0.63-1.17 | 8.56E-01 | 0.93 | 0.66-1.33 | 9.63E-01 |
| N,N-dimethyl-5-aminovalerate | 0.83 | 0.56-1.22 | 8.56E-01 | 0.92 | 0.61-1.39 | 9.63E-01 |
| (14 or 15)-methylpalmitate (a17:0 or i17:0) | 0.85 | 0.55-1.3 | 8.76E-01 | 0.92 | 0.59-1.44 | 9.63E-01 |
| ascorbic acid 3-sulfate* | 0.84 | 0.55-1.3 | 8.76E-01 | 0.93 | 0.65-1.33 | 9.63E-01 |
| 2-hydroxybehenate | 1.17 | 0.78-1.75 | 8.80E-01 | 1.09 | 0.72-1.64 | 9.63E-01 |
| X - 21353 | 0.88 | 0.61-1.27 | 9.10E-01 | 0.93 | 0.66-1.32 | 9.63E-01 |
| N-acetylglutamine | 1.13 | 0.79-1.61 | 9.10E-01 | 1.08 | 0.73-1.58 | 9.63E-01 |
| butyrate/isobutyrate (4:0) | 0.91 | 0.66-1.25 | 9.29E-01 | 1.07 | 0.76-1.49 | 9.63E-01 |
| linolenate [alpha or gamma; (18:3n3 or 6)] | 0.90 | 0.6-1.33 | 9.42E-01 | 0.92 | 0.62-1.37 | 9.63E-01 |
| dimethyl sulfone | 1.10 | 0.77-1.58 | 9.42E-01 | 1.08 | 0.75-1.55 | 9.63E-01 |
| dopamine 3-O-sulfate | 1.08 | 0.8-1.45 | 9.42E-01 | 1.07 | 0.77-1.48 | 9.63E-01 |
| taurodeoxycholic acid 3-sulfate | 1.11 | 0.76-1.63 | 9.42E-01 | 1.08 | 0.74-1.58 | 9.63E-01 |
| X - 17612 | 0.90 | 0.62-1.31 | 9.42E-01 | 0.93 | 0.63-1.36 | 9.63E-01 |
| 1-arachidonoyl-GPI (20:4)* | 0.92 | 0.63-1.34 | 9.48E-01 | 0.93 | 0.65-1.33 | 9.63E-01 |
| 1-oleoylglycerol (18:1) | 1.11 | 0.68-1.8 | 9.56E-01 | 1.10 | 0.7-1.74 | 9.63E-01 |
| deoxycarnitine | 1.06 | 0.8-1.41 | 9.56E-01 | 1.04 | 0.84-1.3 | 9.63E-01 |
| X - 11979 | 1.08 | 0.73-1.61 | 9.58E-01 | 1.09 | 0.7-1.69 | 9.63E-01 |
| lithocholate sulfate (1) | 1.06 | 0.74-1.51 | 9.59E-01 | 1.07 | 0.77-1.48 | 9.63E-01 |
| 3-ureidopropionate | 1.07 | 0.74-1.53 | 9.59E-01 | 1.08 | 0.74-1.58 | 9.63E-01 |
| sphingomyelin (d18:1/21:0, d17:1/22:0, d16:1/23:0)* | 0.92 | 0.56-1.54 | 9.61E-01 | 1.12 | 0.64-1.95 | 9.63E-01 |
| octanoylcarnitine (C8) | 0.96 | 0.71-1.31 | 9.69E-01 | 0.95 | 0.72-1.24 | 9.63E-01 |
| 5-oxoproline | 0.96 | 0.61-1.5 | 9.88E-01 | 0.93 | 0.62-1.37 | 9.63E-01 |
| 7-methylguanine | 0.97 | 0.61-1.54 | 9.89E-01 | 0.91 | 0.56-1.46 | 9.63E-01 |
| sphingomyelin (d18:2/24:1, d18:1/24:2)* | 0.97 | 0.6-1.55 | 9.89E-01 | 0.90 | 0.55-1.48 | 9.63E-01 |
| 4-hydroxyphenylacetylglutamine | 1.02 | 0.71-1.49 | 9.89E-01 | 1.07 | 0.76-1.49 | 9.63E-01 |
| docosahexaenoylcarnitine (C22:6)* | 1.04 | 0.66-1.64 | 9.89E-01 | 1.09 | 0.7-1.68 | 9.63E-01 |
| taurochenodeoxycholic acid 3-sulfate | 1.02 | 0.65-1.61 | 9.89E-01 | 0.91 | 0.6-1.4 | 9.63E-01 |
| margarate (17:0) | 0.97 | 0.62-1.5 | 9.89E-01 | 1.09 | 0.71-1.68 | 9.63E-01 |
| X - 12729 | 1.02 | 0.76-1.38 | 9.89E-01 | 1.06 | 0.79-1.44 | 9.63E-01 |
| X - 17653 | 0.97 | 0.66-1.41 | 9.89E-01 | 1.09 | 0.71-1.66 | 9.63E-01 |
| proline | 1.02 | 0.7-1.48 | 9.91E-01 | 1.09 | 0.72-1.63 | 9.63E-01 |
| X - 21339 | 0.99 | 0.67-1.45 | 9.91E-01 | 1.09 | 0.71-1.68 | 9.63E-01 |
| X - 17654 | 1.00 | 0.69-1.46 | 9.96E-01 | 1.09 | 0.7-1.7 | 9.63E-01 |
| X - 23739 | 0.99 | 0.67-1.48 | 9.96E-01 | 0.94 | 0.66-1.32 | 9.63E-01 |
| X - 12096 | 0.98 | 0.65-1.49 | 9.89E-01 | 1.09 | 0.69-1.72 | 9.66E-01 |
| N-palmitoylserine | 1.11 | 0.85-1.45 | 8.82E-01 | 1.05 | 0.8-1.39 | 9.67E-01 |
| 10-nonadecenoate (19:1n9) | 0.87 | 0.56-1.34 | 9.20E-01 | 0.93 | 0.62-1.39 | 9.67E-01 |
| X - 24588 | 0.89 | 0.6-1.3 | 9.23E-01 | 0.93 | 0.62-1.39 | 9.67E-01 |
| 10-heptadecenoate (17:1n7) | 0.89 | 0.58-1.35 | 9.39E-01 | 0.93 | 0.63-1.38 | 9.67E-01 |
| X - 16935 | 0.93 | 0.69-1.25 | 9.42E-01 | 0.95 | 0.71-1.27 | 9.67E-01 |
| 1-ribosyl-imidazoleacetate* | 0.91 | 0.51-1.6 | 9.59E-01 | 0.92 | 0.57-1.48 | 9.67E-01 |
| 4-allylcatechol sulfate | 0.94 | 0.65-1.37 | 9.59E-01 | 1.07 | 0.73-1.58 | 9.67E-01 |
| cis-3,4-methyleneheptanoylcarnitine | 0.94 | 0.68-1.31 | 9.59E-01 | 0.94 | 0.69-1.29 | 9.67E-01 |
| hexanoylglycine | 1.04 | 0.74-1.46 | 9.76E-01 | 0.93 | 0.64-1.36 | 9.67E-01 |
| X - 25271 | 0.98 | 0.65-1.48 | 9.90E-01 | 1.08 | 0.7-1.68 | 9.67E-01 |
| nonadecanoate (19:0) | 1.00 | 0.69-1.45 | 9.96E-01 | 1.07 | 0.73-1.57 | 9.67E-01 |
| X - 18921 | 1.00 | 0.69-1.44 | 9.96E-01 | 0.94 | 0.66-1.34 | 9.67E-01 |
| sphingomyelin (d18:0/18:0, d19:0/17:0)* | 1.10 | 0.75-1.63 | 9.42E-01 | 1.08 | 0.7-1.66 | 9.67E-01 |
| ethyl alpha-glucopyranoside | 1.22 | 0.84-1.77 | 8.33E-01 | 1.07 | 0.74-1.53 | 9.69E-01 |
| X - 12812 | 0.84 | 0.59-1.19 | 8.42E-01 | 0.94 | 0.65-1.35 | 9.69E-01 |
| (R)-3-hydroxybutyrylcarnitine | 1.15 | 0.81-1.64 | 8.71E-01 | 1.06 | 0.77-1.46 | 9.69E-01 |
| 4-cholesten-3-one | 0.89 | 0.62-1.28 | 9.20E-01 | 0.94 | 0.65-1.36 | 9.69E-01 |
| 1-stearoyl-2-arachidonoyl-GPI (18:0/20:4) | 0.91 | 0.57-1.45 | 9.56E-01 | 0.92 | 0.59-1.45 | 9.69E-01 |
| 4-methylcatechol sulfate | 1.05 | 0.76-1.47 | 9.59E-01 | 1.05 | 0.79-1.41 | 9.69E-01 |
| sphingomyelin (d17:1/14:0, d16:1/15:0)* | 0.91 | 0.56-1.5 | 9.59E-01 | 1.09 | 0.66-1.82 | 9.69E-01 |
| 2,2'-Methylenebis(6-tert-butyl-p-cresol) | 1.07 | 0.72-1.57 | 9.59E-01 | 1.06 | 0.75-1.52 | 9.69E-01 |
| 17alpha-hydroxypregnanolone glucuronide | 1.09 | 0.59-2.02 | 9.68E-01 | 0.90 | 0.48-1.67 | 9.69E-01 |
| 1-linoleoyl-GPE (18:2)* | 1.06 | 0.69-1.63 | 9.69E-01 | 1.09 | 0.68-1.75 | 9.69E-01 |
| campesterol | 0.99 | 0.65-1.52 | 9.96E-01 | 0.93 | 0.61-1.41 | 9.69E-01 |
| alpha-hydroxyisovalerate | 1.21 | 0.75-1.93 | 8.76E-01 | 1.07 | 0.71-1.63 | 9.70E-01 |
| stearidonate (18:4n3) | 1.05 | 0.79-1.39 | 9.59E-01 | 1.05 | 0.78-1.42 | 9.70E-01 |
| erythronate* | 0.98 | 0.68-1.41 | 9.89E-01 | 1.06 | 0.75-1.5 | 9.70E-01 |
| suberoylcarnitine (C8-DC) | 0.98 | 0.65-1.47 | 9.89E-01 | 0.93 | 0.62-1.4 | 9.70E-01 |
| myristate (14:0) | 0.87 | 0.58-1.31 | 9.12E-01 | 0.93 | 0.63-1.4 | 9.70E-01 |
| 6-hydroxyindole sulfate | 0.92 | 0.65-1.29 | 9.42E-01 | 1.06 | 0.73-1.55 | 9.73E-01 |
| 1H-indole-7-acetic acid | 1.06 | 0.74-1.52 | 9.59E-01 | 1.06 | 0.74-1.54 | 9.73E-01 |
| X - 18899 | 0.83 | 0.6-1.14 | 8.14E-01 | 0.95 | 0.68-1.32 | 9.74E-01 |
| linoleoylcarnitine (C18:2)* | 0.84 | 0.56-1.27 | 8.62E-01 | 0.94 | 0.65-1.36 | 9.74E-01 |
| X - 12112 | 1.12 | 0.86-1.46 | 8.63E-01 | 1.05 | 0.79-1.38 | 9.74E-01 |
| 4-hydroxyphenylpyruvate | 0.90 | 0.63-1.26 | 9.20E-01 | 0.95 | 0.67-1.34 | 9.74E-01 |
| X - 23974 | 0.94 | 0.66-1.32 | 9.58E-01 | 0.94 | 0.64-1.37 | 9.74E-01 |
| cis-4-decenoylcarnitine (C10:1) | 1.04 | 0.75-1.43 | 9.81E-01 | 1.05 | 0.79-1.39 | 9.74E-01 |
| N-acetylcitrulline | 1.02 | 0.7-1.49 | 9.89E-01 | 1.06 | 0.74-1.53 | 9.74E-01 |
| N-acetylvaline | 0.98 | 0.72-1.33 | 9.89E-01 | 1.05 | 0.76-1.46 | 9.74E-01 |
| X - 14939 | 0.97 | 0.69-1.38 | 9.89E-01 | 0.95 | 0.69-1.31 | 9.74E-01 |
| N-acetylneuraminate | 1.21 | 0.79-1.85 | 8.62E-01 | 1.07 | 0.67-1.73 | 9.75E-01 |
| X - 17367 | 0.86 | 0.57-1.28 | 8.80E-01 | 0.93 | 0.59-1.48 | 9.75E-01 |
| 3,4-dihydroxybutyrate | 1.10 | 0.83-1.45 | 9.10E-01 | 1.05 | 0.77-1.42 | 9.75E-01 |
| linoleate (18:2n6) | 0.89 | 0.62-1.28 | 9.18E-01 | 0.95 | 0.66-1.35 | 9.75E-01 |
| 2-hydroxybutyrate/2-hydroxyisobutyrate | 0.86 | 0.52-1.43 | 9.37E-01 | 0.93 | 0.57-1.51 | 9.75E-01 |
| X - 23782 | 0.91 | 0.64-1.28 | 9.41E-01 | 0.95 | 0.68-1.34 | 9.75E-01 |
| cysteine s-sulfate | 1.08 | 0.8-1.47 | 9.42E-01 | 1.05 | 0.76-1.45 | 9.75E-01 |
| 12-HETE | 1.11 | 0.75-1.65 | 9.42E-01 | 1.06 | 0.72-1.56 | 9.75E-01 |
| 3-acetylphenol sulfate | 1.13 | 0.67-1.9 | 9.42E-01 | 1.08 | 0.64-1.81 | 9.75E-01 |
| 1-(1-enyl-palmitoyl)-2-arachidonoyl-GPC (P-16:0/20:4)* | 1.12 | 0.73-1.71 | 9.42E-01 | 1.06 | 0.71-1.57 | 9.75E-01 |
| beta-alanine | 1.08 | 0.76-1.54 | 9.48E-01 | 1.05 | 0.76-1.45 | 9.75E-01 |
| 1-palmitoyl-2-stearoyl-GPC (16:0/18:0) | 0.94 | 0.69-1.28 | 9.56E-01 | 0.95 | 0.69-1.31 | 9.75E-01 |
| pantoate | 1.06 | 0.77-1.47 | 9.58E-01 | 1.05 | 0.74-1.5 | 9.75E-01 |
| lanthionine | 1.06 | 0.77-1.46 | 9.58E-01 | 1.05 | 0.77-1.43 | 9.75E-01 |
| arachidoylcarnitine (C20)* | 0.92 | 0.6-1.41 | 9.58E-01 | 0.94 | 0.6-1.46 | 9.75E-01 |
| X - 12283 | 0.94 | 0.68-1.3 | 9.58E-01 | 1.05 | 0.73-1.52 | 9.75E-01 |
| 5,6-dihydrouridine | 1.06 | 0.76-1.47 | 9.59E-01 | 1.06 | 0.74-1.51 | 9.75E-01 |
| N-stearoyltaurine | 0.95 | 0.68-1.32 | 9.61E-01 | 0.95 | 0.67-1.34 | 9.75E-01 |
| X - 17335 | 0.94 | 0.64-1.39 | 9.61E-01 | 0.94 | 0.66-1.36 | 9.75E-01 |
| ascorbic acid 2-sulfate | 1.04 | 0.7-1.53 | 9.89E-01 | 1.06 | 0.71-1.59 | 9.75E-01 |
| 2-hydroxy-3-methylvalerate | 1.04 | 0.63-1.73 | 9.89E-01 | 0.93 | 0.57-1.51 | 9.75E-01 |
| gulonate* | 0.98 | 0.76-1.28 | 9.89E-01 | 1.05 | 0.76-1.44 | 9.75E-01 |
| phenylalanine | 1.02 | 0.7-1.48 | 9.89E-01 | 1.05 | 0.75-1.47 | 9.75E-01 |
| X - 25957 | 0.98 | 0.67-1.43 | 9.89E-01 | 0.94 | 0.64-1.39 | 9.75E-01 |
| tryptophan | 1.02 | 0.66-1.56 | 9.91E-01 | 1.07 | 0.7-1.63 | 9.75E-01 |
| X - 24295 | 1.02 | 0.68-1.52 | 9.91E-01 | 1.06 | 0.71-1.57 | 9.75E-01 |
| 2R,3R-dihydroxybutyrate | 1.01 | 0.64-1.59 | 9.96E-01 | 1.07 | 0.7-1.63 | 9.75E-01 |
| hydroxypalmitoyl sphingomyelin (d18:1/16:0(OH))** | 1.00 | 0.63-1.6 | 9.96E-01 | 1.07 | 0.69-1.66 | 9.75E-01 |
| galactonate | 0.93 | 0.69-1.24 | 9.42E-01 | 0.96 | 0.71-1.3 | 9.75E-01 |
| 3-methoxycatechol sulfate (1) | 0.91 | 0.57-1.44 | 9.56E-01 | 0.94 | 0.58-1.51 | 9.75E-01 |
| 3-hydroxydecanoylcarnitine | 0.93 | 0.64-1.35 | 9.58E-01 | 0.95 | 0.67-1.35 | 9.75E-01 |
| X - 11308 | 0.94 | 0.66-1.34 | 9.59E-01 | 1.06 | 0.7-1.6 | 9.75E-01 |
| glutarylcarnitine (C5-DC) | 1.00 | 0.73-1.38 | 9.96E-01 | 1.04 | 0.77-1.42 | 9.75E-01 |
| X - 15486 | 1.00 | 0.67-1.51 | 9.96E-01 | 0.95 | 0.67-1.36 | 9.75E-01 |
| docosadienoate (22:2n6) | 1.00 | 0.65-1.55 | 9.99E-01 | 0.94 | 0.63-1.43 | 9.75E-01 |
| 3-methoxycatechol sulfate (2) | 0.86 | 0.59-1.28 | 8.88E-01 | 0.95 | 0.63-1.42 | 9.81E-01 |
| 5-methyluridine (ribothymidine) | 0.96 | 0.72-1.28 | 9.69E-01 | 1.04 | 0.77-1.41 | 9.81E-01 |
| 7-alpha-hydroxy-3-oxo-4-cholestenoate (7-Hoca) | 1.04 | 0.77-1.41 | 9.69E-01 | 1.05 | 0.75-1.47 | 9.81E-01 |
| X - 15503 | 0.75 | 0.56-1.02 | 6.16E-01 | 0.95 | 0.66-1.39 | 9.82E-01 |
| beta-cryptoxanthin | 0.77 | 0.55-1.09 | 7.45E-01 | 0.96 | 0.67-1.37 | 9.82E-01 |
| sphingomyelin (d18:2/14:0, d18:1/14:1)* | 0.81 | 0.5-1.32 | 8.62E-01 | 0.94 | 0.54-1.64 | 9.82E-01 |
| pyridoxate | 0.90 | 0.69-1.17 | 8.76E-01 | 1.03 | 0.84-1.26 | 9.82E-01 |
| X - 21821 | 0.86 | 0.57-1.28 | 8.76E-01 | 0.95 | 0.6-1.5 | 9.82E-01 |
| X - 11795 | 1.11 | 0.81-1.54 | 9.16E-01 | 1.04 | 0.76-1.41 | 9.82E-01 |
| X - 25267 | 1.08 | 0.83-1.42 | 9.31E-01 | 0.97 | 0.75-1.26 | 9.82E-01 |
| 1-stearoyl-2-oleoyl-GPS (18:0/18:1) | 1.09 | 0.77-1.53 | 9.42E-01 | 1.05 | 0.71-1.53 | 9.82E-01 |
| isovalerylcarnitine (C5) | 0.92 | 0.65-1.29 | 9.42E-01 | 0.96 | 0.69-1.34 | 9.82E-01 |
| 5alpha-androstan-3alpha,17alpha-diol monosulfate | 0.90 | 0.59-1.39 | 9.42E-01 | 0.95 | 0.61-1.48 | 9.82E-01 |
| tetrahydrocortisol glucuronide | 1.11 | 0.74-1.67 | 9.42E-01 | 1.05 | 0.7-1.58 | 9.82E-01 |
| palmitoyl sphingomyelin (d18:1/16:0) | 1.12 | 0.67-1.87 | 9.48E-01 | 1.06 | 0.65-1.72 | 9.82E-01 |
| X - 15728 | 1.06 | 0.78-1.45 | 9.58E-01 | 1.04 | 0.74-1.46 | 9.82E-01 |
| X - 26054 | 1.08 | 0.72-1.62 | 9.58E-01 | 1.05 | 0.71-1.56 | 9.82E-01 |
| hippurate | 0.93 | 0.63-1.38 | 9.59E-01 | 1.06 | 0.69-1.62 | 9.82E-01 |
| 3-hydroxy-2-ethylpropionate | 0.93 | 0.6-1.44 | 9.59E-01 | 0.95 | 0.58-1.55 | 9.82E-01 |
| 1-palmitoleoyl-GPC (16:1)* | 1.08 | 0.71-1.63 | 9.59E-01 | 1.04 | 0.7-1.54 | 9.82E-01 |
| 4-methoxyphenol sulfate | 0.94 | 0.64-1.38 | 9.59E-01 | 0.95 | 0.61-1.47 | 9.82E-01 |
| 1-palmitoyl-2-arachidonoyl-GPI (16:0/20:4)* | 0.94 | 0.62-1.42 | 9.61E-01 | 0.95 | 0.6-1.49 | 9.82E-01 |
| hydroxyasparagine** | 0.94 | 0.62-1.41 | 9.61E-01 | 0.95 | 0.62-1.46 | 9.82E-01 |
| taurochenodeoxycholate | 1.08 | 0.63-1.86 | 9.67E-01 | 1.07 | 0.62-1.83 | 9.82E-01 |
| 4-guanidinobutanoate | 1.06 | 0.71-1.57 | 9.68E-01 | 1.05 | 0.71-1.57 | 9.82E-01 |
| carotene diol (1) | 0.93 | 0.56-1.55 | 9.69E-01 | 1.06 | 0.66-1.7 | 9.82E-01 |
| N-carbamoylalanine | 1.05 | 0.71-1.56 | 9.70E-01 | 1.05 | 0.69-1.62 | 9.82E-01 |
| carotene diol (3) | 0.96 | 0.66-1.39 | 9.78E-01 | 1.05 | 0.7-1.59 | 9.82E-01 |
| allantoin | 0.96 | 0.71-1.32 | 9.79E-01 | 1.04 | 0.77-1.4 | 9.82E-01 |
| 3-hydroxyhippurate sulfate | 0.96 | 0.66-1.41 | 9.87E-01 | 1.05 | 0.68-1.6 | 9.82E-01 |
| glycolithocholate | 1.02 | 0.69-1.53 | 9.89E-01 | 1.04 | 0.71-1.54 | 9.82E-01 |
| gamma-glutamylvaline | 0.98 | 0.69-1.4 | 9.89E-01 | 1.04 | 0.72-1.51 | 9.82E-01 |
| eicosenoate (20:1) | 0.97 | 0.66-1.45 | 9.89E-01 | 0.95 | 0.65-1.4 | 9.82E-01 |
| 5alpha-androstan-3beta,17beta-diol monosulfate (2) | 1.03 | 0.7-1.51 | 9.89E-01 | 0.95 | 0.65-1.41 | 9.82E-01 |
| N-acetylmethionine | 1.02 | 0.7-1.48 | 9.89E-01 | 1.04 | 0.71-1.52 | 9.82E-01 |
| nicotinamide | 1.02 | 0.71-1.47 | 9.89E-01 | 1.05 | 0.69-1.59 | 9.82E-01 |
| X - 12544 | 0.98 | 0.74-1.3 | 9.89E-01 | 1.04 | 0.76-1.41 | 9.82E-01 |
| X - 21312 | 0.97 | 0.67-1.4 | 9.89E-01 | 1.04 | 0.73-1.49 | 9.82E-01 |
| X - 23583 | 0.97 | 0.66-1.43 | 9.89E-01 | 0.95 | 0.64-1.41 | 9.82E-01 |
| mannonate* | 0.98 | 0.62-1.55 | 9.91E-01 | 0.95 | 0.61-1.49 | 9.82E-01 |
| lithocholate | 0.98 | 0.62-1.57 | 9.91E-01 | 1.06 | 0.65-1.73 | 9.82E-01 |
| X - 13507 | 1.01 | 0.77-1.32 | 9.91E-01 | 1.03 | 0.79-1.35 | 9.82E-01 |
| hexanoylcarnitine (C6) | 1.00 | 0.73-1.37 | 9.96E-01 | 0.97 | 0.74-1.27 | 9.82E-01 |
| 4-hydroxy-2-oxoglutaric acid | 1.00 | 0.71-1.43 | 9.96E-01 | 1.04 | 0.72-1.49 | 9.82E-01 |
| nonanoylcarnitine (C9) | 1.00 | 0.71-1.41 | 9.96E-01 | 1.03 | 0.77-1.39 | 9.82E-01 |
| dihomo-linolenoyl-choline | 1.01 | 0.74-1.37 | 9.96E-01 | 1.04 | 0.73-1.48 | 9.82E-01 |
| 5-methylthioadenosine (MTA) | 1.01 | 0.65-1.56 | 9.96E-01 | 1.05 | 0.67-1.65 | 9.82E-01 |
| stearate (18:0) | 1.00 | 0.67-1.48 | 9.96E-01 | 1.05 | 0.72-1.54 | 9.82E-01 |
| X - 11478 | 0.99 | 0.69-1.43 | 9.96E-01 | 0.96 | 0.67-1.38 | 9.82E-01 |
| X - 11852 | 1.01 | 0.71-1.42 | 9.96E-01 | 1.05 | 0.73-1.49 | 9.82E-01 |
| X - 14056 | 0.99 | 0.67-1.47 | 9.96E-01 | 1.05 | 0.71-1.56 | 9.82E-01 |
| N-acetyltaurine | 1.10 | 0.81-1.5 | 9.20E-01 | 0.97 | 0.7-1.34 | 9.83E-01 |
| palmitate (16:0) | 0.93 | 0.63-1.39 | 9.59E-01 | 0.96 | 0.66-1.4 | 9.84E-01 |
| fumarate | 0.96 | 0.7-1.31 | 9.73E-01 | 0.97 | 0.69-1.36 | 9.86E-01 |
| 1,2,3-benzenetriol sulfate (2) | 0.91 | 0.6-1.36 | 9.42E-01 | 0.96 | 0.63-1.46 | 9.87E-01 |
| X - 17351 | 0.92 | 0.65-1.28 | 9.42E-01 | 1.04 | 0.7-1.53 | 9.87E-01 |
| dihomo-linolenoylcarnitine (C20:3n3 or 6)* | 0.91 | 0.6-1.39 | 9.54E-01 | 0.96 | 0.63-1.46 | 9.87E-01 |
| pipecolate | 1.05 | 0.77-1.42 | 9.67E-01 | 1.03 | 0.77-1.38 | 9.87E-01 |
| glyco-beta-muricholate** | 0.96 | 0.66-1.4 | 9.82E-01 | 0.96 | 0.65-1.42 | 9.87E-01 |
| alpha-ketoglutarate | 1.03 | 0.73-1.44 | 9.89E-01 | 1.03 | 0.73-1.47 | 9.87E-01 |
| glycosyl-N-stearoyl-sphingosine (d18:1/18:0) | 0.99 | 0.67-1.47 | 9.95E-01 | 0.97 | 0.67-1.39 | 9.87E-01 |
| palmitoyl-sphingosine-phosphoethanolamine (d18:1/16:0) | 1.02 | 0.68-1.53 | 9.91E-01 | 0.97 | 0.67-1.39 | 9.88E-01 |
| 2-hydroxynervonate* | 1.17 | 0.78-1.75 | 8.76E-01 | 0.97 | 0.66-1.42 | 9.89E-01 |
| 12,13-DiHOME | 0.91 | 0.67-1.25 | 9.30E-01 | 1.03 | 0.76-1.39 | 9.89E-01 |
| oxindolylalanine | 0.93 | 0.62-1.39 | 9.58E-01 | 1.04 | 0.7-1.53 | 9.89E-01 |
| 4-acetamidobutanoate | 0.93 | 0.64-1.36 | 9.58E-01 | 0.97 | 0.65-1.44 | 9.89E-01 |
| homoarginine | 0.95 | 0.7-1.28 | 9.59E-01 | 1.03 | 0.76-1.38 | 9.89E-01 |
| cystine | 1.07 | 0.65-1.74 | 9.69E-01 | 1.04 | 0.66-1.64 | 9.89E-01 |
| palmitoyl ethanolamide | 1.05 | 0.69-1.61 | 9.79E-01 | 1.04 | 0.69-1.56 | 9.89E-01 |
| docosapentaenoate (n3 DPA; 22:5n3) | 0.97 | 0.63-1.51 | 9.89E-01 | 1.04 | 0.67-1.62 | 9.89E-01 |
| X - 12830 | 0.96 | 0.62-1.49 | 9.89E-01 | 0.96 | 0.62-1.48 | 9.89E-01 |
| X - 24949 | 1.02 | 0.7-1.48 | 9.89E-01 | 1.03 | 0.71-1.49 | 9.89E-01 |
| oleoylcholine | 1.01 | 0.7-1.46 | 9.96E-01 | 1.04 | 0.69-1.57 | 9.89E-01 |
| arachidonoylcholine | 1.01 | 0.71-1.43 | 9.96E-01 | 1.03 | 0.7-1.52 | 9.89E-01 |
| X - 18922 | 1.00 | 0.67-1.51 | 9.96E-01 | 0.97 | 0.66-1.42 | 9.89E-01 |
| aspartate | 1.03 | 0.71-1.48 | 9.89E-01 | 0.97 | 0.68-1.39 | 9.89E-01 |
| X - 25371 | 0.81 | 0.56-1.18 | 8.15E-01 | 0.97 | 0.67-1.4 | 9.90E-01 |
| docosatrienoate (22:3n3) | 0.93 | 0.63-1.37 | 9.58E-01 | 0.97 | 0.67-1.41 | 9.90E-01 |
| eicosanedioate (C20-DC) | 0.93 | 0.62-1.39 | 9.59E-01 | 0.97 | 0.63-1.48 | 9.90E-01 |
| X - 17685 | 1.02 | 0.62-1.66 | 9.91E-01 | 1.04 | 0.64-1.71 | 9.90E-01 |
| sphingomyelin (d17:1/16:0, d18:1/15:0, d16:1/17:0)* | 0.91 | 0.55-1.5 | 9.58E-01 | 1.04 | 0.61-1.78 | 9.90E-01 |
| beta-hydroxyisovalerate | 1.07 | 0.63-1.81 | 9.69E-01 | 1.05 | 0.58-1.92 | 9.90E-01 |
| 2,3-dihydroxyisovalerate | 0.98 | 0.68-1.41 | 9.89E-01 | 0.97 | 0.68-1.39 | 9.90E-01 |
| (N(1) + N(8))-acetylspermidine | 0.98 | 0.67-1.43 | 9.89E-01 | 1.03 | 0.68-1.57 | 9.90E-01 |
| 5-hydroxyindole sulfate | 0.87 | 0.62-1.2 | 8.62E-01 | 0.98 | 0.7-1.36 | 9.90E-01 |
| lignoceroyl sphingomyelin (d18:1/24:0) | 1.14 | 0.77-1.69 | 9.18E-01 | 1.03 | 0.7-1.53 | 9.90E-01 |
| myo-inositol | 0.90 | 0.65-1.25 | 9.21E-01 | 0.98 | 0.7-1.35 | 9.90E-01 |
| X - 12101 | 1.08 | 0.78-1.51 | 9.42E-01 | 1.02 | 0.73-1.43 | 9.90E-01 |
| corticosterone | 1.06 | 0.81-1.4 | 9.52E-01 | 1.02 | 0.75-1.39 | 9.90E-01 |
| X - 11315 | 0.93 | 0.65-1.34 | 9.58E-01 | 1.04 | 0.63-1.7 | 9.90E-01 |
| 9-hydroxystearate | 1.05 | 0.74-1.5 | 9.68E-01 | 0.97 | 0.65-1.44 | 9.90E-01 |
| 1-(1-enyl-palmitoyl)-GPC (P-16:0)* | 0.95 | 0.65-1.41 | 9.76E-01 | 1.03 | 0.7-1.52 | 9.90E-01 |
| X - 25343 | 1.05 | 0.66-1.67 | 9.82E-01 | 1.04 | 0.65-1.65 | 9.90E-01 |
| suberate (C8-DC) | 1.04 | 0.69-1.55 | 9.89E-01 | 0.97 | 0.65-1.46 | 9.90E-01 |
| behenoyl sphingomyelin (d18:1/22:0)* | 0.97 | 0.67-1.4 | 9.89E-01 | 0.97 | 0.65-1.45 | 9.90E-01 |
| 1-methylnicotinamide | 1.02 | 0.73-1.42 | 9.89E-01 | 1.03 | 0.73-1.45 | 9.90E-01 |
| indoleacetoylcarnitine* | 0.99 | 0.66-1.5 | 9.96E-01 | 0.97 | 0.64-1.46 | 9.90E-01 |
| 3-formylindole | 0.87 | 0.59-1.27 | 8.96E-01 | 0.98 | 0.68-1.41 | 9.92E-01 |
| X - 23659 | 0.91 | 0.66-1.26 | 9.40E-01 | 0.98 | 0.7-1.37 | 9.92E-01 |
| sphingomyelin (d18:1/22:2, d18:2/22:1, d16:1/24:2)* | 0.88 | 0.54-1.44 | 9.42E-01 | 0.97 | 0.62-1.53 | 9.92E-01 |
| cholate | 0.92 | 0.64-1.32 | 9.42E-01 | 0.98 | 0.69-1.38 | 9.92E-01 |
| imidazole lactate | 1.10 | 0.71-1.69 | 9.52E-01 | 1.03 | 0.66-1.61 | 9.92E-01 |
| N-stearoyl-sphingadienine (d18:2/18:0)* | 0.93 | 0.63-1.35 | 9.56E-01 | 0.98 | 0.7-1.37 | 9.92E-01 |
| X - 12193 | 1.09 | 0.72-1.63 | 9.56E-01 | 0.97 | 0.64-1.48 | 9.92E-01 |
| tricosanoyl sphingomyelin (d18:1/23:0)* | 0.94 | 0.6-1.47 | 9.69E-01 | 0.97 | 0.62-1.51 | 9.92E-01 |
| pseudouridine | 1.07 | 0.65-1.75 | 9.69E-01 | 1.04 | 0.62-1.74 | 9.92E-01 |
| dihomo-linolenate (20:3n3 or n6) | 0.98 | 0.67-1.44 | 9.89E-01 | 1.02 | 0.71-1.48 | 9.92E-01 |
| X - 18886 | 1.02 | 0.67-1.55 | 9.89E-01 | 1.03 | 0.67-1.58 | 9.92E-01 |
| ceramide (d18:2/24:1, d18:1/24:2)* | 0.98 | 0.64-1.5 | 9.90E-01 | 0.97 | 0.63-1.5 | 9.92E-01 |
| X - 23665 | 1.02 | 0.66-1.57 | 9.91E-01 | 1.03 | 0.69-1.53 | 9.92E-01 |
| 2-hydroxyfluorene sulfate | 1.62 | 1.12-2.34 | 2.74E-01 | 0.98 | 0.65-1.46 | 9.92E-01 |
| branched chain 14:0 dicarboxylic acid** | 0.86 | 0.67-1.09 | 7.97E-01 | 0.99 | 0.7-1.38 | 9.92E-01 |
| threonate | 0.82 | 0.59-1.14 | 8.14E-01 | 1.02 | 0.75-1.37 | 9.92E-01 |
| 3beta-hydroxy-5-cholestenoate | 1.23 | 0.86-1.74 | 8.15E-01 | 1.02 | 0.67-1.55 | 9.92E-01 |
| margaroylcarnitine (C17)* | 0.84 | 0.61-1.17 | 8.33E-01 | 0.98 | 0.73-1.33 | 9.92E-01 |
| 8-methoxykynurenate | 0.83 | 0.57-1.2 | 8.42E-01 | 0.99 | 0.68-1.43 | 9.92E-01 |
| 2-hydroxyarachidate* | 1.21 | 0.82-1.77 | 8.56E-01 | 1.03 | 0.67-1.58 | 9.92E-01 |
| cholesterol sulfate | 1.19 | 0.81-1.74 | 8.62E-01 | 0.99 | 0.69-1.42 | 9.92E-01 |
| sphingomyelin (d17:2/16:0, d18:2/15:0)* | 0.80 | 0.49-1.31 | 8.62E-01 | 1.03 | 0.62-1.73 | 9.92E-01 |
| 5-dodecenoylcarnitine (C12:1) | 1.10 | 0.83-1.45 | 9.18E-01 | 0.99 | 0.73-1.34 | 9.92E-01 |
| N-formylanthranilic acid | 0.89 | 0.62-1.27 | 9.19E-01 | 1.01 | 0.71-1.45 | 9.92E-01 |
| 1-(1-enyl-palmitoyl)-2-palmitoleoyl-GPC (P-16:0/16:1)* | 1.15 | 0.74-1.79 | 9.19E-01 | 1.02 | 0.67-1.55 | 9.92E-01 |
| pentadecanoate (15:0) | 0.87 | 0.56-1.35 | 9.20E-01 | 0.98 | 0.63-1.52 | 9.92E-01 |
| sphingomyelin (d18:1/19:0, d19:1/18:0)* | 0.87 | 0.52-1.44 | 9.40E-01 | 1.03 | 0.61-1.75 | 9.92E-01 |
| X - 24334 | 0.89 | 0.57-1.36 | 9.40E-01 | 0.97 | 0.63-1.51 | 9.92E-01 |
| indolepropionate | 0.92 | 0.67-1.27 | 9.42E-01 | 1.02 | 0.68-1.53 | 9.92E-01 |
| tetrahydrocortisone glucuronide (5) | 0.93 | 0.69-1.25 | 9.42E-01 | 0.98 | 0.71-1.36 | 9.92E-01 |
| choline phosphate | 0.89 | 0.56-1.41 | 9.42E-01 | 1.02 | 0.67-1.56 | 9.92E-01 |
| X - 17325 | 0.91 | 0.6-1.37 | 9.42E-01 | 1.01 | 0.65-1.59 | 9.92E-01 |
| X - 12100 | 0.90 | 0.55-1.47 | 9.52E-01 | 1.02 | 0.62-1.69 | 9.92E-01 |
| N-acetyl-isoputreanine | 1.08 | 0.75-1.57 | 9.54E-01 | 1.01 | 0.71-1.43 | 9.92E-01 |
| isovalerate (i5:0) | 0.93 | 0.64-1.34 | 9.56E-01 | 0.99 | 0.67-1.45 | 9.92E-01 |
| iminodiacetate (IDA) | 1.06 | 0.77-1.45 | 9.58E-01 | 0.99 | 0.72-1.36 | 9.92E-01 |
| 1-linoleoyl-GPG (18:2)* | 0.94 | 0.68-1.31 | 9.58E-01 | 0.98 | 0.72-1.35 | 9.92E-01 |
| 11beta-hydroxyandrosterone glucuronide | 1.07 | 0.75-1.53 | 9.58E-01 | 1.02 | 0.71-1.46 | 9.92E-01 |
| 1-stearoyl-GPG (18:0) | 0.95 | 0.68-1.32 | 9.59E-01 | 0.98 | 0.71-1.36 | 9.92E-01 |
| 2-palmitoyl-GPC (16:0)* | 0.94 | 0.63-1.41 | 9.59E-01 | 0.98 | 0.67-1.46 | 9.92E-01 |
| stearoyl sphingomyelin (d18:1/18:0) | 0.92 | 0.57-1.48 | 9.59E-01 | 0.99 | 0.61-1.59 | 9.92E-01 |
| ribitol | 1.05 | 0.75-1.49 | 9.63E-01 | 1.02 | 0.73-1.43 | 9.92E-01 |
| adipoylcarnitine (C6-DC) | 1.07 | 0.67-1.72 | 9.68E-01 | 1.02 | 0.65-1.58 | 9.92E-01 |
| alpha-ketobutyrate | 0.94 | 0.6-1.47 | 9.68E-01 | 0.98 | 0.64-1.49 | 9.92E-01 |
| inosine | 1.07 | 0.67-1.71 | 9.69E-01 | 1.02 | 0.62-1.68 | 9.92E-01 |
| threonine | 1.06 | 0.68-1.66 | 9.69E-01 | 1.02 | 0.68-1.53 | 9.92E-01 |
| X - 25519 | 1.06 | 0.68-1.64 | 9.69E-01 | 0.98 | 0.6-1.61 | 9.92E-01 |
| X - 26108 | 0.95 | 0.64-1.41 | 9.69E-01 | 0.98 | 0.65-1.46 | 9.92E-01 |
| glycodeoxycholate 3-sulfate | 1.05 | 0.68-1.63 | 9.79E-01 | 1.01 | 0.66-1.57 | 9.92E-01 |
| glycochenodeoxycholate glucuronide (1) | 1.06 | 0.65-1.72 | 9.79E-01 | 0.98 | 0.58-1.67 | 9.92E-01 |
| 1-oleoyl-2-docosahexaenoyl-GPC (18:1/22:6)* | 0.96 | 0.67-1.37 | 9.80E-01 | 1.01 | 0.7-1.46 | 9.92E-01 |
| thymol sulfate | 0.95 | 0.59-1.54 | 9.85E-01 | 1.01 | 0.66-1.55 | 9.92E-01 |
| 9,10-DiHOME | 0.97 | 0.72-1.3 | 9.85E-01 | 1.01 | 0.76-1.34 | 9.92E-01 |
| glutamate | 1.03 | 0.78-1.36 | 9.85E-01 | 1.02 | 0.69-1.5 | 9.92E-01 |
| 1-dihomo-linolenylglycerol (20:3) | 0.97 | 0.67-1.39 | 9.88E-01 | 1.01 | 0.71-1.45 | 9.92E-01 |
| 1-stearoyl-GPI (18:0) | 0.97 | 0.66-1.41 | 9.89E-01 | 1.01 | 0.68-1.51 | 9.92E-01 |
| glutamine_degradant* | 1.03 | 0.66-1.61 | 9.89E-01 | 0.98 | 0.63-1.51 | 9.92E-01 |
| N-acetyl-aspartyl-glutamate (NAAG) | 1.03 | 0.71-1.49 | 9.89E-01 | 0.98 | 0.67-1.46 | 9.92E-01 |
| 2-aminooctanoate | 0.98 | 0.65-1.47 | 9.89E-01 | 0.98 | 0.63-1.51 | 9.92E-01 |
| methionine sulfone | 0.96 | 0.63-1.47 | 9.89E-01 | 0.98 | 0.65-1.48 | 9.92E-01 |
| 1-palmitoleoyl-2-linolenoyl-GPC (16:1/18:3)* | 1.05 | 0.65-1.69 | 9.89E-01 | 1.03 | 0.63-1.68 | 9.92E-01 |
| gamma-glutamylcitrulline* | 1.03 | 0.71-1.48 | 9.89E-01 | 0.98 | 0.7-1.38 | 9.92E-01 |
| N6,N6,N6-trimethyllysine | 0.98 | 0.64-1.5 | 9.89E-01 | 0.98 | 0.65-1.49 | 9.92E-01 |
| arachidonate (20:4n6) | 0.96 | 0.65-1.43 | 9.89E-01 | 0.99 | 0.68-1.42 | 9.92E-01 |
| X - 12410 | 0.98 | 0.72-1.32 | 9.89E-01 | 0.99 | 0.71-1.38 | 9.92E-01 |
| X - 24243 | 0.98 | 0.68-1.4 | 9.89E-01 | 1.01 | 0.71-1.44 | 9.92E-01 |
| X - 24947 | 1.03 | 0.71-1.49 | 9.89E-01 | 0.98 | 0.66-1.47 | 9.92E-01 |
| X - 24970 | 1.03 | 0.71-1.49 | 9.89E-01 | 0.99 | 0.68-1.44 | 9.92E-01 |
| X - 26119 | 1.02 | 0.76-1.36 | 9.89E-01 | 0.99 | 0.73-1.33 | 9.92E-01 |
| 3-(3-amino-3-carboxypropyl)uridine* | 1.02 | 0.68-1.52 | 9.91E-01 | 1.02 | 0.66-1.57 | 9.92E-01 |
| indoleacetylglutamine | 1.01 | 0.67-1.54 | 9.94E-01 | 1.01 | 0.66-1.54 | 9.92E-01 |
| X - 25265 | 1.01 | 0.7-1.46 | 9.95E-01 | 0.99 | 0.69-1.4 | 9.92E-01 |
| orotidine | 1.00 | 0.72-1.38 | 9.96E-01 | 0.99 | 0.7-1.41 | 9.92E-01 |
| 11beta-hydroxyetiocholanolone glucuronide* | 0.99 | 0.67-1.46 | 9.96E-01 | 1.01 | 0.68-1.5 | 9.92E-01 |
| methyl indole-3-acetate | 1.00 | 0.68-1.47 | 9.96E-01 | 1.01 | 0.72-1.42 | 9.92E-01 |
| lactate | 0.99 | 0.64-1.54 | 9.96E-01 | 1.03 | 0.65-1.61 | 9.92E-01 |
| X - 12127 | 0.99 | 0.7-1.42 | 9.96E-01 | 1.01 | 0.73-1.4 | 9.92E-01 |
| X - 23648 | 1.00 | 0.7-1.42 | 9.96E-01 | 1.01 | 0.71-1.45 | 9.92E-01 |
| homocitrulline | 1.00 | 0.72-1.38 | 9.98E-01 | 0.99 | 0.72-1.37 | 9.92E-01 |
| pantothenate | 0.92 | 0.65-1.29 | 9.42E-01 | 1.01 | 0.76-1.34 | 9.93E-01 |
| cis-3,4-methyleneheptanoylglycine | 1.05 | 0.71-1.55 | 9.69E-01 | 1.01 | 0.68-1.5 | 9.93E-01 |
| 5,6-dihydrothymine | 0.97 | 0.6-1.56 | 9.89E-01 | 1.01 | 0.6-1.71 | 9.93E-01 |
| 17alpha-hydroxypregnenolone 3-sulfate | 1.08 | 0.72-1.61 | 9.58E-01 | 0.99 | 0.64-1.53 | 9.95E-01 |
| X - 16580 | 1.06 | 0.74-1.51 | 9.59E-01 | 0.99 | 0.72-1.37 | 9.95E-01 |
| X - 23639 | 1.15 | 0.78-1.71 | 9.01E-01 | 1.01 | 0.66-1.53 | 9.96E-01 |
| vanillactate | 0.95 | 0.63-1.44 | 9.80E-01 | 0.99 | 0.64-1.55 | 9.96E-01 |
| methionine sulfoxide | 0.85 | 0.59-1.24 | 8.62E-01 | 1.00 | 0.7-1.44 | 9.96E-01 |
| sphingomyelin (d18:2/21:0, d16:2/23:0)* | 0.79 | 0.44-1.41 | 8.74E-01 | 1.00 | 0.53-1.87 | 9.96E-01 |
| X - 11880 | 0.88 | 0.62-1.26 | 9.01E-01 | 0.99 | 0.69-1.43 | 9.96E-01 |
| 2-O-methylascorbic acid | 0.88 | 0.6-1.29 | 9.10E-01 | 1.00 | 0.67-1.5 | 9.96E-01 |
| sphingomyelin (d18:1/18:1, d18:2/18:0) | 0.85 | 0.51-1.41 | 9.18E-01 | 0.99 | 0.6-1.64 | 9.96E-01 |
| N2,N2-dimethylguanosine | 0.87 | 0.56-1.36 | 9.27E-01 | 1.00 | 0.6-1.67 | 9.96E-01 |
| xanthine | 0.90 | 0.56-1.45 | 9.53E-01 | 1.00 | 0.63-1.59 | 9.96E-01 |
| N-acetylglucosamine/N-acetylgalactosamine | 1.08 | 0.76-1.54 | 9.54E-01 | 1.00 | 0.71-1.42 | 9.96E-01 |
| 2-stearoyl-GPE (18:0)* | 0.93 | 0.63-1.38 | 9.59E-01 | 1.00 | 0.68-1.46 | 9.96E-01 |
| X - 24418 | 1.06 | 0.72-1.57 | 9.61E-01 | 1.00 | 0.69-1.45 | 9.96E-01 |
| N-delta-acetylornithine | 0.95 | 0.69-1.31 | 9.67E-01 | 1.00 | 0.72-1.38 | 9.96E-01 |
| (16 or 17)-methylstearate (a19:0 or i19:0) | 0.95 | 0.65-1.4 | 9.69E-01 | 1.00 | 0.69-1.46 | 9.96E-01 |
| N-acetylserine | 0.96 | 0.67-1.37 | 9.70E-01 | 1.00 | 0.69-1.44 | 9.96E-01 |
| succinate | 0.97 | 0.69-1.36 | 9.88E-01 | 1.00 | 0.71-1.41 | 9.96E-01 |
| (S)-3-hydroxybutyrylcarnitine | 1.03 | 0.7-1.53 | 9.89E-01 | 1.00 | 0.67-1.5 | 9.96E-01 |
| N6-methyladenosine | 1.02 | 0.72-1.46 | 9.89E-01 | 1.00 | 0.69-1.47 | 9.96E-01 |
| X - 13553 | 0.98 | 0.63-1.52 | 9.89E-01 | 1.00 | 0.63-1.56 | 9.96E-01 |
| adenosine 3',5'-cyclic monophosphate (cAMP) | 0.98 | 0.67-1.44 | 9.91E-01 | 0.99 | 0.67-1.48 | 9.96E-01 |
| N-acetyl-beta-alanine | 0.98 | 0.65-1.5 | 9.91E-01 | 1.01 | 0.64-1.57 | 9.96E-01 |
| 1-linoleoyl-2-linolenoyl-GPC (18:2/18:3)* | 1.00 | 0.57-1.78 | 9.96E-01 | 1.01 | 0.57-1.8 | 9.96E-01 |
| X - 13866 | 0.95 | 0.75-1.21 | 9.56E-01 | 1.00 | 0.77-1.3 | 9.99E-01 |
| X - 21310 | 0.93 | 0.64-1.36 | 9.58E-01 | 1.00 | 0.7-1.43 | 9.99E-01 |
| glucuronide of C10H18O2 (7)* | 0.95 | 0.62-1.47 | 9.82E-01 | 1.00 | 0.62-1.61 | 9.99E-01 |
| taurine | 0.96 | 0.56-1.64 | 9.89E-01 | 1.00 | 0.56-1.81 | 9.99E-01 |
| X - 16397 | 1.03 | 0.66-1.6 | 9.89E-01 | 1.00 | 0.67-1.51 | 9.99E-01 |
| X - 26106 | 0.97 | 0.66-1.41 | 9.89E-01 | 1.00 | 0.72-1.39 | 9.99E-01 |
| 10-undecenoate (11:1n1) | 0.99 | 0.67-1.46 | 9.95E-01 | 1.00 | 0.7-1.43 | 9.99E-01 |

Note: the table shows associations from the competing risk Cox proportional hazards regression analysis where plasms metabolomics level from the Metabolon platform was main exposure and incident lung cancer as the main outcome in ERGO-4 cohort. Model 1 : adjusted for age, sex, cohort, and lipid lowering medication. Model 2: adjusted for model1, smoking status, BMI, alcohol and education. The table is ranked based on the *FDR* values in model 2, using bold text indicates metabolites are surpassing *FDR* adjusted P value treshold (*P*<0.05). ERGO-4 refer to the fourth and fifth visits from the baseline cohort. Abbreviations: BMI, body mass index; HR, hazard ratio; CI, confidence interval; FDR, false discovery rate.

**Supplementary Table 21. Serum metabolites associated with incident prostate cancer from the Nightingale platform.**

| **Metabolite** | **Model 1** | | | **Model 2** | | |
| --- | --- | --- | --- | --- | --- | --- |
|  | **HR** | **95% CI** | ***FDR* value** | **HR** | **95% CI** | ***FDR* value** |
| Citrate | 0.71 | 0.53-0.95 | 2.30E-01 | 0.69 | 0.51-0.94 | 2.61E-01 |
| Cholesteryl esters in medium HDL | 0.67 | 0.51-0.88 | 1.68E-01 | 0.65 | 0.48-0.88 | 2.61E-01 |
| Cholesteryl esters in HDL | 0.65 | 0.48-0.86 | 1.68E-01 | 0.63 | 0.45-0.87 | 2.61E-01 |
| Cholesterol in medium HDL | 0.67 | 0.5-0.88 | 1.68E-01 | 0.65 | 0.47-0.88 | 2.61E-01 |
| HDL cholesterol | 0.64 | 0.48-0.86 | 1.68E-01 | 0.63 | 0.45-0.87 | 2.61E-01 |
| Phospholipids in HDL | 0.67 | 0.49-0.9 | 1.75E-01 | 0.65 | 0.46-0.91 | 2.61E-01 |
| Total lipids in HDL | 0.65 | 0.48-0.89 | 1.68E-01 | 0.64 | 0.45-0.89 | 2.61E-01 |
| Acetoacetate | 0.66 | 0.5-0.89 | 1.68E-01 | 0.67 | 0.5-0.9 | 2.61E-01 |
| Concentration of medium HDL particles | 0.68 | 0.5-0.91 | 1.90E-01 | 0.66 | 0.47-0.92 | 2.61E-01 |
| Free cholesterol in medium HDL | 0.67 | 0.51-0.9 | 1.68E-01 | 0.66 | 0.48-0.9 | 2.61E-01 |
| Total lipids in medium HDL | 0.68 | 0.5-0.92 | 1.90E-01 | 0.66 | 0.47-0.92 | 2.61E-01 |
| Free cholesterol in HDL | 0.66 | 0.49-0.88 | 1.68E-01 | 0.65 | 0.47-0.9 | 2.61E-01 |
| Average diameter for HDL particles | 0.68 | 0.51-0.91 | 1.75E-01 | 0.69 | 0.51-0.93 | 2.61E-01 |
| Phospholipids in large HDL | 0.73 | 0.59-0.92 | 1.68E-01 | 0.74 | 0.58-0.95 | 2.61E-01 |
| 3-Hydroxybutyrate | 0.72 | 0.57-0.9 | 1.68E-01 | 0.73 | 0.58-0.94 | 2.61E-01 |
| Apolipoprotein A1 | 0.66 | 0.48-0.92 | 1.90E-01 | 0.64 | 0.45-0.92 | 2.61E-01 |
| Free cholesterol to total lipids ratio in medium HDL | 0.7 | 0.55-0.91 | 1.68E-01 | 0.71 | 0.54-0.93 | 2.61E-01 |
| Pyruvate | 0.74 | 0.56-0.97 | 2.95E-01 | 0.71 | 0.53-0.95 | 2.97E-01 |
| Ratio of apolipoprotein B to apolipoprotein A1 | 1.41 | 1.05-1.89 | 2.30E-01 | 1.41 | 1.05-1.91 | 3.02E-01 |
| Tyrosine | 0.77 | 0.57-1.05 | 5.72E-01 | 0.72 | 0.53-0.96 | 3.02E-01 |
| Phospholipids in medium HDL | 0.7 | 0.51-0.95 | 2.30E-01 | 0.68 | 0.48-0.96 | 3.02E-01 |
| Total lipids in large HDL | 0.74 | 0.59-0.93 | 1.90E-01 | 0.75 | 0.58-0.96 | 3.02E-01 |
| Concentration of HDL particles | 0.68 | 0.49-0.95 | 2.30E-01 | 0.67 | 0.47-0.96 | 3.02E-01 |
| Cholesteryl esters in large HDL | 0.74 | 0.58-0.94 | 2.06E-01 | 0.75 | 0.57-0.97 | 3.04E-01 |
| Concentration of large HDL particles | 0.75 | 0.59-0.95 | 2.19E-01 | 0.76 | 0.59-0.98 | 3.32E-01 |
| Phospholipids to total lipids ratio in medium HDL | 1.35 | 1.04-1.75 | 2.30E-01 | 1.35 | 1.02-1.78 | 3.32E-01 |
| Cholesterol in large HDL | 0.75 | 0.59-0.95 | 2.19E-01 | 0.76 | 0.58-0.98 | 3.37E-01 |
| Glucose | 0.72 | 0.51-1.04 | 5.18E-01 | 0.67 | 0.46-0.98 | 3.59E-01 |
| Total concentration of lipoprotein particles | 0.71 | 0.51-0.98 | 3.27E-01 | 0.7 | 0.49-0.99 | 4.00E-01 |
| Alanine | 0.8 | 0.61-1.04 | 5.72E-01 | 0.76 | 0.57-1.01 | 4.47E-01 |
| Cholesterol to total lipids ratio in medium HDL | 0.76 | 0.59-0.98 | 3.15E-01 | 0.76 | 0.57-1.01 | 4.47E-01 |
| Phospholipids to total lipids ratio in IDL | 1.3 | 1-1.68 | 3.96E-01 | 1.28 | 1-1.65 | 4.47E-01 |
| Free cholesterol in large HDL | 0.77 | 0.62-0.97 | 2.74E-01 | 0.79 | 0.61-1.01 | 4.73E-01 |
| Lactate | 0.8 | 0.6-1.07 | 5.73E-01 | 0.77 | 0.57-1.04 | 6.11E-01 |
| Free cholesterol to total lipids ratio in very large HDL | 1.24 | 1-1.54 | 3.83E-01 | 1.21 | 0.97-1.51 | 6.11E-01 |
| Cholesteryl esters in very large HDL | 0.74 | 0.55-1 | 3.96E-01 | 0.76 | 0.55-1.05 | 6.11E-01 |
| Cholesteryl esters to total lipids ratio in small LDL | 1.28 | 0.97-1.68 | 5.18E-01 | 1.26 | 0.96-1.67 | 6.11E-01 |
| Acetone | 0.77 | 0.59-1 | 3.96E-01 | 0.79 | 0.61-1.03 | 6.11E-01 |
| Sphingomyelins | 0.75 | 0.57-1 | 3.96E-01 | 0.76 | 0.56-1.04 | 6.11E-01 |
| Ratio of saturated fatty acids to total fatty acids | 0.82 | 0.62-1.1 | 6.06E-01 | 0.79 | 0.59-1.07 | 6.57E-01 |
| Triglycerides to total lipids ratio in small HDL | 1.32 | 0.97-1.81 | 5.18E-01 | 1.32 | 0.93-1.86 | 6.57E-01 |
| Cholesteryl esters in very large VLDL | 1.3 | 0.95-1.78 | 5.72E-01 | 1.29 | 0.93-1.77 | 6.57E-01 |
| Ratio of triglycerides to phosphoglycerides | 1.29 | 0.97-1.73 | 5.18E-01 | 1.28 | 0.94-1.75 | 6.57E-01 |
| Cholesterol in very large VLDL | 1.31 | 0.95-1.8 | 5.73E-01 | 1.29 | 0.93-1.8 | 6.57E-01 |
| Cholesterol to total lipids ratio in medium LDL | 1.22 | 0.95-1.57 | 5.73E-01 | 1.24 | 0.96-1.62 | 6.57E-01 |
| Cholesteryl esters to total lipids ratio in medium HDL | 0.8 | 0.62-1.03 | 5.18E-01 | 0.8 | 0.61-1.05 | 6.57E-01 |
| Albumin | 0.8 | 0.61-1.06 | 5.73E-01 | 0.79 | 0.59-1.06 | 6.57E-01 |
| Glycoprotein acetyls | 1.24 | 0.92-1.68 | 5.87E-01 | 1.26 | 0.94-1.68 | 6.57E-01 |
| Creatinine | 1.25 | 0.93-1.67 | 5.73E-01 | 1.24 | 0.91-1.68 | 6.72E-01 |
| Cholesteryl esters in large VLDL | 1.28 | 0.94-1.76 | 5.73E-01 | 1.27 | 0.92-1.76 | 6.72E-01 |
| VLDL cholesterol | 1.26 | 0.93-1.7 | 5.73E-01 | 1.24 | 0.91-1.69 | 6.72E-01 |
| Free cholesterol in VLDL | 1.26 | 0.93-1.71 | 5.73E-01 | 1.25 | 0.92-1.7 | 6.72E-01 |
| Cholesterol in very large HDL | 0.75 | 0.55-1.03 | 5.18E-01 | 0.77 | 0.55-1.08 | 6.72E-01 |
| Cholesterol in large VLDL | 1.29 | 0.94-1.77 | 5.73E-01 | 1.27 | 0.92-1.77 | 6.72E-01 |
| Phospholipids in VLDL | 1.26 | 0.93-1.7 | 5.73E-01 | 1.24 | 0.91-1.69 | 6.72E-01 |
| Free cholesterol in large VLDL | 1.28 | 0.93-1.77 | 5.73E-01 | 1.27 | 0.91-1.77 | 6.72E-01 |
| Free cholesterol in very large VLDL | 1.3 | 0.93-1.81 | 5.73E-01 | 1.28 | 0.91-1.81 | 6.72E-01 |
| Phospholipids to total lipids ratio in small LDL | 0.8 | 0.61-1.07 | 5.73E-01 | 0.81 | 0.61-1.08 | 6.72E-01 |
| Cholesteryl esters in small HDL | 0.79 | 0.58-1.08 | 5.77E-01 | 0.79 | 0.58-1.09 | 6.72E-01 |
| Cholesterol in small HDL | 0.78 | 0.56-1.09 | 5.73E-01 | 0.78 | 0.56-1.09 | 6.72E-01 |
| Average diameter for VLDL particles | 1.25 | 0.95-1.66 | 5.73E-01 | 1.24 | 0.93-1.65 | 6.72E-01 |
| Free cholesterol in small HDL | 0.79 | 0.55-1.11 | 5.98E-01 | 0.78 | 0.54-1.11 | 6.72E-01 |
| Free cholesterol to total lipids ratio in small LDL | 0.81 | 0.63-1.06 | 5.73E-01 | 0.83 | 0.63-1.08 | 6.72E-01 |
| Phospholipids in very large VLDL | 1.3 | 0.91-1.84 | 5.77E-01 | 1.28 | 0.89-1.84 | 7.00E-01 |
| Phospholipids in large VLDL | 1.28 | 0.91-1.8 | 5.80E-01 | 1.26 | 0.89-1.79 | 7.03E-01 |
| Total lipids in VLDL | 1.24 | 0.92-1.68 | 5.80E-01 | 1.23 | 0.9-1.67 | 7.03E-01 |
| Concentration of very large HDL particles | 0.8 | 0.61-1.05 | 5.73E-01 | 0.83 | 0.62-1.1 | 7.03E-01 |
| Phospholipids to total lipids ratio in very large VLDL | 1.44 | 0.86-2.42 | 5.98E-01 | 1.43 | 0.84-2.43 | 7.03E-01 |
| Cholesteryl esters to total lipids ratio in large HDL | 0.83 | 0.65-1.06 | 5.73E-01 | 0.83 | 0.64-1.09 | 7.03E-01 |
| Total lipids in very large HDL | 0.8 | 0.61-1.06 | 5.73E-01 | 0.83 | 0.62-1.11 | 7.07E-01 |
| Free cholesterol to total lipids ratio in large VLDL | 1.17 | 0.91-1.51 | 6.25E-01 | 1.16 | 0.91-1.48 | 7.10E-01 |
| Cholesteryl esters in VLDL | 1.23 | 0.91-1.66 | 5.98E-01 | 1.21 | 0.89-1.65 | 7.10E-01 |
| Phospholipids to total lipids ratio in large VLDL | 1.34 | 0.9-1.99 | 5.80E-01 | 1.3 | 0.86-1.96 | 7.10E-01 |
| Phospholipids in medium VLDL | 1.22 | 0.9-1.64 | 6.06E-01 | 1.2 | 0.89-1.62 | 7.10E-01 |
| Concentration of medium VLDL particles | 1.22 | 0.9-1.64 | 6.06E-01 | 1.2 | 0.89-1.62 | 7.10E-01 |
| Total lipids in large VLDL | 1.24 | 0.9-1.71 | 5.98E-01 | 1.23 | 0.89-1.7 | 7.10E-01 |
| Concentration of very large VLDL particles | 1.26 | 0.9-1.77 | 5.98E-01 | 1.25 | 0.88-1.76 | 7.10E-01 |
| Cholesteryl esters to total lipids ratio in medium LDL | 1.23 | 0.91-1.68 | 5.98E-01 | 1.22 | 0.89-1.66 | 7.10E-01 |
| Total lipids in very large VLDL | 1.26 | 0.9-1.76 | 5.98E-01 | 1.24 | 0.88-1.75 | 7.10E-01 |
| Total cholines | 0.81 | 0.58-1.13 | 6.24E-01 | 0.81 | 0.58-1.14 | 7.10E-01 |
| Concentration of small HDL particles | 0.81 | 0.58-1.14 | 6.25E-01 | 0.81 | 0.58-1.14 | 7.10E-01 |
| Concentration of small LDL particles | 1.2 | 0.9-1.59 | 6.25E-01 | 1.18 | 0.89-1.56 | 7.17E-01 |
| Free cholesterol in medium VLDL | 1.21 | 0.9-1.63 | 6.25E-01 | 1.19 | 0.89-1.61 | 7.17E-01 |
| Triglycerides to total lipids ratio in medium HDL | 1.25 | 0.9-1.76 | 6.06E-01 | 1.24 | 0.86-1.79 | 7.17E-01 |
| Phospholipids in small HDL | 0.8 | 0.54-1.18 | 6.47E-01 | 0.79 | 0.53-1.17 | 7.17E-01 |
| Total lipids in medium VLDL | 1.21 | 0.9-1.64 | 6.25E-01 | 1.2 | 0.88-1.62 | 7.17E-01 |
| Concentration of large VLDL particles | 1.23 | 0.9-1.69 | 6.06E-01 | 1.22 | 0.88-1.68 | 7.17E-01 |
| Total lipids in small HDL | 0.81 | 0.56-1.18 | 6.51E-01 | 0.8 | 0.54-1.18 | 7.30E-01 |
| Concentration of VLDL particles | 1.2 | 0.89-1.62 | 6.25E-01 | 1.19 | 0.88-1.61 | 7.41E-01 |
| Cholesterol to total lipids ratio in large HDL | 0.84 | 0.65-1.09 | 6.06E-01 | 0.85 | 0.64-1.13 | 7.46E-01 |
| Cholesteryl esters in small VLDL | 1.19 | 0.89-1.6 | 6.25E-01 | 1.18 | 0.87-1.6 | 7.52E-01 |
| Phosphoglycerides | 0.83 | 0.6-1.17 | 6.58E-01 | 0.83 | 0.59-1.17 | 7.52E-01 |
| Triglycerides in VLDL | 1.21 | 0.89-1.65 | 6.25E-01 | 1.19 | 0.87-1.64 | 7.52E-01 |
| Triglycerides in large VLDL | 1.21 | 0.88-1.66 | 6.40E-01 | 1.19 | 0.87-1.63 | 7.52E-01 |
| Free cholesterol to total lipids ratio in medium LDL | 0.83 | 0.62-1.12 | 6.25E-01 | 0.84 | 0.62-1.15 | 7.52E-01 |
| Glutamine | 1.15 | 0.86-1.53 | 6.96E-01 | 1.17 | 0.87-1.57 | 7.52E-01 |
| Ratio of monounsaturated fatty acids to total fatty acids | 1.19 | 0.87-1.62 | 6.47E-01 | 1.18 | 0.87-1.6 | 7.56E-01 |
| Cholesterol in small VLDL | 1.18 | 0.88-1.57 | 6.47E-01 | 1.16 | 0.87-1.56 | 7.68E-01 |
| Concentration of small VLDL particles | 1.18 | 0.88-1.58 | 6.47E-01 | 1.17 | 0.86-1.59 | 7.68E-01 |
| Total lipids in small VLDL | 1.18 | 0.88-1.59 | 6.47E-01 | 1.17 | 0.86-1.59 | 7.68E-01 |
| Phospholipids in very large HDL | 0.86 | 0.68-1.09 | 6.06E-01 | 0.88 | 0.69-1.13 | 7.68E-01 |
| Triglycerides to total lipids ratio in very large HDL | 1.21 | 0.88-1.67 | 6.25E-01 | 1.19 | 0.84-1.66 | 7.68E-01 |
| Cholesterol in medium VLDL | 1.16 | 0.87-1.55 | 6.76E-01 | 1.15 | 0.86-1.54 | 7.68E-01 |
| Cholesteryl esters in small LDL | 1.16 | 0.87-1.55 | 6.76E-01 | 1.15 | 0.86-1.53 | 7.68E-01 |
| Triglycerides in medium VLDL | 1.19 | 0.87-1.63 | 6.55E-01 | 1.17 | 0.85-1.61 | 7.68E-01 |
| Total triglycerides | 1.19 | 0.88-1.62 | 6.47E-01 | 1.17 | 0.85-1.61 | 7.68E-01 |
| Phospholipids to total lipids ratio in medium LDL | 0.85 | 0.63-1.15 | 6.64E-01 | 0.87 | 0.64-1.16 | 7.68E-01 |
| Triglycerides in very large VLDL | 1.22 | 0.86-1.74 | 6.47E-01 | 1.2 | 0.84-1.72 | 7.68E-01 |
| Free cholesterol in chylomicrons and extremely large VLDL | 1.25 | 0.84-1.85 | 6.47E-01 | 1.22 | 0.81-1.83 | 7.68E-01 |
| Cholesteryl esters to total lipids ratio in IDL | 0.86 | 0.67-1.11 | 6.47E-01 | 0.88 | 0.68-1.15 | 7.68E-01 |
| Cholesterol in chylomicrons and extremely large VLDL | 1.22 | 0.85-1.77 | 6.51E-01 | 1.2 | 0.82-1.75 | 7.68E-01 |
| Cholesteryl esters to total lipids ratio in large LDL | 1.16 | 0.88-1.54 | 6.64E-01 | 1.15 | 0.88-1.52 | 7.68E-01 |
| Free cholesterol to total lipids ratio in small HDL | 0.87 | 0.66-1.13 | 6.58E-01 | 0.87 | 0.66-1.15 | 7.68E-01 |
| Concentration of LDL particles | 1.15 | 0.87-1.52 | 6.92E-01 | 1.14 | 0.86-1.51 | 7.81E-01 |
| Phosphatidylcholines | 0.86 | 0.62-1.19 | 6.98E-01 | 0.86 | 0.61-1.2 | 7.81E-01 |
| Phospholipids in small VLDL | 1.16 | 0.87-1.54 | 6.76E-01 | 1.14 | 0.85-1.53 | 7.82E-01 |
| Triglycerides in small HDL | 1.2 | 0.85-1.71 | 6.64E-01 | 1.18 | 0.82-1.71 | 7.82E-01 |
| Cholesterol to total lipids ratio in IDL | 0.86 | 0.67-1.12 | 6.47E-01 | 0.88 | 0.67-1.16 | 7.82E-01 |
| Free cholesterol to total lipids ratio in large LDL | 0.85 | 0.63-1.14 | 6.51E-01 | 0.86 | 0.63-1.19 | 7.82E-01 |
| Cholesteryl esters in chylomicrons and extremely large VLDL | 1.21 | 0.84-1.73 | 6.64E-01 | 1.18 | 0.82-1.7 | 7.82E-01 |
| Cholesteryl esters to total lipids ratio in very large HDL | 0.88 | 0.67-1.17 | 7.32E-01 | 0.89 | 0.68-1.16 | 7.91E-01 |
| Triglycerides to total lipids ratio in large VLDL | 0.89 | 0.69-1.16 | 7.38E-01 | 0.9 | 0.7-1.15 | 7.96E-01 |
| Free cholesterol in small VLDL | 1.15 | 0.86-1.52 | 6.96E-01 | 1.13 | 0.85-1.51 | 8.04E-01 |
| Concentration of medium LDL particles | 1.14 | 0.85-1.53 | 7.11E-01 | 1.13 | 0.85-1.5 | 8.07E-01 |
| Apolipoprotein B | 1.14 | 0.86-1.51 | 7.11E-01 | 1.13 | 0.85-1.5 | 8.07E-01 |
| Concentration of chylomicrons and extremely large VLDL particles | 1.21 | 0.82-1.78 | 6.96E-01 | 1.18 | 0.79-1.76 | 8.07E-01 |
| Free cholesterol in very large HDL | 0.84 | 0.59-1.19 | 6.76E-01 | 0.86 | 0.59-1.24 | 8.16E-01 |
| Triglycerides to total lipids ratio in large HDL | 1.17 | 0.86-1.6 | 6.76E-01 | 1.15 | 0.82-1.61 | 8.21E-01 |
| Triglycerides in small LDL | 1.15 | 0.85-1.56 | 6.98E-01 | 1.13 | 0.83-1.54 | 8.21E-01 |
| Total lipids in chylomicrons and extremely large VLDL | 1.19 | 0.83-1.72 | 6.96E-01 | 1.16 | 0.8-1.7 | 8.21E-01 |
| Cholesteryl esters in medium LDL | 1.13 | 0.84-1.53 | 7.45E-01 | 1.12 | 0.84-1.51 | 8.29E-01 |
| Cholesteryl esters to total lipids ratio in medium VLDL | 0.87 | 0.65-1.17 | 7.00E-01 | 0.89 | 0.65-1.2 | 8.29E-01 |
| Concentration of large LDL particles | 1.12 | 0.85-1.48 | 7.65E-01 | 1.11 | 0.84-1.47 | 8.34E-01 |
| Phospholipids in chylomicrons and extremely large VLDL | 1.22 | 0.79-1.87 | 7.11E-01 | 1.19 | 0.76-1.84 | 8.35E-01 |
| Cholesterol in small LDL | 1.12 | 0.84-1.49 | 7.65E-01 | 1.11 | 0.84-1.47 | 8.37E-01 |
| Total phospholipids in lipoprotein particles | 0.88 | 0.64-1.23 | 7.86E-01 | 0.88 | 0.63-1.23 | 8.37E-01 |
| Phospholipids to total lipids ratio in very small VLDL | 1.12 | 0.88-1.43 | 6.96E-01 | 1.1 | 0.85-1.42 | 8.37E-01 |
| Triglycerides in small VLDL | 1.15 | 0.84-1.59 | 7.30E-01 | 1.13 | 0.81-1.6 | 8.43E-01 |
| Phospholipids to total lipids ratio in small HDL | 0.9 | 0.67-1.22 | 8.10E-01 | 0.9 | 0.68-1.2 | 8.47E-01 |
| Total lipids in small LDL | 1.12 | 0.84-1.48 | 7.86E-01 | 1.1 | 0.84-1.46 | 8.47E-01 |
| Phospholipids to total lipids ratio in small VLDL | 0.86 | 0.6-1.22 | 7.42E-01 | 0.88 | 0.61-1.27 | 8.47E-01 |
| Triglycerides in chylomicrons and extremely large VLDL | 1.18 | 0.78-1.79 | 7.65E-01 | 1.16 | 0.77-1.73 | 8.47E-01 |
| Remnant cholesterol (non-HDL | 1.12 | 0.83-1.51 | 7.86E-01 | 1.11 | 0.82-1.5 | 8.53E-01 |
| Cholesterol to total lipids ratio in small HDL | 0.88 | 0.63-1.22 | 7.65E-01 | 0.89 | 0.65-1.23 | 8.53E-01 |
| Free cholesterol to total lipids ratio in very large VLDL | 1.09 | 0.79-1.51 | 8.79E-01 | 1.12 | 0.8-1.55 | 8.83E-01 |
| Cholesterol in medium LDL | 1.11 | 0.83-1.48 | 8.05E-01 | 1.1 | 0.83-1.46 | 8.87E-01 |
| Cholesterol to total lipids ratio in medium VLDL | 0.88 | 0.64-1.22 | 7.86E-01 | 0.9 | 0.65-1.25 | 8.87E-01 |
| Total esterified cholesterol | 0.9 | 0.67-1.21 | 8.05E-01 | 0.91 | 0.67-1.23 | 8.87E-01 |
| Ratio of polyunsaturated fatty acids to monounsaturated fatty acids | 0.89 | 0.66-1.21 | 7.93E-01 | 0.91 | 0.67-1.23 | 8.87E-01 |
| Cholesteryl esters in IDL | 0.9 | 0.68-1.2 | 7.93E-01 | 0.91 | 0.68-1.22 | 8.87E-01 |
| Total lipids in medium LDL | 1.1 | 0.82-1.46 | 8.35E-01 | 1.09 | 0.82-1.44 | 8.99E-01 |
| Free cholesterol to total lipids ratio in small VLDL | 0.88 | 0.62-1.24 | 7.86E-01 | 0.9 | 0.62-1.29 | 8.99E-01 |
| Monounsaturated fatty acids | 1.12 | 0.8-1.55 | 8.10E-01 | 1.1 | 0.8-1.52 | 8.99E-01 |
| Triglycerides in large HDL | 0.92 | 0.68-1.24 | 8.60E-01 | 0.91 | 0.67-1.24 | 8.99E-01 |
| Cholesteryl esters to total lipids ratio in very large VLDL | 0.88 | 0.62-1.26 | 8.05E-01 | 0.9 | 0.63-1.28 | 8.99E-01 |
| Cholesterol in IDL | 0.9 | 0.67-1.21 | 8.05E-01 | 0.91 | 0.68-1.23 | 8.99E-01 |
| Cholesterol to total lipids ratio in small LDL | 1.06 | 0.8-1.4 | 8.79E-01 | 1.09 | 0.82-1.44 | 9.00E-01 |
| Triglycerides to total lipids ratio in small LDL | 1.13 | 0.81-1.58 | 7.93E-01 | 1.1 | 0.78-1.55 | 9.01E-01 |
| Total cholesterol minus HDL-C | 1.08 | 0.81-1.44 | 8.79E-01 | 1.07 | 0.81-1.43 | 9.36E-01 |
| Leucine | 0.98 | 0.7-1.36 | 9.80E-01 | 0.91 | 0.64-1.31 | 9.36E-01 |
| Linoleic acid | 1.09 | 0.8-1.48 | 8.79E-01 | 1.08 | 0.8-1.47 | 9.36E-01 |
| Average diameter for LDL particles | 0.91 | 0.68-1.24 | 8.50E-01 | 0.93 | 0.69-1.24 | 9.36E-01 |
| Cholesteryl esters to total lipids ratio in very small VLDL | 0.9 | 0.65-1.24 | 8.10E-01 | 0.92 | 0.65-1.29 | 9.36E-01 |
| Cholesteryl esters to total lipids ratio in chylomicrons and extremely large VLDL | 0.89 | 0.61-1.29 | 8.37E-01 | 0.91 | 0.62-1.33 | 9.36E-01 |
| Cholesteryl esters to total lipids ratio in small HDL | 0.91 | 0.67-1.24 | 8.50E-01 | 0.93 | 0.68-1.26 | 9.36E-01 |
| Free cholesterol in IDL | 0.91 | 0.66-1.24 | 8.47E-01 | 0.92 | 0.67-1.27 | 9.36E-01 |
| Total lipids in IDL | 0.92 | 0.69-1.24 | 8.74E-01 | 0.93 | 0.69-1.25 | 9.36E-01 |
| Triglycerides to total lipids ratio in chylomicrons and extremely large VLDL | 1.11 | 0.8-1.54 | 8.46E-01 | 1.09 | 0.79-1.51 | 9.36E-01 |
| Omega-3 fatty acids | 1.08 | 0.8-1.44 | 8.79E-01 | 1.06 | 0.79-1.43 | 9.50E-01 |
| Isoleucine | 0.99 | 0.72-1.37 | 9.97E-01 | 0.93 | 0.67-1.3 | 9.50E-01 |
| Phospholipids in small LDL | 1.06 | 0.81-1.4 | 8.79E-01 | 1.05 | 0.81-1.38 | 9.50E-01 |
| Phospholipids to total lipids ratio in very large HDL | 0.93 | 0.76-1.13 | 7.86E-01 | 0.95 | 0.78-1.17 | 9.50E-01 |
| Total concentration of branched-chain amino acids (leucine + isoleucine + valine) | 1 | 0.73-1.37 | 9.97E-01 | 0.93 | 0.66-1.31 | 9.50E-01 |
| Cholesteryl esters in medium VLDL | 1.06 | 0.79-1.43 | 8.79E-01 | 1.07 | 0.79-1.44 | 9.50E-01 |
| Phospholipids in medium LDL | 1.07 | 0.81-1.41 | 8.79E-01 | 1.06 | 0.81-1.39 | 9.50E-01 |
| Triglycerides in very small VLDL | 1.09 | 0.8-1.48 | 8.74E-01 | 1.07 | 0.77-1.48 | 9.50E-01 |
| Cholesteryl esters in LDL | 1.06 | 0.8-1.4 | 8.79E-01 | 1.06 | 0.8-1.39 | 9.50E-01 |
| Polyunsaturated fatty acids | 1.07 | 0.78-1.47 | 8.79E-01 | 1.06 | 0.78-1.44 | 9.50E-01 |
| Cholesterol to total lipids ratio in very small VLDL | 0.9 | 0.65-1.25 | 8.35E-01 | 0.92 | 0.65-1.3 | 9.50E-01 |
| Ratio of docosahexaenoic acid to total fatty acids | 0.93 | 0.7-1.24 | 8.79E-01 | 0.94 | 0.7-1.26 | 9.50E-01 |
| Triglycerides to total lipids ratio in IDL | 1.11 | 0.81-1.52 | 8.35E-01 | 1.08 | 0.77-1.5 | 9.50E-01 |
| Phospholipids to total lipids ratio in large LDL | 0.94 | 0.71-1.24 | 8.79E-01 | 0.95 | 0.72-1.24 | 9.50E-01 |
| Total cholesterol | 0.93 | 0.69-1.25 | 8.79E-01 | 0.94 | 0.69-1.26 | 9.50E-01 |
| Ratio of omega-3 fatty acids to total fatty acids | 1.06 | 0.79-1.42 | 8.79E-01 | 1.05 | 0.78-1.41 | 9.50E-01 |
| Ratio of omega-6 fatty acids to omega-3 fatty acids | 0.94 | 0.7-1.25 | 8.79E-01 | 0.95 | 0.71-1.28 | 9.50E-01 |
| Concentration of very small VLDL particles | 1.07 | 0.8-1.44 | 8.79E-01 | 1.06 | 0.78-1.43 | 9.50E-01 |
| Ratio of linoleic acid to total fatty acids | 1.02 | 0.76-1.37 | 9.77E-01 | 1.05 | 0.79-1.41 | 9.50E-01 |
| Omega-6 fatty acids | 1.06 | 0.77-1.47 | 8.86E-01 | 1.06 | 0.77-1.46 | 9.50E-01 |
| Phospholipids to total lipids ratio in chylomicrons and extremely large VLDL | 1.09 | 0.75-1.59 | 8.79E-01 | 1.07 | 0.73-1.57 | 9.50E-01 |
| Phenylalanine | 0.99 | 0.72-1.36 | 9.92E-01 | 0.94 | 0.66-1.33 | 9.50E-01 |
| Cholesterol to total lipids ratio in chylomicrons and extremely large VLDL | 0.91 | 0.64-1.3 | 8.79E-01 | 0.94 | 0.66-1.34 | 9.50E-01 |
| Acetate | 0.93 | 0.66-1.31 | 8.79E-01 | 0.94 | 0.67-1.32 | 9.50E-01 |
| Free cholesterol to total lipids ratio in medium VLDL | 0.92 | 0.64-1.3 | 8.79E-01 | 0.93 | 0.66-1.33 | 9.50E-01 |
| Histidine | 1.08 | 0.81-1.44 | 8.79E-01 | 1.05 | 0.79-1.4 | 9.51E-01 |
| Total lipids in very small VLDL | 1.07 | 0.79-1.44 | 8.79E-01 | 1.05 | 0.77-1.43 | 9.52E-01 |
| Valine | 1.02 | 0.74-1.39 | 9.84E-01 | 0.95 | 0.67-1.33 | 9.52E-01 |
| Free cholesterol in very small VLDL | 1.07 | 0.79-1.45 | 8.79E-01 | 1.05 | 0.77-1.44 | 9.52E-01 |
| Phospholipids in very small VLDL | 1.07 | 0.79-1.43 | 8.79E-01 | 1.05 | 0.77-1.43 | 9.52E-01 |
| Total lipids in LDL | 1.04 | 0.78-1.37 | 9.31E-01 | 1.03 | 0.78-1.36 | 9.52E-01 |
| LDL cholesterol | 1.04 | 0.79-1.37 | 9.31E-01 | 1.04 | 0.79-1.37 | 9.52E-01 |
| Triglycerides in medium LDL | 1.07 | 0.79-1.45 | 8.79E-01 | 1.05 | 0.77-1.43 | 9.52E-01 |
| Triglycerides in very large HDL | 1.05 | 0.78-1.42 | 8.95E-01 | 1.04 | 0.77-1.4 | 9.52E-01 |
| Triglycerides to total lipids ratio in very large VLDL | 0.96 | 0.67-1.37 | 9.45E-01 | 0.95 | 0.66-1.35 | 9.52E-01 |
| Docosahexaenoic acid | 0.97 | 0.73-1.29 | 9.43E-01 | 0.97 | 0.72-1.29 | 9.52E-01 |
| Free cholesterol to total lipids ratio in IDL | 0.95 | 0.7-1.28 | 8.95E-01 | 0.96 | 0.71-1.3 | 9.52E-01 |
| Cholesteryl esters to total lipids ratio in large VLDL | 0.94 | 0.67-1.32 | 8.95E-01 | 0.96 | 0.69-1.34 | 9.52E-01 |
| Triglycerides to total lipids ratio in very small VLDL | 1.08 | 0.74-1.57 | 8.79E-01 | 1.05 | 0.71-1.56 | 9.52E-01 |
| Free cholesterol in large LDL | 0.95 | 0.71-1.26 | 8.94E-01 | 0.96 | 0.72-1.28 | 9.52E-01 |
| Phospholipids in IDL | 0.96 | 0.72-1.29 | 9.31E-01 | 0.97 | 0.72-1.3 | 9.52E-01 |
| Total fatty acids | 1.07 | 0.76-1.5 | 8.86E-01 | 1.05 | 0.76-1.46 | 9.52E-01 |
| Triglycerides in medium HDL | 1.07 | 0.74-1.53 | 8.95E-01 | 1.05 | 0.73-1.51 | 9.52E-01 |
| Cholesterol to total lipids ratio in large LDL | 1.02 | 0.76-1.36 | 9.80E-01 | 1.04 | 0.78-1.38 | 9.52E-01 |
| Free cholesterol to total lipids ratio in large HDL | 0.94 | 0.72-1.24 | 8.79E-01 | 0.96 | 0.73-1.28 | 9.52E-01 |
| Cholesterol to total lipids ratio in very large VLDL | 0.93 | 0.65-1.32 | 8.79E-01 | 0.95 | 0.67-1.35 | 9.52E-01 |
| Triglycerides to total lipids ratio in medium VLDL | 1.07 | 0.74-1.55 | 8.94E-01 | 1.05 | 0.72-1.52 | 9.52E-01 |
| Cholesterol to total lipids ratio in small VLDL | 0.94 | 0.66-1.34 | 8.95E-01 | 0.96 | 0.68-1.36 | 9.52E-01 |
| Glycine | 0.95 | 0.69-1.31 | 8.95E-01 | 0.96 | 0.69-1.35 | 9.52E-01 |
| Cholesterol to total lipids ratio in very large HDL | 1.04 | 0.84-1.29 | 8.86E-01 | 1.02 | 0.83-1.26 | 9.63E-01 |
| Triglycerides in HDL | 1.06 | 0.74-1.52 | 9.06E-01 | 1.04 | 0.72-1.5 | 9.63E-01 |
| Clinical LDL cholesterol | 1.02 | 0.77-1.36 | 9.65E-01 | 1.03 | 0.77-1.36 | 9.66E-01 |
| Triglycerides in LDL | 1.05 | 0.78-1.42 | 8.95E-01 | 1.03 | 0.75-1.4 | 9.66E-01 |
| Free cholesterol in LDL | 0.97 | 0.73-1.28 | 9.39E-01 | 0.98 | 0.74-1.29 | 9.66E-01 |
| Cholesterol to total lipids ratio in large VLDL | 1.01 | 0.73-1.4 | 9.97E-01 | 1.02 | 0.75-1.39 | 9.72E-01 |
| Phospholipids in LDL | 1.02 | 0.77-1.35 | 9.77E-01 | 1.02 | 0.77-1.34 | 9.72E-01 |
| Cholesterol in very small VLDL | 1.03 | 0.75-1.41 | 9.65E-01 | 1.02 | 0.74-1.41 | 9.72E-01 |
| Phospholipids to total lipids ratio in large HDL | 1.05 | 0.78-1.42 | 8.95E-01 | 1.02 | 0.75-1.39 | 9.72E-01 |
| Total lipids in lipoprotein particles | 1.03 | 0.75-1.43 | 9.58E-01 | 1.02 | 0.75-1.4 | 9.72E-01 |
| Phospholipids to total lipids ratio in medium VLDL | 0.96 | 0.68-1.35 | 9.33E-01 | 0.97 | 0.69-1.37 | 9.72E-01 |
| Saturated fatty acids | 1 | 0.71-1.41 | 9.97E-01 | 0.98 | 0.7-1.37 | 9.72E-01 |
| Triglycerides to total lipids ratio in medium LDL | 1 | 0.73-1.38 | 9.97E-01 | 0.98 | 0.71-1.35 | 9.72E-01 |
| Triglycerides in IDL | 1.04 | 0.78-1.39 | 9.31E-01 | 1.02 | 0.75-1.39 | 9.82E-01 |
| Triglycerides to total lipids ratio in small VLDL | 1.04 | 0.71-1.53 | 9.52E-01 | 1.02 | 0.69-1.51 | 9.82E-01 |
| Cholesteryl esters in large LDL | 1.01 | 0.77-1.33 | 9.85E-01 | 1.01 | 0.77-1.33 | 9.84E-01 |
| Phospholipids in large LDL | 0.99 | 0.74-1.31 | 9.84E-01 | 0.99 | 0.74-1.31 | 9.89E-01 |
| Ratio of omega-6 fatty acids to total fatty acids | 0.96 | 0.71-1.31 | 9.31E-01 | 0.99 | 0.73-1.34 | 9.89E-01 |
| Free cholesterol to total lipids ratio in very small VLDL | 1.02 | 0.75-1.39 | 9.80E-01 | 1.01 | 0.75-1.37 | 9.89E-01 |
| Free cholesterol to total lipids ratio in chylomicrons and extremely large VLDL | 0.98 | 0.73-1.3 | 9.70E-01 | 0.99 | 0.74-1.33 | 9.91E-01 |
| Free cholesterol in small LDL | 0.99 | 0.75-1.3 | 9.97E-01 | 0.99 | 0.76-1.3 | 9.91E-01 |
| Ratio of polyunsaturated fatty acids to total fatty acids | 0.97 | 0.71-1.33 | 9.52E-01 | 0.99 | 0.73-1.35 | 9.91E-01 |
| Total free cholesterol | 1.01 | 0.75-1.36 | 9.97E-01 | 1.01 | 0.75-1.36 | 9.91E-01 |
| Concentration of IDL particles | 1 | 0.74-1.33 | 9.97E-01 | 0.99 | 0.74-1.33 | 9.91E-01 |
| Triglycerides in large LDL | 1.01 | 0.75-1.36 | 9.85E-01 | 0.99 | 0.73-1.35 | 9.91E-01 |
| Cholesteryl esters to total lipids ratio in small VLDL | 1 | 0.7-1.41 | 9.97E-01 | 1.01 | 0.72-1.41 | 9.91E-01 |
| Triglycerides to total lipids ratio in large LDL | 1.02 | 0.75-1.39 | 9.80E-01 | 0.99 | 0.73-1.36 | 9.91E-01 |
| Free cholesterol in medium LDL | 1 | 0.76-1.32 | 9.97E-01 | 1 | 0.76-1.32 | 9.93E-01 |
| Total lipids in large LDL | 1 | 0.76-1.31 | 9.97E-01 | 1 | 0.76-1.31 | 9.93E-01 |
| Cholesteryl esters in very small VLDL | 1 | 0.73-1.39 | 9.97E-01 | 1 | 0.72-1.39 | 9.93E-01 |
| Degree of unsaturation | 0.99 | 0.74-1.31 | 9.84E-01 | 1 | 0.76-1.32 | 9.97E-01 |
| Cholesterol in large LDL | 1 | 0.76-1.31 | 9.97E-01 | 1 | 0.76-1.32 | 9.97E-01 |

Note: the table shows associations from the competing risk Cox proportional hazards regression analysis where plasms metabolomics level from the Nightingale platform was main exposure and incident prostate cancer as the main outcome in ERGO-4 cohort. Model 1 : adjusted for age, cohort, and lipid lowering medication. Model 2: adjusted for model1, smoking status, BMI, alcohol and education. The table is ranked based on the *FDR* values in model 2, using bold text indicates metabolites are surpassing *FDR* adjusted P value treshold (*P*<0.05). ERGO-4 refer to the fourth and fifth visits from the baseline cohort. Abbreviations: IDL, intermediate-density lipoprotein; LDL, low-density lipoprotein; HDL, high-density lipoprotein; VLDL, very-low-density lipoprotein; BMI, body mass index; HR, hazard ratio; CI, confidence interval; FDR, false discovery rate.

**Supplementary Table 22. Serum metabolites associated with incident prostate cancer from the Metabolon platform.**

| **Metabolite** | **Model 1** | | | **Model 2** | | |
| --- | --- | --- | --- | --- | --- | --- |
|  | **HR** | **95% CI** | ***FDR* value** | **HR** | **95% CI** | ***FDR* value** |
| X - 25419 | 2.07 | 1.42-3 | **3.54E-02** | 2.25 | 1.57-3.22 | **1.00E-02** |
| ceramide (d16:1/24:1, d18:1/22:1)* | 2.47 | 1.56-3.92 | **3.54E-02** | 2.41 | 1.58-3.67 | **2.21E-02** |
| stearate (18:0) | 0.41 | 0.25-0.68 | 7.05E-02 | 0.40 | 0.25-0.64 | **4.86E-02** |
| oleate/vaccenate (18:1) | 0.52 | 0.37-0.72 | **3.54E-02** | 0.53 | 0.38-0.74 | 5.94E-02 |
| X - 17335 | 0.47 | 0.32-0.69 | **3.54E-02** | 0.45 | 0.3-0.7 | 5.94E-02 |
| myristoleoylcarnitine (C14:1)* | 0.48 | 0.32-0.71 | 6.21E-02 | 0.50 | 0.33-0.75 | 8.94E-02 |
| linolenate [alpha or gamma; (18:3n3 or 6)] | 0.51 | 0.35-0.74 | 6.84E-02 | 0.50 | 0.33-0.74 | 8.94E-02 |
| linoleate (18:2n6) | 0.58 | 0.42-0.79 | 7.05E-02 | 0.57 | 0.41-0.78 | 8.94E-02 |
| X - 24531 | 1.64 | 1.24-2.18 | 7.08E-02 | 1.68 | 1.24-2.27 | 8.94E-02 |
| nonadecanoate (19:0) | 0.39 | 0.23-0.69 | 8.32E-02 | 0.37 | 0.2-0.66 | 8.94E-02 |
| pyruvate | 0.49 | 0.31-0.76 | 8.40E-02 | 0.48 | 0.31-0.74 | 8.94E-02 |
| dihomo-linoleate (20:2n6) | 0.46 | 0.29-0.74 | 8.32E-02 | 0.45 | 0.28-0.73 | 9.36E-02 |
| 10-nonadecenoate (19:1n9) | 0.40 | 0.23-0.7 | 8.32E-02 | 0.40 | 0.23-0.7 | 1.04E-01 |
| 10-undecenoate (11:1n1) | 0.51 | 0.34-0.76 | 8.32E-02 | 0.50 | 0.33-0.78 | 1.12E-01 |
| cystine | 0.64 | 0.49-0.83 | 8.32E-02 | 0.65 | 0.5-0.85 | 1.12E-01 |
| palmitate (16:0) | 0.55 | 0.38-0.79 | 8.32E-02 | 0.57 | 0.4-0.81 | 1.12E-01 |
| margarate (17:0) | 0.40 | 0.23-0.71 | 8.40E-02 | 0.37 | 0.2-0.69 | 1.12E-01 |
| 3-hydroxylaurate | 0.41 | 0.23-0.73 | 9.94E-02 | 0.38 | 0.21-0.69 | 1.12E-01 |
| alpha-ketobutyrate | 0.57 | 0.38-0.84 | 1.54E-01 | 0.51 | 0.33-0.78 | 1.12E-01 |
| N,N-dimethylalanine | 1.48 | 1.13-1.95 | 1.58E-01 | 1.60 | 1.19-2.14 | 1.12E-01 |
| myristoylcarnitine (C14) | 0.56 | 0.4-0.8 | 8.32E-02 | 0.58 | 0.4-0.82 | 1.14E-01 |
| 3-hydroxydecanoate | 0.35 | 0.17-0.69 | 9.94E-02 | 0.32 | 0.15-0.67 | 1.23E-01 |
| propionylglycine | 1.78 | 1.22-2.6 | 9.94E-02 | 1.75 | 1.21-2.53 | 1.33E-01 |
| glycosyl-N-(2-hydroxynervonoyl)-sphingosine (d18:1/24:1(2OH))* | 2.03 | 1.31-3.17 | 8.40E-02 | 2.05 | 1.27-3.31 | 1.47E-01 |
| 3-hydroxydodecanedioate* | 0.43 | 0.25-0.73 | 8.41E-02 | 0.38 | 0.2-0.72 | 1.47E-01 |
| phenylacetylglutamine | 2.01 | 1.23-3.3 | 1.58E-01 | 2.05 | 1.26-3.32 | 1.48E-01 |
| X - 14056 | 0.53 | 0.33-0.85 | 1.76E-01 | 0.48 | 0.29-0.79 | 1.48E-01 |
| 10-heptadecenoate (17:1n7) | 0.47 | 0.29-0.76 | 9.92E-02 | 0.48 | 0.29-0.79 | 1.63E-01 |
| alpha-ketoglutarate | 0.65 | 0.48-0.88 | 1.58E-01 | 0.65 | 0.49-0.87 | 1.63E-01 |
| inosine | 0.65 | 0.5-0.85 | 8.32E-02 | 0.66 | 0.5-0.88 | 1.67E-01 |
| X - 21353 | 0.58 | 0.4-0.85 | 1.58E-01 | 0.55 | 0.36-0.83 | 1.67E-01 |
| trans-urocanate | 0.53 | 0.36-0.78 | 8.32E-02 | 0.54 | 0.35-0.83 | 1.67E-01 |
| 2-hydroxy-4-(methylthio)butanoic acid | 0.61 | 0.4-0.95 | 2.71E-01 | 0.52 | 0.33-0.82 | 1.67E-01 |
| isovalerylglycine | 1.91 | 1.29-2.83 | 8.32E-02 | 1.98 | 1.22-3.2 | 1.74E-01 |
| laurylcarnitine (C12) | 0.50 | 0.32-0.78 | 9.94E-02 | 0.50 | 0.31-0.82 | 1.74E-01 |
| 3-methoxycatechol sulfate (2) | 1.63 | 1.19-2.24 | 9.94E-02 | 1.64 | 1.16-2.34 | 1.74E-01 |
| sphingomyelin (d18:0/20:0, d16:0/22:0)* | 1.99 | 1.2-3.3 | 1.76E-01 | 2.03 | 1.22-3.38 | 1.74E-01 |
| 3-hydroxyoctanoylcarnitine (2) | 0.52 | 0.32-0.83 | 1.76E-01 | 0.52 | 0.32-0.83 | 1.74E-01 |
| (16 or 17)-methylstearate (a19:0 or i19:0) | 0.43 | 0.22-0.82 | 1.84E-01 | 0.43 | 0.23-0.79 | 1.74E-01 |
| sphingomyelin (d18:0/18:0, d19:0/17:0)* | 1.65 | 1.1-2.45 | 2.10E-01 | 1.77 | 1.18-2.68 | 1.74E-01 |
| myo-inositol | 0.71 | 0.53-0.95 | 2.46E-01 | 0.66 | 0.49-0.89 | 1.74E-01 |
| ethyl alpha-glucopyranoside | 1.38 | 0.96-1.98 | 4.17E-01 | 1.80 | 1.17-2.77 | 1.81E-01 |
| sphingomyelin (d18:1/22:2, d18:2/22:1, d16:1/24:2)* | 1.83 | 1.2-2.8 | 1.58E-01 | 1.96 | 1.2-3.21 | 1.83E-01 |
| branched-chain, straight-chain, or cyclopropyl 12:1 fatty acid* | 0.60 | 0.4-0.92 | 2.35E-01 | 0.55 | 0.36-0.85 | 1.85E-01 |
| 3-hydroxyoctanoate | 0.42 | 0.23-0.78 | 1.58E-01 | 0.42 | 0.22-0.79 | 1.87E-01 |
| butyrylglycine | 2.24 | 1.24-4.02 | 1.76E-01 | 2.44 | 1.26-4.7 | 1.87E-01 |
| 1,2-dipalmitoyl-GPC (16:0/16:0) | 1.52 | 1.12-2.06 | 1.76E-01 | 1.58 | 1.13-2.22 | 1.87E-01 |
| 3-methyl catechol sulfate (2) | 1.51 | 1.11-2.04 | 1.78E-01 | 1.58 | 1.12-2.22 | 1.90E-01 |
| tetradecadienoate (14:2)* | 0.57 | 0.37-0.87 | 1.84E-01 | 0.58 | 0.38-0.87 | 1.90E-01 |
| sphingomyelin (d18:2/23:1)* | 1.84 | 1.19-2.83 | 1.68E-01 | 1.89 | 1.17-3.06 | 1.93E-01 |
| myristoyl dihydrosphingomyelin (d18:0/14:0)* | 1.55 | 1.1-2.18 | 2.01E-01 | 1.61 | 1.13-2.3 | 1.93E-01 |
| cysteine s-sulfate | 0.57 | 0.37-0.86 | 1.76E-01 | 0.57 | 0.37-0.87 | 1.97E-01 |
| eicosenoate (20:1) | 0.51 | 0.31-0.84 | 1.76E-01 | 0.51 | 0.3-0.85 | 2.11E-01 |
| 6-bromotryptophan | 1.61 | 1.13-2.3 | 1.81E-01 | 1.60 | 1.11-2.29 | 2.11E-01 |
| N-oleoyltaurine | 0.55 | 0.35-0.86 | 1.84E-01 | 0.53 | 0.33-0.86 | 2.11E-01 |
| X - 23583 | 0.54 | 0.34-0.86 | 1.84E-01 | 0.53 | 0.32-0.86 | 2.11E-01 |
| docosahexaenoylcarnitine (C22:6)* | 1.59 | 1.08-2.36 | 2.38E-01 | 1.68 | 1.12-2.52 | 2.32E-01 |
| 4-hydroxyphenylacetylglutamine | 1.49 | 1.1-2.01 | 1.84E-01 | 1.52 | 1.09-2.11 | 2.39E-01 |
| X - 26106 | 0.55 | 0.36-0.86 | 1.76E-01 | 0.54 | 0.34-0.88 | 2.59E-01 |
| laurate (12:0) | 0.50 | 0.28-0.89 | 2.25E-01 | 0.47 | 0.26-0.86 | 2.61E-01 |
| undecenoylcarnitine (C11:1) | 0.59 | 0.4-0.87 | 1.76E-01 | 0.58 | 0.37-0.9 | 2.62E-01 |
| palmitoleoylcarnitine (C16:1)* | 0.55 | 0.34-0.87 | 1.84E-01 | 0.55 | 0.34-0.89 | 2.62E-01 |
| dihomo-linolenate (20:3n3 or n6) | 0.59 | 0.37-0.93 | 2.46E-01 | 0.55 | 0.34-0.89 | 2.62E-01 |
| N-acetylputrescine | 1.50 | 1.06-2.14 | 2.46E-01 | 1.57 | 1.09-2.26 | 2.62E-01 |
| X - 25371 | 1.41 | 1.11-1.79 | 1.51E-01 | 1.36 | 1.05-1.75 | 2.62E-01 |
| hexadecanedioate (C16-DC) | 0.52 | 0.32-0.85 | 1.84E-01 | 0.54 | 0.33-0.9 | 2.62E-01 |
| hypoxanthine | 0.59 | 0.4-0.89 | 1.84E-01 | 0.60 | 0.39-0.92 | 2.62E-01 |
| 1-(1-enyl-stearoyl)-GPE (P-18:0)* | 1.82 | 1.14-2.9 | 1.89E-01 | 1.77 | 1.11-2.84 | 2.62E-01 |
| arachidate (20:0) | 0.48 | 0.27-0.85 | 1.89E-01 | 0.49 | 0.27-0.89 | 2.62E-01 |
| tetradecanedioate (C14-DC) | 0.49 | 0.27-0.87 | 2.10E-01 | 0.50 | 0.28-0.89 | 2.62E-01 |
| 3-hydroxydecanoylcarnitine | 0.57 | 0.37-0.9 | 2.18E-01 | 0.58 | 0.37-0.91 | 2.62E-01 |
| myristate (14:0) | 0.49 | 0.28-0.88 | 2.18E-01 | 0.50 | 0.28-0.89 | 2.62E-01 |
| dodecanedioate (C12-DC) | 0.55 | 0.33-0.91 | 2.38E-01 | 0.53 | 0.31-0.9 | 2.62E-01 |
| 2-hydroxysebacate | 0.61 | 0.4-0.92 | 2.38E-01 | 0.62 | 0.41-0.92 | 2.62E-01 |
| phytanate | 0.57 | 0.35-0.92 | 2.38E-01 | 0.50 | 0.29-0.88 | 2.62E-01 |
| pristanate | 0.66 | 0.46-0.95 | 2.46E-01 | 0.59 | 0.38-0.91 | 2.62E-01 |
| X - 26119 | 1.53 | 1.05-2.23 | 2.71E-01 | 1.60 | 1.08-2.37 | 2.62E-01 |
| gluconate | 0.66 | 0.45-0.97 | 2.84E-01 | 0.62 | 0.43-0.92 | 2.62E-01 |
| X - 15245 | 0.62 | 0.39-0.98 | 3.18E-01 | 0.60 | 0.39-0.92 | 2.62E-01 |
| 3-hydroxyisobutyrate | 0.64 | 0.41-1 | 3.36E-01 | 0.56 | 0.35-0.91 | 2.62E-01 |
| X - 24951 | 0.77 | 0.57-1.04 | 4.20E-01 | 0.67 | 0.48-0.93 | 2.62E-01 |
| X - 17685 | 1.69 | 1.08-2.64 | 2.38E-01 | 1.68 | 1.09-2.61 | 2.63E-01 |
| sphingomyelin (d18:2/21:0, d16:2/23:0)* | 1.89 | 1.18-3.04 | 1.81E-01 | 1.78 | 1.09-2.92 | 2.78E-01 |
| sphingomyelin (d18:2/14:0, d18:1/14:1)* | 1.74 | 1.14-2.67 | 1.84E-01 | 1.69 | 1.08-2.64 | 2.78E-01 |
| 3-hydroxyhexanoylcarnitine (1) | 0.59 | 0.39-0.89 | 1.89E-01 | 0.62 | 0.41-0.93 | 2.78E-01 |
| octadecenedioate (C18:1-DC) | 0.64 | 0.45-0.9 | 1.84E-01 | 0.66 | 0.46-0.94 | 2.79E-01 |
| cystathionine | 1.49 | 1.1-2.01 | 1.84E-01 | 1.46 | 1.05-2.02 | 2.79E-01 |
| alpha-CEHC sulfate | 1.72 | 1.13-2.62 | 1.89E-01 | 1.77 | 1.08-2.9 | 2.79E-01 |
| docosapentaenoate (n3 DPA; 22:5n3) | 0.51 | 0.28-0.9 | 2.38E-01 | 0.47 | 0.25-0.9 | 2.79E-01 |
| 3-hydroxyoctanoylcarnitine (1) | 0.59 | 0.37-0.93 | 2.46E-01 | 0.60 | 0.39-0.93 | 2.79E-01 |
| methyl-4-hydroxybenzoate sulfate | 0.69 | 0.47-1.02 | 3.57E-01 | 0.61 | 0.4-0.94 | 2.79E-01 |
| cholesterol | 1.66 | 1.09-2.52 | 2.21E-01 | 1.76 | 1.08-2.89 | 2.84E-01 |
| palmitoleate (16:1n7) | 0.55 | 0.34-0.87 | 1.84E-01 | 0.58 | 0.36-0.93 | 2.86E-01 |
| cysteine | 0.69 | 0.5-0.96 | 2.73E-01 | 0.67 | 0.47-0.95 | 2.87E-01 |
| (14 or 15)-methylpalmitate (a17:0 or i17:0) | 0.66 | 0.44-0.97 | 2.90E-01 | 0.63 | 0.42-0.94 | 2.87E-01 |
| suberoylcarnitine (C8-DC) | 0.63 | 0.44-0.91 | 2.01E-01 | 0.64 | 0.44-0.95 | 2.90E-01 |
| valine | 1.99 | 1.13-3.51 | 2.21E-01 | 1.83 | 1.08-3.13 | 2.90E-01 |
| oleoyl ethanolamide | 0.51 | 0.31-0.85 | 1.84E-01 | 0.54 | 0.31-0.93 | 2.92E-01 |
| 1-(1-enyl-oleoyl)-GPE (P-18:1)* | 1.56 | 1.06-2.31 | 2.52E-01 | 1.52 | 1.05-2.21 | 2.92E-01 |
| X - 21312 | 1.60 | 1.05-2.44 | 2.73E-01 | 1.59 | 1.05-2.4 | 2.92E-01 |
| eugenol sulfate | 0.69 | 0.47-1 | 3.43E-01 | 0.66 | 0.46-0.95 | 2.92E-01 |
| 3-methoxycatechol sulfate (1) | 1.59 | 1.04-2.43 | 2.74E-01 | 1.60 | 1.05-2.43 | 3.00E-01 |
| X - 15503 | 1.55 | 1.18-2.04 | 8.57E-02 | 1.52 | 1.04-2.22 | 3.07E-01 |
| caprylate (8:0) | 0.62 | 0.42-0.91 | 2.17E-01 | 0.64 | 0.43-0.96 | 3.07E-01 |
| cis-3,4-methyleneheptanoylcarnitine | 0.70 | 0.51-0.98 | 2.90E-01 | 0.65 | 0.44-0.96 | 3.07E-01 |
| anthranilate | 1.75 | 1.09-2.81 | 2.38E-01 | 1.70 | 1.05-2.74 | 3.07E-01 |
| adrenate (22:4n6) | 0.64 | 0.42-0.97 | 2.99E-01 | 0.63 | 0.41-0.96 | 3.07E-01 |
| nicotinamide | 1.54 | 1.08-2.2 | 2.21E-01 | 1.49 | 1.04-2.15 | 3.07E-01 |
| cis-4-decenoate (10:1n6)* | 0.67 | 0.48-0.95 | 2.46E-01 | 0.68 | 0.48-0.97 | 3.14E-01 |
| linoleoyl ethanolamide | 0.69 | 0.49-0.97 | 2.84E-01 | 0.67 | 0.46-0.97 | 3.19E-01 |
| N-methylhydroxyproline** | 1.86 | 1.12-3.09 | 2.21E-01 | 1.83 | 1.05-3.19 | 3.20E-01 |
| spermidine | 1.72 | 1.11-2.68 | 2.18E-01 | 1.64 | 1.03-2.61 | 3.26E-01 |
| 3-indoxyl sulfate | 1.78 | 1.11-2.84 | 2.21E-01 | 1.69 | 1.04-2.74 | 3.26E-01 |
| 2-hydroxyhippurate (salicylurate) | 0.60 | 0.39-0.93 | 2.38E-01 | 0.63 | 0.41-0.97 | 3.26E-01 |
| glucuronate | 1.31 | 1.03-1.66 | 2.72E-01 | 1.36 | 1.02-1.82 | 3.26E-01 |
| 2'-deoxyuridine | 0.74 | 0.54-1.01 | 3.55E-01 | 0.65 | 0.43-0.97 | 3.26E-01 |
| X - 15486 | 0.79 | 0.62-1.01 | 3.57E-01 | 0.73 | 0.55-0.98 | 3.26E-01 |
| isovalerylcarnitine (C5) | 0.67 | 0.42-1.07 | 4.47E-01 | 0.59 | 0.36-0.96 | 3.26E-01 |
| 3beta-hydroxy-5-cholestenoate | 0.59 | 0.38-0.92 | 2.35E-01 | 0.60 | 0.38-0.97 | 3.29E-01 |
| pentadecanoate (15:0) | 0.54 | 0.31-0.97 | 3.09E-01 | 0.54 | 0.31-0.96 | 3.35E-01 |
| glycosyl ceramide (d18:2/24:1, d18:1/24:2)* | 1.41 | 0.94-2.12 | 4.51E-01 | 1.54 | 1.02-2.31 | 3.43E-01 |
| gamma-glutamylglycine | 1.43 | 1-2.05 | 3.36E-01 | 1.66 | 1.03-2.68 | 3.45E-01 |
| myristoleate (14:1n5) | 0.55 | 0.33-0.92 | 2.46E-01 | 0.59 | 0.36-0.98 | 3.52E-01 |
| pregnenetriol disulfate* | 1.57 | 0.98-2.5 | 3.57E-01 | 1.66 | 1.02-2.69 | 3.52E-01 |
| glucose | 0.58 | 0.32-1.02 | 3.57E-01 | 0.53 | 0.29-0.97 | 3.52E-01 |
| 1-methylurate | 1.51 | 1.06-2.15 | 2.38E-01 | 1.47 | 1.02-2.14 | 3.53E-01 |
| vanillactate | 1.40 | 1.05-1.87 | 2.38E-01 | 1.44 | 1.01-2.03 | 3.53E-01 |
| sebacate (C10-DC) | 0.66 | 0.46-0.94 | 2.42E-01 | 0.68 | 0.47-0.99 | 3.53E-01 |
| dopamine 3-O-sulfate | 1.33 | 0.98-1.79 | 3.85E-01 | 1.36 | 1.01-1.84 | 3.53E-01 |
| X - 11795 | 1.52 | 0.94-2.45 | 4.20E-01 | 1.64 | 1.02-2.64 | 3.53E-01 |
| vanillic acid glycine | 1.48 | 1.08-2.03 | 2.03E-01 | 1.43 | 1.01-2.03 | 3.54E-01 |
| dodecenedioate (C12:1-DC)* | 0.59 | 0.37-0.96 | 2.84E-01 | 0.57 | 0.33-0.99 | 3.54E-01 |
| 1-methylnicotinamide | 1.44 | 1.02-2.04 | 3.09E-01 | 1.47 | 1.01-2.15 | 3.54E-01 |
| hexanoylglutamine | 0.50 | 0.26-0.97 | 3.18E-01 | 0.50 | 0.26-0.98 | 3.54E-01 |
| heptenedioate (C7:1-DC)* | 0.65 | 0.43-0.99 | 3.18E-01 | 0.63 | 0.4-0.99 | 3.54E-01 |
| 2-hydroxy-3-methylvalerate | 0.60 | 0.35-1.04 | 3.85E-01 | 0.55 | 0.3-0.98 | 3.54E-01 |
| hexanoylcarnitine (C6) | 0.59 | 0.36-0.96 | 2.84E-01 | 0.60 | 0.37-0.99 | 3.58E-01 |
| erythritol | 1.42 | 1.04-1.94 | 2.51E-01 | 1.38 | 1.01-1.9 | 3.61E-01 |
| 1-palmitoyl-GPE (16:0) | 1.62 | 1.05-2.5 | 2.73E-01 | 1.58 | 1.01-2.49 | 3.64E-01 |
| sphingomyelin (d17:2/16:0, d18:2/15:0)* | 1.90 | 1.09-3.29 | 2.46E-01 | 1.85 | 1.01-3.4 | 3.65E-01 |
| 4-hydroxyphenylacetoylcarnitine | 1.40 | 1.04-1.89 | 2.51E-01 | 1.40 | 1-1.94 | 3.65E-01 |
| (S)-3-hydroxybutyrylcarnitine | 0.56 | 0.32-0.99 | 3.21E-01 | 0.57 | 0.33-0.99 | 3.65E-01 |
| X - 26107 | 0.75 | 0.58-0.97 | 2.73E-01 | 0.77 | 0.59-1 | 3.66E-01 |
| salicylate | 0.56 | 0.33-0.96 | 2.90E-01 | 0.59 | 0.34-1 | 3.66E-01 |
| bilirubin degradation product, C16H18N2O5 (2)** | 0.68 | 0.45-1.04 | 3.91E-01 | 0.63 | 0.4-1 | 3.66E-01 |
| pregnenediol disulfate (C21H34O8S2)* | 1.53 | 0.94-2.47 | 4.20E-01 | 1.59 | 1-2.53 | 3.66E-01 |
| sphingomyelin (d18:2/24:1, d18:1/24:2)* | 1.47 | 0.92-2.35 | 4.67E-01 | 1.70 | 1-2.88 | 3.66E-01 |
| X - 24295 | 0.73 | 0.5-1.08 | 4.68E-01 | 0.69 | 0.48-1 | 3.66E-01 |
| 1-(1-enyl-palmitoyl)-GPE (P-16:0)* | 1.54 | 1.02-2.33 | 3.09E-01 | 1.49 | 1-2.22 | 3.67E-01 |
| hexadecadienoate (16:2n6) | 0.69 | 0.47-1.01 | 3.43E-01 | 0.70 | 0.49-1 | 3.67E-01 |
| X - 14939 | 0.81 | 0.62-1.04 | 4.47E-01 | 0.77 | 0.59-1 | 3.68E-01 |
| lactate | 0.67 | 0.42-1.07 | 4.42E-01 | 0.63 | 0.4-1 | 3.71E-01 |
| X - 17357 | 1.51 | 1.08-2.12 | 2.18E-01 | 1.51 | 0.99-2.3 | 3.75E-01 |
| sphingomyelin (d17:1/14:0, d16:1/15:0)* | 1.48 | 1.06-2.08 | 2.46E-01 | 1.51 | 0.99-2.31 | 3.75E-01 |
| X - 25790 | 0.73 | 0.55-0.97 | 2.71E-01 | 0.70 | 0.48-1.01 | 3.75E-01 |
| sphingomyelin (d18:1/14:0, d16:1/16:0)* | 1.50 | 1.04-2.17 | 2.73E-01 | 1.49 | 0.99-2.24 | 3.75E-01 |
| 6-hydroxyindole sulfate | 1.66 | 1.05-2.64 | 2.73E-01 | 1.58 | 0.99-2.53 | 3.75E-01 |
| N-acetylcitrulline | 1.59 | 1.04-2.42 | 2.74E-01 | 1.59 | 0.98-2.57 | 3.75E-01 |
| 1-(1-enyl-palmitoyl)-GPC (P-16:0)* | 1.76 | 1.03-3.01 | 3.02E-01 | 1.84 | 0.98-3.44 | 3.75E-01 |
| indolin-2-one | 1.52 | 1.02-2.26 | 3.09E-01 | 1.48 | 0.99-2.2 | 3.75E-01 |
| 3-hydroxysebacate | 0.56 | 0.31-0.99 | 3.24E-01 | 0.55 | 0.3-1.02 | 3.75E-01 |
| glycosyl-N-stearoyl-sphingosine (d18:1/18:0) | 1.58 | 0.99-2.53 | 3.43E-01 | 1.67 | 0.99-2.84 | 3.75E-01 |
| glucuronide of C10H18O2 (7)* | 1.55 | 0.99-2.43 | 3.43E-01 | 1.56 | 0.99-2.46 | 3.75E-01 |
| decanoylcarnitine (C10) | 0.64 | 0.41-1.01 | 3.55E-01 | 0.62 | 0.38-1.01 | 3.75E-01 |
| 2-aminophenol sulfate | 1.34 | 0.97-1.85 | 3.90E-01 | 1.40 | 0.99-1.98 | 3.75E-01 |
| X - 19438 | 0.75 | 0.54-1.04 | 4.20E-01 | 0.72 | 0.52-1.01 | 3.75E-01 |
| N-acetyltaurine | 1.33 | 0.93-1.91 | 4.68E-01 | 1.40 | 0.99-1.97 | 3.75E-01 |
| N-formylanthranilic acid | 1.51 | 1.12-2.03 | 1.76E-01 | 1.42 | 0.98-2.06 | 3.89E-01 |
| X - 23665 | 0.55 | 0.31-0.95 | 2.84E-01 | 0.50 | 0.24-1.04 | 3.89E-01 |
| 4-hydroxyphenylacetate | 1.40 | 1.02-1.91 | 2.90E-01 | 1.44 | 0.98-2.12 | 3.89E-01 |
| X - 11849 | 1.49 | 1.02-2.16 | 3.02E-01 | 1.48 | 0.98-2.26 | 3.89E-01 |
| guaiacol sulfate | 1.43 | 1.02-2.02 | 3.09E-01 | 1.44 | 0.98-2.1 | 3.89E-01 |
| 5-dodecenoate (12:1n7) | 0.54 | 0.3-0.98 | 3.18E-01 | 0.58 | 0.32-1.03 | 3.89E-01 |
| N-methylproline | 1.67 | 1.02-2.75 | 3.18E-01 | 1.61 | 0.97-2.67 | 3.89E-01 |
| sphingomyelin (d18:1/20:2, d18:2/20:1, d16:1/22:2)* | 1.58 | 1.01-2.47 | 3.21E-01 | 1.59 | 0.98-2.58 | 3.89E-01 |
| X - 18922 | 0.71 | 0.51-0.99 | 3.21E-01 | 0.70 | 0.48-1.02 | 3.89E-01 |
| glycerophosphorylcholine (GPC) | 1.66 | 1-2.75 | 3.39E-01 | 1.69 | 0.98-2.94 | 3.89E-01 |
| 3-hydroxybutyrate (BHBA) | 0.50 | 0.24-1.02 | 3.53E-01 | 0.49 | 0.23-1.04 | 3.89E-01 |
| p-cresol glucuronide* | 1.59 | 0.97-2.6 | 3.66E-01 | 1.69 | 0.97-2.93 | 3.89E-01 |
| N-acetylaspartate (NAA) | 1.83 | 0.92-3.67 | 4.20E-01 | 1.93 | 0.96-3.87 | 3.89E-01 |
| palmitoyl dihydrosphingomyelin (d18:0/16:0)* | 1.56 | 0.91-2.66 | 4.67E-01 | 1.75 | 0.97-3.14 | 3.89E-01 |
| 2-pyrrolidinone | 1.84 | 0.97-3.51 | 3.66E-01 | 1.86 | 0.96-3.62 | 3.90E-01 |
| sphingomyelin (d18:2/24:2)* | 1.42 | 0.93-2.16 | 4.56E-01 | 1.51 | 0.97-2.34 | 3.91E-01 |
| 2-stearoyl-GPE (18:0)* | 0.71 | 0.49-1.02 | 3.69E-01 | 0.67 | 0.43-1.03 | 3.98E-01 |
| X - 21310 | 1.63 | 0.98-2.72 | 3.57E-01 | 1.59 | 0.97-2.61 | 4.01E-01 |
| 1-palmitoleoyl-GPC (16:1)* | 1.50 | 0.9-2.5 | 4.74E-01 | 1.63 | 0.96-2.75 | 4.01E-01 |
| docosadienoate (22:2n6) | 0.59 | 0.34-1.02 | 3.60E-01 | 0.61 | 0.35-1.04 | 4.03E-01 |
| tridecenedioate (C13:1-DC)* | 0.67 | 0.43-1.02 | 3.66E-01 | 0.64 | 0.4-1.04 | 4.03E-01 |
| oleoylcarnitine (C18:1) | 0.60 | 0.35-1.03 | 3.70E-01 | 0.59 | 0.33-1.05 | 4.09E-01 |
| tartronate (hydroxymalonate) | 2.01 | 0.99-4.06 | 3.43E-01 | 2.03 | 0.94-4.42 | 4.17E-01 |
| X - 25810 | 1.37 | 0.95-1.98 | 4.32E-01 | 1.36 | 0.97-1.91 | 4.20E-01 |
| X - 21733 | 2.03 | 1.04-3.96 | 3.02E-01 | 1.85 | 0.94-3.65 | 4.20E-01 |
| 1-palmitoyl-GPC (16:0) | 1.75 | 1.02-3.01 | 3.20E-01 | 1.77 | 0.94-3.31 | 4.20E-01 |
| X - 24556 | 1.44 | 1.01-2.06 | 3.21E-01 | 1.40 | 0.97-2.02 | 4.20E-01 |
| acetylcarnitine (C2) | 0.64 | 0.4-1.01 | 3.43E-01 | 0.63 | 0.38-1.05 | 4.20E-01 |
| 3-methylhistidine | 1.42 | 0.99-2.02 | 3.43E-01 | 1.42 | 0.96-2.11 | 4.20E-01 |
| suberate (C8-DC) | 0.72 | 0.51-1.03 | 3.87E-01 | 0.72 | 0.5-1.04 | 4.20E-01 |
| 2'-O-methylcytidine | 1.46 | 0.95-2.26 | 4.20E-01 | 1.47 | 0.96-2.25 | 4.20E-01 |
| ergothioneine | 1.54 | 0.94-2.53 | 4.22E-01 | 1.68 | 0.94-3.01 | 4.20E-01 |
| 1-stearoyl-GPG (18:0) | 0.73 | 0.5-1.05 | 4.38E-01 | 0.71 | 0.49-1.04 | 4.20E-01 |
| bilirubin degradation product, C16H18N2O5 (4)** | 0.70 | 0.45-1.07 | 4.53E-01 | 0.65 | 0.4-1.05 | 4.20E-01 |
| X - 17654 | 0.76 | 0.55-1.07 | 4.68E-01 | 0.75 | 0.54-1.03 | 4.20E-01 |
| campesterol | 1.43 | 0.91-2.25 | 4.82E-01 | 1.65 | 0.94-2.9 | 4.20E-01 |
| X - 11979 | 0.74 | 0.5-1.09 | 4.87E-01 | 0.69 | 0.46-1.04 | 4.20E-01 |
| alanine | 0.76 | 0.52-1.1 | 5.13E-01 | 0.70 | 0.47-1.04 | 4.20E-01 |
| retinol (Vitamin A) | 1.40 | 1.01-1.94 | 3.18E-01 | 1.38 | 0.96-2 | 4.21E-01 |
| sphingomyelin (d18:1/19:0, d19:1/18:0)* | 1.59 | 1.01-2.49 | 3.21E-01 | 1.53 | 0.94-2.46 | 4.21E-01 |
| hexadecenedioate (C16:1-DC)* | 0.58 | 0.34-0.99 | 3.23E-01 | 0.63 | 0.37-1.07 | 4.21E-01 |
| X - 16580 | 0.65 | 0.42-0.99 | 3.27E-01 | 0.66 | 0.41-1.06 | 4.21E-01 |
| octadecanedioylcarnitine (C18-DC)* | 0.70 | 0.49-1.01 | 3.57E-01 | 0.69 | 0.45-1.05 | 4.21E-01 |
| stearoyl sphingomyelin (d18:1/18:0) | 1.57 | 0.97-2.52 | 3.66E-01 | 1.52 | 0.94-2.45 | 4.21E-01 |
| palmitoyl ethanolamide | 0.58 | 0.32-1.05 | 3.89E-01 | 0.58 | 0.31-1.08 | 4.21E-01 |
| glycosyl ceramide (d18:1/20:0, d16:1/22:0)* | 1.42 | 0.96-2.12 | 4.20E-01 | 1.45 | 0.95-2.21 | 4.21E-01 |
| linolenoylcarnitine (C18:3)* | 0.67 | 0.43-1.05 | 4.20E-01 | 0.66 | 0.41-1.06 | 4.21E-01 |
| lanthionine | 1.35 | 0.95-1.93 | 4.51E-01 | 1.43 | 0.95-2.16 | 4.21E-01 |
| EDTA | 1.30 | 0.94-1.78 | 4.67E-01 | 1.34 | 0.97-1.87 | 4.21E-01 |
| X - 23587 | 1.36 | 0.93-1.97 | 4.67E-01 | 1.40 | 0.96-2.05 | 4.21E-01 |
| carboxyethyl-GABA | 1.68 | 0.88-3.19 | 4.68E-01 | 1.72 | 0.93-3.18 | 4.21E-01 |
| thioproline | 0.65 | 0.38-1.1 | 4.68E-01 | 0.62 | 0.36-1.07 | 4.21E-01 |
| bilirubin degradation product, C16H18N2O5 (1)** | 0.73 | 0.5-1.07 | 4.68E-01 | 0.69 | 0.46-1.05 | 4.21E-01 |
| isoleucylglycine | 1.36 | 0.93-1.99 | 4.70E-01 | 1.37 | 0.96-1.94 | 4.21E-01 |
| X - 18935 | 1.46 | 0.91-2.33 | 4.70E-01 | 1.48 | 0.95-2.32 | 4.21E-01 |
| gamma-glutamyltryptophan | 0.75 | 0.51-1.09 | 5.08E-01 | 0.71 | 0.49-1.05 | 4.21E-01 |
| dopamine 4-sulfate | 1.45 | 0.97-2.17 | 3.92E-01 | 1.44 | 0.95-2.19 | 4.22E-01 |
| oxalate (ethanedioate) | 2.04 | 0.97-4.26 | 3.57E-01 | 2.13 | 0.89-5.08 | 4.27E-01 |
| X - 12830 | 0.66 | 0.41-1.07 | 4.34E-01 | 0.66 | 0.4-1.07 | 4.27E-01 |
| uridine | 0.78 | 0.57-1.08 | 4.97E-01 | 0.75 | 0.55-1.04 | 4.27E-01 |
| sphingomyelin (d18:1/24:1, d18:2/24:0)* | 1.42 | 0.84-2.4 | 5.88E-01 | 1.63 | 0.93-2.88 | 4.27E-01 |
| phenylacetylglutamate | 1.41 | 0.98-2.01 | 3.58E-01 | 1.40 | 0.95-2.07 | 4.35E-01 |
| 1-palmitoyl-2-docosahexaenoyl-GPC (16:0/22:6) | 1.49 | 0.94-2.36 | 4.37E-01 | 1.49 | 0.94-2.36 | 4.35E-01 |
| caprate (10:0) | 0.54 | 0.26-1.11 | 4.40E-01 | 0.55 | 0.28-1.1 | 4.35E-01 |
| 1-oleoyl-2-arachidonoyl-GPE (18:1/20:4)* | 0.77 | 0.56-1.06 | 4.67E-01 | 0.75 | 0.54-1.05 | 4.43E-01 |
| cis-3,4-methyleneheptanoate | 0.81 | 0.6-1.08 | 5.19E-01 | 0.76 | 0.55-1.05 | 4.51E-01 |
| octadecanedioate (C18-DC) | 0.65 | 0.4-1.05 | 3.97E-01 | 0.63 | 0.36-1.09 | 4.61E-01 |
| 2-aminobutyrate | 0.75 | 0.51-1.1 | 5.17E-01 | 0.73 | 0.5-1.06 | 4.61E-01 |
| X - 11880 | 0.79 | 0.56-1.13 | 5.99E-01 | 0.71 | 0.47-1.07 | 4.61E-01 |
| oleoyl-linoleoyl-glycerol (18:1/18:2) [2] | 0.78 | 0.51-1.2 | 6.51E-01 | 0.71 | 0.47-1.07 | 4.61E-01 |
| X - 12111 | 1.64 | 0.99-2.72 | 3.43E-01 | 1.57 | 0.91-2.7 | 4.66E-01 |
| X - 13729 | 1.39 | 0.93-2.08 | 4.67E-01 | 1.40 | 0.93-2.11 | 4.66E-01 |
| sphingomyelin (d18:1/25:0, d19:0/24:1, d20:1/23:0, d19:1/24:0)* | 1.57 | 0.94-2.6 | 4.20E-01 | 1.60 | 0.9-2.83 | 4.73E-01 |
| 1-oleoyl-2-docosahexaenoyl-GPC (18:1/22:6)* | 1.47 | 0.91-2.38 | 4.68E-01 | 1.50 | 0.92-2.44 | 4.73E-01 |
| X - 23780 | 1.50 | 0.9-2.48 | 4.70E-01 | 1.52 | 0.92-2.51 | 4.73E-01 |
| 3-aminoisobutyrate | 1.42 | 0.91-2.22 | 4.73E-01 | 1.44 | 0.93-2.25 | 4.73E-01 |
| N2,N5-diacetylornithine | 1.35 | 0.89-2.04 | 5.37E-01 | 1.44 | 0.93-2.24 | 4.73E-01 |
| mannitol/sorbitol | 1.33 | 0.97-1.83 | 3.92E-01 | 1.31 | 0.94-1.83 | 4.73E-01 |
| bilirubin degradation product, C17H20N2O5 (2)** | 0.75 | 0.51-1.09 | 4.90E-01 | 0.73 | 0.49-1.07 | 4.73E-01 |
| stachydrine | 1.83 | 0.92-3.66 | 4.20E-01 | 1.80 | 0.88-3.68 | 4.82E-01 |
| adipoylcarnitine (C6-DC) | 0.70 | 0.47-1.03 | 3.85E-01 | 0.69 | 0.44-1.09 | 4.84E-01 |
| X - 25343 | 0.71 | 0.46-1.08 | 4.67E-01 | 0.72 | 0.48-1.08 | 4.89E-01 |
| taurine | 1.34 | 0.91-1.98 | 5.17E-01 | 1.38 | 0.93-2.07 | 4.89E-01 |
| quinolinate | 1.40 | 0.97-2 | 3.85E-01 | 1.36 | 0.93-2 | 4.91E-01 |
| 4-methylhexanoylglutamine | 0.66 | 0.4-1.1 | 4.68E-01 | 0.64 | 0.37-1.11 | 4.91E-01 |
| X - 23659 | 1.29 | 0.91-1.83 | 5.19E-01 | 1.33 | 0.93-1.91 | 4.91E-01 |
| X - 17676 | 1.32 | 1-1.75 | 3.40E-01 | 1.28 | 0.94-1.73 | 4.93E-01 |
| glycerophosphoethanolamine | 1.65 | 0.94-2.92 | 4.20E-01 | 1.56 | 0.89-2.71 | 4.93E-01 |
| X - 18921 | 0.78 | 0.57-1.06 | 4.67E-01 | 0.76 | 0.55-1.07 | 4.93E-01 |
| (2 or 3)-decenoate (10:1n7 or n8) | 0.76 | 0.52-1.1 | 5.17E-01 | 0.74 | 0.51-1.08 | 4.93E-01 |
| palmitoyl-sphingosine-phosphoethanolamine (d18:1/16:0) | 1.23 | 0.88-1.73 | 6.24E-01 | 1.31 | 0.93-1.83 | 4.93E-01 |
| N,N-dimethyl-5-aminovalerate | 1.49 | 1-2.2 | 3.32E-01 | 1.46 | 0.91-2.34 | 4.94E-01 |
| X - 16397 | 0.72 | 0.51-1 | 3.40E-01 | 0.75 | 0.52-1.08 | 4.94E-01 |
| 2-hydroxydecanoate | 0.70 | 0.43-1.12 | 5.12E-01 | 0.68 | 0.42-1.1 | 4.94E-01 |
| 12,13-DiHOME | 0.79 | 0.56-1.12 | 5.79E-01 | 0.75 | 0.52-1.08 | 4.94E-01 |
| isobutyrylglycine | 1.50 | 0.95-2.38 | 4.20E-01 | 1.49 | 0.9-2.49 | 5.01E-01 |
| sphingomyelin (d18:1/17:0, d17:1/18:0, d19:1/16:0) | 1.50 | 0.91-2.48 | 4.68E-01 | 1.53 | 0.89-2.61 | 5.01E-01 |
| X - 12104 | 0.72 | 0.47-1.11 | 5.09E-01 | 0.72 | 0.47-1.1 | 5.01E-01 |
| bilirubin degradation product, C17H20N2O5 (1)** | 0.75 | 0.52-1.1 | 5.17E-01 | 0.74 | 0.5-1.09 | 5.01E-01 |
| succinate | 1.28 | 0.91-1.8 | 5.41E-01 | 1.34 | 0.92-1.94 | 5.01E-01 |
| bilirubin degradation product, C16H18N2O5 (3)** | 0.73 | 0.47-1.14 | 5.48E-01 | 0.68 | 0.42-1.11 | 5.01E-01 |
| 1-linoleoyl-GPG (18:2)* | 0.76 | 0.51-1.13 | 5.48E-01 | 0.73 | 0.49-1.09 | 5.01E-01 |
| X - 22776 | 1.38 | 0.84-2.27 | 5.99E-01 | 1.43 | 0.91-2.25 | 5.01E-01 |
| 3,7-dimethylurate | 1.27 | 0.96-1.69 | 4.34E-01 | 1.23 | 0.94-1.61 | 5.04E-01 |
| N-palmitoylglycine | 0.66 | 0.39-1.13 | 5.02E-01 | 0.63 | 0.34-1.14 | 5.04E-01 |
| X - 24953 | 0.78 | 0.56-1.09 | 5.17E-01 | 0.77 | 0.55-1.08 | 5.04E-01 |
| X - 12798 | 0.80 | 0.58-1.1 | 5.47E-01 | 0.79 | 0.59-1.07 | 5.04E-01 |
| glycine | 1.36 | 0.86-2.16 | 5.88E-01 | 1.46 | 0.9-2.36 | 5.04E-01 |
| 1-stearoyl-2-docosahexaenoyl-GPC (18:0/22:6) | 1.50 | 0.92-2.45 | 4.67E-01 | 1.47 | 0.89-2.44 | 5.09E-01 |
| sphingomyelin (d17:1/16:0, d18:1/15:0, d16:1/17:0)* | 1.44 | 0.92-2.26 | 4.68E-01 | 1.48 | 0.89-2.47 | 5.09E-01 |
| X - 12729 | 1.22 | 0.95-1.57 | 4.70E-01 | 1.21 | 0.95-1.55 | 5.09E-01 |
| docosatrienoate (22:3n6)* | 0.66 | 0.43-1.01 | 3.43E-01 | 0.70 | 0.44-1.11 | 5.11E-01 |
| 2-hydroxyfluorene sulfate | 0.63 | 0.44-0.91 | 2.03E-01 | 0.73 | 0.48-1.1 | 5.13E-01 |
| cysteinylglycine | 0.80 | 0.61-1.06 | 4.68E-01 | 0.81 | 0.62-1.07 | 5.13E-01 |
| 1-methyl-5-imidazoleacetate | 1.36 | 0.93-2.01 | 4.70E-01 | 1.37 | 0.91-2.08 | 5.13E-01 |
| X - 12839 | 0.68 | 0.41-1.11 | 4.73E-01 | 0.68 | 0.41-1.13 | 5.13E-01 |
| X - 25267 | 0.78 | 0.55-1.09 | 5.19E-01 | 0.76 | 0.52-1.09 | 5.13E-01 |
| 6-oxopiperidine-2-carboxylate | 1.39 | 1-1.94 | 3.40E-01 | 1.33 | 0.91-1.94 | 5.20E-01 |
| picolinoylglycine | 1.43 | 0.97-2.12 | 3.91E-01 | 1.43 | 0.89-2.3 | 5.20E-01 |
| ceramide (d18:1/14:0, d16:1/16:0)* | 1.38 | 0.97-1.96 | 3.92E-01 | 1.33 | 0.91-1.95 | 5.20E-01 |
| ascorbic acid 3-sulfate* | 1.39 | 0.94-2.04 | 4.51E-01 | 1.41 | 0.89-2.21 | 5.20E-01 |
| 3-(3-hydroxyphenyl)propionate sulfate | 1.38 | 0.93-2.07 | 4.68E-01 | 1.38 | 0.9-2.11 | 5.20E-01 |
| X - 18888 | 0.75 | 0.52-1.07 | 4.68E-01 | 0.75 | 0.51-1.1 | 5.20E-01 |
| X - 07765 | 1.36 | 0.92-2 | 4.73E-01 | 1.33 | 0.91-1.96 | 5.20E-01 |
| methyl glucopyranoside (alpha + beta) | 1.39 | 0.91-2.12 | 4.83E-01 | 1.37 | 0.9-2.11 | 5.20E-01 |
| pyridoxate | 1.26 | 0.92-1.71 | 5.17E-01 | 1.35 | 0.91-2.02 | 5.20E-01 |
| N,N,N-trimethyl-5-aminovalerate | 0.79 | 0.57-1.08 | 5.17E-01 | 0.78 | 0.56-1.09 | 5.20E-01 |
| stearoylcarnitine (C18) | 0.67 | 0.39-1.16 | 5.23E-01 | 0.62 | 0.33-1.17 | 5.20E-01 |
| 2,6-dihydroxybenzoic acid | 1.35 | 0.89-2.06 | 5.40E-01 | 1.42 | 0.89-2.27 | 5.20E-01 |
| phenylacetate | 1.41 | 0.87-2.31 | 5.45E-01 | 1.44 | 0.88-2.34 | 5.20E-01 |
| arachidonate (20:4n6) | 0.75 | 0.49-1.14 | 5.59E-01 | 0.70 | 0.43-1.12 | 5.20E-01 |
| X - 12216 | 1.34 | 0.87-2.06 | 5.79E-01 | 1.37 | 0.9-2.08 | 5.20E-01 |
| biliverdin | 0.79 | 0.55-1.14 | 5.99E-01 | 0.74 | 0.49-1.11 | 5.20E-01 |
| N-acetyl-isoputreanine | 1.27 | 0.87-1.85 | 6.00E-01 | 1.36 | 0.9-2.04 | 5.20E-01 |
| 1-(1-enyl-palmitoyl)-2-palmitoyl-GPC (P-16:0/16:0)* | 1.31 | 0.81-2.11 | 6.56E-01 | 1.49 | 0.88-2.51 | 5.20E-01 |
| fructose | 0.85 | 0.67-1.09 | 5.88E-01 | 0.83 | 0.64-1.07 | 5.20E-01 |
| urea | 1.37 | 0.96-1.94 | 4.20E-01 | 1.37 | 0.9-2.08 | 5.21E-01 |
| X - 24328 | 1.48 | 0.87-2.51 | 5.23E-01 | 1.46 | 0.88-2.45 | 5.21E-01 |
| sphingomyelin (d18:1/21:0, d17:1/22:0, d16:1/23:0)* | 1.64 | 0.93-2.88 | 4.20E-01 | 1.57 | 0.85-2.9 | 5.22E-01 |
| xanthine | 0.78 | 0.49-1.22 | 6.66E-01 | 0.72 | 0.46-1.13 | 5.22E-01 |
| glycosyl-N-palmitoyl-sphingosine (d18:1/16:0) | 1.27 | 0.82-1.99 | 6.78E-01 | 1.41 | 0.89-2.24 | 5.22E-01 |
| androstenediol (3beta,17beta) disulfate (1) | 1.29 | 0.8-2.07 | 6.81E-01 | 1.51 | 0.86-2.64 | 5.22E-01 |
| 3-hydroxyhexanoate | 0.71 | 0.49-1.04 | 4.19E-01 | 0.76 | 0.52-1.1 | 5.22E-01 |
| gulonate* | 1.37 | 1.01-1.86 | 3.09E-01 | 1.30 | 0.91-1.87 | 5.24E-01 |
| X - 21286 | 1.46 | 0.95-2.24 | 4.22E-01 | 1.35 | 0.9-2.04 | 5.24E-01 |
| X - 24757 | 1.44 | 0.92-2.26 | 4.68E-01 | 1.43 | 0.88-2.34 | 5.24E-01 |
| theophylline | 1.37 | 0.97-1.94 | 3.96E-01 | 1.34 | 0.9-2.01 | 5.26E-01 |
| 9,10-DiHOME | 0.73 | 0.47-1.14 | 5.48E-01 | 0.73 | 0.47-1.13 | 5.29E-01 |
| 4-chlorobenzoic acid | 1.25 | 0.9-1.75 | 5.79E-01 | 1.26 | 0.92-1.73 | 5.29E-01 |
| X - 16087 | 1.32 | 0.98-1.79 | 3.89E-01 | 1.28 | 0.91-1.8 | 5.34E-01 |
| behenoyl dihydrosphingomyelin (d18:0/22:0)* | 1.46 | 0.87-2.45 | 5.23E-01 | 1.49 | 0.86-2.59 | 5.34E-01 |
| 2-hydroxybutyrate/2-hydroxyisobutyrate | 0.71 | 0.39-1.31 | 6.66E-01 | 0.62 | 0.32-1.2 | 5.34E-01 |
| X - 11372 | 0.84 | 0.58-1.21 | 7.13E-01 | 0.75 | 0.5-1.12 | 5.34E-01 |
| ethyl beta-glucopyranoside | 1.47 | 0.9-2.39 | 4.73E-01 | 1.47 | 0.86-2.49 | 5.37E-01 |
| gamma-glutamylhistidine | 1.33 | 0.89-1.98 | 5.47E-01 | 1.37 | 0.88-2.11 | 5.38E-01 |
| alpha-hydroxyisocaproate | 0.81 | 0.54-1.22 | 7.01E-01 | 0.74 | 0.48-1.13 | 5.47E-01 |
| allantoin | 0.83 | 0.6-1.14 | 6.47E-01 | 0.79 | 0.57-1.1 | 5.50E-01 |
| glutamine conjugate of C6H10O2 (2)* | 0.68 | 0.41-1.14 | 5.17E-01 | 0.70 | 0.42-1.16 | 5.58E-01 |
| 1-ribosyl-imidazoleacetate* | 1.21 | 0.94-1.56 | 5.08E-01 | 1.24 | 0.91-1.67 | 5.59E-01 |
| 16-hydroxypalmitate | 0.72 | 0.49-1.07 | 4.67E-01 | 0.75 | 0.49-1.13 | 5.65E-01 |
| X - 12462 | 0.75 | 0.48-1.17 | 5.99E-01 | 0.74 | 0.48-1.14 | 5.65E-01 |
| threonate | 1.56 | 0.91-2.69 | 4.67E-01 | 1.49 | 0.84-2.67 | 5.71E-01 |
| citramalate | 1.28 | 0.93-1.75 | 4.83E-01 | 1.25 | 0.91-1.73 | 5.71E-01 |
| 5alpha-androstan-3beta,17beta-diol disulfate | 1.40 | 0.8-2.45 | 6.44E-01 | 1.56 | 0.82-2.94 | 5.71E-01 |
| palmitoyl sphingomyelin (d18:1/16:0) | 1.31 | 0.8-2.16 | 6.70E-01 | 1.47 | 0.84-2.56 | 5.71E-01 |
| X - 24334 | 0.74 | 0.45-1.21 | 6.25E-01 | 0.68 | 0.39-1.18 | 5.72E-01 |
| (R)-3-hydroxybutyrylcarnitine | 0.68 | 0.42-1.1 | 4.68E-01 | 0.71 | 0.43-1.17 | 5.74E-01 |
| urate | 1.40 | 0.87-2.25 | 5.37E-01 | 1.39 | 0.86-2.24 | 5.74E-01 |
| octadecadienedioate (C18:2-DC)* | 0.72 | 0.44-1.17 | 5.69E-01 | 0.71 | 0.43-1.17 | 5.74E-01 |
| indoleacetoylcarnitine* | 0.77 | 0.51-1.17 | 6.25E-01 | 0.75 | 0.49-1.14 | 5.74E-01 |
| cortolone glucuronide (1) | 1.38 | 0.94-2.04 | 4.56E-01 | 1.37 | 0.86-2.19 | 5.77E-01 |
| 4-guanidinobutanoate | 1.23 | 0.94-1.62 | 5.10E-01 | 1.22 | 0.91-1.63 | 5.77E-01 |
| 3-carboxy-4-methyl-5-propyl-2-furanpropanoate (CMPF) | 1.43 | 0.87-2.35 | 5.23E-01 | 1.43 | 0.85-2.41 | 5.77E-01 |
| 1H-indole-7-acetic acid | 1.59 | 0.84-3.01 | 5.23E-01 | 1.54 | 0.81-2.92 | 5.77E-01 |
| deoxycholate | 1.36 | 0.89-2.08 | 5.23E-01 | 1.33 | 0.87-2.01 | 5.77E-01 |
| X - 12906 | 1.20 | 0.88-1.64 | 6.48E-01 | 1.26 | 0.9-1.75 | 5.77E-01 |
| X - 24588 | 0.84 | 0.61-1.16 | 6.79E-01 | 0.78 | 0.54-1.12 | 5.77E-01 |
| bilirubin degradation product, C17H18N2O4 (2)** | 0.84 | 0.6-1.19 | 7.02E-01 | 0.79 | 0.56-1.12 | 5.77E-01 |
| glutamine conjugate of C7H12O2* | 0.70 | 0.42-1.16 | 5.48E-01 | 0.70 | 0.42-1.18 | 5.79E-01 |
| taurodeoxycholate | 0.80 | 0.56-1.14 | 6.11E-01 | 0.78 | 0.54-1.13 | 5.82E-01 |
| N-acetylglucosaminylasparagine | 1.28 | 0.93-1.76 | 5.09E-01 | 1.24 | 0.9-1.72 | 5.85E-01 |
| homostachydrine* | 0.70 | 0.45-1.08 | 4.67E-01 | 0.73 | 0.46-1.17 | 5.87E-01 |
| 4-hydroxyhippurate | 1.29 | 0.97-1.71 | 4.19E-01 | 1.24 | 0.9-1.7 | 5.89E-01 |
| X - 21470 | 1.38 | 0.79-2.42 | 6.51E-01 | 1.54 | 0.81-2.92 | 5.89E-01 |
| 4-methyl-2-oxopentanoate | 0.78 | 0.49-1.25 | 6.92E-01 | 0.71 | 0.43-1.19 | 5.91E-01 |
| X - 23641 | 0.78 | 0.5-1.22 | 6.70E-01 | 0.74 | 0.47-1.17 | 6.01E-01 |
| dodecadienoate (12:2)* | 0.82 | 0.56-1.19 | 6.81E-01 | 0.78 | 0.54-1.14 | 6.01E-01 |
| X - 12740 | 0.77 | 0.5-1.17 | 6.19E-01 | 0.75 | 0.49-1.16 | 6.05E-01 |
| ascorbic acid 2-sulfate | 1.41 | 0.88-2.25 | 5.23E-01 | 1.39 | 0.84-2.31 | 6.06E-01 |
| N1-Methyl-2-pyridone-5-carboxamide | 1.36 | 0.88-2.09 | 5.45E-01 | 1.38 | 0.84-2.25 | 6.06E-01 |
| X - 23997 | 1.84 | 0.77-4.42 | 5.49E-01 | 1.78 | 0.74-4.29 | 6.06E-01 |
| trans-2-hexenoylglycine | 0.73 | 0.46-1.16 | 5.79E-01 | 0.73 | 0.45-1.18 | 6.06E-01 |
| X - 21319 | 0.84 | 0.65-1.09 | 5.79E-01 | 0.83 | 0.62-1.11 | 6.06E-01 |
| histidine betaine (hercynine)* | 1.34 | 0.8-2.24 | 6.56E-01 | 1.42 | 0.83-2.43 | 6.06E-01 |
| X - 12126 | 1.29 | 0.8-2.1 | 6.81E-01 | 1.34 | 0.85-2.11 | 6.06E-01 |
| cyclo(leu-pro) | 0.89 | 0.6-1.33 | 8.48E-01 | 0.78 | 0.54-1.14 | 6.06E-01 |
| androstenediol (3alpha, 17alpha) monosulfate (2) | 0.73 | 0.45-1.18 | 5.94E-01 | 0.71 | 0.42-1.2 | 6.06E-01 |
| X - 21607 | 0.85 | 0.64-1.12 | 6.49E-01 | 0.83 | 0.62-1.11 | 6.06E-01 |
| 11-ketoetiocholanolone glucuronide | 1.33 | 0.89-2 | 5.47E-01 | 1.32 | 0.86-2.04 | 6.10E-01 |
| creatine | 1.33 | 0.83-2.14 | 6.25E-01 | 1.37 | 0.84-2.25 | 6.10E-01 |
| X - 24546 | 1.33 | 0.77-2.29 | 6.95E-01 | 1.49 | 0.8-2.74 | 6.10E-01 |
| X - 12680 | 0.86 | 0.66-1.14 | 6.81E-01 | 0.82 | 0.61-1.11 | 6.12E-01 |
| sulfate* | 1.25 | 0.9-1.72 | 5.59E-01 | 1.27 | 0.87-1.84 | 6.13E-01 |
| trimethylamine N-oxide | 1.30 | 0.87-1.93 | 5.99E-01 | 1.30 | 0.86-1.95 | 6.13E-01 |
| X - 25422 | 0.79 | 0.59-1.06 | 4.67E-01 | 0.82 | 0.6-1.12 | 6.13E-01 |
| sphingomyelin (d18:1/22:1, d18:2/22:0, d16:1/24:1)* | 1.38 | 0.87-2.19 | 5.45E-01 | 1.36 | 0.84-2.2 | 6.15E-01 |
| 2,2'-Methylenebis(6-tert-butyl-p-cresol) | 0.72 | 0.43-1.2 | 5.99E-01 | 0.73 | 0.44-1.2 | 6.17E-01 |
| 1-(1-enyl-stearoyl)-2-arachidonoyl-GPE (P-18:0/20:4)* | 1.38 | 0.82-2.33 | 6.29E-01 | 1.38 | 0.83-2.3 | 6.17E-01 |
| palmitoylcarnitine (C16) | 0.75 | 0.45-1.24 | 6.55E-01 | 0.71 | 0.41-1.22 | 6.17E-01 |
| X - 21834 | 1.24 | 0.9-1.71 | 5.88E-01 | 1.26 | 0.88-1.8 | 6.18E-01 |
| tetrahydrocortisone glucuronide (5) | 1.46 | 0.93-2.29 | 4.56E-01 | 1.36 | 0.83-2.2 | 6.23E-01 |
| 2,3-dihydroxy-5-methylthio-4-pentenoate (DMTPA)* | 1.25 | 0.93-1.68 | 5.13E-01 | 1.26 | 0.87-1.84 | 6.23E-01 |
| X - 14082 | 1.32 | 0.91-1.9 | 5.17E-01 | 1.30 | 0.86-1.97 | 6.23E-01 |
| S-adenosylhomocysteine (SAH) | 1.25 | 0.9-1.74 | 5.63E-01 | 1.28 | 0.87-1.88 | 6.23E-01 |
| beta-alanine | 0.75 | 0.46-1.21 | 6.32E-01 | 0.71 | 0.41-1.22 | 6.23E-01 |
| tryptophan betaine | 0.80 | 0.56-1.16 | 6.43E-01 | 0.77 | 0.5-1.17 | 6.25E-01 |
| imidazole lactate | 1.17 | 0.83-1.63 | 7.35E-01 | 1.24 | 0.88-1.76 | 6.25E-01 |
| X - 17351 | 1.26 | 0.94-1.7 | 4.83E-01 | 1.21 | 0.89-1.64 | 6.33E-01 |
| 1-palmitoleoyl-2-linolenoyl-GPC (16:1/18:3)* | 0.70 | 0.43-1.14 | 5.20E-01 | 0.73 | 0.45-1.21 | 6.33E-01 |
| X - 21441 | 1.22 | 0.83-1.8 | 7.01E-01 | 1.27 | 0.86-1.88 | 6.33E-01 |
| X - 24243 | 1.45 | 0.85-2.46 | 5.48E-01 | 1.42 | 0.8-2.53 | 6.44E-01 |
| ethylmalonate | 1.26 | 0.88-1.81 | 5.99E-01 | 1.24 | 0.87-1.76 | 6.44E-01 |
| glycochenodeoxycholate glucuronide (1) | 0.76 | 0.5-1.16 | 5.99E-01 | 0.75 | 0.47-1.2 | 6.44E-01 |
| p-cresol sulfate | 1.58 | 0.75-3.3 | 6.25E-01 | 1.58 | 0.74-3.38 | 6.44E-01 |
| O-sulfo-L-tyrosine | 1.27 | 0.85-1.89 | 6.43E-01 | 1.29 | 0.85-1.97 | 6.44E-01 |
| erythronate* | 0.87 | 0.66-1.15 | 7.01E-01 | 0.79 | 0.54-1.16 | 6.44E-01 |
| 5-acetylamino-6-formylamino-3-methyluracil | 1.28 | 0.77-2.13 | 7.14E-01 | 1.37 | 0.82-2.29 | 6.44E-01 |
| hydantoin-5-propionate | 0.87 | 0.63-1.21 | 7.58E-01 | 0.80 | 0.55-1.16 | 6.46E-01 |
| X - 12410 | 1.30 | 0.86-1.96 | 5.99E-01 | 1.29 | 0.84-1.97 | 6.62E-01 |
| 3-hydroxy-2-ethylpropionate | 0.76 | 0.47-1.22 | 6.51E-01 | 0.74 | 0.45-1.22 | 6.62E-01 |
| 2S,3R-dihydroxybutyrate | 0.75 | 0.42-1.34 | 7.07E-01 | 0.68 | 0.35-1.3 | 6.64E-01 |
| 1-stearoyl-GPC (18:0) | 1.48 | 0.87-2.5 | 5.17E-01 | 1.44 | 0.77-2.69 | 6.67E-01 |
| N-acetylglutamine | 0.77 | 0.52-1.15 | 5.95E-01 | 0.80 | 0.54-1.17 | 6.67E-01 |
| tyramine O-sulfate | 1.26 | 0.87-1.82 | 6.25E-01 | 1.24 | 0.86-1.78 | 6.67E-01 |
| malate | 0.80 | 0.53-1.19 | 6.56E-01 | 0.78 | 0.51-1.19 | 6.67E-01 |
| docosatrienoate (22:3n3) | 0.83 | 0.57-1.2 | 7.01E-01 | 0.80 | 0.54-1.17 | 6.67E-01 |
| 1-myristoylglycerol (14:0) | 0.81 | 0.54-1.22 | 7.01E-01 | 0.77 | 0.49-1.2 | 6.67E-01 |
| phenyllactate (PLA) | 0.78 | 0.44-1.37 | 7.42E-01 | 0.69 | 0.37-1.3 | 6.67E-01 |
| prolylglycine | 1.26 | 0.92-1.71 | 5.17E-01 | 1.22 | 0.86-1.74 | 6.67E-01 |
| X - 12306 | 1.21 | 0.94-1.56 | 5.17E-01 | 1.18 | 0.89-1.55 | 6.67E-01 |
| X - 23639 | 0.72 | 0.46-1.12 | 5.17E-01 | 0.74 | 0.44-1.25 | 6.67E-01 |
| gamma-glutamylvaline | 1.39 | 0.89-2.18 | 5.19E-01 | 1.35 | 0.81-2.27 | 6.67E-01 |
| octadecenedioylcarnitine (C18:1-DC)* | 0.78 | 0.55-1.1 | 5.23E-01 | 0.81 | 0.56-1.16 | 6.67E-01 |
| isoeugenol sulfate | 0.79 | 0.56-1.1 | 5.45E-01 | 0.82 | 0.57-1.16 | 6.67E-01 |
| tetradecadienedioate (C14:2-DC)* | 0.73 | 0.46-1.15 | 5.49E-01 | 0.76 | 0.48-1.21 | 6.67E-01 |
| beta-cryptoxanthin | 1.40 | 0.85-2.3 | 5.79E-01 | 1.40 | 0.78-2.48 | 6.67E-01 |
| N6,N6,N6-trimethyllysine | 1.26 | 0.89-1.79 | 5.79E-01 | 1.25 | 0.85-1.83 | 6.67E-01 |
| 5-hydroxyindole sulfate | 1.39 | 0.83-2.32 | 5.99E-01 | 1.35 | 0.8-2.28 | 6.67E-01 |
| X - 12007 | 1.18 | 0.91-1.54 | 5.99E-01 | 1.18 | 0.89-1.58 | 6.67E-01 |
| X - 24306 | 1.30 | 0.87-1.93 | 5.99E-01 | 1.27 | 0.85-1.9 | 6.67E-01 |
| X - 15728 | 0.79 | 0.54-1.15 | 6.24E-01 | 0.79 | 0.53-1.18 | 6.67E-01 |
| 5alpha-pregnan-3beta,20alpha-diol monosulfate (2) | 0.79 | 0.51-1.21 | 6.70E-01 | 0.79 | 0.52-1.19 | 6.67E-01 |
| androstenediol (3beta,17beta) disulfate (2) | 1.47 | 0.71-3.03 | 6.81E-01 | 1.51 | 0.74-3.09 | 6.67E-01 |
| 2,3-dihydroxy-2-methylbutyrate | 1.18 | 0.85-1.62 | 7.01E-01 | 1.21 | 0.86-1.7 | 6.67E-01 |
| N6-methyladenosine | 0.82 | 0.57-1.2 | 7.01E-01 | 0.81 | 0.56-1.17 | 6.67E-01 |
| 2-acetamidophenol sulfate | 1.24 | 0.81-1.89 | 7.06E-01 | 1.30 | 0.83-2.04 | 6.67E-01 |
| X - 13431 | 0.84 | 0.58-1.2 | 7.06E-01 | 0.80 | 0.55-1.17 | 6.67E-01 |
| glutamine_degradant* | 1.37 | 0.72-2.58 | 7.09E-01 | 1.41 | 0.78-2.54 | 6.67E-01 |
| 1-stearoyl-2-linoleoyl-GPE (18:0/18:2)* | 0.81 | 0.53-1.24 | 7.12E-01 | 0.77 | 0.49-1.21 | 6.67E-01 |
| bilirubin degradation product, C17H18N2O4 (3)** | 0.84 | 0.55-1.27 | 7.51E-01 | 0.78 | 0.51-1.2 | 6.67E-01 |
| oleoyl-linoleoyl-glycerol (18:1/18:2) [1] | 0.86 | 0.55-1.34 | 8.13E-01 | 0.79 | 0.52-1.19 | 6.67E-01 |
| S-methylcysteine | 0.76 | 0.47-1.22 | 6.48E-01 | 0.75 | 0.46-1.24 | 6.70E-01 |
| 1-myristoyl-2-palmitoyl-GPC (14:0/16:0) | 1.31 | 0.83-2.06 | 6.48E-01 | 1.28 | 0.83-1.98 | 6.72E-01 |
| X - 11299 | 0.83 | 0.58-1.2 | 7.06E-01 | 0.80 | 0.54-1.18 | 6.72E-01 |
| N1-methylinosine | 0.78 | 0.52-1.17 | 6.25E-01 | 0.77 | 0.49-1.22 | 6.74E-01 |
| N-oleoylserine | 0.77 | 0.51-1.18 | 6.34E-01 | 0.78 | 0.5-1.22 | 6.74E-01 |
| bilirubin (E,Z or Z,E)* | 0.83 | 0.55-1.26 | 7.42E-01 | 0.77 | 0.48-1.23 | 6.74E-01 |
| glycosyl-N-behenoyl-sphingadienine (d18:2/22:0)* | 1.24 | 0.84-1.85 | 6.70E-01 | 1.27 | 0.83-1.95 | 6.76E-01 |
| branched-chain, straight-chain, or cyclopropyl 10:1 fatty acid (1)* | 0.77 | 0.52-1.14 | 5.79E-01 | 0.80 | 0.53-1.2 | 6.79E-01 |
| 2-ketocaprylate | 0.78 | 0.48-1.27 | 7.01E-01 | 0.75 | 0.45-1.25 | 6.81E-01 |
| sphingomyelin (d18:1/20:0, d16:1/22:0)* | 1.26 | 0.9-1.77 | 5.69E-01 | 1.21 | 0.85-1.73 | 6.82E-01 |
| hexadecasphingosine (d16:1)* | 1.45 | 0.84-2.48 | 5.69E-01 | 1.35 | 0.78-2.35 | 6.82E-01 |
| X - 23890 | 1.28 | 0.87-1.87 | 5.99E-01 | 1.29 | 0.81-2.05 | 6.82E-01 |
| methylsuccinate | 1.38 | 0.83-2.3 | 6.11E-01 | 1.32 | 0.79-2.2 | 6.82E-01 |
| X - 11847 | 1.28 | 0.86-1.91 | 6.23E-01 | 1.27 | 0.83-1.94 | 6.82E-01 |
| pantoate | 1.47 | 0.79-2.75 | 6.25E-01 | 1.46 | 0.73-2.91 | 6.82E-01 |
| X - 12015 | 1.36 | 0.82-2.25 | 6.36E-01 | 1.33 | 0.79-2.26 | 6.82E-01 |
| 2-hydroxybehenate | 0.78 | 0.51-1.19 | 6.44E-01 | 0.78 | 0.5-1.22 | 6.82E-01 |
| X - 25520 | 1.22 | 0.87-1.72 | 6.44E-01 | 1.21 | 0.86-1.7 | 6.82E-01 |
| N4-acetylcytidine | 1.24 | 0.85-1.82 | 6.51E-01 | 1.22 | 0.85-1.75 | 6.82E-01 |
| indole-3-carboxylate | 1.26 | 0.84-1.89 | 6.51E-01 | 1.27 | 0.82-1.97 | 6.82E-01 |
| 1-myristoyl-2-arachidonoyl-GPC (14:0/20:4)* | 1.34 | 0.8-2.26 | 6.55E-01 | 1.32 | 0.8-2.2 | 6.82E-01 |
| 1-(1-enyl-palmitoyl)-2-arachidonoyl-GPE (P-16:0/20:4)* | 1.24 | 0.85-1.83 | 6.56E-01 | 1.23 | 0.84-1.8 | 6.82E-01 |
| N1-methyladenosine | 0.81 | 0.54-1.21 | 6.81E-01 | 0.80 | 0.54-1.19 | 6.82E-01 |
| sucrose | 0.82 | 0.57-1.19 | 6.81E-01 | 0.79 | 0.52-1.21 | 6.82E-01 |
| S-methylcysteine sulfoxide | 0.76 | 0.45-1.28 | 6.87E-01 | 0.75 | 0.45-1.26 | 6.82E-01 |
| behenoylcarnitine (C22)* | 0.82 | 0.55-1.22 | 7.01E-01 | 0.80 | 0.53-1.2 | 6.82E-01 |
| N-acetylglucosamine/N-acetylgalactosamine | 1.22 | 0.81-1.86 | 7.13E-01 | 1.29 | 0.81-2.04 | 6.82E-01 |
| isovalerate (i5:0) | 0.89 | 0.65-1.23 | 8.01E-01 | 0.86 | 0.65-1.14 | 6.82E-01 |
| X - 25948 | 0.91 | 0.68-1.21 | 8.16E-01 | 0.85 | 0.64-1.14 | 6.82E-01 |
| glycerate | 1.46 | 0.83-2.56 | 5.79E-01 | 1.37 | 0.77-2.43 | 6.84E-01 |
| X - 24456 | 1.21 | 0.82-1.78 | 7.13E-01 | 1.27 | 0.81-2 | 6.84E-01 |
| propionylcarnitine (C3) | 0.83 | 0.52-1.31 | 7.58E-01 | 0.78 | 0.49-1.24 | 6.84E-01 |
| X - 24475 | 1.39 | 0.85-2.28 | 5.79E-01 | 1.33 | 0.78-2.27 | 6.84E-01 |
| 3-methylglutarylcarnitine (2) | 1.30 | 0.86-1.99 | 6.11E-01 | 1.29 | 0.8-2.09 | 6.84E-01 |
| 13-HODE + 9-HODE | 0.71 | 0.39-1.29 | 6.51E-01 | 0.73 | 0.41-1.31 | 6.84E-01 |
| ursodeoxycholate | 1.41 | 0.76-2.6 | 6.65E-01 | 1.45 | 0.72-2.92 | 6.84E-01 |
| X - 13866 | 0.81 | 0.52-1.26 | 7.13E-01 | 0.80 | 0.52-1.22 | 6.84E-01 |
| alpha-hydroxyisovalerate | 0.76 | 0.42-1.36 | 7.13E-01 | 0.69 | 0.35-1.37 | 6.84E-01 |
| 5-hydroxylysine | 0.81 | 0.57-1.16 | 6.51E-01 | 0.82 | 0.56-1.2 | 6.85E-01 |
| 5-(galactosylhydroxy)-L-lysine | 1.33 | 0.81-2.19 | 6.56E-01 | 1.32 | 0.78-2.25 | 6.85E-01 |
| 2,4-di-tert-butylphenol | 0.80 | 0.51-1.27 | 7.13E-01 | 0.79 | 0.5-1.24 | 6.85E-01 |
| hydroxypalmitoyl sphingomyelin (d18:1/16:0(OH))** | 1.31 | 0.73-2.32 | 7.31E-01 | 1.40 | 0.75-2.63 | 6.85E-01 |
| 2-methoxyhydroquinone sulfate (1) | 1.21 | 0.83-1.78 | 7.01E-01 | 1.23 | 0.83-1.83 | 6.87E-01 |
| X - 12812 | 1.28 | 0.83-1.98 | 6.56E-01 | 1.26 | 0.81-1.96 | 6.88E-01 |
| 1-linoleoyl-2-arachidonoyl-GPC (18:2/20:4n6)* | 1.25 | 0.81-1.93 | 7.01E-01 | 1.32 | 0.78-2.22 | 6.88E-01 |
| margaroylcarnitine (C17)* | 0.83 | 0.57-1.23 | 7.26E-01 | 0.81 | 0.55-1.21 | 6.89E-01 |
| 4-methoxyphenol sulfate | 1.26 | 0.76-2.08 | 7.39E-01 | 1.28 | 0.8-2.02 | 6.89E-01 |
| X - 18913 | 0.82 | 0.52-1.28 | 7.39E-01 | 0.78 | 0.49-1.25 | 6.92E-01 |
| 5-methyluridine (ribothymidine) | 0.89 | 0.67-1.18 | 7.58E-01 | 0.85 | 0.63-1.16 | 6.92E-01 |
| androsterone sulfate | 1.31 | 0.76-2.24 | 7.06E-01 | 1.36 | 0.76-2.42 | 6.94E-01 |
| creatinine | 1.28 | 0.91-1.8 | 5.23E-01 | 1.23 | 0.83-1.82 | 6.96E-01 |
| 1-palmitoyl-2-linoleoyl-GPE (16:0/18:2) | 0.83 | 0.57-1.22 | 7.13E-01 | 0.81 | 0.53-1.22 | 6.96E-01 |
| 3-methoxytyramine sulfate | 1.16 | 0.83-1.62 | 7.42E-01 | 1.21 | 0.84-1.75 | 6.96E-01 |
| 5alpha-pregnan-3beta,20alpha-diol disulfate | 1.18 | 0.79-1.75 | 7.64E-01 | 1.23 | 0.82-1.84 | 6.96E-01 |
| bilirubin (Z,Z) | 0.84 | 0.54-1.3 | 7.71E-01 | 0.77 | 0.47-1.27 | 6.96E-01 |
| fructosyllysine | 0.87 | 0.59-1.27 | 7.84E-01 | 0.81 | 0.53-1.22 | 6.96E-01 |
| bilirubin degradation product, C17H18N2O4 (1)** | 0.86 | 0.57-1.29 | 7.84E-01 | 0.81 | 0.54-1.22 | 6.96E-01 |
| bilirubin (E,E)* | 1.24 | 0.87-1.75 | 6.31E-01 | 1.20 | 0.84-1.69 | 6.99E-01 |
| X - 11478 | 0.88 | 0.68-1.15 | 7.31E-01 | 0.86 | 0.64-1.15 | 6.99E-01 |
| X - 17653 | 0.88 | 0.65-1.19 | 7.50E-01 | 0.85 | 0.61-1.17 | 6.99E-01 |
| 1,7-dimethylurate | 1.32 | 0.86-2.01 | 5.99E-01 | 1.24 | 0.81-1.89 | 7.00E-01 |
| lithocholate | 1.35 | 0.83-2.21 | 6.25E-01 | 1.31 | 0.77-2.25 | 7.00E-01 |
| sphingomyelin (d18:1/20:1, d18:2/20:0)* | 1.39 | 0.81-2.38 | 6.32E-01 | 1.30 | 0.78-2.16 | 7.00E-01 |
| indolepropionate | 1.27 | 0.85-1.89 | 6.36E-01 | 1.23 | 0.82-1.84 | 7.00E-01 |
| dihydrocaffeate sulfate (2) | 1.29 | 0.82-2.02 | 6.56E-01 | 1.26 | 0.8-1.98 | 7.00E-01 |
| 1-arachidonoyl-GPC (20:4n6)* | 1.28 | 0.79-2.05 | 7.01E-01 | 1.32 | 0.76-2.28 | 7.00E-01 |
| 1-oleoyl-GPC (18:1) | 1.22 | 0.79-1.89 | 7.35E-01 | 1.30 | 0.77-2.2 | 7.00E-01 |
| X - 22834 | 0.85 | 0.58-1.23 | 7.44E-01 | 0.83 | 0.58-1.19 | 7.00E-01 |
| X - 11444 | 0.95 | 0.76-1.18 | 8.68E-01 | 0.89 | 0.71-1.12 | 7.00E-01 |
| gamma-glutamylglutamate | 0.92 | 0.62-1.35 | 8.80E-01 | 0.83 | 0.58-1.2 | 7.00E-01 |
| hydroquinone sulfate | 1.18 | 0.87-1.61 | 6.70E-01 | 1.18 | 0.85-1.62 | 7.01E-01 |
| pipecolate | 0.80 | 0.55-1.17 | 6.49E-01 | 0.82 | 0.56-1.21 | 7.01E-01 |
| 5alpha-androstan-3beta,17alpha-diol disulfate | 1.22 | 0.81-1.85 | 7.07E-01 | 1.24 | 0.81-1.89 | 7.01E-01 |
| N-stearoyltaurine | 0.82 | 0.54-1.25 | 7.27E-01 | 0.80 | 0.51-1.25 | 7.01E-01 |
| methylsuccinoylcarnitine | 1.18 | 0.85-1.65 | 7.01E-01 | 1.21 | 0.83-1.77 | 7.02E-01 |
| gamma-tocopherol/beta-tocopherol | 0.79 | 0.47-1.3 | 7.18E-01 | 0.78 | 0.47-1.29 | 7.04E-01 |
| X - 12117 | 0.80 | 0.48-1.32 | 7.42E-01 | 0.76 | 0.43-1.32 | 7.05E-01 |
| taurodeoxycholic acid 3-sulfate | 0.82 | 0.55-1.23 | 7.12E-01 | 0.81 | 0.53-1.24 | 7.06E-01 |
| glycolithocholate sulfate* | 0.86 | 0.64-1.17 | 7.13E-01 | 0.86 | 0.63-1.17 | 7.07E-01 |
| 1-stearoyl-2-arachidonoyl-GPE (18:0/20:4) | 0.79 | 0.47-1.34 | 7.42E-01 | 0.76 | 0.44-1.32 | 7.08E-01 |
| X - 13507 | 1.16 | 0.9-1.48 | 6.44E-01 | 1.14 | 0.88-1.47 | 7.09E-01 |
| X - 21471 | 1.17 | 0.8-1.7 | 7.63E-01 | 1.19 | 0.84-1.7 | 7.09E-01 |
| choline | 0.80 | 0.49-1.31 | 7.40E-01 | 0.77 | 0.46-1.3 | 7.09E-01 |
| succinoyltaurine | 0.83 | 0.53-1.33 | 7.72E-01 | 0.80 | 0.51-1.26 | 7.09E-01 |
| X - 16576 | 1.16 | 0.79-1.7 | 7.72E-01 | 1.20 | 0.83-1.74 | 7.09E-01 |
| X - 19141 | 1.31 | 0.81-2.1 | 6.56E-01 | 1.25 | 0.79-1.98 | 7.12E-01 |
| sphingomyelin (d18:2/23:0, d18:1/23:1, d17:1/24:1)* | 1.27 | 0.81-2 | 6.81E-01 | 1.26 | 0.78-2.04 | 7.12E-01 |
| homoarginine | 0.87 | 0.52-1.45 | 8.59E-01 | 0.77 | 0.45-1.32 | 7.13E-01 |
| carotene diol (3) | 1.35 | 0.74-2.46 | 7.07E-01 | 1.35 | 0.73-2.5 | 7.13E-01 |
| X - 23648 | 0.90 | 0.71-1.14 | 7.37E-01 | 0.87 | 0.66-1.15 | 7.15E-01 |
| gentisate | 1.15 | 0.79-1.67 | 7.86E-01 | 1.20 | 0.82-1.77 | 7.15E-01 |
| 1-linoleoylglycerol (18:2) | 0.84 | 0.52-1.33 | 7.74E-01 | 0.80 | 0.5-1.27 | 7.19E-01 |
| tetrahydrocortisol glucuronide | 1.18 | 0.86-1.64 | 6.95E-01 | 1.18 | 0.84-1.66 | 7.20E-01 |
| 2-hydroxyarachidate* | 0.85 | 0.62-1.16 | 7.01E-01 | 0.85 | 0.6-1.19 | 7.20E-01 |
| phenylpyruvate | 0.85 | 0.59-1.23 | 7.49E-01 | 0.84 | 0.59-1.2 | 7.20E-01 |
| ornithine | 0.85 | 0.59-1.23 | 7.44E-01 | 0.85 | 0.61-1.19 | 7.22E-01 |
| dimethyl sulfone | 1.16 | 0.92-1.47 | 5.99E-01 | 1.13 | 0.88-1.44 | 7.23E-01 |
| N-acetylproline | 1.22 | 0.86-1.71 | 6.51E-01 | 1.19 | 0.82-1.74 | 7.23E-01 |
| thyroxine | 1.21 | 0.87-1.68 | 6.55E-01 | 1.19 | 0.82-1.74 | 7.23E-01 |
| 3-hydroxyhippurate sulfate | 1.20 | 0.87-1.67 | 6.56E-01 | 1.18 | 0.83-1.69 | 7.23E-01 |
| 1-oleoyl-2-linoleoyl-GPE (18:1/18:2)* | 0.84 | 0.61-1.16 | 6.81E-01 | 0.85 | 0.61-1.2 | 7.23E-01 |
| octanoylcarnitine (C8) | 0.82 | 0.55-1.21 | 6.95E-01 | 0.83 | 0.56-1.23 | 7.23E-01 |
| X - 25519 | 0.78 | 0.48-1.26 | 6.95E-01 | 0.79 | 0.48-1.31 | 7.23E-01 |
| X - 23782 | 0.81 | 0.52-1.24 | 7.06E-01 | 0.81 | 0.52-1.27 | 7.23E-01 |
| nonanoylcarnitine (C9) | 0.80 | 0.5-1.27 | 7.13E-01 | 0.78 | 0.46-1.32 | 7.23E-01 |
| glycodeoxycholate 3-sulfate | 0.86 | 0.61-1.21 | 7.42E-01 | 0.85 | 0.59-1.21 | 7.23E-01 |
| lignoceroylcarnitine (C24)* | 0.79 | 0.47-1.33 | 7.42E-01 | 0.76 | 0.42-1.37 | 7.23E-01 |
| X - 21364 | 1.38 | 0.65-2.9 | 7.50E-01 | 1.44 | 0.66-3.11 | 7.23E-01 |
| linoleoylcarnitine (C18:2)* | 0.82 | 0.52-1.3 | 7.58E-01 | 0.79 | 0.48-1.3 | 7.23E-01 |
| GlcNAc sulfate conjugate of C21H34O2 steroid** | 1.20 | 0.75-1.92 | 7.74E-01 | 1.25 | 0.78-2.01 | 7.23E-01 |
| X - 23680 | 0.89 | 0.66-1.22 | 7.86E-01 | 0.85 | 0.59-1.21 | 7.23E-01 |
| X - 24462 | 0.86 | 0.58-1.3 | 7.96E-01 | 0.82 | 0.53-1.25 | 7.23E-01 |
| 3,5-dichloro-2,6-dihydroxybenzoic acid | 0.88 | 0.61-1.26 | 8.01E-01 | 0.83 | 0.55-1.23 | 7.23E-01 |
| 3-methyl-2-oxovalerate | 0.86 | 0.53-1.42 | 8.40E-01 | 0.78 | 0.46-1.32 | 7.23E-01 |
| X - 12844 | 1.23 | 0.86-1.76 | 6.56E-01 | 1.21 | 0.8-1.85 | 7.28E-01 |
| cys-gly, oxidized | 1.28 | 0.8-2.03 | 6.85E-01 | 1.23 | 0.78-1.95 | 7.28E-01 |
| X - 24307 | 1.23 | 0.82-1.83 | 7.01E-01 | 1.22 | 0.79-1.87 | 7.28E-01 |
| 1-palmitoyl-GPI (16:0) | 1.25 | 0.8-1.95 | 7.06E-01 | 1.22 | 0.79-1.89 | 7.28E-01 |
| X - 24411 | 1.37 | 0.72-2.6 | 7.06E-01 | 1.35 | 0.7-2.61 | 7.28E-01 |
| 4-hydroxyphenylpyruvate | 1.26 | 0.78-2.04 | 7.13E-01 | 1.24 | 0.77-2.01 | 7.28E-01 |
| X - 24947 | 1.22 | 0.79-1.88 | 7.35E-01 | 1.22 | 0.79-1.87 | 7.28E-01 |
| cysteine sulfinic acid | 1.16 | 0.74-1.83 | 8.16E-01 | 1.26 | 0.76-2.08 | 7.28E-01 |
| X - 11308 | 0.88 | 0.59-1.3 | 8.17E-01 | 0.82 | 0.53-1.27 | 7.28E-01 |
| 2-hydroxyoctanoate | 0.87 | 0.57-1.33 | 8.17E-01 | 0.81 | 0.52-1.27 | 7.28E-01 |
| X - 11787 | 0.92 | 0.65-1.29 | 8.65E-01 | 0.84 | 0.58-1.23 | 7.28E-01 |
| 1-(1-enyl-palmitoyl)-2-palmitoleoyl-GPC (P-16:0/16:1)* | 1.05 | 0.68-1.63 | 9.50E-01 | 1.23 | 0.78-1.92 | 7.28E-01 |
| cytosine | 1.24 | 0.82-1.87 | 7.01E-01 | 1.22 | 0.79-1.89 | 7.29E-01 |
| X - 23739 | 0.85 | 0.63-1.17 | 7.01E-01 | 0.85 | 0.59-1.22 | 7.29E-01 |
| palmitoylcholine | 1.30 | 0.73-2.29 | 7.35E-01 | 1.29 | 0.73-2.27 | 7.29E-01 |
| 5,6-dihydrothymine | 0.84 | 0.57-1.25 | 7.50E-01 | 0.84 | 0.57-1.24 | 7.29E-01 |
| X - 22162 | 1.19 | 0.78-1.8 | 7.62E-01 | 1.24 | 0.77-1.99 | 7.29E-01 |
| 11beta-hydroxyetiocholanolone glucuronide* | 1.20 | 0.74-1.95 | 7.76E-01 | 1.26 | 0.75-2.11 | 7.29E-01 |
| N-acetyltyrosine | 1.14 | 0.82-1.59 | 7.72E-01 | 1.17 | 0.82-1.67 | 7.30E-01 |
| X - 18886 | 0.86 | 0.58-1.27 | 7.72E-01 | 0.84 | 0.57-1.24 | 7.31E-01 |
| 2-piperidinone | 1.26 | 0.72-2.19 | 7.62E-01 | 1.34 | 0.69-2.6 | 7.33E-01 |
| glycoursodeoxycholic acid sulfate (1) | 1.18 | 0.71-1.98 | 8.17E-01 | 1.30 | 0.72-2.35 | 7.34E-01 |
| N2,N2-dimethylguanosine | 1.24 | 0.86-1.79 | 6.51E-01 | 1.19 | 0.8-1.78 | 7.36E-01 |
| 3-hydroxyphenylacetoylglutamine | 1.23 | 0.8-1.87 | 7.13E-01 | 1.23 | 0.77-1.96 | 7.36E-01 |
| gamma-glutamylglutamine | 1.25 | 0.83-1.89 | 6.81E-01 | 1.22 | 0.78-1.92 | 7.38E-01 |
| 3-phosphoglycerate | 1.18 | 0.83-1.68 | 7.27E-01 | 1.16 | 0.83-1.63 | 7.38E-01 |
| N-stearoyl-sphinganine (d18:0/18:0)* | 1.12 | 0.84-1.49 | 7.81E-01 | 1.13 | 0.86-1.48 | 7.38E-01 |
| citrate | 0.85 | 0.55-1.32 | 7.86E-01 | 0.82 | 0.52-1.29 | 7.38E-01 |
| 1,3,7-trimethylurate | 1.28 | 0.86-1.92 | 6.23E-01 | 1.20 | 0.79-1.84 | 7.40E-01 |
| tetrahydrocortisol sulfate (1) | 1.24 | 0.86-1.8 | 6.44E-01 | 1.17 | 0.82-1.66 | 7.40E-01 |
| gamma-glutamylleucine | 1.27 | 0.87-1.84 | 6.02E-01 | 1.17 | 0.81-1.69 | 7.41E-01 |
| X - 12283 | 1.19 | 0.89-1.59 | 6.31E-01 | 1.14 | 0.84-1.54 | 7.43E-01 |
| N-acetyl-1-methylhistidine* | 1.15 | 0.79-1.68 | 7.87E-01 | 1.19 | 0.79-1.8 | 7.43E-01 |
| androstenediol (3alpha, 17alpha) monosulfate (3) | 1.19 | 0.73-1.95 | 7.96E-01 | 1.25 | 0.75-2.1 | 7.43E-01 |
| catechol glucuronide | 1.23 | 0.85-1.78 | 6.70E-01 | 1.18 | 0.8-1.73 | 7.45E-01 |
| X - 25172 | 0.80 | 0.5-1.3 | 7.35E-01 | 0.81 | 0.51-1.31 | 7.45E-01 |
| 5alpha-pregnan-3beta,20beta-diol monosulfate (1) | 0.85 | 0.58-1.25 | 7.58E-01 | 0.83 | 0.55-1.27 | 7.45E-01 |
| N-acetylthreonine | 0.83 | 0.51-1.34 | 7.72E-01 | 0.81 | 0.5-1.32 | 7.49E-01 |
| oxindolylalanine | 0.85 | 0.53-1.35 | 7.96E-01 | 0.82 | 0.51-1.31 | 7.49E-01 |
| paraxanthine | 1.27 | 0.84-1.92 | 6.55E-01 | 1.20 | 0.79-1.82 | 7.50E-01 |
| 1-lignoceroyl-GPC (24:0) | 1.16 | 0.79-1.7 | 7.72E-01 | 1.21 | 0.78-1.88 | 7.50E-01 |
| 1-arachidonylglycerol (20:4) | 0.82 | 0.5-1.36 | 7.72E-01 | 0.78 | 0.44-1.39 | 7.51E-01 |
| 5-hydroxyhexanoate | 1.16 | 0.78-1.73 | 7.77E-01 | 1.19 | 0.79-1.78 | 7.51E-01 |
| catechol sulfate | 1.21 | 0.85-1.71 | 6.81E-01 | 1.17 | 0.81-1.67 | 7.54E-01 |
| kynurenine | 1.28 | 0.85-1.92 | 6.44E-01 | 1.21 | 0.77-1.92 | 7.55E-01 |
| X - 21442 | 1.26 | 0.74-2.13 | 7.49E-01 | 1.25 | 0.73-2.15 | 7.55E-01 |
| 8-methoxykynurenate | 1.22 | 0.88-1.69 | 6.37E-01 | 1.13 | 0.84-1.53 | 7.57E-01 |
| proline | 1.27 | 0.87-1.85 | 6.10E-01 | 1.17 | 0.8-1.7 | 7.57E-01 |
| 1-palmitoyl-2-oleoyl-GPE (16:0/18:1) | 0.81 | 0.5-1.33 | 7.58E-01 | 0.81 | 0.49-1.33 | 7.57E-01 |
| 5-methylthioadenosine (MTA) | 0.88 | 0.59-1.3 | 8.17E-01 | 0.84 | 0.56-1.27 | 7.57E-01 |
| mannonate* | 0.90 | 0.7-1.17 | 7.72E-01 | 0.87 | 0.62-1.22 | 7.60E-01 |
| X - 25420 | 1.15 | 0.65-2.05 | 8.67E-01 | 1.27 | 0.71-2.26 | 7.60E-01 |
| 1-stearoyl-GPE (18:0) | 1.32 | 0.78-2.24 | 6.81E-01 | 1.27 | 0.71-2.25 | 7.60E-01 |
| X - 24518 | 0.81 | 0.51-1.27 | 7.26E-01 | 0.83 | 0.54-1.29 | 7.60E-01 |
| 1-stearoyl-2-oleoyl-GPC (18:0/18:1) | 0.80 | 0.5-1.29 | 7.31E-01 | 0.83 | 0.53-1.3 | 7.60E-01 |
| X - 21467 | 0.91 | 0.6-1.39 | 8.80E-01 | 0.83 | 0.53-1.3 | 7.61E-01 |
| o-cresol sulfate | 0.99 | 0.7-1.41 | 9.83E-01 | 1.18 | 0.79-1.74 | 7.66E-01 |
| 4-hydroxyglutamate | 1.17 | 0.75-1.83 | 8.01E-01 | 1.17 | 0.79-1.74 | 7.71E-01 |
| 3beta,7alpha-dihydroxy-5-cholestenoate | 0.82 | 0.58-1.16 | 6.51E-01 | 0.87 | 0.62-1.23 | 7.71E-01 |
| hexanoylglycine | 0.80 | 0.49-1.3 | 7.30E-01 | 0.82 | 0.5-1.34 | 7.71E-01 |
| X - 13695 | 1.17 | 0.82-1.66 | 7.42E-01 | 1.17 | 0.79-1.72 | 7.71E-01 |
| 1-linoleoyl-GPE (18:2)* | 0.85 | 0.57-1.26 | 7.58E-01 | 0.84 | 0.54-1.29 | 7.71E-01 |
| 1-palmitoyl-2-arachidonoyl-GPE (16:0/20:4)* | 0.84 | 0.54-1.29 | 7.58E-01 | 0.82 | 0.51-1.33 | 7.71E-01 |
| 2-hydroxypalmitate | 0.81 | 0.45-1.44 | 7.86E-01 | 0.78 | 0.42-1.45 | 7.71E-01 |
| isoursodeoxycholate | 1.15 | 0.73-1.82 | 8.29E-01 | 1.20 | 0.76-1.9 | 7.71E-01 |
| imidazole propionate | 1.17 | 0.78-1.76 | 7.74E-01 | 1.18 | 0.78-1.81 | 7.74E-01 |
| umbelliferone sulfate | 1.21 | 0.75-1.98 | 7.70E-01 | 1.20 | 0.76-1.88 | 7.74E-01 |
| gamma-glutamylisoleucine* | 1.24 | 0.85-1.81 | 6.51E-01 | 1.16 | 0.8-1.7 | 7.75E-01 |
| X - 23636 | 1.23 | 0.78-1.95 | 7.36E-01 | 1.21 | 0.74-1.97 | 7.79E-01 |
| 3-hydroxystachydrine* | 1.29 | 0.73-2.27 | 7.42E-01 | 1.26 | 0.7-2.27 | 7.79E-01 |
| 4-acetamidobutanoate | 1.30 | 0.67-2.5 | 7.72E-01 | 1.30 | 0.67-2.52 | 7.79E-01 |
| 3-methoxytyrosine | 1.28 | 0.68-2.43 | 7.72E-01 | 1.31 | 0.65-2.64 | 7.79E-01 |
| epiandrosterone sulfate | 1.15 | 0.73-1.8 | 8.39E-01 | 1.22 | 0.74-2.02 | 7.79E-01 |
| glycerol 3-phosphate | 0.95 | 0.69-1.29 | 9.15E-01 | 0.87 | 0.61-1.24 | 7.79E-01 |
| glutarylcarnitine (C5-DC) | 0.88 | 0.6-1.28 | 8.01E-01 | 0.86 | 0.57-1.28 | 7.86E-01 |
| 3-methyl-2-oxobutyrate | 0.89 | 0.55-1.44 | 8.68E-01 | 0.83 | 0.52-1.34 | 7.87E-01 |
| isobutyrylcarnitine (C4) | 1.26 | 0.84-1.89 | 6.56E-01 | 1.19 | 0.76-1.86 | 7.87E-01 |
| indoleacetate | 1.17 | 0.88-1.55 | 6.79E-01 | 1.14 | 0.81-1.61 | 7.87E-01 |
| behenoyl sphingomyelin (d18:1/22:0)* | 1.23 | 0.78-1.97 | 7.37E-01 | 1.19 | 0.76-1.84 | 7.87E-01 |
| N-palmitoyl-sphingadienine (d18:2/16:0)* | 1.17 | 0.81-1.7 | 7.49E-01 | 1.14 | 0.81-1.62 | 7.87E-01 |
| stearidonate (18:4n3) | 0.78 | 0.43-1.43 | 7.62E-01 | 0.79 | 0.42-1.47 | 7.87E-01 |
| citrulline | 1.12 | 0.81-1.56 | 8.01E-01 | 1.16 | 0.79-1.71 | 7.87E-01 |
| docosapentaenoate (n6 DPA; 22:5n6) | 0.86 | 0.54-1.35 | 8.11E-01 | 0.83 | 0.52-1.34 | 7.87E-01 |
| pyrraline | 0.92 | 0.64-1.31 | 8.68E-01 | 0.86 | 0.59-1.27 | 7.87E-01 |
| dihomo-linolenoyl-choline | 1.23 | 0.75-2.03 | 7.58E-01 | 1.20 | 0.74-1.93 | 7.89E-01 |
| N-acetylasparagine | 1.17 | 0.79-1.73 | 7.72E-01 | 1.16 | 0.79-1.71 | 7.89E-01 |
| 5,6-dihydrouridine | 1.14 | 0.81-1.6 | 7.72E-01 | 1.16 | 0.79-1.71 | 7.89E-01 |
| phenylacetylcarnitine | 1.13 | 0.82-1.54 | 7.84E-01 | 1.12 | 0.83-1.53 | 7.89E-01 |
| X - 18901 | 0.90 | 0.62-1.31 | 8.59E-01 | 0.86 | 0.58-1.28 | 7.89E-01 |
| lignoceroyl sphingomyelin (d18:1/24:0) | 1.16 | 0.7-1.93 | 8.39E-01 | 1.22 | 0.71-2.1 | 7.95E-01 |
| 17alpha-hydroxypregnanolone glucuronide | 0.77 | 0.5-1.2 | 6.44E-01 | 0.83 | 0.51-1.36 | 7.95E-01 |
| X - 25265 | 0.87 | 0.64-1.17 | 7.26E-01 | 0.89 | 0.66-1.21 | 7.95E-01 |
| 16a-hydroxy DHEA 3-sulfate | 0.86 | 0.62-1.19 | 7.33E-01 | 0.87 | 0.61-1.26 | 7.95E-01 |
| hydroxy-CMPF* | 1.26 | 0.75-2.12 | 7.44E-01 | 1.23 | 0.7-2.15 | 7.95E-01 |
| 3-hydroxyhippurate | 1.17 | 0.81-1.69 | 7.49E-01 | 1.16 | 0.78-1.71 | 7.95E-01 |
| 3-methylglutaconate | 1.24 | 0.74-2.08 | 7.58E-01 | 1.24 | 0.69-2.23 | 7.95E-01 |
| stearoylcholine* | 1.25 | 0.72-2.17 | 7.70E-01 | 1.22 | 0.71-2.08 | 7.95E-01 |
| X - 12707 | 1.23 | 0.7-2.16 | 7.84E-01 | 1.24 | 0.69-2.26 | 7.95E-01 |
| glyco-beta-muricholate** | 0.87 | 0.61-1.26 | 7.84E-01 | 0.87 | 0.61-1.26 | 7.95E-01 |
| asparagine | 0.83 | 0.51-1.37 | 7.86E-01 | 0.83 | 0.5-1.38 | 7.95E-01 |
| cortisone | 1.18 | 0.72-1.94 | 8.16E-01 | 1.24 | 0.69-2.24 | 7.95E-01 |
| androsterone glucuronide | 0.89 | 0.61-1.31 | 8.39E-01 | 0.85 | 0.54-1.33 | 7.95E-01 |
| 1-(1-enyl-palmitoyl)-2-oleoyl-GPC (P-16:0/18:1)* | 1.06 | 0.65-1.71 | 9.53E-01 | 1.21 | 0.72-2.01 | 7.95E-01 |
| X - 21821 | 1.18 | 0.87-1.61 | 6.70E-01 | 1.12 | 0.82-1.51 | 7.97E-01 |
| glutamine | 1.14 | 0.78-1.68 | 8.01E-01 | 1.17 | 0.76-1.81 | 7.98E-01 |
| taurolithocholate 3-sulfate | 0.87 | 0.61-1.23 | 7.64E-01 | 0.87 | 0.59-1.28 | 8.00E-01 |
| pentose acid* | 1.21 | 0.8-1.83 | 7.31E-01 | 1.18 | 0.75-1.84 | 8.01E-01 |
| 2-aminoheptanoate | 1.20 | 0.75-1.92 | 7.72E-01 | 1.18 | 0.74-1.9 | 8.04E-01 |
| retinal | 0.86 | 0.53-1.4 | 8.34E-01 | 0.84 | 0.52-1.37 | 8.04E-01 |
| 2-palmitoleoyl-GPC (16:1)* | 1.11 | 0.67-1.84 | 9.02E-01 | 1.20 | 0.72-1.98 | 8.04E-01 |
| X - 12127 | 0.90 | 0.63-1.3 | 8.55E-01 | 0.87 | 0.58-1.29 | 8.05E-01 |
| arachidonoylcholine | 1.23 | 0.7-2.13 | 7.86E-01 | 1.21 | 0.71-2.07 | 8.12E-01 |
| X - 12101 | 0.84 | 0.57-1.26 | 7.58E-01 | 0.85 | 0.53-1.35 | 8.15E-01 |
| N-acetyl-aspartyl-glutamate (NAAG) | 1.12 | 0.77-1.63 | 8.29E-01 | 1.13 | 0.79-1.61 | 8.17E-01 |
| 1-methyl-5-imidazolelactate | 1.16 | 0.81-1.67 | 7.64E-01 | 1.16 | 0.75-1.79 | 8.22E-01 |
| N6-acetyllysine | 0.84 | 0.5-1.42 | 8.16E-01 | 0.83 | 0.49-1.42 | 8.22E-01 |
| branched chain 14:0 dicarboxylic acid** | 1.34 | 0.75-2.42 | 7.01E-01 | 1.23 | 0.67-2.29 | 8.23E-01 |
| 4-methylguaiacol sulfate | 1.16 | 0.86-1.58 | 7.07E-01 | 1.13 | 0.79-1.6 | 8.23E-01 |
| 2-naphthol sulfate | 0.88 | 0.66-1.17 | 7.35E-01 | 1.15 | 0.76-1.75 | 8.23E-01 |
| 2-hydroxyphenylacetate | 1.24 | 0.72-2.14 | 7.72E-01 | 1.22 | 0.69-2.16 | 8.23E-01 |
| dimethylarginine (SDMA + ADMA) | 0.82 | 0.48-1.38 | 7.74E-01 | 0.82 | 0.47-1.45 | 8.23E-01 |
| lactosyl-N-behenoyl-sphingosine (d18:1/22:0)* | 0.85 | 0.52-1.39 | 8.16E-01 | 0.84 | 0.49-1.42 | 8.23E-01 |
| taurochenodeoxycholate | 0.86 | 0.55-1.35 | 8.16E-01 | 0.85 | 0.53-1.37 | 8.23E-01 |
| cortisol | 1.17 | 0.72-1.91 | 8.25E-01 | 1.20 | 0.71-2.02 | 8.23E-01 |
| 3-methyl catechol sulfate (1) | 1.13 | 0.74-1.72 | 8.55E-01 | 1.17 | 0.74-1.87 | 8.23E-01 |
| X - 26108 | 0.90 | 0.57-1.42 | 8.78E-01 | 0.86 | 0.54-1.35 | 8.23E-01 |
| X - 22509 | 1.21 | 0.73-1.99 | 7.84E-01 | 1.20 | 0.7-2.04 | 8.24E-01 |
| tricosanoyl sphingomyelin (d18:1/23:0)* | 1.20 | 0.7-2.07 | 8.09E-01 | 1.20 | 0.7-2.06 | 8.28E-01 |
| X - 24494 | 1.15 | 0.7-1.9 | 8.48E-01 | 1.19 | 0.71-1.99 | 8.28E-01 |
| iminodiacetate (IDA) | 1.20 | 0.71-2.04 | 8.01E-01 | 1.20 | 0.7-2.04 | 8.28E-01 |
| isoleucine | 1.27 | 0.79-2.04 | 7.01E-01 | 1.17 | 0.73-1.89 | 8.29E-01 |
| N-acetylneuraminate | 0.80 | 0.51-1.27 | 7.13E-01 | 0.85 | 0.51-1.4 | 8.29E-01 |
| N-acetyl-2-aminooctanoate* | 1.20 | 0.8-1.81 | 7.42E-01 | 1.15 | 0.76-1.75 | 8.29E-01 |
| sphingomyelin (d18:2/18:1)* | 1.24 | 0.76-2.01 | 7.49E-01 | 1.19 | 0.7-2.01 | 8.29E-01 |
| linoleoylcholine* | 1.20 | 0.71-2.02 | 8.01E-01 | 1.19 | 0.71-2.01 | 8.29E-01 |
| sphingomyelin (d18:2/16:0, d18:1/16:1)* | 1.24 | 0.66-2.32 | 8.12E-01 | 1.24 | 0.64-2.41 | 8.29E-01 |
| X - 23787 | 1.20 | 0.68-2.14 | 8.25E-01 | 1.23 | 0.65-2.34 | 8.29E-01 |
| 1,2,3-benzenetriol sulfate (2) | 1.16 | 0.71-1.9 | 8.39E-01 | 1.18 | 0.71-1.96 | 8.29E-01 |
| glycine conjugate of C10H14O2 (1)* | 0.94 | 0.67-1.31 | 9.04E-01 | 0.87 | 0.58-1.32 | 8.29E-01 |
| caffeine | 1.26 | 0.82-1.94 | 6.81E-01 | 1.16 | 0.74-1.8 | 8.30E-01 |
| lyxonate | 1.16 | 0.83-1.63 | 7.44E-01 | 1.13 | 0.78-1.63 | 8.30E-01 |
| X - 24970 | 0.90 | 0.6-1.34 | 8.59E-01 | 0.88 | 0.61-1.29 | 8.30E-01 |
| 5-dodecenoylcarnitine (C12:1) | 0.79 | 0.52-1.2 | 6.55E-01 | 0.88 | 0.58-1.32 | 8.32E-01 |
| X - 21383 | 0.87 | 0.54-1.39 | 8.34E-01 | 0.86 | 0.53-1.39 | 8.32E-01 |
| 1-dihomo-linolenylglycerol (20:3) | 0.86 | 0.51-1.46 | 8.48E-01 | 0.85 | 0.52-1.39 | 8.32E-01 |
| 3-bromo-5-chloro-2,6-dihydroxybenzoic acid* | 0.91 | 0.58-1.42 | 8.82E-01 | 0.85 | 0.52-1.4 | 8.32E-01 |
| 3-formylindole | 0.95 | 0.62-1.47 | 9.52E-01 | 0.86 | 0.54-1.37 | 8.32E-01 |
| X - 17354 | 1.23 | 0.73-2.06 | 7.72E-01 | 1.19 | 0.69-2.06 | 8.33E-01 |
| ribitol | 1.22 | 0.75-2.01 | 7.62E-01 | 1.20 | 0.67-2.15 | 8.34E-01 |
| N-acetyl-beta-alanine | 1.14 | 0.75-1.72 | 8.33E-01 | 1.16 | 0.73-1.86 | 8.34E-01 |
| X - 12822 | 0.94 | 0.64-1.37 | 9.24E-01 | 0.88 | 0.59-1.31 | 8.34E-01 |
| glycocholate | 0.87 | 0.53-1.44 | 8.59E-01 | 0.85 | 0.51-1.41 | 8.34E-01 |
| tiglylcarnitine (C5:1-DC) | 1.15 | 0.83-1.58 | 7.49E-01 | 1.12 | 0.78-1.6 | 8.39E-01 |
| cysteinylglycine disulfide* | 0.89 | 0.58-1.37 | 8.59E-01 | 0.87 | 0.57-1.34 | 8.40E-01 |
| sphingosine 1-phosphate | 1.09 | 0.73-1.62 | 8.80E-01 | 1.13 | 0.76-1.67 | 8.40E-01 |
| N-acetylcarnosine | 0.92 | 0.46-1.82 | 9.47E-01 | 0.81 | 0.42-1.58 | 8.40E-01 |
| glycerol | 0.81 | 0.48-1.35 | 7.58E-01 | 0.84 | 0.49-1.46 | 8.42E-01 |
| N-acetylphenylalanine | 0.88 | 0.62-1.26 | 8.01E-01 | 0.90 | 0.64-1.27 | 8.42E-01 |
| phenol glucuronide | 1.14 | 0.77-1.68 | 8.17E-01 | 1.13 | 0.76-1.69 | 8.42E-01 |
| pro-hydroxy-pro | 0.87 | 0.56-1.37 | 8.33E-01 | 0.87 | 0.56-1.35 | 8.42E-01 |
| trans-4-hydroxyproline | 0.90 | 0.62-1.32 | 8.59E-01 | 0.89 | 0.61-1.3 | 8.42E-01 |
| sulfate of piperine metabolite C16H19NO3 (3)* | 0.94 | 0.67-1.32 | 9.11E-01 | 0.88 | 0.59-1.32 | 8.42E-01 |
| theobromine | 1.21 | 0.76-1.92 | 7.58E-01 | 1.15 | 0.73-1.81 | 8.45E-01 |
| N-palmitoyl-heptadecasphingosine (d17:1/16:0)* | 0.91 | 0.58-1.43 | 9.02E-01 | 0.88 | 0.57-1.35 | 8.45E-01 |
| 1-linoleoyl-GPC (18:2) | 1.13 | 0.76-1.67 | 8.39E-01 | 1.17 | 0.7-1.96 | 8.46E-01 |
| docosahexaenoate (DHA; 22:6n3) | 0.88 | 0.53-1.46 | 8.67E-01 | 0.85 | 0.51-1.44 | 8.46E-01 |
| vanillylmandelate (VMA) | 1.20 | 0.78-1.85 | 7.58E-01 | 1.17 | 0.7-1.96 | 8.47E-01 |
| 3,4-dihydroxybutyrate | 0.93 | 0.71-1.22 | 8.59E-01 | 0.91 | 0.65-1.26 | 8.48E-01 |
| sphingomyelin (d18:1/18:1, d18:2/18:0) | 1.25 | 0.71-2.19 | 7.72E-01 | 1.18 | 0.68-2.06 | 8.50E-01 |
| cholate | 1.21 | 0.72-2.03 | 7.86E-01 | 1.19 | 0.67-2.1 | 8.50E-01 |
| homocitrulline | 0.89 | 0.63-1.26 | 8.16E-01 | 0.90 | 0.62-1.29 | 8.50E-01 |
| quinate | 0.89 | 0.63-1.27 | 8.25E-01 | 0.90 | 0.62-1.29 | 8.50E-01 |
| 5-hydroxy-2-methylpyridine sulfate | 0.90 | 0.63-1.28 | 8.40E-01 | 0.89 | 0.61-1.3 | 8.50E-01 |
| X - 12193 | 1.09 | 0.79-1.5 | 8.59E-01 | 1.11 | 0.79-1.56 | 8.50E-01 |
| 1-methyl-4-imidazoleacetate | 1.13 | 0.71-1.8 | 8.65E-01 | 1.15 | 0.71-1.87 | 8.50E-01 |
| N-acetyltryptophan | 1.09 | 0.74-1.6 | 8.80E-01 | 1.12 | 0.76-1.66 | 8.50E-01 |
| phosphoethanolamine | 0.94 | 0.6-1.45 | 9.37E-01 | 0.88 | 0.58-1.35 | 8.50E-01 |
| eicosenoylcarnitine (C20:1)* | 0.88 | 0.59-1.31 | 8.16E-01 | 0.88 | 0.57-1.35 | 8.51E-01 |
| xanthurenate | 1.27 | 0.87-1.86 | 6.11E-01 | 1.12 | 0.75-1.67 | 8.54E-01 |
| 4-methylcatechol sulfate | 1.15 | 0.83-1.6 | 7.49E-01 | 1.10 | 0.78-1.55 | 8.54E-01 |
| 3-indoleglyoxylic acid | 1.19 | 0.78-1.81 | 7.58E-01 | 1.12 | 0.75-1.69 | 8.54E-01 |
| 3b-hydroxy-5-cholenoic acid | 0.84 | 0.56-1.27 | 7.58E-01 | 0.89 | 0.58-1.36 | 8.54E-01 |
| N-formylmethionine | 1.18 | 0.79-1.74 | 7.62E-01 | 1.13 | 0.74-1.71 | 8.54E-01 |
| N-(2-furoyl)glycine | 1.11 | 0.85-1.45 | 7.81E-01 | 1.10 | 0.78-1.54 | 8.54E-01 |
| X - 23644 | 1.17 | 0.77-1.79 | 7.86E-01 | 1.13 | 0.73-1.75 | 8.54E-01 |
| guanidinoacetate | 0.85 | 0.54-1.33 | 7.96E-01 | 0.87 | 0.55-1.4 | 8.54E-01 |
| X - 24728 | 1.14 | 0.76-1.72 | 8.17E-01 | 1.13 | 0.73-1.76 | 8.54E-01 |
| ximenoylcarnitine (C26:1)* | 1.13 | 0.76-1.69 | 8.25E-01 | 1.12 | 0.75-1.69 | 8.54E-01 |
| cholic acid glucuronide | 1.17 | 0.7-1.97 | 8.33E-01 | 1.17 | 0.68-2.03 | 8.54E-01 |
| 3-hydroxy-2-methylpyridine sulfate | 1.11 | 0.79-1.54 | 8.39E-01 | 1.12 | 0.75-1.66 | 8.54E-01 |
| 1-linolenoyl-GPC (18:3)* | 1.17 | 0.69-2 | 8.39E-01 | 1.17 | 0.67-2.03 | 8.54E-01 |
| 1-palmitoyl-2-linoleoyl-GPC (16:0/18:2) | 0.88 | 0.56-1.37 | 8.39E-01 | 0.87 | 0.53-1.42 | 8.54E-01 |
| 3-amino-2-piperidone | 0.89 | 0.61-1.31 | 8.40E-01 | 0.90 | 0.62-1.3 | 8.54E-01 |
| N-palmitoyl-sphingosine (d18:1/16:0) | 1.07 | 0.81-1.43 | 8.67E-01 | 1.08 | 0.81-1.44 | 8.54E-01 |
| 1-linoleoyl-GPI (18:2)* | 1.08 | 0.77-1.51 | 8.76E-01 | 1.10 | 0.79-1.53 | 8.54E-01 |
| X - 26111 | 0.91 | 0.62-1.36 | 8.79E-01 | 0.89 | 0.59-1.35 | 8.54E-01 |
| 5-acetylamino-6-amino-3-methyluracil | 1.07 | 0.74-1.55 | 9.11E-01 | 1.11 | 0.76-1.64 | 8.54E-01 |
| X - 12544 | 0.95 | 0.62-1.45 | 9.47E-01 | 0.88 | 0.55-1.39 | 8.54E-01 |
| X - 26109 | 0.95 | 0.65-1.4 | 9.47E-01 | 0.90 | 0.63-1.29 | 8.54E-01 |
| (2,4 or 2,5)-dimethylphenol sulfate | 0.96 | 0.68-1.37 | 9.53E-01 | 1.12 | 0.76-1.63 | 8.54E-01 |
| 3-ethylcatechol sulfate (1) | 1.03 | 0.69-1.55 | 9.67E-01 | 1.16 | 0.7-1.93 | 8.54E-01 |
| X - 12713 | 1.16 | 0.73-1.84 | 8.26E-01 | 1.14 | 0.72-1.81 | 8.55E-01 |
| X - 17612 | 1.15 | 0.83-1.6 | 7.50E-01 | 1.12 | 0.74-1.68 | 8.57E-01 |
| carotene diol (2) | 1.15 | 0.78-1.69 | 8.01E-01 | 1.13 | 0.73-1.75 | 8.57E-01 |
| X - 12816 | 0.89 | 0.58-1.37 | 8.59E-01 | 0.89 | 0.58-1.37 | 8.57E-01 |
| pantothenate | 1.13 | 0.67-1.9 | 8.77E-01 | 1.17 | 0.65-2.11 | 8.57E-01 |
| X - 21842 | 0.92 | 0.61-1.36 | 8.80E-01 | 0.89 | 0.59-1.35 | 8.57E-01 |
| 17alpha-hydroxypregnenolone 3-sulfate | 1.10 | 0.7-1.73 | 8.97E-01 | 1.14 | 0.7-1.86 | 8.57E-01 |
| 1-stearoyl-2-linoleoyl-GPI (18:0/18:2) | 0.93 | 0.66-1.31 | 9.02E-01 | 0.91 | 0.64-1.29 | 8.57E-01 |
| perfluorooctanoate (PFOA) | 0.92 | 0.6-1.41 | 9.02E-01 | 0.88 | 0.56-1.39 | 8.57E-01 |
| aspartate | 0.94 | 0.57-1.54 | 9.42E-01 | 0.87 | 0.52-1.45 | 8.57E-01 |
| cis-3,4-methyleneheptanoylglycine | 0.98 | 0.75-1.27 | 9.58E-01 | 0.92 | 0.67-1.26 | 8.57E-01 |
| glutamine conjugate of C6H10O2 (1)* | 0.89 | 0.55-1.45 | 8.68E-01 | 0.87 | 0.52-1.46 | 8.57E-01 |
| 3-methylxanthine | 1.13 | 0.72-1.77 | 8.59E-01 | 1.13 | 0.71-1.8 | 8.58E-01 |
| heneicosapentaenoate (21:5n3) | 1.12 | 0.7-1.82 | 8.68E-01 | 1.14 | 0.69-1.89 | 8.58E-01 |
| sulfate of piperine metabolite C18H21NO3 (1)* | 0.92 | 0.67-1.27 | 8.65E-01 | 0.91 | 0.64-1.29 | 8.58E-01 |
| lactosyl-N-palmitoyl-sphingosine (d18:1/16:0) | 1.10 | 0.67-1.8 | 9.04E-01 | 1.15 | 0.69-1.91 | 8.58E-01 |
| 1-stearoyl-2-oleoyl-GPE (18:0/18:1) | 0.91 | 0.54-1.53 | 9.15E-01 | 0.87 | 0.52-1.47 | 8.58E-01 |
| etiocholanolone glucuronide | 1.04 | 0.69-1.56 | 9.58E-01 | 1.11 | 0.75-1.66 | 8.58E-01 |
| N-acetylarginine | 1.16 | 0.75-1.81 | 8.06E-01 | 1.13 | 0.72-1.76 | 8.59E-01 |
| X - 23593 | 0.86 | 0.59-1.26 | 7.72E-01 | 0.89 | 0.58-1.37 | 8.60E-01 |
| 7-alpha-hydroxy-3-oxo-4-cholestenoate (7-Hoca) | 0.88 | 0.51-1.54 | 8.80E-01 | 0.87 | 0.51-1.48 | 8.62E-01 |
| 3-hydroxy-3-methylglutarate | 0.94 | 0.7-1.26 | 8.97E-01 | 0.92 | 0.65-1.28 | 8.62E-01 |
| X - 12738 | 1.15 | 0.75-1.77 | 8.16E-01 | 1.13 | 0.71-1.8 | 8.62E-01 |
| oleoylcholine | 1.16 | 0.66-2.03 | 8.65E-01 | 1.16 | 0.65-2.06 | 8.66E-01 |
| indoleacetylglutamine | 1.16 | 0.86-1.57 | 7.13E-01 | 1.10 | 0.77-1.57 | 8.66E-01 |
| 1-(1-enyl-palmitoyl)-2-arachidonoyl-GPC (P-16:0/20:4)* | 1.08 | 0.68-1.71 | 9.28E-01 | 1.14 | 0.68-1.93 | 8.66E-01 |
| adenosine 3',5'-cyclic monophosphate (cAMP) | 0.91 | 0.6-1.38 | 8.79E-01 | 0.89 | 0.58-1.38 | 8.67E-01 |
| X - 15461 | 1.07 | 0.79-1.46 | 8.73E-01 | 1.08 | 0.79-1.48 | 8.67E-01 |
| N-palmitoyl-sphinganine (d18:0/16:0) | 1.09 | 0.78-1.51 | 8.65E-01 | 1.09 | 0.77-1.56 | 8.68E-01 |
| androstenediol (3beta,17beta) monosulfate (2) | 0.87 | 0.52-1.45 | 8.59E-01 | 0.86 | 0.49-1.53 | 8.68E-01 |
| sphingosine | 1.13 | 0.71-1.8 | 8.65E-01 | 1.12 | 0.72-1.74 | 8.68E-01 |
| X - 24949 | 0.92 | 0.65-1.31 | 8.68E-01 | 0.92 | 0.65-1.29 | 8.68E-01 |
| gamma-glutamylmethionine | 1.17 | 0.77-1.77 | 7.84E-01 | 1.11 | 0.73-1.69 | 8.68E-01 |
| 1-oleoyl-GPE (18:1) | 0.85 | 0.51-1.42 | 8.25E-01 | 0.88 | 0.53-1.47 | 8.70E-01 |
| gamma-glutamylcitrulline* | 1.12 | 0.73-1.74 | 8.59E-01 | 1.12 | 0.71-1.78 | 8.70E-01 |
| 4-hydroxy-2-oxoglutaric acid | 1.12 | 0.69-1.8 | 8.76E-01 | 1.12 | 0.71-1.77 | 8.70E-01 |
| cerotoylcarnitine (C26)* | 0.95 | 0.57-1.59 | 9.58E-01 | 0.88 | 0.53-1.47 | 8.70E-01 |
| N,N-dimethyl-pro-pro | 0.94 | 0.59-1.49 | 9.41E-01 | 0.88 | 0.53-1.46 | 8.70E-01 |
| 4-acetylphenol sulfate | 1.12 | 0.8-1.55 | 8.16E-01 | 1.10 | 0.75-1.6 | 8.72E-01 |
| glycochenodeoxycholate | 0.86 | 0.51-1.44 | 8.39E-01 | 0.87 | 0.51-1.51 | 8.72E-01 |
| X - 13553 | 1.17 | 0.69-1.99 | 8.41E-01 | 1.16 | 0.64-2.09 | 8.72E-01 |
| dihomo-linoleoylcarnitine (C20:2)* | 0.86 | 0.51-1.47 | 8.55E-01 | 0.87 | 0.49-1.53 | 8.72E-01 |
| 3-ureidopropionate | 1.13 | 0.7-1.84 | 8.62E-01 | 1.13 | 0.68-1.88 | 8.72E-01 |
| beta-citrylglutamate | 0.97 | 0.66-1.43 | 9.69E-01 | 0.91 | 0.63-1.32 | 8.72E-01 |
| 3-(4-hydroxyphenyl)lactate | 0.95 | 0.56-1.6 | 9.53E-01 | 0.88 | 0.53-1.47 | 8.73E-01 |
| 1-stearoyl-2-oleoyl-GPI (18:0/18:1)* | 0.91 | 0.61-1.35 | 8.67E-01 | 0.90 | 0.57-1.41 | 8.74E-01 |
| fumarate | 1.06 | 0.85-1.32 | 8.59E-01 | 1.05 | 0.86-1.29 | 8.75E-01 |
| 1-palmitoyl-2-arachidonoyl-GPC (16:0/20:4n6) | 1.09 | 0.66-1.81 | 9.15E-01 | 1.14 | 0.67-1.94 | 8.78E-01 |
| X - 12407 | 0.98 | 0.76-1.25 | 9.58E-01 | 0.94 | 0.74-1.21 | 8.78E-01 |
| glutamate | 0.97 | 0.64-1.48 | 9.67E-01 | 0.91 | 0.61-1.35 | 8.78E-01 |
| X - 24812 | 0.73 | 0.47-1.13 | 5.41E-01 | 0.88 | 0.52-1.5 | 8.83E-01 |
| X - 23666 | 1.13 | 0.86-1.48 | 7.35E-01 | 1.07 | 0.78-1.47 | 8.83E-01 |
| X - 12411 | 1.12 | 0.83-1.51 | 7.86E-01 | 1.08 | 0.76-1.53 | 8.83E-01 |
| methionine | 1.15 | 0.78-1.69 | 7.91E-01 | 1.10 | 0.73-1.65 | 8.83E-01 |
| 1-stearoyl-2-docosahexaenoyl-GPE (18:0/22:6)* | 1.19 | 0.72-1.95 | 8.01E-01 | 1.13 | 0.66-1.95 | 8.83E-01 |
| alpha-tocopherol | 1.16 | 0.72-1.88 | 8.37E-01 | 1.12 | 0.67-1.89 | 8.83E-01 |
| caproate (6:0) | 0.89 | 0.61-1.31 | 8.39E-01 | 0.91 | 0.61-1.36 | 8.83E-01 |
| carotene diol (1) | 1.14 | 0.71-1.85 | 8.55E-01 | 1.14 | 0.65-2 | 8.83E-01 |
| 5-hydroxyindoleacetate | 1.16 | 0.65-2.09 | 8.64E-01 | 1.15 | 0.62-2.16 | 8.83E-01 |
| glucuronide of piperine metabolite C17H21NO3 (4)* | 1.10 | 0.75-1.62 | 8.65E-01 | 1.10 | 0.73-1.64 | 8.83E-01 |
| eicosanedioate (C20-DC) | 1.07 | 0.78-1.48 | 8.80E-01 | 1.07 | 0.78-1.46 | 8.83E-01 |
| thymol sulfate | 1.11 | 0.69-1.77 | 8.88E-01 | 1.12 | 0.7-1.79 | 8.83E-01 |
| X - 17325 | 0.91 | 0.54-1.53 | 9.12E-01 | 0.89 | 0.53-1.5 | 8.83E-01 |
| sulfate of piperine metabolite C18H21NO3 (3)* | 0.94 | 0.65-1.35 | 9.15E-01 | 0.91 | 0.62-1.35 | 8.83E-01 |
| beta-hydroxyisovalerate | 1.07 | 0.73-1.57 | 9.16E-01 | 1.08 | 0.75-1.56 | 8.83E-01 |
| aconitate [cis or trans] | 0.92 | 0.54-1.58 | 9.38E-01 | 0.87 | 0.48-1.59 | 8.83E-01 |
| 2,3-dihydroxypyridine | 0.94 | 0.61-1.45 | 9.41E-01 | 0.91 | 0.6-1.39 | 8.83E-01 |
| 3-carboxy-4-methyl-5-pentyl-2-furanpropionate (3-CMPFP)** | 0.96 | 0.63-1.46 | 9.53E-01 | 0.90 | 0.58-1.42 | 8.83E-01 |
| X - 11483 | 0.96 | 0.66-1.4 | 9.53E-01 | 0.92 | 0.63-1.34 | 8.83E-01 |
| 1-palmitoyl-2-palmitoleoyl-GPC (16:0/16:1)* | 1.05 | 0.61-1.82 | 9.53E-01 | 1.14 | 0.65-1.98 | 8.83E-01 |
| 2-methylbutyrylcarnitine (C5) | 0.98 | 0.72-1.32 | 9.67E-01 | 0.93 | 0.68-1.28 | 8.83E-01 |
| formiminoglutamate | 0.98 | 0.62-1.52 | 9.72E-01 | 0.91 | 0.59-1.39 | 8.83E-01 |
| pregnanediol-3-glucuronide | 1.02 | 0.7-1.5 | 9.73E-01 | 1.09 | 0.74-1.62 | 8.83E-01 |
| X - 12726 | 1.01 | 0.7-1.46 | 9.92E-01 | 0.91 | 0.58-1.41 | 8.83E-01 |
| N6-methyllysine | 0.90 | 0.57-1.42 | 8.78E-01 | 0.91 | 0.58-1.41 | 8.84E-01 |
| argininate* | 1.15 | 0.82-1.61 | 7.58E-01 | 1.08 | 0.75-1.56 | 8.85E-01 |
| 2,3-dihydroxyisovalerate | 1.12 | 0.71-1.75 | 8.65E-01 | 1.11 | 0.69-1.8 | 8.85E-01 |
| 1-oleoyl-GPI (18:1) | 1.06 | 0.72-1.56 | 9.35E-01 | 1.09 | 0.74-1.6 | 8.85E-01 |
| N-acetylglycine | 1.04 | 0.6-1.79 | 9.69E-01 | 1.13 | 0.65-1.97 | 8.85E-01 |
| 2-hydroxynervonate* | 0.88 | 0.65-1.19 | 7.58E-01 | 0.93 | 0.67-1.3 | 8.86E-01 |
| taurocholenate sulfate* | 0.82 | 0.48-1.41 | 7.86E-01 | 0.88 | 0.49-1.6 | 8.86E-01 |
| butyrate/isobutyrate (4:0) | 1.14 | 0.8-1.62 | 7.86E-01 | 1.07 | 0.77-1.5 | 8.86E-01 |
| X - 12221 | 1.13 | 0.78-1.63 | 8.16E-01 | 1.09 | 0.73-1.61 | 8.86E-01 |
| indolelactate | 1.18 | 0.71-1.95 | 8.23E-01 | 1.11 | 0.67-1.86 | 8.86E-01 |
| pseudouridine | 0.88 | 0.59-1.32 | 8.25E-01 | 0.92 | 0.61-1.38 | 8.86E-01 |
| N-stearoyl-sphingosine (d18:1/18:0)* | 1.09 | 0.8-1.5 | 8.57E-01 | 1.06 | 0.8-1.42 | 8.86E-01 |
| pregnenediol sulfate (C21H34O5S)* | 0.87 | 0.51-1.49 | 8.65E-01 | 0.89 | 0.52-1.54 | 8.86E-01 |
| dihydroferulate | 1.11 | 0.73-1.7 | 8.65E-01 | 1.10 | 0.7-1.71 | 8.86E-01 |
| 1-oleoyl-GPG (18:1)* | 0.91 | 0.64-1.31 | 8.65E-01 | 0.93 | 0.64-1.34 | 8.86E-01 |
| gamma-glutamylalanine | 1.14 | 0.66-1.94 | 8.74E-01 | 1.12 | 0.66-1.88 | 8.86E-01 |
| 3-(3-hydroxyphenyl)propionate | 1.09 | 0.71-1.68 | 9.04E-01 | 1.10 | 0.71-1.69 | 8.86E-01 |
| 1-palmitoyl-GPG (16:0)* | 0.92 | 0.59-1.44 | 9.15E-01 | 0.90 | 0.56-1.45 | 8.86E-01 |
| 1-palmitoyl-2-linoleoyl-GPI (16:0/18:2) | 1.07 | 0.7-1.64 | 9.31E-01 | 1.10 | 0.71-1.69 | 8.86E-01 |
| glycohyocholate | 1.04 | 0.69-1.58 | 9.53E-01 | 1.09 | 0.73-1.64 | 8.86E-01 |
| X - 25936 | 1.03 | 0.71-1.48 | 9.67E-01 | 1.08 | 0.74-1.58 | 8.86E-01 |
| sarcosine | 1.14 | 0.63-2.08 | 8.82E-01 | 1.13 | 0.63-2.04 | 8.87E-01 |
| 4-vinylguaiacol sulfate | 0.92 | 0.66-1.28 | 8.65E-01 | 0.93 | 0.64-1.34 | 8.87E-01 |
| 1,2-dilinoleoyl-GPC (18:2/18:2) | 0.91 | 0.64-1.3 | 8.65E-01 | 0.91 | 0.58-1.42 | 8.87E-01 |
| N-acetylisoleucine | 1.14 | 0.76-1.7 | 8.17E-01 | 1.09 | 0.71-1.67 | 8.88E-01 |
| ceramide (d18:1/17:0, d17:1/18:0)* | 1.11 | 0.76-1.63 | 8.59E-01 | 1.07 | 0.76-1.52 | 8.88E-01 |
| 1-arachidonoyl-GPE (20:4n6)* | 0.86 | 0.47-1.55 | 8.64E-01 | 0.88 | 0.47-1.64 | 8.88E-01 |
| 3-methylcytidine | 1.13 | 0.67-1.91 | 8.79E-01 | 1.13 | 0.6-2.13 | 8.88E-01 |
| trigonelline (N'-methylnicotinate) | 1.13 | 0.66-1.91 | 8.80E-01 | 1.12 | 0.63-2.01 | 8.88E-01 |
| 3-hydroxybutyroylglycine** | 1.07 | 0.77-1.48 | 8.91E-01 | 1.08 | 0.74-1.56 | 8.88E-01 |
| betaine | 1.08 | 0.64-1.82 | 9.35E-01 | 1.12 | 0.64-1.94 | 8.88E-01 |
| X - 12100 | 0.93 | 0.57-1.51 | 9.35E-01 | 0.90 | 0.55-1.5 | 8.88E-01 |
| dehydroepiandrosterone sulfate (DHEA-S) | 1.06 | 0.61-1.84 | 9.53E-01 | 1.12 | 0.63-2.02 | 8.88E-01 |
| ceramide (d18:2/24:1, d18:1/24:2)* | 1.08 | 0.79-1.48 | 8.65E-01 | 1.06 | 0.79-1.43 | 8.90E-01 |
| erucate (22:1n9) | 0.83 | 0.43-1.6 | 8.59E-01 | 0.88 | 0.45-1.72 | 8.90E-01 |
| 1-oleoylglycerol (18:1) | 0.93 | 0.58-1.51 | 9.41E-01 | 0.90 | 0.54-1.52 | 8.90E-01 |
| choline phosphate | 1.14 | 0.69-1.88 | 8.59E-01 | 1.10 | 0.67-1.83 | 8.92E-01 |
| lithocholate sulfate (1) | 1.08 | 0.75-1.58 | 8.87E-01 | 1.08 | 0.73-1.59 | 8.92E-01 |
| 1,3-dimethylurate | 1.09 | 0.59-2.01 | 9.41E-01 | 1.11 | 0.63-1.98 | 9.02E-01 |
| 4-ethylphenylsulfate | 1.05 | 0.73-1.51 | 9.47E-01 | 1.08 | 0.73-1.58 | 9.02E-01 |
| arabitol/xylitol | 1.08 | 0.81-1.45 | 8.59E-01 | 1.07 | 0.74-1.55 | 9.03E-01 |
| X - 12851 | 1.04 | 0.77-1.4 | 9.42E-01 | 1.06 | 0.78-1.45 | 9.03E-01 |
| piperine | 1.00 | 0.7-1.42 | 9.93E-01 | 0.92 | 0.6-1.41 | 9.03E-01 |
| adenosine 5'-monophosphate (AMP) | 1.08 | 0.71-1.63 | 9.18E-01 | 1.07 | 0.73-1.57 | 9.04E-01 |
| glucuronide of piperine metabolite C17H21NO3 (3)* | 0.93 | 0.57-1.5 | 9.31E-01 | 0.91 | 0.55-1.51 | 9.04E-01 |
| N6-carbamoylthreonyladenosine | 0.94 | 0.6-1.47 | 9.41E-01 | 0.92 | 0.58-1.45 | 9.04E-01 |
| X - 17346 | 1.12 | 0.78-1.6 | 8.25E-01 | 1.09 | 0.69-1.7 | 9.04E-01 |
| 5-oxoproline | 1.07 | 0.76-1.5 | 9.12E-01 | 1.07 | 0.75-1.53 | 9.04E-01 |
| 2-oleoylglycerol (18:1) | 1.12 | 0.73-1.73 | 8.64E-01 | 1.08 | 0.72-1.62 | 9.05E-01 |
| sulfate of piperine metabolite C16H19NO3 (2)* | 0.98 | 0.69-1.39 | 9.72E-01 | 0.93 | 0.62-1.4 | 9.05E-01 |
| gamma-CEHC | 1.02 | 0.7-1.48 | 9.75E-01 | 0.92 | 0.59-1.44 | 9.05E-01 |
| X - 11632 | 1.05 | 0.71-1.53 | 9.53E-01 | 1.08 | 0.71-1.63 | 9.06E-01 |
| sphinganine-1-phosphate | 1.03 | 0.72-1.47 | 9.61E-01 | 1.07 | 0.74-1.55 | 9.06E-01 |
| 1-(1-enyl-stearoyl)-2-linoleoyl-GPE (P-18:0/18:2)* | 0.92 | 0.58-1.47 | 9.24E-01 | 0.92 | 0.58-1.47 | 9.06E-01 |
| valylglycine | 1.08 | 0.78-1.49 | 8.76E-01 | 1.06 | 0.76-1.49 | 9.08E-01 |
| taurochenodeoxycholic acid 3-sulfate | 1.06 | 0.67-1.66 | 9.50E-01 | 1.09 | 0.68-1.73 | 9.09E-01 |
| N-acetylvaline | 1.13 | 0.77-1.66 | 8.25E-01 | 1.08 | 0.7-1.67 | 9.09E-01 |
| 7-methylxanthine | 1.09 | 0.71-1.67 | 9.10E-01 | 1.08 | 0.68-1.73 | 9.09E-01 |
| N2-acetyl,N6-methyllysine | 1.06 | 0.71-1.6 | 9.35E-01 | 1.08 | 0.7-1.66 | 9.09E-01 |
| N-acetylhistidine | 0.88 | 0.62-1.24 | 7.84E-01 | 0.94 | 0.68-1.32 | 9.09E-01 |
| 12-HETE | 1.10 | 0.67-1.82 | 9.11E-01 | 1.09 | 0.66-1.82 | 9.09E-01 |
| sphinganine | 0.97 | 0.63-1.49 | 9.69E-01 | 0.93 | 0.61-1.42 | 9.09E-01 |
| 5alpha-androstan-3beta,17beta-diol monosulfate (2) | 1.05 | 0.71-1.56 | 9.47E-01 | 1.08 | 0.69-1.69 | 9.13E-01 |
| 1-stearoyl-2-arachidonoyl-GPC (18:0/20:4) | 1.08 | 0.66-1.77 | 9.30E-01 | 1.09 | 0.65-1.82 | 9.13E-01 |
| 2-hydroxystearate | 0.95 | 0.53-1.71 | 9.67E-01 | 0.90 | 0.49-1.65 | 9.13E-01 |
| orotidine | 1.09 | 0.82-1.44 | 8.40E-01 | 1.06 | 0.75-1.5 | 9.14E-01 |
| metabolonic lactone sulfate | 0.98 | 0.69-1.4 | 9.72E-01 | 0.94 | 0.66-1.35 | 9.20E-01 |
| X - 13728 | 1.07 | 0.83-1.4 | 8.59E-01 | 1.05 | 0.78-1.42 | 9.20E-01 |
| cytidine | 1.10 | 0.61-1.99 | 9.30E-01 | 1.11 | 0.58-2.15 | 9.20E-01 |
| arabonate/xylonate | 0.99 | 0.74-1.32 | 9.75E-01 | 0.94 | 0.66-1.35 | 9.20E-01 |
| 11beta-hydroxyandrosterone glucuronide | 1.09 | 0.8-1.49 | 8.48E-01 | 1.06 | 0.74-1.53 | 9.20E-01 |
| 4-ethylcatechol sulfate | 0.91 | 0.61-1.37 | 8.80E-01 | 0.93 | 0.58-1.48 | 9.20E-01 |
| 5alpha-androstan-3alpha,17beta-diol monosulfate (1) | 1.09 | 0.69-1.7 | 9.15E-01 | 1.08 | 0.67-1.72 | 9.20E-01 |
| N-acetylmethionine | 1.09 | 0.66-1.8 | 9.16E-01 | 1.09 | 0.64-1.85 | 9.20E-01 |
| dihomo-linolenoylcarnitine (C20:3n3 or 6)* | 0.91 | 0.53-1.56 | 9.24E-01 | 0.92 | 0.54-1.57 | 9.20E-01 |
| X - 12261 | 0.97 | 0.65-1.44 | 9.67E-01 | 0.94 | 0.62-1.42 | 9.20E-01 |
| 4-vinylcatechol sulfate | 1.00 | 0.62-1.63 | 9.94E-01 | 1.09 | 0.64-1.86 | 9.20E-01 |
| gamma-CEHC glucuronide* | 1.14 | 0.79-1.64 | 8.05E-01 | 1.07 | 0.7-1.62 | 9.22E-01 |
| X - 12112 | 0.89 | 0.59-1.35 | 8.55E-01 | 0.94 | 0.63-1.41 | 9.22E-01 |
| N-carbamoylvaline | 1.05 | 0.72-1.54 | 9.41E-01 | 1.06 | 0.72-1.56 | 9.22E-01 |
| phosphate | 0.89 | 0.57-1.39 | 8.59E-01 | 0.93 | 0.58-1.5 | 9.24E-01 |
| dihydroferulic acid sulfate | 1.08 | 0.72-1.6 | 9.13E-01 | 1.06 | 0.71-1.58 | 9.24E-01 |
| N,N,N-trimethyl-alanylproline betaine (TMAP) | 0.98 | 0.63-1.52 | 9.75E-01 | 0.93 | 0.58-1.49 | 9.24E-01 |
| N-acetylleucine | 1.09 | 0.76-1.58 | 8.68E-01 | 1.06 | 0.72-1.55 | 9.29E-01 |
| dihydroorotate | 0.97 | 0.66-1.43 | 9.67E-01 | 0.95 | 0.65-1.37 | 9.29E-01 |
| 2-methylserine | 0.98 | 0.65-1.48 | 9.73E-01 | 0.93 | 0.59-1.48 | 9.29E-01 |
| X - 17367 | 1.08 | 0.67-1.73 | 9.31E-01 | 1.07 | 0.66-1.75 | 9.30E-01 |
| 3-(3-amino-3-carboxypropyl)uridine* | 0.93 | 0.67-1.29 | 8.82E-01 | 0.95 | 0.67-1.36 | 9.32E-01 |
| tryptophan | 1.00 | 0.63-1.59 | 9.94E-01 | 0.93 | 0.57-1.51 | 9.32E-01 |
| gamma-glutamylphenylalanine | 1.09 | 0.76-1.57 | 8.68E-01 | 1.06 | 0.72-1.56 | 9.33E-01 |
| hyocholate | 1.08 | 0.67-1.73 | 9.30E-01 | 1.07 | 0.65-1.78 | 9.33E-01 |
| X - 23974 | 0.94 | 0.6-1.47 | 9.41E-01 | 0.94 | 0.62-1.44 | 9.33E-01 |
| X - 12847 | 0.88 | 0.54-1.44 | 8.65E-01 | 0.93 | 0.55-1.57 | 9.33E-01 |
| 1-linoleoyl-2-linolenoyl-GPC (18:2/18:3)* | 1.05 | 0.72-1.53 | 9.41E-01 | 1.06 | 0.71-1.58 | 9.33E-01 |
| gamma-glutamylthreonine | 0.95 | 0.66-1.38 | 9.45E-01 | 0.94 | 0.63-1.41 | 9.33E-01 |
| homovanillate (HVA) | 1.07 | 0.68-1.68 | 9.31E-01 | 1.07 | 0.67-1.69 | 9.34E-01 |
| 1-palmitoyl-2-oleoyl-GPC (16:0/18:1) | 1.00 | 0.59-1.71 | 9.98E-01 | 1.08 | 0.63-1.86 | 9.34E-01 |
| cyclo(gly-pro) | 0.91 | 0.61-1.36 | 8.79E-01 | 0.94 | 0.62-1.43 | 9.35E-01 |
| X - 24337 | 0.97 | 0.58-1.61 | 9.69E-01 | 0.93 | 0.55-1.58 | 9.35E-01 |
| X - 17010 | 0.92 | 0.58-1.47 | 9.15E-01 | 0.94 | 0.6-1.47 | 9.36E-01 |
| N-acetylkynurenine (2) | 1.10 | 0.75-1.63 | 8.65E-01 | 1.06 | 0.69-1.64 | 9.36E-01 |
| pregnenetriol sulfate* | 0.91 | 0.58-1.44 | 9.02E-01 | 0.94 | 0.59-1.51 | 9.37E-01 |
| 4-allylphenol sulfate | 1.10 | 0.69-1.75 | 9.04E-01 | 1.06 | 0.69-1.64 | 9.37E-01 |
| X - 10458 | 1.05 | 0.68-1.61 | 9.53E-01 | 1.06 | 0.7-1.59 | 9.37E-01 |
| X - 11381 | 0.97 | 0.67-1.4 | 9.58E-01 | 0.95 | 0.63-1.42 | 9.37E-01 |
| 9-hydroxystearate | 0.92 | 0.65-1.29 | 8.65E-01 | 0.96 | 0.68-1.35 | 9.38E-01 |
| 4-hydroxycoumarin | 1.13 | 0.62-2.07 | 9.02E-01 | 1.07 | 0.62-1.87 | 9.38E-01 |
| kynurenate | 1.03 | 0.73-1.45 | 9.61E-01 | 0.95 | 0.65-1.4 | 9.38E-01 |
| N-stearoyl-sphingadienine (d18:2/18:0)* | 0.99 | 0.67-1.46 | 9.82E-01 | 0.95 | 0.66-1.38 | 9.38E-01 |
| 2-O-methylascorbic acid | 1.08 | 0.73-1.6 | 9.13E-01 | 1.06 | 0.69-1.62 | 9.39E-01 |
| 4-hydroxychlorothalonil | 1.09 | 0.63-1.88 | 9.34E-01 | 1.08 | 0.6-1.95 | 9.39E-01 |
| citraconate/glutaconate | 0.98 | 0.7-1.37 | 9.69E-01 | 0.95 | 0.64-1.41 | 9.39E-01 |
| C-glycosyltryptophan | 1.01 | 0.72-1.43 | 9.78E-01 | 1.05 | 0.71-1.55 | 9.39E-01 |
| X - 24418 | 1.04 | 0.71-1.52 | 9.53E-01 | 1.05 | 0.7-1.57 | 9.40E-01 |
| X - 24811 | 0.90 | 0.62-1.3 | 8.40E-01 | 0.95 | 0.65-1.41 | 9.41E-01 |
| X - 13844 | 1.09 | 0.7-1.7 | 9.03E-01 | 1.06 | 0.64-1.75 | 9.41E-01 |
| 1-stearoyl-2-linoleoyl-GPC (18:0/18:2)* | 1.06 | 0.69-1.62 | 9.47E-01 | 1.06 | 0.65-1.74 | 9.41E-01 |
| orotate | 0.95 | 0.64-1.43 | 9.53E-01 | 0.95 | 0.62-1.45 | 9.42E-01 |
| X - 25957 | 1.06 | 0.66-1.71 | 9.50E-01 | 1.06 | 0.64-1.76 | 9.43E-01 |
| X - 24544 | 0.96 | 0.66-1.39 | 9.53E-01 | 0.95 | 0.62-1.46 | 9.43E-01 |
| cinnamoylglycine | 1.02 | 0.67-1.53 | 9.78E-01 | 1.05 | 0.69-1.6 | 9.43E-01 |
| X - 21736 | 1.04 | 0.75-1.43 | 9.53E-01 | 1.04 | 0.76-1.41 | 9.44E-01 |
| X - 16935 | 0.98 | 0.65-1.49 | 9.78E-01 | 0.95 | 0.59-1.52 | 9.47E-01 |
| 1-palmitoleoylglycerol (16:1)* | 0.95 | 0.57-1.58 | 9.53E-01 | 0.94 | 0.55-1.61 | 9.48E-01 |
| N-palmitoylserine | 1.05 | 0.7-1.58 | 9.49E-01 | 1.05 | 0.69-1.6 | 9.48E-01 |
| 1-linolenoylglycerol (18:3) | 1.11 | 0.72-1.71 | 8.65E-01 | 1.05 | 0.69-1.59 | 9.48E-01 |
| 1-stearoyl-GPI (18:0) | 1.07 | 0.73-1.56 | 9.16E-01 | 1.04 | 0.72-1.49 | 9.48E-01 |
| 1-palmitoyl-2-docosahexaenoyl-GPE (16:0/22:6)* | 1.09 | 0.63-1.89 | 9.28E-01 | 1.07 | 0.59-1.94 | 9.48E-01 |
| X - 21327 | 1.06 | 0.74-1.51 | 9.31E-01 | 1.04 | 0.73-1.47 | 9.48E-01 |
| 1-stearoyl-2-oleoyl-GPS (18:0/18:1) | 1.07 | 0.65-1.75 | 9.41E-01 | 1.05 | 0.64-1.72 | 9.48E-01 |
| 1-methylxanthine | 0.95 | 0.66-1.38 | 9.41E-01 | 0.96 | 0.65-1.41 | 9.48E-01 |
| deoxycarnitine | 1.04 | 0.77-1.4 | 9.41E-01 | 1.03 | 0.75-1.43 | 9.48E-01 |
| 3-acetylphenol sulfate | 1.06 | 0.68-1.65 | 9.41E-01 | 1.06 | 0.63-1.78 | 9.48E-01 |
| gamma-glutamyl-2-aminobutyrate | 1.05 | 0.7-1.59 | 9.47E-01 | 1.05 | 0.66-1.68 | 9.48E-01 |
| 1-(1-enyl-stearoyl)-2-oleoyl-GPE (P-18:0/18:1) | 0.94 | 0.59-1.52 | 9.47E-01 | 0.95 | 0.6-1.51 | 9.48E-01 |
| cis-4-decenoylcarnitine (C10:1) | 1.03 | 0.77-1.37 | 9.53E-01 | 1.03 | 0.77-1.38 | 9.48E-01 |
| X - 18887 | 1.05 | 0.65-1.7 | 9.53E-01 | 1.05 | 0.66-1.66 | 9.48E-01 |
| deoxycholic acid glucuronide | 1.06 | 0.58-1.93 | 9.58E-01 | 1.07 | 0.59-1.95 | 9.48E-01 |
| nervonoylcarnitine (C24:1)* | 1.03 | 0.7-1.53 | 9.61E-01 | 1.04 | 0.7-1.55 | 9.48E-01 |
| uracil | 0.97 | 0.65-1.45 | 9.67E-01 | 0.96 | 0.65-1.42 | 9.48E-01 |
| pimeloylcarnitine/3-methyladipoylcarnitine (C7-DC) | 1.02 | 0.71-1.48 | 9.69E-01 | 1.04 | 0.74-1.47 | 9.48E-01 |
| 3-hydroxypyridine sulfate | 1.03 | 0.67-1.57 | 9.71E-01 | 1.05 | 0.66-1.69 | 9.48E-01 |
| xanthosine | 1.02 | 0.62-1.68 | 9.75E-01 | 0.95 | 0.59-1.52 | 9.48E-01 |
| phenol sulfate | 0.99 | 0.65-1.49 | 9.82E-01 | 0.96 | 0.64-1.45 | 9.48E-01 |
| gamma-carboxyglutamate | 1.01 | 0.74-1.37 | 9.85E-01 | 1.04 | 0.75-1.44 | 9.48E-01 |
| 5alpha-androstan-3alpha,17beta-diol monosulfate (2) | 1.01 | 0.57-1.81 | 9.88E-01 | 0.94 | 0.52-1.7 | 9.48E-01 |
| gamma-glutamyltyrosine | 0.99 | 0.69-1.43 | 9.92E-01 | 0.96 | 0.66-1.39 | 9.48E-01 |
| ferulic acid 4-sulfate | 0.99 | 0.62-1.6 | 9.92E-01 | 0.95 | 0.6-1.51 | 9.48E-01 |
| tyrosine | 0.99 | 0.63-1.58 | 9.93E-01 | 0.95 | 0.59-1.53 | 9.48E-01 |
| X - 21796 | 1.00 | 0.68-1.47 | 9.94E-01 | 0.96 | 0.65-1.41 | 9.48E-01 |
| taurocholate | 1.00 | 0.63-1.58 | 9.98E-01 | 0.96 | 0.6-1.51 | 9.48E-01 |
| ribonate | 1.07 | 0.82-1.4 | 8.59E-01 | 1.03 | 0.78-1.36 | 9.51E-01 |
| (N(1) + N(8))-acetylspermidine | 1.08 | 0.75-1.54 | 9.02E-01 | 1.03 | 0.72-1.48 | 9.51E-01 |
| glycocholenate sulfate* | 0.91 | 0.55-1.5 | 9.04E-01 | 0.95 | 0.55-1.63 | 9.51E-01 |
| 1-palmitoyl-2-oleoyl-GPI (16:0/18:1)* | 0.89 | 0.5-1.59 | 9.04E-01 | 0.95 | 0.54-1.65 | 9.51E-01 |
| phenylalanine | 1.02 | 0.65-1.58 | 9.78E-01 | 0.95 | 0.58-1.56 | 9.51E-01 |
| 3-phenylpropionate (hydrocinnamate) | 1.01 | 0.64-1.58 | 9.92E-01 | 1.04 | 0.67-1.63 | 9.51E-01 |
| lysine | 1.00 | 0.68-1.48 | 9.93E-01 | 0.96 | 0.62-1.49 | 9.51E-01 |
| lactosyl-N-nervonoyl-sphingosine (d18:1/24:1)* | 0.89 | 0.51-1.58 | 9.04E-01 | 0.95 | 0.53-1.71 | 9.53E-01 |
| N-delta-acetylornithine | 0.96 | 0.63-1.46 | 9.53E-01 | 0.96 | 0.6-1.54 | 9.53E-01 |
| andro steroid monosulfate C19H28O6S (1)* | 0.93 | 0.63-1.39 | 9.24E-01 | 0.96 | 0.62-1.49 | 9.53E-01 |
| 2-aminooctanoate | 1.03 | 0.72-1.46 | 9.67E-01 | 1.03 | 0.74-1.43 | 9.53E-01 |
| 1-(1-enyl-palmitoyl)-2-linoleoyl-GPC (P-16:0/18:2)* | 0.93 | 0.59-1.47 | 9.35E-01 | 0.95 | 0.56-1.63 | 9.56E-01 |
| 4-allylcatechol sulfate | 1.06 | 0.71-1.57 | 9.41E-01 | 1.03 | 0.7-1.54 | 9.56E-01 |
| 1-arachidonoyl-GPI (20:4)* | 0.96 | 0.67-1.39 | 9.53E-01 | 0.97 | 0.66-1.41 | 9.56E-01 |
| X - 26097 | 1.01 | 0.73-1.4 | 9.80E-01 | 1.03 | 0.73-1.45 | 9.56E-01 |
| N6,N6-dimethyllysine | 1.04 | 0.64-1.67 | 9.67E-01 | 1.04 | 0.65-1.66 | 9.56E-01 |
| histidine | 0.96 | 0.62-1.47 | 9.53E-01 | 0.96 | 0.62-1.49 | 9.61E-01 |
| X - 13007 | 1.07 | 0.64-1.79 | 9.41E-01 | 1.04 | 0.65-1.67 | 9.61E-01 |
| mannose | 1.02 | 0.72-1.44 | 9.74E-01 | 1.03 | 0.7-1.52 | 9.61E-01 |
| 1-(1-enyl-palmitoyl)-2-linoleoyl-GPE (P-16:0/18:2)* | 0.98 | 0.57-1.7 | 9.82E-01 | 0.96 | 0.56-1.65 | 9.61E-01 |
| acisoga | 0.93 | 0.62-1.4 | 9.15E-01 | 0.97 | 0.63-1.48 | 9.62E-01 |
| nisinate (24:6n3) | 1.06 | 0.7-1.59 | 9.41E-01 | 1.03 | 0.67-1.6 | 9.62E-01 |
| hydroxy-N6,N6,N6-trimethyllysine* | 1.08 | 0.81-1.45 | 8.59E-01 | 1.02 | 0.74-1.41 | 9.63E-01 |
| X - 21258 | 0.93 | 0.61-1.42 | 9.24E-01 | 0.97 | 0.64-1.47 | 9.63E-01 |
| methyl indole-3-acetate | 1.05 | 0.79-1.4 | 9.28E-01 | 1.02 | 0.76-1.39 | 9.63E-01 |
| hypotaurine | 0.95 | 0.68-1.34 | 9.35E-01 | 0.98 | 0.7-1.36 | 9.63E-01 |
| ectoine | 1.07 | 0.6-1.9 | 9.53E-01 | 1.05 | 0.56-1.96 | 9.63E-01 |
| glycine conjugate of C10H12O2* | 1.02 | 0.71-1.48 | 9.72E-01 | 0.97 | 0.63-1.5 | 9.63E-01 |
| arachidoylcarnitine (C20)* | 0.97 | 0.53-1.76 | 9.72E-01 | 0.95 | 0.48-1.89 | 9.63E-01 |
| X - 17438 | 0.98 | 0.67-1.43 | 9.72E-01 | 0.97 | 0.67-1.41 | 9.63E-01 |
| 1-palmitoylglycerol (16:0) | 0.98 | 0.61-1.57 | 9.74E-01 | 0.96 | 0.59-1.58 | 9.63E-01 |
| N2-acetyl,N6,N6-dimethyllysine | 1.01 | 0.65-1.58 | 9.82E-01 | 1.03 | 0.66-1.62 | 9.63E-01 |
| eicosenedioate (C20:1-DC)* | 0.97 | 0.6-1.57 | 9.72E-01 | 0.97 | 0.59-1.58 | 9.63E-01 |
| tartarate | 1.07 | 0.79-1.46 | 8.80E-01 | 1.02 | 0.76-1.38 | 9.63E-01 |
| leucine | 1.15 | 0.74-1.78 | 8.25E-01 | 1.03 | 0.67-1.58 | 9.64E-01 |
| X - 16124 | 1.08 | 0.67-1.75 | 9.28E-01 | 1.03 | 0.64-1.66 | 9.65E-01 |
| butyrylcarnitine (C4) | 0.94 | 0.61-1.46 | 9.41E-01 | 0.97 | 0.65-1.46 | 9.65E-01 |
| perfluorooctanesulfonate (PFOS) | 0.99 | 0.63-1.55 | 9.82E-01 | 0.97 | 0.57-1.63 | 9.65E-01 |
| X - 12096 | 1.09 | 0.66-1.81 | 9.24E-01 | 1.04 | 0.58-1.84 | 9.68E-01 |
| X - 21339 | 1.05 | 0.76-1.44 | 9.41E-01 | 0.98 | 0.69-1.39 | 9.68E-01 |
| threonine | 1.05 | 0.71-1.57 | 9.42E-01 | 1.03 | 0.66-1.61 | 9.68E-01 |
| X - 21410 | 0.95 | 0.6-1.52 | 9.53E-01 | 0.97 | 0.56-1.66 | 9.68E-01 |
| X - 25271 | 0.96 | 0.68-1.36 | 9.53E-01 | 0.98 | 0.66-1.44 | 9.68E-01 |
| X - 23276 | 1.03 | 0.71-1.5 | 9.58E-01 | 1.02 | 0.72-1.46 | 9.68E-01 |
| hippurate | 1.02 | 0.64-1.64 | 9.74E-01 | 1.03 | 0.63-1.7 | 9.68E-01 |
| X - 17690 | 1.02 | 0.65-1.6 | 9.74E-01 | 1.03 | 0.64-1.66 | 9.68E-01 |
| 2-palmitoyl-GPC (16:0)* | 0.98 | 0.59-1.63 | 9.78E-01 | 0.97 | 0.58-1.61 | 9.68E-01 |
| glucuronide of piperine metabolite C17H21NO3 (5)* | 0.99 | 0.65-1.52 | 9.89E-01 | 0.97 | 0.62-1.53 | 9.68E-01 |
| 1-stearoyl-2-arachidonoyl-GPI (18:0/20:4) | 0.99 | 0.6-1.64 | 9.92E-01 | 0.97 | 0.59-1.61 | 9.68E-01 |
| picolinate | 0.99 | 0.57-1.74 | 9.94E-01 | 0.96 | 0.55-1.69 | 9.68E-01 |
| 2-oxoarginine* | 1.12 | 0.72-1.73 | 8.65E-01 | 1.03 | 0.64-1.66 | 9.70E-01 |
| serine | 0.94 | 0.56-1.6 | 9.53E-01 | 0.97 | 0.56-1.69 | 9.73E-01 |
| corticosterone | 0.99 | 0.75-1.31 | 9.83E-01 | 1.02 | 0.73-1.42 | 9.74E-01 |
| X - 11315 | 0.99 | 0.6-1.65 | 9.93E-01 | 0.97 | 0.56-1.67 | 9.76E-01 |
| methionine sulfoxide | 1.06 | 0.77-1.46 | 9.14E-01 | 0.98 | 0.71-1.37 | 9.76E-01 |
| 4-vinylphenol sulfate | 0.97 | 0.66-1.43 | 9.67E-01 | 1.02 | 0.69-1.5 | 9.76E-01 |
| 3-hydroxyoleoylcarnitine | 0.96 | 0.67-1.4 | 9.53E-01 | 0.98 | 0.69-1.4 | 9.77E-01 |
| 1-palmitoyl-2-dihomo-linolenoyl-GPC (16:0/20:3n3 or 6)* | 1.02 | 0.59-1.78 | 9.78E-01 | 1.03 | 0.6-1.76 | 9.78E-01 |
| S-carboxyethylcysteine | 0.93 | 0.52-1.66 | 9.49E-01 | 1.03 | 0.56-1.9 | 9.79E-01 |
| hydroxyasparagine** | 1.04 | 0.73-1.47 | 9.53E-01 | 1.02 | 0.65-1.62 | 9.79E-01 |
| 7-methylguanine | 0.98 | 0.7-1.37 | 9.74E-01 | 0.98 | 0.69-1.41 | 9.79E-01 |
| X - 24344 | 0.95 | 0.64-1.39 | 9.41E-01 | 1.02 | 0.66-1.58 | 9.80E-01 |
| X - 26054 | 0.97 | 0.64-1.49 | 9.69E-01 | 0.98 | 0.65-1.48 | 9.80E-01 |
| eicosapentaenoate (EPA; 20:5n3) | 0.97 | 0.58-1.62 | 9.74E-01 | 0.98 | 0.59-1.63 | 9.80E-01 |
| S-methylmethionine | 1.02 | 0.65-1.59 | 9.78E-01 | 1.02 | 0.64-1.63 | 9.80E-01 |
| N-acetyl-2-aminoadipate | 1.02 | 0.72-1.46 | 9.69E-01 | 0.98 | 0.69-1.41 | 9.80E-01 |
| X - 18345 | 1.02 | 0.74-1.41 | 9.69E-01 | 1.02 | 0.71-1.46 | 9.80E-01 |
| 1-palmitoyl-2-stearoyl-GPC (16:0/18:0) | 1.02 | 0.78-1.33 | 9.67E-01 | 1.01 | 0.77-1.33 | 9.80E-01 |
| X - 11843 | 1.03 | 0.67-1.59 | 9.67E-01 | 0.98 | 0.63-1.54 | 9.80E-01 |
| N-methylpipecolate | 0.98 | 0.67-1.43 | 9.72E-01 | 0.98 | 0.67-1.45 | 9.80E-01 |
| glutarate (C5-DC) | 1.03 | 0.59-1.79 | 9.72E-01 | 1.02 | 0.58-1.79 | 9.80E-01 |
| 1-palmitoyl-2-arachidonoyl-GPI (16:0/20:4)* | 1.02 | 0.57-1.84 | 9.78E-01 | 1.02 | 0.57-1.84 | 9.80E-01 |
| arachidonoylcarnitine (C20:4) | 0.98 | 0.52-1.84 | 9.78E-01 | 0.97 | 0.51-1.87 | 9.80E-01 |
| X - 12013 | 1.00 | 0.63-1.57 | 9.94E-01 | 0.98 | 0.62-1.56 | 9.80E-01 |
| gamma-glutamyl-alpha-lysine | 1.04 | 0.71-1.52 | 9.53E-01 | 0.99 | 0.68-1.44 | 9.81E-01 |
| pregnenolone sulfate | 0.95 | 0.59-1.52 | 9.53E-01 | 1.02 | 0.62-1.68 | 9.82E-01 |
| N-acetylserine | 1.03 | 0.66-1.59 | 9.72E-01 | 0.98 | 0.59-1.64 | 9.83E-01 |
| methionine sulfone | 0.99 | 0.59-1.67 | 9.90E-01 | 1.02 | 0.6-1.72 | 9.83E-01 |
| decadienedioic acid (C10:2-DC)** | 0.97 | 0.64-1.46 | 9.61E-01 | 1.01 | 0.66-1.56 | 9.86E-01 |
| X - 12026 | 1.05 | 0.66-1.65 | 9.53E-01 | 0.99 | 0.6-1.63 | 9.86E-01 |
| N-carbamoylalanine | 1.02 | 0.71-1.45 | 9.75E-01 | 1.01 | 0.71-1.45 | 9.86E-01 |
| X - 21959 | 1.02 | 0.7-1.47 | 9.75E-01 | 0.99 | 0.69-1.41 | 9.89E-01 |
| indolebutyrate | 0.97 | 0.66-1.43 | 9.67E-01 | 1.01 | 0.69-1.48 | 9.91E-01 |
| X - 26058 | 1.10 | 0.67-1.8 | 9.11E-01 | 1.01 | 0.62-1.64 | 9.94E-01 |
| cholesterol sulfate | 0.93 | 0.58-1.49 | 9.34E-01 | 0.99 | 0.58-1.7 | 9.94E-01 |
| N-acetylalanine | 1.04 | 0.67-1.63 | 9.58E-01 | 0.99 | 0.59-1.67 | 9.94E-01 |
| N-acetylglutamate | 0.97 | 0.62-1.51 | 9.67E-01 | 1.01 | 0.67-1.53 | 9.94E-01 |
| arginine | 0.97 | 0.67-1.41 | 9.67E-01 | 1.01 | 0.66-1.53 | 9.94E-01 |
| carnitine | 0.98 | 0.61-1.58 | 9.78E-01 | 0.99 | 0.6-1.63 | 9.94E-01 |
| 1-(1-enyl-palmitoyl)-2-oleoyl-GPE (P-16:0/18:1)* | 0.99 | 0.57-1.72 | 9.87E-01 | 1.01 | 0.58-1.76 | 9.94E-01 |
| 3-hydroxypyridine glucuronide | 1.01 | 0.69-1.46 | 9.90E-01 | 1.01 | 0.67-1.53 | 9.94E-01 |
| X - 18899 | 1.00 | 0.65-1.55 | 9.94E-01 | 0.99 | 0.65-1.52 | 9.94E-01 |
| docosahexaenoylcholine | 1.00 | 0.58-1.73 | 9.98E-01 | 0.99 | 0.59-1.65 | 9.94E-01 |
| X - 11852 | 1.00 | 0.67-1.49 | 9.98E-01 | 1.01 | 0.68-1.48 | 9.94E-01 |
| dimethylglycine | 0.95 | 0.71-1.28 | 9.30E-01 | 1.00 | 0.72-1.38 | 9.96E-01 |
| galactonate | 1.06 | 0.72-1.54 | 9.41E-01 | 1.00 | 0.68-1.48 | 9.96E-01 |
| X - 24422 | 0.95 | 0.64-1.42 | 9.48E-01 | 1.00 | 0.65-1.55 | 9.96E-01 |
| succinylcarnitine (C4-DC) | 1.04 | 0.72-1.51 | 9.53E-01 | 1.01 | 0.67-1.5 | 9.96E-01 |
| delta-CEHC | 1.05 | 0.62-1.77 | 9.53E-01 | 1.00 | 0.57-1.75 | 9.96E-01 |
| X - 11470 | 1.03 | 0.8-1.31 | 9.53E-01 | 1.00 | 0.77-1.29 | 9.96E-01 |
| X - 13658 | 1.03 | 0.79-1.33 | 9.53E-01 | 1.00 | 0.72-1.38 | 9.96E-01 |
| glycoursodeoxycholate | 0.95 | 0.56-1.62 | 9.58E-01 | 1.01 | 0.58-1.75 | 9.96E-01 |
| X - 11850 | 1.03 | 0.69-1.55 | 9.67E-01 | 1.00 | 0.65-1.53 | 9.96E-01 |
| androstenediol (3beta,17beta) monosulfate (1) | 0.96 | 0.57-1.64 | 9.69E-01 | 1.01 | 0.56-1.8 | 9.96E-01 |
| 5alpha-androstan-3alpha,17alpha-diol monosulfate | 1.03 | 0.58-1.82 | 9.74E-01 | 1.01 | 0.58-1.73 | 9.96E-01 |
| X - 23655 | 0.98 | 0.69-1.4 | 9.75E-01 | 1.00 | 0.66-1.49 | 9.96E-01 |
| malonylcarnitine | 0.98 | 0.66-1.47 | 9.75E-01 | 1.00 | 0.67-1.51 | 9.96E-01 |
| 4-cholesten-3-one | 1.01 | 0.68-1.5 | 9.82E-01 | 1.00 | 0.67-1.47 | 9.96E-01 |
| trans-3,4-methyleneheptanoate | 1.01 | 0.69-1.48 | 9.83E-01 | 1.01 | 0.66-1.54 | 9.96E-01 |
| X - 24473 | 1.01 | 0.59-1.73 | 9.88E-01 | 0.99 | 0.57-1.72 | 9.96E-01 |
| glycolithocholate | 1.01 | 0.7-1.45 | 9.92E-01 | 1.00 | 0.69-1.43 | 9.96E-01 |
| maleate | 1.00 | 0.59-1.7 | 9.95E-01 | 1.00 | 0.58-1.7 | 9.96E-01 |
| 1,5-anhydroglucitol (1,5-AG) | 1.00 | 0.64-1.58 | 9.97E-01 | 1.00 | 0.62-1.61 | 9.96E-01 |
| beta-hydroxyisovaleroylcarnitine | 1.06 | 0.78-1.44 | 9.15E-01 | 1.00 | 0.69-1.44 | 9.96E-01 |
| X - 12456 | 1.05 | 0.69-1.58 | 9.53E-01 | 1.00 | 0.66-1.5 | 9.97E-01 |
| 2R,3R-dihydroxybutyrate | 1.05 | 0.75-1.46 | 9.41E-01 | 1.00 | 0.69-1.45 | 9.98E-01 |
| 2-linoleoylglycerol (18:2) | 1.04 | 0.62-1.75 | 9.67E-01 | 1.00 | 0.61-1.64 | 1.00E+00 |
| glycochenodeoxycholate 3-sulfate | 0.97 | 0.66-1.44 | 9.67E-01 | 1.00 | 0.68-1.48 | 1.00E+00 |

Note: the table shows associations from the competing risk Cox proportional hazards regression analysis where plasms metabolomics level from the Metabolon platform was main exposure and incident prostate cancer as the main outcome in ERGO-4 cohort. Model 1 : adjusted for age, cohort, and lipid lowering medication. Model 2: adjusted for model1, smoking status, BMI, alcohol and education. The table is ranked based on the *FDR* values in model 2, using bold text indicates metabolites are surpassing *FDR* adjusted P value treshold (*P*<0.05). ERGO-4 refer to the fourth and fifth visits from the baseline cohort. Abbreviations: BMI, body mass index; HR, hazard ratio; CI, confidence interval; FDR, false discovery rate.

**Supplementary Table 24. Serum metabolites associated with incident breast cancer from the Nightingale platform.**

| **Metabolite** | **Model 1** | | | **Model 2** | | |
| --- | --- | --- | --- | --- | --- | --- |
|  | **HR** | **95% CI** | ***FDR* value** | **HR** | **95% CI** | ***FDR* value** |
| Ratio of polyunsaturated fatty acids to total fatty acids | 0.8 | 0.64-1 | 9.83E-01 | 0.84 | 0.67-1.04 | 9.98E-01 |
| Phospholipids to total lipids ratio in large HDL | 1.29 | 0.98-1.71 | 9.83E-01 | 1.31 | 0.95-1.81 | 9.98E-01 |
| Degree of unsaturation | 0.8 | 0.63-1.02 | 9.83E-01 | 0.82 | 0.65-1.05 | 9.98E-01 |
| Pyruvate | 1.31 | 0.97-1.76 | 9.83E-01 | 1.33 | 0.97-1.82 | 9.98E-01 |
| Ratio of polyunsaturated fatty acids to monounsaturated fatty acids | 0.8 | 0.62-1.02 | 9.83E-01 | 0.83 | 0.66-1.06 | 9.98E-01 |
| Ratio of saturated fatty acids to total fatty acids | 1.22 | 0.92-1.61 | 9.83E-01 | 1.15 | 0.89-1.49 | 9.98E-01 |
| Ratio of omega-6 fatty acids to total fatty acids | 0.83 | 0.65-1.06 | 9.83E-01 | 0.88 | 0.7-1.1 | 9.98E-01 |
| Saturated fatty acids | 1.22 | 0.95-1.56 | 9.83E-01 | 1.15 | 0.91-1.46 | 9.98E-01 |
| Phospholipids to total lipids ratio in very large HDL | 0.8 | 0.65-0.98 | 9.83E-01 | 0.8 | 0.64-1 | 9.98E-01 |
| Ratio of linoleic acid to total fatty acids | 0.83 | 0.63-1.08 | 9.83E-01 | 0.88 | 0.69-1.13 | 9.98E-01 |
| Cholesteryl esters to total lipids ratio in large HDL | 0.83 | 0.64-1.09 | 9.83E-01 | 0.84 | 0.64-1.11 | 9.98E-01 |
| Ratio of monounsaturated fatty acids to total fatty acids | 1.23 | 0.93-1.63 | 9.83E-01 | 1.2 | 0.91-1.57 | 9.98E-01 |
| Monounsaturated fatty acids | 1.21 | 0.93-1.57 | 9.83E-01 | 1.16 | 0.9-1.5 | 9.98E-01 |
| Glutamine | 0.79 | 0.6-1.04 | 9.83E-01 | 0.8 | 0.6-1.08 | 9.98E-01 |
| Cholesterol to total lipids ratio in large HDL | 0.82 | 0.62-1.09 | 9.83E-01 | 0.82 | 0.6-1.11 | 9.98E-01 |
| Cholesterol to total lipids ratio in very large HDL | 1.31 | 0.97-1.76 | 9.83E-01 | 1.31 | 0.96-1.77 | 9.98E-01 |
| Cholesteryl esters to total lipids ratio in very large VLDL | 0.86 | 0.64-1.16 | 9.83E-01 | 0.89 | 0.67-1.19 | 9.98E-01 |
| Phospholipids to total lipids ratio in large LDL | 0.76 | 0.56-1.02 | 9.83E-01 | 0.77 | 0.58-1.02 | 9.98E-01 |
| Glycoprotein acetyls | 1.2 | 0.87-1.67 | 9.83E-01 | 1.21 | 0.86-1.7 | 9.98E-01 |
| Free cholesterol to total lipids ratio in chylomicrons and extremely large VLDL | 0.91 | 0.7-1.19 | 9.83E-01 | 0.94 | 0.73-1.22 | 9.98E-01 |
| Cholesteryl esters to total lipids ratio in very large HDL | 1.34 | 1.01-1.78 | 9.83E-01 | 1.33 | 1-1.78 | 9.98E-01 |
| Cholesteryl esters to total lipids ratio in large VLDL | 0.84 | 0.63-1.13 | 9.83E-01 | 0.88 | 0.65-1.18 | 9.98E-01 |
| Triglycerides in medium LDL | 1.22 | 0.93-1.6 | 9.83E-01 | 1.19 | 0.91-1.55 | 9.98E-01 |
| Cholesterol to total lipids ratio in very large VLDL | 0.87 | 0.64-1.18 | 9.83E-01 | 0.9 | 0.66-1.22 | 9.98E-01 |
| Free cholesterol in small HDL | 1.3 | 0.93-1.82 | 9.83E-01 | 1.26 | 0.9-1.79 | 9.98E-01 |
| Total fatty acids | 1.17 | 0.89-1.52 | 9.83E-01 | 1.12 | 0.86-1.46 | 9.98E-01 |
| Phospholipids in very large HDL | 0.8 | 0.6-1.08 | 9.83E-01 | 0.8 | 0.58-1.1 | 9.98E-01 |
| Triglycerides in LDL | 1.2 | 0.91-1.57 | 9.83E-01 | 1.16 | 0.89-1.52 | 9.98E-01 |
| Cholesteryl esters to total lipids ratio in medium VLDL | 0.84 | 0.62-1.14 | 9.83E-01 | 0.86 | 0.62-1.19 | 9.98E-01 |
| 3-Hydroxybutyrate | 1.27 | 0.99-1.63 | 9.83E-01 | 1.28 | 0.99-1.64 | 9.98E-01 |
| Lactate | 1.23 | 0.93-1.62 | 9.83E-01 | 1.25 | 0.93-1.69 | 9.98E-01 |
| Triglycerides in large LDL | 1.19 | 0.9-1.58 | 9.83E-01 | 1.16 | 0.88-1.53 | 9.98E-01 |
| Triglycerides in large VLDL | 1.11 | 0.82-1.49 | 9.83E-01 | 1.08 | 0.8-1.44 | 9.98E-01 |
| Cholesterol to total lipids ratio in large VLDL | 0.86 | 0.64-1.16 | 9.83E-01 | 0.9 | 0.67-1.21 | 9.98E-01 |
| Total lipids in very large VLDL | 1.09 | 0.82-1.45 | 9.83E-01 | 1.07 | 0.81-1.41 | 9.98E-01 |
| Triglycerides in very large VLDL | 1.1 | 0.82-1.47 | 9.83E-01 | 1.07 | 0.8-1.42 | 9.98E-01 |
| Glucose | 1.21 | 0.96-1.53 | 9.83E-01 | 1.2 | 0.94-1.54 | 9.98E-01 |
| Cholesterol to total lipids ratio in medium VLDL | 0.85 | 0.62-1.17 | 9.83E-01 | 0.87 | 0.63-1.21 | 9.98E-01 |
| Total lipids in lipoprotein particles | 1.14 | 0.87-1.5 | 9.83E-01 | 1.11 | 0.84-1.46 | 9.98E-01 |
| Phospholipids in very large VLDL | 1.08 | 0.82-1.43 | 9.83E-01 | 1.07 | 0.82-1.39 | 9.98E-01 |
| Total triglycerides | 1.11 | 0.84-1.47 | 9.83E-01 | 1.08 | 0.82-1.42 | 9.98E-01 |
| Cholesteryl esters in medium LDL | 1.17 | 0.87-1.58 | 9.83E-01 | 1.15 | 0.85-1.56 | 9.98E-01 |
| Concentration of very large VLDL particles | 1.09 | 0.82-1.46 | 9.83E-01 | 1.07 | 0.81-1.41 | 9.98E-01 |
| Phospholipids in small LDL | 1.14 | 0.85-1.52 | 9.83E-01 | 1.12 | 0.83-1.51 | 9.98E-01 |
| Triglycerides in small LDL | 1.14 | 0.87-1.49 | 9.83E-01 | 1.1 | 0.85-1.44 | 9.98E-01 |
| Cholesteryl esters in large HDL | 0.85 | 0.6-1.22 | 9.83E-01 | 0.85 | 0.58-1.23 | 9.98E-01 |
| Free cholesterol in very large VLDL | 1.08 | 0.82-1.42 | 9.83E-01 | 1.06 | 0.81-1.38 | 9.98E-01 |
| Triglycerides in VLDL | 1.09 | 0.82-1.46 | 9.83E-01 | 1.06 | 0.8-1.41 | 9.98E-01 |
| Total lipids in large VLDL | 1.09 | 0.81-1.46 | 9.83E-01 | 1.07 | 0.8-1.42 | 9.98E-01 |
| Total lipids in medium LDL | 1.17 | 0.86-1.58 | 9.83E-01 | 1.15 | 0.84-1.57 | 9.98E-01 |
| Total lipids in very large HDL | 0.82 | 0.59-1.15 | 9.83E-01 | 0.82 | 0.57-1.17 | 9.98E-01 |
| Triglycerides to total lipids ratio in large VLDL | 1.14 | 0.87-1.51 | 9.83E-01 | 1.1 | 0.83-1.44 | 9.98E-01 |
| Triglycerides in medium VLDL | 1.1 | 0.81-1.48 | 9.83E-01 | 1.07 | 0.8-1.45 | 9.98E-01 |
| Cholesterol in medium LDL | 1.16 | 0.85-1.58 | 9.83E-01 | 1.15 | 0.84-1.57 | 9.98E-01 |
| Cholesterol in large HDL | 0.86 | 0.6-1.22 | 9.83E-01 | 0.85 | 0.58-1.24 | 9.98E-01 |
| Free cholesterol to total lipids ratio in large LDL | 0.86 | 0.66-1.12 | 9.83E-01 | 0.88 | 0.67-1.15 | 9.98E-01 |
| Concentration of large VLDL particles | 1.09 | 0.81-1.46 | 9.83E-01 | 1.06 | 0.8-1.42 | 9.98E-01 |
| Total lipids in small HDL | 1.24 | 0.86-1.78 | 9.83E-01 | 1.2 | 0.82-1.76 | 9.98E-01 |
| Free cholesterol in large VLDL | 1.09 | 0.81-1.45 | 9.83E-01 | 1.07 | 0.8-1.42 | 9.98E-01 |
| Cholesteryl esters in LDL | 1.15 | 0.85-1.56 | 9.83E-01 | 1.14 | 0.84-1.56 | 9.98E-01 |
| Phospholipids to total lipids ratio in medium VLDL | 0.87 | 0.63-1.2 | 9.83E-01 | 0.89 | 0.64-1.24 | 9.98E-01 |
| Phospholipids in small HDL | 1.22 | 0.86-1.74 | 9.83E-01 | 1.18 | 0.82-1.7 | 9.98E-01 |
| Free cholesterol to total lipids ratio in very large HDL | 1.16 | 0.81-1.66 | 9.83E-01 | 1.17 | 0.8-1.71 | 9.98E-01 |
| Total lipids in small LDL | 1.13 | 0.84-1.51 | 9.83E-01 | 1.11 | 0.83-1.5 | 9.98E-01 |
| Total lipids in VLDL | 1.08 | 0.81-1.43 | 9.83E-01 | 1.05 | 0.8-1.38 | 9.98E-01 |
| Acetoacetate | 1.21 | 0.92-1.6 | 9.83E-01 | 1.21 | 0.92-1.59 | 9.98E-01 |
| Citrate | 1.19 | 0.86-1.66 | 9.83E-01 | 1.25 | 0.9-1.74 | 9.98E-01 |
| Ratio of docosahexaenoic acid to total fatty acids | 0.93 | 0.72-1.21 | 9.83E-01 | 0.92 | 0.69-1.22 | 9.98E-01 |
| Sphingomyelins | 1.19 | 0.88-1.6 | 9.83E-01 | 1.18 | 0.86-1.61 | 9.98E-01 |
| Average diameter for VLDL particles | 1.1 | 0.82-1.48 | 9.83E-01 | 1.08 | 0.8-1.46 | 9.98E-01 |
| Cholesteryl esters to total lipids ratio in large LDL | 1.21 | 0.85-1.72 | 9.83E-01 | 1.19 | 0.84-1.7 | 9.98E-01 |
| Triglycerides to total lipids ratio in very large HDL | 1.17 | 0.86-1.6 | 9.83E-01 | 1.16 | 0.85-1.59 | 9.98E-01 |
| Total lipids in LDL | 1.14 | 0.85-1.54 | 9.83E-01 | 1.13 | 0.83-1.54 | 9.98E-01 |
| Acetate | 0.89 | 0.69-1.14 | 9.83E-01 | 0.9 | 0.7-1.15 | 9.98E-01 |
| Cholesteryl esters in large LDL | 1.15 | 0.85-1.54 | 9.83E-01 | 1.14 | 0.83-1.56 | 9.98E-01 |
| Phospholipids in large VLDL | 1.07 | 0.8-1.44 | 9.83E-01 | 1.06 | 0.79-1.41 | 9.98E-01 |
| LDL cholesterol | 1.14 | 0.84-1.54 | 9.83E-01 | 1.13 | 0.83-1.55 | 9.98E-01 |
| Concentration of small HDL particles | 1.25 | 0.85-1.82 | 9.83E-01 | 1.22 | 0.82-1.83 | 9.98E-01 |
| Total lipids in chylomicrons and extremely large VLDL | 1.06 | 0.81-1.4 | 9.83E-01 | 1.04 | 0.8-1.35 | 9.98E-01 |
| Total phospholipids in lipoprotein particles | 1.14 | 0.85-1.52 | 9.83E-01 | 1.1 | 0.82-1.48 | 9.98E-01 |
| Phospholipids in medium LDL | 1.14 | 0.84-1.56 | 9.83E-01 | 1.13 | 0.82-1.56 | 9.98E-01 |
| Cholesteryl esters to total lipids ratio in very small VLDL | 0.85 | 0.62-1.16 | 9.83E-01 | 0.86 | 0.62-1.18 | 9.98E-01 |
| Ratio of triglycerides to phosphoglycerides | 1.07 | 0.8-1.45 | 9.83E-01 | 1.05 | 0.78-1.42 | 9.98E-01 |
| Free cholesterol in very large HDL | 0.82 | 0.59-1.15 | 9.83E-01 | 0.82 | 0.57-1.17 | 9.98E-01 |
| Total lipids in medium VLDL | 1.08 | 0.8-1.44 | 9.83E-01 | 1.06 | 0.79-1.41 | 9.98E-01 |
| Concentration of large HDL particles | 0.88 | 0.62-1.25 | 9.83E-01 | 0.87 | 0.6-1.27 | 9.98E-01 |
| Cholesteryl esters to total lipids ratio in medium LDL | 1.08 | 0.84-1.39 | 9.83E-01 | 1.06 | 0.83-1.36 | 9.98E-01 |
| Cholesteryl esters to total lipids ratio in small VLDL | 0.84 | 0.62-1.14 | 9.83E-01 | 0.87 | 0.64-1.18 | 9.98E-01 |
| Alanine | 1.1 | 0.87-1.38 | 9.83E-01 | 1.1 | 0.85-1.41 | 9.98E-01 |
| Total lipids in large LDL | 1.13 | 0.84-1.52 | 9.83E-01 | 1.13 | 0.83-1.53 | 9.98E-01 |
| Free cholesterol to total lipids ratio in medium LDL | 0.91 | 0.7-1.18 | 9.83E-01 | 0.93 | 0.72-1.2 | 9.98E-01 |
| Cholesterol in very large VLDL | 1.05 | 0.8-1.4 | 9.83E-01 | 1.04 | 0.79-1.37 | 9.98E-01 |
| Triglycerides to total lipids ratio in medium VLDL | 1.11 | 0.8-1.55 | 9.83E-01 | 1.09 | 0.78-1.52 | 9.98E-01 |
| Phospholipids to total lipids ratio in medium HDL | 1.01 | 0.77-1.33 | 9.83E-01 | 0.99 | 0.75-1.3 | 9.98E-01 |
| Cholesterol in large VLDL | 1.06 | 0.8-1.42 | 9.83E-01 | 1.05 | 0.79-1.39 | 9.98E-01 |
| Cholesterol in very large HDL | 0.86 | 0.62-1.19 | 9.83E-01 | 0.85 | 0.59-1.21 | 9.98E-01 |
| Cholesterol in large LDL | 1.13 | 0.84-1.52 | 9.83E-01 | 1.13 | 0.83-1.54 | 9.98E-01 |
| Cholesteryl esters in small LDL | 1.11 | 0.83-1.49 | 9.83E-01 | 1.1 | 0.81-1.48 | 9.98E-01 |
| Cholesteryl esters in very large HDL | 0.86 | 0.62-1.2 | 9.83E-01 | 0.86 | 0.6-1.22 | 9.98E-01 |
| Triglycerides in small HDL | 1.1 | 0.83-1.47 | 9.83E-01 | 1.08 | 0.81-1.43 | 9.98E-01 |
| Cholesterol to total lipids ratio in small VLDL | 0.85 | 0.62-1.17 | 9.83E-01 | 0.88 | 0.63-1.21 | 9.98E-01 |
| Triglycerides in small VLDL | 1.1 | 0.81-1.49 | 9.83E-01 | 1.08 | 0.8-1.45 | 9.98E-01 |
| Phospholipids in VLDL | 1.06 | 0.8-1.4 | 9.83E-01 | 1.04 | 0.79-1.37 | 9.98E-01 |
| Free cholesterol in VLDL | 1.06 | 0.81-1.4 | 9.83E-01 | 1.04 | 0.8-1.36 | 9.98E-01 |
| Cholesterol in small LDL | 1.11 | 0.82-1.5 | 9.83E-01 | 1.1 | 0.81-1.49 | 9.98E-01 |
| Cholesterol in small HDL | 1.24 | 0.85-1.8 | 9.83E-01 | 1.21 | 0.81-1.81 | 9.98E-01 |
| Acetone | 1.18 | 0.93-1.49 | 9.83E-01 | 1.18 | 0.93-1.5 | 9.98E-01 |
| Free cholesterol to total lipids ratio in medium VLDL | 0.88 | 0.64-1.22 | 9.83E-01 | 0.9 | 0.65-1.26 | 9.98E-01 |
| Phospholipids in LDL | 1.12 | 0.83-1.5 | 9.83E-01 | 1.11 | 0.81-1.51 | 9.98E-01 |
| Free cholesterol to total lipids ratio in large HDL | 0.8 | 0.59-1.08 | 9.83E-01 | 0.78 | 0.56-1.1 | 9.98E-01 |
| Triglycerides in IDL | 1.11 | 0.83-1.49 | 9.83E-01 | 1.08 | 0.81-1.45 | 9.98E-01 |
| Free cholesterol in large HDL | 0.87 | 0.61-1.24 | 9.83E-01 | 0.86 | 0.59-1.25 | 9.98E-01 |
| Cholesterol to total lipids ratio in chylomicrons and extremely large VLDL | 0.92 | 0.69-1.21 | 9.83E-01 | 0.95 | 0.73-1.24 | 9.98E-01 |
| Cholesteryl esters to total lipids ratio in chylomicrons and extremely large VLDL | 0.9 | 0.67-1.22 | 9.83E-01 | 0.94 | 0.7-1.25 | 9.98E-01 |
| Total lipids in large HDL | 0.89 | 0.63-1.27 | 9.83E-01 | 0.89 | 0.62-1.28 | 9.98E-01 |
| Ratio of omega-3 fatty acids to total fatty acids | 0.91 | 0.68-1.21 | 9.83E-01 | 0.88 | 0.64-1.2 | 9.98E-01 |
| Cholesterol to total lipids ratio in very small VLDL | 0.85 | 0.62-1.17 | 9.83E-01 | 0.86 | 0.62-1.19 | 9.98E-01 |
| Phospholipids to total lipids ratio in medium LDL | 0.92 | 0.71-1.18 | 9.83E-01 | 0.93 | 0.73-1.18 | 9.98E-01 |
| Isoleucine | 1.04 | 0.8-1.35 | 9.83E-01 | 1.05 | 0.79-1.38 | 9.98E-01 |
| Cholesteryl esters to total lipids ratio in medium HDL | 0.96 | 0.73-1.27 | 9.83E-01 | 0.99 | 0.75-1.3 | 9.98E-01 |
| Cholesteryl esters in chylomicrons and extremely large VLDL | 1.04 | 0.8-1.35 | 9.83E-01 | 1.03 | 0.79-1.33 | 9.98E-01 |
| Cholesterol in chylomicrons and extremely large VLDL | 1.04 | 0.8-1.36 | 9.83E-01 | 1.03 | 0.8-1.33 | 9.98E-01 |
| Concentration of VLDL particles | 1.06 | 0.8-1.4 | 9.83E-01 | 1.04 | 0.79-1.37 | 9.98E-01 |
| Concentration of small VLDL particles | 1.08 | 0.8-1.45 | 9.83E-01 | 1.07 | 0.8-1.42 | 9.98E-01 |
| Free cholesterol in small LDL | 1.1 | 0.81-1.5 | 9.83E-01 | 1.09 | 0.79-1.51 | 9.98E-01 |
| Total free cholesterol | 1.09 | 0.82-1.43 | 9.83E-01 | 1.07 | 0.8-1.42 | 9.98E-01 |
| Average diameter for HDL particles | 0.9 | 0.63-1.28 | 9.83E-01 | 0.9 | 0.62-1.3 | 9.98E-01 |
| Concentration of very large HDL particles | 0.87 | 0.62-1.22 | 9.83E-01 | 0.86 | 0.6-1.24 | 9.98E-01 |
| Total lipids in small VLDL | 1.07 | 0.8-1.44 | 9.83E-01 | 1.06 | 0.8-1.41 | 9.98E-01 |
| Total cholesterol minus HDL-C | 1.08 | 0.81-1.44 | 9.83E-01 | 1.07 | 0.79-1.44 | 9.98E-01 |
| Phosphatidylcholines | 1.14 | 0.84-1.54 | 9.83E-01 | 1.09 | 0.8-1.48 | 9.98E-01 |
| Phosphoglycerides | 1.14 | 0.83-1.57 | 9.83E-01 | 1.1 | 0.8-1.51 | 9.98E-01 |
| Triglycerides in very small VLDL | 1.09 | 0.81-1.47 | 9.83E-01 | 1.06 | 0.79-1.43 | 9.98E-01 |
| Total cholesterol | 1.1 | 0.83-1.47 | 9.83E-01 | 1.09 | 0.81-1.47 | 9.98E-01 |
| Free cholesterol in medium LDL | 1.12 | 0.82-1.53 | 9.83E-01 | 1.12 | 0.8-1.55 | 9.98E-01 |
| Concentration of medium VLDL particles | 1.05 | 0.79-1.41 | 9.83E-01 | 1.04 | 0.78-1.38 | 9.98E-01 |
| Triglycerides to total lipids ratio in very small VLDL | 1.19 | 0.86-1.65 | 9.83E-01 | 1.17 | 0.84-1.63 | 9.98E-01 |
| Total esterified cholesterol | 1.11 | 0.83-1.48 | 9.83E-01 | 1.09 | 0.81-1.48 | 9.98E-01 |
| Phospholipids in medium VLDL | 1.05 | 0.78-1.41 | 9.83E-01 | 1.04 | 0.78-1.38 | 9.98E-01 |
| Leucine | 1.02 | 0.76-1.37 | 9.83E-01 | 1.02 | 0.75-1.39 | 9.98E-01 |
| Phospholipids in large LDL | 1.09 | 0.81-1.47 | 9.83E-01 | 1.08 | 0.8-1.48 | 9.98E-01 |
| Albumin | 1.25 | 0.91-1.71 | 9.83E-01 | 1.28 | 0.92-1.77 | 9.98E-01 |
| Cholesteryl esters in very large VLDL | 1.03 | 0.77-1.38 | 9.83E-01 | 1.02 | 0.77-1.36 | 9.98E-01 |
| Cholesteryl esters in large VLDL | 1.03 | 0.78-1.37 | 9.83E-01 | 1.02 | 0.78-1.35 | 9.98E-01 |
| Cholesteryl esters in small HDL | 1.2 | 0.82-1.77 | 9.83E-01 | 1.19 | 0.79-1.78 | 9.98E-01 |
| Free cholesterol in medium VLDL | 1.04 | 0.78-1.39 | 9.83E-01 | 1.03 | 0.78-1.37 | 9.98E-01 |
| Free cholesterol in LDL | 1.09 | 0.81-1.48 | 9.83E-01 | 1.09 | 0.8-1.5 | 9.98E-01 |
| Triglycerides to total lipids ratio in very large VLDL | 1.09 | 0.76-1.56 | 9.83E-01 | 1.05 | 0.74-1.48 | 9.98E-01 |
| Glycine | 0.88 | 0.67-1.16 | 9.83E-01 | 0.91 | 0.67-1.24 | 9.98E-01 |
| Clinical LDL cholesterol | 1.08 | 0.8-1.46 | 9.83E-01 | 1.08 | 0.79-1.47 | 9.98E-01 |
| Concentration of LDL particles | 1.06 | 0.79-1.42 | 9.83E-01 | 1.05 | 0.78-1.42 | 9.98E-01 |
| Concentration of medium LDL particles | 1.08 | 0.81-1.44 | 9.83E-01 | 1.06 | 0.8-1.41 | 9.98E-01 |
| Cholesterol to total lipids ratio in small LDL | 0.9 | 0.66-1.22 | 9.83E-01 | 0.93 | 0.68-1.26 | 9.98E-01 |
| Total cholines | 1.13 | 0.83-1.55 | 9.83E-01 | 1.1 | 0.8-1.51 | 9.98E-01 |
| Free cholesterol to total lipids ratio in small VLDL | 0.9 | 0.65-1.24 | 9.83E-01 | 0.91 | 0.66-1.27 | 9.98E-01 |
| Phospholipids in large HDL | 0.91 | 0.65-1.29 | 9.83E-01 | 0.91 | 0.64-1.3 | 9.98E-01 |
| Phospholipids in small VLDL | 1.06 | 0.79-1.42 | 9.83E-01 | 1.05 | 0.78-1.4 | 9.98E-01 |
| Concentration of large LDL particles | 1.05 | 0.78-1.4 | 9.83E-01 | 1.05 | 0.78-1.41 | 9.98E-01 |
| Concentration of small LDL particles | 1.06 | 0.79-1.42 | 9.83E-01 | 1.05 | 0.78-1.41 | 9.98E-01 |
| Apolipoprotein B | 1.05 | 0.79-1.39 | 9.83E-01 | 1.04 | 0.78-1.39 | 9.98E-01 |
| Phospholipids to total lipids ratio in small VLDL | 0.91 | 0.66-1.25 | 9.83E-01 | 0.93 | 0.67-1.28 | 9.98E-01 |
| Concentration of chylomicrons and extremely large VLDL particles | 1.06 | 0.8-1.4 | 9.83E-01 | 1.03 | 0.79-1.36 | 9.98E-01 |
| VLDL cholesterol | 1.02 | 0.78-1.35 | 9.83E-01 | 1.01 | 0.77-1.32 | 9.98E-01 |
| Triglycerides to total lipids ratio in large HDL | 1.1 | 0.79-1.52 | 9.83E-01 | 1.09 | 0.78-1.52 | 9.98E-01 |
| Free cholesterol in large LDL | 1.08 | 0.8-1.45 | 9.83E-01 | 1.08 | 0.79-1.47 | 9.98E-01 |
| Triglycerides to total lipids ratio in small VLDL | 1.11 | 0.79-1.54 | 9.83E-01 | 1.08 | 0.77-1.51 | 9.98E-01 |
| Free cholesterol to total lipids ratio in very small VLDL | 0.97 | 0.75-1.25 | 9.83E-01 | 0.98 | 0.76-1.26 | 9.98E-01 |
| Free cholesterol to total lipids ratio in very large VLDL | 0.9 | 0.64-1.28 | 9.83E-01 | 0.94 | 0.67-1.32 | 9.98E-01 |
| Cholesterol to total lipids ratio in medium HDL | 0.98 | 0.74-1.31 | 9.83E-01 | 1 | 0.75-1.34 | 9.98E-01 |
| Total concentration of branched-chain amino acids (leucine + isoleucine + valine) | 1.01 | 0.76-1.33 | 9.83E-01 | 1.01 | 0.75-1.36 | 9.98E-01 |
| Free cholesterol in small VLDL | 1.04 | 0.77-1.39 | 9.83E-01 | 1.03 | 0.77-1.38 | 9.98E-01 |
| Free cholesterol to total lipids ratio in medium HDL | 1.1 | 0.81-1.5 | 9.83E-01 | 1.11 | 0.8-1.54 | 9.98E-01 |
| Free cholesterol to total lipids ratio in small HDL | 1.1 | 0.84-1.44 | 9.83E-01 | 1.12 | 0.84-1.48 | 9.98E-01 |
| Remnant cholesterol (non-HDL | 1.01 | 0.77-1.34 | 9.83E-01 | 1 | 0.76-1.32 | 9.98E-01 |
| Triglycerides in HDL | 1.07 | 0.8-1.43 | 9.83E-01 | 1.04 | 0.78-1.38 | 9.98E-01 |
| Free cholesterol to total lipids ratio in IDL | 0.88 | 0.65-1.19 | 9.83E-01 | 0.91 | 0.67-1.23 | 9.98E-01 |
| Phospholipids to total lipids ratio in very large VLDL | 1 | 0.79-1.26 | 9.87E-01 | 1.02 | 0.8-1.3 | 9.98E-01 |
| Triglycerides to total lipids ratio in small HDL | 1.04 | 0.77-1.38 | 9.83E-01 | 1.02 | 0.77-1.36 | 9.98E-01 |
| Triglycerides to total lipids ratio in small LDL | 1.08 | 0.8-1.47 | 9.83E-01 | 1.06 | 0.78-1.43 | 9.98E-01 |
| Total concentration of lipoprotein particles | 1.16 | 0.82-1.65 | 9.83E-01 | 1.14 | 0.79-1.63 | 9.98E-01 |
| Cholesteryl esters to total lipids ratio in small HDL | 0.96 | 0.72-1.29 | 9.83E-01 | 0.99 | 0.74-1.33 | 9.98E-01 |
| Free cholesterol to total lipids ratio in small LDL | 0.94 | 0.71-1.24 | 9.83E-01 | 0.95 | 0.72-1.26 | 9.98E-01 |
| Cholesterol to total lipids ratio in IDL | 0.9 | 0.68-1.2 | 9.83E-01 | 0.91 | 0.69-1.2 | 9.98E-01 |
| Triglycerides in medium HDL | 1.05 | 0.79-1.41 | 9.83E-01 | 1.02 | 0.77-1.36 | 9.98E-01 |
| Cholesterol in small VLDL | 1.02 | 0.76-1.37 | 9.83E-01 | 1.02 | 0.77-1.35 | 9.98E-01 |
| Phospholipids in chylomicrons and extremely large VLDL | 1.03 | 0.78-1.37 | 9.83E-01 | 1.01 | 0.77-1.34 | 9.98E-01 |
| Triglycerides to total lipids ratio in IDL | 1.11 | 0.81-1.51 | 9.83E-01 | 1.09 | 0.79-1.5 | 9.98E-01 |
| Phospholipids in IDL | 1.02 | 0.76-1.37 | 9.83E-01 | 1.02 | 0.76-1.38 | 9.98E-01 |
| Cholesteryl esters in small VLDL | 1.01 | 0.76-1.35 | 9.83E-01 | 1.01 | 0.77-1.33 | 9.98E-01 |
| Omega-6 fatty acids | 1.05 | 0.8-1.38 | 9.83E-01 | 1.05 | 0.79-1.4 | 9.98E-01 |
| Triglycerides to total lipids ratio in medium LDL | 1.12 | 0.82-1.53 | 9.83E-01 | 1.1 | 0.8-1.51 | 9.98E-01 |
| Cholesteryl esters in VLDL | 0.99 | 0.75-1.31 | 9.83E-01 | 0.99 | 0.75-1.29 | 9.98E-01 |
| Ratio of apolipoprotein B to apolipoprotein A1 | 1 | 0.72-1.37 | 9.87E-01 | 1 | 0.73-1.37 | 9.98E-01 |
| Total lipids in IDL | 1.01 | 0.76-1.35 | 9.83E-01 | 1.01 | 0.75-1.35 | 9.98E-01 |
| Triglycerides in chylomicrons and extremely large VLDL | 1.03 | 0.76-1.38 | 9.83E-01 | 1.01 | 0.77-1.33 | 9.98E-01 |
| Creatinine | 1.07 | 0.78-1.46 | 9.83E-01 | 1.09 | 0.8-1.48 | 9.98E-01 |
| Free cholesterol to total lipids ratio in large VLDL | 0.96 | 0.76-1.23 | 9.83E-01 | 0.99 | 0.78-1.26 | 9.98E-01 |
| Free cholesterol in chylomicrons and extremely large VLDL | 1.02 | 0.77-1.35 | 9.83E-01 | 1 | 0.76-1.32 | 9.98E-01 |
| Free cholesterol in very small VLDL | 0.97 | 0.72-1.31 | 9.83E-01 | 0.97 | 0.72-1.29 | 9.98E-01 |
| Valine | 0.99 | 0.76-1.3 | 9.83E-01 | 0.98 | 0.73-1.32 | 9.98E-01 |
| Ratio of omega-6 fatty acids to omega-3 fatty acids | 1.05 | 0.78-1.41 | 9.83E-01 | 1.1 | 0.8-1.51 | 9.98E-01 |
| Concentration of HDL particles | 1.15 | 0.8-1.63 | 9.83E-01 | 1.12 | 0.78-1.62 | 9.98E-01 |
| Phospholipids to total lipids ratio in chylomicrons and extremely large VLDL | 1.03 | 0.79-1.34 | 9.83E-01 | 1.03 | 0.78-1.34 | 9.98E-01 |
| Phospholipids in very small VLDL | 0.97 | 0.71-1.32 | 9.83E-01 | 0.97 | 0.72-1.3 | 9.98E-01 |
| Cholesterol to total lipids ratio in large LDL | 1.06 | 0.78-1.44 | 9.83E-01 | 1.07 | 0.78-1.47 | 9.98E-01 |
| Total lipids in very small VLDL | 0.98 | 0.72-1.32 | 9.83E-01 | 0.97 | 0.72-1.3 | 9.98E-01 |
| Cholesteryl esters in IDL | 1 | 0.75-1.33 | 9.93E-01 | 0.99 | 0.74-1.33 | 9.98E-01 |
| Triglycerides to total lipids ratio in large LDL | 1.08 | 0.79-1.49 | 9.83E-01 | 1.06 | 0.77-1.47 | 9.98E-01 |
| Cholesterol in IDL | 1 | 0.75-1.33 | 9.87E-01 | 0.99 | 0.73-1.33 | 9.98E-01 |
| Cholesterol in medium VLDL | 0.99 | 0.74-1.32 | 9.83E-01 | 0.98 | 0.73-1.32 | 9.98E-01 |
| Cholesteryl esters in HDL | 1.01 | 0.72-1.41 | 9.83E-01 | 1 | 0.71-1.41 | 9.98E-01 |
| Cholesteryl esters to total lipids ratio in small LDL | 0.95 | 0.71-1.26 | 9.83E-01 | 0.95 | 0.71-1.28 | 9.98E-01 |
| Concentration of very small VLDL particles | 0.98 | 0.72-1.31 | 9.83E-01 | 0.97 | 0.72-1.3 | 9.98E-01 |
| Cholesterol to total lipids ratio in small HDL | 0.99 | 0.75-1.32 | 9.83E-01 | 1.03 | 0.78-1.36 | 9.98E-01 |
| Polyunsaturated fatty acids | 1.03 | 0.78-1.37 | 9.83E-01 | 1.02 | 0.76-1.38 | 9.98E-01 |
| Concentration of IDL particles | 0.97 | 0.74-1.27 | 9.83E-01 | 0.95 | 0.72-1.26 | 9.98E-01 |
| Cholesterol to total lipids ratio in medium LDL | 0.99 | 0.76-1.29 | 9.83E-01 | 0.99 | 0.77-1.28 | 9.98E-01 |
| Linoleic acid | 1.01 | 0.77-1.33 | 9.83E-01 | 1.03 | 0.78-1.36 | 9.98E-01 |
| Cholesteryl esters to total lipids ratio in IDL | 0.94 | 0.71-1.26 | 9.83E-01 | 0.94 | 0.71-1.25 | 9.98E-01 |
| HDL cholesterol | 1.01 | 0.72-1.42 | 9.83E-01 | 1 | 0.71-1.42 | 9.98E-01 |
| Phospholipids to total lipids ratio in small LDL | 1.04 | 0.75-1.43 | 9.83E-01 | 1.03 | 0.74-1.43 | 9.98E-01 |
| Phospholipids to total lipids ratio in IDL | 1.04 | 0.8-1.36 | 9.83E-01 | 1.07 | 0.82-1.39 | 9.98E-01 |
| Free cholesterol in IDL | 0.98 | 0.73-1.32 | 9.83E-01 | 0.98 | 0.72-1.33 | 9.98E-01 |
| Omega-3 fatty acids | 0.97 | 0.72-1.31 | 9.83E-01 | 0.93 | 0.67-1.28 | 9.98E-01 |
| Phenylalanine | 1.01 | 0.76-1.34 | 9.83E-01 | 1.02 | 0.77-1.37 | 9.98E-01 |
| Triglycerides in large HDL | 0.99 | 0.71-1.36 | 9.83E-01 | 0.97 | 0.69-1.34 | 9.98E-01 |
| Tyrosine | 1.01 | 0.79-1.29 | 9.83E-01 | 1 | 0.76-1.31 | 9.98E-01 |
| Docosahexaenoic acid | 1.01 | 0.75-1.35 | 9.83E-01 | 0.98 | 0.71-1.35 | 9.98E-01 |
| Phospholipids to total lipids ratio in very small VLDL | 0.94 | 0.64-1.36 | 9.83E-01 | 0.95 | 0.65-1.38 | 9.98E-01 |
| Histidine | 0.98 | 0.7-1.37 | 9.83E-01 | 0.99 | 0.7-1.39 | 9.98E-01 |
| Phospholipids to total lipids ratio in small HDL | 0.96 | 0.69-1.34 | 9.83E-01 | 0.93 | 0.67-1.29 | 9.98E-01 |
| Cholesteryl esters in medium HDL | 1.05 | 0.74-1.49 | 9.83E-01 | 1.03 | 0.72-1.47 | 9.98E-01 |
| Free cholesterol in medium HDL | 1.08 | 0.76-1.54 | 9.83E-01 | 1.06 | 0.74-1.52 | 9.98E-01 |
| Phospholipids to total lipids ratio in large VLDL | 0.98 | 0.77-1.24 | 9.83E-01 | 0.99 | 0.78-1.25 | 9.98E-01 |
| Triglycerides in very large HDL | 1.01 | 0.72-1.41 | 9.83E-01 | 0.98 | 0.71-1.37 | 9.98E-01 |
| Total lipids in HDL | 1.03 | 0.73-1.47 | 9.83E-01 | 1.01 | 0.71-1.44 | 9.98E-01 |
| Phospholipids in medium HDL | 1.07 | 0.75-1.55 | 9.83E-01 | 1.04 | 0.72-1.5 | 9.98E-01 |
| Cholesterol in medium HDL | 1.05 | 0.74-1.5 | 9.83E-01 | 1.04 | 0.73-1.48 | 9.98E-01 |
| Cholesterol in very small VLDL | 0.92 | 0.68-1.25 | 9.83E-01 | 0.92 | 0.68-1.25 | 9.98E-01 |
| Cholesteryl esters in medium VLDL | 0.93 | 0.68-1.26 | 9.83E-01 | 0.94 | 0.69-1.27 | 9.98E-01 |
| Triglycerides to total lipids ratio in chylomicrons and extremely large VLDL | 1 | 0.75-1.34 | 9.93E-01 | 0.99 | 0.75-1.3 | 9.98E-01 |
| Cholesteryl esters in very small VLDL | 0.9 | 0.66-1.23 | 9.83E-01 | 0.9 | 0.66-1.23 | 9.98E-01 |
| Concentration of medium HDL particles | 1.06 | 0.74-1.52 | 9.83E-01 | 1.03 | 0.71-1.49 | 9.98E-01 |
| Phospholipids in HDL | 1.04 | 0.73-1.48 | 9.83E-01 | 1.01 | 0.71-1.45 | 9.98E-01 |
| Apolipoprotein A1 | 1.08 | 0.76-1.53 | 9.83E-01 | 1.05 | 0.74-1.5 | 9.98E-01 |
| Free cholesterol in HDL | 1.04 | 0.74-1.45 | 9.83E-01 | 1.02 | 0.72-1.44 | 9.98E-01 |
| Total lipids in medium HDL | 1.07 | 0.74-1.53 | 9.83E-01 | 1.04 | 0.72-1.49 | 9.98E-01 |
| Average diameter for LDL particles | 0.98 | 0.76-1.27 | 9.83E-01 | 1 | 0.78-1.29 | 9.98E-01 |
| Triglycerides to total lipids ratio in medium HDL | 1.02 | 0.76-1.36 | 9.83E-01 | 1 | 0.74-1.34 | 1.00E+00 |

Note: the table shows associations from the competing risk Cox proportional hazards regression analysis where plasms metabolomics level from the Nightingale platform was main exposure and incident breast cancer as the main outcome in ERGO-4 cohort. Model 1 : adjusted for age, cohort, and lipid lowering medication. Model 2: adjusted for model1, smoking status, BMI, alcohol and education. The table is ranked based on the *FDR* values in model 2, using bold text indicates metabolites are surpassing *FDR* adjusted P value treshold (*P*<0.05). ERGO-4 refer to the fourth and fifth visits from the baseline cohort. Abbreviations: IDL, intermediate-density lipoprotein; LDL, low-density lipoprotein; HDL, high-density lipoprotein; VLDL, very-low-density lipoprotein; BMI, body mass index; HR, hazard ratio; CI, confidence interval; FDR, false discovery rate.

**Supplementary Table 24. Serum metabolites associated with incident breast cancer from the Metabolon platform.**

| **Metabolite** | **Model 1** | | | **Model 2** | | |
| --- | --- | --- | --- | --- | --- | --- |
|  | **HR** | **95% CI** | ***FDR* value** | **HR** | **95% CI** | ***FDR* value** |
| 7-methylxanthine | 1.93 | 1.26-2.96 | 9.98E-01 | 1.96 | 1.26-3.04 | 7.56E-01 |
| 4-cholesten-3-one | 0.61 | 0.43-0.85 | 9.98E-01 | 0.60 | 0.42-0.86 | 7.56E-01 |
| 5-(galactosylhydroxy)-L-lysine | 1.94 | 1.18-3.18 | 9.98E-01 | 2.07 | 1.25-3.44 | 7.56E-01 |
| 4-acetamidobutanoate | 2.15 | 1.27-3.62 | 9.98E-01 | 2.18 | 1.3-3.66 | 7.56E-01 |
| corticosterone | 0.68 | 0.52-0.9 | 9.98E-01 | 0.68 | 0.52-0.89 | 7.56E-01 |
| X - 21339 | 1.70 | 1.22-2.36 | 9.98E-01 | 1.76 | 1.25-2.46 | 7.56E-01 |
| X - 24812 | 1.42 | 1.06-1.89 | 9.98E-01 | 1.67 | 1.17-2.39 | 7.56E-01 |
| sphingomyelin (d18:1/20:0, d16:1/22:0)* | 1.38 | 1.08-1.77 | 9.98E-01 | 1.40 | 1.1-1.79 | 8.82E-01 |
| X - 12104 | 0.65 | 0.47-0.89 | 9.98E-01 | 0.65 | 0.47-0.89 | 8.82E-01 |
| 3-methylxanthine | 1.59 | 1.13-2.23 | 9.98E-01 | 1.56 | 1.12-2.17 | 8.95E-01 |
| carnitine | 1.08 | 0.78-1.47 | 9.98E-01 | 1.09 | 0.79-1.51 | 9.98E-01 |
| 3-phenylpropionate (hydrocinnamate) | 1.06 | 0.7-1.6 | 9.98E-01 | 1.06 | 0.7-1.6 | 9.98E-01 |
| phenylacetate | 0.86 | 0.55-1.34 | 9.98E-01 | 0.90 | 0.58-1.4 | 9.98E-01 |
| hippurate | 0.96 | 0.72-1.28 | 9.98E-01 | 0.96 | 0.72-1.3 | 9.98E-01 |
| xanthurenate | 0.94 | 0.67-1.31 | 9.98E-01 | 0.93 | 0.65-1.33 | 9.98E-01 |
| suberate (C8-DC) | 0.89 | 0.62-1.27 | 9.98E-01 | 0.89 | 0.63-1.27 | 9.98E-01 |
| 3-methyl-2-oxovalerate | 0.97 | 0.65-1.47 | 9.98E-01 | 0.97 | 0.62-1.51 | 9.98E-01 |
| methionine sulfoxide | 0.82 | 0.58-1.14 | 9.98E-01 | 0.82 | 0.59-1.16 | 9.98E-01 |
| 3-methylhistidine | 0.99 | 0.72-1.35 | 9.98E-01 | 0.99 | 0.71-1.36 | 9.98E-01 |
| 5-hydroxylysine | 0.98 | 0.65-1.46 | 9.98E-01 | 1.00 | 0.69-1.44 | 9.98E-01 |
| 4-guanidinobutanoate | 0.90 | 0.64-1.26 | 9.98E-01 | 0.91 | 0.64-1.29 | 9.98E-01 |
| glucuronate | 1.10 | 0.74-1.64 | 9.98E-01 | 1.10 | 0.74-1.63 | 9.98E-01 |
| glycerol 3-phosphate | 0.80 | 0.49-1.29 | 9.98E-01 | 0.80 | 0.48-1.31 | 9.98E-01 |
| imidazole lactate | 1.11 | 0.73-1.67 | 9.98E-01 | 1.10 | 0.72-1.7 | 9.98E-01 |
| kynurenine | 1.10 | 0.71-1.69 | 9.98E-01 | 1.17 | 0.74-1.85 | 9.98E-01 |
| glycerophosphorylcholine (GPC) | 0.83 | 0.6-1.15 | 9.98E-01 | 0.80 | 0.54-1.17 | 9.98E-01 |
| N-acetylglutamate | 0.99 | 0.67-1.46 | 9.98E-01 | 0.99 | 0.67-1.47 | 9.98E-01 |
| tartarate | 1.05 | 0.74-1.5 | 9.98E-01 | 1.03 | 0.72-1.47 | 9.98E-01 |
| xanthosine | 1.32 | 0.88-1.99 | 9.98E-01 | 1.35 | 0.9-2.04 | 9.98E-01 |
| ribitol | 0.73 | 0.49-1.09 | 9.98E-01 | 0.74 | 0.49-1.1 | 9.98E-01 |
| theophylline | 1.11 | 0.74-1.68 | 9.98E-01 | 1.10 | 0.74-1.63 | 9.98E-01 |
| quinate | 1.11 | 0.82-1.52 | 9.98E-01 | 1.10 | 0.8-1.49 | 9.98E-01 |
| theobromine | 1.54 | 1.01-2.36 | 9.98E-01 | 1.57 | 1.04-2.36 | 9.98E-01 |
| gentisate | 1.41 | 0.91-2.18 | 9.98E-01 | 1.41 | 0.92-2.16 | 9.98E-01 |
| paraxanthine | 1.01 | 0.71-1.45 | 9.98E-01 | 1.02 | 0.72-1.44 | 9.98E-01 |
| indolelactate | 1.20 | 0.83-1.74 | 9.98E-01 | 1.22 | 0.83-1.79 | 9.98E-01 |
| 3-indoxyl sulfate | 0.85 | 0.5-1.43 | 9.98E-01 | 0.86 | 0.5-1.49 | 9.98E-01 |
| gamma-glutamylphenylalanine | 0.94 | 0.66-1.35 | 9.98E-01 | 0.94 | 0.65-1.36 | 9.98E-01 |
| 4-methyl-2-oxopentanoate | 0.95 | 0.6-1.5 | 9.98E-01 | 0.93 | 0.58-1.5 | 9.98E-01 |
| 1,5-anhydroglucitol (1,5-AG) | 0.89 | 0.64-1.24 | 9.98E-01 | 0.89 | 0.63-1.23 | 9.98E-01 |
| 1-stearoyl-2-arachidonoyl-GPI (18:0/20:4) | 0.84 | 0.61-1.16 | 9.98E-01 | 0.81 | 0.59-1.13 | 9.98E-01 |
| sphingosine 1-phosphate | 1.03 | 0.65-1.62 | 9.98E-01 | 1.03 | 0.64-1.65 | 9.98E-01 |
| 1-stearoyl-2-oleoyl-GPS (18:0/18:1) | 1.23 | 0.84-1.8 | 9.98E-01 | 1.25 | 0.85-1.82 | 9.98E-01 |
| 1-stearoyl-GPI (18:0) | 1.08 | 0.79-1.48 | 9.98E-01 | 1.09 | 0.79-1.5 | 9.98E-01 |
| 1,2-dipalmitoyl-GPC (16:0/16:0) | 0.71 | 0.5-1.01 | 9.98E-01 | 0.69 | 0.47-1.01 | 9.98E-01 |
| docosahexaenoate (DHA; 22:6n3) | 0.88 | 0.65-1.2 | 9.98E-01 | 0.84 | 0.61-1.16 | 9.98E-01 |
| 1-myristoyl-2-palmitoyl-GPC (14:0/16:0) | 0.94 | 0.65-1.35 | 9.98E-01 | 0.94 | 0.65-1.37 | 9.98E-01 |
| alpha-hydroxyisocaproate | 1.14 | 0.84-1.55 | 9.98E-01 | 1.12 | 0.8-1.55 | 9.98E-01 |
| maleate | 0.74 | 0.45-1.22 | 9.98E-01 | 0.75 | 0.44-1.26 | 9.98E-01 |
| isovalerate (i5:0) | 0.87 | 0.67-1.13 | 9.98E-01 | 0.88 | 0.67-1.15 | 9.98E-01 |
| 4-acetylphenol sulfate | 1.03 | 0.71-1.48 | 9.98E-01 | 1.02 | 0.71-1.48 | 9.98E-01 |
| 2-hydroxyoctanoate | 1.05 | 0.73-1.52 | 9.98E-01 | 1.04 | 0.69-1.56 | 9.98E-01 |
| 3-hydroxyoctanoate | 1.08 | 0.8-1.44 | 9.98E-01 | 1.03 | 0.77-1.36 | 9.98E-01 |
| phenyllactate (PLA) | 1.01 | 0.73-1.41 | 9.98E-01 | 1.00 | 0.71-1.41 | 9.98E-01 |
| palmitoylcarnitine (C16) | 1.14 | 0.77-1.69 | 9.98E-01 | 1.12 | 0.75-1.68 | 9.98E-01 |
| hexanoylcarnitine (C6) | 1.09 | 0.8-1.48 | 9.98E-01 | 1.07 | 0.78-1.47 | 9.98E-01 |
| N-acetylaspartate (NAA) | 1.05 | 0.7-1.59 | 9.98E-01 | 1.06 | 0.69-1.62 | 9.98E-01 |
| dehydroepiandrosterone sulfate (DHEA-S) | 1.01 | 0.64-1.57 | 9.98E-01 | 0.99 | 0.65-1.51 | 9.98E-01 |
| acetylcarnitine (C2) | 1.15 | 0.86-1.53 | 9.98E-01 | 1.13 | 0.84-1.51 | 9.98E-01 |
| cysteine s-sulfate | 0.64 | 0.42-0.96 | 9.98E-01 | 0.66 | 0.45-0.97 | 9.98E-01 |
| 1-palmitoylglycerol (16:0) | 0.78 | 0.53-1.14 | 9.98E-01 | 0.78 | 0.54-1.13 | 9.98E-01 |
| tartronate (hydroxymalonate) | 1.13 | 0.66-1.92 | 9.98E-01 | 1.12 | 0.7-1.79 | 9.98E-01 |
| oxalate (ethanedioate) | 0.98 | 0.63-1.54 | 9.98E-01 | 0.97 | 0.66-1.44 | 9.98E-01 |
| erythritol | 0.94 | 0.62-1.43 | 9.98E-01 | 0.96 | 0.63-1.46 | 9.98E-01 |
| 2,3-dihydroxypyridine | 0.95 | 0.68-1.33 | 9.98E-01 | 0.95 | 0.68-1.32 | 9.98E-01 |
| iminodiacetate (IDA) | 1.05 | 0.73-1.51 | 9.98E-01 | 1.02 | 0.7-1.48 | 9.98E-01 |
| 1-oleoylglycerol (18:1) | 0.95 | 0.64-1.41 | 9.98E-01 | 0.93 | 0.64-1.36 | 9.98E-01 |
| 3-methyl-2-oxobutyrate | 0.87 | 0.6-1.28 | 9.98E-01 | 0.85 | 0.57-1.28 | 9.98E-01 |
| 2-oleoylglycerol (18:1) | 0.97 | 0.66-1.43 | 9.98E-01 | 0.96 | 0.64-1.44 | 9.98E-01 |
| homoarginine | 1.03 | 0.68-1.57 | 9.98E-01 | 1.05 | 0.67-1.65 | 9.98E-01 |
| homocitrulline | 1.42 | 1.01-2 | 9.98E-01 | 1.44 | 1.04-2 | 9.98E-01 |
| 2-linoleoylglycerol (18:2) | 0.78 | 0.58-1.05 | 9.98E-01 | 0.76 | 0.56-1.03 | 9.98E-01 |
| 3-hydroxydecanoate | 1.05 | 0.74-1.47 | 9.98E-01 | 1.01 | 0.72-1.4 | 9.98E-01 |
| citramalate | 1.42 | 0.97-2.09 | 9.98E-01 | 1.41 | 0.95-2.07 | 9.98E-01 |
| EDTA | 0.86 | 0.58-1.28 | 9.98E-01 | 0.84 | 0.56-1.27 | 9.98E-01 |
| N-acetylglycine | 1.05 | 0.71-1.55 | 9.98E-01 | 1.02 | 0.66-1.56 | 9.98E-01 |
| ribonate | 0.91 | 0.69-1.2 | 9.98E-01 | 0.94 | 0.73-1.22 | 9.98E-01 |
| threonate | 1.01 | 0.63-1.63 | 9.98E-01 | 0.99 | 0.65-1.51 | 9.98E-01 |
| galactonate | 1.01 | 0.69-1.46 | 9.98E-01 | 1.03 | 0.69-1.52 | 9.98E-01 |
| indoleacetate | 0.99 | 0.58-1.69 | 9.98E-01 | 1.03 | 0.59-1.8 | 9.98E-01 |
| 1-linoleoylglycerol (18:2) | 0.77 | 0.54-1.1 | 9.98E-01 | 0.76 | 0.55-1.07 | 9.98E-01 |
| butyrylcarnitine (C4) | 1.05 | 0.75-1.47 | 9.98E-01 | 1.06 | 0.77-1.47 | 9.98E-01 |
| isobutyrylcarnitine (C4) | 0.86 | 0.58-1.26 | 9.98E-01 | 0.88 | 0.59-1.29 | 9.98E-01 |
| glycolithocholate | 0.99 | 0.71-1.38 | 9.98E-01 | 1.03 | 0.73-1.44 | 9.98E-01 |
| androsterone sulfate | 1.01 | 0.7-1.45 | 9.98E-01 | 0.99 | 0.7-1.4 | 9.98E-01 |
| 2-pyrrolidinone | 1.63 | 1.09-2.42 | 9.98E-01 | 1.64 | 1.1-2.44 | 9.98E-01 |
| indolepropionate | 0.83 | 0.58-1.2 | 9.98E-01 | 0.80 | 0.55-1.15 | 9.98E-01 |
| N-(2-furoyl)glycine | 0.89 | 0.69-1.16 | 9.98E-01 | 0.90 | 0.7-1.16 | 9.98E-01 |
| trigonelline (N'-methylnicotinate) | 1.26 | 0.91-1.75 | 9.98E-01 | 1.25 | 0.89-1.75 | 9.98E-01 |
| dodecanedioate (C12-DC) | 0.95 | 0.55-1.64 | 9.98E-01 | 0.92 | 0.51-1.68 | 9.98E-01 |
| N-acetyltyrosine | 0.79 | 0.49-1.26 | 9.98E-01 | 0.75 | 0.46-1.23 | 9.98E-01 |
| 1,3-dimethylurate | 1.01 | 0.67-1.5 | 9.98E-01 | 0.99 | 0.66-1.51 | 9.98E-01 |
| 3-hydroxylaurate | 1.07 | 0.75-1.51 | 9.98E-01 | 1.02 | 0.73-1.43 | 9.98E-01 |
| pyridoxate | 0.91 | 0.58-1.45 | 9.98E-01 | 0.89 | 0.57-1.4 | 9.98E-01 |
| gamma-glutamylvaline | 0.97 | 0.62-1.51 | 9.98E-01 | 1.03 | 0.66-1.59 | 9.98E-01 |
| 3-hydroxysebacate | 1.04 | 0.71-1.52 | 9.98E-01 | 1.01 | 0.69-1.49 | 9.98E-01 |
| 5-hydroxyhexanoate | 1.02 | 0.79-1.32 | 9.98E-01 | 1.04 | 0.8-1.37 | 9.98E-01 |
| propionylglycine | 1.45 | 1.05-2.02 | 9.98E-01 | 1.49 | 1.05-2.11 | 9.98E-01 |
| butyrylglycine | 1.05 | 0.71-1.55 | 9.98E-01 | 1.06 | 0.7-1.62 | 9.98E-01 |
| propionylcarnitine (C3) | 0.95 | 0.66-1.36 | 9.98E-01 | 0.99 | 0.67-1.46 | 9.98E-01 |
| pro-hydroxy-pro | 1.28 | 0.85-1.94 | 9.98E-01 | 1.35 | 0.87-2.08 | 9.98E-01 |
| 3-hydroxy-2-ethylpropionate | 0.77 | 0.53-1.11 | 9.98E-01 | 0.76 | 0.51-1.14 | 9.98E-01 |
| 3-carboxy-4-methyl-5-propyl-2-furanpropanoate (CMPF) | 1.04 | 0.73-1.49 | 9.98E-01 | 1.02 | 0.72-1.45 | 9.98E-01 |
| docosapentaenoate (n3 DPA; 22:5n3) | 0.88 | 0.64-1.21 | 9.98E-01 | 0.82 | 0.59-1.13 | 9.98E-01 |
| docosadienoate (22:2n6) | 0.90 | 0.67-1.21 | 9.98E-01 | 0.84 | 0.62-1.14 | 9.98E-01 |
| adrenate (22:4n6) | 0.86 | 0.63-1.16 | 9.98E-01 | 0.81 | 0.6-1.1 | 9.98E-01 |
| docosatrienoate (22:3n3) | 1.00 | 0.72-1.39 | 9.98E-01 | 1.02 | 0.74-1.4 | 9.98E-01 |
| 10-undecenoate (11:1n1) | 1.31 | 0.93-1.84 | 9.98E-01 | 1.24 | 0.9-1.72 | 9.98E-01 |
| myristoleate (14:1n5) | 1.02 | 0.66-1.57 | 9.98E-01 | 0.97 | 0.63-1.5 | 9.98E-01 |
| 1-methyl-4-imidazoleacetate | 0.87 | 0.5-1.53 | 9.98E-01 | 0.92 | 0.53-1.61 | 9.98E-01 |
| sebacate (C10-DC) | 0.94 | 0.6-1.46 | 9.98E-01 | 0.93 | 0.59-1.46 | 9.98E-01 |
| stearidonate (18:4n3) | 0.80 | 0.58-1.1 | 9.98E-01 | 0.74 | 0.53-1.04 | 9.98E-01 |
| 5-dodecenoate (12:1n7) | 0.98 | 0.63-1.52 | 9.98E-01 | 0.93 | 0.58-1.48 | 9.98E-01 |
| octanoylcarnitine (C8) | 1.02 | 0.73-1.44 | 9.98E-01 | 0.99 | 0.7-1.4 | 9.98E-01 |
| decanoylcarnitine (C10) | 1.02 | 0.77-1.37 | 9.98E-01 | 0.99 | 0.73-1.33 | 9.98E-01 |
| N-acetylglutamine | 0.87 | 0.54-1.4 | 9.98E-01 | 0.84 | 0.5-1.42 | 9.98E-01 |
| N-acetyltryptophan | 1.01 | 0.67-1.54 | 9.98E-01 | 1.01 | 0.66-1.54 | 9.98E-01 |
| N-acetylphenylalanine | 0.98 | 0.69-1.4 | 9.98E-01 | 0.96 | 0.68-1.37 | 9.98E-01 |
| N-acetylasparagine | 0.86 | 0.62-1.18 | 9.98E-01 | 0.84 | 0.6-1.18 | 9.98E-01 |
| 1-palmitoyl-GPC (16:0) | 0.95 | 0.69-1.29 | 9.98E-01 | 0.91 | 0.63-1.32 | 9.98E-01 |
| N-acetylarginine | 0.92 | 0.63-1.35 | 9.98E-01 | 0.91 | 0.63-1.33 | 9.98E-01 |
| piperine | 0.98 | 0.68-1.41 | 9.98E-01 | 0.97 | 0.66-1.42 | 9.98E-01 |
| campesterol | 0.88 | 0.63-1.23 | 9.98E-01 | 0.84 | 0.58-1.22 | 9.98E-01 |
| myristoylcarnitine (C14) | 1.00 | 0.65-1.56 | 9.98E-01 | 0.98 | 0.63-1.52 | 9.98E-01 |
| 1-stearoyl-GPC (18:0) | 0.92 | 0.64-1.33 | 9.98E-01 | 0.90 | 0.6-1.36 | 9.98E-01 |
| 1-oleoyl-GPC (18:1) | 0.98 | 0.68-1.41 | 9.98E-01 | 0.93 | 0.63-1.37 | 9.98E-01 |
| N-acetylthreonine | 1.06 | 0.62-1.8 | 9.98E-01 | 1.04 | 0.62-1.74 | 9.98E-01 |
| N-acetylisoleucine | 0.85 | 0.58-1.24 | 9.98E-01 | 0.86 | 0.58-1.27 | 9.98E-01 |
| 10-nonadecenoate (19:1n9) | 0.95 | 0.69-1.33 | 9.98E-01 | 0.92 | 0.66-1.28 | 9.98E-01 |
| 10-heptadecenoate (17:1n7) | 0.89 | 0.64-1.24 | 9.98E-01 | 0.85 | 0.61-1.19 | 9.98E-01 |
| hyocholate | 1.20 | 0.74-1.93 | 9.98E-01 | 1.18 | 0.75-1.87 | 9.98E-01 |
| epiandrosterone sulfate | 0.95 | 0.66-1.37 | 9.98E-01 | 0.93 | 0.66-1.32 | 9.98E-01 |
| N-acetylhistidine | 1.12 | 0.77-1.64 | 9.98E-01 | 1.10 | 0.72-1.69 | 9.98E-01 |
| gamma-glutamylglycine | 0.96 | 0.68-1.37 | 9.98E-01 | 0.94 | 0.62-1.42 | 9.98E-01 |
| gamma-glutamyltryptophan | 1.10 | 0.75-1.59 | 9.98E-01 | 1.08 | 0.74-1.59 | 9.98E-01 |
| stachydrine | 0.89 | 0.61-1.31 | 9.98E-01 | 0.90 | 0.62-1.32 | 9.98E-01 |
| alpha-hydroxyisovalerate | 1.08 | 0.77-1.51 | 9.98E-01 | 1.03 | 0.73-1.45 | 9.98E-01 |
| gamma-glutamylmethionine | 0.94 | 0.63-1.39 | 9.98E-01 | 0.93 | 0.61-1.42 | 9.98E-01 |
| gamma-glutamylthreonine | 0.95 | 0.63-1.42 | 9.98E-01 | 0.95 | 0.63-1.43 | 9.98E-01 |
| p-cresol sulfate | 0.90 | 0.64-1.26 | 9.98E-01 | 0.92 | 0.65-1.3 | 9.98E-01 |
| erythronate* | 1.04 | 0.74-1.46 | 9.98E-01 | 1.04 | 0.76-1.42 | 9.98E-01 |
| N-acetylproline | 1.11 | 0.81-1.51 | 9.98E-01 | 1.15 | 0.83-1.6 | 9.98E-01 |
| eicosenoate (20:1) | 0.93 | 0.69-1.26 | 9.98E-01 | 0.88 | 0.65-1.19 | 9.98E-01 |
| linolenate [alpha or gamma; (18:3n3 or 6)] | 0.86 | 0.63-1.16 | 9.98E-01 | 0.81 | 0.59-1.12 | 9.98E-01 |
| aconitate [cis or trans] | 0.89 | 0.62-1.29 | 9.98E-01 | 0.89 | 0.63-1.26 | 9.98E-01 |
| stearoylcarnitine (C18) | 1.22 | 0.82-1.82 | 9.98E-01 | 1.22 | 0.79-1.89 | 9.98E-01 |
| laurylcarnitine (C12) | 1.09 | 0.77-1.54 | 9.98E-01 | 1.05 | 0.73-1.51 | 9.98E-01 |
| isovalerylcarnitine (C5) | 0.70 | 0.49-1.01 | 9.98E-01 | 0.70 | 0.48-1.02 | 9.98E-01 |
| 1-linoleoyl-GPC (18:2) | 0.92 | 0.62-1.37 | 9.98E-01 | 0.88 | 0.54-1.43 | 9.98E-01 |
| 1,3,7-trimethylurate | 0.95 | 0.66-1.35 | 9.98E-01 | 0.94 | 0.67-1.31 | 9.98E-01 |
| 3,7-dimethylurate | 1.17 | 0.79-1.73 | 9.98E-01 | 1.18 | 0.79-1.76 | 9.98E-01 |
| 1,7-dimethylurate | 1.08 | 0.73-1.6 | 9.98E-01 | 1.07 | 0.74-1.56 | 9.98E-01 |
| 1-methylurate | 1.57 | 1.08-2.29 | 9.98E-01 | 1.62 | 1.1-2.39 | 9.98E-01 |
| 5-acetylamino-6-formylamino-3-methyluracil | 0.87 | 0.59-1.29 | 9.98E-01 | 0.88 | 0.6-1.29 | 9.98E-01 |
| 5-acetylamino-6-amino-3-methyluracil | 1.17 | 0.83-1.65 | 9.98E-01 | 1.18 | 0.84-1.66 | 9.98E-01 |
| indolebutyrate | 0.90 | 0.6-1.37 | 9.98E-01 | 0.94 | 0.61-1.44 | 9.98E-01 |
| 1-methylxanthine | 1.18 | 0.8-1.74 | 9.98E-01 | 1.18 | 0.8-1.75 | 9.98E-01 |
| N1-methylinosine | 1.13 | 0.7-1.81 | 9.98E-01 | 1.16 | 0.71-1.88 | 9.98E-01 |
| N2,N2-dimethylguanosine | 1.21 | 0.82-1.79 | 9.98E-01 | 1.23 | 0.83-1.83 | 9.98E-01 |
| N4-acetylcytidine | 0.88 | 0.68-1.15 | 9.98E-01 | 0.89 | 0.66-1.18 | 9.98E-01 |
| N6-carbamoylthreonyladenosine | 1.22 | 0.78-1.91 | 9.98E-01 | 1.23 | 0.77-1.95 | 9.98E-01 |
| orotidine | 1.00 | 0.71-1.4 | 9.98E-01 | 1.04 | 0.71-1.52 | 9.98E-01 |
| phenylacetylglutamine | 1.01 | 0.67-1.51 | 9.98E-01 | 1.05 | 0.7-1.58 | 9.98E-01 |
| 4-hydroxyhippurate | 0.91 | 0.56-1.49 | 9.98E-01 | 0.89 | 0.54-1.49 | 9.98E-01 |
| 5,6-dihydrouridine | 1.07 | 0.66-1.71 | 9.98E-01 | 1.09 | 0.66-1.78 | 9.98E-01 |
| 3-(3-amino-3-carboxypropyl)uridine* | 1.05 | 0.74-1.5 | 9.98E-01 | 1.06 | 0.74-1.52 | 9.98E-01 |
| 1-arachidonylglycerol (20:4) | 0.78 | 0.56-1.09 | 9.98E-01 | 0.76 | 0.55-1.06 | 9.98E-01 |
| 1-linolenoylglycerol (18:3) | 1.05 | 0.75-1.48 | 9.98E-01 | 1.04 | 0.73-1.47 | 9.98E-01 |
| 5-methyluridine (ribothymidine) | 0.99 | 0.63-1.56 | 9.98E-01 | 0.99 | 0.64-1.5 | 9.98E-01 |
| isovalerylglycine | 0.79 | 0.53-1.18 | 9.98E-01 | 0.80 | 0.53-1.21 | 9.98E-01 |
| 3-hydroxydodecanedioate* | 0.95 | 0.6-1.49 | 9.98E-01 | 0.93 | 0.59-1.46 | 9.98E-01 |
| 7-methylguanine | 0.83 | 0.55-1.26 | 9.98E-01 | 0.82 | 0.53-1.25 | 9.98E-01 |
| 1-stearoyl-GPE (18:0) | 1.00 | 0.67-1.49 | 9.98E-01 | 0.98 | 0.64-1.48 | 9.98E-01 |
| 1-stearoyl-GPG (18:0) | 0.91 | 0.62-1.34 | 9.98E-01 | 0.93 | 0.64-1.34 | 9.98E-01 |
| 3-methylcytidine | 1.15 | 0.86-1.54 | 9.98E-01 | 1.13 | 0.83-1.53 | 9.98E-01 |
| N1-Methyl-2-pyridone-5-carboxamide | 0.77 | 0.54-1.11 | 9.98E-01 | 0.78 | 0.53-1.16 | 9.98E-01 |
| gamma-glutamylisoleucine* | 0.77 | 0.54-1.08 | 9.98E-01 | 0.79 | 0.56-1.11 | 9.98E-01 |
| oleoylcarnitine (C18:1) | 1.07 | 0.69-1.65 | 9.98E-01 | 1.03 | 0.66-1.6 | 9.98E-01 |
| gamma-glutamyl-2-aminobutyrate | 0.96 | 0.63-1.48 | 9.98E-01 | 0.95 | 0.62-1.46 | 9.98E-01 |
| 2-methylbutyrylcarnitine (C5) | 0.77 | 0.58-1.01 | 9.98E-01 | 0.78 | 0.58-1.06 | 9.98E-01 |
| phenol sulfate | 0.77 | 0.46-1.29 | 9.98E-01 | 0.75 | 0.44-1.29 | 9.98E-01 |
| 1-palmitoleoyl-GPC (16:1)* | 0.87 | 0.61-1.23 | 9.98E-01 | 0.84 | 0.59-1.21 | 9.98E-01 |
| malonylcarnitine | 1.21 | 0.74-1.98 | 9.98E-01 | 1.25 | 0.76-2.04 | 9.98E-01 |
| hexanoylglycine | 0.95 | 0.64-1.43 | 9.98E-01 | 0.92 | 0.6-1.4 | 9.98E-01 |
| glutamine_degradant* | 1.27 | 0.78-2.07 | 9.98E-01 | 1.33 | 0.81-2.18 | 9.98E-01 |
| 2-hydroxy-3-methylvalerate | 0.93 | 0.64-1.36 | 9.98E-01 | 0.91 | 0.61-1.35 | 9.98E-01 |
| homostachydrine* | 0.85 | 0.63-1.15 | 9.98E-01 | 0.83 | 0.61-1.13 | 9.98E-01 |
| 1-arachidonoyl-GPC (20:4n6)* | 0.80 | 0.58-1.1 | 9.98E-01 | 0.75 | 0.53-1.05 | 9.98E-01 |
| 2-palmitoleoyl-GPC (16:1)* | 1.09 | 0.76-1.56 | 9.98E-01 | 1.10 | 0.77-1.57 | 9.98E-01 |
| 2-palmitoyl-GPC (16:0)* | 0.85 | 0.67-1.06 | 9.98E-01 | 0.81 | 0.64-1.04 | 9.98E-01 |
| 1-palmitoyl-GPE (16:0) | 0.96 | 0.64-1.42 | 9.98E-01 | 0.94 | 0.61-1.44 | 9.98E-01 |
| 1-oleoyl-GPE (18:1) | 1.00 | 0.63-1.6 | 9.98E-01 | 0.98 | 0.62-1.57 | 9.98E-01 |
| 1-linoleoyl-GPE (18:2)* | 0.96 | 0.57-1.62 | 9.98E-01 | 0.96 | 0.55-1.67 | 9.98E-01 |
| 1-arachidonoyl-GPE (20:4n6)* | 0.95 | 0.65-1.37 | 9.98E-01 | 0.90 | 0.61-1.33 | 9.98E-01 |
| N-acetylcitrulline | 0.86 | 0.55-1.37 | 9.98E-01 | 0.89 | 0.56-1.41 | 9.98E-01 |
| 2-hydroxypalmitate | 0.79 | 0.6-1.04 | 9.98E-01 | 0.78 | 0.6-1.02 | 9.98E-01 |
| docosapentaenoate (n6 DPA; 22:5n6) | 0.93 | 0.7-1.24 | 9.98E-01 | 0.93 | 0.7-1.25 | 9.98E-01 |
| gulonate* | 0.96 | 0.68-1.35 | 9.98E-01 | 0.97 | 0.69-1.36 | 9.98E-01 |
| isobutyrylglycine | 0.97 | 0.68-1.37 | 9.98E-01 | 0.99 | 0.68-1.44 | 9.98E-01 |
| glutarylcarnitine (C5-DC) | 1.01 | 0.69-1.47 | 9.98E-01 | 1.02 | 0.69-1.53 | 9.98E-01 |
| beta-hydroxyisovaleroylcarnitine | 0.87 | 0.61-1.22 | 9.98E-01 | 0.84 | 0.59-1.18 | 9.98E-01 |
| tiglylcarnitine (C5:1-DC) | 0.88 | 0.6-1.29 | 9.98E-01 | 0.89 | 0.6-1.31 | 9.98E-01 |
| hydroquinone sulfate | 0.91 | 0.58-1.43 | 9.98E-01 | 0.94 | 0.61-1.47 | 9.98E-01 |
| catechol sulfate | 0.89 | 0.53-1.51 | 9.98E-01 | 0.89 | 0.51-1.55 | 9.98E-01 |
| cholesterol sulfate | 1.01 | 0.67-1.54 | 9.98E-01 | 0.99 | 0.63-1.57 | 9.98E-01 |
| 7-alpha-hydroxy-3-oxo-4-cholestenoate (7-Hoca) | 1.14 | 0.68-1.93 | 9.98E-01 | 1.13 | 0.66-1.93 | 9.98E-01 |
| 3beta,7alpha-dihydroxy-5-cholestenoate | 1.39 | 0.94-2.07 | 9.98E-01 | 1.36 | 0.91-2.05 | 9.98E-01 |
| N-acetyl-aspartyl-glutamate (NAAG) | 1.75 | 1.05-2.91 | 9.98E-01 | 1.75 | 1.02-3.02 | 9.98E-01 |
| tetradecanedioate (C14-DC) | 1.03 | 0.67-1.58 | 9.98E-01 | 1.00 | 0.63-1.58 | 9.98E-01 |
| hexadecanedioate (C16-DC) | 0.88 | 0.57-1.36 | 9.98E-01 | 0.84 | 0.54-1.31 | 9.98E-01 |
| octadecanedioate (C18-DC) | 0.82 | 0.54-1.23 | 9.98E-01 | 0.77 | 0.51-1.18 | 9.98E-01 |
| 1-myristoylglycerol (14:0) | 0.67 | 0.47-0.96 | 9.98E-01 | 0.67 | 0.47-0.95 | 9.98E-01 |
| glycerophosphoethanolamine | 1.02 | 0.71-1.49 | 9.98E-01 | 1.01 | 0.66-1.55 | 9.98E-01 |
| 3-(3-hydroxyphenyl)propionate | 1.23 | 0.86-1.76 | 9.98E-01 | 1.24 | 0.87-1.75 | 9.98E-01 |
| ectoine | 0.84 | 0.65-1.08 | 9.98E-01 | 0.85 | 0.65-1.1 | 9.98E-01 |
| 1-palmitoyl-GPI (16:0) | 0.93 | 0.65-1.32 | 9.98E-01 | 0.95 | 0.66-1.36 | 9.98E-01 |
| glycolithocholate sulfate* | 1.08 | 0.75-1.55 | 9.98E-01 | 1.09 | 0.75-1.58 | 9.98E-01 |
| taurolithocholate 3-sulfate | 1.17 | 0.85-1.6 | 9.98E-01 | 1.17 | 0.84-1.64 | 9.98E-01 |
| deoxycarnitine | 1.36 | 0.91-2.03 | 9.98E-01 | 1.41 | 0.91-2.19 | 9.98E-01 |
| 1-ribosyl-imidazoleacetate* | 1.22 | 0.81-1.81 | 9.98E-01 | 1.25 | 0.85-1.84 | 9.98E-01 |
| 3,4-dihydroxybutyrate | 0.80 | 0.5-1.3 | 9.98E-01 | 0.81 | 0.49-1.33 | 9.98E-01 |
| indoleacetylglutamine | 0.95 | 0.64-1.4 | 9.98E-01 | 0.98 | 0.65-1.48 | 9.98E-01 |
| hexanoylglutamine | 1.29 | 0.88-1.89 | 9.98E-01 | 1.27 | 0.86-1.87 | 9.98E-01 |
| N6-acetyllysine | 1.28 | 0.73-2.25 | 9.98E-01 | 1.33 | 0.74-2.39 | 9.98E-01 |
| dihomo-linolenate (20:3n3 or n6) | 0.83 | 0.64-1.08 | 9.98E-01 | 0.81 | 0.62-1.07 | 9.98E-01 |
| mannitol/sorbitol | 1.33 | 0.99-1.79 | 9.98E-01 | 1.33 | 1-1.77 | 9.98E-01 |
| tryptophan betaine | 0.92 | 0.6-1.41 | 9.98E-01 | 0.89 | 0.59-1.34 | 9.98E-01 |
| 4-vinylphenol sulfate | 1.03 | 0.64-1.64 | 9.98E-01 | 1.03 | 0.6-1.75 | 9.98E-01 |
| 4-ethylphenylsulfate | 0.84 | 0.54-1.32 | 9.98E-01 | 0.84 | 0.51-1.38 | 9.98E-01 |
| thymol sulfate | 1.16 | 0.82-1.65 | 9.98E-01 | 1.14 | 0.8-1.61 | 9.98E-01 |
| pyrraline | 0.89 | 0.59-1.36 | 9.98E-01 | 0.89 | 0.59-1.34 | 9.98E-01 |
| 1-oleoyl-GPI (18:1) | 1.07 | 0.84-1.38 | 9.98E-01 | 1.08 | 0.84-1.39 | 9.98E-01 |
| 1-linoleoyl-GPI (18:2)* | 0.98 | 0.67-1.42 | 9.98E-01 | 0.98 | 0.69-1.4 | 9.98E-01 |
| o-cresol sulfate | 0.99 | 0.68-1.43 | 9.98E-01 | 0.99 | 0.62-1.57 | 9.98E-01 |
| dimethylarginine (SDMA + ADMA) | 0.98 | 0.64-1.5 | 9.98E-01 | 0.96 | 0.61-1.52 | 9.98E-01 |
| gamma-glutamylalanine | 0.93 | 0.58-1.5 | 9.98E-01 | 0.92 | 0.55-1.52 | 9.98E-01 |
| N-acetylserine | 1.00 | 0.67-1.48 | 9.98E-01 | 1.01 | 0.68-1.51 | 9.98E-01 |
| 1-stearoyl-2-oleoyl-GPE (18:0/18:1) | 0.96 | 0.64-1.46 | 9.98E-01 | 0.99 | 0.65-1.5 | 9.98E-01 |
| 4-allylphenol sulfate | 0.85 | 0.57-1.29 | 9.98E-01 | 0.84 | 0.54-1.29 | 9.98E-01 |
| 1-stearoyl-2-arachidonoyl-GPC (18:0/20:4) | 0.95 | 0.69-1.33 | 9.98E-01 | 0.91 | 0.65-1.27 | 9.98E-01 |
| 1-palmitoyl-2-linoleoyl-GPE (16:0/18:2) | 1.04 | 0.62-1.73 | 9.98E-01 | 1.09 | 0.68-1.75 | 9.98E-01 |
| sphinganine-1-phosphate | 1.14 | 0.79-1.65 | 9.98E-01 | 1.14 | 0.76-1.71 | 9.98E-01 |
| glycosyl-N-stearoyl-sphingosine (d18:1/18:0) | 1.58 | 1-2.5 | 9.98E-01 | 1.60 | 0.98-2.61 | 9.98E-01 |
| cyclo(leu-pro) | 1.03 | 0.7-1.51 | 9.98E-01 | 1.03 | 0.68-1.56 | 9.98E-01 |
| cyclo(gly-pro) | 0.94 | 0.62-1.43 | 9.98E-01 | 0.96 | 0.63-1.44 | 9.98E-01 |
| succinylcarnitine (C4-DC) | 0.88 | 0.63-1.24 | 9.98E-01 | 0.91 | 0.63-1.3 | 9.98E-01 |
| bilirubin (E,E)* | 1.12 | 0.78-1.61 | 9.98E-01 | 1.06 | 0.74-1.53 | 9.98E-01 |
| bilirubin (E,Z or Z,E)* | 1.35 | 0.93-1.96 | 9.98E-01 | 1.29 | 0.87-1.91 | 9.98E-01 |
| N-methylproline | 0.88 | 0.59-1.3 | 9.98E-01 | 0.89 | 0.59-1.32 | 9.98E-01 |
| beta-cryptoxanthin | 0.94 | 0.66-1.35 | 9.98E-01 | 0.93 | 0.66-1.3 | 9.98E-01 |
| 5alpha-androstan-3beta,17beta-diol disulfate | 1.00 | 0.62-1.6 | 9.98E-01 | 0.96 | 0.59-1.56 | 9.98E-01 |
| 5alpha-pregnan-3beta,20alpha-diol disulfate | 0.81 | 0.58-1.15 | 9.98E-01 | 0.83 | 0.59-1.18 | 9.98E-01 |
| glycocholenate sulfate* | 1.03 | 0.63-1.69 | 9.98E-01 | 1.05 | 0.66-1.68 | 9.98E-01 |
| taurocholenate sulfate* | 1.08 | 0.73-1.6 | 9.98E-01 | 1.09 | 0.73-1.63 | 9.98E-01 |
| androstenediol (3beta,17beta) disulfate (1) | 1.07 | 0.67-1.72 | 9.98E-01 | 1.05 | 0.63-1.73 | 9.98E-01 |
| pregnenediol disulfate (C21H34O8S2)* | 1.03 | 0.62-1.7 | 9.98E-01 | 1.02 | 0.62-1.68 | 9.98E-01 |
| androstenediol (3beta,17beta) disulfate (2) | 0.87 | 0.58-1.33 | 9.98E-01 | 0.87 | 0.57-1.31 | 9.98E-01 |
| 5alpha-androstan-3alpha,17alpha-diol monosulfate | 0.81 | 0.54-1.2 | 9.98E-01 | 0.79 | 0.54-1.15 | 9.98E-01 |
| 5alpha-pregnan-3beta,20beta-diol monosulfate (1) | 0.93 | 0.65-1.34 | 9.98E-01 | 0.93 | 0.64-1.37 | 9.98E-01 |
| 5alpha-pregnan-3beta,20alpha-diol monosulfate (2) | 0.75 | 0.54-1.05 | 9.98E-01 | 0.77 | 0.55-1.08 | 9.98E-01 |
| 5alpha-androstan-3alpha,17beta-diol monosulfate (1) | 1.10 | 0.7-1.73 | 9.98E-01 | 1.09 | 0.7-1.69 | 9.98E-01 |
| 5alpha-androstan-3beta,17alpha-diol disulfate | 0.74 | 0.56-0.97 | 9.98E-01 | 0.71 | 0.53-0.96 | 9.98E-01 |
| 5alpha-androstan-3beta,17beta-diol monosulfate (2) | 1.13 | 0.71-1.79 | 9.98E-01 | 1.10 | 0.69-1.75 | 9.98E-01 |
| androstenediol (3alpha, 17alpha) monosulfate (2) | 0.81 | 0.57-1.16 | 9.98E-01 | 0.80 | 0.56-1.15 | 9.98E-01 |
| androstenediol (3alpha, 17alpha) monosulfate (3) | 0.89 | 0.56-1.4 | 9.98E-01 | 0.89 | 0.57-1.39 | 9.98E-01 |
| androstenediol (3beta,17beta) monosulfate (1) | 1.20 | 0.71-2.02 | 9.98E-01 | 1.17 | 0.7-1.97 | 9.98E-01 |
| androstenediol (3beta,17beta) monosulfate (2) | 1.13 | 0.71-1.77 | 9.98E-01 | 1.11 | 0.71-1.75 | 9.98E-01 |
| 4-hydroxycoumarin | 0.79 | 0.55-1.15 | 9.98E-01 | 0.79 | 0.55-1.14 | 9.98E-01 |
| pregnenediol sulfate (C21H34O5S)* | 0.99 | 0.63-1.56 | 9.98E-01 | 0.99 | 0.63-1.54 | 9.98E-01 |
| gamma-CEHC | 1.01 | 0.63-1.61 | 9.98E-01 | 0.99 | 0.61-1.62 | 9.98E-01 |
| N-acetyl-beta-alanine | 1.26 | 0.82-1.93 | 9.98E-01 | 1.22 | 0.8-1.87 | 9.98E-01 |
| sphingomyelin (d18:1/18:1, d18:2/18:0) | 1.33 | 0.79-2.22 | 9.98E-01 | 1.42 | 0.81-2.49 | 9.98E-01 |
| palmitoyl sphingomyelin (d18:1/16:0) | 1.00 | 0.63-1.56 | 9.98E-01 | 1.00 | 0.65-1.56 | 9.98E-01 |
| cysteine sulfinic acid | 1.03 | 0.57-1.87 | 9.98E-01 | 1.02 | 0.56-1.86 | 9.98E-01 |
| 3-hydroxyhippurate | 1.04 | 0.7-1.55 | 9.98E-01 | 1.05 | 0.72-1.54 | 9.98E-01 |
| 16a-hydroxy DHEA 3-sulfate | 0.98 | 0.63-1.53 | 9.98E-01 | 0.99 | 0.63-1.56 | 9.98E-01 |
| 17alpha-hydroxypregnenolone 3-sulfate | 0.84 | 0.58-1.23 | 9.98E-01 | 0.84 | 0.58-1.21 | 9.98E-01 |
| pregnenolone sulfate | 0.79 | 0.56-1.12 | 9.98E-01 | 0.80 | 0.56-1.15 | 9.98E-01 |
| andro steroid monosulfate C19H28O6S (1)* | 0.93 | 0.6-1.45 | 9.98E-01 | 0.93 | 0.59-1.47 | 9.98E-01 |
| ergothioneine | 0.89 | 0.54-1.48 | 9.98E-01 | 0.84 | 0.51-1.39 | 9.98E-01 |
| 12-HETE | 1.13 | 0.76-1.68 | 9.98E-01 | 1.12 | 0.76-1.66 | 9.98E-01 |
| S-methylmethionine | 1.01 | 0.66-1.56 | 9.98E-01 | 0.99 | 0.62-1.56 | 9.98E-01 |
| indole-3-carboxylate | 0.93 | 0.59-1.46 | 9.98E-01 | 0.95 | 0.59-1.53 | 9.98E-01 |
| 13-HODE + 9-HODE | 0.84 | 0.58-1.22 | 9.98E-01 | 0.78 | 0.52-1.17 | 9.98E-01 |
| tridecenedioate (C13:1-DC)* | 1.18 | 0.77-1.82 | 9.98E-01 | 1.15 | 0.75-1.77 | 9.98E-01 |
| cinnamoylglycine | 1.01 | 0.7-1.44 | 9.98E-01 | 0.99 | 0.69-1.41 | 9.98E-01 |
| cis-4-decenoylcarnitine (C10:1) | 1.03 | 0.73-1.46 | 9.98E-01 | 1.00 | 0.71-1.42 | 9.98E-01 |
| 2S,3R-dihydroxybutyrate | 0.99 | 0.69-1.44 | 9.98E-01 | 0.96 | 0.64-1.45 | 9.98E-01 |
| (16 or 17)-methylstearate (a19:0 or i19:0) | 0.95 | 0.68-1.32 | 9.98E-01 | 0.92 | 0.66-1.28 | 9.98E-01 |
| 2R,3R-dihydroxybutyrate | 0.81 | 0.56-1.17 | 9.98E-01 | 0.80 | 0.54-1.19 | 9.98E-01 |
| 2,3-dihydroxyisovalerate | 1.05 | 0.8-1.39 | 9.98E-01 | 1.03 | 0.73-1.45 | 9.98E-01 |
| 3-methylglutaconate | 0.90 | 0.61-1.33 | 9.98E-01 | 0.90 | 0.61-1.33 | 9.98E-01 |
| cysteinylglycine disulfide* | 0.99 | 0.71-1.39 | 9.98E-01 | 0.98 | 0.7-1.37 | 9.98E-01 |
| isoursodeoxycholate | 1.04 | 0.67-1.59 | 9.98E-01 | 1.06 | 0.68-1.63 | 9.98E-01 |
| formiminoglutamate | 0.86 | 0.57-1.28 | 9.98E-01 | 0.87 | 0.56-1.37 | 9.98E-01 |
| hydantoin-5-propionate | 1.08 | 0.79-1.47 | 9.98E-01 | 1.14 | 0.84-1.55 | 9.98E-01 |
| sulfate* | 0.81 | 0.53-1.25 | 9.98E-01 | 0.83 | 0.53-1.28 | 9.98E-01 |
| 4-hydroxy-2-oxoglutaric acid | 0.94 | 0.64-1.37 | 9.98E-01 | 0.95 | 0.66-1.37 | 9.98E-01 |
| 4-hydroxyglutamate | 1.16 | 0.85-1.59 | 9.98E-01 | 1.26 | 0.86-1.84 | 9.98E-01 |
| pantoate | 1.28 | 0.9-1.83 | 9.98E-01 | 1.27 | 0.92-1.75 | 9.98E-01 |
| gamma-carboxyglutamate | 0.83 | 0.62-1.13 | 9.98E-01 | 0.82 | 0.6-1.13 | 9.98E-01 |
| S-methylcysteine | 0.85 | 0.55-1.29 | 9.98E-01 | 0.80 | 0.5-1.31 | 9.98E-01 |
| androsterone glucuronide | 1.18 | 0.83-1.67 | 9.98E-01 | 1.18 | 0.84-1.64 | 9.98E-01 |
| argininate* | 0.79 | 0.57-1.1 | 9.98E-01 | 0.81 | 0.58-1.14 | 9.98E-01 |
| 2-oxoarginine* | 0.76 | 0.52-1.12 | 9.98E-01 | 0.79 | 0.52-1.2 | 9.98E-01 |
| cis-4-decenoate (10:1n6)* | 1.01 | 0.7-1.46 | 9.98E-01 | 0.99 | 0.68-1.43 | 9.98E-01 |
| 1-lignoceroyl-GPC (24:0) | 0.87 | 0.52-1.47 | 9.98E-01 | 0.81 | 0.44-1.49 | 9.98E-01 |
| 1-(1-enyl-palmitoyl)-GPC (P-16:0)* | 0.78 | 0.55-1.1 | 9.98E-01 | 0.75 | 0.51-1.1 | 9.98E-01 |
| 1-methyl-5-imidazoleacetate | 0.91 | 0.65-1.29 | 9.98E-01 | 0.92 | 0.65-1.3 | 9.98E-01 |
| glycoursodeoxycholate | 0.96 | 0.62-1.5 | 9.98E-01 | 0.95 | 0.6-1.49 | 9.98E-01 |
| S-methylcysteine sulfoxide | 0.74 | 0.52-1.07 | 9.98E-01 | 0.70 | 0.46-1.07 | 9.98E-01 |
| (14 or 15)-methylpalmitate (a17:0 or i17:0) | 1.02 | 0.74-1.4 | 9.98E-01 | 0.99 | 0.71-1.36 | 9.98E-01 |
| eicosanedioate (C20-DC) | 1.08 | 0.74-1.58 | 9.98E-01 | 1.05 | 0.7-1.58 | 9.98E-01 |
| 16-hydroxypalmitate | 0.95 | 0.63-1.43 | 9.98E-01 | 0.89 | 0.58-1.38 | 9.98E-01 |
| oleoyl-linoleoyl-glycerol (18:1/18:2) [1] | 0.92 | 0.62-1.39 | 9.98E-01 | 0.93 | 0.63-1.37 | 9.98E-01 |
| oleoyl-linoleoyl-glycerol (18:1/18:2) [2] | 0.89 | 0.57-1.39 | 9.98E-01 | 0.90 | 0.6-1.36 | 9.98E-01 |
| 1-(1-enyl-palmitoyl)-GPE (P-16:0)* | 0.96 | 0.61-1.49 | 9.98E-01 | 0.92 | 0.57-1.49 | 9.98E-01 |
| 1-(1-enyl-stearoyl)-GPE (P-18:0)* | 0.98 | 0.66-1.47 | 9.98E-01 | 0.96 | 0.62-1.5 | 9.98E-01 |
| N-oleoyltaurine | 1.10 | 0.8-1.5 | 9.98E-01 | 1.09 | 0.81-1.47 | 9.98E-01 |
| linoleoylcarnitine (C18:2)* | 0.92 | 0.59-1.43 | 9.98E-01 | 0.91 | 0.57-1.44 | 9.98E-01 |
| isoleucylglycine | 1.15 | 0.76-1.75 | 9.98E-01 | 1.19 | 0.73-1.93 | 9.98E-01 |
| N-stearoyltaurine | 1.03 | 0.74-1.44 | 9.98E-01 | 1.05 | 0.76-1.45 | 9.98E-01 |
| 2-O-methylascorbic acid | 1.28 | 0.8-2.05 | 9.98E-01 | 1.31 | 0.82-2.1 | 9.98E-01 |
| carboxyethyl-GABA | 1.28 | 0.85-1.93 | 9.98E-01 | 1.32 | 0.85-2.04 | 9.98E-01 |
| beta-citrylglutamate | 1.35 | 0.88-2.06 | 9.98E-01 | 1.33 | 0.83-2.11 | 9.98E-01 |
| trimethylamine N-oxide | 1.27 | 0.94-1.71 | 9.98E-01 | 1.26 | 0.92-1.72 | 9.98E-01 |
| N6-methyllysine | 0.95 | 0.62-1.45 | 9.98E-01 | 0.92 | 0.6-1.42 | 9.98E-01 |
| dihydroferulate | 1.02 | 0.68-1.51 | 9.98E-01 | 1.01 | 0.68-1.51 | 9.98E-01 |
| imidazole propionate | 0.94 | 0.64-1.36 | 9.98E-01 | 0.96 | 0.67-1.38 | 9.98E-01 |
| pregnanediol-3-glucuronide | 0.94 | 0.65-1.38 | 9.98E-01 | 0.96 | 0.66-1.39 | 9.98E-01 |
| valylglycine | 1.15 | 0.76-1.75 | 9.98E-01 | 1.19 | 0.76-1.85 | 9.98E-01 |
| prolylglycine | 0.79 | 0.55-1.14 | 9.98E-01 | 0.81 | 0.56-1.15 | 9.98E-01 |
| N-palmitoylglycine | 0.84 | 0.65-1.09 | 9.98E-01 | 0.83 | 0.63-1.1 | 9.98E-01 |
| mannonate* | 0.96 | 0.64-1.44 | 9.98E-01 | 0.96 | 0.65-1.44 | 9.98E-01 |
| lanthionine | 0.97 | 0.71-1.34 | 9.98E-01 | 1.01 | 0.72-1.4 | 9.98E-01 |
| 2-stearoyl-GPE (18:0)* | 0.87 | 0.68-1.1 | 9.98E-01 | 0.86 | 0.67-1.1 | 9.98E-01 |
| (R)-3-hydroxybutyrylcarnitine | 1.17 | 0.82-1.67 | 9.98E-01 | 1.18 | 0.82-1.7 | 9.98E-01 |
| N-acetylcarnosine | 1.05 | 0.72-1.53 | 9.98E-01 | 1.05 | 0.73-1.51 | 9.98E-01 |
| margaroylcarnitine (C17)* | 1.10 | 0.74-1.65 | 9.98E-01 | 1.08 | 0.7-1.67 | 9.98E-01 |
| histidine betaine (hercynine)* | 0.85 | 0.57-1.25 | 9.98E-01 | 0.82 | 0.55-1.22 | 9.98E-01 |
| glycohyocholate | 1.10 | 0.72-1.68 | 9.98E-01 | 1.08 | 0.68-1.71 | 9.98E-01 |
| retinal | 1.00 | 0.66-1.54 | 9.98E-01 | 0.96 | 0.62-1.46 | 9.98E-01 |
| 2-hydroxydecanoate | 0.95 | 0.71-1.27 | 9.98E-01 | 0.91 | 0.66-1.26 | 9.98E-01 |
| 3-methyl catechol sulfate (2) | 1.23 | 0.87-1.74 | 9.98E-01 | 1.25 | 0.88-1.78 | 9.98E-01 |
| 4-methylcatechol sulfate | 1.12 | 0.71-1.77 | 9.98E-01 | 1.13 | 0.72-1.78 | 9.98E-01 |
| 3-methyl catechol sulfate (1) | 0.95 | 0.68-1.32 | 9.98E-01 | 0.95 | 0.66-1.35 | 9.98E-01 |
| 3b-hydroxy-5-cholenoic acid | 1.06 | 0.75-1.5 | 9.98E-01 | 1.06 | 0.74-1.51 | 9.98E-01 |
| guaiacol sulfate | 0.89 | 0.56-1.43 | 9.98E-01 | 0.91 | 0.56-1.49 | 9.98E-01 |
| 2-aminooctanoate | 0.92 | 0.63-1.36 | 9.98E-01 | 0.90 | 0.6-1.36 | 9.98E-01 |
| gamma-CEHC glucuronide* | 1.25 | 0.76-2.08 | 9.98E-01 | 1.27 | 0.74-2.17 | 9.98E-01 |
| dimethyl sulfone | 0.87 | 0.58-1.3 | 9.98E-01 | 0.89 | 0.59-1.34 | 9.98E-01 |
| 2-piperidinone | 1.00 | 0.64-1.56 | 9.98E-01 | 1.00 | 0.64-1.57 | 9.98E-01 |
| N-acetyl-1-methylhistidine* | 0.87 | 0.61-1.24 | 9.98E-01 | 0.86 | 0.6-1.23 | 9.98E-01 |
| indolin-2-one | 0.89 | 0.63-1.26 | 9.98E-01 | 0.90 | 0.63-1.29 | 9.98E-01 |
| 2-aminophenol sulfate | 0.78 | 0.53-1.16 | 9.98E-01 | 0.77 | 0.51-1.18 | 9.98E-01 |
| 3-acetylphenol sulfate | 1.15 | 0.78-1.69 | 9.98E-01 | 1.15 | 0.77-1.73 | 9.98E-01 |
| sphingomyelin (d18:1/14:0, d16:1/16:0)* | 0.96 | 0.63-1.45 | 9.98E-01 | 0.97 | 0.62-1.51 | 9.98E-01 |
| sphingomyelin (d18:2/16:0, d18:1/16:1)* | 1.21 | 0.76-1.92 | 9.98E-01 | 1.23 | 0.77-1.97 | 9.98E-01 |
| 6-oxopiperidine-2-carboxylate | 1.16 | 0.85-1.58 | 9.98E-01 | 1.23 | 0.9-1.67 | 9.98E-01 |
| N-delta-acetylornithine | 0.97 | 0.71-1.33 | 9.98E-01 | 0.96 | 0.7-1.34 | 9.98E-01 |
| acisoga | 0.97 | 0.69-1.38 | 9.98E-01 | 0.97 | 0.66-1.43 | 9.98E-01 |
| 2-aminoheptanoate | 1.31 | 0.94-1.82 | 9.98E-01 | 1.36 | 0.96-1.92 | 9.98E-01 |
| N-formylanthranilic acid | 1.23 | 0.81-1.87 | 9.98E-01 | 1.24 | 0.81-1.9 | 9.98E-01 |
| N2,N5-diacetylornithine | 0.87 | 0.56-1.37 | 9.98E-01 | 0.84 | 0.52-1.36 | 9.98E-01 |
| 1H-indole-7-acetic acid | 1.01 | 0.67-1.52 | 9.98E-01 | 1.05 | 0.69-1.59 | 9.98E-01 |
| 3-methoxytyramine sulfate | 1.13 | 0.83-1.54 | 9.98E-01 | 1.12 | 0.82-1.52 | 9.98E-01 |
| methionine sulfone | 0.91 | 0.59-1.4 | 9.98E-01 | 0.91 | 0.57-1.44 | 9.98E-01 |
| 1-linolenoyl-GPC (18:3)* | 0.88 | 0.6-1.27 | 9.98E-01 | 0.85 | 0.56-1.3 | 9.98E-01 |
| 1-(1-enyl-oleoyl)-GPE (P-18:1)* | 0.98 | 0.6-1.6 | 9.98E-01 | 0.96 | 0.58-1.57 | 9.98E-01 |
| fructosyllysine | 1.03 | 0.68-1.57 | 9.98E-01 | 1.03 | 0.67-1.6 | 9.98E-01 |
| N-methylpipecolate | 0.84 | 0.58-1.21 | 9.98E-01 | 0.82 | 0.56-1.2 | 9.98E-01 |
| O-sulfo-L-tyrosine | 1.31 | 0.95-1.82 | 9.98E-01 | 1.30 | 0.93-1.82 | 9.98E-01 |
| ferulic acid 4-sulfate | 0.99 | 0.7-1.4 | 9.98E-01 | 0.98 | 0.69-1.39 | 9.98E-01 |
| 3-(3-hydroxyphenyl)propionate sulfate | 1.08 | 0.76-1.52 | 9.98E-01 | 1.08 | 0.76-1.53 | 9.98E-01 |
| 11-ketoetiocholanolone glucuronide | 0.78 | 0.57-1.08 | 9.98E-01 | 0.78 | 0.57-1.07 | 9.98E-01 |
| etiocholanolone glucuronide | 0.88 | 0.6-1.31 | 9.98E-01 | 0.89 | 0.61-1.31 | 9.98E-01 |
| 17alpha-hydroxypregnanolone glucuronide | 1.15 | 0.76-1.75 | 9.98E-01 | 1.17 | 0.77-1.79 | 9.98E-01 |
| 1-oleoyl-GPG (18:1)* | 1.07 | 0.75-1.5 | 9.98E-01 | 1.05 | 0.75-1.49 | 9.98E-01 |
| 1-palmitoyl-GPG (16:0)* | 0.92 | 0.64-1.32 | 9.98E-01 | 0.94 | 0.66-1.34 | 9.98E-01 |
| 9-hydroxystearate | 1.13 | 0.74-1.71 | 9.98E-01 | 1.10 | 0.73-1.65 | 9.98E-01 |
| 3-methylglutarylcarnitine (2) | 1.02 | 0.69-1.53 | 9.98E-01 | 1.05 | 0.68-1.63 | 9.98E-01 |
| methyl glucopyranoside (alpha + beta) | 1.15 | 0.71-1.86 | 9.98E-01 | 1.14 | 0.69-1.87 | 9.98E-01 |
| alpha-CEHC sulfate | 1.16 | 0.8-1.68 | 9.98E-01 | 1.17 | 0.79-1.73 | 9.98E-01 |
| sphingomyelin (d18:2/14:0, d18:1/14:1)* | 1.17 | 0.64-2.14 | 9.98E-01 | 1.20 | 0.62-2.35 | 9.98E-01 |
| sphingomyelin (d18:1/24:1, d18:2/24:0)* | 0.87 | 0.55-1.38 | 9.98E-01 | 0.84 | 0.53-1.33 | 9.98E-01 |
| octadecenedioylcarnitine (C18:1-DC)* | 0.81 | 0.53-1.23 | 9.98E-01 | 0.79 | 0.52-1.2 | 9.98E-01 |
| octadecanedioylcarnitine (C18-DC)* | 0.84 | 0.54-1.31 | 9.98E-01 | 0.86 | 0.55-1.32 | 9.98E-01 |
| 5alpha-androstan-3alpha,17beta-diol monosulfate (2) | 1.23 | 0.69-2.2 | 9.98E-01 | 1.14 | 0.63-2.04 | 9.98E-01 |
| myristoleoylcarnitine (C14:1)* | 1.06 | 0.74-1.53 | 9.98E-01 | 1.02 | 0.7-1.49 | 9.98E-01 |
| 4-hydroxychlorothalonil | 1.22 | 0.75-1.97 | 9.98E-01 | 1.18 | 0.75-1.87 | 9.98E-01 |
| isoeugenol sulfate | 0.76 | 0.52-1.13 | 9.98E-01 | 0.76 | 0.51-1.14 | 9.98E-01 |
| tyramine O-sulfate | 0.94 | 0.63-1.39 | 9.98E-01 | 0.95 | 0.62-1.46 | 9.98E-01 |
| 3-hydroxypyridine sulfate | 1.00 | 0.73-1.36 | 9.98E-01 | 1.00 | 0.73-1.37 | 9.98E-01 |
| 4-methylguaiacol sulfate | 1.22 | 0.77-1.92 | 9.98E-01 | 1.25 | 0.79-1.98 | 9.98E-01 |
| phenylacetylcarnitine | 0.96 | 0.72-1.29 | 9.98E-01 | 1.01 | 0.73-1.39 | 9.98E-01 |
| arabonate/xylonate | 0.79 | 0.57-1.09 | 9.98E-01 | 0.79 | 0.57-1.09 | 9.98E-01 |
| methyl-4-hydroxybenzoate sulfate | 1.49 | 0.83-2.65 | 9.98E-01 | 1.41 | 0.74-2.7 | 9.98E-01 |
| 1-dihomo-linolenylglycerol (20:3) | 0.84 | 0.56-1.27 | 9.98E-01 | 0.84 | 0.56-1.25 | 9.98E-01 |
| 4-vinylguaiacol sulfate | 0.89 | 0.6-1.32 | 9.98E-01 | 0.90 | 0.6-1.34 | 9.98E-01 |
| vanillactate | 0.82 | 0.6-1.13 | 9.98E-01 | 0.82 | 0.59-1.14 | 9.98E-01 |
| eugenol sulfate | 0.74 | 0.5-1.09 | 9.98E-01 | 0.73 | 0.48-1.09 | 9.98E-01 |
| 2-acetamidophenol sulfate | 0.97 | 0.69-1.36 | 9.98E-01 | 0.96 | 0.66-1.39 | 9.98E-01 |
| p-cresol glucuronide* | 1.19 | 0.78-1.82 | 9.98E-01 | 1.24 | 0.83-1.86 | 9.98E-01 |
| 6-hydroxyindole sulfate | 0.81 | 0.48-1.36 | 9.98E-01 | 0.82 | 0.45-1.48 | 9.98E-01 |
| 4-methoxyphenol sulfate | 0.98 | 0.69-1.4 | 9.98E-01 | 1.00 | 0.69-1.46 | 9.98E-01 |
| umbelliferone sulfate | 1.32 | 0.85-2.03 | 9.98E-01 | 1.30 | 0.84-2.02 | 9.98E-01 |
| sphingomyelin (d18:1/20:1, d18:2/20:0)* | 1.02 | 0.64-1.62 | 9.98E-01 | 1.05 | 0.65-1.69 | 9.98E-01 |
| sphingomyelin (d18:1/20:2, d18:2/20:1, d16:1/22:2)* | 1.23 | 0.75-1.99 | 9.98E-01 | 1.25 | 0.75-2.08 | 9.98E-01 |
| behenoyl sphingomyelin (d18:1/22:0)* | 0.86 | 0.58-1.28 | 9.98E-01 | 0.85 | 0.57-1.26 | 9.98E-01 |
| sphingomyelin (d18:1/22:1, d18:2/22:0, d16:1/24:1)* | 1.17 | 0.71-1.93 | 9.98E-01 | 1.17 | 0.69-1.97 | 9.98E-01 |
| sphingomyelin (d18:1/22:2, d18:2/22:1, d16:1/24:2)* | 1.01 | 0.64-1.62 | 9.98E-01 | 1.04 | 0.66-1.66 | 9.98E-01 |
| lignoceroyl sphingomyelin (d18:1/24:0) | 0.96 | 0.6-1.56 | 9.98E-01 | 0.92 | 0.56-1.49 | 9.98E-01 |
| sphingomyelin (d17:1/16:0, d18:1/15:0, d16:1/17:0)* | 1.28 | 0.84-1.97 | 9.98E-01 | 1.38 | 0.87-2.18 | 9.98E-01 |
| dopamine 4-sulfate | 0.84 | 0.56-1.25 | 9.98E-01 | 0.85 | 0.56-1.29 | 9.98E-01 |
| dopamine 3-O-sulfate | 0.87 | 0.55-1.37 | 9.98E-01 | 0.88 | 0.55-1.41 | 9.98E-01 |
| 3-hydroxyhexanoate | 1.12 | 0.79-1.58 | 9.98E-01 | 1.08 | 0.76-1.53 | 9.98E-01 |
| N-carbamoylalanine | 1.30 | 0.9-1.89 | 9.98E-01 | 1.29 | 0.87-1.9 | 9.98E-01 |
| 3beta-hydroxy-5-cholestenoate | 0.91 | 0.61-1.34 | 9.98E-01 | 0.84 | 0.55-1.3 | 9.98E-01 |
| 1,2,3-benzenetriol sulfate (2) | 1.10 | 0.7-1.72 | 9.98E-01 | 1.12 | 0.71-1.76 | 9.98E-01 |
| 3-methoxycatechol sulfate (1) | 1.21 | 0.81-1.8 | 9.98E-01 | 1.26 | 0.84-1.88 | 9.98E-01 |
| 3-methoxycatechol sulfate (2) | 0.88 | 0.61-1.27 | 9.98E-01 | 0.90 | 0.61-1.33 | 9.98E-01 |
| N-acetylkynurenine (2) | 0.93 | 0.63-1.38 | 9.98E-01 | 0.91 | 0.6-1.37 | 9.98E-01 |
| C-glycosyltryptophan | 0.85 | 0.6-1.21 | 9.98E-01 | 0.85 | 0.58-1.24 | 9.98E-01 |
| arabitol/xylitol | 1.04 | 0.8-1.35 | 9.98E-01 | 1.03 | 0.78-1.35 | 9.98E-01 |
| N-acetylglucosamine/N-acetylgalactosamine | 1.27 | 0.91-1.77 | 9.98E-01 | 1.29 | 0.92-1.81 | 9.98E-01 |
| citraconate/glutaconate | 0.88 | 0.6-1.28 | 9.98E-01 | 0.88 | 0.61-1.25 | 9.98E-01 |
| adipoylcarnitine (C6-DC) | 1.04 | 0.75-1.46 | 9.98E-01 | 1.07 | 0.78-1.46 | 9.98E-01 |
| 4-hydroxyphenylacetoylcarnitine | 1.28 | 0.86-1.91 | 9.98E-01 | 1.35 | 0.88-2.07 | 9.98E-01 |
| nonanoylcarnitine (C9) | 1.07 | 0.69-1.67 | 9.98E-01 | 1.05 | 0.68-1.62 | 9.98E-01 |
| suberoylcarnitine (C8-DC) | 1.13 | 0.78-1.65 | 9.98E-01 | 1.15 | 0.79-1.67 | 9.98E-01 |
| glycochenodeoxycholate 3-sulfate | 1.49 | 0.92-2.43 | 9.98E-01 | 1.47 | 0.88-2.46 | 9.98E-01 |
| glycodeoxycholate 3-sulfate | 1.07 | 0.72-1.61 | 9.98E-01 | 1.10 | 0.72-1.69 | 9.98E-01 |
| taurodeoxycholic acid 3-sulfate | 1.04 | 0.7-1.55 | 9.98E-01 | 1.05 | 0.7-1.6 | 9.98E-01 |
| trans-3,4-methyleneheptanoate | 1.57 | 1.07-2.32 | 9.98E-01 | 1.60 | 1.09-2.35 | 9.98E-01 |
| phenol glucuronide | 1.13 | 0.73-1.74 | 9.98E-01 | 1.12 | 0.7-1.8 | 9.98E-01 |
| linoleoyl ethanolamide | 0.96 | 0.75-1.22 | 9.98E-01 | 0.96 | 0.76-1.21 | 9.98E-01 |
| 1,2-dilinoleoyl-GPC (18:2/18:2) | 0.92 | 0.61-1.39 | 9.98E-01 | 0.90 | 0.56-1.42 | 9.98E-01 |
| 1-stearoyl-2-oleoyl-GPC (18:0/18:1) | 1.18 | 0.81-1.72 | 9.98E-01 | 1.15 | 0.78-1.68 | 9.98E-01 |
| 1-palmitoyl-2-arachidonoyl-GPC (16:0/20:4n6) | 0.98 | 0.71-1.36 | 9.98E-01 | 0.92 | 0.66-1.29 | 9.98E-01 |
| 1-palmitoyl-2-docosahexaenoyl-GPC (16:0/22:6) | 1.15 | 0.81-1.64 | 9.98E-01 | 1.12 | 0.77-1.63 | 9.98E-01 |
| 1-stearoyl-2-docosahexaenoyl-GPC (18:0/22:6) | 1.05 | 0.7-1.57 | 9.98E-01 | 1.04 | 0.69-1.57 | 9.98E-01 |
| 1-(1-enyl-stearoyl)-2-oleoyl-GPE (P-18:0/18:1) | 0.79 | 0.52-1.2 | 9.98E-01 | 0.76 | 0.47-1.22 | 9.98E-01 |
| sphingomyelin (d18:1/17:0, d17:1/18:0, d19:1/16:0) | 1.40 | 0.88-2.24 | 9.98E-01 | 1.50 | 0.89-2.53 | 9.98E-01 |
| 1-palmitoyl-2-stearoyl-GPC (16:0/18:0) | 1.07 | 0.75-1.51 | 9.98E-01 | 1.05 | 0.7-1.55 | 9.98E-01 |
| 2-hydroxybutyrate/2-hydroxyisobutyrate | 1.02 | 0.67-1.56 | 9.98E-01 | 1.02 | 0.62-1.68 | 9.98E-01 |
| oleate/vaccenate (18:1) | 0.93 | 0.72-1.2 | 9.98E-01 | 0.89 | 0.69-1.14 | 9.98E-01 |
| 1-palmitoleoylglycerol (16:1)* | 0.84 | 0.58-1.2 | 9.98E-01 | 0.82 | 0.56-1.19 | 9.98E-01 |
| palmitoyl dihydrosphingomyelin (d18:0/16:0)* | 1.50 | 1.01-2.23 | 9.98E-01 | 1.47 | 0.98-2.21 | 9.98E-01 |
| tricosanoyl sphingomyelin (d18:1/23:0)* | 0.95 | 0.66-1.37 | 9.98E-01 | 0.95 | 0.66-1.36 | 9.98E-01 |
| sphingomyelin (d18:2/23:0, d18:1/23:1, d17:1/24:1)* | 1.04 | 0.69-1.57 | 9.98E-01 | 1.07 | 0.69-1.64 | 9.98E-01 |
| sphingomyelin (d18:2/24:1, d18:1/24:2)* | 1.07 | 0.6-1.91 | 9.98E-01 | 1.07 | 0.6-1.89 | 9.98E-01 |
| 1-stearoyl-2-linoleoyl-GPE (18:0/18:2)* | 1.03 | 0.65-1.65 | 9.98E-01 | 1.07 | 0.69-1.65 | 9.98E-01 |
| 1-stearoyl-2-arachidonoyl-GPE (18:0/20:4) | 1.01 | 0.67-1.55 | 9.98E-01 | 1.01 | 0.67-1.53 | 9.98E-01 |
| 1-stearoyl-2-linoleoyl-GPC (18:0/18:2)* | 1.11 | 0.77-1.59 | 9.98E-01 | 1.10 | 0.74-1.63 | 9.98E-01 |
| 1-palmitoyl-2-palmitoleoyl-GPC (16:0/16:1)* | 1.11 | 0.8-1.55 | 9.98E-01 | 1.11 | 0.79-1.55 | 9.98E-01 |
| 1-palmitoyl-2-dihomo-linolenoyl-GPC (16:0/20:3n3 or 6)* | 1.12 | 0.8-1.58 | 9.98E-01 | 1.13 | 0.8-1.6 | 9.98E-01 |
| 1-palmitoyl-2-arachidonoyl-GPE (16:0/20:4)* | 1.00 | 0.61-1.63 | 9.98E-01 | 1.02 | 0.62-1.65 | 9.98E-01 |
| 1-palmitoyl-2-docosahexaenoyl-GPE (16:0/22:6)* | 1.29 | 0.85-1.95 | 9.98E-01 | 1.32 | 0.87-1.99 | 9.98E-01 |
| 1-stearoyl-2-docosahexaenoyl-GPE (18:0/22:6)* | 1.24 | 0.81-1.89 | 9.98E-01 | 1.27 | 0.84-1.93 | 9.98E-01 |
| 1-palmitoyl-2-arachidonoyl-GPI (16:0/20:4)* | 0.79 | 0.54-1.14 | 9.98E-01 | 0.78 | 0.54-1.14 | 9.98E-01 |
| 1-stearoyl-2-linoleoyl-GPI (18:0/18:2) | 0.88 | 0.55-1.4 | 9.98E-01 | 0.86 | 0.55-1.35 | 9.98E-01 |
| gamma-tocopherol/beta-tocopherol | 1.04 | 0.66-1.65 | 9.98E-01 | 1.05 | 0.65-1.69 | 9.98E-01 |
| 1-(1-enyl-stearoyl)-2-arachidonoyl-GPE (P-18:0/20:4)* | 0.89 | 0.63-1.25 | 9.98E-01 | 0.87 | 0.59-1.26 | 9.98E-01 |
| 1-(1-enyl-palmitoyl)-2-arachidonoyl-GPE (P-16:0/20:4)* | 0.78 | 0.56-1.08 | 9.98E-01 | 0.76 | 0.53-1.09 | 9.98E-01 |
| 1-(1-enyl-palmitoyl)-2-oleoyl-GPE (P-16:0/18:1)* | 0.84 | 0.57-1.23 | 9.98E-01 | 0.81 | 0.52-1.25 | 9.98E-01 |
| 1-(1-enyl-palmitoyl)-2-oleoyl-GPC (P-16:0/18:1)* | 0.96 | 0.67-1.39 | 9.98E-01 | 0.92 | 0.65-1.3 | 9.98E-01 |
| 1-(1-enyl-palmitoyl)-2-linoleoyl-GPC (P-16:0/18:2)* | 0.86 | 0.57-1.31 | 9.98E-01 | 0.83 | 0.52-1.32 | 9.98E-01 |
| 1-(1-enyl-palmitoyl)-2-arachidonoyl-GPC (P-16:0/20:4)* | 0.69 | 0.46-1.05 | 9.98E-01 | 0.67 | 0.45-1.01 | 9.98E-01 |
| sphingomyelin (d18:1/21:0, d17:1/22:0, d16:1/23:0)* | 1.05 | 0.7-1.57 | 9.98E-01 | 1.08 | 0.69-1.68 | 9.98E-01 |
| behenoyl dihydrosphingomyelin (d18:0/22:0)* | 0.99 | 0.69-1.42 | 9.98E-01 | 0.97 | 0.65-1.46 | 9.98E-01 |
| sphingomyelin (d18:0/18:0, d19:0/17:0)* | 1.12 | 0.75-1.66 | 9.98E-01 | 1.13 | 0.73-1.74 | 9.98E-01 |
| N-palmitoyl-sphinganine (d18:0/16:0) | 1.02 | 0.79-1.32 | 9.98E-01 | 1.01 | 0.79-1.3 | 9.98E-01 |
| lactosyl-N-palmitoyl-sphingosine (d18:1/16:0) | 0.69 | 0.4-1.19 | 9.98E-01 | 0.66 | 0.36-1.2 | 9.98E-01 |
| myristoyl dihydrosphingomyelin (d18:0/14:0)* | 1.35 | 0.89-2.06 | 9.98E-01 | 1.36 | 0.88-2.12 | 9.98E-01 |
| 5-hydroxyindole sulfate | 0.93 | 0.62-1.39 | 9.98E-01 | 0.96 | 0.64-1.44 | 9.98E-01 |
| phenylacetylglutamate | 0.88 | 0.64-1.2 | 9.98E-01 | 0.91 | 0.65-1.25 | 9.98E-01 |
| 1-palmitoyl-2-oleoyl-GPI (16:0/18:1)* | 1.24 | 0.91-1.69 | 9.98E-01 | 1.24 | 0.9-1.71 | 9.98E-01 |
| 1-(1-enyl-palmitoyl)-2-linoleoyl-GPE (P-16:0/18:2)* | 0.85 | 0.61-1.18 | 9.98E-01 | 0.84 | 0.57-1.23 | 9.98E-01 |
| 1-oleoyl-2-linoleoyl-GPE (18:1/18:2)* | 0.92 | 0.63-1.35 | 9.98E-01 | 0.94 | 0.63-1.4 | 9.98E-01 |
| 1-oleoyl-2-docosahexaenoyl-GPC (18:1/22:6)* | 1.01 | 0.68-1.49 | 9.98E-01 | 0.97 | 0.63-1.48 | 9.98E-01 |
| 1-linoleoyl-2-arachidonoyl-GPC (18:2/20:4n6)* | 0.96 | 0.68-1.36 | 9.98E-01 | 0.91 | 0.65-1.27 | 9.98E-01 |
| 1-myristoyl-2-arachidonoyl-GPC (14:0/20:4)* | 0.86 | 0.57-1.29 | 9.98E-01 | 0.81 | 0.52-1.27 | 9.98E-01 |
| 1-(1-enyl-palmitoyl)-2-palmitoleoyl-GPC (P-16:0/16:1)* | 0.75 | 0.46-1.23 | 9.98E-01 | 0.70 | 0.42-1.17 | 9.98E-01 |
| 1-(1-enyl-palmitoyl)-2-palmitoyl-GPC (P-16:0/16:0)* | 0.88 | 0.57-1.35 | 9.98E-01 | 0.83 | 0.53-1.31 | 9.98E-01 |
| 1-stearoyl-2-oleoyl-GPI (18:0/18:1)* | 1.04 | 0.78-1.38 | 9.98E-01 | 1.02 | 0.75-1.37 | 9.98E-01 |
| 1-oleoyl-2-arachidonoyl-GPE (18:1/20:4)* | 1.17 | 0.73-1.88 | 9.98E-01 | 1.15 | 0.72-1.85 | 9.98E-01 |
| 1-(1-enyl-stearoyl)-2-linoleoyl-GPE (P-18:0/18:2)* | 0.98 | 0.71-1.35 | 9.98E-01 | 0.97 | 0.68-1.39 | 9.98E-01 |
| 1-linoleoyl-GPG (18:2)* | 0.81 | 0.56-1.16 | 9.98E-01 | 0.79 | 0.55-1.13 | 9.98E-01 |
| thioproline | 0.89 | 0.6-1.3 | 9.98E-01 | 0.88 | 0.6-1.31 | 9.98E-01 |
| 2-methylserine | 1.35 | 0.96-1.89 | 9.98E-01 | 1.34 | 0.95-1.89 | 9.98E-01 |
| glycochenodeoxycholate glucuronide (1) | 1.22 | 0.76-1.95 | 9.98E-01 | 1.25 | 0.77-2.04 | 9.98E-01 |
| glycosyl-N-palmitoyl-sphingosine (d18:1/16:0) | 1.12 | 0.62-2 | 9.98E-01 | 1.08 | 0.58-2.02 | 9.98E-01 |
| methylsuccinoylcarnitine | 1.25 | 0.82-1.92 | 9.98E-01 | 1.29 | 0.82-2.03 | 9.98E-01 |
| catechol glucuronide | 1.28 | 0.88-1.88 | 9.98E-01 | 1.29 | 0.89-1.88 | 9.98E-01 |
| ascorbic acid 2-sulfate | 0.88 | 0.64-1.2 | 9.98E-01 | 0.87 | 0.62-1.22 | 9.98E-01 |
| oleoylcholine | 1.05 | 0.75-1.49 | 9.98E-01 | 1.04 | 0.72-1.5 | 9.98E-01 |
| arachidonoylcholine | 0.98 | 0.72-1.35 | 9.98E-01 | 0.96 | 0.69-1.35 | 9.98E-01 |
| docosahexaenoylcholine | 1.11 | 0.75-1.64 | 9.98E-01 | 1.08 | 0.71-1.66 | 9.98E-01 |
| dihomo-linolenoyl-choline | 1.00 | 0.7-1.41 | 9.98E-01 | 1.00 | 0.69-1.46 | 9.98E-01 |
| 1-linoleoyl-2-linolenoyl-GPC (18:2/18:3)* | 0.72 | 0.49-1.05 | 9.98E-01 | 0.69 | 0.45-1.04 | 9.98E-01 |
| 1-palmitoleoyl-2-linolenoyl-GPC (16:1/18:3)* | 0.92 | 0.63-1.34 | 9.98E-01 | 0.91 | 0.62-1.33 | 9.98E-01 |
| hexadecadienoate (16:2n6) | 0.94 | 0.68-1.31 | 9.98E-01 | 0.90 | 0.63-1.28 | 9.98E-01 |
| palmitoleoylcarnitine (C16:1)* | 1.03 | 0.66-1.63 | 9.98E-01 | 0.99 | 0.62-1.58 | 9.98E-01 |
| pimeloylcarnitine/3-methyladipoylcarnitine (C7-DC) | 1.11 | 0.76-1.61 | 9.98E-01 | 1.09 | 0.73-1.63 | 9.98E-01 |
| 4-hydroxyphenylacetylglutamine | 1.11 | 0.71-1.74 | 9.98E-01 | 1.16 | 0.74-1.82 | 9.98E-01 |
| 2,3-dihydroxy-2-methylbutyrate | 1.03 | 0.72-1.46 | 9.98E-01 | 1.05 | 0.74-1.49 | 9.98E-01 |
| 2'-O-methylcytidine | 1.09 | 0.72-1.65 | 9.98E-01 | 1.09 | 0.7-1.68 | 9.98E-01 |
| gamma-glutamyl-alpha-lysine | 0.84 | 0.57-1.24 | 9.98E-01 | 0.84 | 0.58-1.22 | 9.98E-01 |
| perfluorooctanesulfonate (PFOS) | 1.07 | 0.69-1.65 | 9.98E-01 | 1.05 | 0.68-1.63 | 9.98E-01 |
| N-palmitoyl-sphingadienine (d18:2/16:0)* | 1.13 | 0.81-1.59 | 9.98E-01 | 1.13 | 0.81-1.58 | 9.98E-01 |
| lactosyl-N-nervonoyl-sphingosine (d18:1/24:1)* | 0.62 | 0.38-1.02 | 9.98E-01 | 0.59 | 0.34-0.99 | 9.98E-01 |
| lactosyl-N-behenoyl-sphingosine (d18:1/22:0)* | 0.86 | 0.61-1.2 | 9.98E-01 | 0.82 | 0.58-1.14 | 9.98E-01 |
| glycosyl-N-behenoyl-sphingadienine (d18:2/22:0)* | 1.19 | 0.72-1.97 | 9.98E-01 | 1.16 | 0.68-1.97 | 9.98E-01 |
| N-stearoyl-sphingadienine (d18:2/18:0)* | 1.00 | 0.67-1.5 | 9.98E-01 | 1.03 | 0.68-1.56 | 9.98E-01 |
| 2-hydroxybehenate | 1.00 | 0.77-1.29 | 9.98E-01 | 0.99 | 0.77-1.28 | 9.98E-01 |
| 2-hydroxynervonate* | 0.92 | 0.72-1.16 | 9.98E-01 | 0.89 | 0.68-1.15 | 9.98E-01 |
| N-palmitoylserine | 1.24 | 0.94-1.62 | 9.98E-01 | 1.26 | 0.96-1.65 | 9.98E-01 |
| N-oleoylserine | 0.95 | 0.69-1.3 | 9.98E-01 | 0.94 | 0.68-1.3 | 9.98E-01 |
| hexadecasphingosine (d16:1)* | 0.93 | 0.64-1.36 | 9.98E-01 | 0.95 | 0.63-1.41 | 9.98E-01 |
| ceramide (d16:1/24:1, d18:1/22:1)* | 0.87 | 0.6-1.25 | 9.98E-01 | 0.85 | 0.58-1.25 | 9.98E-01 |
| N-palmitoyl-heptadecasphingosine (d17:1/16:0)* | 0.92 | 0.65-1.29 | 9.98E-01 | 0.93 | 0.65-1.33 | 9.98E-01 |
| ceramide (d18:1/14:0, d16:1/16:0)* | 1.06 | 0.71-1.58 | 9.98E-01 | 1.08 | 0.72-1.61 | 9.98E-01 |
| ceramide (d18:1/17:0, d17:1/18:0)* | 0.90 | 0.58-1.4 | 9.98E-01 | 0.94 | 0.57-1.54 | 9.98E-01 |
| ceramide (d18:2/24:1, d18:1/24:2)* | 0.97 | 0.63-1.5 | 9.98E-01 | 0.98 | 0.63-1.52 | 9.98E-01 |
| glycosyl ceramide (d18:2/24:1, d18:1/24:2)* | 1.04 | 0.6-1.82 | 9.98E-01 | 1.02 | 0.57-1.83 | 9.98E-01 |
| glycosyl-N-(2-hydroxynervonoyl)-sphingosine (d18:1/24:1(2OH))* | 0.80 | 0.55-1.16 | 9.98E-01 | 0.81 | 0.54-1.21 | 9.98E-01 |
| stearoylcholine* | 0.99 | 0.7-1.4 | 9.98E-01 | 0.98 | 0.67-1.44 | 9.98E-01 |
| linoleoylcholine* | 1.00 | 0.71-1.41 | 9.98E-01 | 0.99 | 0.68-1.45 | 9.98E-01 |
| docosatrienoate (22:3n6)* | 1.06 | 0.73-1.55 | 9.98E-01 | 1.01 | 0.71-1.43 | 9.98E-01 |
| nisinate (24:6n3) | 1.05 | 0.77-1.45 | 9.98E-01 | 1.02 | 0.75-1.39 | 9.98E-01 |
| sphingomyelin (d18:0/20:0, d16:0/22:0)* | 1.20 | 0.87-1.66 | 9.98E-01 | 1.20 | 0.85-1.69 | 9.98E-01 |
| sphingomyelin (d18:1/19:0, d19:1/18:0)* | 1.28 | 0.72-2.26 | 9.98E-01 | 1.38 | 0.73-2.61 | 9.98E-01 |
| sphingomyelin (d18:2/18:1)* | 1.24 | 0.8-1.93 | 9.98E-01 | 1.29 | 0.78-2.11 | 9.98E-01 |
| sphingomyelin (d18:2/24:2)* | 1.07 | 0.68-1.68 | 9.98E-01 | 1.09 | 0.69-1.73 | 9.98E-01 |
| sphingomyelin (d18:2/21:0, d16:2/23:0)* | 1.16 | 0.73-1.86 | 9.98E-01 | 1.25 | 0.75-2.1 | 9.98E-01 |
| sphingomyelin (d18:2/23:1)* | 1.13 | 0.66-1.93 | 9.98E-01 | 1.18 | 0.67-2.09 | 9.98E-01 |
| sphingomyelin (d18:1/25:0, d19:0/24:1, d20:1/23:0, d19:1/24:0)* | 1.12 | 0.79-1.57 | 9.98E-01 | 1.10 | 0.78-1.53 | 9.98E-01 |
| sphingomyelin (d17:2/16:0, d18:2/15:0)* | 1.03 | 0.66-1.6 | 9.98E-01 | 1.07 | 0.66-1.72 | 9.98E-01 |
| heneicosapentaenoate (21:5n3) | 1.09 | 0.8-1.48 | 9.98E-01 | 1.05 | 0.76-1.45 | 9.98E-01 |
| linolenoylcarnitine (C18:3)* | 0.83 | 0.57-1.21 | 9.98E-01 | 0.82 | 0.55-1.22 | 9.98E-01 |
| behenoylcarnitine (C22)* | 0.95 | 0.6-1.52 | 9.98E-01 | 0.93 | 0.57-1.5 | 9.98E-01 |
| arachidoylcarnitine (C20)* | 1.18 | 0.81-1.73 | 9.98E-01 | 1.15 | 0.78-1.71 | 9.98E-01 |
| lignoceroylcarnitine (C24)* | 1.34 | 0.94-1.9 | 9.98E-01 | 1.33 | 0.9-1.98 | 9.98E-01 |
| cerotoylcarnitine (C26)* | 0.96 | 0.62-1.5 | 9.98E-01 | 0.97 | 0.63-1.48 | 9.98E-01 |
| ximenoylcarnitine (C26:1)* | 1.13 | 0.8-1.59 | 9.98E-01 | 1.11 | 0.76-1.63 | 9.98E-01 |
| arachidonoylcarnitine (C20:4) | 0.93 | 0.63-1.36 | 9.98E-01 | 0.92 | 0.63-1.35 | 9.98E-01 |
| eicosenoylcarnitine (C20:1)* | 1.06 | 0.67-1.65 | 9.98E-01 | 1.02 | 0.64-1.63 | 9.98E-01 |
| dihomo-linoleoylcarnitine (C20:2)* | 0.97 | 0.63-1.48 | 9.98E-01 | 0.94 | 0.61-1.46 | 9.98E-01 |
| dihomo-linolenoylcarnitine (C20:3n3 or 6)* | 0.90 | 0.57-1.42 | 9.98E-01 | 0.90 | 0.56-1.43 | 9.98E-01 |
| docosahexaenoylcarnitine (C22:6)* | 1.30 | 0.9-1.87 | 9.98E-01 | 1.26 | 0.88-1.81 | 9.98E-01 |
| nervonoylcarnitine (C24:1)* | 1.02 | 0.79-1.32 | 9.98E-01 | 0.97 | 0.73-1.3 | 9.98E-01 |
| glycosyl ceramide (d18:1/20:0, d16:1/22:0)* | 1.26 | 0.85-1.87 | 9.98E-01 | 1.24 | 0.81-1.9 | 9.98E-01 |
| N,N,N-trimethyl-5-aminovalerate | 0.87 | 0.6-1.25 | 9.98E-01 | 0.88 | 0.62-1.25 | 9.98E-01 |
| ethyl alpha-glucopyranoside | 0.99 | 0.73-1.34 | 9.98E-01 | 0.87 | 0.56-1.36 | 9.98E-01 |
| carotene diol (1) | 1.04 | 0.7-1.55 | 9.98E-01 | 1.03 | 0.67-1.58 | 9.98E-01 |
| carotene diol (2) | 1.03 | 0.72-1.49 | 9.98E-01 | 1.03 | 0.69-1.54 | 9.98E-01 |
| carotene diol (3) | 1.03 | 0.71-1.5 | 9.98E-01 | 1.03 | 0.71-1.51 | 9.98E-01 |
| cortolone glucuronide (1) | 1.19 | 0.84-1.69 | 9.98E-01 | 1.22 | 0.85-1.75 | 9.98E-01 |
| (N(1) + N(8))-acetylspermidine | 0.82 | 0.55-1.22 | 9.98E-01 | 0.82 | 0.55-1.23 | 9.98E-01 |
| 5-dodecenoylcarnitine (C12:1) | 0.94 | 0.66-1.33 | 9.98E-01 | 0.91 | 0.64-1.3 | 9.98E-01 |
| hydroxy-CMPF* | 1.18 | 0.82-1.71 | 9.98E-01 | 1.16 | 0.8-1.67 | 9.98E-01 |
| 3-hydroxyoleoylcarnitine | 1.16 | 0.74-1.84 | 9.98E-01 | 1.14 | 0.72-1.8 | 9.98E-01 |
| 3-hydroxyphenylacetoylglutamine | 1.07 | 0.69-1.65 | 9.98E-01 | 1.05 | 0.7-1.59 | 9.98E-01 |
| trans-2-hexenoylglycine | 0.93 | 0.6-1.43 | 9.98E-01 | 0.89 | 0.56-1.41 | 9.98E-01 |
| 2-hydroxyarachidate* | 1.04 | 0.81-1.33 | 9.98E-01 | 1.07 | 0.81-1.42 | 9.98E-01 |
| lyxonate | 1.06 | 0.73-1.53 | 9.98E-01 | 1.04 | 0.73-1.46 | 9.98E-01 |
| dodecenedioate (C12:1-DC)* | 1.01 | 0.62-1.64 | 9.98E-01 | 0.98 | 0.6-1.62 | 9.98E-01 |
| hexadecenedioate (C16:1-DC)* | 0.99 | 0.64-1.54 | 9.98E-01 | 0.95 | 0.6-1.51 | 9.98E-01 |
| octadecenedioate (C18:1-DC) | 0.77 | 0.5-1.21 | 9.98E-01 | 0.74 | 0.47-1.17 | 9.98E-01 |
| heptenedioate (C7:1-DC)* | 0.99 | 0.62-1.59 | 9.98E-01 | 0.97 | 0.59-1.58 | 9.98E-01 |
| octadecadienedioate (C18:2-DC)* | 0.98 | 0.62-1.53 | 9.98E-01 | 0.96 | 0.59-1.56 | 9.98E-01 |
| 3-carboxy-4-methyl-5-pentyl-2-furanpropionate (3-CMPFP)** | 0.82 | 0.55-1.22 | 9.98E-01 | 0.81 | 0.54-1.2 | 9.98E-01 |
| glucuronide of C10H18O2 (7)* | 0.80 | 0.55-1.19 | 9.98E-01 | 0.83 | 0.55-1.23 | 9.98E-01 |
| 3-hydroxystachydrine* | 0.96 | 0.64-1.45 | 9.98E-01 | 0.97 | 0.64-1.47 | 9.98E-01 |
| N-acetyl-2-aminooctanoate* | 1.01 | 0.72-1.41 | 9.98E-01 | 0.98 | 0.67-1.44 | 9.98E-01 |
| hydroxyasparagine** | 1.03 | 0.65-1.62 | 9.98E-01 | 1.06 | 0.69-1.64 | 9.98E-01 |
| perfluorooctanoate (PFOA) | 1.08 | 0.68-1.73 | 9.98E-01 | 1.05 | 0.67-1.64 | 9.98E-01 |
| 3-hydroxybutyroylglycine** | 0.91 | 0.63-1.32 | 9.98E-01 | 0.88 | 0.6-1.28 | 9.98E-01 |
| glyco-beta-muricholate** | 0.92 | 0.66-1.29 | 9.98E-01 | 0.94 | 0.68-1.31 | 9.98E-01 |
| N-methylhydroxyproline** | 0.98 | 0.63-1.52 | 9.98E-01 | 0.99 | 0.62-1.57 | 9.98E-01 |
| N,N,N-trimethyl-alanylproline betaine (TMAP) | 1.00 | 0.71-1.41 | 9.98E-01 | 1.00 | 0.71-1.42 | 9.98E-01 |
| 3-formylindole | 0.87 | 0.58-1.32 | 9.98E-01 | 0.88 | 0.58-1.34 | 9.98E-01 |
| gamma-glutamylcitrulline* | 1.41 | 1-1.99 | 9.98E-01 | 1.41 | 0.97-2.06 | 9.98E-01 |
| glycine conjugate of C10H12O2* | 0.99 | 0.68-1.44 | 9.98E-01 | 1.00 | 0.71-1.43 | 9.98E-01 |
| glycine conjugate of C10H14O2 (1)* | 1.07 | 0.74-1.53 | 9.98E-01 | 1.06 | 0.74-1.52 | 9.98E-01 |
| glutamine conjugate of C7H12O2* | 1.15 | 0.81-1.64 | 9.98E-01 | 1.12 | 0.78-1.61 | 9.98E-01 |
| glutamine conjugate of C6H10O2 (1)* | 1.05 | 0.73-1.5 | 9.98E-01 | 1.04 | 0.73-1.47 | 9.98E-01 |
| glutamine conjugate of C6H10O2 (2)* | 1.07 | 0.75-1.51 | 9.98E-01 | 1.05 | 0.75-1.47 | 9.98E-01 |
| dihydroferulic acid sulfate | 1.02 | 0.66-1.56 | 9.98E-01 | 1.01 | 0.65-1.59 | 9.98E-01 |
| sphingomyelin (d17:1/14:0, d16:1/15:0)* | 0.98 | 0.62-1.55 | 9.98E-01 | 1.00 | 0.6-1.68 | 9.98E-01 |
| tetradecadienoate (14:2)* | 0.98 | 0.68-1.41 | 9.98E-01 | 0.93 | 0.64-1.35 | 9.98E-01 |
| N-carbamoylvaline | 1.20 | 0.81-1.77 | 9.98E-01 | 1.21 | 0.81-1.79 | 9.98E-01 |
| 8-methoxykynurenate | 0.87 | 0.61-1.24 | 9.98E-01 | 0.86 | 0.6-1.24 | 9.98E-01 |
| 3-amino-2-piperidone | 0.85 | 0.53-1.37 | 9.98E-01 | 0.90 | 0.56-1.45 | 9.98E-01 |
| N,N-dimethylalanine | 0.82 | 0.51-1.31 | 9.98E-01 | 0.83 | 0.52-1.33 | 9.98E-01 |
| 3-indoleglyoxylic acid | 0.74 | 0.48-1.15 | 9.98E-01 | 0.76 | 0.49-1.17 | 9.98E-01 |
| 2,2'-Methylenebis(6-tert-butyl-p-cresol) | 0.71 | 0.46-1.11 | 9.98E-01 | 0.70 | 0.46-1.08 | 9.98E-01 |
| ethyl beta-glucopyranoside | 0.97 | 0.62-1.52 | 9.98E-01 | 0.92 | 0.59-1.45 | 9.98E-01 |
| 2-hydroxysebacate | 0.93 | 0.71-1.21 | 9.98E-01 | 0.91 | 0.68-1.2 | 9.98E-01 |
| ascorbic acid 3-sulfate* | 1.05 | 0.67-1.64 | 9.98E-01 | 1.04 | 0.68-1.6 | 9.98E-01 |
| 3-hydroxyhippurate sulfate | 1.21 | 0.83-1.78 | 9.98E-01 | 1.22 | 0.84-1.77 | 9.98E-01 |
| 6-bromotryptophan | 1.28 | 0.87-1.88 | 9.98E-01 | 1.28 | 0.89-1.84 | 9.98E-01 |
| delta-CEHC | 1.40 | 0.91-2.15 | 9.98E-01 | 1.41 | 0.89-2.23 | 9.98E-01 |
| N6,N6-dimethyllysine | 0.96 | 0.6-1.53 | 9.98E-01 | 0.94 | 0.59-1.5 | 9.98E-01 |
| dodecadienoate (12:2)* | 0.82 | 0.57-1.2 | 9.98E-01 | 0.79 | 0.54-1.14 | 9.98E-01 |
| indoleacetoylcarnitine* | 1.11 | 0.77-1.62 | 9.98E-01 | 1.15 | 0.8-1.67 | 9.98E-01 |
| N-acetyl-isoputreanine | 1.19 | 0.82-1.73 | 9.98E-01 | 1.18 | 0.8-1.75 | 9.98E-01 |
| glucuronide of piperine metabolite C17H21NO3 (3)* | 1.39 | 0.86-2.24 | 9.98E-01 | 1.42 | 0.88-2.28 | 9.98E-01 |
| glucuronide of piperine metabolite C17H21NO3 (4)* | 1.05 | 0.69-1.59 | 9.98E-01 | 1.04 | 0.67-1.6 | 9.98E-01 |
| glucuronide of piperine metabolite C17H21NO3 (5)* | 1.04 | 0.67-1.61 | 9.98E-01 | 1.01 | 0.65-1.59 | 9.98E-01 |
| sulfate of piperine metabolite C16H19NO3 (2)* | 0.99 | 0.62-1.57 | 9.98E-01 | 0.97 | 0.6-1.55 | 9.98E-01 |
| sulfate of piperine metabolite C16H19NO3 (3)* | 0.93 | 0.6-1.45 | 9.98E-01 | 0.91 | 0.58-1.42 | 9.98E-01 |
| sulfate of piperine metabolite C18H21NO3 (1)* | 0.95 | 0.63-1.43 | 9.98E-01 | 0.92 | 0.61-1.39 | 9.98E-01 |
| sulfate of piperine metabolite C18H21NO3 (3)* | 0.94 | 0.62-1.43 | 9.98E-01 | 0.90 | 0.59-1.37 | 9.98E-01 |
| 2-naphthol sulfate | 0.88 | 0.61-1.28 | 9.98E-01 | 0.83 | 0.5-1.38 | 9.98E-01 |
| (2,4 or 2,5)-dimethylphenol sulfate | 1.05 | 0.75-1.46 | 9.98E-01 | 1.09 | 0.74-1.61 | 9.98E-01 |
| 4-ethylcatechol sulfate | 0.93 | 0.61-1.41 | 9.98E-01 | 0.92 | 0.59-1.42 | 9.98E-01 |
| 11beta-hydroxyandrosterone glucuronide | 1.19 | 0.76-1.86 | 9.98E-01 | 1.22 | 0.75-1.99 | 9.98E-01 |
| 11beta-hydroxyetiocholanolone glucuronide* | 0.86 | 0.63-1.17 | 9.98E-01 | 0.83 | 0.6-1.15 | 9.98E-01 |
| N2-acetyl,N6,N6-dimethyllysine | 0.93 | 0.62-1.39 | 9.98E-01 | 0.90 | 0.59-1.35 | 9.98E-01 |
| N2-acetyl,N6-methyllysine | 0.90 | 0.64-1.26 | 9.98E-01 | 0.87 | 0.62-1.21 | 9.98E-01 |
| cholic acid glucuronide | 1.41 | 0.91-2.18 | 9.98E-01 | 1.39 | 0.91-2.12 | 9.98E-01 |
| deoxycholic acid glucuronide | 1.54 | 0.98-2.41 | 9.98E-01 | 1.53 | 1.01-2.31 | 9.98E-01 |
| 4-allylcatechol sulfate | 0.89 | 0.64-1.23 | 9.98E-01 | 0.87 | 0.63-1.2 | 9.98E-01 |
| 3-hydroxypyridine glucuronide | 0.99 | 0.69-1.42 | 9.98E-01 | 1.00 | 0.71-1.4 | 9.98E-01 |
| 4-methylhexanoylglutamine | 1.33 | 0.94-1.87 | 9.98E-01 | 1.31 | 0.92-1.86 | 9.98E-01 |
| glycoursodeoxycholic acid sulfate (1) | 0.97 | 0.57-1.64 | 9.98E-01 | 0.95 | 0.55-1.65 | 9.98E-01 |
| dihydrocaffeate sulfate (2) | 1.17 | 0.79-1.73 | 9.98E-01 | 1.16 | 0.78-1.73 | 9.98E-01 |
| lithocholate sulfate (1) | 1.02 | 0.74-1.41 | 9.98E-01 | 1.06 | 0.77-1.45 | 9.98E-01 |
| 3-hydroxyhexanoylcarnitine (1) | 1.04 | 0.74-1.47 | 9.98E-01 | 1.03 | 0.73-1.44 | 9.98E-01 |
| 2-ketocaprylate | 1.11 | 0.77-1.59 | 9.98E-01 | 1.08 | 0.74-1.6 | 9.98E-01 |
| 2,6-dihydroxybenzoic acid | 1.05 | 0.68-1.6 | 9.98E-01 | 1.06 | 0.67-1.67 | 9.98E-01 |
| N,N-dimethyl-5-aminovalerate | 0.79 | 0.54-1.14 | 9.98E-01 | 0.78 | 0.53-1.14 | 9.98E-01 |
| tetrahydrocortisol sulfate (1) | 1.25 | 0.81-1.93 | 9.98E-01 | 1.30 | 0.83-2.04 | 9.98E-01 |
| 3-ethylcatechol sulfate (1) | 1.01 | 0.73-1.4 | 9.98E-01 | 0.99 | 0.66-1.49 | 9.98E-01 |
| 2,3-dihydroxy-5-methylthio-4-pentenoate (DMTPA)* | 1.05 | 0.71-1.54 | 9.98E-01 | 1.07 | 0.74-1.56 | 9.98E-01 |
| 5-hydroxy-2-methylpyridine sulfate | 0.85 | 0.59-1.23 | 9.98E-01 | 0.85 | 0.58-1.24 | 9.98E-01 |
| 3-hydroxy-2-methylpyridine sulfate | 0.93 | 0.65-1.33 | 9.98E-01 | 0.95 | 0.67-1.33 | 9.98E-01 |
| hydroxypalmitoyl sphingomyelin (d18:1/16:0(OH))** | 1.08 | 0.69-1.68 | 9.98E-01 | 1.11 | 0.72-1.71 | 9.98E-01 |
| taurochenodeoxycholic acid 3-sulfate | 1.53 | 1.08-2.17 | 9.98E-01 | 1.51 | 1.05-2.17 | 9.98E-01 |
| tetradecadienedioate (C14:2-DC)* | 0.94 | 0.56-1.58 | 9.98E-01 | 0.92 | 0.56-1.51 | 9.98E-01 |
| pregnenetriol sulfate* | 1.04 | 0.65-1.69 | 9.98E-01 | 1.05 | 0.64-1.71 | 9.98E-01 |
| pregnenetriol disulfate* | 1.09 | 0.67-1.77 | 9.98E-01 | 1.10 | 0.67-1.81 | 9.98E-01 |
| eicosenedioate (C20:1-DC)* | 1.40 | 0.97-2.01 | 9.98E-01 | 1.44 | 0.94-2.21 | 9.98E-01 |
| hydroxy-N6,N6,N6-trimethyllysine* | 1.21 | 0.82-1.77 | 9.98E-01 | 1.24 | 0.87-1.77 | 9.98E-01 |
| undecenoylcarnitine (C11:1) | 1.13 | 0.85-1.5 | 9.98E-01 | 1.10 | 0.83-1.48 | 9.98E-01 |
| 3-hydroxydecanoylcarnitine | 1.06 | 0.72-1.55 | 9.98E-01 | 1.00 | 0.69-1.47 | 9.98E-01 |
| palmitoyl-sphingosine-phosphoethanolamine (d18:1/16:0) | 1.31 | 0.7-2.48 | 9.98E-01 | 1.30 | 0.7-2.43 | 9.98E-01 |
| picolinoylglycine | 0.84 | 0.55-1.3 | 9.98E-01 | 0.85 | 0.53-1.38 | 9.98E-01 |
| 4-vinylcatechol sulfate | 1.02 | 0.71-1.48 | 9.98E-01 | 1.02 | 0.66-1.58 | 9.98E-01 |
| succinoyltaurine | 1.00 | 0.75-1.35 | 9.98E-01 | 1.01 | 0.74-1.38 | 9.98E-01 |
| 3,5-dichloro-2,6-dihydroxybenzoic acid | 1.04 | 0.65-1.68 | 9.98E-01 | 1.07 | 0.63-1.8 | 9.98E-01 |
| metabolonic lactone sulfate | 1.06 | 0.67-1.67 | 9.98E-01 | 1.09 | 0.68-1.75 | 9.98E-01 |
| vanillic acid glycine | 1.11 | 0.68-1.81 | 9.98E-01 | 1.12 | 0.68-1.86 | 9.98E-01 |
| 2-hydroxy-4-(methylthio)butanoic acid | 1.17 | 0.82-1.66 | 9.98E-01 | 1.18 | 0.85-1.65 | 9.98E-01 |
| branched chain 14:0 dicarboxylic acid** | 1.02 | 0.66-1.58 | 9.98E-01 | 1.00 | 0.65-1.53 | 9.98E-01 |
| pentose acid* | 0.88 | 0.62-1.26 | 9.98E-01 | 0.85 | 0.59-1.22 | 9.98E-01 |
| 1-methyl-5-imidazolelactate | 0.86 | 0.6-1.23 | 9.98E-01 | 0.84 | 0.57-1.24 | 9.98E-01 |
| (2 or 3)-decenoate (10:1n7 or n8) | 1.08 | 0.72-1.62 | 9.98E-01 | 1.03 | 0.69-1.53 | 9.98E-01 |
| S-carboxyethylcysteine | 0.93 | 0.62-1.39 | 9.98E-01 | 0.92 | 0.61-1.38 | 9.98E-01 |
| branched-chain, straight-chain, or cyclopropyl 10:1 fatty acid (1)* | 0.97 | 0.64-1.47 | 9.98E-01 | 0.90 | 0.56-1.46 | 9.98E-01 |
| 3-bromo-5-chloro-2,6-dihydroxybenzoic acid* | 0.97 | 0.62-1.52 | 9.98E-01 | 0.97 | 0.61-1.54 | 9.98E-01 |
| decadienedioic acid (C10:2-DC)** | 0.90 | 0.57-1.42 | 9.98E-01 | 0.89 | 0.56-1.4 | 9.98E-01 |
| GlcNAc sulfate conjugate of C21H34O2 steroid** | 1.15 | 0.76-1.75 | 9.98E-01 | 1.17 | 0.75-1.82 | 9.98E-01 |
| cis-3,4-methyleneheptanoate | 0.88 | 0.64-1.22 | 9.98E-01 | 0.86 | 0.62-1.2 | 9.98E-01 |
| cis-3,4-methyleneheptanoylcarnitine | 0.87 | 0.61-1.22 | 9.98E-01 | 0.87 | 0.62-1.22 | 9.98E-01 |
| N-acetyl-2-aminoadipate | 1.09 | 0.79-1.49 | 9.98E-01 | 1.11 | 0.82-1.52 | 9.98E-01 |
| 4-chlorobenzoic acid | 0.93 | 0.57-1.51 | 9.98E-01 | 0.95 | 0.59-1.54 | 9.98E-01 |
| 2-methoxyhydroquinone sulfate (1) | 1.11 | 0.78-1.57 | 9.98E-01 | 1.15 | 0.8-1.64 | 9.98E-01 |
| 2,4-di-tert-butylphenol | 0.77 | 0.5-1.19 | 9.98E-01 | 0.76 | 0.49-1.18 | 9.98E-01 |
| 3-hydroxyoctanoylcarnitine (1) | 1.17 | 0.82-1.65 | 9.98E-01 | 1.11 | 0.79-1.57 | 9.98E-01 |
| 3-hydroxyoctanoylcarnitine (2) | 1.20 | 0.82-1.78 | 9.98E-01 | 1.15 | 0.78-1.69 | 9.98E-01 |
| cis-3,4-methyleneheptanoylglycine | 0.90 | 0.61-1.31 | 9.98E-01 | 0.87 | 0.58-1.31 | 9.98E-01 |
| bilirubin degradation product, C16H18N2O5 (1)** | 1.10 | 0.65-1.85 | 9.98E-01 | 1.06 | 0.61-1.85 | 9.98E-01 |
| bilirubin degradation product, C16H18N2O5 (2)** | 1.41 | 0.94-2.1 | 9.98E-01 | 1.37 | 0.89-2.11 | 9.98E-01 |
[truncated: 1,135,953 more chars]
